# Saprophytic to Pathogenic Mycobacteria: Loss of Cytochrome P450s Vis a Vis Their Prominent Involvement in Natural Metabolite Biosynthesis

**DOI:** 10.3390/ijms24010149

**Published:** 2022-12-21

**Authors:** Ntokozo Minenhle Zondo, Tiara Padayachee, David R. Nelson, Khajamohiddin Syed

**Affiliations:** 1 Department of Biochemistry and Microbiology, Faculty of Science and Agriculture, University of Zululand, KwaDlangezwa 3886, South Africa; 2 Department of Microbiology, Immunology and Biochemistry, University of Tennessee Health Science Center, Memphis, TN 38163, USA

**Keywords:** *Mycobacterium tuberculosis*, biosynthetic gene clusters, natural product, secondary metabolism, cytochrome P450 monooxygenases, pathogens, ecological niches, adaptation, complex metabolites

## Abstract

Cytochrome P450 monooxygenases (P450s/CYPs) are ubiquitous enzymes with unique regio- and stereo-selective oxidation activities. Due to these properties, P450s play a key role in the biosynthesis of natural metabolites. Mycobacterial species are well-known producers of complex metabolites that help them survive in diverse ecological niches, including in the host. In this study, a comprehensive analysis of P450s and their role in natural metabolite synthesis in 2666 mycobacterial species was carried out. The study revealed the presence of 62,815 P450s that can be grouped into 182 P450 families and 345 subfamilies. Blooming (the presence of more than one copy of the same gene) and expansion (presence of the same gene in many species) were observed at the family and subfamily levels. CYP135 was the dominant family in mycobacterial species. The mycobacterial species have distinct P450 profiles, indicating that lifestyle impacts P450 content in their genome vis a vis P450s, playing a key role in organisms’ adaptation. Analysis of the P450 profile revealed a gradual loss of P450s from non-pathogenic to pathogenic mycobacteria. Pathogenic mycobacteria have more P450s in biosynthetic gene clusters that produce natural metabolites. This indicates that P450s are recruited for the biosynthesis of unique metabolites, thus helping these pathogens survive in their niches. This study is the first to analyze P450s and their role in natural metabolite synthesis in many mycobacterial species.

## 1. Introduction

Cytochrome P450 monooxygenases (CYPs/P450s) are undoubtedly one of the most extensively studied enzymes due to their role in organisms’ primary and secondary metabolism [1,2,3]. The word “cytochrome” indicates the presence of a chromophore such as heme as a prosthetic group, and “P450” denotes their characteristic absorbance spectrum of 450 nm wavelength when they are reduced and complexed with carbon monoxide [4,5,6,7]. The word “monooxygenase” indicates the incorporation of one oxygen into substrates by these enzymes [4,5,6,7,8]. However, since their discovery, it has been observed that P450s catalyze diverse enzymatic reactions with regio- and stereo-selectivity [9,10,11]. Due to their unique enzymatic properties, P450s were exploited for their biomedical and biotechnological applications [12,13,14,15,16,17].

P450s are ubiquitous, as they have been found in living and non-living entities such as viruses [3,18]. The International P450 Nomenclature Committee developed a nomenclature and annotation method for correctly identifying P450s in species across all the biological kingdoms [19,20,21]. The nomenclature system begins with the prefix “CYP” for cytochrome P450 monooxygenase, followed by an Arabic numeral designating the family, a capital letter representing the subfamily, and an Arabic digit specifying the individual P450 in a family. The annotation criteria include assigning family and subfamily with >40% identity belonging to the same family and all P450s with >55% identity belonging to the same subfamily [19,20,21,22]. All researchers have universally accepted this nomenclature and annotation method; thus, any P450 identified should be subjected to this criterion for its proper identification.

The application of P450 in synthesizing natural metabolites has gained momentum [23,24,25]. Natural products are metabolites, either primary or secondary, produced by organisms. Primary metabolites are involved in organisms’ physiology. In contrast, secondary metabolites, although they have no direct role in organisms’ physiology, tend to play a role indirectly in helping organisms survive. The ongoing genome sequencing rush revealed many P450s from the species across the biological kingdom [3]. It is impossible to clone, express, and characterize many P450s. Due to this hurdle, an in silico analysis of P450s will help understand their role in different biological processes including in the biosynthesis of natural products.

In silico studies based on the genome-wide analysis of P450s and their role in the biosynthesis of natural products revealed the presence of many P450s in biosynthetic gene clusters (BGCs) in bacterial species. BGCs are physical groupings of two or more genes in a genome that is responsible for the biosynthesis of a metabolite [26]. An analysis of BGCs across different bacterial species revealed that *Salinispora* species have the highest percentage of P450s, and *Bacteroidetes* species and cyanobacterial species have the lowest percentage of P450s as part of BGCs [27,28,29,30,31,32,33,34,35,36,37]. The percentage of P450s as part of BGCs from highest to lowest is as follows: *Salinispora* species (47%) > *Streptomyces* species (23% > *Firmicutes* species (18%) > mycobacterial species (15%) > proteobacterial species (12%) > *Bacteroidetes* species = cyanobacterial species (8%).

Mycobacterial P450s gained attention due to their potential as drug targets [38] and for the production of valuable human metabolites [39]. CYP121A1 and CYP128A1 from *Mycobacterium tuberculosis* H37Rv are involved in the biosynthesis of natural metabolites mycocyclosin [37,39,40,41] and sulfomenaquinone [40]. A point to be noted is that *CYP121A1* is essential for the survival of *M. tuberculosis*, indicating that the metabolite it synthesizes is a vital primary metabolite [37,39,40,41]. Menaquinone is a primary metabolite involved in the electron transport in *M. tuberculosis* [40]. CYP139 family members were found to be part of different BGC types, indicating their involvement in the biosynthesis of diverse natural compounds [41]. Based on the results, the authors proposed that these metabolites might provide mycobacterial species with advantageous traits in diverse niches competing with other microbial or viral agents and helping these microbes infect hosts by interfering with the host’s metabolism and immune system [41]. A genome-wide analysis of P450s in 60 mycobacterial species revealed that 15% of P450s belonging to 31 different families are part of BGCs [35]. It is well-known that mycobacterial species have complex metabolites as part of their cellular structure, and some of these metabolites are well-known for their virulence [42,43,44]. Based on P450s and their BGCs, the authors concluded that these P450s of BGCs possibly play a role in the synthesis of complex metabolites [36].

Previously mentioned studies are limited to a single family (CYP121, CYP128, and CYP139) and a few species (60 species). To date, a comprehensive analysis of P450s and their role in natural metabolites biosynthesis in a large number of mycobacterial species has not been carried out. This study aims to address this research gap to understand the role of P450s in natural product biosynthesis concerning different mycobacterial categories.

## 2. Results and Discussion

### 2.1. Saprophytic to Pathogen Life Style Led to the Loss of P450s in Mycobacterial Species

Genome-wide data mining and annotation for P450s in 2666 mycobacterial species revealed the presence of 62,815 P450s and 90 P450 fragments/pseudo genes in their genomes (Table 1 and Table S1). The 2666 mycobacterial species include *Mycobacterium tuberculosis* complex (MTBC) (2128 species), *M. chelonae-abscessus* complex (MCAC) (255 species), *M. avium* complex (MAC) (106 species), Mycobacteria causing leprosy (MCL) (four species), Nontuberculous mycobacteria (NTM) (163 species) and Saprophytes (SAP) (10 species). A comparative analysis with other bacterial species revealed that mycobacterial species have a high average number of P450s (24 P450s) compared to other bacterial species, only exceeded by *Streptomyces* species (27 P450s) (Table 1), which is in agreement with the previously mentioned studies. [36,37]. However, it is worthy of note that only 126 *Streptomyces* species data are available for comparison with 2666 mycobacterial species. Thus, analysis of more *Streptomyces* species probably bridges the gap and shows a similar trend to mycobacterial species. Nonetheless, it is clear that among the bacterial population, actinomycetes have the highest average number of P450s in their genomes (Table 1), indicating that P450s play a key role in their primary and secondary metabolism, including adaptation to diverse ecological niches as described elsewhere [35,36,37]. Among mycobacterial species, *Mycolicibacterium rhodesiae* JS60 has the highest (95 P450s), and *Mycobacterium leprae* Br492 has the lowest number of P450s (3 P450s) in their genomes (Table S1).

An analysis of the P450 profile of six mycobacterial categories revealed a gradual loss of P450s from SAP to MTBC (Table 2 and Table S1). The order is as follows: SAP (35–95:51) > MAC (28–69:48)  = NTM (9–89:48) > MCAC (22–52:26) > MTBC (5–74:20) > MCL (3–7:5), where the minimum and maximum number of P450s are shown, and after the semicolon, the average number of P450s, are shown in parentheses. This suggests that during the progression from a saprophytic to a pathogenic lifestyle, mycobacterial species lost P450s (Table 2 and Table S1). The P450 count differences observed among different mycobacterial groups were found to be statistically significant (Table S2). This phenomenon of the gradual loss of P450s in mycobacterial species from SAP to MTBC/MCL was previously reported [35]. However, in the previous study, only 60 mycobacterial species were analyzed [35]. The observation of the same phenomenon in this study, where many species (2666 species) were examined, strongly supports the hypothesis that mycobacterial species lost P450s in their genomes to adapt to diverse ecological niches. As described elsewhere [35,36,37,41,45], the P450s retained in these species played a crucial role in their adaptation to diverse ecological niches. A detailed analysis of P450 key features in six mycobacterial categories is presented in Table 2.

### 2.2. P450 Family and Subfamily Blooming/Expansion in Mycobacterial Species

**Figure 1:**
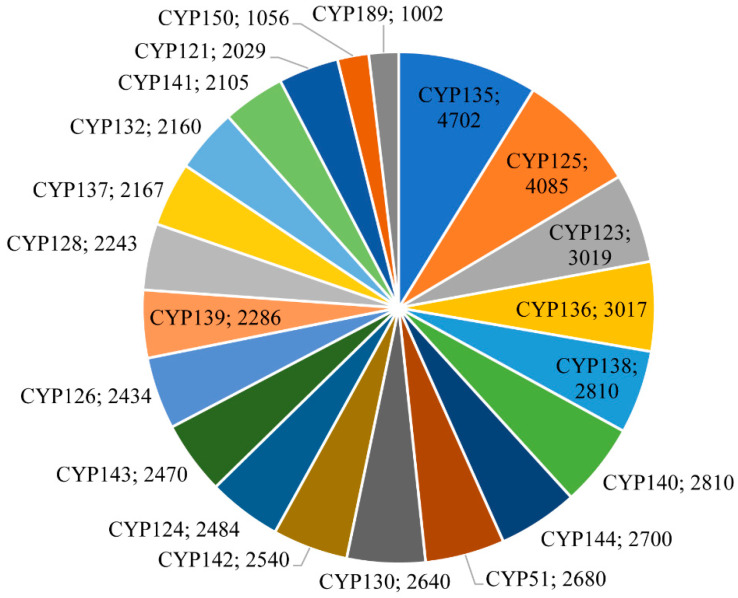
Comparative analysis of P450 families in 2666 mycobacterial species. P450 families with >1000 members were presented in the figure. The P450 family name and number of P450s are shown in the figure. Detailed information on P450 families and subfamilies is presented in Table S3.

Based on the International P450 Nomenclature Committee rules [19,20,22], the percentage identity of >40% for a family and >55% for a subfamily, the 62,815 P450s from 2666 mycobacterial species can be grouped into 182 P450 families and 345 P450 subfamilies (Figure 1 and Table S3). Among mycobacterial species, *M. rhodesiae* JS60 had the highest number of P450s and also had the highest number of P450 families (53) and subfamilies (75) (Table S1). No P450 family was conserved in the mycobacterial species (Table S1). Among 182 P450 families, 21 P450 families contributed 85% of the total P450s in the mycobacterial species (Figure 1 and Table S3), indicating their important role in these species. Among the P450 families, CYP135 is dominant with 4702 members, followed by CYP125 with 4085 members, CYP123 with 3019 members, and CYP136 with 3017 members (Figure 1 and Table S3). It is safe to say that these P450 families bloomed (present more than a member in a species) in mycobacterial species, considering the number of species analyzed in this study. The P450 families CYP138, CYP140, CYP144, CYP51, CYP130, CYP142, CYP124, CYP143, CYP126, CYP139, CYP128, CYP137, CYP132, CYP141, and CYP121 have members between 2000–3000, indicating their expansion in mycobacterial species (Figure 1 and Table S3). Contrary to the P450 families that bloomed/expanded, 43 P450 families had a single member, and 25 P450 families had only two members (Table S3).

Among P450 families, CYP107 had the highest number of subfamilies (15), followed by CYP125 (13 subfamilies) and CYP105 (11 subfamilies) (Table S3). The Blooming/expansion phenomenon is also observed at a P450 subfamily level (Table 3 and Table S3). A P450 subfamily analysis revealed that specific subfamilies were bloomed or expanded in mycobacterial species, indicating a selective preference for distinct subfamilies by the species (Table 3 and Table S3).

A comparative analysis with other bacterial species revealed that mycobacterial species have the highest number of P450 families and subfamilies, but only next to the *Streptomyces* species (Table 1). This suggests that the *Streptomyces* species has the highest P450 family and subfamily diversity compared to mycobacterial species (Table 1).

### 2.3. Different Mycobacterial Categories Have Distinct P450 Profiles

**Figure 2:**
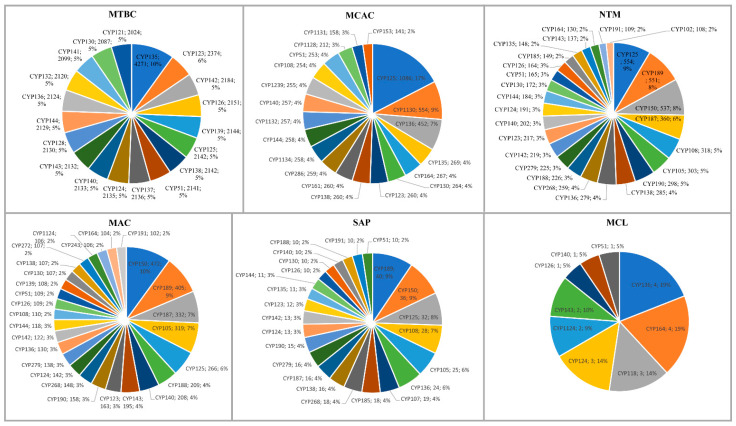
Comparative analysis of P450s families in six mycobacterial categories. The P450 family, members, and their percentage in the total number of P450s in a category are presented in the figure. Abbreviations: MTBC, *Mycobacterium tuberculosis* complex; MCAC, *M. chelonae-abscessus* complex; MAC, *M. avium* complex; MCL, Mycobacteria causing leprosy; NTM, Nontuberculous mycobacteria and SAP, Saprophytes. Detailed information on P450 families with their specific count for each mycobacterial category is presented in Table S4.

P450s play a key role in organisms’ adaptation vis a vis lifestyle impacts of P450 content in their genome [30,32,34,35,36,37]. This phenomenon is evident in mycobacterial species, as mycobacterial categories have distinct P450 profiles (Figure 2 and Table 2 and Table S4). Among mycobacterial categories, NTM had the highest number of P450 families and subfamilies (145 and 261), followed by SAP (66 and 101), MTBC (66 and 95), MAC (59 and 88), MCAC (37 and 48), and MCL (nine families and subfamilies) (Table 2 and Table S4). A point worthy of note is that despite the lower number of species analyzed for NTM (163 species) compared to MTBC (2128 species) and MCAC (255 species), NTM has the highest number of P450 families (145) and subfamilies (261) (Table 2 and Table S4). The same can be seen when comparing them to SAP and MTBC, where only ten species of SAP and 2128 species of MTBC had the same number of P450 families (Table 2 and Table S4). This indicates that NTM had a high P450 family and subfamily diversity among mycobacterial categories, indicative of diverse P450s in the species.

A P450 family conservation analysis revealed different P450 families conserved in different mycobacterial categories. CYP135 is conserved in MTBC, CYP125 is conserved in MCAC, CYP105 and CYP150 are conserved in MAC, CYP189 is conserved in SAP, and CYP164 is conserved in MCL (Tables S1 and S5). No P450 family was conserved in NTM, possibly due to the high P450 family diversity mentioned above. However, CYP125 was the dominant P450 family in NTM (Table 2). The analysis of unique and shared P450 families among mycobacterial categories revealed that NTM has the highest number of unique P450 families (71 families), followed by SAP (23 families), MAC (15 families), MTBC (11 families), and MCAC (six families) (Table 4 and Table S5). No P450 family was unique for MCL (Table 4 and Table S5). As indicated in Table 4, many P450 families are shared among mycobacterial groups. CYP105, CYP125, CYP150, and CYP189 families are dominantly shared across mycobacterial categories (Table 4 and Table S5). This indicates that these P450 families play an important role in mycobacterial species; thus, they are not only retained but also bloomed/expanded (Table 3). MCL shares the CYP136 and CYP164 families with other bacterial groups (Table 4).

### 2.4. CYP121, CYP124, and CYP128 P450s Are Part of the Same Biosynthetic Gene Cluster

**Figure 3:**
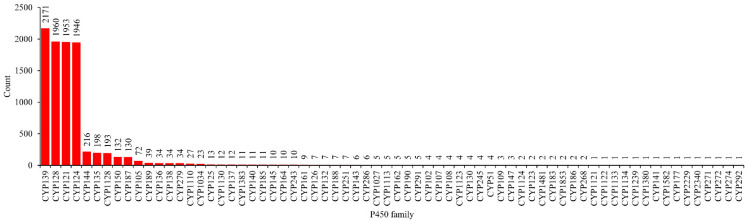
Comparative analysis of P450s involved in natural metabolite biosynthesis in mycobacterial species. The number next to the bars represents the number of members in a P450 family. Detailed information on the P450s part of biosynthetic gene clusters (BGCs), their species names, and BGC types are presented in Table S6. An analysis of the member P450s part BGCs as per their mycobacterial categories is presented in Table S7.

An analysis of P450s involved in natural metabolite biosynthesis in the mycobacterial species revealed that a total of 9399 P450s out of 62,815 (15%) are part of BGCs (Table 1 and Table S6). Mycobacterial species have the highest percentage of P450s that are part of BGCs compared to cyanobacterial species, *Bacteroidetes* species, *Firmicutes* species, and proteobacterial species (Table 1). However, *Streptomyces*-, and *Salinispora*-species had the highest percentage of P450s part of BGCs compared to the mycobacterial species (Table 1). This indicates that actinomycetes have more P450s involved in natural metabolite biosynthesis among bacterial species and that particularly the *Salinispora* species have a larger percentage of P450s as a part of BGCs (Table 1). Among 182 P450 families identified in the mycobacterial species, only 68 P450 families (37%) are part of BGCs (Figure 3 and Table S6).

Among 68 P450 families that are part of BGCs, CYP139 was the most dominant with 2171 members, followed by CYP128 with 1960 members, CYP121 with 1953 members, and CYP124 with 1946 members (Figure 3 and Table S6). In total, these four P450 families contributed 85% of the P450s part of BGCs in mycobacterial species (Figure 3 and Table S6). A point to be noted is that these four P450 families possibly play a key role in natural metabolite biosynthesis, and thus are expanded in mycobacterial species (see Section 2.2). One example is CYP139 BGC clusters that are known to produce metabolites that provide mycobacterial species with advantageous traits in diverse niches competing with other microbial or viral agents, which might help these microbes infect hosts by interfering with the host’s metabolism and immune system [41]. CYP128 was found to play an essential role in producing a metabolite that acts as a negative regulator of virulence [40,46]. CYP121 is a crucial P450 and drug target against pathogenic mycobacterial species and is involved in synthesizing a metabolite named mycocyclosin [38,47,48,49]. CYP124 is a lipid hydroxylase and secondary drug target in pathogenic mycobacterial species [38,50,51]. A recent study reported that CYP128,CYP121, and CYP124 are part of the same BGC, indicating their collective role in synthesizing complex natural metabolites that play an important role in the mycobacterial species [46]. In this current study, we also observed that these three P450s are part of the same BGC (Table S6), further supporting the previous observation and conclusion on the collective role of these P450s in synthesizing natural metabolites in mycobacterial species [46].

Five P450 families: CYP144 with 216 members, CYP135 with 198 members, CYP1128 with 193 members, CYP150 with 132 members, and CYP187 with 130 members, contributed 9% of the P450s part of BGCs in mycobacterial species (Figure 3 and Table S7). The remaining 59 P450 families contributed only 6% of the P450s part of BGCs, indicating their minor role in natural metabolite biosynthesis in mycobacterial species (Figure 3 and Table S7). A point worthy of note is that with regard to CYP135, despite being the most dominant P450 family and CYP144 and CYP150 families being expanded in mycobacterial species (Figure 1), only a fraction of these family members are part of BGCs (Figure 3 and Table S7). Furthermore, the CYP125, CYP123, CYP136, CYP138, and CYP140 families, despite being bloomed/expanded (see Section 2.2), their role in natural metabolite biosynthesis is negligible as only a few members were found to be part of BGCs (Figure 3 and Table S6). This suggests that these P450s might be involved in key primary metabolism and thus are bloomed/expanded in these species. This also supports previous observations that the dominant P450 family may not necessarily play a role in natural metabolite biosynthesis [27,28,32,33,34,35,37]. Detailed information on the P450s part of BGCs, their species name, and BGC type and the similar known-gene cluster is presented in Table S6, and the data on the analysis of P450s that are part of BGCs is shown in Table S7.

### 2.5. More P450s Are Involved in Natural Metabolite Biosynthesis in Pathogenic Mycobacterial Species

**Figure 4:**
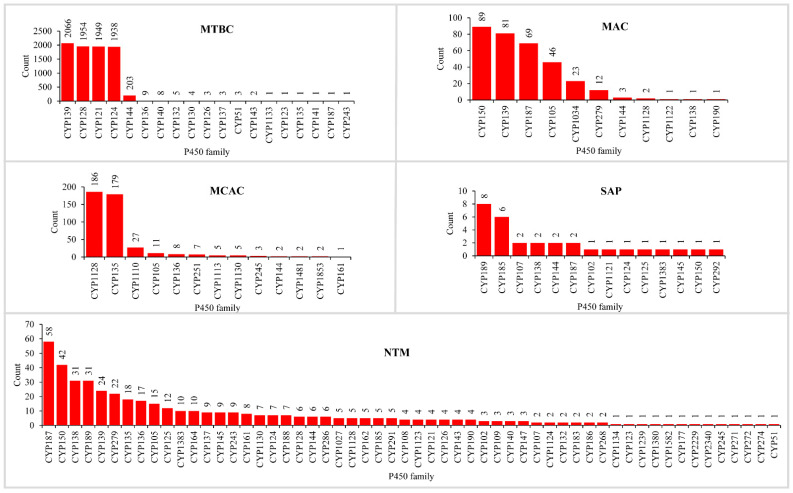
Comparative analysis of P450s that are part of biosynthetic gene clusters (BGCs) in mycobacterial categories. Detailed information on the P450s part of BGCs, their species names, and BGC types are presented in Table S6. A P450 family analysis in individual categories is presented in Table S7. Abbreviations: MTBC, *Mycobacterium tuberculosis* complex; MCAC, *M. chelonae-abscessus* complex; MAC, *M. avium* complex; MCL, Mycobacteria causing leprosy; NTM, Nontuberculous mycobacteria; and SAP, Saprophytes.

The analysis of P450s involved in natural metabolite biosynthesis revealed that more P450s are part of BGCs in pathogenic mycobacteria compared to non-pathogenic bacteria (Figure 4 and Table 2 and Table S7). The number of P450s that are part of BGCs in MTBC was the highest (8153 P450s), followed by NTM (450 P450s), MCAC (438 P450s), MAC (328 P450s), and SAP (30 P450s) (Figure 4 and Table 2 and Table S7). As expected, due to reduced genome size and having few P450s in their genomes, MCL has no P450s as part of BGCs, and thus, we did not include MCL for comparative analysis, which is the same as followed elsewhere [35]. One can argue that the number of species analyzed for MTBC is the highest compared to other categories, and thus one can see the highest number of P450s as part of BGCs (Table 2). To clarify and nullify this argument, we have compared the percentage of P450s part of BGCs in different mycobacterial categories (Table 2). An analysis of the percentage of P450s part of BGCs revealed that, indeed, MTBC has the highest percentage of P450s (19%) part of BGCs, followed by MCAC (6.7%), MAC (6.4%), SAP (5.9%), and NTM (5.8%) (Table 2). This suggests that pathogenic mycobacterial species such as MTBC indeed have more P450s as part of BGCs and, thus, more P450s in these species involved in the biosynthesis of natural metabolites. The point to be noted is that MTBC has more P450 parts of BGCs (Table 2) despite having the lowest number of P450s in their genomes compared to MCAC, MAC, NTM, and SAP (Table 1). This indicates that P450s in MTBC may play a vital role in the biosynthesis of natural metabolites, thus helping these organisms survive in the host, as mentioned in Section 2.4.

Analysis of the P450 families part of BGCs revealed that NTM has the highest number of P450 families part of BGCs, followed by MTBC, SAP, MCAC, and MAC (Figure 4 and Table S7). The number of P450 families part of BGCs followed the same pattern as the number of P450 families in these categories, indicating that diverse P450s are indeed involved in natural metabolite biosynthesis in NTM (Figure 4 and Table 2). A clear picture emerged when we compared the percentage of P450 families as part of BGCs, where NTM still had the highest P450 families as part of the BGCs, followed by MCAC, MTBC, SAP, and MAC (Table 2). The lowest percentage of P450 families part of BGC indicates the blooming/expansion of a particular P450 family. This is true, as few P450 families are populated in BGCs in different mycobacterial categories (Figure 4 and Table S7). Four P450 families such as CYP139, CYP128, CYP121, and CYP124, have contributed 97% of P450 families as part of BGCs in MTBC; CYP150, CYP139, CYP187, and CYP105 families contributed 87% in MAC, and CYP1128 and CYP135 families contributed 83% in MCAC (Figure 4 and Table S7) indicating that these P450 families were preferred in these species possibly due to their importance in the natural product synthesis. Pathogenic mycobacterial species seem to have recruited more P450 families belonging to the same family for natural metabolite biosynthesis. In contrast, the non-pathogenic mycobacterial species have fewer and more diverse P450s for natural metabolite biosynthesis. This suggests that natural metabolites produced by MTBC play a role in their survival in the host (as mentioned in Section 2.4). Thus, P450s play a crucial role in synthesizing these metabolites.

## 3. Materials and Methods

### 3.1. Species and Their Genome Database Information

Mycobacterial species genomes (permanent and finished draft genomes) available for public use at the Joint Genome Institute Integrated Microbial Genomes and Microbiomes (JGI IMG/M) [52] were used in the study (last accessed on April 2022). Information on the species and their genome IDs used in the study is provided in Table S1.

### 3.2. Grouping of Mycobacterial Species

The mycobacterial species were grouped into six categories following the criteria described elsewhere [35]. The six categories include *Mycobacterium tuberculosis* complex (MTBC), *M. chelonae-abscessus* complex (MCAC), *M. avium* complex (MAC), Mycobacteria causing leprosy (MCL), nontuberculous mycobacteria (NTM), and Saprophytes (SAP). Briefly, mycobacterial species are grouped into six categories based on their characteristic features, including ecological niches and the nature and site of infection, as described elsewhere [53]. Also, a taxonomical grouping of mycobacterial species is considered as described elsewhere [54]. Mycobacterial species and their categories are presented in Table S1.

### 3.3. Genome Data Mining and Annotation of P450s

Genome data mining and the identification of P450s in mycobacterial species were carried out following the protocol described elsewhere [28,37,41]. Briefly, each mycobacterial species genome available at JGI IMG/M [52] was searched for P450s using the InterPro code “IPR001128”. The hit protein sequences were then searched for the presence of P450 characteristic motifs such as EXXR and CXG [55,56]. Proteins with one of these motifs or short amino acid length are considered as P450-fragments. P450 fragments were not considered for the final P450 family and subfamily count. Proteins having both motifs were selected for assigning the family and subfamilies. Following the International P450 Nomenclature Committee rule [19,20,22], proteins with >40% identity and >55% identity will be grouped under the same family and subfamily, respectively. P450s with less than 40% identity were assigned to a new P450 family. Mycobacterial species P450s identified in this study and their protein sequences, assigned names, and species are presented in Supplementary Dataset S1. Information on homolog P450s and percentage identity used to assign names for mycobacterial P450s is presented in Table S8.

### 3.4. Identification of P450s Part of BGCs

P450s that are part of BGCs were identified following the method described elsewhere [28,37,41]. Briefly, for each mycobacterial species genome available at JGI IMG/M [52], the BGCs were searched for the presence of P450s using the P450 gene ID. The cluster type is noted if a P450 is found as part of the cluster. The gene cluster sequence was downloaded and submitted to anti-SMASH (antibiotics and Secondary Metabolite Analysis Shell) [57] to find a similar known cluster. The results were recorded in Excel spreadsheets and represented species-wise smBGCs, smBGC type, percentage similarity to known gene clusters, and P450s part of specific BGCs.

### 3.5. P450 Key Features Analysis

All calculations were carried out following the procedure reported previously by our laboratory [29,30]. The average number of P450s was calculated using the formula: Average number of P450s = Number of P450s/Number of species. The percentage of P450s that formed part of BGCs was calculated using the formula: Percentage of P450s part of BGCs = 100 × Number of P450s part of BGCs/Total number of P450s present in species. The P450 family/subfamily is considered bloomed when a member count exceeds the number of species and expands when the member count exceeds >500. The statistical significance of the P450 count among different mycobacterial categories was calculated using Welch’s T-test calculator (https://www.statskingdom.com/150MeanT2uneq.html, accessed on 29 October 2022). The average number of P450s, standard deviation, and sample size were used as inputs. The results for the null hypothesis, *p*-value, T-value, and effect size (d) for different mycobacterial categories were presented in a tabular format (Table S2).

### 3.6. Comparative Analysis of P450s and BGCs Data

For comparative analysis of P450s and BGCs, information for bacterial species belonging to different groups such as phyla, *Firmicutes* [34], *Bacteroidetes* [32], *Proteobacteria* [30], and *Cyanobacteria* [27], and the genera *Streptomyces* [36,37], and *Salinispora* [28] was resourced from published articles.

## 4. Conclusions

Cytochrome P450 monooxygenases (CYPs/P450s) play a key role in synthesizing natural metabolites in organisms. They attribute diversity to the metabolites by performing unique regio- and stereo-selective oxidation reactions. Mycobacterial species have complex metabolites that help them survive in diverse ecological niches. Previous studies limited to a few P450 families or a few species indicated that P450s play a role in synthesizing natural metabolites in mycobacterial species. The availability of many mycobacterial genomes allowed us to look into the P450s role in the biosynthesis of natural metabolites concerning their lifestyle. This study’s results indicated that despite having a low number of P450s, the pathogenic mycobacterial species used most of the available P450s to synthesize natural metabolites. In contrast, non-pathogenic mycobacterial species had fewer P450s playing a role in the biosynthesis of natural metabolites. This suggests that the lifestyle of mycobacterial species changed the P450 profiles vis a vis P450s playing a role in these species’ adaptation to different niches as observed in other bacterial species. Characterizing P450 biosynthetic gene cluster metabolites will provide insights into their role in mycobacterial physiology.

## Figures and Tables

**Table 1:** Comparative analysis of key features of P450s and their association with secondary metabolism in different bacterial species. Abbreviation: No., number of; BGCs: biosynthetic gene clusters.

Category	*Salinispora* species	*Streptomyces* species	Mycobacterial species	Cyanobacterial species	*Bacteroidetes* species	*Firmicutes* species	Alphaproteobacterial species	Betaproteobacterial species	Gammaproteobacterial species	Deltaproteobacterial species	Epsilonproteobacterial species
Species analyzed	126	203	2666	114	334	972	599	513	1261	107	216
Species with P450s	126	203	2666	114	77	229	229	290	169	23	53
Percentage of species with P450s	100	100	100	100	23	24	38	57	13	21	25
No. of P450s	2643	5460	62,815	341	98	712	873	603	277	333	53
No. of families	45	253	182	36	21	14	143	79	81	74	2
No. of subfamilies	103	698	345	79	28	53	214	119	102	171	2
Dominant P450 family	CYP105	CYP107	CYP135	CYP110	CYP1103	CYP107	CYP202	CYP116	CYP133 & CYP107	CYP107	CYP172
Average No. of P450s	21	27	24	3	1	3	4	2	2	14	1
No. of P450s part of BGCs	1236	1231	9399	27	8	126	21	107	49	69	0
No. of P450 families part of BGCs	35	135	68	6	5	10	16	18	22	37	0
Percentage of P450s part of BGCs	47	23	15	8	8	18	2	18	18	21	0
Reference(s)	[28]	[36,37]	This work	[27]	[32]	[34]	[33]	[30]	[29]	[30]	[30]

**Table 2:** Comparative analysis of key features of P450s and their association with secondary metabolism in mycobacterial categories. A statistical analysis of P450 counts among different mycobacterial categories was presented in Table S2. Abbreviations: MTBC, *Mycobacterium tuberculosis* complex; MCAC, *M. chelonae-abscessus* complex; MAC, *M. avium* complex; MCL, Mycobacteria causing leprosy; NTM, nontuberculous mycobacteria; and SAP, Saprophytes; No., number of; BGCs, biosynthetic gene clusters.

Category	MTBC	MCAC	NTM	MAC	SAP	MCL
Total No. of species analysed	2128	255	163	106	10	4
Total No. of P450s	42,917	6519	7760	5093	505	21
P450 fragments/pseudo	2	10	69	3	6	0
Average No. of P450s	20	26	48	48	51	5
Min No. of P450s	5	22	9	28	35	3
Maximum No. of P450s	74	52	89	69	95	7
No. of P450 families	66	37	145	59	66	9
No of P450 subfamilies	95	48	261	88	101	9
Dominant P450 family	CYP135	CYP125	CYP125	CYP150	CYP189	CYP136 & CYP184
No of the P450s part of BGCs	8153	438	450	328	30	0
No. of P450 families part of BGCs	19	13	56	11	15	0
Percentage of P450s part of BGCs	19.0	6.7	5.8	6.4	5.9	0
Percentage of P450 families part of BGCs	28.8	35.1	38.6	18.6	22.7	0

**Table 3:** Analysis of P450 subfamily blooming/expansion in mycobacterial species. Subfamily information for P450 families with >500 members is presented in the table, along with the nature of the subfamily (blooming/expansion). A detailed analysis of P450 subfamilies is shown in Table S3.

P450 Family	Count	Subfamily	Count	Nature of the Subfamily
CYP123	3019	A	2908	Bloomed
CYP125	4085	A	3978	Bloomed
CYP136	3017	A	2687	Bloomed
CYP105	674	Q	377	Expanded
CYP108	714	B	703	Expanded
CYP121	2029	A	2029	Expanded
CYP124	2484	A	2425	Expanded
CYP126	2434	A	2429	Expanded
CYP128	2243	A	2143	Expanded
CYP130	2640	A	2640	Expanded
CYP132	2160	A	2160	Expanded
CYP135	4702	A	2149	Expanded
		B	2543	Expanded
CYP137	2167	A	2167	Expanded
CYP138	2810	A	2733	Expanded
CYP139	2286	A	2286	Expanded
CYP140	2810	A	2401	Expanded
CYP141	2105	A	2105	Expanded
CYP142	2540	A	2507	Expanded
CYP143	2470	A	2387	Expanded
CYP144	2700	A	2421	Expanded
CYP150	1056	A	932	Expanded
CYP164	514	A	505	Expanded
CYP189	1002	A	987	Expanded
CYP51	2680	B	2680	Expanded

**Table 4:** Analysis of unique and shared P450 families among mycobacterial categories. The P450 family dominantly shared between mycobacterial categories is listed in parenthesis. A detailed analysis of P450 families and their member count is presented in Table S5. Abbreviations: MTBC, *Mycobacterium tuberculosis* complex; MCAC, *M. chelonae-abscessus* complex; MAC, *M. avium* complex; MCL, Mycobacteria causing leprosy; NTM, Nontuberculous mycobacteria; and SAP, Saprophytes.

Category	MTBC	MCAC	NTM	MAC	SAP	MCL
MTBC	11	22 (CYP125)	49 (CYP189)	36 (CYP150)	34 (CYP189)	8 (CYP136 & CYP164)
MCAC	22 (CYP125)	6	28 (CYP125)	20 (CYP105)	19 (CYP125)	5 (CYP136 & CYP164)
NTM	49 (CYP189)	28 (CYP125)	71	34 (CYP189)	29 (CYP189)	7 (CYP164)
MAC	36 (CYP150)	20 (CYP105)	34 (CYP189)	15	25 (CYP189)	9 (CYP136 & CYP164)
SAP	34 (CYP189)	19 (CYP125)	29 (CYP189)	27 (CYP189)	23	5 (CYP136)
MCL	8 (CYP136 & CYP164)	5 (CYP136 & CYP164)	7 (CYP164)	9 (CYP136 & CYP164)	5 (CYP136)	0

## Data Availability

Not applicable.

## Supplementary Materials

The following supporting information can be downloaded at: https://www.mdpi.com/article/10.3390/ijms24010149/s1.
