# Supplementary material for: Saprophytic to Pathogenic Mycobacteria: Loss of Cytochrome P450s Vis a Vis Their Prominent Involvement in Natural Metabolite Biosynthesis
Source: Int J Mol Sci. 2022 Dec 21;24(1):149. doi: 10.3390/ijms24010149 (PMC9820752; doi:10.3390/ijms24010149)
Supplement: Supplementary file 1 [file Supplementary Dataset 1.pdf › Supplementary Dataset 1.pdf]

Supplementary Materials

Saprophytic to Pathogenic Mycobacteria: Loss of Cytochrome P450s vis a vis Their Prominent Involvement in Natural Metabolite Biosynthesis

Ntokozo Minenhle Zondo [1,†], Tiara Padayachee [1,†], David R. Nelson [2,*] and Khajamohiddin Syed [1,*]

[1] Department of Biochemistry and Microbiology, Faculty of Science and Agriculture, University of Zululand, KwaDlangezwa 3886, South Africa

[2] Department of Microbiology, Immunology and Biochemistry, University of Tennessee Health Science Center, Memphis, TN 38163, USA

* Correspondence: drnelson1@gmail.com (D.R.N.); khajamohiddinsyed@gmail.com (K.S.); Tel.: +19-014488303 (D.R.N.); +27-035-902-6857 (K.S.)

† These authors contributed equally to this work.

Supplementary Dataset S1. P450s identified and annotated in mycobacterial species are presented with their assigned name, followed by species name, species code, and protein sequence.

>CYP51B1_Mycobacterium africanum 25 2688148616
MSAVALPRVSGGHDEHGHLEEFRTDPIGLMQRVRDECGDVGTFQLAGKQVVLLSGSHANEFFFRAGDDDLDQA
KAYPFMTPIFGEGVVFDASPERRKEMLHNAALRGEQMKGHAATIEDQVRRMIADWGEAGEIDLLDFFAELTIY
TSSACLIGKKFRDQLDGRFAKLYHELERGTDPLAYVDPYLPIESFRRRDEARNGLVALVADIMNGRIANPPTD
KSDRDMLDVLIAVKAETGTPRFSADEITGMFISMMFAGHHTSSGTASWTLIELMRHRDAYAAVIDELDELYGD
GRSVSFHALRQIPQLENVLKETLRLHPPLIILMRVAKGEFEVQGHRIHEGDLVAASPAISNRIPEDFPDPHDF
VPARYEQPRQEDLLNRWTWIPFGAGRHRCVGAAFAIMQIKAIFSVLLREYEFEMAQPPESYRNDHSKMVVQLA
QPACVRYRRRTGV
>CYP51B1_Mycobacterium africanum GM041182 chromosome 651024267
MSAVALPRVSGGHDEHGHLEEFRTDPIGLMQRVRDECGDVGTFQLAGKQVVLLSGSHANEFFFRAGDDDLDQA
KAYPFMTPIFGEGVVFDASPERRKEMLHNAALRGEQMKGHAATIEDQVRRMIADWGEAGEIDLLDFFAELTIY
TSSACLIGKKFRDQLDGRFAKLYHELERGTDPLAYVDPYLPIESFRRRDEARNGLVALVADIMNGRIANPPTD
KSDRDMLDVLIAVKAETGTPRFSADEITGMFISMMFAGHHTSSGTASWTLIELMRHRDAYAAVIDELDELYGD
GRSVSFHALRQIPQLENVLKETLRLHPPLIILMRVAKGEFEVQGHRIHEGDLVAASPAISNRIPEDFPDPHDF
VPARYEQPRQEDLLNRWTWIPFGAGRHRCVGAAFAIMQIKAIFSVLLREYEFEMAQPPESYRNDHSKMVVQLA
QPACVRYRRRTGV
>CYP51B1_Mycobacterium africanum K85 2584195519
MSAVALPRVSGGHDEHGHLEEFRTDPIGLMQRVRDECGDVGTFQLAGKQVVLLSGSHANEFFFRAGDDDLDQA
KAYPFMTPIFGEGVVFDASPERRKEMLHNAALRGEQMKGHAATIEDQVRRMIADWGEAGEIDLLDFFAELTIY
TSSACLIGKKFRDQLDGRFAKLYHELERGTDPLAYVDPYLPIESFRRRDEARNGLVALVADIMNGRIANPPTD
KSDRDMLDVLIAVKAETGTPRFSADEITGMFISMMFAGHHTSSGTASWTLIELMRHRDAYAAVIDELDELYGD
GRSVSFHALRQIPQLENVLKETLRLHPPLIILMRVAKGEFEVQGHRIHEGDLVAASPAISNRIPEDFPDPHDF
VPARYEQPRQEDLLNRWTWIPFGAGRHRCVGAAFAIMQIKAIFSVLLREYEFEMAQPPESYRNDHSKMVVQLA
QPACVRYRRRTGV
>CYP51B1_Mycobacterium tuberculosis K85 646017679
MSAVALPRVSGGHDEHGHLEEFRTDPIGLMQRVRDECGDVGTFQLAGKQVVLLSGSHANEFFFRAGDDDLDQA
KAYPFMTPIFGEGVVFDASPERRKEMLHNAALRGEQMKGHAATIEDQVRRMIADWGEAGEIDLLDFFAELTIY
TSSACLIGKKFRDQLDGRFAKLYHELERGTDPLAYVDPYLPIESFRRRDEARNGLVALVADIMNGRIANPPTD
KSDRDMLDVLIAVKAETGTPRFSADEITGMFISMMFAGHHTSSGTASWTLIELMRHRDAYAAVIDELDELYGD
GRSVSFHALRQIPQLENVLKETLRLHPPLIILMRVAKGEFEVQGHRIHEGDLVAASPAISNRIPEDFPDPHDF
VPARYEQPRQEDLLNRWTWIPFGAGRHRCVGAAFAIMQIKAIFSVLLREYEFEMAQPPESYRNDHSKMVVQLA
QPACVRYRRRTGV
>CYP51B1_Mycobacterium africanum MAL010070 2583718889
MSAVALPRVSGGHDEHGHLEEFRTDPIGLMQRVRDECGDVGTFQLAGKQVVLLSGSHANEFFFRAGDDDLDQA
KAYPFMTPIFGEGVVFDASPERRKEMLHNAALRGEQMKGHAATIEDQVRRMIADWGEAGEIDLLDFFAELTIY
TSSACLIGKKFRDQLDGRFAKLYHELERGTDPLAYVDPYLPIESFRRRDEARNGLVALVADIMNGRIANPPTD
KSDRDMLDVLIAVKAETGTPRFSADEITGMFISMMFAGHHTSSGTASWTLIELMRHRDAYAAVIDELDELYGD
GRSVSFHALRQIPQLENVLKETLRLHPPLIILMRVAKGEFEVQGHRIHEGDLVAASPAISNRIPEDFPDPHDF
VPARYEQPRQEDLLNRWTWIPFGAGRHRCVGAAFAIMQIKAIFSVLLREYEFEMAQPPESYRNDHSKMVVQLA
QPACVRYRRRTGV
>CYP51B1_Mycobacterium africanum MAL010079 2580938237
MSAVALPRVSGGHDEHGHLEEFRTDPIGLMQRVRDECGDVGTFQLAGKQVVLLSGSHANEFFFRAGDDDLDQA
KAYPFMTPIFGEGVVFDASPERRKEMLHNAALRGEQMKGHAATIEDQVRRMIADWGEAGEIDLLDFFAELTIY
TSSACLIGKKFRDQLDGRFAKLYHELERGTDPLAYVDPYLPIESFRRRDEARNGLVALVADIMNGRIANPPTD
KSDRDMLDVLIAVKAETGTPRFSADEITGMFISMMFAGHHTSSGTASWTLIELMRHRDAYAAVIDELDELYGD
GRSVSFHALRQIPQLENVLKETLRLHPPLIILMRVAKGEFEVQGHRIHEGDLVAASPAISNRIPEDFPDPHDF
VPARYEQPRQEDLLNRWTWIPFGAGRHRCVGAAFAIMQIKAIFSVLLREYEFEMAQPPESYRNDHSKMVVQLA
QPACVRYRRRTGV
>CYP51B1_Mycobacterium africanum MAL010074 2582416691
MSAVALPRVSGGHDEHGHLEEFRTDPIGLMQRVRDECGDVGTFQLAGKQVVLLSGSHANEFFFRAGDDDLDQA
KAYPFMTPIFGEGVVFDASPERRKEMLHNAALRGEQMKGHAATIEDQVRRMIADWGEAGEIDLLDFFAELTIY
TSSACLIGKKFRDQLDGRFAKLYHELERGTDPLAYVDPYLPIESFRRRDEARNGLVALVADIMNGRIANPPTD
KSDRDMLDVLIAVKAETGTPRFSADEITGMFISMMFAGHHTSSGTASWTLIELMRHRDAYAAVIDELDELYGD
GRSVSFHALRQIPQLENVLKETLRLHPPLIILMRVAKGEFEVQGHRIHEGDLVAASPAISNRIPEDFPDPHDF

VPARYEQPRQEDLLNRWTWIPFGAGRHRCVGAAFAIMQIKAIFSVLLREYEFEMAQPPESYRNDHSKMVVQLA
QPACVRYRRRTGV
>CYP51B1_Mycobacterium africanum MAL010071 2582017240
MSAVALPRVSGGHDEHGHLEEFRTDPIGLMQRVRDECGDVGTFQLAGKQVVLLSGSHANEFFFRAGDDDLDQA
KAYPFMTPIFGEGVVFDASPERRKEMLHNAALRGEQMKGHAATIEDQVRRMIADWGEAGEIDLLDFFAELTIY
TSSACLIGKKFRDQLDGRFAKLYHELERGTDPLAYVDPYLPIESFRRRDEARNGLVALVADIMNGRIANPPTD
KSDRDMLDVLIAVKAETGTPRFSADEITGMFISMMFAGHHTSSGTASWTLIELMRHRDAYAAVIDELDELYGD
GRSVSFHALRQIPQLENVLKETLRLHPPLIILMRVAKGEFEVQGHRIHEGDLVAASPAISNRIPEDFPDPHDF
VPARYEQPRQEDLLNRWTWIPFGAGRHRCVGAAFAIMQIKAIFSVLLREYEFEMAQPPESYRNDHSKMVVQLA
QPACVRYRRRTGV
>CYP51B1_Mycobacterium africanum MAL010081 2580670020
MSAVALPRVSGGHDEHGHLEEFRTDPIGLMQRVRDECGDVGTFQLAGKQVVLLSGSHANEFFFRAGDDDLDQA
KAYPFMTPIFGEGVVFDASPERRKEMLHNAALRGEQMKGHAATIEDQVRRMIADWGEAGEIDLLDFFAELTIY
TSSACLIGKKFRDQLDGRFAKLYHELERGTDPLAYVDPYLPIESFRRRDEARNGLVALVADIMNGRIANPPTD
KSDRDMLDVLIAVKAETGTPRFSADEITGMFISMMFAGHHTSSGTASWTLIELMRHRDAYAAVIDELDELYGD
GRSVSFHALRQIPQLENVLKETLRLHPPLIILMRVAKGEFEVQGHRIHEGDLVAASPAISNRIPEDFPDPHDF
VPARYEQPRQEDLLNRWTWIPFGAGRHRCVGAAFAIMQIKAIFSVLLREYEFEMAQPPESYRNDHSKMVVQLA
QPACVRYRRRTGV
>CYP51B1_Mycobacterium africanum MAL010084 2581509958
MSAVALPRVSGGHDEHGHLEEFRTDPIGLMQRVRDECGDVGTFQLAGKQVVLLSGSHANEFFFRAGDDDLDQA
KAYPFMTPIFGEGVVFDASPERRKEMLHNAALRGEQMKGHAATIEDQVRRMIADWGEAGEIDLLDFFAELTIY
TSSACLIGKKFRDQLDGRFAKLYHELERGTDPLAYVDPYLPIESFRRRDEARNGLVALVADIMNGRIANPPTD
KSDRDMLDVLIAVKAETGTPRFSADEITGMFISMMFAGHHTSSGTASWTLIELMRHRDAYAAVIDELDELYGD
GRSVSFHALRQIPQLENVLKETLRLHPPLIILMRVAKGEFEVQGHRIHEGDLVAASPAISNRIPEDFPDPHDF
VPARYEQPRQEDLLNRWTWIPFGAGRHRCVGAAFAIMQIKAIFSVLLREYEFEMAQPPESYRNDHSKMVVQLA
QPACVRYRRRTGV
>CYP51B1_Mycobacterium africanum MAL010099 2583722925
MSAVALPRVSGGHDEHGHLEEFRTDPIGLMQRVRDECGDVGTFQLAGKQVVLLSGSHANEFFFRAGDDDLDQA
KAYPFMTPIFGEGVVFDASPERRKEMLHNAALRGEQMKGHAATIEDQVRRMIADWGEAGEIDLLDFFAELTIY
TSSACLIGKKFRDQLDGRFAKLYHELERGTDPLAYVDPYLPIESFRRRDEARNGLVALVADIMNGRIANPPTD
KSDRDMLDVLIAVKAETGTPRFSADEITGMFISMMFAGHHTSSGTASWTLIELMRHRDAYAAVIDELDELYGD
GRSVSFHALRQIPQLENVLKETLRLHPPLIILMRVAKGEFEVQGHRIHEGDLVAASPAISNRIPEDFPDPHDF
VPARYEQPRQEDLLNRWTWIPFGAGRHRCVGAAFAIMQIKAIFSVLLREYEFEMAQPPESYRNDHSKMVVQLA
QPACVRYRRRTGV
>CYP51B1_Mycobacterium africanum MAL010100 2574971511
MSAVALPRVSGGHDEHGHLEEFRTDPIGLMQRVRDECGDVGTFQLAGKQVVLLSGSHANEFFFRAGDDDLDQA
KAYPFMTPIFGEGVVFDASPERRKEMLHNAALRGEQMKGHAATIEDQVRRMIADWGEAGEIDLLDFFAELTIY
TSSACLIGKKFRDQLDGRFAKLYHELERGTDPLAYVDPYLPIESFRRRDEARNGLVALVADIMNGRIANPPTD
KSDRDMLDVLIAVKAETGTPRFSADEITGMFISMMFAGHHTSSGTASWTLIELMRHRDAYAAVIDELDELYGD
GRSVSFHALRQIPQLENVLKETLRLHPPLIILMRVAKGEFEVQGHRIHEGDLVAASPAISNRIPEDFPDPHDF
VPARYEQPRQEDLLNRWTWIPFGAGRHRCVGAAFAIMQIKAIFSVLLREYEFEMAQPPESYRNDHSKMVVQLA
QPACVRYRRRTGV
>CYP51B1_Mycobacterium africanum MAL010102 2577174268
MSAVALPRVSGGHDEHGHLEEFRTDPIGLMQRVRDECGDVGTFQLAGKQVVLLSGSHANEFFFRAGDDDLDQA
KAYPFMTPIFGEGVVFDASPERRKEMLHNAALRGEQMKGHAATIEDQVRRMIADWGEAGEIDLLDFFAELTIY
TSSACLIGKKFRDQLDGRFAKLYHELERGTDPLAYVDPYLPIESFRRRDEARNGLVALVADIMNGRIANPPTD
KSDRDMLDVLIAVKAETGTPRFSADEITGMFISMMFAGHHTSSGTASWTLIELMRHRDAYAAVIDELDELYGD
GRSVSFHALRQIPQLENVLKETLRLHPPLIILMRVAKGEFEVQGHRIHEGDLVAASPAISNRIPEDFPDPHDF
VPARYEQPRQEDLLNRWTWIPFGAGRHRCVGAAFAIMQIKAIFSVLLREYEFEMAQPPESYRNDHSKMVVQLA
QPACVRYRRRTGV
>CYP51B1_Mycobacterium africanum MAL010111 2575938051
MSAVALPRVSGGHDEHGHLEEFRTDPIGLMQRVRDECGDVGTFQLAGKQVVLLSGSHANEFFFRAGDDDLDQA
KAYPFMTPIFGEGVVFDASPERRKEMLHNAALRGEQMKGHAATIEDQVRRMIADWGEAGEIDLLDFFAELTIY
TSSACLIGKKFRDQLDGRFAKLYHELERGTDPLAYVDPYLPIESFRRRDEARNGLVALVADIMNGRIANPPTD
KSDRDMLDVLIAVKAETGTPRFSADEITGMFISMMFAGHHTSSGTASWTLIELMRHRDAYAAVIDELDELYGD
GRSVSFHALRQIPQLENVLKETLRLHPPLIILMRVAKGEFEVQGHRIHEGDLVAASPAISNRIPEDFPDPHDF
VPARYEQPRQEDLLNRWTWIPFGAGRHRCVGAAFAIMQIKAIFSVLLREYEFEMAQPPESYRNDHSKMVVQLA
QPACVRYRRRTGV

>CYP51B1_Mycobacterium africanum MAL010112 2575197673
MSAVALPRVSGGHDEHGHLEEFRTDPIGLMQRVRDECGDVGTFQLAGKQVVLLSGSHANEFFFRAGDDDLDQA
KAYPFMTPIFGEGVVFDASPERRKEMLHNAALRGEQMKGHAATIEDQVRRMIADWGEAGEIDLLDFFAELTIY
TSSACLIGKKFRDQLDGRFAKLYHELERGTDPLAYVDPYLPIESFRRRDEARNGLVALVADIMNGRIANPPTD
KSDRDMLDVLIAVKAETGTPRFSADEITGMFISMMFAGHHTSSGTASWTLIELMRHRDAYAAVIDELDELYGD
GRSVSFHALRQIPQLENVLKETLRLHPPLIILMRVAKGEFEVQGHRIHEGDLVAASPAISNRIPEDFPDPHDF
VPARYEQPRQEDLLNRWTWIPFGAGRHRCVGAAFAIMQIKAIFSVLLREYEFEMAQPPESYRNDHSKMVVQLA
QPACVRYRRRTGV
>CYP51B1_Mycobacterium africanum MAL010118 2581868850
MSAVALPRVSGGHDEHGHLEEFRTDPIGLMQRVRDECGDVGTFQLAGKQVVLLSGSHANEFFFRAGDDDLDQA
KAYPFMTPIFGEGVVFDASPERRKEMLHNAALRGEQMKGHAATIEDQVRRMIADWGEAGEIDLLDFFAELTIY
TSSACLIGKKFRDQLDGRFAKLYHELERGTDPLAYVDPYLPIESFRRRDEARNGLVALVADIMNGRIANPPTD
KSDRDMLDVLIAVKAETGTPRFSADEITGMFISMMFAGHHTSSGTASWTLIELMRHRDAYAAVIDELDELYGD
GRSVSFHALRQIPQLENVLKETLRLHPPLIILMRVAKGEFEVQGHRIHEGDLVAASPAISNRIPEDFPDPHDF
VPARYEQPRQEDLLNRWTWIPFGAGRHRCVGAAFAIMQIKAIFSVLLREYEFEMAQPPESYRNDHSKMVVQLA
QPACVRYRRRTGV
>CYP51B1_Mycobacterium africanum MAL010123 2581806781
MSAVALPRVSGGHDEHGHLEEFRTDPIGLMQRVRDECGDVGTFQLAGKQVVLLSGSHANEFFFRAGDDDLDQA
KAYPFMTPIFGEGVVFDASPERRKEMLHNAALRGEQMKGHAATIEDQVRRMIADWGEAGEIDLLDFFAELTIY
TSSACLIGKKFRDQLDGRFAKLYHELERGTDPLAYVDPYLPIESFRRRDEARNGLVALVADIMNGRIANPPTD
KSDRDMLDVLIAVKAETGTPRFSADEITGMFISMMFAGHHTSSGTASWTLIELMRHRDAYAAVIDELDELYGD
GRSVSFHALRQIPQLENVLKETLRLHPPLIILMRVAKGEFEVQGHRIHEGDLVAASPAISNRIPEDFPDPHDF
VPARYEQPRQEDLLNRWTWIPFGAGRHRCVGAAFAIMQIKAIFSVLLREYEFEMAQPPESYRNDHSKMVVQLA
QPACVRYRRRTGV
>CYP51B1_Mycobacterium africanum MAL010128 2582000981
MSAVALPRVSGGHDEHGHLEEFRTDPIGLMQRVRDECGDVGTFQLAGKQVVLLSGSHANEFFFRAGDDDLDQA
KAYPFMTPIFGEGVVFDASPERRKEMLHNAALRGEQMKGHAATIEDQVRRMIADWGEAGEIDLLDFFAELTIY
TSSACLIGKKFRDQLDGRFAKLYHELERGTDPLAYVDPYLPIESFRRRDEARNGLVALVADIMNGRIANPPTD
KSDRDMLDVLIAVKAETGTPRFSADEITGMFISMMFAGHHTSSGTASWTLIELMRHRDAYAAVIDELDELYGD
GRSVSFHALRQIPQLENVLKETLRLHPPLIILMRVAKGEFEVQGHRIHEGDLVAASPAISNRIPEDFPDPHDF
VPARYEQPRQEDLLNRWTWIPFGAGRHRCVGAAFAIMQIKAIFSVLLREYEFEMAQPPESYRNDHSKMVVQLA
QPACVRYRRRTGV
>CYP51B1_Mycobacterium africanum MAL010129 2581900484
MSAVALPRVSGGHDEHGHLEEFRTDPIGLMQRVRDECGDVGTFQLAGKQVVLLSGSHANEFFFRAGDDDLDQA
KAYPFMTPIFGEGVVFDASPERRKEMLHNAALRGEQMKGHAATIEDQVRRMIADWGEAGEIDLLDFFAELTIY
TSSACLIGKKFRDQLDGRFAKLYHELERGTDPLAYVDPYLPIESFRRRDEARNGLVALVADIMNGRIANPPTD
KSDRDMLDVLIAVKAETGTPRFSADEITGMFISMMFAGHHTSSGTASWTLIELMRHRDAYAAVIDELDELYGD
GRSVSFHALRQIPQLENVLKETLRLHPPLIILMRVAKGEFEVQGHRIHEGDLVAASPAISNRIPEDFPDPHDF
VPARYEQPRQEDLLNRWTWIPFGAGRHRCVGAAFAIMQIKAIFSVLLREYEFEMAQPPESYRNDHSKMVVQLA
QPACVRYRRRTGV
>CYP51B1_Mycobacterium africanum MAL010131 2581377938
MSAVALPRVSGGHDEHGHLEEFRTDPIGLMQRVRDECGDVGTFQLAGKQVVLLSGSHANEFFFRAGDDDLDQA
KAYPFMTPIFGEGVVFDASPERRKEMLHNAALRGEQMKGHAATIEDQVRRMIADWGEAGEIDLLDFFAELTIY
TSSACLIGKKFRDQLDGRFAKLYHELERGTDPLAYVDPYLPIESFRRRDEARNGLVALVADIMNGRIANPPTD
KSDRDMLDVLIAVKAETGTPRFSADEITGMFISMMFAGHHTSSGTASWTLIELMRHRDAYAAVIDELDELYGD
GRSVSFHALRQIPQLENVLKETLRLHPPLIILMRVAKGEFEVQGHRIHEGDLVAASPAISNRIPEDFPDPHDF
VPARYEQPRQEDLLNRWTWIPFGAGRHRCVGAAFAIMQIKAIFSVLLREYEFEMAQPPESYRNDHSKMVVQLA
QPACVRYRRRTGV
>CYP51B1_Mycobacterium africanum MAL010136 2583726978
MSAVALPRVSGGHDEHGHLEEFRTDPIGLMQRVRDECGDVGTFQLAGKQVVLLSGSHANEFFFRAGDDDLDQA
KAYPFMTPIFGEGVVFDASPERRKEMLHNAALRGEQMKGHAATIEDQVRRMIADWGEAGEIDLLDFFAELTIY
TSSACLIGKKFRDQLDGRFAKLYHELERGTDPLAYVDPYLPIESFRRRDEARNGLVALVADIMNGRIANPPTD
KSDRDMLDVLIAVKAETGTPRFSADEITGMFISMMFAGHHTSSGTASWTLIELMRHRDAYAAVIDELDELYGD
GRSVSFHALRQIPQLENVLKETLRLHPPLIILMRVAKGEFEVQGHRIHEGDLVAASPAISNRIPEDFPDPHDF
VPARYEQPRQEDLLNRWTWIPFGAGRHRCVGAAFAIMQIKAIFSVLLREYEFEMAQPPESYRNDHSKMVVQLA
QPACVRYRRRTGV
>CYP51B1_Mycobacterium africanum MAL010137 2575704305

MSAVALPRVSGGHDEHGHLEEFRTDPIGLMQRVRDECGDVGTFQLAGKQVVLLSGSHANEFFFRAGDDDLDQA
KAYPFMTPIFGEGVVFDASPERRKEMLHNAALRGEQMKGHAATIEDQVRRMIADWGEAGEIDLLDFFAELTIY
TSSACLIGKKFRDQLDGRFAKLYHELERGTDPLAYVDPYLPIESFRRRDEARNGLVALVADIMNGRIANPPTD
KSDRDMLDVLIAVKAETGTPRFSADEITGMFISMMFAGHHTSSGTASWTLIELMRHRDAYAAVIDELDELYGD
GRSVSFHALRQIPQLENVLKETLRLHPPLIILMRVAKGEFEVQGHRIHEGDLVAASPAISNRIPEDFPDPHDF
VPARYEQPRQEDLLNRWTWIPFGAGRHRCVGAAFAIMQIKAIFSVLLREYEFEMAQPPESYRNDHSKMVVQLA
QPACVRYRRRTGV
>CYP51B1_Mycobacterium africanum MAL020107 2581929831
MSAVALPRVSGGHDEHGHLEEFRTDPIGLMQRVRDECGDVGTFQLAGKQVVLLSGSHANEFFFRAGDDDLDQA
KAYPFMTPIFGEGVVFDASPERRKEMLHNAALRGEQMKGHAATIEDQVRRMIADWGEAGEIDLLDFFAELTIY
TSSACLIGKKFRDQLDGRFAKLYHELERGTDPLAYVDPYLPIESFRRRDEARNGLVALVADIMNGRIANPPTD
KSDRDMLDVLIAVKAETGTPRFSADEITGMFISMMFAGHHTSSGTASWTLIELMRHRDAYAAVIDELDELYGD
GRSVSFHALRQIPQLENVLKETLRLHPPLIILMRVAKGEFEVQGHRIHEGDLVAASPAISNRIPEDFPDPHDF
VPARYEQPRQEDLLNRWTWIPFGAGRHRCVGAAFAIMQIKAIFSVLLREYEFEMAQPPESYRNDHSKMVVQLA
QPACVRYRRRTGV
>CYP51B1_Mycobacterium africanum MAL020130 2583735078
MSAVALPRVSGGHDEHGHLEEFRTDPIGLMQRVRDECGDVGTFQLAGKQVVLLSGSHANEFFFRAGDDDLDQA
KAYPFMTPIFGEGVVFDASPERRKEMLHNAALRGEQMKGHAATIEDQVRRMIADWGEAGEIDLLDFFAELTIY
TSSACLIGKKFRDQLDGRFAKLYHELERGTDPLAYVDPYLPIESFRRRDEARNGLVALVADIMNGRIANPPTD
KSDRDMLDVLIAVKAETGTPRFSADEITGMFISMMFAGHHTSSGTASWTLIELMRHRDAYAAVIDELDELYGD
GRSVSFHALRQIPQLENVLKETLRLHPPLIILMRVAKGEFEVQGHRIHEGDLVAASPAISNRIPEDFPDPHDF
VPARYEQPRQEDLLNRWTWIPFGAGRHRCVGAAFAIMQIKAIFSVLLREYEFEMAQPPESYRNDHSKMVVQLA
QPACVRYRRRTGV
>CYP51B1_Mycobacterium africanum MAL020148 2581561441
MSAVALPRVSGGHDEHGHLEEFRTDPIGLMQRVRDECGDVGTFQLAGKQVVLLSGSHANEFFFRAGDDDLDQA
KAYPFMTPIFGEGVVFDASPERRKEMLHNAALRGEQMKGHAATIEDQVRRMIADWGEAGEIDLLDFFAELTIY
TSSACLIGKKFRDQLDGRFAKLYHELERGTDPLAYVDPYLPIESFRRRDEARNGLVALVADIMNGRIANPPTD
KSDRDMLDVLIAVKAETGTPRFSADEITGMFISMMFAGHHTSSGTASWTLIELMRHRDAYAAVIDELDELYGD
GRSVSFHALRQIPQLENVLKETLRLHPPLIILMRVAKGEFEVQGHRIHEGDLVAASPAISNRIPEDFPDPHDF
VPARYEQPRQEDLLNRWTWIPFGAGRHRCVGAAFAIMQIKAIFSVLLREYEFEMAQPPESYRNDHSKMVVQLA
QPACVRYRRRTGV
>CYP51B1_Mycobacterium africanum MAL020173 2581365640
MSAVALPRVSGGHDEHGHLEEFRTDPIGLMQRVRDECGDVGTFQLAGKQVVLLSGSHANEFFFRAGDDDLDQA
KAYPFMTPIFGEGVVFDASPERRKEMLHNAALRGEQMKGHAATIEDQVRRMIADWGEAGEIDLLDFFAELTIY
TSSACLIGKKFRDQLDGRFAKLYHELERGTDPLAYVDPYLPIESFRRRDEARNGLVALVADIMNGRIANPPTD
KSDRDMLDVLIAVKAETGTPRFSADEITGMFISMMFAGHHTSSGTASWTLIELMRHRDAYAAVIDELDELYGD
GRSVSFHALRQIPQLENVLKETLRLHPPLIILMRVAKGEFEVQGHRIHEGDLVAASPAISNRIPEDFPDPHDF
VPARYEQPRQEDLLNRWTWIPFGAGRHRCVGAAFAIMQIKAIFSVLLREYEFEMAQPPESYRNDHSKMVVQLA
QPACVRYRRRTGV
>CYP51B1_Mycobacterium africanum MAL020176 2583741586
MSAVALPRVSGGHDEHGHLEEFRTDPIGLMQRVRDECGDVGTFQLAGKQVVLLSGSHANEFFFRAGDDDLDQA
KAYPFMTPIFGEGVVFDASPERRKEMLHNAALRGEQMKGHAATIEDQVRRMIADWGEAGEIDLLDFFAELTIY
TSSACLIGKKFRDQLDGRFAKLYHELERGTDPLAYVDPYLPIESFRRRDEARNGLVALVADIMNGRIANPPTD
KSDRDMLDVLIAVKAETGTPRFSADEITGMFISMMFAGHHTSSGTASWTLIELMRHRDAYAAVIDELDELYGD
GRSVSFHALRQIPQLENVLKETLRLHPPLIILMRVAKGEFEVQGHRIHEGDLVAASPAISNRIPEDFPDPHDF
VPARYEQPRQEDLLNRWTWIPFGAGRHRCVGAAFAIMQIKAIFSVLLREYEFEMAQPPESYRNDHSKMVVQLA
QPACVRYRRRTGV
>CYP51B1_Mycobacterium africanum MAL020185 2580466879
MSAVALPRVSGGHDEHGHLEEFRTDPIGLMQRVRDECGDVGTFQLAGKQVVLLSGSHANEFFFRAGDDDLDQA
KAYPFMTPIFGEGVVFDASPERRKEMLHNAALRGEQMKGHAATIEDQVRRMIADWGEAGEIDLLDFFAELTIY
TSSACLIGKKFRDQLDGRFAKLYHELERGTDPLAYVDPYLPIESFRRRDEARNGLVALVADIMNGRIANPPTD
KSDRDMLDVLIAVKAETGTPRFSADEITGMFISMMFAGHHTSSGTASWTLIELMRHRDAYAAVIDELDELYGD
GRSVSFHALRQIPQLENVLKETLRLHPPLIILMRVAKGEFEVQGHRIHEGDLVAASPAISNRIPEDFPDPHDF
VPARYEQPRQEDLLNRWTWIPFGAGRHRCVGAAFAIMQIKAIFSVLLREYEFEMAQPPESYRNDHSKMVVQLA
QPACVRYRRRTGV
>CYP51B1_Mycobacterium africanum UT307 2687241399
MSAVALPRVSGGHDEHGHLEEFRTDPIGLMQRVRDECGDVGTFQLAGKQVVLLSGSHANEFFFRAGDDDLDQA
KAYPFMTPIFGEGVVFDASPERRKEMLHNAALRGEQMKGHAATIEDQVRRMIADWGEAGEIDLLDFFAELTIY

TSSACLIGKKFRDQLDGRFAKLYHELERGTDPLAYVDPYLPIESFRRRDEARNGLVALVADIMNGRIANPPTD
KSDRDMLDVLIAVKAETGTPRFSADEITGMFISMMFAGHHTSSGTASWTLIELMRHRDAYAAVIDELDELYGD
GRSVSFHALRQIPQLENVLKETLRLHPPLIILMRVAKGEFEVQGHRIHEGDLVAASPAISNRIPEDFPDPHDF
VPARYEQPRQEDLLNRWTWIPFGAGRHRCVGAAFAIMQIKAIFSVLLREYEFEMAQPPESYRNDHSKMVVQLA
QPACVRYRRRTGV
>CYP51B1_Mycobacterium bovis 1595 2638475987
MSAVALPRVSGGHDEHGHLEEFRTDPIGLMQRVRDECGDVGTFQLAGKQVVLLSGSHANEFFFRAGDDDLDQA
KAYPFMTPIFGEGVVFDASPERRKEMLHNAALRGEQMKGHAATIEDQVRRMIADWGEAGEIDLLDFFAELTIY
TSSACLIGKKFRDQLDGRFAKLYHELERGTDPLAYVDPYLPIESFRRRDEARNGLVALVADIMNGRIANPPTD
KSDRDMLDVLIAVKAETGTPRFSADEITGMFISMMFAGHHTSSGTASWTLIELMRHRDAYAAVIDELDELYGD
GRSVSFHALRQIPQLENVLKETLRLHPPLIILMRVAKGEFEVQGHRIHEGDLVAASPAISNRIPEDFPDPHDF
VPARYEQPRQEDLLNRWTWIPFGAGRHRCVGAAFAIMQIKAIFSVLLREYEFEMAQPPESYRNDHSKMVVQLA
QPACVRYRRRTGV
>CYP51B1_Mycobacterium bovis 30 2687956476
MSAVALPRVSGGHDEHGHLEEFRTDPIGLMQRVRDECGDVGTFQLAGKQVVLLSGSHANEFFFRAGDDDLDQA
KAYPFMTPIFGEGVVFDASPERRKEMLHNAALRGEQMKGHAATIEDQVRRMIADWGEAGEIDLLDFFAELTIY
TSSACLIGKKFRDQLDGRFAKLYHELERGTDPLAYVDPYLPIESFRRRDEARNGLVALVADIMNGRIANPPTD
KSDRDMLDVLIAVKAETGTPRFSADEITGMFISMMFAGHHTSSGTASWTLIELMRHRDAYAAVIDELDELYGD
GRSVSFHALRQIPQLENVLKETLRLHPPLIILMRVAKGEFEVQGHRIHEGDLVAASPAISNRIPEDFPDPHDF
VPARYEQPRQEDLLNRWTWIPFGAGRHRCVGAAFAIMQIKAIFSVLLREYEFEMAQPPESYRNDHSKMVVQLA
QPACVRYRRRTGV
>CYP51B1_Mycobacterium bovis AF 2122/97 2638122066
MSAVALPRVSGGHDEHGHLEEFRTDPIGLMQRVRDECGDVGTFQLAGKQVVLLSGSHANEFFFRAGDDDLDQA
KAYPFMTPIFGEGVVFDASPERRKEMLHNAALRGEQMKGHAATIEDQVRRMIADWGEAGEIDLLDFFAELTIY
TSSACLIGKKFRDQLDGRFAKLYHELERGTDPLAYVDPYLPIESFRRRDEARNGLVALVADIMNGRIANPPTD
KSDRDMLDVLIAVKAETGTPRFSADEITGMFISMMFAGHHTSSGTASWTLIELMRHRDAYAAVIDELDELYGD
GRSVSFHALRQIPQLENVLKETLRLHPPLIILMRVAKGEFEVQGHRIHEGDLVAASPAISNRIPEDFPDPHDF
VPARYEQPRQEDLLNRWTWIPFGAGRHRCVGAAFAIMQIKAIFSVLLREYEFEMAQPPESYRNDHSKMVVQLA
QPACVRYRRRTGV
>CYP51B1_Mycobacterium bovis AF2122/97 637138119
MSAVALPRVSGGHDEHGHLEEFRTDPIGLMQRVRDECGDVGTFQLAGKQVVLLSGSHANEFFFRAGDDDLDQA
KAYPFMTPIFGEGVVFDASPERRKEMLHNAALRGEQMKGHAATIEDQVRRMIADWGEAGEIDLLDFFAELTIY
TSSACLIGKKFRDQLDGRFAKLYHELERGTDPLAYVDPYLPIESFRRRDEARNGLVALVADIMNGRIANPPTD
KSDRDMLDVLIAVKAETGTPRFSADEITGMFISMMFAGHHTSSGTASWTLIELMRHRDAYAAVIDELDELYGD
GRSVSFHALRQIPQLENVLKETLRLHPPLIILMRVAKGEFEVQGHRIHEGDLVAASPAISNRIPEDFPDPHDF
VPARYEQPRQEDLLNRWTWIPFGAGRHRCVGAAFAIMQIKAIFSVLLREYEFEMAQPPESYRNDHSKMVVQLA
QPACVRYRRRTGV
>CYP51B1_Mycobacterium bovis AN5 2655179431
MSAVALPRVSGGHDEHGHLEEFRTDPIGLMQRVRDECGDVGTFQLAGKQVVLLSGSHANEFFFRAGDDDLDQA
KAYPFMTPIFGEGVVFDASPERRKEMLHNAALRGEQMKGHAATIEDQVRRMIADWGEAGEIDLLDFFAELTIY
TSSACLIGKKFRDQLDGRFAKLYHELERGTDPLAYVDPYLPIESFRRRDEARNGLVALVADIMNGRIANPPTD
KSDRDMLDVLIAVKAETGTPRFSADEITGMFISMMFAGHHTSSGTASWTLIELMRHRDAYAAVIDELDELYGD
GRSVSFHALRQIPQLENVLKETLRLHPPLIILMRVAKGEFEVQGHRIHEGDLVAASPAISNRIPEDFPDPHDF
VPARYEQPRQEDLLNRWTWIPFGAGRHRCVGAAFAIMQIKAIFSVLLREYEFEMAQPPESYRNDHSKMVVQLA
QPACVRYRRRTGV
>CYP51B1_Mycobacterium bovis B2 7505 2580300442
MSAVALPRVSGGHDEHGHLEEFRTDPIGLMQRVRDECGDVGTFQLAGKQVVLLSGSHANEFFFRAGDDDLDQA
KAYPFMTPIFGEGVVFDASPERRKEMLHNAALRGEQMKGHAATIEDQVRRMIADWGEAGEIDLLDFFAELTIY
TSSACLIGKKFRDQLDGRFAKLYHELERGTDPLAYVDPYLPIESFRRRDEARNGLVALVADIMNGRIANPPTD
KSDRDMLDVLIAVKAETGTPRFSADEITGMFISMMFAGHHTSSGTASWTLIELMRHRDAYAAVIDELDELYGD
GRSVSFHALRQIPQLENVLKETLRLHPPLIILMRVAKGEFEVQGHRIHEGDLVAASPAISNRIPEDFPDPHDF
VPARYEQPRQEDLLNRWTWIPFGAGRHRCVGAAFAIMQIKAIFSVLLREYEFEMAQPPESYRNDHSKMVVQLA
QPACVRYRRRTGV
>CYP51B1_Mycobacterium bovis B-3222 2652354329
MSAVALPRVSGGHDEHGHLEEFRTDPIGLMQRVRDECGDVGTFQLAGKQVVLLSGSHANEFFFRAGDDDLDQA
KAYPFMTPIFGEGVVFDASPERRKEMLHNAALRGEQMKGHAATIEDQVRRMIADWGEAGEIDLLDFFAELTIY
TSSACLIGKKFRDQLDGRFAKLYHELERGTDPLAYVDPYLPIESFRRRDEARNGLVALVADIMNGRIANPPTD
KSDRDMLDVLIAVKAETGTPRFSADEITGMFISMMFAGHHTSSGTASWTLIELMRHRDAYAAVIDELDELYGD

GRSVSFHALRQIPQLENVLKETLRLHPPLIILMRVAKGEFEVQGHRIHEGDLVAASPAISNRIPEDFPDPHDF
VPARYEQPRQEDLLNRWTWIPFGAGRHRCVGAAFAIMQIKAIFSVLLREYEFEMAQPPESYRNDHSKMVVQLA
QPACVRYRRRTGV
>CYP51B1_Mycobacterium bovis BCG 2646606197
MSAVALPRVSGGHDEHGHLEEFRTDPIGLMQRVRDECGDVGTFQLAGKQVVLLSGSHANEFFFRAGDDDLDQA
KAYPFMTPIFGEGVVFDASPERRKEMLHNAALRGEQMKGHAATIEDQVRRMIADWGEAGEIDLLDFFAELTIY
TSSACLIGKKFRDQLDGRFAKLYHELERGTDPLAYVDPYLPIESFRRRDEARNGLVALVADIMNGRIANPPTD
KSDRDMLDVLIAVKAETGTPRFSADEITGMFISMMFAGHHTSSGTASWTLIELMRHRDAYAAVIDELDELYGD
GRSVSFHALRQIPQLENVLKETLRLHPPLIILMRVAKGEFEVQGHRIHEGDLVAASPAISNRIPEDFPDPHDF
VPARYEQPRQEDLLNRWTWIPFGAGRHRCVGAAFAIMQIKAIFSVLLREYEFEMAQPPESYRNDHSKMVVQLA
QPACVRYRRRTGV
>CYP51B1_Mycobacterium bovis BCG 26 2687966017
MSAVALPRVSGGHDEHGHLEEFRTDPIGLMQRVRDECGDVGTFQLAGKQVVLLSGSHANEFFFRAGDDDLDQA
KAYPFMTPIFGEGVVFDASPERRKEMLHNAALRGEQMKGHAATIEDQVRRMIADWGEAGEIDLLDFFAELTIY
TSSACLIGKKFRDQLDGRFAKLYHELERGTDPLAYVDPYLPIESFRRRDEARNGLVALVADIMNGRIANPPTD
KSDRDMLDVLIAVKAETGTPRFSADEITGMFISMMFAGHHTSSGTASWTLIELMRHRDAYAAVIDELDELYGD
GRSVSFHALRQIPQLENVLKETLRLHPPLIILMRVAKGEFEVQGHRIHEGDLVAASPAISNRIPEDFPDPHDF
VPARYEQPRQEDLLNRWTWIPFGAGRHRCVGAAFAIMQIKAIFSVLLREYEFEMAQPPESYRNDHSKMVVQLA
QPACVRYRRRTGV
>CYP51B1_Mycobacterium bovis BCG 3281 (unscreened) 2646007228
MSAVALPRVSGGHDEHGHLEEFRTDPIGLMQRVRDECGDVGTFQLAGKQVVLLSGSHANEFFFRAGDDDLDQA
KAYPFMTPIFGEGVVFDASPERRKEMLHNAALRGEQMKGHAATIEDQVRRMIADWGEAGEIDLLDFFAELTIY
TSSACLIGKKFRDQLDGRFAKLYHELERGTDPLAYVDPYLPIESFRRRDEARNGLVALVADIMNGRIANPPTD
KSDRDMLDVLIAVKAETGTPRFSADEITGMFISMMFAGHHTSSGTASWTLIELMRHRDAYAAVIDELDELYGD
GRSVSFHALRQIPQLENVLKETLRLHPPLIILMRVAKGEFEVQGHRIHEGDLVAASPAISNRIPEDFPDPHDF
VPARYEQPRQEDLLNRWTWIPFGAGRHRCVGAAFAIMQIKAIFSVLLREYEFEMAQPPESYRNDHSKMVVQLA
QPACVRYRRRTGV
>CYP51B1_Mycobacterium bovis BCG Birkhaug 2695555482
MSAVALPRVSGGHDEHGHLEEFRTDPIGLMQRVRDECGDVGTFQLAGKQVVLLSGSHANEFFFRAGDDDLDQA
KAYPFMTPIFGEGVVFDASPERRKEMLHNAALRGEQMKGHAATIEDQVRRMIADWGEAGEIDLLDFFAELTIY
TSSACLIGKKFRDQLDGRFAKLYHELERGTDPLAYVDPYLPIESFRRRDEARNGLVALVADIMNGRIANPPTD
KSDRDMLDVLIAVKAETGTPRFSADEITGMFISMMFAGHHTSSGTASWTLIELMRHRDAYAAVIDELDELYGD
GRSVSFHALRQIPQLENVLKETLRLHPPLIILMRVAKGEFEVQGHRIHEGDLVAASPAISNRIPEDFPDPHDF
VPARYEQPRQEDLLNRWTWIPFGAGRHRCVGAAFAIMQIKAIFSVLLREYEFEMAQPPESYRNDHSKMVVQLA
QPACVRYRRRTGV
>CYP51B1_Mycobacterium bovis BCG China 2547308460
MSAVALPRVSGGHDEHGHLEEFRTDPIGLMQRVRDECGDVGTFQLAGKQVVLLSGSHANEFFFRAGDDDLDQA
KAYPFMTPIFGEGVVFDASPERRKEMLHNAALRGEQMKGHAATIEDQVRRMIADWGEAGEIDLLDFFAELTIY
TSSACLIGKKFRDQLDGRFAKLYHELERGTDPLAYVDPYLPIESFRRRDEARNGLVALVADIMNGRIANPPTD
KSDRDMLDVLIAVKAETGTPRFSADEITGMFISMMFAGHHTSSGTASWTLIELMRHRDAYAAVIDELDELYGD
GRSVSFHALRQIPQLENVLKETLRLHPPLIILMRVAKGEFEVQGHRIHEGDLVAASPAISNRIPEDFPDPHDF
VPARYEQPRQEDLLNRWTWIPFGAGRHRCVGAAFAIMQIKAIFSVLLREYEFEMAQPPESYRNDHSKMVVQLA
QPACVRYRRRTGV
>CYP51B1_Mycobacterium bovis BCG China 2695501569
MSAVALPRVSGGHDEHGHLEEFRTDPIGLMQRVRDECGDVGTFQLAGKQVVLLSGSHANEFFFRAGDDDLDQA
KAYPFMTPIFGEGVVFDASPERRKEMLHNAALRGEQMKGHAATIEDQVRRMIADWGEAGEIDLLDFFAELTIY
TSSACLIGKKFRDQLDGRFAKLYHELERGTDPLAYVDPYLPIESFRRRDEARNGLVALVADIMNGRIANPPTD
KSDRDMLDVLIAVKAETGTPRFSADEITGMFISMMFAGHHTSSGTASWTLIELMRHRDAYAAVIDELDELYGD
GRSVSFHALRQIPQLENVLKETLRLHPPLIILMRVAKGEFEVQGHRIHEGDLVAASPAISNRIPEDFPDPHDF
VPARYEQPRQEDLLNRWTWIPFGAGRHRCVGAAFAIMQIKAIFSVLLREYEFEMAQPPESYRNDHSKMVVQLA
QPACVRYRRRTGV
>CYP51B1_Mycobacterium bovis BCG Connaught 2695541779
MSAVALPRVSGGHDEHGHLEEFRTDPIGLMQRVRDECGDVGTFQLAGKQVVLLSGSHANEFFFRAGDDDLDQA
KAYPFMTPIFGEGVVFDASPERRKEMLHNAALRGEQMKGHAATIEDQVRRMIADWGEAGEIDLLDFFAELTIY
TSSACLIGKKFRDQLDGRFAKLYHELERGTDPLAYVDPYLPIESFRRRDEARNGLVALVADIMNGRIANPPTD
KSDRDMLDVLIAVKAETGTPRFSADEITGMFISMMFAGHHTSSGTASWTLIELMRHRDAYAAVIDELDELYGD
GRSVSFHALRQIPQLENVLKETLRLHPPLIILMRVAKGEFEVQGHRIHEGDLVAASPAISNRIPEDFPDPHDF

VPARYEQPRQEDLLNRWTWIPFGAGRHRCVGAAFAIMQIKAIFSVLLREYEFEMAQPPESYRNDHSKMVVQLA
QPACVRYRRRTGV
>CYP51B1_Mycobacterium bovis BCG Danish 2695497515
MSAVALPRVSGGHDEHGHLEEFRTDPIGLMQRVRDECGDVGTFQLAGKQVVLLSGSHANEFFFRAGDDDLDQA
KAYPFMTPIFGEGVVFDASPERRKEMLHNAALRGEQMKGHAATIEDQVRRMIADWGEAGEIDLLDFFAELTIY
TSSACLIGKKFRDQLDGRFAKLYHELERGTDPLAYVDPYLPIESFRRRDEARNGLVALVADIMNGRIANPPTD
KSDRDMLDVLIAVKAETGTPRFSADEITGMFISMMFAGHHTSSGTASWTLIELMRHRDAYAAVIDELDELYGD
GRSVSFHALRQIPQLENVLKETLRLHPPLIILMRVAKGEFEVQGHRIHEGDLVAASPAISNRIPEDFPDPHDF
VPARYEQPRQEDLLNRWTWIPFGAGRHRCVGAAFAIMQIKAIFSVLLREYEFEMAQPPESYRNDHSKMVVQLA
QPACVRYRRRTGV
>CYP51B1_Mycobacterium bovis BCG Frappier 2695528363
MSAVALPRVSGGHDEHGHLEEFRTDPIGLMQRVRDECGDVGTFQLAGKQVVLLSGSHANEFFFRAGDDDLDQA
KAYPFMTPIFGEGVVFDASPERRKEMLHNAALRGEQMKGHAATIEDQVRRMIADWGEAGEIDLLDFFAELTIY
TSSACLIGKKFRDQLDGRFAKLYHELERGTDPLAYVDPYLPIESFRRRDEARNGLVALVADIMNGRIANPPTD
KSDRDMLDVLIAVKAETGTPRFSADEITGMFISMMFAGHHTSSGTASWTLIELMRHRDAYAAVIDELDELYGD
GRSVSFHALRQIPQLENVLKETLRLHPPLIILMRVAKGEFEVQGHRIHEGDLVAASPAISNRIPEDFPDPHDF
VPARYEQPRQEDLLNRWTWIPFGAGRHRCVGAAFAIMQIKAIFSVLLREYEFEMAQPPESYRNDHSKMVVQLA
QPACVRYRRRTGV
>CYP51B1_Mycobacterium bovis BCG Glaxo 2600681052
MSAVALPRVSGGHDEHGHLEEFRTDPIGLMQRVRDECGDVGTFQLAGKQVVLLSGSHANEFFFRAGDDDLDQA
KAYPFMTPIFGEGVVFDASPERRKEMLHNAALRGEQMKGHAATIEDQVRRMIADWGEAGEIDLLDFFAELTIY
TSSACLIGKKFRDQLDGRFAKLYHELERGTDPLAYVDPYLPIESFRRRDEARNGLVALVADIMNGRIANPPTD
KSDRDMLDVLIAVKAETGTPRFSADEITGMFISMMFAGHHTSSGTASWTLIELMRHRDAYAAVIDELDELYGD
GRSVSFHALRQIPQLENVLKETLRLHPPLIILMRVAKGEFEVQGHRIHEGDLVAASPAISNRIPEDFPDPHDF
VPARYEQPRQEDLLNRWTWIPFGAGRHRCVGAAFAIMQIKAIFSVLLREYEFEMAQPPESYRNDHSKMVVQLA
QPACVRYRRRTGV
>CYP51B1_Mycobacterium bovis BCG Glaxo 2695532412
MSAVALPRVSGGHDEHGHLEEFRTDPIGLMQRVRDECGDVGTFQLAGKQVVLLSGSHANEFFFRAGDDDLDQA
KAYPFMTPIFGEGVVFDASPERRKEMLHNAALRGEQMKGHAATIEDQVRRMIADWGEAGEIDLLDFFAELTIY
TSSACLIGKKFRDQLDGRFAKLYHELERGTDPLAYVDPYLPIESFRRRDEARNGLVALVADIMNGRIANPPTD
KSDRDMLDVLIAVKAETGTPRFSADEITGMFISMMFAGHHTSSGTASWTLIELMRHRDAYAAVIDELDELYGD
GRSVSFHALRQIPQLENVLKETLRLHPPLIILMRVAKGEFEVQGHRIHEGDLVAASPAISNRIPEDFPDPHDF
VPARYEQPRQEDLLNRWTWIPFGAGRHRCVGAAFAIMQIKAIFSVLLREYEFEMAQPPESYRNDHSKMVVQLA
QPACVRYRRRTGV
>CYP51B1_Mycobacterium bovis BCG Glaxo 2600681052
MSAVALPRVSGGHDEHGHLEEFRTDPIGLMQRVRDECGDVGTFQLAGKQVVLLSGSHANEFFFRAGDDDLDQA
KAYPFMTPIFGEGVVFDASPERRKEMLHNAALRGEQMKGHAATIEDQVRRMIADWGEAGEIDLLDFFAELTIY
TSSACLIGKKFRDQLDGRFAKLYHELERGTDPLAYVDPYLPIESFRRRDEARNGLVALVADIMNGRIANPPTD
KSDRDMLDVLIAVKAETGTPRFSADEITGMFISMMFAGHHTSSGTASWTLIELMRHRDAYAAVIDELDELYGD
GRSVSFHALRQIPQLENVLKETLRLHPPLIILMRVAKGEFEVQGHRIHEGDLVAASPAISNRIPEDFPDPHDF
VPARYEQPRQEDLLNRWTWIPFGAGRHRCVGAAFAIMQIKAIFSVLLREYEFEMAQPPESYRNDHSKMVVQLA
QPACVRYRRRTGV
>CYP51B1_Mycobacterium bovis BCG Grappier 2600685019
MSAVALPRVSGGHDEHGHLEEFRTDPIGLMQRVRDECGDVGTFQLAGKQVVLLSGSHANEFFFRAGDDDLDQA
KAYPFMTPIFGEGVVFDASPERRKEMLHNAALRGEQMKGHAATIEDQVRRMIADWGEAGEIDLLDFFAELTIY
TSSACLIGKKFRDQLDGRFAKLYHELERGTDPLAYVDPYLPIESFRRRDEARNGLVALVADIMNGRIANPPTD
KSDRDMLDVLIAVKAETGTPRFSADEITGMFISMMFAGHHTSSGTASWTLIELMRHRDAYAAVIDELDELYGD
GRSVSFHALRQIPQLENVLKETLRLHPPLIILMRVAKGEFEVQGHRIHEGDLVAASPAISNRIPEDFPDPHDF
VPARYEQPRQEDLLNRWTWIPFGAGRHRCVGAAFAIMQIKAIFSVLLREYEFEMAQPPESYRNDHSKMVVQLA
QPACVRYRRRTGV
>CYP51B1_Mycobacterium bovis BCG Japan 2695641980
MSAVALPRVSGGHDEHGHLEEFRTDPIGLMQRVRDECGDVGTFQLAGKQVVLLSGSHANEFFFRAGDDDLDQA
KAYPFMTPIFGEGVVFDASPERRKEMLHNAALRGEQMKGHAATIEDQVRRMIADWGEAGEIDLLDFFAELTIY
TSSACLIGKKFRDQLDGRFAKLYHELERGTDPLAYVDPYLPIESFRRRDEARNGLVALVADIMNGRIANPPTD
KSDRDMLDVLIAVKAETGTPRFSADEITGMFISMMFAGHHTSSGTASWTLIELMRHRDAYAAVIDELDELYGD
GRSVSFHALRQIPQLENVLKETLRLHPPLIILMRVAKGEFEVQGHRIHEGDLVAASPAISNRIPEDFPDPHDF
VPARYEQPRQEDLLNRWTWIPFGAGRHRCVGAAFAIMQIKAIFSVLLREYEFEMAQPPESYRNDHSKMVVQLA
QPACVRYRRRTGV

>CYP51B1_Mycobacterium bovis BCG Korea 1168P 2540802892
MSAVALPRVSGGHDEHGHLEEFRTDPIGLMQRVRDECGDVGTFQLAGKQVVLLSGSHANEFFFRAGDDDLDQA
KAYPFMTPIFGEGVVFDASPERRKEMLHNAALRGEQMKGHAATIEDQVRRMIADWGEAGEIDLLDFFAELTIY
TSSACLIGKKFRDQLDGRFAKLYHELERGTDPLAYVDPYLPIESFRRRDEARNGLVALVADIMNGRIANPPTD
KSDRDMLDVLIAVKAETGTPRFSADEITGMFISMMFAGHHTSSGTASWTLIELMRHRDAYAAVIDELDELYGD
GRSVSFHALRQIPQLENVLKETLRLHPPLIILMRVAKGEFEVQGHRIHEGDLVAASPAISNRIPEDFPDPHDF
VPARYEQPRQEDLLNRWTWIPFGAGRHRCVGAAFAIMQIKAIFSVLLREYEFEMAQPPESYRNDHSKMVVQLA
QPACVRYRRRTGV
>CYP51B1_Mycobacterium bovis BCG Mexico 2511810370
MSAVALPRVSGGHDEHGHLEEFRTDPIGLMQRVRDECGDVGTFQLAGKQVVLLSGSHANEFFFRAGDDDLDQA
KAYPFMTPIFGEGVVFDASPERRKEMLHNAALRGEQMKGHAATIEDQVRRMIADWGEAGEIDLLDFFAELTIY
TSSACLIGKKFRDQLDGRFAKLYHELERGTDPLAYVDPYLPIESFRRRDEARNGLVALVADIMNGRIANPPTD
KSDRDMLDVLIAVKAETGTPRFSADEITGMFISMMFAGHHTSSGTASWTLIELMRHRDAYAAVIDELDELYGD
GRSVSFHALRQIPQLENVLKETLRLHPPLIILMRVAKGEFEVQGHRIHEGDLVAASPAISNRIPEDFPDPHDF
VPARYEQPRQEDLLNRWTWIPFGAGRHRCVGAAFAIMQIKAIFSVLLREYEFEMAQPPESYRNDHSKMVVQLA
QPACVRYRRRTGV
>CYP51B1_Mycobacterium bovis BCG Moreau 2600459504
MSAVALPRVSGGHDEHGHLEEFRTDPIGLMQRVRDECGDVGTFQLAGKQVVLLSGSHANEFFFRAGDDDLDQA
KAYPFMTPIFGEGVVFDASPERRKEMLHNAALRGEQMKGHAATIEDQVRRMIADWGEAGEIDLLDFFAELTIY
TSSACLIGKKFRDQLDGRFAKLYHELERGTDPLAYVDPYLPIESFRRRDEARNGLVALVADIMNGRIANPPTD
KSDRDMLDVLIAVKAETGTPRFSADEITGMFISMMFAGHHTSSGTASWTLIELMRHRDAYAAVIDELDELYGD
GRSVSFHALRQIPQLENVLKETLRLHPPLIILMRVAKGEFEVQGHRIHEGDLVAASPAISNRIPEDFPDPHDF
VPARYEQPRQEDLLNRWTWIPFGAGRHRCVGAAFAIMQIKAIFSVLLREYEFEMAQPPESYRNDHSKMVVQLA
QPACVRYRRRTGV
>CYP51B1_Mycobacterium bovis BCG Moreau 2695509678
MSAVALPRVSGGHDEHGHLEEFRTDPIGLMQRVRDECGDVGTFQLAGKQVVLLSGSHANEFFFRAGDDDLDQA
KAYPFMTPIFGEGVVFDASPERRKEMLHNAALRGEQMKGHAATIEDQVRRMIADWGEAGEIDLLDFFAELTIY
TSSACLIGKKFRDQLDGRFAKLYHELERGTDPLAYVDPYLPIESFRRRDEARNGLVALVADIMNGRIANPPTD
KSDRDMLDVLIAVKAETGTPRFSADEITGMFISMMFAGHHTSSGTASWTLIELMRHRDAYAAVIDELDELYGD
GRSVSFHALRQIPQLENVLKETLRLHPPLIILMRVAKGEFEVQGHRIHEGDLVAASPAISNRIPEDFPDPHDF
VPARYEQPRQEDLLNRWTWIPFGAGRHRCVGAAFAIMQIKAIFSVLLREYEFEMAQPPESYRNDHSKMVVQLA
QPACVRYRRRTGV
>CYP51B1_Mycobacterium bovis BCG Moreau RDJ 2620698775
MSAVALPRVSGGHDEHGHLEEFRTDPIGLMQRVRDECGDVGTFQLAGKQVVLLSGSHANEFFFRAGDDDLDQA
KAYPFMTPIFGEGVVFDASPERRKEMLHNAALRGEQMKGHAATIEDQVRRMIADWGEAGEIDLLDFFAELTIY
TSSACLIGKKFRDQLDGRFAKLYHELERGTDPLAYVDPYLPIESFRRRDEARNGLVALVADIMNGRIANPPTD
KSDRDMLDVLIAVKAETGTPRFSADEITGMFISMMFAGHHTSSGTASWTLIELMRHRDAYAAVIDELDELYGD
GRSVSFHALRQIPQLENVLKETLRLHPPLIILMRVAKGEFEVQGHRIHEGDLVAASPAISNRIPEDFPDPHDF
VPARYEQPRQEDLLNRWTWIPFGAGRHRCVGAAFAIMQIKAIFSVLLREYEFEMAQPPESYRNDHSKMVVQLA
QPACVRYRRRTGV
>CYP51B1_Mycobacterium bovis BCG Pasteur 2695565205
MSAVALPRVSGGHDEHGHLEEFRTDPIGLMQRVRDECGDVGTFQLAGKQVVLLSGSHANEFFFRAGDDDLDQA
KAYPFMTPIFGEGVVFDASPERRKEMLHNAALRGEQMKGHAATIEDQVRRMIADWGEAGEIDLLDFFAELTIY
TSSACLIGKKFRDQLDGRFAKLYHELERGTDPLAYVDPYLPIESFRRRDEARNGLVALVADIMNGRIANPPTD
KSDRDMLDVLIAVKAETGTPRFSADEITGMFISMMFAGHHTSSGTASWTLIELMRHRDAYAAVIDELDELYGD
GRSVSFHALRQIPQLENVLKETLRLHPPLIILMRVAKGEFEVQGHRIHEGDLVAASPAISNRIPEDFPDPHDF
VPARYEQPRQEDLLNRWTWIPFGAGRHRCVGAAFAIMQIKAIFSVLLREYEFEMAQPPESYRNDHSKMVVQLA
QPACVRYRRRTGV
>CYP51B1_Mycobacterium bovis BCG str. Pasteur 1173P2 639829717
MSAVALPRVSGGHDEHGHLEEFRTDPIGLMQRVRDECGDVGTFQLAGKQVVLLSGSHANEFFFRAGDDDLDQA
KAYPFMTPIFGEGVVFDASPERRKEMLHNAALRGEQMKGHAATIEDQVRRMIADWGEAGEIDLLDFFAELTIY
TSSACLIGKKFRDQLDGRFAKLYHELERGTDPLAYVDPYLPIESFRRRDEARNGLVALVADIMNGRIANPPTD
KSDRDMLDVLIAVKAETGTPRFSADEITGMFISMMFAGHHTSSGTASWTLIELMRHRDAYAAVIDELDELYGD
GRSVSFHALRQIPQLENVLKETLRLHPPLIILMRVAKGEFEVQGHRIHEGDLVAASPAISNRIPEDFPDPHDF
VPARYEQPRQEDLLNRWTWIPFGAGRHRCVGAAFAIMQIKAIFSVLLREYEFEMAQPPESYRNDHSKMVVQLA
QPACVRYRRRTGV
>CYP51B1_Mycobacterium bovis BCG Phipps 2600669407

MSAVALPRVSGGHDEHGHLEEFRTDPIGLMQRVRDECGDVGTFQLAGKQVVLLSGSHANEFFFRAGDDDLDQA
KAYPFMTPIFGEGVVFDASPERRKEMLHNAALRGEQMKGHAATIEDQVRRMIADWGEAGEIDLLDFFAELTIY
TSSACLIGKKFRDQLDGRFAKLYHELERGTDPLAYVDPYLPIESFRRRDEARNGLVALVADIMNGRIANPPTD
KSDRDMLDVLIAVKAETGTPRFSADEITGMFISMMFAGHHTSSGTASWTLIELMRHRDAYAAVIDELDELYGD
GRSVSFHALRQIPQLENVLKETLRLHPPLIILMRVAKGEFEVQGHRIHEGDLVAASPAISNRIPEDFPDPHDF
VPARYEQPRQEDLLNRWTWIPFGAGRHRCVGAAFAIMQIKAIFSVLLREYEFEMAQPPESYRNDHSKMVVQLA
QPACVRYRRRTGV
>CYP51B1_Mycobacterium bovis BCG Phipps 2695493471
MSAVALPRVSGGHDEHGHLEEFRTDPIGLMQRVRDECGDVGTFQLAGKQVVLLSGSHANEFFFRAGDDDLDQA
KAYPFMTPIFGEGVVFDASPERRKEMLHNAALRGEQMKGHAATIEDQVRRMIADWGEAGEIDLLDFFAELTIY
TSSACLIGKKFRDQLDGRFAKLYHELERGTDPLAYVDPYLPIESFRRRDEARNGLVALVADIMNGRIANPPTD
KSDRDMLDVLIAVKAETGTPRFSADEITGMFISMMFAGHHTSSGTASWTLIELMRHRDAYAAVIDELDELYGD
GRSVSFHALRQIPQLENVLKETLRLHPPLIILMRVAKGEFEVQGHRIHEGDLVAASPAISNRIPEDFPDPHDF
VPARYEQPRQEDLLNRWTWIPFGAGRHRCVGAAFAIMQIKAIFSVLLREYEFEMAQPPESYRNDHSKMVVQLA
QPACVRYRRRTGV
>CYP51B1_Mycobacterium bovis BCG Prague 2600677529
MSAVALPRVSGGHDEHGHLEEFRTDPIGLMQRVRDECGDVGTFQLAGKQVVLLSGSHANEFFFRAGDDDLDQA
KAYPFMTPIFGEGVVFDASPERRKEMLHNAALRGEQMKGHAATIEDQVRRMIADWGEAGEIDLLDFFAELTIY
TSSACLIGKKFRDQLDGRFAKLYHELERGTDPLAYVDPYLPIESFRRRDEARNGLVALVADIMNGRIANPPTD
KSDRDMLDVLIAVKAETGTPRFSADEITGMFISMMFAGHHTSSGTASWTLIELMRHRDAYAAVIDELDELYGD
GRSVSFHALRQIPQLENVLKETLRLHPPLIILMRVAKGEFEVQGHRIHEGDLVAASPAISNRIPEDFPDPHDF
VPARYEQPRQEDLLNRWTWIPFGAGRHRCVGAAFAIMQIKAIFSVLLREYEFEMAQPPESYRNDHSKMVVQLA
QPACVRYRRRTGV
>CYP51B1_Mycobacterium bovis BCG Prague 2695505616
MSAVALPRVSGGHDEHGHLEEFRTDPIGLMQRVRDECGDVGTFQLAGKQVVLLSGSHANEFFFRAGDDDLDQA
KAYPFMTPIFGEGVVFDASPERRKEMLHNAALRGEQMKGHAATIEDQVRRMIADWGEAGEIDLLDFFAELTIY
TSSACLIGKKFRDQLDGRFAKLYHELERGTDPLAYVDPYLPIESFRRRDEARNGLVALVADIMNGRIANPPTD
KSDRDMLDVLIAVKAETGTPRFSADEITGMFISMMFAGHHTSSGTASWTLIELMRHRDAYAAVIDELDELYGD
GRSVSFHALRQIPQLENVLKETLRLHPPLIILMRVAKGEFEVQGHRIHEGDLVAASPAISNRIPEDFPDPHDF
VPARYEQPRQEDLLNRWTWIPFGAGRHRCVGAAFAIMQIKAIFSVLLREYEFEMAQPPESYRNDHSKMVVQLA
QPACVRYRRRTGV
>CYP51B1_Mycobacterium bovis BCG Russia 2695646048
MSAVALPRVSGGHDEHGHLEEFRTDPIGLMQRVRDECGDVGTFQLAGKQVVLLSGSHANEFFFRAGDDDLDQA
KAYPFMTPIFGEGVVFDASPERRKEMLHNAALRGEQMKGHAATIEDQVRRMIADWGEAGEIDLLDFFAELTIY
TSSACLIGKKFRDQLDGRFAKLYHELERGTDPLAYVDPYLPIESFRRRDEARNGLVALVADIMNGRIANPPTD
KSDRDMLDVLIAVKAETGTPRFSADEITGMFISMMFAGHHTSSGTASWTLIELMRHRDAYAAVIDELDELYGD
GRSVSFHALRQIPQLENVLKETLRLHPPLIILMRVAKGEFEVQGHRIHEGDLVAASPAISNRIPEDFPDPHDF
VPARYEQPRQEDLLNRWTWIPFGAGRHRCVGAAFAIMQIKAIFSVLLREYEFEMAQPPESYRNDHSKMVVQLA
QPACVRYRRRTGV
>CYP51B1_Mycobacterium bovis BCG S4 2715051999
MSAVALPRVSGGHDEHGHLEEFRTDPIGLMQRVRDECGDVGTFQLAGKQVVLLSGSHANEFFFRAGDDDLDQA
KAYPFMTPIFGEGVVFDASPERRKEMLHNAALRGEQMKGHAATIEDQVRRMIADWGEAGEIDLLDFFAELTIY
TSSACLIGKKFRDQLDGRFAKLYHELERGTDPLAYVDPYLPIESFRRRDEARNGLVALVADIMNGRIANPPTD
KSDRDMLDVLIAVKAETGTPRFSADEITGMFISMMFAGHHTSSGTASWTLIELMRHRDAYAAVIDELDELYGD
GRSVSFHALRQIPQLENVLKETLRLHPPLIILMRVAKGEFEVQGHRIHEGDLVAASPAISNRIPEDFPDPHDF
VPARYEQPRQEDLLNRWTWIPFGAGRHRCVGAAFAIMQIKAIFSVLLREYEFEMAQPPESYRNDHSKMVVQLA
QPACVRYRRRTGV
>CYP51B1_Mycobacterium bovis BCG Sweden 2600672565
MSAVALPRVSGGHDEHGHLEEFRTDPIGLMQRVRDECGDVGTFQLAGKQVVLLSGSHANEFFFRAGDDDLDQA
KAYPFMTPIFGEGVVFDASPERRKEMLHNAALRGEQMKGHAATIEDQVRRMIADWGEAGEIDLLDFFAELTIY
TSSACLIGKKFRDQLDGRFAKLYHELERGTDPLAYVDPYLPIESFRRRDEARNGLVALVADIMNGRIANPPTD
KSDRDMLDVLIAVKAETGTPRFSADEITGMFISMMFAGHHTSSGTASWTLIELMRHRDAYAAVIDELDELYGD
GRSVSFHALRQIPQLENVLKETLRLHPPLIILMRVAKGEFEVQGHRIHEGDLVAASPAISNRIPEDFPDPHDF
VPARYEQPRQEDLLNRWTWIPFGAGRHRCVGAAFAIMQIKAIFSVLLREYEFEMAQPPESYRNDHSKMVVQLA
QPACVRYRRRTGV
>CYP51B1_Mycobacterium bovis BCG Sweden 2695652794
MSAVALPRVSGGHDEHGHLEEFRTDPIGLMQRVRDECGDVGTFQLAGKQVVLLSGSHANEFFFRAGDDDLDQA
KAYPFMTPIFGEGVVFDASPERRKEMLHNAALRGEQMKGHAATIEDQVRRMIADWGEAGEIDLLDFFAELTIY

TSSACLIGKKFRDQLDGRFAKLYHELERGTDPLAYVDPYLPIESFRRRDEARNGLVALVADIMNGRIANPPTD
KSDRDMLDVLIAVKAETGTPRFSADEITGMFISMMFAGHHTSSGTASWTLIELMRHRDAYAAVIDELDELYGD
GRSVSFHALRQIPQLENVLKETLRLHPPLIILMRVAKGEFEVQGHRIHEGDLVAASPAISNRIPEDFPDPHDF
VPARYEQPRQEDLLNRWTWIPFGAGRHRCVGAAFAIMQIKAIFSVLLREYEFEMAQPPESYRNDHSKMVVQLA
QPACVRYRRRTGV
>CYP51B1_Mycobacterium bovis BCG Tice 2695631496
MSAVALPRVSGGHDEHGHLEEFRTDPIGLMQRVRDECGDVGTFQLAGKQVVLLSGSHANEFFFRAGDDDLDQA
KAYPFMTPIFGEGVVFDASPERRKEMLHNAALRGEQMKGHAATIEDQVRRMIADWGEAGEIDLLDFFAELTIY
TSSACLIGKKFRDQLDGRFAKLYHELERGTDPLAYVDPYLPIESFRRRDEARNGLVALVADIMNGRIANPPTD
KSDRDMLDVLIAVKAETGTPRFSADEITGMFISMMFAGHHTSSGTASWTLIELMRHRDAYAAVIDELDELYGD
GRSVSFHALRQIPQLENVLKETLRLHPPLIILMRVAKGEFEVQGHRIHEGDLVAASPAISNRIPEDFPDPHDF
VPARYEQPRQEDLLNRWTWIPFGAGRHRCVGAAFAIMQIKAIFSVLLREYEFEMAQPPESYRNDHSKMVVQLA
QPACVRYRRRTGV
>CYP51B1_Mycobacterium bovis BCG str. Tokyo 172 643733603
MSAVALPRVSGGHDEHGHLEEFRTDPIGLMQRVRDECGDVGTFQLAGKQVVLLSGSHANEFFFRAGDDDLDQA
KAYPFMTPIFGEGVVFDASPERRKEMLHNAALRGEQMKGHAATIEDQVRRMIADWGEAGEIDLLDFFAELTIY
TSSACLIGKKFRDQLDGRFAKLYHELERGTDPLAYVDPYLPIESFRRRDEARNGLVALVADIMNGRIANPPTD
KSDRDMLDVLIAVKAETGTPRFSADEITGMFISMMFAGHHTSSGTASWTLIELMRHRDAYAAVIDELDELYGD
GRSVSFHALRQIPQLENVLKETLRLHPPLIILMRVAKGEFEVQGHRIHEGDLVAASPAISNRIPEDFPDPHDF
VPARYEQPRQEDLLNRWTWIPFGAGRHRCVGAAFAIMQIKAIFSVLLREYEFEMAQPPESYRNDHSKMVVQLA
QPACVRYRRRTGV
>CYP51B1_Mycobacterium bovis BCG Tokyo 172 2686790928
MSAVALPRVSGGHDEHGHLEEFRTDPIGLMQRVRDECGDVGTFQLAGKQVVLLSGSHANEFFFRAGDDDLDQA
KAYPFMTPIFGEGVVFDASPERRKEMLHNAALRGEQMKGHAATIEDQVRRMIADWGEAGEIDLLDFFAELTIY
TSSACLIGKKFRDQLDGRFAKLYHELERGTDPLAYVDPYLPIESFRRRDEARNGLVALVADIMNGRIANPPTD
KSDRDMLDVLIAVKAETGTPRFSADEITGMFISMMFAGHHTSSGTASWTLIELMRHRDAYAAVIDELDELYGD
GRSVSFHALRQIPQLENVLKETLRLHPPLIILMRVAKGEFEVQGHRIHEGDLVAASPAISNRIPEDFPDPHDF
VPARYEQPRQEDLLNRWTWIPFGAGRHRCVGAAFAIMQIKAIFSVLLREYEFEMAQPPESYRNDHSKMVVQLA
QPACVRYRRRTGV
>CYP51B1_Mycobacterium bovis BCG-1 2687570516
MSAVALPRVSGGHDEHGHLEEFRTDPIGLMQRVRDECGDVGTFQLAGKQVVLLSGSHANEFFFRAGDDDLDQA
KAYPFMTPIFGEGVVFDASPERRKEMLHNAALRGEQMKGHAATIEDQVRRMIADWGEAGEIDLLDFFAELTIY
TSSACLIGKKFRDQLDGRFAKLYHELERGTDPLAYVDPYLPIESFRRRDEARNGLVALVADIMNGRIANPPTD
KSDRDMLDVLIAVKAETGTPRFSADEITGMFISMMFAGHHTSSGTASWTLIELMRHRDAYAAVIDELDELYGD
GRSVSFHALRQIPQLENVLKETLRLHPPLIILMRVAKGEFEVQGHRIHEGDLVAASPAISNRIPEDFPDPHDF
VPARYEQPRQEDLLNRWTWIPFGAGRHRCVGAAFAIMQIKAIFSVLLREYEFEMAQPPESYRNDHSKMVVQLA
QPACVRYRRRTGV
>CYP51B1_Mycobacterium bovis BCG-1 (Russia) 2688649574
MSAVALPRVSGGHDEHGHLEEFRTDPIGLMQRVRDECGDVGTFQLAGKQVVLLSGSHANEFFFRAGDDDLDQA
KAYPFMTPIFGEGVVFDASPERRKEMLHNAALRGEQMKGHAATIEDQVRRMIADWGEAGEIDLLDFFAELTIY
TSSACLIGKKFRDQLDGRFAKLYHELERGTDPLAYVDPYLPIESFRRRDEARNGLVALVADIMNGRIANPPTD
KSDRDMLDVLIAVKAETGTPRFSADEITGMFISMMFAGHHTSSGTASWTLIELMRHRDAYAAVIDELDELYGD
GRSVSFHALRQIPQLENVLKETLRLHPPLIILMRVAKGEFEVQGHRIHEGDLVAASPAISNRIPEDFPDPHDF
VPARYEQPRQEDLLNRWTWIPFGAGRHRCVGAAFAIMQIKAIFSVLLREYEFEMAQPPESYRNDHSKMVVQLA
QPACVRYRRRTGV
>CYP51B1_Mycobacterium bovis BCG-Denmark TMC 1010 ATCC 35733 2547312619
MSAVALPRVSGGHDEHGHLEEFRTDPIGLMQRVRDECGDVGTFQLAGKQVVLLSGSHANEFFFRAGDDDLDQA
KAYPFMTPIFGEGVVFDASPERRKEMLHNAALRGEQMKGHAATIEDQVRRMIADWGEAGEIDLLDFFAELTIY
TSSACLIGKKFRDQLDGRFAKLYHELERGTDPLAYVDPYLPIESFRRRDEARNGLVALVADIMNGRIANPPTD
KSDRDMLDVLIAVKAETGTPRFSADEITGMFISMMFAGHHTSSGTASWTLIELMRHRDAYAAVIDELDELYGD
GRSVSFHALRQIPQLENVLKETLRLHPPLIILMRVAKGEFEVQGHRIHEGDLVAASPAISNRIPEDFPDPHDF
VPARYEQPRQEDLLNRWTWIPFGAGRHRCVGAAFAIMQIKAIFSVLLREYEFEMAQPPESYRNDHSKMVVQLA
QPACVRYRRRTGV
>CYP51B1_Mycobacterium bovis BCG-Russia TMC 1022 ATCC 35740 2547320905
MSAVALPRVSGGHDEHGHLEEFRTDPIGLMQRVRDECGDVGTFQLAGKQVVLLSGSHANEFFFRAGDDDLDQA
KAYPFMTPIFGEGVVFDASPERRKEMLHNAALRGEQMKGHAATIEDQVRRMIADWGEAGEIDLLDFFAELTIY
TSSACLIGKKFRDQLDGRFAKLYHELERGTDPLAYVDPYLPIESFRRRDEARNGLVALVADIMNGRIANPPTD
KSDRDMLDVLIAVKAETGTPRFSADEITGMFISMMFAGHHTSSGTASWTLIELMRHRDAYAAVIDELDELYGD

GRSVSFHALRQIPQLENVLKETLRLHPPLIILMRVAKGEFEVQGHRIHEGDLVAASPAISNRIPEDFPDPHDF
VPARYEQPRQEDLLNRWTWIPFGAGRHRCVGAAFAIMQIKAIFSVLLREYEFEMAQPPESYRNDHSKMVVQLA
QPACVRYRRRTGV
>CYP51B1_Mycobacterium bovis BCG-Tice TMC 1028 ATCC 35743 2547316791
MSAVALPRVSGGHDEHGHLEEFRTDPIGLMQRVRDECGDVGTFQLAGKQVVLLSGSHANEFFFRAGDDDLDQA
KAYPFMTPIFGEGVVFDASPERRKEMLHNAALRGEQMKGHAATIEDQVRRMIADWGEAGEIDLLDFFAELTIY
TSSACLIGKKFRDQLDGRFAKLYHELERGTDPLAYVDPYLPIESFRRRDEARNGLVALVADIMNGRIANPPTD
KSDRDMLDVLIAVKAETGTPRFSADEITGMFISMMFAGHHTSSGTASWTLIELMRHRDAYAAVIDELDELYGD
GRSVSFHALRQIPQLENVLKETLRLHPPLIILMRVAKGEFEVQGHRIHEGDLVAASPAISNRIPEDFPDPHDF
VPARYEQPRQEDLLNRWTWIPFGAGRHRCVGAAFAIMQIKAIFSVLLREYEFEMAQPPESYRNDHSKMVVQLA
QPACVRYRRRTGV
>CYP51B1_Mycobacterium bovis Bz 31150 2581588666
MSAVALPRVSGGHDEHGHLEEFRTDPIGLMQRVRDECGDVGTFQLAGKQVVLLSGSHANEFFFRAGDDDLDQA
KAYPFMTPIFGEGVVFDASPERRKEMLHNAALRGEQMKGHAATIEDQVRRMIADWGEAGEIDLLDFFAELTIY
TSSACLIGKKFRDQLDGRFAKLYHELERGTDPLAYVDPYLPIESFRRRDEARNGLVALVADIMNGRIANPPTD
KSDRDMLDVLIAVKAETGTPRFSADEITGMFISMMFAGHHTSSGTASWTLIELMRHRDAYAAVIDELDELYGD
GRSVSFHALRQIPQLENVLKETLRLHPPLIILMRVAKGEFEVQGHRIHEGDLVAASPAISNRIPEDFPDPHDF
VPARYEQPRQEDLLNRWTWIPFGAGRHRCVGAAFAIMQIKAIFSVLLREYEFEMAQPPESYRNDHSKMVVQLA
QPACVRYRRRTGV
>CYP51B1_Mycobacterium bovis D 4155 2580743715
MSAVALPRVSGGHDEHGHLEEFRTDPIGLMQRVRDECGDVGTFQLAGKQVVLLSGSHANEFFFRAGDDDLDQA
KAYPFMTPIFGEGVVFDASPERRKEMLHNAALRGEQMKGHAATIEDQVRRMIADWGEAGEIDLLDFFAELTIY
TSSACLIGKKFRDQLDGRFAKLYHELERGTDPLAYVDPYLPIESFRRRDEARNGLVALVADIMNGRIANPPTD
KSDRDMLDVLIAVKAETGTPRFSADEITGMFISMMFAGHHTSSGTASWTLIELMRHRDAYAAVIDELDELYGD
GRSVSFHALRQIPQLENVLKETLRLHPPLIILMRVAKGEFEVQGHRIHEGDLVAASPAISNRIPEDFPDPHDF
VPARYEQPRQEDLLNRWTWIPFGAGRHRCVGAAFAIMQIKAIFSVLLREYEFEMAQPPESYRNDHSKMVVQLA
QPACVRYRRRTGV
>CYP51B1_Mycobacterium bovis Kc 32216 2584107824
MSAVALPRVSGGHDEHGHLEEFRTDPIGLMQRVRDECGDVGTFQLAGKQVVLLSGSHANEFFFRAGDDDLDQA
KAYPFMTPIFGEGVVFDASPERRKEMLHNAALRGEQMKGHAATIEDQVRRMIADWGEAGEIDLLDFFAELTIY
TSSACLIGKKFRDQLDGRFAKLYHELERGTDPLAYVDPYLPIESFRRRDEARNGLVALVADIMNGRIANPPTD
KSDRDMLDVLIAVKAETGTPRFSADEITGMFISMMFAGHHTSSGTASWTLIELMRHRDAYAAVIDELDELYGD
GRSVSFHALRQIPQLENVLKETLRLHPPLIILMRVAKGEFEVQGHRIHEGDLVAASPAISNRIPEDFPDPHDF
VPARYEQPRQEDLLNRWTWIPFGAGRHRCVGAAFAIMQIKAIFSVLLREYEFEMAQPPESYRNDHSKMVVQLA
QPACVRYRRRTGV
>CYP51B1_Mycobacterium bovis Kc 9614 2580125716
MSAVALPRVSGGHDEHGHLEEFRTDPIGLMQRVRDECGDVGTFQLAGKQVVLLSGSHANEFFFRAGDDDLDQA
KAYPFMTPIFGEGVVFDASPERRKEMLHNAALRGEQMKGHAATIEDQVRRMIADWGEAGEIDLLDFFAELTIY
TSSACLIGKKFRDQLDGRFAKLYHELERGTDPLAYVDPYLPIESFRRRDEARNGLVALVADIMNGRIANPPTD
KSDRDMLDVLIAVKAETGTPRFSADEITGMFISMMFAGHHTSSGTASWTLIELMRHRDAYAAVIDELDELYGD
GRSVSFHALRQIPQLENVLKETLRLHPPLIILMRVAKGEFEVQGHRIHEGDLVAASPAISNRIPEDFPDPHDF
VPARYEQPRQEDLLNRWTWIPFGAGRHRCVGAAFAIMQIKAIFSVLLREYEFEMAQPPESYRNDHSKMVVQLA
QPACVRYRRRTGV
>CYP51B1_Mycobacterium bovis MAL010093 2583743219
MSAVALPRVSGGHDEHGHLEEFRTDPIGLMQRVRDECGDVGTFQLAGKQVVLLSGSHANEFFFRAGDDDLDQA
KAYPFMTPIFGEGVVFDASPERRKEMLHNAALRGEQMKGHAATIEDQVRRMIADWGEAGEIDLLDFFAELTIY
TSSACLIGKKFRDQLDGRFAKLYHELERGTDPLAYVDPYLPIESFRRRDEARNGLVALVADIMNGRIANPPTD
KSDRDMLDVLIAVKAETGTPRFSADEITGMFISMMFAGHHTSSGTASWTLIELMRHRDAYAAVIDELDELYGD
GRSVSFHALRQIPQLENVLKETLRLHPPLIILMRVAKGEFEVQGHRIHEGDLVAASPAISNRIPEDFPDPHDF
VPARYEQPRQEDLLNRWTWIPFGAGRHRCVGAAFAIMQIKAIFSVLLREYEFEMAQPPESYRNDHSKMVVQLA
QPACVRYRRRTGV
>CYP51B1_Mycobacterium bovis MB1 2652513000
MSAVALPRVSGGHDEHGHLEEFRTDPIGLMQRVRDECGDVGTFQLAGKQVVLLSGSHANEFFFRAGDDDLDQA
KAYPFMTPIFGEGVVFDASPERRKEMLHNAALRGEQMKGHAATIEDQVRRMIADWGEAGEIDLLDFFAELTIY
TSSACLIGKKFRDQLDGRFAKLYHELERGTDPLAYVDPYLPIESFRRRDEARNGLVALVADIMNGRIANPPTD
KSDRDMLDVLIAVKAETGTPRFSADEITGMFISMMFAGHHTSSGTASWTLIELMRHRDAYAAVIDELDELYGD
GRSVSFHALRQIPQLENVLKETLRLHPPLIILMRVAKGEFEVQGHRIHEGDLVAASPAISNRIPEDFPDPHDF

VPARYEQPRQEDLLNRWTWIPFGAGRHRCVGAAFAIMQIKAIFSVLLREYEFEMAQPPESYRNDHSKMVVQLA
QPACVRYRRRTGV
>CYP51B1_Mycobacterium bovis MB3 2641311709
MSAVALPRVSGGHDEHGHLEEFRTDPIGLMQRVRDECGDVGTFQLAGKQVVLLSGSHANEFFFRAGDDDLDQA
KAYPFMTPIFGEGVVFDASPERRKEMLHNAALRGEQMKGHAATIEDQVRRMIADWGEAGEIDLLDFFAELTIY
TSSACLIGKKFRDQLDGRFAKLYHELERGTDPLAYVDPYLPIESFRRRDEARNGLVALVADIMNGRIANPPTD
KSDRDMLDVLIAVKAETGTPRFSADEITGMFISMMFAGHHTSSGTASWTLIELMRHRDAYAAVIDELDELYGD
GRSVSFHALRQIPQLENVLKETLRLHPPLIILMRVAKGEFEVQGHRIHEGDLVAASPAISNRIPEDFPDPHDF
VPARYEQPRQEDLLNRWTWIPFGAGRHRCVGAAFAIMQIKAIFSVLLREYEFEMAQPPESYRNDHSKMVVQLA
QPACVRYRRRTGV
>CYP51B1_Mycobacterium bovis MB4 2707492837
MSAVALPRVSGGHDEHGHLEEFRTDPIGLMQRVRDECGDVGTFQLAGKQVVLLSGSHANEFFFRAGDDDLDQA
KAYPFMTPIFGEGVVFDASPERRKEMLHNAALRGEQMKGHAATIEDQVRRMIADWGEAGEIDLLDFFAELTIY
TSSACLIGKKFRDQLDGRFAKLYHELERGTDPLAYVDPYLPIESFRRRDEARNGLVALVADIMNGRIANPPTD
KSDRDMLDVLIAVKAETGTPRFSADEITGMFISMMFAGHHTSSGTASWTLIELMRHRDAYAAVIDELDELYGD
GRSVSFHALRQIPQLENVLKETLRLHPPLIILMRVAKGEFEVQGHRIHEGDLVAASPAISNRIPEDFPDPHDF
VPARYEQPRQEDLLNRWTWIPFGAGRHRCVGAAFAIMQIKAIFSVLLREYEFEMAQPPESYRNDHSKMVVQLA
QPACVRYRRRTGV
>CYP51B1_Mycobacterium bovis MbURU-001 2647877475
MSAVALPRVSGGHDEHGHLEEFRTDPIGLMQRVRDECGDVGTFQLAGKQVVLLSGSHANEFFFRAGDDDLDQA
KAYPFMTPIFGEGVVFDASPERRKEMLHNAALRGEQMKGHAATIEDQVRRMIADWGEAGEIDLLDFFAELTIY
TSSACLIGKKFRDQLDGRFAKLYHELERGTDPLAYVDPYLPIESFRRRDEARNGLVALVADIMNGRIANPPTD
KSDRDMLDVLIAVKAETGTPRFSADEITGMFISMMFAGHHTSSGTASWTLIELMRHRDAYAAVIDELDELYGD
GRSVSFHALRQIPQLENVLKETLRLHPPLIILMRVAKGEFEVQGHRIHEGDLVAASPAISNRIPEDFPDPHDF
VPARYEQPRQEDLLNRWTWIPFGAGRHRCVGAAFAIMQIKAIFSVLLREYEFEMAQPPESYRNDHSKMVVQLA
QPACVRYRRRTGV
>CYP51B1_Mycobacterium bovis Mr 4387 2580768721
MSAVALPRVSGGHDEHGHLEEFRTDPIGLMQRVRDECGDVGTFQLAGKQVVLLSGSHANEFFFRAGDDDLDQA
KAYPFMTPIFGEGVVFDASPERRKEMLHNAALRGEQMKGHAATIEDQVRRMIADWGEAGEIDLLDFFAELTIY
TSSACLIGKKFRDQLDGRFAKLYHELERGTDPLAYVDPYLPIESFRRRDEARNGLVALVADIMNGRIANPPTD
KSDRDMLDVLIAVKAETGTPRFSADEITGMFISMMFAGHHTSSGTASWTLIELMRHRDAYAAVIDELDELYGD
GRSVSFHALRQIPQLENVLKETLRLHPPLIILMRVAKGEFEVQGHRIHEGDLVAASPAISNRIPEDFPDPHDF
VPARYEQPRQEDLLNRWTWIPFGAGRHRCVGAAFAIMQIKAIFSVLLREYEFEMAQPPESYRNDHSKMVVQLA
QPACVRYRRRTGV
>CYP51B1_Mycobacterium bovis Wt 21231 2584004136
MSAVALPRVSGGHDEHGHLEEFRTDPIGLMQRVRDECGDVGTFQLAGKQVVLLSGSHANEFFFRAGDDDLDQA
KAYPFMTPIFGEGVVFDASPERRKEMLHNAALRGEQMKGHAATIEDQVRRMIADWGEAGEIDLLDFFAELTIY
TSSACLIGKKFRDQLDGRFAKLYHELERGTDPLAYVDPYLPIESFRRRDEARNGLVALVADIMNGRIANPPTD
KSDRDMLDVLIAVKAETGTPRFSADEITGMFISMMFAGHHTSSGTASWTLIELMRHRDAYAAVIDELDELYGD
GRSVSFHALRQIPQLENVLKETLRLHPPLIILMRVAKGEFEVQGHRIHEGDLVAASPAISNRIPEDFPDPHDF
VPARYEQPRQEDLLNRWTWIPFGAGRHRCVGAAFAIMQIKAIFSVLLREYEFEMAQPPESYRNDHSKMVVQLA
QPACVRYRRRTGV
>CYP51B1_Mycobacterium bovis Wt 21419 2581354562
MSAVALPRVSGGHDEHGHLEEFRTDPIGLMQRVRDECGDVGTFQLAGKQVVLLSGSHANEFFFRAGDDDLDQA
KAYPFMTPIFGEGVVFDASPERRKEMLHNAALRGEQMKGHAATIEDQVRRMIADWGEAGEIDLLDFFAELTIY
TSSACLIGKKFRDQLDGRFAKLYHELERGTDPLAYVDPYLPIESFRRRDEARNGLVALVADIMNGRIANPPTD
KSDRDMLDVLIAVKAETGTPRFSADEITGMFISMMFAGHHTSSGTASWTLIELMRHRDAYAAVIDELDELYGD
GRSVSFHALRQIPQLENVLKETLRLHPPLIILMRVAKGEFEVQGHRIHEGDLVAASPAISNRIPEDFPDPHDF
VPARYEQPRQEDLLNRWTWIPFGAGRHRCVGAAFAIMQIKAIFSVLLREYEFEMAQPPESYRNDHSKMVVQLA
QPACVRYRRRTGV
>CYP51B1_Mycobacterium canettii CIPT 140060008 2541577097
MSAVALPRVSGGHDEHGHLEEFRTDPIGLMQRVRDECGDVGTFQLAGKQVVLLSGSHANEFFFRAGDDDLDQA
KAYPFMTPIFGEGVVFDASPERRKEMLHNAALRGEQMKGHAATIEDQVRRMIADWGEAGEIDLLDFFAELTIY
TSSACLIGKKFRDQLDGRFAKLYHELERGTDPLAYVDPYLPIESFRRRDEARNGLVALVADIMNGRIANPPTD
KSDRDMLDVLIAVKAETGTPRFSADEITGMFISMMFAGHHTSSGTASWTLIELMRHRDAYAAVIDELDELYGD
GRSVSFHALRQIPQLENVLKETLRLHPPLIILMRVAKGEFEVQGHRIHEGDLVAASPAISNRIPEDFPDPHDF
VPARYEQPRQEDLLNRWTWIPFGAGRHRCVGAAFAIMQIKAIFSVLLREYEFEMAQPPESYRNDHSKMVVQLA
QPACVRYRRRTGV

>CYP51B1_Mycobacterium canettii CIPT 140070002 2566979893
MSAVALPRVSGGHDEHGHLEEFRTDPIGLMQRVRDECGDVGTFQLAGKQVVLLSGSHANEFFFRAGDDDLDQA
KAYPFMTPIFGEGVVFDASPERRKEMLHNAALRGEQMKGHAATIEDQVRRMIADWGEAGEIDLLDFFAELTIY
TSSACLIGKKFRDQLDGRFAKLYHELERGTDPLAYVDPYLPIESFRRRDEARNGLVALVADIMNGRIANPPTD
KSDRDMLDVLIAVKAETGTPRFSADEITGMFISMMFAGHHTSSGTASWTLIELMRHRDAYAAVIDELDELYGD
GRSVSFHALRQIPQLENVLKETLRLHPPLIILMRVAKGEFEVQGHRIHEGDLVAASPAISNRIPEDFPDPHDF
VPARYEQPRQEDLLNRWTWIPFGAGRHRCVGAAFAIMQIKAIFSVLLREYEFEMAQPPESYRNDHSKMVVQLA
QPACVRYRRRTGV
>CYP51B1_Mycobacterium caprae MB2 2699761780
MSAVALPRVSGGHDEHGHLEEFRTDPIGLMQRVRDECGDVGTFQLAGKQVVLLSGSHANEFFFRAGDDDLDQA
KAYPFMTPIFGEGVVFDASPERRKEMLHNAALRGEQMKGHAATIEDQVRRMIADWGEAGEIDLLDFFAELTIY
TSSACLIGKKFRDQLDGRFAKLYHELERGTDPLAYVDPYLPIESFRRRDEARNGLVALVADIMNGRIANPPTD
KSDRDMLDVLIAVKAETGTPRFSADEITGMFISMMFAGHHTSSGTASWTLIELMRHRDAYAAVIDELDELYGD
GRSVSFHALRQIPQLENVLKETLRLHPPLIILMRVAKGEFEVQGHRIHEGDLVAASPAISNRIPEDFPDPHDF
VPARYEQPRQEDLLNRWTWIPFGAGRHRCVGAAFAIMQIKAIFSVLLREYEFEMAQPPESYRNDHSKMVVQLA
QPACVRYRRRTGV
>CYP51B1_Mycobacterium tuberculosis BTB08-183 2604562572
MSAVALPRVSGGHDEHGHLEEFRTDPIGLMQRVRDECGDVGTFQLAGKQVVLLSGSHANEFFFRAGDDDLDQA
KAYPFMTPIFGEGVVFDASPERRKEMLHNAALRGEQMKGHAATIEDQVRRMIADWGEAGEIDLLDFFAELTIY
TSSACLIGKKFRDQLDGRFAKLYHELERGTDPLAYVDPYLPIESFRRRDEARNGLVALVADIMNGRIANPPTD
KSDRDMLDVLIAVKAETGTPRFSADEITGMFISMMFAGHHTSSGTASWTLIELMRHRDAYAAVIDELDELYGD
GRSVSFHALRQIPQLENVLKETLRLHPPLIILMRVAKGEFEVQGHRIHEGDLVAASPAISNRIPEDFPDPHDF
VPARYEQPRQEDLLNRWTWIPFGAGRHRCVGAAFAIMQIKAIFSVLLREYEFEMAQPPESYRNDHSKMVVQLA
QPACVRYRRRTGV
>CYP51B1_Mycobacterium tuberculosis 001_13432 2716310099
MSAVALPRVSGGHDEHGHLEEFRTDPIGLMQRVRDECGDVGTFQLAGKQVVLLSGSHANEFFFRAGDDDLDQA
KAYPFMTPIFGEGVVFDASPERRKEMLHNAALRGEQMKGHAATIEDQVRRMIADWGEAGEIDLLDFFAELTIY
TSSACLIGKKFRDQLDGRFAKLYHELERGTDPLAYVDPYLPIESFRRRDEARNGLVALVADIMNGRIANPPTD
KSDRDMLDVLIAVKAETGTPRFSADEITGMFISMMFAGHHTSSGTASWTLIELMRHRDAYAAVIDELDELYGD
GRSVSFHALRQIPQLENVLKETLRLHPPLIILMRVAKGEFEVQGHRIHEGDLVAASPAISNRIPEDFPDPHDF
VPARYEQPRQEDLLNRWTWIPFGAGRHRCVGAAFAIMQIKAIFSVLLREYEFEMAQPPESYRNDHSKMVVQLA
QPACVRYRRRTGV
>CYP51B1_Mycobacterium tuberculosis 02_1987 2577464864
MSAVALPRVSGGHDEHGHLEEFRTDPIGLMQRVRDECGDVGTFQLAGKQVVLLSGSHANEFFFRAGDDDLDQA
KAYPFMTPIFGEGVVFDASPERRKEMLHNAALRGEQMKGHAATIEDQVRRMIADWGEAGEIDLLDFFAELTIY
TSSACLIGKKFRDQLDGRFAKLYHELERGTDPLAYVDPYLPIESFRRRDEARNGLVALVADIMNGRIANPPTD
KSDRDMLDVLIAVKAETGTPRFSADEITGMFISMMFAGHHTSSGTASWTLIELMRHRDAYAAVIDELDELYGD
GRSVSFHALRQIPQLENVLKETLRLHPPLIILMRVAKGEFEVQGHRIHEGDLVAASPAISNRIPEDFPDPHDF
VPARYEQPRQEDLLNRWTWIPFGAGRHRCVGAAFAIMQIKAIFSVLLREYEFEMAQPPESYRNDHSKMVVQLA
QPACVRYRRRTGV
>CYP51B1_Mycobacterium tuberculosis 02_1987 unfinished sequence 643022165
MSAVALPRVSGGHDEHGHLEEFRTDPIGLMQRVRDECGDVGTFQLAGKQVVLLSGSHANEFFFRAGDDDLDQA
KAYPFMTPIFGEGVVFDASPERRKEMLHNAALRGEQMKGHAATIEDQVRRMIADWGEAGEIDLLDFFAELTIY
TSSACLIGKKFRDQLDGRFAKLYHELERGTDPLAYVDPYLPIESFRRRDEARNGLVALVADIMNGRIANPPTD
KSDRDMLDVLIAVKAETGTPRFSADEITGMFISMMFAGHHTSSGTASWTLIELMRHRDAYAAVIDELDELYGD
GRSVSFHALRQIPQLENVLKETLRLHPPLIILMRVAKGEFEVQGHRIHEGDLVAASPAISNRIPEDFPDPHDF
VPARYEQPRQEDLLNRWTWIPFGAGRHRCVGAAFAIMQIKAIFSVLLREYEFEMAQPPESYRNDHSKMVVQLA
QPACVRYRRRTGV
>CYP51B1_Mycobacterium tuberculosis 0A005DS 2720374504
MSAVALPRVSGGHDEHGHLEEFRTDPIGLMQRVRDECGDVGTFQLAGKQVVLLSGSHANEFFFRAGDDDLDQA
KAYPFMTPIFGEGVVFDASPERRKEMLHNAALRGEQMKGHAATIEDQVRRMIADWGEAGEIDLLDFFAELTIY
TSSACLIGKKFRDQLDGRFAKLYHELERGTDPLAYVDPYLPIESFRRRDEARNGLVALVADIMNGRIANPPTD
KSDRDMLDVLIAVKAETGTPRFSADEITGMFISMMFAGHHTSSGTASWTLIELMRHRDAYAAVIDELDELYGD
GRSVSFHALRQIPQLENVLKETLRLHPPLIILMRVAKGEFEVQGHRIHEGDLVAASPAISNRIPEDFPDPHDF
VPARYEQPRQEDLLNRWTWIPFGAGRHRCVGAAFAIMQIKAIFSVLLREYEFEMAQPPESYRNDHSKMVVQLA
QPACVRYRRRTGV
>CYP51B1_Mycobacterium tuberculosis 0A012DS 2718846842

MSAVALPRVSGGHDEHGHLEEFRTDPIGLMQRVRDECGDVGTFQLAGKQVVLLSGSHANEFFFRAGDDDLDQA
KAYPFMTPIFGEGVVFDASPERRKEMLHNAALRGEQMKGHAATIEDQVRRMIADWGEAGEIDLLDFFAELTIY
TSSACLIGKKFRDQLDGRFAKLYHELERGTDPLAYVDPYLPIESFRRRDEARNGLVALVADIMNGRIANPPTD
KSDRDMLDVLIAVKAETGTPRFSADEITGMFISMMFAGHHTSSGTASWTLIELMRHRDAYAAVIDELDELYGD
GRSVSFHALRQIPQLENVLKETLRLHPPLIILMRVAKGEFEVQGHRIHEGDLVAASPAISNRIPEDFPDPHDF
VPARYEQPRQEDLLNRWTWIPFGAGRHRCVGAAFAIMQIKAIFSVLLREYEFEMAQPPESYRNDHSKMVVQLA
QPACVRYRRRTGV
>CYP51B1_Mycobacterium tuberculosis 0A029DS 2722764452
MSAVALPRVSGGHDEHGHLEEFRTDPIGLMQRVRDECGDVGTFQLAGKQVVLLSGSHANEFFFRAGDDDLDQA
KAYPFMTPIFGEGVVFDASPERRKEMLHNAALRGEQMKGHAATIEDQVRRMIADWGEAGEIDLLDFFAELTIY
TSSACLIGKKFRDQLDGRFAKLYHELERGTDPLAYVDPYLPIESFRRRDEARNGLVALVADIMNGRIANPPTD
KSDRDMLDVLIAVKAETGTPRFSADEITGMFISMMFAGHHTSSGTASWTLIELMRHRDAYAAVIDELDELYGD
GRSVSFHALRQIPQLENVLKETLRLHPPLIILMRVAKGEFEVQGHRIHEGDLVAASPAISNRIPEDFPDPHDF
VPARYEQPRQEDLLNRWTWIPFGAGRHRCVGAAFAIMQIKAIFSVLLREYEFEMAQPPESYRNDHSKMVVQLA
QPACVRYRRRTGV
>CYP51B1_Mycobacterium tuberculosis 0A033DS 2730276667
MSAVALPRVSGGHDEHGHLEEFRTDPIGLMQRVRDECGDVGTFQLAGKQVVLLSGSHANEFFFRAGDDDLDQA
KAYPFMTPIFGEGVVFDASPERRKEMLHNAALRGEQMKGHAATIEDQVRRMIADWGEAGEIDLLDFFAELTIY
TSSACLIGKKFRDQLDGRFAKLYHELERGTDPLAYVDPYLPIESFRRRDEARNGLVALVADIMNGRIANPPTD
KSDRDMLDVLIAVKAETGTPRFSADEITGMFISMMFAGHHTSSGTASWTLIELMRHRDAYAAVIDELDELYGD
GRSVSFHALRQIPQLENVLKETLRLHPPLIILMRVAKGEFEVQGHRIHEGDLVAASPAISNRIPEDFPDPHDF
VPARYEQPRQEDLLNRWTWIPFGAGRHRCVGAAFAIMQIKAIFSVLLREYEFEMAQPPESYRNDHSKMVVQLA
QPACVRYRRRTGV
>CYP51B1_Mycobacterium tuberculosis 0A036DS 2720570487
MSAVALPRVSGGHDEHGHLEEFRTDPIGLMQRVRDECGDVGTFQLAGKQVVLLSGSHANEFFFRAGDDDLDQA
KAYPFMTPIFGEGVVFDASPERRKEMLHNAALRGEQMKGHAATIEDQVRRMIADWGEAGEIDLLDFFAELTIY
TSSACLIGKKFRDQLDGRFAKLYHELERGTDPLAYVDPYLPIESFRRRDEARNGLVALVADIMNGRIANPPTD
KSDRDMLDVLIAVKAETGTPRFSADEITGMFISMMFAGHHTSSGTASWTLIELMRHRDAYAAVIDELDELYGD
GRSVSFHALRQIPQLENVLKETLRLHPPLIILMRVAKGEFEVQGHRIHEGDLVAASPAISNRIPEDFPDPHDF
VPARYEQPRQEDLLNRWTWIPFGAGRHRCVGAAFAIMQIKAIFSVLLREYEFEMAQPPESYRNDHSKMVVQLA
QPACVRYRRRTGV
>CYP51B1_Mycobacterium tuberculosis 0A087DS 2723411657
MSAVALPRVSGGHDEHGHLEEFRTDPIGLMQRVRDECGDVGTFQLAGKQVVLLSGSHANEFFFRAGDDDLDQA
KAYPFMTPIFGEGVVFDASPERRKEMLHNAALRGEQMKGHAATIEDQVRRMIADWGEAGEIDLLDFFAELTIY
TSSACLIGKKFRDQLDGRFAKLYHELERGTDPLAYVDPYLPIESFRRRDEARNGLVALVADIMNGRIANPPTD
KSDRDMLDVLIAVKAETGTPRFSADEITGMFISMMFAGHHTSSGTASWTLIELMRHRDAYAAVIDELDELYGD
GRSVSFHALRQIPQLENVLKETLRLHPPLIILMRVAKGEFEVQGHRIHEGDLVAASPAISNRIPEDFPDPHDF
VPARYEQPRQEDLLNRWTWIPFGAGRHRCVGAAFAIMQIKAIFSVLLREYEFEMAQPPESYRNDHSKMVVQLA
QPACVRYRRRTGV
>CYP51B1_Mycobacterium tuberculosis 0A092DS 2720280314
MSAVALPRVSGGHDEHGHLEEFRTDPIGLMQRVRDECGDVGTFQLAGKQVVLLSGSHANEFFFRAGDDDLDQA
KAYPFMTPIFGEGVVFDASPERRKEMLHNAALRGEQMKGHAATIEDQVRRMIADWGEAGEIDLLDFFAELTIY
TSSACLIGKKFRDQLDGRFAKLYHELERGTDPLAYVDPYLPIESFRRRDEARNGLVALVADIMNGRIANPPTD
KSDRDMLDVLIAVKAETGTPRFSADEITGMFISMMFAGHHTSSGTASWTLIELMRHRDAYAAVIDELDELYGD
GRSVSFHALRQIPQLENVLKETLRLHPPLIILMRVAKGEFEVQGHRIHEGDLVAASPAISNRIPEDFPDPHDF
VPARYEQPRQEDLLNRWTWIPFGAGRHRCVGAAFAIMQIKAIFSVLLREYEFEMAQPPESYRNDHSKMVVQLA
QPACVRYRRRTGV
>CYP51B1_Mycobacterium tuberculosis 0A093DS 2719236067
MSAVALPRVSGGHDEHGHLEEFRTDPIGLMQRVRDECGDVGTFQLAGKQVVLLSGSHANEFFFRAGDDDLDQA
KAYPFMTPIFGEGVVFDASPERRKEMLHNAALRGEQMKGHAATIEDQVRRMIADWGEAGEIDLLDFFAELTIY
TSSACLIGKKFRDQLDGRFAKLYHELERGTDPLAYVDPYLPIESFRRRDEARNGLVALVADIMNGRIANPPTD
KSDRDMLDVLIAVKAETGTPRFSADEITGMFISMMFAGHHTSSGTASWTLIELMRHRDAYAAVIDELDELYGD
GRSVSFHALRQIPQLENVLKETLRLHPPLIILMRVAKGEFEVQGHRIHEGDLVAASPAISNRIPEDFPDPHDF
VPARYEQPRQEDLLNRWTWIPFGAGRHRCVGAAFAIMQIKAIFSVLLREYEFEMAQPPESYRNDHSKMVVQLA
QPACVRYRRRTGV
>CYP51B1_Mycobacterium tuberculosis 0A094DS 2719253982
MSAVALPRVSGGHDEHGHLEEFRTDPIGLMQRVRDECGDVGTFQLAGKQVVLLSGSHANEFFFRAGDDDLDQA
KAYPFMTPIFGEGVVFDASPERRKEMLHNAALRGEQMKGHAATIEDQVRRMIADWGEAGEIDLLDFFAELTIY

TSSACLIGKKFRDQLDGRFAKLYHELERGTDPLAYVDPYLPIESFRRRDEARNGLVALVADIMNGRIANPPTD
KSDRDMLDVLIAVKAETGTPRFSADEITGMFISMMFAGHHTSSGTASWTLIELMRHRDAYAAVIDELDELYGD
GRSVSFHALRQIPQLENVLKETLRLHPPLIILMRVAKGEFEVQGHRIHEGDLVAASPAISNRIPEDFPDPHDF
VPARYEQPRQEDLLNRWTWIPFGAGRHRCVGAAFAIMQIKAIFSVLLREYEFEMAQPPESYRNDHSKMVVQLA
QPACVRYRRRTGV
>CYP51B1_Mycobacterium tuberculosis 0A115DS 2723663654
MSAVALPRVSGGHDEHGHLEEFRTDPIGLMQRVRDECGDVGTFQLAGKQVVLLSGSHANEFFFRAGDDDLDQA
KAYPFMTPIFGEGVVFDASPERRKEMLHNAALRGEQMKGHAATIEDQVRRMIADWGEAGEIDLLDFFAELTIY
TSSACLIGKKFRDQLDGRFAKLYHELERGTDPLAYVDPYLPIESFRRRDEARNGLVALVADIMNGRIANPPTD
KSDRDMLDVLIAVKAETGTPRFSADEITGMFISMMFAGHHTSSGTASWTLIELMRHRDAYAAVIDELDELYGD
GRSVSFHALRQIPQLENVLKETLRLHPPLIILMRVAKGEFEVQGHRIHEGDLVAASPAISNRIPEDFPDPHDF
VPARYEQPRQEDLLNRWTWIPFGAGRHRCVGAAFAIMQIKAIFSVLLREYEFEMAQPPESYRNDHSKMVVQLA
QPACVRYRRRTGV
>CYP51B1_Mycobacterium tuberculosis 0A117DS 2719115594
MSAVALPRVSGGHDEHGHLEEFRTDPIGLMQRVRDECGDVGTFQLAGKQVVLLSGSHANEFFFRAGDDDLDQA
KAYPFMTPIFGEGVVFDASPERRKEMLHNAALRGEQMKGHAATIEDQVRRMIADWGEAGEIDLLDFFAELTIY
TSSACLIGKKFRDQLDGRFAKLYHELERGTDPLAYVDPYLPIESFRRRDEARNGLVALVADIMNGRIANPPTD
KSDRDMLDVLIAVKAETGTPRFSADEITGMFISMMFAGHHTSSGTASWTLIELMRHRDAYAAVIDELDELYGD
GRSVSFHALRQIPQLENVLKETLRLHPPLIILMRVAKGEFEVQGHRIHEGDLVAASPAISNRIPEDFPDPHDF
VPARYEQPRQEDLLNRWTWIPFGAGRHRCVGAAFAIMQIKAIFSVLLREYEFEMAQPPESYRNDHSKMVVQLA
QPACVRYRRRTGV
>CYP51B1_Mycobacterium tuberculosis 0B026XDR 2720192686
MSAVALPRVSGGHDEHGHLEEFRTDPIGLMQRVRDECGDVGTFQLAGKQVVLLSGSHANEFFFRAGDDDLDQA
KAYPFMTPIFGEGVVFDASPERRKEMLHNAALRGEQMKGHAATIEDQVRRMIADWGEAGEIDLLDFFAELTIY
TSSACLIGKKFRDQLDGRFAKLYHELERGTDPLAYVDPYLPIESFRRRDEARNGLVALVADIMNGRIANPPTD
KSDRDMLDVLIAVKAETGTPRFSADEITGMFISMMFAGHHTSSGTASWTLIELMRHRDAYAAVIDELDELYGD
GRSVSFHALRQIPQLENVLKETLRLHPPLIILMRVAKGEFEVQGHRIHEGDLVAASPAISNRIPEDFPDPHDF
VPARYEQPRQEDLLNRWTWIPFGAGRHRCVGAAFAIMQIKAIFSVLLREYEFEMAQPPESYRNDHSKMVVQLA
QPACVRYRRRTGV
>CYP51B1_Mycobacterium tuberculosis 0B049XDR 2720263188
MSAVALPRVSGGHDEHGHLEEFRTDPIGLMQRVRDECGDVGTFQLAGKQVVLLSGSHANEFFFRAGDDDLDQA
KAYPFMTPIFGEGVVFDASPERRKEMLHNAALRGEQMKGHAATIEDQVRRMIADWGEAGEIDLLDFFAELTIY
TSSACLIGKKFRDQLDGRFAKLYHELERGTDPLAYVDPYLPIESFRRRDEARNGLVALVADIMNGRIANPPTD
KSDRDMLDVLIAVKAETGTPRFSADEITGMFISMMFAGHHTSSGTASWTLIELMRHRDAYAAVIDELDELYGD
GRSVSFHALRQIPQLENVLKETLRLHPPLIILMRVAKGEFEVQGHRIHEGDLVAASPAISNRIPEDFPDPHDF
VPARYEQPRQEDLLNRWTWIPFGAGRHRCVGAAFAIMQIKAIFSVLLREYEFEMAQPPESYRNDHSKMVVQLA
QPACVR
>CYP51B1_Mycobacterium tuberculosis 0B070XDR 2720986988
MSAVALPRVSGGHDEHGHLEEFRTDPIGLMQRVRDECGDVGTFQLAGKQVVLLSGSHANEFFFRAGDDDLDQA
KAYPFMTPIFGEGVVFDASPERRKEMLHNAALRGEQMKGHAATIEDQVRRMIADWGEAGEIDLLDFFAELTIY
TSSACLIGKKFRDQLDGRFAKLYHELERGTDPLAYVDPYLPIESFRRRDEARNGLVALVADIMNGRIANPPTD
KSDRDMLDVLIAVKAETGTPRFSADEITGMFISMMFAGHHTSSGTASWTLIELMRHRDAYAAVIDELDELYGD
GRSVSFHALRQIPQLENVLKETLRLHPPLIILMRVAKGEFEVQGHRIHEGDLVAASPAISNRIPEDFPDPHDF
VPARYEQPRQEDLLNRWTWIPFGAGRHRCVGAAFAIMQIKAIFSVLLREYEFEMAQPPESYRNDHSKMVVQLA
QPACVRYRRRTGV
>CYP51B1_Mycobacterium tuberculosis 0B076XDR 2720067307
MSAVALPRVSGGHDEHGHLEEFRTDPIGLMQRVRDECGDVGTFQLAGKQVVLLSGSHANEFFFRAGDDDLDQA
KAYPFMTPIFGEGVVFDASPERRKEMLHNAALRGEQMKGHAATIEDQVRRMIADWGEAGEIDLLDFFAELTIY
TSSACLIGKKFRDQLDGRFAKLYHELERGTDPLAYVDPYLPIESFRRRDEARNGLVALVADIMNGRIANPPTD
KSDRDMLDVLIAVKAETGTPRFSADEITGMFISMMFAGHHTSSGTASWTLIELMRHRDAYAAVIDELDELYGD
GRSVSFHALRQIPQLENVLKETLRLHPPLIILMRVAKGEFEVQGHRIHEGDLVAASPAISNRIPEDFPDPHDF
VPARYEQPRQEDLLNRWTWIPFGAGRHRCVGAAFAIMQIKAIFSVLLREYEFEMAQPPESYRNDHSKMVVQLA
QPACVRYRRRTGV
>CYP51B1_Mycobacterium tuberculosis 0B123ND 2720991310
MSAVALPRVSGGHDEHGHLEEFRTDPIGLMQRVRDECGDVGTFQLAGKQVVLLSGSHANEFFFRAGDDDLDQA
KAYPFMTPIFGEGVVFDASPERRKEMLHNAALRGEQMKGHAATIEDQVRRMIADWGEAGEIDLLDFFAELTIY
TSSACLIGKKFRDQLDGRFAKLYHELERGTDPLAYVDPYLPIESFRRRDEARNGLVALVADIMNGRIANPPTD
KSDRDMLDVLIAVKAETGTPRFSADEITGMFISMMFAGHHTSSGTASWTLIELMRHRDAYAAVIDELDELYGD

GRSVSFHALRQIPQLENVLKETLRLHPPLIILMRVAKGEFEVQGHRIHEGDLVAASPAISNRIPEDFPDPHDF
VPARYEQPRQEDLLNRWTWIPFGAGRHRCVGAAFAIMQIKAIFSVLLREYEFEMAQPPESYRNDHSKMVVQLA
QPACVRYRRRTGV
>CYP51B1_Mycobacterium tuberculosis 0B169XDR 2720996055
MSAVALPRVSGGHDEHGHLEEFRTDPIGLMQRVRDECGDVGTFQLAGKQVVLLSGSHANEFFFRAGDDDLDQA
KAYPFMTPIFGEGVVFDASPERRKEMLHNAALRGEQMKGHAATIEDQVRRMIADWGEAGEIDLLDFFAELTIY
TSSACLIGKKFRDQLDGRFAKLYHELERGTDPLAYVDPYLPIESFRRRDEARNGLVALVADIMNGRIANPPTD
KSDRDMLDVLIAVKAETGTPRFSADEITGMFISMMFAGHHTSSGTASWTLIELMRHRDAYAAVIDELDELYGD
GRSVSFHALRQIPQLENVLKETLRLHPPLIILMRVAKGEFEVQGHRIHEGDLVAASPAISNRIPEDFPDPHDF
VPARYEQPRQEDLLNRWTWIPFGAGRHRCVGAAFAIMQIKAIFSVLLREYEFEMAQPPESYRNDHSKMVVQLA
QPACVRYRRRTGV
>CYP51B1_Mycobacterium tuberculosis 0B218DS 2720187971
MSAVALPRVSGGHDEHGHLEEFRTDPIGLMQRVRDECGDVGTFQLAGKQVVLLSGSHANEFFFRAGDDDLDQA
KAYPFMTPIFGEGVVFDASPERRKEMLHNAALRGEQMKGHAATIEDQVRRMIADWGEAGEIDLLDFFAELTIY
TSSACLIGKKFRDQLDGRFAKLYHELERGTDPLAYVDPYLPIESFRRRDEARNGLVALVADIMNGRIANPPTD
KSDRDMLDVLIAVKAETGTPRFSADEITGMFISMMFAGHHTSSGTASWTLIELMRHRDAYAAVIDELDELYGD
GRSVSFHALRQIPQLENVLKETLRLHPPLIILMRVAKGEFEVQGHRIHEGDLVAASPAISNRIPEDFPDPHDF
VPARYEQPRQEDLLNRWTWIPFGAGRHRCVGAAFAIMQIKAIFSVLLREYEFEMAQPPESYRNDHSKMVVQLA
QPACVRYRRRTGV
>CYP51B1_Mycobacterium tuberculosis 0B222DS 2723521510
MSAVALPRVSGGHDEHGHLEEFRTDPIGLMQRVRDECGDVGTFQLAGKQVVLLSGSHANEFFFRAGDDDLDQA
KAYPFMTPIFGEGVVFDASPERRKEMLHNAALRGEQMKGHAATIEDQVRRMIADWGEAGEIDLLDFFAELTIY
TSSACLIGKKFRDQLDGRFAKLYHELERGTDPLAYVDPYLPIESFRRRDEARNGLVALVADIMNGRIANPPTD
KSDRDMLDVLIAVKAETGTPRFSADEITGMFISMMFAGHHTSSGTASWTLIELMRHRDAYAAVIDELDELYGD
GRSVSFHALRQIPQLENVLKETLRLHPPLIILMRVAKGEFEVQGHRIHEGDLVAASPAISNRIPEDFPDPHDF
VPARYEQPRQEDLLNRWTWIPFGAGRHRCVGAAFAIMQIKAIFSVLLREYEFEMAQPPESYRNDHSKMVVQLA
QPACVRYRRRTGV
>CYP51B1_Mycobacterium tuberculosis 0B228DS 2720267964
MSAVALPRVSGGHDEHGHLEEFRTDPIGLMQRVRDECGDVGTFQLAGKQVVLLSGSHANEFFFRAGDDDLDQA
KAYPFMTPIFGEGVVFDASPERRKEMLHNAALRGEQMKGHAATIEDQVRRMIADWGEAGEIDLLDFFAELTIY
TSSACLIGKKFRDQLDGRFAKLYHELERGTDPLAYVDPYLPIESFRRRDEARNGLVALVADIMNGRIANPPTD
KSDRDMLDVLIAVKAETGTPRFSADEITGMFISMMFAGHHTSSGTASWTLIELMRHRDAYAAVIDELDELYGD
GRSVSFHALRQIPQLENVLKETLRLHPPLIILMRVAKGEFEVQGHRIHEGDLVAASPAISNRIPEDFPDPHDF
VPARYEQPRQEDLLNRWTWIPFGAGRHRCVGAAFAIMQIKAIFSVLLREYEFEMAQPPESYRNDHSKMVVQLA
QPACVRYRRRTGV
>CYP51B1_Mycobacterium tuberculosis 0B229DS 2721103627
MSAVALPRVSGGHDEHGHLEEFRTDPIGLMQRVRDECGDVGTFQLAGKQVVLLSGSHANEFFFRAGDDDLDQA
KAYPFMTPIFGEGVVFDASPERRKEMLHNAALRGEQMKGHAATIEDQVRRMIADWGEAGEIDLLDFFAELTIY
TSSACLIGKKFRDQLDGRFAKLYHELERGTDPLAYVDPYLPIESFRRRDEARNGLVALVADIMNGRIANPPTD
KSDRDMLDVLIAVKAETGTPRFSADEITGMFISMMFAGHHTSSGTASWTLIELMRHRDAYAAVIDELDELYGD
GRSVSFHALRQIPQLENVLKETLRLHPPLIILMRVAKGEFEVQGHRIHEGDLVAASPAISNRIPEDFPDPHDF
VPARYEQPRQEDLLNRWTWIPFGAGRHRCVGAAFAIMQIKAIFSVLLREYEFEMAQPPESYRNDHSKMVVQLA
QPACVRYRRRTGV
>CYP51B1_Mycobacterium tuberculosis 0B259XDR 2718552175
MSAVALPRVSGGHDEHGHLEEFRTDPIGLMQRVRDECGDVGTFQLAGKQVVLLSGSHANEFFFRAGDDDLDQA
KAYPFMTPIFGEGVVFDASPERRKEMLHNAALRGEQMKGHAATIEDQVRRMIADWGEAGEIDLLDFFAELTIY
TSSACLIGKKFRDQLDGRFAKLYHELERGTDPLAYVDPYLPIESFRRRDEARNGLVALVADIMNGRIANPPTD
KSDRDMLDVLIAVKAETGTPRFSADEITGMFISMMFAGHHTSSGTASWTLIELMRHRDAYAAVIDELDELYGD
GRSVSFHALRQIPQLENVLKETLRLHPPLIILMRVAKGEFEVQGHRIHEGDLVAASPAISNRIPEDFPDPHDF
VPARYEQPRQEDLLNRWTWIPFGAGRHRCVGAAFAIMQIKAIFSVLLREYEFEMAQPPESYRNDHSKMVVQLA
QPACVRYRRRTGV
>CYP51B1_Mycobacterium tuberculosis 0B329XDR 2719143911
MSAVALPRVSGGHDEHGHLEEFRTDPIGLMQRVRDECGDVGTFQLAGKQVVLLSGSHANEFFFRAGDDDLDQA
KAYPFMTPIFGEGVVFDASPERRKEMLHNAALRGEQMKGHAATIEDQVRRMIADWGEAGEIDLLDFFAELTIY
TSSACLIGKKFRDQLDGRFAKLYHELERGTDPLAYVDPYLPIESFRRRDEARNGLVALVADIMNGRIANPPTD
KSDRDMLDVLIAVKAETGTPRFSADEITGMFISMMFAGHHTSSGTASWTLIELMRHRDAYAAVIDELDELYGD
GRSVSFHALRQIPQLENVLKETLRLHPPLIILMRVAKGEFEVQGHRIHEGDLVAASPAISNRIPEDFPDPHDF

VPARYEQPRQEDLLNRWTWIPFGAGRHRCVGAAFAIMQIKAIFSVLLREYEFEMAQPPESYRNDHSKMVVQLA
QPACVRYRRRTGV
>CYP51B1_Mycobacterium tuberculosis 10060 2631863878
MSAVALPRVSGGHDEHGHLEEFRTDPIGLMQRVRDECGDVGTFQLAGKQVVLLSGSHANEFFFRAGDDDLDQA
KAYPFMTPIFGEGVVFDASPERRKEMLHNAALRGEQMKGHAATIEDQVRRMIADWGEAGEIDLLDFFAELTIY
TSSACLIGKKFRDQLDGRFAKLYHELERGTDPLAYVDPYLPIESFRRRDEARNGLVALVADIMNGRIANPPTD
KSDRDMLDVLIAVKAETGTPRFSADEITGMFISMMFAGHHTSSGTASWTLIELMRHRDAYAAVIDELDELYGD
GRSVSFHALRQIPQLENVLKETLRLHPPLIILMRVAKGEFEVQGHRIHEGDLVAASPAISNRIPEDFPDPHDF
VPARYEQPRQEDLLNRWTWIPFGAGRHRCVGAAFAIMQIKAIFSVLLREYEFEMAQPPESYRNDHSKMVVQLA
QPACVRYRRRTGV
>CYP51B1_Mycobacterium tuberculosis 1010SM 2584794490
MSAVALPRVSGGHDEHGHLEEFRTDPIGLMQRVRDECGDVGTFQLAGKQVVLLSGSHANEFFFRAGDDDLDQA
KAYPFMTPIFGEGVVFDASPERRKEMLHNAALRGEQMKGHAATIEDQVRRMIADWGEAGEIDLLDFFAELTIY
TSSACLIGKKFRDQLDGRFAKLYHELERGTDPLAYVDPYLPIESFRRRDEARNGLVALVADIMNGRIANPPTD
KSDRDMLDVLIAVKAETGTPRFSADEITGMFISMMFAGHHTSSGTASWTLIELMRHRDAYAAVIDELDELYGD
GRSVSFHALRQIPQLENVLKETLRLHPPLIILMRVAKGEFEVQGHRIHEGDLVAASPAISNRIPEDFPDPHDF
VPARYEQPRQEDLLNRWTWIPFGAGRHRCVGAAFAIMQIKAIFSVLLREYEFEMAQPPESYRNDHSKMVVQLA
QPACVRYRRRTGV
>CYP51B1_Mycobacterium tuberculosis 10217 2621700027
MSAVALPRVSGGHDEHGHLEEFRTDPIGLMQRVRDECGDVGTFQLAGKQVVLLSGSHANEFFFRAGDDDLDQA
KAYPFMTPIFGEGVVFDASPERRKEMLHNAALRGEQMKGHAATIEDQVRRMIADWGEAGEIDLLDFFAELTIY
TSSACLIGKKFRDQLDGRFAKLYHELERGTDPLAYVDPYLPIESFRRRDEARNGLVALVADIMNGRIANPPTD
KSDRDMLDVLIAVKAETGTPRFSADEITGMFISMMFAGHHTSSGTASWTLIELMRHRDAYAAVIDELDELYGD
GRSVSFHALRQIPQLENVLKETLRLHPPLIILMRVAKGEFEVQGHRIHEGDLVAASPAISNRIPEDFPDPHDF
VPARYEQPRQEDLLNRWTWIPFGAGRHRCVGAAFAIMQIKAIFSVLLREYEFEMAQPPESYRNDHSKMVVQLA
QPACVRYRRRTGV
>CYP51B1_Mycobacterium tuberculosis 1034 2621342761
MSAVALPRVSGGHDEHGHLEEFRTDPIGLMQRVRDECGDVGTFQLAGKQVVLLSGSHANEFFFRAGDDDLDQA
KAYPFMTPIFGEGVVFDASPERRKEMLHNAALRGEQMKGHAATIEDQVRRMIADWGEAGEIDLLDFFAELTIY
TSSACLIGKKFRDQLDGRFAKLYHELERGTDPLAYVDPYLPIESFRRRDEARNGLVALVADIMNGRIANPPTD
KSDRDMLDVLIAVKAETGTPRFSADEITGMFISMMFAGHHTSSGTASWTLIELMRHRDAYAAVIDELDELYGD
GRSVSFHALRQIPQLENVLKETLRLHPPLIILMRVAKGEFEVQGHRIHEGDLVAASPAISNRIPEDFPDPHDF
VPARYEQPRQEDLLNRWTWIPFGAGRHRCVGAAFAIMQIKAIFSVLLREYEFEMAQPPESYRNDHSKMVVQLA
QPACVRYRRRTGV
>CYP51B1_Mycobacterium tuberculosis 1034 2621342761
MSAVALPRVSGGHDEHGHLEEFRTDPIGLMQRVRDECGDVGTFQLAGKQVVLLSGSHANEFFFRAGDDDLDQA
KAYPFMTPIFGEGVVFDASPERRKEMLHNAALRGEQMKGHAATIEDQVRRMIADWGEAGEIDLLDFFAELTIY
TSSACLIGKKFRDQLDGRFAKLYHELERGTDPLAYVDPYLPIESFRRRDEARNGLVALVADIMNGRIANPPTD
KSDRDMLDVLIAVKAETGTPRFSADEITGMFISMMFAGHHTSSGTASWTLIELMRHRDAYAAVIDELDELYGD
GRSVSFHALRQIPQLENVLKETLRLHPPLIILMRVAKGEFEVQGHRIHEGDLVAASPAISNRIPEDFPDPHDF
VPARYEQPRQEDLLNRWTWIPFGAGRHRCVGAAFAIMQIKAIFSVLLREYEFEMAQPPESYRNDHSKMVVQLA
QPACVRYRRRTGV
>CYP51B1_Mycobacterium tuberculosis 1173CS 2576562554
MSAVALPRVSGGHDEHGHLEEFRTDPIGLMQRVRDECGDVGTFQLAGKQVVLLSGSHANEFFFRAGDDDLDQA
KAYPFMTPIFGEGVVFDASPERRKEMLHNAALRGEQMKGHAATIEDQVRRMIADWGEAGEIDLLDFFAELTIY
TSSACLIGKKFRDQLDGRFAKLYHELERGTDPLAYVDPYLPIESFRRRDEARNGLVALVADIMNGRIANPPTD
KSDRDMLDVLIAVKAETGTPRFSADEITGMFISMMFAGHHTSSGTASWTLIELMRHRDAYAAVIDELDELYGD
GRSVSFHALRQIPQLENVLKETLRLHPPLIILMRVAKGEFEVQGHRIHEGDLVAASPAISNRIPEDFPDPHDF
VPARYEQPRQEDLLNRWTWIPFGAGRHRCVGAAFAIMQIKAIFSVLLREYEFEMAQPPESYRNDHSKMVVQLA
QPACVRYRRRTGV
>CYP51B1_Mycobacterium tuberculosis 1200172 2701678021
MSAVALPRVSGGHDEHGHLEEFRTDPIGLMQRVRDECGDVGTFQLAGKQVVLLSGSHANEFFFRAGDDDLDQA
KAYPFMTPIFGEGVVFDASPERRKEMLHNAALRGEQMKGHAATIEDQVRRMIADWGEAGEIDLLDFFAELTIY
TSSACLIGKKFRDQLDGRFAKLYHELERGTDPLAYVDPYLPIESFRRRDEARNGLVALVADIMNGRIANPPTD
KSDRDMLDVLIAVKAETGTPRFSADEITGMFISMMFAGHHTSSGTASWTLIELMRHRDAYAAVIDELDELYGD
GRSVSFHALRQIPQLENVLKETLRLHPPLIILMRVAKGEFEVQGHRIHEGDLVAASPAISNRIPEDFPDPHDF
VPARYEQPRQEDLLNRWTWIPFGAGRHRCVGAAFAIMQIKAIFSVLLREYEFEMAQPPESYRNDHSKMVVQLA
QPACVRYRRRTGV

>CYP51B1_Mycobacterium tuberculosis 1200782 2659058801
MSAVALPRVSGGHDEHGHLEEFRTDPIGLMQRVRDECGDVGTFQLAGKQVVLLSGSHANEFFFRAGDDDLDQA
KAYPFMTPIFGEGVVFDASPERRKEMLHNAALRGEQMKGHAATIEDQVRRMIADWGEAGEIDLLDFFAELTIY
TSSACLIGKKFRDQLDGRFAKLYHELERGTDPLAYVDPYLPIESFRRRDEARNGLVALVADIMNGRIANPPTD
KSDRDMLDVLIAVKAETGTPRFSADEITGMFISMMFAGHHTSSGTASWTLIELMRHRDAYAAVIDELDELYGD
GRSVSFHALRQIPQLENVLKETLRLHPPLIILMRVAKGEFEVQGHRIHEGDLVAASPAISNRIPEDFPDPHDF
VPARYEQPRQEDLLNRWTWIPFGAGRHRCVGAAFAIMQIKAIFSVLLREYEFEMAQPPESYRNDHSKMVVQLA
QPACVRYRRRTGV
>CYP51B1_Mycobacterium tuberculosis 12974 2621694359
MSAVALPRVSGGHDEHGHLEEFRTDPIGLMQRVRDECGDVGTFQLAGKQVVLLSGSHANEFFFRAGDDDLDQA
KAYPFMTPIFGEGVVFDASPERRKEMLHNAALRGEQMKGHAATIEDQVRRMIADWGEAGEIDLLDFFAELTIY
TSSACLIGKKFRDQLDGRFAKLYHELERGTDPLAYVDPYLPIESFRRRDEARNGLVALVADIMNGRIANPPTD
KSDRDMLDVLIAVKAETGTPRFSADEITGMFISMMFAGHHTSSGTASWTLIELMRHRDAYAAVIDELDELYGD
GRSVSFHALRQIPQLENVLKETLRLHPPLIILMRVAKGEFEVQGHRIHEGDLVAASPAISNRIPEDFPDPHDF
VPARYEQPRQEDLLNRWTWIPFGAGRHRCVGAAFAIMQIKAIFSVLLREYEFEMAQPPESYRNDHSKMVVQLA
QPACVRYRRRTGV
>CYP51B1_Mycobacterium tuberculosis 1300037 2692718234
MSAVALPRVSGGHDEHGHLEEFRTDPIGLMQRVRDECGDVGTFQLAGKQVVLLSGSHANEFFFRAGDDDLDQA
KAYPFMTPIFGEGVVFDASPERRKEMLHNAALRGEQMKGHAATIEDQVRRMIADWGEAGEIDLLDFFAELTIY
TSSACLIGKKFRDQLDGRFAKLYHELERGTDPLAYVDPYLPIESFRRRDEARNGLVALVADIMNGRIANPPTD
KSDRDMLDVLIAVKAETGTPRFSADEITGMFISMMFAGHHTSSGTASWTLIELMRHRDAYAAVIDELDELYGD
GRSVSFHALRQIPQLENVLKETLRLHPPLIILMRVAKGEFEVQGHRIHEGDLVAASPAISNRIPEDFPDPHDF
VPARYEQPRQEDLLNRWTWIPFGAGRHRCVGAAFAIMQIKAIFSVLLREYEFEMAQPPESYRNDHSKMVVQLA
QPACVRYRRRTGV
>CYP51B1_Mycobacterium tuberculosis 1300039 2666673878
MSAVALPRVSGGHDEHGHLEEFRTDPIGLMQRVRDECGDVGTFQLAGKQVVLLSGSHANEFFFRAGDDDLDQA
KAYPFMTPIFGEGVVFDASPERRKEMLHNAALRGEQMKGHAATIEDQVRRMIADWGEAGEIDLLDFFAELTIY
TSSACLIGKKFRDQLDGRFAKLYHELERGTDPLAYVDPYLPIESFRRRDEARNGLVALVADIMNGRIANPPTD
KSDRDMLDVLIAVKAETGTPRFSADEITGMFISMMFAGHHTSSGTASWTLIELMRHRDAYAAVIDELDELYGD
GRSVSFHALRQIPQLENVLKETLRLHPPLIILMRVAKGEFEVQGHRIHEGDLVAASPAISNRIPEDFPDPHDF
VPARYEQPRQEDLLNRWTWIPFGAGRHRCVGAAFAIMQIKAIFSVLLREYEFEMAQPPESYRNDHSKMVVQLA
QPACVRYRRRTGV
>CYP51B1_Mycobacterium tuberculosis 1300215 2701646706
MSAVALPRVSGGHDEHGHLEEFRTDPIGLMQRVRDECGDVGTFQLAGKQVVLLSGSHANEFFFRAGDDDLDQA
KAYPFMTPIFGEGVVFDASPERRKEMLHNAALRGEQMKGHAATIEDQVRRMIADWGEAGEIDLLDFFAELTIY
TSSACLIGKKFRDQLDGRFAKLYHELERGTDPLAYVDPYLPIESFRRRDEARNGLVALVADIMNGRIANPPTD
KSDRDMLDVLIAVKAETGTPRFSADEITGMFISMMFAGHHTSSGTASWTLIELMRHRDAYAAVIDELDELYGD
GRSVSFHALRQIPQLENVLKETLRLHPPLIILMRVAKGEFEVQGHRIHEGDLVAASPAISNRIPEDFPDPHDF
VPARYEQPRQEDLLNRWTWIPFGAGRHRCVGAAFAIMQIKAIFSVLLREYEFEMAQPPESYRNDHSKMVVQLA
QPACVRYRRRTGV
>CYP51B1_Mycobacterium tuberculosis 1300325 2659693478
MSAVALPRVSGGHDEHGHLEEFRTDPIGLMQRVRDECGDVGTFQLAGKQVVLLSGSHANEFFFRAGDDDLDQA
KAYPFMTPIFGEGVVFDASPERRKEMLHNAALRGEQMKGHAATIEDQVRRMIADWGEAGEIDLLDFFAELTIY
TSSACLIGKKFRDQLDGRFAKLYHELERGTDPLAYVDPYLPIESFRRRDEARNGLVALVADIMNGRIANPPTD
KSDRDMLDVLIAVKAETGTPRFSADEITGMFISMMFAGHHTSSGTASWTLIELMRHRDAYAAVIDELDELYGD
GRSVSFHALRQIPQLENVLKETLRLHPPLIILMRVAKGEFEVQGHRIHEGDLVAASPAISNRIPEDFPDPHDF
VPARYEQPRQEDLLNRWTWIPFGAGRHRCVGAAFAIMQIKAIFSVLLREYEFEMAQPPESYRNDHSKMVVQLA
QPACVRYRRRTGV
>CYP51B1_Mycobacterium tuberculosis 1301087 2670247190
MSAVALPRVSGGHDEHGHLEEFRTDPIGLMQRVRDECGDVGTFQLAGKQVVLLSGSHANEFFFRAGDDDLDQA
KAYPFMTPIFGEGVVFDASPERRKEMLHNAALRGEQMKGHAATIEDQVRRMIADWGEAGEIDLLDFFAELTIY
TSSACLIGKKFRDQLDGRFAKLYHELERGTDPLAYVDPYLPIESFRRRDEARNGLVALVADIMNGRIANPPTD
KSDRDMLDVLIAVKAETGTPRFSADEITGMFISMMFAGHHTSSGTASWTLIELMRHRDAYAAVIDELDELYGD
GRSVSFHALRQIPQLENVLKETLRLHPPLIILMRVAKGEFEVQGHRIHEGDLVAASPAISNRIPEDFPDPHDF
VPARYEQPRQEDLLNRWTWIPFGAGRHRCVGAAFAIMQIKAIFSVLLREYEFEMAQPPESYRNDHSKMVVQLA
QPACVRYRRRTGV
>CYP51B1_Mycobacterium tuberculosis 1301088 2669626515

MSAVALPRVSGGHDEHGHLEEFRTDPIGLMQRVRDECGDVGTFQLAGKQVVLLSGSHANEFFFRAGDDDLDQA
KAYPFMTPIFGEGVVFDASPERRKEMLHNAALRGEQMKGHAATIEDQVRRMIADWGEAGEIDLLDFFAELTIY
TSSACLIGKKFRDQLDGRFAKLYHELERGTDPLAYVDPYLPIESFRRRDEARNGLVALVADIMNGRIANPPTD
KSDRDMLDVLIAVKAETGTPRFSADEITGMFISMMFAGHHTSSGTASWTLIELMRHRDAYAAVIDELDELYGD
GRSVSFHALRQIPQLENVLKETLRLHPPLIILMRVAKGEFEVQGHRIHEGDLVAASPAISNRIPEDFPDPHDF
VPARYEQPRQEDLLNRWTWIPFGAGRHRCVGAAFAIMQIKAIFSVLLREYEFEMAQPPESYRNDHSKMVVQLA
QPACVRYRRRTGV
>CYP51B1_Mycobacterium tuberculosis 13-2459 2641680923
MSAVALPRVSGGHDEHGHLEEFRTDPIGLMQRVRDECGDVGTFQLAGKQVVLLSGSHANEFFFRAGDDDLDQA
KAYPFMTPIFGEGVVFDASPERRKEMLHNAALRGEQMKGHAATIEDQVRRMIADWGEAGEIDLLDFFAELTIY
TSSACLIGKKFRDQLDGRFAKLYHELERGTDPLAYVDPYLPIESFRRRDEARNGLVALVADIMNGRIANPPTD
KSDRDMLDVLIAVKAETGTPRFSADEITGMFISMMFAGHHTSSGTASWTLIELMRHRDAYAAVIDELDELYGD
GRSVSFHALRQIPQLENVLKETLRLHPPLIILMRVAKGEFEVQGHRIHEGDLVAASPAISNRIPEDFPDPHDF
VPARYEQPRQEDLLNRWTWIPFGAGRHRCVGAAFAIMQIKAIFSVLLREYEFEMAQPPESYRNDHSKMVVQLA
QPACVRYRRRTGV
>CYP51B1_Mycobacterium tuberculosis 13-3935 2651519437
MSAVALPRVSGGHDEHGHLEEFRTDPIGLMQRVRDECGDVGTFQLAGKQVVLLSGSHANEFFFRAGDDDLDQA
KAYPFMTPIFGEGVVFDASPERRKEMLHNAALRGEQMKGHAATIEDQVRRMIADWGEAGEIDLLDFFAELTIY
TSSACLIGKKFRDQLDGRFAKLYHELERGTDPLAYVDPYLPIESFRRRDEARNGLVALVADIMNGRIANPPTD
KSDRDMLDVLIAVKAETGTPRFSADEITGMFISMMFAGHHTSSGTASWTLIELMRHRDAYAAVIDELDELYGD
GRSVSFHALRQIPQLENVLKETLRLHPPLIILMRVAKGEFEVQGHRIHEGDLVAASPAISNRIPEDFPDPHDF
VPARYEQPRQEDLLNRWTWIPFGAGRHRCVGAAFAIMQIKAIFSVLLREYEFEMAQPPESYRNDHSKMVVQLA
QPACVRYRRRTGV
>CYP51B1_Mycobacterium tuberculosis 13-4152 2635745278
MSAVALPRVSGGHDEHGHLEEFRTDPIGLMQRVRDECGDVGTFQLAGKQVVLLSGSHANEFFFRAGDDDLDQA
KAYPFMTPIFGEGVVFDASPERRKEMLHNAALRGEQMKGHAATIEDQVRRMIADWGEAGEIDLLDFFAELTIY
TSSACLIGKKFRDQLDGRFAKLYHELERGTDPLAYVDPYLPIESFRRRDEARNGLVALVADIMNGRIANPPTD
KSDRDMLDVLIAVKAETGTPRFSADEITGMFISMMFAGHHTSSGTASWTLIELMRHRDAYAAVIDELDELYGD
GRSVSFHALRQIPQLENVLKETLRLHPPLIILMRVAKGEFEVQGHRIHEGDLVAASPAISNRIPEDFPDPHDF
VPARYEQPRQEDLLNRWTWIPFGAGRHRCVGAAFAIMQIKAIFSVLLREYEFEMAQPPESYRNDHSKMVVQLA
QPACVRYRRRTGV
>CYP51B1_Mycobacterium tuberculosis 1429BH 2606489963
MSAVALPRVSGGHDEHGHLEEFRTDPIGLMQRVRDECGDVGTFQLAGKQVVLLSGSHANEFFFRAGDDDLDQA
KAYPFMTPIFGEGVVFDASPERRKEMLHNAALRGEQMKGHAATIEDQVRRMIADWGEAGEIDLLDFFAELTIY
TSSACLIGKKFRDQLDGRFAKLYHELERGTDPLAYVDPYLPIESFRRRDEARNGLVALVADIMNGRIANPPTD
KSDRDMLDVLIAVKAETGTPRFSADEITGMFISMMFAGHHTSSGTASWTLIELMRHRDAYAAVIDELDELYGD
GRSVSFHALRQIPQLENVLKETLRLHPPLIILMRVAKGEFEVQGHRIHEGDLVAASPAISNRIPEDFPDPHDF
VPARYEQPRQEDLLNRWTWIPFGAGRHRCVGAAFAIMQIKAIFSVLLREYEFEMAQPPESYRNDHSKMVVQLA
QPACVRYRRRTGV
>CYP51B1_Mycobacterium tuberculosis 1430BH 2621763772
MSAVALPRVSGGHDEHGHLEEFRTDPIGLMQRVRDECGDVGTFQLAGKQVVLLSGSHANEFFFRAGDDDLDQA
KAYPFMTPIFGEGVVFDASPERRKEMLHNAALRGEQMKGHAATIEDQVRRMIADWGEAGEIDLLDFFAELTIY
TSSACLIGKKFRDQLDGRFAKLYHELERGTDPLAYVDPYLPIESFRRRDEARNGLVALVADIMNGRIANPPTD
KSDRDMLDVLIAVKAETGTPRFSADEITGMFISMMFAGHHTSSGTASWTLIELMRHRDAYAAVIDELDELYGD
GRSVSFHALRQIPQLENVLKETLRLHPPLIILMRVAKGEFEVQGHRIHEGDLVAASPAISNRIPEDFPDPHDF
VPARYEQPRQEDLLNRWTWIPFGAGRHRCVGAAFAIMQIKAIFSVLLREYEFEMAQPPESYRNDHSKMVVQLA
QPACVRYRRRTGV
>CYP51B1_Mycobacterium tuberculosis 1458 2722807292
MSAVALPRVSGGHDEHGHLEEFRTDPIGLMQRVRDECGDVGTFQLAGKQVVLLSGSHANEFFFRAGDDDLDQA
KAYPFMTPIFGEGVVFDASPERRKEMLHNAALRGEQMKGHAATIEDQVRRMIADWGEAGEIDLLDFFAELTIY
TSSACLIGKKFRDQLDGRFAKLYHELERGTDPLAYVDPYLPIESFRRRDEARNGLVALVADIMNGRIANPPTD
KSDRDMLDVLIAVKAETGTPRFSADEITGMFISMMFAGHHTSSGTASWTLIELMRHRDAYAAVIDELDELYGD
GRSVSFHALRQIPQLENVLKETLRLHPPLIILMRVAKGEFEVQGHRIHEGDLVAASPAISNRIPEDFPDPHDF
VPARYEQPRQEDLLNRWTWIPFGAGRHRCVGAAFAIMQIKAIFSVLLREYEFEMAQPPESYRNDHSKMVVQLA
QPACVRYRRRTGV
>CYP51B1_Mycobacterium tuberculosis 1500034 2658285070
MSAVALPRVSGGHDEHGHLEEFRTDPIGLMQRVRDECGDVGTFQLAGKQVVLLSGSHANEFFFRAGDDDLDQA
KAYPFMTPIFGEGVVFDASPERRKEMLHNAALRGEQMKGHAATIEDQVRRMIADWGEAGEIDLLDFFAELTIY

TSSACLIGKKFRDQLDGRFAKLYHELERGTDPLAYVDPYLPIESFRRRDEARNGLVALVADIMNGRIANPPTD
KSDRDMLDVLIAVKAETGTPRFSADEITGMFISMMFAGHHTSSGTASWTLIELMRHRDAYAAVIDELDELYGD
GRSVSFHALRQIPQLENVLKETLRLHPPLIILMRVAKGEFEVQGHRIHEGDLVAASPAISNRIPEDFPDPHDF
VPARYEQPRQEDLLNRWTWIPFGAGRHRCVGAAFAIMQIKAIFSVLLREYEFEMAQPPESYRNDHSKMVVQLA
QPACVRYRRRTGV
>CYP51B1_Mycobacterium tuberculosis 1500176 2665602634
MSAVALPRVSGGHDEHGHLEEFRTDPIGLMQRVRDECGDVGTFQLAGKQVVLLSGSHANEFFFRAGDDDLDQA
KAYPFMTPIFGEGVVFDASPERRKEMLHNAALRGEQMKGHAATIEDQVRRMIADWGEAGEIDLLDFFAELTIY
TSSACLIGKKFRDQLDGRFAKLYHELERGTDPLAYVDPYLPIESFRRRDEARNGLVALVADIMNGRIANPPTD
KSDRDMLDVLIAVKAETGTPRFSADEITGMFISMMFAGHHTSSGTASWTLIELMRHRDAYAAVIDELDELYGD
GRSVSFHALRQIPQLENVLKETLRLHPPLIILMRVAKGEFEVQGHRIHEGDLVAASPAISNRIPEDFPDPHDF
VPARYEQPRQEDLLNRWTWIPFGAGRHRCVGAAFAIMQIKAIFSVLLREYEFEMAQPPESYRNDHSKMVVQLA
QPACVRYRRRTGV
>CYP51B1_Mycobacterium tuberculosis 1500283 2658890902
MSAVALPRVSGGHDEHGHLEEFRTDPIGLMQRVRDECGDVGTFQLAGKQVVLLSGSHANEFFFRAGDDDLDQA
KAYPFMTPIFGEGVVFDASPERRKEMLHNAALRGEQMKGHAATIEDQVRRMIADWGEAGEIDLLDFFAELTIY
TSSACLIGKKFRDQLDGRFAKLYHELERGTDPLAYVDPYLPIESFRRRDEARNGLVALVADIMNGRIANPPTD
KSDRDMLDVLIAVKAETGTPRFSADEITGMFISMMFAGHHTSSGTASWTLIELMRHRDAYAAVIDELDELYGD
GRSVSFHALRQIPQLENVLKETLRLHPPLIILMRVAKGEFEVQGHRIHEGDLVAASPAISNRIPEDFPDPHDF
VPARYEQPRQEDLLNRWTWIPFGAGRHRCVGAAFAIMQIKAIFSVLLREYEFEMAQPPESYRNDHSKMVVQLA
QPACVRYRRRTGV
>CYP51B1_Mycobacterium tuberculosis 1500524 2663438681
MSAVALPRVSGGHDEHGHLEEFRTDPIGLMQRVRDECGDVGTFQLAGKQVVLLSGSHANEFFFRAGDDDLDQA
KAYPFMTPIFGEGVVFDASPERRKEMLHNAALRGEQMKGHAATIEDQVRRMIADWGEAGEIDLLDFFAELTIY
TSSACLIGKKFRDQLDGRFAKLYHELERGTDPLAYVDPYLPIESFRRRDEARNGLVALVADIMNGRIANPPTD
KSDRDMLDVLIAVKAETGTPRFSADEITGMFISMMFAGHHTSSGTASWTLIELMRHRDAYAAVIDELDELYGD
GRSVSFHALRQIPQLENVLKETLRLHPPLIILMRVAKGEFEVQGHRIHEGDLVAASPAISNRIPEDFPDPHDF
VPARYEQPRQEDLLNRWTWIPFGAGRHRCVGAAFAIMQIKAIFSVLLREYEFEMAQPPESYRNDHSKMVVQLA
QPACVRYRRRTGVCPAARGGGPSRAVGSQ
>CYP51B1_Mycobacterium tuberculosis 16954 2606380609
MSAVALPRVSGGHDEHGHLEEFRTDPIGLMQRVRDECGDVGTFQLAGKQVVLLSGSHANEFFFRAGDDDLDQA
KAYPFMTPIFGEGVVFDASPERRKEMLHNAALRGEQMKGHAATIEDQVRRMIADWGEAGEIDLLDFFAELTIY
TSSACLIGKKFRDQLDGRFAKLYHELERGTDPLAYVDPYLPIESFRRRDEARNGLVALVADIMNGRIANPPTD
KSDRDMLDVLIAVKAETGTPRFSADEITGMFISMMFAGHHTSSGTASWTLIELMRHRDAYAAVIDELDELYGD
GRSVSFHALRQIPQLENVLKETLRLHPPLIILMRVAKGEFEVQGHRIHEGDLVAASPAISNRIPEDFPDPHDF
VPARYEQPRQEDLLNRWTWIPFGAGRHRCVGAAFAIMQIKAIFSVLLREYEFEMAQPPESYRNDHSKMVVQLA
QPACVRYRRRTGV
>CYP51B1_Mycobacterium tuberculosis 1700089 2667085293
MSAVALPRVSGGHDEHGHLEEFRTDPIGLMQRVRDECGDVGTFQLAGKQVVLLSGSHANEFFFRAGDDDLDQA
KAYPFMTPIFGEGVVFDASPERRKEMLHNAALRGEQMKGHAATIEDQVRRMIADWGEAGEIDLLDFFAELTIY
TSSACLIGKKFRDQLDGRFAKLYHELERGTDPLAYVDPYLPIESFRRRDEARNGLVALVADIMNGRIANPPTD
KSDRDMLDVLIAVKAETGTPRFSADEITGMFISMMFAGHHTSSGTASWTLIELMRHRDAYAAVIDELDELYGD
GRSVSFHALRQIPQLENVLKETLRLHPPLIILMRVAKGEFEVQGHRIHEGDLVAASPAISNRIPEDFPDPHDF
VPARYEQPRQEDLLNRWTWIPFGAGRHRCVGAAFAIMQIKAIFSVLLREYEFEMAQPPESYRNDHSKMVVQLA
QPACVRYRRRTGV
>CYP51B1_Mycobacterium tuberculosis 1700284 2702166369
MSAVALPRVSGGHDEHGHLEEFRTDPIGLMQRVRDECGDVGTFQLAGKQVVLLSGSHANEFFFRAGDDDLDQA
KAYPFMTPIFGEGVVFDASPERRKEMLHNAALRGEQMKGHAATIEDQVRRMIADWGEAGEIDLLDFFAELTIY
TSSACLIGKKFRDQLDGRFAKLYHELERGTDPLAYVDPYLPIESFRRRDEARNGLVALVADIMNGRIANPPTD
KSDRDMLDVLIAVKAETGTPRFSADEITGMFISMMFAGHHTSSGTASWTLIELMRHRDAYAAVIDELDELYGD
GRSVSFHALRQIPQLENVLKETLRLHPPLIILMRVAKGEFEVQGHRIHEGDLVAASPAISNRIPEDFPDPHDF
VPARYEQPRQEDLLNRWTWIPFGAGRHRCVGAAFAIMQIKAIFSVLLREYEFEMAQPPESYRNDHSKMVVQLA
QPACVRYRRRTGV
>CYP51B1_Mycobacterium tuberculosis 1700302 2661055613
MSAVALPRVSGGHDEHGHLEEFRTDPIGLMQRVRDECGDVGTFQLAGKQVVLLSGSHANEFFFRAGDDDLDQA
KAYPFMTPIFGEGVVFDASPERRKEMLHNAALRGEQMKGHAATIEDQVRRMIADWGEAGEIDLLDFFAELTIY
TSSACLIGKKFRDQLDGRFAKLYHELERGTDPLAYVDPYLPIESFRRRDEARNGLVALVADIMNGRIANPPTD
KSDRDMLDVLIAVKAETGTPRFSADEITGMFISMMFAGHHTSSGTASWTLIELMRHRDAYAAVIDELDELYGD

GRSVSFHALRQIPQLENVLKETLRLHPPLIILMRVAKGEFEVQGHRIHEGDLVAASPAISNRIPEDFPDPHDF
VPARYEQPRQEDLLNRWTWIPFGAGRHRCVGAAFAIMQIKAIFSVLLREYEFEMAQPPESYRNDHSKMVVQLA
QPACVRYRRRTGV
>CYP51B1_Mycobacterium tuberculosis 1700745 2660525625
MSAVALPRVSGGHDEHGHLEEFRTDPIGLMQRVRDECGDVGTFQLAGKQVVLLSGSHANEFFFRAGDDDLDQA
KAYPFMTPIFGEGVVFDASPERRKEMLHNAALRGEQMKGHAATIEDQVRRMIADWGEAGEIDLLDFFAELTIY
TSSACLIGKKFRDQLDGRFAKLYHELERGTDPLAYVDPYLPIESFRRRDEARNGLVALVADIMNGRIANPPTD
KSDRDMLDVLIAVKAETGTPRFSADEITGMFISMMFAGHHTSSGTASWTLIELMRHRDAYAAVIDELDELYGD
GRSVSFHALRQIPQLENVLKETLRLHPPLIILMRVAKGEFEVQGHRIHEGDLVAASPAISNRIPEDFPDPHDF
VPARYEQPRQEDLLNRWTWIPFGAGRHRCVGAAFAIMQIKAIFSVLLREYEFEMAQPPESYRNDHSKMVVQLA
QPACVRYRRRTGV
>CYP51B1_Mycobacterium tuberculosis 1700893 2659619537
MSAVALPRVSGGHDEHGHLEEFRTDPIGLMQRVRDECGDVGTFQLAGKQVVLLSGSHANEFFFRAGDDDLDQA
KAYPFMTPIFGEGVVFDASPERRKEMLHNAALRGEQMKGHAATIEDQVRRMIADWGEAGEIDLLDFFAELTIY
TSSACLIGKKFRDQLDGRFAKLYHELERGTDPLAYVDPYLPIESFRRRDEARNGLVALVADIMNGRIANPPTD
KSDRDMLDVLIAVKAETGTPRFSADEITGMFISMMFAGHHTSSGTASWTLIELMRHRDAYAAVIDELDELYGD
GRSVSFHALRQIPQLENVLKETLRLHPPLIILMRVAKGEFEVQGHRIHEGDLVAASPAISNRIPEDFPDPHDF
VPARYEQPRQEDLLNRWTWIPFGAGRHRCVGAAFAIMQIKAIFSVLLREYEFEMAQPPESYRNDHSKMVVQLA
QPACVRYRRRTGV
>CYP51B1_Mycobacterium tuberculosis 1700938 2691680276
MSAVALPRVSGGHDEHGHLEEFRTDPIGLMQRVRDECGDVGTFQLAGKQVVLLSGSHANEFFFRAGDDDLDQA
KAYPFMTPIFGEGVVFDASPERRKEMLHNAALRGEQMKGHAATIEDQVRRMIADWGEAGEIDLLDFFAELTIY
TSSACLIGKKFRDQLDGRFAKLYHELERGTDPLAYVDPYLPIESFRRRDEARNGLVALVADIMNGRIANPPTD
KSDRDMLDVLIAVKAETGTPRFSADEITGMFISMMFAGHHTSSGTASWTLIELMRHRDAYAAVIDELDELYGD
GRSVSFHALRQIPQLENVLKETLRLHPPLIILMRVAKGEFEVQGHRIHEGDLVAASPAISNRIPEDFPDPHDF
VPARYEQPRQEDLLNRWTWIPFGAGRHRCVGAAFAIMQIKAIFSVLLREYEFEMAQPPESYRNDHSKMVVQLA
QPACVRYRRRTGV
>CYP51B1_Mycobacterium tuberculosis 1755BN 2606406009
MSAVALPRVSGGHDEHGHLEEFRTDPIGLMQRVRDECGDVGTFQLAGKQVVLLSGSHANEFFFRAGDDDLDQA
KAYPFMTPIFGEGVVFDASPERRKEMLHNAALRGEQMKGHAATIEDQVRRMIADWGEAGEIDLLDFFAELTIY
TSSACLIGKKFRDQLDGRFAKLYHELERGTDPLAYVDPYLPIESFRRRDEARNGLVALVADIMNGRIANPPTD
KSDRDMLDVLIAVKAETGTPRFSADEITGMFISMMFAGHHTSSGTASWTLIELMRHRDAYAAVIDELDELYGD
GRSVSFHALRQIPQLENVLKETLRLHPPLIILMRVAKGEFEVQGHRIHEGDLVAASPAISNRIPEDFPDPHDF
VPARYEQPRQEDLLNRWTWIPFGAGRHRCVGAAFAIMQIKAIFSVLLREYEFEMAQPPESYRNDHSKMVVQLA
QPACVRYRRRTGV
>CYP51B1_Mycobacterium tuberculosis 18b 2640956016
MSAVALPRVSGGHDEHGHLEEFRTDPIGLMQRVRDECGDVGTFQLAGKQVVLLSGSHANEFFFRAGDDDLDQA
KAYPFMTPIFGEGVVFDASPERRKEMLHNAALRGEQMKGHAATIEDQVRRMIADWGEAGEIDLLDFFAELTIY
TSSACLIGKKFRDQLDGRFAKLYHELERGTDPLAYVDPYLPIESFRRRDEARNGLVALVADIMNGRIANPPTD
KSDRDMLDVLIAVKAETGTPRFSADEITGMFISMMFAGHHTSSGTASWTLIELMRHRDAYAAVIDELDELYGD
GRSVSFHALRQIPQLENVLKETLRLHPPLIILMRVAKGEFEVQGHRIHEGDLVAASPAISNRIPEDFPDPHDF
VPARYEQPRQEDLLNRWTWIPFGAGRHRCVGAAFAIMQIKAIFSVLLREYEFEMAQPPESYRNDHSKMVVQLA
QPACVRYRRRTGV
>CYP51B1_Mycobacterium tuberculosis 200066 2693443572
MSAVALPRVSGGHDEHGHLEEFRTDPIGLMQRVRDECGDVGTFQLAGKQVVLLSGSHANEFFFRAGDDDLDQA
KAYPFMTPIFGEGVVFDASPERRKEMLHNAALRGEQMKGHAATIEDQVRRMIADWGEAGEIDLLDFFAELTIY
TSSACLIGKKFRDQLDGRFAKLYHELERGTDPLAYVDPYLPIESFRRRDEARNGLVALVADIMNGRIANPPTD
KSDRDMLDVLIAVKAETGTPRFSADEITGMFISMMFAGHHTSSGTASWTLIELMRHRDAYAAVIDELDELYGD
GRSVSFHALRQIPQLENVLKETLRLHPPLIILMRVAKGEFEVQGHRIHEGDLVAASPAISNRIPEDFPDPHDF
VPARYEQPRQEDLLNRWTWIPFGAGRHRCVGAAFAIMQIKAIFSVLLREYEFEMAQPPESYRNDHSKMVVQLA
QPACVRYRRRTGV
>CYP51B1_Mycobacterium tuberculosis 200127 2682897451
MSAVALPRVSGGHDEHGHLEEFRTDPIGLMQRVRDECGDVGTFQLAGKQVVLLSGSHANEFFFRAGDDDLDQA
KAYPFMTPIFGEGVVFDASPERRKEMLHNAALRGEQMKGHAATIEDQVRRMIADWGEAGEIDLLDFFAELTIY
TSSACLIGKKFRDQLDGRFAKLYHELERGTDPLAYVDPYLPIESFRRRDEARNGLVALVADIMNGRIANPPTD
KSDRDMLDVLIAVKAETGTPRFSADEITGMFISMMFAGHHTSSGTASWTLIELMRHRDAYAAVIDELDELYGD
GRSVSFHALRQIPQLENVLKETLRLHPPLIILMRVAKGEFEVQGHRIHEGDLVAASPAISNRIPEDFPDPHDF

VPARYEQPRQEDLLNRWTWIPFGAGRHRCVGAAFAIMQIKAIFSVLLREYEFEMAQPPESYRNDHSKMVVQLA
QPACVRYRRRTGV
>CYP51B1_Mycobacterium tuberculosis 200135 2695841972
MSAVALPRVSGGHDEHGHLEEFRTDPIGLMQRVRDECGDVGTFQLAGKQVVLLSGSHANEFFFRAGDDDLDQA
KAYPFMTPIFGEGVVFDASPERRKEMLHNAALRGEQMKGHAATIEDQVRRMIADWGEAGEIDLLDFFAELTIY
TSSACLIGKKFRDQLDGRFAKLYHELERGTDPLAYVDPYLPIESFRRRDEARNGLVALVADIMNGRIANPPTD
KSDRDMLDVLIAVKAETGTPRFSADEITGMFISMMFAGHHTSSGTASWTLIELMRHRDAYAAVIDELDELYGD
GRSVSFHALRQIPQLENVLKETLRLHPPLIILMRVAKGEFEVQGHRIHEGDLVAASPAISNRIPEDFPDPHDF
VPARYEQPRQEDLLNRWTWIPFGAGRHRCVGAAFAIMQIKAIFSVLLREYEFEMAQPPESYRNDHSKMVVQLA
QPACVRYRRRTGV
>CYP51B1_Mycobacterium tuberculosis 200253 2668687908
MSAVALPRVSGGHDEHGHLEEFRTDPIGLMQRVRDECGDVGTFQLAGKQVVLLSGSHANEFFFRAGDDDLDQA
KAYPFMTPIFGEGVVFDASPERRKEMLHNAALRGEQMKGHAATIEDQVRRMIADWGEAGEIDLLDFFAELTIY
TSSACLIGKKFRDQLDGRFAKLYHELERGTDPLAYVDPYLPIESFRRRDEARNGLVALVADIMNGRIANPPTD
KSDRDMLDVLIAVKAETGTPRFSADEITGMFISMMFAGHHTSSGTASWTLIELMRHRDAYAAVIDELDELYGD
GRSVSFHALRQIPQLENVLKETLRLHPPLIILMRVAKGEFEVQGHRIHEGDLVAASPAISNRIPEDFPDPHDF
VPARYEQPRQEDLLNRWTWIPFGAGRHRCVGAAFAIMQIKAIFSVLLREYEFEMAQPPESYRNDHSKMVVQLA
QPACVRYRRRTGV
>CYP51B1_Mycobacterium tuberculosis 200353 2661494721
MSAVALPRVSGGHDEHGHLEEFRTDPIGLMQRVRDECGDVGTFQLAGKQVVLLSGSHANEFFFRAGDDDLDQA
KAYPFMTPIFGEGVVFDASPERRKEMLHNAALRGEQMKGHAATIEDQVRRMIADWGEAGEIDLLDFFAELTIY
TSSACLIGKKFRDQLDGRFAKLYHELERGTDPLAYVDPYLPIESFRRRDEARNGLVALVADIMNGRIANPPTD
KSDRDMLDVLIAVKAETGTPRFSADEITGMFISMMFAGHHTSSGTASWTLIELMRHRDAYAAVIDELDELYGD
GRSVSFHALRQIPQLENVLKETLRLHPPLIILMRVAKGEFEVQGHRIHEGDLVAASPAISNRIPEDFPDPHDF
VPARYEQPRQEDLLNRWTWIPFGAGRHRCVGAAFAIMQIKAIFSVLLREYEFEMAQPPESYRNDHSKMVVQLA
QPACVRYRRRTGV
>CYP51B1_Mycobacterium tuberculosis 200470 2659554546
MSAVALPRVSGGHDEHGHLEEFRTDPIGLMQRVRDECGDVGTFQLAGKQVVLLSGSHANEFFFRAGDDDLDQA
KAYPFMTPIFGEGVVFDASPERRKEMLHNAALRGEQMKGHAATIEDQVRRMIADWGEAGEIDLLDFFAELTIY
TSSACLIGKKFRDQLDGRFAKLYHELERGTDPLAYVDPYLPIESFRRRDEARNGLVALVADIMNGRIANPPTD
KSDRDMLDVLIAVKAETGTPRFSADEITGMFISMMFAGHHTSSGTASWTLIELMRHRDAYAAVIDELDELYGD
GRSVSFHALRQIPQLENVLKETLRLHPPLIILMRVAKGEFEVQGHRIHEGDLVAASPAISNRIPEDFPDPHDF
VPARYEQPRQEDLLNRWTWIPFGAGRHRCVGAAFAIMQIKAIFSVLLREYEFEMAQPPESYRNDHSKMVVQLA
QPACVRYRRRTGV
>CYP51B1_Mycobacterium tuberculosis 200541 2691882955
MSAVALPRVSGGHDEHGHLEEFRTDPIGLMQRVRDECGDVGTFQLAGKQVVLLSGSHANEFFFRAGDDDLDQA
KAYPFMTPIFGEGVVFDASPERRKEMLHNAALRGEQMKGHAATIEDQVRRMIADWGEAGEIDLLDFFAELTIY
TSSACLIGKKFRDQLDGRFAKLYHELERGTDPLAYVDPYLPIESFRRRDEARNGLVALVADIMNGRIANPPTD
KSDRDMLDVLIAVKAETGTPRFSADEITGMFISMMFAGHHTSSGTASWTLIELMRHRDAYAAVIDELDELYGD
GRSVSFHALRQIPQLENVLKETLRLHPPLIILMRVAKGEFEVQGHRIHEGDLVAASPAISNRIPEDFPDPHDF
VPARYEQPRQEDLLNRWTWIPFGAGRHRCVGAAFAIMQIKAIFSVLLREYEFEMAQPPESYRNDHSKMVVQLA
QPACVRYRRRTGV
>CYP51B1_Mycobacterium tuberculosis 200625 2675459495
MSAVALPRVSGGHDEHGHLEEFRTDPIGLMQRVRDECGDVGTFQLAGKQVVLLSGSHANEFFFRAGDDDLDQA
KAYPFMTPIFGEGVVFDASPERRKEMLHNAALRGEQMKGHAATIEDQVRRMIADWGEAGEIDLLDFFAELTIY
TSSACLIGKKFRDQLDGRFAKLYHELERGTDPLAYVDPYLPIESFRRRDEARNGLVALVADIMNGRIANPPTD
KSDRDMLDVLIAVKAETGTPRFSADEITGMFISMMFAGHHTSSGTASWTLIELMRHRDAYAAVIDELDELYGD
GRSVSFHALRQIPQLENVLKETLRLHPPLIILMRVAKGEFEVQGHRIHEGDLVAASPAISNRIPEDFPDPHDF
VPARYEQPRQEDLLNRWTWIPFGAGRHRCVGAAFAIMQIKAIFSVLLREYEFEMAQPPESYRNDHSKMVVQLA
QPACVRYRRRTGV
>CYP51B1_Mycobacterium tuberculosis 201035 2662922266
MSAVALPRVSGGHDEHGHLEEFRTDPIGLMQRVRDECGDVGTFQLAGKQVVLLSGSHANEFFFRAGDDDLDQA
KAYPFMTPIFGEGVVFDASPERRKEMLHNAALRGEQMKGHAATIEDQVRRMIADWGEAGEIDLLDFFAELTIY
TSSACLIGKKFRDQLDGRFAKLYHELERGTDPLAYVDPYLPIESFRRRDEARNGLVALVADIMNGRIANPPTD
KSDRDMLDVLIAVKAETGTPRFSADEITGMFISMMFAGHHTSSGTASWTLIELMRHRDAYAAVIDELDELYGD
GRSVSFHALRQIPQLENVLKETLRLHPPLIILMRVAKGEFEVQGHRIHEGDLVAASPAISNRIPEDFPDPHDF
VPARYEQPRQEDLLNRWTWIPFGAGRHRCVGAAFAIMQIKAIFSVLLREYEFEMAQPPESYRNDHSKMVVQLA
QPACVRYRRRTGV

>CYP51B1_Mycobacterium tuberculosis 201113 2666293557
MSAVALPRVSGGHDEHGHLEEFRTDPIGLMQRVRDECGDVGTFQLAGKQVVLLSGSHANEFFFRAGDDDLDQA
KAYPFMTPIFGEGVVFDASPERRKEMLHNAALRGEQMKGHAATIEDQVRRMIADWGEAGEIDLLDFFAELTIY
TSSACLIGKKFRDQLDGRFAKLYHELERGTDPLAYVDPYLPIESFRRRDEARNGLVALVADIMNGRIANPPTD
KSDRDMLDVLIAVKAETGTPRFSADEITGMFISMMFAGHHTSSGTASWTLIELMRHRDAYAAVIDELDELYGD
GRSVSFHALRQIPQLENVLKETLRLHPPLIILMRVAKGEFEVQGHRIHEGDLVAASPAISNRIPEDFPDPHDF
VPARYEQPRQEDLLNRWTWIPFGAGRHRCVGAAFAIMQIKAIFSVLLREYEFEMAQPPESYRNDHSKMVVQLA
QPACVRYRRRTGV
>CYP51B1_Mycobacterium tuberculosis 201127 2702146509
MSAVALPRVSGGHDEHGHLEEFRTDPIGLMQRVRDECGDVGTFQLAGKQVVLLSGSHANEFFFRAGDDDLDQA
KAYPFMTPIFGEGVVFDASPERRKEMLHNAALRGEQMKGHAATIEDQVRRMIADWGEAGEIDLLDFFAELTIY
TSSACLIGKKFRDQLDGRFAKLYHELERGTDPLAYVDPYLPIESFRRRDEARNGLVALVADIMNGRIANPPTD
KSDRDMLDVLIAVKAETGTPRFSADEITGMFISMMFAGHHTSSGTASWTLIELMRHRDAYAAVIDELDELYGD
GRSVSFHALRQIPQLENVLKETLRLHPPLIILMRVAKGEFEVQGHRIHEGDLVAASPAISNRIPEDFPDPHDF
VPARYEQPRQEDLLNRWTWIPFGAGRHRCVGAAFAIMQIKAIFSVLLREYEFEMAQPPESYRNDHSKMVVQLA
QPACVRYRRRTGV
>CYP51B1_Mycobacterium tuberculosis 201150 2682906078
MSAVALPRVSGGHDEHGHLEEFRTDPIGLMQRVRDECGDVGTFQLAGKQVVLLSGSHANEFFFRAGDDDLDQA
KAYPFMTPIFGEGVVFDASPERRKEMLHNAALRGEQMKGHAATIEDQVRRMIADWGEAGEIDLLDFFAELTIY
TSSACLIGKKFRDQLDGRFAKLYHELERGTDPLAYVDPYLPIESFRRRDEARNGLVALVADIMNGRIANPPTD
KSDRDMLDVLIAVKAETGTPRFSADEITGMFISMMFAGHHTSSGTASWTLIELMRHRDAYAAVIDELDELYGD
GRSVSFHALRQIPQLENVLKETLRLHPPLIILMRVAKGEFEVQGHRIHEGDLVAASPAISNRIPEDFPDPHDF
VPARYEQPRQEDLLNRWTWIPFGAGRHRCVGAAFAIMQIKAIFSVLLREYEFEMAQPPESYRNDHSKMVVQLA
QPACVRYRRRTGV
>CYP51B1_Mycobacterium tuberculosis 201314 2665806669
MSAVALPRVSGGHDEHGHLEEFRTDPIGLMQRVRDECGDVGTFQLAGKQVVLLSGSHANEFFFRAGDDDLDQA
KAYPFMTPIFGEGVVFDASPERRKEMLHNAALRGEQMKGHAATIEDQVRRMIADWGEAGEIDLLDFFAELTIY
TSSACLIGKKFRDQLDGRFAKLYHELERGTDPLAYVDPYLPIESFRRRDEARNGLVALVADIMNGRIANPPTD
KSDRDMLDVLIAVKAETGTPRFSADEITGMFISMMFAGHHTSSGTASWTLIELMRHRDAYAAVIDELDELYGD
GRSVSFHALRQIPQLENVLKETLRLHPPLIILMRVAKGEFEVQGHRIHEGDLVAASPAISNRIPEDFPDPHDF
VPARYEQPRQEDLLNRWTWIPFGAGRHRCVGAAFAIMQIKAIFSVLLREYEFEMAQPPESYRNDHSKMVVQLA
QPACVRYRRRTGV
>CYP51B1_Mycobacterium tuberculosis 201366 2660484988
MSAVALPRVSGGHDEHGHLEEFRTDPIGLMQRVRDECGDVGTFQLAGKQVVLLSGSHANEFFFRAGDDDLDQA
KAYPFMTPIFGEGVVFDASPERRKEMLHNAALRGEQMKGHAATIEDQVRRMIADWGEAGEIDLLDFFAELTIY
TSSACLIGKKFRDQLDGRFAKLYHELERGTDPLAYVDPYLPIESFRRRDEARNGLVALVADIMNGRIANPPTD
KSDRDMLDVLIAVKAETGTPRFSADEITGMFISMMFAGHHTSSGTASWTLIELMRHRDAYAAVIDELDELYGD
GRSVSFHALRQIPQLENVLKETLRLHPPLIILMRVAKGEFEVQGHRIHEGDLVAASPAISNRIPEDFPDPHDF
VPARYEQPRQEDLLNRWTWIPFGAGRHRCVGAAFAIMQIKAIFSVLLREYEFEMAQPPESYRNDHSKMVVQLA
QPACVRYRRRTGV
>CYP51B1_Mycobacterium tuberculosis 201404 2673357555
MSAVALPRVSGGHDEHGHLEEFRTDPIGLMQRVRDECGDVGTFQLAGKQVVLLSGSHANEFFFRAGDDDLDQA
KAYPFMTPIFGEGVVFDASPERRKEMLHNAALRGEQMKGHAATIEDQVRRMIADWGEAGEIDLLDFFAELTIY
TSSACLIGKKFRDQLDGRFAKLYHELERGTDPLAYVDPYLPIESFRRRDEARNGLVALVADIMNGRIANPPTD
KSDRDMLDVLIAVKAETGTPRFSADEITGMFISMMFAGHHTSSGTASWTLIELMRHRDAYAAVIDELDELYGD
GRSVSFHALRQIPQLENVLKETLRLHPPLIILMRVAKGEFEVQGHRIHEGDLVAASPAISNRIPEDFPDPHDF
VPARYEQPRQEDLLNRWTWIPFGAGRHRCVGAAFAIMQIKAIFSVLLREYEFEMAQPPESYRNDHSKMVVQLA
QPACVRYRRRTGV
>CYP51B1_Mycobacterium tuberculosis 201457 2658053307
MSAVALPRVSGGHDEHGHLEEFRTDPIGLMQRVRDECGDVGTFQLAGKQVVLLSGSHANEFFFRAGDDDLDQA
KAYPFMTPIFGEGVVFDASPERRKEMLHNAALRGEQMKGHAATIEDQVRRMIADWGEAGEIDLLDFFAELTIY
TSSACLIGKKFRDQLDGRFAKLYHELERGTDPLAYVDPYLPIESFRRRDEARNGLVALVADIMNGRIANPPTD
KSDRDMLDVLIAVKAETGTPRFSADEITGMFISMMFAGHHTSSGTASWTLIELMRHRDAYAAVIDELDELYGD
GRSVSFHALRQIPQLENVLKETLRLHPPLIILMRVAKGEFEVQGHRIHEGDLVAASPAISNRIPEDFPDPHDF
VPARYEQPRQEDLLNRWTWIPFGAGRHRCVGAAFAIMQIKAIFSVLLREYEFEMAQPPESYRNDHSKMVVQLA
QPACVRYRRRTGV
>CYP51B1_Mycobacterium tuberculosis 2074CJ 2621725868

MSAVALPRVSGGHDEHGHLEEFRTDPIGLMQRVRDECGDVGTFQLAGKQVVLLSGSHANEFFFRAGDDDLDQA
KAYPFMTPIFGEGVVFDASPERRKEMLHNAALRGEQMKGHAATIEDQVRRMIADWGEAGEIDLLDFFAELTIY
TSSACLIGKKFRDQLDGRFAKLYHELERGTDPLAYVDPYLPIESFRRRDEARNGLVALVADIMNGRIANPPTD
KSDRDMLDVLIAVKAETGTPRFSADEITGMFISMMFAGHHTSSGTASWTLIELMRHRDAYAAVIDELDELYGD
GRSVSFHALRQIPQLENVLKETLRLHPPLIILMRVAKGEFEVQGHRIHEGDLVAASPAISNRIPEDFPDPHDF
VPARYEQPRQEDLLNRWTWIPFGAGRHRCVGAAFAIMQIKAIFSVLLREYEFEMAQPPESYRNDHSKMVVQLA
QPACVRYRRRTGV
>CYP51B1_Mycobacterium tuberculosis 2088HD 2621759165
MSAVALPRVSGGHDEHGHLEEFRTDPIGLMQRVRDECGDVGTFQLAGKQVVLLSGSHANEFFFRAGDDDLDQA
KAYPFMTPIFGEGVVFDASPERRKEMLHNAALRGEQMKGHAATIEDQVRRMIADWGEAGEIDLLDFFAELTIY
TSSACLIGKKFRDQLDGRFAKLYHELERGTDPLAYVDPYLPIESFRRRDEARNGLVALVADIMNGRIANPPTD
KSDRDMLDVLIAVKAETGTPRFSADEITGMFISMMFAGHHTSSGTASWTLIELMRHRDAYAAVIDELDELYGD
GRSVSFHALRQIPQLENVLKETLRLHPPLIILMRVAKGEFEVQGHRIHEGDLVAASPAISNRIPEDFPDPHDF
VPARYEQPRQEDLLNRWTWIPFGAGRHRCVGAAFAIMQIKAIFSVLLREYEFEMAQPPESYRNDHSKMVVQLA
QPACVRYRRRTGV
>CYP51B1_Mycobacterium tuberculosis 2089HD 2621755433
MSAVALPRVSGGHDEHGHLEEFRTDPIGLMQRVRDECGDVGTFQLAGKQVVLLSGSHANEFFFRAGDDDLDQA
KAYPFMTPIFGEGVVFDASPERRKEMLHNAALRGEQMKGHAATIEDQVRRMIADWGEAGEIDLLDFFAELTIY
TSSACLIGKKFRDQLDGRFAKLYHELERGTDPLAYVDPYLPIESFRRRDEARNGLVALVADIMNGRIANPPTD
KSDRDMLDVLIAVKAETGTPRFSADEITGMFISMMFAGHHTSSGTASWTLIELMRHRDAYAAVIDELDELYGD
GRSVSFHALRQIPQLENVLKETLRLHPPLIILMRVAKGEFEVQGHRIHEGDLVAASPAISNRIPEDFPDPHDF
VPARYEQPRQEDLLNRWTWIPFGAGRHRCVGAAFAIMQIKAIFSVLLREYEFEMAQPPESYRNDHSKMVVQLA
QPACVRYRRRTGV
>CYP51B1_Mycobacterium tuberculosis 2091HD 2584729146
MSAVALPRVSGGHDEHGHLEEFRTDPIGLMQRVRDECGDVGTFQLAGKQVVLLSGSHANEFFFRAGDDDLDQA
KAYPFMTPIFGEGVVFDASPERRKEMLHNAALRGEQMKGHAATIEDQVRRMIADWGEAGEIDLLDFFAELTIY
TSSACLIGKKFRDQLDGRFAKLYHELERGTDPLAYVDPYLPIESFRRRDEARNGLVALVADIMNGRIANPPTD
KSDRDMLDVLIAVKAETGTPRFSADEITGMFISMMFAGHHTSSGTASWTLIELMRHRDAYAAVIDELDELYGD
GRSVSFHALRQIPQLENVLKETLRLHPPLIILMRVAKGEFEVQGHRIHEGDLVAASPAISNRIPEDFPDPHDF
VPARYEQPRQEDLLNRWTWIPFGAGRHRCVGAAFAIMQIKAIFSVLLREYEFEMAQPPESYRNDHSKMVVQLA
QPACVRYRRRTGV
>CYP51B1_Mycobacterium tuberculosis 2092HD 2635595682
MSAVALPRVSGGHDEHGHLEEFRTDPIGLMQRVRDECGDVGTFQLAGKQVVLLSGSHANEFFFRAGDDDLDQA
KAYPFMTPIFGEGVVFDASPERRKEMLHNAALRGEQMKGHAATIEDQVRRMIADWGEAGEIDLLDFFAELTIY
TSSACLIGKKFRDQLDGRFAKLYHELERGTDPLAYVDPYLPIESFRRRDEARNGLVALVADIMNGRIANPPTD
KSDRDMLDVLIAVKAETGTPRFSADEITGMFISMMFAGHHTSSGTASWTLIELMRHRDAYAAVIDELDELYGD
GRSVSFHALRQIPQLENVLKETLRLHPPLIILMRVAKGEFEVQGHRIHEGDLVAASPAISNRIPEDFPDPHDF
VPARYEQPRQEDLLNRWTWIPFGAGRHRCVGAAFAIMQIKAIFSVLLREYEFEMAQPPESYRNDHSKMVVQLA
QPACVRYRRRTGV
>CYP51B1_Mycobacterium tuberculosis 2093HD 2621751194
MSAVALPRVSGGHDEHGHLEEFRTDPIGLMQRVRDECGDVGTFQLAGKQVVLLSGSHANEFFFRAGDDDLDQA
KAYPFMTPIFGEGVVFDASPERRKEMLHNAALRGEQMKGHAATIEDQVRRMIADWGEAGEIDLLDFFAELTIY
TSSACLIGKKFRDQLDGRFAKLYHELERGTDPLAYVDPYLPIESFRRRDEARNGLVALVADIMNGRIANPPTD
KSDRDMLDVLIAVKAETGTPRFSADEITGMFISMMFAGHHTSSGTASWTLIELMRHRDAYAAVIDELDELYGD
GRSVSFHALRQIPQLENVLKETLRLHPPLIILMRVAKGEFEVQGHRIHEGDLVAASPAISNRIPEDFPDPHDF
VPARYEQPRQEDLLNRWTWIPFGAGRHRCVGAAFAIMQIKAIFSVLLREYEFEMAQPPESYRNDHSKMVVQLA
QPACVRYRRRTGV
>CYP51B1_Mycobacterium tuberculosis 2094HD 2577146114
MSAVALPRVSGGHDEHGHLEEFRTDPIGLMQRVRDECGDVGTFQLAGKQVVLLSGSHANEFFFRAGDDDLDQA
KAYPFMTPIFGEGVVFDASPERRKEMLHNAALRGEQMKGHAATIEDQVRRMIADWGEAGEIDLLDFFAELTIY
TSSACLIGKKFRDQLDGRFAKLYHELERGTDPLAYVDPYLPIESFRRRDEARNGLVALVADIMNGRIANPPTD
KSDRDMLDVLIAVKAETGTPRFSADEITGMFISMMFAGHHTSSGTASWTLIELMRHRDAYAAVIDELDELYGD
GRSVSFHALRQIPQLENVLKETLRLHPPLIILMRVAKGEFEVQGHRIHEGDLVAASPAISNRIPEDFPDPHDF
VPARYEQPRQEDLLNRWTWIPFGAGRHRCVGAAFAIMQIKAIFSVLLREYEFEMAQPPESYRNDHSKMVVQLA
QPACVRYRRRTGV
>CYP51B1_Mycobacterium tuberculosis 2097HD 2606401658
MSAVALPRVSGGHDEHGHLEEFRTDPIGLMQRVRDECGDVGTFQLAGKQVVLLSGSHANEFFFRAGDDDLDQA
KAYPFMTPIFGEGVVFDASPERRKEMLHNAALRGEQMKGHAATIEDQVRRMIADWGEAGEIDLLDFFAELTIY

TSSACLIGKKFRDQLDGRFAKLYHELERGTDPLAYVDPYLPIESFRRRDEARNGLVALVADIMNGRIANPPTD
KSDRDMLDVLIAVKAETGTPRFSADEITGMFISMMFAGHHTSSGTASWTLIELMRHRDAYAAVIDELDELYGD
GRSVSFHALRQIPQLENVLKETLRLHPPLIILMRVAKGEFEVQGHRIHEGDLVAASPAISNRIPEDFPDPHDF
VPARYEQPRQEDLLNRWTWIPFGAGRHRCVGAAFAIMQIKAIFSVLLREYEFEMAQPPESYRNDHSKMVVQLA
QPACVRYRRRTGV
>CYP51B1_Mycobacterium tuberculosis 2099HD 2606397103
MSAVALPRVSGGHDEHGHLEEFRTDPIGLMQRVRDECGDVGTFQLAGKQVVLLSGSHANEFFFRAGDDDLDQA
KAYPFMTPIFGEGVVFDASPERRKEMLHNAALRGEQMKGHAATIEDQVRRMIADWGEAGEIDLLDFFAELTIY
TSSACLIGKKFRDQLDGRFAKLYHELERGTDPLAYVDPYLPIESFRRRDEARNGLVALVADIMNGRIANPPTD
KSDRDMLDVLIAVKAETGTPRFSADEITGMFISMMFAGHHTSSGTASWTLIELMRHRDAYAAVIDELDELYGD
GRSVSFHALRQIPQLENVLKETLRLHPPLIILMRVAKGEFEVQGHRIHEGDLVAASPAISNRIPEDFPDPHDF
VPARYEQPRQEDLLNRWTWIPFGAGRHRCVGAAFAIMQIKAIFSVLLREYEFEMAQPPESYRNDHSKMVVQLA
QPACVRYRRRTGV
>CYP51B1_Mycobacterium tuberculosis 210 NZ_ADAB01000120 647209221
MSAVALPRVSGGHDEHGHLEEFRTDPIGLMQRVRDECGDVGTFQLAGKQVVLLSGSHANEFFFRAGDDDLDQA
KAYPFMTPIFGEGVVFDASPERRKEMLHNAALRGEQMKGHAATIEDQVRRMIADWGEAGEIDLLDFFAELTIY
TSSACLIGKKFRDQLDGRFAKLYHELERGTDPLAYVDPYLPIESFRRRDEARNGLVALVADIMNGRIANPPTD
KSDRDMLDVLIAVKAETGTPRFSADEITGMFISMMFAGHHTSSGTASWTLIELMRHRDAYAAVIDELDELYGD
GRSVSFHALRQIPQLENVLKETLRLHPPLIILMRVAKGEFEVQGHRIHEGDLVAASPAISNRIPEDFPDPHDF
VPARYEQPRQEDLLNRWTWIPFGAGRHRCVGAAFAIMQIKAIFSVLLREYEFEMAQPPESYRNDHSKMVVQLA
QPACVRYRRRTGV
>CYP51B1_Mycobacterium tuberculosis 2100HD 2606481207
MSAVALPRVSGGHDEHGHLEEFRTDPIGLMQRVRDECGDVGTFQLAGKQVVLLSGSHANEFFFRAGDDDLDQA
KAYPFMTPIFGEGVVFDASPERRKEMLHNAALRGEQMKGHAATIEDQVRRMIADWGEAGEIDLLDFFAELTIY
TSSACLIGKKFRDQLDGRFAKLYHELERGTDPLAYVDPYLPIESFRRRDEARNGLVALVADIMNGRIANPPTD
KSDRDMLDVLIAVKAETGTPRFSADEITGMFISMMFAGHHTSSGTASWTLIELMRHRDAYAAVIDELDELYGD
GRSVSFHALRQIPQLENVLKETLRLHPPLIILMRVAKGEFEVQGHRIHEGDLVAASPAISNRIPEDFPDPHDF
VPARYEQPRQEDLLNRWTWIPFGAGRHRCVGAAFAIMQIKAIFSVLLREYEFEMAQPPESYRNDHSKMVVQLA
QPACVRYRRRTGV
>CYP51B1_Mycobacterium tuberculosis 2101HD 2621742032
MSAVALPRVSGGHDEHGHLEEFRTDPIGLMQRVRDECGDVGTFQLAGKQVVLLSGSHANEFFFRAGDDDLDQA
KAYPFMTPIFGEGVVFDASPERRKEMLHNAALRGEQMKGHAATIEDQVRRMIADWGEAGEIDLLDFFAELTIY
TSSACLIGKKFRDQLDGRFAKLYHELERGTDPLAYVDPYLPIESFRRRDEARNGLVALVADIMNGRIANPPTD
KSDRDMLDVLIAVKAETGTPRFSADEITGMFISMMFAGHHTSSGTASWTLIELMRHRDAYAAVIDELDELYGD
GRSVSFHALRQIPQLENVLKETLRLHPPLIILMRVAKGEFEVQGHRIHEGDLVAASPAISNRIPEDFPDPHDF
VPARYEQPRQEDLLNRWTWIPFGAGRHRCVGAAFAIMQIKAIFSVLLREYEFEMAQPPESYRNDHSKMVVQLA
QPACVRYRRRTGV
>CYP51B1_Mycobacterium tuberculosis 2104HD 2606385028
MSAVALPRVSGGHDEHGHLEEFRTDPIGLMQRVRDECGDVGTFQLAGKQVVLLSGSHANEFFFRAGDDDLDQA
KAYPFMTPIFGEGVVFDASPERRKEMLHNAALRGEQMKGHAATIEDQVRRMIADWGEAGEIDLLDFFAELTIY
TSSACLIGKKFRDQLDGRFAKLYHELERGTDPLAYVDPYLPIESFRRRDEARNGLVALVADIMNGRIANPPTD
KSDRDMLDVLIAVKAETGTPRFSADEITGMFISMMFAGHHTSSGTASWTLIELMRHRDAYAAVIDELDELYGD
GRSVSFHALRQIPQLENVLKETLRLHPPLIILMRVAKGEFEVQGHRIHEGDLVAASPAISNRIPEDFPDPHDF
VPARYEQPRQEDLLNRWTWIPFGAGRHRCVGAAFAIMQIKAIFSVLLREYEFEMAQPPESYRNDHSKMVVQLA
QPACVRYRRRTGV
>CYP51B1_Mycobacterium tuberculosis 2196MM 2621747296
MSAVALPRVSGGHDEHGHLEEFRTDPIGLMQRVRDECGDVGTFQLAGKQVVLLSGSHANEFFFRAGDDDLDQA
KAYPFMTPIFGEGVVFDASPERRKEMLHNAALRGEQMKGHAATIEDQVRRMIADWGEAGEIDLLDFFAELTIY
TSSACLIGKKFRDQLDGRFAKLYHELERGTDPLAYVDPYLPIESFRRRDEARNGLVALVADIMNGRIANPPTD
KSDRDMLDVLIAVKAETGTPRFSADEITGMFISMMFAGHHTSSGTASWTLIELMRHRDAYAAVIDELDELYGD
GRSVSFHALRQIPQLENVLKETLRLHPPLIILMRVAKGEFEVQGHRIHEGDLVAASPAISNRIPEDFPDPHDF
VPARYEQPRQEDLLNRWTWIPFGAGRHRCVGAAFAIMQIKAIFSVLLREYEFEMAQPPESYRNDHSKMVVQLA
QPACVRYRRRTGV
>CYP51B1_Mycobacterium tuberculosis 22103 2688926590
MSAVALPRVSGGHDEHGHLEEFRTDPIGLMQRVRDECGDVGTFQLAGKQVVLLSGSHANEFFFRAGDDDLDQA
KAYPFMTPIFGEGVVFDASPERRKEMLHNAALRGEQMKGHAATIEDQVRRMIADWGEAGEIDLLDFFAELTIY
TSSACLIGKKFRDQLDGRFAKLYHELERGTDPLAYVDPYLPIESFRRRDEARNGLVALVADIMNGRIANPPTD
KSDRDMLDVLIAVKAETGTPRFSADEITGMFISMMFAGHHTSSGTASWTLIELMRHRDAYAAVIDELDELYGD

GRSVSFHALRQIPQLENVLKETLRLHPPLIILMRVAKGEFEVQGHRIHEGDLVAASPAISNRIPEDFPDPHDF
VPARYEQPRQEDLLNRWTWIPFGAGRHRCVGAAFAIMQIKAIFSVLLREYEFEMAQPPESYRNDHSKMVVQLA
QPACVRYRRRTGV
>CYP51B1_Mycobacterium tuberculosis 22115 2688941471
MSAVALPRVSGGHDEHGHLEEFRTDPIGLMQRVRDECGDVGTFQLAGKQVVLLSGSHANEFFFRAGDDDLDQA
KAYPFMTPIFGEGVVFDASPERRKEMLHNAALRGEQMKGHAATIEDQVRRMIADWGEAGEIDLLDFFAELTIY
TSSACLIGKKFRDQLDGRFAKLYHELERGTDPLAYVDPYLPIESFRRRDEARNGLVALVADIMNGRIANPPTD
KSDRDMLDVLIAVKAETGTPRFSADEITGMFISMMFAGHHTSSGTASWTLIELMRHRDAYAAVIDELDELYGD
GRSVSFHALRQIPQLENVLKETLRLHPPLIILMRVAKGEFEVQGHRIHEGDLVAASPAISNRIPEDFPDPHDF
VPARYEQPRQEDLLNRWTWIPFGAGRHRCVGAAFAIMQIKAIFSVLLREYEFEMAQPPESYRNDHSKMVVQLA
QPACVRYRRRTGV
>CYP51B1_Mycobacterium tuberculosis 2228BH 2621734589
MSAVALPRVSGGHDEHGHLEEFRTDPIGLMQRVRDECGDVGTFQLAGKQVVLLSGSHANEFFFRAGDDDLDQA
KAYPFMTPIFGEGVVFDASPERRKEMLHNAALRGEQMKGHAATIEDQVRRMIADWGEAGEIDLLDFFAELTIY
TSSACLIGKKFRDQLDGRFAKLYHELERGTDPLAYVDPYLPIESFRRRDEARNGLVALVADIMNGRIANPPTD
KSDRDMLDVLIAVKAETGTPRFSADEITGMFISMMFAGHHTSSGTASWTLIELMRHRDAYAAVIDELDELYGD
GRSVSFHALRQIPQLENVLKETLRLHPPLIILMRVAKGEFEVQGHRIHEGDLVAASPAISNRIPEDFPDPHDF
VPARYEQPRQEDLLNRWTWIPFGAGRHRCVGAAFAIMQIKAIFSVLLREYEFEMAQPPESYRNDHSKMVVQLA
QPACVRYRRRTGV
>CYP51B1_Mycobacterium tuberculosis 2229BH 2633602287
MSAVALPRVSGGHDEHGHLEEFRTDPIGLMQRVRDECGDVGTFQLAGKQVVLLSGSHANEFFFRAGDDDLDQA
KAYPFMTPIFGEGVVFDASPERRKEMLHNAALRGEQMKGHAATIEDQVRRMIADWGEAGEIDLLDFFAELTIY
TSSACLIGKKFRDQLDGRFAKLYHELERGTDPLAYVDPYLPIESFRRRDEARNGLVALVADIMNGRIANPPTD
KSDRDMLDVLIAVKAETGTPRFSADEITGMFISMMFAGHHTSSGTASWTLIELMRHRDAYAAVIDELDELYGD
GRSVSFHALRQIPQLENVLKETLRLHPPLIILMRVAKGEFEVQGHRIHEGDLVAASPAISNRIPEDFPDPHDF
VPARYEQPRQEDLLNRWTWIPFGAGRHRCVGAAFAIMQIKAIFSVLLREYEFEMAQPPESYRNDHSKMVVQLA
QPACVRYRRRTGV
>CYP51B1_Mycobacterium tuberculosis 2230BH 2621730354
MSAVALPRVSGGHDEHGHLEEFRTDPIGLMQRVRDECGDVGTFQLAGKQVVLLSGSHANEFFFRAGDDDLDQA
KAYPFMTPIFGEGVVFDASPERRKEMLHNAALRGEQMKGHAATIEDQVRRMIADWGEAGEIDLLDFFAELTIY
TSSACLIGKKFRDQLDGRFAKLYHELERGTDPLAYVDPYLPIESFRRRDEARNGLVALVADIMNGRIANPPTD
KSDRDMLDVLIAVKAETGTPRFSADEITGMFISMMFAGHHTSSGTASWTLIELMRHRDAYAAVIDELDELYGD
GRSVSFHALRQIPQLENVLKETLRLHPPLIILMRVAKGEFEVQGHRIHEGDLVAASPAISNRIPEDFPDPHDF
VPARYEQPRQEDLLNRWTWIPFGAGRHRCVGAAFAIMQIKAIFSVLLREYEFEMAQPPESYRNDHSKMVVQLA
QPACVRYRRRTGV
>CYP51B1_Mycobacterium tuberculosis 2231BH 2647566546
MSAVALPRVSGGHDEHGHLEEFRTDPIGLMQRVRDECGDVGTFQLAGKQVVLLSGSHANEFFFRAGDDDLDQA
KAYPFMTPIFGEGVVFDASPERRKEMLHNAALRGEQMKGHAATIEDQVRRMIADWGEAGEIDLLDFFAELTIY
TSSACLIGKKFRDQLDGRFAKLYHELERGTDPLAYVDPYLPIESFRRRDEARNGLVALVADIMNGRIANPPTD
KSDRDMLDVLIAVKAETGTPRFSADEITGMFISMMFAGHHTSSGTASWTLIELMRHRDAYAAVIDELDELYGD
GRSVSFHALRQIPQLENVLKETLRLHPPLIILMRVAKGEFEVQGHRIHEGDLVAASPAISNRIPEDFPDPHDF
VPARYEQPRQEDLLNRWTWIPFGAGRHRCVGAAFAIMQIKAIFSVLLREYEFEMAQPPESYRNDHSKMVVQLA
QPACVRYRRRTGV
>CYP51B1_Mycobacterium tuberculosis 2233BH 2621719398
MSAVALPRVSGGHDEHGHLEEFRTDPIGLMQRVRDECGDVGTFQLAGKQVVLLSGSHANEFFFRAGDDDLDQA
KAYPFMTPIFGEGVVFDASPERRKEMLHNAALRGEQMKGHAATIEDQVRRMIADWGEAGEIDLLDFFAELTIY
TSSACLIGKKFRDQLDGRFAKLYHELERGTDPLAYVDPYLPIESFRRRDEARNGLVALVADIMNGRIANPPTD
KSDRDMLDVLIAVKAETGTPRFSADEITGMFISMMFAGHHTSSGTASWTLIELMRHRDAYAAVIDELDELYGD
GRSVSFHALRQIPQLENVLKETLRLHPPLIILMRVAKGEFEVQGHRIHEGDLVAASPAISNRIPEDFPDPHDF
VPARYEQPRQEDLLNRWTWIPFGAGRHRCVGAAFAIMQIKAIFSVLLREYEFEMAQPPESYRNDHSKMVVQLA
QPACVRYRRRTGV
>CYP51B1_Mycobacterium tuberculosis 2242 2687549710
MSAVALPRVSGGHDEHGHLEEFRTDPIGLMQRVRDECGDVGTFQLAGKQVVLLSGSHANEFFFRAGDDDLDQA
KAYPFMTPIFGEGVVFDASPERRKEMLHNAALRGEQMKGHAATIEDQVRRMIADWGEAGEIDLLDFFAELTIY
TSSACLIGKKFRDQLDGRFAKLYHELERGTDPLAYVDPYLPIESFRRRDEARNGLVALVADIMNGRIANPPTD
KSDRDMLDVLIAVKAETGTPRFSADEITGMFISMMFAGHHTSSGTASWTLIELMRHRDAYAAVIDELDELYGD
GRSVSFHALRQIPQLENVLKETLRLHPPLIILMRVAKGEFEVQGHRIHEGDLVAASPAISNRIPEDFPDPHDF

VPARYEQPRQEDLLNRWTWIPFGAGRHRCVGAAFAIMQIKAIFSVLLREYEFEMAQPPESYRNDHSKMVVQLA
QPACVRYRRRTGV
>CYP51B1_Mycobacterium tuberculosis 2279 2686588880
MSAVALPRVSGGHDEHGHLEEFRTDPIGLMQRVRDECGDVGTFQLAGKQVVLLSGSHANEFFFRAGDDDLDQA
KAYPFMTPIFGEGVVFDASPERRKEMLHNAALRGEQMKGHAATIEDQVRRMIADWGEAGEIDLLDFFAELTIY
TSSACLIGKKFRDQLDGRFAKLYHELERGTDPLAYVDPYLPIESFRRRDEARNGLVALVADIMNGRIANPPTD
KSDRDMLDVLIAVKAETGTPRFSADEITGMFISMMFAGHHTSSGTASWTLIELMRHRDAYAAVIDELDELYGD
GRSVSFHALRQIPQLENVLKETLRLHPPLIILMRVAKGEFEVQGHRIHEGDLVAASPAISNRIPEDFPDPHDF
VPARYEQPRQEDLLNRWTWIPFGAGRHRCVGAAFAIMQIKAIFSVLLREYEFEMAQPPESYRNDHSKMVVQLA
QPACVRYRRRTGV
>CYP51B1_Mycobacterium tuberculosis 2482AR 2606393159
MSAVALPRVSGGHDEHGHLEEFRTDPIGLMQRVRDECGDVGTFQLAGKQVVLLSGSHANEFFFRAGDDDLDQA
KAYPFMTPIFGEGVVFDASPERRKEMLHNAALRGEQMKGHAATIEDQVRRMIADWGEAGEIDLLDFFAELTIY
TSSACLIGKKFRDQLDGRFAKLYHELERGTDPLAYVDPYLPIESFRRRDEARNGLVALVADIMNGRIANPPTD
KSDRDMLDVLIAVKAETGTPRFSADEITGMFISMMFAGHHTSSGTASWTLIELMRHRDAYAAVIDELDELYGD
GRSVSFHALRQIPQLENVLKETLRLHPPLIILMRVAKGEFEVQGHRIHEGDLVAASPAISNRIPEDFPDPHDF
VPARYEQPRQEDLLNRWTWIPFGAGRHRCVGAAFAIMQIKAIFSVLLREYEFEMAQPPESYRNDHSKMVVQLA
QPACVRYRRRTGV
>CYP51B1_Mycobacterium tuberculosis 2483AR 2575165633
MSAVALPRVSGGHDEHGHLEEFRTDPIGLMQRVRDECGDVGTFQLAGKQVVLLSGSHANEFFFRAGDDDLDQA
KAYPFMTPIFGEGVVFDASPERRKEMLHNAALRGEQMKGHAATIEDQVRRMIADWGEAGEIDLLDFFAELTIY
TSSACLIGKKFRDQLDGRFAKLYHELERGTDPLAYVDPYLPIESFRRRDEARNGLVALVADIMNGRIANPPTD
KSDRDMLDVLIAVKAETGTPRFSADEITGMFISMMFAGHHTSSGTASWTLIELMRHRDAYAAVIDELDELYGD
GRSVSFHALRQIPQLENVLKETLRLHPPLIILMRVAKGEFEVQGHRIHEGDLVAASPAISNRIPEDFPDPHDF
VPARYEQPRQEDLLNRWTWIPFGAGRHRCVGAAFAIMQIKAIFSVLLREYEFEMAQPPESYRNDHSKMVVQLA
QPACVRYRRRTGV
>CYP51B1_Mycobacterium tuberculosis 2484AR 2606389259
MSAVALPRVSGGHDEHGHLEEFRTDPIGLMQRVRDECGDVGTFQLAGKQVVLLSGSHANEFFFRAGDDDLDQA
KAYPFMTPIFGEGVVFDASPERRKEMLHNAALRGEQMKGHAATIEDQVRRMIADWGEAGEIDLLDFFAELTIY
TSSACLIGKKFRDQLDGRFAKLYHELERGTDPLAYVDPYLPIESFRRRDEARNGLVALVADIMNGRIANPPTD
KSDRDMLDVLIAVKAETGTPRFSADEITGMFISMMFAGHHTSSGTASWTLIELMRHRDAYAAVIDELDELYGD
GRSVSFHALRQIPQLENVLKETLRLHPPLIILMRVAKGEFEVQGHRIHEGDLVAASPAISNRIPEDFPDPHDF
VPARYEQPRQEDLLNRWTWIPFGAGRHRCVGAAFAIMQIKAIFSVLLREYEFEMAQPPESYRNDHSKMVVQLA
QPACVRYRRRTGV
>CYP51B1_Mycobacterium tuberculosis 2485AR 2606477585
MSAVALPRVSGGHDEHGHLEEFRTDPIGLMQRVRDECGDVGTFQLAGKQVVLLSGSHANEFFFRAGDDDLDQA
KAYPFMTPIFGEGVVFDASPERRKEMLHNAALRGEQMKGHAATIEDQVRRMIADWGEAGEIDLLDFFAELTIY
TSSACLIGKKFRDQLDGRFAKLYHELERGTDPLAYVDPYLPIESFRRRDEARNGLVALVADIMNGRIANPPTD
KSDRDMLDVLIAVKAETGTPRFSADEITGMFISMMFAGHHTSSGTASWTLIELMRHRDAYAAVIDELDELYGD
GRSVSFHALRQIPQLENVLKETLRLHPPLIILMRVAKGEFEVQGHRIHEGDLVAASPAISNRIPEDFPDPHDF
VPARYEQPRQEDLLNRWTWIPFGAGRHRCVGAAFAIMQIKAIFSVLLREYEFEMAQPPESYRNDHSKMVVQLA
QPACVRYRRRTGV
>CYP51B1_Mycobacterium tuberculosis 2489 2621718118
MSAVALPRVSGGHDEHGHLEEFRTDPIGLMQRVRDECGDVGTFQLAGKQVVLLSGSHANEFFFRAGDDDLDQA
KAYPFMTPIFGEGVVFDASPERRKEMLHNAALRGEQMKGHAATIEDQVRRMIADWGEAGEIDLLDFFAELTIY
TSSACLIGKKFRDQLDGRFAKLYHELERGTDPLAYVDPYLPIESFRRRDEARNGLVALVADIMNGRIANPPTD
KSDRDMLDVLIAVKAETGTPRFSADEITGMFISMMFAGHHTSSGTASWTLIELMRHRDAYAAVIDELDELYGD
GRSVSFHALRQIPQLENVLKETLRLHPPLIILMRVAKGEFEVQGHRIHEGDLVAASPAISNRIPEDFPDPHDF
VPARYEQPRQEDLLNRWTWIPFGAGRHRCVGAAFAIMQIKAIFSVLLREYEFEMAQPPESYRNDHSKMVVQLA
QPACVRYRRRTGV
>CYP51B1_Mycobacterium tuberculosis 2541MS 2576374980
MSAVALPRVSGGHDEHGHLEEFRTDPIGLMQRVRDECGDVGTFQLAGKQVVLLSGSHANEFFFRAGDDDLDQA
KAYPFMTPIFGEGVVFDASPERRKEMLHNAALRGEQMKGHAATIEDQVRRMIADWGEAGEIDLLDFFAELTIY
TSSACLIGKKFRDQLDGRFAKLYHELERGTDPLAYVDPYLPIESFRRRDEARNGLVALVADIMNGRIANPPTD
KSDRDMLDVLIAVKAETGTPRFSADEITGMFISMMFAGHHTSSGTASWTLIELMRHRDAYAAVIDELDELYGD
GRSVSFHALRQIPQLENVLKETLRLHPPLIILMRVAKGEFEVQGHRIHEGDLVAASPAISNRIPEDFPDPHDF
VPARYEQPRQEDLLNRWTWIPFGAGRHRCVGAAFAIMQIKAIFSVLLREYEFEMAQPPESYRNDHSKMVVQLA
QPACVRYRRRTGV

>CYP51B1_Mycobacterium tuberculosis 2542MS 2630528122
MSAVALPRVSGGHDEHGHLEEFRTDPIGLMQRVRDECGDVGTFQLAGKQVVLLSGSHANEFFFRAGDDDLDQA
KAYPFMTPIFGEGVVFDASPERRKEMLHNAALRGEQMKGHAATIEDQVRRMIADWGEAGEIDLLDFFAELTIY
TSSACLIGKKFRDQLDGRFAKLYHELERGTDPLAYVDPYLPIESFRRRDEARNGLVALVADIMNGRIANPPTD
KSDRDMLDVLIAVKAETGTPRFSADEITGMFISMMFAGHHTSSGTASWTLIELMRHRDAYAAVIDELDELYGD
GRSVSFHALRQIPQLENVLKETLRLHPPLIILMRVAKGEFEVQGHRIHEGDLVAASPAISNRIPEDFPDPHDF
VPARYEQPRQEDLLNRWTWIPFGAGRHRCVGAAFAIMQIKAIFSVLLREYEFEMAQPPESYRNDHSKMVVQLA
QPACVRYRRRTGV
>CYP51B1_Mycobacterium tuberculosis 2543MS 2606485705
MSAVALPRVSGGHDEHGHLEEFRTDPIGLMQRVRDECGDVGTFQLAGKQVVLLSGSHANEFFFRAGDDDLDQA
KAYPFMTPIFGEGVVFDASPERRKEMLHNAALRGEQMKGHAATIEDQVRRMIADWGEAGEIDLLDFFAELTIY
TSSACLIGKKFRDQLDGRFAKLYHELERGTDPLAYVDPYLPIESFRRRDEARNGLVALVADIMNGRIANPPTD
KSDRDMLDVLIAVKAETGTPRFSADEITGMFISMMFAGHHTSSGTASWTLIELMRHRDAYAAVIDELDELYGD
GRSVSFHALRQIPQLENVLKETLRLHPPLIILMRVAKGEFEVQGHRIHEGDLVAASPAISNRIPEDFPDPHDF
VPARYEQPRQEDLLNRWTWIPFGAGRHRCVGAAFAIMQIKAIFSVLLREYEFEMAQPPESYRNDHSKMVVQLA
QPACVRYRRRTGV
>CYP51B1_Mycobacterium tuberculosis 2995AB 2635709311
MSAVALPRVSGGHDEHGHLEEFRTDPIGLMQRVRDECGDVGTFQLAGKQVVLLSGSHANEFFFRAGDDDLDQA
KAYPFMTPIFGEGVVFDASPERRKEMLHNAALRGEQMKGHAATIEDQVRRMIADWGEAGEIDLLDFFAELTIY
TSSACLIGKKFRDQLDGRFAKLYHELERGTDPLAYVDPYLPIESFRRRDEARNGLVALVADIMNGRIANPPTD
KSDRDMLDVLIAVKAETGTPRFSADEITGMFISMMFAGHHTSSGTASWTLIELMRHRDAYAAVIDELDELYGD
GRSVSFHALRQIPQLENVLKETLRLHPPLIILMRVAKGEFEVQGHRIHEGDLVAASPAISNRIPEDFPDPHDF
VPARYEQPRQEDLLNRWTWIPFGAGRHRCVGAAFAIMQIKAIFSVLLREYEFEMAQPPESYRNDHSKMVVQLA
QPACVRYRRRTGV
>CYP51B1_Mycobacterium tuberculosis 300191 2669250491
MSAVALPRVSGGHDEHGHLEEFRTDPIGLMQRVRDECGDVGTFQLAGKQVVLLSGSHANEFFFRAGDDDLDQA
KAYPFMTPIFGEGVVFDASPERRKEMLHNAALRGEQMKGHAATIEDQVRRMIADWGEAGEIDLLDFFAELTIY
TSSACLIGKKFRDQLDGRFAKLYHELERGTDPLAYVDPYLPIESFRRRDEARNGLVALVADIMNGRIANPPTD
KSDRDMLDVLIAVKAETGTPRFSADEITGMFISMMFAGHHTSSGTASWTLIELMRHRDAYAAVIDELDELYGD
GRSVSFHALRQIPQLENVLKETLRLHPPLIILMRVAKGEFEVQGHRIHEGDLVAASPAISNRIPEDFPDPHDF
VPARYEQPRQEDLLNRWTWIPFGAGRHRCVGAAFAIMQIKAIFSVLLREYEFEMAQPPESYRNDHSKMVVQLA
QPACVRYRRRTGV
>CYP51B1_Mycobacterium tuberculosis 300234 2673508850
MSAVALPRVSGGHDEHGHLEEFRTDPIGLMQRVRDECGDVGTFQLAGKQVVLLSGSHANEFFFRAGDDDLDQA
KAYPFMTPIFGEGVVFDASPERRKEMLHNAALRGEQMKGHAATIEDQVRRMIADWGEAGEIDLLDFFAELTIY
TSSACLIGKKFRDQLDGRFAKLYHELERGTDPLAYVDPYLPIESFRRRDEARNGLVALVADIMNGRIANPPTD
KSDRDMLDVLIAVKAETGTPRFSADEITGMFISMMFAGHHTSSGTASWTLIELMRHRDAYAAVIDELDELYGD
GRSVSFHALRQIPQLENVLKETLRLHPPLIILMRVAKGEFEVQGHRIHEGDLVAASPAISNRIPEDFPDPHDF
VPARYEQPRQEDLLNRWTWIPFGAGRHRCVGAAFAIMQIKAIFSVLLREYEFEMAQPPESYRNDHSKMVVQLA
QPACVRYRRRTGV
>CYP51B1_Mycobacterium tuberculosis 300239 2659962825
MSAVALPRVSGGHDEHGHLEEFRTDPIGLMQRVRDECGDVGTFQLAGKQVVLLSGSHANEFFFRAGDDDLDQA
KAYPFMTPIFGEGVVFDASPERRKEMLHNAALRGEQMKGHAATIEDQVRRMIADWGEAGEIDLLDFFAELTIY
TSSACLIGKKFRDQLDGRFAKLYHELERGTDPLAYVDPYLPIESFRRRDEARNGLVALVADIMNGRIANPPTD
KSDRDMLDVLIAVKAETGTPRFSADEITGMFISMMFAGHHTSSGTASWTLIELMRHRDAYAAVIDELDELYGD
GRSVSFHALRQIPQLENVLKETLRLHPPLIILMRVAKGEFEVQGHRIHEGDLVAASPAISNRIPEDFPDPHDF
VPARYEQPRQEDLLNRWTWIPFGAGRHRCVGAAFAIMQIKAIFSVLLREYEFEMAQPPESYRNDHSKMVVQLA
QPACVRYRRRTGV
>CYP51B1_Mycobacterium tuberculosis 300260 2665929309
MSAVALPRVSGGHDEHGHLEEFRTDPIGLMQRVRDECGDVGTFQLAGKQVVLLSGSHANEFFFRAGDDDLDQA
KAYPFMTPIFGEGVVFDASPERRKEMLHNAALRGEQMKGHAATIEDQVRRMIADWGEAGEIDLLDFFAELTIY
TSSACLIGKKFRDQLDGRFAKLYHELERGTDPLAYVDPYLPIESFRRRDEARNGLVALVADIMNGRIANPPTD
KSDRDMLDVLIAVKAETGTPRFSADEITGMFISMMFAGHHTSSGTASWTLIELMRHRDAYAAVIDELDELYGD
GRSVSFHALRQIPQLENVLKETLRLHPPLIILMRVAKGEFEVQGHRIHEGDLVAASPAISNRIPEDFPDPHDF
VPARYEQPRQEDLLNRWTWIPFGAGRHRCVGAAFAIMQIKAIFSVLLREYEFEMAQPPESYRNDHSKMVVQLA
QPACVRYRRRTGV
>CYP51B1_Mycobacterium tuberculosis 300659 2668867278

MSAVALPRVSGGHDEHGHLEEFRTDPIGLMQRVRDECGDVGTFQLAGKQVVLLSGSHANEFFFRAGDDDLDQA
KAYPFMTPIFGEGVVFDASPERRKEMLHNAALRGEQMKGHAATIEDQVRRMIADWGEAGEIDLLDFFAELTIY
TSSACLIGKKFRDQLDGRFAKLYHELERGTDPLAYVDPYLPIESFRRRDEARNGLVALVADIMNGRIANPPTD
KSDRDMLDVLIAVKAETGTPRFSADEITGMFISMMFAGHHTSSGTASWTLIELMRHRDAYAAVIDELDELYGD
GRSVSFHALRQIPQLENVLKETLRLHPPLIILMRVAKGEFEVQGHRIHEGDLVAASPAISNRIPEDFPDPHDF
VPARYEQPRQEDLLNRWTWIPFGAGRHRCVGAAFAIMQIKAIFSVLLREYEFEMAQPPESYRNDHSKMVVQLA
QPACVRYRRRTGV
>CYP51B1_Mycobacterium tuberculosis 300798 2666227115
MSAVALPRVSGGHDEHGHLEEFRTDPIGLMQRVRDECGDVGTFQLAGKQVVLLSGSHANEFFFRAGDDDLDQA
KAYPFMTPIFGEGVVFDASPERRKEMLHNAALRGEQMKGHAATIEDQVRRMIADWGEAGEIDLLDFFAELTIY
TSSACLIGKKFRDQLDGRFAKLYHELERGTDPLAYVDPYLPIESFRRRDEARNGLVALVADIMNGRIANPPTD
KSDRDMLDVLIAVKAETGTPRFSADEITGMFISMMFAGHHTSSGTASWTLIELMRHRDAYAAVIDELDELYGD
GRSVSFHALRQIPQLENVLKETLRLHPPLIILMRVAKGEFEVQGHRIHEGDLVAASPAISNRIPEDFPDPHDF
VPARYEQPRQEDLLNRWTWIPFGAGRHRCVGAAFAIMQIKAIFSVLLREYEFEMAQPPESYRNDHSKMVVQLA
QPACVRYRRRTGV
>CYP51B1_Mycobacterium tuberculosis 300879 2693685244
MSAVALPRVSGGHDEHGHLEEFRTDPIGLMQRVRDECGDVGTFQLAGKQVVLLSGSHANEFFFRAGDDDLDQA
KAYPFMTPIFGEGVVFDASPERRKEMLHNAALRGEQMKGHAATIEDQVRRMIADWGEAGEIDLLDFFAELTIY
TSSACLIGKKFRDQLDGRFAKLYHELERGTDPLAYVDPYLPIESFRRRDEARNGLVALVADIMNGRIANPPTD
KSDRDMLDVLIAVKAETGTPRFSADEITGMFISMMFAGHHTSSGTASWTLIELMRHRDAYAAVIDELDELYGD
GRSVSFHALRQIPQLENVLKETLRLHPPLIILMRVAKGEFEVQGHRIHEGDLVAASPAISNRIPEDFPDPHDF
VPARYEQPRQEDLLNRWTWIPFGAGRHRCVGAAFAIMQIKAIFSVLLREYEFEMAQPPESYRNDHSKMVVQLA
QPACVRYRRRTGV
>CYP51B1_Mycobacterium tuberculosis 301092 2682910214
MSAVALPRVSGGHDEHGHLEEFRTDPIGLMQRVRDECGDVGTFQLAGKQVVLLSGSHANEFFFRAGDDDLDQA
KAYPFMTPIFGEGVVFDASPERRKEMLHNAALRGEQMKGHAATIEDQVRRMIADWGEAGEIDLLDFFAELTIY
TSSACLIGKKFRDQLDGRFAKLYHELERGTDPLAYVDPYLPIESFRRRDEARNGLVALVADIMNGRIANPPTD
KSDRDMLDVLIAVKAETGTPRFSADEITGMFISMMFAGHHTSSGTASWTLIELMRHRDAYAAVIDELDELYGD
GRSVSFHALRQIPQLENVLKETLRLHPPLIILMRVAKGEFEVQGHRIHEGDLVAASPAISNRIPEDFPDPHDF
VPARYEQPRQEDLLNRWTWIPFGAGRHRCVGAAFAIMQIKAIFSVLLREYEFEMAQPPESYRNDHSKMVVQLA
QPACVRYRRRTGV
>CYP51B1_Mycobacterium tuberculosis 301310 2669037622
MSAVALPRVSGGHDEHGHLEEFRTDPIGLMQRVRDECGDVGTFQLAGKQVVLLSGSHANEFFFRAGDDDLDQA
KAYPFMTPIFGEGVVFDASPERRKEMLHNAALRGEQMKGHAATIEDQVRRMIADWGEAGEIDLLDFFAELTIY
TSSACLIGKKFRDQLDGRFAKLYHELERGTDPLAYVDPYLPIESFRRRDEARNGLVALVADIMNGRIANPPTD
KSDRDMLDVLIAVKAETGTPRFSADEITGMFISMMFAGHHTSSGTASWTLIELMRHRDAYAAVIDELDELYGD
GRSVSFHALRQIPQLENVLKETLRLHPPLIILMRVAKGEFEVQGHRIHEGDLVAASPAISNRIPEDFPDPHDF
VPARYEQPRQEDLLNRWTWIPFGAGRHRCVGAAFAIMQIKAIFSVLLREYEFEMAQPPESYRNDHSKMVVQLA
QPACVRYRRRTGV
>CYP51B1_Mycobacterium tuberculosis 301347 2663322763
MSAVALPRVSGGHDEHGHLEEFRTDPIGLMQRVRDECGDVGTFQLAGKQVVLLSGSHANEFFFRAGDDDLDQA
KAYPFMTPIFGEGVVFDASPERRKEMLHNAALRGEQMKGHAATIEDQVRRMIADWGEAGEIDLLDFFAELTIY
TSSACLIGKKFRDQLDGRFAKLYHELERGTDPLAYVDPYLPIESFRRRDEARNGLVALVADIMNGRIANPPTD
KSDRDMLDVLIAVKAETGTPRFSADEITGMFISMMFAGHHTSSGTASWTLIELMRHRDAYAAVIDELDELYGD
GRSVSFHALRQIPQLENVLKETLRLHPPLIILMRVAKGEFEVQGHRIHEGDLVAASPAISNRIPEDFPDPHDF
VPARYEQPRQEDLLNRWTWIPFGAGRHRCVGAAFAIMQIKAIFSVLLREYEFEMAQPPESYRNDHSKMVVQLA
QPACVRYRRRTGV
>CYP51B1_Mycobacterium tuberculosis 301645 2697028670
MSAVALPRVSGGHDEHGHLEEFRTDPIGLMQRVRDECGDVGTFQLAGKQVVLLSGSHANEFFFRAGDDDLDQA
KAYPFMTPIFGEGVVFDASPERRKEMLHNAALRGEQMKGHAATIEDQVRRMIADWGEAGEIDLLDFFAELTIY
TSSACLIGKKFRDQLDGRFAKLYHELERGTDPLAYVDPYLPIESFRRRDEARNGLVALVADIMNGRIANPPTD
KSDRDMLDVLIAVKAETGTPRFSADEITGMFISMMFAGHHTSSGTASWTLIELMRHRDAYAAVIDELDELYGD
GRSVSFHALRQIPQLENVLKETLRLHPPLIILMRVAKGEFEVQGHRIHEGDLVAASPAISNRIPEDFPDPHDF
VPARYEQPRQEDLLNRWTWIPFGAGRHRCVGAAFAIMQIKAIFSVLLREYEFEMAQPPESYRNDHSKMVVQLA
QPACVRYRRRTGV
>CYP51B1_Mycobacterium tuberculosis 301725 2697162056
MSAVALPRVSGGHDEHGHLEEFRTDPIGLMQRVRDECGDVGTFQLAGKQVVLLSGSHANEFFFRAGDDDLDQA
KAYPFMTPIFGEGVVFDASPERRKEMLHNAALRGEQMKGHAATIEDQVRRMIADWGEAGEIDLLDFFAELTIY

TSSACLIGKKFRDQLDGRFAKLYHELERGTDPLAYVDPYLPIESFRRRDEARNGLVALVADIMNGRIANPPTD
KSDRDMLDVLIAVKAETGTPRFSADEITGMFISMMFAGHHTSSGTASWTLIELMRHRDAYAAVIDELDELYGD
GRSVSFHALRQIPQLENVLKETLRLHPPLIILMRVAKGEFEVQGHRIHEGDLVAASPAISNRIPEDFPDPHDF
VPARYEQPRQEDLLNRWTWIPFGAGRHRCVGAAFAIMQIKAIFSVLLREYEFEMAQPPESYRNDHSKMVVQLA
QPACVRYRRRTGV
>CYP51B1_Mycobacterium tuberculosis 301747 2669901782
MSAVALPRVSGGHDEHGHLEEFRTDPIGLMQRVRDECGDVGTFQLAGKQVVLLSGSHANEFFFRAGDDDLDQA
KAYPFMTPIFGEGVVFDASPERRKEMLHNAALRGEQMKGHAATIEDQVRRMIADWGEAGEIDLLDFFAELTIY
TSSACLIGKKFRDQLDGRFAKLYHELERGTDPLAYVDPYLPIESFRRRDEARNGLVALVADIMNGRIANPPTD
KSDRDMLDVLIAVKAETGTPRFSADEITGMFISMMFAGHHTSSGTASWTLIELMRHRDAYAAVIDELDELYGD
GRSVSFHALRQIPQLENVLKETLRLHPPLIILMRVAKGEFEVQGHRIHEGDLVAASPAISNRIPEDFPDPHDF
VPARYEQPRQEDLLNRWTWIPFGAGRHRCVGAAFAIMQIKAIFSVLLREYEFEMAQPPESYRNDHSKMVVQLA
QPACVRYRRRTGV
>CYP51B1_Mycobacterium tuberculosis 301816 2673023217
MSAVALPRVSGGHDEHGHLEEFRTDPIGLMQRVRDECGDVGTFQLAGKQVVLLSGSHANEFFFRAGDDDLDQA
KAYPFMTPIFGEGVVFDASPERRKEMLHNAALRGEQMKGHAATIEDQVRRMIADWGEAGEIDLLDFFAELTIY
TSSACLIGKKFRDQLDGRFAKLYHELERGTDPLAYVDPYLPIESFRRRDEARNGLVALVADIMNGRIANPPTD
KSDRDMLDVLIAVKAETGTPRFSADEITGMFISMMFAGHHTSSGTASWTLIELMRHRDAYAAVIDELDELYGD
GRSVSFHALRQIPQLENVLKETLRLHPPLIILMRVAKGEFEVQGHRIHEGDLVAASPAISNRIPEDFPDPHDF
VPARYEQPRQEDLLNRWTWIPFGAGRHRCVGAAFAIMQIKAIFSVLLREYEFEMAQPPESYRNDHSKMVVQLA
QPACVRYRRRTGV
>CYP51B1_Mycobacterium tuberculosis 302174 2661456026
MSAVALPRVSGGHDEHGHLEEFRTDPIGLMQRVRDECGDVGTFQLAGKQVVLLSGSHANEFFFRAGDDDLDQA
KAYPFMTPIFGEGVVFDASPERRKEMLHNAALRGEQMKGHAATIEDQVRRMIADWGEAGEIDLLDFFAELTIY
TSSACLIGKKFRDQLDGRFAKLYHELERGTDPLAYVDPYLPIESFRRRDEARNGLVALVADIMNGRIANPPTD
KSDRDMLDVLIAVKAETGTPRFSADEITGMFISMMFAGHHTSSGTASWTLIELMRHRDAYAAVIDELDELYGD
GRSVSFHALRQIPQLENVLKETLRLHPPLIILMRVAKGEFEVQGHRIHEGDLVAASPAISNRIPEDFPDPHDF
VPARYEQPRQEDLLNRWTWIPFGAGRHRCVGAAFAIMQIKAIFSVLLREYEFEMAQPPESYRNDHSKMVVQLA
QPACVRYRRRTGV
>CYP51B1_Mycobacterium tuberculosis 302203 2701651131
MSAVALPRVSGGHDEHGHLEEFRTDPIGLMQRVRDECGDVGTFQLAGKQVVLLSGSHANEFFFRAGDDDLDQA
KAYPFMTPIFGEGVVFDASPERRKEMLHNAALRGEQMKGHAATIEDQVRRMIADWGEAGEIDLLDFFAELTIY
TSSACLIGKKFRDQLDGRFAKLYHELERGTDPLAYVDPYLPIESFRRRDEARNGLVALVADIMNGRIANPPTD
KSDRDMLDVLIAVKAETGTPRFSADEITGMFISMMFAGHHTSSGTASWTLIELMRHRDAYAAVIDELDELYGD
GRSVSFHALRQIPQLENVLKETLRLHPPLIILMRVAKGEFEVQGHRIHEGDLVAASPAISNRIPEDFPDPHDF
VPARYEQPRQEDLLNRWTWIPFGAGRHRCVGAAFAIMQIKAIFSVLLREYEFEMAQPPESYRNDHSKMVVQLA
QPACVRYRRRTGV
>CYP51B1_Mycobacterium tuberculosis 302226 2660593045
MSAVALPRVSGGHDEHGHLEEFRTDPIGLMQRVRDECGDVGTFQLAGKQVVLLSGSHANEFFFRAGDDDLDQA
KAYPFMTPIFGEGVVFDASPERRKEMLHNAALRGEQMKGHAATIEDQVRRMIADWGEAGEIDLLDFFAELTIY
TSSACLIGKKFRDQLDGRFAKLYHELERGTDPLAYVDPYLPIESFRRRDEARNGLVALVADIMNGRIANPPTD
KSDRDMLDVLIAVKAETGTPRFSADEITGMFISMMFAGHHTSSGTASWTLIELMRHRDAYAAVIDELDELYGD
GRSVSFHALRQIPQLENVLKETLRLHPPLIILMRVAKGEFEVQGHRIHEGDLVAASPAISNRIPEDFPDPHDF
VPARYEQPRQEDLLNRWTWIPFGAGRHRCVGAAFAIMQIKAIFSVLLREYEFEMAQPPESYRNDHSKMVVQLA
QPACVRYRRRTGV
>CYP51B1_Mycobacterium tuberculosis 302235 2697890918
MSAVALPRVSGGHDEHGHLEEFRTDPIGLMQRVRDECGDVGTFQLAGKQVVLLSGSHANEFFFRAGDDDLDQA
KAYPFMTPIFGEGVVFDASPERRKEMLHNAALRGEQMKGHAATIEDQVRRMIADWGEAGEIDLLDFFAELTIY
TSSACLIGKKFRDQLDGRFAKLYHELERGTDPLAYVDPYLPIESFRRRDEARNGLVALVADIMNGRIANPPTD
KSDRDMLDVLIAVKAETGTPRFSADEITGMFISMMFAGHHTSSGTASWTLIELMRHRDAYAAVIDELDELYGD
GRSVSFHALRQIPQLENVLKETLRLHPPLIILMRVAKGEFEVQGHRIHEGDLVAASPAISNRIPEDFPDPHDF
VPARYEQPRQEDLLNRWTWIPFGAGRHRCVGAAFAIMQIKAIFSVLLREYEFEMAQPPESYRNDHSKMVVQLA
QPACVRYRRRTGV
>CYP51B1_Mycobacterium tuberculosis 302258 2700039012
MSAVALPRVSGGHDEHGHLEEFRTDPIGLMQRVRDECGDVGTFQLAGKQVVLLSGSHANEFFFRAGDDDLDQA
KAYPFMTPIFGEGVVFDASPERRKEMLHNAALRGEQMKGHAATIEDQVRRMIADWGEAGEIDLLDFFAELTIY
TSSACLIGKKFRDQLDGRFAKLYHELERGTDPLAYVDPYLPIESFRRRDEARNGLVALVADIMNGRIANPPTD
KSDRDMLDVLIAVKAETGTPRFSADEITGMFISMMFAGHHTSSGTASWTLIELMRHRDAYAAVIDELDELYGD

GRSVSFHALRQIPQLENVLKETLRLHPPLIILMRVAKGEFEVQGHRIHEGDLVAASPAISNRIPEDFPDPHDF
VPARYEQPRQEDLLNRWTWIPFGAGRHRCVGAAFAIMQIKAIFSVLLREYEFEMAQPPESYRNDHSKMVVQLA
QPACVRYRRRTGV
>CYP51B1_Mycobacterium tuberculosis 302350 2692571947
MSAVALPRVSGGHDEHGHLEEFRTDPIGLMQRVRDECGDVGTFQLAGKQVVLLSGSHANEFFFRAGDDDLDQA
KAYPFMTPIFGEGVVFDASPERRKEMLHNAALRGEQMKGHAATIEDQVRRMIADWGEAGEIDLLDFFAELTIY
TSSACLIGKKFRDQLDGRFAKLYHELERGTDPLAYVDPYLPIESFRRRDEARNGLVALVADIMNGRIANPPTD
KSDRDMLDVLIAVKAETGTPRFSADEITGMFISMMFAGHHTSSGTASWTLIELMRHRDAYAAVIDELDELYGD
GRSVSFHALRQIPQLENVLKETLRLHPPLIILMRVAKGEFEVQGHRIHEGDLVAASPAISNRIPEDFPDPHDF
VPARYEQPRQEDLLNRWTWIPFGAGRHRCVGAAFAIMQIKAIFSVLLREYEFEMAQPPESYRNDHSKMVVQLA
QPACVRYRRRTGV
>CYP51B1_Mycobacterium tuberculosis 302434 2670955761
MSAVALPRVSGGHDEHGHLEEFRTDPIGLMQRVRDECGDVGTFQLAGKQVVLLSGSHANEFFFRAGDDDLDQA
KAYPFMTPIFGEGVVFDASPERRKEMLHNAALRGEQMKGHAATIEDQVRRMIADWGEAGEIDLLDFFAELTIY
TSSACLIGKKFRDQLDGRFAKLYHELERGTDPLAYVDPYLPIESFRRRDEARNGLVALVADIMNGRIANPPTD
KSDRDMLDVLIAVKAETGTPRFSADEITGMFISMMFAGHHTSSGTASWTLIELMRHRDAYAAVIDELDELYGD
GRSVSFHALRQIPQLENVLKETLRLHPPLIILMRVAKGEFEVQGHRIHEGDLVAASPAISNRIPEDFPDPHDF
VPARYEQPRQEDLLNRWTWIPFGAGRHRCVGAAFAIMQIKAIFSVLLREYEFEMAQPPESYRNDHSKMVVQLA
QPACVRYRRRTGV
>CYP51B1_Mycobacterium tuberculosis 302642 2694442464
MSAVALPRVSGGHDEHGHLEEFRTDPIGLMQRVRDECGDVGTFQLAGKQVVLLSGSHANEFFFRAGDDDLDQA
KAYPFMTPIFGEGVVFDASPERRKEMLHNAALRGEQMKGHAATIEDQVRRMIADWGEAGEIDLLDFFAELTIY
TSSACLIGKKFRDQLDGRFAKLYHELERGTDPLAYVDPYLPIESFRRRDEARNGLVALVADIMNGRIANPPTD
KSDRDMLDVLIAVKAETGTPRFSADEITGMFISMMFAGHHTSSGTASWTLIELMRHRDAYAAVIDELDELYGD
GRSVSFHALRQIPQLENVLKETLRLHPPLIILMRVAKGEFEVQGHRIHEGDLVAASPAISNRIPEDFPDPHDF
VPARYEQPRQEDLLNRWTWIPFGAGRHRCVGAAFAIMQIKAIFSVLLREYEFEMAQPPESYRNDHSKMVVQLA
QPACVRYRRRTGV
>CYP51B1_Mycobacterium tuberculosis 3067CJ 2621736295
MSAVALPRVSGGHDEHGHLEEFRTDPIGLMQRVRDECGDVGTFQLAGKQVVLLSGSHANEFFFRAGDDDLDQA
KAYPFMTPIFGEGVVFDASPERRKEMLHNAALRGEQMKGHAATIEDQVRRMIADWGEAGEIDLLDFFAELTIY
TSSACLIGKKFRDQLDGRFAKLYHELERGTDPLAYVDPYLPIESFRRRDEARNGLVALVADIMNGRIANPPTD
KSDRDMLDVLIAVKAETGTPRFSADEITGMFISMMFAGHHTSSGTASWTLIELMRHRDAYAAVIDELDELYGD
GRSVSFHALRQIPQLENVLKETLRLHPPLIILMRVAKGEFEVQGHRIHEGDLVAASPAISNRIPEDFPDPHDF
VPARYEQPRQEDLLNRWTWIPFGAGRHRCVGAAFAIMQIKAIFSVLLREYEFEMAQPPESYRNDHSKMVVQLA
QPACVRYRRRTGV
>CYP51B1_Mycobacterium tuberculosis 3280CJ 2575656980
MSAVALPRVSGGHDEHGHLEEFRTDPIGLMQRVRDECGDVGTFQLAGKQVVLLSGSHANEFFFRAGDDDLDQA
KAYPFMTPIFGEGVVFDASPERRKEMLHNAALRGEQMKGHAATIEDQVRRMIADWGEAGEIDLLDFFAELTIY
TSSACLIGKKFRDQLDGRFAKLYHELERGTDPLAYVDPYLPIESFRRRDEARNGLVALVADIMNGRIANPPTD
KSDRDMLDVLIAVKAETGTPRFSADEITGMFISMMFAGHHTSSGTASWTLIELMRHRDAYAAVIDELDELYGD
GRSVSFHALRQIPQLENVLKETLRLHPPLIILMRVAKGEFEVQGHRIHEGDLVAASPAISNRIPEDFPDPHDF
VPARYEQPRQEDLLNRWTWIPFGAGRHRCVGAAFAIMQIKAIFSVLLREYEFEMAQPPESYRNDHSKMVVQLA
QPACVRYRRRTGV
>CYP51B1_Mycobacterium tuberculosis 3499MM 2584876127
MSAVALPRVSGGHDEHGHLEEFRTDPIGLMQRVRDECGDVGTFQLAGKQVVLLSGSHANEFFFRAGDDDLDQA
KAYPFMTPIFGEGVVFDASPERRKEMLHNAALRGEQMKGHAATIEDQVRRMIADWGEAGEIDLLDFFAELTIY
TSSACLIGKKFRDQLDGRFAKLYHELERGTDPLAYVDPYLPIESFRRRDEARNGLVALVADIMNGRIANPPTD
KSDRDMLDVLIAVKAETGTPRFSADEITGMFISMMFAGHHTSSGTASWTLIELMRHRDAYAAVIDELDELYGD
GRSVSFHALRQIPQLENVLKETLRLHPPLIILMRVAKGEFEVQGHRIHEGDLVAASPAISNRIPEDFPDPHDF
VPARYEQPRQEDLLNRWTWIPFGAGRHRCVGAAFAIMQIKAIFSVLLREYEFEMAQPPESYRNDHSKMVVQLA
QPACVRYRRRTGV
>CYP51B1_Mycobacterium tuberculosis 37004 2688156908
MSAVALPRVSGGHDEHGHLEEFRTDPIGLMQRVRDECGDVGTFQLAGKQVVLLSGSHANEFFFRAGDDDLDQA
KAYPFMTPIFGEGVVFDASPERRKEMLHNAALRGEQMKGHAATIEDQVRRMIADWGEAGEIDLLDFFAELTIY
TSSACLIGKKFRDQLDGRFAKLYHELERGTDPLAYVDPYLPIESFRRRDEARNGLVALVADIMNGRIANPPTD
KSDRDMLDVLIAVKAETGTPRFSADEITGMFISMMFAGHHTSSGTASWTLIELMRHRDAYAAVIDELDELYGD
GRSVSFHALRQIPQLENVLKETLRLHPPLIILMRVAKGEFEVQGHRIHEGDLVAASPAISNRIPEDFPDPHDF

VPARYEQPRQEDLLNRWTWIPFGAGRHRCVGAAFAIMQIKAIFSVLLREYEFEMAQPPESYRNDHSKMVVQLA
QPACVRYRRRTGV
>CYP51B1_Mycobacterium tuberculosis 38088 2604025510
MSAVALPRVSGGHDEHGHLEEFRTDPIGLMQRVRDECGDVGTFQLAGKQVVLLSGSHANEFFFRAGDDDLDQA
KAYPFMTPIFGEGVVFDASPERRKEMLHNAALRGEQMKGHAATIEDQVRRMIADWGEAGEIDLLDFFAELTIY
TSSACLIGKKFRDQLDGRFAKLYHELERGTDPLAYVDPYLPIESFRRRDEARNGLVALVADIMNGRIANPPTD
KSDRDMLDVLIAVKAETGTPRFSADEITGMFISMMFAGHHTSSGTASWTLIELMRHRDAYAAVIDELDELYGD
GRSVSFHALRQIPQLENVLKETLRLHPPLIILMRVAKGEFEVQGHRIHEGDLVAASPAISNRIPEDFPDPHDF
VPARYEQPRQEDLLNRWTWIPFGAGRHRCVGAAFAIMQIKAIFSVLLREYEFEMAQPPESYRNDHSKMVVQLA
QPACVRYRRRTGV
>CYP51B1_Mycobacterium tuberculosis 38765 2605419400
MSAVALPRVSGGHDEHGHLEEFRTDPIGLMQRVRDECGDVGTFQLAGKQVVLLSGSHANEFFFRAGDDDLDQA
KAYPFMTPIFGEGVVFDASPERRKEMLHNAALRGEQMKGHAATIEDQVRRMIADWGEAGEIDLLDFFAELTIY
TSSACLIGKKFRDQLDGRFAKLYHELERGTDPLAYVDPYLPIESFRRRDEARNGLVALVADIMNGRIANPPTD
KSDRDMLDVLIAVKAETGTPRFSADEITGMFISMMFAGHHTSSGTASWTLIELMRHRDAYAAVIDELDELYGD
GRSVSFHALRQIPQLENVLKETLRLHPPLIILMRVAKGEFEVQGHRIHEGDLVAASPAISNRIPEDFPDPHDF
VPARYEQPRQEDLLNRWTWIPFGAGRHRCVGAAFAIMQIKAIFSVLLREYEFEMAQPPESYRNDHSKMVVQLA
QPACVRYRRRTGV
>CYP51B1_Mycobacterium tuberculosis 400044 2659630557
MSAVALPRVSGGHDEHGHLEEFRTDPIGLMQRVRDECGDVGTFQLAGKQVVLLSGSHANEFFFRAGDDDLDQA
KAYPFMTPIFGEGVVFDASPERRKEMLHNAALRGEQMKGHAATIEDQVRRMIADWGEAGEIDLLDFFAELTIY
TSSACLIGKKFRDQLDGRFAKLYHELERGTDPLAYVDPYLPIESFRRRDEARNGLVALVADIMNGRIANPPTD
KSDRDMLDVLIAVKAETGTPRFSADEITGMFISMMFAGHHTSSGTASWTLIELMRHRDAYAAVIDELDELYGD
GRSVSFHALRQIPQLENVLKETLRLHPPLIILMRVAKGEFEVQGHRIHEGDLVAASPAISNRIPEDFPDPHDF
VPARYEQPRQEDLLNRWTWIPFGAGRHRCVGAAFAIMQIKAIFSVLLREYEFEMAQPPESYRNDHSKMVVQLA
QPACVRYRRRTGV
>CYP51B1_Mycobacterium tuberculosis 400260 2670878367
MSAVALPRVSGGHDEHGHLEEFRTDPIGLMQRVRDECGDVGTFQLAGKQVVLLSGSHANEFFFRAGDDDLDQA
KAYPFMTPIFGEGVVFDASPERRKEMLHNAALRGEQMKGHAATIEDQVRRMIADWGEAGEIDLLDFFAELTIY
TSSACLIGKKFRDQLDGRFAKLYHELERGTDPLAYVDPYLPIESFRRRDEARNGLVALVADIMNGRIANPPTD
KSDRDMLDVLIAVKAETGTPRFSADEITGMFISMMFAGHHTSSGTASWTLIELMRHRDAYAAVIDELDELYGD
GRSVSFHALRQIPQLENVLKETLRLHPPLIILMRVAKGEFEVQGHRIHEGDLVAASPAISNRIPEDFPDPHDF
VPARYEQPRQEDLLNRWTWIPFGAGRHRCVGAAFAIMQIKAIFSVLLREYEFEMAQPPESYRNDHSKMVVQLA
QPACVRYRRRTGV
>CYP51B1_Mycobacterium tuberculosis 400285 2662949872
MSAVALPRVSGGHDEHGHLEEFRTDPIGLMQRVRDECGDVGTFQLAGKQVVLLSGSHANEFFFRAGDDDLDQA
KAYPFMTPIFGEGVVFDASPERRKEMLHNAALRGEQMKGHAATIEDQVRRMIADWGEAGEIDLLDFFAELTIY
TSSACLIGKKFRDQLDGRFAKLYHELERGTDPLAYVDPYLPIESFRRRDEARNGLVALVADIMNGRIANPPTD
KSDRDMLDVLIAVKAETGTPRFSADEITGMFISMMFAGHHTSSGTASWTLIELMRHRDAYAAVIDELDELYGD
GRSVSFHALRQIPQLENVLKETLRLHPPLIILMRVAKGEFEVQGHRIHEGDLVAASPAISNRIPEDFPDPHDF
VPARYEQPRQEDLLNRWTWIPFGAGRHRCVGAAFAIMQIKAIFSVLLREYEFEMAQPPESYRNDHSKMVVQLA
QPACVRYRRRTGV
>CYP51B1_Mycobacterium tuberculosis 400491 2665700275
MSAVALPRVSGGHDEHGHLEEFRTDPIGLMQRVRDECGDVGTFQLAGKQVVLLSGSHANEFFFRAGDDDLDQA
KAYPFMTPIFGEGVVFDASPERRKEMLHNAALRGEQMKGHAATIEDQVRRMIADWGEAGEIDLLDFFAELTIY
TSSACLIGKKFRDQLDGRFAKLYHELERGTDPLAYVDPYLPIESFRRRDEARNGLVALVADIMNGRIANPPTD
KSDRDMLDVLIAVKAETGTPRFSADEITGMFISMMFAGHHTSSGTASWTLIELMRHRDAYAAVIDELDELYGD
GRSVSFHALRQIPQLENVLKETLRLHPPLIILMRVAKGEFEVQGHRIHEGDLVAASPAISNRIPEDFPDPHDF
VPARYEQPRQEDLLNRWTWIPFGAGRHRCVGAAFAIMQIKAIFSVLLREYEFEMAQPPESYRNDHSKMVVQLA
QPACVRYRRRTGV
>CYP51B1_Mycobacterium tuberculosis 400541 2669077619
MSAVALPRVSGGHDEHGHLEEFRTDPIGLMQRVRDECGDVGTFQLAGKQVVLLSGSHANEFFFRAGDDDLDQA
KAYPFMTPIFGEGVVFDASPERRKEMLHNAALRGEQMKGHAATIEDQVRRMIADWGEAGEIDLLDFFAELTIY
TSSACLIGKKFRDQLDGRFAKLYHELERGTDPLAYVDPYLPIESFRRRDEARNGLVALVADIMNGRIANPPTD
KSDRDMLDVLIAVKAETGTPRFSADEITGMFISMMFAGHHTSSGTASWTLIELMRHRDAYAAVIDELDELYGD
GRSVSFHALRQIPQLENVLKETLRLHPPLIILMRVAKGEFEVQGHRIHEGDLVAASPAISNRIPEDFPDPHDF
VPARYEQPRQEDLLNRWTWIPFGAGRHRCVGAAFAIMQIKAIFSVLLREYEFEMAQPPESYRNDHSKMVVQLA
QPACVRYRRRTGV

>CYP51B1_Mycobacterium tuberculosis 400554 2661356963
MSAVALPRVSGGHDEHGHLEEFRTDPIGLMQRVRDECGDVGTFQLAGKQVVLLSGSHANEFFFRAGDDDLDQA
KAYPFMTPIFGEGVVFDASPERRKEMLHNAALRGEQMKGHAATIEDQVRRMIADWGEAGEIDLLDFFAELTIY
TSSACLIGKKFRDQLDGRFAKLYHELERGTDPLAYVDPYLPIESFRRRDEARNGLVALVADIMNGRIANPPTD
KSDRDMLDVLIAVKAETGTPRFSADEITGMFISMMFAGHHTSSGTASWTLIELMRHRDAYAAVIDELDELYGD
GRSVSFHALRQIPQLENVLKETLRLHPPLIILMRVAKGEFEVQGHRIHEGDLVAASPAISNRIPEDFPDPHDF
VPARYEQPRQEDLLNRWTWIPFGAGRHRCVGAAFAIMQIKAIFSVLLREYEFEMAQPPESYRNDHSKMVVQLA
QPACVRYRRRTGV
>CYP51B1_Mycobacterium tuberculosis 400673 2677273307
MSAVALPRVSGGHDEHGHLEEFRTDPIGLMQRVRDECGDVGTFQLAGKQVVLLSGSHANEFFFRAGDDDLDQA
KAYPFMTPIFGEGVVFDASPERRKEMLHNAALRGEQMKGHAATIEDQVRRMIADWGEAGEIDLLDFFAELTIY
TSSACLIGKKFRDQLDGRFAKLYHELERGTDPLAYVDPYLPIESFRRRDEARNGLVALVADIMNGRIANPPTD
KSDRDMLDVLIAVKAETGTPRFSADEITGMFISMMFAGHHTSSGTASWTLIELMRHRDAYAAVIDELDELYGD
GRSVSFHALRQIPQLENVLKETLRLHPPLIILMRVAKGEFEVQGHRIHEGDLVAASPAISNRIPEDFPDPHDF
VPARYEQPRQEDLLNRWTWIPFGAGRHRCVGAAFAIMQIKAIFSVLLREYEFEMAQPPESYRNDHSKMVVQLA
QPACVRYRRRTGV
>CYP51B1_Mycobacterium tuberculosis 400711 2659512994
MSAVALPRVSGGHDEHGHLEEFRTDPIGLMQRVRDECGDVGTFQLAGKQVVLLSGSHANEFFFRAGDDDLDQA
KAYPFMTPIFGEGVVFDASPERRKEMLHNAALRGEQMKGHAATIEDQVRRMIADWGEAGEIDLLDFFAELTIY
TSSACLIGKKFRDQLDGRFAKLYHELERGTDPLAYVDPYLPIESFRRRDEARNGLVALVADIMNGRIANPPTD
KSDRDMLDVLIAVKAETGTPRFSADEITGMFISMMFAGHHTSSGTASWTLIELMRHRDAYAAVIDELDELYGD
GRSVSFHALRQIPQLENVLKETLRLHPPLIILMRVAKGEFEVQGHRIHEGDLVAASPAISNRIPEDFPDPHDF
VPARYEQPRQEDLLNRWTWIPFGAGRHRCVGAAFAIMQIKAIFSVLLREYEFEMAQPPESYRNDHSKMVVQLA
QPACVRYRRRTGV
>CYP51B1_Mycobacterium tuberculosis 400731 2702145735
MSAVALPRVSGGHDEHGHLEEFRTDPIGLMQRVRDECGDVGTFQLAGKQVVLLSGSHANEFFFRAGDDDLDQA
KAYPFMTPIFGEGVVFDASPERRKEMLHNAALRGEQMKGHAATIEDQVRRMIADWGEAGEIDLLDFFAELTIY
TSSACLIGKKFRDQLDGRFAKLYHELERGTDPLAYVDPYLPIESFRRRDEARNGLVALVADIMNGRIANPPTD
KSDRDMLDVLIAVKAETGTPRFSADEITGMFISMMFAGHHTSSGTASWTLIELMRHRDAYAAVIDELDELYGD
GRSVSFHALRQIPQLENVLKETLRLHPPLIILMRVAKGEFEVQGHRIHEGDLVAASPAISNRIPEDFPDPHDF
VPARYEQPRQEDLLNRWTWIPFGAGRHRCVGAAFAIMQIKAIFSVLLREYEFEMAQPPESYRNDHSKMVVQLA
QPACVRYRRRTGV
>CYP51B1_Mycobacterium tuberculosis 400739 2669995279
MSAVALPRVSGGHDEHGHLEEFRTDPIGLMQRVRDECGDVGTFQLAGKQVVLLSGSHANEFFFRAGDDDLDQA
KAYPFMTPIFGEGVVFDASPERRKEMLHNAALRGEQMKGHAATIEDQVRRMIADWGEAGEIDLLDFFAELTIY
TSSACLIGKKFRDQLDGRFAKLYHELERGTDPLAYVDPYLPIESFRRRDEARNGLVALVADIMNGRIANPPTD
KSDRDMLDVLIAVKAETGTPRFSADEITGMFISMMFAGHHTSSGTASWTLIELMRHRDAYAAVIDELDELYGD
GRSVSFHALRQIPQLENVLKETLRLHPPLIILMRVAKGEFEVQGHRIHEGDLVAASPAISNRIPEDFPDPHDF
VPARYEQPRQEDLLNRWTWIPFGAGRHRCVGAAFAIMQIKAIFSVLLREYEFEMAQPPESYRNDHSKMVVQLA
QPACVRYRRRTGV
>CYP51B1_Mycobacterium tuberculosis 400834 2661869798
MSAVALPRVSGGHDEHGHLEEFRTDPIGLMQRVRDECGDVGTFQLAGKQVVLLSGSHANEFFFRAGDDDLDQA
KAYPFMTPIFGEGVVFDASPERRKEMLHNAALRGEQMKGHAATIEDQVRRMIADWGEAGEIDLLDFFAELTIY
TSSACLIGKKFRDQLDGRFAKLYHELERGTDPLAYVDPYLPIESFRRRDEARNGLVALVADIMNGRIANPPTD
KSDRDMLDVLIAVKAETGTPRFSADEITGMFISMMFAGHHTSSGTASWTLIELMRHRDAYAAVIDELDELYGD
GRSVSFHALRQIPQLENVLKETLRLHPPLIILMRVAKGEFEVQGHRIHEGDLVAASPAISNRIPEDFPDPHDF
VPARYEQPRQEDLLNRWTWIPFGAGRHRCVGAAFAIMQIKAIFSVLLREYEFEMAQPPESYRNDHSKMVVQLA
QPACVRYRRRTGV
>CYP51B1_Mycobacterium tuberculosis 400958 2657564180
MSAVALPRVSGGHDEHGHLEEFRTDPIGLMQRVRDECGDVGTFQLAGKQVVLLSGSHANEFFFRAGDDDLDQA
KAYPFMTPIFGEGVVFDASPERRKEMLHNAALRGEQMKGHAATIEDQVRRMIADWGEAGEIDLLDFFAELTIY
TSSACLIGKKFRDQLDGRFAKLYHELERGTDPLAYVDPYLPIESFRRRDEARNGLVALVADIMNGRIANPPTD
KSDRDMLDVLIAVKAETGTPRFSADEITGMFISMMFAGHHTSSGTASWTLIELMRHRDAYAAVIDELDELYGD
GRSVSFHALRQIPQLENVLKETLRLHPPLIILMRVAKGEFEVQGHRIHEGDLVAASPAISNRIPEDFPDPHDF
VPARYEQPRQEDLLNRWTWIPFGAGRHRCVGAAFAIMQIKAIFSVLLREYEFEMAQPPESYRNDHSKMVVQLA
QPACVRYRRRTGV
>CYP51B1_Mycobacterium tuberculosis 400968 2665486034

MSAVALPRVSGGHDEHGHLEEFRTDPIGLMQRVRDECGDVGTFQLAGKQVVLLSGSHANEFFFRAGDDDLDQA
KAYPFMTPIFGEGVVFDASPERRKEMLHNAALRGEQMKGHAATIEDQVRRMIADWGEAGEIDLLDFFAELTIY
TSSACLIGKKFRDQLDGRFAKLYHELERGTDPLAYVDPYLPIESFRRRDEARNGLVALVADIMNGRIANPPTD
KSDRDMLDVLIAVKAETGTPRFSADEITGMFISMMFAGHHTSSGTASWTLIELMRHRDAYAAVIDELDELYGD
GRSVSFHALRQIPQLENVLKETLRLHPPLIILMRVAKGEFEVQGHRIHEGDLVAASPAISNRIPEDFPDPHDF
VPARYEQPRQEDLLNRWTWIPFGAGRHRCVGAAFAIMQIKAIFSVLLREYEFEMAQPPESYRNDHSKMVVQLA
QPACVRYRRRTGV
>CYP51B1_Mycobacterium tuberculosis 401063 2661645625
MSAVALPRVSGGHDEHGHLEEFRTDPIGLMQRVRDECGDVGTFQLAGKQVVLLSGSHANEFFFRAGDDDLDQA
KAYPFMTPIFGEGVVFDASPERRKEMLHNAALRGEQMKGHAATIEDQVRRMIADWGEAGEIDLLDFFAELTIY
TSSACLIGKKFRDQLDGRFAKLYHELERGTDPLAYVDPYLPIESFRRRDEARNGLVALVADIMNGRIANPPTD
KSDRDMLDVLIAVKAETGTPRFSADEITGMFISMMFAGHHTSSGTASWTLIELMRHRDAYAAVIDELDELYGD
GRSVSFHALRQIPQLENVLKETLRLHPPLIILMRVAKGEFEVQGHRIHEGDLVAASPAISNRIPEDFPDPHDF
VPARYEQPRQEDLLNRWTWIPFGAGRHRCVGAAFAIMQIKAIFSVLLREYEFEMAQPPESYRNDHSKMVVQLA
QPACVRYRRRTGV
>CYP51B1_Mycobacterium tuberculosis 401206 2702154104
MSAVALPRVSGGHDEHGHLEEFRTDPIGLMQRVRDECGDVGTFQLAGKQVVLLSGSHANEFFFRAGDDDLDQA
KAYPFMTPIFGEGVVFDASPERRKEMLHNAALRGEQMKGHAATIEDQVRRMIADWGEAGEIDLLDFFAELTIY
TSSACLIGKKFRDQLDGRFAKLYHELERGTDPLAYVDPYLPIESFRRRDEARNGLVALVADIMNGRIANPPTD
KSDRDMLDVLIAVKAETGTPRFSADEITGMFISMMFAGHHTSSGTASWTLIELMRHRDAYAAVIDELDELYGD
GRSVSFHALRQIPQLENVLKETLRLHPPLIILMRVAKGEFEVQGHRIHEGDLVAASPAISNRIPEDFPDPHDF
VPARYEQPRQEDLLNRWTWIPFGAGRHRCVGAAFAIMQIKAIFSVLLREYEFEMAQPPESYRNDHSKMVVQLA
QPACVRYRRRTGV
>CYP51B1_Mycobacterium tuberculosis 401212 2666216815
MSAVALPRVSGGHDEHGHLEEFRTDPIGLMQRVRDECGDVGTFQLAGKQVVLLSGSHANEFFFRAGDDDLDQA
KAYPFMTPIFGEGVVFDASPERRKEMLHNAALRGEQMKGHAATIEDQVRRMIADWGEAGEIDLLDFFAELTIY
TSSACLIGKKFRDQLDGRFAKLYHELERGTDPLAYVDPYLPIESFRRRDEARNGLVALVADIMNGRIANPPTD
KSDRDMLDVLIAVKAETGTPRFSADEITGMFISMMFAGHHTSSGTASWTLIELMRHRDAYAAVIDELDELYGD
GRSVSFHALRQIPQLENVLKETLRLHPPLIILMRVAKGEFEVQGHRIHEGDLVAASPAISNRIPEDFPDPHDF
VPARYEQPRQEDLLNRWTWIPFGAGRHRCVGAAFAIMQIKAIFSVLLREYEFEMAQPPESYRNDHSKMVVQLA
QPACVRYRRRTGV
>CYP51B1_Mycobacterium tuberculosis 401243 2702170630
MSAVALPRVSGGHDEHGHLEEFRTDPIGLMQRVRDECGDVGTFQLAGKQVVLLSGSHANEFFFRAGDDDLDQA
KAYPFMTPIFGEGVVFDASPERRKEMLHNAALRGEQMKGHAATIEDQVRRMIADWGEAGEIDLLDFFAELTIY
TSSACLIGKKFRDQLDGRFAKLYHELERGTDPLAYVDPYLPIESFRRRDEARNGLVALVADIMNGRIANPPTD
KSDRDMLDVLIAVKAETGTPRFSADEITGMFISMMFAGHHTSSGTASWTLIELMRHRDAYAAVIDELDELYGD
GRSVSFHALRQIPQLENVLKETLRLHPPLIILMRVAKGEFEVQGHRIHEGDLVAASPAISNRIPEDFPDPHDF
VPARYEQPRQEDLLNRWTWIPFGAGRHRCVGAAFAIMQIKAIFSVLLREYEFEMAQPPESYRNDHSKMVVQLA
QPACVRYRRRTGV
>CYP51B1_Mycobacterium tuberculosis 401325 2657826033
MSAVALPRVSGGHDEHGHLEEFRTDPIGLMQRVRDECGDVGTFQLAGKQVVLLSGSHANEFFFRAGDDDLDQA
KAYPFMTPIFGEGVVFDASPERRKEMLHNAALRGEQMKGHAATIEDQVRRMIADWGEAGEIDLLDFFAELTIY
TSSACLIGKKFRDQLDGRFAKLYHELERGTDPLAYVDPYLPIESFRRRDEARNGLVALVADIMNGRIANPPTD
KSDRDMLDVLIAVKAETGTPRFSADEITGMFISMMFAGHHTSSGTASWTLIELMRHRDAYAAVIDELDELYGD
GRSVSFHALRQIPQLENVLKETLRLHPPLIILMRVAKGEFEVQGHRIHEGDLVAASPAISNRIPEDFPDPHDF
VPARYEQPRQEDLLNRWTWIPFGAGRHRCVGAAFAIMQIKAIFSVLLREYEFEMAQPPESYRNDHSKMVVQLA
QPACVRYRRRTGV
>CYP51B1_Mycobacterium tuberculosis 401391 2661519270
MSAVALPRVSGGHDEHGHLEEFRTDPIGLMQRVRDECGDVGTFQLAGKQVVLLSGSHANEFFFRAGDDDLDQA
KAYPFMTPIFGEGVVFDASPERRKEMLHNAALRGEQMKGHAATIEDQVRRMIADWGEAGEIDLLDFFAELTIY
TSSACLIGKKFRDQLDGRFAKLYHELERGTDPLAYVDPYLPIESFRRRDEARNGLVALVADIMNGRIANPPTD
KSDRDMLDVLIAVKAETGTPRFSADEITGMFISMMFAGHHTSSGTASWTLIELMRHRDAYAAVIDELDELYGD
GRSVSFHALRQIPQLENVLKETLRLHPPLIILMRVAKGEFEVQGHRIHEGDLVAASPAISNRIPEDFPDPHDF
VPARYEQPRQEDLLNRWTWIPFGAGRHRCVGAAFAIMQIKAIFSVLLREYEFEMAQPPESYRNDHSKMVVQLA
QPACVRYRRRTGV
>CYP51B1_Mycobacterium tuberculosis 401539 2665357055
MSAVALPRVSGGHDEHGHLEEFRTDPIGLMQRVRDECGDVGTFQLAGKQVVLLSGSHANEFFFRAGDDDLDQA
KAYPFMTPIFGEGVVFDASPERRKEMLHNAALRGEQMKGHAATIEDQVRRMIADWGEAGEIDLLDFFAELTIY

TSSACLIGKKFRDQLDGRFAKLYHELERGTDPLAYVDPYLPIESFRRRDEARNGLVALVADIMNGRIANPPTD
KSDRDMLDVLIAVKAETGTPRFSADEITGMFISMMFAGHHTSSGTASWTLIELMRHRDAYAAVIDELDELYGD
GRSVSFHALRQIPQLENVLKETLRLHPPLIILMRVAKGEFEVQGHRIHEGDLVAASPAISNRIPEDFPDPHDF
VPARYEQPRQEDLLNRWTWIPFGAGRHRCVGAAFAIMQIKAIFSVLLREYEFEMAQPPESYRNDHSKMVVQLA
QPACVRYRRRTGV
>CYP51B1_Mycobacterium tuberculosis 401555 2701694949
MSAVALPRVSGGHDEHGHLEEFRTDPIGLMQRVRDECGDVGTFQLAGKQVVLLSGSHANEFFFRAGDDDLDQA
KAYPFMTPIFGEGVVFDASPERRKEMLHNAALRGEQMKGHAATIEDQVRRMIADWGEAGEIDLLDFFAELTIY
TSSACLIGKKFRDQLDGRFAKLYHELERGTDPLAYVDPYLPIESFRRRDEARNGLVALVADIMNGRIANPPTD
KSDRDMLDVLIAVKAETGTPRFSADEITGMFISMMFAGHHTSSGTASWTLIELMRHRDAYAAVIDELDELYGD
GRSVSFHALRQIPQLENVLKETLRLHPPLIILMRVAKGEFEVQGHRIHEGDLVAASPAISNRIPEDFPDPHDF
VPARYEQPRQEDLLNRWTWIPFGAGRHRCVGAAFAIMQIKAIFSVLLREYEFEMAQPPESYRNDHSKMVVQLA
QPACVRYRRRTGV
>CYP51B1_Mycobacterium tuberculosis 401594 2701698800
MSAVALPRVSGGHDEHGHLEEFRTDPIGLMQRVRDECGDVGTFQLAGKQVVLLSGSHANEFFFRAGDDDLDQA
KAYPFMTPIFGEGVVFDASPERRKEMLHNAALRGEQMKGHAATIEDQVRRMIADWGEAGEIDLLDFFAELTIY
TSSACLIGKKFRDQLDGRFAKLYHELERGTDPLAYVDPYLPIESFRRRDEARNGLVALVADIMNGRIANPPTD
KSDRDMLDVLIAVKAETGTPRFSADEITGMFISMMFAGHHTSSGTASWTLIELMRHRDAYAAVIDELDELYGD
GRSVSFHALRQIPQLENVLKETLRLHPPLIILMRVAKGEFEVQGHRIHEGDLVAASPAISNRIPEDFPDPHDF
VPARYEQPRQEDLLNRWTWIPFGAGRHRCVGAAFAIMQIKAIFSVLLREYEFEMAQPPESYRNDHSKMVVQLA
QPACVRYRRRTGV
>CYP51B1_Mycobacterium tuberculosis 401787 2668756364
MSAVALPRVSGGHDEHGHLEEFRTDPIGLMQRVRDECGDVGTFQLAGKQVVLLSGSHANEFFFRAGDDDLDQA
KAYPFMTPIFGEGVVFDASPERRKEMLHNAALRGEQMKGHAATIEDQVRRMIADWGEAGEIDLLDFFAELTIY
TSSACLIGKKFRDQLDGRFAKLYHELERGTDPLAYVDPYLPIESFRRRDEARNGLVALVADIMNGRIANPPTD
KSDRDMLDVLIAVKAETGTPRFSADEITGMFISMMFAGHHTSSGTASWTLIELMRHRDAYAAVIDELDELYGD
GRSVSFHALRQIPQLENVLKETLRLHPPLIILMRVAKGEFEVQGHRIHEGDLVAASPAISNRIPEDFPDPHDF
VPARYEQPRQEDLLNRWTWIPFGAGRHRCVGAAFAIMQIKAIFSVLLREYEFEMAQPPESYRNDHSKMVVQLA
QPACVRYRRRTGV
>CYP51B1_Mycobacterium tuberculosis 402106 2659853953
MSAVALPRVSGGHDEHGHLEEFRTDPIGLMQRVRDECGDVGTFQLAGKQVVLLSGSHANEFFFRAGDDDLDQA
KAYPFMTPIFGEGVVFDASPERRKEMLHNAALRGEQMKGHAATIEDQVRRMIADWGEAGEIDLLDFFAELTIY
TSSACLIGKKFRDQLDGRFAKLYHELERGTDPLAYVDPYLPIESFRRRDEARNGLVALVADIMNGRIANPPTD
KSDRDMLDVLIAVKAETGTPRFSADEITGMFISMMFAGHHTSSGTASWTLIELMRHRDAYAAVIDELDELYGD
GRSVSFHALRQIPQLENVLKETLRLHPPLIILMRVAKGEFEVQGHRIHEGDLVAASPAISNRIPEDFPDPHDF
VPARYEQPRQEDLLNRWTWIPFGAGRHRCVGAAFAIMQIKAIFSVLLREYEFEMAQPPESYRNDHSKMVVQLA
QPACVRYRRRTGV
>CYP51B1_Mycobacterium tuberculosis 402138 2662945829
MSAVALPRVSGGHDEHGHLEEFRTDPIGLMQRVRDECGDVGTFQLAGKQVVLLSGSHANEFFFRAGDDDLDQA
KAYPFMTPIFGEGVVFDASPERRKEMLHNAALRGEQMKGHAATIEDQVRRMIADWGEAGEIDLLDFFAELTIY
TSSACLIGKKFRDQLDGRFAKLYHELERGTDPLAYVDPYLPIESFRRRDEARNGLVALVADIMNGRIANPPTD
KSDRDMLDVLIAVKAETGTPRFSADEITGMFISMMFAGHHTSSGTASWTLIELMRHRDAYAAVIDELDELYGD
GRSVSFHALRQIPQLENVLKETLRLHPPLIILMRVAKGEFEVQGHRIHEGDLVAASPAISNRIPEDFPDPHDF
VPARYEQPRQEDLLNRWTWIPFGAGRHRCVGAAFAIMQIKAIFSVLLREYEFEMAQPPESYRNDHSKMVVQLA
QPACVRYRRRTGV
>CYP51B1_Mycobacterium tuberculosis 402206 2657633495
MSAVALPRVSGGHDEHGHLEEFRTDPIGLMQRVRDECGDVGTFQLAGKQVVLLSGSHANEFFFRAGDDDLDQA
KAYPFMTPIFGEGVVFDASPERRKEMLHNAALRGEQMKGHAATIEDQVRRMIADWGEAGEIDLLDFFAELTIY
TSSACLIGKKFRDQLDGRFAKLYHELERGTDPLAYVDPYLPIESFRRRDEARNGLVALVADIMNGRIANPPTD
KSDRDMLDVLIAVKAETGTPRFSADEITGMFISMMFAGHHTSSGTASWTLIELMRHRDAYAAVIDELDELYGD
GRSVSFHALRQIPQLENVLKETLRLHPPLIILMRVAKGEFEVQGHRIHEGDLVAASPAISNRIPEDFPDPHDF
VPARYEQPRQEDLLNRWTWIPFGAGRHRCVGAAFAIMQIKAIFSVLLREYEFEMAQPPESYRNDHSKMVVQLA
QPACVRYRRRTGV
>CYP51B1_Mycobacterium tuberculosis 402248 2659773418
MSAVALPRVSGGHDEHGHLEEFRTDPIGLMQRVRDECGDVGTFQLAGKQVVLLSGSHANEFFFRAGDDDLDQA
KAYPFMTPIFGEGVVFDASPERRKEMLHNAALRGEQMKGHAATIEDQVRRMIADWGEAGEIDLLDFFAELTIY
TSSACLIGKKFRDQLDGRFAKLYHELERGTDPLAYVDPYLPIESFRRRDEARNGLVALVADIMNGRIANPPTD
KSDRDMLDVLIAVKAETGTPRFSADEITGMFISMMFAGHHTSSGTASWTLIELMRHRDAYAAVIDELDELYGD

GRSVSFHALRQIPQLENVLKETLRLHPPLIILMRVAKGEFEVQGHRIHEGDLVAASPAISNRIPEDFPDPHDF
VPARYEQPRQEDLLNRWTWIPFGAGRHRCVGAAFAIMQIKAIFSVLLREYEFEMAQPPESYRNDHSKMVVQLA
QPACVRYRRRTGV
>CYP51B1_Mycobacterium tuberculosis 402251 2701659134
MSAVALPRVSGGHDEHGHLEEFRTDPIGLMQRVRDECGDVGTFQLAGKQVVLLSGSHANEFFFRAGDDDLDQA
KAYPFMTPIFGEGVVFDASPERRKEMLHNAALRGEQMKGHAATIEDQVRRMIADWGEAGEIDLLDFFAELTIY
TSSACLIGKKFRDQLDGRFAKLYHELERGTDPLAYVDPYLPIESFRRRDEARNGLVALVADIMNGRIANPPTD
KSDRDMLDVLIAVKAETGTPRFSADEITGMFISMMFAGHHTSSGTASWTLIELMRHRDAYAAVIDELDELYGD
GRSVSFHALRQIPQLENVLKETLRLHPPLIILMRVAKGEFEVQGHRIHEGDLVAASPAISNRIPEDFPDPHDF
VPARYEQPRQEDLLNRWTWIPFGAGRHRCVGAAFAIMQIKAIFSVLLREYEFEMAQPPESYRNDHSKMVVQLA
QPACVRYRRRTGV
>CYP51B1_Mycobacterium tuberculosis 402267 2692006190
MSAVALPRVSGGHDEHGHLEEFRTDPIGLMQRVRDECGDVGTFQLAGKQVVLLSGSHANEFFFRAGDDDLDQA
KAYPFMTPIFGEGVVFDASPERRKEMLHNAALRGEQMKGHAATIEDQVRRMIADWGEAGEIDLLDFFAELTIY
TSSACLIGKKFRDQLDGRFAKLYHELERGTDPLAYVDPYLPIESFRRRDEARNGLVALVADIMNGRIANPPTD
KSDRDMLDVLIAVKAETGTPRFSADEITGMFISMMFAGHHTSSGTASWTLIELMRHRDAYAAVIDELDELYGD
GRSVSFHALRQIPQLENVLKETLRLHPPLIILMRVAKGEFEVQGHRIHEGDLVAASPAISNRIPEDFPDPHDF
VPARYEQPRQEDLLNRWTWIPFGAGRHRCVGAAFAIMQIKAIFSVLLREYEFEMAQPPESYRNDHSKMVVQLA
QPACVRYRRRTGV
>CYP51B1_Mycobacterium tuberculosis 402321 2701651713
MSAVALPRVSGGHDEHGHLEEFRTDPIGLMQRVRDECGDVGTFQLAGKQVVLLSGSHANEFFFRAGDDDLDQA
KAYPFMTPIFGEGVVFDASPERRKEMLHNAALRGEQMKGHAATIEDQVRRMIADWGEAGEIDLLDFFAELTIY
TSSACLIGKKFRDQLDGRFAKLYHELERGTDPLAYVDPYLPIESFRRRDEARNGLVALVADIMNGRIANPPTD
KSDRDMLDVLIAVKAETGTPRFSADEITGMFISMMFAGHHTSSGTASWTLIELMRHRDAYAAVIDELDELYGD
GRSVSFHALRQIPQLENVLKETLRLHPPLIILMRVAKGEFEVQGHRIHEGDLVAASPAISNRIPEDFPDPHDF
VPARYEQPRQEDLLNRWTWIPFGAGRHRCVGAAFAIMQIKAIFSVLLREYEFEMAQPPESYRNDHSKMVVQLA
QPACVRYRRRTGV
>CYP51B1_Mycobacterium tuberculosis 402431 2657780848
MSAVALPRVSGGHDEHGHLEEFRTDPIGLMQRVRDECGDVGTFQLAGKQVVLLSGSHANEFFFRAGDDDLDQA
KAYPFMTPIFGEGVVFDASPERRKEMLHNAALRGEQMKGHAATIEDQVRRMIADWGEAGEIDLLDFFAELTIY
TSSACLIGKKFRDQLDGRFAKLYHELERGTDPLAYVDPYLPIESFRRRDEARNGLVALVADIMNGRIANPPTD
KSDRDMLDVLIAVKAETGTPRFSADEITGMFISMMFAGHHTSSGTASWTLIELMRHRDAYAAVIDELDELYGD
GRSVSFHALRQIPQLENVLKETLRLHPPLIILMRVAKGEFEVQGHRIHEGDLVAASPAISNRIPEDFPDPHDF
VPARYEQPRQEDLLNRWTWIPFGAGRHRCVGAAFAIMQIKAIFSVLLREYEFEMAQPPESYRNDHSKMVVQLA
QPACVRYRRRTGV
>CYP51B1_Mycobacterium tuberculosis 402495 2665904067
MSAVALPRVSGGHDEHGHLEEFRTDPIGLMQRVRDECGDVGTFQLAGKQVVLLSGSHANEFFFRAGDDDLDQA
KAYPFMTPIFGEGVVFDASPERRKEMLHNAALRGEQMKGHAATIEDQVRRMIADWGEAGEIDLLDFFAELTIY
TSSACLIGKKFRDQLDGRFAKLYHELERGTDPLAYVDPYLPIESFRRRDEARNGLVALVADIMNGRIANPPTD
KSDRDMLDVLIAVKAETGTPRFSADEITGMFISMMFAGHHTSSGTASWTLIELMRHRDAYAAVIDELDELYGD
GRSVSFHALRQIPQLENVLKETLRLHPPLIILMRVAKGEFEVQGHRIHEGDLVAASPAISNRIPEDFPDPHDF
VPARYEQPRQEDLLNRWTWIPFGAGRHRCVGAAFAIMQIKAIFSVLLREYEFEMAQPPESYRNDHSKMVVQLA
QPACVRYRRRTGV
>CYP51B1_Mycobacterium tuberculosis 402542 2678022896
MSAVALPRVSGGHDEHGHLEEFRTDPIGLMQRVRDECGDVGTFQLAGKQVVLLSGSHANEFFFRAGDDDLDQA
KAYPFMTPIFGEGVVFDASPERRKEMLHNAALRGEQMKGHAATIEDQVRRMIADWGEAGEIDLLDFFAELTIY
TSSACLIGKKFRDQLDGRFAKLYHELERGTDPLAYVDPYLPIESFRRRDEARNGLVALVADIMNGRIANPPTD
KSDRDMLDVLIAVKAETGTPRFSADEITGMFISMMFAGHHTSSGTASWTLIELMRHRDAYAAVIDELDELYGD
GRSVSFHALRQIPQLENVLKETLRLHPPLIILMRVAKGEFEVQGHRIHEGDLVAASPAISNRIPEDFPDPHDF
VPARYEQPRQEDLLNRWTWIPFGAGRHRCVGAAFAIMQIKAIFSVLLREYEFEMAQPPESYRNDHSKMVVQLA
QPACVRYRRRTGV
>CYP51B1_Mycobacterium tuberculosis 402568 2665029148
MSAVALPRVSGGHDEHGHLEEFRTDPIGLMQRVRDECGDVGTFQLAGKQVVLLSGSHANEFFFRAGDDDLDQA
KAYPFMTPIFGEGVVFDASPERRKEMLHNAALRGEQMKGHAATIEDQVRRMIADWGEAGEIDLLDFFAELTIY
TSSACLIGKKFRDQLDGRFAKLYHELERGTDPLAYVDPYLPIESFRRRDEARNGLVALVADIMNGRIANPPTD
KSDRDMLDVLIAVKAETGTPRFSADEITGMFISMMFAGHHTSSGTASWTLIELMRHRDAYAAVIDELDELYGD
GRSVSFHALRQIPQLENVLKETLRLHPPLIILMRVAKGEFEVQGHRIHEGDLVAASPAISNRIPEDFPDPHDF

VPARYEQPRQEDLLNRWTWIPFGAGRHRCVGAAFAIMQIKAIFSVLLREYEFEMAQPPESYRNDHSKMVVQLA
QPACVRYRRRTGV
>CYP51B1_Mycobacterium tuberculosis 402629 2658125770
MSAVALPRVSGGHDEHGHLEEFRTDPIGLMQRVRDECGDVGTFQLAGKQVVLLSGSHANEFFFRAGDDDLDQA
KAYPFMTPIFGEGVVFDASPERRKEMLHNAALRGEQMKGHAATIEDQVRRMIADWGEAGEIDLLDFFAELTIY
TSSACLIGKKFRDQLDGRFAKLYHELERGTDPLAYVDPYLPIESFRRRDEARNGLVALVADIMNGRIANPPTD
KSDRDMLDVLIAVKAETGTPRFSADEITGMFISMMFAGHHTSSGTASWTLIELMRHRDAYAAVIDELDELYGD
GRSVSFHALRQIPQLENVLKETLRLHPPLIILMRVAKGEFEVQGHRIHEGDLVAASPAISNRIPEDFPDPHDF
VPARYEQPRQEDLLNRWTWIPFGAGRHRCVGAAFAIMQIKAIFSVLLREYEFEMAQPPESYRNDHSKMVVQLA
QPACVRYRRRTGV
>CYP51B1_Mycobacterium tuberculosis 402713 2701673881
MSAVALPRVSGGHDEHGHLEEFRTDPIGLMQRVRDECGDVGTFQLAGKQVVLLSGSHANEFFFRAGDDDLDQA
KAYPFMTPIFGEGVVFDASPERRKEMLHNAALRGEQMKGHAATIEDQVRRMIADWGEAGEIDLLDFFAELTIY
TSSACLIGKKFRDQLDGRFAKLYHELERGTDPLAYVDPYLPIESFRRRDEARNGLVALVADIMNGRIANPPTD
KSDRDMLDVLIAVKAETGTPRFSADEITGMFISMMFAGHHTSSGTASWTLIELMRHRDAYAAVIDELDELYGD
GRSVSFHALRQIPQLENVLKETLRLHPPLIILMRVAKGEFEVQGHRIHEGDLVAASPAISNRIPEDFPDPHDF
VPARYEQPRQEDLLNRWTWIPFGAGRHRCVGAAFAIMQIKAIFSVLLREYEFEMAQPPESYRNDHSKMVVQLA
QPACVRYRRRTGV
>CYP51B1_Mycobacterium tuberculosis 402797 2701682129
MSAVALPRVSGGHDEHGHLEEFRTDPIGLMQRVRDECGDVGTFQLAGKQVVLLSGSHANEFFFRAGDDDLDQA
KAYPFMTPIFGEGVVFDASPERRKEMLHNAALRGEQMKGHAATIEDQVRRMIADWGEAGEIDLLDFFAELTIY
TSSACLIGKKFRDQLDGRFAKLYHELERGTDPLAYVDPYLPIESFRRRDEARNGLVALVADIMNGRIANPPTD
KSDRDMLDVLIAVKAETGTPRFSADEITGMFISMMFAGHHTSSGTASWTLIELMRHRDAYAAVIDELDELYGD
GRSVSFHALRQIPQLENVLKETLRLHPPLIILMRVAKGEFEVQGHRIHEGDLVAASPAISNRIPEDFPDPHDF
VPARYEQPRQEDLLNRWTWIPFGAGRHRCVGAAFAIMQIKAIFSVLLREYEFEMAQPPESYRNDHSKMVVQLA
QPACVRYRRRTGV
>CYP51B1_Mycobacterium tuberculosis 43-13838 2702446618
MSAVALPRVSGGHDEHGHLEEFRTDPIGLMQRVRDECGDVGTFQLAGKQVVLLSGSHANEFFFRAGDDDLDQA
KAYPFMTPIFGEGVVFDASPERRKEMLHNAALRGEQMKGHAATIEDQVRRMIADWGEAGEIDLLDFFAELTIY
TSSACLIGKKFRDQLDGRFAKLYHELERGTDPLAYVDPYLPIESFRRRDEARNGLVALVADIMNGRIANPPTD
KSDRDMLDVLIAVKAETGTPRFSADEITGMFISMMFAGHHTSSGTASWTLIELMRHRDAYAAVIDELDELYGD
GRSVSFHALRQIPQLENVLKETLRLHPPLIILMRVAKGEFEVQGHRIHEGDLVAASPAISNRIPEDFPDPHDF
VPARYEQPRQEDLLNRWTWIPFGAGRHRCVGAAFAIMQIKAIFSVLLREYEFEMAQPPESYRNDHSKMVVQLA
QPACVRYRRRTGV
>CYP51B1_Mycobacterium tuberculosis 43-16836 2603127681
MSAVALPRVSGGHDEHGHLEEFRTDPIGLMQRVRDECGDVGTFQLAGKQVVLLSGSHANEFFFRAGDDDLDQA
KAYPFMTPIFGEGVVFDASPERRKEMLHNAALRGEQMKGHAATIEDQVRRMIADWGEAGEIDLLDFFAELTIY
TSSACLIGKKFRDQLDGRFAKLYHELERGTDPLAYVDPYLPIESFRRRDEARNGLVALVADIMNGRIANPPTD
KSDRDMLDVLIAVKAETGTPRFSADEITGMFISMMFAGHHTSSGTASWTLIELMRHRDAYAAVIDELDELYGD
GRSVSFHALRQIPQLENVLKETLRLHPPLIILMRVAKGEFEVQGHRIHEGDLVAASPAISNRIPEDFPDPHDF
VPARYEQPRQEDLLNRWTWIPFGAGRHRCVGAAFAIMQIKAIFSVLLREYEFEMAQPPESYRNDHSKMVVQLA
QPACVRYRRRTGV
>CYP51B1_Mycobacterium tuberculosis 44503 2584615055
MSAVALPRVSGGHDEHGHLEEFRTDPIGLMQRVRDECGDVGTFQLAGKQVVLLSGSHANEFFFRAGDDDLDQA
KAYPFMTPIFGEGVVFDASPERRKEMLHNAALRGEQMKGHAATIEDQVRRMIADWGEAGEIDLLDFFAELTIY
TSSACLIGKKFRDQLDGRFAKLYHELERGTDPLAYVDPYLPIESFRRRDEARNGLVALVADIMNGRIANPPTD
KSDRDMLDVLIAVKAETGTPRFSADEITGMFISMMFAGHHTSSGTASWTLIELMRHRDAYAAVIDELDELYGD
GRSVSFHALRQIPQLENVLKETLRLHPPLIILMRVAKGEFEVQGHRIHEGDLVAASPAISNRIPEDFPDPHDF
VPARYEQPRQEDLLNRWTWIPFGAGRHRCVGAAFAIMQIKAIFSVLLREYEFEMAQPPESYRNDHSKMVVQLA
QPACVRYRRRTGV
>CYP51B1_Mycobacterium tuberculosis 46-5069 2702448648
MSAVALPRVSGGHDEHGHLEEFRTDPIGLMQRVRDECGDVGTFQLAGKQVVLLSGSHANEFFFRAGDDDLDQA
KAYPFMTPIFGEGVVFDASPERRKEMLHNAALRGEQMKGHAATIEDQVRRMIADWGEAGEIDLLDFFAELTIY
TSSACLIGKKFRDQLDGRFAKLYHELERGTDPLAYVDPYLPIESFRRRDEARNGLVALVADIMNGRIANPPTD
KSDRDMLDVLIAVKAETGTPRFSADEITGMFISMMFAGHHTSSGTASWTLIELMRHRDAYAAVIDELDELYGD
GRSVSFHALRQIPQLENVLKETLRLHPPLIILMRVAKGEFEVQGHRIHEGDLVAASPAISNRIPEDFPDPHDF
VPARYEQPRQEDLLNRWTWIPFGAGRHRCVGAAFAIMQIKAIFSVLLREYEFEMAQPPESYRNDHSKMVVQLA
QPACVRYRRRTGV

>CYP51B1_Mycobacterium tuberculosis 49-02 2687652029
MSAVALPRVSGGHDEHGHLEEFRTDPIGLMQRVRDECGDVGTFQLAGKQVVLLSGSHANEFFFRAGDDDLDQA
KAYPFMTPIFGEGVVFDASPERRKEMLHNAALRGEQMKGHAATIEDQVRRMIADWGEAGEIDLLDFFAELTIY
TSSACLIGKKFRDQLDGRFAKLYHELERGTDPLAYVDPYLPIESFRRRDEARNGLVALVADIMNGRIANPPTD
KSDRDMLDVLIAVKAETGTPRFSADEITGMFISMMFAGHHTSSGTASWTLIELMRHRDAYAAVIDELDELYGD
GRSVSFHALRQIPQLENVLKETLRLHPPLIILMRVAKGEFEVQGHRIHEGDLVAASPAISNRIPEDFPDPHDF
VPARYEQPRQEDLLNRWTWIPFGAGRHRCVGAAFAIMQIKAIFSVLLREYEFEMAQPPESYRNDHSKMVVQLA
QPACVRYRRRTGV
>CYP51B1_Mycobacterium tuberculosis 49375 2577432016
MSAVALPRVSGGHDEHGHLEEFRTDPIGLMQRVRDECGDVGTFQLAGKQVVLLSGSHANEFFFRAGDDDLDQA
KAYPFMTPIFGEGVVFDASPERRKEMLHNAALRGEQMKGHAATIEDQVRRMIADWGEAGEIDLLDFFAELTIY
TSSACLIGKKFRDQLDGRFAKLYHELERGTDPLAYVDPYLPIESFRRRDEARNGLVALVADIMNGRIANPPTD
KSDRDMLDVLIAVKAETGTPRFSADEITGMFISMMFAGHHTSSGTASWTLIELMRHRDAYAAVIDELDELYGD
GRSVSFHALRQIPQLENVLKETLRLHPPLIILMRVAKGEFEVQGHRIHEGDLVAASPAISNRIPEDFPDPHDF
VPARYEQPRQEDLLNRWTWIPFGAGRHRCVGAAFAIMQIKAIFSVLLREYEFEMAQPPESYRNDHSKMVVQLA
QPACVRYRRRTGV
>CYP51B1_Mycobacterium tuberculosis 51628 2584639578
MSAVALPRVSGGHDEHGHLEEFRTDPIGLMQRVRDECGDVGTFQLAGKQVVLLSGSHANEFFFRAGDDDLDQA
KAYPFMTPIFGEGVVFDASPERRKEMLHNAALRGEQMKGHAATIEDQVRRMIADWGEAGEIDLLDFFAELTIY
TSSACLIGKKFRDQLDGRFAKLYHELERGTDPLAYVDPYLPIESFRRRDEARNGLVALVADIMNGRIANPPTD
KSDRDMLDVLIAVKAETGTPRFSADEITGMFISMMFAGHHTSSGTASWTLIELMRHRDAYAAVIDELDELYGD
GRSVSFHALRQIPQLENVLKETLRLHPPLIILMRVAKGEFEVQGHRIHEGDLVAASPAISNRIPEDFPDPHDF
VPARYEQPRQEDLLNRWTWIPFGAGRHRCVGAAFAIMQIKAIFSVLLREYEFEMAQPPESYRNDHSKMVVQLA
QPACVRYRRRTGV
>CYP51B1_Mycobacterium tuberculosis 6273 2621710657
MSAVALPRVSGGHDEHGHLEEFRTDPIGLMQRVRDECGDVGTFQLAGKQVVLLSGSHANEFFFRAGDDDLDQA
KAYPFMTPIFGEGVVFDASPERRKEMLHNAALRGEQMKGHAATIEDQVRRMIADWGEAGEIDLLDFFAELTIY
TSSACLIGKKFRDQLDGRFAKLYHELERGTDPLAYVDPYLPIESFRRRDEARNGLVALVADIMNGRIANPPTD
KSDRDMLDVLIAVKAETGTPRFSADEITGMFISMMFAGHHTSSGTASWTLIELMRHRDAYAAVIDELDELYGD
GRSVSFHALRQIPQLENVLKETLRLHPPLIILMRVAKGEFEVQGHRIHEGDLVAASPAISNRIPEDFPDPHDF
VPARYEQPRQEDLLNRWTWIPFGAGRHRCVGAAFAIMQIKAIFSVLLREYEFEMAQPPESYRNDHSKMVVQLA
QPACVRYRRRTGV
>CYP51B1_Mycobacterium tuberculosis 6638 2621702637
MSAVALPRVSGGHDEHGHLEEFRTDPIGLMQRVRDECGDVGTFQLAGKQVVLLSGSHANEFFFRAGDDDLDQA
KAYPFMTPIFGEGVVFDASPERRKEMLHNAALRGEQMKGHAATIEDQVRRMIADWGEAGEIDLLDFFAELTIY
TSSACLIGKKFRDQLDGRFAKLYHELERGTDPLAYVDPYLPIESFRRRDEARNGLVALVADIMNGRIANPPTD
KSDRDMLDVLIAVKAETGTPRFSADEITGMFISMMFAGHHTSSGTASWTLIELMRHRDAYAAVIDELDELYGD
GRSVSFHALRQIPQLENVLKETLRLHPPLIILMRVAKGEFEVQGHRIHEGDLVAASPAISNRIPEDFPDPHDF
VPARYEQPRQEDLLNRWTWIPFGAGRHRCVGAAFAIMQIKAIFSVLLREYEFEMAQPPESYRNDHSKMVVQLA
QPACVRYRRRTGV
>CYP51B1_Mycobacterium tuberculosis 6A024XDR 2720081784
MSAVALPRVSGGHDEHGHLEEFRTDPIGLMQRVRDECGDVGTFQLAGKQVVLLSGSHANEFFFRAGDDDLDQA
KAYPFMTPIFGEGVVFDASPERRKEMLHNAALRGEQMKGHAATIEDQVRRMIADWGEAGEIDLLDFFAELTIY
TSSACLIGKKFRDQLDGRFAKLYHELERGTDPLAYVDPYLPIESFRRRDEARNGLVALVADIMNGRIANPPTD
KSDRDMLDVLIAVKAETGTPRFSADEITGMFISMMFAGHHTSSGTASWTLIELMRHRDAYAAVIDELDELYGD
GRSVSFHALRQIPQLENVLKETLRLHPPLIILMRVAKGEFEVQGHRIHEGDLVAASPAISNRIPEDFPDPHDF
VPARYEQPRQEDLLNRWTWIPFGAGRHRCVGAAFAIMQIKAIFSVLLREYEFEMAQPPESYRNDHSKMVVQLA
QPACVRYRRRTGV
>CYP51B1_Mycobacterium tuberculosis 7057 2621685728
MSAVALPRVSGGHDEHGHLEEFRTDPIGLMQRVRDECGDVGTFQLAGKQVVLLSGSHANEFFFRAGDDDLDQA
KAYPFMTPIFGEGVVFDASPERRKEMLHNAALRGEQMKGHAATIEDQVRRMIADWGEAGEIDLLDFFAELTIY
TSSACLIGKKFRDQLDGRFAKLYHELERGTDPLAYVDPYLPIESFRRRDEARNGLVALVADIMNGRIANPPTD
KSDRDMLDVLIAVKAETGTPRFSADEITGMFISMMFAGHHTSSGTASWTLIELMRHRDAYAAVIDELDELYGD
GRSVSFHALRQIPQLENVLKETLRLHPPLIILMRVAKGEFEVQGHRIHEGDLVAASPAISNRIPEDFPDPHDF
VPARYEQPRQEDLLNRWTWIPFGAGRHRCVGAAFAIMQIKAIFSVLLREYEFEMAQPPESYRNDHSKMVVQLA
QPACVRYRRRTGV
>CYP51B1_Mycobacterium tuberculosis 2540754079

MSAVALPRVSGGHDEHGHLEEFRTDPIGLMQRVRDECGDVGTFQLAGKQVVLLSGSHANEFFFRAGDDDLDQA
KAYPFMTPIFGEGVVFDASPERRKEMLHNAALRGEQMKGHAATIEDQVRRMIADWGEAGEIDLLDFFAELTIY
TSSACLIGKKFRDQLDGRFAKLYHELERGTDPLAYVDPYLPIESFRRRDEARNGLVALVADIMNGRIANPPTD
KSDRDMLDVLIAVKAETGTPRFSADEITGMFISMMFAGHHTSSGTASWTLIELMRHRDAYAAVIDELDELYGD
GRSVSFHALRQIPQLENVLKETLRLHPPLIILMRVAKGEFEVQGHRIHEGDLVAASPAISNRIPEDFPDPHDF
VPARYEQPRQEDLLNRWTWIPFGAGRHRCVGAAFAIMQIKAIFSVLLREYEFEMAQPPESYRNDHSKMVVQLA
QPACVRYRRRTGV
>CYP51B1_Mycobacterium tuberculosis 7837 2621693009
MSAVALPRVSGGHDEHGHLEEFRTDPIGLMQRVRDECGDVGTFQLAGKQVVLLSGSHANEFFFRAGDDDLDQA
KAYPFMTPIFGEGVVFDASPERRKEMLHNAALRGEQMKGHAATIEDQVRRMIADWGEAGEIDLLDFFAELTIY
TSSACLIGKKFRDQLDGRFAKLYHELERGTDPLAYVDPYLPIESFRRRDEARNGLVALVADIMNGRIANPPTD
KSDRDMLDVLIAVKAETGTPRFSADEITGMFISMMFAGHHTSSGTASWTLIELMRHRDAYAAVIDELDELYGD
GRSVSFHALRQIPQLENVLKETLRLHPPLIILMRVAKGEFEVQGHRIHEGDLVAASPAISNRIPEDFPDPHDF
VPARYEQPRQEDLLNRWTWIPFGAGRHRCVGAAFAIMQIKAIFSVLLREYEFEMAQPPESYRNDHSKMVVQLA
QPACVRYRRRTGV
>CYP51B1_Mycobacterium tuberculosis 8442 2606473425
MSAVALPRVSGGHDEHGHLEEFRTDPIGLMQRVRDECGDVGTFQLAGKQVVLLSGSHANEFFFRAGDDDLDQA
KAYPFMTPIFGEGVVFDASPERRKEMLHNAALRGEQMKGHAATIEDQVRRMIADWGEAGEIDLLDFFAELTIY
TSSACLIGKKFRDQLDGRFAKLYHELERGTDPLAYVDPYLPIESFRRRDEARNGLVALVADIMNGRIANPPTD
KSDRDMLDVLIAVKAETGTPRFSADEITGMFISMMFAGHHTSSGTASWTLIELMRHRDAYAAVIDELDELYGD
GRSVSFHALRQIPQLENVLKETLRLHPPLIILMRVAKGEFEVQGHRIHEGDLVAASPAISNRIPEDFPDPHDF
VPARYEQPRQEDLLNRWTWIPFGAGRHRCVGAAFAIMQIKAIFSVLLREYEFEMAQPPESYRNDHSKMVVQLA
QPACVRYRRRTGV
>CYP51B1_Mycobacterium tuberculosis 94_M4241A 2621960352
MSAVALPRVSGGHDEHGHLEEFRTDPIGLMQRVRDECGDVGTFQLAGKQVVLLSGSHANEFFFRAGDDDLDQA
KAYPFMTPIFGEGVVFDASPERRKEMLHNAALRGEQMKGHAATIEDQVRRMIADWGEAGEIDLLDFFAELTIY
TSSACLIGKKFRDQLDGRFAKLYHELERGTDPLAYVDPYLPIESFRRRDEARNGLVALVADIMNGRIANPPTD
KSDRDMLDVLIAVKAETGTPRFSADEITGMFISMMFAGHHTSSGTASWTLIELMRHRDAYAAVIDELDELYGD
GRSVSFHALRQIPQLENVLKETLRLHPPLIILMRVAKGEFEVQGHRIHEGDLVAASPAISNRIPEDFPDPHDF
VPARYEQPRQEDLLNRWTWIPFGAGRHRCVGAAFAIMQIKAIFSVLLREYEFEMAQPPESYRNDHSKMVVQLA
QPACVRYRRRTGV
>CYP51B1_Mycobacterium tuberculosis 9542 2621677791
MSAVALPRVSGGHDEHGHLEEFRTDPIGLMQRVRDECGDVGTFQLAGKQVVLLSGSHANEFFFRAGDDDLDQA
KAYPFMTPIFGEGVVFDASPERRKEMLHNAALRGEQMKGHAATIEDQVRRMIADWGEAGEIDLLDFFAELTIY
TSSACLIGKKFRDQLDGRFAKLYHELERGTDPLAYVDPYLPIESFRRRDEARNGLVALVADIMNGRIANPPTD
KSDRDMLDVLIAVKAETGTPRFSADEITGMFISMMFAGHHTSSGTASWTLIELMRHRDAYAAVIDELDELYGD
GRSVSFHALRQIPQLENVLKETLRLHPPLIILMRVAKGEFEVQGHRIHEGDLVAASPAISNRIPEDFPDPHDF
VPARYEQPRQEDLLNRWTWIPFGAGRHRCVGAAFAIMQIKAIFSVLLREYEFEMAQPPESYRNDHSKMVVQLA
QPACVRYRRRTGV
>CYP51B1_Mycobacterium tuberculosis 96075 2687620462
MSAVALPRVSGGHDEHGHLEEFRTDPIGLMQRVRDECGDVGTFQLAGKQVVLLSGSHANEFFFRAGDDDLDQA
KAYPFMTPIFGEGVVFDASPERRKEMLHNAALRGEQMKGHAATIEDQVRRMIADWGEAGEIDLLDFFAELTIY
TSSACLIGKKFRDQLDGRFAKLYHELERGTDPLAYVDPYLPIESFRRRDEARNGLVALVADIMNGRIANPPTD
KSDRDMLDVLIAVKAETGTPRFSADEITGMFISMMFAGHHTSSGTASWTLIELMRHRDAYAAVIDELDELYGD
GRSVSFHALRQIPQLENVLKETLRLHPPLIILMRVAKGEFEVQGHRIHEGDLVAASPAISNRIPEDFPDPHDF
VPARYEQPRQEDLLNRWTWIPFGAGRHRCVGAAFAIMQIKAIFSVLLREYEFEMAQPPESYRNDHSKMVVQLA
QPACVRYRRRTGV
>CYP51B1_Mycobacterium tuberculosis 96121 2671955737
MSAVALPRVSGGHDEHGHLEEFRTDPIGLMQRVRDECGDVGTFQLAGKQVVLLSGSHANEFFFRAGDDDLDQA
KAYPFMTPIFGEGVVFDASPERRKEMLHNAALRGEQMKGHAATIEDQVRRMIADWGEAGEIDLLDFFAELTIY
TSSACLIGKKFRDQLDGRFAKLYHELERGTDPLAYVDPYLPIESFRRRDEARNGLVALVADIMNGRIANPPTD
KSDRDMLDVLIAVKAETGTPRFSADEITGMFISMMFAGHHTSSGTASWTLIELMRHRDAYAAVIDELDELYGD
GRSVSFHALRQIPQLENVLKETLRLHPPLIILMRVAKGEFEVQGHRIHEGDLVAASPAISNRIPEDFPDPHDF
VPARYEQPRQEDLLNRWTWIPFGAGRHRCVGAAFAIMQIKAIFSVLLREYEFEMAQPPESYRNDHSKMVVQLA
QPACVRYRRRTGV
>CYP51B1_Mycobacterium tuberculosis '98-R604 INH-RIF-EM' sequence
645190780

MSAVALPRVSGGHDEHGHLEEFRTDPIGLMQRVRDECGDVGTFQLAGKQVVLLSGSHANEFFFRAGDDDLDQA
KAYPFMTPIFGEGVVFDASPERRKEMLHNAALRGEQMKGHAATIEDQVRRMIADWGEAGEIDLLDFFAELTIY
TSSACLIGKKFRDQLDGRFAKLYHELERGTDPLAYVDPYLPIESFRRRDEARNGLVALVADIMNGRIANPPTD
KSDRDMLDVLIAVKAETGTPRFSADEITGMFISMMFAGHHTSSGTASWTLIELMRHRDAYAAVIDELDELYGD
GRSVSFHALRQIPQLENVLKETLRLHPPLIILMRVAKGEFEVQGHRIHEGDLVAASPAISNRIPEDFPDPHDF
VPARYEQPRQEDLLNRWTWIPFGAGRHRCVGAAFAIMQIKAIFSVLLREYEFEMAQPPESYRNDHSKMVVQLA
QPACVRYRRRTGV
>CYP51B1_Mycobacterium tuberculosis 9956 2621709924
MSAVALPRVSGGHDEHGHLEEFRTDPIGLMQRVRDECGDVGTFQLAGKQVVLLSGSHANEFFFRAGDDDLDQA
KAYPFMTPIFGEGVVFDASPERRKEMLHNAALRGEQMKGHAATIEDQVRRMIADWGEAGEIDLLDFFAELTIY
TSSACLIGKKFRDQLDGRFAKLYHELERGTDPLAYVDPYLPIESFRRRDEARNGLVALVADIMNGRIANPPTD
KSDRDMLDVLIAVKAETGTPRFSADEITGMFISMMFAGHHTSSGTASWTLIELMRHRDAYAAVIDELDELYGD
GRSVSFHALRQIPQLENVLKETLRLHPPLIILMRVAKGEFEVQGHRIHEGDLVAASPAISNRIPEDFPDPHDF
VPARYEQPRQEDLLNRWTWIPFGAGRHRCVGAAFAIMQIKAIFSVLLREYEFEMAQPPESYRNDHSKMVVQLA
QPACVRYRRRTGV
>CYP51B1_Mycobacterium tuberculosis A00301419 2706602146
MSAVALPRVSGGHDEHGHLEEFRTDPIGLMQRVRDECGDVGTFQLAGKQVVLLSGSHANEFFFRAGDDDLDQA
KAYPFMTPIFGEGVVFDASPERRKEMLHNAALRGEQMKGHAATIEDQVRRMIADWGEAGEIDLLDFFAELTIY
TSSACLIGKKFRDQLDGRFAKLYHELERGTDPLAYVDPYLPIESFRRRDEARNGLVALVADIMNGRIANPPTD
KSDRDMLDVLIAVKAETGTPRFSADEITGMFISMMFAGHHTSSGTASWTLIELMRHRDAYAAVIDELDELYGD
GRSVSFHALRQIPQLENVLKETLRLHPPLIILMRVAKGEFEVQGHRIHEGDLVAASPAISNRIPEDFPDPHDF
VPARYEQPRQEDLLNRWTWIPFGAGRHRCVGAAFAIMQIKAIFSVLLREYEFEMAQPPESYRNDHSKMVVQLA
QPACVRYRRRTGV
>CYP51B1_Mycobacterium tuberculosis A00400237 2677313227
MSAVALPRVSGGHDEHGHLEEFRTDPIGLMQRVRDECGDVGTFQLAGKQVVLLSGSHANEFFFRAGDDDLDQA
KAYPFMTPIFGEGVVFDASPERRKEMLHNAALRGEQMKGHAATIEDQVRRMIADWGEAGEIDLLDFFAELTIY
TSSACLIGKKFRDQLDGRFAKLYHELERGTDPLAYVDPYLPIESFRRRDEARNGLVALVADIMNGRIANPPTD
KSDRDMLDVLIAVKAETGTPRFSADEITGMFISMMFAGHHTSSGTASWTLIELMRHRDAYAAVIDELDELYGD
GRSVSFHALRQIPQLENVLKETLRLHPPLIILMRVAKGEFEVQGHRIHEGDLVAASPAISNRIPEDFPDPHDF
VPARYEQPRQEDLLNRWTWIPFGAGRHRCVGAAFAIMQIKAIFSVLLREYEFEMAQPPESYRNDHSKMVVQLA
QPACVRYRRRTGV
>CYP51B1_Mycobacterium tuberculosis A00400885 2673247031
MSAVALPRVSGGHDEHGHLEEFRTDPIGLMQRVRDECGDVGTFQLAGKQVVLLSGSHANEFFFRAGDDDLDQA
KAYPFMTPIFGEGVVFDASPERRKEMLHNAALRGEQMKGHAATIEDQVRRMIADWGEAGEIDLLDFFAELTIY
TSSACLIGKKFRDQLDGRFAKLYHELERGTDPLAYVDPYLPIESFRRRDEARNGLVALVADIMNGRIANPPTD
KSDRDMLDVLIAVKAETGTPRFSADEITGMFISMMFAGHHTSSGTASWTLIELMRHRDAYAAVIDELDELYGD
GRSVSFHALRQIPQLENVLKETLRLHPPLIILMRVAKGEFEVQGHRIHEGDLVAASPAISNRIPEDFPDPHDF
VPARYEQPRQEDLLNRWTWIPFGAGRHRCVGAAFAIMQIKAIFSVLLREYEFEMAQPPESYRNDHSKMVVQLA
QPACVRYRRRTGV
>CYP51B1_Mycobacterium tuberculosis A00401093 2706594955
MSAVALPRVSGGHDEHGHLEEFRTDPIGLMQRVRDECGDVGTFQLAGKQVVLLSGSHANEFFFRAGDDDLDQA
KAYPFMTPIFGEGVVFDASPERRKEMLHNAALRGEQMKGHAATIEDQVRRMIADWGEAGEIDLLDFFAELTIY
TSSACLIGKKFRDQLDGRFAKLYHELERGTDPLAYVDPYLPIESFRRRDEARNGLVALVADIMNGRIANPPTD
KSDRDMLDVLIAVKAETGTPRFSADEITGMFISMMFAGHHTSSGTASWTLIELMRHRDAYAAVIDELDELYGD
GRSVSFHALRQIPQLENVLKETLRLHPPLIILMRVAKGEFEVQGHRIHEGDLVAASPAISNRIPEDFPDPHDF
VPARYEQPRQEDLLNRWTWIPFGAGRHRCVGAAFAIMQIKAIFSVLLREYEFEMAQPPESYRNDHSKMVVQLA
QPACVRYRRRTGV
>CYP51B1_Mycobacterium tuberculosis A00401371 2683852170
MSAVALPRVSGGHDEHGHLEEFRTDPIGLMQRVRDECGDVGTFQLAGKQVVLLSGSHANEFFFRAGDDDLDQA
KAYPFMTPIFGEGVVFDASPERRKEMLHNAALRGEQMKGHAATIEDQVRRMIADWGEAGEIDLLDFFAELTIY
TSSACLIGKKFRDQLDGRFAKLYHELERGTDPLAYVDPYLPIESFRRRDEARNGLVALVADIMNGRIANPPTD
KSDRDMLDVLIAVKAETGTPRFSADEITGMFISMMFAGHHTSSGTASWTLIELMRHRDAYAAVIDELDELYGD
GRSVSFHALRQIPQLENVLKETLRLHPPLIILMRVAKGEFEVQGHRIHEGDLVAASPAISNRIPEDFPDPHDF
VPARYEQPRQEDLLNRWTWIPFGAGRHRCVGAAFAIMQIKAIFSVLLREYEFEMAQPPESYRNDHSKMVVQLA
QPACVRYRRRTGV
>CYP51B1_Mycobacterium tuberculosis A00500149 2703461659
MSAVALPRVSGGHDEHGHLEEFRTDPIGLMQRVRDECGDVGTFQLAGKQVVLLSGSHANEFFFRAGDDDLDQA
KAYPFMTPIFGEGVVFDASPERRKEMLHNAALRGEQMKGHAATIEDQVRRMIADWGEAGEIDLLDFFAELTIY

TSSACLIGKKFRDQLDGRFAKLYHELERGTDPLAYVDPYLPIESFRRRDEARNGLVALVADIMNGRIANPPTD
KSDRDMLDVLIAVKAETGTPRFSADEITGMFISMMFAGHHTSSGTASWTLIELMRHRDAYAAVIDELDELYGD
GRSVSFHALRQIPQLENVLKETLRLHPPLIILMRVAKGEFEVQGHRIHEGDLVAASPAISNRIPEDFPDPHDF
VPARYEQPRQEDLLNRWTWIPFGAGRHRCVGAAFAIMQIKAIFSVLLREYEFEMAQPPESYRNDHSKMVVQLA
QPACVRYRRRTGV
>CYP51B1_Mycobacterium tuberculosis A09900565 2674124419
MSAVALPRVSGGHDEHGHLEEFRTDPIGLMQRVRDECGDVGTFQLAGKQVVLLSGSHANEFFFRAGDDDLDQA
KAYPFMTPIFGEGVVFDASPERRKEMLHNAALRGEQMKGHAATIEDQVRRMIADWGEAGEIDLLDFFAELTIY
TSSACLIGKKFRDQLDGRFAKLYHELERGTDPLAYVDPYLPIESFRRRDEARNGLVALVADIMNGRIANPPTD
KSDRDMLDVLIAVKAETGTPRFSADEITGMFISMMFAGHHTSSGTASWTLIELMRHRDAYAAVIDELDELYGD
GRSVSFHALRQIPQLENVLKETLRLHPPLIILMRVAKGEFEVQGHRIHEGDLVAASPAISNRIPEDFPDPHDF
VPARYEQPRQEDLLNRWTWIPFGAGRHRCVGAAFAIMQIKAIFSVLLREYEFEMAQPPESYRNDHSKMVVQLA
QPACVRYRRRTGV
>CYP51B1_Mycobacterium tuberculosis A09901284 2706560620
MSAVALPRVSGGHDEHGHLEEFRTDPIGLMQRVRDECGDVGTFQLAGKQVVLLSGSHANEFFFRAGDDDLDQA
KAYPFMTPIFGEGVVFDASPERRKEMLHNAALRGEQMKGHAATIEDQVRRMIADWGEAGEIDLLDFFAELTIY
TSSACLIGKKFRDQLDGRFAKLYHELERGTDPLAYVDPYLPIESFRRRDEARNGLVALVADIMNGRIANPPTD
KSDRDMLDVLIAVKAETGTPRFSADEITGMFISMMFAGHHTSSGTASWTLIELMRHRDAYAAVIDELDELYGD
GRSVSFHALRQIPQLENVLKETLRLHPPLIILMRVAKGEFEVQGHRIHEGDLVAASPAISNRIPEDFPDPHDF
VPARYEQPRQEDLLNRWTWIPFGAGRHRCVGAAFAIMQIKAIFSVLLREYEFEMAQPPESYRNDHSKMVVQLA
QPACVRYRRRTGV
>CYP51B1_Mycobacterium tuberculosis A2 2715347182
MSAVALPRVSGGHDEHGHLEEFRTDPIGLMQRVRDECGDVGTFQLAGKQVVLLSGSHANEFFFRAGDDDLDQA
KAYPFMTPIFGEGVVFDASPERRKEMLHNAALRGEQMKGHAATIEDQVRRMIADWGEAGEIDLLDFFAELTIY
TSSACLIGKKFRDQLDGRFAKLYHELERGTDPLAYVDPYLPIESFRRRDEARNGLVALVADIMNGRIANPPTD
KSDRDMLDVLIAVKAETGTPRFSADEITGMFISMMFAGHHTSSGTASWTLIELMRHRDAYAAVIDELDELYGD
GRSVSFHALRQIPQLENVLKETLRLHPPLIILMRVAKGEFEVQGHRIHEGDLVAASPAISNRIPEDFPDPHDF
VPARYEQPRQEDLLNRWTWIPFGAGRHRCVGAAFAIMQIKAIFSVLLREYEFEMAQPPESYRNDHSKMVVQLA
QPACVRYRRRTGV
>CYP51B1_Mycobacterium tuberculosis A4 2715369132
MSAVALPRVSGGHDEHGHLEEFRTDPIGLMQRVRDECGDVGTFQLAGKQVVLLSGSHANEFFFRAGDDDLDQA
KAYPFMTPIFGEGVVFDASPERRKEMLHNAALRGEQMKGHAATIEDQVRRMIADWGEAGEIDLLDFFAELTIY
TSSACLIGKKFRDQLDGRFAKLYHELERGTDPLAYVDPYLPIESFRRRDEARNGLVALVADIMNGRIANPPTD
KSDRDMLDVLIAVKAETGTPRFSADEITGMFISMMFAGHHTSSGTASWTLIELMRHRDAYAAVIDELDELYGD
GRSVSFHALRQIPQLENVLKETLRLHPPLIILMRVAKGEFEVQGHRIHEGDLVAASPAISNRIPEDFPDPHDF
VPARYEQPRQEDLLNRWTWIPFGAGRHRCVGAAFAIMQIKAIFSVLLREYEFEMAQPPESYRNDHSKMVVQLA
QPACVRYRRRTGV
>CYP51B1_Mycobacterium tuberculosis A70011_1 2662931702
MSAVALPRVSGGHDEHGHLEEFRTDPIGLMQRVRDECGDVGTFQLAGKQVVLLSGSHANEFFFRAGDDDLDQA
KAYPFMTPIFGEGVVFDASPERRKEMLHNAALRGEQMKGHAATIEDQVRRMIADWGEAGEIDLLDFFAELTIY
TSSACLIGKKFRDQLDGRFAKLYHELERGTDPLAYVDPYLPIESFRRRDEARNGLVALVADIMNGRIANPPTD
KSDRDMLDVLIAVKAETGTPRFSADEITGMFISMMFAGHHTSSGTASWTLIELMRHRDAYAAVIDELDELYGD
GRSVSFHALRQIPQLENVLKETLRLHPPLIILMRVAKGEFEVQGHRIHEGDLVAASPAISNRIPEDFPDPHDF
VPARYEQPRQEDLLNRWTWIPFGAGRHRCVGAAFAIMQIKAIFSVLLREYEFEMAQPPESYRNDHSKMVVQLA
QPACVRYRRRTGV
>CYP51B1_Mycobacterium tuberculosis A70011_2 2694048207
MSAVALPRVSGGHDEHGHLEEFRTDPIGLMQRVRDECGDVGTFQLAGKQVVLLSGSHANEFFFRAGDDDLDQA
KAYPFMTPIFGEGVVFDASPERRKEMLHNAALRGEQMKGHAATIEDQVRRMIADWGEAGEIDLLDFFAELTIY
TSSACLIGKKFRDQLDGRFAKLYHELERGTDPLAYVDPYLPIESFRRRDEARNGLVALVADIMNGRIANPPTD
KSDRDMLDVLIAVKAETGTPRFSADEITGMFISMMFAGHHTSSGTASWTLIELMRHRDAYAAVIDELDELYGD
GRSVSFHALRQIPQLENVLKETLRLHPPLIILMRVAKGEFEVQGHRIHEGDLVAASPAISNRIPEDFPDPHDF
VPARYEQPRQEDLLNRWTWIPFGAGRHRCVGAAFAIMQIKAIFSVLLREYEFEMAQPPESYRNDHSKMVVQLA
QPACVRYRRRTGV
>CYP51B1_Mycobacterium tuberculosis A70011_3 2694011337
MSAVALPRVSGGHDEHGHLEEFRTDPIGLMQRVRDECGDVGTFQLAGKQVVLLSGSHANEFFFRAGDDDLDQA
KAYPFMTPIFGEGVVFDASPERRKEMLHNAALRGEQMKGHAATIEDQVRRMIADWGEAGEIDLLDFFAELTIY
TSSACLIGKKFRDQLDGRFAKLYHELERGTDPLAYVDPYLPIESFRRRDEARNGLVALVADIMNGRIANPPTD
KSDRDMLDVLIAVKAETGTPRFSADEITGMFISMMFAGHHTSSGTASWTLIELMRHRDAYAAVIDELDELYGD

GRSVSFHALRQIPQLENVLKETLRLHPPLIILMRVAKGEFEVQGHRIHEGDLVAASPAISNRIPEDFPDPHDF
VPARYEQPRQEDLLNRWTWIPFGAGRHRCVGAAFAIMQIKAIFSVLLREYEFEMAQPPESYRNDHSKMVVQLA
QPACVRYRRRTGV
>CYP51B1_Mycobacterium tuberculosis A70011_4 2659400098
MSAVALPRVSGGHDEHGHLEEFRTDPIGLMQRVRDECGDVGTFQLAGKQVVLLSGSHANEFFFRAGDDDLDQA
KAYPFMTPIFGEGVVFDASPERRKEMLHNAALRGEQMKGHAATIEDQVRRMIADWGEAGEIDLLDFFAELTIY
TSSACLIGKKFRDQLDGRFAKLYHELERGTDPLAYVDPYLPIESFRRRDEARNGLVALVADIMNGRIANPPTD
KSDRDMLDVLIAVKAETGTPRFSADEITGMFISMMFAGHHTSSGTASWTLIELMRHRDAYAAVIDELDELYGD
GRSVSFHALRQIPQLENVLKETLRLHPPLIILMRVAKGEFEVQGHRIHEGDLVAASPAISNRIPEDFPDPHDF
VPARYEQPRQEDLLNRWTWIPFGAGRHRCVGAAFAIMQIKAIFSVLLREYEFEMAQPPESYRNDHSKMVVQLA
QPACVRYRRRTGV
>CYP51B1_Mycobacterium tuberculosis A70011_5 2670886087
MSAVALPRVSGGHDEHGHLEEFRTDPIGLMQRVRDECGDVGTFQLAGKQVVLLSGSHANEFFFRAGDDDLDQA
KAYPFMTPIFGEGVVFDASPERRKEMLHNAALRGEQMKGHAATIEDQVRRMIADWGEAGEIDLLDFFAELTIY
TSSACLIGKKFRDQLDGRFAKLYHELERGTDPLAYVDPYLPIESFRRRDEARNGLVALVADIMNGRIANPPTD
KSDRDMLDVLIAVKAETGTPRFSADEITGMFISMMFAGHHTSSGTASWTLIELMRHRDAYAAVIDELDELYGD
GRSVSFHALRQIPQLENVLKETLRLHPPLIILMRVAKGEFEVQGHRIHEGDLVAASPAISNRIPEDFPDPHDF
VPARYEQPRQEDLLNRWTWIPFGAGRHRCVGAAFAIMQIKAIFSVLLREYEFEMAQPPESYRNDHSKMVVQLA
QPACVRYRRRTGV
>CYP51B1_Mycobacterium tuberculosis A70011_6 2670912080
MSAVALPRVSGGHDEHGHLEEFRTDPIGLMQRVRDECGDVGTFQLAGKQVVLLSGSHANEFFFRAGDDDLDQA
KAYPFMTPIFGEGVVFDASPERRKEMLHNAALRGEQMKGHAATIEDQVRRMIADWGEAGEIDLLDFFAELTIY
TSSACLIGKKFRDQLDGRFAKLYHELERGTDPLAYVDPYLPIESFRRRDEARNGLVALVADIMNGRIANPPTD
KSDRDMLDVLIAVKAETGTPRFSADEITGMFISMMFAGHHTSSGTASWTLIELMRHRDAYAAVIDELDELYGD
GRSVSFHALRQIPQLENVLKETLRLHPPLIILMRVAKGEFEVQGHRIHEGDLVAASPAISNRIPEDFPDPHDF
VPARYEQPRQEDLLNRWTWIPFGAGRHRCVGAAFAIMQIKAIFSVLLREYEFEMAQPPESYRNDHSKMVVQLA
QPACVRYRRRTGV
>CYP51B1_Mycobacterium tuberculosis A70012 2695718251
MSAVALPRVSGGHDEHGHLEEFRTDPIGLMQRVRDECGDVGTFQLAGKQVVLLSGSHANEFFFRAGDDDLDQA
KAYPFMTPIFGEGVVFDASPERRKEMLHNAALRGEQMKGHAATIEDQVRRMIADWGEAGEIDLLDFFAELTIY
TSSACLIGKKFRDQLDGRFAKLYHELERGTDPLAYVDPYLPIESFRRRDEARNGLVALVADIMNGRIANPPTD
KSDRDMLDVLIAVKAETGTPRFSADEITGMFISMMFAGHHTSSGTASWTLIELMRHRDAYAAVIDELDELYGD
GRSVSFHALRQIPQLENVLKETLRLHPPLIILMRVAKGEFEVQGHRIHEGDLVAASPAISNRIPEDFPDPHDF
VPARYEQPRQEDLLNRWTWIPFGAGRHRCVGAAFAIMQIKAIFSVLLREYEFEMAQPPESYRNDHSKMVVQLA
QPACVRYRRRTGV
>CYP51B1_Mycobacterium tuberculosis A70067_2 2696874944
MSAVALPRVSGGHDEHGHLEEFRTDPIGLMQRVRDECGDVGTFQLAGKQVVLLSGSHANEFFFRAGDDDLDQA
KAYPFMTPIFGEGVVFDASPERRKEMLHNAALRGEQMKGHAATIEDQVRRMIADWGEAGEIDLLDFFAELTIY
TSSACLIGKKFRDQLDGRFAKLYHELERGTDPLAYVDPYLPIESFRRRDEARNGLVALVADIMNGRIANPPTD
KSDRDMLDVLIAVKAETGTPRFSADEITGMFISMMFAGHHTSSGTASWTLIELMRHRDAYAAVIDELDELYGD
GRSVSFHALRQIPQLENVLKETLRLHPPLIILMRVAKGEFEVQGHRIHEGDLVAASPAISNRIPEDFPDPHDF
VPARYEQPRQEDLLNRWTWIPFGAGRHRCVGAAFAIMQIKAIFSVLLREYEFEMAQPPESYRNDHSKMVVQLA
QPACVRYRRRTGV
>CYP51B1_Mycobacterium tuberculosis A70086 2691916999
MSAVALPRVSGGHDEHGHLEEFRTDPIGLMQRVRDECGDVGTFQLAGKQVVLLSGSHANEFFFRAGDDDLDQA
KAYPFMTPIFGEGVVFDASPERRKEMLHNAALRGEQMKGHAATIEDQVRRMIADWGEAGEIDLLDFFAELTIY
TSSACLIGKKFRDQLDGRFAKLYHELERGTDPLAYVDPYLPIESFRRRDEARNGLVALVADIMNGRIANPPTD
KSDRDMLDVLIAVKAETGTPRFSADEITGMFISMMFAGHHTSSGTASWTLIELMRHRDAYAAVIDELDELYGD
GRSVSFHALRQIPQLENVLKETLRLHPPLIILMRVAKGEFEVQGHRIHEGDLVAASPAISNRIPEDFPDPHDF
VPARYEQPRQEDLLNRWTWIPFGAGRHRCVGAAFAIMQIKAIFSVLLREYEFEMAQPPESYRNDHSKMVVQLA
QPACVRYRRRTGV
>CYP51B1_Mycobacterium tuberculosis A70088 2670440348
MSAVALPRVSGGHDEHGHLEEFRTDPIGLMQRVRDECGDVGTFQLAGKQVVLLSGSHANEFFFRAGDDDLDQA
KAYPFMTPIFGEGVVFDASPERRKEMLHNAALRGEQMKGHAATIEDQVRRMIADWGEAGEIDLLDFFAELTIY
TSSACLIGKKFRDQLDGRFAKLYHELERGTDPLAYVDPYLPIESFRRRDEARNGLVALVADIMNGRIANPPTD
KSDRDMLDVLIAVKAETGTPRFSADEITGMFISMMFAGHHTSSGTASWTLIELMRHRDAYAAVIDELDELYGD
GRSVSFHALRQIPQLENVLKETLRLHPPLIILMRVAKGEFEVQGHRIHEGDLVAASPAISNRIPEDFPDPHDF

VPARYEQPRQEDLLNRWTWIPFGAGRHRCVGAAFAIMQIKAIFSVLLREYEFEMAQPPESYRNDHSKMVVQLA
QPACVRYRRRTGV
>CYP51B1_Mycobacterium tuberculosis A70136_13046 2677461719
MSAVALPRVSGGHDEHGHLEEFRTDPIGLMQRVRDECGDVGTFQLAGKQVVLLSGSHANEFFFRAGDDDLDQA
KAYPFMTPIFGEGVVFDASPERRKEMLHNAALRGEQMKGHAATIEDQVRRMIADWGEAGEIDLLDFFAELTIY
TSSACLIGKKFRDQLDGRFAKLYHELERGTDPLAYVDPYLPIESFRRRDEARNGLVALVADIMNGRIANPPTD
KSDRDMLDVLIAVKAETGTPRFSADEITGMFISMMFAGHHTSSGTASWTLIELMRHRDAYAAVIDELDELYGD
GRSVSFHALRQIPQLENVLKETLRLHPPLIILMRVAKGEFEVQGHRIHEGDLVAASPAISNRIPEDFPDPHDF
VPARYEQPRQEDLLNRWTWIPFGAGRHRCVGAAFAIMQIKAIFSVLLREYEFEMAQPPESYRNDHSKMVVQLA
QPACVRYRRRTGV
>CYP51B1_Mycobacterium tuberculosis A70136_15354 2698040124
MSAVALPRVSGGHDEHGHLEEFRTDPIGLMQRVRDECGDVGTFQLAGKQVVLLSGSHANEFFFRAGDDDLDQA
KAYPFMTPIFGEGVVFDASPERRKEMLHNAALRGEQMKGHAATIEDQVRRMIADWGEAGEIDLLDFFAELTIY
TSSACLIGKKFRDQLDGRFAKLYHELERGTDPLAYVDPYLPIESFRRRDEARNGLVALVADIMNGRIANPPTD
KSDRDMLDVLIAVKAETGTPRFSADEITGMFISMMFAGHHTSSGTASWTLIELMRHRDAYAAVIDELDELYGD
GRSVSFHALRQIPQLENVLKETLRLHPPLIILMRVAKGEFEVQGHRIHEGDLVAASPAISNRIPEDFPDPHDF
VPARYEQPRQEDLLNRWTWIPFGAGRHRCVGAAFAIMQIKAIFSVLLREYEFEMAQPPESYRNDHSKMVVQLA
QPACVRYRRRTGV
>CYP51B1_Mycobacterium tuberculosis A70136_8962 2669855137
MSAVALPRVSGGHDEHGHLEEFRTDPIGLMQRVRDECGDVGTFQLAGKQVVLLSGSHANEFFFRAGDDDLDQA
KAYPFMTPIFGEGVVFDASPERRKEMLHNAALRGEQMKGHAATIEDQVRRMIADWGEAGEIDLLDFFAELTIY
TSSACLIGKKFRDQLDGRFAKLYHELERGTDPLAYVDPYLPIESFRRRDEARNGLVALVADIMNGRIANPPTD
KSDRDMLDVLIAVKAETGTPRFSADEITGMFISMMFAGHHTSSGTASWTLIELMRHRDAYAAVIDELDELYGD
GRSVSFHALRQIPQLENVLKETLRLHPPLIILMRVAKGEFEVQGHRIHEGDLVAASPAISNRIPEDFPDPHDF
VPARYEQPRQEDLLNRWTWIPFGAGRHRCVGAAFAIMQIKAIFSVLLREYEFEMAQPPESYRNDHSKMVVQLA
QPACVRYRRRTGV
>CYP51B1_Mycobacterium tuberculosis A70196 2694245295
MSAVALPRVSGGHDEHGHLEEFRTDPIGLMQRVRDECGDVGTFQLAGKQVVLLSGSHANEFFFRAGDDDLDQA
KAYPFMTPIFGEGVVFDASPERRKEMLHNAALRGEQMKGHAATIEDQVRRMIADWGEAGEIDLLDFFAELTIY
TSSACLIGKKFRDQLDGRFAKLYHELERGTDPLAYVDPYLPIESFRRRDEARNGLVALVADIMNGRIANPPTD
KSDRDMLDVLIAVKAETGTPRFSADEITGMFISMMFAGHHTSSGTASWTLIELMRHRDAYAAVIDELDELYGD
GRSVSFHALRQIPQLENVLKETLRLHPPLIILMRVAKGEFEVQGHRIHEGDLVAASPAISNRIPEDFPDPHDF
VPARYEQPRQEDLLNRWTWIPFGAGRHRCVGAAFAIMQIKAIFSVLLREYEFEMAQPPESYRNDHSKMVVQLA
QPACVRYRRRTGV
>CYP51B1_Mycobacterium tuberculosis A70170 2673858935
MSAVALPRVSGGHDEHGHLEEFRTDPIGLMQRVRDECGDVGTFQLAGKQVVLLSGSHANEFFFRAGDDDLDQA
KAYPFMTPIFGEGVVFDASPERRKEMLHNAALRGEQMKGHAATIEDQVRRMIADWGEAGEIDLLDFFAELTIY
TSSACLIGKKFRDQLDGRFAKLYHELERGTDPLAYVDPYLPIESFRRRDEARNGLVALVADIMNGRIANPPTD
KSDRDMLDVLIAVKAETGTPRFSADEITGMFISMMFAGHHTSSGTASWTLIELMRHRDAYAAVIDELDELYGD
GRSVSFHALRQIPQLENVLKETLRLHPPLIILMRVAKGEFEVQGHRIHEGDLVAASPAISNRIPEDFPDPHDF
VPARYEQPRQEDLLNRWTWIPFGAGRHRCVGAAFAIMQIKAIFSVLLREYEFEMAQPPESYRNDHSKMVVQLA
QPACVRYRRRTGV
>CYP51B1_Mycobacterium tuberculosis A70250 2693434087
MSAVALPRVSGGHDEHGHLEEFRTDPIGLMQRVRDECGDVGTFQLAGKQVVLLSGSHANEFFFRAGDDDLDQA
KAYPFMTPIFGEGVVFDASPERRKEMLHNAALRGEQMKGHAATIEDQVRRMIADWGEAGEIDLLDFFAELTIY
TSSACLIGKKFRDQLDGRFAKLYHELERGTDPLAYVDPYLPIESFRRRDEARNGLVALVADIMNGRIANPPTD
KSDRDMLDVLIAVKAETGTPRFSADEITGMFISMMFAGHHTSSGTASWTLIELMRHRDAYAAVIDELDELYGD
GRSVSFHALRQIPQLENVLKETLRLHPPLIILMRVAKGEFEVQGHRIHEGDLVAASPAISNRIPEDFPDPHDF
VPARYEQPRQEDLLNRWTWIPFGAGRHRCVGAAFAIMQIKAIFSVLLREYEFEMAQPPESYRNDHSKMVVQLA
QPACVRYRRRTGV
>CYP51B1_Mycobacterium tuberculosis A70260 2673924135
MSAVALPRVSGGHDEHGHLEEFRTDPIGLMQRVRDECGDVGTFQLAGKQVVLLSGSHANEFFFRAGDDDLDQA
KAYPFMTPIFGEGVVFDASPERRKEMLHNAALRGEQMKGHAATIEDQVRRMIADWGEAGEIDLLDFFAELTIY
TSSACLIGKKFRDQLDGRFAKLYHELERGTDPLAYVDPYLPIESFRRRDEARNGLVALVADIMNGRIANPPTD
KSDRDMLDVLIAVKAETGTPRFSADEITGMFISMMFAGHHTSSGTASWTLIELMRHRDAYAAVIDELDELYGD
GRSVSFHALRQIPQLENVLKETLRLHPPLIILMRVAKGEFEVQGHRIHEGDLVAASPAISNRIPEDFPDPHDF
VPARYEQPRQEDLLNRWTWIPFGAGRHRCVGAAFAIMQIKAIFSVLLREYEFEMAQPPESYRNDHSKMVVQLA
QPACVRYRRRTGV

>CYP51B1_Mycobacterium tuberculosis A70376 2659220248
MSAVALPRVSGGHDEHGHLEEFRTDPIGLMQRVRDECGDVGTFQLAGKQVVLLSGSHANEFFFRAGDDDLDQA
KAYPFMTPIFGEGVVFDASPERRKEMLHNAALRGEQMKGHAATIEDQVRRMIADWGEAGEIDLLDFFAELTIY
TSSACLIGKKFRDQLDGRFAKLYHELERGTDPLAYVDPYLPIESFRRRDEARNGLVALVADIMNGRIANPPTD
KSDRDMLDVLIAVKAETGTPRFSADEITGMFISMMFAGHHTSSGTASWTLIELMRHRDAYAAVIDELDELYGD
GRSVSFHALRQIPQLENVLKETLRLHPPLIILMRVAKGEFEVQGHRIHEGDLVAASPAISNRIPEDFPDPHDF
VPARYEQPRQEDLLNRWTWIPFGAGRHRCVGAAFAIMQIKAIFSVLLREYEFEMAQPPESYRNDHSKMVVQLA
QPACVRYRRRTGV
>CYP51B1_Mycobacterium tuberculosis A70329 2659262918
MSAVALPRVSGGHDEHGHLEEFRTDPIGLMQRVRDECGDVGTFQLAGKQVVLLSGSHANEFFFRAGDDDLDQA
KAYPFMTPIFGEGVVFDASPERRKEMLHNAALRGEQMKGHAATIEDQVRRMIADWGEAGEIDLLDFFAELTIY
TSSACLIGKKFRDQLDGRFAKLYHELERGTDPLAYVDPYLPIESFRRRDEARNGLVALVADIMNGRIANPPTD
KSDRDMLDVLIAVKAETGTPRFSADEITGMFISMMFAGHHTSSGTASWTLIELMRHRDAYAAVIDELDELYGD
GRSVSFHALRQIPQLENVLKETLRLHPPLIILMRVAKGEFEVQGHRIHEGDLVAASPAISNRIPEDFPDPHDF
VPARYEQPRQEDLLNRWTWIPFGAGRHRCVGAAFAIMQIKAIFSVLLREYEFEMAQPPESYRNDHSKMVVQLA
QPACVRYRRRTGV
>CYP51B1_Mycobacterium tuberculosis A70387 2702064874
MSAVALPRVSGGHDEHGHLEEFRTDPIGLMQRVRDECGDVGTFQLAGKQVVLLSGSHANEFFFRAGDDDLDQA
KAYPFMTPIFGEGVVFDASPERRKEMLHNAALRGEQMKGHAATIEDQVRRMIADWGEAGEIDLLDFFAELTIY
TSSACLIGKKFRDQLDGRFAKLYHELERGTDPLAYVDPYLPIESFRRRDEARNGLVALVADIMNGRIANPPTD
KSDRDMLDVLIAVKAETGTPRFSADEITGMFISMMFAGHHTSSGTASWTLIELMRHRDAYAAVIDELDELYGD
GRSVSFHALRQIPQLENVLKETLRLHPPLIILMRVAKGEFEVQGHRIHEGDLVAASPAISNRIPEDFPDPHDF
VPARYEQPRQEDLLNRWTWIPFGAGRHRCVGAAFAIMQIKAIFSVLLREYEFEMAQPPESYRNDHSKMVVQLA
QPACVRYRRRTGV
>CYP51B1_Mycobacterium tuberculosis A70416 2702120167
MSAVALPRVSGGHDEHGHLEEFRTDPIGLMQRVRDECGDVGTFQLAGKQVVLLSGSHANEFFFRAGDDDLDQA
KAYPFMTPIFGEGVVFDASPERRKEMLHNAALRGEQMKGHAATIEDQVRRMIADWGEAGEIDLLDFFAELTIY
TSSACLIGKKFRDQLDGRFAKLYHELERGTDPLAYVDPYLPIESFRRRDEARNGLVALVADIMNGRIANPPTD
KSDRDMLDVLIAVKAETGTPRFSADEITGMFISMMFAGHHTSSGTASWTLIELMRHRDAYAAVIDELDELYGD
GRSVSFHALRQIPQLENVLKETLRLHPPLIILMRVAKGEFEVQGHRIHEGDLVAASPAISNRIPEDFPDPHDF
VPARYEQPRQEDLLNRWTWIPFGAGRHRCVGAAFAIMQIKAIFSVLLREYEFEMAQPPESYRNDHSKMVVQLA
QPACVRYRRRTGV
>CYP51B1_Mycobacterium tuberculosis A70441 2673863195
MSAVALPRVSGGHDEHGHLEEFRTDPIGLMQRVRDECGDVGTFQLAGKQVVLLSGSHANEFFFRAGDDDLDQA
KAYPFMTPIFGEGVVFDASPERRKEMLHNAALRGEQMKGHAATIEDQVRRMIADWGEAGEIDLLDFFAELTIY
TSSACLIGKKFRDQLDGRFAKLYHELERGTDPLAYVDPYLPIESFRRRDEARNGLVALVADIMNGRIANPPTD
KSDRDMLDVLIAVKAETGTPRFSADEITGMFISMMFAGHHTSSGTASWTLIELMRHRDAYAAVIDELDELYGD
GRSVSFHALRQIPQLENVLKETLRLHPPLIILMRVAKGEFEVQGHRIHEGDLVAASPAISNRIPEDFPDPHDF
VPARYEQPRQEDLLNRWTWIPFGAGRHRCVGAAFAIMQIKAIFSVLLREYEFEMAQPPESYRNDHSKMVVQLA
QPACVRYRRRTGV
>CYP51B1_Mycobacterium tuberculosis A70448 2696810307
MSAVALPRVSGGHDEHGHLEEFRTDPIGLMQRVRDECGDVGTFQLAGKQVVLLSGSHANEFFFRAGDDDLDQA
KAYPFMTPIFGEGVVFDASPERRKEMLHNAALRGEQMKGHAATIEDQVRRMIADWGEAGEIDLLDFFAELTIY
TSSACLIGKKFRDQLDGRFAKLYHELERGTDPLAYVDPYLPIESFRRRDEARNGLVALVADIMNGRIANPPTD
KSDRDMLDVLIAVKAETGTPRFSADEITGMFISMMFAGHHTSSGTASWTLIELMRHRDAYAAVIDELDELYGD
GRSVSFHALRQIPQLENVLKETLRLHPPLIILMRVAKGEFEVQGHRIHEGDLVAASPAISNRIPEDFPDPHDF
VPARYEQPRQEDLLNRWTWIPFGAGRHRCVGAAFAIMQIKAIFSVLLREYEFEMAQPPESYRNDHSKMVVQLA
QPACVRYRRRTGV
>CYP51B1_Mycobacterium tuberculosis A70451 2696137497
MSAVALPRVSGGHDEHGHLEEFRTDPIGLMQRVRDECGDVGTFQLAGKQVVLLSGSHANEFFFRAGDDDLDQA
KAYPFMTPIFGEGVVFDASPERRKEMLHNAALRGEQMKGHAATIEDQVRRMIADWGEAGEIDLLDFFAELTIY
TSSACLIGKKFRDQLDGRFAKLYHELERGTDPLAYVDPYLPIESFRRRDEARNGLVALVADIMNGRIANPPTD
KSDRDMLDVLIAVKAETGTPRFSADEITGMFISMMFAGHHTSSGTASWTLIELMRHRDAYAAVIDELDELYGD
GRSVSFHALRQIPQLENVLKETLRLHPPLIILMRVAKGEFEVQGHRIHEGDLVAASPAISNRIPEDFPDPHDF
VPARYEQPRQEDLLNRWTWIPFGAGRHRCVGAAFAIMQIKAIFSVLLREYEFEMAQPPESYRNDHSKMVVQLA
QPACVRYRRRTGV
>CYP51B1_Mycobacterium tuberculosis A70458 2697714431

MSAVALPRVSGGHDEHGHLEEFRTDPIGLMQRVRDECGDVGTFQLAGKQVVLLSGSHANEFFFRAGDDDLDQA
KAYPFMTPIFGEGVVFDASPERRKEMLHNAALRGEQMKGHAATIEDQVRRMIADWGEAGEIDLLDFFAELTIY
TSSACLIGKKFRDQLDGRFAKLYHELERGTDPLAYVDPYLPIESFRRRDEARNGLVALVADIMNGRIANPPTD
KSDRDMLDVLIAVKAETGTPRFSADEITGMFISMMFAGHHTSSGTASWTLIELMRHRDAYAAVIDELDELYGD
GRSVSFHALRQIPQLENVLKETLRLHPPLIILMRVAKGEFEVQGHRIHEGDLVAASPAISNRIPEDFPDPHDF
VPARYEQPRQEDLLNRWTWIPFGAGRHRCVGAAFAIMQIKAIFSVLLREYEFEMAQPPESYRNDHSKMVVQLA
QPACVRYRRRTGV
>CYP51B1_Mycobacterium tuberculosis A70480 2669725278
MSAVALPRVSGGHDEHGHLEEFRTDPIGLMQRVRDECGDVGTFQLAGKQVVLLSGSHANEFFFRAGDDDLDQA
KAYPFMTPIFGEGVVFDASPERRKEMLHNAALRGEQMKGHAATIEDQVRRMIADWGEAGEIDLLDFFAELTIY
TSSACLIGKKFRDQLDGRFAKLYHELERGTDPLAYVDPYLPIESFRRRDEARNGLVALVADIMNGRIANPPTD
KSDRDMLDVLIAVKAETGTPRFSADEITGMFISMMFAGHHTSSGTASWTLIELMRHRDAYAAVIDELDELYGD
GRSVSFHALRQIPQLENVLKETLRLHPPLIILMRVAKGEFEVQGHRIHEGDLVAASPAISNRIPEDFPDPHDF
VPARYEQPRQEDLLNRWTWIPFGAGRHRCVGAAFAIMQIKAIFSVLLREYEFEMAQPPESYRNDHSKMVVQLA
QPACVRYRRRTGV
>CYP51B1_Mycobacterium tuberculosis A70490 2666326736
MSAVALPRVSGGHDEHGHLEEFRTDPIGLMQRVRDECGDVGTFQLAGKQVVLLSGSHANEFFFRAGDDDLDQA
KAYPFMTPIFGEGVVFDASPERRKEMLHNAALRGEQMKGHAATIEDQVRRMIADWGEAGEIDLLDFFAELTIY
TSSACLIGKKFRDQLDGRFAKLYHELERGTDPLAYVDPYLPIESFRRRDEARNGLVALVADIMNGRIANPPTD
KSDRDMLDVLIAVKAETGTPRFSADEITGMFISMMFAGHHTSSGTASWTLIELMRHRDAYAAVIDELDELYGD
GRSVSFHALRQIPQLENVLKETLRLHPPLIILMRVAKGEFEVQGHRIHEGDLVAASPAISNRIPEDFPDPHDF
VPARYEQPRQEDLLNRWTWIPFGAGRHRCVGAAFAIMQIKAIFSVLLREYEFEMAQPPESYRNDHSKMVVQLA
QPACVRYRRRTGV
>CYP51B1_Mycobacterium tuberculosis A70501 2665694113
MSAVALPRVSGGHDEHGHLEEFRTDPIGLMQRVRDECGDVGTFQLAGKQVVLLSGSHANEFFFRAGDDDLDQA
KAYPFMTPIFGEGVVFDASPERRKEMLHNAALRGEQMKGHAATIEDQVRRMIADWGEAGEIDLLDFFAELTIY
TSSACLIGKKFRDQLDGRFAKLYHELERGTDPLAYVDPYLPIESFRRRDEARNGLVALVADIMNGRIANPPTD
KSDRDMLDVLIAVKAETGTPRFSADEITGMFISMMFAGHHTSSGTASWTLIELMRHRDAYAAVIDELDELYGD
GRSVSFHALRQIPQLENVLKETLRLHPPLIILMRVAKGEFEVQGHRIHEGDLVAASPAISNRIPEDFPDPHDF
VPARYEQPRQEDLLNRWTWIPFGAGRHRCVGAAFAIMQIKAIFSVLLREYEFEMAQPPESYRNDHSKMVVQLA
QPACVRYRRRTGV
>CYP51B1_Mycobacterium tuberculosis A70547 2659476912
MSAVALPRVSGGHDEHGHLEEFRTDPIGLMQRVRDECGDVGTFQLAGKQVVLLSGSHANEFFFRAGDDDLDQA
KAYPFMTPIFGEGVVFDASPERRKEMLHNAALRGEQMKGHAATIEDQVRRMIADWGEAGEIDLLDFFAELTIY
TSSACLIGKKFRDQLDGRFAKLYHELERGTDPLAYVDPYLPIESFRRRDEARNGLVALVADIMNGRIANPPTD
KSDRDMLDVLIAVKAETGTPRFSADEITGMFISMMFAGHHTSSGTASWTLIELMRHRDAYAAVIDELDELYGD
GRSVSFHALRQIPQLENVLKETLRLHPPLIILMRVAKGEFEVQGHRIHEGDLVAASPAISNRIPEDFPDPHDF
VPARYEQPRQEDLLNRWTWIPFGAGRHRCVGAAFAIMQIKAIFSVLLREYEFEMAQPPESYRNDHSKMVVQLA
QPACVRYRRRTGV
>CYP51B1_Mycobacterium tuberculosis A70555 2668797763
MSAVALPRVSGGHDEHGHLEEFRTDPIGLMQRVRDECGDVGTFQLAGKQVVLLSGSHANEFFFRAGDDDLDQA
KAYPFMTPIFGEGVVFDASPERRKEMLHNAALRGEQMKGHAATIEDQVRRMIADWGEAGEIDLLDFFAELTIY
TSSACLIGKKFRDQLDGRFAKLYHELERGTDPLAYVDPYLPIESFRRRDEARNGLVALVADIMNGRIANPPTD
KSDRDMLDVLIAVKAETGTPRFSADEITGMFISMMFAGHHTSSGTASWTLIELMRHRDAYAAVIDELDELYGD
GRSVSFHALRQIPQLENVLKETLRLHPPLIILMRVAKGEFEVQGHRIHEGDLVAASPAISNRIPEDFPDPHDF
VPARYEQPRQEDLLNRWTWIPFGAGRHRCVGAAFAIMQIKAIFSVLLREYEFEMAQPPESYRNDHSKMVVQLA
QPACVRYRRRTGV
>CYP51B1_Mycobacterium tuberculosis A70582 2657989890
MSAVALPRVSGGHDEHGHLEEFRTDPIGLMQRVRDECGDVGTFQLAGKQVVLLSGSHANEFFFRAGDDDLDQA
KAYPFMTPIFGEGVVFDASPERRKEMLHNAALRGEQMKGHAATIEDQVRRMIADWGEAGEIDLLDFFAELTIY
TSSACLIGKKFRDQLDGRFAKLYHELERGTDPLAYVDPYLPIESFRRRDEARNGLVALVADIMNGRIANPPTD
KSDRDMLDVLIAVKAETGTPRFSADEITGMFISMMFAGHHTSSGTASWTLIELMRHRDAYAAVIDELDELYGD
GRSVSFHALRQIPQLENVLKETLRLHPPLIILMRVAKGEFEVQGHRIHEGDLVAASPAISNRIPEDFPDPHDF
VPARYEQPRQEDLLNRWTWIPFGAGRHRCVGAAFAIMQIKAIFSVLLREYEFEMAQPPESYRNDHSKMVVQLA
QPACVRYRRRTGV
>CYP51B1_Mycobacterium tuberculosis A70596 2668928428
MSAVALPRVSGGHDEHGHLEEFRTDPIGLMQRVRDECGDVGTFQLAGKQVVLLSGSHANEFFFRAGDDDLDQA
KAYPFMTPIFGEGVVFDASPERRKEMLHNAALRGEQMKGHAATIEDQVRRMIADWGEAGEIDLLDFFAELTIY

TSSACLIGKKFRDQLDGRFAKLYHELERGTDPLAYVDPYLPIESFRRRDEARNGLVALVADIMNGRIANPPTD
KSDRDMLDVLIAVKAETGTPRFSADEITGMFISMMFAGHHTSSGTASWTLIELMRHRDAYAAVIDELDELYGD
GRSVSFHALRQIPQLENVLKETLRLHPPLIILMRVAKGEFEVQGHRIHEGDLVAASPAISNRIPEDFPDPHDF
VPARYEQPRQEDLLNRWTWIPFGAGRHRCVGAAFAIMQIKAIFSVLLREYEFEMAQPPESYRNDHSKMVVQLA
QPACVRYRRRTGV
>CYP51B1_Mycobacterium tuberculosis A70645 2666245423
MSAVALPRVSGGHDEHGHLEEFRTDPIGLMQRVRDECGDVGTFQLAGKQVVLLSGSHANEFFFRAGDDDLDQA
KAYPFMTPIFGEGVVFDASPERRKEMLHNAALRGEQMKGHAATIEDQVRRMIADWGEAGEIDLLDFFAELTIY
TSSACLIGKKFRDQLDGRFAKLYHELERGTDPLAYVDPYLPIESFRRRDEARNGLVALVADIMNGRIANPPTD
KSDRDMLDVLIAVKAETGTPRFSADEITGMFISMMFAGHHTSSGTASWTLIELMRHRDAYAAVIDELDELYGD
GRSVSFHALRQIPQLENVLKETLRLHPPLIILMRVAKGEFEVQGHRIHEGDLVAASPAISNRIPEDFPDPHDF
VPARYEQPRQEDLLNRWTWIPFGAGRHRCVGAAFAIMQIKAIFSVLLREYEFEMAQPPESYRNDHSKMVVQLA
QPACVRYRRRTGV
>CYP51B1_Mycobacterium tuberculosis A70655 2670572571
MSAVALPRVSGGHDEHGHLEEFRTDPIGLMQRVRDECGDVGTFQLAGKQVVLLSGSHANEFFFRAGDDDLDQA
KAYPFMTPIFGEGVVFDASPERRKEMLHNAALRGEQMKGHAATIEDQVRRMIADWGEAGEIDLLDFFAELTIY
TSSACLIGKKFRDQLDGRFAKLYHELERGTDPLAYVDPYLPIESFRRRDEARNGLVALVADIMNGRIANPPTD
KSDRDMLDVLIAVKAETGTPRFSADEITGMFISMMFAGHHTSSGTASWTLIELMRHRDAYAAVIDELDELYGD
GRSVSFHALRQIPQLENVLKETLRLHPPLIILMRVAKGEFEVQGHRIHEGDLVAASPAISNRIPEDFPDPHDF
VPARYEQPRQEDLLNRWTWIPFGAGRHRCVGAAFAIMQIKAIFSVLLREYEFEMAQPPESYRNDHSKMVVQLA
QPACVRYRRRTGV
>CYP51B1_Mycobacterium tuberculosis A70659 2694445615
MSAVALPRVSGGHDEHGHLEEFRTDPIGLMQRVRDECGDVGTFQLAGKQVVLLSGSHANEFFFRAGDDDLDQA
KAYPFMTPIFGEGVVFDASPERRKEMLHNAALRGEQMKGHAATIEDQVRRMIADWGEAGEIDLLDFFAELTIY
TSSACLIGKKFRDQLDGRFAKLYHELERGTDPLAYVDPYLPIESFRRRDEARNGLVALVADIMNGRIANPPTD
KSDRDMLDVLIAVKAETGTPRFSADEITGMFISMMFAGHHTSSGTASWTLIELMRHRDAYAAVIDELDELYGD
GRSVSFHALRQIPQLENVLKETLRLHPPLIILMRVAKGEFEVQGHRIHEGDLVAASPAISNRIPEDFPDPHDF
VPARYEQPRQEDLLNRWTWIPFGAGRHRCVGAAFAIMQIKAIFSVLLREYEFEMAQPPESYRNDHSKMVVQLA
QPACVRYRRRTGV
>CYP51B1_Mycobacterium tuberculosis A70661 2661734546
MSAVALPRVSGGHDEHGHLEEFRTDPIGLMQRVRDECGDVGTFQLAGKQVVLLSGSHANEFFFRAGDDDLDQA
KAYPFMTPIFGEGVVFDASPERRKEMLHNAALRGEQMKGHAATIEDQVRRMIADWGEAGEIDLLDFFAELTIY
TSSACLIGKKFRDQLDGRFAKLYHELERGTDPLAYVDPYLPIESFRRRDEARNGLVALVADIMNGRIANPPTD
KSDRDMLDVLIAVKAETGTPRFSADEITGMFISMMFAGHHTSSGTASWTLIELMRHRDAYAAVIDELDELYGD
GRSVSFHALRQIPQLENVLKETLRLHPPLIILMRVAKGEFEVQGHRIHEGDLVAASPAISNRIPEDFPDPHDF
VPARYEQPRQEDLLNRWTWIPFGAGRHRCVGAAFAIMQIKAIFSVLLREYEFEMAQPPESYRNDHSKMVVQLA
QPACVRYRRRTGV
>CYP51B1_Mycobacterium tuberculosis A70730 2697940434
MSAVALPRVSGGHDEHGHLEEFRTDPIGLMQRVRDECGDVGTFQLAGKQVVLLSGSHANEFFFRAGDDDLDQA
KAYPFMTPIFGEGVVFDASPERRKEMLHNAALRGEQMKGHAATIEDQVRRMIADWGEAGEIDLLDFFAELTIY
TSSACLIGKKFRDQLDGRFAKLYHELERGTDPLAYVDPYLPIESFRRRDEARNGLVALVADIMNGRIANPPTD
KSDRDMLDVLIAVKAETGTPRFSADEITGMFISMMFAGHHTSSGTASWTLIELMRHRDAYAAVIDELDELYGD
GRSVSFHALRQIPQLENVLKETLRLHPPLIILMRVAKGEFEVQGHRIHEGDLVAASPAISNRIPEDFPDPHDF
VPARYEQPRQEDLLNRWTWIPFGAGRHRCVGAAFAIMQIKAIFSVLLREYEFEMAQPPESYRNDHSKMVVQLA
QPACVRYRRRTGV
>CYP51B1_Mycobacterium tuberculosis A70757 2670581634
MSAVALPRVSGGHDEHGHLEEFRTDPIGLMQRVRDECGDVGTFQLAGKQVVLLSGSHANEFFFRAGDDDLDQA
KAYPFMTPIFGEGVVFDASPERRKEMLHNAALRGEQMKGHAATIEDQVRRMIADWGEAGEIDLLDFFAELTIY
TSSACLIGKKFRDQLDGRFAKLYHELERGTDPLAYVDPYLPIESFRRRDEARNGLVALVADIMNGRIANPPTD
KSDRDMLDVLIAVKAETGTPRFSADEITGMFISMMFAGHHTSSGTASWTLIELMRHRDAYAAVIDELDELYGD
GRSVSFHALRQIPQLENVLKETLRLHPPLIILMRVAKGEFEVQGHRIHEGDLVAASPAISNRIPEDFPDPHDF
VPARYEQPRQEDLLNRWTWIPFGAGRHRCVGAAFAIMQIKAIFSVLLREYEFEMAQPPESYRNDHSKMVVQLA
QPACVRYRRRTGV
>CYP51B1_Mycobacterium tuberculosis A70762 2672117113
MSAVALPRVSGGHDEHGHLEEFRTDPIGLMQRVRDECGDVGTFQLAGKQVVLLSGSHANEFFFRAGDDDLDQA
KAYPFMTPIFGEGVVFDASPERRKEMLHNAALRGEQMKGHAATIEDQVRRMIADWGEAGEIDLLDFFAELTIY
TSSACLIGKKFRDQLDGRFAKLYHELERGTDPLAYVDPYLPIESFRRRDEARNGLVALVADIMNGRIANPPTD
KSDRDMLDVLIAVKAETGTPRFSADEITGMFISMMFAGHHTSSGTASWTLIELMRHRDAYAAVIDELDELYGD

GRSVSFHALRQIPQLENVLKETLRLHPPLIILMRVAKGEFEVQGHRIHEGDLVAASPAISNRIPEDFPDPHDF
VPARYEQPRQEDLLNRWTWIPFGAGRHRCVGAAFAIMQIKAIFSVLLREYEFEMAQPPESYRNDHSKMVVQLA
QPACVRYRRRTGV
>CYP51B1_Mycobacterium tuberculosis A70763_1 2659948118
MSAVALPRVSGGHDEHGHLEEFRTDPIGLMQRVRDECGDVGTFQLAGKQVVLLSGSHANEFFFRAGDDDLDQA
KAYPFMTPIFGEGVVFDASPERRKEMLHNAALRGEQMKGHAATIEDQVRRMIADWGEAGEIDLLDFFAELTIY
TSSACLIGKKFRDQLDGRFAKLYHELERGTDPLAYVDPYLPIESFRRRDEARNGLVALVADIMNGRIANPPTD
KSDRDMLDVLIAVKAETGTPRFSADEITGMFISMMFAGHHTSSGTASWTLIELMRHRDAYAAVIDELDELYGD
GRSVSFHALRQIPQLENVLKETLRLHPPLIILMRVAKGEFEVQGHRIHEGDLVAASPAISNRIPEDFPDPHDF
VPARYEQPRQEDLLNRWTWIPFGAGRHRCVGAAFAIMQIKAIFSVLLREYEFEMAQPPESYRNDHSKMVVQLA
QPACVRYRRRTGV
>CYP51B1_Mycobacterium tuberculosis A70763_2 2659041183
MSAVALPRVSGGHDEHGHLEEFRTDPIGLMQRVRDECGDVGTFQLAGKQVVLLSGSHANEFFFRAGDDDLDQA
KAYPFMTPIFGEGVVFDASPERRKEMLHNAALRGEQMKGHAATIEDQVRRMIADWGEAGEIDLLDFFAELTIY
TSSACLIGKKFRDQLDGRFAKLYHELERGTDPLAYVDPYLPIESFRRRDEARNGLVALVADIMNGRIANPPTD
KSDRDMLDVLIAVKAETGTPRFSADEITGMFISMMFAGHHTSSGTASWTLIELMRHRDAYAAVIDELDELYGD
GRSVSFHALRQIPQLENVLKETLRLHPPLIILMRVAKGEFEVQGHRIHEGDLVAASPAISNRIPEDFPDPHDF
VPARYEQPRQEDLLNRWTWIPFGAGRHRCVGAAFAIMQIKAIFSVLLREYEFEMAQPPESYRNDHSKMVVQLA
QPACVRYRRRTGV
>CYP51B1_Mycobacterium tuberculosis A70785 2693130043
MSAVALPRVSGGHDEHGHLEEFRTDPIGLMQRVRDECGDVGTFQLAGKQVVLLSGSHANEFFFRAGDDDLDQA
KAYPFMTPIFGEGVVFDASPERRKEMLHNAALRGEQMKGHAATIEDQVRRMIADWGEAGEIDLLDFFAELTIY
TSSACLIGKKFRDQLDGRFAKLYHELERGTDPLAYVDPYLPIESFRRRDEARNGLVALVADIMNGRIANPPTD
KSDRDMLDVLIAVKAETGTPRFSADEITGMFISMMFAGHHTSSGTASWTLIELMRHRDAYAAVIDELDELYGD
GRSVSFHALRQIPQLENVLKETLRLHPPLIILMRVAKGEFEVQGHRIHEGDLVAASPAISNRIPEDFPDPHDF
VPARYEQPRQEDLLNRWTWIPFGAGRHRCVGAAFAIMQIKAIFSVLLREYEFEMAQPPESYRNDHSKMVVQLA
QPACVRYRRRTGV
>CYP51B1_Mycobacterium tuberculosis AH_14271 2716314183
MSAVALPRVSGGHDEHGHLEEFRTDPIGLMQRVRDECGDVGTFQLAGKQVVLLSGSHANEFFFRAGDDDLDQA
KAYPFMTPIFGEGVVFDASPERRKEMLHNAALRGEQMKGHAATIEDQVRRMIADWGEAGEIDLLDFFAELTIY
TSSACLIGKKFRDQLDGRFAKLYHELERGTDPLAYVDPYLPIESFRRRDEARNGLVALVADIMNGRIANPPTD
KSDRDMLDVLIAVKAETGTPRFSADEITGMFISMMFAGHHTSSGTASWTLIELMRHRDAYAAVIDELDELYGD
GRSVSFHALRQIPQLENVLKETLRLHPPLIILMRVAKGEFEVQGHRIHEGDLVAASPAISNRIPEDFPDPHDF
VPARYEQPRQEDLLNRWTWIPFGAGRHRCVGAAFAIMQIKAIFSVLLREYEFEMAQPPESYRNDHSKMVVQLA
QPACVRYRRRTGV
>CYP51B1_Mycobacterium tuberculosis AH26_26663 2714490536
MSAVALPRVSGGHDEHGHLEEFRTDPIGLMQRVRDECGDVGTFQLAGKQVVLLSGSHANEFFFRAGDDDLDQA
KAYPFMTPIFGEGVVFDASPERRKEMLHNAALRGEQMKGHAATIEDQVRRMIADWGEAGEIDLLDFFAELTIY
TSSACLIGKKFRDQLDGRFAKLYHELERGTDPLAYVDPYLPIESFRRRDEARNGLVALVADIMNGRIANPPTD
KSDRDMLDVLIAVKAETGTPRFSADEITGMFISMMFAGHHTSSGTASWTLIELMRHRDAYAAVIDELDELYGD
GRSVSFHALRQIPQLENVLKETLRLHPPLIILMRVAKGEFEVQGHRIHEGDLVAASPAISNRIPEDFPDPHDF
VPARYEQPRQEDLLNRWTWIPFGAGRHRCVGAAFAIMQIKAIFSVLLREYEFEMAQPPESYRNDHSKMVVQLA
QPACVRYRRRTGV
>CYP51B1_Mycobacterium tuberculosis AH26_28866 2724802435
MSAVALPRVSGGHDEHGHLEEFRTDPIGLMQRVRDECGDVGTFQLAGKQVVLLSGSHANEFFFRAGDDDLDQA
KAYPFMTPIFGEGVVFDASPERRKEMLHNAALRGEQMKGHAATIEDQVRRMIADWGEAGEIDLLDFFAELTIY
TSSACLIGKKFRDQLDGRFAKLYHELERGTDPLAYVDPYLPIESFRRRDEARNGLVALVADIMNGRIANPPTD
KSDRDMLDVLIAVKAETGTPRFSADEITGMFISMMFAGHHTSSGTASWTLIELMRHRDAYAAVIDELDELYGD
GRSVSFHALRQIPQLENVLKETLRLHPPLIILMRVAKGEFEVQGHRIHEGDLVAASPAISNRIPEDFPDPHDF
VPARYEQPRQEDLLNRWTWIPFGAGRHRCVGAAFAIMQIKAIFSVLLREYEFEMAQPPESYRNDHSKMVVQLA
QPACVRYRRRTGV
>CYP51B1_Mycobacterium tuberculosis AU_8623 2724799600
MSAVALPRVSGGHDEHGHLEEFRTDPIGLMQRVRDECGDVGTFQLAGKQVVLLSGSHANEFFFRAGDDDLDQA
KAYPFMTPIFGEGVVFDASPERRKEMLHNAALRGEQMKGHAATIEDQVRRMIADWGEAGEIDLLDFFAELTIY
TSSACLIGKKFRDQLDGRFAKLYHELERGTDPLAYVDPYLPIESFRRRDEARNGLVALVADIMNGRIANPPTD
KSDRDMLDVLIAVKAETGTPRFSADEITGMFISMMFAGHHTSSGTASWTLIELMRHRDAYAAVIDELDELYGD
GRSVSFHALRQIPQLENVLKETLRLHPPLIILMRVAKGEFEVQGHRIHEGDLVAASPAISNRIPEDFPDPHDF

VPARYEQPRQEDLLNRWTWIPFGAGRHRCVGAAFAIMQIKAIFSVLLREYEFEMAQPPESYRNDHSKMVVQLA
QPACVRYRRRTGV
>CYP51B1_Mycobacterium tuberculosis B00001265 2703435616
MSAVALPRVSGGHDEHGHLEEFRTDPIGLMQRVRDECGDVGTFQLAGKQVVLLSGSHANEFFFRAGDDDLDQA
KAYPFMTPIFGEGVVFDASPERRKEMLHNAALRGEQMKGHAATIEDQVRRMIADWGEAGEIDLLDFFAELTIY
TSSACLIGKKFRDQLDGRFAKLYHELERGTDPLAYVDPYLPIESFRRRDEARNGLVALVADIMNGRIANPPTD
KSDRDMLDVLIAVKAETGTPRFSADEITGMFISMMFAGHHTSSGTASWTLIELMRHRDAYAAVIDELDELYGD
GRSVSFHALRQIPQLENVLKETLRLHPPLIILMRVAKGEFEVQGHRIHEGDLVAASPAISNRIPEDFPDPHDF
VPARYEQPRQEDLLNRWTWIPFGAGRHRCVGAAFAIMQIKAIFSVLLREYEFEMAQPPESYRNDHSKMVVQLA
QPACVRYRRRTGV
>CYP51B1_Mycobacterium tuberculosis B00600458 2699909193
MSAVALPRVSGGHDEHGHLEEFRTDPIGLMQRVRDECGDVGTFQLAGKQVVLLSGSHANEFFFRAGDDDLDQA
KAYPFMTPIFGEGVVFDASPERRKEMLHNAALRGEQMKGHAATIEDQVRRMIADWGEAGEIDLLDFFAELTIY
TSSACLIGKKFRDQLDGRFAKLYHELERGTDPLAYVDPYLPIESFRRRDEARNGLVALVADIMNGRIANPPTD
KSDRDMLDVLIAVKAETGTPRFSADEITGMFISMMFAGHHTSSGTASWTLIELMRHRDAYAAVIDELDELYGD
GRSVSFHALRQIPQLENVLKETLRLHPPLIILMRVAKGEFEVQGHRIHEGDLVAASPAISNRIPEDFPDPHDF
VPARYEQPRQEDLLNRWTWIPFGAGRHRCVGAAFAIMQIKAIFSVLLREYEFEMAQPPESYRNDHSKMVVQLA
QPACVRYRRRTGV
>CYP51B1_Mycobacterium tuberculosis B00801080 2677958998
MSAVALPRVSGGHDEHGHLEEFRTDPIGLMQRVRDECGDVGTFQLAGKQVVLLSGSHANEFFFRAGDDDLDQA
KAYPFMTPIFGEGVVFDASPERRKEMLHNAALRGEQMKGHAATIEDQVRRMIADWGEAGEIDLLDFFAELTIY
TSSACLIGKKFRDQLDGRFAKLYHELERGTDPLAYVDPYLPIESFRRRDEARNGLVALVADIMNGRIANPPTD
KSDRDMLDVLIAVKAETGTPRFSADEITGMFISMMFAGHHTSSGTASWTLIELMRHRDAYAAVIDELDELYGD
GRSVSFHALRQIPQLENVLKETLRLHPPLIILMRVAKGEFEVQGHRIHEGDLVAASPAISNRIPEDFPDPHDF
VPARYEQPRQEDLLNRWTWIPFGAGRHRCVGAAFAIMQIKAIFSVLLREYEFEMAQPPESYRNDHSKMVVQLA
QPACVRYRRRTGV
>CYP51B1_Mycobacterium tuberculosis B09601851 2703470690
MSAVALPRVSGGHDEHGHLEEFRTDPIGLMQRVRDECGDVGTFQLAGKQVVLLSGSHANEFFFRAGDDDLDQA
KAYPFMTPIFGEGVVFDASPERRKEMLHNAALRGEQMKGHAATIEDQVRRMIADWGEAGEIDLLDFFAELTIY
TSSACLIGKKFRDQLDGRFAKLYHELERGTDPLAYVDPYLPIESFRRRDEARNGLVALVADIMNGRIANPPTD
KSDRDMLDVLIAVKAETGTPRFSADEITGMFISMMFAGHHTSSGTASWTLIELMRHRDAYAAVIDELDELYGD
GRSVSFHALRQIPQLENVLKETLRLHPPLIILMRVAKGEFEVQGHRIHEGDLVAASPAISNRIPEDFPDPHDF
VPARYEQPRQEDLLNRWTWIPFGAGRHRCVGAAFAIMQIKAIFSVLLREYEFEMAQPPESYRNDHSKMVVQLA
QPACVRYRRRTGV
>CYP51B1_Mycobacterium tuberculosis B09601926 2693821502
MSAVALPRVSGGHDEHGHLEEFRTDPIGLMQRVRDECGDVGTFQLAGKQVVLLSGSHANEFFFRAGDDDLDQA
KAYPFMTPIFGEGVVFDASPERRKEMLHNAALRGEQMKGHAATIEDQVRRMIADWGEAGEIDLLDFFAELTIY
TSSACLIGKKFRDQLDGRFAKLYHELERGTDPLAYVDPYLPIESFRRRDEARNGLVALVADIMNGRIANPPTD
KSDRDMLDVLIAVKAETGTPRFSADEITGMFISMMFAGHHTSSGTASWTLIELMRHRDAYAAVIDELDELYGD
GRSVSFHALRQIPQLENVLKETLRLHPPLIILMRVAKGEFEVQGHRIHEGDLVAASPAISNRIPEDFPDPHDF
VPARYEQPRQEDLLNRWTWIPFGAGRHRCVGAAFAIMQIKAIFSVLLREYEFEMAQPPESYRNDHSKMVVQLA
QPACVRYRRRTGV
>CYP51B1_Mycobacterium tuberculosis B09701572 2703454167
MSAVALPRVSGGHDEHGHLEEFRTDPIGLMQRVRDECGDVGTFQLAGKQVVLLSGSHANEFFFRAGDDDLDQA
KAYPFMTPIFGEGVVFDASPERRKEMLHNAALRGEQMKGHAATIEDQVRRMIADWGEAGEIDLLDFFAELTIY
TSSACLIGKKFRDQLDGRFAKLYHELERGTDPLAYVDPYLPIESFRRRDEARNGLVALVADIMNGRIANPPTD
KSDRDMLDVLIAVKAETGTPRFSADEITGMFISMMFAGHHTSSGTASWTLIELMRHRDAYAAVIDELDELYGD
GRSVSFHALRQIPQLENVLKETLRLHPPLIILMRVAKGEFEVQGHRIHEGDLVAASPAISNRIPEDFPDPHDF
VPARYEQPRQEDLLNRWTWIPFGAGRHRCVGAAFAIMQIKAIFSVLLREYEFEMAQPPESYRNDHSKMVVQLA
QPACVRYRRRTGV
>CYP51B1_Mycobacterium tuberculosis B9741 2745704988
MSAVALPRVSGGHDEHGHLEEFRTDPIGLMQRVRDECGDVGTFQLAGKQVVLLSGSHANEFFFRAGDDDLDQA
KAYPFMTPIFGEGVVFDASPERRKEMLHNAALRGEQMKGHAATIEDQVRRMIADWGEAGEIDLLDFFAELTIY
TSSACLIGKKFRDQLDGRFAKLYHELERGTDPLAYVDPYLPIESFRRRDEARNGLVALVADIMNGRIANPPTD
KSDRDMLDVLIAVKAETGTPRFSADEITGMFISMMFAGHHTSSGTASWTLIELMRHRDAYAAVIDELDELYGD
GRSVSFHALRQIPQLENVLKETLRLHPPLIILMRVAKGEFEVQGHRIHEGDLVAASPAISNRIPEDFPDPHDF
VPARYEQPRQEDLLNRWTWIPFGAGRHRCVGAAFAIMQIKAIFSVLLREYEFEMAQPPESYRNDHSKMVVQLA
QPACVRYRRRTGV

>CYP51B1_Mycobacterium tuberculosis BE_10225 2714478013
MSAVALPRVSGGHDEHGHLEEFRTDPIGLMQRVRDECGDVGTFQLAGKQVVLLSGSHANEFFFRAGDDDLDQA
KAYPFMTPIFGEGVVFDASPERRKEMLHNAALRGEQMKGHAATIEDQVRRMIADWGEAGEIDLLDFFAELTIY
TSSACLIGKKFRDQLDGRFAKLYHELERGTDPLAYVDPYLPIESFRRRDEARNGLVALVADIMNGRIANPPTD
KSDRDMLDVLIAVKAETGTPRFSADEITGMFISMMFAGHHTSSGTASWTLIELMRHRDAYAAVIDELDELYGD
GRSVSFHALRQIPQLENVLKETLRLHPPLIILMRVAKGEFEVQGHRIHEGDLVAASPAISNRIPEDFPDPHDF
VPARYEQPRQEDLLNRWTWIPFGAGRHRCVGAAFAIMQIKAIFSVLLREYEFEMAQPPESYRNDHSKMVVQLA
QPACVRYRRRTGV
>CYP51B1_Mycobacterium tuberculosis BE_116771 2724793347
MSAVALPRVSGGHDEHGHLEEFRTDPIGLMQRVRDECGDVGTFQLAGKQVVLLSGSHANEFFFRAGDDDLDQA
KAYPFMTPIFGEGVVFDASPERRKEMLHNAALRGEQMKGHAATIEDQVRRMIADWGEAGEIDLLDFFAELTIY
TSSACLIGKKFRDQLDGRFAKLYHELERGTDPLAYVDPYLPIESFRRRDEARNGLVALVADIMNGRIANPPTD
KSDRDMLDVLIAVKAETGTPRFSADEITGMFISMMFAGHHTSSGTASWTLIELMRHRDAYAAVIDELDELYGD
GRSVSFHALRQIPQLENVLKETLRLHPPLIILMRVAKGEFEVQGHRIHEGDLVAASPAISNRIPEDFPDPHDF
VPARYEQPRQEDLLNRWTWIPFGAGRHRCVGAAFAIMQIKAIFSVLLREYEFEMAQPPESYRNDHSKMVVQLA
QPACVRYRRRTGV
>CYP51B1_Mycobacterium tuberculosis BE_13443 2677590273
MSAVALPRVSGGHDEHGHLEEFRTDPIGLMQRVRDECGDVGTFQLAGKQVVLLSGSHANEFFFRAGDDDLDQA
KAYPFMTPIFGEGVVFDASPERRKEMLHNAALRGEQMKGHAATIEDQVRRMIADWGEAGEIDLLDFFAELTIY
TSSACLIGKKFRDQLDGRFAKLYHELERGTDPLAYVDPYLPIESFRRRDEARNGLVALVADIMNGRIANPPTD
KSDRDMLDVLIAVKAETGTPRFSADEITGMFISMMFAGHHTSSGTASWTLIELMRHRDAYAAVIDELDELYGD
GRSVSFHALRQIPQLENVLKETLRLHPPLIILMRVAKGEFEVQGHRIHEGDLVAASPAISNRIPEDFPDPHDF
VPARYEQPRQEDLLNRWTWIPFGAGRHRCVGAAFAIMQIKAIFSVLLREYEFEMAQPPESYRNDHSKMVVQLA
QPACVRYRRRTGV
>CYP51B1_Mycobacterium tuberculosis BE_14248 2716318157
MSAVALPRVSGGHDEHGHLEEFRTDPIGLMQRVRDECGDVGTFQLAGKQVVLLSGSHANEFFFRAGDDDLDQA
KAYPFMTPIFGEGVVFDASPERRKEMLHNAALRGEQMKGHAATIEDQVRRMIADWGEAGEIDLLDFFAELTIY
TSSACLIGKKFRDQLDGRFAKLYHELERGTDPLAYVDPYLPIESFRRRDEARNGLVALVADIMNGRIANPPTD
KSDRDMLDVLIAVKAETGTPRFSADEITGMFISMMFAGHHTSSGTASWTLIELMRHRDAYAAVIDELDELYGD
GRSVSFHALRQIPQLENVLKETLRLHPPLIILMRVAKGEFEVQGHRIHEGDLVAASPAISNRIPEDFPDPHDF
VPARYEQPRQEDLLNRWTWIPFGAGRHRCVGAAFAIMQIKAIFSVLLREYEFEMAQPPESYRNDHSKMVVQLA
QPACVRYRRRTGV
>CYP51B1_Mycobacterium tuberculosis BE_7556 2675441671
MSAVALPRVSGGHDEHGHLEEFRTDPIGLMQRVRDECGDVGTFQLAGKQVVLLSGSHANEFFFRAGDDDLDQA
KAYPFMTPIFGEGVVFDASPERRKEMLHNAALRGEQMKGHAATIEDQVRRMIADWGEAGEIDLLDFFAELTIY
TSSACLIGKKFRDQLDGRFAKLYHELERGTDPLAYVDPYLPIESFRRRDEARNGLVALVADIMNGRIANPPTD
KSDRDMLDVLIAVKAETGTPRFSADEITGMFISMMFAGHHTSSGTASWTLIELMRHRDAYAAVIDELDELYGD
GRSVSFHALRQIPQLENVLKETLRLHPPLIILMRVAKGEFEVQGHRIHEGDLVAASPAISNRIPEDFPDPHDF
VPARYEQPRQEDLLNRWTWIPFGAGRHRCVGAAFAIMQIKAIFSVLLREYEFEMAQPPESYRNDHSKMVVQLA
QPACVRYRRRTGV
>CYP51B1_Mycobacterium tuberculosis BE3_11657 2716303170
MSAVALPRVSGGHDEHGHLEEFRTDPIGLMQRVRDECGDVGTFQLAGKQVVLLSGSHANEFFFRAGDDDLDQA
KAYPFMTPIFGEGVVFDASPERRKEMLHNAALRGEQMKGHAATIEDQVRRMIADWGEAGEIDLLDFFAELTIY
TSSACLIGKKFRDQLDGRFAKLYHELERGTDPLAYVDPYLPIESFRRRDEARNGLVALVADIMNGRIANPPTD
KSDRDMLDVLIAVKAETGTPRFSADEITGMFISMMFAGHHTSSGTASWTLIELMRHRDAYAAVIDELDELYGD
GRSVSFHALRQIPQLENVLKETLRLHPPLIILMRVAKGEFEVQGHRIHEGDLVAASPAISNRIPEDFPDPHDF
VPARYEQPRQEDLLNRWTWIPFGAGRHRCVGAAFAIMQIKAIFSVLLREYEFEMAQPPESYRNDHSKMVVQLA
QPACVRYRRRTGV
>CYP51B1_Mycobacterium tuberculosis Beijing 2730185371
MSAVALPRVSGGHDEHGHLEEFRTDPIGLMQRVRDECGDVGTFQLAGKQVVLLSGSHANEFFFRAGDDDLDQA
KAYPFMTPIFGEGVVFDASPERRKEMLHNAALRGEQMKGHAATIEDQVRRMIADWGEAGEIDLLDFFAELTIY
TSSACLIGKKFRDQLDGRFAKLYHELERGTDPLAYVDPYLPIESFRRRDEARNGLVALVADIMNGRIANPPTD
KSDRDMLDVLIAVKAETGTPRFSADEITGMFISMMFAGHHTSSGTASWTLIELMRHRDAYAAVIDELDELYGD
GRSVSFHALRQIPQLENVLKETLRLHPPLIILMRVAKGEFEVQGHRIHEGDLVAASPAISNRIPEDFPDPHDF
VPARYEQPRQEDLLNRWTWIPFGAGRHRCVGAAFAIMQIKAIFSVLLREYEFEMAQPPESYRNDHSKMVVQLA
QPACVRYRRRTGV
>CYP51B1_Mycobacterium tuberculosis Beijing 2712473824

MSAVALPRVSGGHDEHGHLEEFRTDPIGLMQRVRDECGDVGTFQLAGKQVVLLSGSHANEFFFRAGDDDLDQA
KAYPFMTPIFGEGVVFDASPERRKEMLHNAALRGEQMKGHAATIEDQVRRMIADWGEAGEIDLLDFFAELTIY
TSSACLIGKKFRDQLDGRFAKLYHELERGTDPLAYVDPYLPIESFRRRDEARNGLVALVADIMNGRIANPPTD
KSDRDMLDVLIAVKAETGTPRFSADEITGMFISMMFAGHHTSSGTASWTLIELMRHRDAYAAVIDELDELYGD
GRSVSFHALRQIPQLENVLKETLRLHPPLIILMRVAKGEFEVQGHRIHEGDLVAASPAISNRIPEDFPDPHDF
VPARYEQPRQEDLLNRWTWIPFGAGRHRCVGAAFAIMQIKAIFSVLLREYEFEMAQPPESYRNDHSKMVVQLA
QPACVRYRRRTGV
>CYP51B1_Mycobacterium tuberculosis Beijing/NITR203 2546187181
MSAVALPRVSGGHDEHGHLEEFRTDPIGLMQRVRDECGDVGTFQLAGKQVVLLSGSHANEFFFRAGDDDLDQA
KAYPFMTPIFGEGVVFDASPERRKEMLHNAALRGEQMKGHAATIEDQVRRMIADWGEAGEIDLLDFFAELTIY
TSSACLIGKKFRDQLDGRFAKLYHELERGTDPLAYVDPYLPIESFRRRDEARNGLVALVADIMNGRIANPPTD
KSDRDMLDVLIAVKAETGTPRFSADEITGMFISMMFAGHHTSSGTASWTLIELMRHRDAYAAVIDELDELYGD
GRSVSFHALRQIPQLENVLKETLRLHPPLIILMRVAKGEFEVQGHRIHEGDLVAASPAISNRIPEDFPDPHDF
VPARYEQPRQEDLLNRWTWIPFGAGRHRCVGAAFAIMQIKAIFSVLLREYEFEMAQPPESYRNDHSKMVVQLA
QPACVRYRRRTGV
>CYP51B1_Mycobacterium tuberculosis 323 2647007464
MSAVALPRVSGGHDEHGHLEEFRTDPIGLMQRVRDECGDVGTFQLAGKQVVLLSGSHANEFFFRAGDDDLDQA
KAYPFMTPIFGEGVVFDASPERRKEMLHNAALRGEQMKGHAATIEDQVRRMIADWGEAGEIDLLDFFAELTIY
TSSACLIGKKFRDQLDGRFAKLYHELERGTDPLAYVDPYLPIESFRRRDEARNGLVALVADIMNGRIANPPTD
KSDRDMLDVLIAVKAETGTPRFSADEITGMFISMMFAGHHTSSGTASWTLIELMRHRDAYAAVIDELDELYGD
GRSVSFHALRQIPQLENVLKETLRLHPPLIILMRVAKGEFEVQGHRIHEGDLVAASPAISNRIPEDFPDPHDF
VPARYEQPRQEDLLNRWTWIPFGAGRHRCVGAAFAIMQIKAIFSVLLREYEFEMAQPPESYRNDHSKMVVQLA
QPACVRYRRRTGV
>CYP51B1_Mycobacterium tuberculosis BS1 2559162528
MSAVALPRVSGGHDEHGHLEEFRTDPIGLMQRVRDECGDVGTFQLAGKQVVLLSGSHANEFFFRAGDDDLDQA
KAYPFMTPIFGEGVVFDASPERRKEMLHNAALRGEQMKGHAATIEDQVRRMIADWGEAGEIDLLDFFAELTIY
TSSACLIGKKFRDQLDGRFAKLYHELERGTDPLAYVDPYLPIESFRRRDEARNGLVALVADIMNGRIANPPTD
KSDRDMLDVLIAVKAETGTPRFSADEITGMFISMMFAGHHTSSGTASWTLIELMRHRDAYAAVIDELDELYGD
GRSVSFHALRQIPQLENVLKETLRLHPPLIILMRVAKGEFEVQGHRIHEGDLVAASPAISNRIPEDFPDPHDF
VPARYEQPRQEDLLNRWTWIPFGAGRHRCVGAAFAIMQIKAIFSVLLREYEFEMAQPPESYRNDHSKMVVQLA
QPACVRYRRRTGV
>CYP51B1_Mycobacterium tuberculosis BT2 2566258046
MSAVALPRVSGGHDEHGHLEEFRTDPIGLMQRVRDECGDVGTFQLAGKQVVLLSGSHANEFFFRAGDDDLDQA
KAYPFMTPIFGEGVVFDASPERRKEMLHNAALRGEQMKGHAATIEDQVRRMIADWGEAGEIDLLDFFAELTIY
TSSACLIGKKFRDQLDGRFAKLYHELERGTDPLAYVDPYLPIESFRRRDEARNGLVALVADIMNGRIANPPTD
KSDRDMLDVLIAVKAETGTPRFSADEITGMFISMMFAGHHTSSGTASWTLIELMRHRDAYAAVIDELDELYGD
GRSVSFHALRQIPQLENVLKETLRLHPPLIILMRVAKGEFEVQGHRIHEGDLVAASPAISNRIPEDFPDPHDF
VPARYEQPRQEDLLNRWTWIPFGAGRHRCVGAAFAIMQIKAIFSVLLREYEFEMAQPPESYRNDHSKMVVQLA
QPACVRYRRRTGV
>CYP51B1_Mycobacterium tuberculosis BT1 2688581050
MSAVALPRVSGGHDEHGHLEEFRTDPIGLMQRVRDECGDVGTFQLAGKQVVLLSGSHANEFFFRAGDDDLDQA
KAYPFMTPIFGEGVVFDASPERRKEMLHNAALRGEQMKGHAATIEDQVRRMIADWGEAGEIDLLDFFAELTIY
TSSACLIGKKFRDQLDGRFAKLYHELERGTDPLAYVDPYLPIESFRRRDEARNGLVALVADIMNGRIANPPTD
KSDRDMLDVLIAVKAETGTPRFSADEITGMFISMMFAGHHTSSGTASWTLIELMRHRDAYAAVIDELDELYGD
GRSVSFHALRQIPQLENVLKETLRLHPPLIILMRVAKGEFEVQGHRIHEGDLVAASPAISNRIPEDFPDPHDF
VPARYEQPRQEDLLNRWTWIPFGAGRHRCVGAAFAIMQIKAIFSVLLREYEFEMAQPPESYRNDHSKMVVQLA
QPACVRYRRRTGV
>CYP51B1_Mycobacterium tuberculosis BTB03-012 2577075035
MSAVALPRVSGGHDEHGHLEEFRTDPIGLMQRVRDECGDVGTFQLAGKQVVLLSGSHANEFFFRAGDDDLDQA
KAYPFMTPIFGEGVVFDASPERRKEMLHNAALRGEQMKGHAATIEDQVRRMIADWGEAGEIDLLDFFAELTIY
TSSACLIGKKFRDQLDGRFAKLYHELERGTDPLAYVDPYLPIESFRRRDEARNGLVALVADIMNGRIANPPTD
KSDRDMLDVLIAVKAETGTPRFSADEITGMFISMMFAGHHTSSGTASWTLIELMRHRDAYAAVIDELDELYGD
GRSVSFHALRQIPQLENVLKETLRLHPPLIILMRVAKGEFEVQGHRIHEGDLVAASPAISNRIPEDFPDPHDF
VPARYEQPRQEDLLNRWTWIPFGAGRHRCVGAAFAIMQIKAIFSVLLREYEFEMAQPPESYRNDHSKMVVQLA
QPACVRYRRRTGV
>CYP51B1_Mycobacterium tuberculosis BTB03-108 2604884572
MSAVALPRVSGGHDEHGHLEEFRTDPIGLMQRVRDECGDVGTFQLAGKQVVLLSGSHANEFFFRAGDDDLDQA
KAYPFMTPIFGEGVVFDASPERRKEMLHNAALRGEQMKGHAATIEDQVRRMIADWGEAGEIDLLDFFAELTIY

TSSACLIGKKFRDQLDGRFAKLYHELERGTDPLAYVDPYLPIESFRRRDEARNGLVALVADIMNGRIANPPTD
KSDRDMLDVLIAVKAETGTPRFSADEITGMFISMMFAGHHTSSGTASWTLIELMRHRDAYAAVIDELDELYGD
GRSVSFHALRQIPQLENVLKETLRLHPPLIILMRVAKGEFEVQGHRIHEGDLVAASPAISNRIPEDFPDPHDF
VPARYEQPRQEDLLNRWTWIPFGAGRHRCVGAAFAIMQIKAIFSVLLREYEFEMAQPPESYRNDHSKMVVQLA
QPACVRYRRRTGV
>CYP51B1_Mycobacterium tuberculosis BTB03-142 2603299378
MSAVALPRVSGGHDEHGHLEEFRTDPIGLMQRVRDECGDVGTFQLAGKQVVLLSGSHANEFFFRAGDDDLDQA
KAYPFMTPIFGEGVVFDASPERRKEMLHNAALRGEQMKGHAATIEDQVRRMIADWGEAGEIDLLDFFAELTIY
TSSACLIGKKFRDQLDGRFAKLYHELERGTDPLAYVDPYLPIESFRRRDEARNGLVALVADIMNGRIANPPTD
KSDRDMLDVLIAVKAETGTPRFSADEITGMFISMMFAGHHTSSGTASWTLIELMRHRDAYAAVIDELDELYGD
GRSVSFHALRQIPQLENVLKETLRLHPPLIILMRVAKGEFEVQGHRIHEGDLVAASPAISNRIPEDFPDPHDF
VPARYEQPRQEDLLNRWTWIPFGAGRHRCVGAAFAIMQIKAIFSVLLREYEFEMAQPPESYRNDHSKMVVQLA
QPACVRYRRRTGV
>CYP51B1_Mycobacterium tuberculosis BTB03-143 2576643964
MSAVALPRVSGGHDEHGHLEEFRTDPIGLMQRVRDECGDVGTFQLAGKQVVLLSGSHANEFFFRAGDDDLDQA
KAYPFMTPIFGEGVVFDASPERRKEMLHNAALRGEQMKGHAATIEDQVRRMIADWGEAGEIDLLDFFAELTIY
TSSACLIGKKFRDQLDGRFAKLYHELERGTDPLAYVDPYLPIESFRRRDEARNGLVALVADIMNGRIANPPTD
KSDRDMLDVLIAVKAETGTPRFSADEITGMFISMMFAGHHTSSGTASWTLIELMRHRDAYAAVIDELDELYGD
GRSVSFHALRQIPQLENVLKETLRLHPPLIILMRVAKGEFEVQGHRIHEGDLVAASPAISNRIPEDFPDPHDF
VPARYEQPRQEDLLNRWTWIPFGAGRHRCVGAAFAIMQIKAIFSVLLREYEFEMAQPPESYRNDHSKMVVQLA
QPACVRYRRRTGV
>CYP51B1_Mycobacterium tuberculosis BTB03-144 2576946783
MSAVALPRVSGGHDEHGHLEEFRTDPIGLMQRVRDECGDVGTFQLAGKQVVLLSGSHANEFFFRAGDDDLDQA
KAYPFMTPIFGEGVVFDASPERRKEMLHNAALRGEQMKGHAATIEDQVRRMIADWGEAGEIDLLDFFAELTIY
TSSACLIGKKFRDQLDGRFAKLYHELERGTDPLAYVDPYLPIESFRRRDEARNGLVALVADIMNGRIANPPTD
KSDRDMLDVLIAVKAETGTPRFSADEITGMFISMMFAGHHTSSGTASWTLIELMRHRDAYAAVIDELDELYGD
GRSVSFHALRQIPQLENVLKETLRLHPPLIILMRVAKGEFEVQGHRIHEGDLVAASPAISNRIPEDFPDPHDF
VPARYEQPRQEDLLNRWTWIPFGAGRHRCVGAAFAIMQIKAIFSVLLREYEFEMAQPPESYRNDHSKMVVQLA
QPACVRYRRRTGV
>CYP51B1_Mycobacterium tuberculosis BTB03-168 2605243692
MSAVALPRVSGGHDEHGHLEEFRTDPIGLMQRVRDECGDVGTFQLAGKQVVLLSGSHANEFFFRAGDDDLDQA
KAYPFMTPIFGEGVVFDASPERRKEMLHNAALRGEQMKGHAATIEDQVRRMIADWGEAGEIDLLDFFAELTIY
TSSACLIGKKFRDQLDGRFAKLYHELERGTDPLAYVDPYLPIESFRRRDEARNGLVALVADIMNGRIANPPTD
KSDRDMLDVLIAVKAETGTPRFSADEITGMFISMMFAGHHTSSGTASWTLIELMRHRDAYAAVIDELDELYGD
GRSVSFHALRQIPQLENVLKETLRLHPPLIILMRVAKGEFEVQGHRIHEGDLVAASPAISNRIPEDFPDPHDF
VPARYEQPRQEDLLNRWTWIPFGAGRHRCVGAAFAIMQIKAIFSVLLREYEFEMAQPPESYRNDHSKMVVQLA
QPACVRYRRRTGV
>CYP51B1_Mycobacterium tuberculosis BTB03-169 2604515466
MSAVALPRVSGGHDEHGHLEEFRTDPIGLMQRVRDECGDVGTFQLAGKQVVLLSGSHANEFFFRAGDDDLDQA
KAYPFMTPIFGEGVVFDASPERRKEMLHNAALRGEQMKGHAATIEDQVRRMIADWGEAGEIDLLDFFAELTIY
TSSACLIGKKFRDQLDGRFAKLYHELERGTDPLAYVDPYLPIESFRRRDEARNGLVALVADIMNGRIANPPTD
KSDRDMLDVLIAVKAETGTPRFSADEITGMFISMMFAGHHTSSGTASWTLIELMRHRDAYAAVIDELDELYGD
GRSVSFHALRQIPQLENVLKETLRLHPPLIILMRVAKGEFEVQGHRIHEGDLVAASPAISNRIPEDFPDPHDF
VPARYEQPRQEDLLNRWTWIPFGAGRHRCVGAAFAIMQIKAIFSVLLREYEFEMAQPPESYRNDHSKMVVQLA
QPACVRYRRRTGV
>CYP51B1_Mycobacterium tuberculosis BTB04-001 2604519614
MSAVALPRVSGGHDEHGHLEEFRTDPIGLMQRVRDECGDVGTFQLAGKQVVLLSGSHANEFFFRAGDDDLDQA
KAYPFMTPIFGEGVVFDASPERRKEMLHNAALRGEQMKGHAATIEDQVRRMIADWGEAGEIDLLDFFAELTIY
TSSACLIGKKFRDQLDGRFAKLYHELERGTDPLAYVDPYLPIESFRRRDEARNGLVALVADIMNGRIANPPTD
KSDRDMLDVLIAVKAETGTPRFSADEITGMFISMMFAGHHTSSGTASWTLIELMRHRDAYAAVIDELDELYGD
GRSVSFHALRQIPQLENVLKETLRLHPPLIILMRVAKGEFEVQGHRIHEGDLVAASPAISNRIPEDFPDPHDF
VPARYEQPRQEDLLNRWTWIPFGAGRHRCVGAAFAIMQIKAIFSVLLREYEFEMAQPPESYRNDHSKMVVQLA
QPACVRYRRRTGV
>CYP51B1_Mycobacterium tuberculosis BTB04-011 2604170605
MSAVALPRVSGGHDEHGHLEEFRTDPIGLMQRVRDECGDVGTFQLAGKQVVLLSGSHANEFFFRAGDDDLDQA
KAYPFMTPIFGEGVVFDASPERRKEMLHNAALRGEQMKGHAATIEDQVRRMIADWGEAGEIDLLDFFAELTIY
TSSACLIGKKFRDQLDGRFAKLYHELERGTDPLAYVDPYLPIESFRRRDEARNGLVALVADIMNGRIANPPTD
KSDRDMLDVLIAVKAETGTPRFSADEITGMFISMMFAGHHTSSGTASWTLIELMRHRDAYAAVIDELDELYGD

GRSVSFHALRQIPQLENVLKETLRLHPPLIILMRVAKGEFEVQGHRIHEGDLVAASPAISNRIPEDFPDPHDF
VPARYEQPRQEDLLNRWTWIPFGAGRHRCVGAAFAIMQIKAIFSVLLREYEFEMAQPPESYRNDHSKMVVQLA
QPACVRYRRRTGV
>CYP51B1_Mycobacterium tuberculosis BTB04-097 2605247812
MSAVALPRVSGGHDEHGHLEEFRTDPIGLMQRVRDECGDVGTFQLAGKQVVLLSGSHANEFFFRAGDDDLDQA
KAYPFMTPIFGEGVVFDASPERRKEMLHNAALRGEQMKGHAATIEDQVRRMIADWGEAGEIDLLDFFAELTIY
TSSACLIGKKFRDQLDGRFAKLYHELERGTDPLAYVDPYLPIESFRRRDEARNGLVALVADIMNGRIANPPTD
KSDRDMLDVLIAVKAETGTPRFSADEITGMFISMMFAGHHTSSGTASWTLIELMRHRDAYAAVIDELDELYGD
GRSVSFHALRQIPQLENVLKETLRLHPPLIILMRVAKGEFEVQGHRIHEGDLVAASPAISNRIPEDFPDPHDF
VPARYEQPRQEDLLNRWTWIPFGAGRHRCVGAAFAIMQIKAIFSVLLREYEFEMAQPPESYRNDHSKMVVQLA
QPACVRYRRRTGV
>CYP51B1_Mycobacterium tuberculosis BTB04-120 2604174305
MSAVALPRVSGGHDEHGHLEEFRTDPIGLMQRVRDECGDVGTFQLAGKQVVLLSGSHANEFFFRAGDDDLDQA
KAYPFMTPIFGEGVVFDASPERRKEMLHNAALRGEQMKGHAATIEDQVRRMIADWGEAGEIDLLDFFAELTIY
TSSACLIGKKFRDQLDGRFAKLYHELERGTDPLAYVDPYLPIESFRRRDEARNGLVALVADIMNGRIANPPTD
KSDRDMLDVLIAVKAETGTPRFSADEITGMFISMMFAGHHTSSGTASWTLIELMRHRDAYAAVIDELDELYGD
GRSVSFHALRQIPQLENVLKETLRLHPPLIILMRVAKGEFEVQGHRIHEGDLVAASPAISNRIPEDFPDPHDF
VPARYEQPRQEDLLNRWTWIPFGAGRHRCVGAAFAIMQIKAIFSVLLREYEFEMAQPPESYRNDHSKMVVQLA
QPACVRYRRRTGV
>CYP51B1_Mycobacterium tuberculosis BTB04-128 2603476338
MSAVALPRVSGGHDEHGHLEEFRTDPIGLMQRVRDECGDVGTFQLAGKQVVLLSGSHANEFFFRAGDDDLDQA
KAYPFMTPIFGEGVVFDASPERRKEMLHNAALRGEQMKGHAATIEDQVRRMIADWGEAGEIDLLDFFAELTIY
TSSACLIGKKFRDQLDGRFAKLYHELERGTDPLAYVDPYLPIESFRRRDEARNGLVALVADIMNGRIANPPTD
KSDRDMLDVLIAVKAETGTPRFSADEITGMFISMMFAGHHTSSGTASWTLIELMRHRDAYAAVIDELDELYGD
GRSVSFHALRQIPQLENVLKETLRLHPPLIILMRVAKGEFEVQGHRIHEGDLVAASPAISNRIPEDFPDPHDF
VPARYEQPRQEDLLNRWTWIPFGAGRHRCVGAAFAIMQIKAIFSVLLREYEFEMAQPPESYRNDHSKMVVQLA
QPACVRYRRRTGV
>CYP51B1_Mycobacterium tuberculosis BTB04-158 2605587225
MSAVALPRVSGGHDEHGHLEEFRTDPIGLMQRVRDECGDVGTFQLAGKQVVLLSGSHANEFFFRAGDDDLDQA
KAYPFMTPIFGEGVVFDASPERRKEMLHNAALRGEQMKGHAATIEDQVRRMIADWGEAGEIDLLDFFAELTIY
TSSACLIGKKFRDQLDGRFAKLYHELERGTDPLAYVDPYLPIESFRRRDEARNGLVALVADIMNGRIANPPTD
KSDRDMLDVLIAVKAETGTPRFSADEITGMFISMMFAGHHTSSGTASWTLIELMRHRDAYAAVIDELDELYGD
GRSVSFHALRQIPQLENVLKETLRLHPPLIILMRVAKGEFEVQGHRIHEGDLVAASPAISNRIPEDFPDPHDF
VPARYEQPRQEDLLNRWTWIPFGAGRHRCVGAAFAIMQIKAIFSVLLREYEFEMAQPPESYRNDHSKMVVQLA
QPACVRYRRRTGV
>CYP51B1_Mycobacterium tuberculosis BTB04-172 2604523706
MSAVALPRVSGGHDEHGHLEEFRTDPIGLMQRVRDECGDVGTFQLAGKQVVLLSGSHANEFFFRAGDDDLDQA
KAYPFMTPIFGEGVVFDASPERRKEMLHNAALRGEQMKGHAATIEDQVRRMIADWGEAGEIDLLDFFAELTIY
TSSACLIGKKFRDQLDGRFAKLYHELERGTDPLAYVDPYLPIESFRRRDEARNGLVALVADIMNGRIANPPTD
KSDRDMLDVLIAVKAETGTPRFSADEITGMFISMMFAGHHTSSGTASWTLIELMRHRDAYAAVIDELDELYGD
GRSVSFHALRQIPQLENVLKETLRLHPPLIILMRVAKGEFEVQGHRIHEGDLVAASPAISNRIPEDFPDPHDF
VPARYEQPRQEDLLNRWTWIPFGAGRHRCVGAAFAIMQIKAIFSVLLREYEFEMAQPPESYRNDHSKMVVQLA
QPACVRYRRRTGV
>CYP51B1_Mycobacterium tuberculosis BTB04-416 2603306402
MSAVALPRVSGGHDEHGHLEEFRTDPIGLMQRVRDECGDVGTFQLAGKQVVLLSGSHANEFFFRAGDDDLDQA
KAYPFMTPIFGEGVVFDASPERRKEMLHNAALRGEQMKGHAATIEDQVRRMIADWGEAGEIDLLDFFAELTIY
TSSACLIGKKFRDQLDGRFAKLYHELERGTDPLAYVDPYLPIESFRRRDEARNGLVALVADIMNGRIANPPTD
KSDRDMLDVLIAVKAETGTPRFSADEITGMFISMMFAGHHTSSGTASWTLIELMRHRDAYAAVIDELDELYGD
GRSVSFHALRQIPQLENVLKETLRLHPPLIILMRVAKGEFEVQGHRIHEGDLVAASPAISNRIPEDFPDPHDF
VPARYEQPRQEDLLNRWTWIPFGAGRHRCVGAAFAIMQIKAIFSVLLREYEFEMAQPPESYRNDHSKMVVQLA
QPACVRYRRRTGV
>CYP51B1_Mycobacterium tuberculosis BTB04-452 2576008267
MSAVALPRVSGGHDEHGHLEEFRTDPIGLMQRVRDECGDVGTFQLAGKQVVLLSGSHANEFFFRAGDDDLDQA
KAYPFMTPIFGEGVVFDASPERRKEMLHNAALRGEQMKGHAATIEDQVRRMIADWGEAGEIDLLDFFAELTIY
TSSACLIGKKFRDQLDGRFAKLYHELERGTDPLAYVDPYLPIESFRRRDEARNGLVALVADIMNGRIANPPTD
KSDRDMLDVLIAVKAETGTPRFSADEITGMFISMMFAGHHTSSGTASWTLIELMRHRDAYAAVIDELDELYGD
GRSVSFHALRQIPQLENVLKETLRLHPPLIILMRVAKGEFEVQGHRIHEGDLVAASPAISNRIPEDFPDPHDF

VPARYEQPRQEDLLNRWTWIPFGAGRHRCVGAAFAIMQIKAIFSVLLREYEFEMAQPPESYRNDHSKMVVQLA
QPACVRYRRRTGV
>CYP51B1_Mycobacterium tuberculosis BTB05-371 2605591337
MSAVALPRVSGGHDEHGHLEEFRTDPIGLMQRVRDECGDVGTFQLAGKQVVLLSGSHANEFFFRAGDDDLDQA
KAYPFMTPIFGEGVVFDASPERRKEMLHNAALRGEQMKGHAATIEDQVRRMIADWGEAGEIDLLDFFAELTIY
TSSACLIGKKFRDQLDGRFAKLYHELERGTDPLAYVDPYLPIESFRRRDEARNGLVALVADIMNGRIANPPTD
KSDRDMLDVLIAVKAETGTPRFSADEITGMFISMMFAGHHTSSGTASWTLIELMRHRDAYAAVIDELDELYGD
GRSVSFHALRQIPQLENVLKETLRLHPPLIILMRVAKGEFEVQGHRIHEGDLVAASPAISNRIPEDFPDPHDF
VPARYEQPRQEDLLNRWTWIPFGAGRHRCVGAAFAIMQIKAIFSVLLREYEFEMAQPPESYRNDHSKMVVQLA
QPACVRYRRRTGV
>CYP51B1_Mycobacterium tuberculosis BTB05-481 2603313998
MSAVALPRVSGGHDEHGHLEEFRTDPIGLMQRVRDECGDVGTFQLAGKQVVLLSGSHANEFFFRAGDDDLDQA
KAYPFMTPIFGEGVVFDASPERRKEMLHNAALRGEQMKGHAATIEDQVRRMIADWGEAGEIDLLDFFAELTIY
TSSACLIGKKFRDQLDGRFAKLYHELERGTDPLAYVDPYLPIESFRRRDEARNGLVALVADIMNGRIANPPTD
KSDRDMLDVLIAVKAETGTPRFSADEITGMFISMMFAGHHTSSGTASWTLIELMRHRDAYAAVIDELDELYGD
GRSVSFHALRQIPQLENVLKETLRLHPPLIILMRVAKGEFEVQGHRIHEGDLVAASPAISNRIPEDFPDPHDF
VPARYEQPRQEDLLNRWTWIPFGAGRHRCVGAAFAIMQIKAIFSVLLREYEFEMAQPPESYRNDHSKMVVQLA
QPACVRYRRRTGV
>CYP51B1_Mycobacterium tuberculosis BTB05-552 2548032177
MSAVALPRVSGGHDEHGHLEEFRTDPIGLMQRVRDECGDVGTFQLAGKQVVLLSGSHANEFFFRAGDDDLDQA
KAYPFMTPIFGEGVVFDASPERRKEMLHNAALRGEQMKGHAATIEDQVRRMIADWGEAGEIDLLDFFAELTIY
TSSACLIGKKFRDQLDGRFAKLYHELERGTDPLAYVDPYLPIESFRRRDEARNGLVALVADIMNGRIANPPTD
KSDRDMLDVLIAVKAETGTPRFSADEITGMFISMMFAGHHTSSGTASWTLIELMRHRDAYAAVIDELDELYGD
GRSVSFHALRQIPQLENVLKETLRLHPPLIILMRVAKGEFEVQGHRIHEGDLVAASPAISNRIPEDFPDPHDF
VPARYEQPRQEDLLNRWTWIPFGAGRHRCVGAAFAIMQIKAIFSVLLREYEFEMAQPPESYRNDHSKMVVQLA
QPACVRYRRRTGV
>CYP51B1_Mycobacterium tuberculosis BTB05-559 2548036440
MSAVALPRVSGGHDEHGHLEEFRTDPIGLMQRVRDECGDVGTFQLAGKQVVLLSGSHANEFFFRAGDDDLDQA
KAYPFMTPIFGEGVVFDASPERRKEMLHNAALRGEQMKGHAATIEDQVRRMIADWGEAGEIDLLDFFAELTIY
TSSACLIGKKFRDQLDGRFAKLYHELERGTDPLAYVDPYLPIESFRRRDEARNGLVALVADIMNGRIANPPTD
KSDRDMLDVLIAVKAETGTPRFSADEITGMFISMMFAGHHTSSGTASWTLIELMRHRDAYAAVIDELDELYGD
GRSVSFHALRQIPQLENVLKETLRLHPPLIILMRVAKGEFEVQGHRIHEGDLVAASPAISNRIPEDFPDPHDF
VPARYEQPRQEDLLNRWTWIPFGAGRHRCVGAAFAIMQIKAIFSVLLREYEFEMAQPPESYRNDHSKMVVQLA
QPACVRYRRRTGV
>CYP51B1_Mycobacterium tuberculosis BTB05-650 2604181630
MSAVALPRVSGGHDEHGHLEEFRTDPIGLMQRVRDECGDVGTFQLAGKQVVLLSGSHANEFFFRAGDDDLDQA
KAYPFMTPIFGEGVVFDASPERRKEMLHNAALRGEQMKGHAATIEDQVRRMIADWGEAGEIDLLDFFAELTIY
TSSACLIGKKFRDQLDGRFAKLYHELERGTDPLAYVDPYLPIESFRRRDEARNGLVALVADIMNGRIANPPTD
KSDRDMLDVLIAVKAETGTPRFSADEITGMFISMMFAGHHTSSGTASWTLIELMRHRDAYAAVIDELDELYGD
GRSVSFHALRQIPQLENVLKETLRLHPPLIILMRVAKGEFEVQGHRIHEGDLVAASPAISNRIPEDFPDPHDF
VPARYEQPRQEDLLNRWTWIPFGAGRHRCVGAAFAIMQIKAIFSVLLREYEFEMAQPPESYRNDHSKMVVQLA
QPACVRYRRRTGV
>CYP51B1_Mycobacterium tuberculosis BTB05-660 2604531931
MSAVALPRVSGGHDEHGHLEEFRTDPIGLMQRVRDECGDVGTFQLAGKQVVLLSGSHANEFFFRAGDDDLDQA
KAYPFMTPIFGEGVVFDASPERRKEMLHNAALRGEQMKGHAATIEDQVRRMIADWGEAGEIDLLDFFAELTIY
TSSACLIGKKFRDQLDGRFAKLYHELERGTDPLAYVDPYLPIESFRRRDEARNGLVALVADIMNGRIANPPTD
KSDRDMLDVLIAVKAETGTPRFSADEITGMFISMMFAGHHTSSGTASWTLIELMRHRDAYAAVIDELDELYGD
GRSVSFHALRQIPQLENVLKETLRLHPPLIILMRVAKGEFEVQGHRIHEGDLVAASPAISNRIPEDFPDPHDF
VPARYEQPRQEDLLNRWTWIPFGAGRHRCVGAAFAIMQIKAIFSVLLREYEFEMAQPPESYRNDHSKMVVQLA
QPACVRYRRRTGV
>CYP51B1_Mycobacterium tuberculosis BTB06-001 2578106284
MSAVALPRVSGGHDEHGHLEEFRTDPIGLMQRVRDECGDVGTFQLAGKQVVLLSGSHANEFFFRAGDDDLDQA
KAYPFMTPIFGEGVVFDASPERRKEMLHNAALRGEQMKGHAATIEDQVRRMIADWGEAGEIDLLDFFAELTIY
TSSACLIGKKFRDQLDGRFAKLYHELERGTDPLAYVDPYLPIESFRRRDEARNGLVALVADIMNGRIANPPTD
KSDRDMLDVLIAVKAETGTPRFSADEITGMFISMMFAGHHTSSGTASWTLIELMRHRDAYAAVIDELDELYGD
GRSVSFHALRQIPQLENVLKETLRLHPPLIILMRVAKGEFEVQGHRIHEGDLVAASPAISNRIPEDFPDPHDF
VPARYEQPRQEDLLNRWTWIPFGAGRHRCVGAAFAIMQIKAIFSVLLREYEFEMAQPPESYRNDHSKMVVQLA
QPACVRYRRRTGV

>CYP51B1_Mycobacterium tuberculosis BTB06-340 2603480464
MSAVALPRVSGGHDEHGHLEEFRTDPIGLMQRVRDECGDVGTFQLAGKQVVLLSGSHANEFFFRAGDDDLDQA
KAYPFMTPIFGEGVVFDASPERRKEMLHNAALRGEQMKGHAATIEDQVRRMIADWGEAGEIDLLDFFAELTIY
TSSACLIGKKFRDQLDGRFAKLYHELERGTDPLAYVDPYLPIESFRRRDEARNGLVALVADIMNGRIANPPTD
KSDRDMLDVLIAVKAETGTPRFSADEITGMFISMMFAGHHTSSGTASWTLIELMRHRDAYAAVIDELDELYGD
GRSVSFHALRQIPQLENVLKETLRLHPPLIILMRVAKGEFEVQGHRIHEGDLVAASPAISNRIPEDFPDPHDF
VPARYEQPRQEDLLNRWTWIPFGAGRHRCVGAAFAIMQIKAIFSVLLREYEFEMAQPPESYRNDHSKMVVQLA
QPACVRYRRRTGV
>CYP51B1_Mycobacterium tuberculosis BTB06-494 2605797765
MSAVALPRVSGGHDEHGHLEEFRTDPIGLMQRVRDECGDVGTFQLAGKQVVLLSGSHANEFFFRAGDDDLDQA
KAYPFMTPIFGEGVVFDASPERRKEMLHNAALRGEQMKGHAATIEDQVRRMIADWGEAGEIDLLDFFAELTIY
TSSACLIGKKFRDQLDGRFAKLYHELERGTDPLAYVDPYLPIESFRRRDEARNGLVALVADIMNGRIANPPTD
KSDRDMLDVLIAVKAETGTPRFSADEITGMFISMMFAGHHTSSGTASWTLIELMRHRDAYAAVIDELDELYGD
GRSVSFHALRQIPQLENVLKETLRLHPPLIILMRVAKGEFEVQGHRIHEGDLVAASPAISNRIPEDFPDPHDF
VPARYEQPRQEDLLNRWTWIPFGAGRHRCVGAAFAIMQIKAIFSVLLREYEFEMAQPPESYRNDHSKMVVQLA
QPACVRYRRRTGV
>CYP51B1_Mycobacterium tuberculosis BTB07-001 2604355789
MSAVALPRVSGGHDEHGHLEEFRTDPIGLMQRVRDECGDVGTFQLAGKQVVLLSGSHANEFFFRAGDDDLDQA
KAYPFMTPIFGEGVVFDASPERRKEMLHNAALRGEQMKGHAATIEDQVRRMIADWGEAGEIDLLDFFAELTIY
TSSACLIGKKFRDQLDGRFAKLYHELERGTDPLAYVDPYLPIESFRRRDEARNGLVALVADIMNGRIANPPTD
KSDRDMLDVLIAVKAETGTPRFSADEITGMFISMMFAGHHTSSGTASWTLIELMRHRDAYAAVIDELDELYGD
GRSVSFHALRQIPQLENVLKETLRLHPPLIILMRVAKGEFEVQGHRIHEGDLVAASPAISNRIPEDFPDPHDF
VPARYEQPRQEDLLNRWTWIPFGAGRHRCVGAAFAIMQIKAIFSVLLREYEFEMAQPPESYRNDHSKMVVQLA
QPACVRYRRRTGV
>CYP51B1_Mycobacterium tuberculosis BTB07-123 2604716367
MSAVALPRVSGGHDEHGHLEEFRTDPIGLMQRVRDECGDVGTFQLAGKQVVLLSGSHANEFFFRAGDDDLDQA
KAYPFMTPIFGEGVVFDASPERRKEMLHNAALRGEQMKGHAATIEDQVRRMIADWGEAGEIDLLDFFAELTIY
TSSACLIGKKFRDQLDGRFAKLYHELERGTDPLAYVDPYLPIESFRRRDEARNGLVALVADIMNGRIANPPTD
KSDRDMLDVLIAVKAETGTPRFSADEITGMFISMMFAGHHTSSGTASWTLIELMRHRDAYAAVIDELDELYGD
GRSVSFHALRQIPQLENVLKETLRLHPPLIILMRVAKGEFEVQGHRIHEGDLVAASPAISNRIPEDFPDPHDF
VPARYEQPRQEDLLNRWTWIPFGAGRHRCVGAAFAIMQIKAIFSVLLREYEFEMAQPPESYRNDHSKMVVQLA
QPACVRYRRRTGV
>CYP51B1_Mycobacterium tuberculosis BTB07-168 2604043100
MSAVALPRVSGGHDEHGHLEEFRTDPIGLMQRVRDECGDVGTFQLAGKQVVLLSGSHANEFFFRAGDDDLDQA
KAYPFMTPIFGEGVVFDASPERRKEMLHNAALRGEQMKGHAATIEDQVRRMIADWGEAGEIDLLDFFAELTIY
TSSACLIGKKFRDQLDGRFAKLYHELERGTDPLAYVDPYLPIESFRRRDEARNGLVALVADIMNGRIANPPTD
KSDRDMLDVLIAVKAETGTPRFSADEITGMFISMMFAGHHTSSGTASWTLIELMRHRDAYAAVIDELDELYGD
GRSVSFHALRQIPQLENVLKETLRLHPPLIILMRVAKGEFEVQGHRIHEGDLVAASPAISNRIPEDFPDPHDF
VPARYEQPRQEDLLNRWTWIPFGAGRHRCVGAAFAIMQIKAIFSVLLREYEFEMAQPPESYRNDHSKMVVQLA
QPACVRYRRRTGV
>CYP51B1_Mycobacterium tuberculosis BTB07-170 2605075266
MSAVALPRVSGGHDEHGHLEEFRTDPIGLMQRVRDECGDVGTFQLAGKQVVLLSGSHANEFFFRAGDDDLDQA
KAYPFMTPIFGEGVVFDASPERRKEMLHNAALRGEQMKGHAATIEDQVRRMIADWGEAGEIDLLDFFAELTIY
TSSACLIGKKFRDQLDGRFAKLYHELERGTDPLAYVDPYLPIESFRRRDEARNGLVALVADIMNGRIANPPTD
KSDRDMLDVLIAVKAETGTPRFSADEITGMFISMMFAGHHTSSGTASWTLIELMRHRDAYAAVIDELDELYGD
GRSVSFHALRQIPQLENVLKETLRLHPPLIILMRVAKGEFEVQGHRIHEGDLVAASPAISNRIPEDFPDPHDF
VPARYEQPRQEDLLNRWTWIPFGAGRHRCVGAAFAIMQIKAIFSVLLREYEFEMAQPPESYRNDHSKMVVQLA
QPACVRYRRRTGV
>CYP51B1_Mycobacterium tuberculosis BTB07-206 2574692765
MSAVALPRVSGGHDEHGHLEEFRTDPIGLMQRVRDECGDVGTFQLAGKQVVLLSGSHANEFFFRAGDDDLDQA
KAYPFMTPIFGEGVVFDASPERRKEMLHNAALRGEQMKGHAATIEDQVRRMIADWGEAGEIDLLDFFAELTIY
TSSACLIGKKFRDQLDGRFAKLYHELERGTDPLAYVDPYLPIESFRRRDEARNGLVALVADIMNGRIANPPTD
KSDRDMLDVLIAVKAETGTPRFSADEITGMFISMMFAGHHTSSGTASWTLIELMRHRDAYAAVIDELDELYGD
GRSVSFHALRQIPQLENVLKETLRLHPPLIILMRVAKGEFEVQGHRIHEGDLVAASPAISNRIPEDFPDPHDF
VPARYEQPRQEDLLNRWTWIPFGAGRHRCVGAAFAIMQIKAIFSVLLREYEFEMAQPPESYRNDHSKMVVQLA
QPACVRYRRRTGV
>CYP51B1_Mycobacterium tuberculosis BTB07-234 2605798998

MSAVALPRVSGGHDEHGHLEEFRTDPIGLMQRVRDECGDVGTFQLAGKQVVLLSGSHANEFFFRAGDDDLDQA
KAYPFMTPIFGEGVVFDASPERRKEMLHNAALRGEQMKGHAATIEDQVRRMIADWGEAGEIDLLDFFAELTIY
TSSACLIGKKFRDQLDGRFAKLYHELERGTDPLAYVDPYLPIESFRRRDEARNGLVALVADIMNGRIANPPTD
KSDRDMLDVLIAVKAETGTPRFSADEITGMFISMMFAGHHTSSGTASWTLIELMRHRDAYAAVIDELDELYGD
GRSVSFHALRQIPQLENVLKETLRLHPPLIILMRVAKGEFEVQGHRIHEGDLVAASPAISNRIPEDFPDPHDF
VPARYEQPRQEDLLNRWTWIPFGAGRHRCVGAAFAIMQIKAIFSVLLREYEFEMAQPPESYRNDHSKMVVQLA
QPACVRYRRRTGV
>CYP51B1_Mycobacterium tuberculosis BTB07-246 2584677637
MSAVALPRVSGGHDEHGHLEEFRTDPIGLMQRVRDECGDVGTFQLAGKQVVLLSGSHANEFFFRAGDDDLDQA
KAYPFMTPIFGEGVVFDASPERRKEMLHNAALRGEQMKGHAATIEDQVRRMIADWGEAGEIDLLDFFAELTIY
TSSACLIGKKFRDQLDGRFAKLYHELERGTDPLAYVDPYLPIESFRRRDEARNGLVALVADIMNGRIANPPTD
KSDRDMLDVLIAVKAETGTPRFSADEITGMFISMMFAGHHTSSGTASWTLIELMRHRDAYAAVIDELDELYGD
GRSVSFHALRQIPQLENVLKETLRLHPPLIILMRVAKGEFEVQGHRIHEGDLVAASPAISNRIPEDFPDPHDF
VPARYEQPRQEDLLNRWTWIPFGAGRHRCVGAAFAIMQIKAIFSVLLREYEFEMAQPPESYRNDHSKMVVQLA
QPACVRYRRRTGV
>CYP51B1_Mycobacterium tuberculosis BTB07-283 2604364029
MSAVALPRVSGGHDEHGHLEEFRTDPIGLMQRVRDECGDVGTFQLAGKQVVLLSGSHANEFFFRAGDDDLDQA
KAYPFMTPIFGEGVVFDASPERRKEMLHNAALRGEQMKGHAATIEDQVRRMIADWGEAGEIDLLDFFAELTIY
TSSACLIGKKFRDQLDGRFAKLYHELERGTDPLAYVDPYLPIESFRRRDEARNGLVALVADIMNGRIANPPTD
KSDRDMLDVLIAVKAETGTPRFSADEITGMFISMMFAGHHTSSGTASWTLIELMRHRDAYAAVIDELDELYGD
GRSVSFHALRQIPQLENVLKETLRLHPPLIILMRVAKGEFEVQGHRIHEGDLVAASPAISNRIPEDFPDPHDF
VPARYEQPRQEDLLNRWTWIPFGAGRHRCVGAAFAIMQIKAIFSVLLREYEFEMAQPPESYRNDHSKMVVQLA
QPACVRYRRRTGV
>CYP51B1_Mycobacterium tuberculosis BTB07-275 2604359665
MSAVALPRVSGGHDEHGHLEEFRTDPIGLMQRVRDECGDVGTFQLAGKQVVLLSGSHANEFFFRAGDDDLDQA
KAYPFMTPIFGEGVVFDASPERRKEMLHNAALRGEQMKGHAATIEDQVRRMIADWGEAGEIDLLDFFAELTIY
TSSACLIGKKFRDQLDGRFAKLYHELERGTDPLAYVDPYLPIESFRRRDEARNGLVALVADIMNGRIANPPTD
KSDRDMLDVLIAVKAETGTPRFSADEITGMFISMMFAGHHTSSGTASWTLIELMRHRDAYAAVIDELDELYGD
GRSVSFHALRQIPQLENVLKETLRLHPPLIILMRVAKGEFEVQGHRIHEGDLVAASPAISNRIPEDFPDPHDF
VPARYEQPRQEDLLNRWTWIPFGAGRHRCVGAAFAIMQIKAIFSVLLREYEFEMAQPPESYRNDHSKMVVQLA
QPACVRYRRRTGV
>CYP51B1_Mycobacterium tuberculosis BTB07-314 2604935146
MSAVALPRVSGGHDEHGHLEEFRTDPIGLMQRVRDECGDVGTFQLAGKQVVLLSGSHANEFFFRAGDDDLDQA
KAYPFMTPIFGEGVVFDASPERRKEMLHNAALRGEQMKGHAATIEDQVRRMIADWGEAGEIDLLDFFAELTIY
TSSACLIGKKFRDQLDGRFAKLYHELERGTDPLAYVDPYLPIESFRRRDEARNGLVALVADIMNGRIANPPTD
KSDRDMLDVLIAVKAETGTPRFSADEITGMFISMMFAGHHTSSGTASWTLIELMRHRDAYAAVIDELDELYGD
GRSVSFHALRQIPQLENVLKETLRLHPPLIILMRVAKGEFEVQGHRIHEGDLVAASPAISNRIPEDFPDPHDF
VPARYEQPRQEDLLNRWTWIPFGAGRHRCVGAAFAIMQIKAIFSVLLREYEFEMAQPPESYRNDHSKMVVQLA
QPACVRYRRRTGV
>CYP51B1_Mycobacterium tuberculosis BTB07-323 2603484580
MSAVALPRVSGGHDEHGHLEEFRTDPIGLMQRVRDECGDVGTFQLAGKQVVLLSGSHANEFFFRAGDDDLDQA
KAYPFMTPIFGEGVVFDASPERRKEMLHNAALRGEQMKGHAATIEDQVRRMIADWGEAGEIDLLDFFAELTIY
TSSACLIGKKFRDQLDGRFAKLYHELERGTDPLAYVDPYLPIESFRRRDEARNGLVALVADIMNGRIANPPTD
KSDRDMLDVLIAVKAETGTPRFSADEITGMFISMMFAGHHTSSGTASWTLIELMRHRDAYAAVIDELDELYGD
GRSVSFHALRQIPQLENVLKETLRLHPPLIILMRVAKGEFEVQGHRIHEGDLVAASPAISNRIPEDFPDPHDF
VPARYEQPRQEDLLNRWTWIPFGAGRHRCVGAAFAIMQIKAIFSVLLREYEFEMAQPPESYRNDHSKMVVQLA
QPACVRYRRRTGV
>CYP51B1_Mycobacterium tuberculosis BTB07-324 2603488693
MSAVALPRVSGGHDEHGHLEEFRTDPIGLMQRVRDECGDVGTFQLAGKQVVLLSGSHANEFFFRAGDDDLDQA
KAYPFMTPIFGEGVVFDASPERRKEMLHNAALRGEQMKGHAATIEDQVRRMIADWGEAGEIDLLDFFAELTIY
TSSACLIGKKFRDQLDGRFAKLYHELERGTDPLAYVDPYLPIESFRRRDEARNGLVALVADIMNGRIANPPTD
KSDRDMLDVLIAVKAETGTPRFSADEITGMFISMMFAGHHTSSGTASWTLIELMRHRDAYAAVIDELDELYGD
GRSVSFHALRQIPQLENVLKETLRLHPPLIILMRVAKGEFEVQGHRIHEGDLVAASPAISNRIPEDFPDPHDF
VPARYEQPRQEDLLNRWTWIPFGAGRHRCVGAAFAIMQIKAIFSVLLREYEFEMAQPPESYRNDHSKMVVQLA
QPACVRYRRRTGV
>CYP51B1_Mycobacterium tuberculosis BTB07-325 2578097300
MSAVALPRVSGGHDEHGHLEEFRTDPIGLMQRVRDECGDVGTFQLAGKQVVLLSGSHANEFFFRAGDDDLDQA
KAYPFMTPIFGEGVVFDASPERRKEMLHNAALRGEQMKGHAATIEDQVRRMIADWGEAGEIDLLDFFAELTIY

TSSACLIGKKFRDQLDGRFAKLYHELERGTDPLAYVDPYLPIESFRRRDEARNGLVALVADIMNGRIANPPTD
KSDRDMLDVLIAVKAETGTPRFSADEITGMFISMMFAGHHTSSGTASWTLIELMRHRDAYAAVIDELDELYGD
GRSVSFHALRQIPQLENVLKETLRLHPPLIILMRVAKGEFEVQGHRIHEGDLVAASPAISNRIPEDFPDPHDF
VPARYEQPRQEDLLNRWTWIPFGAGRHRCVGAAFAIMQIKAIFSVLLREYEFEMAQPPESYRNDHSKMVVQLA
QPACVRYRRRTGV
>CYP51B1_Mycobacterium tuberculosis BTB08-001 2603492807
MSAVALPRVSGGHDEHGHLEEFRTDPIGLMQRVRDECGDVGTFQLAGKQVVLLSGSHANEFFFRAGDDDLDQA
KAYPFMTPIFGEGVVFDASPERRKEMLHNAALRGEQMKGHAATIEDQVRRMIADWGEAGEIDLLDFFAELTIY
TSSACLIGKKFRDQLDGRFAKLYHELERGTDPLAYVDPYLPIESFRRRDEARNGLVALVADIMNGRIANPPTD
KSDRDMLDVLIAVKAETGTPRFSADEITGMFISMMFAGHHTSSGTASWTLIELMRHRDAYAAVIDELDELYGD
GRSVSFHALRQIPQLENVLKETLRLHPPLIILMRVAKGEFEVQGHRIHEGDLVAASPAISNRIPEDFPDPHDF
VPARYEQPRQEDLLNRWTWIPFGAGRHRCVGAAFAIMQIKAIFSVLLREYEFEMAQPPESYRNDHSKMVVQLA
QPACVRYRRRTGV
>CYP51B1_Mycobacterium tuberculosis BTB08-022 2577658886
MSAVALPRVSGGHDEHGHLEEFRTDPIGLMQRVRDECGDVGTFQLAGKQVVLLSGSHANEFFFRAGDDDLDQA
KAYPFMTPIFGEGVVFDASPERRKEMLHNAALRGEQMKGHAATIEDQVRRMIADWGEAGEIDLLDFFAELTIY
TSSACLIGKKFRDQLDGRFAKLYHELERGTDPLAYVDPYLPIESFRRRDEARNGLVALVADIMNGRIANPPTD
KSDRDMLDVLIAVKAETGTPRFSADEITGMFISMMFAGHHTSSGTASWTLIELMRHRDAYAAVIDELDELYGD
GRSVSFHALRQIPQLENVLKETLRLHPPLIILMRVAKGEFEVQGHRIHEGDLVAASPAISNRIPEDFPDPHDF
VPARYEQPRQEDLLNRWTWIPFGAGRHRCVGAAFAIMQIKAIFSVLLREYEFEMAQPPESYRNDHSKMVVQLA
QPACVRYRRRTGV
>CYP51B1_Mycobacterium tuberculosis BTB08-093 2604208017
MSAVALPRVSGGHDEHGHLEEFRTDPIGLMQRVRDECGDVGTFQLAGKQVVLLSGSHANEFFFRAGDDDLDQA
KAYPFMTPIFGEGVVFDASPERRKEMLHNAALRGEQMKGHAATIEDQVRRMIADWGEAGEIDLLDFFAELTIY
TSSACLIGKKFRDQLDGRFAKLYHELERGTDPLAYVDPYLPIESFRRRDEARNGLVALVADIMNGRIANPPTD
KSDRDMLDVLIAVKAETGTPRFSADEITGMFISMMFAGHHTSSGTASWTLIELMRHRDAYAAVIDELDELYGD
GRSVSFHALRQIPQLENVLKETLRLHPPLIILMRVAKGEFEVQGHRIHEGDLVAASPAISNRIPEDFPDPHDF
VPARYEQPRQEDLLNRWTWIPFGAGRHRCVGAAFAIMQIKAIFSVLLREYEFEMAQPPESYRNDHSKMVVQLA
QPACVRYRRRTGV
>CYP51B1_Mycobacterium tuberculosis BTB08-148 2576607632
MSAVALPRVSGGHDEHGHLEEFRTDPIGLMQRVRDECGDVGTFQLAGKQVVLLSGSHANEFFFRAGDDDLDQA
KAYPFMTPIFGEGVVFDASPERRKEMLHNAALRGEQMKGHAATIEDQVRRMIADWGEAGEIDLLDFFAELTIY
TSSACLIGKKFRDQLDGRFAKLYHELERGTDPLAYVDPYLPIESFRRRDEARNGLVALVADIMNGRIANPPTD
KSDRDMLDVLIAVKAETGTPRFSADEITGMFISMMFAGHHTSSGTASWTLIELMRHRDAYAAVIDELDELYGD
GRSVSFHALRQIPQLENVLKETLRLHPPLIILMRVAKGEFEVQGHRIHEGDLVAASPAISNRIPEDFPDPHDF
VPARYEQPRQEDLLNRWTWIPFGAGRHRCVGAAFAIMQIKAIFSVLLREYEFEMAQPPESYRNDHSKMVVQLA
QPACVRYRRRTGV
>CYP51B1_Mycobacterium tuberculosis BTB08-183 2604562572
MSAVALPRVSGGHDEHGHLEEFRTDPIGLMQRVRDECGDVGTFQLAGKQVVLLSGSHANEFFFRAGDDDLDQA
KAYPFMTPIFGEGVVFDASPERRKEMLHNAALRGEQMKGHAATIEDQVRRMIADWGEAGEIDLLDFFAELTIY
TSSACLIGKKFRDQLDGRFAKLYHELERGTDPLAYVDPYLPIESFRRRDEARNGLVALVADIMNGRIANPPTD
KSDRDMLDVLIAVKAETGTPRFSADEITGMFISMMFAGHHTSSGTASWTLIELMRHRDAYAAVIDELDELYGD
GRSVSFHALRQIPQLENVLKETLRLHPPLIILMRVAKGEFEVQGHRIHEGDLVAASPAISNRIPEDFPDPHDF
VPARYEQPRQEDLLNRWTWIPFGAGRHRCVGAAFAIMQIKAIFSVLLREYEFEMAQPPESYRNDHSKMVVQLA
QPACVRYRRRTGV
>CYP51B1_Mycobacterium tuberculosis BTB08-221 2605286537
MSAVALPRVSGGHDEHGHLEEFRTDPIGLMQRVRDECGDVGTFQLAGKQVVLLSGSHANEFFFRAGDDDLDQA
KAYPFMTPIFGEGVVFDASPERRKEMLHNAALRGEQMKGHAATIEDQVRRMIADWGEAGEIDLLDFFAELTIY
TSSACLIGKKFRDQLDGRFAKLYHELERGTDPLAYVDPYLPIESFRRRDEARNGLVALVADIMNGRIANPPTD
KSDRDMLDVLIAVKAETGTPRFSADEITGMFISMMFAGHHTSSGTASWTLIELMRHRDAYAAVIDELDELYGD
GRSVSFHALRQIPQLENVLKETLRLHPPLIILMRVAKGEFEVQGHRIHEGDLVAASPAISNRIPEDFPDPHDF
VPARYEQPRQEDLLNRWTWIPFGAGRHRCVGAAFAIMQIKAIFSVLLREYEFEMAQPPESYRNDHSKMVVQLA
QPACVRYRRRTGV
>CYP51B1_Mycobacterium tuberculosis BTB08-283 2604564176
MSAVALPRVSGGHDEHGHLEEFRTDPIGLMQRVRDECGDVGTFQLAGKQVVLLSGSHANEFFFRAGDDDLDQA
KAYPFMTPIFGEGVVFDASPERRKEMLHNAALRGEQMKGHAATIEDQVRRMIADWGEAGEIDLLDFFAELTIY
TSSACLIGKKFRDQLDGRFAKLYHELERGTDPLAYVDPYLPIESFRRRDEARNGLVALVADIMNGRIANPPTD
KSDRDMLDVLIAVKAETGTPRFSADEITGMFISMMFAGHHTSSGTASWTLIELMRHRDAYAAVIDELDELYGD

GRSVSFHALRQIPQLENVLKETLRLHPPLIILMRVAKGEFEVQGHRIHEGDLVAASPAISNRIPEDFPDPHDF
VPARYEQPRQEDLLNRWTWIPFGAGRHRCVGAAFAIMQIKAIFSVLLREYEFEMAQPPESYRNDHSKMVVQLA
QPACVRYRRRTGV
>CYP51B1_Mycobacterium tuberculosis BTB08-288 2604214405
MSAVALPRVSGGHDEHGHLEEFRTDPIGLMQRVRDECGDVGTFQLAGKQVVLLSGSHANEFFFRAGDDDLDQA
KAYPFMTPIFGEGVVFDASPERRKEMLHNAALRGEQMKGHAATIEDQVRRMIADWGEAGEIDLLDFFAELTIY
TSSACLIGKKFRDQLDGRFAKLYHELERGTDPLAYVDPYLPIESFRRRDEARNGLVALVADIMNGRIANPPTD
KSDRDMLDVLIAVKAETGTPRFSADEITGMFISMMFAGHHTSSGTASWTLIELMRHRDAYAAVIDELDELYGD
GRSVSFHALRQIPQLENVLKETLRLHPPLIILMRVAKGEFEVQGHRIHEGDLVAASPAISNRIPEDFPDPHDF
VPARYEQPRQEDLLNRWTWIPFGAGRHRCVGAAFAIMQIKAIFSVLLREYEFEMAQPPESYRNDHSKMVVQLA
QPACVRYRRRTGV
>CYP51B1_Mycobacterium tuberculosis BTB08-356 2605639615
MSAVALPRVSGGHDEHGHLEEFRTDPIGLMQRVRDECGDVGTFQLAGKQVVLLSGSHANEFFFRAGDDDLDQA
KAYPFMTPIFGEGVVFDASPERRKEMLHNAALRGEQMKGHAATIEDQVRRMIADWGEAGEIDLLDFFAELTIY
TSSACLIGKKFRDQLDGRFAKLYHELERGTDPLAYVDPYLPIESFRRRDEARNGLVALVADIMNGRIANPPTD
KSDRDMLDVLIAVKAETGTPRFSADEITGMFISMMFAGHHTSSGTASWTLIELMRHRDAYAAVIDELDELYGD
GRSVSFHALRQIPQLENVLKETLRLHPPLIILMRVAKGEFEVQGHRIHEGDLVAASPAISNRIPEDFPDPHDF
VPARYEQPRQEDLLNRWTWIPFGAGRHRCVGAAFAIMQIKAIFSVLLREYEFEMAQPPESYRNDHSKMVVQLA
QPACVRYRRRTGV
>CYP51B1_Mycobacterium tuberculosis BTB08-309 2604941713
MSAVALPRVSGGHDEHGHLEEFRTDPIGLMQRVRDECGDVGTFQLAGKQVVLLSGSHANEFFFRAGDDDLDQA
KAYPFMTPIFGEGVVFDASPERRKEMLHNAALRGEQMKGHAATIEDQVRRMIADWGEAGEIDLLDFFAELTIY
TSSACLIGKKFRDQLDGRFAKLYHELERGTDPLAYVDPYLPIESFRRRDEARNGLVALVADIMNGRIANPPTD
KSDRDMLDVLIAVKAETGTPRFSADEITGMFISMMFAGHHTSSGTASWTLIELMRHRDAYAAVIDELDELYGD
GRSVSFHALRQIPQLENVLKETLRLHPPLIILMRVAKGEFEVQGHRIHEGDLVAASPAISNRIPEDFPDPHDF
VPARYEQPRQEDLLNRWTWIPFGAGRHRCVGAAFAIMQIKAIFSVLLREYEFEMAQPPESYRNDHSKMVVQLA
QPACVRYRRRTGV
>CYP51B1_Mycobacterium tuberculosis BTB08-362 2575156163
MSAVALPRVSGGHDEHGHLEEFRTDPIGLMQRVRDECGDVGTFQLAGKQVVLLSGSHANEFFFRAGDDDLDQA
KAYPFMTPIFGEGVVFDASPERRKEMLHNAALRGEQMKGHAATIEDQVRRMIADWGEAGEIDLLDFFAELTIY
TSSACLIGKKFRDQLDGRFAKLYHELERGTDPLAYVDPYLPIESFRRRDEARNGLVALVADIMNGRIANPPTD
KSDRDMLDVLIAVKAETGTPRFSADEITGMFISMMFAGHHTSSGTASWTLIELMRHRDAYAAVIDELDELYGD
GRSVSFHALRQIPQLENVLKETLRLHPPLIILMRVAKGEFEVQGHRIHEGDLVAASPAISNRIPEDFPDPHDF
VPARYEQPRQEDLLNRWTWIPFGAGRHRCVGAAFAIMQIKAIFSVLLREYEFEMAQPPESYRNDHSKMVVQLA
QPACVRYRRRTGV
>CYP51B1_Mycobacterium tuberculosis BTB08-394 2603328208
MSAVALPRVSGGHDEHGHLEEFRTDPIGLMQRVRDECGDVGTFQLAGKQVVLLSGSHANEFFFRAGDDDLDQA
KAYPFMTPIFGEGVVFDASPERRKEMLHNAALRGEQMKGHAATIEDQVRRMIADWGEAGEIDLLDFFAELTIY
TSSACLIGKKFRDQLDGRFAKLYHELERGTDPLAYVDPYLPIESFRRRDEARNGLVALVADIMNGRIANPPTD
KSDRDMLDVLIAVKAETGTPRFSADEITGMFISMMFAGHHTSSGTASWTLIELMRHRDAYAAVIDELDELYGD
GRSVSFHALRQIPQLENVLKETLRLHPPLIILMRVAKGEFEVQGHRIHEGDLVAASPAISNRIPEDFPDPHDF
VPARYEQPRQEDLLNRWTWIPFGAGRHRCVGAAFAIMQIKAIFSVLLREYEFEMAQPPESYRNDHSKMVVQLA
QPACVRYRRRTGV
>CYP51B1_Mycobacterium tuberculosis BTB08-395 2603332305
MSAVALPRVSGGHDEHGHLEEFRTDPIGLMQRVRDECGDVGTFQLAGKQVVLLSGSHANEFFFRAGDDDLDQA
KAYPFMTPIFGEGVVFDASPERRKEMLHNAALRGEQMKGHAATIEDQVRRMIADWGEAGEIDLLDFFAELTIY
TSSACLIGKKFRDQLDGRFAKLYHELERGTDPLAYVDPYLPIESFRRRDEARNGLVALVADIMNGRIANPPTD
KSDRDMLDVLIAVKAETGTPRFSADEITGMFISMMFAGHHTSSGTASWTLIELMRHRDAYAAVIDELDELYGD
GRSVSFHALRQIPQLENVLKETLRLHPPLIILMRVAKGEFEVQGHRIHEGDLVAASPAISNRIPEDFPDPHDF
VPARYEQPRQEDLLNRWTWIPFGAGRHRCVGAAFAIMQIKAIFSVLLREYEFEMAQPPESYRNDHSKMVVQLA
QPACVRYRRRTGV
>CYP51B1_Mycobacterium tuberculosis BTB09-001 2604568714
MSAVALPRVSGGHDEHGHLEEFRTDPIGLMQRVRDECGDVGTFQLAGKQVVLLSGSHANEFFFRAGDDDLDQA
KAYPFMTPIFGEGVVFDASPERRKEMLHNAALRGEQMKGHAATIEDQVRRMIADWGEAGEIDLLDFFAELTIY
TSSACLIGKKFRDQLDGRFAKLYHELERGTDPLAYVDPYLPIESFRRRDEARNGLVALVADIMNGRIANPPTD
KSDRDMLDVLIAVKAETGTPRFSADEITGMFISMMFAGHHTSSGTASWTLIELMRHRDAYAAVIDELDELYGD
GRSVSFHALRQIPQLENVLKETLRLHPPLIILMRVAKGEFEVQGHRIHEGDLVAASPAISNRIPEDFPDPHDF

VPARYEQPRQEDLLNRWTWIPFGAGRHRCVGAAFAIMQIKAIFSVLLREYEFEMAQPPESYRNDHSKMVVQLA
QPACVRYRRRTGV
>CYP51B1_Mycobacterium tuberculosis BTB09-058 2578110348
MSAVALPRVSGGHDEHGHLEEFRTDPIGLMQRVRDECGDVGTFQLAGKQVVLLSGSHANEFFFRAGDDDLDQA
KAYPFMTPIFGEGVVFDASPERRKEMLHNAALRGEQMKGHAATIEDQVRRMIADWGEAGEIDLLDFFAELTIY
TSSACLIGKKFRDQLDGRFAKLYHELERGTDPLAYVDPYLPIESFRRRDEARNGLVALVADIMNGRIANPPTD
KSDRDMLDVLIAVKAETGTPRFSADEITGMFISMMFAGHHTSSGTASWTLIELMRHRDAYAAVIDELDELYGD
GRSVSFHALRQIPQLENVLKETLRLHPPLIILMRVAKGEFEVQGHRIHEGDLVAASPAISNRIPEDFPDPHDF
VPARYEQPRQEDLLNRWTWIPFGAGRHRCVGAAFAIMQIKAIFSVLLREYEFEMAQPPESYRNDHSKMVVQLA
QPACVRYRRRTGV
>CYP51B1_Mycobacterium tuberculosis BTB09-085 2603165955
MSAVALPRVSGGHDEHGHLEEFRTDPIGLMQRVRDECGDVGTFQLAGKQVVLLSGSHANEFFFRAGDDDLDQA
KAYPFMTPIFGEGVVFDASPERRKEMLHNAALRGEQMKGHAATIEDQVRRMIADWGEAGEIDLLDFFAELTIY
TSSACLIGKKFRDQLDGRFAKLYHELERGTDPLAYVDPYLPIESFRRRDEARNGLVALVADIMNGRIANPPTD
KSDRDMLDVLIAVKAETGTPRFSADEITGMFISMMFAGHHTSSGTASWTLIELMRHRDAYAAVIDELDELYGD
GRSVSFHALRQIPQLENVLKETLRLHPPLIILMRVAKGEFEVQGHRIHEGDLVAASPAISNRIPEDFPDPHDF
VPARYEQPRQEDLLNRWTWIPFGAGRHRCVGAAFAIMQIKAIFSVLLREYEFEMAQPPESYRNDHSKMVVQLA
QPACVRYRRRTGV
>CYP51B1_Mycobacterium tuberculosis BTB09-086 2605641552
MSAVALPRVSGGHDEHGHLEEFRTDPIGLMQRVRDECGDVGTFQLAGKQVVLLSGSHANEFFFRAGDDDLDQA
KAYPFMTPIFGEGVVFDASPERRKEMLHNAALRGEQMKGHAATIEDQVRRMIADWGEAGEIDLLDFFAELTIY
TSSACLIGKKFRDQLDGRFAKLYHELERGTDPLAYVDPYLPIESFRRRDEARNGLVALVADIMNGRIANPPTD
KSDRDMLDVLIAVKAETGTPRFSADEITGMFISMMFAGHHTSSGTASWTLIELMRHRDAYAAVIDELDELYGD
GRSVSFHALRQIPQLENVLKETLRLHPPLIILMRVAKGEFEVQGHRIHEGDLVAASPAISNRIPEDFPDPHDF
VPARYEQPRQEDLLNRWTWIPFGAGRHRCVGAAFAIMQIKAIFSVLLREYEFEMAQPPESYRNDHSKMVVQLA
QPACVRYRRRTGV
>CYP51B1_Mycobacterium tuberculosis BTB09-091 2604947374
MSAVALPRVSGGHDEHGHLEEFRTDPIGLMQRVRDECGDVGTFQLAGKQVVLLSGSHANEFFFRAGDDDLDQA
KAYPFMTPIFGEGVVFDASPERRKEMLHNAALRGEQMKGHAATIEDQVRRMIADWGEAGEIDLLDFFAELTIY
TSSACLIGKKFRDQLDGRFAKLYHELERGTDPLAYVDPYLPIESFRRRDEARNGLVALVADIMNGRIANPPTD
KSDRDMLDVLIAVKAETGTPRFSADEITGMFISMMFAGHHTSSGTASWTLIELMRHRDAYAAVIDELDELYGD
GRSVSFHALRQIPQLENVLKETLRLHPPLIILMRVAKGEFEVQGHRIHEGDLVAASPAISNRIPEDFPDPHDF
VPARYEQPRQEDLLNRWTWIPFGAGRHRCVGAAFAIMQIKAIFSVLLREYEFEMAQPPESYRNDHSKMVVQLA
QPACVRYRRRTGV
>CYP51B1_Mycobacterium tuberculosis BTB09-151 2603496630
MSAVALPRVSGGHDEHGHLEEFRTDPIGLMQRVRDECGDVGTFQLAGKQVVLLSGSHANEFFFRAGDDDLDQA
KAYPFMTPIFGEGVVFDASPERRKEMLHNAALRGEQMKGHAATIEDQVRRMIADWGEAGEIDLLDFFAELTIY
TSSACLIGKKFRDQLDGRFAKLYHELERGTDPLAYVDPYLPIESFRRRDEARNGLVALVADIMNGRIANPPTD
KSDRDMLDVLIAVKAETGTPRFSADEITGMFISMMFAGHHTSSGTASWTLIELMRHRDAYAAVIDELDELYGD
GRSVSFHALRQIPQLENVLKETLRLHPPLIILMRVAKGEFEVQGHRIHEGDLVAASPAISNRIPEDFPDPHDF
VPARYEQPRQEDLLNRWTWIPFGAGRHRCVGAAFAIMQIKAIFSVLLREYEFEMAQPPESYRNDHSKMVVQLA
QPACVRYRRRTGV
>CYP51B1_Mycobacterium tuberculosis BTB09-157 2603501042
MSAVALPRVSGGHDEHGHLEEFRTDPIGLMQRVRDECGDVGTFQLAGKQVVLLSGSHANEFFFRAGDDDLDQA
KAYPFMTPIFGEGVVFDASPERRKEMLHNAALRGEQMKGHAATIEDQVRRMIADWGEAGEIDLLDFFAELTIY
TSSACLIGKKFRDQLDGRFAKLYHELERGTDPLAYVDPYLPIESFRRRDEARNGLVALVADIMNGRIANPPTD
KSDRDMLDVLIAVKAETGTPRFSADEITGMFISMMFAGHHTSSGTASWTLIELMRHRDAYAAVIDELDELYGD
GRSVSFHALRQIPQLENVLKETLRLHPPLIILMRVAKGEFEVQGHRIHEGDLVAASPAISNRIPEDFPDPHDF
VPARYEQPRQEDLLNRWTWIPFGAGRHRCVGAAFAIMQIKAIFSVLLREYEFEMAQPPESYRNDHSKMVVQLA
QPACVRYRRRTGV
>CYP51B1_Mycobacterium tuberculosis BTB09-184 2604951496
MSAVALPRVSGGHDEHGHLEEFRTDPIGLMQRVRDECGDVGTFQLAGKQVVLLSGSHANEFFFRAGDDDLDQA
KAYPFMTPIFGEGVVFDASPERRKEMLHNAALRGEQMKGHAATIEDQVRRMIADWGEAGEIDLLDFFAELTIY
TSSACLIGKKFRDQLDGRFAKLYHELERGTDPLAYVDPYLPIESFRRRDEARNGLVALVADIMNGRIANPPTD
KSDRDMLDVLIAVKAETGTPRFSADEITGMFISMMFAGHHTSSGTASWTLIELMRHRDAYAAVIDELDELYGD
GRSVSFHALRQIPQLENVLKETLRLHPPLIILMRVAKGEFEVQGHRIHEGDLVAASPAISNRIPEDFPDPHDF
VPARYEQPRQEDLLNRWTWIPFGAGRHRCVGAAFAIMQIKAIFSVLLREYEFEMAQPPESYRNDHSKMVVQLA
QPACVRYRRRTGV

>CYP51B1_Mycobacterium tuberculosis BTB09-230 2604955238
MSAVALPRVSGGHDEHGHLEEFRTDPIGLMQRVRDECGDVGTFQLAGKQVVLLSGSHANEFFFRAGDDDLDQA
KAYPFMTPIFGEGVVFDASPERRKEMLHNAALRGEQMKGHAATIEDQVRRMIADWGEAGEIDLLDFFAELTIY
TSSACLIGKKFRDQLDGRFAKLYHELERGTDPLAYVDPYLPIESFRRRDEARNGLVALVADIMNGRIANPPTD
KSDRDMLDVLIAVKAETGTPRFSADEITGMFISMMFAGHHTSSGTASWTLIELMRHRDAYAAVIDELDELYGD
GRSVSFHALRQIPQLENVLKETLRLHPPLIILMRVAKGEFEVQGHRIHEGDLVAASPAISNRIPEDFPDPHDF
VPARYEQPRQEDLLNRWTWIPFGAGRHRCVGAAFAIMQIKAIFSVLLREYEFEMAQPPESYRNDHSKMVVQLA
QPACVRYRRRTGV
>CYP51B1_Mycobacterium tuberculosis BTB09-377 2604962195
MSAVALPRVSGGHDEHGHLEEFRTDPIGLMQRVRDECGDVGTFQLAGKQVVLLSGSHANEFFFRAGDDDLDQA
KAYPFMTPIFGEGVVFDASPERRKEMLHNAALRGEQMKGHAATIEDQVRRMIADWGEAGEIDLLDFFAELTIY
TSSACLIGKKFRDQLDGRFAKLYHELERGTDPLAYVDPYLPIESFRRRDEARNGLVALVADIMNGRIANPPTD
KSDRDMLDVLIAVKAETGTPRFSADEITGMFISMMFAGHHTSSGTASWTLIELMRHRDAYAAVIDELDELYGD
GRSVSFHALRQIPQLENVLKETLRLHPPLIILMRVAKGEFEVQGHRIHEGDLVAASPAISNRIPEDFPDPHDF
VPARYEQPRQEDLLNRWTWIPFGAGRHRCVGAAFAIMQIKAIFSVLLREYEFEMAQPPESYRNDHSKMVVQLA
QPACVRYRRRTGV
>CYP51B1_Mycobacterium tuberculosis BTB09-382 2603504861
MSAVALPRVSGGHDEHGHLEEFRTDPIGLMQRVRDECGDVGTFQLAGKQVVLLSGSHANEFFFRAGDDDLDQA
KAYPFMTPIFGEGVVFDASPERRKEMLHNAALRGEQMKGHAATIEDQVRRMIADWGEAGEIDLLDFFAELTIY
TSSACLIGKKFRDQLDGRFAKLYHELERGTDPLAYVDPYLPIESFRRRDEARNGLVALVADIMNGRIANPPTD
KSDRDMLDVLIAVKAETGTPRFSADEITGMFISMMFAGHHTSSGTASWTLIELMRHRDAYAAVIDELDELYGD
GRSVSFHALRQIPQLENVLKETLRLHPPLIILMRVAKGEFEVQGHRIHEGDLVAASPAISNRIPEDFPDPHDF
VPARYEQPRQEDLLNRWTWIPFGAGRHRCVGAAFAIMQIKAIFSVLLREYEFEMAQPPESYRNDHSKMVVQLA
QPACVRYRRRTGV
>CYP51B1_Mycobacterium tuberculosis BTB09-485 2604575137
MSAVALPRVSGGHDEHGHLEEFRTDPIGLMQRVRDECGDVGTFQLAGKQVVLLSGSHANEFFFRAGDDDLDQA
KAYPFMTPIFGEGVVFDASPERRKEMLHNAALRGEQMKGHAATIEDQVRRMIADWGEAGEIDLLDFFAELTIY
TSSACLIGKKFRDQLDGRFAKLYHELERGTDPLAYVDPYLPIESFRRRDEARNGLVALVADIMNGRIANPPTD
KSDRDMLDVLIAVKAETGTPRFSADEITGMFISMMFAGHHTSSGTASWTLIELMRHRDAYAAVIDELDELYGD
GRSVSFHALRQIPQLENVLKETLRLHPPLIILMRVAKGEFEVQGHRIHEGDLVAASPAISNRIPEDFPDPHDF
VPARYEQPRQEDLLNRWTWIPFGAGRHRCVGAAFAIMQIKAIFSVLLREYEFEMAQPPESYRNDHSKMVVQLA
QPACVRYRRRTGV
>CYP51B1_Mycobacterium tuberculosis BTB09-554 2605293696
MSAVALPRVSGGHDEHGHLEEFRTDPIGLMQRVRDECGDVGTFQLAGKQVVLLSGSHANEFFFRAGDDDLDQA
KAYPFMTPIFGEGVVFDASPERRKEMLHNAALRGEQMKGHAATIEDQVRRMIADWGEAGEIDLLDFFAELTIY
TSSACLIGKKFRDQLDGRFAKLYHELERGTDPLAYVDPYLPIESFRRRDEARNGLVALVADIMNGRIANPPTD
KSDRDMLDVLIAVKAETGTPRFSADEITGMFISMMFAGHHTSSGTASWTLIELMRHRDAYAAVIDELDELYGD
GRSVSFHALRQIPQLENVLKETLRLHPPLIILMRVAKGEFEVQGHRIHEGDLVAASPAISNRIPEDFPDPHDF
VPARYEQPRQEDLLNRWTWIPFGAGRHRCVGAAFAIMQIKAIFSVLLREYEFEMAQPPESYRNDHSKMVVQLA
QPACVRYRRRTGV
>CYP51B1_Mycobacterium tuberculosis BTB09-565 2578061404
MSAVALPRVSGGHDEHGHLEEFRTDPIGLMQRVRDECGDVGTFQLAGKQVVLLSGSHANEFFFRAGDDDLDQA
KAYPFMTPIFGEGVVFDASPERRKEMLHNAALRGEQMKGHAATIEDQVRRMIADWGEAGEIDLLDFFAELTIY
TSSACLIGKKFRDQLDGRFAKLYHELERGTDPLAYVDPYLPIESFRRRDEARNGLVALVADIMNGRIANPPTD
KSDRDMLDVLIAVKAETGTPRFSADEITGMFISMMFAGHHTSSGTASWTLIELMRHRDAYAAVIDELDELYGD
GRSVSFHALRQIPQLENVLKETLRLHPPLIILMRVAKGEFEVQGHRIHEGDLVAASPAISNRIPEDFPDPHDF
VPARYEQPRQEDLLNRWTWIPFGAGRHRCVGAAFAIMQIKAIFSVLLREYEFEMAQPPESYRNDHSKMVVQLA
QPACVRYRRRTGV
>CYP51B1_Mycobacterium tuberculosis BTB10-092 2605294815
MSAVALPRVSGGHDEHGHLEEFRTDPIGLMQRVRDECGDVGTFQLAGKQVVLLSGSHANEFFFRAGDDDLDQA
KAYPFMTPIFGEGVVFDASPERRKEMLHNAALRGEQMKGHAATIEDQVRRMIADWGEAGEIDLLDFFAELTIY
TSSACLIGKKFRDQLDGRFAKLYHELERGTDPLAYVDPYLPIESFRRRDEARNGLVALVADIMNGRIANPPTD
KSDRDMLDVLIAVKAETGTPRFSADEITGMFISMMFAGHHTSSGTASWTLIELMRHRDAYAAVIDELDELYGD
GRSVSFHALRQIPQLENVLKETLRLHPPLIILMRVAKGEFEVQGHRIHEGDLVAASPAISNRIPEDFPDPHDF
VPARYEQPRQEDLLNRWTWIPFGAGRHRCVGAAFAIMQIKAIFSVLLREYEFEMAQPPESYRNDHSKMVVQLA
QPACVRYRRRTGV
>CYP51B1_Mycobacterium tuberculosis BTB10-097 2604576523

MSAVALPRVSGGHDEHGHLEEFRTDPIGLMQRVRDECGDVGTFQLAGKQVVLLSGSHANEFFFRAGDDDLDQA
KAYPFMTPIFGEGVVFDASPERRKEMLHNAALRGEQMKGHAATIEDQVRRMIADWGEAGEIDLLDFFAELTIY
TSSACLIGKKFRDQLDGRFAKLYHELERGTDPLAYVDPYLPIESFRRRDEARNGLVALVADIMNGRIANPPTD
KSDRDMLDVLIAVKAETGTPRFSADEITGMFISMMFAGHHTSSGTASWTLIELMRHRDAYAAVIDELDELYGD
GRSVSFHALRQIPQLENVLKETLRLHPPLIILMRVAKGEFEVQGHRIHEGDLVAASPAISNRIPEDFPDPHDF
VPARYEQPRQEDLLNRWTWIPFGAGRHRCVGAAFAIMQIKAIFSVLLREYEFEMAQPPESYRNDHSKMVVQLA
QPACVRYRRRTGV
>CYP51B1_Mycobacterium tuberculosis BTB10-120 2605649810
MSAVALPRVSGGHDEHGHLEEFRTDPIGLMQRVRDECGDVGTFQLAGKQVVLLSGSHANEFFFRAGDDDLDQA
KAYPFMTPIFGEGVVFDASPERRKEMLHNAALRGEQMKGHAATIEDQVRRMIADWGEAGEIDLLDFFAELTIY
TSSACLIGKKFRDQLDGRFAKLYHELERGTDPLAYVDPYLPIESFRRRDEARNGLVALVADIMNGRIANPPTD
KSDRDMLDVLIAVKAETGTPRFSADEITGMFISMMFAGHHTSSGTASWTLIELMRHRDAYAAVIDELDELYGD
GRSVSFHALRQIPQLENVLKETLRLHPPLIILMRVAKGEFEVQGHRIHEGDLVAASPAISNRIPEDFPDPHDF
VPARYEQPRQEDLLNRWTWIPFGAGRHRCVGAAFAIMQIKAIFSVLLREYEFEMAQPPESYRNDHSKMVVQLA
QPACVRYRRRTGV
>CYP51B1_Mycobacterium tuberculosis BTB10-253 2604580649
MSAVALPRVSGGHDEHGHLEEFRTDPIGLMQRVRDECGDVGTFQLAGKQVVLLSGSHANEFFFRAGDDDLDQA
KAYPFMTPIFGEGVVFDASPERRKEMLHNAALRGEQMKGHAATIEDQVRRMIADWGEAGEIDLLDFFAELTIY
TSSACLIGKKFRDQLDGRFAKLYHELERGTDPLAYVDPYLPIESFRRRDEARNGLVALVADIMNGRIANPPTD
KSDRDMLDVLIAVKAETGTPRFSADEITGMFISMMFAGHHTSSGTASWTLIELMRHRDAYAAVIDELDELYGD
GRSVSFHALRQIPQLENVLKETLRLHPPLIILMRVAKGEFEVQGHRIHEGDLVAASPAISNRIPEDFPDPHDF
VPARYEQPRQEDLLNRWTWIPFGAGRHRCVGAAFAIMQIKAIFSVLLREYEFEMAQPPESYRNDHSKMVVQLA
QPACVRYRRRTGV
>CYP51B1_Mycobacterium tuberculosis BTB10-257 2605298928
MSAVALPRVSGGHDEHGHLEEFRTDPIGLMQRVRDECGDVGTFQLAGKQVVLLSGSHANEFFFRAGDDDLDQA
KAYPFMTPIFGEGVVFDASPERRKEMLHNAALRGEQMKGHAATIEDQVRRMIADWGEAGEIDLLDFFAELTIY
TSSACLIGKKFRDQLDGRFAKLYHELERGTDPLAYVDPYLPIESFRRRDEARNGLVALVADIMNGRIANPPTD
KSDRDMLDVLIAVKAETGTPRFSADEITGMFISMMFAGHHTSSGTASWTLIELMRHRDAYAAVIDELDELYGD
GRSVSFHALRQIPQLENVLKETLRLHPPLIILMRVAKGEFEVQGHRIHEGDLVAASPAISNRIPEDFPDPHDF
VPARYEQPRQEDLLNRWTWIPFGAGRHRCVGAAFAIMQIKAIFSVLLREYEFEMAQPPESYRNDHSKMVVQLA
QPACVRYRRRTGV
>CYP51B1_Mycobacterium tuberculosis BTB10-258 2603167258
MSAVALPRVSGGHDEHGHLEEFRTDPIGLMQRVRDECGDVGTFQLAGKQVVLLSGSHANEFFFRAGDDDLDQA
KAYPFMTPIFGEGVVFDASPERRKEMLHNAALRGEQMKGHAATIEDQVRRMIADWGEAGEIDLLDFFAELTIY
TSSACLIGKKFRDQLDGRFAKLYHELERGTDPLAYVDPYLPIESFRRRDEARNGLVALVADIMNGRIANPPTD
KSDRDMLDVLIAVKAETGTPRFSADEITGMFISMMFAGHHTSSGTASWTLIELMRHRDAYAAVIDELDELYGD
GRSVSFHALRQIPQLENVLKETLRLHPPLIILMRVAKGEFEVQGHRIHEGDLVAASPAISNRIPEDFPDPHDF
VPARYEQPRQEDLLNRWTWIPFGAGRHRCVGAAFAIMQIKAIFSVLLREYEFEMAQPPESYRNDHSKMVVQLA
QPACVRYRRRTGV
>CYP51B1_Mycobacterium tuberculosis BTB10-295 2603340229
MSAVALPRVSGGHDEHGHLEEFRTDPIGLMQRVRDECGDVGTFQLAGKQVVLLSGSHANEFFFRAGDDDLDQA
KAYPFMTPIFGEGVVFDASPERRKEMLHNAALRGEQMKGHAATIEDQVRRMIADWGEAGEIDLLDFFAELTIY
TSSACLIGKKFRDQLDGRFAKLYHELERGTDPLAYVDPYLPIESFRRRDEARNGLVALVADIMNGRIANPPTD
KSDRDMLDVLIAVKAETGTPRFSADEITGMFISMMFAGHHTSSGTASWTLIELMRHRDAYAAVIDELDELYGD
GRSVSFHALRQIPQLENVLKETLRLHPPLIILMRVAKGEFEVQGHRIHEGDLVAASPAISNRIPEDFPDPHDF
VPARYEQPRQEDLLNRWTWIPFGAGRHRCVGAAFAIMQIKAIFSVLLREYEFEMAQPPESYRNDHSKMVVQLA
QPACVRYRRRTGV
>CYP51B1_Mycobacterium tuberculosis BTB10-305 2604963527
MSAVALPRVSGGHDEHGHLEEFRTDPIGLMQRVRDECGDVGTFQLAGKQVVLLSGSHANEFFFRAGDDDLDQA
KAYPFMTPIFGEGVVFDASPERRKEMLHNAALRGEQMKGHAATIEDQVRRMIADWGEAGEIDLLDFFAELTIY
TSSACLIGKKFRDQLDGRFAKLYHELERGTDPLAYVDPYLPIESFRRRDEARNGLVALVADIMNGRIANPPTD
KSDRDMLDVLIAVKAETGTPRFSADEITGMFISMMFAGHHTSSGTASWTLIELMRHRDAYAAVIDELDELYGD
GRSVSFHALRQIPQLENVLKETLRLHPPLIILMRVAKGEFEVQGHRIHEGDLVAASPAISNRIPEDFPDPHDF
VPARYEQPRQEDLLNRWTWIPFGAGRHRCVGAAFAIMQIKAIFSVLLREYEFEMAQPPESYRNDHSKMVVQLA
QPACVRYRRRTGV
>CYP51B1_Mycobacterium tuberculosis BTB10-357 2584630905
MSAVALPRVSGGHDEHGHLEEFRTDPIGLMQRVRDECGDVGTFQLAGKQVVLLSGSHANEFFFRAGDDDLDQA
KAYPFMTPIFGEGVVFDASPERRKEMLHNAALRGEQMKGHAATIEDQVRRMIADWGEAGEIDLLDFFAELTIY

TSSACLIGKKFRDQLDGRFAKLYHELERGTDPLAYVDPYLPIESFRRRDEARNGLVALVADIMNGRIANPPTD
KSDRDMLDVLIAVKAETGTPRFSADEITGMFISMMFAGHHTSSGTASWTLIELMRHRDAYAAVIDELDELYGD
GRSVSFHALRQIPQLENVLKETLRLHPPLIILMRVAKGEFEVQGHRIHEGDLVAASPAISNRIPEDFPDPHDF
VPARYEQPRQEDLLNRWTWIPFGAGRHRCVGAAFAIMQIKAIFSVLLREYEFEMAQPPESYRNDHSKMVVQLA
QPACVRYRRRTGV
>CYP51B1_Mycobacterium tuberculosis BTB10-367 2604588888
MSAVALPRVSGGHDEHGHLEEFRTDPIGLMQRVRDECGDVGTFQLAGKQVVLLSGSHANEFFFRAGDDDLDQA
KAYPFMTPIFGEGVVFDASPERRKEMLHNAALRGEQMKGHAATIEDQVRRMIADWGEAGEIDLLDFFAELTIY
TSSACLIGKKFRDQLDGRFAKLYHELERGTDPLAYVDPYLPIESFRRRDEARNGLVALVADIMNGRIANPPTD
KSDRDMLDVLIAVKAETGTPRFSADEITGMFISMMFAGHHTSSGTASWTLIELMRHRDAYAAVIDELDELYGD
GRSVSFHALRQIPQLENVLKETLRLHPPLIILMRVAKGEFEVQGHRIHEGDLVAASPAISNRIPEDFPDPHDF
VPARYEQPRQEDLLNRWTWIPFGAGRHRCVGAAFAIMQIKAIFSVLLREYEFEMAQPPESYRNDHSKMVVQLA
QPACVRYRRRTGV
>CYP51B1_Mycobacterium tuberculosis BTB10-426 2603884456
MSAVALPRVSGGHDEHGHLEEFRTDPIGLMQRVRDECGDVGTFQLAGKQVVLLSGSHANEFFFRAGDDDLDQA
KAYPFMTPIFGEGVVFDASPERRKEMLHNAALRGEQMKGHAATIEDQVRRMIADWGEAGEIDLLDFFAELTIY
TSSACLIGKKFRDQLDGRFAKLYHELERGTDPLAYVDPYLPIESFRRRDEARNGLVALVADIMNGRIANPPTD
KSDRDMLDVLIAVKAETGTPRFSADEITGMFISMMFAGHHTSSGTASWTLIELMRHRDAYAAVIDELDELYGD
GRSVSFHALRQIPQLENVLKETLRLHPPLIILMRVAKGEFEVQGHRIHEGDLVAASPAISNRIPEDFPDPHDF
VPARYEQPRQEDLLNRWTWIPFGAGRHRCVGAAFAIMQIKAIFSVLLREYEFEMAQPPESYRNDHSKMVVQLA
QPACVRYRRRTGV
>CYP51B1_Mycobacterium tuberculosis 2242 2687549710
MSAVALPRVSGGHDEHGHLEEFRTDPIGLMQRVRDECGDVGTFQLAGKQVVLLSGSHANEFFFRAGDDDLDQA
KAYPFMTPIFGEGVVFDASPERRKEMLHNAALRGEQMKGHAATIEDQVRRMIADWGEAGEIDLLDFFAELTIY
TSSACLIGKKFRDQLDGRFAKLYHELERGTDPLAYVDPYLPIESFRRRDEARNGLVALVADIMNGRIANPPTD
KSDRDMLDVLIAVKAETGTPRFSADEITGMFISMMFAGHHTSSGTASWTLIELMRHRDAYAAVIDELDELYGD
GRSVSFHALRQIPQLENVLKETLRLHPPLIILMRVAKGEFEVQGHRIHEGDLVAASPAISNRIPEDFPDPHDF
VPARYEQPRQEDLLNRWTWIPFGAGRHRCVGAAFAIMQIKAIFSVLLREYEFEMAQPPESYRNDHSKMVVQLA
QPACVRYRRRTGV
>CYP51B1_Mycobacterium tuberculosis BTB10-487 2576966417
MSAVALPRVSGGHDEHGHLEEFRTDPIGLMQRVRDECGDVGTFQLAGKQVVLLSGSHANEFFFRAGDDDLDQA
KAYPFMTPIFGEGVVFDASPERRKEMLHNAALRGEQMKGHAATIEDQVRRMIADWGEAGEIDLLDFFAELTIY
TSSACLIGKKFRDQLDGRFAKLYHELERGTDPLAYVDPYLPIESFRRRDEARNGLVALVADIMNGRIANPPTD
KSDRDMLDVLIAVKAETGTPRFSADEITGMFISMMFAGHHTSSGTASWTLIELMRHRDAYAAVIDELDELYGD
GRSVSFHALRQIPQLENVLKETLRLHPPLIILMRVAKGEFEVQGHRIHEGDLVAASPAISNRIPEDFPDPHDF
VPARYEQPRQEDLLNRWTWIPFGAGRHRCVGAAFAIMQIKAIFSVLLREYEFEMAQPPESYRNDHSKMVVQLA
QPACVRYRRRTGV
>CYP51B1_Mycobacterium tuberculosis BTB10-472 2605314194
MSAVALPRVSGGHDEHGHLEEFRTDPIGLMQRVRDECGDVGTFQLAGKQVVLLSGSHANEFFFRAGDDDLDQA
KAYPFMTPIFGEGVVFDASPERRKEMLHNAALRGEQMKGHAATIEDQVRRMIADWGEAGEIDLLDFFAELTIY
TSSACLIGKKFRDQLDGRFAKLYHELERGTDPLAYVDPYLPIESFRRRDEARNGLVALVADIMNGRIANPPTD
KSDRDMLDVLIAVKAETGTPRFSADEITGMFISMMFAGHHTSSGTASWTLIELMRHRDAYAAVIDELDELYGD
GRSVSFHALRQIPQLENVLKETLRLHPPLIILMRVAKGEFEVQGHRIHEGDLVAASPAISNRIPEDFPDPHDF
VPARYEQPRQEDLLNRWTWIPFGAGRHRCVGAAFAIMQIKAIFSVLLREYEFEMAQPPESYRNDHSKMVVQLA
QPACVRYRRRTGV
>CYP51B1_Mycobacterium tuberculosis BTB11-027 2603344618
MSAVALPRVSGGHDEHGHLEEFRTDPIGLMQRVRDECGDVGTFQLAGKQVVLLSGSHANEFFFRAGDDDLDQA
KAYPFMTPIFGEGVVFDASPERRKEMLHNAALRGEQMKGHAATIEDQVRRMIADWGEAGEIDLLDFFAELTIY
TSSACLIGKKFRDQLDGRFAKLYHELERGTDPLAYVDPYLPIESFRRRDEARNGLVALVADIMNGRIANPPTD
KSDRDMLDVLIAVKAETGTPRFSADEITGMFISMMFAGHHTSSGTASWTLIELMRHRDAYAAVIDELDELYGD
GRSVSFHALRQIPQLENVLKETLRLHPPLIILMRVAKGEFEVQGHRIHEGDLVAASPAISNRIPEDFPDPHDF
VPARYEQPRQEDLLNRWTWIPFGAGRHRCVGAAFAIMQIKAIFSVLLREYEFEMAQPPESYRNDHSKMVVQLA
QPACVRYRRRTGV
>CYP51B1_Mycobacterium tuberculosis BTB11-047 2604220103
MSAVALPRVSGGHDEHGHLEEFRTDPIGLMQRVRDECGDVGTFQLAGKQVVLLSGSHANEFFFRAGDDDLDQA
KAYPFMTPIFGEGVVFDASPERRKEMLHNAALRGEQMKGHAATIEDQVRRMIADWGEAGEIDLLDFFAELTIY
TSSACLIGKKFRDQLDGRFAKLYHELERGTDPLAYVDPYLPIESFRRRDEARNGLVALVADIMNGRIANPPTD
KSDRDMLDVLIAVKAETGTPRFSADEITGMFISMMFAGHHTSSGTASWTLIELMRHRDAYAAVIDELDELYGD

GRSVSFHALRQIPQLENVLKETLRLHPPLIILMRVAKGEFEVQGHRIHEGDLVAASPAISNRIPEDFPDPHDF
VPARYEQPRQEDLLNRWTWIPFGAGRHRCVGAAFAIMQIKAIFSVLLREYEFEMAQPPESYRNDHSKMVVQLA
QPACVRYRRRTGV
>CYP51B1_Mycobacterium tuberculosis BTB11-112 2604227133
MSAVALPRVSGGHDEHGHLEEFRTDPIGLMQRVRDECGDVGTFQLAGKQVVLLSGSHANEFFFRAGDDDLDQA
KAYPFMTPIFGEGVVFDASPERRKEMLHNAALRGEQMKGHAATIEDQVRRMIADWGEAGEIDLLDFFAELTIY
TSSACLIGKKFRDQLDGRFAKLYHELERGTDPLAYVDPYLPIESFRRRDEARNGLVALVADIMNGRIANPPTD
KSDRDMLDVLIAVKAETGTPRFSADEITGMFISMMFAGHHTSSGTASWTLIELMRHRDAYAAVIDELDELYGD
GRSVSFHALRQIPQLENVLKETLRLHPPLIILMRVAKGEFEVQGHRIHEGDLVAASPAISNRIPEDFPDPHDF
VPARYEQPRQEDLLNRWTWIPFGAGRHRCVGAAFAIMQIKAIFSVLLREYEFEMAQPPESYRNDHSKMVVQLA
QPACVRYRRRTGV
>CYP51B1_Mycobacterium tuberculosis BTB11-133 2604230428
MSAVALPRVSGGHDEHGHLEEFRTDPIGLMQRVRDECGDVGTFQLAGKQVVLLSGSHANEFFFRAGDDDLDQA
KAYPFMTPIFGEGVVFDASPERRKEMLHNAALRGEQMKGHAATIEDQVRRMIADWGEAGEIDLLDFFAELTIY
TSSACLIGKKFRDQLDGRFAKLYHELERGTDPLAYVDPYLPIESFRRRDEARNGLVALVADIMNGRIANPPTD
KSDRDMLDVLIAVKAETGTPRFSADEITGMFISMMFAGHHTSSGTASWTLIELMRHRDAYAAVIDELDELYGD
GRSVSFHALRQIPQLENVLKETLRLHPPLIILMRVAKGEFEVQGHRIHEGDLVAASPAISNRIPEDFPDPHDF
VPARYEQPRQEDLLNRWTWIPFGAGRHRCVGAAFAIMQIKAIFSVLLREYEFEMAQPPESYRNDHSKMVVQLA
QPACVRYRRRTGV
>CYP51B1_Mycobacterium tuberculosis BTB11-139 2603348718
MSAVALPRVSGGHDEHGHLEEFRTDPIGLMQRVRDECGDVGTFQLAGKQVVLLSGSHANEFFFRAGDDDLDQA
KAYPFMTPIFGEGVVFDASPERRKEMLHNAALRGEQMKGHAATIEDQVRRMIADWGEAGEIDLLDFFAELTIY
TSSACLIGKKFRDQLDGRFAKLYHELERGTDPLAYVDPYLPIESFRRRDEARNGLVALVADIMNGRIANPPTD
KSDRDMLDVLIAVKAETGTPRFSADEITGMFISMMFAGHHTSSGTASWTLIELMRHRDAYAAVIDELDELYGD
GRSVSFHALRQIPQLENVLKETLRLHPPLIILMRVAKGEFEVQGHRIHEGDLVAASPAISNRIPEDFPDPHDF
VPARYEQPRQEDLLNRWTWIPFGAGRHRCVGAAFAIMQIKAIFSVLLREYEFEMAQPPESYRNDHSKMVVQLA
QPACVRYRRRTGV
>CYP51B1_Mycobacterium tuberculosis BTB11-160 2603888544
MSAVALPRVSGGHDEHGHLEEFRTDPIGLMQRVRDECGDVGTFQLAGKQVVLLSGSHANEFFFRAGDDDLDQA
KAYPFMTPIFGEGVVFDASPERRKEMLHNAALRGEQMKGHAATIEDQVRRMIADWGEAGEIDLLDFFAELTIY
TSSACLIGKKFRDQLDGRFAKLYHELERGTDPLAYVDPYLPIESFRRRDEARNGLVALVADIMNGRIANPPTD
KSDRDMLDVLIAVKAETGTPRFSADEITGMFISMMFAGHHTSSGTASWTLIELMRHRDAYAAVIDELDELYGD
GRSVSFHALRQIPQLENVLKETLRLHPPLIILMRVAKGEFEVQGHRIHEGDLVAASPAISNRIPEDFPDPHDF
VPARYEQPRQEDLLNRWTWIPFGAGRHRCVGAAFAIMQIKAIFSVLLREYEFEMAQPPESYRNDHSKMVVQLA
QPACVRYRRRTGV
>CYP51B1_Mycobacterium tuberculosis BTB11-192 2605653618
MSAVALPRVSGGHDEHGHLEEFRTDPIGLMQRVRDECGDVGTFQLAGKQVVLLSGSHANEFFFRAGDDDLDQA
KAYPFMTPIFGEGVVFDASPERRKEMLHNAALRGEQMKGHAATIEDQVRRMIADWGEAGEIDLLDFFAELTIY
TSSACLIGKKFRDQLDGRFAKLYHELERGTDPLAYVDPYLPIESFRRRDEARNGLVALVADIMNGRIANPPTD
KSDRDMLDVLIAVKAETGTPRFSADEITGMFISMMFAGHHTSSGTASWTLIELMRHRDAYAAVIDELDELYGD
GRSVSFHALRQIPQLENVLKETLRLHPPLIILMRVAKGEFEVQGHRIHEGDLVAASPAISNRIPEDFPDPHDF
VPARYEQPRQEDLLNRWTWIPFGAGRHRCVGAAFAIMQIKAIFSVLLREYEFEMAQPPESYRNDHSKMVVQLA
QPACVRYRRRTGV
>CYP51B1_Mycobacterium tuberculosis BTB11-207 2605658037
MSAVALPRVSGGHDEHGHLEEFRTDPIGLMQRVRDECGDVGTFQLAGKQVVLLSGSHANEFFFRAGDDDLDQA
KAYPFMTPIFGEGVVFDASPERRKEMLHNAALRGEQMKGHAATIEDQVRRMIADWGEAGEIDLLDFFAELTIY
TSSACLIGKKFRDQLDGRFAKLYHELERGTDPLAYVDPYLPIESFRRRDEARNGLVALVADIMNGRIANPPTD
KSDRDMLDVLIAVKAETGTPRFSADEITGMFISMMFAGHHTSSGTASWTLIELMRHRDAYAAVIDELDELYGD
GRSVSFHALRQIPQLENVLKETLRLHPPLIILMRVAKGEFEVQGHRIHEGDLVAASPAISNRIPEDFPDPHDF
VPARYEQPRQEDLLNRWTWIPFGAGRHRCVGAAFAIMQIKAIFSVLLREYEFEMAQPPESYRNDHSKMVVQLA
QPACVRYRRRTGV
>CYP51B1_Mycobacterium tuberculosis BTB11-210 2604593015
MSAVALPRVSGGHDEHGHLEEFRTDPIGLMQRVRDECGDVGTFQLAGKQVVLLSGSHANEFFFRAGDDDLDQA
KAYPFMTPIFGEGVVFDASPERRKEMLHNAALRGEQMKGHAATIEDQVRRMIADWGEAGEIDLLDFFAELTIY
TSSACLIGKKFRDQLDGRFAKLYHELERGTDPLAYVDPYLPIESFRRRDEARNGLVALVADIMNGRIANPPTD
KSDRDMLDVLIAVKAETGTPRFSADEITGMFISMMFAGHHTSSGTASWTLIELMRHRDAYAAVIDELDELYGD
GRSVSFHALRQIPQLENVLKETLRLHPPLIILMRVAKGEFEVQGHRIHEGDLVAASPAISNRIPEDFPDPHDF

VPARYEQPRQEDLLNRWTWIPFGAGRHRCVGAAFAIMQIKAIFSVLLREYEFEMAQPPESYRNDHSKMVVQLA
QPACVRYRRRTGV
>CYP51B1_Mycobacterium tuberculosis BTB11-214 2576720588
MSAVALPRVSGGHDEHGHLEEFRTDPIGLMQRVRDECGDVGTFQLAGKQVVLLSGSHANEFFFRAGDDDLDQA
KAYPFMTPIFGEGVVFDASPERRKEMLHNAALRGEQMKGHAATIEDQVRRMIADWGEAGEIDLLDFFAELTIY
TSSACLIGKKFRDQLDGRFAKLYHELERGTDPLAYVDPYLPIESFRRRDEARNGLVALVADIMNGRIANPPTD
KSDRDMLDVLIAVKAETGTPRFSADEITGMFISMMFAGHHTSSGTASWTLIELMRHRDAYAAVIDELDELYGD
GRSVSFHALRQIPQLENVLKETLRLHPPLIILMRVAKGEFEVQGHRIHEGDLVAASPAISNRIPEDFPDPHDF
VPARYEQPRQEDLLNRWTWIPFGAGRHRCVGAAFAIMQIKAIFSVLLREYEFEMAQPPESYRNDHSKMVVQLA
QPACVRYRRRTGV
>CYP51B1_Mycobacterium tuberculosis BTB11-289 2604236552
MSAVALPRVSGGHDEHGHLEEFRTDPIGLMQRVRDECGDVGTFQLAGKQVVLLSGSHANEFFFRAGDDDLDQA
KAYPFMTPIFGEGVVFDASPERRKEMLHNAALRGEQMKGHAATIEDQVRRMIADWGEAGEIDLLDFFAELTIY
TSSACLIGKKFRDQLDGRFAKLYHELERGTDPLAYVDPYLPIESFRRRDEARNGLVALVADIMNGRIANPPTD
KSDRDMLDVLIAVKAETGTPRFSADEITGMFISMMFAGHHTSSGTASWTLIELMRHRDAYAAVIDELDELYGD
GRSVSFHALRQIPQLENVLKETLRLHPPLIILMRVAKGEFEVQGHRIHEGDLVAASPAISNRIPEDFPDPHDF
VPARYEQPRQEDLLNRWTWIPFGAGRHRCVGAAFAIMQIKAIFSVLLREYEFEMAQPPESYRNDHSKMVVQLA
QPACVRYRRRTGV
>CYP51B1_Mycobacterium tuberculosis BTB11-299 2604240918
MSAVALPRVSGGHDEHGHLEEFRTDPIGLMQRVRDECGDVGTFQLAGKQVVLLSGSHANEFFFRAGDDDLDQA
KAYPFMTPIFGEGVVFDASPERRKEMLHNAALRGEQMKGHAATIEDQVRRMIADWGEAGEIDLLDFFAELTIY
TSSACLIGKKFRDQLDGRFAKLYHELERGTDPLAYVDPYLPIESFRRRDEARNGLVALVADIMNGRIANPPTD
KSDRDMLDVLIAVKAETGTPRFSADEITGMFISMMFAGHHTSSGTASWTLIELMRHRDAYAAVIDELDELYGD
GRSVSFHALRQIPQLENVLKETLRLHPPLIILMRVAKGEFEVQGHRIHEGDLVAASPAISNRIPEDFPDPHDF
VPARYEQPRQEDLLNRWTWIPFGAGRHRCVGAAFAIMQIKAIFSVLLREYEFEMAQPPESYRNDHSKMVVQLA
QPACVRYRRRTGV
>CYP51B1_Mycobacterium tuberculosis BTB11-300 2605662179
MSAVALPRVSGGHDEHGHLEEFRTDPIGLMQRVRDECGDVGTFQLAGKQVVLLSGSHANEFFFRAGDDDLDQA
KAYPFMTPIFGEGVVFDASPERRKEMLHNAALRGEQMKGHAATIEDQVRRMIADWGEAGEIDLLDFFAELTIY
TSSACLIGKKFRDQLDGRFAKLYHELERGTDPLAYVDPYLPIESFRRRDEARNGLVALVADIMNGRIANPPTD
KSDRDMLDVLIAVKAETGTPRFSADEITGMFISMMFAGHHTSSGTASWTLIELMRHRDAYAAVIDELDELYGD
GRSVSFHALRQIPQLENVLKETLRLHPPLIILMRVAKGEFEVQGHRIHEGDLVAASPAISNRIPEDFPDPHDF
VPARYEQPRQEDLLNRWTWIPFGAGRHRCVGAAFAIMQIKAIFSVLLREYEFEMAQPPESYRNDHSKMVVQLA
QPACVRYRRRTGV
>CYP51B1_Mycobacterium tuberculosis BTB11-371 2604599117
MSAVALPRVSGGHDEHGHLEEFRTDPIGLMQRVRDECGDVGTFQLAGKQVVLLSGSHANEFFFRAGDDDLDQA
KAYPFMTPIFGEGVVFDASPERRKEMLHNAALRGEQMKGHAATIEDQVRRMIADWGEAGEIDLLDFFAELTIY
TSSACLIGKKFRDQLDGRFAKLYHELERGTDPLAYVDPYLPIESFRRRDEARNGLVALVADIMNGRIANPPTD
KSDRDMLDVLIAVKAETGTPRFSADEITGMFISMMFAGHHTSSGTASWTLIELMRHRDAYAAVIDELDELYGD
GRSVSFHALRQIPQLENVLKETLRLHPPLIILMRVAKGEFEVQGHRIHEGDLVAASPAISNRIPEDFPDPHDF
VPARYEQPRQEDLLNRWTWIPFGAGRHRCVGAAFAIMQIKAIFSVLLREYEFEMAQPPESYRNDHSKMVVQLA
QPACVRYRRRTGV
>CYP51B1_Mycobacterium tuberculosis BTB12-001 2575015271
MSAVALPRVSGGHDEHGHLEEFRTDPIGLMQRVRDECGDVGTFQLAGKQVVLLSGSHANEFFFRAGDDDLDQA
KAYPFMTPIFGEGVVFDASPERRKEMLHNAALRGEQMKGHAATIEDQVRRMIADWGEAGEIDLLDFFAELTIY
TSSACLIGKKFRDQLDGRFAKLYHELERGTDPLAYVDPYLPIESFRRRDEARNGLVALVADIMNGRIANPPTD
KSDRDMLDVLIAVKAETGTPRFSADEITGMFISMMFAGHHTSSGTASWTLIELMRHRDAYAAVIDELDELYGD
GRSVSFHALRQIPQLENVLKETLRLHPPLIILMRVAKGEFEVQGHRIHEGDLVAASPAISNRIPEDFPDPHDF
VPARYEQPRQEDLLNRWTWIPFGAGRHRCVGAAFAIMQIKAIFSVLLREYEFEMAQPPESYRNDHSKMVVQLA
QPACVRYRRRTGV
>CYP51B1_Mycobacterium tuberculosis BTB12-002 2603352827
MSAVALPRVSGGHDEHGHLEEFRTDPIGLMQRVRDECGDVGTFQLAGKQVVLLSGSHANEFFFRAGDDDLDQA
KAYPFMTPIFGEGVVFDASPERRKEMLHNAALRGEQMKGHAATIEDQVRRMIADWGEAGEIDLLDFFAELTIY
TSSACLIGKKFRDQLDGRFAKLYHELERGTDPLAYVDPYLPIESFRRRDEARNGLVALVADIMNGRIANPPTD
KSDRDMLDVLIAVKAETGTPRFSADEITGMFISMMFAGHHTSSGTASWTLIELMRHRDAYAAVIDELDELYGD
GRSVSFHALRQIPQLENVLKETLRLHPPLIILMRVAKGEFEVQGHRIHEGDLVAASPAISNRIPEDFPDPHDF
VPARYEQPRQEDLLNRWTWIPFGAGRHRCVGAAFAIMQIKAIFSVLLREYEFEMAQPPESYRNDHSKMVVQLA
QPACVRYRRRTGV

>CYP51B1_Mycobacterium tuberculosis BTB12-094 2603900847
MSAVALPRVSGGHDEHGHLEEFRTDPIGLMQRVRDECGDVGTFQLAGKQVVLLSGSHANEFFFRAGDDDLDQA
KAYPFMTPIFGEGVVFDASPERRKEMLHNAALRGEQMKGHAATIEDQVRRMIADWGEAGEIDLLDFFAELTIY
TSSACLIGKKFRDQLDGRFAKLYHELERGTDPLAYVDPYLPIESFRRRDEARNGLVALVADIMNGRIANPPTD
KSDRDMLDVLIAVKAETGTPRFSADEITGMFISMMFAGHHTSSGTASWTLIELMRHRDAYAAVIDELDELYGD
GRSVSFHALRQIPQLENVLKETLRLHPPLIILMRVAKGEFEVQGHRIHEGDLVAASPAISNRIPEDFPDPHDF
VPARYEQPRQEDLLNRWTWIPFGAGRHRCVGAAFAIMQIKAIFSVLLREYEFEMAQPPESYRNDHSKMVVQLA
QPACVRYRRRTGV
>CYP51B1_Mycobacterium tuberculosis BTB12-211 2603171670
MSAVALPRVSGGHDEHGHLEEFRTDPIGLMQRVRDECGDVGTFQLAGKQVVLLSGSHANEFFFRAGDDDLDQA
KAYPFMTPIFGEGVVFDASPERRKEMLHNAALRGEQMKGHAATIEDQVRRMIADWGEAGEIDLLDFFAELTIY
TSSACLIGKKFRDQLDGRFAKLYHELERGTDPLAYVDPYLPIESFRRRDEARNGLVALVADIMNGRIANPPTD
KSDRDMLDVLIAVKAETGTPRFSADEITGMFISMMFAGHHTSSGTASWTLIELMRHRDAYAAVIDELDELYGD
GRSVSFHALRQIPQLENVLKETLRLHPPLIILMRVAKGEFEVQGHRIHEGDLVAASPAISNRIPEDFPDPHDF
VPARYEQPRQEDLLNRWTWIPFGAGRHRCVGAAFAIMQIKAIFSVLLREYEFEMAQPPESYRNDHSKMVVQLA
QPACVRYRRRTGV
>CYP51B1_Mycobacterium tuberculosis BTB12-305 2603904965
MSAVALPRVSGGHDEHGHLEEFRTDPIGLMQRVRDECGDVGTFQLAGKQVVLLSGSHANEFFFRAGDDDLDQA
KAYPFMTPIFGEGVVFDASPERRKEMLHNAALRGEQMKGHAATIEDQVRRMIADWGEAGEIDLLDFFAELTIY
TSSACLIGKKFRDQLDGRFAKLYHELERGTDPLAYVDPYLPIESFRRRDEARNGLVALVADIMNGRIANPPTD
KSDRDMLDVLIAVKAETGTPRFSADEITGMFISMMFAGHHTSSGTASWTLIELMRHRDAYAAVIDELDELYGD
GRSVSFHALRQIPQLENVLKETLRLHPPLIILMRVAKGEFEVQGHRIHEGDLVAASPAISNRIPEDFPDPHDF
VPARYEQPRQEDLLNRWTWIPFGAGRHRCVGAAFAIMQIKAIFSVLLREYEFEMAQPPESYRNDHSKMVVQLA
QPACVRYRRRTGV
>CYP51B1_Mycobacterium tuberculosis BTB12-314 2577059418
MSAVALPRVSGGHDEHGHLEEFRTDPIGLMQRVRDECGDVGTFQLAGKQVVLLSGSHANEFFFRAGDDDLDQA
KAYPFMTPIFGEGVVFDASPERRKEMLHNAALRGEQMKGHAATIEDQVRRMIADWGEAGEIDLLDFFAELTIY
TSSACLIGKKFRDQLDGRFAKLYHELERGTDPLAYVDPYLPIESFRRRDEARNGLVALVADIMNGRIANPPTD
KSDRDMLDVLIAVKAETGTPRFSADEITGMFISMMFAGHHTSSGTASWTLIELMRHRDAYAAVIDELDELYGD
GRSVSFHALRQIPQLENVLKETLRLHPPLIILMRVAKGEFEVQGHRIHEGDLVAASPAISNRIPEDFPDPHDF
VPARYEQPRQEDLLNRWTWIPFGAGRHRCVGAAFAIMQIKAIFSVLLREYEFEMAQPPESYRNDHSKMVVQLA
QPACVRYRRRTGV
>CYP51B1_Mycobacterium tuberculosis BTB12-315 2604967933
MSAVALPRVSGGHDEHGHLEEFRTDPIGLMQRVRDECGDVGTFQLAGKQVVLLSGSHANEFFFRAGDDDLDQA
KAYPFMTPIFGEGVVFDASPERRKEMLHNAALRGEQMKGHAATIEDQVRRMIADWGEAGEIDLLDFFAELTIY
TSSACLIGKKFRDQLDGRFAKLYHELERGTDPLAYVDPYLPIESFRRRDEARNGLVALVADIMNGRIANPPTD
KSDRDMLDVLIAVKAETGTPRFSADEITGMFISMMFAGHHTSSGTASWTLIELMRHRDAYAAVIDELDELYGD
GRSVSFHALRQIPQLENVLKETLRLHPPLIILMRVAKGEFEVQGHRIHEGDLVAASPAISNRIPEDFPDPHDF
VPARYEQPRQEDLLNRWTWIPFGAGRHRCVGAAFAIMQIKAIFSVLLREYEFEMAQPPESYRNDHSKMVVQLA
QPACVRYRRRTGV
>CYP51B1_Mycobacterium tuberculosis BTB12-384 2584972890
MSAVALPRVSGGHDEHGHLEEFRTDPIGLMQRVRDECGDVGTFQLAGKQVVLLSGSHANEFFFRAGDDDLDQA
KAYPFMTPIFGEGVVFDASPERRKEMLHNAALRGEQMKGHAATIEDQVRRMIADWGEAGEIDLLDFFAELTIY
TSSACLIGKKFRDQLDGRFAKLYHELERGTDPLAYVDPYLPIESFRRRDEARNGLVALVADIMNGRIANPPTD
KSDRDMLDVLIAVKAETGTPRFSADEITGMFISMMFAGHHTSSGTASWTLIELMRHRDAYAAVIDELDELYGD
GRSVSFHALRQIPQLENVLKETLRLHPPLIILMRVAKGEFEVQGHRIHEGDLVAASPAISNRIPEDFPDPHDF
VPARYEQPRQEDLLNRWTWIPFGAGRHRCVGAAFAIMQIKAIFSVLLREYEFEMAQPPESYRNDHSKMVVQLA
QPACVRYRRRTGV
>CYP51B1_Mycobacterium tuberculosis BTB12-400 2576685740
MSAVALPRVSGGHDEHGHLEEFRTDPIGLMQRVRDECGDVGTFQLAGKQVVLLSGSHANEFFFRAGDDDLDQA
KAYPFMTPIFGEGVVFDASPERRKEMLHNAALRGEQMKGHAATIEDQVRRMIADWGEAGEIDLLDFFAELTIY
TSSACLIGKKFRDQLDGRFAKLYHELERGTDPLAYVDPYLPIESFRRRDEARNGLVALVADIMNGRIANPPTD
KSDRDMLDVLIAVKAETGTPRFSADEITGMFISMMFAGHHTSSGTASWTLIELMRHRDAYAAVIDELDELYGD
GRSVSFHALRQIPQLENVLKETLRLHPPLIILMRVAKGEFEVQGHRIHEGDLVAASPAISNRIPEDFPDPHDF
VPARYEQPRQEDLLNRWTWIPFGAGRHRCVGAAFAIMQIKAIFSVLLREYEFEMAQPPESYRNDHSKMVVQLA
QPACVRYRRRTGV
>CYP51B1_Mycobacterium tuberculosis BTB12-449 2577097458

MSAVALPRVSGGHDEHGHLEEFRTDPIGLMQRVRDECGDVGTFQLAGKQVVLLSGSHANEFFFRAGDDDLDQA
KAYPFMTPIFGEGVVFDASPERRKEMLHNAALRGEQMKGHAATIEDQVRRMIADWGEAGEIDLLDFFAELTIY
TSSACLIGKKFRDQLDGRFAKLYHELERGTDPLAYVDPYLPIESFRRRDEARNGLVALVADIMNGRIANPPTD
KSDRDMLDVLIAVKAETGTPRFSADEITGMFISMMFAGHHTSSGTASWTLIELMRHRDAYAAVIDELDELYGD
GRSVSFHALRQIPQLENVLKETLRLHPPLIILMRVAKGEFEVQGHRIHEGDLVAASPAISNRIPEDFPDPHDF
VPARYEQPRQEDLLNRWTWIPFGAGRHRCVGAAFAIMQIKAIFSVLLREYEFEMAQPPESYRNDHSKMVVQLA
QPACVRYRRRTGV
>CYP51B1_Mycobacterium tuberculosis BTB13-063 2576195578
MSAVALPRVSGGHDEHGHLEEFRTDPIGLMQRVRDECGDVGTFQLAGKQVVLLSGSHANEFFFRAGDDDLDQA
KAYPFMTPIFGEGVVFDASPERRKEMLHNAALRGEQMKGHAATIEDQVRRMIADWGEAGEIDLLDFFAELTIY
TSSACLIGKKFRDQLDGRFAKLYHELERGTDPLAYVDPYLPIESFRRRDEARNGLVALVADIMNGRIANPPTD
KSDRDMLDVLIAVKAETGTPRFSADEITGMFISMMFAGHHTSSGTASWTLIELMRHRDAYAAVIDELDELYGD
GRSVSFHALRQIPQLENVLKETLRLHPPLIILMRVAKGEFEVQGHRIHEGDLVAASPAISNRIPEDFPDPHDF
VPARYEQPRQEDLLNRWTWIPFGAGRHRCVGAAFAIMQIKAIFSVLLREYEFEMAQPPESYRNDHSKMVVQLA
QPACVRYRRRTGV
>CYP51B1_Mycobacterium tuberculosis BTB13-089 2603365225
MSAVALPRVSGGHDEHGHLEEFRTDPIGLMQRVRDECGDVGTFQLAGKQVVLLSGSHANEFFFRAGDDDLDQA
KAYPFMTPIFGEGVVFDASPERRKEMLHNAALRGEQMKGHAATIEDQVRRMIADWGEAGEIDLLDFFAELTIY
TSSACLIGKKFRDQLDGRFAKLYHELERGTDPLAYVDPYLPIESFRRRDEARNGLVALVADIMNGRIANPPTD
KSDRDMLDVLIAVKAETGTPRFSADEITGMFISMMFAGHHTSSGTASWTLIELMRHRDAYAAVIDELDELYGD
GRSVSFHALRQIPQLENVLKETLRLHPPLIILMRVAKGEFEVQGHRIHEGDLVAASPAISNRIPEDFPDPHDF
VPARYEQPRQEDLLNRWTWIPFGAGRHRCVGAAFAIMQIKAIFSVLLREYEFEMAQPPESYRNDHSKMVVQLA
QPACVRYRRRTGV
>CYP51B1_Mycobacterium tuberculosis BTB13-127 2603182493
MSAVALPRVSGGHDEHGHLEEFRTDPIGLMQRVRDECGDVGTFQLAGKQVVLLSGSHANEFFFRAGDDDLDQA
KAYPFMTPIFGEGVVFDASPERRKEMLHNAALRGEQMKGHAATIEDQVRRMIADWGEAGEIDLLDFFAELTIY
TSSACLIGKKFRDQLDGRFAKLYHELERGTDPLAYVDPYLPIESFRRRDEARNGLVALVADIMNGRIANPPTD
KSDRDMLDVLIAVKAETGTPRFSADEITGMFISMMFAGHHTSSGTASWTLIELMRHRDAYAAVIDELDELYGD
GRSVSFHALRQIPQLENVLKETLRLHPPLIILMRVAKGEFEVQGHRIHEGDLVAASPAISNRIPEDFPDPHDF
VPARYEQPRQEDLLNRWTWIPFGAGRHRCVGAAFAIMQIKAIFSVLLREYEFEMAQPPESYRNDHSKMVVQLA
QPACVRYRRRTGV
>CYP51B1_Mycobacterium tuberculosis BTB13-128 2574882937
MSAVALPRVSGGHDEHGHLEEFRTDPIGLMQRVRDECGDVGTFQLAGKQVVLLSGSHANEFFFRAGDDDLDQA
KAYPFMTPIFGEGVVFDASPERRKEMLHNAALRGEQMKGHAATIEDQVRRMIADWGEAGEIDLLDFFAELTIY
TSSACLIGKKFRDQLDGRFAKLYHELERGTDPLAYVDPYLPIESFRRRDEARNGLVALVADIMNGRIANPPTD
KSDRDMLDVLIAVKAETGTPRFSADEITGMFISMMFAGHHTSSGTASWTLIELMRHRDAYAAVIDELDELYGD
GRSVSFHALRQIPQLENVLKETLRLHPPLIILMRVAKGEFEVQGHRIHEGDLVAASPAISNRIPEDFPDPHDF
VPARYEQPRQEDLLNRWTWIPFGAGRHRCVGAAFAIMQIKAIFSVLLREYEFEMAQPPESYRNDHSKMVVQLA
QPACVRYRRRTGV
>CYP51B1_Mycobacterium tuberculosis BTB13-206 2584747099
MSAVALPRVSGGHDEHGHLEEFRTDPIGLMQRVRDECGDVGTFQLAGKQVVLLSGSHANEFFFRAGDDDLDQA
KAYPFMTPIFGEGVVFDASPERRKEMLHNAALRGEQMKGHAATIEDQVRRMIADWGEAGEIDLLDFFAELTIY
TSSACLIGKKFRDQLDGRFAKLYHELERGTDPLAYVDPYLPIESFRRRDEARNGLVALVADIMNGRIANPPTD
KSDRDMLDVLIAVKAETGTPRFSADEITGMFISMMFAGHHTSSGTASWTLIELMRHRDAYAAVIDELDELYGD
GRSVSFHALRQIPQLENVLKETLRLHPPLIILMRVAKGEFEVQGHRIHEGDLVAASPAISNRIPEDFPDPHDF
VPARYEQPRQEDLLNRWTWIPFGAGRHRCVGAAFAIMQIKAIFSVLLREYEFEMAQPPESYRNDHSKMVVQLA
QPACVRYRRRTGV
>CYP51B1_Mycobacterium tuberculosis BTB13-222 2576430202
MSAVALPRVSGGHDEHGHLEEFRTDPIGLMQRVRDECGDVGTFQLAGKQVVLLSGSHANEFFFRAGDDDLDQA
KAYPFMTPIFGEGVVFDASPERRKEMLHNAALRGEQMKGHAATIEDQVRRMIADWGEAGEIDLLDFFAELTIY
TSSACLIGKKFRDQLDGRFAKLYHELERGTDPLAYVDPYLPIESFRRRDEARNGLVALVADIMNGRIANPPTD
KSDRDMLDVLIAVKAETGTPRFSADEITGMFISMMFAGHHTSSGTASWTLIELMRHRDAYAAVIDELDELYGD
GRSVSFHALRQIPQLENVLKETLRLHPPLIILMRVAKGEFEVQGHRIHEGDLVAASPAISNRIPEDFPDPHDF
VPARYEQPRQEDLLNRWTWIPFGAGRHRCVGAAFAIMQIKAIFSVLLREYEFEMAQPPESYRNDHSKMVVQLA
QPACVRYRRRTGV
>CYP51B1_Mycobacterium tuberculosis BTB13-276 2603188124
MSAVALPRVSGGHDEHGHLEEFRTDPIGLMQRVRDECGDVGTFQLAGKQVVLLSGSHANEFFFRAGDDDLDQA
KAYPFMTPIFGEGVVFDASPERRKEMLHNAALRGEQMKGHAATIEDQVRRMIADWGEAGEIDLLDFFAELTIY

TSSACLIGKKFRDQLDGRFAKLYHELERGTDPLAYVDPYLPIESFRRRDEARNGLVALVADIMNGRIANPPTD
KSDRDMLDVLIAVKAETGTPRFSADEITGMFISMMFAGHHTSSGTASWTLIELMRHRDAYAAVIDELDELYGD
GRSVSFHALRQIPQLENVLKETLRLHPPLIILMRVAKGEFEVQGHRIHEGDLVAASPAISNRIPEDFPDPHDF
VPARYEQPRQEDLLNRWTWIPFGAGRHRCVGAAFAIMQIKAIFSVLLREYEFEMAQPPESYRNDHSKMVVQLA
QPACVRYRRRTGV
>CYP51B1_Mycobacterium tuberculosis C unfinished sequence 638725976
MSAVALPRVSGGHDEHGHLEEFRTDPIGLMQRVRDECGDVGTFQLAGKQVVLLSGSHANEFFFRAGDDDLDQA
KAYPFMTPIFGEGVVFDASPERRKEMLHNAALRGEQMKGHAATIEDQVRRMIADWGEAGEIDLLDFFAELTIY
TSSACLIGKKFRDQLDGRFAKLYHELERGTDPLAYVDPYLPIESFRRRDEARNGLVALVADIMNGRIANPPTD
KSDRDMLDVLIAVKAETGTPRFSADEITGMFISMMFAGHHTSSGTASWTLIELMRHRDAYAAVIDELDELYGD
GRSVSFHALRQIPQLENVLKETLRLHPPLIILMRVAKGEFEVQGHRIHEGDLVAASPAISNRIPEDFPDPHDF
VPARYEQPRQEDLLNRWTWIPFGAGRHRCVGAAFAIMQIKAIFSVLLREYEFEMAQPPESYRNDHSKMVVQLA
QPACVRYRRRTGV
>CYP51B1_Mycobacterium tuberculosis C_10367 2675220386
MSAVALPRVSGGHDEHGHLEEFRTDPIGLMQRVRDECGDVGTFQLAGKQVVLLSGSHANEFFFRAGDDDLDQA
KAYPFMTPIFGEGVVFDASPERRKEMLHNAALRGEQMKGHAATIEDQVRRMIADWGEAGEIDLLDFFAELTIY
TSSACLIGKKFRDQLDGRFAKLYHELERGTDPLAYVDPYLPIESFRRRDEARNGLVALVADIMNGRIANPPTD
KSDRDMLDVLIAVKAETGTPRFSADEITGMFISMMFAGHHTSSGTASWTLIELMRHRDAYAAVIDELDELYGD
GRSVSFHALRQIPQLENVLKETLRLHPPLIILMRVAKGEFEVQGHRIHEGDLVAASPAISNRIPEDFPDPHDF
VPARYEQPRQEDLLNRWTWIPFGAGRHRCVGAAFAIMQIKAIFSVLLREYEFEMAQPPESYRNDHSKMVVQLA
QPACVRYRRRTGV
>CYP51B1_Mycobacterium tuberculosis C_14229 2677392273
MSAVALPRVSGGHDEHGHLEEFRTDPIGLMQRVRDECGDVGTFQLAGKQVVLLSGSHANEFFFRAGDDDLDQA
KAYPFMTPIFGEGVVFDASPERRKEMLHNAALRGEQMKGHAATIEDQVRRMIADWGEAGEIDLLDFFAELTIY
TSSACLIGKKFRDQLDGRFAKLYHELERGTDPLAYVDPYLPIESFRRRDEARNGLVALVADIMNGRIANPPTD
KSDRDMLDVLIAVKAETGTPRFSADEITGMFISMMFAGHHTSSGTASWTLIELMRHRDAYAAVIDELDELYGD
GRSVSFHALRQIPQLENVLKETLRLHPPLIILMRVAKGEFEVQGHRIHEGDLVAASPAISNRIPEDFPDPHDF
VPARYEQPRQEDLLNRWTWIPFGAGRHRCVGAAFAIMQIKAIFSVLLREYEFEMAQPPESYRNDHSKMVVQLA
QPACVRYRRRTGV
>CYP51B1_Mycobacterium tuberculosis C_913 2724805295
MSAVALPRVSGGHDEHGHLEEFRTDPIGLMQRVRDECGDVGTFQLAGKQVVLLSGSHANEFFFRAGDDDLDQA
KAYPFMTPIFGEGVVFDASPERRKEMLHNAALRGEQMKGHAATIEDQVRRMIADWGEAGEIDLLDFFAELTIY
TSSACLIGKKFRDQLDGRFAKLYHELERGTDPLAYVDPYLPIESFRRRDEARNGLVALVADIMNGRIANPPTD
KSDRDMLDVLIAVKAETGTPRFSADEITGMFISMMFAGHHTSSGTASWTLIELMRHRDAYAAVIDELDELYGD
GRSVSFHALRQIPQLENVLKETLRLHPPLIILMRVAKGEFEVQGHRIHEGDLVAASPAISNRIPEDFPDPHDF
VPARYEQPRQEDLLNRWTWIPFGAGRHRCVGAAFAIMQIKAIFSVLLREYEFEMAQPPESYRNDHSKMVVQLA
QPACVRYRRRTGV
>CYP51B1_Mycobacterium tuberculosis C00401055 2703448756
MSAVALPRVSGGHDEHGHLEEFRTDPIGLMQRVRDECGDVGTFQLAGKQVVLLSGSHANEFFFRAGDDDLDQA
KAYPFMTPIFGEGVVFDASPERRKEMLHNAALRGEQMKGHAATIEDQVRRMIADWGEAGEIDLLDFFAELTIY
TSSACLIGKKFRDQLDGRFAKLYHELERGTDPLAYVDPYLPIESFRRRDEARNGLVALVADIMNGRIANPPTD
KSDRDMLDVLIAVKAETGTPRFSADEITGMFISMMFAGHHTSSGTASWTLIELMRHRDAYAAVIDELDELYGD
GRSVSFHALRQIPQLENVLKETLRLHPPLIILMRVAKGEFEVQGHRIHEGDLVAASPAISNRIPEDFPDPHDF
VPARYEQPRQEDLLNRWTWIPFGAGRHRCVGAAFAIMQIKAIFSVLLREYEFEMAQPPESYRNDHSKMVVQLA
QPACVRYRRRTGV
>CYP51B1_Mycobacterium tuberculosis C09400866 2703446418
MSAVALPRVSGGHDEHGHLEEFRTDPIGLMQRVRDECGDVGTFQLAGKQVVLLSGSHANEFFFRAGDDDLDQA
KAYPFMTPIFGEGVVFDASPERRKEMLHNAALRGEQMKGHAATIEDQVRRMIADWGEAGEIDLLDFFAELTIY
TSSACLIGKKFRDQLDGRFAKLYHELERGTDPLAYVDPYLPIESFRRRDEARNGLVALVADIMNGRIANPPTD
KSDRDMLDVLIAVKAETGTPRFSADEITGMFISMMFAGHHTSSGTASWTLIELMRHRDAYAAVIDELDELYGD
GRSVSFHALRQIPQLENVLKETLRLHPPLIILMRVAKGEFEVQGHRIHEGDLVAASPAISNRIPEDFPDPHDF
VPARYEQPRQEDLLNRWTWIPFGAGRHRCVGAAFAIMQIKAIFSVLLREYEFEMAQPPESYRNDHSKMVVQLA
QPACVRYRRRTGV
>CYP51B1_Mycobacterium tuberculosis C09601061 2699440025
MSAVALPRVSGGHDEHGHLEEFRTDPIGLMQRVRDECGDVGTFQLAGKQVVLLSGSHANEFFFRAGDDDLDQA
KAYPFMTPIFGEGVVFDASPERRKEMLHNAALRGEQMKGHAATIEDQVRRMIADWGEAGEIDLLDFFAELTIY
TSSACLIGKKFRDQLDGRFAKLYHELERGTDPLAYVDPYLPIESFRRRDEARNGLVALVADIMNGRIANPPTD
KSDRDMLDVLIAVKAETGTPRFSADEITGMFISMMFAGHHTSSGTASWTLIELMRHRDAYAAVIDELDELYGD

GRSVSFHALRQIPQLENVLKETLRLHPPLIILMRVAKGEFEVQGHRIHEGDLVAASPAISNRIPEDFPDPHDF
VPARYEQPRQEDLLNRWTWIPFGAGRHRCVGAAFAIMQIKAIFSVLLREYEFEMAQPPESYRNDHSKMVVQLA
QPACVRYRRRTGV
>CYP51B1_Mycobacterium tuberculosis C130 2685358091
MSAVALPRVSGGHDEHGHLEEFRTDPIGLMQRVRDECGDVGTFQLAGKQVVLLSGSHANEFFFRAGDDDLDQA
KAYPFMTPIFGEGVVFDASPERRKEMLHNAALRGEQMKGHAATIEDQVRRMIADWGEAGEIDLLDFFAELTIY
TSSACLIGKKFRDQLDGRFAKLYHELERGTDPLAYVDPYLPIESFRRRDEARNGLVALVADIMNGRIANPPTD
KSDRDMLDVLIAVKAETGTPRFSADEITGMFISMMFAGHHTSSGTASWTLIELMRHRDAYAAVIDELDELYGD
GRSVSFHALRQIPQLENVLKETLRLHPPLIILMRVAKGEFEVQGHRIHEGDLVAASPAISNRIPEDFPDPHDF
VPARYEQPRQEDLLNRWTWIPFGAGRHRCVGAAFAIMQIKAIFSVLLREYEFEMAQPPESYRNDHSKMVVQLA
QPACVRYRRRTGV
>CYP51B1_Mycobacterium tuberculosis C2 2622032051
MSAVALPRVSGGHDEHGHLEEFRTDPIGLMQRVRDECGDVGTFQLAGKQVVLLSGSHANEFFFRAGDDDLDQA
KAYPFMTPIFGEGVVFDASPERRKEMLHNAALRGEQMKGHAATIEDQVRRMIADWGEAGEIDLLDFFAELTIY
TSSACLIGKKFRDQLDGRFAKLYHELERGTDPLAYVDPYLPIESFRRRDEARNGLVALVADIMNGRIANPPTD
KSDRDMLDVLIAVKAETGTPRFSADEITGMFISMMFAGHHTSSGTASWTLIELMRHRDAYAAVIDELDELYGD
GRSVSFHALRQIPQLENVLKETLRLHPPLIILMRVAKGEFEVQGHRIHEGDLVAASPAISNRIPEDFPDPHDF
VPARYEQPRQEDLLNRWTWIPFGAGRHRCVGAAFAIMQIKAIFSVLLREYEFEMAQPPESYRNDHSKMVVQLA
QPACVRYRRRTGV
>CYP51B1_Mycobacterium tuberculosis C2 2622245912
MSAVALPRVSGGHDEHGHLEEFRTDPIGLMQRVRDECGDVGTFQLAGKQVVLLSGSHANEFFFRAGDDDLDQA
KAYPFMTPIFGEGVVFDASPERRKEMLHNAALRGEQMKGHAATIEDQVRRMIADWGEAGEIDLLDFFAELTIY
TSSACLIGKKFRDQLDGRFAKLYHELERGTDPLAYVDPYLPIESFRRRDEARNGLVALVADIMNGRIANPPTD
KSDRDMLDVLIAVKAETGTPRFSADEITGMFISMMFAGHHTSSGTASWTLIELMRHRDAYAAVIDELDELYGD
GRSVSFHALRQIPQLENVLKETLRLHPPLIILMRVAKGEFEVQGHRIHEGDLVAASPAISNRIPEDFPDPHDF
VPARYEQPRQEDLLNRWTWIPFGAGRHRCVGAAFAIMQIKAIFSVLLREYEFEMAQPPESYRNDHSKMVVQLA
QPACVRYRRRTGV
>CYP51B1_Mycobacterium tuberculosis C24_20545 2714480690
MSAVALPRVSGGHDEHGHLEEFRTDPIGLMQRVRDECGDVGTFQLAGKQVVLLSGSHANEFFFRAGDDDLDQA
KAYPFMTPIFGEGVVFDASPERRKEMLHNAALRGEQMKGHAATIEDQVRRMIADWGEAGEIDLLDFFAELTIY
TSSACLIGKKFRDQLDGRFAKLYHELERGTDPLAYVDPYLPIESFRRRDEARNGLVALVADIMNGRIANPPTD
KSDRDMLDVLIAVKAETGTPRFSADEITGMFISMMFAGHHTSSGTASWTLIELMRHRDAYAAVIDELDELYGD
GRSVSFHALRQIPQLENVLKETLRLHPPLIILMRVAKGEFEVQGHRIHEGDLVAASPAISNRIPEDFPDPHDF
VPARYEQPRQEDLLNRWTWIPFGAGRHRCVGAAFAIMQIKAIFSVLLREYEFEMAQPPESYRNDHSKMVVQLA
QPACVRYRRRTGV
>CYP51B1_Mycobacterium tuberculosis C28_9319 2714484759
MSAVALPRVSGGHDEHGHLEEFRTDPIGLMQRVRDECGDVGTFQLAGKQVVLLSGSHANEFFFRAGDDDLDQA
KAYPFMTPIFGEGVVFDASPERRKEMLHNAALRGEQMKGHAATIEDQVRRMIADWGEAGEIDLLDFFAELTIY
TSSACLIGKKFRDQLDGRFAKLYHELERGTDPLAYVDPYLPIESFRRRDEARNGLVALVADIMNGRIANPPTD
KSDRDMLDVLIAVKAETGTPRFSADEITGMFISMMFAGHHTSSGTASWTLIELMRHRDAYAAVIDELDELYGD
GRSVSFHALRQIPQLENVLKETLRLHPPLIILMRVAKGEFEVQGHRIHEGDLVAASPAISNRIPEDFPDPHDF
VPARYEQPRQEDLLNRWTWIPFGAGRHRCVGAAFAIMQIKAIFSVLLREYEFEMAQPPESYRNDHSKMVVQLA
QPACVRYRRRTGV
>CYP51B1_Mycobacterium tuberculosis C28_9904 2714494804
MSAVALPRVSGGHDEHGHLEEFRTDPIGLMQRVRDECGDVGTFQLAGKQVVLLSGSHANEFFFRAGDDDLDQA
KAYPFMTPIFGEGVVFDASPERRKEMLHNAALRGEQMKGHAATIEDQVRRMIADWGEAGEIDLLDFFAELTIY
TSSACLIGKKFRDQLDGRFAKLYHELERGTDPLAYVDPYLPIESFRRRDEARNGLVALVADIMNGRIANPPTD
KSDRDMLDVLIAVKAETGTPRFSADEITGMFISMMFAGHHTSSGTASWTLIELMRHRDAYAAVIDELDELYGD
GRSVSFHALRQIPQLENVLKETLRLHPPLIILMRVAKGEFEVQGHRIHEGDLVAASPAISNRIPEDFPDPHDF
VPARYEQPRQEDLLNRWTWIPFGAGRHRCVGAAFAIMQIKAIFSVLLREYEFEMAQPPESYRNDHSKMVVQLA
QPACVRYRRRTGV
>CYP51B1_Mycobacterium tuberculosis C30_19588 2716321397
MSAVALPRVSGGHDEHGHLEEFRTDPIGLMQRVRDECGDVGTFQLAGKQVVLLSGSHANEFFFRAGDDDLDQA
KAYPFMTPIFGEGVVFDASPERRKEMLHNAALRGEQMKGHAATIEDQVRRMIADWGEAGEIDLLDFFAELTIY
TSSACLIGKKFRDQLDGRFAKLYHELERGTDPLAYVDPYLPIESFRRRDEARNGLVALVADIMNGRIANPPTD
KSDRDMLDVLIAVKAETGTPRFSADEITGMFISMMFAGHHTSSGTASWTLIELMRHRDAYAAVIDELDELYGD
GRSVSFHALRQIPQLENVLKETLRLHPPLIILMRVAKGEFEVQGHRIHEGDLVAASPAISNRIPEDFPDPHDF

VPARYEQPRQEDLLNRWTWIPFGAGRHRCVGAAFAIMQIKAIFSVLLREYEFEMAQPPESYRNDHSKMVVQLA
QPACVRYRRRTGV
>CYP51B1_Mycobacterium tuberculosis C34_13853 2716329792
MSAVALPRVSGGHDEHGHLEEFRTDPIGLMQRVRDECGDVGTFQLAGKQVVLLSGSHANEFFFRAGDDDLDQA
KAYPFMTPIFGEGVVFDASPERRKEMLHNAALRGEQMKGHAATIEDQVRRMIADWGEAGEIDLLDFFAELTIY
TSSACLIGKKFRDQLDGRFAKLYHELERGTDPLAYVDPYLPIESFRRRDEARNGLVALVADIMNGRIANPPTD
KSDRDMLDVLIAVKAETGTPRFSADEITGMFISMMFAGHHTSSGTASWTLIELMRHRDAYAAVIDELDELYGD
GRSVSFHALRQIPQLENVLKETLRLHPPLIILMRVAKGEFEVQGHRIHEGDLVAASPAISNRIPEDFPDPHDF
VPARYEQPRQEDLLNRWTWIPFGAGRHRCVGAAFAIMQIKAIFSVLLREYEFEMAQPPESYRNDHSKMVVQLA
QPACVRYRRRTGV
>CYP51B1_Mycobacterium tuberculosis C4_16679 2716345914
MSAVALPRVSGGHDEHGHLEEFRTDPIGLMQRVRDECGDVGTFQLAGKQVVLLSGSHANEFFFRAGDDDLDQA
KAYPFMTPIFGEGVVFDASPERRKEMLHNAALRGEQMKGHAATIEDQVRRMIADWGEAGEIDLLDFFAELTIY
TSSACLIGKKFRDQLDGRFAKLYHELERGTDPLAYVDPYLPIESFRRRDEARNGLVALVADIMNGRIANPPTD
KSDRDMLDVLIAVKAETGTPRFSADEITGMFISMMFAGHHTSSGTASWTLIELMRHRDAYAAVIDELDELYGD
GRSVSFHALRQIPQLENVLKETLRLHPPLIILMRVAKGEFEVQGHRIHEGDLVAASPAISNRIPEDFPDPHDF
VPARYEQPRQEDLLNRWTWIPFGAGRHRCVGAAFAIMQIKAIFSVLLREYEFEMAQPPESYRNDHSKMVVQLA
QPACVRYRRRTGV
>CYP51B1_Mycobacterium tuberculosis C49_20090 2716352232
MSAVALPRVSGGHDEHGHLEEFRTDPIGLMQRVRDECGDVGTFQLAGKQVVLLSGSHANEFFFRAGDDDLDQA
KAYPFMTPIFGEGVVFDASPERRKEMLHNAALRGEQMKGHAATIEDQVRRMIADWGEAGEIDLLDFFAELTIY
TSSACLIGKKFRDQLDGRFAKLYHELERGTDPLAYVDPYLPIESFRRRDEARNGLVALVADIMNGRIANPPTD
KSDRDMLDVLIAVKAETGTPRFSADEITGMFISMMFAGHHTSSGTASWTLIELMRHRDAYAAVIDELDELYGD
GRSVSFHALRQIPQLENVLKETLRLHPPLIILMRVAKGEFEVQGHRIHEGDLVAASPAISNRIPEDFPDPHDF
VPARYEQPRQEDLLNRWTWIPFGAGRHRCVGAAFAIMQIKAIFSVLLREYEFEMAQPPESYRNDHSKMVVQLA
QPACVRYRRRTGV
>CYP51B1_Mycobacterium tuberculosis C53_20899 2678509534
MSAVALPRVSGGHDEHGHLEEFRTDPIGLMQRVRDECGDVGTFQLAGKQVVLLSGSHANEFFFRAGDDDLDQA
KAYPFMTPIFGEGVVFDASPERRKEMLHNAALRGEQMKGHAATIEDQVRRMIADWGEAGEIDLLDFFAELTIY
TSSACLIGKKFRDQLDGRFAKLYHELERGTDPLAYVDPYLPIESFRRRDEARNGLVALVADIMNGRIANPPTD
KSDRDMLDVLIAVKAETGTPRFSADEITGMFISMMFAGHHTSSGTASWTLIELMRHRDAYAAVIDELDELYGD
GRSVSFHALRQIPQLENVLKETLRLHPPLIILMRVAKGEFEVQGHRIHEGDLVAASPAISNRIPEDFPDPHDF
VPARYEQPRQEDLLNRWTWIPFGAGRHRCVGAAFAIMQIKAIFSVLLREYEFEMAQPPESYRNDHSKMVVQLA
QPACVRYRRRTGV
>CYP51B1_Mycobacterium tuberculosis CCDC5079 651083575
MSAVALPRVSGGHDEHGHLEEFRTDPIGLMQRVRDECGDVGTFQLAGKQVVLLSGSHANEFFFRAGDDDLDQA
KAYPFMTPIFGEGVVFDASPERRKEMLHNAALRGEQMKGHAATIEDQVRRMIADWGEAGEIDLLDFFAELTIY
TSSACLIGKKFRDQLDGRFAKLYHELERGTDPLAYVDPYLPIESFRRRDEARNGLVALVADIMNGRIANPPTD
KSDRDMLDVLIAVKAETGTPRFSADEITGMFISMMFAGHHTSSGTASWTLIELMRHRDAYAAVIDELDELYGD
GRSVSFHALRQIPQLENVLKETLRLHPPLIILMRVAKGEFEVQGHRIHEGDLVAASPAISNRIPEDFPDPHDF
VPARYEQPRQEDLLNRWTWIPFGAGRHRCVGAAFAIMQIKAIFSVLLREYEFEMAQPPESYRNDHSKMVVQLA
QPACVRYRRRTGV
>CYP51B1_Mycobacterium tuberculosis CCDC5079 2688523413
MSAVALPRVSGGHDEHGHLEEFRTDPIGLMQRVRDECGDVGTFQLAGKQVVLLSGSHANEFFFRAGDDDLDQA
KAYPFMTPIFGEGVVFDASPERRKEMLHNAALRGEQMKGHAATIEDQVRRMIADWGEAGEIDLLDFFAELTIY
TSSACLIGKKFRDQLDGRFAKLYHELERGTDPLAYVDPYLPIESFRRRDEARNGLVALVADIMNGRIANPPTD
KSDRDMLDVLIAVKAETGTPRFSADEITGMFISMMFAGHHTSSGTASWTLIELMRHRDAYAAVIDELDELYGD
GRSVSFHALRQIPQLENVLKETLRLHPPLIILMRVAKGEFEVQGHRIHEGDLVAASPAISNRIPEDFPDPHDF
VPARYEQPRQEDLLNRWTWIPFGAGRHRCVGAAFAIMQIKAIFSVLLREYEFEMAQPPESYRNDHSKMVVQLA
QPACVRYRRRTGV
>CYP51B1_Mycobacterium tuberculosis CCDC5180 651087262
MSAVALPRVSGGHDEHGHLEEFRTDPIGLMQRVRDECGDVGTFQLAGKQVVLLSGSHANEFFFRAGDDDLDQA
KAYPFMTPIFGEGVVFDASPERRKEMLHNAALRGEQMKGHAATIEDQVRRMIADWGEAGEIDLLDFFAELTIY
TSSACLIGKKFRDQLDGRFAKLYHELERGTDPLAYVDPYLPIESFRRRDEARNGLVALVADIMNGRIANPPTD
KSDRDMLDVLIAVKAETGTPRFSADEITGMFISMMFAGHHTSSGTASWTLIELMRHRDAYAAVIDELDELYGD
GRSVSFHALRQIPQLENVLKETLRLHPPLIILMRVAKGEFEVQGHRIHEGDLVAASPAISNRIPEDFPDPHDF
VPARYEQPRQEDLLNRWTWIPFGAGRHRCVGAAFAIMQIKAIFSVLLREYEFEMAQPPESYRNDHSKMVVQLA
QPACVRYRRRTGV

>CYP51B1_Mycobacterium tuberculosis CCDC5180 2687608672
MSAVALPRVSGGHDEHGHLEEFRTDPIGLMQRVRDECGDVGTFQLAGKQVVLLSGSHANEFFFRAGDDDLDQA
KAYPFMTPIFGEGVVFDASPERRKEMLHNAALRGEQMKGHAATIEDQVRRMIADWGEAGEIDLLDFFAELTIY
TSSACLIGKKFRDQLDGRFAKLYHELERGTDPLAYVDPYLPIESFRRRDEARNGLVALVADIMNGRIANPPTD
KSDRDMLDVLIAVKAETGTPRFSADEITGMFISMMFAGHHTSSGTASWTLIELMRHRDAYAAVIDELDELYGD
GRSVSFHALRQIPQLENVLKETLRLHPPLIILMRVAKGEFEVQGHRIHEGDLVAASPAISNRIPEDFPDPHDF
VPARYEQPRQEDLLNRWTWIPFGAGRHRCVGAAFAIMQIKAIFSVLLREYEFEMAQPPESYRNDHSKMVVQLA
QPACVRYRRRTGV
>CYP51B1_Mycobacterium tuberculosis CDC1551 637095079
MSAVALPRVSGGHDEHGHLEEFRTDPIGLMQRVRDECGDVGTFQLAGKQVVLLSGSHANEFFFRAGDDDLDQA
KAYPFMTPIFGEGVVFDASPERRKEMLHNAALRGEQMKGHAATIEDQVRRMIADWGEAGEIDLLDFFAELTIY
TSSACLIGKKFRDQLDGRFAKLYHELERGTDPLAYVDPYLPIESFRRRDEARNGLVALVADIMNGRIANPPTD
KSDRDMLDVLIAVKAETGTPRFSADEITGMFISMMFAGHHTSSGTASWTLIELMRHRDAYAAVIDELDELYGD
GRSVSFHALRQIPQLENVLKETLRLHPPLIILMRVAKGEFEVQGHRIHEGDLVAASPAISNRIPEDFPDPHDF
VPARYEQPRQEDLLNRWTWIPFGAGRHRCVGAAFAIMQIKAIFSVLLREYEFEMAQPPESYRNDHSKMVVQLA
QPACVRYRRRTGV
>CYP51B1_Mycobacterium tuberculosis cdc1551 2605054195
MSAVALPRVSGGHDEHGHLEEFRTDPIGLMQRVRDECGDVGTFQLAGKQVVLLSGSHANEFFFRAGDDDLDQA
KAYPFMTPIFGEGVVFDASPERRKEMLHNAALRGEQMKGHAATIEDQVRRMIADWGEAGEIDLLDFFAELTIY
TSSACLIGKKFRDQLDGRFAKLYHELERGTDPLAYVDPYLPIESFRRRDEARNGLVALVADIMNGRIANPPTD
KSDRDMLDVLIAVKAETGTPRFSADEITGMFISMMFAGHHTSSGTASWTLIELMRHRDAYAAVIDELDELYGD
GRSVSFHALRQIPQLENVLKETLRLHPPLIILMRVAKGEFEVQGHRIHEGDLVAASPAISNRIPEDFPDPHDF
VPARYEQPRQEDLLNRWTWIPFGAGRHRCVGAAFAIMQIKAIFSVLLREYEFEMAQPPESYRNDHSKMVVQLA
QPACVRYRRRTGV
>CYP51B1_Mycobacterium tuberculosis CDC1551A 2537734329
MSAVALPRVSGGHDEHGHLEEFRTDPIGLMQRVRDECGDVGTFQLAGKQVVLLSGSHANEFFFRAGDDDLDQA
KAYPFMTPIFGEGVVFDASPERRKEMLHNAALRGEQMKGHAATIEDQVRRMIADWGEAGEIDLLDFFAELTIY
TSSACLIGKKFRDQLDGRFAKLYHELERGTDPLAYVDPYLPIESFRRRDEARNGLVALVADIMNGRIANPPTD
KSDRDMLDVLIAVKAETGTPRFSADEITGMFISMMFAGHHTSSGTASWTLIELMRHRDAYAAVIDELDELYGD
GRSVSFHALRQIPQLENVLKETLRLHPPLIILMRVAKGEFEVQGHRIHEGDLVAASPAISNRIPEDFPDPHDF
VPARYEQPRQEDLLNRWTWIPFGAGRHRCVGAAFAIMQIKAIFSVLLREYEFEMAQPPESYRNDHSKMVVQLA
QPACVRYRRRTGV
>CYP51B1_Mycobacterium tuberculosis CPHL_A 2577095666
MSAVALPRVSGGHDEHGHLEEFRTDPIGLMQRVRDECGDVGTFQLAGKQVVLLSGSHANEFFFRAGDDDLDQA
KAYPFMTPIFGEGVVFDASPERRKEMLHNAALRGEQMKGHAATIEDQVRRMIADWGEAGEIDLLDFFAELTIY
TSSACLIGKKFRDQLDGRFAKLYHELERGTDPLAYVDPYLPIESFRRRDEARNGLVALVADIMNGRIANPPTD
KSDRDMLDVLIAVKAETGTPRFSADEITGMFISMMFAGHHTSSGTASWTLIELMRHRDAYAAVIDELDELYGD
GRSVSFHALRQIPQLENVLKETLRLHPPLIILMRVAKGEFEVQGHRIHEGDLVAASPAISNRIPEDFPDPHDF
VPARYEQPRQEDLLNRWTWIPFGAGRHRCVGAAFAIMQIKAIFSVLLREYEFEMAQPPESYRNDHSKMVVQLA
QPACVRYRRRTGV
>CYP51B1_Mycobacterium tuberculosis CPHL_A 646013454
MSAVALPRVSGGHDEHGHLEEFRTDPIGLMQRVRDECGDVGTFQLAGKQVVLLSGSHANEFFFRAGDDDLDQA
KAYPFMTPIFGEGVVFDASPERRKEMLHNAALRGEQMKGHAATIEDQVRRMIADWGEAGEIDLLDFFAELTIY
TSSACLIGKKFRDQLDGRFAKLYHELERGTDPLAYVDPYLPIESFRRRDEARNGLVALVADIMNGRIANPPTD
KSDRDMLDVLIAVKAETGTPRFSADEITGMFISMMFAGHHTSSGTASWTLIELMRHRDAYAAVIDELDELYGD
GRSVSFHALRQIPQLENVLKETLRLHPPLIILMRVAKGEFEVQGHRIHEGDLVAASPAISNRIPEDFPDPHDF
VPARYEQPRQEDLLNRWTWIPFGAGRHRCVGAAFAIMQIKAIFSVLLREYEFEMAQPPESYRNDHSKMVVQLA
QPACVRYRRRTGV
>CYP51B1_Mycobacterium tuberculosis CSF3053 2702454544
MSAVALPRVSGGHDEHGHLEEFRTDPIGLMQRVRDECGDVGTFQLAGKQVVLLSGSHANEFFFRAGDDDLDQA
KAYPFMTPIFGEGVVFDASPERRKEMLHNAALRGEQMKGHAATIEDQVRRMIADWGEAGEIDLLDFFAELTIY
TSSACLIGKKFRDQLDGRFAKLYHELERGTDPLAYVDPYLPIESFRRRDEARNGLVALVADIMNGRIANPPTD
KSDRDMLDVLIAVKAETGTPRFSADEITGMFISMMFAGHHTSSGTASWTLIELMRHRDAYAAVIDELDELYGD
GRSVSFHALRQIPQLENVLKETLRLHPPLIILMRVAKGEFEVQGHRIHEGDLVAASPAISNRIPEDFPDPHDF
VPARYEQPRQEDLLNRWTWIPFGAGRHRCVGAAFAIMQIKAIFSVLLREYEFEMAQPPESYRNDHSKMVVQLA
QPACVRYRRRTGV
>CYP51B1_Mycobacterium tuberculosis CTRI-2 2511735160

MSAVALPRVSGGHDEHGHLEEFRTDPIGLMQRVRDECGDVGTFQLAGKQVVLLSGSHANEFFFRAGDDDLDQA
KAYPFMTPIFGEGVVFDASPERRKEMLHNAALRGEQMKGHAATIEDQVRRMIADWGEAGEIDLLDFFAELTIY
TSSACLIGKKFRDQLDGRFAKLYHELERGTDPLAYVDPYLPIESFRRRDEARNGLVALVADIMNGRIANPPTD
KSDRDMLDVLIAVKAETGTPRFSADEITGMFISMMFAGHHTSSGTASWTLIELMRHRDAYAAVIDELDELYGD
GRSVSFHALRQIPQLENVLKETLRLHPPLIILMRVAKGEFEVQGHRIHEGDLVAASPAISNRIPEDFPDPHDF
VPARYEQPRQEDLLNRWTWIPFGAGRHRCVGAAFAIMQIKAIFSVLLREYEFEMAQPPESYRNDHSKMVVQLA
QPACVRYRRRTGV
>CYP51B1_Mycobacterium tuberculosis CTRI-4 2547879800
MSAVALPRVSGGHDEHGHLEEFRTDPIGLMQRVRDECGDVGTFQLAGKQVVLLSGSHANEFFFRAGDDDLDQA
KAYPFMTPIFGEGVVFDASPERRKEMLHNAALRGEQMKGHAATIEDQVRRMIADWGEAGEIDLLDFFAELTIY
TSSACLIGKKFRDQLDGRFAKLYHELERGTDPLAYVDPYLPIESFRRRDEARNGLVALVADIMNGRIANPPTD
KSDRDMLDVLIAVKAETGTPRFSADEITGMFISMMFAGHHTSSGTASWTLIELMRHRDAYAAVIDELDELYGD
GRSVSFHALRQIPQLENVLKETLRLHPPLIILMRVAKGEFEVQGHRIHEGDLVAASPAISNRIPEDFPDPHDF
VPARYEQPRQEDLLNRWTWIPFGAGRHRCVGAAFAIMQIKAIFSVLLREYEFEMAQPPESYRNDHSKMVVQLA
QPACVRYRRRTGV
>CYP51B1_Mycobacterium tuberculosis CWCFVRF MDRTB 670 2621955651
MSAVALPRVSGGHDEHGHLEEFRTDPIGLMQRVRDECGDVGTFQLAGKQVVLLSGSHANEFFFRAGDDDLDQA
KAYPFMTPIFGEGVVFDASPERRKEMLHNAALRGEQMKGHAATIEDQVRRMIADWGEAGEIDLLDFFAELTIY
TSSACLIGKKFRDQLDGRFAKLYHELERGTDPLAYVDPYLPIESFRRRDEARNGLVALVADIMNGRIANPPTD
KSDRDMLDVLIAVKAETGTPRFSADEITGMFISMMFAGHHTSSGTASWTLIELMRHRDAYAAVIDELDELYGD
GRSVSFHALRQIPQLENVLKETLRLHPPLIILMRVAKGEFEVQGHRIHEGDLVAASPAISNRIPEDFPDPHDF
VPARYEQPRQEDLLNRWTWIPFGAGRHRCVGAAFAIMQIKAIFSVLLREYEFEMAQPPESYRNDHSKMVVQLA
QPACVRYRRRTGV
>CYP51B1_Mycobacterium tuberculosis D00000071 2675425039
MSAVALPRVSGGHDEHGHLEEFRTDPIGLMQRVRDECGDVGTFQLAGKQVVLLSGSHANEFFFRAGDDDLDQA
KAYPFMTPIFGEGVVFDASPERRKEMLHNAALRGEQMKGHAATIEDQVRRMIADWGEAGEIDLLDFFAELTIY
TSSACLIGKKFRDQLDGRFAKLYHELERGTDPLAYVDPYLPIESFRRRDEARNGLVALVADIMNGRIANPPTD
KSDRDMLDVLIAVKAETGTPRFSADEITGMFISMMFAGHHTSSGTASWTLIELMRHRDAYAAVIDELDELYGD
GRSVSFHALRQIPQLENVLKETLRLHPPLIILMRVAKGEFEVQGHRIHEGDLVAASPAISNRIPEDFPDPHDF
VPARYEQPRQEDLLNRWTWIPFGAGRHRCVGAAFAIMQIKAIFSVLLREYEFEMAQPPESYRNDHSKMVVQLA
QPACVRYRRRTGV
>CYP51B1_Mycobacterium tuberculosis D00000538 2672976518
MSAVALPRVSGGHDEHGHLEEFRTDPIGLMQRVRDECGDVGTFQLAGKQVVLLSGSHANEFFFRAGDDDLDQA
KAYPFMTPIFGEGVVFDASPERRKEMLHNAALRGEQMKGHAATIEDQVRRMIADWGEAGEIDLLDFFAELTIY
TSSACLIGKKFRDQLDGRFAKLYHELERGTDPLAYVDPYLPIESFRRRDEARNGLVALVADIMNGRIANPPTD
KSDRDMLDVLIAVKAETGTPRFSADEITGMFISMMFAGHHTSSGTASWTLIELMRHRDAYAAVIDELDELYGD
GRSVSFHALRQIPQLENVLKETLRLHPPLIILMRVAKGEFEVQGHRIHEGDLVAASPAISNRIPEDFPDPHDF
VPARYEQPRQEDLLNRWTWIPFGAGRHRCVGAAFAIMQIKAIFSVLLREYEFEMAQPPESYRNDHSKMVVQLA
QPACVRYRRRTGV
>CYP51B1_Mycobacterium tuberculosis D00501624 2703465746
MSAVALPRVSGGHDEHGHLEEFRTDPIGLMQRVRDECGDVGTFQLAGKQVVLLSGSHANEFFFRAGDDDLDQA
KAYPFMTPIFGEGVVFDASPERRKEMLHNAALRGEQMKGHAATIEDQVRRMIADWGEAGEIDLLDFFAELTIY
TSSACLIGKKFRDQLDGRFAKLYHELERGTDPLAYVDPYLPIESFRRRDEARNGLVALVADIMNGRIANPPTD
KSDRDMLDVLIAVKAETGTPRFSADEITGMFISMMFAGHHTSSGTASWTLIELMRHRDAYAAVIDELDELYGD
GRSVSFHALRQIPQLENVLKETLRLHPPLIILMRVAKGEFEVQGHRIHEGDLVAASPAISNRIPEDFPDPHDF
VPARYEQPRQEDLLNRWTWIPFGAGRHRCVGAAFAIMQIKAIFSVLLREYEFEMAQPPESYRNDHSKMVVQLA
QPACVRYRRRTGV
>CYP51B1_Mycobacterium tuberculosis D00700688 2703457124
MSAVALPRVSGGHDEHGHLEEFRTDPIGLMQRVRDECGDVGTFQLAGKQVVLLSGSHANEFFFRAGDDDLDQA
KAYPFMTPIFGEGVVFDASPERRKEMLHNAALRGEQMKGHAATIEDQVRRMIADWGEAGEIDLLDFFAELTIY
TSSACLIGKKFRDQLDGRFAKLYHELERGTDPLAYVDPYLPIESFRRRDEARNGLVALVADIMNGRIANPPTD
KSDRDMLDVLIAVKAETGTPRFSADEITGMFISMMFAGHHTSSGTASWTLIELMRHRDAYAAVIDELDELYGD
GRSVSFHALRQIPQLENVLKETLRLHPPLIILMRVAKGEFEVQGHRIHEGDLVAASPAISNRIPEDFPDPHDF
VPARYEQPRQEDLLNRWTWIPFGAGRHRCVGAAFAIMQIKAIFSVLLREYEFEMAQPPESYRNDHSKMVVQLA
QPACVRYRRRTGV
>CYP51B1_Mycobacterium tuberculosis DK9897 2721364021
MSAVALPRVSGGHDEHGHLEEFRTDPIGLMQRVRDECGDVGTFQLAGKQVVLLSGSHANEFFFRAGDDDLDQA
KAYPFMTPIFGEGVVFDASPERRKEMLHNAALRGEQMKGHAATIEDQVRRMIADWGEAGEIDLLDFFAELTIY

TSSACLIGKKFRDQLDGRFAKLYHELERGTDPLAYVDPYLPIESFRRRDEARNGLVALVADIMNGRIANPPTD
KSDRDMLDVLIAVKAETGTPRFSADEITGMFISMMFAGHHTSSGTASWTLIELMRHRDAYAAVIDELDELYGD
GRSVSFHALRQIPQLENVLKETLRLHPPLIILMRVAKGEFEVQGHRIHEGDLVAASPAISNRIPEDFPDPHDF
VPARYEQPRQEDLLNRWTWIPFGAGRHRCVGAAFAIMQIKAIFSVLLREYEFEMAQPPESYRNDHSKMVVQLA
QPACVRYRRRTGV
>CYP51B1_Mycobacterium tuberculosis E09700455 2674938333
MSAVALPRVSGGHDEHGHLEEFRTDPIGLMQRVRDECGDVGTFQLAGKQVVLLSGSHANEFFFRAGDDDLDQA
KAYPFMTPIFGEGVVFDASPERRKEMLHNAALRGEQMKGHAATIEDQVRRMIADWGEAGEIDLLDFFAELTIY
TSSACLIGKKFRDQLDGRFAKLYHELERGTDPLAYVDPYLPIESFRRRDEARNGLVALVADIMNGRIANPPTD
KSDRDMLDVLIAVKAETGTPRFSADEITGMFISMMFAGHHTSSGTASWTLIELMRHRDAYAAVIDELDELYGD
GRSVSFHALRQIPQLENVLKETLRLHPPLIILMRVAKGEFEVQGHRIHEGDLVAASPAISNRIPEDFPDPHDF
VPARYEQPRQEDLLNRWTWIPFGAGRHRCVGAAFAIMQIKAIFSVLLREYEFEMAQPPESYRNDHSKMVVQLA
QPACVRYRRRTGV
>CYP51B1_Mycobacterium tuberculosis E09701106 2703431210
MSAVALPRVSGGHDEHGHLEEFRTDPIGLMQRVRDECGDVGTFQLAGKQVVLLSGSHANEFFFRAGDDDLDQA
KAYPFMTPIFGEGVVFDASPERRKEMLHNAALRGEQMKGHAATIEDQVRRMIADWGEAGEIDLLDFFAELTIY
TSSACLIGKKFRDQLDGRFAKLYHELERGTDPLAYVDPYLPIESFRRRDEARNGLVALVADIMNGRIANPPTD
KSDRDMLDVLIAVKAETGTPRFSADEITGMFISMMFAGHHTSSGTASWTLIELMRHRDAYAAVIDELDELYGD
GRSVSFHALRQIPQLENVLKETLRLHPPLIILMRVAKGEFEVQGHRIHEGDLVAASPAISNRIPEDFPDPHDF
VPARYEQPRQEDLLNRWTWIPFGAGRHRCVGAAFAIMQIKAIFSVLLREYEFEMAQPPESYRNDHSKMVVQLA
QPACVRYRRRTGV
>CYP51B1_Mycobacterium tuberculosis E09701191 2703442099
MSAVALPRVSGGHDEHGHLEEFRTDPIGLMQRVRDECGDVGTFQLAGKQVVLLSGSHANEFFFRAGDDDLDQA
KAYPFMTPIFGEGVVFDASPERRKEMLHNAALRGEQMKGHAATIEDQVRRMIADWGEAGEIDLLDFFAELTIY
TSSACLIGKKFRDQLDGRFAKLYHELERGTDPLAYVDPYLPIESFRRRDEARNGLVALVADIMNGRIANPPTD
KSDRDMLDVLIAVKAETGTPRFSADEITGMFISMMFAGHHTSSGTASWTLIELMRHRDAYAAVIDELDELYGD
GRSVSFHALRQIPQLENVLKETLRLHPPLIILMRVAKGEFEVQGHRIHEGDLVAASPAISNRIPEDFPDPHDF
VPARYEQPRQEDLLNRWTWIPFGAGRHRCVGAAFAIMQIKAIFSVLLREYEFEMAQPPESYRNDHSKMVVQLA
QPACVRYRRRTGV
>CYP51B1_Mycobacterium tuberculosis E09701891 2706580682
MSAVALPRVSGGHDEHGHLEEFRTDPIGLMQRVRDECGDVGTFQLAGKQVVLLSGSHANEFFFRAGDDDLDQA
KAYPFMTPIFGEGVVFDASPERRKEMLHNAALRGEQMKGHAATIEDQVRRMIADWGEAGEIDLLDFFAELTIY
TSSACLIGKKFRDQLDGRFAKLYHELERGTDPLAYVDPYLPIESFRRRDEARNGLVALVADIMNGRIANPPTD
KSDRDMLDVLIAVKAETGTPRFSADEITGMFISMMFAGHHTSSGTASWTLIELMRHRDAYAAVIDELDELYGD
GRSVSFHALRQIPQLENVLKETLRLHPPLIILMRVAKGEFEVQGHRIHEGDLVAASPAISNRIPEDFPDPHDF
VPARYEQPRQEDLLNRWTWIPFGAGRHRCVGAAFAIMQIKAIFSVLLREYEFEMAQPPESYRNDHSKMVVQLA
QPACVRYRRRTGV
>CYP51B1_Mycobacterium tuberculosis E09801032 2699038592
MSAVALPRVSGGHDEHGHLEEFRTDPIGLMQRVRDECGDVGTFQLAGKQVVLLSGSHANEFFFRAGDDDLDQA
KAYPFMTPIFGEGVVFDASPERRKEMLHNAALRGEQMKGHAATIEDQVRRMIADWGEAGEIDLLDFFAELTIY
TSSACLIGKKFRDQLDGRFAKLYHELERGTDPLAYVDPYLPIESFRRRDEARNGLVALVADIMNGRIANPPTD
KSDRDMLDVLIAVKAETGTPRFSADEITGMFISMMFAGHHTSSGTASWTLIELMRHRDAYAAVIDELDELYGD
GRSVSFHALRQIPQLENVLKETLRLHPPLIILMRVAKGEFEVQGHRIHEGDLVAASPAISNRIPEDFPDPHDF
VPARYEQPRQEDLLNRWTWIPFGAGRHRCVGAAFAIMQIKAIFSVLLREYEFEMAQPPESYRNDHSKMVVQLA
QPACVRYRRRTGV
>CYP51B1_Mycobacterium tuberculosis E186hv 2633781615
MSAVALPRVSGGHDEHGHLEEFRTDPIGLMQRVRDECGDVGTFQLAGKQVVLLSGSHANEFFFRAGDDDLDQA
KAYPFMTPIFGEGVVFDASPERRKEMLHNAALRGEQMKGHAATIEDQVRRMIADWGEAGEIDLLDFFAELTIY
TSSACLIGKKFRDQLDGRFAKLYHELERGTDPLAYVDPYLPIESFRRRDEARNGLVALVADIMNGRIANPPTD
KSDRDMLDVLIAVKAETGTPRFSADEITGMFISMMFAGHHTSSGTASWTLIELMRHRDAYAAVIDELDELYGD
GRSVSFHALRQIPQLENVLKETLRLHPPLIILMRVAKGEFEVQGHRIHEGDLVAASPAISNRIPEDFPDPHDF
VPARYEQPRQEDLLNRWTWIPFGAGRHRCVGAAFAIMQIKAIFSVLLREYEFEMAQPPESYRNDHSKMVVQLA
QPACVRYRRRTGV
>CYP51B1_Mycobacterium tuberculosis EAI/OSDD271 2554691220
MSAVALPRVSGGHDEHGHLEEFRTDPIGLMQRVRDECGDVGTFQLAGKQVVLLSGSHANEFFFRAGDDDLDQA
KAYPFMTPIFGEGVVFDASPERRKEMLHNAALRGEQMKGHAATIEDQVRRMIADWGEAGEIDLLDFFAELTIY
TSSACLIGKKFRDQLDGRFAKLYHELERGTDPLAYVDPYLPIESFRRRDEARNGLVALVADIMNGRIANPPTD
KSDRDMLDVLIAVKAETGTPRFSADEITGMFISMMFAGHHTSSGTASWTLIELMRHRDAYAAVIDELDELYGD

GRSVSFHALRQIPQLENVLKETLRLHPPLIILMRVAKGEFEVQGHRIHEGDLVAASPAISNRIPEDFPDPHDF
VPARYEQPRQEDLLNRWTWIPFGAGRHRCVGAAFAIMQIKAIFSVLLREYEFEMAQPPESYRNDHSKMVVQLA
QPACVRYRRRTGV
>CYP51B1_Mycobacterium tuberculosis EAI5 2555959524
MSAVALPRVSGGHDEHGHLEEFRTDPIGLMQRVRDECGDVGTFQLAGKQVVLLSGSHANEFFFRAGDDDLDQA
KAYPFMTPIFGEGVVFDASPERRKEMLHNAALRGEQMKGHAATIEDQVRRMIADWGEAGEIDLLDFFAELTIY
TSSACLIGKKFRDQLDGRFAKLYHELERGTDPLAYVDPYLPIESFRRRDEARNGLVALVADIMNGRIANPPTD
KSDRDMLDVLIAVKAETGTPRFSADEITGMFISMMFAGHHTSSGTASWTLIELMRHRDAYAAVIDELDELYGD
GRSVSFHALRQIPQLENVLKETLRLHPPLIILMRVAKGEFEVQGHRIHEGDLVAASPAISNRIPEDFPDPHDF
VPARYEQPRQEDLLNRWTWIPFGAGRHRCVGAAFAIMQIKAIFSVLLREYEFEMAQPPESYRNDHSKMVVQLA
QPACVRYRRRTGV
>CYP51B1_Mycobacterium tuberculosis EAI5/NITR206 2546205189
MSAVALPRVSGGHDEHGHLEEFRTDPIGLMQRVRDECGDVGTFQLAGKQVVLLSGSHANEFFFRAGDDDLDQA
KAYPFMTPIFGEGVVFDASPERRKEMLHNAALRGEQMKGHAATIEDQVRRMIADWGEAGEIDLLDFFAELTIY
TSSACLIGKKFRDQLDGRFAKLYHELERGTDPLAYVDPYLPIESFRRRDEARNGLVALVADIMNGRIANPPTD
KSDRDMLDVLIAVKAETGTPRFSADEITGMFISMMFAGHHTSSGTASWTLIELMRHRDAYAAVIDELDELYGD
GRSVSFHALRQIPQLENVLKETLRLHPPLIILMRVAKGEFEVQGHRIHEGDLVAASPAISNRIPEDFPDPHDF
VPARYEQPRQEDLLNRWTWIPFGAGRHRCVGAAFAIMQIKAIFSVLLREYEFEMAQPPESYRNDHSKMVVQLA
QPACVRYRRRTGV
>CYP51B1_Mycobacterium tuberculosis EAS054 2621965947
MSAVALPRVSGGHDEHGHLEEFRTDPIGLMQRVRDECGDVGTFQLAGKQVVLLSGSHANEFFFRAGDDDLDQA
KAYPFMTPIFGEGVVFDASPERRKEMLHNAALRGEQMKGHAATIEDQVRRMIADWGEAGEIDLLDFFAELTIY
TSSACLIGKKFRDQLDGRFAKLYHELERGTDPLAYVDPYLPIESFRRRDEARNGLVALVADIMNGRIANPPTD
KSDRDMLDVLIAVKAETGTPRFSADEITGMFISMMFAGHHTSSGTASWTLIELMRHRDAYAAVIDELDELYGD
GRSVSFHALRQIPQLENVLKETLRLHPPLIILMRVAKGEFEVQGHRIHEGDLVAASPAISNRIPEDFPDPHDF
VPARYEQPRQEDLLNRWTWIPFGAGRHRCVGAAFAIMQIKAIFSVLLREYEFEMAQPPESYRNDHSKMVVQLA
QPACVRYRRRTGV
>CYP51B1_Mycobacterium tuberculosis EAS054 unfinished sequence 643030825
MSAVALPRVSGGHDEHGHLEEFRTDPIGLMQRVRDECGDVGTFQLAGKQVVLLSGSHANEFFFRAGDDDLDQA
KAYPFMTPIFGEGVVFDASPERRKEMLHNAALRGEQMKGHAATIEDQVRRMIADWGEAGEIDLLDFFAELTIY
TSSACLIGKKFRDQLDGRFAKLYHELERGTDPLAYVDPYLPIESFRRRDEARNGLVALVADIMNGRIANPPTD
KSDRDMLDVLIAVKAETGTPRFSADEITGMFISMMFAGHHTSSGTASWTLIELMRHRDAYAAVIDELDELYGD
GRSVSFHALRQIPQLENVLKETLRLHPPLIILMRVAKGEFEVQGHRIHEGDLVAASPAISNRIPEDFPDPHDF
VPARYEQPRQEDLLNRWTWIPFGAGRHRCVGAAFAIMQIKAIFSVLLREYEFEMAQPPESYRNDHSKMVVQLA
QPACVRYRRRTGV
>CYP51B1_Mycobacterium tuberculosis EE0502463 2674543579
MSAVALPRVSGGHDEHGHLEEFRTDPIGLMQRVRDECGDVGTFQLAGKQVVLLSGSHANEFFFRAGDDDLDQA
KAYPFMTPIFGEGVVFDASPERRKEMLHNAALRGEQMKGHAATIEDQVRRMIADWGEAGEIDLLDFFAELTIY
TSSACLIGKKFRDQLDGRFAKLYHELERGTDPLAYVDPYLPIESFRRRDEARNGLVALVADIMNGRIANPPTD
KSDRDMLDVLIAVKAETGTPRFSADEITGMFISMMFAGHHTSSGTASWTLIELMRHRDAYAAVIDELDELYGD
GRSVSFHALRQIPQLENVLKETLRLHPPLIILMRVAKGEFEVQGHRIHEGDLVAASPAISNRIPEDFPDPHDF
VPARYEQPRQEDLLNRWTWIPFGAGRHRCVGAAFAIMQIKAIFSVLLREYEFEMAQPPESYRNDHSKMVVQLA
QPACVRYRRRTGV
>CYP51B1_Mycobacterium tuberculosis EE0503811 2683839909
MSAVALPRVSGGHDEHGHLEEFRTDPIGLMQRVRDECGDVGTFQLAGKQVVLLSGSHANEFFFRAGDDDLDQA
KAYPFMTPIFGEGVVFDASPERRKEMLHNAALRGEQMKGHAATIEDQVRRMIADWGEAGEIDLLDFFAELTIY
TSSACLIGKKFRDQLDGRFAKLYHELERGTDPLAYVDPYLPIESFRRRDEARNGLVALVADIMNGRIANPPTD
KSDRDMLDVLIAVKAETGTPRFSADEITGMFISMMFAGHHTSSGTASWTLIELMRHRDAYAAVIDELDELYGD
GRSVSFHALRQIPQLENVLKETLRLHPPLIILMRVAKGEFEVQGHRIHEGDLVAASPAISNRIPEDFPDPHDF
VPARYEQPRQEDLLNRWTWIPFGAGRHRCVGAAFAIMQIKAIFSVLLREYEFEMAQPPESYRNDHSKMVVQLA
QPACVRYRRRTGV
>CYP51B1_Mycobacterium tuberculosis EE0506178 2683844678
MSAVALPRVSGGHDEHGHLEEFRTDPIGLMQRVRDECGDVGTFQLAGKQVVLLSGSHANEFFFRAGDDDLDQA
KAYPFMTPIFGEGVVFDASPERRKEMLHNAALRGEQMKGHAATIEDQVRRMIADWGEAGEIDLLDFFAELTIY
TSSACLIGKKFRDQLDGRFAKLYHELERGTDPLAYVDPYLPIESFRRRDEARNGLVALVADIMNGRIANPPTD
KSDRDMLDVLIAVKAETGTPRFSADEITGMFISMMFAGHHTSSGTASWTLIELMRHRDAYAAVIDELDELYGD
GRSVSFHALRQIPQLENVLKETLRLHPPLIILMRVAKGEFEVQGHRIHEGDLVAASPAISNRIPEDFPDPHDF

VPARYEQPRQEDLLNRWTWIPFGAGRHRCVGAAFAIMQIKAIFSVLLREYEFEMAQPPESYRNDHSKMVVQLA
QPACVRYRRRTGV
>CYP51B1_Mycobacterium tuberculosis EE0506784 2683834941
MSAVALPRVSGGHDEHGHLEEFRTDPIGLMQRVRDECGDVGTFQLAGKQVVLLSGSHANEFFFRAGDDDLDQA
KAYPFMTPIFGEGVVFDASPERRKEMLHNAALRGEQMKGHAATIEDQVRRMIADWGEAGEIDLLDFFAELTIY
TSSACLIGKKFRDQLDGRFAKLYHELERGTDPLAYVDPYLPIESFRRRDEARNGLVALVADIMNGRIANPPTD
KSDRDMLDVLIAVKAETGTPRFSADEITGMFISMMFAGHHTSSGTASWTLIELMRHRDAYAAVIDELDELYGD
GRSVSFHALRQIPQLENVLKETLRLHPPLIILMRVAKGEFEVQGHRIHEGDLVAASPAISNRIPEDFPDPHDF
VPARYEQPRQEDLLNRWTWIPFGAGRHRCVGAAFAIMQIKAIFSVLLREYEFEMAQPPESYRNDHSKMVVQLA
QPACVRYRRRTGV
>CYP51B1_Mycobacterium tuberculosis Erdman ATCC 35801 2540619011
MSAVALPRVSGGHDEHGHLEEFRTDPIGLMQRVRDECGDVGTFQLAGKQVVLLSGSHANEFFFRAGDDDLDQA
KAYPFMTPIFGEGVVFDASPERRKEMLHNAALRGEQMKGHAATIEDQVRRMIADWGEAGEIDLLDFFAELTIY
TSSACLIGKKFRDQLDGRFAKLYHELERGTDPLAYVDPYLPIESFRRRDEARNGLVALVADIMNGRIANPPTD
KSDRDMLDVLIAVKAETGTPRFSADEITGMFISMMFAGHHTSSGTASWTLIELMRHRDAYAAVIDELDELYGD
GRSVSFHALRQIPQLENVLKETLRLHPPLIILMRVAKGEFEVQGHRIHEGDLVAASPAISNRIPEDFPDPHDF
VPARYEQPRQEDLLNRWTWIPFGAGRHRCVGAAFAIMQIKAIFSVLLREYEFEMAQPPESYRNDHSKMVVQLA
QPACVRYRRRTGV
>CYP51B1_Mycobacterium tuberculosis F00001728 2692181875
MSAVALPRVSGGHDEHGHLEEFRTDPIGLMQRVRDECGDVGTFQLAGKQVVLLSGSHANEFFFRAGDDDLDQA
KAYPFMTPIFGEGVVFDASPERRKEMLHNAALRGEQMKGHAATIEDQVRRMIADWGEAGEIDLLDFFAELTIY
TSSACLIGKKFRDQLDGRFAKLYHELERGTDPLAYVDPYLPIESFRRRDEARNGLVALVADIMNGRIANPPTD
KSDRDMLDVLIAVKAETGTPRFSADEITGMFISMMFAGHHTSSGTASWTLIELMRHRDAYAAVIDELDELYGD
GRSVSFHALRQIPQLENVLKETLRLHPPLIILMRVAKGEFEVQGHRIHEGDLVAASPAISNRIPEDFPDPHDF
VPARYEQPRQEDLLNRWTWIPFGAGRHRCVGAAFAIMQIKAIFSVLLREYEFEMAQPPESYRNDHSKMVVQLA
QPACVRYRRRTGV
>CYP51B1_Mycobacterium tuberculosis F00002017 2703543386
MSAVALPRVSGGHDEHGHLEEFRTDPIGLMQRVRDECGDVGTFQLAGKQVVLLSGSHANEFFFRAGDDDLDQA
KAYPFMTPIFGEGVVFDASPERRKEMLHNAALRGEQMKGHAATIEDQVRRMIADWGEAGEIDLLDFFAELTIY
TSSACLIGKKFRDQLDGRFAKLYHELERGTDPLAYVDPYLPIESFRRRDEARNGLVALVADIMNGRIANPPTD
KSDRDMLDVLIAVKAETGTPRFSADEITGMFISMMFAGHHTSSGTASWTLIELMRHRDAYAAVIDELDELYGD
GRSVSFHALRQIPQLENVLKETLRLHPPLIILMRVAKGEFEVQGHRIHEGDLVAASPAISNRIPEDFPDPHDF
VPARYEQPRQEDLLNRWTWIPFGAGRHRCVGAAFAIMQIKAIFSVLLREYEFEMAQPPESYRNDHSKMVVQLA
QPACVRYRRRTGV
>CYP51B1_Mycobacterium tuberculosis F00201178 2703619103
MSAVALPRVSGGHDEHGHLEEFRTDPIGLMQRVRDECGDVGTFQLAGKQVVLLSGSHANEFFFRAGDDDLDQA
KAYPFMTPIFGEGVVFDASPERRKEMLHNAALRGEQMKGHAATIEDQVRRMIADWGEAGEIDLLDFFAELTIY
TSSACLIGKKFRDQLDGRFAKLYHELERGTDPLAYVDPYLPIESFRRRDEARNGLVALVADIMNGRIANPPTD
KSDRDMLDVLIAVKAETGTPRFSADEITGMFISMMFAGHHTSSGTASWTLIELMRHRDAYAAVIDELDELYGD
GRSVSFHALRQIPQLENVLKETLRLHPPLIILMRVAKGEFEVQGHRIHEGDLVAASPAISNRIPEDFPDPHDF
VPARYEQPRQEDLLNRWTWIPFGAGRHRCVGAAFAIMQIKAIFSVLLREYEFEMAQPPESYRNDHSKMVVQLA
QPACVRYRRRTGV
>CYP51B1_Mycobacterium tuberculosis F09900854 2703617595
MSAVALPRVSGGHDEHGHLEEFRTDPIGLMQRVRDECGDVGTFQLAGKQVVLLSGSHANEFFFRAGDDDLDQA
KAYPFMTPIFGEGVVFDASPERRKEMLHNAALRGEQMKGHAATIEDQVRRMIADWGEAGEIDLLDFFAELTIY
TSSACLIGKKFRDQLDGRFAKLYHELERGTDPLAYVDPYLPIESFRRRDEARNGLVALVADIMNGRIANPPTD
KSDRDMLDVLIAVKAETGTPRFSADEITGMFISMMFAGHHTSSGTASWTLIELMRHRDAYAAVIDELDELYGD
GRSVSFHALRQIPQLENVLKETLRLHPPLIILMRVAKGEFEVQGHRIHEGDLVAASPAISNRIPEDFPDPHDF
VPARYEQPRQEDLLNRWTWIPFGAGRHRCVGAAFAIMQIKAIFSVLLREYEFEMAQPPESYRNDHSKMVVQLA
QPACVRYRRRTGV
>CYP51B1_Mycobacterium tuberculosis F1 2688961962
MSAVALPRVSGGHDEHGHLEEFRTDPIGLMQRVRDECGDVGTFQLAGKQVVLLSGSHANEFFFRAGDDDLDQA
KAYPFMTPIFGEGVVFDASPERRKEMLHNAALRGEQMKGHAATIEDQVRRMIADWGEAGEIDLLDFFAELTIY
TSSACLIGKKFRDQLDGRFAKLYHELERGTDPLAYVDPYLPIESFRRRDEARNGLVALVADIMNGRIANPPTD
KSDRDMLDVLIAVKAETGTPRFSADEITGMFISMMFAGHHTSSGTASWTLIELMRHRDAYAAVIDELDELYGD
GRSVSFHALRQIPQLENVLKETLRLHPPLIILMRVAKGEFEVQGHRIHEGDLVAASPAISNRIPEDFPDPHDF
VPARYEQPRQEDLLNRWTWIPFGAGRHRCVGAAFAIMQIKAIFSVLLREYEFEMAQPPESYRNDHSKMVVQLA
QPACVRYRRRTGV

>CYP51B1_Mycobacterium tuberculosis F11 2603381707
MSAVALPRVSGGHDEHGHLEEFRTDPIGLMQRVRDECGDVGTFQLAGKQVVLLSGSHANEFFFRAGDDDLDQA
KAYPFMTPIFGEGVVFDASPERRKEMLHNAALRGEQMKGHAATIEDQVRRMIADWGEAGEIDLLDFFAELTIY
TSSACLIGKKFRDQLDGRFAKLYHELERGTDPLAYVDPYLPIESFRRRDEARNGLVALVADIMNGRIANPPTD
KSDRDMLDVLIAVKAETGTPRFSADEITGMFISMMFAGHHTSSGTASWTLIELMRHRDAYAAVIDELDELYGD
GRSVSFHALRQIPQLENVLKETLRLHPPLIILMRVAKGEFEVQGHRIHEGDLVAASPAISNRIPEDFPDPHDF
VPARYEQPRQEDLLNRWTWIPFGAGRHRCVGAAFAIMQIKAIFSVLLREYEFEMAQPPESYRNDHSKMVVQLA
QPACVRYRRRTGV
>CYP51B1_Mycobacterium tuberculosis F11 640605525
MSAVALPRVSGGHDEHGHLEEFRTDPIGLMQRVRDECGDVGTFQLAGKQVVLLSGSHANEFFFRAGDDDLDQA
KAYPFMTPIFGEGVVFDASPERRKEMLHNAALRGEQMKGHAATIEDQVRRMIADWGEAGEIDLLDFFAELTIY
TSSACLIGKKFRDQLDGRFAKLYHELERGTDPLAYVDPYLPIESFRRRDEARNGLVALVADIMNGRIANPPTD
KSDRDMLDVLIAVKAETGTPRFSADEITGMFISMMFAGHHTSSGTASWTLIELMRHRDAYAAVIDELDELYGD
GRSVSFHALRQIPQLENVLKETLRLHPPLIILMRVAKGEFEVQGHRIHEGDLVAASPAISNRIPEDFPDPHDF
VPARYEQPRQEDLLNRWTWIPFGAGRHRCVGAAFAIMQIKAIFSVLLREYEFEMAQPPESYRNDHSKMVVQLA
QPACVRYRRRTGV
>CYP51B1_Mycobacterium tuberculosis F28 2688937238
MSAVALPRVSGGHDEHGHLEEFRTDPIGLMQRVRDECGDVGTFQLAGKQVVLLSGSHANEFFFRAGDDDLDQA
KAYPFMTPIFGEGVVFDASPERRKEMLHNAALRGEQMKGHAATIEDQVRRMIADWGEAGEIDLLDFFAELTIY
TSSACLIGKKFRDQLDGRFAKLYHELERGTDPLAYVDPYLPIESFRRRDEARNGLVALVADIMNGRIANPPTD
KSDRDMLDVLIAVKAETGTPRFSADEITGMFISMMFAGHHTSSGTASWTLIELMRHRDAYAAVIDELDELYGD
GRSVSFHALRQIPQLENVLKETLRLHPPLIILMRVAKGEFEVQGHRIHEGDLVAASPAISNRIPEDFPDPHDF
VPARYEQPRQEDLLNRWTWIPFGAGRHRCVGAAFAIMQIKAIFSVLLREYEFEMAQPPESYRNDHSKMVVQLA
QPACVRYRRRTGV
>CYP51B1_Mycobacterium tuberculosis FJ05194 2545501361
MSAVALPRVSGGHDEHGHLEEFRTDPIGLMQRVRDECGDVGTFQLAGKQVVLLSGSHANEFFFRAGDDDLDQA
KAYPFMTPIFGEGVVFDASPERRKEMLHNAALRGEQMKGHAATIEDQVRRMIADWGEAGEIDLLDFFAELTIY
TSSACLIGKKFRDQLDGRFAKLYHELERGTDPLAYVDPYLPIESFRRRDEARNGLVALVADIMNGRIANPPTD
KSDRDMLDVLIAVKAETGTPRFSADEITGMFISMMFAGHHTSSGTASWTLIELMRHRDAYAAVIDELDELYGD
GRSVSFHALRQIPQLENVLKETLRLHPPLIILMRVAKGEFEVQGHRIHEGDLVAASPAISNRIPEDFPDPHDF
VPARYEQPRQEDLLNRWTWIPFGAGRHRCVGAAFAIMQIKAIFSVLLREYEFEMAQPPESYRNDHSKMVVQLA
QPACVRYRRRTGV
>CYP51B1_Mycobacterium tuberculosis G-12-005 2577284123
MSAVALPRVSGGHDEHGHLEEFRTDPIGLMQRVRDECGDVGTFQLAGKQVVLLSGSHANEFFFRAGDDDLDQA
KAYPFMTPIFGEGVVFDASPERRKEMLHNAALRGEQMKGHAATIEDQVRRMIADWGEAGEIDLLDFFAELTIY
TSSACLIGKKFRDQLDGRFAKLYHELERGTDPLAYVDPYLPIESFRRRDEARNGLVALVADIMNGRIANPPTD
KSDRDMLDVLIAVKAETGTPRFSADEITGMFISMMFAGHHTSSGTASWTLIELMRHRDAYAAVIDELDELYGD
GRSVSFHALRQIPQLENVLKETLRLHPPLIILMRVAKGEFEVQGHRIHEGDLVAASPAISNRIPEDFPDPHDF
VPARYEQPRQEDLLNRWTWIPFGAGRHRCVGAAFAIMQIKAIFSVLLREYEFEMAQPPESYRNDHSKMVVQLA
QPACVRYRRRTGV
>CYP51B1_Mycobacterium tuberculosis GM 1503 2577895909
MSAVALPRVSGGHDEHGHLEEFRTDPIGLMQRVRDECGDVGTFQLAGKQVVLLSGSHANEFFFRAGDDDLDQA
KAYPFMTPIFGEGVVFDASPERRKEMLHNAALRGEQMKGHAATIEDQVRRMIADWGEAGEIDLLDFFAELTIY
TSSACLIGKKFRDQLDGRFAKLYHELERGTDPLAYVDPYLPIESFRRRDEARNGLVALVADIMNGRIANPPTD
KSDRDMLDVLIAVKAETGTPRFSADEITGMFISMMFAGHHTSSGTASWTLIELMRHRDAYAAVIDELDELYGD
GRSVSFHALRQIPQLENVLKETLRLHPPLIILMRVAKGEFEVQGHRIHEGDLVAASPAISNRIPEDFPDPHDF
VPARYEQPRQEDLLNRWTWIPFGAGRHRCVGAAFAIMQIKAIFSVLLREYEFEMAQPPESYRNDHSKMVVQLA
QPACVRYRRRTGV
>CYP51B1_Mycobacterium tuberculosis GM 1503 unfinished sequence 643044133
MSAVALPRVSGGHDEHGHLEEFRTDPIGLMQRVRDECGDVGTFQLAGKQVVLLSGSHANEFFFRAGDDDLDQA
KAYPFMTPIFGEGVVFDASPERRKEMLHNAALRGEQMKGHAATIEDQVRRMIADWGEAGEIDLLDFFAELTIY
TSSACLIGKKFRDQLDGRFAKLYHELERGTDPLAYVDPYLPIESFRRRDEARNGLVALVADIMNGRIANPPTD
KSDRDMLDVLIAVKAETGTPRFSADEITGMFISMMFAGHHTSSGTASWTLIELMRHRDAYAAVIDELDELYGD
GRSVSFHALRQIPQLENVLKETLRLHPPLIILMRVAKGEFEVQGHRIHEGDLVAASPAISNRIPEDFPDPHDF
VPARYEQPRQEDLLNRWTWIPFGAGRHRCVGAAFAIMQIKAIFSVLLREYEFEMAQPPESYRNDHSKMVVQLA
QPACVRYRRRTGV
>CYP51B1_Mycobacterium tuberculosis GuangZ0019 2546457521

MSAVALPRVSGGHDEHGHLEEFRTDPIGLMQRVRDECGDVGTFQLAGKQVVLLSGSHANEFFFRAGDDDLDQA
KAYPFMTPIFGEGVVFDASPERRKEMLHNAALRGEQMKGHAATIEDQVRRMIADWGEAGEIDLLDFFAELTIY
TSSACLIGKKFRDQLDGRFAKLYHELERGTDPLAYVDPYLPIESFRRRDEARNGLVALVADIMNGRIANPPTD
KSDRDMLDVLIAVKAETGTPRFSADEITGMFISMMFAGHHTSSGTASWTLIELMRHRDAYAAVIDELDELYGD
GRSVSFHALRQIPQLENVLKETLRLHPPLIILMRVAKGEFEVQGHRIHEGDLVAASPAISNRIPEDFPDPHDF
VPARYEQPRQEDLLNRWTWIPFGAGRHRCVGAAFAIMQIKAIFSVLLREYEFEMAQPPESYRNDHSKMVVQLA
QPACVRYRRRTGV
>CYP51B1_Mycobacterium tuberculosis H_13559 2716355543
MSAVALPRVSGGHDEHGHLEEFRTDPIGLMQRVRDECGDVGTFQLAGKQVVLLSGSHANEFFFRAGDDDLDQA
KAYPFMTPIFGEGVVFDASPERRKEMLHNAALRGEQMKGHAATIEDQVRRMIADWGEAGEIDLLDFFAELTIY
TSSACLIGKKFRDQLDGRFAKLYHELERGTDPLAYVDPYLPIESFRRRDEARNGLVALVADIMNGRIANPPTD
KSDRDMLDVLIAVKAETGTPRFSADEITGMFISMMFAGHHTSSGTASWTLIELMRHRDAYAAVIDELDELYGD
GRSVSFHALRQIPQLENVLKETLRLHPPLIILMRVAKGEFEVQGHRIHEGDLVAASPAISNRIPEDFPDPHDF
VPARYEQPRQEDLLNRWTWIPFGAGRHRCVGAAFAIMQIKAIFSVLLREYEFEMAQPPESYRNDHSKMVVQLA
QPACVRYRRRTGV
>CYP51B1_Mycobacterium tuberculosis H_13571 2716360143
MSAVALPRVSGGHDEHGHLEEFRTDPIGLMQRVRDECGDVGTFQLAGKQVVLLSGSHANEFFFRAGDDDLDQA
KAYPFMTPIFGEGVVFDASPERRKEMLHNAALRGEQMKGHAATIEDQVRRMIADWGEAGEIDLLDFFAELTIY
TSSACLIGKKFRDQLDGRFAKLYHELERGTDPLAYVDPYLPIESFRRRDEARNGLVALVADIMNGRIANPPTD
KSDRDMLDVLIAVKAETGTPRFSADEITGMFISMMFAGHHTSSGTASWTLIELMRHRDAYAAVIDELDELYGD
GRSVSFHALRQIPQLENVLKETLRLHPPLIILMRVAKGEFEVQGHRIHEGDLVAASPAISNRIPEDFPDPHDF
VPARYEQPRQEDLLNRWTWIPFGAGRHRCVGAAFAIMQIKAIFSVLLREYEFEMAQPPESYRNDHSKMVVQLA
QPACVRYRRRTGV
>CYP51B1_Mycobacterium tuberculosis H_7300 2678875554
MSAVALPRVSGGHDEHGHLEEFRTDPIGLMQRVRDECGDVGTFQLAGKQVVLLSGSHANEFFFRAGDDDLDQA
KAYPFMTPIFGEGVVFDASPERRKEMLHNAALRGEQMKGHAATIEDQVRRMIADWGEAGEIDLLDFFAELTIY
TSSACLIGKKFRDQLDGRFAKLYHELERGTDPLAYVDPYLPIESFRRRDEARNGLVALVADIMNGRIANPPTD
KSDRDMLDVLIAVKAETGTPRFSADEITGMFISMMFAGHHTSSGTASWTLIELMRHRDAYAAVIDELDELYGD
GRSVSFHALRQIPQLENVLKETLRLHPPLIILMRVAKGEFEVQGHRIHEGDLVAASPAISNRIPEDFPDPHDF
VPARYEQPRQEDLLNRWTWIPFGAGRHRCVGAAFAIMQIKAIFSVLLREYEFEMAQPPESYRNDHSKMVVQLA
QPACVRYRRRTGV
>CYP51B1_Mycobacterium tuberculosis H00200930 2698022978
MSAVALPRVSGGHDEHGHLEEFRTDPIGLMQRVRDECGDVGTFQLAGKQVVLLSGSHANEFFFRAGDDDLDQA
KAYPFMTPIFGEGVVFDASPERRKEMLHNAALRGEQMKGHAATIEDQVRRMIADWGEAGEIDLLDFFAELTIY
TSSACLIGKKFRDQLDGRFAKLYHELERGTDPLAYVDPYLPIESFRRRDEARNGLVALVADIMNGRIANPPTD
KSDRDMLDVLIAVKAETGTPRFSADEITGMFISMMFAGHHTSSGTASWTLIELMRHRDAYAAVIDELDELYGD
GRSVSFHALRQIPQLENVLKETLRLHPPLIILMRVAKGEFEVQGHRIHEGDLVAASPAISNRIPEDFPDPHDF
VPARYEQPRQEDLLNRWTWIPFGAGRHRCVGAAFAIMQIKAIFSVLLREYEFEMAQPPESYRNDHSKMVVQLA
QPACVRYRRRTGV
>CYP51B1_Mycobacterium tuberculosis H09601792 2674052086
MSAVALPRVSGGHDEHGHLEEFRTDPIGLMQRVRDECGDVGTFQLAGKQVVLLSGSHANEFFFRAGDDDLDQA
KAYPFMTPIFGEGVVFDASPERRKEMLHNAALRGEQMKGHAATIEDQVRRMIADWGEAGEIDLLDFFAELTIY
TSSACLIGKKFRDQLDGRFAKLYHELERGTDPLAYVDPYLPIESFRRRDEARNGLVALVADIMNGRIANPPTD
KSDRDMLDVLIAVKAETGTPRFSADEITGMFISMMFAGHHTSSGTASWTLIELMRHRDAYAAVIDELDELYGD
GRSVSFHALRQIPQLENVLKETLRLHPPLIILMRVAKGEFEVQGHRIHEGDLVAASPAISNRIPEDFPDPHDF
VPARYEQPRQEDLLNRWTWIPFGAGRHRCVGAAFAIMQIKAIFSVLLREYEFEMAQPPESYRNDHSKMVVQLA
QPACVRYRRRTGV
>CYP51B1_Mycobacterium tuberculosis H1249 2622250975
MSAVALPRVSGGHDEHGHLEEFRTDPIGLMQRVRDECGDVGTFQLAGKQVVLLSGSHANEFFFRAGDDDLDQA
KAYPFMTPIFGEGVVFDASPERRKEMLHNAALRGEQMKGHAATIEDQVRRMIADWGEAGEIDLLDFFAELTIY
TSSACLIGKKFRDQLDGRFAKLYHELERGTDPLAYVDPYLPIESFRRRDEARNGLVALVADIMNGRIANPPTD
KSDRDMLDVLIAVKAETGTPRFSADEITGMFISMMFAGHHTSSGTASWTLIELMRHRDAYAAVIDELDELYGD
GRSVSFHALRQIPQLENVLKETLRLHPPLIILMRVAKGEFEVQGHRIHEGDLVAASPAISNRIPEDFPDPHDF
VPARYEQPRQEDLLNRWTWIPFGAGRHRCVGAAFAIMQIKAIFSVLLREYEFEMAQPPESYRNDHSKMVVQLA
QPACVRYRRRTGV
>CYP51B1_Mycobacterium tuberculosis H1329 2622254629
MSAVALPRVSGGHDEHGHLEEFRTDPIGLMQRVRDECGDVGTFQLAGKQVVLLSGSHANEFFFRAGDDDLDQA
KAYPFMTPIFGEGVVFDASPERRKEMLHNAALRGEQMKGHAATIEDQVRRMIADWGEAGEIDLLDFFAELTIY

TSSACLIGKKFRDQLDGRFAKLYHELERGTDPLAYVDPYLPIESFRRRDEARNGLVALVADIMNGRIANPPTD
KSDRDMLDVLIAVKAETGTPRFSADEITGMFISMMFAGHHTSSGTASWTLIELMRHRDAYAAVIDELDELYGD
GRSVSFHALRQIPQLENVLKETLRLHPPLIILMRVAKGEFEVQGHRIHEGDLVAASPAISNRIPEDFPDPHDF
VPARYEQPRQEDLLNRWTWIPFGAGRHRCVGAAFAIMQIKAIFSVLLREYEFEMAQPPESYRNDHSKMVVQLA
QPACVRYRRRTGV
>CYP51B1_Mycobacterium tuberculosis H1578 2574789849
MSAVALPRVSGGHDEHGHLEEFRTDPIGLMQRVRDECGDVGTFQLAGKQVVLLSGSHANEFFFRAGDDDLDQA
KAYPFMTPIFGEGVVFDASPERRKEMLHNAALRGEQMKGHAATIEDQVRRMIADWGEAGEIDLLDFFAELTIY
TSSACLIGKKFRDQLDGRFAKLYHELERGTDPLAYVDPYLPIESFRRRDEARNGLVALVADIMNGRIANPPTD
KSDRDMLDVLIAVKAETGTPRFSADEITGMFISMMFAGHHTSSGTASWTLIELMRHRDAYAAVIDELDELYGD
GRSVSFHALRQIPQLENVLKETLRLHPPLIILMRVAKGEFEVQGHRIHEGDLVAASPAISNRIPEDFPDPHDF
VPARYEQPRQEDLLNRWTWIPFGAGRHRCVGAAFAIMQIKAIFSVLLREYEFEMAQPPESYRNDHSKMVVQLA
QPACVRYRRRTGV
>CYP51B1_Mycobacterium tuberculosis H1580 2622258741
MSAVALPRVSGGHDEHGHLEEFRTDPIGLMQRVRDECGDVGTFQLAGKQVVLLSGSHANEFFFRAGDDDLDQA
KAYPFMTPIFGEGVVFDASPERRKEMLHNAALRGEQMKGHAATIEDQVRRMIADWGEAGEIDLLDFFAELTIY
TSSACLIGKKFRDQLDGRFAKLYHELERGTDPLAYVDPYLPIESFRRRDEARNGLVALVADIMNGRIANPPTD
KSDRDMLDVLIAVKAETGTPRFSADEITGMFISMMFAGHHTSSGTASWTLIELMRHRDAYAAVIDELDELYGD
GRSVSFHALRQIPQLENVLKETLRLHPPLIILMRVAKGEFEVQGHRIHEGDLVAASPAISNRIPEDFPDPHDF
VPARYEQPRQEDLLNRWTWIPFGAGRHRCVGAAFAIMQIKAIFSVLLREYEFEMAQPPESYRNDHSKMVVQLA
QPACVRYRRRTGV
>CYP51B1_Mycobacterium tuberculosis H1589 2622262641
MSAVALPRVSGGHDEHGHLEEFRTDPIGLMQRVRDECGDVGTFQLAGKQVVLLSGSHANEFFFRAGDDDLDQA
KAYPFMTPIFGEGVVFDASPERRKEMLHNAALRGEQMKGHAATIEDQVRRMIADWGEAGEIDLLDFFAELTIY
TSSACLIGKKFRDQLDGRFAKLYHELERGTDPLAYVDPYLPIESFRRRDEARNGLVALVADIMNGRIANPPTD
KSDRDMLDVLIAVKAETGTPRFSADEITGMFISMMFAGHHTSSGTASWTLIELMRHRDAYAAVIDELDELYGD
GRSVSFHALRQIPQLENVLKETLRLHPPLIILMRVAKGEFEVQGHRIHEGDLVAASPAISNRIPEDFPDPHDF
VPARYEQPRQEDLLNRWTWIPFGAGRHRCVGAAFAIMQIKAIFSVLLREYEFEMAQPPESYRNDHSKMVVQLA
QPACVRYRRRTGV
>CYP51B1_Mycobacterium tuberculosis H1856 2622267037
MSAVALPRVSGGHDEHGHLEEFRTDPIGLMQRVRDECGDVGTFQLAGKQVVLLSGSHANEFFFRAGDDDLDQA
KAYPFMTPIFGEGVVFDASPERRKEMLHNAALRGEQMKGHAATIEDQVRRMIADWGEAGEIDLLDFFAELTIY
TSSACLIGKKFRDQLDGRFAKLYHELERGTDPLAYVDPYLPIESFRRRDEARNGLVALVADIMNGRIANPPTD
KSDRDMLDVLIAVKAETGTPRFSADEITGMFISMMFAGHHTSSGTASWTLIELMRHRDAYAAVIDELDELYGD
GRSVSFHALRQIPQLENVLKETLRLHPPLIILMRVAKGEFEVQGHRIHEGDLVAASPAISNRIPEDFPDPHDF
VPARYEQPRQEDLLNRWTWIPFGAGRHRCVGAAFAIMQIKAIFSVLLREYEFEMAQPPESYRNDHSKMVVQLA
QPACVRYRRRTGV
>CYP51B1_Mycobacterium tuberculosis H1996 2622270597
MSAVALPRVSGGHDEHGHLEEFRTDPIGLMQRVRDECGDVGTFQLAGKQVVLLSGSHANEFFFRAGDDDLDQA
KAYPFMTPIFGEGVVFDASPERRKEMLHNAALRGEQMKGHAATIEDQVRRMIADWGEAGEIDLLDFFAELTIY
TSSACLIGKKFRDQLDGRFAKLYHELERGTDPLAYVDPYLPIESFRRRDEARNGLVALVADIMNGRIANPPTD
KSDRDMLDVLIAVKAETGTPRFSADEITGMFISMMFAGHHTSSGTASWTLIELMRHRDAYAAVIDELDELYGD
GRSVSFHALRQIPQLENVLKETLRLHPPLIILMRVAKGEFEVQGHRIHEGDLVAASPAISNRIPEDFPDPHDF
VPARYEQPRQEDLLNRWTWIPFGAGRHRCVGAAFAIMQIKAIFSVLLREYEFEMAQPPESYRNDHSKMVVQLA
QPACVRYRRRTGV
>CYP51B1_Mycobacterium tuberculosis H2264 2622283292
MSAVALPRVSGGHDEHGHLEEFRTDPIGLMQRVRDECGDVGTFQLAGKQVVLLSGSHANEFFFRAGDDDLDQA
KAYPFMTPIFGEGVVFDASPERRKEMLHNAALRGEQMKGHAATIEDQVRRMIADWGEAGEIDLLDFFAELTIY
TSSACLIGKKFRDQLDGRFAKLYHELERGTDPLAYVDPYLPIESFRRRDEARNGLVALVADIMNGRIANPPTD
KSDRDMLDVLIAVKAETGTPRFSADEITGMFISMMFAGHHTSSGTASWTLIELMRHRDAYAAVIDELDELYGD
GRSVSFHALRQIPQLENVLKETLRLHPPLIILMRVAKGEFEVQGHRIHEGDLVAASPAISNRIPEDFPDPHDF
VPARYEQPRQEDLLNRWTWIPFGAGRHRCVGAAFAIMQIKAIFSVLLREYEFEMAQPPESYRNDHSKMVVQLA
QPACVRYRRRTGV
>CYP51B1_Mycobacterium tuberculosis H2300 2622290269
MSAVALPRVSGGHDEHGHLEEFRTDPIGLMQRVRDECGDVGTFQLAGKQVVLLSGSHANEFFFRAGDDDLDQA
KAYPFMTPIFGEGVVFDASPERRKEMLHNAALRGEQMKGHAATIEDQVRRMIADWGEAGEIDLLDFFAELTIY
TSSACLIGKKFRDQLDGRFAKLYHELERGTDPLAYVDPYLPIESFRRRDEARNGLVALVADIMNGRIANPPTD
KSDRDMLDVLIAVKAETGTPRFSADEITGMFISMMFAGHHTSSGTASWTLIELMRHRDAYAAVIDELDELYGD

GRSVSFHALRQIPQLENVLKETLRLHPPLIILMRVAKGEFEVQGHRIHEGDLVAASPAISNRIPEDFPDPHDF
VPARYEQPRQEDLLNRWTWIPFGAGRHRCVGAAFAIMQIKAIFSVLLREYEFEMAQPPESYRNDHSKMVVQLA
QPACVRYRRRTGV
>CYP51B1_Mycobacterium tuberculosis H2375 2647413359
MSAVALPRVSGGHDEHGHLEEFRTDPIGLMQRVRDECGDVGTFQLAGKQVVLLSGSHANEFFFRAGDDDLDQA
KAYPFMTPIFGEGVVFDASPERRKEMLHNAALRGEQMKGHAATIEDQVRRMIADWGEAGEIDLLDFFAELTIY
TSSACLIGKKFRDQLDGRFAKLYHELERGTDPLAYVDPYLPIESFRRRDEARNGLVALVADIMNGRIANPPTD
KSDRDMLDVLIAVKAETGTPRFSADEITGMFISMMFAGHHTSSGTASWTLIELMRHRDAYAAVIDELDELYGD
GRSVSFHALRQIPQLENVLKETLRLHPPLIILMRVAKGEFEVQGHRIHEGDLVAASPAISNRIPEDFPDPHDF
VPARYEQPRQEDLLNRWTWIPFGAGRHRCVGAAFAIMQIKAIFSVLLREYEFEMAQPPESYRNDHSKMVVQLA
QPACVRYRRRTGV
>CYP51B1_Mycobacterium tuberculosis H2398 2577687170
MSAVALPRVSGGHDEHGHLEEFRTDPIGLMQRVRDECGDVGTFQLAGKQVVLLSGSHANEFFFRAGDDDLDQA
KAYPFMTPIFGEGVVFDASPERRKEMLHNAALRGEQMKGHAATIEDQVRRMIADWGEAGEIDLLDFFAELTIY
TSSACLIGKKFRDQLDGRFAKLYHELERGTDPLAYVDPYLPIESFRRRDEARNGLVALVADIMNGRIANPPTD
KSDRDMLDVLIAVKAETGTPRFSADEITGMFISMMFAGHHTSSGTASWTLIELMRHRDAYAAVIDELDELYGD
GRSVSFHALRQIPQLENVLKETLRLHPPLIILMRVAKGEFEVQGHRIHEGDLVAASPAISNRIPEDFPDPHDF
VPARYEQPRQEDLLNRWTWIPFGAGRHRCVGAAFAIMQIKAIFSVLLREYEFEMAQPPESYRNDHSKMVVQLA
QPACVRYRRRTGV
>CYP51B1_Mycobacterium tuberculosis H2438 2584964775
MSAVALPRVSGGHDEHGHLEEFRTDPIGLMQRVRDECGDVGTFQLAGKQVVLLSGSHANEFFFRAGDDDLDQA
KAYPFMTPIFGEGVVFDASPERRKEMLHNAALRGEQMKGHAATIEDQVRRMIADWGEAGEIDLLDFFAELTIY
TSSACLIGKKFRDQLDGRFAKLYHELERGTDPLAYVDPYLPIESFRRRDEARNGLVALVADIMNGRIANPPTD
KSDRDMLDVLIAVKAETGTPRFSADEITGMFISMMFAGHHTSSGTASWTLIELMRHRDAYAAVIDELDELYGD
GRSVSFHALRQIPQLENVLKETLRLHPPLIILMRVAKGEFEVQGHRIHEGDLVAASPAISNRIPEDFPDPHDF
VPARYEQPRQEDLLNRWTWIPFGAGRHRCVGAAFAIMQIKAIFSVLLREYEFEMAQPPESYRNDHSKMVVQLA
QPACVRYRRRTGV
>CYP51B1_Mycobacterium tuberculosis H2463 2622294601
MSAVALPRVSGGHDEHGHLEEFRTDPIGLMQRVRDECGDVGTFQLAGKQVVLLSGSHANEFFFRAGDDDLDQA
KAYPFMTPIFGEGVVFDASPERRKEMLHNAALRGEQMKGHAATIEDQVRRMIADWGEAGEIDLLDFFAELTIY
TSSACLIGKKFRDQLDGRFAKLYHELERGTDPLAYVDPYLPIESFRRRDEARNGLVALVADIMNGRIANPPTD
KSDRDMLDVLIAVKAETGTPRFSADEITGMFISMMFAGHHTSSGTASWTLIELMRHRDAYAAVIDELDELYGD
GRSVSFHALRQIPQLENVLKETLRLHPPLIILMRVAKGEFEVQGHRIHEGDLVAASPAISNRIPEDFPDPHDF
VPARYEQPRQEDLLNRWTWIPFGAGRHRCVGAAFAIMQIKAIFSVLLREYEFEMAQPPESYRNDHSKMVVQLA
QPACVRYRRRTGV
>CYP51B1_Mycobacterium tuberculosis H2581 2576391778
MSAVALPRVSGGHDEHGHLEEFRTDPIGLMQRVRDECGDVGTFQLAGKQVVLLSGSHANEFFFRAGDDDLDQA
KAYPFMTPIFGEGVVFDASPERRKEMLHNAALRGEQMKGHAATIEDQVRRMIADWGEAGEIDLLDFFAELTIY
TSSACLIGKKFRDQLDGRFAKLYHELERGTDPLAYVDPYLPIESFRRRDEARNGLVALVADIMNGRIANPPTD
KSDRDMLDVLIAVKAETGTPRFSADEITGMFISMMFAGHHTSSGTASWTLIELMRHRDAYAAVIDELDELYGD
GRSVSFHALRQIPQLENVLKETLRLHPPLIILMRVAKGEFEVQGHRIHEGDLVAASPAISNRIPEDFPDPHDF
VPARYEQPRQEDLLNRWTWIPFGAGRHRCVGAAFAIMQIKAIFSVLLREYEFEMAQPPESYRNDHSKMVVQLA
QPACVRYRRRTGV
>CYP51B1_Mycobacterium tuberculosis H2754 2622298897
MSAVALPRVSGGHDEHGHLEEFRTDPIGLMQRVRDECGDVGTFQLAGKQVVLLSGSHANEFFFRAGDDDLDQA
KAYPFMTPIFGEGVVFDASPERRKEMLHNAALRGEQMKGHAATIEDQVRRMIADWGEAGEIDLLDFFAELTIY
TSSACLIGKKFRDQLDGRFAKLYHELERGTDPLAYVDPYLPIESFRRRDEARNGLVALVADIMNGRIANPPTD
KSDRDMLDVLIAVKAETGTPRFSADEITGMFISMMFAGHHTSSGTASWTLIELMRHRDAYAAVIDELDELYGD
GRSVSFHALRQIPQLENVLKETLRLHPPLIILMRVAKGEFEVQGHRIHEGDLVAASPAISNRIPEDFPDPHDF
VPARYEQPRQEDLLNRWTWIPFGAGRHRCVGAAFAIMQIKAIFSVLLREYEFEMAQPPESYRNDHSKMVVQLA
QPACVRYRRRTGV
>CYP51B1_Mycobacterium tuberculosis H2760 2622299527
MSAVALPRVSGGHDEHGHLEEFRTDPIGLMQRVRDECGDVGTFQLAGKQVVLLSGSHANEFFFRAGDDDLDQA
KAYPFMTPIFGEGVVFDASPERRKEMLHNAALRGEQMKGHAATIEDQVRRMIADWGEAGEIDLLDFFAELTIY
TSSACLIGKKFRDQLDGRFAKLYHELERGTDPLAYVDPYLPIESFRRRDEARNGLVALVADIMNGRIANPPTD
KSDRDMLDVLIAVKAETGTPRFSADEITGMFISMMFAGHHTSSGTASWTLIELMRHRDAYAAVIDELDELYGD
GRSVSFHALRQIPQLENVLKETLRLHPPLIILMRVAKGEFEVQGHRIHEGDLVAASPAISNRIPEDFPDPHDF

VPARYEQPRQEDLLNRWTWIPFGAGRHRCVGAAFAIMQIKAIFSVLLREYEFEMAQPPESYRNDHSKMVVQLA
QPACVRYRRRTGV
>CYP51B1_Mycobacterium tuberculosis H3005 2622309284
MSAVALPRVSGGHDEHGHLEEFRTDPIGLMQRVRDECGDVGTFQLAGKQVVLLSGSHANEFFFRAGDDDLDQA
KAYPFMTPIFGEGVVFDASPERRKEMLHNAALRGEQMKGHAATIEDQVRRMIADWGEAGEIDLLDFFAELTIY
TSSACLIGKKFRDQLDGRFAKLYHELERGTDPLAYVDPYLPIESFRRRDEARNGLVALVADIMNGRIANPPTD
KSDRDMLDVLIAVKAETGTPRFSADEITGMFISMMFAGHHTSSGTASWTLIELMRHRDAYAAVIDELDELYGD
GRSVSFHALRQIPQLENVLKETLRLHPPLIILMRVAKGEFEVQGHRIHEGDLVAASPAISNRIPEDFPDPHDF
VPARYEQPRQEDLLNRWTWIPFGAGRHRCVGAAFAIMQIKAIFSVLLREYEFEMAQPPESYRNDHSKMVVQLA
QPACVRYRRRTGV
>CYP51B1_Mycobacterium tuberculosis H3033 2622312017
MSAVALPRVSGGHDEHGHLEEFRTDPIGLMQRVRDECGDVGTFQLAGKQVVLLSGSHANEFFFRAGDDDLDQA
KAYPFMTPIFGEGVVFDASPERRKEMLHNAALRGEQMKGHAATIEDQVRRMIADWGEAGEIDLLDFFAELTIY
TSSACLIGKKFRDQLDGRFAKLYHELERGTDPLAYVDPYLPIESFRRRDEARNGLVALVADIMNGRIANPPTD
KSDRDMLDVLIAVKAETGTPRFSADEITGMFISMMFAGHHTSSGTASWTLIELMRHRDAYAAVIDELDELYGD
GRSVSFHALRQIPQLENVLKETLRLHPPLIILMRVAKGEFEVQGHRIHEGDLVAASPAISNRIPEDFPDPHDF
VPARYEQPRQEDLLNRWTWIPFGAGRHRCVGAAFAIMQIKAIFSVLLREYEFEMAQPPESYRNDHSKMVVQLA
QPACVRYRRRTGV
>CYP51B1_Mycobacterium tuberculosis H3094 2622317270
MSAVALPRVSGGHDEHGHLEEFRTDPIGLMQRVRDECGDVGTFQLAGKQVVLLSGSHANEFFFRAGDDDLDQA
KAYPFMTPIFGEGVVFDASPERRKEMLHNAALRGEQMKGHAATIEDQVRRMIADWGEAGEIDLLDFFAELTIY
TSSACLIGKKFRDQLDGRFAKLYHELERGTDPLAYVDPYLPIESFRRRDEARNGLVALVADIMNGRIANPPTD
KSDRDMLDVLIAVKAETGTPRFSADEITGMFISMMFAGHHTSSGTASWTLIELMRHRDAYAAVIDELDELYGD
GRSVSFHALRQIPQLENVLKETLRLHPPLIILMRVAKGEFEVQGHRIHEGDLVAASPAISNRIPEDFPDPHDF
VPARYEQPRQEDLLNRWTWIPFGAGRHRCVGAAFAIMQIKAIFSVLLREYEFEMAQPPESYRNDHSKMVVQLA
QPACVRYRRRTGV
>CYP51B1_Mycobacterium tuberculosis H3360 2622323619
MSAVALPRVSGGHDEHGHLEEFRTDPIGLMQRVRDECGDVGTFQLAGKQVVLLSGSHANEFFFRAGDDDLDQA
KAYPFMTPIFGEGVVFDASPERRKEMLHNAALRGEQMKGHAATIEDQVRRMIADWGEAGEIDLLDFFAELTIY
TSSACLIGKKFRDQLDGRFAKLYHELERGTDPLAYVDPYLPIESFRRRDEARNGLVALVADIMNGRIANPPTD
KSDRDMLDVLIAVKAETGTPRFSADEITGMFISMMFAGHHTSSGTASWTLIELMRHRDAYAAVIDELDELYGD
GRSVSFHALRQIPQLENVLKETLRLHPPLIILMRVAKGEFEVQGHRIHEGDLVAASPAISNRIPEDFPDPHDF
VPARYEQPRQEDLLNRWTWIPFGAGRHRCVGAAFAIMQIKAIFSVLLREYEFEMAQPPESYRNDHSKMVVQLA
QPACVRYRRRTGV
>CYP51B1_Mycobacterium tuberculosis H3361 2575022922
MSAVALPRVSGGHDEHGHLEEFRTDPIGLMQRVRDECGDVGTFQLAGKQVVLLSGSHANEFFFRAGDDDLDQA
KAYPFMTPIFGEGVVFDASPERRKEMLHNAALRGEQMKGHAATIEDQVRRMIADWGEAGEIDLLDFFAELTIY
TSSACLIGKKFRDQLDGRFAKLYHELERGTDPLAYVDPYLPIESFRRRDEARNGLVALVADIMNGRIANPPTD
KSDRDMLDVLIAVKAETGTPRFSADEITGMFISMMFAGHHTSSGTASWTLIELMRHRDAYAAVIDELDELYGD
GRSVSFHALRQIPQLENVLKETLRLHPPLIILMRVAKGEFEVQGHRIHEGDLVAASPAISNRIPEDFPDPHDF
VPARYEQPRQEDLLNRWTWIPFGAGRHRCVGAAFAIMQIKAIFSVLLREYEFEMAQPPESYRNDHSKMVVQLA
QPACVRYRRRTGV
>CYP51B1_Mycobacterium tuberculosis H3367 2622324455
MSAVALPRVSGGHDEHGHLEEFRTDPIGLMQRVRDECGDVGTFQLAGKQVVLLSGSHANEFFFRAGDDDLDQA
KAYPFMTPIFGEGVVFDASPERRKEMLHNAALRGEQMKGHAATIEDQVRRMIADWGEAGEIDLLDFFAELTIY
TSSACLIGKKFRDQLDGRFAKLYHELERGTDPLAYVDPYLPIESFRRRDEARNGLVALVADIMNGRIANPPTD
KSDRDMLDVLIAVKAETGTPRFSADEITGMFISMMFAGHHTSSGTASWTLIELMRHRDAYAAVIDELDELYGD
GRSVSFHALRQIPQLENVLKETLRLHPPLIILMRVAKGEFEVQGHRIHEGDLVAASPAISNRIPEDFPDPHDF
VPARYEQPRQEDLLNRWTWIPFGAGRHRCVGAAFAIMQIKAIFSVLLREYEFEMAQPPESYRNDHSKMVVQLA
QPACVRYRRRTGV
>CYP51B1_Mycobacterium tuberculosis H37Ra 640601446
MSAVALPRVSGGHDEHGHLEEFRTDPIGLMQRVRDECGDVGTFQLAGKQVVLLSGSHANEFFFRAGDDDLDQA
KAYPFMTPIFGEGVVFDASPERRKEMLHNAALRGEQMKGHAATIEDQVRRMIADWGEAGEIDLLDFFAELTIY
TSSACLIGKKFRDQLDGRFAKLYHELERGTDPLAYVDPYLPIESFRRRDEARNGLVALVADIMNGRIANPPTD
KSDRDMLDVLIAVKAETGTPRFSADEITGMFISMMFAGHHTSSGTASWTLIELMRHRDAYAAVIDELDELYGD
GRSVSFHALRQIPQLENVLKETLRLHPPLIILMRVAKGEFEVQGHRIHEGDLVAASPAISNRIPEDFPDPHDF
VPARYEQPRQEDLLNRWTWIPFGAGRHRCVGAAFAIMQIKAIFSVLLREYEFEMAQPPESYRNDHSKMVVQLA
QPACVRYRRRTGV

>CYP51B1_Mycobacterium tuberculosis H37Ra unfinished sequence 641814530
MSAVALPRVSGGHDEHGHLEEFRTDPIGLMQRVRDECGDVGTFQLAGKQVVLLSGSHANEFFFRAGDDDLDQA
KAYPFMTPIFGEGVVFDASPERRKEMLHNAALRGEQMKGHAATIEDQVRRMIADWGEAGEIDLLDFFAELTIY
TSSACLIGKKFRDQLDGRFAKLYHELERGTDPLAYVDPYLPIESFRRRDEARNGLVALVADIMNGRIANPPTD
KSDRDMLDVLIAVKAETGTPRFSADEITGMFISMMFAGHHTSSGTASWTLIELMRHRDAYAAVIDELDELYGD
GRSVSFHALRQIPQLENVLKETLRLHPPLIILMRVAKGEFEVQGHRIHEGDLVAASPAISNRIPEDFPDPHDF
VPARYEQPRQEDLLNRWTWIPFGAGRHRCVGAAFAIMQIKAIFSVLLREYEFEMAQPPESYRNDHSKMVVQLA
QPACVRYRRRTGV
>CYP51B1_Mycobacterium tuberculosis H37Rv 2604654998
MSAVALPRVSGGHDEHGHLEEFRTDPIGLMQRVRDECGDVGTFQLAGKQVVLLSGSHANEFFFRAGDDDLDQA
KAYPFMTPIFGEGVVFDASPERRKEMLHNAALRGEQMKGHAATIEDQVRRMIADWGEAGEIDLLDFFAELTIY
TSSACLIGKKFRDQLDGRFAKLYHELERGTDPLAYVDPYLPIESFRRRDEARNGLVALVADIMNGRIANPPTD
KSDRDMLDVLIAVKAETGTPRFSADEITGMFISMMFAGHHTSSGTASWTLIELMRHRDAYAAVIDELDELYGD
GRSVSFHALRQIPQLENVLKETLRLHPPLIILMRVAKGEFEVQGHRIHEGDLVAASPAISNRIPEDFPDPHDF
VPARYEQPRQEDLLNRWTWIPFGAGRHRCVGAAFAIMQIKAIFSVLLREYEFEMAQPPESYRNDHSKMVVQLA
QPACVRYRRRTGV
>CYP51B1_Mycobacterium tuberculosis H37Rv 637025954
MSAVALPRVSGGHDEHGHLEEFRTDPIGLMQRVRDECGDVGTFQLAGKQVVLLSGSHANEFFFRAGDDDLDQA
KAYPFMTPIFGEGVVFDASPERRKEMLHNAALRGEQMKGHAATIEDQVRRMIADWGEAGEIDLLDFFAELTIY
TSSACLIGKKFRDQLDGRFAKLYHELERGTDPLAYVDPYLPIESFRRRDEARNGLVALVADIMNGRIANPPTD
KSDRDMLDVLIAVKAETGTPRFSADEITGMFISMMFAGHHTSSGTASWTLIELMRHRDAYAAVIDELDELYGD
GRSVSFHALRQIPQLENVLKETLRLHPPLIILMRVAKGEFEVQGHRIHEGDLVAASPAISNRIPEDFPDPHDF
VPARYEQPRQEDLLNRWTWIPFGAGRHRCVGAAFAIMQIKAIFSVLLREYEFEMAQPPESYRNDHSKMVVQLA
QPACVRYRRRTGV
>CYP51B1_Mycobacterium tuberculosis H37Rv 2527055930
MSAVALPRVSGGHDEHGHLEEFRTDPIGLMQRVRDECGDVGTFQLAGKQVVLLSGSHANEFFFRAGDDDLDQA
KAYPFMTPIFGEGVVFDASPERRKEMLHNAALRGEQMKGHAATIEDQVRRMIADWGEAGEIDLLDFFAELTIY
TSSACLIGKKFRDQLDGRFAKLYHELERGTDPLAYVDPYLPIESFRRRDEARNGLVALVADIMNGRIANPPTD
KSDRDMLDVLIAVKAETGTPRFSADEITGMFISMMFAGHHTSSGTASWTLIELMRHRDAYAAVIDELDELYGD
GRSVSFHALRQIPQLENVLKETLRLHPPLIILMRVAKGEFEVQGHRIHEGDLVAASPAISNRIPEDFPDPHDF
VPARYEQPRQEDLLNRWTWIPFGAGRHRCVGAAFAIMQIKAIFSVLLREYEFEMAQPPESYRNDHSKMVVQLA
QPACVRYRRRTGV
>CYP51B1_Mycobacterium tuberculosis H37Rv 2633793563
MSAVALPRVSGGHDEHGHLEEFRTDPIGLMQRVRDECGDVGTFQLAGKQVVLLSGSHANEFFFRAGDDDLDQA
KAYPFMTPIFGEGVVFDASPERRKEMLHNAALRGEQMKGHAATIEDQVRRMIADWGEAGEIDLLDFFAELTIY
TSSACLIGKKFRDQLDGRFAKLYHELERGTDPLAYVDPYLPIESFRRRDEARNGLVALVADIMNGRIANPPTD
KSDRDMLDVLIAVKAETGTPRFSADEITGMFISMMFAGHHTSSGTASWTLIELMRHRDAYAAVIDELDELYGD
GRSVSFHALRQIPQLENVLKETLRLHPPLIILMRVAKGEFEVQGHRIHEGDLVAASPAISNRIPEDFPDPHDF
VPARYEQPRQEDLLNRWTWIPFGAGRHRCVGAAFAIMQIKAIFSVLLREYEFEMAQPPESYRNDHSKMVVQLA
QPACVRYRRRTGV
>CYP51B1_Mycobacterium tuberculosis H37RvAE 2603094659
MSAVALPRVSGGHDEHGHLEEFRTDPIGLMQRVRDECGDVGTFQLAGKQVVLLSGSHANEFFFRAGDDDLDQA
KAYPFMTPIFGEGVVFDASPERRKEMLHNAALRGEQMKGHAATIEDQVRRMIADWGEAGEIDLLDFFAELTIY
TSSACLIGKKFRDQLDGRFAKLYHELERGTDPLAYVDPYLPIESFRRRDEARNGLVALVADIMNGRIANPPTD
KSDRDMLDVLIAVKAETGTPRFSADEITGMFISMMFAGHHTSSGTASWTLIELMRHRDAYAAVIDELDELYGD
GRSVSFHALRQIPQLENVLKETLRLHPPLIILMRVAKGEFEVQGHRIHEGDLVAASPAISNRIPEDFPDPHDF
VPARYEQPRQEDLLNRWTWIPFGAGRHRCVGAAFAIMQIKAIFSVLLREYEFEMAQPPESYRNDHSKMVVQLA
QPACVRYRRRTGV
>CYP51B1_Mycobacterium tuberculosis H37RvCO 2547163207
MSAVALPRVSGGHDEHGHLEEFRTDPIGLMQRVRDECGDVGTFQLAGKQVVLLSGSHANEFFFRAGDDDLDQA
KAYPFMTPIFGEGVVFDASPERRKEMLHNAALRGEQMKGHAATIEDQVRRMIADWGEAGEIDLLDFFAELTIY
TSSACLIGKKFRDQLDGRFAKLYHELERGTDPLAYVDPYLPIESFRRRDEARNGLVALVADIMNGRIANPPTD
KSDRDMLDVLIAVKAETGTPRFSADEITGMFISMMFAGHHTSSGTASWTLIELMRHRDAYAAVIDELDELYGD
GRSVSFHALRQIPQLENVLKETLRLHPPLIILMRVAKGEFEVQGHRIHEGDLVAASPAISNRIPEDFPDPHDF
VPARYEQPRQEDLLNRWTWIPFGAGRHRCVGAAFAIMQIKAIFSVLLREYEFEMAQPPESYRNDHSKMVVQLA
QPACVRYRRRTGV
>CYP51B1_Mycobacterium tuberculosis H37RvLP 2603098790

MSAVALPRVSGGHDEHGHLEEFRTDPIGLMQRVRDECGDVGTFQLAGKQVVLLSGSHANEFFFRAGDDDLDQA
KAYPFMTPIFGEGVVFDASPERRKEMLHNAALRGEQMKGHAATIEDQVRRMIADWGEAGEIDLLDFFAELTIY
TSSACLIGKKFRDQLDGRFAKLYHELERGTDPLAYVDPYLPIESFRRRDEARNGLVALVADIMNGRIANPPTD
KSDRDMLDVLIAVKAETGTPRFSADEITGMFISMMFAGHHTSSGTASWTLIELMRHRDAYAAVIDELDELYGD
GRSVSFHALRQIPQLENVLKETLRLHPPLIILMRVAKGEFEVQGHRIHEGDLVAASPAISNRIPEDFPDPHDF
VPARYEQPRQEDLLNRWTWIPFGAGRHRCVGAAFAIMQIKAIFSVLLREYEFEMAQPPESYRNDHSKMVVQLA
QPACVRYRRRTGV
>CYP51B1_Mycobacterium tuberculosis H37RvMA 2603102969
MSAVALPRVSGGHDEHGHLEEFRTDPIGLMQRVRDECGDVGTFQLAGKQVVLLSGSHANEFFFRAGDDDLDQA
KAYPFMTPIFGEGVVFDASPERRKEMLHNAALRGEQMKGHAATIEDQVRRMIADWGEAGEIDLLDFFAELTIY
TSSACLIGKKFRDQLDGRFAKLYHELERGTDPLAYVDPYLPIESFRRRDEARNGLVALVADIMNGRIANPPTD
KSDRDMLDVLIAVKAETGTPRFSADEITGMFISMMFAGHHTSSGTASWTLIELMRHRDAYAAVIDELDELYGD
GRSVSFHALRQIPQLENVLKETLRLHPPLIILMRVAKGEFEVQGHRIHEGDLVAASPAISNRIPEDFPDPHDF
VPARYEQPRQEDLLNRWTWIPFGAGRHRCVGAAFAIMQIKAIFSVLLREYEFEMAQPPESYRNDHSKMVVQLA
QPACVRYRRRTGV
>CYP51B1_Mycobacterium tuberculosis H37RvSiena 2647320001
MSAVALPRVSGGHDEHGHLEEFRTDPIGLMQRVRDECGDVGTFQLAGKQVVLLSGSHANEFFFRAGDDDLDQA
KAYPFMTPIFGEGVVFDASPERRKEMLHNAALRGEQMKGHAATIEDQVRRMIADWGEAGEIDLLDFFAELTIY
TSSACLIGKKFRDQLDGRFAKLYHELERGTDPLAYVDPYLPIESFRRRDEARNGLVALVADIMNGRIANPPTD
KSDRDMLDVLIAVKAETGTPRFSADEITGMFISMMFAGHHTSSGTASWTLIELMRHRDAYAAVIDELDELYGD
GRSVSFHALRQIPQLENVLKETLRLHPPLIILMRVAKGEFEVQGHRIHEGDLVAASPAISNRIPEDFPDPHDF
VPARYEQPRQEDLLNRWTWIPFGAGRHRCVGAAFAIMQIKAIFSVLLREYEFEMAQPPESYRNDHSKMVVQLA
QPACVRYRRRTGV
>CYP51B1_Mycobacterium tuberculosis H3986 2575490126
MSAVALPRVSGGHDEHGHLEEFRTDPIGLMQRVRDECGDVGTFQLAGKQVVLLSGSHANEFFFRAGDDDLDQA
KAYPFMTPIFGEGVVFDASPERRKEMLHNAALRGEQMKGHAATIEDQVRRMIADWGEAGEIDLLDFFAELTIY
TSSACLIGKKFRDQLDGRFAKLYHELERGTDPLAYVDPYLPIESFRRRDEARNGLVALVADIMNGRIANPPTD
KSDRDMLDVLIAVKAETGTPRFSADEITGMFISMMFAGHHTSSGTASWTLIELMRHRDAYAAVIDELDELYGD
GRSVSFHALRQIPQLENVLKETLRLHPPLIILMRVAKGEFEVQGHRIHEGDLVAASPAISNRIPEDFPDPHDF
VPARYEQPRQEDLLNRWTWIPFGAGRHRCVGAAFAIMQIKAIFSVLLREYEFEMAQPPESYRNDHSKMVVQLA
QPACVRYRRRTGV
>CYP51B1_Mycobacterium tuberculosis H55_24991 2716364464
MSAVALPRVSGGHDEHGHLEEFRTDPIGLMQRVRDECGDVGTFQLAGKQVVLLSGSHANEFFFRAGDDDLDQA
KAYPFMTPIFGEGVVFDASPERRKEMLHNAALRGEQMKGHAATIEDQVRRMIADWGEAGEIDLLDFFAELTIY
TSSACLIGKKFRDQLDGRFAKLYHELERGTDPLAYVDPYLPIESFRRRDEARNGLVALVADIMNGRIANPPTD
KSDRDMLDVLIAVKAETGTPRFSADEITGMFISMMFAGHHTSSGTASWTLIELMRHRDAYAAVIDELDELYGD
GRSVSFHALRQIPQLENVLKETLRLHPPLIILMRVAKGEFEVQGHRIHEGDLVAASPAISNRIPEDFPDPHDF
VPARYEQPRQEDLLNRWTWIPFGAGRHRCVGAAFAIMQIKAIFSVLLREYEFEMAQPPESYRNDHSKMVVQLA
QPACVRYRRRTGV
>CYP51B1_Mycobacterium tuberculosis H6_10443 2716368739
MSAVALPRVSGGHDEHGHLEEFRTDPIGLMQRVRDECGDVGTFQLAGKQVVLLSGSHANEFFFRAGDDDLDQA
KAYPFMTPIFGEGVVFDASPERRKEMLHNAALRGEQMKGHAATIEDQVRRMIADWGEAGEIDLLDFFAELTIY
TSSACLIGKKFRDQLDGRFAKLYHELERGTDPLAYVDPYLPIESFRRRDEARNGLVALVADIMNGRIANPPTD
KSDRDMLDVLIAVKAETGTPRFSADEITGMFISMMFAGHHTSSGTASWTLIELMRHRDAYAAVIDELDELYGD
GRSVSFHALRQIPQLENVLKETLRLHPPLIILMRVAKGEFEVQGHRIHEGDLVAASPAISNRIPEDFPDPHDF
VPARYEQPRQEDLLNRWTWIPFGAGRHRCVGAAFAIMQIKAIFSVLLREYEFEMAQPPESYRNDHSKMVVQLA
QPACVRYRRRTGV
>CYP51B1_Mycobacterium tuberculosis H6_12226 2716373153
MSAVALPRVSGGHDEHGHLEEFRTDPIGLMQRVRDECGDVGTFQLAGKQVVLLSGSHANEFFFRAGDDDLDQA
KAYPFMTPIFGEGVVFDASPERRKEMLHNAALRGEQMKGHAATIEDQVRRMIADWGEAGEIDLLDFFAELTIY
TSSACLIGKKFRDQLDGRFAKLYHELERGTDPLAYVDPYLPIESFRRRDEARNGLVALVADIMNGRIANPPTD
KSDRDMLDVLIAVKAETGTPRFSADEITGMFISMMFAGHHTSSGTASWTLIELMRHRDAYAAVIDELDELYGD
GRSVSFHALRQIPQLENVLKETLRLHPPLIILMRVAKGEFEVQGHRIHEGDLVAASPAISNRIPEDFPDPHDF
VPARYEQPRQEDLLNRWTWIPFGAGRHRCVGAAFAIMQIKAIFSVLLREYEFEMAQPPESYRNDHSKMVVQLA
QPACVRYRRRTGV
>CYP51B1_Mycobacterium tuberculosis H6_7420 2716301238
MSAVALPRVSGGHDEHGHLEEFRTDPIGLMQRVRDECGDVGTFQLAGKQVVLLSGSHANEFFFRAGDDDLDQA
KAYPFMTPIFGEGVVFDASPERRKEMLHNAALRGEQMKGHAATIEDQVRRMIADWGEAGEIDLLDFFAELTIY

TSSACLIGKKFRDQLDGRFAKLYHELERGTDPLAYVDPYLPIESFRRRDEARNGLVALVADIMNGRIANPPTD
KSDRDMLDVLIAVKAETGTPRFSADEITGMFISMMFAGHHTSSGTASWTLIELMRHRDAYAAVIDELDELYGD
GRSVSFHALRQIPQLENVLKETLRLHPPLIILMRVAKGEFEVQGHRIHEGDLVAASPAISNRIPEDFPDPHDF
VPARYEQPRQEDLLNRWTWIPFGAGRHRCVGAAFAIMQIKAIFSVLLREYEFEMAQPPESYRNDHSKMVVQLA
QPACVRYRRRTGV
>CYP51B1_Mycobacterium tuberculosis str. Haarlem unfinished sequence
641782213
MSAVALPRVSGGHDEHGHLEEFRTDPIGLMQRVRDECGDVGTFQLAGKQVVLLSGSHANEFFFRAGDDDLDQA
KAYPFMTPIFGEGVVFDASPERRKEMLHNAALRGEQMKGHAATIEDQVRRMIADWGEAGEIDLLDFFAELTIY
TSSACLIGKKFRDQLDGRFAKLYHELERGTDPLAYVDPYLPIESFRRRDEARNGLVALVADIMNGRIANPPTD
KSDRDMLDVLIAVKAETGTPRFSADEITGMFISMMFAGHHTSSGTASWTLIELMRHRDAYAAVIDELDELYGD
GRSVSFHALRQIPQLENVLKETLRLHPPLIILMRVAKGEFEVQGHRIHEGDLVAASPAISNRIPEDFPDPHDF
VPARYEQPRQEDLLNRWTWIPFGAGRHRCVGAAFAIMQIKAIFSVLLREYEFEMAQPPESYRNDHSKMVVQLA
QPACVRYRRRTGV
>CYP51B1_Mycobacterium tuberculosis HM 2621345331
MSAVALPRVSGGHDEHGHLEEFRTDPIGLMQRVRDECGDVGTFQLAGKQVVLLSGSHANEFFFRAGDDDLDQA
KAYPFMTPIFGEGVVFDASPERRKEMLHNAALRGEQMKGHAATIEDQVRRMIADWGEAGEIDLLDFFAELTIY
TSSACLIGKKFRDQLDGRFAKLYHELERGTDPLAYVDPYLPIESFRRRDEARNGLVALVADIMNGRIANPPTD
KSDRDMLDVLIAVKAETGTPRFSADEITGMFISMMFAGHHTSSGTASWTLIELMRHRDAYAAVIDELDELYGD
GRSVSFHALRQIPQLENVLKETLRLHPPLIILMRVAKGEFEVQGHRIHEGDLVAASPAISNRIPEDFPDPHDF
VPARYEQPRQEDLLNRWTWIPFGAGRHRCVGAAFAIMQIKAIFSVLLREYEFEMAQPPESYRNDHSKMVVQLA
QPACVRYRRRTGV
>CYP51B1_Mycobacterium tuberculosis HN878 2547958785
MSAVALPRVSGGHDEHGHLEEFRTDPIGLMQRVRDECGDVGTFQLAGKQVVLLSGSHANEFFFRAGDDDLDQA
KAYPFMTPIFGEGVVFDASPERRKEMLHNAALRGEQMKGHAATIEDQVRRMIADWGEAGEIDLLDFFAELTIY
TSSACLIGKKFRDQLDGRFAKLYHELERGTDPLAYVDPYLPIESFRRRDEARNGLVALVADIMNGRIANPPTD
KSDRDMLDVLIAVKAETGTPRFSADEITGMFISMMFAGHHTSSGTASWTLIELMRHRDAYAAVIDELDELYGD
GRSVSFHALRQIPQLENVLKETLRLHPPLIILMRVAKGEFEVQGHRIHEGDLVAASPAISNRIPEDFPDPHDF
VPARYEQPRQEDLLNRWTWIPFGAGRHRCVGAAFAIMQIKAIFSVLLREYEFEMAQPPESYRNDHSKMVVQLA
QPACVRYRRRTGV
>CYP51B1_Mycobacterium tuberculosis I_15762 2716377076
MSAVALPRVSGGHDEHGHLEEFRTDPIGLMQRVRDECGDVGTFQLAGKQVVLLSGSHANEFFFRAGDDDLDQA
KAYPFMTPIFGEGVVFDASPERRKEMLHNAALRGEQMKGHAATIEDQVRRMIADWGEAGEIDLLDFFAELTIY
TSSACLIGKKFRDQLDGRFAKLYHELERGTDPLAYVDPYLPIESFRRRDEARNGLVALVADIMNGRIANPPTD
KSDRDMLDVLIAVKAETGTPRFSADEITGMFISMMFAGHHTSSGTASWTLIELMRHRDAYAAVIDELDELYGD
GRSVSFHALRQIPQLENVLKETLRLHPPLIILMRVAKGEFEVQGHRIHEGDLVAASPAISNRIPEDFPDPHDF
VPARYEQPRQEDLLNRWTWIPFGAGRHRCVGAAFAIMQIKAIFSVLLREYEFEMAQPPESYRNDHSKMVVQLA
QPACVRYRRRTGV
>CYP51B1_Mycobacterium tuberculosis I0002353-6 2722598948
MSAVALPRVSGGHDEHGHLEEFRTDPIGLMQRVRDECGDVGTFQLAGKQVVLLSGSHANEFFFRAGDDDLDQA
KAYPFMTPIFGEGVVFDASPERRKEMLHNAALRGEQMKGHAATIEDQVRRMIADWGEAGEIDLLDFFAELTIY
TSSACLIGKKFRDQLDGRFAKLYHELERGTDPLAYVDPYLPIESFRRRDEARNGLVALVADIMNGRIANPPTD
KSDRDMLDVLIAVKAETGTPRFSADEITGMFISMMFAGHHTSSGTASWTLIELMRHRDAYAAVIDELDELYGD
GRSVSFHALRQIPQLENVLKETLRLHPPLIILMRVAKGEFEVQGHRIHEGDLVAASPAISNRIPEDFPDPHDF
VPARYEQPRQEDLLNRWTWIPFGAGRHRCVGAAFAIMQIKAIFSVLLREYEFEMAQPPESYRNDHSKMVVQLA
QPACVRYRRRTGV
>CYP51B1_Mycobacterium tuberculosis I0002801-4 2720705213
MSAVALPRVSGGHDEHGHLEEFRTDPIGLMQRVRDECGDVGTFQLAGKQVVLLSGSHANEFFFRAGDDDLDQA
KAYPFMTPIFGEGVVFDASPERRKEMLHNAALRGEQMKGHAATIEDQVRRMIADWGEAGEIDLLDFFAELTIY
TSSACLIGKKFRDQLDGRFAKLYHELERGTDPLAYVDPYLPIESFRRRDEARNGLVALVADIMNGRIANPPTD
KSDRDMLDVLIAVKAETGTPRFSADEITGMFISMMFAGHHTSSGTASWTLIELMRHRDAYAAVIDELDELYGD
GRSVSFHALRQIPQLENVLKETLRLHPPLIILMRVAKGEFEVQGHRIHEGDLVAASPAISNRIPEDFPDPHDF
VPARYEQPRQEDLLNRWTWIPFGAGRHRCVGAAFAIMQIKAIFSVLLREYEFEMAQPPESYRNDHSKMVVQLA
QPACVRYRRRTGV
>CYP51B1_Mycobacterium tuberculosis I0004000-1 2720701098
MSAVALPRVSGGHDEHGHLEEFRTDPIGLMQRVRDECGDVGTFQLAGKQVVLLSGSHANEFFFRAGDDDLDQA
KAYPFMTPIFGEGVVFDASPERRKEMLHNAALRGEQMKGHAATIEDQVRRMIADWGEAGEIDLLDFFAELTIY
TSSACLIGKKFRDQLDGRFAKLYHELERGTDPLAYVDPYLPIESFRRRDEARNGLVALVADIMNGRIANPPTD

KSDRDMLDVLIAVKAETGTPRFSADEITGMFISMMFAGHHTSSGTASWTLIELMRHRDAYAAVIDELDELYGD
GRSVSFHALRQIPQLENVLKETLRLHPPLIILMRVAKGEFEVQGHRIHEGDLVAASPAISNRIPEDFPDPHDF
VPARYEQPRQEDLLNRWTWIPFGAGRHRCVGAAFAIMQIKAIFSVLLREYEFEMAQPPESYRNDHSKMVVQLA
QPACVRYRRRTGV
>CYP51B1_Mycobacterium tuberculosis I0004241-1 2721355807
MSAVALPRVSGGHDEHGHLEEFRTDPIGLMQRVRDECGDVGTFQLAGKQVVLLSGSHANEFFFRAGDDDLDQA
KAYPFMTPIFGEGVVFDASPERRKEMLHNAALRGEQMKGHAATIEDQVRRMIADWGEAGEIDLLDFFAELTIY
TSSACLIGKKFRDQLDGRFAKLYHELERGTDPLAYVDPYLPIESFRRRDEARNGLVALVADIMNGRIANPPTD
KSDRDMLDVLIAVKAETGTPRFSADEITGMFISMMFAGHHTSSGTASWTLIELMRHRDAYAAVIDELDELYGD
GRSVSFHALRQIPQLENVLKETLRLHPPLIILMRVAKGEFEVQGHRIHEGDLVAASPAISNRIPEDFPDPHDF
VPARYEQPRQEDLLNRWTWIPFGAGRHRCVGAAFAIMQIKAIFSVLLREYEFEMAQPPESYRNDHSKMVVQLA
QPACVRYRRRTGV
>CYP51B1_Mycobacterium tuberculosis I00200020 2703653349
MSAVALPRVSGGHDEHGHLEEFRTDPIGLMQRVRDECGDVGTFQLAGKQVVLLSGSHANEFFFRAGDDDLDQA
KAYPFMTPIFGEGVVFDASPERRKEMLHNAALRGEQMATPPPSKIKSDG
>CYP51B1_Mycobacterium tuberculosis I00200020 2703653350
MIADWGEAGEIDLLDFFAELTIYTSSACLIGKKFRDQLDGRFAKLYHELERGTDPLAYVDPYLPIESFRRRDE
ARNGLVALVADIMNGRIANPPTDKSDRDMLDVLIAVKAETGTPRFSADEITGMFISMMFAGHHTSSGTASWTL
IELMRHRDAYAAVIDELDELYGDGRSVSFHALRQIPQLENVLKETLRLHPPLIILMRVAKGEFEVQGHRIHEG
DLVAASPAISNRIPEDFPDPHDFVPARYEQPRQEDLLNRWTWIPFGAGRHRCVGAAFAIMQIKAIFSVLLREY
EFEMAQPPESYRNDHSKMVVQLAQPACVRYRRRTGV
>CYP51B1_Mycobacterium tuberculosis I00300092 2703530615
MIADWGEAGEIDLLDFFAELTIYTSSACLIGKKFRDQLDGRFAKLYHELERGTDPLAYVDPYLPIESFRRRDE
ARNGLVALVADIMNGRIANPPTDKSDRDMLDVLIAVKAETGTPRFSADEITGMFISMMFAGHHTSSGTASWTL
IELMRHRDAYAAVIDELDELYGDGRSVSFHALRQIPQLENVLKETLRLHPPLIILMRVAKGEFEVQGHRIHEG
DLVAASPAISNRIPEDFPDPHDFVPARYEQPRQEDLLNRWTWIPFGAGRHRCVGAAFAIMQIKAIFSVLLREY
EFEMAQPPESYRNDHSKMVVQLAQPACVRYRRRTGV
>CYP51B1_Mycobacterium tuberculosis I00300092 2703530616
MSAVALPRVSGGHDEHGHLEEFRTDPIGLMQRVRDECGDVGTFQLAGKQVVLLSGSHANEFFFRAGDDDLDQA
KAYPFMTPIFGEGVVFDASPERRKEMLHNAALRGEQMATPPPSKIKSDG
>CYP51B1_Mycobacterium tuberculosis I00300818 2683861539
MIADWGEAGEIDLLDFFAELTIYTSSACLIGKKFRDQLDGRFAKLYHELERGTDPLAYVDPYLPIESFRRRDE
ARNGLVALVADIMNGRIANPPTDKSDRDMLDVLIAVKAETGTPRFSADEITGMFISMMFAGHHTSSGTASWTL
IELMRHRDAYAAVIDELDELYGDGRSVSFHALRQIPQLENVLKETLRLHPPLIILMRVAKGEFEVQGHRIHEG
DLVAASPAISNRIPEDFPDPHDFVPARYEQPRQEDLLNRWTWIPFGAGRHRCVGAAFAIMQIKAIFSVLLREY
EFEMAQPPESYRNDHSKMVVQLAQPACVRYRRRTGV
>CYP51B1_Mycobacterium tuberculosis I00300818 2683861540
MSAVALPRVSGGHDEHGHLEEFRTDPIGLMQRVRDECGDVGTFQLAGKQVVLLSGSHANEFFFRAGDDDLDQA
KAYPFMTPIFGEGVVFDASPERRKEMLHNAALRGEQMATPPPSKIKSDG
>CYP51B1_Mycobacterium tuberculosis I00601066 2706475396
MSAVALPRVSGGHDEHGHLEEFRTDPIGLMQRVRDECGDVGTFQLAGKQVVLLSGSHANEFFFRAGDDDLDQA
KAYPFMTPIFGEGVVFDASPERRKEMLHNAALRGEQMATPPPSKIKSDG
>CYP51B1_Mycobacterium tuberculosis I00601066 2706475397
MIADWGEAGEIDLLDFFAELTIYTSSACLIGKKFRDQLDGRFAKLYHELERGTDPLAYVDPYLPIESFRRRDE
ARNGLVALVADIMNGRIANPPTDKSDRDMLDVLIAVKAETGTPRFSADEITGMFISMMFAGHHTSSGTASWTL
IELMRHRDAYAAVIDELDELYGDGRSVSFHALRQIPQLENVLKETLRLHPPLIILMRVAKGEFEVQGHRIHEG
DLVAASPAISNRIPEDFPDPHDFVPARYEQPRQEDLLNRWTWIPFGAGRHRCVGAAFAIMQIKAIFSVLLREY
EFEMAQPPESYRNDHSKMVVQLAQPACVRYRRRTGV
>CYP51B1_Mycobacterium tuberculosis I09400880 2671966249
MSAVALPRVSGGHDEHGHLEEFRTDPIGLMQRVRDECGDVGTFQLAGKQVVLLSGSHANEFFFRAGDDDLDQA
KAYPFMTPIFGEGVVFDASPERRKEMLHNAALRGEQMATPPPSKIKSDG
>CYP51B1_Mycobacterium tuberculosis I09400880 2671966250
MIADWGEAGEIDLLDFFAELTIYTSSACLIGKKFRDQLDGRFAKLYHELERGTDPLAYVDPYLPIESFRRRDE
ARNGLVALVADIMNGRIANPPTDKSDRDMLDVLIAVKAETGTPRFSADEITGMFISMMFAGHHTSSGTASWTL
IELMRHRDAYAAVIDELDELYGDGRSVSFHALRQIPQLENVLKETLRLHPPLIILMRVAKGEFEVQGHRIHEG
DLVAASPAISNRIPEDFPDPHDFVPARYEQPRQEDLLNRWTWIPFGAGRHRCVGAAFAIMQIKAIFSVLLREY
EFEMAQPPESYRNDHSKMVVQLAQPACVRYRRRTGV
>CYP51B1_Mycobacterium tuberculosis I09400970 2703638475

MSAVALPRVSGGHDEHGHLEEFRTDPIGLMQRVRDECGDVGTFQLAGKQVVLLSGSHANEFFFRAGDDDLDQA
KAYPFMTPIFGEGVVFDASPERRKEMLHNAALRGEQMATPPPSKIKSDG
>CYP51B1_Mycobacterium tuberculosis I09400970 2703638476
MIADWGEAGEIDLLDFFAELTIYTSSACLIGKKFRDQLDGRFAKLYHELERGTDPLAYVDPYLPIESFRRRDE
ARNGLVALVADIMNGRIANPPTDKSDRDMLDVLIAVKAETGTPRFSADEITGMFISMMFAGHHTSSGTASWTL
IELMRHRDAYAAVIDELDELYGDGRSVSFHALRQIPQLENVLKETLRLHPPLIILMRVAKGEFEVQGHRIHEG
DLVAASPAISNRIPEDFPDPHDFVPARYEQPRQEDLLNRWTWIPFGAGRHRCVGAAFAIMQIKAIFSVLLREY
EFEMAQPPESYRNDHSKMVVQLAQPACVRYRRRTGV
>CYP51B1_Mycobacterium tuberculosis I09601853 2697442238
MIADWGEAGEIDLLDFFAELTIYTSSACLIGKKFRDQLDGRFAKLYHELERGTDPLAYVDPYLPIESFRRRDE
ARNGLVALVADIMNGRIANPPTDKSDRDMLDVLIAVKAETGTPRFSADEITGMFISMMFAGHHTSSGTASWTL
IELMRHRDAYAAVIDELDELYGDGRSVSFHALRQIPQLENVLKETLRLHPPLIILMRVAKGEFEVQGHRIHEG
DLVAASPAISNRIPEDFPDPHDFVPARYEQPRQEDLLNRWTWIPFGAGRHRCVGAAFAIMQIKAIFSVLLREY
EFEMAQPPESYRNDHSKMVVQLAQPACVRYRRRTGV
>CYP51B1_Mycobacterium tuberculosis I09601853 2697442239
MSAVALPRVSGGHDEHGHLEEFRTDPIGLMQRVRDECGDVGTFQLAGKQVVLLSGSHANEFFFRAGDDDLDQA
KAYPFMTPIFGEGVVFDASPERRKEMLHNAALRGEQMATPPPSKIKSDG
>CYP51B1_Mycobacterium tuberculosis I09702330 2703634258
MSAVALPRVSGGHDEHGHLEEFRTDPIGLMQRVRDECGDVGTFQLAGKQVVLLSGSHANEFFFRAGDDDLDQA
KAYPFMTPIFGEGVVFDASPERRKEMLHNAALRGEQMATPPPSKIKSDG
>CYP51B1_Mycobacterium tuberculosis I09702330 2703634259
MIADWGEAGEIDLLDFFAELTIYTSSACLIGKKFRDQLDGRFAKLYHELERGTDPLAYVDPYLPIESFRRRDE
ARNGLVALVADIMNGRIANPPTDKSDRDMLDVLIAVKAETGTPRFSADEITGMFISMMFAGHHTSSGTASWTL
IELMRHRDAYAAVIDELDELYGDGRSVSFHALRQIPQLENVLKETLRLHPPLIILMRVAKGEFEVQGHRIHEG
DLVAASPAISNRIPEDFPDPHDFVPARYEQPRQEDLLNRWTWIPFGAGRHRCVGAAFAIMQIKAIFSVLLREY
EFEMAQPPESYRNDHSKMVVQLAQPACVRYRRRTGV
>CYP51B1_Mycobacterium tuberculosis I09800059 2703527026
MSAVALPRVSGGHDEHGHLEEFRTDPIGLMQRVRDECGDVGTFQLAGKQVVLLSGSHANEFFFRAGDDDLDQA
KAYPFMTPIFGEGVVFDASPERRKEMLHNAALRGEQMATPPPSKIKSDG
>CYP51B1_Mycobacterium tuberculosis I09800059 2703527027
MIADWGEAGEIDLLDFFAELTIYTSSACLIGKKFRDQLDGRFAKLYHELERGTDPLAYVDPYLPIESFRRRDE
ARNGLVALVADIMNGRIANPPTDKSDRDMLDVLIAVKAETGTPRFSADEITGMFISMMFAGHHTSSGTASWTL
IELMRHRDAYAAVIDELDELYGDGRSVSFHALRQIPQLENVLKETLRLHPPLIILMRVAKGEFEVQGHRIHEG
DLVAASPAISNRIPEDFPDPHDFVPARYEQPRQEDLLNRWTWIPFGAGRHRCVGAAFAIMQIKAIFSVLLREY
EFEMAQPPESYRNDHSKMVVQLAQPACVRYRRRTGV
>CYP51B1_Mycobacterium tuberculosis IEXDR1 2628595322
MSAVALPRVSGGHDEHGHLEEFRTDPIGLMQRVRDECGDVGTFQLAGKQVVLLSGSHANEFFFRAGDDDLDQA
KAYPFMTPIFGEGVVFDASPERRKEMLHNAALRGEQMKGHAATIEDQVRRMIADWGEAGEIDLLDFFAELTIY
TSSACLIGKKFRDQLDGRFAKLYHELERGTDPLAYVDPYLPIESFRRRDEARNGLVALVADIMNGRIANPPTD
KSDRDMLDVLIAVKAETGTPRFSADEITGMFISMMFAGHHTSSGTASWTLIELMRHRDAYAAVIDELDELYGD
GRSVSFHALRQIPQLENVLKETLRLHPPLIILMRVAKGEFEVQGHRIHEGDLVAASPAISNRIPEDFPDPHDF
VPARYEQPRQEDLLNRWTWIPFGAGRHRCVGAAFAIMQIKAIFSVLLREYEFEMAQPPESYRNDHSKMVVQLA
QPACVRYRRRTGV
>CYP51B1_Mycobacterium tuberculosis INS_MDR 2621350868
MSAVALPRVSGGHDEHGHLEEFRTDPIGLMQRVRDECGDVGTFQLAGKQVVLLSGSHANEFFFRAGDDDLDQA
KAYPFMTPIFGEGVVFDASPERRKEMLHNAALRGEQMKGHAATIEDQVRRMIADWGEAGEIDLLDFFAELTIY
TSSACLIGKKFRDQLDGRFAKLYHELERGTDPLAYVDPYLPIESFRRRDEARNGLVALVADIMNGRIANPPTD
KSDRDMLDVLIAVKAETGTPRFSADEITGMFISMMFAGHHTSSGTASWTLIELMRHRDAYAAVIDELDELYGD
GRSVSFHALRQIPQLENVLKETLRLHPPLIILMRVAKGEFEVQGHRIHEGDLVAASPAISNRIPEDFPDPHDF
VPARYEQPRQEDLLNRWTWIPFGAGRHRCVGAAFAIMQIKAIFSVLLREYEFEMAQPPESYRNDHSKMVVQLA
QPACVRYRRRTGV
>CYP51B1_Mycobacterium tuberculosis INS_SEN 2621355259
MSAVALPRVSGGHDEHGHLEEFRTDPIGLMQRVRDECGDVGTFQLAGKQVVLLSGSHANEFFFRAGDDDLDQA
KAYPFMTPIFGEGVVFDASPERRKEMLHNAALRGEQMKGHAATIEDQVRRMIADWGEAGEIDLLDFFAELTIY
TSSACLIGKKFRDQLDGRFAKLYHELERGTDPLAYVDPYLPIESFRRRDEARNGLVALVADIMNGRIANPPTD
KSDRDMLDVLIAVKAETGTPRFSADEITGMFISMMFAGHHTSSGTASWTLIELMRHRDAYAAVIDELDELYGD
GRSVSFHALRQIPQLENVLKETLRLHPPLIILMRVAKGEFEVQGHRIHEGDLVAASPAISNRIPEDFPDPHDF

VPARYEQPRQEDLLNRWTWIPFGAGRHRCVGAAFAIMQIKAIFSVLLREYEFEMAQPPESYRNDHSKMVVQLA
QPACVRYRRRTGV
>CYP51B1_Mycobacterium tuberculosis INS_XDR 2573559818
MSAVALPRVSGGHDEHGHLEEFRTDPIGLMQRVRDECGDVGTFQLAGKQVVLLSGSHANEFFFRAGDDDLDQA
KAYPFMTPIFGEGVVFDASPERRKEMLHNAALRGEQMKGHAATIEDQVRRMIADWGEAGEIDLLDFFAELTIY
TSSACLIGKKFRDQLDGRFAKLYHELERGTDPLAYVDPYLPIESFRRRDEARNGLVALVADIMNGRIANPPTD
KSDRDMLDVLIAVKAETGTPRFSADEITGMFISMMFAGHHTSSGTASWTLIELMRHRDAYAAVIDELDELYGD
GRSVSFHALRQIPQLENVLKETLRLHPPLIILMRVAKGEFEVQGHRIHEGDLVAASPAISNRIPEDFPDPHDF
VPARYEQPRQEDLLNRWTWIPFGAGRHRCVGAAFAIMQIKAIFSVLLREYEFEMAQPPESYRNDHSKMVVQLA
QPACVRYRRRTGV
>CYP51B1_Mycobacterium tuberculosis J09400698 2675374182
MSAVALPRVSGGHDEHGHLEEFRTDPIGLMQRVRDECGDVGTFQLAGKQVVLLSGSHANEFFFRAGDDDLDQA
KAYPFMTPIFGEGVVFDASPERRKEMLHNAALRGEQMKGHAATIEDQVRRMIADWGEAGEIDLLDFFAELTIY
TSSACLIGKKFRDQLDGRFAKLYHELERGTDPLAYVDPYLPIESFRRRDEARNGLVALVADIMNGRIANPPTD
KSDRDMLDVLIAVKAETGTPRFSADEITGMFISMMFAGHHTSSGTASWTLIELMRHRDAYAAVIDELDELYGD
GRSVSFHALRQIPQLENVLKETLRLHPPLIILMRVAKGEFEVQGHRIHEGDLVAASPAISNRIPEDFPDPHDF
VPARYEQPRQEDLLNRWTWIPFGAGRHRCVGAAFAIMQIKAIFSVLLREYEFEMAQPPESYRNDHSKMVVQLA
QPACVRYRRRTGV
>CYP51B1_Mycobacterium tuberculosis J09401337 2697651857
MSAVALPRVSGGHDEHGHLEEFRTDPIGLMQRVRDECGDVGTFQLAGKQVVLLSGSHANEFFFRAGDDDLDQA
KAYPFMTPIFGEGVVFDASPERRKEMLHNAALRGEQMKGHAATIEDQVRRMIADWGEAGEIDLLDFFAELTIY
TSSACLIGKKFRDQLDGRFAKLYHELERGTDPLAYVDPYLPIESFRRRDEARNGLVALVADIMNGRIANPPTD
KSDRDMLDVLIAVKAETGTPRFSADEITGMFISMMFAGHHTSSGTASWTLIELMRHRDAYAAVIDELDELYGD
GRSVSFHALRQIPQLENVLKETLRLHPPLIILMRVAKGEFEVQGHRIHEGDLVAASPAISNRIPEDFPDPHDF
VPARYEQPRQEDLLNRWTWIPFGAGRHRCVGAAFAIMQIKAIFSVLLREYEFEMAQPPESYRNDHSKMVVQLA
QPACVRYRRRTGV
>CYP51B1_Mycobacterium tuberculosis J09402203 2673322775
MSAVALPRVSGGHDEHGHLEEFRTDPIGLMQRVRDECGDVGTFQLAGKQVVLLSGSHANEFFFRAGDDDLDQA
KAYPFMTPIFGEGVVFDASPERRKEMLHNAALRGEQMKGHAATIEDQVRRMIADWGEAGEIDLLDFFAELTIY
TSSACLIGKKFRDQLDGRFAKLYHELERGTDPLAYVDPYLPIESFRRRDEARNGLVALVADIMNGRIANPPTD
KSDRDMLDVLIAVKAETGTPRFSADEITGMFISMMFAGHHTSSGTASWTLIELMRHRDAYAAVIDELDELYGD
GRSVSFHALRQIPQLENVLKETLRLHPPLIILMRVAKGEFEVQGHRIHEGDLVAASPAISNRIPEDFPDPHDF
VPARYEQPRQEDLLNRWTWIPFGAGRHRCVGAAFAIMQIKAIFSVLLREYEFEMAQPPESYRNDHSKMVVQLA
QPACVRYRRRTGV
>CYP51B1_Mycobacterium tuberculosis J09500385 2703483023
MSAVALPRVSGGHDEHGHLEEFRTDPIGLMQRVRDECGDVGTFQLAGKQVVLLSGSHANEFFFRAGDDDLDQA
KAYPFMTPIFGEGVVFDASPERRKEMLHNAALRGEQMKGHAATIEDQVRRMIADWGEAGEIDLLDFFAELTIY
TSSACLIGKKFRDQLDGRFAKLYHELERGTDPLAYVDPYLPIESFRRRDEARNGLVALVADIMNGRIANPPTD
KSDRDMLDVLIAVKAETGTPRFSADEITGMFISMMFAGHHTSSGTASWTLIELMRHRDAYAAVIDELDELYGD
GRSVSFHALRQIPQLENVLKETLRLHPPLIILMRVAKGEFEVQGHRIHEGDLVAASPAISNRIPEDFPDPHDF
VPARYEQPRQEDLLNRWTWIPFGAGRHRCVGAAFAIMQIKAIFSVLLREYEFEMAQPPESYRNDHSKMVVQLA
QPACVRYRRRTGV
>CYP51B1_Mycobacterium tuberculosis J09500392 2703489052
MSAVALPRVSGGHDEHGHLEEFRTDPIGLMQRVRDECGDVGTFQLAGKQVVLLSGSHANEFFFRAGDDDLDQA
KAYPFMTPIFGEGVVFDASPERRKEMLHNAALRGEQMKGHAATIEDQVRRMIADWGEAGEIDLLDFFAELTIY
TSSACLIGKKFRDQLDGRFAKLYHELERGTDPLAYVDPYLPIESFRRRDEARNGLVALVADIMNGRIANPPTD
KSDRDMLDVLIAVKAETGTPRFSADEITGMFISMMFAGHHTSSGTASWTLIELMRHRDAYAAVIDELDELYGD
GRSVSFHALRQIPQLENVLKETLRLHPPLIILMRVAKGEFEVQGHRIHEGDLVAASPAISNRIPEDFPDPHDF
VPARYEQPRQEDLLNRWTWIPFGAGRHRCVGAAFAIMQIKAIFSVLLREYEFEMAQPPESYRNDHSKMVVQLA
QPACVRYRRRTGV
>CYP51B1_Mycobacterium tuberculosis J09602061 2703490698
MIADWGEAGEIDLLDFFAELTIYTSSACLIGKKFRDQLDGRFAKLYHELERGTDPLAYVDPYLPIESFRRRDE
ARNGLVALVADIMNGRIANPPTDKSDRDMLDVLIAVKAETGTPRFSADEITGMFISMMFAGHHTSSGTASWTL
IELMRHRDAYAAVIDELDELYGDGRSVSFHALRQIPQLENVLKETLRLHPPLIILMRVAKGEFEVQGHRIHEG
DLVAASPAISNRIPEDFPDPHDFVPARYEQPRQEDLLNRWTWIPFGAGRHRCVGAAFAIMQIKAIFSVLLREY
EFEMAQPPESYRNDHSKMVVQLAQPACVRYRRRTGV
>CYP51B1_Mycobacterium tuberculosis J09602061 2703490699

MSAVALPRVSGGHDEHGHLEEFRTDPIGLMQRVRDECGDVGTFQLAGKQVVLLSGSHANEFFFRAGDDDLDQA
KAYPFMTPIFGEGVVFDASPERRKEMLHNAALRGEQMATPPPSKIKSDG
>CYP51B1_Mycobacterium tuberculosis J09700140 2703474793
MSAVALPRVSGGHDEHGHLEEFRTDPIGLMQRVRDECGDVGTFQLAGKQVVLLSGSHANEFFFRAGDDDLDQA
KAYPFMTPIFGEGVVFDASPERRKEMLHNAALRGEQMATPPPSKIKSDG
>CYP51B1_Mycobacterium tuberculosis J09700140 2703474794
MIADWGEAGEIDLLDFFAELTIYTSSACLIGKKFRDQLDGRFAKLYHELERGTDPLAYVDPYLPIESFRRRDE
ARNGLVALVADIMNGRIANPPTDKSDRDMLDVLIAVKAETGTPRFSADEITGMFISMMFAGHHTSSGTASWTL
IELMRHRDAYAAVIDELDELYGDGRSVSFHALRQIPQLENVLKETLRLHPPLIILMRVAKGEFEVQGHRIHEG
DLVAASPAISNRIPEDFPDPHDFVPARYEQPRQEDLLNRWTWIPFGAGRHRCVGAAFAIMQIKAIFSVLLREY
EFEMAQPPESYRNDHSKMVVQLAQPACVRYRRRTGV
>CYP51B1_Mycobacterium tuberculosis J09700837 2706609289
MSAVALPRVSGGHDEHGHLEEFRTDPIGLMQRVRDECGDVGTFQLAGKQVVLLSGSHANEFFFRAGDDDLDQA
KAYPFMTPIFGEGVVFDASPERRKEMLHNAALRGEQMKGHAATIEDQVRRMIADWGEAGEIDLLDFFAELTIY
TSSACLIGKKFRDQLDGRFAKLYHELERGTDPLAYVDPYLPIESFRRRDEARNGLVALVADIMNGRIANPPTD
KSDRDMLDVLIAVKAETGTPRFSADEITGMFISMMFAGHHTSSGTASWTLIELMRHRDAYAAVIDELDELYGD
GRSVSFHALRQIPQLENVLKETLRLHPPLIILMRVAKGEFEVQGHRIHEGDLVAASPAISNRIPEDFPDPHDF
VPARYEQPRQEDLLNRWTWIPFGAGRHRCVGAAFAIMQIKAIFSVLLREYEFEMAQPPESYRNDHSKMVVQLA
QPACVRYRRRTGV
>CYP51B1_Mycobacterium tuberculosis J09701231 2703496079
MSAVALPRVSGGHDEHGHLEEFRTDPIGLMQRVRDECGDVGTFQLAGKQVVLLSGSHANEFFFRAGDDDLDQA
KAYPFMTPIFGEGVVFDASPERRKEMLHNAALRGEQMKGHAATIEDQVRRMIADWGEAGEIDLLDFFAELTIY
TSSACLIGKKFRDQLDGRFAKLYHELERGTDPLAYVDPYLPIESFRRRDEARNGLVALVADIMNGRIANPPTD
KSDRDMLDVLIAVKAETGTPRFSADEITGMFISMMFAGHHTSSGTASWTLIELMRHRDAYAAVIDELDELYGD
GRSVSFHALRQIPQLENVLKETLRLHPPLIILMRVAKGEFEVQGHRIHEGDLVAASPAISNRIPEDFPDPHDF
VPARYEQPRQEDLLNRWTWIPFGAGRHRCVGAAFAIMQIKAIFSVLLREYEFEMAQPPESYRNDHSKMVVQLA
QPACVRYRRRTGV
>CYP51B1_Mycobacterium tuberculosis J09701362 2706574477
MSAVALPRVSGGHDEHGHLEEFRTDPIGLMQRVRDECGDVGTFQLAGKQVVLLSGSHANEFFFRAGDDDLDQA
KAYPFMTPIFGEGVVFDASPERRKEMLHNAALRGEQMKGHAATIEDQVRRMIADWGEAGEIDLLDFFAELTIY
TSSACLIGKKFRDQLDGRFAKLYHELERGTDPLAYVDPYLPIESFRRRDEARNGLVALVADIMNGRIANPPTD
KSDRDMLDVLIAVKAETGTPRFSADEITGMFISMMFAGHHTSSGTASWTLIELMRHRDAYAAVIDELDELYGD
GRSVSFHALRQIPQLENVLKETLRLHPPLIILMRVAKGEFEVQGHRIHEGDLVAASPAISNRIPEDFPDPHDF
VPARYEQPRQEDLLNRWTWIPFGAGRHRCVGAAFAIMQIKAIFSVLLREYEFEMAQPPESYRNDHSKMVVQLA
QPACVRYRRRTGV
>CYP51B1_Mycobacterium tuberculosis J09800554 2693206144
MSAVALPRVSGGHDEHGHLEEFRTDPIGLMQRVRDECGDVGTFQLAGKQVVLLSGSHANEFFFRAGDDDLDQA
KAYPFMTPIFGEGVVFDASPERRKEMLHNAALRGEQMKGHAATIEDQVRRMIADWGEAGEIDLLDFFAELTIY
TSSACLIGKKFRDQLDGRFAKLYHELERGTDPLAYVDPYLPIESFRRRDEARNGLVALVADIMNGRIANPPTD
KSDRDMLDVLIAVKAETGTPRFSADEITGMFISMMFAGHHTSSGTASWTLIELMRHRDAYAAVIDELDELYGD
GRSVSFHALRQIPQLENVLKETLRLHPPLIILMRVAKGEFEVQGHRIHEGDLVAASPAISNRIPEDFPDPHDF
VPARYEQPRQEDLLNRWTWIPFGAGRHRCVGAAFAIMQIKAIFSVLLREYEFEMAQPPESYRNDHSKMVVQLA
QPACVRYRRRTGV
>CYP51B1_Mycobacterium tuberculosis J09800628 2673569036
MSAVALPRVSGGHDEHGHLEEFRTDPIGLMQRVRDECGDVGTFQLAGKQVVLLSGSHANEFFFRAGDDDLDQA
KAYPFMTPIFGEGVVFDASPERRKEMLHNAALRGEQMKGHAATIEDQVRRMIADWGEAGEIDLLDFFAELTIY
TSSACLIGKKFRDQLDGRFAKLYHELERGTDPLAYVDPYLPIESFRRRDEARNGLVALVADIMNGRIANPPTD
KSDRDMLDVLIAVKAETGTPRFSADEITGMFISMMFAGHHTSSGTASWTLIELMRHRDAYAAVIDELDELYGD
GRSVSFHALRQIPQLENVLKETLRLHPPLIILMRVAKGEFEVQGHRIHEGDLVAASPAISNRIPEDFPDPHDF
VPARYEQPRQEDLLNRWTWIPFGAGRHRCVGAAFAIMQIKAIFSVLLREYEFEMAQPPESYRNDHSKMVVQLA
QPACVRYRRRTGV
>CYP51B1_Mycobacterium tuberculosis J09800745 2703500492
MSAVALPRVSGGHDEHGHLEEFRTDPIGLMQRVRDECGDVGTFQLAGKQVVLLSGSHANEFFFRAGDDDLDQA
KAYPFMTPIFGEGVVFDASPERRKEMLHNAALRGEQMATPPPSKIKSDG
>CYP51B1_Mycobacterium tuberculosis J09800745 2703500493
MIADWGEAGEIDLLDFFAELTIYTSSACLIGKKFRDQLDGRFAKLYHELERGTDPLAYVDPYLPIESFRRRDE
ARNGLVALVADIMNGRIANPPTDKSDRDMLDVLIAVKAETGTPRFSADEITGMFISMMFAGHHTSSGTASWTL
IELMRHRDAYAAVIDELDELYGDGRSVSFHALRQIPQLENVLKETLRLHPPLIILMRVAKGEFEVQGHRIHEG

DLVAASPAISNRIPEDFPDPHDFVPARYEQPRQEDLLNRWTWIPFGAGRHRCVGAAFAIMQIKAIFSVLLREY
EFEMAQPPESYRNDHSKMVVQLAQPACVRYRRRTGV
>CYP51B1_Mycobacterium tuberculosis J09900137 2703504240
MSAVALPRVSGGHDEHGHLEEFRTDPIGLMQRVRDECGDVGTFQLAGKQVVLLSGSHANEFFFRAGDDDLDQA
KAYPFMTPIFGEGVVFDASPERRKEMLHNAALRGEQMKGHAATIEDQVRRMIADWGEAGEIDLLDFFAELTIY
TSSACLIGKKFRDQLDGRFAKLYHELERGTDPLAYVDPYLPIESFRRRDEARNGLVALVADIMNGRIANPPTD
KSDRDMLDVLIAVKAETGTPRFSADEITGMFISMMFAGHHTSSGTASWTLIELMRHRDAYAAVIDELDELYGD
GRSVSFHALRQIPQLENVLKETLRLHPPLIILMRVAKGEFEVQGHRIHEGDLVAASPAISNRIPEDFPDPHDF
VPARYEQPRQEDLLNRWTWIPFGAGRHRCVGAAFAIMQIKAIFSVLLREYEFEMAQPPESYRNDHSKMVVQLA
QPACVRYRRRTGV
>CYP51B1_Mycobacterium tuberculosis J09900221 2683848946
MSAVALPRVSGGHDEHGHLEEFRTDPIGLMQRVRDECGDVGTFQLAGKQVVLLSGSHANEFFFRAGDDDLDQA
KAYPFMTPIFGEGVVFDASPERRKEMLHNAALRGEQMKGHAATIEDQVRRMIADWGEAGEIDLLDFFAELTIY
TSSACLIGKKFRDQLDGRFAKLYHELERGTDPLAYVDPYLPIESFRRRDEARNGLVALVADIMNGRIANPPTD
KSDRDMLDVLIAVKAETGTPRFSADEITGMFISMMFAGHHTSSGTASWTLIELMRHRDAYAAVIDELDELYGD
GRSVSFHALRQIPQLENVLKETLRLHPPLIILMRVAKGEFEVQGHRIHEGDLVAASPAISNRIPEDFPDPHDF
VPARYEQPRQEDLLNRWTWIPFGAGRHRCVGAAFAIMQIKAIFSVLLREYEFEMAQPPESYRNDHSKMVVQLA
QPACVRYRRRTGV
>CYP51B1_Mycobacterium tuberculosis J09900583 2696310273
MSAVALPRVSGGHDEHGHLEEFRTDPIGLMQRVRDECGDVGTFQLAGKQVVLLSGSHANEFFFRAGDDDLDQA
KAYPFMTPIFGEGVVFDASPERRKEMLHNAALRGEQMKGHAATIEDQVRRMIADWGEAGEIDLLDFFAELTIY
TSSACLIGKKFRDQLDGRFAKLYHELERGTDPLAYVDPYLPIESFRRRDEARNGLVALVADIMNGRIANPPTD
KSDRDMLDVLIAVKAETGTPRFSADEITGMFISMMFAGHHTSSGTASWTLIELMRHRDAYAAVIDELDELYGD
GRSVSFHALRQIPQLENVLKETLRLHPPLIILMRVAKGEFEVQGHRIHEGDLVAASPAISNRIPEDFPDPHDF
VPARYEQPRQEDLLNRWTWIPFGAGRHRCVGAAFAIMQIKAIFSVLLREYEFEMAQPPESYRNDHSKMVVQLA
QPACVRYRRRTGV
>CYP51B1_Mycobacterium tuberculosis J09902073 2675870902
MSAVALPRVSGGHDEHGHLEEFRTDPIGLMQRVRDECGDVGTFQLAGKQVVLLSGSHANEFFFRAGDDDLDQA
KAYPFMTPIFGEGVVFDASPERRKEMLHNAALRGEQMKGHAATIEDQVRRMIADWGEAGEIDLLDFFAELTIY
TSSACLIGKKFRDQLDGRFAKLYHELERGTDPLAYVDPYLPIESFRRRDEARNGLVALVADIMNGRIANPPTD
KSDRDMLDVLIAVKAETGTPRFSADEITGMFISMMFAGHHTSSGTASWTLIELMRHRDAYAAVIDELDELYGD
GRSVSFHALRQIPQLENVLKETLRLHPPLIILMRVAKGEFEVQGHRIHEGDLVAASPAISNRIPEDFPDPHDF
VPARYEQPRQEDLLNRWTWIPFGAGRHRCVGAAFAIMQIKAIFSVLLREYEFEMAQPPESYRNDHSKMVVQLA
QPACVRYRRRTGV
>CYP51B1_Mycobacterium tuberculosis K00101539 2706622218
MSAVALPRVSGGHDEHGHLEEFRTDPIGLMQRVRDECGDVGTFQLAGKQVVLLSGSHANEFFFRAGDDDLDQA
KAYPFMTPIFGEGVVFDASPERRKEMLHNAALRGEQMKGHAATIEDQVRRMIADWGEAGEIDLLDFFAELTIY
TSSACLIGKKFRDQLDGRFAKLYHELERGTDPLAYVDPYLPIESFRRRDEARNGLVALVADIMNGRIANPPTD
KSDRDMLDVLIAVKAETGTPRFSADEITGMFISMMFAGHHTSSGTASWTLIELMRHRDAYAAVIDELDELYGD
GRSVSFHALRQIPQLENVLKETLRLHPPLIILMRVAKGEFEVQGHRIHEGDLVAASPAISNRIPEDFPDPHDF
VPARYEQPRQEDLLNRWTWIPFGAGRHRCVGAAFAIMQIKAIFSVLLREYEFEMAQPPESYRNDHSKMVVQLA
QPACVRYRRRTGV
>CYP51B1_Mycobacterium tuberculosis K00200539 2706591583
MSAVALPRVSGGHDEHGHLEEFRTDPIGLMQRVRDECGDVGTFQLAGKQVVLLSGSHANEFFFRAGDDDLDQA
KAYPFMTPIFGEGVVFDASPERRKEMLHNAALRGEQMKGHAATIEDQVRRMIADWGEAGEIDLLDFFAELTIY
TSSACLIGKKFRDQLDGRFAKLYHELERGTDPLAYVDPYLPIESFRRRDEARNGLVALVADIMNGRIANPPTD
KSDRDMLDVLIAVKAETGTPRFSADEITGMFISMMFAGHHTSSGTASWTLIELMRHRDAYAAVIDELDELYGD
GRSVSFHALRQIPQLENVLKETLRLHPPLIILMRVAKGEFEVQGHRIHEGDLVAASPAISNRIPEDFPDPHDF
VPARYEQPRQEDLLNRWTWIPFGAGRHRCVGAAFAIMQIKAIFSVLLREYEFEMAQPPESYRNDHSKMVVQLA
QPACVRYRRRTGV
>CYP51B1_Mycobacterium tuberculosis K00201415 2703670637
MSAVALPRVSGGHDEHGHLEEFRTDPIGLMQRVRDECGDVGTFQLAGKQVVLLSGSHANEFFFRAGDDDLDQA
KAYPFMTPIFGEGVVFDASPERRKEMLHNAALRGEQMKGHAATIEDQVRRMIADWGEAGEIDLLDFFAELTIY
TSSACLIGKKFRDQLDGRFAKLYHELERGTDPLAYVDPYLPIESFRRRDEARNGLVALVADIMNGRIANPPTD
KSDRDMLDVLIAVKAETGTPRFSADEITGMFISMMFAGHHTSSGTASWTLIELMRHRDAYAAVIDELDELYGD
GRSVSFHALRQIPQLENVLKETLRLHPPLIILMRVAKGEFEVQGHRIHEGDLVAASPAISNRIPEDFPDPHDF
VPARYEQPRQEDLLNRWTWIPFGAGRHRCVGAAFAIMQIKAIFSVLLREYEFEMAQPPESYRNDHSKMVVQLA
QPACVRYRRRTGV

>CYP51B1_Mycobacterium tuberculosis K00500041 2683878628
MSAVALPRVSGGHDEHGHLEEFRTDPIGLMQRVRDECGDVGTFQLAGKQVVLLSGSHANEFFFRAGDDDLDQA
KAYPFMTPIFGEGVVFDASPERRKEMLHNAALRGEQMKGHAATIEDQVRRMIADWGEAGEIDLLDFFAELTIY
TSSACLIGKKFRDQLDGRFAKLYHELERGTDPLAYVDPYLPIESFRRRDEARNGLVALVADIMNGRIANPPTD
KSDRDMLDVLIAVKAETGTPRFSADEITGMFISMMFAGHHTSSGTASWTLIELMRHRDAYAAVIDELDELYGD
GRSVSFHALRQIPQLENVLKETLRLHPPLIILMRVAKGEFEVQGHRIHEGDLVAASPAISNRIPEDFPDPHDF
VPARYEQPRQEDLLNRWTWIPFGAGRHRCVGAAFAIMQIKAIFSVLLREYEFEMAQPPESYRNDHSKMVVQLA
QPACVRYRRRTGV
>CYP51B1_Mycobacterium tuberculosis K00500133 2696814387
MSAVALPRVSGGHDEHGHLEEFRTDPIGLMQRVRDECGDVGTFQLAGKQVVLLSGSHANEFFFRAGDDDLDQA
KAYPFMTPIFGEGVVFDASPERRKEMLHNAALRGEQMKGHAATIEDQVRRMIADWGEAGEIDLLDFFAELTIY
TSSACLIGKKFRDQLDGRFAKLYHELERGTDPLAYVDPYLPIESFRRRDEARNGLVALVADIMNGRIANPPTD
KSDRDMLDVLIAVKAETGTPRFSADEITGMFISMMFAGHHTSSGTASWTLIELMRHRDAYAAVIDELDELYGD
GRSVSFHALRQIPQLENVLKETLRLHPPLIILMRVAKGEFEVQGHRIHEGDLVAASPAISNRIPEDFPDPHDF
VPARYEQPRQEDLLNRWTWIPFGAGRHRCVGAAFAIMQIKAIFSVLLREYEFEMAQPPESYRNDHSKMVVQLA
QPACVRYRRRTGV
>CYP51B1_Mycobacterium tuberculosis K00700186 2693223339
MSAVALPRVSGGHDEHGHLEEFRTDPIGLMQRVRDECGDVGTFQLAGKQVVLLSGSHANEFFFRAGDDDLDQA
KAYPFMTPIFGEGVVFDASPERRKEMLHNAALRGEQMKGHAATIEDQVRRMIADWGEAGEIDLLDFFAELTIY
TSSACLIGKKFRDQLDGRFAKLYHELERGTDPLAYVDPYLPIESFRRRDEARNGLVALVADIMNGRIANPPTD
KSDRDMLDVLIAVKAETGTPRFSADEITGMFISMMFAGHHTSSGTASWTLIELMRHRDAYAAVIDELDELYGD
GRSVSFHALRQIPQLENVLKETLRLHPPLIILMRVAKGEFEVQGHRIHEGDLVAASPAISNRIPEDFPDPHDF
VPARYEQPRQEDLLNRWTWIPFGAGRHRCVGAAFAIMQIKAIFSVLLREYEFEMAQPPESYRNDHSKMVVQLA
QPACVRYRRRTGV
>CYP51B1_Mycobacterium tuberculosis K09600911 2699416371
MSAVALPRVSGGHDEHGHLEEFRTDPIGLMQRVRDECGDVGTFQLAGKQVVLLSGSHANEFFFRAGDDDLDQA
KAYPFMTPIFGEGVVFDASPERRKEMLHNAALRGEQMKGHAATIEDQVRRMIADWGEAGEIDLLDFFAELTIY
TSSACLIGKKFRDQLDGRFAKLYHELERGTDPLAYVDPYLPIESFRRRDEARNGLVALVADIMNGRIANPPTD
KSDRDMLDVLIAVKAETGTPRFSADEITGMFISMMFAGHHTSSGTASWTLIELMRHRDAYAAVIDELDELYGD
GRSVSFHALRQIPQLENVLKETLRLHPPLIILMRVAKGEFEVQGHRIHEGDLVAASPAISNRIPEDFPDPHDF
VPARYEQPRQEDLLNRWTWIPFGAGRHRCVGAAFAIMQIKAIFSVLLREYEFEMAQPPESYRNDHSKMVVQLA
QPACVRYRRRTGV
>CYP51B1_Mycobacterium tuberculosis K09700503 2703628526
MSAVALPRVSGGHDEHGHLEEFRTDPIGLMQRVRDECGDVGTFQLAGKQVVLLSGSHANEFFFRAGDDDLDQA
KAYPFMTPIFGEGVVFDASPERRKEMLHNAALRGEQMKGHAATIEDQVRRMIADWGEAGEIDLLDFFAELTIY
TSSACLIGKKFRDQLDGRFAKLYHELERGTDPLAYVDPYLPIESFRRRDEARNGLVALVADIMNGRIANPPTD
KSDRDMLDVLIAVKAETGTPRFSADEITGMFISMMFAGHHTSSGTASWTLIELMRHRDAYAAVIDELDELYGD
GRSVSFHALRQIPQLENVLKETLRLHPPLIILMRVAKGEFEVQGHRIHEGDLVAASPAISNRIPEDFPDPHDF
VPARYEQPRQEDLLNRWTWIPFGAGRHRCVGAAFAIMQIKAIFSVLLREYEFEMAQPPESYRNDHSKMVVQLA
QPACVRYRRRTGV
>CYP51B1_Mycobacterium tuberculosis K09900646 2703648173
MSAVALPRVSGGHDEHGHLEEFRTDPIGLMQRVRDECGDVGTFQLAGKQVVLLSGSHANEFFFRAGDDDLDQA
KAYPFMTPIFGEGVVFDASPERRKEMLHNAALRGEQMKGHAATIEDQVRRMIADWGEAGEIDLLDFFAELTIY
TSSACLIGKKFRDQLDGRFAKLYHELERGTDPLAYVDPYLPIESFRRRDEARNGLVALVADIMNGRIANPPTD
KSDRDMLDVLIAVKAETGTPRFSADEITGMFISMMFAGHHTSSGTASWTLIELMRHRDAYAAVIDELDELYGD
GRSVSFHALRQIPQLENVLKETLRLHPPLIILMRVAKGEFEVQGHRIHEGDLVAASPAISNRIPEDFPDPHDF
VPARYEQPRQEDLLNRWTWIPFGAGRHRCVGAAFAIMQIKAIFSVLLREYEFEMAQPPESYRNDHSKMVVQLA
QPACVRYRRRTGV
>CYP51B1_Mycobacterium tuberculosis K09901478 2675142360
MSAVALPRVSGGHDEHGHLEEFRTDPIGLMQRVRDECGDVGTFQLAGKQVVLLSGSHANEFFFRAGDDDLDQA
KAYPFMTPIFGEGVVFDASPERRKEMLHNAALRGEQMKGHAATIEDQVRRMIADWGEAGEIDLLDFFAELTIY
TSSACLIGKKFRDQLDGRFAKLYHELERGTDPLAYVDPYLPIESFRRRDEARNGLVALVADIMNGRIANPPTD
KSDRDMLDVLIAVKAETGTPRFSADEITGMFISMMFAGHHTSSGTASWTLIELMRHRDAYAAVIDELDELYGD
GRSVSFHALRQIPQLENVLKETLRLHPPLIILMRVAKGEFEVQGHRIHEGDLVAASPAISNRIPEDFPDPHDF
VPARYEQPRQEDLLNRWTWIPFGAGRHRCVGAAFAIMQIKAIFSVLLREYEFEMAQPPESYRNDHSKMVVQLA
QPACVRYRRRTGV
>CYP51B1_Mycobacterium tuberculosis K09901972 2703664189

MSAVALPRVSGGHDEHGHLEEFRTDPIGLMQRVRDECGDVGTFQLAGKQVVLLSGSHANEFFFRAGDDDLDQA
KAYPFMTPIFGEGVVFDASPERRKEMLHNAALRGEQMKGHAATIEDQVRRMIADWGEAGEIDLLDFFAELTIY
TSSACLIGKKFRDQLDGRFAKLYHELERGTDPLAYVDPYLPIESFRRRDEARNGLVALVADIMNGRIANPPTD
KSDRDMLDVLIAVKAETGTPRFSADEITGMFISMMFAGHHTSSGTASWTLIELMRHRDAYAAVIDELDELYGD
GRSVSFHALRQIPQLENVLKETLRLHPPLIILMRVAKGEFEVQGHRIHEGDLVAASPAISNRIPEDFPDPHDF
VPARYEQPRQEDLLNRWTWIPFGAGRHRCVGAAFAIMQIKAIFSVLLREYEFEMAQPPESYRNDHSKMVVQLA
QPACVRYRRRTGV
>CYP51B1_Mycobacterium tuberculosis KI_19771 2716379718
MSAVALPRVSGGHDEHGHLEEFRTDPIGLMQRVRDECGDVGTFQLAGKQVVLLSGSHANEFFFRAGDDDLDQA
KAYPFMTPIFGEGVVFDASPERRKEMLHNAALRGEQMKGHAATIEDQVRRMIADWGEAGEIDLLDFFAELTIY
TSSACLIGKKFRDQLDGRFAKLYHELERGTDPLAYVDPYLPIESFRRRDEARNGLVALVADIMNGRIANPPTD
KSDRDMLDVLIAVKAETGTPRFSADEITGMFISMMFAGHHTSSGTASWTLIELMRHRDAYAAVIDELDELYGD
GRSVSFHALRQIPQLENVLKETLRLHPPLIILMRVAKGEFEVQGHRIHEGDLVAASPAISNRIPEDFPDPHDF
VPARYEQPRQEDLLNRWTWIPFGAGRHRCVGAAFAIMQIKAIFSVLLREYEFEMAQPPESYRNDHSKMVVQLA
QPACVRYRRRTGV
>CYP51B1_Mycobacterium tuberculosis KT-0001 2578082013
MSAVALPRVSGGHDEHGHLEEFRTDPIGLMQRVRDECGDVGTFQLAGKQVVLLSGSHANEFFFRAGDDDLDQA
KAYPFMTPIFGEGVVFDASPERRKEMLHNAALRGEQMKGHAATIEDQVRRMIADWGEAGEIDLLDFFAELTIY
TSSACLIGKKFRDQLDGRFAKLYHELERGTDPLAYVDPYLPIESFRRRDEARNGLVALVADIMNGRIANPPTD
KSDRDMLDVLIAVKAETGTPRFSADEITGMFISMMFAGHHTSSGTASWTLIELMRHRDAYAAVIDELDELYGD
GRSVSFHALRQIPQLENVLKETLRLHPPLIILMRVAKGEFEVQGHRIHEGDLVAASPAISNRIPEDFPDPHDF
VPARYEQPRQEDLLNRWTWIPFGAGRHRCVGAAFAIMQIKAIFSVLLREYEFEMAQPPESYRNDHSKMVVQLA
QPACVRYRRRTGV
>CYP51B1_Mycobacterium tuberculosis KT-0003 2576202280
MSAVALPRVSGGHDEHGHLEEFRTDPIGLMQRVRDECGDVGTFQLAGKQVVLLSGSHANEFFFRAGDDDLDQA
KAYPFMTPIFGEGVVFDASPERRKEMLHNAALRGEQMKGHAATIEDQVRRMIADWGEAGEIDLLDFFAELTIY
TSSACLIGKKFRDQLDGRFAKLYHELERGTDPLAYVDPYLPIESFRRRDEARNGLVALVADIMNGRIANPPTD
KSDRDMLDVLIAVKAETGTPRFSADEITGMFISMMFAGHHTSSGTASWTLIELMRHRDAYAAVIDELDELYGD
GRSVSFHALRQIPQLENVLKETLRLHPPLIILMRVAKGEFEVQGHRIHEGDLVAASPAISNRIPEDFPDPHDF
VPARYEQPRQEDLLNRWTWIPFGAGRHRCVGAAFAIMQIKAIFSVLLREYEFEMAQPPESYRNDHSKMVVQLA
QPACVRYRRRTGV
>CYP51B1_Mycobacterium tuberculosis KT-0004 2575258136
MSAVALPRVSGGHDEHGHLEEFRTDPIGLMQRVRDECGDVGTFQLAGKQVVLLSGSHANEFFFRAGDDDLDQA
KAYPFMTPIFGEGVVFDASPERRKEMLHNAALRGEQMKGHAATIEDQVRRMIADWGEAGEIDLLDFFAELTIY
TSSACLIGKKFRDQLDGRFAKLYHELERGTDPLAYVDPYLPIESFRRRDEARNGLVALVADIMNGRIANPPTD
KSDRDMLDVLIAVKAETGTPRFSADEITGMFISMMFAGHHTSSGTASWTLIELMRHRDAYAAVIDELDELYGD
GRSVSFHALRQIPQLENVLKETLRLHPPLIILMRVAKGEFEVQGHRIHEGDLVAASPAISNRIPEDFPDPHDF
VPARYEQPRQEDLLNRWTWIPFGAGRHRCVGAAFAIMQIKAIFSVLLREYEFEMAQPPESYRNDHSKMVVQLA
QPACVRYRRRTGV
>CYP51B1_Mycobacterium tuberculosis KT-0005 2653108360
MSAVALPRVSGGHDEHGHLEEFRTDPIGLMQRVRDECGDVGTFQLAGKQVVLLSGSHANEFFFRAGDDDLDQA
KAYPFMTPIFGEGVVFDASPERRKEMLHNAALRGEQMKGHAATIEDQVRRMIADWGEAGEIDLLDFFAELTIY
TSSACLIGKKFRDQLDGRFAKLYHELERGTDPLAYVDPYLPIESFRRRDEARNGLVALVADIMNGRIANPPTD
KSDRDMLDVLIAVKAETGTPRFSADEITGMFISMMFAGHHTSSGTASWTLIELMRHRDAYAAVIDELDELYGD
GRSVSFHALRQIPQLENVLKETLRLHPPLIILMRVAKGEFEVQGHRIHEGDLVAASPAISNRIPEDFPDPHDF
VPARYEQPRQEDLLNRWTWIPFGAGRHRCVGAAFAIMQIKAIFSVLLREYEFEMAQPPESYRNDHSKMVVQLA
QPACVRYRRRTGV
>CYP51B1_Mycobacterium tuberculosis KT-0010 2604609148
MSAVALPRVSGGHDEHGHLEEFRTDPIGLMQRVRDECGDVGTFQLAGKQVVLLSGSHANEFFFRAGDDDLDQA
KAYPFMTPIFGEGVVFDASPERRKEMLHNAALRGEQMKGHAATIEDQVRRMIADWGEAGEIDLLDFFAELTIY
TSSACLIGKKFRDQLDGRFAKLYHELERGTDPLAYVDPYLPIESFRRRDEARNGLVALVADIMNGRIANPPTD
KSDRDMLDVLIAVKAETGTPRFSADEITGMFISMMFAGHHTSSGTASWTLIELMRHRDAYAAVIDELDELYGD
GRSVSFHALRQIPQLENVLKETLRLHPPLIILMRVAKGEFEVQGHRIHEGDLVAASPAISNRIPEDFPDPHDF
VPARYEQPRQEDLLNRWTWIPFGAGRHRCVGAAFAIMQIKAIFSVLLREYEFEMAQPPESYRNDHSKMVVQLA
QPACVRYRRRTGV
>CYP51B1_Mycobacterium tuberculosis KT-0011 2584910169
MSAVALPRVSGGHDEHGHLEEFRTDPIGLMQRVRDECGDVGTFQLAGKQVVLLSGSHANEFFFRAGDDDLDQA
KAYPFMTPIFGEGVVFDASPERRKEMLHNAALRGEQMKGHAATIEDQVRRMIADWGEAGEIDLLDFFAELTIY

TSSACLIGKKFRDQLDGRFAKLYHELERGTDPLAYVDPYLPIESFRRRDEARNGLVALVADIMNGRIANPPTD
KSDRDMLDVLIAVKAETGTPRFSADEITGMFISMMFAGHHTSSGTASWTLIELMRHRDAYAAVIDELDELYGD
GRSVSFHALRQIPQLENVLKETLRLHPPLIILMRVAKGEFEVQGHRIHEGDLVAASPAISNRIPEDFPDPHDF
VPARYEQPRQEDLLNRWTWIPFGAGRHRCVGAAFAIMQIKAIFSVLLREYEFEMAQPPESYRNDHSKMVVQLA
QPACVRYRRRTGV
>CYP51B1_Mycobacterium tuberculosis KT-0012 2605316062
MSAVALPRVSGGHDEHGHLEEFRTDPIGLMQRVRDECGDVGTFQLAGKQVVLLSGSHANEFFFRAGDDDLDQA
KAYPFMTPIFGEGVVFDASPERRKEMLHNAALRGEQMKGHAATIEDQVRRMIADWGEAGEIDLLDFFAELTIY
TSSACLIGKKFRDQLDGRFAKLYHELERGTDPLAYVDPYLPIESFRRRDEARNGLVALVADIMNGRIANPPTD
KSDRDMLDVLIAVKAETGTPRFSADEITGMFISMMFAGHHTSSGTASWTLIELMRHRDAYAAVIDELDELYGD
GRSVSFHALRQIPQLENVLKETLRLHPPLIILMRVAKGEFEVQGHRIHEGDLVAASPAISNRIPEDFPDPHDF
VPARYEQPRQEDLLNRWTWIPFGAGRHRCVGAAFAIMQIKAIFSVLLREYEFEMAQPPESYRNDHSKMVVQLA
QPACVRYRRRTGV
>CYP51B1_Mycobacterium tuberculosis KT-0018 2604974746
MSAVALPRVSGGHDEHGHLEEFRTDPIGLMQRVRDECGDVGTFQLAGKQVVLLSGSHANEFFFRAGDDDLDQA
KAYPFMTPIFGEGVVFDASPERRKEMLHNAALRGEQMKGHAATIEDQVRRMIADWGEAGEIDLLDFFAELTIY
TSSACLIGKKFRDQLDGRFAKLYHELERGTDPLAYVDPYLPIESFRRRDEARNGLVALVADIMNGRIANPPTD
KSDRDMLDVLIAVKAETGTPRFSADEITGMFISMMFAGHHTSSGTASWTLIELMRHRDAYAAVIDELDELYGD
GRSVSFHALRQIPQLENVLKETLRLHPPLIILMRVAKGEFEVQGHRIHEGDLVAASPAISNRIPEDFPDPHDF
VPARYEQPRQEDLLNRWTWIPFGAGRHRCVGAAFAIMQIKAIFSVLLREYEFEMAQPPESYRNDHSKMVVQLA
QPACVRYRRRTGV
>CYP51B1_Mycobacterium tuberculosis KT-0019 2584844661
MSAVALPRVSGGHDEHGHLEEFRTDPIGLMQRVRDECGDVGTFQLAGKQVVLLSGSHANEFFFRAGDDDLDQA
KAYPFMTPIFGEGVVFDASPERRKEMLHNAALRGEQMKGHAATIEDQVRRMIADWGEAGEIDLLDFFAELTIY
TSSACLIGKKFRDQLDGRFAKLYHELERGTDPLAYVDPYLPIESFRRRDEARNGLVALVADIMNGRIANPPTD
KSDRDMLDVLIAVKAETGTPRFSADEITGMFISMMFAGHHTSSGTASWTLIELMRHRDAYAAVIDELDELYGD
GRSVSFHALRQIPQLENVLKETLRLHPPLIILMRVAKGEFEVQGHRIHEGDLVAASPAISNRIPEDFPDPHDF
VPARYEQPRQEDLLNRWTWIPFGAGRHRCVGAAFAIMQIKAIFSVLLREYEFEMAQPPESYRNDHSKMVVQLA
QPACVRYRRRTGV
>CYP51B1_Mycobacterium tuberculosis KT-0020 2604245041
MSAVALPRVSGGHDEHGHLEEFRTDPIGLMQRVRDECGDVGTFQLAGKQVVLLSGSHANEFFFRAGDDDLDQA
KAYPFMTPIFGEGVVFDASPERRKEMLHNAALRGEQMKGHAATIEDQVRRMIADWGEAGEIDLLDFFAELTIY
TSSACLIGKKFRDQLDGRFAKLYHELERGTDPLAYVDPYLPIESFRRRDEARNGLVALVADIMNGRIANPPTD
KSDRDMLDVLIAVKAETGTPRFSADEITGMFISMMFAGHHTSSGTASWTLIELMRHRDAYAAVIDELDELYGD
GRSVSFHALRQIPQLENVLKETLRLHPPLIILMRVAKGEFEVQGHRIHEGDLVAASPAISNRIPEDFPDPHDF
VPARYEQPRQEDLLNRWTWIPFGAGRHRCVGAAFAIMQIKAIFSVLLREYEFEMAQPPESYRNDHSKMVVQLA
QPACVRYRRRTGV
>CYP51B1_Mycobacterium tuberculosis KT-0024 2577112760
MSAVALPRVSGGHDEHGHLEEFRTDPIGLMQRVRDECGDVGTFQLAGKQVVLLSGSHANEFFFRAGDDDLDQA
KAYPFMTPIFGEGVVFDASPERRKEMLHNAALRGEQMKGHAATIEDQVRRMIADWGEAGEIDLLDFFAELTIY
TSSACLIGKKFRDQLDGRFAKLYHELERGTDPLAYVDPYLPIESFRRRDEARNGLVALVADIMNGRIANPPTD
KSDRDMLDVLIAVKAETGTPRFSADEITGMFISMMFAGHHTSSGTASWTLIELMRHRDAYAAVIDELDELYGD
GRSVSFHALRQIPQLENVLKETLRLHPPLIILMRVAKGEFEVQGHRIHEGDLVAASPAISNRIPEDFPDPHDF
VPARYEQPRQEDLLNRWTWIPFGAGRHRCVGAAFAIMQIKAIFSVLLREYEFEMAQPPESYRNDHSKMVVQLA
QPACVRYRRRTGV
>CYP51B1_Mycobacterium tuberculosis KT-0025 2603371487
MSAVALPRVSGGHDEHGHLEEFRTDPIGLMQRVRDECGDVGTFQLAGKQVVLLSGSHANEFFFRAGDDDLDQA
KAYPFMTPIFGEGVVFDASPERRKEMLHNAALRGEQMKGHAATIEDQVRRMIADWGEAGEIDLLDFFAELTIY
TSSACLIGKKFRDQLDGRFAKLYHELERGTDPLAYVDPYLPIESFRRRDEARNGLVALVADIMNGRIANPPTD
KSDRDMLDVLIAVKAETGTPRFSADEITGMFISMMFAGHHTSSGTASWTLIELMRHRDAYAAVIDELDELYGD
GRSVSFHALRQIPQLENVLKETLRLHPPLIILMRVAKGEFEVQGHRIHEGDLVAASPAISNRIPEDFPDPHDF
VPARYEQPRQEDLLNRWTWIPFGAGRHRCVGAAFAIMQIKAIFSVLLREYEFEMAQPPESYRNDHSKMVVQLA
QPACVRYRRRTGV
>CYP51B1_Mycobacterium tuberculosis KT-0026 2633893244
MSAVALPRVSGGHDEHGHLEEFRTDPIGLMQRVRDECGDVGTFQLAGKQVVLLSGSHANEFFFRAGDDDLDQA
KAYPFMTPIFGEGVVFDASPERRKEMLHNAALRGEQMKGHAATIEDQVRRMIADWGEAGEIDLLDFFAELTIY
TSSACLIGKKFRDQLDGRFAKLYHELERGTDPLAYVDPYLPIESFRRRDEARNGLVALVADIMNGRIANPPTD
KSDRDMLDVLIAVKAETGTPRFSADEITGMFISMMFAGHHTSSGTASWTLIELMRHRDAYAAVIDELDELYGD

GRSVSFHALRQIPQLENVLKETLRLHPPLIILMRVAKGEFEVQGHRIHEGDLVAASPAISNRIPEDFPDPHDF
VPARYEQPRQEDLLNRWTWIPFGAGRHRCVGAAFAIMQIKAIFSVLLREYEFEMAQPPESYRNDHSKMVVQLA
QPACVRYRRRTGV
>CYP51B1_Mycobacterium tuberculosis KT-0029 2604251882
MSAVALPRVSGGHDEHGHLEEFRTDPIGLMQRVRDECGDVGTFQLAGKQVVLLSGSHANEFFFRAGDDDLDQA
KAYPFMTPIFGEGVVFDASPERRKEMLHNAALRGEQMKGHAATIEDQVRRMIADWGEAGEIDLLDFFAELTIY
TSSACLIGKKFRDQLDGRFAKLYHELERGTDPLAYVDPYLPIESFRRRDEARNGLVALVADIMNGRIANPPTD
KSDRDMLDVLIAVKAETGTPRFSADEITGMFISMMFAGHHTSSGTASWTLIELMRHRDAYAAVIDELDELYGD
GRSVSFHALRQIPQLENVLKETLRLHPPLIILMRVAKGEFEVQGHRIHEGDLVAASPAISNRIPEDFPDPHDF
VPARYEQPRQEDLLNRWTWIPFGAGRHRCVGAAFAIMQIKAIFSVLLREYEFEMAQPPESYRNDHSKMVVQLA
QPACVRYRRRTGV
>CYP51B1_Mycobacterium tuberculosis KT-0031 2604978807
MSAVALPRVSGGHDEHGHLEEFRTDPIGLMQRVRDECGDVGTFQLAGKQVVLLSGSHANEFFFRAGDDDLDQA
KAYPFMTPIFGEGVVFDASPERRKEMLHNAALRGEQMKGHAATIEDQVRRMIADWGEAGEIDLLDFFAELTIY
TSSACLIGKKFRDQLDGRFAKLYHELERGTDPLAYVDPYLPIESFRRRDEARNGLVALVADIMNGRIANPPTD
KSDRDMLDVLIAVKAETGTPRFSADEITGMFISMMFAGHHTSSGTASWTLIELMRHRDAYAAVIDELDELYGD
GRSVSFHALRQIPQLENVLKETLRLHPPLIILMRVAKGEFEVQGHRIHEGDLVAASPAISNRIPEDFPDPHDF
VPARYEQPRQEDLLNRWTWIPFGAGRHRCVGAAFAIMQIKAIFSVLLREYEFEMAQPPESYRNDHSKMVVQLA
QPACVRYRRRTGV
>CYP51B1_Mycobacterium tuberculosis KT-0032 2604613377
MSAVALPRVSGGHDEHGHLEEFRTDPIGLMQRVRDECGDVGTFQLAGKQVVLLSGSHANEFFFRAGDDDLDQA
KAYPFMTPIFGEGVVFDASPERRKEMLHNAALRGEQMKGHAATIEDQVRRMIADWGEAGEIDLLDFFAELTIY
TSSACLIGKKFRDQLDGRFAKLYHELERGTDPLAYVDPYLPIESFRRRDEARNGLVALVADIMNGRIANPPTD
KSDRDMLDVLIAVKAETGTPRFSADEITGMFISMMFAGHHTSSGTASWTLIELMRHRDAYAAVIDELDELYGD
GRSVSFHALRQIPQLENVLKETLRLHPPLIILMRVAKGEFEVQGHRIHEGDLVAASPAISNRIPEDFPDPHDF
VPARYEQPRQEDLLNRWTWIPFGAGRHRCVGAAFAIMQIKAIFSVLLREYEFEMAQPPESYRNDHSKMVVQLA
QPACVRYRRRTGV
>CYP51B1_Mycobacterium tuberculosis KT-0033 2603194847
MSAVALPRVSGGHDEHGHLEEFRTDPIGLMQRVRDECGDVGTFQLAGKQVVLLSGSHANEFFFRAGDDDLDQA
KAYPFMTPIFGEGVVFDASPERRKEMLHNAALRGEQMKGHAATIEDQVRRMIADWGEAGEIDLLDFFAELTIY
TSSACLIGKKFRDQLDGRFAKLYHELERGTDPLAYVDPYLPIESFRRRDEARNGLVALVADIMNGRIANPPTD
KSDRDMLDVLIAVKAETGTPRFSADEITGMFISMMFAGHHTSSGTASWTLIELMRHRDAYAAVIDELDELYGD
GRSVSFHALRQIPQLENVLKETLRLHPPLIILMRVAKGEFEVQGHRIHEGDLVAASPAISNRIPEDFPDPHDF
VPARYEQPRQEDLLNRWTWIPFGAGRHRCVGAAFAIMQIKAIFSVLLREYEFEMAQPPESYRNDHSKMVVQLA
QPACVRYRRRTGV
>CYP51B1_Mycobacterium tuberculosis KT-0034 2575871757
MSAVALPRVSGGHDEHGHLEEFRTDPIGLMQRVRDECGDVGTFQLAGKQVVLLSGSHANEFFFRAGDDDLDQA
KAYPFMTPIFGEGVVFDASPERRKEMLHNAALRGEQMKGHAATIEDQVRRMIADWGEAGEIDLLDFFAELTIY
TSSACLIGKKFRDQLDGRFAKLYHELERGTDPLAYVDPYLPIESFRRRDEARNGLVALVADIMNGRIANPPTD
KSDRDMLDVLIAVKAETGTPRFSADEITGMFISMMFAGHHTSSGTASWTLIELMRHRDAYAAVIDELDELYGD
GRSVSFHALRQIPQLENVLKETLRLHPPLIILMRVAKGEFEVQGHRIHEGDLVAASPAISNRIPEDFPDPHDF
VPARYEQPRQEDLLNRWTWIPFGAGRHRCVGAAFAIMQIKAIFSVLLREYEFEMAQPPESYRNDHSKMVVQLA
QPACVRYRRRTGV
>CYP51B1_Mycobacterium tuberculosis KT-0027 2577996213
MSAVALPRVSGGHDEHGHLEEFRTDPIGLMQRVRDECGDVGTFQLAGKQVVLLSGSHANEFFFRAGDDDLDQA
KAYPFMTPIFGEGVVFDASPERRKEMLHNAALRGEQMKGHAATIEDQVRRMIADWGEAGEIDLLDFFAELTIY
TSSACLIGKKFRDQLDGRFAKLYHELERGTDPLAYVDPYLPIESFRRRDEARNGLVALVADIMNGRIANPPTD
KSDRDMLDVLIAVKAETGTPRFSADEITGMFISMMFAGHHTSSGTASWTLIELMRHRDAYAAVIDELDELYGD
GRSVSFHALRQIPQLENVLKETLRLHPPLIILMRVAKGEFEVQGHRIHEGDLVAASPAISNRIPEDFPDPHDF
VPARYEQPRQEDLLNRWTWIPFGAGRHRCVGAAFAIMQIKAIFSVLLREYEFEMAQPPESYRNDHSKMVVQLA
QPACVRYRRRTGV
>CYP51B1_Mycobacterium tuberculosis KT-0037 2576525037
MSAVALPRVSGGHDEHGHLEEFRTDPIGLMQRVRDECGDVGTFQLAGKQVVLLSGSHANEFFFRAGDDDLDQA
KAYPFMTPIFGEGVVFDASPERRKEMLHNAALRGEQMKGHAATIEDQVRRMIADWGEAGEIDLLDFFAELTIY
TSSACLIGKKFRDQLDGRFAKLYHELERGTDPLAYVDPYLPIESFRRRDEARNGLVALVADIMNGRIANPPTD
KSDRDMLDVLIAVKAETGTPRFSADEITGMFISMMFAGHHTSSGTASWTLIELMRHRDAYAAVIDELDELYGD
GRSVSFHALRQIPQLENVLKETLRLHPPLIILMRVAKGEFEVQGHRIHEGDLVAASPAISNRIPEDFPDPHDF

VPARYEQPRQEDLLNRWTWIPFGAGRHRCVGAAFAIMQIKAIFSVLLREYEFEMAQPPESYRNDHSKMVVQLA
QPACVRYRRRTGV
>CYP51B1_Mycobacterium tuberculosis KT-0040 2575963786
MSAVALPRVSGGHDEHGHLEEFRTDPIGLMQRVRDECGDVGTFQLAGKQVVLLSGSHANEFFFRAGDDDLDQA
KAYPFMTPIFGEGVVFDASPERRKEMLHNAALRGEQMKGHAATIEDQVRRMIADWGEAGEIDLLDFFAELTIY
TSSACLIGKKFRDQLDGRFAKLYHELERGTDPLAYVDPYLPIESFRRRDEARNGLVALVADIMNGRIANPPTD
KSDRDMLDVLIAVKAETGTPRFSADEITGMFISMMFAGHHTSSGTASWTLIELMRHRDAYAAVIDELDELYGD
GRSVSFHALRQIPQLENVLKETLRLHPPLIILMRVAKGEFEVQGHRIHEGDLVAASPAISNRIPEDFPDPHDF
VPARYEQPRQEDLLNRWTWIPFGAGRHRCVGAAFAIMQIKAIFSVLLREYEFEMAQPPESYRNDHSKMVVQLA
QPACVRYRRRTGV
>CYP51B1_Mycobacterium tuberculosis KT-0041 2584682625
MSAVALPRVSGGHDEHGHLEEFRTDPIGLMQRVRDECGDVGTFQLAGKQVVLLSGSHANEFFFRAGDDDLDQA
KAYPFMTPIFGEGVVFDASPERRKEMLHNAALRGEQMKGHAATIEDQVRRMIADWGEAGEIDLLDFFAELTIY
TSSACLIGKKFRDQLDGRFAKLYHELERGTDPLAYVDPYLPIESFRRRDEARNGLVALVADIMNGRIANPPTD
KSDRDMLDVLIAVKAETGTPRFSADEITGMFISMMFAGHHTSSGTASWTLIELMRHRDAYAAVIDELDELYGD
GRSVSFHALRQIPQLENVLKETLRLHPPLIILMRVAKGEFEVQGHRIHEGDLVAASPAISNRIPEDFPDPHDF
VPARYEQPRQEDLLNRWTWIPFGAGRHRCVGAAFAIMQIKAIFSVLLREYEFEMAQPPESYRNDHSKMVVQLA
QPACVRYRRRTGV
>CYP51B1_Mycobacterium tuberculosis KT-0042 2574946249
MSAVALPRVSGGHDEHGHLEEFRTDPIGLMQRVRDECGDVGTFQLAGKQVVLLSGSHANEFFFRAGDDDLDQA
KAYPFMTPIFGEGVVFDASPERRKEMLHNAALRGEQMKGHAATIEDQVRRMIADWGEAGEIDLLDFFAELTIY
TSSACLIGKKFRDQLDGRFAKLYHELERGTDPLAYVDPYLPIESFRRRDEARNGLVALVADIMNGRIANPPTD
KSDRDMLDVLIAVKAETGTPRFSADEITGMFISMMFAGHHTSSGTASWTLIELMRHRDAYAAVIDELDELYGD
GRSVSFHALRQIPQLENVLKETLRLHPPLIILMRVAKGEFEVQGHRIHEGDLVAASPAISNRIPEDFPDPHDF
VPARYEQPRQEDLLNRWTWIPFGAGRHRCVGAAFAIMQIKAIFSVLLREYEFEMAQPPESYRNDHSKMVVQLA
QPACVRYRRRTGV
>CYP51B1_Mycobacterium tuberculosis KT-0051 2575097194
MSAVALPRVSGGHDEHGHLEEFRTDPIGLMQRVRDECGDVGTFQLAGKQVVLLSGSHANEFFFRAGDDDLDQA
KAYPFMTPIFGEGVVFDASPERRKEMLHNAALRGEQMKGHAATIEDQVRRMIADWGEAGEIDLLDFFAELTIY
TSSACLIGKKFRDQLDGRFAKLYHELERGTDPLAYVDPYLPIESFRRRDEARNGLVALVADIMNGRIANPPTD
KSDRDMLDVLIAVKAETGTPRFSADEITGMFISMMFAGHHTSSGTASWTLIELMRHRDAYAAVIDELDELYGD
GRSVSFHALRQIPQLENVLKETLRLHPPLIILMRVAKGEFEVQGHRIHEGDLVAASPAISNRIPEDFPDPHDF
VPARYEQPRQEDLLNRWTWIPFGAGRHRCVGAAFAIMQIKAIFSVLLREYEFEMAQPPESYRNDHSKMVVQLA
QPACVRYRRRTGV
>CYP51B1_Mycobacterium tuberculosis KT-0056 2576711681
MSAVALPRVSGGHDEHGHLEEFRTDPIGLMQRVRDECGDVGTFQLAGKQVVLLSGSHANEFFFRAGDDDLDQA
KAYPFMTPIFGEGVVFDASPERRKEMLHNAALRGEQMKGHAATIEDQVRRMIADWGEAGEIDLLDFFAELTIY
TSSACLIGKKFRDQLDGRFAKLYHELERGTDPLAYVDPYLPIESFRRRDEARNGLVALVADIMNGRIANPPTD
KSDRDMLDVLIAVKAETGTPRFSADEITGMFISMMFAGHHTSSGTASWTLIELMRHRDAYAAVIDELDELYGD
GRSVSFHALRQIPQLENVLKETLRLHPPLIILMRVAKGEFEVQGHRIHEGDLVAASPAISNRIPEDFPDPHDF
VPARYEQPRQEDLLNRWTWIPFGAGRHRCVGAAFAIMQIKAIFSVLLREYEFEMAQPPESYRNDHSKMVVQLA
QPACVRYRRRTGV
>CYP51B1_Mycobacterium tuberculosis KT-0057 2584725064
MSAVALPRVSGGHDEHGHLEEFRTDPIGLMQRVRDECGDVGTFQLAGKQVVLLSGSHANEFFFRAGDDDLDQA
KAYPFMTPIFGEGVVFDASPERRKEMLHNAALRGEQMKGHAATIEDQVRRMIADWGEAGEIDLLDFFAELTIY
TSSACLIGKKFRDQLDGRFAKLYHELERGTDPLAYVDPYLPIESFRRRDEARNGLVALVADIMNGRIANPPTD
KSDRDMLDVLIAVKAETGTPRFSADEITGMFISMMFAGHHTSSGTASWTLIELMRHRDAYAAVIDELDELYGD
GRSVSFHALRQIPQLENVLKETLRLHPPLIILMRVAKGEFEVQGHRIHEGDLVAASPAISNRIPEDFPDPHDF
VPARYEQPRQEDLLNRWTWIPFGAGRHRCVGAAFAIMQIKAIFSVLLREYEFEMAQPPESYRNDHSKMVVQLA
QPACVRYRRRTGV
>CYP51B1_Mycobacterium tuberculosis KT-0060 2604620437
MSAVALPRVSGGHDEHGHLEEFRTDPIGLMQRVRDECGDVGTFQLAGKQVVLLSGSHANEFFFRAGDDDLDQA
KAYPFMTPIFGEGVVFDASPERRKEMLHNAALRGEQMKGHAATIEDQVRRMIADWGEAGEIDLLDFFAELTIY
TSSACLIGKKFRDQLDGRFAKLYHELERGTDPLAYVDPYLPIESFRRRDEARNGLVALVADIMNGRIANPPTD
KSDRDMLDVLIAVKAETGTPRFSADEITGMFISMMFAGHHTSSGTASWTLIELMRHRDAYAAVIDELDELYGD
GRSVSFHALRQIPQLENVLKETLRLHPPLIILMRVAKGEFEVQGHRIHEGDLVAASPAISNRIPEDFPDPHDF
VPARYEQPRQEDLLNRWTWIPFGAGRHRCVGAAFAIMQIKAIFSVLLREYEFEMAQPPESYRNDHSKMVVQLA
QPACVRYRRRTGV

>CYP51B1_Mycobacterium tuberculosis KT-0067 2574706473
MSAVALPRVSGGHDEHGHLEEFRTDPIGLMQRVRDECGDVGTFQLAGKQVVLLSGSHANEFFFRAGDDDLDQA
KAYPFMTPIFGEGVVFDASPERRKEMLHNAALRGEQMKGHAATIEDQVRRMIADWGEAGEIDLLDFFAELTIY
TSSACLIGKKFRDQLDGRFAKLYHELERGTDPLAYVDPYLPIESFRRRDEARNGLVALVADIMNGRIANPPTD
KSDRDMLDVLIAVKAETGTPRFSADEITGMFISMMFAGHHTSSGTASWTLIELMRHRDAYAAVIDELDELYGD
GRSVSFHALRQIPQLENVLKETLRLHPPLIILMRVAKGEFEVQGHRIHEGDLVAASPAISNRIPEDFPDPHDF
VPARYEQPRQEDLLNRWTWIPFGAGRHRCVGAAFAIMQIKAIFSVLLREYEFEMAQPPESYRNDHSKMVVQLA
QPACVRYRRRTGV
>CYP51B1_Mycobacterium tuberculosis KT-0069 2574914064
MSAVALPRVSGGHDEHGHLEEFRTDPIGLMQRVRDECGDVGTFQLAGKQVVLLSGSHANEFFFRAGDDDLDQA
KAYPFMTPIFGEGVVFDASPERRKEMLHNAALRGEQMKGHAATIEDQVRRMIADWGEAGEIDLLDFFAELTIY
TSSACLIGKKFRDQLDGRFAKLYHELERGTDPLAYVDPYLPIESFRRRDEARNGLVALVADIMNGRIANPPTD
KSDRDMLDVLIAVKAETGTPRFSADEITGMFISMMFAGHHTSSGTASWTLIELMRHRDAYAAVIDELDELYGD
GRSVSFHALRQIPQLENVLKETLRLHPPLIILMRVAKGEFEVQGHRIHEGDLVAASPAISNRIPEDFPDPHDF
VPARYEQPRQEDLLNRWTWIPFGAGRHRCVGAAFAIMQIKAIFSVLLREYEFEMAQPPESYRNDHSKMVVQLA
QPACVRYRRRTGV
>CYP51B1_Mycobacterium tuberculosis KT-0070 2577654477
MSAVALPRVSGGHDEHGHLEEFRTDPIGLMQRVRDECGDVGTFQLAGKQVVLLSGSHANEFFFRAGDDDLDQA
KAYPFMTPIFGEGVVFDASPERRKEMLHNAALRGEQMKGHAATIEDQVRRMIADWGEAGEIDLLDFFAELTIY
TSSACLIGKKFRDQLDGRFAKLYHELERGTDPLAYVDPYLPIESFRRRDEARNGLVALVADIMNGRIANPPTD
KSDRDMLDVLIAVKAETGTPRFSADEITGMFISMMFAGHHTSSGTASWTLIELMRHRDAYAAVIDELDELYGD
GRSVSFHALRQIPQLENVLKETLRLHPPLIILMRVAKGEFEVQGHRIHEGDLVAASPAISNRIPEDFPDPHDF
VPARYEQPRQEDLLNRWTWIPFGAGRHRCVGAAFAIMQIKAIFSVLLREYEFEMAQPPESYRNDHSKMVVQLA
QPACVRYRRRTGV
>CYP51B1_Mycobacterium tuberculosis KT-0072 2576100350
MSAVALPRVSGGHDEHGHLEEFRTDPIGLMQRVRDECGDVGTFQLAGKQVVLLSGSHANEFFFRAGDDDLDQA
KAYPFMTPIFGEGVVFDASPERRKEMLHNAALRGEQMKGHAATIEDQVRRMIADWGEAGEIDLLDFFAELTIY
TSSACLIGKKFRDQLDGRFAKLYHELERGTDPLAYVDPYLPIESFRRRDEARNGLVALVADIMNGRIANPPTD
KSDRDMLDVLIAVKAETGTPRFSADEITGMFISMMFAGHHTSSGTASWTLIELMRHRDAYAAVIDELDELYGD
GRSVSFHALRQIPQLENVLKETLRLHPPLIILMRVAKGEFEVQGHRIHEGDLVAASPAISNRIPEDFPDPHDF
VPARYEQPRQEDLLNRWTWIPFGAGRHRCVGAAFAIMQIKAIFSVLLREYEFEMAQPPESYRNDHSKMVVQLA
QPACVRYRRRTGV
>CYP51B1_Mycobacterium tuberculosis KT-0081 2605673114
MSAVALPRVSGGHDEHGHLEEFRTDPIGLMQRVRDECGDVGTFQLAGKQVVLLSGSHANEFFFRAGDDDLDQA
KAYPFMTPIFGEGVVFDASPERRKEMLHNAALRGEQMKGHAATIEDQVRRMIADWGEAGEIDLLDFFAELTIY
TSSACLIGKKFRDQLDGRFAKLYHELERGTDPLAYVDPYLPIESFRRRDEARNGLVALVADIMNGRIANPPTD
KSDRDMLDVLIAVKAETGTPRFSADEITGMFISMMFAGHHTSSGTASWTLIELMRHRDAYAAVIDELDELYGD
GRSVSFHALRQIPQLENVLKETLRLHPPLIILMRVAKGEFEVQGHRIHEGDLVAASPAISNRIPEDFPDPHDF
VPARYEQPRQEDLLNRWTWIPFGAGRHRCVGAAFAIMQIKAIFSVLLREYEFEMAQPPESYRNDHSKMVVQLA
QPACVRYRRRTGV
>CYP51B1_Mycobacterium tuberculosis KT-0082 2604982894
MSAVALPRVSGGHDEHGHLEEFRTDPIGLMQRVRDECGDVGTFQLAGKQVVLLSGSHANEFFFRAGDDDLDQA
KAYPFMTPIFGEGVVFDASPERRKEMLHNAALRGEQMKGHAATIEDQVRRMIADWGEAGEIDLLDFFAELTIY
TSSACLIGKKFRDQLDGRFAKLYHELERGTDPLAYVDPYLPIESFRRRDEARNGLVALVADIMNGRIANPPTD
KSDRDMLDVLIAVKAETGTPRFSADEITGMFISMMFAGHHTSSGTASWTLIELMRHRDAYAAVIDELDELYGD
GRSVSFHALRQIPQLENVLKETLRLHPPLIILMRVAKGEFEVQGHRIHEGDLVAASPAISNRIPEDFPDPHDF
VPARYEQPRQEDLLNRWTWIPFGAGRHRCVGAAFAIMQIKAIFSVLLREYEFEMAQPPESYRNDHSKMVVQLA
QPACVRYRRRTGV
>CYP51B1_Mycobacterium tuberculosis KT-0086 2574779416
MSAVALPRVSGGHDEHGHLEEFRTDPIGLMQRVRDECGDVGTFQLAGKQVVLLSGSHANEFFFRAGDDDLDQA
KAYPFMTPIFGEGVVFDASPERRKEMLHNAALRGEQMKGHAATIEDQVRRMIADWGEAGEIDLLDFFAELTIY
TSSACLIGKKFRDQLDGRFAKLYHELERGTDPLAYVDPYLPIESFRRRDEARNGLVALVADIMNGRIANPPTD
KSDRDMLDVLIAVKAETGTPRFSADEITGMFISMMFAGHHTSSGTASWTLIELMRHRDAYAAVIDELDELYGD
GRSVSFHALRQIPQLENVLKETLRLHPPLIILMRVAKGEFEVQGHRIHEGDLVAASPAISNRIPEDFPDPHDF
VPARYEQPRQEDLLNRWTWIPFGAGRHRCVGAAFAIMQIKAIFSVLLREYEFEMAQPPESYRNDHSKMVVQLA
QPACVRYRRRTGV
>CYP51B1_Mycobacterium tuberculosis KT-0087 2574837373

MSAVALPRVSGGHDEHGHLEEFRTDPIGLMQRVRDECGDVGTFQLAGKQVVLLSGSHANEFFFRAGDDDLDQA
KAYPFMTPIFGEGVVFDASPERRKEMLHNAALRGEQMKGHAATIEDQVRRMIADWGEAGEIDLLDFFAELTIY
TSSACLIGKKFRDQLDGRFAKLYHELERGTDPLAYVDPYLPIESFRRRDEARNGLVALVADIMNGRIANPPTD
KSDRDMLDVLIAVKAETGTPRFSADEITGMFISMMFAGHHTSSGTASWTLIELMRHRDAYAAVIDELDELYGD
GRSVSFHALRQIPQLENVLKETLRLHPPLIILMRVAKGEFEVQGHRIHEGDLVAASPAISNRIPEDFPDPHDF
VPARYEQPRQEDLLNRWTWIPFGAGRHRCVGAAFAIMQIKAIFSVLLREYEFEMAQPPESYRNDHSKMVVQLA
QPACVRYRRRTGV
>CYP51B1_Mycobacterium tuberculosis KT-0091 2575469661
MSAVALPRVSGGHDEHGHLEEFRTDPIGLMQRVRDECGDVGTFQLAGKQVVLLSGSHANEFFFRAGDDDLDQA
KAYPFMTPIFGEGVVFDASPERRKEMLHNAALRGEQMKGHAATIEDQVRRMIADWGEAGEIDLLDFFAELTIY
TSSACLIGKKFRDQLDGRFAKLYHELERGTDPLAYVDPYLPIESFRRRDEARNGLVALVADIMNGRIANPPTD
KSDRDMLDVLIAVKAETGTPRFSADEITGMFISMMFAGHHTSSGTASWTLIELMRHRDAYAAVIDELDELYGD
GRSVSFHALRQIPQLENVLKETLRLHPPLIILMRVAKGEFEVQGHRIHEGDLVAASPAISNRIPEDFPDPHDF
VPARYEQPRQEDLLNRWTWIPFGAGRHRCVGAAFAIMQIKAIFSVLLREYEFEMAQPPESYRNDHSKMVVQLA
QPACVRYRRRTGV
>CYP51B1_Mycobacterium tuberculosis KT-0096 2578190713
MSAVALPRVSGGHDEHGHLEEFRTDPIGLMQRVRDECGDVGTFQLAGKQVVLLSGSHANEFFFRAGDDDLDQA
KAYPFMTPIFGEGVVFDASPERRKEMLHNAALRGEQMKGHAATIEDQVRRMIADWGEAGEIDLLDFFAELTIY
TSSACLIGKKFRDQLDGRFAKLYHELERGTDPLAYVDPYLPIESFRRRDEARNGLVALVADIMNGRIANPPTD
KSDRDMLDVLIAVKAETGTPRFSADEITGMFISMMFAGHHTSSGTASWTLIELMRHRDAYAAVIDELDELYGD
GRSVSFHALRQIPQLENVLKETLRLHPPLIILMRVAKGEFEVQGHRIHEGDLVAASPAISNRIPEDFPDPHDF
VPARYEQPRQEDLLNRWTWIPFGAGRHRCVGAAFAIMQIKAIFSVLLREYEFEMAQPPESYRNDHSKMVVQLA
QPACVRYRRRTGV
>CYP51B1_Mycobacterium tuberculosis KT-0098 2584877510
MSAVALPRVSGGHDEHGHLEEFRTDPIGLMQRVRDECGDVGTFQLAGKQVVLLSGSHANEFFFRAGDDDLDQA
KAYPFMTPIFGEGVVFDASPERRKEMLHNAALRGEQMKGHAATIEDQVRRMIADWGEAGEIDLLDFFAELTIY
TSSACLIGKKFRDQLDGRFAKLYHELERGTDPLAYVDPYLPIESFRRRDEARNGLVALVADIMNGRIANPPTD
KSDRDMLDVLIAVKAETGTPRFSADEITGMFISMMFAGHHTSSGTASWTLIELMRHRDAYAAVIDELDELYGD
GRSVSFHALRQIPQLENVLKETLRLHPPLIILMRVAKGEFEVQGHRIHEGDLVAASPAISNRIPEDFPDPHDF
VPARYEQPRQEDLLNRWTWIPFGAGRHRCVGAAFAIMQIKAIFSVLLREYEFEMAQPPESYRNDHSKMVVQLA
QPACVRYRRRTGV
>CYP51B1_Mycobacterium tuberculosis KT-0100 2577732541
MSAVALPRVSGGHDEHGHLEEFRTDPIGLMQRVRDECGDVGTFQLAGKQVVLLSGSHANEFFFRAGDDDLDQA
KAYPFMTPIFGEGVVFDASPERRKEMLHNAALRGEQMKGHAATIEDQVRRMIADWGEAGEIDLLDFFAELTIY
TSSACLIGKKFRDQLDGRFAKLYHELERGTDPLAYVDPYLPIESFRRRDEARNGLVALVADIMNGRIANPPTD
KSDRDMLDVLIAVKAETGTPRFSADEITGMFISMMFAGHHTSSGTASWTLIELMRHRDAYAAVIDELDELYGD
GRSVSFHALRQIPQLENVLKETLRLHPPLIILMRVAKGEFEVQGHRIHEGDLVAASPAISNRIPEDFPDPHDF
VPARYEQPRQEDLLNRWTWIPFGAGRHRCVGAAFAIMQIKAIFSVLLREYEFEMAQPPESYRNDHSKMVVQLA
QPACVRYRRRTGV
>CYP51B1_Mycobacterium tuberculosis KT-0108 2575417918
MSAVALPRVSGGHDEHGHLEEFRTDPIGLMQRVRDECGDVGTFQLAGKQVVLLSGSHANEFFFRAGDDDLDQA
KAYPFMTPIFGEGVVFDASPERRKEMLHNAALRGEQMKGHAATIEDQVRRMIADWGEAGEIDLLDFFAELTIY
TSSACLIGKKFRDQLDGRFAKLYHELERGTDPLAYVDPYLPIESFRRRDEARNGLVALVADIMNGRIANPPTD
KSDRDMLDVLIAVKAETGTPRFSADEITGMFISMMFAGHHTSSGTASWTLIELMRHRDAYAAVIDELDELYGD
GRSVSFHALRQIPQLENVLKETLRLHPPLIILMRVAKGEFEVQGHRIHEGDLVAASPAISNRIPEDFPDPHDF
VPARYEQPRQEDLLNRWTWIPFGAGRHRCVGAAFAIMQIKAIFSVLLREYEFEMAQPPESYRNDHSKMVVQLA
QPACVRYRRRTGV
>CYP51B1_Mycobacterium tuberculosis KT-0109 2584999550
MSAVALPRVSGGHDEHGHLEEFRTDPIGLMQRVRDECGDVGTFQLAGKQVVLLSGSHANEFFFRAGDDDLDQA
KAYPFMTPIFGEGVVFDASPERRKEMLHNAALRGEQMKGHAATIEDQVRRMIADWGEAGEIDLLDFFAELTIY
TSSACLIGKKFRDQLDGRFAKLYHELERGTDPLAYVDPYLPIESFRRRDEARNGLVALVADIMNGRIANPPTD
KSDRDMLDVLIAVKAETGTPRFSADEITGMFISMMFAGHHTSSGTASWTLIELMRHRDAYAAVIDELDELYGD
GRSVSFHALRQIPQLENVLKETLRLHPPLIILMRVAKGEFEVQGHRIHEGDLVAASPAISNRIPEDFPDPHDF
VPARYEQPRQEDLLNRWTWIPFGAGRHRCVGAAFAIMQIKAIFSVLLREYEFEMAQPPESYRNDHSKMVVQLA
QPACVRYRRRTGV
>CYP51B1_Mycobacterium tuberculosis KT-0110 2576254666
MSAVALPRVSGGHDEHGHLEEFRTDPIGLMQRVRDECGDVGTFQLAGKQVVLLSGSHANEFFFRAGDDDLDQA
KAYPFMTPIFGEGVVFDASPERRKEMLHNAALRGEQMKGHAATIEDQVRRMIADWGEAGEIDLLDFFAELTIY

TSSACLIGKKFRDQLDGRFAKLYHELERGTDPLAYVDPYLPIESFRRRDEARNGLVALVADIMNGRIANPPTD
KSDRDMLDVLIAVKAETGTPRFSADEITGMFISMMFAGHHTSSGTASWTLIELMRHRDAYAAVIDELDELYGD
GRSVSFHALRQIPQLENVLKETLRLHPPLIILMRVAKGEFEVQGHRIHEGDLVAASPAISNRIPEDFPDPHDF
VPARYEQPRQEDLLNRWTWIPFGAGRHRCVGAAFAIMQIKAIFSVLLREYEFEMAQPPESYRNDHSKMVVQLA
QPACVRYRRRTGV
>CYP51B1_Mycobacterium tuberculosis KT-0133 2638406436
MSAVALPRVSGGHDEHGHLEEFRTDPIGLMQRVRDECGDVGTFQLAGKQVVLLSGSHANEFFFRAGDDDLDQA
KAYPFMTPIFGEGVVFDASPERRKEMLHNAALRGEQMKGHAATIEDQVRRMIADWGEAGEIDLLDFFAELTIY
TSSACLIGKKFRDQLDGRFAKLYHELERGTDPLAYVDPYLPIESFRRRDEARNGLVALVADIMNGRIANPPTD
KSDRDMLDVLIAVKAETGTPRFSADEITGMFISMMFAGHHTSSGTASWTLIELMRHRDAYAAVIDELDELYGD
GRSVSFHALRQIPQLENVLKETLRLHPPLIILMRVAKGEFEVQGHRIHEGDLVAASPAISNRIPEDFPDPHDF
VPARYEQPRQEDLLNRWTWIPFGAGRHRCVGAAFAIMQIKAIFSVLLREYEFEMAQPPESYRNDHSKMVVQLA
QPACVRYRRRTGV
>CYP51B1_Mycobacterium tuberculosis KT-0140 2634023958
MSAVALPRVSGGHDEHGHLEEFRTDPIGLMQRVRDECGDVGTFQLAGKQVVLLSGSHANEFFFRAGDDDLDQA
KAYPFMTPIFGEGVVFDASPERRKEMLHNAALRGEQMKGHAATIEDQVRRMIADWGEAGEIDLLDFFAELTIY
TSSACLIGKKFRDQLDGRFAKLYHELERGTDPLAYVDPYLPIESFRRRDEARNGLVALVADIMNGRIANPPTD
KSDRDMLDVLIAVKAETGTPRFSADEITGMFISMMFAGHHTSSGTASWTLIELMRHRDAYAAVIDELDELYGD
GRSVSFHALRQIPQLENVLKETLRLHPPLIILMRVAKGEFEVQGHRIHEGDLVAASPAISNRIPEDFPDPHDF
VPARYEQPRQEDLLNRWTWIPFGAGRHRCVGAAFAIMQIKAIFSVLLREYEFEMAQPPESYRNDHSKMVVQLA
QPACVRYRRRTGV
>CYP51B1_Mycobacterium tuberculosis KT-0181 2651506943
MSAVALPRVSGGHDEHGHLEEFRTDPIGLMQRVRDECGDVGTFQLAGKQVVLLSGSHANEFFFRAGDDDLDQA
KAYPFMTPIFGEGVVFDASPERRKEMLHNAALRGEQMKGHAATIEDQVRRMIADWGEAGEIDLLDFFAELTIY
TSSACLIGKKFRDQLDGRFAKLYHELERGTDPLAYVDPYLPIESFRRRDEARNGLVALVADIMNGRIANPPTD
KSDRDMLDVLIAVKAETGTPRFSADEITGMFISMMFAGHHTSSGTASWTLIELMRHRDAYAAVIDELDELYGD
GRSVSFHALRQIPQLENVLKETLRLHPPLIILMRVAKGEFEVQGHRIHEGDLVAASPAISNRIPEDFPDPHDF
VPARYEQPRQEDLLNRWTWIPFGAGRHRCVGAAFAIMQIKAIFSVLLREYEFEMAQPPESYRNDHSKMVVQLA
QPACVRYRRRTGV
>CYP51B1_Mycobacterium tuberculosis KT-0182 2637785584
MSAVALPRVSGGHDEHGHLEEFRTDPIGLMQRVRDECGDVGTFQLAGKQVVLLSGSHANEFFFRAGDDDLDQA
KAYPFMTPIFGEGVVFDASPERRKEMLHNAALRGEQMKGHAATIEDQVRRMIADWGEAGEIDLLDFFAELTIY
TSSACLIGKKFRDQLDGRFAKLYHELERGTDPLAYVDPYLPIESFRRRDEARNGLVALVADIMNGRIANPPTD
KSDRDMLDVLIAVKAETGTPRFSADEITGMFISMMFAGHHTSSGTASWTLIELMRHRDAYAAVIDELDELYGD
GRSVSFHALRQIPQLENVLKETLRLHPPLIILMRVAKGEFEVQGHRIHEGDLVAASPAISNRIPEDFPDPHDF
VPARYEQPRQEDLLNRWTWIPFGAGRHRCVGAAFAIMQIKAIFSVLLREYEFEMAQPPESYRNDHSKMVVQLA
QPACVRYRRRTGV
>CYP51B1_Mycobacterium tuberculosis KT-0183 2651182047
MSAVALPRVSGGHDEHGHLEEFRTDPIGLMQRVRDECGDVGTFQLAGKQVVLLSGSHANEFFFRAGDDDLDQA
KAYPFMTPIFGEGVVFDASPERRKEMLHNAALRGEQMKGHAATIEDQVRRMIADWGEAGEIDLLDFFAELTIY
TSSACLIGKKFRDQLDGRFAKLYHELERGTDPLAYVDPYLPIESFRRRDEARNGLVALVADIMNGRIANPPTD
KSDRDMLDVLIAVKAETGTPRFSADEITGMFISMMFAGHHTSSGTASWTLIELMRHRDAYAAVIDELDELYGD
GRSVSFHALRQIPQLENVLKETLRLHPPLIILMRVAKGEFEVQGHRIHEGDLVAASPAISNRIPEDFPDPHDF
VPARYEQPRQEDLLNRWTWIPFGAGRHRCVGAAFAIMQIKAIFSVLLREYEFEMAQPPESYRNDHSKMVVQLA
QPACVRYRRRTGV
>CYP51B1_Mycobacterium tuberculosis KT-0184 2633865059
MSAVALPRVSGGHDEHGHLEEFRTDPIGLMQRVRDECGDVGTFQLAGKQVVLLSGSHANEFFFRAGDDDLDQA
KAYPFMTPIFGEGVVFDASPERRKEMLHNAALRGEQMKGHAATIEDQVRRMIADWGEAGEIDLLDFFAELTIY
TSSACLIGKKFRDQLDGRFAKLYHELERGTDPLAYVDPYLPIESFRRRDEARNGLVALVADIMNGRIANPPTD
KSDRDMLDVLIAVKAETGTPRFSADEITGMFISMMFAGHHTSSGTASWTLIELMRHRDAYAAVIDELDELYGD
GRSVSFHALRQIPQLENVLKETLRLHPPLIILMRVAKGEFEVQGHRIHEGDLVAASPAISNRIPEDFPDPHDF
VPARYEQPRQEDLLNRWTWIPFGAGRHRCVGAAFAIMQIKAIFSVLLREYEFEMAQPPESYRNDHSKMVVQLA
QPACVRYRRRTGV
>CYP51B1_Mycobacterium tuberculosis KT-0185 2648915106
MSAVALPRVSGGHDEHGHLEEFRTDPIGLMQRVRDECGDVGTFQLAGKQVVLLSGSHANEFFFRAGDDDLDQA
KAYPFMTPIFGEGVVFDASPERRKEMLHNAALRGEQMKGHAATIEDQVRRMIADWGEAGEIDLLDFFAELTIY
TSSACLIGKKFRDQLDGRFAKLYHELERGTDPLAYVDPYLPIESFRRRDEARNGLVALVADIMNGRIANPPTD
KSDRDMLDVLIAVKAETGTPRFSADEITGMFISMMFAGHHTSSGTASWTLIELMRHRDAYAAVIDELDELYGD

GRSVSFHALRQIPQLENVLKETLRLHPPLIILMRVAKGEFEVQGHRIHEGDLVAASPAISNRIPEDFPDPHDF
VPARYEQPRQEDLLNRWTWIPFGAGRHRCVGAAFAIMQIKAIFSVLLREYEFEMAQPPESYRNDHSKMVVQLA
QPACVRYRRRTGV
>CYP51B1_Mycobacterium tuberculosis KT-0186 2637694106
MSAVALPRVSGGHDEHGHLEEFRTDPIGLMQRVRDECGDVGTFQLAGKQVVLLSGSHANEFFFRAGDDDLDQA
KAYPFMTPIFGEGVVFDASPERRKEMLHNAALRGEQMKGHAATIEDQVRRMIADWGEAGEIDLLDFFAELTIY
TSSACLIGKKFRDQLDGRFAKLYHELERGTDPLAYVDPYLPIESFRRRDEARNGLVALVADIMNGRIANPPTD
KSDRDMLDVLIAVKAETGTPRFSADEITGMFISMMFAGHHTSSGTASWTLIELMRHRDAYAAVIDELDELYGD
GRSVSFHALRQIPQLENVLKETLRLHPPLIILMRVAKGEFEVQGHRIHEGDLVAASPAISNRIPEDFPDPHDF
VPARYEQPRQEDLLNRWTWIPFGAGRHRCVGAAFAIMQIKAIFSVLLREYEFEMAQPPESYRNDHSKMVVQLA
QPACVRYRRRTGV
>CYP51B1_Mycobacterium tuberculosis KT-0187 2637194983
MSAVALPRVSGGHDEHGHLEEFRTDPIGLMQRVRDECGDVGTFQLAGKQVVLLSGSHANEFFFRAGDDDLDQA
KAYPFMTPIFGEGVVFDASPERRKEMLHNAALRGEQMKGHAATIEDQVRRMIADWGEAGEIDLLDFFAELTIY
TSSACLIGKKFRDQLDGRFAKLYHELERGTDPLAYVDPYLPIESFRRRDEARNGLVALVADIMNGRIANPPTD
KSDRDMLDVLIAVKAETGTPRFSADEITGMFISMMFAGHHTSSGTASWTLIELMRHRDAYAAVIDELDELYGD
GRSVSFHALRQIPQLENVLKETLRLHPPLIILMRVAKGEFEVQGHRIHEGDLVAASPAISNRIPEDFPDPHDF
VPARYEQPRQEDLLNRWTWIPFGAGRHRCVGAAFAIMQIKAIFSVLLREYEFEMAQPPESYRNDHSKMVVQLA
QPACVRYRRRTGV
>CYP51B1_Mycobacterium tuberculosis KT-0188 2638159895
MSAVALPRVSGGHDEHGHLEEFRTDPIGLMQRVRDECGDVGTFQLAGKQVVLLSGSHANEFFFRAGDDDLDQA
KAYPFMTPIFGEGVVFDASPERRKEMLHNAALRGEQMKGHAATIEDQVRRMIADWGEAGEIDLLDFFAELTIY
TSSACLIGKKFRDQLDGRFAKLYHELERGTDPLAYVDPYLPIESFRRRDEARNGLVALVADIMNGRIANPPTD
KSDRDMLDVLIAVKAETGTPRFSADEITGMFISMMFAGHHTSSGTASWTLIELMRHRDAYAAVIDELDELYGD
GRSVSFHALRQIPQLENVLKETLRLHPPLIILMRVAKGEFEVQGHRIHEGDLVAASPAISNRIPEDFPDPHDF
VPARYEQPRQEDLLNRWTWIPFGAGRHRCVGAAFAIMQIKAIFSVLLREYEFEMAQPPESYRNDHSKMVVQLA
QPACVRYRRRTGV
>CYP51B1_Mycobacterium tuberculosis KT-0189 2649390570
MSAVALPRVSGGHDEHGHLEEFRTDPIGLMQRVRDECGDVGTFQLAGKQVVLLSGSHANEFFFRAGDDDLDQA
KAYPFMTPIFGEGVVFDASPERRKEMLHNAALRGEQMKGHAATIEDQVRRMIADWGEAGEIDLLDFFAELTIY
TSSACLIGKKFRDQLDGRFAKLYHELERGTDPLAYVDPYLPIESFRRRDEARNGLVALVADIMNGRIANPPTD
KSDRDMLDVLIAVKAETGTPRFSADEITGMFISMMFAGHHTSSGTASWTLIELMRHRDAYAAVIDELDELYGD
GRSVSFHALRQIPQLENVLKETLRLHPPLIILMRVAKGEFEVQGHRIHEGDLVAASPAISNRIPEDFPDPHDF
VPARYEQPRQEDLLNRWTWIPFGAGRHRCVGAAFAIMQIKAIFSVLLREYEFEMAQPPESYRNDHSKMVVQLA
QPACVRYRRRTGV
>CYP51B1_Mycobacterium tuberculosis KT-0190 2640471431
MSAVALPRVSGGHDEHGHLEEFRTDPIGLMQRVRDECGDVGTFQLAGKQVVLLSGSHANEFFFRAGDDDLDQA
KAYPFMTPIFGEGVVFDASPERRKEMLHNAALRGEQMKGHAATIEDQVRRMIADWGEAGEIDLLDFFAELTIY
TSSACLIGKKFRDQLDGRFAKLYHELERGTDPLAYVDPYLPIESFRRRDEARNGLVALVADIMNGRIANPPTD
KSDRDMLDVLIAVKAETGTPRFSADEITGMFISMMFAGHHTSSGTASWTLIELMRHRDAYAAVIDELDELYGD
GRSVSFHALRQIPQLENVLKETLRLHPPLIILMRVAKGEFEVQGHRIHEGDLVAASPAISNRIPEDFPDPHDF
VPARYEQPRQEDLLNRWTWIPFGAGRHRCVGAAFAIMQIKAIFSVLLREYEFEMAQPPESYRNDHSKMVVQLA
QPACVRYRRRTGV
>CYP51B1_Mycobacterium tuberculosis KT-0191 2633811442
MSAVALPRVSGGHDEHGHLEEFRTDPIGLMQRVRDECGDVGTFQLAGKQVVLLSGSHANEFFFRAGDDDLDQA
KAYPFMTPIFGEGVVFDASPERRKEMLHNAALRGEQMKGHAATIEDQVRRMIADWGEAGEIDLLDFFAELTIY
TSSACLIGKKFRDQLDGRFAKLYHELERGTDPLAYVDPYLPIESFRRRDEARNGLVALVADIMNGRIANPPTD
KSDRDMLDVLIAVKAETGTPRFSADEITGMFISMMFAGHHTSSGTASWTLIELMRHRDAYAAVIDELDELYGD
GRSVSFHALRQIPQLENVLKETLRLHPPLIILMRVAKGEFEVQGHRIHEGDLVAASPAISNRIPEDFPDPHDF
VPARYEQPRQEDLLNRWTWIPFGAGRHRCVGAAFAIMQIKAIFSVLLREYEFEMAQPPESYRNDHSKMVVQLA
QPACVRYRRRTGV
>CYP51B1_Mycobacterium tuberculosis KT-0193 2633033347
MSAVALPRVSGGHDEHGHLEEFRTDPIGLMQRVRDECGDVGTFQLAGKQVVLLSGSHANEFFFRAGDDDLDQA
KAYPFMTPIFGEGVVFDASPERRKEMLHNAALRGEQMKGHAATIEDQVRRMIADWGEAGEIDLLDFFAELTIY
TSSACLIGKKFRDQLDGRFAKLYHELERGTDPLAYVDPYLPIESFRRRDEARNGLVALVADIMNGRIANPPTD
KSDRDMLDVLIAVKAETGTPRFSADEITGMFISMMFAGHHTSSGTASWTLIELMRHRDAYAAVIDELDELYGD
GRSVSFHALRQIPQLENVLKETLRLHPPLIILMRVAKGEFEVQGHRIHEGDLVAASPAISNRIPEDFPDPHDF

VPARYEQPRQEDLLNRWTWIPFGAGRHRCVGAAFAIMQIKAIFSVLLREYEFEMAQPPESYRNDHSKMVVQLA
QPACVRYRRRTGV
>CYP51B1_Mycobacterium tuberculosis KT-0192 2636425854
MSAVALPRVSGGHDEHGHLEEFRTDPIGLMQRVRDECGDVGTFQLAGKQVVLLSGSHANEFFFRAGDDDLDQA
KAYPFMTPIFGEGVVFDASPERRKEMLHNAALRGEQMKGHAATIEDQVRRMIADWGEAGEIDLLDFFAELTIY
TSSACLIGKKFRDQLDGRFAKLYHELERGTDPLAYVDPYLPIESFRRRDEARNGLVALVADIMNGRIANPPTD
KSDRDMLDVLIAVKAETGTPRFSADEITGMFISMMFAGHHTSSGTASWTLIELMRHRDAYAAVIDELDELYGD
GRSVSFHALRQIPQLENVLKETLRLHPPLIILMRVAKGEFEVQGHRIHEGDLVAASPAISNRIPEDFPDPHDF
VPARYEQPRQEDLLNRWTWIPFGAGRHRCVGAAFAIMQIKAIFSVLLREYEFEMAQPPESYRNDHSKMVVQLA
QPACVRYRRRTGV
>CYP51B1_Mycobacterium tuberculosis KT-0195 2632820356
MSAVALPRVSGGHDEHGHLEEFRTDPIGLMQRVRDECGDVGTFQLAGKQVVLLSGSHANEFFFRAGDDDLDQA
KAYPFMTPIFGEGVVFDASPERRKEMLHNAALRGEQMKGHAATIEDQVRRMIADWGEAGEIDLLDFFAELTIY
TSSACLIGKKFRDQLDGRFAKLYHELERGTDPLAYVDPYLPIESFRRRDEARNGLVALVADIMNGRIANPPTD
KSDRDMLDVLIAVKAETGTPRFSADEITGMFISMMFAGHHTSSGTASWTLIELMRHRDAYAAVIDELDELYGD
GRSVSFHALRQIPQLENVLKETLRLHPPLIILMRVAKGEFEVQGHRIHEGDLVAASPAISNRIPEDFPDPHDF
VPARYEQPRQEDLLNRWTWIPFGAGRHRCVGAAFAIMQIKAIFSVLLREYEFEMAQPPESYRNDHSKMVVQLA
QPACVRYRRRTGV
>CYP51B1_Mycobacterium tuberculosis KT-0196 2652491651
MSAVALPRVSGGHDEHGHLEEFRTDPIGLMQRVRDECGDVGTFQLAGKQVVLLSGSHANEFFFRAGDDDLDQA
KAYPFMTPIFGEGVVFDASPERRKEMLHNAALRGEQMKGHAATIEDQVRRMIADWGEAGEIDLLDFFAELTIY
TSSACLIGKKFRDQLDGRFAKLYHELERGTDPLAYVDPYLPIESFRRRDEARNGLVALVADIMNGRIANPPTD
KSDRDMLDVLIAVKAETGTPRFSADEITGMFISMMFAGHHTSSGTASWTLIELMRHRDAYAAVIDELDELYGD
GRSVSFHALRQIPQLENVLKETLRLHPPLIILMRVAKGEFEVQGHRIHEGDLVAASPAISNRIPEDFPDPHDF
VPARYEQPRQEDLLNRWTWIPFGAGRHRCVGAAFAIMQIKAIFSVLLREYEFEMAQPPESYRNDHSKMVVQLA
QPACVRYRRRTGV
>CYP51B1_Mycobacterium tuberculosis KT-0197 2631948631
MSAVALPRVSGGHDEHGHLEEFRTDPIGLMQRVRDECGDVGTFQLAGKQVVLLSGSHANEFFFRAGDDDLDQA
KAYPFMTPIFGEGVVFDASPERRKEMLHNAALRGEQMKGHAATIEDQVRRMIADWGEAGEIDLLDFFAELTIY
TSSACLIGKKFRDQLDGRFAKLYHELERGTDPLAYVDPYLPIESFRRRDEARNGLVALVADIMNGRIANPPTD
KSDRDMLDVLIAVKAETGTPRFSADEITGMFISMMFAGHHTSSGTASWTLIELMRHRDAYAAVIDELDELYGD
GRSVSFHALRQIPQLENVLKETLRLHPPLIILMRVAKGEFEVQGHRIHEGDLVAASPAISNRIPEDFPDPHDF
VPARYEQPRQEDLLNRWTWIPFGAGRHRCVGAAFAIMQIKAIFSVLLREYEFEMAQPPESYRNDHSKMVVQLA
QPACVRYRRRTGV
>CYP51B1_Mycobacterium tuberculosis KT-0198 2635486707
MSAVALPRVSGGHDEHGHLEEFRTDPIGLMQRVRDECGDVGTFQLAGKQVVLLSGSHANEFFFRAGDDDLDQA
KAYPFMTPIFGEGVVFDASPERRKEMLHNAALRGEQMKGHAATIEDQVRRMIADWGEAGEIDLLDFFAELTIY
TSSACLIGKKFRDQLDGRFAKLYHELERGTDPLAYVDPYLPIESFRRRDEARNGLVALVADIMNGRIANPPTD
KSDRDMLDVLIAVKAETGTPRFSADEITGMFISMMFAGHHTSSGTASWTLIELMRHRDAYAAVIDELDELYGD
GRSVSFHALRQIPQLENVLKETLRLHPPLIILMRVAKGEFEVQGHRIHEGDLVAASPAISNRIPEDFPDPHDF
VPARYEQPRQEDLLNRWTWIPFGAGRHRCVGAAFAIMQIKAIFSVLLREYEFEMAQPPESYRNDHSKMVVQLA
QPACVRYRRRTGV
>CYP51B1_Mycobacterium tuberculosis KT-0199 2632695865
MSAVALPRVSGGHDEHGHLEEFRTDPIGLMQRVRDECGDVGTFQLAGKQVVLLSGSHANEFFFRAGDDDLDQA
KAYPFMTPIFGEGVVFDASPERRKEMLHNAALRGEQMKGHAATIEDQVRRMIADWGEAGEIDLLDFFAELTIY
TSSACLIGKKFRDQLDGRFAKLYHELERGTDPLAYVDPYLPIESFRRRDEARNGLVALVADIMNGRIANPPTD
KSDRDMLDVLIAVKAETGTPRFSADEITGMFISMMFAGHHTSSGTASWTLIELMRHRDAYAAVIDELDELYGD
GRSVSFHALRQIPQLENVLKETLRLHPPLIILMRVAKGEFEVQGHRIHEGDLVAASPAISNRIPEDFPDPHDF
VPARYEQPRQEDLLNRWTWIPFGAGRHRCVGAAFAIMQIKAIFSVLLREYEFEMAQPPESYRNDHSKMVVQLA
QPACVRYRRRTGV
>CYP51B1_Mycobacterium tuberculosis KT-0200 2633378680
MSAVALPRVSGGHDEHGHLEEFRTDPIGLMQRVRDECGDVGTFQLAGKQVVLLSGSHANEFFFRAGDDDLDQA
KAYPFMTPIFGEGVVFDASPERRKEMLHNAALRGEQMKGHAATIEDQVRRMIADWGEAGEIDLLDFFAELTIY
TSSACLIGKKFRDQLDGRFAKLYHELERGTDPLAYVDPYLPIESFRRRDEARNGLVALVADIMNGRIANPPTD
KSDRDMLDVLIAVKAETGTPRFSADEITGMFISMMFAGHHTSSGTASWTLIELMRHRDAYAAVIDELDELYGD
GRSVSFHALRQIPQLENVLKETLRLHPPLIILMRVAKGEFEVQGHRIHEGDLVAASPAISNRIPEDFPDPHDF
VPARYEQPRQEDLLNRWTWIPFGAGRHRCVGAAFAIMQIKAIFSVLLREYEFEMAQPPESYRNDHSKMVVQLA
QPACVRYRRRTGV

>CYP51B1_Mycobacterium tuberculosis KT-0201 2640535908
MSAVALPRVSGGHDEHGHLEEFRTDPIGLMQRVRDECGDVGTFQLAGKQVVLLSGSHANEFFFRAGDDDLDQA
KAYPFMTPIFGEGVVFDASPERRKEMLHNAALRGEQMKGHAATIEDQVRRMIADWGEAGEIDLLDFFAELTIY
TSSACLIGKKFRDQLDGRFAKLYHELERGTDPLAYVDPYLPIESFRRRDEARNGLVALVADIMNGRIANPPTD
KSDRDMLDVLIAVKAETGTPRFSADEITGMFISMMFAGHHTSSGTASWTLIELMRHRDAYAAVIDELDELYGD
GRSVSFHALRQIPQLENVLKETLRLHPPLIILMRVAKGEFEVQGHRIHEGDLVAASPAISNRIPEDFPDPHDF
VPARYEQPRQEDLLNRWTWIPFGAGRHRCVGAAFAIMQIKAIFSVLLREYEFEMAQPPESYRNDHSKMVVQLA
QPACVRYRRRTGV
>CYP51B1_Mycobacterium tuberculosis KT-0202 2632681048
MSAVALPRVSGGHDEHGHLEEFRTDPIGLMQRVRDECGDVGTFQLAGKQVVLLSGSHANEFFFRAGDDDLDQA
KAYPFMTPIFGEGVVFDASPERRKEMLHNAALRGEQMKGHAATIEDQVRRMIADWGEAGEIDLLDFFAELTIY
TSSACLIGKKFRDQLDGRFAKLYHELERGTDPLAYVDPYLPIESFRRRDEARNGLVALVADIMNGRIANPPTD
KSDRDMLDVLIAVKAETGTPRFSADEITGMFISMMFAGHHTSSGTASWTLIELMRHRDAYAAVIDELDELYGD
GRSVSFHALRQIPQLENVLKETLRLHPPLIILMRVAKGEFEVQGHRIHEGDLVAASPAISNRIPEDFPDPHDF
VPARYEQPRQEDLLNRWTWIPFGAGRHRCVGAAFAIMQIKAIFSVLLREYEFEMAQPPESYRNDHSKMVVQLA
QPACVRYRRRTGV
>CYP51B1_Mycobacterium tuberculosis KT-0203 2636464383
MSAVALPRVSGGHDEHGHLEEFRTDPIGLMQRVRDECGDVGTFQLAGKQVVLLSGSHANEFFFRAGDDDLDQA
KAYPFMTPIFGEGVVFDASPERRKEMLHNAALRGEQMKGHAATIEDQVRRMIADWGEAGEIDLLDFFAELTIY
TSSACLIGKKFRDQLDGRFAKLYHELERGTDPLAYVDPYLPIESFRRRDEARNGLVALVADIMNGRIANPPTD
KSDRDMLDVLIAVKAETGTPRFSADEITGMFISMMFAGHHTSSGTASWTLIELMRHRDAYAAVIDELDELYGD
GRSVSFHALRQIPQLENVLKETLRLHPPLIILMRVAKGEFEVQGHRIHEGDLVAASPAISNRIPEDFPDPHDF
VPARYEQPRQEDLLNRWTWIPFGAGRHRCVGAAFAIMQIKAIFSVLLREYEFEMAQPPESYRNDHSKMVVQLA
QPACVRYRRRTGV
>CYP51B1_Mycobacterium tuberculosis KT-0204 2633022760
MSAVALPRVSGGHDEHGHLEEFRTDPIGLMQRVRDECGDVGTFQLAGKQVVLLSGSHANEFFFRAGDDDLDQA
KAYPFMTPIFGEGVVFDASPERRKEMLHNAALRGEQMKGHAATIEDQVRRMIADWGEAGEIDLLDFFAELTIY
TSSACLIGKKFRDQLDGRFAKLYHELERGTDPLAYVDPYLPIESFRRRDEARNGLVALVADIMNGRIANPPTD
KSDRDMLDVLIAVKAETGTPRFSADEITGMFISMMFAGHHTSSGTASWTLIELMRHRDAYAAVIDELDELYGD
GRSVSFHALRQIPQLENVLKETLRLHPPLIILMRVAKGEFEVQGHRIHEGDLVAASPAISNRIPEDFPDPHDF
VPARYEQPRQEDLLNRWTWIPFGAGRHRCVGAAFAIMQIKAIFSVLLREYEFEMAQPPESYRNDHSKMVVQLA
QPACVRYRRRTGV
>CYP51B1_Mycobacterium tuberculosis KZN 1435 644878479
MSAVALPRVSGGHDEHGHLEEFRTDPIGLMQRVRDECGDVGTFQLAGKQVVLLSGSHANEFFFRAGDDDLDQA
KAYPFMTPIFGEGVVFDASPERRKEMLHNAALRGEQMKGHAATIEDQVRRMIADWGEAGEIDLLDFFAELTIY
TSSACLIGKKFRDQLDGRFAKLYHELERGTDPLAYVDPYLPIESFRRRDEARNGLVALVADIMNGRIANPPTD
KSDRDMLDVLIAVKAETGTPRFSADEITGMFISMMFAGHHTSSGTASWTLIELMRHRDAYAAVIDELDELYGD
GRSVSFHALRQIPQLENVLKETLRLHPPLIILMRVAKGEFEVQGHRIHEGDLVAASPAISNRIPEDFPDPHDF
VPARYEQPRQEDLLNRWTWIPFGAGRHRCVGAAFAIMQIKAIFSVLLREYEFEMAQPPESYRNDHSKMVVQLA
QPACVRYRRRTGV
>CYP51B1_Mycobacterium tuberculosis KZN 4207 NZ_ACVS02000012 647085348
MSAVALPRVSGGHDEHGHLEEFRTDPIGLMQRVRDECGDVGTFQLAGKQVVLLSGSHANEFFFRAGDDDLDQA
KAYPFMTPIFGEGVVFDASPERRKEMLHNAALRGEQMKGHAATIEDQVRRMIADWGEAGEIDLLDFFAELTIY
TSSACLIGKKFRDQLDGRFAKLYHELERGTDPLAYVDPYLPIESFRRRDEARNGLVALVADIMNGRIANPPTD
KSDRDMLDVLIAVKAETGTPRFSADEITGMFISMMFAGHHTSSGTASWTLIELMRHRDAYAAVIDELDELYGD
GRSVSFHALRQIPQLENVLKETLRLHPPLIILMRVAKGEFEVQGHRIHEGDLVAASPAISNRIPEDFPDPHDF
VPARYEQPRQEDLLNRWTWIPFGAGRHRCVGAAFAIMQIKAIFSVLLREYEFEMAQPPESYRNDHSKMVVQLA
QPACVRYRRRTGV
>CYP51B1_Mycobacterium tuberculosis KZN 4207 (DS) 2511551738
MSAVALPRVSGGHDEHGHLEEFRTDPIGLMQRVRDECGDVGTFQLAGKQVVLLSGSHANEFFFRAGDDDLDQA
KAYPFMTPIFGEGVVFDASPERRKEMLHNAALRGEQMKGHAATIEDQVRRMIADWGEAGEIDLLDFFAELTIY
TSSACLIGKKFRDQLDGRFAKLYHELERGTDPLAYVDPYLPIESFRRRDEARNGLVALVADIMNGRIANPPTD
KSDRDMLDVLIAVKAETGTPRFSADEITGMFISMMFAGHHTSSGTASWTLIELMRHRDAYAAVIDELDELYGD
GRSVSFHALRQIPQLENVLKETLRLHPPLIILMRVAKGEFEVQGHRIHEGDLVAASPAISNRIPEDFPDPHDF
VPARYEQPRQEDLLNRWTWIPFGAGRHRCVGAAFAIMQIKAIFSVLLREYEFEMAQPPESYRNDHSKMVVQLA
QPACVRYRRRTGV
>CYP51B1_Mycobacterium tuberculosis KZN 605 645121980

MSAVALPRVSGGHDEHGHLEEFRTDPIGLMQRVRDECGDVGTFQLAGKQVVLLSGSHANEFFFRAGDDDLDQA
KAYPFMTPIFGEGVVFDASPERRKEMLHNAALRGEQMKGHAATIEDQVRRMIADWGEAGEIDLLDFFAELTIY
TSSACLIGKKFRDQLDGRFAKLYHELERGTDPLAYVDPYLPIESFRRRDEARNGLVALVADIMNGRIANPPTD
KSDRDMLDVLIAVKAETGTPRFSADEITGMFISMMFAGHHTSSGTASWTLIELMRHRDAYAAVIDELDELYGD
GRSVSFHALRQIPQLENVLKETLRLHPPLIILMRVAKGEFEVQGHRIHEGDLVAASPAISNRIPEDFPDPHDF
VPARYEQPRQEDLLNRWTWIPFGAGRHRCVGAAFAIMQIKAIFSVLLREYEFEMAQPPESYRNDHSKMVVQLA
QPACVRYRRRTGV
>CYP51B1_Mycobacterium tuberculosis KZN R506 NZ_ACVU02000020 648335022
MSAVALPRVSGGHDEHGHLEEFRTDPIGLMQRVRDECGDVGTFQLAGKQVVLLSGSHANEFFFRAGDDDLDQA
KAYPFMTPIFGEGVVFDASPERRKEMLHNAALRGEQMKGHAATIEDQVRRMIADWGEAGEIDLLDFFAELTIY
TSSACLIGKKFRDQLDGRFAKLYHELERGTDPLAYVDPYLPIESFRRRDEARNGLVALVADIMNGRIANPPTD
KSDRDMLDVLIAVKAETGTPRFSADEITGMFISMMFAGHHTSSGTASWTLIELMRHRDAYAAVIDELDELYGD
GRSVSFHALRQIPQLENVLKETLRLHPPLIILMRVAKGEFEVQGHRIHEGDLVAASPAISNRIPEDFPDPHDF
VPARYEQPRQEDLLNRWTWIPFGAGRHRCVGAAFAIMQIKAIFSVLLREYEFEMAQPPESYRNDHSKMVVQLA
QPACVRYRRRTGV
>CYP51B1_Mycobacterium tuberculosis KZN V2475 NZ_ACVT02000021 647089549
MSAVALPRVSGGHDEHGHLEEFRTDPIGLMQRVRDECGDVGTFQLAGKQVVLLSGSHANEFFFRAGDDDLDQA
KAYPFMTPIFGEGVVFDASPERRKEMLHNAALRGEQMKGHAATIEDQVRRMIADWGEAGEIDLLDFFAELTIY
TSSACLIGKKFRDQLDGRFAKLYHELERGTDPLAYVDPYLPIESFRRRDEARNGLVALVADIMNGRIANPPTD
KSDRDMLDVLIAVKAETGTPRFSADEITGMFISMMFAGHHTSSGTASWTLIELMRHRDAYAAVIDELDELYGD
GRSVSFHALRQIPQLENVLKETLRLHPPLIILMRVAKGEFEVQGHRIHEGDLVAASPAISNRIPEDFPDPHDF
VPARYEQPRQEDLLNRWTWIPFGAGRHRCVGAAFAIMQIKAIFSVLLREYEFEMAQPPESYRNDHSKMVVQLA
QPACVRYRRRTGV
>CYP51B1_Mycobacterium tuberculosis L_13621 2716385820
MSAVALPRVSGGHDEHGHLEEFRTDPIGLMQRVRDECGDVGTFQLAGKQVVLLSGSHANEFFFRAGDDDLDQA
KAYPFMTPIFGEGVVFDASPERRKEMLHNAALRGEQMKGHAATIEDQVRRMIADWGEAGEIDLLDFFAELTIY
TSSACLIGKKFRDQLDGRFAKLYHELERGTDPLAYVDPYLPIESFRRRDEARNGLVALVADIMNGRIANPPTD
KSDRDMLDVLIAVKAETGTPRFSADEITGMFISMMFAGHHTSSGTASWTLIELMRHRDAYAAVIDELDELYGD
GRSVSFHALRQIPQLENVLKETLRLHPPLIILMRVAKGEFEVQGHRIHEGDLVAASPAISNRIPEDFPDPHDF
VPARYEQPRQEDLLNRWTWIPFGAGRHRCVGAAFAIMQIKAIFSVLLREYEFEMAQPPESYRNDHSKMVVQLA
QPACVRYRRRTGV
>CYP51B1_Mycobacterium tuberculosis L00201493 2703660174
MSAVALPRVSGGHDEHGHLEEFRTDPIGLMQRVRDECGDVGTFQLAGKQVVLLSGSHANEFFFRAGDDDLDQA
KAYPFMTPIFGEGVVFDASPERRKEMLHNAALRGEQMKGHAATIEDQVRRMIADWGEAGEIDLLDFFAELTIY
TSSACLIGKKFRDQLDGRFAKLYHELERGTDPLAYVDPYLPIESFRRRDEARNGLVALVADIMNGRIANPPTD
KSDRDMLDVLIAVKAETGTPRFSADEITGMFISMMFAGHHTSSGTASWTLIELMRHRDAYAAVIDELDELYGD
GRSVSFHALRQIPQLENVLKETLRLHPPLIILMRVAKGEFEVQGHRIHEGDLVAASPAISNRIPEDFPDPHDF
VPARYEQPRQEDLLNRWTWIPFGAGRHRCVGAAFAIMQIKAIFSVLLREYEFEMAQPPESYRNDHSKMVVQLA
QPACVRYRRRTGV
>CYP51B1_Mycobacterium tuberculosis L00401786 2703678131
MSAVALPRVSGGHDEHGHLEEFRTDPIGLMQRVRDECGDVGTFQLAGKQVVLLSGSHANEFFFRAGDDDLDQA
KAYPFMTPIFGEGVVFDASPERRKEMLHNAALRGEQMKGHAATIEDQVRRMIADWGEAGEIDLLDFFAELTIY
TSSACLIGKKFRDQLDGRFAKLYHELERGTDPLAYVDPYLPIESFRRRDEARNGLVALVADIMNGRIANPPTD
KSDRDMLDVLIAVKAETGTPRFSADEITGMFISMMFAGHHTSSGTASWTLIELMRHRDAYAAVIDELDELYGD
GRSVSFHALRQIPQLENVLKETLRLHPPLIILMRVAKGEFEVQGHRIHEGDLVAASPAISNRIPEDFPDPHDF
VPARYEQPRQEDLLNRWTWIPFGAGRHRCVGAAFAIMQIKAIFSVLLREYEFEMAQPPESYRNDHSKMVVQLA
QPACVRYRRRTGV
>CYP51B1_Mycobacterium tuberculosis M00018020 2693106966
MSAVALPRVSGGHDEHGHLEEFRTDPIGLMQRVRDECGDVGTFQLAGKQVVLLSGSHANEFFFRAGDDDLDQA
KAYPFMTPIFGEGVVFDASPERRKEMLHNAALRGEQMKGHAATIEDQVRRMIADWGEAGEIDLLDFFAELTIY
TSSACLIGKKFRDQLDGRFAKLYHELERGTDPLAYVDPYLPIESFRRRDEARNGLVALVADIMNGRIANPPTD
KSDRDMLDVLIAVKAETGTPRFSADEITGMFISMMFAGHHTSSGTASWTLIELMRHRDAYAAVIDELDELYGD
GRSVSFHALRQIPQLENVLKETLRLHPPLIILMRVAKGEFEVQGHRIHEGDLVAASPAISNRIPEDFPDPHDF
VPARYEQPRQEDLLNRWTWIPFGAGRHRCVGAAFAIMQIKAIFSVLLREYEFEMAQPPESYRNDHSKMVVQLA
QPACVRYRRRTGV
>CYP51B1_Mycobacterium tuberculosis M0002959-6 2720400374
MSAVALPRVSGGHDEHGHLEEFRTDPIGLMQRVRDECGDVGTFQLAGKQVVLLSGSHANEFFFRAGDDDLDQA
KAYPFMTPIFGEGVVFDASPERRKEMLHNAALRGEQMKGHAATIEDQVRRMIADWGEAGEIDLLDFFAELTIY

TSSACLIGKKFRDQLDGRFAKLYHELERGTDPLAYVDPYLPIESFRRRDEARNGLVALVADIMNGRIANPPTD
KSDRDMLDVLIAVKAETGTPRFSADEITGMFISMMFAGHHTSSGTASWTLIELMRHRDAYAAVIDELDELYGD
GRSVSFHALRQIPQLENVLKETLRLHPPLIILMRVAKGEFEVQGHRIHEGDLVAASPAISNRIPEDFPDPHDF
VPARYEQPRQEDLLNRWTWIPFGAGRHRCVGAAFAIMQIKAIFSVLLREYEFEMAQPPESYRNDHSKMVVQLA
QPACVRYRRRTGV
>CYP51B1_Mycobacterium tuberculosis M00102058 2706548488
MSAVALPRVSGGHDEHGHLEEFRTDPIGLMQRVRDECGDVGTFQLAGKQVVLLSGSHANEFFFRAGDDDLDQA
KAYPFMTPIFGEGVVFDASPERRKEMLHNAALRGEQMKGHAATIEDQVRRMIADWGEAGEIDLLDFFAELTIY
TSSACLIGKKFRDQLDGRFAKLYHELERGTDPLAYVDPYLPIESFRRRDEARNGLVALVADIMNGRIANPPTD
KSDRDMLDVLIAVKAETGTPRFSADEITGMFISMMFAGHHTSSGTASWTLIELMRHRDAYAAVIDELDELYGD
GRSVSFHALRQIPQLENVLKETLRLHPPLIILMRVAKGEFEVQGHRIHEGDLVAASPAISNRIPEDFPDPHDF
VPARYEQPRQEDLLNRWTWIPFGAGRHRCVGAAFAIMQIKAIFSVLLREYEFEMAQPPESYRNDHSKMVVQLA
QPACVRYRRRTGV
>CYP51B1_Mycobacterium tuberculosis M0018684-2 2730246922
MSAVALPRVSGGHDEHGHLEEFRTDPIGLMQRVRDECGDVGTFQLAGKQVVLLSGSHANEFFFRAGDDDLDQA
KAYPFMTPIFGEGVVFDASPERRKEMLHNAALRGEQMKGHAATIEDQVRRMIADWGEAGEIDLLDFFAELTIY
TSSACLIGKKFRDQLDGRFAKLYHELERGTDPLAYVDPYLPIESFRRRDEARNGLVALVADIMNGRIANPPTD
KSDRDMLDVLIAVKAETGTPRFSADEITGMFISMMFAGHHTSSGTASWTLIELMRHRDAYAAVIDELDELYGD
GRSVSFHALRQIPQLENVLKETLRLHPPLIILMRVAKGEFEVQGHRIHEGDLVAASPAISNRIPEDFPDPHDF
VPARYEQPRQEDLLNRWTWIPFGAGRHRCVGAAFAIMQIKAIFSVLLREYEFEMAQPPESYRNDHSKMVVQLA
QPACVRYRRRTGV
>CYP51B1_Mycobacterium tuberculosis M00201208 2683863968
MSAVALPRVSGGHDEHGHLEEFRTDPIGLMQRVRDECGDVGTFQLAGKQVVLLSGSHANEFFFRAGDDDLDQA
KAYPFMTPIFGEGVVFDASPERRKEMLHNAALRGEQMKGHAATIEDQVRRMIADWGEAGEIDLLDFFAELTIY
TSSACLIGKKFRDQLDGRFAKLYHELERGTD
>CYP51B1_Mycobacterium tuberculosis M00201208 2683863969
LRLHPPLIILMRVAKGEFEVQGHRIHEGDLVAASPAISNRIPEDFPDPHDFVPARYEQPRQEDLLNRWTWIPF
GAGRHRCVGAAFAIMQIKAIFSVLLREYEFEMAQPPESYRNDHSKMVVQLAQPACVRYRRRTGV
>CYP51B1_Mycobacterium tuberculosis M09400328 2703673679
MSAVALPRVSGGHDEHGHLEEFRTDPIGLMQRVRDECGDVGTFQLAGKQVVLLSGSHANEFFFRAGDDDLDQA
KAYPFMTPIFGEGVVFDASPERRKEMLHNAALRGEQMKGHAATIEDQVRRMIADWGEAGEIDLLDFFAELTIY
TSSACLIGKKFRDQLDGRFAKLYHELERGTDPLAYVDPYLPIESFRRRDEARNGLVALVADIMNGRIANPPTD
KSDRDMLDVLIAVKAETGTPRFSADEITGMFISMMFAGHHTSSGTASWTLIELMRHRDAYAAVIDELDELYGD
GRSVSFHALRQIPQLENVLKETLRLHPPLIILMRVAKGEFEVQGHRIHEGDLVAASPAISNRIPEDFPDPHDF
VPARYEQPRQEDLLNRWTWIPFGAGRHRCVGAAFAIMQIKAIFSVLLREYEFEMAQPPESYRNDHSKMVVQLA
QPACVRYRRRTGV
>CYP51B1_Mycobacterium tuberculosis M09400374 2699618176
MSAVALPRVSGGHDEHGHLEEFRTDPIGLMQRVRDECGDVGTFQLAGKQVVLLSGSHANEFFFRAGDDDLDQA
KAYPFMTPIFGEGVVFDASPERRKEMLHNAALRGEQMKGHAATIEDQVRRMIADWGEAGEIDLLDFFAELTIY
TSSACLIGKKFRDQLDGRFAKLYHELERGTDPLAYVDPYLPIESFRRRDEARNGLVALVADIMNGRIANPPTD
KSDRDMLDVLIAVKAETGTPRFSADEITGMFISMMFAGHHTSSGTASWTLIELMRHRDAYAAVIDELDELYGD
GRSVSFHALRQIPQLENVLKETLRLHPPLIILMRVAKGEFEVQGHRIHEGDLVAASPAISNRIPEDFPDPHDF
VPARYEQPRQEDLLNRWTWIPFGAGRHRCVGAAFAIMQIKAIFSVLLREYEFEMAQPPESYRNDHSKMVVQLA
QPACVRYRRRTGV
>CYP51B1_Mycobacterium tuberculosis M09400397 2697406646
MSAVALPRVSGGHDEHGHLEEFRTDPIGLMQRVRDECGDVGTFQLAGKQVVLLSGSHANEFFFRAGDDDLDQA
KAYPFMTPIFGEGVVFDASPERRKEMLHNAALRGEQMKGHAATIEDQVRRMIADWGEAGEIDLLDFFAELTIY
TSSACLIGKKFRDQLDGRFAKLYHELERGTDPLAYVDPYLPIESFRRRDEARNGLVALVADIMNGRIANPPTD
KSDRDMLDVLIAVKAETGTPRFSADEITGMFISMMFAGHHTSSGTASWTLIELMRHRDAYAAVIDELDELYGD
GRSVSFHALRQIPQLENVLKETLRLHPPLIILMRVAKGEFEVQGHRIHEGDLVAASPAISNRIPEDFPDPHDF
VPARYEQPRQEDLLNRWTWIPFGAGRHRCVGAAFAIMQIKAIFSVLLREYEFEMAQPPESYRNDHSKMVVQLA
QPACVRYRRRTGV
>CYP51B1_Mycobacterium tuberculosis M09400937 2703682101
MSAVALPRVSGGHDEHGHLEEFRTDPIGLMQRVRDECGDVGTFQLAGKQVVLLSGSHANEFFFRAGDDDLDQA
KAYPFMTPIFGEGVVFDASPERRKEMLHNAALRGEQMKGHAATIEDQVRRMIADWGEAGEIDLLDFFAELTIY
TSSACLIGKKFRDQLDGRFAKLYHELERGTDPLAYVDPYLPIESFRRRDEARNGLVALVADIMNGRIANPPTD
KSDRDMLDVLIAVKAETGTPRFSADEITGMFISMMFAGHHTSSGTASWTLIELMRHRDAYAAVIDELDELYGD
GRSVSFHALRQIPQLENVLKETLRLHPPLIILMRVAKGEFEVQGHRIHEGDLVAASPAISNRIPEDFPDPHDF

VPARYEQPRQEDLLNRWTWIPFGAGRHRCVGAAFAIMQIKAIFSVLLREYEFEMAQPPESYRNDHSKMVVQLA
QPACVRYRRRTGV
>CYP51B1_Mycobacterium tuberculosis M09401471 2703641663
MSAVALPRVSGGHDEHGHLEEFRTDPIGLMQRVRDECGDVGTFQLAGKQVVLLSGSHANEFFFRAGDDDLDQA
KAYPFMTPIFGEGVVFDASPERRKEMLHNAALRGEQMKGHAATIEDQVRRMIADWGEAGEIDLLDFFAELTIY
TSSACLIGKKFRDQLDGRFAKLYHELERGTDPLAYVDPYLPIESFRRRDEARNGLVALVADIMNGRIANPPTD
KSDRDMLDVLIAVKAETGTPRFSADEITGMFISMMFAGHHTSSGTASWTLIELMRHRDAYAAVIDELDELYGD
GRSVSFHALRQIPQLENVLKETLRLHPPLIILMRVAKGEFEVQGHRIHEGDLVAASPAISNRIPEDFPDPHDF
VPARYEQPRQEDLLNRWTWIPFGAGRHRCVGAAFAIMQIKAIFSVLLREYEFEMAQPPESYRNDHSKMVVQLA
QPACVRYRRRTGV
>CYP51B1_Mycobacterium tuberculosis M09402043 2697734442
MSAVALPRVSGGHDEHGHLEEFRTDPIGLMQRVRDECGDVGTFQLAGKQVVLLSGSHANEFFFRAGDDDLDQA
KAYPFMTPIFGEGVVFDASPERRKEMLHNAALRGEQMKGHAATIEDQVRRMIADWGEAGEIDLLDFFAELTIY
TSSACLIGKKFRDQLDGRFAKLYHELERGTDPLAYVDPYLPIESFRRRDEARNGLVALVADIMNGRIANPPTD
KSDRDMLDVLIAVKAETGTPRFSADEITGMFISMMFAGHHTSSGTASWTLIELMRHRDAYAAVIDELDELYGD
GRSVSFHALRQIPQLENVLKETLRLHPPLIILMRVAKGEFEVQGHRIHEGDLVAASPAISNRIPEDFPDPHDF
VPARYEQPRQEDLLNRWTWIPFGAGRHRCVGAAFAIMQIKAIFSVLLREYEFEMAQPPESYRNDHSKMVVQLA
QPACVRYRRRTGV
>CYP51B1_Mycobacterium tuberculosis M09500081 2703586466
MSAVALPRVSGGHDEHGHLEEFRTDPIGLMQRVRDECGDVGTFQLAGKQVVLLSGSHANEFFFRAGDDDLDQA
KAYPFMTPIFGEGVVFDASPERRKEMLHNAALRGEQMKGHAATIEDQVRRMIADWGEAGEIDLLDFFAELTIY
TSSACLIGKKFRDQLDGRFAKLYHELERGTDPLAYVDPYLPIESFRRRDEARNGLVALVADIMNGRIANPPTD
KSDRDMLDVLIAVKAETGTPRFSADEITGMFISMMFAGHHTSSGTASWTLIELMRHRDAYAAVIDELDELYGD
GRSVSFHALRQIPQLENVLKETLRLHPPLIILMRVAKGEFEVQGHRIHEGDLVAASPAISNRIPEDFPDPHDF
VPARYEQPRQEDLLNRWTWIPFGAGRHRCVGAAFAIMQIKAIFSVLLREYEFEMAQPPESYRNDHSKMVVQLA
QPACVRYRRRTGV
>CYP51B1_Mycobacterium tuberculosis M09502139 2703654981
MSAVALPRVSGGHDEHGHLEEFRTDPIGLMQRVRDECGDVGTFQLAGKQVVLLSGSHANEFFFRAGDDDLDQA
KAYPFMTPIFGEGVVFDASPERRKEMLHNAALRGEQMKGHAATIEDQVRRMIADWGEAGEIDLLDFFAELTIY
TSSACLIGKKFRDQLDGRFAKLYHELERGTDPLAYVDPYLPIESFRRRDEARNGLVALVADIMNGRIANPPTD
KSDRDMLDVLIAVKAETGTPRFSADEITGMFISMMFAGHHTSSGTASWTLIELMRHRDAYAAVIDELDELYGD
GRSVSFHALRQIPQLENVLKETLRLHPPLIILMRVAKGEFEVQGHRIHEGDLVAASPAISNRIPEDFPDPHDF
VPARYEQPRQEDLLNRWTWIPFGAGRHRCVGAAFAIMQIKAIFSVLLREYEFEMAQPPESYRNDHSKMVVQLA
QPACVRYRRRTGV
>CYP51B1_Mycobacterium tuberculosis M1004 2577268512
MSAVALPRVSGGHDEHGHLEEFRTDPIGLMQRVRDECGDVGTFQLAGKQVVLLSGSHANEFFFRAGDDDLDQA
KAYPFMTPIFGEGVVFDASPERRKEMLHNAALRGEQMKGHAATIEDQVRRMIADWGEAGEIDLLDFFAELTIY
TSSACLIGKKFRDQLDGRFAKLYHELERGTDPLAYVDPYLPIESFRRRDEARNGLVALVADIMNGRIANPPTD
KSDRDMLDVLIAVKAETGTPRFSADEITGMFISMMFAGHHTSSGTASWTLIELMRHRDAYAAVIDELDELYGD
GRSVSFHALRQIPQLENVLKETLRLHPPLIILMRVAKGEFEVQGHRIHEGDLVAASPAISNRIPEDFPDPHDF
VPARYEQPRQEDLLNRWTWIPFGAGRHRCVGAAFAIMQIKAIFSVLLREYEFEMAQPPESYRNDHSKMVVQLA
QPACVRYRRRTGV
>CYP51B1_Mycobacterium tuberculosis M1007 2576553015
MSAVALPRVSGGHDEHGHLEEFRTDPIGLMQRVRDECGDVGTFQLAGKQVVLLSGSHANEFFFRAGDDDLDQA
KAYPFMTPIFGEGVVFDASPERRKEMLHNAALRGEQMKGHAATIEDQVRRMIADWGEAGEIDLLDFFAELTIY
TSSACLIGKKFRDQLDGRFAKLYHELERGTDPLAYVDPYLPIESFRRRDEARNGLVALVADIMNGRIANPPTD
KSDRDMLDVLIAVKAETGTPRFSADEITGMFISMMFAGHHTSSGTASWTLIELMRHRDAYAAVIDELDELYGD
GRSVSFHALRQIPQLENVLKETLRLHPPLIILMRVAKGEFEVQGHRIHEGDLVAASPAISNRIPEDFPDPHDF
VPARYEQPRQEDLLNRWTWIPFGAGRHRCVGAAFAIMQIKAIFSVLLREYEFEMAQPPESYRNDHSKMVVQLA
QPACVRYRRRTGV
>CYP51B1_Mycobacterium tuberculosis M1008 2576570721
MSAVALPRVSGGHDEHGHLEEFRTDPIGLMQRVRDECGDVGTFQLAGKQVVLLSGSHANEFFFRAGDDDLDQA
KAYPFMTPIFGEGVVFDASPERRKEMLHNAALRGEQMKGHAATIEDQVRRMIADWGEAGEIDLLDFFAELTIY
TSSACLIGKKFRDQLDGRFAKLYHELERGTDPLAYVDPYLPIESFRRRDEARNGLVALVADIMNGRIANPPTD
KSDRDMLDVLIAVKAETGTPRFSADEITGMFISMMFAGHHTSSGTASWTLIELMRHRDAYAAVIDELDELYGD
GRSVSFHALRQIPQLENVLKETLRLHPPLIILMRVAKGEFEVQGHRIHEGDLVAASPAISNRIPEDFPDPHDF
VPARYEQPRQEDLLNRWTWIPFGAGRHRCVGAAFAIMQIKAIFSVLLREYEFEMAQPPESYRNDHSKMVVQLA
QPACVRYRRRTGV

>CYP51B1_Mycobacterium tuberculosis M1009 2622329155
MSAVALPRVSGGHDEHGHLEEFRTDPIGLMQRVRDECGDVGTFQLAGKQVVLLSGSHANEFFFRAGDDDLDQA
KAYPFMTPIFGEGVVFDASPERRKEMLHNAALRGEQMKGHAATIEDQVRRMIADWGEAGEIDLLDFFAELTIY
TSSACLIGKKFRDQLDGRFAKLYHELERGTDPLAYVDPYLPIESFRRRDEARNGLVALVADIMNGRIANPPTD
KSDRDMLDVLIAVKAETGTPRFSADEITGMFISMMFAGHHTSSGTASWTLIELMRHRDAYAAVIDELDELYGD
GRSVSFHALRQIPQLENVLKETLRLHPPLIILMRVAKGEFEVQGHRIHEGDLVAASPAISNRIPEDFPDPHDF
VPARYEQPRQEDLLNRWTWIPFGAGRHRCVGAAFAIMQIKAIFSVLLREYEFEMAQPPESYRNDHSKMVVQLA
QPACVRYRRRTGV
>CYP51B1_Mycobacterium tuberculosis M1010 2622333262
MSAVALPRVSGGHDEHGHLEEFRTDPIGLMQRVRDECGDVGTFQLAGKQVVLLSGSHANEFFFRAGDDDLDQA
KAYPFMTPIFGEGVVFDASPERRKEMLHNAALRGEQMKGHAATIEDQVRRMIADWGEAGEIDLLDFFAELTIY
TSSACLIGKKFRDQLDGRFAKLYHELERGTDPLAYVDPYLPIESFRRRDEARNGLVALVADIMNGRIANPPTD
KSDRDMLDVLIAVKAETGTPRFSADEITGMFISMMFAGHHTSSGTASWTLIELMRHRDAYAAVIDELDELYGD
GRSVSFHALRQIPQLENVLKETLRLHPPLIILMRVAKGEFEVQGHRIHEGDLVAASPAISNRIPEDFPDPHDF
VPARYEQPRQEDLLNRWTWIPFGAGRHRCVGAAFAIMQIKAIFSVLLREYEFEMAQPPESYRNDHSKMVVQLA
QPACVRYRRRTGV
>CYP51B1_Mycobacterium tuberculosis M1011 2622340156
MSAVALPRVSGGHDEHGHLEEFRTDPIGLMQRVRDECGDVGTFQLAGKQVVLLSGSHANEFFFRAGDDDLDQA
KAYPFMTPIFGEGVVFDASPERRKEMLHNAALRGEQMKGHAATIEDQVRRMIADWGEAGEIDLLDFFAELTIY
TSSACLIGKKFRDQLDGRFAKLYHELERGTDPLAYVDPYLPIESFRRRDEARNGLVALVADIMNGRIANPPTD
KSDRDMLDVLIAVKAETGTPRFSADEITGMFISMMFAGHHTSSGTASWTLIELMRHRDAYAAVIDELDELYGD
GRSVSFHALRQIPQLENVLKETLRLHPPLIILMRVAKGEFEVQGHRIHEGDLVAASPAISNRIPEDFPDPHDF
VPARYEQPRQEDLLNRWTWIPFGAGRHRCVGAAFAIMQIKAIFSVLLREYEFEMAQPPESYRNDHSKMVVQLA
QPACVRYRRRTGV
>CYP51B1_Mycobacterium tuberculosis M1012 2622344112
MSAVALPRVSGGHDEHGHLEEFRTDPIGLMQRVRDECGDVGTFQLAGKQVVLLSGSHANEFFFRAGDDDLDQA
KAYPFMTPIFGEGVVFDASPERRKEMLHNAALRGEQMKGHAATIEDQVRRMIADWGEAGEIDLLDFFAELTIY
TSSACLIGKKFRDQLDGRFAKLYHELERGTDPLAYVDPYLPIESFRRRDEARNGLVALVADIMNGRIANPPTD
KSDRDMLDVLIAVKAETGTPRFSADEITGMFISMMFAGHHTSSGTASWTLIELMRHRDAYAAVIDELDELYGD
GRSVSFHALRQIPQLENVLKETLRLHPPLIILMRVAKGEFEVQGHRIHEGDLVAASPAISNRIPEDFPDPHDF
VPARYEQPRQEDLLNRWTWIPFGAGRHRCVGAAFAIMQIKAIFSVLLREYEFEMAQPPESYRNDHSKMVVQLA
QPACVRYRRRTGV
>CYP51B1_Mycobacterium tuberculosis M1021 2622345340
MSAVALPRVSGGHDEHGHLEEFRTDPIGLMQRVRDECGDVGTFQLAGKQVVLLSGSHANEFFFRAGDDDLDQA
KAYPFMTPIFGEGVVFDASPERRKEMLHNAALRGEQMKGHAATIEDQVRRMIADWGEAGEIDLLDFFAELTIY
TSSACLIGKKFRDQLDGRFAKLYHELERGTDPLAYVDPYLPIESFRRRDEARNGLVALVADIMNGRIANPPTD
KSDRDMLDVLIAVKAETGTPRFSADEITGMFISMMFAGHHTSSGTASWTLIELMRHRDAYAAVIDELDELYGD
GRSVSFHALRQIPQLENVLKETLRLHPPLIILMRVAKGEFEVQGHRIHEGDLVAASPAISNRIPEDFPDPHDF
VPARYEQPRQEDLLNRWTWIPFGAGRHRCVGAAFAIMQIKAIFSVLLREYEFEMAQPPESYRNDHSKMVVQLA
QPACVRYRRRTGV
>CYP51B1_Mycobacterium tuberculosis M1022 2622349746
MSAVALPRVSGGHDEHGHLEEFRTDPIGLMQRVRDECGDVGTFQLAGKQVVLLSGSHANEFFFRAGDDDLDQA
KAYPFMTPIFGEGVVFDASPERRKEMLHNAALRGEQMKGHAATIEDQVRRMIADWGEAGEIDLLDFFAELTIY
TSSACLIGKKFRDQLDGRFAKLYHELERGTDPLAYVDPYLPIESFRRRDEARNGLVALVADIMNGRIANPPTD
KSDRDMLDVLIAVKAETGTPRFSADEITGMFISMMFAGHHTSSGTASWTLIELMRHRDAYAAVIDELDELYGD
GRSVSFHALRQIPQLENVLKETLRLHPPLIILMRVAKGEFEVQGHRIHEGDLVAASPAISNRIPEDFPDPHDF
VPARYEQPRQEDLLNRWTWIPFGAGRHRCVGAAFAIMQIKAIFSVLLREYEFEMAQPPESYRNDHSKMVVQLA
QPACVRYRRRTGV
>CYP51B1_Mycobacterium tuberculosis M1023 2622353858
MSAVALPRVSGGHDEHGHLEEFRTDPIGLMQRVRDECGDVGTFQLAGKQVVLLSGSHANEFFFRAGDDDLDQA
KAYPFMTPIFGEGVVFDASPERRKEMLHNAALRGEQMKGHAATIEDQVRRMIADWGEAGEIDLLDFFAELTIY
TSSACLIGKKFRDQLDGRFAKLYHELERGTDPLAYVDPYLPIESFRRRDEARNGLVALVADIMNGRIANPPTD
KSDRDMLDVLIAVKAETGTPRFSADEITGMFISMMFAGHHTSSGTASWTLIELMRHRDAYAAVIDELDELYGD
GRSVSFHALRQIPQLENVLKETLRLHPPLIILMRVAKGEFEVQGHRIHEGDLVAASPAISNRIPEDFPDPHDF
VPARYEQPRQEDLLNRWTWIPFGAGRHRCVGAAFAIMQIKAIFSVLLREYEFEMAQPPESYRNDHSKMVVQLA
QPACVRYRRRTGV
>CYP51B1_Mycobacterium tuberculosis M1024 2622789833

MSAVALPRVSGGHDEHGHLEEFRTDPIGLMQRVRDECGDVGTFQLAGKQVVLLSGSHANEFFFRAGDDDLDQA
KAYPFMTPIFGEGVVFDASPERRKEMLHNAALRGEQMKGHAATIEDQVRRMIADWGEAGEIDLLDFFAELTIY
TSSACLIGKKFRDQLDGRFAKLYHELERGTDPLAYVDPYLPIESFRRRDEARNGLVALVADIMNGRIANPPTD
KSDRDMLDVLIAVKAETGTPRFSADEITGMFISMMFAGHHTSSGTASWTLIELMRHRDAYAAVIDELDELYGD
GRSVSFHALRQIPQLENVLKETLRLHPPLIILMRVAKGEFEVQGHRIHEGDLVAASPAISNRIPEDFPDPHDF
VPARYEQPRQEDLLNRWTWIPFGAGRHRCVGAAFAIMQIKAIFSVLLREYEFEMAQPPESYRNDHSKMVVQLA
QPACVRYRRRTGV
>CYP51B1_Mycobacterium tuberculosis M1025 2578158305
MSAVALPRVSGGHDEHGHLEEFRTDPIGLMQRVRDECGDVGTFQLAGKQVVLLSGSHANEFFFRAGDDDLDQA
KAYPFMTPIFGEGVVFDASPERRKEMLHNAALRGEQMKGHAATIEDQVRRMIADWGEAGEIDLLDFFAELTIY
TSSACLIGKKFRDQLDGRFAKLYHELERGTDPLAYVDPYLPIESFRRRDEARNGLVALVADIMNGRIANPPTD
KSDRDMLDVLIAVKAETGTPRFSADEITGMFISMMFAGHHTSSGTASWTLIELMRHRDAYAAVIDELDELYGD
GRSVSFHALRQIPQLENVLKETLRLHPPLIILMRVAKGEFEVQGHRIHEGDLVAASPAISNRIPEDFPDPHDF
VPARYEQPRQEDLLNRWTWIPFGAGRHRCVGAAFAIMQIKAIFSVLLREYEFEMAQPPESYRNDHSKMVVQLA
QPACVRYRRRTGV
>CYP51B1_Mycobacterium tuberculosis M1026 2622357662
MSAVALPRVSGGHDEHGHLEEFRTDPIGLMQRVRDECGDVGTFQLAGKQVVLLSGSHANEFFFRAGDDDLDQA
KAYPFMTPIFGEGVVFDASPERRKEMLHNAALRGEQMKGHAATIEDQVRRMIADWGEAGEIDLLDFFAELTIY
TSSACLIGKKFRDQLDGRFAKLYHELERGTDPLAYVDPYLPIESFRRRDEARNGLVALVADIMNGRIANPPTD
KSDRDMLDVLIAVKAETGTPRFSADEITGMFISMMFAGHHTSSGTASWTLIELMRHRDAYAAVIDELDELYGD
GRSVSFHALRQIPQLENVLKETLRLHPPLIILMRVAKGEFEVQGHRIHEGDLVAASPAISNRIPEDFPDPHDF
VPARYEQPRQEDLLNRWTWIPFGAGRHRCVGAAFAIMQIKAIFSVLLREYEFEMAQPPESYRNDHSKMVVQLA
QPACVRYRRRTGV
>CYP51B1_Mycobacterium tuberculosis M1029 2622364840
MSAVALPRVSGGHDEHGHLEEFRTDPIGLMQRVRDECGDVGTFQLAGKQVVLLSGSHANEFFFRAGDDDLDQA
KAYPFMTPIFGEGVVFDASPERRKEMLHNAALRGEQMKGHAATIEDQVRRMIADWGEAGEIDLLDFFAELTIY
TSSACLIGKKFRDQLDGRFAKLYHELERGTDPLAYVDPYLPIESFRRRDEARNGLVALVADIMNGRIANPPTD
KSDRDMLDVLIAVKAETGTPRFSADEITGMFISMMFAGHHTSSGTASWTLIELMRHRDAYAAVIDELDELYGD
GRSVSFHALRQIPQLENVLKETLRLHPPLIILMRVAKGEFEVQGHRIHEGDLVAASPAISNRIPEDFPDPHDF
VPARYEQPRQEDLLNRWTWIPFGAGRHRCVGAAFAIMQIKAIFSVLLREYEFEMAQPPESYRNDHSKMVVQLA
QPACVRYRRRTGV
>CYP51B1_Mycobacterium tuberculosis M1030 2622369101
MSAVALPRVSGGHDEHGHLEEFRTDPIGLMQRVRDECGDVGTFQLAGKQVVLLSGSHANEFFFRAGDDDLDQA
KAYPFMTPIFGEGVVFDASPERRKEMLHNAALRGEQMKGHAATIEDQVRRMIADWGEAGEIDLLDFFAELTIY
TSSACLIGKKFRDQLDGRFAKLYHELERGTDPLAYVDPYLPIESFRRRDEARNGLVALVADIMNGRIANPPTD
KSDRDMLDVLIAVKAETGTPRFSADEITGMFISMMFAGHHTSSGTASWTLIELMRHRDAYAAVIDELDELYGD
GRSVSFHALRQIPQLENVLKETLRLHPPLIILMRVAKGEFEVQGHRIHEGDLVAASPAISNRIPEDFPDPHDF
VPARYEQPRQEDLLNRWTWIPFGAGRHRCVGAAFAIMQIKAIFSVLLREYEFEMAQPPESYRNDHSKMVVQLA
QPACVRYRRRTGV
>CYP51B1_Mycobacterium tuberculosis M1034 2622373171
MSAVALPRVSGGHDEHGHLEEFRTDPIGLMQRVRDECGDVGTFQLAGKQVVLLSGSHANEFFFRAGDDDLDQA
KAYPFMTPIFGEGVVFDASPERRKEMLHNAALRGEQMKGHAATIEDQVRRMIADWGEAGEIDLLDFFAELTIY
TSSACLIGKKFRDQLDGRFAKLYHELERGTDPLAYVDPYLPIESFRRRDEARNGLVALVADIMNGRIANPPTD
KSDRDMLDVLIAVKAETGTPRFSADEITGMFISMMFAGHHTSSGTASWTLIELMRHRDAYAAVIDELDELYGD
GRSVSFHALRQIPQLENVLKETLRLHPPLIILMRVAKGEFEVQGHRIHEGDLVAASPAISNRIPEDFPDPHDF
VPARYEQPRQEDLLNRWTWIPFGAGRHRCVGAAFAIMQIKAIFSVLLREYEFEMAQPPESYRNDHSKMVVQLA
QPACVRYRRRTGV
>CYP51B1_Mycobacterium tuberculosis M1210 2622374514
MSAVALPRVSGGHDEHGHLEEFRTDPIGLMQRVRDECGDVGTFQLAGKQVVLLSGSHANEFFFRAGDDDLDQA
KAYPFMTPIFGEGVVFDASPERRKEMLHNAALRGEQMKGHAATIEDQVRRMIADWGEAGEIDLLDFFAELTIY
TSSACLIGKKFRDQLDGRFAKLYHELERGTDPLAYVDPYLPIESFRRRDEARNGLVALVADIMNGRIANPPTD
KSDRDMLDVLIAVKAETGTPRFSADEITGMFISMMFAGHHTSSGTASWTLIELMRHRDAYAAVIDELDELYGD
GRSVSFHALRQIPQLENVLKETLRLHPPLIILMRVAKGEFEVQGHRIHEGDLVAASPAISNRIPEDFPDPHDF
VPARYEQPRQEDLLNRWTWIPFGAGRHRCVGAAFAIMQIKAIFSVLLREYEFEMAQPPESYRNDHSKMVVQLA
QPACVRYRRRTGV
>CYP51B1_Mycobacterium tuberculosis M1212 2622378629
MSAVALPRVSGGHDEHGHLEEFRTDPIGLMQRVRDECGDVGTFQLAGKQVVLLSGSHANEFFFRAGDDDLDQA
KAYPFMTPIFGEGVVFDASPERRKEMLHNAALRGEQMKGHAATIEDQVRRMIADWGEAGEIDLLDFFAELTIY

TSSACLIGKKFRDQLDGRFAKLYHELERGTDPLAYVDPYLPIESFRRRDEARNGLVALVADIMNGRIANPPTD
KSDRDMLDVLIAVKAETGTPRFSADEITGMFISMMFAGHHTSSGTASWTLIELMRHRDAYAAVIDELDELYGD
GRSVSFHALRQIPQLENVLKETLRLHPPLIILMRVAKGEFEVQGHRIHEGDLVAASPAISNRIPEDFPDPHDF
VPARYEQPRQEDLLNRWTWIPFGAGRHRCVGAAFAIMQIKAIFSVLLREYEFEMAQPPESYRNDHSKMVVQLA
QPACVRYRRRTGV
>CYP51B1_Mycobacterium tuberculosis M1213 2575560412
MSAVALPRVSGGHDEHGHLEEFRTDPIGLMQRVRDECGDVGTFQLAGKQVVLLSGSHANEFFFRAGDDDLDQA
KAYPFMTPIFGEGVVFDASPERRKEMLHNAALRGEQMKGHAATIEDQVRRMIADWGEAGEIDLLDFFAELTIY
TSSACLIGKKFRDQLDGRFAKLYHELERGTDPLAYVDPYLPIESFRRRDEARNGLVALVADIMNGRIANPPTD
KSDRDMLDVLIAVKAETGTPRFSADEITGMFISMMFAGHHTSSGTASWTLIELMRHRDAYAAVIDELDELYGD
GRSVSFHALRQIPQLENVLKETLRLHPPLIILMRVAKGEFEVQGHRIHEGDLVAASPAISNRIPEDFPDPHDF
VPARYEQPRQEDLLNRWTWIPFGAGRHRCVGAAFAIMQIKAIFSVLLREYEFEMAQPPESYRNDHSKMVVQLA
QPACVRYRRRTGV
>CYP51B1_Mycobacterium tuberculosis M1215 2622384804
MSAVALPRVSGGHDEHGHLEEFRTDPIGLMQRVRDECGDVGTFQLAGKQVVLLSGSHANEFFFRAGDDDLDQA
KAYPFMTPIFGEGVVFDASPERRKEMLHNAALRGEQMKGHAATIEDQVRRMIADWGEAGEIDLLDFFAELTIY
TSSACLIGKKFRDQLDGRFAKLYHELERGTDPLAYVDPYLPIESFRRRDEARNGLVALVADIMNGRIANPPTD
KSDRDMLDVLIAVKAETGTPRFSADEITGMFISMMFAGHHTSSGTASWTLIELMRHRDAYAAVIDELDELYGD
GRSVSFHALRQIPQLENVLKETLRLHPPLIILMRVAKGEFEVQGHRIHEGDLVAASPAISNRIPEDFPDPHDF
VPARYEQPRQEDLLNRWTWIPFGAGRHRCVGAAFAIMQIKAIFSVLLREYEFEMAQPPESYRNDHSKMVVQLA
QPACVRYRRRTGV
>CYP51B1_Mycobacterium tuberculosis M1221 2575518517
MSAVALPRVSGGHDEHGHLEEFRTDPIGLMQRVRDECGDVGTFQLAGKQVVLLSGSHANEFFFRAGDDDLDQA
KAYPFMTPIFGEGVVFDASPERRKEMLHNAALRGEQMKGHAATIEDQVRRMIADWGEAGEIDLLDFFAELTIY
TSSACLIGKKFRDQLDGRFAKLYHELERGTDPLAYVDPYLPIESFRRRDEARNGLVALVADIMNGRIANPPTD
KSDRDMLDVLIAVKAETGTPRFSADEITGMFISMMFAGHHTSSGTASWTLIELMRHRDAYAAVIDELDELYGD
GRSVSFHALRQIPQLENVLKETLRLHPPLIILMRVAKGEFEVQGHRIHEGDLVAASPAISNRIPEDFPDPHDF
VPARYEQPRQEDLLNRWTWIPFGAGRHRCVGAAFAIMQIKAIFSVLLREYEFEMAQPPESYRNDHSKMVVQLA
QPACVRYRRRTGV
>CYP51B1_Mycobacterium tuberculosis M1232 2622389403
MSAVALPRVSGGHDEHGHLEEFRTDPIGLMQRVRDECGDVGTFQLAGKQVVLLSGSHANEFFFRAGDDDLDQA
KAYPFMTPIFGEGVVFDASPERRKEMLHNAALRGEQMKGHAATIEDQVRRMIADWGEAGEIDLLDFFAELTIY
TSSACLIGKKFRDQLDGRFAKLYHELERGTDPLAYVDPYLPIESFRRRDEARNGLVALVADIMNGRIANPPTD
KSDRDMLDVLIAVKAETGTPRFSADEITGMFISMMFAGHHTSSGTASWTLIELMRHRDAYAAVIDELDELYGD
GRSVSFHALRQIPQLENVLKETLRLHPPLIILMRVAKGEFEVQGHRIHEGDLVAASPAISNRIPEDFPDPHDF
VPARYEQPRQEDLLNRWTWIPFGAGRHRCVGAAFAIMQIKAIFSVLLREYEFEMAQPPESYRNDHSKMVVQLA
QPACVRYRRRTGV
>CYP51B1_Mycobacterium tuberculosis M1233 2575666763
MSAVALPRVSGGHDEHGHLEEFRTDPIGLMQRVRDECGDVGTFQLAGKQVVLLSGSHANEFFFRAGDDDLDQA
KAYPFMTPIFGEGVVFDASPERRKEMLHNAALRGEQMKGHAATIEDQVRRMIADWGEAGEIDLLDFFAELTIY
TSSACLIGKKFRDQLDGRFAKLYHELERGTDPLAYVDPYLPIESFRRRDEARNGLVALVADIMNGRIANPPTD
KSDRDMLDVLIAVKAETGTPRFSADEITGMFISMMFAGHHTSSGTASWTLIELMRHRDAYAAVIDELDELYGD
GRSVSFHALRQIPQLENVLKETLRLHPPLIILMRVAKGEFEVQGHRIHEGDLVAASPAISNRIPEDFPDPHDF
VPARYEQPRQEDLLNRWTWIPFGAGRHRCVGAAFAIMQIKAIFSVLLREYEFEMAQPPESYRNDHSKMVVQLA
QPACVRYRRRTGV
>CYP51B1_Mycobacterium tuberculosis M1236 2622393808
MSAVALPRVSGGHDEHGHLEEFRTDPIGLMQRVRDECGDVGTFQLAGKQVVLLSGSHANEFFFRAGDDDLDQA
KAYPFMTPIFGEGVVFDASPERRKEMLHNAALRGEQMKGHAATIEDQVRRMIADWGEAGEIDLLDFFAELTIY
TSSACLIGKKFRDQLDGRFAKLYHELERGTDPLAYVDPYLPIESFRRRDEARNGLVALVADIMNGRIANPPTD
KSDRDMLDVLIAVKAETGTPRFSADEITGMFISMMFAGHHTSSGTASWTLIELMRHRDAYAAVIDELDELYGD
GRSVSFHALRQIPQLENVLKETLRLHPPLIILMRVAKGEFEVQGHRIHEGDLVAASPAISNRIPEDFPDPHDF
VPARYEQPRQEDLLNRWTWIPFGAGRHRCVGAAFAIMQIKAIFSVLLREYEFEMAQPPESYRNDHSKMVVQLA
QPACVRYRRRTGV
>CYP51B1_Mycobacterium tuberculosis M1253 2622395082
MSAVALPRVSGGHDEHGHLEEFRTDPIGLMQRVRDECGDVGTFQLAGKQVVLLSGSHANEFFFRAGDDDLDQA
KAYPFMTPIFGEGVVFDASPERRKEMLHNAALRGEQMKGHAATIEDQVRRMIADWGEAGEIDLLDFFAELTIY
TSSACLIGKKFRDQLDGRFAKLYHELERGTDPLAYVDPYLPIESFRRRDEARNGLVALVADIMNGRIANPPTD
KSDRDMLDVLIAVKAETGTPRFSADEITGMFISMMFAGHHTSSGTASWTLIELMRHRDAYAAVIDELDELYGD

GRSVSFHALRQIPQLENVLKETLRLHPPLIILMRVAKGEFEVQGHRIHEGDLVAASPAISNRIPEDFPDPHDF
VPARYEQPRQEDLLNRWTWIPFGAGRHRCVGAAFAIMQIKAIFSVLLREYEFEMAQPPESYRNDHSKMVVQLA
QPACVRYRRRTGV
>CYP51B1_Mycobacterium tuberculosis M1271 2622405319
MSAVALPRVSGGHDEHGHLEEFRTDPIGLMQRVRDECGDVGTFQLAGKQVVLLSGSHANEFFFRAGDDDLDQA
KAYPFMTPIFGEGVVFDASPERRKEMLHNAALRGEQMKGHAATIEDQVRRMIADWGEAGEIDLLDFFAELTIY
TSSACLIGKKFRDQLDGRFAKLYHELERGTDPLAYVDPYLPIESFRRRDEARNGLVALVADIMNGRIANPPTD
KSDRDMLDVLIAVKAETGTPRFSADEITGMFISMMFAGHHTSSGTASWTLIELMRHRDAYAAVIDELDELYGD
GRSVSFHALRQIPQLENVLKETLRLHPPLIILMRVAKGEFEVQGHRIHEGDLVAASPAISNRIPEDFPDPHDF
VPARYEQPRQEDLLNRWTWIPFGAGRHRCVGAAFAIMQIKAIFSVLLREYEFEMAQPPESYRNDHSKMVVQLA
QPACVRYRRRTGV
>CYP51B1_Mycobacterium tuberculosis M1272 2622407452
MSAVALPRVSGGHDEHGHLEEFRTDPIGLMQRVRDECGDVGTFQLAGKQVVLLSGSHANEFFFRAGDDDLDQA
KAYPFMTPIFGEGVVFDASPERRKEMLHNAALRGEQMKGHAATIEDQVRRMIADWGEAGEIDLLDFFAELTIY
TSSACLIGKKFRDQLDGRFAKLYHELERGTDPLAYVDPYLPIESFRRRDEARNGLVALVADIMNGRIANPPTD
KSDRDMLDVLIAVKAETGTPRFSADEITGMFISMMFAGHHTSSGTASWTLIELMRHRDAYAAVIDELDELYGD
GRSVSFHALRQIPQLENVLKETLRLHPPLIILMRVAKGEFEVQGHRIHEGDLVAASPAISNRIPEDFPDPHDF
VPARYEQPRQEDLLNRWTWIPFGAGRHRCVGAAFAIMQIKAIFSVLLREYEFEMAQPPESYRNDHSKMVVQLA
QPACVRYRRRTGV
>CYP51B1_Mycobacterium tuberculosis M1273 2622411560
MSAVALPRVSGGHDEHGHLEEFRTDPIGLMQRVRDECGDVGTFQLAGKQVVLLSGSHANEFFFRAGDDDLDQA
KAYPFMTPIFGEGVVFDASPERRKEMLHNAALRGEQMKGHAATIEDQVRRMIADWGEAGEIDLLDFFAELTIY
TSSACLIGKKFRDQLDGRFAKLYHELERGTDPLAYVDPYLPIESFRRRDEARNGLVALVADIMNGRIANPPTD
KSDRDMLDVLIAVKAETGTPRFSADEITGMFISMMFAGHHTSSGTASWTLIELMRHRDAYAAVIDELDELYGD
GRSVSFHALRQIPQLENVLKETLRLHPPLIILMRVAKGEFEVQGHRIHEGDLVAASPAISNRIPEDFPDPHDF
VPARYEQPRQEDLLNRWTWIPFGAGRHRCVGAAFAIMQIKAIFSVLLREYEFEMAQPPESYRNDHSKMVVQLA
QPACVRYRRRTGV
>CYP51B1_Mycobacterium tuberculosis M1283 2577802565
MSAVALPRVSGGHDEHGHLEEFRTDPIGLMQRVRDECGDVGTFQLAGKQVVLLSGSHANEFFFRAGDDDLDQA
KAYPFMTPIFGEGVVFDASPERRKEMLHNAALRGEQMKGHAATIEDQVRRMIADWGEAGEIDLLDFFAELTIY
TSSACLIGKKFRDQLDGRFAKLYHELERGTDPLAYVDPYLPIESFRRRDEARNGLVALVADIMNGRIANPPTD
KSDRDMLDVLIAVKAETGTPRFSADEITGMFISMMFAGHHTSSGTASWTLIELMRHRDAYAAVIDELDELYGD
GRSVSFHALRQIPQLENVLKETLRLHPPLIILMRVAKGEFEVQGHRIHEGDLVAASPAISNRIPEDFPDPHDF
VPARYEQPRQEDLLNRWTWIPFGAGRHRCVGAAFAIMQIKAIFSVLLREYEFEMAQPPESYRNDHSKMVVQLA
QPACVRYRRRTGV
>CYP51B1_Mycobacterium tuberculosis M1287 2622422390
MSAVALPRVSGGHDEHGHLEEFRTDPIGLMQRVRDECGDVGTFQLAGKQVVLLSGSHANEFFFRAGDDDLDQA
KAYPFMTPIFGEGVVFDASPERRKEMLHNAALRGEQMKGHAATIEDQVRRMIADWGEAGEIDLLDFFAELTIY
TSSACLIGKKFRDQLDGRFAKLYHELERGTDPLAYVDPYLPIESFRRRDEARNGLVALVADIMNGRIANPPTD
KSDRDMLDVLIAVKAETGTPRFSADEITGMFISMMFAGHHTSSGTASWTLIELMRHRDAYAAVIDELDELYGD
GRSVSFHALRQIPQLENVLKETLRLHPPLIILMRVAKGEFEVQGHRIHEGDLVAASPAISNRIPEDFPDPHDF
VPARYEQPRQEDLLNRWTWIPFGAGRHRCVGAAFAIMQIKAIFSVLLREYEFEMAQPPESYRNDHSKMVVQLA
QPACVRYRRRTGV
>CYP51B1_Mycobacterium tuberculosis M1288 2622423918
MSAVALPRVSGGHDEHGHLEEFRTDPIGLMQRVRDECGDVGTFQLAGKQVVLLSGSHANEFFFRAGDDDLDQA
KAYPFMTPIFGEGVVFDASPERRKEMLHNAALRGEQMKGHAATIEDQVRRMIADWGEAGEIDLLDFFAELTIY
TSSACLIGKKFRDQLDGRFAKLYHELERGTDPLAYVDPYLPIESFRRRDEARNGLVALVADIMNGRIANPPTD
KSDRDMLDVLIAVKAETGTPRFSADEITGMFISMMFAGHHTSSGTASWTLIELMRHRDAYAAVIDELDELYGD
GRSVSFHALRQIPQLENVLKETLRLHPPLIILMRVAKGEFEVQGHRIHEGDLVAASPAISNRIPEDFPDPHDF
VPARYEQPRQEDLLNRWTWIPFGAGRHRCVGAAFAIMQIKAIFSVLLREYEFEMAQPPESYRNDHSKMVVQLA
QPACVRYRRRTGV
>CYP51B1_Mycobacterium tuberculosis M1295 2622436324
MSAVALPRVSGGHDEHGHLEEFRTDPIGLMQRVRDECGDVGTFQLAGKQVVLLSGSHANEFFFRAGDDDLDQA
KAYPFMTPIFGEGVVFDASPERRKEMLHNAALRGEQMKGHAATIEDQVRRMIADWGEAGEIDLLDFFAELTIY
TSSACLIGKKFRDQLDGRFAKLYHELERGTDPLAYVDPYLPIESFRRRDEARNGLVALVADIMNGRIANPPTD
KSDRDMLDVLIAVKAETGTPRFSADEITGMFISMMFAGHHTSSGTASWTLIELMRHRDAYAAVIDELDELYGD
GRSVSFHALRQIPQLENVLKETLRLHPPLIILMRVAKGEFEVQGHRIHEGDLVAASPAISNRIPEDFPDPHDF

VPARYEQPRQEDLLNRWTWIPFGAGRHRCVGAAFAIMQIKAIFSVLLREYEFEMAQPPESYRNDHSKMVVQLA
QPACVRYRRRTGV
>CYP51B1_Mycobacterium tuberculosis M1300 2622444579
MSAVALPRVSGGHDEHGHLEEFRTDPIGLMQRVRDECGDVGTFQLAGKQVVLLSGSHANEFFFRAGDDDLDQA
KAYPFMTPIFGEGVVFDASPERRKEMLHNAALRGEQMKGHAATIEDQVRRMIADWGEAGEIDLLDFFAELTIY
TSSACLIGKKFRDQLDGRFAKLYHELERGTDPLAYVDPYLPIESFRRRDEARNGLVALVADIMNGRIANPPTD
KSDRDMLDVLIAVKAETGTPRFSADEITGMFISMMFAGHHTSSGTASWTLIELMRHRDAYAAVIDELDELYGD
GRSVSFHALRQIPQLENVLKETLRLHPPLIILMRVAKGEFEVQGHRIHEGDLVAASPAISNRIPEDFPDPHDF
VPARYEQPRQEDLLNRWTWIPFGAGRHRCVGAAFAIMQIKAIFSVLLREYEFEMAQPPESYRNDHSKMVVQLA
QPACVRYRRRTGV
>CYP51B1_Mycobacterium tuberculosis M1304 2622448687
MSAVALPRVSGGHDEHGHLEEFRTDPIGLMQRVRDECGDVGTFQLAGKQVVLLSGSHANEFFFRAGDDDLDQA
KAYPFMTPIFGEGVVFDASPERRKEMLHNAALRGEQMKGHAATIEDQVRRMIADWGEAGEIDLLDFFAELTIY
TSSACLIGKKFRDQLDGRFAKLYHELERGTDPLAYVDPYLPIESFRRRDEARNGLVALVADIMNGRIANPPTD
KSDRDMLDVLIAVKAETGTPRFSADEITGMFISMMFAGHHTSSGTASWTLIELMRHRDAYAAVIDELDELYGD
GRSVSFHALRQIPQLENVLKETLRLHPPLIILMRVAKGEFEVQGHRIHEGDLVAASPAISNRIPEDFPDPHDF
VPARYEQPRQEDLLNRWTWIPFGAGRHRCVGAAFAIMQIKAIFSVLLREYEFEMAQPPESYRNDHSKMVVQLA
QPACVRYRRRTGV
>CYP51B1_Mycobacterium tuberculosis M1307 2641871441
MSAVALPRVSGGHDEHGHLEEFRTDPIGLMQRVRDECGDVGTFQLAGKQVVLLSGSHANEFFFRAGDDDLDQA
KAYPFMTPIFGEGVVFDASPERRKEMLHNAALRGEQMKGHAATIEDQVRRMIADWGEAGEIDLLDFFAELTIY
TSSACLIGKKFRDQLDGRFAKLYHELERGTDPLAYVDPYLPIESFRRRDEARNGLVALVADIMNGRIANPPTD
KSDRDMLDVLIAVKAETGTPRFSADEITGMFISMMFAGHHTSSGTASWTLIELMRHRDAYAAVIDELDELYGD
GRSVSFHALRQIPQLENVLKETLRLHPPLIILMRVAKGEFEVQGHRIHEGDLVAASPAISNRIPEDFPDPHDF
VPARYEQPRQEDLLNRWTWIPFGAGRHRCVGAAFAIMQIKAIFSVLLREYEFEMAQPPESYRNDHSKMVVQLA
QPACVRYRRRTGV
>CYP51B1_Mycobacterium tuberculosis M1310 2622459523
MSAVALPRVSGGHDEHGHLEEFRTDPIGLMQRVRDECGDVGTFQLAGKQVVLLSGSHANEFFFRAGDDDLDQA
KAYPFMTPIFGEGVVFDASPERRKEMLHNAALRGEQMKGHAATIEDQVRRMIADWGEAGEIDLLDFFAELTIY
TSSACLIGKKFRDQLDGRFAKLYHELERGTDPLAYVDPYLPIESFRRRDEARNGLVALVADIMNGRIANPPTD
KSDRDMLDVLIAVKAETGTPRFSADEITGMFISMMFAGHHTSSGTASWTLIELMRHRDAYAAVIDELDELYGD
GRSVSFHALRQIPQLENVLKETLRLHPPLIILMRVAKGEFEVQGHRIHEGDLVAASPAISNRIPEDFPDPHDF
VPARYEQPRQEDLLNRWTWIPFGAGRHRCVGAAFAIMQIKAIFSVLLREYEFEMAQPPESYRNDHSKMVVQLA
QPACVRYRRRTGV
>CYP51B1_Mycobacterium tuberculosis M1315 2622460752
MSAVALPRVSGGHDEHGHLEEFRTDPIGLMQRVRDECGDVGTFQLAGKQVVLLSGSHANEFFFRAGDDDLDQA
KAYPFMTPIFGEGVVFDASPERRKEMLHNAALRGEQMKGHAATIEDQVRRMIADWGEAGEIDLLDFFAELTIY
TSSACLIGKKFRDQLDGRFAKLYHELERGTDPLAYVDPYLPIESFRRRDEARNGLVALVADIMNGRIANPPTD
KSDRDMLDVLIAVKAETGTPRFSADEITGMFISMMFAGHHTSSGTASWTLIELMRHRDAYAAVIDELDELYGD
GRSVSFHALRQIPQLENVLKETLRLHPPLIILMRVAKGEFEVQGHRIHEGDLVAASPAISNRIPEDFPDPHDF
VPARYEQPRQEDLLNRWTWIPFGAGRHRCVGAAFAIMQIKAIFSVLLREYEFEMAQPPESYRNDHSKMVVQLA
QPACVRYRRRTGV
>CYP51B1_Mycobacterium tuberculosis M1317 2622465149
MSAVALPRVSGGHDEHGHLEEFRTDPIGLMQRVRDECGDVGTFQLAGKQVVLLSGSHANEFFFRAGDDDLDQA
KAYPFMTPIFGEGVVFDASPERRKEMLHNAALRGEQMKGHAATIEDQVRRMIADWGEAGEIDLLDFFAELTIY
TSSACLIGKKFRDQLDGRFAKLYHELERGTDPLAYVDPYLPIESFRRRDEARNGLVALVADIMNGRIANPPTD
KSDRDMLDVLIAVKAETGTPRFSADEITGMFISMMFAGHHTSSGTASWTLIELMRHRDAYAAVIDELDELYGD
GRSVSFHALRQIPQLENVLKETLRLHPPLIILMRVAKGEFEVQGHRIHEGDLVAASPAISNRIPEDFPDPHDF
VPARYEQPRQEDLLNRWTWIPFGAGRHRCVGAAFAIMQIKAIFSVLLREYEFEMAQPPESYRNDHSKMVVQLA
QPACVRYRRRTGV
>CYP51B1_Mycobacterium tuberculosis M1319 2622469265
MSAVALPRVSGGHDEHGHLEEFRTDPIGLMQRVRDECGDVGTFQLAGKQVVLLSGSHANEFFFRAGDDDLDQA
KAYPFMTPIFGEGVVFDASPERRKEMLHNAALRGEQMKGHAATIEDQVRRMIADWGEAGEIDLLDFFAELTIY
TSSACLIGKKFRDQLDGRFAKLYHELERGTDPLAYVDPYLPIESFRRRDEARNGLVALVADIMNGRIANPPTD
KSDRDMLDVLIAVKAETGTPRFSADEITGMFISMMFAGHHTSSGTASWTLIELMRHRDAYAAVIDELDELYGD
GRSVSFHALRQIPQLENVLKETLRLHPPLIILMRVAKGEFEVQGHRIHEGDLVAASPAISNRIPEDFPDPHDF
VPARYEQPRQEDLLNRWTWIPFGAGRHRCVGAAFAIMQIKAIFSVLLREYEFEMAQPPESYRNDHSKMVVQLA
QPACVRYRRRTGV

>CYP51B1_Mycobacterium tuberculosis M1324 2622473351
MSAVALPRVSGGHDEHGHLEEFRTDPIGLMQRVRDECGDVGTFQLAGKQVVLLSGSHANEFFFRAGDDDLDQA
KAYPFMTPIFGEGVVFDASPERRKEMLHNAALRGEQMKGHAATIEDQVRRMIADWGEAGEIDLLDFFAELTIY
TSSACLIGKKFRDQLDGRFAKLYHELERGTDPLAYVDPYLPIESFRRRDEARNGLVALVADIMNGRIANPPTD
KSDRDMLDVLIAVKAETGTPRFSADEITGMFISMMFAGHHTSSGTASWTLIELMRHRDAYAAVIDELDELYGD
GRSVSFHALRQIPQLENVLKETLRLHPPLIILMRVAKGEFEVQGHRIHEGDLVAASPAISNRIPEDFPDPHDF
VPARYEQPRQEDLLNRWTWIPFGAGRHRCVGAAFAIMQIKAIFSVLLREYEFEMAQPPESYRNDHSKMVVQLA
QPACVRYRRRTGV
>CYP51B1_Mycobacterium tuberculosis M1325 2622477180
MSAVALPRVSGGHDEHGHLEEFRTDPIGLMQRVRDECGDVGTFQLAGKQVVLLSGSHANEFFFRAGDDDLDQA
KAYPFMTPIFGEGVVFDASPERRKEMLHNAALRGEQMKGHAATIEDQVRRMIADWGEAGEIDLLDFFAELTIY
TSSACLIGKKFRDQLDGRFAKLYHELERGTDPLAYVDPYLPIESFRRRDEARNGLVALVADIMNGRIANPPTD
KSDRDMLDVLIAVKAETGTPRFSADEITGMFISMMFAGHHTSSGTASWTLIELMRHRDAYAAVIDELDELYGD
GRSVSFHALRQIPQLENVLKETLRLHPPLIILMRVAKGEFEVQGHRIHEGDLVAASPAISNRIPEDFPDPHDF
VPARYEQPRQEDLLNRWTWIPFGAGRHRCVGAAFAIMQIKAIFSVLLREYEFEMAQPPESYRNDHSKMVVQLA
QPACVRYRRRTGV
>CYP51B1_Mycobacterium tuberculosis M1340 2584622731
MSAVALPRVSGGHDEHGHLEEFRTDPIGLMQRVRDECGDVGTFQLAGKQVVLLSGSHANEFFFRAGDDDLDQA
KAYPFMTPIFGEGVVFDASPERRKEMLHNAALRGEQMKGHAATIEDQVRRMIADWGEAGEIDLLDFFAELTIY
TSSACLIGKKFRDQLDGRFAKLYHELERGTDPLAYVDPYLPIESFRRRDEARNGLVALVADIMNGRIANPPTD
KSDRDMLDVLIAVKAETGTPRFSADEITGMFISMMFAGHHTSSGTASWTLIELMRHRDAYAAVIDELDELYGD
GRSVSFHALRQIPQLENVLKETLRLHPPLIILMRVAKGEFEVQGHRIHEGDLVAASPAISNRIPEDFPDPHDF
VPARYEQPRQEDLLNRWTWIPFGAGRHRCVGAAFAIMQIKAIFSVLLREYEFEMAQPPESYRNDHSKMVVQLA
QPACVRYRRRTGV
>CYP51B1_Mycobacterium tuberculosis M1373 2622481312
MSAVALPRVSGGHDEHGHLEEFRTDPIGLMQRVRDECGDVGTFQLAGKQVVLLSGSHANEFFFRAGDDDLDQA
KAYPFMTPIFGEGVVFDASPERRKEMLHNAALRGEQMKGHAATIEDQVRRMIADWGEAGEIDLLDFFAELTIY
TSSACLIGKKFRDQLDGRFAKLYHELERGTDPLAYVDPYLPIESFRRRDEARNGLVALVADIMNGRIANPPTD
KSDRDMLDVLIAVKAETGTPRFSADEITGMFISMMFAGHHTSSGTASWTLIELMRHRDAYAAVIDELDELYGD
GRSVSFHALRQIPQLENVLKETLRLHPPLIILMRVAKGEFEVQGHRIHEGDLVAASPAISNRIPEDFPDPHDF
VPARYEQPRQEDLLNRWTWIPFGAGRHRCVGAAFAIMQIKAIFSVLLREYEFEMAQPPESYRNDHSKMVVQLA
QPACVRYRRRTGV
>CYP51B1_Mycobacterium tuberculosis M1381 2622489856
MSAVALPRVSGGHDEHGHLEEFRTDPIGLMQRVRDECGDVGTFQLAGKQVVLLSGSHANEFFFRAGDDDLDQA
KAYPFMTPIFGEGVVFDASPERRKEMLHNAALRGEQMKGHAATIEDQVRRMIADWGEAGEIDLLDFFAELTIY
TSSACLIGKKFRDQLDGRFAKLYHELERGTDPLAYVDPYLPIESFRRRDEARNGLVALVADIMNGRIANPPTD
KSDRDMLDVLIAVKAETGTPRFSADEITGMFISMMFAGHHTSSGTASWTLIELMRHRDAYAAVIDELDELYGD
GRSVSFHALRQIPQLENVLKETLRLHPPLIILMRVAKGEFEVQGHRIHEGDLVAASPAISNRIPEDFPDPHDF
VPARYEQPRQEDLLNRWTWIPFGAGRHRCVGAAFAIMQIKAIFSVLLREYEFEMAQPPESYRNDHSKMVVQLA
QPACVRYRRRTGV
>CYP51B1_Mycobacterium tuberculosis M1384 2622496656
MSAVALPRVSGGHDEHGHLEEFRTDPIGLMQRVRDECGDVGTFQLAGKQVVLLSGSHANEFFFRAGDDDLDQA
KAYPFMTPIFGEGVVFDASPERRKEMLHNAALRGEQMKGHAATIEDQVRRMIADWGEAGEIDLLDFFAELTIY
TSSACLIGKKFRDQLDGRFAKLYHELERGTDPLAYVDPYLPIESFRRRDEARNGLVALVADIMNGRIANPPTD
KSDRDMLDVLIAVKAETGTPRFSADEITGMFISMMFAGHHTSSGTASWTLIELMRHRDAYAAVIDELDELYGD
GRSVSFHALRQIPQLENVLKETLRLHPPLIILMRVAKGEFEVQGHRIHEGDLVAASPAISNRIPEDFPDPHDF
VPARYEQPRQEDLLNRWTWIPFGAGRHRCVGAAFAIMQIKAIFSVLLREYEFEMAQPPESYRNDHSKMVVQLA
QPACVRYRRRTGV
>CYP51B1_Mycobacterium tuberculosis M1389 2622497314
MSAVALPRVSGGHDEHGHLEEFRTDPIGLMQRVRDECGDVGTFQLAGKQVVLLSGSHANEFFFRAGDDDLDQA
KAYPFMTPIFGEGVVFDASPERRKEMLHNAALRGEQMKGHAATIEDQVRRMIADWGEAGEIDLLDFFAELTIY
TSSACLIGKKFRDQLDGRFAKLYHELERGTDPLAYVDPYLPIESFRRRDEARNGLVALVADIMNGRIANPPTD
KSDRDMLDVLIAVKAETGTPRFSADEITGMFISMMFAGHHTSSGTASWTLIELMRHRDAYAAVIDELDELYGD
GRSVSFHALRQIPQLENVLKETLRLHPPLIILMRVAKGEFEVQGHRIHEGDLVAASPAISNRIPEDFPDPHDF
VPARYEQPRQEDLLNRWTWIPFGAGRHRCVGAAFAIMQIKAIFSVLLREYEFEMAQPPESYRNDHSKMVVQLA
QPACVRYRRRTGV
>CYP51B1_Mycobacterium tuberculosis M1392 2622791860

MSAVALPRVSGGHDEHGHLEEFRTDPIGLMQRVRDECGDVGTFQLAGKQVVLLSGSHANEFFFRAGDDDLDQA
KAYPFMTPIFGEGVVFDASPERRKEMLHNAALRGEQMKGHAATIEDQVRRMIADWGEAGEIDLLDFFAELTIY
TSSACLIGKKFRDQLDGRFAKLYHELERGTDPLAYVDPYLPIESFRRRDEARNGLVALVADIMNGRIANPPTD
KSDRDMLDVLIAVKAETGTPRFSADEITGMFISMMFAGHHTSSGTASWTLIELMRHRDAYAAVIDELDELYGD
GRSVSFHALRQIPQLENVLKETLRLHPPLIILMRVAKGEFEVQGHRIHEGDLVAASPAISNRIPEDFPDPHDF
VPARYEQPRQEDLLNRWTWIPFGAGRHRCVGAAFAIMQIKAIFSVLLREYEFEMAQPPESYRNDHSKMVVQLA
QPACVRYRRRTGV
>CYP51B1_Mycobacterium tuberculosis M1397 2622508877
MSAVALPRVSGGHDEHGHLEEFRTDPIGLMQRVRDECGDVGTFQLAGKQVVLLSGSHANEFFFRAGDDDLDQA
KAYPFMTPIFGEGVVFDASPERRKEMLHNAALRGEQMKGHAATIEDQVRRMIADWGEAGEIDLLDFFAELTIY
TSSACLIGKKFRDQLDGRFAKLYHELERGTDPLAYVDPYLPIESFRRRDEARNGLVALVADIMNGRIANPPTD
KSDRDMLDVLIAVKAETGTPRFSADEITGMFISMMFAGHHTSSGTASWTLIELMRHRDAYAAVIDELDELYGD
GRSVSFHALRQIPQLENVLKETLRLHPPLIILMRVAKGEFEVQGHRIHEGDLVAASPAISNRIPEDFPDPHDF
VPARYEQPRQEDLLNRWTWIPFGAGRHRCVGAAFAIMQIKAIFSVLLREYEFEMAQPPESYRNDHSKMVVQLA
QPACVRYRRRTGV
>CYP51B1_Mycobacterium tuberculosis M1400 2622510158
MSAVALPRVSGGHDEHGHLEEFRTDPIGLMQRVRDECGDVGTFQLAGKQVVLLSGSHANEFFFRAGDDDLDQA
KAYPFMTPIFGEGVVFDASPERRKEMLHNAALRGEQMKGHAATIEDQVRRMIADWGEAGEIDLLDFFAELTIY
TSSACLIGKKFRDQLDGRFAKLYHELERGTDPLAYVDPYLPIESFRRRDEARNGLVALVADIMNGRIANPPTD
KSDRDMLDVLIAVKAETGTPRFSADEITGMFISMMFAGHHTSSGTASWTLIELMRHRDAYAAVIDELDELYGD
GRSVSFHALRQIPQLENVLKETLRLHPPLIILMRVAKGEFEVQGHRIHEGDLVAASPAISNRIPEDFPDPHDF
VPARYEQPRQEDLLNRWTWIPFGAGRHRCVGAAFAIMQIKAIFSVLLREYEFEMAQPPESYRNDHSKMVVQLA
QPACVRYRRRTGV
>CYP51B1_Mycobacterium tuberculosis M1402 2622517280
MSAVALPRVSGGHDEHGHLEEFRTDPIGLMQRVRDECGDVGTFQLAGKQVVLLSGSHANEFFFRAGDDDLDQA
KAYPFMTPIFGEGVVFDASPERRKEMLHNAALRGEQMKGHAATIEDQVRRMIADWGEAGEIDLLDFFAELTIY
TSSACLIGKKFRDQLDGRFAKLYHELERGTDPLAYVDPYLPIESFRRRDEARNGLVALVADIMNGRIANPPTD
KSDRDMLDVLIAVKAETGTPRFSADEITGMFISMMFAGHHTSSGTASWTLIELMRHRDAYAAVIDELDELYGD
GRSVSFHALRQIPQLENVLKETLRLHPPLIILMRVAKGEFEVQGHRIHEGDLVAASPAISNRIPEDFPDPHDF
VPARYEQPRQEDLLNRWTWIPFGAGRHRCVGAAFAIMQIKAIFSVLLREYEFEMAQPPESYRNDHSKMVVQLA
QPACVRYRRRTGV
>CYP51B1_Mycobacterium tuberculosis M1404 2622518383
MSAVALPRVSGGHDEHGHLEEFRTDPIGLMQRVRDECGDVGTFQLAGKQVVLLSGSHANEFFFRAGDDDLDQA
KAYPFMTPIFGEGVVFDASPERRKEMLHNAALRGEQMKGHAATIEDQVRRMIADWGEAGEIDLLDFFAELTIY
TSSACLIGKKFRDQLDGRFAKLYHELERGTDPLAYVDPYLPIESFRRRDEARNGLVALVADIMNGRIANPPTD
KSDRDMLDVLIAVKAETGTPRFSADEITGMFISMMFAGHHTSSGTASWTLIELMRHRDAYAAVIDELDELYGD
GRSVSFHALRQIPQLENVLKETLRLHPPLIILMRVAKGEFEVQGHRIHEGDLVAASPAISNRIPEDFPDPHDF
VPARYEQPRQEDLLNRWTWIPFGAGRHRCVGAAFAIMQIKAIFSVLLREYEFEMAQPPESYRNDHSKMVVQLA
QPACVRYRRRTGV
>CYP51B1_Mycobacterium tuberculosis M1410 2622522489
MSAVALPRVSGGHDEHGHLEEFRTDPIGLMQRVRDECGDVGTFQLAGKQVVLLSGSHANEFFFRAGDDDLDQA
KAYPFMTPIFGEGVVFDASPERRKEMLHNAALRGEQMKGHAATIEDQVRRMIADWGEAGEIDLLDFFAELTIY
TSSACLIGKKFRDQLDGRFAKLYHELERGTDPLAYVDPYLPIESFRRRDEARNGLVALVADIMNGRIANPPTD
KSDRDMLDVLIAVKAETGTPRFSADEITGMFISMMFAGHHTSSGTASWTLIELMRHRDAYAAVIDELDELYGD
GRSVSFHALRQIPQLENVLKETLRLHPPLIILMRVAKGEFEVQGHRIHEGDLVAASPAISNRIPEDFPDPHDF
VPARYEQPRQEDLLNRWTWIPFGAGRHRCVGAAFAIMQIKAIFSVLLREYEFEMAQPPESYRNDHSKMVVQLA
QPACVRYRRRTGV
>CYP51B1_Mycobacterium tuberculosis M1415 2584982127
MSAVALPRVSGGHDEHGHLEEFRTDPIGLMQRVRDECGDVGTFQLAGKQVVLLSGSHANEFFFRAGDDDLDQA
KAYPFMTPIFGEGVVFDASPERRKEMLHNAALRGEQMKGHAATIEDQVRRMIADWGEAGEIDLLDFFAELTIY
TSSACLIGKKFRDQLDGRFAKLYHELERGTDPLAYVDPYLPIESFRRRDEARNGLVALVADIMNGRIANPPTD
KSDRDMLDVLIAVKAETGTPRFSADEITGMFISMMFAGHHTSSGTASWTLIELMRHRDAYAAVIDELDELYGD
GRSVSFHALRQIPQLENVLKETLRLHPPLIILMRVAKGEFEVQGHRIHEGDLVAASPAISNRIPEDFPDPHDF
VPARYEQPRQEDLLNRWTWIPFGAGRHRCVGAAFAIMQIKAIFSVLLREYEFEMAQPPESYRNDHSKMVVQLA
QPACVRYRRRTGV
>CYP51B1_Mycobacterium tuberculosis M1416 2622526908
MSAVALPRVSGGHDEHGHLEEFRTDPIGLMQRVRDECGDVGTFQLAGKQVVLLSGSHANEFFFRAGDDDLDQA
KAYPFMTPIFGEGVVFDASPERRKEMLHNAALRGEQMKGHAATIEDQVRRMIADWGEAGEIDLLDFFAELTIY

TSSACLIGKKFRDQLDGRFAKLYHELERGTDPLAYVDPYLPIESFRRRDEARNGLVALVADIMNGRIANPPTD
KSDRDMLDVLIAVKAETGTPRFSADEITGMFISMMFAGHHTSSGTASWTLIELMRHRDAYAAVIDELDELYGD
GRSVSFHALRQIPQLENVLKETLRLHPPLIILMRVAKGEFEVQGHRIHEGDLVAASPAISNRIPEDFPDPHDF
VPARYEQPRQEDLLNRWTWIPFGAGRHRCVGAAFAIMQIKAIFSVLLREYEFEMAQPPESYRNDHSKMVVQLA
QPACVRYRRRTGV
>CYP51B1_Mycobacterium tuberculosis M1417 2622531004
MSAVALPRVSGGHDEHGHLEEFRTDPIGLMQRVRDECGDVGTFQLAGKQVVLLSGSHANEFFFRAGDDDLDQA
KAYPFMTPIFGEGVVFDASPERRKEMLHNAALRGEQMKGHAATIEDQVRRMIADWGEAGEIDLLDFFAELTIY
TSSACLIGKKFRDQLDGRFAKLYHELERGTDPLAYVDPYLPIESFRRRDEARNGLVALVADIMNGRIANPPTD
KSDRDMLDVLIAVKAETGTPRFSADEITGMFISMMFAGHHTSSGTASWTLIELMRHRDAYAAVIDELDELYGD
GRSVSFHALRQIPQLENVLKETLRLHPPLIILMRVAKGEFEVQGHRIHEGDLVAASPAISNRIPEDFPDPHDF
VPARYEQPRQEDLLNRWTWIPFGAGRHRCVGAAFAIMQIKAIFSVLLREYEFEMAQPPESYRNDHSKMVVQLA
QPACVRYRRRTGV
>CYP51B1_Mycobacterium tuberculosis M1418 2577008672
MSAVALPRVSGGHDEHGHLEEFRTDPIGLMQRVRDECGDVGTFQLAGKQVVLLSGSHANEFFFRAGDDDLDQA
KAYPFMTPIFGEGVVFDASPERRKEMLHNAALRGEQMKGHAATIEDQVRRMIADWGEAGEIDLLDFFAELTIY
TSSACLIGKKFRDQLDGRFAKLYHELERGTDPLAYVDPYLPIESFRRRDEARNGLVALVADIMNGRIANPPTD
KSDRDMLDVLIAVKAETGTPRFSADEITGMFISMMFAGHHTSSGTASWTLIELMRHRDAYAAVIDELDELYGD
GRSVSFHALRQIPQLENVLKETLRLHPPLIILMRVAKGEFEVQGHRIHEGDLVAASPAISNRIPEDFPDPHDF
VPARYEQPRQEDLLNRWTWIPFGAGRHRCVGAAFAIMQIKAIFSVLLREYEFEMAQPPESYRNDHSKMVVQLA
QPACVRYRRRTGV
>CYP51B1_Mycobacterium tuberculosis M1422 2622535102
MSAVALPRVSGGHDEHGHLEEFRTDPIGLMQRVRDECGDVGTFQLAGKQVVLLSGSHANEFFFRAGDDDLDQA
KAYPFMTPIFGEGVVFDASPERRKEMLHNAALRGEQMKGHAATIEDQVRRMIADWGEAGEIDLLDFFAELTIY
TSSACLIGKKFRDQLDGRFAKLYHELERGTDPLAYVDPYLPIESFRRRDEARNGLVALVADIMNGRIANPPTD
KSDRDMLDVLIAVKAETGTPRFSADEITGMFISMMFAGHHTSSGTASWTLIELMRHRDAYAAVIDELDELYGD
GRSVSFHALRQIPQLENVLKETLRLHPPLIILMRVAKGEFEVQGHRIHEGDLVAASPAISNRIPEDFPDPHDF
VPARYEQPRQEDLLNRWTWIPFGAGRHRCVGAAFAIMQIKAIFSVLLREYEFEMAQPPESYRNDHSKMVVQLA
QPACVRYRRRTGV
>CYP51B1_Mycobacterium tuberculosis M1424 2622538528
MSAVALPRVSGGHDEHGHLEEFRTDPIGLMQRVRDECGDVGTFQLAGKQVVLLSGSHANEFFFRAGDDDLDQA
KAYPFMTPIFGEGVVFDASPERRKEMLHNAALRGEQMKGHAATIEDQVRRMIADWGEAGEIDLLDFFAELTIY
TSSACLIGKKFRDQLDGRFAKLYHELERGTDPLAYVDPYLPIESFRRRDEARNGLVALVADIMNGRIANPPTD
KSDRDMLDVLIAVKAETGTPRFSADEITGMFISMMFAGHHTSSGTASWTLIELMRHRDAYAAVIDELDELYGD
GRSVSFHALRQIPQLENVLKETLRLHPPLIILMRVAKGEFEVQGHRIHEGDLVAASPAISNRIPEDFPDPHDF
VPARYEQPRQEDLLNRWTWIPFGAGRHRCVGAAFAIMQIKAIFSVLLREYEFEMAQPPESYRNDHSKMVVQLA
QPACVRYRRRTGV
>CYP51B1_Mycobacterium tuberculosis M1425 2622543318
MSAVALPRVSGGHDEHGHLEEFRTDPIGLMQRVRDECGDVGTFQLAGKQVVLLSGSHANEFFFRAGDDDLDQA
KAYPFMTPIFGEGVVFDASPERRKEMLHNAALRGEQMKGHAATIEDQVRRMIADWGEAGEIDLLDFFAELTIY
TSSACLIGKKFRDQLDGRFAKLYHELERGTDPLAYVDPYLPIESFRRRDEARNGLVALVADIMNGRIANPPTD
KSDRDMLDVLIAVKAETGTPRFSADEITGMFISMMFAGHHTSSGTASWTLIELMRHRDAYAAVIDELDELYGD
GRSVSFHALRQIPQLENVLKETLRLHPPLIILMRVAKGEFEVQGHRIHEGDLVAASPAISNRIPEDFPDPHDF
VPARYEQPRQEDLLNRWTWIPFGAGRHRCVGAAFAIMQIKAIFSVLLREYEFEMAQPPESYRNDHSKMVVQLA
QPACVRYRRRTGV
>CYP51B1_Mycobacterium tuberculosis M1429 2622549408
MSAVALPRVSGGHDEHGHLEEFRTDPIGLMQRVRDECGDVGTFQLAGKQVVLLSGSHANEFFFRAGDDDLDQA
KAYPFMTPIFGEGVVFDASPERRKEMLHNAALRGEQMKGHAATIEDQVRRMIADWGEAGEIDLLDFFAELTIY
TSSACLIGKKFRDQLDGRFAKLYHELERGTDPLAYVDPYLPIESFRRRDEARNGLVALVADIMNGRIANPPTD
KSDRDMLDVLIAVKAETGTPRFSADEITGMFISMMFAGHHTSSGTASWTLIELMRHRDAYAAVIDELDELYGD
GRSVSFHALRQIPQLENVLKETLRLHPPLIILMRVAKGEFEVQGHRIHEGDLVAASPAISNRIPEDFPDPHDF
VPARYEQPRQEDLLNRWTWIPFGAGRHRCVGAAFAIMQIKAIFSVLLREYEFEMAQPPESYRNDHSKMVVQLA
QPACVRYRRRTGV
>CYP51B1_Mycobacterium tuberculosis M1431 2622551572
MSAVALPRVSGGHDEHGHLEEFRTDPIGLMQRVRDECGDVGTFQLAGKQVVLLSGSHANEFFFRAGDDDLDQA
KAYPFMTPIFGEGVVFDASPERRKEMLHNAALRGEQMKGHAATIEDQVRRMIADWGEAGEIDLLDFFAELTIY
TSSACLIGKKFRDQLDGRFAKLYHELERGTDPLAYVDPYLPIESFRRRDEARNGLVALVADIMNGRIANPPTD
KSDRDMLDVLIAVKAETGTPRFSADEITGMFISMMFAGHHTSSGTASWTLIELMRHRDAYAAVIDELDELYGD

GRSVSFHALRQIPQLENVLKETLRLHPPLIILMRVAKGEFEVQGHRIHEGDLVAASPAISNRIPEDFPDPHDF
VPARYEQPRQEDLLNRWTWIPFGAGRHRCVGAAFAIMQIKAIFSVLLREYEFEMAQPPESYRNDHSKMVVQLA
QPACVRYRRRTGV
>CYP51B1_Mycobacterium tuberculosis M1438 2574662345
MSAVALPRVSGGHDEHGHLEEFRTDPIGLMQRVRDECGDVGTFQLAGKQVVLLSGSHANEFFFRAGDDDLDQA
KAYPFMTPIFGEGVVFDASPERRKEMLHNAALRGEQMKGHAATIEDQVRRMIADWGEAGEIDLLDFFAELTIY
TSSACLIGKKFRDQLDGRFAKLYHELERGTDPLAYVDPYLPIESFRRRDEARNGLVALVADIMNGRIANPPTD
KSDRDMLDVLIAVKAETGTPRFSADEITGMFISMMFAGHHTSSGTASWTLIELMRHRDAYAAVIDELDELYGD
GRSVSFHALRQIPQLENVLKETLRLHPPLIILMRVAKGEFEVQGHRIHEGDLVAASPAISNRIPEDFPDPHDF
VPARYEQPRQEDLLNRWTWIPFGAGRHRCVGAAFAIMQIKAIFSVLLREYEFEMAQPPESYRNDHSKMVVQLA
QPACVRYRRRTGV
>CYP51B1_Mycobacterium tuberculosis M1439 2622555683
MSAVALPRVSGGHDEHGHLEEFRTDPIGLMQRVRDECGDVGTFQLAGKQVVLLSGSHANEFFFRAGDDDLDQA
KAYPFMTPIFGEGVVFDASPERRKEMLHNAALRGEQMKGHAATIEDQVRRMIADWGEAGEIDLLDFFAELTIY
TSSACLIGKKFRDQLDGRFAKLYHELERGTDPLAYVDPYLPIESFRRRDEARNGLVALVADIMNGRIANPPTD
KSDRDMLDVLIAVKAETGTPRFSADEITGMFISMMFAGHHTSSGTASWTLIELMRHRDAYAAVIDELDELYGD
GRSVSFHALRQIPQLENVLKETLRLHPPLIILMRVAKGEFEVQGHRIHEGDLVAASPAISNRIPEDFPDPHDF
VPARYEQPRQEDLLNRWTWIPFGAGRHRCVGAAFAIMQIKAIFSVLLREYEFEMAQPPESYRNDHSKMVVQLA
QPACVRYRRRTGV
>CYP51B1_Mycobacterium tuberculosis M1441 2622559787
MSAVALPRVSGGHDEHGHLEEFRTDPIGLMQRVRDECGDVGTFQLAGKQVVLLSGSHANEFFFRAGDDDLDQA
KAYPFMTPIFGEGVVFDASPERRKEMLHNAALRGEQMKGHAATIEDQVRRMIADWGEAGEIDLLDFFAELTIY
TSSACLIGKKFRDQLDGRFAKLYHELERGTDPLAYVDPYLPIESFRRRDEARNGLVALVADIMNGRIANPPTD
KSDRDMLDVLIAVKAETGTPRFSADEITGMFISMMFAGHHTSSGTASWTLIELMRHRDAYAAVIDELDELYGD
GRSVSFHALRQIPQLENVLKETLRLHPPLIILMRVAKGEFEVQGHRIHEGDLVAASPAISNRIPEDFPDPHDF
VPARYEQPRQEDLLNRWTWIPFGAGRHRCVGAAFAIMQIKAIFSVLLREYEFEMAQPPESYRNDHSKMVVQLA
QPACVRYRRRTGV
>CYP51B1_Mycobacterium tuberculosis M1444 2576014815
MSAVALPRVSGGHDEHGHLEEFRTDPIGLMQRVRDECGDVGTFQLAGKQVVLLSGSHANEFFFRAGDDDLDQA
KAYPFMTPIFGEGVVFDASPERRKEMLHNAALRGEQMKGHAATIEDQVRRMIADWGEAGEIDLLDFFAELTIY
TSSACLIGKKFRDQLDGRFAKLYHELERGTDPLAYVDPYLPIESFRRRDEARNGLVALVADIMNGRIANPPTD
KSDRDMLDVLIAVKAETGTPRFSADEITGMFISMMFAGHHTSSGTASWTLIELMRHRDAYAAVIDELDELYGD
GRSVSFHALRQIPQLENVLKETLRLHPPLIILMRVAKGEFEVQGHRIHEGDLVAASPAISNRIPEDFPDPHDF
VPARYEQPRQEDLLNRWTWIPFGAGRHRCVGAAFAIMQIKAIFSVLLREYEFEMAQPPESYRNDHSKMVVQLA
QPACVRYRRRTGV
>CYP51B1_Mycobacterium tuberculosis M1448 2622563906
MSAVALPRVSGGHDEHGHLEEFRTDPIGLMQRVRDECGDVGTFQLAGKQVVLLSGSHANEFFFRAGDDDLDQA
KAYPFMTPIFGEGVVFDASPERRKEMLHNAALRGEQMKGHAATIEDQVRRMIADWGEAGEIDLLDFFAELTIY
TSSACLIGKKFRDQLDGRFAKLYHELERGTDPLAYVDPYLPIESFRRRDEARNGLVALVADIMNGRIANPPTD
KSDRDMLDVLIAVKAETGTPRFSADEITGMFISMMFAGHHTSSGTASWTLIELMRHRDAYAAVIDELDELYGD
GRSVSFHALRQIPQLENVLKETLRLHPPLIILMRVAKGEFEVQGHRIHEGDLVAASPAISNRIPEDFPDPHDF
VPARYEQPRQEDLLNRWTWIPFGAGRHRCVGAAFAIMQIKAIFSVLLREYEFEMAQPPESYRNDHSKMVVQLA
QPACVRYRRRTGV
>CYP51B1_Mycobacterium tuberculosis M1449 2575360853
MSAVALPRVSGGHDEHGHLEEFRTDPIGLMQRVRDECGDVGTFQLAGKQVVLLSGSHANEFFFRAGDDDLDQA
KAYPFMTPIFGEGVVFDASPERRKEMLHNAALRGEQMKGHAATIEDQVRRMIADWGEAGEIDLLDFFAELTIY
TSSACLIGKKFRDQLDGRFAKLYHELERGTDPLAYVDPYLPIESFRRRDEARNGLVALVADIMNGRIANPPTD
KSDRDMLDVLIAVKAETGTPRFSADEITGMFISMMFAGHHTSSGTASWTLIELMRHRDAYAAVIDELDELYGD
GRSVSFHALRQIPQLENVLKETLRLHPPLIILMRVAKGEFEVQGHRIHEGDLVAASPAISNRIPEDFPDPHDF
VPARYEQPRQEDLLNRWTWIPFGAGRHRCVGAAFAIMQIKAIFSVLLREYEFEMAQPPESYRNDHSKMVVQLA
QPACVRYRRRTGV
>CYP51B1_Mycobacterium tuberculosis M1452 2622567705
MSAVALPRVSGGHDEHGHLEEFRTDPIGLMQRVRDECGDVGTFQLAGKQVVLLSGSHANEFFFRAGDDDLDQA
KAYPFMTPIFGEGVVFDASPERRKEMLHNAALRGEQMKGHAATIEDQVRRMIADWGEAGEIDLLDFFAELTIY
TSSACLIGKKFRDQLDGRFAKLYHELERGTDPLAYVDPYLPIESFRRRDEARNGLVALVADIMNGRIANPPTD
KSDRDMLDVLIAVKAETGTPRFSADEITGMFISMMFAGHHTSSGTASWTLIELMRHRDAYAAVIDELDELYGD
GRSVSFHALRQIPQLENVLKETLRLHPPLIILMRVAKGEFEVQGHRIHEGDLVAASPAISNRIPEDFPDPHDF

VPARYEQPRQEDLLNRWTWIPFGAGRHRCVGAAFAIMQIKAIFSVLLREYEFEMAQPPESYRNDHSKMVVQLA
QPACVRYRRRTGV
>CYP51B1_Mycobacterium tuberculosis M1456 2622571810
MSAVALPRVSGGHDEHGHLEEFRTDPIGLMQRVRDECGDVGTFQLAGKQVVLLSGSHANEFFFRAGDDDLDQA
KAYPFMTPIFGEGVVFDASPERRKEMLHNAALRGEQMKGHAATIEDQVRRMIADWGEAGEIDLLDFFAELTIY
TSSACLIGKKFRDQLDGRFAKLYHELERGTDPLAYVDPYLPIESFRRRDEARNGLVALVADIMNGRIANPPTD
KSDRDMLDVLIAVKAETGTPRFSADEITGMFISMMFAGHHTSSGTASWTLIELMRHRDAYAAVIDELDELYGD
GRSVSFHALRQIPQLENVLKETLRLHPPLIILMRVAKGEFEVQGHRIHEGDLVAASPAISNRIPEDFPDPHDF
VPARYEQPRQEDLLNRWTWIPFGAGRHRCVGAAFAIMQIKAIFSVLLREYEFEMAQPPESYRNDHSKMVVQLA
QPACVRYRRRTGV
>CYP51B1_Mycobacterium tuberculosis M1458 2622575902
MSAVALPRVSGGHDEHGHLEEFRTDPIGLMQRVRDECGDVGTFQLAGKQVVLLSGSHANEFFFRAGDDDLDQA
KAYPFMTPIFGEGVVFDASPERRKEMLHNAALRGEQMKGHAATIEDQVRRMIADWGEAGEIDLLDFFAELTIY
TSSACLIGKKFRDQLDGRFAKLYHELERGTDPLAYVDPYLPIESFRRRDEARNGLVALVADIMNGRIANPPTD
KSDRDMLDVLIAVKAETGTPRFSADEITGMFISMMFAGHHTSSGTASWTLIELMRHRDAYAAVIDELDELYGD
GRSVSFHALRQIPQLENVLKETLRLHPPLIILMRVAKGEFEVQGHRIHEGDLVAASPAISNRIPEDFPDPHDF
VPARYEQPRQEDLLNRWTWIPFGAGRHRCVGAAFAIMQIKAIFSVLLREYEFEMAQPPESYRNDHSKMVVQLA
QPACVRYRRRTGV
>CYP51B1_Mycobacterium tuberculosis M1461 2622584465
MSAVALPRVSGGHDEHGHLEEFRTDPIGLMQRVRDECGDVGTFQLAGKQVVLLSGSHANEFFFRAGDDDLDQA
KAYPFMTPIFGEGVVFDASPERRKEMLHNAALRGEQMKGHAATIEDQVRRMIADWGEAGEIDLLDFFAELTIY
TSSACLIGKKFRDQLDGRFAKLYHELERGTDPLAYVDPYLPIESFRRRDEARNGLVALVADIMNGRIANPPTD
KSDRDMLDVLIAVKAETGTPRFSADEITGMFISMMFAGHHTSSGTASWTLIELMRHRDAYAAVIDELDELYGD
GRSVSFHALRQIPQLENVLKETLRLHPPLIILMRVAKGEFEVQGHRIHEGDLVAASPAISNRIPEDFPDPHDF
VPARYEQPRQEDLLNRWTWIPFGAGRHRCVGAAFAIMQIKAIFSVLLREYEFEMAQPPESYRNDHSKMVVQLA
QPACVRYRRRTGV
>CYP51B1_Mycobacterium tuberculosis M1475 2584767431
MSAVALPRVSGGHDEHGHLEEFRTDPIGLMQRVRDECGDVGTFQLAGKQVVLLSGSHANEFFFRAGDDDLDQA
KAYPFMTPIFGEGVVFDASPERRKEMLHNAALRGEQMKGHAATIEDQVRRMIADWGEAGEIDLLDFFAELTIY
TSSACLIGKKFRDQLDGRFAKLYHELERGTDPLAYVDPYLPIESFRRRDEARNGLVALVADIMNGRIANPPTD
KSDRDMLDVLIAVKAETGTPRFSADEITGMFISMMFAGHHTSSGTASWTLIELMRHRDAYAAVIDELDELYGD
GRSVSFHALRQIPQLENVLKETLRLHPPLIILMRVAKGEFEVQGHRIHEGDLVAASPAISNRIPEDFPDPHDF
VPARYEQPRQEDLLNRWTWIPFGAGRHRCVGAAFAIMQIKAIFSVLLREYEFEMAQPPESYRNDHSKMVVQLA
QPACVRYRRRTGV
>CYP51B1_Mycobacterium tuberculosis M1481 2575251468
MSAVALPRVSGGHDEHGHLEEFRTDPIGLMQRVRDECGDVGTFQLAGKQVVLLSGSHANEFFFRAGDDDLDQA
KAYPFMTPIFGEGVVFDASPERRKEMLHNAALRGEQMKGHAATIEDQVRRMIADWGEAGEIDLLDFFAELTIY
TSSACLIGKKFRDQLDGRFAKLYHELERGTDPLAYVDPYLPIESFRRRDEARNGLVALVADIMNGRIANPPTD
KSDRDMLDVLIAVKAETGTPRFSADEITGMFISMMFAGHHTSSGTASWTLIELMRHRDAYAAVIDELDELYGD
GRSVSFHALRQIPQLENVLKETLRLHPPLIILMRVAKGEFEVQGHRIHEGDLVAASPAISNRIPEDFPDPHDF
VPARYEQPRQEDLLNRWTWIPFGAGRHRCVGAAFAIMQIKAIFSVLLREYEFEMAQPPESYRNDHSKMVVQLA
QPACVRYRRRTGV
>CYP51B1_Mycobacterium tuberculosis M1520 2622592721
MSAVALPRVSGGHDEHGHLEEFRTDPIGLMQRVRDECGDVGTFQLAGKQVVLLSGSHANEFFFRAGDDDLDQA
KAYPFMTPIFGEGVVFDASPERRKEMLHNAALRGEQMKGHAATIEDQVRRMIADWGEAGEIDLLDFFAELTIY
TSSACLIGKKFRDQLDGRFAKLYHELERGTDPLAYVDPYLPIESFRRRDEARNGLVALVADIMNGRIANPPTD
KSDRDMLDVLIAVKAETGTPRFSADEITGMFISMMFAGHHTSSGTASWTLIELMRHRDAYAAVIDELDELYGD
GRSVSFHALRQIPQLENVLKETLRLHPPLIILMRVAKGEFEVQGHRIHEGDLVAASPAISNRIPEDFPDPHDF
VPARYEQPRQEDLLNRWTWIPFGAGRHRCVGAAFAIMQIKAIFSVLLREYEFEMAQPPESYRNDHSKMVVQLA
QPACVRYRRRTGV
>CYP51B1_Mycobacterium tuberculosis M1533 2622596527
MSAVALPRVSGGHDEHGHLEEFRTDPIGLMQRVRDECGDVGTFQLAGKQVVLLSGSHANEFFFRAGDDDLDQA
KAYPFMTPIFGEGVVFDASPERRKEMLHNAALRGEQMKGHAATIEDQVRRMIADWGEAGEIDLLDFFAELTIY
TSSACLIGKKFRDQLDGRFAKLYHELERGTDPLAYVDPYLPIESFRRRDEARNGLVALVADIMNGRIANPPTD
KSDRDMLDVLIAVKAETGTPRFSADEITGMFISMMFAGHHTSSGTASWTLIELMRHRDAYAAVIDELDELYGD
GRSVSFHALRQIPQLENVLKETLRLHPPLIILMRVAKGEFEVQGHRIHEGDLVAASPAISNRIPEDFPDPHDF
VPARYEQPRQEDLLNRWTWIPFGAGRHRCVGAAFAIMQIKAIFSVLLREYEFEMAQPPESYRNDHSKMVVQLA
QPACVRYRRRTGV

>CYP51B1_Mycobacterium tuberculosis M1560 2622606499
MSAVALPRVSGGHDEHGHLEEFRTDPIGLMQRVRDECGDVGTFQLAGKQVVLLSGSHANEFFFRAGDDDLDQA
KAYPFMTPIFGEGVVFDASPERRKEMLHNAALRGEQMKGHAATIEDQVRRMIADWGEAGEIDLLDFFAELTIY
TSSACLIGKKFRDQLDGRFAKLYHELERGTDPLAYVDPYLPIESFRRRDEARNGLVALVADIMNGRIANPPTD
KSDRDMLDVLIAVKAETGTPRFSADEITGMFISMMFAGHHTSSGTASWTLIELMRHRDAYAAVIDELDELYGD
GRSVSFHALRQIPQLENVLKETLRLHPPLIILMRVAKGEFEVQGHRIHEGDLVAASPAISNRIPEDFPDPHDF
VPARYEQPRQEDLLNRWTWIPFGAGRHRCVGAAFAIMQIKAIFSVLLREYEFEMAQPPESYRNDHSKMVVQLA
QPACVRYRRRTGV
>CYP51B1_Mycobacterium tuberculosis M1570 2622608934
MSAVALPRVSGGHDEHGHLEEFRTDPIGLMQRVRDECGDVGTFQLAGKQVVLLSGSHANEFFFRAGDDDLDQA
KAYPFMTPIFGEGVVFDASPERRKEMLHNAALRGEQMKGHAATIEDQVRRMIADWGEAGEIDLLDFFAELTIY
TSSACLIGKKFRDQLDGRFAKLYHELERGTDPLAYVDPYLPIESFRRRDEARNGLVALVADIMNGRIANPPTD
KSDRDMLDVLIAVKAETGTPRFSADEITGMFISMMFAGHHTSSGTASWTLIELMRHRDAYAAVIDELDELYGD
GRSVSFHALRQIPQLENVLKETLRLHPPLIILMRVAKGEFEVQGHRIHEGDLVAASPAISNRIPEDFPDPHDF
VPARYEQPRQEDLLNRWTWIPFGAGRHRCVGAAFAIMQIKAIFSVLLREYEFEMAQPPESYRNDHSKMVVQLA
QPACVRYRRRTGV
>CYP51B1_Mycobacterium tuberculosis M1615 2622615951
MSAVALPRVSGGHDEHGHLEEFRTDPIGLMQRVRDECGDVGTFQLAGKQVVLLSGSHANEFFFRAGDDDLDQA
KAYPFMTPIFGEGVVFDASPERRKEMLHNAALRGEQMKGHAATIEDQVRRMIADWGEAGEIDLLDFFAELTIY
TSSACLIGKKFRDQLDGRFAKLYHELERGTDPLAYVDPYLPIESFRRRDEARNGLVALVADIMNGRIANPPTD
KSDRDMLDVLIAVKAETGTPRFSADEITGMFISMMFAGHHTSSGTASWTLIELMRHRDAYAAVIDELDELYGD
GRSVSFHALRQIPQLENVLKETLRLHPPLIILMRVAKGEFEVQGHRIHEGDLVAASPAISNRIPEDFPDPHDF
VPARYEQPRQEDLLNRWTWIPFGAGRHRCVGAAFAIMQIKAIFSVLLREYEFEMAQPPESYRNDHSKMVVQLA
QPACVRYRRRTGV
>CYP51B1_Mycobacterium tuberculosis M1700 2584916419
MSAVALPRVSGGHDEHGHLEEFRTDPIGLMQRVRDECGDVGTFQLAGKQVVLLSGSHANEFFFRAGDDDLDQA
KAYPFMTPIFGEGVVFDASPERRKEMLHNAALRGEQMKGHAATIEDQVRRMIADWGEAGEIDLLDFFAELTIY
TSSACLIGKKFRDQLDGRFAKLYHELERGTDPLAYVDPYLPIESFRRRDEARNGLVALVADIMNGRIANPPTD
KSDRDMLDVLIAVKAETGTPRFSADEITGMFISMMFAGHHTSSGTASWTLIELMRHRDAYAAVIDELDELYGD
GRSVSFHALRQIPQLENVLKETLRLHPPLIILMRVAKGEFEVQGHRIHEGDLVAASPAISNRIPEDFPDPHDF
VPARYEQPRQEDLLNRWTWIPFGAGRHRCVGAAFAIMQIKAIFSVLLREYEFEMAQPPESYRNDHSKMVVQLA
QPACVRYRRRTGV
>CYP51B1_Mycobacterium tuberculosis M1703 2584733413
MSAVALPRVSGGHDEHGHLEEFRTDPIGLMQRVRDECGDVGTFQLAGKQVVLLSGSHANEFFFRAGDDDLDQA
KAYPFMTPIFGEGVVFDASPERRKEMLHNAALRGEQMKGHAATIEDQVRRMIADWGEAGEIDLLDFFAELTIY
TSSACLIGKKFRDQLDGRFAKLYHELERGTDPLAYVDPYLPIESFRRRDEARNGLVALVADIMNGRIANPPTD
KSDRDMLDVLIAVKAETGTPRFSADEITGMFISMMFAGHHTSSGTASWTLIELMRHRDAYAAVIDELDELYGD
GRSVSFHALRQIPQLENVLKETLRLHPPLIILMRVAKGEFEVQGHRIHEGDLVAASPAISNRIPEDFPDPHDF
VPARYEQPRQEDLLNRWTWIPFGAGRHRCVGAAFAIMQIKAIFSVLLREYEFEMAQPPESYRNDHSKMVVQLA
QPACVRYRRRTGV
>CYP51B1_Mycobacterium tuberculosis M1726 2622630044
MSAVALPRVSGGHDEHGHLEEFRTDPIGLMQRVRDECGDVGTFQLAGKQVVLLSGSHANEFFFRAGDDDLDQA
KAYPFMTPIFGEGVVFDASPERRKEMLHNAALRGEQMKGHAATIEDQVRRMIADWGEAGEIDLLDFFAELTIY
TSSACLIGKKFRDQLDGRFAKLYHELERGTDPLAYVDPYLPIESFRRRDEARNGLVALVADIMNGRIANPPTD
KSDRDMLDVLIAVKAETGTPRFSADEITGMFISMMFAGHHTSSGTASWTLIELMRHRDAYAAVIDELDELYGD
GRSVSFHALRQIPQLENVLKETLRLHPPLIILMRVAKGEFEVQGHRIHEGDLVAASPAISNRIPEDFPDPHDF
VPARYEQPRQEDLLNRWTWIPFGAGRHRCVGAAFAIMQIKAIFSVLLREYEFEMAQPPESYRNDHSKMVVQLA
QPACVRYRRRTGV
>CYP51B1_Mycobacterium tuberculosis M1728 2622633866
MSAVALPRVSGGHDEHGHLEEFRTDPIGLMQRVRDECGDVGTFQLAGKQVVLLSGSHANEFFFRAGDDDLDQA
KAYPFMTPIFGEGVVFDASPERRKEMLHNAALRGEQMKGHAATIEDQVRRMIADWGEAGEIDLLDFFAELTIY
TSSACLIGKKFRDQLDGRFAKLYHELERGTDPLAYVDPYLPIESFRRRDEARNGLVALVADIMNGRIANPPTD
KSDRDMLDVLIAVKAETGTPRFSADEITGMFISMMFAGHHTSSGTASWTLIELMRHRDAYAAVIDELDELYGD
GRSVSFHALRQIPQLENVLKETLRLHPPLIILMRVAKGEFEVQGHRIHEGDLVAASPAISNRIPEDFPDPHDF
VPARYEQPRQEDLLNRWTWIPFGAGRHRCVGAAFAIMQIKAIFSVLLREYEFEMAQPPESYRNDHSKMVVQLA
QPACVRYRRRTGV
>CYP51B1_Mycobacterium tuberculosis M1734 2622637989

MSAVALPRVSGGHDEHGHLEEFRTDPIGLMQRVRDECGDVGTFQLAGKQVVLLSGSHANEFFFRAGDDDLDQA
KAYPFMTPIFGEGVVFDASPERRKEMLHNAALRGEQMKGHAATIEDQVRRMIADWGEAGEIDLLDFFAELTIY
TSSACLIGKKFRDQLDGRFAKLYHELERGTDPLAYVDPYLPIESFRRRDEARNGLVALVADIMNGRIANPPTD
KSDRDMLDVLIAVKAETGTPRFSADEITGMFISMMFAGHHTSSGTASWTLIELMRHRDAYAAVIDELDELYGD
GRSVSFHALRQIPQLENVLKETLRLHPPLIILMRVAKGEFEVQGHRIHEGDLVAASPAISNRIPEDFPDPHDF
VPARYEQPRQEDLLNRWTWIPFGAGRHRCVGAAFAIMQIKAIFSVLLREYEFEMAQPPESYRNDHSKMVVQLA
QPACVRYRRRTGV
>CYP51B1_Mycobacterium tuberculosis M1782 2622646545
MSAVALPRVSGGHDEHGHLEEFRTDPIGLMQRVRDECGDVGTFQLAGKQVVLLSGSHANEFFFRAGDDDLDQA
KAYPFMTPIFGEGVVFDASPERRKEMLHNAALRGEQMKGHAATIEDQVRRMIADWGEAGEIDLLDFFAELTIY
TSSACLIGKKFRDQLDGRFAKLYHELERGTDPLAYVDPYLPIESFRRRDEARNGLVALVADIMNGRIANPPTD
KSDRDMLDVLIAVKAETGTPRFSADEITGMFISMMFAGHHTSSGTASWTLIELMRHRDAYAAVIDELDELYGD
GRSVSFHALRQIPQLENVLKETLRLHPPLIILMRVAKGEFEVQGHRIHEGDLVAASPAISNRIPEDFPDPHDF
VPARYEQPRQEDLLNRWTWIPFGAGRHRCVGAAFAIMQIKAIFSVLLREYEFEMAQPPESYRNDHSKMVVQLA
QPACVRYRRRTGV
>CYP51B1_Mycobacterium tuberculosis M1816 2622653515
MSAVALPRVSGGHDEHGHLEEFRTDPIGLMQRVRDECGDVGTFQLAGKQVVLLSGSHANEFFFRAGDDDLDQA
KAYPFMTPIFGEGVVFDASPERRKEMLHNAALRGEQMKGHAATIEDQVRRMIADWGEAGEIDLLDFFAELTIY
TSSACLIGKKFRDQLDGRFAKLYHELERGTDPLAYVDPYLPIESFRRRDEARNGLVALVADIMNGRIANPPTD
KSDRDMLDVLIAVKAETGTPRFSADEITGMFISMMFAGHHTSSGTASWTLIELMRHRDAYAAVIDELDELYGD
GRSVSFHALRQIPQLENVLKETLRLHPPLIILMRVAKGEFEVQGHRIHEGDLVAASPAISNRIPEDFPDPHDF
VPARYEQPRQEDLLNRWTWIPFGAGRHRCVGAAFAIMQIKAIFSVLLREYEFEMAQPPESYRNDHSKMVVQLA
QPACVRYRRRTGV
>CYP51B1_Mycobacterium tuberculosis M1822 2622654832
MSAVALPRVSGGHDEHGHLEEFRTDPIGLMQRVRDECGDVGTFQLAGKQVVLLSGSHANEFFFRAGDDDLDQA
KAYPFMTPIFGEGVVFDASPERRKEMLHNAALRGEQMKGHAATIEDQVRRMIADWGEAGEIDLLDFFAELTIY
TSSACLIGKKFRDQLDGRFAKLYHELERGTDPLAYVDPYLPIESFRRRDEARNGLVALVADIMNGRIANPPTD
KSDRDMLDVLIAVKAETGTPRFSADEITGMFISMMFAGHHTSSGTASWTLIELMRHRDAYAAVIDELDELYGD
GRSVSFHALRQIPQLENVLKETLRLHPPLIILMRVAKGEFEVQGHRIHEGDLVAASPAISNRIPEDFPDPHDF
VPARYEQPRQEDLLNRWTWIPFGAGRHRCVGAAFAIMQIKAIFSVLLREYEFEMAQPPESYRNDHSKMVVQLA
QPACVRYRRRTGV
>CYP51B1_Mycobacterium tuberculosis M1848 2574772142
MSAVALPRVSGGHDEHGHLEEFRTDPIGLMQRVRDECGDVGTFQLAGKQVVLLSGSHANEFFFRAGDDDLDQA
KAYPFMTPIFGEGVVFDASPERRKEMLHNAALRGEQMKGHAATIEDQVRRMIADWGEAGEIDLLDFFAELTIY
TSSACLIGKKFRDQLDGRFAKLYHELERGTDPLAYVDPYLPIESFRRRDEARNGLVALVADIMNGRIANPPTD
KSDRDMLDVLIAVKAETGTPRFSADEITGMFISMMFAGHHTSSGTASWTLIELMRHRDAYAAVIDELDELYGD
GRSVSFHALRQIPQLENVLKETLRLHPPLIILMRVAKGEFEVQGHRIHEGDLVAASPAISNRIPEDFPDPHDF
VPARYEQPRQEDLLNRWTWIPFGAGRHRCVGAAFAIMQIKAIFSVLLREYEFEMAQPPESYRNDHSKMVVQLA
QPACVRYRRRTGV
>CYP51B1_Mycobacterium tuberculosis M1893 2622661289
MSAVALPRVSGGHDEHGHLEEFRTDPIGLMQRVRDECGDVGTFQLAGKQVVLLSGSHANEFFFRAGDDDLDQA
KAYPFMTPIFGEGVVFDASPERRKEMLHNAALRGEQMKGHAATIEDQVRRMIADWGEAGEIDLLDFFAELTIY
TSSACLIGKKFRDQLDGRFAKLYHELERGTDPLAYVDPYLPIESFRRRDEARNGLVALVADIMNGRIANPPTD
KSDRDMLDVLIAVKAETGTPRFSADEITGMFISMMFAGHHTSSGTASWTLIELMRHRDAYAAVIDELDELYGD
GRSVSFHALRQIPQLENVLKETLRLHPPLIILMRVAKGEFEVQGHRIHEGDLVAASPAISNRIPEDFPDPHDF
VPARYEQPRQEDLLNRWTWIPFGAGRHRCVGAAFAIMQIKAIFSVLLREYEFEMAQPPESYRNDHSKMVVQLA
QPACVRYRRRTGV
>CYP51B1_Mycobacterium tuberculosis M1895 2622665928
MSAVALPRVSGGHDEHGHLEEFRTDPIGLMQRVRDECGDVGTFQLAGKQVVLLSGSHANEFFFRAGDDDLDQA
KAYPFMTPIFGEGVVFDASPERRKEMLHNAALRGEQMKGHAATIEDQVRRMIADWGEAGEIDLLDFFAELTIY
TSSACLIGKKFRDQLDGRFAKLYHELERGTDPLAYVDPYLPIESFRRRDEARNGLVALVADIMNGRIANPPTD
KSDRDMLDVLIAVKAETGTPRFSADEITGMFISMMFAGHHTSSGTASWTLIELMRHRDAYAAVIDELDELYGD
GRSVSFHALRQIPQLENVLKETLRLHPPLIILMRVAKGEFEVQGHRIHEGDLVAASPAISNRIPEDFPDPHDF
VPARYEQPRQEDLLNRWTWIPFGAGRHRCVGAAFAIMQIKAIFSVLLREYEFEMAQPPESYRNDHSKMVVQLA
QPACVRYRRRTGV
>CYP51B1_Mycobacterium tuberculosis M1914 2622666706
MSAVALPRVSGGHDEHGHLEEFRTDPIGLMQRVRDECGDVGTFQLAGKQVVLLSGSHANEFFFRAGDDDLDQA
KAYPFMTPIFGEGVVFDASPERRKEMLHNAALRGEQMKGHAATIEDQVRRMIADWGEAGEIDLLDFFAELTIY

TSSACLIGKKFRDQLDGRFAKLYHELERGTDPLAYVDPYLPIESFRRRDEARNGLVALVADIMNGRIANPPTD
KSDRDMLDVLIAVKAETGTPRFSADEITGMFISMMFAGHHTSSGTASWTLIELMRHRDAYAAVIDELDELYGD
GRSVSFHALRQIPQLENVLKETLRLHPPLIILMRVAKGEFEVQGHRIHEGDLVAASPAISNRIPEDFPDPHDF
VPARYEQPRQEDLLNRWTWIPFGAGRHRCVGAAFAIMQIKAIFSVLLREYEFEMAQPPESYRNDHSKMVVQLA
QPACVRYRRRTGV
>CYP51B1_Mycobacterium tuberculosis M1926 2622674137
MSAVALPRVSGGHDEHGHLEEFRTDPIGLMQRVRDECGDVGTFQLAGKQVVLLSGSHANEFFFRAGDDDLDQA
KAYPFMTPIFGEGVVFDASPERRKEMLHNAALRGEQMKGHAATIEDQVRRMIADWGEAGEIDLLDFFAELTIY
TSSACLIGKKFRDQLDGRFAKLYHELERGTDPLAYVDPYLPIESFRRRDEARNGLVALVADIMNGRIANPPTD
KSDRDMLDVLIAVKAETGTPRFSADEITGMFISMMFAGHHTSSGTASWTLIELMRHRDAYAAVIDELDELYGD
GRSVSFHALRQIPQLENVLKETLRLHPPLIILMRVAKGEFEVQGHRIHEGDLVAASPAISNRIPEDFPDPHDF
VPARYEQPRQEDLLNRWTWIPFGAGRHRCVGAAFAIMQIKAIFSVLLREYEFEMAQPPESYRNDHSKMVVQLA
QPACVRYRRRTGV
>CYP51B1_Mycobacterium tuberculosis M1928 2622678062
MSAVALPRVSGGHDEHGHLEEFRTDPIGLMQRVRDECGDVGTFQLAGKQVVLLSGSHANEFFFRAGDDDLDQA
KAYPFMTPIFGEGVVFDASPERRKEMLHNAALRGEQMKGHAATIEDQVRRMIADWGEAGEIDLLDFFAELTIY
TSSACLIGKKFRDQLDGRFAKLYHELERGTDPLAYVDPYLPIESFRRRDEARNGLVALVADIMNGRIANPPTD
KSDRDMLDVLIAVKAETGTPRFSADEITGMFISMMFAGHHTSSGTASWTLIELMRHRDAYAAVIDELDELYGD
GRSVSFHALRQIPQLENVLKETLRLHPPLIILMRVAKGEFEVQGHRIHEGDLVAASPAISNRIPEDFPDPHDF
VPARYEQPRQEDLLNRWTWIPFGAGRHRCVGAAFAIMQIKAIFSVLLREYEFEMAQPPESYRNDHSKMVVQLA
QPACVRYRRRTGV
>CYP51B1_Mycobacterium tuberculosis M1929 2622682504
MSAVALPRVSGGHDEHGHLEEFRTDPIGLMQRVRDECGDVGTFQLAGKQVVLLSGSHANEFFFRAGDDDLDQA
KAYPFMTPIFGEGVVFDASPERRKEMLHNAALRGEQMKGHAATIEDQVRRMIADWGEAGEIDLLDFFAELTIY
TSSACLIGKKFRDQLDGRFAKLYHELERGTDPLAYVDPYLPIESFRRRDEARNGLVALVADIMNGRIANPPTD
KSDRDMLDVLIAVKAETGTPRFSADEITGMFISMMFAGHHTSSGTASWTLIELMRHRDAYAAVIDELDELYGD
GRSVSFHALRQIPQLENVLKETLRLHPPLIILMRVAKGEFEVQGHRIHEGDLVAASPAISNRIPEDFPDPHDF
VPARYEQPRQEDLLNRWTWIPFGAGRHRCVGAAFAIMQIKAIFSVLLREYEFEMAQPPESYRNDHSKMVVQLA
QPACVRYRRRTGV
>CYP51B1_Mycobacterium tuberculosis M1932 2577250910
MSAVALPRVSGGHDEHGHLEEFRTDPIGLMQRVRDECGDVGTFQLAGKQVVLLSGSHANEFFFRAGDDDLDQA
KAYPFMTPIFGEGVVFDASPERRKEMLHNAALRGEQMKGHAATIEDQVRRMIADWGEAGEIDLLDFFAELTIY
TSSACLIGKKFRDQLDGRFAKLYHELERGTDPLAYVDPYLPIESFRRRDEARNGLVALVADIMNGRIANPPTD
KSDRDMLDVLIAVKAETGTPRFSADEITGMFISMMFAGHHTSSGTASWTLIELMRHRDAYAAVIDELDELYGD
GRSVSFHALRQIPQLENVLKETLRLHPPLIILMRVAKGEFEVQGHRIHEGDLVAASPAISNRIPEDFPDPHDF
VPARYEQPRQEDLLNRWTWIPFGAGRHRCVGAAFAIMQIKAIFSVLLREYEFEMAQPPESYRNDHSKMVVQLA
QPACVRYRRRTGV
>CYP51B1_Mycobacterium tuberculosis M1948 2622683578
MSAVALPRVSGGHDEHGHLEEFRTDPIGLMQRVRDECGDVGTFQLAGKQVVLLSGSHANEFFFRAGDDDLDQA
KAYPFMTPIFGEGVVFDASPERRKEMLHNAALRGEQMKGHAATIEDQVRRMIADWGEAGEIDLLDFFAELTIY
TSSACLIGKKFRDQLDGRFAKLYHELERGTDPLAYVDPYLPIESFRRRDEARNGLVALVADIMNGRIANPPTD
KSDRDMLDVLIAVKAETGTPRFSADEITGMFISMMFAGHHTSSGTASWTLIELMRHRDAYAAVIDELDELYGD
GRSVSFHALRQIPQLENVLKETLRLHPPLIILMRVAKGEFEVQGHRIHEGDLVAASPAISNRIPEDFPDPHDF
VPARYEQPRQEDLLNRWTWIPFGAGRHRCVGAAFAIMQIKAIFSVLLREYEFEMAQPPESYRNDHSKMVVQLA
QPACVRYRRRTGV
>CYP51B1_Mycobacterium tuberculosis M1956 2584632737
MSAVALPRVSGGHDEHGHLEEFRTDPIGLMQRVRDECGDVGTFQLAGKQVVLLSGSHANEFFFRAGDDDLDQA
KAYPFMTPIFGEGVVFDASPERRKEMLHNAALRGEQMKGHAATIEDQVRRMIADWGEAGEIDLLDFFAELTIY
TSSACLIGKKFRDQLDGRFAKLYHELERGTDPLAYVDPYLPIESFRRRDEARNGLVALVADIMNGRIANPPTD
KSDRDMLDVLIAVKAETGTPRFSADEITGMFISMMFAGHHTSSGTASWTLIELMRHRDAYAAVIDELDELYGD
GRSVSFHALRQIPQLENVLKETLRLHPPLIILMRVAKGEFEVQGHRIHEGDLVAASPAISNRIPEDFPDPHDF
VPARYEQPRQEDLLNRWTWIPFGAGRHRCVGAAFAIMQIKAIFSVLLREYEFEMAQPPESYRNDHSKMVVQLA
QPACVRYRRRTGV
>CYP51B1_Mycobacterium tuberculosis M1959 2622692233
MSAVALPRVSGGHDEHGHLEEFRTDPIGLMQRVRDECGDVGTFQLAGKQVVLLSGSHANEFFFRAGDDDLDQA
KAYPFMTPIFGEGVVFDASPERRKEMLHNAALRGEQMKGHAATIEDQVRRMIADWGEAGEIDLLDFFAELTIY
TSSACLIGKKFRDQLDGRFAKLYHELERGTDPLAYVDPYLPIESFRRRDEARNGLVALVADIMNGRIANPPTD
KSDRDMLDVLIAVKAETGTPRFSADEITGMFISMMFAGHHTSSGTASWTLIELMRHRDAYAAVIDELDELYGD

GRSVSFHALRQIPQLENVLKETLRLHPPLIILMRVAKGEFEVQGHRIHEGDLVAASPAISNRIPEDFPDPHDF
VPARYEQPRQEDLLNRWTWIPFGAGRHRCVGAAFAIMQIKAIFSVLLREYEFEMAQPPESYRNDHSKMVVQLA
QPACVRYRRRTGV
>CYP51B1_Mycobacterium tuberculosis M1959 2622695343
MSAVALPRVSGGHDEHGHLEEFRTDPIGLMQRVRDECGDVGTFQLAGKQVVLLSGSHANEFFFRAGDDDLDQA
KAYPFMTPIFGEGVVFDASPERRKEMLHNAALRGEQMKGHAATIEDQVRRMIADWGEAGEIDLLDFFAELTIY
TSSACLIGKKFRDQLDGRFAKLYHELERGTDPLAYVDPYLPIESFRRRDEARN
>CYP51B1_Mycobacterium tuberculosis M1961 2584689675
MSAVALPRVSGGHDEHGHLEEFRTDPIGLMQRVRDECGDVGTFQLAGKQVVLLSGSHANEFFFRAGDDDLDQA
KAYPFMTPIFGEGVVFDASPERRKEMLHNAALRGEQMKGHAATIEDQVRRMIADWGEAGEIDLLDFFAELTIY
TSSACLIGKKFRDQLDGRFAKLYHELERGTDPLAYVDPYLPIESFRRRDEARNGLVALVADIMNGRIANPPTD
KSDRDMLDVLIAVKAETGTPRFSADEITGMFISMMFAGHHTSSGTASWTLIELMRHRDAYAAVIDELDELYGD
GRSVSFHALRQIPQLENVLKETLRLHPPLIILMRVAKGEFEVQGHRIHEGDLVAASPAISNRIPEDFPDPHDF
VPARYEQPRQEDLLNRWTWIPFGAGRHRCVGAAFAIMQIKAIFSVLLREYEFEMAQPPESYRNDHSKMVVQLA
QPACVRYRRRTGV
>CYP51B1_Mycobacterium tuberculosis M1967 2622699073
MSAVALPRVSGGHDEHGHLEEFRTDPIGLMQRVRDECGDVGTFQLAGKQVVLLSGSHANEFFFRAGDDDLDQA
KAYPFMTPIFGEGVVFDASPERRKEMLHNAALRGEQMKGHAATIEDQVRRMIADWGEAGEIDLLDFFAELTIY
TSSACLIGKKFRDQLDGRFAKLYHELERGTDPLAYVDPYLPIESFRRRDEARNGLVALVADIMNGRIANPPTD
KSDRDMLDVLIAVKAETGTPRFSADEITGMFISMMFAGHHTSSGTASWTLIELMRHRDAYAAVIDELDELYGD
GRSVSFHALRQIPQLENVLKETLRLHPPLIILMRVAKGEFEVQGHRIHEGDLVAASPAISNRIPEDFPDPHDF
VPARYEQPRQEDLLNRWTWIPFGAGRHRCVGAAFAIMQIKAIFSVLLREYEFEMAQPPESYRNDHSKMVVQLA
QPACVRYRRRTGV
>CYP51B1_Mycobacterium tuberculosis M1973 2622703149
MSAVALPRVSGGHDEHGHLEEFRTDPIGLMQRVRDECGDVGTFQLAGKQVVLLSGSHANEFFFRAGDDDLDQA
KAYPFMTPIFGEGVVFDASPERRKEMLHNAALRGEQMKGHAATIEDQVRRMIADWGEAGEIDLLDFFAELTIY
TSSACLIGKKFRDQLDGRFAKLYHELERGTDPLAYVDPYLPIESFRRRDEARNGLVALVADIMNGRIANPPTD
KSDRDMLDVLIAVKAETGTPRFSADEITGMFISMMFAGHHTSSGTASWTLIELMRHRDAYAAVIDELDELYGD
GRSVSFHALRQIPQLENVLKETLRLHPPLIILMRVAKGEFEVQGHRIHEGDLVAASPAISNRIPEDFPDPHDF
VPARYEQPRQEDLLNRWTWIPFGAGRHRCVGAAFAIMQIKAIFSVLLREYEFEMAQPPESYRNDHSKMVVQLA
QPACVRYRRRTGV
>CYP51B1_Mycobacterium tuberculosis M1976 2622707080
MSAVALPRVSGGHDEHGHLEEFRTDPIGLMQRVRDECGDVGTFQLAGKQVVLLSGSHANEFFFRAGDDDLDQA
KAYPFMTPIFGEGVVFDASPERRKEMLHNAALRGEQMKGHAATIEDQVRRMIADWGEAGEIDLLDFFAELTIY
TSSACLIGKKFRDQLDGRFAKLYHELERGTDPLAYVDPYLPIESFRRRDEARNGLVALVADIMNGRIANPPTD
KSDRDMLDVLIAVKAETGTPRFSADEITGMFISMMFAGHHTSSGTASWTLIELMRHRDAYAAVIDELDELYGD
GRSVSFHALRQIPQLENVLKETLRLHPPLIILMRVAKGEFEVQGHRIHEGDLVAASPAISNRIPEDFPDPHDF
VPARYEQPRQEDLLNRWTWIPFGAGRHRCVGAAFAIMQIKAIFSVLLREYEFEMAQPPESYRNDHSKMVVQLA
QPACVRYRRRTGV
>CYP51B1_Mycobacterium tuberculosis M1978 2578088823
MSAVALPRVSGGHDEHGHLEEFRTDPIGLMQRVRDECGDVGTFQLAGKQVVLLSGSHANEFFFRAGDDDLDQA
KAYPFMTPIFGEGVVFDASPERRKEMLHNAALRGEQMKGHAATIEDQVRRMIADWGEAGEIDLLDFFAELTIY
TSSACLIGKKFRDQLDGRFAKLYHELERGTDPLAYVDPYLPIESFRRRDEARNGLVALVADIMNGRIANPPTD
KSDRDMLDVLIAVKAETGTPRFSADEITGMFISMMFAGHHTSSGTASWTLIELMRHRDAYAAVIDELDELYGD
GRSVSFHALRQIPQLENVLKETLRLHPPLIILMRVAKGEFEVQGHRIHEGDLVAASPAISNRIPEDFPDPHDF
VPARYEQPRQEDLLNRWTWIPFGAGRHRCVGAAFAIMQIKAIFSVLLREYEFEMAQPPESYRNDHSKMVVQLA
QPACVRYRRRTGV
>CYP51B1_Mycobacterium tuberculosis M1979 2622711565
MSAVALPRVSGGHDEHGHLEEFRTDPIGLMQRVRDECGDVGTFQLAGKQVVLLSGSHANEFFFRAGDDDLDQA
KAYPFMTPIFGEGVVFDASPERRKEMLHNAALRGEQMKGHAATIEDQVRRMIADWGEAGEIDLLDFFAELTIY
TSSACLIGKKFRDQLDGRFAKLYHELERGTDPLAYVDPYLPIESFRRRDEARNGLVALVADIMNGRIANPPTD
KSDRDMLDVLIAVKAETGTPRFSADEITGMFISMMFAGHHTSSGTASWTLIELMRHRDAYAAVIDELDELYGD
GRSVSFHALRQIPQLENVLKETLRLHPPLIILMRVAKGEFEVQGHRIHEGDLVAASPAISNRIPEDFPDPHDF
VPARYEQPRQEDLLNRWTWIPFGAGRHRCVGAAFAIMQIKAIFSVLLREYEFEMAQPPESYRNDHSKMVVQLA
QPACVRYRRRTGV
>CYP51B1_Mycobacterium tuberculosis M2003 2622712970
MSAVALPRVSGGHDEHGHLEEFRTDPIGLMQRVRDECGDVGTFQLAGKQVVLLSGSHANEFFFRAGDDDLDQA
KAYPFMTPIFGEGVVFDASPERRKEMLHNAALRGEQMKGHAATIEDQVRRMIADWGEAGEIDLLDFFAELTIY

TSSACLIGKKFRDQLDGRFAKLYHELERGTDPLAYVDPYLPIESFRRRDEARNGLVALVADIMNGRIANPPTD
KSDRDMLDVLIAVKAETGTPRFSADEITGMFISMMFAGHHTSSGTASWTLIELMRHRDAYAAVIDELDELYGD
GRSVSFHALRQIPQLENVLKETLRLHPPLIILMRVAKGEFEVQGHRIHEGDLVAASPAISNRIPEDFPDPHDF
VPARYEQPRQEDLLNRWTWIPFGAGRHRCVGAAFAIMQIKAIFSVLLREYEFEMAQPPESYRNDHSKMVVQLA
QPACVRYRRRTGV
>CYP51B1_Mycobacterium tuberculosis M2010 2622802847
MSAVALPRVSGGHDEHGHLEEFRTDPIGLMQRVRDECGDVGTFQLAGKQVVLLSGSHANEFFFRAGDDDLDQA
KAYPFMTPIFGEGVVFDASPERRKEMLHNAALRGEQMKGHAATIEDQVRRMIADWGEAGEIDLLDFFAELTIY
TSSACLIGKKFRDQLDGRFAKLYHELERGTDPLAYVDPYLPIESFRRRDEARNGLVALVADIMNGRIANPPTD
KSDRDMLDVLIAVKAETGTPRFSADEITGMFISMMFAGHHTSSGTASWTLIELMRHRDAYAAVIDELDELYGD
GRSVSFHALRQIPQLENVLKETLRLHPPLIILMRVAKGEFEVQGHRIHEGDLVAASPAISNRIPEDFPDPHDF
VPARYEQPRQEDLLNRWTWIPFGAGRHRCVGAAFAIMQIKAIFSVLLREYEFEMAQPPESYRNDHSKMVVQLA
QPACVRYRRRTGV
>CYP51B1_Mycobacterium tuberculosis M2020 2622804163
MSAVALPRVSGGHDEHGHLEEFRTDPIGLMQRVRDECGDVGTFQLAGKQVVLLSGSHANEFFFRAGDDDLDQA
KAYPFMTPIFGEGVVFDASPERRKEMLHNAALRGEQMKGHAATIEDQVRRMIADWGEAGEIDLLDFFAELTIY
TSSACLIGKKFRDQLDGRFAKLYHELERGTDPLAYVDPYLPIESFRRRDEARNGLVALVADIMNGRIANPPTD
KSDRDMLDVLIAVKAETGTPRFSADEITGMFISMMFAGHHTSSGTASWTLIELMRHRDAYAAVIDELDELYGD
GRSVSFHALRQIPQLENVLKETLRLHPPLIILMRVAKGEFEVQGHRIHEGDLVAASPAISNRIPEDFPDPHDF
VPARYEQPRQEDLLNRWTWIPFGAGRHRCVGAAFAIMQIKAIFSVLLREYEFEMAQPPESYRNDHSKMVVQLA
QPACVRYRRRTGV
>CYP51B1_Mycobacterium tuberculosis M2021 2622808166
MSAVALPRVSGGHDEHGHLEEFRTDPIGLMQRVRDECGDVGTFQLAGKQVVLLSGSHANEFFFRAGDDDLDQA
KAYPFMTPIFGEGVVFDASPERRKEMLHNAALRGEQMKGHAATIEDQVRRMIADWGEAGEIDLLDFFAELTIY
TSSACLIGKKFRDQLDGRFAKLYHELERGTDPLAYVDPYLPIESFRRRDEARNGLVALVADIMNGRIANPPTD
KSDRDMLDVLIAVKAETGTPRFSADEITGMFISMMFAGHHTSSGTASWTLIELMRHRDAYAAVIDELDELYGD
GRSVSFHALRQIPQLENVLKETLRLHPPLIILMRVAKGEFEVQGHRIHEGDLVAASPAISNRIPEDFPDPHDF
VPARYEQPRQEDLLNRWTWIPFGAGRHRCVGAAFAIMQIKAIFSVLLREYEFEMAQPPESYRNDHSKMVVQLA
QPACVRYRRRTGV
>CYP51B1_Mycobacterium tuberculosis M2032 (re-annotation Oct 2015)
2639513573
MSAVALPRVSGGHDEHGHLEEFRTDPIGLMQRVRDECGDVGTFQLAGKQVVLLSGSHANEFFFRAGDDDLDQA
KAYPFMTPIFGEGVVFDASPERRKEMLHNAALRGEQMKGHAATIEDQVRRMIADWGEAGEIDLLDFFAELTIY
TSSACLIGKKFRDQLDGRFAKLYHELERGTDPLAYVDPYLPIESFRRRDEARNGLVALVADIMNGRIANPPTD
KSDRDMLDVLIAVKAETGTPRFSADEITGMFISMMFAGHHTSSGTASWTLIELMRHRDAYAAVIDELDELYGD
GRSVSFHALRQIPQLENVLKETLRLHPPLIILMRVAKGEFEVQGHRIHEGDLVAASPAISNRIPEDFPDPHDF
VPARYEQPRQEDLLNRWTWIPFGAGRHRCVGAAFAIMQIKAIFSVLLREYEFEMAQPPESYRNDHSKMVVQLA
QPACVRYRRRTGV
>CYP51B1_Mycobacterium tuberculosis M2084 2629497432
MSAVALPRVSGGHDEHGHLEEFRTDPIGLMQRVRDECGDVGTFQLAGKQVVLLSGSHANEFFFRAGDDDLDQA
KAYPFMTPIFGEGVVFDASPERRKEMLHNAALRGEQMKGHAATIEDQVRRMIADWGEAGEIDLLDFFAELTIY
TSSACLIGKKFRDQLDGRFAKLYHELERGTDPLAYVDPYLPIESFRRRDEARNGLVALVADIMNGRIANPPTD
KSDRDMLDVLIAVKAETGTPRFSADEITGMFISMMFAGHHTSSGTASWTLIELMRHRDAYAAVIDELDELYGD
GRSVSFHALRQIPQLENVLKETLRLHPPLIILMRVAKGEFEVQGHRIHEGDLVAASPAISNRIPEDFPDPHDF
VPARYEQPRQEDLLNRWTWIPFGAGRHRCVGAAFAIMQIKAIFSVLLREYEFEMAQPPESYRNDHSKMVVQLA
QPACVRYRRRTGV
>CYP51B1_Mycobacterium tuberculosis M2085 2577906893
MSAVALPRVSGGHDEHGHLEEFRTDPIGLMQRVRDECGDVGTFQLAGKQVVLLSGSHANEFFFRAGDDDLDQA
KAYPFMTPIFGEGVVFDASPERRKEMLHNAALRGEQMKGHAATIEDQVRRMIADWGEAGEIDLLDFFAELTIY
TSSACLIGKKFRDQLDGRFAKLYHELERGTDPLAYVDPYLPIESFRRRDEARNGLVALVADIMNGRIANPPTD
KSDRDMLDVLIAVKAETGTPRFSADEITGMFISMMFAGHHTSSGTASWTLIELMRHRDAYAAVIDELDELYGD
GRSVSFHALRQIPQLENVLKETLRLHPPLIILMRVAKGEFEVQGHRIHEGDLVAASPAISNRIPEDFPDPHDF
VPARYEQPRQEDLLNRWTWIPFGAGRHRCVGAAFAIMQIKAIFSVLLREYEFEMAQPPESYRNDHSKMVVQLA
QPACVRYRRRTGV
>CYP51B1_Mycobacterium tuberculosis M2086 2632818886
MSAVALPRVSGGHDEHGHLEEFRTDPIGLMQRVRDECGDVGTFQLAGKQVVLLSGSHANEFFFRAGDDDLDQA
KAYPFMTPIFGEGVVFDASPERRKEMLHNAALRGEQMKGHAATIEDQVRRMIADWGEAGEIDLLDFFAELTIY
TSSACLIGKKFRDQLDGRFAKLYHELERGTDPLAYVDPYLPIESFRRRDEARNGLVALVADIMNGRIANPPTD

KSDRDMLDVLIAVKAETGTPRFSADEITGMFISMMFAGHHTSSGTASWTLIELMRHRDAYAAVIDELDELYGD
GRSVSFHALRQIPQLENVLKETLRLHPPLIILMRVAKGEFEVQGHRIHEGDLVAASPAISNRIPEDFPDPHDF
VPARYEQPRQEDLLNRWTWIPFGAGRHRCVGAAFAIMQIKAIFSVLLREYEFEMAQPPESYRNDHSKMVVQLA
QPACVRYRRRTGV
>CYP51B1_Mycobacterium tuberculosis M2113 2577067746
MSAVALPRVSGGHDEHGHLEEFRTDPIGLMQRVRDECGDVGTFQLAGKQVVLLSGSHANEFFFRAGDDDLDQA
KAYPFMTPIFGEGVVFDASPERRKEMLHNAALRGEQMKGHAATIEDQVRRMIADWGEAGEIDLLDFFAELTIY
TSSACLIGKKFRDQLDGRFAKLYHELERGTDPLAYVDPYLPIESFRRRDEARNGLVALVADIMNGRIANPPTD
KSDRDMLDVLIAVKAETGTPRFSADEITGMFISMMFAGHHTSSGTASWTLIELMRHRDAYAAVIDELDELYGD
GRSVSFHALRQIPQLENVLKETLRLHPPLIILMRVAKGEFEVQGHRIHEGDLVAASPAISNRIPEDFPDPHDF
VPARYEQPRQEDLLNRWTWIPFGAGRHRCVGAAFAIMQIKAIFSVLLREYEFEMAQPPESYRNDHSKMVVQLA
QPACVRYRRRTGV
>CYP51B1_Mycobacterium tuberculosis M2116 2578032853
MSAVALPRVSGGHDEHGHLEEFRTDPIGLMQRVRDECGDVGTFQLAGKQVVLLSGSHANEFFFRAGDDDLDQA
KAYPFMTPIFGEGVVFDASPERRKEMLHNAALRGEQMKGHAATIEDQVRRMIADWGEAGEIDLLDFFAELTIY
TSSACLIGKKFRDQLDGRFAKLYHELERGTDPLAYVDPYLPIESFRRRDEARNGLVALVADIMNGRIANPPTD
KSDRDMLDVLIAVKAETGTPRFSADEITGMFISMMFAGHHTSSGTASWTLIELMRHRDAYAAVIDELDELYGD
GRSVSFHALRQIPQLENVLKETLRLHPPLIILMRVAKGEFEVQGHRIHEGDLVAASPAISNRIPEDFPDPHDF
VPARYEQPRQEDLLNRWTWIPFGAGRHRCVGAAFAIMQIKAIFSVLLREYEFEMAQPPESYRNDHSKMVVQLA
QPACVRYRRRTGV
>CYP51B1_Mycobacterium tuberculosis M2128 2577613017
MSAVALPRVSGGHDEHGHLEEFRTDPIGLMQRVRDECGDVGTFQLAGKQVVLLSGSHANEFFFRAGDDDLDQA
KAYPFMTPIFGEGVVFDASPERRKEMLHNAALRGEQMKGHAATIEDQVRRMIADWGEAGEIDLLDFFAELTIY
TSSACLIGKKFRDQLDGRFAKLYHELERGTDPLAYVDPYLPIESFRRRDEARNGLVALVADIMNGRIANPPTD
KSDRDMLDVLIAVKAETGTPRFSADEITGMFISMMFAGHHTSSGTASWTLIELMRHRDAYAAVIDELDELYGD
GRSVSFHALRQIPQLENVLKETLRLHPPLIILMRVAKGEFEVQGHRIHEGDLVAASPAISNRIPEDFPDPHDF
VPARYEQPRQEDLLNRWTWIPFGAGRHRCVGAAFAIMQIKAIFSVLLREYEFEMAQPPESYRNDHSKMVVQLA
QPACVRYRRRTGV
>CYP51B1_Mycobacterium tuberculosis M2129 2584994413
MSAVALPRVSGGHDEHGHLEEFRTDPIGLMQRVRDECGDVGTFQLAGKQVVLLSGSHANEFFFRAGDDDLDQA
KAYPFMTPIFGEGVVFDASPERRKEMLHNAALRGEQMKGHAATIEDQVRRMIADWGEAGEIDLLDFFAELTIY
TSSACLIGKKFRDQLDGRFAKLYHELERGTDPLAYVDPYLPIESFRRRDEARNGLVALVADIMNGRIANPPTD
KSDRDMLDVLIAVKAETGTPRFSADEITGMFISMMFAGHHTSSGTASWTLIELMRHRDAYAAVIDELDELYGD
GRSVSFHALRQIPQLENVLKETLRLHPPLIILMRVAKGEFEVQGHRIHEGDLVAASPAISNRIPEDFPDPHDF
VPARYEQPRQEDLLNRWTWIPFGAGRHRCVGAAFAIMQIKAIFSVLLREYEFEMAQPPESYRNDHSKMVVQLA
QPACVRYRRRTGV
>CYP51B1_Mycobacterium tuberculosis M2130 2633918050
MSAVALPRVSGGHDEHGHLEEFRTDPIGLMQRVRDECGDVGTFQLAGKQVVLLSGSHANEFFFRAGDDDLDQA
KAYPFMTPIFGEGVVFDASPERRKEMLHNAALRGEQMKGHAATIEDQVRRMIADWGEAGEIDLLDFFAELTIY
TSSACLIGKKFRDQLDGRFAKLYHELERGTDPLAYVDPYLPIESFRRRDEARNGLVALVADIMNGRIANPPTD
KSDRDMLDVLIAVKAETGTPRFSADEITGMFISMMFAGHHTSSGTASWTLIELMRHRDAYAAVIDELDELYGD
GRSVSFHALRQIPQLENVLKETLRLHPPLIILMRVAKGEFEVQGHRIHEGDLVAASPAISNRIPEDFPDPHDF
VPARYEQPRQEDLLNRWTWIPFGAGRHRCVGAAFAIMQIKAIFSVLLREYEFEMAQPPESYRNDHSKMVVQLA
QPACVRYRRRTGV
>CYP51B1_Mycobacterium tuberculosis M2131 2576734378
MSAVALPRVSGGHDEHGHLEEFRTDPIGLMQRVRDECGDVGTFQLAGKQVVLLSGSHANEFFFRAGDDDLDQA
KAYPFMTPIFGEGVVFDASPERRKEMLHNAALRGEQMKGHAATIEDQVRRMIADWGEAGEIDLLDFFAELTIY
TSSACLIGKKFRDQLDGRFAKLYHELERGTDPLAYVDPYLPIESFRRRDEARNGLVALVADIMNGRIANPPTD
KSDRDMLDVLIAVKAETGTPRFSADEITGMFISMMFAGHHTSSGTASWTLIELMRHRDAYAAVIDELDELYGD
GRSVSFHALRQIPQLENVLKETLRLHPPLIILMRVAKGEFEVQGHRIHEGDLVAASPAISNRIPEDFPDPHDF
VPARYEQPRQEDLLNRWTWIPFGAGRHRCVGAAFAIMQIKAIFSVLLREYEFEMAQPPESYRNDHSKMVVQLA
QPACVRYRRRTGV
>CYP51B1_Mycobacterium tuberculosis M2132 2642160906
MSAVALPRVSGGHDEHGHLEEFRTDPIGLMQRVRDECGDVGTFQLAGKQVVLLSGSHANEFFFRAGDDDLDQA
KAYPFMTPIFGEGVVFDASPERRKEMLHNAALRGEQMKGHAATIEDQVRRMIADWGEAGEIDLLDFFAELTIY
TSSACLIGKKFRDQLDGRFAKLYHELERGTDPLAYVDPYLPIESFRRRDEARNGLVALVADIMNGRIANPPTD
KSDRDMLDVLIAVKAETGTPRFSADEITGMFISMMFAGHHTSSGTASWTLIELMRHRDAYAAVIDELDELYGD
GRSVSFHALRQIPQLENVLKETLRLHPPLIILMRVAKGEFEVQGHRIHEGDLVAASPAISNRIPEDFPDPHDF

VPARYEQPRQEDLLNRWTWIPFGAGRHRCVGAAFAIMQIKAIFSVLLREYEFEMAQPPESYRNDHSKMVVQLA
QPACVRYRRRTGV
>CYP51B1_Mycobacterium tuberculosis M2134 2637399775
MSAVALPRVSGGHDEHGHLEEFRTDPIGLMQRVRDECGDVGTFQLAGKQVVLLSGSHANEFFFRAGDDDLDQA
KAYPFMTPIFGEGVVFDASPERRKEMLHNAALRGEQMKGHAATIEDQVRRMIADWGEAGEIDLLDFFAELTIY
TSSACLIGKKFRDQLDGRFAKLYHELERGTDPLAYVDPYLPIESFRRRDEARNGLVALVADIMNGRIANPPTD
KSDRDMLDVLIAVKAETGTPRFSADEITGMFISMMFAGHHTSSGTASWTLIELMRHRDAYAAVIDELDELYGD
GRSVSFHALRQIPQLENVLKETLRLHPPLIILMRVAKGEFEVQGHRIHEGDLVAASPAISNRIPEDFPDPHDF
VPARYEQPRQEDLLNRWTWIPFGAGRHRCVGAAFAIMQIKAIFSVLLREYEFEMAQPPESYRNDHSKMVVQLA
QPACVRYRRRTGV
>CYP51B1_Mycobacterium tuberculosis M2136 2575987047
MSAVALPRVSGGHDEHGHLEEFRTDPIGLMQRVRDECGDVGTFQLAGKQVVLLSGSHANEFFFRAGDDDLDQA
KAYPFMTPIFGEGVVFDASPERRKEMLHNAALRGEQMKGHAATIEDQVRRMIADWGEAGEIDLLDFFAELTIY
TSSACLIGKKFRDQLDGRFAKLYHELERGTDPLAYVDPYLPIESFRRRDEARNGLVALVADIMNGRIANPPTD
KSDRDMLDVLIAVKAETGTPRFSADEITGMFISMMFAGHHTSSGTASWTLIELMRHRDAYAAVIDELDELYGD
GRSVSFHALRQIPQLENVLKETLRLHPPLIILMRVAKGEFEVQGHRIHEGDLVAASPAISNRIPEDFPDPHDF
VPARYEQPRQEDLLNRWTWIPFGAGRHRCVGAAFAIMQIKAIFSVLLREYEFEMAQPPESYRNDHSKMVVQLA
QPACVRYRRRTGV
>CYP51B1_Mycobacterium tuberculosis M2137 2577496479
MSAVALPRVSGGHDEHGHLEEFRTDPIGLMQRVRDECGDVGTFQLAGKQVVLLSGSHANEFFFRAGDDDLDQA
KAYPFMTPIFGEGVVFDASPERRKEMLHNAALRGEQMKGHAATIEDQVRRMIADWGEAGEIDLLDFFAELTIY
TSSACLIGKKFRDQLDGRFAKLYHELERGTDPLAYVDPYLPIESFRRRDEARNGLVALVADIMNGRIANPPTD
KSDRDMLDVLIAVKAETGTPRFSADEITGMFISMMFAGHHTSSGTASWTLIELMRHRDAYAAVIDELDELYGD
GRSVSFHALRQIPQLENVLKETLRLHPPLIILMRVAKGEFEVQGHRIHEGDLVAASPAISNRIPEDFPDPHDF
VPARYEQPRQEDLLNRWTWIPFGAGRHRCVGAAFAIMQIKAIFSVLLREYEFEMAQPPESYRNDHSKMVVQLA
QPACVRYRRRTGV
>CYP51B1_Mycobacterium tuberculosis M2138 2651636325
MSAVALPRVSGGHDEHGHLEEFRTDPIGLMQRVRDECGDVGTFQLAGKQVVLLSGSHANEFFFRAGDDDLDQA
KAYPFMTPIFGEGVVFDASPERRKEMLHNAALRGEQMKGHAATIEDQVRRMIADWGEAGEIDLLDFFAELTIY
TSSACLIGKKFRDQLDGRFAKLYHELERGTDPLAYVDPYLPIESFRRRDEARNGLVALVADIMNGRIANPPTD
KSDRDMLDVLIAVKAETGTPRFSADEITGMFISMMFAGHHTSSGTASWTLIELMRHRDAYAAVIDELDELYGD
GRSVSFHALRQIPQLENVLKETLRLHPPLIILMRVAKGEFEVQGHRIHEGDLVAASPAISNRIPEDFPDPHDF
VPARYEQPRQEDLLNRWTWIPFGAGRHRCVGAAFAIMQIKAIFSVLLREYEFEMAQPPESYRNDHSKMVVQLA
QPACVRYRRRTGV
>CYP51B1_Mycobacterium tuberculosis M2139 2635727996
MSAVALPRVSGGHDEHGHLEEFRTDPIGLMQRVRDECGDVGTFQLAGKQVVLLSGSHANEFFFRAGDDDLDQA
KAYPFMTPIFGEGVVFDASPERRKEMLHNAALRGEQMKGHAATIEDQVRRMIADWGEAGEIDLLDFFAELTIY
TSSACLIGKKFRDQLDGRFAKLYHELERGTDPLAYVDPYLPIESFRRRDEARNGLVALVADIMNGRIANPPTD
KSDRDMLDVLIAVKAETGTPRFSADEITGMFISMMFAGHHTSSGTASWTLIELMRHRDAYAAVIDELDELYGD
GRSVSFHALRQIPQLENVLKETLRLHPPLIILMRVAKGEFEVQGHRIHEGDLVAASPAISNRIPEDFPDPHDF
VPARYEQPRQEDLLNRWTWIPFGAGRHRCVGAAFAIMQIKAIFSVLLREYEFEMAQPPESYRNDHSKMVVQLA
QPACVRYRRRTGV
>CYP51B1_Mycobacterium tuberculosis M2140 2630871703
MSAVALPRVSGGHDEHGHLEEFRTDPIGLMQRVRDECGDVGTFQLAGKQVVLLSGSHANEFFFRAGDDDLDQA
KAYPFMTPIFGEGVVFDASPERRKEMLHNAALRGEQMKGHAATIEDQVRRMIADWGEAGEIDLLDFFAELTIY
TSSACLIGKKFRDQLDGRFAKLYHELERGTDPLAYVDPYLPIESFRRRDEARNGLVALVADIMNGRIANPPTD
KSDRDMLDVLIAVKAETGTPRFSADEITGMFISMMFAGHHTSSGTASWTLIELMRHRDAYAAVIDELDELYGD
GRSVSFHALRQIPQLENVLKETLRLHPPLIILMRVAKGEFEVQGHRIHEGDLVAASPAISNRIPEDFPDPHDF
VPARYEQPRQEDLLNRWTWIPFGAGRHRCVGAAFAIMQIKAIFSVLLREYEFEMAQPPESYRNDHSKMVVQLA
QPACVRYRRRTGV
>CYP51B1_Mycobacterium tuberculosis M2141 2633453552
MSAVALPRVSGGHDEHGHLEEFRTDPIGLMQRVRDECGDVGTFQLAGKQVVLLSGSHANEFFFRAGDDDLDQA
KAYPFMTPIFGEGVVFDASPERRKEMLHNAALRGEQMKGHAATIEDQVRRMIADWGEAGEIDLLDFFAELTIY
TSSACLIGKKFRDQLDGRFAKLYHELERGTDPLAYVDPYLPIESFRRRDEARNGLVALVADIMNGRIANPPTD
KSDRDMLDVLIAVKAETGTPRFSADEITGMFISMMFAGHHTSSGTASWTLIELMRHRDAYAAVIDELDELYGD
GRSVSFHALRQIPQLENVLKETLRLHPPLIILMRVAKGEFEVQGHRIHEGDLVAASPAISNRIPEDFPDPHDF
VPARYEQPRQEDLLNRWTWIPFGAGRHRCVGAAFAIMQIKAIFSVLLREYEFEMAQPPESYRNDHSKMVVQLA
QPACVRYRRRTGV

>CYP51B1_Mycobacterium tuberculosis M2142 2637755253
MSAVALPRVSGGHDEHGHLEEFRTDPIGLMQRVRDECGDVGTFQLAGKQVVLLSGSHANEFFFRAGDDDLDQA
KAYPFMTPIFGEGVVFDASPERRKEMLHNAALRGEQMKGHAATIEDQVRRMIADWGEAGEIDLLDFFAELTIY
TSSACLIGKKFRDQLDGRFAKLYHELERGTDPLAYVDPYLPIESFRRRDEARNGLVALVADIMNGRIANPPTD
KSDRDMLDVLIAVKAETGTPRFSADEITGMFISMMFAGHHTSSGTASWTLIELMRHRDAYAAVIDELDELYGD
GRSVSFHALRQIPQLENVLKETLRLHPPLIILMRVAKGEFEVQGHRIHEGDLVAASPAISNRIPEDFPDPHDF
VPARYEQPRQEDLLNRWTWIPFGAGRHRCVGAAFAIMQIKAIFSVLLREYEFEMAQPPESYRNDHSKMVVQLA
QPACVRYRRRTGV
>CYP51B1_Mycobacterium tuberculosis M2144 2646083469
MSAVALPRVSGGHDEHGHLEEFRTDPIGLMQRVRDECGDVGTFQLAGKQVVLLSGSHANEFFFRAGDDDLDQA
KAYPFMTPIFGEGVVFDASPERRKEMLHNAALRGEQMKGHAATIEDQVRRMIADWGEAGEIDLLDFFAELTIY
TSSACLIGKKFRDQLDGRFAKLYHELERGTDPLAYVDPYLPIESFRRRDEARNGLVALVADIMNGRIANPPTD
KSDRDMLDVLIAVKAETGTPRFSADEITGMFISMMFAGHHTSSGTASWTLIELMRHRDAYAAVIDELDELYGD
GRSVSFHALRQIPQLENVLKETLRLHPPLIILMRVAKGEFEVQGHRIHEGDLVAASPAISNRIPEDFPDPHDF
VPARYEQPRQEDLLNRWTWIPFGAGRHRCVGAAFAIMQIKAIFSVLLREYEFEMAQPPESYRNDHSKMVVQLA
QPACVRYRRRTGV
>CYP51B1_Mycobacterium tuberculosis M2145 2638529290
MSAVALPRVSGGHDEHGHLEEFRTDPIGLMQRVRDECGDVGTFQLAGKQVVLLSGSHANEFFFRAGDDDLDQA
KAYPFMTPIFGEGVVFDASPERRKEMLHNAALRGEQMKGHAATIEDQVRRMIADWGEAGEIDLLDFFAELTIY
TSSACLIGKKFRDQLDGRFAKLYHELERGTDPLAYVDPYLPIESFRRRDEARNGLVALVADIMNGRIANPPTD
KSDRDMLDVLIAVKAETGTPRFSADEITGMFISMMFAGHHTSSGTASWTLIELMRHRDAYAAVIDELDELYGD
GRSVSFHALRQIPQLENVLKETLRLHPPLIILMRVAKGEFEVQGHRIHEGDLVAASPAISNRIPEDFPDPHDF
VPARYEQPRQEDLLNRWTWIPFGAGRHRCVGAAFAIMQIKAIFSVLLREYEFEMAQPPESYRNDHSKMVVQLA
QPACVRYRRRTGV
>CYP51B1_Mycobacterium tuberculosis M2192 2631899313
MSAVALPRVSGGHDEHGHLEEFRTDPIGLMQRVRDECGDVGTFQLAGKQVVLLSGSHANEFFFRAGDDDLDQA
KAYPFMTPIFGEGVVFDASPERRKEMLHNAALRGEQMKGHAATIEDQVRRMIADWGEAGEIDLLDFFAELTIY
TSSACLIGKKFRDQLDGRFAKLYHELERGTDPLAYVDPYLPIESFRRRDEARNGLVALVADIMNGRIANPPTD
KSDRDMLDVLIAVKAETGTPRFSADEITGMFISMMFAGHHTSSGTASWTLIELMRHRDAYAAVIDELDELYGD
GRSVSFHALRQIPQLENVLKETLRLHPPLIILMRVAKGEFEVQGHRIHEGDLVAASPAISNRIPEDFPDPHDF
VPARYEQPRQEDLLNRWTWIPFGAGRHRCVGAAFAIMQIKAIFSVLLREYEFEMAQPPESYRNDHSKMVVQLA
QPACVRYRRRTGV
>CYP51B1_Mycobacterium tuberculosis M2198 2628353415
MSAVALPRVSGGHDEHGHLEEFRTDPIGLMQRVRDECGDVGTFQLAGKQVVLLSGSHANEFFFRAGDDDLDQA
KAYPFMTPIFGEGVVFDASPERRKEMLHNAALRGEQMKGHAATIEDQVRRMIADWGEAGEIDLLDFFAELTIY
TSSACLIGKKFRDQLDGRFAKLYHELERGTDPLAYVDPYLPIESFRRRDEARNGLVALVADIMNGRIANPPTD
KSDRDMLDVLIAVKAETGTPRFSADEITGMFISMMFAGHHTSSGTASWTLIELMRHRDAYAAVIDELDELYGD
GRSVSFHALRQIPQLENVLKETLRLHPPLIILMRVAKGEFEVQGHRIHEGDLVAASPAISNRIPEDFPDPHDF
VPARYEQPRQEDLLNRWTWIPFGAGRHRCVGAAFAIMQIKAIFSVLLREYEFEMAQPPESYRNDHSKMVVQLA
QPACVRYRRRTGV
>CYP51B1_Mycobacterium tuberculosis M2203 2584651859
MSAVALPRVSGGHDEHGHLEEFRTDPIGLMQRVRDECGDVGTFQLAGKQVVLLSGSHANEFFFRAGDDDLDQA
KAYPFMTPIFGEGVVFDASPERRKEMLHNAALRGEQMKGHAATIEDQVRRMIADWGEAGEIDLLDFFAELTIY
TSSACLIGKKFRDQLDGRFAKLYHELERGTDPLAYVDPYLPIESFRRRDEARNGLVALVADIMNGRIANPPTD
KSDRDMLDVLIAVKAETGTPRFSADEITGMFISMMFAGHHTSSGTASWTLIELMRHRDAYAAVIDELDELYGD
GRSVSFHALRQIPQLENVLKETLRLHPPLIILMRVAKGEFEVQGHRIHEGDLVAASPAISNRIPEDFPDPHDF
VPARYEQPRQEDLLNRWTWIPFGAGRHRCVGAAFAIMQIKAIFSVLLREYEFEMAQPPESYRNDHSKMVVQLA
QPACVRYRRRTGV
>CYP51B1_Mycobacterium tuberculosis M2204 2638691782
MSAVALPRVSGGHDEHGHLEEFRTDPIGLMQRVRDECGDVGTFQLAGKQVVLLSGSHANEFFFRAGDDDLDQA
KAYPFMTPIFGEGVVFDASPERRKEMLHNAALRGEQMKGHAATIEDQVRRMIADWGEAGEIDLLDFFAELTIY
TSSACLIGKKFRDQLDGRFAKLYHELERGTDPLAYVDPYLPIESFRRRDEARNGLVALVADIMNGRIANPPTD
KSDRDMLDVLIAVKAETGTPRFSADEITGMFISMMFAGHHTSSGTASWTLIELMRHRDAYAAVIDELDELYGD
GRSVSFHALRQIPQLENVLKETLRLHPPLIILMRVAKGEFEVQGHRIHEGDLVAASPAISNRIPEDFPDPHDF
VPARYEQPRQEDLLNRWTWIPFGAGRHRCVGAAFAIMQIKAIFSVLLREYEFEMAQPPESYRNDHSKMVVQLA
QPACVRYRRRTGV
>CYP51B1_Mycobacterium tuberculosis M2206 2635610768

MSAVALPRVSGGHDEHGHLEEFRTDPIGLMQRVRDECGDVGTFQLAGKQVVLLSGSHANEFFFRAGDDDLDQA
KAYPFMTPIFGEGVVFDASPERRKEMLHNAALRGEQMKGHAATIEDQVRRMIADWGEAGEIDLLDFFAELTIY
TSSACLIGKKFRDQLDGRFAKLYHELERGTDPLAYVDPYLPIESFRRRDEARNGLVALVADIMNGRIANPPTD
KSDRDMLDVLIAVKAETGTPRFSADEITGMFISMMFAGHHTSSGTASWTLIELMRHRDAYAAVIDELDELYGD
GRSVSFHALRQIPQLENVLKETLRLHPPLIILMRVAKGEFEVQGHRIHEGDLVAASPAISNRIPEDFPDPHDF
VPARYEQPRQEDLLNRWTWIPFGAGRHRCVGAAFAIMQIKAIFSVLLREYEFEMAQPPESYRNDHSKMVVQLA
QPACVRYRRRTGV
>CYP51B1_Mycobacterium tuberculosis M2211 2630022083
MSAVALPRVSGGHDEHGHLEEFRTDPIGLMQRVRDECGDVGTFQLAGKQVVLLSGSHANEFFFRAGDDDLDQA
KAYPFMTPIFGEGVVFDASPERRKEMLHNAALRGEQMKGHAATIEDQVRRMIADWGEAGEIDLLDFFAELTIY
TSSACLIGKKFRDQLDGRFAKLYHELERGTDPLAYVDPYLPIESFRRRDEARNGLVALVADIMNGRIANPPTD
KSDRDMLDVLIAVKAETGTPRFSADEITGMFISMMFAGHHTSSGTASWTLIELMRHRDAYAAVIDELDELYGD
GRSVSFHALRQIPQLENVLKETLRLHPPLIILMRVAKGEFEVQGHRIHEGDLVAASPAISNRIPEDFPDPHDF
VPARYEQPRQEDLLNRWTWIPFGAGRHRCVGAAFAIMQIKAIFSVLLREYEFEMAQPPESYRNDHSKMVVQLA
QPACVRYRRRTGV
>CYP51B1_Mycobacterium tuberculosis M2218 2628257301
MSAVALPRVSGGHDEHGHLEEFRTDPIGLMQRVRDECGDVGTFQLAGKQVVLLSGSHANEFFFRAGDDDLDQA
KAYPFMTPIFGEGVVFDASPERRKEMLHNAALRGEQMKGHAATIEDQVRRMIADWGEAGEIDLLDFFAELTIY
TSSACLIGKKFRDQLDGRFAKLYHELERGTDPLAYVDPYLPIESFRRRDEARNGLVALVADIMNGRIANPPTD
KSDRDMLDVLIAVKAETGTPRFSADEITGMFISMMFAGHHTSSGTASWTLIELMRHRDAYAAVIDELDELYGD
GRSVSFHALRQIPQLENVLKETLRLHPPLIILMRVAKGEFEVQGHRIHEGDLVAASPAISNRIPEDFPDPHDF
VPARYEQPRQEDLLNRWTWIPFGAGRHRCVGAAFAIMQIKAIFSVLLREYEFEMAQPPESYRNDHSKMVVQLA
QPACVRYRRRTGV
>CYP51B1_Mycobacterium tuberculosis M2259 2628937852
MSAVALPRVSGGHDEHGHLEEFRTDPIGLMQRVRDECGDVGTFQLAGKQVVLLSGSHANEFFFRAGDDDLDQA
KAYPFMTPIFGEGVVFDASPERRKEMLHNAALRGEQMKGHAATIEDQVRRMIADWGEAGEIDLLDFFAELTIY
TSSACLIGKKFRDQLDGRFAKLYHELERGTDPLAYVDPYLPIESFRRRDEARNGLVALVADIMNGRIANPPTD
KSDRDMLDVLIAVKAETGTPRFSADEITGMFISMMFAGHHTSSGTASWTLIELMRHRDAYAAVIDELDELYGD
GRSVSFHALRQIPQLENVLKETLRLHPPLIILMRVAKGEFEVQGHRIHEGDLVAASPAISNRIPEDFPDPHDF
VPARYEQPRQEDLLNRWTWIPFGAGRHRCVGAAFAIMQIKAIFSVLLREYEFEMAQPPESYRNDHSKMVVQLA
QPACVRYRRRTGV
>CYP51B1_Mycobacterium tuberculosis M2278 2641740776
MSAVALPRVSGGHDEHGHLEEFRTDPIGLMQRVRDECGDVGTFQLAGKQVVLLSGSHANEFFFRAGDDDLDQA
KAYPFMTPIFGEGVVFDASPERRKEMLHNAALRGEQMKGHAATIEDQVRRMIADWGEAGEIDLLDFFAELTIY
TSSACLIGKKFRDQLDGRFAKLYHELERGTDPLAYVDPYLPIESFRRRDEARNGLVALVADIMNGRIANPPTD
KSDRDMLDVLIAVKAETGTPRFSADEITGMFISMMFAGHHTSSGTASWTLIELMRHRDAYAAVIDELDELYGD
GRSVSFHALRQIPQLENVLKETLRLHPPLIILMRVAKGEFEVQGHRIHEGDLVAASPAISNRIPEDFPDPHDF
VPARYEQPRQEDLLNRWTWIPFGAGRHRCVGAAFAIMQIKAIFSVLLREYEFEMAQPPESYRNDHSKMVVQLA
QPACVRYRRRTGV
>CYP51B1_Mycobacterium tuberculosis M2342 2637502217
MSAVALPRVSGGHDEHGHLEEFRTDPIGLMQRVRDECGDVGTFQLAGKQVVLLSGSHANEFFFRAGDDDLDQA
KAYPFMTPIFGEGVVFDASPERRKEMLHNAALRGEQMKGHAATIEDQVRRMIADWGEAGEIDLLDFFAELTIY
TSSACLIGKKFRDQLDGRFAKLYHELERGTDPLAYVDPYLPIESFRRRDEARNGLVALVADIMNGRIANPPTD
KSDRDMLDVLIAVKAETGTPRFSADEITGMFISMMFAGHHTSSGTASWTLIELMRHRDAYAAVIDELDELYGD
GRSVSFHALRQIPQLENVLKETLRLHPPLIILMRVAKGEFEVQGHRIHEGDLVAASPAISNRIPEDFPDPHDF
VPARYEQPRQEDLLNRWTWIPFGAGRHRCVGAAFAIMQIKAIFSVLLREYEFEMAQPPESYRNDHSKMVVQLA
QPACVRYRRRTGV
>CYP51B1_Mycobacterium tuberculosis M2343 2575348638
MSAVALPRVSGGHDEHGHLEEFRTDPIGLMQRVRDECGDVGTFQLAGKQVVLLSGSHANEFFFRAGDDDLDQA
KAYPFMTPIFGEGVVFDASPERRKEMLHNAALRGEQMKGHAATIEDQVRRMIADWGEAGEIDLLDFFAELTIY
TSSACLIGKKFRDQLDGRFAKLYHELERGTDPLAYVDPYLPIESFRRRDEARNGLVALVADIMNGRIANPPTD
KSDRDMLDVLIAVKAETGTPRFSADEITGMFISMMFAGHHTSSGTASWTLIELMRHRDAYAAVIDELDELYGD
GRSVSFHALRQIPQLENVLKETLRLHPPLIILMRVAKGEFEVQGHRIHEGDLVAASPAISNRIPEDFPDPHDF
VPARYEQPRQEDLLNRWTWIPFGAGRHRCVGAAFAIMQIKAIFSVLLREYEFEMAQPPESYRNDHSKMVVQLA
QPACVRYRRRTGV
>CYP51B1_Mycobacterium tuberculosis M2346 2584926806
MSAVALPRVSGGHDEHGHLEEFRTDPIGLMQRVRDECGDVGTFQLAGKQVVLLSGSHANEFFFRAGDDDLDQA
KAYPFMTPIFGEGVVFDASPERRKEMLHNAALRGEQMKGHAATIEDQVRRMIADWGEAGEIDLLDFFAELTIY

TSSACLIGKKFRDQLDGRFAKLYHELERGTDPLAYVDPYLPIESFRRRDEARNGLVALVADIMNGRIANPPTD
KSDRDMLDVLIAVKAETGTPRFSADEITGMFISMMFAGHHTSSGTASWTLIELMRHRDAYAAVIDELDELYGD
GRSVSFHALRQIPQLENVLKETLRLHPPLIILMRVAKGEFEVQGHRIHEGDLVAASPAISNRIPEDFPDPHDF
VPARYEQPRQEDLLNRWTWIPFGAGRHRCVGAAFAIMQIKAIFSVLLREYEFEMAQPPESYRNDHSKMVVQLA
QPACVRYRRRTGV
>CYP51B1_Mycobacterium tuberculosis M2350 2629405972
MSAVALPRVSGGHDEHGHLEEFRTDPIGLMQRVRDECGDVGTFQLAGKQVVLLSGSHANEFFFRAGDDDLDQA
KAYPFMTPIFGEGVVFDASPERRKEMLHNAALRGEQMKGHAATIEDQVRRMIADWGEAGEIDLLDFFAELTIY
TSSACLIGKKFRDQLDGRFAKLYHELERGTDPLAYVDPYLPIESFRRRDEARNGLVALVADIMNGRIANPPTD
KSDRDMLDVLIAVKAETGTPRFSADEITGMFISMMFAGHHTSSGTASWTLIELMRHRDAYAAVIDELDELYGD
GRSVSFHALRQIPQLENVLKETLRLHPPLIILMRVAKGEFEVQGHRIHEGDLVAASPAISNRIPEDFPDPHDF
VPARYEQPRQEDLLNRWTWIPFGAGRHRCVGAAFAIMQIKAIFSVLLREYEFEMAQPPESYRNDHSKMVVQLA
QPACVRYRRRTGV
>CYP51B1_Mycobacterium tuberculosis M2352 2631507476
MSAVALPRVSGGHDEHGHLEEFRTDPIGLMQRVRDECGDVGTFQLAGKQVVLLSGSHANEFFFRAGDDDLDQA
KAYPFMTPIFGEGVVFDASPERRKEMLHNAALRGEQMKGHAATIEDQVRRMIADWGEAGEIDLLDFFAELTIY
TSSACLIGKKFRDQLDGRFAKLYHELERGTDPLAYVDPYLPIESFRRRDEARNGLVALVADIMNGRIANPPTD
KSDRDMLDVLIAVKAETGTPRFSADEITGMFISMMFAGHHTSSGTASWTLIELMRHRDAYAAVIDELDELYGD
GRSVSFHALRQIPQLENVLKETLRLHPPLIILMRVAKGEFEVQGHRIHEGDLVAASPAISNRIPEDFPDPHDF
VPARYEQPRQEDLLNRWTWIPFGAGRHRCVGAAFAIMQIKAIFSVLLREYEFEMAQPPESYRNDHSKMVVQLA
QPACVRYRRRTGV
>CYP51B1_Mycobacterium tuberculosis M2360 2630253249
MSAVALPRVSGGHDEHGHLEEFRTDPIGLMQRVRDECGDVGTFQLAGKQVVLLSGSHANEFFFRAGDDDLDQA
KAYPFMTPIFGEGVVFDASPERRKEMLHNAALRGEQMKGHAATIEDQVRRMIADWGEAGEIDLLDFFAELTIY
TSSACLIGKKFRDQLDGRFAKLYHELERGTDPLAYVDPYLPIESFRRRDEARNGLVALVADIMNGRIANPPTD
KSDRDMLDVLIAVKAETGTPRFSADEITGMFISMMFAGHHTSSGTASWTLIELMRHRDAYAAVIDELDELYGD
GRSVSFHALRQIPQLENVLKETLRLHPPLIILMRVAKGEFEVQGHRIHEGDLVAASPAISNRIPEDFPDPHDF
VPARYEQPRQEDLLNRWTWIPFGAGRHRCVGAAFAIMQIKAIFSVLLREYEFEMAQPPESYRNDHSKMVVQLA
QPACVRYRRRTGV
>CYP51B1_Mycobacterium tuberculosis M2361 2630883281
MSAVALPRVSGGHDEHGHLEEFRTDPIGLMQRVRDECGDVGTFQLAGKQVVLLSGSHANEFFFRAGDDDLDQA
KAYPFMTPIFGEGVVFDASPERRKEMLHNAALRGEQMKGHAATIEDQVRRMIADWGEAGEIDLLDFFAELTIY
TSSACLIGKKFRDQLDGRFAKLYHELERGTDPLAYVDPYLPIESFRRRDEARNGLVALVADIMNGRIANPPTD
KSDRDMLDVLIAVKAETGTPRFSADEITGMFISMMFAGHHTSSGTASWTLIELMRHRDAYAAVIDELDELYGD
GRSVSFHALRQIPQLENVLKETLRLHPPLIILMRVAKGEFEVQGHRIHEGDLVAASPAISNRIPEDFPDPHDF
VPARYEQPRQEDLLNRWTWIPFGAGRHRCVGAAFAIMQIKAIFSVLLREYEFEMAQPPESYRNDHSKMVVQLA
QPACVRYRRRTGV
>CYP51B1_Mycobacterium tuberculosis M2384 (re-annotation Oct 2015)
2639248534
MSAVALPRVSGGHDEHGHLEEFRTDPIGLMQRVRDECGDVGTFQLAGKQVVLLSGSHANEFFFRAGDDDLDQA
KAYPFMTPIFGEGVVFDASPERRKEMLHNAALRGEQMKGHAATIEDQVRRMIADWGEAGEIDLLDFFAELTIY
TSSACLIGKKFRDQLDGRFAKLYHELERGTDPLAYVDPYLPIESFRRRDEARNGLVALVADIMNGRIANPPTD
KSDRDMLDVLIAVKAETGTPRFSADEITGMFISMMFAGHHTSSGTASWTLIELMRHRDAYAAVIDELDELYGD
GRSVSFHALRQIPQLENVLKETLRLHPPLIILMRVAKGEFEVQGHRIHEGDLVAASPAISNRIPEDFPDPHDF
VPARYEQPRQEDLLNRWTWIPFGAGRHRCVGAAFAIMQIKAIFSVLLREYEFEMAQPPESYRNDHSKMVVQLA
QPACVRYRRRTGV
>CYP51B1_Mycobacterium tuberculosis M2416 2574830102
MSAVALPRVSGGHDEHGHLEEFRTDPIGLMQRVRDECGDVGTFQLAGKQVVLLSGSHANEFFFRAGDDDLDQA
KAYPFMTPIFGEGVVFDASPERRKEMLHNAALRGEQMKGHAATIEDQVRRMIADWGEAGEIDLLDFFAELTIY
TSSACLIGKKFRDQLDGRFAKLYHELERGTDPLAYVDPYLPIESFRRRDEARNGLVALVADIMNGRIANPPTD
KSDRDMLDVLIAVKAETGTPRFSADEITGMFISMMFAGHHTSSGTASWTLIELMRHRDAYAAVIDELDELYGD
GRSVSFHALRQIPQLENVLKETLRLHPPLIILMRVAKGEFEVQGHRIHEGDLVAASPAISNRIPEDFPDPHDF
VPARYEQPRQEDLLNRWTWIPFGAGRHRCVGAAFAIMQIKAIFSVLLREYEFEMAQPPESYRNDHSKMVVQLA
QPACVRYRRRTGV
>CYP51B1_Mycobacterium tuberculosis M2435 2633804184
MSAVALPRVSGGHDEHGHLEEFRTDPIGLMQRVRDECGDVGTFQLAGKQVVLLSGSHANEFFFRAGDDDLDQA
KAYPFMTPIFGEGVVFDASPERRKEMLHNAALRGEQMKGHAATIEDQVRRMIADWGEAGEIDLLDFFAELTIY
TSSACLIGKKFRDQLDGRFAKLYHELERGTDPLAYVDPYLPIESFRRRDEARNGLVALVADIMNGRIANPPTD

KSDRDMLDVLIAVKAETGTPRFSADEITGMFISMMFAGHHTSSGTASWTLIELMRHRDAYAAVIDELDELYGD
GRSVSFHALRQIPQLENVLKETLRLHPPLIILMRVAKGEFEVQGHRIHEGDLVAASPAISNRIPEDFPDPHDF
VPARYEQPRQEDLLNRWTWIPFGAGRHRCVGAAFAIMQIKAIFSVLLREYEFEMAQPPESYRNDHSKMVVQLA
QPACVRYRRRTGV
>CYP51B1_Mycobacterium tuberculosis M2442 2638660823
MSAVALPRVSGGHDEHGHLEEFRTDPIGLMQRVRDECGDVGTFQLAGKQVVLLSGSHANEFFFRAGDDDLDQA
KAYPFMTPIFGEGVVFDASPERRKEMLHNAALRGEQMKGHAATIEDQVRRMIADWGEAGEIDLLDFFAELTIY
TSSACLIGKKFRDQLDGRFAKLYHELERGTDPLAYVDPYLPIESFRRRDEARNGLVALVADIMNGRIANPPTD
KSDRDMLDVLIAVKAETGTPRFSADEITGMFISMMFAGHHTSSGTASWTLIELMRHRDAYAAVIDELDELYGD
GRSVSFHALRQIPQLENVLKETLRLHPPLIILMRVAKGEFEVQGHRIHEGDLVAASPAISNRIPEDFPDPHDF
VPARYEQPRQEDLLNRWTWIPFGAGRHRCVGAAFAIMQIKAIFSVLLREYEFEMAQPPESYRNDHSKMVVQLA
QPACVRYRRRTGV
>CYP51B1_Mycobacterium tuberculosis M2459 2641352037
MSAVALPRVSGGHDEHGHLEEFRTDPIGLMQRVRDECGDVGTFQLAGKQVVLLSGSHANEFFFRAGDDDLDQA
KAYPFMTPIFGEGVVFDASPERRKEMLHNAALRGEQMKGHAATIEDQVRRMIADWGEAGEIDLLDFFAELTIY
TSSACLIGKKFRDQLDGRFAKLYHELERGTDPLAYVDPYLPIESFRRRDEARNGLVALVADIMNGRIANPPTD
KSDRDMLDVLIAVKAETGTPRFSADEITGMFISMMFAGHHTSSGTASWTLIELMRHRDAYAAVIDELDELYGD
GRSVSFHALRQIPQLENVLKETLRLHPPLIILMRVAKGEFEVQGHRIHEGDLVAASPAISNRIPEDFPDPHDF
VPARYEQPRQEDLLNRWTWIPFGAGRHRCVGAAFAIMQIKAIFSVLLREYEFEMAQPPESYRNDHSKMVVQLA
QPACVRYRRRTGV
>CYP51B1_Mycobacterium tuberculosis M2898 2631645428
MSAVALPRVSGGHDEHGHLEEFRTDPIGLMQRVRDECGDVGTFQLAGKQVVLLSGSHANEFFFRAGDDDLDQA
KAYPFMTPIFGEGVVFDASPERRKEMLHNAALRGEQMKGHAATIEDQVRRMIADWGEAGEIDLLDFFAELTIY
TSSACLIGKKFRDQLDGRFAKLYHELERGTDPLAYVDPYLPIESFRRRDEARNGLVALVADIMNGRIANPPTD
KSDRDMLDVLIAVKAETGTPRFSADEITGMFISMMFAGHHTSSGTASWTLIELMRHRDAYAAVIDELDELYGD
GRSVSFHALRQIPQLENVLKETLRLHPPLIILMRVAKGEFEVQGHRIHEGDLVAASPAISNRIPEDFPDPHDF
VPARYEQPRQEDLLNRWTWIPFGAGRHRCVGAAFAIMQIKAIFSVLLREYEFEMAQPPESYRNDHSKMVVQLA
QPACVRYRRRTGV
>CYP51B1_Mycobacterium tuberculosis M984 2637248292
MSAVALPRVSGGHDEHGHLEEFRTDPIGLMQRVRDECGDVGTFQLAGKQVVLLSGSHANEFFFRAGDDDLDQA
KAYPFMTPIFGEGVVFDASPERRKEMLHNAALRGEQMKGHAATIEDQVRRMIADWGEAGEIDLLDFFAELTIY
TSSACLIGKKFRDQLDGRFAKLYHELERGTDPLAYVDPYLPIESFRRRDEARNGLVALVADIMNGRIANPPTD
KSDRDMLDVLIAVKAETGTPRFSADEITGMFISMMFAGHHTSSGTASWTLIELMRHRDAYAAVIDELDELYGD
GRSVSFHALRQIPQLENVLKETLRLHPPLIILMRVAKGEFEVQGHRIHEGDLVAASPAISNRIPEDFPDPHDF
VPARYEQPRQEDLLNRWTWIPFGAGRHRCVGAAFAIMQIKAIFSVLLREYEFEMAQPPESYRNDHSKMVVQLA
QPACVRYRRRTGV
>CYP51B1_Mycobacterium tuberculosis M987 2631482056
MSAVALPRVSGGHDEHGHLEEFRTDPIGLMQRVRDECGDVGTFQLAGKQVVLLSGSHANEFFFRAGDDDLDQA
KAYPFMTPIFGEGVVFDASPERRKEMLHNAALRGEQMKGHAATIEDQVRRMIADWGEAGEIDLLDFFAELTIY
TSSACLIGKKFRDQLDGRFAKLYHELERGTDPLAYVDPYLPIESFRRRDEARNGLVALVADIMNGRIANPPTD
KSDRDMLDVLIAVKAETGTPRFSADEITGMFISMMFAGHHTSSGTASWTLIELMRHRDAYAAVIDELDELYGD
GRSVSFHALRQIPQLENVLKETLRLHPPLIILMRVAKGEFEVQGHRIHEGDLVAASPAISNRIPEDFPDPHDF
VPARYEQPRQEDLLNRWTWIPFGAGRHRCVGAAFAIMQIKAIFSVLLREYEFEMAQPPESYRNDHSKMVVQLA
QPACVRYRRRTGV
>CYP51B1_Mycobacterium tuberculosis M990 2633028900
MSAVALPRVSGGHDEHGHLEEFRTDPIGLMQRVRDECGDVGTFQLAGKQVVLLSGSHANEFFFRAGDDDLDQA
KAYPFMTPIFGEGVVFDASPERRKEMLHNAALRGEQMKGHAATIEDQVRRMIADWGEAGEIDLLDFFAELTIY
TSSACLIGKKFRDQLDGRFAKLYHELERGTDPLAYVDPYLPIESFRRRDEARNGLVALVADIMNGRIANPPTD
KSDRDMLDVLIAVKAETGTPRFSADEITGMFISMMFAGHHTSSGTASWTLIELMRHRDAYAAVIDELDELYGD
GRSVSFHALRQIPQLENVLKETLRLHPPLIILMRVAKGEFEVQGHRIHEGDLVAASPAISNRIPEDFPDPHDF
VPARYEQPRQEDLLNRWTWIPFGAGRHRCVGAAFAIMQIKAIFSVLLREYEFEMAQPPESYRNDHSKMVVQLA
QPACVRYRRRTGV
>CYP51B1_Mycobacterium tuberculosis M991 2629954105
MSAVALPRVSGGHDEHGHLEEFRTDPIGLMQRVRDECGDVGTFQLAGKQVVLLSGSHANEFFFRAGDDDLDQA
KAYPFMTPIFGEGVVFDASPERRKEMLHNAALRGEQMKGHAATIEDQVRRMIADWGEAGEIDLLDFFAELTIY
TSSACLIGKKFRDQLDGRFAKLYHELERGTDPLAYVDPYLPIESFRRRDEARNGLVALVADIMNGRIANPPTD
KSDRDMLDVLIAVKAETGTPRFSADEITGMFISMMFAGHHTSSGTASWTLIELMRHRDAYAAVIDELDELYGD
GRSVSFHALRQIPQLENVLKETLRLHPPLIILMRVAKGEFEVQGHRIHEGDLVAASPAISNRIPEDFPDPHDF

VPARYEQPRQEDLLNRWTWIPFGAGRHRCVGAAFAIMQIKAIFSVLLREYEFEMAQPPESYRNDHSKMVVQLA
QPACVRYRRRTGV
>CYP51B1_Mycobacterium tuberculosis M992 2635648031
MSAVALPRVSGGHDEHGHLEEFRTDPIGLMQRVRDECGDVGTFQLAGKQVVLLSGSHANEFFFRAGDDDLDQA
KAYPFMTPIFGEGVVFDASPERRKEMLHNAALRGEQMKGHAATIEDQVRRMIADWGEAGEIDLLDFFAELTIY
TSSACLIGKKFRDQLDGRFAKLYHELERGTDPLAYVDPYLPIESFRRRDEARNGLVALVADIMNGRIANPPTD
KSDRDMLDVLIAVKAETGTPRFSADEITGMFISMMFAGHHTSSGTASWTLIELMRHRDAYAAVIDELDELYGD
GRSVSFHALRQIPQLENVLKETLRLHPPLIILMRVAKGEFEVQGHRIHEGDLVAASPAISNRIPEDFPDPHDF
VPARYEQPRQEDLLNRWTWIPFGAGRHRCVGAAFAIMQIKAIFSVLLREYEFEMAQPPESYRNDHSKMVVQLA
QPACVRYRRRTGV
>CYP51B1_Mycobacterium tuberculosis MAL010075 2621976201
MSAVALPRVSGGHDEHGHLEEFRTDPIGLMQRVRDECGDVGTFQLAGKQVVLLSGSHANEFFFRAGDDDLDQA
KAYPFMTPIFGEGVVFDASPERRKEMLHNAALRGEQMKGHAATIEDQVRRMIADWGEAGEIDLLDFFAELTIY
TSSACLIGKKFRDQLDGRFAKLYHELERGTDPLAYVDPYLPIESFRRRDEARNGLVALVADIMNGRIANPPTD
KSDRDMLDVLIAVKAETGTPRFSADEITGMFISMMFAGHHTSSGTASWTLIELMRHRDAYAAVIDELDELYGD
GRSVSFHALRQIPQLENVLKETLRLHPPLIILMRVAKGEFEVQGHRIHEGDLVAASPAISNRIPEDFPDPHDF
VPARYEQPRQEDLLNRWTWIPFGAGRHRCVGAAFAIMQIKAIFSVLLREYEFEMAQPPESYRNDHSKMVVQLA
QPACVRYRRRTGV
>CYP51B1_Mycobacterium tuberculosis MAL010077 2621980615
MSAVALPRVSGGHDEHGHLEEFRTDPIGLMQRVRDECGDVGTFQLAGKQVVLLSGSHANEFFFRAGDDDLDQA
KAYPFMTPIFGEGVVFDASPERRKEMLHNAALRGEQMKGHAATIEDQVRRMIADWGEAGEIDLLDFFAELTIY
TSSACLIGKKFRDQLDGRFAKLYHELERGTDPLAYVDPYLPIESFRRRDEARNGLVALVADIMNGRIANPPTD
KSDRDMLDVLIAVKAETGTPRFSADEITGMFISMMFAGHHTSSGTASWTLIELMRHRDAYAAVIDELDELYGD
GRSVSFHALRQIPQLENVLKETLRLHPPLIILMRVAKGEFEVQGHRIHEGDLVAASPAISNRIPEDFPDPHDF
VPARYEQPRQEDLLNRWTWIPFGAGRHRCVGAAFAIMQIKAIFSVLLREYEFEMAQPPESYRNDHSKMVVQLA
QPACVRYRRRTGV
>CYP51B1_Mycobacterium tuberculosis MAL010103 2576152731
MSAVALPRVSGGHDEHGHLEEFRTDPIGLMQRVRDECGDVGTFQLAGKQVVLLSGSHANEFFFRAGDDDLDQA
KAYPFMTPIFGEGVVFDASPERRKEMLHNAALRGEQMKGHAATIEDQVRRMIADWGEAGEIDLLDFFAELTIY
TSSACLIGKKFRDQLDGRFAKLYHELERGTDPLAYVDPYLPIESFRRRDEARNGLVALVADIMNGRIANPPTD
KSDRDMLDVLIAVKAETGTPRFSADEITGMFISMMFAGHHTSSGTASWTLIELMRHRDAYAAVIDELDELYGD
GRSVSFHALRQIPQLENVLKETLRLHPPLIILMRVAKGEFEVQGHRIHEGDLVAASPAISNRIPEDFPDPHDF
VPARYEQPRQEDLLNRWTWIPFGAGRHRCVGAAFAIMQIKAIFSVLLREYEFEMAQPPESYRNDHSKMVVQLA
QPACVRYRRRTGV
>CYP51B1_Mycobacterium tuberculosis MAL010119 2621985959
MSAVALPRVSGGHDEHGHLEEFRTDPIGLMQRVRDECGDVGTFQLAGKQVVLLSGSHANEFFFRAGDDDLDQA
KAYPFMTPIFGEGVVFDASPERRKEMLHNAALRGEQMKGHAATIEDQVRRMIADWGEAGEIDLLDFFAELTIY
TSSACLIGKKFRDQLDGRFAKLYHELERGTDPLAYVDPYLPIESFRRRDEARNGLVALVADIMNGRIANPPTD
KSDRDMLDVLIAVKAETGTPRFSADEITGMFISMMFAGHHTSSGTASWTLIELMRHRDAYAAVIDELDELYGD
GRSVSFHALRQIPQLENVLKETLRLHPPLIILMRVAKGEFEVQGHRIHEGDLVAASPAISNRIPEDFPDPHDF
VPARYEQPRQEDLLNRWTWIPFGAGRHRCVGAAFAIMQIKAIFSVLLREYEFEMAQPPESYRNDHSKMVVQLA
QPACVRYRRRTGV
>CYP51B1_Mycobacterium tuberculosis MAL010121 2603131909
MSAVALPRVSGGHDEHGHLEEFRTDPIGLMQRVRDECGDVGTFQLAGKQVVLLSGSHANEFFFRAGDDDLDQA
KAYPFMTPIFGEGVVFDASPERRKEMLHNAALRGEQMKGHAATIEDQVRRMIADWGEAGEIDLLDFFAELTIY
TSSACLIGKKFRDQLDGRFAKLYHELERGTDPLAYVDPYLPIESFRRRDEARNGLVALVADIMNGRIANPPTD
KSDRDMLDVLIAVKAETGTPRFSADEITGMFISMMFAGHHTSSGTASWTLIELMRHRDAYAAVIDELDELYGD
GRSVSFHALRQIPQLENVLKETLRLHPPLIILMRVAKGEFEVQGHRIHEGDLVAASPAISNRIPEDFPDPHDF
VPARYEQPRQEDLLNRWTWIPFGAGRHRCVGAAFAIMQIKAIFSVLLREYEFEMAQPPESYRNDHSKMVVQLA
QPACVRYRRRTGV
>CYP51B1_Mycobacterium tuberculosis MAL010133 2575283864
MSAVALPRVSGGHDEHGHLEEFRTDPIGLMQRVRDECGDVGTFQLAGKQVVLLSGSHANEFFFRAGDDDLDQA
KAYPFMTPIFGEGVVFDASPERRKEMLHNAALRGEQMKGHAATIEDQVRRMIADWGEAGEIDLLDFFAELTIY
TSSACLIGKKFRDQLDGRFAKLYHELERGTDPLAYVDPYLPIESFRRRDEARNGLVALVADIMNGRIANPPTD
KSDRDMLDVLIAVKAETGTPRFSADEITGMFISMMFAGHHTSSGTASWTLIELMRHRDAYAAVIDELDELYGD
GRSVSFHALRQIPQLENVLKETLRLHPPLIILMRVAKGEFEVQGHRIHEGDLVAASPAISNRIPEDFPDPHDF
VPARYEQPRQEDLLNRWTWIPFGAGRHRCVGAAFAIMQIKAIFSVLLREYEFEMAQPPESYRNDHSKMVVQLA
QPACVRYRRRTGV

>CYP51B1_Mycobacterium tuberculosis MAL020120 2577194450
MSAVALPRVSGGHDEHGHLEEFRTDPIGLMQRVRDECGDVGTFQLAGKQVVLLSGSHANEFFFRAGDDDLDQA
KAYPFMTPIFGEGVVFDASPERRKEMLHNAALRGEQMKGHAATIEDQVRRMIADWGEAGEIDLLDFFAELTIY
TSSACLIGKKFRDQLDGRFAKLYHELERGTDPLAYVDPYLPIESFRRRDEARNGLVALVADIMNGRIANPPTD
KSDRDMLDVLIAVKAETGTPRFSADEITGMFISMMFAGHHTSSGTASWTLIELMRHRDAYAAVIDELDELYGD
GRSVSFHALRQIPQLENVLKETLRLHPPLIILMRVAKGEFEVQGHRIHEGDLVAASPAISNRIPEDFPDPHDF
VPARYEQPRQEDLLNRWTWIPFGAGRHRCVGAAFAIMQIKAIFSVLLREYEFEMAQPPESYRNDHSKMVVQLA
QPACVRYRRRTGV
>CYP51B1_Mycobacterium tuberculosis MAL020131 2574755104
MSAVALPRVSGGHDEHGHLEEFRTDPIGLMQRVRDECGDVGTFQLAGKQVVLLSGSHANEFFFRAGDDDLDQA
KAYPFMTPIFGEGVVFDASPERRKEMLHNAALRGEQMKGHAATIEDQVRRMIADWGEAGEIDLLDFFAELTIY
TSSACLIGKKFRDQLDGRFAKLYHELERGTDPLAYVDPYLPIESFRRRDEARNGLVALVADIMNGRIANPPTD
KSDRDMLDVLIAVKAETGTPRFSADEITGMFISMMFAGHHTSSGTASWTLIELMRHRDAYAAVIDELDELYGD
GRSVSFHALRQIPQLENVLKETLRLHPPLIILMRVAKGEFEVQGHRIHEGDLVAASPAISNRIPEDFPDPHDF
VPARYEQPRQEDLLNRWTWIPFGAGRHRCVGAAFAIMQIKAIFSVLLREYEFEMAQPPESYRNDHSKMVVQLA
QPACVRYRRRTGV
>CYP51B1_Mycobacterium tuberculosis MAL020139 2621988554
MSAVALPRVSGGHDEHGHLEEFRTDPIGLMQRVRDECGDVGTFQLAGKQVVLLSGSHANEFFFRAGDDDLDQA
KAYPFMTPIFGEGVVFDASPERRKEMLHNAALRGEQMKGHAATIEDQVRRMIADWGEAGEIDLLDFFAELTIY
TSSACLIGKKFRDQLDGRFAKLYHELERGTDPLAYVDPYLPIESFRRRDEARNGLVALVADIMNGRIANPPTD
KSDRDMLDVLIAVKAETGTPRFSADEITGMFISMMFAGHHTSSGTASWTLIELMRHRDAYAAVIDELDELYGD
GRSVSFHALRQIPQLENVLKETLRLHPPLIILMRVAKGEFEVQGHRIHEGDLVAASPAISNRIPEDFPDPHDF
VPARYEQPRQEDLLNRWTWIPFGAGRHRCVGAAFAIMQIKAIFSVLLREYEFEMAQPPESYRNDHSKMVVQLA
QPACVRYRRRTGV
>CYP51B1_Mycobacterium tuberculosis MAL020144 2576702113
MSAVALPRVSGGHDEHGHLEEFRTDPIGLMQRVRDECGDVGTFQLAGKQVVLLSGSHANEFFFRAGDDDLDQA
KAYPFMTPIFGEGVVFDASPERRKEMLHNAALRGEQMKGHAATIEDQVRRMIADWGEAGEIDLLDFFAELTIY
TSSACLIGKKFRDQLDGRFAKLYHELERGTDPLAYVDPYLPIESFRRRDEARNGLVALVADIMNGRIANPPTD
KSDRDMLDVLIAVKAETGTPRFSADEITGMFISMMFAGHHTSSGTASWTLIELMRHRDAYAAVIDELDELYGD
GRSVSFHALRQIPQLENVLKETLRLHPPLIILMRVAKGEFEVQGHRIHEGDLVAASPAISNRIPEDFPDPHDF
VPARYEQPRQEDLLNRWTWIPFGAGRHRCVGAAFAIMQIKAIFSVLLREYEFEMAQPPESYRNDHSKMVVQLA
QPACVRYRRRTGV
>CYP51B1_Mycobacterium tuberculosis MAL020145 2584765823
MSAVALPRVSGGHDEHGHLEEFRTDPIGLMQRVRDECGDVGTFQLAGKQVVLLSGSHANEFFFRAGDDDLDQA
KAYPFMTPIFGEGVVFDASPERRKEMLHNAALRGEQMKGHAATIEDQVRRMIADWGEAGEIDLLDFFAELTIY
TSSACLIGKKFRDQLDGRFAKLYHELERGTDPLAYVDPYLPIESFRRRDEARNGLVALVADIMNGRIANPPTD
KSDRDMLDVLIAVKAETGTPRFSADEITGMFISMMFAGHHTSSGTASWTLIELMRHRDAYAAVIDELDELYGD
GRSVSFHALRQIPQLENVLKETLRLHPPLIILMRVAKGEFEVQGHRIHEGDLVAASPAISNRIPEDFPDPHDF
VPARYEQPRQEDLLNRWTWIPFGAGRHRCVGAAFAIMQIKAIFSVLLREYEFEMAQPPESYRNDHSKMVVQLA
QPACVRYRRRTGV
>CYP51B1_Mycobacterium tuberculosis MAL020162 2575534616
MSAVALPRVSGGHDEHGHLEEFRTDPIGLMQRVRDECGDVGTFQLAGKQVVLLSGSHANEFFFRAGDDDLDQA
KAYPFMTPIFGEGVVFDASPERRKEMLHNAALRGEQMKGHAATIEDQVRRMIADWGEAGEIDLLDFFAELTIY
TSSACLIGKKFRDQLDGRFAKLYHELERGTDPLAYVDPYLPIESFRRRDEARNGLVALVADIMNGRIANPPTD
KSDRDMLDVLIAVKAETGTPRFSADEITGMFISMMFAGHHTSSGTASWTLIELMRHRDAYAAVIDELDELYGD
GRSVSFHALRQIPQLENVLKETLRLHPPLIILMRVAKGEFEVQGHRIHEGDLVAASPAISNRIPEDFPDPHDF
VPARYEQPRQEDLLNRWTWIPFGAGRHRCVGAAFAIMQIKAIFSVLLREYEFEMAQPPESYRNDHSKMVVQLA
QPACVRYRRRTGV
>CYP51B1_Mycobacterium tuberculosis MAL020165 2622000244
MSAVALPRVSGGHDEHGHLEEFRTDPIGLMQRVRDECGDVGTFQLAGKQVVLLSGSHANEFFFRAGDDDLDQA
KAYPFMTPIFGEGVVFDASPERRKEMLHNAALRGEQMKGHAATIEDQVRRMIADWGEAGEIDLLDFFAELTIY
TSSACLIGKKFRDQLDGRFAKLYHELERGTDPLAYVDPYLPIESFRRRDEARNGLVALVADIMNGRIANPPTD
KSDRDMLDVLIAVKAETGTPRFSADEITGMFISMMFAGHHTSSGTASWTLIELMRHRDAYAAVIDELDELYGD
GRSVSFHALRQIPQLENVLKETLRLHPPLIILMRVAKGEFEVQGHRIHEGDLVAASPAISNRIPEDFPDPHDF
VPARYEQPRQEDLLNRWTWIPFGAGRHRCVGAAFAIMQIKAIFSVLLREYEFEMAQPPESYRNDHSKMVVQLA
QPACVRYRRRTGV
>CYP51B1_Mycobacterium tuberculosis MAL020179 2576600786

MSAVALPRVSGGHDEHGHLEEFRTDPIGLMQRVRDECGDVGTFQLAGKQVVLLSGSHANEFFFRAGDDDLDQA
KAYPFMTPIFGEGVVFDASPERRKEMLHNAALRGEQMKGHAATIEDQVRRMIADWGEAGEIDLLDFFAELTIY
TSSACLIGKKFRDQLDGRFAKLYHELERGTDPLAYVDPYLPIESFRRRDEARNGLVALVADIMNGRIANPPTD
KSDRDMLDVLIAVKAETGTPRFSADEITGMFISMMFAGHHTSSGTASWTLIELMRHRDAYAAVIDELDELYGD
GRSVSFHALRQIPQLENVLKETLRLHPPLIILMRVAKGEFEVQGHRIHEGDLVAASPAISNRIPEDFPDPHDF
VPARYEQPRQEDLLNRWTWIPFGAGRHRCVGAAFAIMQIKAIFSVLLREYEFEMAQPPESYRNDHSKMVVQLA
QPACVRYRRRTGV
>CYP51B1_Mycobacterium tuberculosis MAL020181 2574681570
MSAVALPRVSGGHDEHGHLEEFRTDPIGLMQRVRDECGDVGTFQLAGKQVVLLSGSHANEFFFRAGDDDLDQA
KAYPFMTPIFGEGVVFDASPERRKEMLHNAALRGEQMKGHAATIEDQVRRMIADWGEAGEIDLLDFFAELTIY
TSSACLIGKKFRDQLDGRFAKLYHELERGTDPLAYVDPYLPIESFRRRDEARNGLVALVADIMNGRIANPPTD
KSDRDMLDVLIAVKAETGTPRFSADEITGMFISMMFAGHHTSSGTASWTLIELMRHRDAYAAVIDELDELYGD
GRSVSFHALRQIPQLENVLKETLRLHPPLIILMRVAKGEFEVQGHRIHEGDLVAASPAISNRIPEDFPDPHDF
VPARYEQPRQEDLLNRWTWIPFGAGRHRCVGAAFAIMQIKAIFSVLLREYEFEMAQPPESYRNDHSKMVVQLA
QPACVRYRRRTGV
>CYP51B1_Mycobacterium tuberculosis Manu ancestor SIT523 2648997395
MSAVALPRVSGGHDEHGHLEEFRTDPIGLMQRVRDECGDVGTFQLAGKQVVLLSGSHANEFFFRAGDDDLDQA
KAYPFMTPIFGEGVVFDASPERRKEMLHNAALRGEQMKGHAATIEDQVRRMIADWGEAGEIDLLDFFAELTIY
TSSACLIGKKFRDQLDGRFAKLYHELERGTDPLAYVDPYLPIESFRRRDEARNGLVALVADIMNGRIANPPTD
KSDRDMLDVLIAVKAETGTPRFSADEITGMFISMMFAGHHTSSGTASWTLIELMRHRDAYAAVIDELDELYGD
GRSVSFHALRQIPQLENVLKETLRLHPPLIILMRVAKGEFEVQGHRIHEGDLVAASPAISNRIPEDFPDPHDF
VPARYEQPRQEDLLNRWTWIPFGAGRHRCVGAAFAIMQIKAIFSVLLREYEFEMAQPPESYRNDHSKMVVQLA
QPACVRYRRRTGV
>CYP51B1_Mycobacterium tuberculosis MD13744 2628658901
MSAVALPRVSGGHDEHGHLEEFRTDPIGLMQRVRDECGDVGTFQLAGKQVVLLSGSHANEFFFRAGDDDLDQA
KAYPFMTPIFGEGVVFDASPERRKEMLHNAALRGEQMKGHAATIEDQVRRMIADWGEAGEIDLLDFFAELTIY
TSSACLIGKKFRDQLDGRFAKLYHELERGTDPLAYVDPYLPIESFRRRDEARNGLVALVADIMNGRIANPPTD
KSDRDMLDVLIAVKAETGTPRFSADEITGMFISMMFAGHHTSSGTASWTLIELMRHRDAYAAVIDELDELYGD
GRSVSFHALRQIPQLENVLKETLRLHPPLIILMRVAKGEFEVQGHRIHEGDLVAASPAISNRIPEDFPDPHDF
VPARYEQPRQEDLLNRWTWIPFGAGRHRCVGAAFAIMQIKAIFSVLLREYEFEMAQPPESYRNDHSKMVVQLA
QPACVRYRRRTGV
>CYP51B1_Mycobacterium tuberculosis MD13874 2622194827
MSAVALPRVSGGHDEHGHLEEFRTDPIGLMQRVRDECGDVGTFQLAGKQVVLLSGSHANEFFFRAGDDDLDQA
KAYPFMTPIFGEGVVFDASPERRKEMLHNAALRGEQMKGHAATIEDQVRRMIADWGEAGEIDLLDFFAELTIY
TSSACLIGKKFRDQLDGRFAKLYHELERGTDPLAYVDPYLPIESFRRRDEARNGLVALVADIMNGRIANPPTD
KSDRDMLDVLIAVKAETGTPRFSADEITGMFISMMFAGHHTSSGTASWTLIELMRHRDAYAAVIDELDELYGD
GRSVSFHALRQIPQLENVLKETLRLHPPLIILMRVAKGEFEVQGHRIHEGDLVAASPAISNRIPEDFPDPHDF
VPARYEQPRQEDLLNRWTWIPFGAGRHRCVGAAFAIMQIKAIFSVLLREYEFEMAQPPESYRNDHSKMVVQLA
QPACVRYRRRTGV
>CYP51B1_Mycobacterium tuberculosis MD13878 2575054532
MSAVALPRVSGGHDEHGHLEEFRTDPIGLMQRVRDECGDVGTFQLAGKQVVLLSGSHANEFFFRAGDDDLDQA
KAYPFMTPIFGEGVVFDASPERRKEMLHNAALRGEQMKGHAATIEDQVRRMIADWGEAGEIDLLDFFAELTIY
TSSACLIGKKFRDQLDGRFAKLYHELERGTDPLAYVDPYLPIESFRRRDEARNGLVALVADIMNGRIANPPTD
KSDRDMLDVLIAVKAETGTPRFSADEITGMFISMMFAGHHTSSGTASWTLIELMRHRDAYAAVIDELDELYGD
GRSVSFHALRQIPQLENVLKETLRLHPPLIILMRVAKGEFEVQGHRIHEGDLVAASPAISNRIPEDFPDPHDF
VPARYEQPRQEDLLNRWTWIPFGAGRHRCVGAAFAIMQIKAIFSVLLREYEFEMAQPPESYRNDHSKMVVQLA
QPACVRYRRRTGV
>CYP51B1_Mycobacterium tuberculosis MD14002 2622211330
MSAVALPRVSGGHDEHGHLEEFRTDPIGLMQRVRDECGDVGTFQLAGKQVVLLSGSHANEFFFRAGDDDLDQA
KAYPFMTPIFGEGVVFDASPERRKEMLHNAALRGEQMKGHAATIEDQVRRMIADWGEAGEIDLLDFFAELTIY
TSSACLIGKKFRDQLDGRFAKLYHELERGTDPLAYVDPYLPIESFRRRDEARNGLVALVADIMNGRIANPPTD
KSDRDMLDVLIAVKAETGTPRFSADEITGMFISMMFAGHHTSSGTASWTLIELMRHRDAYAAVIDELDELYGD
GRSVSFHALRQIPQLENVLKETLRLHPPLIILMRVAKGEFEVQGHRIHEGDLVAASPAISNRIPEDFPDPHDF
VPARYEQPRQEDLLNRWTWIPFGAGRHRCVGAAFAIMQIKAIFSVLLREYEFEMAQPPESYRNDHSKMVVQLA
QPACVRYRRRTGV
>CYP51B1_Mycobacterium tuberculosis MD14435 2577824767
MSAVALPRVSGGHDEHGHLEEFRTDPIGLMQRVRDECGDVGTFQLAGKQVVLLSGSHANEFFFRAGDDDLDQA
KAYPFMTPIFGEGVVFDASPERRKEMLHNAALRGEQMKGHAATIEDQVRRMIADWGEAGEIDLLDFFAELTIY

TSSACLIGKKFRDQLDGRFAKLYHELERGTDPLAYVDPYLPIESFRRRDEARNGLVALVADIMNGRIANPPTD
KSDRDMLDVLIAVKAETGTPRFSADEITGMFISMMFAGHHTSSGTASWTLIELMRHRDAYAAVIDELDELYGD
GRSVSFHALRQIPQLENVLKETLRLHPPLIILMRVAKGEFEVQGHRIHEGDLVAASPAISNRIPEDFPDPHDF
VPARYEQPRQEDLLNRWTWIPFGAGRHRCVGAAFAIMQIKAIFSVLLREYEFEMAQPPESYRNDHSKMVVQLA
QPACVRYRRRTGV
>CYP51B1_Mycobacterium tuberculosis MD14437 2622223936
MSAVALPRVSGGHDEHGHLEEFRTDPIGLMQRVRDECGDVGTFQLAGKQVVLLSGSHANEFFFRAGDDDLDQA
KAYPFMTPIFGEGVVFDASPERRKEMLHNAALRGEQMKGHAATIEDQVRRMIADWGEAGEIDLLDFFAELTIY
TSSACLIGKKFRDQLDGRFAKLYHELERGTDPLAYVDPYLPIESFRRRDEARNGLVALVADIMNGRIANPPTD
KSDRDMLDVLIAVKAETGTPRFSADEITGMFISMMFAGHHTSSGTASWTLIELMRHRDAYAAVIDELDELYGD
GRSVSFHALRQIPQLENVLKETLRLHPPLIILMRVAKGEFEVQGHRIHEGDLVAASPAISNRIPEDFPDPHDF
VPARYEQPRQEDLLNRWTWIPFGAGRHRCVGAAFAIMQIKAIFSVLLREYEFEMAQPPESYRNDHSKMVVQLA
QPACVRYRRRTGV
>CYP51B1_Mycobacterium tuberculosis MD14620 2622179383
MSAVALPRVSGGHDEHGHLEEFRTDPIGLMQRVRDECGDVGTFQLAGKQVVLLSGSHANEFFFRAGDDDLDQA
KAYPFMTPIFGEGVVFDASPERRKEMLHNAALRGEQMKGHAATIEDQVRRMIADWGEAGEIDLLDFFAELTIY
TSSACLIGKKFRDQLDGRFAKLYHELERGTDPLAYVDPYLPIESFRRRDEARNGLVALVADIMNGRIANPPTD
KSDRDMLDVLIAVKAETGTPRFSADEITGMFISMMFAGHHTSSGTASWTLIELMRHRDAYAAVIDELDELYGD
GRSVSFHALRQIPQLENVLKETLRLHPPLIILMRVAKGEFEVQGHRIHEGDLVAASPAISNRIPEDFPDPHDF
VPARYEQPRQEDLLNRWTWIPFGAGRHRCVGAAFAIMQIKAIFSVLLREYEFEMAQPPESYRNDHSKMVVQLA
QPACVRYRRRTGV
>CYP51B1_Mycobacterium tuberculosis MD14621 2622198789
MSAVALPRVSGGHDEHGHLEEFRTDPIGLMQRVRDECGDVGTFQLAGKQVVLLSGSHANEFFFRAGDDDLDQA
KAYPFMTPIFGEGVVFDASPERRKEMLHNAALRGEQMKGHAATIEDQVRRMIADWGEAGEIDLLDFFAELTIY
TSSACLIGKKFRDQLDGRFAKLYHELERGTDPLAYVDPYLPIESFRRRDEARNGLVALVADIMNGRIANPPTD
KSDRDMLDVLIAVKAETGTPRFSADEITGMFISMMFAGHHTSSGTASWTLIELMRHRDAYAAVIDELDELYGD
GRSVSFHALRQIPQLENVLKETLRLHPPLIILMRVAKGEFEVQGHRIHEGDLVAASPAISNRIPEDFPDPHDF
VPARYEQPRQEDLLNRWTWIPFGAGRHRCVGAAFAIMQIKAIFSVLLREYEFEMAQPPESYRNDHSKMVVQLA
QPACVRYRRRTGV
>CYP51B1_Mycobacterium tuberculosis MD14709 2622215483
MSAVALPRVSGGHDEHGHLEEFRTDPIGLMQRVRDECGDVGTFQLAGKQVVLLSGSHANEFFFRAGDDDLDQA
KAYPFMTPIFGEGVVFDASPERRKEMLHNAALRGEQMKGHAATIEDQVRRMIADWGEAGEIDLLDFFAELTIY
TSSACLIGKKFRDQLDGRFAKLYHELERGTDPLAYVDPYLPIESFRRRDEARNGLVALVADIMNGRIANPPTD
KSDRDMLDVLIAVKAETGTPRFSADEITGMFISMMFAGHHTSSGTASWTLIELMRHRDAYAAVIDELDELYGD
GRSVSFHALRQIPQLENVLKETLRLHPPLIILMRVAKGEFEVQGHRIHEGDLVAASPAISNRIPEDFPDPHDF
VPARYEQPRQEDLLNRWTWIPFGAGRHRCVGAAFAIMQIKAIFSVLLREYEFEMAQPPESYRNDHSKMVVQLA
QPACVRYRRRTGV
>CYP51B1_Mycobacterium tuberculosis MD14720 2622219776
MSAVALPRVSGGHDEHGHLEEFRTDPIGLMQRVRDECGDVGTFQLAGKQVVLLSGSHANEFFFRAGDDDLDQA
KAYPFMTPIFGEGVVFDASPERRKEMLHNAALRGEQMKGHAATIEDQVRRMIADWGEAGEIDLLDFFAELTIY
TSSACLIGKKFRDQLDGRFAKLYHELERGTDPLAYVDPYLPIESFRRRDEARNGLVALVADIMNGRIANPPTD
KSDRDMLDVLIAVKAETGTPRFSADEITGMFISMMFAGHHTSSGTASWTLIELMRHRDAYAAVIDELDELYGD
GRSVSFHALRQIPQLENVLKETLRLHPPLIILMRVAKGEFEVQGHRIHEGDLVAASPAISNRIPEDFPDPHDF
VPARYEQPRQEDLLNRWTWIPFGAGRHRCVGAAFAIMQIKAIFSVLLREYEFEMAQPPESYRNDHSKMVVQLA
QPACVRYRRRTGV
>CYP51B1_Mycobacterium tuberculosis MD14825 2632259323
MSAVALPRVSGGHDEHGHLEEFRTDPIGLMQRVRDECGDVGTFQLAGKQVVLLSGSHANEFFFRAGDDDLDQA
KAYPFMTPIFGEGVVFDASPERRKEMLHNAALRGEQMKGHAATIEDQVRRMIADWGEAGEIDLLDFFAELTIY
TSSACLIGKKFRDQLDGRFAKLYHELERGTDPLAYVDPYLPIESFRRRDEARNGLVALVADIMNGRIANPPTD
KSDRDMLDVLIAVKAETGTPRFSADEITGMFISMMFAGHHTSSGTASWTLIELMRHRDAYAAVIDELDELYGD
GRSVSFHALRQIPQLENVLKETLRLHPPLIILMRVAKGEFEVQGHRIHEGDLVAASPAISNRIPEDFPDPHDF
VPARYEQPRQEDLLNRWTWIPFGAGRHRCVGAAFAIMQIKAIFSVLLREYEFEMAQPPESYRNDHSKMVVQLA
QPACVRYRRRTGV
>CYP51B1_Mycobacterium tuberculosis MD14832 2622203006
MSAVALPRVSGGHDEHGHLEEFRTDPIGLMQRVRDECGDVGTFQLAGKQVVLLSGSHANEFFFRAGDDDLDQA
KAYPFMTPIFGEGVVFDASPERRKEMLHNAALRGEQMKGHAATIEDQVRRMIADWGEAGEIDLLDFFAELTIY
TSSACLIGKKFRDQLDGRFAKLYHELERGTDPLAYVDPYLPIESFRRRDEARNGLVALVADIMNGRIANPPTD
KSDRDMLDVLIAVKAETGTPRFSADEITGMFISMMFAGHHTSSGTASWTLIELMRHRDAYAAVIDELDELYGD

GRSVSFHALRQIPQLENVLKETLRLHPPLIILMRVAKGEFEVQGHRIHEGDLVAASPAISNRIPEDFPDPHDF
VPARYEQPRQEDLLNRWTWIPFGAGRHRCVGAAFAIMQIKAIFSVLLREYEFEMAQPPESYRNDHSKMVVQLA
QPACVRYRRRTGV
>CYP51B1_Mycobacterium tuberculosis MD14839 2636806377
MSAVALPRVSGGHDEHGHLEEFRTDPIGLMQRVRDECGDVGTFQLAGKQVVLLSGSHANEFFFRAGDDDLDQA
KAYPFMTPIFGEGVVFDASPERRKEMLHNAALRGEQMKGHAATIEDQVRRMIADWGEAGEIDLLDFFAELTIY
TSSACLIGKKFRDQLDGRFAKLYHELERGTDPLAYVDPYLPIESFRRRDEARNGLVALVADIMNGRIANPPTD
KSDRDMLDVLIAVKAETGTPRFSADEITGMFISMMFAGHHTSSGTASWTLIELMRHRDAYAAVIDELDELYGD
GRSVSFHALRQIPQLENVLKETLRLHPPLIILMRVAKGEFEVQGHRIHEGDLVAASPAISNRIPEDFPDPHDF
VPARYEQPRQEDLLNRWTWIPFGAGRHRCVGAAFAIMQIKAIFSVLLREYEFEMAQPPESYRNDHSKMVVQLA
QPACVRYRRRTGV
>CYP51B1_Mycobacterium tuberculosis MD14844 2622183432
MSAVALPRVSGGHDEHGHLEEFRTDPIGLMQRVRDECGDVGTFQLAGKQVVLLSGSHANEFFFRAGDDDLDQA
KAYPFMTPIFGEGVVFDASPERRKEMLHNAALRGEQMKGHAATIEDQVRRMIADWGEAGEIDLLDFFAELTIY
TSSACLIGKKFRDQLDGRFAKLYHELERGTDPLAYVDPYLPIESFRRRDEARNGLVALVADIMNGRIANPPTD
KSDRDMLDVLIAVKAETGTPRFSADEITGMFISMMFAGHHTSSGTASWTLIELMRHRDAYAAVIDELDELYGD
GRSVSFHALRQIPQLENVLKETLRLHPPLIILMRVAKGEFEVQGHRIHEGDLVAASPAISNRIPEDFPDPHDF
VPARYEQPRQEDLLNRWTWIPFGAGRHRCVGAAFAIMQIKAIFSVLLREYEFEMAQPPESYRNDHSKMVVQLA
QPACVRYRRRTGV
>CYP51B1_Mycobacterium tuberculosis MD14847 2622187624
MSAVALPRVSGGHDEHGHLEEFRTDPIGLMQRVRDECGDVGTFQLAGKQVVLLSGSHANEFFFRAGDDDLDQA
KAYPFMTPIFGEGVVFDASPERRKEMLHNAALRGEQMKGHAATIEDQVRRMIADWGEAGEIDLLDFFAELTIY
TSSACLIGKKFRDQLDGRFAKLYHELERGTDPLAYVDPYLPIESFRRRDEARNGLVALVADIMNGRIANPPTD
KSDRDMLDVLIAVKAETGTPRFSADEITGMFISMMFAGHHTSSGTASWTLIELMRHRDAYAAVIDELDELYGD
GRSVSFHALRQIPQLENVLKETLRLHPPLIILMRVAKGEFEVQGHRIHEGDLVAASPAISNRIPEDFPDPHDF
VPARYEQPRQEDLLNRWTWIPFGAGRHRCVGAAFAIMQIKAIFSVLLREYEFEMAQPPESYRNDHSKMVVQLA
QPACVRYRRRTGV
>CYP51B1_Mycobacterium tuberculosis MD15050 2584948326
MSAVALPRVSGGHDEHGHLEEFRTDPIGLMQRVRDECGDVGTFQLAGKQVVLLSGSHANEFFFRAGDDDLDQA
KAYPFMTPIFGEGVVFDASPERRKEMLHNAALRGEQMKGHAATIEDQVRRMIADWGEAGEIDLLDFFAELTIY
TSSACLIGKKFRDQLDGRFAKLYHELERGTDPLAYVDPYLPIESFRRRDEARNGLVALVADIMNGRIANPPTD
KSDRDMLDVLIAVKAETGTPRFSADEITGMFISMMFAGHHTSSGTASWTLIELMRHRDAYAAVIDELDELYGD
GRSVSFHALRQIPQLENVLKETLRLHPPLIILMRVAKGEFEVQGHRIHEGDLVAASPAISNRIPEDFPDPHDF
VPARYEQPRQEDLLNRWTWIPFGAGRHRCVGAAFAIMQIKAIFSVLLREYEFEMAQPPESYRNDHSKMVVQLA
QPACVRYRRRTGV
>CYP51B1_Mycobacterium tuberculosis MD15212 2577521834
MSAVALPRVSGGHDEHGHLEEFRTDPIGLMQRVRDECGDVGTFQLAGKQVVLLSGSHANEFFFRAGDDDLDQA
KAYPFMTPIFGEGVVFDASPERRKEMLHNAALRGEQMKGHAATIEDQVRRMIADWGEAGEIDLLDFFAELTIY
TSSACLIGKKFRDQLDGRFAKLYHELERGTDPLAYVDPYLPIESFRRRDEARNGLVALVADIMNGRIANPPTD
KSDRDMLDVLIAVKAETGTPRFSADEITGMFISMMFAGHHTSSGTASWTLIELMRHRDAYAAVIDELDELYGD
GRSVSFHALRQIPQLENVLKETLRLHPPLIILMRVAKGEFEVQGHRIHEGDLVAASPAISNRIPEDFPDPHDF
VPARYEQPRQEDLLNRWTWIPFGAGRHRCVGAAFAIMQIKAIFSVLLREYEFEMAQPPESYRNDHSKMVVQLA
QPACVRYRRRTGV
>CYP51B1_Mycobacterium tuberculosis MD15226 2622144803
MSAVALPRVSGGHDEHGHLEEFRTDPIGLMQRVRDECGDVGTFQLAGKQVVLLSGSHANEFFFRAGDDDLDQA
KAYPFMTPIFGEGVVFDASPERRKEMLHNAALRGEQMKGHAATIEDQVRRMIADWGEAGEIDLLDFFAELTIY
TSSACLIGKKFRDQLDGRFAKLYHELERGTDPLAYVDPYLPIESFRRRDEARNGLVALVADIMNGRIANPPTD
KSDRDMLDVLIAVKAETGTPRFSADEITGMFISMMFAGHHTSSGTASWTLIELMRHRDAYAAVIDELDELYGD
GRSVSFHALRQIPQLENVLKETLRLHPPLIILMRVAKGEFEVQGHRIHEGDLVAASPAISNRIPEDFPDPHDF
VPARYEQPRQEDLLNRWTWIPFGAGRHRCVGAAFAIMQIKAIFSVLLREYEFEMAQPPESYRNDHSKMVVQLA
QPACVRYRRRTGV
>CYP51B1_Mycobacterium tuberculosis MD15430 2622161762
MSAVALPRVSGGHDEHGHLEEFRTDPIGLMQRVRDECGDVGTFQLAGKQVVLLSGSHANEFFFRAGDDDLDQA
KAYPFMTPIFGEGVVFDASPERRKEMLHNAALRGEQMKGHAATIEDQVRRMIADWGEAGEIDLLDFFAELTIY
TSSACLIGKKFRDQLDGRFAKLYHELERGTDPLAYVDPYLPIESFRRRDEARNGLVALVADIMNGRIANPPTD
KSDRDMLDVLIAVKAETGTPRFSADEITGMFISMMFAGHHTSSGTASWTLIELMRHRDAYAAVIDELDELYGD
GRSVSFHALRQIPQLENVLKETLRLHPPLIILMRVAKGEFEVQGHRIHEGDLVAASPAISNRIPEDFPDPHDF

VPARYEQPRQEDLLNRWTWIPFGAGRHRCVGAAFAIMQIKAIFSVLLREYEFEMAQPPESYRNDHSKMVVQLA
QPACVRYRRRTGV
>CYP51B1_Mycobacterium tuberculosis MD15584 2622232312
MSAVALPRVSGGHDEHGHLEEFRTDPIGLMQRVRDECGDVGTFQLAGKQVVLLSGSHANEFFFRAGDDDLDQA
KAYPFMTPIFGEGVVFDASPERRKEMLHNAALRGEQMKGHAATIEDQVRRMIADWGEAGEIDLLDFFAELTIY
TSSACLIGKKFRDQLDGRFAKLYHELERGTDPLAYVDPYLPIESFRRRDEARNGLVALVADIMNGRIANPPTD
KSDRDMLDVLIAVKAETGTPRFSADEITGMFISMMFAGHHTSSGTASWTLIELMRHRDAYAAVIDELDELYGD
GRSVSFHALRQIPQLENVLKETLRLHPPLIILMRVAKGEFEVQGHRIHEGDLVAASPAISNRIPEDFPDPHDF
VPARYEQPRQEDLLNRWTWIPFGAGRHRCVGAAFAIMQIKAIFSVLLREYEFEMAQPPESYRNDHSKMVVQLA
QPACVRYRRRTGV
>CYP51B1_Mycobacterium tuberculosis MD15594 2622082653
MSAVALPRVSGGHDEHGHLEEFRTDPIGLMQRVRDECGDVGTFQLAGKQVVLLSGSHANEFFFRAGDDDLDQA
KAYPFMTPIFGEGVVFDASPERRKEMLHNAALRGEQMKGHAATIEDQVRRMIADWGEAGEIDLLDFFAELTIY
TSSACLIGKKFRDQLDGRFAKLYHELERGTDPLAYVDPYLPIESFRRRDEARNGLVALVADIMNGRIANPPTD
KSDRDMLDVLIAVKAETGTPRFSADEITGMFISMMFAGHHTSSGTASWTLIELMRHRDAYAAVIDELDELYGD
GRSVSFHALRQIPQLENVLKETLRLHPPLIILMRVAKGEFEVQGHRIHEGDLVAASPAISNRIPEDFPDPHDF
VPARYEQPRQEDLLNRWTWIPFGAGRHRCVGAAFAIMQIKAIFSVLLREYEFEMAQPPESYRNDHSKMVVQLA
QPACVRYRRRTGV
>CYP51B1_Mycobacterium tuberculosis MD15597 2584952158
MSAVALPRVSGGHDEHGHLEEFRTDPIGLMQRVRDECGDVGTFQLAGKQVVLLSGSHANEFFFRAGDDDLDQA
KAYPFMTPIFGEGVVFDASPERRKEMLHNAALRGEQMKGHAATIEDQVRRMIADWGEAGEIDLLDFFAELTIY
TSSACLIGKKFRDQLDGRFAKLYHELERGTDPLAYVDPYLPIESFRRRDEARNGLVALVADIMNGRIANPPTD
KSDRDMLDVLIAVKAETGTPRFSADEITGMFISMMFAGHHTSSGTASWTLIELMRHRDAYAAVIDELDELYGD
GRSVSFHALRQIPQLENVLKETLRLHPPLIILMRVAKGEFEVQGHRIHEGDLVAASPAISNRIPEDFPDPHDF
VPARYEQPRQEDLLNRWTWIPFGAGRHRCVGAAFAIMQIKAIFSVLLREYEFEMAQPPESYRNDHSKMVVQLA
QPACVRYRRRTGV
>CYP51B1_Mycobacterium tuberculosis MD15751 2634008415
MSAVALPRVSGGHDEHGHLEEFRTDPIGLMQRVRDECGDVGTFQLAGKQVVLLSGSHANEFFFRAGDDDLDQA
KAYPFMTPIFGEGVVFDASPERRKEMLHNAALRGEQMKGHAATIEDQVRRMIADWGEAGEIDLLDFFAELTIY
TSSACLIGKKFRDQLDGRFAKLYHELERGTDPLAYVDPYLPIESFRRRDEARNGLVALVADIMNGRIANPPTD
KSDRDMLDVLIAVKAETGTPRFSADEITGMFISMMFAGHHTSSGTASWTLIELMRHRDAYAAVIDELDELYGD
GRSVSFHALRQIPQLENVLKETLRLHPPLIILMRVAKGEFEVQGHRIHEGDLVAASPAISNRIPEDFPDPHDF
VPARYEQPRQEDLLNRWTWIPFGAGRHRCVGAAFAIMQIKAIFSVLLREYEFEMAQPPESYRNDHSKMVVQLA
QPACVRYRRRTGV
>CYP51B1_Mycobacterium tuberculosis MD15758 2622236289
MSAVALPRVSGGHDEHGHLEEFRTDPIGLMQRVRDECGDVGTFQLAGKQVVLLSGSHANEFFFRAGDDDLDQA
KAYPFMTPIFGEGVVFDASPERRKEMLHNAALRGEQMKGHAATIEDQVRRMIADWGEAGEIDLLDFFAELTIY
TSSACLIGKKFRDQLDGRFAKLYHELERGTDPLAYVDPYLPIESFRRRDEARNGLVALVADIMNGRIANPPTD
KSDRDMLDVLIAVKAETGTPRFSADEITGMFISMMFAGHHTSSGTASWTLIELMRHRDAYAAVIDELDELYGD
GRSVSFHALRQIPQLENVLKETLRLHPPLIILMRVAKGEFEVQGHRIHEGDLVAASPAISNRIPEDFPDPHDF
VPARYEQPRQEDLLNRWTWIPFGAGRHRCVGAAFAIMQIKAIFSVLLREYEFEMAQPPESYRNDHSKMVVQLA
QPACVRYRRRTGV
>CYP51B1_Mycobacterium tuberculosis MD15762 2622244729
MSAVALPRVSGGHDEHGHLEEFRTDPIGLMQRVRDECGDVGTFQLAGKQVVLLSGSHANEFFFRAGDDDLDQA
KAYPFMTPIFGEGVVFDASPERRKEMLHNAALRGEQMKGHAATIEDQVRRMIADWGEAGEIDLLDFFAELTIY
TSSACLIGKKFRDQLDGRFAKLYHELERGTDPLAYVDPYLPIESFRRRDEARNGLVALVADIMNGRIANPPTD
KSDRDMLDVLIAVKAETGTPRFSADEITGMFISMMFAGHHTSSGTASWTLIELMRHRDAYAAVIDELDELYGD
GRSVSFHALRQIPQLENVLKETLRLHPPLIILMRVAKGEFEVQGHRIHEGDLVAASPAISNRIPEDFPDPHDF
VPARYEQPRQEDLLNRWTWIPFGAGRHRCVGAAFAIMQIKAIFSVLLREYEFEMAQPPESYRNDHSKMVVQLA
QPACVRYRRRTGV
>CYP51B1_Mycobacterium tuberculosis MD15765 2622240528
MSAVALPRVSGGHDEHGHLEEFRTDPIGLMQRVRDECGDVGTFQLAGKQVVLLSGSHANEFFFRAGDDDLDQA
KAYPFMTPIFGEGVVFDASPERRKEMLHNAALRGEQMKGHAATIEDQVRRMIADWGEAGEIDLLDFFAELTIY
TSSACLIGKKFRDQLDGRFAKLYHELERGTDPLAYVDPYLPIESFRRRDEARNGLVALVADIMNGRIANPPTD
KSDRDMLDVLIAVKAETGTPRFSADEITGMFISMMFAGHHTSSGTASWTLIELMRHRDAYAAVIDELDELYGD
GRSVSFHALRQIPQLENVLKETLRLHPPLIILMRVAKGEFEVQGHRIHEGDLVAASPAISNRIPEDFPDPHDF
VPARYEQPRQEDLLNRWTWIPFGAGRHRCVGAAFAIMQIKAIFSVLLREYEFEMAQPPESYRNDHSKMVVQLA
QPACVRYRRRTGV

>CYP51B1_Mycobacterium tuberculosis MD15766 2622140998
MSAVALPRVSGGHDEHGHLEEFRTDPIGLMQRVRDECGDVGTFQLAGKQVVLLSGSHANEFFFRAGDDDLDQA
KAYPFMTPIFGEGVVFDASPERRKEMLHNAALRGEQMKGHAATIEDQVRRMIADWGEAGEIDLLDFFAELTIY
TSSACLIGKKFRDQLDGRFAKLYHELERGTDPLAYVDPYLPIESFRRRDEARNGLVALVADIMNGRIANPPTD
KSDRDMLDVLIAVKAETGTPRFSADEITGMFISMMFAGHHTSSGTASWTLIELMRHRDAYAAVIDELDELYGD
GRSVSFHALRQIPQLENVLKETLRLHPPLIILMRVAKGEFEVQGHRIHEGDLVAASPAISNRIPEDFPDPHDF
VPARYEQPRQEDLLNRWTWIPFGAGRHRCVGAAFAIMQIKAIFSVLLREYEFEMAQPPESYRNDHSKMVVQLA
QPACVRYRRRTGV
>CYP51B1_Mycobacterium tuberculosis MD15770 2622169948
MSAVALPRVSGGHDEHGHLEEFRTDPIGLMQRVRDECGDVGTFQLAGKQVVLLSGSHANEFFFRAGDDDLDQA
KAYPFMTPIFGEGVVFDASPERRKEMLHNAALRGEQMKGHAATIEDQVRRMIADWGEAGEIDLLDFFAELTIY
TSSACLIGKKFRDQLDGRFAKLYHELERGTDPLAYVDPYLPIESFRRRDEARNGLVALVADIMNGRIANPPTD
KSDRDMLDVLIAVKAETGTPRFSADEITGMFISMMFAGHHTSSGTASWTLIELMRHRDAYAAVIDELDELYGD
GRSVSFHALRQIPQLENVLKETLRLHPPLIILMRVAKGEFEVQGHRIHEGDLVAASPAISNRIPEDFPDPHDF
VPARYEQPRQEDLLNRWTWIPFGAGRHRCVGAAFAIMQIKAIFSVLLREYEFEMAQPPESYRNDHSKMVVQLA
QPACVRYRRRTGV
>CYP51B1_Mycobacterium tuberculosis MD15774 2630816964
MSAVALPRVSGGHDEHGHLEEFRTDPIGLMQRVRDECGDVGTFQLAGKQVVLLSGSHANEFFFRAGDDDLDQA
KAYPFMTPIFGEGVVFDASPERRKEMLHNAALRGEQMKGHAATIEDQVRRMIADWGEAGEIDLLDFFAELTIY
TSSACLIGKKFRDQLDGRFAKLYHELERGTDPLAYVDPYLPIESFRRRDEARNGLVALVADIMNGRIANPPTD
KSDRDMLDVLIAVKAETGTPRFSADEITGMFISMMFAGHHTSSGTASWTLIELMRHRDAYAAVIDELDELYGD
GRSVSFHALRQIPQLENVLKETLRLHPPLIILMRVAKGEFEVQGHRIHEGDLVAASPAISNRIPEDFPDPHDF
VPARYEQPRQEDLLNRWTWIPFGAGRHRCVGAAFAIMQIKAIFSVLLREYEFEMAQPPESYRNDHSKMVVQLA
QPACVRYRRRTGV
>CYP51B1_Mycobacterium tuberculosis MD15855 2577447170
MSAVALPRVSGGHDEHGHLEEFRTDPIGLMQRVRDECGDVGTFQLAGKQVVLLSGSHANEFFFRAGDDDLDQA
KAYPFMTPIFGEGVVFDASPERRKEMLHNAALRGEQMKGHAATIEDQVRRMIADWGEAGEIDLLDFFAELTIY
TSSACLIGKKFRDQLDGRFAKLYHELERGTDPLAYVDPYLPIESFRRRDEARNGLVALVADIMNGRIANPPTD
KSDRDMLDVLIAVKAETGTPRFSADEITGMFISMMFAGHHTSSGTASWTLIELMRHRDAYAAVIDELDELYGD
GRSVSFHALRQIPQLENVLKETLRLHPPLIILMRVAKGEFEVQGHRIHEGDLVAASPAISNRIPEDFPDPHDF
VPARYEQPRQEDLLNRWTWIPFGAGRHRCVGAAFAIMQIKAIFSVLLREYEFEMAQPPESYRNDHSKMVVQLA
QPACVRYRRRTGV
>CYP51B1_Mycobacterium tuberculosis MD15956 2584944106
MSAVALPRVSGGHDEHGHLEEFRTDPIGLMQRVRDECGDVGTFQLAGKQVVLLSGSHANEFFFRAGDDDLDQA
KAYPFMTPIFGEGVVFDASPERRKEMLHNAALRGEQMKGHAATIEDQVRRMIADWGEAGEIDLLDFFAELTIY
TSSACLIGKKFRDQLDGRFAKLYHELERGTDPLAYVDPYLPIESFRRRDEARNGLVALVADIMNGRIANPPTD
KSDRDMLDVLIAVKAETGTPRFSADEITGMFISMMFAGHHTSSGTASWTLIELMRHRDAYAAVIDELDELYGD
GRSVSFHALRQIPQLENVLKETLRLHPPLIILMRVAKGEFEVQGHRIHEGDLVAASPAISNRIPEDFPDPHDF
VPARYEQPRQEDLLNRWTWIPFGAGRHRCVGAAFAIMQIKAIFSVLLREYEFEMAQPPESYRNDHSKMVVQLA
QPACVRYRRRTGV
>CYP51B1_Mycobacterium tuberculosis MD15958 2636730708
MSAVALPRVSGGHDEHGHLEEFRTDPIGLMQRVRDECGDVGTFQLAGKQVVLLSGSHANEFFFRAGDDDLDQA
KAYPFMTPIFGEGVVFDASPERRKEMLHNAALRGEQMKGHAATIEDQVRRMIADWGEAGEIDLLDFFAELTIY
TSSACLIGKKFRDQLDGRFAKLYHELERGTDPLAYVDPYLPIESFRRRDEARNGLVALVADIMNGRIANPPTD
KSDRDMLDVLIAVKAETGTPRFSADEITGMFISMMFAGHHTSSGTASWTLIELMRHRDAYAAVIDELDELYGD
GRSVSFHALRQIPQLENVLKETLRLHPPLIILMRVAKGEFEVQGHRIHEGDLVAASPAISNRIPEDFPDPHDF
VPARYEQPRQEDLLNRWTWIPFGAGRHRCVGAAFAIMQIKAIFSVLLREYEFEMAQPPESYRNDHSKMVVQLA
QPACVRYRRRTGV
>CYP51B1_Mycobacterium tuberculosis MD15963 2622173975
MSAVALPRVSGGHDEHGHLEEFRTDPIGLMQRVRDECGDVGTFQLAGKQVVLLSGSHANEFFFRAGDDDLDQA
KAYPFMTPIFGEGVVFDASPERRKEMLHNAALRGEQMKGHAATIEDQVRRMIADWGEAGEIDLLDFFAELTIY
TSSACLIGKKFRDQLDGRFAKLYHELERGTDPLAYVDPYLPIESFRRRDEARNGLVALVADIMNGRIANPPTD
KSDRDMLDVLIAVKAETGTPRFSADEITGMFISMMFAGHHTSSGTASWTLIELMRHRDAYAAVIDELDELYGD
GRSVSFHALRQIPQLENVLKETLRLHPPLIILMRVAKGEFEVQGHRIHEGDLVAASPAISNRIPEDFPDPHDF
VPARYEQPRQEDLLNRWTWIPFGAGRHRCVGAAFAIMQIKAIFSVLLREYEFEMAQPPESYRNDHSKMVVQLA
QPACVRYRRRTGV
>CYP51B1_Mycobacterium tuberculosis MD15973 2622207713

MSAVALPRVSGGHDEHGHLEEFRTDPIGLMQRVRDECGDVGTFQLAGKQVVLLSGSHANEFFFRAGDDDLDQA
KAYPFMTPIFGEGVVFDASPERRKEMLHNAALRGEQMKGHAATIEDQVRRMIADWGEAGEIDLLDFFAELTIY
TSSACLIGKKFRDQLDGRFAKLYHELERGTDPLAYVDPYLPIESFRRRDEARNGLVALVADIMNGRIANPPTD
KSDRDMLDVLIAVKAETGTPRFSADEITGMFISMMFAGHHTSSGTASWTLIELMRHRDAYAAVIDELDELYGD
GRSVSFHALRQIPQLENVLKETLRLHPPLIILMRVAKGEFEVQGHRIHEGDLVAASPAISNRIPEDFPDPHDF
VPARYEQPRQEDLLNRWTWIPFGAGRHRCVGAAFAIMQIKAIFSVLLREYEFEMAQPPESYRNDHSKMVVQLA
QPACVRYRRRTGV
>CYP51B1_Mycobacterium tuberculosis MD15974 2577201621
MSAVALPRVSGGHDEHGHLEEFRTDPIGLMQRVRDECGDVGTFQLAGKQVVLLSGSHANEFFFRAGDDDLDQA
KAYPFMTPIFGEGVVFDASPERRKEMLHNAALRGEQMKGHAATIEDQVRRMIADWGEAGEIDLLDFFAELTIY
TSSACLIGKKFRDQLDGRFAKLYHELERGTDPLAYVDPYLPIESFRRRDEARNGLVALVADIMNGRIANPPTD
KSDRDMLDVLIAVKAETGTPRFSADEITGMFISMMFAGHHTSSGTASWTLIELMRHRDAYAAVIDELDELYGD
GRSVSFHALRQIPQLENVLKETLRLHPPLIILMRVAKGEFEVQGHRIHEGDLVAASPAISNRIPEDFPDPHDF
VPARYEQPRQEDLLNRWTWIPFGAGRHRCVGAAFAIMQIKAIFSVLLREYEFEMAQPPESYRNDHSKMVVQLA
QPACVRYRRRTGV
>CYP51B1_Mycobacterium tuberculosis MD15977 2574562927
MSAVALPRVSGGHDEHGHLEEFRTDPIGLMQRVRDECGDVGTFQLAGKQVVLLSGSHANEFFFRAGDDDLDQA
KAYPFMTPIFGEGVVFDASPERRKEMLHNAALRGEQMKGHAATIEDQVRRMIADWGEAGEIDLLDFFAELTIY
TSSACLIGKKFRDQLDGRFAKLYHELERGTDPLAYVDPYLPIESFRRRDEARNGLVALVADIMNGRIANPPTD
KSDRDMLDVLIAVKAETGTPRFSADEITGMFISMMFAGHHTSSGTASWTLIELMRHRDAYAAVIDELDELYGD
GRSVSFHALRQIPQLENVLKETLRLHPPLIILMRVAKGEFEVQGHRIHEGDLVAASPAISNRIPEDFPDPHDF
VPARYEQPRQEDLLNRWTWIPFGAGRHRCVGAAFAIMQIKAIFSVLLREYEFEMAQPPESYRNDHSKMVVQLA
QPACVRYRRRTGV
>CYP51B1_Mycobacterium tuberculosis MD16257 2622178077
MSAVALPRVSGGHDEHGHLEEFRTDPIGLMQRVRDECGDVGTFQLAGKQVVLLSGSHANEFFFRAGDDDLDQA
KAYPFMTPIFGEGVVFDASPERRKEMLHNAALRGEQMKGHAATIEDQVRRMIADWGEAGEIDLLDFFAELTIY
TSSACLIGKKFRDQLDGRFAKLYHELERGTDPLAYVDPYLPIESFRRRDEARNGLVALVADIMNGRIANPPTD
KSDRDMLDVLIAVKAETGTPRFSADEITGMFISMMFAGHHTSSGTASWTLIELMRHRDAYAAVIDELDELYGD
GRSVSFHALRQIPQLENVLKETLRLHPPLIILMRVAKGEFEVQGHRIHEGDLVAASPAISNRIPEDFPDPHDF
VPARYEQPRQEDLLNRWTWIPFGAGRHRCVGAAFAIMQIKAIFSVLLREYEFEMAQPPESYRNDHSKMVVQLA
QPACVRYRRRTGV
>CYP51B1_Mycobacterium tuberculosis MD16261 2622099482
MSAVALPRVSGGHDEHGHLEEFRTDPIGLMQRVRDECGDVGTFQLAGKQVVLLSGSHANEFFFRAGDDDLDQA
KAYPFMTPIFGEGVVFDASPERRKEMLHNAALRGEQMKGHAATIEDQVRRMIADWGEAGEIDLLDFFAELTIY
TSSACLIGKKFRDQLDGRFAKLYHELERGTDPLAYVDPYLPIESFRRRDEARNGLVALVADIMNGRIANPPTD
KSDRDMLDVLIAVKAETGTPRFSADEITGMFISMMFAGHHTSSGTASWTLIELMRHRDAYAAVIDELDELYGD
GRSVSFHALRQIPQLENVLKETLRLHPPLIILMRVAKGEFEVQGHRIHEGDLVAASPAISNRIPEDFPDPHDF
VPARYEQPRQEDLLNRWTWIPFGAGRHRCVGAAFAIMQIKAIFSVLLREYEFEMAQPPESYRNDHSKMVVQLA
QPACVRYRRRTGV
>CYP51B1_Mycobacterium tuberculosis MD16265 2577425287
MSAVALPRVSGGHDEHGHLEEFRTDPIGLMQRVRDECGDVGTFQLAGKQVVLLSGSHANEFFFRAGDDDLDQA
KAYPFMTPIFGEGVVFDASPERRKEMLHNAALRGEQMKGHAATIEDQVRRMIADWGEAGEIDLLDFFAELTIY
TSSACLIGKKFRDQLDGRFAKLYHELERGTDPLAYVDPYLPIESFRRRDEARNGLVALVADIMNGRIANPPTD
KSDRDMLDVLIAVKAETGTPRFSADEITGMFISMMFAGHHTSSGTASWTLIELMRHRDAYAAVIDELDELYGD
GRSVSFHALRQIPQLENVLKETLRLHPPLIILMRVAKGEFEVQGHRIHEGDLVAASPAISNRIPEDFPDPHDF
VPARYEQPRQEDLLNRWTWIPFGAGRHRCVGAAFAIMQIKAIFSVLLREYEFEMAQPPESYRNDHSKMVVQLA
QPACVRYRRRTGV
>CYP51B1_Mycobacterium tuberculosis MD16277 2576172580
MSAVALPRVSGGHDEHGHLEEFRTDPIGLMQRVRDECGDVGTFQLAGKQVVLLSGSHANEFFFRAGDDDLDQA
KAYPFMTPIFGEGVVFDASPERRKEMLHNAALRGEQMKGHAATIEDQVRRMIADWGEAGEIDLLDFFAELTIY
TSSACLIGKKFRDQLDGRFAKLYHELERGTDPLAYVDPYLPIESFRRRDEARNGLVALVADIMNGRIANPPTD
KSDRDMLDVLIAVKAETGTPRFSADEITGMFISMMFAGHHTSSGTASWTLIELMRHRDAYAAVIDELDELYGD
GRSVSFHALRQIPQLENVLKETLRLHPPLIILMRVAKGEFEVQGHRIHEGDLVAASPAISNRIPEDFPDPHDF
VPARYEQPRQEDLLNRWTWIPFGAGRHRCVGAAFAIMQIKAIFSVLLREYEFEMAQPPESYRNDHSKMVVQLA
QPACVRYRRRTGV
>CYP51B1_Mycobacterium tuberculosis MD16553 2574587643
MSAVALPRVSGGHDEHGHLEEFRTDPIGLMQRVRDECGDVGTFQLAGKQVVLLSGSHANEFFFRAGDDDLDQA
KAYPFMTPIFGEGVVFDASPERRKEMLHNAALRGEQMKGHAATIEDQVRRMIADWGEAGEIDLLDFFAELTIY

TSSACLIGKKFRDQLDGRFAKLYHELERGTDPLAYVDPYLPIESFRRRDEARNGLVALVADIMNGRIANPPTD
KSDRDMLDVLIAVKAETGTPRFSADEITGMFISMMFAGHHTSSGTASWTLIELMRHRDAYAAVIDELDELYGD
GRSVSFHALRQIPQLENVLKETLRLHPPLIILMRVAKGEFEVQGHRIHEGDLVAASPAISNRIPEDFPDPHDF
VPARYEQPRQEDLLNRWTWIPFGAGRHRCVGAAFAIMQIKAIFSVLLREYEFEMAQPPESYRNDHSKMVVQLA
QPACVRYRRRTGV
>CYP51B1_Mycobacterium tuberculosis MD16555 2584869321
MSAVALPRVSGGHDEHGHLEEFRTDPIGLMQRVRDECGDVGTFQLAGKQVVLLSGSHANEFFFRAGDDDLDQA
KAYPFMTPIFGEGVVFDASPERRKEMLHNAALRGEQMKGHAATIEDQVRRMIADWGEAGEIDLLDFFAELTIY
TSSACLIGKKFRDQLDGRFAKLYHELERGTDPLAYVDPYLPIESFRRRDEARNGLVALVADIMNGRIANPPTD
KSDRDMLDVLIAVKAETGTPRFSADEITGMFISMMFAGHHTSSGTASWTLIELMRHRDAYAAVIDELDELYGD
GRSVSFHALRQIPQLENVLKETLRLHPPLIILMRVAKGEFEVQGHRIHEGDLVAASPAISNRIPEDFPDPHDF
VPARYEQPRQEDLLNRWTWIPFGAGRHRCVGAAFAIMQIKAIFSVLLREYEFEMAQPPESYRNDHSKMVVQLA
QPACVRYRRRTGV
>CYP51B1_Mycobacterium tuberculosis MD16568 2622148952
MSAVALPRVSGGHDEHGHLEEFRTDPIGLMQRVRDECGDVGTFQLAGKQVVLLSGSHANEFFFRAGDDDLDQA
KAYPFMTPIFGEGVVFDASPERRKEMLHNAALRGEQMKGHAATIEDQVRRMIADWGEAGEIDLLDFFAELTIY
TSSACLIGKKFRDQLDGRFAKLYHELERGTDPLAYVDPYLPIESFRRRDEARNGLVALVADIMNGRIANPPTD
KSDRDMLDVLIAVKAETGTPRFSADEITGMFISMMFAGHHTSSGTASWTLIELMRHRDAYAAVIDELDELYGD
GRSVSFHALRQIPQLENVLKETLRLHPPLIILMRVAKGEFEVQGHRIHEGDLVAASPAISNRIPEDFPDPHDF
VPARYEQPRQEDLLNRWTWIPFGAGRHRCVGAAFAIMQIKAIFSVLLREYEFEMAQPPESYRNDHSKMVVQLA
QPACVRYRRRTGV
>CYP51B1_Mycobacterium tuberculosis MD16571 2622153182
MSAVALPRVSGGHDEHGHLEEFRTDPIGLMQRVRDECGDVGTFQLAGKQVVLLSGSHANEFFFRAGDDDLDQA
KAYPFMTPIFGEGVVFDASPERRKEMLHNAALRGEQMKGHAATIEDQVRRMIADWGEAGEIDLLDFFAELTIY
TSSACLIGKKFRDQLDGRFAKLYHELERGTDPLAYVDPYLPIESFRRRDEARNGLVALVADIMNGRIANPPTD
KSDRDMLDVLIAVKAETGTPRFSADEITGMFISMMFAGHHTSSGTASWTLIELMRHRDAYAAVIDELDELYGD
GRSVSFHALRQIPQLENVLKETLRLHPPLIILMRVAKGEFEVQGHRIHEGDLVAASPAISNRIPEDFPDPHDF
VPARYEQPRQEDLLNRWTWIPFGAGRHRCVGAAFAIMQIKAIFSVLLREYEFEMAQPPESYRNDHSKMVVQLA
QPACVRYRRRTGV
>CYP51B1_Mycobacterium tuberculosis MD16577 2584933171
MSAVALPRVSGGHDEHGHLEEFRTDPIGLMQRVRDECGDVGTFQLAGKQVVLLSGSHANEFFFRAGDDDLDQA
KAYPFMTPIFGEGVVFDASPERRKEMLHNAALRGEQMKGHAATIEDQVRRMIADWGEAGEIDLLDFFAELTIY
TSSACLIGKKFRDQLDGRFAKLYHELERGTDPLAYVDPYLPIESFRRRDEARNGLVALVADIMNGRIANPPTD
KSDRDMLDVLIAVKAETGTPRFSADEITGMFISMMFAGHHTSSGTASWTLIELMRHRDAYAAVIDELDELYGD
GRSVSFHALRQIPQLENVLKETLRLHPPLIILMRVAKGEFEVQGHRIHEGDLVAASPAISNRIPEDFPDPHDF
VPARYEQPRQEDLLNRWTWIPFGAGRHRCVGAAFAIMQIKAIFSVLLREYEFEMAQPPESYRNDHSKMVVQLA
QPACVRYRRRTGV
>CYP51B1_Mycobacterium tuberculosis MD16728 2577696959
MSAVALPRVSGGHDEHGHLEEFRTDPIGLMQRVRDECGDVGTFQLAGKQVVLLSGSHANEFFFRAGDDDLDQA
KAYPFMTPIFGEGVVFDASPERRKEMLHNAALRGEQMKGHAATIEDQVRRMIADWGEAGEIDLLDFFAELTIY
TSSACLIGKKFRDQLDGRFAKLYHELERGTDPLAYVDPYLPIESFRRRDEARNGLVALVADIMNGRIANPPTD
KSDRDMLDVLIAVKAETGTPRFSADEITGMFISMMFAGHHTSSGTASWTLIELMRHRDAYAAVIDELDELYGD
GRSVSFHALRQIPQLENVLKETLRLHPPLIILMRVAKGEFEVQGHRIHEGDLVAASPAISNRIPEDFPDPHDF
VPARYEQPRQEDLLNRWTWIPFGAGRHRCVGAAFAIMQIKAIFSVLLREYEFEMAQPPESYRNDHSKMVVQLA
QPACVRYRRRTGV
>CYP51B1_Mycobacterium tuberculosis MD16732 2622103232
MSAVALPRVSGGHDEHGHLEEFRTDPIGLMQRVRDECGDVGTFQLAGKQVVLLSGSHANEFFFRAGDDDLDQA
KAYPFMTPIFGEGVVFDASPERRKEMLHNAALRGEQMKGHAATIEDQVRRMIADWGEAGEIDLLDFFAELTIY
TSSACLIGKKFRDQLDGRFAKLYHELERGTDPLAYVDPYLPIESFRRRDEARNGLVALVADIMNGRIANPPTD
KSDRDMLDVLIAVKAETGTPRFSADEITGMFISMMFAGHHTSSGTASWTLIELMRHRDAYAAVIDELDELYGD
GRSVSFHALRQIPQLENVLKETLRLHPPLIILMRVAKGEFEVQGHRIHEGDLVAASPAISNRIPEDFPDPHDF
VPARYEQPRQEDLLNRWTWIPFGAGRHRCVGAAFAIMQIKAIFSVLLREYEFEMAQPPESYRNDHSKMVVQLA
QPACVRYRRRTGV
>CYP51B1_Mycobacterium tuberculosis MD16764 2622066009
MSAVALPRVSGGHDEHGHLEEFRTDPIGLMQRVRDECGDVGTFQLAGKQVVLLSGSHANEFFFRAGDDDLDQA
KAYPFMTPIFGEGVVFDASPERRKEMLHNAALRGEQMKGHAATIEDQVRRMIADWGEAGEIDLLDFFAELTIY
TSSACLIGKKFRDQLDGRFAKLYHELERGTDPLAYVDPYLPIESFRRRDEARNGLVALVADIMNGRIANPPTD
KSDRDMLDVLIAVKAETGTPRFSADEITGMFISMMFAGHHTSSGTASWTLIELMRHRDAYAAVIDELDELYGD

GRSVSFHALRQIPQLENVLKETLRLHPPLIILMRVAKGEFEVQGHRIHEGDLVAASPAISNRIPEDFPDPHDF
VPARYEQPRQEDLLNRWTWIPFGAGRHRCVGAAFAIMQIKAIFSVLLREYEFEMAQPPESYRNDHSKMVVQLA
QPACVRYRRRTGV
>CYP51B1_Mycobacterium tuberculosis MD16573 2622228070
MSAVALPRVSGGHDEHGHLEEFRTDPIGLMQRVRDECGDVGTFQLAGKQVVLLSGSHANEFFFRAGDDDLDQA
KAYPFMTPIFGEGVVFDASPERRKEMLHNAALRGEQMKGHAATIEDQVRRMIADWGEAGEIDLLDFFAELTIY
TSSACLIGKKFRDQLDGRFAKLYHELERGTDPLAYVDPYLPIESFRRRDEARNGLVALVADIMNGRIANPPTD
KSDRDMLDVLIAVKAETGTPRFSADEITGMFISMMFAGHHTSSGTASWTLIELMRHRDAYAAVIDELDELYGD
GRSVSFHALRQIPQLENVLKETLRLHPPLIILMRVAKGEFEVQGHRIHEGDLVAASPAISNRIPEDFPDPHDF
VPARYEQPRQEDLLNRWTWIPFGAGRHRCVGAAFAIMQIKAIFSVLLREYEFEMAQPPESYRNDHSKMVVQLA
QPACVRYRRRTGV
>CYP51B1_Mycobacterium tuberculosis MD16765 2622129556
MSAVALPRVSGGHDEHGHLEEFRTDPIGLMQRVRDECGDVGTFQLAGKQVVLLSGSHANEFFFRAGDDDLDQA
KAYPFMTPIFGEGVVFDASPERRKEMLHNAALRGEQMKGHAATIEDQVRRMIADWGEAGEIDLLDFFAELTIY
TSSACLIGKKFRDQLDGRFAKLYHELERGTDPLAYVDPYLPIESFRRRDEARNGLVALVADIMNGRIANPPTD
KSDRDMLDVLIAVKAETGTPRFSADEITGMFISMMFAGHHTSSGTASWTLIELMRHRDAYAAVIDELDELYGD
GRSVSFHALRQIPQLENVLKETLRLHPPLIILMRVAKGEFEVQGHRIHEGDLVAASPAISNRIPEDFPDPHDF
VPARYEQPRQEDLLNRWTWIPFGAGRHRCVGAAFAIMQIKAIFSVLLREYEFEMAQPPESYRNDHSKMVVQLA
QPACVRYRRRTGV
>CYP51B1_Mycobacterium tuberculosis MD16775 2575990974
MSAVALPRVSGGHDEHGHLEEFRTDPIGLMQRVRDECGDVGTFQLAGKQVVLLSGSHANEFFFRAGDDDLDQA
KAYPFMTPIFGEGVVFDASPERRKEMLHNAALRGEQMKGHAATIEDQVRRMIADWGEAGEIDLLDFFAELTIY
TSSACLIGKKFRDQLDGRFAKLYHELERGTDPLAYVDPYLPIESFRRRDEARNGLVALVADIMNGRIANPPTD
KSDRDMLDVLIAVKAETGTPRFSADEITGMFISMMFAGHHTSSGTASWTLIELMRHRDAYAAVIDELDELYGD
GRSVSFHALRQIPQLENVLKETLRLHPPLIILMRVAKGEFEVQGHRIHEGDLVAASPAISNRIPEDFPDPHDF
VPARYEQPRQEDLLNRWTWIPFGAGRHRCVGAAFAIMQIKAIFSVLLREYEFEMAQPPESYRNDHSKMVVQLA
QPACVRYRRRTGV
>CYP51B1_Mycobacterium tuberculosis MD17006 2622156905
MSAVALPRVSGGHDEHGHLEEFRTDPIGLMQRVRDECGDVGTFQLAGKQVVLLSGSHANEFFFRAGDDDLDQA
KAYPFMTPIFGEGVVFDASPERRKEMLHNAALRGEQMKGHAATIEDQVRRMIADWGEAGEIDLLDFFAELTIY
TSSACLIGKKFRDQLDGRFAKLYHELERGTDPLAYVDPYLPIESFRRRDEARNGLVALVADIMNGRIANPPTD
KSDRDMLDVLIAVKAETGTPRFSADEITGMFISMMFAGHHTSSGTASWTLIELMRHRDAYAAVIDELDELYGD
GRSVSFHALRQIPQLENVLKETLRLHPPLIILMRVAKGEFEVQGHRIHEGDLVAASPAISNRIPEDFPDPHDF
VPARYEQPRQEDLLNRWTWIPFGAGRHRCVGAAFAIMQIKAIFSVLLREYEFEMAQPPESYRNDHSKMVVQLA
QPACVRYRRRTGV
>CYP51B1_Mycobacterium tuberculosis MD17021 2622104742
MSAVALPRVSGGHDEHGHLEEFRTDPIGLMQRVRDECGDVGTFQLAGKQVVLLSGSHANEFFFRAGDDDLDQA
KAYPFMTPIFGEGVVFDASPERRKEMLHNAALRGEQMKGHAATIEDQVRRMIADWGEAGEIDLLDFFAELTIY
TSSACLIGKKFRDQLDGRFAKLYHELERGTDPLAYVDPYLPIESFRRRDEARNGLVALVADIMNGRIANPPTD
KSDRDMLDVLIAVKAETGTPRFSADEITGMFISMMFAGHHTSSGTASWTLIELMRHRDAYAAVIDELDELYGD
GRSVSFHALRQIPQLENVLKETLRLHPPLIILMRVAKGEFEVQGHRIHEGDLVAASPAISNRIPEDFPDPHDF
VPARYEQPRQEDLLNRWTWIPFGAGRHRCVGAAFAIMQIKAIFSVLLREYEFEMAQPPESYRNDHSKMVVQLA
QPACVRYRRRTGV
>CYP51B1_Mycobacterium tuberculosis MD17232 2622165669
MSAVALPRVSGGHDEHGHLEEFRTDPIGLMQRVRDECGDVGTFQLAGKQVVLLSGSHANEFFFRAGDDDLDQA
KAYPFMTPIFGEGVVFDASPERRKEMLHNAALRGEQMKGHAATIEDQVRRMIADWGEAGEIDLLDFFAELTIY
TSSACLIGKKFRDQLDGRFAKLYHELERGTDPLAYVDPYLPIESFRRRDEARNGLVALVADIMNGRIANPPTD
KSDRDMLDVLIAVKAETGTPRFSADEITGMFISMMFAGHHTSSGTASWTLIELMRHRDAYAAVIDELDELYGD
GRSVSFHALRQIPQLENVLKETLRLHPPLIILMRVAKGEFEVQGHRIHEGDLVAASPAISNRIPEDFPDPHDF
VPARYEQPRQEDLLNRWTWIPFGAGRHRCVGAAFAIMQIKAIFSVLLREYEFEMAQPPESYRNDHSKMVVQLA
QPACVRYRRRTGV
>CYP51B1_Mycobacterium tuberculosis MD17240 2622121049
MSAVALPRVSGGHDEHGHLEEFRTDPIGLMQRVRDECGDVGTFQLAGKQVVLLSGSHANEFFFRAGDDDLDQA
KAYPFMTPIFGEGVVFDASPERRKEMLHNAALRGEQMKGHAATIEDQVRRMIADWGEAGEIDLLDFFAELTIY
TSSACLIGKKFRDQLDGRFAKLYHELERGTDPLAYVDPYLPIESFRRRDEARNGLVALVADIMNGRIANPPTD
KSDRDMLDVLIAVKAETGTPRFSADEITGMFISMMFAGHHTSSGTASWTLIELMRHRDAYAAVIDELDELYGD
GRSVSFHALRQIPQLENVLKETLRLHPPLIILMRVAKGEFEVQGHRIHEGDLVAASPAISNRIPEDFPDPHDF

VPARYEQPRQEDLLNRWTWIPFGAGRHRCVGAAFAIMQIKAIFSVLLREYEFEMAQPPESYRNDHSKMVVQLA
QPACVRYRRRTGV
>CYP51B1_Mycobacterium tuberculosis MD17503 2622111302
MSAVALPRVSGGHDEHGHLEEFRTDPIGLMQRVRDECGDVGTFQLAGKQVVLLSGSHANEFFFRAGDDDLDQA
KAYPFMTPIFGEGVVFDASPERRKEMLHNAALRGEQMKGHAATIEDQVRRMIADWGEAGEIDLLDFFAELTIY
TSSACLIGKKFRDQLDGRFAKLYHELERGTDPLAYVDPYLPIESFRRRDEARNGLVALVADIMNGRIANPPTD
KSDRDMLDVLIAVKAETGTPRFSADEITGMFISMMFAGHHTSSGTASWTLIELMRHRDAYAAVIDELDELYGD
GRSVSFHALRQIPQLENVLKETLRLHPPLIILMRVAKGEFEVQGHRIHEGDLVAASPAISNRIPEDFPDPHDF
VPARYEQPRQEDLLNRWTWIPFGAGRHRCVGAAFAIMQIKAIFSVLLREYEFEMAQPPESYRNDHSKMVVQLA
QPACVRYRRRTGV
>CYP51B1_Mycobacterium tuberculosis MD17517 2577634919
MSAVALPRVSGGHDEHGHLEEFRTDPIGLMQRVRDECGDVGTFQLAGKQVVLLSGSHANEFFFRAGDDDLDQA
KAYPFMTPIFGEGVVFDASPERRKEMLHNAALRGEQMKGHAATIEDQVRRMIADWGEAGEIDLLDFFAELTIY
TSSACLIGKKFRDQLDGRFAKLYHELERGTDPLAYVDPYLPIESFRRRDEARNGLVALVADIMNGRIANPPTD
KSDRDMLDVLIAVKAETGTPRFSADEITGMFISMMFAGHHTSSGTASWTLIELMRHRDAYAAVIDELDELYGD
GRSVSFHALRQIPQLENVLKETLRLHPPLIILMRVAKGEFEVQGHRIHEGDLVAASPAISNRIPEDFPDPHDF
VPARYEQPRQEDLLNRWTWIPFGAGRHRCVGAAFAIMQIKAIFSVLLREYEFEMAQPPESYRNDHSKMVVQLA
QPACVRYRRRTGV
>CYP51B1_Mycobacterium tuberculosis MD17613 2622119902
MSAVALPRVSGGHDEHGHLEEFRTDPIGLMQRVRDECGDVGTFQLAGKQVVLLSGSHANEFFFRAGDDDLDQA
KAYPFMTPIFGEGVVFDASPERRKEMLHNAALRGEQMKGHAATIEDQVRRMIADWGEAGEIDLLDFFAELTIY
TSSACLIGKKFRDQLDGRFAKLYHELERGTDPLAYVDPYLPIESFRRRDEARNGLVALVADIMNGRIANPPTD
KSDRDMLDVLIAVKAETGTPRFSADEITGMFISMMFAGHHTSSGTASWTLIELMRHRDAYAAVIDELDELYGD
GRSVSFHALRQIPQLENVLKETLRLHPPLIILMRVAKGEFEVQGHRIHEGDLVAASPAISNRIPEDFPDPHDF
VPARYEQPRQEDLLNRWTWIPFGAGRHRCVGAAFAIMQIKAIFSVLLREYEFEMAQPPESYRNDHSKMVVQLA
QPACVRYRRRTGV
>CYP51B1_Mycobacterium tuberculosis MD17614 2630667190
MSAVALPRVSGGHDEHGHLEEFRTDPIGLMQRVRDECGDVGTFQLAGKQVVLLSGSHANEFFFRAGDDDLDQA
KAYPFMTPIFGEGVVFDASPERRKEMLHNAALRGEQMKGHAATIEDQVRRMIADWGEAGEIDLLDFFAELTIY
TSSACLIGKKFRDQLDGRFAKLYHELERGTDPLAYVDPYLPIESFRRRDEARNGLVALVADIMNGRIANPPTD
KSDRDMLDVLIAVKAETGTPRFSADEITGMFISMMFAGHHTSSGTASWTLIELMRHRDAYAAVIDELDELYGD
GRSVSFHALRQIPQLENVLKETLRLHPPLIILMRVAKGEFEVQGHRIHEGDLVAASPAISNRIPEDFPDPHDF
VPARYEQPRQEDLLNRWTWIPFGAGRHRCVGAAFAIMQIKAIFSVLLREYEFEMAQPPESYRNDHSKMVVQLA
QPACVRYRRRTGV
>CYP51B1_Mycobacterium tuberculosis MD17615 2584814993
MSAVALPRVSGGHDEHGHLEEFRTDPIGLMQRVRDECGDVGTFQLAGKQVVLLSGSHANEFFFRAGDDDLDQA
KAYPFMTPIFGEGVVFDASPERRKEMLHNAALRGEQMKGHAATIEDQVRRMIADWGEAGEIDLLDFFAELTIY
TSSACLIGKKFRDQLDGRFAKLYHELERGTDPLAYVDPYLPIESFRRRDEARNGLVALVADIMNGRIANPPTD
KSDRDMLDVLIAVKAETGTPRFSADEITGMFISMMFAGHHTSSGTASWTLIELMRHRDAYAAVIDELDELYGD
GRSVSFHALRQIPQLENVLKETLRLHPPLIILMRVAKGEFEVQGHRIHEGDLVAASPAISNRIPEDFPDPHDF
VPARYEQPRQEDLLNRWTWIPFGAGRHRCVGAAFAIMQIKAIFSVLLREYEFEMAQPPESYRNDHSKMVVQLA
QPACVRYRRRTGV
>CYP51B1_Mycobacterium tuberculosis MD17646 2649130591
MSAVALPRVSGGHDEHGHLEEFRTDPIGLMQRVRDECGDVGTFQLAGKQVVLLSGSHANEFFFRAGDDDLDQA
KAYPFMTPIFGEGVVFDASPERRKEMLHNAALRGEQMKGHAATIEDQVRRMIADWGEAGEIDLLDFFAELTIY
TSSACLIGKKFRDQLDGRFAKLYHELERGTDPLAYVDPYLPIESFRRRDEARNGLVALVADIMNGRIANPPTD
KSDRDMLDVLIAVKAETGTPRFSADEITGMFISMMFAGHHTSSGTASWTLIELMRHRDAYAAVIDELDELYGD
GRSVSFHALRQIPQLENVLKETLRLHPPLIILMRVAKGEFEVQGHRIHEGDLVAASPAISNRIPEDFPDPHDF
VPARYEQPRQEDLLNRWTWIPFGAGRHRCVGAAFAIMQIKAIFSVLLREYEFEMAQPPESYRNDHSKMVVQLA
QPACVRYRRRTGV
>CYP51B1_Mycobacterium tuberculosis MD17647 2622115862
MSAVALPRVSGGHDEHGHLEEFRTDPIGLMQRVRDECGDVGTFQLAGKQVVLLSGSHANEFFFRAGDDDLDQA
KAYPFMTPIFGEGVVFDASPERRKEMLHNAALRGEQMKGHAATIEDQVRRMIADWGEAGEIDLLDFFAELTIY
TSSACLIGKKFRDQLDGRFAKLYHELERGTDPLAYVDPYLPIESFRRRDEARNGLVALVADIMNGRIANPPTD
KSDRDMLDVLIAVKAETGTPRFSADEITGMFISMMFAGHHTSSGTASWTLIELMRHRDAYAAVIDELDELYGD
GRSVSFHALRQIPQLENVLKETLRLHPPLIILMRVAKGEFEVQGHRIHEGDLVAASPAISNRIPEDFPDPHDF
VPARYEQPRQEDLLNRWTWIPFGAGRHRCVGAAFAIMQIKAIFSVLLREYEFEMAQPPESYRNDHSKMVVQLA
QPACVRYRRRTGV

>CYP51B1_Mycobacterium tuberculosis MD17656 2622134022
MSAVALPRVSGGHDEHGHLEEFRTDPIGLMQRVRDECGDVGTFQLAGKQVVLLSGSHANEFFFRAGDDDLDQA
KAYPFMTPIFGEGVVFDASPERRKEMLHNAALRGEQMKGHAATIEDQVRRMIADWGEAGEIDLLDFFAELTIY
TSSACLIGKKFRDQLDGRFAKLYHELERGTDPLAYVDPYLPIESFRRRDEARNGLVALVADIMNGRIANPPTD
KSDRDMLDVLIAVKAETGTPRFSADEITGMFISMMFAGHHTSSGTASWTLIELMRHRDAYAAVIDELDELYGD
GRSVSFHALRQIPQLENVLKETLRLHPPLIILMRVAKGEFEVQGHRIHEGDLVAASPAISNRIPEDFPDPHDF
VPARYEQPRQEDLLNRWTWIPFGAGRHRCVGAAFAIMQIKAIFSVLLREYEFEMAQPPESYRNDHSKMVVQLA
QPACVRYRRRTGV
>CYP51B1_Mycobacterium tuberculosis MD17748 2622036915
MSAVALPRVSGGHDEHGHLEEFRTDPIGLMQRVRDECGDVGTFQLAGKQVVLLSGSHANEFFFRAGDDDLDQA
KAYPFMTPIFGEGVVFDASPERRKEMLHNAALRGEQMKGHAATIEDQVRRMIADWGEAGEIDLLDFFAELTIY
TSSACLIGKKFRDQLDGRFAKLYHELERGTDPLAYVDPYLPIESFRRRDEARNGLVALVADIMNGRIANPPTD
KSDRDMLDVLIAVKAETGTPRFSADEITGMFISMMFAGHHTSSGTASWTLIELMRHRDAYAAVIDELDELYGD
GRSVSFHALRQIPQLENVLKETLRLHPPLIILMRVAKGEFEVQGHRIHEGDLVAASPAISNRIPEDFPDPHDF
VPARYEQPRQEDLLNRWTWIPFGAGRHRCVGAAFAIMQIKAIFSVLLREYEFEMAQPPESYRNDHSKMVVQLA
QPACVRYRRRTGV
>CYP51B1_Mycobacterium tuberculosis MD17749 2622128201
MSAVALPRVSGGHDEHGHLEEFRTDPIGLMQRVRDECGDVGTFQLAGKQVVLLSGSHANEFFFRAGDDDLDQA
KAYPFMTPIFGEGVVFDASPERRKEMLHNAALRGEQMKGHAATIEDQVRRMIADWGEAGEIDLLDFFAELTIY
TSSACLIGKKFRDQLDGRFAKLYHELERGTDPLAYVDPYLPIESFRRRDEARNGLVALVADIMNGRIANPPTD
KSDRDMLDVLIAVKAETGTPRFSADEITGMFISMMFAGHHTSSGTASWTLIELMRHRDAYAAVIDELDELYGD
GRSVSFHALRQIPQLENVLKETLRLHPPLIILMRVAKGEFEVQGHRIHEGDLVAASPAISNRIPEDFPDPHDF
VPARYEQPRQEDLLNRWTWIPFGAGRHRCVGAAFAIMQIKAIFSVLLREYEFEMAQPPESYRNDHSKMVVQLA
QPACVRYRRRTGV
>CYP51B1_Mycobacterium tuberculosis MD17888 2575087562
MSAVALPRVSGGHDEHGHLEEFRTDPIGLMQRVRDECGDVGTFQLAGKQVVLLSGSHANEFFFRAGDDDLDQA
KAYPFMTPIFGEGVVFDASPERRKEMLHNAALRGEQMKGHAATIEDQVRRMIADWGEAGEIDLLDFFAELTIY
TSSACLIGKKFRDQLDGRFAKLYHELERGTDPLAYVDPYLPIESFRRRDEARNGLVALVADIMNGRIANPPTD
KSDRDMLDVLIAVKAETGTPRFSADEITGMFISMMFAGHHTSSGTASWTLIELMRHRDAYAAVIDELDELYGD
GRSVSFHALRQIPQLENVLKETLRLHPPLIILMRVAKGEFEVQGHRIHEGDLVAASPAISNRIPEDFPDPHDF
VPARYEQPRQEDLLNRWTWIPFGAGRHRCVGAAFAIMQIKAIFSVLLREYEFEMAQPPESYRNDHSKMVVQLA
QPACVRYRRRTGV
>CYP51B1_Mycobacterium tuberculosis MD17890 2633506032
MSAVALPRVSGGHDEHGHLEEFRTDPIGLMQRVRDECGDVGTFQLAGKQVVLLSGSHANEFFFRAGDDDLDQA
KAYPFMTPIFGEGVVFDASPERRKEMLHNAALRGEQMKGHAATIEDQVRRMIADWGEAGEIDLLDFFAELTIY
TSSACLIGKKFRDQLDGRFAKLYHELERGTDPLAYVDPYLPIESFRRRDEARNGLVALVADIMNGRIANPPTD
KSDRDMLDVLIAVKAETGTPRFSADEITGMFISMMFAGHHTSSGTASWTLIELMRHRDAYAAVIDELDELYGD
GRSVSFHALRQIPQLENVLKETLRLHPPLIILMRVAKGEFEVQGHRIHEGDLVAASPAISNRIPEDFPDPHDF
VPARYEQPRQEDLLNRWTWIPFGAGRHRCVGAAFAIMQIKAIFSVLLREYEFEMAQPPESYRNDHSKMVVQLA
QPACVRYRRRTGV
>CYP51B1_Mycobacterium tuberculosis MD17898 2622003850
MSAVALPRVSGGHDEHGHLEEFRTDPIGLMQRVRDECGDVGTFQLAGKQVVLLSGSHANEFFFRAGDDDLDQA
KAYPFMTPIFGEGVVFDASPERRKEMLHNAALRGEQMKGHAATIEDQVRRMIADWGEAGEIDLLDFFAELTIY
TSSACLIGKKFRDQLDGRFAKLYHELERGTDPLAYVDPYLPIESFRRRDEARNGLVALVADIMNGRIANPPTD
KSDRDMLDVLIAVKAETGTPRFSADEITGMFISMMFAGHHTSSGTASWTLIELMRHRDAYAAVIDELDELYGD
GRSVSFHALRQIPQLENVLKETLRLHPPLIILMRVAKGEFEVQGHRIHEGDLVAASPAISNRIPEDFPDPHDF
VPARYEQPRQEDLLNRWTWIPFGAGRHRCVGAAFAIMQIKAIFSVLLREYEFEMAQPPESYRNDHSKMVVQLA
QPACVRYRRRTGV
>CYP51B1_Mycobacterium tuberculosis MD17901 2631968794
MSAVALPRVSGGHDEHGHLEEFRTDPIGLMQRVRDECGDVGTFQLAGKQVVLLSGSHANEFFFRAGDDDLDQA
KAYPFMTPIFGEGVVFDASPERRKEMLHNAALRGEQMKGHAATIEDQVRRMIADWGEAGEIDLLDFFAELTIY
TSSACLIGKKFRDQLDGRFAKLYHELERGTDPLAYVDPYLPIESFRRRDEARNGLVALVADIMNGRIANPPTD
KSDRDMLDVLIAVKAETGTPRFSADEITGMFISMMFAGHHTSSGTASWTLIELMRHRDAYAAVIDELDELYGD
GRSVSFHALRQIPQLENVLKETLRLHPPLIILMRVAKGEFEVQGHRIHEGDLVAASPAISNRIPEDFPDPHDF
VPARYEQPRQEDLLNRWTWIPFGAGRHRCVGAAFAIMQIKAIFSVLLREYEFEMAQPPESYRNDHSKMVVQLA
QPACVRYRRRTGV
>CYP51B1_Mycobacterium tuberculosis MD17902 2575140965

MSAVALPRVSGGHDEHGHLEEFRTDPIGLMQRVRDECGDVGTFQLAGKQVVLLSGSHANEFFFRAGDDDLDQA
KAYPFMTPIFGEGVVFDASPERRKEMLHNAALRGEQMKGHAATIEDQVRRMIADWGEAGEIDLLDFFAELTIY
TSSACLIGKKFRDQLDGRFAKLYHELERGTDPLAYVDPYLPIESFRRRDEARNGLVALVADIMNGRIANPPTD
KSDRDMLDVLIAVKAETGTPRFSADEITGMFISMMFAGHHTSSGTASWTLIELMRHRDAYAAVIDELDELYGD
GRSVSFHALRQIPQLENVLKETLRLHPPLIILMRVAKGEFEVQGHRIHEGDLVAASPAISNRIPEDFPDPHDF
VPARYEQPRQEDLLNRWTWIPFGAGRHRCVGAAFAIMQIKAIFSVLLREYEFEMAQPPESYRNDHSKMVVQLA
QPACVRYRRRTGV
>CYP51B1_Mycobacterium tuberculosis MD17903 2622076802
MSAVALPRVSGGHDEHGHLEEFRTDPIGLMQRVRDECGDVGTFQLAGKQVVLLSGSHANEFFFRAGDDDLDQA
KAYPFMTPIFGEGVVFDASPERRKEMLHNAALRGEQMKGHAATIEDQVRRMIADWGEAGEIDLLDFFAELTIY
TSSACLIGKKFRDQLDGRFAKLYHELERGTDPLAYVDPYLPIESFRRRDEARNGLVALVADIMNGRIANPPTD
KSDRDMLDVLIAVKAETGTPRFSADEITGMFISMMFAGHHTSSGTASWTLIELMRHRDAYAAVIDELDELYGD
GRSVSFHALRQIPQLENVLKETLRLHPPLIILMRVAKGEFEVQGHRIHEGDLVAASPAISNRIPEDFPDPHDF
VPARYEQPRQEDLLNRWTWIPFGAGRHRCVGAAFAIMQIKAIFSVLLREYEFEMAQPPESYRNDHSKMVVQLA
QPACVRYRRRTGV
>CYP51B1_Mycobacterium tuberculosis MD17904 2622007920
MSAVALPRVSGGHDEHGHLEEFRTDPIGLMQRVRDECGDVGTFQLAGKQVVLLSGSHANEFFFRAGDDDLDQA
KAYPFMTPIFGEGVVFDASPERRKEMLHNAALRGEQMKGHAATIEDQVRRMIADWGEAGEIDLLDFFAELTIY
TSSACLIGKKFRDQLDGRFAKLYHELERGTDPLAYVDPYLPIESFRRRDEARNGLVALVADIMNGRIANPPTD
KSDRDMLDVLIAVKAETGTPRFSADEITGMFISMMFAGHHTSSGTASWTLIELMRHRDAYAAVIDELDELYGD
GRSVSFHALRQIPQLENVLKETLRLHPPLIILMRVAKGEFEVQGHRIHEGDLVAASPAISNRIPEDFPDPHDF
VPARYEQPRQEDLLNRWTWIPFGAGRHRCVGAAFAIMQIKAIFSVLLREYEFEMAQPPESYRNDHSKMVVQLA
QPACVRYRRRTGV
>CYP51B1_Mycobacterium tuberculosis MD17973 2622070099
MSAVALPRVSGGHDEHGHLEEFRTDPIGLMQRVRDECGDVGTFQLAGKQVVLLSGSHANEFFFRAGDDDLDQA
KAYPFMTPIFGEGVVFDASPERRKEMLHNAALRGEQMKGHAATIEDQVRRMIADWGEAGEIDLLDFFAELTIY
TSSACLIGKKFRDQLDGRFAKLYHELERGTDPLAYVDPYLPIESFRRRDEARNGLVALVADIMNGRIANPPTD
KSDRDMLDVLIAVKAETGTPRFSADEITGMFISMMFAGHHTSSGTASWTLIELMRHRDAYAAVIDELDELYGD
GRSVSFHALRQIPQLENVLKETLRLHPPLIILMRVAKGEFEVQGHRIHEGDLVAASPAISNRIPEDFPDPHDF
VPARYEQPRQEDLLNRWTWIPFGAGRHRCVGAAFAIMQIKAIFSVLLREYEFEMAQPPESYRNDHSKMVVQLA
QPACVRYRRRTGV
>CYP51B1_Mycobacterium tuberculosis MD18090 2622091020
MSAVALPRVSGGHDEHGHLEEFRTDPIGLMQRVRDECGDVGTFQLAGKQVVLLSGSHANEFFFRAGDDDLDQA
KAYPFMTPIFGEGVVFDASPERRKEMLHNAALRGEQMKGHAATIEDQVRRMIADWGEAGEIDLLDFFAELTIY
TSSACLIGKKFRDQLDGRFAKLYHELERGTDPLAYVDPYLPIESFRRRDEARNGLVALVADIMNGRIANPPTD
KSDRDMLDVLIAVKAETGTPRFSADEITGMFISMMFAGHHTSSGTASWTLIELMRHRDAYAAVIDELDELYGD
GRSVSFHALRQIPQLENVLKETLRLHPPLIILMRVAKGEFEVQGHRIHEGDLVAASPAISNRIPEDFPDPHDF
VPARYEQPRQEDLLNRWTWIPFGAGRHRCVGAAFAIMQIKAIFSVLLREYEFEMAQPPESYRNDHSKMVVQLA
QPACVRYRRRTGV
>CYP51B1_Mycobacterium tuberculosis MD18096 2576276054
MSAVALPRVSGGHDEHGHLEEFRTDPIGLMQRVRDECGDVGTFQLAGKQVVLLSGSHANEFFFRAGDDDLDQA
KAYPFMTPIFGEGVVFDASPERRKEMLHNAALRGEQMKGHAATIEDQVRRMIADWGEAGEIDLLDFFAELTIY
TSSACLIGKKFRDQLDGRFAKLYHELERGTDPLAYVDPYLPIESFRRRDEARNGLVALVADIMNGRIANPPTD
KSDRDMLDVLIAVKAETGTPRFSADEITGMFISMMFAGHHTSSGTASWTLIELMRHRDAYAAVIDELDELYGD
GRSVSFHALRQIPQLENVLKETLRLHPPLIILMRVAKGEFEVQGHRIHEGDLVAASPAISNRIPEDFPDPHDF
VPARYEQPRQEDLLNRWTWIPFGAGRHRCVGAAFAIMQIKAIFSVLLREYEFEMAQPPESYRNDHSKMVVQLA
QPACVRYRRRTGV
>CYP51B1_Mycobacterium tuberculosis MD18112 2622074592
MSAVALPRVSGGHDEHGHLEEFRTDPIGLMQRVRDECGDVGTFQLAGKQVVLLSGSHANEFFFRAGDDDLDQA
KAYPFMTPIFGEGVVFDASPERRKEMLHNAALRGEQMKGHAATIEDQVRRMIADWGEAGEIDLLDFFAELTIY
TSSACLIGKKFRDQLDGRFAKLYHELERGTDPLAYVDPYLPIESFRRRDEARNGLVALVADIMNGRIANPPTD
KSDRDMLDVLIAVKAETGTPRFSADEITGMFISMMFAGHHTSSGTASWTLIELMRHRDAYAAVIDELDELYGD
GRSVSFHALRQIPQLENVLKETLRLHPPLIILMRVAKGEFEVQGHRIHEGDLVAASPAISNRIPEDFPDPHDF
VPARYEQPRQEDLLNRWTWIPFGAGRHRCVGAAFAIMQIKAIFSVLLREYEFEMAQPPESYRNDHSKMVVQLA
QPACVRYRRRTGV
>CYP51B1_Mycobacterium tuberculosis MD18115 2631906331
MSAVALPRVSGGHDEHGHLEEFRTDPIGLMQRVRDECGDVGTFQLAGKQVVLLSGSHANEFFFRAGDDDLDQA
KAYPFMTPIFGEGVVFDASPERRKEMLHNAALRGEQMKGHAATIEDQVRRMIADWGEAGEIDLLDFFAELTIY

TSSACLIGKKFRDQLDGRFAKLYHELERGTDPLAYVDPYLPIESFRRRDEARNGLVALVADIMNGRIANPPTD
KSDRDMLDVLIAVKAETGTPRFSADEITGMFISMMFAGHHTSSGTASWTLIELMRHRDAYAAVIDELDELYGD
GRSVSFHALRQIPQLENVLKETLRLHPPLIILMRVAKGEFEVQGHRIHEGDLVAASPAISNRIPEDFPDPHDF
VPARYEQPRQEDLLNRWTWIPFGAGRHRCVGAAFAIMQIKAIFSVLLREYEFEMAQPPESYRNDHSKMVVQLA
QPACVRYRRRTGV
>CYP51B1_Mycobacterium tuberculosis MD18116 2622086429
MSAVALPRVSGGHDEHGHLEEFRTDPIGLMQRVRDECGDVGTFQLAGKQVVLLSGSHANEFFFRAGDDDLDQA
KAYPFMTPIFGEGVVFDASPERRKEMLHNAALRGEQMKGHAATIEDQVRRMIADWGEAGEIDLLDFFAELTIY
TSSACLIGKKFRDQLDGRFAKLYHELERGTDPLAYVDPYLPIESFRRRDEARNGLVALVADIMNGRIANPPTD
KSDRDMLDVLIAVKAETGTPRFSADEITGMFISMMFAGHHTSSGTASWTLIELMRHRDAYAAVIDELDELYGD
GRSVSFHALRQIPQLENVLKETLRLHPPLIILMRVAKGEFEVQGHRIHEGDLVAASPAISNRIPEDFPDPHDF
VPARYEQPRQEDLLNRWTWIPFGAGRHRCVGAAFAIMQIKAIFSVLLREYEFEMAQPPESYRNDHSKMVVQLA
QPACVRYRRRTGV
>CYP51B1_Mycobacterium tuberculosis MD18314 2622095069
MSAVALPRVSGGHDEHGHLEEFRTDPIGLMQRVRDECGDVGTFQLAGKQVVLLSGSHANEFFFRAGDDDLDQA
KAYPFMTPIFGEGVVFDASPERRKEMLHNAALRGEQMKGHAATIEDQVRRMIADWGEAGEIDLLDFFAELTIY
TSSACLIGKKFRDQLDGRFAKLYHELERGTDPLAYVDPYLPIESFRRRDEARNGLVALVADIMNGRIANPPTD
KSDRDMLDVLIAVKAETGTPRFSADEITGMFISMMFAGHHTSSGTASWTLIELMRHRDAYAAVIDELDELYGD
GRSVSFHALRQIPQLENVLKETLRLHPPLIILMRVAKGEFEVQGHRIHEGDLVAASPAISNRIPEDFPDPHDF
VPARYEQPRQEDLLNRWTWIPFGAGRHRCVGAAFAIMQIKAIFSVLLREYEFEMAQPPESYRNDHSKMVVQLA
QPACVRYRRRTGV
>CYP51B1_Mycobacterium tuberculosis MD18320 2622012166
MSAVALPRVSGGHDEHGHLEEFRTDPIGLMQRVRDECGDVGTFQLAGKQVVLLSGSHANEFFFRAGDDDLDQA
KAYPFMTPIFGEGVVFDASPERRKEMLHNAALRGEQMKGHAATIEDQVRRMIADWGEAGEIDLLDFFAELTIY
TSSACLIGKKFRDQLDGRFAKLYHELERGTDPLAYVDPYLPIESFRRRDEARNGLVALVADIMNGRIANPPTD
KSDRDMLDVLIAVKAETGTPRFSADEITGMFISMMFAGHHTSSGTASWTLIELMRHRDAYAAVIDELDELYGD
GRSVSFHALRQIPQLENVLKETLRLHPPLIILMRVAKGEFEVQGHRIHEGDLVAASPAISNRIPEDFPDPHDF
VPARYEQPRQEDLLNRWTWIPFGAGRHRCVGAAFAIMQIKAIFSVLLREYEFEMAQPPESYRNDHSKMVVQLA
QPACVRYRRRTGV
>CYP51B1_Mycobacterium tuberculosis MD18472 2622053636
MSAVALPRVSGGHDEHGHLEEFRTDPIGLMQRVRDECGDVGTFQLAGKQVVLLSGSHANEFFFRAGDDDLDQA
KAYPFMTPIFGEGVVFDASPERRKEMLHNAALRGEQMKGHAATIEDQVRRMIADWGEAGEIDLLDFFAELTIY
TSSACLIGKKFRDQLDGRFAKLYHELERGTDPLAYVDPYLPIESFRRRDEARNGLVALVADIMNGRIANPPTD
KSDRDMLDVLIAVKAETGTPRFSADEITGMFISMMFAGHHTSSGTASWTLIELMRHRDAYAAVIDELDELYGD
GRSVSFHALRQIPQLENVLKETLRLHPPLIILMRVAKGEFEVQGHRIHEGDLVAASPAISNRIPEDFPDPHDF
VPARYEQPRQEDLLNRWTWIPFGAGRHRCVGAAFAIMQIKAIFSVLLREYEFEMAQPPESYRNDHSKMVVQLA
QPACVRYRRRTGV
>CYP51B1_Mycobacterium tuberculosis MD18478 2577882333
MSAVALPRVSGGHDEHGHLEEFRTDPIGLMQRVRDECGDVGTFQLAGKQVVLLSGSHANEFFFRAGDDDLDQA
KAYPFMTPIFGEGVVFDASPERRKEMLHNAALRGEQMKGHAATIEDQVRRMIADWGEAGEIDLLDFFAELTIY
TSSACLIGKKFRDQLDGRFAKLYHELERGTDPLAYVDPYLPIESFRRRDEARNGLVALVADIMNGRIANPPTD
KSDRDMLDVLIAVKAETGTPRFSADEITGMFISMMFAGHHTSSGTASWTLIELMRHRDAYAAVIDELDELYGD
GRSVSFHALRQIPQLENVLKETLRLHPPLIILMRVAKGEFEVQGHRIHEGDLVAASPAISNRIPEDFPDPHDF
VPARYEQPRQEDLLNRWTWIPFGAGRHRCVGAAFAIMQIKAIFSVLLREYEFEMAQPPESYRNDHSKMVVQLA
QPACVRYRRRTGV
>CYP51B1_Mycobacterium tuberculosis MD18486 2622014018
MSAVALPRVSGGHDEHGHLEEFRTDPIGLMQRVRDECGDVGTFQLAGKQVVLLSGSHANEFFFRAGDDDLDQA
KAYPFMTPIFGEGVVFDASPERRKEMLHNAALRGEQMKGHAATIEDQVRRMIADWGEAGEIDLLDFFAELTIY
TSSACLIGKKFRDQLDGRFAKLYHELERGTDPLAYVDPYLPIESFRRRDEARNGLVALVADIMNGRIANPPTD
KSDRDMLDVLIAVKAETGTPRFSADEITGMFISMMFAGHHTSSGTASWTLIELMRHRDAYAAVIDELDELYGD
GRSVSFHALRQIPQLENVLKETLRLHPPLIILMRVAKGEFEVQGHRIHEGDLVAASPAISNRIPEDFPDPHDF
VPARYEQPRQEDLLNRWTWIPFGAGRHRCVGAAFAIMQIKAIFSVLLREYEFEMAQPPESYRNDHSKMVVQLA
QPACVRYRRRTGV
>CYP51B1_Mycobacterium tuberculosis MD18489 2622020252
MSAVALPRVSGGHDEHGHLEEFRTDPIGLMQRVRDECGDVGTFQLAGKQVVLLSGSHANEFFFRAGDDDLDQA
KAYPFMTPIFGEGVVFDASPERRKEMLHNAALRGEQMKGHAATIEDQVRRMIADWGEAGEIDLLDFFAELTIY
TSSACLIGKKFRDQLDGRFAKLYHELERGTDPLAYVDPYLPIESFRRRDEARNGLVALVADIMNGRIANPPTD
KSDRDMLDVLIAVKAETGTPRFSADEITGMFISMMFAGHHTSSGTASWTLIELMRHRDAYAAVIDELDELYGD

GRSVSFHALRQIPQLENVLKETLRLHPPLIILMRVAKGEFEVQGHRIHEGDLVAASPAISNRIPEDFPDPHDF
VPARYEQPRQEDLLNRWTWIPFGAGRHRCVGAAFAIMQIKAIFSVLLREYEFEMAQPPESYRNDHSKMVVQLA
QPACVRYRRRTGV
>CYP51B1_Mycobacterium tuberculosis MD18498 2574990235
MSAVALPRVSGGHDEHGHLEEFRTDPIGLMQRVRDECGDVGTFQLAGKQVVLLSGSHANEFFFRAGDDDLDQA
KAYPFMTPIFGEGVVFDASPERRKEMLHNAALRGEQMKGHAATIEDQVRRMIADWGEAGEIDLLDFFAELTIY
TSSACLIGKKFRDQLDGRFAKLYHELERGTDPLAYVDPYLPIESFRRRDEARNGLVALVADIMNGRIANPPTD
KSDRDMLDVLIAVKAETGTPRFSADEITGMFISMMFAGHHTSSGTASWTLIELMRHRDAYAAVIDELDELYGD
GRSVSFHALRQIPQLENVLKETLRLHPPLIILMRVAKGEFEVQGHRIHEGDLVAASPAISNRIPEDFPDPHDF
VPARYEQPRQEDLLNRWTWIPFGAGRHRCVGAAFAIMQIKAIFSVLLREYEFEMAQPPESYRNDHSKMVVQLA
QPACVRYRRRTGV
>CYP51B1_Mycobacterium tuberculosis MD19042 2622026097
MSAVALPRVSGGHDEHGHLEEFRTDPIGLMQRVRDECGDVGTFQLAGKQVVLLSGSHANEFFFRAGDDDLDQA
KAYPFMTPIFGEGVVFDASPERRKEMLHNAALRGEQMKGHAATIEDQVRRMIADWGEAGEIDLLDFFAELTIY
TSSACLIGKKFRDQLDGRFAKLYHELERGTDPLAYVDPYLPIESFRRRDEARNGLVALVADIMNGRIANPPTD
KSDRDMLDVLIAVKAETGTPRFSADEITGMFISMMFAGHHTSSGTASWTLIELMRHRDAYAAVIDELDELYGD
GRSVSFHALRQIPQLENVLKETLRLHPPLIILMRVAKGEFEVQGHRIHEGDLVAASPAISNRIPEDFPDPHDF
VPARYEQPRQEDLLNRWTWIPFGAGRHRCVGAAFAIMQIKAIFSVLLREYEFEMAQPPESYRNDHSKMVVQLA
QPACVRYRRRTGV
>CYP51B1_Mycobacterium tuberculosis MD19043 2574763670
MSAVALPRVSGGHDEHGHLEEFRTDPIGLMQRVRDECGDVGTFQLAGKQVVLLSGSHANEFFFRAGDDDLDQA
KAYPFMTPIFGEGVVFDASPERRKEMLHNAALRGEQMKGHAATIEDQVRRMIADWGEAGEIDLLDFFAELTIY
TSSACLIGKKFRDQLDGRFAKLYHELERGTDPLAYVDPYLPIESFRRRDEARNGLVALVADIMNGRIANPPTD
KSDRDMLDVLIAVKAETGTPRFSADEITGMFISMMFAGHHTSSGTASWTLIELMRHRDAYAAVIDELDELYGD
GRSVSFHALRQIPQLENVLKETLRLHPPLIILMRVAKGEFEVQGHRIHEGDLVAASPAISNRIPEDFPDPHDF
VPARYEQPRQEDLLNRWTWIPFGAGRHRCVGAAFAIMQIKAIFSVLLREYEFEMAQPPESYRNDHSKMVVQLA
QPACVRYRRRTGV
>CYP51B1_Mycobacterium tuberculosis MD19051 2622062037
MSAVALPRVSGGHDEHGHLEEFRTDPIGLMQRVRDECGDVGTFQLAGKQVVLLSGSHANEFFFRAGDDDLDQA
KAYPFMTPIFGEGVVFDASPERRKEMLHNAALRGEQMKGHAATIEDQVRRMIADWGEAGEIDLLDFFAELTIY
TSSACLIGKKFRDQLDGRFAKLYHELERGTDPLAYVDPYLPIESFRRRDEARNGLVALVADIMNGRIANPPTD
KSDRDMLDVLIAVKAETGTPRFSADEITGMFISMMFAGHHTSSGTASWTLIELMRHRDAYAAVIDELDELYGD
GRSVSFHALRQIPQLENVLKETLRLHPPLIILMRVAKGEFEVQGHRIHEGDLVAASPAISNRIPEDFPDPHDF
VPARYEQPRQEDLLNRWTWIPFGAGRHRCVGAAFAIMQIKAIFSVLLREYEFEMAQPPESYRNDHSKMVVQLA
QPACVRYRRRTGV
>CYP51B1_Mycobacterium tuberculosis MD19323 2622057776
MSAVALPRVSGGHDEHGHLEEFRTDPIGLMQRVRDECGDVGTFQLAGKQVVLLSGSHANEFFFRAGDDDLDQA
KAYPFMTPIFGEGVVFDASPERRKEMLHNAALRGEQMKGHAATIEDQVRRMIADWGEAGEIDLLDFFAELTIY
TSSACLIGKKFRDQLDGRFAKLYHELERGTDPLAYVDPYLPIESFRRRDEARNGLVALVADIMNGRIANPPTD
KSDRDMLDVLIAVKAETGTPRFSADEITGMFISMMFAGHHTSSGTASWTLIELMRHRDAYAAVIDELDELYGD
GRSVSFHALRQIPQLENVLKETLRLHPPLIILMRVAKGEFEVQGHRIHEGDLVAASPAISNRIPEDFPDPHDF
VPARYEQPRQEDLLNRWTWIPFGAGRHRCVGAAFAIMQIKAIFSVLLREYEFEMAQPPESYRNDHSKMVVQLA
QPACVRYRRRTGV
>CYP51B1_Mycobacterium tuberculosis MD19339 2622045019
MSAVALPRVSGGHDEHGHLEEFRTDPIGLMQRVRDECGDVGTFQLAGKQVVLLSGSHANEFFFRAGDDDLDQA
KAYPFMTPIFGEGVVFDASPERRKEMLHNAALRGEQMKGHAATIEDQVRRMIADWGEAGEIDLLDFFAELTIY
TSSACLIGKKFRDQLDGRFAKLYHELERGTDPLAYVDPYLPIESFRRRDEARNGLVALVADIMNGRIANPPTD
KSDRDMLDVLIAVKAETGTPRFSADEITGMFISMMFAGHHTSSGTASWTLIELMRHRDAYAAVIDELDELYGD
GRSVSFHALRQIPQLENVLKETLRLHPPLIILMRVAKGEFEVQGHRIHEGDLVAASPAISNRIPEDFPDPHDF
VPARYEQPRQEDLLNRWTWIPFGAGRHRCVGAAFAIMQIKAIFSVLLREYEFEMAQPPESYRNDHSKMVVQLA
QPACVRYRRRTGV
>CYP51B1_Mycobacterium tuberculosis MD19591 2622049553
MSAVALPRVSGGHDEHGHLEEFRTDPIGLMQRVRDECGDVGTFQLAGKQVVLLSGSHANEFFFRAGDDDLDQA
KAYPFMTPIFGEGVVFDASPERRKEMLHNAALRGEQMKGHAATIEDQVRRMIADWGEAGEIDLLDFFAELTIY
TSSACLIGKKFRDQLDGRFAKLYHELERGTDPLAYVDPYLPIESFRRRDEARNGLVALVADIMNGRIANPPTD
KSDRDMLDVLIAVKAETGTPRFSADEITGMFISMMFAGHHTSSGTASWTLIELMRHRDAYAAVIDELDELYGD
GRSVSFHALRQIPQLENVLKETLRLHPPLIILMRVAKGEFEVQGHRIHEGDLVAASPAISNRIPEDFPDPHDF

VPARYEQPRQEDLLNRWTWIPFGAGRHRCVGAAFAIMQIKAIFSVLLREYEFEMAQPPESYRNDHSKMVVQLA
QPACVRYRRRTGV
>CYP51B1_Mycobacterium tuberculosis MD19693 2622024575
MSAVALPRVSGGHDEHGHLEEFRTDPIGLMQRVRDECGDVGTFQLAGKQVVLLSGSHANEFFFRAGDDDLDQA
KAYPFMTPIFGEGVVFDASPERRKEMLHNAALRGEQMKGHAATIEDQVRRMIADWGEAGEIDLLDFFAELTIY
TSSACLIGKKFRDQLDGRFAKLYHELERGTDPLAYVDPYLPIESFRRRDEARNGLVALVADIMNGRIANPPTD
KSDRDMLDVLIAVKAETGTPRFSADEITGMFISMMFAGHHTSSGTASWTLIELMRHRDAYAAVIDELDELYGD
GRSVSFHALRQIPQLENVLKETLRLHPPLIILMRVAKGEFEVQGHRIHEGDLVAASPAISNRIPEDFPDPHDF
VPARYEQPRQEDLLNRWTWIPFGAGRHRCVGAAFAIMQIKAIFSVLLREYEFEMAQPPESYRNDHSKMVVQLA
QPACVRYRRRTGV
>CYP51B1_Mycobacterium tuberculosis MD19964 2575187686
MSAVALPRVSGGHDEHGHLEEFRTDPIGLMQRVRDECGDVGTFQLAGKQVVLLSGSHANEFFFRAGDDDLDQA
KAYPFMTPIFGEGVVFDASPERRKEMLHNAALRGEQMKGHAATIEDQVRRMIADWGEAGEIDLLDFFAELTIY
TSSACLIGKKFRDQLDGRFAKLYHELERGTDPLAYVDPYLPIESFRRRDEARNGLVALVADIMNGRIANPPTD
KSDRDMLDVLIAVKAETGTPRFSADEITGMFISMMFAGHHTSSGTASWTLIELMRHRDAYAAVIDELDELYGD
GRSVSFHALRQIPQLENVLKETLRLHPPLIILMRVAKGEFEVQGHRIHEGDLVAASPAISNRIPEDFPDPHDF
VPARYEQPRQEDLLNRWTWIPFGAGRHRCVGAAFAIMQIKAIFSVLLREYEFEMAQPPESYRNDHSKMVVQLA
QPACVRYRRRTGV
>CYP51B1_Mycobacterium tuberculosis MD20344 2577510709
MSAVALPRVSGGHDEHGHLEEFRTDPIGLMQRVRDECGDVGTFQLAGKQVVLLSGSHANEFFFRAGDDDLDQA
KAYPFMTPIFGEGVVFDASPERRKEMLHNAALRGEQMKGHAATIEDQVRRMIADWGEAGEIDLLDFFAELTIY
TSSACLIGKKFRDQLDGRFAKLYHELERGTDPLAYVDPYLPIESFRRRDEARNGLVALVADIMNGRIANPPTD
KSDRDMLDVLIAVKAETGTPRFSADEITGMFISMMFAGHHTSSGTASWTLIELMRHRDAYAAVIDELDELYGD
GRSVSFHALRQIPQLENVLKETLRLHPPLIILMRVAKGEFEVQGHRIHEGDLVAASPAISNRIPEDFPDPHDF
VPARYEQPRQEDLLNRWTWIPFGAGRHRCVGAAFAIMQIKAIFSVLLREYEFEMAQPPESYRNDHSKMVVQLA
QPACVRYRRRTGV
>CYP51B1_Mycobacterium tuberculosis MD20845 2622041242
MSAVALPRVSGGHDEHGHLEEFRTDPIGLMQRVRDECGDVGTFQLAGKQVVLLSGSHANEFFFRAGDDDLDQA
KAYPFMTPIFGEGVVFDASPERRKEMLHNAALRGEQMKGHAATIEDQVRRMIADWGEAGEIDLLDFFAELTIY
TSSACLIGKKFRDQLDGRFAKLYHELERGTDPLAYVDPYLPIESFRRRDEARNGLVALVADIMNGRIANPPTD
KSDRDMLDVLIAVKAETGTPRFSADEITGMFISMMFAGHHTSSGTASWTLIELMRHRDAYAAVIDELDELYGD
GRSVSFHALRQIPQLENVLKETLRLHPPLIILMRVAKGEFEVQGHRIHEGDLVAASPAISNRIPEDFPDPHDF
VPARYEQPRQEDLLNRWTWIPFGAGRHRCVGAAFAIMQIKAIFSVLLREYEFEMAQPPESYRNDHSKMVVQLA
QPACVRYRRRTGV
>CYP51B1_Mycobacterium tuberculosis MT433 2646889934
MSAVALPRVSGGHDEHGHLEEFRTDPIGLMQRVRDECGDVGTFQLAGKQVVLLSGSHANEFFFRAGDDDLDQA
KAYPFMTPIFGEGVVFDASPERRKEMLHNAALRGEQMKGHAATIEDQVRRMIADWGEAGEIDLLDFFAELTIY
TSSACLIGKKFRDQLDGRFAKLYHELERGTDPLAYVDPYLPIESFRRRDEARNGLVALVADIMNGRIANPPTD
KSDRDMLDVLIAVKAETGTPRFSADEITGMFISMMFAGHHTSSGTASWTLIELMRHRDAYAAVIDELDELYGD
GRSVSFHALRQIPQLENVLKETLRLHPPLIILMRVAKGEFEVQGHRIHEGDLVAASPAISNRIPEDFPDPHDF
VPARYEQPRQEDLLNRWTWIPFGAGRHRCVGAAFAIMQIKAIFSVLLREYEFEMAQPPESYRNDHSKMVVQLA
QPACVRYRRRTGV
>CYP51B1_Mycobacterium tuberculosis Mtb194 2606422546
MSAVALPRVSGGHDEHGHLEEFRTDPIGLMQRVRDECGDVGTFQLAGKQVVLLSGSHANEFFFRAGDDDLDQA
KAYPFMTPIFGEGVVFDASPERRKEMLHNAALRGEQMKGHAATIEDQVRRMIADWGEAGEIDLLDFFAELTIY
TSSACLIGKKFRDQLDGRFAKLYHELERGTDPLAYVDPYLPIESFRRRDEARNGLVALVADIMNGRIANPPTD
KSDRDMLDVLIAVKAETGTPRFSADEITGMFISMMFAGHHTSSGTASWTLIELMRHRDAYAAVIDELDELYGD
GRSVSFHALRQIPQLENVLKETLRLHPPLIILMRVAKGEFEVQGHRIHEGDLVAASPAISNRIPEDFPDPHDF
VPARYEQPRQEDLLNRWTWIPFGAGRHRCVGAAFAIMQIKAIFSVLLREYEFEMAQPPESYRNDHSKMVVQLA
QPACVRYRRRTGV
>CYP51B1_Mycobacterium tuberculosis MTB221/11 2702377313
MSAVALPRVSGGHDEHGHLEEFRTDPIGLMQRVRDECGDVGTFQLAGKQVVLLSGSHANEFFFRAGDDDLDQA
KAYPFMTPIFGEGVVFDASPERRKEMLHNAALRGEQMKGHAATIEDQVRRMIADWGEAGEIDLLDFFAELTIY
TSSACLIGKKFRDQLDGRFAKLYHELERGTDPLAYVDPYLPIESFRRRDEARNGLVALVADIMNGRIANPPTD
KSDRDMLDVLIAVKAETGTPRFSADEITGMFISMMFAGHHTSSGTASWTLIELMRHRDAYAAVIDELDELYGD
GRSVSFHALRQIPQLENVLKETLRLHPPLIILMRVAKGEFEVQGHRIHEGDLVAASPAISNRIPEDFPDPHDF
VPARYEQPRQEDLLNRWTWIPFGAGRHRCVGAAFAIMQIKAIFSVLLREYEFEMAQPPESYRNDHSKMVVQLA
QPACVRYRRRTGV

>CYP51B1_Mycobacterium tuberculosis Mtb293 2645902118
MSAVALPRVSGGHDEHGHLEEFRTDPIGLMQRVRDECGDVGTFQLAGKQVVLLSGSHANEFFFRAGDDDLDQA
KAYPFMTPIFGEGVVFDASPERRKEMLHNAALRGEQMKGHAATIEDQVRRMIADWGEAGEIDLLDFFAELTIY
TSSACLIGKKFRDQLDGRFAKLYHELERGTDPLAYVDPYLPIESFRRRDEARNGLVALVADIMNGRIANPPTD
KSDRDMLDVLIAVKAETGTPRFSADEITGMFISMMFAGHHTSSGTASWTLIELMRHRDAYAAVIDELDELYGD
GRSVSFHALRQIPQLENVLKETLRLHPPLIILMRVAKGEFEVQGHRIHEGDLVAASPAISNRIPEDFPDPHDF
VPARYEQPRQEDLLNRWTWIPFGAGRHRCVGAAFAIMQIKAIFSVLLREYEFEMAQPPESYRNDHSKMVVQLA
QPACVRYRRRTGV
>CYP51B1_Mycobacterium tuberculosis Mtb43 2654752669
MSAVALPRVSGGHDEHGHLEEFRTDPIGLMQRVRDECGDVGTFQLAGKQVVLLSGSHANEFFFRAGDDDLDQA
KAYPFMTPIFGEGVVFDASPERRKEMLHNAALRGEQMKGHAATIEDQVRRMIADWGEAGEIDLLDFFAELTIY
TSSACLIGKKFRDQLDGRFAKLYHELERGTDPLAYVDPYLPIESFRRRDEARNGLVALVADIMNGRIANPPTD
KSDRDMLDVLIAVKAETGTPRFSADEITGMFISMMFAGHHTSSGTASWTLIELMRHRDAYAAVIDELDELYGD
GRSVSFHALRQIPQLENVLKETLRLHPPLIILMRVAKGEFEVQGHRIHEGDLVAASPAISNRIPEDFPDPHDF
VPARYEQPRQEDLLNRWTWIPFGAGRHRCVGAAFAIMQIKAIFSVLLREYEFEMAQPPESYRNDHSKMVVQLA
QPACVRYRRRTGV
>CYP51B1_Mycobacterium tuberculosis MTB-476 2573575659
MSAVALPRVSGGHDEHGHLEEFRTDPIGLMQRVRDECGDVGTFQLAGKQVVLLSGSHANEFFFRAGDDDLDQA
KAYPFMTPIFGEGVVFDASPERRKEMLHNAALRGEQMKGHAATIEDQVRRMIADWGEAGEIDLLDFFAELTIY
TSSACLIGKKFRDQLDGRFAKLYHELERGTDPLAYVDPYLPIESFRRRDEARNGLVALVADIMNGRIANPPTD
KSDRDMLDVLIAVKAETGTPRFSADEITGMFISMMFAGHHTSSGTASWTLIELMRHRDAYAAVIDELDELYGD
GRSVSFHALRQIPQLENVLKETLRLHPPLIILMRVAKGEFEVQGHRIHEGDLVAASPAISNRIPEDFPDPHDF
VPARYEQPRQEDLLNRWTWIPFGAGRHRCVGAAFAIMQIKAIFSVLLREYEFEMAQPPESYRNDHSKMVVQLA
QPACVRYRRRTGV
>CYP51B1_Mycobacterium tuberculosis MTB-489 2621358175
MSAVALPRVSGGHDEHGHLEEFRTDPIGLMQRVRDECGDVGTFQLAGKQVVLLSGSHANEFFFRAGDDDLDQA
KAYPFMTPIFGEGVVFDASPERRKEMLHNAALRGEQMKGHAATIEDQVRRMIADWGEAGEIDLLDFFAELTIY
TSSACLIGKKFRDQLDGRFAKLYHELERGTDPLAYVDPYLPIESFRRRDEARNGLVALVADIMNGRIANPPTD
KSDRDMLDVLIAVKAETGTPRFSADEITGMFISMMFAGHHTSSGTASWTLIELMRHRDAYAAVIDELDELYGD
GRSVSFHALRQIPQLENVLKETLRLHPPLIILMRVAKGEFEVQGHRIHEGDLVAASPAISNRIPEDFPDPHDF
VPARYEQPRQEDLLNRWTWIPFGAGRHRCVGAAFAIMQIKAIFSVLLREYEFEMAQPPESYRNDHSKMVVQLA
QPACVRYRRRTGV
>CYP51B1_Mycobacterium tuberculosis Mtb526 2641991003
MNGRIANPPTDKSDRDMLDVLIAVKAETGTPRFSADEITGMFISMMFAGHHTSSGTASWTLIELMRHRDAYAA
VIDELDELYGDGRSVSFHALRQIPQLENVLKETLRLHPPLIILMRVAKGEFEVQGHRIHEGDLVAASPAISNR
IPEDFPDPHDFVPARYEQPRQEDLLNRWTWIPFGAGRHRCVGAAFAIMQIKAIFSVLLREYEFEMAQPPESYR
NDHSKMVVQLAQPACVRYRRRTGV
>CYP51B1_Mycobacterium tuberculosis Mtb562 2649309597
MSAVALPRVSGGHDEHGHLEEFRTDPIGLMQRVRDECGDVGTFQLAGKQVVLLSGSHANEFFFRAGDDDLDQA
KAYPFMTPIFGEGVVFDASPERRKEMLHNAALRGEQMKGHAATIEDQVRRMIADWGEAGEIDLLDFFAELTIY
TSSACLIGKKFRDQLDGRFAKLYHELERGTDPLAYVDPYLPIESFRRRDEARNGLVALVADIMNGRIANPPTD
KSDRDMLDVLIAVKAETGTPRFSADEITGMFISMMFAGHHTSSGTASWTLIELMRHRDAYAAVIDELDELYGD
GRSVSFHALRQIPQLENVLKETLRLHPPLIILMRVAKGEFEVQGHRIHEGDLVAASPAISNRIPEDFPDPHDF
VPARYEQPRQEDLLNRWTWIPFGAGRHRCVGAAFAIMQIKAIFSVLLREYEFEMAQPPESYRNDHSKMVVQLA
QPACVRYRRRTGV
>CYP51B1_Mycobacterium tuberculosis Mtb940 2606427006
MSAVALPRVSGGHDEHGHLEEFRTDPIGLMQRVRDECGDVGTFQLAGKQVVLLSGSHANEFFFRAGDDDLDQA
KAYPFMTPIFGEGVVFDASPERRKEMLHNAALRGEQMKGHAATIEDQVRRMIADWGEAGEIDLLDFFAELTIY
TSSACLIGKKFRDQLDGRFAKLYHELERGTDPLAYVDPYLPIESFRRRDEARNGLVALVADIMNGRIANPPTD
KSDRDMLDVLIAVKAETGTPRFSADEITGMFISMMFAGHHTSSGTASWTLIELMRHRDAYAAVIDELDELYGD
GRSVSFHALRQIPQLENVLKETLRLHPPLIILMRVAKGEFEVQGHRIHEGDLVAASPAISNRIPEDFPDPHDF
VPARYEQPRQEDLLNRWTWIPFGAGRHRCVGAAFAIMQIKAIFSVLLREYEFEMAQPPESYRNDHSKMVVQLA
QPACVRYRRRTGV
>CYP51B1_Mycobacterium tuberculosis Mtb984 2647498561
MSAVALPRVSGGHDEHGHLEEFRTDPIGLMQRVRDECGDVGTFQLAGKQVVLLSGSHANEFFFRAGDDDLDQA
KAYPFMTPIFGEGVVFDASPERRKEMLHNAALRGEQMKGHAATIEDQVRRMIADWGEAGEIDLLDFFAELTIY
TSSACLIGKKFRDQLDGRFAKLYHELERGTDPLAYVDPYLPIESFRRRDEARNGLVALVADIMNGRIANPPTD
KSDRDMLDVLIAVKAETGTPRFSADEITGMFISMMFAGHHTSSGTASWTLIELMRHRDAYAAVIDELDELYGD

GRSVSFHALRQIPQLENVLKETLRLHPPLIILMRVAKGEFEVQGHRIHEGDLVAASPAISNRIPEDFPDPHDF
VPARYEQPRQEDLLNRWTWIPFGAGRHRCVGAAFAIMQIKAIFSVLLREYEFEMAQPPESYRNDHSKMVVQLA
QPACVRYRRRTGV
>CYP51B1_Mycobacterium tuberculosis MTBR1/09 2667026841
MSAVALPRVSGGHDEHGHLEEFRTDPIGLMQRVRDECGDVGTFQLAGKQVVLLSGSHANEFFFRAGDDDLDQA
KAYPFMTPIFGEGVVFDASPERRKEMLHNAALRGEQMKGHAATIEDQVRRMIADWGEAGEIDLLDFFAELTIY
TSSACLIGKKFRDQLDGRFAKLYHELERGTDPLAYVDPYLPIESFRRRDEARNGLVALVADIMNGRIANPPTD
KSDRDMLDVLIAVKAETGTPRFSADEITGMFISMMFAGHHTSSGTASWTLIELMRHRDAYAAVIDELDELYGD
GRSVSFHALRQIPQLENVLKETLRLHPPLIILMRVAKGEFEVQGHRIHEGDLVAASPAISNRIPEDFPDPHDF
VPARYEQPRQEDLLNRWTWIPFGAGRHRCVGAAFAIMQIKAIFSVLLREYEFEMAQPPESYRNDHSKMVVQLA
QPACVRYRRRTGV
>CYP51B1_Mycobacterium tuberculosis MTBR2/09 2660406442
MSAVALPRVSGGHDEHGHLEEFRTDPIGLMQRVRDECGDVGTFQLAGKQVVLLSGSHANEFFFRAGDDDLDQA
KAYPFMTPIFGEGVVFDASPERRKEMLHNAALRGEQMKGHAATIEDQVRRMIADWGEAGEIDLLDFFAELTIY
TSSACLIGKKFRDQLDGRFAKLYHELERGTDPLAYVDPYLPIESFRRRDEARNGLVALVADIMNGRIANPPTD
KSDRDMLDVLIAVKAETGTPRFSADEITGMFISMMFAGHHTSSGTASWTLIELMRHRDAYAAVIDELDELYGD
GRSVSFHALRQIPQLENVLKETLRLHPPLIILMRVAKGEFEVQGHRIHEGDLVAASPAISNRIPEDFPDPHDF
VPARYEQPRQEDLLNRWTWIPFGAGRHRCVGAAFAIMQIKAIFSVLLREYEFEMAQPPESYRNDHSKMVVQLA
QPACVRYRRRTGV
>CYP51B1_Mycobacterium tuberculosis MTBR3/09 2703076544
MSAVALPRVSGGHDEHGHLEEFRTDPIGLMQRVRDECGDVGTFQLAGKQVVLLSGSHANEFFFRAGDDDLDQA
KAYPFMTPIFGEGVVFDASPERRKEMLHNAALRGEQMKGHAATIEDQVRRMIADWGEAGEIDLLDFFAELTIY
TSSACLIGKKFRDQLDGRFAKLYHELERGTDPLAYVDPYLPIESFRRRDEARNGLVALVADIMNGRIANPPTD
KSDRDMLDVLIAVKAETGTPRFSADEITGMFISMMFAGHHTSSGTASWTLIELMRHRDAYAAVIDELDELYGD
GRSVSFHALRQIPQLENVLKETLRLHPPLIILMRVAKGEFEVQGHRIHEGDLVAASPAISNRIPEDFPDPHDF
VPARYEQPRQEDLLNRWTWIPFGAGRHRCVGAAFAIMQIKAIFSVLLREYEFEMAQPPESYRNDHSKMVVQLA
QPACVRYRRRTGV
>CYP51B1_Mycobacterium tuberculosis MtURU-002 2630826359
MSAVALPRVSGGHDEHGHLEEFRTDPIGLMQRVRDECGDVGTFQLAGKQVVLLSGSHANEFFFRAGDDDLDQA
KAYPFMTPIFGEGVVFDASPERRKEMLHNAALRGEQMKGHAATIEDQVRRMIADWGEAGEIDLLDFFAELTIY
TSSACLIGKKFRDQLDGRFAKLYHELERGTDPLAYVDPYLPIESFRRRDEARNGLVALVADIMNGRIANPPTD
KSDRDMLDVLIAVKAETGTPRFSADEITGMFISMMFAGHHTSSGTASWTLIELMRHRDAYAAVIDELDELYGD
GRSVSFHALRQIPQLENVLKETLRLHPPLIILMRVAKGEFEVQGHRIHEGDLVAASPAISNRIPEDFPDPHDF
VPARYEQPRQEDLLNRWTWIPFGAGRHRCVGAAFAIMQIKAIFSVLLREYEFEMAQPPESYRNDHSKMVVQLA
QPACVRYRRRTGV
>CYP51B1_Mycobacterium tuberculosis N00001996 2706568904
MSAVALPRVSGGHDEHGHLEEFRTDPIGLMQRVRDECGDVGTFQLAGKQVVLLSGSHANEFFFRAGDDDLDQA
KAYPFMTPIFGEGVVFDASPERRKEMLHNAALRGEQMKGHAATIEDQVRRMIADWGEAGEIDLLDFFAELTIY
TSSACLIGKKFRDQLDGRFAKLYHELERGTDPLAYVDPYLPIESFRRRDEARNGLVALVADIMNGRIANPPTD
KSDRDMLDVLIAVKAETGTPRFSADEITGMFISMMFAGHHTSSGTASWTLIELMRHRDAYAAVIDELDELYGD
GRSVSFHALRQIPQLENVLKETLRLHPPLIILMRVAKGEFEVQGHRIHEGDLVAASPAISNRIPEDFPDPHDF
VPARYEQPRQEDLLNRWTWIPFGAGRHRCVGAAFAIMQIKAIFSVLLREYEFEMAQPPESYRNDHSKMVVQLA
QPACVRYRRRTGV
>CYP51B1_Mycobacterium tuberculosis N00200616 2693858725
MSAVALPRVSGGHDEHGHLEEFRTDPIGLMQRVRDECGDVGTFQLAGKQVVLLSGSHANEFFFRAGDDDLDQA
KAYPFMTPIFGEGVVFDASPERRKEMLHNAALRGEQMKGHAATIEDQVRRMIADWGEAGEIDLLDFFAELTIY
TSSACLIGKKFRDQLDGRFAKLYHELERGTDPLAYVDPYLPIESFRRRDEARNGLVALVADIMNGRIANPPTD
KSDRDMLDVLIAVKAETGTPRFSADEITGMFISMMFAGHHTSSGTASWTLIELMRHRDAYAAVIDELDELYGD
GRSVSFHALRQIPQLENVLKETLRLHPPLIILMRVAKGEFEVQGHRIHEGDLVAASPAISNRIPEDFPDPHDF
VPARYEQPRQEDLLNRWTWIPFGAGRHRCVGAAFAIMQIKAIFSVLLREYEFEMAQPPESYRNDHSKMVVQLA
QPACVRYRRRTGV
>CYP51B1_Mycobacterium tuberculosis N00302086 2683865944
MSAVALPRVSGGHDEHGHLEEFRTDPIGLMQRVRDECGDVGTFQLAGKQVVLLSGSHANEFFFRAGDDDLDQA
KAYPFMTPIFGEGVVFDASPERRKEMLHNAALRGEQMKGHAATIEDQVRRMIADWGEAGEIDLLDFFAELTIY
TSSACLIGKKFRDQLDGRFAKLYHELERGTDPLAYVDPYLPIESFRRRDEARNGLVALVADIMNGRIANPPTD
KSDRDMLDVLIAVKAETGTPRFSADEITGMFISMMFAGHHTSSGTASWTLIELMRHRDAYAAVIDELDELYGD
GRSVSFHALRQIPQLENVLKETLRLHPPLIILMRVAKGEFEVQGHRIHEGDLVAASPAISNRIPEDFPDPHDF

VPARYEQPRQEDLLNRWTWIPFGAGRHRCVGAAFAIMQIKAIFSVLLREYEFEMAQPPESYRNDHSKMVVQLA
QPACVRYRRRTGV
>CYP51B1_Mycobacterium tuberculosis N00400624 2703602956
MSAVALPRVSGGHDEHGHLEEFRTDPIGLMQRVRDECGDVGTFQLAGKQVVLLSGSHANEFFFRAGDDDLDQA
KAYPFMTPIFGEGVVFDASPERRKEMLHNAALRGEQMKGHAATIEDQVRRMIADWGEAGEIDLLDFFAELTIY
TSSACLIGKKFRDQLDGRFAKLYHELERGTDPLAYVDPYLPIESFRRRDEARNGLVALVADIMNGRIANPPTD
KSDRDMLDVLIAVKAETGTPRFSADEITGMFISMMFAGHHTSSGTASWTLIELMRHRDAYAAVIDELDELYGD
GRSVSFHALRQIPQLENVLKETLRLHPPLIILMRVAKGEFEVQGHRIHEGDLVAASPAISNRIPEDFPDPHDF
VPARYEQPRQEDLLNRWTWIPFGAGRHRCVGAAFAIMQIKAIFSVLLREYEFEMAQPPESYRNDHSKMVVQLA
QPACVRYRRRTGV
>CYP51B1_Mycobacterium tuberculosis N00400960 2698903289
MSAVALPRVSGGHDEHGHLEEFRTDPIGLMQRVRDECGDVGTFQLAGKQVVLLSGSHANEFFFRAGDDDLDQA
KAYPFMTPIFGEGVVFDASPERRKEMLHNAALRGEQMKGHAATIEDQVRRMIADWGEAGEIDLLDFFAELTIY
TSSACLIGKKFRDQLDGRFAKLYHELERGTDPLAYVDPYLPIESFRRRDEARNGLVALVADIMNGRIANPPTD
KSDRDMLDVLIAVKAETGTPRFSADEITGMFISMMFAGHHTSSGTASWTLIELMRHRDAYAAVIDELDELYGD
GRSVSFHALRQIPQLENVLKETLRLHPPLIILMRVAKGEFEVQGHRIHEGDLVAASPAISNRIPEDFPDPHDF
VPARYEQPRQEDLLNRWTWIPFGAGRHRCVGAAFAIMQIKAIFSVLLREYEFEMAQPPESYRNDHSKMVVQLA
QPACVRYRRRTGV
>CYP51B1_Mycobacterium tuberculosis N00401131 2683871546
MSAVALPRVSGGHDEHGHLEEFRTDPIGLMQRVRDECGDVGTFQLAGKQVVLLSGSHANEFFFRAGDDDLDQA
KAYPFMTPIFGEGVVFDASPERRKEMLHNAALRGEQMKGHAATIEDQVRRMIADWGEAGEIDLLDFFAELTIY
TSSACLIGKKFRDQLDGRFAKLYHELERGTDPLAYVDPYLPIESFRRRDEARNGLVALVADIMNGRIANPPTD
KSDRDMLDVLIAVKAETGTPRFSADEITGMFISMMFAGHHTSSGTASWTLIELMRHRDAYAAVIDELDELYGD
GRSVSFHALRQIPQLENVLKETLRLHPPLIILMRVAKGEFEVQGHRIHEGDLVAASPAISNRIPEDFPDPHDF
VPARYEQPRQEDLLNRWTWIPFGAGRHRCVGAAFAIMQIKAIFSVLLREYEFEMAQPPESYRNDHSKMVVQLA
QPACVRYRRRTGV
>CYP51B1_Mycobacterium tuberculosis N00500166 2675247262
MSAVALPRVSGGHDEHGHLEEFRTDPIGLMQRVRDECGDVGTFQLAGKQVVLLSGSHANEFFFRAGDDDLDQA
KAYPFMTPIFGEGVVFDASPERRKEMLHNAALRGEQMKGHAATIEDQVRRMIADWGEAGEIDLLDFFAELTIY
TSSACLIGKKFRDQLDGRFAKLYHELERGTDPLAYVDPYLPIESFRRRDEARNGLVALVADIMNGRIANPPTD
KSDRDMLDVLIAVKAETGTPRFSADEITGMFISMMFAGHHTSSGTASWTLIELMRHRDAYAAVIDELDELYGD
GRSVSFHALRQIPQLENVLKETLRLHPPLIILMRVAKGEFEVQGHRIHEGDLVAASPAISNRIPEDFPDPHDF
VPARYEQPRQEDLLNRWTWIPFGAGRHRCVGAAFAIMQIKAIFSVLLREYEFEMAQPPESYRNDHSKMVVQLA
QPACVRYRRRTGV
>CYP51B1_Mycobacterium tuberculosis N00500759 2692378266
MSAVALPRVSGGHDEHGHLEEFRTDPIGLMQRVRDECGDVGTFQLAGKQVVLLSGSHANEFFFRAGDDDLDQA
KAYPFMTPIFGEGVVFDASPERRKEMLHNAALRGEQMKGHAATIEDQVRRMIADWGEAGEIDLLDFFAELTIY
TSSACLIGKKFRDQLDGRFAKLYHELERGTDPLAYVDPYLPIESFRRRDEARNGLVALVADIMNGRIANPPTD
KSDRDMLDVLIAVKAETGTPRFSADEITGMFISMMFAGHHTSSGTASWTLIELMRHRDAYAAVIDELDELYGD
GRSVSFHALRQIPQLENVLKETLRLHPPLIILMRVAKGEFEVQGHRIHEGDLVAASPAISNRIPEDFPDPHDF
VPARYEQPRQEDLLNRWTWIPFGAGRHRCVGAAFAIMQIKAIFSVLLREYEFEMAQPPESYRNDHSKMVVQLA
QPACVRYRRRTGV
>CYP51B1_Mycobacterium tuberculosis N00501578 2697273587
MSAVALPRVSGGHDEHGHLEEFRTDPIGLMQRVRDECGDVGTFQLAGKQVVLLSGSHANEFFFRAGDDDLDQA
KAYPFMTPIFGEGVVFDASPERRKEMLHNAALRGEQMKGHAATIEDQVRRMIADWGEAGEIDLLDFFAELTIY
TSSACLIGKKFRDQLDGRFAKLYHELERGTDPLAYVDPYLPIESFRRRDEARNGLVALVADIMNGRIANPPTD
KSDRDMLDVLIAVKAETGTPRFSADEITGMFISMMFAGHHTSSGTASWTLIELMRHRDAYAAVIDELDELYGD
GRSVSFHALRQIPQLENVLKETLRLHPPLIILMRVAKGEFEVQGHRIHEGDLVAASPAISNRIPEDFPDPHDF
VPARYEQPRQEDLLNRWTWIPFGAGRHRCVGAAFAIMQIKAIFSVLLREYEFEMAQPPESYRNDHSKMVVQLA
QPACVRYRRRTGV
>CYP51B1_Mycobacterium tuberculosis N00501741 2672455304
MSAVALPRVSGGHDEHGHLEEFRTDPIGLMQRVRDECGDVGTFQLAGKQVVLLSGSHANEFFFRAGDDDLDQA
KAYPFMTPIFGEGVVFDASPERRKEMLHNAALRGEQMKGHAATIEDQVRRMIADWGEAGEIDLLDFFAELTIY
TSSACLIGKKFRDQLDGRFAKLYHELERGTDPLAYVDPYLPIESFRRRDEARNGLVALVADIMNGRIANPPTD
KSDRDMLDVLIAVKAETGTPRFSADEITGMFISMMFAGHHTSSGTASWTLIELMRHRDAYAAVIDELDELYGD
GRSVSFHALRQIPQLENVLKETLRLHPPLIILMRVAKGEFEVQGHRIHEGDLVAASPAISNRIPEDFPDPHDF
VPARYEQPRQEDLLNRWTWIPFGAGRHRCVGAAFAIMQIKAIFSVLLREYEFEMAQPPESYRNDHSKMVVQLA
QPACVRYRRRTGV

>CYP51B1_Mycobacterium tuberculosis N00701874 2697197226
MSAVALPRVSGGHDEHGHLEEFRTDPIGLMQRVRDECGDVGTFQLAGKQVVLLSGSHANEFFFRAGDDDLDQA
KAYPFMTPIFGEGVVFDASPERRKEMLHNAALRGEQMKGHAATIEDQVRRMIADWGEAGEIDLLDFFAELTIY
TSSACLIGKKFRDQLDGRFAKLYHELERGTDPLAYVDPYLPIESFRRRDEARNGLVALVADIMNGRIANPPTD
KSDRDMLDVLIAVKAETGTPRFSADEITGMFISMMFAGHHTSSGTASWTLIELMRHRDAYAAVIDELDELYGD
GRSVSFHALRQIPQLENVLKETLRLHPPLIILMRVAKGEFEVQGHRIHEGDLVAASPAISNRIPEDFPDPHDF
VPARYEQPRQEDLLNRWTWIPFGAGRHRCVGAAFAIMQIKAIFSVLLREYEFEMAQPPESYRNDHSKMVVQLA
QPACVRYRRRTGV
>CYP51B1_Mycobacterium tuberculosis N00800620 2696316736
MSAVALPRVSGGHDEHGHLEEFRTDPIGLMQRVRDECGDVGTFQLAGKQVVLLSGSHANEFFFRAGDDDLDQA
KAYPFMTPIFGEGVVFDASPERRKEMLHNAALRGEQMKGHAATIEDQVRRMIADWGEAGEIDLLDFFAELTIY
TSSACLIGKKFRDQLDGRFAKLYHELERGTDPLAYVDPYLPIESFRRRDEARNGLVALVADIMNGRIANPPTD
KSDRDMLDVLIAVKAETGTPRFSADEITGMFISMMFAGHHTSSGTASWTLIELMRHRDAYAAVIDELDELYGD
GRSVSFHALRQIPQLENVLKETLRLHPPLIILMRVAKGEFEVQGHRIHEGDLVAASPAISNRIPEDFPDPHDF
VPARYEQPRQEDLLNRWTWIPFGAGRHRCVGAAFAIMQIKAIFSVLLREYEFEMAQPPESYRNDHSKMVVQLA
QPACVRYRRRTGV
>CYP51B1_Mycobacterium tuberculosis N00800922 2694131489
MSAVALPRVSGGHDEHGHLEEFRTDPIGLMQRVRDECGDVGTFQLAGKQVVLLSGSHANEFFFRAGDDDLDQA
KAYPFMTPIFGEGVVFDASPERRKEMLHNAALRGEQMKGHAATIEDQVRRMIADWGEAGEIDLLDFFAELTIY
TSSACLIGKKFRDQLDGRFAKLYHELERGTDPLAYVDPYLPIESFRRRDEARNGLVALVADIMNGRIANPPTD
KSDRDMLDVLIAVKAETGTPRFSADEITGMFISMMFAGHHTSSGTASWTLIELMRHRDAYAAVIDELDELYGD
GRSVSFHALRQIPQLENVLKETLRLHPPLIILMRVAKGEFEVQGHRIHEGDLVAASPAISNRIPEDFPDPHDF
VPARYEQPRQEDLLNRWTWIPFGAGRHRCVGAAFAIMQIKAIFSVLLREYEFEMAQPPESYRNDHSKMVVQLA
QPACVRYRRRTGV
>CYP51B1_Mycobacterium tuberculosis N09501026 2706615568
MSAVALPRVSGGHDEHGHLEEFRTDPIGLMQRVRDECGDVGTFQLAGKQVVLLSGSHANEFFFRAGDDDLDQA
KAYPFMTPIFGEGVVFDASPERRKEMLHNAALRGEQMKGHAATIEDQVRRMIADWGEAGEIDLLDFFAELTIY
TSSACLIGKKFRDQLDGRFAKLYHELERGTDPLAYVDPYLPIESFRRRDEARNGLVALVADIMNGRIANPPTD
KSDRDMLDVLIAVKAETGTPRFSADEITGMFISMMFAGHHTSSGTASWTLIELMRHRDAYAAVIDELDELYGD
GRSVSFHALRQIPQLENVLKETLRLHPPLIILMRVAKGEFEVQGHRIHEGDLVAASPAISNRIPEDFPDPHDF
VPARYEQPRQEDLLNRWTWIPFGAGRHRCVGAAFAIMQIKAIFSVLLREYEFEMAQPPESYRNDHSKMVVQLA
QPACVRYRRRTGV
>CYP51B1_Mycobacterium tuberculosis N09900612 2699157393
MSAVALPRVSGGHDEHGHLEEFRTDPIGLMQRVRDECGDVGTFQLAGKQVVLLSGSHANEFFFRAGDDDLDQA
KAYPFMTPIFGEGVVFDASPERRKEMLHNAALRGEQMKGHAATIEDQVRRMIADWGEAGEIDLLDFFAELTIY
TSSACLIGKKFRDQLDGRFAKLYHELERGTDPLAYVDPYLPIESFRRRDEARNGLVALVADIMNGRIANPPTD
KSDRDMLDVLIAVKAETGTPRFSADEITGMFISMMFAGHHTSSGTASWTLIELMRHRDAYAAVIDELDELYGD
GRSVSFHALRQIPQLENVLKETLRLHPPLIILMRVAKGEFEVQGHRIHEGDLVAASPAISNRIPEDFPDPHDF
VPARYEQPRQEDLLNRWTWIPFGAGRHRCVGAAFAIMQIKAIFSVLLREYEFEMAQPPESYRNDHSKMVVQLA
QPACVRYRRRTGV
>CYP51B1_Mycobacterium tuberculosis N09900805 2706612128
MSAVALPRVSGGHDEHGHLEEFRTDPIGLMQRVRDECGDVGTFQLAGKQVVLLSGSHANEFFFRAGDDDLDQA
KAYPFMTPIFGEGVVFDASPERRKEMLHNAALRGEQMKGHAATIEDQVRRMIADWGEAGEIDLLDFFAELTIY
TSSACLIGKKFRDQLDGRFAKLYHELERGTDPLAYVDPYLPIESFRRRDEARNGLVALVADIMNGRIANPPTD
KSDRDMLDVLIAVKAETGTPRFSADEITGMFISMMFAGHHTSSGTASWTLIELMRHRDAYAAVIDELDELYGD
GRSVSFHALRQIPQLENVLKETLRLHPPLIILMRVAKGEFEVQGHRIHEGDLVAASPAISNRIPEDFPDPHDF
VPARYEQPRQEDLLNRWTWIPFGAGRHRCVGAAFAIMQIKAIFSVLLREYEFEMAQPPESYRNDHSKMVVQLA
QPACVRYRRRTGV
>CYP51B1_Mycobacterium tuberculosis N09900855 2695858995
MSAVALPRVSGGHDEHGHLEEFRTDPIGLMQRVRDECGDVGTFQLAGKQVVLLSGSHANEFFFRAGDDDLDQA
KAYPFMTPIFGEGVVFDASPERRKEMLHNAALRGEQMKGHAATIEDQVRRMIADWGEAGEIDLLDFFAELTIY
TSSACLIGKKFRDQLDGRFAKLYHELERGTDPLAYVDPYLPIESFRRRDEARNGLVALVADIMNGRIANPPTD
KSDRDMLDVLIAVKAETGTPRFSADEITGMFISMMFAGHHTSSGTASWTLIELMRHRDAYAAVIDELDELYGD
GRSVSFHALRQIPQLENVLKETLRLHPPLIILMRVAKGEFEVQGHRIHEGDLVAASPAISNRIPEDFPDPHDF
VPARYEQPRQEDLLNRWTWIPFGAGRHRCVGAAFAIMQIKAIFSVLLREYEFEMAQPPESYRNDHSKMVVQLA
QPACVRYRRRTGV
>CYP51B1_Mycobacterium tuberculosis N09901607 2703611882

MSAVALPRVSGGHDEHGHLEEFRTDPIGLMQRVRDECGDVGTFQLAGKQVVLLSGSHANEFFFRAGDDDLDQA
KAYPFMTPIFGEGVVFDASPERRKEMLHNAALRGEQMKGHAATIEDQVRRMIADWGEAGEIDLLDFFAELTIY
TSSACLIGKKFRDQLDGRFAKLYHELERGTDPLAYVDPYLPIESFRRRDEARNGLVALVADIMNGRIANPPTD
KSDRDMLDVLIAVKAETGTPRFSADEITGMFISMMFAGHHTSSGTASWTLIELMRHRDAYAAVIDELDELYGD
GRSVSFHALRQIPQLENVLKETLRLHPPLIILMRVAKGEFEVQGHRIHEGDLVAASPAISNRIPEDFPDPHDF
VPARYEQPRQEDLLNRWTWIPFGAGRHRCVGAAFAIMQIKAIFSVLLREYEFEMAQPPESYRNDHSKMVVQLA
QPACVRYRRRTGV
>CYP51B1_Mycobacterium tuberculosis N09902308 2697092438
MSAVALPRVSGGHDEHGHLEEFRTDPIGLMQRVRDECGDVGTFQLAGKQVVLLSGSHANEFFFRAGDDDLDQA
KAYPFMTPIFGEGVVFDASPERRKEMLHNAALRGEQMKGHAATIEDQVRRMIADWGEAGEIDLLDFFAELTIY
TSSACLIGKKFRDQLDGRFAKLYHELERGTDPLAYVDPYLPIESFRRRDEARNGLVALVADIMNGRIANPPTD
KSDRDMLDVLIAVKAETGTPRFSADEITGMFISMMFAGHHTSSGTASWTLIELMRHRDAYAAVIDELDELYGD
GRSVSFHALRQIPQLENVLKETLRLHPPLIILMRVAKGEFEVQGHRIHEGDLVAASPAISNRIPEDFPDPHDF
VPARYEQPRQEDLLNRWTWIPFGAGRHRCVGAAFAIMQIKAIFSVLLREYEFEMAQPPESYRNDHSKMVVQLA
QPACVRYRRRTGV
>CYP51B1_Mycobacterium tuberculosis NA-A0008 2551861848
MSAVALPRVSGGHDEHGHLEEFRTDPIGLMQRVRDECGDVGTFQLAGKQVVLLSGSHANEFFFRAGDDDLDQA
KAYPFMTPIFGEGVVFDASPERRKEMLHNAALRGEQMKGHAATIEDQVRRMIADWGEAGEIDLLDFFAELTIY
TSSACLIGKKFRDQLDGRFAKLYHELERGTDPLAYVDPYLPIESFRRRDEARNGLVALVADIMNGRIANPPTD
KSDRDMLDVLIAVKAETGTPRFSADEITGMFISMMFAGHHTSSGTASWTLIELMRHRDAYAAVIDELDELYGD
GRSVSFHALRQIPQLENVLKETLRLHPPLIILMRVAKGEFEVQGHRIHEGDLVAASPAISNRIPEDFPDPHDF
VPARYEQPRQEDLLNRWTWIPFGAGRHRCVGAAFAIMQIKAIFSVLLREYEFEMAQPPESYRNDHSKMVVQLA
QPACVRYRRRTGV
>CYP51B1_Mycobacterium tuberculosis NA-A0009 2551813793
MSAVALPRVSGGHDEHGHLEEFRTDPIGLMQRVRDECGDVGTFQLAGKQVVLLSGSHANEFFFRAGDDDLDQA
KAYPFMTPIFGEGVVFDASPERRKEMLHNAALRGEQMKGHAATIEDQVRRMIADWGEAGEIDLLDFFAELTIY
TSSACLIGKKFRDQLDGRFAKLYHELERGTDPLAYVDPYLPIESFRRRDEARNGLVALVADIMNGRIANPPTD
KSDRDMLDVLIAVKAETGTPRFSADEITGMFISMMFAGHHTSSGTASWTLIELMRHRDAYAAVIDELDELYGD
GRSVSFHALRQIPQLENVLKETLRLHPPLIILMRVAKGEFEVQGHRIHEGDLVAASPAISNRIPEDFPDPHDF
VPARYEQPRQEDLLNRWTWIPFGAGRHRCVGAAFAIMQIKAIFSVLLREYEFEMAQPPESYRNDHSKMVVQLA
QPACVRYRRRTGV
>CYP51B1_Mycobacterium tuberculosis NCGM2209 2549400820
MSAVALPRVSGGHDEHGHLEEFRTDPIGLMQRVRDECGDVGTFQLAGKQVVLLSGSHANEFFFRAGDDDLDQA
KAYPFMTPIFGEGVVFDASPERRKEMLHNAALRGEQMKGHAATIEDQVRRMIADWGEAGEIDLLDFFAELTIY
TSSACLIGKKFRDQLDGRFAKLYHELERGTDPLAYVDPYLPIESFRRRDEARNGLVALVADIMNGRIANPPTD
KSDRDMLDVLIAVKAETGTPRFSADEITGMFISMMFAGHHTSSGTASWTLIELMRHRDAYAAVIDELDELYGD
GRSVSFHALRQIPQLENVLKETLRLHPPLIILMRVAKGEFEVQGHRIHEGDLVAASPAISNRIPEDFPDPHDF
VPARYEQPRQEDLLNRWTWIPFGAGRHRCVGAAFAIMQIKAIFSVLLREYEFEMAQPPESYRNDHSKMVVQLA
QPACVRYRRRTGV
>CYP51B1_Mycobacterium tuberculosis NRITLD09 2584829543
MSAVALPRVSGGHDEHGHLEEFRTDPIGLMQRVRDECGDVGTFQLAGKQVVLLSGSHANEFFFRAGDDDLDQA
KAYPFMTPIFGEGVVFDASPERRKEMLHNAALRGEQMKGHAATIEDQVRRMIADWGEAGEIDLLDFFAELTIY
TSSACLIGKKFRDQLDGRFAKLYHELERGTDPLAYVDPYLPIESFRRRDEARNGLVALVADIMNGRIANPPTD
KSDRDMLDVLIAVKAETGTPRFSADEITGMFISMMFAGHHTSSGTASWTLIELMRHRDAYAAVIDELDELYGD
GRSVSFHALRQIPQLENVLKETLRLHPPLIILMRVAKGEFEVQGHRIHEGDLVAASPAISNRIPEDFPDPHDF
VPARYEQPRQEDLLNRWTWIPFGAGRHRCVGAAFAIMQIKAIFSVLLREYEFEMAQPPESYRNDHSKMVVQLA
QPACVRYRRRTGV
>CYP51B1_Mycobacterium tuberculosis NRITLD10 2638348455
MSAVALPRVSGGHDEHGHLEEFRTDPIGLMQRVRDECGDVGTFQLAGKQVVLLSGSHANEFFFRAGDDDLDQA
KAYPFMTPIFGEGVVFDASPERRKEMLHNAALRGEQMKGHAATIEDQVRRMIADWGEAGEIDLLDFFAELTIY
TSSACLIGKKFRDQLDGRFAKLYHELERGTDPLAYVDPYLPIESFRRRDEARNGLVALVADIMNGRIANPPTD
KSDRDMLDVLIAVKAETGTPRFSADEITGMFISMMFAGHHTSSGTASWTLIELMRHRDAYAAVIDELDELYGD
GRSVSFHALRQIPQLENVLKETLRLHPPLIILMRVAKGEFEVQGHRIHEGDLVAASPAISNRIPEDFPDPHDF
VPARYEQPRQEDLLNRWTWIPFGAGRHRCVGAAFAIMQIKAIFSVLLREYEFEMAQPPESYRNDHSKMVVQLA
QPACVRYRRRTGV
>CYP51B1_Mycobacterium tuberculosis NRITLD11 2638494388
MSAVALPRVSGGHDEHGHLEEFRTDPIGLMQRVRDECGDVGTFQLAGKQVVLLSGSHANEFFFRAGDDDLDQA
KAYPFMTPIFGEGVVFDASPERRKEMLHNAALRGEQMKGHAATIEDQVRRMIADWGEAGEIDLLDFFAELTIY

TSSACLIGKKFRDQLDGRFAKLYHELERGTDPLAYVDPYLPIESFRRRDEARNGLVALVADIMNGRIANPPTD
KSDRDMLDVLIAVKAETGTPRFSADEITGMFISMMFAGHHTSSGTASWTLIELMRHRDAYAAVIDELDELYGD
GRSVSFHALRQIPQLENVLKETLRLHPPLIILMRVAKGEFEVQGHRIHEGDLVAASPAISNRIPEDFPDPHDF
VPARYEQPRQEDLLNRWTWIPFGAGRHRCVGAAFAIMQIKAIFSVLLREYEFEMAQPPESYRNDHSKMVVQLA
QPACVRYRRRTGV
>CYP51B1_Mycobacterium tuberculosis NRITLD12 2574657474
MSAVALPRVSGGHDEHGHLEEFRTDPIGLMQRVRDECGDVGTFQLAGKQVVLLSGSHANEFFFRAGDDDLDQA
KAYPFMTPIFGEGVVFDASPERRKEMLHNAALRGEQMKGHAATIEDQVRRMIADWGEAGEIDLLDFFAELTIY
TSSACLIGKKFRDQLDGRFAKLYHELERGTDPLAYVDPYLPIESFRRRDEARNGLVALVADIMNGRIANPPTD
KSDRDMLDVLIAVKAETGTPRFSADEITGMFISMMFAGHHTSSGTASWTLIELMRHRDAYAAVIDELDELYGD
GRSVSFHALRQIPQLENVLKETLRLHPPLIILMRVAKGEFEVQGHRIHEGDLVAASPAISNRIPEDFPDPHDF
VPARYEQPRQEDLLNRWTWIPFGAGRHRCVGAAFAIMQIKAIFSVLLREYEFEMAQPPESYRNDHSKMVVQLA
QPACVRYRRRTGV
>CYP51B1_Mycobacterium tuberculosis NRITLD13 2641027930
MSAVALPRVSGGHDEHGHLEEFRTDPIGLMQRVRDECGDVGTFQLAGKQVVLLSGSHANEFFFRAGDDDLDQA
KAYPFMTPIFGEGVVFDASPERRKEMLHNAALRGEQMKGHAATIEDQVRRMIADWGEAGEIDLLDFFAELTIY
TSSACLIGKKFRDQLDGRFAKLYHELERGTDPLAYVDPYLPIESFRRRDEARNGLVALVADIMNGRIANPPTD
KSDRDMLDVLIAVKAETGTPRFSADEITGMFISMMFAGHHTSSGTASWTLIELMRHRDAYAAVIDELDELYGD
GRSVSFHALRQIPQLENVLKETLRLHPPLIILMRVAKGEFEVQGHRIHEGDLVAASPAISNRIPEDFPDPHDF
VPARYEQPRQEDLLNRWTWIPFGAGRHRCVGAAFAIMQIKAIFSVLLREYEFEMAQPPESYRNDHSKMVVQLA
QPACVRYRRRTGV
>CYP51B1_Mycobacterium tuberculosis NRITLD14 2577921840
MSAVALPRVSGGHDEHGHLEEFRTDPIGLMQRVRDECGDVGTFQLAGKQVVLLSGSHANEFFFRAGDDDLDQA
KAYPFMTPIFGEGVVFDASPERRKEMLHNAALRGEQMKGHAATIEDQVRRMIADWGEAGEIDLLDFFAELTIY
TSSACLIGKKFRDQLDGRFAKLYHELERGTDPLAYVDPYLPIESFRRRDEARNGLVALVADIMNGRIANPPTD
KSDRDMLDVLIAVKAETGTPRFSADEITGMFISMMFAGHHTSSGTASWTLIELMRHRDAYAAVIDELDELYGD
GRSVSFHALRQIPQLENVLKETLRLHPPLIILMRVAKGEFEVQGHRIHEGDLVAASPAISNRIPEDFPDPHDF
VPARYEQPRQEDLLNRWTWIPFGAGRHRCVGAAFAIMQIKAIFSVLLREYEFEMAQPPESYRNDHSKMVVQLA
QPACVRYRRRTGV
>CYP51B1_Mycobacterium tuberculosis NRITLD15 2578092791
MSAVALPRVSGGHDEHGHLEEFRTDPIGLMQRVRDECGDVGTFQLAGKQVVLLSGSHANEFFFRAGDDDLDQA
KAYPFMTPIFGEGVVFDASPERRKEMLHNAALRGEQMKGHAATIEDQVRRMIADWGEAGEIDLLDFFAELTIY
TSSACLIGKKFRDQLDGRFAKLYHELERGTDPLAYVDPYLPIESFRRRDEARNGLVALVADIMNGRIANPPTD
KSDRDMLDVLIAVKAETGTPRFSADEITGMFISMMFAGHHTSSGTASWTLIELMRHRDAYAAVIDELDELYGD
GRSVSFHALRQIPQLENVLKETLRLHPPLIILMRVAKGEFEVQGHRIHEGDLVAASPAISNRIPEDFPDPHDF
VPARYEQPRQEDLLNRWTWIPFGAGRHRCVGAAFAIMQIKAIFSVLLREYEFEMAQPPESYRNDHSKMVVQLA
QPACVRYRRRTGV
>CYP51B1_Mycobacterium tuberculosis NRITLD16 2635672074
MSAVALPRVSGGHDEHGHLEEFRTDPIGLMQRVRDECGDVGTFQLAGKQVVLLSGSHANEFFFRAGDDDLDQA
KAYPFMTPIFGEGVVFDASPERRKEMLHNAALRGEQMKGHAATIEDQVRRMIADWGEAGEIDLLDFFAELTIY
TSSACLIGKKFRDQLDGRFAKLYHELERGTDPLAYVDPYLPIESFRRRDEARNGLVALVADIMNGRIANPPTD
KSDRDMLDVLIAVKAETGTPRFSADEITGMFISMMFAGHHTSSGTASWTLIELMRHRDAYAAVIDELDELYGD
GRSVSFHALRQIPQLENVLKETLRLHPPLIILMRVAKGEFEVQGHRIHEGDLVAASPAISNRIPEDFPDPHDF
VPARYEQPRQEDLLNRWTWIPFGAGRHRCVGAAFAIMQIKAIFSVLLREYEFEMAQPPESYRNDHSKMVVQLA
QPACVRYRRRTGV
>CYP51B1_Mycobacterium tuberculosis NRITLD18 2655329115
MSAVALPRVSGGHDEHGHLEEFRTDPIGLMQRVRDECGDVGTFQLAGKQVVLLSGSHANEFFFRAGDDDLDQA
KAYPFMTPIFGEGVVFDASPERRKEMLHNAALRGEQMKGHAATIEDQVRRMIADWGEAGEIDLLDFFAELTIY
TSSACLIGKKFRDQLDGRFAKLYHELERGTDPLAYVDPYLPIESFRRRDEARNGLVALVADIMNGRIANPPTD
KSDRDMLDVLIAVKAETGTPRFSADEITGMFISMMFAGHHTSSGTASWTLIELMRHRDAYAAVIDELDELYGD
GRSVSFHALRQIPQLENVLKETLRLHPPLIILMRVAKGEFEVQGHRIHEGDLVAASPAISNRIPEDFPDPHDF
VPARYEQPRQEDLLNRWTWIPFGAGRHRCVGAAFAIMQIKAIFSVLLREYEFEMAQPPESYRNDHSKMVVQLA
QPACVRYRRRTGV
>CYP51B1_Mycobacterium tuberculosis NRITLD19 2637462701
MSAVALPRVSGGHDEHGHLEEFRTDPIGLMQRVRDECGDVGTFQLAGKQVVLLSGSHANEFFFRAGDDDLDQA
KAYPFMTPIFGEGVVFDASPERRKEMLHNAALRGEQMKGHAATIEDQVRRMIADWGEAGEIDLLDFFAELTIY
TSSACLIGKKFRDQLDGRFAKLYHELERGTDPLAYVDPYLPIESFRRRDEARNGLVALVADIMNGRIANPPTD
KSDRDMLDVLIAVKAETGTPRFSADEITGMFISMMFAGHHTSSGTASWTLIELMRHRDAYAAVIDELDELYGD

GRSVSFHALRQIPQLENVLKETLRLHPPLIILMRVAKGEFEVQGHRIHEGDLVAASPAISNRIPEDFPDPHDF
VPARYEQPRQEDLLNRWTWIPFGAGRHRCVGAAFAIMQIKAIFSVLLREYEFEMAQPPESYRNDHSKMVVQLA
QPACVRYRRRTGV
>CYP51B1_Mycobacterium tuberculosis NRITLD20 2635665124
MSAVALPRVSGGHDEHGHLEEFRTDPIGLMQRVRDECGDVGTFQLAGKQVVLLSGSHANEFFFRAGDDDLDQA
KAYPFMTPIFGEGVVFDASPERRKEMLHNAALRGEQMKGHAATIEDQVRRMIADWGEAGEIDLLDFFAELTIY
TSSACLIGKKFRDQLDGRFAKLYHELERGTDPLAYVDPYLPIESFRRRDEARNGLVALVADIMNGRIANPPTD
KSDRDMLDVLIAVKAETGTPRFSADEITGMFISMMFAGHHTSSGTASWTLIELMRHRDAYAAVIDELDELYGD
GRSVSFHALRQIPQLENVLKETLRLHPPLIILMRVAKGEFEVQGHRIHEGDLVAASPAISNRIPEDFPDPHDF
VPARYEQPRQEDLLNRWTWIPFGAGRHRCVGAAFAIMQIKAIFSVLLREYEFEMAQPPESYRNDHSKMVVQLA
QPACVRYRRRTGV
>CYP51B1_Mycobacterium tuberculosis NRITLD21 2630536148
MSAVALPRVSGGHDEHGHLEEFRTDPIGLMQRVRDECGDVGTFQLAGKQVVLLSGSHANEFFFRAGDDDLDQA
KAYPFMTPIFGEGVVFDASPERRKEMLHNAALRGEQMKGHAATIEDQVRRMIADWGEAGEIDLLDFFAELTIY
TSSACLIGKKFRDQLDGRFAKLYHELERGTDPLAYVDPYLPIESFRRRDEARNGLVALVADIMNGRIANPPTD
KSDRDMLDVLIAVKAETGTPRFSADEITGMFISMMFAGHHTSSGTASWTLIELMRHRDAYAAVIDELDELYGD
GRSVSFHALRQIPQLENVLKETLRLHPPLIILMRVAKGEFEVQGHRIHEGDLVAASPAISNRIPEDFPDPHDF
VPARYEQPRQEDLLNRWTWIPFGAGRHRCVGAAFAIMQIKAIFSVLLREYEFEMAQPPESYRNDHSKMVVQLA
QPACVRYRRRTGV
>CYP51B1_Mycobacterium tuberculosis NRITLD28 2655077586
MSAVALPRVSGGHDEHGHLEEFRTDPIGLMQRVRDECGDVGTFQLAGKQVVLLSGSHANEFFFRAGDDDLDQA
KAYPFMTPIFGEGVVFDASPERRKEMLHNAALRGEQMKGHAATIEDQVRRMIADWGEAGEIDLLDFFAELTIY
TSSACLIGKKFRDQLDGRFAKLYHELERGTDPLAYVDPYLPIESFRRRDEARNGLVALVADIMNGRIANPPTD
KSDRDMLDVLIAVKAETGTPRFSADEITGMFISMMFAGHHTSSGTASWTLIELMRHRDAYAAVIDELDELYGD
GRSVSFHALRQIPQLENVLKETLRLHPPLIILMRVAKGEFEVQGHRIHEGDLVAASPAISNRIPEDFPDPHDF
VPARYEQPRQEDLLNRWTWIPFGAGRHRCVGAAFAIMQIKAIFSVLLREYEFEMAQPPESYRNDHSKMVVQLA
QPACVRYRRRTGV
>CYP51B1_Mycobacterium tuberculosis NRITLD31 2635569628
MSAVALPRVSGGHDEHGHLEEFRTDPIGLMQRVRDECGDVGTFQLAGKQVVLLSGSHANEFFFRAGDDDLDQA
KAYPFMTPIFGEGVVFDASPERRKEMLHNAALRGEQMKGHAATIEDQVRRMIADWGEAGEIDLLDFFAELTIY
TSSACLIGKKFRDQLDGRFAKLYHELERGTDPLAYVDPYLPIESFRRRDEARNGLVALVADIMNGRIANPPTD
KSDRDMLDVLIAVKAETGTPRFSADEITGMFISMMFAGHHTSSGTASWTLIELMRHRDAYAAVIDELDELYGD
GRSVSFHALRQIPQLENVLKETLRLHPPLIILMRVAKGEFEVQGHRIHEGDLVAASPAISNRIPEDFPDPHDF
VPARYEQPRQEDLLNRWTWIPFGAGRHRCVGAAFAIMQIKAIFSVLLREYEFEMAQPPESYRNDHSKMVVQLA
QPACVRYRRRTGV
>CYP51B1_Mycobacterium tuberculosis NRITLD34 2630806237
MSAVALPRVSGGHDEHGHLEEFRTDPIGLMQRVRDECGDVGTFQLAGKQVVLLSGSHANEFFFRAGDDDLDQA
KAYPFMTPIFGEGVVFDASPERRKEMLHNAALRGEQMKGHAATIEDQVRRMIADWGEAGEIDLLDFFAELTIY
TSSACLIGKKFRDQLDGRFAKLYHELERGTDPLAYVDPYLPIESFRRRDEARNGLVALVADIMNGRIANPPTD
KSDRDMLDVLIAVKAETGTPRFSADEITGMFISMMFAGHHTSSGTASWTLIELMRHRDAYAAVIDELDELYGD
GRSVSFHALRQIPQLENVLKETLRLHPPLIILMRVAKGEFEVQGHRIHEGDLVAASPAISNRIPEDFPDPHDF
VPARYEQPRQEDLLNRWTWIPFGAGRHRCVGAAFAIMQIKAIFSVLLREYEFEMAQPPESYRNDHSKMVVQLA
QPACVRYRRRTGV
>CYP51B1_Mycobacterium tuberculosis NRITLD37 2647425582
MSAVALPRVSGGHDEHGHLEEFRTDPIGLMQRVRDECGDVGTFQLAGKQVVLLSGSHANEFFFRAGDDDLDQA
KAYPFMTPIFGEGVVFDASPERRKEMLHNAALRGEQMKGHAATIEDQVRRMIADWGEAGEIDLLDFFAELTIY
TSSACLIGKKFRDQLDGRFAKLYHELERGTDPLAYVDPYLPIESFRRRDEARNGLVALVADIMNGRIANPPTD
KSDRDMLDVLIAVKAETGTPRFSADEITGMFISMMFAGHHTSSGTASWTLIELMRHRDAYAAVIDELDELYGD
GRSVSFHALRQIPQLENVLKETLRLHPPLIILMRVAKGEFEVQGHRIHEGDLVAASPAISNRIPEDFPDPHDF
VPARYEQPRQEDLLNRWTWIPFGAGRHRCVGAAFAIMQIKAIFSVLLREYEFEMAQPPESYRNDHSKMVVQLA
QPACVRYRRRTGV
>CYP51B1_Mycobacterium tuberculosis NRITLD38 2652370125
MSAVALPRVSGGHDEHGHLEEFRTDPIGLMQRVRDECGDVGTFQLAGKQVVLLSGSHANEFFFRAGDDDLDQA
KAYPFMTPIFGEGVVFDASPERRKEMLHNAALRGEQMKGHAATIEDQVRRMIADWGEAGEIDLLDFFAELTIY
TSSACLIGKKFRDQLDGRFAKLYHELERGTDPLAYVDPYLPIESFRRRDEARNGLVALVADIMNGRIANPPTD
KSDRDMLDVLIAVKAETGTPRFSADEITGMFISMMFAGHHTSSGTASWTLIELMRHRDAYAAVIDELDELYGD
GRSVSFHALRQIPQLENVLKETLRLHPPLIILMRVAKGEFEVQGHRIHEGDLVAASPAISNRIPEDFPDPHDF

VPARYEQPRQEDLLNRWTWIPFGAGRHRCVGAAFAIMQIKAIFSVLLREYEFEMAQPPESYRNDHSKMVVQLA
QPACVRYRRRTGV
>CYP51B1_Mycobacterium tuberculosis NRITLD45 2633492626
MSAVALPRVSGGHDEHGHLEEFRTDPIGLMQRVRDECGDVGTFQLAGKQVVLLSGSHANEFFFRAGDDDLDQA
KAYPFMTPIFGEGVVFDASPERRKEMLHNAALRGEQMKGHAATIEDQVRRMIADWGEAGEIDLLDFFAELTIY
TSSACLIGKKFRDQLDGRFAKLYHELERGTDPLAYVDPYLPIESFRRRDEARNGLVALVADIMNGRIANPPTD
KSDRDMLDVLIAVKAETGTPRFSADEITGMFISMMFAGHHTSSGTASWTLIELMRHRDAYAAVIDELDELYGD
GRSVSFHALRQIPQLENVLKETLRLHPPLIILMRVAKGEFEVQGHRIHEGDLVAASPAISNRIPEDFPDPHDF
VPARYEQPRQEDLLNRWTWIPFGAGRHRCVGAAFAIMQIKAIFSVLLREYEFEMAQPPESYRNDHSKMVVQLA
QPACVRYRRRTGV
>CYP51B1_Mycobacterium tuberculosis NRITLD47 2632799164
MSAVALPRVSGGHDEHGHLEEFRTDPIGLMQRVRDECGDVGTFQLAGKQVVLLSGSHANEFFFRAGDDDLDQA
KAYPFMTPIFGEGVVFDASPERRKEMLHNAALRGEQMKGHAATIEDQVRRMIADWGEAGEIDLLDFFAELTIY
TSSACLIGKKFRDQLDGRFAKLYHELERGTDPLAYVDPYLPIESFRRRDEARNGLVALVADIMNGRIANPPTD
KSDRDMLDVLIAVKAETGTPRFSADEITGMFISMMFAGHHTSSGTASWTLIELMRHRDAYAAVIDELDELYGD
GRSVSFHALRQIPQLENVLKETLRLHPPLIILMRVAKGEFEVQGHRIHEGDLVAASPAISNRIPEDFPDPHDF
VPARYEQPRQEDLLNRWTWIPFGAGRHRCVGAAFAIMQIKAIFSVLLREYEFEMAQPPESYRNDHSKMVVQLA
QPACVRYRRRTGV
>CYP51B1_Mycobacterium tuberculosis NRITLD49 2642027946
MSAVALPRVSGGHDEHGHLEEFRTDPIGLMQRVRDECGDVGTFQLAGKQVVLLSGSHANEFFFRAGDDDLDQA
KAYPFMTPIFGEGVVFDASPERRKEMLHNAALRGEQMKGHAATIEDQVRRMIADWGEAGEIDLLDFFAELTIY
TSSACLIGKKFRDQLDGRFAKLYHELERGTDPLAYVDPYLPIESFRRRDEARNGLVALVADIMNGRIANPPTD
KSDRDMLDVLIAVKAETGTPRFSADEITGMFISMMFAGHHTSSGTASWTLIELMRHRDAYAAVIDELDELYGD
GRSVSFHALRQIPQLENVLKETLRLHPPLIILMRVAKGEFEVQGHRIHEGDLVAASPAISNRIPEDFPDPHDF
VPARYEQPRQEDLLNRWTWIPFGAGRHRCVGAAFAIMQIKAIFSVLLREYEFEMAQPPESYRNDHSKMVVQLA
QPACVRYRRRTGV
>CYP51B1_Mycobacterium tuberculosis NRITLD50 2630496413
MSAVALPRVSGGHDEHGHLEEFRTDPIGLMQRVRDECGDVGTFQLAGKQVVLLSGSHANEFFFRAGDDDLDQA
KAYPFMTPIFGEGVVFDASPERRKEMLHNAALRGEQMKGHAATIEDQVRRMIADWGEAGEIDLLDFFAELTIY
TSSACLIGKKFRDQLDGRFAKLYHELERGTDPLAYVDPYLPIESFRRRDEARNGLVALVADIMNGRIANPPTD
KSDRDMLDVLIAVKAETGTPRFSADEITGMFISMMFAGHHTSSGTASWTLIELMRHRDAYAAVIDELDELYGD
GRSVSFHALRQIPQLENVLKETLRLHPPLIILMRVAKGEFEVQGHRIHEGDLVAASPAISNRIPEDFPDPHDF
VPARYEQPRQEDLLNRWTWIPFGAGRHRCVGAAFAIMQIKAIFSVLLREYEFEMAQPPESYRNDHSKMVVQLA
QPACVRYRRRTGV
>CYP51B1_Mycobacterium tuberculosis NRITLD51 2648301940
MSAVALPRVSGGHDEHGHLEEFRTDPIGLMQRVRDECGDVGTFQLAGKQVVLLSGSHANEFFFRAGDDDLDQA
KAYPFMTPIFGEGVVFDASPERRKEMLHNAALRGEQMKGHAATIEDQVRRMIADWGEAGEIDLLDFFAELTIY
TSSACLIGKKFRDQLDGRFAKLYHELERGTDPLAYVDPYLPIESFRRRDEARNGLVALVADIMNGRIANPPTD
KSDRDMLDVLIAVKAETGTPRFSADEITGMFISMMFAGHHTSSGTASWTLIELMRHRDAYAAVIDELDELYGD
GRSVSFHALRQIPQLENVLKETLRLHPPLIILMRVAKGEFEVQGHRIHEGDLVAASPAISNRIPEDFPDPHDF
VPARYEQPRQEDLLNRWTWIPFGAGRHRCVGAAFAIMQIKAIFSVLLREYEFEMAQPPESYRNDHSKMVVQLA
QPACVRYRRRTGV
>CYP51B1_Mycobacterium tuberculosis NRITLD53 2632107897
MSAVALPRVSGGHDEHGHLEEFRTDPIGLMQRVRDECGDVGTFQLAGKQVVLLSGSHANEFFFRAGDDDLDQA
KAYPFMTPIFGEGVVFDASPERRKEMLHNAALRGEQMKGHAATIEDQVRRMIADWGEAGEIDLLDFFAELTIY
TSSACLIGKKFRDQLDGRFAKLYHELERGTDPLAYVDPYLPIESFRRRDEARNGLVALVADIMNGRIANPPTD
KSDRDMLDVLIAVKAETGTPRFSADEITGMFISMMFAGHHTSSGTASWTLIELMRHRDAYAAVIDELDELYGD
GRSVSFHALRQIPQLENVLKETLRLHPPLIILMRVAKGEFEVQGHRIHEGDLVAASPAISNRIPEDFPDPHDF
VPARYEQPRQEDLLNRWTWIPFGAGRHRCVGAAFAIMQIKAIFSVLLREYEFEMAQPPESYRNDHSKMVVQLA
QPACVRYRRRTGV
>CYP51B1_Mycobacterium tuberculosis NRITLD55 2629966975
MSAVALPRVSGGHDEHGHLEEFRTDPIGLMQRVRDECGDVGTFQLAGKQVVLLSGSHANEFFFRAGDDDLDQA
KAYPFMTPIFGEGVVFDASPERRKEMLHNAALRGEQMKGHAATIEDQVRRMIADWGEAGEIDLLDFFAELTIY
TSSACLIGKKFRDQLDGRFAKLYHELERGTDPLAYVDPYLPIESFRRRDEARNGLVALVADIMNGRIANPPTD
KSDRDMLDVLIAVKAETGTPRFSADEITGMFISMMFAGHHTSSGTASWTLIELMRHRDAYAAVIDELDELYGD
GRSVSFHALRQIPQLENVLKETLRLHPPLIILMRVAKGEFEVQGHRIHEGDLVAASPAISNRIPEDFPDPHDF
VPARYEQPRQEDLLNRWTWIPFGAGRHRCVGAAFAIMQIKAIFSVLLREYEFEMAQPPESYRNDHSKMVVQLA
QPACVRYRRRTGV

>CYP51B1_Mycobacterium tuberculosis NRITLD56 2577471150
MSAVALPRVSGGHDEHGHLEEFRTDPIGLMQRVRDECGDVGTFQLAGKQVVLLSGSHANEFFFRAGDDDLDQA
KAYPFMTPIFGEGVVFDASPERRKEMLHNAALRGEQMKGHAATIEDQVRRMIADWGEAGEIDLLDFFAELTIY
TSSACLIGKKFRDQLDGRFAKLYHELERGTDPLAYVDPYLPIESFRRRDEARNGLVALVADIMNGRIANPPTD
KSDRDMLDVLIAVKAETGTPRFSADEITGMFISMMFAGHHTSSGTASWTLIELMRHRDAYAAVIDELDELYGD
GRSVSFHALRQIPQLENVLKETLRLHPPLIILMRVAKGEFEVQGHRIHEGDLVAASPAISNRIPEDFPDPHDF
VPARYEQPRQEDLLNRWTWIPFGAGRHRCVGAAFAIMQIKAIFSVLLREYEFEMAQPPESYRNDHSKMVVQLA
QPACVRYRRRTGV
>CYP51B1_Mycobacterium tuberculosis NRITLD57 2575386016
MSAVALPRVSGGHDEHGHLEEFRTDPIGLMQRVRDECGDVGTFQLAGKQVVLLSGSHANEFFFRAGDDDLDQA
KAYPFMTPIFGEGVVFDASPERRKEMLHNAALRGEQMKGHAATIEDQVRRMIADWGEAGEIDLLDFFAELTIY
TSSACLIGKKFRDQLDGRFAKLYHELERGTDPLAYVDPYLPIESFRRRDEARNGLVALVADIMNGRIANPPTD
KSDRDMLDVLIAVKAETGTPRFSADEITGMFISMMFAGHHTSSGTASWTLIELMRHRDAYAAVIDELDELYGD
GRSVSFHALRQIPQLENVLKETLRLHPPLIILMRVAKGEFEVQGHRIHEGDLVAASPAISNRIPEDFPDPHDF
VPARYEQPRQEDLLNRWTWIPFGAGRHRCVGAAFAIMQIKAIFSVLLREYEFEMAQPPESYRNDHSKMVVQLA
QPACVRYRRRTGV
>CYP51B1_Mycobacterium tuberculosis NRITLD59 2632425183
MSAVALPRVSGGHDEHGHLEEFRTDPIGLMQRVRDECGDVGTFQLAGKQVVLLSGSHANEFFFRAGDDDLDQA
KAYPFMTPIFGEGVVFDASPERRKEMLHNAALRGEQMKGHAATIEDQVRRMIADWGEAGEIDLLDFFAELTIY
TSSACLIGKKFRDQLDGRFAKLYHELERGTDPLAYVDPYLPIESFRRRDEARNGLVALVADIMNGRIANPPTD
KSDRDMLDVLIAVKAETGTPRFSADEITGMFISMMFAGHHTSSGTASWTLIELMRHRDAYAAVIDELDELYGD
GRSVSFHALRQIPQLENVLKETLRLHPPLIILMRVAKGEFEVQGHRIHEGDLVAASPAISNRIPEDFPDPHDF
VPARYEQPRQEDLLNRWTWIPFGAGRHRCVGAAFAIMQIKAIFSVLLREYEFEMAQPPESYRNDHSKMVVQLA
QPACVRYRRRTGV
>CYP51B1_Mycobacterium tuberculosis NRITLD60 2628775681
MSAVALPRVSGGHDEHGHLEEFRTDPIGLMQRVRDECGDVGTFQLAGKQVVLLSGSHANEFFFRAGDDDLDQA
KAYPFMTPIFGEGVVFDASPERRKEMLHNAALRGEQMKGHAATIEDQVRRMIADWGEAGEIDLLDFFAELTIY
TSSACLIGKKFRDQLDGRFAKLYHELERGTDPLAYVDPYLPIESFRRRDEARNGLVALVADIMNGRIANPPTD
KSDRDMLDVLIAVKAETGTPRFSADEITGMFISMMFAGHHTSSGTASWTLIELMRHRDAYAAVIDELDELYGD
GRSVSFHALRQIPQLENVLKETLRLHPPLIILMRVAKGEFEVQGHRIHEGDLVAASPAISNRIPEDFPDPHDF
VPARYEQPRQEDLLNRWTWIPFGAGRHRCVGAAFAIMQIKAIFSVLLREYEFEMAQPPESYRNDHSKMVVQLA
QPACVRYRRRTGV
>CYP51B1_Mycobacterium tuberculosis NZ494 2746194886
MSAVALPRVSGGHDEHGHLEEFRTDPIGLMQRVRDECGDVGTFQLAGKQVVLLSGSHANEFFFRAGDDDLDQA
KAYPFMTPIFGEGVVFDASPERRKEMLHNAALRGEQMKGHAATIEDQVRRMIADWGEAGEIDLLDFFAELTIY
TSSACLIGKKFRDQLDGRFAKLYHELERGTDPLAYVDPYLPIESFRRRDEARNGLVALVADIMNGRIANPPTD
KSDRDMLDVLIAVKAETGTPRFSADEITGMFISMMFAGHHTSSGTASWTLIELMRHRDAYAAVIDELDELYGD
GRSVSFHALRQIPQLENVLKETLRLHPPLIILMRVAKGEFEVQGHRIHEGDLVAASPAISNRIPEDFPDPHDF
VPARYEQPRQEDLLNRWTWIPFGAGRHRCVGAAFAIMQIKAIFSVLLREYEFEMAQPPESYRNDHSKMVVQLA
QPACVRYRRRTGV
>CYP51B1_Mycobacterium tuberculosis O00100977 2692176721
MSAVALPRVSGGHDEHGHLEEFRTDPIGLMQRVRDECGDVGTFQLAGKQVVLLSGSHANEFFFRAGDDDLDQA
KAYPFMTPIFGEGVVFDASPERRKEMLHNAALRGEQMKGHAATIEDQVRRMIADWGEAGEIDLLDFFAELTIY
TSSACLIGKKFRDQLDGRFAKLYHELERGTDPLAYVDPYLPIESFRRRDEARNGLVALVADIMNGRIANPPTD
KSDRDMLDVLIAVKAETGTPRFSADEITGMFISMMFAGHHTSSGTASWTLIELMRHRDAYAAVIDELDELYGD
GRSVSFHALRQIPQLENVLKETLRLHPPLIILMRVAKGEFEVQGHRIHEGDLVAASPAISNRIPEDFPDPHDF
VPARYEQPRQEDLLNRWTWIPFGAGRHRCVGAAFAIMQIKAIFSVLLREYEFEMAQPPESYRNDHSKMVVQLA
QPACVRYRRRTGV
>CYP51B1_Mycobacterium tuberculosis NZXDR1 2628819915
MSAVALPRVSGGHDEHGHLEEFRTDPIGLMQRVRDECGDVGTFQLAGKQVVLLSGSHANEFFFRAGDDDLDQA
KAYPFMTPIFGEGVVFDASPERRKEMLHNAALRGEQMKGHAATIEDQVRRMIADWGEAGEIDLLDFFAELTIY
TSSACLIGKKFRDQLDGRFAKLYHELERGTDPLAYVDPYLPIESFRRRDEARNGLVALVADIMNGRIANPPTD
KSDRDMLDVLIAVKAETGTPRFSADEITGMFISMMFAGHHTSSGTASWTLIELMRHRDAYAAVIDELDELYGD
GRSVSFHALRQIPQLENVLKETLRLHPPLIILMRVAKGEFEVQGHRIHEGDLVAASPAISNRIPEDFPDPHDF
VPARYEQPRQEDLLNRWTWIPFGAGRHRCVGAAFAIMQIKAIFSVLLREYEFEMAQPPESYRNDHSKMVVQLA
QPACVRYRRRTGV
>CYP51B1_Mycobacterium tuberculosis O00200127 2671984125

MSAVALPRVSGGHDEHGHLEEFRTDPIGLMQRVRDECGDVGTFQLAGKQVVLLSGSHANEFFFRAGDDDLDQA
KAYPFMTPIFGEGVVFDASPERRKEMLHNAALRGEQMKGHAATIEDQVRRMIADWGEAGEIDLLDFFAELTIY
TSSACLIGKKFRDQLDGRFAKLYHELERGTDPLAYVDPYLPIESFRRRDEARNGLVALVADIMNGRIANPPTD
KSDRDMLDVLIAVKAETGTPRFSADEITGMFISMMFAGHHTSSGTASWTLIELMRHRDAYAAVIDELDELYGD
GRSVSFHALRQIPQLENVLKETLRLHPPLIILMRVAKGEFEVQGHRIHEGDLVAASPAISNRIPEDFPDPHDF
VPARYEQPRQEDLLNRWTWIPFGAGRHRCVGAAFAIMQIKAIFSVLLREYEFEMAQPPESYRNDHSKMVVQLA
QPACVRYRRRTGV
>CYP51B1_Mycobacterium tuberculosis O00401640 2673980679
MSAVALPRVSGGHDEHGHLEEFRTDPIGLMQRVRDECGDVGTFQLAGKQVVLLSGSHANEFFFRAGDDDLDQA
KAYPFMTPIFGEGVVFDASPERRKEMLHNAALRGEQMKGHAATIEDQVRRMIADWGEAGEIDLLDFFAELTIY
TSSACLIGKKFRDQLDGRFAKLYHELERGTDPLAYVDPYLPIESFRRRDEARNGLVALVADIMNGRIANPPTD
KSDRDMLDVLIAVKAETGTPRFSADEITGMFISMMFAGHHTSSGTASWTLIELMRHRDAYAAVIDELDELYGD
GRSVSFHALRQIPQLENVLKETLRLHPPLIILMRVAKGEFEVQGHRIHEGDLVAASPAISNRIPEDFPDPHDF
VPARYEQPRQEDLLNRWTWIPFGAGRHRCVGAAFAIMQIKAIFSVLLREYEFEMAQPPESYRNDHSKMVVQLA
QPACVRYRRRTGV
>CYP51B1_Mycobacterium tuberculosis O09401777 2697084848
MSAVALPRVSGGHDEHGHLEEFRTDPIGLMQRVRDECGDVGTFQLAGKQVVLLSGSHANEFFFRAGDDDLDQA
KAYPFMTPIFGEGVVFDASPERRKEMLHNAALRGEQMKGHAATIEDQVRRMIADWGEAGEIDLLDFFAELTIY
TSSACLIGKKFRDQLDGRFAKLYHELERGTDPLAYVDPYLPIESFRRRDEARNGLVALVADIMNGRIANPPTD
KSDRDMLDVLIAVKAETGTPRFSADEITGMFISMMFAGHHTSSGTASWTLIELMRHRDAYAAVIDELDELYGD
GRSVSFHALRQIPQLENVLKETLRLHPPLIILMRVAKGEFEVQGHRIHEGDLVAASPAISNRIPEDFPDPHDF
VPARYEQPRQEDLLNRWTWIPFGAGRHRCVGAAFAIMQIKAIFSVLLREYEFEMAQPPESYRNDHSKMVVQLA
QPACVRYRRRTGV
>CYP51B1_Mycobacterium tuberculosis O09501089 2698764077
MSAVALPRVSGGHDEHGHLEEFRTDPIGLMQRVRDECGDVGTFQLAGKQVVLLSGSHANEFFFRAGDDDLDQA
KAYPFMTPIFGEGVVFDASPERRKEMLHNAALRGEQMKGHAATIEDQVRRMIADWGEAGEIDLLDFFAELTIY
TSSACLIGKKFRDQLDGRFAKLYHELERGTDPLAYVDPYLPIESFRRRDEARNGLVALVADIMNGRIANPPTD
KSDRDMLDVLIAVKAETGTPRFSADEITGMFISMMFAGHHTSSGTASWTLIELMRHRDAYAAVIDELDELYGD
GRSVSFHALRQIPQLENVLKETLRLHPPLIILMRVAKGEFEVQGHRIHEGDLVAASPAISNRIPEDFPDPHDF
VPARYEQPRQEDLLNRWTWIPFGAGRHRCVGAAFAIMQIKAIFSVLLREYEFEMAQPPESYRNDHSKMVVQLA
QPACVRYRRRTGV
>CYP51B1_Mycobacterium tuberculosis O09501127 2694333383
MSAVALPRVSGGHDEHGHLEEFRTDPIGLMQRVRDECGDVGTFQLAGKQVVLLSGSHANEFFFRAGDDDLDQA
KAYPFMTPIFGEGVVFDASPERRKEMLHNAALRGEQMKGHAATIEDQVRRMIADWGEAGEIDLLDFFAELTIY
TSSACLIGKKFRDQLDGRFAKLYHELERGTDPLAYVDPYLPIESFRRRDEARNGLVALVADIMNGRIANPPTD
KSDRDMLDVLIAVKAETGTPRFSADEITGMFISMMFAGHHTSSGTASWTLIELMRHRDAYAAVIDELDELYGD
GRSVSFHALRQIPQLENVLKETLRLHPPLIILMRVAKGEFEVQGHRIHEGDLVAASPAISNRIPEDFPDPHDF
VPARYEQPRQEDLLNRWTWIPFGAGRHRCVGAAFAIMQIKAIFSVLLREYEFEMAQPPESYRNDHSKMVVQLA
QPACVRYRRRTGV
>CYP51B1_Mycobacterium tuberculosis O09501596 2675611876
MSAVALPRVSGGHDEHGHLEEFRTDPIGLMQRVRDECGDVGTFQLAGKQVVLLSGSHANEFFFRAGDDDLDQA
KAYPFMTPIFGEGVVFDASPERRKEMLHNAALRGEQMKGHAATIEDQVRRMIADWGEAGEIDLLDFFAELTIY
TSSACLIGKKFRDQLDGRFAKLYHELERGTDPLAYVDPYLPIESFRRRDEARNGLVALVADIMNGRIANPPTD
KSDRDMLDVLIAVKAETGTPRFSADEITGMFISMMFAGHHTSSGTASWTLIELMRHRDAYAAVIDELDELYGD
GRSVSFHALRQIPQLENVLKETLRLHPPLIILMRVAKGEFEVQGHRIHEGDLVAASPAISNRIPEDFPDPHDF
VPARYEQPRQEDLLNRWTWIPFGAGRHRCVGAAFAIMQIKAIFSVLLREYEFEMAQPPESYRNDHSKMVVQLA
QPACVRYRRRTGV
>CYP51B1_Mycobacterium tuberculosis O09700898 2703509882
MSAVALPRVSGGHDEHGHLEEFRTDPIGLMQRVRDECGDVGTFQLAGKQVVLLSGSHANEFFFRAGDDDLDQA
KAYPFMTPIFGEGVVFDASPERRKEMLHNAALRGEQMKGHAATIEDQVRRMIADWGEAGEIDLLDFFAELTIY
TSSACLIGKKFRDQLDGRFAKLYHELERGTDPLAYVDPYLPIESFRRRDEARNGLVALVADIMNGRIANPPTD
KSDRDMLDVLIAVKAETGTPRFSADEITGMFISMMFAGHHTSSGTASWTLIELMRHRDAYAAVIDELDELYGD
GRSVSFHALRQIPQLENVLKETLRLHPPLIILMRVAKGEFEVQGHRIHEGDLVAASPAISNRIPEDFPDPHDF
VPARYEQPRQEDLLNRWTWIPFGAGRHRCVGAAFAIMQIKAIFSVLLREYEFEMAQPPESYRNDHSKMVVQLA
QPACVRYRRRTGV
>CYP51B1_Mycobacterium tuberculosis O09701563 2696018519
MSAVALPRVSGGHDEHGHLEEFRTDPIGLMQRVRDECGDVGTFQLAGKQVVLLSGSHANEFFFRAGDDDLDQA
KAYPFMTPIFGEGVVFDASPERRKEMLHNAALRGEQMKGHAATIEDQVRRMIADWGEAGEIDLLDFFAELTIY

TSSACLIGKKFRDQLDGRFAKLYHELERGTDPLAYVDPYLPIESFRRRDEARNGLVALVADIMNGRIANPPTD
KSDRDMLDVLIAVKAETGTPRFSADEITGMFISMMFAGHHTSSGTASWTLIELMRHRDAYAAVIDELDELYGD
GRSVSFHALRQIPQLENVLKETLRLHPPLIILMRVAKGEFEVQGHRIHEGDLVAASPAISNRIPEDFPDPHDF
VPARYEQPRQEDLLNRWTWIPFGAGRHRCVGAAFAIMQIKAIFSVLLREYEFEMAQPPESYRNDHSKMVVQLA
QPACVRYRRRTGV
>CYP51B1_Mycobacterium tuberculosis O09801157 2706551905
MSAVALPRVSGGHDEHGHLEEFRTDPIGLMQRVRDECGDVGTFQLAGKQVVLLSGSHANEFFFRAGDDDLDQA
KAYPFMTPIFGEGVVFDASPERRKEMLHNAALRGEQMKGHAATIEDQVRRMIADWGEAGEIDLLDFFAELTIY
TSSACLIGKKFRDQLDGRFAKLYHELERGTDPLAYVDPYLPIESFRRRDEARNGLVALVADIMNGRIANPPTD
KSDRDMLDVLIAVKAETGTPRFSADEITGMFISMMFAGHHTSSGTASWTLIELMRHRDAYAAVIDELDELYGD
GRSVSFHALRQIPQLENVLKETLRLHPPLIILMRVAKGEFEVQGHRIHEGDLVAASPAISNRIPEDFPDPHDF
VPARYEQPRQEDLLNRWTWIPFGAGRHRCVGAAFAIMQIKAIFSVLLREYEFEMAQPPESYRNDHSKMVVQLA
QPACVRYRRRTGV
>CYP51B1_Mycobacterium tuberculosis O09801359 2696573366
MSAVALPRVSGGHDEHGHLEEFRTDPIGLMQRVRDECGDVGTFQLAGKQVVLLSGSHANEFFFRAGDDDLDQA
KAYPFMTPIFGEGVVFDASPERRKEMLHNAALRGEQMKGHAATIEDQVRRMIADWGEAGEIDLLDFFAELTIY
TSSACLIGKKFRDQLDGRFAKLYHELERGTDPLAYVDPYLPIESFRRRDEARNGLVALVADIMNGRIANPPTD
KSDRDMLDVLIAVKAETGTPRFSADEITGMFISMMFAGHHTSSGTASWTLIELMRHRDAYAAVIDELDELYGD
GRSVSFHALRQIPQLENVLKETLRLHPPLIILMRVAKGEFEVQGHRIHEGDLVAASPAISNRIPEDFPDPHDF
VPARYEQPRQEDLLNRWTWIPFGAGRHRCVGAAFAIMQIKAIFSVLLREYEFEMAQPPESYRNDHSKMVVQLA
QPACVRYRRRTGV
>CYP51B1_Mycobacterium tuberculosis OFXR-10 2578231598
MSAVALPRVSGGHDEHGHLEEFRTDPIGLMQRVRDECGDVGTFQLAGKQVVLLSGSHANEFFFRAGDDDLDQA
KAYPFMTPIFGEGVVFDASPERRKEMLHNAALRGEQMKGHAATIEDQVRRMIADWGEAGEIDLLDFFAELTIY
TSSACLIGKKFRDQLDGRFAKLYHELERGTDPLAYVDPYLPIESFRRRDEARNGLVALVADIMNGRIANPPTD
KSDRDMLDVLIAVKAETGTPRFSADEITGMFISMMFAGHHTSSGTASWTLIELMRHRDAYAAVIDELDELYGD
GRSVSFHALRQIPQLENVLKETLRLHPPLIILMRVAKGEFEVQGHRIHEGDLVAASPAISNRIPEDFPDPHDF
VPARYEQPRQEDLLNRWTWIPFGAGRHRCVGAAFAIMQIKAIFSVLLREYEFEMAQPPESYRNDHSKMVVQLA
QPACVRYRRRTGV
>CYP51B1_Mycobacterium tuberculosis OFXR-13 2576535834
MSAVALPRVSGGHDEHGHLEEFRTDPIGLMQRVRDECGDVGTFQLAGKQVVLLSGSHANEFFFRAGDDDLDQA
KAYPFMTPIFGEGVVFDASPERRKEMLHNAALRGEQMKGHAATIEDQVRRMIADWGEAGEIDLLDFFAELTIY
TSSACLIGKKFRDQLDGRFAKLYHELERGTDPLAYVDPYLPIESFRRRDEARNGLVALVADIMNGRIANPPTD
KSDRDMLDVLIAVKAETGTPRFSADEITGMFISMMFAGHHTSSGTASWTLIELMRHRDAYAAVIDELDELYGD
GRSVSFHALRQIPQLENVLKETLRLHPPLIILMRVAKGEFEVQGHRIHEGDLVAASPAISNRIPEDFPDPHDF
VPARYEQPRQEDLLNRWTWIPFGAGRHRCVGAAFAIMQIKAIFSVLLREYEFEMAQPPESYRNDHSKMVVQLA
QPACVRYRRRTGV
>CYP51B1_Mycobacterium tuberculosis OFXR-15 2576479639
MSAVALPRVSGGHDEHGHLEEFRTDPIGLMQRVRDECGDVGTFQLAGKQVVLLSGSHANEFFFRAGDDDLDQA
KAYPFMTPIFGEGVVFDASPERRKEMLHNAALRGEQMKGHAATIEDQVRRMIADWGEAGEIDLLDFFAELTIY
TSSACLIGKKFRDQLDGRFAKLYHELERGTDPLAYVDPYLPIESFRRRDEARNGLVALVADIMNGRIANPPTD
KSDRDMLDVLIAVKAETGTPRFSADEITGMFISMMFAGHHTSSGTASWTLIELMRHRDAYAAVIDELDELYGD
GRSVSFHALRQIPQLENVLKETLRLHPPLIILMRVAKGEFEVQGHRIHEGDLVAASPAISNRIPEDFPDPHDF
VPARYEQPRQEDLLNRWTWIPFGAGRHRCVGAAFAIMQIKAIFSVLLREYEFEMAQPPESYRNDHSKMVVQLA
QPACVRYRRRTGV
>CYP51B1_Mycobacterium tuberculosis OFXR-26 2576997972
MSAVALPRVSGGHDEHGHLEEFRTDPIGLMQRVRDECGDVGTFQLAGKQVVLLSGSHANEFFFRAGDDDLDQA
KAYPFMTPIFGEGVVFDASPERRKEMLHNAALRGEQMKGHAATIEDQVRRMIADWGEAGEIDLLDFFAELTIY
TSSACLIGKKFRDQLDGRFAKLYHELERGTDPLAYVDPYLPIESFRRRDEARNGLVALVADIMNGRIANPPTD
KSDRDMLDVLIAVKAETGTPRFSADEITGMFISMMFAGHHTSSGTASWTLIELMRHRDAYAAVIDELDELYGD
GRSVSFHALRQIPQLENVLKETLRLHPPLIILMRVAKGEFEVQGHRIHEGDLVAASPAISNRIPEDFPDPHDF
VPARYEQPRQEDLLNRWTWIPFGAGRHRCVGAAFAIMQIKAIFSVLLREYEFEMAQPPESYRNDHSKMVVQLA
QPACVRYRRRTGV
>CYP51B1_Mycobacterium tuberculosis OFXR-27 2603147213
MSAVALPRVSGGHDEHGHLEEFRTDPIGLMQRVRDECGDVGTFQLAGKQVVLLSGSHANEFFFRAGDDDLDQA
KAYPFMTPIFGEGVVFDASPERRKEMLHNAALRGEQMKGHAATIEDQVRRMIADWGEAGEIDLLDFFAELTIY
TSSACLIGKKFRDQLDGRFAKLYHELERGTDPLAYVDPYLPIESFRRRDEARNGLVALVADIMNGRIANPPTD
KSDRDMLDVLIAVKAETGTPRFSADEITGMFISMMFAGHHTSSGTASWTLIELMRHRDAYAAVIDELDELYGD

GRSVSFHALRQIPQLENVLKETLRLHPPLIILMRVAKGEFEVQGHRIHEGDLVAASPAISNRIPEDFPDPHDF
VPARYEQPRQEDLLNRWTWIPFGAGRHRCVGAAFAIMQIKAIFSVLLREYEFEMAQPPESYRNDHSKMVVQLA
QPACVRYRRRTGV
>CYP51B1_Mycobacterium tuberculosis OFXR-28 2604337887
MSAVALPRVSGGHDEHGHLEEFRTDPIGLMQRVRDECGDVGTFQLAGKQVVLLSGSHANEFFFRAGDDDLDQA
KAYPFMTPIFGEGVVFDASPERRKEMLHNAALRGEQMKGHAATIEDQVRRMIADWGEAGEIDLLDFFAELTIY
TSSACLIGKKFRDQLDGRFAKLYHELERGTDPLAYVDPYLPIESFRRRDEARNGLVALVADIMNGRIANPPTD
KSDRDMLDVLIAVKAETGTPRFSADEITGMFISMMFAGHHTSSGTASWTLIELMRHRDAYAAVIDELDELYGD
GRSVSFHALRQIPQLENVLKETLRLHPPLIILMRVAKGEFEVQGHRIHEGDLVAASPAISNRIPEDFPDPHDF
VPARYEQPRQEDLLNRWTWIPFGAGRHRCVGAAFAIMQIKAIFSVLLREYEFEMAQPPESYRNDHSKMVVQLA
QPACVRYRRRTGV
>CYP51B1_Mycobacterium tuberculosis OFXR-3 2576472146
MSAVALPRVSGGHDEHGHLEEFRTDPIGLMQRVRDECGDVGTFQLAGKQVVLLSGSHANEFFFRAGDDDLDQA
KAYPFMTPIFGEGVVFDASPERRKEMLHNAALRGEQMKGHAATIEDQVRRMIADWGEAGEIDLLDFFAELTIY
TSSACLIGKKFRDQLDGRFAKLYHELERGTDPLAYVDPYLPIESFRRRDEARNGLVALVADIMNGRIANPPTD
KSDRDMLDVLIAVKAETGTPRFSADEITGMFISMMFAGHHTSSGTASWTLIELMRHRDAYAAVIDELDELYGD
GRSVSFHALRQIPQLENVLKETLRLHPPLIILMRVAKGEFEVQGHRIHEGDLVAASPAISNRIPEDFPDPHDF
VPARYEQPRQEDLLNRWTWIPFGAGRHRCVGAAFAIMQIKAIFSVLLREYEFEMAQPPESYRNDHSKMVVQLA
QPACVRYRRRTGV
>CYP51B1_Mycobacterium tuberculosis OFXR-33 2577372397
MSAVALPRVSGGHDEHGHLEEFRTDPIGLMQRVRDECGDVGTFQLAGKQVVLLSGSHANEFFFRAGDDDLDQA
KAYPFMTPIFGEGVVFDASPERRKEMLHNAALRGEQMKGHAATIEDQVRRMIADWGEAGEIDLLDFFAELTIY
TSSACLIGKKFRDQLDGRFAKLYHELERGTDPLAYVDPYLPIESFRRRDEARNGLVALVADIMNGRIANPPTD
KSDRDMLDVLIAVKAETGTPRFSADEITGMFISMMFAGHHTSSGTASWTLIELMRHRDAYAAVIDELDELYGD
GRSVSFHALRQIPQLENVLKETLRLHPPLIILMRVAKGEFEVQGHRIHEGDLVAASPAISNRIPEDFPDPHDF
VPARYEQPRQEDLLNRWTWIPFGAGRHRCVGAAFAIMQIKAIFSVLLREYEFEMAQPPESYRNDHSKMVVQLA
QPACVRYRRRTGV
>CYP51B1_Mycobacterium tuberculosis OFXR-6 2575326364
MSAVALPRVSGGHDEHGHLEEFRTDPIGLMQRVRDECGDVGTFQLAGKQVVLLSGSHANEFFFRAGDDDLDQA
KAYPFMTPIFGEGVVFDASPERRKEMLHNAALRGEQMKGHAATIEDQVRRMIADWGEAGEIDLLDFFAELTIY
TSSACLIGKKFRDQLDGRFAKLYHELERGTDPLAYVDPYLPIESFRRRDEARNGLVALVADIMNGRIANPPTD
KSDRDMLDVLIAVKAETGTPRFSADEITGMFISMMFAGHHTSSGTASWTLIELMRHRDAYAAVIDELDELYGD
GRSVSFHALRQIPQLENVLKETLRLHPPLIILMRVAKGEFEVQGHRIHEGDLVAASPAISNRIPEDFPDPHDF
VPARYEQPRQEDLLNRWTWIPFGAGRHRCVGAAFAIMQIKAIFSVLLREYEFEMAQPPESYRNDHSKMVVQLA
QPACVRYRRRTGV
>CYP51B1_Mycobacterium tuberculosis OFXR-7 2575618310
MSAVALPRVSGGHDEHGHLEEFRTDPIGLMQRVRDECGDVGTFQLAGKQVVLLSGSHANEFFFRAGDDDLDQA
KAYPFMTPIFGEGVVFDASPERRKEMLHNAALRGEQMKGHAATIEDQVRRMIADWGEAGEIDLLDFFAELTIY
TSSACLIGKKFRDQLDGRFAKLYHELERGTDPLAYVDPYLPIESFRRRDEARNGLVALVADIMNGRIANPPTD
KSDRDMLDVLIAVKAETGTPRFSADEITGMFISMMFAGHHTSSGTASWTLIELMRHRDAYAAVIDELDELYGD
GRSVSFHALRQIPQLENVLKETLRLHPPLIILMRVAKGEFEVQGHRIHEGDLVAASPAISNRIPEDFPDPHDF
VPARYEQPRQEDLLNRWTWIPFGAGRHRCVGAAFAIMQIKAIFSVLLREYEFEMAQPPESYRNDHSKMVVQLA
QPACVRYRRRTGV
>CYP51B1_Mycobacterium tuberculosis OFXR-8 2575466460
MSAVALPRVSGGHDEHGHLEEFRTDPIGLMQRVRDECGDVGTFQLAGKQVVLLSGSHANEFFFRAGDDDLDQA
KAYPFMTPIFGEGVVFDASPERRKEMLHNAALRGEQMKGHAATIEDQVRRMIADWGEAGEIDLLDFFAELTIY
TSSACLIGKKFRDQLDGRFAKLYHELERGTDPLAYVDPYLPIESFRRRDEARNGLVALVADIMNGRIANPPTD
KSDRDMLDVLIAVKAETGTPRFSADEITGMFISMMFAGHHTSSGTASWTLIELMRHRDAYAAVIDELDELYGD
GRSVSFHALRQIPQLENVLKETLRLHPPLIILMRVAKGEFEVQGHRIHEGDLVAASPAISNRIPEDFPDPHDF
VPARYEQPRQEDLLNRWTWIPFGAGRHRCVGAAFAIMQIKAIFSVLLREYEFEMAQPPESYRNDHSKMVVQLA
QPACVRYRRRTGV
>CYP51B1_Mycobacterium tuberculosis OM-V02_005 2570712315
MSAVALPRVSGGHDEHGHLEEFRTDPIGLMQRVRDECGDVGTFQLAGKQVVLLSGSHANEFFFRAGDDDLDQA
KAYPFMTPIFGEGVVFDASPERRKEMLHNAALRGEQMKGHAATIEDQVRRMIADWGEAGEIDLLDFFAELTIY
TSSACLIGKKFRDQLDGRFAKLYHELERGTDPLAYVDPYLPIESFRRRDEARNGLVALVADIMNGRIANPPTD
KSDRDMLDVLIAVKAETGTPRFSADEITGMFISMMFAGHHTSSGTASWTLIELMRHRDAYAAVIDELDELYGD
GRSVSFHALRQIPQLENVLKETLRLHPPLIILMRVAKGEFEVQGHRIHEGDLVAASPAISNRIPEDFPDPHDF

VPARYEQPRQEDLLNRWTWIPFGAGRHRCVGAAFAIMQIKAIFSVLLREYEFEMAQPPESYRNDHSKMVVQLA
QPACVRYRRRTGV
>CYP51B1_Mycobacterium tuberculosis OSDD105 2603137659
MSAVALPRVSGGHDEHGHLEEFRTDPIGLMQRVRDECGDVGTFQLAGKQVVLLSGSHANEFFFRAGDDDLDQA
KAYPFMTPIFGEGVVFDASPERRKEMLHNAALRGEQMKGHAATIEDQVRRMIADWGEAGEIDLLDFFAELTIY
TSSACLIGKKFRDQLDGRFAKLYHELERGTDPLAYVDPYLPIESFRRRDEARNGLVALVADIMNGRIANPPTD
KSDRDMLDVLIAVKAETGTPRFSADEITGMFISMMFAGHHTSSGTASWTLIELMRHRDAYAAVIDELDELYGD
GRSVSFHALRQIPQLENVLKETLRLHPPLIILMRVAKGEFEVQGHRIHEGDLVAASPAISNRIPEDFPDPHDF
VPARYEQPRQEDLLNRWTWIPFGAGRHRCVGAAFAIMQIKAIFSVLLREYEFEMAQPPESYRNDHSKMVVQLA
QPACVRYRRRTGV
>CYP51B1_Mycobacterium tuberculosis OSDD493 2603142434
MSAVALPRVSGGHDEHGHLEEFRTDPIGLMQRVRDECGDVGTFQLAGKQVVLLSGSHANEFFFRAGDDDLDQA
KAYPFMTPIFGEGVVFDASPERRKEMLHNAALRGEQMKGHAATIEDQVRRMIADWGEAGEIDLLDFFAELTIY
TSSACLIGKKFRDQLDGRFAKLYHELERGTDPLAYVDPYLPIESFRRRDEARNGLVALVADIMNGRIANPPTD
KSDRDMLDVLIAVKAETGTPRFSADEITGMFISMMFAGHHTSSGTASWTLIELMRHRDAYAAVIDELDELYGD
GRSVSFHALRQIPQLENVLKETLRLHPPLIILMRVAKGEFEVQGHRIHEGDLVAASPAISNRIPEDFPDPHDF
VPARYEQPRQEDLLNRWTWIPFGAGRHRCVGAAFAIMQIKAIFSVLLREYEFEMAQPPESYRNDHSKMVVQLA
QPACVRYRRRTGV
>CYP51B1_Mycobacterium tuberculosis P00301553 2697963476
MSAVALPRVSGGHDEHGHLEEFRTDPIGLMQRVRDECGDVGTFQLAGKQVVLLSGSHANEFFFRAGDDDLDQA
KAYPFMTPIFGEGVVFDASPERRKEMLHNAALRGEQMKGHAATIEDQVRRMIADWGEAGEIDLLDFFAELTIY
TSSACLIGKKFRDQLDGRFAKLYHELERGTDPLAYVDPYLPIESFRRRDEARNGLVALVADIMNGRIANPPTD
KSDRDMLDVLIAVKAETGTPRFSADEITGMFISMMFAGHHTSSGTASWTLIELMRHRDAYAAVIDELDELYGD
GRSVSFHALRQIPQLENVLKETLRLHPPLIILMRVAKGEFEVQGHRIHEGDLVAASPAISNRIPEDFPDPHDF
VPARYEQPRQEDLLNRWTWIPFGAGRHRCVGAAFAIMQIKAIFSVLLREYEFEMAQPPESYRNDHSKMVVQLA
QPACVRYRRRTGV
>CYP51B1_Mycobacterium tuberculosis P00401210 2674505844
MSAVALPRVSGGHDEHGHLEEFRTDPIGLMQRVRDECGDVGTFQLAGKQVVLLSGSHANEFFFRAGDDDLDQA
KAYPFMTPIFGEGVVFDASPERRKEMLHNAALRGEQMKGHAATIEDQVRRMIADWGEAGEIDLLDFFAELTIY
TSSACLIGKKFRDQLDGRFAKLYHELERGTDPLAYVDPYLPIESFRRRDEARNGLVALVADIMNGRIANPPTD
KSDRDMLDVLIAVKAETGTPRFSADEITGMFISMMFAGHHTSSGTASWTLIELMRHRDAYAAVIDELDELYGD
GRSVSFHALRQIPQLENVLKETLRLHPPLIILMRVAKGEFEVQGHRIHEGDLVAASPAISNRIPEDFPDPHDF
VPARYEQPRQEDLLNRWTWIPFGAGRHRCVGAAFAIMQIKAIFSVLLREYEFEMAQPPESYRNDHSKMVVQLA
QPACVRYRRRTGV
>CYP51B1_Mycobacterium tuberculosis P00401720 2692507452
MSAVALPRVSGGHDEHGHLEEFRTDPIGLMQRVRDECGDVGTFQLAGKQVVLLSGSHANEFFFRAGDDDLDQA
KAYPFMTPIFGEGVVFDASPERRKEMLHNAALRGEQMKGHAATIEDQVRRMIADWGEAGEIDLLDFFAELTIY
TSSACLIGKKFRDQLDGRFAKLYHELERGTDPLAYVDPYLPIESFRRRDEARNGLVALVADIMNGRIANPPTD
KSDRDMLDVLIAVKAETGTPRFSADEITGMFISMMFAGHHTSSGTASWTLIELMRHRDAYAAVIDELDELYGD
GRSVSFHALRQIPQLENVLKETLRLHPPLIILMRVAKGEFEVQGHRIHEGDLVAASPAISNRIPEDFPDPHDF
VPARYEQPRQEDLLNRWTWIPFGAGRHRCVGAAFAIMQIKAIFSVLLREYEFEMAQPPESYRNDHSKMVVQLA
QPACVRYRRRTGV
>CYP51B1_Mycobacterium tuberculosis P00601463 2697654369
MSAVALPRVSGGHDEHGHLEEFRTDPIGLMQRVRDECGDVGTFQLAGKQVVLLSGSHANEFFFRAGDDDLDQA
KAYPFMTPIFGEGVVFDASPERRKEMLHNAALRGEQMKGHAATIEDQVRRMIADWGEAGEIDLLDFFAELTIY
TSSACLIGKKFRDQLDGRFAKLYHELERGTDPLAYVDPYLPIESFRRRDEARNGLVALVADIMNGRIANPPTD
KSDRDMLDVLIAVKAETGTPRFSADEITGMFISMMFAGHHTSSGTASWTLIELMRHRDAYAAVIDELDELYGD
GRSVSFHALRQIPQLENVLKETLRLHPPLIILMRVAKGEFEVQGHRIHEGDLVAASPAISNRIPEDFPDPHDF
VPARYEQPRQEDLLNRWTWIPFGAGRHRCVGAAFAIMQIKAIFSVLLREYEFEMAQPPESYRNDHSKMVVQLA
QPACVRYRRRTGV
>CYP51B1_Mycobacterium tuberculosis P09501076 2698017850
MSAVALPRVSGGHDEHGHLEEFRTDPIGLMQRVRDECGDVGTFQLAGKQVVLLSGSHANEFFFRAGDDDLDQA
KAYPFMTPIFGEGVVFDASPERRKEMLHNAALRGEQMKGHAATIEDQVRRMIADWGEAGEIDLLDFFAELTIY
TSSACLIGKKFRDQLDGRFAKLYHELERGTDPLAYVDPYLPIESFRRRDEARNGLVALVADIMNGRIANPPTD
KSDRDMLDVLIAVKAETGTPRFSADEITGMFISMMFAGHHTSSGTASWTLIELMRHRDAYAAVIDELDELYGD
GRSVSFHALRQIPQLENVLKETLRLHPPLIILMRVAKGEFEVQGHRIHEGDLVAASPAISNRIPEDFPDPHDF
VPARYEQPRQEDLLNRWTWIPFGAGRHRCVGAAFAIMQIKAIFSVLLREYEFEMAQPPESYRNDHSKMVVQLA
QPACVRYRRRTGV

>CYP51B1_Mycobacterium tuberculosis P09501164 2699226876
MSAVALPRVSGGHDEHGHLEEFRTDPIGLMQRVRDECGDVGTFQLAGKQVVLLSGSHANEFFFRAGDDDLDQA
KAYPFMTPIFGEGVVFDASPERRKEMLHNAALRGEQMKGHAATIEDQVRRMIADWGEAGEIDLLDFFAELTIY
TSSACLIGKKFRDQLDGRFAKLYHELERGTDPLAYVDPYLPIESFRRRDEARNGLVALVADIMNGRIANPPTD
KSDRDMLDVLIAVKAETGTPRFSADEITGMFISMMFAGHHTSSGTASWTLIELMRHRDAYAAVIDELDELYGD
GRSVSFHALRQIPQLENVLKETLRLHPPLIILMRVAKGEFEVQGHRIHEGDLVAASPAISNRIPEDFPDPHDF
VPARYEQPRQEDLLNRWTWIPFGAGRHRCVGAAFAIMQIKAIFSVLLREYEFEMAQPPESYRNDHSKMVVQLA
QPACVRYRRRTGV
>CYP51B1_Mycobacterium tuberculosis P09501258 2673319208
MSAVALPRVSGGHDEHGHLEEFRTDPIGLMQRVRDECGDVGTFQLAGKQVVLLSGSHANEFFFRAGDDDLDQA
KAYPFMTPIFGEGVVFDASPERRKEMLHNAALRGEQMKGHAATIEDQVRRMIADWGEAGEIDLLDFFAELTIY
TSSACLIGKKFRDQLDGRFAKLYHELERGTDPLAYVDPYLPIESFRRRDEARNGLVALVADIMNGRIANPPTD
KSDRDMLDVLIAVKAETGTPRFSADEITGMFISMMFAGHHTSSGTASWTLIELMRHRDAYAAVIDELDELYGD
GRSVSFHALRQIPQLENVLKETLRLHPPLIILMRVAKGEFEVQGHRIHEGDLVAASPAISNRIPEDFPDPHDF
VPARYEQPRQEDLLNRWTWIPFGAGRHRCVGAAFAIMQIKAIFSVLLREYEFEMAQPPESYRNDHSKMVVQLA
QPACVRYRRRTGV
>CYP51B1_Mycobacterium tuberculosis P09501329 2692823963
MSAVALPRVSGGHDEHGHLEEFRTDPIGLMQRVRDECGDVGTFQLAGKQVVLLSGSHANEFFFRAGDDDLDQA
KAYPFMTPIFGEGVVFDASPERRKEMLHNAALRGEQMKGHAATIEDQVRRMIADWGEAGEIDLLDFFAELTIY
TSSACLIGKKFRDQLDGRFAKLYHELERGTDPLAYVDPYLPIESFRRRDEARNGLVALVADIMNGRIANPPTD
KSDRDMLDVLIAVKAETGTPRFSADEITGMFISMMFAGHHTSSGTASWTLIELMRHRDAYAAVIDELDELYGD
GRSVSFHALRQIPQLENVLKETLRLHPPLIILMRVAKGEFEVQGHRIHEGDLVAASPAISNRIPEDFPDPHDF
VPARYEQPRQEDLLNRWTWIPFGAGRHRCVGAAFAIMQIKAIFSVLLREYEFEMAQPPESYRNDHSKMVVQLA
QPACVRYRRRTGV
>CYP51B1_Mycobacterium tuberculosis P09501583 2698980614
MSAVALPRVSGGHDEHGHLEEFRTDPIGLMQRVRDECGDVGTFQLAGKQVVLLSGSHANEFFFRAGDDDLDQA
KAYPFMTPIFGEGVVFDASPERRKEMLHNAALRGEQMKGHAATIEDQVRRMIADWGEAGEIDLLDFFAELTIY
TSSACLIGKKFRDQLDGRFAKLYHELERGTDPLAYVDPYLPIESFRRRDEARNGLVALVADIMNGRIANPPTD
KSDRDMLDVLIAVKAETGTPRFSADEITGMFISMMFAGHHTSSGTASWTLIELMRHRDAYAAVIDELDELYGD
GRSVSFHALRQIPQLENVLKETLRLHPPLIILMRVAKGEFEVQGHRIHEGDLVAASPAISNRIPEDFPDPHDF
VPARYEQPRQEDLLNRWTWIPFGAGRHRCVGAAFAIMQIKAIFSVLLREYEFEMAQPPESYRNDHSKMVVQLA
QPACVRYRRRTGV
>CYP51B1_Mycobacterium tuberculosis P09501896 2694503680
MSAVALPRVSGGHDEHGHLEEFRTDPIGLMQRVRDECGDVGTFQLAGKQVVLLSGSHANEFFFRAGDDDLDQA
KAYPFMTPIFGEGVVFDASPERRKEMLHNAALRGEQMKGHAATIEDQVRRMIADWGEAGEIDLLDFFAELTIY
TSSACLIGKKFRDQLDGRFAKLYHELERGTDPLAYVDPYLPIESFRRRDEARNGLVALVADIMNGRIANPPTD
KSDRDMLDVLIAVKAETGTPRFSADEITGMFISMMFAGHHTSSGTASWTLIELMRHRDAYAAVIDELDELYGD
GRSVSFHALRQIPQLENVLKETLRLHPPLIILMRVAKGEFEVQGHRIHEGDLVAASPAISNRIPEDFPDPHDF
VPARYEQPRQEDLLNRWTWIPFGAGRHRCVGAAFAIMQIKAIFSVLLREYEFEMAQPPESYRNDHSKMVVQLA
QPACVRYRRRTGV
>CYP51B1_Mycobacterium tuberculosis P09502221 2678550939
MSAVALPRVSGGHDEHGHLEEFRTDPIGLMQRVRDECGDVGTFQLAGKQVVLLSGSHANEFFFRAGDDDLDQA
KAYPFMTPIFGEGVVFDASPERRKEMLHNAALRGEQMKGHAATIEDQVRRMIADWGEAGEIDLLDFFAELTIY
TSSACLIGKKFRDQLDGRFAKLYHELERGTDPLAYVDPYLPIESFRRRDEARNGLVALVADIMNGRIANPPTD
KSDRDMLDVLIAVKAETGTPRFSADEITGMFISMMFAGHHTSSGTASWTLIELMRHRDAYAAVIDELDELYGD
GRSVSFHALRQIPQLENVLKETLRLHPPLIILMRVAKGEFEVQGHRIHEGDLVAASPAISNRIPEDFPDPHDF
VPARYEQPRQEDLLNRWTWIPFGAGRHRCVGAAFAIMQIKAIFSVLLREYEFEMAQPPESYRNDHSKMVVQLA
QPACVRYRRRTGV
>CYP51B1_Mycobacterium tuberculosis P09600222 2690928307
MSAVALPRVSGGHDEHGHLEEFRTDPIGLMQRVRDECGDVGTFQLAGKQVVLLSGSHANEFFFRAGDDDLDQA
KAYPFMTPIFGEGVVFDASPERRKEMLHNAALRGEQMKGHAATIEDQVRRMIADWGEAGEIDLLDFFAELTIY
TSSACLIGKKFRDQLDGRFAKLYHELERGTDPLAYVDPYLPIESFRRRDEARNGLVALVADIMNGRIANPPTD
KSDRDMLDVLIAVKAETGTPRFSADEITGMFISMMFAGHHTSSGTASWTLIELMRHRDAYAAVIDELDELYGD
GRSVSFHALRQIPQLENVLKETLRLHPPLIILMRVAKGEFEVQGHRIHEGDLVAASPAISNRIPEDFPDPHDF
VPARYEQPRQEDLLNRWTWIPFGAGRHRCVGAAFAIMQIKAIFSVLLREYEFEMAQPPESYRNDHSKMVVQLA
QPACVRYRRRTGV
>CYP51B1_Mycobacterium tuberculosis P09701236 2692981980

MSAVALPRVSGGHDEHGHLEEFRTDPIGLMQRVRDECGDVGTFQLAGKQVVLLSGSHANEFFFRAGDDDLDQA
KAYPFMTPIFGEGVVFDASPERRKEMLHNAALRGEQMKGHAATIEDQVRRMIADWGEAGEIDLLDFFAELTIY
TSSACLIGKKFRDQLDGRFAKLYHELERGTDPLAYVDPYLPIESFRRRDEARNGLVALVADIMNGRIANPPTD
KSDRDMLDVLIAVKAETGTPRFSADEITGMFISMMFAGHHTSSGTASWTLIELMRHRDAYAAVIDELDELYGD
GRSVSFHALRQIPQLENVLKETLRLHPPLIILMRVAKGEFEVQGHRIHEGDLVAASPAISNRIPEDFPDPHDF
VPARYEQPRQEDLLNRWTWIPFGAGRHRCVGAAFAIMQIKAIFSVLLREYEFEMAQPPESYRNDHSKMVVQLA
QPACVRYRRRTGV
>CYP51B1_Mycobacterium tuberculosis PanR0201 2555577898
MSAVALPRVSGGHDEHGHLEEFRTDPIGLMQRVRDECGDVGTFQLAGKQVVLLSGSHANEFFFRAGDDDLDQA
KAYPFMTPIFGEGVVFDASPERRKEMLHNAALRGEQMKGHAATIEDQVRRMIADWGEAGEIDLLDFFAELTIY
TSSACLIGKKFRDQLDGRFAKLYHELERGTDPLAYVDPYLPIESFRRRDEARNGLVALVADIMNGRIANPPTD
KSDRDMLDVLIAVKAETGTPRFSADEITGMFISMMFAGHHTSSGTASWTLIELMRHRDAYAAVIDELDELYGD
GRSVSFHALRQIPQLENVLKETLRLHPPLIILMRVAKGEFEVQGHRIHEGDLVAASPAISNRIPEDFPDPHDF
VPARYEQPRQEDLLNRWTWIPFGAGRHRCVGAAFAIMQIKAIFSVLLREYEFEMAQPPESYRNDHSKMVVQLA
QPACVRYRRRTGV
>CYP51B1_Mycobacterium tuberculosis PanR0202 2598066482
MSAVALPRVSGGHDEHGHLEEFRTDPIGLMQRVRDECGDVGTFQLAGKQVVLLSGSHANEFFFRAGDDDLDQA
KAYPFMTPIFGEGVVFDASPERRKEMLHNAALRGEQMKGHAATIEDQVRRMIADWGEAGEIDLLDFFAELTIY
TSSACLIGKKFRDQLDGRFAKLYHELERGTDPLAYVDPYLPIESFRRRDEARNGLVALVADIMNGRIANPPTD
KSDRDMLDVLIAVKAETGTPRFSADEITGMFISMMFAGHHTSSGTASWTLIELMRHRDAYAAVIDELDELYGD
GRSVSFHALRQIPQLENVLKETLRLHPPLIILMRVAKGEFEVQGHRIHEGDLVAASPAISNRIPEDFPDPHDF
VPARYEQPRQEDLLNRWTWIPFGAGRHRCVGAAFAIMQIKAIFSVLLREYEFEMAQPPESYRNDHSKMVVQLA
QPACVRYRRRTGV
>CYP51B1_Mycobacterium tuberculosis PanR0203 2555147527
MSAVALPRVSGGHDEHGHLEEFRTDPIGLMQRVRDECGDVGTFQLAGKQVVLLSGSHANEFFFRAGDDDLDQA
KAYPFMTPIFGEGVVFDASPERRKEMLHNAALRGEQMKGHAATIEDQVRRMIADWGEAGEIDLLDFFAELTIY
TSSACLIGKKFRDQLDGRFAKLYHELERGTDPLAYVDPYLPIESFRRRDEARNGLVALVADIMNGRIANPPTD
KSDRDMLDVLIAVKAETGTPRFSADEITGMFISMMFAGHHTSSGTASWTLIELMRHRDAYAAVIDELDELYGD
GRSVSFHALRQIPQLENVLKETLRLHPPLIILMRVAKGEFEVQGHRIHEGDLVAASPAISNRIPEDFPDPHDF
VPARYEQPRQEDLLNRWTWIPFGAGRHRCVGAAFAIMQIKAIFSVLLREYEFEMAQPPESYRNDHSKMVVQLA
QPACVRYRRRTGV
>CYP51B1_Mycobacterium tuberculosis PanR0205 2555139128
MSAVALPRVSGGHDEHGHLEEFRTDPIGLMQRVRDECGDVGTFQLAGKQVVLLSGSHANEFFFRAGDDDLDQA
KAYPFMTPIFGEGVVFDASPERRKEMLHNAALRGEQMKGHAATIEDQVRRMIADWGEAGEIDLLDFFAELTIY
TSSACLIGKKFRDQLDGRFAKLYHELERGTDPLAYVDPYLPIESFRRRDEARNGLVALVADIMNGRIANPPTD
KSDRDMLDVLIAVKAETGTPRFSADEITGMFISMMFAGHHTSSGTASWTLIELMRHRDAYAAVIDELDELYGD
GRSVSFHALRQIPQLENVLKETLRLHPPLIILMRVAKGEFEVQGHRIHEGDLVAASPAISNRIPEDFPDPHDF
VPARYEQPRQEDLLNRWTWIPFGAGRHRCVGAAFAIMQIKAIFSVLLREYEFEMAQPPESYRNDHSKMVVQLA
QPACVRYRRRTGV
>CYP51B1_Mycobacterium tuberculosis PanR0206 2555143346
MSAVALPRVSGGHDEHGHLEEFRTDPIGLMQRVRDECGDVGTFQLAGKQVVLLSGSHANEFFFRAGDDDLDQA
KAYPFMTPIFGEGVVFDASPERRKEMLHNAALRGEQMKGHAATIEDQVRRMIADWGEAGEIDLLDFFAELTIY
TSSACLIGKKFRDQLDGRFAKLYHELERGTDPLAYVDPYLPIESFRRRDEARNGLVALVADIMNGRIANPPTD
KSDRDMLDVLIAVKAETGTPRFSADEITGMFISMMFAGHHTSSGTASWTLIELMRHRDAYAAVIDELDELYGD
GRSVSFHALRQIPQLENVLKETLRLHPPLIILMRVAKGEFEVQGHRIHEGDLVAASPAISNRIPEDFPDPHDF
VPARYEQPRQEDLLNRWTWIPFGAGRHRCVGAAFAIMQIKAIFSVLLREYEFEMAQPPESYRNDHSKMVVQLA
QPACVRYRRRTGV
>CYP51B1_Mycobacterium tuberculosis PanR0207 2555159323
MSAVALPRVSGGHDEHGHLEEFRTDPIGLMQRVRDECGDVGTFQLAGKQVVLLSGSHANEFFFRAGDDDLDQA
KAYPFMTPIFGEGVVFDASPERRKEMLHNAALRGEQMKGHAATIEDQVRRMIADWGEAGEIDLLDFFAELTIY
TSSACLIGKKFRDQLDGRFAKLYHELERGTDPLAYVDPYLPIESFRRRDEARNGLVALVADIMNGRIANPPTD
KSDRDMLDVLIAVKAETGTPRFSADEITGMFISMMFAGHHTSSGTASWTLIELMRHRDAYAAVIDELDELYGD
GRSVSFHALRQIPQLENVLKETLRLHPPLIILMRVAKGEFEVQGHRIHEGDLVAASPAISNRIPEDFPDPHDF
VPARYEQPRQEDLLNRWTWIPFGAGRHRCVGAAFAIMQIKAIFSVLLREYEFEMAQPPESYRNDHSKMVVQLA
QPACVRYRRRTGV
>CYP51B1_Mycobacterium tuberculosis PanR0208 2555151721
MSAVALPRVSGGHDEHGHLEEFRTDPIGLMQRVRDECGDVGTFQLAGKQVVLLSGSHANEFFFRAGDDDLDQA
KAYPFMTPIFGEGVVFDASPERRKEMLHNAALRGEQMKGHAATIEDQVRRMIADWGEAGEIDLLDFFAELTIY

TSSACLIGKKFRDQLDGRFAKLYHELERGTDPLAYVDPYLPIESFRRRDEARNGLVALVADIMNGRIANPPTD
KSDRDMLDVLIAVKAETGTPRFSADEITGMFISMMFAGHHTSSGTASWTLIELMRHRDAYAAVIDELDELYGD
GRSVSFHALRQIPQLENVLKETLRLHPPLIILMRVAKGEFEVQGHRIHEGDLVAASPAISNRIPEDFPDPHDF
VPARYEQPRQEDLLNRWTWIPFGAGRHRCVGAAFAIMQIKAIFSVLLREYEFEMAQPPESYRNDHSKMVVQLA
QPACVRYRRRTGV
>CYP51B1_Mycobacterium tuberculosis PanR0209 2555163488
MSAVALPRVSGGHDEHGHLEEFRTDPIGLMQRVRDECGDVGTFQLAGKQVVLLSGSHANEFFFRAGDDDLDQA
KAYPFMTPIFGEGVVFDASPERRKEMLHNAALRGEQMKGHAATIEDQVRRMIADWGEAGEIDLLDFFAELTIY
TSSACLIGKKFRDQLDGRFAKLYHELERGTDPLAYVDPYLPIESFRRRDEARNGLVALVADIMNGRIANPPTD
KSDRDMLDVLIAVKAETGTPRFSADEITGMFISMMFAGHHTSSGTASWTLIELMRHRDAYAAVIDELDELYGD
GRSVSFHALRQIPQLENVLKETLRLHPPLIILMRVAKGEFEVQGHRIHEGDLVAASPAISNRIPEDFPDPHDF
VPARYEQPRQEDLLNRWTWIPFGAGRHRCVGAAFAIMQIKAIFSVLLREYEFEMAQPPESYRNDHSKMVVQLA
QPACVRYRRRTGV
>CYP51B1_Mycobacterium tuberculosis PanR0301 2555281388
MSAVALPRVSGGHDEHGHLEEFRTDPIGLMQRVRDECGDVGTFQLAGKQVVLLSGSHANEFFFRAGDDDLDQA
KAYPFMTPIFGEGVVFDASPERRKEMLHNAALRGEQMKGHAATIEDQVRRMIADWGEAGEIDLLDFFAELTIY
TSSACLIGKKFRDQLDGRFAKLYHELERGTDPLAYVDPYLPIESFRRRDEARNGLVALVADIMNGRIANPPTD
KSDRDMLDVLIAVKAETGTPRFSADEITGMFISMMFAGHHTSSGTASWTLIELMRHRDAYAAVIDELDELYGD
GRSVSFHALRQIPQLENVLKETLRLHPPLIILMRVAKGEFEVQGHRIHEGDLVAASPAISNRIPEDFPDPHDF
VPARYEQPRQEDLLNRWTWIPFGAGRHRCVGAAFAIMQIKAIFSVLLREYEFEMAQPPESYRNDHSKMVVQLA
QPACVRYRRRTGV
>CYP51B1_Mycobacterium tuberculosis PanR0304 2555277120
MSAVALPRVSGGHDEHGHLEEFRTDPIGLMQRVRDECGDVGTFQLAGKQVVLLSGSHANEFFFRAGDDDLDQA
KAYPFMTPIFGEGVVFDASPERRKEMLHNAALRGEQMKGHAATIEDQVRRMIADWGEAGEIDLLDFFAELTIY
TSSACLIGKKFRDQLDGRFAKLYHELERGTDPLAYVDPYLPIESFRRRDEARNGLVALVADIMNGRIANPPTD
KSDRDMLDVLIAVKAETGTPRFSADEITGMFISMMFAGHHTSSGTASWTLIELMRHRDAYAAVIDELDELYGD
GRSVSFHALRQIPQLENVLKETLRLHPPLIILMRVAKGEFEVQGHRIHEGDLVAASPAISNRIPEDFPDPHDF
VPARYEQPRQEDLLNRWTWIPFGAGRHRCVGAAFAIMQIKAIFSVLLREYEFEMAQPPESYRNDHSKMVVQLA
QPACVRYRRRTGV
>CYP51B1_Mycobacterium tuberculosis PanR0306 2555302499
MSAVALPRVSGGHDEHGHLEEFRTDPIGLMQRVRDECGDVGTFQLAGKQVVLLSGSHANEFFFRAGDDDLDQA
KAYPFMTPIFGEGVVFDASPERRKEMLHNAALRGEQMKGHAATIEDQVRRMIADWGEAGEIDLLDFFAELTIY
TSSACLIGKKFRDQLDGRFAKLYHELERGTDPLAYVDPYLPIESFRRRDEARNGLVALVADIMNGRIANPPTD
KSDRDMLDVLIAVKAETGTPRFSADEITGMFISMMFAGHHTSSGTASWTLIELMRHRDAYAAVIDELDELYGD
GRSVSFHALRQIPQLENVLKETLRLHPPLIILMRVAKGEFEVQGHRIHEGDLVAASPAISNRIPEDFPDPHDF
VPARYEQPRQEDLLNRWTWIPFGAGRHRCVGAAFAIMQIKAIFSVLLREYEFEMAQPPESYRNDHSKMVVQLA
QPACVRYRRRTGV
>CYP51B1_Mycobacterium tuberculosis PanR0307 2555289926
MSAVALPRVSGGHDEHGHLEEFRTDPIGLMQRVRDECGDVGTFQLAGKQVVLLSGSHANEFFFRAGDDDLDQA
KAYPFMTPIFGEGVVFDASPERRKEMLHNAALRGEQMKGHAATIEDQVRRMIADWGEAGEIDLLDFFAELTIY
TSSACLIGKKFRDQLDGRFAKLYHELERGTDPLAYVDPYLPIESFRRRDEARNGLVALVADIMNGRIANPPTD
KSDRDMLDVLIAVKAETGTPRFSADEITGMFISMMFAGHHTSSGTASWTLIELMRHRDAYAAVIDELDELYGD
GRSVSFHALRQIPQLENVLKETLRLHPPLIILMRVAKGEFEVQGHRIHEGDLVAASPAISNRIPEDFPDPHDF
VPARYEQPRQEDLLNRWTWIPFGAGRHRCVGAAFAIMQIKAIFSVLLREYEFEMAQPPESYRNDHSKMVVQLA
QPACVRYRRRTGV
>CYP51B1_Mycobacterium tuberculosis PanR0308 2555272919
MSAVALPRVSGGHDEHGHLEEFRTDPIGLMQRVRDECGDVGTFQLAGKQVVLLSGSHANEFFFRAGDDDLDQA
KAYPFMTPIFGEGVVFDASPERRKEMLHNAALRGEQMKGHAATIEDQVRRMIADWGEAGEIDLLDFFAELTIY
TSSACLIGKKFRDQLDGRFAKLYHELERGTDPLAYVDPYLPIESFRRRDEARNGLVALVADIMNGRIANPPTD
KSDRDMLDVLIAVKAETGTPRFSADEITGMFISMMFAGHHTSSGTASWTLIELMRHRDAYAAVIDELDELYGD
GRSVSFHALRQIPQLENVLKETLRLHPPLIILMRVAKGEFEVQGHRIHEGDLVAASPAISNRIPEDFPDPHDF
VPARYEQPRQEDLLNRWTWIPFGAGRHRCVGAAFAIMQIKAIFSVLLREYEFEMAQPPESYRNDHSKMVVQLA
QPACVRYRRRTGV
>CYP51B1_Mycobacterium tuberculosis PanR0309 2555294151
MSAVALPRVSGGHDEHGHLEEFRTDPIGLMQRVRDECGDVGTFQLAGKQVVLLSGSHANEFFFRAGDDDLDQA
KAYPFMTPIFGEGVVFDASPERRKEMLHNAALRGEQMKGHAATIEDQVRRMIADWGEAGEIDLLDFFAELTIY
TSSACLIGKKFRDQLDGRFAKLYHELERGTDPLAYVDPYLPIESFRRRDEARNGLVALVADIMNGRIANPPTD
KSDRDMLDVLIAVKAETGTPRFSADEITGMFISMMFAGHHTSSGTASWTLIELMRHRDAYAAVIDELDELYGD

GRSVSFHALRQIPQLENVLKETLRLHPPLIILMRVAKGEFEVQGHRIHEGDLVAASPAISNRIPEDFPDPHDF
VPARYEQPRQEDLLNRWTWIPFGAGRHRCVGAAFAIMQIKAIFSVLLREYEFEMAQPPESYRNDHSKMVVQLA
QPACVRYRRRTGV
>CYP51B1_Mycobacterium tuberculosis PanR0311 2555285632
MSAVALPRVSGGHDEHGHLEEFRTDPIGLMQRVRDECGDVGTFQLAGKQVVLLSGSHANEFFFRAGDDDLDQA
KAYPFMTPIFGEGVVFDASPERRKEMLHNAALRGEQMKGHAATIEDQVRRMIADWGEAGEIDLLDFFAELTIY
TSSACLIGKKFRDQLDGRFAKLYHELERGTDPLAYVDPYLPIESFRRRDEARNGLVALVADIMNGRIANPPTD
KSDRDMLDVLIAVKAETGTPRFSADEITGMFISMMFAGHHTSSGTASWTLIELMRHRDAYAAVIDELDELYGD
GRSVSFHALRQIPQLENVLKETLRLHPPLIILMRVAKGEFEVQGHRIHEGDLVAASPAISNRIPEDFPDPHDF
VPARYEQPRQEDLLNRWTWIPFGAGRHRCVGAAFAIMQIKAIFSVLLREYEFEMAQPPESYRNDHSKMVVQLA
QPACVRYRRRTGV
>CYP51B1_Mycobacterium tuberculosis PanR0313 2555345150
MSAVALPRVSGGHDEHGHLEEFRTDPIGLMQRVRDECGDVGTFQLAGKQVVLLSGSHANEFFFRAGDDDLDQA
KAYPFMTPIFGEGVVFDASPERRKEMLHNAALRGEQMKGHAATIEDQVRRMIADWGEAGEIDLLDFFAELTIY
TSSACLIGKKFRDQLDGRFAKLYHELERGTDPLAYVDPYLPIESFRRRDEARNGLVALVADIMNGRIANPPTD
KSDRDMLDVLIAVKAETGTPRFSADEITGMFISMMFAGHHTSSGTASWTLIELMRHRDAYAAVIDELDELYGD
GRSVSFHALRQIPQLENVLKETLRLHPPLIILMRVAKGEFEVQGHRIHEGDLVAASPAISNRIPEDFPDPHDF
VPARYEQPRQEDLLNRWTWIPFGAGRHRCVGAAFAIMQIKAIFSVLLREYEFEMAQPPESYRNDHSKMVVQLA
QPACVRYRRRTGV
>CYP51B1_Mycobacterium tuberculosis PanR0314 2555306743
MSAVALPRVSGGHDEHGHLEEFRTDPIGLMQRVRDECGDVGTFQLAGKQVVLLSGSHANEFFFRAGDDDLDQA
KAYPFMTPIFGEGVVFDASPERRKEMLHNAALRGEQMKGHAATIEDQVRRMIADWGEAGEIDLLDFFAELTIY
TSSACLIGKKFRDQLDGRFAKLYHELERGTDPLAYVDPYLPIESFRRRDEARNGLVALVADIMNGRIANPPTD
KSDRDMLDVLIAVKAETGTPRFSADEITGMFISMMFAGHHTSSGTASWTLIELMRHRDAYAAVIDELDELYGD
GRSVSFHALRQIPQLENVLKETLRLHPPLIILMRVAKGEFEVQGHRIHEGDLVAASPAISNRIPEDFPDPHDF
VPARYEQPRQEDLLNRWTWIPFGAGRHRCVGAAFAIMQIKAIFSVLLREYEFEMAQPPESYRNDHSKMVVQLA
QPACVRYRRRTGV
>CYP51B1_Mycobacterium tuberculosis PanR0315 2555310947
MSAVALPRVSGGHDEHGHLEEFRTDPIGLMQRVRDECGDVGTFQLAGKQVVLLSGSHANEFFFRAGDDDLDQA
KAYPFMTPIFGEGVVFDASPERRKEMLHNAALRGEQMKGHAATIEDQVRRMIADWGEAGEIDLLDFFAELTIY
TSSACLIGKKFRDQLDGRFAKLYHELERGTDPLAYVDPYLPIESFRRRDEARNGLVALVADIMNGRIANPPTD
KSDRDMLDVLIAVKAETGTPRFSADEITGMFISMMFAGHHTSSGTASWTLIELMRHRDAYAAVIDELDELYGD
GRSVSFHALRQIPQLENVLKETLRLHPPLIILMRVAKGEFEVQGHRIHEGDLVAASPAISNRIPEDFPDPHDF
VPARYEQPRQEDLLNRWTWIPFGAGRHRCVGAAFAIMQIKAIFSVLLREYEFEMAQPPESYRNDHSKMVVQLA
QPACVRYRRRTGV
>CYP51B1_Mycobacterium tuberculosis PanR0316 2555328369
MSAVALPRVSGGHDEHGHLEEFRTDPIGLMQRVRDECGDVGTFQLAGKQVVLLSGSHANEFFFRAGDDDLDQA
KAYPFMTPIFGEGVVFDASPERRKEMLHNAALRGEQMKGHAATIEDQVRRMIADWGEAGEIDLLDFFAELTIY
TSSACLIGKKFRDQLDGRFAKLYHELERGTDPLAYVDPYLPIESFRRRDEARNGLVALVADIMNGRIANPPTD
KSDRDMLDVLIAVKAETGTPRFSADEITGMFISMMFAGHHTSSGTASWTLIELMRHRDAYAAVIDELDELYGD
GRSVSFHALRQIPQLENVLKETLRLHPPLIILMRVAKGEFEVQGHRIHEGDLVAASPAISNRIPEDFPDPHDF
VPARYEQPRQEDLLNRWTWIPFGAGRHRCVGAAFAIMQIKAIFSVLLREYEFEMAQPPESYRNDHSKMVVQLA
QPACVRYRRRTGV
>CYP51B1_Mycobacterium tuberculosis PanR0317 2555559834
MSAVALPRVSGGHDEHGHLEEFRTDPIGLMQRVRDECGDVGTFQLAGKQVVLLSGSHANEFFFRAGDDDLDQA
KAYPFMTPIFGEGVVFDASPERRKEMLHNAALRGEQMKGHAATIEDQVRRMIADWGEAGEIDLLDFFAELTIY
TSSACLIGKKFRDQLDGRFAKLYHELERGTDPLAYVDPYLPIESFRRRDEARNGLVALVADIMNGRIANPPTD
KSDRDMLDVLIAVKAETGTPRFSADEITGMFISMMFAGHHTSSGTASWTLIELMRHRDAYAAVIDELDELYGD
GRSVSFHALRQIPQLENVLKETLRLHPPLIILMRVAKGEFEVQGHRIHEGDLVAASPAISNRIPEDFPDPHDF
VPARYEQPRQEDLLNRWTWIPFGAGRHRCVGAAFAIMQIKAIFSVLLREYEFEMAQPPESYRNDHSKMVVQLA
QPACVRYRRRTGV
>CYP51B1_Mycobacterium tuberculosis PanR0401 2555598820
MSAVALPRVSGGHDEHGHLEEFRTDPIGLMQRVRDECGDVGTFQLAGKQVVLLSGSHANEFFFRAGDDDLDQA
KAYPFMTPIFGEGVVFDASPERRKEMLHNAALRGEQMKGHAATIEDQVRRMIADWGEAGEIDLLDFFAELTIY
TSSACLIGKKFRDQLDGRFAKLYHELERGTDPLAYVDPYLPIESFRRRDEARNGLVALVADIMNGRIANPPTD
KSDRDMLDVLIAVKAETGTPRFSADEITGMFISMMFAGHHTSSGTASWTLIELMRHRDAYAAVIDELDELYGD
GRSVSFHALRQIPQLENVLKETLRLHPPLIILMRVAKGEFEVQGHRIHEGDLVAASPAISNRIPEDFPDPHDF

VPARYEQPRQEDLLNRWTWIPFGAGRHRCVGAAFAIMQIKAIFSVLLREYEFEMAQPPESYRNDHSKMVVQLA
QPACVRYRRRTGV
>CYP51B1_Mycobacterium tuberculosis PanR0402 2555551311
MSAVALPRVSGGHDEHGHLEEFRTDPIGLMQRVRDECGDVGTFQLAGKQVVLLSGSHANEFFFRAGDDDLDQA
KAYPFMTPIFGEGVVFDASPERRKEMLHNAALRGEQMKGHAATIEDQVRRMIADWGEAGEIDLLDFFAELTIY
TSSACLIGKKFRDQLDGRFAKLYHELERGTDPLAYVDPYLPIESFRRRDEARNGLVALVADIMNGRIANPPTD
KSDRDMLDVLIAVKAETGTPRFSADEITGMFISMMFAGHHTSSGTASWTLIELMRHRDAYAAVIDELDELYGD
GRSVSFHALRQIPQLENVLKETLRLHPPLIILMRVAKGEFEVQGHRIHEGDLVAASPAISNRIPEDFPDPHDF
VPARYEQPRQEDLLNRWTWIPFGAGRHRCVGAAFAIMQIKAIFSVLLREYEFEMAQPPESYRNDHSKMVVQLA
QPACVRYRRRTGV
>CYP51B1_Mycobacterium africanum MAL010120 2580365188
MSAVALLRVSGGHDEHGHLEEFRTDPIGLMQRVRDECGDVGTFQLAGKQVVLLSGSHANEFFFRAGDDDLDQA
KAYPFMTPIFGEGVVFDASPERRKEMLHNAALRGEQMKGHAATIEDQVRRMIADWGEAGEIDLLDFFAELTIY
TSSACLIGKKFRDQLDGRFAKLYHELERGTDPLAYVDPYLPIESFRRRDEARNGLVALVADIMNGRIANPPTD
KSDRDMLDVLIAVKAETGTPRFSADEITGMFISMMFAGHHTSSGTASWTLIELMRHRDAYAAVIDELDELYGD
GRSVSFHALRQIPQLENVLKETLRLHPPLIILMRVAKGEFEVQGHRIHEGDLVAASPAISNRIPEDFPDPHDF
VPARYEQPRQEDLLNRWTWIPFGAGRHRCVGAAFAIMQIKAIFSVLLREYEFEMAQPPESYRNDHSKMVVQLA
QPACVRYRRRTGV
>CYP51B1_Mycobacterium africanum MAL020135 2583731157
MSAVALLRVSGGHDEHGHLEEFRTDPIGLMQRVRDECGDVGTFQLAGKQVVLLSGSHANEFFFRAGDDDLDQA
KAYPFMTPIFGEGVVFDASPERRKEMLHNAALRGEQMKGHAATIEDQVRRMIADWGEAGEIDLLDFFAELTIY
TSSACLIGKKFRDQLDGRFAKLYHELERGTDPLAYVDPYLPIESFRRRDEARNGLVALVADIMNGRIANPPTD
KSDRDMLDVLIAVKAETGTPRFSADEITGMFISMMFAGHHTSSGTASWTLIELMRHRDAYAAVIDELDELYGD
GRSVSFHALRQIPQLENVLKETLRLHPPLIILMRVAKGEFEVQGHRIHEGDLVAASPAISNRIPEDFPDPHDF
VPARYEQPRQEDLLNRWTWIPFGAGRHRCVGAAFAIMQIKAIFSVLLREYEFEMAQPPESYRNDHSKMVVQLA
QPACVRYRRRTGV
>CYP51B1_Mycobacterium canettii CIPT 140070008 2541568820
MSAVALPRVSGGHDEHGHLEEFRTDPIGLMQRVRDECGDVGTFQLAGKQVVLLSGSHANEFFFRAGDDDLDQA
KAYPFMTPIFGEGVVFDASPERRKEMLHNAALRGEQMKGHAATIEDQVRRMIADWGEAGEIDLLDFFAELTIY
TSSACLIGKKFRDQLDGRFAKLYHELERGTDPLAYVDPYLPIESFRRRDEARNGLVALVADIMNGRIANPPTD
KSDRDMLDVLIAVKAETGTPRFSADEITGMFISMMFAGHHTSSGTASWTLIELMRHRDAYAAVIDELDELYGD
GRSVSFHALHQIPQLENVLKETLRLHPPLIILMRVAKGEFEVQGHRIHEGDLVAASPAISNRIPEDFPDPHDF
VPARYEQPRQEDLLNRWTWIPFGAGRHRCVGAAFAIMQIKAIFSVLLREYEFEMAQPPESYRNDHSKMVVQLA
QPACVRYRRRTGV
>CYP51B1_Mycobacterium canettii CIPT 140070013 2566975628
MSAVALPRVSGGHDEHGHLEEFRTDPIGLMQRVRDECGDVGTFQLAGKQVVLLSGSHANEFFFRAGDDDLDQA
KAYPFMTPIFGEGVVFDASPERRKEMLHNAALRGEQMKGHAATIEDQVRRMIADWGEAGEIDLLDFFAELTIY
TSSACLIGKKFRDQLDGRFAKLYHELERGTDPLAYVDPYLPIESFRRRDEARNGLVALVADIMNGRIANPPTD
KSDRDMLDVLIAVKAETGTPRFSADEITGMFISMMFAGHHTSSGTASWTLIELMRHRDAYAAVIDELDELYGD
GRSVSFHALHQIPQLENVLKETLRLHPPLIILMRVAKGEFEVQGHRIHEGDLVAASPAISNRIPEDFPDPHDF
VPARYEQPRQEDLLNRWTWIPFGAGRHRCVGAAFAIMQIKAIFSVLLREYEFEMAQPPESYRNDHSKMVVQLA
QPACVRYRRRTGV
>CYP51B1_Mycobacterium caprae Allgaeu 2730170435
MSAVALPRVSGGHDEHGHLEEFRTDPIGLMQRVRDECGDVGTFQLAGKQVVLLSGSHANEFFFRAGDDDLDQA
KAYPFMTPIFGEGVVFDASPERRKEMLHNAALRGEQMKGHAATIEGQVRRMIADWGEAGEIDLLDFFAELTIY
TSSACLIGKKFRDQLDGRFAKLYHELERGTDPLAYVDPYLPIESFRRRDEARNGLVALVADIMNGRIANPPTD
KSDRDMLDVLIAVKAETGTPRFSADEITGMFISMMFAGHHTSSGTASWTLIELMRHRDAYAAVIDELDELYGD
GRSVSFHALRQIPQLENVLKETLRLHPPLIILMRVAKGEFEVQGHRIHEGDLVAASPAISNRIPEDFPDPHDF
VPARYEQPRQEDLLNRWTWIPFGAGRHRCVGAAFAIMQIKAIFSVLLREYEFEMAQPPESYRNDHSKMVVQLA
QPACVRYRRRTGV
>CYP51B1_Mycobacterium tuberculosis 1615 2577956531
MSAVALPRVSGGHDEHGHLEEFRTDPIGLMQRVRDECGDVGTFQLAGKQVVLLSGSHANEFFFRAGDDDLDQA
KAYPFMTPIFGEGVVFDASPERRKEMLHNAALRGEQMKGHAATIEDQVRRMIADWGEAAEIDLLDFFAELTIY
TSSACLIGKKFRDQLDGRFAKLYHELERGTDPLAYVDPYLPIESFRRRDEARNGLVALVADIMNGRIANPPTD
KSDRDMLDVLIAVKAETGTPRFSADEITGMFISMMFAGHHTSSGTASWTLIELMRHRDAYAAVIDELDELYGD
GRSVSFHALRQIPQLENVLKETLRLHPPLIILMRVAKGEFEVQGHRIHEGDLVAASPAISNRIPEDFPDPHDF
VPARYEQPRQEDLLNRWTWIPFGAGRHRCVGAAFAIMQIKAIFSVLLREYEFEMAQPPESYRNDHSKMVVQLA
QPACVRYRRRTGV

>CYP51B1_Mycobacterium tuberculosis 16955 2575106333
MSAVALPRVSGGHDEHGHLEEFRTDPIGLMQRVRDECGDVGTFQLAGKQVVLLSGSHANEFFFRAGDDDLDQA
KAYPFMTPIFGEGVVFDASPERRKEMLHNAALRGEQMKGHAATIEDQVRRMIADWGEAAEIDLLDFFAELTIY
TSSACLIGKKFRDQLDGRFAKLYHELERGTDPLAYVDPYLPIESFRRRDEARNGLVALVADIMNGRIANPPTD
KSDRDMLDVLIAVKAETGTPRFSADEITGMFISMMFAGHHTSSGTASWTLIELMRHRDAYAAVIDELDELYGD
GRSVSFHALRQIPQLENVLKETLRLHPPLIILMRVAKGEFEVQGHRIHEGDLVAASPAISNRIPEDFPDPHDF
VPARYEQPRQEDLLNRWTWIPFGAGRHRCVGAAFAIMQIKAIFSVLLREYEFEMAQPPESYRNDHSKMVVQLA
QPACVRYRRRTGV
>CYP51B1_Mycobacterium tuberculosis 200105 2678011138
MSAVALPRVSGGHDEHGHLEEFRTDPIGLMQRVRDECGDVGTFQLAGKQVVLLSGSHANEFFFRAGDDDLDQA
KAYPFMTPIFGEGVVFDASPERRKEMLHNAALRGEQMKGHAATIEDQVRRMIADWGEAAEIDLLDFFAELTIY
TSSACLIGKKFRDQLDGRFAKLYHELERGTDPLAYVDPYLPIESFRRRDEARNGLVALVADIMNGRIANPPTD
KSDRDMLDVLIAVKAETGTPRFSADEITGMFISMMFAGHHTSSGTASWTLIELMRHRDAYAAVIDELDELYGD
GRSVSFHALRQIPQLENVLKETLRLHPPLIILMRVAKGEFEVQGHRIHEGDLVAASPAISNRIPEDFPDPHDF
VPARYEQPRQEDLLNRWTWIPFGAGRHRCVGAAFAIMQIKAIFSVLLREYEFEMAQPPESYRNDHSKMVVQLA
QPACVRYRRRTGV
>CYP51B1_Mycobacterium tuberculosis 201009 2693265199
MSAVALPRVSGGHDEHGHLEEFRTDPIGLMQRVRDECGDVGTFQLAGKQVVLLSGSHANEFFFRAGDDDLDQA
KAYPFMTPIFGEGVVFDASPERRKEMLHNAALRGEQMKGHAATIEDQVRRMIADWGEAAEIDLLDFFAELTIY
TSSACLIGKKFRDQLDGRFAKLYHELERGTDPLAYVDPYLPIESFRRRDEARNGLVALVADIMNGRIANPPTD
KSDRDMLDVLIAVKAETGTPRFSADEITGMFISMMFAGHHTSSGTASWTLIELMRHRDAYAAVIDELDELYGD
GRSVSFHALRQIPQLENVLKETLRLHPPLIILMRVAKGEFEVQGHRIHEGDLVAASPAISNRIPEDFPDPHDF
VPARYEQPRQEDLLNRWTWIPFGAGRHRCVGAAFAIMQIKAIFSVLLREYEFEMAQPPESYRNDHSKMVVQLA
QPACVRYRRRTGV
>CYP51B1_Mycobacterium tuberculosis 26105 2688176697
MSAVALPRVSGGHDEHGHLEEFRTDPIGLMQRVRDECGDVGTFQLAGKQVVLLSGSHANEFFFRAGDDDLDQA
KAYPFMTPIFGEGVVFDASPERRKEMLHNAALRGEQMKGHAATIEDQVRRMIADWGEAAEIDLLDFFAELTIY
TSSACLIGKKFRDQLDGRFAKLYHELERGTDPLAYVDPYLPIESFRRRDEARNGLVALVADIMNGRIANPPTD
KSDRDMLDVLIAVKAETGTPRFSADEITGMFISMMFAGHHTSSGTASWTLIELMRHRDAYAAVIDELDELYGD
GRSVSFHALRQIPQLENVLKETLRLHPPLIILMRVAKGEFEVQGHRIHEGDLVAASPAISNRIPEDFPDPHDF
VPARYEQPRQEDLLNRWTWIPFGAGRHRCVGAAFAIMQIKAIFSVLLREYEFEMAQPPESYRNDHSKMVVQLA
QPACVRYRRRTGV
>CYP51B1_Mycobacterium tuberculosis 401107 2696799389
MSAVALPRVSGGHDEHGHLEEFRTDPIGLMQRVRDECGDVGTFQLAGKQVVLLSGSHANEFFFRAGDDDLDQA
KAYPFMTPIFGEGVVFDASPERRKEMLHNAALRGEQMKGHAATIEDQVRRMIADWGEAAEIDLLDFFAELTIY
TSSACLIGKKFRDQLDGRFAKLYHELERGTDPLAYVDPYLPIESFRRRDEARNGLVALVADIMNGRIANPPTD
KSDRDMLDVLIAVKAETGTPRFSADEITGMFISMMFAGHHTSSGTASWTLIELMRHRDAYAAVIDELDELYGD
GRSVSFHALRQIPQLENVLKETLRLHPPLIILMRVAKGEFEVQGHRIHEGDLVAASPAISNRIPEDFPDPHDF
VPARYEQPRQEDLLNRWTWIPFGAGRHRCVGAAFAIMQIKAIFSVLLREYEFEMAQPPESYRNDHSKMVVQLA
QPACVRYRRRTGV
>CYP51B1_Mycobacterium tuberculosis 402538 2701669702
MSAVALPRVSGGHDEHGHLEEFRTDPIGLMQRVRDECGDVGTFQLAGKQVVLLSGSHANEFFFRAGDDDLDQA
KAYPFMTPIFGEGVVFDASPERRKEMLHNAALRGEQMKGHAATIEDQVRRMIADWGEAAEIDLLDFFAELTIY
TSSACLIGKKFRDQLDGRFAKLYHELERGTDPLAYVDPYLPIESFRRRDEARNGLVALVADIMNGRIANPPTD
KSDRDMLDVLIAVKAETGTPRFSADEITGMFISMMFAGHHTSSGTASWTLIELMRHRDAYAAVIDELDELYGD
GRSVSFHALRQIPQLENVLKETLRLHPPLIILMRVAKGEFEVQGHRIHEGDLVAASPAISNRIPEDFPDPHDF
VPARYEQPRQEDLLNRWTWIPFGAGRHRCVGAAFAIMQIKAIFSVLLREYEFEMAQPPESYRNDHSKMVVQLA
QPACVRYRRRTGV
>CYP51B1_Mycobacterium tuberculosis 6605 2621684715
MSAVALPRVSGGHDEHGHLEEFRTDPIGLMQRVRDECGDVGTFQLAGKQVVLLSGSHANEFFFRAGDDDLDQA
KAYPFMTPIFGEGVVFDASPERRKEMLHNAALRGEQMKGHAATIEDQVRRMIADWGEAAEIDLLDFFAELTIY
TSSACLIGKKFRDQLDGRFAKLYHELERGTDPLAYVDPYLPIESFRRRDEARNGLVALVADIMNGRIANPPTD
KSDRDMLDVLIAVKAETGTPRFSADEITGMFISMMFAGHHTSSGTASWTLIELMRHRDAYAAVIDELDELYGD
GRSVSFHALRQIPQLENVLKETLRLHPPLIILMRVAKGEFEVQGHRIHEGDLVAASPAISNRIPEDFPDPHDF
VPARYEQPRQEDLLNRWTWIPFGAGRHRCVGAAFAIMQIKAIFSVLLREYEFEMAQPPESYRNDHSKMVVQLA
QPACVRYRRRTGV
>CYP51B1_Mycobacterium tuberculosis A70067_1 2693752373

MSAVALPRVSGGHDEHGHLEEFRTDPIGLMQRVRDECGDVGTFQLAGKQVVLLSGSHANEFFFRAGDDDLDQA
KAYPFMTPIFGEGVVFDASPERRKEMLHNAALRGEQMKGHAATIEDQVRRMIADWGEAAEIDLLDFFAELTIY
TSSACLIGKKFRDQLDGRFAKLYHELERGTDPLAYVDPYLPIESFRRRDEARNGLVALVADIMNGRIANPPTD
KSDRDMLDVLIAVKAETGTPRFSADEITGMFISMMFAGHHTSSGTASWTLIELMRHRDAYAAVIDELDELYGD
GRSVSFHALRQIPQLENVLKETLRLHPPLIILMRVAKGEFEVQGHRIHEGDLVAASPAISNRIPEDFPDPHDF
VPARYEQPRQEDLLNRWTWIPFGAGRHRCVGAAFAIMQIKAIFSVLLREYEFEMAQPPESYRNDHSKMVVQLA
QPACVRYRRRTGV
>CYP51B1_Mycobacterium tuberculosis A70144_1 2662243123
MSAVALPRVSGGHDEHGHLEEFRTDPIGLMQRVRDECGDVGTFQLAGKQVVLLSGSHANEFFFRAGDDDLDQA
KAYPFMTPIFGEGVVFDASPERRKEMLHNAALRGEQMKGHAATIEDQVRRMIADWGEAAEIDLLDFFAELTIY
TSSACLIGKKFRDQLDGRFAKLYHELERGTDPLAYVDPYLPIESFRRRDEARNGLVALVADIMNGRIANPPTD
KSDRDMLDVLIAVKAETGTPRFSADEITGMFISMMFAGHHTSSGTASWTLIELMRHRDAYAAVIDELDELYGD
GRSVSFHALRQIPQLENVLKETLRLHPPLIILMRVAKGEFEVQGHRIHEGDLVAASPAISNRIPEDFPDPHDF
VPARYEQPRQEDLLNRWTWIPFGAGRHRCVGAAFAIMQIKAIFSVLLREYEFEMAQPPESYRNDHSKMVVQLA
QPACVRYRRRTGV
>CYP51B1_Mycobacterium tuberculosis A70144_2 2695925228
MSAVALPRVSGGHDEHGHLEEFRTDPIGLMQRVRDECGDVGTFQLAGKQVVLLSGSHANEFFFRAGDDDLDQA
KAYPFMTPIFGEGVVFDASPERRKEMLHNAALRGEQMKGHAATIEDQVRRMIADWGEAAEIDLLDFFAELTIY
TSSACLIGKKFRDQLDGRFAKLYHELERGTDPLAYVDPYLPIESFRRRDEARNGLVALVADIMNGRIANPPTD
KSDRDMLDVLIAVKAETGTPRFSADEITGMFISMMFAGHHTSSGTASWTLIELMRHRDAYAAVIDELDELYGD
GRSVSFHALRQIPQLENVLKETLRLHPPLIILMRVAKGEFEVQGHRIHEGDLVAASPAISNRIPEDFPDPHDF
VPARYEQPRQEDLLNRWTWIPFGAGRHRCVGAAFAIMQIKAIFSVLLREYEFEMAQPPESYRNDHSKMVVQLA
QPACVRYRRRTGV
>CYP51B1_Mycobacterium tuberculosis A70280 2665655117
MSAVALPRVSGGHDEHGHLEEFRTDPIGLMQRVRDECGDVGTFQLAGKQVVLLSGSHANEFFFRAGDDDLDQA
KAYPFMTPIFGEGVVFDASPERRKEMLHNAALRGEQMKGHAATIEDQVRRMIADWGEAAEIDLLDFFAELTIY
TSSACLIGKKFRDQLDGRFAKLYHELERGTDPLAYVDPYLPIESFRRRDEARNGLVALVADIMNGRIANPPTD
KSDRDMLDVLIAVKAETGTPRFSADEITGMFISMMFAGHHTSSGTASWTLIELMRHRDAYAAVIDELDELYGD
GRSVSFHALRQIPQLENVLKETLRLHPPLIILMRVAKGEFEVQGHRIHEGDLVAASPAISNRIPEDFPDPHDF
VPARYEQPRQEDLLNRWTWIPFGAGRHRCVGAAFAIMQIKAIFSVLLREYEFEMAQPPESYRNDHSKMVVQLA
QPACVRYRRRTGV
>CYP51B1_Mycobacterium tuberculosis A70428 2663814404
MSAVALPRVSGGHDEHGHLEEFRTDPIGLMQRVRDECGDVGTFQLAGKQVVLLSGSHANEFFFRAGDDDLDQA
KAYPFMTPIFGEGVVFDASPERRKEMLHNAALRGEQMKGHAATIEDQVRRMIADWGEAAEIDLLDFFAELTIY
TSSACLIGKKFRDQLDGRFAKLYHELERGTDPLAYVDPYLPIESFRRRDEARNGLVALVADIMNGRIANPPTD
KSDRDMLDVLIAVKAETGTPRFSADEITGMFISMMFAGHHTSSGTASWTLIELMRHRDAYAAVIDELDELYGD
GRSVSFHALRQIPQLENVLKETLRLHPPLIILMRVAKGEFEVQGHRIHEGDLVAASPAISNRIPEDFPDPHDF
VPARYEQPRQEDLLNRWTWIPFGAGRHRCVGAAFAIMQIKAIFSVLLREYEFEMAQPPESYRNDHSKMVVQLA
QPACVRYRRRTGV
>CYP51B1_Mycobacterium tuberculosis A70620 2702088419
MSAVALPRVSGGHDEHGHLEEFRTDPIGLMQRVRDECGDVGTFQLAGKQVVLLSGSHANEFFFRAGDDDLDQA
KAYPFMTPILGEGVVFDASPERRKEMLHNAALRGEQMKGHAATIEDQVRRMIADWGEAGEIDLLDFFAELTIY
TSSACLIGKKFRDQLDGRFAKLYHELERGTDPLAYVDPYLPIESFRRRDEARNGLVALVADIMNGRIANPPTD
KSDRDMLDVLIAVKAETGTPRFSADEITGMFISMMFAGHHTSSGTASWTLIELMRHRDAYAAVIDELDELYGD
GRSVSFHALRQIPQLENVLKETLRLHPPLIILMRVAKGEFEVQGHRIHEGDLVAASPAISNRIPEDFPDPHDF
VPARYEQPRQEDLLNRWTWIPFGAGRHRCVGAAFAIMQIKAIFSVLLREYEFEMAQPPESYRNDHSKMVVQLA
QPACVRYRRRTGV
>CYP51B1_Mycobacterium tuberculosis A70769 2659135637
MSAVALPRVSGGHDEHGHLEEFRTDPIGLMQRVRDECGDVGTFQLAGKQVVLLSGSHANEFFFRAGDDDLDQA
KAYPFMTPIFGEGVVFDASPERRKEMLHNAALRGEQMKGHAATIEDQVRRMIADWGEAAEIDLLDFFAELTIY
TSSACLIGKKFRDQLDGRFAKLYHELERGTDPLAYVDPYLPIESFRRRDEARNGLVALVADIMNGRIANPPTD
KSDRDMLDVLIAVKAETGTPRFSADEITGMFISMMFAGHHTSSGTASWTLIELMRHRDAYAAVIDELDELYGD
GRSVSFHALRQIPQLENVLKETLRLHPPLIILMRVAKGEFEVQGHRIHEGDLVAASPAISNRIPEDFPDPHDF
VPARYEQPRQEDLLNRWTWIPFGAGRHRCVGAAFAIMQIKAIFSVLLREYEFEMAQPPESYRNDHSKMVVQLA
QPACVRYRRRTGV
>CYP51B1_Mycobacterium tuberculosis A70780 2682778291
MSAVALPRVSGGHDEHGHLEEFRTDPIGLMQRVRDECGDVGTFQLAGKQVVLLSGSHANEFFFRAGDDDLDQA
KAYPFMTPIFGEGVVFDASPERRKEMLHNAALRGEQMKGHAATIEDQVRRMIADWGEAAEIDLLDFFAELTIY

TSSACLIGKKFRDQLDGRFAKLYHELERGTDPLAYVDPYLPIESFRRRDEARNGLVALVADIMNGRIANPPTD
KSDRDMLDVLIAVKAETGTPRFSADEITGMFISMMFAGHHTSSGTASWTLIELMRHRDAYAAVIDELDELYGD
GRSVSFHALRQIPQLENVLKETLRLHPPLIILMRVAKGEFEVQGHRIHEGDLVAASPAISNRIPEDFPDPHDF
VPARYEQPRQEDLLNRWTWIPFGAGRHRCVGAAFAIMQIKAIFSVLLREYEFEMAQPPESYRNDHSKMVVQLA
QPACVRYRRRTGV
>CYP51B1_Mycobacterium tuberculosis Aethiop_vetus_229 2746213151
MSAVALPRVSGGHDEHGHLEEFRTDPIGLMQRVRDECGDVGTFQLAGKQVVLLSGSHANEFFFRAGDDDLDQA
KAYPFVTPIFGEGVVFDASPERRKEMLHNAALRGEQMKGHAATIEDQVRRMIADWGEAGEIDLLDFFAELTIY
TSSACLIGKKFRDQLDGRFAKLYHELERGTDPLAYVDPYLPIESFRRRDEARNGLVALVADIMNGRIANPPTD
KSDRDMLDVLIAVKAETGTPRFSADEITGMFISMMFAGHHTSSGTASWTLIELMRHRDAYAAVIDELDELYGD
GRSVSFHALRQIPQLENVLKETLRLHPPLIILMRVAKGEFEVQGHRIHEGDLVAASPAISNRIPEDFPDPHDF
VPARYEQPRQEDLLNRWTWIPFGAGRHRCVGAAFAIMQIKAIFSVLLREYEFEMAQPPESYRNDHSKMVVQLA
QPACVRYRRRTGV
>CYP51B1_Mycobacterium tuberculosis Aethiop_vetus_230 2742638281
MSAVALPRVSGGHDEHGHLEEFRTDPIGLMQRVRDECGDVGTFQLAGKQVVLLSGSHANEFFFRAGDDDLDQA
KAYPFVTPIFGEGVVFDASPERRKEMLHNAALRGEQMKGHAATIEDQVRRMIADWGEAGEIDLLDFFAELTIY
TSSACLIGKKFRDQLDGRFAKLYHELERGTDPLAYVDPYLPIESFRRRDEARNGLVALVADIMNGRIANPPTD
KSDRDMLDVLIAVKAETGTPRFSADEITGMFISMMFAGHHTSSGTASWTLIELMRHRDAYAAVIDELDELYGD
GRSVSFHALRQIPQLENVLKETLRLHPPLIILMRVAKGEFEVQGHRIHEGDLVAASPAISNRIPEDFPDPHDF
VPARYEQPRQEDLLNRWTWIPFGAGRHRCVGAAFAIMQIKAIFSVLLREYEFEMAQPPESYRNDHSKMVVQLA
QPACVRYRRRTGV
>CYP51B1_Mycobacterium tuberculosis Aethiop_vetus_232 2746222227
MSAVALPRVSGGHDEHGHLEEFRTDPIGLMQRVRDECGDVGTFQLAGKQVVLLSGSHANEFFFRAGDDDLDQA
KAYPFVTPIFGEGVVFDASPERRKEMLHNAALRGEQMKGHAATIEDQVRRMIADWGEAGEIDLLDFFAELTIY
TSSACLIGKKFRDQLDGRFAKLYHELERGTDPLAYVDPYLPIESFRRRDEARNGLVALVADIMNGRIANPPTD
KSDRDMLDVLIAVKAETGTPRFSADEITGMFISMMFAGHHTSSGTASWTLIELMRHRDAYAAVIDELDELYGD
GRSVSFHALRQIPQLENVLKETLRLHPPLIILMRVAKGEFEVQGHRIHEGDLVAASPAISNRIPEDFPDPHDF
VPARYEQPRQEDLLNRWTWIPFGAGRHRCVGAAFAIMQIKAIFSVLLREYEFEMAQPPESYRNDHSKMVVQLA
QPACVRYRRRTGV
>CYP51B1_Mycobacterium tuberculosis Aethiop_vetus_233 2746226061
MSAVALPRVSGGHDEHGHLEEFRTDPIGLMQRVRDECGDVGTFQLAGKQVVLLSGSHANEFFFRAGDDDLDQA
KAYPFVTPIFGEGVVFDASPERRKEMLHNAALRGEQMKGHAATIEDQVRRMIADWGEAGEIDLLDFFAELTIY
TSSACLIGKKFRDQLDGRFAKLYHELERGTDPLAYVDPYLPIESFRRRDEARNGLVALVADIMNGRIANPPTD
KSDRDMLDVLIAVKAETGTPRFSADEITGMFISMMFAGHHTSSGTASWTLIELMRHRDAYAAVIDELDELYGD
GRSVSFHALRQIPQLENVLKETLRLHPPLIILMRVAKGEFEVQGHRIHEGDLVAASPAISNRIPEDFPDPHDF
VPARYEQPRQEDLLNRWTWIPFGAGRHRCVGAAFAIMQIKAIFSVLLREYEFEMAQPPESYRNDHSKMVVQLA
QPACVRYRRRTGV
>CYP51B1_Mycobacterium tuberculosis Aethiop_vetus_234 2746217282
MSAVALPRVSGGHDEHGHLEEFRTDPIGLMQRVRDECGDVGTFQLAGKQVVLLSGSHANEFFFRAGDDDLDQA
KAYPFVTPIFGEGVVFDASPERRKEMLHNAALRGEQMKGHAATIEDQVRRMIADWGEAGEIDLLDFFAELTIY
TSSACLIGKKFRDQLDGRFAKLYHELERGTDPLAYVDPYLPIESFRRRDEARNGLVALVADIMNGRIANPPTD
KSDRDMLDVLIAVKAETGTPRFSADEITGMFISMMFAGHHTSSGTASWTLIELMRHRDAYAAVIDELDELYGD
GRSVSFHALRQIPQLENVLKETLRLHPPLIILMRVAKGEFEVQGHRIHEGDLVAASPAISNRIPEDFPDPHDF
VPARYEQPRQEDLLNRWTWIPFGAGRHRCVGAAFAIMQIKAIFSVLLREYEFEMAQPPESYRNDHSKMVVQLA
QPACVRYRRRTGV
>CYP51B1_Mycobacterium tuberculosis BTB04-388 2604526992
MSAVALPRVSGGHDEHGHLEEFRTDPIGLMQRVRDECGDVGTFQLAGKQVVLLSGSHANEFFFRAGDDDLDQA
KAYPFMTPIFGEGVVFDASPERRKEMLHNAALRGEQMKGHAATIEDQVRRMIADWGEAAEIDLLDFFAELTIY
TSSACLIGKKFRDQLDGRFAKLYHELERGTDPLAYVDPYLPIESFRRRDEARNGLVALVADIMNGRIANPPTD
KSDRDMLDVLIAVKAETGTPRFSADEITGMFISMMFAGHHTSSGTASWTLIELMRHRDAYAAVIDELDELYGD
GRSVSFHALRQIPQLENVLKETLRLHPPLIILMRVAKGEFEVQGHRIHEGDLVAASPAISNRIPEDFPDPHDF
VPARYEQPRQEDLLNRWTWIPFGAGRHRCVGAAFAIMQIKAIFSVLLREYEFEMAQPPESYRNDHSKMVVQLA
QPACVRYRRRTGV
>CYP51B1_Mycobacterium tuberculosis BTB05-001 2603307615
MSAVALPRVSGGHDEHGHLEEFRTDPIGLMQRVRDECGDVGTFQLAGKQVVLLSGSHANEFFFRAGDDDLDQA
KAYPFMTPIFGEGVVFDASPERRKEMLHNAALRGEQMKGHAATIEDQVRRMIADWGEAAEIDLLDFFAELTIY
TSSACLIGKKFRDQLDGRFAKLYHELERGTDPLAYVDPYLPIESFRRRDEARNGLVALVADIMNGRIANPPTD
KSDRDMLDVLIAVKAETGTPRFSADEITGMFISMMFAGHHTSSGTASWTLIELMRHRDAYAAVIDELDELYGD

GRSVSFHALRQIPQLENVLKETLRLHPPLIILMRVAKGEFEVQGHRIHEGDLVAASPAISNRIPEDFPDPHDF
VPARYEQPRQEDLLNRWTWIPFGAGRHRCVGAAFAIMQIKAIFSVLLREYEFEMAQPPESYRNDHSKMVVQLA
QPACVRYRRRTGV
>CYP51B1_Mycobacterium tuberculosis BTB05-013 2576563889
MSAVALPRVSGGHDEHGHLEEFRTDPIGLMQRVRDECGDVGTFQLAGKQVVLLSGSHANEFFFRAGDDDLDQA
KAYPFMTPILGEGVVFDASPERRKEMLHNAALRGEQMKGHAATIEDQVRRMIADWGEAGEIDLLDFFAELTIY
TSSACLIGKKFRDQLDGRFAKLYHELERGTDPLAYVDPYLPIESFRRRDEARNGLVALVADIMNGRIANPPTD
KSDRDMLDVLIAVKAETGTPRFSADEITGMFISMMFAGHHTSSGTASWTLIELMRHRDAYAAVIDELDELYGD
GRSVSFHALRQIPQLENVLKETLRLHPPLIILMRVAKGEFEVQGHRIHEGDLVAASPAISNRIPEDFPDPHDF
VPARYEQPRQEDLLNRWTWIPFGAGRHRCVGAAFAIMQIKAIFSVLLREYEFEMAQPPESYRNDHSKMVVQLA
QPACVRYRRRTGV
>CYP51B1_Mycobacterium tuberculosis BTB05-285 2577184506
MSAVALPRVSGGHDEHGHLEEFRTDPIGLMQRVRDECGDVGTFQLAGKQVVLLSGSHANEFFFRAGDDDLDQA
KAYPFMTPIFGEGVVFDASPERRKEMLHNAALRGEQMKGHAATIEDQVRRMIADWGEAAEIDLLDFFAELTIY
TSSACLIGKKFRDQLDGRFAKLYHELERGTDPLAYVDPYLPIESFRRRDEARNGLVALVADIMNGRIANPPTD
KSDRDMLDVLIAVKAETGTPRFSADEITGMFISMMFAGHHTSSGTASWTLIELMRHRDAYAAVIDELDELYGD
GRSVSFHALRQIPQLENVLKETLRLHPPLIILMRVAKGEFEVQGHRIHEGDLVAASPAISNRIPEDFPDPHDF
VPARYEQPRQEDLLNRWTWIPFGAGRHRCVGAAFAIMQIKAIFSVLLREYEFEMAQPPESYRNDHSKMVVQLA
QPACVRYRRRTGV
>CYP51B1_Mycobacterium tuberculosis BTB05-348 2605254719
MSAVALPRVSGGHDEHGHLEEFRTDPIGLMQRVRDECGDVGTFQLAGKQVVLLSGSHANEFFFRAGDDDLDQA
KAYPFMTPIFGEGVVFDASPERRKEMLHNAALRGEQMKGHAATIEDQVRRMIADWGEAAEIDLLDFFAELTIY
TSSACLIGKKFRDQLDGRFAKLYHELERGTDPLAYVDPYLPIESFRRRDEARNGLVALVADIMNGRIANPPTD
KSDRDMLDVLIAVKAETGTPRFSADEITGMFISMMFAGHHTSSGTASWTLIELMRHRDAYAAVIDELDELYGD
GRSVSFHALRQIPQLENVLKETLRLHPPLIILMRVAKGEFEVQGHRIHEGDLVAASPAISNRIPEDFPDPHDF
VPARYEQPRQEDLLNRWTWIPFGAGRHRCVGAAFAIMQIKAIFSVLLREYEFEMAQPPESYRNDHSKMVVQLA
QPACVRYRRRTGV
>CYP51B1_Mycobacterium tuberculosis BTB06-467 2604888991
MSAVALPRVSGGHDEHGHLEEFRTDPIGLMQRVRDECGDVGTFQLAGKQVVLLSGSHANEFFFRAGDDDLDQA
KAYPFMTPIFGEGVVFDASPERRKEMLHNAALRGEQMKGHAATIEDQVRRMIADWGEAAEIDLLDFFAELTIY
TSSACLIGKKFRDQLDGRFAKLYHELERGTDPLAYVDPYLPIESFRRRDEARNGLVALVADIMNGRIANPPTD
KSDRDMLDVLIAVKAETGTPRFSADEITGMFISMMFAGHHTSSGTASWTLIELMRHRDAYAAVIDELDELYGD
GRSVSFHALRQIPQLENVLKETLRLHPPLIILMRVAKGEFEVQGHRIHEGDLVAASPAISNRIPEDFPDPHDF
VPARYEQPRQEDLLNRWTWIPFGAGRHRCVGAAFAIMQIKAIFSVLLREYEFEMAQPPESYRNDHSKMVVQLA
QPACVRYRRRTGV
>CYP51B1_Mycobacterium tuberculosis BTB07-070 2605071577
MSAVALPRVSGGHDEHGHLEEFRTDPIGLMQRVRDECGDVGTFQLAGKQVVLLSGSHANEFFFRAGDDDLDQA
KAYPFMTPILGEGVVFDASPERRKEMLHNAALRGEQMKGHAATIEDQVRRMIADWGEAGEIDLLDFFAELTIY
TSSACLIGKKFRDQLDGRFAKLYHELERGTDPLAYVDPYLPIESFRRRDEARNGLVALVADIMNGRIANPPTD
KSDRDMLDVLIAVKAETGTPRFSADEITGMFISMMFAGHHTSSGTASWTLIELMRHRDAYAAVIDELDELYGD
GRSVSFHALRQIPQLENVLKETLRLHPPLIILMRVAKGEFEVQGHRIHEGDLVAASPAISNRIPEDFPDPHDF
VPARYEQPRQEDLLNRWTWIPFGAGRHRCVGAAFAIMQIKAIFSVLLREYEFEMAQPPESYRNDHSKMVVQLA
QPACVRYRRRTGV
>CYP51B1_Mycobacterium tuberculosis BTB07-034 2577454034
MSAVALPRVSGGHDEHGHLEEFRTDPIGLMQRVRDECGDVGTFQLAGKQVVLLSGSHANEFFFRAGDDDLDQA
KAYPFMTPILGEGVVFDASPERRKEMLHNAALRGEQMKGHAATIEDQVRRMIADWGEAGEIDLLDFFAELTIY
TSSACLIGKKFRDQLDGRFAKLYHELERGTDPLAYVDPYLPIESFRRRDEARNGLVALVADIMNGRIANPPTD
KSDRDMLDVLIAVKAETGTPRFSADEITGMFISMMFAGHHTSSGTASWTLIELMRHRDAYAAVIDELDELYGD
GRSVSFHALRQIPQLENVLKETLRLHPPLIILMRVAKGEFEVQGHRIHEGDLVAASPAISNRIPEDFPDPHDF
VPARYEQPRQEDLLNRWTWIPFGAGRHRCVGAAFAIMQIKAIFSVLLREYEFEMAQPPESYRNDHSKMVVQLA
QPACVRYRRRTGV
>CYP51B1_Mycobacterium tuberculosis BTB07-126 2603459889
MSAVALPRVSGGHDEHGHLEEFRTDPIGLMQRVRDECGDVGTFQLAGKQVVLLSGSHANEFFFRAGDDDLDQA
KAYPFMTPILGEGVVFDASPERRKEMLHNAALRGEQMKGHAATIEDQVRRMIADWGEAGEIDLLDFFAELTIY
TSSACLIGKKFRDQLDGRFAKLYHELERGTDPLAYVDPYLPIESFRRRDEARNGLVALVADIMNGRIANPPTD
KSDRDMLDVLIAVKAETGTPRFSADEITGMFISMMFAGHHTSSGTASWTLIELMRHRDAYAAVIDELDELYGD
GRSVSFHALRQIPQLENVLKETLRLHPPLIILMRVAKGEFEVQGHRIHEGDLVAASPAISNRIPEDFPDPHDF

VPARYEQPRQEDLLNRWTWIPFGAGRHRCVGAAFAIMQIKAIFSVLLREYEFEMAQPPESYRNDHSKMVVQLA
QPACVRYRRRTGV
>CYP51B1_Mycobacterium tuberculosis BTB07-243 2603466521
MSAVALPRVSGGHDEHGHLEEFRTDPIGLMQRVRDECGDVGTFQLAGKQVVLLSGSHANEFFFRAGDDDLDQA
KAYPFMTPILGEGVVFDASPERRKEMLHNAALRGEQMKGHAATIEDQVRRMIADWGEAGEIDLLDFFAELTIY
TSSACLIGKKFRDQLDGRFAKLYHELERGTDPLAYVDPYLPIESFRRRDEARNGLVALVADIMNGRIANPPTD
KSDRDMLDVLIAVKAETGTPRFSADEITGMFISMMFAGHHTSSGTASWTLIELMRHRDAYAAVIDELDELYGD
GRSVSFHALRQIPQLENVLKETLRLHPPLIILMRVAKGEFEVQGHRIHEGDLVAASPAISNRIPEDFPDPHDF
VPARYEQPRQEDLLNRWTWIPFGAGRHRCVGAAFAIMQIKAIFSVLLREYEFEMAQPPESYRNDHSKMVVQLA
QPACVRYRRRTGV
>CYP51B1_Mycobacterium tuberculosis BTB07-254 2584993181
MSAVALPRVSGGHDEHGHLEEFRTDPIGLMQRVRDECGDVGTFQLAGKQVVLLSGSHANEFFFRAGDDDLDQA
KAYPFMTPILGEGVVFDASPERRKEMLHNAALRGEQMKGHAATIEDQVRRMIADWGEAGEIDLLDFFAELTIY
TSSACLIGKKFRDQLDGRFAKLYHELERGTDPLAYVDPYLPIESFRRRDEARNGLVALVADIMNGRIANPPTD
KSDRDMLDVLIAVKAETGTPRFSADEITGMFISMMFAGHHTSSGTASWTLIELMRHRDAYAAVIDELDELYGD
GRSVSFHALRQIPQLENVLKETLRLHPPLIILMRVAKGEFEVQGHRIHEGDLVAASPAISNRIPEDFPDPHDF
VPARYEQPRQEDLLNRWTWIPFGAGRHRCVGAAFAIMQIKAIFSVLLREYEFEMAQPPESYRNDHSKMVVQLA
QPACVRYRRRTGV
>CYP51B1_Mycobacterium tuberculosis BTB07-261 2605079795
MSAVALPRVSGGHDEHGHLEEFRTDPIGLMQRVRDECGDVGTFQLAGKQVVLLSGSHANEFFFRAGDDDLDQA
KAYPFMTPILGEGVVFDASPERRKEMLHNAALRGEQMKGHAATIEDQVRRMIADWGEAGEIDLLDFFAELTIY
TSSACLIGKKFRDQLDGRFAKLYHELERGTDPLAYVDPYLPIESFRRRDEARNGLVALVADIMNGRIANPPTD
KSDRDMLDVLIAVKAETGTPRFSADEITGMFISMMFAGHHTSSGTASWTLIELMRHRDAYAAVIDELDELYGD
GRSVSFHALRQIPQLENVLKETLRLHPPLIILMRVAKGEFEVQGHRIHEGDLVAASPAISNRIPEDFPDPHDF
VPARYEQPRQEDLLNRWTWIPFGAGRHRCVGAAFAIMQIKAIFSVLLREYEFEMAQPPESYRNDHSKMVVQLA
QPACVRYRRRTGV
>CYP51B1_Mycobacterium tuberculosis BTB07-299 2603159336
MSAVALPRVSGGHDEHGHLEEFRTDPIGLMQRVRDECGDVGTFQLAGKQVVLLSGSHANEFFFRAGDDDLDQA
KAYPFMTPILGEGVVFDASPERRKEMLHNAALRGEQMKGHAATIEDQVRRMIADWGEAGEIDLLDFFAELTIY
TSSACLIGKKFRDQLDGRFAKLYHELERGTDPLAYVDPYLPIESFRRRDEARNGLVALVADIMNGRIANPPTD
KSDRDMLDVLIAVKAETGTPRFSADEITGMFISMMFAGHHTSSGTASWTLIELMRHRDAYAAVIDELDELYGD
GRSVSFHALRQIPQLENVLKETLRLHPPLIILMRVAKGEFEVQGHRIHEGDLVAASPAISNRIPEDFPDPHDF
VPARYEQPRQEDLLNRWTWIPFGAGRHRCVGAAFAIMQIKAIFSVLLREYEFEMAQPPESYRNDHSKMVVQLA
QPACVRYRRRTGV
>CYP51B1_Mycobacterium tuberculosis BTB07-300 2604555848
MSAVALPRVSGGHDEHGHLEEFRTDPIGLMQRVRDECGDVGTFQLAGKQVVLLSGSHANEFFFRAGDDDLDQA
KAYPFMTPILGEGVVFDASPERRKEMLHNAALRGEQMKGHAATIEDQVRRMIADWGEAGEIDLLDFFAELTIY
TSSACLIGKKFRDQLDGRFAKLYHELERGTDPLAYVDPYLPIESFRRRDEARNGLVALVADIMNGRIANPPTD
KSDRDMLDVLIAVKAETGTPRFSADEITGMFISMMFAGHHTSSGTASWTLIELMRHRDAYAAVIDELDELYGD
GRSVSFHALRQIPQLENVLKETLRLHPPLIILMRVAKGEFEVQGHRIHEGDLVAASPAISNRIPEDFPDPHDF
VPARYEQPRQEDLLNRWTWIPFGAGRHRCVGAAFAIMQIKAIFSVLLREYEFEMAQPPESYRNDHSKMVVQLA
QPACVRYRRRTGV
>CYP51B1_Mycobacterium tuberculosis BTB07-327 2605282400
MSAVALPRVSGGHDEHGHLEEFRTDPIGLMQRVRDECGDVGTFQLAGKQVVLLSGSHANEFFFRAGDDDLDQA
KAYPFMTPIFGEGVVFDASPERRKEMLHNAALRGEQMKGHAATIEDQVRRMIADWGEAAEIDLLDFFAELTIY
TSSACLIGKKFRDQLDGRFAKLYHELERGTDPLAYVDPYLPIESFRRRDEARNGLVALVADIMNGRIANPPTD
KSDRDMLDVLIAVKAETGTPRFSADEITGMFISMMFAGHHTSSGTASWTLIELMRHRDAYAAVIDELDELYGD
GRSVSFHALRQIPQLENVLKETLRLHPPLIILMRVAKGEFEVQGHRIHEGDLVAASPAISNRIPEDFPDPHDF
VPARYEQPRQEDLLNRWTWIPFGAGRHRCVGAAFAIMQIKAIFSVLLREYEFEMAQPPESYRNDHSKMVVQLA
QPACVRYRRRTGV
>CYP51B1_Mycobacterium tuberculosis BTB07-354 2605633293
MSAVALPRVSGGHDEHGHLEEFRTDPIGLMQRVRDECGDVGTFQLAGKQVVLLSGSHANEFFFRAGDDDLDQA
KAYPFMTPIFGEGVVFDASPERRKEMLHNAALRGEQMKGHAATIEDQVRRMIADWGEAAEIDLLDFFAELTIY
TSSACLIGKKFRDQLDGRFAKLYHELERGTDPLAYVDPYLPIESFRRRDEARNGLVALVADIMNGRIANPPTD
KSDRDMLDVLIAVKAETGTPRFSADEITGMFISMMFAGHHTSSGTASWTLIELMRHRDAYAAVIDELDELYGD
GRSVSFHALRQIPQLENVLKETLRLHPPLIILMRVAKGEFEVQGHRIHEGDLVAASPAISNRIPEDFPDPHDF
VPARYEQPRQEDLLNRWTWIPFGAGRHRCVGAAFAIMQIKAIFSVLLREYEFEMAQPPESYRNDHSKMVVQLA
QPACVRYRRRTGV

>CYP51B1_Mycobacterium tuberculosis BTB08-014 2603315569
MSAVALPRVSGGHDEHGHLEEFRTDPIGLMQRVRDECGDVGTFQLAGKQVVLLSGSHANEFFFRAGDDDLDQA
KAYPFMTPILGEGVVFDASPERRKEMLHNAALRGEQMKGHAATIEDQVRRMIADWGEAGEIDLLDFFAELTIY
TSSACLIGKKFRDQLDGRFAKLYHELERGTDPLAYVDPYLPIESFRRRDEARNGLVALVADIMNGRIANPPTD
KSDRDMLDVLIAVKAETGTPRFSADEITGMFISMMFAGHHTSSGTASWTLIELMRHRDAYAAVIDELDELYGD
GRSVSFHALRQIPQLENVLKETLRLHPPLIILMRVAKGEFEVQGHRIHEGDLVAASPAISNRIPEDFPDPHDF
VPARYEQPRQEDLLNRWTWIPFGAGRHRCVGAAFAIMQIKAIFSVLLREYEFEMAQPPESYRNDHSKMVVQLA
QPACVRYRRRTGV
>CYP51B1_Mycobacterium tuberculosis BTB08-043 2603323250
MSAVALPRVSGGHDEHGHLEEFRTDPIGLMQRVRDECGDVGTFQLAGKQVVLLSGSHANEFFFRAGDDDLDQA
KAYPFMTPIFGEGVVFDASPERRKEMLHNAALRGEQMKGHAATIEDQVRRMIADWGEAAEIDLLDFFAELTIY
TSSACLIGKKFRDQLDGRFAKLYHELERGTDPLAYVDPYLPIESFRRRDEARNGLVALVADIMNGRIANPPTD
KSDRDMLDVLIAVKAETGTPRFSADEITGMFISMMFAGHHTSSGTASWTLIELMRHRDAYAAVIDELDELYGD
GRSVSFHALRQIPQLENVLKETLRLHPPLIILMRVAKGEFEVQGHRIHEGDLVAASPAISNRIPEDFPDPHDF
VPARYEQPRQEDLLNRWTWIPFGAGRHRCVGAAFAIMQIKAIFSVLLREYEFEMAQPPESYRNDHSKMVVQLA
QPACVRYRRRTGV
>CYP51B1_Mycobacterium tuberculosis BTB08-072 2603323992
MSAVALPRVSGGHDEHGHLEEFRTDPIGLMQRVRDECGDVGTFQLAGKQVVLLSGSHANEFFFRAGDDDLDQA
KAYPFMTPILGEGVVFDASPERRKEMLHNAALRGEQMKGHAATIEDQVRRMIADWGEAGEIDLLDFFAELTIY
TSSACLIGKKFRDQLDGRFAKLYHELERGTDPLAYVDPYLPIESFRRRDEARNGLVALVADIMNGRIANPPTD
KSDRDMLDVLIAVKAETGTPRFSADEITGMFISMMFAGHHTSSGTASWTLIELMRHRDAYAAVIDELDELYGD
GRSVSFHALRQIPQLENVLKETLRLHPPLIILMRVAKGEFEVQGHRIHEGDLVAASPAISNRIPEDFPDPHDF
VPARYEQPRQEDLLNRWTWIPFGAGRHRCVGAAFAIMQIKAIFSVLLREYEFEMAQPPESYRNDHSKMVVQLA
QPACVRYRRRTGV
>CYP51B1_Mycobacterium tuberculosis BTB09-036 2604945778
MSAVALPRVSGGHDEHGHLEEFRTDPIGLMQRVRDECGDVGTFQLAGKQVVLLSGSHANEFFFRAGDDDLDQA
KAYPFMTPIFGEGVVFDASPERRKEMLHNAALRGEQMKGHAATIEDQVRRMIADWGEAAEIDLLDFFAELTIY
TSSACLIGKKFRDQLDGRFAKLYHELERGTDPLAYVDPYLPIESFRRRDEARNGLVALVADIMNGRIANPPTD
KSDRDMLDVLIAVKAETGTPRFSADEITGMFISMMFAGHHTSSGTASWTLIELMRHRDAYAAVIDELDELYGD
GRSVSFHALRQIPQLENVLKETLRLHPPLIILMRVAKGEFEVQGHRIHEGDLVAASPAISNRIPEDFPDPHDF
VPARYEQPRQEDLLNRWTWIPFGAGRHRCVGAAFAIMQIKAIFSVLLREYEFEMAQPPESYRNDHSKMVVQLA
QPACVRYRRRTGV
>CYP51B1_Mycobacterium tuberculosis BTB09-453 2604218563
MSAVALPRVSGGHDEHGHLEEFRTDPIGLMQRVRDECGDVGTFQLAGKQVVLLSGSHANEFFFRAGDDDLDQA
KAYPFMTPIFGEGVVFDASPERRKEMLHNAALRGEQMKGHAATIEDQVRRMIADWGEAAEIDLLDFFAELTIY
TSSACLIGKKFRDQLDGRFAKLYHELERGTDPLAYVDPYLPIESFRRRDEARNGLVALVADIMNGRIANPPTD
KSDRDMLDVLIAVKAETGTPRFSADEITGMFISMMFAGHHTSSGTASWTLIELMRHRDAYAAVIDELDELYGD
GRSVSFHALRQIPQLENVLKETLRLHPPLIILMRVAKGEFEVQGHRIHEGDLVAASPAISNRIPEDFPDPHDF
VPARYEQPRQEDLLNRWTWIPFGAGRHRCVGAAFAIMQIKAIFSVLLREYEFEMAQPPESYRNDHSKMVVQLA
QPACVRYRRRTGV
>CYP51B1_Mycobacterium tuberculosis BTB09-553 2603508975
MSAVALPRVSGGHDEHGHLEEFRTDPIGLMQRVRDECGDVGTFQLAGKQVVLLSGSHANEFFFRAGDDDLDQA
KAYPFMTPILGEGVVFDASPERRKEMLHNAALRGEQMKGHAATIEDQVRRMIADWGEAGEIDLLDFFAELTIY
TSSACLIGKKFRDQLDGRFAKLYHELERGTDPLAYVDPYLPIESFRRRDEARNGLVALVADIMNGRIANPPTD
KSDRDMLDVLIAVKAETGTPRFSADEITGMFISMMFAGHHTSSGTASWTLIELMRHRDAYAAVIDELDELYGD
GRSVSFHALRQIPQLENVLKETLRLHPPLIILMRVAKGEFEVQGHRIHEGDLVAASPAISNRIPEDFPDPHDF
VPARYEQPRQEDLLNRWTWIPFGAGRHRCVGAAFAIMQIKAIFSVLLREYEFEMAQPPESYRNDHSKMVVQLA
QPACVRYRRRTGV
>CYP51B1_Mycobacterium tuberculosis BTB10-001 2605645678
MSAVALPRVSGGHDEHGHLEEFRTDPIGLMQRVRDECGDVGTFQLAGKQVVLLSGSHANEFFFRAGDDDLDQA
KAYPFMTPIFGEGVVFDASPERRKEMLHNAALRGEQMKGHAATIEDQVRRMIADWGEAAEIDLLDFFAELTIY
TSSACLIGKKFRDQLDGRFAKLYHELERGTDPLAYVDPYLPIESFRRRDEARNGLVALVADIMNGRIANPPTD
KSDRDMLDVLIAVKAETGTPRFSADEITGMFISMMFAGHHTSSGTASWTLIELMRHRDAYAAVIDELDELYGD
GRSVSFHALRQIPQLENVLKETLRLHPPLIILMRVAKGEFEVQGHRIHEGDLVAASPAISNRIPEDFPDPHDF
VPARYEQPRQEDLLNRWTWIPFGAGRHRCVGAAFAIMQIKAIFSVLLREYEFEMAQPPESYRNDHSKMVVQLA
QPACVRYRRRTGV
>CYP51B1_Mycobacterium tuberculosis BTB10-005 2603336429

MSAVALPRVSGGHDEHGHLEEFRTDPIGLMQRVRDECGDVGTFQLAGKQVVLLSGSHANEFFFRAGDDDLDQA
KAYPFMTPILGEGVVFDASPERRKEMLHNAALRGEQMKGHAATIEDQVRRMIADWGEAGEIDLLDFFAELTIY
TSSACLIGKKFRDQLDGRFAKLYHELERGTDPLAYVDPYLPIESFRRRDEARNGLVALVADIMNGRIANPPTD
KSDRDMLDVLIAVKAETGTPRFSADEITGMFISMMFAGHHTSSGTASWTLIELMRHRDAYAAVIDELDELYGD
GRSVSFHALRQIPQLENVLKETLRLHPPLIILMRVAKGEFEVQGHRIHEGDLVAASPAISNRIPEDFPDPHDF
VPARYEQPRQEDLLNRWTWIPFGAGRHRCVGAAFAIMQIKAIFSVLLREYEFEMAQPPESYRNDHSKMVVQLA
QPACVRYRRRTGV
>CYP51B1_Mycobacterium tuberculosis BTB10-005 2603336429
MSAVALPRVSGGHDEHGHLEEFRTDPIGLMQRVRDECGDVGTFQLAGKQVVLLSGSHANEFFFRAGDDDLDQA
KAYPFMTPILGEGVVFDASPERRKEMLHNAALRGEQMKGHAATIEDQVRRMIADWGEAGEIDLLDFFAELTIY
TSSACLIGKKFRDQLDGRFAKLYHELERGTDPLAYVDPYLPIESFRRRDEARNGLVALVADIMNGRIANPPTD
KSDRDMLDVLIAVKAETGTPRFSADEITGMFISMMFAGHHTSSGTASWTLIELMRHRDAYAAVIDELDELYGD
GRSVSFHALRQIPQLENVLKETLRLHPPLIILMRVAKGEFEVQGHRIHEGDLVAASPAISNRIPEDFPDPHDF
VPARYEQPRQEDLLNRWTWIPFGAGRHRCVGAAFAIMQIKAIFSVLLREYEFEMAQPPESYRNDHSKMVVQLA
QPACVRYRRRTGV
>CYP51B1_Mycobacterium tuberculosis BTB10-142 2603880007
MSAVALPRVSGGHDEHGHLEEFRTDPIGLMQRVRDECGDVGTFQLAGKQVVLLSGSHANEFFFRAGDDDLDQA
KAYPFMTPIFGEGVVFDASPERRKEMLHNAALRGEQMKGHAATIEDQVRRMIADWGEAAEIDLLDFFAELTIY
TSSACLIGKKFRDQLDGRFAKLYHELERGTDPLAYVDPYLPIESFRRRDEARNGLVALVADIMNGRIANPPTD
KSDRDMLDVLIAVKAETGTPRFSADEITGMFISMMFAGHHTSSGTASWTLIELMRHRDAYAAVIDELDELYGD
GRSVSFHALRQIPQLENVLKETLRLHPPLIILMRVAKGEFEVQGHRIHEGDLVAASPAISNRIPEDFPDPHDF
VPARYEQPRQEDLLNRWTWIPFGAGRHRCVGAAFAIMQIKAIFSVLLREYEFEMAQPPESYRNDHSKMVVQLA
QPACVRYRRRTGV
>CYP51B1_Mycobacterium tuberculosis BTB10-277 2604584750
MSAVALPRVSGGHDEHGHLEEFRTDPIGLMQRVRDECGDVGTFQLAGKQVVLLSGSHANEFFFRAGDDDLDQA
KAYPFMTPIFGEGVVFDASPERRKEMLHNAALRGEQMKGHAATIEDQVRRMIADWGEAAEIDLLDFFAELTIY
TSSACLIGKKFRDQLDGRFAKLYHELERGTDPLAYVDPYLPIESFRRRDEARNGLVALVADIMNGRIANPPTD
KSDRDMLDVLIAVKAETGTPRFSADEITGMFISMMFAGHHTSSGTASWTLIELMRHRDAYAAVIDELDELYGD
GRSVSFHALRQIPQLENVLKETLRLHPPLIILMRVAKGEFEVQGHRIHEGDLVAASPAISNRIPEDFPDPHDF
VPARYEQPRQEDLLNRWTWIPFGAGRHRCVGAAFAIMQIKAIFSVLLREYEFEMAQPPESYRNDHSKMVVQLA
QPACVRYRRRTGV
>CYP51B1_Mycobacterium tuberculosis BTB10-308 2576292646
MSAVALPRVSGGHDEHGHLEEFRTDPIGLMQRVRDECGDVGTFQLAGKQVVLLSGSHANEFFFRAGDDDLDQA
KAYPFMTPILGEGVVFDASPERRKEMLHNAALRGEQMKGHAATIEDQVRRMIADWGEAGEIDLLDFFAELTIY
TSSACLIGKKFRDQLDGRFAKLYHELERGTDPLAYVDPYLPIESFRRRDEARNGLVALVADIMNGRIANPPTD
KSDRDMLDVLIAVKAETGTPRFSADEITGMFISMMFAGHHTSSGTASWTLIELMRHRDAYAAVIDELDELYGD
GRSVSFHALRQIPQLENVLKETLRLHPPLIILMRVAKGEFEVQGHRIHEGDLVAASPAISNRIPEDFPDPHDF
VPARYEQPRQEDLLNRWTWIPFGAGRHRCVGAAFAIMQIKAIFSVLLREYEFEMAQPPESYRNDHSKMVVQLA
QPACVRYRRRTGV
>CYP51B1_Mycobacterium tuberculosis BTB10-406 2605303046
MSAVALPRVSGGHDEHGHLEEFRTDPIGLMQRVRDECGDVGTFQLAGKQVVLLSGSHANEFFFRAGDDDLDQA
KAYPFMTPILGEGVVFDASPERRKEMLHNAALRGEQMKGHAATIEDQVRRMIADWGEAGEIDLLDFFAELTIY
TSSACLIGKKFRDQLDGRFAKLYHELERGTDPLAYVDPYLPIESFRRRDEARNGLVALVADIMNGRIANPPTD
KSDRDMLDVLIAVKAETGTPRFSADEITGMFISMMFAGHHTSSGTASWTLIELMRHRDAYAAVIDELDELYGD
GRSVSFHALRQIPQLENVLKETLRLHPPLIILMRVAKGEFEVQGHRIHEGDLVAASPAISNRIPEDFPDPHDF
VPARYEQPRQEDLLNRWTWIPFGAGRHRCVGAAFAIMQIKAIFSVLLREYEFEMAQPPESYRNDHSKMVVQLA
QPACVRYRRRTGV
>CYP51B1_Mycobacterium tuberculosis BTB10-422 2605309739
MSAVALPRVSGGHDEHGHLEEFRTDPIGLMQRVRDECGDVGTFQLAGKQVVLLSGSHANEFFFRAGDDDLDQA
KAYPFMTPIFGEGVVFDASPERRKEMLHNAALRGEQMKGHAATIEDQVRRMIADWGEAAEIDLLDFFAELTIY
TSSACLIGKKFRDQLDGRFAKLYHELERGTDPLAYVDPYLPIESFRRRDEARNGLVALVADIMNGRIANPPTD
KSDRDMLDVLIAVKAETGTPRFSADEITGMFISMMFAGHHTSSGTASWTLIELMRHRDAYAAVIDELDELYGD
GRSVSFHALRQIPQLENVLKETLRLHPPLIILMRVAKGEFEVQGHRIHEGDLVAASPAISNRIPEDFPDPHDF
VPARYEQPRQEDLLNRWTWIPFGAGRHRCVGAAFAIMQIKAIFSVLLREYEFEMAQPPESYRNDHSKMVVQLA
QPACVRYRRRTGV
>CYP51B1_Mycobacterium tuberculosis BTB11-001 2575519792
MSAVALPRVSGGHDEHGHLEEFRTDPIGLMQRVRDECGDVGTFQLAGKQVVLLSGSHANEFFFRAGDDDLDQA
KAYPFMTPIFGEGVVFDASPERRKEMLHNAALRGEQMKGHAATIEDQVRRMIADWGEAAEIDLLDFFAELTIY

TSSACLIGKKFRDQLDGRFAKLYHELERGTDPLAYVDPYLPIESFRRRDEARNGLVALVADIMNGRIANPPTD
KSDRDMLDVLIAVKAETGTPRFSADEITGMFISMMFAGHHTSSGTASWTLIELMRHRDAYAAVIDELDELYGD
GRSVSFHALRQIPQLENVLKETLRLHPPLIILMRVAKGEFEVQGHRIHEGDLVAASPAISNRIPEDFPDPHDF
VPARYEQPRQEDLLNRWTWIPFGAGRHRCVGAAFAIMQIKAIFSVLLREYEFEMAQPPESYRNDHSKMVVQLA
QPACVRYRRRTGV
>CYP51B1_Mycobacterium tuberculosis BTB11-195 2604232704
MSAVALPRVSGGHDEHGHLEEFRTDPIGLMQRVRDECGDVGTFQLAGKQVVLLSGSHANEFFFRAGDDDLDQA
KAYPFMTPILGEGVVFDASPERRKEMLHNAALRGEQMKGHAATIEDQVRRMIADWGEAGEIDLLDFFAELTIY
TSSACLIGKKFRDQLDGRFAKLYHELERGTDPLAYVDPYLPIESFRRRDEARNGLVALVADIMNGRIANPPTD
KSDRDMLDVLIAVKAETGTPRFSADEITGMFISMMFAGHHTSSGTASWTLIELMRHRDAYAAVIDELDELYGD
GRSVSFHALRQIPQLENVLKETLRLHPPLIILMRVAKGEFEVQGHRIHEGDLVAASPAISNRIPEDFPDPHDF
VPARYEQPRQEDLLNRWTWIPFGAGRHRCVGAAFAIMQIKAIFSVLLREYEFEMAQPPESYRNDHSKMVVQLA
QPACVRYRRRTGV
>CYP51B1_Mycobacterium tuberculosis BTB11-236 2578170081
MSAVALPRVSGGHDEHGHLEEFRTDPIGLMQRVRDECGDVGTFQLAGKQVVLLSGSHANEFFFRAGDDDLDQA
KAYPFMTPILGEGVVFDASPERRKEMLHNAALRGEQMKGHAATIEDQVRRMIADWGEAGEIDLLDFFAELTIY
TSSACLIGKKFRDQLDGRFAKLYHELERGTDPLAYVDPYLPIESFRRRDEARNGLVALVADIMNGRIANPPTD
KSDRDMLDVLIAVKAETGTPRFSADEITGMFISMMFAGHHTSSGTASWTLIELMRHRDAYAAVIDELDELYGD
GRSVSFHALRQIPQLENVLKETLRLHPPLIILMRVAKGEFEVQGHRIHEGDLVAASPAISNRIPEDFPDPHDF
VPARYEQPRQEDLLNRWTWIPFGAGRHRCVGAAFAIMQIKAIFSVLLREYEFEMAQPPESYRNDHSKMVVQLA
QPACVRYRRRTGV
>CYP51B1_Mycobacterium tuberculosis BTB11-250 2603892658
MSAVALPRVSGGHDEHGHLEEFRTDPIGLMQRVRDECGDVGTFQLAGKQVVLLSGSHANEFFFRAGDDDLDQA
KAYPFMTPILGEGVVFDASPERRKEMLHNAALRGEQMKGHAATIEDQVRRMIADWGEAGEIDLLDFFAELTIY
TSSACLIGKKFRDQLDGRFAKLYHELERGTDPLAYVDPYLPIESFRRRDEARNGLVALVADIMNGRIANPPTD
KSDRDMLDVLIAVKAETGTPRFSADEITGMFISMMFAGHHTSSGTASWTLIELMRHRDAYAAVIDELDELYGD
GRSVSFHALRQIPQLENVLKETLRLHPPLIILMRVAKGEFEVQGHRIHEGDLVAASPAISNRIPEDFPDPHDF
VPARYEQPRQEDLLNRWTWIPFGAGRHRCVGAAFAIMQIKAIFSVLLREYEFEMAQPPESYRNDHSKMVVQLA
QPACVRYRRRTGV
>CYP51B1_Mycobacterium tuberculosis BTB11-343 2575646971
MSAVALPRVSGGHDEHGHLEEFRTDPIGLMQRVRDECGDVGTFQLAGKQVVLLSGSHANEFFFRAGDDDLDQA
KAYPFMTPIFGEGVVFDASPERRKEMLHNAALRGEQMKGHAATIEDQVRRMIADWGEAGEIDLLDFFAELTIY
TSSACLIGKKFRDQLDGRFAKLYHELERGTDPLAYVDPYLPIESFRRRDEARNGLVALVADIMNGRIANPPTD
KSDRDMLDVLIGVKAETGTPRFSADEITGMFISMMFAGHHTSSGTASWTLIELMRHRDAYAAVIDELDELYGD
GRSVSFHALRQIPQLENVLKETLRLHPPLIILMRVAKGEFEVQGHRIHEGDLVAASPAISNRIPEDFPDPHDF
VPARYEQPRQEDLLNRWTWIPFGAGRHRCVGAAFAIMQIKAIFSVLLREYEFEMAQPPESYRNDHSKMVVQLA
QPACVRYRRRTGV
>CYP51B1_Mycobacterium tuberculosis BTB12-046 2604600957
MSAVALPRVSGGHDEHGHLEEFRTDPIGLMQRVRDECGDVGTFQLAGKQVVLLSGSHANEFFFRAGDDDLDQA
KAYPFMTPILGEGVVFDASPERRKEMLHNAALRGEQMKGHAATIEDQVRRMIADWGEAGEIDLLDFFAELTIY
TSSACLIGKKFRDQLDGRFAKLYHELERGTDPLAYVDPYLPIESFRRRDEARNGLVALVADIMNGRIANPPTD
KSDRDMLDVLIAVKAETGTPRFSADEITGMFISMMFAGHHTSSGTASWTLIELMRHRDAYAAVIDELDELYGD
GRSVSFHALRQIPQLENVLKETLRLHPPLIILMRVAKGEFEVQGHRIHEGDLVAASPAISNRIPEDFPDPHDF
VPARYEQPRQEDLLNRWTWIPFGAGRHRCVGAAFAIMQIKAIFSVLLREYEFEMAQPPESYRNDHSKMVVQLA
QPACVRYRRRTGV
>CYP51B1_Mycobacterium tuberculosis BTB12-054 2603896758
MSAVALPRVSGGHDEHGHLEEFRTDPIGLMQRVRDECGDVGTFQLAGKQVVLLSGSHANEFFFRAGDDDLDQA
KAYPFMTPILGEGVVFDASPERRKEMLHNAALRGEQMKGHAATIEDQVRRMIADWGEAGEIDLLDFFAELTIY
TSSACLIGKKFRDQLDGRFAKLYHELERGTDPLAYVDPYLPIESFRRRDEARNGLVALVADIMNGRIANPPTD
KSDRDMLDVLIAVKAETGTPRFSADEITGMFISMMFAGHHTSSGTASWTLIELMRHRDAYAAVIDELDELYGD
GRSVSFHALRQIPQLENVLKETLRLHPPLIILMRVAKGEFEVQGHRIHEGDLVAASPAISNRIPEDFPDPHDF
VPARYEQPRQEDLLNRWTWIPFGAGRHRCVGAAFAIMQIKAIFSVLLREYEFEMAQPPESYRNDHSKMVVQLA
QPACVRYRRRTGV
>CYP51B1_Mycobacterium tuberculosis BTB12-162 2604604588
MSAVALPRVSGGHDEHGHLEEFRTDPIGLMQRVRDECGDVGTFQLAGKQVVLLSGSHANEFFFRAGDDDLDQA
KAYPFMTPIFGEGVVFDASPERRKEMLHNAALRGEQMKGHAATIEDQVRRMIADWGEAAEIDLLDFFAELTIY
TSSACLIGKKFRDQLDGRFAKLYHELERGTDPLAYVDPYLPIESFRRRDEARNGLVALVADIMNGRIANPPTD
KSDRDMLDVLIAVKAETGTPRFSADEITGMFISMMFAGHHTSSGTASWTLIELMRHRDAYAAVIDELDELYGD

GRSVSFHALRQIPQLENVLKETLRLHPPLIILMRVAKGEFEVQGHRIHEGDLVAASPAISNRIPEDFPDPHDF
VPARYEQPRQEDLLNRWTWIPFGAGRHRCVGAAFAIMQIKAIFSVLLREYEFEMAQPPESYRNDHSKMVVQLA
QPACVRYRRRTGV
>CYP51B1_Mycobacterium tuberculosis BTB12-206 2584889762
MSAVALPRVSGGHDEHGHLEEFRTDPIGLMQRVRDECGDVGTFQLAGKQVVLLSGSHANEFFFRAGDDDLDQA
KAYPFMTPILGEGVVFDASPERRKEMLHNAALRGEQMKGHAATIEDQVRRMIADWGEAGEIDLLDFFAELTIY
TSSACLIGKKFRDQLDGRFAKLYHELERGTDPLAYVDPYLPIESFRRRDEARNGLVALVADIMNGRIANPPTD
KSDRDMLDVLIAVKAETGTPRFSADEITGMFISMMFAGHHTSSGTASWTLIELMRHRDAYAAVIDELDELYGD
GRSVSFHALRQIPQLENVLKETLRLHPPLIILMRVAKGEFEVQGHRIHEGDLVAASPAISNRIPEDFPDPHDF
VPARYEQPRQEDLLNRWTWIPFGAGRHRCVGAAFAIMQIKAIFSVLLREYEFEMAQPPESYRNDHSKMVVQLA
QPACVRYRRRTGV
>CYP51B1_Mycobacterium tuberculosis BTB12-294 2605666753
MSAVALPRVSGGHDEHGHLEEFRTDPIGLMQRVRDECGDVGTFQLAGKQVVLLSGSHANEFFFRAGDDDLDQA
KAYPFMTPIFGEGVVFDASPERRKEMLHNAALRGEQMKGHAATIEDQVRRMIADWGEAAEIDLLDFFAELTIY
TSSACLIGKKFRDQLDGRFAKLYHELERGTDPLAYVDPYLPIESFRRRDEARNGLVALVADIMNGRIANPPTD
KSDRDMLDVLIAVKAETGTPRFSADEITGMFISMMFAGHHTSSGTASWTLIELMRHRDAYAAVIDELDELYGD
GRSVSFHALRQIPQLENVLKETLRLHPPLIILMRVAKGEFEVQGHRIHEGDLVAASPAISNRIPEDFPDPHDF
VPARYEQPRQEDLLNRWTWIPFGAGRHRCVGAAFAIMQIKAIFSVLLREYEFEMAQPPESYRNDHSKMVVQLA
QPACVRYRRRTGV
>CYP51B1_Mycobacterium tuberculosis BTB12-304 2603175798
MSAVALPRVSGGHDEHGHLEEFRTDPIGLMQRVRDECGDVGTFQLAGKQVVLLSGSHANEFFFRAGDDDLDQA
KAYPFMTPILGEGVVFDASPERRKEMLHNAALRGEQMKGHAATIEDQVRRMIADWGEAGEIDLLDFFAELTIY
TSSACLIGKKFRDQLDGRFAKLYHELERGTDPLAYVDPYLPIESFRRRDEARNGLVALVADIMNGRIANPPTD
KSDRDMLDVLIAVKAETGTPRFSADEITGMFISMMFAGHHTSSGTASWTLIELMRHRDAYAAVIDELDELYGD
GRSVSFHALRQIPQLENVLKETLRLHPPLIILMRVAKGEFEVQGHRIHEGDLVAASPAISNRIPEDFPDPHDF
VPARYEQPRQEDLLNRWTWIPFGAGRHRCVGAAFAIMQIKAIFSVLLREYEFEMAQPPESYRNDHSKMVVQLA
QPACVRYRRRTGV
>CYP51B1_Mycobacterium tuberculosis BTB12-313 2603356951
MSAVALPRVSGGHDEHGHLEEFRTDPIGLMQRVRDECGDVGTFQLAGKQVVLLSGSHANEFFFRAGDDDLDQA
KAYPFMTPIFGEGVVFDASPERRKEMLHNAALRGEQMKGHAATIEDQVRRMIADWGEAAEIDLLDFFAELTIY
TSSACLIGKKFRDQLDGRFAKLYHELERGTDPLAYVDPYLPIESFRRRDEARNGLVALVADIMNGRIANPPTD
KSDRDMLDVLIAVKAETGTPRFSADEITGMFISMMFAGHHTSSGTASWTLIELMRHRDAYAAVIDELDELYGD
GRSVSFHALRQIPQLENVLKETLRLHPPLIILMRVAKGEFEVQGHRIHEGDLVAASPAISNRIPEDFPDPHDF
VPARYEQPRQEDLLNRWTWIPFGAGRHRCVGAAFAIMQIKAIFSVLLREYEFEMAQPPESYRNDHSKMVVQLA
QPACVRYRRRTGV
>CYP51B1_Mycobacterium tuberculosis BTB13-001 2603361095
MSAVALPRVSGGHDEHGHLEEFRTDPIGLMQRVRDECGDVGTFQLAGKQVVLLSGSHANEFFFRAGDDDLDQA
KAYPFMTPIFGEGVVFDASPERRKEMLHNAALRGEQMKGHAATIEDQVRRMIADWGEAAEIDLLDFFAELTIY
TSSACLIGKKFRDQLDGRFAKLYHELERGTDPLAYVDPYLPIESFRRRDEARNGLVALVADIMNGRIANPPTD
KSDRDMLDVLIAVKAETGTPRFSADEITGMFISMMFAGHHTSSGTASWTLIELMRHRDAYAAVIDELDELYGD
GRSVSFHALRQIPQLENVLKETLRLHPPLIILMRVAKGEFEVQGHRIHEGDLVAASPAISNRIPEDFPDPHDF
VPARYEQPRQEDLLNRWTWIPFGAGRHRCVGAAFAIMQIKAIFSVLLREYEFEMAQPPESYRNDHSKMVVQLA
QPACVRYRRRTGV
>CYP51B1_Mycobacterium tuberculosis BTB13-156 2603185628
MSAVALPRVSGGHDEHGHLEEFRTDPIGLMQRVRDECGDVGTFQLAGKQVVLLSGSHANEFFFRAGDDDLDQA
KAYPFMTPIFGEGVVFDASPERRKEMLHNAALRGEQMKGHAATIEDQVRRMIADWGEAAEIDLLDFFAELTIY
TSSACLIGKKFRDQLDGRFAKLYHELERGTDPLAYVDPYLPIESFRRRDEARNGLVALVADIMNGRIANPPTD
KSDRDMLDVLIAVKAETGTPRFSADEITGMFISMMFAGHHTSSGTASWTLIELMRHRDAYAAVIDELDELYGD
GRSVSFHALRQIPQLENVLKETLRLHPPLIILMRVAKGEFEVQGHRIHEGDLVAASPAISNRIPEDFPDPHDF
VPARYEQPRQEDLLNRWTWIPFGAGRHRCVGAAFAIMQIKAIFSVLLREYEFEMAQPPESYRNDHSKMVVQLA
QPACVRYRRRTGV
>CYP51B1_Mycobacterium tuberculosis CAS/NITR204 2546201177
MSAVALPRVSGGHDEHGHLEEFRTDPIGLMQRVRDECGDVGTFQLAGKQVVLLSGSHANEFFFRAGDDDLDQA
KAYPFMTPIFGEGVVFDASPERRKEMLHNAALRGEQMKGHAATIEDQVRRMIADWGEAAEIDLLDFFAELTIY
TSSACLIGKKFRDQLDGRFAKLYHELERGTDPLAYVDPYLPIESFRRRDEARNGLVALVADIMNGRIANPPTD
KSDRDMLDVLIAVKAETGTPRFSADEITGMFISMMFAGHHTSSGTASWTLIELMRHRDAYAAVIDELDELYGD
GRSVSFHALRQIPQLENVLKETLRLHPPLIILMRVAKGEFEVQGHRIHEGDLVAASPAISNRIPEDFPDPHDF

VPARYEQPRQEDLLNRWTWIPFGAGRHRCVGAAFAIMQIKAIFSVLLREYEFEMAQPPESYRNDHSKMVVQLA
QPACVRYRRRTGV
>CYP51B1_Mycobacterium tuberculosis East African-Indian NZ3MDR1
2633042241
MSAVALPRVSGGHDEHGHLEEFRTDPIGLMQRVRDECGDVGTFQLAGKQVVLLSGSHANEFFFRAGDDDLDQA
KAYPFMTPIFGEGVVFDASPERRKEMLHNAALRGEQMKGHAATIEDQVRRMIADWGEAAEIDLLDFFAELTIY
TSSACLIGKKFRDQLDGRFAKLYHELERGTDPLAYVDPYLPIESFRRRDEARNGLVALVADIMNGRIANPPTD
KSDRDMLDVLIAVKAETGTPRFSADEITGMFISMMFAGHHTSSGTASWTLIELMRHRDAYAAVIDELDELYGD
GRSVSFHALRQIPQLENVLKETLRLHPPLIILMRVAKGEFEVQGHRIHEGDLVAASPAISNRIPEDFPDPHDF
VPARYEQPRQEDLLNRWTWIPFGAGRHRCVGAAFAIMQIKAIFSVLLREYEFEMAQPPESYRNDHSKMVVQLA
QPACVRYRRRTGV
>CYP51B1_Mycobacterium tuberculosis G00000978 2678225661
MSAVALPRVSGGHDEHGHLEEFRTDPIGLMQRVRDECGDVGTFQLAGKQVVLLSGSHANEFFFRAGDDDLDQA
KAYPFMTPIFGEGVVFDASPERRKEMLHNAALRGEQMKGHAATIEDQVRRMIADWGEAAEIDLLDFFAELTIY
TSSACLIGKKFRDQLDGRFAKLYHELERGTDPLAYVDPYLPIESFRRRDEARNGLVALVADIMNGRIANPPTD
KSDRDMLDVLIAVKAETGTPRFSADEITGMFISMMFAGHHTSSGTASWTLIELMRHRDAYAAVIDELDELYGD
GRSVSFHALRQIPQLENVLKETLRLHPPLIILMRVAKGEFEVQGHRIHEGDLVAASPAISNRIPEDFPDPHDF
VPARYEQPRQEDLLNRWTWIPFGAGRHRCVGAAFAIMQIKAIFSVLLREYEFEMAQPPESYRNDHSKMVVQLA
QPACVRYRRRTGV
>CYP51B1_Mycobacterium tuberculosis G00001233 2692052715
MSAVALPRVSGGHDEHGHLEEFRTDPIGLMQRVRDECGDVGTFQLAGKQVVLLSGSHANEFFFRAGDDDLDQA
KAYPFMTPIFGEGVVFDASPERRKEMLHNAALRGEQMKGHAATIEDQVRRMIADWGEAAEIDLLDFFAELTIY
TSSACLIGKKFRDQLDGRFAKLYHELERGTDPLAYVDPYLPIESFRRRDEARNGLVALVADIMNGRIANPPTD
KSDRDMLDVLIAVKAETGTPRFSADEITGMFISMMFAGHHTSSGTASWTLIELMRHRDAYAAVIDELDELYGD
GRSVSFHALRQIPQLENVLKETLRLHPPLIILMRVAKGEFEVQGHRIHEGDLVAASPAISNRIPEDFPDPHDF
VPARYEQPRQEDLLNRWTWIPFGAGRHRCVGAAFAIMQIKAIFSVLLREYEFEMAQPPESYRNDHSKMVVQLA
QPACVRYRRRTGV
>CYP51B1_Mycobacterium tuberculosis G00101715 2697046005
MSAVALPRVSGGHDEHGHLEEFRTDPIGLMQRVRDECGDVGTFQLAGKQVVLLSGSHANEFFFRAGDDDLDQA
KAYPFMTPIFGEGVVFDASPERRKEMLHNAALRGEQMKGHAATIEDQVRRMIADWGEAAEIDLLDFFAELTIY
TSSACLIGKKFRDQLDGRFAKLYHELERGTDPLAYVDPYLPIESFRRRDEARNGLVALVADIMNGRIANPPTD
KSDRDMLDVLIAVKAETGTPRFSADEITGMFISMMFAGHHTSSGTASWTLIELMRHRDAYAAVIDELDELYGD
GRSVSFHALRQIPQLENVLKETLRLHPPLIILMRVAKGEFEVQGHRIHEGDLVAASPAISNRIPEDFPDPHDF
VPARYEQPRQEDLLNRWTWIPFGAGRHRCVGAAFAIMQIKAIFSVLLREYEFEMAQPPESYRNDHSKMVVQLA
QPACVRYRRRTGV
>CYP51B1_Mycobacterium tuberculosis G09801536 2703624957
MSAVALPRVSGGHDEHGHLEEFRTDPIGLMQRVRDECGDVGTFQLAGKQVVLLSGSHANEFFFRAGDDDLDQA
KAYPFMTPIFGEGVVFDASPERRKEMLHNAALRGEQMKGHAATIEDQVRRMIADWGEAAEIDLLDFFAELTIY
TSSACLIGKKFRDQLDGRFAKLYHELERGTDPLAYVDPYLPIESFRRRDEARNGLVALVADIMNGRIANPPTD
KSDRDMLDVLIAVKAETGTPRFSADEITGMFISMMFAGHHTSSGTASWTLIELMRHRDAYAAVIDELDELYGD
GRSVSFHALRQIPQLENVLKETLRLHPPLIILMRVAKGEFEVQGHRIHEGDLVAASPAISNRIPEDFPDPHDF
VPARYEQPRQEDLLNRWTWIPFGAGRHRCVGAAFAIMQIKAIFSVLLREYEFEMAQPPESYRNDHSKMVVQLA
QPACVRYRRRTGV
>CYP51B1_Mycobacterium tuberculosis G09901357 2706554251
MSAVALPRVSGGHDEHGHLEEFRTDPIGLMQRVRDECGDVGTFQLAGKQVVLLSGSHANEFFFRAGDDDLDQA
KAYPFMTPIFGEGVVFDASPERRKEMLHNAALRGEQMKGHAATIEDQVRRMIADWGEAAEIDLLDFFAELTIY
TSSACLIGKKFRDQLDGRFAKLYHELERGTDPLAYVDPYLPIESFRRRDEARNGLVALVADIMNGRIANPPTD
KSDRDMLDVLIAVKAETGTPRFSADEITGMFISMMFAGHHTSSGTASWTLIELMRHRDAYAAVIDELDELYGD
GRSVSFHALRQIPQLENVLKETLRLHPPLIILMRVAKGEFEVQGHRIHEGDLVAASPAISNRIPEDFPDPHDF
VPARYEQPRQEDLLNRWTWIPFGAGRHRCVGAAFAIMQIKAIFSVLLREYEFEMAQPPESYRNDHSKMVVQLA
QPACVRYRRRTGV
>CYP51B1_Mycobacterium tuberculosis H1964 2646372598
MSAVALPRVSGGHDEHGHLEEFRTDPIGLMQRVRDECGDVGTFQLAGKQVVLLSGSHANEFFFRAGDDDLDQA
KAYPFMTPIFGEGVVFDASPERRKEMLHNAALRGEQMKGHAATIEDQVRRMIADWGEAAEIDLLDFFAELTIY
TSSACLIGKKFRDQLDGRFAKLYHELERGTDPLAYVDPYLPIESFRRRDEARNGLVALVADIMNGRIANPPTD
KSDRDMLDVLIAVKAETGTPRFSADEITGMFISMMFAGHHTSSGTASWTLIELMRHRDAYAAVIDELDELYGD
GRSVSFHALRQIPQLENVLKETLRLHPPLIILMRVAKGEFEVQGHRIHEGDLVAASPAISNRIPEDFPDPHDF

VPARYEQPRQEDLLNRWTWIPFGAGRHRCVGAAFAIMQIKAIFSVLLREYEFEMAQPPESYRNDHSKMVVQLA
QPACVRYRRRTGV
>CYP51B1_Mycobacterium tuberculosis H2101 2622278092
MSAVALPRVSGGHDEHGHLEEFRTDPIGLMQRVRDECGDVGTFQLAGKQVVLLSGSHANEFFFRAGDDDLDQA
KAYPFMTPIFGEGVVFDASPERRKEMLHNAALRGEQMKGHAATIEDQVRRMIADWGEAAEIDLLDFFAELTIY
TSSACLIGKKFRDQLDGRFAKLYHELERGTDPLAYVDPYLPIESFRRRDEARNGLVALVADIMNGRIANPPTD
KSDRDMLDVLIAVKAETGTPRFSADEITGMFISMMFAGHHTSSGTASWTLIELMRHRDAYAAVIDELDELYGD
GRSVSFHALRQIPQLENVLKETLRLHPPLIILMRVAKGEFEVQGHRIHEGDLVAASPAISNRIPEDFPDPHDF
VPARYEQPRQEDLLNRWTWIPFGAGRHRCVGAAFAIMQIKAIFSVLLREYEFEMAQPPESYRNDHSKMVVQLA
QPACVRYRRRTGV
>CYP51B1_Mycobacterium tuberculosis H2102 2622282076
MSAVALPRVSGGHDEHGHLEEFRTDPIGLMQRVRDECGDVGTFQLAGKQVVLLSGSHANEFFFRAGDDDLDQA
KAYPFMTPIFGEGVVFDASPERRKEMLHNAALRGEQMKGHAATIEDQVRRMIADWGEAGEIDLLDFFAELTIY
TSSACLIGKKFRDQLDGRFAKLYHELERGTDPLAYVDPYLPIESLRRRDEARNGLVALVADIMNGRIANPPTD
KSDRDMLDVLIAVKAETGTPRFSADEITGMFISMMFAGHHTSSGTASWTLIELMRHRDAYAAVIDELDELYGD
GRSVSFHALRQIPQLENVLKETLRLHPPLIILMRVAKGEFEVQGHRIHEGDLVAASPAISNRIPEDFPDPHDF
VPARYEQPRQEDLLNRWTWIPFGAGRHRCVGAAFAIMQIKAIFSVLLREYEFEMAQPPESYRNDHSKMVVQLA
QPACVRYRRRTGV
>CYP51B1_Mycobacterium tuberculosis H3004 2622305491
MSAVALPRVSGGHDEHGHLEEFRTDPIGLMQRVRDECGDVGTFQLAGKQVVLLSGSHANEFFFRAGDDDLDQA
KAYPFMTPIFGEGVVFDASPERRKEMLHNAALRGEQMKGHAATIEDQVRRMIADWGEAGEIDLLDFFAELTIY
TSSACLIGKKFRDQLDGRFAKLYHELERGTDPLAYVDPYLPIESLRRRDEARNGLVALVADIMNGRIANPPTD
KSDRDMLDVLIAVKAETGTPRFSADEITGMFISMMFAGHHTSSGTASWTLIELMRHRDAYAAVIDELDELYGD
GRSVSFHALRQIPQLENVLKETLRLHPPLIILMRVAKGEFEVQGHRIHEGDLVAASPAISNRIPEDFPDPHDF
VPARYEQPRQEDLLNRWTWIPFGAGRHRCVGAAFAIMQIKAIFSVLLREYEFEMAQPPESYRNDHSKMVVQLA
QPACVRYRRRTGV
>CYP51B1_Mycobacterium tuberculosis KT-0194 2631396386
MSAVALPRVSGGHDEHGHLEEFRTDPIGLMQRVRDECGDVGTFQLAGKQVVLLSGSHANEFFFRAGDDDLDQA
KAYPFMTPIFGEGVVFDASPERRKEMLHNAALRGEQMKGHAATIEDQVRRMIADWGEAAEIDLLDFFAELTIY
TSSACLIGKKFRDQLDGRFAKLYHELERGTDPLAYVDPYLPIESFRRRDEARNGLVALVADIMNGRIANPPTD
KSDRDMLDVLIAVKAETGTPRFSADEITGMFISMMFAGHHTSSGTASWTLIELMRHRDAYAAVIDELDELYGD
GRSVSFHALRQIPQLENVLKETLRLHPPLIILMRVAKGEFEVQGHRIHEGDLVAASPAISNRIPEDFPDPHDF
VPARYEQPRQEDLLNRWTWIPFGAGRHRCVGAAFAIMQIKAIFSVLLREYEFEMAQPPESYRNDHSKMVVQLA
QPACVRYRRRTGV
>CYP51B1_Mycobacterium tuberculosis M1017 2577886747
MSAVALPRVSGGHDEHGHLEEFRTDPIGLMQRVRDECGDVGTFQLAGKQVVLLSGSHANEFFFRAGDDDLDQA
KAYPFMTPIFGEGVVFDASPERRKEMLHNAALRGEQMKGHAATIEDQVRRMIADWGEAAEIDLLDFFAELTIY
TSSACLIGKKFRDQLDGRFAKLYHELERGTDPLAYVDPYLPIESFRRRDEARNGLVALVADIMNGRIANPPTD
KSDRDMLDVLIAVKAETGTPRFSADEITGMFISMMFAGHHTSSGTASWTLIELMRHRDAYAAVIDELDELYGD
GRSVSFHALRQIPQLENVLKETLRLHPPLIILMRVAKGEFEVQGHRIHEGDLVAASPAISNRIPEDFPDPHDF
VPARYEQPRQEDLLNRWTWIPFGAGRHRCVGAAFAIMQIKAIFSVLLREYEFEMAQPPESYRNDHSKMVVQLA
QPACVRYRRRTGV
>CYP51B1_Mycobacterium tuberculosis M1270 2622399205
MSAVALPRVSGGHDEHGHLEEFRTDPIGLMQRVRDECGDVGTFQLAGKQVVLLSGSHANEFFFRAGDDDLDQA
KAYPFMTPIFGEGVVFDASPERRKEMLHNAALRGEQMKGHAATIEDQVRRMIADWGEAAEIDLLDFFAELTIY
TSSACLIGKKFRDQLDGRFAKLYHELERGTDPLAYVDPYLPIESFRRRDEARNGLVALVADIMNGRIANPPTD
KSDRDMLDVLIAVKAETGTPRFSADEITGMFISMMFAGHHTSSGTASWTLIELMRHRDAYAAVIDELDELYGD
GRSVSFHALRQIPQLENVLKETLRLHPPLIILMRVAKGEFEVQGHRIHEGDLVAASPAISNRIPEDFPDPHDF
VPARYEQPRQEDLLNRWTWIPFGAGRHRCVGAAFAIMQIKAIFSVLLREYEFEMAQPPESYRNDHSKMVVQLA
QPACVRYRRRTGV
>CYP51B1_Mycobacterium tuberculosis M1274 2622418520
MSAVALPRVSGGHDEHGHLEEFRTDPIGLMQRVRDECGDVGTFQLAGKQVVLLSGSHANEFFFRAGDDDLDQA
KAYPFMTPIFGEGVVFDASPERRKEMLHNAALRGEQMKGHAATIEDQVRRMIADWGEAAEIDLLDFFAELTIY
TSSACLIGKKFRDQLDGRFAKLYHELERGTDPLAYVDPYLPIESFRRRDEARNGLVALVADIMNGRIANPPTD
KSDRDMLDVLIAVKAETGTPRFSADEITGMFISMMFAGHHTSSGTASWTLIELMRHRDAYAAVIDELDELYGD
GRSVSFHALRQIPQLENVLKETLRLHPPLIILMRVAKGEFEVQGHRIHEGDLVAASPAISNRIPEDFPDPHDF
VPARYEQPRQEDLLNRWTWIPFGAGRHRCVGAAFAIMQIKAIFSVLLREYEFEMAQPPESYRNDHSKMVVQLA
QPACVRYRRRTGV

>CYP51B1_Mycobacterium tuberculosis M1292 2622430794
MSAVALPRVSGGHDEHGHLEEFRTDPIGLMQRVRDECGDVGTFQLAGKQVVLLSGSHANEFFFRAGDDDLDQA
KAYPFMTPIFGEGVVFDASPERRKEMLHNAALRGEQMKGHAATIEDQVRRMIADWGEAAEIDLLDFFAELTIY
TSSACLIGKKFRDQLDGRFAKLYHELERGTDPLAYVDPYLPIESFRRRDEARNGLVALVADIMNGRIANPPTD
KSDRDMLDVLIAVKAETGTPRFSADEITGMFISMMFAGHHTSSGTASWTLIELMRHRDAYAAVIDELDELYGD
GRSVSFHALRQIPQLENVLKETLRLHPPLIILMRVAKGEFEVQGHRIHEGDLVAASPAISNRIPEDFPDPHDF
VPARYEQPRQEDLLNRWTWIPFGAGRHRCVGAAFAIMQIKAIFSVLLREYEFEMAQPPESYRNDHSKMVVQLA
QPACVRYRRRTGV
>CYP51B1_Mycobacterium tuberculosis M1294 2622434957
MSAVALPRVSGGHDEHGHLEEFRTDPIGLMQRVRDECGDVGTFQLAGKQVVLLSGSHANEFFFRAGDDDLDQA
KAYPFMTPIFGEGVVFDASPERRKEMLHNAALRGEQMKGHAATIEDQVRRMIADWGEAAEIDLLDFFAELTIY
TSSACLIGKKFRDQLDGRFAKLYHELERGTDPLAYVDPYLPIESFRRRDEARNGLVALVADIMNGRIANPPTD
KSDRDMLDVLIAVKAETGTPRFSADEITGMFISMMFAGHHTSSGTASWTLIELMRHRDAYAAVIDELDELYGD
GRSVSFHALRQIPQLENVLKETLRLHPPLIILMRVAKGEFEVQGHRIHEGDLVAASPAISNRIPEDFPDPHDF
VPARYEQPRQEDLLNRWTWIPFGAGRHRCVGAAFAIMQIKAIFSVLLREYEFEMAQPPESYRNDHSKMVVQLA
QPACVRYRRRTGV
>CYP51B1_Mycobacterium tuberculosis M13 2622439914
MSAVALPRVSGGHDEHGHLEEFRTDPIGLMQRVRDECGDVGTFQLAGKQVVLLSGSHANEFFFRAGDDDLDQA
KAYPFMTPIFGEGVVFDASPERRKEMLHNAALRGEQMKGHAATIEDQVRRMIADWGEAGEIDLLDFFAELTIY
TSSACLIGKKFRDQLDGRFAKLYHELERGTDLLAYVDPYLPIESFRRRDEARNGLVALVADIMNGRIANPPTD
KSDRDMLDVLIAVKAETGTPRFSADEITGMFISMMFAGHHTSSGTASWTLIELMRHRDAYAAVIDELDELYGD
GRSVSFHALRQIPQLENVLKETLRLHPPLIILMRVAKGEFEVQGHRIHEGDLVAASPAISNRIPEDFPDPHDF
VPARYEQPRQEDLLNRWTWIPFGAGRHRCVGAAFAIMQIKAIFSVLLREYEFEMAQPPESYRNDHSKMVVQLA
QPACVRYRRRTGV
>CYP51B1_Mycobacterium tuberculosis M1309 2622452812
MSAVALPRVSGGHDEHGHLEEFRTDPIGLMQRVRDECGDVGTFQLAGKQVVLLSGSHANEFFFRAGDDDLDQA
KAYPFMTPIFGEGVVFDASPERRKEMLHNAALRGEQMKGHAATIEDQVRRMIADWGEAAEIDLLDFFAELTIY
TSSACLIGKKFRDQLDGRFAKLYHELERGTDPLAYVDPYLPIESFRRRDEARNGLVALVADIMNGRIANPPTD
KSDRDMLDVLIAVKAETGTPRFSADEITGMFISMMFAGHHTSSGTASWTLIELMRHRDAYAAVIDELDELYGD
GRSVSFHALRQIPQLENVLKETLRLHPPLIILMRVAKGEFEVQGHRIHEGDLVAASPAISNRIPEDFPDPHDF
VPARYEQPRQEDLLNRWTWIPFGAGRHRCVGAAFAIMQIKAIFSVLLREYEFEMAQPPESYRNDHSKMVVQLA
QPACVRYRRRTGV
>CYP51B1_Mycobacterium tuberculosis M1374 2622485747
MSAVALPRVSGGHDEHGHLEEFRTDPIGLMQRVRDECGDVGTFQLAGKQVVLLSGSHANEFFFRAGDDDLDQA
KAYPFMTPIFGEGVVFDASPERRKEMLHNAALRGEQMKGHAATIEDQVRRMIADWGEAAEIDLLDFFAELTIY
TSSACLIGKKFRDQLDGRFAKLYHELERGTDPLAYVDPYLPIESFRRRDEARNGLVALVADIMNGRIANPPTD
KSDRDMLDVLIAVKAETGTPRFSADEITGMFISMMFAGHHTSSGTASWTLIELMRHRDAYAAVIDELDELYGD
GRSVSFHALRQIPQLENVLKETLRLHPPLIILMRVAKGEFEVQGHRIHEGDLVAASPAISNRIPEDFPDPHDF
VPARYEQPRQEDLLNRWTWIPFGAGRHRCVGAAFAIMQIKAIFSVLLREYEFEMAQPPESYRNDHSKMVVQLA
QPACVRYRRRTGV
>CYP51B1_Mycobacterium tuberculosis M1393 2622502219
MSAVALPRVSGGHDEHGHLEEFRTDPIGLMQRVRDECGDVGTFQLAGKQVVLLSGSHANEFFFRAGDDDLDQA
KAYPFMTPIFGEGVVFDASPERRKEMLHNAALRGEQMKGHAATIEDQVRRMIADWGEAAEIDLLDFFAELTIY
TSSACLIGKKFRDQLDGRFAKLYHELERGTDPLAYVDPYLPIESFRRRDEARNGLVALVADIMNGRIANPPTD
KSDRDMLDVLIAVKAETGTPRFSADEITGMFISMMFAGHHTSSGTASWTLIELMRHRDAYAAVIDELDELYGD
GRSVSFHALRQIPQLENVLKETLRLHPPLIILMRVAKGEFEVQGHRIHEGDLVAASPAISNRIPEDFPDPHDF
VPARYEQPRQEDLLNRWTWIPFGAGRHRCVGAAFAIMQIKAIFSVLLREYEFEMAQPPESYRNDHSKMVVQLA
QPACVRYRRRTGV
>CYP51B1_Mycobacterium tuberculosis M1459 2622580025
MSAVALPRVSGGHDEHGHLEEFRTDPIGLMQRVRDECGDVGTFQLAGKQVVLLSGSHANEFFFRAGDDDLDQA
KAYPFMTPIFGEGVVFDASPERRKEMLHNAALRGEQMKGHAATIEDQVRRMIADWGEAAEIDLLDFFAELTIY
TSSACLIGKKFRDQLDGRFAKLYHELERGTDPLAYVDPYLPIESFRRRDEARNGLVALVADIMNGRIANPPTD
KSDRDMLDVLIAVKAETGTPRFSADEITGMFISMMFAGHHTSSGTASWTLIELMRHRDAYAAVIDELDELYGD
GRSVSFHALRQIPQLENVLKETLRLHPPLIILMRVAKGEFEVQGHRIHEGDLVAASPAISNRIPEDFPDPHDF
VPARYEQPRQEDLLNRWTWIPFGAGRHRCVGAAFAIMQIKAIFSVLLREYEFEMAQPPESYRNDHSKMVVQLA
QPACVRYRRRTGV
>CYP51B1_Mycobacterium tuberculosis M1488 2622588278

MSAVALPRVSGGHDEHGHLEEFRTDPIGLMQRVRDECGDVGTFQLAGKQVVLLSGSHANEFFFRAGDDDLDQA
KAYPFMTPIFGEGVVFDASPERRKEMLHNAALRGEQMKGHAATIEDQVRRMIADWGEAAEIDLLDFFAELTIY
TSSACLIGKKFRDQLDGRFAKLYHELERGTDPLAYVDPYLPIESFRRRDEARNGLVALVADIMNGRIANPPTD
KSDRDMLDVLIAVKAETGTPRFSADEITGMFISMMFAGHHTSSGTASWTLIELMRHRDAYAAVIDELDELYGD
GRSVSFHALRQIPQLENVLKETLRLHPPLIILMRVAKGEFEVQGHRIHEGDLVAASPAISNRIPEDFPDPHDF
VPARYEQPRQEDLLNRWTWIPFGAGRHRCVGAAFAIMQIKAIFSVLLREYEFEMAQPPESYRNDHSKMVVQLA
QPACVRYRRRTGV
>CYP51B1_Mycobacterium tuberculosis M1545 2622600941
MSAVALPRVSGGHDEHGHLEEFRTDPIGLMQRVRDECGDVGTFQLAGKQVVLLSGSHANEFFFRAGDDDLDQA
KAYPFMTPIFGEGVVFDASPERRKEMLHNAALRGEQMKGHAATIEDQVRRMIADWGEAAEIDLLDFFAELTIY
TSSACLIGKKFRDQLDGRFAKLYHELERGTDPLAYVDPYLPIESFRRRDEARNGLVALVADIMNGRIANPPTD
KSDRDMLDVLIAVKAETGTPRFSADEITGMFISMMFAGHHTSSGTASWTLIELMRHRDAYAAVIDELDELYGD
GRSVSFHALRQIPQLENVLKETLRLHPPLIILMRVAKGEFEVQGHRIHEGDLVAASPAISNRIPEDFPDPHDF
VPARYEQPRQEDLLNRWTWIPFGAGRHRCVGAAFAIMQIKAIFSVLLREYEFEMAQPPESYRNDHSKMVVQLA
QPACVRYRRRTGV
>CYP51B1_Mycobacterium tuberculosis M1559 2575796963
MSAVALPRVSGGHDEHGHLEEFRTDPIGLMQRVRDECGDVGTFQLAGKQVVLLSGSHANEFFFRAGDDDLDQA
KAYPFMTPIFGEGVVFDASPERRKEMLHNAALRGEQMKGHAATIEDQVRRMIADWGEAAEIDLLDFFAELTIY
TSSACLIGKKFRDQLDGRFAKLYHELERGTDPLAYVDPYLPIESFRRRDEARNGLVALVADIMNGRIANPPTD
KSDRDMLDVLIAVKAETGTPRFSADEITGMFISMMFAGHHTSSGTASWTLIELMRHRDAYAAVIDELDELYGD
GRSVSFHALRQIPQLENVLKETLRLHPPLIILMRVAKGEFEVQGHRIHEGDLVAASPAISNRIPEDFPDPHDF
VPARYEQPRQEDLLNRWTWIPFGAGRHRCVGAAFAIMQIKAIFSVLLREYEFEMAQPPESYRNDHSKMVVQLA
QPACVRYRRRTGV
>CYP51B1_Mycobacterium tuberculosis M1638 2622620303
MSAVALPRVSGGHDEHGHLEEFRTDPIGLMQRVRDECGDVGTFQLAGKQVVLLSGSHANEFFFRAGDDDLDQA
KAYPFMTPIFGEGVVFDASPERRKEMLHNAALRGEQMKGHAATIEDQVRRMIADWGEAAEIDLLDFFAELTIY
TSSACLIGKKFRDQLDGRFAKLYHELERGTDPLAYVDPYLPIESFRRRDEARNGLVALVADIMNGRIANPPTD
KSDRDMLDVLIAVKAETGTPRFSADEITGMFISMMFAGHHTSSGTASWTLIELMRHRDAYAAVIDELDELYGD
GRSVSFHALRQIPQLENVLKETLRLHPPLIILMRVAKGEFEVQGHRIHEGDLVAASPAISNRIPEDFPDPHDF
VPARYEQPRQEDLLNRWTWIPFGAGRHRCVGAAFAIMQIKAIFSVLLREYEFEMAQPPESYRNDHSKMVVQLA
QPACVRYRRRTGV
>CYP51B1_Mycobacterium tuberculosis M1702 2622624460
MSAVALPRVSGGHDEHGHLEEFRTDPIGLMQRVRDECGDVGTFQLAGKQVVLLSGSHANEFFFRAGDDDLDQA
KAYPFMTPIFGEGVVFDASPERRKEMLHNAALRGEQMKGHAATIEDQVRRMIADWGEAAEIDLLDFFAELTIY
TSSACLIGKKFRDQLDGRFAKLYHELERGTDPLAYVDPYLPIESFRRRDEARNGLVALVADIMNGRIANPPTD
KSDRDMLDVLIAVKAETGTPRFSADEITGMFISMMFAGHHTSSGTASWTLIELMRHRDAYAAVIDELDELYGD
GRSVSFHALRQIPQLENVLKETLRLHPPLIILMRVAKGEFEVQGHRIHEGDLVAASPAISNRIPEDFPDPHDF
VPARYEQPRQEDLLNRWTWIPFGAGRHRCVGAAFAIMQIKAIFSVLLREYEFEMAQPPESYRNDHSKMVVQLA
QPACVRYRRRTGV
>CYP51B1_Mycobacterium tuberculosis M1704 2622628701
MSAVALPRVSGGHDEHGHLEEFRTDPIGLMQRVRDECGDVGTFQLAGKQVVLLSGSHANEFFFRAGDDDLDQA
KAYPFMTPIFGEGVVFDASPERRKEMLHNAALRGEQMKGHAATIEDQVRRMIADWGEAAEIDLLDFFAELTIY
TSSACLIGKKFRDQLDGRFAKLYHELERGTDPLAYVDPYLPIESFRRRDEARNGLVALVADIMNGRIANPPTD
KSDRDMLDVLIAVKAETGTPRFSADEITGMFISMMFAGHHTSSGTASWTLIELMRHRDAYAAVIDELDELYGD
GRSVSFHALRQIPQLENVLKETLRLHPPLIILMRVAKGEFEVQGHRIHEGDLVAASPAISNRIPEDFPDPHDF
VPARYEQPRQEDLLNRWTWIPFGAGRHRCVGAAFAIMQIKAIFSVLLREYEFEMAQPPESYRNDHSKMVVQLA
QPACVRYRRRTGV
>CYP51B1_Mycobacterium tuberculosis M1759 2630121935
MSAVALPRVSGGHDEHGHLEEFRTDPIGLMQRVRDECGDVGTFQLAGKQVVLLSGSHANEFFFRAGDDDLDQA
KAYPFMTPIFGEGVVFDASPERRKEMLHNAALRGEQMKGHAATIEDQVRRMIADWGEAAEIDLLDFFAELTIY
TSSACLIGKKFRDQLDGRFAKLYHELERGTDPLAYVDPYLPIESFRRRDEARNGLVALVADIMNGRIANPPTD
KSDRDMLDVLIAVKAETGTPRFSADEITGMFISMMFAGHHTSSGTASWTLIELMRHRDAYAAVIDELDELYGD
GRSVSFHALRQIPQLENVLKETLRLHPPLIILMRVAKGEFEVQGHRIHEGDLVAASPAISNRIPEDFPDPHDF
VPARYEQPRQEDLLNRWTWIPFGAGRHRCVGAAFAIMQIKAIFSVLLREYEFEMAQPPESYRNDHSKMVVQLA
QPACVRYRRRTGV
>CYP51B1_Mycobacterium tuberculosis M1762 2574903455
MSAVALPRVSGGHDEHGHLEEFRTDPIGLMQRVRDECGDVGTFQLAGKQVVLLSGSHANEFFFRAGDDDLDQA
KAYPFMTPIFGEGVVFDASPERRKEMLHNAALRGEQMKGHAATIEDQVRRMIADWGEAAEIDLLDFFAELTIY

TSSACLIGKKFRDQLDGRFAKLYHELERGTDPLAYVDPYLPIESFRRRDEARNGLVALVADIMNGRIANPPTD
KSDRDMLDVLIAVKAETGTPRFSADEITGMFISMMFAGHHTSSGTASWTLIELMRHRDAYAAVIDELDELYGD
GRSVSFHALRQIPQLENVLKETLRLHPPLIILMRVAKGEFEVQGHRIHEGDLVAASPAISNRIPEDFPDPHDF
VPARYEQPRQEDLLNRWTWIPFGAGRHRCVGAAFAIMQIKAIFSVLLREYEFEMAQPPESYRNDHSKMVVQLA
QPACVRYRRRTGV
>CYP51B1_Mycobacterium tuberculosis M1780 2622645292
MSAVALPRVSGGHDEHGHLEEFRTDPIGLMQRVRDECGDVGTFQLAGKQVVLLSGSHANEFFFRAGDDDLDQA
KAYPFMTPIFGEGVVFDASPERRKEMLHNAALRGEQMKGHAATIEDQVRRMIADWGEAAEIDLLDFFAELTIY
TSSACLIGKKFRDQLDGRFAKLYHELERGTDPLAYVDPYLPIESFRRRDEARNGLVALVADIMNGRIANPPTD
KSDRDMLDVLIAVKAETGTPRFSADEITGMFISMMFAGHHTSSGTASWTLIELMRHRDAYAAVIDELDELYGD
GRSVSFHALRQIPQLENVLKETLRLHPPLIILMRVAKGEFEVQGHRIHEGDLVAASPAISNRIPEDFPDPHDF
VPARYEQPRQEDLLNRWTWIPFGAGRHRCVGAAFAIMQIKAIFSVLLREYEFEMAQPPESYRNDHSKMVVQLA
QPACVRYRRRTGV
>CYP51B1_Mycobacterium tuberculosis M1787 2579819694
MSAVALPRVSGGHDEHGHLEEFRTDPIGLMQRVRDECGDVGTFQLAGKQVVLLSGSHANEFFFRAGDDDLDQA
KAYPFMTPIFGEGVVFDASPERRKEMLHNAALRGEQMKGHAATIEDQVRRMIADWGEAAEIDLLDFFAELTIY
TSSACLIGKKFRDQLDGRFAKLYHELERGTDPLAYVDPYLPIESFRRRDEARNGLVALVADIMNGRIANPPTD
KSDRDMLDVLIAVKAETGTPRFSADEITGMFISMMFAGHHTSSGTASWTLIELMRHRDAYAAVIDELDELYGD
GRSVSFHALRQIPQLENVLKETLRLHPPLIILMRVAKGEFEVQGHRIHEGDLVAASPAISNRIPEDFPDPHDF
VPARYEQPRQEDLLNRWTWIPFGAGRHRCVGAAFAIMQIKAIFSVLLREYEFEMAQPPESYRNDHSKMVVQLA
QPACVRYRRRTGV
>CYP51B1_Mycobacterium tuberculosis M1913 2576930170
MSAVALPRVSGGHDEHGHLEEFRTDPIGLMQRVRDECGDVGTFQLAGKQVVLLSGSHANEFFFRAGDDDLDQA
KAYPFMTPIFGEGVVFDASPERRKEMLHNAALRGEQMKGHAATIEDQVRRMIADWGEAAEIDLLDFFAELTIY
TSSACLIGKKFRDQLDGRFAKLYHELERGTDPLAYVDPYLPIESFRRRDEARNGLVALVADIMNGRIANPPTD
KSDRDMLDVLIAVKAETGTPRFSADEITGMFISMMFAGHHTSSGTASWTLIELMRHRDAYAAVIDELDELYGD
GRSVSFHALRQIPQLENVLKETLRLHPPLIILMRVAKGEFEVQGHRIHEGDLVAASPAISNRIPEDFPDPHDF
VPARYEQPRQEDLLNRWTWIPFGAGRHRCVGAAFAIMQIKAIFSVLLREYEFEMAQPPESYRNDHSKMVVQLA
QPACVRYRRRTGV
>CYP51B1_Mycobacterium tuberculosis M1955 2622690920
MSAVALPRVSGGHDEHGHLEEFRTDPIGLMQRVRDECGDVGTFQLAGKQVVLLSGSHANEFFFRAGDDDLDQA
KAYPFMTPIFGEGVVFDASPERRKEMLHNAALRGEQMKGHAATIEDQVRRMIADWGEAAEIDLLDFFAELTIY
TSSACLIGKKFRDQLDGRFAKLYHELERGTDPLAYVDPYLPIESFRRRDEARNGLVALVADIMNGRIANPPTD
KSDRDMLDVLIAVKAETGTPRFSADEITGMFISMMFAGHHTSSGTASWTLIELMRHRDAYAAVIDELDELYGD
GRSVSFHALRQIPQLENVLKETLRLHPPLIILMRVAKGEFEVQGHRIHEGDLVAASPAISNRIPEDFPDPHDF
VPARYEQPRQEDLLNRWTWIPFGAGRHRCVGAAFAIMQIKAIFSVLLREYEFEMAQPPESYRNDHSKMVVQLA
QPACVRYRRRTGV
>CYP51B1_Mycobacterium tuberculosis M2006 2577305526
MSAVALPRVSGGHDEHGHLEEFRTDPIGLMQRVRDECGDVGTFQLAGKQVVLLSGSHANEFFFRAGDDDLDQA
KAYPFMTPIFGEGVVFDASPERRKEMLHNAALRGEQMKGHAATIEDQVRRMIADWGEAAEIDLLDFFAELTIY
TSSACLIGKKFRDQLDGRFAKLYHELERGTDPLAYVDPYLPIESFRRRDEARNGLVALVADIMNGRIANPPTD
KSDRDMLDVLIAVKAETGTPRFSADEITGMFISMMFAGHHTSSGTASWTLIELMRHRDAYAAVIDELDELYGD
GRSVSFHALRQIPQLENVLKETLRLHPPLIILMRVAKGEFEVQGHRIHEGDLVAASPAISNRIPEDFPDPHDF
VPARYEQPRQEDLLNRWTWIPFGAGRHRCVGAAFAIMQIKAIFSVLLREYEFEMAQPPESYRNDHSKMVVQLA
QPACVRYRRRTGV
>CYP51B1_Mycobacterium tuberculosis M2007 2622798742
MSAVALPRVSGGHDEHGHLEEFRTDPIGLMQRVRDECGDVGTFQLAGKQVVLLSGSHANEFFFRAGDDDLDQA
KAYPFMTPIFGEGVVFDASPERRKEMLHNAALRGEQMKGHAATIEDQVRRMIADWGEAAEIDLLDFFAELTIY
TSSACLIGKKFRDQLDGRFAKLYHELERGTDPLAYVDPYLPIESFRRRDEARNGLVALVADIMNGRIANPPTD
KSDRDMLDVLIAVKAETGTPRFSADEITGMFISMMFAGHHTSSGTASWTLIELMRHRDAYAAVIDELDELYGD
GRSVSFHALRQIPQLENVLKETLRLHPPLIILMRVAKGEFEVQGHRIHEGDLVAASPAISNRIPEDFPDPHDF
VPARYEQPRQEDLLNRWTWIPFGAGRHRCVGAAFAIMQIKAIFSVLLREYEFEMAQPPESYRNDHSKMVVQLA
QPACVRYRRRTGV
>CYP51B1_Mycobacterium tuberculosis M2191 2642225291
MSAVALPRVSGGHDEHGHLEEFRTDPIGLMQRVRDECGDVGTFQLAGKQVVLLSGSHANEFFFRAGDDDLDQA
KAYPFMTPIFGEGVVFDASPERRKEMLHNAALRGEQMKGHAATIEDQVRRMIADWGEAAEIDLLDFFAELTIY
TSSACLIGKKFRDQLDGRFAKLYHELERGTDPLAYVDPYLPIESFRRRDEARNGLVALVADIMNGRIANPPTD
KSDRDMLDVLIAVKAETGTPRFSADEITGMFISMMFAGHHTSSGTASWTLIELMRHRDAYAAVIDELDELYGD

GRSVSFHALRQIPQLENVLKETLRLHPPLIILMRVAKGEFEVQGHRIHEGDLVAASPAISNRIPEDFPDPHDF
VPARYEQPRQEDLLNRWTWIPFGAGRHRCVGAAFAIMQIKAIFSVLLREYEFEMAQPPESYRNDHSKMVVQLA
QPACVRYRRRTGV
>CYP51B1_Mycobacterium tuberculosis M2193 2631475733
MSAVALPRVSGGHDEHGHLEEFRTDPIGLMQRVRDECGDVGTFQLAGKQVVLLSGSHANEFFFRAGDDDLDQA
KAYPFMTPIFGEGVVFDASPERRKEMLHNAALRGEQMKGHAATIEDQVRRMIADWGEAAEIDLLDFFAELTIY
TSSACLIGKKFRDQLDGRFAKLYHELERGTDPLAYVDPYLPIESFRRRDEARNGLVALVADIMNGRIANPPTD
KSDRDMLDVLIAVKAETGTPRFSADEITGMFISMMFAGHHTSSGTASWTLIELMRHRDAYAAVIDELDELYGD
GRSVSFHALRQIPQLENVLKETLRLHPPLIILMRVAKGEFEVQGHRIHEGDLVAASPAISNRIPEDFPDPHDF
VPARYEQPRQEDLLNRWTWIPFGAGRHRCVGAAFAIMQIKAIFSVLLREYEFEMAQPPESYRNDHSKMVVQLA
QPACVRYRRRTGV
>CYP51B1_Mycobacterium tuberculosis M2199 2632047189
MSAVALPRVSGGHDEHGHLEEFRTDPIGLMQRVRDECGDVGTFQLAGKQVVLLSGSHANEFFFRAGDDDLDQA
KAYPFMTPIFGEGVVFDASPERRKEMLHNAALRGEQMKGHAATIEDQVRRMIADWGEAGEIDLLDFFAELTIY
TSSACLIGKKFRDQLDGRFAKLYHELERGTDPLAYVDPYLPIESFRRRDEARNGLVALVADIMNGRIANPPTD
KSDRDMLDVLIAVKAETGTPRFSADEITGMFISMMFAGHHTSSGTASWTLIELMRHRDAYAAVIDELDELYGD
GRSVSFHALRQIPQLENVLKETLRLHPPLIILMRVAKGEFEVQGHRIHEGDLVAASPAISNRIPEDFPDPHDF
VPARYEQPRQEDLLNRWTWIPFGAGRHRCVGAAFAIMQIKAIFSVLLREYEFEMGQPPESYRNDHSKMVVQLA
QPACVRYRRRTGV
>CYP51B1_Mycobacterium tuberculosis M2248 2577221695
MSAVALPRVSGGHDEHGHLEEFRTDPIGLMQRVRDECGDVGTFQLAGKQVVLLSGSHANEFFFRAGDDDLDQA
KAYPFMTPIFGEGVVFDASPERRKEMLHNAALRGEQMKGHAATIEDQVRRMIADWGEAAEIDLLDFFAELTIY
TSSACLIGKKFRDQLDGRFAKLYHELERGTDPLAYVDPYLPIESFRRRDEARNGLVALVADIMNGRIANPPTD
KSDRDMLDVLIAVKAETGTPRFSADEITGMFISMMFAGHHTSSGTASWTLIELMRHRDAYAAVIDELDELYGD
GRSVSFHALRQIPQLENVLKETLRLHPPLIILMRVAKGEFEVQGHRIHEGDLVAASPAISNRIPEDFPDPHDF
VPARYEQPRQEDLLNRWTWIPFGAGRHRCVGAAFAIMQIKAIFSVLLREYEFEMAQPPESYRNDHSKMVVQLA
QPACVRYRRRTGV
>CYP51B1_Mycobacterium tuberculosis M2249 2577921329
MSAVALPRVSGGHDEHGHLEEFRTDPIGLMQRVRDECGDVGTFQLAGKQVVLLSGSHANEFFFRAGDDDLDQA
KAYPFMTPIFGEGVVFDASPERRKEMLHNAALRGEQMKGHAATIEDQVRRMIADWGEAAEIDLLDFFAELTIY
TSSACLIGKKFRDQLDGRFAKLYHELERGTDPLAYVDPYLPIESFRRRDEARNGLVALVADIMNGRIANPPTD
KSDRDMLDVLIAVKAETGTPRFSADEITGMFISMMFAGHHTSSGTASWTLIELMRHRDAYAAVIDELDELYGD
GRSVSFHALRQIPQLENVLKETLRLHPPLIILMRVAKGEFEVQGHRIHEGDLVAASPAISNRIPEDFPDPHDF
VPARYEQPRQEDLLNRWTWIPFGAGRHRCVGAAFAIMQIKAIFSVLLREYEFEMAQPPESYRNDHSKMVVQLA
QPACVRYRRRTGV
>CYP51B1_Mycobacterium tuberculosis M2276 2633232788
MSAVALPRVSGGHDEHGHLEEFRTDPIGLMQRVRDECGDVGTFQLAGKQVVLLSGSHANEFFFRAGDDDLDQA
KAYPFMTPIFGEGVVFDASPERRKEMLHNAALRGEQMKGHAATIEDQVRRMIADWGEAAEIDLLDFFAELTIY
TSSACLIGKKFRDQLDGRFAKLYHELERGTDPLAYVDPYLPIESFRRRDEARNGLVALVADIMNGRIANPPTD
KSDRDMLDVLIAVKAETGTPRFSADEITGMFISMMFAGHHTSSGTASWTLIELMRHRDAYAAVIDELDELYGD
GRSVSFHALRQIPQLENVLKETLRLHPPLIILMRVAKGEFEVQGHRIHEGDLVAASPAISNRIPEDFPDPHDF
VPARYEQPRQEDLLNRWTWIPFGAGRHRCVGAAFAIMQIKAIFSVLLREYEFEMAQPPESYRNDHSKMVVQLA
QPACVRYRRRTGV
>CYP51B1_Mycobacterium tuberculosis M2277 2636831957
MSAVALPRVSGGHDEHGHLEEFRTDPIGLMQRVRDECGDVGTFQLAGKQVVLLSGSHANEFFFRAGDDDLDQA
KAYPFMTPIFGEGVVFDASPERRKEMLHNAALRGEQMKGHAATIEDQVRRMIADWGEAAEIDLLDFFAELTIY
TSSACLIGKKFRDQLDGRFAKLYHELERGTDPLAYVDPYLPIESFRRRDEARNGLVALVADIMNGRIANPPTD
KSDRDMLDVLIAVKAETGTPRFSADEITGMFISMMFAGHHTSSGTASWTLIELMRHRDAYAAVIDELDELYGD
GRSVSFHALRQIPQLENVLKETLRLHPPLIILMRVAKGEFEVQGHRIHEGDLVAASPAISNRIPEDFPDPHDF
VPARYEQPRQEDLLNRWTWIPFGAGRHRCVGAAFAIMQIKAIFSVLLREYEFEMAQPPESYRNDHSKMVVQLA
QPACVRYRRRTGV
>CYP51B1_Mycobacterium tuberculosis M2323 2629770754
MSAVALPRVSGGHDEHGHLEEFRTDPIGLMQRVRDECGDVGTFQLAGKQVVLLSGSHANEFFFRAGDDDLDQA
KAYPFMTPIFGEGVVFDASPERRKEMLHNAALRGEQMKGHAATIEDQVRRMIADWGEAAEIDLLDFFAELTIY
TSSACLIGKKFRDQLDGRFAKLYHELERGTDPLAYVDPYLPIESFRRRDEARNGLVALVADIMNGRIANPPTD
KSDRDMLDVLIAVKAETGTPRFSADEITGMFISMMFAGHHTSSGTASWTLIELMRHRDAYAAVIDELDELYGD
GRSVSFHALRQIPQLENVLKETLRLHPPLIILMRVAKGEFEVQGHRIHEGDLVAASPAISNRIPEDFPDPHDF

VPARYEQPRQEDLLNRWTWIPFGAGRHRCVGAAFAIMQIKAIFSVLLREYEFEMAQPPESYRNDHSKMVVQLA
QPACVRYRRRTGV
>CYP51B1_Mycobacterium tuberculosis M2324 2629230984
MSAVALPRVSGGHDEHGHLEEFRTDPIGLMQRVRDECGDVGTFQLAGKQVVLLSGSHANEFFFRAGDDDLDQA
KAYPFMTPIFGEGVVFDASPERRKEMLHNAALRGEQMKGHAATIEDQVRRMIADWGEAAEIDLLDFFAELTIY
TSSACLIGKKFRDQLDGRFAKLYHELERGTDPLAYVDPYLPIESFRRRDEARNGLVALVADIMNGRIANPPTD
KSDRDMLDVLIAVKAETGTPRFSADEITGMFISMMFAGHHTSSGTASWTLIELMRHRDAYAAVIDELDELYGD
GRSVSFHALRQIPQLENVLKETLRLHPPLIILMRVAKGEFEVQGHRIHEGDLVAASPAISNRIPEDFPDPHDF
VPARYEQPRQEDLLNRWTWIPFGAGRHRCVGAAFAIMQIKAIFSVLLREYEFEMAQPPESYRNDHSKMVVQLA
QPACVRYRRRTGV
>CYP51B1_Mycobacterium tuberculosis M2376 2642112271
MSAVALPRVSGGHDEHGHLEEFRTDPIGLMQRVRDECGDVGTFQLAGKQVVLLSGSHANEFFFRAGDDDLDQA
KAYPFMTPIFGEGVVFDASPERRKEMLHNAALRGEQMKGHAATIEDQVRRMIADWGEAAEIDLLDFFAELTIY
TSSACLIGKKFRDQLDGRFAKLYHELERGTDPLAYVDPYLPIESFRRRDEARNGLVALVADIMNGRIANPPTD
KSDRDMLDVLIAVKAETGTPRFSADEITGMFISMMFAGHHTSSGTASWTLIELMRHRDAYAAVIDELDELYGD
GRSVSFHALRQIPQLENVLKETLRLHPPLIILMRVAKGEFEVQGHRIHEGDLVAASPAISNRIPEDFPDPHDF
VPARYEQPRQEDLLNRWTWIPFGAGRHRCVGAAFAIMQIKAIFSVLLREYEFEMAQPPESYRNDHSKMVVQLA
QPACVRYRRRTGV
>CYP51B1_Mycobacterium tuberculosis M2402 2574855703
MSAVALPRVSGGHDEHGHLEEFRTDPIGLMQRVRDECGDVGTFQLAGKQVVLLSGSHANEFFFRAGDDDLDQA
KAYPFMTPIFGEGVVFDASPERRKEMLHNAALRGEQMKGHAATIEDQVRRMIADWGEAAEIDLLDFFAELTIY
TSSACLIGKKFRDQLDGRFAKLYHELERGTDPLAYVDPYLPIESFRRRDEARNGLVALVADIMNGRIANPPTD
KSDRDMLDVLIAVKAETGTPRFSADEITGMFISMMFAGHHTSSGTASWTLIELMRHRDAYAAVIDELDELYGD
GRSVSFHALRQIPQLENVLKETLRLHPPLIILMRVAKGEFEVQGHRIHEGDLVAASPAISNRIPEDFPDPHDF
VPARYEQPRQEDLLNRWTWIPFGAGRHRCVGAAFAIMQIKAIFSVLLREYEFEMAQPPESYRNDHSKMVVQLA
QPACVRYRRRTGV
>CYP51B1_Mycobacterium tuberculosis M2440 2637264791
MSAVALPRVSGGHDEHGHLEEFRTDPIGLMQRVRDECGDVGTFQLAGKQVVLLSGSHANEFFFRAGDDDLDQA
KAYPFMTPIFGEGVVFDASPERRKEMLHNAALRGEQMKGHAATIEDQVRRMIADWGEAAEIDLLDFFAELTIY
TSSACLIGKKFRDQLDGRFAKLYHELERGTDPLAYVDPYLPIESFRRRDEARNGLVALVADIMNGRIANPPTD
KSDRDMLDVLIAVKAETGTPRFSADEITGMFISMMFAGHHTSSGTASWTLIELMRHRDAYAAVIDELDELYGD
GRSVSFHALRQIPQLENVLKETLRLHPPLIILMRVAKGEFEVQGHRIHEGDLVAASPAISNRIPEDFPDPHDF
VPARYEQPRQEDLLNRWTWIPFGAGRHRCVGAAFAIMQIKAIFSVLLREYEFEMAQPPESYRNDHSKMVVQLA
QPACVRYRRRTGV
>CYP51B1_Mycobacterium tuberculosis M2479 2577723535
MSAVALPRVSGGHDEHGHLEEFRTDPIGLMQRVRDECGDVGTFQLAGKQVVLLSGSHANEFFFRAGDDDLDQA
KAYPFMTPIFGEGVVFDASPERRKEMLHNAALRGEQMKGHAATIEDQVRRMIADWGEAAEIDLLDFFAELTIY
TSSACLIGKKFRDQLDGRFAKLYHELERGTDPLAYVDPYLPIESFRRRDEARNGLVALVADIMNGRIANPPTD
KSDRDMLDVLIAVKAETGTPRFSADEITGMFISMMFAGHHTSSGTASWTLIELMRHRDAYAAVIDELDELYGD
GRSVSFHALRQIPQLENVLKETLRLHPPLIILMRVAKGEFEVQGHRIHEGDLVAASPAISNRIPEDFPDPHDF
VPARYEQPRQEDLLNRWTWIPFGAGRHRCVGAAFAIMQIKAIFSVLLREYEFEMAQPPESYRNDHSKMVVQLA
QPACVRYRRRTGV
>CYP51B1_Mycobacterium tuberculosis M2508 2577238330
MSAVALPRVSGGHDEHGHLEEFRTDPIGLMQRVRDECGDVGTFQLAGKQVVLLSGSHANEFFFRAGDDDLDQA
KAYPFMTPIFGEGVVFDASPERRKEMLHNAALRGEQMKGHAATIEDQVRRMIADWGEAAEIDLLDFFAELTIY
TSSACLIGKKFRDQLDGRFAKLYHELERGTDPLAYVDPYLPIESFRRRDEARNGLVALVADIMNGRIANPPTD
KSDRDMLDVLIAVKAETGTPRFSADEITGMFISMMFAGHHTSSGTASWTLIELMRHRDAYAAVIDELDELYGD
GRSVSFHALRQIPQLENVLKETLRLHPPLIILMRVAKGEFEVQGHRIHEGDLVAASPAISNRIPEDFPDPHDF
VPARYEQPRQEDLLNRWTWIPFGAGRHRCVGAAFAIMQIKAIFSVLLREYEFEMAQPPESYRNDHSKMVVQLA
QPACVRYRRRTGV
>CYP51B1_Mycobacterium tuberculosis M2828 2630587300
MSAVALPRVSGGHDEHGHLEEFRTDPIGLMQRVRDECGDVGTFQLAGKQVVLLSGSHANEFFFRAGDDDLDQA
KAYPFMTPIFGEGVVFDASPERRKEMLHNAALRGEQMKGHAATIEDQVRRMIADWGEAAEIDLLDFFAELTIY
TSSACLIGKKFRDQLDGRFAKLYHELERGTDPLAYVDPYLPIESFRRRDEARNGLVALVADIMNGRIANPPTD
KSDRDMLDVLIAVKAETGTPRFSADEITGMFISMMFAGHHTSSGTASWTLIELMRHRDAYAAVIDELDELYGD
GRSVSFHALRQIPQLENVLKETLRLHPPLIILMRVAKGEFEVQGHRIHEGDLVAASPAISNRIPEDFPDPHDF
VPARYEQPRQEDLLNRWTWIPFGAGRHRCVGAAFAIMQIKAIFSVLLREYEFEMAQPPESYRNDHSKMVVQLA
QPACVRYRRRTGV

>CYP51B1_Mycobacterium tuberculosis M995 2579811185
MSAVALPRVSGGHDEHGHLEEFRTDPIGLMQRVRDECGDVGTFQLAGKQVVLLSGSHANEFFFRAGDDDLDQA
KAYPFMTPIFGEGVVFDASPERRKEMLHNAALRGEQMKGHAATIEDQVRRMIADWGEAGEIDLLDFFAELTIY
TSSACLIGKKFRDQLDGRFAKLYHELERGTDPLAYVDPYLPIESFRRRDEARNGLVALVADIMNGRIANPPTD
KSDRDMLDVLIAVKAETGTPRFSADEITGMFISMMFAGHHTSSGTASWTLIELMRHRDAYAAVIDELDELYGD
GRSVSFHALRQIPQLENVLKETLRLHPPLIILMRVAKGEFEVQGHRIHEGDLVTASPAISNRIPEDFPDPHDF
VPARYEQPRQEDLLNRWTWIPFGAGRHRCVGAAFAIMQIKAIFSVLLREYEFEMAQPPESYRNDHSKMVVQLA
QPACVRYRRRTGV
>CYP51B1_Mycobacterium tuberculosis MAL010109 2576456375
MSAVALPRVSGGHDEHGHLEEFRTDPIGLMQRVRDECGDVGTFQLAGKQVVLLSGSHANEFFFRAGDDDLDQA
KAYPFMTPIFGEGVVFDASPERRKEMLHNAALRGEQMKGHAATIEDQVRRMIADWGEAGEIDLLDFFAELTIY
TSSACLIGKKFRDQLDGRFAKLYHELERGTDPLAYVDPYLPIESFRRRDEARNGLVALVADIMNGRIANPPTD
KSDRDMLDVLIGVKAETGTPRFSADEITGMFISMMFAGHHTSSGTASWTLIELMRHRDAYAAVIDELDELYGD
GRSVSFHALRQIPQLENVLKETLRLHPPLIILMRVAKGEFEVQGHRIHEGDLVAASPAISNRIPEDFPDPHDF
VPARYEQPRQEDLLNRWTWIPFGAGRHRCVGAAFAIMQIKAIFSVLLREYEFEMAQPPESYRNDHSKMVVQLA
QPACVRYRRRTGV
>CYP51B1_Mycobacterium tuberculosis MAL020151 2621994030
MSAVALPRVSGGHDEHGHLEEFRTDPIGLMQRVRDECGDVGTFQLAGKQVVLLSGSHANEFFFRAGDDDLDQA
KAYPFMTPIFGEGVVFDASPERRKEMLHNAALRGEQMKGHAATIEDQVRRMIADWGEAGEIDLLDFFAELTIY
TSSACLIGKKFRDQLDGRFAKLYHELERGTDPLAYVDPYLPIESFRRRDEARNGLVALVADIMNGRIANPPTD
KSDRDMLDVLIGVKAETGTPRFSADEITGMFISMMFAGHHTSSGTASWTLIELMRHRDAYAAVIDELDELYGD
GRSVSFHALRQIPQLENVLKETLRLHPPLIILMRVAKGEFEVQGHRIHEGDLVAASPAISNRIPEDFPDPHDF
VPARYEQPRQEDLLNRWTWIPFGAGRHRCVGAAFAIMQIKAIFSVLLREYEFEMAQPPESYRNDHSKMVVQLA
QPACVRYRRRTGV
>CYP51B1_Mycobacterium tuberculosis N00000064 2699012075
MSAVALPRVSGGHDEHGHLEEFRTDPIGLMQRVRDECGDVGTFQLAGKQVVLLSGSHANEFFFRAGDDDLDQA
KAYPFMTPILGEGVVFDASPERRKEMLHNAALRGEQMKGHAATIEDQVRRMIADWGEAGEIDLLDFFAELTIY
TSSACLIGKKFRDQLDGRFAKLYHELERGTDPLAYVDPYLPIESFRRRDEARNGLVALVADIMNGRIANPPTD
KSDRDMLDVLIAVKAETGTPRFSADEITGMFISMMFAGHHTSSGTASWTLIELMRHRDAYAAVIDELDELYGD
GRSVSFHALRQIPQLENVLKETLRLHPPLIILMRVAKGEFEVQGHRIHEGDLVAASPAISNRIPEDFPDPHDF
VPARYEQPRQEDLLNRWTWIPFGAGRHRCVGAAFAIMQIKAIFSVLLREYEFEMAQPPESYRNDHSKMVVQLA
QPACVRYRRRTGV
>CYP51B1_Mycobacterium tuberculosis N09901829 2706577157
MSAVALPRVSGGHDEHGHLEEFRTDPIGLMQRVRDECGDVGTFQLAGKQVVLLSGSHANEFFFRAGDDDLDQA
KAYPFMTPILGEGVVFDASPERRKEMLHNAALRGEQMKGHAATIEDQVRRMIADWGEAGEIDLLDFFAELTIY
TSSACLIGKKFRDQLDGRFAKLYHELERGTDPLAYVDPYLPIESFRRRDEARNGLVALVADIMNGRIANPPTD
KSDRDMLDVLIAVKAETGTPRFSADEITGMFISMMFAGHHTSSGTASWTLIELMRHRDAYAAVIDELDELYGD
GRSVSFHALRQIPQLENVLKETLRLHPPLIILMRVAKGEFEVQGHRIHEGDLVAASPAISNRIPEDFPDPHDF
VPARYEQPRQEDLLNRWTWIPFGAGRHRCVGAAFAIMQIKAIFSVLLREYEFEMAQPPESYRNDHSKMVVQLA
QPACVRYRRRTGV
>CYP51B1_Mycobacterium tuberculosis NRITLD17 2633876163
MSAVALPRVSGGHDEHGHLEEFRTDPIGLMQRVRDECGDVGTFQLAGKQVVLLSGSHANEFFFRAGDDDLDQA
KAYPFMTPIFGEGVVFDASPERRKEMLHNAALRGEQMKGHAATIEDQVRRMIADWGEAAEIDLLDFFAELTIY
TSSACLIGKKFRDQLDGRFAKLYHELERGTDPLAYVDPYLPIESFRRRDEARNGLVALVADIMNGRIANPPTD
KSDRDMLDVLIAVKAETGTPRFSADEITGMFISMMFAGHHTSSGTASWTLIELMRHRDAYAAVIDELDELYGD
GRSVSFHALRQIPQLENVLKETLRLHPPLIILMRVAKGEFEVQGHRIHEGDLVAASPAISNRIPEDFPDPHDF
VPARYEQPRQEDLLNRWTWIPFGAGRHRCVGAAFAIMQIKAIFSVLLREYEFEMAQPPESYRNDHSKMVVQLA
QPACVRYRRRTGV
>CYP51B1_Mycobacterium tuberculosis NRITLD33 2633750529
MSAVALPRVSGGHDEHGHLEEFRTDPIGLMQRVRDECGDVGTFQLAGKQVVLLSGSHANEFFFRAGDDDLDQA
KAYPFMTPIFGEGVVFDASPERRKEMLHNAALRGEQMKGHAATIEDQVRRMIADWGEAAEIDLLDFFAELTIY
TSSACLIGKKFRDQLDGRFAKLYHELERGTDPLAYVDPYLPIESFRRRDEARNGLVALVADIMNGRIANPPTD
KSDRDMLDVLIAVKAETGTPRFSADEITGMFISMMFAGHHTSSGTASWTLIELMRHRDAYAAVIDELDELYGD
GRSVSFHALRQIPQLENVLKETLRLHPPLIILMRVAKGEFEVQGHRIHEGDLVAASPAISNRIPEDFPDPHDF
VPARYEQPRQEDLLNRWTWIPFGAGRHRCVGAAFAIMQIKAIFSVLLREYEFEMAQPPESYRNDHSKMVVQLA
QPACVRYRRRTGV
>CYP51B1_Mycobacterium tuberculosis NRITLD44 2575709189

MSAVALPRVSGGHDEHGHLEEFRTDPIGLMQRVRDECGDVGTFQLAGKQVVLLSGPHANEFFFRAGDDDLDQA
KAYPFMTPIFGEGVVFDASPERRKEMLHNAALRGEQMKGHAATIEDQVRRMIADWGEAGEIDLLDFFAELTIY
TSSACLIGKKFRDQLDGRFAKLYHELERGTDPLAYVDPYLPIESFRRRDEARNGLVALVADIMNGRIANPPTD
KSDRDMLDVLIAVKAETGTPRFSADEITGMFISMMFAGHHTSSGTASWTLIELMRHRDAYAAVIDELDELYGD
GRSVSFHALRQIPQLENVLKETLRLHPPLIILMRVAKGEFEVQGHRIHEGDLVAASPAISNRIPEDFPDPHDF
VPARYEQPRQEDLLNRWTWIPFGAGRHRCVGAAFAIMQIKAIFSVLLREYEFEMAQPPESYRNDHSKMVVQLA
QPACVRYRRRTGV
>CYP51B1_Mycobacterium tuberculosis NRITLD54 2632340765
MSAVALPRVSGGHDEHGHLEEFRTDPIGLMQRVRDECGDVGTFQLAGKQVVLLSGSHANEFFFRAGDDDLDQA
KAYPFMTPIFGEGVVFDASPERRKEMLHNAALRGEQMKGHAATIEDQVRRMIADWGEAAEIDLLDFFAELTIY
TSSACLIGKKFRDQLDGRFAKLYHELERGTDPLAYVDPYLPIESFRRRDEARNGLVALVADIMNGRIANPPTD
KSDRDMLDVLIAVKAETGTPRFSADEITGMFISMMFAGHHTSSGTASWTLIELMRHRDAYAAVIDELDELYGD
GRSVSFHALRQIPQLENVLKETLRLHPPLIILMRVAKGEFEVQGHRIHEGDLVAASPAISNRIPEDFPDPHDF
VPARYEQPRQEDLLNRWTWIPFGAGRHRCVGAAFAIMQIKAIFSVLLREYEFEMAQPPESYRNDHSKMVVQLA
QPACVRYRRRTGV
>CYP51B1_Mycobacterium tuberculosis O09700920 2675698308
MSAVALPRVSGGHDEHGHLEEFRTDPIGLMQRGRDECGDVGTFQLAGKQVVLLSGSHANEFFFRAGDDDLDQA
KAYPFMTPIFGEGVVFDASPERRKEMLHNAALRGEQMKGHAATIEDQVRRMIADWGEAGEIDLLDFFAELTIY
TSSACLIGKKFRDQLDGRFAKLYHELERGTDPLAYVDPYLPIESFRRRDEARNGLVALVADIMNGRIANPPTD
KSDRDMLDVLIAVKAETGTPRFSADEITGMFISMMFAGHHTSSGTASWTLIELMRHRDAYAAVIDELDELYGD
GRSVSFHALRQIPQLENVLKETLRLHPPLIILMRVAKGEFEVQGHRIHEGDLVAASPAISNRIPEDFPDPHDF
VPARYEQPRQEDLLNRWTWIPFGAGRHRCVGAAFAIMQIKAIFSVLLREYEFEMAQPPESYRNDHSKMVVQLA
QPACVRYRRRTGV
>CYP51B1_Mycobacterium tuberculosis OFXR-19 2576863736
MSAVALPRVSGGHDEHGHLEEFRTDPIGLMQRVRDECGDVGTFQLAGKQVVLLSGSHANEFFFRAGDDDLDQA
KAYPFMTPIFGEGVVFDASPERRKEMLHNAALRGEQMKGHAATIEDQVRRMIADWGEAGEIDLLDFFAELTIY
TSSACLIGKKFRDQLDGRFAKLYHELERGTDPLAYVDPYLPIESFRRRDEARNGLVALVADIMNGRIANPPTD
KSDRDMLDVLIGVKAETGTPRFSADEITGMFISMMFAGHHTSSGTASWTLIELMRHRDAYAAVIDELDELYGD
GRSVSFHALRQIPQLENVLKETLRLHPPLIILMRVAKGEFEVQGHRIHEGDLVAASPAISNRIPEDFPDPHDF
VPARYEQPRQEDLLNRWTWIPFGAGRHRCVGAAFAIMQIKAIFSVLLREYEFEMAQPPESYRNDHSKMVVQLA
QPACVRYRRRTGV
>CYP51B1_Mycobacterium tuberculosis OSDD071 2549404987
MSAVALPRVSGGHDEHGHLEEFRTDPIGLMQRVRDECGDVGTFQLAGKQVVLLSGSHANEFFFRAGDDDLDQA
KAYPFMTPIFGEGVVFDASPERRKEMLHNAALRGEQMKGHAATIEDQVRRMIADWGEAAEIDLLDFFAELTIY
TSSACLIGKKFRDQLDGRFAKLYHELERGTDPLAYVDPYLPIESFRRRDEARNGLVALVADIMNGRIANPPTD
KSDRDMLDVLIAVKAETGTPRFSADEITGMFISMMFAGHHTSSGTASWTLIELMRHRDAYAAVIDELDELYGD
GRSVSFHALRQIPQLENVLKETLRLHPPLIILMRVAKGEFEVQGHRIHEGDLVAASPAISNRIPEDFPDPHDF
VPARYEQPRQEDLLNRWTWIPFGAGRHRCVGAAFAIMQIKAIFSVLLREYEFEMAQPPESYRNDHSKMVVQLA
QPACVRYRRRTGV
>CYP51B1_Mycobacterium tuberculosis OSDD504 2549408568
MSAVALPRVSGGHDEHGHLEEFRTDPIGLMQRVRDECGDVGTFQLAGKQVVLLSGSHANEFFFRAGDDDLDQA
KAYPFMTPIFGEGVVFDASPERRKEMLHNAALRGEQMKGHAATIEDQVRRMIADWGEAAEIDLLDFFAELTIY
TSSACLIGKKFRDQLDGRFAKLYHELERGTDPLAYVDPYLPIESFRRRDEARNGLVALVADIMNGRIANPPTD
KSDRDMLDVLIAVKAETGTPRFSADEITGMFISMMFAGHHTSSGTASWTLIELMRHRDAYAAVIDELDELYGD
GRSVSFHALRQIPQLENVLKETLRLHPPLIILMRVAKGEFEVQGHRIHEGDLVAASPAISNRIPEDFPDPHDF
VPARYEQPRQEDLLNRWTWIPFGAGRHRCVGAAFAIMQIKAIFSVLLREYEFEMAQPPESYRNDHSKMVVQLA
QPACVRYRRRTGV
>CYP51B1_Mycobacterium tuberculosis OSDD518 2549416516
MSAVALPRVSGGHDEHGHLEEFRTDPIGLMQRVRDECGDVGTFQLAGKQVVLLSGSHANEFFFRAGDDDLDQA
KAYPFMTPIFGEGVVFDASPERRKEMLHNAALRGEQMKGHAATIEDQVRRMIADWGEAAEIDLLDFFAELTIY
TSSACLIGKKFRDQLDGRFAKLYHELERGTDPLAYVDPYLPIESFRRRDEARNGLVALVADIMNGRIANPPTD
KSDRDMLDVLIAVKAETGTPRFSADEITGMFISMMFAGHHTSSGTASWTLIELMRHRDAYAAVIDELDELYGD
GRSVSFHALRQIPQLENVLKETLRLHPPLIILMRVAKGEFEVQGHRIHEGDLVAASPAISNRIPEDFPDPHDF
VPARYEQPRQEDLLNRWTWIPFGAGRHRCVGAAFAIMQIKAIFSVLLREYEFEMAQPPESYRNDHSKMVVQLA
QPACVRYRRRTGV
>CYP51B1_Mycobacterium tuberculosis Haarlem3/NITR202 2546191262
MSAVALPRVSGGHDEHGHLEEFRTDPIGLMQRVRDECGDVGTFQLAGKQVVLLSGSHANEFFFRAGDDDLDQA
KAYPFMTPIFGEGVVFDASPERRKEMLHNAALRGEQMKGHAATIEDQVRRMIADWGEAGEIDLLDFFAELTIY

TSSACLIGKKFRDQLDGRFAKLYHELERGTDPLAYVDPYLPIESFRRRDEARNGLVALVADIMNGRIANPPTD
KSDRDMLDVLIAVKAETGTPRFSADEITGMFISMMFAGHHTSSGTAXWTLIELMRHRDAYAAVIDELDELYGD
GRSVSFHALRQIPQLENVLKETLRLXPPLIILMRVAKGEFEVQGHRIHEGDLVAASPAISNRIPEDFPDPHDF
VPARYEQPRQEDLLNRWTWIPFGAGRHRCVGAAFAIMQIKAIFSVLLREYEFEMAQPPESYRNDHSKMVVQLA
QPACVRYRRRTGV
>CYP51B1_Mycobacterium tuberculosis 1500108 2671008001
MSAVALPRVSGGHDEHGHLEEFRTDPIGLMQRVRDECGDVGTFQLAGKQVVLLSGSHANEFFFRAGDDDLDQA
KAYPFMTPIFGEGVVFDASPERRKEMLHNAALRGEQMKGHAATIEDQVRRMIADWGEAGEIDLLDFFAELTIY
TSSACLIGKKFRDQLDGRFAKLYHELERGTDPLAYVDPYLPIESFRRRDEARNGLVALVADIMNGRIANPPTD
KSDRDMLDVLIAVKAETGTPRFSADEITGMFISMMFAGHHTSSGTASWTLIELMRHRDAYAAVIDELDELYGD
GRSVSFHALRQIPQLENVLKETLRLHPPLIILMRVAKGEFECKATGFMRAIWWRPPRRSPTGSPKTSPIPTTS
CQHDTSSRARKICSTAGRGFRSAPAGIVAWGRRSPSCRSKRSSRCCCASMSLRWRNRQKAIVTTIRRWWCSWP
SPLACATAGEREFNDGLSSRSRPGSVPRTRDVRAGGTRVFPGAQARPGRNPRPRAARGSPRRDQACRVGLSHP
GTVHPRNGRVTVAGYPRDELEDVVHRWLQANRTAERRGDWTLLADFYTDDATYGWNVGPNEDVMCVGIDEIRD
IALGQEMDGLQGWRYPYQRVVIDEKQGEVVGFWKQVATDANGAEQEVYGIGGSWFRYAGGGKWNWQRDFFDFG
HVSALYLELIKAGKLSPGMQKRIERAVSGNKVPGYYPLGKTPVPLW
>CYP51B1_Mycobacterium canettii CIPT 140070010 2540553597
MSAVALPRVSGGHDEHGHLEEFRTDPIGLMQRVRDECGDVGTFQLAGKQVVLLSGSHANEFFFRAGDDDLDQA
KAYPFMTPIFGEGVVFDASPERRKEMLHNAALRGEQMKGHAATIEDQVRRMIADWGEAGEIDLLDFFAELTIY
TSSACLIGKKFRDQLDGRFAKLYHELERGTDPLAYVDPYLPIESFRRRDEARNGLVALVADIMNGRIANPPTD
KSHRDMLDVLIAVKDETGTPRFSADEITGMFISMMFAGHHTSSGTASWTLIELMRHRDAYAAVIDELDELYGD
GRSVSFHALRQIPRLENVLKETLRLHPPLIILMRVAKGEFEVQGHRIHEGDLVAASPAISNRIPEDFPDPDDF
VPARYEQPRQEDLLNRWTWIPFGAGRHRCVGAAFAIMQIKAIFSVLLREYEFEMAQPPESYRNDHSKMVVQLA
QPACVRYRRRTGA
>CYP51B1_Mycobacterium canettii CIPT 140070005 2566984181
MSAVALPRVSGGHDEHGHLEEFRTDPIGLMQRVRDECGDVGTFQLAGKQVVLLSGAQANEFFFRAGDDDLDQA
KAYPFMTPIFGEGVVFDASPERRKEMLHNAALRGEQMKGHAATIEDQVRRMIADWGEAGEIDLLDFFAELTIY
TSSACLIGKKFRDQLDARFAKLYHELERGTDPLAYVDPYLPIESFRRRDEARNGLVALVAGIMNGRIANPPTD
KSDRDMLDVLIAVKDETGTPRFSADEITGMFISMMFAGHHTSSGTASWTLIELMRHRDAYAAVIDELDELYGD
GRSVSFHALRQIPRLENVLKETLRLHPPLIILMRVAKGEFEVQGHRIHEGDLVAASPAISNRIPEDFPDPDDF
VPARYEQPRQEDLLNRWTWIPFGAGRHRCVGAAFAIMQIKAIFSVLLREYEFEMAQPPESYRNDHSKMVVQLA
QPACVRYRRRTGV
>CYP51B1_Mycobacterium tuberculosis Mtb526 2641991002
MSAVALPRVSGGHDEHGHLEEFRTDPIGLMQRVRDECGDVGTFQLAGKQVVLLSGSHANEFFFRAGDDDLDQA
KAYPFMTPIFGEGVVFDASPERRKEMLHNAALRGEQMKGHAATIEDQVRRMIADWGEAGEIDLLDFFAELTIY
TSSACLIGKKFRDQLDGRFAKLYHELERGTDPLALRRPVSADRELPSPRRSPQWSGGTGCGHHERPDRQPTHR
QERP
>CYP51B1_Mycobacterium tuberculosis 94_M4241A unfinished sequence
643018022
MSAVALPRVSGGHDEHGHLEEFRTDPIGLMQRVRDECGDVGTFQLAGKQVVLLSGSHANEFFFRAGDDDLDQA
KAYPFMTPIFGEGVVFDASPERRKEMLHNAALRGEQMKGHAATIEDQVRRMIADWGEAGEIDLLDFFAELTIY
TSSACLIGKKFRDQLDGRFAKLYHELERGTDPLAYVDPYLPIESFRRRDEARNGLVALVADIMNGRIANPPTD
KSDRDMLDVLIAVKAETGTPRFSADEITGMFISMMFAGHHTSSGTASWTLIELMRHRDAYAAVIDELDELYGD
GRSVSFHALRQIPQLENVLKETLRLHPPLIILMRVAKGEFEVQGHRIHEPGGVVPRYFSNRIPEDFPDPHDFV
PARYEQPRQEDLLNRWTWIPFGAGRHRCVGAAFAIMQIKAIFSVLLREYEFEMAQPPESYRNDHSKMVVQLAQ
PACVRYRRRTGV
>CYP51B1_Mycobacterium canettii CIPT 140010059 651038103
MSAVALPRVSGGHDEHGHLEEFRTDPIGLMQRVRDECGDVGTFQLAGKQVVLLSGAQANEFFFRAGDDDLDQA
KAYPFMKPIFGEGVVFDASPERRKEMLHNAALRGEQMKGHAATIEDQVRRMIADWGEAGEIDLLDFFAELTIY
TSSACLIGKKFRDQLDRRFAKLYHELERGTDPLAYVDPYLPIESFRRRDEARKGLVALVADIMNGRIANPPTD
KSDRDMLDVLIAVKDETGTPRFSADEITGMFISMMFAGHHTSSGTASWTLIELMRHRDAYAAVINELDELYGD
GRSVSFHALRQIPRLENVLKETLRLHPPLIILMRVAKGEFEVQGHRIHEGDLVAASPAISNRIPEDFPDPDDF
VPARYEQPRQEDLLNRWTWIPFGAGRHRCVGAAFAIMQIKAIFSVLLREYEFEMAQPPESYRNDHSKMVVQLA
QPACVRYRLRSGTGG
>CYP51B1_Mycobacterium canettii CIPT 140070007 2566971291
MSAVALPRVSGGHDEHGHLEEFRTDPIGLMQRVRDECGDVGTFQLAGKQVVLLSGAQANEFFFRAGDDDLDQA
KAYPFMKPIFGEGVVFDASPERRKEMLHNAALRGEQMKGHAATIEDQVRRMIADWGEAGEIDLLDFFAELTIY
TSSACLIGKKFRDQLDRRFAKLYHELERGTDPLAYVDPYLPIESFRRRDEARKGLVALVADIMNGRIANPPTD

KSDRDMLDVLIAVKDETGTPRFSADEITGMFISMMFAGHHTSSGTASWTLIELMRHRDAYAAVINELDELYGD
GRSVSFHALRQIPRLENVLKETLRLHPPLIILMRVAKGEFEVQGHRIHEGDLVAASPAISNRIPEDFPDPDDF
VPARYEQPRQEDLLNRWTWIPFGAGRHRCVGAAFAIMQIKAIFSVLLREYEFEMAQPPESYRNDHSKMVVQLA
QPACVRYRLRSGTGG
>CYP51B1_Mycobacterium canettii CIPT 140070017 2541572985
MSAVALPRVSGGHDEHGHLEEFRTDPIGLMQRVRDECGDVGTFQLAGKQVVLLSGAQANEFFFRAGDDDLDQA
KAYPFMKPIFGEGVVFDASPERRKEMLHNAALRGEQMKGHAATIEDQVRRMIADWGEAGEIDLLDFFAELTIY
TSSACLIGKKFRDQLDRRFAKLYHELERGTDPLAYVDPYLPIESFRRRDEARKGLVALVADIMNGRIANPPTD
KSDRDMLDVLIAVKDETGTPRFSADEITGMFISMMFAGHHTSSGTASWTLIELMRHRDAYAAVINELDELYGD
GRSVSFHALRQIPRLENVLKETLRLHPPLIILMRVAKGEFEVQGHRIHEGDLVAASPAISNRIPEDFPDPDDF
VPARYEQPRQEDLLNRWTWIPFGAGRHRCVGAAFAIMQIKAIFSVLLREYEFEMAQPPESYRNDHSKMVVQLA
QPACVRYRLRSGTGG
>CYP51B1_Mycobacterium tuberculosis PanR0403 2555542592
MSAVALPRVSGGHDEHGHLEEFRTDPIGLMQRVRDECGDVGTFQLAGKQVVLLSGSHANEFFFRAGDDDLDQA
KAYPFMTPIFGEGVVFDASPERRKEMLHNAALRGEQMKGHAATIEDQVRRMIADWGEAGEIDLLDFFAELTIY
TSSACLIGKKFRDQLDGRFAKLYHELERGTDPLAYVDPYLPIESFRRRDEARNGLVALVADIMNGRIANPPTD
KSDRDMLDVLIAVKAETGTPRFSADEITGMFISMMFAGHHTSSGTASWTLIELMRHRDAYAAVIDELDELYGD
GRSVSFHALRQIPQLENVLKETLRLHPPLIILMRVAKGEFEVQGHRIHEGDLVAASPAISNRIPEDFPDPHDF
VPARYEQPRQEDLLNRWTWIPFGAGRHRCVGAAFAIMQIKAIFSVLLREYEFEMAQPPESYRNDHSKMVVQLA
QPACVRYRRRTGV
>CYP51B1_Mycobacterium tuberculosis PanR0404 2555555561
MSAVALPRVSGGHDEHGHLEEFRTDPIGLMQRVRDECGDVGTFQLAGKQVVLLSGSHANEFFFRAGDDDLDQA
KAYPFMTPIFGEGVVFDASPERRKEMLHNAALRGEQMKGHAATIEDQVRRMIADWGEAGEIDLLDFFAELTIY
TSSACLIGKKFRDQLDGRFAKLYHELERGTDPLAYVDPYLPIESFRRRDEARNGLVALVADIMNGRIANPPTD
KSDRDMLDVLIAVKAETGTPRFSADEITGMFISMMFAGHHTSSGTASWTLIELMRHRDAYAAVIDELDELYGD
GRSVSFHALRQIPQLENVLKETLRLHPPLIILMRVAKGEFEVQGHRIHEGDLVAASPAISNRIPEDFPDPHDF
VPARYEQPRQEDLLNRWTWIPFGAGRHRCVGAAFAIMQIKAIFSVLLREYEFEMAQPPESYRNDHSKMVVQLA
QPACVRYRRRTGV
>CYP51B1_Mycobacterium tuberculosis PanR0405 2555515048
MSAVALPRVSGGHDEHGHLEEFRTDPIGLMQRVRDECGDVGTFQLAGKQVVLLSGSHANEFFFRAGDDDLDQA
KAYPFMTPIFGEGVVFDASPERRKEMLHNAALRGEQMKGHAATIEDQVRRMIADWGEAGEIDLLDFFAELTIY
TSSACLIGKKFRDQLDGRFAKLYHELERGTDPLAYVDPYLPIESFRRRDEARNGLVALVADIMNGRIANPPTD
KSDRDMLDVLIAVKAETGTPRFSADEITGMFISMMFAGHHTSSGTASWTLIELMRHRDAYAAVIDELDELYGD
GRSVSFHALRQIPQLENVLKETLRLHPPLIILMRVAKGEFEVQGHRIHEGDLVAASPAISNRIPEDFPDPHDF
VPARYEQPRQEDLLNRWTWIPFGAGRHRCVGAAFAIMQIKAIFSVLLREYEFEMAQPPESYRNDHSKMVVQLA
QPACVRYRRRTGV
>CYP51B1_Mycobacterium tuberculosis PanR0407 2555546872
MSAVALPRVSGGHDEHGHLEEFRTDPIGLMQRVRDECGDVGTFQLAGKQVVLLSGSHANEFFFRAGDDDLDQA
KAYPFMTPIFGEGVVFDASPERRKEMLHNAALRGEQMKGHAATIEDQVRRMIADWGEAGEIDLLDFFAELTIY
TSSACLIGKKFRDQLDGRFAKLYHELERGTDPLAYVDPYLPIESFRRRDEARNGLVALVADIMNGRIANPPTD
KSDRDMLDVLIAVKAETGTPRFSADEITGMFISMMFAGHHTSSGTASWTLIELMRHRDAYAAVIDELDELYGD
GRSVSFHALRQIPQLENVLKETLRLHPPLIILMRVAKGEFEVQGHRIHEGDLVAASPAISNRIPEDFPDPHDF
VPARYEQPRQEDLLNRWTWIPFGAGRHRCVGAAFAIMQIKAIFSVLLREYEFEMAQPPESYRNDHSKMVVQLA
QPACVRYRRRTGV
>CYP51B1_Mycobacterium tuberculosis PanR0409 2555586256
MSAVALPRVSGGHDEHGHLEEFRTDPIGLMQRVRDECGDVGTFQLAGKQVVLLSGSHANEFFFRAGDDDLDQA
KAYPFMTPIFGEGVVFDASPERRKEMLHNAALRGEQMKGHAATIEDQVRRMIADWGEAGEIDLLDFFAELTIY
TSSACLIGKKFRDQLDGRFAKLYHELERGTDPLAYVDPYLPIESFRRRDEARNGLVALVADIMNGRIANPPTD
KSDRDMLDVLIAVKAETGTPRFSADEITGMFISMMFAGHHTSSGTASWTLIELMRHRDAYAAVIDELDELYGD
GRSVSFHALRQIPQLENVLKETLRLHPPLIILMRVAKGEFEVQGHRIHEGDLVAASPAISNRIPEDFPDPHDF
VPARYEQPRQEDLLNRWTWIPFGAGRHRCVGAAFAIMQIKAIFSVLLREYEFEMAQPPESYRNDHSKMVVQLA
QPACVRYRRRTGV
>CYP51B1_Mycobacterium tuberculosis PanR0410 2555582092
MSAVALPRVSGGHDEHGHLEEFRTDPIGLMQRVRDECGDVGTFQLAGKQVVLLSGSHANEFFFRAGDDDLDQA
KAYPFMTPIFGEGVVFDASPERRKEMLHNAALRGEQMKGHAATIEDQVRRMIADWGEAGEIDLLDFFAELTIY
TSSACLIGKKFRDQLDGRFAKLYHELERGTDPLAYVDPYLPIESFRRRDEARNGLVALVADIMNGRIANPPTD
KSDRDMLDVLIAVKAETGTPRFSADEITGMFISMMFAGHHTSSGTASWTLIELMRHRDAYAAVIDELDELYGD
GRSVSFHALRQIPQLENVLKETLRLHPPLIILMRVAKGEFEVQGHRIHEGDLVAASPAISNRIPEDFPDPHDF

VPARYEQPRQEDLLNRWTWIPFGAGRHRCVGAAFAIMQIKAIFSVLLREYEFEMAQPPESYRNDHSKMVVQLA
QPACVRYRRRTGV
>CYP51B1_Mycobacterium tuberculosis PanR0411 2555519239
MSAVALPRVSGGHDEHGHLEEFRTDPIGLMQRVRDECGDVGTFQLAGKQVVLLSGSHANEFFFRAGDDDLDQA
KAYPFMTPIFGEGVVFDASPERRKEMLHNAALRGEQMKGHAATIEDQVRRMIADWGEAGEIDLLDFFAELTIY
TSSACLIGKKFRDQLDGRFAKLYHELERGTDPLAYVDPYLPIESFRRRDEARNGLVALVADIMNGRIANPPTD
KSDRDMLDVLIAVKAETGTPRFSADEITGMFISMMFAGHHTSSGTASWTLIELMRHRDAYAAVIDELDELYGD
GRSVSFHALRQIPQLENVLKETLRLHPPLIILMRVAKGEFEVQGHRIHEGDLVAASPAISNRIPEDFPDPHDF
VPARYEQPRQEDLLNRWTWIPFGAGRHRCVGAAFAIMQIKAIFSVLLREYEFEMAQPPESYRNDHSKMVVQLA
QPACVRYRRRTGV
>CYP51B1_Mycobacterium tuberculosis PanR0412 2555590441
MSAVALPRVSGGHDEHGHLEEFRTDPIGLMQRVRDECGDVGTFQLAGKQVVLLSGSHANEFFFRAGDDDLDQA
KAYPFMTPIFGEGVVFDASPERRKEMLHNAALRGEQMKGHAATIEDQVRRMIADWGEAGEIDLLDFFAELTIY
TSSACLIGKKFRDQLDGRFAKLYHELERGTDPLAYVDPYLPIESFRRRDEARNGLVALVADIMNGRIANPPTD
KSDRDMLDVLIAVKAETGTPRFSADEITGMFISMMFAGHHTSSGTASWTLIELMRHRDAYAAVIDELDELYGD
GRSVSFHALRQIPQLENVLKETLRLHPPLIILMRVAKGEFEVQGHRIHEGDLVAASPAISNRIPEDFPDPHDF
VPARYEQPRQEDLLNRWTWIPFGAGRHRCVGAAFAIMQIKAIFSVLLREYEFEMAQPPESYRNDHSKMVVQLA
QPACVRYRRRTGV
>CYP51B1_Mycobacterium tuberculosis PanR0501 2555332569
MSAVALPRVSGGHDEHGHLEEFRTDPIGLMQRVRDECGDVGTFQLAGKQVVLLSGSHANEFFFRAGDDDLDQA
KAYPFMTPIFGEGVVFDASPERRKEMLHNAALRGEQMKGHAATIEDQVRRMIADWGEAGEIDLLDFFAELTIY
TSSACLIGKKFRDQLDGRFAKLYHELERGTDPLAYVDPYLPIESFRRRDEARNGLVALVADIMNGRIANPPTD
KSDRDMLDVLIAVKAETGTPRFSADEITGMFISMMFAGHHTSSGTASWTLIELMRHRDAYAAVIDELDELYGD
GRSVSFHALRQIPQLENVLKETLRLHPPLIILMRVAKGEFEVQGHRIHEGDLVAASPAISNRIPEDFPDPHDF
VPARYEQPRQEDLLNRWTWIPFGAGRHRCVGAAFAIMQIKAIFSVLLREYEFEMAQPPESYRNDHSKMVVQLA
QPACVRYRRRTGV
>CYP51B1_Mycobacterium tuberculosis PanR0503 2555349324
MSAVALPRVSGGHDEHGHLEEFRTDPIGLMQRVRDECGDVGTFQLAGKQVVLLSGSHANEFFFRAGDDDLDQA
KAYPFMTPIFGEGVVFDASPERRKEMLHNAALRGEQMKGHAATIEDQVRRMIADWGEAGEIDLLDFFAELTIY
TSSACLIGKKFRDQLDGRFAKLYHELERGTDPLAYVDPYLPIESFRRRDEARNGLVALVADIMNGRIANPPTD
KSDRDMLDVLIAVKAETGTPRFSADEITGMFISMMFAGHHTSSGTASWTLIELMRHRDAYAAVIDELDELYGD
GRSVSFHALRQIPQLENVLKETLRLHPPLIILMRVAKGEFEVQGHRIHEGDLVAASPAISNRIPEDFPDPHDF
VPARYEQPRQEDLLNRWTWIPFGAGRHRCVGAAFAIMQIKAIFSVLLREYEFEMAQPPESYRNDHSKMVVQLA
QPACVRYRRRTGV
>CYP51B1_Mycobacterium tuberculosis PanR0505 2555353537
MSAVALPRVSGGHDEHGHLEEFRTDPIGLMQRVRDECGDVGTFQLAGKQVVLLSGSHANEFFFRAGDDDLDQA
KAYPFMTPIFGEGVVFDASPERRKEMLHNAALRGEQMKGHAATIEDQVRRMIADWGEAGEIDLLDFFAELTIY
TSSACLIGKKFRDQLDGRFAKLYHELERGTDPLAYVDPYLPIESFRRRDEARNGLVALVADIMNGRIANPPTD
KSDRDMLDVLIAVKAETGTPRFSADEITGMFISMMFAGHHTSSGTASWTLIELMRHRDAYAAVIDELDELYGD
GRSVSFHALRQIPQLENVLKETLRLHPPLIILMRVAKGEFEVQGHRIHEGDLVAASPAISNRIPEDFPDPHDF
VPARYEQPRQEDLLNRWTWIPFGAGRHRCVGAAFAIMQIKAIFSVLLREYEFEMAQPPESYRNDHSKMVVQLA
QPACVRYRRRTGV
>CYP51B1_Mycobacterium tuberculosis PanR0601 2555319582
MSAVALPRVSGGHDEHGHLEEFRTDPIGLMQRVRDECGDVGTFQLAGKQVVLLSGSHANEFFFRAGDDDLDQA
KAYPFMTPIFGEGVVFDASPERRKEMLHNAALRGEQMKGHAATIEDQVRRMIADWGEAGEIDLLDFFAELTIY
TSSACLIGKKFRDQLDGRFAKLYHELERGTDPLAYVDPYLPIESFRRRDEARNGLVALVADIMNGRIANPPTD
KSDRDMLDVLIAVKAETGTPRFSADEITGMFISMMFAGHHTSSGTASWTLIELMRHRDAYAAVIDELDELYGD
GRSVSFHALRQIPQLENVLKETLRLHPPLIILMRVAKGEFEVQGHRIHEGDLVAASPAISNRIPEDFPDPHDF
VPARYEQPRQEDLLNRWTWIPFGAGRHRCVGAAFAIMQIKAIFSVLLREYEFEMAQPPESYRNDHSKMVVQLA
QPACVRYRRRTGV
>CYP51B1_Mycobacterium tuberculosis PanR0602 2555336785
MSAVALPRVSGGHDEHGHLEEFRTDPIGLMQRVRDECGDVGTFQLAGKQVVLLSGSHANEFFFRAGDDDLDQA
KAYPFMTPIFGEGVVFDASPERRKEMLHNAALRGEQMKGHAATIEDQVRRMIADWGEAGEIDLLDFFAELTIY
TSSACLIGKKFRDQLDGRFAKLYHELERGTDPLAYVDPYLPIESFRRRDEARNGLVALVADIMNGRIANPPTD
KSDRDMLDVLIAVKAETGTPRFSADEITGMFISMMFAGHHTSSGTASWTLIELMRHRDAYAAVIDELDELYGD
GRSVSFHALRQIPQLENVLKETLRLHPPLIILMRVAKGEFEVQGHRIHEGDLVAASPAISNRIPEDFPDPHDF
VPARYEQPRQEDLLNRWTWIPFGAGRHRCVGAAFAIMQIKAIFSVLLREYEFEMAQPPESYRNDHSKMVVQLA
QPACVRYRRRTGV

>CYP51B1_Mycobacterium tuberculosis PanR0603 2555315249
MSAVALPRVSGGHDEHGHLEEFRTDPIGLMQRVRDECGDVGTFQLAGKQVVLLSGSHANEFFFRAGDDDLDQA
KAYPFMTPIFGEGVVFDASPERRKEMLHNAALRGEQMKGHAATIEDQVRRMIADWGEAGEIDLLDFFAELTIY
TSSACLIGKKFRDQLDGRFAKLYHELERGTDPLAYVDPYLPIESFRRRDEARNGLVALVADIMNGRIANPPTD
KSDRDMLDVLIAVKAETGTPRFSADEITGMFISMMFAGHHTSSGTASWTLIELMRHRDAYAAVIDELDELYGD
GRSVSFHALRQIPQLENVLKETLRLHPPLIILMRVAKGEFEVQGHRIHEGDLVAASPAISNRIPEDFPDPHDF
VPARYEQPRQEDLLNRWTWIPFGAGRHRCVGAAFAIMQIKAIFSVLLREYEFEMAQPPESYRNDHSKMVVQLA
QPACVRYRRRTGV
>CYP51B1_Mycobacterium tuberculosis PanR0604 2555324173
MSAVALPRVSGGHDEHGHLEEFRTDPIGLMQRVRDECGDVGTFQLAGKQVVLLSGSHANEFFFRAGDDDLDQA
KAYPFMTPIFGEGVVFDASPERRKEMLHNAALRGEQMKGHAATIEDQVRRMIADWGEAGEIDLLDFFAELTIY
TSSACLIGKKFRDQLDGRFAKLYHELERGTDPLAYVDPYLPIESFRRRDEARNGLVALVADIMNGRIANPPTD
KSDRDMLDVLIAVKAETGTPRFSADEITGMFISMMFAGHHTSSGTASWTLIELMRHRDAYAAVIDELDELYGD
GRSVSFHALRQIPQLENVLKETLRLHPPLIILMRVAKGEFEVQGHRIHEGDLVAASPAISNRIPEDFPDPHDF
VPARYEQPRQEDLLNRWTWIPFGAGRHRCVGAAFAIMQIKAIFSVLLREYEFEMAQPPESYRNDHSKMVVQLA
QPACVRYRRRTGV
>CYP51B1_Mycobacterium tuberculosis PanR0605 2555340976
MSAVALPRVSGGHDEHGHLEEFRTDPIGLMQRVRDECGDVGTFQLAGKQVVLLSGSHANEFFFRAGDDDLDQA
KAYPFMTPIFGEGVVFDASPERRKEMLHNAALRGEQMKGHAATIEDQVRRMIADWGEAGEIDLLDFFAELTIY
TSSACLIGKKFRDQLDGRFAKLYHELERGTDPLAYVDPYLPIESFRRRDEARNGLVALVADIMNGRIANPPTD
KSDRDMLDVLIAVKAETGTPRFSADEITGMFISMMFAGHHTSSGTASWTLIELMRHRDAYAAVIDELDELYGD
GRSVSFHALRQIPQLENVLKETLRLHPPLIILMRVAKGEFEVQGHRIHEGDLVAASPAISNRIPEDFPDPHDF
VPARYEQPRQEDLLNRWTWIPFGAGRHRCVGAAFAIMQIKAIFSVLLREYEFEMAQPPESYRNDHSKMVVQLA
QPACVRYRRRTGV
>CYP51B1_Mycobacterium tuberculosis PanR0606 2555357768
MSAVALPRVSGGHDEHGHLEEFRTDPIGLMQRVRDECGDVGTFQLAGKQVVLLSGSHANEFFFRAGDDDLDQA
KAYPFMTPIFGEGVVFDASPERRKEMLHNAALRGEQMKGHAATIEDQVRRMIADWGEAGEIDLLDFFAELTIY
TSSACLIGKKFRDQLDGRFAKLYHELERGTDPLAYVDPYLPIESFRRRDEARNGLVALVADIMNGRIANPPTD
KSDRDMLDVLIAVKAETGTPRFSADEITGMFISMMFAGHHTSSGTASWTLIELMRHRDAYAAVIDELDELYGD
GRSVSFHALRQIPQLENVLKETLRLHPPLIILMRVAKGEFEVQGHRIHEGDLVAASPAISNRIPEDFPDPHDF
VPARYEQPRQEDLLNRWTWIPFGAGRHRCVGAAFAIMQIKAIFSVLLREYEFEMAQPPESYRNDHSKMVVQLA
QPACVRYRRRTGV
>CYP51B1_Mycobacterium tuberculosis PanR0607 2555408225
MSAVALPRVSGGHDEHGHLEEFRTDPIGLMQRVRDECGDVGTFQLAGKQVVLLSGSHANEFFFRAGDDDLDQA
KAYPFMTPIFGEGVVFDASPERRKEMLHNAALRGEQMKGHAATIEDQVRRMIADWGEAGEIDLLDFFAELTIY
TSSACLIGKKFRDQLDGRFAKLYHELERGTDPLAYVDPYLPIESFRRRDEARNGLVALVADIMNGRIANPPTD
KSDRDMLDVLIAVKAETGTPRFSADEITGMFISMMFAGHHTSSGTASWTLIELMRHRDAYAAVIDELDELYGD
GRSVSFHALRQIPQLENVLKETLRLHPPLIILMRVAKGEFEVQGHRIHEGDLVAASPAISNRIPEDFPDPHDF
VPARYEQPRQEDLLNRWTWIPFGAGRHRCVGAAFAIMQIKAIFSVLLREYEFEMAQPPESYRNDHSKMVVQLA
QPACVRYRRRTGV
>CYP51B1_Mycobacterium tuberculosis PanR0609 2555404053
MSAVALPRVSGGHDEHGHLEEFRTDPIGLMQRVRDECGDVGTFQLAGKQVVLLSGSHANEFFFRAGDDDLDQA
KAYPFMTPIFGEGVVFDASPERRKEMLHNAALRGEQMKGHAATIEDQVRRMIADWGEAGEIDLLDFFAELTIY
TSSACLIGKKFRDQLDGRFAKLYHELERGTDPLAYVDPYLPIESFRRRDEARNGLVALVADIMNGRIANPPTD
KSDRDMLDVLIAVKAETGTPRFSADEITGMFISMMFAGHHTSSGTASWTLIELMRHRDAYAAVIDELDELYGD
GRSVSFHALRQIPQLENVLKETLRLHPPLIILMRVAKGEFEVQGHRIHEGDLVAASPAISNRIPEDFPDPHDF
VPARYEQPRQEDLLNRWTWIPFGAGRHRCVGAAFAIMQIKAIFSVLLREYEFEMAQPPESYRNDHSKMVVQLA
QPACVRYRRRTGV
>CYP51B1_Mycobacterium tuberculosis PanR0610 2555395633
MSAVALPRVSGGHDEHGHLEEFRTDPIGLMQRVRDECGDVGTFQLAGKQVVLLSGSHANEFFFRAGDDDLDQA
KAYPFMTPIFGEGVVFDASPERRKEMLHNAALRGEQMKGHAATIEDQVRRMIADWGEAGEIDLLDFFAELTIY
TSSACLIGKKFRDQLDGRFAKLYHELERGTDPLAYVDPYLPIESFRRRDEARNGLVALVADIMNGRIANPPTD
KSDRDMLDVLIAVKAETGTPRFSADEITGMFISMMFAGHHTSSGTASWTLIELMRHRDAYAAVIDELDELYGD
GRSVSFHALRQIPQLENVLKETLRLHPPLIILMRVAKGEFEVQGHRIHEGDLVAASPAISNRIPEDFPDPHDF
VPARYEQPRQEDLLNRWTWIPFGAGRHRCVGAAFAIMQIKAIFSVLLREYEFEMAQPPESYRNDHSKMVVQLA
QPACVRYRRRTGV
>CYP51B1_Mycobacterium tuberculosis PanR0611 2555412410

MSAVALPRVSGGHDEHGHLEEFRTDPIGLMQRVRDECGDVGTFQLAGKQVVLLSGSHANEFFFRAGDDDLDQA
KAYPFMTPIFGEGVVFDASPERRKEMLHNAALRGEQMKGHAATIEDQVRRMIADWGEAGEIDLLDFFAELTIY
TSSACLIGKKFRDQLDGRFAKLYHELERGTDPLAYVDPYLPIESFRRRDEARNGLVALVADIMNGRIANPPTD
KSDRDMLDVLIAVKAETGTPRFSADEITGMFISMMFAGHHTSSGTASWTLIELMRHRDAYAAVIDELDELYGD
GRSVSFHALRQIPQLENVLKETLRLHPPLIILMRVAKGEFEVQGHRIHEGDLVAASPAISNRIPEDFPDPHDF
VPARYEQPRQEDLLNRWTWIPFGAGRHRCVGAAFAIMQIKAIFSVLLREYEFEMAQPPESYRNDHSKMVVQLA
QPACVRYRRRTGV
>CYP51B1_Mycobacterium tuberculosis PanR0702 2555416650
MSAVALPRVSGGHDEHGHLEEFRTDPIGLMQRVRDECGDVGTFQLAGKQVVLLSGSHANEFFFRAGDDDLDQA
KAYPFMTPIFGEGVVFDASPERRKEMLHNAALRGEQMKGHAATIEDQVRRMIADWGEAGEIDLLDFFAELTIY
TSSACLIGKKFRDQLDGRFAKLYHELERGTDPLAYVDPYLPIESFRRRDEARNGLVALVADIMNGRIANPPTD
KSDRDMLDVLIAVKAETGTPRFSADEITGMFISMMFAGHHTSSGTASWTLIELMRHRDAYAAVIDELDELYGD
GRSVSFHALRQIPQLENVLKETLRLHPPLIILMRVAKGEFEVQGHRIHEGDLVAASPAISNRIPEDFPDPHDF
VPARYEQPRQEDLLNRWTWIPFGAGRHRCVGAAFAIMQIKAIFSVLLREYEFEMAQPPESYRNDHSKMVVQLA
QPACVRYRRRTGV
>CYP51B1_Mycobacterium tuberculosis PanR0704 2555527585
MSAVALPRVSGGHDEHGHLEEFRTDPIGLMQRVRDECGDVGTFQLAGKQVVLLSGSHANEFFFRAGDDDLDQA
KAYPFMTPIFGEGVVFDASPERRKEMLHNAALRGEQMKGHAATIEDQVRRMIADWGEAGEIDLLDFFAELTIY
TSSACLIGKKFRDQLDGRFAKLYHELERGTDPLAYVDPYLPIESFRRRDEARNGLVALVADIMNGRIANPPTD
KSDRDMLDVLIAVKAETGTPRFSADEITGMFISMMFAGHHTSSGTASWTLIELMRHRDAYAAVIDELDELYGD
GRSVSFHALRQIPQLENVLKETLRLHPPLIILMRVAKGEFEVQGHRIHEGDLVAASPAISNRIPEDFPDPHDF
VPARYEQPRQEDLLNRWTWIPFGAGRHRCVGAAFAIMQIKAIFSVLLREYEFEMAQPPESYRNDHSKMVVQLA
QPACVRYRRRTGV
>CYP51B1_Mycobacterium tuberculosis PanR0707 2555387174
MSAVALPRVSGGHDEHGHLEEFRTDPIGLMQRVRDECGDVGTFQLAGKQVVLLSGSHANEFFFRAGDDDLDQA
KAYPFMTPIFGEGVVFDASPERRKEMLHNAALRGEQMKGHAATIEDQVRRMIADWGEAGEIDLLDFFAELTIY
TSSACLIGKKFRDQLDGRFAKLYHELERGTDPLAYVDPYLPIESFRRRDEARNGLVALVADIMNGRIANPPTD
KSDRDMLDVLIAVKAETGTPRFSADEITGMFISMMFAGHHTSSGTASWTLIELMRHRDAYAAVIDELDELYGD
GRSVSFHALRQIPQLENVLKETLRLHPPLIILMRVAKGEFEVQGHRIHEGDLVAASPAISNRIPEDFPDPHDF
VPARYEQPRQEDLLNRWTWIPFGAGRHRCVGAAFAIMQIKAIFSVLLREYEFEMAQPPESYRNDHSKMVVQLA
QPACVRYRRRTGV
>CYP51B1_Mycobacterium tuberculosis PanR0708 2555370407
MSAVALPRVSGGHDEHGHLEEFRTDPIGLMQRVRDECGDVGTFQLAGKQVVLLSGSHANEFFFRAGDDDLDQA
KAYPFMTPIFGEGVVFDASPERRKEMLHNAALRGEQMKGHAATIEDQVRRMIADWGEAGEIDLLDFFAELTIY
TSSACLIGKKFRDQLDGRFAKLYHELERGTDPLAYVDPYLPIESFRRRDEARNGLVALVADIMNGRIANPPTD
KSDRDMLDVLIAVKAETGTPRFSADEITGMFISMMFAGHHTSSGTASWTLIELMRHRDAYAAVIDELDELYGD
GRSVSFHALRQIPQLENVLKETLRLHPPLIILMRVAKGEFEVQGHRIHEGDLVAASPAISNRIPEDFPDPHDF
VPARYEQPRQEDLLNRWTWIPFGAGRHRCVGAAFAIMQIKAIFSVLLREYEFEMAQPPESYRNDHSKMVVQLA
QPACVRYRRRTGV
>CYP51B1_Mycobacterium tuberculosis PanR0801 2555378724
MSAVALPRVSGGHDEHGHLEEFRTDPIGLMQRVRDECGDVGTFQLAGKQVVLLSGSHANEFFFRAGDDDLDQA
KAYPFMTPIFGEGVVFDASPERRKEMLHNAALRGEQMKGHAATIEDQVRRMIADWGEAGEIDLLDFFAELTIY
TSSACLIGKKFRDQLDGRFAKLYHELERGTDPLAYVDPYLPIESFRRRDEARNGLVALVADIMNGRIANPPTD
KSDRDMLDVLIAVKAETGTPRFSADEITGMFISMMFAGHHTSSGTASWTLIELMRHRDAYAAVIDELDELYGD
GRSVSFHALRQIPQLENVLKETLRLHPPLIILMRVAKGEFEVQGHRIHEGDLVAASPAISNRIPEDFPDPHDF
VPARYEQPRQEDLLNRWTWIPFGAGRHRCVGAAFAIMQIKAIFSVLLREYEFEMAQPPESYRNDHSKMVVQLA
QPACVRYRRRTGV
>CYP51B1_Mycobacterium tuberculosis PanR0802 2555523413
MSAVALPRVSGGHDEHGHLEEFRTDPIGLMQRVRDECGDVGTFQLAGKQVVLLSGSHANEFFFRAGDDDLDQA
KAYPFMTPIFGEGVVFDASPERRKEMLHNAALRGEQMKGHAATIEDQVRRMIADWGEAGEIDLLDFFAELTIY
TSSACLIGKKFRDQLDGRFAKLYHELERGTDPLAYVDPYLPIESFRRRDEARNGLVALVADIMNGRIANPPTD
KSDRDMLDVLIAVKAETGTPRFSADEITGMFISMMFAGHHTSSGTASWTLIELMRHRDAYAAVIDELDELYGD
GRSVSFHALRQIPQLENVLKETLRLHPPLIILMRVAKGEFEVQGHRIHEGDLVAASPAISNRIPEDFPDPHDF
VPARYEQPRQEDLLNRWTWIPFGAGRHRCVGAAFAIMQIKAIFSVLLREYEFEMAQPPESYRNDHSKMVVQLA
QPACVRYRRRTGV
>CYP51B1_Mycobacterium tuberculosis PanR0803 2555366257
MSAVALPRVSGGHDEHGHLEEFRTDPIGLMQRVRDECGDVGTFQLAGKQVVLLSGSHANEFFFRAGDDDLDQA
KAYPFMTPIFGEGVVFDASPERRKEMLHNAALRGEQMKGHAATIEDQVRRMIADWGEAGEIDLLDFFAELTIY

TSSACLIGKKFRDQLDGRFAKLYHELERGTDPLAYVDPYLPIESFRRRDEARNGLVALVADIMNGRIANPPTD
KSDRDMLDVLIAVKAETGTPRFSADEITGMFISMMFAGHHTSSGTASWTLIELMRHRDAYAAVIDELDELYGD
GRSVSFHALRQIPQLENVLKETLRLHPPLIILMRVAKGEFEVQGHRIHEGDLVAASPAISNRIPEDFPDPHDF
VPARYEQPRQEDLLNRWTWIPFGAGRHRCVGAAFAIMQIKAIFSVLLREYEFEMAQPPESYRNDHSKMVVQLA
QPACVRYRRRTGV
>CYP51B1_Mycobacterium tuberculosis PanR0804 2555391404
MSAVALPRVSGGHDEHGHLEEFRTDPIGLMQRVRDECGDVGTFQLAGKQVVLLSGSHANEFFFRAGDDDLDQA
KAYPFMTPIFGEGVVFDASPERRKEMLHNAALRGEQMKGHAATIEDQVRRMIADWGEAGEIDLLDFFAELTIY
TSSACLIGKKFRDQLDGRFAKLYHELERGTDPLAYVDPYLPIESFRRRDEARNGLVALVADIMNGRIANPPTD
KSDRDMLDVLIAVKAETGTPRFSADEITGMFISMMFAGHHTSSGTASWTLIELMRHRDAYAAVIDELDELYGD
GRSVSFHALRQIPQLENVLKETLRLHPPLIILMRVAKGEFEVQGHRIHEGDLVAASPAISNRIPEDFPDPHDF
VPARYEQPRQEDLLNRWTWIPFGAGRHRCVGAAFAIMQIKAIFSVLLREYEFEMAQPPESYRNDHSKMVVQLA
QPACVRYRRRTGV
>CYP51B1_Mycobacterium tuberculosis PanR0805 2555361966
MSAVALPRVSGGHDEHGHLEEFRTDPIGLMQRVRDECGDVGTFQLAGKQVVLLSGSHANEFFFRAGDDDLDQA
KAYPFMTPIFGEGVVFDASPERRKEMLHNAALRGEQMKGHAATIEDQVRRMIADWGEAGEIDLLDFFAELTIY
TSSACLIGKKFRDQLDGRFAKLYHELERGTDPLAYVDPYLPIESFRRRDEARNGLVALVADIMNGRIANPPTD
KSDRDMLDVLIAVKAETGTPRFSADEITGMFISMMFAGHHTSSGTASWTLIELMRHRDAYAAVIDELDELYGD
GRSVSFHALRQIPQLENVLKETLRLHPPLIILMRVAKGEFEVQGHRIHEGDLVAASPAISNRIPEDFPDPHDF
VPARYEQPRQEDLLNRWTWIPFGAGRHRCVGAAFAIMQIKAIFSVLLREYEFEMAQPPESYRNDHSKMVVQLA
QPACVRYRRRTGV
>CYP51B1_Mycobacterium tuberculosis PanR0902 2555382935
MSAVALPRVSGGHDEHGHLEEFRTDPIGLMQRVRDECGDVGTFQLAGKQVVLLSGSHANEFFFRAGDDDLDQA
KAYPFMTPIFGEGVVFDASPERRKEMLHNAALRGEQMKGHAATIEDQVRRMIADWGEAGEIDLLDFFAELTIY
TSSACLIGKKFRDQLDGRFAKLYHELERGTDPLAYVDPYLPIESFRRRDEARNGLVALVADIMNGRIANPPTD
KSDRDMLDVLIAVKAETGTPRFSADEITGMFISMMFAGHHTSSGTASWTLIELMRHRDAYAAVIDELDELYGD
GRSVSFHALRQIPQLENVLKETLRLHPPLIILMRVAKGEFEVQGHRIHEGDLVAASPAISNRIPEDFPDPHDF
VPARYEQPRQEDLLNRWTWIPFGAGRHRCVGAAFAIMQIKAIFSVLLREYEFEMAQPPESYRNDHSKMVVQLA
QPACVRYRRRTGV
>CYP51B1_Mycobacterium tuberculosis PanR0903 2555374552
MSAVALPRVSGGHDEHGHLEEFRTDPIGLMQRVRDECGDVGTFQLAGKQVVLLSGSHANEFFFRAGDDDLDQA
KAYPFMTPIFGEGVVFDASPERRKEMLHNAALRGEQMKGHAATIEDQVRRMIADWGEAGEIDLLDFFAELTIY
TSSACLIGKKFRDQLDGRFAKLYHELERGTDPLAYVDPYLPIESFRRRDEARNGLVALVADIMNGRIANPPTD
KSDRDMLDVLIAVKAETGTPRFSADEITGMFISMMFAGHHTSSGTASWTLIELMRHRDAYAAVIDELDELYGD
GRSVSFHALRQIPQLENVLKETLRLHPPLIILMRVAKGEFEVQGHRIHEGDLVAASPAISNRIPEDFPDPHDF
VPARYEQPRQEDLLNRWTWIPFGAGRHRCVGAAFAIMQIKAIFSVLLREYEFEMAQPPESYRNDHSKMVVQLA
QPACVRYRRRTGV
>CYP51B1_Mycobacterium tuberculosis PanR0904 2555441691
MSAVALPRVSGGHDEHGHLEEFRTDPIGLMQRVRDECGDVGTFQLAGKQVVLLSGSHANEFFFRAGDDDLDQA
KAYPFMTPIFGEGVVFDASPERRKEMLHNAALRGEQMKGHAATIEDQVRRMIADWGEAGEIDLLDFFAELTIY
TSSACLIGKKFRDQLDGRFAKLYHELERGTDPLAYVDPYLPIESFRRRDEARNGLVALVADIMNGRIANPPTD
KSDRDMLDVLIAVKAETGTPRFSADEITGMFISMMFAGHHTSSGTASWTLIELMRHRDAYAAVIDELDELYGD
GRSVSFHALRQIPQLENVLKETLRLHPPLIILMRVAKGEFEVQGHRIHEGDLVAASPAISNRIPEDFPDPHDF
VPARYEQPRQEDLLNRWTWIPFGAGRHRCVGAAFAIMQIKAIFSVLLREYEFEMAQPPESYRNDHSKMVVQLA
QPACVRYRRRTGV
>CYP51B1_Mycobacterium tuberculosis PanR0906 2555445874
MSAVALPRVSGGHDEHGHLEEFRTDPIGLMQRVRDECGDVGTFQLAGKQVVLLSGSHANEFFFRAGDDDLDQA
KAYPFMTPIFGEGVVFDASPERRKEMLHNAALRGEQMKGHAATIEDQVRRMIADWGEAGEIDLLDFFAELTIY
TSSACLIGKKFRDQLDGRFAKLYHELERGTDPLAYVDPYLPIESFRRRDEARNGLVALVADIMNGRIANPPTD
KSDRDMLDVLIAVKAETGTPRFSADEITGMFISMMFAGHHTSSGTASWTLIELMRHRDAYAAVIDELDELYGD
GRSVSFHALRQIPQLENVLKETLRLHPPLIILMRVAKGEFEVQGHRIHEGDLVAASPAISNRIPEDFPDPHDF
VPARYEQPRQEDLLNRWTWIPFGAGRHRCVGAAFAIMQIKAIFSVLLREYEFEMAQPPESYRNDHSKMVVQLA
QPACVRYRRRTGV
>CYP51B1_Mycobacterium tuberculosis PanR0907 2555454246
MSAVALPRVSGGHDEHGHLEEFRTDPIGLMQRVRDECGDVGTFQLAGKQVVLLSGSHANEFFFRAGDDDLDQA
KAYPFMTPIFGEGVVFDASPERRKEMLHNAALRGEQMKGHAATIEDQVRRMIADWGEAGEIDLLDFFAELTIY
TSSACLIGKKFRDQLDGRFAKLYHELERGTDPLAYVDPYLPIESFRRRDEARNGLVALVADIMNGRIANPPTD
KSDRDMLDVLIAVKAETGTPRFSADEITGMFISMMFAGHHTSSGTASWTLIELMRHRDAYAAVIDELDELYGD

GRSVSFHALRQIPQLENVLKETLRLHPPLIILMRVAKGEFEVQGHRIHEGDLVAASPAISNRIPEDFPDPHDF
VPARYEQPRQEDLLNRWTWIPFGAGRHRCVGAAFAIMQIKAIFSVLLREYEFEMAQPPESYRNDHSKMVVQLA
QPACVRYRRRTGV
>CYP51B1_Mycobacterium tuberculosis PanR0908 2555420892
MSAVALPRVSGGHDEHGHLEEFRTDPIGLMQRVRDECGDVGTFQLAGKQVVLLSGSHANEFFFRAGDDDLDQA
KAYPFMTPIFGEGVVFDASPERRKEMLHNAALRGEQMKGHAATIEDQVRRMIADWGEAGEIDLLDFFAELTIY
TSSACLIGKKFRDQLDGRFAKLYHELERGTDPLAYVDPYLPIESFRRRDEARNGLVALVADIMNGRIANPPTD
KSDRDMLDVLIAVKAETGTPRFSADEITGMFISMMFAGHHTSSGTASWTLIELMRHRDAYAAVIDELDELYGD
GRSVSFHALRQIPQLENVLKETLRLHPPLIILMRVAKGEFEVQGHRIHEGDLVAASPAISNRIPEDFPDPHDF
VPARYEQPRQEDLLNRWTWIPFGAGRHRCVGAAFAIMQIKAIFSVLLREYEFEMAQPPESYRNDHSKMVVQLA
QPACVRYRRRTGV
>CYP51B1_Mycobacterium tuberculosis PanR0909 2555429129
MSAVALPRVSGGHDEHGHLEEFRTDPIGLMQRVRDECGDVGTFQLAGKQVVLLSGSHANEFFFRAGDDDLDQA
KAYPFMTPIFGEGVVFDASPERRKEMLHNAALRGEQMKGHAATIEDQVRRMIADWGEAGEIDLLDFFAELTIY
TSSACLIGKKFRDQLDGRFAKLYHELERGTDPLAYVDPYLPIESFRRRDEARNGLVALVADIMNGRIANPPTD
KSDRDMLDVLIAVKAETGTPRFSADEITGMFISMMFAGHHTSSGTASWTLIELMRHRDAYAAVIDELDELYGD
GRSVSFHALRQIPQLENVLKETLRLHPPLIILMRVAKGEFEVQGHRIHEGDLVAASPAISNRIPEDFPDPHDF
VPARYEQPRQEDLLNRWTWIPFGAGRHRCVGAAFAIMQIKAIFSVLLREYEFEMAQPPESYRNDHSKMVVQLA
QPACVRYRRRTGV
>CYP51B1_Mycobacterium tuberculosis PanR1005 2555594618
MSAVALPRVSGGHDEHGHLEEFRTDPIGLMQRVRDECGDVGTFQLAGKQVVLLSGSHANEFFFRAGDDDLDQA
KAYPFMTPIFGEGVVFDASPERRKEMLHNAALRGEQMKGHAATIEDQVRRMIADWGEAGEIDLLDFFAELTIY
TSSACLIGKKFRDQLDGRFAKLYHELERGTDPLAYVDPYLPIESFRRRDEARNGLVALVADIMNGRIANPPTD
KSDRDMLDVLIAVKAETGTPRFSADEITGMFISMMFAGHHTSSGTASWTLIELMRHRDAYAAVIDELDELYGD
GRSVSFHALRQIPQLENVLKETLRLHPPLIILMRVAKGEFEVQGHRIHEGDLVAASPAISNRIPEDFPDPHDF
VPARYEQPRQEDLLNRWTWIPFGAGRHRCVGAAFAIMQIKAIFSVLLREYEFEMAQPPESYRNDHSKMVVQLA
QPACVRYRRRTGV
>CYP51B1_Mycobacterium tuberculosis PanR1006 2555450034
MSAVALPRVSGGHDEHGHLEEFRTDPIGLMQRVRDECGDVGTFQLAGKQVVLLSGSHANEFFFRAGDDDLDQA
KAYPFMTPIFGEGVVFDASPERRKEMLHNAALRGEQMKGHAATIEDQVRRMIADWGEAGEIDLLDFFAELTIY
TSSACLIGKKFRDQLDGRFAKLYHELERGTDPLAYVDPYLPIESFRRRDEARNGLVALVADIMNGRIANPPTD
KSDRDMLDVLIAVKAETGTPRFSADEITGMFISMMFAGHHTSSGTASWTLIELMRHRDAYAAVIDELDELYGD
GRSVSFHALRQIPQLENVLKETLRLHPPLIILMRVAKGEFEVQGHRIHEGDLVAASPAISNRIPEDFPDPHDF
VPARYEQPRQEDLLNRWTWIPFGAGRHRCVGAAFAIMQIKAIFSVLLREYEFEMAQPPESYRNDHSKMVVQLA
QPACVRYRRRTGV
>CYP51B1_Mycobacterium tuberculosis PanR1007 2555433345
MSAVALPRVSGGHDEHGHLEEFRTDPIGLMQRVRDECGDVGTFQLAGKQVVLLSGSHANEFFFRAGDDDLDQA
KAYPFMTPIFGEGVVFDASPERRKEMLHNAALRGEQMKGHAATIEDQVRRMIADWGEAGEIDLLDFFAELTIY
TSSACLIGKKFRDQLDGRFAKLYHELERGTDPLAYVDPYLPIESFRRRDEARNGLVALVADIMNGRIANPPTD
KSDRDMLDVLIAVKAETGTPRFSADEITGMFISMMFAGHHTSSGTASWTLIELMRHRDAYAAVIDELDELYGD
GRSVSFHALRQIPQLENVLKETLRLHPPLIILMRVAKGEFEVQGHRIHEGDLVAASPAISNRIPEDFPDPHDF
VPARYEQPRQEDLLNRWTWIPFGAGRHRCVGAAFAIMQIKAIFSVLLREYEFEMAQPPESYRNDHSKMVVQLA
QPACVRYRRRTGV
>CYP51B1_Mycobacterium tuberculosis PanR1101 2555437471
MSAVALPRVSGGHDEHGHLEEFRTDPIGLMQRVRDECGDVGTFQLAGKQVVLLSGSHANEFFFRAGDDDLDQA
KAYPFMTPIFGEGVVFDASPERRKEMLHNAALRGEQMKGHAATIEDQVRRMIADWGEAGEIDLLDFFAELTIY
TSSACLIGKKFRDQLDGRFAKLYHELERGTDPLAYVDPYLPIESFRRRDEARNGLVALVADIMNGRIANPPTD
KSDRDMLDVLIAVKAETGTPRFSADEITGMFISMMFAGHHTSSGTASWTLIELMRHRDAYAAVIDELDELYGD
GRSVSFHALRQIPQLENVLKETLRLHPPLIILMRVAKGEFEVQGHRIHEGDLVAASPAISNRIPEDFPDPHDF
VPARYEQPRQEDLLNRWTWIPFGAGRHRCVGAAFAIMQIKAIFSVLLREYEFEMAQPPESYRNDHSKMVVQLA
QPACVRYRRRTGV
>CYP51B1_Mycobacterium tuberculosis PR05 2554700254
MSAVALPRVSGGHDEHGHLEEFRTDPIGLMQRVRDECGDVGTFQLAGKQVVLLSGSHANEFFFRAGDDDLDQA
KAYPFMTPIFGEGVVFDASPERRKEMLHNAALRGEQMKGHAATIEDQVRRMIADWGEAGEIDLLDFFAELTIY
TSSACLIGKKFRDQLDGRFAKLYHELERGTDPLAYVDPYLPIESFRRRDEARNGLVALVADIMNGRIANPPTD
KSDRDMLDVLIAVKAETGTPRFSADEITGMFISMMFAGHHTSSGTASWTLIELMRHRDAYAAVIDELDELYGD
GRSVSFHALRQIPQLENVLKETLRLHPPLIILMRVAKGEFEVQGHRIHEGDLVAASPAISNRIPEDFPDPHDF

VPARYEQPRQEDLLNRWTWIPFGAGRHRCVGAAFAIMQIKAIFSVLLREYEFEMAQPPESYRNDHSKMVVQLA
QPACVRYRRRTGV
>CYP51B1_Mycobacterium tuberculosis PR08 2687727573
MSAVALPRVSGGHDEHGHLEEFRTDPIGLMQRVRDECGDVGTFQLAGKQVVLLSGSHANEFFFRAGDDDLDQA
KAYPFMTPIFGEGVVFDASPERRKEMLHNAALRGEQMKGHAATIEDQVRRMIADWGEAGEIDLLDFFAELTIY
TSSACLIGKKFRDQLDGRFAKLYHELERGTDPLAYVDPYLPIESFRRRDEARNGLVALVADIMNGRIANPPTD
KSDRDMLDVLIAVKAETGTPRFSADEITGMFISMMFAGHHTSSGTASWTLIELMRHRDAYAAVIDELDELYGD
GRSVSFHALRQIPQLENVLKETLRLHPPLIILMRVAKGEFEVQGHRIHEGDLVAASPAISNRIPEDFPDPHDF
VPARYEQPRQEDLLNRWTWIPFGAGRHRCVGAAFAIMQIKAIFSVLLREYEFEMAQPPESYRNDHSKMVVQLA
QPACVRYRRRTGV
>CYP51B1_Mycobacterium tuberculosis PR10 2721359918
MSAVALPRVSGGHDEHGHLEEFRTDPIGLMQRVRDECGDVGTFQLAGKQVVLLSGSHANEFFFRAGDDDLDQA
KAYPFMTPIFGEGVVFDASPERRKEMLHNAALRGEQMKGHAATIEDQVRRMIADWGEAGEIDLLDFFAELTIY
TSSACLIGKKFRDQLDGRFAKLYHELERGTDPLAYVDPYLPIESFRRRDEARNGLVALVADIMNGRIANPPTD
KSDRDMLDVLIAVKAETGTPRFSADEITGMFISMMFAGHHTSSGTASWTLIELMRHRDAYAAVIDELDELYGD
GRSVSFHALRQIPQLENVLKETLRLHPPLIILMRVAKGEFEVQGHRIHEGDLVAASPAISNRIPEDFPDPHDF
VPARYEQPRQEDLLNRWTWIPFGAGRHRCVGAAFAIMQIKAIFSVLLREYEFEMAQPPESYRNDHSKMVVQLA
QPACVRYRRRTGV
>CYP51B1_Mycobacterium tuberculosis Q00501124 2693592061
MSAVALPRVSGGHDEHGHLEEFRTDPIGLMQRVRDECGDVGTFQLAGKQVVLLSGSHANEFFFRAGDDDLDQA
KAYPFMTPIFGEGVVFDASPERRKEMLHNAALRGEQMKGHAATIEDQVRRMIADWGEAGEIDLLDFFAELTIY
TSSACLIGKKFRDQLDGRFAKLYHELERGTDPLAYVDPYLPIESFRRRDEARNGLVALVADIMNGRIANPPTD
KSDRDMLDVLIAVKAETGTPRFSADEITGMFISMMFAGHHTSSGTASWTLIELMRHRDAYAAVIDELDELYGD
GRSVSFHALRQIPQLENVLKETLRLHPPLIILMRVAKGEFEVQGHRIHEGDLVAASPAISNRIPEDFPDPHDF
VPARYEQPRQEDLLNRWTWIPFGAGRHRCVGAAFAIMQIKAIFSVLLREYEFEMAQPPESYRNDHSKMVVQLA
QPACVRYRRRTGV
>CYP51B1_Mycobacterium tuberculosis Q09600228 2694128026
MSAVALPRVSGGHDEHGHLEEFRTDPIGLMQRVRDECGDVGTFQLAGKQVVLLSGSHANEFFFRAGDDDLDQA
KAYPFMTPIFGEGVVFDASPERRKEMLHNAALRGEQMKGHAATIEDQVRRMIADWGEAGEIDLLDFFAELTIY
TSSACLIGKKFRDQLDGRFAKLYHELERGTDPLAYVDPYLPIESFRRRDEARNGLVALVADIMNGRIANPPTD
KSDRDMLDVLIAVKAETGTPRFSADEITGMFISMMFAGHHTSSGTASWTLIELMRHRDAYAAVIDELDELYGD
GRSVSFHALRQIPQLENVLKETLRLHPPLIILMRVAKGEFEVQGHRIHEGDLVAASPAISNRIPEDFPDPHDF
VPARYEQPRQEDLLNRWTWIPFGAGRHRCVGAAFAIMQIKAIFSVLLREYEFEMAQPPESYRNDHSKMVVQLA
QPACVRYRRRTGV
>CYP51B1_Mycobacterium tuberculosis Q09600750 2697730898
MSAVALPRVSGGHDEHGHLEEFRTDPIGLMQRVRDECGDVGTFQLAGKQVVLLSGSHANEFFFRAGDDDLDQA
KAYPFMTPIFGEGVVFDASPERRKEMLHNAALRGEQMKGHAATIEDQVRRMIADWGEAGEIDLLDFFAELTIY
TSSACLIGKKFRDQLDGRFAKLYHELERGTDPLAYVDPYLPIESFRRRDEARNGLVALVADIMNGRIANPPTD
KSDRDMLDVLIAVKAETGTPRFSADEITGMFISMMFAGHHTSSGTASWTLIELMRHRDAYAAVIDELDELYGD
GRSVSFHALRQIPQLENVLKETLRLHPPLIILMRVAKGEFEVQGHRIHEGDLVAASPAISNRIPEDFPDPHDF
VPARYEQPRQEDLLNRWTWIPFGAGRHRCVGAAFAIMQIKAIFSVLLREYEFEMAQPPESYRNDHSKMVVQLA
QPACVRYRRRTGV
>CYP51B1_Mycobacterium tuberculosis Q09701888 2672029889
MSAVALPRVSGGHDEHGHLEEFRTDPIGLMQRVRDECGDVGTFQLAGKQVVLLSGSHANEFFFRAGDDDLDQA
KAYPFMTPIFGEGVVFDASPERRKEMLHNAALRGEQMKGHAATIEDQVRRMIADWGEAGEIDLLDFFAELTIY
TSSACLIGKKFRDQLDGRFAKLYHELERGTDPLAYVDPYLPIESFRRRDEARNGLVALVADIMNGRIANPPTD
KSDRDMLDVLIAVKAETGTPRFSADEITGMFISMMFAGHHTSSGTASWTLIELMRHRDAYAAVIDELDELYGD
GRSVSFHALRQIPQLENVLKETLRLHPPLIILMRVAKGEFEVQGHRIHEGDLVAASPAISNRIPEDFPDPHDF
VPARYEQPRQEDLLNRWTWIPFGAGRHRCVGAAFAIMQIKAIFSVLLREYEFEMAQPPESYRNDHSKMVVQLA
QPACVRYRRRTGV
>CYP51B1_Mycobacterium tuberculosis Q09802318 2674283673
MSAVALPRVSGGHDEHGHLEEFRTDPIGLMQRVRDECGDVGTFQLAGKQVVLLSGSHANEFFFRAGDDDLDQA
KAYPFMTPIFGEGVVFDASPERRKEMLHNAALRGEQMKGHAATIEDQVRRMIADWGEAGEIDLLDFFAELTIY
TSSACLIGKKFRDQLDGRFAKLYHELERGTDPLAYVDPYLPIESFRRRDEARNGLVALVADIMNGRIANPPTD
KSDRDMLDVLIAVKAETGTPRFSADEITGMFISMMFAGHHTSSGTASWTLIELMRHRDAYAAVIDELDELYGD
GRSVSFHALRQIPQLENVLKETLRLHPPLIILMRVAKGEFEVQGHRIHEGDLVAASPAISNRIPEDFPDPHDF
VPARYEQPRQEDLLNRWTWIPFGAGRHRCVGAAFAIMQIKAIFSVLLREYEFEMAQPPESYRNDHSKMVVQLA
QPACVRYRRRTGV

>CYP51B1_Mycobacterium tuberculosis Q09900060 2692036964
MSAVALPRVSGGHDEHGHLEEFRTDPIGLMQRVRDECGDVGTFQLAGKQVVLLSGSHANEFFFRAGDDDLDQA
KAYPFMTPIFGEGVVFDASPERRKEMLHNAALRGEQMKGHAATIEDQVRRMIADWGEAGEIDLLDFFAELTIY
TSSACLIGKKFRDQLDGRFAKLYHELERGTDPLAYVDPYLPIESFRRRDEARNGLVALVADIMNGRIANPPTD
KSDRDMLDVLIAVKAETGTPRFSADEITGMFISMMFAGHHTSSGTASWTLIELMRHRDAYAAVIDELDELYGD
GRSVSFHALRQIPQLENVLKETLRLHPPLIILMRVAKGEFEVQGHRIHEGDLVAASPAISNRIPEDFPDPHDF
VPARYEQPRQEDLLNRWTWIPFGAGRHRCVGAAFAIMQIKAIFSVLLREYEFEMAQPPESYRNDHSKMVVQLA
QPACVRYRRRTGV
>CYP51B1_Mycobacterium tuberculosis Q09900131 2706565246
MSAVALPRVSGGHDEHGHLEEFRTDPIGLMQRVRDECGDVGTFQLAGKQVVLLSGSHANEFFFRAGDDDLDQA
KAYPFMTPIFGEGVVFDASPERRKEMLHNAALRGEQMKGHAATIEDQVRRMIADWGEAGEIDLLDFFAELTIY
TSSACLIGKKFRDQLDGRFAKLYHELERGTDPLAYVDPYLPIESFRRRDEARNGLVALVADIMNGRIANPPTD
KSDRDMLDVLIAVKAETGTPRFSADEITGMFISMMFAGHHTSSGTASWTLIELMRHRDAYAAVIDELDELYGD
GRSVSFHALRQIPQLENVLKETLRLHPPLIILMRVAKGEFEVQGHRIHEGDLVAASPAISNRIPEDFPDPHDF
VPARYEQPRQEDLLNRWTWIPFGAGRHRCVGAAFAIMQIKAIFSVLLREYEFEMAQPPESYRNDHSKMVVQLA
QPACVRYRRRTGV
>CYP51B1_Mycobacterium tuberculosis Q09900809 2683857382
MSAVALPRVSGGHDEHGHLEEFRTDPIGLMQRVRDECGDVGTFQLAGKQVVLLSGSHANEFFFRAGDDDLDQA
KAYPFMTPIFGEGVVFDASPERRKEMLHNAALRGEQMKGHAATIEDQVRRMIADWGEAGEIDLLDFFAELTIY
TSSACLIGKKFRDQLDGRFAKLYHELERGTDPLAYVDPYLPIESFRRRDEARNGLVALVADIMNGRIANPPTD
KSDRDMLDVLIAVKAETGTPRFSADEITGMFISMMFAGHHTSSGTASWTLIELMRHRDAYAAVIDELDELYGD
GRSVSFHALRQIPQLENVLKETLRLHPPLIILMRVAKGEFEVQGHRIHEGDLVAASPAISNRIPEDFPDPHDF
VPARYEQPRQEDLLNRWTWIPFGAGRHRCVGAAFAIMQIKAIFSVLLREYEFEMAQPPESYRNDHSKMVVQLA
QPACVRYRRRTGV
>CYP51B1_Mycobacterium tuberculosis Q09901139 2672328975
MSAVALPRVSGGHDEHGHLEEFRTDPIGLMQRVRDECGDVGTFQLAGKQVVLLSGSHANEFFFRAGDDDLDQA
KAYPFMTPIFGEGVVFDASPERRKEMLHNAALRGEQMKGHAATIEDQVRRMIADWGEAGEIDLLDFFAELTIY
TSSACLIGKKFRDQLDGRFAKLYHELERGTDPLAYVDPYLPIESFRRRDEARNGLVALVADIMNGRIANPPTD
KSDRDMLDVLIAVKAETGTPRFSADEITGMFISMMFAGHHTSSGTASWTLIELMRHRDAYAAVIDELDELYGD
GRSVSFHALRQIPQLENVLKETLRLHPPLIILMRVAKGEFEVQGHRIHEGDLVAASPAISNRIPEDFPDPHDF
VPARYEQPRQEDLLNRWTWIPFGAGRHRCVGAAFAIMQIKAIFSVLLREYEFEMAQPPESYRNDHSKMVVQLA
QPACVRYRRRTGV
>CYP51B1_Mycobacterium tuberculosis Q09901557 2683874615
MSAVALPRVSGGHDEHGHLEEFRTDPIGLMQRVRDECGDVGTFQLAGKQVVLLSGSHANEFFFRAGDDDLDQA
KAYPFMTPIFGEGVVFDASPERRKEMLHNAALRGEQMKGHAATIEDQVRRMIADWGEAGEIDLLDFFAELTIY
TSSACLIGKKFRDQLDGRFAKLYHELERGTDPLAYVDPYLPIESFRRRDEARNGLVALVADIMNGRIANPPTD
KSDRDMLDVLIAVKAETGTPRFSADEITGMFISMMFAGHHTSSGTASWTLIELMRHRDAYAAVIDELDELYGD
GRSVSFHALRQIPQLENVLKETLRLHPPLIILMRVAKGEFEVQGHRIHEGDLVAASPAISNRIPEDFPDPHDF
VPARYEQPRQEDLLNRWTWIPFGAGRHRCVGAAFAIMQIKAIFSVLLREYEFEMAQPPESYRNDHSKMVVQLA
QPACVRYRRRTGV
>CYP51B1_Mycobacterium tuberculosis Q09901859 2703539935
MSAVALPRVSGGHDEHGHLEEFRTDPIGLMQRVRDECGDVGTFQLAGKQVVLLSGSHANEFFFRAGDDDLDQA
KAYPFMTPIFGEGVVFDASPERRKEMLHNAALRGEQMKGHAATIEDQVRRMIADWGEAGEIDLLDFFAELTIY
TSSACLIGKKFRDQLDGRFAKLYHELERGTDPLAYVDPYLPIESFRRRDEARNGLVALVADIMNGRIANPPTD
KSDRDMLDVLIAVKAETGTPRFSADEITGMFISMMFAGHHTSSGTASWTLIELMRHRDAYAAVIDELDELYGD
GRSVSFHALRQIPQLENVLKETLRLHPPLIILMRVAKGEFEVQGHRIHEGDLVAASPAISNRIPEDFPDPHDF
VPARYEQPRQEDLLNRWTWIPFGAGRHRCVGAAFAIMQIKAIFSVLLREYEFEMAQPPESYRNDHSKMVVQLA
QPACVRYRRRTGV
>CYP51B1_Mycobacterium tuberculosis R00018412 2683885039
MSAVALPRVSGGHDEHGHLEEFRTDPIGLMQRVRDECGDVGTFQLAGKQVVLLSGSHANEFFFRAGDDDLDQA
KAYPFMTPIFGEGVVFDASPERRKEMLHNAALRGEQMKGHAATIEDQVRRMIADWGEAGEIDLLDFFAELTIY
TSSACLIGKKFRDQLDGRFAKLYHELERGTDPLAYVDPYLPIESFRRRDEARNGLVALVADIMNGRIANPPTD
KSDRDMLDVLIAVKAETGTPRFSADEITGMFISMMFAGHHTSSGTASWTLIELMRHRDAYAAVIDELDELYGD
GRSVSFHALRQIPQLENVLKETLRLHPPLIILMRVAKGEFEVQGHRIHEGDLVAASPAISNRIPEDFPDPHDF
VPARYEQPRQEDLLNRWTWIPFGAGRHRCVGAAFAIMQIKAIFSVLLREYEFEMAQPPESYRNDHSKMVVQLA
QPACVRYRRRTGV
>CYP51B1_Mycobacterium tuberculosis R00301759 2703547708

MSAVALPRVSGGHDEHGHLEEFRTDPIGLMQRVRDECGDVGTFQLAGKQVVLLSGSHANEFFFRAGDDDLDQA
KAYPFMTPIFGEGVVFDASPERRKEMLHNAALRGEQMKGHAATIEDQVRRMIADWGEAGEIDLLDFFAELTIY
TSSACLIGKKFRDQLDGRFAKLYHELERGTDPLAYVDPYLPIESFRRRDEARNGLVALVADIMNGRIANPPTD
KSDRDMLDVLIAVKAETGTPRFSADEITGMFISMMFAGHHTSSGTASWTLIELMRHRDAYAAVIDELDELYGD
GRSVSFHALRQIPQLENVLKETLRLHPPLIILMRVAKGEFEVQGHRIHEGDLVAASPAISNRIPEDFPDPHDF
VPARYEQPRQEDLLNRWTWIPFGAGRHRCVGAAFAIMQIKAIFSVLLREYEFEMAQPPESYRNDHSKMVVQLA
QPACVRYRRRTGV
>CYP51B1_Mycobacterium tuberculosis R00401166 2703535332
MSAVALPRVSGGHDEHGHLEEFRTDPIGLMQRVRDECGDVGTFQLAGKQVVLLSGSHANEFFFRAGDDDLDQA
KAYPFMTPIFGEGVVFDASPERRKEMLHNAALRGEQMKGHAATIEDQVRRMIADWGEAGEIDLLDFFAELTIY
TSSACLIGKKFRDQLDGRFAKLYHELERGTDPLAYVDPYLPIESFRRRDEARNGLVALVADIMNGRIANPPTD
KSDRDMLDVLIAVKAETGTPRFSADEITGMFISMMFAGHHTSSGTASWTLIELMRHRDAYAAVIDELDELYGD
GRSVSFHALRQIPQLENVLKETLRLHPPLIILMRVAKGEFEVQGHRIHEGDLVAASPAISNRIPEDFPDPHDF
VPARYEQPRQEDLLNRWTWIPFGAGRHRCVGAAFAIMQIKAIFSVLLREYEFEMAQPPESYRNDHSKMVVQLA
QPACVRYRRRTGV
>CYP51B1_Mycobacterium tuberculosis R09401256 2675741840
MSAVALPRVSGGHDEHGHLEEFRTDPIGLMQRVRDECGDVGTFQLAGKQVVLLSGSHANEFFFRAGDDDLDQA
KAYPFMTPIFGEGVVFDASPERRKEMLHNAALRGEQMKGHAATIEDQVRRMIADWGEAGEIDLLDFFAELTIY
TSSACLIGKKFRDQLDGRFAKLYHELERGTDPLAYVDPYLPIESFRRRDEARNGLVALVADIMNGRIANPPTD
KSDRDMLDVLIAVKAETGTPRFSADEITGMFISMMFAGHHTSSGTASWTLIELMRHRDAYAAVIDELDELYGD
GRSVSFHALRQIPQLENVLKETLRLHPPLIILMRVAKGEFEVQGHRIHEGDLVAASPAISNRIPEDFPDPHDF
VPARYEQPRQEDLLNRWTWIPFGAGRHRCVGAAFAIMQIKAIFSVLLREYEFEMAQPPESYRNDHSKMVVQLA
QPACVRYRRRTGV
>CYP51B1_Mycobacterium tuberculosis R09401839 2696525230
MSAVALPRVSGGHDEHGHLEEFRTDPIGLMQRVRDECGDVGTFQLAGKQVVLLSGSHANEFFFRAGDDDLDQA
KAYPFMTPIFGEGVVFDASPERRKEMLHNAALRGEQMKGHAATIEDQVRRMIADWGEAGEIDLLDFFAELTIY
TSSACLIGKKFRDQLDGRFAKLYHELERGTDPLAYVDPYLPIESFRRRDEARNGLVALVADIMNGRIANPPTD
KSDRDMLDVLIAVKAETGTPRFSADEITGMFISMMFAGHHTSSGTASWTLIELMRHRDAYAAVIDELDELYGD
GRSVSFHALRQIPQLENVLKETLRLHPPLIILMRVAKGEFEVQGHRIHEGDLVAASPAISNRIPEDFPDPHDF
VPARYEQPRQEDLLNRWTWIPFGAGRHRCVGAAFAIMQIKAIFSVLLREYEFEMAQPPESYRNDHSKMVVQLA
QPACVRYRRRTGV
>CYP51B1_Mycobacterium tuberculosis R09401899 2694455402
MSAVALPRVSGGHDEHGHLEEFRTDPIGLMQRVRDECGDVGTFQLAGKQVVLLSGSHANEFFFRAGDDDLDQA
KAYPFMTPIFGEGVVFDASPERRKEMLHNAALRGEQMKGHAATIEDQVRRMIADWGEAGEIDLLDFFAELTIY
TSSACLIGKKFRDQLDGRFAKLYHELERGTDPLAYVDPYLPIESFRRRDEARNGLVALVADIMNGRIANPPTD
KSDRDMLDVLIAVKAETGTPRFSADEITGMFISMMFAGHHTSSGTASWTLIELMRHRDAYAAVIDELDELYGD
GRSVSFHALRQIPQLENVLKETLRLHPPLIILMRVAKGEFEVQGHRIHEGDLVAASPAISNRIPEDFPDPHDF
VPARYEQPRQEDLLNRWTWIPFGAGRHRCVGAAFAIMQIKAIFSVLLREYEFEMAQPPESYRNDHSKMVVQLA
QPACVRYRRRTGV
>CYP51B1_Mycobacterium tuberculosis R09402296 2694338487
MSAVALPRVSGGHDEHGHLEEFRTDPIGLMQRVRDECGDVGTFQLAGKQVVLLSGSHANEFFFRAGDDDLDQA
KAYPFMTPIFGEGVVFDASPERRKEMLHNAALRGEQMKGHAATIEDQVRRMIADWGEAGEIDLLDFFAELTIY
TSSACLIGKKFRDQLDGRFAKLYHELERGTDPLAYVDPYLPIESFRRRDEARNGLVALVADIMNGRIANPPTD
KSDRDMLDVLIAVKAETGTPRFSADEITGMFISMMFAGHHTSSGTASWTLIELMRHRDAYAAVIDELDELYGD
GRSVSFHALRQIPQLENVLKETLRLHPPLIILMRVAKGEFEVQGHRIHEGDLVAASPAISNRIPEDFPDPHDF
VPARYEQPRQEDLLNRWTWIPFGAGRHRCVGAAFAIMQIKAIFSVLLREYEFEMAQPPESYRNDHSKMVVQLA
QPACVRYRRRTGV
>CYP51B1_Mycobacterium tuberculosis R09500613 2703560940
MSAVALPRVSGGHDEHGHLEEFRTDPIGLMQRVRDECGDVGTFQLAGKQVVLLSGSHANEFFFRAGDDDLDQA
KAYPFMTPIFGEGVVFDASPERRKEMLHNAALRGEQMKGHAATIEDQVRRMIADWGEAGEIDLLDFFAELTIY
TSSACLIGKKFRDQLDGRFAKLYHELERGTDPLAYVDPYLPIESFRRRDEARNGLVALVADIMNGRIANPPTD
KSDRDMLDVLIAVKAETGTPRFSADEITGMFISMMFAGHHTSSGTASWTLIELMRHRDAYAAVIDELDELYGD
GRSVSFHALRQIPQLENVLKETLRLHPPLIILMRVAKGEFEVQGHRIHEGDLVAASPAISNRIPEDFPDPHDF
VPARYEQPRQEDLLNRWTWIPFGAGRHRCVGAAFAIMQIKAIFSVLLREYEFEMAQPPESYRNDHSKMVVQLA
QPACVRYRRRTGV
>CYP51B1_Mycobacterium tuberculosis R09500614 2692673081
MSAVALPRVSGGHDEHGHLEEFRTDPIGLMQRVRDECGDVGTFQLAGKQVVLLSGSHANEFFFRAGDDDLDQA
KAYPFMTPIFGEGVVFDASPERRKEMLHNAALRGEQMKGHAATIEDQVRRMIADWGEAGEIDLLDFFAELTIY

TSSACLIGKKFRDQLDGRFAKLYHELERGTDPLAYVDPYLPIESFRRRDEARNGLVALVADIMNGRIANPPTD
KSDRDMLDVLIAVKAETGTPRFSADEITGMFISMMFAGHHTSSGTASWTLIELMRHRDAYAAVIDELDELYGD
GRSVSFHALRQIPQLENVLKETLRLHPPLIILMRVAKGEFEVQGHRIHEGDLVAASPAISNRIPEDFPDPHDF
VPARYEQPRQEDLLNRWTWIPFGAGRHRCVGAAFAIMQIKAIFSVLLREYEFEMAQPPESYRNDHSKMVVQLA
QPACVRYRRRTGV
>CYP51B1_Mycobacterium tuberculosis R09601315 2703553020
MSAVALPRVSGGHDEHGHLEEFRTDPIGLMQRVRDECGDVGTFQLAGKQVVLLSGSHANEFFFRAGDDDLDQA
KAYPFMTPIFGEGVVFDASPERRKEMLHNAALRGEQMKGHAATIEDQVRRMIADWGEAGEIDLLDFFAELTIY
TSSACLIGKKFRDQLDGRFAKLYHELERGTDPLAYVDPYLPIESFRRRDEARNGLVALVADIMNGRIANPPTD
KSDRDMLDVLIAVKAETGTPRFSADEITGMFISMMFAGHHTSSGTASWTLIELMRHRDAYAAVIDELDELYGD
GRSVSFHALRQIPQLENVLKETLRLHPPLIILMRVAKGEFEVQGHRIHEGDLVAASPAISNRIPEDFPDPHDF
VPARYEQPRQEDLLNRWTWIPFGAGRHRCVGAAFAIMQIKAIFSVLLREYEFEMAQPPESYRNDHSKMVVQLA
QPACVRYRRRTGV
>CYP51B1_Mycobacterium tuberculosis R09801836 2696872180
MSAVALPRVSGGHDEHGHLEEFRTDPIGLMQRVRDECGDVGTFQLAGKQVVLLSGSHANEFFFRAGDDDLDQA
KAYPFMTPIFGEGVVFDASPERRKEMLHNAALRGEQMKGHAATIEDQVRRMIADWGEAGEIDLLDFFAELTIY
TSSACLIGKKFRDQLDGRFAKLYHELERGTDPLAYVDPYLPIESFRRRDEARNGLVALVADIMNGRIANPPTD
KSDRDMLDVLIAVKAETGTPRFSADEITGMFISMMFAGHHTSSGTASWTLIELMRHRDAYAAVIDELDELYGD
GRSVSFHALRQIPQLENVLKETLRLHPPLIILMRVAKGEFEVQGHRIHEGDLVAASPAISNRIPEDFPDPHDF
VPARYEQPRQEDLLNRWTWIPFGAGRHRCVGAAFAIMQIKAIFSVLLREYEFEMAQPPESYRNDHSKMVVQLA
QPACVRYRRRTGV
>CYP51B1_Mycobacterium tuberculosis R1207 2547950368
MSAVALPRVSGGHDEHGHLEEFRTDPIGLMQRVRDECGDVGTFQLAGKQVVLLSGSHANEFFFRAGDDDLDQA
KAYPFMTPIFGEGVVFDASPERRKEMLHNAALRGEQMKGHAATIEDQVRRMIADWGEAGEIDLLDFFAELTIY
TSSACLIGKKFRDQLDGRFAKLYHELERGTDPLAYVDPYLPIESFRRRDEARNGLVALVADIMNGRIANPPTD
KSDRDMLDVLIAVKAETGTPRFSADEITGMFISMMFAGHHTSSGTASWTLIELMRHRDAYAAVIDELDELYGD
GRSVSFHALRQIPQLENVLKETLRLHPPLIILMRVAKGEFEVQGHRIHEGDLVAASPAISNRIPEDFPDPHDF
VPARYEQPRQEDLLNRWTWIPFGAGRHRCVGAAFAIMQIKAIFSVLLREYEFEMAQPPESYRNDHSKMVVQLA
QPACVRYRRRTGV
>CYP51B1_Mycobacterium tuberculosis RGTB327 2512818385
MSAVALPRVSGGHDEHGHLEEFRTDPIGLMQRVRDECGDVGTFQLAGKQVVLLSGSHANEFFFRAGDDDLDQA
KAYPFMTPIFGEGVVFDASPERRKEMLHNAALRGEQMKGHAATIEDQVRRMIADWGEAGEIDLLDFFAELTIY
TSSACLIGKKFRDQLDGRFAKLYHELERGTDPLAYVDPYLPIESFRRRDEARNGLVALVADIMNGRIANPPTD
KSDRDMLDVLIAVKAETGTPRFSADEITGMFISMMFAGHHTSSGTASWTLIELMRHRDAYAAVIDELDELYGD
GRSVSFHALRQIPQLENVLKETLRLHPPLIILMRVAKGEFEVQGHRIHEGDLVAASPAISNRIPEDFPDPHDF
VPARYEQPRQEDLLNRWTWIPFGAGRHRCVGAAFAIMQIKAIFSVLLREYEFEMAQPPESYRNDHSKMVVQLA
QPACVRYRRRTGV
>CYP51B1_Mycobacterium tuberculosis RGTB423 2514117291
MSAVALPRVSGGHDEHGHLEEFRTDPIGLMQRVRDECGDVGTFQLAGKQVVLLSGSHANEFFFRAGDDDLDQA
KAYPFMTPIFGEGVVFDASPERRKEMLHNAALRGEQMKGHAATIEDQVRRMIADWGEAGEIDLLDFFAELTIY
TSSACLIGKKFRDQLDGRFAKLYHELERGTDPLAYVDPYLPIESFRRRDEARNGLVALVADIMNGRIANPPTD
KSDRDMLDVLIAVKAETGTPRFSADEITGMFISMMFAGHHTSSGTASWTLIELMRHRDAYAAVIDELDELYGD
GRSVSFHALRQIPQLENVLKETLRLHPPLIILMRVAKGEFEVQGHRIHEGDLVAASPAISNRIPEDFPDPHDF
VPARYEQPRQEDLLNRWTWIPFGAGRHRCVGAAFAIMQIKAIFSVLLREYEFEMAQPPESYRNDHSKMVVQLA
QPACVRYRRRTGV
>CYP51B1_Mycobacterium tuberculosis S00600143 2674728121
MSAVALPRVSGGHDEHGHLEEFRTDPIGLMQRVRDECGDVGTFQLAGKQVVLLSGSHANEFFFRAGDDDLDQA
KAYPFMTPIFGEGVVFDASPERRKEMLHNAALRGEQMKGHAATIEDQVRRMIADWGEAGEIDLLDFFAELTIY
TSSACLIGKKFRDQLDGRFAKLYHELERGTDPLAYVDPYLPIESFRRRDEARNGLVALVADIMNGRIANPPTD
KSDRDMLDVLIAVKAETGTPRFSADEITGMFISMMFAGHHTSSGTASWTLIELMRHRDAYAAVIDELDELYGD
GRSVSFHALRQIPQLENVLKETLRLHPPLIILMRVAKGEFEVQGHRIHEGDLVAASPAISNRIPEDFPDPHDF
VPARYEQPRQEDLLNRWTWIPFGAGRHRCVGAAFAIMQIKAIFSVLLREYEFEMAQPPESYRNDHSKMVVQLA
QPACVRYRRRTGV
>CYP51B1_Mycobacterium tuberculosis S00600351 2703566209
MSAVALPRVSGGHDEHGHLEEFRTDPIGLMQRVRDECGDVGTFQLAGKQVVLLSGSHANEFFFRAGDDDLDQA
KAYPFMTPIFGEGVVFDASPERRKEMLHNAALRGEQMKGHAATIEDQVRRMIADWGEAGEIDLLDFFAELTIY
TSSACLIGKKFRDQLDGRFAKLYHELERGTDPLAYVDPYLPIESFRRRDEARNGLVALVADIMNGRIANPPTD
KSDRDMLDVLIAVKAETGTPRFSADEITGMFISMMFAGHHTSSGTASWTLIELMRHRDAYAAVIDELDELYGD

GRSVSFHALRQIPQLENVLKETLRLHPPLIILMRVAKGEFEVQGHRIHEGDLVAASPAISNRIPEDFPDPHDF
VPARYEQPRQEDLLNRWTWIPFGAGRHRCVGAAFAIMQIKAIFSVLLREYEFEMAQPPESYRNDHSKMVVQLA
QPACVRYRRRTGV
>CYP51B1_Mycobacterium tuberculosis S96-129 2548027875
MSAVALPRVSGGHDEHGHLEEFRTDPIGLMQRVRDECGDVGTFQLAGKQVVLLSGSHANEFFFRAGDDDLDQA
KAYPFMTPIFGEGVVFDASPERRKEMLHNAALRGEQMKGHAATIEDQVRRMIADWGEAGEIDLLDFFAELTIY
TSSACLIGKKFRDQLDGRFAKLYHELERGTDPLAYVDPYLPIESFRRRDEARNGLVALVADIMNGRIANPPTD
KSDRDMLDVLIAVKAETGTPRFSADEITGMFISMMFAGHHTSSGTASWTLIELMRHRDAYAAVIDELDELYGD
GRSVSFHALRQIPQLENVLKETLRLHPPLIILMRVAKGEFEVQGHRIHEGDLVAASPAISNRIPEDFPDPHDF
VPARYEQPRQEDLLNRWTWIPFGAGRHRCVGAAFAIMQIKAIFSVLLREYEFEMAQPPESYRNDHSKMVVQLA
QPACVRYRRRTGV
>CYP51B1_Mycobacterium tuberculosis SAWC507 2703599948
MSAVALPRVSGGHDEHGHLEEFRTDPIGLMQRVRDECGDVGTFQLAGKQVVLLSGSHANEFFFRAGDDDLDQA
KAYPFMTPIFGEGVVFDASPERRKEMLHNAALRGEQMKGHAATIEDQVRRMIADWGEAGEIDLLDFFAELTIY
TSSACLIGKKFRDQLDGRFAKLYHELERGTDPLAYVDPYLPIESFRRRDEARNGLVALVADIMNGRIANPPTD
KSDRDMLDVLIAVKAETGTPRFSADEITGMFISMMFAGHHTSSGTASWTLIELMRHRDAYAAVIDELDELYGD
GRSVSFHALRQIPQLENVLKETLRLHPPLIILMRVAKGEFEVQGHRIHEGDLVAASPAISNRIPEDFPDPHDF
VPARYEQPRQEDLLNRWTWIPFGAGRHRCVGAAFAIMQIKAIFSVLLREYEFEMAQPPESYRNDHSKMVVQLA
QPACVRYRRRTGV
>CYP51B1_Mycobacterium tuberculosis SCAID 187 2650945899
MSAVALPRVSGGHDEHGHLEEFRTDPIGLMQRVRDECGDVGTFQLAGKQVVLLSGSHANEFFFRAGDDDLDQA
KAYPFMTPIFGEGVVFDASPERRKEMLHNAALRGEQMKGHAATIEDQVRRMIADWGEAGEIDLLDFFAELTIY
TSSACLIGKKFRDQLDGRFAKLYHELERGTDPLAYVDPYLPIESFRRRDEARNGLVALVADIMNGRIANPPTD
KSDRDMLDVLIAVKAETGTPRFSADEITGMFISMMFAGHHTSSGTASWTLIELMRHRDAYAAVIDELDELYGD
GRSVSFHALRQIPQLENVLKETLRLHPPLIILMRVAKGEFEVQGHRIHEGDLVAASPAISNRIPEDFPDPHDF
VPARYEQPRQEDLLNRWTWIPFGAGRHRCVGAAFAIMQIKAIFSVLLREYEFEMAQPPESYRNDHSKMVVQLA
QPACVRYRRRTGV
>CYP51B1_Mycobacterium tuberculosis SH1-874 2698761313
MSAVALPRVSGGHDEHGHLEEFRTDPIGLMQRVRDECGDVGTFQLAGKQVVLLSGSHANEFFFRAGDDDLDQA
KAYPFMTPIFGEGVVFDASPERRKEMLHNAALRGEQMKGHAATIEDQVRRMIADWGEAGEIDLLDFFAELTIY
TSSACLIGKKFRDQLDGRFAKLYHELERGTDPLAYVDPYLPIESFRRRDEARNGLVALVADIMNGRIANPPTD
KSDRDMLDVLIAVKAETGTPRFSADEITGMFISMMFAGHHTSSGTASWTLIELMRHRDAYAAVIDELDELYGD
GRSVSFHALRQIPQLENVLKETLRLHPPLIILMRVAKGEFEVQGHRIHEGDLVAASPAISNRIPEDFPDPHDF
VPARYEQPRQEDLLNRWTWIPFGAGRHRCVGAAFAIMQIKAIFSVLLREYEFEMAQPPESYRNDHSKMVVQLA
QPACVRYRRRTGV
>CYP51B1_Mycobacterium tuberculosis SH5-875 2697439114
MSAVALPRVSGGHDEHGHLEEFRTDPIGLMQRVRDECGDVGTFQLAGKQVVLLSGSHANEFFFRAGDDDLDQA
KAYPFMTPIFGEGVVFDASPERRKEMLHNAALRGEQMKGHAATIEDQVRRMIADWGEAGEIDLLDFFAELTIY
TSSACLIGKKFRDQLDGRFAKLYHELERGTDPLAYVDPYLPIESFRRRDEARNGLVALVADIMNGRIANPPTD
KSDRDMLDVLIAVKAETGTPRFSADEITGMFISMMFAGHHTSSGTASWTLIELMRHRDAYAAVIDELDELYGD
GRSVSFHALRQIPQLENVLKETLRLHPPLIILMRVAKGEFEVQGHRIHEGDLVAASPAISNRIPEDFPDPHDF
VPARYEQPRQEDLLNRWTWIPFGAGRHRCVGAAFAIMQIKAIFSVLLREYEFEMAQPPESYRNDHSKMVVQLA
QPACVRYRRRTGV
>CYP51B1_Mycobacterium tuberculosis SIT745/EAI1-MYS 2730911412
MSAVALPRVSGGHDEHGHLEEFRTDPIGLMQRVRDECGDVGTFQLAGKQVVLLSGSHANEFFFRAGDDDLDQA
KAYPFMTPIFGEGVVFDASPERRKEMLHNAALRGEQMKGHAATIEDQVRRMIADWGEAGEIDLLDFFAELTIY
TSSACLIGKKFRDQLDGRFAKLYHELERGTDPLAYVDPYLPIESFRRRDEARNGLVALVADIMNGRIANPPTD
KSDRDMLDVLIAVKAETGTPRFSADEITGMFISMMFAGHHTSSGTASWTLIELMRHRDAYAAVIDELDELYGD
GRSVSFHALRQIPQLENVLKETLRLHPPLIILMRVAKGEFEVQGHRIHEGDLVAASPAISNRIPEDFPDPHDF
VPARYEQPRQEDLLNRWTWIPFGAGRHRCVGAAFAIMQIKAIFSVLLREYEFEMAQPPESYRNDHSKMVVQLA
QPACVRYRRRTGV
>CYP51B1_Mycobacterium tuberculosis SK-B 2576738757
MSAVALPRVSGGHDEHGHLEEFRTDPIGLMQRVRDECGDVGTFQLAGKQVVLLSGSHANEFFFRAGDDDLDQA
KAYPFMTPIFGEGVVFDASPERRKEMLHNAALRGEQMKGHAATIEDQVRRMIADWGEAGEIDLLDFFAELTIY
TSSACLIGKKFRDQLDGRFAKLYHELERGTDPLAYVDPYLPIESFRRRDEARNGLVALVADIMNGRIANPPTD
KSDRDMLDVLIAVKAETGTPRFSADEITGMFISMMFAGHHTSSGTASWTLIELMRHRDAYAAVIDELDELYGD
GRSVSFHALRQIPQLENVLKETLRLHPPLIILMRVAKGEFEVQGHRIHEGDLVAASPAISNRIPEDFPDPHDF

VPARYEQPRQEDLLNRWTWIPFGAGRHRCVGAAFAIMQIKAIFSVLLREYEFEMAQPPESYRNDHSKMVVQLA
QPACVRYRRRTGV
>CYP51B1_Mycobacterium tuberculosis SK-C 2592446234
MSAVALPRVSGGHDEHGHLEEFRTDPIGLMQRVRDECGDVGTFQLAGKQVVLLSGSHANEFFFRAGDDDLDQA
KAYPFMTPIFGEGVVFDASPERRKEMLHNAALRGEQMKGHAATIEDQVRRMIADWGEAGEIDLLDFFAELTIY
TSSACLIGKKFRDQLDGRFAKLYHELERGTDPLAYVDPYLPIESFRRRDEARNGLVALVADIMNGRIANPPTD
KSDRDMLDVLIAVKAETGTPRFSADEITGMFISMMFAGHHTSSGTASWTLIELMRHRDAYAAVIDELDELYGD
GRSVSFHALRQIPQLENVLKETLRLHPPLIILMRVAKGEFEVQGHRIHEGDLVAASPAISNRIPEDFPDPHDF
VPARYEQPRQEDLLNRWTWIPFGAGRHRCVGAAFAIMQIKAIFSVLLREYEFEMAQPPESYRNDHSKMVVQLA
QPACVRYRRRTGV
>CYP51B1_Mycobacterium tuberculosis SK-E 2584655898
MSAVALPRVSGGHDEHGHLEEFRTDPIGLMQRVRDECGDVGTFQLAGKQVVLLSGSHANEFFFRAGDDDLDQA
KAYPFMTPIFGEGVVFDASPERRKEMLHNAALRGEQMKGHAATIEDQVRRMIADWGEAGEIDLLDFFAELTIY
TSSACLIGKKFRDQLDGRFAKLYHELERGTDPLAYVDPYLPIESFRRRDEARNGLVALVADIMNGRIANPPTD
KSDRDMLDVLIAVKAETGTPRFSADEITGMFISMMFAGHHTSSGTASWTLIELMRHRDAYAAVIDELDELYGD
GRSVSFHALRQIPQLENVLKETLRLHPPLIILMRVAKGEFEVQGHRIHEGDLVAASPAISNRIPEDFPDPHDF
VPARYEQPRQEDLLNRWTWIPFGAGRHRCVGAAFAIMQIKAIFSVLLREYEFEMAQPPESYRNDHSKMVVQLA
QPACVRYRRRTGV
>CYP51B1_Mycobacterium tuberculosis 2554287016
MSAVALPRVSGGHDEHGHLEEFRTDPIGLMQRVRDECGDVGTFQLAGKQVVLLSGSHANEFFFRAGDDDLDQA
KAYPFMTPIFGEGVVFDASPERRKEMLHNAALRGEQMKGHAATIEDQVRRMIADWGEAGEIDLLDFFAELTIY
TSSACLIGKKFRDQLDGRFAKLYHELERGTDPLAYVDPYLPIESFRRRDEARNGLVALVADIMNGRIANPPTD
KSDRDMLDVLIAVKAETGTPRFSADEITGMFISMMFAGHHTSSGTASWTLIELMRHRDAYAAVIDELDELYGD
GRSVSFHALRQIPQLENVLKETLRLHPPLIILMRVAKGEFEVQGHRIHEGDLVAASPAISNRIPEDFPDPHDF
VPARYEQPRQEDLLNRWTWIPFGAGRHRCVGAAFAIMQIKAIFSVLLREYEFEMAQPPESYRNDHSKMVVQLA
QPACVRYRRRTGV
>CYP51B1_Mycobacterium tuberculosis str. Beijing/DS6701 2631271913
MSAVALPRVSGGHDEHGHLEEFRTDPIGLMQRVRDECGDVGTFQLAGKQVVLLSGSHANEFFFRAGDDDLDQA
KAYPFMTPIFGEGVVFDASPERRKEMLHNAALRGEQMKGHAATIEDQVRRMIADWGEAGEIDLLDFFAELTIY
TSSACLIGKKFRDQLDGRFAKLYHELERGTDPLAYVDPYLPIESFRRRDEARNGLVALVADIMNGRIANPPTD
KSDRDMLDVLIAVKAETGTPRFSADEITGMFISMMFAGHHTSSGTASWTLIELMRHRDAYAAVIDELDELYGD
GRSVSFHALRQIPQLENVLKETLRLHPPLIILMRVAKGEFEVQGHRIHEGDLVAASPAISNRIPEDFPDPHDF
VPARYEQPRQEDLLNRWTWIPFGAGRHRCVGAAFAIMQIKAIFSVLLREYEFEMAQPPESYRNDHSKMVVQLA
QPACVRYRRRTGV
>CYP51B1_Mycobacterium tuberculosis str. Kurono 2648143054
MSAVALPRVSGGHDEHGHLEEFRTDPIGLMQRVRDECGDVGTFQLAGKQVVLLSGSHANEFFFRAGDDDLDQA
KAYPFMTPIFGEGVVFDASPERRKEMLHNAALRGEQMKGHAATIEDQVRRMIADWGEAGEIDLLDFFAELTIY
TSSACLIGKKFRDQLDGRFAKLYHELERGTDPLAYVDPYLPIESFRRRDEARNGLVALVADIMNGRIANPPTD
KSDRDMLDVLIAVKAETGTPRFSADEITGMFISMMFAGHHTSSGTASWTLIELMRHRDAYAAVIDELDELYGD
GRSVSFHALRQIPQLENVLKETLRLHPPLIILMRVAKGEFEVQGHRIHEGDLVAASPAISNRIPEDFPDPHDF
VPARYEQPRQEDLLNRWTWIPFGAGRHRCVGAAFAIMQIKAIFSVLLREYEFEMAQPPESYRNDHSKMVVQLA
QPACVRYRRRTGV
>CYP51B1_Mycobacterium tuberculosis SUMu001 cont1.20 648442365
MSAVALPRVSGGHDEHGHLEEFRTDPIGLMQRVRDECGDVGTFQLAGKQVVLLSGSHANEFFFRAGDDDLDQA
KAYPFMTPIFGEGVVFDASPERRKEMLHNAALRGEQMKGHAATIEDQVRRMIADWGEAGEIDLLDFFAELTIY
TSSACLIGKKFRDQLDGRFAKLYHELERGTDPLAYVDPYLPIESFRRRDEARNGLVALVADIMNGRIANPPTD
KSDRDMLDVLIAVKAETGTPRFSADEITGMFISMMFAGHHTSSGTASWTLIELMRHRDAYAAVIDELDELYGD
GRSVSFHALRQIPQLENVLKETLRLHPPLIILMRVAKGEFEVQGHRIHEGDLVAASPAISNRIPEDFPDPHDF
VPARYEQPRQEDLLNRWTWIPFGAGRHRCVGAAFAIMQIKAIFSVLLREYEFEMAQPPESYRNDHSKMVVQLA
QPACVRYRRRTGV
>CYP51B1_Mycobacterium tuberculosis SUMu002 NZ_ADHR01000136 648448659
MSAVALPRVSGGHDEHGHLEEFRTDPIGLMQRVRDECGDVGTFQLAGKQVVLLSGSHANEFFFRAGDDDLDQA
KAYPFMTPIFGEGVVFDASPERRKEMLHNAALRGEQMKGHAATIEDQVRRMIADWGEAGEIDLLDFFAELTIY
TSSACLIGKKFRDQLDGRFAKLYHELERGTDPLAYVDPYLPIESFRRRDEARNGLVALVADIMNGRIANPPTD
KSDRDMLDVLIAVKAETGTPRFSADEITGMFISMMFAGHHTSSGTASWTLIELMRHRDAYAAVIDELDELYGD
GRSVSFHALRQIPQLENVLKETLRLHPPLIILMRVAKGEFEVQGHRIHEGDLVAASPAISNRIPEDFPDPHDF
VPARYEQPRQEDLLNRWTWIPFGAGRHRCVGAAFAIMQIKAIFSVLLREYEFEMAQPPESYRNDHSKMVVQLA
QPACVRYRRRTGV

>CYP51B1_Mycobacterium tuberculosis SUMu003 NZ_ADHS01000027 648450706
MSAVALPRVSGGHDEHGHLEEFRTDPIGLMQRVRDECGDVGTFQLAGKQVVLLSGSHANEFFFRAGDDDLDQA
KAYPFMTPIFGEGVVFDASPERRKEMLHNAALRGEQMKGHAATIEDQVRRMIADWGEAGEIDLLDFFAELTIY
TSSACLIGKKFRDQLDGRFAKLYHELERGTDPLAYVDPYLPIESFRRRDEARNGLVALVADIMNGRIANPPTD
KSDRDMLDVLIAVKAETGTPRFSADEITGMFISMMFAGHHTSSGTASWTLIELMRHRDAYAAVIDELDELYGD
GRSVSFHALRQIPQLENVLKETLRLHPPLIILMRVAKGEFEVQGHRIHEGDLVAASPAISNRIPEDFPDPHDF
VPARYEQPRQEDLLNRWTWIPFGAGRHRCVGAAFAIMQIKAIFSVLLREYEFEMAQPPESYRNDHSKMVVQLA
QPACVRYRRRTGV
>CYP51B1_Mycobacterium tuberculosis SUMu004 NZ_ADHT01000036 648455124
MSAVALPRVSGGHDEHGHLEEFRTDPIGLMQRVRDECGDVGTFQLAGKQVVLLSGSHANEFFFRAGDDDLDQA
KAYPFMTPIFGEGVVFDASPERRKEMLHNAALRGEQMKGHAATIEDQVRRMIADWGEAGEIDLLDFFAELTIY
TSSACLIGKKFRDQLDGRFAKLYHELERGTDPLAYVDPYLPIESFRRRDEARNGLVALVADIMNGRIANPPTD
KSDRDMLDVLIAVKAETGTPRFSADEITGMFISMMFAGHHTSSGTASWTLIELMRHRDAYAAVIDELDELYGD
GRSVSFHALRQIPQLENVLKETLRLHPPLIILMRVAKGEFEVQGHRIHEGDLVAASPAISNRIPEDFPDPHDF
VPARYEQPRQEDLLNRWTWIPFGAGRHRCVGAAFAIMQIKAIFSVLLREYEFEMAQPPESYRNDHSKMVVQLA
QPACVRYRRRTGV
>CYP51B1_Mycobacterium tuberculosis SUMu005 NZ_ADHU01000037 648459528
MSAVALPRVSGGHDEHGHLEEFRTDPIGLMQRVRDECGDVGTFQLAGKQVVLLSGSHANEFFFRAGDDDLDQA
KAYPFMTPIFGEGVVFDASPERRKEMLHNAALRGEQMKGHAATIEDQVRRMIADWGEAGEIDLLDFFAELTIY
TSSACLIGKKFRDQLDGRFAKLYHELERGTDPLAYVDPYLPIESFRRRDEARNGLVALVADIMNGRIANPPTD
KSDRDMLDVLIAVKAETGTPRFSADEITGMFISMMFAGHHTSSGTASWTLIELMRHRDAYAAVIDELDELYGD
GRSVSFHALRQIPQLENVLKETLRLHPPLIILMRVAKGEFEVQGHRIHEGDLVAASPAISNRIPEDFPDPHDF
VPARYEQPRQEDLLNRWTWIPFGAGRHRCVGAAFAIMQIKAIFSVLLREYEFEMAQPPESYRNDHSKMVVQLA
QPACVRYRRRTGV
>CYP51B1_Mycobacterium tuberculosis SUMu006 NZ_ADHV01000035 648463939
MSAVALPRVSGGHDEHGHLEEFRTDPIGLMQRVRDECGDVGTFQLAGKQVVLLSGSHANEFFFRAGDDDLDQA
KAYPFMTPIFGEGVVFDASPERRKEMLHNAALRGEQMKGHAATIEDQVRRMIADWGEAGEIDLLDFFAELTIY
TSSACLIGKKFRDQLDGRFAKLYHELERGTDPLAYVDPYLPIESFRRRDEARNGLVALVADIMNGRIANPPTD
KSDRDMLDVLIAVKAETGTPRFSADEITGMFISMMFAGHHTSSGTASWTLIELMRHRDAYAAVIDELDELYGD
GRSVSFHALRQIPQLENVLKETLRLHPPLIILMRVAKGEFEVQGHRIHEGDLVAASPAISNRIPEDFPDPHDF
VPARYEQPRQEDLLNRWTWIPFGAGRHRCVGAAFAIMQIKAIFSVLLREYEFEMAQPPESYRNDHSKMVVQLA
QPACVRYRRRTGV
>CYP51B1_Mycobacterium tuberculosis SUMu007 NZ_ADHW01000035 648468208
MSAVALPRVSGGHDEHGHLEEFRTDPIGLMQRVRDECGDVGTFQLAGKQVVLLSGSHANEFFFRAGDDDLDQA
KAYPFMTPIFGEGVVFDASPERRKEMLHNAALRGEQMKGHAATIEDQVRRMIADWGEAGEIDLLDFFAELTIY
TSSACLIGKKFRDQLDGRFAKLYHELERGTDPLAYVDPYLPIESFRRRDEARNGLVALVADIMNGRIANPPTD
KSDRDMLDVLIAVKAETGTPRFSADEITGMFISMMFAGHHTSSGTASWTLIELMRHRDAYAAVIDELDELYGD
GRSVSFHALRQIPQLENVLKETLRLHPPLIILMRVAKGEFEVQGHRIHEGDLVAASPAISNRIPEDFPDPHDF
VPARYEQPRQEDLLNRWTWIPFGAGRHRCVGAAFAIMQIKAIFSVLLREYEFEMAQPPESYRNDHSKMVVQLA
QPACVRYRRRTGV
>CYP51B1_Mycobacterium tuberculosis SUMu008 NZ_ADHX01000050 648472442
MSAVALPRVSGGHDEHGHLEEFRTDPIGLMQRVRDECGDVGTFQLAGKQVVLLSGSHANEFFFRAGDDDLDQA
KAYPFMTPIFGEGVVFDASPERRKEMLHNAALRGEQMKGHAATIEDQVRRMIADWGEAGEIDLLDFFAELTIY
TSSACLIGKKFRDQLDGRFAKLYHELERGTDPLAYVDPYLPIESFRRRDEARNGLVALVADIMNGRIANPPTD
KSDRDMLDVLIAVKAETGTPRFSADEITGMFISMMFAGHHTSSGTASWTLIELMRHRDAYAAVIDELDELYGD
GRSVSFHALRQIPQLENVLKETLRLHPPLIILMRVAKGEFEVQGHRIHEGDLVAASPAISNRIPEDFPDPHDF
VPARYEQPRQEDLLNRWTWIPFGAGRHRCVGAAFAIMQIKAIFSVLLREYEFEMAQPPESYRNDHSKMVVQLA
QPACVRYRRRTGV
>CYP51B1_Mycobacterium tuberculosis SUMu009 NZ_ADHY01000020 648475959
MSAVALPRVSGGHDEHGHLEEFRTDPIGLMQRVRDECGDVGTFQLAGKQVVLLSGSHANEFFFRAGDDDLDQA
KAYPFMTPIFGEGVVFDASPERRKEMLHNAALRGEQMKGHAATIEDQVRRMIADWGEAGEIDLLDFFAELTIY
TSSACLIGKKFRDQLDGRFAKLYHELERGTDPLAYVDPYLPIESFRRRDEARNGLVALVADIMNGRIANPPTD
KSDRDMLDVLIAVKAETGTPRFSADEITGMFISMMFAGHHTSSGTASWTLIELMRHRDAYAAVIDELDELYGD
GRSVSFHALRQIPQLENVLKETLRLHPPLIILMRVAKGEFEVQGHRIHEGDLVAASPAISNRIPEDFPDPHDF
VPARYEQPRQEDLLNRWTWIPFGAGRHRCVGAAFAIMQIKAIFSVLLREYEFEMAQPPESYRNDHSKMVVQLA
QPACVRYRRRTGV
>CYP51B1_Mycobacterium tuberculosis SUMu010 NZ_ADHZ01000017 648480200

MSAVALPRVSGGHDEHGHLEEFRTDPIGLMQRVRDECGDVGTFQLAGKQVVLLSGSHANEFFFRAGDDDLDQA
KAYPFMTPIFGEGVVFDASPERRKEMLHNAALRGEQMKGHAATIEDQVRRMIADWGEAGEIDLLDFFAELTIY
TSSACLIGKKFRDQLDGRFAKLYHELERGTDPLAYVDPYLPIESFRRRDEARNGLVALVADIMNGRIANPPTD
KSDRDMLDVLIAVKAETGTPRFSADEITGMFISMMFAGHHTSSGTASWTLIELMRHRDAYAAVIDELDELYGD
GRSVSFHALRQIPQLENVLKETLRLHPPLIILMRVAKGEFEVQGHRIHEGDLVAASPAISNRIPEDFPDPHDF
VPARYEQPRQEDLLNRWTWIPFGAGRHRCVGAAFAIMQIKAIFSVLLREYEFEMAQPPESYRNDHSKMVVQLA
QPACVRYRRRTGV
>CYP51B1_Mycobacterium tuberculosis SUMu011 NZ_ADIA01000025 648484487
MSAVALPRVSGGHDEHGHLEEFRTDPIGLMQRVRDECGDVGTFQLAGKQVVLLSGSHANEFFFRAGDDDLDQA
KAYPFMTPIFGEGVVFDASPERRKEMLHNAALRGEQMKGHAATIEDQVRRMIADWGEAGEIDLLDFFAELTIY
TSSACLIGKKFRDQLDGRFAKLYHELERGTDPLAYVDPYLPIESFRRRDEARNGLVALVADIMNGRIANPPTD
KSDRDMLDVLIAVKAETGTPRFSADEITGMFISMMFAGHHTSSGTASWTLIELMRHRDAYAAVIDELDELYGD
GRSVSFHALRQIPQLENVLKETLRLHPPLIILMRVAKGEFEVQGHRIHEGDLVAASPAISNRIPEDFPDPHDF
VPARYEQPRQEDLLNRWTWIPFGAGRHRCVGAAFAIMQIKAIFSVLLREYEFEMAQPPESYRNDHSKMVVQLA
QPACVRYRRRTGV
>CYP51B1_Mycobacterium tuberculosis SUMu012 NZ_ADIB01000056 648489039
MSAVALPRVSGGHDEHGHLEEFRTDPIGLMQRVRDECGDVGTFQLAGKQVVLLSGSHANEFFFRAGDDDLDQA
KAYPFMTPIFGEGVVFDASPERRKEMLHNAALRGEQMKGHAATIEDQVRRMIADWGEAGEIDLLDFFAELTIY
TSSACLIGKKFRDQLDGRFAKLYHELERGTDPLAYVDPYLPIESFRRRDEARNGLVALVADIMNGRIANPPTD
KSDRDMLDVLIAVKAETGTPRFSADEITGMFISMMFAGHHTSSGTASWTLIELMRHRDAYAAVIDELDELYGD
GRSVSFHALRQIPQLENVLKETLRLHPPLIILMRVAKGEFEVQGHRIHEGDLVAASPAISNRIPEDFPDPHDF
VPARYEQPRQEDLLNRWTWIPFGAGRHRCVGAAFAIMQIKAIFSVLLREYEFEMAQPPESYRNDHSKMVVQLA
QPACVRYRRRTGV
>CYP51B1_Mycobacterium tuberculosis T00200958 2672894842
MSAVALPRVSGGHDEHGHLEEFRTDPIGLMQRVRDECGDVGTFQLAGKQVVLLSGSHANEFFFRAGDDDLDQA
KAYPFMTPIFGEGVVFDASPERRKEMLHNAALRGEQMKGHAATIEDQVRRMIADWGEAGEIDLLDFFAELTIY
TSSACLIGKKFRDQLDGRFAKLYHELERGTDPLAYVDPYLPIESFRRRDEARNGLVALVADIMNGRIANPPTD
KSDRDMLDVLIAVKAETGTPRFSADEITGMFISMMFAGHHTSSGTASWTLIELMRHRDAYAAVIDELDELYGD
GRSVSFHALRQIPQLENVLKETLRLHPPLIILMRVAKGEFEVQGHRIHEGDLVAASPAISNRIPEDFPDPHDF
VPARYEQPRQEDLLNRWTWIPFGAGRHRCVGAAFAIMQIKAIFSVLLREYEFEMAQPPESYRNDHSKMVVQLA
QPACVRYRRRTGV
>CYP51B1_Mycobacterium tuberculosis T09601545 2703557456
MSAVALPRVSGGHDEHGHLEEFRTDPIGLMQRVRDECGDVGTFQLAGKQVVLLSGSHANEFFFRAGDDDLDQA
KAYPFMTPIFGEGVVFDASPERRKEMLHNAALRGEQMKGHAATIEDQVRRMIADWGEAGEIDLLDFFAELTIY
TSSACLIGKKFRDQLDGRFAKLYHELERGTDPLAYVDPYLPIESFRRRDEARNGLVALVADIMNGRIANPPTD
KSDRDMLDVLIAVKAETGTPRFSADEITGMFISMMFAGHHTSSGTASWTLIELMRHRDAYAAVIDELDELYGD
GRSVSFHALRQIPQLENVLKETLRLHPPLIILMRVAKGEFEVQGHRIHEGDLVAASPAISNRIPEDFPDPHDF
VPARYEQPRQEDLLNRWTWIPFGAGRHRCVGAAFAIMQIKAIFSVLLREYEFEMAQPPESYRNDHSKMVVQLA
QPACVRYRRRTGV
>CYP51B1_Mycobacterium tuberculosis T09800777 2703578360
MSAVALPRVSGGHDEHGHLEEFRTDPIGLMQRVRDECGDVGTFQLAGKQVVLLSGSHANEFFFRAGDDDLDQA
KAYPFMTPIFGEGVVFDASPERRKEMLHNAALRGEQMKGHAATIEDQVRRMIADWGEAGEIDLLDFFAELTIY
TSSACLIGKKFRDQLDGRFAKLYHELERGTDPLAYVDPYLPIESFRRRDEARNGLVALVADIMNGRIANPPTD
KSDRDMLDVLIAVKAETGTPRFSADEITGMFISMMFAGHHTSSGTASWTLIELMRHRDAYAAVIDELDELYGD
GRSVSFHALRQIPQLENVLKETLRLHPPLIILMRVAKGEFEVQGHRIHEGDLVAASPAISNRIPEDFPDPHDF
VPARYEQPRQEDLLNRWTWIPFGAGRHRCVGAAFAIMQIKAIFSVLLREYEFEMAQPPESYRNDHSKMVVQLA
QPACVRYRRRTGV
>CYP51B1_Mycobacterium tuberculosis T09800928 2703581637
MSAVALPRVSGGHDEHGHLEEFRTDPIGLMQRVRDECGDVGTFQLAGKQVVLLSGSHANEFFFRAGDDDLDQA
KAYPFMTPIFGEGVVFDASPERRKEMLHNAALRGEQMKGHAATIEDQVRRMIADWGEAGEIDLLDFFAELTIY
TSSACLIGKKFRDQLDGRFAKLYHELERGTDPLAYVDPYLPIESFRRRDEARNGLVALVADIMNGRIANPPTD
KSDRDMLDVLIAVKAETGTPRFSADEITGMFISMMFAGHHTSSGTASWTLIELMRHRDAYAAVIDELDELYGD
GRSVSFHALRQIPQLENVLKETLRLHPPLIILMRVAKGEFEVQGHRIHEGDLVAASPAISNRIPEDFPDPHDF
VPARYEQPRQEDLLNRWTWIPFGAGRHRCVGAAFAIMQIKAIFSVLLREYEFEMAQPPESYRNDHSKMVVQLA
QPACVRYRRRTGV
>CYP51B1_Mycobacterium tuberculosis T09900050 2674943666
MSAVALPRVSGGHDEHGHLEEFRTDPIGLMQRVRDECGDVGTFQLAGKQVVLLSGSHANEFFFRAGDDDLDQA
KAYPFMTPIFGEGVVFDASPERRKEMLHNAALRGEQMKGHAATIEDQVRRMIADWGEAGEIDLLDFFAELTIY

TSSACLIGKKFRDQLDGRFAKLYHELERGTDPLAYVDPYLPIESFRRRDEARNGLVALVADIMNGRIANPPTD
KSDRDMLDVLIAVKAETGTPRFSADEITGMFISMMFAGHHTSSGTASWTLIELMRHRDAYAAVIDELDELYGD
GRSVSFHALRQIPQLENVLKETLRLHPPLIILMRVAKGEFEVQGHRIHEGDLVAASPAISNRIPEDFPDPHDF
VPARYEQPRQEDLLNRWTWIPFGAGRHRCVGAAFAIMQIKAIFSVLLREYEFEMAQPPESYRNDHSKMVVQLA
QPACVRYRRRTGV
>CYP51B1_Mycobacterium tuberculosis T09900518 2674818452
MSAVALPRVSGGHDEHGHLEEFRTDPIGLMQRVRDECGDVGTFQLAGKQVVLLSGSHANEFFFRAGDDDLDQA
KAYPFMTPIFGEGVVFDASPERRKEMLHNAALRGEQMKGHAATIEDQVRRMIADWGEAGEIDLLDFFAELTIY
TSSACLIGKKFRDQLDGRFAKLYHELERGTDPLAYVDPYLPIESFRRRDEARNGLVALVADIMNGRIANPPTD
KSDRDMLDVLIAVKAETGTPRFSADEITGMFISMMFAGHHTSSGTASWTLIELMRHRDAYAAVIDELDELYGD
GRSVSFHALRQIPQLENVLKETLRLHPPLIILMRVAKGEFEVQGHRIHEGDLVAASPAISNRIPEDFPDPHDF
VPARYEQPRQEDLLNRWTWIPFGAGRHRCVGAAFAIMQIKAIFSVLLREYEFEMAQPPESYRNDHSKMVVQLA
QPACVRYRRRTGV
>CYP51B1_Mycobacterium tuberculosis T09901127 2703573533
MSAVALPRVSGGHDEHGHLEEFRTDPIGLMQRVRDECGDVGTFQLAGKQVVLLSGSHANEFFFRAGDDDLDQA
KAYPFMTPIFGEGVVFDASPERRKEMLHNAALRGEQMKGHAATIEDQVRRMIADWGEAGEIDLLDFFAELTIY
TSSACLIGKKFRDQLDGRFAKLYHELERGTDPLAYVDPYLPIESFRRRDEARNGLVALVADIMNGRIANPPTD
KSDRDMLDVLIAVKAETGTPRFSADEITGMFISMMFAGHHTSSGTASWTLIELMRHRDAYAAVIDELDELYGD
GRSVSFHALRQIPQLENVLKETLRLHPPLIILMRVAKGEFEVQGHRIHEGDLVAASPAISNRIPEDFPDPHDF
VPARYEQPRQEDLLNRWTWIPFGAGRHRCVGAAFAIMQIKAIFSVLLREYEFEMAQPPESYRNDHSKMVVQLA
QPACVRYRRRTGV
>CYP51B1_Mycobacterium tuberculosis T17 2621970987
MSAVALPRVSGGHDEHGHLEEFRTDPIGLMQRVRDECGDVGTFQLAGKQVVLLSGSHANEFFFRAGDDDLDQA
KAYPFMTPIFGEGVVFDASPERRKEMLHNAALRGEQMKGHAATIEDQVRRMIADWGEAGEIDLLDFFAELTIY
TSSACLIGKKFRDQLDGRFAKLYHELERGTDPLAYVDPYLPIESFRRRDEARNGLVALVADIMNGRIANPPTD
KSDRDMLDVLIAVKAETGTPRFSADEITGMFISMMFAGHHTSSGTASWTLIELMRHRDAYAAVIDELDELYGD
GRSVSFHALRQIPQLENVLKETLRLHPPLIILMRVAKGEFEVQGHRIHEGDLVAASPAISNRIPEDFPDPHDF
VPARYEQPRQEDLLNRWTWIPFGAGRHRCVGAAFAIMQIKAIFSVLLREYEFEMAQPPESYRNDHSKMVVQLA
QPACVRYRRRTGV
>CYP51B1_Mycobacterium tuberculosis T17 unfinished sequence 643048618
MSAVALPRVSGGHDEHGHLEEFRTDPIGLMQRVRDECGDVGTFQLAGKQVVLLSGSHANEFFFRAGDDDLDQA
KAYPFMTPIFGEGVVFDASPERRKEMLHNAALRGEQMKGHAATIEDQVRRMIADWGEAGEIDLLDFFAELTIY
TSSACLIGKKFRDQLDGRFAKLYHELERGTDPLAYVDPYLPIESFRRRDEARNGLVALVADIMNGRIANPPTD
KSDRDMLDVLIAVKAETGTPRFSADEITGMFISMMFAGHHTSSGTASWTLIELMRHRDAYAAVIDELDELYGD
GRSVSFHALRQIPQLENVLKETLRLHPPLIILMRVAKGEFEVQGHRIHEGDLVAASPAISNRIPEDFPDPHDF
VPARYEQPRQEDLLNRWTWIPFGAGRHRCVGAAFAIMQIKAIFSVLLREYEFEMAQPPESYRNDHSKMVVQLA
QPACVRYRRRTGV
>CYP51B1_Mycobacterium tuberculosis T46 2574760327
MSAVALPRVSGGHDEHGHLEEFRTDPIGLMQRVRDECGDVGTFQLAGKQVVLLSGSHANEFFFRAGDDDLDQA
KAYPFMTPIFGEGVVFDASPERRKEMLHNAALRGEQMKGHAATIEDQVRRMIADWGEAGEIDLLDFFAELTIY
TSSACLIGKKFRDQLDGRFAKLYHELERGTDPLAYVDPYLPIESFRRRDEARNGLVALVADIMNGRIANPPTD
KSDRDMLDVLIAVKAETGTPRFSADEITGMFISMMFAGHHTSSGTASWTLIELMRHRDAYAAVIDELDELYGD
GRSVSFHALRQIPQLENVLKETLRLHPPLIILMRVAKGEFEVQGHRIHEGDLVAASPAISNRIPEDFPDPHDF
VPARYEQPRQEDLLNRWTWIPFGAGRHRCVGAAFAIMQIKAIFSVLLREYEFEMAQPPESYRNDHSKMVVQLA
QPACVRYRRRTGV
>CYP51B1_Mycobacterium tuberculosis T46 646009270
MSAVALPRVSGGHDEHGHLEEFRTDPIGLMQRVRDECGDVGTFQLAGKQVVLLSGSHANEFFFRAGDDDLDQA
KAYPFMTPIFGEGVVFDASPERRKEMLHNAALRGEQMKGHAATIEDQVRRMIADWGEAGEIDLLDFFAELTIY
TSSACLIGKKFRDQLDGRFAKLYHELERGTDPLAYVDPYLPIESFRRRDEARNGLVALVADIMNGRIANPPTD
KSDRDMLDVLIAVKAETGTPRFSADEITGMFISMMFAGHHTSSGTASWTLIELMRHRDAYAAVIDELDELYGD
GRSVSFHALRQIPQLENVLKETLRLHPPLIILMRVAKGEFEVQGHRIHEGDLVAASPAISNRIPEDFPDPHDF
VPARYEQPRQEDLLNRWTWIPFGAGRHRCVGAAFAIMQIKAIFSVLLREYEFEMAQPPESYRNDHSKMVVQLA
QPACVRYRRRTGV
>CYP51B1_Mycobacterium tuberculosis T85 2621972378
MSAVALPRVSGGHDEHGHLEEFRTDPIGLMQRVRDECGDVGTFQLAGKQVVLLSGSHANEFFFRAGDDDLDQA
KAYPFMTPIFGEGVVFDASPERRKEMLHNAALRGEQMKGHAATIEDQVRRMIADWGEAGEIDLLDFFAELTIY
TSSACLIGKKFRDQLDGRFAKLYHELERGTDPLAYVDPYLPIESFRRRDEARNGLVALVADIMNGRIANPPTD
KSDRDMLDVLIAVKAETGTPRFSADEITGMFISMMFAGHHTSSGTASWTLIELMRHRDAYAAVIDELDELYGD

GRSVSFHALRQIPQLENVLKETLRLHPPLIILMRVAKGEFEVQGHRIHEGDLVAASPAISNRIPEDFPDPHDF
VPARYEQPRQEDLLNRWTWIPFGAGRHRCVGAAFAIMQIKAIFSVLLREYEFEMAQPPESYRNDHSKMVVQLA
QPACVRYRRRTGV
>CYP51B1_Mycobacterium tuberculosis T85 unfinished sequence 643034988
MSAVALPRVSGGHDEHGHLEEFRTDPIGLMQRVRDECGDVGTFQLAGKQVVLLSGSHANEFFFRAGDDDLDQA
KAYPFMTPIFGEGVVFDASPERRKEMLHNAALRGEQMKGHAATIEDQVRRMIADWGEAGEIDLLDFFAELTIY
TSSACLIGKKFRDQLDGRFAKLYHELERGTDPLAYVDPYLPIESFRRRDEARNGLVALVADIMNGRIANPPTD
KSDRDMLDVLIAVKAETGTPRFSADEITGMFISMMFAGHHTSSGTASWTLIELMRHRDAYAAVIDELDELYGD
GRSVSFHALRQIPQLENVLKETLRLHPPLIILMRVAKGEFEVQGHRIHEGDLVAASPAISNRIPEDFPDPHDF
VPARYEQPRQEDLLNRWTWIPFGAGRHRCVGAAFAIMQIKAIFSVLLREYEFEMAQPPESYRNDHSKMVVQLA
QPACVRYRRRTGV
>CYP51B1_Mycobacterium tuberculosis T92 unfinished sequence 643027192
MSAVALPRVSGGHDEHGHLEEFRTDPIGLMQRVRDECGDVGTFQLAGKQVVLLSGSHANEFFFRAGDDDLDQA
KAYPFMTPIFGEGVVFDASPERRKEMLHNAALRGEQMKGHAATIEDQVRRMIADWGEAGEIDLLDFFAELTIY
TSSACLIGKKFRDQLDGRFAKLYHELERGTDPLAYVDPYLPIESFRRRDEARNGLVALVADIMNGRIANPPTD
KSDRDMLDVLIAVKAETGTPRFSADEITGMFISMMFAGHHTSSGTASWTLIELMRHRDAYAAVIDELDELYGD
GRSVSFHALRQIPQLENVLKETLRLHPPLIILMRVAKGEFEVQGHRIHEGDLVAASPAISNRIPEDFPDPHDF
VPARYEQPRQEDLLNRWTWIPFGAGRHRCVGAAFAIMQIKAIFSVLLREYEFEMAQPPESYRNDHSKMVVQLA
QPACVRYRRRTGV
>CYP51B1_Mycobacterium tuberculosis T92 2584887346
MSAVALPRVSGGHDEHGHLEEFRTDPIGLMQRVRDECGDVGTFQLAGKQVVLLSGSHANEFFFRAGDDDLDQA
KAYPFMTPIFGEGVVFDASPERRKEMLHNAALRGEQMKGHAATIEDQVRRMIADWGEAGEIDLLDFFAELTIY
TSSACLIGKKFRDQLDGRFAKLYHELERGTDPLAYVDPYLPIESFRRRDEARNGLVALVADIMNGRIANPPTD
KSDRDMLDVLIAVKAETGTPRFSADEITGMFISMMFAGHHTSSGTASWTLIELMRHRDAYAAVIDELDELYGD
GRSVSFHALRQIPQLENVLKETLRLHPPLIILMRVAKGEFEVQGHRIHEGDLVAASPAISNRIPEDFPDPHDF
VPARYEQPRQEDLLNRWTWIPFGAGRHRCVGAAFAIMQIKAIFSVLLREYEFEMAQPPESYRNDHSKMVVQLA
QPACVRYRRRTGV
>CYP51B1_Mycobacterium tuberculosis tahitimassiliensis MT11 2691047151
MSAVALPRVSGGHDEHGHLEEFRTDPIGLMQRVRDECGDVGTFQLAGKQVVLLSGSHANEFFFRAGDDDLDQA
KAYPFMTPIFGEGVVFDASPERRKEMLHNAALRGEQMKGHAATIEDQVRRMIADWGEAGEIDLLDFFAELTIY
TSSACLIGKKFRDQLDGRFAKLYHELERGTDPLAYVDPYLPIESFRRRDEARNGLVALVADIMNGRIANPPTD
KSDRDMLDVLIAVKAETGTPRFSADEITGMFISMMFAGHHTSSGTASWTLIELMRHRDAYAAVIDELDELYGD
GRSVSFHALRQIPQLENVLKETLRLHPPLIILMRVAKGEFEVQGHRIHEGDLVAASPAISNRIPEDFPDPHDF
VPARYEQPRQEDLLNRWTWIPFGAGRHRCVGAAFAIMQIKAIFSVLLREYEFEMAQPPESYRNDHSKMVVQLA
QPACVRYRRRTGV
>CYP51B1_Mycobacterium tuberculosis tahitimassiliensis MT43 2691023620
MSAVALPRVSGGHDEHGHLEEFRTDPIGLMQRVRDECGDVGTFQLAGKQVVLLSGSHANEFFFRAGDDDLDQA
KAYPFMTPIFGEGVVFDASPERRKEMLHNAALRGEQMKGHAATIEDQVRRMIADWGEAGEIDLLDFFAELTIY
TSSACLIGKKFRDQLDGRFAKLYHELERGTDPLAYVDPYLPIESFRRRDEARNGLVALVADIMNGRIANPPTD
KSDRDMLDVLIAVKAETGTPRFSADEITGMFISMMFAGHHTSSGTASWTLIELMRHRDAYAAVIDELDELYGD
GRSVSFHALRQIPQLENVLKETLRLHPPLIILMRVAKGEFEVQGHRIHEGDLVAASPAISNRIPEDFPDPHDF
VPARYEQPRQEDLLNRWTWIPFGAGRHRCVGAAFAIMQIKAIFSVLLREYEFEMAQPPESYRNDHSKMVVQLA
QPACVRYRRRTGV
>CYP51B1_Mycobacterium tuberculosis TB_RSA01 2584710334
MSAVALPRVSGGHDEHGHLEEFRTDPIGLMQRVRDECGDVGTFQLAGKQVVLLSGSHANEFFFRAGDDDLDQA
KAYPFMTPIFGEGVVFDASPERRKEMLHNAALRGEQMKGHAATIEDQVRRMIADWGEAGEIDLLDFFAELTIY
TSSACLIGKKFRDQLDGRFAKLYHELERGTDPLAYVDPYLPIESFRRRDEARNGLVALVADIMNGRIANPPTD
KSDRDMLDVLIAVKAETGTPRFSADEITGMFISMMFAGHHTSSGTASWTLIELMRHRDAYAAVIDELDELYGD
GRSVSFHALRQIPQLENVLKETLRLHPPLIILMRVAKGEFEVQGHRIHEGDLVAASPAISNRIPEDFPDPHDF
VPARYEQPRQEDLLNRWTWIPFGAGRHRCVGAAFAIMQIKAIFSVLLREYEFEMAQPPESYRNDHSKMVVQLA
QPACVRYRRRTGV
>CYP51B1_Mycobacterium tuberculosis TB_RSA02 2603933820
MSAVALPRVSGGHDEHGHLEEFRTDPIGLMQRVRDECGDVGTFQLAGKQVVLLSGSHANEFFFRAGDDDLDQA
KAYPFMTPIFGEGVVFDASPERRKEMLHNAALRGEQMKGHAATIEDQVRRMIADWGEAGEIDLLDFFAELTIY
TSSACLIGKKFRDQLDGRFAKLYHELERGTDPLAYVDPYLPIESFRRRDEARNGLVALVADIMNGRIANPPTD
KSDRDMLDVLIAVKAETGTPRFSADEITGMFISMMFAGHHTSSGTASWTLIELMRHRDAYAAVIDELDELYGD
GRSVSFHALRQIPQLENVLKETLRLHPPLIILMRVAKGEFEVQGHRIHEGDLVAASPAISNRIPEDFPDPHDF

VPARYEQPRQEDLLNRWTWIPFGAGRHRCVGAAFAIMQIKAIFSVLLREYEFEMAQPPESYRNDHSKMVVQLA
QPACVRYRRRTGV
>CYP51B1_Mycobacterium tuberculosis TB_RSA03 2574699670
MSAVALPRVSGGHDEHGHLEEFRTDPIGLMQRVRDECGDVGTFQLAGKQVVLLSGSHANEFFFRAGDDDLDQA
KAYPFMTPIFGEGVVFDASPERRKEMLHNAALRGEQMKGHAATIEDQVRRMIADWGEAGEIDLLDFFAELTIY
TSSACLIGKKFRDQLDGRFAKLYHELERGTDPLAYVDPYLPIESFRRRDEARNGLVALVADIMNGRIANPPTD
KSDRDMLDVLIAVKAETGTPRFSADEITGMFISMMFAGHHTSSGTASWTLIELMRHRDAYAAVIDELDELYGD
GRSVSFHALRQIPQLENVLKETLRLHPPLIILMRVAKGEFEVQGHRIHEGDLVAASPAISNRIPEDFPDPHDF
VPARYEQPRQEDLLNRWTWIPFGAGRHRCVGAAFAIMQIKAIFSVLLREYEFEMAQPPESYRNDHSKMVVQLA
QPACVRYRRRTGV
>CYP51B1_Mycobacterium tuberculosis TB_RSA04 2603229363
MSAVALPRVSGGHDEHGHLEEFRTDPIGLMQRVRDECGDVGTFQLAGKQVVLLSGSHANEFFFRAGDDDLDQA
KAYPFMTPIFGEGVVFDASPERRKEMLHNAALRGEQMKGHAATIEDQVRRMIADWGEAGEIDLLDFFAELTIY
TSSACLIGKKFRDQLDGRFAKLYHELERGTDPLAYVDPYLPIESFRRRDEARNGLVALVADIMNGRIANPPTD
KSDRDMLDVLIAVKAETGTPRFSADEITGMFISMMFAGHHTSSGTASWTLIELMRHRDAYAAVIDELDELYGD
GRSVSFHALRQIPQLENVLKETLRLHPPLIILMRVAKGEFEVQGHRIHEGDLVAASPAISNRIPEDFPDPHDF
VPARYEQPRQEDLLNRWTWIPFGAGRHRCVGAAFAIMQIKAIFSVLLREYEFEMAQPPESYRNDHSKMVVQLA
QPACVRYRRRTGV
>CYP51B1_Mycobacterium tuberculosis TB_RSA06 2575959206
MSAVALPRVSGGHDEHGHLEEFRTDPIGLMQRVRDECGDVGTFQLAGKQVVLLSGSHANEFFFRAGDDDLDQA
KAYPFMTPIFGEGVVFDASPERRKEMLHNAALRGEQMKGHAATIEDQVRRMIADWGEAGEIDLLDFFAELTIY
TSSACLIGKKFRDQLDGRFAKLYHELERGTDPLAYVDPYLPIESFRRRDEARNGLVALVADIMNGRIANPPTD
KSDRDMLDVLIAVKAETGTPRFSADEITGMFISMMFAGHHTSSGTASWTLIELMRHRDAYAAVIDELDELYGD
GRSVSFHALRQIPQLENVLKETLRLHPPLIILMRVAKGEFEVQGHRIHEGDLVAASPAISNRIPEDFPDPHDF
VPARYEQPRQEDLLNRWTWIPFGAGRHRCVGAAFAIMQIKAIFSVLLREYEFEMAQPPESYRNDHSKMVVQLA
QPACVRYRRRTGV
>CYP51B1_Mycobacterium tuberculosis TB_RSA07 2577871676
MSAVALPRVSGGHDEHGHLEEFRTDPIGLMQRVRDECGDVGTFQLAGKQVVLLSGSHANEFFFRAGDDDLDQA
KAYPFMTPIFGEGVVFDASPERRKEMLHNAALRGEQMKGHAATIEDQVRRMIADWGEAGEIDLLDFFAELTIY
TSSACLIGKKFRDQLDGRFAKLYHELERGTDPLAYVDPYLPIESFRRRDEARNGLVALVADIMNGRIANPPTD
KSDRDMLDVLIAVKAETGTPRFSADEITGMFISMMFAGHHTSSGTASWTLIELMRHRDAYAAVIDELDELYGD
GRSVSFHALRQIPQLENVLKETLRLHPPLIILMRVAKGEFEVQGHRIHEGDLVAASPAISNRIPEDFPDPHDF
VPARYEQPRQEDLLNRWTWIPFGAGRHRCVGAAFAIMQIKAIFSVLLREYEFEMAQPPESYRNDHSKMVVQLA
QPACVRYRRRTGV
>CYP51B1_Mycobacterium tuberculosis TB_RSA08 2603385510
MSAVALPRVSGGHDEHGHLEEFRTDPIGLMQRVRDECGDVGTFQLAGKQVVLLSGSHANEFFFRAGDDDLDQA
KAYPFMTPIFGEGVVFDASPERRKEMLHNAALRGEQMKGHAATIEDQVRRMIADWGEAGEIDLLDFFAELTIY
TSSACLIGKKFRDQLDGRFAKLYHELERGTDPLAYVDPYLPIESFRRRDEARNGLVALVADIMNGRIANPPTD
KSDRDMLDVLIAVKAETGTPRFSADEITGMFISMMFAGHHTSSGTASWTLIELMRHRDAYAAVIDELDELYGD
GRSVSFHALRQIPQLENVLKETLRLHPPLIILMRVAKGEFEVQGHRIHEGDLVAASPAISNRIPEDFPDPHDF
VPARYEQPRQEDLLNRWTWIPFGAGRHRCVGAAFAIMQIKAIFSVLLREYEFEMAQPPESYRNDHSKMVVQLA
QPACVRYRRRTGV
>CYP51B1_Mycobacterium tuberculosis TB_RSA09 2576885425
MSAVALPRVSGGHDEHGHLEEFRTDPIGLMQRVRDECGDVGTFQLAGKQVVLLSGSHANEFFFRAGDDDLDQA
KAYPFMTPIFGEGVVFDASPERRKEMLHNAALRGEQMKGHAATIEDQVRRMIADWGEAGEIDLLDFFAELTIY
TSSACLIGKKFRDQLDGRFAKLYHELERGTDPLAYVDPYLPIESFRRRDEARNGLVALVADIMNGRIANPPTD
KSDRDMLDVLIAVKAETGTPRFSADEITGMFISMMFAGHHTSSGTASWTLIELMRHRDAYAAVIDELDELYGD
GRSVSFHALRQIPQLENVLKETLRLHPPLIILMRVAKGEFEVQGHRIHEGDLVAASPAISNRIPEDFPDPHDF
VPARYEQPRQEDLLNRWTWIPFGAGRHRCVGAAFAIMQIKAIFSVLLREYEFEMAQPPESYRNDHSKMVVQLA
QPACVRYRRRTGV
>CYP51B1_Mycobacterium tuberculosis TB_RSA10 2605000817
MSAVALPRVSGGHDEHGHLEEFRTDPIGLMQRVRDECGDVGTFQLAGKQVVLLSGSHANEFFFRAGDDDLDQA
KAYPFMTPIFGEGVVFDASPERRKEMLHNAALRGEQMKGHAATIEDQVRRMIADWGEAGEIDLLDFFAELTIY
TSSACLIGKKFRDQLDGRFAKLYHELERGTDPLAYVDPYLPIESFRRRDEARNGLVALVADIMNGRIANPPTD
KSDRDMLDVLIAVKAETGTPRFSADEITGMFISMMFAGHHTSSGTASWTLIELMRHRDAYAAVIDELDELYGD
GRSVSFHALRQIPQLENVLKETLRLHPPLIILMRVAKGEFEVQGHRIHEGDLVAASPAISNRIPEDFPDPHDF
VPARYEQPRQEDLLNRWTWIPFGAGRHRCVGAAFAIMQIKAIFSVLLREYEFEMAQPPESYRNDHSKMVVQLA
QPACVRYRRRTGV

>CYP51B1_Mycobacterium tuberculosis TB_RSA100 2603389900
MSAVALPRVSGGHDEHGHLEEFRTDPIGLMQRVRDECGDVGTFQLAGKQVVLLSGSHANEFFFRAGDDDLDQA
KAYPFMTPIFGEGVVFDASPERRKEMLHNAALRGEQMKGHAATIEDQVRRMIADWGEAGEIDLLDFFAELTIY
TSSACLIGKKFRDQLDGRFAKLYHELERGTDPLAYVDPYLPIESFRRRDEARNGLVALVADIMNGRIANPPTD
KSDRDMLDVLIAVKAETGTPRFSADEITGMFISMMFAGHHTSSGTASWTLIELMRHRDAYAAVIDELDELYGD
GRSVSFHALRQIPQLENVLKETLRLHPPLIILMRVAKGEFEVQGHRIHEGDLVAASPAISNRIPEDFPDPHDF
VPARYEQPRQEDLLNRWTWIPFGAGRHRCVGAAFAIMQIKAIFSVLLREYEFEMAQPPESYRNDHSKMVVQLA
QPACVRYRRRTGV
>CYP51B1_Mycobacterium tuberculosis TB_RSA101 2605345105
MSAVALPRVSGGHDEHGHLEEFRTDPIGLMQRVRDECGDVGTFQLAGKQVVLLSGSHANEFFFRAGDDDLDQA
KAYPFMTPIFGEGVVFDASPERRKEMLHNAALRGEQMKGHAATIEDQVRRMIADWGEAGEIDLLDFFAELTIY
TSSACLIGKKFRDQLDGRFAKLYHELERGTDPLAYVDPYLPIESFRRRDEARNGLVALVADIMNGRIANPPTD
KSDRDMLDVLIAVKAETGTPRFSADEITGMFISMMFAGHHTSSGTASWTLIELMRHRDAYAAVIDELDELYGD
GRSVSFHALRQIPQLENVLKETLRLHPPLIILMRVAKGEFEVQGHRIHEGDLVAASPAISNRIPEDFPDPHDF
VPARYEQPRQEDLLNRWTWIPFGAGRHRCVGAAFAIMQIKAIFSVLLREYEFEMAQPPESYRNDHSKMVVQLA
QPACVRYRRRTGV
>CYP51B1_Mycobacterium tuberculosis TB_RSA102 2575861687
MSAVALPRVSGGHDEHGHLEEFRTDPIGLMQRVRDECGDVGTFQLAGKQVVLLSGSHANEFFFRAGDDDLDQA
KAYPFMTPIFGEGVVFDASPERRKEMLHNAALRGEQMKGHAATIEDQVRRMIADWGEAGEIDLLDFFAELTIY
TSSACLIGKKFRDQLDGRFAKLYHELERGTDPLAYVDPYLPIESFRRRDEARNGLVALVADIMNGRIANPPTD
KSDRDMLDVLIAVKAETGTPRFSADEITGMFISMMFAGHHTSSGTASWTLIELMRHRDAYAAVIDELDELYGD
GRSVSFHALRQIPQLENVLKETLRLHPPLIILMRVAKGEFEVQGHRIHEGDLVAASPAISNRIPEDFPDPHDF
VPARYEQPRQEDLLNRWTWIPFGAGRHRCVGAAFAIMQIKAIFSVLLREYEFEMAQPPESYRNDHSKMVVQLA
QPACVRYRRRTGV
>CYP51B1_Mycobacterium tuberculosis TB_RSA103 2605348025
MSAVALPRVSGGHDEHGHLEEFRTDPIGLMQRVRDECGDVGTFQLAGKQVVLLSGSHANEFFFRAGDDDLDQA
KAYPFMTPIFGEGVVFDASPERRKEMLHNAALRGEQMKGHAATIEDQVRRMIADWGEAGEIDLLDFFAELTIY
TSSACLIGKKFRDQLDGRFAKLYHELERGTDPLAYVDPYLPIESFRRRDEARNGLVALVADIMNGRIANPPTD
KSDRDMLDVLIAVKAETGTPRFSADEITGMFISMMFAGHHTSSGTASWTLIELMRHRDAYAAVIDELDELYGD
GRSVSFHALRQIPQLENVLKETLRLHPPLIILMRVAKGEFEVQGHRIHEGDLVAASPAISNRIPEDFPDPHDF
VPARYEQPRQEDLLNRWTWIPFGAGRHRCVGAAFAIMQIKAIFSVLLREYEFEMAQPPESYRNDHSKMVVQLA
QPACVRYRRRTGV
>CYP51B1_Mycobacterium tuberculosis TB_RSA104 2584805043
MSAVALPRVSGGHDEHGHLEEFRTDPIGLMQRVRDECGDVGTFQLAGKQVVLLSGSHANEFFFRAGDDDLDQA
KAYPFMTPIFGEGVVFDASPERRKEMLHNAALRGEQMKGHAATIEDQVRRMIADWGEAGEIDLLDFFAELTIY
TSSACLIGKKFRDQLDGRFAKLYHELERGTDPLAYVDPYLPIESFRRRDEARNGLVALVADIMNGRIANPPTD
KSDRDMLDVLIAVKAETGTPRFSADEITGMFISMMFAGHHTSSGTASWTLIELMRHRDAYAAVIDELDELYGD
GRSVSFHALRQIPQLENVLKETLRLHPPLIILMRVAKGEFEVQGHRIHEGDLVAASPAISNRIPEDFPDPHDF
VPARYEQPRQEDLLNRWTWIPFGAGRHRCVGAAFAIMQIKAIFSVLLREYEFEMAQPPESYRNDHSKMVVQLA
QPACVRYRRRTGV
>CYP51B1_Mycobacterium tuberculosis TB_RSA105 2603937642
MSAVALPRVSGGHDEHGHLEEFRTDPIGLMQRVRDECGDVGTFQLAGKQVVLLSGSHANEFFFRAGDDDLDQA
KAYPFMTPIFGEGVVFDASPERRKEMLHNAALRGEQMKGHAATIEDQVRRMIADWGEAGEIDLLDFFAELTIY
TSSACLIGKKFRDQLDGRFAKLYHELERGTDPLAYVDPYLPIESFRRRDEARNGLVALVADIMNGRIANPPTD
KSDRDMLDVLIAVKAETGTPRFSADEITGMFISMMFAGHHTSSGTASWTLIELMRHRDAYAAVIDELDELYGD
GRSVSFHALRQIPQLENVLKETLRLHPPLIILMRVAKGEFEVQGHRIHEGDLVAASPAISNRIPEDFPDPHDF
VPARYEQPRQEDLLNRWTWIPFGAGRHRCVGAAFAIMQIKAIFSVLLREYEFEMAQPPESYRNDHSKMVVQLA
QPACVRYRRRTGV
>CYP51B1_Mycobacterium tuberculosis TB_RSA106 2603942048
MSAVALPRVSGGHDEHGHLEEFRTDPIGLMQRVRDECGDVGTFQLAGKQVVLLSGSHANEFFFRAGDDDLDQA
KAYPFMTPIFGEGVVFDASPERRKEMLHNAALRGEQMKGHAATIEDQVRRMIADWGEAGEIDLLDFFAELTIY
TSSACLIGKKFRDQLDGRFAKLYHELERGTDPLAYVDPYLPIESFRRRDEARNGLVALVADIMNGRIANPPTD
KSDRDMLDVLIAVKAETGTPRFSADEITGMFISMMFAGHHTSSGTASWTLIELMRHRDAYAAVIDELDELYGD
GRSVSFHALRQIPQLENVLKETLRLHPPLIILMRVAKGEFEVQGHRIHEGDLVAASPAISNRIPEDFPDPHDF
VPARYEQPRQEDLLNRWTWIPFGAGRHRCVGAAFAIMQIKAIFSVLLREYEFEMAQPPESYRNDHSKMVVQLA
QPACVRYRRRTGV
>CYP51B1_Mycobacterium tuberculosis TB_RSA107 2584896253

MSAVALPRVSGGHDEHGHLEEFRTDPIGLMQRVRDECGDVGTFQLAGKQVVLLSGSHANEFFFRAGDDDLDQA
KAYPFMTPIFGEGVVFDASPERRKEMLHNAALRGEQMKGHAATIEDQVRRMIADWGEAGEIDLLDFFAELTIY
TSSACLIGKKFRDQLDGRFAKLYHELERGTDPLAYVDPYLPIESFRRRDEARNGLVALVADIMNGRIANPPTD
KSDRDMLDVLIAVKAETGTPRFSADEITGMFISMMFAGHHTSSGTASWTLIELMRHRDAYAAVIDELDELYGD
GRSVSFHALRQIPQLENVLKETLRLHPPLIILMRVAKGEFEVQGHRIHEGDLVAASPAISNRIPEDFPDPHDF
VPARYEQPRQEDLLNRWTWIPFGAGRHRCVGAAFAIMQIKAIFSVLLREYEFEMAQPPESYRNDHSKMVVQLA
QPACVRYRRRTGV
>CYP51B1_Mycobacterium tuberculosis TB_RSA108 2603946588
MSAVALPRVSGGHDEHGHLEEFRTDPIGLMQRVRDECGDVGTFQLAGKQVVLLSGSHANEFFFRAGDDDLDQA
KAYPFMTPIFGEGVVFDASPERRKEMLHNAALRGEQMKGHAATIEDQVRRMIADWGEAGEIDLLDFFAELTIY
TSSACLIGKKFRDQLDGRFAKLYHELERGTDPLAYVDPYLPIESFRRRDEARNGLVALVADIMNGRIANPPTD
KSDRDMLDVLIAVKAETGTPRFSADEITGMFISMMFAGHHTSSGTASWTLIELMRHRDAYAAVIDELDELYGD
GRSVSFHALRQIPQLENVLKETLRLHPPLIILMRVAKGEFEVQGHRIHEGDLVAASPAISNRIPEDFPDPHDF
VPARYEQPRQEDLLNRWTWIPFGAGRHRCVGAAFAIMQIKAIFSVLLREYEFEMAQPPESYRNDHSKMVVQLA
QPACVRYRRRTGV
>CYP51B1_Mycobacterium tuberculosis TB_RSA109 2605707935
MSAVALPRVSGGHDEHGHLEEFRTDPIGLMQRVRDECGDVGTFQLAGKQVVLLSGSHANEFFFRAGDDDLDQA
KAYPFMTPIFGEGVVFDASPERRKEMLHNAALRGEQMKGHAATIEDQVRRMIADWGEAGEIDLLDFFAELTIY
TSSACLIGKKFRDQLDGRFAKLYHELERGTDPLAYVDPYLPIESFRRRDEARNGLVALVADIMNGRIANPPTD
KSDRDMLDVLIAVKAETGTPRFSADEITGMFISMMFAGHHTSSGTASWTLIELMRHRDAYAAVIDELDELYGD
GRSVSFHALRQIPQLENVLKETLRLHPPLIILMRVAKGEFEVQGHRIHEGDLVAASPAISNRIPEDFPDPHDF
VPARYEQPRQEDLLNRWTWIPFGAGRHRCVGAAFAIMQIKAIFSVLLREYEFEMAQPPESYRNDHSKMVVQLA
QPACVRYRRRTGV
>CYP51B1_Mycobacterium tuberculosis TB_RSA11 2603237285
MSAVALPRVSGGHDEHGHLEEFRTDPIGLMQRVRDECGDVGTFQLAGKQVVLLSGSHANEFFFRAGDDDLDQA
KAYPFMTPIFGEGVVFDASPERRKEMLHNAALRGEQMKGHAATIEDQVRRMIADWGEAGEIDLLDFFAELTIY
TSSACLIGKKFRDQLDGRFAKLYHELERGTDPLAYVDPYLPIESFRRRDEARNGLVALVADIMNGRIANPPTD
KSDRDMLDVLIAVKAETGTPRFSADEITGMFISMMFAGHHTSSGTASWTLIELMRHRDAYAAVIDELDELYGD
GRSVSFHALRQIPQLENVLKETLRLHPPLIILMRVAKGEFEVQGHRIHEGDLVAASPAISNRIPEDFPDPHDF
VPARYEQPRQEDLLNRWTWIPFGAGRHRCVGAAFAIMQIKAIFSVLLREYEFEMAQPPESYRNDHSKMVVQLA
QPACVRYRRRTGV
>CYP51B1_Mycobacterium tuberculosis TB_RSA110 2604289983
MSAVALPRVSGGHDEHGHLEEFRTDPIGLMQRVRDECGDVGTFQLAGKQVVLLSGSHANEFFFRAGDDDLDQA
KAYPFMTPIFGEGVVFDASPERRKEMLHNAALRGEQMKGHAATIEDQVRRMIADWGEAGEIDLLDFFAELTIY
TSSACLIGKKFRDQLDGRFAKLYHELERGTDPLAYVDPYLPIESFRRRDEARNGLVALVADIMNGRIANPPTD
KSDRDMLDVLIAVKAETGTPRFSADEITGMFISMMFAGHHTSSGTASWTLIELMRHRDAYAAVIDELDELYGD
GRSVSFHALRQIPQLENVLKETLRLHPPLIILMRVAKGEFEVQGHRIHEGDLVAASPAISNRIPEDFPDPHDF
VPARYEQPRQEDLLNRWTWIPFGAGRHRCVGAAFAIMQIKAIFSVLLREYEFEMAQPPESYRNDHSKMVVQLA
QPACVRYRRRTGV
>CYP51B1_Mycobacterium tuberculosis TB_RSA111 2574613703
MSAVALPRVSGGHDEHGHLEEFRTDPIGLMQRVRDECGDVGTFQLAGKQVVLLSGSHANEFFFRAGDDDLDQA
KAYPFMTPIFGEGVVFDASPERRKEMLHNAALRGEQMKGHAATIEDQVRRMIADWGEAGEIDLLDFFAELTIY
TSSACLIGKKFRDQLDGRFAKLYHELERGTDPLAYVDPYLPIESFRRRDEARNGLVALVADIMNGRIANPPTD
KSDRDMLDVLIAVKAETGTPRFSADEITGMFISMMFAGHHTSSGTASWTLIELMRHRDAYAAVIDELDELYGD
GRSVSFHALRQIPQLENVLKETLRLHPPLIILMRVAKGEFEVQGHRIHEGDLVAASPAISNRIPEDFPDPHDF
VPARYEQPRQEDLLNRWTWIPFGAGRHRCVGAAFAIMQIKAIFSVLLREYEFEMAQPPESYRNDHSKMVVQLA
QPACVRYRRRTGV
>CYP51B1_Mycobacterium tuberculosis TB_RSA113 2603951262
MSAVALPRVSGGHDEHGHLEEFRTDPIGLMQRVRDECGDVGTFQLAGKQVVLLSGSHANEFFFRAGDDDLDQA
KAYPFMTPIFGEGVVFDASPERRKEMLHNAALRGEQMKGHAATIEDQVRRMIADWGEAGEIDLLDFFAELTIY
TSSACLIGKKFRDQLDGRFAKLYHELERGTDPLAYVDPYLPIESFRRRDEARNGLVALVADIMNGRIANPPTD
KSDRDMLDVLIAVKAETGTPRFSADEITGMFISMMFAGHHTSSGTASWTLIELMRHRDAYAAVIDELDELYGD
GRSVSFHALRQIPQLENVLKETLRLHPPLIILMRVAKGEFEVQGHRIHEGDLVAASPAISNRIPEDFPDPHDF
VPARYEQPRQEDLLNRWTWIPFGAGRHRCVGAAFAIMQIKAIFSVLLREYEFEMAQPPESYRNDHSKMVVQLA
QPACVRYRRRTGV
>CYP51B1_Mycobacterium tuberculosis TB_RSA114 2603395440
MSAVALPRVSGGHDEHGHLEEFRTDPIGLMQRVRDECGDVGTFQLAGKQVVLLSGSHANEFFFRAGDDDLDQA
KAYPFMTPIFGEGVVFDASPERRKEMLHNAALRGEQMKGHAATIEDQVRRMIADWGEAGEIDLLDFFAELTIY

TSSACLIGKKFRDQLDGRFAKLYHELERGTDPLAYVDPYLPIESFRRRDEARNGLVALVADIMNGRIANPPTD
KSDRDMLDVLIAVKAETGTPRFSADEITGMFISMMFAGHHTSSGTASWTLIELMRHRDAYAAVIDELDELYGD
GRSVSFHALRQIPQLENVLKETLRLHPPLIILMRVAKGEFEVQGHRIHEGDLVAASPAISNRIPEDFPDPHDF
VPARYEQPRQEDLLNRWTWIPFGAGRHRCVGAAFAIMQIKAIFSVLLREYEFEMAQPPESYRNDHSKMVVQLA
QPACVRYRRRTGV
>CYP51B1_Mycobacterium tuberculosis TB_RSA117 2605007171
MSAVALPRVSGGHDEHGHLEEFRTDPIGLMQRVRDECGDVGTFQLAGKQVVLLSGSHANEFFFRAGDDDLDQA
KAYPFMTPIFGEGVVFDASPERRKEMLHNAALRGEQMKGHAATIEDQVRRMIADWGEAGEIDLLDFFAELTIY
TSSACLIGKKFRDQLDGRFAKLYHELERGTDPLAYVDPYLPIESFRRRDEARNGLVALVADIMNGRIANPPTD
KSDRDMLDVLIAVKAETGTPRFSADEITGMFISMMFAGHHTSSGTASWTLIELMRHRDAYAAVIDELDELYGD
GRSVSFHALRQIPQLENVLKETLRLHPPLIILMRVAKGEFEVQGHRIHEGDLVAASPAISNRIPEDFPDPHDF
VPARYEQPRQEDLLNRWTWIPFGAGRHRCVGAAFAIMQIKAIFSVLLREYEFEMAQPPESYRNDHSKMVVQLA
QPACVRYRRRTGV
>CYP51B1_Mycobacterium tuberculosis TB_RSA118 2575428769
MSAVALPRVSGGHDEHGHLEEFRTDPIGLMQRVRDECGDVGTFQLAGKQVVLLSGSHANEFFFRAGDDDLDQA
KAYPFMTPIFGEGVVFDASPERRKEMLHNAALRGEQMKGHAATIEDQVRRMIADWGEAGEIDLLDFFAELTIY
TSSACLIGKKFRDQLDGRFAKLYHELERGTDPLAYVDPYLPIESFRRRDEARNGLVALVADIMNGRIANPPTD
KSDRDMLDVLIAVKAETGTPRFSADEITGMFISMMFAGHHTSSGTASWTLIELMRHRDAYAAVIDELDELYGD
GRSVSFHALRQIPQLENVLKETLRLHPPLIILMRVAKGEFEVQGHRIHEGDLVAASPAISNRIPEDFPDPHDF
VPARYEQPRQEDLLNRWTWIPFGAGRHRCVGAAFAIMQIKAIFSVLLREYEFEMAQPPESYRNDHSKMVVQLA
QPACVRYRRRTGV
>CYP51B1_Mycobacterium tuberculosis TB_RSA119 2603399787
MSAVALPRVSGGHDEHGHLEEFRTDPIGLMQRVRDECGDVGTFQLAGKQVVLLSGSHANEFFFRAGDDDLDQA
KAYPFMTPIFGEGVVFDASPERRKEMLHNAALRGEQMKGHAATIEDQVRRMIADWGEAGEIDLLDFFAELTIY
TSSACLIGKKFRDQLDGRFAKLYHELERGTDPLAYVDPYLPIESFRRRDEARNGLVALVADIMNGRIANPPTD
KSDRDMLDVLIAVKAETGTPRFSADEITGMFISMMFAGHHTSSGTASWTLIELMRHRDAYAAVIDELDELYGD
GRSVSFHALRQIPQLENVLKETLRLHPPLIILMRVAKGEFEVQGHRIHEGDLVAASPAISNRIPEDFPDPHDF
VPARYEQPRQEDLLNRWTWIPFGAGRHRCVGAAFAIMQIKAIFSVLLREYEFEMAQPPESYRNDHSKMVVQLA
QPACVRYRRRTGV
>CYP51B1_Mycobacterium tuberculosis TB_RSA12 2577088760
MSAVALPRVSGGHDEHGHLEEFRTDPIGLMQRVRDECGDVGTFQLAGKQVVLLSGSHANEFFFRAGDDDLDQA
KAYPFMTPIFGEGVVFDASPERRKEMLHNAALRGEQMKGHAATIEDQVRRMIADWGEAGEIDLLDFFAELTIY
TSSACLIGKKFRDQLDGRFAKLYHELERGTDPLAYVDPYLPIESFRRRDEARNGLVALVADIMNGRIANPPTD
KSDRDMLDVLIAVKAETGTPRFSADEITGMFISMMFAGHHTSSGTASWTLIELMRHRDAYAAVIDELDELYGD
GRSVSFHALRQIPQLENVLKETLRLHPPLIILMRVAKGEFEVQGHRIHEGDLVAASPAISNRIPEDFPDPHDF
VPARYEQPRQEDLLNRWTWIPFGAGRHRCVGAAFAIMQIKAIFSVLLREYEFEMAQPPESYRNDHSKMVVQLA
QPACVRYRRRTGV
>CYP51B1_Mycobacterium tuberculosis TB_RSA120 2577399999
MSAVALPRVSGGHDEHGHLEEFRTDPIGLMQRVRDECGDVGTFQLAGKQVVLLSGSHANEFFFRAGDDDLDQA
KAYPFMTPIFGEGVVFDASPERRKEMLHNAALRGEQMKGHAATIEDQVRRMIADWGEAGEIDLLDFFAELTIY
TSSACLIGKKFRDQLDGRFAKLYHELERGTDPLAYVDPYLPIESFRRRDEARNGLVALVADIMNGRIANPPTD
KSDRDMLDVLIAVKAETGTPRFSADEITGMFISMMFAGHHTSSGTASWTLIELMRHRDAYAAVIDELDELYGD
GRSVSFHALRQIPQLENVLKETLRLHPPLIILMRVAKGEFEVQGHRIHEGDLVAASPAISNRIPEDFPDPHDF
VPARYEQPRQEDLLNRWTWIPFGAGRHRCVGAAFAIMQIKAIFSVLLREYEFEMAQPPESYRNDHSKMVVQLA
QPACVRYRRRTGV
>CYP51B1_Mycobacterium tuberculosis TB_RSA121 2584706504
MSAVALPRVSGGHDEHGHLEEFRTDPIGLMQRVRDECGDVGTFQLAGKQVVLLSGSHANEFFFRAGDDDLDQA
KAYPFMTPIFGEGVVFDASPERRKEMLHNAALRGEQMKGHAATIEDQVRRMIADWGEAGEIDLLDFFAELTIY
TSSACLIGKKFRDQLDGRFAKLYHELERGTDPLAYVDPYLPIESFRRRDEARNGLVALVADIMNGRIANPPTD
KSDRDMLDVLIAVKAETGTPRFSADEITGMFISMMFAGHHTSSGTASWTLIELMRHRDAYAAVIDELDELYGD
GRSVSFHALRQIPQLENVLKETLRLHPPLIILMRVAKGEFEVQGHRIHEGDLVAASPAISNRIPEDFPDPHDF
VPARYEQPRQEDLLNRWTWIPFGAGRHRCVGAAFAIMQIKAIFSVLLREYEFEMAQPPESYRNDHSKMVVQLA
QPACVRYRRRTGV
>CYP51B1_Mycobacterium tuberculosis TB_RSA122 2604663239
MSAVALPRVSGGHDEHGHLEEFRTDPIGLMQRVRDECGDVGTFQLAGKQVVLLSGSHANEFFFRAGDDDLDQA
KAYPFMTPIFGEGVVFDASPERRKEMLHNAALRGEQMKGHAATIEDQVRRMIADWGEAGEIDLLDFFAELTIY
TSSACLIGKKFRDQLDGRFAKLYHELERGTDPLAYVDPYLPIESFRRRDEARNGLVALVADIMNGRIANPPTD
KSDRDMLDVLIAVKAETGTPRFSADEITGMFISMMFAGHHTSSGTASWTLIELMRHRDAYAAVIDELDELYGD

GRSVSFHALRQIPQLENVLKETLRLHPPLIILMRVAKGEFEVQGHRIHEGDLVAASPAISNRIPEDFPDPHDF
VPARYEQPRQEDLLNRWTWIPFGAGRHRCVGAAFAIMQIKAIFSVLLREYEFEMAQPPESYRNDHSKMVVQLA
QPACVRYRRRTGV
>CYP51B1_Mycobacterium tuberculosis TB_RSA123 2578004733
MSAVALPRVSGGHDEHGHLEEFRTDPIGLMQRVRDECGDVGTFQLAGKQVVLLSGSHANEFFFRAGDDDLDQA
KAYPFMTPIFGEGVVFDASPERRKEMLHNAALRGEQMKGHAATIEDQVRRMIADWGEAGEIDLLDFFAELTIY
TSSACLIGKKFRDQLDGRFAKLYHELERGTDPLAYVDPYLPIESFRRRDEARNGLVALVADIMNGRIANPPTD
KSDRDMLDVLIAVKAETGTPRFSADEITGMFISMMFAGHHTSSGTASWTLIELMRHRDAYAAVIDELDELYGD
GRSVSFHALRQIPQLENVLKETLRLHPPLIILMRVAKGEFEVQGHRIHEGDLVAASPAISNRIPEDFPDPHDF
VPARYEQPRQEDLLNRWTWIPFGAGRHRCVGAAFAIMQIKAIFSVLLREYEFEMAQPPESYRNDHSKMVVQLA
QPACVRYRRRTGV
>CYP51B1_Mycobacterium tuberculosis TB_RSA124 2578199508
MSAVALPRVSGGHDEHGHLEEFRTDPIGLMQRVRDECGDVGTFQLAGKQVVLLSGSHANEFFFRAGDDDLDQA
KAYPFMTPIFGEGVVFDASPERRKEMLHNAALRGEQMKGHAATIEDQVRRMIADWGEAGEIDLLDFFAELTIY
TSSACLIGKKFRDQLDGRFAKLYHELERGTDPLAYVDPYLPIESFRRRDEARNGLVALVADIMNGRIANPPTD
KSDRDMLDVLIAVKAETGTPRFSADEITGMFISMMFAGHHTSSGTASWTLIELMRHRDAYAAVIDELDELYGD
GRSVSFHALRQIPQLENVLKETLRLHPPLIILMRVAKGEFEVQGHRIHEGDLVAASPAISNRIPEDFPDPHDF
VPARYEQPRQEDLLNRWTWIPFGAGRHRCVGAAFAIMQIKAIFSVLLREYEFEMAQPPESYRNDHSKMVVQLA
QPACVRYRRRTGV
>CYP51B1_Mycobacterium tuberculosis TB_RSA125 2603241687
MSAVALPRVSGGHDEHGHLEEFRTDPIGLMQRVRDECGDVGTFQLAGKQVVLLSGSHANEFFFRAGDDDLDQA
KAYPFMTPIFGEGVVFDASPERRKEMLHNAALRGEQMKGHAATIEDQVRRMIADWGEAGEIDLLDFFAELTIY
TSSACLIGKKFRDQLDGRFAKLYHELERGTDPLAYVDPYLPIESFRRRDEARNGLVALVADIMNGRIANPPTD
KSDRDMLDVLIAVKAETGTPRFSADEITGMFISMMFAGHHTSSGTASWTLIELMRHRDAYAAVIDELDELYGD
GRSVSFHALRQIPQLENVLKETLRLHPPLIILMRVAKGEFEVQGHRIHEGDLVAASPAISNRIPEDFPDPHDF
VPARYEQPRQEDLLNRWTWIPFGAGRHRCVGAAFAIMQIKAIFSVLLREYEFEMAQPPESYRNDHSKMVVQLA
QPACVRYRRRTGV
>CYP51B1_Mycobacterium tuberculosis TB_RSA126 2605712626
MSAVALPRVSGGHDEHGHLEEFRTDPIGLMQRVRDECGDVGTFQLAGKQVVLLSGSHANEFFFRAGDDDLDQA
KAYPFMTPIFGEGVVFDASPERRKEMLHNAALRGEQMKGHAATIEDQVRRMIADWGEAGEIDLLDFFAELTIY
TSSACLIGKKFRDQLDGRFAKLYHELERGTDPLAYVDPYLPIESFRRRDEARNGLVALVADIMNGRIANPPTD
KSDRDMLDVLIAVKAETGTPRFSADEITGMFISMMFAGHHTSSGTASWTLIELMRHRDAYAAVIDELDELYGD
GRSVSFHALRQIPQLENVLKETLRLHPPLIILMRVAKGEFEVQGHRIHEGDLVAASPAISNRIPEDFPDPHDF
VPARYEQPRQEDLLNRWTWIPFGAGRHRCVGAAFAIMQIKAIFSVLLREYEFEMAQPPESYRNDHSKMVVQLA
QPACVRYRRRTGV
>CYP51B1_Mycobacterium tuberculosis TB_RSA127 2577552694
MSAVALPRVSGGHDEHGHLEEFRTDPIGLMQRVRDECGDVGTFQLAGKQVVLLSGSHANEFFFRAGDDDLDQA
KAYPFMTPIFGEGVVFDASPERRKEMLHNAALRGEQMKGHAATIEDQVRRMIADWGEAGEIDLLDFFAELTIY
TSSACLIGKKFRDQLDGRFAKLYHELERGTDPLAYVDPYLPIESFRRRDEARNGLVALVADIMNGRIANPPTD
KSDRDMLDVLIAVKAETGTPRFSADEITGMFISMMFAGHHTSSGTASWTLIELMRHRDAYAAVIDELDELYGD
GRSVSFHALRQIPQLENVLKETLRLHPPLIILMRVAKGEFEVQGHRIHEGDLVAASPAISNRIPEDFPDPHDF
VPARYEQPRQEDLLNRWTWIPFGAGRHRCVGAAFAIMQIKAIFSVLLREYEFEMAQPPESYRNDHSKMVVQLA
QPACVRYRRRTGV
>CYP51B1_Mycobacterium tuberculosis TB_RSA128 2603956870
MSAVALPRVSGGHDEHGHLEEFRTDPIGLMQRVRDECGDVGTFQLAGKQVVLLSGSHANEFFFRAGDDDLDQA
KAYPFMTPIFGEGVVFDASPERRKEMLHNAALRGEQMKGHAATIEDQVRRMIADWGEAGEIDLLDFFAELTIY
TSSACLIGKKFRDQLDGRFAKLYHELERGTDPLAYVDPYLPIESFRRRDEARNGLVALVADIMNGRIANPPTD
KSDRDMLDVLIAVKAETGTPRFSADEITGMFISMMFAGHHTSSGTASWTLIELMRHRDAYAAVIDELDELYGD
GRSVSFHALRQIPQLENVLKETLRLHPPLIILMRVAKGEFEVQGHRIHEGDLVAASPAISNRIPEDFPDPHDF
VPARYEQPRQEDLLNRWTWIPFGAGRHRCVGAAFAIMQIKAIFSVLLREYEFEMAQPPESYRNDHSKMVVQLA
QPACVRYRRRTGV
>CYP51B1_Mycobacterium tuberculosis TB_RSA129 2605718189
MSAVALPRVSGGHDEHGHLEEFRTDPIGLMQRVRDECGDVGTFQLAGKQVVLLSGSHANEFFFRAGDDDLDQA
KAYPFMTPIFGEGVVFDASPERRKEMLHNAALRGEQMKGHAATIEDQVRRMIADWGEAGEIDLLDFFAELTIY
TSSACLIGKKFRDQLDGRFAKLYHELERGTDPLAYVDPYLPIESFRRRDEARNGLVALVADIMNGRIANPPTD
KSDRDMLDVLIAVKAETGTPRFSADEITGMFISMMFAGHHTSSGTASWTLIELMRHRDAYAAVIDELDELYGD
GRSVSFHALRQIPQLENVLKETLRLHPPLIILMRVAKGEFEVQGHRIHEGDLVAASPAISNRIPEDFPDPHDF

VPARYEQPRQEDLLNRWTWIPFGAGRHRCVGAAFAIMQIKAIFSVLLREYEFEMAQPPESYRNDHSKMVVQLA
QPACVRYRRRTGV
>CYP51B1_Mycobacterium tuberculosis TB_RSA13 2603247403
MSAVALPRVSGGHDEHGHLEEFRTDPIGLMQRVRDECGDVGTFQLAGKQVVLLSGSHANEFFFRAGDDDLDQA
KAYPFMTPIFGEGVVFDASPERRKEMLHNAALRGEQMKGHAATIEDQVRRMIADWGEAGEIDLLDFFAELTIY
TSSACLIGKKFRDQLDGRFAKLYHELERGTDPLAYVDPYLPIESFRRRDEARNGLVALVADIMNGRIANPPTD
KSDRDMLDVLIAVKAETGTPRFSADEITGMFISMMFAGHHTSSGTASWTLIELMRHRDAYAAVIDELDELYGD
GRSVSFHALRQIPQLENVLKETLRLHPPLIILMRVAKGEFEVQGHRIHEGDLVAASPAISNRIPEDFPDPHDF
VPARYEQPRQEDLLNRWTWIPFGAGRHRCVGAAFAIMQIKAIFSVLLREYEFEMAQPPESYRNDHSKMVVQLA
QPACVRYRRRTGV
>CYP51B1_Mycobacterium tuberculosis TB_RSA130 2605352460
MSAVALPRVSGGHDEHGHLEEFRTDPIGLMQRVRDECGDVGTFQLAGKQVVLLSGSHANEFFFRAGDDDLDQA
KAYPFMTPIFGEGVVFDASPERRKEMLHNAALRGEQMKGHAATIEDQVRRMIADWGEAGEIDLLDFFAELTIY
TSSACLIGKKFRDQLDGRFAKLYHELERGTDPLAYVDPYLPIESFRRRDEARNGLVALVADIMNGRIANPPTD
KSDRDMLDVLIAVKAETGTPRFSADEITGMFISMMFAGHHTSSGTASWTLIELMRHRDAYAAVIDELDELYGD
GRSVSFHALRQIPQLENVLKETLRLHPPLIILMRVAKGEFEVQGHRIHEGDLVAASPAISNRIPEDFPDPHDF
VPARYEQPRQEDLLNRWTWIPFGAGRHRCVGAAFAIMQIKAIFSVLLREYEFEMAQPPESYRNDHSKMVVQLA
QPACVRYRRRTGV
>CYP51B1_Mycobacterium tuberculosis TB_RSA131 2603402241
MSAVALPRVSGGHDEHGHLEEFRTDPIGLMQRVRDECGDVGTFQLAGKQVVLLSGSHANEFFFRAGDDDLDQA
KAYPFMTPIFGEGVVFDASPERRKEMLHNAALRGEQMKGHAATIEDQVRRMIADWGEAGEIDLLDFFAELTIY
TSSACLIGKKFRDQLDGRFAKLYHELERGTDPLAYVDPYLPIESFRRRDEARNGLVALVADIMNGRIANPPTD
KSDRDMLDVLIAVKAETGTPRFSADEITGMFISMMFAGHHTSSGTASWTLIELMRHRDAYAAVIDELDELYGD
GRSVSFHALRQIPQLENVLKETLRLHPPLIILMRVAKGEFEVQGHRIHEGDLVAASPAISNRIPEDFPDPHDF
VPARYEQPRQEDLLNRWTWIPFGAGRHRCVGAAFAIMQIKAIFSVLLREYEFEMAQPPESYRNDHSKMVVQLA
QPACVRYRRRTGV
>CYP51B1_Mycobacterium tuberculosis TB_RSA132 2577989844
MSAVALPRVSGGHDEHGHLEEFRTDPIGLMQRVRDECGDVGTFQLAGKQVVLLSGSHANEFFFRAGDDDLDQA
KAYPFMTPIFGEGVVFDASPERRKEMLHNAALRGEQMKGHAATIEDQVRRMIADWGEAGEIDLLDFFAELTIY
TSSACLIGKKFRDQLDGRFAKLYHELERGTDPLAYVDPYLPIESFRRRDEARNGLVALVADIMNGRIANPPTD
KSDRDMLDVLIAVKAETGTPRFSADEITGMFISMMFAGHHTSSGTASWTLIELMRHRDAYAAVIDELDELYGD
GRSVSFHALRQIPQLENVLKETLRLHPPLIILMRVAKGEFEVQGHRIHEGDLVAASPAISNRIPEDFPDPHDF
VPARYEQPRQEDLLNRWTWIPFGAGRHRCVGAAFAIMQIKAIFSVLLREYEFEMAQPPESYRNDHSKMVVQLA
QPACVRYRRRTGV
>CYP51B1_Mycobacterium tuberculosis TB_RSA133 2605359069
MSAVALPRVSGGHDEHGHLEEFRTDPIGLMQRVRDECGDVGTFQLAGKQVVLLSGSHANEFFFRAGDDDLDQA
KAYPFMTPIFGEGVVFDASPERRKEMLHNAALRGEQMKGHAATIEDQVRRMIADWGEAGEIDLLDFFAELTIY
TSSACLIGKKFRDQLDGRFAKLYHELERGTDPLAYVDPYLPIESFRRRDEARNGLVALVADIMNGRIANPPTD
KSDRDMLDVLIAVKAETGTPRFSADEITGMFISMMFAGHHTSSGTASWTLIELMRHRDAYAAVIDELDELYGD
GRSVSFHALRQIPQLENVLKETLRLHPPLIILMRVAKGEFEVQGHRIHEGDLVAASPAISNRIPEDFPDPHDF
VPARYEQPRQEDLLNRWTWIPFGAGRHRCVGAAFAIMQIKAIFSVLLREYEFEMAQPPESYRNDHSKMVVQLA
QPACVRYRRRTGV
>CYP51B1_Mycobacterium tuberculosis TB_RSA134 2575626532
MSAVALPRVSGGHDEHGHLEEFRTDPIGLMQRVRDECGDVGTFQLAGKQVVLLSGSHANEFFFRAGDDDLDQA
KAYPFMTPIFGEGVVFDASPERRKEMLHNAALRGEQMKGHAATIEDQVRRMIADWGEAGEIDLLDFFAELTIY
TSSACLIGKKFRDQLDGRFAKLYHELERGTDPLAYVDPYLPIESFRRRDEARNGLVALVADIMNGRIANPPTD
KSDRDMLDVLIAVKAETGTPRFSADEITGMFISMMFAGHHTSSGTASWTLIELMRHRDAYAAVIDELDELYGD
GRSVSFHALRQIPQLENVLKETLRLHPPLIILMRVAKGEFEVQGHRIHEGDLVAASPAISNRIPEDFPDPHDF
VPARYEQPRQEDLLNRWTWIPFGAGRHRCVGAAFAIMQIKAIFSVLLREYEFEMAQPPESYRNDHSKMVVQLA
QPACVRYRRRTGV
>CYP51B1_Mycobacterium tuberculosis TB_RSA135 2603960577
MSAVALPRVSGGHDEHGHLEEFRTDPIGLMQRVRDECGDVGTFQLAGKQVVLLSGSHANEFFFRAGDDDLDQA
KAYPFMTPIFGEGVVFDASPERRKEMLHNAALRGEQMKGHAATIEDQVRRMIADWGEAGEIDLLDFFAELTIY
TSSACLIGKKFRDQLDGRFAKLYHELERGTDPLAYVDPYLPIESFRRRDEARNGLVALVADIMNGRIANPPTD
KSDRDMLDVLIAVKAETGTPRFSADEITGMFISMMFAGHHTSSGTASWTLIELMRHRDAYAAVIDELDELYGD
GRSVSFHALRQIPQLENVLKETLRLHPPLIILMRVAKGEFEVQGHRIHEGDLVAASPAISNRIPEDFPDPHDF
VPARYEQPRQEDLLNRWTWIPFGAGRHRCVGAAFAIMQIKAIFSVLLREYEFEMAQPPESYRNDHSKMVVQLA
QPACVRYRRRTGV

>CYP51B1_Mycobacterium tuberculosis TB_RSA136 2576445822
MSAVALPRVSGGHDEHGHLEEFRTDPIGLMQRVRDECGDVGTFQLAGKQVVLLSGSHANEFFFRAGDDDLDQA
KAYPFMTPIFGEGVVFDASPERRKEMLHNAALRGEQMKGHAATIEDQVRRMIADWGEAGEIDLLDFFAELTIY
TSSACLIGKKFRDQLDGRFAKLYHELERGTDPLAYVDPYLPIESFRRRDEARNGLVALVADIMNGRIANPPTD
KSDRDMLDVLIAVKAETGTPRFSADEITGMFISMMFAGHHTSSGTASWTLIELMRHRDAYAAVIDELDELYGD
GRSVSFHALRQIPQLENVLKETLRLHPPLIILMRVAKGEFEVQGHRIHEGDLVAASPAISNRIPEDFPDPHDF
VPARYEQPRQEDLLNRWTWIPFGAGRHRCVGAAFAIMQIKAIFSVLLREYEFEMAQPPESYRNDHSKMVVQLA
QPACVRYRRRTGV
>CYP51B1_Mycobacterium tuberculosis TB_RSA137 2603962350
MSAVALPRVSGGHDEHGHLEEFRTDPIGLMQRVRDECGDVGTFQLAGKQVVLLSGSHANEFFFRAGDDDLDQA
KAYPFMTPIFGEGVVFDASPERRKEMLHNAALRGEQMKGHAATIEDQVRRMIADWGEAGEIDLLDFFAELTIY
TSSACLIGKKFRDQLDGRFAKLYHELERGTDPLAYVDPYLPIESFRRRDEARNGLVALVADIMNGRIANPPTD
KSDRDMLDVLIAVKAETGTPRFSADEITGMFISMMFAGHHTSSGTASWTLIELMRHRDAYAAVIDELDELYGD
GRSVSFHALRQIPQLENVLKETLRLHPPLIILMRVAKGEFEVQGHRIHEGDLVAASPAISNRIPEDFPDPHDF
VPARYEQPRQEDLLNRWTWIPFGAGRHRCVGAAFAIMQIKAIFSVLLREYEFEMAQPPESYRNDHSKMVVQLA
QPACVRYRRRTGV
>CYP51B1_Mycobacterium tuberculosis TB_RSA138 2577900048
MSAVALPRVSGGHDEHGHLEEFRTDPIGLMQRVRDECGDVGTFQLAGKQVVLLSGSHANEFFFRAGDDDLDQA
KAYPFMTPIFGEGVVFDASPERRKEMLHNAALRGEQMKGHAATIEDQVRRMIADWGEAGEIDLLDFFAELTIY
TSSACLIGKKFRDQLDGRFAKLYHELERGTDPLAYVDPYLPIESFRRRDEARNGLVALVADIMNGRIANPPTD
KSDRDMLDVLIAVKAETGTPRFSADEITGMFISMMFAGHHTSSGTASWTLIELMRHRDAYAAVIDELDELYGD
GRSVSFHALRQIPQLENVLKETLRLHPPLIILMRVAKGEFEVQGHRIHEGDLVAASPAISNRIPEDFPDPHDF
VPARYEQPRQEDLLNRWTWIPFGAGRHRCVGAAFAIMQIKAIFSVLLREYEFEMAQPPESYRNDHSKMVVQLA
QPACVRYRRRTGV
>CYP51B1_Mycobacterium tuberculosis TB_RSA139 2605363629
MSAVALPRVSGGHDEHGHLEEFRTDPIGLMQRVRDECGDVGTFQLAGKQVVLLSGSHANEFFFRAGDDDLDQA
KAYPFMTPIFGEGVVFDASPERRKEMLHNAALRGEQMKGHAATIEDQVRRMIADWGEAGEIDLLDFFAELTIY
TSSACLIGKKFRDQLDGRFAKLYHELERGTDPLAYVDPYLPIESFRRRDEARNGLVALVADIMNGRIANPPTD
KSDRDMLDVLIAVKAETGTPRFSADEITGMFISMMFAGHHTSSGTASWTLIELMRHRDAYAAVIDELDELYGD
GRSVSFHALRQIPQLENVLKETLRLHPPLIILMRVAKGEFEVQGHRIHEGDLVAASPAISNRIPEDFPDPHDF
VPARYEQPRQEDLLNRWTWIPFGAGRHRCVGAAFAIMQIKAIFSVLLREYEFEMAQPPESYRNDHSKMVVQLA
QPACVRYRRRTGV
>CYP51B1_Mycobacterium tuberculosis TB_RSA14 2605364464
MSAVALPRVSGGHDEHGHLEEFRTDPIGLMQRVRDECGDVGTFQLAGKQVVLLSGSHANEFFFRAGDDDLDQA
KAYPFMTPIFGEGVVFDASPERRKEMLHNAALRGEQMKGHAATIEDQVRRMIADWGEAGEIDLLDFFAELTIY
TSSACLIGKKFRDQLDGRFAKLYHELERGTDPLAYVDPYLPIESFRRRDEARNGLVALVADIMNGRIANPPTD
KSDRDMLDVLIAVKAETGTPRFSADEITGMFISMMFAGHHTSSGTASWTLIELMRHRDAYAAVIDELDELYGD
GRSVSFHALRQIPQLENVLKETLRLHPPLIILMRVAKGEFEVQGHRIHEGDLVAASPAISNRIPEDFPDPHDF
VPARYEQPRQEDLLNRWTWIPFGAGRHRCVGAAFAIMQIKAIFSVLLREYEFEMAQPPESYRNDHSKMVVQLA
QPACVRYRRRTGV
>CYP51B1_Mycobacterium tuberculosis TB_RSA140 2576162926
MSAVALPRVSGGHDEHGHLEEFRTDPIGLMQRVRDECGDVGTFQLAGKQVVLLSGSHANEFFFRAGDDDLDQA
KAYPFMTPIFGEGVVFDASPERRKEMLHNAALRGEQMKGHAATIEDQVRRMIADWGEAGEIDLLDFFAELTIY
TSSACLIGKKFRDQLDGRFAKLYHELERGTDPLAYVDPYLPIESFRRRDEARNGLVALVADIMNGRIANPPTD
KSDRDMLDVLIAVKAETGTPRFSADEITGMFISMMFAGHHTSSGTASWTLIELMRHRDAYAAVIDELDELYGD
GRSVSFHALRQIPQLENVLKETLRLHPPLIILMRVAKGEFEVQGHRIHEGDLVAASPAISNRIPEDFPDPHDF
VPARYEQPRQEDLLNRWTWIPFGAGRHRCVGAAFAIMQIKAIFSVLLREYEFEMAQPPESYRNDHSKMVVQLA
QPACVRYRRRTGV
>CYP51B1_Mycobacterium tuberculosis TB_RSA141 2603467822
MSAVALPRVSGGHDEHGHLEEFRTDPIGLMQRVRDECGDVGTFQLAGKQVVLLSGSHANEFFFRAGDDDLDQA
KAYPFMTPIFGEGVVFDASPERRKEMLHNAALRGEQMKGHAATIEDQVRRMIADWGEAGEIDLLDFFAELTIY
TSSACLIGKKFRDQLDGRFAKLYHELERGTDPLAYVDPYLPIESFRRRDEARNGLVALVADIMNGRIANPPTD
KSDRDMLDVLIAVKAETGTPRFSADEITGMFISMMFAGHHTSSGTASWTLIELMRHRDAYAAVIDELDELYGD
GRSVSFHALRQIPQLENVLKETLRLHPPLIILMRVAKGEFEVQGHRIHEGDLVAASPAISNRIPEDFPDPHDF
VPARYEQPRQEDLLNRWTWIPFGAGRHRCVGAAFAIMQIKAIFSVLLREYEFEMAQPPESYRNDHSKMVVQLA
QPACVRYRRRTGV
>CYP51B1_Mycobacterium tuberculosis TB_RSA142 2603474737

MSAVALPRVSGGHDEHGHLEEFRTDPIGLMQRVRDECGDVGTFQLAGKQVVLLSGSHANEFFFRAGDDDLDQA
KAYPFMTPIFGEGVVFDASPERRKEMLHNAALRGEQMKGHAATIEDQVRRMIADWGEAGEIDLLDFFAELTIY
TSSACLIGKKFRDQLDGRFAKLYHELERGTDPLAYVDPYLPIESFRRRDEARNGLVALVADIMNGRIANPPTD
KSDRDMLDVLIAVKAETGTPRFSADEITGMFISMMFAGHHTSSGTASWTLIELMRHRDAYAAVIDELDELYGD
GRSVSFHALRQIPQLENVLKETLRLHPPLIILMRVAKGEFEVQGHRIHEGDLVAASPAISNRIPEDFPDPHDF
VPARYEQPRQEDLLNRWTWIPFGAGRHRCVGAAFAIMQIKAIFSVLLREYEFEMAQPPESYRNDHSKMVVQLA
QPACVRYRRRTGV
>CYP51B1_Mycobacterium tuberculosis TB_RSA143 2604426976
MSAVALPRVSGGHDEHGHLEEFRTDPIGLMQRVRDECGDVGTFQLAGKQVVLLSGSHANEFFFRAGDDDLDQA
KAYPFMTPIFGEGVVFDASPERRKEMLHNAALRGEQMKGHAATIEDQVRRMIADWGEAGEIDLLDFFAELTIY
TSSACLIGKKFRDQLDGRFAKLYHELERGTDPLAYVDPYLPIESFRRRDEARNGLVALVADIMNGRIANPPTD
KSDRDMLDVLIAVKAETGTPRFSADEITGMFISMMFAGHHTSSGTASWTLIELMRHRDAYAAVIDELDELYGD
GRSVSFHALRQIPQLENVLKETLRLHPPLIILMRVAKGEFEVQGHRIHEGDLVAASPAISNRIPEDFPDPHDF
VPARYEQPRQEDLLNRWTWIPFGAGRHRCVGAAFAIMQIKAIFSVLLREYEFEMAQPPESYRNDHSKMVVQLA
QPACVRYRRRTGV
>CYP51B1_Mycobacterium tuberculosis TB_RSA144 2604773787
MSAVALPRVSGGHDEHGHLEEFRTDPIGLMQRVRDECGDVGTFQLAGKQVVLLSGSHANEFFFRAGDDDLDQA
KAYPFMTPIFGEGVVFDASPERRKEMLHNAALRGEQMKGHAATIEDQVRRMIADWGEAGEIDLLDFFAELTIY
TSSACLIGKKFRDQLDGRFAKLYHELERGTDPLAYVDPYLPIESFRRRDEARNGLVALVADIMNGRIANPPTD
KSDRDMLDVLIAVKAETGTPRFSADEITGMFISMMFAGHHTSSGTASWTLIELMRHRDAYAAVIDELDELYGD
GRSVSFHALRQIPQLENVLKETLRLHPPLIILMRVAKGEFEVQGHRIHEGDLVAASPAISNRIPEDFPDPHDF
VPARYEQPRQEDLLNRWTWIPFGAGRHRCVGAAFAIMQIKAIFSVLLREYEFEMAQPPESYRNDHSKMVVQLA
QPACVRYRRRTGV
>CYP51B1_Mycobacterium tuberculosis TB_RSA145 2605009058
MSAVALPRVSGGHDEHGHLEEFRTDPIGLMQRVRDECGDVGTFQLAGKQVVLLSGSHANEFFFRAGDDDLDQA
KAYPFMTPIFGEGVVFDASPERRKEMLHNAALRGEQMKGHAATIEDQVRRMIADWGEAGEIDLLDFFAELTIY
TSSACLIGKKFRDQLDGRFAKLYHELERGTDPLAYVDPYLPIESFRRRDEARNGLVALVADIMNGRIANPPTD
KSDRDMLDVLIAVKAETGTPRFSADEITGMFISMMFAGHHTSSGTASWTLIELMRHRDAYAAVIDELDELYGD
GRSVSFHALRQIPQLENVLKETLRLHPPLIILMRVAKGEFEVQGHRIHEGDLVAASPAISNRIPEDFPDPHDF
VPARYEQPRQEDLLNRWTWIPFGAGRHRCVGAAFAIMQIKAIFSVLLREYEFEMAQPPESYRNDHSKMVVQLA
QPACVRYRRRTGV
>CYP51B1_Mycobacterium tuberculosis TB_RSA146 2604296925
MSAVALPRVSGGHDEHGHLEEFRTDPIGLMQRVRDECGDVGTFQLAGKQVVLLSGSHANEFFFRAGDDDLDQA
KAYPFMTPIFGEGVVFDASPERRKEMLHNAALRGEQMKGHAATIEDQVRRMIADWGEAGEIDLLDFFAELTIY
TSSACLIGKKFRDQLDGRFAKLYHELERGTDPLAYVDPYLPIESFRRRDEARNGLVALVADIMNGRIANPPTD
KSDRDMLDVLIAVKAETGTPRFSADEITGMFISMMFAGHHTSSGTASWTLIELMRHRDAYAAVIDELDELYGD
GRSVSFHALRQIPQLENVLKETLRLHPPLIILMRVAKGEFEVQGHRIHEGDLVAASPAISNRIPEDFPDPHDF
VPARYEQPRQEDLLNRWTWIPFGAGRHRCVGAAFAIMQIKAIFSVLLREYEFEMAQPPESYRNDHSKMVVQLA
QPACVRYRRRTGV
>CYP51B1_Mycobacterium tuberculosis TB_RSA147 2603252625
MSAVALPRVSGGHDEHGHLEEFRTDPIGLMQRVRDECGDVGTFQLAGKQVVLLSGSHANEFFFRAGDDDLDQA
KAYPFMTPIFGEGVVFDASPERRKEMLHNAALRGEQMKGHAATIEDQVRRMIADWGEAGEIDLLDFFAELTIY
TSSACLIGKKFRDQLDGRFAKLYHELERGTDPLAYVDPYLPIESFRRRDEARNGLVALVADIMNGRIANPPTD
KSDRDMLDVLIAVKAETGTPRFSADEITGMFISMMFAGHHTSSGTASWTLIELMRHRDAYAAVIDELDELYGD
GRSVSFHALRQIPQLENVLKETLRLHPPLIILMRVAKGEFEVQGHRIHEGDLVAASPAISNRIPEDFPDPHDF
VPARYEQPRQEDLLNRWTWIPFGAGRHRCVGAAFAIMQIKAIFSVLLREYEFEMAQPPESYRNDHSKMVVQLA
QPACVRYRRRTGV
>CYP51B1_Mycobacterium tuberculosis TB_RSA148 2575294427
MSAVALPRVSGGHDEHGHLEEFRTDPIGLMQRVRDECGDVGTFQLAGKQVVLLSGSHANEFFFRAGDDDLDQA
KAYPFMTPIFGEGVVFDASPERRKEMLHNAALRGEQMKGHAATIEDQVRRMIADWGEAGEIDLLDFFAELTIY
TSSACLIGKKFRDQLDGRFAKLYHELERGTDPLAYVDPYLPIESFRRRDEARNGLVALVADIMNGRIANPPTD
KSDRDMLDVLIAVKAETGTPRFSADEITGMFISMMFAGHHTSSGTASWTLIELMRHRDAYAAVIDELDELYGD
GRSVSFHALRQIPQLENVLKETLRLHPPLIILMRVAKGEFEVQGHRIHEGDLVAASPAISNRIPEDFPDPHDF
VPARYEQPRQEDLLNRWTWIPFGAGRHRCVGAAFAIMQIKAIFSVLLREYEFEMAQPPESYRNDHSKMVVQLA
QPACVRYRRRTGV
>CYP51B1_Mycobacterium tuberculosis TB_RSA15 2575770982
MSAVALPRVSGGHDEHGHLEEFRTDPIGLMQRVRDECGDVGTFQLAGKQVVLLSGSHANEFFFRAGDDDLDQA
KAYPFMTPIFGEGVVFDASPERRKEMLHNAALRGEQMKGHAATIEDQVRRMIADWGEAGEIDLLDFFAELTIY

TSSACLIGKKFRDQLDGRFAKLYHELERGTDPLAYVDPYLPIESFRRRDEARNGLVALVADIMNGRIANPPTD
KSDRDMLDVLIAVKAETGTPRFSADEITGMFISMMFAGHHTSSGTASWTLIELMRHRDAYAAVIDELDELYGD
GRSVSFHALRQIPQLENVLKETLRLHPPLIILMRVAKGEFEVQGHRIHEGDLVAASPAISNRIPEDFPDPHDF
VPARYEQPRQEDLLNRWTWIPFGAGRHRCVGAAFAIMQIKAIFSVLLREYEFEMAQPPESYRNDHSKMVVQLA
QPACVRYRRRTGV
>CYP51B1_Mycobacterium tuberculosis TB_RSA151 2605015296
MSAVALPRVSGGHDEHGHLEEFRTDPIGLMQRVRDECGDVGTFQLAGKQVVLLSGSHANEFFFRAGDDDLDQA
KAYPFMTPIFGEGVVFDASPERRKEMLHNAALRGEQMKGHAATIEDQVRRMIADWGEAGEIDLLDFFAELTIY
TSSACLIGKKFRDQLDGRFAKLYHELERGTDPLAYVDPYLPIESFRRRDEARNGLVALVADIMNGRIANPPTD
KSDRDMLDVLIAVKAETGTPRFSADEITGMFISMMFAGHHTSSGTASWTLIELMRHRDAYAAVIDELDELYGD
GRSVSFHALRQIPQLENVLKETLRLHPPLIILMRVAKGEFEVQGHRIHEGDLVAASPAISNRIPEDFPDPHDF
VPARYEQPRQEDLLNRWTWIPFGAGRHRCVGAAFAIMQIKAIFSVLLREYEFEMAQPPESYRNDHSKMVVQLA
QPACVRYRRRTGV
>CYP51B1_Mycobacterium tuberculosis TB_RSA152 2603253345
MSAVALPRVSGGHDEHGHLEEFRTDPIGLMQRVRDECGDVGTFQLAGKQVVLLSGSHANEFFFRAGDDDLDQA
KAYPFMTPIFGEGVVFDASPERRKEMLHNAALRGEQMKGHAATIEDQVRRMIADWGEAGEIDLLDFFAELTIY
TSSACLIGKKFRDQLDGRFAKLYHELERGTDPLAYVDPYLPIESFRRRDEARNGLVALVADIMNGRIANPPTD
KSDRDMLDVLIAVKAETGTPRFSADEITGMFISMMFAGHHTSSGTASWTLIELMRHRDAYAAVIDELDELYGD
GRSVSFHALRQIPQLENVLKETLRLHPPLIILMRVAKGEFEVQGHRIHEGDLVAASPAISNRIPEDFPDPHDF
VPARYEQPRQEDLLNRWTWIPFGAGRHRCVGAAFAIMQIKAIFSVLLREYEFEMAQPPESYRNDHSKMVVQLA
QPACVRYRRRTGV
>CYP51B1_Mycobacterium tuberculosis TB_RSA153 2603408342
MSAVALPRVSGGHDEHGHLEEFRTDPIGLMQRVRDECGDVGTFQLAGKQVVLLSGSHANEFFFRAGDDDLDQA
KAYPFMTPIFGEGVVFDASPERRKEMLHNAALRGEQMKGHAATIEDQVRRMIADWGEAGEIDLLDFFAELTIY
TSSACLIGKKFRDQLDGRFAKLYHELERGTDPLAYVDPYLPIESFRRRDEARNGLVALVADIMNGRIANPPTD
KSDRDMLDVLIAVKAETGTPRFSADEITGMFISMMFAGHHTSSGTASWTLIELMRHRDAYAAVIDELDELYGD
GRSVSFHALRQIPQLENVLKETLRLHPPLIILMRVAKGEFEVQGHRIHEGDLVAASPAISNRIPEDFPDPHDF
VPARYEQPRQEDLLNRWTWIPFGAGRHRCVGAAFAIMQIKAIFSVLLREYEFEMAQPPESYRNDHSKMVVQLA
QPACVRYRRRTGV
>CYP51B1_Mycobacterium tuberculosis TB_RSA154 2605372705
MSAVALPRVSGGHDEHGHLEEFRTDPIGLMQRVRDECGDVGTFQLAGKQVVLLSGSHANEFFFRAGDDDLDQA
KAYPFMTPIFGEGVVFDASPERRKEMLHNAALRGEQMKGHAATIEDQVRRMIADWGEAGEIDLLDFFAELTIY
TSSACLIGKKFRDQLDGRFAKLYHELERGTDPLAYVDPYLPIESFRRRDEARNGLVALVADIMNGRIANPPTD
KSDRDMLDVLIAVKAETGTPRFSADEITGMFISMMFAGHHTSSGTASWTLIELMRHRDAYAAVIDELDELYGD
GRSVSFHALRQIPQLENVLKETLRLHPPLIILMRVAKGEFEVQGHRIHEGDLVAASPAISNRIPEDFPDPHDF
VPARYEQPRQEDLLNRWTWIPFGAGRHRCVGAAFAIMQIKAIFSVLLREYEFEMAQPPESYRNDHSKMVVQLA
QPACVRYRRRTGV
>CYP51B1_Mycobacterium tuberculosis TB_RSA155 2605020217
MSAVALPRVSGGHDEHGHLEEFRTDPIGLMQRVRDECGDVGTFQLAGKQVVLLSGSHANEFFFRAGDDDLDQA
KAYPFMTPIFGEGVVFDASPERRKEMLHNAALRGEQMKGHAATIEDQVRRMIADWGEAGEIDLLDFFAELTIY
TSSACLIGKKFRDQLDGRFAKLYHELERGTDPLAYVDPYLPIESFRRRDEARNGLVALVADIMNGRIANPPTD
KSDRDMLDVLIAVKAETGTPRFSADEITGMFISMMFAGHHTSSGTASWTLIELMRHRDAYAAVIDELDELYGD
GRSVSFHALRQIPQLENVLKETLRLHPPLIILMRVAKGEFEVQGHRIHEGDLVAASPAISNRIPEDFPDPHDF
VPARYEQPRQEDLLNRWTWIPFGAGRHRCVGAAFAIMQIKAIFSVLLREYEFEMAQPPESYRNDHSKMVVQLA
QPACVRYRRRTGV
>CYP51B1_Mycobacterium tuberculosis TB_RSA156 2605376381
MSAVALPRVSGGHDEHGHLEEFRTDPIGLMQRVRDECGDVGTFQLAGKQVVLLSGSHANEFFFRAGDDDLDQA
KAYPFMTPIFGEGVVFDASPERRKEMLHNAALRGEQMKGHAATIEDQVRRMIADWGEAGEIDLLDFFAELTIY
TSSACLIGKKFRDQLDGRFAKLYHELERGTDPLAYVDPYLPIESFRRRDEARNGLVALVADIMNGRIANPPTD
KSDRDMLDVLIAVKAETGTPRFSADEITGMFISMMFAGHHTSSGTASWTLIELMRHRDAYAAVIDELDELYGD
GRSVSFHALRQIPQLENVLKETLRLHPPLIILMRVAKGEFEVQGHRIHEGDLVAASPAISNRIPEDFPDPHDF
VPARYEQPRQEDLLNRWTWIPFGAGRHRCVGAAFAIMQIKAIFSVLLREYEFEMAQPPESYRNDHSKMVVQLA
QPACVRYRRRTGV
>CYP51B1_Mycobacterium tuberculosis TB_RSA157 2603410475
MSAVALPRVSGGHDEHGHLEEFRTDPIGLMQRVRDECGDVGTFQLAGKQVVLLSGSHANEFFFRAGDDDLDQA
KAYPFMTPIFGEGVVFDASPERRKEMLHNAALRGEQMKGHAATIEDQVRRMIADWGEAGEIDLLDFFAELTIY
TSSACLIGKKFRDQLDGRFAKLYHELERGTDPLAYVDPYLPIESFRRRDEARNGLVALVADIMNGRIANPPTD
KSDRDMLDVLIAVKAETGTPRFSADEITGMFISMMFAGHHTSSGTASWTLIELMRHRDAYAAVIDELDELYGD

GRSVSFHALRQIPQLENVLKETLRLHPPLIILMRVAKGEFEVQGHRIHEGDLVAASPAISNRIPEDFPDPHDF
VPARYEQPRQEDLLNRWTWIPFGAGRHRCVGAAFAIMQIKAIFSVLLREYEFEMAQPPESYRNDHSKMVVQLA
QPACVRYRRRTGV
>CYP51B1_Mycobacterium tuberculosis TB_RSA158 2605722337
MSAVALPRVSGGHDEHGHLEEFRTDPIGLMQRVRDECGDVGTFQLAGKQVVLLSGSHANEFFFRAGDDDLDQA
KAYPFMTPIFGEGVVFDASPERRKEMLHNAALRGEQMKGHAATIEDQVRRMIADWGEAGEIDLLDFFAELTIY
TSSACLIGKKFRDQLDGRFAKLYHELERGTDPLAYVDPYLPIESFRRRDEARNGLVALVADIMNGRIANPPTD
KSDRDMLDVLIAVKAETGTPRFSADEITGMFISMMFAGHHTSSGTASWTLIELMRHRDAYAAVIDELDELYGD
GRSVSFHALRQIPQLENVLKETLRLHPPLIILMRVAKGEFEVQGHRIHEGDLVAASPAISNRIPEDFPDPHDF
VPARYEQPRQEDLLNRWTWIPFGAGRHRCVGAAFAIMQIKAIFSVLLREYEFEMAQPPESYRNDHSKMVVQLA
QPACVRYRRRTGV
>CYP51B1_Mycobacterium tuberculosis TB_RSA159 2605021381
MSAVALPRVSGGHDEHGHLEEFRTDPIGLMQRVRDECGDVGTFQLAGKQVVLLSGSHANEFFFRAGDDDLDQA
KAYPFMTPIFGEGVVFDASPERRKEMLHNAALRGEQMKGHAATIEDQVRRMIADWGEAGEIDLLDFFAELTIY
TSSACLIGKKFRDQLDGRFAKLYHELERGTDPLAYVDPYLPIESFRRRDEARNGLVALVADIMNGRIANPPTD
KSDRDMLDVLIAVKAETGTPRFSADEITGMFISMMFAGHHTSSGTASWTLIELMRHRDAYAAVIDELDELYGD
GRSVSFHALRQIPQLENVLKETLRLHPPLIILMRVAKGEFEVQGHRIHEGDLVAASPAISNRIPEDFPDPHDF
VPARYEQPRQEDLLNRWTWIPFGAGRHRCVGAAFAIMQIKAIFSVLLREYEFEMAQPPESYRNDHSKMVVQLA
QPACVRYRRRTGV
>CYP51B1_Mycobacterium tuberculosis TB_RSA16 2604667028
MSAVALPRVSGGHDEHGHLEEFRTDPIGLMQRVRDECGDVGTFQLAGKQVVLLSGSHANEFFFRAGDDDLDQA
KAYPFMTPIFGEGVVFDASPERRKEMLHNAALRGEQMKGHAATIEDQVRRMIADWGEAGEIDLLDFFAELTIY
TSSACLIGKKFRDQLDGRFAKLYHELERGTDPLAYVDPYLPIESFRRRDEARNGLVALVADIMNGRIANPPTD
KSDRDMLDVLIAVKAETGTPRFSADEITGMFISMMFAGHHTSSGTASWTLIELMRHRDAYAAVIDELDELYGD
GRSVSFHALRQIPQLENVLKETLRLHPPLIILMRVAKGEFEVQGHRIHEGDLVAASPAISNRIPEDFPDPHDF
VPARYEQPRQEDLLNRWTWIPFGAGRHRCVGAAFAIMQIKAIFSVLLREYEFEMAQPPESYRNDHSKMVVQLA
QPACVRYRRRTGV
>CYP51B1_Mycobacterium tuberculosis TB_RSA160 2604298868
MSAVALPRVSGGHDEHGHLEEFRTDPIGLMQRVRDECGDVGTFQLAGKQVVLLSGSHANEFFFRAGDDDLDQA
KAYPFMTPIFGEGVVFDASPERRKEMLHNAALRGEQMKGHAATIEDQVRRMIADWGEAGEIDLLDFFAELTIY
TSSACLIGKKFRDQLDGRFAKLYHELERGTDPLAYVDPYLPIESFRRRDEARNGLVALVADIMNGRIANPPTD
KSDRDMLDVLIAVKAETGTPRFSADEITGMFISMMFAGHHTSSGTASWTLIELMRHRDAYAAVIDELDELYGD
GRSVSFHALRQIPQLENVLKETLRLHPPLIILMRVAKGEFEVQGHRIHEGDLVAASPAISNRIPEDFPDPHDF
VPARYEQPRQEDLLNRWTWIPFGAGRHRCVGAAFAIMQIKAIFSVLLREYEFEMAQPPESYRNDHSKMVVQLA
QPACVRYRRRTGV
>CYP51B1_Mycobacterium tuberculosis TB_RSA161 2577568842
MSAVALPRVSGGHDEHGHLEEFRTDPIGLMQRVRDECGDVGTFQLAGKQVVLLSGSHANEFFFRAGDDDLDQA
KAYPFMTPIFGEGVVFDASPERRKEMLHNAALRGEQMKGHAATIEDQVRRMIADWGEAGEIDLLDFFAELTIY
TSSACLIGKKFRDQLDGRFAKLYHELERGTDPLAYVDPYLPIESFRRRDEARNGLVALVADIMNGRIANPPTD
KSDRDMLDVLIAVKAETGTPRFSADEITGMFISMMFAGHHTSSGTASWTLIELMRHRDAYAAVIDELDELYGD
GRSVSFHALRQIPQLENVLKETLRLHPPLIILMRVAKGEFEVQGHRIHEGDLVAASPAISNRIPEDFPDPHDF
VPARYEQPRQEDLLNRWTWIPFGAGRHRCVGAAFAIMQIKAIFSVLLREYEFEMAQPPESYRNDHSKMVVQLA
QPACVRYRRRTGV
>CYP51B1_Mycobacterium tuberculosis TB_RSA162 2604673924
MSAVALPRVSGGHDEHGHLEEFRTDPIGLMQRVRDECGDVGTFQLAGKQVVLLSGSHANEFFFRAGDDDLDQA
KAYPFMTPIFGEGVVFDASPERRKEMLHNAALRGEQMKGHAATIEDQVRRMIADWGEAGEIDLLDFFAELTIY
TSSACLIGKKFRDQLDGRFAKLYHELERGTDPLAYVDPYLPIESFRRRDEARNGLVALVADIMNGRIANPPTD
KSDRDMLDVLIAVKAETGTPRFSADEITGMFISMMFAGHHTSSGTASWTLIELMRHRDAYAAVIDELDELYGD
GRSVSFHALRQIPQLENVLKETLRLHPPLIILMRVAKGEFEVQGHRIHEGDLVAASPAISNRIPEDFPDPHDF
VPARYEQPRQEDLLNRWTWIPFGAGRHRCVGAAFAIMQIKAIFSVLLREYEFEMAQPPESYRNDHSKMVVQLA
QPACVRYRRRTGV
>CYP51B1_Mycobacterium tuberculosis TB_RSA163 2576881350
MSAVALPRVSGGHDEHGHLEEFRTDPIGLMQRVRDECGDVGTFQLAGKQVVLLSGSHANEFFFRAGDDDLDQA
KAYPFMTPIFGEGVVFDASPERRKEMLHNAALRGEQMKGHAATIEDQVRRMIADWGEAGEIDLLDFFAELTIY
TSSACLIGKKFRDQLDGRFAKLYHELERGTDPLAYVDPYLPIESFRRRDEARNGLVALVADIMNGRIANPPTD
KSDRDMLDVLIAVKAETGTPRFSADEITGMFISMMFAGHHTSSGTASWTLIELMRHRDAYAAVIDELDELYGD
GRSVSFHALRQIPQLENVLKETLRLHPPLIILMRVAKGEFEVQGHRIHEGDLVAASPAISNRIPEDFPDPHDF

VPARYEQPRQEDLLNRWTWIPFGAGRHRCVGAAFAIMQIKAIFSVLLREYEFEMAQPPESYRNDHSKMVVQLA
QPACVRYRRRTGV
>CYP51B1_Mycobacterium tuberculosis TB_RSA164 2605384079
MSAVALPRVSGGHDEHGHLEEFRTDPIGLMQRVRDECGDVGTFQLAGKQVVLLSGSHANEFFFRAGDDDLDQA
KAYPFMTPIFGEGVVFDASPERRKEMLHNAALRGEQMKGHAATIEDQVRRMIADWGEAGEIDLLDFFAELTIY
TSSACLIGKKFRDQLDGRFAKLYHELERGTDPLAYVDPYLPIESFRRRDEARNGLVALVADIMNGRIANPPTD
KSDRDMLDVLIAVKAETGTPRFSADEITGMFISMMFAGHHTSSGTASWTLIELMRHRDAYAAVIDELDELYGD
GRSVSFHALRQIPQLENVLKETLRLHPPLIILMRVAKGEFEVQGHRIHEGDLVAASPAISNRIPEDFPDPHDF
VPARYEQPRQEDLLNRWTWIPFGAGRHRCVGAAFAIMQIKAIFSVLLREYEFEMAQPPESYRNDHSKMVVQLA
QPACVRYRRRTGV
>CYP51B1_Mycobacterium tuberculosis TB_RSA165 2575409081
MSAVALPRVSGGHDEHGHLEEFRTDPIGLMQRVRDECGDVGTFQLAGKQVVLLSGSHANEFFFRAGDDDLDQA
KAYPFMTPIFGEGVVFDASPERRKEMLHNAALRGEQMKGHAATIEDQVRRMIADWGEAGEIDLLDFFAELTIY
TSSACLIGKKFRDQLDGRFAKLYHELERGTDPLAYVDPYLPIESFRRRDEARNGLVALVADIMNGRIANPPTD
KSDRDMLDVLIAVKAETGTPRFSADEITGMFISMMFAGHHTSSGTASWTLIELMRHRDAYAAVIDELDELYGD
GRSVSFHALRQIPQLENVLKETLRLHPPLIILMRVAKGEFEVQGHRIHEGDLVAASPAISNRIPEDFPDPHDF
VPARYEQPRQEDLLNRWTWIPFGAGRHRCVGAAFAIMQIKAIFSVLLREYEFEMAQPPESYRNDHSKMVVQLA
QPACVRYRRRTGV
>CYP51B1_Mycobacterium tuberculosis TB_RSA166 2584714421
MSAVALPRVSGGHDEHGHLEEFRTDPIGLMQRVRDECGDVGTFQLAGKQVVLLSGSHANEFFFRAGDDDLDQA
KAYPFMTPIFGEGVVFDASPERRKEMLHNAALRGEQMKGHAATIEDQVRRMIADWGEAGEIDLLDFFAELTIY
TSSACLIGKKFRDQLDGRFAKLYHELERGTDPLAYVDPYLPIESFRRRDEARNGLVALVADIMNGRIANPPTD
KSDRDMLDVLIAVKAETGTPRFSADEITGMFISMMFAGHHTSSGTASWTLIELMRHRDAYAAVIDELDELYGD
GRSVSFHALRQIPQLENVLKETLRLHPPLIILMRVAKGEFEVQGHRIHEGDLVAASPAISNRIPEDFPDPHDF
VPARYEQPRQEDLLNRWTWIPFGAGRHRCVGAAFAIMQIKAIFSVLLREYEFEMAQPPESYRNDHSKMVVQLA
QPACVRYRRRTGV
>CYP51B1_Mycobacterium tuberculosis TB_RSA167 2603966456
MSAVALPRVSGGHDEHGHLEEFRTDPIGLMQRVRDECGDVGTFQLAGKQVVLLSGSHANEFFFRAGDDDLDQA
KAYPFMTPIFGEGVVFDASPERRKEMLHNAALRGEQMKGHAATIEDQVRRMIADWGEAGEIDLLDFFAELTIY
TSSACLIGKKFRDQLDGRFAKLYHELERGTDPLAYVDPYLPIESFRRRDEARNGLVALVADIMNGRIANPPTD
KSDRDMLDVLIAVKAETGTPRFSADEITGMFISMMFAGHHTSSGTASWTLIELMRHRDAYAAVIDELDELYGD
GRSVSFHALRQIPQLENVLKETLRLHPPLIILMRVAKGEFEVQGHRIHEGDLVAASPAISNRIPEDFPDPHDF
VPARYEQPRQEDLLNRWTWIPFGAGRHRCVGAAFAIMQIKAIFSVLLREYEFEMAQPPESYRNDHSKMVVQLA
QPACVRYRRRTGV
>CYP51B1_Mycobacterium tuberculosis TB_RSA168 2605723986
MSAVALPRVSGGHDEHGHLEEFRTDPIGLMQRVRDECGDVGTFQLAGKQVVLLSGSHANEFFFRAGDDDLDQA
KAYPFMTPIFGEGVVFDASPERRKEMLHNAALRGEQMKGHAATIEDQVRRMIADWGEAGEIDLLDFFAELTIY
TSSACLIGKKFRDQLDGRFAKLYHELERGTDPLAYVDPYLPIESFRRRDEARNGLVALVADIMNGRIANPPTD
KSDRDMLDVLIAVKAETGTPRFSADEITGMFISMMFAGHHTSSGTASWTLIELMRHRDAYAAVIDELDELYGD
GRSVSFHALRQIPQLENVLKETLRLHPPLIILMRVAKGEFEVQGHRIHEGDLVAASPAISNRIPEDFPDPHDF
VPARYEQPRQEDLLNRWTWIPFGAGRHRCVGAAFAIMQIKAIFSVLLREYEFEMAQPPESYRNDHSKMVVQLA
QPACVRYRRRTGV
>CYP51B1_Mycobacterium tuberculosis TB_RSA169 2603258781
MSAVALPRVSGGHDEHGHLEEFRTDPIGLMQRVRDECGDVGTFQLAGKQVVLLSGSHANEFFFRAGDDDLDQA
KAYPFMTPIFGEGVVFDASPERRKEMLHNAALRGEQMKGHAATIEDQVRRMIADWGEAGEIDLLDFFAELTIY
TSSACLIGKKFRDQLDGRFAKLYHELERGTDPLAYVDPYLPIESFRRRDEARNGLVALVADIMNGRIANPPTD
KSDRDMLDVLIAVKAETGTPRFSADEITGMFISMMFAGHHTSSGTASWTLIELMRHRDAYAAVIDELDELYGD
GRSVSFHALRQIPQLENVLKETLRLHPPLIILMRVAKGEFEVQGHRIHEGDLVAASPAISNRIPEDFPDPHDF
VPARYEQPRQEDLLNRWTWIPFGAGRHRCVGAAFAIMQIKAIFSVLLREYEFEMAQPPESYRNDHSKMVVQLA
QPACVRYRRRTGV
>CYP51B1_Mycobacterium tuberculosis TB_RSA17 2603262005
MSAVALPRVSGGHDEHGHLEEFRTDPIGLMQRVRDECGDVGTFQLAGKQVVLLSGSHANEFFFRAGDDDLDQA
KAYPFMTPIFGEGVVFDASPERRKEMLHNAALRGEQMKGHAATIEDQVRRMIADWGEAGEIDLLDFFAELTIY
TSSACLIGKKFRDQLDGRFAKLYHELERGTDPLAYVDPYLPIESFRRRDEARNGLVALVADIMNGRIANPPTD
KSDRDMLDVLIAVKAETGTPRFSADEITGMFISMMFAGHHTSSGTASWTLIELMRHRDAYAAVIDELDELYGD
GRSVSFHALRQIPQLENVLKETLRLHPPLIILMRVAKGEFEVQGHRIHEGDLVAASPAISNRIPEDFPDPHDF
VPARYEQPRQEDLLNRWTWIPFGAGRHRCVGAAFAIMQIKAIFSVLLREYEFEMAQPPESYRNDHSKMVVQLA
QPACVRYRRRTGV

>CYP51B1_Mycobacterium tuberculosis TB_RSA170 2605727792
MSAVALPRVSGGHDEHGHLEEFRTDPIGLMQRVRDECGDVGTFQLAGKQVVLLSGSHANEFFFRAGDDDLDQA
KAYPFMTPIFGEGVVFDASPERRKEMLHNAALRGEQMKGHAATIEDQVRRMIADWGEAGEIDLLDFFAELTIY
TSSACLIGKKFRDQLDGRFAKLYHELERGTDPLAYVDPYLPIESFRRRDEARNGLVALVADIMNGRIANPPTD
KSDRDMLDVLIAVKAETGTPRFSADEITGMFISMMFAGHHTSSGTASWTLIELMRHRDAYAAVIDELDELYGD
GRSVSFHALRQIPQLENVLKETLRLHPPLIILMRVAKGEFEVQGHRIHEGDLVAASPAISNRIPEDFPDPHDF
VPARYEQPRQEDLLNRWTWIPFGAGRHRCVGAAFAIMQIKAIFSVLLREYEFEMAQPPESYRNDHSKMVVQLA
QPACVRYRRRTGV
>CYP51B1_Mycobacterium tuberculosis TB_RSA171 2603416205
MSAVALPRVSGGHDEHGHLEEFRTDPIGLMQRVRDECGDVGTFQLAGKQVVLLSGSHANEFFFRAGDDDLDQA
KAYPFMTPIFGEGVVFDASPERRKEMLHNAALRGEQMKGHAATIEDQVRRMIADWGEAGEIDLLDFFAELTIY
TSSACLIGKKFRDQLDGRFAKLYHELERGTDPLAYVDPYLPIESFRRRDEARNGLVALVADIMNGRIANPPTD
KSDRDMLDVLIAVKAETGTPRFSADEITGMFISMMFAGHHTSSGTASWTLIELMRHRDAYAAVIDELDELYGD
GRSVSFHALRQIPQLENVLKETLRLHPPLIILMRVAKGEFEVQGHRIHEGDLVAASPAISNRIPEDFPDPHDF
VPARYEQPRQEDLLNRWTWIPFGAGRHRCVGAAFAIMQIKAIFSVLLREYEFEMAQPPESYRNDHSKMVVQLA
QPACVRYRRRTGV
>CYP51B1_Mycobacterium tuberculosis TB_RSA172 2604304131
MSAVALPRVSGGHDEHGHLEEFRTDPIGLMQRVRDECGDVGTFQLAGKQVVLLSGSHANEFFFRAGDDDLDQA
KAYPFMTPIFGEGVVFDASPERRKEMLHNAALRGEQMKGHAATIEDQVRRMIADWGEAGEIDLLDFFAELTIY
TSSACLIGKKFRDQLDGRFAKLYHELERGTDPLAYVDPYLPIESFRRRDEARNGLVALVADIMNGRIANPPTD
KSDRDMLDVLIAVKAETGTPRFSADEITGMFISMMFAGHHTSSGTASWTLIELMRHRDAYAAVIDELDELYGD
GRSVSFHALRQIPQLENVLKETLRLHPPLIILMRVAKGEFEVQGHRIHEGDLVAASPAISNRIPEDFPDPHDF
VPARYEQPRQEDLLNRWTWIPFGAGRHRCVGAAFAIMQIKAIFSVLLREYEFEMAQPPESYRNDHSKMVVQLA
QPACVRYRRRTGV
>CYP51B1_Mycobacterium tuberculosis TB_RSA173 2574938447
MSAVALPRVSGGHDEHGHLEEFRTDPIGLMQRVRDECGDVGTFQLAGKQVVLLSGSHANEFFFRAGDDDLDQA
KAYPFMTPIFGEGVVFDASPERRKEMLHNAALRGEQMKGHAATIEDQVRRMIADWGEAGEIDLLDFFAELTIY
TSSACLIGKKFRDQLDGRFAKLYHELERGTDPLAYVDPYLPIESFRRRDEARNGLVALVADIMNGRIANPPTD
KSDRDMLDVLIAVKAETGTPRFSADEITGMFISMMFAGHHTSSGTASWTLIELMRHRDAYAAVIDELDELYGD
GRSVSFHALRQIPQLENVLKETLRLHPPLIILMRVAKGEFEVQGHRIHEGDLVAASPAISNRIPEDFPDPHDF
VPARYEQPRQEDLLNRWTWIPFGAGRHRCVGAAFAIMQIKAIFSVLLREYEFEMAQPPESYRNDHSKMVVQLA
QPACVRYRRRTGV
>CYP51B1_Mycobacterium tuberculosis TB_RSA174 2575141769
MSAVALPRVSGGHDEHGHLEEFRTDPIGLMQRVRDECGDVGTFQLAGKQVVLLSGSHANEFFFRAGDDDLDQA
KAYPFMTPIFGEGVVFDASPERRKEMLHNAALRGEQMKGHAATIEDQVRRMIADWGEAGEIDLLDFFAELTIY
TSSACLIGKKFRDQLDGRFAKLYHELERGTDPLAYVDPYLPIESFRRRDEARNGLVALVADIMNGRIANPPTD
KSDRDMLDVLIAVKAETGTPRFSADEITGMFISMMFAGHHTSSGTASWTLIELMRHRDAYAAVIDELDELYGD
GRSVSFHALRQIPQLENVLKETLRLHPPLIILMRVAKGEFEVQGHRIHEGDLVAASPAISNRIPEDFPDPHDF
VPARYEQPRQEDLLNRWTWIPFGAGRHRCVGAAFAIMQIKAIFSVLLREYEFEMAQPPESYRNDHSKMVVQLA
QPACVRYRRRTGV
>CYP51B1_Mycobacterium tuberculosis TB_RSA175 2605387628
MSAVALPRVSGGHDEHGHLEEFRTDPIGLMQRVRDECGDVGTFQLAGKQVVLLSGSHANEFFFRAGDDDLDQA
KAYPFMTPIFGEGVVFDASPERRKEMLHNAALRGEQMKGHAATIEDQVRRMIADWGEAGEIDLLDFFAELTIY
TSSACLIGKKFRDQLDGRFAKLYHELERGTDPLAYVDPYLPIESFRRRDEARNGLVALVADIMNGRIANPPTD
KSDRDMLDVLIAVKAETGTPRFSADEITGMFISMMFAGHHTSSGTASWTLIELMRHRDAYAAVIDELDELYGD
GRSVSFHALRQIPQLENVLKETLRLHPPLIILMRVAKGEFEVQGHRIHEGDLVAASPAISNRIPEDFPDPHDF
VPARYEQPRQEDLLNRWTWIPFGAGRHRCVGAAFAIMQIKAIFSVLLREYEFEMAQPPESYRNDHSKMVVQLA
QPACVRYRRRTGV
>CYP51B1_Mycobacterium tuberculosis TB_RSA176 2603971140
MSAVALPRVSGGHDEHGHLEEFRTDPIGLMQRVRDECGDVGTFQLAGKQVVLLSGSHANEFFFRAGDDDLDQA
KAYPFMTPIFGEGVVFDASPERRKEMLHNAALRGEQMKGHAATIEDQVRRMIADWGEAGEIDLLDFFAELTIY
TSSACLIGKKFRDQLDGRFAKLYHELERGTDPLAYVDPYLPIESFRRRDEARNGLVALVADIMNGRIANPPTD
KSDRDMLDVLIAVKAETGTPRFSADEITGMFISMMFAGHHTSSGTASWTLIELMRHRDAYAAVIDELDELYGD
GRSVSFHALRQIPQLENVLKETLRLHPPLIILMRVAKGEFEVQGHRIHEGDLVAASPAISNRIPEDFPDPHDF
VPARYEQPRQEDLLNRWTWIPFGAGRHRCVGAAFAIMQIKAIFSVLLREYEFEMAQPPESYRNDHSKMVVQLA
QPACVRYRRRTGV
>CYP51B1_Mycobacterium tuberculosis TB_RSA177 2605390682

MSAVALPRVSGGHDEHGHLEEFRTDPIGLMQRVRDECGDVGTFQLAGKQVVLLSGSHANEFFFRAGDDDLDQA
KAYPFMTPIFGEGVVFDASPERRKEMLHNAALRGEQMKGHAATIEDQVRRMIADWGEAGEIDLLDFFAELTIY
TSSACLIGKKFRDQLDGRFAKLYHELERGTDPLAYVDPYLPIESFRRRDEARNGLVALVADIMNGRIANPPTD
KSDRDMLDVLIAVKAETGTPRFSADEITGMFISMMFAGHHTSSGTASWTLIELMRHRDAYAAVIDELDELYGD
GRSVSFHALRQIPQLENVLKETLRLHPPLIILMRVAKGEFEVQGHRIHEGDLVAASPAISNRIPEDFPDPHDF
VPARYEQPRQEDLLNRWTWIPFGAGRHRCVGAAFAIMQIKAIFSVLLREYEFEMAQPPESYRNDHSKMVVQLA
QPACVRYRRRTGV
>CYP51B1_Mycobacterium tuberculosis TB_RSA178 2577037102
MSAVALPRVSGGHDEHGHLEEFRTDPIGLMQRVRDECGDVGTFQLAGKQVVLLSGSHANEFFFRAGDDDLDQA
KAYPFMTPIFGEGVVFDASPERRKEMLHNAALRGEQMKGHAATIEDQVRRMIADWGEAGEIDLLDFFAELTIY
TSSACLIGKKFRDQLDGRFAKLYHELERGTDPLAYVDPYLPIESFRRRDEARNGLVALVADIMNGRIANPPTD
KSDRDMLDVLIAVKAETGTPRFSADEITGMFISMMFAGHHTSSGTASWTLIELMRHRDAYAAVIDELDELYGD
GRSVSFHALRQIPQLENVLKETLRLHPPLIILMRVAKGEFEVQGHRIHEGDLVAASPAISNRIPEDFPDPHDF
VPARYEQPRQEDLLNRWTWIPFGAGRHRCVGAAFAIMQIKAIFSVLLREYEFEMAQPPESYRNDHSKMVVQLA
QPACVRYRRRTGV
>CYP51B1_Mycobacterium tuberculosis TB_RSA179 2603977500
MSAVALPRVSGGHDEHGHLEEFRTDPIGLMQRVRDECGDVGTFQLAGKQVVLLSGSHANEFFFRAGDDDLDQA
KAYPFMTPIFGEGVVFDASPERRKEMLHNAALRGEQMKGHAATIEDQVRRMIADWGEAGEIDLLDFFAELTIY
TSSACLIGKKFRDQLDGRFAKLYHELERGTDPLAYVDPYLPIESFRRRDEARNGLVALVADIMNGRIANPPTD
KSDRDMLDVLIAVKAETGTPRFSADEITGMFISMMFAGHHTSSGTASWTLIELMRHRDAYAAVIDELDELYGD
GRSVSFHALRQIPQLENVLKETLRLHPPLIILMRVAKGEFEVQGHRIHEGDLVAASPAISNRIPEDFPDPHDF
VPARYEQPRQEDLLNRWTWIPFGAGRHRCVGAAFAIMQIKAIFSVLLREYEFEMAQPPESYRNDHSKMVVQLA
QPACVRYRRRTGV
>CYP51B1_Mycobacterium tuberculosis TB_RSA18 2576588209
MSAVALPRVSGGHDEHGHLEEFRTDPIGLMQRVRDECGDVGTFQLAGKQVVLLSGSHANEFFFRAGDDDLDQA
KAYPFMTPIFGEGVVFDASPERRKEMLHNAALRGEQMKGHAATIEDQVRRMIADWGEAGEIDLLDFFAELTIY
TSSACLIGKKFRDQLDGRFAKLYHELERGTDPLAYVDPYLPIESFRRRDEARNGLVALVADIMNGRIANPPTD
KSDRDMLDVLIAVKAETGTPRFSADEITGMFISMMFAGHHTSSGTASWTLIELMRHRDAYAAVIDELDELYGD
GRSVSFHALRQIPQLENVLKETLRLHPPLIILMRVAKGEFEVQGHRIHEGDLVAASPAISNRIPEDFPDPHDF
VPARYEQPRQEDLLNRWTWIPFGAGRHRCVGAAFAIMQIKAIFSVLLREYEFEMAQPPESYRNDHSKMVVQLA
QPACVRYRRRTGV
>CYP51B1_Mycobacterium tuberculosis TB_RSA180 2603981607
MSAVALPRVSGGHDEHGHLEEFRTDPIGLMQRVRDECGDVGTFQLAGKQVVLLSGSHANEFFFRAGDDDLDQA
KAYPFMTPIFGEGVVFDASPERRKEMLHNAALRGEQMKGHAATIEDQVRRMIADWGEAGEIDLLDFFAELTIY
TSSACLIGKKFRDQLDGRFAKLYHELERGTDPLAYVDPYLPIESFRRRDEARNGLVALVADIMNGRIANPPTD
KSDRDMLDVLIAVKAETGTPRFSADEITGMFISMMFAGHHTSSGTASWTLIELMRHRDAYAAVIDELDELYGD
GRSVSFHALRQIPQLENVLKETLRLHPPLIILMRVAKGEFEVQGHRIHEGDLVAASPAISNRIPEDFPDPHDF
VPARYEQPRQEDLLNRWTWIPFGAGRHRCVGAAFAIMQIKAIFSVLLREYEFEMAQPPESYRNDHSKMVVQLA
QPACVRYRRRTGV
>CYP51B1_Mycobacterium tuberculosis TB_RSA181 2603421655
MSAVALPRVSGGHDEHGHLEEFRTDPIGLMQRVRDECGDVGTFQLAGKQVVLLSGSHANEFFFRAGDDDLDQA
KAYPFMTPIFGEGVVFDASPERRKEMLHNAALRGEQMKGHAATIEDQVRRMIADWGEAGEIDLLDFFAELTIY
TSSACLIGKKFRDQLDGRFAKLYHELERGTDPLAYVDPYLPIESFRRRDEARNGLVALVADIMNGRIANPPTD
KSDRDMLDVLIAVKAETGTPRFSADEITGMFISMMFAGHHTSSGTASWTLIELMRHRDAYAAVIDELDELYGD
GRSVSFHALRQIPQLENVLKETLRLHPPLIILMRVAKGEFEVQGHRIHEGDLVAASPAISNRIPEDFPDPHDF
VPARYEQPRQEDLLNRWTWIPFGAGRHRCVGAAFAIMQIKAIFSVLLREYEFEMAQPPESYRNDHSKMVVQLA
QPACVRYRRRTGV
>CYP51B1_Mycobacterium tuberculosis TB_RSA182 2604306738
MSAVALPRVSGGHDEHGHLEEFRTDPIGLMQRVRDECGDVGTFQLAGKQVVLLSGSHANEFFFRAGDDDLDQA
KAYPFMTPIFGEGVVFDASPERRKEMLHNAALRGEQMKGHAATIEDQVRRMIADWGEAGEIDLLDFFAELTIY
TSSACLIGKKFRDQLDGRFAKLYHELERGTDPLAYVDPYLPIESFRRRDEARNGLVALVADIMNGRIANPPTD
KSDRDMLDVLIAVKAETGTPRFSADEITGMFISMMFAGHHTSSGTASWTLIELMRHRDAYAAVIDELDELYGD
GRSVSFHALRQIPQLENVLKETLRLHPPLIILMRVAKGEFEVQGHRIHEGDLVAASPAISNRIPEDFPDPHDF
VPARYEQPRQEDLLNRWTWIPFGAGRHRCVGAAFAIMQIKAIFSVLLREYEFEMAQPPESYRNDHSKMVVQLA
QPACVRYRRRTGV
>CYP51B1_Mycobacterium tuberculosis TB_RSA184 2603268641
MSAVALPRVSGGHDEHGHLEEFRTDPIGLMQRVRDECGDVGTFQLAGKQVVLLSGSHANEFFFRAGDDDLDQA
KAYPFMTPIFGEGVVFDASPERRKEMLHNAALRGEQMKGHAATIEDQVRRMIADWGEAGEIDLLDFFAELTIY

TSSACLIGKKFRDQLDGRFAKLYHELERGTDPLAYVDPYLPIESFRRRDEARNGLVALVADIMNGRIANPPTD
KSDRDMLDVLIAVKAETGTPRFSADEITGMFISMMFAGHHTSSGTASWTLIELMRHRDAYAAVIDELDELYGD
GRSVSFHALRQIPQLENVLKETLRLHPPLIILMRVAKGEFEVQGHRIHEGDLVAASPAISNRIPEDFPDPHDF
VPARYEQPRQEDLLNRWTWIPFGAGRHRCVGAAFAIMQIKAIFSVLLREYEFEMAQPPESYRNDHSKMVVQLA
QPACVRYRRRTGV
>CYP51B1_Mycobacterium tuberculosis TB_RSA189 2604675546
MSAVALPRVSGGHDEHGHLEEFRTDPIGLMQRVRDECGDVGTFQLAGKQVVLLSGSHANEFFFRAGDDDLDQA
KAYPFMTPIFGEGVVFDASPERRKEMLHNAALRGEQMKGHAATIEDQVRRMIADWGEAGEIDLLDFFAELTIY
TSSACLIGKKFRDQLDGRFAKLYHELERGTDPLAYVDPYLPIESFRRRDEARNGLVALVADIMNGRIANPPTD
KSDRDMLDVLIAVKAETGTPRFSADEITGMFISMMFAGHHTSSGTASWTLIELMRHRDAYAAVIDELDELYGD
GRSVSFHALRQIPQLENVLKETLRLHPPLIILMRVAKGEFEVQGHRIHEGDLVAASPAISNRIPEDFPDPHDF
VPARYEQPRQEDLLNRWTWIPFGAGRHRCVGAAFAIMQIKAIFSVLLREYEFEMAQPPESYRNDHSKMVVQLA
QPACVRYRRRTGV
>CYP51B1_Mycobacterium tuberculosis TB_RSA19 2603983230
MSAVALPRVSGGHDEHGHLEEFRTDPIGLMQRVRDECGDVGTFQLAGKQVVLLSGSHANEFFFRAGDDDLDQA
KAYPFMTPIFGEGVVFDASPERRKEMLHNAALRGEQMKGHAATIEDQVRRMIADWGEAGEIDLLDFFAELTIY
TSSACLIGKKFRDQLDGRFAKLYHELERGTDPLAYVDPYLPIESFRRRDEARNGLVALVADIMNGRIANPPTD
KSDRDMLDVLIAVKAETGTPRFSADEITGMFISMMFAGHHTSSGTASWTLIELMRHRDAYAAVIDELDELYGD
GRSVSFHALRQIPQLENVLKETLRLHPPLIILMRVAKGEFEVQGHRIHEGDLVAASPAISNRIPEDFPDPHDF
VPARYEQPRQEDLLNRWTWIPFGAGRHRCVGAAFAIMQIKAIFSVLLREYEFEMAQPPESYRNDHSKMVVQLA
QPACVRYRRRTGV
>CYP51B1_Mycobacterium tuberculosis TB_RSA190 2603987043
MSAVALPRVSGGHDEHGHLEEFRTDPIGLMQRVRDECGDVGTFQLAGKQVVLLSGSHANEFFFRAGDDDLDQA
KAYPFMTPIFGEGVVFDASPERRKEMLHNAALRGEQMKGHAATIEDQVRRMIADWGEAGEIDLLDFFAELTIY
TSSACLIGKKFRDQLDGRFAKLYHELERGTDPLAYVDPYLPIESFRRRDEARNGLVALVADIMNGRIANPPTD
KSDRDMLDVLIAVKAETGTPRFSADEITGMFISMMFAGHHTSSGTASWTLIELMRHRDAYAAVIDELDELYGD
GRSVSFHALRQIPQLENVLKETLRLHPPLIILMRVAKGEFEVQGHRIHEGDLVAASPAISNRIPEDFPDPHDF
VPARYEQPRQEDLLNRWTWIPFGAGRHRCVGAAFAIMQIKAIFSVLLREYEFEMAQPPESYRNDHSKMVVQLA
QPACVRYRRRTGV
>CYP51B1_Mycobacterium tuberculosis TB_RSA191 2604310847
MSAVALPRVSGGHDEHGHLEEFRTDPIGLMQRVRDECGDVGTFQLAGKQVVLLSGSHANEFFFRAGDDDLDQA
KAYPFMTPIFGEGVVFDASPERRKEMLHNAALRGEQMKGHAATIEDQVRRMIADWGEAGEIDLLDFFAELTIY
TSSACLIGKKFRDQLDGRFAKLYHELERGTDPLAYVDPYLPIESFRRRDEARNGLVALVADIMNGRIANPPTD
KSDRDMLDVLIAVKAETGTPRFSADEITGMFISMMFAGHHTSSGTASWTLIELMRHRDAYAAVIDELDELYGD
GRSVSFHALRQIPQLENVLKETLRLHPPLIILMRVAKGEFEVQGHRIHEGDLVAASPAISNRIPEDFPDPHDF
VPARYEQPRQEDLLNRWTWIPFGAGRHRCVGAAFAIMQIKAIFSVLLREYEFEMAQPPESYRNDHSKMVVQLA
QPACVRYRRRTGV
>CYP51B1_Mycobacterium tuberculosis TB_RSA192 2603270535
MSAVALPRVSGGHDEHGHLEEFRTDPIGLMQRVRDECGDVGTFQLAGKQVVLLSGSHANEFFFRAGDDDLDQA
KAYPFMTPIFGEGVVFDASPERRKEMLHNAALRGEQMKGHAATIEDQVRRMIADWGEAGEIDLLDFFAELTIY
TSSACLIGKKFRDQLDGRFAKLYHELERGTDPLAYVDPYLPIESFRRRDEARNGLVALVADIMNGRIANPPTD
KSDRDMLDVLIAVKAETGTPRFSADEITGMFISMMFAGHHTSSGTASWTLIELMRHRDAYAAVIDELDELYGD
GRSVSFHALRQIPQLENVLKETLRLHPPLIILMRVAKGEFEVQGHRIHEGDLVAASPAISNRIPEDFPDPHDF
VPARYEQPRQEDLLNRWTWIPFGAGRHRCVGAAFAIMQIKAIFSVLLREYEFEMAQPPESYRNDHSKMVVQLA
QPACVRYRRRTGV
>CYP51B1_Mycobacterium tuberculosis TB_RSA193 2605025176
MSAVALPRVSGGHDEHGHLEEFRTDPIGLMQRVRDECGDVGTFQLAGKQVVLLSGSHANEFFFRAGDDDLDQA
KAYPFMTPIFGEGVVFDASPERRKEMLHNAALRGEQMKGHAATIEDQVRRMIADWGEAGEIDLLDFFAELTIY
TSSACLIGKKFRDQLDGRFAKLYHELERGTDPLAYVDPYLPIESFRRRDEARNGLVALVADIMNGRIANPPTD
KSDRDMLDVLIAVKAETGTPRFSADEITGMFISMMFAGHHTSSGTASWTLIELMRHRDAYAAVIDELDELYGD
GRSVSFHALRQIPQLENVLKETLRLHPPLIILMRVAKGEFEVQGHRIHEGDLVAASPAISNRIPEDFPDPHDF
VPARYEQPRQEDLLNRWTWIPFGAGRHRCVGAAFAIMQIKAIFSVLLREYEFEMAQPPESYRNDHSKMVVQLA
QPACVRYRRRTGV
>CYP51B1_Mycobacterium tuberculosis TB_RSA194 2574783478
MSAVALPRVSGGHDEHGHLEEFRTDPIGLMQRVRDECGDVGTFQLAGKQVVLLSGSHANEFFFRAGDDDLDQA
KAYPFMTPIFGEGVVFDASPERRKEMLHNAALRGEQMKGHAATIEDQVRRMIADWGEAGEIDLLDFFAELTIY
TSSACLIGKKFRDQLDGRFAKLYHELERGTDPLAYVDPYLPIESFRRRDEARNGLVALVADIMNGRIANPPTD
KSDRDMLDVLIAVKAETGTPRFSADEITGMFISMMFAGHHTSSGTASWTLIELMRHRDAYAAVIDELDELYGD

GRSVSFHALRQIPQLENVLKETLRLHPPLIILMRVAKGEFEVQGHRIHEGDLVAASPAISNRIPEDFPDPHDF
VPARYEQPRQEDLLNRWTWIPFGAGRHRCVGAAFAIMQIKAIFSVLLREYEFEMAQPPESYRNDHSKMVVQLA
QPACVRYRRRTGV
>CYP51B1_Mycobacterium tuberculosis TB_RSA195 2576398779
MSAVALPRVSGGHDEHGHLEEFRTDPIGLMQRVRDECGDVGTFQLAGKQVVLLSGSHANEFFFRAGDDDLDQA
KAYPFMTPIFGEGVVFDASPERRKEMLHNAALRGEQMKGHAATIEDQVRRMIADWGEAGEIDLLDFFAELTIY
TSSACLIGKKFRDQLDGRFAKLYHELERGTDPLAYVDPYLPIESFRRRDEARNGLVALVADIMNGRIANPPTD
KSDRDMLDVLIAVKAETGTPRFSADEITGMFISMMFAGHHTSSGTASWTLIELMRHRDAYAAVIDELDELYGD
GRSVSFHALRQIPQLENVLKETLRLHPPLIILMRVAKGEFEVQGHRIHEGDLVAASPAISNRIPEDFPDPHDF
VPARYEQPRQEDLLNRWTWIPFGAGRHRCVGAAFAIMQIKAIFSVLLREYEFEMAQPPESYRNDHSKMVVQLA
QPACVRYRRRTGV
>CYP51B1_Mycobacterium tuberculosis TB_RSA196 2603994096
MSAVALPRVSGGHDEHGHLEEFRTDPIGLMQRVRDECGDVGTFQLAGKQVVLLSGSHANEFFFRAGDDDLDQA
KAYPFMTPIFGEGVVFDASPERRKEMLHNAALRGEQMKGHAATIEDQVRRMIADWGEAGEIDLLDFFAELTIY
TSSACLIGKKFRDQLDGRFAKLYHELERGTDPLAYVDPYLPIESFRRRDEARNGLVALVADIMNGRIANPPTD
KSDRDMLDVLIAVKAETGTPRFSADEITGMFISMMFAGHHTSSGTASWTLIELMRHRDAYAAVIDELDELYGD
GRSVSFHALRQIPQLENVLKETLRLHPPLIILMRVAKGEFEVQGHRIHEGDLVAASPAISNRIPEDFPDPHDF
VPARYEQPRQEDLLNRWTWIPFGAGRHRCVGAAFAIMQIKAIFSVLLREYEFEMAQPPESYRNDHSKMVVQLA
QPACVRYRRRTGV
>CYP51B1_Mycobacterium tuberculosis TB_RSA197 2604314658
MSAVALPRVSGGHDEHGHLEEFRTDPIGLMQRVRDECGDVGTFQLAGKQVVLLSGSHANEFFFRAGDDDLDQA
KAYPFMTPIFGEGVVFDASPERRKEMLHNAALRGEQMKGHAATIEDQVRRMIADWGEAGEIDLLDFFAELTIY
TSSACLIGKKFRDQLDGRFAKLYHELERGTDPLAYVDPYLPIESFRRRDEARNGLVALVADIMNGRIANPPTD
KSDRDMLDVLIAVKAETGTPRFSADEITGMFISMMFAGHHTSSGTASWTLIELMRHRDAYAAVIDELDELYGD
GRSVSFHALRQIPQLENVLKETLRLHPPLIILMRVAKGEFEVQGHRIHEGDLVAASPAISNRIPEDFPDPHDF
VPARYEQPRQEDLLNRWTWIPFGAGRHRCVGAAFAIMQIKAIFSVLLREYEFEMAQPPESYRNDHSKMVVQLA
QPACVRYRRRTGV
>CYP51B1_Mycobacterium tuberculosis TB_RSA199 2576062634
MSAVALPRVSGGHDEHGHLEEFRTDPIGLMQRVRDECGDVGTFQLAGKQVVLLSGSHANEFFFRAGDDDLDQA
KAYPFMTPIFGEGVVFDASPERRKEMLHNAALRGEQMKGHAATIEDQVRRMIADWGEAGEIDLLDFFAELTIY
TSSACLIGKKFRDQLDGRFAKLYHELERGTDPLAYVDPYLPIESFRRRDEARNGLVALVADIMNGRIANPPTD
KSDRDMLDVLIAVKAETGTPRFSADEITGMFISMMFAGHHTSSGTASWTLIELMRHRDAYAAVIDELDELYGD
GRSVSFHALRQIPQLENVLKETLRLHPPLIILMRVAKGEFEVQGHRIHEGDLVAASPAISNRIPEDFPDPHDF
VPARYEQPRQEDLLNRWTWIPFGAGRHRCVGAAFAIMQIKAIFSVLLREYEFEMAQPPESYRNDHSKMVVQLA
QPACVRYRRRTGV
>CYP51B1_Mycobacterium tuberculosis TB_RSA20 2604318768
MSAVALPRVSGGHDEHGHLEEFRTDPIGLMQRVRDECGDVGTFQLAGKQVVLLSGSHANEFFFRAGDDDLDQA
KAYPFMTPIFGEGVVFDASPERRKEMLHNAALRGEQMKGHAATIEDQVRRMIADWGEAGEIDLLDFFAELTIY
TSSACLIGKKFRDQLDGRFAKLYHELERGTDPLAYVDPYLPIESFRRRDEARNGLVALVADIMNGRIANPPTD
KSDRDMLDVLIAVKAETGTPRFSADEITGMFISMMFAGHHTSSGTASWTLIELMRHRDAYAAVIDELDELYGD
GRSVSFHALRQIPQLENVLKETLRLHPPLIILMRVAKGEFEVQGHRIHEGDLVAASPAISNRIPEDFPDPHDF
VPARYEQPRQEDLLNRWTWIPFGAGRHRCVGAAFAIMQIKAIFSVLLREYEFEMAQPPESYRNDHSKMVVQLA
QPACVRYRRRTGV
>CYP51B1_Mycobacterium tuberculosis TB_RSA200 2605732864
MSAVALPRVSGGHDEHGHLEEFRTDPIGLMQRVRDECGDVGTFQLAGKQVVLLSGSHANEFFFRAGDDDLDQA
KAYPFMTPIFGEGVVFDASPERRKEMLHNAALRGEQMKGHAATIEDQVRRMIADWGEAGEIDLLDFFAELTIY
TSSACLIGKKFRDQLDGRFAKLYHELERGTDPLAYVDPYLPIESFRRRDEARNGLVALVADIMNGRIANPPTD
KSDRDMLDVLIAVKAETGTPRFSADEITGMFISMMFAGHHTSSGTASWTLIELMRHRDAYAAVIDELDELYGD
GRSVSFHALRQIPQLENVLKETLRLHPPLIILMRVAKGEFEVQGHRIHEGDLVAASPAISNRIPEDFPDPHDF
VPARYEQPRQEDLLNRWTWIPFGAGRHRCVGAAFAIMQIKAIFSVLLREYEFEMAQPPESYRNDHSKMVVQLA
QPACVRYRRRTGV
>CYP51B1_Mycobacterium tuberculosis TB_RSA21 2577811119
MSAVALPRVSGGHDEHGHLEEFRTDPIGLMQRVRDECGDVGTFQLAGKQVVLLSGSHANEFFFRAGDDDLDQA
KAYPFMTPIFGEGVVFDASPERRKEMLHNAALRGEQMKGHAATIEDQVRRMIADWGEAGEIDLLDFFAELTIY
TSSACLIGKKFRDQLDGRFAKLYHELERGTDPLAYVDPYLPIESFRRRDEARNGLVALVADIMNGRIANPPTD
KSDRDMLDVLIAVKAETGTPRFSADEITGMFISMMFAGHHTSSGTASWTLIELMRHRDAYAAVIDELDELYGD
GRSVSFHALRQIPQLENVLKETLRLHPPLIILMRVAKGEFEVQGHRIHEGDLVAASPAISNRIPEDFPDPHDF

VPARYEQPRQEDLLNRWTWIPFGAGRHRCVGAAFAIMQIKAIFSVLLREYEFEMAQPPESYRNDHSKMVVQLA
QPACVRYRRRTGV
>CYP51B1_Mycobacterium tuberculosis TB_RSA22 2603274346
MSAVALPRVSGGHDEHGHLEEFRTDPIGLMQRVRDECGDVGTFQLAGKQVVLLSGSHANEFFFRAGDDDLDQA
KAYPFMTPIFGEGVVFDASPERRKEMLHNAALRGEQMKGHAATIEDQVRRMIADWGEAGEIDLLDFFAELTIY
TSSACLIGKKFRDQLDGRFAKLYHELERGTDPLAYVDPYLPIESFRRRDEARNGLVALVADIMNGRIANPPTD
KSDRDMLDVLIAVKAETGTPRFSADEITGMFISMMFAGHHTSSGTASWTLIELMRHRDAYAAVIDELDELYGD
GRSVSFHALRQIPQLENVLKETLRLHPPLIILMRVAKGEFEVQGHRIHEGDLVAASPAISNRIPEDFPDPHDF
VPARYEQPRQEDLLNRWTWIPFGAGRHRCVGAAFAIMQIKAIFSVLLREYEFEMAQPPESYRNDHSKMVVQLA
QPACVRYRRRTGV
>CYP51B1_Mycobacterium tuberculosis TB_RSA23 2604680914
MSAVALPRVSGGHDEHGHLEEFRTDPIGLMQRVRDECGDVGTFQLAGKQVVLLSGSHANEFFFRAGDDDLDQA
KAYPFMTPIFGEGVVFDASPERRKEMLHNAALRGEQMKGHAATIEDQVRRMIADWGEAGEIDLLDFFAELTIY
TSSACLIGKKFRDQLDGRFAKLYHELERGTDPLAYVDPYLPIESFRRRDEARNGLVALVADIMNGRIANPPTD
KSDRDMLDVLIAVKAETGTPRFSADEITGMFISMMFAGHHTSSGTASWTLIELMRHRDAYAAVIDELDELYGD
GRSVSFHALRQIPQLENVLKETLRLHPPLIILMRVAKGEFEVQGHRIHEGDLVAASPAISNRIPEDFPDPHDF
VPARYEQPRQEDLLNRWTWIPFGAGRHRCVGAAFAIMQIKAIFSVLLREYEFEMAQPPESYRNDHSKMVVQLA
QPACVRYRRRTGV
>CYP51B1_Mycobacterium tuberculosis TB_RSA24 2603279343
MSAVALPRVSGGHDEHGHLEEFRTDPIGLMQRVRDECGDVGTFQLAGKQVVLLSGSHANEFFFRAGDDDLDQA
KAYPFMTPIFGEGVVFDASPERRKEMLHNAALRGEQMKGHAATIEDQVRRMIADWGEAGEIDLLDFFAELTIY
TSSACLIGKKFRDQLDGRFAKLYHELERGTDPLAYVDPYLPIESFRRRDEARNGLVALVADIMNGRIANPPTD
KSDRDMLDVLIAVKAETGTPRFSADEITGMFISMMFAGHHTSSGTASWTLIELMRHRDAYAAVIDELDELYGD
GRSVSFHALRQIPQLENVLKETLRLHPPLIILMRVAKGEFEVQGHRIHEGDLVAASPAISNRIPEDFPDPHDF
VPARYEQPRQEDLLNRWTWIPFGAGRHRCVGAAFAIMQIKAIFSVLLREYEFEMAQPPESYRNDHSKMVVQLA
QPACVRYRRRTGV
>CYP51B1_Mycobacterium tuberculosis TB_RSA25 2584736123
MSAVALPRVSGGHDEHGHLEEFRTDPIGLMQRVRDECGDVGTFQLAGKQVVLLSGSHANEFFFRAGDDDLDQA
KAYPFMTPIFGEGVVFDASPERRKEMLHNAALRGEQMKGHAATIEDQVRRMIADWGEAGEIDLLDFFAELTIY
TSSACLIGKKFRDQLDGRFAKLYHELERGTDPLAYVDPYLPIESFRRRDEARNGLVALVADIMNGRIANPPTD
KSDRDMLDVLIAVKAETGTPRFSADEITGMFISMMFAGHHTSSGTASWTLIELMRHRDAYAAVIDELDELYGD
GRSVSFHALRQIPQLENVLKETLRLHPPLIILMRVAKGEFEVQGHRIHEGDLVAASPAISNRIPEDFPDPHDF
VPARYEQPRQEDLLNRWTWIPFGAGRHRCVGAAFAIMQIKAIFSVLLREYEFEMAQPPESYRNDHSKMVVQLA
QPACVRYRRRTGV
>CYP51B1_Mycobacterium tuberculosis TB_RSA26 2604325623
MSAVALPRVSGGHDEHGHLEEFRTDPIGLMQRVRDECGDVGTFQLAGKQVVLLSGSHANEFFFRAGDDDLDQA
KAYPFMTPIFGEGVVFDASPERRKEMLHNAALRGEQMKGHAATIEDQVRRMIADWGEAGEIDLLDFFAELTIY
TSSACLIGKKFRDQLDGRFAKLYHELERGTDPLAYVDPYLPIESFRRRDEARNGLVALVADIMNGRIANPPTD
KSDRDMLDVLIAVKAETGTPRFSADEITGMFISMMFAGHHTSSGTASWTLIELMRHRDAYAAVIDELDELYGD
GRSVSFHALRQIPQLENVLKETLRLHPPLIILMRVAKGEFEVQGHRIHEGDLVAASPAISNRIPEDFPDPHDF
VPARYEQPRQEDLLNRWTWIPFGAGRHRCVGAAFAIMQIKAIFSVLLREYEFEMAQPPESYRNDHSKMVVQLA
QPACVRYRRRTGV
>CYP51B1_Mycobacterium tuberculosis TB_RSA27 2605393562
MSAVALPRVSGGHDEHGHLEEFRTDPIGLMQRVRDECGDVGTFQLAGKQVVLLSGSHANEFFFRAGDDDLDQA
KAYPFMTPIFGEGVVFDASPERRKEMLHNAALRGEQMKGHAATIEDQVRRMIADWGEAGEIDLLDFFAELTIY
TSSACLIGKKFRDQLDGRFAKLYHELERGTDPLAYVDPYLPIESFRRRDEARNGLVALVADIMNGRIANPPTD
KSDRDMLDVLIAVKAETGTPRFSADEITGMFISMMFAGHHTSSGTASWTLIELMRHRDAYAAVIDELDELYGD
GRSVSFHALRQIPQLENVLKETLRLHPPLIILMRVAKGEFEVQGHRIHEGDLVAASPAISNRIPEDFPDPHDF
VPARYEQPRQEDLLNRWTWIPFGAGRHRCVGAAFAIMQIKAIFSVLLREYEFEMAQPPESYRNDHSKMVVQLA
QPACVRYRRRTGV
>CYP51B1_Mycobacterium tuberculosis TB_RSA28 2605400197
MSAVALPRVSGGHDEHGHLEEFRTDPIGLMQRVRDECGDVGTFQLAGKQVVLLSGSHANEFFFRAGDDDLDQA
KAYPFMTPIFGEGVVFDASPERRKEMLHNAALRGEQMKGHAATIEDQVRRMIADWGEAGEIDLLDFFAELTIY
TSSACLIGKKFRDQLDGRFAKLYHELERGTDPLAYVDPYLPIESFRRRDEARNGLVALVADIMNGRIANPPTD
KSDRDMLDVLIAVKAETGTPRFSADEITGMFISMMFAGHHTSSGTASWTLIELMRHRDAYAAVIDELDELYGD
GRSVSFHALRQIPQLENVLKETLRLHPPLIILMRVAKGEFEVQGHRIHEGDLVAASPAISNRIPEDFPDPHDF
VPARYEQPRQEDLLNRWTWIPFGAGRHRCVGAAFAIMQIKAIFSVLLREYEFEMAQPPESYRNDHSKMVVQLA
QPACVRYRRRTGV

>CYP51B1_Mycobacterium tuberculosis TB_RSA29 2605401537
MSAVALPRVSGGHDEHGHLEEFRTDPIGLMQRVRDECGDVGTFQLAGKQVVLLSGSHANEFFFRAGDDDLDQA
KAYPFMTPIFGEGVVFDASPERRKEMLHNAALRGEQMKGHAATIEDQVRRMIADWGEAGEIDLLDFFAELTIY
TSSACLIGKKFRDQLDGRFAKLYHELERGTDPLAYVDPYLPIESFRRRDEARNGLVALVADIMNGRIANPPTD
KSDRDMLDVLIAVKAETGTPRFSADEITGMFISMMFAGHHTSSGTASWTLIELMRHRDAYAAVIDELDELYGD
GRSVSFHALRQIPQLENVLKETLRLHPPLIILMRVAKGEFEVQGHRIHEGDLVAASPAISNRIPEDFPDPHDF
VPARYEQPRQEDLLNRWTWIPFGAGRHRCVGAAFAIMQIKAIFSVLLREYEFEMAQPPESYRNDHSKMVVQLA
QPACVRYRRRTGV
>CYP51B1_Mycobacterium tuberculosis TB_RSA30 2603998863
MSAVALPRVSGGHDEHGHLEEFRTDPIGLMQRVRDECGDVGTFQLAGKQVVLLSGSHANEFFFRAGDDDLDQA
KAYPFMTPIFGEGVVFDASPERRKEMLHNAALRGEQMKGHAATIEDQVRRMIADWGEAGEIDLLDFFAELTIY
TSSACLIGKKFRDQLDGRFAKLYHELERGTDPLAYVDPYLPIESFRRRDEARNGLVALVADIMNGRIANPPTD
KSDRDMLDVLIAVKAETGTPRFSADEITGMFISMMFAGHHTSSGTASWTLIELMRHRDAYAAVIDELDELYGD
GRSVSFHALRQIPQLENVLKETLRLHPPLIILMRVAKGEFEVQGHRIHEGDLVAASPAISNRIPEDFPDPHDF
VPARYEQPRQEDLLNRWTWIPFGAGRHRCVGAAFAIMQIKAIFSVLLREYEFEMAQPPESYRNDHSKMVVQLA
QPACVRYRRRTGV
>CYP51B1_Mycobacterium tuberculosis TB_RSA31 2605737378
MSAVALPRVSGGHDEHGHLEEFRTDPIGLMQRVRDECGDVGTFQLAGKQVVLLSGSHANEFFFRAGDDDLDQA
KAYPFMTPIFGEGVVFDASPERRKEMLHNAALRGEQMKGHAATIEDQVRRMIADWGEAGEIDLLDFFAELTIY
TSSACLIGKKFRDQLDGRFAKLYHELERGTDPLAYVDPYLPIESFRRRDEARNGLVALVADIMNGRIANPPTD
KSDRDMLDVLIAVKAETGTPRFSADEITGMFISMMFAGHHTSSGTASWTLIELMRHRDAYAAVIDELDELYGD
GRSVSFHALRQIPQLENVLKETLRLHPPLIILMRVAKGEFEVQGHRIHEGDLVAASPAISNRIPEDFPDPHDF
VPARYEQPRQEDLLNRWTWIPFGAGRHRCVGAAFAIMQIKAIFSVLLREYEFEMAQPPESYRNDHSKMVVQLA
QPACVRYRRRTGV
>CYP51B1_Mycobacterium tuberculosis TB_RSA32 2578012653
MSAVALPRVSGGHDEHGHLEEFRTDPIGLMQRVRDECGDVGTFQLAGKQVVLLSGSHANEFFFRAGDDDLDQA
KAYPFMTPIFGEGVVFDASPERRKEMLHNAALRGEQMKGHAATIEDQVRRMIADWGEAGEIDLLDFFAELTIY
TSSACLIGKKFRDQLDGRFAKLYHELERGTDPLAYVDPYLPIESFRRRDEARNGLVALVADIMNGRIANPPTD
KSDRDMLDVLIAVKAETGTPRFSADEITGMFISMMFAGHHTSSGTASWTLIELMRHRDAYAAVIDELDELYGD
GRSVSFHALRQIPQLENVLKETLRLHPPLIILMRVAKGEFEVQGHRIHEGDLVAASPAISNRIPEDFPDPHDF
VPARYEQPRQEDLLNRWTWIPFGAGRHRCVGAAFAIMQIKAIFSVLLREYEFEMAQPPESYRNDHSKMVVQLA
QPACVRYRRRTGV
>CYP51B1_Mycobacterium tuberculosis TB_RSA33 2605029292
MSAVALPRVSGGHDEHGHLEEFRTDPIGLMQRVRDECGDVGTFQLAGKQVVLLSGSHANEFFFRAGDDDLDQA
KAYPFMTPIFGEGVVFDASPERRKEMLHNAALRGEQMKGHAATIEDQVRRMIADWGEAGEIDLLDFFAELTIY
TSSACLIGKKFRDQLDGRFAKLYHELERGTDPLAYVDPYLPIESFRRRDEARNGLVALVADIMNGRIANPPTD
KSDRDMLDVLIAVKAETGTPRFSADEITGMFISMMFAGHHTSSGTASWTLIELMRHRDAYAAVIDELDELYGD
GRSVSFHALRQIPQLENVLKETLRLHPPLIILMRVAKGEFEVQGHRIHEGDLVAASPAISNRIPEDFPDPHDF
VPARYEQPRQEDLLNRWTWIPFGAGRHRCVGAAFAIMQIKAIFSVLLREYEFEMAQPPESYRNDHSKMVVQLA
QPACVRYRRRTGV
>CYP51B1_Mycobacterium tuberculosis TB_RSA34 2604684122
MSAVALPRVSGGHDEHGHLEEFRTDPIGLMQRVRDECGDVGTFQLAGKQVVLLSGSHANEFFFRAGDDDLDQA
KAYPFMTPIFGEGVVFDASPERRKEMLHNAALRGEQMKGHAATIEDQVRRMIADWGEAGEIDLLDFFAELTIY
TSSACLIGKKFRDQLDGRFAKLYHELERGTDPLAYVDPYLPIESFRRRDEARNGLVALVADIMNGRIANPPTD
KSDRDMLDVLIAVKAETGTPRFSADEITGMFISMMFAGHHTSSGTASWTLIELMRHRDAYAAVIDELDELYGD
GRSVSFHALRQIPQLENVLKETLRLHPPLIILMRVAKGEFEVQGHRIHEGDLVAASPAISNRIPEDFPDPHDF
VPARYEQPRQEDLLNRWTWIPFGAGRHRCVGAAFAIMQIKAIFSVLLREYEFEMAQPPESYRNDHSKMVVQLA
QPACVRYRRRTGV
>CYP51B1_Mycobacterium tuberculosis TB_RSA35 2605740043
MSAVALPRVSGGHDEHGHLEEFRTDPIGLMQRVRDECGDVGTFQLAGKQVVLLSGSHANEFFFRAGDDDLDQA
KAYPFMTPIFGEGVVFDASPERRKEMLHNAALRGEQMKGHAATIEDQVRRMIADWGEAGEIDLLDFFAELTIY
TSSACLIGKKFRDQLDGRFAKLYHELERGTDPLAYVDPYLPIESFRRRDEARNGLVALVADIMNGRIANPPTD
KSDRDMLDVLIAVKAETGTPRFSADEITGMFISMMFAGHHTSSGTASWTLIELMRHRDAYAAVIDELDELYGD
GRSVSFHALRQIPQLENVLKETLRLHPPLIILMRVAKGEFEVQGHRIHEGDLVAASPAISNRIPEDFPDPHDF
VPARYEQPRQEDLLNRWTWIPFGAGRHRCVGAAFAIMQIKAIFSVLLREYEFEMAQPPESYRNDHSKMVVQLA
QPACVRYRRRTGV
>CYP51B1_Mycobacterium tuberculosis TB_RSA36 2603999727

MSAVALPRVSGGHDEHGHLEEFRTDPIGLMQRVRDECGDVGTFQLAGKQVVLLSGSHANEFFFRAGDDDLDQA
KAYPFMTPIFGEGVVFDASPERRKEMLHNAALRGEQMKGHAATIEDQVRRMIADWGEAGEIDLLDFFAELTIY
TSSACLIGKKFRDQLDGRFAKLYHELERGTDPLAYVDPYLPIESFRRRDEARNGLVALVADIMNGRIANPPTD
KSDRDMLDVLIAVKAETGTPRFSADEITGMFISMMFAGHHTSSGTASWTLIELMRHRDAYAAVIDELDELYGD
GRSVSFHALRQIPQLENVLKETLRLHPPLIILMRVAKGEFEVQGHRIHEGDLVAASPAISNRIPEDFPDPHDF
VPARYEQPRQEDLLNRWTWIPFGAGRHRCVGAAFAIMQIKAIFSVLLREYEFEMAQPPESYRNDHSKMVVQLA
QPACVRYRRRTGV
>CYP51B1_Mycobacterium tuberculosis TB_RSA37 2603424101
MSAVALPRVSGGHDEHGHLEEFRTDPIGLMQRVRDECGDVGTFQLAGKQVVLLSGSHANEFFFRAGDDDLDQA
KAYPFMTPIFGEGVVFDASPERRKEMLHNAALRGEQMKGHAATIEDQVRRMIADWGEAGEIDLLDFFAELTIY
TSSACLIGKKFRDQLDGRFAKLYHELERGTDPLAYVDPYLPIESFRRRDEARNGLVALVADIMNGRIANPPTD
KSDRDMLDVLIAVKAETGTPRFSADEITGMFISMMFAGHHTSSGTASWTLIELMRHRDAYAAVIDELDELYGD
GRSVSFHALRQIPQLENVLKETLRLHPPLIILMRVAKGEFEVQGHRIHEGDLVAASPAISNRIPEDFPDPHDF
VPARYEQPRQEDLLNRWTWIPFGAGRHRCVGAAFAIMQIKAIFSVLLREYEFEMAQPPESYRNDHSKMVVQLA
QPACVRYRRRTGV
>CYP51B1_Mycobacterium tuberculosis TB_RSA38 2604687868
MSAVALPRVSGGHDEHGHLEEFRTDPIGLMQRVRDECGDVGTFQLAGKQVVLLSGSHANEFFFRAGDDDLDQA
KAYPFMTPIFGEGVVFDASPERRKEMLHNAALRGEQMKGHAATIEDQVRRMIADWGEAGEIDLLDFFAELTIY
TSSACLIGKKFRDQLDGRFAKLYHELERGTDPLAYVDPYLPIESFRRRDEARNGLVALVADIMNGRIANPPTD
KSDRDMLDVLIAVKAETGTPRFSADEITGMFISMMFAGHHTSSGTASWTLIELMRHRDAYAAVIDELDELYGD
GRSVSFHALRQIPQLENVLKETLRLHPPLIILMRVAKGEFEVQGHRIHEGDLVAASPAISNRIPEDFPDPHDF
VPARYEQPRQEDLLNRWTWIPFGAGRHRCVGAAFAIMQIKAIFSVLLREYEFEMAQPPESYRNDHSKMVVQLA
QPACVRYRRRTGV
>CYP51B1_Mycobacterium tuberculosis TB_RSA39 2604003535
MSAVALPRVSGGHDEHGHLEEFRTDPIGLMQRVRDECGDVGTFQLAGKQVVLLSGSHANEFFFRAGDDDLDQA
KAYPFMTPIFGEGVVFDASPERRKEMLHNAALRGEQMKGHAATIEDQVRRMIADWGEAGEIDLLDFFAELTIY
TSSACLIGKKFRDQLDGRFAKLYHELERGTDPLAYVDPYLPIESFRRRDEARNGLVALVADIMNGRIANPPTD
KSDRDMLDVLIAVKAETGTPRFSADEITGMFISMMFAGHHTSSGTASWTLIELMRHRDAYAAVIDELDELYGD
GRSVSFHALRQIPQLENVLKETLRLHPPLIILMRVAKGEFEVQGHRIHEGDLVAASPAISNRIPEDFPDPHDF
VPARYEQPRQEDLLNRWTWIPFGAGRHRCVGAAFAIMQIKAIFSVLLREYEFEMAQPPESYRNDHSKMVVQLA
QPACVRYRRRTGV
>CYP51B1_Mycobacterium tuberculosis TB_RSA40 2604009313
MSAVALPRVSGGHDEHGHLEEFRTDPIGLMQRVRDECGDVGTFQLAGKQVVLLSGSHANEFFFRAGDDDLDQA
KAYPFMTPIFGEGVVFDASPERRKEMLHNAALRGEQMKGHAATIEDQVRRMIADWGEAGEIDLLDFFAELTIY
TSSACLIGKKFRDQLDGRFAKLYHELERGTDPLAYVDPYLPIESFRRRDEARNGLVALVADIMNGRIANPPTD
KSDRDMLDVLIAVKAETGTPRFSADEITGMFISMMFAGHHTSSGTASWTLIELMRHRDAYAAVIDELDELYGD
GRSVSFHALRQIPQLENVLKETLRLHPPLIILMRVAKGEFEVQGHRIHEGDLVAASPAISNRIPEDFPDPHDF
VPARYEQPRQEDLLNRWTWIPFGAGRHRCVGAAFAIMQIKAIFSVLLREYEFEMAQPPESYRNDHSKMVVQLA
QPACVRYRRRTGV
>CYP51B1_Mycobacterium tuberculosis TB_RSA41 2605747672
MSAVALPRVSGGHDEHGHLEEFRTDPIGLMQRVRDECGDVGTFQLAGKQVVLLSGSHANEFFFRAGDDDLDQA
KAYPFMTPIFGEGVVFDASPERRKEMLHNAALRGEQMKGHAATIEDQVRRMIADWGEAGEIDLLDFFAELTIY
TSSACLIGKKFRDQLDGRFAKLYHELERGTDPLAYVDPYLPIESFRRRDEARNGLVALVADIMNGRIANPPTD
KSDRDMLDVLIAVKAETGTPRFSADEITGMFISMMFAGHHTSSGTASWTLIELMRHRDAYAAVIDELDELYGD
GRSVSFHALRQIPQLENVLKETLRLHPPLIILMRVAKGEFEVQGHRIHEGDLVAASPAISNRIPEDFPDPHDF
VPARYEQPRQEDLLNRWTWIPFGAGRHRCVGAAFAIMQIKAIFSVLLREYEFEMAQPPESYRNDHSKMVVQLA
QPACVRYRRRTGV
>CYP51B1_Mycobacterium tuberculosis TB_RSA42 2605033392
MSAVALPRVSGGHDEHGHLEEFRTDPIGLMQRVRDECGDVGTFQLAGKQVVLLSGSHANEFFFRAGDDDLDQA
KAYPFMTPIFGEGVVFDASPERRKEMLHNAALRGEQMKGHAATIEDQVRRMIADWGEAGEIDLLDFFAELTIY
TSSACLIGKKFRDQLDGRFAKLYHELERGTDPLAYVDPYLPIESFRRRDEARNGLVALVADIMNGRIANPPTD
KSDRDMLDVLIAVKAETGTPRFSADEITGMFISMMFAGHHTSSGTASWTLIELMRHRDAYAAVIDELDELYGD
GRSVSFHALRQIPQLENVLKETLRLHPPLIILMRVAKGEFEVQGHRIHEGDLVAASPAISNRIPEDFPDPHDF
VPARYEQPRQEDLLNRWTWIPFGAGRHRCVGAAFAIMQIKAIFSVLLREYEFEMAQPPESYRNDHSKMVVQLA
QPACVRYRRRTGV
>CYP51B1_Mycobacterium tuberculosis TB_RSA43 2603429517
MSAVALPRVSGGHDEHGHLEEFRTDPIGLMQRVRDECGDVGTFQLAGKQVVLLSGSHANEFFFRAGDDDLDQA
KAYPFMTPIFGEGVVFDASPERRKEMLHNAALRGEQMKGHAATIEDQVRRMIADWGEAGEIDLLDFFAELTIY

TSSACLIGKKFRDQLDGRFAKLYHELERGTDPLAYVDPYLPIESFRRRDEARNGLVALVADIMNGRIANPPTD
KSDRDMLDVLIAVKAETGTPRFSADEITGMFISMMFAGHHTSSGTASWTLIELMRHRDAYAAVIDELDELYGD
GRSVSFHALRQIPQLENVLKETLRLHPPLIILMRVAKGEFEVQGHRIHEGDLVAASPAISNRIPEDFPDPHDF
VPARYEQPRQEDLLNRWTWIPFGAGRHRCVGAAFAIMQIKAIFSVLLREYEFEMAQPPESYRNDHSKMVVQLA
QPACVRYRRRTGV
>CYP51B1_Mycobacterium tuberculosis TB_RSA44 2604327284
MSAVALPRVSGGHDEHGHLEEFRTDPIGLMQRVRDECGDVGTFQLAGKQVVLLSGSHANEFFFRAGDDDLDQA
KAYPFMTPIFGEGVVFDASPERRKEMLHNAALRGEQMKGHAATIEDQVRRMIADWGEAGEIDLLDFFAELTIY
TSSACLIGKKFRDQLDGRFAKLYHELERGTDPLAYVDPYLPIESFRRRDEARNGLVALVADIMNGRIANPPTD
KSDRDMLDVLIAVKAETGTPRFSADEITGMFISMMFAGHHTSSGTASWTLIELMRHRDAYAAVIDELDELYGD
GRSVSFHALRQIPQLENVLKETLRLHPPLIILMRVAKGEFEVQGHRIHEGDLVAASPAISNRIPEDFPDPHDF
VPARYEQPRQEDLLNRWTWIPFGAGRHRCVGAAFAIMQIKAIFSVLLREYEFEMAQPPESYRNDHSKMVVQLA
QPACVRYRRRTGV
>CYP51B1_Mycobacterium tuberculosis TB_RSA45 2584743023
MSAVALPRVSGGHDEHGHLEEFRTDPIGLMQRVRDECGDVGTFQLAGKQVVLLSGSHANEFFFRAGDDDLDQA
KAYPFMTPIFGEGVVFDASPERRKEMLHNAALRGEQMKGHAATIEDQVRRMIADWGEAGEIDLLDFFAELTIY
TSSACLIGKKFRDQLDGRFAKLYHELERGTDPLAYVDPYLPIESFRRRDEARNGLVALVADIMNGRIANPPTD
KSDRDMLDVLIAVKAETGTPRFSADEITGMFISMMFAGHHTSSGTASWTLIELMRHRDAYAAVIDELDELYGD
GRSVSFHALRQIPQLENVLKETLRLHPPLIILMRVAKGEFEVQGHRIHEGDLVAASPAISNRIPEDFPDPHDF
VPARYEQPRQEDLLNRWTWIPFGAGRHRCVGAAFAIMQIKAIFSVLLREYEFEMAQPPESYRNDHSKMVVQLA
QPACVRYRRRTGV
>CYP51B1_Mycobacterium tuberculosis TB_RSA46 2584865000
MSAVALPRVSGGHDEHGHLEEFRTDPIGLMQRVRDECGDVGTFQLAGKQVVLLSGSHANEFFFRAGDDDLDQA
KAYPFMTPIFGEGVVFDASPERRKEMLHNAALRGEQMKGHAATIEDQVRRMIADWGEAGEIDLLDFFAELTIY
TSSACLIGKKFRDQLDGRFAKLYHELERGTDPLAYVDPYLPIESFRRRDEARNGLVALVADIMNGRIANPPTD
KSDRDMLDVLIAVKAETGTPRFSADEITGMFISMMFAGHHTSSGTASWTLIELMRHRDAYAAVIDELDELYGD
GRSVSFHALRQIPQLENVLKETLRLHPPLIILMRVAKGEFEVQGHRIHEGDLVAASPAISNRIPEDFPDPHDF
VPARYEQPRQEDLLNRWTWIPFGAGRHRCVGAAFAIMQIKAIFSVLLREYEFEMAQPPESYRNDHSKMVVQLA
QPACVRYRRRTGV
>CYP51B1_Mycobacterium tuberculosis TB_RSA48 2604432726
MSAVALPRVSGGHDEHGHLEEFRTDPIGLMQRVRDECGDVGTFQLAGKQVVLLSGSHANEFFFRAGDDDLDQA
KAYPFMTPIFGEGVVFDASPERRKEMLHNAALRGEQMKGHAATIEDQVRRMIADWGEAGEIDLLDFFAELTIY
TSSACLIGKKFRDQLDGRFAKLYHELERGTDPLAYVDPYLPIESFRRRDEARNGLVALVADIMNGRIANPPTD
KSDRDMLDVLIAVKAETGTPRFSADEITGMFISMMFAGHHTSSGTASWTLIELMRHRDAYAAVIDELDELYGD
GRSVSFHALRQIPQLENVLKETLRLHPPLIILMRVAKGEFEVQGHRIHEGDLVAASPAISNRIPEDFPDPHDF
VPARYEQPRQEDLLNRWTWIPFGAGRHRCVGAAFAIMQIKAIFSVLLREYEFEMAQPPESYRNDHSKMVVQLA
QPACVRYRRRTGV
>CYP51B1_Mycobacterium tuberculosis TB_RSA49 2605878785
MSAVALPRVSGGHDEHGHLEEFRTDPIGLMQRVRDECGDVGTFQLAGKQVVLLSGSHANEFFFRAGDDDLDQA
KAYPFMTPIFGEGVVFDASPERRKEMLHNAALRGEQMKGHAATIEDQVRRMIADWGEAGEIDLLDFFAELTIY
TSSACLIGKKFRDQLDGRFAKLYHELERGTDPLAYVDPYLPIESFRRRDEARNGLVALVADIMNGRIANPPTD
KSDRDMLDVLIAVKAETGTPRFSADEITGMFISMMFAGHHTSSGTASWTLIELMRHRDAYAAVIDELDELYGD
GRSVSFHALRQIPQLENVLKETLRLHPPLIILMRVAKGEFEVQGHRIHEGDLVAASPAISNRIPEDFPDPHDF
VPARYEQPRQEDLLNRWTWIPFGAGRHRCVGAAFAIMQIKAIFSVLLREYEFEMAQPPESYRNDHSKMVVQLA
QPACVRYRRRTGV
>CYP51B1_Mycobacterium tuberculosis TB_RSA50 2604775662
MSAVALPRVSGGHDEHGHLEEFRTDPIGLMQRVRDECGDVGTFQLAGKQVVLLSGSHANEFFFRAGDDDLDQA
KAYPFMTPIFGEGVVFDASPERRKEMLHNAALRGEQMKGHAATIEDQVRRMIADWGEAGEIDLLDFFAELTIY
TSSACLIGKKFRDQLDGRFAKLYHELERGTDPLAYVDPYLPIESFRRRDEARNGLVALVADIMNGRIANPPTD
KSDRDMLDVLIAVKAETGTPRFSADEITGMFISMMFAGHHTSSGTASWTLIELMRHRDAYAAVIDELDELYGD
GRSVSFHALRQIPQLENVLKETLRLHPPLIILMRVAKGEFEVQGHRIHEGDLVAASPAISNRIPEDFPDPHDF
VPARYEQPRQEDLLNRWTWIPFGAGRHRCVGAAFAIMQIKAIFSVLLREYEFEMAQPPESYRNDHSKMVVQLA
QPACVRYRRRTGV
>CYP51B1_Mycobacterium tuberculosis TB_RSA51 2577688194
MSAVALPRVSGGHDEHGHLEEFRTDPIGLMQRVRDECGDVGTFQLAGKQVVLLSGSHANEFFFRAGDDDLDQA
KAYPFMTPIFGEGVVFDASPERRKEMLHNAALRGEQMKGHAATIEDQVRRMIADWGEAGEIDLLDFFAELTIY
TSSACLIGKKFRDQLDGRFAKLYHELERGTDPLAYVDPYLPIESFRRRDEARNGLVALVADIMNGRIANPPTD
KSDRDMLDVLIAVKAETGTPRFSADEITGMFISMMFAGHHTSSGTASWTLIELMRHRDAYAAVIDELDELYGD

GRSVSFHALRQIPQLENVLKETLRLHPPLIILMRVAKGEFEVQGHRIHEGDLVAASPAISNRIPEDFPDPHDF
VPARYEQPRQEDLLNRWTWIPFGAGRHRCVGAAFAIMQIKAIFSVLLREYEFEMAQPPESYRNDHSKMVVQLA
QPACVRYRRRTGV
>CYP51B1_Mycobacterium tuberculosis TB_RSA52 2603283020
MSAVALPRVSGGHDEHGHLEEFRTDPIGLMQRVRDECGDVGTFQLAGKQVVLLSGSHANEFFFRAGDDDLDQA
KAYPFMTPIFGEGVVFDASPERRKEMLHNAALRGEQMKGHAATIEDQVRRMIADWGEAGEIDLLDFFAELTIY
TSSACLIGKKFRDQLDGRFAKLYHELERGTDPLAYVDPYLPIESFRRRDEARNGLVALVADIMNGRIANPPTD
KSDRDMLDVLIAVKAETGTPRFSADEITGMFISMMFAGHHTSSGTASWTLIELMRHRDAYAAVIDELDELYGD
GRSVSFHALRQIPQLENVLKETLRLHPPLIILMRVAKGEFEVQGHRIHEGDLVAASPAISNRIPEDFPDPHDF
VPARYEQPRQEDLLNRWTWIPFGAGRHRCVGAAFAIMQIKAIFSVLLREYEFEMAQPPESYRNDHSKMVVQLA
QPACVRYRRRTGV
>CYP51B1_Mycobacterium tuberculosis TB_RSA53 2603431106
MSAVALPRVSGGHDEHGHLEEFRTDPIGLMQRVRDECGDVGTFQLAGKQVVLLSGSHANEFFFRAGDDDLDQA
KAYPFMTPIFGEGVVFDASPERRKEMLHNAALRGEQMKGHAATIEDQVRRMIADWGEAGEIDLLDFFAELTIY
TSSACLIGKKFRDQLDGRFAKLYHELERGTDPLAYVDPYLPIESFRRRDEARNGLVALVADIMNGRIANPPTD
KSDRDMLDVLIAVKAETGTPRFSADEITGMFISMMFAGHHTSSGTASWTLIELMRHRDAYAAVIDELDELYGD
GRSVSFHALRQIPQLENVLKETLRLHPPLIILMRVAKGEFEVQGHRIHEGDLVAASPAISNRIPEDFPDPHDF
VPARYEQPRQEDLLNRWTWIPFGAGRHRCVGAAFAIMQIKAIFSVLLREYEFEMAQPPESYRNDHSKMVVQLA
QPACVRYRRRTGV
>CYP51B1_Mycobacterium tuberculosis TB_RSA54 2605754040
MSAVALPRVSGGHDEHGHLEEFRTDPIGLMQRVRDECGDVGTFQLAGKQVVLLSGSHANEFFFRAGDDDLDQA
KAYPFMTPIFGEGVVFDASPERRKEMLHNAALRGEQMKGHAATIEDQVRRMIADWGEAGEIDLLDFFAELTIY
TSSACLIGKKFRDQLDGRFAKLYHELERGTDPLAYVDPYLPIESFRRRDEARNGLVALVADIMNGRIANPPTD
KSDRDMLDVLIAVKAETGTPRFSADEITGMFISMMFAGHHTSSGTASWTLIELMRHRDAYAAVIDELDELYGD
GRSVSFHALRQIPQLENVLKETLRLHPPLIILMRVAKGEFEVQGHRIHEGDLVAASPAISNRIPEDFPDPHDF
VPARYEQPRQEDLLNRWTWIPFGAGRHRCVGAAFAIMQIKAIFSVLLREYEFEMAQPPESYRNDHSKMVVQLA
QPACVRYRRRTGV
>CYP51B1_Mycobacterium tuberculosis TB_RSA55 2604691988
MSAVALPRVSGGHDEHGHLEEFRTDPIGLMQRVRDECGDVGTFQLAGKQVVLLSGSHANEFFFRAGDDDLDQA
KAYPFMTPIFGEGVVFDASPERRKEMLHNAALRGEQMKGHAATIEDQVRRMIADWGEAGEIDLLDFFAELTIY
TSSACLIGKKFRDQLDGRFAKLYHELERGTDPLAYVDPYLPIESFRRRDEARNGLVALVADIMNGRIANPPTD
KSDRDMLDVLIAVKAETGTPRFSADEITGMFISMMFAGHHTSSGTASWTLIELMRHRDAYAAVIDELDELYGD
GRSVSFHALRQIPQLENVLKETLRLHPPLIILMRVAKGEFEVQGHRIHEGDLVAASPAISNRIPEDFPDPHDF
VPARYEQPRQEDLLNRWTWIPFGAGRHRCVGAAFAIMQIKAIFSVLLREYEFEMAQPPESYRNDHSKMVVQLA
QPACVRYRRRTGV
>CYP51B1_Mycobacterium tuberculosis TB_RSA56 2604695800
MSAVALPRVSGGHDEHGHLEEFRTDPIGLMQRVRDECGDVGTFQLAGKQVVLLSGSHANEFFFRAGDDDLDQA
KAYPFMTPIFGEGVVFDASPERRKEMLHNAALRGEQMKGHAATIEDQVRRMIADWGEAGEIDLLDFFAELTIY
TSSACLIGKKFRDQLDGRFAKLYHELERGTDPLAYVDPYLPIESFRRRDEARNGLVALVADIMNGRIANPPTD
KSDRDMLDVLIAVKAETGTPRFSADEITGMFISMMFAGHHTSSGTASWTLIELMRHRDAYAAVIDELDELYGD
GRSVSFHALRQIPQLENVLKETLRLHPPLIILMRVAKGEFEVQGHRIHEGDLVAASPAISNRIPEDFPDPHDF
VPARYEQPRQEDLLNRWTWIPFGAGRHRCVGAAFAIMQIKAIFSVLLREYEFEMAQPPESYRNDHSKMVVQLA
QPACVRYRRRTGV
>CYP51B1_Mycobacterium tuberculosis TB_RSA57 2605755074
MSAVALPRVSGGHDEHGHLEEFRTDPIGLMQRVRDECGDVGTFQLAGKQVVLLSGSHANEFFFRAGDDDLDQA
KAYPFMTPIFGEGVVFDASPERRKEMLHNAALRGEQMKGHAATIEDQVRRMIADWGEAGEIDLLDFFAELTIY
TSSACLIGKKFRDQLDGRFAKLYHELERGTDPLAYVDPYLPIESFRRRDEARNGLVALVADIMNGRIANPPTD
KSDRDMLDVLIAVKAETGTPRFSADEITGMFISMMFAGHHTSSGTASWTLIELMRHRDAYAAVIDELDELYGD
GRSVSFHALRQIPQLENVLKETLRLHPPLIILMRVAKGEFEVQGHRIHEGDLVAASPAISNRIPEDFPDPHDF
VPARYEQPRQEDLLNRWTWIPFGAGRHRCVGAAFAIMQIKAIFSVLLREYEFEMAQPPESYRNDHSKMVVQLA
QPACVRYRRRTGV
>CYP51B1_Mycobacterium tuberculosis TB_RSA58 2604333722
MSAVALPRVSGGHDEHGHLEEFRTDPIGLMQRVRDECGDVGTFQLAGKQVVLLSGSHANEFFFRAGDDDLDQA
KAYPFMTPIFGEGVVFDASPERRKEMLHNAALRGEQMKGHAATIEDQVRRMIADWGEAGEIDLLDFFAELTIY
TSSACLIGKKFRDQLDGRFAKLYHELERGTDPLAYVDPYLPIESFRRRDEARNGLVALVADIMNGRIANPPTD
KSDRDMLDVLIAVKAETGTPRFSADEITGMFISMMFAGHHTSSGTASWTLIELMRHRDAYAAVIDELDELYGD
GRSVSFHALRQIPQLENVLKETLRLHPPLIILMRVAKGEFEVQGHRIHEGDLVAASPAISNRIPEDFPDPHDF

VPARYEQPRQEDLLNRWTWIPFGAGRHRCVGAAFAIMQIKAIFSVLLREYEFEMAQPPESYRNDHSKMVVQLA
QPACVRYRRRTGV
>CYP51B1_Mycobacterium tuberculosis TB_RSA59 2576633576
MSAVALPRVSGGHDEHGHLEEFRTDPIGLMQRVRDECGDVGTFQLAGKQVVLLSGSHANEFFFRAGDDDLDQA
KAYPFMTPIFGEGVVFDASPERRKEMLHNAALRGEQMKGHAATIEDQVRRMIADWGEAGEIDLLDFFAELTIY
TSSACLIGKKFRDQLDGRFAKLYHELERGTDPLAYVDPYLPIESFRRRDEARNGLVALVADIMNGRIANPPTD
KSDRDMLDVLIAVKAETGTPRFSADEITGMFISMMFAGHHTSSGTASWTLIELMRHRDAYAAVIDELDELYGD
GRSVSFHALRQIPQLENVLKETLRLHPPLIILMRVAKGEFEVQGHRIHEGDLVAASPAISNRIPEDFPDPHDF
VPARYEQPRQEDLLNRWTWIPFGAGRHRCVGAAFAIMQIKAIFSVLLREYEFEMAQPPESYRNDHSKMVVQLA
QPACVRYRRRTGV
>CYP51B1_Mycobacterium tuberculosis TB_RSA60 2604017446
MSAVALPRVSGGHDEHGHLEEFRTDPIGLMQRVRDECGDVGTFQLAGKQVVLLSGSHANEFFFRAGDDDLDQA
KAYPFMTPIFGEGVVFDASPERRKEMLHNAALRGEQMKGHAATIEDQVRRMIADWGEAGEIDLLDFFAELTIY
TSSACLIGKKFRDQLDGRFAKLYHELERGTDPLAYVDPYLPIESFRRRDEARNGLVALVADIMNGRIANPPTD
KSDRDMLDVLIAVKAETGTPRFSADEITGMFISMMFAGHHTSSGTASWTLIELMRHRDAYAAVIDELDELYGD
GRSVSFHALRQIPQLENVLKETLRLHPPLIILMRVAKGEFEVQGHRIHEGDLVAASPAISNRIPEDFPDPHDF
VPARYEQPRQEDLLNRWTWIPFGAGRHRCVGAAFAIMQIKAIFSVLLREYEFEMAQPPESYRNDHSKMVVQLA
QPACVRYRRRTGV
>CYP51B1_Mycobacterium tuberculosis TB_RSA61 2605759463
MSAVALPRVSGGHDEHGHLEEFRTDPIGLMQRVRDECGDVGTFQLAGKQVVLLSGSHANEFFFRAGDDDLDQA
KAYPFMTPIFGEGVVFDASPERRKEMLHNAALRGEQMKGHAATIEDQVRRMIADWGEAGEIDLLDFFAELTIY
TSSACLIGKKFRDQLDGRFAKLYHELERGTDPLAYVDPYLPIESFRRRDEARNGLVALVADIMNGRIANPPTD
KSDRDMLDVLIAVKAETGTPRFSADEITGMFISMMFAGHHTSSGTASWTLIELMRHRDAYAAVIDELDELYGD
GRSVSFHALRQIPQLENVLKETLRLHPPLIILMRVAKGEFEVQGHRIHEGDLVAASPAISNRIPEDFPDPHDF
VPARYEQPRQEDLLNRWTWIPFGAGRHRCVGAAFAIMQIKAIFSVLLREYEFEMAQPPESYRNDHSKMVVQLA
QPACVRYRRRTGV
>CYP51B1_Mycobacterium tuberculosis TB_RSA62 2584902013
MSAVALPRVSGGHDEHGHLEEFRTDPIGLMQRVRDECGDVGTFQLAGKQVVLLSGSHANEFFFRAGDDDLDQA
KAYPFMTPIFGEGVVFDASPERRKEMLHNAALRGEQMKGHAATIEDQVRRMIADWGEAGEIDLLDFFAELTIY
TSSACLIGKKFRDQLDGRFAKLYHELERGTDPLAYVDPYLPIESFRRRDEARNGLVALVADIMNGRIANPPTD
KSDRDMLDVLIAVKAETGTPRFSADEITGMFISMMFAGHHTSSGTASWTLIELMRHRDAYAAVIDELDELYGD
GRSVSFHALRQIPQLENVLKETLRLHPPLIILMRVAKGEFEVQGHRIHEGDLVAASPAISNRIPEDFPDPHDF
VPARYEQPRQEDLLNRWTWIPFGAGRHRCVGAAFAIMQIKAIFSVLLREYEFEMAQPPESYRNDHSKMVVQLA
QPACVRYRRRTGV
>CYP51B1_Mycobacterium tuberculosis TB_RSA63 2605765692
MSAVALPRVSGGHDEHGHLEEFRTDPIGLMQRVRDECGDVGTFQLAGKQVVLLSGSHANEFFFRAGDDDLDQA
KAYPFMTPIFGEGVVFDASPERRKEMLHNAALRGEQMKGHAATIEDQVRRMIADWGEAGEIDLLDFFAELTIY
TSSACLIGKKFRDQLDGRFAKLYHELERGTDPLAYVDPYLPIESFRRRDEARNGLVALVADIMNGRIANPPTD
KSDRDMLDVLIAVKAETGTPRFSADEITGMFISMMFAGHHTSSGTASWTLIELMRHRDAYAAVIDELDELYGD
GRSVSFHALRQIPQLENVLKETLRLHPPLIILMRVAKGEFEVQGHRIHEGDLVAASPAISNRIPEDFPDPHDF
VPARYEQPRQEDLLNRWTWIPFGAGRHRCVGAAFAIMQIKAIFSVLLREYEFEMAQPPESYRNDHSKMVVQLA
QPACVRYRRRTGV
>CYP51B1_Mycobacterium tuberculosis TB_RSA64 2579801281
MSAVALPRVSGGHDEHGHLEEFRTDPIGLMQRVRDECGDVGTFQLAGKQVVLLSGSHANEFFFRAGDDDLDQA
KAYPFMTPIFGEGVVFDASPERRKEMLHNAALRGEQMKGHAATIEDQVRRMIADWGEAGEIDLLDFFAELTIY
TSSACLIGKKFRDQLDGRFAKLYHELERGTDPLAYVDPYLPIESFRRRDEARNGLVALVADIMNGRIANPPTD
KSDRDMLDVLIAVKAETGTPRFSADEITGMFISMMFAGHHTSSGTASWTLIELMRHRDAYAAVIDELDELYGD
GRSVSFHALRQIPQLENVLKETLRLHPPLIILMRVAKGEFEVQGHRIHEGDLVAASPAISNRIPEDFPDPHDF
VPARYEQPRQEDLLNRWTWIPFGAGRHRCVGAAFAIMQIKAIFSVLLREYEFEMAQPPESYRNDHSKMVVQLA
QPACVRYRRRTGV
>CYP51B1_Mycobacterium tuberculosis TB_RSA66 2576612090
MSAVALPRVSGGHDEHGHLEEFRTDPIGLMQRVRDECGDVGTFQLAGKQVVLLSGSHANEFFFRAGDDDLDQA
KAYPFMTPIFGEGVVFDASPERRKEMLHNAALRGEQMKGHAATIEDQVRRMIADWGEAGEIDLLDFFAELTIY
TSSACLIGKKFRDQLDGRFAKLYHELERGTDPLAYVDPYLPIESFRRRDEARNGLVALVADIMNGRIANPPTD
KSDRDMLDVLIAVKAETGTPRFSADEITGMFISMMFAGHHTSSGTASWTLIELMRHRDAYAAVIDELDELYGD
GRSVSFHALRQIPQLENVLKETLRLHPPLIILMRVAKGEFEVQGHRIHEGDLVAASPAISNRIPEDFPDPHDF
VPARYEQPRQEDLLNRWTWIPFGAGRHRCVGAAFAIMQIKAIFSVLLREYEFEMAQPPESYRNDHSKMVVQLA
QPACVRYRRRTGV

>CYP51B1_Mycobacterium tuberculosis TB_RSA67 2576050889
MSAVALPRVSGGHDEHGHLEEFRTDPIGLMQRVRDECGDVGTFQLAGKQVVLLSGSHANEFFFRAGDDDLDQA
KAYPFMTPIFGEGVVFDASPERRKEMLHNAALRGEQMKGHAATIEDQVRRMIADWGEAGEIDLLDFFAELTIY
TSSACLIGKKFRDQLDGRFAKLYHELERGTDPLAYVDPYLPIESFRRRDEARNGLVALVADIMNGRIANPPTD
KSDRDMLDVLIAVKAETGTPRFSADEITGMFISMMFAGHHTSSGTASWTLIELMRHRDAYAAVIDELDELYGD
GRSVSFHALRQIPQLENVLKETLRLHPPLIILMRVAKGEFEVQGHRIHEGDLVAASPAISNRIPEDFPDPHDF
VPARYEQPRQEDLLNRWTWIPFGAGRHRCVGAAFAIMQIKAIFSVLLREYEFEMAQPPESYRNDHSKMVVQLA
QPACVRYRRRTGV
>CYP51B1_Mycobacterium tuberculosis TB_RSA68 2574875227
MSAVALPRVSGGHDEHGHLEEFRTDPIGLMQRVRDECGDVGTFQLAGKQVVLLSGSHANEFFFRAGDDDLDQA
KAYPFMTPIFGEGVVFDASPERRKEMLHNAALRGEQMKGHAATIEDQVRRMIADWGEAGEIDLLDFFAELTIY
TSSACLIGKKFRDQLDGRFAKLYHELERGTDPLAYVDPYLPIESFRRRDEARNGLVALVADIMNGRIANPPTD
KSDRDMLDVLIAVKAETGTPRFSADEITGMFISMMFAGHHTSSGTASWTLIELMRHRDAYAAVIDELDELYGD
GRSVSFHALRQIPQLENVLKETLRLHPPLIILMRVAKGEFEVQGHRIHEGDLVAASPAISNRIPEDFPDPHDF
VPARYEQPRQEDLLNRWTWIPFGAGRHRCVGAAFAIMQIKAIFSVLLREYEFEMAQPPESYRNDHSKMVVQLA
QPACVRYRRRTGV
>CYP51B1_Mycobacterium tuberculosis TB_RSA69 2603435213
MSAVALPRVSGGHDEHGHLEEFRTDPIGLMQRVRDECGDVGTFQLAGKQVVLLSGSHANEFFFRAGDDDLDQA
KAYPFMTPIFGEGVVFDASPERRKEMLHNAALRGEQMKGHAATIEDQVRRMIADWGEAGEIDLLDFFAELTIY
TSSACLIGKKFRDQLDGRFAKLYHELERGTDPLAYVDPYLPIESFRRRDEARNGLVALVADIMNGRIANPPTD
KSDRDMLDVLIAVKAETGTPRFSADEITGMFISMMFAGHHTSSGTASWTLIELMRHRDAYAAVIDELDELYGD
GRSVSFHALRQIPQLENVLKETLRLHPPLIILMRVAKGEFEVQGHRIHEGDLVAASPAISNRIPEDFPDPHDF
VPARYEQPRQEDLLNRWTWIPFGAGRHRCVGAAFAIMQIKAIFSVLLREYEFEMAQPPESYRNDHSKMVVQLA
QPACVRYRRRTGV
>CYP51B1_Mycobacterium tuberculosis TB_RSA70 2577640999
MSAVALPRVSGGHDEHGHLEEFRTDPIGLMQRVRDECGDVGTFQLAGKQVVLLSGSHANEFFFRAGDDDLDQA
KAYPFMTPIFGEGVVFDASPERRKEMLHNAALRGEQMKGHAATIEDQVRRMIADWGEAGEIDLLDFFAELTIY
TSSACLIGKKFRDQLDGRFAKLYHELERGTDPLAYVDPYLPIESFRRRDEARNGLVALVADIMNGRIANPPTD
KSDRDMLDVLIAVKAETGTPRFSADEITGMFISMMFAGHHTSSGTASWTLIELMRHRDAYAAVIDELDELYGD
GRSVSFHALRQIPQLENVLKETLRLHPPLIILMRVAKGEFEVQGHRIHEGDLVAASPAISNRIPEDFPDPHDF
VPARYEQPRQEDLLNRWTWIPFGAGRHRCVGAAFAIMQIKAIFSVLLREYEFEMAQPPESYRNDHSKMVVQLA
QPACVRYRRRTGV
>CYP51B1_Mycobacterium tuberculosis TB_RSA71 2605408693
MSAVALPRVSGGHDEHGHLEEFRTDPIGLMQRVRDECGDVGTFQLAGKQVVLLSGSHANEFFFRAGDDDLDQA
KAYPFMTPIFGEGVVFDASPERRKEMLHNAALRGEQMKGHAATIEDQVRRMIADWGEAGEIDLLDFFAELTIY
TSSACLIGKKFRDQLDGRFAKLYHELERGTDPLAYVDPYLPIESFRRRDEARNGLVALVADIMNGRIANPPTD
KSDRDMLDVLIAVKAETGTPRFSADEITGMFISMMFAGHHTSSGTASWTLIELMRHRDAYAAVIDELDELYGD
GRSVSFHALRQIPQLENVLKETLRLHPPLIILMRVAKGEFEVQGHRIHEGDLVAASPAISNRIPEDFPDPHDF
VPARYEQPRQEDLLNRWTWIPFGAGRHRCVGAAFAIMQIKAIFSVLLREYEFEMAQPPESYRNDHSKMVVQLA
QPACVRYRRRTGV
>CYP51B1_Mycobacterium tuberculosis TB_RSA72 2605039211
MSAVALPRVSGGHDEHGHLEEFRTDPIGLMQRVRDECGDVGTFQLAGKQVVLLSGSHANEFFFRAGDDDLDQA
KAYPFMTPIFGEGVVFDASPERRKEMLHNAALRGEQMKGHAATIEDQVRRMIADWGEAGEIDLLDFFAELTIY
TSSACLIGKKFRDQLDGRFAKLYHELERGTDPLAYVDPYLPIESFRRRDEARNGLVALVADIMNGRIANPPTD
KSDRDMLDVLIAVKAETGTPRFSADEITGMFISMMFAGHHTSSGTASWTLIELMRHRDAYAAVIDELDELYGD
GRSVSFHALRQIPQLENVLKETLRLHPPLIILMRVAKGEFEVQGHRIHEGDLVAASPAISNRIPEDFPDPHDF
VPARYEQPRQEDLLNRWTWIPFGAGRHRCVGAAFAIMQIKAIFSVLLREYEFEMAQPPESYRNDHSKMVVQLA
QPACVRYRRRTGV
>CYP51B1_Mycobacterium tuberculosis TB_RSA73 2604702724
MSAVALPRVSGGHDEHGHLEEFRTDPIGLMQRVRDECGDVGTFQLAGKQVVLLSGSHANEFFFRAGDDDLDQA
KAYPFMTPIFGEGVVFDASPERRKEMLHNAALRGEQMKGHAATIEDQVRRMIADWGEAGEIDLLDFFAELTIY
TSSACLIGKKFRDQLDGRFAKLYHELERGTDPLAYVDPYLPIESFRRRDEARNGLVALVADIMNGRIANPPTD
KSDRDMLDVLIAVKAETGTPRFSADEITGMFISMMFAGHHTSSGTASWTLIELMRHRDAYAAVIDELDELYGD
GRSVSFHALRQIPQLENVLKETLRLHPPLIILMRVAKGEFEVQGHRIHEGDLVAASPAISNRIPEDFPDPHDF
VPARYEQPRQEDLLNRWTWIPFGAGRHRCVGAAFAIMQIKAIFSVLLREYEFEMAQPPESYRNDHSKMVVQLA
QPACVRYRRRTGV
>CYP51B1_Mycobacterium tuberculosis TB_RSA74 2578072861

MSAVALPRVSGGHDEHGHLEEFRTDPIGLMQRVRDECGDVGTFQLAGKQVVLLSGSHANEFFFRAGDDDLDQA
KAYPFMTPIFGEGVVFDASPERRKEMLHNAALRGEQMKGHAATIEDQVRRMIADWGEAGEIDLLDFFAELTIY
TSSACLIGKKFRDQLDGRFAKLYHELERGTDPLAYVDPYLPIESFRRRDEARNGLVALVADIMNGRIANPPTD
KSDRDMLDVLIAVKAETGTPRFSADEITGMFISMMFAGHHTSSGTASWTLIELMRHRDAYAAVIDELDELYGD
GRSVSFHALRQIPQLENVLKETLRLHPPLIILMRVAKGEFEVQGHRIHEGDLVAASPAISNRIPEDFPDPHDF
VPARYEQPRQEDLLNRWTWIPFGAGRHRCVGAAFAIMQIKAIFSVLLREYEFEMAQPPESYRNDHSKMVVQLA
QPACVRYRRRTGV
>CYP51B1_Mycobacterium tuberculosis TB_RSA75 2605768805
MSAVALPRVSGGHDEHGHLEEFRTDPIGLMQRVRDECGDVGTFQLAGKQVVLLSGSHANEFFFRAGDDDLDQA
KAYPFMTPIFGEGVVFDASPERRKEMLHNAALRGEQMKGHAATIEDQVRRMIADWGEAGEIDLLDFFAELTIY
TSSACLIGKKFRDQLDGRFAKLYHELERGTDPLAYVDPYLPIESFRRRDEARNGLVALVADIMNGRIANPPTD
KSDRDMLDVLIAVKAETGTPRFSADEITGMFISMMFAGHHTSSGTASWTLIELMRHRDAYAAVIDELDELYGD
GRSVSFHALRQIPQLENVLKETLRLHPPLIILMRVAKGEFEVQGHRIHEGDLVAASPAISNRIPEDFPDPHDF
VPARYEQPRQEDLLNRWTWIPFGAGRHRCVGAAFAIMQIKAIFSVLLREYEFEMAQPPESYRNDHSKMVVQLA
QPACVRYRRRTGV
>CYP51B1_Mycobacterium tuberculosis TB_RSA77 2577202930
MSAVALPRVSGGHDEHGHLEEFRTDPIGLMQRVRDECGDVGTFQLAGKQVVLLSGSHANEFFFRAGDDDLDQA
KAYPFMTPIFGEGVVFDASPERRKEMLHNAALRGEQMKGHAATIEDQVRRMIADWGEAGEIDLLDFFAELTIY
TSSACLIGKKFRDQLDGRFAKLYHELERGTDPLAYVDPYLPIESFRRRDEARNGLVALVADIMNGRIANPPTD
KSDRDMLDVLIAVKAETGTPRFSADEITGMFISMMFAGHHTSSGTASWTLIELMRHRDAYAAVIDELDELYGD
GRSVSFHALRQIPQLENVLKETLRLHPPLIILMRVAKGEFEVQGHRIHEGDLVAASPAISNRIPEDFPDPHDF
VPARYEQPRQEDLLNRWTWIPFGAGRHRCVGAAFAIMQIKAIFSVLLREYEFEMAQPPESYRNDHSKMVVQLA
QPACVRYRRRTGV
>CYP51B1_Mycobacterium tuberculosis TB_RSA78 2578052464
MSAVALPRVSGGHDEHGHLEEFRTDPIGLMQRVRDECGDVGTFQLAGKQVVLLSGSHANEFFFRAGDDDLDQA
KAYPFMTPIFGEGVVFDASPERRKEMLHNAALRGEQMKGHAATIEDQVRRMIADWGEAGEIDLLDFFAELTIY
TSSACLIGKKFRDQLDGRFAKLYHELERGTDPLAYVDPYLPIESFRRRDEARNGLVALVADIMNGRIANPPTD
KSDRDMLDVLIAVKAETGTPRFSADEITGMFISMMFAGHHTSSGTASWTLIELMRHRDAYAAVIDELDELYGD
GRSVSFHALRQIPQLENVLKETLRLHPPLIILMRVAKGEFEVQGHRIHEGDLVAASPAISNRIPEDFPDPHDF
VPARYEQPRQEDLLNRWTWIPFGAGRHRCVGAAFAIMQIKAIFSVLLREYEFEMAQPPESYRNDHSKMVVQLA
QPACVRYRRRTGV
>CYP51B1_Mycobacterium tuberculosis TB_RSA79 2584922382
MSAVALPRVSGGHDEHGHLEEFRTDPIGLMQRVRDECGDVGTFQLAGKQVVLLSGSHANEFFFRAGDDDLDQA
KAYPFMTPIFGEGVVFDASPERRKEMLHNAALRGEQMKGHAATIEDQVRRMIADWGEAGEIDLLDFFAELTIY
TSSACLIGKKFRDQLDGRFAKLYHELERGTDPLAYVDPYLPIESFRRRDEARNGLVALVADIMNGRIANPPTD
KSDRDMLDVLIAVKAETGTPRFSADEITGMFISMMFAGHHTSSGTASWTLIELMRHRDAYAAVIDELDELYGD
GRSVSFHALRQIPQLENVLKETLRLHPPLIILMRVAKGEFEVQGHRIHEGDLVAASPAISNRIPEDFPDPHDF
VPARYEQPRQEDLLNRWTWIPFGAGRHRCVGAAFAIMQIKAIFSVLLREYEFEMAQPPESYRNDHSKMVVQLA
QPACVRYRRRTGV
>CYP51B1_Mycobacterium tuberculosis TB_RSA80 2605041928
MSAVALPRVSGGHDEHGHLEEFRTDPIGLMQRVRDECGDVGTFQLAGKQVVLLSGSHANEFFFRAGDDDLDQA
KAYPFMTPIFGEGVVFDASPERRKEMLHNAALRGEQMKGHAATIEDQVRRMIADWGEAGEIDLLDFFAELTIY
TSSACLIGKKFRDQLDGRFAKLYHELERGTDPLAYVDPYLPIESFRRRDEARNGLVALVADIMNGRIANPPTD
KSDRDMLDVLIAVKAETGTPRFSADEITGMFISMMFAGHHTSSGTASWTLIELMRHRDAYAAVIDELDELYGD
GRSVSFHALRQIPQLENVLKETLRLHPPLIILMRVAKGEFEVQGHRIHEGDLVAASPAISNRIPEDFPDPHDF
VPARYEQPRQEDLLNRWTWIPFGAGRHRCVGAAFAIMQIKAIFSVLLREYEFEMAQPPESYRNDHSKMVVQLA
QPACVRYRRRTGV
>CYP51B1_Mycobacterium tuberculosis TB_RSA81 2603286969
MSAVALPRVSGGHDEHGHLEEFRTDPIGLMQRVRDECGDVGTFQLAGKQVVLLSGSHANEFFFRAGDDDLDQA
KAYPFMTPIFGEGVVFDASPERRKEMLHNAALRGEQMKGHAATIEDQVRRMIADWGEAGEIDLLDFFAELTIY
TSSACLIGKKFRDQLDGRFAKLYHELERGTDPLAYVDPYLPIESFRRRDEARNGLVALVADIMNGRIANPPTD
KSDRDMLDVLIAVKAETGTPRFSADEITGMFISMMFAGHHTSSGTASWTLIELMRHRDAYAAVIDELDELYGD
GRSVSFHALRQIPQLENVLKETLRLHPPLIILMRVAKGEFEVQGHRIHEGDLVAASPAISNRIPEDFPDPHDF
VPARYEQPRQEDLLNRWTWIPFGAGRHRCVGAAFAIMQIKAIFSVLLREYEFEMAQPPESYRNDHSKMVVQLA
QPACVRYRRRTGV
>CYP51B1_Mycobacterium tuberculosis TB_RSA82 2576582198
MSAVALPRVSGGHDEHGHLEEFRTDPIGLMQRVRDECGDVGTFQLAGKQVVLLSGSHANEFFFRAGDDDLDQA
KAYPFMTPIFGEGVVFDASPERRKEMLHNAALRGEQMKGHAATIEDQVRRMIADWGEAGEIDLLDFFAELTIY

TSSACLIGKKFRDQLDGRFAKLYHELERGTDPLAYVDPYLPIESFRRRDEARNGLVALVADIMNGRIANPPTD
KSDRDMLDVLIAVKAETGTPRFSADEITGMFISMMFAGHHTSSGTASWTLIELMRHRDAYAAVIDELDELYGD
GRSVSFHALRQIPQLENVLKETLRLHPPLIILMRVAKGEFEVQGHRIHEGDLVAASPAISNRIPEDFPDPHDF
VPARYEQPRQEDLLNRWTWIPFGAGRHRCVGAAFAIMQIKAIFSVLLREYEFEMAQPPESYRNDHSKMVVQLA
QPACVRYRRRTGV
>CYP51B1_Mycobacterium tuberculosis TB_RSA83 2576161193
MSAVALPRVSGGHDEHGHLEEFRTDPIGLMQRVRDECGDVGTFQLAGKQVVLLSGSHANEFFFRAGDDDLDQA
KAYPFMTPIFGEGVVFDASPERRKEMLHNAALRGEQMKGHAATIEDQVRRMIADWGEAGEIDLLDFFAELTIY
TSSACLIGKKFRDQLDGRFAKLYHELERGTDPLAYVDPYLPIESFRRRDEARNGLVALVADIMNGRIANPPTD
KSDRDMLDVLIAVKAETGTPRFSADEITGMFISMMFAGHHTSSGTASWTLIELMRHRDAYAAVIDELDELYGD
GRSVSFHALRQIPQLENVLKETLRLHPPLIILMRVAKGEFEVQGHRIHEGDLVAASPAISNRIPEDFPDPHDF
VPARYEQPRQEDLLNRWTWIPFGAGRHRCVGAAFAIMQIKAIFSVLLREYEFEMAQPPESYRNDHSKMVVQLA
QPACVRYRRRTGV
>CYP51B1_Mycobacterium tuberculosis TB_RSA84 2603441118
MSAVALPRVSGGHDEHGHLEEFRTDPIGLMQRVRDECGDVGTFQLAGKQVVLLSGSHANEFFFRAGDDDLDQA
KAYPFMTPIFGEGVVFDASPERRKEMLHNAALRGEQMKGHAATIEDQVRRMIADWGEAGEIDLLDFFAELTIY
TSSACLIGKKFRDQLDGRFAKLYHELERGTDPLAYVDPYLPIESFRRRDEARNGLVALVADIMNGRIANPPTD
KSDRDMLDVLIAVKAETGTPRFSADEITGMFISMMFAGHHTSSGTASWTLIELMRHRDAYAAVIDELDELYGD
GRSVSFHALRQIPQLENVLKETLRLHPPLIILMRVAKGEFEVQGHRIHEGDLVAASPAISNRIPEDFPDPHDF
VPARYEQPRQEDLLNRWTWIPFGAGRHRCVGAAFAIMQIKAIFSVLLREYEFEMAQPPESYRNDHSKMVVQLA
QPACVRYRRRTGV
>CYP51B1_Mycobacterium tuberculosis TB_RSA85 2604018499
MSAVALPRVSGGHDEHGHLEEFRTDPIGLMQRVRDECGDVGTFQLAGKQVVLLSGSHANEFFFRAGDDDLDQA
KAYPFMTPIFGEGVVFDASPERRKEMLHNAALRGEQMKGHAATIEDQVRRMIADWGEAGEIDLLDFFAELTIY
TSSACLIGKKFRDQLDGRFAKLYHELERGTDPLAYVDPYLPIESFRRRDEARNGLVALVADIMNGRIANPPTD
KSDRDMLDVLIAVKAETGTPRFSADEITGMFISMMFAGHHTSSGTASWTLIELMRHRDAYAAVIDELDELYGD
GRSVSFHALRQIPQLENVLKETLRLHPPLIILMRVAKGEFEVQGHRIHEGDLVAASPAISNRIPEDFPDPHDF
VPARYEQPRQEDLLNRWTWIPFGAGRHRCVGAAFAIMQIKAIFSVLLREYEFEMAQPPESYRNDHSKMVVQLA
QPACVRYRRRTGV
>CYP51B1_Mycobacterium tuberculosis TB_RSA86 2603291064
MSAVALPRVSGGHDEHGHLEEFRTDPIGLMQRVRDECGDVGTFQLAGKQVVLLSGSHANEFFFRAGDDDLDQA
KAYPFMTPIFGEGVVFDASPERRKEMLHNAALRGEQMKGHAATIEDQVRRMIADWGEAGEIDLLDFFAELTIY
TSSACLIGKKFRDQLDGRFAKLYHELERGTDPLAYVDPYLPIESFRRRDEARNGLVALVADIMNGRIANPPTD
KSDRDMLDVLIAVKAETGTPRFSADEITGMFISMMFAGHHTSSGTASWTLIELMRHRDAYAAVIDELDELYGD
GRSVSFHALRQIPQLENVLKETLRLHPPLIILMRVAKGEFEVQGHRIHEGDLVAASPAISNRIPEDFPDPHDF
VPARYEQPRQEDLLNRWTWIPFGAGRHRCVGAAFAIMQIKAIFSVLLREYEFEMAQPPESYRNDHSKMVVQLA
QPACVRYRRRTGV
>CYP51B1_Mycobacterium tuberculosis TB_RSA87 2605046020
MSAVALPRVSGGHDEHGHLEEFRTDPIGLMQRVRDECGDVGTFQLAGKQVVLLSGSHANEFFFRAGDDDLDQA
KAYPFMTPIFGEGVVFDASPERRKEMLHNAALRGEQMKGHAATIEDQVRRMIADWGEAGEIDLLDFFAELTIY
TSSACLIGKKFRDQLDGRFAKLYHELERGTDPLAYVDPYLPIESFRRRDEARNGLVALVADIMNGRIANPPTD
KSDRDMLDVLIAVKAETGTPRFSADEITGMFISMMFAGHHTSSGTASWTLIELMRHRDAYAAVIDELDELYGD
GRSVSFHALRQIPQLENVLKETLRLHPPLIILMRVAKGEFEVQGHRIHEGDLVAASPAISNRIPEDFPDPHDF
VPARYEQPRQEDLLNRWTWIPFGAGRHRCVGAAFAIMQIKAIFSVLLREYEFEMAQPPESYRNDHSKMVVQLA
QPACVRYRRRTGV
>CYP51B1_Mycobacterium tuberculosis TB_RSA88 2604704303
MSAVALPRVSGGHDEHGHLEEFRTDPIGLMQRVRDECGDVGTFQLAGKQVVLLSGSHANEFFFRAGDDDLDQA
KAYPFMTPIFGEGVVFDASPERRKEMLHNAALRGEQMKGHAATIEDQVRRMIADWGEAGEIDLLDFFAELTIY
TSSACLIGKKFRDQLDGRFAKLYHELERGTDPLAYVDPYLPIESFRRRDEARNGLVALVADIMNGRIANPPTD
KSDRDMLDVLIAVKAETGTPRFSADEITGMFISMMFAGHHTSSGTASWTLIELMRHRDAYAAVIDELDELYGD
GRSVSFHALRQIPQLENVLKETLRLHPPLIILMRVAKGEFEVQGHRIHEGDLVAASPAISNRIPEDFPDPHDF
VPARYEQPRQEDLLNRWTWIPFGAGRHRCVGAAFAIMQIKAIFSVLLREYEFEMAQPPESYRNDHSKMVVQLA
QPACVRYRRRTGV
>CYP51B1_Mycobacterium tuberculosis TB_RSA89 2603445121
MSAVALPRVSGGHDEHGHLEEFRTDPIGLMQRVRDECGDVGTFQLAGKQVVLLSGSHANEFFFRAGDDDLDQA
KAYPFMTPIFGEGVVFDASPERRKEMLHNAALRGEQMKGHAATIEDQVRRMIADWGEAGEIDLLDFFAELTIY
TSSACLIGKKFRDQLDGRFAKLYHELERGTDPLAYVDPYLPIESFRRRDEARNGLVALVADIMNGRIANPPTD
KSDRDMLDVLIAVKAETGTPRFSADEITGMFISMMFAGHHTSSGTASWTLIELMRHRDAYAAVIDELDELYGD

GRSVSFHALRQIPQLENVLKETLRLHPPLIILMRVAKGEFEVQGHRIHEGDLVAASPAISNRIPEDFPDPHDF
VPARYEQPRQEDLLNRWTWIPFGAGRHRCVGAAFAIMQIKAIFSVLLREYEFEMAQPPESYRNDHSKMVVQLA
QPACVRYRRRTGV
>CYP51B1_Mycobacterium tuberculosis TB_RSA91 2603447504
MSAVALPRVSGGHDEHGHLEEFRTDPIGLMQRVRDECGDVGTFQLAGKQVVLLSGSHANEFFFRAGDDDLDQA
KAYPFMTPIFGEGVVFDASPERRKEMLHNAALRGEQMKGHAATIEDQVRRMIADWGEAGEIDLLDFFAELTIY
TSSACLIGKKFRDQLDGRFAKLYHELERGTDPLAYVDPYLPIESFRRRDEARNGLVALVADIMNGRIANPPTD
KSDRDMLDVLIAVKAETGTPRFSADEITGMFISMMFAGHHTSSGTASWTLIELMRHRDAYAAVIDELDELYGD
GRSVSFHALRQIPQLENVLKETLRLHPPLIILMRVAKGEFEVQGHRIHEGDLVAASPAISNRIPEDFPDPHDF
VPARYEQPRQEDLLNRWTWIPFGAGRHRCVGAAFAIMQIKAIFSVLLREYEFEMAQPPESYRNDHSKMVVQLA
QPACVRYRRRTGV
>CYP51B1_Mycobacterium tuberculosis TB_RSA92 2605050114
MSAVALPRVSGGHDEHGHLEEFRTDPIGLMQRVRDECGDVGTFQLAGKQVVLLSGSHANEFFFRAGDDDLDQA
KAYPFMTPIFGEGVVFDASPERRKEMLHNAALRGEQMKGHAATIEDQVRRMIADWGEAGEIDLLDFFAELTIY
TSSACLIGKKFRDQLDGRFAKLYHELERGTDPLAYVDPYLPIESFRRRDEARNGLVALVADIMNGRIANPPTD
KSDRDMLDVLIAVKAETGTPRFSADEITGMFISMMFAGHHTSSGTASWTLIELMRHRDAYAAVIDELDELYGD
GRSVSFHALRQIPQLENVLKETLRLHPPLIILMRVAKGEFEVQGHRIHEGDLVAASPAISNRIPEDFPDPHDF
VPARYEQPRQEDLLNRWTWIPFGAGRHRCVGAAFAIMQIKAIFSVLLREYEFEMAQPPESYRNDHSKMVVQLA
QPACVRYRRRTGV
>CYP51B1_Mycobacterium tuberculosis TB_RSA93 2605414377
MSAVALPRVSGGHDEHGHLEEFRTDPIGLMQRVRDECGDVGTFQLAGKQVVLLSGSHANEFFFRAGDDDLDQA
KAYPFMTPIFGEGVVFDASPERRKEMLHNAALRGEQMKGHAATIEDQVRRMIADWGEAGEIDLLDFFAELTIY
TSSACLIGKKFRDQLDGRFAKLYHELERGTDPLAYVDPYLPIESFRRRDEARNGLVALVADIMNGRIANPPTD
KSDRDMLDVLIAVKAETGTPRFSADEITGMFISMMFAGHHTSSGTASWTLIELMRHRDAYAAVIDELDELYGD
GRSVSFHALRQIPQLENVLKETLRLHPPLIILMRVAKGEFEVQGHRIHEGDLVAASPAISNRIPEDFPDPHDF
VPARYEQPRQEDLLNRWTWIPFGAGRHRCVGAAFAIMQIKAIFSVLLREYEFEMAQPPESYRNDHSKMVVQLA
QPACVRYRRRTGV
>CYP51B1_Mycobacterium tuberculosis TB_RSA94 2605771650
MSAVALPRVSGGHDEHGHLEEFRTDPIGLMQRVRDECGDVGTFQLAGKQVVLLSGSHANEFFFRAGDDDLDQA
KAYPFMTPIFGEGVVFDASPERRKEMLHNAALRGEQMKGHAATIEDQVRRMIADWGEAGEIDLLDFFAELTIY
TSSACLIGKKFRDQLDGRFAKLYHELERGTDPLAYVDPYLPIESFRRRDEARNGLVALVADIMNGRIANPPTD
KSDRDMLDVLIAVKAETGTPRFSADEITGMFISMMFAGHHTSSGTASWTLIELMRHRDAYAAVIDELDELYGD
GRSVSFHALRQIPQLENVLKETLRLHPPLIILMRVAKGEFEVQGHRIHEGDLVAASPAISNRIPEDFPDPHDF
VPARYEQPRQEDLLNRWTWIPFGAGRHRCVGAAFAIMQIKAIFSVLLREYEFEMAQPPESYRNDHSKMVVQLA
QPACVRYRRRTGV
>CYP51B1_Mycobacterium tuberculosis TB_RSA95 2603298259
MSAVALPRVSGGHDEHGHLEEFRTDPIGLMQRVRDECGDVGTFQLAGKQVVLLSGSHANEFFFRAGDDDLDQA
KAYPFMTPIFGEGVVFDASPERRKEMLHNAALRGEQMKGHAATIEDQVRRMIADWGEAGEIDLLDFFAELTIY
TSSACLIGKKFRDQLDGRFAKLYHELERGTDPLAYVDPYLPIESFRRRDEARNGLVALVADIMNGRIANPPTD
KSDRDMLDVLIAVKAETGTPRFSADEITGMFISMMFAGHHTSSGTASWTLIELMRHRDAYAAVIDELDELYGD
GRSVSFHALRQIPQLENVLKETLRLHPPLIILMRVAKGEFEVQGHRIHEGDLVAASPAISNRIPEDFPDPHDF
VPARYEQPRQEDLLNRWTWIPFGAGRHRCVGAAFAIMQIKAIFSVLLREYEFEMAQPPESYRNDHSKMVVQLA
QPACVRYRRRTGV
>CYP51B1_Mycobacterium tuberculosis TB_RSA96 2574737153
MSAVALPRVSGGHDEHGHLEEFRTDPIGLMQRVRDECGDVGTFQLAGKQVVLLSGSHANEFFFRAGDDDLDQA
KAYPFMTPIFGEGVVFDASPERRKEMLHNAALRGEQMKGHAATIEDQVRRMIADWGEAGEIDLLDFFAELTIY
TSSACLIGKKFRDQLDGRFAKLYHELERGTDPLAYVDPYLPIESFRRRDEARNGLVALVADIMNGRIANPPTD
KSDRDMLDVLIAVKAETGTPRFSADEITGMFISMMFAGHHTSSGTASWTLIELMRHRDAYAAVIDELDELYGD
GRSVSFHALRQIPQLENVLKETLRLHPPLIILMRVAKGEFEVQGHRIHEGDLVAASPAISNRIPEDFPDPHDF
VPARYEQPRQEDLLNRWTWIPFGAGRHRCVGAAFAIMQIKAIFSVLLREYEFEMAQPPESYRNDHSKMVVQLA
QPACVRYRRRTGV
>CYP51B1_Mycobacterium tuberculosis TB_RSA97 2576584908
MSAVALPRVSGGHDEHGHLEEFRTDPIGLMQRVRDECGDVGTFQLAGKQVVLLSGSHANEFFFRAGDDDLDQA
KAYPFMTPIFGEGVVFDASPERRKEMLHNAALRGEQMKGHAATIEDQVRRMIADWGEAGEIDLLDFFAELTIY
TSSACLIGKKFRDQLDGRFAKLYHELERGTDPLAYVDPYLPIESFRRRDEARNGLVALVADIMNGRIANPPTD
KSDRDMLDVLIAVKAETGTPRFSADEITGMFISMMFAGHHTSSGTASWTLIELMRHRDAYAAVIDELDELYGD
GRSVSFHALRQIPQLENVLKETLRLHPPLIILMRVAKGEFEVQGHRIHEGDLVAASPAISNRIPEDFPDPHDF

VPARYEQPRQEDLLNRWTWIPFGAGRHRCVGAAFAIMQIKAIFSVLLREYEFEMAQPPESYRNDHSKMVVQLA
QPACVRYRRRTGV
>CYP51B1_Mycobacterium tuberculosis TB282 2719879871
MSAVALPRVSGGHDEHGHLEEFRTDPIGLMQRVRDECGDVGTFQLAGKQVVLLSGSHANEFFFRAGDDDLDQA
KAYPFMTPIFGEGVVFDASPERRKEMLHNAALRGEQMKGHAATIEDQVRRMIADWGEAGEIDLLDFFAELTIY
TSSACLIGKKFRDQLDGRFAKLYHELERGTDPLAYVDPYLPIESFRRRDEARNGLVALVADIMNGRIANPPTD
KSDRDMLDVLIAVKAETGTPRFSADEITGMFISMMFAGHHTSSGTASWTLIELMRHRDAYAAVIDELDELYGD
GRSVSFHALRQIPQLENVLKETLRLHPPLIILMRVAKGEFEVQGHRIHEGDLVAASPAISNRIPEDFPDPHDF
VPARYEQPRQEDLLNRWTWIPFGAGRHRCVGAAFAIMQIKAIFSVLLREYEFEMAQPPESYRNDHSKMVVQLA
QPACVRYRRRTGV
>CYP51B1_Mycobacterium tuberculosis TBR10 2579812851
MSAVALPRVSGGHDEHGHLEEFRTDPIGLMQRVRDECGDVGTFQLAGKQVVLLSGSHANEFFFRAGDDDLDQA
KAYPFMTPIFGEGVVFDASPERRKEMLHNAALRGEQMKGHAATIEDQVRRMIADWGEAGEIDLLDFFAELTIY
TSSACLIGKKFRDQLDGRFAKLYHELERGTDPLAYVDPYLPIESFRRRDEARNGLVALVADIMNGRIANPPTD
KSDRDMLDVLIAVKAETGTPRFSADEITGMFISMMFAGHHTSSGTASWTLIELMRHRDAYAAVIDELDELYGD
GRSVSFHALRQIPQLENVLKETLRLHPPLIILMRVAKGEFEVQGHRIHEGDLVAASPAISNRIPEDFPDPHDF
VPARYEQPRQEDLLNRWTWIPFGAGRHRCVGAAFAIMQIKAIFSVLLREYEFEMAQPPESYRNDHSKMVVQLA
QPACVRYRRRTGV
>CYP51B1_Mycobacterium tuberculosis TBR-103 2667095189
MSAVALPRVSGGHDEHGHLEEFRTDPIGLMQRVRDECGDVGTFQLAGKQVVLLSGSHANEFFFRAGDDDLDQA
KAYPFMTPIFGEGVVFDASPERRKEMLHNAALRGEQMKGHAATIEDQVRRMIADWGEAGEIDLLDFFAELTIY
TSSACLIGKKFRDQLDGRFAKLYHELERGTDPLAYVDPYLPIESFRRRDEARNGLVALVADIMNGRIANPPTD
KSDRDMLDVLIAVKAETGTPRFSADEITGMFISMMFAGHHTSSGTASWTLIELMRHRDAYAAVIDELDELYGD
GRSVSFHALRQIPQLENVLKETLRLHPPLIILMRVAKGEFEVQGHRIHEGDLVAASPAISNRIPEDFPDPHDF
VPARYEQPRQEDLLNRWTWIPFGAGRHRCVGAAFAIMQIKAIFSVLLREYEFEMAQPPESYRNDHSKMVVQLA
QPACVRYRRRTGV
>CYP51B1_Mycobacterium tuberculosis TBR-152 2696496930
MSAVALPRVSGGHDEHGHLEEFRTDPIGLMQRVRDECGDVGTFQLAGKQVVLLSGSHANEFFFRAGDDDLDQA
KAYPFMTPIFGEGVVFDASPERRKEMLHNAALRGEQMKGHAATIEDQVRRMIADWGEAGEIDLLDFFAELTIY
TSSACLIGKKFRDQLDGRFAKLYHELERGTDPLAYVDPYLPIESFRRRDEARNGLVALVADIMNGRIANPPTD
KSDRDMLDVLIAVKAETGTPRFSADEITGMFISMMFAGHHTSSGTASWTLIELMRHRDAYAAVIDELDELYGD
GRSVSFHALRQIPQLENVLKETLRLHPPLIILMRVAKGEFEVQGHRIHEGDLVAASPAISNRIPEDFPDPHDF
VPARYEQPRQEDLLNRWTWIPFGAGRHRCVGAAFAIMQIKAIFSVLLREYEFEMAQPPESYRNDHSKMVVQLA
QPACVRYRRRTGV
>CYP51B1_Mycobacterium tuberculosis TBR-175 2663316638
MSAVALPRVSGGHDEHGHLEEFRTDPIGLMQRVRDECGDVGTFQLAGKQVVLLSGSHANEFFFRAGDDDLDQA
KAYPFMTPIFGEGVVFDASPERRKEMLHNAALRGEQMKGHAATIEDQVRRMIADWGEAGEIDLLDFFAELTIY
TSSACLIGKKFRDQLDGRFAKLYHELERGTDPLAYVDPYLPIESFRRRDEARNGLVALVADIMNGRIANPPTD
KSDRDMLDVLIAVKAETGTPRFSADEITGMFISMMFAGHHTSSGTASWTLIELMRHRDAYAAVIDELDELYGD
GRSVSFHALRQIPQLENVLKETLRLHPPLIILMRVAKGEFEVQGHRIHEGDLVAASPAISNRIPEDFPDPHDF
VPARYEQPRQEDLLNRWTWIPFGAGRHRCVGAAFAIMQIKAIFSVLLREYEFEMAQPPESYRNDHSKMVVQLA
QPACVRYRRRTGV
>CYP51B1_Mycobacterium tuberculosis TBR26 2577794326
MSAVALPRVSGGHDEHGHLEEFRTDPIGLMQRVRDECGDVGTFQLAGKQVVLLSGSHANEFFFRAGDDDLDQA
KAYPFMTPIFGEGVVFDASPERRKEMLHNAALRGEQMKGHAATIEDQVRRMIADWGEAGEIDLLDFFAELTIY
TSSACLIGKKFRDQLDGRFAKLYHELERGTDPLAYVDPYLPIESFRRRDEARNGLVALVADIMNGRIANPPTD
KSDRDMLDVLIAVKAETGTPRFSADEITGMFISMMFAGHHTSSGTASWTLIELMRHRDAYAAVIDELDELYGD
GRSVSFHALRQIPQLENVLKETLRLHPPLIILMRVAKGEFEVQGHRIHEGDLVAASPAISNRIPEDFPDPHDF
VPARYEQPRQEDLLNRWTWIPFGAGRHRCVGAAFAIMQIKAIFSVLLREYEFEMAQPPESYRNDHSKMVVQLA
QPACVRYRRRTGV
>CYP51B1_Mycobacterium tuberculosis TBR28 2575383285
MSAVALPRVSGGHDEHGHLEEFRTDPIGLMQRVRDECGDVGTFQLAGKQVVLLSGSHANEFFFRAGDDDLDQA
KAYPFMTPIFGEGVVFDASPERRKEMLHNAALRGEQMKGHAATIEDQVRRMIADWGEAGEIDLLDFFAELTIY
TSSACLIGKKFRDQLDGRFAKLYHELERGTDPLAYVDPYLPIESFRRRDEARNGLVALVADIMNGRIANPPTD
KSDRDMLDVLIAVKAETGTPRFSADEITGMFISMMFAGHHTSSGTASWTLIELMRHRDAYAAVIDELDELYGD
GRSVSFHALRQIPQLENVLKETLRLHPPLIILMRVAKGEFEVQGHRIHEGDLVAASPAISNRIPEDFPDPHDF
VPARYEQPRQEDLLNRWTWIPFGAGRHRCVGAAFAIMQIKAIFSVLLREYEFEMAQPPESYRNDHSKMVVQLA
QPACVRYRRRTGV

>CYP51B1_Mycobacterium tuberculosis TBR31 2574636634
MSAVALPRVSGGHDEHGHLEEFRTDPIGLMQRVRDECGDVGTFQLAGKQVVLLSGSHANEFFFRAGDDDLDQA
KAYPFMTPIFGEGVVFDASPERRKEMLHNAALRGEQMKGHAATIEDQVRRMIADWGEAGEIDLLDFFAELTIY
TSSACLIGKKFRDQLDGRFAKLYHELERGTDPLAYVDPYLPIESFRRRDEARNGLVALVADIMNGRIANPPTD
KSDRDMLDVLIAVKAETGTPRFSADEITGMFISMMFAGHHTSSGTASWTLIELMRHRDAYAAVIDELDELYGD
GRSVSFHALRQIPQLENVLKETLRLHPPLIILMRVAKGEFEVQGHRIHEGDLVAASPAISNRIPEDFPDPHDF
VPARYEQPRQEDLLNRWTWIPFGAGRHRCVGAAFAIMQIKAIFSVLLREYEFEMAQPPESYRNDHSKMVVQLA
QPACVRYRRRTGV
>CYP51B1_Mycobacterium tuberculosis TBR37 2576759914
MSAVALPRVSGGHDEHGHLEEFRTDPIGLMQRVRDECGDVGTFQLAGKQVVLLSGSHANEFFFRAGDDDLDQA
KAYPFMTPIFGEGVVFDASPERRKEMLHNAALRGEQMKGHAATIEDQVRRMIADWGEAGEIDLLDFFAELTIY
TSSACLIGKKFRDQLDGRFAKLYHELERGTDPLAYVDPYLPIESFRRRDEARNGLVALVADIMNGRIANPPTD
KSDRDMLDVLIAVKAETGTPRFSADEITGMFISMMFAGHHTSSGTASWTLIELMRHRDAYAAVIDELDELYGD
GRSVSFHALRQIPQLENVLKETLRLHPPLIILMRVAKGEFEVQGHRIHEGDLVAASPAISNRIPEDFPDPHDF
VPARYEQPRQEDLLNRWTWIPFGAGRHRCVGAAFAIMQIKAIFSVLLREYEFEMAQPPESYRNDHSKMVVQLA
QPACVRYRRRTGV
>CYP51B1_Mycobacterium tuberculosis TBR41 2575890177
MSAVALPRVSGGHDEHGHLEEFRTDPIGLMQRVRDECGDVGTFQLAGKQVVLLSGSHANEFFFRAGDDDLDQA
KAYPFMTPIFGEGVVFDASPERRKEMLHNAALRGEQMKGHAATIEDQVRRMIADWGEAGEIDLLDFFAELTIY
TSSACLIGKKFRDQLDGRFAKLYHELERGTDPLAYVDPYLPIESFRRRDEARNGLVALVADIMNGRIANPPTD
KSDRDMLDVLIAVKAETGTPRFSADEITGMFISMMFAGHHTSSGTASWTLIELMRHRDAYAAVIDELDELYGD
GRSVSFHALRQIPQLENVLKETLRLHPPLIILMRVAKGEFEVQGHRIHEGDLVAASPAISNRIPEDFPDPHDF
VPARYEQPRQEDLLNRWTWIPFGAGRHRCVGAAFAIMQIKAIFSVLLREYEFEMAQPPESYRNDHSKMVVQLA
QPACVRYRRRTGV
>CYP51B1_Mycobacterium tuberculosis TBR50 2576155192
MSAVALPRVSGGHDEHGHLEEFRTDPIGLMQRVRDECGDVGTFQLAGKQVVLLSGSHANEFFFRAGDDDLDQA
KAYPFMTPIFGEGVVFDASPERRKEMLHNAALRGEQMKGHAATIEDQVRRMIADWGEAGEIDLLDFFAELTIY
TSSACLIGKKFRDQLDGRFAKLYHELERGTDPLAYVDPYLPIESFRRRDEARNGLVALVADIMNGRIANPPTD
KSDRDMLDVLIAVKAETGTPRFSADEITGMFISMMFAGHHTSSGTASWTLIELMRHRDAYAAVIDELDELYGD
GRSVSFHALRQIPQLENVLKETLRLHPPLIILMRVAKGEFEVQGHRIHEGDLVAASPAISNRIPEDFPDPHDF
VPARYEQPRQEDLLNRWTWIPFGAGRHRCVGAAFAIMQIKAIFSVLLREYEFEMAQPPESYRNDHSKMVVQLA
QPACVRYRRRTGV
>CYP51B1_Mycobacterium tuberculosis TBR51 2584610675
MSAVALPRVSGGHDEHGHLEEFRTDPIGLMQRVRDECGDVGTFQLAGKQVVLLSGSHANEFFFRAGDDDLDQA
KAYPFMTPIFGEGVVFDASPERRKEMLHNAALRGEQMKGHAATIEDQVRRMIADWGEAGEIDLLDFFAELTIY
TSSACLIGKKFRDQLDGRFAKLYHELERGTDPLAYVDPYLPIESFRRRDEARNGLVALVADIMNGRIANPPTD
KSDRDMLDVLIAVKAETGTPRFSADEITGMFISMMFAGHHTSSGTASWTLIELMRHRDAYAAVIDELDELYGD
GRSVSFHALRQIPQLENVLKETLRLHPPLIILMRVAKGEFEVQGHRIHEGDLVAASPAISNRIPEDFPDPHDF
VPARYEQPRQEDLLNRWTWIPFGAGRHRCVGAAFAIMQIKAIFSVLLREYEFEMAQPPESYRNDHSKMVVQLA
QPACVRYRRRTGV
>CYP51B1_Mycobacterium tuberculosis TBR55 2575229645
MSAVALPRVSGGHDEHGHLEEFRTDPIGLMQRVRDECGDVGTFQLAGKQVVLLSGSHANEFFFRAGDDDLDQA
KAYPFMTPIFGEGVVFDASPERRKEMLHNAALRGEQMKGHAATIEDQVRRMIADWGEAGEIDLLDFFAELTIY
TSSACLIGKKFRDQLDGRFAKLYHELERGTDPLAYVDPYLPIESFRRRDEARNGLVALVADIMNGRIANPPTD
KSDRDMLDVLIAVKAETGTPRFSADEITGMFISMMFAGHHTSSGTASWTLIELMRHRDAYAAVIDELDELYGD
GRSVSFHALRQIPQLENVLKETLRLHPPLIILMRVAKGEFEVQGHRIHEGDLVAASPAISNRIPEDFPDPHDF
VPARYEQPRQEDLLNRWTWIPFGAGRHRCVGAAFAIMQIKAIFSVLLREYEFEMAQPPESYRNDHSKMVVQLA
QPACVRYRRRTGV
>CYP51B1_Mycobacterium tuberculosis TBR58 2574878182
MSAVALPRVSGGHDEHGHLEEFRTDPIGLMQRVRDECGDVGTFQLAGKQVVLLSGSHANEFFFRAGDDDLDQA
KAYPFMTPIFGEGVVFDASPERRKEMLHNAALRGEQMKGHAATIEDQVRRMIADWGEAGEIDLLDFFAELTIY
TSSACLIGKKFRDQLDGRFAKLYHELERGTDPLAYVDPYLPIESFRRRDEARNGLVALVADIMNGRIANPPTD
KSDRDMLDVLIAVKAETGTPRFSADEITGMFISMMFAGHHTSSGTASWTLIELMRHRDAYAAVIDELDELYGD
GRSVSFHALRQIPQLENVLKETLRLHPPLIILMRVAKGEFEVQGHRIHEGDLVAASPAISNRIPEDFPDPHDF
VPARYEQPRQEDLLNRWTWIPFGAGRHRCVGAAFAIMQIKAIFSVLLREYEFEMAQPPESYRNDHSKMVVQLA
QPACVRYRRRTGV
>CYP51B1_Mycobacterium tuberculosis TBR65 2574884706

MSAVALPRVSGGHDEHGHLEEFRTDPIGLMQRVRDECGDVGTFQLAGKQVVLLSGSHANEFFFRAGDDDLDQA
KAYPFMTPIFGEGVVFDASPERRKEMLHNAALRGEQMKGHAATIEDQVRRMIADWGEAGEIDLLDFFAELTIY
TSSACLIGKKFRDQLDGRFAKLYHELERGTDPLAYVDPYLPIESFRRRDEARNGLVALVADIMNGRIANPPTD
KSDRDMLDVLIAVKAETGTPRFSADEITGMFISMMFAGHHTSSGTASWTLIELMRHRDAYAAVIDELDELYGD
GRSVSFHALRQIPQLENVLKETLRLHPPLIILMRVAKGEFEVQGHRIHEGDLVAASPAISNRIPEDFPDPHDF
VPARYEQPRQEDLLNRWTWIPFGAGRHRCVGAAFAIMQIKAIFSVLLREYEFEMAQPPESYRNDHSKMVVQLA
QPACVRYRRRTGV
>CYP51B1_Mycobacterium tuberculosis TBR7 2575980009
MSAVALPRVSGGHDEHGHLEEFRTDPIGLMQRVRDECGDVGTFQLAGKQVVLLSGSHANEFFFRAGDDDLDQA
KAYPFMTPIFGEGVVFDASPERRKEMLHNAALRGEQMKGHAATIEDQVRRMIADWGEAGEIDLLDFFAELTIY
TSSACLIGKKFRDQLDGRFAKLYHELERGTDPLAYVDPYLPIESFRRRDEARNGLVALVADIMNGRIANPPTD
KSDRDMLDVLIAVKAETGTPRFSADEITGMFISMMFAGHHTSSGTASWTLIELMRHRDAYAAVIDELDELYGD
GRSVSFHALRQIPQLENVLKETLRLHPPLIILMRVAKGEFEVQGHRIHEGDLVAASPAISNRIPEDFPDPHDF
VPARYEQPRQEDLLNRWTWIPFGAGRHRCVGAAFAIMQIKAIFSVLLREYEFEMAQPPESYRNDHSKMVVQLA
QPACVRYRRRTGV
>CYP51B1_Mycobacterium tuberculosis TKK_02_0001 2592441794
MSAVALPRVSGGHDEHGHLEEFRTDPIGLMQRVRDECGDVGTFQLAGKQVVLLSGSHANEFFFRAGDDDLDQA
KAYPFMTPIFGEGVVFDASPERRKEMLHNAALRGEQMKGHAATIEDQVRRMIADWGEAGEIDLLDFFAELTIY
TSSACLIGKKFRDQLDGRFAKLYHELERGTDPLAYVDPYLPIESFRRRDEARNGLVALVADIMNGRIANPPTD
KSDRDMLDVLIAVKAETGTPRFSADEITGMFISMMFAGHHTSSGTASWTLIELMRHRDAYAAVIDELDELYGD
GRSVSFHALRQIPQLENVLKETLRLHPPLIILMRVAKGEFEVQGHRIHEGDLVAASPAISNRIPEDFPDPHDF
VPARYEQPRQEDLLNRWTWIPFGAGRHRCVGAAFAIMQIKAIFSVLLREYEFEMAQPPESYRNDHSKMVVQLA
QPACVRYRRRTGV
>CYP51B1_Mycobacterium tuberculosis TKK_02_0002 2584800089
MSAVALPRVSGGHDEHGHLEEFRTDPIGLMQRVRDECGDVGTFQLAGKQVVLLSGSHANEFFFRAGDDDLDQA
KAYPFMTPIFGEGVVFDASPERRKEMLHNAALRGEQMKGHAATIEDQVRRMIADWGEAGEIDLLDFFAELTIY
TSSACLIGKKFRDQLDGRFAKLYHELERGTDPLAYVDPYLPIESFRRRDEARNGLVALVADIMNGRIANPPTD
KSDRDMLDVLIAVKAETGTPRFSADEITGMFISMMFAGHHTSSGTASWTLIELMRHRDAYAAVIDELDELYGD
GRSVSFHALRQIPQLENVLKETLRLHPPLIILMRVAKGEFEVQGHRIHEGDLVAASPAISNRIPEDFPDPHDF
VPARYEQPRQEDLLNRWTWIPFGAGRHRCVGAAFAIMQIKAIFSVLLREYEFEMAQPPESYRNDHSKMVVQLA
QPACVRYRRRTGV
>CYP51B1_Mycobacterium tuberculosis TKK_02_0003 2592437710
MSAVALPRVSGGHDEHGHLEEFRTDPIGLMQRVRDECGDVGTFQLAGKQVVLLSGSHANEFFFRAGDDDLDQA
KAYPFMTPIFGEGVVFDASPERRKEMLHNAALRGEQMKGHAATIEDQVRRMIADWGEAGEIDLLDFFAELTIY
TSSACLIGKKFRDQLDGRFAKLYHELERGTDPLAYVDPYLPIESFRRRDEARNGLVALVADIMNGRIANPPTD
KSDRDMLDVLIAVKAETGTPRFSADEITGMFISMMFAGHHTSSGTASWTLIELMRHRDAYAAVIDELDELYGD
GRSVSFHALRQIPQLENVLKETLRLHPPLIILMRVAKGEFEVQGHRIHEGDLVAASPAISNRIPEDFPDPHDF
VPARYEQPRQEDLLNRWTWIPFGAGRHRCVGAAFAIMQIKAIFSVLLREYEFEMAQPPESYRNDHSKMVVQLA
QPACVRYRRRTGV
>CYP51B1_Mycobacterium tuberculosis TKK_02_0006 2592433680
MSAVALPRVSGGHDEHGHLEEFRTDPIGLMQRVRDECGDVGTFQLAGKQVVLLSGSHANEFFFRAGDDDLDQA
KAYPFMTPIFGEGVVFDASPERRKEMLHNAALRGEQMKGHAATIEDQVRRMIADWGEAGEIDLLDFFAELTIY
TSSACLIGKKFRDQLDGRFAKLYHELERGTDPLAYVDPYLPIESFRRRDEARNGLVALVADIMNGRIANPPTD
KSDRDMLDVLIAVKAETGTPRFSADEITGMFISMMFAGHHTSSGTASWTLIELMRHRDAYAAVIDELDELYGD
GRSVSFHALRQIPQLENVLKETLRLHPPLIILMRVAKGEFEVQGHRIHEGDLVAASPAISNRIPEDFPDPHDF
VPARYEQPRQEDLLNRWTWIPFGAGRHRCVGAAFAIMQIKAIFSVLLREYEFEMAQPPESYRNDHSKMVVQLA
QPACVRYRRRTGV
>CYP51B1_Mycobacterium tuberculosis TKK_02_0012 2592431995
MSAVALPRVSGGHDEHGHLEEFRTDPIGLMQRVRDECGDVGTFQLAGKQVVLLSGSHANEFFFRAGDDDLDQA
KAYPFMTPIFGEGVVFDASPERRKEMLHNAALRGEQMKGHAATIEDQVRRMIADWGEAGEIDLLDFFAELTIY
TSSACLIGKKFRDQLDGRFAKLYHELERGTDPLAYVDPYLPIESFRRRDEARNGLVALVADIMNGRIANPPTD
KSDRDMLDVLIAVKAETGTPRFSADEITGMFISMMFAGHHTSSGTASWTLIELMRHRDAYAAVIDELDELYGD
GRSVSFHALRQIPQLENVLKETLRLHPPLIILMRVAKGEFEVQGHRIHEGDLVAASPAISNRIPEDFPDPHDF
VPARYEQPRQEDLLNRWTWIPFGAGRHRCVGAAFAIMQIKAIFSVLLREYEFEMAQPPESYRNDHSKMVVQLA
QPACVRYRRRTGV
>CYP51B1_Mycobacterium tuberculosis TKK_02_0013 2592427922
MSAVALPRVSGGHDEHGHLEEFRTDPIGLMQRVRDECGDVGTFQLAGKQVVLLSGSHANEFFFRAGDDDLDQA
KAYPFMTPIFGEGVVFDASPERRKEMLHNAALRGEQMKGHAATIEDQVRRMIADWGEAGEIDLLDFFAELTIY

TSSACLIGKKFRDQLDGRFAKLYHELERGTDPLAYVDPYLPIESFRRRDEARNGLVALVADIMNGRIANPPTD
KSDRDMLDVLIAVKAETGTPRFSADEITGMFISMMFAGHHTSSGTASWTLIELMRHRDAYAAVIDELDELYGD
GRSVSFHALRQIPQLENVLKETLRLHPPLIILMRVAKGEFEVQGHRIHEGDLVAASPAISNRIPEDFPDPHDF
VPARYEQPRQEDLLNRWTWIPFGAGRHRCVGAAFAIMQIKAIFSVLLREYEFEMAQPPESYRNDHSKMVVQLA
QPACVRYRRRTGV
>CYP51B1_Mycobacterium tuberculosis TKK_02_0014 2584941129
MSAVALPRVSGGHDEHGHLEEFRTDPIGLMQRVRDECGDVGTFQLAGKQVVLLSGSHANEFFFRAGDDDLDQA
KAYPFMTPIFGEGVVFDASPERRKEMLHNAALRGEQMKGHAATIEDQVRRMIADWGEAGEIDLLDFFAELTIY
TSSACLIGKKFRDQLDGRFAKLYHELERGTDPLAYVDPYLPIESFRRRDEARNGLVALVADIMNGRIANPPTD
KSDRDMLDVLIAVKAETGTPRFSADEITGMFISMMFAGHHTSSGTASWTLIELMRHRDAYAAVIDELDELYGD
GRSVSFHALRQIPQLENVLKETLRLHPPLIILMRVAKGEFEVQGHRIHEGDLVAASPAISNRIPEDFPDPHDF
VPARYEQPRQEDLLNRWTWIPFGAGRHRCVGAAFAIMQIKAIFSVLLREYEFEMAQPPESYRNDHSKMVVQLA
QPACVRYRRRTGV
>CYP51B1_Mycobacterium tuberculosis TKK_02_0015 2574728090
MSAVALPRVSGGHDEHGHLEEFRTDPIGLMQRVRDECGDVGTFQLAGKQVVLLSGSHANEFFFRAGDDDLDQA
KAYPFMTPIFGEGVVFDASPERRKEMLHNAALRGEQMKGHAATIEDQVRRMIADWGEAGEIDLLDFFAELTIY
TSSACLIGKKFRDQLDGRFAKLYHELERGTDPLAYVDPYLPIESFRRRDEARNGLVALVADIMNGRIANPPTD
KSDRDMLDVLIAVKAETGTPRFSADEITGMFISMMFAGHHTSSGTASWTLIELMRHRDAYAAVIDELDELYGD
GRSVSFHALRQIPQLENVLKETLRLHPPLIILMRVAKGEFEVQGHRIHEGDLVAASPAISNRIPEDFPDPHDF
VPARYEQPRQEDLLNRWTWIPFGAGRHRCVGAAFAIMQIKAIFSVLLREYEFEMAQPPESYRNDHSKMVVQLA
QPACVRYRRRTGV
>CYP51B1_Mycobacterium tuberculosis TKK_02_0016 2592423853
MSAVALPRVSGGHDEHGHLEEFRTDPIGLMQRVRDECGDVGTFQLAGKQVVLLSGSHANEFFFRAGDDDLDQA
KAYPFMTPIFGEGVVFDASPERRKEMLHNAALRGEQMKGHAATIEDQVRRMIADWGEAGEIDLLDFFAELTIY
TSSACLIGKKFRDQLDGRFAKLYHELERGTDPLAYVDPYLPIESFRRRDEARNGLVALVADIMNGRIANPPTD
KSDRDMLDVLIAVKAETGTPRFSADEITGMFISMMFAGHHTSSGTASWTLIELMRHRDAYAAVIDELDELYGD
GRSVSFHALRQIPQLENVLKETLRLHPPLIILMRVAKGEFEVQGHRIHEGDLVAASPAISNRIPEDFPDPHDF
VPARYEQPRQEDLLNRWTWIPFGAGRHRCVGAAFAIMQIKAIFSVLLREYEFEMAQPPESYRNDHSKMVVQLA
QPACVRYRRRTGV
>CYP51B1_Mycobacterium tuberculosis TKK_02_0017 2592420550
MSAVALPRVSGGHDEHGHLEEFRTDPIGLMQRVRDECGDVGTFQLAGKQVVLLSGSHANEFFFRAGDDDLDQA
KAYPFMTPIFGEGVVFDASPERRKEMLHNAALRGEQMKGHAATIEDQVRRMIADWGEAGEIDLLDFFAELTIY
TSSACLIGKKFRDQLDGRFAKLYHELERGTDPLAYVDPYLPIESFRRRDEARNGLVALVADIMNGRIANPPTD
KSDRDMLDVLIAVKAETGTPRFSADEITGMFISMMFAGHHTSSGTASWTLIELMRHRDAYAAVIDELDELYGD
GRSVSFHALRQIPQLENVLKETLRLHPPLIILMRVAKGEFEVQGHRIHEGDLVAASPAISNRIPEDFPDPHDF
VPARYEQPRQEDLLNRWTWIPFGAGRHRCVGAAFAIMQIKAIFSVLLREYEFEMAQPPESYRNDHSKMVVQLA
QPACVRYRRRTGV
>CYP51B1_Mycobacterium tuberculosis TKK_02_0018 2592413274
MSAVALPRVSGGHDEHGHLEEFRTDPIGLMQRVRDECGDVGTFQLAGKQVVLLSGSHANEFFFRAGDDDLDQA
KAYPFMTPIFGEGVVFDASPERRKEMLHNAALRGEQMKGHAATIEDQVRRMIADWGEAGEIDLLDFFAELTIY
TSSACLIGKKFRDQLDGRFAKLYHELERGTDPLAYVDPYLPIESFRRRDEARNGLVALVADIMNGRIANPPTD
KSDRDMLDVLIAVKAETGTPRFSADEITGMFISMMFAGHHTSSGTASWTLIELMRHRDAYAAVIDELDELYGD
GRSVSFHALRQIPQLENVLKETLRLHPPLIILMRVAKGEFEVQGHRIHEGDLVAASPAISNRIPEDFPDPHDF
VPARYEQPRQEDLLNRWTWIPFGAGRHRCVGAAFAIMQIKAIFSVLLREYEFEMAQPPESYRNDHSKMVVQLA
QPACVRYRRRTGV
>CYP51B1_Mycobacterium tuberculosis TKK_02_0019 2575328412
MSAVALPRVSGGHDEHGHLEEFRTDPIGLMQRVRDECGDVGTFQLAGKQVVLLSGSHANEFFFRAGDDDLDQA
KAYPFMTPIFGEGVVFDASPERRKEMLHNAALRGEQMKGHAATIEDQVRRMIADWGEAGEIDLLDFFAELTIY
TSSACLIGKKFRDQLDGRFAKLYHELERGTDPLAYVDPYLPIESFRRRDEARNGLVALVADIMNGRIANPPTD
KSDRDMLDVLIAVKAETGTPRFSADEITGMFISMMFAGHHTSSGTASWTLIELMRHRDAYAAVIDELDELYGD
GRSVSFHALRQIPQLENVLKETLRLHPPLIILMRVAKGEFEVQGHRIHEGDLVAASPAISNRIPEDFPDPHDF
VPARYEQPRQEDLLNRWTWIPFGAGRHRCVGAAFAIMQIKAIFSVLLREYEFEMAQPPESYRNDHSKMVVQLA
QPACVRYRRRTGV
>CYP51B1_Mycobacterium tuberculosis TKK_02_0020 2592411632
MSAVALPRVSGGHDEHGHLEEFRTDPIGLMQRVRDECGDVGTFQLAGKQVVLLSGSHANEFFFRAGDDDLDQA
KAYPFMTPIFGEGVVFDASPERRKEMLHNAALRGEQMKGHAATIEDQVRRMIADWGEAGEIDLLDFFAELTIY
TSSACLIGKKFRDQLDGRFAKLYHELERGTDPLAYVDPYLPIESFRRRDEARNGLVALVADIMNGRIANPPTD
KSDRDMLDVLIAVKAETGTPRFSADEITGMFISMMFAGHHTSSGTASWTLIELMRHRDAYAAVIDELDELYGD

GRSVSFHALRQIPQLENVLKETLRLHPPLIILMRVAKGEFEVQGHRIHEGDLVAASPAISNRIPEDFPDPHDF
VPARYEQPRQEDLLNRWTWIPFGAGRHRCVGAAFAIMQIKAIFSVLLREYEFEMAQPPESYRNDHSKMVVQLA
QPACVRYRRRTGV
>CYP51B1_Mycobacterium tuberculosis TKK_02_0021 2592407562
MSAVALPRVSGGHDEHGHLEEFRTDPIGLMQRVRDECGDVGTFQLAGKQVVLLSGSHANEFFFRAGDDDLDQA
KAYPFMTPIFGEGVVFDASPERRKEMLHNAALRGEQMKGHAATIEDQVRRMIADWGEAGEIDLLDFFAELTIY
TSSACLIGKKFRDQLDGRFAKLYHELERGTDPLAYVDPYLPIESFRRRDEARNGLVALVADIMNGRIANPPTD
KSDRDMLDVLIAVKAETGTPRFSADEITGMFISMMFAGHHTSSGTASWTLIELMRHRDAYAAVIDELDELYGD
GRSVSFHALRQIPQLENVLKETLRLHPPLIILMRVAKGEFEVQGHRIHEGDLVAASPAISNRIPEDFPDPHDF
VPARYEQPRQEDLLNRWTWIPFGAGRHRCVGAAFAIMQIKAIFSVLLREYEFEMAQPPESYRNDHSKMVVQLA
QPACVRYRRRTGV
>CYP51B1_Mycobacterium tuberculosis TKK_02_0022 2592401496
MSAVALPRVSGGHDEHGHLEEFRTDPIGLMQRVRDECGDVGTFQLAGKQVVLLSGSHANEFFFRAGDDDLDQA
KAYPFMTPIFGEGVVFDASPERRKEMLHNAALRGEQMKGHAATIEDQVRRMIADWGEAGEIDLLDFFAELTIY
TSSACLIGKKFRDQLDGRFAKLYHELERGTDPLAYVDPYLPIESFRRRDEARNGLVALVADIMNGRIANPPTD
KSDRDMLDVLIAVKAETGTPRFSADEITGMFISMMFAGHHTSSGTASWTLIELMRHRDAYAAVIDELDELYGD
GRSVSFHALRQIPQLENVLKETLRLHPPLIILMRVAKGEFEVQGHRIHEGDLVAASPAISNRIPEDFPDPHDF
VPARYEQPRQEDLLNRWTWIPFGAGRHRCVGAAFAIMQIKAIFSVLLREYEFEMAQPPESYRNDHSKMVVQLA
QPACVRYRRRTGV
>CYP51B1_Mycobacterium tuberculosis TKK_02_0025 2592396982
MSAVALPRVSGGHDEHGHLEEFRTDPIGLMQRVRDECGDVGTFQLAGKQVVLLSGSHANEFFFRAGDDDLDQA
KAYPFMTPIFGEGVVFDASPERRKEMLHNAALRGEQMKGHAATIEDQVRRMIADWGEAGEIDLLDFFAELTIY
TSSACLIGKKFRDQLDGRFAKLYHELERGTDPLAYVDPYLPIESFRRRDEARNGLVALVADIMNGRIANPPTD
KSDRDMLDVLIAVKAETGTPRFSADEITGMFISMMFAGHHTSSGTASWTLIELMRHRDAYAAVIDELDELYGD
GRSVSFHALRQIPQLENVLKETLRLHPPLIILMRVAKGEFEVQGHRIHEGDLVAASPAISNRIPEDFPDPHDF
VPARYEQPRQEDLLNRWTWIPFGAGRHRCVGAAFAIMQIKAIFSVLLREYEFEMAQPPESYRNDHSKMVVQLA
QPACVRYRRRTGV
>CYP51B1_Mycobacterium tuberculosis TKK_02_0027 2592392627
MSAVALPRVSGGHDEHGHLEEFRTDPIGLMQRVRDECGDVGTFQLAGKQVVLLSGSHANEFFFRAGDDDLDQA
KAYPFMTPIFGEGVVFDASPERRKEMLHNAALRGEQMKGHAATIEDQVRRMIADWGEAGEIDLLDFFAELTIY
TSSACLIGKKFRDQLDGRFAKLYHELERGTDPLAYVDPYLPIESFRRRDEARNGLVALVADIMNGRIANPPTD
KSDRDMLDVLIAVKAETGTPRFSADEITGMFISMMFAGHHTSSGTASWTLIELMRHRDAYAAVIDELDELYGD
GRSVSFHALRQIPQLENVLKETLRLHPPLIILMRVAKGEFEVQGHRIHEGDLVAASPAISNRIPEDFPDPHDF
VPARYEQPRQEDLLNRWTWIPFGAGRHRCVGAAFAIMQIKAIFSVLLREYEFEMAQPPESYRNDHSKMVVQLA
QPACVRYRRRTGV
>CYP51B1_Mycobacterium tuberculosis TKK_02_0033 2592391305
MSAVALPRVSGGHDEHGHLEEFRTDPIGLMQRVRDECGDVGTFQLAGKQVVLLSGSHANEFFFRAGDDDLDQA
KAYPFMTPIFGEGVVFDASPERRKEMLHNAALRGEQMKGHAATIEDQVRRMIADWGEAGEIDLLDFFAELTIY
TSSACLIGKKFRDQLDGRFAKLYHELERGTDPLAYVDPYLPIESFRRRDEARNGLVALVADIMNGRIANPPTD
KSDRDMLDVLIAVKAETGTPRFSADEITGMFISMMFAGHHTSSGTASWTLIELMRHRDAYAAVIDELDELYGD
GRSVSFHALRQIPQLENVLKETLRLHPPLIILMRVAKGEFEVQGHRIHEGDLVAASPAISNRIPEDFPDPHDF
VPARYEQPRQEDLLNRWTWIPFGAGRHRCVGAAFAIMQIKAIFSVLLREYEFEMAQPPESYRNDHSKMVVQLA
QPACVRYRRRTGV
>CYP51B1_Mycobacterium tuberculosis TKK_02_0034 2592387201
MSAVALPRVSGGHDEHGHLEEFRTDPIGLMQRVRDECGDVGTFQLAGKQVVLLSGSHANEFFFRAGDDDLDQA
KAYPFMTPIFGEGVVFDASPERRKEMLHNAALRGEQMKGHAATIEDQVRRMIADWGEAGEIDLLDFFAELTIY
TSSACLIGKKFRDQLDGRFAKLYHELERGTDPLAYVDPYLPIESFRRRDEARNGLVALVADIMNGRIANPPTD
KSDRDMLDVLIAVKAETGTPRFSADEITGMFISMMFAGHHTSSGTASWTLIELMRHRDAYAAVIDELDELYGD
GRSVSFHALRQIPQLENVLKETLRLHPPLIILMRVAKGEFEVQGHRIHEGDLVAASPAISNRIPEDFPDPHDF
VPARYEQPRQEDLLNRWTWIPFGAGRHRCVGAAFAIMQIKAIFSVLLREYEFEMAQPPESYRNDHSKMVVQLA
QPACVRYRRRTGV
>CYP51B1_Mycobacterium tuberculosis TKK_02_0036 2592381213
MSAVALPRVSGGHDEHGHLEEFRTDPIGLMQRVRDECGDVGTFQLAGKQVVLLSGSHANEFFFRAGDDDLDQA
KAYPFMTPIFGEGVVFDASPERRKEMLHNAALRGEQMKGHAATIEDQVRRMIADWGEAGEIDLLDFFAELTIY
TSSACLIGKKFRDQLDGRFAKLYHELERGTDPLAYVDPYLPIESFRRRDEARNGLVALVADIMNGRIANPPTD
KSDRDMLDVLIAVKAETGTPRFSADEITGMFISMMFAGHHTSSGTASWTLIELMRHRDAYAAVIDELDELYGD
GRSVSFHALRQIPQLENVLKETLRLHPPLIILMRVAKGEFEVQGHRIHEGDLVAASPAISNRIPEDFPDPHDF

VPARYEQPRQEDLLNRWTWIPFGAGRHRCVGAAFAIMQIKAIFSVLLREYEFEMAQPPESYRNDHSKMVVQLA
QPACVRYRRRTGV
>CYP51B1_Mycobacterium tuberculosis TKK_02_0038 2577653187
MSAVALPRVSGGHDEHGHLEEFRTDPIGLMQRVRDECGDVGTFQLAGKQVVLLSGSHANEFFFRAGDDDLDQA
KAYPFMTPIFGEGVVFDASPERRKEMLHNAALRGEQMKGHAATIEDQVRRMIADWGEAGEIDLLDFFAELTIY
TSSACLIGKKFRDQLDGRFAKLYHELERGTDPLAYVDPYLPIESFRRRDEARNGLVALVADIMNGRIANPPTD
KSDRDMLDVLIAVKAETGTPRFSADEITGMFISMMFAGHHTSSGTASWTLIELMRHRDAYAAVIDELDELYGD
GRSVSFHALRQIPQLENVLKETLRLHPPLIILMRVAKGEFEVQGHRIHEGDLVAASPAISNRIPEDFPDPHDF
VPARYEQPRQEDLLNRWTWIPFGAGRHRCVGAAFAIMQIKAIFSVLLREYEFEMAQPPESYRNDHSKMVVQLA
QPACVRYRRRTGV
>CYP51B1_Mycobacterium tuberculosis TKK_02_0039 2592379056
MSAVALPRVSGGHDEHGHLEEFRTDPIGLMQRVRDECGDVGTFQLAGKQVVLLSGSHANEFFFRAGDDDLDQA
KAYPFMTPIFGEGVVFDASPERRKEMLHNAALRGEQMKGHAATIEDQVRRMIADWGEAGEIDLLDFFAELTIY
TSSACLIGKKFRDQLDGRFAKLYHELERGTDPLAYVDPYLPIESFRRRDEARNGLVALVADIMNGRIANPPTD
KSDRDMLDVLIAVKAETGTPRFSADEITGMFISMMFAGHHTSSGTASWTLIELMRHRDAYAAVIDELDELYGD
GRSVSFHALRQIPQLENVLKETLRLHPPLIILMRVAKGEFEVQGHRIHEGDLVAASPAISNRIPEDFPDPHDF
VPARYEQPRQEDLLNRWTWIPFGAGRHRCVGAAFAIMQIKAIFSVLLREYEFEMAQPPESYRNDHSKMVVQLA
QPACVRYRRRTGV
>CYP51B1_Mycobacterium tuberculosis TKK_02_0045 2576368005
MSAVALPRVSGGHDEHGHLEEFRTDPIGLMQRVRDECGDVGTFQLAGKQVVLLSGSHANEFFFRAGDDDLDQA
KAYPFMTPIFGEGVVFDASPERRKEMLHNAALRGEQMKGHAATIEDQVRRMIADWGEAGEIDLLDFFAELTIY
TSSACLIGKKFRDQLDGRFAKLYHELERGTDPLAYVDPYLPIESFRRRDEARNGLVALVADIMNGRIANPPTD
KSDRDMLDVLIAVKAETGTPRFSADEITGMFISMMFAGHHTSSGTASWTLIELMRHRDAYAAVIDELDELYGD
GRSVSFHALRQIPQLENVLKETLRLHPPLIILMRVAKGEFEVQGHRIHEGDLVAASPAISNRIPEDFPDPHDF
VPARYEQPRQEDLLNRWTWIPFGAGRHRCVGAAFAIMQIKAIFSVLLREYEFEMAQPPESYRNDHSKMVVQLA
QPACVRYRRRTGV
>CYP51B1_Mycobacterium tuberculosis TKK_02_0046 2592372533
MSAVALPRVSGGHDEHGHLEEFRTDPIGLMQRVRDECGDVGTFQLAGKQVVLLSGSHANEFFFRAGDDDLDQA
KAYPFMTPIFGEGVVFDASPERRKEMLHNAALRGEQMKGHAATIEDQVRRMIADWGEAGEIDLLDFFAELTIY
TSSACLIGKKFRDQLDGRFAKLYHELERGTDPLAYVDPYLPIESFRRRDEARNGLVALVADIMNGRIANPPTD
KSDRDMLDVLIAVKAETGTPRFSADEITGMFISMMFAGHHTSSGTASWTLIELMRHRDAYAAVIDELDELYGD
GRSVSFHALRQIPQLENVLKETLRLHPPLIILMRVAKGEFEVQGHRIHEGDLVAASPAISNRIPEDFPDPHDF
VPARYEQPRQEDLLNRWTWIPFGAGRHRCVGAAFAIMQIKAIFSVLLREYEFEMAQPPESYRNDHSKMVVQLA
QPACVRYRRRTGV
>CYP51B1_Mycobacterium tuberculosis TKK_02_0052 2592368897
MSAVALPRVSGGHDEHGHLEEFRTDPIGLMQRVRDECGDVGTFQLAGKQVVLLSGSHANEFFFRAGDDDLDQA
KAYPFMTPIFGEGVVFDASPERRKEMLHNAALRGEQMKGHAATIEDQVRRMIADWGEAGEIDLLDFFAELTIY
TSSACLIGKKFRDQLDGRFAKLYHELERGTDPLAYVDPYLPIESFRRRDEARNGLVALVADIMNGRIANPPTD
KSDRDMLDVLIAVKAETGTPRFSADEITGMFISMMFAGHHTSSGTASWTLIELMRHRDAYAAVIDELDELYGD
GRSVSFHALRQIPQLENVLKETLRLHPPLIILMRVAKGEFEVQGHRIHEGDLVAASPAISNRIPEDFPDPHDF
VPARYEQPRQEDLLNRWTWIPFGAGRHRCVGAAFAIMQIKAIFSVLLREYEFEMAQPPESYRNDHSKMVVQLA
QPACVRYRRRTGV
>CYP51B1_Mycobacterium tuberculosis TKK_02_0061 2592366286
MSAVALPRVSGGHDEHGHLEEFRTDPIGLMQRVRDECGDVGTFQLAGKQVVLLSGSHANEFFFRAGDDDLDQA
KAYPFMTPIFGEGVVFDASPERRKEMLHNAALRGEQMKGHAATIEDQVRRMIADWGEAGEIDLLDFFAELTIY
TSSACLIGKKFRDQLDGRFAKLYHELERGTDPLAYVDPYLPIESFRRRDEARNGLVALVADIMNGRIANPPTD
KSDRDMLDVLIAVKAETGTPRFSADEITGMFISMMFAGHHTSSGTASWTLIELMRHRDAYAAVIDELDELYGD
GRSVSFHALRQIPQLENVLKETLRLHPPLIILMRVAKGEFEVQGHRIHEGDLVAASPAISNRIPEDFPDPHDF
VPARYEQPRQEDLLNRWTWIPFGAGRHRCVGAAFAIMQIKAIFSVLLREYEFEMAQPPESYRNDHSKMVVQLA
QPACVRYRRRTGV
>CYP51B1_Mycobacterium tuberculosis TKK_02_0063 2592356190
MSAVALPRVSGGHDEHGHLEEFRTDPIGLMQRVRDECGDVGTFQLAGKQVVLLSGSHANEFFFRAGDDDLDQA
KAYPFMTPIFGEGVVFDASPERRKEMLHNAALRGEQMKGHAATIEDQVRRMIADWGEAGEIDLLDFFAELTIY
TSSACLIGKKFRDQLDGRFAKLYHELERGTDPLAYVDPYLPIESFRRRDEARNGLVALVADIMNGRIANPPTD
KSDRDMLDVLIAVKAETGTPRFSADEITGMFISMMFAGHHTSSGTASWTLIELMRHRDAYAAVIDELDELYGD
GRSVSFHALRQIPQLENVLKETLRLHPPLIILMRVAKGEFEVQGHRIHEGDLVAASPAISNRIPEDFPDPHDF
VPARYEQPRQEDLLNRWTWIPFGAGRHRCVGAAFAIMQIKAIFSVLLREYEFEMAQPPESYRNDHSKMVVQLA
QPACVRYRRRTGV

>CYP51B1_Mycobacterium tuberculosis TKK_02_0067 2592352116
MSAVALPRVSGGHDEHGHLEEFRTDPIGLMQRVRDECGDVGTFQLAGKQVVLLSGSHANEFFFRAGDDDLDQA
KAYPFMTPIFGEGVVFDASPERRKEMLHNAALRGEQMKGHAATIEDQVRRMIADWGEAGEIDLLDFFAELTIY
TSSACLIGKKFRDQLDGRFAKLYHELERGTDPLAYVDPYLPIESFRRRDEARNGLVALVADIMNGRIANPPTD
KSDRDMLDVLIAVKAETGTPRFSADEITGMFISMMFAGHHTSSGTASWTLIELMRHRDAYAAVIDELDELYGD
GRSVSFHALRQIPQLENVLKETLRLHPPLIILMRVAKGEFEVQGHRIHEGDLVAASPAISNRIPEDFPDPHDF
VPARYEQPRQEDLLNRWTWIPFGAGRHRCVGAAFAIMQIKAIFSVLLREYEFEMAQPPESYRNDHSKMVVQLA
QPACVRYRRRTGV
>CYP51B1_Mycobacterium tuberculosis TKK_02_0068 2592351235
MSAVALPRVSGGHDEHGHLEEFRTDPIGLMQRVRDECGDVGTFQLAGKQVVLLSGSHANEFFFRAGDDDLDQA
KAYPFMTPIFGEGVVFDASPERRKEMLHNAALRGEQMKGHAATIEDQVRRMIADWGEAGEIDLLDFFAELTIY
TSSACLIGKKFRDQLDGRFAKLYHELERGTDPLAYVDPYLPIESFRRRDEARNGLVALVADIMNGRIANPPTD
KSDRDMLDVLIAVKAETGTPRFSADEITGMFISMMFAGHHTSSGTASWTLIELMRHRDAYAAVIDELDELYGD
GRSVSFHALRQIPQLENVLKETLRLHPPLIILMRVAKGEFEVQGHRIHEGDLVAASPAISNRIPEDFPDPHDF
VPARYEQPRQEDLLNRWTWIPFGAGRHRCVGAAFAIMQIKAIFSVLLREYEFEMAQPPESYRNDHSKMVVQLA
QPACVRYRRRTGV
>CYP51B1_Mycobacterium tuberculosis TKK_02_0069 2592343248
MSAVALPRVSGGHDEHGHLEEFRTDPIGLMQRVRDECGDVGTFQLAGKQVVLLSGSHANEFFFRAGDDDLDQA
KAYPFMTPIFGEGVVFDASPERRKEMLHNAALRGEQMKGHAATIEDQVRRMIADWGEAGEIDLLDFFAELTIY
TSSACLIGKKFRDQLDGRFAKLYHELERGTDPLAYVDPYLPIESFRRRDEARNGLVALVADIMNGRIANPPTD
KSDRDMLDVLIAVKAETGTPRFSADEITGMFISMMFAGHHTSSGTASWTLIELMRHRDAYAAVIDELDELYGD
GRSVSFHALRQIPQLENVLKETLRLHPPLIILMRVAKGEFEVQGHRIHEGDLVAASPAISNRIPEDFPDPHDF
VPARYEQPRQEDLLNRWTWIPFGAGRHRCVGAAFAIMQIKAIFSVLLREYEFEMAQPPESYRNDHSKMVVQLA
QPACVRYRRRTGV
>CYP51B1_Mycobacterium tuberculosis TKK_02_0070 2592339855
MSAVALPRVSGGHDEHGHLEEFRTDPIGLMQRVRDECGDVGTFQLAGKQVVLLSGSHANEFFFRAGDDDLDQA
KAYPFMTPIFGEGVVFDASPERRKEMLHNAALRGEQMKGHAATIEDQVRRMIADWGEAGEIDLLDFFAELTIY
TSSACLIGKKFRDQLDGRFAKLYHELERGTDPLAYVDPYLPIESFRRRDEARNGLVALVADIMNGRIANPPTD
KSDRDMLDVLIAVKAETGTPRFSADEITGMFISMMFAGHHTSSGTASWTLIELMRHRDAYAAVIDELDELYGD
GRSVSFHALRQIPQLENVLKETLRLHPPLIILMRVAKGEFEVQGHRIHEGDLVAASPAISNRIPEDFPDPHDF
VPARYEQPRQEDLLNRWTWIPFGAGRHRCVGAAFAIMQIKAIFSVLLREYEFEMAQPPESYRNDHSKMVVQLA
QPACVRYRRRTGV
>CYP51B1_Mycobacterium tuberculosis TKK_02_0071 2574862664
MSAVALPRVSGGHDEHGHLEEFRTDPIGLMQRVRDECGDVGTFQLAGKQVVLLSGSHANEFFFRAGDDDLDQA
KAYPFMTPIFGEGVVFDASPERRKEMLHNAALRGEQMKGHAATIEDQVRRMIADWGEAGEIDLLDFFAELTIY
TSSACLIGKKFRDQLDGRFAKLYHELERGTDPLAYVDPYLPIESFRRRDEARNGLVALVADIMNGRIANPPTD
KSDRDMLDVLIAVKAETGTPRFSADEITGMFISMMFAGHHTSSGTASWTLIELMRHRDAYAAVIDELDELYGD
GRSVSFHALRQIPQLENVLKETLRLHPPLIILMRVAKGEFEVQGHRIHEGDLVAASPAISNRIPEDFPDPHDF
VPARYEQPRQEDLLNRWTWIPFGAGRHRCVGAAFAIMQIKAIFSVLLREYEFEMAQPPESYRNDHSKMVVQLA
QPACVRYRRRTGV
>CYP51B1_Mycobacterium tuberculosis TKK_02_0073 2592336215
MSAVALPRVSGGHDEHGHLEEFRTDPIGLMQRVRDECGDVGTFQLAGKQVVLLSGSHANEFFFRAGDDDLDQA
KAYPFMTPIFGEGVVFDASPERRKEMLHNAALRGEQMKGHAATIEDQVRRMIADWGEAGEIDLLDFFAELTIY
TSSACLIGKKFRDQLDGRFAKLYHELERGTDPLAYVDPYLPIESFRRRDEARNGLVALVADIMNGRIANPPTD
KSDRDMLDVLIAVKAETGTPRFSADEITGMFISMMFAGHHTSSGTASWTLIELMRHRDAYAAVIDELDELYGD
GRSVSFHALRQIPQLENVLKETLRLHPPLIILMRVAKGEFEVQGHRIHEGDLVAASPAISNRIPEDFPDPHDF
VPARYEQPRQEDLLNRWTWIPFGAGRHRCVGAAFAIMQIKAIFSVLLREYEFEMAQPPESYRNDHSKMVVQLA
QPACVRYRRRTGV
>CYP51B1_Mycobacterium tuberculosis TKK_02_0077 2592331691
MSAVALPRVSGGHDEHGHLEEFRTDPIGLMQRVRDECGDVGTFQLAGKQVVLLSGSHANEFFFRAGDDDLDQA
KAYPFMTPIFGEGVVFDASPERRKEMLHNAALRGEQMKGHAATIEDQVRRMIADWGEAGEIDLLDFFAELTIY
TSSACLIGKKFRDQLDGRFAKLYHELERGTDPLAYVDPYLPIESFRRRDEARNGLVALVADIMNGRIANPPTD
KSDRDMLDVLIAVKAETGTPRFSADEITGMFISMMFAGHHTSSGTASWTLIELMRHRDAYAAVIDELDELYGD
GRSVSFHALRQIPQLENVLKETLRLHPPLIILMRVAKGEFEVQGHRIHEGDLVAASPAISNRIPEDFPDPHDF
VPARYEQPRQEDLLNRWTWIPFGAGRHRCVGAAFAIMQIKAIFSVLLREYEFEMAQPPESYRNDHSKMVVQLA
QPACVRYRRRTGV
>CYP51B1_Mycobacterium tuberculosis TKK_03_0012 2649436074

MSAVALPRVSGGHDEHGHLEEFRTDPIGLMQRVRDECGDVGTFQLAGKQVVLLSGSHANEFFFRAGDDDLDQA
KAYPFMTPIFGEGVVFDASPERRKEMLHNAALRGEQMKGHAATIEDQVRRMIADWGEAGEIDLLDFFAELTIY
TSSACLIGKKFRDQLDGRFAKLYHELERGTDPLAYVDPYLPIESFRRRDEARNGLVALVADIMNGRIANPPTD
KSDRDMLDVLIAVKAETGTPRFSADEITGMFISMMFAGHHTSSGTASWTLIELMRHRDAYAAVIDELDELYGD
GRSVSFHALRQIPQLENVLKETLRLHPPLIILMRVAKGEFEVQGHRIHEGDLVAASPAISNRIPEDFPDPHDF
VPARYEQPRQEDLLNRWTWIPFGAGRHRCVGAAFAIMQIKAIFSVLLREYEFEMAQPPESYRNDHSKMVVQLA
QPACVRYRRRTGV
>CYP51B1_Mycobacterium tuberculosis TKK_03_0015 2649276431
MSAVALPRVSGGHDEHGHLEEFRTDPIGLMQRVRDECGDVGTFQLAGKQVVLLSGSHANEFFFRAGDDDLDQA
KAYPFMTPIFGEGVVFDASPERRKEMLHNAALRGEQMKGHAATIEDQVRRMIADWGEAGEIDLLDFFAELTIY
TSSACLIGKKFRDQLDGRFAKLYHELERGTDPLAYVDPYLPIESFRRRDEARNGLVALVADIMNGRIANPPTD
KSDRDMLDVLIAVKAETGTPRFSADEITGMFISMMFAGHHTSSGTASWTLIELMRHRDAYAAVIDELDELYGD
GRSVSFHALRQIPQLENVLKETLRLHPPLIILMRVAKGEFEVQGHRIHEGDLVAASPAISNRIPEDFPDPHDF
VPARYEQPRQEDLLNRWTWIPFGAGRHRCVGAAFAIMQIKAIFSVLLREYEFEMAQPPESYRNDHSKMVVQLA
QPACVRYRRRTGV
>CYP51B1_Mycobacterium tuberculosis TKK_03_0018 2592330353
MSAVALPRVSGGHDEHGHLEEFRTDPIGLMQRVRDECGDVGTFQLAGKQVVLLSGSHANEFFFRAGDDDLDQA
KAYPFMTPIFGEGVVFDASPERRKEMLHNAALRGEQMKGHAATIEDQVRRMIADWGEAGEIDLLDFFAELTIY
TSSACLIGKKFRDQLDGRFAKLYHELERGTDPLAYVDPYLPIESFRRRDEARNGLVALVADIMNGRIANPPTD
KSDRDMLDVLIAVKAETGTPRFSADEITGMFISMMFAGHHTSSGTASWTLIELMRHRDAYAAVIDELDELYGD
GRSVSFHALRQIPQLENVLKETLRLHPPLIILMRVAKGEFEVQGHRIHEGDLVAASPAISNRIPEDFPDPHDF
VPARYEQPRQEDLLNRWTWIPFGAGRHRCVGAAFAIMQIKAIFSVLLREYEFEMAQPPESYRNDHSKMVVQLA
QPACVRYRRRTGV
>CYP51B1_Mycobacterium tuberculosis TKK_03_0020 2592323507
MSAVALPRVSGGHDEHGHLEEFRTDPIGLMQRVRDECGDVGTFQLAGKQVVLLSGSHANEFFFRAGDDDLDQA
KAYPFMTPIFGEGVVFDASPERRKEMLHNAALRGEQMKGHAATIEDQVRRMIADWGEAGEIDLLDFFAELTIY
TSSACLIGKKFRDQLDGRFAKLYHELERGTDPLAYVDPYLPIESFRRRDEARNGLVALVADIMNGRIANPPTD
KSDRDMLDVLIAVKAETGTPRFSADEITGMFISMMFAGHHTSSGTASWTLIELMRHRDAYAAVIDELDELYGD
GRSVSFHALRQIPQLENVLKETLRLHPPLIILMRVAKGEFEVQGHRIHEGDLVAASPAISNRIPEDFPDPHDF
VPARYEQPRQEDLLNRWTWIPFGAGRHRCVGAAFAIMQIKAIFSVLLREYEFEMAQPPESYRNDHSKMVVQLA
QPACVRYRRRTGV
>CYP51B1_Mycobacterium tuberculosis TKK_03_0021 2633286481
MSAVALPRVSGGHDEHGHLEEFRTDPIGLMQRVRDECGDVGTFQLAGKQVVLLSGSHANEFFFRAGDDDLDQA
KAYPFMTPIFGEGVVFDASPERRKEMLHNAALRGEQMKGHAATIEDQVRRMIADWGEAGEIDLLDFFAELTIY
TSSACLIGKKFRDQLDGRFAKLYHELERGTDPLAYVDPYLPIESFRRRDEARNGLVALVADIMNGRIANPPTD
KSDRDMLDVLIAVKAETGTPRFSADEITGMFISMMFAGHHTSSGTASWTLIELMRHRDAYAAVIDELDELYGD
GRSVSFHALRQIPQLENVLKETLRLHPPLIILMRVAKGEFEVQGHRIHEGDLVAASPAISNRIPEDFPDPHDF
VPARYEQPRQEDLLNRWTWIPFGAGRHRCVGAAFAIMQIKAIFSVLLREYEFEMAQPPESYRNDHSKMVVQLA
QPACVRYRRRTGV
>CYP51B1_Mycobacterium tuberculosis TKK_03_0022 2592322296
MSAVALPRVSGGHDEHGHLEEFRTDPIGLMQRVRDECGDVGTFQLAGKQVVLLSGSHANEFFFRAGDDDLDQA
KAYPFMTPIFGEGVVFDASPERRKEMLHNAALRGEQMKGHAATIEDQVRRMIADWGEAGEIDLLDFFAELTIY
TSSACLIGKKFRDQLDGRFAKLYHELERGTDPLAYVDPYLPIESFRRRDEARNGLVALVADIMNGRIANPPTD
KSDRDMLDVLIAVKAETGTPRFSADEITGMFISMMFAGHHTSSGTASWTLIELMRHRDAYAAVIDELDELYGD
GRSVSFHALRQIPQLENVLKETLRLHPPLIILMRVAKGEFEVQGHRIHEGDLVAASPAISNRIPEDFPDPHDF
VPARYEQPRQEDLLNRWTWIPFGAGRHRCVGAAFAIMQIKAIFSVLLREYEFEMAQPPESYRNDHSKMVVQLA
QPACVRYRRRTGV
>CYP51B1_Mycobacterium tuberculosis TKK_03_0023 2641037142
MSAVALPRVSGGHDEHGHLEEFRTDPIGLMQRVRDECGDVGTFQLAGKQVVLLSGSHANEFFFRAGDDDLDQA
KAYPFMTPIFGEGVVFDASPERRKEMLHNAALRGEQMKGHAATIEDQVRRMIADWGEAGEIDLLDFFAELTIY
TSSACLIGKKFRDQLDGRFAKLYHELERGTDPLAYVDPYLPIESFRRRDEARNGLVALVADIMNGRIANPPTD
KSDRDMLDVLIAVKAETGTPRFSADEITGMFISMMFAGHHTSSGTASWTLIELMRHRDAYAAVIDELDELYGD
GRSVSFHALRQIPQLENVLKETLRLHPPLIILMRVAKGEFEVQGHRIHEGDLVAASPAISNRIPEDFPDPHDF
VPARYEQPRQEDLLNRWTWIPFGAGRHRCVGAAFAIMQIKAIFSVLLREYEFEMAQPPESYRNDHSKMVVQLA
QPACVRYRRRTGV
>CYP51B1_Mycobacterium tuberculosis TKK_03_0024 2577167558
MSAVALPRVSGGHDEHGHLEEFRTDPIGLMQRVRDECGDVGTFQLAGKQVVLLSGSHANEFFFRAGDDDLDQA
KAYPFMTPIFGEGVVFDASPERRKEMLHNAALRGEQMKGHAATIEDQVRRMIADWGEAGEIDLLDFFAELTIY

TSSACLIGKKFRDQLDGRFAKLYHELERGTDPLAYVDPYLPIESFRRRDEARNGLVALVADIMNGRIANPPTD
KSDRDMLDVLIAVKAETGTPRFSADEITGMFISMMFAGHHTSSGTASWTLIELMRHRDAYAAVIDELDELYGD
GRSVSFHALRQIPQLENVLKETLRLHPPLIILMRVAKGEFEVQGHRIHEGDLVAASPAISNRIPEDFPDPHDF
VPARYEQPRQEDLLNRWTWIPFGAGRHRCVGAAFAIMQIKAIFSVLLREYEFEMAQPPESYRNDHSKMVVQLA
QPACVRYRRRTGV
>CYP51B1_Mycobacterium tuberculosis TKK_03_0025 2592315358
MSAVALPRVSGGHDEHGHLEEFRTDPIGLMQRVRDECGDVGTFQLAGKQVVLLSGSHANEFFFRAGDDDLDQA
KAYPFMTPIFGEGVVFDASPERRKEMLHNAALRGEQMKGHAATIEDQVRRMIADWGEAGEIDLLDFFAELTIY
TSSACLIGKKFRDQLDGRFAKLYHELERGTDPLAYVDPYLPIESFRRRDEARNGLVALVADIMNGRIANPPTD
KSDRDMLDVLIAVKAETGTPRFSADEITGMFISMMFAGHHTSSGTASWTLIELMRHRDAYAAVIDELDELYGD
GRSVSFHALRQIPQLENVLKETLRLHPPLIILMRVAKGEFEVQGHRIHEGDLVAASPAISNRIPEDFPDPHDF
VPARYEQPRQEDLLNRWTWIPFGAGRHRCVGAAFAIMQIKAIFSVLLREYEFEMAQPPESYRNDHSKMVVQLA
QPACVRYRRRTGV
>CYP51B1_Mycobacterium tuberculosis TKK_03_0026 2574802326
MSAVALPRVSGGHDEHGHLEEFRTDPIGLMQRVRDECGDVGTFQLAGKQVVLLSGSHANEFFFRAGDDDLDQA
KAYPFMTPIFGEGVVFDASPERRKEMLHNAALRGEQMKGHAATIEDQVRRMIADWGEAGEIDLLDFFAELTIY
TSSACLIGKKFRDQLDGRFAKLYHELERGTDPLAYVDPYLPIESFRRRDEARNGLVALVADIMNGRIANPPTD
KSDRDMLDVLIAVKAETGTPRFSADEITGMFISMMFAGHHTSSGTASWTLIELMRHRDAYAAVIDELDELYGD
GRSVSFHALRQIPQLENVLKETLRLHPPLIILMRVAKGEFEVQGHRIHEGDLVAASPAISNRIPEDFPDPHDF
VPARYEQPRQEDLLNRWTWIPFGAGRHRCVGAAFAIMQIKAIFSVLLREYEFEMAQPPESYRNDHSKMVVQLA
QPACVRYRRRTGV
>CYP51B1_Mycobacterium tuberculosis TKK_03_0027 2592311280
MSAVALPRVSGGHDEHGHLEEFRTDPIGLMQRVRDECGDVGTFQLAGKQVVLLSGSHANEFFFRAGDDDLDQA
KAYPFMTPIFGEGVVFDASPERRKEMLHNAALRGEQMKGHAATIEDQVRRMIADWGEAGEIDLLDFFAELTIY
TSSACLIGKKFRDQLDGRFAKLYHELERGTDPLAYVDPYLPIESFRRRDEARNGLVALVADIMNGRIANPPTD
KSDRDMLDVLIAVKAETGTPRFSADEITGMFISMMFAGHHTSSGTASWTLIELMRHRDAYAAVIDELDELYGD
GRSVSFHALRQIPQLENVLKETLRLHPPLIILMRVAKGEFEVQGHRIHEGDLVAASPAISNRIPEDFPDPHDF
VPARYEQPRQEDLLNRWTWIPFGAGRHRCVGAAFAIMQIKAIFSVLLREYEFEMAQPPESYRNDHSKMVVQLA
QPACVRYRRRTGV
>CYP51B1_Mycobacterium tuberculosis TKK_03_0028 2575968037
MSAVALPRVSGGHDEHGHLEEFRTDPIGLMQRVRDECGDVGTFQLAGKQVVLLSGSHANEFFFRAGDDDLDQA
KAYPFMTPIFGEGVVFDASPERRKEMLHNAALRGEQMKGHAATIEDQVRRMIADWGEAGEIDLLDFFAELTIY
TSSACLIGKKFRDQLDGRFAKLYHELERGTDPLAYVDPYLPIESFRRRDEARNGLVALVADIMNGRIANPPTD
KSDRDMLDVLIAVKAETGTPRFSADEITGMFISMMFAGHHTSSGTASWTLIELMRHRDAYAAVIDELDELYGD
GRSVSFHALRQIPQLENVLKETLRLHPPLIILMRVAKGEFEVQGHRIHEGDLVAASPAISNRIPEDFPDPHDF
VPARYEQPRQEDLLNRWTWIPFGAGRHRCVGAAFAIMQIKAIFSVLLREYEFEMAQPPESYRNDHSKMVVQLA
QPACVRYRRRTGV
>CYP51B1_Mycobacterium tuberculosis TKK_03_0029 2592307191
MSAVALPRVSGGHDEHGHLEEFRTDPIGLMQRVRDECGDVGTFQLAGKQVVLLSGSHANEFFFRAGDDDLDQA
KAYPFMTPIFGEGVVFDASPERRKEMLHNAALRGEQMKGHAATIEDQVRRMIADWGEAGEIDLLDFFAELTIY
TSSACLIGKKFRDQLDGRFAKLYHELERGTDPLAYVDPYLPIESFRRRDEARNGLVALVADIMNGRIANPPTD
KSDRDMLDVLIAVKAETGTPRFSADEITGMFISMMFAGHHTSSGTASWTLIELMRHRDAYAAVIDELDELYGD
GRSVSFHALRQIPQLENVLKETLRLHPPLIILMRVAKGEFEVQGHRIHEGDLVAASPAISNRIPEDFPDPHDF
VPARYEQPRQEDLLNRWTWIPFGAGRHRCVGAAFAIMQIKAIFSVLLREYEFEMAQPPESYRNDHSKMVVQLA
QPACVRYRRRTGV
>CYP51B1_Mycobacterium tuberculosis TKK_03_0030 2592302342
MSAVALPRVSGGHDEHGHLEEFRTDPIGLMQRVRDECGDVGTFQLAGKQVVLLSGSHANEFFFRAGDDDLDQA
KAYPFMTPIFGEGVVFDASPERRKEMLHNAALRGEQMKGHAATIEDQVRRMIADWGEAGEIDLLDFFAELTIY
TSSACLIGKKFRDQLDGRFAKLYHELERGTDPLAYVDPYLPIESFRRRDEARNGLVALVADIMNGRIANPPTD
KSDRDMLDVLIAVKAETGTPRFSADEITGMFISMMFAGHHTSSGTASWTLIELMRHRDAYAAVIDELDELYGD
GRSVSFHALRQIPQLENVLKETLRLHPPLIILMRVAKGEFEVQGHRIHEGDLVAASPAISNRIPEDFPDPHDF
VPARYEQPRQEDLLNRWTWIPFGAGRHRCVGAAFAIMQIKAIFSVLLREYEFEMAQPPESYRNDHSKMVVQLA
QPACVRYRRRTGV
>CYP51B1_Mycobacterium tuberculosis TKK_03_0031 2577815480
MSAVALPRVSGGHDEHGHLEEFRTDPIGLMQRVRDECGDVGTFQLAGKQVVLLSGSHANEFFFRAGDDDLDQA
KAYPFMTPIFGEGVVFDASPERRKEMLHNAALRGEQMKGHAATIEDQVRRMIADWGEAGEIDLLDFFAELTIY
TSSACLIGKKFRDQLDGRFAKLYHELERGTDPLAYVDPYLPIESFRRRDEARNGLVALVADIMNGRIANPPTD
KSDRDMLDVLIAVKAETGTPRFSADEITGMFISMMFAGHHTSSGTASWTLIELMRHRDAYAAVIDELDELYGD

GRSVSFHALRQIPQLENVLKETLRLHPPLIILMRVAKGEFEVQGHRIHEGDLVAASPAISNRIPEDFPDPHDF
VPARYEQPRQEDLLNRWTWIPFGAGRHRCVGAAFAIMQIKAIFSVLLREYEFEMAQPPESYRNDHSKMVVQLA
QPACVRYRRRTGV
>CYP51B1_Mycobacterium tuberculosis TKK_03_0034 2592294934
MSAVALPRVSGGHDEHGHLEEFRTDPIGLMQRVRDECGDVGTFQLAGKQVVLLSGSHANEFFFRAGDDDLDQA
KAYPFMTPIFGEGVVFDASPERRKEMLHNAALRGEQMKGHAATIEDQVRRMIADWGEAGEIDLLDFFAELTIY
TSSACLIGKKFRDQLDGRFAKLYHELERGTDPLAYVDPYLPIESFRRRDEARNGLVALVADIMNGRIANPPTD
KSDRDMLDVLIAVKAETGTPRFSADEITGMFISMMFAGHHTSSGTASWTLIELMRHRDAYAAVIDELDELYGD
GRSVSFHALRQIPQLENVLKETLRLHPPLIILMRVAKGEFEVQGHRIHEGDLVAASPAISNRIPEDFPDPHDF
VPARYEQPRQEDLLNRWTWIPFGAGRHRCVGAAFAIMQIKAIFSVLLREYEFEMAQPPESYRNDHSKMVVQLA
QPACVRYRRRTGV
>CYP51B1_Mycobacterium tuberculosis TKK_03_0035 2635881731
MSAVALPRVSGGHDEHGHLEEFRTDPIGLMQRVRDECGDVGTFQLAGKQVVLLSGSHANEFFFRAGDDDLDQA
KAYPFMTPIFGEGVVFDASPERRKEMLHNAALRGEQMKGHAATIEDQVRRMIADWGEAGEIDLLDFFAELTIY
TSSACLIGKKFRDQLDGRFAKLYHELERGTDPLAYVDPYLPIESFRRRDEARNGLVALVADIMNGRIANPPTD
KSDRDMLDVLIAVKAETGTPRFSADEITGMFISMMFAGHHTSSGTASWTLIELMRHRDAYAAVIDELDELYGD
GRSVSFHALRQIPQLENVLKETLRLHPPLIILMRVAKGEFEVQGHRIHEGDLVAASPAISNRIPEDFPDPHDF
VPARYEQPRQEDLLNRWTWIPFGAGRHRCVGAAFAIMQIKAIFSVLLREYEFEMAQPPESYRNDHSKMVVQLA
QPACVRYRRRTGV
>CYP51B1_Mycobacterium tuberculosis TKK_03_0036 2592290567
MSAVALPRVSGGHDEHGHLEEFRTDPIGLMQRVRDECGDVGTFQLAGKQVVLLSGSHANEFFFRAGDDDLDQA
KAYPFMTPIFGEGVVFDASPERRKEMLHNAALRGEQMKGHAATIEDQVRRMIADWGEAGEIDLLDFFAELTIY
TSSACLIGKKFRDQLDGRFAKLYHELERGTDPLAYVDPYLPIESFRRRDEARNGLVALVADIMNGRIANPPTD
KSDRDMLDVLIAVKAETGTPRFSADEITGMFISMMFAGHHTSSGTASWTLIELMRHRDAYAAVIDELDELYGD
GRSVSFHALRQIPQLENVLKETLRLHPPLIILMRVAKGEFEVQGHRIHEGDLVAASPAISNRIPEDFPDPHDF
VPARYEQPRQEDLLNRWTWIPFGAGRHRCVGAAFAIMQIKAIFSVLLREYEFEMAQPPESYRNDHSKMVVQLA
QPACVRYRRRTGV
>CYP51B1_Mycobacterium tuberculosis TKK_03_0038 2628222854
MSAVALPRVSGGHDEHGHLEEFRTDPIGLMQRVRDECGDVGTFQLAGKQVVLLSGSHANEFFFRAGDDDLDQA
KAYPFMTPIFGEGVVFDASPERRKEMLHNAALRGEQMKGHAATIEDQVRRMIADWGEAGEIDLLDFFAELTIY
TSSACLIGKKFRDQLDGRFAKLYHELERGTDPLAYVDPYLPIESFRRRDEARNGLVALVADIMNGRIANPPTD
KSDRDMLDVLIAVKAETGTPRFSADEITGMFISMMFAGHHTSSGTASWTLIELMRHRDAYAAVIDELDELYGD
GRSVSFHALRQIPQLENVLKETLRLHPPLIILMRVAKGEFEVQGHRIHEGDLVAASPAISNRIPEDFPDPHDF
VPARYEQPRQEDLLNRWTWIPFGAGRHRCVGAAFAIMQIKAIFSVLLREYEFEMAQPPESYRNDHSKMVVQLA
QPACVRYRRRTGV
>CYP51B1_Mycobacterium tuberculosis TKK_03_0039 2638562330
MSAVALPRVSGGHDEHGHLEEFRTDPIGLMQRVRDECGDVGTFQLAGKQVVLLSGSHANEFFFRAGDDDLDQA
KAYPFMTPIFGEGVVFDASPERRKEMLHNAALRGEQMKGHAATIEDQVRRMIADWGEAGEIDLLDFFAELTIY
TSSACLIGKKFRDQLDGRFAKLYHELERGTDPLAYVDPYLPIESFRRRDEARNGLVALVADIMNGRIANPPTD
KSDRDMLDVLIAVKAETGTPRFSADEITGMFISMMFAGHHTSSGTASWTLIELMRHRDAYAAVIDELDELYGD
GRSVSFHALRQIPQLENVLKETLRLHPPLIILMRVAKGEFEVQGHRIHEGDLVAASPAISNRIPEDFPDPHDF
VPARYEQPRQEDLLNRWTWIPFGAGRHRCVGAAFAIMQIKAIFSVLLREYEFEMAQPPESYRNDHSKMVVQLA
QPACVRYRRRTGV
>CYP51B1_Mycobacterium tuberculosis TKK_03_0040 2584673555
MSAVALPRVSGGHDEHGHLEEFRTDPIGLMQRVRDECGDVGTFQLAGKQVVLLSGSHANEFFFRAGDDDLDQA
KAYPFMTPIFGEGVVFDASPERRKEMLHNAALRGEQMKGHAATIEDQVRRMIADWGEAGEIDLLDFFAELTIY
TSSACLIGKKFRDQLDGRFAKLYHELERGTDPLAYVDPYLPIESFRRRDEARNGLVALVADIMNGRIANPPTD
KSDRDMLDVLIAVKAETGTPRFSADEITGMFISMMFAGHHTSSGTASWTLIELMRHRDAYAAVIDELDELYGD
GRSVSFHALRQIPQLENVLKETLRLHPPLIILMRVAKGEFEVQGHRIHEGDLVAASPAISNRIPEDFPDPHDF
VPARYEQPRQEDLLNRWTWIPFGAGRHRCVGAAFAIMQIKAIFSVLLREYEFEMAQPPESYRNDHSKMVVQLA
QPACVRYRRRTGV
>CYP51B1_Mycobacterium tuberculosis TKK_03_0042 2575857901
MSAVALPRVSGGHDEHGHLEEFRTDPIGLMQRVRDECGDVGTFQLAGKQVVLLSGSHANEFFFRAGDDDLDQA
KAYPFMTPIFGEGVVFDASPERRKEMLHNAALRGEQMKGHAATIEDQVRRMIADWGEAGEIDLLDFFAELTIY
TSSACLIGKKFRDQLDGRFAKLYHELERGTDPLAYVDPYLPIESFRRRDEARNGLVALVADIMNGRIANPPTD
KSDRDMLDVLIAVKAETGTPRFSADEITGMFISMMFAGHHTSSGTASWTLIELMRHRDAYAAVIDELDELYGD
GRSVSFHALRQIPQLENVLKETLRLHPPLIILMRVAKGEFEVQGHRIHEGDLVAASPAISNRIPEDFPDPHDF

VPARYEQPRQEDLLNRWTWIPFGAGRHRCVGAAFAIMQIKAIFSVLLREYEFEMAQPPESYRNDHSKMVVQLA
QPACVRYRRRTGV
>CYP51B1_Mycobacterium tuberculosis TKK_03_0043 2592286780
MSAVALPRVSGGHDEHGHLEEFRTDPIGLMQRVRDECGDVGTFQLAGKQVVLLSGSHANEFFFRAGDDDLDQA
KAYPFMTPIFGEGVVFDASPERRKEMLHNAALRGEQMKGHAATIEDQVRRMIADWGEAGEIDLLDFFAELTIY
TSSACLIGKKFRDQLDGRFAKLYHELERGTDPLAYVDPYLPIESFRRRDEARNGLVALVADIMNGRIANPPTD
KSDRDMLDVLIAVKAETGTPRFSADEITGMFISMMFAGHHTSSGTASWTLIELMRHRDAYAAVIDELDELYGD
GRSVSFHALRQIPQLENVLKETLRLHPPLIILMRVAKGEFEVQGHRIHEGDLVAASPAISNRIPEDFPDPHDF
VPARYEQPRQEDLLNRWTWIPFGAGRHRCVGAAFAIMQIKAIFSVLLREYEFEMAQPPESYRNDHSKMVVQLA
QPACVRYRRRTGV
>CYP51B1_Mycobacterium tuberculosis TKK_03_0044 2629572026
MSAVALPRVSGGHDEHGHLEEFRTDPIGLMQRVRDECGDVGTFQLAGKQVVLLSGSHANEFFFRAGDDDLDQA
KAYPFMTPIFGEGVVFDASPERRKEMLHNAALRGEQMKGHAATIEDQVRRMIADWGEAGEIDLLDFFAELTIY
TSSACLIGKKFRDQLDGRFAKLYHELERGTDPLAYVDPYLPIESFRRRDEARNGLVALVADIMNGRIANPPTD
KSDRDMLDVLIAVKAETGTPRFSADEITGMFISMMFAGHHTSSGTASWTLIELMRHRDAYAAVIDELDELYGD
GRSVSFHALRQIPQLENVLKETLRLHPPLIILMRVAKGEFEVQGHRIHEGDLVAASPAISNRIPEDFPDPHDF
VPARYEQPRQEDLLNRWTWIPFGAGRHRCVGAAFAIMQIKAIFSVLLREYEFEMAQPPESYRNDHSKMVVQLA
QPACVRYRRRTGV
>CYP51B1_Mycobacterium tuberculosis TKK_03_0045 2592282697
MSAVALPRVSGGHDEHGHLEEFRTDPIGLMQRVRDECGDVGTFQLAGKQVVLLSGSHANEFFFRAGDDDLDQA
KAYPFMTPIFGEGVVFDASPERRKEMLHNAALRGEQMKGHAATIEDQVRRMIADWGEAGEIDLLDFFAELTIY
TSSACLIGKKFRDQLDGRFAKLYHELERGTDPLAYVDPYLPIESFRRRDEARNGLVALVADIMNGRIANPPTD
KSDRDMLDVLIAVKAETGTPRFSADEITGMFISMMFAGHHTSSGTASWTLIELMRHRDAYAAVIDELDELYGD
GRSVSFHALRQIPQLENVLKETLRLHPPLIILMRVAKGEFEVQGHRIHEGDLVAASPAISNRIPEDFPDPHDF
VPARYEQPRQEDLLNRWTWIPFGAGRHRCVGAAFAIMQIKAIFSVLLREYEFEMAQPPESYRNDHSKMVVQLA
QPACVRYRRRTGV
>CYP51B1_Mycobacterium tuberculosis TKK_03_0047 2630104534
MSAVALPRVSGGHDEHGHLEEFRTDPIGLMQRVRDECGDVGTFQLAGKQVVLLSGSHANEFFFRAGDDDLDQA
KAYPFMTPIFGEGVVFDASPERRKEMLHNAALRGEQMKGHAATIEDQVRRMIADWGEAGEIDLLDFFAELTIY
TSSACLIGKKFRDQLDGRFAKLYHELERGTDPLAYVDPYLPIESFRRRDEARNGLVALVADIMNGRIANPPTD
KSDRDMLDVLIAVKAETGTPRFSADEITGMFISMMFAGHHTSSGTASWTLIELMRHRDAYAAVIDELDELYGD
GRSVSFHALRQIPQLENVLKETLRLHPPLIILMRVAKGEFEVQGHRIHEGDLVAASPAISNRIPEDFPDPHDF
VPARYEQPRQEDLLNRWTWIPFGAGRHRCVGAAFAIMQIKAIFSVLLREYEFEMAQPPESYRNDHSKMVVQLA
QPACVRYRRRTGV
>CYP51B1_Mycobacterium tuberculosis TKK_03_0050 2648063184
MSAVALPRVSGGHDEHGHLEEFRTDPIGLMQRVRDECGDVGTFQLAGKQVVLLSGSHANEFFFRAGDDDLDQA
KAYPFMTPIFGEGVVFDASPERRKEMLHNAALRGEQMKGHAATIEDQVRRMIADWGEAGEIDLLDFFAELTIY
TSSACLIGKKFRDQLDGRFAKLYHELERGTDPLAYVDPYLPIESFRRRDEARNGLVALVADIMNGRIANPPTD
KSDRDMLDVLIAVKAETGTPRFSADEITGMFISMMFAGHHTSSGTASWTLIELMRHRDAYAAVIDELDELYGD
GRSVSFHALRQIPQLENVLKETLRLHPPLIILMRVAKGEFEVQGHRIHEGDLVAASPAISNRIPEDFPDPHDF
VPARYEQPRQEDLLNRWTWIPFGAGRHRCVGAAFAIMQIKAIFSVLLREYEFEMAQPPESYRNDHSKMVVQLA
QPACVRYRRRTGV
>CYP51B1_Mycobacterium tuberculosis TKK_03_0058 2646420237
MSAVALPRVSGGHDEHGHLEEFRTDPIGLMQRVRDECGDVGTFQLAGKQVVLLSGSHANEFFFRAGDDDLDQA
KAYPFMTPIFGEGVVFDASPERRKEMLHNAALRGEQMKGHAATIEDQVRRMIADWGEAGEIDLLDFFAELTIY
TSSACLIGKKFRDQLDGRFAKLYHELERGTDPLAYVDPYLPIESFRRRDEARNGLVALVADIMNGRIANPPTD
KSDRDMLDVLIAVKAETGTPRFSADEITGMFISMMFAGHHTSSGTASWTLIELMRHRDAYAAVIDELDELYGD
GRSVSFHALRQIPQLENVLKETLRLHPPLIILMRVAKGEFEVQGHRIHEGDLVAASPAISNRIPEDFPDPHDF
VPARYEQPRQEDLLNRWTWIPFGAGRHRCVGAAFAIMQIKAIFSVLLREYEFEMAQPPESYRNDHSKMVVQLA
QPACVRYRRRTGV
>CYP51B1_Mycobacterium tuberculosis TKK_03_0059 2584669473
MSAVALPRVSGGHDEHGHLEEFRTDPIGLMQRVRDECGDVGTFQLAGKQVVLLSGSHANEFFFRAGDDDLDQA
KAYPFMTPIFGEGVVFDASPERRKEMLHNAALRGEQMKGHAATIEDQVRRMIADWGEAGEIDLLDFFAELTIY
TSSACLIGKKFRDQLDGRFAKLYHELERGTDPLAYVDPYLPIESFRRRDEARNGLVALVADIMNGRIANPPTD
KSDRDMLDVLIAVKAETGTPRFSADEITGMFISMMFAGHHTSSGTASWTLIELMRHRDAYAAVIDELDELYGD
GRSVSFHALRQIPQLENVLKETLRLHPPLIILMRVAKGEFEVQGHRIHEGDLVAASPAISNRIPEDFPDPHDF
VPARYEQPRQEDLLNRWTWIPFGAGRHRCVGAAFAIMQIKAIFSVLLREYEFEMAQPPESYRNDHSKMVVQLA
QPACVRYRRRTGV

>CYP51B1_Mycobacterium tuberculosis TKK_03_0062 2640932577
MSAVALPRVSGGHDEHGHLEEFRTDPIGLMQRVRDECGDVGTFQLAGKQVVLLSGSHANEFFFRAGDDDLDQA
KAYPFMTPIFGEGVVFDASPERRKEMLHNAALRGEQMKGHAATIEDQVRRMIADWGEAGEIDLLDFFAELTIY
TSSACLIGKKFRDQLDGRFAKLYHELERGTDPLAYVDPYLPIESFRRRDEARNGLVALVADIMNGRIANPPTD
KSDRDMLDVLIAVKAETGTPRFSADEITGMFISMMFAGHHTSSGTASWTLIELMRHRDAYAAVIDELDELYGD
GRSVSFHALRQIPQLENVLKETLRLHPPLIILMRVAKGEFEVQGHRIHEGDLVAASPAISNRIPEDFPDPHDF
VPARYEQPRQEDLLNRWTWIPFGAGRHRCVGAAFAIMQIKAIFSVLLREYEFEMAQPPESYRNDHSKMVVQLA
QPACVRYRRRTGV
>CYP51B1_Mycobacterium tuberculosis TKK_03_0063 2630159194
MSAVALPRVSGGHDEHGHLEEFRTDPIGLMQRVRDECGDVGTFQLAGKQVVLLSGSHANEFFFRAGDDDLDQA
KAYPFMTPIFGEGVVFDASPERRKEMLHNAALRGEQMKGHAATIEDQVRRMIADWGEAGEIDLLDFFAELTIY
TSSACLIGKKFRDQLDGRFAKLYHELERGTDPLAYVDPYLPIESFRRRDEARNGLVALVADIMNGRIANPPTD
KSDRDMLDVLIAVKAETGTPRFSADEITGMFISMMFAGHHTSSGTASWTLIELMRHRDAYAAVIDELDELYGD
GRSVSFHALRQIPQLENVLKETLRLHPPLIILMRVAKGEFEVQGHRIHEGDLVAASPAISNRIPEDFPDPHDF
VPARYEQPRQEDLLNRWTWIPFGAGRHRCVGAAFAIMQIKAIFSVLLREYEFEMAQPPESYRNDHSKMVVQLA
QPACVRYRRRTGV
>CYP51B1_Mycobacterium tuberculosis TKK_03_0064 2647359009
MSAVALPRVSGGHDEHGHLEEFRTDPIGLMQRVRDECGDVGTFQLAGKQVVLLSGSHANEFFFRAGDDDLDQA
KAYPFMTPIFGEGVVFDASPERRKEMLHNAALRGEQMKGHAATIEDQVRRMIADWGEAGEIDLLDFFAELTIY
TSSACLIGKKFRDQLDGRFAKLYHELERGTDPLAYVDPYLPIESFRRRDEARNGLVALVADIMNGRIANPPTD
KSDRDMLDVLIAVKAETGTPRFSADEITGMFISMMFAGHHTSSGTASWTLIELMRHRDAYAAVIDELDELYGD
GRSVSFHALRQIPQLENVLKETLRLHPPLIILMRVAKGEFEVQGHRIHEGDLVAASPAISNRIPEDFPDPHDF
VPARYEQPRQEDLLNRWTWIPFGAGRHRCVGAAFAIMQIKAIFSVLLREYEFEMAQPPESYRNDHSKMVVQLA
QPACVRYRRRTGV
>CYP51B1_Mycobacterium tuberculosis TKK_03_0065 2629870507
MSAVALPRVSGGHDEHGHLEEFRTDPIGLMQRVRDECGDVGTFQLAGKQVVLLSGSHANEFFFRAGDDDLDQA
KAYPFMTPIFGEGVVFDASPERRKEMLHNAALRGEQMKGHAATIEDQVRRMIADWGEAGEIDLLDFFAELTIY
TSSACLIGKKFRDQLDGRFAKLYHELERGTDPLAYVDPYLPIESFRRRDEARNGLVALVADIMNGRIANPPTD
KSDRDMLDVLIAVKAETGTPRFSADEITGMFISMMFAGHHTSSGTASWTLIELMRHRDAYAAVIDELDELYGD
GRSVSFHALRQIPQLENVLKETLRLHPPLIILMRVAKGEFEVQGHRIHEGDLVAASPAISNRIPEDFPDPHDF
VPARYEQPRQEDLLNRWTWIPFGAGRHRCVGAAFAIMQIKAIFSVLLREYEFEMAQPPESYRNDHSKMVVQLA
QPACVRYRRRTGV
>CYP51B1_Mycobacterium tuberculosis TKK_03_0072 2632966307
MSAVALPRVSGGHDEHGHLEEFRTDPIGLMQRVRDECGDVGTFQLAGKQVVLLSGSHANEFFFRAGDDDLDQA
KAYPFMTPIFGEGVVFDASPERRKEMLHNAALRGEQMKGHAATIEDQVRRMIADWGEAGEIDLLDFFAELTIY
TSSACLIGKKFRDQLDGRFAKLYHELERGTDPLAYVDPYLPIESFRRRDEARNGLVALVADIMNGRIANPPTD
KSDRDMLDVLIAVKAETGTPRFSADEITGMFISMMFAGHHTSSGTASWTLIELMRHRDAYAAVIDELDELYGD
GRSVSFHALRQIPQLENVLKETLRLHPPLIILMRVAKGEFEVQGHRIHEGDLVAASPAISNRIPEDFPDPHDF
VPARYEQPRQEDLLNRWTWIPFGAGRHRCVGAAFAIMQIKAIFSVLLREYEFEMAQPPESYRNDHSKMVVQLA
QPACVRYRRRTGV
>CYP51B1_Mycobacterium tuberculosis TKK_03_0075 2635588629
MSAVALPRVSGGHDEHGHLEEFRTDPIGLMQRVRDECGDVGTFQLAGKQVVLLSGSHANEFFFRAGDDDLDQA
KAYPFMTPIFGEGVVFDASPERRKEMLHNAALRGEQMKGHAATIEDQVRRMIADWGEAGEIDLLDFFAELTIY
TSSACLIGKKFRDQLDGRFAKLYHELERGTDPLAYVDPYLPIESFRRRDEARNGLVALVADIMNGRIANPPTD
KSDRDMLDVLIAVKAETGTPRFSADEITGMFISMMFAGHHTSSGTASWTLIELMRHRDAYAAVIDELDELYGD
GRSVSFHALRQIPQLENVLKETLRLHPPLIILMRVAKGEFEVQGHRIHEGDLVAASPAISNRIPEDFPDPHDF
VPARYEQPRQEDLLNRWTWIPFGAGRHRCVGAAFAIMQIKAIFSVLLREYEFEMAQPPESYRNDHSKMVVQLA
QPACVRYRRRTGV
>CYP51B1_Mycobacterium tuberculosis TKK_03_0078 2584665391
MSAVALPRVSGGHDEHGHLEEFRTDPIGLMQRVRDECGDVGTFQLAGKQVVLLSGSHANEFFFRAGDDDLDQA
KAYPFMTPIFGEGVVFDASPERRKEMLHNAALRGEQMKGHAATIEDQVRRMIADWGEAGEIDLLDFFAELTIY
TSSACLIGKKFRDQLDGRFAKLYHELERGTDPLAYVDPYLPIESFRRRDEARNGLVALVADIMNGRIANPPTD
KSDRDMLDVLIAVKAETGTPRFSADEITGMFISMMFAGHHTSSGTASWTLIELMRHRDAYAAVIDELDELYGD
GRSVSFHALRQIPQLENVLKETLRLHPPLIILMRVAKGEFEVQGHRIHEGDLVAASPAISNRIPEDFPDPHDF
VPARYEQPRQEDLLNRWTWIPFGAGRHRCVGAAFAIMQIKAIFSVLLREYEFEMAQPPESYRNDHSKMVVQLA
QPACVRYRRRTGV
>CYP51B1_Mycobacterium tuberculosis TKK_03_0081 2592279057

MSAVALPRVSGGHDEHGHLEEFRTDPIGLMQRVRDECGDVGTFQLAGKQVVLLSGSHANEFFFRAGDDDLDQA
KAYPFMTPIFGEGVVFDASPERRKEMLHNAALRGEQMKGHAATIEDQVRRMIADWGEAGEIDLLDFFAELTIY
TSSACLIGKKFRDQLDGRFAKLYHELERGTDPLAYVDPYLPIESFRRRDEARNGLVALVADIMNGRIANPPTD
KSDRDMLDVLIAVKAETGTPRFSADEITGMFISMMFAGHHTSSGTASWTLIELMRHRDAYAAVIDELDELYGD
GRSVSFHALRQIPQLENVLKETLRLHPPLIILMRVAKGEFEVQGHRIHEGDLVAASPAISNRIPEDFPDPHDF
VPARYEQPRQEDLLNRWTWIPFGAGRHRCVGAAFAIMQIKAIFSVLLREYEFEMAQPPESYRNDHSKMVVQLA
QPACVRYRRRTGV
>CYP51B1_Mycobacterium tuberculosis TKK_03_0082 2592274547
MSAVALPRVSGGHDEHGHLEEFRTDPIGLMQRVRDECGDVGTFQLAGKQVVLLSGSHANEFFFRAGDDDLDQA
KAYPFMTPIFGEGVVFDASPERRKEMLHNAALRGEQMKGHAATIEDQVRRMIADWGEAGEIDLLDFFAELTIY
TSSACLIGKKFRDQLDGRFAKLYHELERGTDPLAYVDPYLPIESFRRRDEARNGLVALVADIMNGRIANPPTD
KSDRDMLDVLIAVKAETGTPRFSADEITGMFISMMFAGHHTSSGTASWTLIELMRHRDAYAAVIDELDELYGD
GRSVSFHALRQIPQLENVLKETLRLHPPLIILMRVAKGEFEVQGHRIHEGDLVAASPAISNRIPEDFPDPHDF
VPARYEQPRQEDLLNRWTWIPFGAGRHRCVGAAFAIMQIKAIFSVLLREYEFEMAQPPESYRNDHSKMVVQLA
QPACVRYRRRTGV
>CYP51B1_Mycobacterium tuberculosis TKK_03_0083 2577672260
MSAVALPRVSGGHDEHGHLEEFRTDPIGLMQRVRDECGDVGTFQLAGKQVVLLSGSHANEFFFRAGDDDLDQA
KAYPFMTPIFGEGVVFDASPERRKEMLHNAALRGEQMKGHAATIEDQVRRMIADWGEAGEIDLLDFFAELTIY
TSSACLIGKKFRDQLDGRFAKLYHELERGTDPLAYVDPYLPIESFRRRDEARNGLVALVADIMNGRIANPPTD
KSDRDMLDVLIAVKAETGTPRFSADEITGMFISMMFAGHHTSSGTASWTLIELMRHRDAYAAVIDELDELYGD
GRSVSFHALRQIPQLENVLKETLRLHPPLIILMRVAKGEFEVQGHRIHEGDLVAASPAISNRIPEDFPDPHDF
VPARYEQPRQEDLLNRWTWIPFGAGRHRCVGAAFAIMQIKAIFSVLLREYEFEMAQPPESYRNDHSKMVVQLA
QPACVRYRRRTGV
>CYP51B1_Mycobacterium tuberculosis TKK_03_0089 2642264259
MSAVALPRVSGGHDEHGHLEEFRTDPIGLMQRVRDECGDVGTFQLAGKQVVLLSGSHANEFFFRAGDDDLDQA
KAYPFMTPIFGEGVVFDASPERRKEMLHNAALRGEQMKGHAATIEDQVRRMIADWGEAGEIDLLDFFAELTIY
TSSACLIGKKFRDQLDGRFAKLYHELERGTDPLAYVDPYLPIESFRRRDEARNGLVALVADIMNGRIANPPTD
KSDRDMLDVLIAVKAETGTPRFSADEITGMFISMMFAGHHTSSGTASWTLIELMRHRDAYAAVIDELDELYGD
GRSVSFHALRQIPQLENVLKETLRLHPPLIILMRVAKGEFEVQGHRIHEGDLVAASPAISNRIPEDFPDPHDF
VPARYEQPRQEDLLNRWTWIPFGAGRHRCVGAAFAIMQIKAIFSVLLREYEFEMAQPPESYRNDHSKMVVQLA
QPACVRYRRRTGV
>CYP51B1_Mycobacterium tuberculosis TKK_03_0090 2576707898
MSAVALPRVSGGHDEHGHLEEFRTDPIGLMQRVRDECGDVGTFQLAGKQVVLLSGSHANEFFFRAGDDDLDQA
KAYPFMTPIFGEGVVFDASPERRKEMLHNAALRGEQMKGHAATIEDQVRRMIADWGEAGEIDLLDFFAELTIY
TSSACLIGKKFRDQLDGRFAKLYHELERGTDPLAYVDPYLPIESFRRRDEARNGLVALVADIMNGRIANPPTD
KSDRDMLDVLIAVKAETGTPRFSADEITGMFISMMFAGHHTSSGTASWTLIELMRHRDAYAAVIDELDELYGD
GRSVSFHALRQIPQLENVLKETLRLHPPLIILMRVAKGEFEVQGHRIHEGDLVAASPAISNRIPEDFPDPHDF
VPARYEQPRQEDLLNRWTWIPFGAGRHRCVGAAFAIMQIKAIFSVLLREYEFEMAQPPESYRNDHSKMVVQLA
QPACVRYRRRTGV
>CYP51B1_Mycobacterium tuberculosis TKK_03_0092 2637410471
MSAVALPRVSGGHDEHGHLEEFRTDPIGLMQRVRDECGDVGTFQLAGKQVVLLSGSHANEFFFRAGDDDLDQA
KAYPFMTPIFGEGVVFDASPERRKEMLHNAALRGEQMKGHAATIEDQVRRMIADWGEAGEIDLLDFFAELTIY
TSSACLIGKKFRDQLDGRFAKLYHELERGTDPLAYVDPYLPIESFRRRDEARNGLVALVADIMNGRIANPPTD
KSDRDMLDVLIAVKAETGTPRFSADEITGMFISMMFAGHHTSSGTASWTLIELMRHRDAYAAVIDELDELYGD
GRSVSFHALRQIPQLENVLKETLRLHPPLIILMRVAKGEFEVQGHRIHEGDLVAASPAISNRIPEDFPDPHDF
VPARYEQPRQEDLLNRWTWIPFGAGRHRCVGAAFAIMQIKAIFSVLLREYEFEMAQPPESYRNDHSKMVVQLA
QPACVRYRRRTGV
>CYP51B1_Mycobacterium tuberculosis TKK_03_0094 2578245643
MSAVALPRVSGGHDEHGHLEEFRTDPIGLMQRVRDECGDVGTFQLAGKQVVLLSGSHANEFFFRAGDDDLDQA
KAYPFMTPIFGEGVVFDASPERRKEMLHNAALRGEQMKGHAATIEDQVRRMIADWGEAGEIDLLDFFAELTIY
TSSACLIGKKFRDQLDGRFAKLYHELERGTDPLAYVDPYLPIESFRRRDEARNGLVALVADIMNGRIANPPTD
KSDRDMLDVLIAVKAETGTPRFSADEITGMFISMMFAGHHTSSGTASWTLIELMRHRDAYAAVIDELDELYGD
GRSVSFHALRQIPQLENVLKETLRLHPPLIILMRVAKGEFEVQGHRIHEGDLVAASPAISNRIPEDFPDPHDF
VPARYEQPRQEDLLNRWTWIPFGAGRHRCVGAAFAIMQIKAIFSVLLREYEFEMAQPPESYRNDHSKMVVQLA
QPACVRYRRRTGV
>CYP51B1_Mycobacterium tuberculosis TKK_03_0096 2578194310
MSAVALPRVSGGHDEHGHLEEFRTDPIGLMQRVRDECGDVGTFQLAGKQVVLLSGSHANEFFFRAGDDDLDQA
KAYPFMTPIFGEGVVFDASPERRKEMLHNAALRGEQMKGHAATIEDQVRRMIADWGEAGEIDLLDFFAELTIY

TSSACLIGKKFRDQLDGRFAKLYHELERGTDPLAYVDPYLPIESFRRRDEARNGLVALVADIMNGRIANPPTD
KSDRDMLDVLIAVKAETGTPRFSADEITGMFISMMFAGHHTSSGTASWTLIELMRHRDAYAAVIDELDELYGD
GRSVSFHALRQIPQLENVLKETLRLHPPLIILMRVAKGEFEVQGHRIHEGDLVAASPAISNRIPEDFPDPHDF
VPARYEQPRQEDLLNRWTWIPFGAGRHRCVGAAFAIMQIKAIFSVLLREYEFEMAQPPESYRNDHSKMVVQLA
QPACVRYRRRTGV
>CYP51B1_Mycobacterium tuberculosis TKK_03_0098 2647499994
MSAVALPRVSGGHDEHGHLEEFRTDPIGLMQRVRDECGDVGTFQLAGKQVVLLSGSHANEFFFRAGDDDLDQA
KAYPFMTPIFGEGVVFDASPERRKEMLHNAALRGEQMKGHAATIEDQVRRMIADWGEAGEIDLLDFFAELTIY
TSSACLIGKKFRDQLDGRFAKLYHELERGTDPLAYVDPYLPIESFRRRDEARNGLVALVADIMNGRIANPPTD
KSDRDMLDVLIAVKAETGTPRFSADEITGMFISMMFAGHHTSSGTASWTLIELMRHRDAYAAVIDELDELYGD
GRSVSFHALRQIPQLENVLKETLRLHPPLIILMRVAKGEFEVQGHRIHEGDLVAASPAISNRIPEDFPDPHDF
VPARYEQPRQEDLLNRWTWIPFGAGRHRCVGAAFAIMQIKAIFSVLLREYEFEMAQPPESYRNDHSKMVVQLA
QPACVRYRRRTGV
>CYP51B1_Mycobacterium tuberculosis TKK_03_0099 2575805248
MSAVALPRVSGGHDEHGHLEEFRTDPIGLMQRVRDECGDVGTFQLAGKQVVLLSGSHANEFFFRAGDDDLDQA
KAYPFMTPIFGEGVVFDASPERRKEMLHNAALRGEQMKGHAATIEDQVRRMIADWGEAGEIDLLDFFAELTIY
TSSACLIGKKFRDQLDGRFAKLYHELERGTDPLAYVDPYLPIESFRRRDEARNGLVALVADIMNGRIANPPTD
KSDRDMLDVLIAVKAETGTPRFSADEITGMFISMMFAGHHTSSGTASWTLIELMRHRDAYAAVIDELDELYGD
GRSVSFHALRQIPQLENVLKETLRLHPPLIILMRVAKGEFEVQGHRIHEGDLVAASPAISNRIPEDFPDPHDF
VPARYEQPRQEDLLNRWTWIPFGAGRHRCVGAAFAIMQIKAIFSVLLREYEFEMAQPPESYRNDHSKMVVQLA
QPACVRYRRRTGV
>CYP51B1_Mycobacterium tuberculosis TKK_03_0100 2641477368
MSAVALPRVSGGHDEHGHLEEFRTDPIGLMQRVRDECGDVGTFQLAGKQVVLLSGSHANEFFFRAGDDDLDQA
KAYPFMTPIFGEGVVFDASPERRKEMLHNAALRGEQMKGHAATIEDQVRRMIADWGEAGEIDLLDFFAELTIY
TSSACLIGKKFRDQLDGRFAKLYHELERGTDPLAYVDPYLPIESFRRRDEARNGLVALVADIMNGRIANPPTD
KSDRDMLDVLIAVKAETGTPRFSADEITGMFISMMFAGHHTSSGTASWTLIELMRHRDAYAAVIDELDELYGD
GRSVSFHALRQIPQLENVLKETLRLHPPLIILMRVAKGEFEVQGHRIHEGDLVAASPAISNRIPEDFPDPHDF
VPARYEQPRQEDLLNRWTWIPFGAGRHRCVGAAFAIMQIKAIFSVLLREYEFEMAQPPESYRNDHSKMVVQLA
QPACVRYRRRTGV
>CYP51B1_Mycobacterium tuberculosis TKK_03_0101 2630765785
MSAVALPRVSGGHDEHGHLEEFRTDPIGLMQRVRDECGDVGTFQLAGKQVVLLSGSHANEFFFRAGDDDLDQA
KAYPFMTPIFGEGVVFDASPERRKEMLHNAALRGEQMKGHAATIEDQVRRMIADWGEAGEIDLLDFFAELTIY
TSSACLIGKKFRDQLDGRFAKLYHELERGTDPLAYVDPYLPIESFRRRDEARNGLVALVADIMNGRIANPPTD
KSDRDMLDVLIAVKAETGTPRFSADEITGMFISMMFAGHHTSSGTASWTLIELMRHRDAYAAVIDELDELYGD
GRSVSFHALRQIPQLENVLKETLRLHPPLIILMRVAKGEFEVQGHRIHEGDLVAASPAISNRIPEDFPDPHDF
VPARYEQPRQEDLLNRWTWIPFGAGRHRCVGAAFAIMQIKAIFSVLLREYEFEMAQPPESYRNDHSKMVVQLA
QPACVRYRRRTGV
>CYP51B1_Mycobacterium tuberculosis TKK_03_0102 2632402171
MSAVALPRVSGGHDEHGHLEEFRTDPIGLMQRVRDECGDVGTFQLAGKQVVLLSGSHANEFFFRAGDDDLDQA
KAYPFMTPIFGEGVVFDASPERRKEMLHNAALRGEQMKGHAATIEDQVRRMIADWGEAGEIDLLDFFAELTIY
TSSACLIGKKFRDQLDGRFAKLYHELERGTDPLAYVDPYLPIESFRRRDEARNGLVALVADIMNGRIANPPTD
KSDRDMLDVLIAVKAETGTPRFSADEITGMFISMMFAGHHTSSGTASWTLIELMRHRDAYAAVIDELDELYGD
GRSVSFHALRQIPQLENVLKETLRLHPPLIILMRVAKGEFEVQGHRIHEGDLVAASPAISNRIPEDFPDPHDF
VPARYEQPRQEDLLNRWTWIPFGAGRHRCVGAAFAIMQIKAIFSVLLREYEFEMAQPPESYRNDHSKMVVQLA
QPACVRYRRRTGV
>CYP51B1_Mycobacterium tuberculosis TKK_03_0103 2577795971
MSAVALPRVSGGHDEHGHLEEFRTDPIGLMQRVRDECGDVGTFQLAGKQVVLLSGSHANEFFFRAGDDDLDQA
KAYPFMTPIFGEGVVFDASPERRKEMLHNAALRGEQMKGHAATIEDQVRRMIADWGEAGEIDLLDFFAELTIY
TSSACLIGKKFRDQLDGRFAKLYHELERGTDPLAYVDPYLPIESFRRRDEARNGLVALVADIMNGRIANPPTD
KSDRDMLDVLIAVKAETGTPRFSADEITGMFISMMFAGHHTSSGTASWTLIELMRHRDAYAAVIDELDELYGD
GRSVSFHALRQIPQLENVLKETLRLHPPLIILMRVAKGEFEVQGHRIHEGDLVAASPAISNRIPEDFPDPHDF
VPARYEQPRQEDLLNRWTWIPFGAGRHRCVGAAFAIMQIKAIFSVLLREYEFEMAQPPESYRNDHSKMVVQLA
QPACVRYRRRTGV
>CYP51B1_Mycobacterium tuberculosis TKK_03_0105 2637958096
MSAVALPRVSGGHDEHGHLEEFRTDPIGLMQRVRDECGDVGTFQLAGKQVVLLSGSHANEFFFRAGDDDLDQA
KAYPFMTPIFGEGVVFDASPERRKEMLHNAALRGEQMKGHAATIEDQVRRMIADWGEAGEIDLLDFFAELTIY
TSSACLIGKKFRDQLDGRFAKLYHELERGTDPLAYVDPYLPIESFRRRDEARNGLVALVADIMNGRIANPPTD
KSDRDMLDVLIAVKAETGTPRFSADEITGMFISMMFAGHHTSSGTASWTLIELMRHRDAYAAVIDELDELYGD

GRSVSFHALRQIPQLENVLKETLRLHPPLIILMRVAKGEFEVQGHRIHEGDLVAASPAISNRIPEDFPDPHDF
VPARYEQPRQEDLLNRWTWIPFGAGRHRCVGAAFAIMQIKAIFSVLLREYEFEMAQPPESYRNDHSKMVVQLA
QPACVRYRRRTGV
>CYP51B1_Mycobacterium tuberculosis TKK_03_0106 2629880420
MSAVALPRVSGGHDEHGHLEEFRTDPIGLMQRVRDECGDVGTFQLAGKQVVLLSGSHANEFFFRAGDDDLDQA
KAYPFMTPIFGEGVVFDASPERRKEMLHNAALRGEQMKGHAATIEDQVRRMIADWGEAGEIDLLDFFAELTIY
TSSACLIGKKFRDQLDGRFAKLYHELERGTDPLAYVDPYLPIESFRRRDEARNGLVALVADIMNGRIANPPTD
KSDRDMLDVLIAVKAETGTPRFSADEITGMFISMMFAGHHTSSGTASWTLIELMRHRDAYAAVIDELDELYGD
GRSVSFHALRQIPQLENVLKETLRLHPPLIILMRVAKGEFEVQGHRIHEGDLVAASPAISNRIPEDFPDPHDF
VPARYEQPRQEDLLNRWTWIPFGAGRHRCVGAAFAIMQIKAIFSVLLREYEFEMAQPPESYRNDHSKMVVQLA
QPACVRYRRRTGV
>CYP51B1_Mycobacterium tuberculosis TKK_03_0108 2628383783
MSAVALPRVSGGHDEHGHLEEFRTDPIGLMQRVRDECGDVGTFQLAGKQVVLLSGSHANEFFFRAGDDDLDQA
KAYPFMTPIFGEGVVFDASPERRKEMLHNAALRGEQMKGHAATIEDQVRRMIADWGEAGEIDLLDFFAELTIY
TSSACLIGKKFRDQLDGRFAKLYHELERGTDPLAYVDPYLPIESFRRRDEARNGLVALVADIMNGRIANPPTD
KSDRDMLDVLIAVKAETGTPRFSADEITGMFISMMFAGHHTSSGTASWTLIELMRHRDAYAAVIDELDELYGD
GRSVSFHALRQIPQLENVLKETLRLHPPLIILMRVAKGEFEVQGHRIHEGDLVAASPAISNRIPEDFPDPHDF
VPARYEQPRQEDLLNRWTWIPFGAGRHRCVGAAFAIMQIKAIFSVLLREYEFEMAQPPESYRNDHSKMVVQLA
QPACVRYRRRTGV
>CYP51B1_Mycobacterium tuberculosis TKK_03_0109 2575454276
MSAVALPRVSGGHDEHGHLEEFRTDPIGLMQRVRDECGDVGTFQLAGKQVVLLSGSHANEFFFRAGDDDLDQA
KAYPFMTPIFGEGVVFDASPERRKEMLHNAALRGEQMKGHAATIEDQVRRMIADWGEAGEIDLLDFFAELTIY
TSSACLIGKKFRDQLDGRFAKLYHELERGTDPLAYVDPYLPIESFRRRDEARNGLVALVADIMNGRIANPPTD
KSDRDMLDVLIAVKAETGTPRFSADEITGMFISMMFAGHHTSSGTASWTLIELMRHRDAYAAVIDELDELYGD
GRSVSFHALRQIPQLENVLKETLRLHPPLIILMRVAKGEFEVQGHRIHEGDLVAASPAISNRIPEDFPDPHDF
VPARYEQPRQEDLLNRWTWIPFGAGRHRCVGAAFAIMQIKAIFSVLLREYEFEMAQPPESYRNDHSKMVVQLA
QPACVRYRRRTGV
>CYP51B1_Mycobacterium tuberculosis TKK_03_0111 2630498020
MSAVALPRVSGGHDEHGHLEEFRTDPIGLMQRVRDECGDVGTFQLAGKQVVLLSGSHANEFFFRAGDDDLDQA
KAYPFMTPIFGEGVVFDASPERRKEMLHNAALRGEQMKGHAATIEDQVRRMIADWGEAGEIDLLDFFAELTIY
TSSACLIGKKFRDQLDGRFAKLYHELERGTDPLAYVDPYLPIESFRRRDEARNGLVALVADIMNGRIANPPTD
KSDRDMLDVLIAVKAETGTPRFSADEITGMFISMMFAGHHTSSGTASWTLIELMRHRDAYAAVIDELDELYGD
GRSVSFHALRQIPQLENVLKETLRLHPPLIILMRVAKGEFEVQGHRIHEGDLVAASPAISNRIPEDFPDPHDF
VPARYEQPRQEDLLNRWTWIPFGAGRHRCVGAAFAIMQIKAIFSVLLREYEFEMAQPPESYRNDHSKMVVQLA
QPACVRYRRRTGV
>CYP51B1_Mycobacterium tuberculosis TKK_03_0112 2631641574
MSAVALPRVSGGHDEHGHLEEFRTDPIGLMQRVRDECGDVGTFQLAGKQVVLLSGSHANEFFFRAGDDDLDQA
KAYPFMTPIFGEGVVFDASPERRKEMLHNAALRGEQMKGHAATIEDQVRRMIADWGEAGEIDLLDFFAELTIY
TSSACLIGKKFRDQLDGRFAKLYHELERGTDPLAYVDPYLPIESFRRRDEARNGLVALVADIMNGRIANPPTD
KSDRDMLDVLIAVKAETGTPRFSADEITGMFISMMFAGHHTSSGTASWTLIELMRHRDAYAAVIDELDELYGD
GRSVSFHALRQIPQLENVLKETLRLHPPLIILMRVAKGEFEVQGHRIHEGDLVAASPAISNRIPEDFPDPHDF
VPARYEQPRQEDLLNRWTWIPFGAGRHRCVGAAFAIMQIKAIFSVLLREYEFEMAQPPESYRNDHSKMVVQLA
QPACVRYRRRTGV
>CYP51B1_Mycobacterium tuberculosis TKK_03_0113 2637033451
MSAVALPRVSGGHDEHGHLEEFRTDPIGLMQRVRDECGDVGTFQLAGKQVVLLSGSHANEFFFRAGDDDLDQA
KAYPFMTPIFGEGVVFDASPERRKEMLHNAALRGEQMKGHAATIEDQVRRMIADWGEAGEIDLLDFFAELTIY
TSSACLIGKKFRDQLDGRFAKLYHELERGTDPLAYVDPYLPIESFRRRDEARNGLVALVADIMNGRIANPPTD
KSDRDMLDVLIAVKAETGTPRFSADEITGMFISMMFAGHHTSSGTASWTLIELMRHRDAYAAVIDELDELYGD
GRSVSFHALRQIPQLENVLKETLRLHPPLIILMRVAKGEFEVQGHRIHEGDLVAASPAISNRIPEDFPDPHDF
VPARYEQPRQEDLLNRWTWIPFGAGRHRCVGAAFAIMQIKAIFSVLLREYEFEMAQPPESYRNDHSKMVVQLA
QPACVRYRRRTGV
>CYP51B1_Mycobacterium tuberculosis TKK_03_0114 2641772365
MSAVALPRVSGGHDEHGHLEEFRTDPIGLMQRVRDECGDVGTFQLAGKQVVLLSGSHANEFFFRAGDDDLDQA
KAYPFMTPIFGEGVVFDASPERRKEMLHNAALRGEQMKGHAATIEDQVRRMIADWGEAGEIDLLDFFAELTIY
TSSACLIGKKFRDQLDGRFAKLYHELERGTDPLAYVDPYLPIESFRRRDEARNGLVALVADIMNGRIANPPTD
KSDRDMLDVLIAVKAETGTPRFSADEITGMFISMMFAGHHTSSGTASWTLIELMRHRDAYAAVIDELDELYGD
GRSVSFHALRQIPQLENVLKETLRLHPPLIILMRVAKGEFEVQGHRIHEGDLVAASPAISNRIPEDFPDPHDF

VPARYEQPRQEDLLNRWTWIPFGAGRHRCVGAAFAIMQIKAIFSVLLREYEFEMAQPPESYRNDHSKMVVQLA
QPACVRYRRRTGV
>CYP51B1_Mycobacterium tuberculosis TKK_03_0115 2638670743
MSAVALPRVSGGHDEHGHLEEFRTDPIGLMQRVRDECGDVGTFQLAGKQVVLLSGSHANEFFFRAGDDDLDQA
KAYPFMTPIFGEGVVFDASPERRKEMLHNAALRGEQMKGHAATIEDQVRRMIADWGEAGEIDLLDFFAELTIY
TSSACLIGKKFRDQLDGRFAKLYHELERGTDPLAYVDPYLPIESFRRRDEARNGLVALVADIMNGRIANPPTD
KSDRDMLDVLIAVKAETGTPRFSADEITGMFISMMFAGHHTSSGTASWTLIELMRHRDAYAAVIDELDELYGD
GRSVSFHALRQIPQLENVLKETLRLHPPLIILMRVAKGEFEVQGHRIHEGDLVAASPAISNRIPEDFPDPHDF
VPARYEQPRQEDLLNRWTWIPFGAGRHRCVGAAFAIMQIKAIFSVLLREYEFEMAQPPESYRNDHSKMVVQLA
QPACVRYRRRTGV
>CYP51B1_Mycobacterium tuberculosis TKK_03_0116 2576935423
MSAVALPRVSGGHDEHGHLEEFRTDPIGLMQRVRDECGDVGTFQLAGKQVVLLSGSHANEFFFRAGDDDLDQA
KAYPFMTPIFGEGVVFDASPERRKEMLHNAALRGEQMKGHAATIEDQVRRMIADWGEAGEIDLLDFFAELTIY
TSSACLIGKKFRDQLDGRFAKLYHELERGTDPLAYVDPYLPIESFRRRDEARNGLVALVADIMNGRIANPPTD
KSDRDMLDVLIAVKAETGTPRFSADEITGMFISMMFAGHHTSSGTASWTLIELMRHRDAYAAVIDELDELYGD
GRSVSFHALRQIPQLENVLKETLRLHPPLIILMRVAKGEFEVQGHRIHEGDLVAASPAISNRIPEDFPDPHDF
VPARYEQPRQEDLLNRWTWIPFGAGRHRCVGAAFAIMQIKAIFSVLLREYEFEMAQPPESYRNDHSKMVVQLA
QPACVRYRRRTGV
>CYP51B1_Mycobacterium tuberculosis TKK_03_0118 2577935409
MSAVALPRVSGGHDEHGHLEEFRTDPIGLMQRVRDECGDVGTFQLAGKQVVLLSGSHANEFFFRAGDDDLDQA
KAYPFMTPIFGEGVVFDASPERRKEMLHNAALRGEQMKGHAATIEDQVRRMIADWGEAGEIDLLDFFAELTIY
TSSACLIGKKFRDQLDGRFAKLYHELERGTDPLAYVDPYLPIESFRRRDEARNGLVALVADIMNGRIANPPTD
KSDRDMLDVLIAVKAETGTPRFSADEITGMFISMMFAGHHTSSGTASWTLIELMRHRDAYAAVIDELDELYGD
GRSVSFHALRQIPQLENVLKETLRLHPPLIILMRVAKGEFEVQGHRIHEGDLVAASPAISNRIPEDFPDPHDF
VPARYEQPRQEDLLNRWTWIPFGAGRHRCVGAAFAIMQIKAIFSVLLREYEFEMAQPPESYRNDHSKMVVQLA
QPACVRYRRRTGV
>CYP51B1_Mycobacterium tuberculosis TKK_03_0149 2649203472
MSAVALPRVSGGHDEHGHLEEFRTDPIGLMQRVRDECGDVGTFQLAGKQVVLLSGSHANEFFFRAGDDDLDQA
KAYPFMTPIFGEGVVFDASPERRKEMLHNAALRGEQMKGHAATIEDQVRRMIADWGEAGEIDLLDFFAELTIY
TSSACLIGKKFRDQLDGRFAKLYHELERGTDPLAYVDPYLPIESFRRRDEARNGLVALVADIMNGRIANPPTD
KSDRDMLDVLIAVKAETGTPRFSADEITGMFISMMFAGHHTSSGTASWTLIELMRHRDAYAAVIDELDELYGD
GRSVSFHALRQIPQLENVLKETLRLHPPLIILMRVAKGEFEVQGHRIHEGDLVAASPAISNRIPEDFPDPHDF
VPARYEQPRQEDLLNRWTWIPFGAGRHRCVGAAFAIMQIKAIFSVLLREYEFEMAQPPESYRNDHSKMVVQLA
QPACVRYRRRTGV
>CYP51B1_Mycobacterium tuberculosis TKK_03_0150 2631319671
MSAVALPRVSGGHDEHGHLEEFRTDPIGLMQRVRDECGDVGTFQLAGKQVVLLSGSHANEFFFRAGDDDLDQA
KAYPFMTPIFGEGVVFDASPERRKEMLHNAALRGEQMKGHAATIEDQVRRMIADWGEAGEIDLLDFFAELTIY
TSSACLIGKKFRDQLDGRFAKLYHELERGTDPLAYVDPYLPIESFRRRDEARNGLVALVADIMNGRIANPPTD
KSDRDMLDVLIAVKAETGTPRFSADEITGMFISMMFAGHHTSSGTASWTLIELMRHRDAYAAVIDELDELYGD
GRSVSFHALRQIPQLENVLKETLRLHPPLIILMRVAKGEFEVQGHRIHEGDLVAASPAISNRIPEDFPDPHDF
VPARYEQPRQEDLLNRWTWIPFGAGRHRCVGAAFAIMQIKAIFSVLLREYEFEMAQPPESYRNDHSKMVVQLA
QPACVRYRRRTGV
>CYP51B1_Mycobacterium tuberculosis TKK_03_0153 2633661238
MSAVALPRVSGGHDEHGHLEEFRTDPIGLMQRVRDECGDVGTFQLAGKQVVLLSGSHANEFFFRAGDDDLDQA
KAYPFMTPIFGEGVVFDASPERRKEMLHNAALRGEQMKGHAATIEDQVRRMIADWGEAGEIDLLDFFAELTIY
TSSACLIGKKFRDQLDGRFAKLYHELERGTDPLAYVDPYLPIESFRRRDEARNGLVALVADIMNGRIANPPTD
KSDRDMLDVLIAVKAETGTPRFSADEITGMFISMMFAGHHTSSGTASWTLIELMRHRDAYAAVIDELDELYGD
GRSVSFHALRQIPQLENVLKETLRLHPPLIILMRVAKGEFEVQGHRIHEGDLVAASPAISNRIPEDFPDPHDF
VPARYEQPRQEDLLNRWTWIPFGAGRHRCVGAAFAIMQIKAIFSVLLREYEFEMAQPPESYRNDHSKMVVQLA
QPACVRYRRRTGV
>CYP51B1_Mycobacterium tuberculosis TKK_03_0154 2633687903
MSAVALPRVSGGHDEHGHLEEFRTDPIGLMQRVRDECGDVGTFQLAGKQVVLLSGSHANEFFFRAGDDDLDQA
KAYPFMTPIFGEGVVFDASPERRKEMLHNAALRGEQMKGHAATIEDQVRRMIADWGEAGEIDLLDFFAELTIY
TSSACLIGKKFRDQLDGRFAKLYHELERGTDPLAYVDPYLPIESFRRRDEARNGLVALVADIMNGRIANPPTD
KSDRDMLDVLIAVKAETGTPRFSADEITGMFISMMFAGHHTSSGTASWTLIELMRHRDAYAAVIDELDELYGD
GRSVSFHALRQIPQLENVLKETLRLHPPLIILMRVAKGEFEVQGHRIHEGDLVAASPAISNRIPEDFPDPHDF
VPARYEQPRQEDLLNRWTWIPFGAGRHRCVGAAFAIMQIKAIFSVLLREYEFEMAQPPESYRNDHSKMVVQLA
QPACVRYRRRTGV

>CYP51B1_Mycobacterium tuberculosis TKK_03_0156 2575102584
MSAVALPRVSGGHDEHGHLEEFRTDPIGLMQRVRDECGDVGTFQLAGKQVVLLSGSHANEFFFRAGDDDLDQA
KAYPFMTPIFGEGVVFDASPERRKEMLHNAALRGEQMKGHAATIEDQVRRMIADWGEAGEIDLLDFFAELTIY
TSSACLIGKKFRDQLDGRFAKLYHELERGTDPLAYVDPYLPIESFRRRDEARNGLVALVADIMNGRIANPPTD
KSDRDMLDVLIAVKAETGTPRFSADEITGMFISMMFAGHHTSSGTASWTLIELMRHRDAYAAVIDELDELYGD
GRSVSFHALRQIPQLENVLKETLRLHPPLIILMRVAKGEFEVQGHRIHEGDLVAASPAISNRIPEDFPDPHDF
VPARYEQPRQEDLLNRWTWIPFGAGRHRCVGAAFAIMQIKAIFSVLLREYEFEMAQPPESYRNDHSKMVVQLA
QPACVRYRRRTGV
>CYP51B1_Mycobacterium tuberculosis TKK_03_0158 2576931361
MSAVALPRVSGGHDEHGHLEEFRTDPIGLMQRVRDECGDVGTFQLAGKQVVLLSGSHANEFFFRAGDDDLDQA
KAYPFMTPIFGEGVVFDASPERRKEMLHNAALRGEQMKGHAATIEDQVRRMIADWGEAGEIDLLDFFAELTIY
TSSACLIGKKFRDQLDGRFAKLYHELERGTDPLAYVDPYLPIESFRRRDEARNGLVALVADIMNGRIANPPTD
KSDRDMLDVLIAVKAETGTPRFSADEITGMFISMMFAGHHTSSGTASWTLIELMRHRDAYAAVIDELDELYGD
GRSVSFHALRQIPQLENVLKETLRLHPPLIILMRVAKGEFEVQGHRIHEGDLVAASPAISNRIPEDFPDPHDF
VPARYEQPRQEDLLNRWTWIPFGAGRHRCVGAAFAIMQIKAIFSVLLREYEFEMAQPPESYRNDHSKMVVQLA
QPACVRYRRRTGV
>CYP51B1_Mycobacterium tuberculosis TKK_03_0159 2637667316
MSAVALPRVSGGHDEHGHLEEFRTDPIGLMQRVRDECGDVGTFQLAGKQVVLLSGSHANEFFFRAGDDDLDQA
KAYPFMTPIFGEGVVFDASPERRKEMLHNAALRGEQMKGHAATIEDQVRRMIADWGEAGEIDLLDFFAELTIY
TSSACLIGKKFRDQLDGRFAKLYHELERGTDPLAYVDPYLPIESFRRRDEARNGLVALVADIMNGRIANPPTD
KSDRDMLDVLIAVKAETGTPRFSADEITGMFISMMFAGHHTSSGTASWTLIELMRHRDAYAAVIDELDELYGD
GRSVSFHALRQIPQLENVLKETLRLHPPLIILMRVAKGEFEVQGHRIHEGDLVAASPAISNRIPEDFPDPHDF
VPARYEQPRQEDLLNRWTWIPFGAGRHRCVGAAFAIMQIKAIFSVLLREYEFEMAQPPESYRNDHSKMVVQLA
QPACVRYRRRTGV
>CYP51B1_Mycobacterium tuberculosis TKK_03_0160 2647436313
MSAVALPRVSGGHDEHGHLEEFRTDPIGLMQRVRDECGDVGTFQLAGKQVVLLSGSHANEFFFRAGDDDLDQA
KAYPFMTPIFGEGVVFDASPERRKEMLHNAALRGEQMKGHAATIEDQVRRMIADWGEAGEIDLLDFFAELTIY
TSSACLIGKKFRDQLDGRFAKLYHELERGTDPLAYVDPYLPIESFRRRDEARNGLVALVADIMNGRIANPPTD
KSDRDMLDVLIAVKAETGTPRFSADEITGMFISMMFAGHHTSSGTASWTLIELMRHRDAYAAVIDELDELYGD
GRSVSFHALRQIPQLENVLKETLRLHPPLIILMRVAKGEFEVQGHRIHEGDLVAASPAISNRIPEDFPDPHDF
VPARYEQPRQEDLLNRWTWIPFGAGRHRCVGAAFAIMQIKAIFSVLLREYEFEMAQPPESYRNDHSKMVVQLA
QPACVRYRRRTGV
>CYP51B1_Mycobacterium tuberculosis TKK_04_0001 2592270462
MSAVALPRVSGGHDEHGHLEEFRTDPIGLMQRVRDECGDVGTFQLAGKQVVLLSGSHANEFFFRAGDDDLDQA
KAYPFMTPIFGEGVVFDASPERRKEMLHNAALRGEQMKGHAATIEDQVRRMIADWGEAGEIDLLDFFAELTIY
TSSACLIGKKFRDQLDGRFAKLYHELERGTDPLAYVDPYLPIESFRRRDEARNGLVALVADIMNGRIANPPTD
KSDRDMLDVLIAVKAETGTPRFSADEITGMFISMMFAGHHTSSGTASWTLIELMRHRDAYAAVIDELDELYGD
GRSVSFHALRQIPQLENVLKETLRLHPPLIILMRVAKGEFEVQGHRIHEGDLVAASPAISNRIPEDFPDPHDF
VPARYEQPRQEDLLNRWTWIPFGAGRHRCVGAAFAIMQIKAIFSVLLREYEFEMAQPPESYRNDHSKMVVQLA
QPACVRYRRRTGV
>CYP51B1_Mycobacterium tuberculosis TKK_04_0002 2584966093
MSAVALPRVSGGHDEHGHLEEFRTDPIGLMQRVRDECGDVGTFQLAGKQVVLLSGSHANEFFFRAGDDDLDQA
KAYPFMTPIFGEGVVFDASPERRKEMLHNAALRGEQMKGHAATIEDQVRRMIADWGEAGEIDLLDFFAELTIY
TSSACLIGKKFRDQLDGRFAKLYHELERGTDPLAYVDPYLPIESFRRRDEARNGLVALVADIMNGRIANPPTD
KSDRDMLDVLIAVKAETGTPRFSADEITGMFISMMFAGHHTSSGTASWTLIELMRHRDAYAAVIDELDELYGD
GRSVSFHALRQIPQLENVLKETLRLHPPLIILMRVAKGEFEVQGHRIHEGDLVAASPAISNRIPEDFPDPHDF
VPARYEQPRQEDLLNRWTWIPFGAGRHRCVGAAFAIMQIKAIFSVLLREYEFEMAQPPESYRNDHSKMVVQLA
QPACVRYRRRTGV
>CYP51B1_Mycobacterium tuberculosis TKK_04_0003 2576387997
MSAVALPRVSGGHDEHGHLEEFRTDPIGLMQRVRDECGDVGTFQLAGKQVVLLSGSHANEFFFRAGDDDLDQA
KAYPFMTPIFGEGVVFDASPERRKEMLHNAALRGEQMKGHAATIEDQVRRMIADWGEAGEIDLLDFFAELTIY
TSSACLIGKKFRDQLDGRFAKLYHELERGTDPLAYVDPYLPIESFRRRDEARNGLVALVADIMNGRIANPPTD
KSDRDMLDVLIAVKAETGTPRFSADEITGMFISMMFAGHHTSSGTASWTLIELMRHRDAYAAVIDELDELYGD
GRSVSFHALRQIPQLENVLKETLRLHPPLIILMRVAKGEFEVQGHRIHEGDLVAASPAISNRIPEDFPDPHDF
VPARYEQPRQEDLLNRWTWIPFGAGRHRCVGAAFAIMQIKAIFSVLLREYEFEMAQPPESYRNDHSKMVVQLA
QPACVRYRRRTGV
>CYP51B1_Mycobacterium tuberculosis TKK_04_0005 2592266373

MSAVALPRVSGGHDEHGHLEEFRTDPIGLMQRVRDECGDVGTFQLAGKQVVLLSGSHANEFFFRAGDDDLDQA
KAYPFMTPIFGEGVVFDASPERRKEMLHNAALRGEQMKGHAATIEDQVRRMIADWGEAGEIDLLDFFAELTIY
TSSACLIGKKFRDQLDGRFAKLYHELERGTDPLAYVDPYLPIESFRRRDEARNGLVALVADIMNGRIANPPTD
KSDRDMLDVLIAVKAETGTPRFSADEITGMFISMMFAGHHTSSGTASWTLIELMRHRDAYAAVIDELDELYGD
GRSVSFHALRQIPQLENVLKETLRLHPPLIILMRVAKGEFEVQGHRIHEGDLVAASPAISNRIPEDFPDPHDF
VPARYEQPRQEDLLNRWTWIPFGAGRHRCVGAAFAIMQIKAIFSVLLREYEFEMAQPPESYRNDHSKMVVQLA
QPACVRYRRRTGV
>CYP51B1_Mycobacterium tuberculosis TKK_04_0006 2592264435
MSAVALPRVSGGHDEHGHLEEFRTDPIGLMQRVRDECGDVGTFQLAGKQVVLLSGSHANEFFFRAGDDDLDQA
KAYPFMTPIFGEGVVFDASPERRKEMLHNAALRGEQMKGHAATIEDQVRRMIADWGEAGEIDLLDFFAELTIY
TSSACLIGKKFRDQLDGRFAKLYHELERGTDPLAYVDPYLPIESFRRRDEARNGLVALVADIMNGRIANPPTD
KSDRDMLDVLIAVKAETGTPRFSADEITGMFISMMFAGHHTSSGTASWTLIELMRHRDAYAAVIDELDELYGD
GRSVSFHALRQIPQLENVLKETLRLHPPLIILMRVAKGEFEVQGHRIHEGDLVAASPAISNRIPEDFPDPHDF
VPARYEQPRQEDLLNRWTWIPFGAGRHRCVGAAFAIMQIKAIFSVLLREYEFEMAQPPESYRNDHSKMVVQLA
QPACVRYRRRTGV
>CYP51B1_Mycobacterium tuberculosis TKK_04_0007 2592258228
MSAVALPRVSGGHDEHGHLEEFRTDPIGLMQRVRDECGDVGTFQLAGKQVVLLSGSHANEFFFRAGDDDLDQA
KAYPFMTPIFGEGVVFDASPERRKEMLHNAALRGEQMKGHAATIEDQVRRMIADWGEAGEIDLLDFFAELTIY
TSSACLIGKKFRDQLDGRFAKLYHELERGTDPLAYVDPYLPIESFRRRDEARNGLVALVADIMNGRIANPPTD
KSDRDMLDVLIAVKAETGTPRFSADEITGMFISMMFAGHHTSSGTASWTLIELMRHRDAYAAVIDELDELYGD
GRSVSFHALRQIPQLENVLKETLRLHPPLIILMRVAKGEFEVQGHRIHEGDLVAASPAISNRIPEDFPDPHDF
VPARYEQPRQEDLLNRWTWIPFGAGRHRCVGAAFAIMQIKAIFSVLLREYEFEMAQPPESYRNDHSKMVVQLA
QPACVRYRRRTGV
>CYP51B1_Mycobacterium tuberculosis TKK_04_0008 2592257338
MSAVALPRVSGGHDEHGHLEEFRTDPIGLMQRVRDECGDVGTFQLAGKQVVLLSGSHANEFFFRAGDDDLDQA
KAYPFMTPIFGEGVVFDASPERRKEMLHNAALRGEQMKGHAATIEDQVRRMIADWGEAGEIDLLDFFAELTIY
TSSACLIGKKFRDQLDGRFAKLYHELERGTDPLAYVDPYLPIESFRRRDEARNGLVALVADIMNGRIANPPTD
KSDRDMLDVLIAVKAETGTPRFSADEITGMFISMMFAGHHTSSGTASWTLIELMRHRDAYAAVIDELDELYGD
GRSVSFHALRQIPQLENVLKETLRLHPPLIILMRVAKGEFEVQGHRIHEGDLVAASPAISNRIPEDFPDPHDF
VPARYEQPRQEDLLNRWTWIPFGAGRHRCVGAAFAIMQIKAIFSVLLREYEFEMAQPPESYRNDHSKMVVQLA
QPACVRYRRRTGV
>CYP51B1_Mycobacterium tuberculosis TKK_04_0013 2592252532
MSAVALPRVSGGHDEHGHLEEFRTDPIGLMQRVRDECGDVGTFQLAGKQVVLLSGSHANEFFFRAGDDDLDQA
KAYPFMTPIFGEGVVFDASPERRKEMLHNAALRGEQMKGHAATIEDQVRRMIADWGEAGEIDLLDFFAELTIY
TSSACLIGKKFRDQLDGRFAKLYHELERGTDPLAYVDPYLPIESFRRRDEARNGLVALVADIMNGRIANPPTD
KSDRDMLDVLIAVKAETGTPRFSADEITGMFISMMFAGHHTSSGTASWTLIELMRHRDAYAAVIDELDELYGD
GRSVSFHALRQIPQLENVLKETLRLHPPLIILMRVAKGEFEVQGHRIHEGDLVAASPAISNRIPEDFPDPHDF
VPARYEQPRQEDLLNRWTWIPFGAGRHRCVGAAFAIMQIKAIFSVLLREYEFEMAQPPESYRNDHSKMVVQLA
QPACVRYRRRTGV
>CYP51B1_Mycobacterium tuberculosis TKK_04_0014 2592245959
MSAVALPRVSGGHDEHGHLEEFRTDPIGLMQRVRDECGDVGTFQLAGKQVVLLSGSHANEFFFRAGDDDLDQA
KAYPFMTPIFGEGVVFDASPERRKEMLHNAALRGEQMKGHAATIEDQVRRMIADWGEAGEIDLLDFFAELTIY
TSSACLIGKKFRDQLDGRFAKLYHELERGTDPLAYVDPYLPIESFRRRDEARNGLVALVADIMNGRIANPPTD
KSDRDMLDVLIAVKAETGTPRFSADEITGMFISMMFAGHHTSSGTASWTLIELMRHRDAYAAVIDELDELYGD
GRSVSFHALRQIPQLENVLKETLRLHPPLIILMRVAKGEFEVQGHRIHEGDLVAASPAISNRIPEDFPDPHDF
VPARYEQPRQEDLLNRWTWIPFGAGRHRCVGAAFAIMQIKAIFSVLLREYEFEMAQPPESYRNDHSKMVVQLA
QPACVRYRRRTGV
>CYP51B1_Mycobacterium tuberculosis TKK_04_0015 2592241878
MSAVALPRVSGGHDEHGHLEEFRTDPIGLMQRVRDECGDVGTFQLAGKQVVLLSGSHANEFFFRAGDDDLDQA
KAYPFMTPIFGEGVVFDASPERRKEMLHNAALRGEQMKGHAATIEDQVRRMIADWGEAGEIDLLDFFAELTIY
TSSACLIGKKFRDQLDGRFAKLYHELERGTDPLAYVDPYLPIESFRRRDEARNGLVALVADIMNGRIANPPTD
KSDRDMLDVLIAVKAETGTPRFSADEITGMFISMMFAGHHTSSGTASWTLIELMRHRDAYAAVIDELDELYGD
GRSVSFHALRQIPQLENVLKETLRLHPPLIILMRVAKGEFEVQGHRIHEGDLVAASPAISNRIPEDFPDPHDF
VPARYEQPRQEDLLNRWTWIPFGAGRHRCVGAAFAIMQIKAIFSVLLREYEFEMAQPPESYRNDHSKMVVQLA
QPACVRYRRRTGV
>CYP51B1_Mycobacterium tuberculosis TKK_04_0017 2592237795
MSAVALPRVSGGHDEHGHLEEFRTDPIGLMQRVRDECGDVGTFQLAGKQVVLLSGSHANEFFFRAGDDDLDQA
KAYPFMTPIFGEGVVFDASPERRKEMLHNAALRGEQMKGHAATIEDQVRRMIADWGEAGEIDLLDFFAELTIY

TSSACLIGKKFRDQLDGRFAKLYHELERGTDPLAYVDPYLPIESFRRRDEARNGLVALVADIMNGRIANPPTD
KSDRDMLDVLIAVKAETGTPRFSADEITGMFISMMFAGHHTSSGTASWTLIELMRHRDAYAAVIDELDELYGD
GRSVSFHALRQIPQLENVLKETLRLHPPLIILMRVAKGEFEVQGHRIHEGDLVAASPAISNRIPEDFPDPHDF
VPARYEQPRQEDLLNRWTWIPFGAGRHRCVGAAFAIMQIKAIFSVLLREYEFEMAQPPESYRNDHSKMVVQLA
QPACVRYRRRTGV
>CYP51B1_Mycobacterium tuberculosis TKK_04_0018 2592233721
MSAVALPRVSGGHDEHGHLEEFRTDPIGLMQRVRDECGDVGTFQLAGKQVVLLSGSHANEFFFRAGDDDLDQA
KAYPFMTPIFGEGVVFDASPERRKEMLHNAALRGEQMKGHAATIEDQVRRMIADWGEAGEIDLLDFFAELTIY
TSSACLIGKKFRDQLDGRFAKLYHELERGTDPLAYVDPYLPIESFRRRDEARNGLVALVADIMNGRIANPPTD
KSDRDMLDVLIAVKAETGTPRFSADEITGMFISMMFAGHHTSSGTASWTLIELMRHRDAYAAVIDELDELYGD
GRSVSFHALRQIPQLENVLKETLRLHPPLIILMRVAKGEFEVQGHRIHEGDLVAASPAISNRIPEDFPDPHDF
VPARYEQPRQEDLLNRWTWIPFGAGRHRCVGAAFAIMQIKAIFSVLLREYEFEMAQPPESYRNDHSKMVVQLA
QPACVRYRRRTGV
>CYP51B1_Mycobacterium tuberculosis TKK_04_0019 2575131740
MSAVALPRVSGGHDEHGHLEEFRTDPIGLMQRVRDECGDVGTFQLAGKQVVLLSGSHANEFFFRAGDDDLDQA
KAYPFMTPIFGEGVVFDASPERRKEMLHNAALRGEQMKGHAATIEDQVRRMIADWGEAGEIDLLDFFAELTIY
TSSACLIGKKFRDQLDGRFAKLYHELERGTDPLAYVDPYLPIESFRRRDEARNGLVALVADIMNGRIANPPTD
KSDRDMLDVLIAVKAETGTPRFSADEITGMFISMMFAGHHTSSGTASWTLIELMRHRDAYAAVIDELDELYGD
GRSVSFHALRQIPQLENVLKETLRLHPPLIILMRVAKGEFEVQGHRIHEGDLVAASPAISNRIPEDFPDPHDF
VPARYEQPRQEDLLNRWTWIPFGAGRHRCVGAAFAIMQIKAIFSVLLREYEFEMAQPPESYRNDHSKMVVQLA
QPACVRYRRRTGV
>CYP51B1_Mycobacterium tuberculosis TKK_04_0020 2592229649
MSAVALPRVSGGHDEHGHLEEFRTDPIGLMQRVRDECGDVGTFQLAGKQVVLLSGSHANEFFFRAGDDDLDQA
KAYPFMTPIFGEGVVFDASPERRKEMLHNAALRGEQMKGHAATIEDQVRRMIADWGEAGEIDLLDFFAELTIY
TSSACLIGKKFRDQLDGRFAKLYHELERGTDPLAYVDPYLPIESFRRRDEARNGLVALVADIMNGRIANPPTD
KSDRDMLDVLIAVKAETGTPRFSADEITGMFISMMFAGHHTSSGTASWTLIELMRHRDAYAAVIDELDELYGD
GRSVSFHALRQIPQLENVLKETLRLHPPLIILMRVAKGEFEVQGHRIHEGDLVAASPAISNRIPEDFPDPHDF
VPARYEQPRQEDLLNRWTWIPFGAGRHRCVGAAFAIMQIKAIFSVLLREYEFEMAQPPESYRNDHSKMVVQLA
QPACVRYRRRTGV
>CYP51B1_Mycobacterium tuberculosis TKK_04_0021 2575541919
MSAVALPRVSGGHDEHGHLEEFRTDPIGLMQRVRDECGDVGTFQLAGKQVVLLSGSHANEFFFRAGDDDLDQA
KAYPFMTPIFGEGVVFDASPERRKEMLHNAALRGEQMKGHAATIEDQVRRMIADWGEAGEIDLLDFFAELTIY
TSSACLIGKKFRDQLDGRFAKLYHELERGTDPLAYVDPYLPIESFRRRDEARNGLVALVADIMNGRIANPPTD
KSDRDMLDVLIAVKAETGTPRFSADEITGMFISMMFAGHHTSSGTASWTLIELMRHRDAYAAVIDELDELYGD
GRSVSFHALRQIPQLENVLKETLRLHPPLIILMRVAKGEFEVQGHRIHEGDLVAASPAISNRIPEDFPDPHDF
VPARYEQPRQEDLLNRWTWIPFGAGRHRCVGAAFAIMQIKAIFSVLLREYEFEMAQPPESYRNDHSKMVVQLA
QPACVRYRRRTGV
>CYP51B1_Mycobacterium tuberculosis TKK_04_0022 2592225577
MSAVALPRVSGGHDEHGHLEEFRTDPIGLMQRVRDECGDVGTFQLAGKQVVLLSGSHANEFFFRAGDDDLDQA
KAYPFMTPIFGEGVVFDASPERRKEMLHNAALRGEQMKGHAATIEDQVRRMIADWGEAGEIDLLDFFAELTIY
TSSACLIGKKFRDQLDGRFAKLYHELERGTDPLAYVDPYLPIESFRRRDEARNGLVALVADIMNGRIANPPTD
KSDRDMLDVLIAVKAETGTPRFSADEITGMFISMMFAGHHTSSGTASWTLIELMRHRDAYAAVIDELDELYGD
GRSVSFHALRQIPQLENVLKETLRLHPPLIILMRVAKGEFEVQGHRIHEGDLVAASPAISNRIPEDFPDPHDF
VPARYEQPRQEDLLNRWTWIPFGAGRHRCVGAAFAIMQIKAIFSVLLREYEFEMAQPPESYRNDHSKMVVQLA
QPACVRYRRRTGV
>CYP51B1_Mycobacterium tuberculosis TKK_04_0023 2592221505
MSAVALPRVSGGHDEHGHLEEFRTDPIGLMQRVRDECGDVGTFQLAGKQVVLLSGSHANEFFFRAGDDDLDQA
KAYPFMTPIFGEGVVFDASPERRKEMLHNAALRGEQMKGHAATIEDQVRRMIADWGEAGEIDLLDFFAELTIY
TSSACLIGKKFRDQLDGRFAKLYHELERGTDPLAYVDPYLPIESFRRRDEARNGLVALVADIMNGRIANPPTD
KSDRDMLDVLIAVKAETGTPRFSADEITGMFISMMFAGHHTSSGTASWTLIELMRHRDAYAAVIDELDELYGD
GRSVSFHALRQIPQLENVLKETLRLHPPLIILMRVAKGEFEVQGHRIHEGDLVAASPAISNRIPEDFPDPHDF
VPARYEQPRQEDLLNRWTWIPFGAGRHRCVGAAFAIMQIKAIFSVLLREYEFEMAQPPESYRNDHSKMVVQLA
QPACVRYRRRTGV
>CYP51B1_Mycobacterium tuberculosis TKK_04_0024 2576076926
MSAVALPRVSGGHDEHGHLEEFRTDPIGLMQRVRDECGDVGTFQLAGKQVVLLSGSHANEFFFRAGDDDLDQA
KAYPFMTPIFGEGVVFDASPERRKEMLHNAALRGEQMKGHAATIEDQVRRMIADWGEAGEIDLLDFFAELTIY
TSSACLIGKKFRDQLDGRFAKLYHELERGTDPLAYVDPYLPIESFRRRDEARNGLVALVADIMNGRIANPPTD
KSDRDMLDVLIAVKAETGTPRFSADEITGMFISMMFAGHHTSSGTASWTLIELMRHRDAYAAVIDELDELYGD

GRSVSFHALRQIPQLENVLKETLRLHPPLIILMRVAKGEFEVQGHRIHEGDLVAASPAISNRIPEDFPDPHDF
VPARYEQPRQEDLLNRWTWIPFGAGRHRCVGAAFAIMQIKAIFSVLLREYEFEMAQPPESYRNDHSKMVVQLA
QPACVRYRRRTGV
>CYP51B1_Mycobacterium tuberculosis TKK_04_0029 2592577506
MSAVALPRVSGGHDEHGHLEEFRTDPIGLMQRVRDECGDVGTFQLAGKQVVLLSGSHANEFFFRAGDDDLDQA
KAYPFMTPIFGEGVVFDASPERRKEMLHNAALRGEQMKGHAATIEDQVRRMIADWGEAGEIDLLDFFAELTIY
TSSACLIGKKFRDQLDGRFAKLYHELERGTDPLAYVDPYLPIESFRRRDEARNGLVALVADIMNGRIANPPTD
KSDRDMLDVLIAVKAETGTPRFSADEITGMFISMMFAGHHTSSGTASWTLIELMRHRDAYAAVIDELDELYGD
GRSVSFHALRQIPQLENVLKETLRLHPPLIILMRVAKGEFEVQGHRIHEGDLVAASPAISNRIPEDFPDPHDF
VPARYEQPRQEDLLNRWTWIPFGAGRHRCVGAAFAIMQIKAIFSVLLREYEFEMAQPPESYRNDHSKMVVQLA
QPACVRYRRRTGV
>CYP51B1_Mycobacterium tuberculosis TKK_04_0030 2592573732
MSAVALPRVSGGHDEHGHLEEFRTDPIGLMQRVRDECGDVGTFQLAGKQVVLLSGSHANEFFFRAGDDDLDQA
KAYPFMTPIFGEGVVFDASPERRKEMLHNAALRGEQMKGHAATIEDQVRRMIADWGEAGEIDLLDFFAELTIY
TSSACLIGKKFRDQLDGRFAKLYHELERGTDPLAYVDPYLPIESFRRRDEARNGLVALVADIMNGRIANPPTD
KSDRDMLDVLIAVKAETGTPRFSADEITGMFISMMFAGHHTSSGTASWTLIELMRHRDAYAAVIDELDELYGD
GRSVSFHALRQIPQLENVLKETLRLHPPLIILMRVAKGEFEVQGHRIHEGDLVAASPAISNRIPEDFPDPHDF
VPARYEQPRQEDLLNRWTWIPFGAGRHRCVGAAFAIMQIKAIFSVLLREYEFEMAQPPESYRNDHSKMVVQLA
QPACVRYRRRTGV
>CYP51B1_Mycobacterium tuberculosis TKK_04_0031 2592569626
MSAVALPRVSGGHDEHGHLEEFRTDPIGLMQRVRDECGDVGTFQLAGKQVVLLSGSHANEFFFRAGDDDLDQA
KAYPFMTPIFGEGVVFDASPERRKEMLHNAALRGEQMKGHAATIEDQVRRMIADWGEAGEIDLLDFFAELTIY
TSSACLIGKKFRDQLDGRFAKLYHELERGTDPLAYVDPYLPIESFRRRDEARNGLVALVADIMNGRIANPPTD
KSDRDMLDVLIAVKAETGTPRFSADEITGMFISMMFAGHHTSSGTASWTLIELMRHRDAYAAVIDELDELYGD
GRSVSFHALRQIPQLENVLKETLRLHPPLIILMRVAKGEFEVQGHRIHEGDLVAASPAISNRIPEDFPDPHDF
VPARYEQPRQEDLLNRWTWIPFGAGRHRCVGAAFAIMQIKAIFSVLLREYEFEMAQPPESYRNDHSKMVVQLA
QPACVRYRRRTGV
>CYP51B1_Mycobacterium tuberculosis TKK_04_0033 2578176887
MSAVALPRVSGGHDEHGHLEEFRTDPIGLMQRVRDECGDVGTFQLAGKQVVLLSGSHANEFFFRAGDDDLDQA
KAYPFMTPIFGEGVVFDASPERRKEMLHNAALRGEQMKGHAATIEDQVRRMIADWGEAGEIDLLDFFAELTIY
TSSACLIGKKFRDQLDGRFAKLYHELERGTDPLAYVDPYLPIESFRRRDEARNGLVALVADIMNGRIANPPTD
KSDRDMLDVLIAVKAETGTPRFSADEITGMFISMMFAGHHTSSGTASWTLIELMRHRDAYAAVIDELDELYGD
GRSVSFHALRQIPQLENVLKETLRLHPPLIILMRVAKGEFEVQGHRIHEGDLVAASPAISNRIPEDFPDPHDF
VPARYEQPRQEDLLNRWTWIPFGAGRHRCVGAAFAIMQIKAIFSVLLREYEFEMAQPPESYRNDHSKMVVQLA
QPACVRYRRRTGV
>CYP51B1_Mycobacterium tuberculosis TKK_04_0034 2592565566
MSAVALPRVSGGHDEHGHLEEFRTDPIGLMQRVRDECGDVGTFQLAGKQVVLLSGSHANEFFFRAGDDDLDQA
KAYPFMTPIFGEGVVFDASPERRKEMLHNAALRGEQMKGHAATIEDQVRRMIADWGEAGEIDLLDFFAELTIY
TSSACLIGKKFRDQLDGRFAKLYHELERGTDPLAYVDPYLPIESFRRRDEARNGLVALVADIMNGRIANPPTD
KSDRDMLDVLIAVKAETGTPRFSADEITGMFISMMFAGHHTSSGTASWTLIELMRHRDAYAAVIDELDELYGD
GRSVSFHALRQIPQLENVLKETLRLHPPLIILMRVAKGEFEVQGHRIHEGDLVAASPAISNRIPEDFPDPHDF
VPARYEQPRQEDLLNRWTWIPFGAGRHRCVGAAFAIMQIKAIFSVLLREYEFEMAQPPESYRNDHSKMVVQLA
QPACVRYRRRTGV
>CYP51B1_Mycobacterium tuberculosis TKK_04_0036 2592561481
MSAVALPRVSGGHDEHGHLEEFRTDPIGLMQRVRDECGDVGTFQLAGKQVVLLSGSHANEFFFRAGDDDLDQA
KAYPFMTPIFGEGVVFDASPERRKEMLHNAALRGEQMKGHAATIEDQVRRMIADWGEAGEIDLLDFFAELTIY
TSSACLIGKKFRDQLDGRFAKLYHELERGTDPLAYVDPYLPIESFRRRDEARNGLVALVADIMNGRIANPPTD
KSDRDMLDVLIAVKAETGTPRFSADEITGMFISMMFAGHHTSSGTASWTLIELMRHRDAYAAVIDELDELYGD
GRSVSFHALRQIPQLENVLKETLRLHPPLIILMRVAKGEFEVQGHRIHEGDLVAASPAISNRIPEDFPDPHDF
VPARYEQPRQEDLLNRWTWIPFGAGRHRCVGAAFAIMQIKAIFSVLLREYEFEMAQPPESYRNDHSKMVVQLA
QPACVRYRRRTGV
>CYP51B1_Mycobacterium tuberculosis TKK_04_0037 2592557405
MSAVALPRVSGGHDEHGHLEEFRTDPIGLMQRVRDECGDVGTFQLAGKQVVLLSGSHANEFFFRAGDDDLDQA
KAYPFMTPIFGEGVVFDASPERRKEMLHNAALRGEQMKGHAATIEDQVRRMIADWGEAGEIDLLDFFAELTIY
TSSACLIGKKFRDQLDGRFAKLYHELERGTDPLAYVDPYLPIESFRRRDEARNGLVALVADIMNGRIANPPTD
KSDRDMLDVLIAVKAETGTPRFSADEITGMFISMMFAGHHTSSGTASWTLIELMRHRDAYAAVIDELDELYGD
GRSVSFHALRQIPQLENVLKETLRLHPPLIILMRVAKGEFEVQGHRIHEGDLVAASPAISNRIPEDFPDPHDF

VPARYEQPRQEDLLNRWTWIPFGAGRHRCVGAAFAIMQIKAIFSVLLREYEFEMAQPPESYRNDHSKMVVQLA
QPACVRYRRRTGV
>CYP51B1_Mycobacterium tuberculosis TKK_04_0038 2584838563
MSAVALPRVSGGHDEHGHLEEFRTDPIGLMQRVRDECGDVGTFQLAGKQVVLLSGSHANEFFFRAGDDDLDQA
KAYPFMTPIFGEGVVFDASPERRKEMLHNAALRGEQMKGHAATIEDQVRRMIADWGEAGEIDLLDFFAELTIY
TSSACLIGKKFRDQLDGRFAKLYHELERGTDPLAYVDPYLPIESFRRRDEARNGLVALVADIMNGRIANPPTD
KSDRDMLDVLIAVKAETGTPRFSADEITGMFISMMFAGHHTSSGTASWTLIELMRHRDAYAAVIDELDELYGD
GRSVSFHALRQIPQLENVLKETLRLHPPLIILMRVAKGEFEVQGHRIHEGDLVAASPAISNRIPEDFPDPHDF
VPARYEQPRQEDLLNRWTWIPFGAGRHRCVGAAFAIMQIKAIFSVLLREYEFEMAQPPESYRNDHSKMVVQLA
QPACVRYRRRTGV
>CYP51B1_Mycobacterium tuberculosis TKK_04_0039 2592555084
MSAVALPRVSGGHDEHGHLEEFRTDPIGLMQRVRDECGDVGTFQLAGKQVVLLSGSHANEFFFRAGDDDLDQA
KAYPFMTPIFGEGVVFDASPERRKEMLHNAALRGEQMKGHAATIEDQVRRMIADWGEAGEIDLLDFFAELTIY
TSSACLIGKKFRDQLDGRFAKLYHELERGTDPLAYVDPYLPIESFRRRDEARNGLVALVADIMNGRIANPPTD
KSDRDMLDVLIAVKAETGTPRFSADEITGMFISMMFAGHHTSSGTASWTLIELMRHRDAYAAVIDELDELYGD
GRSVSFHALRQIPQLENVLKETLRLHPPLIILMRVAKGEFEVQGHRIHEGDLVAASPAISNRIPEDFPDPHDF
VPARYEQPRQEDLLNRWTWIPFGAGRHRCVGAAFAIMQIKAIFSVLLREYEFEMAQPPESYRNDHSKMVVQLA
QPACVRYRRRTGV
>CYP51B1_Mycobacterium tuberculosis TKK_04_0040 2576939522
MSAVALPRVSGGHDEHGHLEEFRTDPIGLMQRVRDECGDVGTFQLAGKQVVLLSGSHANEFFFRAGDDDLDQA
KAYPFMTPIFGEGVVFDASPERRKEMLHNAALRGEQMKGHAATIEDQVRRMIADWGEAGEIDLLDFFAELTIY
TSSACLIGKKFRDQLDGRFAKLYHELERGTDPLAYVDPYLPIESFRRRDEARNGLVALVADIMNGRIANPPTD
KSDRDMLDVLIAVKAETGTPRFSADEITGMFISMMFAGHHTSSGTASWTLIELMRHRDAYAAVIDELDELYGD
GRSVSFHALRQIPQLENVLKETLRLHPPLIILMRVAKGEFEVQGHRIHEGDLVAASPAISNRIPEDFPDPHDF
VPARYEQPRQEDLLNRWTWIPFGAGRHRCVGAAFAIMQIKAIFSVLLREYEFEMAQPPESYRNDHSKMVVQLA
QPACVRYRRRTGV
>CYP51B1_Mycobacterium tuberculosis TKK_04_0042 2584905795
MSAVALPRVSGGHDEHGHLEEFRTDPIGLMQRVRDECGDVGTFQLAGKQVVLLSGSHANEFFFRAGDDDLDQA
KAYPFMTPIFGEGVVFDASPERRKEMLHNAALRGEQMKGHAATIEDQVRRMIADWGEAGEIDLLDFFAELTIY
TSSACLIGKKFRDQLDGRFAKLYHELERGTDPLAYVDPYLPIESFRRRDEARNGLVALVADIMNGRIANPPTD
KSDRDMLDVLIAVKAETGTPRFSADEITGMFISMMFAGHHTSSGTASWTLIELMRHRDAYAAVIDELDELYGD
GRSVSFHALRQIPQLENVLKETLRLHPPLIILMRVAKGEFEVQGHRIHEGDLVAASPAISNRIPEDFPDPHDF
VPARYEQPRQEDLLNRWTWIPFGAGRHRCVGAAFAIMQIKAIFSVLLREYEFEMAQPPESYRNDHSKMVVQLA
QPACVRYRRRTGV
>CYP51B1_Mycobacterium tuberculosis TKK_04_0043 2592550954
MSAVALPRVSGGHDEHGHLEEFRTDPIGLMQRVRDECGDVGTFQLAGKQVVLLSGSHANEFFFRAGDDDLDQA
KAYPFMTPIFGEGVVFDASPERRKEMLHNAALRGEQMKGHAATIEDQVRRMIADWGEAGEIDLLDFFAELTIY
TSSACLIGKKFRDQLDGRFAKLYHELERGTDPLAYVDPYLPIESFRRRDEARNGLVALVADIMNGRIANPPTD
KSDRDMLDVLIAVKAETGTPRFSADEITGMFISMMFAGHHTSSGTASWTLIELMRHRDAYAAVIDELDELYGD
GRSVSFHALRQIPQLENVLKETLRLHPPLIILMRVAKGEFEVQGHRIHEGDLVAASPAISNRIPEDFPDPHDF
VPARYEQPRQEDLLNRWTWIPFGAGRHRCVGAAFAIMQIKAIFSVLLREYEFEMAQPPESYRNDHSKMVVQLA
QPACVRYRRRTGV
>CYP51B1_Mycobacterium tuberculosis TKK_04_0044 2575934748
MSAVALPRVSGGHDEHGHLEEFRTDPIGLMQRVRDECGDVGTFQLAGKQVVLLSGSHANEFFFRAGDDDLDQA
KAYPFMTPIFGEGVVFDASPERRKEMLHNAALRGEQMKGHAATIEDQVRRMIADWGEAGEIDLLDFFAELTIY
TSSACLIGKKFRDQLDGRFAKLYHELERGTDPLAYVDPYLPIESFRRRDEARNGLVALVADIMNGRIANPPTD
KSDRDMLDVLIAVKAETGTPRFSADEITGMFISMMFAGHHTSSGTASWTLIELMRHRDAYAAVIDELDELYGD
GRSVSFHALRQIPQLENVLKETLRLHPPLIILMRVAKGEFEVQGHRIHEGDLVAASPAISNRIPEDFPDPHDF
VPARYEQPRQEDLLNRWTWIPFGAGRHRCVGAAFAIMQIKAIFSVLLREYEFEMAQPPESYRNDHSKMVVQLA
QPACVRYRRRTGV
>CYP51B1_Mycobacterium tuberculosis TKK_04_0045 2584857157
MSAVALPRVSGGHDEHGHLEEFRTDPIGLMQRVRDECGDVGTFQLAGKQVVLLSGSHANEFFFRAGDDDLDQA
KAYPFMTPIFGEGVVFDASPERRKEMLHNAALRGEQMKGHAATIEDQVRRMIADWGEAGEIDLLDFFAELTIY
TSSACLIGKKFRDQLDGRFAKLYHELERGTDPLAYVDPYLPIESFRRRDEARNGLVALVADIMNGRIANPPTD
KSDRDMLDVLIAVKAETGTPRFSADEITGMFISMMFAGHHTSSGTASWTLIELMRHRDAYAAVIDELDELYGD
GRSVSFHALRQIPQLENVLKETLRLHPPLIILMRVAKGEFEVQGHRIHEGDLVAASPAISNRIPEDFPDPHDF
VPARYEQPRQEDLLNRWTWIPFGAGRHRCVGAAFAIMQIKAIFSVLLREYEFEMAQPPESYRNDHSKMVVQLA
QPACVRYRRRTGV

>CYP51B1_Mycobacterium tuberculosis TKK_04_0046 2592545196
MSAVALPRVSGGHDEHGHLEEFRTDPIGLMQRVRDECGDVGTFQLAGKQVVLLSGSHANEFFFRAGDDDLDQA
KAYPFMTPIFGEGVVFDASPERRKEMLHNAALRGEQMKGHAATIEDQVRRMIADWGEAGEIDLLDFFAELTIY
TSSACLIGKKFRDQLDGRFAKLYHELERGTDPLAYVDPYLPIESFRRRDEARNGLVALVADIMNGRIANPPTD
KSDRDMLDVLIAVKAETGTPRFSADEITGMFISMMFAGHHTSSGTASWTLIELMRHRDAYAAVIDELDELYGD
GRSVSFHALRQIPQLENVLKETLRLHPPLIILMRVAKGEFEVQGHRIHEGDLVAASPAISNRIPEDFPDPHDF
VPARYEQPRQEDLLNRWTWIPFGAGRHRCVGAAFAIMQIKAIFSVLLREYEFEMAQPPESYRNDHSKMVVQLA
QPACVRYRRRTGV
>CYP51B1_Mycobacterium tuberculosis TKK_04_0047 2592541105
MSAVALPRVSGGHDEHGHLEEFRTDPIGLMQRVRDECGDVGTFQLAGKQVVLLSGSHANEFFFRAGDDDLDQA
KAYPFMTPIFGEGVVFDASPERRKEMLHNAALRGEQMKGHAATIEDQVRRMIADWGEAGEIDLLDFFAELTIY
TSSACLIGKKFRDQLDGRFAKLYHELERGTDPLAYVDPYLPIESFRRRDEARNGLVALVADIMNGRIANPPTD
KSDRDMLDVLIAVKAETGTPRFSADEITGMFISMMFAGHHTSSGTASWTLIELMRHRDAYAAVIDELDELYGD
GRSVSFHALRQIPQLENVLKETLRLHPPLIILMRVAKGEFEVQGHRIHEGDLVAASPAISNRIPEDFPDPHDF
VPARYEQPRQEDLLNRWTWIPFGAGRHRCVGAAFAIMQIKAIFSVLLREYEFEMAQPPESYRNDHSKMVVQLA
QPACVRYRRRTGV
>CYP51B1_Mycobacterium tuberculosis TKK_04_0048 2592539464
MSAVALPRVSGGHDEHGHLEEFRTDPIGLMQRVRDECGDVGTFQLAGKQVVLLSGSHANEFFFRAGDDDLDQA
KAYPFMTPIFGEGVVFDASPERRKEMLHNAALRGEQMKGHAATIEDQVRRMIADWGEAGEIDLLDFFAELTIY
TSSACLIGKKFRDQLDGRFAKLYHELERGTDPLAYVDPYLPIESFRRRDEARNGLVALVADIMNGRIANPPTD
KSDRDMLDVLIAVKAETGTPRFSADEITGMFISMMFAGHHTSSGTASWTLIELMRHRDAYAAVIDELDELYGD
GRSVSFHALRQIPQLENVLKETLRLHPPLIILMRVAKGEFEVQGHRIHEGDLVAASPAISNRIPEDFPDPHDF
VPARYEQPRQEDLLNRWTWIPFGAGRHRCVGAAFAIMQIKAIFSVLLREYEFEMAQPPESYRNDHSKMVVQLA
QPACVRYRRRTGV
>CYP51B1_Mycobacterium tuberculosis TKK_04_0051 2576380548
MSAVALPRVSGGHDEHGHLEEFRTDPIGLMQRVRDECGDVGTFQLAGKQVVLLSGSHANEFFFRAGDDDLDQA
KAYPFMTPIFGEGVVFDASPERRKEMLHNAALRGEQMKGHAATIEDQVRRMIADWGEAGEIDLLDFFAELTIY
TSSACLIGKKFRDQLDGRFAKLYHELERGTDPLAYVDPYLPIESFRRRDEARNGLVALVADIMNGRIANPPTD
KSDRDMLDVLIAVKAETGTPRFSADEITGMFISMMFAGHHTSSGTASWTLIELMRHRDAYAAVIDELDELYGD
GRSVSFHALRQIPQLENVLKETLRLHPPLIILMRVAKGEFEVQGHRIHEGDLVAASPAISNRIPEDFPDPHDF
VPARYEQPRQEDLLNRWTWIPFGAGRHRCVGAAFAIMQIKAIFSVLLREYEFEMAQPPESYRNDHSKMVVQLA
QPACVRYRRRTGV
>CYP51B1_Mycobacterium tuberculosis TKK_04_0054 2577385898
MSAVALPRVSGGHDEHGHLEEFRTDPIGLMQRVRDECGDVGTFQLAGKQVVLLSGSHANEFFFRAGDDDLDQA
KAYPFMTPIFGEGVVFDASPERRKEMLHNAALRGEQMKGHAATIEDQVRRMIADWGEAGEIDLLDFFAELTIY
TSSACLIGKKFRDQLDGRFAKLYHELERGTDPLAYVDPYLPIESFRRRDEARNGLVALVADIMNGRIANPPTD
KSDRDMLDVLIAVKAETGTPRFSADEITGMFISMMFAGHHTSSGTASWTLIELMRHRDAYAAVIDELDELYGD
GRSVSFHALRQIPQLENVLKETLRLHPPLIILMRVAKGEFEVQGHRIHEGDLVAASPAISNRIPEDFPDPHDF
VPARYEQPRQEDLLNRWTWIPFGAGRHRCVGAAFAIMQIKAIFSVLLREYEFEMAQPPESYRNDHSKMVVQLA
QPACVRYRRRTGV
>CYP51B1_Mycobacterium tuberculosis TKK_04_0059 2630636330
MSAVALPRVSGGHDEHGHLEEFRTDPIGLMQRVRDECGDVGTFQLAGKQVVLLSGSHANEFFFRAGDDDLDQA
KAYPFMTPIFGEGVVFDASPERRKEMLHNAALRGEQMKGHAATIEDQVRRMIADWGEAGEIDLLDFFAELTIY
TSSACLIGKKFRDQLDGRFAKLYHELERGTDPLAYVDPYLPIESFRRRDEARNGLVALVADIMNGRIANPPTD
KSDRDMLDVLIAVKAETGTPRFSADEITGMFISMMFAGHHTSSGTASWTLIELMRHRDAYAAVIDELDELYGD
GRSVSFHALRQIPQLENVLKETLRLHPPLIILMRVAKGEFEVQGHRIHEGDLVAASPAISNRIPEDFPDPHDF
VPARYEQPRQEDLLNRWTWIPFGAGRHRCVGAAFAIMQIKAIFSVLLREYEFEMAQPPESYRNDHSKMVVQLA
QPACVRYRRRTGV
>CYP51B1_Mycobacterium tuberculosis TKK_04_0060 2584842632
MSAVALPRVSGGHDEHGHLEEFRTDPIGLMQRVRDECGDVGTFQLAGKQVVLLSGSHANEFFFRAGDDDLDQA
KAYPFMTPIFGEGVVFDASPERRKEMLHNAALRGEQMKGHAATIEDQVRRMIADWGEAGEIDLLDFFAELTIY
TSSACLIGKKFRDQLDGRFAKLYHELERGTDPLAYVDPYLPIESFRRRDEARNGLVALVADIMNGRIANPPTD
KSDRDMLDVLIAVKAETGTPRFSADEITGMFISMMFAGHHTSSGTASWTLIELMRHRDAYAAVIDELDELYGD
GRSVSFHALRQIPQLENVLKETLRLHPPLIILMRVAKGEFEVQGHRIHEGDLVAASPAISNRIPEDFPDPHDF
VPARYEQPRQEDLLNRWTWIPFGAGRHRCVGAAFAIMQIKAIFSVLLREYEFEMAQPPESYRNDHSKMVVQLA
QPACVRYRRRTGV
>CYP51B1_Mycobacterium tuberculosis TKK_04_0061 2628540203

MSAVALPRVSGGHDEHGHLEEFRTDPIGLMQRVRDECGDVGTFQLAGKQVVLLSGSHANEFFFRAGDDDLDQA
KAYPFMTPIFGEGVVFDASPERRKEMLHNAALRGEQMKGHAATIEDQVRRMIADWGEAGEIDLLDFFAELTIY
TSSACLIGKKFRDQLDGRFAKLYHELERGTDPLAYVDPYLPIESFRRRDEARNGLVALVADIMNGRIANPPTD
KSDRDMLDVLIAVKAETGTPRFSADEITGMFISMMFAGHHTSSGTASWTLIELMRHRDAYAAVIDELDELYGD
GRSVSFHALRQIPQLENVLKETLRLHPPLIILMRVAKGEFEVQGHRIHEGDLVAASPAISNRIPEDFPDPHDF
VPARYEQPRQEDLLNRWTWIPFGAGRHRCVGAAFAIMQIKAIFSVLLREYEFEMAQPPESYRNDHSKMVVQLA
QPACVRYRRRTGV
>CYP51B1_Mycobacterium tuberculosis TKK_04_0062 2633632190
MSAVALPRVSGGHDEHGHLEEFRTDPIGLMQRVRDECGDVGTFQLAGKQVVLLSGSHANEFFFRAGDDDLDQA
KAYPFMTPIFGEGVVFDASPERRKEMLHNAALRGEQMKGHAATIEDQVRRMIADWGEAGEIDLLDFFAELTIY
TSSACLIGKKFRDQLDGRFAKLYHELERGTDPLAYVDPYLPIESFRRRDEARNGLVALVADIMNGRIANPPTD
KSDRDMLDVLIAVKAETGTPRFSADEITGMFISMMFAGHHTSSGTASWTLIELMRHRDAYAAVIDELDELYGD
GRSVSFHALRQIPQLENVLKETLRLHPPLIILMRVAKGEFEVQGHRIHEGDLVAASPAISNRIPEDFPDPHDF
VPARYEQPRQEDLLNRWTWIPFGAGRHRCVGAAFAIMQIKAIFSVLLREYEFEMAQPPESYRNDHSKMVVQLA
QPACVRYRRRTGV
>CYP51B1_Mycobacterium tuberculosis TKK_04_0063 2637011686
MSAVALPRVSGGHDEHGHLEEFRTDPIGLMQRVRDECGDVGTFQLAGKQVVLLSGSHANEFFFRAGDDDLDQA
KAYPFMTPIFGEGVVFDASPERRKEMLHNAALRGEQMKGHAATIEDQVRRMIADWGEAGEIDLLDFFAELTIY
TSSACLIGKKFRDQLDGRFAKLYHELERGTDPLAYVDPYLPIESFRRRDEARNGLVALVADIMNGRIANPPTD
KSDRDMLDVLIAVKAETGTPRFSADEITGMFISMMFAGHHTSSGTASWTLIELMRHRDAYAAVIDELDELYGD
GRSVSFHALRQIPQLENVLKETLRLHPPLIILMRVAKGEFEVQGHRIHEGDLVAASPAISNRIPEDFPDPHDF
VPARYEQPRQEDLLNRWTWIPFGAGRHRCVGAAFAIMQIKAIFSVLLREYEFEMAQPPESYRNDHSKMVVQLA
QPACVRYRRRTGV
>CYP51B1_Mycobacterium tuberculosis TKK_04_0064 2575922025
MSAVALPRVSGGHDEHGHLEEFRTDPIGLMQRVRDECGDVGTFQLAGKQVVLLSGSHANEFFFRAGDDDLDQA
KAYPFMTPIFGEGVVFDASPERRKEMLHNAALRGEQMKGHAATIEDQVRRMIADWGEAGEIDLLDFFAELTIY
TSSACLIGKKFRDQLDGRFAKLYHELERGTDPLAYVDPYLPIESFRRRDEARNGLVALVADIMNGRIANPPTD
KSDRDMLDVLIAVKAETGTPRFSADEITGMFISMMFAGHHTSSGTASWTLIELMRHRDAYAAVIDELDELYGD
GRSVSFHALRQIPQLENVLKETLRLHPPLIILMRVAKGEFEVQGHRIHEGDLVAASPAISNRIPEDFPDPHDF
VPARYEQPRQEDLLNRWTWIPFGAGRHRCVGAAFAIMQIKAIFSVLLREYEFEMAQPPESYRNDHSKMVVQLA
QPACVRYRRRTGV
>CYP51B1_Mycobacterium tuberculosis TKK_04_0066 2584696441
MSAVALPRVSGGHDEHGHLEEFRTDPIGLMQRVRDECGDVGTFQLAGKQVVLLSGSHANEFFFRAGDDDLDQA
KAYPFMTPIFGEGVVFDASPERRKEMLHNAALRGEQMKGHAATIEDQVRRMIADWGEAGEIDLLDFFAELTIY
TSSACLIGKKFRDQLDGRFAKLYHELERGTDPLAYVDPYLPIESFRRRDEARNGLVALVADIMNGRIANPPTD
KSDRDMLDVLIAVKAETGTPRFSADEITGMFISMMFAGHHTSSGTASWTLIELMRHRDAYAAVIDELDELYGD
GRSVSFHALRQIPQLENVLKETLRLHPPLIILMRVAKGEFEVQGHRIHEGDLVAASPAISNRIPEDFPDPHDF
VPARYEQPRQEDLLNRWTWIPFGAGRHRCVGAAFAIMQIKAIFSVLLREYEFEMAQPPESYRNDHSKMVVQLA
QPACVRYRRRTGV
>CYP51B1_Mycobacterium tuberculosis TKK_04_0067 2584978340
MSAVALPRVSGGHDEHGHLEEFRTDPIGLMQRVRDECGDVGTFQLAGKQVVLLSGSHANEFFFRAGDDDLDQA
KAYPFMTPIFGEGVVFDASPERRKEMLHNAALRGEQMKGHAATIEDQVRRMIADWGEAGEIDLLDFFAELTIY
TSSACLIGKKFRDQLDGRFAKLYHELERGTDPLAYVDPYLPIESFRRRDEARNGLVALVADIMNGRIANPPTD
KSDRDMLDVLIAVKAETGTPRFSADEITGMFISMMFAGHHTSSGTASWTLIELMRHRDAYAAVIDELDELYGD
GRSVSFHALRQIPQLENVLKETLRLHPPLIILMRVAKGEFEVQGHRIHEGDLVAASPAISNRIPEDFPDPHDF
VPARYEQPRQEDLLNRWTWIPFGAGRHRCVGAAFAIMQIKAIFSVLLREYEFEMAQPPESYRNDHSKMVVQLA
QPACVRYRRRTGV
>CYP51B1_Mycobacterium tuberculosis TKK_04_0068 2649199360
MSAVALPRVSGGHDEHGHLEEFRTDPIGLMQRVRDECGDVGTFQLAGKQVVLLSGSHANEFFFRAGDDDLDQA
KAYPFMTPIFGEGVVFDASPERRKEMLHNAALRGEQMKGHAATIEDQVRRMIADWGEAGEIDLLDFFAELTIY
TSSACLIGKKFRDQLDGRFAKLYHELERGTDPLAYVDPYLPIESFRRRDEARNGLVALVADIMNGRIANPPTD
KSDRDMLDVLIAVKAETGTPRFSADEITGMFISMMFAGHHTSSGTASWTLIELMRHRDAYAAVIDELDELYGD
GRSVSFHALRQIPQLENVLKETLRLHPPLIILMRVAKGEFEVQGHRIHEGDLVAASPAISNRIPEDFPDPHDF
VPARYEQPRQEDLLNRWTWIPFGAGRHRCVGAAFAIMQIKAIFSVLLREYEFEMAQPPESYRNDHSKMVVQLA
QPACVRYRRRTGV
>CYP51B1_Mycobacterium tuberculosis TKK_04_0069 2647568141
MSAVALPRVSGGHDEHGHLEEFRTDPIGLMQRVRDECGDVGTFQLAGKQVVLLSGSHANEFFFRAGDDDLDQA
KAYPFMTPIFGEGVVFDASPERRKEMLHNAALRGEQMKGHAATIEDQVRRMIADWGEAGEIDLLDFFAELTIY

TSSACLIGKKFRDQLDGRFAKLYHELERGTDPLAYVDPYLPIESFRRRDEARNGLVALVADIMNGRIANPPTD
KSDRDMLDVLIAVKAETGTPRFSADEITGMFISMMFAGHHTSSGTASWTLIELMRHRDAYAAVIDELDELYGD
GRSVSFHALRQIPQLENVLKETLRLHPPLIILMRVAKGEFEVQGHRIHEGDLVAASPAISNRIPEDFPDPHDF
VPARYEQPRQEDLLNRWTWIPFGAGRHRCVGAAFAIMQIKAIFSVLLREYEFEMAQPPESYRNDHSKMVVQLA
QPACVRYRRRTGV
>CYP51B1_Mycobacterium tuberculosis TKK_04_0070 2629711731
MSAVALPRVSGGHDEHGHLEEFRTDPIGLMQRVRDECGDVGTFQLAGKQVVLLSGSHANEFFFRAGDDDLDQA
KAYPFMTPIFGEGVVFDASPERRKEMLHNAALRGEQMKGHAATIEDQVRRMIADWGEAGEIDLLDFFAELTIY
TSSACLIGKKFRDQLDGRFAKLYHELERGTDPLAYVDPYLPIESFRRRDEARNGLVALVADIMNGRIANPPTD
KSDRDMLDVLIAVKAETGTPRFSADEITGMFISMMFAGHHTSSGTASWTLIELMRHRDAYAAVIDELDELYGD
GRSVSFHALRQIPQLENVLKETLRLHPPLIILMRVAKGEFEVQGHRIHEGDLVAASPAISNRIPEDFPDPHDF
VPARYEQPRQEDLLNRWTWIPFGAGRHRCVGAAFAIMQIKAIFSVLLREYEFEMAQPPESYRNDHSKMVVQLA
QPACVRYRRRTGV
>CYP51B1_Mycobacterium tuberculosis TKK_04_0071 2631962138
MSAVALPRVSGGHDEHGHLEEFRTDPIGLMQRVRDECGDVGTFQLAGKQVVLLSGSHANEFFFRAGDDDLDQA
KAYPFMTPIFGEGVVFDASPERRKEMLHNAALRGEQMKGHAATIEDQVRRMIADWGEAGEIDLLDFFAELTIY
TSSACLIGKKFRDQLDGRFAKLYHELERGTDPLAYVDPYLPIESFRRRDEARNGLVALVADIMNGRIANPPTD
KSDRDMLDVLIAVKAETGTPRFSADEITGMFISMMFAGHHTSSGTASWTLIELMRHRDAYAAVIDELDELYGD
GRSVSFHALRQIPQLENVLKETLRLHPPLIILMRVAKGEFEVQGHRIHEGDLVAASPAISNRIPEDFPDPHDF
VPARYEQPRQEDLLNRWTWIPFGAGRHRCVGAAFAIMQIKAIFSVLLREYEFEMAQPPESYRNDHSKMVVQLA
QPACVRYRRRTGV
>CYP51B1_Mycobacterium tuberculosis TKK_04_0072 2584702177
MSAVALPRVSGGHDEHGHLEEFRTDPIGLMQRVRDECGDVGTFQLAGKQVVLLSGSHANEFFFRAGDDDLDQA
KAYPFMTPIFGEGVVFDASPERRKEMLHNAALRGEQMKGHAATIEDQVRRMIADWGEAGEIDLLDFFAELTIY
TSSACLIGKKFRDQLDGRFAKLYHELERGTDPLAYVDPYLPIESFRRRDEARNGLVALVADIMNGRIANPPTD
KSDRDMLDVLIAVKAETGTPRFSADEITGMFISMMFAGHHTSSGTASWTLIELMRHRDAYAAVIDELDELYGD
GRSVSFHALRQIPQLENVLKETLRLHPPLIILMRVAKGEFEVQGHRIHEGDLVAASPAISNRIPEDFPDPHDF
VPARYEQPRQEDLLNRWTWIPFGAGRHRCVGAAFAIMQIKAIFSVLLREYEFEMAQPPESYRNDHSKMVVQLA
QPACVRYRRRTGV
>CYP51B1_Mycobacterium tuberculosis TKK_04_0074 2632877825
MSAVALPRVSGGHDEHGHLEEFRTDPIGLMQRVRDECGDVGTFQLAGKQVVLLSGSHANEFFFRAGDDDLDQA
KAYPFMTPIFGEGVVFDASPERRKEMLHNAALRGEQMKGHAATIEDQVRRMIADWGEAGEIDLLDFFAELTIY
TSSACLIGKKFRDQLDGRFAKLYHELERGTDPLAYVDPYLPIESFRRRDEARNGLVALVADIMNGRIANPPTD
KSDRDMLDVLIAVKAETGTPRFSADEITGMFISMMFAGHHTSSGTASWTLIELMRHRDAYAAVIDELDELYGD
GRSVSFHALRQIPQLENVLKETLRLHPPLIILMRVAKGEFEVQGHRIHEGDLVAASPAISNRIPEDFPDPHDF
VPARYEQPRQEDLLNRWTWIPFGAGRHRCVGAAFAIMQIKAIFSVLLREYEFEMAQPPESYRNDHSKMVVQLA
QPACVRYRRRTGV
>CYP51B1_Mycobacterium tuberculosis TKK_04_0075 2574927636
MSAVALPRVSGGHDEHGHLEEFRTDPIGLMQRVRDECGDVGTFQLAGKQVVLLSGSHANEFFFRAGDDDLDQA
KAYPFMTPIFGEGVVFDASPERRKEMLHNAALRGEQMKGHAATIEDQVRRMIADWGEAGEIDLLDFFAELTIY
TSSACLIGKKFRDQLDGRFAKLYHELERGTDPLAYVDPYLPIESFRRRDEARNGLVALVADIMNGRIANPPTD
KSDRDMLDVLIAVKAETGTPRFSADEITGMFISMMFAGHHTSSGTASWTLIELMRHRDAYAAVIDELDELYGD
GRSVSFHALRQIPQLENVLKETLRLHPPLIILMRVAKGEFEVQGHRIHEGDLVAASPAISNRIPEDFPDPHDF
VPARYEQPRQEDLLNRWTWIPFGAGRHRCVGAAFAIMQIKAIFSVLLREYEFEMAQPPESYRNDHSKMVVQLA
QPACVRYRRRTGV
>CYP51B1_Mycobacterium tuberculosis TKK_04_0078 2630531880
MSAVALPRVSGGHDEHGHLEEFRTDPIGLMQRVRDECGDVGTFQLAGKQVVLLSGSHANEFFFRAGDDDLDQA
KAYPFMTPIFGEGVVFDASPERRKEMLHNAALRGEQMKGHAATIEDQVRRMIADWGEAGEIDLLDFFAELTIY
TSSACLIGKKFRDQLDGRFAKLYHELERGTDPLAYVDPYLPIESFRRRDEARNGLVALVADIMNGRIANPPTD
KSDRDMLDVLIAVKAETGTPRFSADEITGMFISMMFAGHHTSSGTASWTLIELMRHRDAYAAVIDELDELYGD
GRSVSFHALRQIPQLENVLKETLRLHPPLIILMRVAKGEFEVQGHRIHEGDLVAASPAISNRIPEDFPDPHDF
VPARYEQPRQEDLLNRWTWIPFGAGRHRCVGAAFAIMQIKAIFSVLLREYEFEMAQPPESYRNDHSKMVVQLA
QPACVRYRRRTGV
>CYP51B1_Mycobacterium tuberculosis TKK_04_0079 2638052036
MSAVALPRVSGGHDEHGHLEEFRTDPIGLMQRVRDECGDVGTFQLAGKQVVLLSGSHANEFFFRAGDDDLDQA
KAYPFMTPIFGEGVVFDASPERRKEMLHNAALRGEQMKGHAATIEDQVRRMIADWGEAGEIDLLDFFAELTIY
TSSACLIGKKFRDQLDGRFAKLYHELERGTDPLAYVDPYLPIESFRRRDEARNGLVALVADIMNGRIANPPTD
KSDRDMLDVLIAVKAETGTPRFSADEITGMFISMMFAGHHTSSGTASWTLIELMRHRDAYAAVIDELDELYGD

GRSVSFHALRQIPQLENVLKETLRLHPPLIILMRVAKGEFEVQGHRIHEGDLVAASPAISNRIPEDFPDPHDF
VPARYEQPRQEDLLNRWTWIPFGAGRHRCVGAAFAIMQIKAIFSVLLREYEFEMAQPPESYRNDHSKMVVQLA
QPACVRYRRRTGV
>CYP51B1_Mycobacterium tuberculosis TKK_04_0080 2574733096
MSAVALPRVSGGHDEHGHLEEFRTDPIGLMQRVRDECGDVGTFQLAGKQVVLLSGSHANEFFFRAGDDDLDQA
KAYPFMTPIFGEGVVFDASPERRKEMLHNAALRGEQMKGHAATIEDQVRRMIADWGEAGEIDLLDFFAELTIY
TSSACLIGKKFRDQLDGRFAKLYHELERGTDPLAYVDPYLPIESFRRRDEARNGLVALVADIMNGRIANPPTD
KSDRDMLDVLIAVKAETGTPRFSADEITGMFISMMFAGHHTSSGTASWTLIELMRHRDAYAAVIDELDELYGD
GRSVSFHALRQIPQLENVLKETLRLHPPLIILMRVAKGEFEVQGHRIHEGDLVAASPAISNRIPEDFPDPHDF
VPARYEQPRQEDLLNRWTWIPFGAGRHRCVGAAFAIMQIKAIFSVLLREYEFEMAQPPESYRNDHSKMVVQLA
QPACVRYRRRTGV
>CYP51B1_Mycobacterium tuberculosis TKK_04_0081 2630852405
MSAVALPRVSGGHDEHGHLEEFRTDPIGLMQRVRDECGDVGTFQLAGKQVVLLSGSHANEFFFRAGDDDLDQA
KAYPFMTPIFGEGVVFDASPERRKEMLHNAALRGEQMKGHAATIEDQVRRMIADWGEAGEIDLLDFFAELTIY
TSSACLIGKKFRDQLDGRFAKLYHELERGTDPLAYVDPYLPIESFRRRDEARNGLVALVADIMNGRIANPPTD
KSDRDMLDVLIAVKAETGTPRFSADEITGMFISMMFAGHHTSSGTASWTLIELMRHRDAYAAVIDELDELYGD
GRSVSFHALRQIPQLENVLKETLRLHPPLIILMRVAKGEFEVQGHRIHEGDLVAASPAISNRIPEDFPDPHDF
VPARYEQPRQEDLLNRWTWIPFGAGRHRCVGAAFAIMQIKAIFSVLLREYEFEMAQPPESYRNDHSKMVVQLA
QPACVRYRRRTGV
>CYP51B1_Mycobacterium tuberculosis TKK_04_0082 2584761693
MSAVALPRVSGGHDEHGHLEEFRTDPIGLMQRVRDECGDVGTFQLAGKQVVLLSGSHANEFFFRAGDDDLDQA
KAYPFMTPIFGEGVVFDASPERRKEMLHNAALRGEQMKGHAATIEDQVRRMIADWGEAGEIDLLDFFAELTIY
TSSACLIGKKFRDQLDGRFAKLYHELERGTDPLAYVDPYLPIESFRRRDEARNGLVALVADIMNGRIANPPTD
KSDRDMLDVLIAVKAETGTPRFSADEITGMFISMMFAGHHTSSGTASWTLIELMRHRDAYAAVIDELDELYGD
GRSVSFHALRQIPQLENVLKETLRLHPPLIILMRVAKGEFEVQGHRIHEGDLVAASPAISNRIPEDFPDPHDF
VPARYEQPRQEDLLNRWTWIPFGAGRHRCVGAAFAIMQIKAIFSVLLREYEFEMAQPPESYRNDHSKMVVQLA
QPACVRYRRRTGV
>CYP51B1_Mycobacterium tuberculosis TKK_04_0083 2629195125
MSAVALPRVSGGHDEHGHLEEFRTDPIGLMQRVRDECGDVGTFQLAGKQVVLLSGSHANEFFFRAGDDDLDQA
KAYPFMTPIFGEGVVFDASPERRKEMLHNAALRGEQMKGHAATIEDQVRRMIADWGEAGEIDLLDFFAELTIY
TSSACLIGKKFRDQLDGRFAKLYHELERGTDPLAYVDPYLPIESFRRRDEARNGLVALVADIMNGRIANPPTD
KSDRDMLDVLIAVKAETGTPRFSADEITGMFISMMFAGHHTSSGTASWTLIELMRHRDAYAAVIDELDELYGD
GRSVSFHALRQIPQLENVLKETLRLHPPLIILMRVAKGEFEVQGHRIHEGDLVAASPAISNRIPEDFPDPHDF
VPARYEQPRQEDLLNRWTWIPFGAGRHRCVGAAFAIMQIKAIFSVLLREYEFEMAQPPESYRNDHSKMVVQLA
QPACVRYRRRTGV
>CYP51B1_Mycobacterium tuberculosis TKK_04_0084 2630289848
MSAVALPRVSGGHDEHGHLEEFRTDPIGLMQRVRDECGDVGTFQLAGKQVVLLSGSHANEFFFRAGDDDLDQA
KAYPFMTPIFGEGVVFDASPERRKEMLHNAALRGEQMKGHAATIEDQVRRMIADWGEAGEIDLLDFFAELTIY
TSSACLIGKKFRDQLDGRFAKLYHELERGTDPLAYVDPYLPIESFRRRDEARNGLVALVADIMNGRIANPPTD
KSDRDMLDVLIAVKAETGTPRFSADEITGMFISMMFAGHHTSSGTASWTLIELMRHRDAYAAVIDELDELYGD
GRSVSFHALRQIPQLENVLKETLRLHPPLIILMRVAKGEFEVQGHRIHEGDLVAASPAISNRIPEDFPDPHDF
VPARYEQPRQEDLLNRWTWIPFGAGRHRCVGAAFAIMQIKAIFSVLLREYEFEMAQPPESYRNDHSKMVVQLA
QPACVRYRRRTGV
>CYP51B1_Mycobacterium tuberculosis TKK_04_0085 2629667363
MSAVALPRVSGGHDEHGHLEEFRTDPIGLMQRVRDECGDVGTFQLAGKQVVLLSGSHANEFFFRAGDDDLDQA
KAYPFMTPIFGEGVVFDASPERRKEMLHNAALRGEQMKGHAATIEDQVRRMIADWGEAGEIDLLDFFAELTIY
TSSACLIGKKFRDQLDGRFAKLYHELERGTDPLAYVDPYLPIESFRRRDEARNGLVALVADIMNGRIANPPTD
KSDRDMLDVLIAVKAETGTPRFSADEITGMFISMMFAGHHTSSGTASWTLIELMRHRDAYAAVIDELDELYGD
GRSVSFHALRQIPQLENVLKETLRLHPPLIILMRVAKGEFEVQGHRIHEGDLVAASPAISNRIPEDFPDPHDF
VPARYEQPRQEDLLNRWTWIPFGAGRHRCVGAAFAIMQIKAIFSVLLREYEFEMAQPPESYRNDHSKMVVQLA
QPACVRYRRRTGV
>CYP51B1_Mycobacterium tuberculosis TKK_04_0086 2632054415
MSAVALPRVSGGHDEHGHLEEFRTDPIGLMQRVRDECGDVGTFQLAGKQVVLLSGSHANEFFFRAGDDDLDQA
KAYPFMTPIFGEGVVFDASPERRKEMLHNAALRGEQMKGHAATIEDQVRRMIADWGEAGEIDLLDFFAELTIY
TSSACLIGKKFRDQLDGRFAKLYHELERGTDPLAYVDPYLPIESFRRRDEARNGLVALVADIMNGRIANPPTD
KSDRDMLDVLIAVKAETGTPRFSADEITGMFISMMFAGHHTSSGTASWTLIELMRHRDAYAAVIDELDELYGD
GRSVSFHALRQIPQLENVLKETLRLHPPLIILMRVAKGEFEVQGHRIHEGDLVAASPAISNRIPEDFPDPHDF

VPARYEQPRQEDLLNRWTWIPFGAGRHRCVGAAFAIMQIKAIFSVLLREYEFEMAQPPESYRNDHSKMVVQLA
QPACVRYRRRTGV
>CYP51B1_Mycobacterium tuberculosis TKK_04_0089 2628941795
MSAVALPRVSGGHDEHGHLEEFRTDPIGLMQRVRDECGDVGTFQLAGKQVVLLSGSHANEFFFRAGDDDLDQA
KAYPFMTPIFGEGVVFDASPERRKEMLHNAALRGEQMKGHAATIEDQVRRMIADWGEAGEIDLLDFFAELTIY
TSSACLIGKKFRDQLDGRFAKLYHELERGTDPLAYVDPYLPIESFRRRDEARNGLVALVADIMNGRIANPPTD
KSDRDMLDVLIAVKAETGTPRFSADEITGMFISMMFAGHHTSSGTASWTLIELMRHRDAYAAVIDELDELYGD
GRSVSFHALRQIPQLENVLKETLRLHPPLIILMRVAKGEFEVQGHRIHEGDLVAASPAISNRIPEDFPDPHDF
VPARYEQPRQEDLLNRWTWIPFGAGRHRCVGAAFAIMQIKAIFSVLLREYEFEMAQPPESYRNDHSKMVVQLA
QPACVRYRRRTGV
>CYP51B1_Mycobacterium tuberculosis TKK_04_0090 2628512543
MSAVALPRVSGGHDEHGHLEEFRTDPIGLMQRVRDECGDVGTFQLAGKQVVLLSGSHANEFFFRAGDDDLDQA
KAYPFMTPIFGEGVVFDASPERRKEMLHNAALRGEQMKGHAATIEDQVRRMIADWGEAGEIDLLDFFAELTIY
TSSACLIGKKFRDQLDGRFAKLYHELERGTDPLAYVDPYLPIESFRRRDEARNGLVALVADIMNGRIANPPTD
KSDRDMLDVLIAVKAETGTPRFSADEITGMFISMMFAGHHTSSGTASWTLIELMRHRDAYAAVIDELDELYGD
GRSVSFHALRQIPQLENVLKETLRLHPPLIILMRVAKGEFEVQGHRIHEGDLVAASPAISNRIPEDFPDPHDF
VPARYEQPRQEDLLNRWTWIPFGAGRHRCVGAAFAIMQIKAIFSVLLREYEFEMAQPPESYRNDHSKMVVQLA
QPACVRYRRRTGV
>CYP51B1_Mycobacterium tuberculosis TKK_04_0094 2584975143
MSAVALPRVSGGHDEHGHLEEFRTDPIGLMQRVRDECGDVGTFQLAGKQVVLLSGSHANEFFFRAGDDDLDQA
KAYPFMTPIFGEGVVFDASPERRKEMLHNAALRGEQMKGHAATIEDQVRRMIADWGEAGEIDLLDFFAELTIY
TSSACLIGKKFRDQLDGRFAKLYHELERGTDPLAYVDPYLPIESFRRRDEARNGLVALVADIMNGRIANPPTD
KSDRDMLDVLIAVKAETGTPRFSADEITGMFISMMFAGHHTSSGTASWTLIELMRHRDAYAAVIDELDELYGD
GRSVSFHALRQIPQLENVLKETLRLHPPLIILMRVAKGEFEVQGHRIHEGDLVAASPAISNRIPEDFPDPHDF
VPARYEQPRQEDLLNRWTWIPFGAGRHRCVGAAFAIMQIKAIFSVLLREYEFEMAQPPESYRNDHSKMVVQLA
QPACVRYRRRTGV
>CYP51B1_Mycobacterium tuberculosis TKK_04_0095 2633730246
MSAVALPRVSGGHDEHGHLEEFRTDPIGLMQRVRDECGDVGTFQLAGKQVVLLSGSHANEFFFRAGDDDLDQA
KAYPFMTPIFGEGVVFDASPERRKEMLHNAALRGEQMKGHAATIEDQVRRMIADWGEAGEIDLLDFFAELTIY
TSSACLIGKKFRDQLDGRFAKLYHELERGTDPLAYVDPYLPIESFRRRDEARNGLVALVADIMNGRIANPPTD
KSDRDMLDVLIAVKAETGTPRFSADEITGMFISMMFAGHHTSSGTASWTLIELMRHRDAYAAVIDELDELYGD
GRSVSFHALRQIPQLENVLKETLRLHPPLIILMRVAKGEFEVQGHRIHEGDLVAASPAISNRIPEDFPDPHDF
VPARYEQPRQEDLLNRWTWIPFGAGRHRCVGAAFAIMQIKAIFSVLLREYEFEMAQPPESYRNDHSKMVVQLA
QPACVRYRRRTGV
>CYP51B1_Mycobacterium tuberculosis TKK_04_0096 2633263785
MSAVALPRVSGGHDEHGHLEEFRTDPIGLMQRVRDECGDVGTFQLAGKQVVLLSGSHANEFFFRAGDDDLDQA
KAYPFMTPIFGEGVVFDASPERRKEMLHNAALRGEQMKGHAATIEDQVRRMIADWGEAGEIDLLDFFAELTIY
TSSACLIGKKFRDQLDGRFAKLYHELERGTDPLAYVDPYLPIESFRRRDEARNGLVALVADIMNGRIANPPTD
KSDRDMLDVLIAVKAETGTPRFSADEITGMFISMMFAGHHTSSGTASWTLIELMRHRDAYAAVIDELDELYGD
GRSVSFHALRQIPQLENVLKETLRLHPPLIILMRVAKGEFEVQGHRIHEGDLVAASPAISNRIPEDFPDPHDF
VPARYEQPRQEDLLNRWTWIPFGAGRHRCVGAAFAIMQIKAIFSVLLREYEFEMAQPPESYRNDHSKMVVQLA
QPACVRYRRRTGV
>CYP51B1_Mycobacterium tuberculosis TKK_04_0097 2629446172
MSAVALPRVSGGHDEHGHLEEFRTDPIGLMQRVRDECGDVGTFQLAGKQVVLLSGSHANEFFFRAGDDDLDQA
KAYPFMTPIFGEGVVFDASPERRKEMLHNAALRGEQMKGHAATIEDQVRRMIADWGEAGEIDLLDFFAELTIY
TSSACLIGKKFRDQLDGRFAKLYHELERGTDPLAYVDPYLPIESFRRRDEARNGLVALVADIMNGRIANPPTD
KSDRDMLDVLIAVKAETGTPRFSADEITGMFISMMFAGHHTSSGTASWTLIELMRHRDAYAAVIDELDELYGD
GRSVSFHALRQIPQLENVLKETLRLHPPLIILMRVAKGEFEVQGHRIHEGDLVAASPAISNRIPEDFPDPHDF
VPARYEQPRQEDLLNRWTWIPFGAGRHRCVGAAFAIMQIKAIFSVLLREYEFEMAQPPESYRNDHSKMVVQLA
QPACVRYRRRTGV
>CYP51B1_Mycobacterium tuberculosis TKK_04_0098 2652555687
MSAVALPRVSGGHDEHGHLEEFRTDPIGLMQRVRDECGDVGTFQLAGKQVVLLSGSHANEFFFRAGDDDLDQA
KAYPFMTPIFGEGVVFDASPERRKEMLHNAALRGEQMKGHAATIEDQVRRMIADWGEAGEIDLLDFFAELTIY
TSSACLIGKKFRDQLDGRFAKLYHELERGTDPLAYVDPYLPIESFRRRDEARNGLVALVADIMNGRIANPPTD
KSDRDMLDVLIAVKAETGTPRFSADEITGMFISMMFAGHHTSSGTASWTLIELMRHRDAYAAVIDELDELYGD
GRSVSFHALRQIPQLENVLKETLRLHPPLIILMRVAKGEFEVQGHRIHEGDLVAASPAISNRIPEDFPDPHDF
VPARYEQPRQEDLLNRWTWIPFGAGRHRCVGAAFAIMQIKAIFSVLLREYEFEMAQPPESYRNDHSKMVVQLA
QPACVRYRRRTGV

>CYP51B1_Mycobacterium tuberculosis TKK_04_0099 2628931647
MSAVALPRVSGGHDEHGHLEEFRTDPIGLMQRVRDECGDVGTFQLAGKQVVLLSGSHANEFFFRAGDDDLDQA
KAYPFMTPIFGEGVVFDASPERRKEMLHNAALRGEQMKGHAATIEDQVRRMIADWGEAGEIDLLDFFAELTIY
TSSACLIGKKFRDQLDGRFAKLYHELERGTDPLAYVDPYLPIESFRRRDEARNGLVALVADIMNGRIANPPTD
KSDRDMLDVLIAVKAETGTPRFSADEITGMFISMMFAGHHTSSGTASWTLIELMRHRDAYAAVIDELDELYGD
GRSVSFHALRQIPQLENVLKETLRLHPPLIILMRVAKGEFEVQGHRIHEGDLVAASPAISNRIPEDFPDPHDF
VPARYEQPRQEDLLNRWTWIPFGAGRHRCVGAAFAIMQIKAIFSVLLREYEFEMAQPPESYRNDHSKMVVQLA
QPACVRYRRRTGV
>CYP51B1_Mycobacterium tuberculosis TKK_04_0103 2576248189
MSAVALPRVSGGHDEHGHLEEFRTDPIGLMQRVRDECGDVGTFQLAGKQVVLLSGSHANEFFFRAGDDDLDQA
KAYPFMTPIFGEGVVFDASPERRKEMLHNAALRGEQMKGHAATIEDQVRRMIADWGEAGEIDLLDFFAELTIY
TSSACLIGKKFRDQLDGRFAKLYHELERGTDPLAYVDPYLPIESFRRRDEARNGLVALVADIMNGRIANPPTD
KSDRDMLDVLIAVKAETGTPRFSADEITGMFISMMFAGHHTSSGTASWTLIELMRHRDAYAAVIDELDELYGD
GRSVSFHALRQIPQLENVLKETLRLHPPLIILMRVAKGEFEVQGHRIHEGDLVAASPAISNRIPEDFPDPHDF
VPARYEQPRQEDLLNRWTWIPFGAGRHRCVGAAFAIMQIKAIFSVLLREYEFEMAQPPESYRNDHSKMVVQLA
QPACVRYRRRTGV
>CYP51B1_Mycobacterium tuberculosis TKK_04_0104 2636779516
MSAVALPRVSGGHDEHGHLEEFRTDPIGLMQRVRDECGDVGTFQLAGKQVVLLSGSHANEFFFRAGDDDLDQA
KAYPFMTPIFGEGVVFDASPERRKEMLHNAALRGEQMKGHAATIEDQVRRMIADWGEAGEIDLLDFFAELTIY
TSSACLIGKKFRDQLDGRFAKLYHELERGTDPLAYVDPYLPIESFRRRDEARNGLVALVADIMNGRIANPPTD
KSDRDMLDVLIAVKAETGTPRFSADEITGMFISMMFAGHHTSSGTASWTLIELMRHRDAYAAVIDELDELYGD
GRSVSFHALRQIPQLENVLKETLRLHPPLIILMRVAKGEFEVQGHRIHEGDLVAASPAISNRIPEDFPDPHDF
VPARYEQPRQEDLLNRWTWIPFGAGRHRCVGAAFAIMQIKAIFSVLLREYEFEMAQPPESYRNDHSKMVVQLA
QPACVRYRRRTGV
>CYP51B1_Mycobacterium tuberculosis TKK_04_0105 2632884303
MSAVALPRVSGGHDEHGHLEEFRTDPIGLMQRVRDECGDVGTFQLAGKQVVLLSGSHANEFFFRAGDDDLDQA
KAYPFMTPIFGEGVVFDASPERRKEMLHNAALRGEQMKGHAATIEDQVRRMIADWGEAGEIDLLDFFAELTIY
TSSACLIGKKFRDQLDGRFAKLYHELERGTDPLAYVDPYLPIESFRRRDEARNGLVALVADIMNGRIANPPTD
KSDRDMLDVLIAVKAETGTPRFSADEITGMFISMMFAGHHTSSGTASWTLIELMRHRDAYAAVIDELDELYGD
GRSVSFHALRQIPQLENVLKETLRLHPPLIILMRVAKGEFEVQGHRIHEGDLVAASPAISNRIPEDFPDPHDF
VPARYEQPRQEDLLNRWTWIPFGAGRHRCVGAAFAIMQIKAIFSVLLREYEFEMAQPPESYRNDHSKMVVQLA
QPACVRYRRRTGV
>CYP51B1_Mycobacterium tuberculosis TKK_04_0106 2640718148
MSAVALPRVSGGHDEHGHLEEFRTDPIGLMQRVRDECGDVGTFQLAGKQVVLLSGSHANEFFFRAGDDDLDQA
KAYPFMTPIFGEGVVFDASPERRKEMLHNAALRGEQMKGHAATIEDQVRRMIADWGEAGEIDLLDFFAELTIY
TSSACLIGKKFRDQLDGRFAKLYHELERGTDPLAYVDPYLPIESFRRRDEARNGLVALVADIMNGRIANPPTD
KSDRDMLDVLIAVKAETGTPRFSADEITGMFISMMFAGHHTSSGTASWTLIELMRHRDAYAAVIDELDELYGD
GRSVSFHALRQIPQLENVLKETLRLHPPLIILMRVAKGEFEVQGHRIHEGDLVAASPAISNRIPEDFPDPHDF
VPARYEQPRQEDLLNRWTWIPFGAGRHRCVGAAFAIMQIKAIFSVLLREYEFEMAQPPESYRNDHSKMVVQLA
QPACVRYRRRTGV
>CYP51B1_Mycobacterium tuberculosis TKK_04_0107 2633919036
MSAVALPRVSGGHDEHGHLEEFRTDPIGLMQRVRDECGDVGTFQLAGKQVVLLSGSHANEFFFRAGDDDLDQA
KAYPFMTPIFGEGVVFDASPERRKEMLHNAALRGEQMKGHAATIEDQVRRMIADWGEAGEIDLLDFFAELTIY
TSSACLIGKKFRDQLDGRFAKLYHELERGTDPLAYVDPYLPIESFRRRDEARNGLVALVADIMNGRIANPPTD
KSDRDMLDVLIAVKAETGTPRFSADEITGMFISMMFAGHHTSSGTASWTLIELMRHRDAYAAVIDELDELYGD
GRSVSFHALRQIPQLENVLKETLRLHPPLIILMRVAKGEFEVQGHRIHEGDLVAASPAISNRIPEDFPDPHDF
VPARYEQPRQEDLLNRWTWIPFGAGRHRCVGAAFAIMQIKAIFSVLLREYEFEMAQPPESYRNDHSKMVVQLA
QPACVRYRRRTGV
>CYP51B1_Mycobacterium tuberculosis TKK_04_0108 2576668557
MSAVALPRVSGGHDEHGHLEEFRTDPIGLMQRVRDECGDVGTFQLAGKQVVLLSGSHANEFFFRAGDDDLDQA
KAYPFMTPIFGEGVVFDASPERRKEMLHNAALRGEQMKGHAATIEDQVRRMIADWGEAGEIDLLDFFAELTIY
TSSACLIGKKFRDQLDGRFAKLYHELERGTDPLAYVDPYLPIESFRRRDEARNGLVALVADIMNGRIANPPTD
KSDRDMLDVLIAVKAETGTPRFSADEITGMFISMMFAGHHTSSGTASWTLIELMRHRDAYAAVIDELDELYGD
GRSVSFHALRQIPQLENVLKETLRLHPPLIILMRVAKGEFEVQGHRIHEGDLVAASPAISNRIPEDFPDPHDF
VPARYEQPRQEDLLNRWTWIPFGAGRHRCVGAAFAIMQIKAIFSVLLREYEFEMAQPPESYRNDHSKMVVQLA
QPACVRYRRRTGV
>CYP51B1_Mycobacterium tuberculosis TKK_04_0109 2628662945

MSAVALPRVSGGHDEHGHLEEFRTDPIGLMQRVRDECGDVGTFQLAGKQVVLLSGSHANEFFFRAGDDDLDQA
KAYPFMTPIFGEGVVFDASPERRKEMLHNAALRGEQMKGHAATIEDQVRRMIADWGEAGEIDLLDFFAELTIY
TSSACLIGKKFRDQLDGRFAKLYHELERGTDPLAYVDPYLPIESFRRRDEARNGLVALVADIMNGRIANPPTD
KSDRDMLDVLIAVKAETGTPRFSADEITGMFISMMFAGHHTSSGTASWTLIELMRHRDAYAAVIDELDELYGD
GRSVSFHALRQIPQLENVLKETLRLHPPLIILMRVAKGEFEVQGHRIHEGDLVAASPAISNRIPEDFPDPHDF
VPARYEQPRQEDLLNRWTWIPFGAGRHRCVGAAFAIMQIKAIFSVLLREYEFEMAQPPESYRNDHSKMVVQLA
QPACVRYRRRTGV
>CYP51B1_Mycobacterium tuberculosis TKK_04_0112 2631499839
MSAVALPRVSGGHDEHGHLEEFRTDPIGLMQRVRDECGDVGTFQLAGKQVVLLSGSHANEFFFRAGDDDLDQA
KAYPFMTPIFGEGVVFDASPERRKEMLHNAALRGEQMKGHAATIEDQVRRMIADWGEAGEIDLLDFFAELTIY
TSSACLIGKKFRDQLDGRFAKLYHELERGTDPLAYVDPYLPIESFRRRDEARNGLVALVADIMNGRIANPPTD
KSDRDMLDVLIAVKAETGTPRFSADEITGMFISMMFAGHHTSSGTASWTLIELMRHRDAYAAVIDELDELYGD
GRSVSFHALRQIPQLENVLKETLRLHPPLIILMRVAKGEFEVQGHRIHEGDLVAASPAISNRIPEDFPDPHDF
VPARYEQPRQEDLLNRWTWIPFGAGRHRCVGAAFAIMQIKAIFSVLLREYEFEMAQPPESYRNDHSKMVVQLA
QPACVRYRRRTGV
>CYP51B1_Mycobacterium tuberculosis TKK_04_0113 2630867451
MSAVALPRVSGGHDEHGHLEEFRTDPIGLMQRVRDECGDVGTFQLAGKQVVLLSGSHANEFFFRAGDDDLDQA
KAYPFMTPIFGEGVVFDASPERRKEMLHNAALRGEQMKGHAATIEDQVRRMIADWGEAGEIDLLDFFAELTIY
TSSACLIGKKFRDQLDGRFAKLYHELERGTDPLAYVDPYLPIESFRRRDEARNGLVALVADIMNGRIANPPTD
KSDRDMLDVLIAVKAETGTPRFSADEITGMFISMMFAGHHTSSGTASWTLIELMRHRDAYAAVIDELDELYGD
GRSVSFHALRQIPQLENVLKETLRLHPPLIILMRVAKGEFEVQGHRIHEGDLVAASPAISNRIPEDFPDPHDF
VPARYEQPRQEDLLNRWTWIPFGAGRHRCVGAAFAIMQIKAIFSVLLREYEFEMAQPPESYRNDHSKMVVQLA
QPACVRYRRRTGV
>CYP51B1_Mycobacterium tuberculosis TKK_04_0114 2641088430
MSAVALPRVSGGHDEHGHLEEFRTDPIGLMQRVRDECGDVGTFQLAGKQVVLLSGSHANEFFFRAGDDDLDQA
KAYPFMTPIFGEGVVFDASPERRKEMLHNAALRGEQMKGHAATIEDQVRRMIADWGEAGEIDLLDFFAELTIY
TSSACLIGKKFRDQLDGRFAKLYHELERGTDPLAYVDPYLPIESFRRRDEARNGLVALVADIMNGRIANPPTD
KSDRDMLDVLIAVKAETGTPRFSADEITGMFISMMFAGHHTSSGTASWTLIELMRHRDAYAAVIDELDELYGD
GRSVSFHALRQIPQLENVLKETLRLHPPLIILMRVAKGEFEVQGHRIHEGDLVAASPAISNRIPEDFPDPHDF
VPARYEQPRQEDLLNRWTWIPFGAGRHRCVGAAFAIMQIKAIFSVLLREYEFEMAQPPESYRNDHSKMVVQLA
QPACVRYRRRTGV
>CYP51B1_Mycobacterium tuberculosis TKK_04_0117 2576667310
MSAVALPRVSGGHDEHGHLEEFRTDPIGLMQRVRDECGDVGTFQLAGKQVVLLSGSHANEFFFRAGDDDLDQA
KAYPFMTPIFGEGVVFDASPERRKEMLHNAALRGEQMKGHAATIEDQVRRMIADWGEAGEIDLLDFFAELTIY
TSSACLIGKKFRDQLDGRFAKLYHELERGTDPLAYVDPYLPIESFRRRDEARNGLVALVADIMNGRIANPPTD
KSDRDMLDVLIAVKAETGTPRFSADEITGMFISMMFAGHHTSSGTASWTLIELMRHRDAYAAVIDELDELYGD
GRSVSFHALRQIPQLENVLKETLRLHPPLIILMRVAKGEFEVQGHRIHEGDLVAASPAISNRIPEDFPDPHDF
VPARYEQPRQEDLLNRWTWIPFGAGRHRCVGAAFAIMQIKAIFSVLLREYEFEMAQPPESYRNDHSKMVVQLA
QPACVRYRRRTGV
>CYP51B1_Mycobacterium tuberculosis TKK_04_0118 2646191814
MSAVALPRVSGGHDEHGHLEEFRTDPIGLMQRVRDECGDVGTFQLAGKQVVLLSGSHANEFFFRAGDDDLDQA
KAYPFMTPIFGEGVVFDASPERRKEMLHNAALRGEQMKGHAATIEDQVRRMIADWGEAGEIDLLDFFAELTIY
TSSACLIGKKFRDQLDGRFAKLYHELERGTDPLAYVDPYLPIESFRRRDEARNGLVALVADIMNGRIANPPTD
KSDRDMLDVLIAVKAETGTPRFSADEITGMFISMMFAGHHTSSGTASWTLIELMRHRDAYAAVIDELDELYGD
GRSVSFHALRQIPQLENVLKETLRLHPPLIILMRVAKGEFEVQGHRIHEGDLVAASPAISNRIPEDFPDPHDF
VPARYEQPRQEDLLNRWTWIPFGAGRHRCVGAAFAIMQIKAIFSVLLREYEFEMAQPPESYRNDHSKMVVQLA
QPACVRYRRRTGV
>CYP51B1_Mycobacterium tuberculosis TKK_04_0120 2574641187
MSAVALPRVSGGHDEHGHLEEFRTDPIGLMQRVRDECGDVGTFQLAGKQVVLLSGSHANEFFFRAGDDDLDQA
KAYPFMTPIFGEGVVFDASPERRKEMLHNAALRGEQMKGHAATIEDQVRRMIADWGEAGEIDLLDFFAELTIY
TSSACLIGKKFRDQLDGRFAKLYHELERGTDPLAYVDPYLPIESFRRRDEARNGLVALVADIMNGRIANPPTD
KSDRDMLDVLIAVKAETGTPRFSADEITGMFISMMFAGHHTSSGTASWTLIELMRHRDAYAAVIDELDELYGD
GRSVSFHALRQIPQLENVLKETLRLHPPLIILMRVAKGEFEVQGHRIHEGDLVAASPAISNRIPEDFPDPHDF
VPARYEQPRQEDLLNRWTWIPFGAGRHRCVGAAFAIMQIKAIFSVLLREYEFEMAQPPESYRNDHSKMVVQLA
QPACVRYRRRTGV
>CYP51B1_Mycobacterium tuberculosis TKK_04_0122 2630308458
MSAVALPRVSGGHDEHGHLEEFRTDPIGLMQRVRDECGDVGTFQLAGKQVVLLSGSHANEFFFRAGDDDLDQA
KAYPFMTPIFGEGVVFDASPERRKEMLHNAALRGEQMKGHAATIEDQVRRMIADWGEAGEIDLLDFFAELTIY

TSSACLIGKKFRDQLDGRFAKLYHELERGTDPLAYVDPYLPIESFRRRDEARNGLVALVADIMNGRIANPPTD
KSDRDMLDVLIAVKAETGTPRFSADEITGMFISMMFAGHHTSSGTASWTLIELMRHRDAYAAVIDELDELYGD
GRSVSFHALRQIPQLENVLKETLRLHPPLIILMRVAKGEFEVQGHRIHEGDLVAASPAISNRIPEDFPDPHDF
VPARYEQPRQEDLLNRWTWIPFGAGRHRCVGAAFAIMQIKAIFSVLLREYEFEMAQPPESYRNDHSKMVVQLA
QPACVRYRRRTGV
>CYP51B1_Mycobacterium tuberculosis TKK_04_0123 2630356207
MSAVALPRVSGGHDEHGHLEEFRTDPIGLMQRVRDECGDVGTFQLAGKQVVLLSGSHANEFFFRAGDDDLDQA
KAYPFMTPIFGEGVVFDASPERRKEMLHNAALRGEQMKGHAATIEDQVRRMIADWGEAGEIDLLDFFAELTIY
TSSACLIGKKFRDQLDGRFAKLYHELERGTDPLAYVDPYLPIESFRRRDEARNGLVALVADIMNGRIANPPTD
KSDRDMLDVLIAVKAETGTPRFSADEITGMFISMMFAGHHTSSGTASWTLIELMRHRDAYAAVIDELDELYGD
GRSVSFHALRQIPQLENVLKETLRLHPPLIILMRVAKGEFEVQGHRIHEGDLVAASPAISNRIPEDFPDPHDF
VPARYEQPRQEDLLNRWTWIPFGAGRHRCVGAAFAIMQIKAIFSVLLREYEFEMAQPPESYRNDHSKMVVQLA
QPACVRYRRRTGV
>CYP51B1_Mycobacterium tuberculosis TKK_04_0124 2656052448
MSAVALPRVSGGHDEHGHLEEFRTDPIGLMQRVRDECGDVGTFQLAGKQVVLLSGSHANEFFFRAGDDDLDQA
KAYPFMTPIFGEGVVFDASPERRKEMLHNAALRGEQMKGHAATIEDQVRRMIADWGEAGEIDLLDFFAELTIY
TSSACLIGKKFRDQLDGRFAKLYHELERGTDPLAYVDPYLPIESFRRRDEARNGLVALVADIMNGRIANPPTD
KSDRDMLDVLIAVKAETGTPRFSADEITGMFISMMFAGHHTSSGTASWTLIELMRHRDAYAAVIDELDELYGD
GRSVSFHALRQIPQLENVLKETLRLHPPLIILMRVAKGEFEVQGHRIHEGDLVAASPAISNRIPEDFPDPHDF
VPARYEQPRQEDLLNRWTWIPFGAGRHRCVGAAFAIMQIKAIFSVLLREYEFEMAQPPESYRNDHSKMVVQLA
QPACVRYRRRTGV
>CYP51B1_Mycobacterium tuberculosis TKK_04_0125 2652673450
MSAVALPRVSGGHDEHGHLEEFRTDPIGLMQRVRDECGDVGTFQLAGKQVVLLSGSHANEFFFRAGDDDLDQA
KAYPFMTPIFGEGVVFDASPERRKEMLHNAALRGEQMKGHAATIEDQVRRMIADWGEAGEIDLLDFFAELTIY
TSSACLIGKKFRDQLDGRFAKLYHELERGTDPLAYVDPYLPIESFRRRDEARNGLVALVADIMNGRIANPPTD
KSDRDMLDVLIAVKAETGTPRFSADEITGMFISMMFAGHHTSSGTASWTLIELMRHRDAYAAVIDELDELYGD
GRSVSFHALRQIPQLENVLKETLRLHPPLIILMRVAKGEFEVQGHRIHEGDLVAASPAISNRIPEDFPDPHDF
VPARYEQPRQEDLLNRWTWIPFGAGRHRCVGAAFAIMQIKAIFSVLLREYEFEMAQPPESYRNDHSKMVVQLA
QPACVRYRRRTGV
>CYP51B1_Mycobacterium tuberculosis TKK_04_0126 2638210594
MSAVALPRVSGGHDEHGHLEEFRTDPIGLMQRVRDECGDVGTFQLAGKQVVLLSGSHANEFFFRAGDDDLDQA
KAYPFMTPIFGEGVVFDASPERRKEMLHNAALRGEQMKGHAATIEDQVRRMIADWGEAGEIDLLDFFAELTIY
TSSACLIGKKFRDQLDGRFAKLYHELERGTDPLAYVDPYLPIESFRRRDEARNGLVALVADIMNGRIANPPTD
KSDRDMLDVLIAVKAETGTPRFSADEITGMFISMMFAGHHTSSGTASWTLIELMRHRDAYAAVIDELDELYGD
GRSVSFHALRQIPQLENVLKETLRLHPPLIILMRVAKGEFEVQGHRIHEGDLVAASPAISNRIPEDFPDPHDF
VPARYEQPRQEDLLNRWTWIPFGAGRHRCVGAAFAIMQIKAIFSVLLREYEFEMAQPPESYRNDHSKMVVQLA
QPACVRYRRRTGV
>CYP51B1_Mycobacterium tuberculosis TKK_04_0129 2584778006
MSAVALPRVSGGHDEHGHLEEFRTDPIGLMQRVRDECGDVGTFQLAGKQVVLLSGSHANEFFFRAGDDDLDQA
KAYPFMTPIFGEGVVFDASPERRKEMLHNAALRGEQMKGHAATIEDQVRRMIADWGEAGEIDLLDFFAELTIY
TSSACLIGKKFRDQLDGRFAKLYHELERGTDPLAYVDPYLPIESFRRRDEARNGLVALVADIMNGRIANPPTD
KSDRDMLDVLIAVKAETGTPRFSADEITGMFISMMFAGHHTSSGTASWTLIELMRHRDAYAAVIDELDELYGD
GRSVSFHALRQIPQLENVLKETLRLHPPLIILMRVAKGEFEVQGHRIHEGDLVAASPAISNRIPEDFPDPHDF
VPARYEQPRQEDLLNRWTWIPFGAGRHRCVGAAFAIMQIKAIFSVLLREYEFEMAQPPESYRNDHSKMVVQLA
QPACVRYRRRTGV
>CYP51B1_Mycobacterium tuberculosis TKK_04_0130 2632002919
MSAVALPRVSGGHDEHGHLEEFRTDPIGLMQRVRDECGDVGTFQLAGKQVVLLSGSHANEFFFRAGDDDLDQA
KAYPFMTPIFGEGVVFDASPERRKEMLHNAALRGEQMKGHAATIEDQVRRMIADWGEAGEIDLLDFFAELTIY
TSSACLIGKKFRDQLDGRFAKLYHELERGTDPLAYVDPYLPIESFRRRDEARNGLVALVADIMNGRIANPPTD
KSDRDMLDVLIAVKAETGTPRFSADEITGMFISMMFAGHHTSSGTASWTLIELMRHRDAYAAVIDELDELYGD
GRSVSFHALRQIPQLENVLKETLRLHPPLIILMRVAKGEFEVQGHRIHEGDLVAASPAISNRIPEDFPDPHDF
VPARYEQPRQEDLLNRWTWIPFGAGRHRCVGAAFAIMQIKAIFSVLLREYEFEMAQPPESYRNDHSKMVVQLA
QPACVRYRRRTGV
>CYP51B1_Mycobacterium tuberculosis TKK_04_0131 2632917120
MSAVALPRVSGGHDEHGHLEEFRTDPIGLMQRVRDECGDVGTFQLAGKQVVLLSGSHANEFFFRAGDDDLDQA
KAYPFMTPIFGEGVVFDASPERRKEMLHNAALRGEQMKGHAATIEDQVRRMIADWGEAGEIDLLDFFAELTIY
TSSACLIGKKFRDQLDGRFAKLYHELERGTDPLAYVDPYLPIESFRRRDEARNGLVALVADIMNGRIANPPTD
KSDRDMLDVLIAVKAETGTPRFSADEITGMFISMMFAGHHTSSGTASWTLIELMRHRDAYAAVIDELDELYGD

GRSVSFHALRQIPQLENVLKETLRLHPPLIILMRVAKGEFEVQGHRIHEGDLVAASPAISNRIPEDFPDPHDF
VPARYEQPRQEDLLNRWTWIPFGAGRHRCVGAAFAIMQIKAIFSVLLREYEFEMAQPPESYRNDHSKMVVQLA
QPACVRYRRRTGV
>CYP51B1_Mycobacterium tuberculosis TKK_04_0132 2577517651
MSAVALPRVSGGHDEHGHLEEFRTDPIGLMQRVRDECGDVGTFQLAGKQVVLLSGSHANEFFFRAGDDDLDQA
KAYPFMTPIFGEGVVFDASPERRKEMLHNAALRGEQMKGHAATIEDQVRRMIADWGEAGEIDLLDFFAELTIY
TSSACLIGKKFRDQLDGRFAKLYHELERGTDPLAYVDPYLPIESFRRRDEARNGLVALVADIMNGRIANPPTD
KSDRDMLDVLIAVKAETGTPRFSADEITGMFISMMFAGHHTSSGTASWTLIELMRHRDAYAAVIDELDELYGD
GRSVSFHALRQIPQLENVLKETLRLHPPLIILMRVAKGEFEVQGHRIHEGDLVAASPAISNRIPEDFPDPHDF
VPARYEQPRQEDLLNRWTWIPFGAGRHRCVGAAFAIMQIKAIFSVLLREYEFEMAQPPESYRNDHSKMVVQLA
QPACVRYRRRTGV
>CYP51B1_Mycobacterium tuberculosis TKK_04_0134 2642226350
MSAVALPRVSGGHDEHGHLEEFRTDPIGLMQRVRDECGDVGTFQLAGKQVVLLSGSHANEFFFRAGDDDLDQA
KAYPFMTPIFGEGVVFDASPERRKEMLHNAALRGEQMKGHAATIEDQVRRMIADWGEAGEIDLLDFFAELTIY
TSSACLIGKKFRDQLDGRFAKLYHELERGTDPLAYVDPYLPIESFRRRDEARNGLVALVADIMNGRIANPPTD
KSDRDMLDVLIAVKAETGTPRFSADEITGMFISMMFAGHHTSSGTASWTLIELMRHRDAYAAVIDELDELYGD
GRSVSFHALRQIPQLENVLKETLRLHPPLIILMRVAKGEFEVQGHRIHEGDLVAASPAISNRIPEDFPDPHDF
VPARYEQPRQEDLLNRWTWIPFGAGRHRCVGAAFAIMQIKAIFSVLLREYEFEMAQPPESYRNDHSKMVVQLA
QPACVRYRRRTGV
>CYP51B1_Mycobacterium tuberculosis TKK_04_0136 2649373836
MSAVALPRVSGGHDEHGHLEEFRTDPIGLMQRVRDECGDVGTFQLAGKQVVLLSGSHANEFFFRAGDDDLDQA
KAYPFMTPIFGEGVVFDASPERRKEMLHNAALRGEQMKGHAATIEDQVRRMIADWGEAGEIDLLDFFAELTIY
TSSACLIGKKFRDQLDGRFAKLYHELERGTDPLAYVDPYLPIESFRRRDEARNGLVALVADIMNGRIANPPTD
KSDRDMLDVLIAVKAETGTPRFSADEITGMFISMMFAGHHTSSGTASWTLIELMRHRDAYAAVIDELDELYGD
GRSVSFHALRQIPQLENVLKETLRLHPPLIILMRVAKGEFEVQGHRIHEGDLVAASPAISNRIPEDFPDPHDF
VPARYEQPRQEDLLNRWTWIPFGAGRHRCVGAAFAIMQIKAIFSVLLREYEFEMAQPPESYRNDHSKMVVQLA
QPACVRYRRRTGV
>CYP51B1_Mycobacterium tuberculosis TKK_04_0137 2621796724
MSAVALPRVSGGHDEHGHLEEFRTDPIGLMQRVRDECGDVGTFQLAGKQVVLLSGSHANEFFFRAGDDDLDQA
KAYPFMTPIFGEGVVFDASPERRKEMLHNAALRGEQMKGHAATIEDQVRRMIADWGEAGEIDLLDFFAELTIY
TSSACLIGKKFRDQLDGRFAKLYHELERGTDPLAYVDPYLPIESFRRRDEARNGLVALVADIMNGRIANPPTD
KSDRDMLDVLIAVKAETGTPRFSADEITGMFISMMFAGHHTSSGTASWTLIELMRHRDAYAAVIDELDELYGD
GRSVSFHALRQIPQLENVLKETLRLHPPLIILMRVAKGEFEVQGHRIHEGDLVAASPAISNRIPEDFPDPHDF
VPARYEQPRQEDLLNRWTWIPFGAGRHRCVGAAFAIMQIKAIFSVLLREYEFEMAQPPESYRNDHSKMVVQLA
QPACVRYRRRTGV
>CYP51B1_Mycobacterium tuberculosis TKK_04_0140 2576494920
MSAVALPRVSGGHDEHGHLEEFRTDPIGLMQRVRDECGDVGTFQLAGKQVVLLSGSHANEFFFRAGDDDLDQA
KAYPFMTPIFGEGVVFDASPERRKEMLHNAALRGEQMKGHAATIEDQVRRMIADWGEAGEIDLLDFFAELTIY
TSSACLIGKKFRDQLDGRFAKLYHELERGTDPLAYVDPYLPIESFRRRDEARNGLVALVADIMNGRIANPPTD
KSDRDMLDVLIAVKAETGTPRFSADEITGMFISMMFAGHHTSSGTASWTLIELMRHRDAYAAVIDELDELYGD
GRSVSFHALRQIPQLENVLKETLRLHPPLIILMRVAKGEFEVQGHRIHEGDLVAASPAISNRIPEDFPDPHDF
VPARYEQPRQEDLLNRWTWIPFGAGRHRCVGAAFAIMQIKAIFSVLLREYEFEMAQPPESYRNDHSKMVVQLA
QPACVRYRRRTGV
>CYP51B1_Mycobacterium tuberculosis TKK_04_0141 2621768780
MSAVALPRVSGGHDEHGHLEEFRTDPIGLMQRVRDECGDVGTFQLAGKQVVLLSGSHANEFFFRAGDDDLDQA
KAYPFMTPIFGEGVVFDASPERRKEMLHNAALRGEQMKGHAATIEDQVRRMIADWGEAGEIDLLDFFAELTIY
TSSACLIGKKFRDQLDGRFAKLYHELERGTDPLAYVDPYLPIESFRRRDEARNGLVALVADIMNGRIANPPTD
KSDRDMLDVLIAVKAETGTPRFSADEITGMFISMMFAGHHTSSGTASWTLIELMRHRDAYAAVIDELDELYGD
GRSVSFHALRQIPQLENVLKETLRLHPPLIILMRVAKGEFEVQGHRIHEGDLVAASPAISNRIPEDFPDPHDF
VPARYEQPRQEDLLNRWTWIPFGAGRHRCVGAAFAIMQIKAIFSVLLREYEFEMAQPPESYRNDHSKMVVQLA
QPACVRYRRRTGV
>CYP51B1_Mycobacterium tuberculosis TKK_04_0145 2621774020
MSAVALPRVSGGHDEHGHLEEFRTDPIGLMQRVRDECGDVGTFQLAGKQVVLLSGSHANEFFFRAGDDDLDQA
KAYPFMTPIFGEGVVFDASPERRKEMLHNAALRGEQMKGHAATIEDQVRRMIADWGEAGEIDLLDFFAELTIY
TSSACLIGKKFRDQLDGRFAKLYHELERGTDPLAYVDPYLPIESFRRRDEARNGLVALVADIMNGRIANPPTD
KSDRDMLDVLIAVKAETGTPRFSADEITGMFISMMFAGHHTSSGTASWTLIELMRHRDAYAAVIDELDELYGD
GRSVSFHALRQIPQLENVLKETLRLHPPLIILMRVAKGEFEVQGHRIHEGDLVAASPAISNRIPEDFPDPHDF

VPARYEQPRQEDLLNRWTWIPFGAGRHRCVGAAFAIMQIKAIFSVLLREYEFEMAQPPESYRNDHSKMVVQLA
QPACVRYRRRTGV
>CYP51B1_Mycobacterium tuberculosis TKK_04_0149 2621781612
MSAVALPRVSGGHDEHGHLEEFRTDPIGLMQRVRDECGDVGTFQLAGKQVVLLSGSHANEFFFRAGDDDLDQA
KAYPFMTPIFGEGVVFDASPERRKEMLHNAALRGEQMKGHAATIEDQVRRMIADWGEAGEIDLLDFFAELTIY
TSSACLIGKKFRDQLDGRFAKLYHELERGTDPLAYVDPYLPIESFRRRDEARNGLVALVADIMNGRIANPPTD
KSDRDMLDVLIAVKAETGTPRFSADEITGMFISMMFAGHHTSSGTASWTLIELMRHRDAYAAVIDELDELYGD
GRSVSFHALRQIPQLENVLKETLRLHPPLIILMRVAKGEFEVQGHRIHEGDLVAASPAISNRIPEDFPDPHDF
VPARYEQPRQEDLLNRWTWIPFGAGRHRCVGAAFAIMQIKAIFSVLLREYEFEMAQPPESYRNDHSKMVVQLA
QPACVRYRRRTGV
>CYP51B1_Mycobacterium tuberculosis TKK_04_0150 2621788619
MSAVALPRVSGGHDEHGHLEEFRTDPIGLMQRVRDECGDVGTFQLAGKQVVLLSGSHANEFFFRAGDDDLDQA
KAYPFMTPIFGEGVVFDASPERRKEMLHNAALRGEQMKGHAATIEDQVRRMIADWGEAGEIDLLDFFAELTIY
TSSACLIGKKFRDQLDGRFAKLYHELERGTDPLAYVDPYLPIESFRRRDEARNGLVALVADIMNGRIANPPTD
KSDRDMLDVLIAVKAETGTPRFSADEITGMFISMMFAGHHTSSGTASWTLIELMRHRDAYAAVIDELDELYGD
GRSVSFHALRQIPQLENVLKETLRLHPPLIILMRVAKGEFEVQGHRIHEGDLVAASPAISNRIPEDFPDPHDF
VPARYEQPRQEDLLNRWTWIPFGAGRHRCVGAAFAIMQIKAIFSVLLREYEFEMAQPPESYRNDHSKMVVQLA
QPACVRYRRRTGV
>CYP51B1_Mycobacterium tuberculosis TKK_04_0153 2621792960
MSAVALPRVSGGHDEHGHLEEFRTDPIGLMQRVRDECGDVGTFQLAGKQVVLLSGSHANEFFFRAGDDDLDQA
KAYPFMTPIFGEGVVFDASPERRKEMLHNAALRGEQMKGHAATIEDQVRRMIADWGEAGEIDLLDFFAELTIY
TSSACLIGKKFRDQLDGRFAKLYHELERGTDPLAYVDPYLPIESFRRRDEARNGLVALVADIMNGRIANPPTD
KSDRDMLDVLIAVKAETGTPRFSADEITGMFISMMFAGHHTSSGTASWTLIELMRHRDAYAAVIDELDELYGD
GRSVSFHALRQIPQLENVLKETLRLHPPLIILMRVAKGEFEVQGHRIHEGDLVAASPAISNRIPEDFPDPHDF
VPARYEQPRQEDLLNRWTWIPFGAGRHRCVGAAFAIMQIKAIFSVLLREYEFEMAQPPESYRNDHSKMVVQLA
QPACVRYRRRTGV
>CYP51B1_Mycobacterium tuberculosis TKK_04_0155 2621800869
MSAVALPRVSGGHDEHGHLEEFRTDPIGLMQRVRDECGDVGTFQLAGKQVVLLSGSHANEFFFRAGDDDLDQA
KAYPFMTPIFGEGVVFDASPERRKEMLHNAALRGEQMKGHAATIEDQVRRMIADWGEAGEIDLLDFFAELTIY
TSSACLIGKKFRDQLDGRFAKLYHELERGTDPLAYVDPYLPIESFRRRDEARNGLVALVADIMNGRIANPPTD
KSDRDMLDVLIAVKAETGTPRFSADEITGMFISMMFAGHHTSSGTASWTLIELMRHRDAYAAVIDELDELYGD
GRSVSFHALRQIPQLENVLKETLRLHPPLIILMRVAKGEFEVQGHRIHEGDLVAASPAISNRIPEDFPDPHDF
VPARYEQPRQEDLLNRWTWIPFGAGRHRCVGAAFAIMQIKAIFSVLLREYEFEMAQPPESYRNDHSKMVVQLA
QPACVRYRRRTGV
>CYP51B1_Mycobacterium tuberculosis TKK_04_0157 2574842306
MSAVALPRVSGGHDEHGHLEEFRTDPIGLMQRVRDECGDVGTFQLAGKQVVLLSGSHANEFFFRAGDDDLDQA
KAYPFMTPIFGEGVVFDASPERRKEMLHNAALRGEQMKGHAATIEDQVRRMIADWGEAGEIDLLDFFAELTIY
TSSACLIGKKFRDQLDGRFAKLYHELERGTDPLAYVDPYLPIESFRRRDEARNGLVALVADIMNGRIANPPTD
KSDRDMLDVLIAVKAETGTPRFSADEITGMFISMMFAGHHTSSGTASWTLIELMRHRDAYAAVIDELDELYGD
GRSVSFHALRQIPQLENVLKETLRLHPPLIILMRVAKGEFEVQGHRIHEGDLVAASPAISNRIPEDFPDPHDF
VPARYEQPRQEDLLNRWTWIPFGAGRHRCVGAAFAIMQIKAIFSVLLREYEFEMAQPPESYRNDHSKMVVQLA
QPACVRYRRRTGV
>CYP51B1_Mycobacterium tuberculosis TKK_04_0158 2631440184
MSAVALPRVSGGHDEHGHLEEFRTDPIGLMQRVRDECGDVGTFQLAGKQVVLLSGSHANEFFFRAGDDDLDQA
KAYPFMTPIFGEGVVFDASPERRKEMLHNAALRGEQMKGHAATIEDQVRRMIADWGEAGEIDLLDFFAELTIY
TSSACLIGKKFRDQLDGRFAKLYHELERGTDPLAYVDPYLPIESFRRRDEARNGLVALVADIMNGRIANPPTD
KSDRDMLDVLIAVKAETGTPRFSADEITGMFISMMFAGHHTSSGTASWTLIELMRHRDAYAAVIDELDELYGD
GRSVSFHALRQIPQLENVLKETLRLHPPLIILMRVAKGEFEVQGHRIHEGDLVAASPAISNRIPEDFPDPHDF
VPARYEQPRQEDLLNRWTWIPFGAGRHRCVGAAFAIMQIKAIFSVLLREYEFEMAQPPESYRNDHSKMVVQLA
QPACVRYRRRTGV
>CYP51B1_Mycobacterium tuberculosis TKK_04_0159 2621802540
MSAVALPRVSGGHDEHGHLEEFRTDPIGLMQRVRDECGDVGTFQLAGKQVVLLSGSHANEFFFRAGDDDLDQA
KAYPFMTPIFGEGVVFDASPERRKEMLHNAALRGEQMKGHAATIEDQVRRMIADWGEAGEIDLLDFFAELTIY
TSSACLIGKKFRDQLDGRFAKLYHELERGTDPLAYVDPYLPIESFRRRDEARNGLVALVADIMNGRIANPPTD
KSDRDMLDVLIAVKAETGTPRFSADEITGMFISMMFAGHHTSSGTASWTLIELMRHRDAYAAVIDELDELYGD
GRSVSFHALRQIPQLENVLKETLRLHPPLIILMRVAKGEFEVQGHRIHEGDLVAASPAISNRIPEDFPDPHDF
VPARYEQPRQEDLLNRWTWIPFGAGRHRCVGAAFAIMQIKAIFSVLLREYEFEMAQPPESYRNDHSKMVVQLA
QPACVRYRRRTGV

>CYP51B1_Mycobacterium tuberculosis TKK_05MA_0001 2621809185
MSAVALPRVSGGHDEHGHLEEFRTDPIGLMQRVRDECGDVGTFQLAGKQVVLLSGSHANEFFFRAGDDDLDQA
KAYPFMTPIFGEGVVFDASPERRKEMLHNAALRGEQMKGHAATIEDQVRRMIADWGEAGEIDLLDFFAELTIY
TSSACLIGKKFRDQLDGRFAKLYHELERGTDPLAYVDPYLPIESFRRRDEARNGLVALVADIMNGRIANPPTD
KSDRDMLDVLIAVKAETGTPRFSADEITGMFISMMFAGHHTSSGTASWTLIELMRHRDAYAAVIDELDELYGD
GRSVSFHALRQIPQLENVLKETLRLHPPLIILMRVAKGEFEVQGHRIHEGDLVAASPAISNRIPEDFPDPHDF
VPARYEQPRQEDLLNRWTWIPFGAGRHRCVGAAFAIMQIKAIFSVLLREYEFEMAQPPESYRNDHSKMVVQLA
QPACVRYRRRTGV
>CYP51B1_Mycobacterium tuberculosis TKK_05MA_0004 2621813285
MSAVALPRVSGGHDEHGHLEEFRTDPIGLMQRVRDECGDVGTFQLAGKQVVLLSGSHANEFFFRAGDDDLDQA
KAYPFMTPIFGEGVVFDASPERRKEMLHNAALRGEQMKGHAATIEDQVRRMIADWGEAGEIDLLDFFAELTIY
TSSACLIGKKFRDQLDGRFAKLYHELERGTDPLAYVDPYLPIESFRRRDEARNGLVALVADIMNGRIANPPTD
KSDRDMLDVLIAVKAETGTPRFSADEITGMFISMMFAGHHTSSGTASWTLIELMRHRDAYAAVIDELDELYGD
GRSVSFHALRQIPQLENVLKETLRLHPPLIILMRVAKGEFEVQGHRIHEGDLVAASPAISNRIPEDFPDPHDF
VPARYEQPRQEDLLNRWTWIPFGAGRHRCVGAAFAIMQIKAIFSVLLREYEFEMAQPPESYRNDHSKMVVQLA
QPACVRYRRRTGV
>CYP51B1_Mycobacterium tuberculosis TKK_05MA_0009 2584786453
MSAVALPRVSGGHDEHGHLEEFRTDPIGLMQRVRDECGDVGTFQLAGKQVVLLSGSHANEFFFRAGDDDLDQA
KAYPFMTPIFGEGVVFDASPERRKEMLHNAALRGEQMKGHAATIEDQVRRMIADWGEAGEIDLLDFFAELTIY
TSSACLIGKKFRDQLDGRFAKLYHELERGTDPLAYVDPYLPIESFRRRDEARNGLVALVADIMNGRIANPPTD
KSDRDMLDVLIAVKAETGTPRFSADEITGMFISMMFAGHHTSSGTASWTLIELMRHRDAYAAVIDELDELYGD
GRSVSFHALRQIPQLENVLKETLRLHPPLIILMRVAKGEFEVQGHRIHEGDLVAASPAISNRIPEDFPDPHDF
VPARYEQPRQEDLLNRWTWIPFGAGRHRCVGAAFAIMQIKAIFSVLLREYEFEMAQPPESYRNDHSKMVVQLA
QPACVRYRRRTGV
>CYP51B1_Mycobacterium tuberculosis TKK_05MA_0010 2621815039
MSAVALPRVSGGHDEHGHLEEFRTDPIGLMQRVRDECGDVGTFQLAGKQVVLLSGSHANEFFFRAGDDDLDQA
KAYPFMTPIFGEGVVFDASPERRKEMLHNAALRGEQMKGHAATIEDQVRRMIADWGEAGEIDLLDFFAELTIY
TSSACLIGKKFRDQLDGRFAKLYHELERGTDPLAYVDPYLPIESFRRRDEARNGLVALVADIMNGRIANPPTD
KSDRDMLDVLIAVKAETGTPRFSADEITGMFISMMFAGHHTSSGTASWTLIELMRHRDAYAAVIDELDELYGD
GRSVSFHALRQIPQLENVLKETLRLHPPLIILMRVAKGEFEVQGHRIHEGDLVAASPAISNRIPEDFPDPHDF
VPARYEQPRQEDLLNRWTWIPFGAGRHRCVGAAFAIMQIKAIFSVLLREYEFEMAQPPESYRNDHSKMVVQLA
QPACVRYRRRTGV
>CYP51B1_Mycobacterium tuberculosis TKK_05MA_0011 2621818527
MSAVALPRVSGGHDEHGHLEEFRTDPIGLMQRVRDECGDVGTFQLAGKQVVLLSGSHANEFFFRAGDDDLDQA
KAYPFMTPIFGEGVVFDASPERRKEMLHNAALRGEQMKGHAATIEDQVRRMIADWGEAGEIDLLDFFAELTIY
TSSACLIGKKFRDQLDGRFAKLYHELERGTDPLAYVDPYLPIESFRRRDEARNGLVALVADIMNGRIANPPTD
KSDRDMLDVLIAVKAETGTPRFSADEITGMFISMMFAGHHTSSGTASWTLIELMRHRDAYAAVIDELDELYGD
GRSVSFHALRQIPQLENVLKETLRLHPPLIILMRVAKGEFEVQGHRIHEGDLVAASPAISNRIPEDFPDPHDF
VPARYEQPRQEDLLNRWTWIPFGAGRHRCVGAAFAIMQIKAIFSVLLREYEFEMAQPPESYRNDHSKMVVQLA
QPACVRYRRRTGV
>CYP51B1_Mycobacterium tuberculosis TKK_05MA_0012 2577217304
MSAVALPRVSGGHDEHGHLEEFRTDPIGLMQRVRDECGDVGTFQLAGKQVVLLSGSHANEFFFRAGDDDLDQA
KAYPFMTPIFGEGVVFDASPERRKEMLHNAALRGEQMKGHAATIEDQVRRMIADWGEAGEIDLLDFFAELTIY
TSSACLIGKKFRDQLDGRFAKLYHELERGTDPLAYVDPYLPIESFRRRDEARNGLVALVADIMNGRIANPPTD
KSDRDMLDVLIAVKAETGTPRFSADEITGMFISMMFAGHHTSSGTASWTLIELMRHRDAYAAVIDELDELYGD
GRSVSFHALRQIPQLENVLKETLRLHPPLIILMRVAKGEFEVQGHRIHEGDLVAASPAISNRIPEDFPDPHDF
VPARYEQPRQEDLLNRWTWIPFGAGRHRCVGAAFAIMQIKAIFSVLLREYEFEMAQPPESYRNDHSKMVVQLA
QPACVRYRRRTGV
>CYP51B1_Mycobacterium tuberculosis TKK_05MA_0016 2621823218
MSAVALPRVSGGHDEHGHLEEFRTDPIGLMQRVRDECGDVGTFQLAGKQVVLLSGSHANEFFFRAGDDDLDQA
KAYPFMTPIFGEGVVFDASPERRKEMLHNAALRGEQMKGHAATIEDQVRRMIADWGEAGEIDLLDFFAELTIY
TSSACLIGKKFRDQLDGRFAKLYHELERGTDPLAYVDPYLPIESFRRRDEARNGLVALVADIMNGRIANPPTD
KSDRDMLDVLIAVKAETGTPRFSADEITGMFISMMFAGHHTSSGTASWTLIELMRHRDAYAAVIDELDELYGD
GRSVSFHALRQIPQLENVLKETLRLHPPLIILMRVAKGEFEVQGHRIHEGDLVAASPAISNRIPEDFPDPHDF
VPARYEQPRQEDLLNRWTWIPFGAGRHRCVGAAFAIMQIKAIFSVLLREYEFEMAQPPESYRNDHSKMVVQLA
QPACVRYRRRTGV
>CYP51B1_Mycobacterium tuberculosis TKK_05MA_0017 2621830054

MSAVALPRVSGGHDEHGHLEEFRTDPIGLMQRVRDECGDVGTFQLAGKQVVLLSGSHANEFFFRAGDDDLDQA
KAYPFMTPIFGEGVVFDASPERRKEMLHNAALRGEQMKGHAATIEDQVRRMIADWGEAGEIDLLDFFAELTIY
TSSACLIGKKFRDQLDGRFAKLYHELERGTDPLAYVDPYLPIESFRRRDEARNGLVALVADIMNGRIANPPTD
KSDRDMLDVLIAVKAETGTPRFSADEITGMFISMMFAGHHTSSGTASWTLIELMRHRDAYAAVIDELDELYGD
GRSVSFHALRQIPQLENVLKETLRLHPPLIILMRVAKGEFEVQGHRIHEGDLVAASPAISNRIPEDFPDPHDF
VPARYEQPRQEDLLNRWTWIPFGAGRHRCVGAAFAIMQIKAIFSVLLREYEFEMAQPPESYRNDHSKMVVQLA
QPACVRYRRRTGV
>CYP51B1_Mycobacterium tuberculosis TKK_05MA_0018 2621831263
MSAVALPRVSGGHDEHGHLEEFRTDPIGLMQRVRDECGDVGTFQLAGKQVVLLSGSHANEFFFRAGDDDLDQA
KAYPFMTPIFGEGVVFDASPERRKEMLHNAALRGEQMKGHAATIEDQVRRMIADWGEAGEIDLLDFFAELTIY
TSSACLIGKKFRDQLDGRFAKLYHELERGTDPLAYVDPYLPIESFRRRDEARNGLVALVADIMNGRIANPPTD
KSDRDMLDVLIAVKAETGTPRFSADEITGMFISMMFAGHHTSSGTASWTLIELMRHRDAYAAVIDELDELYGD
GRSVSFHALRQIPQLENVLKETLRLHPPLIILMRVAKGEFEVQGHRIHEGDLVAASPAISNRIPEDFPDPHDF
VPARYEQPRQEDLLNRWTWIPFGAGRHRCVGAAFAIMQIKAIFSVLLREYEFEMAQPPESYRNDHSKMVVQLA
QPACVRYRRRTGV
>CYP51B1_Mycobacterium tuberculosis TKK_05MA_0020 2574801038
MSAVALPRVSGGHDEHGHLEEFRTDPIGLMQRVRDECGDVGTFQLAGKQVVLLSGSHANEFFFRAGDDDLDQA
KAYPFMTPIFGEGVVFDASPERRKEMLHNAALRGEQMKGHAATIEDQVRRMIADWGEAGEIDLLDFFAELTIY
TSSACLIGKKFRDQLDGRFAKLYHELERGTDPLAYVDPYLPIESFRRRDEARNGLVALVADIMNGRIANPPTD
KSDRDMLDVLIAVKAETGTPRFSADEITGMFISMMFAGHHTSSGTASWTLIELMRHRDAYAAVIDELDELYGD
GRSVSFHALRQIPQLENVLKETLRLHPPLIILMRVAKGEFEVQGHRIHEGDLVAASPAISNRIPEDFPDPHDF
VPARYEQPRQEDLLNRWTWIPFGAGRHRCVGAAFAIMQIKAIFSVLLREYEFEMAQPPESYRNDHSKMVVQLA
QPACVRYRRRTGV
>CYP51B1_Mycobacterium tuberculosis TKK_05MA_0025 2575673109
MSAVALPRVSGGHDEHGHLEEFRTDPIGLMQRVRDECGDVGTFQLAGKQVVLLSGSHANEFFFRAGDDDLDQA
KAYPFMTPIFGEGVVFDASPERRKEMLHNAALRGEQMKGHAATIEDQVRRMIADWGEAGEIDLLDFFAELTIY
TSSACLIGKKFRDQLDGRFAKLYHELERGTDPLAYVDPYLPIESFRRRDEARNGLVALVADIMNGRIANPPTD
KSDRDMLDVLIAVKAETGTPRFSADEITGMFISMMFAGHHTSSGTASWTLIELMRHRDAYAAVIDELDELYGD
GRSVSFHALRQIPQLENVLKETLRLHPPLIILMRVAKGEFEVQGHRIHEGDLVAASPAISNRIPEDFPDPHDF
VPARYEQPRQEDLLNRWTWIPFGAGRHRCVGAAFAIMQIKAIFSVLLREYEFEMAQPPESYRNDHSKMVVQLA
QPACVRYRRRTGV
>CYP51B1_Mycobacterium tuberculosis TKK_05MA_0026 2621838081
MSAVALPRVSGGHDEHGHLEEFRTDPIGLMQRVRDECGDVGTFQLAGKQVVLLSGSHANEFFFRAGDDDLDQA
KAYPFMTPIFGEGVVFDASPERRKEMLHNAALRGEQMKGHAATIEDQVRRMIADWGEAGEIDLLDFFAELTIY
TSSACLIGKKFRDQLDGRFAKLYHELERGTDPLAYVDPYLPIESFRRRDEARNGLVALVADIMNGRIANPPTD
KSDRDMLDVLIAVKAETGTPRFSADEITGMFISMMFAGHHTSSGTASWTLIELMRHRDAYAAVIDELDELYGD
GRSVSFHALRQIPQLENVLKETLRLHPPLIILMRVAKGEFEVQGHRIHEGDLVAASPAISNRIPEDFPDPHDF
VPARYEQPRQEDLLNRWTWIPFGAGRHRCVGAAFAIMQIKAIFSVLLREYEFEMAQPPESYRNDHSKMVVQLA
QPACVRYRRRTGV
>CYP51B1_Mycobacterium tuberculosis TKK_05MA_0031 2621839743
MSAVALPRVSGGHDEHGHLEEFRTDPIGLMQRVRDECGDVGTFQLAGKQVVLLSGSHANEFFFRAGDDDLDQA
KAYPFMTPIFGEGVVFDASPERRKEMLHNAALRGEQMKGHAATIEDQVRRMIADWGEAGEIDLLDFFAELTIY
TSSACLIGKKFRDQLDGRFAKLYHELERGTDPLAYVDPYLPIESFRRRDEARNGLVALVADIMNGRIANPPTD
KSDRDMLDVLIAVKAETGTPRFSADEITGMFISMMFAGHHTSSGTASWTLIELMRHRDAYAAVIDELDELYGD
GRSVSFHALRQIPQLENVLKETLRLHPPLIILMRVAKGEFEVQGHRIHEGDLVAASPAISNRIPEDFPDPHDF
VPARYEQPRQEDLLNRWTWIPFGAGRHRCVGAAFAIMQIKAIFSVLLREYEFEMAQPPESYRNDHSKMVVQLA
QPACVRYRRRTGV
>CYP51B1_Mycobacterium tuberculosis TKK_05MA_0033 2577592176
MSAVALPRVSGGHDEHGHLEEFRTDPIGLMQRVRDECGDVGTFQLAGKQVVLLSGSHANEFFFRAGDDDLDQA
KAYPFMTPIFGEGVVFDASPERRKEMLHNAALRGEQMKGHAATIEDQVRRMIADWGEAGEIDLLDFFAELTIY
TSSACLIGKKFRDQLDGRFAKLYHELERGTDPLAYVDPYLPIESFRRRDEARNGLVALVADIMNGRIANPPTD
KSDRDMLDVLIAVKAETGTPRFSADEITGMFISMMFAGHHTSSGTASWTLIELMRHRDAYAAVIDELDELYGD
GRSVSFHALRQIPQLENVLKETLRLHPPLIILMRVAKGEFEVQGHRIHEGDLVAASPAISNRIPEDFPDPHDF
VPARYEQPRQEDLLNRWTWIPFGAGRHRCVGAAFAIMQIKAIFSVLLREYEFEMAQPPESYRNDHSKMVVQLA
QPACVRYRRRTGV
>CYP51B1_Mycobacterium tuberculosis TKK_05MA_0035 2621846732
MSAVALPRVSGGHDEHGHLEEFRTDPIGLMQRVRDECGDVGTFQLAGKQVVLLSGSHANEFFFRAGDDDLDQA
KAYPFMTPIFGEGVVFDASPERRKEMLHNAALRGEQMKGHAATIEDQVRRMIADWGEAGEIDLLDFFAELTIY

TSSACLIGKKFRDQLDGRFAKLYHELERGTDPLAYVDPYLPIESFRRRDEARNGLVALVADIMNGRIANPPTD
KSDRDMLDVLIAVKAETGTPRFSADEITGMFISMMFAGHHTSSGTASWTLIELMRHRDAYAAVIDELDELYGD
GRSVSFHALRQIPQLENVLKETLRLHPPLIILMRVAKGEFEVQGHRIHEGDLVAASPAISNRIPEDFPDPHDF
VPARYEQPRQEDLLNRWTWIPFGAGRHRCVGAAFAIMQIKAIFSVLLREYEFEMAQPPESYRNDHSKMVVQLA
QPACVRYRRRTGV
>CYP51B1_Mycobacterium tuberculosis TKK_05MA_0036 2621848558
MSAVALPRVSGGHDEHGHLEEFRTDPIGLMQRVRDECGDVGTFQLAGKQVVLLSGSHANEFFFRAGDDDLDQA
KAYPFMTPIFGEGVVFDASPERRKEMLHNAALRGEQMKGHAATIEDQVRRMIADWGEAGEIDLLDFFAELTIY
TSSACLIGKKFRDQLDGRFAKLYHELERGTDPLAYVDPYLPIESFRRRDEARNGLVALVADIMNGRIANPPTD
KSDRDMLDVLIAVKAETGTPRFSADEITGMFISMMFAGHHTSSGTASWTLIELMRHRDAYAAVIDELDELYGD
GRSVSFHALRQIPQLENVLKETLRLHPPLIILMRVAKGEFEVQGHRIHEGDLVAASPAISNRIPEDFPDPHDF
VPARYEQPRQEDLLNRWTWIPFGAGRHRCVGAAFAIMQIKAIFSVLLREYEFEMAQPPESYRNDHSKMVVQLA
QPACVRYRRRTGV
>CYP51B1_Mycobacterium tuberculosis TKK_05MA_0037 2632131920
MSAVALPRVSGGHDEHGHLEEFRTDPIGLMQRVRDECGDVGTFQLAGKQVVLLSGSHANEFFFRAGDDDLDQA
KAYPFMTPIFGEGVVFDASPERRKEMLHNAALRGEQMKGHAATIEDQVRRMIADWGEAGEIDLLDFFAELTIY
TSSACLIGKKFRDQLDGRFAKLYHELERGTDPLAYVDPYLPIESFRRRDEARNGLVALVADIMNGRIANPPTD
KSDRDMLDVLIAVKAETGTPRFSADEITGMFISMMFAGHHTSSGTASWTLIELMRHRDAYAAVIDELDELYGD
GRSVSFHALRQIPQLENVLKETLRLHPPLIILMRVAKGEFEVQGHRIHEGDLVAASPAISNRIPEDFPDPHDF
VPARYEQPRQEDLLNRWTWIPFGAGRHRCVGAAFAIMQIKAIFSVLLREYEFEMAQPPESYRNDHSKMVVQLA
QPACVRYRRRTGV
>CYP51B1_Mycobacterium tuberculosis TKK_05MA_0040 2584750730
MSAVALPRVSGGHDEHGHLEEFRTDPIGLMQRVRDECGDVGTFQLAGKQVVLLSGSHANEFFFRAGDDDLDQA
KAYPFMTPIFGEGVVFDASPERRKEMLHNAALRGEQMKGHAATIEDQVRRMIADWGEAGEIDLLDFFAELTIY
TSSACLIGKKFRDQLDGRFAKLYHELERGTDPLAYVDPYLPIESFRRRDEARNGLVALVADIMNGRIANPPTD
KSDRDMLDVLIAVKAETGTPRFSADEITGMFISMMFAGHHTSSGTASWTLIELMRHRDAYAAVIDELDELYGD
GRSVSFHALRQIPQLENVLKETLRLHPPLIILMRVAKGEFEVQGHRIHEGDLVAASPAISNRIPEDFPDPHDF
VPARYEQPRQEDLLNRWTWIPFGAGRHRCVGAAFAIMQIKAIFSVLLREYEFEMAQPPESYRNDHSKMVVQLA
QPACVRYRRRTGV
>CYP51B1_Mycobacterium tuberculosis TKK_05MA_0044 2621855052
MSAVALPRVSGGHDEHGHLEEFRTDPIGLMQRVRDECGDVGTFQLAGKQVVLLSGSHANEFFFRAGDDDLDQA
KAYPFMTPIFGEGVVFDASPERRKEMLHNAALRGEQMKGHAATIEDQVRRMIADWGEAGEIDLLDFFAELTIY
TSSACLIGKKFRDQLDGRFAKLYHELERGTDPLAYVDPYLPIESFRRRDEARNGLVALVADIMNGRIANPPTD
KSDRDMLDVLIAVKAETGTPRFSADEITGMFISMMFAGHHTSSGTASWTLIELMRHRDAYAAVIDELDELYGD
GRSVSFHALRQIPQLENVLKETLRLHPPLIILMRVAKGEFEVQGHRIHEGDLVAASPAISNRIPEDFPDPHDF
VPARYEQPRQEDLLNRWTWIPFGAGRHRCVGAAFAIMQIKAIFSVLLREYEFEMAQPPESYRNDHSKMVVQLA
QPACVRYRRRTGV
>CYP51B1_Mycobacterium tuberculosis TKK_05MA_0046 2621858093
MSAVALPRVSGGHDEHGHLEEFRTDPIGLMQRVRDECGDVGTFQLAGKQVVLLSGSHANEFFFRAGDDDLDQA
KAYPFMTPIFGEGVVFDASPERRKEMLHNAALRGEQMKGHAATIEDQVRRMIADWGEAGEIDLLDFFAELTIY
TSSACLIGKKFRDQLDGRFAKLYHELERGTDPLAYVDPYLPIESFRRRDEARNGLVALVADIMNGRIANPPTD
KSDRDMLDVLIAVKAETGTPRFSADEITGMFISMMFAGHHTSSGTASWTLIELMRHRDAYAAVIDELDELYGD
GRSVSFHALRQIPQLENVLKETLRLHPPLIILMRVAKGEFEVQGHRIHEGDLVAASPAISNRIPEDFPDPHDF
VPARYEQPRQEDLLNRWTWIPFGAGRHRCVGAAFAIMQIKAIFSVLLREYEFEMAQPPESYRNDHSKMVVQLA
QPACVRYRRRTGV
>CYP51B1_Mycobacterium tuberculosis TKK_05MA_0048 2621859849
MSAVALPRVSGGHDEHGHLEEFRTDPIGLMQRVRDECGDVGTFQLAGKQVVLLSGSHANEFFFRAGDDDLDQA
KAYPFMTPIFGEGVVFDASPERRKEMLHNAALRGEQMKGHAATIEDQVRRMIADWGEAGEIDLLDFFAELTIY
TSSACLIGKKFRDQLDGRFAKLYHELERGTDPLAYVDPYLPIESFRRRDEARNGLVALVADIMNGRIANPPTD
KSDRDMLDVLIAVKAETGTPRFSADEITGMFISMMFAGHHTSSGTASWTLIELMRHRDAYAAVIDELDELYGD
GRSVSFHALRQIPQLENVLKETLRLHPPLIILMRVAKGEFEVQGHRIHEGDLVAASPAISNRIPEDFPDPHDF
VPARYEQPRQEDLLNRWTWIPFGAGRHRCVGAAFAIMQIKAIFSVLLREYEFEMAQPPESYRNDHSKMVVQLA
QPACVRYRRRTGV
>CYP51B1_Mycobacterium tuberculosis TKK_05MA_0051 2621864012
MSAVALPRVSGGHDEHGHLEEFRTDPIGLMQRVRDECGDVGTFQLAGKQVVLLSGSHANEFFFRAGDDDLDQA
KAYPFMTPIFGEGVVFDASPERRKEMLHNAALRGEQMKGHAATIEDQVRRMIADWGEAGEIDLLDFFAELTIY
TSSACLIGKKFRDQLDGRFAKLYHELERGTDPLAYVDPYLPIESFRRRDEARNGLVALVADIMNGRIANPPTD
KSDRDMLDVLIAVKAETGTPRFSADEITGMFISMMFAGHHTSSGTASWTLIELMRHRDAYAAVIDELDELYGD

GRSVSFHALRQIPQLENVLKETLRLHPPLIILMRVAKGEFEVQGHRIHEGDLVAASPAISNRIPEDFPDPHDF
VPARYEQPRQEDLLNRWTWIPFGAGRHRCVGAAFAIMQIKAIFSVLLREYEFEMAQPPESYRNDHSKMVVQLA
QPACVRYRRRTGV
>CYP51B1_Mycobacterium tuberculosis TKK_05MA_0052 2577128607
MSAVALPRVSGGHDEHGHLEEFRTDPIGLMQRVRDECGDVGTFQLAGKQVVLLSGSHANEFFFRAGDDDLDQA
KAYPFMTPIFGEGVVFDASPERRKEMLHNAALRGEQMKGHAATIEDQVRRMIADWGEAGEIDLLDFFAELTIY
TSSACLIGKKFRDQLDGRFAKLYHELERGTDPLAYVDPYLPIESFRRRDEARNGLVALVADIMNGRIANPPTD
KSDRDMLDVLIAVKAETGTPRFSADEITGMFISMMFAGHHTSSGTASWTLIELMRHRDAYAAVIDELDELYGD
GRSVSFHALRQIPQLENVLKETLRLHPPLIILMRVAKGEFEVQGHRIHEGDLVAASPAISNRIPEDFPDPHDF
VPARYEQPRQEDLLNRWTWIPFGAGRHRCVGAAFAIMQIKAIFSVLLREYEFEMAQPPESYRNDHSKMVVQLA
QPACVRYRRRTGV
>CYP51B1_Mycobacterium tuberculosis TKK_05MA_0055 2628475236
MSAVALPRVSGGHDEHGHLEEFRTDPIGLMQRVRDECGDVGTFQLAGKQVVLLSGSHANEFFFRAGDDDLDQA
KAYPFMTPIFGEGVVFDASPERRKEMLHNAALRGEQMKGHAATIEDQVRRMIADWGEAGEIDLLDFFAELTIY
TSSACLIGKKFRDQLDGRFAKLYHELERGTDPLAYVDPYLPIESFRRRDEARNGLVALVADIMNGRIANPPTD
KSDRDMLDVLIAVKAETGTPRFSADEITGMFISMMFAGHHTSSGTASWTLIELMRHRDAYAAVIDELDELYGD
GRSVSFHALRQIPQLENVLKETLRLHPPLIILMRVAKGEFEVQGHRIHEGDLVAASPAISNRIPEDFPDPHDF
VPARYEQPRQEDLLNRWTWIPFGAGRHRCVGAAFAIMQIKAIFSVLLREYEFEMAQPPESYRNDHSKMVVQLA
QPACVRYRRRTGV
>CYP51B1_Mycobacterium tuberculosis TKK_05MA_0056 2621871400
MSAVALPRVSGGHDEHGHLEEFRTDPIGLMQRVRDECGDVGTFQLAGKQVVLLSGSHANEFFFRAGDDDLDQA
KAYPFMTPIFGEGVVFDASPERRKEMLHNAALRGEQMKGHAATIEDQVRRMIADWGEAGEIDLLDFFAELTIY
TSSACLIGKKFRDQLDGRFAKLYHELERGTDPLAYVDPYLPIESFRRRDEARNGLVALVADIMNGRIANPPTD
KSDRDMLDVLIAVKAETGTPRFSADEITGMFISMMFAGHHTSSGTASWTLIELMRHRDAYAAVIDELDELYGD
GRSVSFHALRQIPQLENVLKETLRLHPPLIILMRVAKGEFEVQGHRIHEGDLVAASPAISNRIPEDFPDPHDF
VPARYEQPRQEDLLNRWTWIPFGAGRHRCVGAAFAIMQIKAIFSVLLREYEFEMAQPPESYRNDHSKMVVQLA
QPACVRYRRRTGV
>CYP51B1_Mycobacterium tuberculosis TKK_05MA_0058 2621873356
MSAVALPRVSGGHDEHGHLEEFRTDPIGLMQRVRDECGDVGTFQLAGKQVVLLSGSHANEFFFRAGDDDLDQA
KAYPFMTPIFGEGVVFDASPERRKEMLHNAALRGEQMKGHAATIEDQVRRMIADWGEAGEIDLLDFFAELTIY
TSSACLIGKKFRDQLDGRFAKLYHELERGTDPLAYVDPYLPIESFRRRDEARNGLVALVADIMNGRIANPPTD
KSDRDMLDVLIAVKAETGTPRFSADEITGMFISMMFAGHHTSSGTASWTLIELMRHRDAYAAVIDELDELYGD
GRSVSFHALRQIPQLENVLKETLRLHPPLIILMRVAKGEFEVQGHRIHEGDLVAASPAISNRIPEDFPDPHDF
VPARYEQPRQEDLLNRWTWIPFGAGRHRCVGAAFAIMQIKAIFSVLLREYEFEMAQPPESYRNDHSKMVVQLA
QPACVRYRRRTGV
>CYP51B1_Mycobacterium tuberculosis TKK_05MA_2005 2621879449
MSAVALPRVSGGHDEHGHLEEFRTDPIGLMQRVRDECGDVGTFQLAGKQVVLLSGSHANEFFFRAGDDDLDQA
KAYPFMTPIFGEGVVFDASPERRKEMLHNAALRGEQMKGHAATIEDQVRRMIADWGEAGEIDLLDFFAELTIY
TSSACLIGKKFRDQLDGRFAKLYHELERGTDPLAYVDPYLPIESFRRRDEARNGLVALVADIMNGRIANPPTD
KSDRDMLDVLIAVKAETGTPRFSADEITGMFISMMFAGHHTSSGTASWTLIELMRHRDAYAAVIDELDELYGD
GRSVSFHALRQIPQLENVLKETLRLHPPLIILMRVAKGEFEVQGHRIHEGDLVAASPAISNRIPEDFPDPHDF
VPARYEQPRQEDLLNRWTWIPFGAGRHRCVGAAFAIMQIKAIFSVLLREYEFEMAQPPESYRNDHSKMVVQLA
QPACVRYRRRTGV
>CYP51B1_Mycobacterium tuberculosis TKK_05MA_2008 2621883606
MSAVALPRVSGGHDEHGHLEEFRTDPIGLMQRVRDECGDVGTFQLAGKQVVLLSGSHANEFFFRAGDDDLDQA
KAYPFMTPIFGEGVVFDASPERRKEMLHNAALRGEQMKGHAATIEDQVRRMIADWGEAGEIDLLDFFAELTIY
TSSACLIGKKFRDQLDGRFAKLYHELERGTDPLAYVDPYLPIESFRRRDEARNGLVALVADIMNGRIANPPTD
KSDRDMLDVLIAVKAETGTPRFSADEITGMFISMMFAGHHTSSGTASWTLIELMRHRDAYAAVIDELDELYGD
GRSVSFHALRQIPQLENVLKETLRLHPPLIILMRVAKGEFEVQGHRIHEGDLVAASPAISNRIPEDFPDPHDF
VPARYEQPRQEDLLNRWTWIPFGAGRHRCVGAAFAIMQIKAIFSVLLREYEFEMAQPPESYRNDHSKMVVQLA
QPACVRYRRRTGV
>CYP51B1_Mycobacterium tuberculosis TKK_05MA_2015 2576573643
MSAVALPRVSGGHDEHGHLEEFRTDPIGLMQRVRDECGDVGTFQLAGKQVVLLSGSHANEFFFRAGDDDLDQA
KAYPFMTPIFGEGVVFDASPERRKEMLHNAALRGEQMKGHAATIEDQVRRMIADWGEAGEIDLLDFFAELTIY
TSSACLIGKKFRDQLDGRFAKLYHELERGTDPLAYVDPYLPIESFRRRDEARNGLVALVADIMNGRIANPPTD
KSDRDMLDVLIAVKAETGTPRFSADEITGMFISMMFAGHHTSSGTASWTLIELMRHRDAYAAVIDELDELYGD
GRSVSFHALRQIPQLENVLKETLRLHPPLIILMRVAKGEFEVQGHRIHEGDLVAASPAISNRIPEDFPDPHDF

VPARYEQPRQEDLLNRWTWIPFGAGRHRCVGAAFAIMQIKAIFSVLLREYEFEMAQPPESYRNDHSKMVVQLA
QPACVRYRRRTGV
>CYP51B1_Mycobacterium tuberculosis TKK_05SA_0010 2621885496
MSAVALPRVSGGHDEHGHLEEFRTDPIGLMQRVRDECGDVGTFQLAGKQVVLLSGSHANEFFFRAGDDDLDQA
KAYPFMTPIFGEGVVFDASPERRKEMLHNAALRGEQMKGHAATIEDQVRRMIADWGEAGEIDLLDFFAELTIY
TSSACLIGKKFRDQLDGRFAKLYHELERGTDPLAYVDPYLPIESFRRRDEARNGLVALVADIMNGRIANPPTD
KSDRDMLDVLIAVKAETGTPRFSADEITGMFISMMFAGHHTSSGTASWTLIELMRHRDAYAAVIDELDELYGD
GRSVSFHALRQIPQLENVLKETLRLHPPLIILMRVAKGEFEVQGHRIHEGDLVAASPAISNRIPEDFPDPHDF
VPARYEQPRQEDLLNRWTWIPFGAGRHRCVGAAFAIMQIKAIFSVLLREYEFEMAQPPESYRNDHSKMVVQLA
QPACVRYRRRTGV
>CYP51B1_Mycobacterium tuberculosis TKK_05SA_0011 2621892615
MSAVALPRVSGGHDEHGHLEEFRTDPIGLMQRVRDECGDVGTFQLAGKQVVLLSGSHANEFFFRAGDDDLDQA
KAYPFMTPIFGEGVVFDASPERRKEMLHNAALRGEQMKGHAATIEDQVRRMIADWGEAGEIDLLDFFAELTIY
TSSACLIGKKFRDQLDGRFAKLYHELERGTDPLAYVDPYLPIESFRRRDEARNGLVALVADIMNGRIANPPTD
KSDRDMLDVLIAVKAETGTPRFSADEITGMFISMMFAGHHTSSGTASWTLIELMRHRDAYAAVIDELDELYGD
GRSVSFHALRQIPQLENVLKETLRLHPPLIILMRVAKGEFEVQGHRIHEGDLVAASPAISNRIPEDFPDPHDF
VPARYEQPRQEDLLNRWTWIPFGAGRHRCVGAAFAIMQIKAIFSVLLREYEFEMAQPPESYRNDHSKMVVQLA
QPACVRYRRRTGV
>CYP51B1_Mycobacterium tuberculosis TKK_05SA_0012 2575599868
MSAVALPRVSGGHDEHGHLEEFRTDPIGLMQRVRDECGDVGTFQLAGKQVVLLSGSHANEFFFRAGDDDLDQA
KAYPFMTPIFGEGVVFDASPERRKEMLHNAALRGEQMKGHAATIEDQVRRMIADWGEAGEIDLLDFFAELTIY
TSSACLIGKKFRDQLDGRFAKLYHELERGTDPLAYVDPYLPIESFRRRDEARNGLVALVADIMNGRIANPPTD
KSDRDMLDVLIAVKAETGTPRFSADEITGMFISMMFAGHHTSSGTASWTLIELMRHRDAYAAVIDELDELYGD
GRSVSFHALRQIPQLENVLKETLRLHPPLIILMRVAKGEFEVQGHRIHEGDLVAASPAISNRIPEDFPDPHDF
VPARYEQPRQEDLLNRWTWIPFGAGRHRCVGAAFAIMQIKAIFSVLLREYEFEMAQPPESYRNDHSKMVVQLA
QPACVRYRRRTGV
>CYP51B1_Mycobacterium tuberculosis TKK_05SA_0014 2577856532
MSAVALPRVSGGHDEHGHLEEFRTDPIGLMQRVRDECGDVGTFQLAGKQVVLLSGSHANEFFFRAGDDDLDQA
KAYPFMTPIFGEGVVFDASPERRKEMLHNAALRGEQMKGHAATIEDQVRRMIADWGEAGEIDLLDFFAELTIY
TSSACLIGKKFRDQLDGRFAKLYHELERGTDPLAYVDPYLPIESFRRRDEARNGLVALVADIMNGRIANPPTD
KSDRDMLDVLIAVKAETGTPRFSADEITGMFISMMFAGHHTSSGTASWTLIELMRHRDAYAAVIDELDELYGD
GRSVSFHALRQIPQLENVLKETLRLHPPLIILMRVAKGEFEVQGHRIHEGDLVAASPAISNRIPEDFPDPHDF
VPARYEQPRQEDLLNRWTWIPFGAGRHRCVGAAFAIMQIKAIFSVLLREYEFEMAQPPESYRNDHSKMVVQLA
QPACVRYRRRTGV
>CYP51B1_Mycobacterium tuberculosis TKK_05SA_0016 2574983791
MSAVALPRVSGGHDEHGHLEEFRTDPIGLMQRVRDECGDVGTFQLAGKQVVLLSGSHANEFFFRAGDDDLDQA
KAYPFMTPIFGEGVVFDASPERRKEMLHNAALRGEQMKGHAATIEDQVRRMIADWGEAGEIDLLDFFAELTIY
TSSACLIGKKFRDQLDGRFAKLYHELERGTDPLAYVDPYLPIESFRRRDEARNGLVALVADIMNGRIANPPTD
KSDRDMLDVLIAVKAETGTPRFSADEITGMFISMMFAGHHTSSGTASWTLIELMRHRDAYAAVIDELDELYGD
GRSVSFHALRQIPQLENVLKETLRLHPPLIILMRVAKGEFEVQGHRIHEGDLVAASPAISNRIPEDFPDPHDF
VPARYEQPRQEDLLNRWTWIPFGAGRHRCVGAAFAIMQIKAIFSVLLREYEFEMAQPPESYRNDHSKMVVQLA
QPACVRYRRRTGV
>CYP51B1_Mycobacterium tuberculosis TKK_05SA_0017 2578039586
MSAVALPRVSGGHDEHGHLEEFRTDPIGLMQRVRDECGDVGTFQLAGKQVVLLSGSHANEFFFRAGDDDLDQA
KAYPFMTPIFGEGVVFDASPERRKEMLHNAALRGEQMKGHAATIEDQVRRMIADWGEAGEIDLLDFFAELTIY
TSSACLIGKKFRDQLDGRFAKLYHELERGTDPLAYVDPYLPIESFRRRDEARNGLVALVADIMNGRIANPPTD
KSDRDMLDVLIAVKAETGTPRFSADEITGMFISMMFAGHHTSSGTASWTLIELMRHRDAYAAVIDELDELYGD
GRSVSFHALRQIPQLENVLKETLRLHPPLIILMRVAKGEFEVQGHRIHEGDLVAASPAISNRIPEDFPDPHDF
VPARYEQPRQEDLLNRWTWIPFGAGRHRCVGAAFAIMQIKAIFSVLLREYEFEMAQPPESYRNDHSKMVVQLA
QPACVRYRRRTGV
>CYP51B1_Mycobacterium tuberculosis TKK_05SA_0018 2621895111
MSAVALPRVSGGHDEHGHLEEFRTDPIGLMQRVRDECGDVGTFQLAGKQVVLLSGSHANEFFFRAGDDDLDQA
KAYPFMTPIFGEGVVFDASPERRKEMLHNAALRGEQMKGHAATIEDQVRRMIADWGEAGEIDLLDFFAELTIY
TSSACLIGKKFRDQLDGRFAKLYHELERGTDPLAYVDPYLPIESFRRRDEARNGLVALVADIMNGRIANPPTD
KSDRDMLDVLIAVKAETGTPRFSADEITGMFISMMFAGHHTSSGTASWTLIELMRHRDAYAAVIDELDELYGD
GRSVSFHALRQIPQLENVLKETLRLHPPLIILMRVAKGEFEVQGHRIHEGDLVAASPAISNRIPEDFPDPHDF
VPARYEQPRQEDLLNRWTWIPFGAGRHRCVGAAFAIMQIKAIFSVLLREYEFEMAQPPESYRNDHSKMVVQLA
QPACVRYRRRTGV

>CYP51B1_Mycobacterium tuberculosis TKK_05SA_0019 2577033173
MSAVALPRVSGGHDEHGHLEEFRTDPIGLMQRVRDECGDVGTFQLAGKQVVLLSGSHANEFFFRAGDDDLDQA
KAYPFMTPIFGEGVVFDASPERRKEMLHNAALRGEQMKGHAATIEDQVRRMIADWGEAGEIDLLDFFAELTIY
TSSACLIGKKFRDQLDGRFAKLYHELERGTDPLAYVDPYLPIESFRRRDEARNGLVALVADIMNGRIANPPTD
KSDRDMLDVLIAVKAETGTPRFSADEITGMFISMMFAGHHTSSGTASWTLIELMRHRDAYAAVIDELDELYGD
GRSVSFHALRQIPQLENVLKETLRLHPPLIILMRVAKGEFEVQGHRIHEGDLVAASPAISNRIPEDFPDPHDF
VPARYEQPRQEDLLNRWTWIPFGAGRHRCVGAAFAIMQIKAIFSVLLREYEFEMAQPPESYRNDHSKMVVQLA
QPACVRYRRRTGV
>CYP51B1_Mycobacterium tuberculosis TKK_05SA_0020 2584782415
MSAVALPRVSGGHDEHGHLEEFRTDPIGLMQRVRDECGDVGTFQLAGKQVVLLSGSHANEFFFRAGDDDLDQA
KAYPFMTPIFGEGVVFDASPERRKEMLHNAALRGEQMKGHAATIEDQVRRMIADWGEAGEIDLLDFFAELTIY
TSSACLIGKKFRDQLDGRFAKLYHELERGTDPLAYVDPYLPIESFRRRDEARNGLVALVADIMNGRIANPPTD
KSDRDMLDVLIAVKAETGTPRFSADEITGMFISMMFAGHHTSSGTASWTLIELMRHRDAYAAVIDELDELYGD
GRSVSFHALRQIPQLENVLKETLRLHPPLIILMRVAKGEFEVQGHRIHEGDLVAASPAISNRIPEDFPDPHDF
VPARYEQPRQEDLLNRWTWIPFGAGRHRCVGAAFAIMQIKAIFSVLLREYEFEMAQPPESYRNDHSKMVVQLA
QPACVRYRRRTGV
>CYP51B1_Mycobacterium tuberculosis TKK_05SA_0021 2621897422
MSAVALPRVSGGHDEHGHLEEFRTDPIGLMQRVRDECGDVGTFQLAGKQVVLLSGSHANEFFFRAGDDDLDQA
KAYPFMTPIFGEGVVFDASPERRKEMLHNAALRGEQMKGHAATIEDQVRRMIADWGEAGEIDLLDFFAELTIY
TSSACLIGKKFRDQLDGRFAKLYHELERGTDPLAYVDPYLPIESFRRRDEARNGLVALVADIMNGRIANPPTD
KSDRDMLDVLIAVKAETGTPRFSADEITGMFISMMFAGHHTSSGTASWTLIELMRHRDAYAAVIDELDELYGD
GRSVSFHALRQIPQLENVLKETLRLHPPLIILMRVAKGEFEVQGHRIHEGDLVAASPAISNRIPEDFPDPHDF
VPARYEQPRQEDLLNRWTWIPFGAGRHRCVGAAFAIMQIKAIFSVLLREYEFEMAQPPESYRNDHSKMVVQLA
QPACVRYRRRTGV
>CYP51B1_Mycobacterium tuberculosis TKK_05SA_0023 2621901499
MSAVALPRVSGGHDEHGHLEEFRTDPIGLMQRVRDECGDVGTFQLAGKQVVLLSGSHANEFFFRAGDDDLDQA
KAYPFMTPIFGEGVVFDASPERRKEMLHNAALRGEQMKGHAATIEDQVRRMIADWGEAGEIDLLDFFAELTIY
TSSACLIGKKFRDQLDGRFAKLYHELERGTDPLAYVDPYLPIESFRRRDEARNGLVALVADIMNGRIANPPTD
KSDRDMLDVLIAVKAETGTPRFSADEITGMFISMMFAGHHTSSGTASWTLIELMRHRDAYAAVIDELDELYGD
GRSVSFHALRQIPQLENVLKETLRLHPPLIILMRVAKGEFEVQGHRIHEGDLVAASPAISNRIPEDFPDPHDF
VPARYEQPRQEDLLNRWTWIPFGAGRHRCVGAAFAIMQIKAIFSVLLREYEFEMAQPPESYRNDHSKMVVQLA
QPACVRYRRRTGV
>CYP51B1_Mycobacterium tuberculosis TKK_05SA_0024 2630456383
MSAVALPRVSGGHDEHGHLEEFRTDPIGLMQRVRDECGDVGTFQLAGKQVVLLSGSHANEFFFRAGDDDLDQA
KAYPFMTPIFGEGVVFDASPERRKEMLHNAALRGEQMKGHAATIEDQVRRMIADWGEAGEIDLLDFFAELTIY
TSSACLIGKKFRDQLDGRFAKLYHELERGTDPLAYVDPYLPIESFRRRDEARNGLVALVADIMNGRIANPPTD
KSDRDMLDVLIAVKAETGTPRFSADEITGMFISMMFAGHHTSSGTASWTLIELMRHRDAYAAVIDELDELYGD
GRSVSFHALRQIPQLENVLKETLRLHPPLIILMRVAKGEFEVQGHRIHEGDLVAASPAISNRIPEDFPDPHDF
VPARYEQPRQEDLLNRWTWIPFGAGRHRCVGAAFAIMQIKAIFSVLLREYEFEMAQPPESYRNDHSKMVVQLA
QPACVRYRRRTGV
>CYP51B1_Mycobacterium tuberculosis TKK_05SA_0025 2621906178
MSAVALPRVSGGHDEHGHLEEFRTDPIGLMQRVRDECGDVGTFQLAGKQVVLLSGSHANEFFFRAGDDDLDQA
KAYPFMTPIFGEGVVFDASPERRKEMLHNAALRGEQMKGHAATIEDQVRRMIADWGEAGEIDLLDFFAELTIY
TSSACLIGKKFRDQLDGRFAKLYHELERGTDPLAYVDPYLPIESFRRRDEARNGLVALVADIMNGRIANPPTD
KSDRDMLDVLIAVKAETGTPRFSADEITGMFISMMFAGHHTSSGTASWTLIELMRHRDAYAAVIDELDELYGD
GRSVSFHALRQIPQLENVLKETLRLHPPLIILMRVAKGEFEVQGHRIHEGDLVAASPAISNRIPEDFPDPHDF
VPARYEQPRQEDLLNRWTWIPFGAGRHRCVGAAFAIMQIKAIFSVLLREYEFEMAQPPESYRNDHSKMVVQLA
QPACVRYRRRTGV
>CYP51B1_Mycobacterium tuberculosis TKK_05SA_0026 2621909555
MSAVALPRVSGGHDEHGHLEEFRTDPIGLMQRVRDECGDVGTFQLAGKQVVLLSGSHANEFFFRAGDDDLDQA
KAYPFMTPIFGEGVVFDASPERRKEMLHNAALRGEQMKGHAATIEDQVRRMIADWGEAGEIDLLDFFAELTIY
TSSACLIGKKFRDQLDGRFAKLYHELERGTDPLAYVDPYLPIESFRRRDEARNGLVALVADIMNGRIANPPTD
KSDRDMLDVLIAVKAETGTPRFSADEITGMFISMMFAGHHTSSGTASWTLIELMRHRDAYAAVIDELDELYGD
GRSVSFHALRQIPQLENVLKETLRLHPPLIILMRVAKGEFEVQGHRIHEGDLVAASPAISNRIPEDFPDPHDF
VPARYEQPRQEDLLNRWTWIPFGAGRHRCVGAAFAIMQIKAIFSVLLREYEFEMAQPPESYRNDHSKMVVQLA
QPACVRYRRRTGV
>CYP51B1_Mycobacterium tuberculosis TKK_05SA_0031 2621916831

MSAVALPRVSGGHDEHGHLEEFRTDPIGLMQRVRDECGDVGTFQLAGKQVVLLSGSHANEFFFRAGDDDLDQA
KAYPFMTPIFGEGVVFDASPERRKEMLHNAALRGEQMKGHAATIEDQVRRMIADWGEAGEIDLLDFFAELTIY
TSSACLIGKKFRDQLDGRFAKLYHELERGTDPLAYVDPYLPIESFRRRDEARNGLVALVADIMNGRIANPPTD
KSDRDMLDVLIAVKAETGTPRFSADEITGMFISMMFAGHHTSSGTASWTLIELMRHRDAYAAVIDELDELYGD
GRSVSFHALRQIPQLENVLKETLRLHPPLIILMRVAKGEFEVQGHRIHEGDLVAASPAISNRIPEDFPDPHDF
VPARYEQPRQEDLLNRWTWIPFGAGRHRCVGAAFAIMQIKAIFSVLLREYEFEMAQPPESYRNDHSKMVVQLA
QPACVRYRRRTGV
>CYP51B1_Mycobacterium tuberculosis TKK_05SA_0036 2621919984
MSAVALPRVSGGHDEHGHLEEFRTDPIGLMQRVRDECGDVGTFQLAGKQVVLLSGSHANEFFFRAGDDDLDQA
KAYPFMTPIFGEGVVFDASPERRKEMLHNAALRGEQMKGHAATIEDQVRRMIADWGEAGEIDLLDFFAELTIY
TSSACLIGKKFRDQLDGRFAKLYHELERGTDPLAYVDPYLPIESFRRRDEARNGLVALVADIMNGRIANPPTD
KSDRDMLDVLIAVKAETGTPRFSADEITGMFISMMFAGHHTSSGTASWTLIELMRHRDAYAAVIDELDELYGD
GRSVSFHALRQIPQLENVLKETLRLHPPLIILMRVAKGEFEVQGHRIHEGDLVAASPAISNRIPEDFPDPHDF
VPARYEQPRQEDLLNRWTWIPFGAGRHRCVGAAFAIMQIKAIFSVLLREYEFEMAQPPESYRNDHSKMVVQLA
QPACVRYRRRTGV
>CYP51B1_Mycobacterium tuberculosis TKK_05SA_0041 2621925360
MSAVALPRVSGGHDEHGHLEEFRTDPIGLMQRVRDECGDVGTFQLAGKQVVLLSGSHANEFFFRAGDDDLDQA
KAYPFMTPIFGEGVVFDASPERRKEMLHNAALRGEQMKGHAATIEDQVRRMIADWGEAGEIDLLDFFAELTIY
TSSACLIGKKFRDQLDGRFAKLYHELERGTDPLAYVDPYLPIESFRRRDEARNGLVALVADIMNGRIANPPTD
KSDRDMLDVLIAVKAETGTPRFSADEITGMFISMMFAGHHTSSGTASWTLIELMRHRDAYAAVIDELDELYGD
GRSVSFHALRQIPQLENVLKETLRLHPPLIILMRVAKGEFEVQGHRIHEGDLVAASPAISNRIPEDFPDPHDF
VPARYEQPRQEDLLNRWTWIPFGAGRHRCVGAAFAIMQIKAIFSVLLREYEFEMAQPPESYRNDHSKMVVQLA
QPACVRYRRRTGV
>CYP51B1_Mycobacterium tuberculosis TKK_05SA_0042 2584862843
MSAVALPRVSGGHDEHGHLEEFRTDPIGLMQRVRDECGDVGTFQLAGKQVVLLSGSHANEFFFRAGDDDLDQA
KAYPFMTPIFGEGVVFDASPERRKEMLHNAALRGEQMKGHAATIEDQVRRMIADWGEAGEIDLLDFFAELTIY
TSSACLIGKKFRDQLDGRFAKLYHELERGTDPLAYVDPYLPIESFRRRDEARNGLVALVADIMNGRIANPPTD
KSDRDMLDVLIAVKAETGTPRFSADEITGMFISMMFAGHHTSSGTASWTLIELMRHRDAYAAVIDELDELYGD
GRSVSFHALRQIPQLENVLKETLRLHPPLIILMRVAKGEFEVQGHRIHEGDLVAASPAISNRIPEDFPDPHDF
VPARYEQPRQEDLLNRWTWIPFGAGRHRCVGAAFAIMQIKAIFSVLLREYEFEMAQPPESYRNDHSKMVVQLA
QPACVRYRRRTGV
>CYP51B1_Mycobacterium tuberculosis TKK_05SA_0043 2621929168
MSAVALPRVSGGHDEHGHLEEFRTDPIGLMQRVRDECGDVGTFQLAGKQVVLLSGSHANEFFFRAGDDDLDQA
KAYPFMTPIFGEGVVFDASPERRKEMLHNAALRGEQMKGHAATIEDQVRRMIADWGEAGEIDLLDFFAELTIY
TSSACLIGKKFRDQLDGRFAKLYHELERGTDPLAYVDPYLPIESFRRRDEARNGLVALVADIMNGRIANPPTD
KSDRDMLDVLIAVKAETGTPRFSADEITGMFISMMFAGHHTSSGTASWTLIELMRHRDAYAAVIDELDELYGD
GRSVSFHALRQIPQLENVLKETLRLHPPLIILMRVAKGEFEVQGHRIHEGDLVAASPAISNRIPEDFPDPHDF
VPARYEQPRQEDLLNRWTWIPFGAGRHRCVGAAFAIMQIKAIFSVLLREYEFEMAQPPESYRNDHSKMVVQLA
QPACVRYRRRTGV
>CYP51B1_Mycobacterium tuberculosis TKK_05SA_0044 2621930251
MSAVALPRVSGGHDEHGHLEEFRTDPIGLMQRVRDECGDVGTFQLAGKQVVLLSGSHANEFFFRAGDDDLDQA
KAYPFMTPIFGEGVVFDASPERRKEMLHNAALRGEQMKGHAATIEDQVRRMIADWGEAGEIDLLDFFAELTIY
TSSACLIGKKFRDQLDGRFAKLYHELERGTDPLAYVDPYLPIESFRRRDEARNGLVALVADIMNGRIANPPTD
KSDRDMLDVLIAVKAETGTPRFSADEITGMFISMMFAGHHTSSGTASWTLIELMRHRDAYAAVIDELDELYGD
GRSVSFHALRQIPQLENVLKETLRLHPPLIILMRVAKGEFEVQGHRIHEGDLVAASPAISNRIPEDFPDPHDF
VPARYEQPRQEDLLNRWTWIPFGAGRHRCVGAAFAIMQIKAIFSVLLREYEFEMAQPPESYRNDHSKMVVQLA
QPACVRYRRRTGV
>CYP51B1_Mycobacterium tuberculosis TKK_05SA_0046 2621937538
MSAVALPRVSGGHDEHGHLEEFRTDPIGLMQRVRDECGDVGTFQLAGKQVVLLSGSHANEFFFRAGDDDLDQA
KAYPFMTPIFGEGVVFDASPERRKEMLHNAALRGEQMKGHAATIEDQVRRMIADWGEAGEIDLLDFFAELTIY
TSSACLIGKKFRDQLDGRFAKLYHELERGTDPLAYVDPYLPIESFRRRDEARNGLVALVADIMNGRIANPPTD
KSDRDMLDVLIAVKAETGTPRFSADEITGMFISMMFAGHHTSSGTASWTLIELMRHRDAYAAVIDELDELYGD
GRSVSFHALRQIPQLENVLKETLRLHPPLIILMRVAKGEFEVQGHRIHEGDLVAASPAISNRIPEDFPDPHDF
VPARYEQPRQEDLLNRWTWIPFGAGRHRCVGAAFAIMQIKAIFSVLLREYEFEMAQPPESYRNDHSKMVVQLA
QPACVRYRRRTGV
>CYP51B1_Mycobacterium tuberculosis TKK_05SA_0047 2621941659
MSAVALPRVSGGHDEHGHLEEFRTDPIGLMQRVRDECGDVGTFQLAGKQVVLLSGSHANEFFFRAGDDDLDQA
KAYPFMTPIFGEGVVFDASPERRKEMLHNAALRGEQMKGHAATIEDQVRRMIADWGEAGEIDLLDFFAELTIY

TSSACLIGKKFRDQLDGRFAKLYHELERGTDPLAYVDPYLPIESFRRRDEARNGLVALVADIMNGRIANPPTD
KSDRDMLDVLIAVKAETGTPRFSADEITGMFISMMFAGHHTSSGTASWTLIELMRHRDAYAAVIDELDELYGD
GRSVSFHALRQIPQLENVLKETLRLHPPLIILMRVAKGEFEVQGHRIHEGDLVAASPAISNRIPEDFPDPHDF
VPARYEQPRQEDLLNRWTWIPFGAGRHRCVGAAFAIMQIKAIFSVLLREYEFEMAQPPESYRNDHSKMVVQLA
QPACVRYRRRTGV
>CYP51B1_Mycobacterium tuberculosis TKK_05SA_0048 2575791780
MSAVALPRVSGGHDEHGHLEEFRTDPIGLMQRVRDECGDVGTFQLAGKQVVLLSGSHANEFFFRAGDDDLDQA
KAYPFMTPIFGEGVVFDASPERRKEMLHNAALRGEQMKGHAATIEDQVRRMIADWGEAGEIDLLDFFAELTIY
TSSACLIGKKFRDQLDGRFAKLYHELERGTDPLAYVDPYLPIESFRRRDEARNGLVALVADIMNGRIANPPTD
KSDRDMLDVLIAVKAETGTPRFSADEITGMFISMMFAGHHTSSGTASWTLIELMRHRDAYAAVIDELDELYGD
GRSVSFHALRQIPQLENVLKETLRLHPPLIILMRVAKGEFEVQGHRIHEGDLVAASPAISNRIPEDFPDPHDF
VPARYEQPRQEDLLNRWTWIPFGAGRHRCVGAAFAIMQIKAIFSVLLREYEFEMAQPPESYRNDHSKMVVQLA
QPACVRYRRRTGV
>CYP51B1_Mycobacterium tuberculosis TKK_05SA_0052 2621942888
MSAVALPRVSGGHDEHGHLEEFRTDPIGLMQRVRDECGDVGTFQLAGKQVVLLSGSHANEFFFRAGDDDLDQA
KAYPFMTPIFGEGVVFDASPERRKEMLHNAALRGEQMKGHAATIEDQVRRMIADWGEAGEIDLLDFFAELTIY
TSSACLIGKKFRDQLDGRFAKLYHELERGTDPLAYVDPYLPIESFRRRDEARNGLVALVADIMNGRIANPPTD
KSDRDMLDVLIAVKAETGTPRFSADEITGMFISMMFAGHHTSSGTASWTLIELMRHRDAYAAVIDELDELYGD
GRSVSFHALRQIPQLENVLKETLRLHPPLIILMRVAKGEFEVQGHRIHEGDLVAASPAISNRIPEDFPDPHDF
VPARYEQPRQEDLLNRWTWIPFGAGRHRCVGAAFAIMQIKAIFSVLLREYEFEMAQPPESYRNDHSKMVVQLA
QPACVRYRRRTGV
>CYP51B1_Mycobacterium tuberculosis TKK_05SA_0054 2621948797
MSAVALPRVSGGHDEHGHLEEFRTDPIGLMQRVRDECGDVGTFQLAGKQVVLLSGSHANEFFFRAGDDDLDQA
KAYPFMTPIFGEGVVFDASPERRKEMLHNAALRGEQMKGHAATIEDQVRRMIADWGEAGEIDLLDFFAELTIY
TSSACLIGKKFRDQLDGRFAKLYHELERGTDPLAYVDPYLPIESFRRRDEARNGLVALVADIMNGRIANPPTD
KSDRDMLDVLIAVKAETGTPRFSADEITGMFISMMFAGHHTSSGTASWTLIELMRHRDAYAAVIDELDELYGD
GRSVSFHALRQIPQLENVLKETLRLHPPLIILMRVAKGEFEVQGHRIHEGDLVAASPAISNRIPEDFPDPHDF
VPARYEQPRQEDLLNRWTWIPFGAGRHRCVGAAFAIMQIKAIFSVLLREYEFEMAQPPESYRNDHSKMVVQLA
QPACVRYRRRTGV
>CYP51B1_Mycobacterium tuberculosis TKK_05SA_0055 2621952788
MSAVALPRVSGGHDEHGHLEEFRTDPIGLMQRVRDECGDVGTFQLAGKQVVLLSGSHANEFFFRAGDDDLDQA
KAYPFMTPIFGEGVVFDASPERRKEMLHNAALRGEQMKGHAATIEDQVRRMIADWGEAGEIDLLDFFAELTIY
TSSACLIGKKFRDQLDGRFAKLYHELERGTDPLAYVDPYLPIESFRRRDEARNGLVALVADIMNGRIANPPTD
KSDRDMLDVLIAVKAETGTPRFSADEITGMFISMMFAGHHTSSGTASWTLIELMRHRDAYAAVIDELDELYGD
GRSVSFHALRQIPQLENVLKETLRLHPPLIILMRVAKGEFEVQGHRIHEGDLVAASPAISNRIPEDFPDPHDF
VPARYEQPRQEDLLNRWTWIPFGAGRHRCVGAAFAIMQIKAIFSVLLREYEFEMAQPPESYRNDHSKMVVQLA
QPACVRYRRRTGV
>CYP51B1_Mycobacterium tuberculosis TKK_05SA_0058 2575025982
MSAVALPRVSGGHDEHGHLEEFRTDPIGLMQRVRDECGDVGTFQLAGKQVVLLSGSHANEFFFRAGDDDLDQA
KAYPFMTPIFGEGVVFDASPERRKEMLHNAALRGEQMKGHAATIEDQVRRMIADWGEAGEIDLLDFFAELTIY
TSSACLIGKKFRDQLDGRFAKLYHELERGTDPLAYVDPYLPIESFRRRDEARNGLVALVADIMNGRIANPPTD
KSDRDMLDVLIAVKAETGTPRFSADEITGMFISMMFAGHHTSSGTASWTLIELMRHRDAYAAVIDELDELYGD
GRSVSFHALRQIPQLENVLKETLRLHPPLIILMRVAKGEFEVQGHRIHEGDLVAASPAISNRIPEDFPDPHDF
VPARYEQPRQEDLLNRWTWIPFGAGRHRCVGAAFAIMQIKAIFSVLLREYEFEMAQPPESYRNDHSKMVVQLA
QPACVRYRRRTGV
>CYP51B1_Mycobacterium tuberculosis TKK-01-0006 2577239837
MSAVALPRVSGGHDEHGHLEEFRTDPIGLMQRVRDECGDVGTFQLAGKQVVLLSGSHANEFFFRAGDDDLDQA
KAYPFMTPIFGEGVVFDASPERRKEMLHNAALRGEQMKGHAATIEDQVRRMIADWGEAGEIDLLDFFAELTIY
TSSACLIGKKFRDQLDGRFAKLYHELERGTDPLAYVDPYLPIESFRRRDEARNGLVALVADIMNGRIANPPTD
KSDRDMLDVLIAVKAETGTPRFSADEITGMFISMMFAGHHTSSGTASWTLIELMRHRDAYAAVIDELDELYGD
GRSVSFHALRQIPQLENVLKETLRLHPPLIILMRVAKGEFEVQGHRIHEGDLVAASPAISNRIPEDFPDPHDF
VPARYEQPRQEDLLNRWTWIPFGAGRHRCVGAAFAIMQIKAIFSVLLREYEFEMAQPPESYRNDHSKMVVQLA
QPACVRYRRRTGV
>CYP51B1_Mycobacterium tuberculosis TKK-01-0010 2575494440
MSAVALPRVSGGHDEHGHLEEFRTDPIGLMQRVRDECGDVGTFQLAGKQVVLLSGSHANEFFFRAGDDDLDQA
KAYPFMTPIFGEGVVFDASPERRKEMLHNAALRGEQMKGHAATIEDQVRRMIADWGEAGEIDLLDFFAELTIY
TSSACLIGKKFRDQLDGRFAKLYHELERGTDPLAYVDPYLPIESFRRRDEARNGLVALVADIMNGRIANPPTD
KSDRDMLDVLIAVKAETGTPRFSADEITGMFISMMFAGHHTSSGTASWTLIELMRHRDAYAAVIDELDELYGD

GRSVSFHALRQIPQLENVLKETLRLHPPLIILMRVAKGEFEVQGHRIHEGDLVAASPAISNRIPEDFPDPHDF
VPARYEQPRQEDLLNRWTWIPFGAGRHRCVGAAFAIMQIKAIFSVLLREYEFEMAQPPESYRNDHSKMVVQLA
QPACVRYRRRTGV
>CYP51B1_Mycobacterium tuberculosis TKK-01-0011 2575539865
MSAVALPRVSGGHDEHGHLEEFRTDPIGLMQRVRDECGDVGTFQLAGKQVVLLSGSHANEFFFRAGDDDLDQA
KAYPFMTPIFGEGVVFDASPERRKEMLHNAALRGEQMKGHAATIEDQVRRMIADWGEAGEIDLLDFFAELTIY
TSSACLIGKKFRDQLDGRFAKLYHELERGTDPLAYVDPYLPIESFRRRDEARNGLVALVADIMNGRIANPPTD
KSDRDMLDVLIAVKAETGTPRFSADEITGMFISMMFAGHHTSSGTASWTLIELMRHRDAYAAVIDELDELYGD
GRSVSFHALRQIPQLENVLKETLRLHPPLIILMRVAKGEFEVQGHRIHEGDLVAASPAISNRIPEDFPDPHDF
VPARYEQPRQEDLLNRWTWIPFGAGRHRCVGAAFAIMQIKAIFSVLLREYEFEMAQPPESYRNDHSKMVVQLA
QPACVRYRRRTGV
>CYP51B1_Mycobacterium tuberculosis TKK-01-0019 2575785977
MSAVALPRVSGGHDEHGHLEEFRTDPIGLMQRVRDECGDVGTFQLAGKQVVLLSGSHANEFFFRAGDDDLDQA
KAYPFMTPIFGEGVVFDASPERRKEMLHNAALRGEQMKGHAATIEDQVRRMIADWGEAGEIDLLDFFAELTIY
TSSACLIGKKFRDQLDGRFAKLYHELERGTDPLAYVDPYLPIESFRRRDEARNGLVALVADIMNGRIANPPTD
KSDRDMLDVLIAVKAETGTPRFSADEITGMFISMMFAGHHTSSGTASWTLIELMRHRDAYAAVIDELDELYGD
GRSVSFHALRQIPQLENVLKETLRLHPPLIILMRVAKGEFEVQGHRIHEGDLVAASPAISNRIPEDFPDPHDF
VPARYEQPRQEDLLNRWTWIPFGAGRHRCVGAAFAIMQIKAIFSVLLREYEFEMAQPPESYRNDHSKMVVQLA
QPACVRYRRRTGV
>CYP51B1_Mycobacterium tuberculosis TKK-01-0021 2584832729
MSAVALPRVSGGHDEHGHLEEFRTDPIGLMQRVRDECGDVGTFQLAGKQVVLLSGSHANEFFFRAGDDDLDQA
KAYPFMTPIFGEGVVFDASPERRKEMLHNAALRGEQMKGHAATIEDQVRRMIADWGEAGEIDLLDFFAELTIY
TSSACLIGKKFRDQLDGRFAKLYHELERGTDPLAYVDPYLPIESFRRRDEARNGLVALVADIMNGRIANPPTD
KSDRDMLDVLIAVKAETGTPRFSADEITGMFISMMFAGHHTSSGTASWTLIELMRHRDAYAAVIDELDELYGD
GRSVSFHALRQIPQLENVLKETLRLHPPLIILMRVAKGEFEVQGHRIHEGDLVAASPAISNRIPEDFPDPHDF
VPARYEQPRQEDLLNRWTWIPFGAGRHRCVGAAFAIMQIKAIFSVLLREYEFEMAQPPESYRNDHSKMVVQLA
QPACVRYRRRTGV
>CYP51B1_Mycobacterium tuberculosis TKK-01-0025 2578181013
MSAVALPRVSGGHDEHGHLEEFRTDPIGLMQRVRDECGDVGTFQLAGKQVVLLSGSHANEFFFRAGDDDLDQA
KAYPFMTPIFGEGVVFDASPERRKEMLHNAALRGEQMKGHAATIEDQVRRMIADWGEAGEIDLLDFFAELTIY
TSSACLIGKKFRDQLDGRFAKLYHELERGTDPLAYVDPYLPIESFRRRDEARNGLVALVADIMNGRIANPPTD
KSDRDMLDVLIAVKAETGTPRFSADEITGMFISMMFAGHHTSSGTASWTLIELMRHRDAYAAVIDELDELYGD
GRSVSFHALRQIPQLENVLKETLRLHPPLIILMRVAKGEFEVQGHRIHEGDLVAASPAISNRIPEDFPDPHDF
VPARYEQPRQEDLLNRWTWIPFGAGRHRCVGAAFAIMQIKAIFSVLLREYEFEMAQPPESYRNDHSKMVVQLA
QPACVRYRRRTGV
>CYP51B1_Mycobacterium tuberculosis TKK-01-0026 2578007344
MSAVALPRVSGGHDEHGHLEEFRTDPIGLMQRVRDECGDVGTFQLAGKQVVLLSGSHANEFFFRAGDDDLDQA
KAYPFMTPIFGEGVVFDASPERRKEMLHNAALRGEQMKGHAATIEDQVRRMIADWGEAGEIDLLDFFAELTIY
TSSACLIGKKFRDQLDGRFAKLYHELERGTDPLAYVDPYLPIESFRRRDEARNGLVALVADIMNGRIANPPTD
KSDRDMLDVLIAVKAETGTPRFSADEITGMFISMMFAGHHTSSGTASWTLIELMRHRDAYAAVIDELDELYGD
GRSVSFHALRQIPQLENVLKETLRLHPPLIILMRVAKGEFEVQGHRIHEGDLVAASPAISNRIPEDFPDPHDF
VPARYEQPRQEDLLNRWTWIPFGAGRHRCVGAAFAIMQIKAIFSVLLREYEFEMAQPPESYRNDHSKMVVQLA
QPACVRYRRRTGV
>CYP51B1_Mycobacterium tuberculosis TKK-01-0032 2584826645
MSAVALPRVSGGHDEHGHLEEFRTDPIGLMQRVRDECGDVGTFQLAGKQVVLLSGSHANEFFFRAGDDDLDQA
KAYPFMTPIFGEGVVFDASPERRKEMLHNAALRGEQMKGHAATIEDQVRRMIADWGEAGEIDLLDFFAELTIY
TSSACLIGKKFRDQLDGRFAKLYHELERGTDPLAYVDPYLPIESFRRRDEARNGLVALVADIMNGRIANPPTD
KSDRDMLDVLIAVKAETGTPRFSADEITGMFISMMFAGHHTSSGTASWTLIELMRHRDAYAAVIDELDELYGD
GRSVSFHALRQIPQLENVLKETLRLHPPLIILMRVAKGEFEVQGHRIHEGDLVAASPAISNRIPEDFPDPHDF
VPARYEQPRQEDLLNRWTWIPFGAGRHRCVGAAFAIMQIKAIFSVLLREYEFEMAQPPESYRNDHSKMVVQLA
QPACVRYRRRTGV
>CYP51B1_Mycobacterium tuberculosis TKK-01-0035 2579824159
MSAVALPRVSGGHDEHGHLEEFRTDPIGLMQRVRDECGDVGTFQLAGKQVVLLSGSHANEFFFRAGDDDLDQA
KAYPFMTPIFGEGVVFDASPERRKEMLHNAALRGEQMKGHAATIEDQVRRMIADWGEAGEIDLLDFFAELTIY
TSSACLIGKKFRDQLDGRFAKLYHELERGTDPLAYVDPYLPIESFRRRDEARNGLVALVADIMNGRIANPPTD
KSDRDMLDVLIAVKAETGTPRFSADEITGMFISMMFAGHHTSSGTASWTLIELMRHRDAYAAVIDELDELYGD
GRSVSFHALRQIPQLENVLKETLRLHPPLIILMRVAKGEFEVQGHRIHEGDLVAASPAISNRIPEDFPDPHDF

VPARYEQPRQEDLLNRWTWIPFGAGRHRCVGAAFAIMQIKAIFSVLLREYEFEMAQPPESYRNDHSKMVVQLA
QPACVRYRRRTGV
>CYP51B1_Mycobacterium tuberculosis TKK-01-0037 2577860878
MSAVALPRVSGGHDEHGHLEEFRTDPIGLMQRVRDECGDVGTFQLAGKQVVLLSGSHANEFFFRAGDDDLDQA
KAYPFMTPIFGEGVVFDASPERRKEMLHNAALRGEQMKGHAATIEDQVRRMIADWGEAGEIDLLDFFAELTIY
TSSACLIGKKFRDQLDGRFAKLYHELERGTDPLAYVDPYLPIESFRRRDEARNGLVALVADIMNGRIANPPTD
KSDRDMLDVLIAVKAETGTPRFSADEITGMFISMMFAGHHTSSGTASWTLIELMRHRDAYAAVIDELDELYGD
GRSVSFHALRQIPQLENVLKETLRLHPPLIILMRVAKGEFEVQGHRIHEGDLVAASPAISNRIPEDFPDPHDF
VPARYEQPRQEDLLNRWTWIPFGAGRHRCVGAAFAIMQIKAIFSVLLREYEFEMAQPPESYRNDHSKMVVQLA
QPACVRYRRRTGV
>CYP51B1_Mycobacterium tuberculosis TKK-01-0038 2584644964
MSAVALPRVSGGHDEHGHLEEFRTDPIGLMQRVRDECGDVGTFQLAGKQVVLLSGSHANEFFFRAGDDDLDQA
KAYPFMTPIFGEGVVFDASPERRKEMLHNAALRGEQMKGHAATIEDQVRRMIADWGEAGEIDLLDFFAELTIY
TSSACLIGKKFRDQLDGRFAKLYHELERGTDPLAYVDPYLPIESFRRRDEARNGLVALVADIMNGRIANPPTD
KSDRDMLDVLIAVKAETGTPRFSADEITGMFISMMFAGHHTSSGTASWTLIELMRHRDAYAAVIDELDELYGD
GRSVSFHALRQIPQLENVLKETLRLHPPLIILMRVAKGEFEVQGHRIHEGDLVAASPAISNRIPEDFPDPHDF
VPARYEQPRQEDLLNRWTWIPFGAGRHRCVGAAFAIMQIKAIFSVLLREYEFEMAQPPESYRNDHSKMVVQLA
QPACVRYRRRTGV
>CYP51B1_Mycobacterium tuberculosis TKK-01-0041 2575829105
MSAVALPRVSGGHDEHGHLEEFRTDPIGLMQRVRDECGDVGTFQLAGKQVVLLSGSHANEFFFRAGDDDLDQA
KAYPFMTPIFGEGVVFDASPERRKEMLHNAALRGEQMKGHAATIEDQVRRMIADWGEAGEIDLLDFFAELTIY
TSSACLIGKKFRDQLDGRFAKLYHELERGTDPLAYVDPYLPIESFRRRDEARNGLVALVADIMNGRIANPPTD
KSDRDMLDVLIAVKAETGTPRFSADEITGMFISMMFAGHHTSSGTASWTLIELMRHRDAYAAVIDELDELYGD
GRSVSFHALRQIPQLENVLKETLRLHPPLIILMRVAKGEFEVQGHRIHEGDLVAASPAISNRIPEDFPDPHDF
VPARYEQPRQEDLLNRWTWIPFGAGRHRCVGAAFAIMQIKAIFSVLLREYEFEMAQPPESYRNDHSKMVVQLA
QPACVRYRRRTGV
>CYP51B1_Mycobacterium tuberculosis TKK-01-0047 2577322034
MSAVALPRVSGGHDEHGHLEEFRTDPIGLMQRVRDECGDVGTFQLAGKQVVLLSGSHANEFFFRAGDDDLDQA
KAYPFMTPIFGEGVVFDASPERRKEMLHNAALRGEQMKGHAATIEDQVRRMIADWGEAGEIDLLDFFAELTIY
TSSACLIGKKFRDQLDGRFAKLYHELERGTDPLAYVDPYLPIESFRRRDEARNGLVALVADIMNGRIANPPTD
KSDRDMLDVLIAVKAETGTPRFSADEITGMFISMMFAGHHTSSGTASWTLIELMRHRDAYAAVIDELDELYGD
GRSVSFHALRQIPQLENVLKETLRLHPPLIILMRVAKGEFEVQGHRIHEGDLVAASPAISNRIPEDFPDPHDF
VPARYEQPRQEDLLNRWTWIPFGAGRHRCVGAAFAIMQIKAIFSVLLREYEFEMAQPPESYRNDHSKMVVQLA
QPACVRYRRRTGV
>CYP51B1_Mycobacterium tuberculosis TKK-01-0053 2575413864
MSAVALPRVSGGHDEHGHLEEFRTDPIGLMQRVRDECGDVGTFQLAGKQVVLLSGSHANEFFFRAGDDDLDQA
KAYPFMTPIFGEGVVFDASPERRKEMLHNAALRGEQMKGHAATIEDQVRRMIADWGEAGEIDLLDFFAELTIY
TSSACLIGKKFRDQLDGRFAKLYHELERGTDPLAYVDPYLPIESFRRRDEARNGLVALVADIMNGRIANPPTD
KSDRDMLDVLIAVKAETGTPRFSADEITGMFISMMFAGHHTSSGTASWTLIELMRHRDAYAAVIDELDELYGD
GRSVSFHALRQIPQLENVLKETLRLHPPLIILMRVAKGEFEVQGHRIHEGDLVAASPAISNRIPEDFPDPHDF
VPARYEQPRQEDLLNRWTWIPFGAGRHRCVGAAFAIMQIKAIFSVLLREYEFEMAQPPESYRNDHSKMVVQLA
QPACVRYRRRTGV
>CYP51B1_Mycobacterium tuberculosis V_13678 2716390667
MSAVALPRVSGGHDEHGHLEEFRTDPIGLMQRVRDECGDVGTFQLAGKQVVLLSGSHANEFFFRAGDDDLDQA
KAYPFMTPIFGEGVVFDASPERRKEMLHNAALRGEQMKGHAATIEDQVRRMIADWGEAGEIDLLDFFAELTIY
TSSACLIGKKFRDQLDGRFAKLYHELERGTDPLAYVDPYLPIESFRRRDEARNGLVALVADIMNGRIANPPTD
KSDRDMLDVLIAVKAETGTPRFSADEITGMFISMMFAGHHTSSGTASWTLIELMRHRDAYAAVIDELDELYGD
GRSVSFHALRQIPQLENVLKETLRLHPPLIILMRVAKGEFEVQGHRIHEGDLVAASPAISNRIPEDFPDPHDF
VPARYEQPRQEDLLNRWTWIPFGAGRHRCVGAAFAIMQIKAIFSVLLREYEFEMAQPPESYRNDHSKMVVQLA
QPACVRYRRRTGV
>CYP51B1_Mycobacterium tuberculosis V09501561 2675728463
MSAVALPRVSGGHDEHGHLEEFRTDPIGLMQRVRDECGDVGTFQLAGKQVVLLSGSHANEFFFRAGDDDLDQA
KAYPFMTPIFGEGVVFDASPERRKEMLHNAALRGEQMKGHAATIEDQVRRMIADWGEAGEIDLLDFFAELTIY
TSSACLIGKKFRDQLDGRFAKLYHELERGTDPLAYVDPYLPIESFRRRDEARNGLVALVADIMNGRIANPPTD
KSDRDMLDVLIAVKAETGTPRFSADEITGMFISMMFAGHHTSSGTASWTLIELMRHRDAYAAVIDELDELYGD
GRSVSFHALRQIPQLENVLKETLRLHPPLIILMRVAKGEFEVQGHRIHEGDLVAASPAISNRIPEDFPDPHDF
VPARYEQPRQEDLLNRWTWIPFGAGRHRCVGAAFAIMQIKAIFSVLLREYEFEMAQPPESYRNDHSKMVVQLA
QPACVRYRRRTGV

>CYP51B1_Mycobacterium tuberculosis V09601310 2706587076
MSAVALPRVSGGHDEHGHLEEFRTDPIGLMQRVRDECGDVGTFQLAGKQVVLLSGSHANEFFFRAGDDDLDQA
KAYPFMTPIFGEGVVFDASPERRKEMLHNAALRGEQMKGHAATIEDQVRRMIADWGEAGEIDLLDFFAELTIY
TSSACLIGKKFRDQLDGRFAKLYHELERGTDPLAYVDPYLPIESFRRRDEARNGLVALVADIMNGRIANPPTD
KSDRDMLDVLIAVKAETGTPRFSADEITGMFISMMFAGHHTSSGTASWTLIELMRHRDAYAAVIDELDELYGD
GRSVSFHALRQIPQLENVLKETLRLHPPLIILMRVAKGEFEVQGHRIHEGDLVAASPAISNRIPEDFPDPHDF
VPARYEQPRQEDLLNRWTWIPFGAGRHRCVGAAFAIMQIKAIFSVLLREYEFEMAQPPESYRNDHSKMVVQLA
QPACVRYRRRTGV
>CYP51B1_Mycobacterium tuberculosis VRF CWCF XDRTB 1028 2598812180
MSAVALPRVSGGHDEHGHLEEFRTDPIGLMQRVRDECGDVGTFQLAGKQVVLLSGSHANEFFFRAGDDDLDQA
KAYPFMTPIFGEGVVFDASPERRKEMLHNAALRGEQMKGHAATIEDQVRRMIADWGEAGEIDLLDFFAELTIY
TSSACLIGKKFRDQLDGRFAKLYHELERGTDPLAYVDPYLPIESFRRRDEARNGLVALVADIMNGRIANPPTD
KSDRDMLDVLIAVKAETGTPRFSADEITGMFISMMFAGHHTSSGTASWTLIELMRHRDAYAAVIDELDELYGD
GRSVSFHALRQIPQLENVLKETLRLHPPLIILMRVAKGEFEVQGHRIHEGDLVAASPAISNRIPEDFPDPHDF
VPARYEQPRQEDLLNRWTWIPFGAGRHRCVGAAFAIMQIKAIFSVLLREYEFEMAQPPESYRNDHSKMVVQLA
QPACVRYRRRTGV
>CYP51B1_Mycobacterium tuberculosis VRFCWCF XDRTB 232 2638414902
MSAVALPRVSGGHDEHGHLEEFRTDPIGLMQRVRDECGDVGTFQLAGKQVVLLSGSHANEFFFRAGDDDLDQA
KAYPFMTPIFGEGVVFDASPERRKEMLHNAALRGEQMKGHAATIEDQVRRMIADWGEAGEIDLLDFFAELTIY
TSSACLIGKKFRDQLDGRFAKLYHELERGTDPLAYVDPYLPIESFRRRDEARNGLVALVADIMNGRIANPPTD
KSDRDMLDVLIAVKAETGTPRFSADEITGMFISMMFAGHHTSSGTASWTLIELMRHRDAYAAVIDELDELYGD
GRSVSFHALRQIPQLENVLKETLRLHPPLIILMRVAKGEFEVQGHRIHEGDLVAASPAISNRIPEDFPDPHDF
VPARYEQPRQEDLLNRWTWIPFGAGRHRCVGAAFAIMQIKAIFSVLLREYEFEMAQPPESYRNDHSKMVVQLA
QPACVRYRRRTGV
>CYP51B1_Mycobacterium tuberculosis W06 2691072262
MSAVALPRVSGGHDEHGHLEEFRTDPIGLMQRVRDECGDVGTFQLAGKQVVLLSGSHANEFFFRAGDDDLDQA
KAYPFMTPIFGEGVVFDASPERRKEMLHNAALRGEQMKGHAATIEDQVRRMIADWGEAGEIDLLDFFAELTIY
TSSACLIGKKFRDQLDGRFAKLYHELERGTDPLAYVDPYLPIESFRRRDEARNGLVALVADIMNGRIANPPTD
KSDRDMLDVLIAVKAETGTPRFSADEITGMFISMMFAGHHTSSGTASWTLIELMRHRDAYAAVIDELDELYGD
GRSVSFHALRQIPQLENVLKETLRLHPPLIILMRVAKGEFEVQGHRIHEGDLVAASPAISNRIPEDFPDPHDF
VPARYEQPRQEDLLNRWTWIPFGAGRHRCVGAAFAIMQIKAIFSVLLREYEFEMAQPPESYRNDHSKMVVQLA
QPACVRYRRRTGV
>CYP51B1_Mycobacterium tuberculosis W09900252 2703608681
MSAVALPRVSGGHDEHGHLEEFRTDPIGLMQRVRDECGDVGTFQLAGKQVVLLSGSHANEFFFRAGDDDLDQA
KAYPFMTPIFGEGVVFDASPERRKEMLHNAALRGEQMKGHAATIEDQVRRMIADWGEAGEIDLLDFFAELTIY
TSSACLIGKKFRDQLDGRFAKLYHELERGTDPLAYVDPYLPIESFRRRDEARNGLVALVADIMNGRIANPPTD
KSDRDMLDVLIAVKAETGTPRFSADEITGMFISMMFAGHHTSSGTASWTLIELMRHRDAYAAVIDELDELYGD
GRSVSFHALRQIPQLENVLKETLRLHPPLIILMRVAKGEFEVQGHRIHEGDLVAASPAISNRIPEDFPDPHDF
VPARYEQPRQEDLLNRWTWIPFGAGRHRCVGAAFAIMQIKAIFSVLLREYEFEMAQPPESYRNDHSKMVVQLA
QPACVRYRRRTGV
>CYP51B1_Mycobacterium tuberculosis W09900339 2678684111
MSAVALPRVSGGHDEHGHLEEFRTDPIGLMQRVRDECGDVGTFQLAGKQVVLLSGSHANEFFFRAGDDDLDQA
KAYPFMTPIFGEGVVFDASPERRKEMLHNAALRGEQMKGHAATIEDQVRRMIADWGEAGEIDLLDFFAELTIY
TSSACLIGKKFRDQLDGRFAKLYHELERGTDPLAYVDPYLPIESFRRRDEARNGLVALVADIMNGRIANPPTD
KSDRDMLDVLIAVKAETGTPRFSADEITGMFISMMFAGHHTSSGTASWTLIELMRHRDAYAAVIDELDELYGD
GRSVSFHALRQIPQLENVLKETLRLHPPLIILMRVAKGEFEVQGHRIHEGDLVAASPAISNRIPEDFPDPHDF
VPARYEQPRQEDLLNRWTWIPFGAGRHRCVGAAFAIMQIKAIFSVLLREYEFEMAQPPESYRNDHSKMVVQLA
QPACVRYRRRTGV
>CYP51B1_Mycobacterium tuberculosis W09900422 2703514159
MSAVALPRVSGGHDEHGHLEEFRTDPIGLMQRVRDECGDVGTFQLAGKQVVLLSGSHANEFFFRAGDDDLDQA
KAYPFMTPIFGEGVVFDASPERRKEMLHNAALRGEQMKGHAATIEDQVRRMIADWGEAGEIDLLDFFAELTIY
TSSACLIGKKFRDQLDGRFAKLYHELERGTDPLAYVDPYLPIESFRRRDEARNGLVALVADIMNGRIANPPTD
KSDRDMLDVLIAVKAETGTPRFSADEITGMFISMMFAGHHTSSGTASWTLIELMRHRDAYAAVIDELDELYGD
GRSVSFHALRQIPQLENVLKETLRLHPPLIILMRVAKGEFEVQGHRIHEGDLVAASPAISNRIPEDFPDPHDF
VPARYEQPRQEDLLNRWTWIPFGAGRHRCVGAAFAIMQIKAIFSVLLREYEFEMAQPPESYRNDHSKMVVQLA
QPACVRYRRRTGV
>CYP51B1_Mycobacterium tuberculosis W09900423 2703518424

MSAVALPRVSGGHDEHGHLEEFRTDPIGLMQRVRDECGDVGTFQLAGKQVVLLSGSHANEFFFRAGDDDLDQA
KAYPFMTPIFGEGVVFDASPERRKEMLHNAALRGEQMKGHAATIEDQVRRMIADWGEAGEIDLLDFFAELTIY
TSSACLIGKKFRDQLDGRFAKLYHELERGTDPLAYVDPYLPIESFRRRDEARNGLVALVADIMNGRIANPPTD
KSDRDMLDVLIAVKAETGTPRFSADEITGMFISMMFAGHHTSSGTASWTLIELMRHRDAYAAVIDELDELYGD
GRSVSFHALRQIPQLENVLKETLRLHPPLIILMRVAKGEFEVQGHRIHEGDLVAASPAISNRIPEDFPDPHDF
VPARYEQPRQEDLLNRWTWIPFGAGRHRCVGAAFAIMQIKAIFSVLLREYEFEMAQPPESYRNDHSKMVVQLA
QPACVRYRRRTGV
>CYP51B1_Mycobacterium tuberculosis W09900434 2674704794
MSAVALPRVSGGHDEHGHLEEFRTDPIGLMQRVRDECGDVGTFQLAGKQVVLLSGSHANEFFFRAGDDDLDQA
KAYPFMTPIFGEGVVFDASPERRKEMLHNAALRGEQMKGHAATIEDQVRRMIADWGEAGEIDLLDFFAELTIY
TSSACLIGKKFRDQLDGRFAKLYHELERGTDPLAYVDPYLPIESFRRRDEARNGLVALVADIMNGRIANPPTD
KSDRDMLDVLIAVKAETGTPRFSADEITGMFISMMFAGHHTSSGTASWTLIELMRHRDAYAAVIDELDELYGD
GRSVSFHALRQIPQLENVLKETLRLHPPLIILMRVAKGEFEVQGHRIHEGDLVAASPAISNRIPEDFPDPHDF
VPARYEQPRQEDLLNRWTWIPFGAGRHRCVGAAFAIMQIKAIFSVLLREYEFEMAQPPESYRNDHSKMVVQLA
QPACVRYRRRTGV
>CYP51B1_Mycobacterium tuberculosis W09900442 2672218166
MSAVALPRVSGGHDEHGHLEEFRTDPIGLMQRVRDECGDVGTFQLAGKQVVLLSGSHANEFFFRAGDDDLDQA
KAYPFMTPIFGEGVVFDASPERRKEMLHNAALRGEQMKGHAATIEDQVRRMIADWGEAGEIDLLDFFAELTIY
TSSACLIGKKFRDQLDGRFAKLYHELERGTDPLAYVDPYLPIESFRRRDEARNGLVALVADIMNGRIANPPTD
KSDRDMLDVLIAVKAETGTPRFSADEITGMFISMMFAGHHTSSGTASWTLIELMRHRDAYAAVIDELDELYGD
GRSVSFHALRQIPQLENVLKETLRLHPPLIILMRVAKGEFEVQGHRIHEGDLVAASPAISNRIPEDFPDPHDF
VPARYEQPRQEDLLNRWTWIPFGAGRHRCVGAAFAIMQIKAIFSVLLREYEFEMAQPPESYRNDHSKMVVQLA
QPACVRYRRRTGV
>CYP51B1_Mycobacterium tuberculosis W146 2742392631
MSAVALPRVSGGHDEHGHLEEFRTDPIGLMQRVRDECGDVGTFQLAGKQVVLLSGSHANEFFFRAGDDDLDQA
KAYPFMTPIFGEGVVFDASPERRKEMLHNAALRGEQMKGHAATIEDQVRRMIADWGEAGEIDLLDFFAELTIY
TSSACLIGKKFRDQLDGRFAKLYHELERGTDPLAYVDPYLPIESFRRRDEARNGLVALVADIMNGRIANPPTD
KSDRDMLDVLIAVKAETGTPRFSADEITGMFISMMFAGHHTSSGTASWTLIELMRHRDAYAAVIDELDELYGD
GRSVSFHALRQIPQLENVLKETLRLHPPLIILMRVAKGEFEVQGHRIHEGDLVAASPAISNRIPEDFPDPHDF
VPARYEQPRQEDLLNRWTWIPFGAGRHRCVGAAFAIMQIKAIFSVLLREYEFEMAQPPESYRNDHSKMVVQLA
QPACVRYRRRTGV
>CYP51B1_Mycobacterium tuberculosis WX1 2560452753
MSAVALPRVSGGHDEHGHLEEFRTDPIGLMQRVRDECGDVGTFQLAGKQVVLLSGSHANEFFFRAGDDDLDQA
KAYPFMTPIFGEGVVFDASPERRKEMLHNAALRGEQMKGHAATIEDQVRRMIADWGEAGEIDLLDFFAELTIY
TSSACLIGKKFRDQLDGRFAKLYHELERGTDPLAYVDPYLPIESFRRRDEARNGLVALVADIMNGRIANPPTD
KSDRDMLDVLIAVKAETGTPRFSADEITGMFISMMFAGHHTSSGTASWTLIELMRHRDAYAAVIDELDELYGD
GRSVSFHALRQIPQLENVLKETLRLHPPLIILMRVAKGEFEVQGHRIHEGDLVAASPAISNRIPEDFPDPHDF
VPARYEQPRQEDLLNRWTWIPFGAGRHRCVGAAFAIMQIKAIFSVLLREYEFEMAQPPESYRNDHSKMVVQLA
QPACVRYRRRTGV
>CYP51B1_Mycobacterium tuberculosis WX3 2560461158
MSAVALPRVSGGHDEHGHLEEFRTDPIGLMQRVRDECGDVGTFQLAGKQVVLLSGSHANEFFFRAGDDDLDQA
KAYPFMTPIFGEGVVFDASPERRKEMLHNAALRGEQMKGHAATIEDQVRRMIADWGEAGEIDLLDFFAELTIY
TSSACLIGKKFRDQLDGRFAKLYHELERGTDPLAYVDPYLPIESFRRRDEARNGLVALVADIMNGRIANPPTD
KSDRDMLDVLIAVKAETGTPRFSADEITGMFISMMFAGHHTSSGTASWTLIELMRHRDAYAAVIDELDELYGD
GRSVSFHALRQIPQLENVLKETLRLHPPLIILMRVAKGEFEVQGHRIHEGDLVAASPAISNRIPEDFPDPHDF
VPARYEQPRQEDLLNRWTWIPFGAGRHRCVGAAFAIMQIKAIFSVLLREYEFEMAQPPESYRNDHSKMVVQLA
QPACVRYRRRTGV
>CYP51B1_Mycobacterium tuberculosis X00800532 2703522544
MSAVALPRVSGGHDEHGHLEEFRTDPIGLMQRVRDECGDVGTFQLAGKQVVLLSGSHANEFFFRAGDDDLDQA
KAYPFMTPIFGEGVVFDASPERRKEMLHNAALRGEQMKGHAATIEDQVRRMIADWGEAGEIDLLDFFAELTIY
TSSACLIGKKFRDQLDGRFAKLYHELERGTDPLAYVDPYLPIESFRRRDEARNGLVALVADIMNGRIANPPTD
KSDRDMLDVLIAVKAETGTPRFSADEITGMFISMMFAGHHTSSGTASWTLIELMRHRDAYAAVIDELDELYGD
GRSVSFHALRQIPQLENVLKETLRLHPPLIILMRVAKGEFEVQGHRIHEGDLVAASPAISNRIPEDFPDPHDF
VPARYEQPRQEDLLNRWTWIPFGAGRHRCVGAAFAIMQIKAIFSVLLREYEFEMAQPPESYRNDHSKMVVQLA
QPACVRYRRRTGV
>CYP51B1_Mycobacterium tuberculosis X00800994 2674720901
MSAVALPRVSGGHDEHGHLEEFRTDPIGLMQRVRDECGDVGTFQLAGKQVVLLSGSHANEFFFRAGDDDLDQA
KAYPFMTPIFGEGVVFDASPERRKEMLHNAALRGEQMKGHAATIEDQVRRMIADWGEAGEIDLLDFFAELTIY

TSSACLIGKKFRDQLDGRFAKLYHELERGTDPLAYVDPYLPIESFRRRDEARNGLVALVADIMNGRIANPPTD
KSDRDMLDVLIAVKAETGTPRFSADEITGMFISMMFAGHHTSSGTASWTLIELMRHRDAYAAVIDELDELYGD
GRSVSFHALRQIPQLENVLKETLRLHPPLIILMRVAKGEFEVQGHRIHEGDLVAASPAISNRIPEDFPDPHDF
VPARYEQPRQEDLLNRWTWIPFGAGRHRCVGAAFAIMQIKAIFSVLLREYEFEMAQPPESYRNDHSKMVVQLA
QPACVRYRRRTGV
>CYP51B1_Mycobacterium tuberculosis X122 2547954570
MSAVALPRVSGGHDEHGHLEEFRTDPIGLMQRVRDECGDVGTFQLAGKQVVLLSGSHANEFFFRAGDDDLDQA
KAYPFMTPIFGEGVVFDASPERRKEMLHNAALRGEQMKGHAATIEDQVRRMIADWGEAGEIDLLDFFAELTIY
TSSACLIGKKFRDQLDGRFAKLYHELERGTDPLAYVDPYLPIESFRRRDEARNGLVALVADIMNGRIANPPTD
KSDRDMLDVLIAVKAETGTPRFSADEITGMFISMMFAGHHTSSGTASWTLIELMRHRDAYAAVIDELDELYGD
GRSVSFHALRQIPQLENVLKETLRLHPPLIILMRVAKGEFEVQGHRIHEGDLVAASPAISNRIPEDFPDPHDF
VPARYEQPRQEDLLNRWTWIPFGAGRHRCVGAAFAIMQIKAIFSVLLREYEFEMAQPPESYRNDHSKMVVQLA
QPACVRYRRRTGV
>CYP51B1_Mycobacterium tuberculosis XDR KZN 1435 2603932160
MSAVALPRVSGGHDEHGHLEEFRTDPIGLMQRVRDECGDVGTFQLAGKQVVLLSGSHANEFFFRAGDDDLDQA
KAYPFMTPIFGEGVVFDASPERRKEMLHNAALRGEQMKGHAATIEDQVRRMIADWGEAGEIDLLDFFAELTIY
TSSACLIGKKFRDQLDGRFAKLYHELERGTDPLAYVDPYLPIESFRRRDEARNGLVALVADIMNGRIANPPTD
KSDRDMLDVLIAVKAETGTPRFSADEITGMFISMMFAGHHTSSGTASWTLIELMRHRDAYAAVIDELDELYGD
GRSVSFHALRQIPQLENVLKETLRLHPPLIILMRVAKGEFEVQGHRIHEGDLVAASPAISNRIPEDFPDPHDF
VPARYEQPRQEDLLNRWTWIPFGAGRHRCVGAAFAIMQIKAIFSVLLREYEFEMAQPPESYRNDHSKMVVQLA
QPACVRYRRRTGV
>CYP51B1_Mycobacterium tuberculosis XDR KZN 4207 2604996415
MSAVALPRVSGGHDEHGHLEEFRTDPIGLMQRVRDECGDVGTFQLAGKQVVLLSGSHANEFFFRAGDDDLDQA
KAYPFMTPIFGEGVVFDASPERRKEMLHNAALRGEQMKGHAATIEDQVRRMIADWGEAGEIDLLDFFAELTIY
TSSACLIGKKFRDQLDGRFAKLYHELERGTDPLAYVDPYLPIESFRRRDEARNGLVALVADIMNGRIANPPTD
KSDRDMLDVLIAVKAETGTPRFSADEITGMFISMMFAGHHTSSGTASWTLIELMRHRDAYAAVIDELDELYGD
GRSVSFHALRQIPQLENVLKETLRLHPPLIILMRVAKGEFEVQGHRIHEGDLVAASPAISNRIPEDFPDPHDF
VPARYEQPRQEDLLNRWTWIPFGAGRHRCVGAAFAIMQIKAIFSVLLREYEFEMAQPPESYRNDHSKMVVQLA
QPACVRYRRRTGV
>CYP51B1_Mycobacterium tuberculosis XDR KZN 605 2604661560
MSAVALPRVSGGHDEHGHLEEFRTDPIGLMQRVRDECGDVGTFQLAGKQVVLLSGSHANEFFFRAGDDDLDQA
KAYPFMTPIFGEGVVFDASPERRKEMLHNAALRGEQMKGHAATIEDQVRRMIADWGEAGEIDLLDFFAELTIY
TSSACLIGKKFRDQLDGRFAKLYHELERGTDPLAYVDPYLPIESFRRRDEARNGLVALVADIMNGRIANPPTD
KSDRDMLDVLIAVKAETGTPRFSADEITGMFISMMFAGHHTSSGTASWTLIELMRHRDAYAAVIDELDELYGD
GRSVSFHALRQIPQLENVLKETLRLHPPLIILMRVAKGEFEVQGHRIHEGDLVAASPAISNRIPEDFPDPHDF
VPARYEQPRQEDLLNRWTWIPFGAGRHRCVGAAFAIMQIKAIFSVLLREYEFEMAQPPESYRNDHSKMVVQLA
QPACVRYRRRTGV
>CYP51B1_Mycobacterium tuberculosis XDR1219 2560447990
MSAVALPRVSGGHDEHGHLEEFRTDPIGLMQRVRDECGDVGTFQLAGKQVVLLSGSHANEFFFRAGDDDLDQA
KAYPFMTPIFGEGVVFDASPERRKEMLHNAALRGEQMKGHAATIEDQVRRMIADWGEAGEIDLLDFFAELTIY
TSSACLIGKKFRDQLDGRFAKLYHELERGTDPLAYVDPYLPIESFRRRDEARNGLVALVADIMNGRIANPPTD
KSDRDMLDVLIAVKAETGTPRFSADEITGMFISMMFAGHHTSSGTASWTLIELMRHRDAYAAVIDELDELYGD
GRSVSFHALRQIPQLENVLKETLRLHPPLIILMRVAKGEFEVQGHRIHEGDLVAASPAISNRIPEDFPDPHDF
VPARYEQPRQEDLLNRWTWIPFGAGRHRCVGAAFAIMQIKAIFSVLLREYEFEMAQPPESYRNDHSKMVVQLA
QPACVRYRRRTGV
>CYP51B1_Mycobacterium tuberculosis XDR1221 2560455212
MSAVALPRVSGGHDEHGHLEEFRTDPIGLMQRVRDECGDVGTFQLAGKQVVLLSGSHANEFFFRAGDDDLDQA
KAYPFMTPIFGEGVVFDASPERRKEMLHNAALRGEQMKGHAATIEDQVRRMIADWGEAGEIDLLDFFAELTIY
TSSACLIGKKFRDQLDGRFAKLYHELERGTDPLAYVDPYLPIESFRRRDEARNGLVALVADIMNGRIANPPTD
KSDRDMLDVLIAVKAETGTPRFSADEITGMFISMMFAGHHTSSGTASWTLIELMRHRDAYAAVIDELDELYGD
GRSVSFHALRQIPQLENVLKETLRLHPPLIILMRVAKGEFEVQGHRIHEGDLVAASPAISNRIPEDFPDPHDF
VPARYEQPRQEDLLNRWTWIPFGAGRHRCVGAAFAIMQIKAIFSVLLREYEFEMAQPPESYRNDHSKMVVQLA
QPACVRYRRRTGV
>CYP51B1_Mycobacterium tuberculosis XTB13-081 2576700560
MSAVALPRVSGGHDEHGHLEEFRTDPIGLMQRVRDECGDVGTFQLAGKQVVLLSGSHANEFFFRAGDDDLDQA
KAYPFMTPIFGEGVVFDASPERRKEMLHNAALRGEQMKGHAATIEDQVRRMIADWGEAGEIDLLDFFAELTIY
TSSACLIGKKFRDQLDGRFAKLYHELERGTDPLAYVDPYLPIESFRRRDEARNGLVALVADIMNGRIANPPTD
KSDRDMLDVLIAVKAETGTPRFSADEITGMFISMMFAGHHTSSGTASWTLIELMRHRDAYAAVIDELDELYGD

GRSVSFHALRQIPQLENVLKETLRLHPPLIILMRVAKGEFEVQGHRIHEGDLVAASPAISNRIPEDFPDPHDF
VPARYEQPRQEDLLNRWTWIPFGAGRHRCVGAAFAIMQIKAIFSVLLREYEFEMAQPPESYRNDHSKMVVQLA
QPACVRYRRRTGV
>CYP51B1_Mycobacterium tuberculosis XTB13-082 2584721037
MSAVALPRVSGGHDEHGHLEEFRTDPIGLMQRVRDECGDVGTFQLAGKQVVLLSGSHANEFFFRAGDDDLDQA
KAYPFMTPIFGEGVVFDASPERRKEMLHNAALRGEQMKGHAATIEDQVRRMIADWGEAGEIDLLDFFAELTIY
TSSACLIGKKFRDQLDGRFAKLYHELERGTDPLAYVDPYLPIESFRRRDEARNGLVALVADIMNGRIANPPTD
KSDRDMLDVLIAVKAETGTPRFSADEITGMFISMMFAGHHTSSGTASWTLIELMRHRDAYAAVIDELDELYGD
GRSVSFHALRQIPQLENVLKETLRLHPPLIILMRVAKGEFEVQGHRIHEGDLVAASPAISNRIPEDFPDPHDF
VPARYEQPRQEDLLNRWTWIPFGAGRHRCVGAAFAIMQIKAIFSVLLREYEFEMAQPPESYRNDHSKMVVQLA
QPACVRYRRRTGV
>CYP51B1_Mycobacterium tuberculosis XTB13-083 2621360866
MSAVALPRVSGGHDEHGHLEEFRTDPIGLMQRVRDECGDVGTFQLAGKQVVLLSGSHANEFFFRAGDDDLDQA
KAYPFMTPIFGEGVVFDASPERRKEMLHNAALRGEQMKGHAATIEDQVRRMIADWGEAGEIDLLDFFAELTIY
TSSACLIGKKFRDQLDGRFAKLYHELERGTDPLAYVDPYLPIESFRRRDEARNGLVALVADIMNGRIANPPTD
KSDRDMLDVLIAVKAETGTPRFSADEITGMFISMMFAGHHTSSGTASWTLIELMRHRDAYAAVIDELDELYGD
GRSVSFHALRQIPQLENVLKETLRLHPPLIILMRVAKGEFEVQGHRIHEGDLVAASPAISNRIPEDFPDPHDF
VPARYEQPRQEDLLNRWTWIPFGAGRHRCVGAAFAIMQIKAIFSVLLREYEFEMAQPPESYRNDHSKMVVQLA
QPACVRYRRRTGV
>CYP51B1_Mycobacterium tuberculosis XTB13-084 2621366317
MSAVALPRVSGGHDEHGHLEEFRTDPIGLMQRVRDECGDVGTFQLAGKQVVLLSGSHANEFFFRAGDDDLDQA
KAYPFMTPIFGEGVVFDASPERRKEMLHNAALRGEQMKGHAATIEDQVRRMIADWGEAGEIDLLDFFAELTIY
TSSACLIGKKFRDQLDGRFAKLYHELERGTDPLAYVDPYLPIESFRRRDEARNGLVALVADIMNGRIANPPTD
KSDRDMLDVLIAVKAETGTPRFSADEITGMFISMMFAGHHTSSGTASWTLIELMRHRDAYAAVIDELDELYGD
GRSVSFHALRQIPQLENVLKETLRLHPPLIILMRVAKGEFEVQGHRIHEGDLVAASPAISNRIPEDFPDPHDF
VPARYEQPRQEDLLNRWTWIPFGAGRHRCVGAAFAIMQIKAIFSVLLREYEFEMAQPPESYRNDHSKMVVQLA
QPACVRYRRRTGV
>CYP51B1_Mycobacterium tuberculosis XTB13-086 2584754948
MSAVALPRVSGGHDEHGHLEEFRTDPIGLMQRVRDECGDVGTFQLAGKQVVLLSGSHANEFFFRAGDDDLDQA
KAYPFMTPIFGEGVVFDASPERRKEMLHNAALRGEQMKGHAATIEDQVRRMIADWGEAGEIDLLDFFAELTIY
TSSACLIGKKFRDQLDGRFAKLYHELERGTDPLAYVDPYLPIESFRRRDEARNGLVALVADIMNGRIANPPTD
KSDRDMLDVLIAVKAETGTPRFSADEITGMFISMMFAGHHTSSGTASWTLIELMRHRDAYAAVIDELDELYGD
GRSVSFHALRQIPQLENVLKETLRLHPPLIILMRVAKGEFEVQGHRIHEGDLVAASPAISNRIPEDFPDPHDF
VPARYEQPRQEDLLNRWTWIPFGAGRHRCVGAAFAIMQIKAIFSVLLREYEFEMAQPPESYRNDHSKMVVQLA
QPACVRYRRRTGV
>CYP51B1_Mycobacterium tuberculosis XTB13-088 2584643410
MSAVALPRVSGGHDEHGHLEEFRTDPIGLMQRVRDECGDVGTFQLAGKQVVLLSGSHANEFFFRAGDDDLDQA
KAYPFMTPIFGEGVVFDASPERRKEMLHNAALRGEQMKGHAATIEDQVRRMIADWGEAGEIDLLDFFAELTIY
TSSACLIGKKFRDQLDGRFAKLYHELERGTDPLAYVDPYLPIESFRRRDEARNGLVALVADIMNGRIANPPTD
KSDRDMLDVLIAVKAETGTPRFSADEITGMFISMMFAGHHTSSGTASWTLIELMRHRDAYAAVIDELDELYGD
GRSVSFHALRQIPQLENVLKETLRLHPPLIILMRVAKGEFEVQGHRIHEGDLVAASPAISNRIPEDFPDPHDF
VPARYEQPRQEDLLNRWTWIPFGAGRHRCVGAAFAIMQIKAIFSVLLREYEFEMAQPPESYRNDHSKMVVQLA
QPACVRYRRRTGV
>CYP51B1_Mycobacterium tuberculosis XTB13-089 2621370643
MSAVALPRVSGGHDEHGHLEEFRTDPIGLMQRVRDECGDVGTFQLAGKQVVLLSGSHANEFFFRAGDDDLDQA
KAYPFMTPIFGEGVVFDASPERRKEMLHNAALRGEQMKGHAATIEDQVRRMIADWGEAGEIDLLDFFAELTIY
TSSACLIGKKFRDQLDGRFAKLYHELERGTDPLAYVDPYLPIESFRRRDEARNGLVALVADIMNGRIANPPTD
KSDRDMLDVLIAVKAETGTPRFSADEITGMFISMMFAGHHTSSGTASWTLIELMRHRDAYAAVIDELDELYGD
GRSVSFHALRQIPQLENVLKETLRLHPPLIILMRVAKGEFEVQGHRIHEGDLVAASPAISNRIPEDFPDPHDF
VPARYEQPRQEDLLNRWTWIPFGAGRHRCVGAAFAIMQIKAIFSVLLREYEFEMAQPPESYRNDHSKMVVQLA
QPACVRYRRRTGV
>CYP51B1_Mycobacterium tuberculosis XTB13-091 2621376117
MSAVALPRVSGGHDEHGHLEEFRTDPIGLMQRVRDECGDVGTFQLAGKQVVLLSGSHANEFFFRAGDDDLDQA
KAYPFMTPIFGEGVVFDASPERRKEMLHNAALRGEQMKGHAATIEDQVRRMIADWGEAGEIDLLDFFAELTIY
TSSACLIGKKFRDQLDGRFAKLYHELERGTDPLAYVDPYLPIESFRRRDEARNGLVALVADIMNGRIANPPTD
KSDRDMLDVLIAVKAETGTPRFSADEITGMFISMMFAGHHTSSGTASWTLIELMRHRDAYAAVIDELDELYGD
GRSVSFHALRQIPQLENVLKETLRLHPPLIILMRVAKGEFEVQGHRIHEGDLVAASPAISNRIPEDFPDPHDF

VPARYEQPRQEDLLNRWTWIPFGAGRHRCVGAAFAIMQIKAIFSVLLREYEFEMAQPPESYRNDHSKMVVQLA
QPACVRYRRRTGV
>CYP51B1_Mycobacterium tuberculosis XTB13-092 2584738939
MSAVALPRVSGGHDEHGHLEEFRTDPIGLMQRVRDECGDVGTFQLAGKQVVLLSGSHANEFFFRAGDDDLDQA
KAYPFMTPIFGEGVVFDASPERRKEMLHNAALRGEQMKGHAATIEDQVRRMIADWGEAGEIDLLDFFAELTIY
TSSACLIGKKFRDQLDGRFAKLYHELERGTDPLAYVDPYLPIESFRRRDEARNGLVALVADIMNGRIANPPTD
KSDRDMLDVLIAVKAETGTPRFSADEITGMFISMMFAGHHTSSGTASWTLIELMRHRDAYAAVIDELDELYGD
GRSVSFHALRQIPQLENVLKETLRLHPPLIILMRVAKGEFEVQGHRIHEGDLVAASPAISNRIPEDFPDPHDF
VPARYEQPRQEDLLNRWTWIPFGAGRHRCVGAAFAIMQIKAIFSVLLREYEFEMAQPPESYRNDHSKMVVQLA
QPACVRYRRRTGV
>CYP51B1_Mycobacterium tuberculosis XTB13-093 2576515696
MSAVALPRVSGGHDEHGHLEEFRTDPIGLMQRVRDECGDVGTFQLAGKQVVLLSGSHANEFFFRAGDDDLDQA
KAYPFMTPIFGEGVVFDASPERRKEMLHNAALRGEQMKGHAATIEDQVRRMIADWGEAGEIDLLDFFAELTIY
TSSACLIGKKFRDQLDGRFAKLYHELERGTDPLAYVDPYLPIESFRRRDEARNGLVALVADIMNGRIANPPTD
KSDRDMLDVLIAVKAETGTPRFSADEITGMFISMMFAGHHTSSGTASWTLIELMRHRDAYAAVIDELDELYGD
GRSVSFHALRQIPQLENVLKETLRLHPPLIILMRVAKGEFEVQGHRIHEGDLVAASPAISNRIPEDFPDPHDF
VPARYEQPRQEDLLNRWTWIPFGAGRHRCVGAAFAIMQIKAIFSVLLREYEFEMAQPPESYRNDHSKMVVQLA
QPACVRYRRRTGV
>CYP51B1_Mycobacterium tuberculosis XTB13-094 2576001109
MSAVALPRVSGGHDEHGHLEEFRTDPIGLMQRVRDECGDVGTFQLAGKQVVLLSGSHANEFFFRAGDDDLDQA
KAYPFMTPIFGEGVVFDASPERRKEMLHNAALRGEQMKGHAATIEDQVRRMIADWGEAGEIDLLDFFAELTIY
TSSACLIGKKFRDQLDGRFAKLYHELERGTDPLAYVDPYLPIESFRRRDEARNGLVALVADIMNGRIANPPTD
KSDRDMLDVLIAVKAETGTPRFSADEITGMFISMMFAGHHTSSGTASWTLIELMRHRDAYAAVIDELDELYGD
GRSVSFHALRQIPQLENVLKETLRLHPPLIILMRVAKGEFEVQGHRIHEGDLVAASPAISNRIPEDFPDPHDF
VPARYEQPRQEDLLNRWTWIPFGAGRHRCVGAAFAIMQIKAIFSVLLREYEFEMAQPPESYRNDHSKMVVQLA
QPACVRYRRRTGV
>CYP51B1_Mycobacterium tuberculosis XTB13-095 2621380008
MSAVALPRVSGGHDEHGHLEEFRTDPIGLMQRVRDECGDVGTFQLAGKQVVLLSGSHANEFFFRAGDDDLDQA
KAYPFMTPIFGEGVVFDASPERRKEMLHNAALRGEQMKGHAATIEDQVRRMIADWGEAGEIDLLDFFAELTIY
TSSACLIGKKFRDQLDGRFAKLYHELERGTDPLAYVDPYLPIESFRRRDEARNGLVALVADIMNGRIANPPTD
KSDRDMLDVLIAVKAETGTPRFSADEITGMFISMMFAGHHTSSGTASWTLIELMRHRDAYAAVIDELDELYGD
GRSVSFHALRQIPQLENVLKETLRLHPPLIILMRVAKGEFEVQGHRIHEGDLVAASPAISNRIPEDFPDPHDF
VPARYEQPRQEDLLNRWTWIPFGAGRHRCVGAAFAIMQIKAIFSVLLREYEFEMAQPPESYRNDHSKMVVQLA
QPACVRYRRRTGV
>CYP51B1_Mycobacterium tuberculosis XTB13-096 2576206568
MSAVALPRVSGGHDEHGHLEEFRTDPIGLMQRVRDECGDVGTFQLAGKQVVLLSGSHANEFFFRAGDDDLDQA
KAYPFMTPIFGEGVVFDASPERRKEMLHNAALRGEQMKGHAATIEDQVRRMIADWGEAGEIDLLDFFAELTIY
TSSACLIGKKFRDQLDGRFAKLYHELERGTDPLAYVDPYLPIESFRRRDEARNGLVALVADIMNGRIANPPTD
KSDRDMLDVLIAVKAETGTPRFSADEITGMFISMMFAGHHTSSGTASWTLIELMRHRDAYAAVIDELDELYGD
GRSVSFHALRQIPQLENVLKETLRLHPPLIILMRVAKGEFEVQGHRIHEGDLVAASPAISNRIPEDFPDPHDF
VPARYEQPRQEDLLNRWTWIPFGAGRHRCVGAAFAIMQIKAIFSVLLREYEFEMAQPPESYRNDHSKMVVQLA
QPACVRYRRRTGV
>CYP51B1_Mycobacterium tuberculosis XTB13-097 2606431896
MSAVALPRVSGGHDEHGHLEEFRTDPIGLMQRVRDECGDVGTFQLAGKQVVLLSGSHANEFFFRAGDDDLDQA
KAYPFMTPIFGEGVVFDASPERRKEMLHNAALRGEQMKGHAATIEDQVRRMIADWGEAGEIDLLDFFAELTIY
TSSACLIGKKFRDQLDGRFAKLYHELERGTDPLAYVDPYLPIESFRRRDEARNGLVALVADIMNGRIANPPTD
KSDRDMLDVLIAVKAETGTPRFSADEITGMFISMMFAGHHTSSGTASWTLIELMRHRDAYAAVIDELDELYGD
GRSVSFHALRQIPQLENVLKETLRLHPPLIILMRVAKGEFEVQGHRIHEGDLVAASPAISNRIPEDFPDPHDF
VPARYEQPRQEDLLNRWTWIPFGAGRHRCVGAAFAIMQIKAIFSVLLREYEFEMAQPPESYRNDHSKMVVQLA
QPACVRYRRRTGV
>CYP51B1_Mycobacterium tuberculosis XTB13-098 2606311642
MSAVALPRVSGGHDEHGHLEEFRTDPIGLMQRVRDECGDVGTFQLAGKQVVLLSGSHANEFFFRAGDDDLDQA
KAYPFMTPIFGEGVVFDASPERRKEMLHNAALRGEQMKGHAATIEDQVRRMIADWGEAGEIDLLDFFAELTIY
TSSACLIGKKFRDQLDGRFAKLYHELERGTDPLAYVDPYLPIESFRRRDEARNGLVALVADIMNGRIANPPTD
KSDRDMLDVLIAVKAETGTPRFSADEITGMFISMMFAGHHTSSGTASWTLIELMRHRDAYAAVIDELDELYGD
GRSVSFHALRQIPQLENVLKETLRLHPPLIILMRVAKGEFEVQGHRIHEGDLVAASPAISNRIPEDFPDPHDF
VPARYEQPRQEDLLNRWTWIPFGAGRHRCVGAAFAIMQIKAIFSVLLREYEFEMAQPPESYRNDHSKMVVQLA
QPACVRYRRRTGV

>CYP51B1_Mycobacterium tuberculosis XTB13-100 2584920626
MSAVALPRVSGGHDEHGHLEEFRTDPIGLMQRVRDECGDVGTFQLAGKQVVLLSGSHANEFFFRAGDDDLDQA
KAYPFMTPIFGEGVVFDASPERRKEMLHNAALRGEQMKGHAATIEDQVRRMIADWGEAGEIDLLDFFAELTIY
TSSACLIGKKFRDQLDGRFAKLYHELERGTDPLAYVDPYLPIESFRRRDEARNGLVALVADIMNGRIANPPTD
KSDRDMLDVLIAVKAETGTPRFSADEITGMFISMMFAGHHTSSGTASWTLIELMRHRDAYAAVIDELDELYGD
GRSVSFHALRQIPQLENVLKETLRLHPPLIILMRVAKGEFEVQGHRIHEGDLVAASPAISNRIPEDFPDPHDF
VPARYEQPRQEDLLNRWTWIPFGAGRHRCVGAAFAIMQIKAIFSVLLREYEFEMAQPPESYRNDHSKMVVQLA
QPACVRYRRRTGV
>CYP51B1_Mycobacterium tuberculosis XTB13-102 2621381493
MSAVALPRVSGGHDEHGHLEEFRTDPIGLMQRVRDECGDVGTFQLAGKQVVLLSGSHANEFFFRAGDDDLDQA
KAYPFMTPIFGEGVVFDASPERRKEMLHNAALRGEQMKGHAATIEDQVRRMIADWGEAGEIDLLDFFAELTIY
TSSACLIGKKFRDQLDGRFAKLYHELERGTDPLAYVDPYLPIESFRRRDEARNGLVALVADIMNGRIANPPTD
KSDRDMLDVLIAVKAETGTPRFSADEITGMFISMMFAGHHTSSGTASWTLIELMRHRDAYAAVIDELDELYGD
GRSVSFHALRQIPQLENVLKETLRLHPPLIILMRVAKGEFEVQGHRIHEGDLVAASPAISNRIPEDFPDPHDF
VPARYEQPRQEDLLNRWTWIPFGAGRHRCVGAAFAIMQIKAIFSVLLREYEFEMAQPPESYRNDHSKMVVQLA
QPACVRYRRRTGV
>CYP51B1_Mycobacterium tuberculosis XTB13-103 2621385611
MSAVALPRVSGGHDEHGHLEEFRTDPIGLMQRVRDECGDVGTFQLAGKQVVLLSGSHANEFFFRAGDDDLDQA
KAYPFMTPIFGEGVVFDASPERRKEMLHNAALRGEQMKGHAATIEDQVRRMIADWGEAGEIDLLDFFAELTIY
TSSACLIGKKFRDQLDGRFAKLYHELERGTDPLAYVDPYLPIESFRRRDEARNGLVALVADIMNGRIANPPTD
KSDRDMLDVLIAVKAETGTPRFSADEITGMFISMMFAGHHTSSGTASWTLIELMRHRDAYAAVIDELDELYGD
GRSVSFHALRQIPQLENVLKETLRLHPPLIILMRVAKGEFEVQGHRIHEGDLVAASPAISNRIPEDFPDPHDF
VPARYEQPRQEDLLNRWTWIPFGAGRHRCVGAAFAIMQIKAIFSVLLREYEFEMAQPPESYRNDHSKMVVQLA
QPACVRYRRRTGV
>CYP51B1_Mycobacterium tuberculosis XTB13-104 2621391434
MSAVALPRVSGGHDEHGHLEEFRTDPIGLMQRVRDECGDVGTFQLAGKQVVLLSGSHANEFFFRAGDDDLDQA
KAYPFMTPIFGEGVVFDASPERRKEMLHNAALRGEQMKGHAATIEDQVRRMIADWGEAGEIDLLDFFAELTIY
TSSACLIGKKFRDQLDGRFAKLYHELERGTDPLAYVDPYLPIESFRRRDEARNGLVALVADIMNGRIANPPTD
KSDRDMLDVLIAVKAETGTPRFSADEITGMFISMMFAGHHTSSGTASWTLIELMRHRDAYAAVIDELDELYGD
GRSVSFHALRQIPQLENVLKETLRLHPPLIILMRVAKGEFEVQGHRIHEGDLVAASPAISNRIPEDFPDPHDF
VPARYEQPRQEDLLNRWTWIPFGAGRHRCVGAAFAIMQIKAIFSVLLREYEFEMAQPPESYRNDHSKMVVQLA
QPACVRYRRRTGV
>CYP51B1_Mycobacterium tuberculosis XTB13-105 2621393802
MSAVALPRVSGGHDEHGHLEEFRTDPIGLMQRVRDECGDVGTFQLAGKQVVLLSGSHANEFFFRAGDDDLDQA
KAYPFMTPIFGEGVVFDASPERRKEMLHNAALRGEQMKGHAATIEDQVRRMIADWGEAGEIDLLDFFAELTIY
TSSACLIGKKFRDQLDGRFAKLYHELERGTDPLAYVDPYLPIESFRRRDEARNGLVALVADIMNGRIANPPTD
KSDRDMLDVLIAVKAETGTPRFSADEITGMFISMMFAGHHTSSGTASWTLIELMRHRDAYAAVIDELDELYGD
GRSVSFHALRQIPQLENVLKETLRLHPPLIILMRVAKGEFEVQGHRIHEGDLVAASPAISNRIPEDFPDPHDF
VPARYEQPRQEDLLNRWTWIPFGAGRHRCVGAAFAIMQIKAIFSVLLREYEFEMAQPPESYRNDHSKMVVQLA
QPACVRYRRRTGV
>CYP51B1_Mycobacterium tuberculosis XTB13-107 2621397627
MSAVALPRVSGGHDEHGHLEEFRTDPIGLMQRVRDECGDVGTFQLAGKQVVLLSGSHANEFFFRAGDDDLDQA
KAYPFMTPIFGEGVVFDASPERRKEMLHNAALRGEQMKGHAATIEDQVRRMIADWGEAGEIDLLDFFAELTIY
TSSACLIGKKFRDQLDGRFAKLYHELERGTDPLAYVDPYLPIESFRRRDEARNGLVALVADIMNGRIANPPTD
KSDRDMLDVLIAVKAETGTPRFSADEITGMFISMMFAGHHTSSGTASWTLIELMRHRDAYAAVIDELDELYGD
GRSVSFHALRQIPQLENVLKETLRLHPPLIILMRVAKGEFEVQGHRIHEGDLVAASPAISNRIPEDFPDPHDF
VPARYEQPRQEDLLNRWTWIPFGAGRHRCVGAAFAIMQIKAIFSVLLREYEFEMAQPPESYRNDHSKMVVQLA
QPACVRYRRRTGV
>CYP51B1_Mycobacterium tuberculosis XTB13-108 2621404554
MSAVALPRVSGGHDEHGHLEEFRTDPIGLMQRVRDECGDVGTFQLAGKQVVLLSGSHANEFFFRAGDDDLDQA
KAYPFMTPIFGEGVVFDASPERRKEMLHNAALRGEQMKGHAATIEDQVRRMIADWGEAGEIDLLDFFAELTIY
TSSACLIGKKFRDQLDGRFAKLYHELERGTDPLAYVDPYLPIESFRRRDEARNGLVALVADIMNGRIANPPTD
KSDRDMLDVLIAVKAETGTPRFSADEITGMFISMMFAGHHTSSGTASWTLIELMRHRDAYAAVIDELDELYGD
GRSVSFHALRQIPQLENVLKETLRLHPPLIILMRVAKGEFEVQGHRIHEGDLVAASPAISNRIPEDFPDPHDF
VPARYEQPRQEDLLNRWTWIPFGAGRHRCVGAAFAIMQIKAIFSVLLREYEFEMAQPPESYRNDHSKMVVQLA
QPACVRYRRRTGV
>CYP51B1_Mycobacterium tuberculosis XTB13-110 2584936588

MSAVALPRVSGGHDEHGHLEEFRTDPIGLMQRVRDECGDVGTFQLAGKQVVLLSGSHANEFFFRAGDDDLDQA
KAYPFMTPIFGEGVVFDASPERRKEMLHNAALRGEQMKGHAATIEDQVRRMIADWGEAGEIDLLDFFAELTIY
TSSACLIGKKFRDQLDGRFAKLYHELERGTDPLAYVDPYLPIESFRRRDEARNGLVALVADIMNGRIANPPTD
KSDRDMLDVLIAVKAETGTPRFSADEITGMFISMMFAGHHTSSGTASWTLIELMRHRDAYAAVIDELDELYGD
GRSVSFHALRQIPQLENVLKETLRLHPPLIILMRVAKGEFEVQGHRIHEGDLVAASPAISNRIPEDFPDPHDF
VPARYEQPRQEDLLNRWTWIPFGAGRHRCVGAAFAIMQIKAIFSVLLREYEFEMAQPPESYRNDHSKMVVQLA
QPACVRYRRRTGV
>CYP51B1_Mycobacterium tuberculosis XTB13-111 2606409720
MSAVALPRVSGGHDEHGHLEEFRTDPIGLMQRVRDECGDVGTFQLAGKQVVLLSGSHANEFFFRAGDDDLDQA
KAYPFMTPIFGEGVVFDASPERRKEMLHNAALRGEQMKGHAATIEDQVRRMIADWGEAGEIDLLDFFAELTIY
TSSACLIGKKFRDQLDGRFAKLYHELERGTDPLAYVDPYLPIESFRRRDEARNGLVALVADIMNGRIANPPTD
KSDRDMLDVLIAVKAETGTPRFSADEITGMFISMMFAGHHTSSGTASWTLIELMRHRDAYAAVIDELDELYGD
GRSVSFHALRQIPQLENVLKETLRLHPPLIILMRVAKGEFEVQGHRIHEGDLVAASPAISNRIPEDFPDPHDF
VPARYEQPRQEDLLNRWTWIPFGAGRHRCVGAAFAIMQIKAIFSVLLREYEFEMAQPPESYRNDHSKMVVQLA
QPACVRYRRRTGV
>CYP51B1_Mycobacterium tuberculosis XTB13-112 2621408727
MSAVALPRVSGGHDEHGHLEEFRTDPIGLMQRVRDECGDVGTFQLAGKQVVLLSGSHANEFFFRAGDDDLDQA
KAYPFMTPIFGEGVVFDASPERRKEMLHNAALRGEQMKGHAATIEDQVRRMIADWGEAGEIDLLDFFAELTIY
TSSACLIGKKFRDQLDGRFAKLYHELERGTDPLAYVDPYLPIESFRRRDEARNGLVALVADIMNGRIANPPTD
KSDRDMLDVLIAVKAETGTPRFSADEITGMFISMMFAGHHTSSGTASWTLIELMRHRDAYAAVIDELDELYGD
GRSVSFHALRQIPQLENVLKETLRLHPPLIILMRVAKGEFEVQGHRIHEGDLVAASPAISNRIPEDFPDPHDF
VPARYEQPRQEDLLNRWTWIPFGAGRHRCVGAAFAIMQIKAIFSVLLREYEFEMAQPPESYRNDHSKMVVQLA
QPACVRYRRRTGV
>CYP51B1_Mycobacterium tuberculosis XTB13-113 2584818956
MSAVALPRVSGGHDEHGHLEEFRTDPIGLMQRVRDECGDVGTFQLAGKQVVLLSGSHANEFFFRAGDDDLDQA
KAYPFMTPIFGEGVVFDASPERRKEMLHNAALRGEQMKGHAATIEDQVRRMIADWGEAGEIDLLDFFAELTIY
TSSACLIGKKFRDQLDGRFAKLYHELERGTDPLAYVDPYLPIESFRRRDEARNGLVALVADIMNGRIANPPTD
KSDRDMLDVLIAVKAETGTPRFSADEITGMFISMMFAGHHTSSGTASWTLIELMRHRDAYAAVIDELDELYGD
GRSVSFHALRQIPQLENVLKETLRLHPPLIILMRVAKGEFEVQGHRIHEGDLVAASPAISNRIPEDFPDPHDF
VPARYEQPRQEDLLNRWTWIPFGAGRHRCVGAAFAIMQIKAIFSVLLREYEFEMAQPPESYRNDHSKMVVQLA
QPACVRYRRRTGV
>CYP51B1_Mycobacterium tuberculosis XTB13-114 2574796693
MSAVALPRVSGGHDEHGHLEEFRTDPIGLMQRVRDECGDVGTFQLAGKQVVLLSGSHANEFFFRAGDDDLDQA
KAYPFMTPIFGEGVVFDASPERRKEMLHNAALRGEQMKGHAATIEDQVRRMIADWGEAGEIDLLDFFAELTIY
TSSACLIGKKFRDQLDGRFAKLYHELERGTDPLAYVDPYLPIESFRRRDEARNGLVALVADIMNGRIANPPTD
KSDRDMLDVLIAVKAETGTPRFSADEITGMFISMMFAGHHTSSGTASWTLIELMRHRDAYAAVIDELDELYGD
GRSVSFHALRQIPQLENVLKETLRLHPPLIILMRVAKGEFEVQGHRIHEGDLVAASPAISNRIPEDFPDPHDF
VPARYEQPRQEDLLNRWTWIPFGAGRHRCVGAAFAIMQIKAIFSVLLREYEFEMAQPPESYRNDHSKMVVQLA
QPACVRYRRRTGV
>CYP51B1_Mycobacterium tuberculosis XTB13-115 2621411220
MSAVALPRVSGGHDEHGHLEEFRTDPIGLMQRVRDECGDVGTFQLAGKQVVLLSGSHANEFFFRAGDDDLDQA
KAYPFMTPIFGEGVVFDASPERRKEMLHNAALRGEQMKGHAATIEDQVRRMIADWGEAGEIDLLDFFAELTIY
TSSACLIGKKFRDQLDGRFAKLYHELERGTDPLAYVDPYLPIESFRRRDEARNGLVALVADIMNGRIANPPTD
KSDRDMLDVLIAVKAETGTPRFSADEITGMFISMMFAGHHTSSGTASWTLIELMRHRDAYAAVIDELDELYGD
GRSVSFHALRQIPQLENVLKETLRLHPPLIILMRVAKGEFEVQGHRIHEGDLVAASPAISNRIPEDFPDPHDF
VPARYEQPRQEDLLNRWTWIPFGAGRHRCVGAAFAIMQIKAIFSVLLREYEFEMAQPPESYRNDHSKMVVQLA
QPACVRYRRRTGV
>CYP51B1_Mycobacterium tuberculosis XTB13-116 2621416847
MSAVALPRVSGGHDEHGHLEEFRTDPIGLMQRVRDECGDVGTFQLAGKQVVLLSGSHANEFFFRAGDDDLDQA
KAYPFMTPIFGEGVVFDASPERRKEMLHNAALRGEQMKGHAATIEDQVRRMIADWGEAGEIDLLDFFAELTIY
TSSACLIGKKFRDQLDGRFAKLYHELERGTDPLAYVDPYLPIESFRRRDEARNGLVALVADIMNGRIANPPTD
KSDRDMLDVLIAVKAETGTPRFSADEITGMFISMMFAGHHTSSGTASWTLIELMRHRDAYAAVIDELDELYGD
GRSVSFHALRQIPQLENVLKETLRLHPPLIILMRVAKGEFEVQGHRIHEGDLVAASPAISNRIPEDFPDPHDF
VPARYEQPRQEDLLNRWTWIPFGAGRHRCVGAAFAIMQIKAIFSVLLREYEFEMAQPPESYRNDHSKMVVQLA
QPACVRYRRRTGV
>CYP51B1_Mycobacterium tuberculosis XTB13-117 2621421049
MSAVALPRVSGGHDEHGHLEEFRTDPIGLMQRVRDECGDVGTFQLAGKQVVLLSGSHANEFFFRAGDDDLDQA
KAYPFMTPIFGEGVVFDASPERRKEMLHNAALRGEQMKGHAATIEDQVRRMIADWGEAGEIDLLDFFAELTIY

TSSACLIGKKFRDQLDGRFAKLYHELERGTDPLAYVDPYLPIESFRRRDEARNGLVALVADIMNGRIANPPTD
KSDRDMLDVLIAVKAETGTPRFSADEITGMFISMMFAGHHTSSGTASWTLIELMRHRDAYAAVIDELDELYGD
GRSVSFHALRQIPQLENVLKETLRLHPPLIILMRVAKGEFEVQGHRIHEGDLVAASPAISNRIPEDFPDPHDF
VPARYEQPRQEDLLNRWTWIPFGAGRHRCVGAAFAIMQIKAIFSVLLREYEFEMAQPPESYRNDHSKMVVQLA
QPACVRYRRRTGV
>CYP51B1_Mycobacterium tuberculosis XTB13-119 2606433460
MSAVALPRVSGGHDEHGHLEEFRTDPIGLMQRVRDECGDVGTFQLAGKQVVLLSGSHANEFFFRAGDDDLDQA
KAYPFMTPIFGEGVVFDASPERRKEMLHNAALRGEQMKGHAATIEDQVRRMIADWGEAGEIDLLDFFAELTIY
TSSACLIGKKFRDQLDGRFAKLYHELERGTDPLAYVDPYLPIESFRRRDEARNGLVALVADIMNGRIANPPTD
KSDRDMLDVLIAVKAETGTPRFSADEITGMFISMMFAGHHTSSGTASWTLIELMRHRDAYAAVIDELDELYGD
GRSVSFHALRQIPQLENVLKETLRLHPPLIILMRVAKGEFEVQGHRIHEGDLVAASPAISNRIPEDFPDPHDF
VPARYEQPRQEDLLNRWTWIPFGAGRHRCVGAAFAIMQIKAIFSVLLREYEFEMAQPPESYRNDHSKMVVQLA
QPACVRYRRRTGV
>CYP51B1_Mycobacterium tuberculosis XTB13-120 2621425167
MSAVALPRVSGGHDEHGHLEEFRTDPIGLMQRVRDECGDVGTFQLAGKQVVLLSGSHANEFFFRAGDDDLDQA
KAYPFMTPIFGEGVVFDASPERRKEMLHNAALRGEQMKGHAATIEDQVRRMIADWGEAGEIDLLDFFAELTIY
TSSACLIGKKFRDQLDGRFAKLYHELERGTDPLAYVDPYLPIESFRRRDEARNGLVALVADIMNGRIANPPTD
KSDRDMLDVLIAVKAETGTPRFSADEITGMFISMMFAGHHTSSGTASWTLIELMRHRDAYAAVIDELDELYGD
GRSVSFHALRQIPQLENVLKETLRLHPPLIILMRVAKGEFEVQGHRIHEGDLVAASPAISNRIPEDFPDPHDF
VPARYEQPRQEDLLNRWTWIPFGAGRHRCVGAAFAIMQIKAIFSVLLREYEFEMAQPPESYRNDHSKMVVQLA
QPACVRYRRRTGV
>CYP51B1_Mycobacterium tuberculosis XTB13-121 2577163194
MSAVALPRVSGGHDEHGHLEEFRTDPIGLMQRVRDECGDVGTFQLAGKQVVLLSGSHANEFFFRAGDDDLDQA
KAYPFMTPIFGEGVVFDASPERRKEMLHNAALRGEQMKGHAATIEDQVRRMIADWGEAGEIDLLDFFAELTIY
TSSACLIGKKFRDQLDGRFAKLYHELERGTDPLAYVDPYLPIESFRRRDEARNGLVALVADIMNGRIANPPTD
KSDRDMLDVLIAVKAETGTPRFSADEITGMFISMMFAGHHTSSGTASWTLIELMRHRDAYAAVIDELDELYGD
GRSVSFHALRQIPQLENVLKETLRLHPPLIILMRVAKGEFEVQGHRIHEGDLVAASPAISNRIPEDFPDPHDF
VPARYEQPRQEDLLNRWTWIPFGAGRHRCVGAAFAIMQIKAIFSVLLREYEFEMAQPPESYRNDHSKMVVQLA
QPACVRYRRRTGV
>CYP51B1_Mycobacterium tuberculosis XTB13-122 2621429290
MSAVALPRVSGGHDEHGHLEEFRTDPIGLMQRVRDECGDVGTFQLAGKQVVLLSGSHANEFFFRAGDDDLDQA
KAYPFMTPIFGEGVVFDASPERRKEMLHNAALRGEQMKGHAATIEDQVRRMIADWGEAGEIDLLDFFAELTIY
TSSACLIGKKFRDQLDGRFAKLYHELERGTDPLAYVDPYLPIESFRRRDEARNGLVALVADIMNGRIANPPTD
KSDRDMLDVLIAVKAETGTPRFSADEITGMFISMMFAGHHTSSGTASWTLIELMRHRDAYAAVIDELDELYGD
GRSVSFHALRQIPQLENVLKETLRLHPPLIILMRVAKGEFEVQGHRIHEGDLVAASPAISNRIPEDFPDPHDF
VPARYEQPRQEDLLNRWTWIPFGAGRHRCVGAAFAIMQIKAIFSVLLREYEFEMAQPPESYRNDHSKMVVQLA
QPACVRYRRRTGV
>CYP51B1_Mycobacterium tuberculosis XTB13-123 2578238836
MSAVALPRVSGGHDEHGHLEEFRTDPIGLMQRVRDECGDVGTFQLAGKQVVLLSGSHANEFFFRAGDDDLDQA
KAYPFMTPIFGEGVVFDASPERRKEMLHNAALRGEQMKGHAATIEDQVRRMIADWGEAGEIDLLDFFAELTIY
TSSACLIGKKFRDQLDGRFAKLYHELERGTDPLAYVDPYLPIESFRRRDEARNGLVALVADIMNGRIANPPTD
KSDRDMLDVLIAVKAETGTPRFSADEITGMFISMMFAGHHTSSGTASWTLIELMRHRDAYAAVIDELDELYGD
GRSVSFHALRQIPQLENVLKETLRLHPPLIILMRVAKGEFEVQGHRIHEGDLVAASPAISNRIPEDFPDPHDF
VPARYEQPRQEDLLNRWTWIPFGAGRHRCVGAAFAIMQIKAIFSVLLREYEFEMAQPPESYRNDHSKMVVQLA
QPACVRYRRRTGV
>CYP51B1_Mycobacterium tuberculosis XTB13-124 2621430227
MSAVALPRVSGGHDEHGHLEEFRTDPIGLMQRVRDECGDVGTFQLAGKQVVLLSGSHANEFFFRAGDDDLDQA
KAYPFMTPIFGEGVVFDASPERRKEMLHNAALRGEQMKGHAATIEDQVRRMIADWGEAGEIDLLDFFAELTIY
TSSACLIGKKFRDQLDGRFAKLYHELERGTDPLAYVDPYLPIESFRRRDEARNGLVALVADIMNGRIANPPTD
KSDRDMLDVLIAVKAETGTPRFSADEITGMFISMMFAGHHTSSGTASWTLIELMRHRDAYAAVIDELDELYGD
GRSVSFHALRQIPQLENVLKETLRLHPPLIILMRVAKGEFEVQGHRIHEGDLVAASPAISNRIPEDFPDPHDF
VPARYEQPRQEDLLNRWTWIPFGAGRHRCVGAAFAIMQIKAIFSVLLREYEFEMAQPPESYRNDHSKMVVQLA
QPACVRYRRRTGV
>CYP51B1_Mycobacterium tuberculosis XTB13-125 2621436375
MSAVALPRVSGGHDEHGHLEEFRTDPIGLMQRVRDECGDVGTFQLAGKQVVLLSGSHANEFFFRAGDDDLDQA
KAYPFMTPIFGEGVVFDASPERRKEMLHNAALRGEQMKGHAATIEDQVRRMIADWGEAGEIDLLDFFAELTIY
TSSACLIGKKFRDQLDGRFAKLYHELERGTDPLAYVDPYLPIESFRRRDEARNGLVALVADIMNGRIANPPTD
KSDRDMLDVLIAVKAETGTPRFSADEITGMFISMMFAGHHTSSGTASWTLIELMRHRDAYAAVIDELDELYGD

GRSVSFHALRQIPQLENVLKETLRLHPPLIILMRVAKGEFEVQGHRIHEGDLVAASPAISNRIPEDFPDPHDF
VPARYEQPRQEDLLNRWTWIPFGAGRHRCVGAAFAIMQIKAIFSVLLREYEFEMAQPPESYRNDHSKMVVQLA
QPACVRYRRRTGV
>CYP51B1_Mycobacterium tuberculosis XTB13-126 2606437541
MSAVALPRVSGGHDEHGHLEEFRTDPIGLMQRVRDECGDVGTFQLAGKQVVLLSGSHANEFFFRAGDDDLDQA
KAYPFMTPIFGEGVVFDASPERRKEMLHNAALRGEQMKGHAATIEDQVRRMIADWGEAGEIDLLDFFAELTIY
TSSACLIGKKFRDQLDGRFAKLYHELERGTDPLAYVDPYLPIESFRRRDEARNGLVALVADIMNGRIANPPTD
KSDRDMLDVLIAVKAETGTPRFSADEITGMFISMMFAGHHTSSGTASWTLIELMRHRDAYAAVIDELDELYGD
GRSVSFHALRQIPQLENVLKETLRLHPPLIILMRVAKGEFEVQGHRIHEGDLVAASPAISNRIPEDFPDPHDF
VPARYEQPRQEDLLNRWTWIPFGAGRHRCVGAAFAIMQIKAIFSVLLREYEFEMAQPPESYRNDHSKMVVQLA
QPACVRYRRRTGV
>CYP51B1_Mycobacterium tuberculosis XTB13-127 2575364928
MSAVALPRVSGGHDEHGHLEEFRTDPIGLMQRVRDECGDVGTFQLAGKQVVLLSGSHANEFFFRAGDDDLDQA
KAYPFMTPIFGEGVVFDASPERRKEMLHNAALRGEQMKGHAATIEDQVRRMIADWGEAGEIDLLDFFAELTIY
TSSACLIGKKFRDQLDGRFAKLYHELERGTDPLAYVDPYLPIESFRRRDEARNGLVALVADIMNGRIANPPTD
KSDRDMLDVLIAVKAETGTPRFSADEITGMFISMMFAGHHTSSGTASWTLIELMRHRDAYAAVIDELDELYGD
GRSVSFHALRQIPQLENVLKETLRLHPPLIILMRVAKGEFEVQGHRIHEGDLVAASPAISNRIPEDFPDPHDF
VPARYEQPRQEDLLNRWTWIPFGAGRHRCVGAAFAIMQIKAIFSVLLREYEFEMAQPPESYRNDHSKMVVQLA
QPACVRYRRRTGV
>CYP51B1_Mycobacterium tuberculosis XTB13-128 2621438777
MSAVALPRVSGGHDEHGHLEEFRTDPIGLMQRVRDECGDVGTFQLAGKQVVLLSGSHANEFFFRAGDDDLDQA
KAYPFMTPIFGEGVVFDASPERRKEMLHNAALRGEQMKGHAATIEDQVRRMIADWGEAGEIDLLDFFAELTIY
TSSACLIGKKFRDQLDGRFAKLYHELERGTDPLAYVDPYLPIESFRRRDEARNGLVALVADIMNGRIANPPTD
KSDRDMLDVLIAVKAETGTPRFSADEITGMFISMMFAGHHTSSGTASWTLIELMRHRDAYAAVIDELDELYGD
GRSVSFHALRQIPQLENVLKETLRLHPPLIILMRVAKGEFEVQGHRIHEGDLVAASPAISNRIPEDFPDPHDF
VPARYEQPRQEDLLNRWTWIPFGAGRHRCVGAAFAIMQIKAIFSVLLREYEFEMAQPPESYRNDHSKMVVQLA
QPACVRYRRRTGV
>CYP51B1_Mycobacterium tuberculosis XTB13-131 2575700016
MSAVALPRVSGGHDEHGHLEEFRTDPIGLMQRVRDECGDVGTFQLAGKQVVLLSGSHANEFFFRAGDDDLDQA
KAYPFMTPIFGEGVVFDASPERRKEMLHNAALRGEQMKGHAATIEDQVRRMIADWGEAGEIDLLDFFAELTIY
TSSACLIGKKFRDQLDGRFAKLYHELERGTDPLAYVDPYLPIESFRRRDEARNGLVALVADIMNGRIANPPTD
KSDRDMLDVLIAVKAETGTPRFSADEITGMFISMMFAGHHTSSGTASWTLIELMRHRDAYAAVIDELDELYGD
GRSVSFHALRQIPQLENVLKETLRLHPPLIILMRVAKGEFEVQGHRIHEGDLVAASPAISNRIPEDFPDPHDF
VPARYEQPRQEDLLNRWTWIPFGAGRHRCVGAAFAIMQIKAIFSVLLREYEFEMAQPPESYRNDHSKMVVQLA
QPACVRYRRRTGV
>CYP51B1_Mycobacterium tuberculosis XTB13-132 2606442578
MSAVALPRVSGGHDEHGHLEEFRTDPIGLMQRVRDECGDVGTFQLAGKQVVLLSGSHANEFFFRAGDDDLDQA
KAYPFMTPIFGEGVVFDASPERRKEMLHNAALRGEQMKGHAATIEDQVRRMIADWGEAGEIDLLDFFAELTIY
TSSACLIGKKFRDQLDGRFAKLYHELERGTDPLAYVDPYLPIESFRRRDEARNGLVALVADIMNGRIANPPTD
KSDRDMLDVLIAVKAETGTPRFSADEITGMFISMMFAGHHTSSGTASWTLIELMRHRDAYAAVIDELDELYGD
GRSVSFHALRQIPQLENVLKETLRLHPPLIILMRVAKGEFEVQGHRIHEGDLVAASPAISNRIPEDFPDPHDF
VPARYEQPRQEDLLNRWTWIPFGAGRHRCVGAAFAIMQIKAIFSVLLREYEFEMAQPPESYRNDHSKMVVQLA
QPACVRYRRRTGV
>CYP51B1_Mycobacterium tuberculosis XTB13-133 2621445740
MSAVALPRVSGGHDEHGHLEEFRTDPIGLMQRVRDECGDVGTFQLAGKQVVLLSGSHANEFFFRAGDDDLDQA
KAYPFMTPIFGEGVVFDASPERRKEMLHNAALRGEQMKGHAATIEDQVRRMIADWGEAGEIDLLDFFAELTIY
TSSACLIGKKFRDQLDGRFAKLYHELERGTDPLAYVDPYLPIESFRRRDEARNGLVALVADIMNGRIANPPTD
KSDRDMLDVLIAVKAETGTPRFSADEITGMFISMMFAGHHTSSGTASWTLIELMRHRDAYAAVIDELDELYGD
GRSVSFHALRQIPQLENVLKETLRLHPPLIILMRVAKGEFEVQGHRIHEGDLVAASPAISNRIPEDFPDPHDF
VPARYEQPRQEDLLNRWTWIPFGAGRHRCVGAAFAIMQIKAIFSVLLREYEFEMAQPPESYRNDHSKMVVQLA
QPACVRYRRRTGV
>CYP51B1_Mycobacterium tuberculosis XTB13-134 2621449826
MSAVALPRVSGGHDEHGHLEEFRTDPIGLMQRVRDECGDVGTFQLAGKQVVLLSGSHANEFFFRAGDDDLDQA
KAYPFMTPIFGEGVVFDASPERRKEMLHNAALRGEQMKGHAATIEDQVRRMIADWGEAGEIDLLDFFAELTIY
TSSACLIGKKFRDQLDGRFAKLYHELERGTDPLAYVDPYLPIESFRRRDEARNGLVALVADIMNGRIANPPTD
KSDRDMLDVLIAVKAETGTPRFSADEITGMFISMMFAGHHTSSGTASWTLIELMRHRDAYAAVIDELDELYGD
GRSVSFHALRQIPQLENVLKETLRLHPPLIILMRVAKGEFEVQGHRIHEGDLVAASPAISNRIPEDFPDPHDF

VPARYEQPRQEDLLNRWTWIPFGAGRHRCVGAAFAIMQIKAIFSVLLREYEFEMAQPPESYRNDHSKMVVQLA
QPACVRYRRRTGV
>CYP51B1_Mycobacterium tuberculosis XTB13-135 2606318543
MSAVALPRVSGGHDEHGHLEEFRTDPIGLMQRVRDECGDVGTFQLAGKQVVLLSGSHANEFFFRAGDDDLDQA
KAYPFMTPIFGEGVVFDASPERRKEMLHNAALRGEQMKGHAATIEDQVRRMIADWGEAGEIDLLDFFAELTIY
TSSACLIGKKFRDQLDGRFAKLYHELERGTDPLAYVDPYLPIESFRRRDEARNGLVALVADIMNGRIANPPTD
KSDRDMLDVLIAVKAETGTPRFSADEITGMFISMMFAGHHTSSGTASWTLIELMRHRDAYAAVIDELDELYGD
GRSVSFHALRQIPQLENVLKETLRLHPPLIILMRVAKGEFEVQGHRIHEGDLVAASPAISNRIPEDFPDPHDF
VPARYEQPRQEDLLNRWTWIPFGAGRHRCVGAAFAIMQIKAIFSVLLREYEFEMAQPPESYRNDHSKMVVQLA
QPACVRYRRRTGV
>CYP51B1_Mycobacterium tuberculosis XTB13-136 2576745589
MSAVALPRVSGGHDEHGHLEEFRTDPIGLMQRVRDECGDVGTFQLAGKQVVLLSGSHANEFFFRAGDDDLDQA
KAYPFMTPIFGEGVVFDASPERRKEMLHNAALRGEQMKGHAATIEDQVRRMIADWGEAGEIDLLDFFAELTIY
TSSACLIGKKFRDQLDGRFAKLYHELERGTDPLAYVDPYLPIESFRRRDEARNGLVALVADIMNGRIANPPTD
KSDRDMLDVLIAVKAETGTPRFSADEITGMFISMMFAGHHTSSGTASWTLIELMRHRDAYAAVIDELDELYGD
GRSVSFHALRQIPQLENVLKETLRLHPPLIILMRVAKGEFEVQGHRIHEGDLVAASPAISNRIPEDFPDPHDF
VPARYEQPRQEDLLNRWTWIPFGAGRHRCVGAAFAIMQIKAIFSVLLREYEFEMAQPPESYRNDHSKMVVQLA
QPACVRYRRRTGV
>CYP51B1_Mycobacterium tuberculosis XTB13-137 2621453985
MSAVALPRVSGGHDEHGHLEEFRTDPIGLMQRVRDECGDVGTFQLAGKQVVLLSGSHANEFFFRAGDDDLDQA
KAYPFMTPIFGEGVVFDASPERRKEMLHNAALRGEQMKGHAATIEDQVRRMIADWGEAGEIDLLDFFAELTIY
TSSACLIGKKFRDQLDGRFAKLYHELERGTDPLAYVDPYLPIESFRRRDEARNGLVALVADIMNGRIANPPTD
KSDRDMLDVLIAVKAETGTPRFSADEITGMFISMMFAGHHTSSGTASWTLIELMRHRDAYAAVIDELDELYGD
GRSVSFHALRQIPQLENVLKETLRLHPPLIILMRVAKGEFEVQGHRIHEGDLVAASPAISNRIPEDFPDPHDF
VPARYEQPRQEDLLNRWTWIPFGAGRHRCVGAAFAIMQIKAIFSVLLREYEFEMAQPPESYRNDHSKMVVQLA
QPACVRYRRRTGV
>CYP51B1_Mycobacterium tuberculosis XTB13-138 2621455542
MSAVALPRVSGGHDEHGHLEEFRTDPIGLMQRVRDECGDVGTFQLAGKQVVLLSGSHANEFFFRAGDDDLDQA
KAYPFMTPIFGEGVVFDASPERRKEMLHNAALRGEQMKGHAATIEDQVRRMIADWGEAGEIDLLDFFAELTIY
TSSACLIGKKFRDQLDGRFAKLYHELERGTDPLAYVDPYLPIESFRRRDEARNGLVALVADIMNGRIANPPTD
KSDRDMLDVLIAVKAETGTPRFSADEITGMFISMMFAGHHTSSGTASWTLIELMRHRDAYAAVIDELDELYGD
GRSVSFHALRQIPQLENVLKETLRLHPPLIILMRVAKGEFEVQGHRIHEGDLVAASPAISNRIPEDFPDPHDF
VPARYEQPRQEDLLNRWTWIPFGAGRHRCVGAAFAIMQIKAIFSVLLREYEFEMAQPPESYRNDHSKMVVQLA
QPACVRYRRRTGV
>CYP51B1_Mycobacterium tuberculosis XTB13-140 2621462224
MSAVALPRVSGGHDEHGHLEEFRTDPIGLMQRVRDECGDVGTFQLAGKQVVLLSGSHANEFFFRAGDDDLDQA
KAYPFMTPIFGEGVVFDASPERRKEMLHNAALRGEQMKGHAATIEDQVRRMIADWGEAGEIDLLDFFAELTIY
TSSACLIGKKFRDQLDGRFAKLYHELERGTDPLAYVDPYLPIESFRRRDEARNGLVALVADIMNGRIANPPTD
KSDRDMLDVLIAVKAETGTPRFSADEITGMFISMMFAGHHTSSGTASWTLIELMRHRDAYAAVIDELDELYGD
GRSVSFHALRQIPQLENVLKETLRLHPPLIILMRVAKGEFEVQGHRIHEGDLVAASPAISNRIPEDFPDPHDF
VPARYEQPRQEDLLNRWTWIPFGAGRHRCVGAAFAIMQIKAIFSVLLREYEFEMAQPPESYRNDHSKMVVQLA
QPACVRYRRRTGV
>CYP51B1_Mycobacterium tuberculosis XTB13-141 2606320310
MSAVALPRVSGGHDEHGHLEEFRTDPIGLMQRVRDECGDVGTFQLAGKQVVLLSGSHANEFFFRAGDDDLDQA
KAYPFMTPIFGEGVVFDASPERRKEMLHNAALRGEQMKGHAATIEDQVRRMIADWGEAGEIDLLDFFAELTIY
TSSACLIGKKFRDQLDGRFAKLYHELERGTDPLAYVDPYLPIESFRRRDEARNGLVALVADIMNGRIANPPTD
KSDRDMLDVLIAVKAETGTPRFSADEITGMFISMMFAGHHTSSGTASWTLIELMRHRDAYAAVIDELDELYGD
GRSVSFHALRQIPQLENVLKETLRLHPPLIILMRVAKGEFEVQGHRIHEGDLVAASPAISNRIPEDFPDPHDF
VPARYEQPRQEDLLNRWTWIPFGAGRHRCVGAAFAIMQIKAIFSVLLREYEFEMAQPPESYRNDHSKMVVQLA
QPACVRYRRRTGV
>CYP51B1_Mycobacterium tuberculosis XTB13-142 2621466343
MSAVALPRVSGGHDEHGHLEEFRTDPIGLMQRVRDECGDVGTFQLAGKQVVLLSGSHANEFFFRAGDDDLDQA
KAYPFMTPIFGEGVVFDASPERRKEMLHNAALRGEQMKGHAATIEDQVRRMIADWGEAGEIDLLDFFAELTIY
TSSACLIGKKFRDQLDGRFAKLYHELERGTDPLAYVDPYLPIESFRRRDEARNGLVALVADIMNGRIANPPTD
KSDRDMLDVLIAVKAETGTPRFSADEITGMFISMMFAGHHTSSGTASWTLIELMRHRDAYAAVIDELDELYGD
GRSVSFHALRQIPQLENVLKETLRLHPPLIILMRVAKGEFEVQGHRIHEGDLVAASPAISNRIPEDFPDPHDF
VPARYEQPRQEDLLNRWTWIPFGAGRHRCVGAAFAIMQIKAIFSVLLREYEFEMAQPPESYRNDHSKMVVQLA
QPACVRYRRRTGV

>CYP51B1_Mycobacterium tuberculosis XTB13-143 2576318861
MSAVALPRVSGGHDEHGHLEEFRTDPIGLMQRVRDECGDVGTFQLAGKQVVLLSGSHANEFFFRAGDDDLDQA
KAYPFMTPIFGEGVVFDASPERRKEMLHNAALRGEQMKGHAATIEDQVRRMIADWGEAGEIDLLDFFAELTIY
TSSACLIGKKFRDQLDGRFAKLYHELERGTDPLAYVDPYLPIESFRRRDEARNGLVALVADIMNGRIANPPTD
KSDRDMLDVLIAVKAETGTPRFSADEITGMFISMMFAGHHTSSGTASWTLIELMRHRDAYAAVIDELDELYGD
GRSVSFHALRQIPQLENVLKETLRLHPPLIILMRVAKGEFEVQGHRIHEGDLVAASPAISNRIPEDFPDPHDF
VPARYEQPRQEDLLNRWTWIPFGAGRHRCVGAAFAIMQIKAIFSVLLREYEFEMAQPPESYRNDHSKMVVQLA
QPACVRYRRRTGV
>CYP51B1_Mycobacterium tuberculosis XTB13-144 2606445749
MSAVALPRVSGGHDEHGHLEEFRTDPIGLMQRVRDECGDVGTFQLAGKQVVLLSGSHANEFFFRAGDDDLDQA
KAYPFMTPIFGEGVVFDASPERRKEMLHNAALRGEQMKGHAATIEDQVRRMIADWGEAGEIDLLDFFAELTIY
TSSACLIGKKFRDQLDGRFAKLYHELERGTDPLAYVDPYLPIESFRRRDEARNGLVALVADIMNGRIANPPTD
KSDRDMLDVLIAVKAETGTPRFSADEITGMFISMMFAGHHTSSGTASWTLIELMRHRDAYAAVIDELDELYGD
GRSVSFHALRQIPQLENVLKETLRLHPPLIILMRVAKGEFEVQGHRIHEGDLVAASPAISNRIPEDFPDPHDF
VPARYEQPRQEDLLNRWTWIPFGAGRHRCVGAAFAIMQIKAIFSVLLREYEFEMAQPPESYRNDHSKMVVQLA
QPACVRYRRRTGV
>CYP51B1_Mycobacterium tuberculosis XTB13-145 2621470460
MSAVALPRVSGGHDEHGHLEEFRTDPIGLMQRVRDECGDVGTFQLAGKQVVLLSGSHANEFFFRAGDDDLDQA
KAYPFMTPIFGEGVVFDASPERRKEMLHNAALRGEQMKGHAATIEDQVRRMIADWGEAGEIDLLDFFAELTIY
TSSACLIGKKFRDQLDGRFAKLYHELERGTDPLAYVDPYLPIESFRRRDEARNGLVALVADIMNGRIANPPTD
KSDRDMLDVLIAVKAETGTPRFSADEITGMFISMMFAGHHTSSGTASWTLIELMRHRDAYAAVIDELDELYGD
GRSVSFHALRQIPQLENVLKETLRLHPPLIILMRVAKGEFEVQGHRIHEGDLVAASPAISNRIPEDFPDPHDF
VPARYEQPRQEDLLNRWTWIPFGAGRHRCVGAAFAIMQIKAIFSVLLREYEFEMAQPPESYRNDHSKMVVQLA
QPACVRYRRRTGV
>CYP51B1_Mycobacterium tuberculosis XTB13-146 2621474582
MSAVALPRVSGGHDEHGHLEEFRTDPIGLMQRVRDECGDVGTFQLAGKQVVLLSGSHANEFFFRAGDDDLDQA
KAYPFMTPIFGEGVVFDASPERRKEMLHNAALRGEQMKGHAATIEDQVRRMIADWGEAGEIDLLDFFAELTIY
TSSACLIGKKFRDQLDGRFAKLYHELERGTDPLAYVDPYLPIESFRRRDEARNGLVALVADIMNGRIANPPTD
KSDRDMLDVLIAVKAETGTPRFSADEITGMFISMMFAGHHTSSGTASWTLIELMRHRDAYAAVIDELDELYGD
GRSVSFHALRQIPQLENVLKETLRLHPPLIILMRVAKGEFEVQGHRIHEGDLVAASPAISNRIPEDFPDPHDF
VPARYEQPRQEDLLNRWTWIPFGAGRHRCVGAAFAIMQIKAIFSVLLREYEFEMAQPPESYRNDHSKMVVQLA
QPACVRYRRRTGV
>CYP51B1_Mycobacterium tuberculosis XTB13-147 2621478671
MSAVALPRVSGGHDEHGHLEEFRTDPIGLMQRVRDECGDVGTFQLAGKQVVLLSGSHANEFFFRAGDDDLDQA
KAYPFMTPIFGEGVVFDASPERRKEMLHNAALRGEQMKGHAATIEDQVRRMIADWGEAGEIDLLDFFAELTIY
TSSACLIGKKFRDQLDGRFAKLYHELERGTDPLAYVDPYLPIESFRRRDEARNGLVALVADIMNGRIANPPTD
KSDRDMLDVLIAVKAETGTPRFSADEITGMFISMMFAGHHTSSGTASWTLIELMRHRDAYAAVIDELDELYGD
GRSVSFHALRQIPQLENVLKETLRLHPPLIILMRVAKGEFEVQGHRIHEGDLVAASPAISNRIPEDFPDPHDF
VPARYEQPRQEDLLNRWTWIPFGAGRHRCVGAAFAIMQIKAIFSVLLREYEFEMAQPPESYRNDHSKMVVQLA
QPACVRYRRRTGV
>CYP51B1_Mycobacterium tuberculosis XTB13-150 2606326998
MSAVALPRVSGGHDEHGHLEEFRTDPIGLMQRVRDECGDVGTFQLAGKQVVLLSGSHANEFFFRAGDDDLDQA
KAYPFMTPIFGEGVVFDASPERRKEMLHNAALRGEQMKGHAATIEDQVRRMIADWGEAGEIDLLDFFAELTIY
TSSACLIGKKFRDQLDGRFAKLYHELERGTDPLAYVDPYLPIESFRRRDEARNGLVALVADIMNGRIANPPTD
KSDRDMLDVLIAVKAETGTPRFSADEITGMFISMMFAGHHTSSGTASWTLIELMRHRDAYAAVIDELDELYGD
GRSVSFHALRQIPQLENVLKETLRLHPPLIILMRVAKGEFEVQGHRIHEGDLVAASPAISNRIPEDFPDPHDF
VPARYEQPRQEDLLNRWTWIPFGAGRHRCVGAAFAIMQIKAIFSVLLREYEFEMAQPPESYRNDHSKMVVQLA
QPACVRYRRRTGV
>CYP51B1_Mycobacterium tuberculosis XTB13-152 2606449559
MSAVALPRVSGGHDEHGHLEEFRTDPIGLMQRVRDECGDVGTFQLAGKQVVLLSGSHANEFFFRAGDDDLDQA
KAYPFMTPIFGEGVVFDASPERRKEMLHNAALRGEQMKGHAATIEDQVRRMIADWGEAGEIDLLDFFAELTIY
TSSACLIGKKFRDQLDGRFAKLYHELERGTDPLAYVDPYLPIESFRRRDEARNGLVALVADIMNGRIANPPTD
KSDRDMLDVLIAVKAETGTPRFSADEITGMFISMMFAGHHTSSGTASWTLIELMRHRDAYAAVIDELDELYGD
GRSVSFHALRQIPQLENVLKETLRLHPPLIILMRVAKGEFEVQGHRIHEGDLVAASPAISNRIPEDFPDPHDF
VPARYEQPRQEDLLNRWTWIPFGAGRHRCVGAAFAIMQIKAIFSVLLREYEFEMAQPPESYRNDHSKMVVQLA
QPACVRYRRRTGV
>CYP51B1_Mycobacterium tuberculosis XTB13-153 2606331126

MSAVALPRVSGGHDEHGHLEEFRTDPIGLMQRVRDECGDVGTFQLAGKQVVLLSGSHANEFFFRAGDDDLDQA
KAYPFMTPIFGEGVVFDASPERRKEMLHNAALRGEQMKGHAATIEDQVRRMIADWGEAGEIDLLDFFAELTIY
TSSACLIGKKFRDQLDGRFAKLYHELERGTDPLAYVDPYLPIESFRRRDEARNGLVALVADIMNGRIANPPTD
KSDRDMLDVLIAVKAETGTPRFSADEITGMFISMMFAGHHTSSGTASWTLIELMRHRDAYAAVIDELDELYGD
GRSVSFHALRQIPQLENVLKETLRLHPPLIILMRVAKGEFEVQGHRIHEGDLVAASPAISNRIPEDFPDPHDF
VPARYEQPRQEDLLNRWTWIPFGAGRHRCVGAAFAIMQIKAIFSVLLREYEFEMAQPPESYRNDHSKMVVQLA
QPACVRYRRRTGV
>CYP51B1_Mycobacterium tuberculosis XTB13-154 2621482798
MSAVALPRVSGGHDEHGHLEEFRTDPIGLMQRVRDECGDVGTFQLAGKQVVLLSGSHANEFFFRAGDDDLDQA
KAYPFMTPIFGEGVVFDASPERRKEMLHNAALRGEQMKGHAATIEDQVRRMIADWGEAGEIDLLDFFAELTIY
TSSACLIGKKFRDQLDGRFAKLYHELERGTDPLAYVDPYLPIESFRRRDEARNGLVALVADIMNGRIANPPTD
KSDRDMLDVLIAVKAETGTPRFSADEITGMFISMMFAGHHTSSGTASWTLIELMRHRDAYAAVIDELDELYGD
GRSVSFHALRQIPQLENVLKETLRLHPPLIILMRVAKGEFEVQGHRIHEGDLVAASPAISNRIPEDFPDPHDF
VPARYEQPRQEDLLNRWTWIPFGAGRHRCVGAAFAIMQIKAIFSVLLREYEFEMAQPPESYRNDHSKMVVQLA
QPACVRYRRRTGV
>CYP51B1_Mycobacterium tuberculosis XTB13-155 2621486948
MSAVALPRVSGGHDEHGHLEEFRTDPIGLMQRVRDECGDVGTFQLAGKQVVLLSGSHANEFFFRAGDDDLDQA
KAYPFMTPIFGEGVVFDASPERRKEMLHNAALRGEQMKGHAATIEDQVRRMIADWGEAGEIDLLDFFAELTIY
TSSACLIGKKFRDQLDGRFAKLYHELERGTDPLAYVDPYLPIESFRRRDEARNGLVALVADIMNGRIANPPTD
KSDRDMLDVLIAVKAETGTPRFSADEITGMFISMMFAGHHTSSGTASWTLIELMRHRDAYAAVIDELDELYGD
GRSVSFHALRQIPQLENVLKETLRLHPPLIILMRVAKGEFEVQGHRIHEGDLVAASPAISNRIPEDFPDPHDF
VPARYEQPRQEDLLNRWTWIPFGAGRHRCVGAAFAIMQIKAIFSVLLREYEFEMAQPPESYRNDHSKMVVQLA
QPACVRYRRRTGV
>CYP51B1_Mycobacterium tuberculosis XTB13-156 2576650700
MSAVALPRVSGGHDEHGHLEEFRTDPIGLMQRVRDECGDVGTFQLAGKQVVLLSGSHANEFFFRAGDDDLDQA
KAYPFMTPIFGEGVVFDASPERRKEMLHNAALRGEQMKGHAATIEDQVRRMIADWGEAGEIDLLDFFAELTIY
TSSACLIGKKFRDQLDGRFAKLYHELERGTDPLAYVDPYLPIESFRRRDEARNGLVALVADIMNGRIANPPTD
KSDRDMLDVLIAVKAETGTPRFSADEITGMFISMMFAGHHTSSGTASWTLIELMRHRDAYAAVIDELDELYGD
GRSVSFHALRQIPQLENVLKETLRLHPPLIILMRVAKGEFEVQGHRIHEGDLVAASPAISNRIPEDFPDPHDF
VPARYEQPRQEDLLNRWTWIPFGAGRHRCVGAAFAIMQIKAIFSVLLREYEFEMAQPPESYRNDHSKMVVQLA
QPACVRYRRRTGV
>CYP51B1_Mycobacterium tuberculosis XTB13-158 2621488514
MSAVALPRVSGGHDEHGHLEEFRTDPIGLMQRVRDECGDVGTFQLAGKQVVLLSGSHANEFFFRAGDDDLDQA
KAYPFMTPIFGEGVVFDASPERRKEMLHNAALRGEQMKGHAATIEDQVRRMIADWGEAGEIDLLDFFAELTIY
TSSACLIGKKFRDQLDGRFAKLYHELERGTDPLAYVDPYLPIESFRRRDEARNGLVALVADIMNGRIANPPTD
KSDRDMLDVLIAVKAETGTPRFSADEITGMFISMMFAGHHTSSGTASWTLIELMRHRDAYAAVIDELDELYGD
GRSVSFHALRQIPQLENVLKETLRLHPPLIILMRVAKGEFEVQGHRIHEGDLVAASPAISNRIPEDFPDPHDF
VPARYEQPRQEDLLNRWTWIPFGAGRHRCVGAAFAIMQIKAIFSVLLREYEFEMAQPPESYRNDHSKMVVQLA
QPACVRYRRRTGV
>CYP51B1_Mycobacterium tuberculosis XTB13-159 2621492640
MSAVALPRVSGGHDEHGHLEEFRTDPIGLMQRVRDECGDVGTFQLAGKQVVLLSGSHANEFFFRAGDDDLDQA
KAYPFMTPIFGEGVVFDASPERRKEMLHNAALRGEQMKGHAATIEDQVRRMIADWGEAGEIDLLDFFAELTIY
TSSACLIGKKFRDQLDGRFAKLYHELERGTDPLAYVDPYLPIESFRRRDEARNGLVALVADIMNGRIANPPTD
KSDRDMLDVLIAVKAETGTPRFSADEITGMFISMMFAGHHTSSGTASWTLIELMRHRDAYAAVIDELDELYGD
GRSVSFHALRQIPQLENVLKETLRLHPPLIILMRVAKGEFEVQGHRIHEGDLVAASPAISNRIPEDFPDPHDF
VPARYEQPRQEDLLNRWTWIPFGAGRHRCVGAAFAIMQIKAIFSVLLREYEFEMAQPPESYRNDHSKMVVQLA
QPACVRYRRRTGV
>CYP51B1_Mycobacterium tuberculosis XTB13-161 2577625458
MSAVALPRVSGGHDEHGHLEEFRTDPIGLMQRVRDECGDVGTFQLAGKQVVLLSGSHANEFFFRAGDDDLDQA
KAYPFMTPIFGEGVVFDASPERRKEMLHNAALRGEQMKGHAATIEDQVRRMIADWGEAGEIDLLDFFAELTIY
TSSACLIGKKFRDQLDGRFAKLYHELERGTDPLAYVDPYLPIESFRRRDEARNGLVALVADIMNGRIANPPTD
KSDRDMLDVLIAVKAETGTPRFSADEITGMFISMMFAGHHTSSGTASWTLIELMRHRDAYAAVIDELDELYGD
GRSVSFHALRQIPQLENVLKETLRLHPPLIILMRVAKGEFEVQGHRIHEGDLVAASPAISNRIPEDFPDPHDF
VPARYEQPRQEDLLNRWTWIPFGAGRHRCVGAAFAIMQIKAIFSVLLREYEFEMAQPPESYRNDHSKMVVQLA
QPACVRYRRRTGV
>CYP51B1_Mycobacterium tuberculosis XTB13-162 2577878251
MSAVALPRVSGGHDEHGHLEEFRTDPIGLMQRVRDECGDVGTFQLAGKQVVLLSGSHANEFFFRAGDDDLDQA
KAYPFMTPIFGEGVVFDASPERRKEMLHNAALRGEQMKGHAATIEDQVRRMIADWGEAGEIDLLDFFAELTIY

TSSACLIGKKFRDQLDGRFAKLYHELERGTDPLAYVDPYLPIESFRRRDEARNGLVALVADIMNGRIANPPTD
KSDRDMLDVLIAVKAETGTPRFSADEITGMFISMMFAGHHTSSGTASWTLIELMRHRDAYAAVIDELDELYGD
GRSVSFHALRQIPQLENVLKETLRLHPPLIILMRVAKGEFEVQGHRIHEGDLVAASPAISNRIPEDFPDPHDF
VPARYEQPRQEDLLNRWTWIPFGAGRHRCVGAAFAIMQIKAIFSVLLREYEFEMAQPPESYRNDHSKMVVQLA
QPACVRYRRRTGV
>CYP51B1_Mycobacterium tuberculosis XTB13-165 2606456120
MSAVALPRVSGGHDEHGHLEEFRTDPIGLMQRVRDECGDVGTFQLAGKQVVLLSGSHANEFFFRAGDDDLDQA
KAYPFMTPIFGEGVVFDASPERRKEMLHNAALRGEQMKGHAATIEDQVRRMIADWGEAGEIDLLDFFAELTIY
TSSACLIGKKFRDQLDGRFAKLYHELERGTDPLAYVDPYLPIESFRRRDEARNGLVALVADIMNGRIANPPTD
KSDRDMLDVLIAVKAETGTPRFSADEITGMFISMMFAGHHTSSGTASWTLIELMRHRDAYAAVIDELDELYGD
GRSVSFHALRQIPQLENVLKETLRLHPPLIILMRVAKGEFEVQGHRIHEGDLVAASPAISNRIPEDFPDPHDF
VPARYEQPRQEDLLNRWTWIPFGAGRHRCVGAAFAIMQIKAIFSVLLREYEFEMAQPPESYRNDHSKMVVQLA
QPACVRYRRRTGV
>CYP51B1_Mycobacterium tuberculosis XTB13-167 2577392608
MSAVALPRVSGGHDEHGHLEEFRTDPIGLMQRVRDECGDVGTFQLAGKQVVLLSGSHANEFFFRAGDDDLDQA
KAYPFMTPIFGEGVVFDASPERRKEMLHNAALRGEQMKGHAATIEDQVRRMIADWGEAGEIDLLDFFAELTIY
TSSACLIGKKFRDQLDGRFAKLYHELERGTDPLAYVDPYLPIESFRRRDEARNGLVALVADIMNGRIANPPTD
KSDRDMLDVLIAVKAETGTPRFSADEITGMFISMMFAGHHTSSGTASWTLIELMRHRDAYAAVIDELDELYGD
GRSVSFHALRQIPQLENVLKETLRLHPPLIILMRVAKGEFEVQGHRIHEGDLVAASPAISNRIPEDFPDPHDF
VPARYEQPRQEDLLNRWTWIPFGAGRHRCVGAAFAIMQIKAIFSVLLREYEFEMAQPPESYRNDHSKMVVQLA
QPACVRYRRRTGV
>CYP51B1_Mycobacterium tuberculosis XTB13-168 2621496282
MSAVALPRVSGGHDEHGHLEEFRTDPIGLMQRVRDECGDVGTFQLAGKQVVLLSGSHANEFFFRAGDDDLDQA
KAYPFMTPIFGEGVVFDASPERRKEMLHNAALRGEQMKGHAATIEDQVRRMIADWGEAGEIDLLDFFAELTIY
TSSACLIGKKFRDQLDGRFAKLYHELERGTDPLAYVDPYLPIESFRRRDEARNGLVALVADIMNGRIANPPTD
KSDRDMLDVLIAVKAETGTPRFSADEITGMFISMMFAGHHTSSGTASWTLIELMRHRDAYAAVIDELDELYGD
GRSVSFHALRQIPQLENVLKETLRLHPPLIILMRVAKGEFEVQGHRIHEGDLVAASPAISNRIPEDFPDPHDF
VPARYEQPRQEDLLNRWTWIPFGAGRHRCVGAAFAIMQIKAIFSVLLREYEFEMAQPPESYRNDHSKMVVQLA
QPACVRYRRRTGV
>CYP51B1_Mycobacterium tuberculosis XTB13-169 2621501494
MSAVALPRVSGGHDEHGHLEEFRTDPIGLMQRVRDECGDVGTFQLAGKQVVLLSGSHANEFFFRAGDDDLDQA
KAYPFMTPIFGEGVVFDASPERRKEMLHNAALRGEQMKGHAATIEDQVRRMIADWGEAGEIDLLDFFAELTIY
TSSACLIGKKFRDQLDGRFAKLYHELERGTDPLAYVDPYLPIESFRRRDEARNGLVALVADIMNGRIANPPTD
KSDRDMLDVLIAVKAETGTPRFSADEITGMFISMMFAGHHTSSGTASWTLIELMRHRDAYAAVIDELDELYGD
GRSVSFHALRQIPQLENVLKETLRLHPPLIILMRVAKGEFEVQGHRIHEGDLVAASPAISNRIPEDFPDPHDF
VPARYEQPRQEDLLNRWTWIPFGAGRHRCVGAAFAIMQIKAIFSVLLREYEFEMAQPPESYRNDHSKMVVQLA
QPACVRYRRRTGV
>CYP51B1_Mycobacterium tuberculosis XTB13-172 2621504963
MSAVALPRVSGGHDEHGHLEEFRTDPIGLMQRVRDECGDVGTFQLAGKQVVLLSGSHANEFFFRAGDDDLDQA
KAYPFMTPIFGEGVVFDASPERRKEMLHNAALRGEQMKGHAATIEDQVRRMIADWGEAGEIDLLDFFAELTIY
TSSACLIGKKFRDQLDGRFAKLYHELERGTDPLAYVDPYLPIESFRRRDEARNGLVALVADIMNGRIANPPTD
KSDRDMLDVLIAVKAETGTPRFSADEITGMFISMMFAGHHTSSGTASWTLIELMRHRDAYAAVIDELDELYGD
GRSVSFHALRQIPQLENVLKETLRLHPPLIILMRVAKGEFEVQGHRIHEGDLVAASPAISNRIPEDFPDPHDF
VPARYEQPRQEDLLNRWTWIPFGAGRHRCVGAAFAIMQIKAIFSVLLREYEFEMAQPPESYRNDHSKMVVQLA
QPACVRYRRRTGV
>CYP51B1_Mycobacterium tuberculosis XTB13-173 2621511926
MSAVALPRVSGGHDEHGHLEEFRTDPIGLMQRVRDECGDVGTFQLAGKQVVLLSGSHANEFFFRAGDDDLDQA
KAYPFMTPIFGEGVVFDASPERRKEMLHNAALRGEQMKGHAATIEDQVRRMIADWGEAGEIDLLDFFAELTIY
TSSACLIGKKFRDQLDGRFAKLYHELERGTDPLAYVDPYLPIESFRRRDEARNGLVALVADIMNGRIANPPTD
KSDRDMLDVLIAVKAETGTPRFSADEITGMFISMMFAGHHTSSGTASWTLIELMRHRDAYAAVIDELDELYGD
GRSVSFHALRQIPQLENVLKETLRLHPPLIILMRVAKGEFEVQGHRIHEGDLVAASPAISNRIPEDFPDPHDF
VPARYEQPRQEDLLNRWTWIPFGAGRHRCVGAAFAIMQIKAIFSVLLREYEFEMAQPPESYRNDHSKMVVQLA
QPACVRYRRRTGV
>CYP51B1_Mycobacterium tuberculosis XTB13-175 2584685802
MSAVALPRVSGGHDEHGHLEEFRTDPIGLMQRVRDECGDVGTFQLAGKQVVLLSGSHANEFFFRAGDDDLDQA
KAYPFMTPIFGEGVVFDASPERRKEMLHNAALRGEQMKGHAATIEDQVRRMIADWGEAGEIDLLDFFAELTIY
TSSACLIGKKFRDQLDGRFAKLYHELERGTDPLAYVDPYLPIESFRRRDEARNGLVALVADIMNGRIANPPTD
KSDRDMLDVLIAVKAETGTPRFSADEITGMFISMMFAGHHTSSGTASWTLIELMRHRDAYAAVIDELDELYGD

GRSVSFHALRQIPQLENVLKETLRLHPPLIILMRVAKGEFEVQGHRIHEGDLVAASPAISNRIPEDFPDPHDF
VPARYEQPRQEDLLNRWTWIPFGAGRHRCVGAAFAIMQIKAIFSVLLREYEFEMAQPPESYRNDHSKMVVQLA
QPACVRYRRRTGV
>CYP51B1_Mycobacterium tuberculosis XTB13-176 2606334880
MSAVALPRVSGGHDEHGHLEEFRTDPIGLMQRVRDECGDVGTFQLAGKQVVLLSGSHANEFFFRAGDDDLDQA
KAYPFMTPIFGEGVVFDASPERRKEMLHNAALRGEQMKGHAATIEDQVRRMIADWGEAGEIDLLDFFAELTIY
TSSACLIGKKFRDQLDGRFAKLYHELERGTDPLAYVDPYLPIESFRRRDEARNGLVALVADIMNGRIANPPTD
KSDRDMLDVLIAVKAETGTPRFSADEITGMFISMMFAGHHTSSGTASWTLIELMRHRDAYAAVIDELDELYGD
GRSVSFHALRQIPQLENVLKETLRLHPPLIILMRVAKGEFEVQGHRIHEGDLVAASPAISNRIPEDFPDPHDF
VPARYEQPRQEDLLNRWTWIPFGAGRHRCVGAAFAIMQIKAIFSVLLREYEFEMAQPPESYRNDHSKMVVQLA
QPACVRYRRRTGV
>CYP51B1_Mycobacterium tuberculosis XTB13-177 2621513266
MSAVALPRVSGGHDEHGHLEEFRTDPIGLMQRVRDECGDVGTFQLAGKQVVLLSGSHANEFFFRAGDDDLDQA
KAYPFMTPIFGEGVVFDASPERRKEMLHNAALRGEQMKGHAATIEDQVRRMIADWGEAGEIDLLDFFAELTIY
TSSACLIGKKFRDQLDGRFAKLYHELERGTDPLAYVDPYLPIESFRRRDEARNGLVALVADIMNGRIANPPTD
KSDRDMLDVLIAVKAETGTPRFSADEITGMFISMMFAGHHTSSGTASWTLIELMRHRDAYAAVIDELDELYGD
GRSVSFHALRQIPQLENVLKETLRLHPPLIILMRVAKGEFEVQGHRIHEGDLVAASPAISNRIPEDFPDPHDF
VPARYEQPRQEDLLNRWTWIPFGAGRHRCVGAAFAIMQIKAIFSVLLREYEFEMAQPPESYRNDHSKMVVQLA
QPACVRYRRRTGV
>CYP51B1_Mycobacterium tuberculosis XTB13-178 2621517111
MSAVALPRVSGGHDEHGHLEEFRTDPIGLMQRVRDECGDVGTFQLAGKQVVLLSGSHANEFFFRAGDDDLDQA
KAYPFMTPIFGEGVVFDASPERRKEMLHNAALRGEQMKGHAATIEDQVRRMIADWGEAGEIDLLDFFAELTIY
TSSACLIGKKFRDQLDGRFAKLYHELERGTDPLAYVDPYLPIESFRRRDEARNGLVALVADIMNGRIANPPTD
KSDRDMLDVLIAVKAETGTPRFSADEITGMFISMMFAGHHTSSGTASWTLIELMRHRDAYAAVIDELDELYGD
GRSVSFHALRQIPQLENVLKETLRLHPPLIILMRVAKGEFEVQGHRIHEGDLVAASPAISNRIPEDFPDPHDF
VPARYEQPRQEDLLNRWTWIPFGAGRHRCVGAAFAIMQIKAIFSVLLREYEFEMAQPPESYRNDHSKMVVQLA
QPACVRYRRRTGV
>CYP51B1_Mycobacterium tuberculosis XTB13-179 2606338481
MSAVALPRVSGGHDEHGHLEEFRTDPIGLMQRVRDECGDVGTFQLAGKQVVLLSGSHANEFFFRAGDDDLDQA
KAYPFMTPIFGEGVVFDASPERRKEMLHNAALRGEQMKGHAATIEDQVRRMIADWGEAGEIDLLDFFAELTIY
TSSACLIGKKFRDQLDGRFAKLYHELERGTDPLAYVDPYLPIESFRRRDEARNGLVALVADIMNGRIANPPTD
KSDRDMLDVLIAVKAETGTPRFSADEITGMFISMMFAGHHTSSGTASWTLIELMRHRDAYAAVIDELDELYGD
GRSVSFHALRQIPQLENVLKETLRLHPPLIILMRVAKGEFEVQGHRIHEGDLVAASPAISNRIPEDFPDPHDF
VPARYEQPRQEDLLNRWTWIPFGAGRHRCVGAAFAIMQIKAIFSVLLREYEFEMAQPPESYRNDHSKMVVQLA
QPACVRYRRRTGV
>CYP51B1_Mycobacterium tuberculosis XTB13-180 2621524086
MSAVALPRVSGGHDEHGHLEEFRTDPIGLMQRVRDECGDVGTFQLAGKQVVLLSGSHANEFFFRAGDDDLDQA
KAYPFMTPIFGEGVVFDASPERRKEMLHNAALRGEQMKGHAATIEDQVRRMIADWGEAGEIDLLDFFAELTIY
TSSACLIGKKFRDQLDGRFAKLYHELERGTDPLAYVDPYLPIESFRRRDEARNGLVALVADIMNGRIANPPTD
KSDRDMLDVLIAVKAETGTPRFSADEITGMFISMMFAGHHTSSGTASWTLIELMRHRDAYAAVIDELDELYGD
GRSVSFHALRQIPQLENVLKETLRLHPPLIILMRVAKGEFEVQGHRIHEGDLVAASPAISNRIPEDFPDPHDF
VPARYEQPRQEDLLNRWTWIPFGAGRHRCVGAAFAIMQIKAIFSVLLREYEFEMAQPPESYRNDHSKMVVQLA
QPACVRYRRRTGV
>CYP51B1_Mycobacterium tuberculosis XTB13-182 2621528317
MSAVALPRVSGGHDEHGHLEEFRTDPIGLMQRVRDECGDVGTFQLAGKQVVLLSGSHANEFFFRAGDDDLDQA
KAYPFMTPIFGEGVVFDASPERRKEMLHNAALRGEQMKGHAATIEDQVRRMIADWGEAGEIDLLDFFAELTIY
TSSACLIGKKFRDQLDGRFAKLYHELERGTDPLAYVDPYLPIESFRRRDEARNGLVALVADIMNGRIANPPTD
KSDRDMLDVLIAVKAETGTPRFSADEITGMFISMMFAGHHTSSGTASWTLIELMRHRDAYAAVIDELDELYGD
GRSVSFHALRQIPQLENVLKETLRLHPPLIILMRVAKGEFEVQGHRIHEGDLVAASPAISNRIPEDFPDPHDF
VPARYEQPRQEDLLNRWTWIPFGAGRHRCVGAAFAIMQIKAIFSVLLREYEFEMAQPPESYRNDHSKMVVQLA
QPACVRYRRRTGV
>CYP51B1_Mycobacterium tuberculosis XTB13-183 2606342197
MSAVALPRVSGGHDEHGHLEEFRTDPIGLMQRVRDECGDVGTFQLAGKQVVLLSGSHANEFFFRAGDDDLDQA
KAYPFMTPIFGEGVVFDASPERRKEMLHNAALRGEQMKGHAATIEDQVRRMIADWGEAGEIDLLDFFAELTIY
TSSACLIGKKFRDQLDGRFAKLYHELERGTDPLAYVDPYLPIESFRRRDEARNGLVALVADIMNGRIANPPTD
KSDRDMLDVLIAVKAETGTPRFSADEITGMFISMMFAGHHTSSGTASWTLIELMRHRDAYAAVIDELDELYGD
GRSVSFHALRQIPQLENVLKETLRLHPPLIILMRVAKGEFEVQGHRIHEGDLVAASPAISNRIPEDFPDPHDF

VPARYEQPRQEDLLNRWTWIPFGAGRHRCVGAAFAIMQIKAIFSVLLREYEFEMAQPPESYRNDHSKMVVQLA
QPACVRYRRRTGV
>CYP51B1_Mycobacterium tuberculosis XTB13-184 2621532381
MSAVALPRVSGGHDEHGHLEEFRTDPIGLMQRVRDECGDVGTFQLAGKQVVLLSGSHANEFFFRAGDDDLDQA
KAYPFMTPIFGEGVVFDASPERRKEMLHNAALRGEQMKGHAATIEDQVRRMIADWGEAGEIDLLDFFAELTIY
TSSACLIGKKFRDQLDGRFAKLYHELERGTDPLAYVDPYLPIESFRRRDEARNGLVALVADIMNGRIANPPTD
KSDRDMLDVLIAVKAETGTPRFSADEITGMFISMMFAGHHTSSGTASWTLIELMRHRDAYAAVIDELDELYGD
GRSVSFHALRQIPQLENVLKETLRLHPPLIILMRVAKGEFEVQGHRIHEGDLVAASPAISNRIPEDFPDPHDF
VPARYEQPRQEDLLNRWTWIPFGAGRHRCVGAAFAIMQIKAIFSVLLREYEFEMAQPPESYRNDHSKMVVQLA
QPACVRYRRRTGV
>CYP51B1_Mycobacterium tuberculosis XTB13-186 2606345068
MSAVALPRVSGGHDEHGHLEEFRTDPIGLMQRVRDECGDVGTFQLAGKQVVLLSGSHANEFFFRAGDDDLDQA
KAYPFMTPIFGEGVVFDASPERRKEMLHNAALRGEQMKGHAATIEDQVRRMIADWGEAGEIDLLDFFAELTIY
TSSACLIGKKFRDQLDGRFAKLYHELERGTDPLAYVDPYLPIESFRRRDEARNGLVALVADIMNGRIANPPTD
KSDRDMLDVLIAVKAETGTPRFSADEITGMFISMMFAGHHTSSGTASWTLIELMRHRDAYAAVIDELDELYGD
GRSVSFHALRQIPQLENVLKETLRLHPPLIILMRVAKGEFEVQGHRIHEGDLVAASPAISNRIPEDFPDPHDF
VPARYEQPRQEDLLNRWTWIPFGAGRHRCVGAAFAIMQIKAIFSVLLREYEFEMAQPPESYRNDHSKMVVQLA
QPACVRYRRRTGV
>CYP51B1_Mycobacterium tuberculosis XTB13-187 2621536203
MSAVALPRVSGGHDEHGHLEEFRTDPIGLMQRVRDECGDVGTFQLAGKQVVLLSGSHANEFFFRAGDDDLDQA
KAYPFMTPIFGEGVVFDASPERRKEMLHNAALRGEQMKGHAATIEDQVRRMIADWGEAGEIDLLDFFAELTIY
TSSACLIGKKFRDQLDGRFAKLYHELERGTDPLAYVDPYLPIESFRRRDEARNGLVALVADIMNGRIANPPTD
KSDRDMLDVLIAVKAETGTPRFSADEITGMFISMMFAGHHTSSGTASWTLIELMRHRDAYAAVIDELDELYGD
GRSVSFHALRQIPQLENVLKETLRLHPPLIILMRVAKGEFEVQGHRIHEGDLVAASPAISNRIPEDFPDPHDF
VPARYEQPRQEDLLNRWTWIPFGAGRHRCVGAAFAIMQIKAIFSVLLREYEFEMAQPPESYRNDHSKMVVQLA
QPACVRYRRRTGV
>CYP51B1_Mycobacterium tuberculosis XTB13-188 2621541249
MSAVALPRVSGGHDEHGHLEEFRTDPIGLMQRVRDECGDVGTFQLAGKQVVLLSGSHANEFFFRAGDDDLDQA
KAYPFMTPIFGEGVVFDASPERRKEMLHNAALRGEQMKGHAATIEDQVRRMIADWGEAGEIDLLDFFAELTIY
TSSACLIGKKFRDQLDGRFAKLYHELERGTDPLAYVDPYLPIESFRRRDEARNGLVALVADIMNGRIANPPTD
KSDRDMLDVLIAVKAETGTPRFSADEITGMFISMMFAGHHTSSGTASWTLIELMRHRDAYAAVIDELDELYGD
GRSVSFHALRQIPQLENVLKETLRLHPPLIILMRVAKGEFEVQGHRIHEGDLVAASPAISNRIPEDFPDPHDF
VPARYEQPRQEDLLNRWTWIPFGAGRHRCVGAAFAIMQIKAIFSVLLREYEFEMAQPPESYRNDHSKMVVQLA
QPACVRYRRRTGV
>CYP51B1_Mycobacterium tuberculosis XTB13-190 2621542131
MSAVALPRVSGGHDEHGHLEEFRTDPIGLMQRVRDECGDVGTFQLAGKQVVLLSGSHANEFFFRAGDDDLDQA
KAYPFMTPIFGEGVVFDASPERRKEMLHNAALRGEQMKGHAATIEDQVRRMIADWGEAGEIDLLDFFAELTIY
TSSACLIGKKFRDQLDGRFAKLYHELERGTDPLAYVDPYLPIESFRRRDEARNGLVALVADIMNGRIANPPTD
KSDRDMLDVLIAVKAETGTPRFSADEITGMFISMMFAGHHTSSGTASWTLIELMRHRDAYAAVIDELDELYGD
GRSVSFHALRQIPQLENVLKETLRLHPPLIILMRVAKGEFEVQGHRIHEGDLVAASPAISNRIPEDFPDPHDF
VPARYEQPRQEDLLNRWTWIPFGAGRHRCVGAAFAIMQIKAIFSVLLREYEFEMAQPPESYRNDHSKMVVQLA
QPACVRYRRRTGV
>CYP51B1_Mycobacterium tuberculosis XTB13-191 2621546226
MSAVALPRVSGGHDEHGHLEEFRTDPIGLMQRVRDECGDVGTFQLAGKQVVLLSGSHANEFFFRAGDDDLDQA
KAYPFMTPIFGEGVVFDASPERRKEMLHNAALRGEQMKGHAATIEDQVRRMIADWGEAGEIDLLDFFAELTIY
TSSACLIGKKFRDQLDGRFAKLYHELERGTDPLAYVDPYLPIESFRRRDEARNGLVALVADIMNGRIANPPTD
KSDRDMLDVLIAVKAETGTPRFSADEITGMFISMMFAGHHTSSGTASWTLIELMRHRDAYAAVIDELDELYGD
GRSVSFHALRQIPQLENVLKETLRLHPPLIILMRVAKGEFEVQGHRIHEGDLVAASPAISNRIPEDFPDPHDF
VPARYEQPRQEDLLNRWTWIPFGAGRHRCVGAAFAIMQIKAIFSVLLREYEFEMAQPPESYRNDHSKMVVQLA
QPACVRYRRRTGV
>CYP51B1_Mycobacterium tuberculosis XTB13-192 2606349194
MSAVALPRVSGGHDEHGHLEEFRTDPIGLMQRVRDECGDVGTFQLAGKQVVLLSGSHANEFFFRAGDDDLDQA
KAYPFMTPIFGEGVVFDASPERRKEMLHNAALRGEQMKGHAATIEDQVRRMIADWGEAGEIDLLDFFAELTIY
TSSACLIGKKFRDQLDGRFAKLYHELERGTDPLAYVDPYLPIESFRRRDEARNGLVALVADIMNGRIANPPTD
KSDRDMLDVLIAVKAETGTPRFSADEITGMFISMMFAGHHTSSGTASWTLIELMRHRDAYAAVIDELDELYGD
GRSVSFHALRQIPQLENVLKETLRLHPPLIILMRVAKGEFEVQGHRIHEGDLVAASPAISNRIPEDFPDPHDF
VPARYEQPRQEDLLNRWTWIPFGAGRHRCVGAAFAIMQIKAIFSVLLREYEFEMAQPPESYRNDHSKMVVQLA
QPACVRYRRRTGV

>CYP51B1_Mycobacterium tuberculosis XTB13-193 2606353297
MSAVALPRVSGGHDEHGHLEEFRTDPIGLMQRVRDECGDVGTFQLAGKQVVLLSGSHANEFFFRAGDDDLDQA
KAYPFMTPIFGEGVVFDASPERRKEMLHNAALRGEQMKGHAATIEDQVRRMIADWGEAGEIDLLDFFAELTIY
TSSACLIGKKFRDQLDGRFAKLYHELERGTDPLAYVDPYLPIESFRRRDEARNGLVALVADIMNGRIANPPTD
KSDRDMLDVLIAVKAETGTPRFSADEITGMFISMMFAGHHTSSGTASWTLIELMRHRDAYAAVIDELDELYGD
GRSVSFHALRQIPQLENVLKETLRLHPPLIILMRVAKGEFEVQGHRIHEGDLVAASPAISNRIPEDFPDPHDF
VPARYEQPRQEDLLNRWTWIPFGAGRHRCVGAAFAIMQIKAIFSVLLREYEFEMAQPPESYRNDHSKMVVQLA
QPACVRYRRRTGV
>CYP51B1_Mycobacterium tuberculosis XTB13-194 2584656745
MSAVALPRVSGGHDEHGHLEEFRTDPIGLMQRVRDECGDVGTFQLAGKQVVLLSGSHANEFFFRAGDDDLDQA
KAYPFMTPIFGEGVVFDASPERRKEMLHNAALRGEQMKGHAATIEDQVRRMIADWGEAGEIDLLDFFAELTIY
TSSACLIGKKFRDQLDGRFAKLYHELERGTDPLAYVDPYLPIESFRRRDEARNGLVALVADIMNGRIANPPTD
KSDRDMLDVLIAVKAETGTPRFSADEITGMFISMMFAGHHTSSGTASWTLIELMRHRDAYAAVIDELDELYGD
GRSVSFHALRQIPQLENVLKETLRLHPPLIILMRVAKGEFEVQGHRIHEGDLVAASPAISNRIPEDFPDPHDF
VPARYEQPRQEDLLNRWTWIPFGAGRHRCVGAAFAIMQIKAIFSVLLREYEFEMAQPPESYRNDHSKMVVQLA
QPACVRYRRRTGV
>CYP51B1_Mycobacterium tuberculosis XTB13-195 2576082470
MSAVALPRVSGGHDEHGHLEEFRTDPIGLMQRVRDECGDVGTFQLAGKQVVLLSGSHANEFFFRAGDDDLDQA
KAYPFMTPIFGEGVVFDASPERRKEMLHNAALRGEQMKGHAATIEDQVRRMIADWGEAGEIDLLDFFAELTIY
TSSACLIGKKFRDQLDGRFAKLYHELERGTDPLAYVDPYLPIESFRRRDEARNGLVALVADIMNGRIANPPTD
KSDRDMLDVLIAVKAETGTPRFSADEITGMFISMMFAGHHTSSGTASWTLIELMRHRDAYAAVIDELDELYGD
GRSVSFHALRQIPQLENVLKETLRLHPPLIILMRVAKGEFEVQGHRIHEGDLVAASPAISNRIPEDFPDPHDF
VPARYEQPRQEDLLNRWTWIPFGAGRHRCVGAAFAIMQIKAIFSVLLREYEFEMAQPPESYRNDHSKMVVQLA
QPACVRYRRRTGV
>CYP51B1_Mycobacterium tuberculosis XTB13-196 2621550337
MSAVALPRVSGGHDEHGHLEEFRTDPIGLMQRVRDECGDVGTFQLAGKQVVLLSGSHANEFFFRAGDDDLDQA
KAYPFMTPIFGEGVVFDASPERRKEMLHNAALRGEQMKGHAATIEDQVRRMIADWGEAGEIDLLDFFAELTIY
TSSACLIGKKFRDQLDGRFAKLYHELERGTDPLAYVDPYLPIESFRRRDEARNGLVALVADIMNGRIANPPTD
KSDRDMLDVLIAVKAETGTPRFSADEITGMFISMMFAGHHTSSGTASWTLIELMRHRDAYAAVIDELDELYGD
GRSVSFHALRQIPQLENVLKETLRLHPPLIILMRVAKGEFEVQGHRIHEGDLVAASPAISNRIPEDFPDPHDF
VPARYEQPRQEDLLNRWTWIPFGAGRHRCVGAAFAIMQIKAIFSVLLREYEFEMAQPPESYRNDHSKMVVQLA
QPACVRYRRRTGV
>CYP51B1_Mycobacterium tuberculosis XTB13-197 2621554814
MSAVALPRVSGGHDEHGHLEEFRTDPIGLMQRVRDECGDVGTFQLAGKQVVLLSGSHANEFFFRAGDDDLDQA
KAYPFMTPIFGEGVVFDASPERRKEMLHNAALRGEQMKGHAATIEDQVRRMIADWGEAGEIDLLDFFAELTIY
TSSACLIGKKFRDQLDGRFAKLYHELERGTDPLAYVDPYLPIESFRRRDEARNGLVALVADIMNGRIANPPTD
KSDRDMLDVLIAVKAETGTPRFSADEITGMFISMMFAGHHTSSGTASWTLIELMRHRDAYAAVIDELDELYGD
GRSVSFHALRQIPQLENVLKETLRLHPPLIILMRVAKGEFEVQGHRIHEGDLVAASPAISNRIPEDFPDPHDF
VPARYEQPRQEDLLNRWTWIPFGAGRHRCVGAAFAIMQIKAIFSVLLREYEFEMAQPPESYRNDHSKMVVQLA
QPACVRYRRRTGV
>CYP51B1_Mycobacterium tuberculosis XTB13-198 2584881299
MSAVALPRVSGGHDEHGHLEEFRTDPIGLMQRVRDECGDVGTFQLAGKQVVLLSGSHANEFFFRAGDDDLDQA
KAYPFMTPIFGEGVVFDASPERRKEMLHNAALRGEQMKGHAATIEDQVRRMIADWGEAGEIDLLDFFAELTIY
TSSACLIGKKFRDQLDGRFAKLYHELERGTDPLAYVDPYLPIESFRRRDEARNGLVALVADIMNGRIANPPTD
KSDRDMLDVLIAVKAETGTPRFSADEITGMFISMMFAGHHTSSGTASWTLIELMRHRDAYAAVIDELDELYGD
GRSVSFHALRQIPQLENVLKETLRLHPPLIILMRVAKGEFEVQGHRIHEGDLVAASPAISNRIPEDFPDPHDF
VPARYEQPRQEDLLNRWTWIPFGAGRHRCVGAAFAIMQIKAIFSVLLREYEFEMAQPPESYRNDHSKMVVQLA
QPACVRYRRRTGV
>CYP51B1_Mycobacterium tuberculosis XTB13-199 2577843985
MSAVALPRVSGGHDEHGHLEEFRTDPIGLMQRVRDECGDVGTFQLAGKQVVLLSGSHANEFFFRAGDDDLDQA
KAYPFMTPIFGEGVVFDASPERRKEMLHNAALRGEQMKGHAATIEDQVRRMIADWGEAGEIDLLDFFAELTIY
TSSACLIGKKFRDQLDGRFAKLYHELERGTDPLAYVDPYLPIESFRRRDEARNGLVALVADIMNGRIANPPTD
KSDRDMLDVLIAVKAETGTPRFSADEITGMFISMMFAGHHTSSGTASWTLIELMRHRDAYAAVIDELDELYGD
GRSVSFHALRQIPQLENVLKETLRLHPPLIILMRVAKGEFEVQGHRIHEGDLVAASPAISNRIPEDFPDPHDF
VPARYEQPRQEDLLNRWTWIPFGAGRHRCVGAAFAIMQIKAIFSVLLREYEFEMAQPPESYRNDHSKMVVQLA
QPACVRYRRRTGV
>CYP51B1_Mycobacterium tuberculosis XTB13-200 2575945950

MSAVALPRVSGGHDEHGHLEEFRTDPIGLMQRVRDECGDVGTFQLAGKQVVLLSGSHANEFFFRAGDDDLDQA
KAYPFMTPIFGEGVVFDASPERRKEMLHNAALRGEQMKGHAATIEDQVRRMIADWGEAGEIDLLDFFAELTIY
TSSACLIGKKFRDQLDGRFAKLYHELERGTDPLAYVDPYLPIESFRRRDEARNGLVALVADIMNGRIANPPTD
KSDRDMLDVLIAVKAETGTPRFSADEITGMFISMMFAGHHTSSGTASWTLIELMRHRDAYAAVIDELDELYGD
GRSVSFHALRQIPQLENVLKETLRLHPPLIILMRVAKGEFEVQGHRIHEGDLVAASPAISNRIPEDFPDPHDF
VPARYEQPRQEDLLNRWTWIPFGAGRHRCVGAAFAIMQIKAIFSVLLREYEFEMAQPPESYRNDHSKMVVQLA
QPACVRYRRRTGV
>CYP51B1_Mycobacterium tuberculosis XTB13-201 2629789651
MSAVALPRVSGGHDEHGHLEEFRTDPIGLMQRVRDECGDVGTFQLAGKQVVLLSGSHANEFFFRAGDDDLDQA
KAYPFMTPIFGEGVVFDASPERRKEMLHNAALRGEQMKGHAATIEDQVRRMIADWGEAGEIDLLDFFAELTIY
TSSACLIGKKFRDQLDGRFAKLYHELERGTDPLAYVDPYLPIESFRRRDEARNGLVALVADIMNGRIANPPTD
KSDRDMLDVLIAVKAETGTPRFSADEITGMFISMMFAGHHTSSGTASWTLIELMRHRDAYAAVIDELDELYGD
GRSVSFHALRQIPQLENVLKETLRLHPPLIILMRVAKGEFEVQGHRIHEGDLVAASPAISNRIPEDFPDPHDF
VPARYEQPRQEDLLNRWTWIPFGAGRHRCVGAAFAIMQIKAIFSVLLREYEFEMAQPPESYRNDHSKMVVQLA
QPACVRYRRRTGV
>CYP51B1_Mycobacterium tuberculosis XTB13-203 2584810733
MSAVALPRVSGGHDEHGHLEEFRTDPIGLMQRVRDECGDVGTFQLAGKQVVLLSGSHANEFFFRAGDDDLDQA
KAYPFMTPIFGEGVVFDASPERRKEMLHNAALRGEQMKGHAATIEDQVRRMIADWGEAGEIDLLDFFAELTIY
TSSACLIGKKFRDQLDGRFAKLYHELERGTDPLAYVDPYLPIESFRRRDEARNGLVALVADIMNGRIANPPTD
KSDRDMLDVLIAVKAETGTPRFSADEITGMFISMMFAGHHTSSGTASWTLIELMRHRDAYAAVIDELDELYGD
GRSVSFHALRQIPQLENVLKETLRLHPPLIILMRVAKGEFEVQGHRIHEGDLVAASPAISNRIPEDFPDPHDF
VPARYEQPRQEDLLNRWTWIPFGAGRHRCVGAAFAIMQIKAIFSVLLREYEFEMAQPPESYRNDHSKMVVQLA
QPACVRYRRRTGV
>CYP51B1_Mycobacterium tuberculosis XTB13-204 2621559658
MSAVALPRVSGGHDEHGHLEEFRTDPIGLMQRVRDECGDVGTFQLAGKQVVLLSGSHANEFFFRAGDDDLDQA
KAYPFMTPIFGEGVVFDASPERRKEMLHNAALRGEQMKGHAATIEDQVRRMIADWGEAGEIDLLDFFAELTIY
TSSACLIGKKFRDQLDGRFAKLYHELERGTDPLAYVDPYLPIESFRRRDEARNGLVALVADIMNGRIANPPTD
KSDRDMLDVLIAVKAETGTPRFSADEITGMFISMMFAGHHTSSGTASWTLIELMRHRDAYAAVIDELDELYGD
GRSVSFHALRQIPQLENVLKETLRLHPPLIILMRVAKGEFEVQGHRIHEGDLVAASPAISNRIPEDFPDPHDF
VPARYEQPRQEDLLNRWTWIPFGAGRHRCVGAAFAIMQIKAIFSVLLREYEFEMAQPPESYRNDHSKMVVQLA
QPACVRYRRRTGV
>CYP51B1_Mycobacterium tuberculosis XTB13-205 2621562257
MSAVALPRVSGGHDEHGHLEEFRTDPIGLMQRVRDECGDVGTFQLAGKQVVLLSGSHANEFFFRAGDDDLDQA
KAYPFMTPIFGEGVVFDASPERRKEMLHNAALRGEQMKGHAATIEDQVRRMIADWGEAGEIDLLDFFAELTIY
TSSACLIGKKFRDQLDGRFAKLYHELERGTDPLAYVDPYLPIESFRRRDEARNGLVALVADIMNGRIANPPTD
KSDRDMLDVLIAVKAETGTPRFSADEITGMFISMMFAGHHTSSGTASWTLIELMRHRDAYAAVIDELDELYGD
GRSVSFHALRQIPQLENVLKETLRLHPPLIILMRVAKGEFEVQGHRIHEGDLVAASPAISNRIPEDFPDPHDF
VPARYEQPRQEDLLNRWTWIPFGAGRHRCVGAAFAIMQIKAIFSVLLREYEFEMAQPPESYRNDHSKMVVQLA
QPACVRYRRRTGV
>CYP51B1_Mycobacterium tuberculosis XTB13-206 2621566829
MSAVALPRVSGGHDEHGHLEEFRTDPIGLMQRVRDECGDVGTFQLAGKQVVLLSGSHANEFFFRAGDDDLDQA
KAYPFMTPIFGEGVVFDASPERRKEMLHNAALRGEQMKGHAATIEDQVRRMIADWGEAGEIDLLDFFAELTIY
TSSACLIGKKFRDQLDGRFAKLYHELERGTDPLAYVDPYLPIESFRRRDEARNGLVALVADIMNGRIANPPTD
KSDRDMLDVLIAVKAETGTPRFSADEITGMFISMMFAGHHTSSGTASWTLIELMRHRDAYAAVIDELDELYGD
GRSVSFHALRQIPQLENVLKETLRLHPPLIILMRVAKGEFEVQGHRIHEGDLVAASPAISNRIPEDFPDPHDF
VPARYEQPRQEDLLNRWTWIPFGAGRHRCVGAAFAIMQIKAIFSVLLREYEFEMAQPPESYRNDHSKMVVQLA
QPACVRYRRRTGV
>CYP51B1_Mycobacterium tuberculosis XTB13-208 2606458101
MSAVALPRVSGGHDEHGHLEEFRTDPIGLMQRVRDECGDVGTFQLAGKQVVLLSGSHANEFFFRAGDDDLDQA
KAYPFMTPIFGEGVVFDASPERRKEMLHNAALRGEQMKGHAATIEDQVRRMIADWGEAGEIDLLDFFAELTIY
TSSACLIGKKFRDQLDGRFAKLYHELERGTDPLAYVDPYLPIESFRRRDEARNGLVALVADIMNGRIANPPTD
KSDRDMLDVLIAVKAETGTPRFSADEITGMFISMMFAGHHTSSGTASWTLIELMRHRDAYAAVIDELDELYGD
GRSVSFHALRQIPQLENVLKETLRLHPPLIILMRVAKGEFEVQGHRIHEGDLVAASPAISNRIPEDFPDPHDF
VPARYEQPRQEDLLNRWTWIPFGAGRHRCVGAAFAIMQIKAIFSVLLREYEFEMAQPPESYRNDHSKMVVQLA
QPACVRYRRRTGV
>CYP51B1_Mycobacterium tuberculosis XTB13-209 2575058627
MSAVALPRVSGGHDEHGHLEEFRTDPIGLMQRVRDECGDVGTFQLAGKQVVLLSGSHANEFFFRAGDDDLDQA
KAYPFMTPIFGEGVVFDASPERRKEMLHNAALRGEQMKGHAATIEDQVRRMIADWGEAGEIDLLDFFAELTIY

TSSACLIGKKFRDQLDGRFAKLYHELERGTDPLAYVDPYLPIESFRRRDEARNGLVALVADIMNGRIANPPTD
KSDRDMLDVLIAVKAETGTPRFSADEITGMFISMMFAGHHTSSGTASWTLIELMRHRDAYAAVIDELDELYGD
GRSVSFHALRQIPQLENVLKETLRLHPPLIILMRVAKGEFEVQGHRIHEGDLVAASPAISNRIPEDFPDPHDF
VPARYEQPRQEDLLNRWTWIPFGAGRHRCVGAAFAIMQIKAIFSVLLREYEFEMAQPPESYRNDHSKMVVQLA
QPACVRYRRRTGV
>CYP51B1_Mycobacterium tuberculosis XTB13-210 2621570954
MSAVALPRVSGGHDEHGHLEEFRTDPIGLMQRVRDECGDVGTFQLAGKQVVLLSGSHANEFFFRAGDDDLDQA
KAYPFMTPIFGEGVVFDASPERRKEMLHNAALRGEQMKGHAATIEDQVRRMIADWGEAGEIDLLDFFAELTIY
TSSACLIGKKFRDQLDGRFAKLYHELERGTDPLAYVDPYLPIESFRRRDEARNGLVALVADIMNGRIANPPTD
KSDRDMLDVLIAVKAETGTPRFSADEITGMFISMMFAGHHTSSGTASWTLIELMRHRDAYAAVIDELDELYGD
GRSVSFHALRQIPQLENVLKETLRLHPPLIILMRVAKGEFEVQGHRIHEGDLVAASPAISNRIPEDFPDPHDF
VPARYEQPRQEDLLNRWTWIPFGAGRHRCVGAAFAIMQIKAIFSVLLREYEFEMAQPPESYRNDHSKMVVQLA
QPACVRYRRRTGV
>CYP51B1_Mycobacterium tuberculosis XTB13-211 2621575072
MSAVALPRVSGGHDEHGHLEEFRTDPIGLMQRVRDECGDVGTFQLAGKQVVLLSGSHANEFFFRAGDDDLDQA
KAYPFMTPIFGEGVVFDASPERRKEMLHNAALRGEQMKGHAATIEDQVRRMIADWGEAGEIDLLDFFAELTIY
TSSACLIGKKFRDQLDGRFAKLYHELERGTDPLAYVDPYLPIESFRRRDEARNGLVALVADIMNGRIANPPTD
KSDRDMLDVLIAVKAETGTPRFSADEITGMFISMMFAGHHTSSGTASWTLIELMRHRDAYAAVIDELDELYGD
GRSVSFHALRQIPQLENVLKETLRLHPPLIILMRVAKGEFEVQGHRIHEGDLVAASPAISNRIPEDFPDPHDF
VPARYEQPRQEDLLNRWTWIPFGAGRHRCVGAAFAIMQIKAIFSVLLREYEFEMAQPPESYRNDHSKMVVQLA
QPACVRYRRRTGV
>CYP51B1_Mycobacterium tuberculosis XTB13-212 2621578856
MSAVALPRVSGGHDEHGHLEEFRTDPIGLMQRVRDECGDVGTFQLAGKQVVLLSGSHANEFFFRAGDDDLDQA
KAYPFMTPIFGEGVVFDASPERRKEMLHNAALRGEQMKGHAATIEDQVRRMIADWGEAGEIDLLDFFAELTIY
TSSACLIGKKFRDQLDGRFAKLYHELERGTDPLAYVDPYLPIESFRRRDEARNGLVALVADIMNGRIANPPTD
KSDRDMLDVLIAVKAETGTPRFSADEITGMFISMMFAGHHTSSGTASWTLIELMRHRDAYAAVIDELDELYGD
GRSVSFHALRQIPQLENVLKETLRLHPPLIILMRVAKGEFEVQGHRIHEGDLVAASPAISNRIPEDFPDPHDF
VPARYEQPRQEDLLNRWTWIPFGAGRHRCVGAAFAIMQIKAIFSVLLREYEFEMAQPPESYRNDHSKMVVQLA
QPACVRYRRRTGV
>CYP51B1_Mycobacterium tuberculosis XTB13-213 2621583244
MSAVALPRVSGGHDEHGHLEEFRTDPIGLMQRVRDECGDVGTFQLAGKQVVLLSGSHANEFFFRAGDDDLDQA
KAYPFMTPIFGEGVVFDASPERRKEMLHNAALRGEQMKGHAATIEDQVRRMIADWGEAGEIDLLDFFAELTIY
TSSACLIGKKFRDQLDGRFAKLYHELERGTDPLAYVDPYLPIESFRRRDEARNGLVALVADIMNGRIANPPTD
KSDRDMLDVLIAVKAETGTPRFSADEITGMFISMMFAGHHTSSGTASWTLIELMRHRDAYAAVIDELDELYGD
GRSVSFHALRQIPQLENVLKETLRLHPPLIILMRVAKGEFEVQGHRIHEGDLVAASPAISNRIPEDFPDPHDF
VPARYEQPRQEDLLNRWTWIPFGAGRHRCVGAAFAIMQIKAIFSVLLREYEFEMAQPPESYRNDHSKMVVQLA
QPACVRYRRRTGV
>CYP51B1_Mycobacterium tuberculosis XTB13-214 2577973989
MSAVALPRVSGGHDEHGHLEEFRTDPIGLMQRVRDECGDVGTFQLAGKQVVLLSGSHANEFFFRAGDDDLDQA
KAYPFMTPIFGEGVVFDASPERRKEMLHNAALRGEQMKGHAATIEDQVRRMIADWGEAGEIDLLDFFAELTIY
TSSACLIGKKFRDQLDGRFAKLYHELERGTDPLAYVDPYLPIESFRRRDEARNGLVALVADIMNGRIANPPTD
KSDRDMLDVLIAVKAETGTPRFSADEITGMFISMMFAGHHTSSGTASWTLIELMRHRDAYAAVIDELDELYGD
GRSVSFHALRQIPQLENVLKETLRLHPPLIILMRVAKGEFEVQGHRIHEGDLVAASPAISNRIPEDFPDPHDF
VPARYEQPRQEDLLNRWTWIPFGAGRHRCVGAAFAIMQIKAIFSVLLREYEFEMAQPPESYRNDHSKMVVQLA
QPACVRYRRRTGV
>CYP51B1_Mycobacterium tuberculosis XTB13-216 2621589832
MSAVALPRVSGGHDEHGHLEEFRTDPIGLMQRVRDECGDVGTFQLAGKQVVLLSGSHANEFFFRAGDDDLDQA
KAYPFMTPIFGEGVVFDASPERRKEMLHNAALRGEQMKGHAATIEDQVRRMIADWGEAGEIDLLDFFAELTIY
TSSACLIGKKFRDQLDGRFAKLYHELERGTDPLAYVDPYLPIESFRRRDEARNGLVALVADIMNGRIANPPTD
KSDRDMLDVLIAVKAETGTPRFSADEITGMFISMMFAGHHTSSGTASWTLIELMRHRDAYAAVIDELDELYGD
GRSVSFHALRQIPQLENVLKETLRLHPPLIILMRVAKGEFEVQGHRIHEGDLVAASPAISNRIPEDFPDPHDF
VPARYEQPRQEDLLNRWTWIPFGAGRHRCVGAAFAIMQIKAIFSVLLREYEFEMAQPPESYRNDHSKMVVQLA
QPACVRYRRRTGV
>CYP51B1_Mycobacterium tuberculosis XTB13-217 2621592609
MSAVALPRVSGGHDEHGHLEEFRTDPIGLMQRVRDECGDVGTFQLAGKQVVLLSGSHANEFFFRAGDDDLDQA
KAYPFMTPIFGEGVVFDASPERRKEMLHNAALRGEQMKGHAATIEDQVRRMIADWGEAGEIDLLDFFAELTIY
TSSACLIGKKFRDQLDGRFAKLYHELERGTDPLAYVDPYLPIESFRRRDEARNGLVALVADIMNGRIANPPTD
KSDRDMLDVLIAVKAETGTPRFSADEITGMFISMMFAGHHTSSGTASWTLIELMRHRDAYAAVIDELDELYGD

GRSVSFHALRQIPQLENVLKETLRLHPPLIILMRVAKGEFEVQGHRIHEGDLVAASPAISNRIPEDFPDPHDF
VPARYEQPRQEDLLNRWTWIPFGAGRHRCVGAAFAIMQIKAIFSVLLREYEFEMAQPPESYRNDHSKMVVQLA
QPACVRYRRRTGV
>CYP51B1_Mycobacterium tuberculosis XTB13-218 2606356818
MSAVALPRVSGGHDEHGHLEEFRTDPIGLMQRVRDECGDVGTFQLAGKQVVLLSGSHANEFFFRAGDDDLDQA
KAYPFMTPIFGEGVVFDASPERRKEMLHNAALRGEQMKGHAATIEDQVRRMIADWGEAGEIDLLDFFAELTIY
TSSACLIGKKFRDQLDGRFAKLYHELERGTDPLAYVDPYLPIESFRRRDEARNGLVALVADIMNGRIANPPTD
KSDRDMLDVLIAVKAETGTPRFSADEITGMFISMMFAGHHTSSGTASWTLIELMRHRDAYAAVIDELDELYGD
GRSVSFHALRQIPQLENVLKETLRLHPPLIILMRVAKGEFEVQGHRIHEGDLVAASPAISNRIPEDFPDPHDF
VPARYEQPRQEDLLNRWTWIPFGAGRHRCVGAAFAIMQIKAIFSVLLREYEFEMAQPPESYRNDHSKMVVQLA
QPACVRYRRRTGV
>CYP51B1_Mycobacterium tuberculosis XTB13-219 2621597817
MSAVALPRVSGGHDEHGHLEEFRTDPIGLMQRVRDECGDVGTFQLAGKQVVLLSGSHANEFFFRAGDDDLDQA
KAYPFMTPIFGEGVVFDASPERRKEMLHNAALRGEQMKGHAATIEDQVRRMIADWGEAGEIDLLDFFAELTIY
TSSACLIGKKFRDQLDGRFAKLYHELERGTDPLAYVDPYLPIESFRRRDEARNGLVALVADIMNGRIANPPTD
KSDRDMLDVLIAVKAETGTPRFSADEITGMFISMMFAGHHTSSGTASWTLIELMRHRDAYAAVIDELDELYGD
GRSVSFHALRQIPQLENVLKETLRLHPPLIILMRVAKGEFEVQGHRIHEGDLVAASPAISNRIPEDFPDPHDF
VPARYEQPRQEDLLNRWTWIPFGAGRHRCVGAAFAIMQIKAIFSVLLREYEFEMAQPPESYRNDHSKMVVQLA
QPACVRYRRRTGV
>CYP51B1_Mycobacterium tuberculosis PanR0703 2555399832
MSAVALPRVSGGHDEHGHLEEFRTDPIGLMQRVRDECGDVGTFQLAGKQVVLLSGSHANEFFFRAGDDDLDQA
KAYPFMTPIFGEGVVFDASPERRKEMLHNAALRGEQMKGHAATIEDQVRRMIADWGEAGEIDLLDFFAELTIY
TSSACLIGKKFRDQLDGRFAKLYHELERGTDPLAYVDPYLPIESFRRRDEARNGLVALVADIMNGRIANPPTD
KSDRDMLDVLIAVKAETGTPRFSADEITGMFISMMFAGHHTSSGTASWTLIELMRHRDAYAAVIDELDELYGD
GRSVSFHALRQIPQLENVLKETLRLHPPLIILMRVAKGEFEVQGHRIHEGDLVAASPAISNRIPEDFPDPHDF
VPARYEQPRQEDLLNRWTWIPFGAGRHRCVGAAFAIMQIKAIFSVLLREYEFEMAQPPESYRNDHSKMVVQLA
QPACVRYRRRTGV
>CYP51B1_Mycobacterium tuberculosis XTB13-225 2621599812
MSAVALPRVSGGHDEHGHLEEFRTDPIGLMQRVRDECGDVGTFQLAGKQVVLLSGSHANEFFFRAGDDDLDQA
KAYPFMTPIFGEGVVFDASPERRKEMLHNAALRGEQMKGHAATIEDQVRRMIADWGEAGEIDLLDFFAELTIY
TSSACLIGKKFRDQLDGRFAKLYHELERGTDPLAYVDPYLPIESFRRRDEARNGLVALVADIMNGRIANPPTD
KSDRDMLDVLIAVKAETGTPRFSADEITGMFISMMFAGHHTSSGTASWTLIELMRHRDAYAAVIDELDELYGD
GRSVSFHALRQIPQLENVLKETLRLHPPLIILMRVAKGEFEVQGHRIHEGDLVAASPAISNRIPEDFPDPHDF
VPARYEQPRQEDLLNRWTWIPFGAGRHRCVGAAFAIMQIKAIFSVLLREYEFEMAQPPESYRNDHSKMVVQLA
QPACVRYRRRTGV
>CYP51B1_Mycobacterium tuberculosis XTB13-227 2621603906
MSAVALPRVSGGHDEHGHLEEFRTDPIGLMQRVRDECGDVGTFQLAGKQVVLLSGSHANEFFFRAGDDDLDQA
KAYPFMTPIFGEGVVFDASPERRKEMLHNAALRGEQMKGHAATIEDQVRRMIADWGEAGEIDLLDFFAELTIY
TSSACLIGKKFRDQLDGRFAKLYHELERGTDPLAYVDPYLPIESFRRRDEARNGLVALVADIMNGRIANPPTD
KSDRDMLDVLIAVKAETGTPRFSADEITGMFISMMFAGHHTSSGTASWTLIELMRHRDAYAAVIDELDELYGD
GRSVSFHALRQIPQLENVLKETLRLHPPLIILMRVAKGEFEVQGHRIHEGDLVAASPAISNRIPEDFPDPHDF
VPARYEQPRQEDLLNRWTWIPFGAGRHRCVGAAFAIMQIKAIFSVLLREYEFEMAQPPESYRNDHSKMVVQLA
QPACVRYRRRTGV
>CYP51B1_Mycobacterium tuberculosis XTB13-228 2621607560
MSAVALPRVSGGHDEHGHLEEFRTDPIGLMQRVRDECGDVGTFQLAGKQVVLLSGSHANEFFFRAGDDDLDQA
KAYPFMTPIFGEGVVFDASPERRKEMLHNAALRGEQMKGHAATIEDQVRRMIADWGEAGEIDLLDFFAELTIY
TSSACLIGKKFRDQLDGRFAKLYHELERGTDPLAYVDPYLPIESFRRRDEARNGLVALVADIMNGRIANPPTD
KSDRDMLDVLIAVKAETGTPRFSADEITGMFISMMFAGHHTSSGTASWTLIELMRHRDAYAAVIDELDELYGD
GRSVSFHALRQIPQLENVLKETLRLHPPLIILMRVAKGEFEVQGHRIHEGDLVAASPAISNRIPEDFPDPHDF
VPARYEQPRQEDLLNRWTWIPFGAGRHRCVGAAFAIMQIKAIFSVLLREYEFEMAQPPESYRNDHSKMVVQLA
QPACVRYRRRTGV
>CYP51B1_Mycobacterium tuberculosis XTB13-229 2575654282
MSAVALPRVSGGHDEHGHLEEFRTDPIGLMQRVRDECGDVGTFQLAGKQVVLLSGSHANEFFFRAGDDDLDQA
KAYPFMTPIFGEGVVFDASPERRKEMLHNAALRGEQMKGHAATIEDQVRRMIADWGEAGEIDLLDFFAELTIY
TSSACLIGKKFRDQLDGRFAKLYHELERGTDPLAYVDPYLPIESFRRRDEARNGLVALVADIMNGRIANPPTD
KSDRDMLDVLIAVKAETGTPRFSADEITGMFISMMFAGHHTSSGTASWTLIELMRHRDAYAAVIDELDELYGD
GRSVSFHALRQIPQLENVLKETLRLHPPLIILMRVAKGEFEVQGHRIHEGDLVAASPAISNRIPEDFPDPHDF

VPARYEQPRQEDLLNRWTWIPFGAGRHRCVGAAFAIMQIKAIFSVLLREYEFEMAQPPESYRNDHSKMVVQLA
QPACVRYRRRTGV
>CYP51B1_Mycobacterium tuberculosis XTB13-230 2621612151
MSAVALPRVSGGHDEHGHLEEFRTDPIGLMQRVRDECGDVGTFQLAGKQVVLLSGSHANEFFFRAGDDDLDQA
KAYPFMTPIFGEGVVFDASPERRKEMLHNAALRGEQMKGHAATIEDQVRRMIADWGEAGEIDLLDFFAELTIY
TSSACLIGKKFRDQLDGRFAKLYHELERGTDPLAYVDPYLPIESFRRRDEARNGLVALVADIMNGRIANPPTD
KSDRDMLDVLIAVKAETGTPRFSADEITGMFISMMFAGHHTSSGTASWTLIELMRHRDAYAAVIDELDELYGD
GRSVSFHALRQIPQLENVLKETLRLHPPLIILMRVAKGEFEVQGHRIHEGDLVAASPAISNRIPEDFPDPHDF
VPARYEQPRQEDLLNRWTWIPFGAGRHRCVGAAFAIMQIKAIFSVLLREYEFEMAQPPESYRNDHSKMVVQLA
QPACVRYRRRTGV
>CYP51B1_Mycobacterium tuberculosis XTB13-231 2606363549
MSAVALPRVSGGHDEHGHLEEFRTDPIGLMQRVRDECGDVGTFQLAGKQVVLLSGSHANEFFFRAGDDDLDQA
KAYPFMTPIFGEGVVFDASPERRKEMLHNAALRGEQMKGHAATIEDQVRRMIADWGEAGEIDLLDFFAELTIY
TSSACLIGKKFRDQLDGRFAKLYHELERGTDPLAYVDPYLPIESFRRRDEARNGLVALVADIMNGRIANPPTD
KSDRDMLDVLIAVKAETGTPRFSADEITGMFISMMFAGHHTSSGTASWTLIELMRHRDAYAAVIDELDELYGD
GRSVSFHALRQIPQLENVLKETLRLHPPLIILMRVAKGEFEVQGHRIHEGDLVAASPAISNRIPEDFPDPHDF
VPARYEQPRQEDLLNRWTWIPFGAGRHRCVGAAFAIMQIKAIFSVLLREYEFEMAQPPESYRNDHSKMVVQLA
QPACVRYRRRTGV
>CYP51B1_Mycobacterium tuberculosis XTB13-232 2621619142
MSAVALPRVSGGHDEHGHLEEFRTDPIGLMQRVRDECGDVGTFQLAGKQVVLLSGSHANEFFFRAGDDDLDQA
KAYPFMTPIFGEGVVFDASPERRKEMLHNAALRGEQMKGHAATIEDQVRRMIADWGEAGEIDLLDFFAELTIY
TSSACLIGKKFRDQLDGRFAKLYHELERGTDPLAYVDPYLPIESFRRRDEARNGLVALVADIMNGRIANPPTD
KSDRDMLDVLIAVKAETGTPRFSADEITGMFISMMFAGHHTSSGTASWTLIELMRHRDAYAAVIDELDELYGD
GRSVSFHALRQIPQLENVLKETLRLHPPLIILMRVAKGEFEVQGHRIHEGDLVAASPAISNRIPEDFPDPHDF
VPARYEQPRQEDLLNRWTWIPFGAGRHRCVGAAFAIMQIKAIFSVLLREYEFEMAQPPESYRNDHSKMVVQLA
QPACVRYRRRTGV
>CYP51B1_Mycobacterium tuberculosis XTB13-233 2621620117
MSAVALPRVSGGHDEHGHLEEFRTDPIGLMQRVRDECGDVGTFQLAGKQVVLLSGSHANEFFFRAGDDDLDQA
KAYPFMTPIFGEGVVFDASPERRKEMLHNAALRGEQMKGHAATIEDQVRRMIADWGEAGEIDLLDFFAELTIY
TSSACLIGKKFRDQLDGRFAKLYHELERGTDPLAYVDPYLPIESFRRRDEARNGLVALVADIMNGRIANPPTD
KSDRDMLDVLIAVKAETGTPRFSADEITGMFISMMFAGHHTSSGTASWTLIELMRHRDAYAAVIDELDELYGD
GRSVSFHALRQIPQLENVLKETLRLHPPLIILMRVAKGEFEVQGHRIHEGDLVAASPAISNRIPEDFPDPHDF
VPARYEQPRQEDLLNRWTWIPFGAGRHRCVGAAFAIMQIKAIFSVLLREYEFEMAQPPESYRNDHSKMVVQLA
QPACVRYRRRTGV
>CYP51B1_Mycobacterium tuberculosis XTB13-234 2606365727
MSAVALPRVSGGHDEHGHLEEFRTDPIGLMQRVRDECGDVGTFQLAGKQVVLLSGSHANEFFFRAGDDDLDQA
KAYPFMTPIFGEGVVFDASPERRKEMLHNAALRGEQMKGHAATIEDQVRRMIADWGEAGEIDLLDFFAELTIY
TSSACLIGKKFRDQLDGRFAKLYHELERGTDPLAYVDPYLPIESFRRRDEARNGLVALVADIMNGRIANPPTD
KSDRDMLDVLIAVKAETGTPRFSADEITGMFISMMFAGHHTSSGTASWTLIELMRHRDAYAAVIDELDELYGD
GRSVSFHALRQIPQLENVLKETLRLHPPLIILMRVAKGEFEVQGHRIHEGDLVAASPAISNRIPEDFPDPHDF
VPARYEQPRQEDLLNRWTWIPFGAGRHRCVGAAFAIMQIKAIFSVLLREYEFEMAQPPESYRNDHSKMVVQLA
QPACVRYRRRTGV
>CYP51B1_Mycobacterium tuberculosis XTB13-235 2621625756
MSAVALPRVSGGHDEHGHLEEFRTDPIGLMQRVRDECGDVGTFQLAGKQVVLLSGSHANEFFFRAGDDDLDQA
KAYPFMTPIFGEGVVFDASPERRKEMLHNAALRGEQMKGHAATIEDQVRRMIADWGEAGEIDLLDFFAELTIY
TSSACLIGKKFRDQLDGRFAKLYHELERGTDPLAYVDPYLPIESFRRRDEARNGLVALVADIMNGRIANPPTD
KSDRDMLDVLIAVKAETGTPRFSADEITGMFISMMFAGHHTSSGTASWTLIELMRHRDAYAAVIDELDELYGD
GRSVSFHALRQIPQLENVLKETLRLHPPLIILMRVAKGEFEVQGHRIHEGDLVAASPAISNRIPEDFPDPHDF
VPARYEQPRQEDLLNRWTWIPFGAGRHRCVGAAFAIMQIKAIFSVLLREYEFEMAQPPESYRNDHSKMVVQLA
QPACVRYRRRTGV
>CYP51B1_Mycobacterium tuberculosis XTB13-237 2621628637
MSAVALPRVSGGHDEHGHLEEFRTDPIGLMQRVRDECGDVGTFQLAGKQVVLLSGSHANEFFFRAGDDDLDQA
KAYPFMTPIFGEGVVFDASPERRKEMLHNAALRGEQMKGHAATIEDQVRRMIADWGEAGEIDLLDFFAELTIY
TSSACLIGKKFRDQLDGRFAKLYHELERGTDPLAYVDPYLPIESFRRRDEARNGLVALVADIMNGRIANPPTD
KSDRDMLDVLIAVKAETGTPRFSADEITGMFISMMFAGHHTSSGTASWTLIELMRHRDAYAAVIDELDELYGD
GRSVSFHALRQIPQLENVLKETLRLHPPLIILMRVAKGEFEVQGHRIHEGDLVAASPAISNRIPEDFPDPHDF
VPARYEQPRQEDLLNRWTWIPFGAGRHRCVGAAFAIMQIKAIFSVLLREYEFEMAQPPESYRNDHSKMVVQLA
QPACVRYRRRTGV

>CYP51B1_Mycobacterium tuberculosis XTB13-238 2584821086
MSAVALPRVSGGHDEHGHLEEFRTDPIGLMQRVRDECGDVGTFQLAGKQVVLLSGSHANEFFFRAGDDDLDQA
KAYPFMTPIFGEGVVFDASPERRKEMLHNAALRGEQMKGHAATIEDQVRRMIADWGEAGEIDLLDFFAELTIY
TSSACLIGKKFRDQLDGRFAKLYHELERGTDPLAYVDPYLPIESFRRRDEARNGLVALVADIMNGRIANPPTD
KSDRDMLDVLIAVKAETGTPRFSADEITGMFISMMFAGHHTSSGTASWTLIELMRHRDAYAAVIDELDELYGD
GRSVSFHALRQIPQLENVLKETLRLHPPLIILMRVAKGEFEVQGHRIHEGDLVAASPAISNRIPEDFPDPHDF
VPARYEQPRQEDLLNRWTWIPFGAGRHRCVGAAFAIMQIKAIFSVLLREYEFEMAQPPESYRNDHSKMVVQLA
QPACVRYRRRTGV
>CYP51B1_Mycobacterium tuberculosis XTB13-239 2621635285
MSAVALPRVSGGHDEHGHLEEFRTDPIGLMQRVRDECGDVGTFQLAGKQVVLLSGSHANEFFFRAGDDDLDQA
KAYPFMTPIFGEGVVFDASPERRKEMLHNAALRGEQMKGHAATIEDQVRRMIADWGEAGEIDLLDFFAELTIY
TSSACLIGKKFRDQLDGRFAKLYHELERGTDPLAYVDPYLPIESFRRRDEARNGLVALVADIMNGRIANPPTD
KSDRDMLDVLIAVKAETGTPRFSADEITGMFISMMFAGHHTSSGTASWTLIELMRHRDAYAAVIDELDELYGD
GRSVSFHALRQIPQLENVLKETLRLHPPLIILMRVAKGEFEVQGHRIHEGDLVAASPAISNRIPEDFPDPHDF
VPARYEQPRQEDLLNRWTWIPFGAGRHRCVGAAFAIMQIKAIFSVLLREYEFEMAQPPESYRNDHSKMVVQLA
QPACVRYRRRTGV
>CYP51B1_Mycobacterium tuberculosis XTB13-240 2621638335
MSAVALPRVSGGHDEHGHLEEFRTDPIGLMQRVRDECGDVGTFQLAGKQVVLLSGSHANEFFFRAGDDDLDQA
KAYPFMTPIFGEGVVFDASPERRKEMLHNAALRGEQMKGHAATIEDQVRRMIADWGEAGEIDLLDFFAELTIY
TSSACLIGKKFRDQLDGRFAKLYHELERGTDPLAYVDPYLPIESFRRRDEARNGLVALVADIMNGRIANPPTD
KSDRDMLDVLIAVKAETGTPRFSADEITGMFISMMFAGHHTSSGTASWTLIELMRHRDAYAAVIDELDELYGD
GRSVSFHALRQIPQLENVLKETLRLHPPLIILMRVAKGEFEVQGHRIHEGDLVAASPAISNRIPEDFPDPHDF
VPARYEQPRQEDLLNRWTWIPFGAGRHRCVGAAFAIMQIKAIFSVLLREYEFEMAQPPESYRNDHSKMVVQLA
QPACVRYRRRTGV
>CYP51B1_Mycobacterium tuberculosis XTB13-241 2576542844
MSAVALPRVSGGHDEHGHLEEFRTDPIGLMQRVRDECGDVGTFQLAGKQVVLLSGSHANEFFFRAGDDDLDQA
KAYPFMTPIFGEGVVFDASPERRKEMLHNAALRGEQMKGHAATIEDQVRRMIADWGEAGEIDLLDFFAELTIY
TSSACLIGKKFRDQLDGRFAKLYHELERGTDPLAYVDPYLPIESFRRRDEARNGLVALVADIMNGRIANPPTD
KSDRDMLDVLIAVKAETGTPRFSADEITGMFISMMFAGHHTSSGTASWTLIELMRHRDAYAAVIDELDELYGD
GRSVSFHALRQIPQLENVLKETLRLHPPLIILMRVAKGEFEVQGHRIHEGDLVAASPAISNRIPEDFPDPHDF
VPARYEQPRQEDLLNRWTWIPFGAGRHRCVGAAFAIMQIKAIFSVLLREYEFEMAQPPESYRNDHSKMVVQLA
QPACVRYRRRTGV
>CYP51B1_Mycobacterium tuberculosis XTB13-242 2606369207
MSAVALPRVSGGHDEHGHLEEFRTDPIGLMQRVRDECGDVGTFQLAGKQVVLLSGSHANEFFFRAGDDDLDQA
KAYPFMTPIFGEGVVFDASPERRKEMLHNAALRGEQMKGHAATIEDQVRRMIADWGEAGEIDLLDFFAELTIY
TSSACLIGKKFRDQLDGRFAKLYHELERGTDPLAYVDPYLPIESFRRRDEARNGLVALVADIMNGRIANPPTD
KSDRDMLDVLIAVKAETGTPRFSADEITGMFISMMFAGHHTSSGTASWTLIELMRHRDAYAAVIDELDELYGD
GRSVSFHALRQIPQLENVLKETLRLHPPLIILMRVAKGEFEVQGHRIHEGDLVAASPAISNRIPEDFPDPHDF
VPARYEQPRQEDLLNRWTWIPFGAGRHRCVGAAFAIMQIKAIFSVLLREYEFEMAQPPESYRNDHSKMVVQLA
QPACVRYRRRTGV
>CYP51B1_Mycobacterium tuberculosis XTB13-244 2621640660
MSAVALPRVSGGHDEHGHLEEFRTDPIGLMQRVRDECGDVGTFQLAGKQVVLLSGSHANEFFFRAGDDDLDQA
KAYPFMTPIFGEGVVFDASPERRKEMLHNAALRGEQMKGHAATIEDQVRRMIADWGEAGEIDLLDFFAELTIY
TSSACLIGKKFRDQLDGRFAKLYHELERGTDPLAYVDPYLPIESFRRRDEARNGLVALVADIMNGRIANPPTD
KSDRDMLDVLIAVKAETGTPRFSADEITGMFISMMFAGHHTSSGTASWTLIELMRHRDAYAAVIDELDELYGD
GRSVSFHALRQIPQLENVLKETLRLHPPLIILMRVAKGEFEVQGHRIHEGDLVAASPAISNRIPEDFPDPHDF
VPARYEQPRQEDLLNRWTWIPFGAGRHRCVGAAFAIMQIKAIFSVLLREYEFEMAQPPESYRNDHSKMVVQLA
QPACVRYRRRTGV
>CYP51B1_Mycobacterium tuberculosis XTB13-245 2621646491
MSAVALPRVSGGHDEHGHLEEFRTDPIGLMQRVRDECGDVGTFQLAGKQVVLLSGSHANEFFFRAGDDDLDQA
KAYPFMTPIFGEGVVFDASPERRKEMLHNAALRGEQMKGHAATIEDQVRRMIADWGEAGEIDLLDFFAELTIY
TSSACLIGKKFRDQLDGRFAKLYHELERGTDPLAYVDPYLPIESFRRRDEARNGLVALVADIMNGRIANPPTD
KSDRDMLDVLIAVKAETGTPRFSADEITGMFISMMFAGHHTSSGTASWTLIELMRHRDAYAAVIDELDELYGD
GRSVSFHALRQIPQLENVLKETLRLHPPLIILMRVAKGEFEVQGHRIHEGDLVAASPAISNRIPEDFPDPHDF
VPARYEQPRQEDLLNRWTWIPFGAGRHRCVGAAFAIMQIKAIFSVLLREYEFEMAQPPESYRNDHSKMVVQLA
QPACVRYRRRTGV
>CYP51B1_Mycobacterium tuberculosis XTB13-246 2621649197

MSAVALPRVSGGHDEHGHLEEFRTDPIGLMQRVRDECGDVGTFQLAGKQVVLLSGSHANEFFFRAGDDDLDQA
KAYPFMTPIFGEGVVFDASPERRKEMLHNAALRGEQMKGHAATIEDQVRRMIADWGEAGEIDLLDFFAELTIY
TSSACLIGKKFRDQLDGRFAKLYHELERGTDPLAYVDPYLPIESFRRRDEARNGLVALVADIMNGRIANPPTD
KSDRDMLDVLIAVKAETGTPRFSADEITGMFISMMFAGHHTSSGTASWTLIELMRHRDAYAAVIDELDELYGD
GRSVSFHALRQIPQLENVLKETLRLHPPLIILMRVAKGEFEVQGHRIHEGDLVAASPAISNRIPEDFPDPHDF
VPARYEQPRQEDLLNRWTWIPFGAGRHRCVGAAFAIMQIKAIFSVLLREYEFEMAQPPESYRNDHSKMVVQLA
QPACVRYRRRTGV
>CYP51B1_Mycobacterium tuberculosis XTB13-247 2606375732
MSAVALPRVSGGHDEHGHLEEFRTDPIGLMQRVRDECGDVGTFQLAGKQVVLLSGSHANEFFFRAGDDDLDQA
KAYPFMTPIFGEGVVFDASPERRKEMLHNAALRGEQMKGHAATIEDQVRRMIADWGEAGEIDLLDFFAELTIY
TSSACLIGKKFRDQLDGRFAKLYHELERGTDPLAYVDPYLPIESFRRRDEARNGLVALVADIMNGRIANPPTD
KSDRDMLDVLIAVKAETGTPRFSADEITGMFISMMFAGHHTSSGTASWTLIELMRHRDAYAAVIDELDELYGD
GRSVSFHALRQIPQLENVLKETLRLHPPLIILMRVAKGEFEVQGHRIHEGDLVAASPAISNRIPEDFPDPHDF
VPARYEQPRQEDLLNRWTWIPFGAGRHRCVGAAFAIMQIKAIFSVLLREYEFEMAQPPESYRNDHSKMVVQLA
QPACVRYRRRTGV
>CYP51B1_Mycobacterium tuberculosis XTB13-249 2621653293
MSAVALPRVSGGHDEHGHLEEFRTDPIGLMQRVRDECGDVGTFQLAGKQVVLLSGSHANEFFFRAGDDDLDQA
KAYPFMTPIFGEGVVFDASPERRKEMLHNAALRGEQMKGHAATIEDQVRRMIADWGEAGEIDLLDFFAELTIY
TSSACLIGKKFRDQLDGRFAKLYHELERGTDPLAYVDPYLPIESFRRRDEARNGLVALVADIMNGRIANPPTD
KSDRDMLDVLIAVKAETGTPRFSADEITGMFISMMFAGHHTSSGTASWTLIELMRHRDAYAAVIDELDELYGD
GRSVSFHALRQIPQLENVLKETLRLHPPLIILMRVAKGEFEVQGHRIHEGDLVAASPAISNRIPEDFPDPHDF
VPARYEQPRQEDLLNRWTWIPFGAGRHRCVGAAFAIMQIKAIFSVLLREYEFEMAQPPESYRNDHSKMVVQLA
QPACVRYRRRTGV
>CYP51B1_Mycobacterium tuberculosis XTB13-250 2621658006
MSAVALPRVSGGHDEHGHLEEFRTDPIGLMQRVRDECGDVGTFQLAGKQVVLLSGSHANEFFFRAGDDDLDQA
KAYPFMTPIFGEGVVFDASPERRKEMLHNAALRGEQMKGHAATIEDQVRRMIADWGEAGEIDLLDFFAELTIY
TSSACLIGKKFRDQLDGRFAKLYHELERGTDPLAYVDPYLPIESFRRRDEARNGLVALVADIMNGRIANPPTD
KSDRDMLDVLIAVKAETGTPRFSADEITGMFISMMFAGHHTSSGTASWTLIELMRHRDAYAAVIDELDELYGD
GRSVSFHALRQIPQLENVLKETLRLHPPLIILMRVAKGEFEVQGHRIHEGDLVAASPAISNRIPEDFPDPHDF
VPARYEQPRQEDLLNRWTWIPFGAGRHRCVGAAFAIMQIKAIFSVLLREYEFEMAQPPESYRNDHSKMVVQLA
QPACVRYRRRTGV
>CYP51B1_Mycobacterium tuberculosis XTB13-251 2575098073
MSAVALPRVSGGHDEHGHLEEFRTDPIGLMQRVRDECGDVGTFQLAGKQVVLLSGSHANEFFFRAGDDDLDQA
KAYPFMTPIFGEGVVFDASPERRKEMLHNAALRGEQMKGHAATIEDQVRRMIADWGEAGEIDLLDFFAELTIY
TSSACLIGKKFRDQLDGRFAKLYHELERGTDPLAYVDPYLPIESFRRRDEARNGLVALVADIMNGRIANPPTD
KSDRDMLDVLIAVKAETGTPRFSADEITGMFISMMFAGHHTSSGTASWTLIELMRHRDAYAAVIDELDELYGD
GRSVSFHALRQIPQLENVLKETLRLHPPLIILMRVAKGEFEVQGHRIHEGDLVAASPAISNRIPEDFPDPHDF
VPARYEQPRQEDLLNRWTWIPFGAGRHRCVGAAFAIMQIKAIFSVLLREYEFEMAQPPESYRNDHSKMVVQLA
QPACVRYRRRTGV
>CYP51B1_Mycobacterium tuberculosis XTB13-252 2584699309
MSAVALPRVSGGHDEHGHLEEFRTDPIGLMQRVRDECGDVGTFQLAGKQVVLLSGSHANEFFFRAGDDDLDQA
KAYPFMTPIFGEGVVFDASPERRKEMLHNAALRGEQMKGHAATIEDQVRRMIADWGEAGEIDLLDFFAELTIY
TSSACLIGKKFRDQLDGRFAKLYHELERGTDPLAYVDPYLPIESFRRRDEARNGLVALVADIMNGRIANPPTD
KSDRDMLDVLIAVKAETGTPRFSADEITGMFISMMFAGHHTSSGTASWTLIELMRHRDAYAAVIDELDELYGD
GRSVSFHALRQIPQLENVLKETLRLHPPLIILMRVAKGEFEVQGHRIHEGDLVAASPAISNRIPEDFPDPHDF
VPARYEQPRQEDLLNRWTWIPFGAGRHRCVGAAFAIMQIKAIFSVLLREYEFEMAQPPESYRNDHSKMVVQLA
QPACVRYRRRTGV
>CYP51B1_Mycobacterium tuberculosis XTB13-253 2606462231
MSAVALPRVSGGHDEHGHLEEFRTDPIGLMQRVRDECGDVGTFQLAGKQVVLLSGSHANEFFFRAGDDDLDQA
KAYPFMTPIFGEGVVFDASPERRKEMLHNAALRGEQMKGHAATIEDQVRRMIADWGEAGEIDLLDFFAELTIY
TSSACLIGKKFRDQLDGRFAKLYHELERGTDPLAYVDPYLPIESFRRRDEARNGLVALVADIMNGRIANPPTD
KSDRDMLDVLIAVKAETGTPRFSADEITGMFISMMFAGHHTSSGTASWTLIELMRHRDAYAAVIDELDELYGD
GRSVSFHALRQIPQLENVLKETLRLHPPLIILMRVAKGEFEVQGHRIHEGDLVAASPAISNRIPEDFPDPHDF
VPARYEQPRQEDLLNRWTWIPFGAGRHRCVGAAFAIMQIKAIFSVLLREYEFEMAQPPESYRNDHSKMVVQLA
QPACVRYRRRTGV
>CYP51B1_Mycobacterium tuberculosis XTB13-255 2578212190
MSAVALPRVSGGHDEHGHLEEFRTDPIGLMQRVRDECGDVGTFQLAGKQVVLLSGSHANEFFFRAGDDDLDQA
KAYPFMTPIFGEGVVFDASPERRKEMLHNAALRGEQMKGHAATIEDQVRRMIADWGEAGEIDLLDFFAELTIY

TSSACLIGKKFRDQLDGRFAKLYHELERGTDPLAYVDPYLPIESFRRRDEARNGLVALVADIMNGRIANPPTD
KSDRDMLDVLIAVKAETGTPRFSADEITGMFISMMFAGHHTSSGTASWTLIELMRHRDAYAAVIDELDELYGD
GRSVSFHALRQIPQLENVLKETLRLHPPLIILMRVAKGEFEVQGHRIHEGDLVAASPAISNRIPEDFPDPHDF
VPARYEQPRQEDLLNRWTWIPFGAGRHRCVGAAFAIMQIKAIFSVLLREYEFEMAQPPESYRNDHSKMVVQLA
QPACVRYRRRTGV
>CYP51B1_Mycobacterium tuberculosis XTB13-256 2606465710
MSAVALPRVSGGHDEHGHLEEFRTDPIGLMQRVRDECGDVGTFQLAGKQVVLLSGSHANEFFFRAGDDDLDQA
KAYPFMTPIFGEGVVFDASPERRKEMLHNAALRGEQMKGHAATIEDQVRRMIADWGEAGEIDLLDFFAELTIY
TSSACLIGKKFRDQLDGRFAKLYHELERGTDPLAYVDPYLPIESFRRRDEARNGLVALVADIMNGRIANPPTD
KSDRDMLDVLIAVKAETGTPRFSADEITGMFISMMFAGHHTSSGTASWTLIELMRHRDAYAAVIDELDELYGD
GRSVSFHALRQIPQLENVLKETLRLHPPLIILMRVAKGEFEVQGHRIHEGDLVAASPAISNRIPEDFPDPHDF
VPARYEQPRQEDLLNRWTWIPFGAGRHRCVGAAFAIMQIKAIFSVLLREYEFEMAQPPESYRNDHSKMVVQLA
QPACVRYRRRTGV
>CYP51B1_Mycobacterium tuberculosis XTB13-262 2621661538
MSAVALPRVSGGHDEHGHLEEFRTDPIGLMQRVRDECGDVGTFQLAGKQVVLLSGSHANEFFFRAGDDDLDQA
KAYPFMTPIFGEGVVFDASPERRKEMLHNAALRGEQMKGHAATIEDQVRRMIADWGEAGEIDLLDFFAELTIY
TSSACLIGKKFRDQLDGRFAKLYHELERGTDPLAYVDPYLPIESFRRRDEARNGLVALVADIMNGRIANPPTD
KSDRDMLDVLIAVKAETGTPRFSADEITGMFISMMFAGHHTSSGTASWTLIELMRHRDAYAAVIDELDELYGD
GRSVSFHALRQIPQLENVLKETLRLHPPLIILMRVAKGEFEVQGHRIHEGDLVAASPAISNRIPEDFPDPHDF
VPARYEQPRQEDLLNRWTWIPFGAGRHRCVGAAFAIMQIKAIFSVLLREYEFEMAQPPESYRNDHSKMVVQLA
QPACVRYRRRTGV
>CYP51B1_Mycobacterium tuberculosis XTB13-265 2621666095
MSAVALPRVSGGHDEHGHLEEFRTDPIGLMQRVRDECGDVGTFQLAGKQVVLLSGSHANEFFFRAGDDDLDQA
KAYPFMTPIFGEGVVFDASPERRKEMLHNAALRGEQMKGHAATIEDQVRRMIADWGEAGEIDLLDFFAELTIY
TSSACLIGKKFRDQLDGRFAKLYHELERGTDPLAYVDPYLPIESFRRRDEARNGLVALVADIMNGRIANPPTD
KSDRDMLDVLIAVKAETGTPRFSADEITGMFISMMFAGHHTSSGTASWTLIELMRHRDAYAAVIDELDELYGD
GRSVSFHALRQIPQLENVLKETLRLHPPLIILMRVAKGEFEVQGHRIHEGDLVAASPAISNRIPEDFPDPHDF
VPARYEQPRQEDLLNRWTWIPFGAGRHRCVGAAFAIMQIKAIFSVLLREYEFEMAQPPESYRNDHSKMVVQLA
QPACVRYRRRTGV
>CYP51B1_Mycobacterium tuberculosis XTB13-290 2584958334
MSAVALPRVSGGHDEHGHLEEFRTDPIGLMQRVRDECGDVGTFQLAGKQVVLLSGSHANEFFFRAGDDDLDQA
KAYPFMTPIFGEGVVFDASPERRKEMLHNAALRGEQMKGHAATIEDQVRRMIADWGEAGEIDLLDFFAELTIY
TSSACLIGKKFRDQLDGRFAKLYHELERGTDPLAYVDPYLPIESFRRRDEARNGLVALVADIMNGRIANPPTD
KSDRDMLDVLIAVKAETGTPRFSADEITGMFISMMFAGHHTSSGTASWTLIELMRHRDAYAAVIDELDELYGD
GRSVSFHALRQIPQLENVLKETLRLHPPLIILMRVAKGEFEVQGHRIHEGDLVAASPAISNRIPEDFPDPHDF
VPARYEQPRQEDLLNRWTWIPFGAGRHRCVGAAFAIMQIKAIFSVLLREYEFEMAQPPESYRNDHSKMVVQLA
QPACVRYRRRTGV
>CYP51B1_Mycobacterium tuberculosis XTB13-278 2621669498
MSAVALPRVSGGHDEHGHLEEFRTDPIGLMQRVRDECGDVGTFQLAGKQVVLLSGSHANEFFFRAGDDDLDQA
KAYPFMTPIFGEGVVFDASPERRKEMLHNAALRGEQMKGHAATIEDQVRRMIADWGEAGEIDLLDFFAELTIY
TSSACLIGKKFRDQLDGRFAKLYHELERGTDPLAYVDPYLPIESFRRRDEARNGLVALVADIMNGRIANPPTD
KSDRDMLDVLIAVKAETGTPRFSADEITGMFISMMFAGHHTSSGTASWTLIELMRHRDAYAAVIDELDELYGD
GRSVSFHALRQIPQLENVLKETLRLHPPLIILMRVAKGEFEVQGHRIHEGDLVAASPAISNRIPEDFPDPHDF
VPARYEQPRQEDLLNRWTWIPFGAGRHRCVGAAFAIMQIKAIFSVLLREYEFEMAQPPESYRNDHSKMVVQLA
QPACVRYRRRTGV
>CYP51B1_Mycobacterium tuberculosis XTB13-299 2621674302
MSAVALPRVSGGHDEHGHLEEFRTDPIGLMQRVRDECGDVGTFQLAGKQVVLLSGSHANEFFFRAGDDDLDQA
KAYPFMTPIFGEGVVFDASPERRKEMLHNAALRGEQMKGHAATIEDQVRRMIADWGEAGEIDLLDFFAELTIY
TSSACLIGKKFRDQLDGRFAKLYHELERGTDPLAYVDPYLPIESFRRRDEARNGLVALVADIMNGRIANPPTD
KSDRDMLDVLIAVKAETGTPRFSADEITGMFISMMFAGHHTSSGTASWTLIELMRHRDAYAAVIDELDELYGD
GRSVSFHALRQIPQLENVLKETLRLHPPLIILMRVAKGEFEVQGHRIHEGDLVAASPAISNRIPEDFPDPHDF
VPARYEQPRQEDLLNRWTWIPFGAGRHRCVGAAFAIMQIKAIFSVLLREYEFEMAQPPESYRNDHSKMVVQLA
QPACVRYRRRTGV
>CYP51B1_Mycobacterium tuberculosis Y00015573 2703568940
MSAVALPRVSGGHDEHGHLEEFRTDPIGLMQRVRDECGDVGTFQLAGKQVVLLSGSHANEFFFRAGDDDLDQA
KAYPFMTPIFGEGVVFDASPERRKEMLHNAALRGEQMKGHAATIEDQVRRMIADWGEAGEIDLLDFFAELTIY
TSSACLIGKKFRDQLDGRFAKLYHELERGTDPLAYVDPYLPIESFRRRDEARNGLVALVADIMNGRIANPPTD
KSDRDMLDVLIAVKAETGTPRFSADEITGMFISMMFAGHHTSSGTASWTLIELMRHRDAYAAVIDELDELYGD

GRSVSFHALRQIPQLENVLKETLRLHPPLIILMRVAKGEFEVQGHRIHEGDLVAASPAISNRIPEDFPDPHDF
VPARYEQPRQEDLLNRWTWIPFGAGRHRCVGAAFAIMQIKAIFSVLLREYEFEMAQPPESYRNDHSKMVVQLA
QPACVRYRRRTGV
>CYP51B1_Mycobacterium tuberculosis Y00015898 2693632417
MSAVALPRVSGGHDEHGHLEEFRTDPIGLMQRVRDECGDVGTFQLAGKQVVLLSGSHANEFFFRAGDDDLDQA
KAYPFMTPIFGEGVVFDASPERRKEMLHNAALRGEQMKGHAATIEDQVRRMIADWGEAGEIDLLDFFAELTIY
TSSACLIGKKFRDQLDGRFAKLYHELERGTDPLAYVDPYLPIESFRRRDEARNGLVALVADIMNGRIANPPTD
KSDRDMLDVLIAVKAETGTPRFSADEITGMFISMMFAGHHTSSGTASWTLIELMRHRDAYAAVIDELDELYGD
GRSVSFHALRQIPQLENVLKETLRLHPPLIILMRVAKGEFEVQGHRIHEGDLVAASPAISNRIPEDFPDPHDF
VPARYEQPRQEDLLNRWTWIPFGAGRHRCVGAAFAIMQIKAIFSVLLREYEFEMAQPPESYRNDHSKMVVQLA
QPACVRYRRRTGV
>CYP51B1_Mycobacterium tuberculosis Y09400034 2703595022
MLHNAALRGEQMKGHAATIEDQVRRMIADWGEAGEIDLLDFFAELTIYTSSACLIGKKFRDQLDGRFAKLYHE
LERGTDPLAYVDPYLPIESFRRRDEARNGLVALVADIMNGRIANPPTDKSDRDMLDVLIAVKAETGTPRFSAD
EITGMFISMMFAGHHTSSGTASWTLIELMRHRDAYAAVIDELDELYGDGRSVSFHALRQIPQLENVLKETLRL
HPPLIILMRVAKGEFEVQGHRIHEGDLVAASPAISNRIPEDFPDPHDFVPARYEQPRQEDLLNRWTWIPFGAG
RHRCVGAAFAIMQIKAIFSVLLREYEFEMAQPPESYRNDHSKMVVQLAQPACVRYRRRTGV
>CYP51B1_Mycobacterium tuberculosis Y09500720 2703590757
MSAVALPRVSGGHDEHGHLEEFRTDPIGLMQRVRDECGDVGTFQLAGKQVVLLSGSHANEFFFRAGDDDLDQA
KAYPFMTPIFGEGVVFDASPERRKEMLHNAALRGEQMKGHAATIEDQVRRMIADWGEAGEIDLLDFFAELTIY
TSSACLIGKKFRDQLDGRFAKLYHELERGTDPLAYVDPYLPIESFRRRDEARNGLVALVADIMNGRIANPPTD
KSDRDMLDVLIAVKAETGTPRFSADEITGMFISMMFAGHHTSSGTASWTLIELMRHRDAYAAVIDELDELYGD
GRSVSFHALRQIPQLENVLKETLRLHPPLIILMRVAKGEFEVQGHRIHEGDLVAASPAISNRIPEDFPDPHDF
VPARYEQPRQEDLLNRWTWIPFGAGRHRCVGAAFAIMQIKAIFSVLLREYEFEMAQPPESYRNDHSKMVVQLA
QPACVRYRRRTGV
>CYP51B1_Mycobacterium tuberculosis Z09600572 2678089182
MSAVALPRVSGGHDEHGHLEEFRTDPIGLMQRVRDECGDVGTFQLAGKQVVLLSGSHANEFFFRAGDDDLDQA
KAYPFMTPIFGEGVVFDASPERRKEMLHNAALRGEQMKGHAATIEDQVRRMIADWGEAGEIDLLDFFAELTIY
TSSACLIGKKFRDQLDGRFAKLYHELERGTDPLAYVDPYLPIESFRRRDEARNGLVALVADIMNGRIANPPTD
KSDRDMLDVLIAVKAETGTPRFSADEITGMFISMMFAGHHTSSGTASWTLIELMRHRDAYAAVIDELDELYGD
GRSVSFHALRQIPQLENVLKETLRLHPPLIILMRVAKGEFEVQGHRIHEGDLVAASPAISNRIPEDFPDPHDF
VPARYEQPRQEDLLNRWTWIPFGAGRHRCVGAAFAIMQIKAIFSVLLREYEFEMAQPPESYRNDHSKMVVQLA
QPACVRYRRRTGV
>CYP51B1_Mycobacterium tuberculosis Z09901262 2672879618
MSAVALPRVSGGHDEHGHLEEFRTDPIGLMQRVRDECGDVGTFQLAGKQVVLLSGSHANEFFFRAGDDDLDQA
KAYPFMTPIFGEGVVFDASPERRKEMLHNAALRGEQMKGHAATIEDQVRRMIADWGEAGEIDLLDFFAELTIY
TSSACLIGKKFRDQLDGRFAKLYHELERGTDPLAYVDPYLPIESFRRRDEARNGLVALVADIMNGRIANPPTD
KSDRDMLDVLIAVKAETGTPRFSADEITGMFISMMFAGHHTSSGTASWTLIELMRHRDAYAAVIDELDELYGD
GRSVSFHALRQIPQLENVLKETLRLHPPLIILMRVAKGEFEVQGHRIHEGDLVAASPAISNRIPEDFPDPHDF
VPARYEQPRQEDLLNRWTWIPFGAGRHRCVGAAFAIMQIKAIFSVLLREYEFEMAQPPESYRNDHSKMVVQLA
QPACVRYRRRTGV
>CYP51B1_Mycobacterium tuberculosis ZMC13-264 2720688365
MSAVALPRVSGGHDEHGHLEEFRTDPIGLMQRVRDECGDVGTFQLAGKQVVLLSGSHANEFFFRAGDDDLDQA
KAYPFMTPIFGEGVVFDASPERRKEMLHNAALRGEQMKGHAATIEDQVRRMIADWGEAGEIDLLDFFAELTIY
TSSACLIGKKFRDQLDGRFAKLYHELERGTDPLAYVDPYLPIESFRRRDEARNGLVALVADIMNGRIANPPTD
KSDRDMLDVLIAVKAETGTPRFSADEITGMFISMMFAGHHTSSGTASWTLIELMRHRDAYAAVIDELDELYGD
GRSVSFHALRQIPQLENVLKETLRLHPPLIILMRVAKGEFEVQGHRIHEGDLVAASPAISNRIPEDFPDPHDF
VPARYEQPRQEDLLNRWTWIPFGAGRHRCVGAAFAIMQIKAIFSVLLREYEFEMAQPPESYRNDHSKMVVQLA
QPACVRYRRRTGV
>CYP51B1_Mycobacterium tuberculosis ZMC13-88 2722798632
MSAVALPRVSGGHDEHGHLEEFRTDPIGLMQRVRDECGDVGTFQLAGKQVVLLSGSHANEFFFRAGDDDLDQA
KAYPFMTPIFGEGVVFDASPERRKEMLHNAALRGEQMKGHAATIEDQVRRMIADWGEAGEIDLLDFFAELTIY
TSSACLIGKKFRDQLDGRFAKLYHELERGTDPLAYVDPYLPIESFRRRDEARNGLVALVADIMNGRIANPPTD
KSDRDMLDVLIAVKAETGTPRFSADEITGMFISMMFAGHHTSSGTASWTLIELMRHRDAYAAVIDELDELYGD
GRSVSFHALRQIPQLENVLKETLRLHPPLIILMRVAKGEFEVQGHRIHEGDLVAASPAISNRIPEDFPDPHDF
VPARYEQPRQEDLLNRWTWIPFGAGRHRCVGAAFAIMQIKAIFSVLLREYEFEMAQPPESYRNDHSKMVVQLA
QPACVRYRRRTGV
>CYP51B1_Mycobacterium tuberculosis ZT272 2648009283

MSAVALPRVSGGHDEHGHLEEFRTDPIGLMQRVRDECGDVGTFQLAGKQVVLLSGSHANEFFFRAGDDDLDQA
KAYPFMTPIFGEGVVFDASPERRKEMLHNAALRGEQMKGHAATIEDQVRRMIADWGEAGEIDLLDFFAELTIY
TSSACLIGKKFRDQLDGRFAKLYHELERGTDPLAYVDPYLPIESFRRRDEARNGLVALVADIMNGRIANPPTD
KSDRDMLDVLIAVKAETGTPRFSADEITGMFISMMFAGHHTSSGTASWTLIELMRHRDAYAAVIDELDELYGD
GRSVSFHALRQIPQLENVLKETLRLHPPLIILMRVAKGEFEVQGHRIHEGDLVAASPAISNRIPEDFPDPHDF
VPARYEQPRQEDLLNRWTWIPFGAGRHRCVGAAFAIMQIKAIFSVLLREYEFEMAQPPESYRNDHSKMVVQLA
QPACVRYRRRTGV
>CYP51B1_Mycobacterium tuberculosis TKK-01-0061 2575154113
MSAVALPRVSGGHDEHGHLEEFRTDPIGLMQRVRDECGDVGTFQLAGKQVVLLSGSHANEFFFRAGDDDLDQA
KAYPFMTPIFGEGVVFDASPERRKEMLHNAALRGEQMKGHAATIEDQVRRMIADWGEAGEIDLLDFFAELTIY
TSSACLIGKKFRDQLDGRFAKLYHELERGTDPLAYVDPYLPIESFRRRDEARNGLVALVADIMNGRIANPPTD
KSDRDMLDVLIAVKAETGTPRFSADEITGMFISMMFAGHHTSSGTASWTLIELMRHRDAYAAVIDELDELYGD
GRSVSFHALRQIPQLENVLKETLRLHPPLIILMRVAKGEFEVQGHRIHEGDLVAASPAISNRIPEDFPDPHDF
VPARYEQPRQEDLLNRWTWIPFGAGRHRCVGAAFAIMQIKAIFSVLLREYEFEMAQPPESYRNDHSKMVVQLA
QPACVRYRRRTGV
>CYP51B1_Mycobacterium tuberculosis TKK-01-0064 2584624579
MSAVALPRVSGGHDEHGHLEEFRTDPIGLMQRVRDECGDVGTFQLAGKQVVLLSGSHANEFFFRAGDDDLDQA
KAYPFMTPIFGEGVVFDASPERRKEMLHNAALRGEQMKGHAATIEDQVRRMIADWGEAGEIDLLDFFAELTIY
TSSACLIGKKFRDQLDGRFAKLYHELERGTDPLAYVDPYLPIESFRRRDEARNGLVALVADIMNGRIANPPTD
KSDRDMLDVLIAVKAETGTPRFSADEITGMFISMMFAGHHTSSGTASWTLIELMRHRDAYAAVIDELDELYGD
GRSVSFHALRQIPQLENVLKETLRLHPPLIILMRVAKGEFEVQGHRIHEGDLVAASPAISNRIPEDFPDPHDF
VPARYEQPRQEDLLNRWTWIPFGAGRHRCVGAAFAIMQIKAIFSVLLREYEFEMAQPPESYRNDHSKMVVQLA
QPACVRYRRRTGV
>CYP51B1_Mycobacterium tuberculosis TKK-01-0065 2584899778
MSAVALPRVSGGHDEHGHLEEFRTDPIGLMQRVRDECGDVGTFQLAGKQVVLLSGSHANEFFFRAGDDDLDQA
KAYPFMTPIFGEGVVFDASPERRKEMLHNAALRGEQMKGHAATIEDQVRRMIADWGEAGEIDLLDFFAELTIY
TSSACLIGKKFRDQLDGRFAKLYHELERGTDPLAYVDPYLPIESFRRRDEARNGLVALVADIMNGRIANPPTD
KSDRDMLDVLIAVKAETGTPRFSADEITGMFISMMFAGHHTSSGTASWTLIELMRHRDAYAAVIDELDELYGD
GRSVSFHALRQIPQLENVLKETLRLHPPLIILMRVAKGEFEVQGHRIHEGDLVAASPAISNRIPEDFPDPHDF
VPARYEQPRQEDLLNRWTWIPFGAGRHRCVGAAFAIMQIKAIFSVLLREYEFEMAQPPESYRNDHSKMVVQLA
QPACVRYRRRTGV
>CYP51B1_Mycobacterium tuberculosis TKK-01-0068 2577749575
MSAVALPRVSGGHDEHGHLEEFRTDPIGLMQRVRDECGDVGTFQLAGKQVVLLSGSHANEFFFRAGDDDLDQA
KAYPFMTPIFGEGVVFDASPERRKEMLHNAALRGEQMKGHAATIEDQVRRMIADWGEAGEIDLLDFFAELTIY
TSSACLIGKKFRDQLDGRFAKLYHELERGTDPLAYVDPYLPIESFRRRDEARNGLVALVADIMNGRIANPPTD
KSDRDMLDVLIAVKAETGTPRFSADEITGMFISMMFAGHHTSSGTASWTLIELMRHRDAYAAVIDELDELYGD
GRSVSFHALRQIPQLENVLKETLRLHPPLIILMRVAKGEFEVQGHRIHEGDLVAASPAISNRIPEDFPDPHDF
VPARYEQPRQEDLLNRWTWIPFGAGRHRCVGAAFAIMQIKAIFSVLLREYEFEMAQPPESYRNDHSKMVVQLA
QPACVRYRRRTGV
>CYP51B1_Mycobacterium tuberculosis TKK-01-0075 2575507170
MSAVALPRVSGGHDEHGHLEEFRTDPIGLMQRVRDECGDVGTFQLAGKQVVLLSGSHANEFFFRAGDDDLDQA
KAYPFMTPIFGEGVVFDASPERRKEMLHNAALRGEQMKGHAATIEDQVRRMIADWGEAGEIDLLDFFAELTIY
TSSACLIGKKFRDQLDGRFAKLYHELERGTDPLAYVDPYLPIESFRRRDEARNGLVALVADIMNGRIANPPTD
KSDRDMLDVLIAVKAETGTPRFSADEITGMFISMMFAGHHTSSGTASWTLIELMRHRDAYAAVIDELDELYGD
GRSVSFHALRQIPQLENVLKETLRLHPPLIILMRVAKGEFEVQGHRIHEGDLVAASPAISNRIPEDFPDPHDF
VPARYEQPRQEDLLNRWTWIPFGAGRHRCVGAAFAIMQIKAIFSVLLREYEFEMAQPPESYRNDHSKMVVQLA
QPACVRYRRRTGV
>CYP51B1_Mycobacterium tuberculosis TKK-01-0077 2575448344
MSAVALPRVSGGHDEHGHLEEFRTDPIGLMQRVRDECGDVGTFQLAGKQVVLLSGSHANEFFFRAGDDDLDQA
KAYPFMTPIFGEGVVFDASPERRKEMLHNAALRGEQMKGHAATIEDQVRRMIADWGEAGEIDLLDFFAELTIY
TSSACLIGKKFRDQLDGRFAKLYHELERGTDPLAYVDPYLPIESFRRRDEARNGLVALVADIMNGRIANPPTD
KSDRDMLDVLIAVKAETGTPRFSADEITGMFISMMFAGHHTSSGTASWTLIELMRHRDAYAAVIDELDELYGD
GRSVSFHALRQIPQLENVLKETLRLHPPLIILMRVAKGEFEVQGHRIHEGDLVAASPAISNRIPEDFPDPHDF
VPARYEQPRQEDLLNRWTWIPFGAGRHRCVGAAFAIMQIKAIFSVLLREYEFEMAQPPESYRNDHSKMVVQLA
QPACVRYRRRTGV
>CYP51B1_Mycobacterium tuberculosis TKK-01-0079 2577257097
MSAVALPRVSGGHDEHGHLEEFRTDPIGLMQRVRDECGDVGTFQLAGKQVVLLSGSHANEFFFRAGDDDLDQA
KAYPFMTPIFGEGVVFDASPERRKEMLHNAALRGEQMKGHAATIEDQVRRMIADWGEAGEIDLLDFFAELTIY

TSSACLIGKKFRDQLDGRFAKLYHELERGTDPLAYVDPYLPIESFRRRDEARNGLVALVADIMNGRIANPPTD
KSDRDMLDVLIAVKAETGTPRFSADEITGMFISMMFAGHHTSSGTASWTLIELMRHRDAYAAVIDELDELYGD
GRSVSFHALRQIPQLENVLKETLRLHPPLIILMRVAKGEFEVQGHRIHEGDLVAASPAISNRIPEDFPDPHDF
VPARYEQPRQEDLLNRWTWIPFGAGRHRCVGAAFAIMQIKAIFSVLLREYEFEMAQPPESYRNDHSKMVVQLA
QPACVRYRRRTGV
>CYP51B1_Mycobacterium tuberculosis TKK-01-0085 2574604178
MSAVALPRVSGGHDEHGHLEEFRTDPIGLMQRVRDECGDVGTFQLAGKQVVLLSGSHANEFFFRAGDDDLDQA
KAYPFMTPIFGEGVVFDASPERRKEMLHNAALRGEQMKGHAATIEDQVRRMIADWGEAGEIDLLDFFAELTIY
TSSACLIGKKFRDQLDGRFAKLYHELERGTDPLAYVDPYLPIESFRRRDEARNGLVALVADIMNGRIANPPTD
KSDRDMLDVLIAVKAETGTPRFSADEITGMFISMMFAGHHTSSGTASWTLIELMRHRDAYAAVIDELDELYGD
GRSVSFHALRQIPQLENVLKETLRLHPPLIILMRVAKGEFEVQGHRIHEGDLVAASPAISNRIPEDFPDPHDF
VPARYEQPRQEDLLNRWTWIPFGAGRHRCVGAAFAIMQIKAIFSVLLREYEFEMAQPPESYRNDHSKMVVQLA
QPACVRYRRRTGV
>CYP51B1_Mycobacterium tuberculosis TKK-01-0090 2575204392
MSAVALPRVSGGHDEHGHLEEFRTDPIGLMQRVRDECGDVGTFQLAGKQVVLLSGSHANEFFFRAGDDDLDQA
KAYPFMTPIFGEGVVFDASPERRKEMLHNAALRGEQMKGHAATIEDQVRRMIADWGEAGEIDLLDFFAELTIY
TSSACLIGKKFRDQLDGRFAKLYHELERGTDPLAYVDPYLPIESFRRRDEARNGLVALVADIMNGRIANPPTD
KSDRDMLDVLIAVKAETGTPRFSADEITGMFISMMFAGHHTSSGTASWTLIELMRHRDAYAAVIDELDELYGD
GRSVSFHALRQIPQLENVLKETLRLHPPLIILMRVAKGEFEVQGHRIHEGDLVAASPAISNRIPEDFPDPHDF
VPARYEQPRQEDLLNRWTWIPFGAGRHRCVGAAFAIMQIKAIFSVLLREYEFEMAQPPESYRNDHSKMVVQLA
QPACVRYRRRTGV
>CYP51B1_Mycobacterium tuberculosis TKK-01-0091 2577023832
MSAVALPRVSGGHDEHGHLEEFRTDPIGLMQRVRDECGDVGTFQLAGKQVVLLSGSHANEFFFRAGDDDLDQA
KAYPFMTPIFGEGVVFDASPERRKEMLHNAALRGEQMKGHAATIEDQVRRMIADWGEAGEIDLLDFFAELTIY
TSSACLIGKKFRDQLDGRFAKLYHELERGTDPLAYVDPYLPIESFRRRDEARNGLVALVADIMNGRIANPPTD
KSDRDMLDVLIAVKAETGTPRFSADEITGMFISMMFAGHHTSSGTASWTLIELMRHRDAYAAVIDELDELYGD
GRSVSFHALRQIPQLENVLKETLRLHPPLIILMRVAKGEFEVQGHRIHEGDLVAASPAISNRIPEDFPDPHDF
VPARYEQPRQEDLLNRWTWIPFGAGRHRCVGAAFAIMQIKAIFSVLLREYEFEMAQPPESYRNDHSKMVVQLA
QPACVRYRRRTGV
>CYP51B1_Mycobacterium tuberculosis TRUG0001 2603909092
MSAVALPRVSGGHDEHGHLEEFRTDPIGLMQRVRDECGDVGTFQLAGKQVVLLSGSHANEFFFRAGDDDLDQA
KAYPFMTPIFGEGVVFDASPERRKEMLHNAALRGEQMKGHAATIEDQVRRMIADWGEAGEIDLLDFFAELTIY
TSSACLIGKKFRDQLDGRFAKLYHELERGTDPLAYVDPYLPIESFRRRDEARNGLVALVADIMNGRIANPPTD
KSDRDMLDVLIAVKAETGTPRFSADEITGMFISMMFAGHHTSSGTASWTLIELMRHRDAYAAVIDELDELYGD
GRSVSFHALRQIPQLENVLKETLRLHPPLIILMRVAKGEFEVQGHRIHEGDLVAASPAISNRIPEDFPDPHDF
VPARYEQPRQEDLLNRWTWIPFGAGRHRCVGAAFAIMQIKAIFSVLLREYEFEMAQPPESYRNDHSKMVVQLA
QPACVRYRRRTGV
>CYP51B1_Mycobacterium tuberculosis TRUG0002 2604984091
MSAVALPRVSGGHDEHGHLEEFRTDPIGLMQRVRDECGDVGTFQLAGKQVVLLSGSHANEFFFRAGDDDLDQA
KAYPFMTPIFGEGVVFDASPERRKEMLHNAALRGEQMKGHAATIEDQVRRMIADWGEAGEIDLLDFFAELTIY
TSSACLIGKKFRDQLDGRFAKLYHELERGTDPLAYVDPYLPIESFRRRDEARNGLVALVADIMNGRIANPPTD
KSDRDMLDVLIAVKAETGTPRFSADEITGMFISMMFAGHHTSSGTASWTLIELMRHRDAYAAVIDELDELYGD
GRSVSFHALRQIPQLENVLKETLRLHPPLIILMRVAKGEFEVQGHRIHEGDLVAASPAISNRIPEDFPDPHDF
VPARYEQPRQEDLLNRWTWIPFGAGRHRCVGAAFAIMQIKAIFSVLLREYEFEMAQPPESYRNDHSKMVVQLA
QPACVRYRRRTGV
>CYP51B1_Mycobacterium tuberculosis TRUG0003 2605674594
MSAVALPRVSGGHDEHGHLEEFRTDPIGLMQRVRDECGDVGTFQLAGKQVVLLSGSHANEFFFRAGDDDLDQA
KAYPFMTPIFGEGVVFDASPERRKEMLHNAALRGEQMKGHAATIEDQVRRMIADWGEAGEIDLLDFFAELTIY
TSSACLIGKKFRDQLDGRFAKLYHELERGTDPLAYVDPYLPIESFRRRDEARNGLVALVADIMNGRIANPPTD
KSDRDMLDVLIAVKAETGTPRFSADEITGMFISMMFAGHHTSSGTASWTLIELMRHRDAYAAVIDELDELYGD
GRSVSFHALRQIPQLENVLKETLRLHPPLIILMRVAKGEFEVQGHRIHEGDLVAASPAISNRIPEDFPDPHDF
VPARYEQPRQEDLLNRWTWIPFGAGRHRCVGAAFAIMQIKAIFSVLLREYEFEMAQPPESYRNDHSKMVVQLA
QPACVRYRRRTGV
>CYP51B1_Mycobacterium tuberculosis TRUG0004 2574674720
MSAVALPRVSGGHDEHGHLEEFRTDPIGLMQRVRDECGDVGTFQLAGKQVVLLSGSHANEFFFRAGDDDLDQA
KAYPFMTPIFGEGVVFDASPERRKEMLHNAALRGEQMKGHAATIEDQVRRMIADWGEAGEIDLLDFFAELTIY
TSSACLIGKKFRDQLDGRFAKLYHELERGTDPLAYVDPYLPIESFRRRDEARNGLVALVADIMNGRIANPPTD
KSDRDMLDVLIAVKAETGTPRFSADEITGMFISMMFAGHHTSSGTASWTLIELMRHRDAYAAVIDELDELYGD

GRSVSFHALRQIPQLENVLKETLRLHPPLIILMRVAKGEFEVQGHRIHEGDLVAASPAISNRIPEDFPDPHDF
VPARYEQPRQEDLLNRWTWIPFGAGRHRCVGAAFAIMQIKAIFSVLLREYEFEMAQPPESYRNDHSKMVVQLA
QPACVRYRRRTGV
>CYP51B1_Mycobacterium tuberculosis TRUG0009 2605319195
MSAVALPRVSGGHDEHGHLEEFRTDPIGLMQRVRDECGDVGTFQLAGKQVVLLSGSHANEFFFRAGDDDLDQA
KAYPFMTPIFGEGVVFDASPERRKEMLHNAALRGEQMKGHAATIEDQVRRMIADWGEAGEIDLLDFFAELTIY
TSSACLIGKKFRDQLDGRFAKLYHELERGTDPLAYVDPYLPIESFRRRDEARNGLVALVADIMNGRIANPPTD
KSDRDMLDVLIAVKAETGTPRFSADEITGMFISMMFAGHHTSSGTASWTLIELMRHRDAYAAVIDELDELYGD
GRSVSFHALRQIPQLENVLKETLRLHPPLIILMRVAKGEFEVQGHRIHEGDLVAASPAISNRIPEDFPDPHDF
VPARYEQPRQEDLLNRWTWIPFGAGRHRCVGAAFAIMQIKAIFSVLLREYEFEMAQPPESYRNDHSKMVVQLA
QPACVRYRRRTGV
>CYP51B1_Mycobacterium tuberculosis TRUG0015 2604252942
MSAVALPRVSGGHDEHGHLEEFRTDPIGLMQRVRDECGDVGTFQLAGKQVVLLSGSHANEFFFRAGDDDLDQA
KAYPFMTPIFGEGVVFDASPERRKEMLHNAALRGEQMKGHAATIEDQVRRMIADWGEAGEIDLLDFFAELTIY
TSSACLIGKKFRDQLDGRFAKLYHELERGTDPLAYVDPYLPIESFRRRDEARNGLVALVADIMNGRIANPPTD
KSDRDMLDVLIAVKAETGTPRFSADEITGMFISMMFAGHHTSSGTASWTLIELMRHRDAYAAVIDELDELYGD
GRSVSFHALRQIPQLENVLKETLRLHPPLIILMRVAKGEFEVQGHRIHEGDLVAASPAISNRIPEDFPDPHDF
VPARYEQPRQEDLLNRWTWIPFGAGRHRCVGAAFAIMQIKAIFSVLLREYEFEMAQPPESYRNDHSKMVVQLA
QPACVRYRRRTGV
>CYP51B1_Mycobacterium tuberculosis TRUG0017 2604623098
MSAVALPRVSGGHDEHGHLEEFRTDPIGLMQRVRDECGDVGTFQLAGKQVVLLSGSHANEFFFRAGDDDLDQA
KAYPFMTPIFGEGVVFDASPERRKEMLHNAALRGEQMKGHAATIEDQVRRMIADWGEAGEIDLLDFFAELTIY
TSSACLIGKKFRDQLDGRFAKLYHELERGTDPLAYVDPYLPIESFRRRDEARNGLVALVADIMNGRIANPPTD
KSDRDMLDVLIAVKAETGTPRFSADEITGMFISMMFAGHHTSSGTASWTLIELMRHRDAYAAVIDELDELYGD
GRSVSFHALRQIPQLENVLKETLRLHPPLIILMRVAKGEFEVQGHRIHEGDLVAASPAISNRIPEDFPDPHDF
VPARYEQPRQEDLLNRWTWIPFGAGRHRCVGAAFAIMQIKAIFSVLLREYEFEMAQPPESYRNDHSKMVVQLA
QPACVRYRRRTGV
>CYP51B1_Mycobacterium tuberculosis TRUG0020 2604626637
MSAVALPRVSGGHDEHGHLEEFRTDPIGLMQRVRDECGDVGTFQLAGKQVVLLSGSHANEFFFRAGDDDLDQA
KAYPFMTPIFGEGVVFDASPERRKEMLHNAALRGEQMKGHAATIEDQVRRMIADWGEAGEIDLLDFFAELTIY
TSSACLIGKKFRDQLDGRFAKLYHELERGTDPLAYVDPYLPIESFRRRDEARNGLVALVADIMNGRIANPPTD
KSDRDMLDVLIAVKAETGTPRFSADEITGMFISMMFAGHHTSSGTASWTLIELMRHRDAYAAVIDELDELYGD
GRSVSFHALRQIPQLENVLKETLRLHPPLIILMRVAKGEFEVQGHRIHEGDLVAASPAISNRIPEDFPDPHDF
VPARYEQPRQEDLLNRWTWIPFGAGRHRCVGAAFAIMQIKAIFSVLLREYEFEMAQPPESYRNDHSKMVVQLA
QPACVRYRRRTGV
>CYP51B1_Mycobacterium tuberculosis TRUG0022 2604632171
MSAVALPRVSGGHDEHGHLEEFRTDPIGLMQRVRDECGDVGTFQLAGKQVVLLSGSHANEFFFRAGDDDLDQA
KAYPFMTPIFGEGVVFDASPERRKEMLHNAALRGEQMKGHAATIEDQVRRMIADWGEAGEIDLLDFFAELTIY
TSSACLIGKKFRDQLDGRFAKLYHELERGTDPLAYVDPYLPIESFRRRDEARNGLVALVADIMNGRIANPPTD
KSDRDMLDVLIAVKAETGTPRFSADEITGMFISMMFAGHHTSSGTASWTLIELMRHRDAYAAVIDELDELYGD
GRSVSFHALRQIPQLENVLKETLRLHPPLIILMRVAKGEFEVQGHRIHEGDLVAASPAISNRIPEDFPDPHDF
VPARYEQPRQEDLLNRWTWIPFGAGRHRCVGAAFAIMQIKAIFSVLLREYEFEMAQPPESYRNDHSKMVVQLA
QPACVRYRRRTGV
>CYP51B1_Mycobacterium tuberculosis TRUG0024 2605678418
MSAVALPRVSGGHDEHGHLEEFRTDPIGLMQRVRDECGDVGTFQLAGKQVVLLSGSHANEFFFRAGDDDLDQA
KAYPFMTPIFGEGVVFDASPERRKEMLHNAALRGEQMKGHAATIEDQVRRMIADWGEAGEIDLLDFFAELTIY
TSSACLIGKKFRDQLDGRFAKLYHELERGTDPLAYVDPYLPIESFRRRDEARNGLVALVADIMNGRIANPPTD
KSDRDMLDVLIAVKAETGTPRFSADEITGMFISMMFAGHHTSSGTASWTLIELMRHRDAYAAVIDELDELYGD
GRSVSFHALRQIPQLENVLKETLRLHPPLIILMRVAKGEFEVQGHRIHEGDLVAASPAISNRIPEDFPDPHDF
VPARYEQPRQEDLLNRWTWIPFGAGRHRCVGAAFAIMQIKAIFSVLLREYEFEMAQPPESYRNDHSKMVVQLA
QPACVRYRRRTGV
>CYP51B1_Mycobacterium tuberculosis TRUG0030 2604638114
MSAVALPRVSGGHDEHGHLEEFRTDPIGLMQRVRDECGDVGTFQLAGKQVVLLSGSHANEFFFRAGDDDLDQA
KAYPFMTPIFGEGVVFDASPERRKEMLHNAALRGEQMKGHAATIEDQVRRMIADWGEAGEIDLLDFFAELTIY
TSSACLIGKKFRDQLDGRFAKLYHELERGTDPLAYVDPYLPIESFRRRDEARNGLVALVADIMNGRIANPPTD
KSDRDMLDVLIAVKAETGTPRFSADEITGMFISMMFAGHHTSSGTASWTLIELMRHRDAYAAVIDELDELYGD
GRSVSFHALRQIPQLENVLKETLRLHPPLIILMRVAKGEFEVQGHRIHEGDLVAASPAISNRIPEDFPDPHDF

VPARYEQPRQEDLLNRWTWIPFGAGRHRCVGAAFAIMQIKAIFSVLLREYEFEMAQPPESYRNDHSKMVVQLA
QPACVRYRRRTGV
>CYP51B1_Mycobacterium tuberculosis TRUG0034 2604645224
MSAVALPRVSGGHDEHGHLEEFRTDPIGLMQRVRDECGDVGTFQLAGKQVVLLSGSHANEFFFRAGDDDLDQA
KAYPFMTPIFGEGVVFDASPERRKEMLHNAALRGEQMKGHAATIEDQVRRMIADWGEAGEIDLLDFFAELTIY
TSSACLIGKKFRDQLDGRFAKLYHELERGTDPLAYVDPYLPIESFRRRDEARNGLVALVADIMNGRIANPPTD
KSDRDMLDVLIAVKAETGTPRFSADEITGMFISMMFAGHHTSSGTASWTLIELMRHRDAYAAVIDELDELYGD
GRSVSFHALRQIPQLENVLKETLRLHPPLIILMRVAKGEFEVQGHRIHEGDLVAASPAISNRIPEDFPDPHDF
VPARYEQPRQEDLLNRWTWIPFGAGRHRCVGAAFAIMQIKAIFSVLLREYEFEMAQPPESYRNDHSKMVVQLA
QPACVRYRRRTGV
>CYP51B1_Mycobacterium tuberculosis TRUG0036 2603196400
MSAVALPRVSGGHDEHGHLEEFRTDPIGLMQRVRDECGDVGTFQLAGKQVVLLSGSHANEFFFRAGDDDLDQA
KAYPFMTPIFGEGVVFDASPERRKEMLHNAALRGEQMKGHAATIEDQVRRMIADWGEAGEIDLLDFFAELTIY
TSSACLIGKKFRDQLDGRFAKLYHELERGTDPLAYVDPYLPIESFRRRDEARNGLVALVADIMNGRIANPPTD
KSDRDMLDVLIAVKAETGTPRFSADEITGMFISMMFAGHHTSSGTASWTLIELMRHRDAYAAVIDELDELYGD
GRSVSFHALRQIPQLENVLKETLRLHPPLIILMRVAKGEFEVQGHRIHEGDLVAASPAISNRIPEDFPDPHDF
VPARYEQPRQEDLLNRWTWIPFGAGRHRCVGAAFAIMQIKAIFSVLLREYEFEMAQPPESYRNDHSKMVVQLA
QPACVRYRRRTGV
>CYP51B1_Mycobacterium tuberculosis TRUG0037 2576104702
MSAVALPRVSGGHDEHGHLEEFRTDPIGLMQRVRDECGDVGTFQLAGKQVVLLSGSHANEFFFRAGDDDLDQA
KAYPFMTPIFGEGVVFDASPERRKEMLHNAALRGEQMKGHAATIEDQVRRMIADWGEAGEIDLLDFFAELTIY
TSSACLIGKKFRDQLDGRFAKLYHELERGTDPLAYVDPYLPIESFRRRDEARNGLVALVADIMNGRIANPPTD
KSDRDMLDVLIAVKAETGTPRFSADEITGMFISMMFAGHHTSSGTASWTLIELMRHRDAYAAVIDELDELYGD
GRSVSFHALRQIPQLENVLKETLRLHPPLIILMRVAKGEFEVQGHRIHEGDLVAASPAISNRIPEDFPDPHDF
VPARYEQPRQEDLLNRWTWIPFGAGRHRCVGAAFAIMQIKAIFSVLLREYEFEMAQPPESYRNDHSKMVVQLA
QPACVRYRRRTGV
>CYP51B1_Mycobacterium tuberculosis TRUG0040 2576985687
MSAVALPRVSGGHDEHGHLEEFRTDPIGLMQRVRDECGDVGTFQLAGKQVVLLSGSHANEFFFRAGDDDLDQA
KAYPFMTPIFGEGVVFDASPERRKEMLHNAALRGEQMKGHAATIEDQVRRMIADWGEAGEIDLLDFFAELTIY
TSSACLIGKKFRDQLDGRFAKLYHELERGTDPLAYVDPYLPIESFRRRDEARNGLVALVADIMNGRIANPPTD
KSDRDMLDVLIAVKAETGTPRFSADEITGMFISMMFAGHHTSSGTASWTLIELMRHRDAYAAVIDELDELYGD
GRSVSFHALRQIPQLENVLKETLRLHPPLIILMRVAKGEFEVQGHRIHEGDLVAASPAISNRIPEDFPDPHDF
VPARYEQPRQEDLLNRWTWIPFGAGRHRCVGAAFAIMQIKAIFSVLLREYEFEMAQPPESYRNDHSKMVVQLA
QPACVRYRRRTGV
>CYP51B1_Mycobacterium tuberculosis TRUG0041 2605686890
MSAVALPRVSGGHDEHGHLEEFRTDPIGLMQRVRDECGDVGTFQLAGKQVVLLSGSHANEFFFRAGDDDLDQA
KAYPFMTPIFGEGVVFDASPERRKEMLHNAALRGEQMKGHAATIEDQVRRMIADWGEAGEIDLLDFFAELTIY
TSSACLIGKKFRDQLDGRFAKLYHELERGTDPLAYVDPYLPIESFRRRDEARNGLVALVADIMNGRIANPPTD
KSDRDMLDVLIAVKAETGTPRFSADEITGMFISMMFAGHHTSSGTASWTLIELMRHRDAYAAVIDELDELYGD
GRSVSFHALRQIPQLENVLKETLRLHPPLIILMRVAKGEFEVQGHRIHEGDLVAASPAISNRIPEDFPDPHDF
VPARYEQPRQEDLLNRWTWIPFGAGRHRCVGAAFAIMQIKAIFSVLLREYEFEMAQPPESYRNDHSKMVVQLA
QPACVRYRRRTGV
>CYP51B1_Mycobacterium tuberculosis TRUG0045 2603912851
MSAVALPRVSGGHDEHGHLEEFRTDPIGLMQRVRDECGDVGTFQLAGKQVVLLSGSHANEFFFRAGDDDLDQA
KAYPFMTPIFGEGVVFDASPERRKEMLHNAALRGEQMKGHAATIEDQVRRMIADWGEAGEIDLLDFFAELTIY
TSSACLIGKKFRDQLDGRFAKLYHELERGTDPLAYVDPYLPIESFRRRDEARNGLVALVADIMNGRIANPPTD
KSDRDMLDVLIAVKAETGTPRFSADEITGMFISMMFAGHHTSSGTASWTLIELMRHRDAYAAVIDELDELYGD
GRSVSFHALRQIPQLENVLKETLRLHPPLIILMRVAKGEFEVQGHRIHEGDLVAASPAISNRIPEDFPDPHDF
VPARYEQPRQEDLLNRWTWIPFGAGRHRCVGAAFAIMQIKAIFSVLLREYEFEMAQPPESYRNDHSKMVVQLA
QPACVRYRRRTGV
>CYP51B1_Mycobacterium tuberculosis TRUG0046 2604258655
MSAVALPRVSGGHDEHGHLEEFRTDPIGLMQRVRDECGDVGTFQLAGKQVVLLSGSHANEFFFRAGDDDLDQA
KAYPFMTPIFGEGVVFDASPERRKEMLHNAALRGEQMKGHAATIEDQVRRMIADWGEAGEIDLLDFFAELTIY
TSSACLIGKKFRDQLDGRFAKLYHELERGTDPLAYVDPYLPIESFRRRDEARNGLVALVADIMNGRIANPPTD
KSDRDMLDVLIAVKAETGTPRFSADEITGMFISMMFAGHHTSSGTASWTLIELMRHRDAYAAVIDELDELYGD
GRSVSFHALRQIPQLENVLKETLRLHPPLIILMRVAKGEFEVQGHRIHEGDLVAASPAISNRIPEDFPDPHDF
VPARYEQPRQEDLLNRWTWIPFGAGRHRCVGAAFAIMQIKAIFSVLLREYEFEMAQPPESYRNDHSKMVVQLA
QPACVRYRRRTGV

>CYP51B1_Mycobacterium tuberculosis TRUG0047 2605323667
MSAVALPRVSGGHDEHGHLEEFRTDPIGLMQRVRDECGDVGTFQLAGKQVVLLSGSHANEFFFRAGDDDLDQA
KAYPFMTPIFGEGVVFDASPERRKEMLHNAALRGEQMKGHAATIEDQVRRMIADWGEAGEIDLLDFFAELTIY
TSSACLIGKKFRDQLDGRFAKLYHELERGTDPLAYVDPYLPIESFRRRDEARNGLVALVADIMNGRIANPPTD
KSDRDMLDVLIAVKAETGTPRFSADEITGMFISMMFAGHHTSSGTASWTLIELMRHRDAYAAVIDELDELYGD
GRSVSFHALRQIPQLENVLKETLRLHPPLIILMRVAKGEFEVQGHRIHEGDLVAASPAISNRIPEDFPDPHDF
VPARYEQPRQEDLLNRWTWIPFGAGRHRCVGAAFAIMQIKAIFSVLLREYEFEMAQPPESYRNDHSKMVVQLA
QPACVRYRRRTGV
>CYP51B1_Mycobacterium tuberculosis TRUG0049 2605692318
MSAVALPRVSGGHDEHGHLEEFRTDPIGLMQRVRDECGDVGTFQLAGKQVVLLSGSHANEFFFRAGDDDLDQA
KAYPFMTPIFGEGVVFDASPERRKEMLHNAALRGEQMKGHAATIEDQVRRMIADWGEAGEIDLLDFFAELTIY
TSSACLIGKKFRDQLDGRFAKLYHELERGTDPLAYVDPYLPIESFRRRDEARNGLVALVADIMNGRIANPPTD
KSDRDMLDVLIAVKAETGTPRFSADEITGMFISMMFAGHHTSSGTASWTLIELMRHRDAYAAVIDELDELYGD
GRSVSFHALRQIPQLENVLKETLRLHPPLIILMRVAKGEFEVQGHRIHEGDLVAASPAISNRIPEDFPDPHDF
VPARYEQPRQEDLLNRWTWIPFGAGRHRCVGAAFAIMQIKAIFSVLLREYEFEMAQPPESYRNDHSKMVVQLA
QPACVRYRRRTGV
>CYP51B1_Mycobacterium tuberculosis TRUG0051 2605327734
MSAVALPRVSGGHDEHGHLEEFRTDPIGLMQRVRDECGDVGTFQLAGKQVVLLSGSHANEFFFRAGDDDLDQA
KAYPFMTPIFGEGVVFDASPERRKEMLHNAALRGEQMKGHAATIEDQVRRMIADWGEAGEIDLLDFFAELTIY
TSSACLIGKKFRDQLDGRFAKLYHELERGTDPLAYVDPYLPIESFRRRDEARNGLVALVADIMNGRIANPPTD
KSDRDMLDVLIAVKAETGTPRFSADEITGMFISMMFAGHHTSSGTASWTLIELMRHRDAYAAVIDELDELYGD
GRSVSFHALRQIPQLENVLKETLRLHPPLIILMRVAKGEFEVQGHRIHEGDLVAASPAISNRIPEDFPDPHDF
VPARYEQPRQEDLLNRWTWIPFGAGRHRCVGAAFAIMQIKAIFSVLLREYEFEMAQPPESYRNDHSKMVVQLA
QPACVRYRRRTGV
>CYP51B1_Mycobacterium tuberculosis TRUG0052 2604261433
MSAVALPRVSGGHDEHGHLEEFRTDPIGLMQRVRDECGDVGTFQLAGKQVVLLSGSHANEFFFRAGDDDLDQA
KAYPFMTPIFGEGVVFDASPERRKEMLHNAALRGEQMKGHAATIEDQVRRMIADWGEAGEIDLLDFFAELTIY
TSSACLIGKKFRDQLDGRFAKLYHELERGTDPLAYVDPYLPIESFRRRDEARNGLVALVADIMNGRIANPPTD
KSDRDMLDVLIAVKAETGTPRFSADEITGMFISMMFAGHHTSSGTASWTLIELMRHRDAYAAVIDELDELYGD
GRSVSFHALRQIPQLENVLKETLRLHPPLIILMRVAKGEFEVQGHRIHEGDLVAASPAISNRIPEDFPDPHDF
VPARYEQPRQEDLLNRWTWIPFGAGRHRCVGAAFAIMQIKAIFSVLLREYEFEMAQPPESYRNDHSKMVVQLA
QPACVRYRRRTGV
>CYP51B1_Mycobacterium tuberculosis TRUG0053 2604988490
MSAVALPRVSGGHDEHGHLEEFRTDPIGLMQRVRDECGDVGTFQLAGKQVVLLSGSHANEFFFRAGDDDLDQA
KAYPFMTPIFGEGVVFDASPERRKEMLHNAALRGEQMKGHAATIEDQVRRMIADWGEAGEIDLLDFFAELTIY
TSSACLIGKKFRDQLDGRFAKLYHELERGTDPLAYVDPYLPIESFRRRDEARNGLVALVADIMNGRIANPPTD
KSDRDMLDVLIAVKAETGTPRFSADEITGMFISMMFAGHHTSSGTASWTLIELMRHRDAYAAVIDELDELYGD
GRSVSFHALRQIPQLENVLKETLRLHPPLIILMRVAKGEFEVQGHRIHEGDLVAASPAISNRIPEDFPDPHDF
VPARYEQPRQEDLLNRWTWIPFGAGRHRCVGAAFAIMQIKAIFSVLLREYEFEMAQPPESYRNDHSKMVVQLA
QPACVRYRRRTGV
>CYP51B1_Mycobacterium tuberculosis TRUG0054 2604646852
MSAVALPRVSGGHDEHGHLEEFRTDPIGLMQRVRDECGDVGTFQLAGKQVVLLSGSHANEFFFRAGDDDLDQA
KAYPFMTPIFGEGVVFDASPERRKEMLHNAALRGEQMKGHAATIEDQVRRMIADWGEAGEIDLLDFFAELTIY
TSSACLIGKKFRDQLDGRFAKLYHELERGTDPLAYVDPYLPIESFRRRDEARNGLVALVADIMNGRIANPPTD
KSDRDMLDVLIAVKAETGTPRFSADEITGMFISMMFAGHHTSSGTASWTLIELMRHRDAYAAVIDELDELYGD
GRSVSFHALRQIPQLENVLKETLRLHPPLIILMRVAKGEFEVQGHRIHEGDLVAASPAISNRIPEDFPDPHDF
VPARYEQPRQEDLLNRWTWIPFGAGRHRCVGAAFAIMQIKAIFSVLLREYEFEMAQPPESYRNDHSKMVVQLA
QPACVRYRRRTGV
>CYP51B1_Mycobacterium tuberculosis TRUG0056 2605331858
MSAVALPRVSGGHDEHGHLEEFRTDPIGLMQRVRDECGDVGTFQLAGKQVVLLSGSHANEFFFRAGDDDLDQA
KAYPFMTPIFGEGVVFDASPERRKEMLHNAALRGEQMKGHAATIEDQVRRMIADWGEAGEIDLLDFFAELTIY
TSSACLIGKKFRDQLDGRFAKLYHELERGTDPLAYVDPYLPIESFRRRDEARNGLVALVADIMNGRIANPPTD
KSDRDMLDVLIAVKAETGTPRFSADEITGMFISMMFAGHHTSSGTASWTLIELMRHRDAYAAVIDELDELYGD
GRSVSFHALRQIPQLENVLKETLRLHPPLIILMRVAKGEFEVQGHRIHEGDLVAASPAISNRIPEDFPDPHDF
VPARYEQPRQEDLLNRWTWIPFGAGRHRCVGAAFAIMQIKAIFSVLLREYEFEMAQPPESYRNDHSKMVVQLA
QPACVRYRRRTGV
>CYP51B1_Mycobacterium tuberculosis TRUG0057 2604267859

MSAVALPRVSGGHDEHGHLEEFRTDPIGLMQRVRDECGDVGTFQLAGKQVVLLSGSHANEFFFRAGDDDLDQA
KAYPFMTPIFGEGVVFDASPERRKEMLHNAALRGEQMKGHAATIEDQVRRMIADWGEAGEIDLLDFFAELTIY
TSSACLIGKKFRDQLDGRFAKLYHELERGTDPLAYVDPYLPIESFRRRDEARNGLVALVADIMNGRIANPPTD
KSDRDMLDVLIAVKAETGTPRFSADEITGMFISMMFAGHHTSSGTASWTLIELMRHRDAYAAVIDELDELYGD
GRSVSFHALRQIPQLENVLKETLRLHPPLIILMRVAKGEFEVQGHRIHEGDLVAASPAISNRIPEDFPDPHDF
VPARYEQPRQEDLLNRWTWIPFGAGRHRCVGAAFAIMQIKAIFSVLLREYEFEMAQPPESYRNDHSKMVVQLA
QPACVRYRRRTGV
>CYP51B1_Mycobacterium tuberculosis TRUG0058 2603202587
MSAVALPRVSGGHDEHGHLEEFRTDPIGLMQRVRDECGDVGTFQLAGKQVVLLSGSHANEFFFRAGDDDLDQA
KAYPFMTPIFGEGVVFDASPERRKEMLHNAALRGEQMKGHAATIEDQVRRMIADWGEAGEIDLLDFFAELTIY
TSSACLIGKKFRDQLDGRFAKLYHELERGTDPLAYVDPYLPIESFRRRDEARNGLVALVADIMNGRIANPPTD
KSDRDMLDVLIAVKAETGTPRFSADEITGMFISMMFAGHHTSSGTASWTLIELMRHRDAYAAVIDELDELYGD
GRSVSFHALRQIPQLENVLKETLRLHPPLIILMRVAKGEFEVQGHRIHEGDLVAASPAISNRIPEDFPDPHDF
VPARYEQPRQEDLLNRWTWIPFGAGRHRCVGAAFAIMQIKAIFSVLLREYEFEMAQPPESYRNDHSKMVVQLA
QPACVRYRRRTGV
>CYP51B1_Mycobacterium tuberculosis TRUG0060 2604271019
MSAVALPRVSGGHDEHGHLEEFRTDPIGLMQRVRDECGDVGTFQLAGKQVVLLSGSHANEFFFRAGDDDLDQA
KAYPFMTPIFGEGVVFDASPERRKEMLHNAALRGEQMKGHAATIEDQVRRMIADWGEAGEIDLLDFFAELTIY
TSSACLIGKKFRDQLDGRFAKLYHELERGTDPLAYVDPYLPIESFRRRDEARNGLVALVADIMNGRIANPPTD
KSDRDMLDVLIAVKAETGTPRFSADEITGMFISMMFAGHHTSSGTASWTLIELMRHRDAYAAVIDELDELYGD
GRSVSFHALRQIPQLENVLKETLRLHPPLIILMRVAKGEFEVQGHRIHEGDLVAASPAISNRIPEDFPDPHDF
VPARYEQPRQEDLLNRWTWIPFGAGRHRCVGAAFAIMQIKAIFSVLLREYEFEMAQPPESYRNDHSKMVVQLA
QPACVRYRRRTGV
>CYP51B1_Mycobacterium tuberculosis TRUG0061 2603204319
MSAVALPRVSGGHDEHGHLEEFRTDPIGLMQRVRDECGDVGTFQLAGKQVVLLSGSHANEFFFRAGDDDLDQA
KAYPFMTPIFGEGVVFDASPERRKEMLHNAALRGEQMKGHAATIEDQVRRMIADWGEAGEIDLLDFFAELTIY
TSSACLIGKKFRDQLDGRFAKLYHELERGTDPLAYVDPYLPIESFRRRDEARNGLVALVADIMNGRIANPPTD
KSDRDMLDVLIAVKAETGTPRFSADEITGMFISMMFAGHHTSSGTASWTLIELMRHRDAYAAVIDELDELYGD
GRSVSFHALRQIPQLENVLKETLRLHPPLIILMRVAKGEFEVQGHRIHEGDLVAASPAISNRIPEDFPDPHDF
VPARYEQPRQEDLLNRWTWIPFGAGRHRCVGAAFAIMQIKAIFSVLLREYEFEMAQPPESYRNDHSKMVVQLA
QPACVRYRRRTGV
>CYP51B1_Mycobacterium tuberculosis TRUG0068 2604653623
MSAVALPRVSGGHDEHGHLEEFRTDPIGLMQRVRDECGDVGTFQLAGKQVVLLSGSHANEFFFRAGDDDLDQA
KAYPFMTPIFGEGVVFDASPERRKEMLHNAALRGEQMKGHAATIEDQVRRMIADWGEAGEIDLLDFFAELTIY
TSSACLIGKKFRDQLDGRFAKLYHELERGTDPLAYVDPYLPIESFRRRDEARNGLVALVADIMNGRIANPPTD
KSDRDMLDVLIAVKAETGTPRFSADEITGMFISMMFAGHHTSSGTASWTLIELMRHRDAYAAVIDELDELYGD
GRSVSFHALRQIPQLENVLKETLRLHPPLIILMRVAKGEFEVQGHRIHEGDLVAASPAISNRIPEDFPDPHDF
VPARYEQPRQEDLLNRWTWIPFGAGRHRCVGAAFAIMQIKAIFSVLLREYEFEMAQPPESYRNDHSKMVVQLA
QPACVRYRRRTGV
>CYP51B1_Mycobacterium tuberculosis TRUG0070 2575284700
MSAVALPRVSGGHDEHGHLEEFRTDPIGLMQRVRDECGDVGTFQLAGKQVVLLSGSHANEFFFRAGDDDLDQA
KAYPFMTPIFGEGVVFDASPERRKEMLHNAALRGEQMKGHAATIEDQVRRMIADWGEAGEIDLLDFFAELTIY
TSSACLIGKKFRDQLDGRFAKLYHELERGTDPLAYVDPYLPIESFRRRDEARNGLVALVADIMNGRIANPPTD
KSDRDMLDVLIAVKAETGTPRFSADEITGMFISMMFAGHHTSSGTASWTLIELMRHRDAYAAVIDELDELYGD
GRSVSFHALRQIPQLENVLKETLRLHPPLIILMRVAKGEFEVQGHRIHEGDLVAASPAISNRIPEDFPDPHDF
VPARYEQPRQEDLLNRWTWIPFGAGRHRCVGAAFAIMQIKAIFSVLLREYEFEMAQPPESYRNDHSKMVVQLA
QPACVRYRRRTGV
>CYP51B1_Mycobacterium tuberculosis TRUG0071 2603919240
MSAVALPRVSGGHDEHGHLEEFRTDPIGLMQRVRDECGDVGTFQLAGKQVVLLSGSHANEFFFRAGDDDLDQA
KAYPFMTPIFGEGVVFDASPERRKEMLHNAALRGEQMKGHAATIEDQVRRMIADWGEAGEIDLLDFFAELTIY
TSSACLIGKKFRDQLDGRFAKLYHELERGTDPLAYVDPYLPIESFRRRDEARNGLVALVADIMNGRIANPPTD
KSDRDMLDVLIAVKAETGTPRFSADEITGMFISMMFAGHHTSSGTASWTLIELMRHRDAYAAVIDELDELYGD
GRSVSFHALRQIPQLENVLKETLRLHPPLIILMRVAKGEFEVQGHRIHEGDLVAASPAISNRIPEDFPDPHDF
VPARYEQPRQEDLLNRWTWIPFGAGRHRCVGAAFAIMQIKAIFSVLLREYEFEMAQPPESYRNDHSKMVVQLA
QPACVRYRRRTGV
>CYP51B1_Mycobacterium tuberculosis TRUG0076 2576072728
MSAVALPRVSGGHDEHGHLEEFRTDPIGLMQRVRDECGDVGTFQLAGKQVVLLSGSHANEFFFRAGDDDLDQA
KAYPFMTPIFGEGVVFDASPERRKEMLHNAALRGEQMKGHAATIEDQVRRMIADWGEAGEIDLLDFFAELTIY

TSSACLIGKKFRDQLDGRFAKLYHELERGTDPLAYVDPYLPIESFRRRDEARNGLVALVADIMNGRIANPPTD
KSDRDMLDVLIAVKAETGTPRFSADEITGMFISMMFAGHHTSSGTASWTLIELMRHRDAYAAVIDELDELYGD
GRSVSFHALRQIPQLENVLKETLRLHPPLIILMRVAKGEFEVQGHRIHEGDLVAASPAISNRIPEDFPDPHDF
VPARYEQPRQEDLLNRWTWIPFGAGRHRCVGAAFAIMQIKAIFSVLLREYEFEMAQPPESYRNDHSKMVVQLA
QPACVRYRRRTGV
>CYP51B1_Mycobacterium tuberculosis TRUG0078 2603208722
MSAVALPRVSGGHDEHGHLEEFRTDPIGLMQRVRDECGDVGTFQLAGKQVVLLSGSHANEFFFRAGDDDLDQA
KAYPFMTPIFGEGVVFDASPERRKEMLHNAALRGEQMKGHAATIEDQVRRMIADWGEAGEIDLLDFFAELTIY
TSSACLIGKKFRDQLDGRFAKLYHELERGTDPLAYVDPYLPIESFRRRDEARNGLVALVADIMNGRIANPPTD
KSDRDMLDVLIAVKAETGTPRFSADEITGMFISMMFAGHHTSSGTASWTLIELMRHRDAYAAVIDELDELYGD
GRSVSFHALRQIPQLENVLKETLRLHPPLIILMRVAKGEFEVQGHRIHEGDLVAASPAISNRIPEDFPDPHDF
VPARYEQPRQEDLLNRWTWIPFGAGRHRCVGAAFAIMQIKAIFSVLLREYEFEMAQPPESYRNDHSKMVVQLA
QPACVRYRRRTGV
>CYP51B1_Mycobacterium tuberculosis TRUG0080 2584771503
MSAVALPRVSGGHDEHGHLEEFRTDPIGLMQRVRDECGDVGTFQLAGKQVVLLSGSHANEFFFRAGDDDLDQA
KAYPFMTPIFGEGVVFDASPERRKEMLHNAALRGEQMKGHAATIEDQVRRMIADWGEAGEIDLLDFFAELTIY
TSSACLIGKKFRDQLDGRFAKLYHELERGTDPLAYVDPYLPIESFRRRDEARNGLVALVADIMNGRIANPPTD
KSDRDMLDVLIAVKAETGTPRFSADEITGMFISMMFAGHHTSSGTASWTLIELMRHRDAYAAVIDELDELYGD
GRSVSFHALRQIPQLENVLKETLRLHPPLIILMRVAKGEFEVQGHRIHEGDLVAASPAISNRIPEDFPDPHDF
VPARYEQPRQEDLLNRWTWIPFGAGRHRCVGAAFAIMQIKAIFSVLLREYEFEMAQPPESYRNDHSKMVVQLA
QPACVRYRRRTGV
>CYP51B1_Mycobacterium tuberculosis TRUG0083 2584848722
MSAVALPRVSGGHDEHGHLEEFRTDPIGLMQRVRDECGDVGTFQLAGKQVVLLSGSHANEFFFRAGDDDLDQA
KAYPFMTPIFGEGVVFDASPERRKEMLHNAALRGEQMKGHAATIEDQVRRMIADWGEAGEIDLLDFFAELTIY
TSSACLIGKKFRDQLDGRFAKLYHELERGTDPLAYVDPYLPIESFRRRDEARNGLVALVADIMNGRIANPPTD
KSDRDMLDVLIAVKAETGTPRFSADEITGMFISMMFAGHHTSSGTASWTLIELMRHRDAYAAVIDELDELYGD
GRSVSFHALRQIPQLENVLKETLRLHPPLIILMRVAKGEFEVQGHRIHEGDLVAASPAISNRIPEDFPDPHDF
VPARYEQPRQEDLLNRWTWIPFGAGRHRCVGAAFAIMQIKAIFSVLLREYEFEMAQPPESYRNDHSKMVVQLA
QPACVRYRRRTGV
>CYP51B1_Mycobacterium tuberculosis TRUG0084 2603378456
MSAVALPRVSGGHDEHGHLEEFRTDPIGLMQRVRDECGDVGTFQLAGKQVVLLSGSHANEFFFRAGDDDLDQA
KAYPFMTPIFGEGVVFDASPERRKEMLHNAALRGEQMKGHAATIEDQVRRMIADWGEAGEIDLLDFFAELTIY
TSSACLIGKKFRDQLDGRFAKLYHELERGTDPLAYVDPYLPIESFRRRDEARNGLVALVADIMNGRIANPPTD
KSDRDMLDVLIAVKAETGTPRFSADEITGMFISMMFAGHHTSSGTASWTLIELMRHRDAYAAVIDELDELYGD
GRSVSFHALRQIPQLENVLKETLRLHPPLIILMRVAKGEFEVQGHRIHEGDLVAASPAISNRIPEDFPDPHDF
VPARYEQPRQEDLLNRWTWIPFGAGRHRCVGAAFAIMQIKAIFSVLLREYEFEMAQPPESYRNDHSKMVVQLA
QPACVRYRRRTGV
>CYP51B1_Mycobacterium tuberculosis TRUG0086 2605695172
MSAVALPRVSGGHDEHGHLEEFRTDPIGLMQRVRDECGDVGTFQLAGKQVVLLSGSHANEFFFRAGDDDLDQA
KAYPFMTPIFGEGVVFDASPERRKEMLHNAALRGEQMKGHAATIEDQVRRMIADWGEAGEIDLLDFFAELTIY
TSSACLIGKKFRDQLDGRFAKLYHELERGTDPLAYVDPYLPIESFRRRDEARNGLVALVADIMNGRIANPPTD
KSDRDMLDVLIAVKAETGTPRFSADEITGMFISMMFAGHHTSSGTASWTLIELMRHRDAYAAVIDELDELYGD
GRSVSFHALRQIPQLENVLKETLRLHPPLIILMRVAKGEFEVQGHRIHEGDLVAASPAISNRIPEDFPDPHDF
VPARYEQPRQEDLLNRWTWIPFGAGRHRCVGAAFAIMQIKAIFSVLLREYEFEMAQPPESYRNDHSKMVVQLA
QPACVRYRRRTGV
>CYP51B1_Mycobacterium tuberculosis TRUG0085 2577314297
MSAVALPRVSGGHDEHGHLEEFRTDPIGLMQRVRDECGDVGTFQLAGKQVVLLSGSHANEFFFRAGDDDLDQA
KAYPFMTPIFGEGVVFDASPERRKEMLHNAALRGEQMKGHAATIEDQVRRMIADWGEAGEIDLLDFFAELTIY
TSSACLIGKKFRDQLDGRFAKLYHELERGTDPLAYVDPYLPIESFRRRDEARNGLVALVADIMNGRIANPPTD
KSDRDMLDVLIAVKAETGTPRFSADEITGMFISMMFAGHHTSSGTASWTLIELMRHRDAYAAVIDELDELYGD
GRSVSFHALRQIPQLENVLKETLRLHPPLIILMRVAKGEFEVQGHRIHEGDLVAASPAISNRIPEDFPDPHDF
VPARYEQPRQEDLLNRWTWIPFGAGRHRCVGAAFAIMQIKAIFSVLLREYEFEMAQPPESYRNDHSKMVVQLA
QPACVRYRRRTGV
>CYP51B1_Mycobacterium tuberculosis TRUG0089 2605698480
MSAVALPRVSGGHDEHGHLEEFRTDPIGLMQRVRDECGDVGTFQLAGKQVVLLSGSHANEFFFRAGDDDLDQA
KAYPFMTPIFGEGVVFDASPERRKEMLHNAALRGEQMKGHAATIEDQVRRMIADWGEAGEIDLLDFFAELTIY
TSSACLIGKKFRDQLDGRFAKLYHELERGTDPLAYVDPYLPIESFRRRDEARNGLVALVADIMNGRIANPPTD
KSDRDMLDVLIAVKAETGTPRFSADEITGMFISMMFAGHHTSSGTASWTLIELMRHRDAYAAVIDELDELYGD

GRSVSFHALRQIPQLENVLKETLRLHPPLIILMRVAKGEFEVQGHRIHEGDLVAASPAISNRIPEDFPDPHDF
VPARYEQPRQEDLLNRWTWIPFGAGRHRCVGAAFAIMQIKAIFSVLLREYEFEMAQPPESYRNDHSKMVVQLA
QPACVRYRRRTGV
>CYP51B1_Mycobacterium tuberculosis TRUG0090 2605703396
MSAVALPRVSGGHDEHGHLEEFRTDPIGLMQRVRDECGDVGTFQLAGKQVVLLSGSHANEFFFRAGDDDLDQA
KAYPFMTPIFGEGVVFDASPERRKEMLHNAALRGEQMKGHAATIEDQVRRMIADWGEAGEIDLLDFFAELTIY
TSSACLIGKKFRDQLDGRFAKLYHELERGTDPLAYVDPYLPIESFRRRDEARNGLVALVADIMNGRIANPPTD
KSDRDMLDVLIAVKAETGTPRFSADEITGMFISMMFAGHHTSSGTASWTLIELMRHRDAYAAVIDELDELYGD
GRSVSFHALRQIPQLENVLKETLRLHPPLIILMRVAKGEFEVQGHRIHEGDLVAASPAISNRIPEDFPDPHDF
VPARYEQPRQEDLLNRWTWIPFGAGRHRCVGAAFAIMQIKAIFSVLLREYEFEMAQPPESYRNDHSKMVVQLA
QPACVRYRRRTGV
>CYP51B1_Mycobacterium tuberculosis TRUG0091 2604273800
MSAVALPRVSGGHDEHGHLEEFRTDPIGLMQRVRDECGDVGTFQLAGKQVVLLSGSHANEFFFRAGDDDLDQA
KAYPFMTPIFGEGVVFDASPERRKEMLHNAALRGEQMKGHAATIEDQVRRMIADWGEAGEIDLLDFFAELTIY
TSSACLIGKKFRDQLDGRFAKLYHELERGTDPLAYVDPYLPIESFRRRDEARNGLVALVADIMNGRIANPPTD
KSDRDMLDVLIAVKAETGTPRFSADEITGMFISMMFAGHHTSSGTASWTLIELMRHRDAYAAVIDELDELYGD
GRSVSFHALRQIPQLENVLKETLRLHPPLIILMRVAKGEFEVQGHRIHEGDLVAASPAISNRIPEDFPDPHDF
VPARYEQPRQEDLLNRWTWIPFGAGRHRCVGAAFAIMQIKAIFSVLLREYEFEMAQPPESYRNDHSKMVVQLA
QPACVRYRRRTGV
>CYP51B1_Mycobacterium tuberculosis TRUG0092 2604280027
MSAVALPRVSGGHDEHGHLEEFRTDPIGLMQRVRDECGDVGTFQLAGKQVVLLSGSHANEFFFRAGDDDLDQA
KAYPFMTPIFGEGVVFDASPERRKEMLHNAALRGEQMKGHAATIEDQVRRMIADWGEAGEIDLLDFFAELTIY
TSSACLIGKKFRDQLDGRFAKLYHELERGTDPLAYVDPYLPIESFRRRDEARNGLVALVADIMNGRIANPPTD
KSDRDMLDVLIAVKAETGTPRFSADEITGMFISMMFAGHHTSSGTASWTLIELMRHRDAYAAVIDELDELYGD
GRSVSFHALRQIPQLENVLKETLRLHPPLIILMRVAKGEFEVQGHRIHEGDLVAASPAISNRIPEDFPDPHDF
VPARYEQPRQEDLLNRWTWIPFGAGRHRCVGAAFAIMQIKAIFSVLLREYEFEMAQPPESYRNDHSKMVVQLA
QPACVRYRRRTGV
>CYP51B1_Mycobacterium tuberculosis TRUG0093 2603212854
MSAVALPRVSGGHDEHGHLEEFRTDPIGLMQRVRDECGDVGTFQLAGKQVVLLSGSHANEFFFRAGDDDLDQA
KAYPFMTPIFGEGVVFDASPERRKEMLHNAALRGEQMKGHAATIEDQVRRMIADWGEAGEIDLLDFFAELTIY
TSSACLIGKKFRDQLDGRFAKLYHELERGTDPLAYVDPYLPIESFRRRDEARNGLVALVADIMNGRIANPPTD
KSDRDMLDVLIAVKAETGTPRFSADEITGMFISMMFAGHHTSSGTASWTLIELMRHRDAYAAVIDELDELYGD
GRSVSFHALRQIPQLENVLKETLRLHPPLIILMRVAKGEFEVQGHRIHEGDLVAASPAISNRIPEDFPDPHDF
VPARYEQPRQEDLLNRWTWIPFGAGRHRCVGAAFAIMQIKAIFSVLLREYEFEMAQPPESYRNDHSKMVVQLA
QPACVRYRRRTGV
>CYP51B1_Mycobacterium tuberculosis TRUG0095 2584953856
MSAVALPRVSGGHDEHGHLEEFRTDPIGLMQRVRDECGDVGTFQLAGKQVVLLSGSHANEFFFRAGDDDLDQA
KAYPFMTPIFGEGVVFDASPERRKEMLHNAALRGEQMKGHAATIEDQVRRMIADWGEAGEIDLLDFFAELTIY
TSSACLIGKKFRDQLDGRFAKLYHELERGTDPLAYVDPYLPIESFRRRDEARNGLVALVADIMNGRIANPPTD
KSDRDMLDVLIAVKAETGTPRFSADEITGMFISMMFAGHHTSSGTASWTLIELMRHRDAYAAVIDELDELYGD
GRSVSFHALRQIPQLENVLKETLRLHPPLIILMRVAKGEFEVQGHRIHEGDLVAASPAISNRIPEDFPDPHDF
VPARYEQPRQEDLLNRWTWIPFGAGRHRCVGAAFAIMQIKAIFSVLLREYEFEMAQPPESYRNDHSKMVVQLA
QPACVRYRRRTGV
>CYP51B1_Mycobacterium tuberculosis TRUG0097 2603223896
MSAVALPRVSGGHDEHGHLEEFRTDPIGLMQRVRDECGDVGTFQLAGKQVVLLSGSHANEFFFRAGDDDLDQA
KAYPFMTPIFGEGVVFDASPERRKEMLHNAALRGEQMKGHAATIEDQVRRMIADWGEAGEIDLLDFFAELTIY
TSSACLIGKKFRDQLDGRFAKLYHELERGTDPLAYVDPYLPIESFRRRDEARNGLVALVADIMNGRIANPPTD
KSDRDMLDVLIAVKAETGTPRFSADEITGMFISMMFAGHHTSSGTASWTLIELMRHRDAYAAVIDELDELYGD
GRSVSFHALRQIPQLENVLKETLRLHPPLIILMRVAKGEFEVQGHRIHEGDLVAASPAISNRIPEDFPDPHDF
VPARYEQPRQEDLLNRWTWIPFGAGRHRCVGAAFAIMQIKAIFSVLLREYEFEMAQPPESYRNDHSKMVVQLA
QPACVRYRRRTGV
>CYP51B1_Mycobacterium tuberculosis TRUG0098 2574904751
MSAVALPRVSGGHDEHGHLEEFRTDPIGLMQRVRDECGDVGTFQLAGKQVVLLSGSHANEFFFRAGDDDLDQA
KAYPFMTPIFGEGVVFDASPERRKEMLHNAALRGEQMKGHAATIEDQVRRMIADWGEAGEIDLLDFFAELTIY
TSSACLIGKKFRDQLDGRFAKLYHELERGTDPLAYVDPYLPIESFRRRDEARNGLVALVADIMNGRIANPPTD
KSDRDMLDVLIAVKAETGTPRFSADEITGMFISMMFAGHHTSSGTASWTLIELMRHRDAYAAVIDELDELYGD
GRSVSFHALRQIPQLENVLKETLRLHPPLIILMRVAKGEFEVQGHRIHEGDLVAASPAISNRIPEDFPDPHDF

VPARYEQPRQEDLLNRWTWIPFGAGRHRCVGAAFAIMQIKAIFSVLLREYEFEMAQPPESYRNDHSKMVVQLA
QPACVRYRRRTGV
>CYP51B1_Mycobacterium tuberculosis TRUG0101 2576676985
MSAVALPRVSGGHDEHGHLEEFRTDPIGLMQRVRDECGDVGTFQLAGKQVVLLSGSHANEFFFRAGDDDLDQA
KAYPFMTPIFGEGVVFDASPERRKEMLHNAALRGEQMKGHAATIEDQVRRMIADWGEAGEIDLLDFFAELTIY
TSSACLIGKKFRDQLDGRFAKLYHELERGTDPLAYVDPYLPIESFRRRDEARNGLVALVADIMNGRIANPPTD
KSDRDMLDVLIAVKAETGTPRFSADEITGMFISMMFAGHHTSSGTASWTLIELMRHRDAYAAVIDELDELYGD
GRSVSFHALRQIPQLENVLKETLRLHPPLIILMRVAKGEFEVQGHRIHEGDLVAASPAISNRIPEDFPDPHDF
VPARYEQPRQEDLLNRWTWIPFGAGRHRCVGAAFAIMQIKAIFSVLLREYEFEMAQPPESYRNDHSKMVVQLA
QPACVRYRRRTGV
>CYP51B1_Mycobacterium tuberculosis TRUG0105 2603925600
MSAVALPRVSGGHDEHGHLEEFRTDPIGLMQRVRDECGDVGTFQLAGKQVVLLSGSHANEFFFRAGDDDLDQA
KAYPFMTPIFGEGVVFDASPERRKEMLHNAALRGEQMKGHAATIEDQVRRMIADWGEAGEIDLLDFFAELTIY
TSSACLIGKKFRDQLDGRFAKLYHELERGTDPLAYVDPYLPIESFRRRDEARNGLVALVADIMNGRIANPPTD
KSDRDMLDVLIAVKAETGTPRFSADEITGMFISMMFAGHHTSSGTASWTLIELMRHRDAYAAVIDELDELYGD
GRSVSFHALRQIPQLENVLKETLRLHPPLIILMRVAKGEFEVQGHRIHEGDLVAASPAISNRIPEDFPDPHDF
VPARYEQPRQEDLLNRWTWIPFGAGRHRCVGAAFAIMQIKAIFSVLLREYEFEMAQPPESYRNDHSKMVVQLA
QPACVRYRRRTGV
>CYP51B1_Mycobacterium tuberculosis TRUG0106 2584787818
MSAVALPRVSGGHDEHGHLEEFRTDPIGLMQRVRDECGDVGTFQLAGKQVVLLSGSHANEFFFRAGDDDLDQA
KAYPFMTPIFGEGVVFDASPERRKEMLHNAALRGEQMKGHAATIEDQVRRMIADWGEAGEIDLLDFFAELTIY
TSSACLIGKKFRDQLDGRFAKLYHELERGTDPLAYVDPYLPIESFRRRDEARNGLVALVADIMNGRIANPPTD
KSDRDMLDVLIAVKAETGTPRFSADEITGMFISMMFAGHHTSSGTASWTLIELMRHRDAYAAVIDELDELYGD
GRSVSFHALRQIPQLENVLKETLRLHPPLIILMRVAKGEFEVQGHRIHEGDLVAASPAISNRIPEDFPDPHDF
VPARYEQPRQEDLLNRWTWIPFGAGRHRCVGAAFAIMQIKAIFSVLLREYEFEMAQPPESYRNDHSKMVVQLA
QPACVRYRRRTGV
>CYP51B1_Mycobacterium tuberculosis TRUG0111 2604992596
MSAVALPRVSGGHDEHGHLEEFRTDPIGLMQRVRDECGDVGTFQLAGKQVVLLSGSHANEFFFRAGDDDLDQA
KAYPFMTPIFGEGVVFDASPERRKEMLHNAALRGEQMKGHAATIEDQVRRMIADWGEAGEIDLLDFFAELTIY
TSSACLIGKKFRDQLDGRFAKLYHELERGTDPLAYVDPYLPIESFRRRDEARNGLVALVADIMNGRIANPPTD
KSDRDMLDVLIAVKAETGTPRFSADEITGMFISMMFAGHHTSSGTASWTLIELMRHRDAYAAVIDELDELYGD
GRSVSFHALRQIPQLENVLKETLRLHPPLIILMRVAKGEFEVQGHRIHEGDLVAASPAISNRIPEDFPDPHDF
VPARYEQPRQEDLLNRWTWIPFGAGRHRCVGAAFAIMQIKAIFSVLLREYEFEMAQPPESYRNDHSKMVVQLA
QPACVRYRRRTGV
>CYP51B1_Mycobacterium tuberculosis TRUG0113 2604282023
MSAVALPRVSGGHDEHGHLEEFRTDPIGLMQRVRDECGDVGTFQLAGKQVVLLSGSHANEFFFRAGDDDLDQA
KAYPFMTPIFGEGVVFDASPERRKEMLHNAALRGEQMKGHAATIEDQVRRMIADWGEAGEIDLLDFFAELTIY
TSSACLIGKKFRDQLDGRFAKLYHELERGTDPLAYVDPYLPIESFRRRDEARNGLVALVADIMNGRIANPPTD
KSDRDMLDVLIAVKAETGTPRFSADEITGMFISMMFAGHHTSSGTASWTLIELMRHRDAYAAVIDELDELYGD
GRSVSFHALRQIPQLENVLKETLRLHPPLIILMRVAKGEFEVQGHRIHEGDLVAASPAISNRIPEDFPDPHDF
VPARYEQPRQEDLLNRWTWIPFGAGRHRCVGAAFAIMQIKAIFSVLLREYEFEMAQPPESYRNDHSKMVVQLA
QPACVRYRRRTGV
>CYP51B1_Mycobacterium tuberculosis TRUG0114 2603227301
MSAVALPRVSGGHDEHGHLEEFRTDPIGLMQRVRDECGDVGTFQLAGKQVVLLSGSHANEFFFRAGDDDLDQA
KAYPFMTPIFGEGVVFDASPERRKEMLHNAALRGEQMKGHAATIEDQVRRMIADWGEAGEIDLLDFFAELTIY
TSSACLIGKKFRDQLDGRFAKLYHELERGTDPLAYVDPYLPIESFRRRDEARNGLVALVADIMNGRIANPPTD
KSDRDMLDVLIAVKAETGTPRFSADEITGMFISMMFAGHHTSSGTASWTLIELMRHRDAYAAVIDELDELYGD
GRSVSFHALRQIPQLENVLKETLRLHPPLIILMRVAKGEFEVQGHRIHEGDLVAASPAISNRIPEDFPDPHDF
VPARYEQPRQEDLLNRWTWIPFGAGRHRCVGAAFAIMQIKAIFSVLLREYEFEMAQPPESYRNDHSKMVVQLA
QPACVRYRRRTGV
>CYP51B1_Mycobacterium tuberculosis TRUG0116 2584616907
MSAVALPRVSGGHDEHGHLEEFRTDPIGLMQRVRDECGDVGTFQLAGKQVVLLSGSHANEFFFRAGDDDLDQA
KAYPFMTPIFGEGVVFDASPERRKEMLHNAALRGEQMKGHAATIEDQVRRMIADWGEAGEIDLLDFFAELTIY
TSSACLIGKKFRDQLDGRFAKLYHELERGTDPLAYVDPYLPIESFRRRDEARNGLVALVADIMNGRIANPPTD
KSDRDMLDVLIAVKAETGTPRFSADEITGMFISMMFAGHHTSSGTASWTLIELMRHRDAYAAVIDELDELYGD
GRSVSFHALRQIPQLENVLKETLRLHPPLIILMRVAKGEFEVQGHRIHEGDLVAASPAISNRIPEDFPDPHDF
VPARYEQPRQEDLLNRWTWIPFGAGRHRCVGAAFAIMQIKAIFSVLLREYEFEMAQPPESYRNDHSKMVVQLA
QPACVRYRRRTGV

>CYP51B1_Mycobacterium tuberculosis TRUG0117 2584986491
MSAVALPRVSGGHDEHGHLEEFRTDPIGLMQRVRDECGDVGTFQLAGKQVVLLSGSHANEFFFRAGDDDLDQA
KAYPFMTPIFGEGVVFDASPERRKEMLHNAALRGEQMKGHAATIEDQVRRMIADWGEAGEIDLLDFFAELTIY
TSSACLIGKKFRDQLDGRFAKLYHELERGTDPLAYVDPYLPIESFRRRDEARNGLVALVADIMNGRIANPPTD
KSDRDMLDVLIAVKAETGTPRFSADEITGMFISMMFAGHHTSSGTASWTLIELMRHRDAYAAVIDELDELYGD
GRSVSFHALRQIPQLENVLKETLRLHPPLIILMRVAKGEFEVQGHRIHEGDLVAASPAISNRIPEDFPDPHDF
VPARYEQPRQEDLLNRWTWIPFGAGRHRCVGAAFAIMQIKAIFSVLLREYEFEMAQPPESYRNDHSKMVVQLA
QPACVRYRRRTGV
>CYP51B1_Mycobacterium tuberculosis TRUG0124 2584855103
MSAVALPRVSGGHDEHGHLEEFRTDPIGLMQRVRDECGDVGTFQLAGKQVVLLSGSHANEFFFRAGDDDLDQA
KAYPFMTPIFGEGVVFDASPERRKEMLHNAALRGEQMKGHAATIEDQVRRMIADWGEAGEIDLLDFFAELTIY
TSSACLIGKKFRDQLDGRFAKLYHELERGTDPLAYVDPYLPIESFRRRDEARNGLVALVADIMNGRIANPPTD
KSDRDMLDVLIAVKAETGTPRFSADEITGMFISMMFAGHHTSSGTASWTLIELMRHRDAYAAVIDELDELYGD
GRSVSFHALRQIPQLENVLKETLRLHPPLIILMRVAKGEFEVQGHRIHEGDLVAASPAISNRIPEDFPDPHDF
VPARYEQPRQEDLLNRWTWIPFGAGRHRCVGAAFAIMQIKAIFSVLLREYEFEMAQPPESYRNDHSKMVVQLA
QPACVRYRRRTGV
>CYP51B1_Mycobacterium tuberculosis TRUG0125 2604288252
MSAVALPRVSGGHDEHGHLEEFRTDPIGLMQRVRDECGDVGTFQLAGKQVVLLSGSHANEFFFRAGDDDLDQA
KAYPFMTPIFGEGVVFDASPERRKEMLHNAALRGEQMKGHAATIEDQVRRMIADWGEAGEIDLLDFFAELTIY
TSSACLIGKKFRDQLDGRFAKLYHELERGTDPLAYVDPYLPIESFRRRDEARNGLVALVADIMNGRIANPPTD
KSDRDMLDVLIAVKAETGTPRFSADEITGMFISMMFAGHHTSSGTASWTLIELMRHRDAYAAVIDELDELYGD
GRSVSFHALRQIPQLENVLKETLRLHPPLIILMRVAKGEFEVQGHRIHEGDLVAASPAISNRIPEDFPDPHDF
VPARYEQPRQEDLLNRWTWIPFGAGRHRCVGAAFAIMQIKAIFSVLLREYEFEMAQPPESYRNDHSKMVVQLA
QPACVRYRRRTGV
>CYP51B1_Mycobacterium tuberculosis U09801187 2706598269
MSAVALPRVSGGHDEHGHLEEFRTDPIGLMQRVRDECGDVGTFQLAGKQVVLLSGSHANEFFFRAGDDDLDQA
KAYPFMTPIFGEGVVFDASPERRKEMLHNAALRGEQMKGHAATIEDQVRRMIADWGEAGEIDLLDFFAELTIY
TSSACLIGKKFRDQLDGRFAKLYHELERGTDPLAYVDPYLPIESFRRRDEARNGLVALVADIMNGRIANPPTD
KSDRDMLDVLIAVKAETGTPRFSADEITGMFISMMFAGHHTSSGTASWTLIELMRHRDAYAAVIDELDELYGD
GRSVSFHALRQIPQLENVLKETLRLHPPLIILMRVAKGEFEVQGHRIHEGDLVAASPAISNRIPEDFPDPHDF
VPARYEQPRQEDLLNRWTWIPFGAGRHRCVGAAFAIMQIKAIFSVLLREYEFEMAQPPESYRNDHSKMVVQLA
QPACVRYRRRTGV
>CYP51B1_Mycobacterium tuberculosis U09801472 2699525320
MSAVALPRVSGGHDEHGHLEEFRTDPIGLMQRVRDECGDVGTFQLAGKQVVLLSGSHANEFFFRAGDDDLDQA
KAYPFMTPIFGEGVVFDASPERRKEMLHNAALRGEQMKGHAATIEDQVRRMIADWGEAGEIDLLDFFAELTIY
TSSACLIGKKFRDQLDGRFAKLYHELERGTDPLAYVDPYLPIESFRRRDEARNGLVALVADIMNGRIANPPTD
KSDRDMLDVLIAVKAETGTPRFSADEITGMFISMMFAGHHTSSGTASWTLIELMRHRDAYAAVIDELDELYGD
GRSVSFHALRQIPQLENVLKETLRLHPPLIILMRVAKGEFEVQGHRIHEGDLVAASPAISNRIPEDFPDPHDF
VPARYEQPRQEDLLNRWTWIPFGAGRHRCVGAAFAIMQIKAIFSVLLREYEFEMAQPPESYRNDHSKMVVQLA
QPACVRYRRRTGV
>CYP51B1_Mycobacterium tuberculosis Uganda 1 2575403526
MSAVALPRVSGGHDEHGHLEEFRTDPIGLMQRVRDECGDVGTFQLAGKQVVLLSGSHANEFFFRAGDDDLDQA
KAYPFMTPIFGEGVVFDASPERRKEMLHNAALRGEQMKGHAATIEDQVRRMIADWGEAGEIDLLDFFAELTIY
TSSACLIGKKFRDQLDGRFAKLYHELERGTDPLAYVDPYLPIESFRRRDEARNGLVALVADIMNGRIANPPTD
KSDRDMLDVLIAVKAETGTPRFSADEITGMFISMMFAGHHTSSGTASWTLIELMRHRDAYAAVIDELDELYGD
GRSVSFHALRQIPQLENVLKETLRLHPPLIILMRVAKGEFEVQGHRIHEGDLVAASPAISNRIPEDFPDPHDF
VPARYEQPRQEDLLNRWTWIPFGAGRHRCVGAAFAIMQIKAIFSVLLREYEFEMAQPPESYRNDHSKMVVQLA
QPACVRYRRRTGV
>CYP51B1_Mycobacterium tuberculosis UG-C 2575307019
MSAVALPRVSGGHDEHGHLEEFRTDPIGLMQRVRDECGDVGTFQLAGKQVVLLSGSHANEFFFRAGDDDLDQA
KAYPFMTPIFGEGVVFDASPERRKEMLHNAALRGEQMKGHAATIEDQVRRMIADWGEAGEIDLLDFFAELTIY
TSSACLIGKKFRDQLDGRFAKLYHELERGTDPLAYVDPYLPIESFRRRDEARNGLVALVADIMNGRIANPPTD
KSDRDMLDVLIAVKAETGTPRFSADEITGMFISMMFAGHHTSSGTASWTLIELMRHRDAYAAVIDELDELYGD
GRSVSFHALRQIPQLENVLKETLRLHPPLIILMRVAKGEFEVQGHRIHEGDLVAASPAISNRIPEDFPDPHDF
VPARYEQPRQEDLLNRWTWIPFGAGRHRCVGAAFAIMQIKAIFSVLLREYEFEMAQPPESYRNDHSKMVVQLA
QPACVRYRRRTGV
>CYP51B1_Mycobacterium tuberculosis UM 1072388579 2553260949

MSAVALPRVSGGHDEHGHLEEFRTDPIGLMQRVRDECGDVGTFQLAGKQVVLLSGSHANEFFFRAGDDDLDQA
KAYPFMTPIFGEGVVFDASPERRKEMLHNAALRGEQMKGHAATIEDQVRRMIADWGEAGEIDLLDFFAELTIY
TSSACLIGKKFRDQLDGRFAKLYHELERGTDPLAYVDPYLPIESFRRRDEARNGLVALVADIMNGRIANPPTD
KSDRDMLDVLIAVKAETGTPRFSADEITGMFISMMFAGHHTSSGTASWTLIELMRHRDAYAAVIDELDELYGD
GRSVSFHALRQIPQLENVLKETLRLHPPLIILMRVAKGEFEVQGHRIHEGDLVAASPAISNRIPEDFPDPHDF
VPARYEQPRQEDLLNRWTWIPFGAGRHRCVGAAFAIMQIKAIFSVLLREYEFEMAQPPESYRNDHSKMVVQLA
QPACVRYRRRTGV
>CYP51B1_Mycobacterium tuberculosis UM-CSF01 2724872266
MSAVALPRVSGGHDEHGHLEEFRTDPIGLMQRVRDECGDVGTFQLAGKQVVLLSGSHANEFFFRAGDDDLDQA
KAYPFMTPIFGEGVVFDASPERRKEMLHNAALRGEQMKGHAATIEDQVRRMIADWGEAGEIDLLDFFAELTIY
TSSACLIGKKFRDQLDGRFAKLYHELERGTDPLAYVDPYLPIESFRRRDEARNGLVALVADIMNGRIANPPTD
KSDRDMLDVLIAVKAETGTPRFSADEITGMFISMMFAGHHTSSGTASWTLIELMRHRDAYAAVIDELDELYGD
GRSVSFHALRQIPQLENVLKETLRLHPPLIILMRVAKGEFEVQGHRIHEGDLVAASPAISNRIPEDFPDPHDF
VPARYEQPRQEDLLNRWTWIPFGAGRHRCVGAAFAIMQIKAIFSVLLREYEFEMAQPPESYRNDHSKMVVQLA
QPACVRYRRRTGV
>CYP51B1_Mycobacterium tuberculosis UM-CSF04 2746037725
MSAVALPRVSGGHDEHGHLEEFRTDPIGLMQRVRDECGDVGTFQLAGKQVVLLSGSHANEFFFRAGDDDLDQA
KAYPFMTPIFGEGVVFDASPERRKEMLHNAALRGEQMKGHAATIEDQVRRMIADWGEAGEIDLLDFFAELTIY
TSSACLIGKKFRDQLDGRFAKLYHELERGTDPLAYVDPYLPIESFRRRDEARNGLVALVADIMNGRIANPPTD
KSDRDMLDVLIAVKAETGTPRFSADEITGMFISMMFAGHHTSSGTASWTLIELMRHRDAYAAVIDELDELYGD
GRSVSFHALRQIPQLENVLKETLRLHPPLIILMRVAKGEFEVQGHRIHEGDLVAASPAISNRIPEDFPDPHDF
VPARYEQPRQEDLLNRWTWIPFGAGRHRCVGAAFAIMQIKAIFSVLLREYEFEMAQPPESYRNDHSKMVVQLA
QPACVRYRRRTGV
>CYP51B1_Mycobacterium tuberculosis UM-CSF05 2724876416
MSAVALPRVSGGHDEHGHLEEFRTDPIGLMQRVRDECGDVGTFQLAGKQVVLLSGSHANEFFFRAGDDDLDQA
KAYPFMTPIFGEGVVFDASPERRKEMLHNAALRGEQMKGHAATIEDQVRRMIADWGEAGEIDLLDFFAELTIY
TSSACLIGKKFRDQLDGRFAKLYHELERGTDPLAYVDPYLPIESFRRRDEARNGLVALVADIMNGRIANPPTD
KSDRDMLDVLIAVKAETGTPRFSADEITGMFISMMFAGHHTSSGTASWTLIELMRHRDAYAAVIDELDELYGD
GRSVSFHALRQIPQLENVLKETLRLHPPLIILMRVAKGEFEVQGHRIHEGDLVAASPAISNRIPEDFPDPHDF
VPARYEQPRQEDLLNRWTWIPFGAGRHRCVGAAFAIMQIKAIFSVLLREYEFEMAQPPESYRNDHSKMVVQLA
QPACVRYRRRTGV
>CYP51B1_Mycobacterium tuberculosis UM-CSF06 2746042885
MSAVALPRVSGGHDEHGHLEEFRTDPIGLMQRVRDECGDVGTFQLAGKQVVLLSGSHANEFFFRAGDDDLDQA
KAYPFMTPIFGEGVVFDASPERRKEMLHNAALRGEQMKGHAATIEDQVRRMIADWGEAGEIDLLDFFAELTIY
TSSACLIGKKFRDQLDGRFAKLYHELERGTDPLAYVDPYLPIESFRRRDEARNGLVALVADIMNGRIANPPTD
KSDRDMLDVLIAVKAETGTPRFSADEITGMFISMMFAGHHTSSGTASWTLIELMRHRDAYAAVIDELDELYGD
GRSVSFHALRQIPQLENVLKETLRLHPPLIILMRVAKGEFEVQGHRIHEGDLVAASPAISNRIPEDFPDPHDF
VPARYEQPRQEDLLNRWTWIPFGAGRHRCVGAAFAIMQIKAIFSVLLREYEFEMAQPPESYRNDHSKMVVQLA
QPACVRYRRRTGV
>CYP51B1_Mycobacterium tuberculosis UM-CSF08 2746043962
MSAVALPRVSGGHDEHGHLEEFRTDPIGLMQRVRDECGDVGTFQLAGKQVVLLSGSHANEFFFRAGDDDLDQA
KAYPFMTPIFGEGVVFDASPERRKEMLHNAALRGEQMKGHAATIEDQVRRMIADWGEAGEIDLLDFFAELTIY
TSSACLIGKKFRDQLDGRFAKLYHELERGTDPLAYVDPYLPIESFRRRDEARNGLVALVADIMNGRIANPPTD
KSDRDMLDVLIAVKAETGTPRFSADEITGMFISMMFAGHHTSSGTASWTLIELMRHRDAYAAVIDELDELYGD
GRSVSFHALRQIPQLENVLKETLRLHPPLIILMRVAKGEFEVQGHRIHEGDLVAASPAISNRIPEDFPDPHDF
VPARYEQPRQEDLLNRWTWIPFGAGRHRCVGAAFAIMQIKAIFSVLLREYEFEMAQPPESYRNDHSKMVVQLA
QPACVRYRRRTGV
>CYP51B1_Mycobacterium tuberculosis UM-CSF09 2746047721
MSAVALPRVSGGHDEHGHLEEFRTDPIGLMQRVRDECGDVGTFQLAGKQVVLLSGSHANEFFFRAGDDDLDQA
KAYPFMTPIFGEGVVFDASPERRKEMLHNAALRGEQMKGHAATIEDQVRRMIADWGEAGEIDLLDFFAELTIY
TSSACLIGKKFRDQLDGRFAKLYHELERGTDPLAYVDPYLPIESFRRRDEARNGLVALVADIMNGRIANPPTD
KSDRDMLDVLIAVKAETGTPRFSADEITGMFISMMFAGHHTSSGTASWTLIELMRHRDAYAAVIDELDELYGD
GRSVSFHALRQIPQLENVLKETLRLHPPLIILMRVAKGEFEVQGHRIHEGDLVAASPAISNRIPEDFPDPHDF
VPARYEQPRQEDLLNRWTWIPFGAGRHRCVGAAFAIMQIKAIFSVLLREYEFEMAQPPESYRNDHSKMVVQLA
QPACVRYRRRTGV
>CYP51B1_Mycobacterium tuberculosis UM-CSF15 2746054544
MSAVALPRVSGGHDEHGHLEEFRTDPIGLMQRVRDECGDVGTFQLAGKQVVLLSGSHANEFFFRAGDDDLDQA
KAYPFMTPIFGEGVVFDASPERRKEMLHNAALRGEQMKGHAATIEDQVRRMIADWGEAGEIDLLDFFAELTIY

TSSACLIGKKFRDQLDGRFAKLYHELERGTDPLAYVDPYLPIESFRRRDEARNGLVALVADIMNGRIANPPTD
KSDRDMLDVLIAVKAETGTPRFSADEITGMFISMMFAGHHTSSGTASWTLIELMRHRDAYAAVIDELDELYGD
GRSVSFHALRQIPQLENVLKETLRLHPPLIILMRVAKGEFEVQGHRIHEGDLVAASPAISNRIPEDFPDPHDF
VPARYEQPRQEDLLNRWTWIPFGAGRHRCVGAAFAIMQIKAIFSVLLREYEFEMAQPPESYRNDHSKMVVQLA
QPACVRYRRRTGV
>CYP51B1_Mycobacterium tuberculosis UM-CSF17 2746057113
MSAVALPRVSGGHDEHGHLEEFRTDPIGLMQRVRDECGDVGTFQLAGKQVVLLSGSHANEFFFRAGDDDLDQA
KAYPFMTPIFGEGVVFDASPERRKEMLHNAALRGEQMKGHAATIEDQVRRMIADWGEAGEIDLLDFFAELTIY
TSSACLIGKKFRDQLDGRFAKLYHELERGTDPLAYVDPYLPIESFRRRDEARNGLVALVADIMNGRIANPPTD
KSDRDMLDVLIAVKAETGTPRFSADEITGMFISMMFAGHHTSSGTASWTLIELMRHRDAYAAVIDELDELYGD
GRSVSFHALRQIPQLENVLKETLRLHPPLIILMRVAKGEFEVQGHRIHEGDLVAASPAISNRIPEDFPDPHDF
VPARYEQPRQEDLLNRWTWIPFGAGRHRCVGAAFAIMQIKAIFSVLLREYEFEMAQPPESYRNDHSKMVVQLA
QPACVRYRRRTGV
>CYP51B1_Mycobacterium marinum M 641720184
MTTAIVPRVSGGEEEHGHLEEFRTDPIGLMQRVRDECGDVGWFQLANKHVVLLSGAKANEFFFRSSDEELDQA
EAYPFMTPIFGKGVVFDASPERRKEMLHNSALRGEHMKGHATTIEREVHRMIENWGQEGEIDLLEFFAELTIY
TSTSCLIGTKFRNQLDSRFAHFYHELERGTDPLCYVDPYLPIESFRRRDEARKGLVALVQDIMHQRVANPPTD
KRDRDMLDVLVSITDEQGNPRFCADEVTGMFISLMFAGHHTSSGTSAWTLIELLRHPDAYAAVIDELDELYAD
GQPVSFHALRQIPRLENVLKETLRLHPPLIILMRVAKGEFQVEGYPIHEGELVAASPAISNRIAEDFPDPDEF
VPERYQEPRQEDLINRWTWIPFGAGRHRCVGAAFATMQIKAIFSVLLREYEFEMAQPADSYRNDHSKMVVQLA
RPARVRYRRRKMSDNRGH
>CYP51B1_Mycobacterium marinum MB2 2546371393
MTTAIVPRVSGGEEEHGHLEEFRTDPIGLMQRVRDECGDVGWFQLANKHVVLLSGAKANEFFFRSSDEELDQA
EAYPFMTPIFGKGVVFDASPERRKEMLHNSALRGEHMKGHATTIEREVHRMIENWGQEGEIDLLEFFAELTIY
TSTSCLIGTKFRNQLDSRFAHFYHELERGTDPLCYVDPYLPIESFRRRDEARKGLVALVQDIMHQRVANPPTD
KRDRDMLDVLVSITDEQGNPRFCADEVTGMFISLMFAGHHTSSGTSAWTLIELLRHPDAYAAVIDELDELYAD
GQPVSFHALRQIPRLENVLKETLRLHPPLIILMRVAKGEFQVEGYPIHEGELVAASPAISNRIAEDFPDPDEF
VPERYQEPRQEDLINRWTWIPFGAGRHRCVGAAFATMQIKAIFSVLLREYEFEMAQPADSYRNDHSKMVVQLA
RPARVRYRRRKMSDNRGH
>CYP51B1_Mycobacterium microti 12 2689335166
MSAVALPRVSGGHDEHGHLEEFRTDPIGLMQRVRDECGDVGTFQLAGKQVVLLSGSHANEFFFRAGDDDLDQA
KAYPFMTPIFGEGVVFDASPERRKEMLHNAALRGEQMKGHAATIEDQVRRMIADWGEAGEIDLLDFFAELTIY
TSSACLIGKKFRDQLDGRFAKLYHELERGTDPLAYVDPYLPIESFRRRDEARNGLVALVADIMNGRIANPPTD
KSDRDMLDVLIAVKAETGTPRFSADEITGMFISMMFAGHHTSSGTASWTLIELMRHRDAYAAVIDELDELYGD
GRSVSFHALRQIPQLENVLKETLRLHPPLIILMRVAKGEFEVQGHRIHEGDLVAASPAISNRIPEDFPDPHDF
VPARYEQPRQEDLLNRWTWIPFGAGRHRCVGAAFAIMQIKAIFSVLLREYEFEMAQPPESYRNDHSKMVVQLA
QPACVRYRRRTGV
>CYP51B1_Mycobacterium mungi BM22813 2746181483
MSAVALPRVSGGHDEHGHLEEFRTDPIGLMQRVRDECGDVGTFQLAGKQVVLLSGSHANEFFFRAGDDDLDQA
KAYPFMTPIFGEGVVFDASPERRKEMLHNAALRGEQMKGHAATIEDQVRRMIADWGEAGEIDLLDFFAELTIY
TSSACLIGKKFRDQLDGRFAKLYHELERGTDPLAYVDPYLPIESFRRRDEARNGLVALVADIMNGRIANPPTD
KSDRDMLDVLIAVKAETGTPRFSADEITGMFISMMFAGHHTSSGTASWTLIELMRHRDAYAAVIDELDELYGD
GRSVSFHALRQIPQLENVLKETLRLHPPLIILMRVAKGEFEVQGHRIHEGDLVAASPAISNRIPEDFPDPHDF
VPARYEQPRQEDLLNRWTWIPFGAGRHRCVGAAFAIMQIKAIFSVLLREYEFEMAQPPESYRNDHSKMVVQLA
QPACVRYRRRTGV
>CYP51B1_Mycobacterium tuberculosis TB_RSA149 2576983565
MSAVALPRVSGGHDEHGHLEEFRTDPIGLMQRVRDECGDVGTFQLAGKQVVLLSGSHANEFFFRAGDDDLDQA
KAYPFMTPIFGEGVVFDASPERRKEMLHNAALRGEQMKGHAATIEDQVRRMIADWGEAAEIDLLDFFAELTIY
TSSACLIGKKFRDQLDGRFAKLYHELERGTDPLAYVDPYLPIESFRRRDEARNGLVALVADIMNGRIANPPTD
KSDRDMLDVLIAVKAETGTPRFSADEITGMFISMMFAGHHTSSGTASWTLIELMRHRDAYAAVIDELDELYGD
GRSVSFHALRQIPQLENVLKETLRLHPPLIILMRVAKGEFEVQGHRIHEGDLVAASPAISNRIPEDFPDPHDF
VPARYEQPRQEDLLNRWTWIPFGAGRHRCVGAAFAIMQIKAIFSVLLREYEFEMAQPPESYRNDHSKMVVQLA
QPACVRYRRRTGV
>CYP51B1_Mycobacterium tuberculosis TB_RSA150 2605368870
MSAVALPRVSGGHDEHGHLEEFRTDPIGLMQRVRDECGDVGTFQLAGKQVVLLSGSHANEFFFRAGDDDLDQA
KAYPFMTPIFGEGVVFDASPERRKEMLHNAALRGEQMKGHAATIEDQVRRMIADWGEAAEIDLLDFFAELTIY
TSSACLIGKKFRDQLDGRFAKLYHELERGTDPLAYVDPYLPIESFRRRDEARNGLVALVADIMNGRIANPPTD
KSDRDMLDVLIAVKAETGTPRFSADEITGMFISMMFAGHHTSSGTASWTLIELMRHRDAYAAVIDELDELYGD

GRSVSFHALRQIPQLENVLKETLRLHPPLIILMRVAKGEFEVQGHRIHEGDLVAASPAISNRIPEDFPDPHDF
VPARYEQPRQEDLLNRWTWIPFGAGRHRCVGAAFAIMQIKAIFSVLLREYEFEMAQPPESYRNDHSKMVVQLA
QPACVRYRRRTGV
>CYP51B1_Mycobacterium tuberculosis TB_RSA76 2576320418
MSAVALPRVSGGHDEHGHLEEFRTDPIGLMQRVRDECGDVGTFQLAGKQVVLLSGSHANEFFFRAGDDDLDQA
KAYPFMTPIFGEGVVFDASPERRKEMLHNAALRGEQMKGHAATIEDQVRRMIADWGEAAEIDLLDFFAELTIY
TSSACLIGKKFRDQLDGRFAKLYHELERGTDPLAYVDPYLPIESFRRRDEARNGLVALVADIMNGRIANPPTD
KSDRDMLDVLIAVKAETGTPRFSADEITGMFISMMFAGHHTSSGTASWTLIELMRHRDAYAAVIDELDELYGD
GRSVSFHALRQIPQLENVLKETLRLHPPLIILMRVAKGEFEVQGHRIHEGDLVAASPAISNRIPEDFPDPHDF
VPARYEQPRQEDLLNRWTWIPFGAGRHRCVGAAFAIMQIKAIFSVLLREYEFEMAQPPESYRNDHSKMVVQLA
QPACVRYRRRTGV
>CYP51B1_Mycobacterium tuberculosis TB_RSA90 2575476243
MSAVALPRVSGGHDEHGHLEEFRTDPIGLMQRVRDECGDVGTFQLAGKQVVLLSGSHANEFFFRAGDDDLDQA
KAYPFMTPIFGEGVVFDASPERRKEMLHNAALRGEQMKGHAATIEDQVRRMIADWGEAAEIDLLDFFAELTIY
TSSACLIGKKFRDQLDGRFAKLYHELERGTDPLAYVDPYLPIESFRRRDEARNGLVALVADIMNGRIANPPTD
KSDRDMLDVLIAVKAETGTPRFSADEITGMFISMMFAGHHTSSGTASWTLIELMRHRDAYAAVIDELDELYGD
GRSVSFHALRQIPQLENVLKETLRLHPPLIILMRVAKGEFEVQGHRIHEGDLVAASPAISNRIPEDFPDPHDF
VPARYEQPRQEDLLNRWTWIPFGAGRHRCVGAAFAIMQIKAIFSVLLREYEFEMAQPPESYRNDHSKMVVQLA
QPACVRYRRRTGV
>CYP51B1_Mycobacterium tuberculosis TB_RSA98 2603451587
MSAVALPRVSGGHDEHGHLEEFRTDPIGLMQRVRDECGDVGTFQLAGKQVVLLSGSHANEFFFRAGDDDLDQA
KAYPFMTPIFGEGVVFDASPERRKEMLHNAALRGEQMKGHAATIEDQVRRMIADWGEAAEIDLLDFFAELTIY
TSSACLIGKKFRDQLDGRFAKLYHELERGTDPLAYVDPYLPIESFRRRDEARNGLVALVADIMNGRIANPPTD
KSDRDMLDVLIAVKAETGTPRFSADEITGMFISMMFAGHHTSSGTASWTLIELMRHRDAYAAVIDELDELYGD
GRSVSFHALRQIPQLENVLKETLRLHPPLIILMRVAKGEFEVQGHRIHEGDLVAASPAISNRIPEDFPDPHDF
VPARYEQPRQEDLLNRWTWIPFGAGRHRCVGAAFAIMQIKAIFSVLLREYEFEMAQPPESYRNDHSKMVVQLA
QPACVRYRRRTGV
>CYP51B1_Mycobacterium tuberculosis TB_RSA99 2575613208
MSAVALPRVSGGHDEHGHLEEFRTDPIGLMQRVRDECGDVGTFQLAGKQVVLLSGSHANEFFFRAGDDDLDQA
KAYPFMTPIFGEGVVFDASPERRKEMLHNAALRGEQMKGHAATIEDQVRRMIADWGEAAEIDLLDFFAELTIY
TSSACLIGKKFRDQLDGRFAKLYHELERGTDPLAYVDPYLPIESFRRRDEARNGLVALVADIMNGRIANPPTD
KSDRDMLDVLIAVKAETGTPRFSADEITGMFISMMFAGHHTSSGTASWTLIELMRHRDAYAAVIDELDELYGD
GRSVSFHALRQIPQLENVLKETLRLHPPLIILMRVAKGEFEVQGHRIHEGDLVAASPAISNRIPEDFPDPHDF
VPARYEQPRQEDLLNRWTWIPFGAGRHRCVGAAFAIMQIKAIFSVLLREYEFEMAQPPESYRNDHSKMVVQLA
QPACVRYRRRTGV
>CYP51B1_Mycobacterium tuberculosis TKK_02_0062 2592360287
MSAVALPRVSGGHDEHGHLEEFRTDPIGLMQRVRDECGDVGTFQLAGKQVVLLSGSHANEFFFRAGDDDLDQA
KAYPFMTPIFGEGVVFDASPERRKEMLHNAALRGEQMKGHAATIEDQVRRMIADWGEAAEIDLLDFFAELTIY
TSSACLIGKKFRDQLDGRFAKLYHELERGTDPLAYVDPYLPIESFRRRDEARNGLVALVADIMNGRIANPPTD
KSDRDMLDVLIAVKAETGTPRFSADEITGMFISMMFAGHHTSSGTASWTLIELMRHRDAYAAVIDELDELYGD
GRSVSFHALRQIPQLENVLKETLRLHPPLIILMRVAKGEFEVQGHRIHEGDLVAASPAISNRIPEDFPDPHDF
VPARYEQPRQEDLLNRWTWIPFGAGRHRCVGAAFAIMQIKAIFSVLLREYEFEMAQPPESYRNDHSKMVVQLA
QPACVRYRRRTGV
>CYP51B1_Mycobacterium tuberculosis TKK_03_0033 2592299020
MSAVALPRVSGGHDEHGHLEEFRTDPIGLMQRVRDECGDVGTFQLAGKQVVLLSGSHANEFFFRAGDDDLDQA
KAYPFMTPIFGEGVVFDASPERRKEMLHNAALRGEQMKGHAATIEDQVRRMIADWGEAAEIDLLDFFAELTIY
TSSACLIGKKFRDQLDGRFAKLYHELERGTDPLAYVDPYLPIESFRRRDEARNGLVALVADIMNGRIANPPTD
KSDRDMLDVLIAVKAETGTPRFSADEITGMFISMMFAGHHTSSGTASWTLIELMRHRDAYAAVIDELDELYGD
GRSVSFHALRQIPQLENVLKETLRLHPPLIILMRVAKGEFEVQGHRIHEGDLVAASPAISNRIPEDFPDPHDF
VPARYEQPRQEDLLNRWTWIPFGAGRHRCVGAAFAIMQIKAIFSVLLREYEFEMAQPPESYRNDHSKMVVQLA
QPACVRYRRRTGV
>CYP51B1_Mycobacterium tuberculosis TKK_03_0037 2652969365
MSAVALPRVSGGHDEHGHLEEFRTDPIGLMQRVRDECGDVGTFQLAGKQVVLLSGSHANEFFFRAGDDDLDQA
KAYPFMTPIFGEGVVFDASPERRKEMLHNAALRGEQMKGHAATIEDQVRRMIADWGEAAEIDLLDFFAELTIY
TSSACLIGKKFRDQLDGRFAKLYHELERGTDPLAYVDPYLPIESFRRRDEARNGLVALVADIMNGRIANPPTD
KSDRDMLDVLIAVKAETGTPRFSADEITGMFISMMFAGHHTSSGTASWTLIELMRHRDAYAAVIDELDELYGD
GRSVSFHALRQIPQLENVLKETLRLHPPLIILMRVAKGEFEVQGHRIHEGDLVAASPAISNRIPEDFPDPHDF

VPARYEQPRQEDLLNRWTWIPFGAGRHRCVGAAFAIMQIKAIFSVLLREYEFEMAQPPESYRNDHSKMVVQLA
QPACVRYRRRTGV
>CYP51B1_Mycobacterium tuberculosis TKK_04_0139 2621768031
MSAVALPRVSGGHDEHGHLEEFRTDPIGLMQRVRDECGDVGTFQLAGKQVVLLSGSHANEFFFRAGDDDLDQA
KAYPFMTPIFGEGVVFDASPERRKEMLHNAALRGEQMKGHAATIEDQVRRMIADWGEAAEIDLLDFFAELTIY
TSSACLIGKKFRDQLDGRFAKLYHELERGTDPLAYVDPYLPIESFRRRDEARNGLVALVADIMNGRIANPPTD
KSDRDMLDVLIAVKAETGTPRFSADEITGMFISMMFAGHHTSSGTASWTLIELMRHRDAYAAVIDELDELYGD
GRSVSFHALRQIPQLENVLKETLRLHPPLIILMRVAKGEFEVQGHRIHEGDLVAASPAISNRIPEDFPDPHDF
VPARYEQPRQEDLLNRWTWIPFGAGRHRCVGAAFAIMQIKAIFSVLLREYEFEMAQPPESYRNDHSKMVVQLA
QPACVRYRRRTGV
>CYP51B1_Mycobacterium tuberculosis TKK_04_0148 2621780574
MSAVALPRVSGGHDEHGHLEEFRTDPIGLMQRVRDECGDVGTFQLAGKQVVLLSGSHANEFFFRAGDDDLDQA
KAYPFMTPIFGEGVVFDASPERRKEMLHNAALRGEQMKGHAATIEDQVRRMIADWGEAAEIDLLDFFAELTIY
TSSACLIGKKFRDQLDGRFAKLYHELERGTDPLAYVDPYLPIESFRRRDEARNGLVALVADIMNGRIANPPTD
KSDRDMLDVLIAVKAETGTPRFSADEITGMFISMMFAGHHTSSGTASWTLIELMRHRDAYAAVIDELDELYGD
GRSVSFHALRQIPQLENVLKETLRLHPPLIILMRVAKGEFEVQGHRIHEGDLVAASPAISNRIPEDFPDPHDF
VPARYEQPRQEDLLNRWTWIPFGAGRHRCVGAAFAIMQIKAIFSVLLREYEFEMAQPPESYRNDHSKMVVQLA
QPACVRYRRRTGV
>CYP51B1_Mycobacterium tuberculosis TKK_05SA_0050 2577384624
MSAVALPRVSGGHDEHGHLEEFRTDPIGLMQRVRDECGDVGTFQLAGKQVVLLSGSHANEFFFRAGDDDLDQA
KAYPFMTPIFGEGVVFDASPERRKEMLHNAALRGEQMKGHAATIEDQVRRMIADWGEAAEIDLLDFFAELTIY
TSSACLIGKKFRDQLDGRFAKLYHELERGTDPLAYVDPYLPIESFRRRDEARNGLVALVADIMNGRIANPPTD
KSDRDMLDVLIAVKAETGTPRFSADEITGMFISMMFAGHHTSSGTASWTLIELMRHRDAYAAVIDELDELYGD
GRSVSFHALRQIPQLENVLKETLRLHPPLIILMRVAKGEFEVQGHRIHEGDLVAASPAISNRIPEDFPDPHDF
VPARYEQPRQEDLLNRWTWIPFGAGRHRCVGAAFAIMQIKAIFSVLLREYEFEMAQPPESYRNDHSKMVVQLA
QPACVRYRRRTGV
>CYP51B1_Mycobacterium orygis 112400015 2546435233
MSAVALPRVSGGHDEHGHLEEFRTDPIGLMQRVRDECGDVGTFQLAGKQVVLLSGSHANEFFFRAGDDDLDQA
KAYPFMTPIFGEGVVFDASPERRKEMLHNAALRGEQMKGHAATIEDQVRRMIADWGEAGEIDLLDFFAELTIY
TSSACLIGKKFRDQLDGRFAKLYHELERGTDPLAYVDPYLPIESFRRRDEARNGLVALVADIMNGRIANPPTD
KSDRDMLDVLIAVKAETGTPRFSADEITGMFISMMFAGHHTSSGTASWTLIELMRHRDAYAAMIDELDELYGD
GRSVSFHALRQIPQLENVLKETLRLHPPLIILMRVAKGEFEVQGHRIHEGDLVAASPAISNRIPEDFPDPHDF
VPARYEQPRQEDLLNRWTWIPFGAGRHRCVGAAFAIMQIKAIFSVLLREYEFEMAQPPESYRNDHSKMVVQLA
QPACVRYRRRTGV
>CYP51B1_Mycobacterium tuberculosis TRUG0016 2603376221
MSAVALPRVSGGHDEHGHLEEFRTDPIGLMQRVRDECGDVGTFQLAGKQVVLLSGSHANEFFFRAGDDDLDQA
KAYPFMTPIFGEGVVFDASPERRKEMLHNAALRGEQMKGHAATIEDQVRRMIADWGEAAEIDLLDFFAELTIY
TSSACLIGKKFRDQLDGRFAKLYHELERGTDPLAYVDPYLPIESFRRRDEARNGLVALVADIMNGRIANPPTD
KSDRDMLDVLIAVKAETGTPRFSADEITGMFISMMFAGHHTSSGTASWTLIELMRHRDAYAAVIDELDELYGD
GRSVSFHALRQIPQLENVLKETLRLHPPLIILMRVAKGEFEVQGHRIHEGDLVAASPAISNRIPEDFPDPHDF
VPARYEQPRQEDLLNRWTWIPFGAGRHRCVGAAFAIMQIKAIFSVLLREYEFEMAQPPESYRNDHSKMVVQLA
QPACVRYRRRTGV
>CYP51B1_Mycobacterium tuberculosis TRUG0026 2604635296
MSAVALPRVSGGHDEHGHLEEFRTDPIGLMQRVRDECGDVGTFQLAGKQVVLLSGSHANEFFFRAGDDDLDQA
KAYPFMTPIFGEGVVFDASPERRKEMLHNAALRGEQMKGHAATIEDQVRRMIADWGEAAEIDLLDFFAELTIY
TSSACLIGKKFRDQLDGRFAKLYHELERGTDPLAYVDPYLPIESFRRRDEARNGLVALVADIMNGRIANPPTD
KSDRDMLDVLIAVKAETGTPRFSADEITGMFISMMFAGHHTSSGTASWTLIELMRHRDAYAAVIDELDELYGD
GRSVSFHALRQIPQLENVLKETLRLHPPLIILMRVAKGEFEVQGHRIHEGDLVAASPAISNRIPEDFPDPHDF
VPARYEQPRQEDLLNRWTWIPFGAGRHRCVGAAFAIMQIKAIFSVLLREYEFEMAQPPESYRNDHSKMVVQLA
QPACVRYRRRTGV
>CYP51B1_Mycobacterium tuberculosis TRUG0038 2605684831
MSAVALPRVSGGHDEHGHLEEFRTDPIGLMQRVRDECGDVGTFQLAGKQVVLLSGSHANEFFFRAGDDDLDQA
KAYPFMTPILGEGVVFDASPERRKEMLHNAALRGEQMKGHAATIEDQVRRMIADWGEAGEIDLLDFFAELTIY
TSSACLIGKKFRDQLDGRFAKLYHELERGTDPLAYVDPYLPIESFRRRDEARNGLVALVADIMNGRIANPPTD
KSDRDMLDVLIAVKAETGTPRFSADEITGMFISMMFAGHHTSSGTASWTLIELMRHRDAYAAVIDELDELYGD
GRSVSFHALRQIPQLENVLKETLRLHPPLIILMRVAKGEFEVQGHRIHEGDLVAASPAISNRIPEDFPDPHDF
VPARYEQPRQEDLLNRWTWIPFGAGRHRCVGAAFAIMQIKAIFSVLLREYEFEMAQPPESYRNDHSKMVVQLA
QPACVRYRRRTGV

>CYP51B1_Mycobacterium tuberculosis TRUG0059 2605337399
MSAVALPRVSGGHDEHGHLEEFRTDPIGLMQRVRDECGDVGTFQLAGKQVVLLSGSHANEFFFRAGDDDLDQA
KAYPFMTPIFGEGVVFDASPERRKEMLHNAALRGEQMKGHAATIEDQVRRMIADWGEAAEIDLLDFFAELTIY
TSSACLIGKKFRDQLDGRFAKLYHELERGTDPLAYVDPYLPIESFRRRDEARNGLVALVADIMNGRIANPPTD
KSDRDMLDVLIAVKAETGTPRFSADEITGMFISMMFAGHHTSSGTASWTLIELMRHRDAYAAVIDELDELYGD
GRSVSFHALRQIPQLENVLKETLRLHPPLIILMRVAKGEFEVQGHRIHEGDLVAASPAISNRIPEDFPDPHDF
VPARYEQPRQEDLLNRWTWIPFGAGRHRCVGAAFAIMQIKAIFSVLLREYEFEMAQPPESYRNDHSKMVVQLA
QPACVRYRRRTGV
>CYP51B1_Mycobacterium tuberculosis TRUG0072 2584663444
MSAVALPRVSGGHDEHGHLEEFRTDPIGLMQRVRDECGDVGTFQLAGKQVVLLSGSHANEFFFRAGDDDLDQA
KAYPFMTPIFGEGVVFDASPERRKEMLHNAALRGEQMKGHAATIEDQVRRMIADWGEAAEIDLLDFFAELTIY
TSSACLIGKKFRDQLDGRFAKLYHELERGTDPLAYVDPYLPIESFRRRDEARNGLVALVADIMNGRIANPPTD
KSDRDMLDVLIAVKAETGTPRFSADEITGMFISMMFAGHHTSSGTASWTLIELMRHRDAYAAVIDELDELYGD
GRSVSFHALRQIPQLENVLKETLRLHPPLIILMRVAKGEFEVQGHRIHEGDLVAASPAISNRIPEDFPDPHDF
VPARYEQPRQEDLLNRWTWIPFGAGRHRCVGAAFAIMQIKAIFSVLLREYEFEMAQPPESYRNDHSKMVVQLA
QPACVRYRRRTGV
>CYP51B1_Mycobacterium tuberculosis TRUG0081 2603921200
MSAVALPRVSGGHDEHGHLEEFRTDPIGLMQRVRDECGDVGTFQLAGKQVVLLSGSHANEFFFRAGDDDLDQA
KAYPFMTPILGEGVVFDASPERRKEMLHNAALRGEQMKGHAATIEDQVRRMIADWGEAGEIDLLDFFAELTIY
TSSACLIGKKFRDQLDGRFAKLYHELERGTDPLAYVDPYLPIESFRRRDEARNGLVALVADIMNGRIANPPTD
KSDRDMLDVLIAVKAETGTPRFSADEITGMFISMMFAGHHTSSGTASWTLIELMRHRDAYAAVIDELDELYGD
GRSVSFHALRQIPQLENVLKETLRLHPPLIILMRVAKGEFEVQGHRIHEGDLVAASPAISNRIPEDFPDPHDF
VPARYEQPRQEDLLNRWTWIPFGAGRHRCVGAAFAIMQIKAIFSVLLREYEFEMAQPPESYRNDHSKMVVQLA
QPACVRYRRRTGV
>CYP51B1_Mycobacterium tuberculosis TRUG0088 2575640712
MSAVALPRVSGGHDEHGHLEEFRTDPIGLMQRVRDECGDVGTFQLAGKQVVLLSGSHANEFFFRAGDDDLDQA
KAYPFMTPIFGEGVVFDASPERRKEMLHNAALRGEQMKGHAATIEDQVRRMIADWGEAAEIDLLDFFAELTIY
TSSACLIGKKFRDQLDGRFAKLYHELERGTDPLAYVDPYLPIESFRRRDEARNGLVALVADIMNGRIANPPTD
KSDRDMLDVLIAVKAETGTPRFSADEITGMFISMMFAGHHTSSGTASWTLIELMRHRDAYAAVIDELDELYGD
GRSVSFHALRQIPQLENVLKETLRLHPPLIILMRVAKGEFEVQGHRIHEGDLVAASPAISNRIPEDFPDPHDF
VPARYEQPRQEDLLNRWTWIPFGAGRHRCVGAAFAIMQIKAIFSVLLREYEFEMAQPPESYRNDHSKMVVQLA
QPACVRYRRRTGV
>CYP51B1_Mycobacterium tuberculosis TRUG0094 2603219578
MSAVALPRVSGGHDEHGHLEEFRTDPIGLMQRVRDECGDVGTFQLAGKQVVLLSGSHANEFFFRAGDDDLDQA
KAYPFMTPIFGEGVVFDASPERRKEMLHNAALRGEQMKGHAATIEDQVRRMIADWGEAAEIDLLDFFAELTIY
TSSACLIGKKFRDQLDGRFAKLYHELERGTDPLAYVDPYLPIESFRRRDEARNGLVALVADIMNGRIANPPTD
KSDRDMLDVLIAVKAETGTPRFSADEITGMFISMMFAGHHTSSGTASWTLIELMRHRDAYAAVIDELDELYGD
GRSVSFHALRQIPQLENVLKETLRLHPPLIILMRVAKGEFEVQGHRIHEGDLVAASPAISNRIPEDFPDPHDF
VPARYEQPRQEDLLNRWTWIPFGAGRHRCVGAAFAIMQIKAIFSVLLREYEFEMAQPPESYRNDHSKMVVQLA
QPACVRYRRRTGV
>CYP51B1_Mycobacterium tuberculosis TRUG0107 2576512075
MSAVALPRVSGGHDEHGHLEEFRTDPIGLMQRVRDECGDVGTFQLAGKQVVLLSGSHANEFFFRAGDDDLDQA
KAYPFMTPIFGEGVVFDASPERRKEMLHNAALRGEQMKGHAATIEDQVRRMIADWGEAAEIDLLDFFAELTIY
TSSACLIGKKFRDQLDGRFAKLYHELERGTDPLAYVDPYLPIESFRRRDEARNGLVALVADIMNGRIANPPTD
KSDRDMLDVLIAVKAETGTPRFSADEITGMFISMMFAGHHTSSGTASWTLIELMRHRDAYAAVIDELDELYGD
GRSVSFHALRQIPQLENVLKETLRLHPPLIILMRVAKGEFEVQGHRIHEGDLVAASPAISNRIPEDFPDPHDF
VPARYEQPRQEDLLNRWTWIPFGAGRHRCVGAAFAIMQIKAIFSVLLREYEFEMAQPPESYRNDHSKMVVQLA
QPACVRYRRRTGV
>CYP51B1_Mycobacterium tuberculosis TRUG0119 2605342710
MSAVALPRVSGGHDEHGHLEEFRTDPIGLMQRVRDECGDVGTFQLAGKQVVLLSGSHANEFFFRAGDDDLDQA
KAYPFMTPIFGEGVVFDASPERRKEMLHNAALRGEQMKGHAATIEDQVRRMIADWGEAAEIDLLDFFAELTIY
TSSACLIGKKFRDQLDGRFAKLYHELERGTDPLAYVDPYLPIESFRRRDEARNGLVALVADIMNGRIANPPTD
KSDRDMLDVLIAVKAETGTPRFSADEITGMFISMMFAGHHTSSGTASWTLIELMRHRDAYAAVIDELDELYGD
GRSVSFHALRQIPQLENVLKETLRLHPPLIILMRVAKGEFEVQGHRIHEGDLVAASPAISNRIPEDFPDPHDF
VPARYEQPRQEDLLNRWTWIPFGAGRHRCVGAAFAIMQIKAIFSVLLREYEFEMAQPPESYRNDHSKMVVQLA
QPACVRYRRRTGV
>CYP51B1_Mycobacterium tuberculosis UG-D 2584796564

MSAVALPRVSGGHDEHGHLEEFRTDPIGLMQRVRDECGDVGTFQLAGKQVVLLSGSHANEFFFRAGDDDLDQA
KAYPFMTPIFGEGVVFDASPERRKEMLHNAALRGEQMKGHAATIEDQVRRMIADWGEAAEIDLLDFFAELTIY
TSSACLIGKKFRDQLDGRFAKLYHELERGTDPLAYVDPYLPIESFRRRDEARNGLVALVADIMNGRIANPPTD
KSDRDMLDVLIAVKAETGTPRFSADEITGMFISMMFAGHHTSSGTASWTLIELMRHRDAYAAVIDELDELYGD
GRSVSFHALRQIPQLENVLKETLRLHPPLIILMRVAKGEFEVQGHRIHEGDLVAASPAISNRIPEDFPDPHDF
VPARYEQPRQEDLLNRWTWIPFGAGRHRCVGAAFAIMQIKAIFSVLLREYEFEMAQPPESYRNDHSKMVVQLA
QPACVRYRRRTGV
>CYP51B1_Mycobacterium tuberculosis VRFCWCF MDRTB 17 2646580223
MSAVALPRVSGGHDEHGHLEEFRTDPIGLMQRVRDECGDVGTFQLAGKQVVLLSGSHANEFFFRAGDDDLDQA
KAYPFMTPIFGEGVVFDASPERRKEMLHNAALRGEQMKGHAATIEDQVRRMIADWGEAGEIDLLDFFAELTIY
TSSACLIGKKFRDQLDGRFAKLYHELERGTDPLAYVDPYLPIESFRRRDEARNGLVALVADIMNGRIANPPTD
KSDRDMLDVLIAVKAETGTPRFSADEITGMFISMMFAGHHTSSGTASWTLIELMRHRDAYAAVIDELDELYGD
GRSVSFHALRQIPQLENVLKETLRLHPPLIILMRVPRASSRCKATGFMRAIWWRPPRRSPTGSPKTSPIPTTS
CQHDTSSRARKICSTAGRGFRSAPAGIVAWGRRSPSCRSKRSSRCCCASMSLRWRNRQKAIVTTIRRWWCSWP
SPLACATAGEREFNDGLSSRSRPGSVPRTRDVRAGGTRVFPGAQARPGRNPRPRAARGSPRRDQACRVGLSHP
GTVHPRNGRVTVAGYPRDELEDVVHRWLQANRTAERRGDWTLLADFYTDDATYGWNVGPNEDVMCVGIDEIRD
IALGQEMDGLQGWRYPYQRVVIDEKQGEVVGFWKQVATDANGAEQEVYGIGGSWFRYAGGGKWNWQRDFFDFG
HVSALYLELIKAGKLSPGMQKRIERAVSGNKVPGYYPLGKTPVPLW
>CYP51B1_Mycobacterium tuberculosis VRFCWCF MRTB180 2715342543
MSAVALPRVSGGHDEHGHLEEFRTDPIGLMQRVRDECGDVGTFQLAGKQVVLLSGSHANEFFFRAGDDDLDQA
KAYPFMTPIFGEGVVFDASPERRKEMLHNAALRGEQMKGHAATIEDQVRRMIADWGEAGEIDLLDFFAELTIY
TSSACLIGKKFRDQLDGRFAKLYHELERGTDPLAYVDPYLPIESFRRRDEARNGLVALVADIMNGRIANPPTD
KSDRDMLDVLIAVKAETGTPRFSADEITGMFISMMFAGHHTSSGTASWTLIELMRHRDAYAAVIDELDELYGD
GRSVSFHALRQIPQLENVLKETLRLHPPLIILMRVPRASSRCKATGFMRAIWWRPPRRSPTGSPKTSPIPTTS
CQHDTSSRARKICSTAGRGFRSAPAGIVAWGRRSPSCRSKRSSRCCCASMSLRWRNRQKAIVTTIRRWWCSWP
SPLACATAGEREFNDGLSSRSRPGSVPRTRDVRAGGTRVFPGAQARPGRNPRPRAARGSPRRDQACRVGLSHP
GTVHPRNGRVTVAGYPRDELEDVVHRWLQANRTAERRGDWTLLADFYTDDATYGWNVGPNEDVMCVGIDEIRD
IALGQEMDGLQGWRYPYQRVVIDEKQGEVVGFWKQVATDANGAEQEVYGIGGSWFRYAGGGKWNWQRDFFDFG
HVSALYLELIKAGKLSPGMQKRIERAVSGNKVPGYYPLGKTPVPLW
>CYP51B1_Mycobacterium tuberculosis VRFCWCF PRTB19 2651419163
MSAVALPRVSGGHDEHGHLEEFRTDPIGLMQRVRDECGDVGTFQLAGKQVVLLSGSHANEFFFRAGDDDLDQA
KAYPFMTPIFGEGVVFDASPERRKEMLHNAALRGEQMKGHAATIEDQVRRMIADWGEAGEIDLLDFFAELTIY
TSSACLIGKKFRDQLDGRFAKLYHELERGTDPLAYVDPYLPIESFRRRDEARNGLVALVADIMNGRIANPPTD
KSDRDMLDVLIAVKAETGTPRFSADEITGMFISMMFAGHHTSSGTASWTLIELMRHRDAYAAVIDELDELYGD
GRSVSFHALRQIPQLENVLKETLRLHPPLIILMRVPRASSRCKATGFMRAIWWRPPRRSPTGSPKTSPIPTTS
CQHDTSSRARKICSTAGRGFRSAPAGIVAWGRRSPSCRSKRSSRCCCASMSLRWRNRQKAIVTTIRRWWCSWP
SPLACATAGEREFNDGLSSRSRPGSVPRTRDVRAGGTRVFPGAQARPGRNPRPRAARGSPRRDQACRVGLSHP
GTVHPRNGRVTVAGYPRDELEDVVHRWLQANRTAERRGDWTLLADFYTDDATYGWNVGPNEDVMCVGIDEIRD
IALGQEMDGLQGWRYPYQRVVIDEKQGEVVGFWKQVATDANGAEQEVYGIGGSWFRYAGGGKWNWQRDFFDFG
HVSALYLELIKAGKLSPGMQKRIERAVSGNKVPGYYPLGKTPVPLW
>CYP51B1_Mycobacterium marinum Europe 2543279484
MTTAIVPRVSGGEEEHGHLEEFRTDPIGLMQRVRDECGDVGWFQLANKHVVLLSGAKANEFFFRSSDEELDQA
EAYPFMTPIFGKGVVFDASPERRKEMLHNSALRGEHMKGHATTIEREVHRMIENWGQEGEIDLLEFFAELTIY
TSTSCLIGTKFRNQLDSRFAYFYHELERGTDPLCYADPYLPIESFRRRDEARKGLVALVQEIMHQRVANPPTD
KRDRDMLDVLVSITDEQGNPRFCADEVTGMFISLMFAGHHTSSGTSAWTLIELLRHPDAYAAVIDELDELYAD
GQPVSFHALRQIPRLENVLKETLRLHPPLIILMRVAKGEFEVEGYPIHEGELVAASPAISNRIAEDFPDPDEF
VPERYQEPRQEDLINRWTWIPFGAGRHRCVGAAFATMQIKAIFSVLLREYEFEMAQPADSYRNDHSKMVVQLA
RPARVRYRRRKMSDNRGH
>CYP51B1_Mycobacterium tuberculosis XTB13-223 2606414616
MTTSTVVPRVSGGEEEHGHLEEFRTDPIGLMQRVRDECGDVGWFQLVDKHVILLSGAQANEFFFRSADEDLDQ
AEAYPFMTPIFGKGVVFDASPERRKEMLHNSALRGEQMKGHASTIEGEVKKMIADWGDEGEIELLDFFAELTI
YTSTACLIGLKFREQLDHRFAEYYHDLERGTDPLCYVDPYLPIESFKRRDEARVKLVALVQEIMDQRLANPPK
DKADRDMLDVLVSIKDEDGKPRFSADEITGMFISLMFAGHHTSSGTSAWTLIELIRHPDVYAEVLAELEELYA
DGQEVSFHALRSIPKLDNVVKETLRLHPPLIILMRVAKGEFEVEGFPIHEGDYVAASPAISNRIPEDFPDPDA
FKPDRYNKPEQADIVNRWTWIPFGAGRHRCVGAAFAQMQIKAIFSVLLREYDFEMAQPADSYRNDHSKMVVQL
ARPAKVRYRKRNA
>CYP51B1_Mycobacterium ulcerans Agy99 642567643

MTTAIVPRVSGGEEEHGHLEEFRTDPIGLMQRVRDECGDVGWFQLANKHVVLLSGAKANEFFFRSSDEELDQA
EAYPFMTPIFGKGVVFDASPERRKEMLHNSALRGEHMKGHATTIEREVHRMIENWGQEGEIDLLEFFAELTSY
TSTSCLIGTKFRNQLDSRFAHFCHELERGTDPLCYVDPYLPIESFRRRDEARKGLVALVQDIMHQRVANPPTD
KRDRDMLDVLVSITDEQGNPRFCTDEVTGMFISLMFAGHHTSSGTSAWTLIELLRHPDAYAAVIDELDELYAD
GQLVSFHALRQIPRLENVLKETLRLHPPLIILMRVAKGEFQVEGYPIHEGELVAASPAISNRIAEDFPDPDEF
VPERYQEPRQEDLINRWTWIPFGAGRHRCVGAAFATMQIKAIFSVLLREYEFEMAQPADSYRNDHSKMVVQLA
RPARVRYRRRKLSDNRGH
>CYP51B1_Mycobacterium smegmatis INHR2 2687287129
VTTLHDVPRVSGGEEEHGHLEEFRTDPIGLMKRVRSECGDVGWFQLADKQVVLLSGAEANEFFFRSSDSELNQ
AEAYPFMTPIFGEGVVFDADPERRAEMLHNTALRGEQMKGHAATIENEVRRMVESWGDEGEIDLLEFFAELTI
YTSTACLIGVKFRNQLDKRFADYYHLLERGTDPLCYVDPYLPIESFRIRDEARANLVELVQEVMNGRIANPPK
DKSDRDLLDVLVSIKDEDGTPRFSANEVTGMFISLMFAGHHTSSGTASWTLIELLRHPEFYAKVQAELDDLYA
DGQEISFHALRQIPNLDNALKETLRLHPPLIILMRVAQDEFEVAGRPIHKGQMVAASPAISNRIPEDFPDPDT
FDPDRYDKPRQEDLINRWTWIPFGAGKHRCVGAAFAQMQIKAIFSVLLRDFEFEMAQPSESYRNDHSKMVVQL
ARPAKVRYRRRVR
>CYP51B1_Mycobacterium smegmatis str. MC2 155 639744279
MTTLHDVPRVSGGEEEHGHLEEFRTDPIGLMKRVRSECGDVGWFQLADKQVVLLSGAEANEFFFRSSDSELNQ
AEAYPFMTPIFGEGVVFDADPERRAEMLHNTALRGEQMKGHAATIENEVRRMVESWGDEGEIDLLEFFAELTI
YTSTACLIGVKFRNQLDKRFADYYHLLERGTDPLCYVDPYLPIESFRIRDEARANLVELVQEVMNGRIANPPK
DKSDRDLLDVLVSIKDEDGTPRFSANEVTGMFISLMFAGHHTSSGTASWTLIELLRHPEFYAKVQAELDDLYA
DGQEISFHALRQIPNLDNALKETLRLHPPLIILMRVAQDEFEVAGRPIHKGQMVAASPAISNRIPEDFPDPDT
FDPDRYDKPRQEDLINRWTWIPFGAGKHRCVGAAFAQMQIKAIFSVLLRDFEFEMAQPSESYRNDHSKMVVQL
ARPAKVRYRRRVR
>CYP51B1_Mycobacterium smegmatis MC2 155 2518780060
MTTLHDVPRVSGGEEEHGHLEEFRTDPIGLMKRVRSECGDVGWFQLADKQVVLLSGAEANEFFFRSSDSELNQ
AEAYPFMTPIFGEGVVFDADPERRAEMLHNTALRGEQMKGHAATIENEVRRMVESWGDEGEIDLLEFFAELTI
YTSTACLIGVKFRNQLDKRFADYYHLLERGTDPLCYVDPYLPIESFRIRDEARANLVELVQEVMNGRIANPPK
DKSDRDLLDVLVSIKDEDGTPRFSANEVTGMFISLMFAGHHTSSGTASWTLIELLRHPEFYAKVQAELDDLYA
DGQEISFHALRQIPNLDNALKETLRLHPPLIILMRVAQDEFEVAGRPIHKGQMVAASPAISNRIPEDFPDPDT
FDPDRYDKPRQEDLINRWTWIPFGAGKHRCVGAAFAQMQIKAIFSVLLRDFEFEMAQPSESYRNDHSKMVVQL
ARPAKVRYRRRVR
>CYP51B1_Mycobacterium smegmatis MC2 155 2689265304
VTTLHDVPRVSGGEEEHGHLEEFRTDPIGLMKRVRSECGDVGWFQLADKQVVLLSGAEANEFFFRSSDSELNQ
AEAYPFMTPIFGEGVVFDADPERRAEMLHNTALRGEQMKGHAATIENEVRRMVESWGDEGEIDLLEFFAELTI
YTSTACLIGVKFRNQLDKRFADYYHLLERGTDPLCYVDPYLPIESFRIRDEARANLVELVQEVMNGRIANPPK
DKSDRDLLDVLVSIKDEDGTPRFSANEVTGMFISLMFAGHHTSSGTASWTLIELLRHPEFYAKVQAELDDLYA
DGQEISFHALRQIPNLDNALKETLRLHPPLIILMRVAQDEFEVAGRPIHKGQMVAASPAISNRIPEDFPDPDT
FDPDRYDKPRQEDLINRWTWIPFGAGKHRCVGAAFAQMQIKAIFSVLLRDFEFEMAQPSESYRNDHSKMVVQL
ARPAKVRYRRRVR
>CYP51B1_Mycobacterium smegmatis MC2 51 2586876612
VTTLHDVPRVSGGEEEHGHLEEFRTDPIGLMKRVRSECGDVGWFQLADKQVVLLSGAEANEFFFRSSDSELNQ
AEAYPFMTPIFGEGVVFDADPERRAEMLHNTALRGEQMKGHAATIENEVRRMVESWGDEGEIDLLEFFAELTI
YTSTACLIGVKFRNQLDKRFADYYHLLERGTDPLCYVDPYLPIESFRIRDEARANLVELVQEVMNGRIANPPK
DKSDRDLLDVLVSIKDEDGTPRFSANEVTGMFISLMFAGHHTSSGTASWTLIELLRHPEFYAKVQAELDDLYA
DGQEISFHALRQIPNLDNALKETLRLHPPLIILMRVAQDEFEVAGRPIHKGQMVAASPAISNRIPEDFPDPDT
FDPDRYDKPRQEDLINRWTWIPFGAGKHRCVGAAFAQMQIKAIFSVLLRDFEFEMAQPSESYRNDHSKMVVQL
ARPAKVRYRRRVR
>CYP51B1_Mycobacterium smegmatis INHR1 2689420686
VTTLHDVPRVSGGEEEHGHLEEFRTDPIGLMKRVRSECGDVGWFQLADKQVVLLSGAEANEFFFRSSDSELNQ
AEAYPFMTPIFGEGVVFDADPERRAEMLHNTALRGEQMKGHAATIENEVRRMVESWGDEGEIDLLEFFAELTI
YTSTACLIGVKFRNQLDKRFADYYHLLERGTDPLCYVDPYLPIESFRIRDEARANLVELVQEVMNGRIANPPK
DKSDRDLLDVLVSIKDEDGTPRFSANEVTGMFISLMFAGHHTSSGTASWTLIELLRHPEFYAKVQAELDDLYA
DGQEISFHALRQIPNLDNALKETLRLHPPLIILMRVAQDEFEVAGRPIHKGQMVAASPAISNRIPEDFPDPDT
FDPDRYDKPRQEDLINRWTWIPFGAGKHRCVGAAFAQMQIKAIFSVLLRDFEFEMAQPSESYRNDHSKMVVQL
ARPAKVRYRRRVR
>CYP51B1_Mycobacterium austroafricanum DSM 44191 2649760175
MTAVKEVPRVSGGEEEHGHLEEFRTDPIGLMKRVREECGDVGWFQLADKQVILLSGAEANEFFFRSSDSELNQ
AEAYPFMTPIFGEGVVFDADPERRAEMLHNTALRGEHMKGHATTIEAEVRKMIEGWGESGEIDLLEFFAELTI

YTSTACLIGLKFRNQLDSRFANYYHLLERGTDPLCYVDPYLPIESFRIRDEARAGLVELVQDVMHGRIANPPK
DKSDRDMLDVLVSIKDEDGNPRFTANEITGMFISLMFAGHHTSSGTSSWTLIELLRHPEFYAKVQQELDDLYA
DGQEVSFHALRQIPNLDNALKETLRLHPPLIILMRVAQDEFEVAGYPIHKGQMVAASPAISNRIPEDFPNPDD
FDPDRYEKPRQEDLINRWTWIPFGAGKHRCVGAAFAQMQIKAIFSVLLREYEFEMAQPPESYQNDHSKMVVQL
ARPAKVRYRRRVRD
>CYP51B1_Mycobacterium ulcerans Harvey 2567100968
MTTAIVPRVSGGEEEHGHLEEFRTDPIGLMQRVRDECGDVGWFQLANKHVVLLSGAKANEFFFRSSDEELDQA
EAYPFMTPIFGKGVVFDASPERRKEMLHNSALRGEHMKGHATTIEREVHRMIENWGQEGEIDLLEFFAELTSY
TSTSCLIGTKFRNQLDSRFAHFYHELERGTDPLCYVDPYLPIENFRRRDEARKGLVALVQDIMHQRVANPPTD
KRDRDMLDVLVSITDEQGNPRFCTDEVTGMFISLMFAGHHTSSGTSAWTLIELLRHPDAYAAVIDELDELYAD
GQPVSFHALRQIPRLENVLKETLRLHPPLIILMRVAKGEFQVEGYPIHEGELVAASPAISNRIAEDFPDPDEF
VPERYQEPRQEDLINRWTWIPFGAGRHRCVGAAFATMQIKAIFSVLLREYEFEMAQPADSYRNDHSKMVVQLA
RPARVRYRRRKLSDNRGH
>CYP51B1_Mycobacterium sp. MAC_011194_8550 2582208400
MTTSTVVPRVSGGEEEHGHLEEFRTDPIGLMQRVRDECGDVGWFQLVDKHVILLSGAQANEFFFRSADEDLDQ
AEAYPFMTPIFGKGVVFDASPERRKEMLHNSALRGEQMKGHASTIEGEVKKMIADWGDEGEIELLDFFAELTI
YTSTACLIGLKFREQLDHRFAEYYHDLERGTDPLCYVDPYLPIESFKRRDEARVKLVALVQEIMDQRLANPPK
DKADRDMLDVLVSIKDEDGKPRFSADEITGMFISLMFAGHHTSSGTSAWTLIELIRHPDVYAEVLAELEELYA
DGQEVSFHALRSIPKLDNVVKETLRLHPPLIILMRVAKGEFEVEGFPIHEGDYVAASPAISNRIPEDFPDPDA
FKPDRYNKPEQADIVNRWTWIPFGAGRHRCVGAAFAQMQIKAIFSVLLREYDFEMAQPADSYRNDHSKMVVQL
ARPAKVRYRKRNA
>CYP51B1_Mycobacterium sp. MAC_080597_8934 2576975589
MTTSTVVPRVSGGEEEHGHLEEFRTDPIGLMQRVRDECGDVGWFQLVDKHVILLSGAQANEFFFRSADEDLDQ
AEAYPFMTPIFGKGVVFDASPERRKEMLHNSALRGEQMKGHASTIEGEVKKMIADWGDEGEIELLDFFAELTI
YTSTACLIGLKFREQLDHRFAEYYHDLERGTDPLCYVDPYLPIESFKRRDEARVKLVALVQEIMDQRLANPPK
DKADRDMLDVLVSIKDEDGKPRFSADEITGMFISLMFAGHHTSSGTSAWTLIELIRHPDVYAEVLAELEELYA
DGQEVSFHALRSIPKLDNVVKETLRLHPPLIILMRVAKGEFEVEGFPIHEGDYVAASPAISNRIPEDFPDPDA
FKPDRYNKPEQADIVNRWTWIPFGAGRHRCVGAAFAQMQIKAIFSVLLREYDFEMAQPADSYRNDHSKMVVQL
ARPAKVRYRKRNA
>CYP51B1_Mycobacterium avium 05-4293 2581913849
MTTSTVVPRVSGGEEEHGHLEEFRTDPIGLMQRVRDECGDVGWFQLVDKHVILLSGAQANEFFFRSADEDLDQ
AEAYPFMTPIFGKGVVFDASPERRKEMLHNSALRGEQMKGHASTIEGEVKKMIADWGDEGEIELLDFFAELTI
YTSTACLIGLKFREQLDHRFAEYYHDLERGTDPLCYVDPYLPIESFKRRDEARVKLVALVQEIMDQRLANPPK
DKADRDMLDVLVSIKDEDGKPRFSADEITGMFISLMFAGHHTSSGTSAWTLIELIRHPDVYAEVLAELEELYA
DGQEVSFHALRSIPKLDNVVKETLRLHPPLIILMRVAKGEFEVEGFPIHEGDYVAASPAISNRIPEDFPDPDA
FKPDRYNKPEQADIVNRWTWIPFGAGRHRCVGAAFAQMQIKAIFSVLLREYDFEMAQPADSYRNDHSKMVVQL
ARPAKVRYRKRNA
>CYP51B1_Mycobacterium avium 104 639734091
MTTSTVVPRVSGGEEEHGHLEEFRTDPIGLMQRVRDECGDVGWFQLVDKHVILLSGAQANEFFFRSADEDLDQ
AEAYPFMTPIFGKGVVFDASPERRKEMLHNSALRGEQMKGHASTIEGEVKKMIADWGDEGEIELLDFFAELTI
YTSTACLIGLKFREQLDHRFAEYYHDLERGTDPLCYVDPYLPIESFKRRDEARVKLVALVQEIMDQRLANPPK
DKADRDMLDVLVSIKDEDGKPRFSADEITGMFISLMFAGHHTSSGTSAWTLIELIRHPDVYAEVLAELEELYA
DGQEVSFHALRSIPKLDNVVKETLRLHPPLIILMRVAKGEFEVEGFPIHEGDYVAASPAISNRIPEDFPDPDA
FKPDRYNKPEQADIVNRWTWIPFGAGRHRCVGAAFAQMQIKAIFSVLLREYDFEMAQPADSYRNDHSKMVVQL
ARPAKVRYRKRNA
>CYP51B1_Mycobacterium avium 10-5560 2576191731
MTTSTVVPRVSGGEEEHGHLEEFRTDPIGLMQRVRDECGDVGWFQLVDKHVILLSGAQANEFFFRSADEDLDQ
AEAYPFMTPIFGKGVVFDASPERRKEMLHNSALRGEQMKGHASTIEGEVKKMIADWGDEGEIELLDFFAELTI
YTSTACLIGLKFREQLDHRFAEYYHDLERGTDPLCYVDPYLPIESFKRRDEARVKLVALVQEIMDQRLANPPK
DKADRDMLDVLVSIKDEDGKPRFSADEITGMFISLMFAGHHTSSGTSAWTLIELIRHPDVYAEVLAELEELYA
DGQEVSFHALRSIPKLDNVVKETLRLHPPLIILMRVAKGEFEVEGFPIHEGDYVAASPAISNRIPEDFPDPDA
FKPDRYNKPEQADIVNRWTWIPFGAGRHRCVGAAFAQMQIKAIFSVLLREYDFEMAQPADSYRNDHSKMVVQL
ARPAKVRYRKRNA
>CYP51B1_Mycobacterium avium 10-5581 2572768830
MTTSTVVPRVSGGEEEHGHLEEFRTDPIGLMQRVRDECGDVGWFQLVDKHVILLSGAQANEFFFRSADEDLDQ
AEAYPFMTPIFGKGVVFDASPERRKEMLHNSALRGEQMKGHASTIEGEVKKMIADWGDEGEIELLDFFAELTI
YTSTACLIGLKFREQLDHRFAEYYHDLERGTDPLCYVDPYLPIESFKRRDEARVKLVALVQEIMDQRLANPPK
DKADRDMLDVLVSIKDEDGKPRFSADEITGMFISLMFAGHHTSSGTSAWTLIELIRHPDVYAEVLAELEELYA

DGQEVSFHALRSIPKLDNVVKETLRLHPPLIILMRVAKGEFEVEGFPIHEGDYVAASPAISNRIPEDFPDPDA
FKPDRYNKPEQADIVNRWTWIPFGAGRHRCVGAAFAQMQIKAIFSVLLREYDFEMAQPADSYRNDHSKMVVQL
ARPAKVRYRKRNA
>CYP51B1_Mycobacterium avium 11-0986 2583773091
MTTSTVVPRVSGGEEEHGHLEEFRTDPIGLMQRVRDECGDVGWFQLVDKHVILLSGAQANEFFFRSADEDLDQ
AEAYPFMTPIFGKGVVFDASPERRKEMLHNSALRGEQMKGHASTIEGEVKKMIADWGDEGEIELLDFFAELTI
YTSTACLIGLKFREQLDHRFAEYYHDLERGTDPLCYVDPYLPIESFKRRDEARVKLVALVQEIMDQRLANPPK
DKADRDMLDVLVSIKDEDGKPRFSADEITGMFISLMFAGHHTSSGTSAWTLIELIRHPDVYAEVLAELEELYA
DGQEVSFHALRSIPKLDNVVKETLRLHPPLIILMRVAKGEFEVEGFPIHEGDYVAASPAISNRIPEDFPDPDA
FKPDRYNKPEQADIVNRWTWIPFGAGRHRCVGAAFAQMQIKAIFSVLLREYDFEMAQPADSYRNDHSKMVVQL
ARPAKVRYRKRNA
>CYP51B1_Mycobacterium avium 2285 (R) 2629821987
MTTSTVVPRVSGGEEEHGHLEEFRTDPIGLMQRVRDECGDVGWFQLVDKHVILLSGAQANEFFFRSADEDLDQ
AEAYPFMTPIFGKGVVFDASPERRKEMLHNSALRGEQMKGHASTIEGEVKKMIADWGDEGEIELLDFFAELTI
YTSTACLIGLKFREQLDHRFAEYYHDLERGTDPLCYVDPYLPIESFKRRDEARVKLVALVQEIMDQRLANPPK
DKADRDMLDVLVSIKDEDGKPRFSADEITGMFISLMFAGHHTSSGTSAWTLIELIRHPDVYAEVLAELEELYA
DGQEVSFHALRSIPKLDNVVKETLRLHPPLIILMRVAKGEFEVEGFPIHEGDYVAASPAISNRIPEDFPDPDA
FKPDRYNKPEQADIVNRWTWIPFGAGRHRCVGAAFAQMQIKAIFSVLLREYDFEMAQPADSYRNDHSKMVVQL
ARPAKVRYRKRNA
>CYP51B1_Mycobacterium avium 2285 (S) 2647943184
MTTSTVVPRVSGGEEEHGHLEEFRTDPIGLMQRVRDECGDVGWFQLVDKHVILLSGAQANEFFFRSADEDLDQ
AEAYPFMTPIFGKGVVFDASPERRKEMLHNSALRGEQMKGHASTIEGEVKKMIADWGDEGEIELLDFFAELTI
YTSTACLIGLKFREQLDHRFAEYYHDLERGTDPLCYVDPYLPIESFKRRDEARVKLVALVQEIMDQRLANPPK
DKADRDMLDVLVSIKDEDGKPRFSADEITGMFISLMFAGHHTSSGTSAWTLIELIRHPDVYAEVLAELEELYA
DGQEVSFHALRSIPKLDNVVKETLRLHPPLIILMRVAKGEFEVEGFPIHEGDYVAASPAISNRIPEDFPDPDA
FKPDRYNKPEQADIVNRWTWIPFGAGRHRCVGAAFAQMQIKAIFSVLLREYDFEMAQPADSYRNDHSKMVVQL
ARPAKVRYRKRNA
>CYP51B1_Mycobacterium avium avium 10-9275 2577506326
MTTSTVVPRVSGGEEEHGHLEEFRTDPIGLMQRVRDECGDVGWFQLVDKHVILLSGAQANEFFFRSADEDLDQ
AEAYPFMTPIFGKGVVFDASPERRKEMLHNSALRGEQMKGHASTIEGEVKKMIADWGDEGEIELLDFFAELTI
YTSTACLIGLKFREQLDHRFAEYYHDLERGTDPLCYVDPYLPIESFKRRDEARVKLVALVQEIMDQRLANPPK
DKADRDMLDVLVSIKDEDGKPRFSADEITGMFISLMFAGHHTSSGTSAWTLIELIRHPDVYAEVLAELEELYA
DGQEVSFHALRSIPKLDNVVKETLRLHPPLIILMRVAKGEFEVEGFPIHEGDYVAASPAISNRIPEDFPDPDA
FKPDRYNKPEQADIVNRWTWIPFGAGRHRCVGAAFAQMQIKAIFSVLLREYDFEMAQPADSYRNDHSKMVVQL
ARPAKVRYRKRNA
>CYP51B1_Mycobacterium avium paratuberculosis 11-1786 2592475783
PYLPIESFKRRDEARVKLVALVQEIMDQRLANPPKDKADRDMLDVLVSIKDEDGKPRFSADEITGMFISLMFA
GHHTSSGTSAWTLIELIRHPDVYAEVLAELEELYADGQEVSFHALRSIPKLDNVVKETLRLHPPLIILMRVAK
GEFEVEGFPIHEGDYVAASPAISNRIPEDFPDPDAFKPDRYNKPEQADIVNRWTWIPFGAGRHRCVGAAFAQM
QIKAIFSVLLREYDFEMAQPADSYRNDHSKMVVQLARPAKVRYRKRNA
>CYP51B1_Mycobacterium avium hominissuis MAH 27-1 2581110015
MTTSTVVPRVSGGEEEHGHLEEFRTDPIGLMQRVRDECGDVGWFQLVDKHVILLSGAQANEFFFRSADEDLDQ
AEAYPFMTPIFGKGVVFDASPERRKEMLHNSALRGEQMKGHASTIEGEVKKMIADWGDEGEIELLDFFAELTI
YTSTACLIGLKFREQLDHRFAEYYHDLERGTDPLCYVDPYLPIESFKRRDEARVKLVALVQEIMDQRLANPPK
DKADRDMLDVLVSIKDEDGKPRFSADEITGMFISLMFAGHHTSSGTSAWTLIELIRHPDVYAEVLAELEELYA
DGQEVSFHALRSIPKLDNVVKETLRLHPPLIILMRVAKGEFEVEGFPIHEGDYVAASPAISNRIPEDFPDPDA
FKPDRYNKPEQADIVNRWTWIPFGAGRHRCVGAAFAQMQIKAIFSVLLREYDFEMAQPADSYRNDHSKMVVQL
ARPAKVRYRKRNA
>CYP51B1_Mycobacterium avium hominissuis MAH-P-9060-06 2725096702
MTTSTVVPRVSGGEEEHGHLEEFRTDPIGLMQRVRDECGDVGWFQLVDKHVILLSGAQANEFFFRSADEDLDQ
AEAYPFMTPIFGKGVVFDASPERRKEMLHNSALRGEQMKGHASTIEGEVKKMIADWGDEGEIELLDFFAELTI
YTSTACLIGLKFREQLDHRFAEYYHDLERGTDPLCYVDPYLPIESFKRRDEARVKLVALVQEIMDQRLANPPK
DKADRDMLDVLVSIKDEDGKPRFSADEITGMFISLMFAGHHTSSGTSAWTLIELIRHPDVYAEVLAELEELYA
DGQEVSFHALRSIPKLDNVVKETLRLHPPLIILMRVAKGEFEVEGFPIHEGDYVAASPAISNRIPEDFPDPDA
FKPDRYNKPEQADIVNRWTWIPFGAGRHRCVGAAFAQMQIKAIFSVLLREYDFEMAQPADSYRNDHSKMVVQL
ARPAKVRYRKRNA
>CYP51B1_Mycobacterium avium hominissuis MAH-P-8933-06 2725099777

MTTSTVVPRVSGGEEEHGHLEEFRTDPIGLMQRVRDECGDVGWFQLVDKHVILLSGAQANEFFFRSADEDLDQ
AEAYPFMTPIFGKGVVFDASPERRKEMLHNSALRGEQMKGHASTIEGEVKKMIADWGDEGEIELLDFFAELTI
YTSTACLIGLKFREQLDHRFAEYYHDLERGTDPLCYVDPYLPIESFKRRDEARVKLVALVQEIMDQRLANPPK
DKADRDMLDVLVSIKDEDGKPRFSADEITGMFISLMFAGHHTSSGTSAWTLIELIRHPDVYAEVLAELEELYA
DGQEVSFHALRSIPKLDNVVKETLRLHPPLIILMRVAKGEFEVEGFPIHEGDYVAASPAISNRIPEDFPDPDA
FKPDRYNKPEQADIVNRWTWIPFGAGRHRCVGAAFAQMQIKAIFSVLLREYDFEMAQPADSYRNDHSKMVVQL
ARPAKVRYRKRNA
>CYP51B1_Mycobacterium avium hominissuis MAH-P-772-08 2725125655
MTTSTVVPRVSGGEEEHGHLEEFRTDPIGLMQRVRDECGDVGWFQLVDKHVILLSGAQANEFFFRSADEDLDQ
AEAYPFMTPIFGKGVVFDASPERRKEMLHNSALRGEQMKGHASTIEGEVKKMIADWGDEGEIELLDFFAELTI
YTSTACLIGLKFREQLDHRFAEYYHDLERGTDPLCYVDPYLPIESFKRRDEARVKLVALVQEIMDQRLANPPK
DKADRDMLDVLVSIKDEDGKPRFSADEITGMFISLMFAGHHTSSGTSAWTLIELIRHPDVYAEVLAELEELYA
DGQEVSFHALRSIPKLDNVVKETLRLHPPLIILMRVAKGEFEVEGFPIHEGDYVAASPAISNRIPEDFPDPDA
FKPDRYNKPEQADIVNRWTWIPFGAGRHRCVGAAFAQMQIKAIFSVLLREYDFEMAQPADSYRNDHSKMVVQL
ARPAKVRYRKRNA
>CYP51B1_Mycobacterium avium hominissuis MAH-P-7673-04 2725152086
MTTSTVVPRVSGGEEEHGHLEEFRTDPIGLMQRVRDECGDVGWFQLVDKHVILLSGAQANEFFFRSADEDLDQ
AEAYPFMTPIFGKGVVFDASPERRKEMLHNSALRGEQMKGHASTIEGEVKKMIADWGDEGEIELLDFFAELTI
YTSTACLIGLKFREQLDHRFAEYYHDLERGTDPLCYVDPYLPIESFKRRDEARVKLVALVQEIMDQRLANPPK
DKADRDMLDVLVSIKDEDGKPRFSADEITGMFISLMFAGHHTSSGTSAWTLIELIRHPDVYAEVLAELEELYA
DGQEVSFHALRSIPKLDNVVKETLRLHPPLIILMRVAKGEFEVEGFPIHEGDYVAASPAISNRIPEDFPDPDA
FKPDRYNKPEQADIVNRWTWIPFGAGRHRCVGAAFAQMQIKAIFSVLLREYDFEMAQPADSYRNDHSKMVVQL
ARPAKVRYRKRNA
>CYP51B1_Mycobacterium avium hominissuis MAH-P-709-08 2725122232
MTTSTVVPRVSGGEEEHGHLEEFRTDPIGLMQRVRDECGDVGWFQLVDKHVILLSGAQANEFFFRSADEDLDQ
AEAYPFMTPIFGKGVVFDASPERRKEMLHNSALRGEQMKGHASTIEGEVKKMIADWGDEGEIELLDFFAELTI
YTSTACLIGLKFREQLDHRFAEYYHDLERGTDPLCYVDPYLPIESFKRRDEARVKLVALVQEIMDQRLANPPK
DKADRDMLDVLVSIKDEDGKPRFSADEITGMFISLMFAGHHTSSGTSAWTLIELIRHPDVYAEVLAELEELYA
DGQEVSFHALRSIPKLDNVVKETLRLHPPLIILMRVAKGEFEVEGFPIHEGDYVAASPAISNRIPEDFPDPDA
FKPDRYNKPEQADIVNRWTWIPFGAGRHRCVGAAFAQMQIKAIFSVLLREYDFEMAQPADSYRNDHSKMVVQL
ARPAKVRYRKRNA
>CYP51B1_Mycobacterium avium hominissuis MAH-P-589-08 2685182095
MTTSTVVPRVSGGEEEHGHLEEFRTDPIGLMQRVRDECGDVGWFQLVDKHVILLSGAQANEFFFRSADEDLDQ
AEAYPFMTPIFGKGVVFDASPERRKEMLHNSALRGEQMKGHASTIEGEVKKMIADWGDEGEIELLDFFAELTI
YTSTACLIGLKFREQLDHRFAEYYHDLERGTDPLCYVDPYLPIESFKRRDEARVKLVALVQEIMDQRLANPPK
DKADRDMLDVLVSIKDEDGKPRFSADEITGMFISLMFAGHHTSSGTSAWTLIELIRHPDVYAEVLAELEELYA
DGQEVSFHALRSIPKLDNVVKETLRLHPPLIILMRVAKGEFEVEGFPIHEGDYVAASPAISNRIPEDFPDPDA
FKPDRYNKPEQADIVNRWTWIPFGAGRHRCVGAAFAQMQIKAIFSVLLREYDFEMAQPADSYRNDHSKMVVQL
ARPAKVRYRKRNA
>CYP51B1_Mycobacterium avium hominissuis MAH-P-4023-08 2725143770
MTTSTVVPRVSGGEEEHGHLEEFRTDPIGLMQRVRDECGDVGWFQLVDKHVILLSGAQANEFFFRSADEDLDQ
AEAYPFMTPIFGKGVVFDASPERRKEMLHNSALRGEQMKGHASTIEGEVKKMIADWGDEGEIELLDFFAELTI
YTSTACLIGLKFREQLDHRFAEYYHDLERGTDPLCYVDPYLPIESFKRRDEARVKLVALVQEIMDQRLANPPK
DKADRDMLDVLVSIKDEDGKPRFSADEITGMFISLMFAGHHTSSGTSAWTLIELIRHPDVYAEVLAELEELYA
DGQEVSFHALRSIPKLDNVVKETLRLHPPLIILMRVAKGEFEVEGFPIHEGDYVAASPAISNRIPEDFPDPDA
FKPDRYNKPEQADIVNRWTWIPFGAGRHRCVGAAFAQMQIKAIFSVLLREYDFEMAQPADSYRNDHSKMVVQL
ARPAKVRYRKRNA
>CYP51B1_Mycobacterium avium hominissuis MAH-P-2014-08 2725130611
MTTSTVVPRVSGGEEEHGHLEEFRTDPIGLMQRVRDECGDVGWFQLVDKHVILLSGAQANEFFFRSADEDLDQ
AEAYPFMTPIFGKGVVFDASPERRKEMLHNSALRGEQMKGHASTIEGEVKKMIADWGDEGEIELLDFFAELTI
YTSTACLIGLKFREQLDHRFAEYYHDLERGTDPLCYVDPYLPIESFKRRDEARVKLVALVQEIMDQRLANPPK
DKADRDMLDVLVSIKDEDGKPRFSADEITGMFISLMFAGHHTSSGTSAWTLIELIRHPDVYAEVLAELEELYA
DGQEVSFHALRSIPKLDNVVKETLRLHPPLIILMRVAKGEFEVEGFPIHEGDYVAASPAISNRIPEDFPDPDA
FKPDRYNKPEQADIVNRWTWIPFGAGRHRCVGAAFAQMQIKAIFSVLLREYDFEMAQPADSYRNDHSKMVVQL
ARPAKVRYRKRNA
>CYP51B1_Mycobacterium avium hominissuis MAH-P-11082-03 2725057398
MTTSTVVPRVSGGEEEHGHLEEFRTDPIGLMQRVRDECGDVGWFQLVDKHVILLSGAQANEFFFRSADEDLDQ
AEAYPFMTPIFGKGVVFDASPERRKEMLHNSALRGEQMKGHASTIEGEVKKMIADWGDEGEIELLDFFAELTI

YTSTACLIGLKFREQLDHRFAEYYHDLERGTDPLCYVDPYLPIESFKRRDEARVKLVALVQEIMDQRLANPPK
DKADRDMLDVLVSIKDEDGKPRFSADEITGMFISLMFAGHHTSSGTSAWTLIELIRHPDVYAEVLAELEELYA
DGQEVSFHALRSIPKLDNVVKETLRLHPPLIILMRVAKGEFEVEGFPIHEGDYVAASPAISNRIPEDFPDPDA
FKPDRYNKPEQADIVNRWTWIPFGAGRHRCVGAAFAQMQIKAIFSVLLREYDFEMAQPADSYRNDHSKMVVQL
ARPAKVRYRKRNA
>CYP51B1_Mycobacterium avium hominissuis MAHP-10058-06 2678802717
MTTSTVVPRVSGGEEEHGHLEEFRTDPIGLMQRVRDECGDVGWFQLVDKHVILLSGAQANEFFFRSADEDLDQ
AEAYPFMTPIFGKGVVFDASPERRKEMLHNSALRGEQMKGHASTIEGEVKKMIADWGDEGEIELLDFFAELTI
YTSTACLIGLKFREQLDHRFAEYYHDLERGTDPLCYVDPYLPIESFKRRDEARVKLVALVQEIMDQRLANPPK
DKADRDMLDVLVSIKDEDGKPRFSADEITGMFISLMFAGHHTSSGTSAWTLIELIRHPDVYAEVLAELEELYA
DGQEVSFHALRSIPKLDNVVKETLRLHPPLIILMRVAKGEFEVEGFPIHEGDYVAASPAISNRIPEDFPDPDA
FKPDRYNKPEQADIVNRWTWIPFGAGRHRCVGAAFAQMQIKAIFSVLLREYDFEMAQPADSYRNDHSKMVVQL
ARPAKVRYRKRNA
>CYP51B1_Mycobacterium avium hominissuis MAH-P-0913 2725078126
MTTSTVVPRVSGGEEEHGHLEEFRTDPIGLMQRVRDECGDVGWFQLVDKHVILLSGAQANEFFFRSADEDLDQ
AEAYPFMTPIFGKGVVFDASPERRKEMLHNSALRGEQMKGHASTIEGEVKKMIADWGDEGEIELLDFFAELTI
YTSTACLIGLKFREQLDHRFAEYYHDLERGTDPLCYVDPYLPIESFKRRDEARVKLVALVQEIMDQRLANPPK
DKADRDMLDVLVSIKDEDGKPRFSADEITGMFISLMFAGHHTSSGTSAWTLIELIRHPDVYAEVLAELEELYA
DGQEVSFHALRSIPKLDNVVKETLRLHPPLIILMRVAKGEFEVEGFPIHEGDYVAASPAISNRIPEDFPDPDA
FKPDRYNKPEQADIVNRWTWIPFGAGRHRCVGAAFAQMQIKAIFSVLLREYDFEMAQPADSYRNDHSKMVVQL
ARPAKVRYRKRNA
>CYP51B1_Mycobacterium avium hominissuis MAHP-04-13 2725073750
MTTSTVVPRVSGGEEEHGHLEEFRTDPIGLMQRVRDECGDVGWFQLVDKHVILLSGAQANEFFFRSADEDLDQ
AEAYPFMTPIFGKGVVFDASPERRKEMLHNSALRGEQMKGHASTIEGEVKKMIADWGDEGEIELLDFFAELTI
YTSTACLIGLKFREQLDHRFAEYYHDLERGTDPLCYVDPYLPIESFKRRDEARVKLVALVQEIMDQRLANPPK
DKADRDMLDVLVSIKDEDGKPRFSADEITGMFISLMFAGHHTSSGTSAWTLIELIRHPDVYAEVLAELEELYA
DGQEVSFHALRSIPKLDNVVKETLRLHPPLIILMRVAKGEFEVEGFPIHEGDYVAASPAISNRIPEDFPDPDA
FKPDRYNKPEQADIVNRWTWIPFGAGRHRCVGAAFAQMQIKAIFSVLLREYDFEMAQPADSYRNDHSKMVVQL
ARPAKVRYRKRNA
>CYP51B1_Mycobacterium avium hominissuis MAH-E-89-1 2724956969
MTTSTVVPRVSGGEEEHGHLEEFRTDPIGLMQRVRDECGDVGWFQLVDKHVILLSGAQANEFFFRSADEDLDQ
AEAYPFMTPIFGKGVVFDASPERRKEMLHNSALRGEQMKGHASTIEGEVKKMIADWGDEGEIELLDFFAELTI
YTSTACLIGLKFREQLDHRFAEYYHDLERGTDPLCYVDPYLPIESFKRRDEARVKLVALVQEIMDQRLANPPK
DKADRDMLDVLVSIKDEDGKPRFSADEITGMFISLMFAGHHTSSGTSAWTLIELIRHPDVYAEVLAELEELYA
DGQEVSFHALRSIPKLDNVVKETLRLHPPLIILMRVAKGEFEVEGFPIHEGDYVAASPAISNRIPEDFPDPDA
FKPDRYNKPEQADIVNRWTWIPFGAGRHRCVGAAFAQMQIKAIFSVLLREYDFEMAQPADSYRNDHSKMVVQL
ARPAKVRYRKRNA
>CYP51B1_Mycobacterium avium hominissuis MAH-E-82-7 2725028685
MTTSTVVPRVSGGEEEHGHLEEFRTDPIGLMQRVRDECGDVGWFQLVDKHVILLSGAQANEFFFRSADEDLDQ
AEAYPFMTPIFGKGVVFDASPERRKEMLHNSALRGEQMKGHASTIEGEVKKMIADWGDEGEIELLDFFAELTI
YTSTACLIGLKFREQLDHRFAEYYHDLERGTDPLCYVDPYLPIESFKRRDEARVKLVALVQEIMDQRLANPPK
DKADRDMLDVLVSIKDEDGKPRFSADEITGMFISLMFAGHHTSSGTSAWTLIELIRHPDVYAEVLAELEELYA
DGQEVSFHALRSIPKLDNVVKETLRLHPPLIILMRVAKGEFEVEGFPIHEGDYVAASPAISNRIPEDFPDPDA
FKPDRYNKPEQADIVNRWTWIPFGAGRHRCVGAAFAQMQIKAIFSVLLREYDFEMAQPADSYRNDHSKMVVQL
ARPAKVRYRKRNA
>CYP51B1_Mycobacterium avium hominissuis MAH-E-83-1 2725033868
MTTSTVVPRVSGGEEEHGHLEEFRTDPIGLMQRVRDECGDVGWFQLVDKHVILLSGAQANEFFFRSADEDLDQ
AEAYPFMTPIFGKGVVFDASPERRKEMLHNSALRGEQMKGHASTIEGEVKKMIADWGDEGEIELLDFFAELTI
YTSTACLIGLKFREQLDHRFAEYYHDLERGTDPLCYVDPYLPIESFKRRDEARVKLVALVQEIMDQRLANPPK
DKADRDMLDVLVSIKDEDGKPRFSADEITGMFISLMFAGHHTSSGTSAWTLIELIRHPDVYAEVLAELEELYA
DGQEVSFHALRSIPKLDNVVKETLRLHPPLIILMRVAKGEFEVEGFPIHEGDYVAASPAISNRIPEDFPDPDA
FKPDRYNKPEQADIVNRWTWIPFGAGRHRCVGAAFAQMQIKAIFSVLLREYDFEMAQPADSYRNDHSKMVVQL
ARPAKVRYRKRNA
>CYP51B1_Mycobacterium avium hominissuis MAH-E-63-1 2724972537
MTTSTVVPRVSGGEEEHGHLEEFRTDPIGLMQRVRDECGDVGWFQLVDKHVILLSGAQANEFFFRSADEDLDQ
AEAYPFMTPIFGKGVVFDASPERRKEMLHNSALRGEQMKGHASTIEGEVKKMIADWGDEGEIELLDFFAELTI
YTSTACLIGLKFREQLDHRFAEYYHDLERGTDPLCYVDPYLPIESFKRRDEARVKLVALVQEIMDQRLANPPK
DKADRDMLDVLVSIKDEDGKPRFSADEITGMFISLMFAGHHTSSGTSAWTLIELIRHPDVYAEVLAELEELYA

DGQEVSFHALRSIPKLDNVVKETLRLHPPLIILMRVAKGEFEVEGFPIHEGDYVAASPAISNRIPEDFPDPDA
FKPDRYNKPEQADIVNRWTWIPFGAGRHRCVGAAFAQMQIKAIFSVLLREYDFEMAQPADSYRNDHSKMVVQL
ARPAKVRYRKRNA
>CYP51B1_Mycobacterium avium hominissuis MAH-E-61-1y 2725042418
MTTSTVVPRVSGGEEEHGHLEEFRTDPIGLMQRVRDECGDVGWFQLVDKHVILLSGAQANEFFFRSADEDLDQ
AEAYPFMTPIFGKGVVFDASPERRKEMLHNSALRGEQMKGHASTIEGEVKKMIADWGDEGEIELLDFFAELTI
YTSTACLIGLKFREQLDHRFAEYYHDLERGTDPLCYVDPYLPIESFKRRDEARVKLVALVQEIMDQRLANPPK
DKADRDMLDVLVSIKDEDGKPRFSADEITGMFISLMFAGHHTSSGTSAWTLIELIRHPDVYAEVLAELEELYA
DGQEVSFHALRSIPKLDNVVKETLRLHPPLIILMRVAKGEFEVEGFPIHEGDYVAASPAISNRIPEDFPDPDA
FKPDRYNKPEQADIVNRWTWIPFGAGRHRCVGAAFAQMQIKAIFSVLLREYDFEMAQPADSYRNDHSKMVVQL
ARPAKVRYRKRNA
>CYP51B1_Mycobacterium avium hominissuis MAH-E-61-1t 2725038922
MTTSTVVPRVSGGEEEHGHLEEFRTDPIGLMQRVRDECGDVGWFQLVDKHVILLSGAQANEFFFRSADEDLDQ
AEAYPFMTPIFGKGVVFDASPERRKEMLHNSALRGEQMKGHASTIEGEVKKMIADWGDEGEIELLDFFAELTI
YTSTACLIGLKFREQLDHRFAEYYHDLERGTDPLCYVDPYLPIESFKRRDEARVKLVALVQEIMDQRLANPPK
DKADRDMLDVLVSIKDEDGKPRFSADEITGMFISLMFAGHHTSSGTSAWTLIELIRHPDVYAEVLAELEELYA
DGQEVSFHALRSIPKLDNVVKETLRLHPPLIILMRVAKGEFEVEGFPIHEGDYVAASPAISNRIPEDFPDPDA
FKPDRYNKPEQADIVNRWTWIPFGAGRHRCVGAAFAQMQIKAIFSVLLREYDFEMAQPADSYRNDHSKMVVQL
ARPAKVRYRKRNA
>CYP51B1_Mycobacterium avium hominissuis MAH-E-57-3 2724964294
MTTSTVVPRVSGGEEEHGHLEEFRTDPIGLMQRVRDECGDVGWFQLVDKHVILLSGAQANEFFFRSADEDLDQ
AEAYPFMTPIFGKGVVFDASPERRKEMLHNSALRGEQMKGHASTIEGEVKKMIADWGDEGEIELLDFFAELTI
YTSTACLIGLKFREQLDHRFAEYYHDLERGTDPLCYVDPYLPIESFKRRDEARVKLVALVQEIMDQRLANPPK
DKADRDMLDVLVSIKDEDGKPRFSADEITGMFISLMFAGHHTSSGTSAWTLIELIRHPDVYAEVLAELEELYA
DGQEVSFHALRSIPKLDNVVKETLRLHPPLIILMRVAKGEFEVEGFPIHEGDYVAASPAISNRIPEDFPDPDA
FKPDRYNKPEQADIVNRWTWIPFGAGRHRCVGAAFAQMQIKAIFSVLLREYDFEMAQPADSYRNDHSKMVVQL
ARPAKVRYRKRNA
>CYP51B1_Mycobacterium avium hominissuis MAHE-22-1 2724961801
MTTSTVVPRVSGGEEEHGHLEEFRTDPIGLMQRVRDECGDVGWFQLVDKHVILLSGAQANEFFFRSADEDLDQ
AEAYPFMTPIFGKGVVFDASPERRKEMLHNSALRGEQMKGHASTIEGEVKKMIADWGDEGEIELLDFFAELTI
YTSTACLIGLKFREQLDHRFAEYYHDLERGTDPLCYVDPYLPIESFKRRDEARVKLVALVQEIMDQRLANPPK
DKADRDMLDVLVSIKDEDGKPRFSADEITGMFISLMFAGHHTSSGTSAWTLIELIRHPDVYAEVLAELEELYA
DGQEVSFHALRSIPKLDNVVKETLRLHPPLIILMRVAKGEFEVEGFPIHEGDYVAASPAISNRIPEDFPDPDA
FKPDRYNKPEQADIVNRWTWIPFGAGRHRCVGAAFAQMQIKAIFSVLLREYDFEMAQPADSYRNDHSKMVVQL
ARPAKVRYRKRNA
>CYP51B1_Mycobacterium avium hominissuis MAH-E-104-1 2724985027
MTTSTVVPRVSGGEEEHGHLEEFRTDPIGLMQRVRDECGDVGWFQLVDKHVILLSGAQANEFFFRSADEDLDQ
AEAYPFMTPIFGKGVVFDASPERRKEMLHNSALRGEQMKGHASTIEGEVKKMIADWGDEGEIELLDFFAELTI
YTSTACLIGLKFREQLDHRFAEYYHDLERGTDPLCYVDPYLPIESFKRRDEARVKLVALVQEIMDQRLANPPK
DKADRDMLDVLVSIKDEDGKPRFSADEITGMFISLMFAGHHTSSGTSAWTLIELIRHPDVYAEVLAELEELYA
DGQEVSFHALRSIPKLDNVVKETLRLHPPLIILMRVAKGEFEVEGFPIHEGDYVAASPAISNRIPEDFPDPDA
FKPDRYNKPEQADIVNRWTWIPFGAGRHRCVGAAFAQMQIKAIFSVLLREYDFEMAQPADSYRNDHSKMVVQL
ARPAKVRYRKRNA
>CYP51B1_Mycobacterium avium avium A5 2580781807
MTTSTVVPRVSGGEEEHGHLEEFRTDPIGLMQRVRDECGDVGWFQLVDKHVILLSGAQANEFFFRSADEDLDQ
AEAYPFMTPIFGKGVVFDASPERRKEMLHNSALRGEQMKGHASTIEGEVKKMIADWGDEGEIELLDFFAELTI
YTSTACLIGLKFREQLDHRFAEYYHDLERGTDPLCYVDPYLPIESFKRRDEARVKLVALVQEIMDQRLANPPK
DKADRDMLDVLVSIKDEDGKPRFSADEITGMFISLMFAGHHTSSGTSAWTLIELIRHPDVYAEVLAELEELYA
DGQEVSFHALRSIPKLDNVVKETLRLHPPLIILMRVAKGEFEVEGFPIHEGDYVAASPAISNRIPEDFPDPDA
FKPDRYNKPEQADIVNRWTWIPFGAGRHRCVGAAFAQMQIKAIFSVLLREYDFEMAQPADSYRNDHSKMVVQL
ARPAKVRYRKRNA
>CYP51B1_Mycobacterium avium hominissuis 10-4249 2580526358
MTTSTVVPRVSGGEEEHGHLEEFRTDPIGLMQRVRDECGDVGWFQLVDKHVILLSGAQANEFFFRSADEDLDQ
AEAYPFMTPIFGKGVVFDASPERRKEMLHNSALRGEQMKGHASTIEGEVKKMIADWGDEGEIELLDFFAELTI
YTSTACLIGLKFREQLDHRFAEYYHDLERGTDPLCYVDPYLPIESFKRRDEARVKLVALVQEIMDQRLANPPK
DKADRDMLDVLVSIKDEDGKPRFSADEITGMFISLMFAGHHTSSGTSAWTLIELIRHPDVYAEVLAELEELYA
DGQEVSFHALRSIPKLDNVVKETLRLHPPLIILMRVAKGEFEVEGFPIHEGDYVAASPAISNRIPEDFPDPDA

FKPDRYNKPEQADIVNRWTWIPFGAGRHRCVGAAFAQMQIKAIFSVLLREYDFEMAQPADSYRNDHSKMVVQL
ARPAKVRYRKRNA
>CYP51B1_Mycobacterium avium 101 2580004075
MTTSTVVPRVSGGEEEHGHLEEFRTDPIGLMQRVRDECGDVGWFQLVDKHVILLSGAQANEFFFRSADEDLDQ
AEAYPFMTPIFGKGVVFDASPERRKEMLHNSALRGEQMKGHASTIEGEVKKMIADWGDEGEIELLDFFAELTI
YTSTACLIGLKFREQLDHRFAEYYHDLERGTDPLCYVDPYLPIESFKRRDEARVKLVALVQEIMDQRLANPPK
DKADRDMLDVLVSIKDEDGKPRFSADEITGMFISLMFAGHHTSSGTSAWTLIELIRHPDVYAEVLAELEELYA
DGQEVSFHALRSIPKLDNVVKETLRLHPPLIILMRVAKGEFEVEGFPIHEGDYVAASPAISNRIPEDFPDPDA
FKPDRYNKPEQADIVNRWTWIPFGAGRHRCVGAAFAQMQIKAIFSVLLREYDFEMAQPADSYRNDHSKMVVQL
ARPAKVRYRKRNA
>CYP51B1_Mycobacterium avium 100 2580972468
MTTSTVVPRVSGGEEEHGHLEEFRTDPIGLMQRVRDECGDVGWFQLVDKHVILLSGAQANEFFFRSADEDLDQ
AEAYPFMTPIFGKGVVFDASPERRKEMLHNSALRGEQMKGHASTIEGEVKKMIADWGDEGEIELLDFFAELTI
YTSTACLIGLKFREQLDHRFAEYYHDLERGTDPLCYVDPYLPIESFKRRDEARVKLVALVQEIMDQRLANPPK
DKADRDMLDVLVSIKDEDGKPRFSADEITGMFISLMFAGHHTSSGTSAWTLIELIRHPDVYAEVLAELEELYA
DGQEVSFHALRSIPKLDNVVKETLRLHPPLIILMRVAKGEFEVEGFPIHEGDYVAASPAISNRIPEDFPDPDA
FKPDRYNKPEQADIVNRWTWIPFGAGRHRCVGAAFAQMQIKAIFSVLLREYDFEMAQPADSYRNDHSKMVVQL
ARPAKVRYRKRNA
>CYP51B1_Mycobacterium avium avium DT 78 2549385113
MTTSTVVPRVSGGEEEHGHLEEFRTDPIGLMQRVRDECGDVGWFQLVDKHVILLSGAQANEFFFRSADEDLDQ
AEAYPFMTPIFGKGVVFDASPERRKEMLHNSALRGEQMKGHASTIEGEVKKMIADWGDEGEIELLDFFAELTI
YTSTACLIGLKFREQLDHRFAEYYHDLERGTDPLCYVDPYLPIESFKRRDEARVKLVALVQEIMDQRLANPPK
DKADRDMLDVLVSIKDEDGKPRFSADEITGMFISLMFAGHHTSSGTSAWTLIELIRHPDVYAEVLAELEELYA
DGQEVSFHALRSIPKLDNVVKETLRLHPPLIILMRVAKGEFEVEGFPIHEGDYVAASPAISNRIPEDFPDPDA
FKPDRYNKPEQADIVNRWTWIPFGAGRHRCVGAAFAQMQIKAIFSVLLREYDFEMAQPADSYRND
>CYP51B1_Mycobacterium avium avium DJO-44271 2628524828
MTTSTVVPRVSGGEEEHGHLEEFRTDPIGLMQRVRDECGDVGWFQLVDKHVILLSGAQANEFFFRSADEDLDQ
AEAYPFMTPIFGKGVVFDASPERRKEMLHNSALRGEQMKGHASTIEGEVKKMIADWGDEGEIELLDFFAELTI
YTSTACLIGLKFREQLDHRFAEYYHDLERGTDPLCYVDPYLPIESFKRRDEARVKLVALVQEIMDQRLANPPK
DKADRDMLDVLVSIKDEDGKPRFSADEITGMFISLMFAGHHTSSGTSAWTLIELIRHPDVYAEVLAELEELYA
DGQEVSFHALRSIPKLDNVVKETLRLHPPLIILMRVAKGEFEVEGFPIHEGDYVAASPAISNRIPEDFPDPDA
FKPDRYNKPEQADIVNRWTWIPFGAGRHRCVGAAFAQMQIKAIFSVLLREYDFEMAQPADSYRNDHSKMVVQL
ARPAKVRYRKRNA
>CYP51B1_Mycobacterium avium subsp. avium ATCC 25291 645423488
MTTSTVVPRVSGGEEEHGHLEEFRTDPIGLMQRVRDECGDVGWFQLVDKHVILLSGAQANEFFFRSADEDLDQ
AEAYPFMTPIFGKGVVFDASPERRKEMLHNSALRGEQMKGHASTIEGEVKKMIADWGDEGEIELLDFFAELTI
YTSTACLIGLKFREQLDHRFAEYYHDLERGTDPLCYVDPYLPIESFKRRDEARVKLVALVQEIMDQRLANPPK
DKADRDMLDVLVSIKDEDGKPRFSADEITGMFISLMFAGHHTSSGTSAWTLIELIRHPDVYAEVLAELEELYA
DGQEVSFHALRSIPKLDNVVKETLRLHPPLIILMRVAKGEFEVEGFPIHEGDYVAASPAISNRIPEDFPDPDA
FKPDRYNKPEQADIVNRWTWIPFGAGRHRCVGAAFAQMQIKAIFSVLLREYDFEMAQPADSYRNDHSKMVVQL
ARPAKVRYRKRNA
>CYP51B1_Mycobacterium avium 09-5983 2573431594
MTTSTVVPRVSGGEEQHGHLEEFRTDPIGLMQRVRDECGDVGWFQLVDKHVILLSGAQANEFFFRSADEDLDQ
AEAYPFMTPIFGKGVVFDASPERRKEMLHNSALRGEQMKGHASTIEGEVKKMIADWGDEGEIELLDFFAELTI
YTSTACLIGLKFREQLDHRFAEYYHDLERGTDPLCYVDPYLPIESFKRRDEARVKLVALVQEIMDQRLANPPK
DKADRDMLDVLVSIKDEDGKPRFSADEITGMFISLMFAGHHTSSGTSAWTLIELIRHPDVYAEVLAELEELYA
DGQEVSFHALRSIPKLDNVVKETLRLHPPLIILMRVAKGEFEVEGFPIHEGDYVAASPAISNRIPEDFPDPDA
FKPDRYNKPEQADIVNRWTWIPFGAGRHRCVGAAFAQMQIKAIFSVLLREYDFEMAQPADSYRNDHSKMVVQL
ARPAKVRYRKRNA
>CYP51B1_Mycobacterium avium 2285 (R) 2567081981
MVPRVSGGEEEHGHLEEFRTDPIGLMQRVRDECGDVGWFQLVDKHVILLSGAQANEFFFRSADEDLDQAEAYP
FMTPIFGKGVVFDASPERRKEMLHNSALRGEQMKGHASTIEGEVKKMIADWGDEGEIELLDFFAELTIYTSTA
CLIGLKFREQLDHRFAEYYHDLERGTDPLCYVDPYLPIESFKRRDEARVKLVALVQEIMDQRLANPPKDKADR
DMLDVLVSIKDEDGKPRFSADEITGMFISLMFAGHHTSSGTSAWTLIELIRHPDVYAEVLAELEELYADGQEV
SFHALRSIPKLDNVVKETLRLHPPLIILMRVAKGEFEVEGFPIHEGDYVAASPAISNRIPEDFPDPDAFKPDR
YNKPEQADIVNRWTWIPFGAGRHRCVGAAFAQMQIKAIFSVLLREYDFEMAQPADSYRNDHSKMVVQLARPAK
VRYRKRNA
>CYP51B1_Mycobacterium avium avium 3388 2581395783

MTTSTVVPRVSGGEEQHGHLEEFRTDPIGLMQRVRDECGDVGWFQLVDKHVILLSGAQANEFFFRSADEDLDQ
AEAYPFMTPIFGKGVVFDASPERRKEMLHNSALRGEQMKGHASTIEGEVKKMIADWGDEGEIELLDFFAELTI
YTSTACLIGLKFREQLDHRFAEYYHDLERGTDPLCYVDPYLPIESFKRRDEARVKLVALVQEIMDQRLANPPK
DKADRDMLDVLVSIKDEDGKPRFSADEITGMFISLMFAGHHTSSGTSAWTLIELIRHPDVYAEVLAELEELYA
DGQEVSFHALRSIPKLDNVVKETLRLHPPLIILMRVAKGEFEVEGFPIHEGDYVAASPAISNRIPEDFPDPDA
FKPDRYNKPEQADIVNRWTWIPFGAGRHRCVGAAFAQMQIKAIFSVLLREYDFEMAQPADSYRNDHSKMVVQL
ARPAKVRYRKRNA
>CYP51B1_Mycobacterium avium paratuberculosis 08-8281 2574999332
MTTSTVVPRISGGEEEHGHLEEFRTDPIGLMQRVRDECGDVGWFQLVDKHVILLSGAQANEFFFRSADEDLDQ
AEAYPFMTPIFGKGVVFDASPERRKEMLHNSALRGEQMKGHASTIEGEVKKMIADWGDEGEIELLDFFAELTI
YTSTACLIGLKFREQLDHRFAEYYHDLERGTDPLCYVDPYLPIESFKRRDEARVKLVALVQEIMDQRLANPPK
DKADRDMLDVLVSIKDEDGKPRFSADEITGMFISLMFAGHHTSSGTSAWTLIELIRHPDVYAEVLAELEELYA
DGQEVSFHALRSIPKLDNVVKETLRLHPPLIILMRVAKGEFEVEGFPIHEGDYVAASPAISNRIPEDFPDPDA
FKPDRYNKPEQADIVNRWTWIPFGAGRHRCVGAAFAQMQIKAIFSVLLREYDFEMAQPADSYRNDHSKMVVQL
ARPAKVRYRKRNA
>CYP51B1_Mycobacterium avium paratuberculosis S5 2543325905
MTTSTVVPRISGGEEEHGHLEEFRTDPIGLMQRVRDECGDVGWFQLVDKHVILLSGAQANEFFFRSADEDLDQ
AEAYPFMTPIFGKGVVFDASPERRKEMLHNSALRGEQMKGHASTIEGEVKKMIADWGDEGEIELLDFFAELTI
YTSTACLIGLKFREQLDHRFAEYYHDLERGTDPLCYVDPYLPIESFKRRDEARVKLVALVQEIMDQRLANPPK
DKADRDMLDVLVSIKDEDGKPRFSADEITGMFISLMFAGHHTSSGTSAWTLIELIRHPDVYAEVLAELEELYA
DGQEVSFHALRSIPKLDNVVKETLRLHPPLIILMRVAKGEFEVEGFPIHEGDYVAASPAISNRIPEDFPDPDA
FKPDRYNKPEQADIVNRWTWIPFGAGRHRCVGAAFAQMQIKAIFSVLLREYDFEMAQPADSYRNDHSKMVVQL
ARPAKVRYRKRNA
>CYP51B1_Mycobacterium avium paratuberculosis S397 2534967933
MTTSTVVPRISGGEEEHGHLEEFRTDPIGLMQRVRDECGDVGWFQLVDKHVILLSGAQANEFFFRSADEDLDQ
AEAYPFMTPIFGKGVVFDASPERRKEMLHNSALRGEQMKGHASTIEGEVKKMIADWGDEGEIELLDFFAELTI
YTSTACLIGLKFREQLDHRFAEYYHDLERGTDPLCYVDPYLPIESFKRRDEARVKLVALVQEIMDQRLANPPK
DKADRDMLDVLVSIKDEDGKPRFSADEITGMFISLMFAGHHTSSGTSAWTLIELIRHPDVYAEVLAELEELYA
DGQEVSFHALRSIPKLDNVVKETLRLHPPLIILMRVAKGEFEVEGFPIHEGDYVAASPAISNRIPEDFPDPDA
FKPDRYNKPEQADIVNRWTWIPFGAGRHRCVGAAFAQMQIKAIFSVLLREYDFEMAQPADSYRNDHSKMVVQL
ARPAKVRYRKRNA
>CYP51B1_Mycobacterium avium paratuberculosis Pt164 2548544957
MTTSTVVPRISGGEEEHGHLEEFRTDPIGLMQRVRDECGDVGWFQLVDKHVILLSGAQANEFFFRSADEDLDQ
AEAYPFMTPIFGKGVVFDASPERRKEMLHNSALRGEQMKGHASTIEGEVKKMIADWGDEGEIELLDFFAELTI
YTSTACLIGLKFREQLDHRFAEYYHDLERGTDPLCYVDPYLPIESFKRRDEARVKLVALVQEIMDQRLANPPK
DKADRDMLDVLVSIKDEDGKPRFSADEITGMFISLMFAGHHTSSGTSAWTLIELIRHPDVYAEVLAELEELYA
DGQEVSFHALRSIPKLDNVVKETLRLHPPLIILMRVAKGEFEVEGFPIHEGDYVAASPAISNRIPEDFPDPDA
FKPDRYNKPEQADIVNRWTWIPFGAGRHRCVGAAFAQMQIKAIFSVLLREYDFEMAQPADSYRNDHSKMVVQL
ARPAKVRYRKRNA
>CYP51B1_Mycobacterium avium paratuberculosis Pt155 2548538512
MTTSTVVPRISGGEEEHGHLEEFRTDPIGLMQRVRDECGDVGWFQLVDKHVILLSGAQANEFFFRSADEDLDQ
AEAYPFMTPIFGKGVVFDASPERRKEMLHNSALRGEQMKGHASTIEGEVKKMIADWGDEGEIELLDFFAELTI
YTSTACLIGLKFREQLDHRFAEYYHDLERGTDPLCYVDPYLPIESFKRRDEARVKLVALVQEIMDQRLANPPK
DKADRDMLDVLVSIKDEDGKPRFSADEITGMFISLMFAGHHTSSGTSAWTLIELIRHPDVYAEVLAELEELYA
DGQEVSFHALRSIPKLDNVVKETLRLHPPLIILMRVAKGEFEVEGFPIHEGDYVAASPAISNRIPEDFPDPDA
FKPDRYNKPEQADIVNRWTWIPFGAGRHRCVGAAFAQMQIKAIFSVLLREYDFEMAQPADSYRNDHSKMVVQL
ARPAKVRYRKRNA
>CYP51B1_Mycobacterium avium paratuberculosis Pt154 2548533346
MTTSTVVPRISGGEEEHGHLEEFRTDPIGLMQRVRDECGDVGWFQLVDKHVILLSGAQANEFFFRSADEDLDQ
AEAYPFMTPIFGKGVVFDASPERRKEMLHNSALRGEQMKGHASTIEGEVKKMIADWGDEGEIELLDFFAELTI
YTSTACLIGLKFREQLDHRFAEYYHDLERGTDPLCYVDPYLPIESFKRRDEARVKLVALVQEIMDQRLANPPK
DKADRDMLDVLVSIKDEDGKPRFSADEITGMFISLMFAGHHTSSGTSAWTLIELIRHPDVYAEVLAELEELYA
DGQEVSFHALRSIPKLDNVVKETLRLHPPLIILMRVAKGEFEVEGFPIHEGDYVAASPAISNRIPEDFPDPDA
FKPDRYNKPEQADIVNRWTWIPFGAGRHRCVGAAFAQMQIKAIFSVLLREYDFEMAQPADSYRNDHSKMVVQL
ARPAKVRYRKRNA
>CYP51B1_Mycobacterium avium paratuberculosis Pt146 2548528723
MTTSTVVPRISGGEEEHGHLEEFRTDPIGLMQRVRDECGDVGWFQLVDKHVILLSGAQANEFFFRSADEDLDQ
AEAYPFMTPIFGKGVVFDASPERRKEMLHNSALRGEQMKGHASTIEGEVKKMIADWGDEGEIELLDFFAELTI

YTSTACLIGLKFREQLDHRFAEYYHDLERGTDPLCYVDPYLPIESFKRRDEARVKLVALVQEIMDQRLANPPK
DKADRDMLDVLVSIKDEDGKPRFSADEITGMFISLMFAGHHTSSGTSAWTLIELIRHPDVYAEVLAELEELYA
DGQEVSFHALRSIPKLDNVVKETLRLHPPLIILMRVAKGEFEVEGFPIHEGDYVAASPAISNRIPEDFPDPDA
FKPDRYNKPEQADIVNRWTWIPFGAGRHRCVGAAFAQMQIKAIFSVLLREYDFEMAQPADSYRNDHSKMVVQL
ARPAKVRYRKRNA
>CYP51B1_Mycobacterium avium paratuberculosis Pt145 2548524414
MTTSTVVPRISGGEEEHGHLEEFRTDPIGLMQRVRDECGDVGWFQLVDKHVILLSGAQANEFFFRSADEDLDQ
AEAYPFMTPIFGKGVVFDASPERRKEMLHNSALRGEQMKGHASTIEGEVKKMIADWGDEGEIELLDFFAELTI
YTSTACLIGLKFREQLDHRFAEYYHDLERGTDPLCYVDPYLPIESFKRRDEARVKLVALVQEIMDQRLANPPK
DKADRDMLDVLVSIKDEDGKPRFSADEITGMFISLMFAGHHTSSGTSAWTLIELIRHPDVYAEVLAELEELYA
DGQEVSFHALRSIPKLDNVVKETLRLHPPLIILMRVAKGEFEVEGFPIHEGDYVAASPAISNRIPEDFPDPDA
FKPDRYNKPEQADIVNRWTWIPFGAGRHRCVGAAFAQMQIKAIFSVLLREYDFEMAQPADSYRNDHSKMVVQL
ARPAKVRYRKRNA
>CYP51B1_Mycobacterium avium paratuberculosis Pt144 2548519720
MTTSTVVPRISGGEEEHGHLEEFRTDPIGLMQRVRDECGDVGWFQLVDKHVILLSGAQANEFFFRSADEDLDQ
AEAYPFMTPIFGKGVVFDASPERRKEMLHNSALRGEQMKGHASTIEGEVKKMIADWGDEGEIELLDFFAELTI
YTSTACLIGLKFREQLDHRFAEYYHDLERGTDPLCYVDPYLPIESFKRRDEARVKLVALVQEIMDQRLANPPK
DKADRDMLDVLVSIKDEDGKPRFSADEITGMFISLMFAGHHTSSGTSAWTLIELIRHPDVYAEVLAELEELYA
DGQEVSFHALRSIPKLDNVVKETLRLHPPLIILMRVAKGEFEVEGFPIHEGDYVAASPAISNRIPEDFPDPDA
FKPDRYNKPEQADIVNRWTWIPFGAGRHRCVGAAFAQMQIKAIFSVLLREYDFEMAQPADSYRNDHSKMVVQL
ARPAKVRYRKRNA
>CYP51B1_Mycobacterium avium paratuberculosis MAP4 2555736398
MTTSTVVPRISGGEEEHGHLEEFRTDPIGLMQRVRDECGDVGWFQLVDKHVILLSGAQANEFFFRSADEDLDQ
AEAYPFMTPIFGKGVVFDASPERRKEMLHNSALRGEQMKGHASTIEGEVKKMIADWGDEGEIELLDFFAELTI
YTSTACLIGLKFREQLDHRFAEYYHDLERGTDPLCYVDPYLPIESFKRRDEARVKLVALVQEIMDQRLANPPK
DKADRDMLDVLVSIKDEDGKPRFSADEITGMFISLMFAGHHTSSGTSAWTLIELIRHPDVYAEVLAELEELYA
DGQEVSFHALRSIPKLDNVVKETLRLHPPLIILMRVAKGEFEVEGFPIHEGDYVAASPAISNRIPEDFPDPDA
FKPDRYNKPEQADIVNRWTWIPFGAGRHRCVGAAFAQMQIKAIFSVLLREYDFEMAQPADSYRNDHSKMVVQL
ARPAKVRYRKRNA
>CYP51B1_Mycobacterium avium paratuberculosis Pt139 2548512333
MTTSTVVPRISGGEEEHGHLEEFRTDPIGLMQRVRDECGDVGWFQLVDKHVILLSGAQANEFFFRSADEDLDQ
AEAYPFMTPIFGKGVVFDASPERRKEMLHNSALRGEQMKGHASTIEGEVKKMIADWGDEGEIELLDFFAELTI
YTSTACLIGLKFREQLDHRFAEYYHDLERGTDPLCYVDPYLPIESFKRRDEARVKLVALVQEIMDQRLANPPK
DKADRDMLDVLVSIKDEDGKPRFSADEITGMFISLMFAGHHTSSGTSAWTLIELIRHPDVYAEVLAELEELYA
DGQEVSFHALRSIPKLDNVVKETLRLHPPLIILMRVAKGEFEVEGFPIHEGDYVAASPAISNRIPEDFPDPDA
FKPDRYNKPEQADIVNRWTWIPFGAGRHRCVGAAFAQMQIKAIFSVLLREYDFEMAQPADSYRNDHSKMVVQL
ARPAKVRYRKRNA
>CYP51B1_Mycobacterium avium paratuberculosis MAP/TANUVAS/TN/India/2008
2719419536
MTTSTVVPRISGGEEEHGHLEEFRTDPIGLMQRVRDECGDVGWFQLVDKHVILLSGAQANEFFFRSADEDLDQ
AEAYPFMTPIFGKGVVFDASPERRKEMLHNSALRGEQMKGHASTIEGEVKKMIADWGDEGEIELLDFFAELTI
YTSTACLIGLKFREQLDHRFAEYYHDLERGTDPLCYVDPYLPIESFKRRDEARVKLVALVQEIMDQRLANPPK
DKADRDMLDVLVSIKDEDGKPRFSADEITGMFISLMFAGHHTSSGTSAWTLIELIRHPDVYAEVLAELEELYA
DGQEVSFHALRSIPKLDNVVKETLRLHPPLIILMRVAKGEFEVEGFPIHEGDYVAASPAISNRIPEDFPDPDA
FKPDRYNKPEQADIVNRWTWIPFGAGRHRCVGAAFAQMQIKAIFSVLLREYDFEMAQPADSYRNDHSKMVVQL
ARPAKVRYRKRNA
>CYP51B1_Mycobacterium avium paratuberculosis M. ap E93 2689466402
MTTSTVVPRISGGEEEHGHLEEFRTDPIGLMQRVRDECGDVGWFQLVDKHVILLSGAQANEFFFRSADEDLDQ
AEAYPFMTPIFGKGVVFDASPERRKEMLHNSALRGEQMKGHASTIEGEVKKMIADWGDEGEIELLDFFAELTI
YTSTACLIGLKFREQLDHRFAEYYHDLERGTDPLCYVDPYLPIESFKRRDEARVKLVALVQEIMDQRLANPPK
DKADRDMLDVLVSIKDEDGKPRFSADEITGMFISLMFAGHHTSSGTSAWTLIELIRHPDVYAEVLAELEELYA
DGQEVSFHALRSIPKLDNVVKETLRLHPPLIILMRVAKGEFEVEGFPIHEGDYVAASPAISNRIPEDFPDPDA
FKPDRYNKPEQADIVNRWTWIPFGAGRHRCVGAAFAQMQIKAIFSVLLREYDFEMAQPADSYRNDHSKMVVQL
ARPAKVRYRKRNA
>CYP51B1_Mycobacterium avium subsp. paratuberculosis K-10 637133547
MTTSTVVPRISGGEEEHGHLEEFRTDPIGLMQRVRDECGDVGWFQLVDKHVILLSGAQANEFFFRSADEDLDQ
AEAYPFMTPIFGKGVVFDASPERRKEMLHNSALRGEQMKGHASTIEGEVKKMIADWGDEGEIELLDFFAELTI
YTSTACLIGLKFREQLDHRFAEYYHDLERGTDPLCYVDPYLPIESFKRRDEARVKLVALVQEIMDQRLANPPK

DKADRDMLDVLVSIKDEDGKPRFSADEITGMFISLMFAGHHTSSGTSAWTLIELIRHPDVYAEVLAELEELYA
DGQEVSFHALRSIPKLDNVVKETLRLHPPLIILMRVAKGEFEVEGFPIHEGDYVAASPAISNRIPEDFPDPDA
FKPDRYNKPEQADIVNRWTWIPFGAGRHRCVGAAFAQMQIKAIFSVLLREYDFEMAQPADSYRNDHSKMVVQL
ARPAKVRYRKRNA
>CYP51B1_Mycobacterium avium paratuberculosis JTC 1285 2549370479
MTTSTVVPRISGGEEEHGHLEEFRTDPIGLMQRVRDECGDVGWFQLVDKHVILLSGAQANEFFFRSADEDLDQ
AEAYPFMTPIFGKGVVFDASPERRKEMLHNSALRGEQMKGHASTIEGEVKKMIADWGDEGEIELLDFFAELTI
YTSTACLIGLKFREQLDHRFAEYYHDLERGTDPLCYVDPYLPIESFKRRDEARVKLVALVQEIMDQRLANPPK
DKADRDMLDVLVSIKDEDGKPRFSADEITGMFISLMFAGHHTSSGTSAWTLIELIRHPDVYAEVLAELEELYA
DGQEVSFHALRSIPKLDNVVKETLRLHPPLIILMRVAKGEFEVEGFPIHEGDYVAASPAISNRIPEDFPDPDA
FKPDRYNKPEQADIVNRWTWIPFGAGRHRCVGAAFAQMQIKAIFSVLLREYDFEMAQPADSYRNDHSKMVVQL
ARPAKVRYRKRNA
>CYP51B1_Mycobacterium avium paratuberculosis JQ6 2549398420
MTTSTVVPRISGGEEEHGHLEEFRTDPIGLMQRVRDECGDVGWFQLVDKHVILLSGAQANEFFFRSADEDLDQ
AEAYPFMTPIFGKGVVFDASPERRKEMLHNSALRGEQMKGHASTIEGEVKKMIADWGDEGEIELLDFFAELTI
YTSTACLIGLKFREQLDHRFAEYYHDLERGTDPLCYVDPYLPIESFKRRDEARVKLVALVQEIMDQRLANPPK
DKADRDMLDVLVSIKDEDGKPRFSADEITGMFISLMFAGHHTSSGTSAWTLIELIRHPDVYAEVLAELEELYA
DGQEVSFHALRSIPKLDNVVKETLRLHPPLIILMRVAKGEFEVEGFPIHEGDYVAASPAISNRIPEDFPDPDA
FKPDRYNKPEQADIVNRWTWIPFGAGRHRCVGAAFAQMQIKAIFSVLLREYDFEMAQPADSYRNDHSKMVVQL
ARPAKVRYRKRNA
>CYP51B1_Mycobacterium avium paratuberculosis JQ5 2549361940
MTTSTVVPRISGGEEEHGHLEEFRTDPIGLMQRVRDECGDVGWFQLVDKHVILLSGAQANEFFFRSADEDLDQ
AEAYPFMTPIFGKGVVFDASPERRKEMLHNSALRGEQMKGHASTIEGEVKKMIADWGDEGEIELLDFFAELTI
YTSTACLIGLKFREQLDHRFAEYYHDLERGTDPLCYVDPYLPIESFKRRDEARVKLVALVQEIMDQRLANPPK
DKADRDMLDVLVSIKDEDGKPRFSADEITGMFISLMFAGHHTSSGTSAWTLIELIRHPDVYAEVLAELEELYA
DGQEVSFHALRSIPKLDNVVKETLRLHPPLIILMRVAKGEFEVEGFPIHEGDYVAASPAISNRIPEDFPDPDA
FKPDRYNKPEQADIVNRWTWIPFGAGRHRCVGAAFAQMQIKAIFSVLLREYDFEMAQPADSYRNDHSKMVVQL
ARPAKVRYRKRNA
>CYP51B1_Mycobacterium avium paratuberculosis Env 210 2549380529
MTTSTVVPRISGGEEEHGHLEEFRTDPIGLMQRVRDECGDVGWFQLVDKHVILLSGAQANEFFFRSADEDLDQ
AEAYPFMTPIFGKGVVFDASPERRKEMLHNSALRGEQMKGHASTIEGEVKKMIADWGDEGEIELLDFFAELTI
YTSTACLIGLKFREQLDHRFAEYYHDLERGTDPLCYVDPYLPIESFKRRDEARVKLVALVQEIMDQRLANPPK
DKADRDMLDVLVSIKDEDGKPRFSADEITGMFISLMFAGHHTSSGTSAWTLIELIRHPDVYAEVLAELEELYA
DGQEVSFHALRSIPKLDNVVKETLRLHPPLIILMRVAKGEFEVEGFPIHEGDYVAASPAISNRIPEDFPDPDA
FKPDRYNKPEQADIVNRWTWIPFGAGRHRCVGAAFAQMQIKAIFSVLLREYDFEMAQPADSYRNDHSKMVVQL
ARPAKVRYRKRNA
>CYP51B1_Mycobacterium avium paratuberculosis E1 2688293808
MTTSTVVPRISGGEEEHGHLEEFRTDPIGLMQRVRDECGDVGWFQLVDKHVILLSGAQANEFFFRSADEDLDQ
AEAYPFMTPIFGKGVVFDASPERRKEMLHNSALRGEQMKGHASTIEGEVKKMIADWGDEGEIELLDFFAELTI
YTSTACLIGLKFREQLDHRFAEYYHDLERGTDPLCYVDPYLPIESFKRRDEARVKLVALVQEIMDQRLANPPK
DKADRDMLDVLVSIKDEDGKPRFSADEITGMFISLMFAGHHTSSGTSAWTLIELIRHPDVYAEVLAELEELYA
DGQEVSFHALRSIPKLDNVVKETLRLHPPLIILMRVAKGEFEVEGFPIHEGDYVAASPAISNRIPEDFPDPDA
FKPDRYNKPEQADIVNRWTWIPFGAGRHRCVGAAFAQMQIKAIFSVLLREYDFEMAQPADSYRNDHSKMVVQL
ARPAKVRYRKRNA
>CYP51B1_Mycobacterium avium paratuberculosis DT 3 2549378016
MTTSTVVPRISGGEEEHGHLEEFRTDPIGLMQRVRDECGDVGWFQLVDKHVILLSGAQANEFFFRSADEDLDQ
AEAYPFMTPIFGKGVVFDASPERRKEMLHNSALRGEQMKGHASTIEGEVKKMIADWGDEGEIELLDFFAELTI
YTSTACLIGLKFREQLDHRFAEYYHDLERGTDPLCYVDPYLPIESFKRRDEARVKLVALVQEIMDQRLANPPK
DKADRDMLDVLVSIKDEDGKPRFSADEITGMFISLMFAGHHTSSGTSAWTLIELIRHPDVYAEVLAELEELYA
DGQEVSFHALRSIPKLDNVVKETLRLHPPLIILMRVAKGEFEVEGFPIHEGDYVAASPAISNRIPEDFPDPDA
FKPDRYNKPEQADIVNRWTWIPFGAGRHRCVGAAFAQMQIKAIFSVLLREYDFEMAQPADSYRNDHSKMVVQL
ARPAKVRYRKRNA
>CYP51B1_Mycobacterium avium paratuberculosis CLIJ644 2547367996
MTTSTVVPRISGGEEEHGHLEEFRTDPIGLMQRVRDECGDVGWFQLVDKHVILLSGAQANEFFFRSADEDLDQ
AEAYPFMTPIFGKGVVFDASPERRKEMLHNSALRGEQMKGHASTIEGEVKKMIADWGDEGEIELLDFFAELTI
YTSTACLIGLKFREQLDHRFAEYYHDLERGTDPLCYVDPYLPIESFKRRDEARVKLVALVQEIMDQRLANPPK
DKADRDMLDVLVSIKDEDGKPRFSADEITGMFISLMFAGHHTSSGTSAWTLIELIRHPDVYAEVLAELEELYA
DGQEVSFHALRSIPKLDNVVKETLRLHPPLIILMRVAKGEFEVEGFPIHEGDYVAASPAISNRIPEDFPDPDA

FKPDRYNKPEQADIVNRWTWIPFGAGRHRCVGAAFAQMQIKAIFSVLLREYDFEMAQPADSYRNDHSKMVVQL
ARPAKVRYRKRNA
>CYP51B1_Mycobacterium avium paratuberculosis CLIJ623 2548575636
MTTSTVVPRISGGEEEHGHLEEFRTDPIGLMQRVRDECGDVGWFQLVDKHVILLSGAQANEFFFRSADEDLDQ
AEAYPFMTPIFGKGVVFDASPERRKEMLHNSALRGEQMKGHASTIEGEVKKMIADWGDEGEIELLDFFAELTI
YTSTACLIGLKFREQLDHRFAEYYHDLERGTDPLCYVDPYLPIESFKRRDEARVKLVALVQEIMDQRLANPPK
DKADRDMLDVLVSIKDEDGKPRFSADEITGMFISLMFAGHHTSSGTSAWTLIELIRHPDVYAEVLAELEELYA
DGQEVSFHALRSIPKLDNVVKETLRLHPPLIILMRVAKGEFEVEGFPIHEGDYVAASPAISNRIPEDFPDPDA
FKPDRYNKPEQADIVNRWTWIPFGAGRHRCVGAAFAQMQIKAIFSVLLREYDFEMAQPADSYRNDHSKMVVQL
ARPAKVRYRKRNA
>CYP51B1_Mycobacterium avium paratuberculosis CLIJ361 2548596985
MTTSTVVPRISGGEEEHGHLEEFRTDPIGLMQRVRDECGDVGWFQLVDKHVILLSGAQANEFFFRSADEDLDQ
AEAYPFMTPIFGKGVVFDASPERRKEMLHNSALRGEQMKGHASTIEGEVKKMIADWGDEGEIELLDFFAELTI
YTSTACLIGLKFREQLDHRFAEYYHDLERGTDPLCYVDPYLPIESFKRRDEARVKLVALVQEIMDQRLANPPK
DKADRDMLDVLVSIKDEDGKPRFSADEITGMFISLMFAGHHTSSGTSAWTLIELIRHPDVYAEVLAELEELYA
DGQEVSFHALRSIPKLDNVVKETLRLHPPLIILMRVAKGEFEVEGFPIHEGDYVAASPAISNRIPEDFPDPDA
FKPDRYNKPEQADIVNRWTWIPFGAGRHRCVGAAFAQMQIKAIFSVLLREYDFEMAQPADSYRNDHSKMVVQL
ARPAKVRYRKRNA
>CYP51B1_Mycobacterium avium paratuberculosis ATCC 19698 2549000321
MTTSTVVPRISGGEEEHGHLEEFRTDPIGLMQRVRDECGDVGWFQLVDKHVILLSGAQANEFFFRSADEDLDQ
AEAYPFMTPIFGKGVVFDASPERRKEMLHNSALRGEQMKGHASTIEGEVKKMIADWGDEGEIELLDFFAELTI
YTSTACLIGLKFREQLDHRFAEYYHDLERGTDPLCYVDPYLPIESFKRRDEARVKLVALVQEIMDQRLANPPK
DKADRDMLDVLVSIKDEDGKPRFSADEITGMFISLMFAGHHTSSGTSAWTLIELIRHPDVYAEVLAELEELYA
DGQEVSFHALRSIPKLDNVVKETLRLHPPLIILMRVAKGEFEVEGFPIHEGDYVAASPAISNRIPEDFPDPDA
FKPDRYNKPEQADIVNRWTWIPFGAGRHRCVGAAFAQMQIKAIFSVLLREYDFEMAQPADSYRNDHSKMVVQL
ARPAKVRYRKRNA
>CYP51B1_Mycobacterium avium paratuberculosis 1281 2549366407
MTTSTVVPRISGGEEEHGHLEEFRTDPIGLMQRVRDECGDVGWFQLVDKHVILLSGAQANEFFFRSADEDLDQ
AEAYPFMTPIFGKGVVFDASPERRKEMLHNSALRGEQMKGHASTIEGEVKKMIADWGDEGEIELLDFFAELTI
YTSTACLIGLKFREQLDHRFAEYYHDLERGTDPLCYVDPYLPIESFKRRDEARVKLVALVQEIMDQRLANPPK
DKADRDMLDVLVSIKDEDGKPRFSADEITGMFISLMFAGHHTSSGTSAWTLIELIRHPDVYAEVLAELEELYA
DGQEVSFHALRSIPKLDNVVKETLRLHPPLIILMRVAKGEFEVEGFPIHEGDYVAASPAISNRIPEDFPDPDA
FKPDRYNKPEQADIVNRWTWIPFGAGRHRCVGAAFAQMQIKAIFSVLLREYDFEMAQPADSYRNDHSKMVVQL
ARPAKVRYRKRNA
>CYP51B1_Mycobacterium avium paratuberculosis 4B 2550742056
MTTSTVVPRISGGEEEHGHLEEFRTDPIGLMQRVRDECGDVGWFQLVDKHVILLSGAQANEFFFRSADEDLDQ
AEAYPFMTPIFGKGVVFDASPERRKEMLHNSALRGEQMKGHASTIEGEVKKMIADWGDEGEIELLDFFAELTI
YTSTACLIGLKFREQLDHRFAEYYHDLERGTDPLCYVDPYLPIESFKRRDEARVKLVALVQEIMDQRLANPPK
DKADRDMLDVLVSIKDEDGKPRFSADEITGMFISLMFAGHHTSSGTSAWTLIELIRHPDVYAEVLAELEELYA
DGQEVSFHALRSIPKLDNVVKETLRLHPPLIILMRVAKGEFEVEGFPIHEGDYVAASPAISNRIPEDFPDPDA
FKPDRYNKPEQADIVNRWTWIPFGAGRHRCVGAAFAQMQIKAIFSVLLREYDFEMAQPADSYRNDHSKMVVQL
ARPAKVRYRKRNA
>CYP51B1_Mycobacterium avium paratuberculosis 10-8425 2592482287
MTTSTVVPRISGGEEEHGHLEEFRTDPIGLMQRVRDECGDVGWFQLVDKHVILLSGAQANEFFFRSADEDLDQ
AEAYPFMTPIFGKGVVFDASPERRKEMLHNSALRGEQMKGHASTIEGEVKKMIADWGDEGEIELLDFFAELTI
YTSTACLIGLKFREQLDHRFAEYYHDLERGTDPLCYVDPYLPIESFKRRDEARVKLVALVQEIMDQRLANPPK
DKADRDMLDVLVSIKDEDGKPRFSADEITGMFISLMFAGHHTSSGTSAWTLIELIRHPDVYAEVLAELEELYA
DGQEVSFHALRSIPKLDNVVKETLRLHPPLIILMRVAKGEFEVEGFPIHEGDYVAASPAISNRIPEDFPDPDA
FKPDRYNKPEQADIVNRWTWIPFGAGRHRCVGAAFAQMQIKAIFSVLLREYDFEMAQPADSYRNDHSKMVVQL
ARPAKVRYRKRNA
>CYP51B1_Mycobacterium avium paratuberculosis 10-5975 2576285804
MTTSTVVPRISGGEEEHGHLEEFRTDPIGLMQRVRDECGDVGWFQLVDKHVILLSGAQANEFFFRSADEDLDQ
AEAYPFMTPIFGKGVVFDASPERRKEMLHNSALRGEQMKGHASTIEGEVKKMIADWGDEGEIELLDFFAELTI
YTSTACLIGLKFREQLDHRFAEYYHDLERGTDPLCYVDPYLPIESFKRRDEARVKLVALVQEIMDQRLANPPK
DKADRDMLDVLVSIKDEDGKPRFSADEITGMFISLMFAGHHTSSGTSAWTLIELIRHPDVYAEVLAELEELYA
DGQEVSFHALRSIPKLDNVVKETLRLHPPLIILMRVAKGEFEVEGFPIHEGDYVAASPAISNRIPEDFPDPDA
FKPDRYNKPEQADIVNRWTWIPFGAGRHRCVGAAFAQMQIKAIFSVLLREYDFEMAQPADSYRNDHSKMVVQL
ARPAKVRYRKRNA

>CYP51B1_Mycobacterium avium paratuberculosis 10-5864 2570866629
MTTSTVVPRISGGEEEHGHLEEFRTDPIGLMQRVRDECGDVGWFQLVDKHVILLSGAQANEFFFRSADEDLDQ
AEAYPFMTPIFGKGVVFDASPERRKEMLHNSALRGEQMKGHASTIEGEVKKMIADWGDEGEIELLDFFAELTI
YTSTACLIGLKFREQLDHRFAEYYHDLERGTDPLCYVDPYLPIESFKRRDEARVKLVALVQEIMDQRLANPPK
DKADRDMLDVLVSIKDEDGKPRFSADEITGMFISLMFAGHHTSSGTSAWTLIELIRHPDVYAEVLAELEELYA
DGQEVSFHALRSIPKLDNVVKETLRLHPPLIILMRVAKGEFEVEGFPIHEGDYVAASPAISNRIPEDFPDPDA
FKPDRYNKPEQADIVNRWTWIPFGAGRHRCVGAAFAQMQIKAIFSVLLREYDFEMAQPADSYRNDHSKMVVQL
ARPAKVRYRKRNA
>CYP51B1_Mycobacterium avium paratuberculosis 10-4404 2592486056
MTTSTVVPRISGGEEEHGHLEEFRTDPIGLMQRVRDECGDVGWFQLVDKHVILLSGAQANEFFFRSADEDLDQ
AEAYPFMTPIFGKGVVFDASPERRKEMLHNSALRGEQMKGHASTIEGEVKKMIADWGDEGEIELLDFFAELTI
YTSTACLIGLKFREQLDHRFAEYYHDLERGTDPLCYVDPYLPIESFKRRDEARVKLVALVQEIMDQRLANPPK
DKADRDMLDVLVSIKDEDGKPRFSADEITGMFISLMFAGHHTSSGTSAWTLIELIRHPDVYAEVLAELEELYA
DGQEVSFHALRSIPKLDNVVKETLRLHPPLIILMRVAKGEFEVEGFPIHEGDYVAASPAISNRIPEDFPDPDA
FKPDRYNKPEQADIVNRWTWIPFGAGRHRCVGAAFAQMQIKAIFSVLLREYDFEMAQPADSYRNDHSKMVVQL
ARPAKVRYRKRNA
>CYP51B1_Mycobacterium avium paratuberculosis 08-8281 2574999332
MTTSTVVPRISGGEEEHGHLEEFRTDPIGLMQRVRDECGDVGWFQLVDKHVILLSGAQANEFFFRSADEDLDQ
AEAYPFMTPIFGKGVVFDASPERRKEMLHNSALRGEQMKGHASTIEGEVKKMIADWGDEGEIELLDFFAELTI
YTSTACLIGLKFREQLDHRFAEYYHDLERGTDPLCYVDPYLPIESFKRRDEARVKLVALVQEIMDQRLANPPK
DKADRDMLDVLVSIKDEDGKPRFSADEITGMFISLMFAGHHTSSGTSAWTLIELIRHPDVYAEVLAELEELYA
DGQEVSFHALRSIPKLDNVVKETLRLHPPLIILMRVAKGEFEVEGFPIHEGDYVAASPAISNRIPEDFPDPDA
FKPDRYNKPEQADIVNRWTWIPFGAGRHRCVGAAFAQMQIKAIFSVLLREYDFEMAQPADSYRNDHSKMVVQL
ARPAKVRYRKRNA
>CYP51B1_Mycobacterium avium MAV_120709_2344 2582389324
MTTSTVVPRVSGGEEEHGHLEEFRTDPIGLMQRVRDECGDVGWFQLVDKHVILLSGAQANEFFFRSADEDLDQ
AEAYPFMTPIFGKGVVFDASPERRKEMLHNSALRGEQMKGHASTIEGEVKKMIADWGDEGEIELLDFFAELTI
YTSTACLIGLKFREQLDHRFAEYYHDLERGTDPLCYVDPYLPIESFKRRDEARVKLVALVQEIMDQRLANPPK
DKADRDMLDVLVSIKDEDGKPRFSADEITGMFISLMFAGHHTSSGTSAWTLIELIRHPDVYAEVLAELEELYA
DGHEVSFHALRSIPKLDNVVKETLRLHPPLIILMRVAKGEFEVEGFPIHEGDYVAASPAISNRIPEDFPDPDA
FKPDRYNKPEQADIVNRWTWIPFGAGRHRCVGAAFAQMQIKAIFSVLLREYDFEMAQPADSYRNDHSKMVVQL
ARPAKVRYRKRNA
>CYP51B1_Mycobacterium avium hominissuis MAHP-883 2725150282
MTTSTVVPRVSGGEEEHGHLEEFRTDPIGLMQRVRDECGDVGWFQLVDKHVILLSGAQANEFFFRSADEDLDQ
AEAYPFMTPIFGKGVVFDASPERRKEMLHNSALRGEQMKGHASTIEGEVKKMIADWGDEGEIELLDFFAELTI
YTSTACLIGLKFREQLDHRFAEYYHDLERGTDPLCYVDPYLPIESFKRRDEARVKLVALVQEIMDQRLANPPK
DKADRDMLDVLVSIKDEDGKPRFSADEITGMFISLMFAGHHTSSGTSAWTLIELIRHPDVYAEVLAELEELYA
DGHEVSFHALRSIPKLDNVVKETLRLHPPLIILMRVAKGEFEVEGFPIHEGDYVAASPAISNRIPEDFPDPDA
FKPDRYNKPEQADIVNRWTWIPFGAGRHRCVGAAFAQMQIKAIFSVLLREYDFEMAQPADSYRNDHSKMVVQL
ARPAKVRYRKRNA
>CYP51B1_Mycobacterium avium hominissuis MAH-P-528-08 2725114252
MTTSTVVPRVSGGEEEHGHLEEFRTDPIGLMQRVRDECGDVGWFQLVDKHVILLSGAQANEFFFRSADEDLDQ
AEAYPFMTPIFGKGVVFDASPERRKEMLHNSALRGEQMKGHASTIEGEVKKMIADWGDEGEIELLDFFAELTI
YTSTACLIGLKFREQLDHRFAEYYHDLERGTDPLCYVDPYLPIESFKRRDEARVKLVALVQEIMDQRLANPPK
DKADRDMLDVLVSIKDEDGKPRFSADEITGMFISLMFAGHHTSSGTSAWTLIELIRHPDVYAEVLAELEELYA
DGHEVSFHALRSIPKLDNVVKETLRLHPPLIILMRVAKGEFEVEGFPIHEGDYVAASPAISNRIPEDFPDPDA
FKPDRYNKPEQADIVNRWTWIPFGAGRHRCVGAAFAQMQIKAIFSVLLREYDFEMAQPADSYRNDHSKMVVQL
ARPAKVRYRKRNA
>CYP51B1_Mycobacterium avium hominissuis MAH-P-3449-08 2725139425
MTTSTVVPRVSGGEEEHGHLEEFRTDPIGLMQRVRDECGDVGWFQLVDKHVILLSGAQANEFFFRSADEDLDQ
AEAYPFMTPIFGKGVVFDASPERRKEMLHNSALRGEQMKGHASTIEGEVKKMIADWGDEGEIELLDFFAELTI
YTSTACLIGLKFREQLDHRFAEYYHDLERGTDPLCYVDPYLPIESFKRRDEARVKLVALVQEIMDQRLANPPK
DKADRDMLDVLVSIKDEDGKPRFSADEITGMFISLMFAGHHTSSGTSAWTLIELIRHPDVYAEVLAELEELYA
DGHEVSFHALRSIPKLDNVVKETLRLHPPLIILMRVAKGEFEVEGFPIHEGDYVAASPAISNRIPEDFPDPDA
FKPDRYNKPEQADIVNRWTWIPFGAGRHRCVGAAFAQMQIKAIFSVLLREYDFEMAQPADSYRNDHSKMVVQL
ARPAKVRYRKRNA
>CYP51B1_Mycobacterium avium hominissuis MAH-P-10203-06 2678753649

MTTSTVVPRVSGGEEEHGHLEEFRTDPIGLMQRVRDECGDVGWFQLVDKHVILLSGAQANEFFFRSADEDLDQ
AEAYPFMTPIFGKGVVFDASPERRKEMLHNSALRGEQMKGHASTIEGEVKKMIADWGDEGEIELLDFFAELTI
YTSTACLIGLKFREQLDHRFAEYYHDLERGTDPLCYVDPYLPIESFKRRDEARVKLVALVQEIMDQRLANPPK
DKADRDMLDVLVSIKDEDGKPRFSADEITGMFISLMFAGHHTSSGTSAWTLIELIRHPDVYAEVLAELEELYA
DGHEVSFHALRSIPKLDNVVKETLRLHPPLIILMRVAKGEFEVEGFPIHEGDYVAASPAISNRIPEDFPDPDA
FKPDRYNKPEQADIVNRWTWIPFGAGRHRCVGAAFAQMQIKAIFSVLLREYDFEMAQPADSYRNDHSKMVVQL
ARPAKVRYRKRNA
>CYP51B1_Mycobacterium avium hominissuis MAH-P-10091-06 2678123630
MTTSTVVPRVSGGEEEHGHLEEFRTDPIGLMQRVRDECGDVGWFQLVDKHVILLSGAQANEFFFRSADEDLDQ
AEAYPFMTPIFGKGVVFDASPERRKEMLHNSALRGEQMKGHASTIEGEVKKMIADWGDEGEIELLDFFAELTI
YTSTACLIGLKFREQLDHRFAEYYHDLERGTDPLCYVDPYLPIESFKRRDEARVKLVALVQEIMDQRLANPPK
DKADRDMLDVLVSIKDEDGKPRFSADEITGMFISLMFAGHHTSSGTSAWTLIELIRHPDVYAEVLAELEELYA
DGHEVSFHALRSIPKLDNVVKETLRLHPPLIILMRVAKGEFEVEGFPIHEGDYVAASPAISNRIPEDFPDPDA
FKPDRYNKPEQADIVNRWTWIPFGAGRHRCVGAAFAQMQIKAIFSVLLREYDFEMAQPADSYRNDHSKMVVQL
ARPAKVRYRKRNA
>CYP51B1_Mycobacterium avium hominissuis MAH-E-96-2 2724973824
MTTSTVVPRVSGGEEEHGHLEEFRTDPIGLMQRVRDECGDVGWFQLVDKHVILLSGAQANEFFFRSADEDLDQ
AEAYPFMTPIFGKGVVFDASPERRKEMLHNSALRGEQMKGHASTIEGEVKKMIADWGDEGEIELLDFFAELTI
YTSTACLIGLKFREQLDHRFAEYYHDLERGTDPLCYVDPYLPIESFKRRDEARVKLVALVQEIMDQRLANPPK
DKADRDMLDVLVSIKDEDGKPRFSADEITGMFISLMFAGHHTSSGTSAWTLIELIRHPDVYAEVLAELEELYA
DGHEVSFHALRSIPKLDNVVKETLRLHPPLIILMRVAKGEFEVEGFPIHEGDYVAASPAISNRIPEDFPDPDA
FKPDRYNKPEQADIVNRWTWIPFGAGRHRCVGAAFAQMQIKAIFSVLLREYDFEMAQPADSYRNDHSKMVVQL
ARPAKVRYRKRNA
>CYP51B1_Mycobacterium avium hominissuis MAH-E-88-1 2724953458
MTTSTVVPRVSGGEEEHGHLEEFRTDPIGLMQRVRDECGDVGWFQLVDKHVILLSGAQANEFFFRSADEDLDQ
AEAYPFMTPIFGKGVVFDASPERRKEMLHNSALRGEQMKGHASTIEGEVKKMIADWGDEGEIELLDFFAELTI
YTSTACLIGLKFREQLDHRFAEYYHDLERGTDPLCYVDPYLPIESFKRRDEARVKLVALVQEIMDQRLANPPK
DKADRDMLDVLVSIKDEDGKPRFSADEITGMFISLMFAGHHTSSGTSAWTLIELIRHPDVYAEVLAELEELYA
DGHEVSFHALRSIPKLDNVVKETLRLHPPLIILMRVAKGEFEVEGFPIHEGDYVAASPAISNRIPEDFPDPDA
FKPDRYNKPEQADIVNRWTWIPFGAGRHRCVGAAFAQMQIKAIFSVLLREYDFEMAQPADSYRNDHSKMVVQL
ARPAKVRYRKRNA
>CYP51B1_Mycobacterium avium hominissuis MAH-E-3044 2725109675
MTTSTVVPRVSGGEEEHGHLEEFRTDPIGLMQRVRDECGDVGWFQLVDKHVILLSGAQANEFFFRSADEDLDQ
AEAYPFMTPIFGKGVVFDASPERRKEMLHNSALRGEQMKGHASTIEGEVKKMIADWGDEGEIELLDFFAELTI
YTSTACLIGLKFREQLDHRFAEYYHDLERGTDPLCYVDPYLPIESFKRRDEARVKLVALVQEIMDQRLANPPK
DKADRDMLDVLVSIKDEDGKPRFSADEITGMFISLMFAGHHTSSGTSAWTLIELIRHPDVYAEVLAELEELYA
DGHEVSFHALRSIPKLDNVVKETLRLHPPLIILMRVAKGEFEVEGFPIHEGDYVAASPAISNRIPEDFPDPDA
FKPDRYNKPEQADIVNRWTWIPFGAGRHRCVGAAFAQMQIKAIFSVLLREYDFEMAQPADSYRNDHSKMVVQL
ARPAKVRYRKRNA
>CYP51B1_Mycobacterium avium hominissuis MAH-E-2514 2725106454
MTTSTVVPRVSGGEEEHGHLEEFRTDPIGLMQRVRDECGDVGWFQLVDKHVILLSGAQANEFFFRSADEDLDQ
AEAYPFMTPIFGKGVVFDASPERRKEMLHNSALRGEQMKGHASTIEGEVKKMIADWGDEGEIELLDFFAELTI
YTSTACLIGLKFREQLDHRFAEYYHDLERGTDPLCYVDPYLPIESFKRRDEARVKLVALVQEIMDQRLANPPK
DKADRDMLDVLVSIKDEDGKPRFSADEITGMFISLMFAGHHTSSGTSAWTLIELIRHPDVYAEVLAELEELYA
DGHEVSFHALRSIPKLDNVVKETLRLHPPLIILMRVAKGEFEVEGFPIHEGDYVAASPAISNRIPEDFPDPDA
FKPDRYNKPEQADIVNRWTWIPFGAGRHRCVGAAFAQMQIKAIFSVLLREYDFEMAQPADSYRNDHSKMVVQL
ARPAKVRYRKRNA
>CYP51B1_Mycobacterium avium hominissuis MAH-E-149-7 2725067131
MTTSTVVPRVSGGEEEHGHLEEFRTDPIGLMQRVRDECGDVGWFQLVDKHVILLSGAQANEFFFRSADEDLDQ
AEAYPFMTPIFGKGVVFDASPERRKEMLHNSALRGEQMKGHASTIEGEVKKMIADWGDEGEIELLDFFAELTI
YTSTACLIGLKFREQLDHRFAEYYHDLERGTDPLCYVDPYLPIESFKRRDEARVKLVALVQEIMDQRLANPPK
DKADRDMLDVLVSIKDEDGKPRFSADEITGMFISLMFAGHHTSSGTSAWTLIELIRHPDVYAEVLAELEELYA
DGHEVSFHALRSIPKLDNVVKETLRLHPPLIILMRVAKGEFEVEGFPIHEGDYVAASPAISNRIPEDFPDPDA
FKPDRYNKPEQADIVNRWTWIPFGAGRHRCVGAAFAQMQIKAIFSVLLREYDFEMAQPADSYRNDHSKMVVQL
ARPAKVRYRKRNA
>CYP51B1_Mycobacterium avium hominissuis MAH-E-149-3 2725063465
MTTSTVVPRVSGGEEEHGHLEEFRTDPIGLMQRVRDECGDVGWFQLVDKHVILLSGAQANEFFFRSADEDLDQ
AEAYPFMTPIFGKGVVFDASPERRKEMLHNSALRGEQMKGHASTIEGEVKKMIADWGDEGEIELLDFFAELTI

YTSTACLIGLKFREQLDHRFAEYYHDLERGTDPLCYVDPYLPIESFKRRDEARVKLVALVQEIMDQRLANPPK
DKADRDMLDVLVSIKDEDGKPRFSADEITGMFISLMFAGHHTSSGTSAWTLIELIRHPDVYAEVLAELEELYA
DGHEVSFHALRSIPKLDNVVKETLRLHPPLIILMRVAKGEFEVEGFPIHEGDYVAASPAISNRIPEDFPDPDA
FKPDRYNKPEQADIVNRWTWIPFGAGRHRCVGAAFAQMQIKAIFSVLLREYDFEMAQPADSYRNDHSKMVVQL
ARPAKVRYRKRNA
>CYP51B1_Mycobacterium avium hominissuis MAH-E-149-2 2725049999
MTTSTVVPRVSGGEEEHGHLEEFRTDPIGLMQRVRDECGDVGWFQLVDKHVILLSGAQANEFFFRSADEDLDQ
AEAYPFMTPIFGKGVVFDASPERRKEMLHNSALRGEQMKGHASTIEGEVKKMIADWGDEGEIELLDFFAELTI
YTSTACLIGLKFREQLDHRFAEYYHDLERGTDPLCYVDPYLPIESFKRRDEARVKLVALVQEIMDQRLANPPK
DKADRDMLDVLVSIKDEDGKPRFSADEITGMFISLMFAGHHTSSGTSAWTLIELIRHPDVYAEVLAELEELYA
DGHEVSFHALRSIPKLDNVVKETLRLHPPLIILMRVAKGEFEVEGFPIHEGDYVAASPAISNRIPEDFPDPDA
FKPDRYNKPEQADIVNRWTWIPFGAGRHRCVGAAFAQMQIKAIFSVLLREYDFEMAQPADSYRNDHSKMVVQL
ARPAKVRYRKRNA
>CYP51B1_Mycobacterium avium hominissuis MAH-E-118 2678565924
MTTSTVVPRVSGGEEEHGHLEEFRTDPIGLMQRVRDECGDVGWFQLVDKHVILLSGAQANEFFFRSADEDLDQ
AEAYPFMTPIFGKGVVFDASPERRKEMLHNSALRGEQMKGHASTIEGEVKKMIADWGDEGEIELLDFFAELTI
YTSTACLIGLKFREQLDHRFAEYYHDLERGTDPLCYVDPYLPIESFKRRDEARVKLVALVQEIMDQRLANPPK
DKADRDMLDVLVSIKDEDGKPRFSADEITGMFISLMFAGHHTSSGTSAWTLIELIRHPDVYAEVLAELEELYA
DGHEVSFHALRSIPKLDNVVKETLRLHPPLIILMRVAKGEFEVEGFPIHEGDYVAASPAISNRIPEDFPDPDA
FKPDRYNKPEQADIVNRWTWIPFGAGRHRCVGAAFAQMQIKAIFSVLLREYDFEMAQPADSYRNDHSKMVVQL
ARPAKVRYRKRNA
>CYP51B1_Mycobacterium avium hominissuis MAH-E-106 2678352575
MTTSTVVPRVSGGEEQHGHLEEFRTDPIGLMQRVRDECGDVGWFQLVDKHVILLSGAQANEFFFRSADEDLDQ
AEAYPFMTPIFGKGVVFDASPERRKEMLHNSALRGEQMKGHASTIEGEVKKMIADWGDEGEIELLDFFAELTI
YTSTACLIGLKFREQLDHRFAEYYHDLERGTDPLCYVDPYLPIESFKRRDEARVKLVALVQEIMDQRLANPPK
DKADRDMLDVLVSIKDEDGKPRFSADEITGMFISLMFAGHHTSSGTSAWTLIELIRHPDVYAEVLAELEELYA
DGQEVSFHALRSIPKLDNVVKETLRLHPPLIILMRVAKGEFEVEGFPIHEGDYVAASPAISNRIPEDFPDPDA
FKPDRYNKPEQADIVNRWTWIPFGAGRHRCVGAAFAQMQIKAIFSVLLREYDFEMAQPADSYRNDHSKMVVQL
ARPAKVRYRKRNA
>CYP51B1_Mycobacterium avium hominissuis MAH-E-101-6 2724982014
MTTSTVVPRVSGGEEEHGHLEEFRTDPIGLMQRVRDECGDVGWFQLVDKHVILLSGAQANEFFFRSADEDLDQ
AEAYPFMTPIFGKGVVFDASPERRKEMLHNSALRGEQMKGHASTIEGEVKKMIADWGDEGEIELLDFFAELTI
YTSTACLIGLKFREQLDHRFAEYYHDLERGTDPLCYVDPYLPIESFKRRDEARVKLVALVQEIMDQRLANPPK
DKADRDMLDVLVSIKDEDGKPRFSADEITGMFISLMFAGHHTSSGTSAWTLIELIRHPDVYAEVLAELEELYA
DGHEVSFHALRSIPKLDNVVKETLRLHPPLIILMRVAKGEFEVEGFPIHEGDYVAASPAISNRIPEDFPDPDA
FKPDRYNKPEQADIVNRWTWIPFGAGRHRCVGAAFAQMQIKAIFSVLLREYDFEMAQPADSYRNDHSKMVVQL
ARPAKVRYRKRNA
>CYP51B1_Mycobacterium avium hominissuis MAH 2721 2573740872
MTTSTVVPRVSGGEEEHGHLEEFRTDPIGLMQRVRDECGDVGWFQLVDKHVILLSGAQANEFFFRSADEDLDQ
AEAYPFMTPIFGKGVVFDASPERRKEMLHNSALRGEQMKGHASTIEGEVKKMIADWGDEGEIELLDFFAELTI
YTSTACLIGLKFREQLDHRFAEYYHDLERGTDPLCYVDPYLPIESFKRRDEARVKLVALVQEIMDQRLANPPK
DKADRDMLDVLVSIKDEDGKPRFSADEITGMFISLMFAGHHTSSGTSAWTLIELIRHPDVYAEVLAELEELYA
DGHEVSFHALRSIPKLDNVVKETLRLHPPLIILMRVAKGEFEVEGFPIHEGDYVAASPAISNRIPEDFPDPDA
FKPDRYNKPEQADIVNRWTWIPFGAGRHRCVGAAFAQMQIKAIFSVLLREYDFEMAQPADSYRNDHSKMVVQL
ARPAKVRYRKRNA
>CYP51B1_Mycobacterium avium hominissuis 10-5606 2582116813
MTTSTVVPRVSGGEEQHGHLEEFRTDPIGLMQRVRDECGDVGWFQLVDKHVILLSGAQANEFFFRSADEDLDQ
AEAYPFMTPIFGKGVVFDASPERRKEMLHNSALRGEQMKGHASTIEGEVKKMIADWGDEGEIELLDFFAELTI
YTSTACLIGLKFREQLDHRFAEYYHDLERGTDPLCYVDPYLPIESFKRRDEARVKLVALVQEIMDQRLANPPK
DKADRDMLDVLVSIKDEDGKPRFSADEITGMFISLMFAGHHTSSGTSAWTLIELIRHPDVYAEVLAELEELYA
DGQEVSFHALRSIPKLDNVVKETLRLHPPLIILMRVAKGEFEVEGFPIHEGDYVAASPAISNRIPEDFPDPDA
FKPDRYNKPEQADIVNRWTWIPFGAGRHRCVGAAFAQMQIKAIFSVLLREYDFEMAQPADSYRNDHSKMVVQL
ARPAKVRYRKRNA
>CYP51B1_Mycobacterium avium hominissuis MAH-E-108 2724992347
MTTSTVVPRVSGGEEEHGHLEEFRTDPIGLMQRVRDECGDVGWFQLVDKHVILLSGAQANEFFFRSADEDLDQ
AEAYPFMTPIFGKGVVFDASPERRKEMLHNSALRGEQMKGHASTIEGEVKKMIADWGDEGEIELLDFFAELTI
YTSTACLIGLKFREQLDHRFAEYYHDLERGTDPLCYVDPYLPIESFKRRDEARVKLVALVQEIMDQRLANPPK
DKADRDMLDVLVSIKDEDGKPRFSADEITGMFISLMFAGHHTSSGTSAWTLIELIRHPDVYAEVLAELEELYA

DGHEVSFHALRSIPKLDNVVKETLRLHPPLIILMRVAKGEFEVEGFPIHEGDYVAASPAISNRIPEDFPDPDA
FKPDRYNKPEQADIVNRWTWIPFGAGRHRCVGAAFAQMQIKAIFSVLLREYDF
>CYP51B1_Mycobacterium avium MAV_120809_2495 2580742796
MVPRVSGGEEEHGHLEEFRTDPIGLMQRVRDECGDVGWFQLVDKHVILLSGAQANEFFFRSADEDLDQAEAYP
FMTPIFGKGVVFDASPERRKEMLHNSALRGEQMKGHASTIEGEVKKMIADWGDEGEIELLDFFAELTIYTSTA
CLIGLKFREQLDHRFAEYYHDLERGTDPLCYVDPYLPIESFKRRDEARVKLVALVQEIMDQRLANPPKDKADR
DMLDVLVSIKDEDGKPRFSADEITGMFISLMFAGHHTSSGTSAWTLIELIRHPDVYAEVLAELEELYADGHEV
SFHALRSIPKLDNVVKETLRLHPPLIILMRVAKGEFEVEGFPIHEGDYVAASPAISNRIPEDFPDPDAFKPDR
YNKPEQADIVNRWTWIPFGAGRHRCVGAAFAQMQIKAIFSVLLREYDFEMAQPADSYRNDHSKMVVQLARPAK
VRYRKRNA
>CYP51B1_Mycobacterium avium paratuberculosis 11-1786 2592477832
MTTSTVVPRISGGEEEHGHLEEFRTDPIGLMQRVRDECGDVGWFQLVDKHVILLSGAQANEFFFRSADEDLDQ
AEAYPFMTPIFGKGVVFDASPERRKEMLHNSALRGEQMKGHASTIEGEVKKMIADWGDEGEIELLDFFAELTI
YTSTACLIGLKFREQLDHRFAEYYHDLERGTDPLCYVDPYLPIESFKRRDEARVKLVALVQEIMDQRL
>CYP102B13_Mycobacterium vaccae 95051 2689431763
MAIVGDRRSFPKLPHPPRRVPILGDVFGFSTDILTQPPAAMVGPVFEFRFLGARYVVAAGPEAVVELSDESRF
CKHIGPDIEALRIMGGDGLFTAYNDEPNWQIAHDVLMPAFSRQAMRDYHAVMFDVADELLARWDARADTGELI
DVPADTTRVTLETIGRCAAGHPFGCFDSEDPHPFVEHMVAGLEGSDRLGVLRATFLPGAVARRAERAVRYHAR
QLHAIADEIIGERVREGLGRHDDLLELMLVSELEPANIRYQLINFLVAGHETTSGALSFALYFLSVHPHVAER
ARAEIAEVWGDDDRPDFELIAKLRYVRRVFDEALRLQPTVPGYYRAAREDTVLAGVHPMRKGDWVLALTMGLH
RDRRWGEDPDAFDPDRFAAEQVKARPAGLYKPFGTGPRSCIGRQFALHEAVLLLAVLLRRYDLTPDPDYRLEV
AQRLTLMPKDFRLTLTRRG
>CYP102G37_Mycobacterium obuense UC1 2638292914
MQWMMAQAKELGPIMRLRIANTNTIVVSDGDLVAELSDTARFCKSVYTELVQLRDIGGDGLFTAFNDEPNWRK
AHNILLPAFSLEAMSQYHATMVDVARKLLDRWDRYAAAASVVEIPEDMTRLTFDTIGLCGFGFDLESFQRETP
HPFVEAMSRALHHAQGLAATLPIMNAFKRTSGEQYERDIALMHKVVDDVVRQRLESGDNHTDDLLGRMLNTAD
KDTGQRLDPENVRNQVITFLVAGHETTSGAMSFALYYLAKNPAVLARAQAEVDALWGTSDDIDPSYTDIGRLT
YVQQILNETLRLWPTAPGYAVEPFEDTVIGGRYAVKRGDVITILTPPLHRSPEWGDSVEAFDPERFNTENVSR
RPMHLYKPFGNGERACIGRQFALHEATLVLAMIIHRYRLNDYSNYQLKITEALTIKPGGLTLKLARRLPADRS
VRPADTGRHASTVVAQPGNAVELGTTLSVLHGSNLGTSARLARDITDRATQAGYNATLGSLNDAVGQLRSDSV
VLVVASSYNGKPTDDADEFLPWLEFVTPGSLDGLRYAVLGVGDRNWAATYQHVPATIDARLSAAGGHRLIDRG
AADVAGDFTGEVERWNTDLWATILPRDGITAEPPDEHDAGLTIESAVWSLDQDLQSRHGLEPMTVVSAHELVD
TTHPLGRSKMFLRLELPAGTTYRTADHLALLPQNPAELIARVQRRFGTDLTELVILHRHGRVPGHIPTERPIT
LGEVFSRYVELQSPATIEQIETMIARCSCPPERTALQKLVDDADEFAARITTARVSLLDLLESYRSVELGVDT
FLAMLPALAPRRYSVSSSPAAQPGHIDLMVAAVDAPHLSGEGTYRGTASTYVRQLQQGDTVLGRVLPCSDDFR
LENDQPAIVISAGTGLAPFRGVIQDRAHSADAAPLLCYFGCDHPDVDFLHREELEEAESNGVVDLRPTYFAAP
EDNCAFVQDRIRKEGREVWRMLEEEQRHLRVCGDGARLVPGVTRALLDVYRTQSGNDDEERALAWFEALTASG
RLVVDAWSSK
>CYP102L2_Mycobacterium haemophilum DSM 44634 ATCC 29548 2646678420
MIQAEATPIPHPRFRLPVLGDLLTIDFARPTQGLAAETRKLGSGIFEQRIFKFPVIVLADTALIADVNNEDLW
EKHLGPSLYKLRSVAGDGLFTAYNDEPNWRKAHNILMPAFTKTAMLNYHDSMTGTVQELLDTWKTNGANQSWI
DIATEANRLTIEIIARAGLDHSFTKLSDRSGNPFIAAVLRELAYANRRTDAIPFYEKLLGAKCKAQHYGDKVY
VRQQVIDIIDARRRNPHASNRTDMLDIMLHTTDPETGEQLDNDNIVNQILTLLVAGSETSANTIAFALHYLSA
HPDIAAQAQVEIDQRWPSSDFPNIQFHDIAKLRYLRRIVDETLRLWPVAPGYFRRARHDSTIGNGKYPFKAGD
WVFVLLVAAHRDPAWGADADEFNPDRFLPENLRKLPPYTYKPFGTGPRACMGRQFALHEIVLTLAAILHQYDL
QPRPGYQLKVSETLTLKPAGLHLRLHRRR
>CYP102L2_Mycobacterium haemophilum UC1 2675199852
MIQAEATPIPHPRFRLPVLGDLLTIDFARPTQGLAAETRKLGSGIFEQRIFKFPVIVLADTALIADVNNEDLW
EKHLGPSLYKLRSVAGDGLFTAYNDEPNWRKAHNILMPAFTKTAMLNYHDSMTGTVQELLDTWKTNGANQSWI
DIATEANRLTIEIIARAGLDHSFTKLSDRSGNPFIAAVLRELAYANRRTDAIPFYEKLLGAKRKAQHYGDKVH
VRQQVIDIIDARRRNPHASNRTDMLDIMLHTTDPETGEQLDNDNIVNQILTLLVAGSETSANTIAFALHYLSA
HPDIAAQAQAEIDQRWPSSDFPNIQFHDIAKLRYLRRIVDETLRLWPVAPGYFRRARHDSTIGNGKYPFKAGD
WVFVLLVAAHRDPVWGADADGFNPDRFLPENLRKLPPYTYKPFGTGPRACMGRQFALHEIVLTLAAILHQYDL
QPRPGYQLKVSETLTLKPAGLYLRLHRRPDRSYC
>CYP102L2_Mycobacterium haemophilum UC2 2672790673
MIQAEATPIPHPRFRLPVLGDLLTIDFARPTQGLAAETRKLGSGIFEQRIFKFPVIVLADTALIADVNNEDLW
EKHLGPSLYKLRSVAGDGLFTAYNDEPNWRKAHNILMPAFTKTAMLNYHDSMTGTVQELLDTWKTNGANQSWI
DIATEANRLTIEIIARAGLDHSFTKLSDRSGNPFIAAVLRELAYANRRTDAIPFYEKLLGAKRKAQHYGDKVH

VRQQVIDIIDARRRNPHASNRTDMLDIMLHTTDPETGEQLDNDNIVNQILTLLVAGSETSANTIAFALHYLSA
HPDIAAQAQAEIDQRWPSSDFPNIQFHDIAKLRYLRRIVDETLRLWPVAPGYFRRARHDSTIGNGKYPFKAGD
WVFVLLVAAHRDPVWGADADGFNPDRFLPENLRKLPPYTYKPFGTGPRACMGRQFALHEIVLTLAAILHQYDL
QPRPGYQLKVSETLTLKPAGLYLRLHRRPDRSYC
>CYP102L2_Mycobacterium haemophilum UC3 2714153722
MIQAEATPIPHPRFRLPVLGDLLTIDFARPTQGLAAETRKLGSGIFEQRIFKFPVIVLADTALIADVNNEDLW
EKHLGPSLYKLRSVAGDGLFTAYNDEPNWRKAHNILMPAFTKTAMLNYHDSMTGTVQELLDTWKTNGANQSWI
DIATEANRLTIEIIARAGLDHSFTKLSDRSGNPFIAAVLRELAYANRRTDAIPFYEKLLGAKRKAQHYGDKVH
VRQQVIDIIDARRRNPHASNRTDMLDIMLHTTDPETGEQLDNDNIVNQILTLLVAGSETSANTIAFALHYLSA
HPDIAAQAQAEIDQRWPSSDFPNIQFHDIAKLRYLRRIVDETLRLWPVAPGYFRRARHDSTIGNGKYPFKAGD
WVFVLLVAAHRDPVWGADADGFNPDRFLPENLRKLPPYTYKPFGTGPRACMGRQFALHEIVLTLAAILHQYDL
QPRPGYQLKVSETLTLKPAGLYLRLHRRPDRSYC
>CYP102L2_Mycobacterium haemophilum UC4 2714149502
MIQAEATPIPHPRFRLPVLGDLLTIDFARPTQGLAAETRKLGSGIFEQRIFKFPVIVLADTALIADVNNEDLW
EKHLGPSLYKLRSVAGDGLFTAYNDEPNWRKAHNILMPAFTKTAMLNYHDSMTGTVQELLDTWKTNGANQSWI
DIATEANRLTIEIIARAGLDHSFTKLSDRSGNPFIAAVLRELAYANRRTDAIPFYEKLLGAKRKAQHYGDKVH
VRQQVIDIIDARRRNPHASNRTDMLDIMLHTTDPETGEQLDNDNIVNQILTLLVAGSETSANTIAFALHYLSA
HPDIAAQAQAEIDQRWPSSDFPNIQFHDIAKLRYLRRIVDETLRLWPVAPGYFRRARHDSTIGNGKYPFKAGD
WVFVLLVAAHRDPVWGADADGFNPDRFLPENLRKLPPYTYKPFGTGPRACMGRQFALHEIVLTLAAILHQYDL
QPRPGYQLKVSETLTLKPAGLYLRLHRRPDRSYC
>CYP102L3_Mycobacterium haemophilum DSM 44634 ATCC 29548 2646680714
MTLATATPIPHPRFRLPIVGDLLTTDFAKPAQGLAAEFGKLGSGIVEQRIFGLSIIALADPALIDEVNDETVW
EKHIGPVMRKLRLLMGDGLFTADNEEPNWRKAHNILMPAFTKTAMMAYHDTMAATVRELVEFWDTDSAGQSWI
DIPAQANRLTIEIIARAGLGYSFSKLSDPGDNAFVAAVIRELSYFRLTARTDAIPFYDKLFGQKHHQQHVADN
AYIRRQVCDIIEARRRNPTAGPRDDMLDIMLHNTDPDTGEHLDTDNIINQILTLLVAGSETSANAIAFALHYL
SIHPDVAARARREVDQRWPDRGIPDVSFGDIAKLRYLRRIVDETLRLWPVAPGYLRKARRDTTLENGKYSFRQ
GDWVLVLLPAAHRDRAWGPDAHDFNPDRFLPENLRKLPPYTYKPFGTGPRACIGRQFALHEMVLALAMIVHQF
TVEPHPGYQLEVAETMTLKPKNLRLRLHRRR
>CYP102L3_Mycobacterium haemophilum UC1 2675200090
MTLATATPIPHPCFRLPIVGDLLTTDFAKPAQGLAAEFGKLGSGIVEQRIFGLSIIALADPALIDEVNDETVW
EKHIGPVMRKLRLLMGDGLFTADNEEPNWRKAHNILMPAFTKTAMMAYHDTMAATVRELVEFWDTDSAGQSWI
NIPAQANRLTIEIIARAGLGYSFSKLSDLSDNAFVAAVIRELSYFRLTARTDAIPFYGKLFGQKHHQQHVADN
AYIRRQVCDIIEARRRNPTAGPRDDMLDIMLHNTDPDTGEHLDTDNISNQILTLLVAGSETSANAIAFALHYL
SIHPDVAARARREVDQRWPDRGIPDVLFGDIAKLRYLRRIVDETLRLWPVAPGYLRKARCGTTLGNGKYSFRQ
GDWVLVLLPAAHRDRAWGPDAHDFNPDRFLPENLRKLPPYTYKPFGTGPRACIGRQFALHEMVLALAMIVHQF
TVEPHPGYQLEVAETMTLKPKNLRLRLHRRR
>CYP102L3_Mycobacterium haemophilum UC2 2672790911
MTLATATPIPHPCFRLPIVGDLLTTDFAKPAQGLAAEFGKLGSGIVEQRIFGLSIIALADPALIDEVNDETVW
EKHIGPVMRKLRLLMGDGLFTADNEEPNWRKAHNILMPAFTKTAMMAYHDTMAATVRELVEFWDTDSAGQSWI
NIPAQANRLTIEIIARAGLGYSFSKLSDLSDNAFVAAVIRELSYFRLTARTDAIPFYGKLFGQKHHQQHVADN
AYIRRQVCDIIEARRRNPTAGPRDDMLDIMLHNTDPDTGEHLDTDNISNQILTLLVAGSETSANAIAFALHYL
SIHPDVAARARREVDQRWPDRGIPDVLFGDIAKLRYLRRIVDETLRLWPVAPGYLRKARCGTTLGNGKYSFRQ
GDWVLVLLPAAHRDRAWGPDAHDFNPDRFLPENLRKLPPYTYKPFGTGPRACIGRQFALHEMVLALAMIVHQF
TVEPHPGYQLEVAETMTLKPKNLRLRLHRRR
>CYP102L3_Mycobacterium haemophilum UC3 2714154197
MTLATATPIPHPCFRLPIVGDLLTTDFAKPAQGLAAEFGKLGSGIVEQRIFGLSIIALADPALIDEVNDETVW
EKHIGPVMRKLRLLMGDGLFTADNEEPNWRKAHNILMPAFTKTAMMAYHDTMAATVRELVEFWDTDSAGQSWI
NIPAQANRLTIEIIARAGLGYSFSKLSDLSDNAFVAAVIRELSYFRLTARTDAIPFYGKLFGQKHHQQHVADN
AYIRRQVCDIIEARRRNPTAGPRDDMLDIMLHNTDPDTGEHLDTDNISNQILTLLVAGSETSANAIAFALHYL
SIHPDVAARARREVDQRWPDRGIPDVLFGDIAKLRYLRRIVDETLRLWPVAPGYLRKARCGTTLGNGKYSFRQ
GDWVLVLLPAAHRDRAWGPDAHDFNPDRFLPENLRKLPPYTYKPFGTGPRACIGRQFALHEMVLALAMIVHQF
TVEPHPGYQLEVAETMTLKPKNLRLRLHRRR
>CYP102L3_Mycobacterium haemophilum UC4 2714151610
MTLATATPIPHPCFRLPIVGDLLTTDFAKPAQGLAAEFGKLGSGIVEQRIFGLSIIALADPALIDEVNDETVW
EKHIGPVMRKLRLLMGDGLFTADNEEPNWRKAHNILMPAFTKTAMMAYHDTMAATVRELVEFWDTDSAGQSWI
NIPAQANRLTIEIIARAGLGYSFSKLSDLSDNAFVAAVIRELSYFRLTARTDAIPFYGKLFGQKHHQQHVADN
AYIRRQVCDIIEARRRNPTAGPRDDMLDIMLHNTDPDTGEHLDTDNISNQILTLLVAGSETSANAIAFALHYL
SIHPDVAARARREVDQRWPDRGIPDVLFGDIAKLRYLRRIVDETLRLWPVAPGYLRKARCGTTLGNGKYSFRQ

GDWVLVLLPAAHRDRAWGPDAHDFNPDRFLPENLRKLPPYTYKPFGTGPRACIGRQFALHEMVLALAMIVHQF
TVEPHPGYQLEVAETMTLKPKNLRLRLHRRR
>CYP102L4P_Mycobacterium haemophilum DSM 44634 ATCC 29548 2646677760
MRSLAGDGLFTAFNDEPNWSKAHNILMPAFTKAAMASYHASMLDTAGELVSTWSQHAADGTWVDIPGEANRLT
TEMVARAGLGYSFTKLSDTSESPFNTAFLRGLKYAIGKVRTDALPLYEQLLGRRGRNQHQRDTQYLREQVAAI
IEARRNSDSRDDAAKDMLDIMLHETDPDTGERLDEANIVNQVMTLLIAGSETSANTIAFALHYLSTNPGMAAA
ARAEVDQRWPGRDIPEVSFDDVAKLRYLRRIVDETLRIWPIAPGYFR
>CYP102L4P_Mycobacterium haemophilum UC1 2675201963
MMTKTSPHASVLPRPRFRLPVMGDLLTLNFREPVMGTAREMDAYGGIVEKRIFNLRLIVISRTDLIDEVNDES
RWEKHVGHVLLNMRSLAGDGLFTAFNDEPNWSKAHNILMPAFTKAAMASYHASMLDTAGELVSTWSQHAADGT
WVDIPGEANRLTTEMVARAGLGYSFTKLSDTSESPFNTAFLRGLKYAIGKVRTDALPLYEQLLGRRGRNQHQR
DTQYLREQVAAIIEARRNSDSRDDAAKDMLDIMLHETDPDTGERLDEANIVNQVMTLLIAGSETSANTIAFAL
HYLATNPGMAAAARAEVDQRWPDRDIPEVSFDDVAKLRYLRRIVDETLRLWPIAPGYFRQAKTDTTLGEGRYR
FTAGDRVFVFLVAAHRDRPWGDDADQFNPDRFLPENIRKLPPRIYKPFGTGSRACIGRQFALHEILLTLAMIL
HQFTLEPYPGYQLQVSETVSLKPKDLRLRLHRRP
>CYP102L4P_Mycobacterium haemophilum UC2 2672791400
MMTKTSPHASVLPRPRFRLPVMGDLLTLNFREPVMGTAREMDAYGGIVEKRIFNLRLIVISRTDLIDEVNDES
RWEKHVGHVLLNMRSLAGDGLFTAFNDEPNWSKAHNILMPAFTKAAMASYHASMLDTAGELVSTWSQHAADGT
WVDIPGEANRLTTEMVARAGLGYSFTKLSDTSESPFNTAFLRGLKYAIGKVRTDALPLYEQLLGRRGRNQHQR
DTQYLREQVAAIIEARRNSDSRDDAAKDMLDIMLHETDPDTGERLDEANIVNQVMTLLIAGSETSANTIAFAL
HYLATNPGMAAAARAEVDQRWPDRDIPEVSFDDVAKLRYLRRIVDETLRLWPIAPGYFRQAKTDTTLGEGRYR
FTAGDRVFVFLVAAHRDRPWGDDADQFNPDRFLPENIRKLPPRIYKPFGTGSRACIGRQFALHEILLTLAMIL
HQFTLEPYPGYQLQVSETVSLKPKDLRLRLHRRP
>CYP102L4P_Mycobacterium haemophilum UC3 2714154686
MMTKTSPHASVLPRPRFRLPVMGDLLTLNFREPVMGTAREMDAYGGIVEKRIFNLRLIVISRTDLIDEVNDES
RWEKHVGHVLLNMRSLAGDGLFTAFNDEPNWSKAHNILMPAFTKAAMASYHASMLDTAGELVSTWSQHAADGT
WVDIPGEANRLTTEMVARAGLGYSFTKLSDTSESPFNTAFLRGLKYAIGKVRTDALPLYEQLLGRRGRNQHQR
DTQYLREQVAAIIEARRNSDSRDDAAKDMLDIMLHETDPDTGERLDEANIVNQVMTLLIAGSETSANTIAFAL
HYLATNPGMAAAARAEVDQRWPDRDIPEVSFDDVAKLRYLRRIVDETLRLWPIAPGYFRQAKTDTTLGEGRYR
FTAGDRVFVFLVAAHRDRPWGDDADQFNPDRFLPENIRKLPPRIYKPFGTGSRACIGRQFALHEILLTLAMIL
HQFTLEPYPGYQLQVSETVSLKPKDLRLRLHRRP
>CYP102L4P_Mycobacterium haemophilum UC4 2714149997
MMTKTSPHASVLPRPRFRLPVMGDLLTLNFREPVMGTAREMDAYGGIVEKRIFNLRLIVISRTDLIDEVNDES
RWEKHVGHVLLNMRSLAGDGLFTAFNDEPNWSKAHNILMPAFTKAAMASYHASMLDTAGELVSTWSQHAADGT
WVDIPGEANRLTTEMVARAGLGYSFTKLSDTSESPFNTAFLRGLKYAIGKVRTDALPLYEQLLGRRGRNQHQR
DTQYLREQVAAIIEARRNSDSRDDAAKDMLDIMLHETDPDTGERLDEANIVNQVMTLLIAGSETSANTIAFAL
HYLATNPGMAAAARAEVDQRWPDRDIPEVSFDDVAKLRYLRRIVDETLRLWPIAPGYFRQAKTDTTLGEGRYR
FTAGDRVFVFLVAAHRDRPWGDDADQFNPDRFLPENIRKLPPRIYKPFGTGSRACIGRQFALHEILLTLAMIL
HQFTLEPYPGYQLQVSETVSLKPKDLRLRLHRRP
>CYP102L1_Mycobacterium abscessus bolletii 50594 2562499526
MTNTRASTHGTAPLPHPRFRLPILGDLASVDFAQPIQGLAREARRHHGIFEQRIGDFPVVVVDGPELIEEINN
EELWEKNVGPTLHKLRSVAGDGLFTAYNTEDNWRKAHDILMPAFTKQAMTNYHASIVDTVHELVTVWNSHAKN
KSWVDVPFDLNRLTIEIISRSGFGYSFSSLADPRENPFLSAVLRELQYANRRTDSIPLYEQFLGGARRREHVA
DKRAIRAQIDKIIDARRAESRTGQSPDILDIMLTSSDPLTGEKLDNNNIGNQILTFLVAGSETSANAIAFALH
FLSTHPDIAAKARAEVDASWPARAFPAFAFDQIAKLRYLRLVVDETLRLWPVAPGYFRQAKQDTTIGEGRYSF
KEKDWVFVNLLAAQRHPSWGPDADQFNPDRFLTENRRKLPTHVHRPFGVGARACIGRQFAQHEILIALAAILH
QYELTPRPGYKLKVSETLTLKPSALELGLTKRT
>CYP102L1_Mycobacterium abscessus 159 2550355962
MTNTRASTHGTAPLPHPRFRLPILGDLASVDFAQPIQGLAREARRHHGIFEQRIGDFPVVVVDGPELIEEINN
EELWEKNVGPTLHKLRSVAGDGLFTAYNTEDNWRKAHDILMPAFTKQAMTNYHASIVDTVHELVTVWNSHAKN
KSWVDVPFDLNRLTIEIISRSGFGYSFSSLADPRENPFLSAVLRELQYANRRTDSIPLYEQFLGGARRGHVA
DKRAIRAQIDKIIDARRAESRTGQSPDILDIMLTSSDPLTGEKLDNNNIGNQILTFLVAGSETSANAIAFALH
FLSTHPDIAAKARAEVDASWPARAFPAFAFDQIAKLRYLRLVVDETLRLWPVAPGYFRQAKQDTTIGEGRYSF
KEKDWVFVNLLAAQRHPSWGPDADQFNPDRFLTENRRKLPTHVHRPFGVGARACIGRQFAQHEILIALAAILH
QYELTPRPGYKLKVSETLTLKPSALELGLTKRT
>CYP102L1_Mycobacterium abscessus M148 2550891505
MTNTRASTHGTAPLPHPRFRLPILGDLASVDFAQPIQGLAREARRHHGIFEQRIGDFPVVVVDGPELIEEINN
EELWEKNVGPTLHKLRSVAGDGLFTAYNTEDNWRKAHDILMPAFTKQAMTNYHASIVDTVHELVTVWNSHAKN

KSWVDVPFDLNRLTIEIISRSGFGYSFSSLADPRENPFLSAVLRELQYANRRTDSIPLYEQFLGGARRRGHVA
DKRAIRAQIDKIIDARRAESRTGQSPDILDIMLTSSDPLTGEKLDNNNIGNQILTFLVAGSETSANAIAFALH
FLSTHPDIAAKARAEVDASWPARAFPAFAFDQIAKLRYLRLVVDETLRLWPVAPGYFRQAKQDTTIGEGRYSF
KEKDWVFVNLLAAQRHPSWGPDADQFNPDRFLTENRRKLPTHVHRPFGVGARACIGRQFAQHEILIALAAILH
QYELTPRPGYKLKVSETLTLKPSALELGLTKRT
>CYP102L1_Mycobacterium abscessus M154 2550877935
MTNTRASTHGTAPLPHPRFRLPILGDLASVDFAQPIQGLAREARRHHGIFEQRIGDFPVVVVDGPELIEEINN
EELWEKNVGPTLHKLRSVAGDGLFTAYNTEDNWRKAHDILMPAFTKQAMTNYHASIVDTVHELVTVWNSHAKN
KSWVDVPFDLNRLTIEIISRSGFGYSFSSLADPRENPFLSAVLRELQYANRRTDSIPLYEQFLGGARRRGHVA
DKRAIRAQIDKIIDARRAESRTGQSPDILDIMLTSSDPLTGEKLDNNNIGNQILTFLVAGSETSANAIAFALH
FLSTHPDIAAKARAEVDASWPARAFPAFAFDQIAKLRYLRLVVDETLRLWPVAPGYFRQAKQDTTIGEGRYSF
KEKDWVFVNLLAAQRHPSWGPDADQFNPDRFLTENRRKLPTHVHRPFGVGARACIGRQFAQHEILIALAAILH
QYELTPRPGYKLKVSETLTLKPSALELGLTKRT
>CYP102L1_Mycobacterium abscessus M156 2550872626
MTNTRASTHGTAPLPHPRFRLPILGDLASVDFAQPIQGLAREARRHHGIFEQRIGDFPVVVVDGPELIEEINN
EELWEKNVGPTLHKLRSVAGDGLFTAYNTEDNWRKAHDILMPAFTKQAMTNYHASIVDTVHELVTVWNSHAKN
KSWVDVPFDLNRLTIEIISRSGFGYSFSSLADPRENPFLSAVLRELQYANRRTDSIPLYEQFLGGARRRGHVA
DKRAIRAQIDKIIDARRAESRTGQSPDILDIMLTSSDPLTGEKLDNNNIGNQILTFLVAGSETSANAIAFALH
FLSTHPDIAAKARAEVDASWPARAFPAFAFDQIAKLRYLRLVVDETLRLWPVAPGYFRQAKQDTTIGEGRYSF
KEKDWVFVNLLAAQRHPSWGPDADQFNPDRFLTENRRKLPTHVHRPFGVGARACIGRQFAQHEILIALAAILH
QYELTPRPGYKLKVSETLTLKPSALELGLTKRT
>CYP102L1_Mycobacterium abscessus M172 2550353308
MTNTRASTHGTAPLPHPRFRLPILGDLASVDFAQPIQGLAREARRHHGIFEQRIGDFPVVVVDGPELIEEINN
EELWEKNVGPTLHKLRSVAGDGLFTAYNTEDNWRKAHDILMPAFTKQAMTNYHASIVDTVHELVTVWNSHAKN
KSWVDVPFDLNRLTIEIISRSGFGYSFSSLADPRENPFLSAVLRELQYANRRTDSIPLYEQFLGGARRRGHVA
DKRAIRAQIDKIIDARRAESRTGQSPDILDIMLTSSDPLTGEKLDNNNIGNQILTFLVAGSETSANAIAFALH
FLSTHPDIAAKARAEVDASWPARAFPAFAFDQIAKLRYLRLVVDETLRLWPVAPGYFRQAKQDTTIGEGRYSF
KEKDWVFVNLLAAQRHPSWGPDADQFNPDRFLTENRRKLPTHVHRPFGVGARACIGRQFAQHEILIALAAILH
QYELTPRPGYKLKVSETLTLKPSALELGLTKRT
>CYP102L1_Mycobacterium abscessus PAP043 2661833779
MTNTRASTHGTAPLPHPRFRLPILGDLASVDFAQPIQGLAREARRHHGIFEQRIGDFPVVVVDGPELIEEINN
EELWEKNVGPTLHKLRSVAGDGLFTAYNTEDNWRKAHDILMPAFTKQAMTNYHASIVDTVHELVTVWNSHAKN
KSWVDVPFDLNRLTIEIISRSGFGYSFSSLADPRENPFLSAVLRELQYANRRTDSIPLYEQFLGGARRRGHVA
DKRAIRAQIDKIIDARRAESRTGQSPDILDIMLTSSDPLTGEKLDNNNIGNQILTFLVAGSETSANAIAFALH
FLSTHPDIAAKARAEVDASWPARAFPAFAFDQIAKLRYLRLVVDETLRLWPVAPGYFRQAKQDTTIGEGRYSF
KEKDWVFVNLLAAQRHPSWGPDADQFNPDRFLTENRRKLPTHVHRPFGVGARACIGRQFAQHEILIALAAILH
QYELTPRPGYKLKVSETLTLKPSALELGLTKRT
>CYP102L1_Mycobacterium abscessus PAP070 2696450471
MTNTRASTHGTAPLPHPRFRLPILGDLASVDFAQPIQGLAREARRHHGIFEQRIGDFPVVVVDGPELIEEINN
EELWEKNVGPTLHKLRSVAGDGLFTAYNTEDNWRKAHDILMPAFTKQAMTNYHASIVDTVHELVTVWNSHAKN
KSWVDVPFDLNRLTIEIISRSGFGYSFSSLADPRENPFLSAVLRELQYANRRTDSIPLYEQFLGGARRRGHVA
DKRAIRAQIDKIIDARRAESRTGQSPDILDIMLTSSDPLTGEKLDNNNIGNQILTFLVAGSETSANAIAFALH
FLSTHPDIAAKARAEVDASWPARAFPAFAFDQIAKLRYLRLVVDETLRLWPVAPGYFRQAKQDTTIGEGRYSF
KEKDWVFVNLLAAQRHPSWGPDADQFNPDRFLTENRRKLPTHVHRPFGVGARACIGRQFAQHEILIALAAILH
QYELTPRPGYKLKVSETLTLKPSALELGLTKRT
>CYP102L1_Mycobacterium abscessus PAP088 2669839431
MTNTRASTHGTAPLPHPRFRLPILGDLASVDFAQPIQGLAREARRHHGIFEQRIGDFPVVVVDGPELIEEINN
EELWEKNVGPTLHKLRSVAGDGLFTAYNTEDNWRKAHDILMPAFTKQAMTNYHASIVDTVHELVTVWNSHAKN
KSWVDVPFDLNRLTIEIISRSGFGYSFSSLADPRENPFLSAVLRELQYANRRTDSIPLYEQFLGGARRRGHVA
DKRAIRAQIDKIIDARRAESRTGQSPDILDIMLTSSDPLTGEKLDNNNIGNQILTFLVAGSETSANAIAFALH
FLSTHPDIAAKARAEVDASWPARAFPAFAFDQIAKLRYLRLVVDETLRLWPVAPGYFRQAKQDTTIGEGRYSF
KEKDWVFVNLLAAQRHPSWGPDADQFNPDRFLTENRRKLPTHVHRPFGVGARACIGRQFAQHEILIALAAILH
QYELTPRPGYKLKVSETLTLKPSALELGLTKRT
>CYP102U1_Mycobacterium sp. NRRL B-3805 2686848599
MTEFAPSLPPTLDDIVSAEGHAFPAGAELAGRPYALPIDLLGERYGPIFYADFNGFRKLYVCSMDLVDELCDE
ARFAKNITQTLDRVRPLAGDGLFTAYRGEPNWQKAHDVLLPGFSYAGLRSYHEAMLDINAELISRWDARVGQR
RVNVSDDLQKLAMDTVALAGFGARFASFDHDELAPIPQCFMSALTEAVTHGETAEFIAGRTELHNYFDELITG
HRAAGTGADLLYVMLGQDGSSALDTANIRNQIMTFLIAGQLTTSELMPNTVYNLIHHPAALDRVRREVDAVFG

TDDDYLPSYDDIGKLGYLRQAISETLRLSPPVLTFDRMALADTVIGGKYPIKAGEAVTVLVGALHRQPQWGDN
VELFDPDRFDPDQVAGRSSALFKPFGTGERSCIGRQFALHEATMLIARLVHRYRLVDSQHYVLQWESSLSRRP
IGFEVDLVRRTATERTSTASAVAAPTVSDGTSASSPVRAGTTLAVLHGSNLGTCRALATQLAEEAADIGCTTT
VGPLDDAAHGLPDADVILIVASSYNGQPTDDAKQFTTWLFGDQAAMSADTRFAVLGVGDHNWAETYQAVPIAI
DDRLTALGAQALVPRGAADTSGDMTGALEEFTSALWSAMSELFGDPDAAPLTDSDEPLYDLTLITGPITAAID
ARFAVTPMTVVLNDELVGNDNSLGQGKRYVRVTLPDDVDYSTGDHLTVLADNPPEAVEAAVNLLGIDPQLRLS
INPRRTSRRLIALDREVSVSELLTHFVELRKPATRSQLRKLAAANPCRPEQERLQALAESDDPCPLSPIECLQ
EFPACALTGAELLEMLEPMTPRHYSIASSSRLSPKEVALIVSVLDAPARSGRGLFKGVASNHLADIAPGACVR
ARVDPARQAFRAGADPEKNVILVSAGTGVAPFCGFLGDRLAAKQSGAPFAPALCFFGVRDPDVDYIFRDLFED
AERQGIVSMRPAFSRAPQAGVRYVQDRIAADADDVWALIGDPAKDTHVFVCGDGAKMAPAVREAFRDIYRART
GADDDGAREWLEVMVASDHYVEDVWAG
>CYP102U1_Mycobacterium sp. VKM Ac-1816D 2553519802
MTEFAPSLPPTLDDIVSAEGHAFPAGAELAGRPYALPIDLLGERYGPIFYADFNGFRKLYVCSMDLVDELCDE
ARFAKNITQTLDRVRPLAGDGLFTAYRGEPNWQKAHDVLLPGFSYAGLRSYHEAMLDINAELISRWDARVGQR
RVNVSDDLQKLAMDTVALAGFGARFASFDHDELAPIPQCFMSALTEAVTHGETAEFIAGRTELHNYFDELITG
HRAAGTGADLLYVMLGQDGSSALDTANIRNQIMTFLIAGQLTTSELMPNTVYNLIHHPAALDRVRREVDAVFG
TDDDYLPSYDDIGKLGYLRQAISETLRLSPPVLTFDRMALADTVIGGKYPIKAGEAVTVLVGALHRQPQWGDN
VELFDPDRFDPDQVAGRSSALFKPFGTGERSCIGRQFALHEATMLIARLVHRYRLVDSQHYVLQWESSLSRRP
IGFEVDLVRRTATERTSTASAVAAPTVSDGTSASSPVRAGTTLAVLHGSNLGTCRALATQLAEEAADIGCTTT
VGPLDDAAHGLPDADVILIVASSYNGQPTDDAKQFTTWLFGDQAAMSADTRFAVLGVGDHNWAETYQAVPIAI
DDRLTALGAQALVPRGAADTSGDMTGALEEFTSALWSAMSELFGDPDAAPLTDSDEPLYDLTLITGPITAAID
ARFAVTPMTVVLNDELVGNDNSLGQGKRYVRVTLPDDVDYSTGDHLTVLADNPPEAVEAAVNLLGIDPQLRLS
INPRRTSRRLIALDREVSVSELLTHFVELRKPATRSQLRKLAAANPCRPEQERLQALAESDDPCPLSPIECLQ
EFPACALTGAELLEMLEPMTPRHYSIASSSRLSPKEVALIVSVLDAPARSGRGLFKGVASNHLADIAPGACVR
ARVDPARQAFRAGADPEKNVILVSAGTGVAPFCGFLGDRLAAKQSGAPFAPALCFFGVRDPDVDYIFRDLFED
AERQGIVSMRPAFSRAPQAGVRYVQDRIAADADDVWALIGDPAKDTHVFVCGDGAKMAPAVREAFRDIYRART
GADDDGAREWLEVMVASDHYVEDVWAG
>CYP102U1_Mycobacterium neoaurum MN2 2746456295
MTEFAPSLPPTLDDIVSAEGHAFPAGAELAGRPYALPIDLLGERYGPIFYADFNGFRKLYVCSMDLVDELCDE
ARFAKNITQTLDRVRPLAGDGLFTAYRGEPNWQKAHDVLLPGFSYAGLRSYHEAMLDINAELISRWDARVGQR
RVNVSDDLQKLAMDTVALAGFGARFASFDHDELAPIPQCFMSALTEAVTHGETAEFIAGRTELHNYFDELITG
HRAAGTGADLLYVMLGQDGSSALDTANIRNQIMTFLIAGQLTTSELMPNTVYNLIHHPAALDRVRREVDAVFG
TDDDYLPSYDDIGKLGYLRQAISETLRLSPPVLTFDRMALADTVIGGKYPIKAGEAVTVLVGALHRQPQWGDN
VELFDPDRFDPDQVAGRSSALFKPFGTGERSCIGRQFALHEATMLIARLVHRYRLVDSQHYVLQWESSLSRRP
IGFEVDLVRRTATERTSTASAVAAPTVSDGTSASSPVRAGTTLAVLHGSNLGTCRALATQLAEEAADIGCTTT
VGPLDDAAHGLPDADVILIVASSYNGQPTDDAKQFTTWLFGDQAAMSADTRFAVLGVGDHNWAETYQAVPIAI
DDRLTALGAQALVPRGAADTSGDMTGALEEFTSALWSAMSELFGDPDAAPLTDSDEPLYDLTLITGPITAAID
ARFAVTPMTVVLNDELVGNDNSLGQGKRYVRVTLPDDVDYSTGDHLTVLADNPPEAVEAAVNLLGIDPQLRLS
INPRRTSRRLIALDREVSVSELLTHFVELRKPATRSQLRKLAAANPCRPEQERLQALAESDDPCPLSPIECLQ
EFPACALTGAELLEMLEPMTPRHYSIASSSRLSPKEVALIVSVLDAPARSGRGLFKGVASNHLADIAPGACVR
ARVDPARQAFRAGADPEKNVILVSAGTGVAPFCGFLGDRLAAKQSGAPFAPALCFFGVRDPDVDYIFRDLFED
AERQGIVSMRPAFSRAPQAGVRYVQDRIAADADDVWALIGDPAKDTHVFVCGDGAKMAPAVREAFRDIYRART
GADDDGAREWLEVMVASDHYVEDVWAG
>CYP102U1_Mycobacterium neoaurum MN4 2630069607
MTEFAPSLPPTLDDIVSAEGHAFPAGAELAGRPYALPIDLLGERYGPIFYADFNGFRKLYVCSMDLVDELCDE
ARFAKNITQTLDRVRPLAGDGLFTAYRGEPNWQKAHDVLLPGFSYAGLRSYHEAMLDINAELISRWDARVGQR
RVNVSDDLQKLAMDTVALAGFGARFASFDHDELAPIPQCFMSALTEAVTHGETAEFIAGRTELHNYFDELITG
HRAAGTGADLLYVMLGQDGSSALDTANIRNQIMTFLIAGQLTTSELMPNTVYNLIHHPAALDRVRREVDAVFG
TDDDYLPSYDDIGKLGYLRQAISETLRLSPPVLTFDRMALADTVIGGKYPIKAGEAVTVLVGALHRQPQWGDN
VELFDPDRFDPDQVAGRSSALFKPFGTGERSCIGRQFALHEATMLIARLVHRYRLVDSQHYVLQWESSLSRRP
IGFEVDLVRRTATERTSTASAVAAPTVSDGTSASSPVRAGTTLAVLHGSNLGTCRALATQLAEEAADIGCTTT
VGPLDDAAHGLPDADVILIVASSYNGQPTDDAKQFTTWLFGDQAAMSADTRFAVLGVGDHNWAETYQAVPIAI
DDRLTALGAQALVPRGAADTSGDMTGALEEFTSALWSAMSELFGDPDAAPLTDSDEPLYDLTLITGPITAAID
ARFAVTPMTVVLNDELVGNDNSLGQGKRYVRVTLPDDVDYSTGDHLTVLADNPPEAVEAAVNLLGIDPQLRLS
INPRRTSRRLIALDREVSVSELLTHFVELRKPATRSQLRKLAAANPCRPEQERLQALAESDDPCPLSPIECLQ
EFPACALTGAELLEMLEPMTPRHYSIASSSRLSPKEVALIVSVLDAPARSGRGLFKGVASNHLADIAPGACVR
ARVDPARQAFRAGADPEKNVILVSAGTGVAPFCGFLGDRLAAKQSGAPFAPALCFFGVRDPDVDYIFRDLFED

AERQGIVSMRPAFSRAPQAGVRYVQDRIAADADDVWALIGDPAKDTHVFVCGDGAKMAPAVREAFRDIYRART
GADDDGAREWLEVMVASDHYVEDVWAG
>CYP102U1_Mycobacterium sp. VKM Ac-1815D 2553173725
MTEFAPSLPPTLDDIVSAEGHAFPAGAELAGRPYALPIDLLGERYGPIFYADFNGFRKLYVCSMDLVDELCDE
ARFAKNITQTLDRVRPLAGDGLFTAYRGEPNWQKAHDVLLPGFSYAGLRSYHEAMLDINAELISRWDARVGQR
RVNVSDDLQKLAMDTVALAGFGARFASFDHDELAPIPQCFMSALTEAVTHGETAEFIAGRTELHNYFDELITG
HRAAGTGADLLYVMLGQDGSSALDTANIRNQIMTFLIAGQLTTSELMPNTVYNLIHHPAALDRVRREVDAVFG
TDDDYLPSYDDIGKLGYLRQAISETLRLSPPVLTFDRMALADTVIGGKYPIKAGEAVTVLVGALHRQPQWGDN
VELFDPDRFDPDQVAGRSSALFKPFGTGERSCIGRQFALHEATMLIARLVHRYRLVDSQHYVLQWESSLSRRP
IGFEVDLVRRTATERTSTASAVAAPTVSDGTSASSPVRAGTTLAVLHGSNLGTCRALATQLAEEAADIGCTTT
VGPLDDAAHGLPDADVILIVASSYNGQPTDDAKQFTTWLFGDQAAMSADTRFAVLGVGDHNWAETYQAVPIAI
DDRLTALGAQALVPRGAADTSGDMTGALEEFTSALWSAMSELFGDPDAAPLTDSDEPLYDLTLITGPITAAID
ARFAVTPMTVVLNDELVGNDNSLGQGKRYVRVTLPDDVDYSTGDHLTVLADNPPEAVEAAVNLLGIDPQLRLS
INPRRTSRRLIALDREVSVSELLTHFVELRKPATRSQLRKLAAANPCRPEQERLQALAESDDPCPLSPIECLQ
EFPACALTGAELLEMLEPMTPRHYSIASSSRLSPKEVALIVSVLDAPARSGRGLFKGVASNHLADIAPGACVR
ARVDPARQAFRAGADPEKNVILVSAGTGVAPFCGFLGDRLAAKQSGAPFAPALCFFGVRDPDVDYIFRDLFED
AERQGIVSMRPAFSRAPQAGVRYVQDRIAADADDVWALIGDPAKDTHVFVCGDGAKMAPAVREAFRDIYRART
GADDDGAREWLEVMVASDHYVEDVWAG
>CYP102U3_Mycobacterium sp. QIA-37 2687869917
MSSDIRVVEPGEYTSPARPPEIEGIPCADGRPLPPGPVPGRPYALPADLLVEEFGPLFYADFGVSRRLYACSL
ALVEELCDESRFIKGITDRLDRFRPLAGDGLFTAYPGEPNWQKAHDVLLPGFSFSGLRNYHPAMLDINRQLLA
RWDASAGEHLVDVAEDLGKLAMDTVGLAGFGARFDSYQREGLAAIPASFAVALHQMLAPGGENSDLFAAEQHK
LHTFIDELIARHDDGADEHENLLGLMLAPGTPQTPALELSSVHSQVLTFLIAGQDTTSTVMPTALYSLVKNPA
VLHRAQAEVDALFGPGDEHTPTFDEIGKLRYIRQIVDETLRLSPPVREFDRMATEDTLLAGRYPVRKGEVVTV
LTTALHRQPQWGDNVETFDPDRFSPERSAKRPVNLFKPFGTGARSCIGRQFALHEATMAIATLVHRYRFIDSE
HYQLRTQSDLIRKPVGFRIGLARRTSQDRQHESADTTTPTPQESRQLAVTAAPGSSLTIFHGSNLGTCRALAH
QLAEEASDLGYRTTVAPLNEATHALPSEGATVVVASSYNGQPTDDARQFLTWLDSAEARADGGLRYAVLGVGD
RNWAETYQAVPRKIDERLAALGGTPIVSRCEADTSGDFAGSVESFSQALWTSLGAVASADTPTGTLEGPLYEL
RAIDGPVTAAIDARFEVIPMTVLENRELVGANNPLGQAKRLVRVALPADVEYHTGDHLTVLADNQPEVVDKAG
ELLGLILDRRISINTRRNSRRAIALDREVSVRELLTHFVELRKPATRTQLLRLAAANPCPPERSALEALAEHP
ETRSLGIAGCLMEFPATTLSRAELLELFEPMTPRHYSIASSARQSPGIVELVVSVLDAPARSGFGDYQGVASN
YLANITPGQQIRARVDQARQAFRAGANPARNVILVSAGTGVAPFRGFVGDRLAAQRAGEPYAPALCFFGVRHP
EVDYLFRDEFAAAEQSGVVHMRPAFSRASENGIKYVQDRIAADADDVWDLLGDPAKQTHVYVCGDGGKMAPAV
REAFLDIYRKHTGTTEPQARNWLTGLVEADRYVEDVWTE
>CYP102U3_Mycobacterium abscessus MAB_082312_2273 2587329381
MSSDIRVVEPGEYTSPARPPEIEGIPCADGRPLPPGPVPGRPYALPADLLVEEFGPLFYADFGVSRRLYACSL
ALVEELCDESRFIKGITDRLDRFRPLAGDGLFTAYPGEPNWQKAHDVLLPGFSFSGLRNYHPAMLDINRQLLA
RWDASAGEHLVDVAEDLGKLAMDTVGLAGFGARFDSYQREGLAAIPASFAVALHQMLAPEGAHSDLFAAEQQK
LHTFIDELIARQREGADEHENLLGLMLAPGTPQSPALELSSVHSQVLTFLIAGQDTTSTVMPTALYSLVKNPA
VLHRAQAEVDALFGPEDDHTPTFDEIGKLRYIRQIVDETLRLSPPVREFDRMATEDTLLAGRYPVRKGEVVTV
LTTALHRQPQWGDNVETFDPDRFSPERSAKRLVNLFKPFGTGARSCIGRQFALHEATMALATLVHRYRFIDSE
HYQLRTQSDLIRKPVGFRIGLARRTSQDRQHESAATATPSPQASRQLAVTAVPGSALIVFHGSNLGTCRALAH
QLAEEASDLGYRTTVAPLNEATHALPSESAMVVVASSYNGQPTDDARQFLTWLDSAEARADGGLRYAILGVGD
RNWAETYQAVPRKIDERLAALGGTPIVSRCEADTSGDFAGSVESFSQALWTSLGTVASTDTPSGTLEGPLYEL
RAIDGPVTAAIDARFEVIPMTVLENRELVGTNNPLGQAKRLVRVALPADVEYHTGDHLTVLADNQPEVVDKAG
ELLGLILDRRISINTRRNSRRAIALDREVSVRELLTHFVELRKPATRTQLLRLAAANPCPPERSALEALAEHP
ETRSLGIAGCLMEFPATTLSRAELLELFEPMTPRHYSIASSARQSPGIVELVVSVLDAPARSGFGDYQGVASN
YLANITPGQQIRARVDQARQAFRAGANPARNVILVSAGTGVAPFRGFVGDRLAAQRAGEPYAPALCFFGVRHP
EVDYLFRDEFAAAEQSGVVHMRPAFSRASENGIKYVQDRIAADADDVWDLLGDPAKQTHVYVCGDGGKMAPAV
REAFLDIYRKHTGATEPQARNWLTGLVEADRYVEDVWTE
>CYP105BP1_Mycobacterium sp. EPa45 2652904819
VTDHVDVDLPTMPAPRAPRCPLDDPPAEFAAWRDNPGLRQAINLFTGQPAWVASRYEDIRAAVTDPRMSANTLP
ESVKPPGEEDDMPVFFARTDDPEHNRLRRMLTMDFTVRRAKEMAPDIQQLVDGFLDKMIAAGPPRDLVRDFAL
PVPSLVICLLLGVPYEDHEYFEEHSVAGLDVKTSDEQRTASIMTMIGYITELLQRKKDEPGDDLLSRLMTHNV
ADGEISVMTAAMTGWVMLQAGHETTASMIALGTLVLLKNPDAFARLRDSDDPTVRLQIIDELLRYLTIVHSGL
VDRIATEDFELGGQLVRKGEHVMMNLTAGNYDPAFVENPERFDIDRNPRGHLAFGYGVHQCVGQNLARTELDI
ALTTLARRLPTLRLAVPEDELDFMNDQAIWRLKELPVTW
>CYP105Q4_Mycobacterium marinum M 641720010

MSDTLASPSPETASGIPDYPMSRSAGCPFAPPPGVMALAAAKPLTRVRIWDGSTPWLITGYEQVRELFSDSRV
SVDDRLPGFPHWNAGMLSTVHKRPRSVFTADGEEHTRFRRMLSKPFTFKRVEALRPTIQQITDEHIDAMLAGP
QPADLVAKLALPVPSLVISQLLGVPYEDAEMFQHHANVGLARYATGADTVKGAMSLHKYLAELVEAKMANPAE
DAVSDLAERVKAGELSVKEAAQLGTGLLIAGHETTANMIGLGVLALLVNPDQAGILRDAQDPKIVANAVEELL
RYLSIIQNGQRRVAHEDIHIGGETIRAGEGIIIDLAPANWDAHAFTEPDRLYLHRAGAERNVAFGYGRHQCVG
QQLARAELQIVYRTLLQRIPTLTLATALEDVPFKDDRLAYGVYELPVTW
>CYP105Q4_Mycobacterium marinum MB2 2546371232
MSDTLASPSPETASGIPDYPMSRSAGCPFAPPPGVMALAAAKPLTRVRIWDGSTPWLITGYEQVRELFSDSRV
SVDDRLPGFPHWNAGMLSTVHKRPRSVFTADGEEHTRFRRMLSKPFTFKRVEALRPTIQQITDEHIDAMLAGP
QPADLVAKLALPVPSLVISQLLGVPYEDAEMFQHHANVGLARYATGADTVKGAMSLHKYLAELVEAKMANPAE
DAVSDLAERVKAGELSVKEAAQLGTGLLIAGHETTANMIGLGVLALLVNPDQAGILRDAHDPKIVANAVEELL
RYLSIIQNGQRRVAHEDIHIGGETIRAGEGIIIDLAPANWDAHAFTEPDRLYLHRAGAERNVAFGYGRHQCVG
QQLARAELQIVYRTLLQRIPTLTLATALEDVPFKDDRLAYGVYELPVTW
>CYP105Q4_Mycobacterium liflandii 128FXT 2563570988
MSDTLASPSPETAAGIPDYPMSRSAGCPFAPPPGVMSLAAAKPLTRVRIWDGSTPWLITGYEQVRELFSDSRV
SVDDRLPGFPHWNAGMLSTVHKRPRSVFTADGEEHTRFRRMLSKPFTFKRVEALRPTIQQITDEHIDAMLAGP
QPADLVAKLALPVPSLVISQLLGVPYEDAEMFQHHANVGLARYATGADTVKGAMSLHKYLAELVEAKMANPAE
DAVSDLAERVKAGELSVKEAAQLGTGLLIAGHETTANMIGLGVLALLVNPDQAGILRDAHDPKIVANAVEELL
RYLSIIQNGQRRVAHEDIHIGGETIRAGEGIIIDLAPANWDAHAFTEPDRLYLHRAGAERNVAFGYGRHQCVG
QQLARAELQIVYRTLLQRIPTLTLATALEDVPFKDDRLAYGVYELPVTW
>CYP105Q4_Mycobacterium marinum Europe 2543277937
MSDTLASPSPETAAGIPDYPMSRSAGCPFAPPPGVMALAAARPLTRVRIWDGSTPWLITGYEQVRELFSDSRV
SVDDRLPGFPHWNAGMLSTVHKRPRSVFTADGEEHTRFRRMLSKPFTFKRVEALRPTIQQITDEHIDAMLAGP
QPADLVAKLALPVPSLVISQLLGVPYEDAEMFQHHANVGLARYATGADTVKGAMSLHKYLAELVEAKMANPAE
DAVSDLAERAKAGELSVKEAAQLGTGLLIAGHETTANMIGLGVLALLVNPDQAGILRDAHDPKIVANAVEELL
RYLSIIQNGQRRVAHEDIHIGGETIRAGEGIIIDLAPANWDAHAFAEPDRLYLHRAGAERNVAFGYGRHQCVG
QQLARAELQIVYRTLLQRIPTLTLATALEDVPFKDDRLAYGVYELPVTW
>CYP105Q4_Mycobacterium ulcerans Agy99 642567525
MSDTLASPSPETAAGIPDYPMSRSAGCPFAPPPGVMSLAAAKPLTRVRIWDGSAPWLITGYEQVRELFSGSRV
SVDDRVPGFPHWNAGMLSTVHKRPRSVFTADGEEHTRFRRMLSKPFTFKRVEALRPTIQQITDEHIDAMLAGP
QPADLVAKLALPVPSLVISQLLGVPYEDAEMFQHHANVGLARYATGADTVKGAMSLHKYLAELVEAKMANPAE
DAVSDLAERVKAGELSVKEAAQLGTGLLIAGHETTANMIGLGVLALLVNPDQAGILRDAHDPKIVANAVEELL
RYLSIIQNGQRRVAHEDIHIGGETIRAGEGIIIDLAPANWDAHAFTEPDRLYLHRAGAERNVAFGYGRHQCVG
QQLARTELQIVYRTLLQRIPTLTLATALEDVPFKDDRLAYGVYELPVTW
>CYP105Q6_Mycobacterium austroafricanum DSM 44191 2649756909
MTETLAQEAVSVPEYPMERTAGCPFAPPQQMLEMNQVKPLSRVRIWNGTTPWLVTGHEVARTLFADSRVSVDD
RREGFPHWNEHMLSTVDKRPRSVFTSDAEEHTRFRRMLSKPFTFRRVEALRPVIQQVTDECIDEILAGPQPAD
MVAKLALPVPTRVISDMLGVPYEDHEFFQEHANAGLARYAAADAMQKGAMSLHQYLINLVEEKQAHPAEDAVS
DLAERVTAGEISVKEAAQLGTGLLIAGHETTANMIGIGICALLENPEQAALLRDSDDPKFIANAVEELMRYLS
IIQNGQRRVATEDIEIGGETIRAGEGIILDLAPANWDARAFPEPDKLDLTRDATQQLGFGYGRHQCVGQQLAR
AELQIVFHTLLRRIPTMKPAIPLEEVPFKHDRLAYGVYELPVTW
>CYP105Q7_Mycobacterium smegmatis INHR2 2687286149
MSETLTQPSATDIPGYPMERAAACPFAPPPQMLDMNKAKGLSRVRIWDGSTPWLITGHEEARALFADSRVSVD
DRRPGFPHWNEHMLATVHKRPRSVFTSDAEEHTRFRRMLSKPFTFRRVEGLRPAIQKITDECIDAILAGPQPA
DIVDKLALPVPTVVISEMLGVPYEDHEFFQEHANAGLARYAAADAMQKGAMSLHQYLIDLIEKKQAEPAEDAV
SDLAERVTAGELSVKEAAQLGTGLLIAGHETTANMIGIGILALLENPEQADFLRNAEDPKVIANAVEELMRYL
SIIQTGQRRVAVEDIEIGGETIKAGEGIIIDLVPANWDAKAFPEPDKLDLTRDAGQQLGFGYGRHQCVGQQLA
RAELQIVFHTLLRRIPTLRLAIPLEEVPFKHDRLAYGVYELPVAW
>CYP105Q7_Mycobacterium smegmatis str. MC2 155 639743288
MSETLTQPSATDIPGYPMERAAACPFAPPPQMLDMNKAKGLSRVRIWDGSTPWLITGHEEARALFADSRVSVD
DRRPGFPHWNEHMLATVHKRPRSVFTSDAEEHTRFRRMLSKPFTFRRVEGLRPAIQKITDECIDAILAGPQPA
DIVDKLALPVPTVVISEMLGVPYEDHEFFQEHANAGLARYAAADAMQKGAMSLHQYLIDLIEKKQAEPAEDAV
SDLAERVTAGELSVKEAAQLGTGLLIAGHETTANMIGIGILALLENPEQADFLRNAEDPKVIANAVEELMRYL
SIIQTGQRRVAVEDIEIGGETIKAGEGIIIDLVPANWDAKAFPEPDKLDLTRDAGQQLGFGYGRHQCVGQQLA
RAELQIVFHTLLRRIPTLRLAIPLEEVPFKHDRLAYGVYELPVAW
>CYP105Q7_Mycobacterium smegmatis MC2 155 2518779088
MSETLTQPSATDIPGYPMERAAACPFAPPPQMLDMNKAKGLSRVRIWDGSTPWLITGHEEARALFADSRVSVD
DRRPGFPHWNEHMLATVHKRPRSVFTSDAEEHTRFRRMLSKPFTFRRVEGLRPAIQKITDECIDAILAGPQPA

DIVDKLALPVPTVVISEMLGVPYEDHEFFQEHANAGLARYAAADAMQKGAMSLHQYLIDLIEKKQAEPAEDAV
SDLAERVTAGELSVKEAAQLGTGLLIAGHETTANMIGIGILALLENPEQADFLRNAEDPKVIANAVEELMRYL
SIIQTGQRRVAVEDIEIGGETIKAGEGIIIDLVPANWDAKAFPEPDKLDLTRDAGQQLGFGYGRHQCVGQQLA
RAELQIVFHTLLRRIPTLRLAIPLEEVPFKHDRLAYGVYELPVAW
>CYP105Q7_Mycobacterium smegmatis MC2 155 2689264324
MSETLTQPSATDIPGYPMERAAACPFAPPPQMLDMNKAKGLSRVRIWDGSTPWLITGHEEARALFADSRVSVD
DRRPGFPHWNEHMLATVHKRPRSVFTSDAEEHTRFRRMLSKPFTFRRVEGLRPAIQKITDECIDAILAGPQPA
DIVDKLALPVPTVVISEMLGVPYEDHEFFQEHANAGLARYAAADAMQKGAMSLHQYLIDLIEKKQAEPAEDAV
SDLAERVTAGELSVKEAAQLGTGLLIAGHETTANMIGIGILALLENPEQADFLRNAEDPKVIANAVEELMRYL
SIIQTGQRRVAVEDIEIGGETIKAGEGIIIDLVPANWDAKAFPEPDKLDLTRDAGQQLGFGYGRHQCVGQQLA
RAELQIVFHTLLRRIPTLRLAIPLEEVPFKHDRLAYGVYELPVAW
>CYP105Q7_Mycobacterium smegmatis MC2 51 2586872883
MSETLTQPSATDIPGYPMERAAACPFAPPPQMLDMNKAKGLSRVRIWDGSTPWLITGHEEARALFADSRVSVD
DRRPGFPHWNEHMLATVHKRPRSVFTSDAEEHTRFRRMLSKPFTFRRVEGLRPAIQKITDECIDAILAGPQPA
DIVDKLALPVPTVVISEMLGVPYEDHEFFQEHANAGLARYAAADAMQKGAMSLHQYLIDLIEKKQAEPAEDAV
SDLAERVTAGELSVKEAAQLGTGLLIAGHETTANMIGIGILALLENPEQADFLRNAEDPKVIANAVEELMRYL
SIIQTGQRRVAVEDIEIGGETIKAGEGIIIDLVPANWDAKAFPEPDKLDLTRDAGQQLGFGYGRHQCVGQQLA
RAELQIVFHTLLRRIPTLRLAIPLEEVPFKHDRLAYGVYELPVAW
>CYP105Q7_Mycobacterium smegmatis INHR1 2689419707
MSETLTQPSATDIPGYPMERAAACPFAPPPQMLDMNKAKGLSRVRIWDGSTPWLITGHEEARALFADSRVSVD
DRRPGFPHWNEHMLATVHKRPRSVFTSDAEEHTRFRRMLSKPFTFRRVEGLRPAIQKITDECIDAILAGPQPA
DIVDKLALPVPTVVISEMLGVPYEDHEFFQEHANAGLARYAAADAMQKGAMSLHQYLIDLIEKKQAEPAEDAV
SDLAERVTAGELSVKEAAQLGTGLLIAGHETTANMIGIGILALLENPEQADFLRNAEDPKVIANAVEELMRYL
SIIQTGQRRVAVEDIEIGGETIKAGEGIIIDLVPANWDAKAFPEPDKLDLTRDAGQQLGFGYGRHQCVGQQLA
RAELQIVFHTLLRRIPTLRLAIPLEEVPFKHDRLAYGVYELPVAW
>CYP105Q7_Mycobacterium goodii X7B 2652639207
MSETLTQPSATDIPGYPMERAAACPFAPPPQMLEMNKAKGLSRVRIWDGSTPWLITGHEEARVLFADSRVSVD
DRRPGFPHWNEHMLATVHKRPRSVFTSDAEEHTRFRRMLSKPFTFRRVEGLRPAIQKITDECIDAILAGPQPA
DIVDKLALPVPTVVISEMLGVPYEDHEFFQEHANAGLARYAAADAMQKGAMSLHEYLINLIEKKQAEPAEDAV
SDLAERVSAGELSVKEAAQLGTGLLIAGHETTANMIGIGILALLENPEQADFLRNAEDPKVIANAVEELMRYL
SIIQTGQRRVAVEDIEIGGETIRAGEGIIIDLVPANWDAKAFSEPEKLDLTRDAGQQLGFGFGRHQCVGQQLA
RAELQIVFHTLLRRIPTLRLAIPLEEVPFKHDRLAYGVYELPVAW
>CYP105S1_Mycobacterium smegmatis INHR2 2687282192
MTQAQALPPLHIRRDAFDPTPELGEIRAGEGVHVTVNPFGMQVYLVTRHEDVKTVLSDHERFSNSRPPGFVLP
GAPQISAEEQASNRAGNLLGLDPPEHQRLRRMLTPEFTIRRIKRLEPRIVEIVDAHLDAMESAGPPADIVADF
ALPIPSLVICELLGVPYEDRTDFQQRSARQLDLSAPMPERLELQRQGRAYMRGLVERSRTRPGDDILGMLVRE
HGTELTDDELIGIAGLLLLAGHETTSNMLGLGVLALLRHPDQLACVRDDPDAVGPAIEELLRWLSIVSTALPR
ITTTDVELAGVTIPAGHLVFASLPAGNRDPEFIDDPDTLDIRRGAPGHLAFGHGVHHCLGAPLARMEMRIALP
ALLRRFPTLALAEPFEDVRWRPFHFIYGLQSLAVAW
>CYP105S1_Mycobacterium smegmatis str. MC2 155 639739316
MTQAQALPPLHIRRDAFDPTPELGEIRAGEGVHVTVNPFGMQVYLVTRHEDVKTVLSDHERFSNSRPPGFVLP
GAPQISAEEQASNRAGNLLGLDPPEHQRLRRMLTPEFTIRRIKRLEPRIVEIVDAHLDAMESAGPPADIVADF
ALPIPSLVICELLGVPYEDRTDFQQRSARQLDLSAPMPERLELQRQGRAYMRGLVERSRTRPGDDILGMLVRE
HGTELTDDELIGIAGLLLLAGHETTSNMLGLGVLALLRHPDQLACVRDDPDAVGPAIEELLRWLSIVSTALPR
ITTTDVELAGVTIPAGHLVFASLPAGNRDPEFIDDPDTLDIRRGAPGHLAFGHGVHHCLGAPLARMEMRIALP
ALLRRFPTLALAEPFEDVRWRPFHFIYGLQSLAVAW
>CYP105S1_Mycobacterium smegmatis MC2 155 2518775107
MTQAQALPPLHIRRDAFDPTPELGEIRAGEGVHVTVNPFGMQVYLVTRHEDVKTVLSDHERFSNSRPPGFVLP
GAPQISAEEQASNRAGNLLGLDPPEHQRLRRMLTPEFTIRRIKRLEPRIVEIVDAHLDAMESAGPPADIVADF
ALPIPSLVICELLGVPYEDRTDFQQRSARQLDLSAPMPERLELQRQGRAYMRGLVERSRTRPGDDILGMLVRE
HGTELTDDELIGIAGLLLLAGHETTSNMLGLGVLALLRHPDQLACVRDDPDAVGPAIEELLRWLSIVSTALPR
ITTTDVELAGVTIPAGHLVFASLPAGNRDPEFIDDPDTLDIRRGAPGHLAFGHGVHHCLGAPLARMEMRIALP
ALLRRFPTLALAEPFEDVRWRPFHFIYGLQSLAVAW
>CYP105S1_Mycobacterium smegmatis MC2 155 2689260366
MTQAQALPPLHIRRDAFDPTPELGEIRAGEGVHVTVNPFGMQVYLVTRHEDVKTVLSDHERFSNSRPPGFVLP
GAPQISAEEQASNRAGNLLGLDPPEHQRLRRMLTPEFTIRRIKRLEPRIVEIVDAHLDAMESAGPPADIVADF
ALPIPSLVICELLGVPYEDRTDFQQRSARQLDLSAPMPERLELQRQGRAYMRGLVERSRTRPGDDILGMLVRE
HGTELTDDELIGIAGLLLLAGHETTSNMLGLGVLALLRHPDQLACVRDDPDAVGPAIEELLRWLSIVSTALPR

ITTTDVELAGVTIPAGHLVFASLPAGNRDPEFIDDPDTLDIRRGAPGHLAFGHGVHHCLGAPLARMEMRIALP
ALLRRFPTLALAEPFEDVRWRPFHFIYGLQSLAVAW
>CYP105S1_Mycobacterium smegmatis MC2 51 2586872451
MTQAQALPPLHIRRDAFDPTPELGEIRAGEGVHVTVNPFGMQVYLVTRHEDVKTVLSDHERFSNSRPPGFVLP
GAPQISAEEQASNRAGNLLGLDPPEHQRLRRMLTPEFTIRRIKRLEPRIVEIVDAHLDAMESAGPPADIVADF
ALPIPSLVICELLGVPYEDRTDFQQRSARQLDLSAPMPERLELQRQGRAYMRGLVERSRTRPGDDILGMLVRE
HGTELTDDELIGIAGLLLLAGHETTSNMLGLGVLALLRHPDQLACVRDDPDAVGPAIEELLRWLSIVSTALPR
ITTTDVELAGVTIPAGHLVFASLPAGNRDPEFIDDPDTLDIRRGAPGHLAFGHGVHHCLGAPLARMEMRIALP
ALLRRFPTLALAEPFEDVRWRPFHFIYGLQSLAVAW
>CYP105S1_Mycobacterium smegmatis INHR1 2689415750
MTQAQALPPLHIRRDAFDPTPELGEIRAGEGVHVTVNPFGMQVYLVTRHEDVKTVLSDHERFSNSRPPGFVLP
GAPQISAEEQASNRAGNLLGLDPPEHQRLRRMLTPEFTIRRIKRLEPRIVEIVDAHLDAMESAGPPADIVADF
ALPIPSLVICELLGVPYEDRTDFQQRSARQLDLSAPMPERLELQRQGRAYMRGLVERSRTRPGDDILGMLVRE
HGTELTDDELIGIAGLLLLAGHETTSNMLGLGVLALLRHPDQLACVRDDPDAVGPAIEELLRWLSIVSTALPR
ITTTDVELAGVTIPAGHLVFASLPAGNRDPEFIDDPDTLDIRRGAPGHLAFGHGVHHCLGAPLARMEMRIALP
ALLRRFPTLALAEPFEDVRWRPFHFIYGLQSLAVAW
>CYP107AA1_Mycobacterium smegmatis INHR2 2687284509
MLRLGNSFVQNPHEVYDRLRRSGPVQRVEMWGGVPVWLVTRYQEARNLLTDPRIGKDGAAASALFPPGTDGSI
GTVLGDNMLFRDPPDHTRLRRFVTSAFTAHAVRRLRPTIAGFADALLDDIAASVPGQVDLLQAFAQPLPVQVI
GELLGVPERDRELFAALVVPIFTSTDTTVLRRAQKELTQLLTDMLAEKRQSPADDVLSSLVHRRDGTDQLSEA
ELLGTAFLLIVAGYETTVNLLANGILALLRNPEQLRAVRADRSLLPRAVEEALRFESPLNTATVRYTSAPVTV
GDVEIPSGELVVIGLLAANHDDEQFPDAHRFDVSRTHNRHLAFGYGVHHCVGAPLARMEAEIGFDRLLSRFEV
MELVDSGPPRYRPSTLMRGVERLPVILGYPHDIASTMREWSGSLPSSGEADSSFAH
>CYP107AA1_Mycobacterium smegmatis str. MC2 155 639741609
MLRLGNSFVQNPHEVYDRLRRSGPVQRVEMWGGVPVWLVTRYQEARNLLTDPRIGKDGAAASALFPPGTDGSI
GTVLGDNMLFRDPPDHTRLRRFVTSAFTAHAVRRLRPTIAGFADALLDDIAASVPGQVDLLQAFAQPLPVQVI
GELLGVPERDRELFAALVVPIFTSTDTTVLRRAQKELTQLLTDMLAEKRQSPADDVLSSLVHRRDGTDQLSEA
ELLGTAFLLIVAGYETTVNLLANGILALLRNPEQLRAVRADRSLLPRAVEEALRFESPLNTATVRYTSAPVTV
GDVEIPSGELVVIGLLAANHDDEQFPDAHRFDVSRTHNRHLAFGYGVHHCVGAPLARMEAEIGFDRLLSRFEV
MELVDSGPPRYRPSTLMRGVERLPVILGYPHDIASTMREWSGSLPSSGEADSSFAH
>CYP107AA1_Mycobacterium smegmatis MC2 155 2518777410
MLRLGNSFVQNPHEVYDRLRRSGPVQRVEMWGGVPVWLVTRYQEARNLLTDPRIGKDGAAASALFPPGTDGSI
GTVLGDNMLFRDPPDHTRLRRFVTSAFTAHAVRRLRPTIAGFADALLDDIAASVPGQVDLLQAFAQPLPVQVI
GELLGVPERDRELFAALVVPIFTSTDTTVLRRAQKELTQLLTDMLAEKRQSPADDVLSSLVHRRDGTDQLSEA
ELLGTAFLLIVAGYETTVNLLANGILALLRNPEQLRAVRADRSLLPRAVEEALRFESPLNTATVRYTSAPVTV
GDVEIPSGELVVIGLLAANHDDEQFPDAHRFDVSRTHNRHLAFGYGVHHCVGAPLARMEAEIGFDRLLSRFEV
MELVDSGPPRYRPSTLMRGVERLPVILGYPHDIASTMREWSGSLPSSGEADSSFAH
>CYP107AA1_Mycobacterium smegmatis MC2 155 2689262683
MLRLGNSFVQNPHEVYDRLRRSGPVQRVEMWGGVPVWLVTRYQEARNLLTDPRIGKDGAAASALFPPGTDGSI
GTVLGDNMLFRDPPDHTRLRRFVTSAFTAHAVRRLRPTIAGFADALLDDIAASVPGQVDLLQAFAQPLPVQVI
GELLGVPERDRELFAALVVPIFTSTDTTVLRRAQKELTQLLTDMLAEKRQSPADDVLSSLVHRRDGTDQLSEA
ELLGTAFLLIVAGYETTVNLLANGILALLRNPEQLRAVRADRSLLPRAVEEALRFESPLNTATVRYTSAPVTV
GDVEIPSGELVVIGLLAANHDDEQFPDAHRFDVSRTHNRHLAFGYGVHHCVGAPLARMEAEIGFDRLLSRFEV
MELVDSGPPRYRPSTLMRGVERLPVILGYPHDIASTMREWSGSLPSSGEADSSFAH
>CYP107AA1_Mycobacterium smegmatis MC2 51 2586877428
MLRLGNSFVQNPHEVYDRLRRSGPVQRVEMWGGVPVWLVTRYQEARNLLTDPRIGKDGAAASALFPPGTDGSI
GTVLGDNMLFRDPPDHTRLRRFVTSAFTAHAVRRLRPTIAGFADALLDDIAASVPGQVDLLQAFAQPLPVQVI
GELLGVPERDRELFAALVVPIFTSTDTTVLRRAQKELTQLLTDMLAEKRQSPADDVLSSLVHRRDGTDQLSEA
ELLGTAFLLIVAGYETTVNLLANGILALLRNPEQLRAVRADRSLLPRAVEEALRFESPLNTATVRYTSAPVTV
GDVEIPSGELVVIGLLAANHDDEQFPDAHRFDVSRTHNRHLAFGYGVHHCVGAPLARMEAEIGFDRLLSRFEV
MELVDSGPPRYRPSTLMRGVERLPVILGYPHDIASTMREWSGSLPSSGEADSSFAH
>CYP107AA1_Mycobacterium smegmatis INHR1 2689418067
MLRLGNSFVQNPHEVYDRLRRSGPVQRVEMWGGVPVWLVTRYQEARNLLTDPRIGKDGAAASALFPPGTDGSI
GTVLGDNMLFRDPPDHTRLRRFVTSAFTAHAVRRLRPTIAGFADALLDDIAASVPGQVDLLQAFAQPLPVQVI
GELLGVPERDRELFAALVVPIFTSTDTTVLRRAQKELTQLLTDMLAEKRQSPADDVLSSLVHRRDGTDQLSEA
ELLGTAFLLIVAGYETTVNLLANGILALLRNPEQLRAVRADRSLLPRAVEEALRFESPLNTATVRYTSAPVTV
GDVEIPSGELVVIGLLAANHDDEQFPDAHRFDVSRTHNRHLAFGYGVHHCVGAPLARMEAEIGFDRLLSRFEV
MELVDSGPPRYRPSTLMRGVERLPVILGYPHDIASTMREWSGSLPSSGEADSSFAH

>CYP107AB1P_Mycobacterium smegmatis INHR2 2687283421
MSVTVEEKAESGRATIEILNAVIDEKIREPGEDLLSGMIGASVDGHGLTRDELMAMCFLLITAGYETTVNLIG
NGTLALIDNPSQLEKVRENPDLTAGAVEEILRFDGPVNIATWRYATADIDVDGVVIPANEQIFLSLLSANRDT
GRFENADRFDIERNTRGHIAFGHGIHYCLGAPLARMEGVTAIGRIVQRYDSITLDPTAELRYHNGTLMHGLKS
LPVRLTRVPQPRP
>CYP107AB1P_Mycobacterium smegmatis INHR2 2687283422
MILDEQFAQDPEGLYRMLRSEAPVCEVELIGGVRGWLVTRYADVMALLKDPRVSKDHTSALPRLAPDRVRPYI
SPQLHNHMLNLDPPEHTRLRRLVVQAFTPKALARMQPVIDAIADELLDDIDLRSGDEPIDLMADYAEPLPIQV
IAELLGVAVEYA
>CYP107AB1P_Mycobacterium smegmatis MC2 155 2518776331
MSVTVEEKAESGRATIEILNAVIDEKIREPGEDLLSGMIGASVDGHGLTRDELMAMCFLLITAGYETTVNLIG
NGTLALIDNPSQLEKVRENPDLTAGAVEEILRFDGPVNIATWRYATADIDVDGVVIPANEQIFLSLLSANRDT
GRFENADRFDIERNTRGHIAFGHGIHYCLGAPLARMEGVTAIGRIVQRYDSITLDPTAELRYHNGTLMHGLKS
LPVRLTRVPQPRP
>CYP107AB1P_Mycobacterium smegmatis MC2 155 2518776332
MSRAASSGVGDSADEGVDLAVAGHRVGKRPPELDCFETGRPRGARPLQQRQIGQQDRAVDHIARRHGTHTSAS
SPAANGPVNRRVYTYEVRPIPGGSRWCGEALIDNMILDEQFAQDPEGLYRMLRSEAPVCEVELIGGVRGWLVT
RYADVMALLKDPRVSKDHTSALPRLAPDRVRPYISPQLHNHMLNLDPPEHTRLRRLVVQAFTPKALARMQPVI
DAIADELLDDIDLRSGDEPIDLMADYAEPLPIQVIAELLGVAVEYA
>CYP107AB1P_Mycobacterium smegmatis MC2 155 2689261595
MSVTVEEKAESGRATIEILNAVIDEKIREPGEDLLSGMIGASVDGHGLTRDELMAMCFLLITAGYETTVNLIG
NGTLALIDNPSQLEKVRENPDLTAGAVEEILRFDGPVNIATWRYATADIDVDGVVIPANEQIFLSLLSANRDT
GRFENADRFDIERNTRGHIAFGHGIHYCLGAPLARMEGVTAIGRIVQRYDSITLDPTAELRYHNGTLMHGLKS
LPVRLTRVPQPRP
>CYP107AB1P_Mycobacterium smegmatis MC2 155 2689261596
MILDEQFAQDPEGLYRMLRSEAPVCEVELIGGVRGWLVTRYADVMALLKDPRVSKDHTSALPRLAPDRVRPYI
SPQLHNHMLNLDPPEHTRLRRLVVQAFTPKALARMQPVIDAIADELLDDIDLRSGDEPIDLMADYAEPLPIQV
IAELLGVAVEYA
>CYP107AB1P_Mycobacterium smegmatis MC2 51 2586874352
MILDEQFAQDPEGLYRMLRSEAPVCEVELIGGVRGWLVTRYADVMALLKDPRVSKDHTSALPRLAPDRVRPYI
SPQLHNHMLNLDPPEHTRLRRLVVQAFTPKALARMQPVIDAIADELLDDIDLRSGDEPIDLMADYAEPLPIQV
IAELLGVAVEYA
>CYP107AB1P_Mycobacterium smegmatis MC2 51 2586874353
MSVTVEEKAESGRATIEILNAVIDEKIREPGEDLLSGMIGASVDGHGLTRDELMAMCFLLITAGYETTVNLIG
NGTLALIDNPSQLEKVRENPDLTAGAVEEILRFDGPVNIATWRYATADIDVDGVVIPANEQIFLSLLSANRDT
GRFENADRFDIERNTRGHIAFGHGIHYCLGAPLARMEGVTAIGRIVQRYDSITLDPTAELRYHNGTLMHGLKS
LPVRLTRVPQPRP
>CYP107AB1P_Mycobacterium smegmatis INHR1 2689416979
MSVTVEEKAESGRATIEILNAVIDEKIREPGEDLLSGMIGASVDGHGLTRDELMAMCFLLITAGYETTVNLIG
NGTLALIDNPSQLEKVRENPDLTAGAVEEILRFDGPVNIATWRYATADIDVDGVVIPANEQIFLSLLSANRDT
GRFENADRFDIERNTRGHIAFGHGIHYCLGAPLARMEGVTAIGRIVQRYDSITLDPTAELRYHNGTLMHGLKS
LPVRLTRVPQPRP
>CYP107AB1P_Mycobacterium smegmatis INHR1 2689416980
MILDEQFAQDPEGLYRMLRSEAPVCEVELIGGVRGWLVTRYADVMALLKDPRVSKDHTSALPRLAPDRVRPYI
SPQLHNHMLNLDPPEHTRLRRLVVQAFTPKALARMQPVIDAIADELLDDIDLRSGDEPIDLMADYAEPLPIQV
IAELLGVAVEYA
>CYP107AB1P_Mycobacterium smegmatis str. MC2 155 640741236
LIDNMILDEQFAQDPEGLYRMLRSEAPVCEVELIGGVRGWLVTRYADVMALLKDPRVSKDHTSALPRLAPDRV
RPYISPQLHNHMLNLDPPEHTRLRRLVVQAFTPKALARMQPVIDAIADELLDDIDLRSGDEPIDLMADYAEPL
PIQVIAELLGVAVEYAPFRAAVTPLLMSVTVEEKAESGRATIEILNAVIDEKIREPGEDLLSGMIGASVDGHG
LTRDELMAMCFLLITAGYETTVNLIGNGTLALIDNPSQLEKVRENPDLTAGAVEEILRFDGPVNIATWRYATA
DIDVDGVVIPANEQIFLSLLSANRDTGRFENADRFDIERNTRGHIAFGHGIHYCLGAPLARMEGVTAIGRIVQ
RYDSITLDPTAELRYHNGTLMHGLKSLPVRLTRVPQPRP
>CYP107DH1_Mycobacterium kansasii 662 2567129487
MISHPITVGTAEFNADQHRYYDWMRRNAPVYRGRLALRPPDRDVYFISRYQDCVDVVTDPRIRRVVEGAEHLP
LPEAIRMLTTGTMVYQDDPAHLRLRKLVSRPFTPRAIARLGDRVQTVTRVVLDGFKAGQRIDLQRDYALPIPT
TVISEMVGIPAEDRSAFYDYIDSLFELMLAGGTEGAAQRMDEFVDCLRTLVQQRRGDPGQDIITAMIEASEDG
DRLSDDEIVAMVFLLITGDYQTTPNVITNGIAALLSHREQLSLLQRRPELIGSAVEEVLRYTGTVGSTEATTF

AAEDIVVHGVTIPRGAMVVPLLTSANRDPAEFDDPDRFDITRKPNNHLAFSRGSHFCLGSHLARMETRIAIAG
LITRFPGLTLAIAPEELRLEPIPLLNRYAELPVMLG
>CYP107DH1_Mycobacterium kansasii ATCC 12478 2563576958
MISHPITVGTAEFNADQHRYYDWMRRNAPVYRGRLALRPPDRDVYFISRYQDCVDVVTDPRIRRVVEGAEHLP
LPEAIRMLTTGTMVYQDDPAHLRLRKLVSRPFTPRAIARLGDRVQTVTRVVLDGFKAGQRIDLQRDYALPIPT
TVISEMVGIPAEDRSAFYDYIDSLFELMLAGGTEGAAQRMDEFVDCLRTLVQQRRGDPGQDIITAMIEASEDG
DRLSDDEIVAMVFLLITGDYQTTPNVITNGIAALLSHREQLSLLQRRPELIGSAVEEVLRYTGTVGSTEATTF
AAEDIVVHGVTIPRGAMVVPLLTSANRDPAEFDDPDRFDITRKPNNHLAFSRGSHFCLGSHLARMETRIAIAG
LITRFPGLTLAIAPEELRLEPIPLLNRYAELPVMLG
>CYP107DH1_Mycobacterium kansasii SMC1 2587479956
MISHPITVGTAEFNADQHRYYDWMRRNAPVYRGRLALRPPDRDVYFISRYQDCVDVVTDPRIRRVVEGAEHLP
LPEAIRMLTTGTMVYQDDPAHLRLRKLVSRPFTPRAIARLGDRVQTVTRVVLDGFKAGQRIDLQRDYALPIPT
TVISEMVGIPAEDRSAFYDYIDSLFELMLAGGTEGAAQRMDEFVDCLRTLVQQRRGDPGQDIITAMIEASEDG
DRLSDDEIVAMVFLLITGDYQTTPNVITNGIAALLSHREQLSLLQRRPELIGSAVEEVLRYTGTVGSTEATTF
AAEDIVVHGVTIPRGAMVVPLLTSANRDPAEFDDPDRFDITRKPNNHLAFSRGSHFCLGSHLARMETRIAIAG
LITRFPGLTLAIAPEELRLEPIPLLNRYAELPVMLG
>CYP107DH1_Mycobacterium kansasii 1010001495 2740573765
LISHPITVGTAEFNADQHRYYDWMRRNAPVYRGRLALRPPDRDVYFISRYQDCVDVVTDPRIRRVVEGAEHLP
LPEAIRMLTTGTMVYQDDPAHLRLRKLVSRPFTPRAIARLGDRVQTVTRVVLDGFKAGQRIDLQRDYALPIPT
TVISEMVGIPAEDRSAFYDYIDSLFELMLAGGTEGAAQRMDEFVDCLRTLVQQRRGDPGQDIITAMIEASEDG
DRLSDDEIVAMVFLLITGDYQTTPNVITNGIAALLSHREQLSLLQRRPELIGSAVEEVLRYTGTVGSTEATTF
AAEDIVVHGVTIPRGAMVVPLLTSANRDPAEFDDPDRFDITRKPNNHLAFSRGSHFCLGSHLARMETRIAIAG
LITRFPGLTLAIAPEELRLEPIPLLNRYAELPVMLG
>CYP107DH1_Mycobacterium kansasii 662 2636648687
LISHPITVGTAEFNADQHRYYDWMRRNAPVYRGRLALRPPDRDVYFISRYQDCVDVVTDPRIRRVVEGAEHLP
LPEAIRMLTTGTMVYQDDPAHLRLRKLVSRPFTPRAIARLGDRVQTVTRVVLDGFKAGQRIDLQRDYALPIPT
TVISEMVGIPAEDRSAFYDYIDSLFELMLAGGTEGAAQRMDEFVDCLRTLVQQRRGDPGQDIITAMIEASEDG
DRLSDDEIVAMVFLLITGDYQTTPNVITNGIAALLSHREQLSLLQRRPELIGSAVEEVLRYTGTVGSTEATTF
AAEDIVVHGVTIPRGAMVVPLLTSANRDPAEFDDPDRFDITRKPNNHLAFSRGSHFCLGSHLARMETRIAIAG
LITRFPGLTLAIAPEELRLEPIPLLNRYAELPVMLG
>CYP107DH1_Mycobacterium kansasii 824 2567232348
MGTAEFNADQHRYYDWMRRNAPVYRGRLALRPPDRDVYFISRYQDCVDVVTDPRIRRVVEGAEHLPLPEAIRM
LTTGTMVYQDDPAHLRLRKLVSRPFTPRAIARLGDRVQTVTRVVLDGFKAGQRIDLQRDYALPIPTTVISEMV
GIPAEDRSAFYDYIDSLFELMLAGGTEGAAQRMDEFVDCLRTLVQQRRGDPGQDIITAMIEASEDGDRLSDDE
IVAMVFLLITGDYQTTPNVITNGIAALLSHREQLSLLQRRPELIGSAVEEVLRYTGTVGSTEATTFAAEDIVV
HGVTIPRGAMVVPLLTSANRDPAEFDDPDRFDITRKPNNHLAFSRGSHFCLGSHLARMETRIAIAGLITRFPG
LTLAIAPEELRLEPIPLLNRYAELPVMLG
>CYP107DH1_Mycobacterium kansasii 824 2638026404
LISHPITVGTAEFNADQHRYYDWMRRNAPVYRGRLALRPPDRDVYFISRYQDCVDVVTDPRIRRVVEGAEHLP
LPEAIRMLTTGTMVYQDDPAHLRLRKLVSRPFTPRAIARLGDRVQTVTRVVLDGFKAGQRIDLQRDYALPIPT
TVISEMVGIPAEDRSAFYDYIDSLFELMLAGGTEGAAQRMDEFVDCLRTLVQQRRGDPGQDIITAMIEASEDG
DRLSDDEIVAMVFLLITGDYQTTPNVITNGIAALLSHREQLSLLQRRPELIGSAVEEVLRYTGTVGSTEATTF
AAEDIVVHGVTIPRGAMVVPLLTSANRDPAEFDDPDRFDITRKPNNHLAFSRGSHFCLGSHLARMETRIAIAG
LITRFPGLTLAIAPEELRLEPIPLLNRYAELPVMLG
>CYP107DS1_Mycobacterium abscessus 5S-1215 2526932624
MAEHEALTVLDDAFFDNPWPVYNQLRAVGPVHRIRLPNGLLAWLVIDYHMGKQVLADHRLSKDETSSSRAARS
QGIDTGWQDAAGPRPLRMNLLNTDPPEHTRLRALVNKAFTPAASARMRPAIEEVAKDLVQSLVDGPKTVDLLA
NYATPLPITVICRLLGVPEKDQETFGSLIVTIMDETAVADGEPSRLARVQLGEYLGKLIADRRAQRKSADTTD
SDLLDRLITAQVDQDRLSDMELISTIGLLLVAGYDTTVHLIANSIFRLATDASLLHRIHSNPDDIGPFIEEML
RWEGPGHTATLRHTISPITLGDKEIGAGEFLIVSVAAANRDPAAYADPEEFHLDRKEHGHLSFGHGIHYCVGA
PLARLEAEIAVATLAHNFPKLSLASAPDTLKWRRSMIIRGRTELPVQLF
>CYP107DS1_Mycobacterium abscessus 5S-1212 2526947963
MAEHEALTVLDDAFFDNPWPVYNQLRAVGPVHRIRLPNGLLAWLVIDYHMGKQVLADHRLSKDETSSSRAARS
QGIDTGWQDAAGPRPLRMNLLNTDPPEHTRLRALVNKAFTPAASARMRPAIEEVAKDLVQSLVDGPKTVDLLA
NYATPLPITVICRLLGVPEKDQETFGSLIVTIMDETAVADGEPSRLARVQLGEYLGKLIADRRAQRKSADTTD
SDLLDRLITAQVDQDRLSDMELISTIGLLLVAGYDTTVHLIANSIFRLATDASLLHRIHSNPDDIGPFIEEML
RWEGPGHTATLRHTISPITLGDKEIGAGEFLIVSVAAANRDPAAYADPEEFHLDRKEHGHLSFGHGIHYCVGA
PLARLEAEIAVATLAHNFPKLSLASAPDTLKWRRSMIIRGRTELPVQLF

>CYP107DS1_Mycobacterium abscessus 5S-0921 2526940932
MAEHEALTVLDDAFFDNPWPVYNQLRAVGPVHRIRLPNGLLAWLVIDYHMGKQVLADHRLSKDETSSSRAARS
QGIDTGWQDAAGPRPLRMNLLNTDPPEHTRLRALVNKAFTPAASARMRPAIEEVAKDLVQSLVDGPKTVDLLA
NYATPLPITVICRLLGVPEKDQETFGSLIVTIMDETAVADGEPSRLARVQLGEYLGKLIADRRAQRKSADTTD
SDLLDRLITAQVDQDRLSDMELISTIGLLLVAGYDTTVHLIANSIFRLATDASLLHRIHSNPDDIGPFIEEML
RWEGPGHTATLRHTISPITLGDKEIGAGEFLIVSVAAANRDPAAYADPEEFHLDRKEHGHLSFGHGIHYCVGA
PLARLEAEIAVATLAHNFPKLSLASAPDTLKWRRSMIIRGRTELPVQLF
>CYP107DS1_Mycobacterium abscessus 5S-0817 2537758918
MAEHEALTVLDDAFFDNPWPVYNQLRAVGPVHRIRLPNGLLAWLVIDYHMGKQVLADHRLSKDETSSSRAARS
QGIDTGWQDAAGPRPLRMNLLNTDPPEHTRLRALVNKAFTPAASARMRPAIEEVAKDLVQSLVDGPKTVDLLA
NYATPLPITVICRLLGVPEKDQETFGSLIVTIMDETAVADGEPSRLARVQLGEYLGKLIADRRAQRKSADTTD
SDLLDRLITAQVDQDRLSDMELISTIGLLLVAGYDTTVHLIANSIFRLATDASLLHRIHSNPDDIGPFIEEML
RWEGPGHTATLRHTISPITLGDKEIGAGEFLIVSVAAANRDPAAYADPEEFHLDRKEHGHLSFGHGIHYCVGA
PLARLEAEIAVATLAHNFPKLSLASAPDTLKWRRSMIIRGRTELPVQLF
>CYP107DS1_Mycobacterium abscessus 5S-0708 2526522789
MAEHEALTVLDDAFFDNPWPVYNQLRAVGPVHRIRLPNGLLAWLVIDYHMGKQVLADHRLSKDETSSSRAARS
QGIDTGWQDAAGPRPLRMNLLNTDPPEHTRLRALVNKAFTPAASARMRPAIEEVAKDLVQSLVDGPKTVDLLA
NYATPLPITVICRLLGVPEKDQETFGSLIVTIMDETAVADGEPSRLARVQLGEYLGKLIADRRAQRKSADTTD
SDLLDRLITAQVDQDRLSDMELISTIGLLLVAGYDTTVHLIANSIFRLATDASLLHRIHSNPDDIGPFIEEML
RWEGPGHTATLRHTISPITLGDKEIGAGEFLIVSVAAANRDPAAYADPEEFHLDRKEHGHLSFGHGIHYCVGA
PLARLEAEIAVATLAHNFPKLSLASAPDTLKWRRSMIIRGRTELPVQLF
>CYP107DS1_Mycobacterium abscessus 5S-0422 2526499402
MAEHEALTVLDDAFFDNPWPVYNQLRAVGPVHRIRLPNGLLAWLVIDYHMGKQVLADHRLSKDETSSSRAARS
QGIDTGWQDAAGPRPLRMNLLNTDPPEHTRLRALVNKAFTPAASARMRPAIEEVAKDLVQSLVDGPKTVDLLA
NYATPLPITVICRLLGVPEKDQETFGSLIVTIMDETAVADGEPSRLARVQLGEYLGKLIADRRAQRKSADTTD
SDLLDRLITAQVDQDRLSDMELISTIGLLLVAGYDTTVHLIANSIFRLATDASLLHRIHSNPDDIGPFIEEML
RWEGPGHTATLRHTISPITLGDKEIGAGEFLIVSVAAANRDPAAYADPEEFHLDRKEHGHLSFGHGIHYCVGA
PLARLEAEIAVATLAHNFPKLSLASAPDTLKWRRSMIIRGRTELPVQLF
>CYP107DS1_Mycobacterium abscessus 5S-0421 2526504253
MAEHEALTVLDDAFFDNPWPVYNQLRAVGPVHRIRLPNGLLAWLVIDYHMGKQVLADHRLSKDETSSSRAARS
QGIDTGWQDAAGPRPLRMNLLNTDPPEHTRLRALVNKAFTPAASARMRPAIEEVAKDLVQSLVDGPKTVDLLA
NYATPLPITVICRLLGVPEKDQETFGSLIVTIMDETAVADGEPSRLARVQLGEYLGKLIADRRAQRKSADTTD
SDLLDRLITAQVDQDRLSDMELISTIGLLLVAGYDTTVHLIANSIFRLATDASLLHRIHSNPDDIGPFIEEML
RWEGPGHTATLRHTISPITLGDKEIGAGEFLIVSVAAANRDPAAYADPEEFHLDRKEHGHLSFGHGIHYCVGA
PLARLEAEIAVATLAHNFPKLSLASAPDTLKWRRSMIIRGRTELPVQLF
>CYP107DS1_Mycobacterium abscessus 5S-0304 2526509425
MAEHEALTVLDDAFFDNPWPVYNQLRAVGPVHRIRLPNGLLAWLVIDYHMGKQVLADHRLSKDETSSSRAARS
QGIDTGWQDAAGPRPLRMNLLNTDPPEHTRLRALVNKAFTPAASARMRPAIEEVAKDLVQSLVDGPKTVDLLA
NYATPLPITVICRLLGVPEKDQETFGSLIVTIMDETAVADGEPSRLARVQLGEYLGKLIADRRAQRKSADTTD
SDLLDRLITAQVDQDRLSDMELISTIGLLLVAGYDTTVHLIANSIFRLATDASLLHRIHSNPDDIGPFIEEML
RWEGPGHTATLRHTISPITLGDKEIGAGEFLIVSVAAANRDPAAYADPEEFHLDRKEHGHLSFGHGIHYCVGA
PLARLEAEIAVATLAHNFPKLSLASAPDTLKWRRSMIIRGRTELPVQLF
>CYP107DS1_Mycobacterium abscessus PAP093 2662571587
GHGIHYCVGAPLARLEAEIAVATLAHNFPKLSLASAPDTLKWRRSMIIRGRTELPVQLF
>CYP107DS1_Mycobacterium massiliense 1513 2567111758
MAEHEALTVLDDAFFDNPWPVYNQLRAVGPVHRIRLPNGLLAWLVIDYHMGKQVLADHRLSKDETSSSRAARS
QGIDTGWQDAAGPRPLRMNLLNTDPPEHTRLRALVNKAFTPAASARMRPAIEEVAKDLVQSLVDGPKTVDLLA
NYATPLPITVICRLLGVPEKDQETFGSLIVTIMDETAVADGEPSRLARVQLGEYLGKLIADRRAQRKSADTTD
SDLLDRLITAQVDQDRLSDMELISTIGLLLVAGYDTTVHLIANSIFRLATDASLLHRIHSNPDDIGPFIEEML
RWEGPGHTATLRHTISPITLGDKEIGAGEFLIVSVAAANRDPAAYADPEEFHLDRKEHGHLSFGHGIHYCVGA
PLARLEAEIAVATLAHNFPKLSLASAPDTLKWRRSMIIRGRTELPVQLF
>CYP107DS1_Mycobacterium abscessus PAP_CF_004d 2670653486
MAEHEALTVLDDAFFDNPWPVYNQLRAVGPIHRIRLPNGLLAWLVIDYHMGKQVLADHRLSKDETSSSRAARS
QGIDTGWQDAAGPRPLRMNLLNTDPPEHTRLRALVNKAFTPAASARMRPAIEEVAKDLVQSLVDGPKTVDLLA
NYATPLPITVICRLLGVPEKDQETFGSLIVTIMDETAVADGEPSRLARVQLGEYLGKLIADRRAQRKSADTTD
SDLLDRLITAQVDQDRLSDMELISTIGLLLVAGYDTTVHLIANSIFRLATDASLLHRIHSNPDDIGPFIEEML
RWEGPGHTATLRHTISPITLGDKEIGAGEFLIVSVAAANRDPAAYADPEEFHLDRKEHGHLSFGHGIHYCVGA
PLARLEAEIAVATLAHNFPKLSLASAPDTLKWRRSMIIRGRTELPVQLF

>CYP107DS1_Mycobacterium abscessus PAP067 2665425374
MAEHEALTVLDDAFFDNPWPVYNQLRAVGPIHRIRLPNGLLAWLVIDYHMGKQVLADHRLSKDETSSSRAARS
QGIDTGWQDAAGPRPLRMNLLNTDPPEHTRLRALVNKAFTPAASARMRPAIEEVAKDLVQSLVDGPKTVDLLA
NYATPLPITVICRLLGVPEKDQETFGSLIVTIMDETAVADGEPSRLARVQLGEYLGKLIADRRAQRKSADTTD
SDLLDRLITAQVDQDRLSDMELISTIGLLLVAGYDTTVHLIANSIFRLATDASLLHRIHSNPDDIGPFIEEML
RWEGPGHTATLRHTISPITLGDKEIGAGEFLIVSVAAANRDPAAYADPEEFHLDRKEHGHLSFGHGIHYCVGA
PLARLEAEIAVATLAHNFPKLSLASAPDTLKWRRSMIIRGRTELPVQLF
>CYP107DS1_Mycobacterium abscessus PAP068 2697975256
MAEHEALTVLDDAFFDNPWPVYNQLRAVGPIHRIRLPNGLLAWLVIDYHMGKQVLADHRLSKDETSSSRAARS
QGIDTGWQDAAGPRPLRMNLLNTDPPEHTRLRALVNKAFTPAASARMRPAIEEVAKDLVQSLVDGPKTVDLLA
NYATPLPITVICRLLGVPEKDQETFGSLIVTIMDETAVADGEPSRLARVQLGEYLGKLIADRRAQRKSADTTD
SDLLDRLITAQVDQDRLSDMELISTIGLLLVAGYDTTVHLIANSIFRLATDASLLHRIHSNPDDIGPFIEEML
RWEGPGHTATLRHTISPITLGDKEIGAGEFLIVSVAAANRDPAAYADPEEFHLDRKEHGHLSFGHGIHYCVGA
PLARLEAEIAVATLAHNFPKLSLASAPDTLKWRRSMIIRGRTELPVQLF
>CYP107DS1_Mycobacterium abscessus PAP069 2673112091
MAEHEALTVLDDAFFDNPWPVYNQLRAVGPIHRIRLPNGLLAWLVIDYHMGKQVLADHRLSKDETSSSRAARS
QGIDTGWQDAAGPRPLRMNLLNTDPPEHTRLRALVNKAFTPAASARMRPAIEEVAKDLVQSLVDGPKTVDLLA
NYATPLPITVICRLLGVPEKDQETFGSLIVTIMDETAVADGEPSRLARVQLGEYLGKLIADRRAQRKSADTTD
SDLLDRLITAQVDQDRLSDMELISTIGLLLVAGYDTTVHLIANSIFRLATDASLLHRIHSNPDDIGPFIEEML
RWEGPGHTATLRHTISPITLGDKEIGAGEFLIVSVAAANRDPAAYADPEEFHLDRKEHGHLSFGHGIHYCVGA
PLARLEAEIAVATLAHNFPKLSLASAPDTLKWRRSMIIRGRTELPVQLF
>CYP107DS1_Mycobacterium abscessus PAP010 2660698663
MAEHEALTVLDDAFFDNPWPVYNQLRAVGPIHRIRLPNGLLAWLVIDYHMGKQVLADHRLSKDETSSSRAARS
QGIDTGWQDAAGPRPLRMNLLNTDPPEHTRLRALVNKAFTPAASARMRPAIEDVAKDLVQSLVDGPKTVDLLA
NYATPLPITVICRLLGVPEKDQETFGSLIVTIMDETAVADGEPSRLARVQLGEYLGKLIADRRAQRKSADTTD
SDLLDRLITAQVDQDRLSDMELISTIGLLLVAGYDTTVHLIANSIFRLATDASLLHRIHSNPDDIGPFIEEML
RWEGPGHTATLRHTISPITLGDKEIGAGEFLIVSVAAANRDPAAYADPEEFHLDRKEHGHLSFGHGIHYCVGA
PLARLEAEIAVATLAHNFPKLSLASAPDTLKWRRSMIIRGRTELPVQLF
>CYP107DS1_Mycobacterium abscessus PAP022 2696660208
MAEHEALTVLDDAFFDNPWPVYNQLRAVGPIHRIRLPNGLLAWLVIDYHMGKQVLADHRLSKDETSSSRAARS
QGIDTGWQDAAGPRPLRMNLLNTDPPEHTRLRALVNKAFTPAASARMRPAIEDVAKDLVQSLVDGPKTVDLLA
NYATPLPITVICRLLGVPEKDQETFGSLIVTIMDETAVADGEPSRLARVQLGEYLGKLIADRRAQRKSADTTD
SDLLDRLITAQVDQDRLSDMELISTIGLLLVAGYDTTVHLIANSIFRLATDASLLHRIHSNPDDIGPFIEEML
RWEGPGHTATLRHTISPITLGDKEIGAGEFLIVSVAAANRDPAAYADPEEFHLDRKEHGHLSFGHGIHYCVGA
PLARLEAEIAVATLAHNFPKLSLASAPDTLKWRRSMIIRGRTELPVQLF
>CYP107DS1_Mycobacterium abscessus PAP138 2696401844
MAEHEALTVLDDAFFDNPWPVYNQLRAVGPIHRIRLPNGLLAWLVIDYHMGKQVLADHRLSKDETSSSRAARS
QGIDTGWQDAAGPRPLRMNLLNTDPPEHTRLRALVNKAFTPAASARMRPAIEDVAKDLVQSLVDGPKTVDLLA
NYATPLPITVICRLLGVPEKDQETFGSLIVTIMDETAVADGEPSRLARVQLGEYLGKLIADRRAQRKSADTTD
SDLLDRLITAQVDQDRLSDMELISTIGLLLVAGYDTTVHLIANSIFRLATDASLLHRIHSNPDDIGPFIEEML
RWEGPGHTATLRHTISPITLGDKEIGAGEFLIVSVAAANRDPAAYADPEEFHLDRKEHGHLSFGHGIHYCVGA
PLARLEAEIAVATLAHNFPKLSLASAPDTLKWRRSMIIRGRTELPVQLF
>CYP107DS1_Mycobacterium abscessus PAP140 2662327518
MAEHEALTVLDDAFFDNPWPVYNQLRAVGPIHRIRLPNGLLAWLVIDYHMGKQVLADHRLSKDETSSSRAARS
QGIDTGWQDAAGPRPLRMNLLNTDPPEHTRLRALVNKAFTPAASARMRPAIEDVAKDLVQSLVDGPKTVDLLA
NYATPLPITVICRLLGVPEKDQETFGSLIVTIMDETAVADGEPSRLARVQLGEYLGKLIADRRAQRKSADTTD
SDLLDRLITAQVDQDRLSDMELISTIGLLLVAGYDTTVHLIANSIFRLATDASLLHRIHSNPDDIGPFIEEML
RWEGPGHTATLRHTISPITLGDKEIGAGEFLIVSVAAANRDPAAYADPEEFHLDRKEHGHLSFGHGIHYCVGA
PLARLEAEIAVATLAHNFPKLSLASAPDTLKWRRSMIIRGRTELPVQLF
>CYP107DS1_Mycobacterium abscessus PAP_CF_011g 2696828289
MAEHEALTVLDDAFFDNPWPVYNQLRAVGPIHRIRLPNGLLAWLVIDYHMGKQVLADHRLSKDETSSSRAARS
QGIDTGWQDAAGPRPLRMNLLNTDPPEHTRLRALVNKAFTPAASARMRPAIEDVAKDLVQSLVDGPKTVDLLA
NYATPLPITVICQLLGVPEKDQETFGSLIVTIMDETAVADGEPSRLARVQLGEYLGKLIADRRAQRKSADTTD
SDLLDRLITAQVDQDRLSDMELISTIGLLLVAGYDTTVHLIANSIFRLATDASLLHRIHSNPDDIGPFIEEML
RWEGPGHTATLRHTISPITLGDKAIGAGEFLIVSVAAANRDPAAYADPEEFHLDRKEHGHLSFGHGIHYCVGA
PLARLEAEIAVATLAHNFPKLSLASAPDTLKWRRSMIIRGRTELPVQLF
>CYP107DS1_Mycobacterium abscessus PAP036 2669490205

MAEHEALTVLDDAFFDNPWPVYNQLRAVGPIHRIRLPNGLLAWLVIDYHMGKQVLADHRLSKDETSSSRAARS
QGIDTGWQDAAGPRPLRMNLLNTDPPEHTRLRALVNKAFTPAASARMRPAIEDVAKDLVQSLVDGPKTVDLLA
NYATPLPITVICQLLGVPEKDQETFGSLIVTIMDETAVADGEPSRLARVQLGEYLGKLIADRRAQRKSADTTD
SDLLDRLITAQVDQDRLSDMELISTIGLLLVAGYDTTVHLIANSIFRLATDASLLHRIHSNPDDIGPFIEEML
RWEGPGHTATLRHTISPITLGDKAIGAGEFLIVSVAAANRDPAAYADPEEFHLDRKEHGHLSFGHGIHYCVGA
PLARLEAEIAVATLAHNFPKLSLASAPDTLKWRRSMIIRGRTELPVQLF
>CYP107DS1_Mycobacterium abscessus PAP040 2694031145
MAEHEALTVLDDAFFDNPWPVYNQLRAVGPIHRIRLPNGLLAWLVIDYHMGKQVLADHRLSKDETSSSRAARS
QGIDTGWQDAAGPRPLRMNLLNTDPPEHTRLRALVNKAFTPAASARMRPAIEDVAKDLVQSLVDGPKTVDLLA
NYATPLPITVICQLLGVPEKDQETFGSLIVTIMDETAVADGEPSRLARVQLGEYLGKLIADRRAQRKSADTTD
SDLLDRLITAQVDQDRLSDMELISTIGLLLVAGYDTTVHLIANSIFRLATDASLLHRIHSNPDDIGPFIEEML
RWEGPGHTATLRHTISPITLGDKAIGAGEFLIVSVAAANRDPAAYADPEEFHLDRKEHGHLSFGHGIHYCVGA
PLARLEAEIAVATLAHNFPKLSLASAPDTLKWRRSMIIRGRTELPVQLF
>CYP107DS1_Mycobacterium abscessus PAP041 2666779782
MAEHEALTVLDDAFFDNPWPVYNQLRAVGPIHRIRLPNGLLAWLVIDYHMGKQVLADHRLSKDETSSSRAARS
QGIDTGWQDAAGPRPLRMNLLNTDPPEHTRLRALVNKAFTPAASARMRPAIEDVAKDLVQSLVDGPKTVDLLA
NYATPLPITVICQLLGVPEKDQETFGSLIVTIMDETAVADGEPSRLARVQLGEYLGKLIADRRAQRKSADTTD
SDLLDRLITAQVDQDRLSDMELISTIGLLLVAGYDTTVHLIANSIFRLATDASLLHRIHSNPDDIGPFIEEML
RWEGPGHTATLRHTISPITLGDKAIGAGEFLIVSVAAANRDPAAYADPEEFHLDRKEHGHLSFGHGIHYCVGA
PLARLEAEIAVATLAHNFPKLSLASAPDTLKWRRSMIIRGRTELPVQLF
>CYP107DS1_Mycobacterium abscessus PAP076 2692376281
MAEHEALTVLDDAFFDNPWPVYNQLRAVGPIHRIRLPNGLLAWLVIDYHMGKQVLADHRLSKDETSSSRAARS
QGIDTGWQDAAGPRPLRMNLLNTDPPEHTRLRALVNKAFTPAASARMRPAIEDVAKDLVQSLVDGPKTVDLLA
NYATPLPITVICQLLGVPEKDQETFGSLIVTIMDETAVADGEPSRLARVQLGEYLGKLIADRRAQRKSADTTD
SDLLDRLITAQVDQDRLSDMELISTIGLLLVAGYDTTVHLIANSIFRLATDASLLHRIHSNPDDIGPFIEEML
RWEGPGHTATLRHTISPITLGDKAIGAGEFLIVSVAAANRDPAAYADPEEFHLDRKEHGHLSFGHGIHYCVGA
PLARLEAEIAVATLAHNFPKLSLASAPDTLKWRRSMIIRGRTELPVQLF
>CYP107DS1_Mycobacterium abscessus PAP082 2660749948
MAEHEALTVLDDAFFDNPWPVYNQLRAVGPIHRIRLPNGLLAWLVIDYHMGKQVLADHRLSKDETSSSRAARS
QGIDTGWQDAAGPRPLRMNLLNTDPPEHTRLRALVNKAFTPAASARMRPAIEDVAKDLVQSLVDGPKTVDLLA
NYATPLPITVICQLLGVPEKDQETFGSLIVTIMDETAVADGEPSRLARVQLGEYLGKLIADRRAQRKSADTTD
SDLLDRLITAQVDQDRLSDMELISTIGLLLVAGYDTTVHLIANSIFRLATDASLLHRIHSNPDDIGPFIEEML
RWEGPGHTATLRHTISPITLGDKAIGAGEFLIVSVAAANRDPAAYADPEEFHLDRKEHGHLSFGHGIHYCVGA
PLARLEAEIAVATLAHNFPKLSLASAPDTLKWRRSMIIRGRTELPVQLF
>CYP107DS1_Mycobacterium abscessus PAP170 2695754983
MAEHEALTVLDDAFFDNPWPVYNQLRAVGPIHRIRLPNGLLAWLVIDYHMGKQVLADHRLSKDETSSSRAARS
QGIDTGWQDAAGPRPLRMNLLNTDPPEHTRLRALVNKAFTPAASARMRPAIEDVAKDLVQSLVDGPKTVDLLA
NYATPLPITVICQLLGVPEKDQETFGSLIVTIMDETAVADGEPSRLARVQLGEYLGKLIADRRAQRKSADTTD
SDLLDRLITAQVDQDRLSDMELISTIGLLLVAGYDTTVHLIANSIFRLATDASLLHRIHSNPDDIGPFIEEML
RWEGPGHTATLRHTISPITLGDKAIGAGEFLIVSVAAANRDPAAYADPEEFHLDRKEHGHLSFGHGIHYCVGA
PLARLEAEIAVATLAHNFPKLSLASAPDTLKWRRSMIIRGRTELPVQLF
>CYP107DS1_Mycobacterium abscessus FLAC004 2687565464
MAEHDALTVLDDVFFDNPWPVYNQLRAVGPIHRIRLPNGLLAWLVIDYHMGKQVLADHRLSKDETSSSRAARS
QGIDTGWQDAAGPRPLRMNLLNTDPPEHTRLRALVNKAFTPAASARMRPAIEDVAKELVQSLVDGPKTVDLLA
NYATPLPITVICRLLGVPEKDQETFGSLIVTIMDETAVADGEPSRLARVQLGEYLGKLIADRRAQRKSADTTD
SDLLDRLITAQVDQDRLSDMELISTIGLLLVAGYDTTVHLIANSIFRLATDASLLHRIHSNPDDIGPFIEEML
RWEGPGHTATLRHTISPITLGDKEIGAGEFLIVSVAAANRDPAAYADPEEFHLDRKEHGHLSFGHGIHYCVGA
PLARLEAEIAVATLAHNFPKLSLASAPDTLKWRRSMIIRGRTELPVQLF
>CYP107DS1_Mycobacterium abscessus FLAC007 2688468897
MAEHDALTVLDDVFFDNPWPVYNQLRAVGPIHRIRLPNGLLAWLVIDYHMGKQVLADHRLSKDETSSSRAARS
QGIDTGWQDAAGPRPLRMNLLNTDPPEHTRLRALVNKAFTPAASARMRPAIEDVAKELVQSLVDGPKTVDLLA
NYATPLPITVICRLLGVPEKDQETFGSLIVTIMDETAVADGEPSRLARVQLGEYLGKLIADRRAQRKSADTTD
SDLLDRLITAQVDQDRLSDMELISTIGLLLVAGYDTTVHLIANSIFRLATDASLLHRIHSNPDDIGPFIEEML
RWEGPGHTATLRHTISPITLGDKEIGAGEFLIVSVAAANRDPAAYADPEEFHLDRKEHGHLSFGHGIHYCVGA
PLARLEAEIAVATLAHNFPKLSLASAPDTLKWRRSMIIRGRTELPVQLF
>CYP107DS1_Mycobacterium abscessus FLAC008 2678622039
MAEHDALTVLDDVFFDNPWPVYNQLRAVGPIHRIRLPNGLLAWLVIDYHMGKQVLADHRLSKDETSSSRAARS
QGIDTGWQDAAGPRPLRMNLLNTDPPEHTRLRALVNKAFTPAASARMRPAIEDVAKELVQSLVDGPKTVDLLA

NYATPLPITVICRLLGVPEKDQETFGSLIVTIMDETAVADGEPSRLARVQLGEYLGKLIADRRAQRKSADTTD
SDLLDRLITAQVDQDRLSDMELISTIGLLLVAGYDTTVHLIANSIFRLATDASLLHRIHSNPDDIGPFIEEML
RWEGPGHTATLRHTISPITLGDKEIGAGEFLIVSVAAANRDPAAYADPEEFHLDRKEHGHLSFGHGIHYCVGA
PLARLEAEIAVATLAHNFPKLSLASAPDTLKWRRSMIIRGRTELPVQLF
>CYP107DS1_Mycobacterium abscessus FLAC045 2686919338
MAEHDALTVLDDVFFDNPWPVYNQLRAVGPIHRIRLPNGLLAWLVIDYHMGKQVLADHRLSKDETSSSRAARS
QGIDTGWQDAAGPRPLRMNLLNTDPPEHTRLRALVNKAFTPAASARMRPAIEDVAKELVQSLVDGPKTVDLLA
NYATPLPITVICRLLGVPEKDQETFGSLIVTIMDETAVADGEPSRLARVQLGEYLGKLIADRRAQRKSADTTD
SDLLDRLITAQVDQDRLSDMELISTIGLLLVAGYDTTVHLIANSIFRLATDASLLHRIHSNPDDIGPFIEEML
RWEGPGHTATLRHTISPITLGDKEIGAGEFLIVSVAAANRDPAAYADPEEFHLDRKEHGHLSFGHGIHYCVGA
PLARLEAEIAVATLAHNFPKLSLASAPDTLKWRRSMIIRGRTELPVQLF
>CYP107DS1_Mycobacterium abscessus PAP_CF_014c 2677683238
MAEHDALTVLDDVFFDNPWPVYNQLRAVGPIHRIRLPNGLLAWLVIDYHMGKQVLADHRLSKDETSSSRAARS
QGIDTGWQDAAGPRPLRMNLLNTDPPEHTRLRALVNKAFTPAASARMRPAIEDVAKELVQSLVDGPKTVDLLA
NYATPLPITVICRLLGVPEKDQETFGSLIVTIMDETAVADGEPSRLARVQLGEYLGKLIADRRAQRKSADTTD
SDLLDRLITAQVDQDRLSDMELISTIGLLLVAGYDTTVHLIANSIFRLATDASLLHRIHSNPDDIGPFIEEML
RWEGPGHTATLRHTISPITLGDKEIGAGEFLIVSVAAANRDPAAYADPEEFHLDRKEHGHLSFGHGIHYCVGA
PLARLEAEIAVATLAHNFPKLSLASAPDTLKWRRSMIIRGRTELPVQLF
>CYP107DS1_Mycobacterium abscessus PAP_CF_019g 2698580594
MAEHDALTVLDDVFFDNPWPVYNQLRAVGPIHRIRLPNGLLAWLVIDYHMGKQVLADHRLSKDETSSSRAARS
QGIDTGWQDAAGPRPLRMNLLNTDPPEHTRLRALVNKAFTPAASARMRPAIEDVAKELVQSLVDGPKTVDLLA
NYATPLPITVICRLLGVPEKDQETFGSLIVTIMDETAVADGEPSRLARVQLGEYLGKLIADRRAQRKSADTTD
SDLLDRLITAQVDQDRLSDMELISTIGLLLVAGYDTTVHLIANSIFRLATDASLLHRIHSNPDDIGPFIEEML
RWEGPGHTATLRHTISPITLGDKEIGAGEFLIVSVAAANRDPAAYADPEEFHLDRKEHGHLSFGHGIHYCVGA
PLARLEAEIAVATLAHNFPKLSLASAPDTLKWRRSMIIRGRTELPVQLF
>CYP107DS1_Mycobacterium abscessus PAP_CF_024g 2665058563
MAEHDALTVLDDVFFDNPWPVYNQLRAVGPIHRIRLPNGLLAWLVIDYHMGKQVLADHRLSKDETSSSRAARS
QGIDTGWQDAAGPRPLRMNLLNTDPPEHTRLRALVNKAFTPAASARMRPAIEDVAKELVQSLVDGPKTVDLLA
NYATPLPITVICRLLGVPEKDQETFGSLIVTIMDETAVADGEPSRLARVQLGEYLGKLIADRRAQRKSADTTD
SDLLDRLITAQVDQDRLSDMELISTIGLLLVAGYDTTVHLIANSIFRLATDASLLHRIHSNPDDIGPFIEEML
RWEGPGHTATLRHTISPITLGDKEIGAGEFLIVSVAAANRDPAAYADPEEFHLDRKEHGHLSFGHGIHYCVGA
PLARLEAEIAVATLAHNFPKLSLASAPDTLKWRRSMIIRGRTELPVQLF
>CYP107DS1_Mycobacterium abscessus PAP_CF_024h 2697920118
MAEHDALTVLDDVFFDNPWPVYNQLRAVGPIHRIRLPNGLLAWLVIDYHMGKQVLADHRLSKDETSSSRAARS
QGIDTGWQDAAGPRPLRMNLLNTDPPEHTRLRALVNKAFTPAASARMRPAIEDVAKELVQSLVDGPKTVDLLA
NYATPLPITVICRLLGVPEKDQETFGSLIVTIMDETAVADGEPSRLARVQLGEYLGKLIADRRAQRKSADTTD
SDLLDRLITAQVDQDRLSDMELISTIGLLLVAGYDTTVHLIANSIFRLATDASLLHRIHSNPDDIGPFIEEML
RWEGPGHTATLRHTISPITLGDKEIGAGEFLIVSVAAANRDPAAYADPEEFHLDRKEHGHLSFGHGIHYCVGA
PLARLEAEIAVATLAHNFPKLSLASAPDTLKWRRSMIIRGRTELPVQLF
>CYP107DS1_Mycobacterium abscessus PAP023 2702203294
MAEHDALTVLDDVFFDNPWPVYNQLRAVGPIHRIRLPNGLLAWLVIDYHMGKQVLADHRLSKDETSSSRAARS
QGIDTGWQDAAGPRPLRMNLLNTDPPEHTRLRALVNKAFTPAASARMRPAIEDVAKELVQSLVDGPKTVDLLA
NYATPLPITVICRLLGVPEKDQETFGSLIVTIMDETAVADGEPSRLARVQLGEYLGKLIADRRAQRKSADTTD
SDLLDRLITAQVDQDRLSDMELISTIGLLLVAGYDTTVHLIANSIFRLATDASLLHRIHSNPDDIGPFIEEML
RWEGPGHTATLRHTISPITLGDKEIGAGEFLIVSVAAANRDPAAYADPEEFHLDRKEHGHLSFGHGIHYCVGA
PLARLEAEIAVATLAHNFPKLSLASAPDTLKWRRSMIIRGRTELPVQLF
>CYP107DS1_Mycobacterium abscessus PAP039 2661753584
MAEHDALTVLDDVFFDNPWPVYNQLRAVGPIHRIRLPNGLLAWLVIDYHMGKQVLADHRLSKDETSSSRAARS
QGIDTGWQDAAGPRPLRMNLLNTDPPEHTRLRALVNKAFTPAASARMRPAIEDVAKELVQSLVDGPKTVDLLA
NYATPLPITVICRLLGVPEKDQETFGSLIVTIMDETAVADGEPSRLARVQLGEYLGKLIADRRAQRKSADTTD
SDLLDRLITAQVDQDRLSDMELISTIGLLLVAGYDTTVHLIANSIFRLATDASLLHRIHSNPDDIGPFIEEML
RWEGPGHTATLRHTISPITLGDKEIGAGEFLIVSVAAANRDPAAYADPEEFHLDRKEHGHLSFGHGIHYCVGA
PLARLEAEIAVATLAHNFPKLSLASAPDTLKWRRSMIIRGRTELPVQLF
>CYP107DS1_Mycobacterium abscessus PAP048 2670747098
MAEHDALTVLDDVFFDNPWPVYNQLRAVGPIHRIRLPNGLLAWLVIDYHMGKQVLADHRLSKDETSSSRAARS
QGIDTGWQDAAGPRPLRMNLLNTDPPEHTRLRALVNKAFTPAASARMRPAIEDVAKELVQSLVDGPKTVDLLA
NYATPLPITVICRLLGVPEKDQETFGSLIVTIMDETAVADGEPSRLARVQLGEYLGKLIADRRAQRKSADTTD
SDLLDRLITAQVDQDRLSDMELISTIGLLLVAGYDTTVHLIANSIFRLATDASLLHRIHSNPDDIGPFIEEML

RWEGPGHTATLRHTISPITLGDKEIGAGEFLIVSVAAANRDPAAYADPEEFHLDRKEHGHLSFGHGIHYCVGA
PLARLEAEIAVATLAHNFPKLSLASAPDTLKWRRSMIIRGRTELPVQLF
>CYP107DS1_Mycobacterium abscessus PAP049 2675332682
MAEHDALTVLDDVFFDNPWPVYNQLRAVGPIHRIRLPNGLLAWLVIDYHMGKQVLADHRLSKDETSSSRAARS
QGIDTGWQDAAGPRPLRMNLLNTDPPEHTRLRALVNKAFTPAASARMRPAIEDVAKELVQSLVDGPKTVDLLA
NYATPLPITVICRLLGVPEKDQETFGSLIVTIMDETAVADGEPSRLARVQLGEYLGKLIADRRAQRKSADTTD
SDLLDRLITAQVDQDRLSDMELISTIGLLLVAGYDTTVHLIANSIFRLATDASLLHRIHSNPDDIGPFIEEML
RWEGPGHTATLRHTISPITLGDKEIGAGEFLIVSVAAANRDPAAYADPEEFHLDRKEHGHLSFGHGIHYCVGA
PLARLEAEIAVATLAHNFPKLSLASAPDTLKWRRSMIIRGRTELPVQLF
>CYP107DS1_Mycobacterium abscessus PAP055 2682879513
MAEHDALTVLDDVFFDNPWPVYNQLRAVGPIHRIRLPNGLLAWLVIDYHMGKQVLADHRLSKDETSSSRAARS
QGIDTGWQDAAGPRPLRMNLLNTDPPEHTRLRALVNKAFTPAASARMRPAIEDVAKELVQSLVDGPKTVDLLA
NYATPLPITVICRLLGVPEKDQETFGSLIVTIMDETAVADGEPSRLARVQLGEYLGKLIADRRAQRKSADTTD
SDLLDRLITAQVDQDRLSDMELISTIGLLLVAGYDTTVHLIANSIFRLATDASLLHRIHSNPDDIGPFIEEML
RWEGPGHTATLRHTISPITLGDKEIGAGEFLIVSVAAANRDPAAYADPEEFHLDRKEHGHLSFGHGIHYCVGA
PLARLEAEIAVATLAHNFPKLSLASAPDTLKWRRSMIIRGRTELPVQLF
>CYP107DS1_Mycobacterium abscessus PAP057 2659849962
MAEHDALTVLDDVFFDNPWPVYNQLRAVGPIHRIRLPNGLLAWLVIDYHMGKQVLADHRLSKDETSSSRAARS
QGIDTGWQDAAGPRPLRMNLLNTDPPEHTRLRALVNKAFTPAASARMRPAIEDVAKELVQSLVDGPKTVDLLA
NYATPLPITVICRLLGVPEKDQETFGSLIVTIMDETAVADGEPSRLARVQLGEYLGKLIADRRAQRKSADTTD
SDLLDRLITAQVDQDRLSDMELISTIGLLLVAGYDTTVHLIANSIFRLATDASLLHRIHSNPDDIGPFIEEML
RWEGPGHTATLRHTISPITLGDKEIGAGEFLIVSVAAANRDPAAYADPEEFHLDRKEHGHLSFGHGIHYCVGA
PLARLEAEIAVATLAHNFPKLSLASAPDTLKWRRSMIIRGRTELPVQLF
>CYP107DS1_Mycobacterium abscessus PAP058 2662904213
MAEHDALTVLDDVFFDNPWPVYNQLRAVGPIHRIRLPNGLLAWLVIDYHMGKQVLADHRLSKDETSSSRAARS
QGIDTGWQDAAGPRPLRMNLLNTDPPEHTRLRALVNKAFTPAASARMRPAIEDVAKELVQSLVDGPKTVDLLA
NYATPLPITVICRLLGVPEKDQETFGSLIVTIMDETAVADGEPSRLARVQLGEYLGKLIADRRAQRKSADTTD
SDLLDRLITAQVDQDRLSDMELISTIGLLLVAGYDTTVHLIANSIFRLATDASLLHRIHSNPDDIGPFIEEML
RWEGPGHTATLRHTISPITLGDKEIGAGEFLIVSVAAANRDPAAYADPEEFHLDRKEHGHLSFGHGIHYCVGA
PLARLEAEIAVATLAHNFPKLSLASAPDTLKWRRSMIIRGRTELPVQLF
>CYP107DS1_Mycobacterium abscessus PAP059 2692161657
MAEHDALTVLDDVFFDNPWPVYNQLRAVGPIHRIRLPNGLLAWLVIDYHMGKQVLADHRLSKDETSSSRAARS
QGIDTGWQDAAGPRPLRMNLLNTDPPEHTRLRALVNKAFTPAASARMRPAIEDVAKELVQSLVDGPKTVDLLA
NYATPLPITVICRLLGVPEKDQETFGSLIVTIMDETAVADGEPSRLARVQLGEYLGKLIADRRAQRKSADTTD
SDLLDRLITAQVDQDRLSDMELISTIGLLLVAGYDTTVHLIANSIFRLATDASLLHRIHSNPDDIGPFIEEML
RWEGPGHTATLRHTISPITLGDKEIGAGEFLIVSVAAANRDPAAYADPEEFHLDRKEHGHLSFGHGIHYCVGA
PLARLEAEIAVATLAHNFPKLSLASAPDTLKWRRSMIIRGRTELPVQLF
>CYP107DS1_Mycobacterium abscessus PAP072 2672054835
MAEHDALTVLDDVFFDNPWPVYNQLRAVGPIHRIRLPNGLLAWLVIDYHMGKQVLADHRLSKDETSSSRAARS
QGIDTGWQDAAGPRPLRMNLLNTDPPEHTRLRALVNKAFTPAASARMRPAIEDVAKELVQSLVDGPKTVDLLA
NYATPLPITVICRLLGVPEKDQETFGSLIVTIMDETAVADGEPSRLARVQLGEYLGKLIADRRAQRKSADTTD
SDLLDRLITAQVDQDRLSDMELISTIGLLLVAGYDTTVHLIANSIFRLATDASLLHRIHSNPDDIGPFIEEML
RWEGPGHTATLRHTISPITLGDKEIGAGEFLIVSVAAANRDPAAYADPEEFHLDRKEHGHLSFGHGIHYCVGA
PLARLEAEIAVATLAHNFPKLSLASAPDTLKWRRSMIIRGRTELPVQLF
>CYP107DS1_Mycobacterium abscessus PAP073 2663428576
MAEHDALTVLDDVFFDNPWPVYNQLRAVGPIHRIRLPNGLLAWLVIDYHMGKQVLADHRLSKDETSSSRAARS
QGIDTGWQDAAGPRPLRMNLLNTDPPEHTRLRALVNKAFTPAASARMRPAIEDVAKELVQSLVDGPKTVDLLA
NYATPLPITVICRLLGVPEKDQETFGSLIVTIMDETAVADGEPSRLARVQLGEYLGKLIADRRAQRKSADTTD
SDLLDRLITAQVDQDRLSDMELISTIGLLLVAGYDTTVHLIANSIFRLATDASLLHRIHSNPDDIGPFIEEML
RWEGPGHTATLRHTISPITLGDKEIGAGEFLIVSVAAANRDPAAYADPEEFHLDRKEHGHLSFGHGIHYCVGA
PLARLEAEIAVATLAHNFPKLSLASAPDTLKWRRSMIIRGRTELPVQLF
>CYP107DS1_Mycobacterium abscessus PAP074 2665664170
MAEHDALTVLDDVFFDNPWPVYNQLRAVGPIHRIRLPNGLLAWLVIDYHMGKQVLADHRLSKDETSSSRAARS
QGIDTGWQDAAGPRPLRMNLLNTDPPEHTRLRALVNKAFTPAASARMRPAIEDVAKELVQSLVDGPKTVDLLA
NYATPLPITVICRLLGVPEKDQETFGSLIVTIMDETAVADGEPSRLARVQLGEYLGKLIADRRAQRKSADTTD
SDLLDRLITAQVDQDRLSDMELISTIGLLLVAGYDTTVHLIANSIFRLATDASLLHRIHSNPDDIGPFIEEML
RWEGPGHTATLRHTISPITLGDKEIGAGEFLIVSVAAANRDPAAYADPEEFHLDRKEHGHLSFGHGIHYCVGA
PLARLEAEIAVATLAHNFPKLSLASAPDTLKWRRSMIIRGRTELPVQLF

>CYP107DS1_Mycobacterium abscessus PAP078 2666566536
MAEHDALTVLDDVFFDNPWPVYNQLRAVGPIHRIRLPNGLLAWLVIDYHMGKQVLADHRLSKDETSSSRAARS
QGIDTGWQDAAGPRPLRMNLLNTDPPEHTRLRALVNKAFTPAASARMRPAIEDVAKELVQSLVDGPKTVDLLA
NYATPLPITVICRLLGVPEKDQETFGSLIVTIMDETAVADGEPSRLARVQLGEYLGKLIADRRAQRKSADTTD
SDLLDRLITAQVDQDRLSDMELISTIGLLLVAGYDTTVHLIANSIFRLATDASLLHRIHSNPDDIGPFIEEML
RWEGPGHTATLRHTISPITLGDKEIGAGEFLIVSVAAANRDPAAYADPEEFHLDRKEHGHLSFGHGIHYCVGA
PLARLEAEIAVATLAHNFPKLSLASAPDTLKWRRSMIIRGRTELPVQLF
>CYP107DS1_Mycobacterium abscessus PAP080 2669573285
MAEHDALTVLDDVFFDNPWPVYNQLRAVGPIHRIRLPNGLLAWLVIDYHMGKQVLADHRLSKDETSSSRAARS
QGIDTGWQDAAGPRPLRMNLLNTDPPEHTRLRALVNKAFTPAASARMRPAIEDVAKELVQSLVDGPKTVDLLA
NYATPLPITVICRLLGVPEKDQETFGSLIVTIMDETAVADGEPSRLARVQLGEYLGKLIADRRAQRKSADTTD
SDLLDRLITAQVDQDRLSDMELISTIGLLLVAGYDTTVHLIANSIFRLATDASLLHRIHSNPDDIGPFIEEML
RWEGPGHTATLRHTISPITLGDKEIGAGEFLIVSVAAANRDPAAYADPEEFHLDRKEHGHLSFGHGIHYCVGA
PLARLEAEIAVATLAHNFPKLSLASAPDTLKWRRSMIIRGRTELPVQLF
>CYP107DS1_Mycobacterium abscessus PAP081 2670851859
MAEHDALTVLDDVFFDNPWPVYNQLRAVGPIHRIRLPNGLLAWLVIDYHMGKQVLADHRLSKDETSSSRAARS
QGIDTGWQDAAGPRPLRMNLLNTDPPEHTRLRALVNKAFTPAASARMRPAIEDVAKELVQSLVDGPKTVDLLA
NYATPLPITVICRLLGVPEKDQETFGSLIVTIMDETAVADGEPSRLARVQLGEYLGKLIADRRAQRKSADTTD
SDLLDRLITAQVDQDRLSDMELISTIGLLLVAGYDTTVHLIANSIFRLATDASLLHRIHSNPDDIGPFIEEML
RWEGPGHTATLRHTISPITLGDKEIGAGEFLIVSVAAANRDPAAYADPEEFHLDRKEHGHLSFGHGIHYCVGA
PLARLEAEIAVATLAHNFPKLSLASAPDTLKWRRSMIIRGRTELPVQLF
>CYP107DS1_Mycobacterium abscessus PAP084 2663343469
MAEHDALTVLDDVFFDNPWPVYNQLRAVGPIHRIRLPNGLLAWLVIDYHMGKQVLADHRLSKDETSSSRAARS
QGIDTGWQDAAGPRPLRMNLLNTDPPEHTRLRALVNKAFTPAASARMRPAIEDVAKELVQSLVDGPKTVDLLA
NYATPLPITVICRLLGVPEKDQETFGSLIVTIMDETAVADGEPSRLARVQLGEYLGKLIADRRAQRKSADTTD
SDLLDRLITAQVDQDRLSDMELISTIGLLLVAGYDTTVHLIANSIFRLATDASLLHRIHSNPDDIGPFIEEML
RWEGPGHTATLRHTISPITLGDKEIGAGEFLIVSVAAANRDPAAYADPEEFHLDRKEHGHLSFGHGIHYCVGA
PLARLEAEIAVATLAHNFPKLSLASAPDTLKWRRSMIIRGRTELPVQLF
>CYP107DS1_Mycobacterium abscessus PAP086 2661435489
MAEHDALTVLDDVFFDNPWPVYNQLRAVGPIHRIRLPNGLLAWLVIDYHMGKQVLADHRLSKDETSSSRAARS
QGIDTGWQDAAGPRPLRMNLLNTDPPEHTRLRALVNKAFTPAASARMRPAIEDVAKELVQSLVDGPKTVDLLA
NYATPLPITVICRLLGVPEKDQETFGSLIVTIMDETAVADGEPSRLARVQLGEYLGKLIADRRAQRKSADTTD
SDLLDRLITAQVDQDRLSDMELISTIGLLLVAGYDTTVHLIANSIFRLATDASLLHRIHSNPDDIGPFIEEML
RWEGPGHTATLRHTISPITLGDKEIGAGEFLIVSVAAANRDPAAYADPEEFHLDRKEHGHLSFGHGIHYCVGA
PLARLEAEIAVATLAHNFPKLSLASAPDTLKWRRSMIIRGRTELPVQLF
>CYP107DS1_Mycobacterium abscessus PAP087 2660983255
MAEHDALTVLDDVFFDNPWPVYNQLRAVGPIHRIRLPNGLLAWLVIDYHMGKQVLADHRLSKDETSSSRAARS
QGIDTGWQDAAGPRPLRMNLLNTDPPEHTRLRALVNKAFTPAASARMRPAIEDVAKELVQSLVDGPKTVDLLA
NYATPLPITVICRLLGVPEKDQETFGSLIVTIMDETAVADGEPSRLARVQLGEYLGKLIADRRAQRKSADTTD
SDLLDRLITAQVDQDRLSDMELISTIGLLLVAGYDTTVHLIANSIFRLATDASLLHRIHSNPDDIGPFIEEML
RWEGPGHTATLRHTISPITLGDKEIGAGEFLIVSVAAANRDPAAYADPEEFHLDRKEHGHLSFGHGIHYCVGA
PLARLEAEIAVATLAHNFPKLSLASAPDTLKWRRSMIIRGRTELPVQLF
>CYP107DS1_Mycobacterium abscessus PAP088 2669841739
MAEHDALTVLDDVFFDNPWPVYNQLRAVGPIHRIRLPNGLLAWLVIDYHMGKQVLADHRLSKDETSSSRAARS
QGIDTGWQDAAGPRPLRMNLLNTDPPEHTRLRALVNKAFTPAASARMRPAIEDVAKELVQSLVDGPKTVDLLA
NYATPLPITVICRLLGVPEKDQETFGSLIVTIMDETAVADGEPSRLARVQLGEYLGKLIADRRAQRKSADTTD
SDLLDRLITAQVDQDRLSDMELISTIGLLLVAGYDTTVHLIANSIFRLATDASLLHRIHSNPDDIGPFIEEML
RWEGPGHTATLRHTISPITLGDKEIGAGEFLIVSVAAANRDPAAYADPEEFHLDRKEHGHLSFGHGIHYCVGA
PLARLEAEIAVATLAHNFPKLSLASAPDTLKWRRSMIIRGRTELPVQLF
>CYP107DS1_Mycobacterium abscessus PAP091 2660672096
MAEHDALTVLDDVFFDNPWPVYNQLRAVGPIHRIRLPNGLLAWLVIDYHMGKQVLADHRLSKDETSSSRAARS
QGIDTGWQDAAGPRPLRMNLLNTDPPEHTRLRALVNKAFTPAASARMRPAIEDVAKELVQSLVDGPKTVDLLA
NYATPLPITVICRLLGVPEKDQETFGSLIVTIMDETAVADGEPSRLARVQLGEYLGKLIADRRAQRKSADTTD
SDLLDRLITAQVDQDRLSDMELISTIGLLLVAGYDTTVHLIANSIFRLATDASLLHRIHSNPDDIGPFIEEML
RWEGPGHTATLRHTISPITLGDKEIGAGEFLIVSVAAANRDPAAYADPEEFHLDRKEHGHLSFGHGIHYCVGA
PLARLEAEIAVATLAHNFPKLSLASAPDTLKWRRSMIIRGRTELPVQLF
>CYP107DS1_Mycobacterium abscessus PAP092 2694232842

MAEHDALTVLDDVFFDNPWPVYNQLRAVGPIHRIRLPNGLLAWLVIDYHMGKQVLADHRLSKDETSSSRAARS
QGIDTGWQDAAGPRPLRMNLLNTDPPEHTRLRALVNKAFTPAASARMRPAIEDVAKELVQSLVDGPKTVDLLA
NYATPLPITVICRLLGVPEKDQETFGSLIVTIMDETAVADGEPSRLARVQLGEYLGKLIADRRAQRKSADTTD
SDLLDRLITAQVDQDRLSDMELISTIGLLLVAGYDTTVHLIANSIFRLATDASLLHRIHSNPDDIGPFIEEML
RWEGPGHTATLRHTISPITLGDKEIGAGEFLIVSVAAANRDPAAYADPEEFHLDRKEHGHLSFGHGIHYCVGA
PLARLEAEIAVATLAHNFPKLSLASAPDTLKWRRSMIIRGRTELPVQLF
>CYP107DS1_Mycobacterium abscessus PAP094 2669205881
MAEHDALTVLDDVFFDNPWPVYNQLRAVGPIHRIRLPNGLLAWLVIDYHMGKQVLADHRLSKDETSSSRAARS
QGIDTGWQDAAGPRPLRMNLLNTDPPEHTRLRALVNKAFTPAASARMRPAIEDVAKELVQSLVDGPKTVDLLA
NYATPLPITVICRLLGVPEKDQETFGSLIVTIMDETAVADGEPSRLARVQLGEYLGKLIADRRAQRKSADTTD
SDLLDRLITAQVDQDRLSDMELISTIGLLLVAGYDTTVHLIANSIFRLATDASLLHRIHSNPDDIGPFIEEML
RWEGPGHTATLRHTISPITLGDKEIGAGEFLIVSVAAANRDPAAYADPEEFHLDRKEHGHLSFGHGIHYCVGA
PLARLEAEIAVATLAHNFPKLSLASAPDTLKWRRSMIIRGRTELPVQLF
>CYP107DS1_Mycobacterium abscessus PAP095 2667070800
MAEHDALTVLDDVFFDNPWPVYNQLRAVGPIHRIRLPNGLLAWLVIDYHMGKQVLADHRLSKDETSSSRAARS
QGIDTGWQDAAGPRPLRMNLLNTDPPEHTRLRALVNKAFTPAASARMRPAIEDVAKELVQSLVDGPKTVDLLA
NYATPLPITVICRLLGVPEKDQETFGSLIVTIMDETAVADGEPSRLARVQLGEYLGKLIADRRAQRKSADTTD
SDLLDRLITAQVDQDRLSDMELISTIGLLLVAGYDTTVHLIANSIFRLATDASLLHRIHSNPDDIGPFIEEML
RWEGPGHTATLRHTISPITLGDKEIGAGEFLIVSVAAANRDPAAYADPEEFHLDRKEHGHLSFGHGIHYCVGA
PLARLEAEIAVATLAHNFPKLSLASAPDTLKWRRSMIIRGRTELPVQLF
>CYP107DS1_Mycobacterium abscessus PAP100 2661917863
MAEHDALTVLDDVFFDNPWPVYNQLRAVGPIHRIRLPNGLLAWLVIDYHMGKQVLADHRLSKDETSSSRAARS
QGIDTGWQDAAGPRPLRMNLLNTDPPEHTRLRALVNKAFTPAASARMRPAIEDVAKELVQSLVDGPKTVDLLA
NYATPLPITVICRLLGVPEKDQETFGSLIVTIMDETAVADGEPSRLARVQLGEYLGKLIADRRAQRKSADTTD
SDLLDRLITAQVDQDRLSDMELISTIGLLLVAGYDTTVHLIANSIFRLATDASLLHRIHSNPDDIGPFIEEML
RWEGPGHTATLRHTISPITLGDKEIGAGEFLIVSVAAANRDPAAYADPEEFHLDRKEHGHLSFGHGIHYCVGA
PLARLEAEIAVATLAHNFPKLSLASAPDTLKWRRSMIIRGRTELPVQLF
>CYP107DS1_Mycobacterium abscessus PAP103 2697229282
MAEHDALTVLDDVFFDNPWPVYNQLRAVGPIHRIRLPNGLLAWLVIDYHMGKQVLADHRLSKDETSSSRAARS
QGIDTGWQDAAGPRPLRMNLLNTDPPEHTRLRALVNKAFTPAASARMRPAIEDVAKELVQSLVDGPKTVDLLA
NYATPLPITVICRLLGVPEKDQETFGSLIVTIMDETAVADGEPSRLARVQLGEYLGKLIADRRAQRKSADTTD
SDLLDRLITAQVDQDRLSDMELISTIGLLLVAGYDTTVHLIANSIFRLATDASLLHRIHSNPDDIGPFIEEML
RWEGPGHTATLRHTISPITLGDKEIGAGEFLIVSVAAANRDPAAYADPEEFHLDRKEHGHLSFGHGIHYCVGA
PLARLEAEIAVATLAHNFPKLSLASAPDTLKWRRSMIIRGRTELPVQLF
>CYP107DS1_Mycobacterium abscessus PAP104 2694260707
MAEHDALTVLDDVFFDNPWPVYNQLRAVGPIHRIRLPNGLLAWLVIDYHMGKQVLADHRLSKDETSSSRAARS
QGIDTGWQDAAGPRPLRMNLLNTDPPEHTRLRALVNKAFTPAASARMRPAIEDVAKELVQSLVDGPKTVDLLA
NYATPLPITVICRLLGVPEKDQETFGSLIVTIMDETAVADGEPSRLARVQLGEYLGKLIADRRAQRKSADTTD
SDLLDRLITAQVDQDRLSDMELISTIGLLLVAGYDTTVHLIANSIFRLATDASLLHRIHSNPDDIGPFIEEML
RWEGPGHTATLRHTISPITLGDKEIGAGEFLIVSVAAANRDPAAYADPEEFHLDRKEHGHLSFGHGIHYCVGA
PLARLEAEIAVATLAHNFPKLSLASAPDTLKWRRSMIIRGRTELPVQLF
>CYP107DS1_Mycobacterium abscessus PAP107 2666667868
MAEHDALTVLDDVFFDNPWPVYNQLRAVGPIHRIRLPNGLLAWLVIDYHMGKQVLADHRLSKDETSSSRAARS
QGIDTGWQDAAGPRPLRMNLLNTDPPEHTRLRALVNKAFTPAASARMRPAIEDVAKELVQSLVDGPKTVDLLA
NYATPLPITVICRLLGVPEKDQETFGSLIVTIMDETAVADGEPSRLARVQLGEYLGKLIADRRAQRKSADTTD
SDLLDRLITAQVDQDRLSDMELISTIGLLLVAGYDTTVHLIANSIFRLATDASLLHRIHSNPDDIGPFIEEML
RWEGPGHTATLRHTISPITLGDKEIGAGEFLIVSVAAANRDPAAYADPEEFHLDRKEHGHLSFGHGIHYCVGA
PLARLEAEIAVATLAHNFPKLSLASAPDTLKWRRSMIIRGRTELPVQLF
>CYP107DS1_Mycobacterium abscessus PAP108 2693717357
MAEHDALTVLDDVFFDNPWPVYNQLRAVGPIHRIRLPNGLLAWLVIDYHMGKQVLADHRLSKDETSSSRAARS
QGIDTGWQDAAGPRPLRMNLLNTDPPEHTRLRALVNKAFTPAASARMRPAIEDVAKELVQSLVDGPKTVDLLA
NYATPLPITVICRLLGVPEKDQETFGSLIVTIMDETAVADGEPSRLARVQLGEYLGKLIADRRAQRKSADTTD
SDLLDRLITAQVDQDRLSDMELISTIGLLLVAGYDTTVHLIANSIFRLATDASLLHRIHSNPDDIGPFIEEML
RWEGPGHTATLRHTISPITLGDKEIGAGEFLIVSVAAANRDPAAYADPEEFHLDRKEHGHLSFGHGIHYCVGA
PLARLEAEIAVATLAHNFPKLSLASAPDTLKWRRSMIIRGRTELPVQLF
>CYP107DS1_Mycobacterium abscessus PAP109 2691745850
MAEHDALTVLDDVFFDNPWPVYNQLRAVGPIHRIRLPNGLLAWLVIDYHMGKQVLADHRLSKDETSSSRAARS
QGIDTGWQDAAGPRPLRMNLLNTDPPEHTRLRALVNKAFTPAASARMRPAIEDVAKELVQSLVDGPKTVDLLA

NYATPLPITVICRLLGVPEKDQETFGSLIVTIMDETAVADGEPSRLARVQLGEYLGKLIADRRAQRKSADTTD
SDLLDRLITAQVDQDRLSDMELISTIGLLLVAGYDTTVHLIANSIFRLATDASLLHRIHSNPDDIGPFIEEML
RWEGPGHTATLRHTISPITLGDKEIGAGEFLIVSVAAANRDPAAYADPEEFHLDRKEHGHLSFGHGIHYCVGA
PLARLEAEIAVATLAHNFPKLSLASAPDTLKWRRSMIIRGRTELPVQLF
>CYP107DS1_Mycobacterium abscessus PAP111 2667269131
MAEHDALTVLDDVFFDNPWPVYNQLRAVGPIHRIRLPNGLLAWLVIDYHMGKQVLADHRLSKDETSSSRAARS
QGIDTGWQDAAGPRPLRMNLLNTDPPEHTRLRALVNKAFTPAASARMRPAIEDVAKELVQSLVDGPKTVDLLA
NYATPLPITVICRLLGVPEKDQETFGSLIVTIMDETAVADGEPSRLARVQLGEYLGKLIADRRAQRKSADTTD
SDLLDRLITAQVDQDRLSDMELISTIGLLLVAGYDTTVHLIANSIFRLATDASLLHRIHSNPDDIGPFIEEML
RWEGPGHTATLRHTISPITLGDKEIGAGEFLIVSVAAANRDPAAYADPEEFHLDRKEHGHLSFGHGIHYCVGA
PLARLEAEIAVATLAHNFPKLSLASAPDTLKWRRSMIIRGRTELPVQLF
>CYP107DS1_Mycobacterium abscessus PAP112 2666684348
MAEHDALTVLDDVFFDNPWPVYNQLRAVGPIHRIRLPNGLLAWLVIDYHMGKQVLADHRLSKDETSSSRAARS
QGIDTGWQDAAGPRPLRMNLLNTDPPEHTRLRALVNKAFTPAASARMRPAIEDVAKELVQSLVDGPKTVDLLA
NYATPLPITVICRLLGVPEKDQETFGSLIVTIMDETAVADGEPSRLARVQLGEYLGKLIADRRAQRKSADTTD
SDLLDRLITAQVDQDRLSDMELISTIGLLLVAGYDTTVHLIANSIFRLATDASLLHRIHSNPDDIGPFIEEML
RWEGPGHTATLRHTISPITLGDKEIGAGEFLIVSVAAANRDPAAYADPEEFHLDRKEHGHLSFGHGIHYCVGA
PLARLEAEIAVATLAHNFPKLSLASAPDTLKWRRSMIIRGRTELPVQLF
>CYP107DS1_Mycobacterium abscessus PAP114 2693461645
MAEHDALTVLDDVFFDNPWPVYNQLRAVGPIHRIRLPNGLLAWLVIDYHMGKQVLADHRLSKDETSSSRAARS
QGIDTGWQDAAGPRPLRMNLLNTDPPEHTRLRALVNKAFTPAASARMRPAIEDVAKELVQSLVDGPKTVDLLA
NYATPLPITVICRLLGVPEKDQETFGSLIVTIMDETAVADGEPSRLARVQLGEYLGKLIADRRAQRKSADTTD
SDLLDRLITAQVDQDRLSDMELISTIGLLLVAGYDTTVHLIANSIFRLATDASLLHRIHSNPDDIGPFIEEML
RWEGPGHTATLRHTISPITLGDKEIGAGEFLIVSVAAANRDPAAYADPEEFHLDRKEHGHLSFGHGIHYCVGA
PLARLEAEIAVATLAHNFPKLSLASAPDTLKWRRSMIIRGRTELPVQLF
>CYP107DS1_Mycobacterium abscessus PAP116 2691895988
MAEHDALTVLDDVFFDNPWPVYNQLRAVGPIHRIRLPNGLLAWLVIDYHMGKQVLADHRLSKDETSSSRAARS
QGIDTGWQDAAGPRPLRMNLLNTDPPEHTRLRALVNKAFTPAASARMRPAIEDVAKELVQSLVDGPKTVDLLA
NYATPLPITVICRLLGVPEKDQETFGSLIVTIMDETAVADGEPSRLARVQLGEYLGKLIADRRAQRKSADTTD
SDLLDRLITAQVDQDRLSDMELISTIGLLLVAGYDTTVHLIANSIFRLATDASLLHRIHSNPDDIGPFIEEML
RWEGPGHTATLRHTISPITLGDKEIGAGEFLIVSVAAANRDPAAYADPEEFHLDRKEHGHLSFGHGIHYCVGA
PLARLEAEIAVATLAHNFPKLSLASAPDTLKWRRSMIIRGRTELPVQLF
>CYP107DS1_Mycobacterium abscessus PAP118 2670473639
MAEHDALTVLDDVFFDNPWPVYNQLRAVGPIHRIRLPNGLLAWLVIDYHMGKQVLADHRLSKDETSSSRAARS
QGIDTGWQDAAGPRPLRMNLLNTDPPEHTRLRALVNKAFTPAASARMRPAIEDVAKELVQSLVDGPKTVDLLA
NYATPLPITVICRLLGVPEKDQETFGSLIVTIMDETAVADGEPSRLARVQLGEYLGKLIADRRAQRKSADTTD
SDLLDRLITAQVDQDRLSDMELISTIGLLLVAGYDTTVHLIANSIFRLATDASLLHRIHSNPDDIGPFIEEML
RWEGPGHTATLRHTISPITLGDKEIGAGEFLIVSVAAANRDPAAYADPEEFHLDRKEHGHLSFGHGIHYCVGA
PLARLEAEIAVATLAHNFPKLSLASAPDTLKWRRSMIIRGRTELPVQLF
>CYP107DS1_Mycobacterium abscessus PAP119 2664917472
MAEHDALTVLDDVFFDNPWPVYNQLRAVGPIHRIRLPNGLLAWLVIDYHMGKQVLADHRLSKDETSSSRAARS
QGIDTGWQDAAGPRPLRMNLLNTDPPEHTRLRALVNKAFTPAASARMRPAIEDVAKELVQSLVDGPKTVDLLA
NYATPLPITVICRLLGVPEKDQETFGSLIVTIMDETAVADGEPSRLARVQLGEYLGKLIADRRAQRKSADTTD
SDLLDRLITAQVDQDRLSDMELISTIGLLLVAGYDTTVHLIANSIFRLATDASLLHRIHSNPDDIGPFIEEML
RWEGPGHTATLRHTISPITLGDKEIGAGEFLIVSVAAANRDPAAYADPEEFHLDRKEHGHLSFGHGIHYCVGA
PLARLEAEIAVATLAHNFPKLSLASAPDTLKWRRSMIIRGRTELPVQLF
>CYP107DS1_Mycobacterium abscessus PAP123 2660995736
MAEHDALTVLDDVFFDNPWPVYNQLRAVGPIHRIRLPNGLLAWLVIDYHMGKQVLADHRLSKDETSSSRAARS
QGIDTGWQDAAGPRPLRMNLLNTDPPEHTRLRALVNKAFTPAASARMRPAIEDVAKELVQSLVDGPKTVDLLA
NYATPLPITVICRLLGVPEKDQETFGSLIVTIMDETAVADGEPSRLARVQLGEYLGKLIADRRAQRKSADTTD
SDLLDRLITAQVDQDRLSDMELISTIGLLLVAGYDTTVHLIANSIFRLATDASLLHRIHSNPDDIGPFIEEML
RWEGPGHTATLRHTISPITLGDKEIGAGEFLIVSVAAANRDPAAYADPEEFHLDRKEHGHLSFGHGIHYCVGA
PLARLEAEIAVATLAHNFPKLSLASAPDTLKWRRSMIIRGRTELPVQLF
>CYP107DS1_Mycobacterium abscessus PAP124 2697001180
MAEHDALTVLDDVFFDNPWPVYNQLRAVGPIHRIRLPNGLLAWLVIDYHMGKQVLADHRLSKDETSSSRAARS
QGIDTGWQDAAGPRPLRMNLLNTDPPEHTRLRALVNKAFTPAASARMRPAIEDVAKELVQSLVDGPKTVDLLA
NYATPLPITVICRLLGVPEKDQETFGSLIVTIMDETAVADGEPSRLARVQLGEYLGKLIADRRAQRKSADTTD
SDLLDRLITAQVDQDRLSDMELISTIGLLLVAGYDTTVHLIANSIFRLATDASLLHRIHSNPDDIGPFIEEML

RWEGPGHTATLRHTISPITLGDKEIGAGEFLIVSVAAANRDPAAYADPEEFHLDRKEHGHLSFGHGIHYCVGA
PLARLEAEIAVATLAHNFPKLSLASAPDTLKWRRSMIIRGRTELPVQLF
>CYP107DS1_Mycobacterium abscessus PAP125 2698565936
MAEHDALTVLDDVFFDNPWPVYNQLRAVGPIHRIRLPNGLLAWLVIDYHMGKQVLADHRLSKDETSSSRAARS
QGIDTGWQDAAGPRPLRMNLLNTDPPEHTRLRALVNKAFTPAASARMRPAIEDVAKELVQSLVDGPKTVDLLA
NYATPLPITVICRLLGVPEKDQETFGSLIVTIMDETAVADGEPSRLARVQLGEYLGKLIADRRAQRKSADTTD
SDLLDRLITAQVDQDRLSDMELISTIGLLLVAGYDTTVHLIANSIFRLATDASLLHRIHSNPDDIGPFIEEML
RWEGPGHTATLRHTISPITLGDKEIGAGEFLIVSVAAANRDPAAYADPEEFHLDRKEHGHLSFGHGIHYCVGA
PLARLEAEIAVATLAHNFPKLSLASAPDTLKWRRSMIIRGRTELPVQLF
>CYP107DS1_Mycobacterium abscessus PAP126 2665846370
MAEHDALTVLDDVFFDNPWPVYNQLRAVGPIHRIRLPNGLLAWLVIDYHMGKQVLADHRLSKDETSSSRAARS
QGIDTGWQDAAGPRPLRMNLLNTDPPEHTRLRALVNKAFTPAASARMRPAIEDVAKELVQSLVDGPKTVDLLA
NYATPLPITVICRLLGVPEKDQETFGSLIVTIMDETAVADGEPSRLARVQLGEYLGKLIADRRAQRKSADTTD
SDLLDRLITAQVDQDRLSDMELISTIGLLLVAGYDTTVHLIANSIFRLATDASLLHRIHSNPDDIGPFIEEML
RWEGPGHTATLRHTISPITLGDKEIGAGEFLIVSVAAANRDPAAYADPEEFHLDRKEHGHLSFGHGIHYCVGA
PLARLEAEIAVATLAHNFPKLSLASAPDTLKWRRSMIIRGRTELPVQLF
>CYP107DS1_Mycobacterium abscessus PAP128 2692929056
MAEHDALTVLDDVFFDNPWPVYNQLRAVGPIHRIRLPNGLLAWLVIDYHMGKQVLADHRLSKDETSSSRAARS
QGIDTGWQDAAGPRPLRMNLLNTDPPEHTRLRALVNKAFTPAASARMRPAIEDVAKELVQSLVDGPKTVDLLA
NYATPLPITVICRLLGVPEKDQETFGSLIVTIMDETAVADGEPSRLARVQLGEYLGKLIADRRAQRKSADTTD
SDLLDRLITAQVDQDRLSDMELISTIGLLLVAGYDTTVHLIANSIFRLATDASLLHRIHSNPDDIGPFIEEML
RWEGPGHTATLRHTISPITLGDKEIGAGEFLIVSVAAANRDPAAYADPEEFHLDRKEHGHLSFGHGIHYCVGA
PLARLEAEIAVATLAHNFPKLSLASAPDTLKWRRSMIIRGRTELPVQLF
>CYP107DS1_Mycobacterium abscessus PAP136 2659909999
MAEHDALTVLDDVFFDNPWPVYNQLRAVGPIHRIRLPNGLLAWLVIDYHMGKQVLADHRLSKDETSSSRAARS
QGIDTGWQDAAGPRPLRMNLLNTDPPEHTRLRALVNKAFTPAASARMRPAIEDVAKELVQSLVDGPKTVDLLA
NYATPLPITVICRLLGVPEKDQETFGSLIVTIMDETAVADGEPSRLARVQLGEYLGKLIADRRAQRKSADTTD
SDLLDRLITAQVDQDRLSDMELISTIGLLLVAGYDTTVHLIANSIFRLATDASLLHRIHSNPDDIGPFIEEML
RWEGPGHTATLRHTISPITLGDKEIGAGEFLIVSVAAANRDPAAYADPEEFHLDRKEHGHLSFGHGIHYCVGA
PLARLEAEIAVATLAHNFPKLSLASAPDTLKWRRSMIIRGRTELPVQLF
>CYP107DS1_Mycobacterium abscessus PAP137 2665109553
MAEHDALTVLDDVFFDNPWPVYNQLRAVGPIHRIRLPNGLLAWLVIDYHMGKQVLADHRLSKDETSSSRAARS
QGIDTGWQDAAGPRPLRMNLLNTDPPEHTRLRALVNKAFTPAASARMRPAIEDVAKELVQSLVDGPKTVDLLA
NYATPLPITVICRLLGVPEKDQETFGSLIVTIMDETAVADGEPSRLARVQLGEYLGKLIADRRAQRKSADTTD
SDLLDRLITAQVDQDRLSDMELISTIGLLLVAGYDTTVHLIANSIFRLATDASLLHRIHSNPDDIGPFIEEML
RWEGPGHTATLRHTISPITLGDKEIGAGEFLIVSVAAANRDPAAYADPEEFHLDRKEHGHLSFGHGIHYCVGA
PLARLEAEIAVATLAHNFPKLSLASAPDTLKWRRSMIIRGRTELPVQLF
>CYP107DS1_Mycobacterium abscessus PAP141 2674027671
MAEHDALTVLDDVFFDNPWPVYNQLRAVGPIHRIRLPNGLLAWLVIDYHMGKQVLADHRLSKDETSSSRAARS
QGIDTGWQDAAGPRPLRMNLLNTDPPEHTRLRALVNKAFTPAASARMRPAIEDVAKELVQSLVDGPKTVDLLA
NYATPLPITVICRLLGVPEKDQETFGSLIVTIMDETAVADGEPSRLARVQLGEYLGKLIADRRAQRKSADTTD
SDLLDRLITAQVDQDRLSDMELISTIGLLLVAGYDTTVHLIANSIFRLATDASLLHRIHSNPDDIGPFIEEML
RWEGPGHTATLRHTISPITLGDKEIGAGEFLIVSVAAANRDPAAYADPEEFHLDRKEHGHLSFGHGIHYCVGA
PLARLEAEIAVATLAHNFPKLSLASAPDTLKWRRSMIIRGRTELPVQLF
>CYP107DS1_Mycobacterium abscessus PAP143 2662193552
MAEHDALTVLDDVFFDNPWPVYNQLRAVGPIHRIRLPNGLLAWLVIDYHMGKQVLADHRLSKDETSSSRAARS
QGIDTGWQDAAGPRPLRMNLLNTDPPEHTRLRALVNKAFTPAASARMRPAIEDVAKELVQSLVDGPKTVDLLA
NYATPLPITVICRLLGVPEKDQETFGSLIVTIMDETAVADGEPSRLARVQLGEYLGKLIADRRAQRKSADTTD
SDLLDRLITAQVDQDRLSDMELISTIGLLLVAGYDTTVHLIANSIFRLATDASLLHRIHSNPDDIGPFIEEML
RWEGPGHTATLRHTISPITLGDKEIGAGEFLIVSVAAANRDPAAYADPEEFHLDRKEHGHLSFGHGIHYCVGA
PLARLEAEIAVATLAHNFPKLSLASAPDTLKWRRSMIIRGRTELPVQLF
>CYP107DS1_Mycobacterium abscessus PAP144 2665022517
MAEHDALTVLDDVFFDNPWPVYNQLRAVGPIHRIRLPNGLLAWLVIDYHMGKQVLADHRLSKDETSSSRAARS
QGIDTGWQDAAGPRPLRMNLLNTDPPEHTRLRALVNKAFTPAASARMRPAIEDVAKELVQSLVDGPKTVDLLA
NYATPLPITVICRLLGVPEKDQETFGSLIVTIMDETAVADGEPSRLARVQLGEYLGKLIADRRAQRKSADTTD
SDLLDRLITAQVDQDRLSDMELISTIGLLLVAGYDTTVHLIANSIFRLATDASLLHRIHSNPDDIGPFIEEML
RWEGPGHTATLRHTISPITLGDKEIGAGEFLIVSVAAANRDPAAYADPEEFHLDRKEHGHLSFGHGIHYCVGA
PLARLEAEIAVATLAHNFPKLSLASAPDTLKWRRSMIIRGRTELPVQLF

>CYP107DS1_Mycobacterium abscessus PAP145 2692656579
MAEHDALTVLDDVFFDNPWPVYNQLRAVGPIHRIRLPNGLLAWLVIDYHMGKQVLADHRLSKDETSSSRAARS
QGIDTGWQDAAGPRPLRMNLLNTDPPEHTRLRALVNKAFTPAASARMRPAIEDVAKELVQSLVDGPKTVDLLA
NYATPLPITVICRLLGVPEKDQETFGSLIVTIMDETAVADGEPSRLARVQLGEYLGKLIADRRAQRKSADTTD
SDLLDRLITAQVDQDRLSDMELISTIGLLLVAGYDTTVHLIANSIFRLATDASLLHRIHSNPDDIGPFIEEML
RWEGPGHTATLRHTISPITLGDKEIGAGEFLIVSVAAANRDPAAYADPEEFHLDRKEHGHLSFGHGIHYCVGA
PLARLEAEIAVATLAHNFPKLSLASAPDTLKWRRSMIIRGRTELPVQLF
>CYP107DS1_Mycobacterium abscessus PAP147 2697352511
MAEHDALTVLDDVFFDNPWPVYNQLRAVGPIHRIRLPNGLLAWLVIDYHMGKQVLADHRLSKDETSSSRAARS
QGIDTGWQDAAGPRPLRMNLLNTDPPEHTRLRALVNKAFTPAASARMRPAIEDVAKELVQSLVDGPKTVDLLA
NYATPLPITVICRLLGVPEKDQETFGSLIVTIMDETAVADGEPSRLARVQLGEYLGKLIADRRAQRKSADTTD
SDLLDRLITAQVDQDRLSDMELISTIGLLLVAGYDTTVHLIANSIFRLATDASLLHRIHSNPDDIGPFIEEML
RWEGPGHTATLRHTISPITLGDKEIGAGEFLIVSVAAANRDPAAYADPEEFHLDRKEHGHLSFGHGIHYCVGA
PLARLEAEIAVATLAHNFPKLSLASAPDTLKWRRSMIIRGRTELPVQLF
>CYP107DS1_Mycobacterium abscessus PAP148 2661933217
MAEHDALTVLDDVFFDNPWPVYNQLRAVGPIHRIRLPNGLLAWLVIDYHMGKQVLADHRLSKDETSSSRAARS
QGIDTGWQDAAGPRPLRMNLLNTDPPEHTRLRALVNKAFTPAASARMRPAIEDVAKELVQSLVDGPKTVDLLA
NYATPLPITVICRLLGVPEKDQETFGSLIVTIMDETAVADGEPSRLARVQLGEYLGKLIADRRAQRKSADTTD
SDLLDRLITAQVDQDRLSDMELISTIGLLLVAGYDTTVHLIANSIFRLATDASLLHRIHSNPDDIGPFIEEML
RWEGPGHTATLRHTISPITLGDKEIGAGEFLIVSVAAANRDPAAYADPEEFHLDRKEHGHLSFGHGIHYCVGA
PLARLEAEIAVATLAHNFPKLSLASAPDTLKWRRSMIIRGRTELPVQLF
>CYP107DS1_Mycobacterium abscessus PAP149 2682895301
MAEHDALTVLDDVFFDNPWPVYNQLRAVGPIHRIRLPNGLLAWLVIDYHMGKQVLADHRLSKDETSSSRAARS
QGIDTGWQDAAGPRPLRMNLLNTDPPEHTRLRALVNKAFTPAASARMRPAIEDVAKELVQSLVDGPKTVDLLA
NYATPLPITVICRLLGVPEKDQETFGSLIVTIMDETAVADGEPSRLARVQLGEYLGKLIADRRAQRKSADTTD
SDLLDRLITAQVDQDRLSDMELISTIGLLLVAGYDTTVHLIANSIFRLATDASLLHRIHSNPDDIGPFIEEML
RWEGPGHTATLRHTISPITLGDKEIGAGEFLIVSVAAANRDPAAYADPEEFHLDRKEHGHLSFGHGIHYCVGA
PLARLEAEIAVATLAHNFPKLSLASAPDTLKWRRSMIIRGRTELPVQLF
>CYP107DS1_Mycobacterium abscessus PAP150 2662344860
MAEHDALTVLDDVFFDNPWPVYNQLRAVGPIHRIRLPNGLLAWLVIDYHMGKQVLADHRLSKDETSSSRAARS
QGIDTGWQDAAGPRPLRMNLLNTDPPEHTRLRALVNKAFTPAASARMRPAIEDVAKELVQSLVDGPKTVDLLA
NYATPLPITVICRLLGVPEKDQETFGSLIVTIMDETAVADGEPSRLARVQLGEYLGKLIADRRAQRKSADTTD
SDLLDRLITAQVDQDRLSDMELISTIGLLLVAGYDTTVHLIANSIFRLATDASLLHRIHSNPDDIGPFIEEML
RWEGPGHTATLRHTISPITLGDKEIGAGEFLIVSVAAANRDPAAYADPEEFHLDRKEHGHLSFGHGIHYCVGA
PLARLEAEIAVATLAHNFPKLSLASAPDTLKWRRSMIIRGRTELPVQLF
>CYP107DS1_Mycobacterium abscessus PAP151 2660864410
MAEHDALTVLDDVFFDNPWPVYNQLRAVGPIHRIRLPNGLLAWLVIDYHMGKQVLADHRLSKDETSSSRAARS
QGIDTGWQDAAGPRPLRMNLLNTDPPEHTRLRALVNKAFTPAASARMRPAIEDVAKELVQSLVDGPKTVDLLA
NYATPLPITVICRLLGVPEKDQETFGSLIVTIMDETAVADGEPSRLARVQLGEYLGKLIADRRAQRKSADTTD
SDLLDRLITAQVDQDRLSDMELISTIGLLLVAGYDTTVHLIANSIFRLATDASLLHRIHSNPDDIGPFIEEML
RWEGPGHTATLRHTISPITLGDKEIGAGEFLIVSVAAANRDPAAYADPEEFHLDRKEHGHLSFGHGIHYCVGA
PLARLEAEIAVATLAHNFPKLSLASAPDTLKWRRSMIIRGRTELPVQLF
>CYP107DS1_Mycobacterium abscessus PAP159 2660312206
MAEHDALTVLDDVFFDNPWPVYNQLRAVGPIHRIRLPNGLLAWLVIDYHMGKQVLADHRLSKDETSSSRAARS
QGIDTGWQDAAGPRPLRMNLLNTDPPEHTRLRALVNKAFTPAASARMRPAIEDVAKELVQSLVDGPKTVDLLA
NYATPLPITVICRLLGVPEKDQETFGSLIVTIMDETAVADGEPSRLARVQLGEYLGKLIADRRAQRKSADTTD
SDLLDRLITAQVDQDRLSDMELISTIGLLLVAGYDTTVHLIANSIFRLATDASLLHRIHSNPDDIGPFIEEML
RWEGPGHTATLRHTISPITLGDKEIGAGEFLIVSVAAANRDPAAYADPEEFHLDRKEHGHLSFGHGIHYCVGA
PLARLEAEIAVATLAHNFPKLSLASAPDTLKWRRSMIIRGRTELPVQLF
>CYP107DS1_Mycobacterium abscessus PAP164 2670488003
MAEHDALTVLDDVFFDNPWPVYNQLRAVGPIHRIRLPNGLLAWLVIDYHMGKQVLADHRLSKDETSSSRAARS
QGIDTGWQDAAGPRPLRMNLLNTDPPEHTRLRALVNKAFTPAASARMRPAIEDVAKELVQSLVDGPKTVDLLA
NYATPLPITVICRLLGVPEKDQETFGSLIVTIMDETAVADGEPSRLARVQLGEYLGKLIADRRAQRKSADTTD
SDLLDRLITAQVDQDRLSDMELISTIGLLLVAGYDTTVHLIANSIFRLATDASLLHRIHSNPDDIGPFIEEML
RWEGPGHTATLRHTISPITLGDKEIGAGEFLIVSVAAANRDPAAYADPEEFHLDRKEHGHLSFGHGIHYCVGA
PLARLEAEIAVATLAHNFPKLSLASAPDTLKWRRSMIIRGRTELPVQLF
>CYP107DS1_Mycobacterium abscessus PAP167 2675568975

MAEHDALTVLDDVFFDNPWPVYNQLRAVGPIHRIRLPNGLLAWLVIDYHMGKQVLADHRLSKDETSSSRAARS
QGIDTGWQDAAGPRPLRMNLLNTDPPEHTRLRALVNKAFTPAASARMRPAIEDVAKELVQSLVDGPKTVDLLA
NYATPLPITVICRLLGVPEKDQETFGSLIVTIMDETAVADGEPSRLARVQLGEYLGKLIADRRAQRKSADTTD
SDLLDRLITAQVDQDRLSDMELISTIGLLLVAGYDTTVHLIANSIFRLATDASLLHRIHSNPDDIGPFIEEML
RWEGPGHTATLRHTISPITLGDKEIGAGEFLIVSVAAANRDPAAYADPEEFHLDRKEHGHLSFGHGIHYCVGA
PLARLEAEIAVATLAHNFPKLSLASAPDTLKWRRSMIIRGRTELPVQLF
>CYP107DS1_Mycobacterium abscessus PAP168 2690832570
MAEHDALTVLDDVFFDNPWPVYNQLRAVGPIHRIRLPNGLLAWLVIDYHMGKQVLADHRLSKDETSSSRAARS
QGIDTGWQDAAGPRPLRMNLLNTDPPEHTRLRALVNKAFTPAASARMRPAIEDVAKELVQSLVDGPKTVDLLA
NYATPLPITVICRLLGVPEKDQETFGSLIVTIMDETAVADGEPSRLARVQLGEYLGKLIADRRAQRKSADTTD
SDLLDRLITAQVDQDRLSDMELISTIGLLLVAGYDTTVHLIANSIFRLATDASLLHRIHSNPDDIGPFIEEML
RWEGPGHTATLRHTISPITLGDKEIGAGEFLIVSVAAANRDPAAYADPEEFHLDRKEHGHLSFGHGIHYCVGA
PLARLEAEIAVATLAHNFPKLSLASAPDTLKWRRSMIIRGRTELPVQLF
>CYP107DS1_Mycobacterium abscessus PAP169 2671020361
MAEHDALTVLDDVFFDNPWPVYNQLRAVGPIHRIRLPNGLLAWLVIDYHMGKQVLADHRLSKDETSSSRAARS
QGIDTGWQDAAGPRPLRMNLLNTDPPEHTRLRALVNKAFTPAASARMRPAIEDVAKELVQSLVDGPKTVDLLA
NYATPLPITVICRLLGVPEKDQETFGSLIVTIMDETAVADGEPSRLARVQLGEYLGKLIADRRAQRKSADTTD
SDLLDRLITAQVDQDRLSDMELISTIGLLLVAGYDTTVHLIANSIFRLATDASLLHRIHSNPDDIGPFIEEML
RWEGPGHTATLRHTISPITLGDKEIGAGEFLIVSVAAANRDPAAYADPEEFHLDRKEHGHLSFGHGIHYCVGA
PLARLEAEIAVATLAHNFPKLSLASAPDTLKWRRSMIIRGRTELPVQLF
>CYP107DS1_Mycobacterium abscessus UC22 2629320944
MAEHDALTVLDDVFFDNPWPVYNQLRAVGPIHRIRLPNGLLAWLVIDYHMGKQVLADHRLSKDETSSSRAARS
QGIDTGWQDAAGPRPLRMNLLNTDPPEHTRLRALVNKAFTPAASARMRPAIEDVAKELVQSLVDGPKTVDLLA
NYATPLPITVICRLLGVPEKDQETFGSLIVTIMDETAVADGEPSRLARVQLGEYLGKLIADRRAQRKSADTTD
SDLLDRLITAQVDQDRLSDMELISTIGLLLVAGYDTTVHLIANSIFRLATDASLLHRIHSNPDDIGPFIEEML
RWEGPGHTATLRHTISPITLGDKEIGAGEFLIVSVAAANRDPAAYADPEEFHLDRKEHGHLSFGHGIHYCVGA
PLARLEAEIAVATLAHNFPKLSLASAPDTLKWRRSMIIRGRTELPVQLF
>CYP107DS1_Mycobacterium abscessus PAP105 2659576888
MAEHDALTVLDDVFFDNPWPVYNQLRAVGPIHRIRLPNGLLAWLVIDYHMGKQVLADHRLSKDETSSSRAARS
QGIDTGWQDAAGPRPLRMNLLNTDPPEHTRLRALVNKAFTPAASARMRPAIEDVAKELVQSLVDGPKTVDLLA
NYATPLPITVICRLLGVPEKDQETFGSLIVTIMDETAVADGEPSRLARVQLGEYLGKLIADRRAQRKSADTTD
SDLLDRLITAQVDQDRLSDMELISTIGLLLVAGYDTTVHLIANSIFRLATDASLLHRIHSNPDDIGPFIEEML
RWEGPGHTATLRHTISPITLGDKEIGAGEFLIVSVAAANRDPAAYADPEEFHLDRKEHGHLSFGHGIHYCVGA
PLARLEAEIAVATLAHNFPKLSLASAPDTLKWRRSMIIRGRTELPVQLF
>CYP107DS1_Mycobacterium abscessus PAP003 2666181811
MAEHEALTVLDDAFFDNPWPVYNQLRAVGPIHRIRLPNGLLAWLVIDYHMGKQVLADHRLSKDETSSSRAARS
QGIDTGWQDAAGPRPLRMNLLNTDPPEHTRLRALVNKAFTPAASARMRPAIEDVAKELVQSLVDGSKTVDLLA
NYATPLPITVICRLLGVPEKDQETFGSLIVTIMDETAVADGEPSRLARVQLGEYLGKLIADRRAQRNSADTTD
SDLLDRLITAQVDQDRLSDMELISTIGLLLVAGYDTTVHLIANSIFRLATDASLLHRIHSNPDDIGPFIEEML
RWEGPGHTATLRHTINPITLGDKEIGAGEFLIVSVAAANRDPAAYADPEEFHLDRKEHGHLSFGHGIHYCVGA
PLARLEAEIAVATLAHNFPKLSLATAPDTLKWRRSMIIRGRTELPVQLF
>CYP107DS1_Mycobacterium abscessus PAP071 2662356339
MAEHEALTVLDDAFFDNPWPVYNQLRAVGPIHRIRLPNGLLAWLVIDYHMGKQVLADHRLSKDETSSSRAARS
QGIDTGWQDAAGPRPLRMNLLNTDPPEHTRLRALVNKAFTPAASARMRPAIEDVAKELVQSLVDGSKTVDLLA
NYATPLPITVICRLLGVPEKDQETFGSLIVTIMDETAVADGEPSRLARVQLGEYLGKLIADRRAQRNSADTTD
SDLLDRLITAQVDQDRLSDMELISTIGLLLVAGYDTTVHLIANSIFRLATDASLLHRIHSNPDDIGPFIEEML
RWEGPGHTATLRHTINPITLGDKEIGAGEFLIVSVAAANRDPAAYADPEEFHLDRKEHGHLSFGHGIHYCVGA
PLARLEAEIAVATLAHNFPKLSLATAPDTLKWRRSMIIRGRTELPVQLF
>CYP107DZ1_Mycobacterium vaccae 95051 2689432168
MSKVIETLERTDLDTREMLDNPHARFAALRAAQPVSWATATGLLQGKGGYLLTRYDDVMTVHSDERFSTDVLK
NSAAGKFIWLLPPSLRMLAQTMVFKDDPDHKRLRTLVHKAFTPKLVTTMAPDIAKIADQLADQVAAKREVDLV
HEFAVKLPLAVIATMLGVADEDRDMFHTLVEKMGNQQGSPLRQYPTARRLAKLFEALIEDRRLKPDEGLISEL
VRANEGGDRLSHRELVSMVFLLLLAGHDTTANLIGSSVLALIEHPDQLELLRAHPELLETTAVEELLRFTSPV
ADGAARFAMADMEIRGVPIPRGSQVLGSITSANRDEAVFDRPGQLDLTRKPNRHLAFAFGIHYCLGHQLARLE
GRIALAALLERFPEWELAVPRESLRYKPTVSLRGLTKLPTRMY
>CYP107DZ2_Mycobacterium sp. UM_11 2714017807
MTAQAGCDEFTDLEDPIMFSDPYPRYAELRRSAPVSRARAPLITGGKGKGYMLTRYEDVLAMHADPRFSSDVM
RHDPVRRVRWLIPKPIRLLTETMATKDDPDHQRLRRLVHKAFTPKLVAGLADEITRIALELIEQLARAGEVDL

VSDFAVPFPLTVIARLLGVKDDERKEFHVLCMRLTQSSASGPRGMFGVITAAARLTDLFERLADERRLDPDDG
LISQLVRATHEGDKLSDREAIAMIFLLLLAGHDTTSNLIGNSVVALLDHPDQLALLRAQPEMIDAGIEELLRF
TAPVAVGAPRVALEDVDIAGTRISKGSRVFGMLISANRDETVFPDADELNLSRSPNKHLAFASGAHYCLGHHL
ARLEGRIALTELLQRFDHLELTVPRESLRLRRIVTLRGLESLPMRVS
>CYP107DZ2_Mycobacterium sp. UM_3 2743664981
MTAQAGCDEFTDLEDPIMFSDPYPRYAELRRSAPVSRARAPLITGGKGKGYMLTRYEDVLAMHADPRFSSDVM
RHDPVRRVRWLIPKPIRLLTETMATKDDPDHQRLRRLVHKAFTPKLVAGLADEITRIALELIEQLARAGEVDL
VSDFAVPFPLTVIARLLGVKDDERKEFHVLCMRLTQSSASGPRGMFGVITAAARLTDLFERLADERRLDPDDG
LISQLVRATHEGDKLSDREAIAMIFLLLLAGHDTTSNLIGNSVVALLDHPDQLALLRAQPEMIDAGIEELLRF
TAPVAVGAPRVALEDVDIAGTRISKGSRVFGMLISANRDETVFPDADELNLSRSPNKHLAFASGAHYCLGHHL
ARLEGRIALTELLQRFDHLELTVPRESLRLRRIVTLRGLESLPMRVS
>CYP107KF2_Mycobacterium triplex DSM 44626 2621130229
VTTVQFNPLAPEMAVDPYPAYQDLRENSPVHEIEGLGIYVLSRYADVAEFYKDRRLFMRYSDRETLRYGPEVV
DEPFYGYFIQMAHVADAPMHTELRRMFQAAFTPNRVTALRDNIKRIAETLIDKHISDGGMEFISSFAEPFPRI
VIGQMIGLPAEDNEMIGKWALELGPAFEFLPMSPETKEIVNDRVVRLMEYFIDLADKRAVEPVDDLFTAMVQA
AENSDGKITREAVAANAVLLYIGGHETTGGALGLSLLALHRNPGQLERLLGDPTLVPRAAEELLRFDTSAQGT
GRISGEDVVYGDIEIPSGSMILAYIGSANRDAVVYENPNVLDLGRQPGARLLAFGPGPHQCVGHALTRLELEV
FLEVLIERFPRHHLATLDPQFRTFPLLRGIVNMELNW
>CYP108B1_Mycobacterium smegmatis INHR2 2687282853
MSTPVINDAARVLAEPRAYADEPRLHAALAELRSQTPVAYVDVPGYYPFWAITKHADVMAIERDNELFINAPR
PMLITKEKDDLAKANLAAGGGIRTLIHMDDPLHRDIRKIGADWFRPKAMRALKERVDELAKIYVDKLVEKGPE
CDFVQEVAVNYPLYVILSLLGLPESDFDRMLKLTQELFGNDDDEMGRGSSAEELNAVILDFFNYFTELTADRR
ANPTEDLASAIANAKLNGEYLNDVDCLSYYVIVASAGHDTTSAAISGGLLALTENQDQLARLKADMSLMPLAT
EEIIRWSAPVKEFMRTATRDTEVRGVPIKEGESVLLSYVSANRDEEIFENADKFDVGRDPNKHLSFGYGVHFC
LGAALARMEINSFFTELIPRLESIELAGDPEFMATTFVGGLKHLPIRYSVR
>CYP108B1_Mycobacterium smegmatis str. MC2 155 639739966
MFLTPVKFSPSEVPDMSTPVINDAARVLAEPRAYADEPRLHAALAELRSQTPVAYVDVPGYYPFWAITKHADV
MAIERDNELFINAPRPMLITKEKDDLAKANLAAGGGIRTLIHMDDPLHRDIRKIGADWFRPKAMRALKERVDE
LAKIYVDKLVEKGPECDFVQEVAVNYPLYVILSLLGLPESDFDRMLKLTQELFGNDDDEMGRGSSAEELNAVI
LDFFNYFTELTADRRANPTEDLASAIANAKLNGEYLNDVDCLSYYVIVASAGHDTTSAAISGGLLALTENQDQ
LARLKADMSLMPLATEEIIRWSAPVKEFMRTATRDTEVRGVPIKEGESVLLSYVSANRDEEIFENADKFDVGR
DPNKHLSFGYGVHFCLGAALARMEINSFFTELIPRLESIELAGDPEFMATTFVGGLKHLPIRYSVR
>CYP108B1_Mycobacterium smegmatis MC2 155 2518775753
MSTPVINDAARVLAEPRAYADEPRLHAALAELRSQTPVAYVDVPGYYPFWAITKHADVMAIERDNELFINAPR
PMLITKEKDDLAKANLAAGGGIRTLIHMDDPLHRDIRKIGADWFRPKAMRALKERVDELAKIYVDKLVEKGPE
CDFVQEVAVNYPLYVILSLLGLPESDFDRMLKLTQELFGNDDDEMGRGSSAEELNAVILDFFNYFTELTADRR
ANPTEDLASAIANAKLNGEYLNDVDCLSYYVIVASAGHDTTSAAISGGLLALTENQDQLARLKADMSLMPLAT
EEIIRWSAPVKEFMRTATRDTEVRGVPIKEGESVLLSYVSANRDEEIFENADKFDVGRDPNKHLSFGYGVHFC
LGAALARMEINSFFTELIPRLESIELAGDPEFMATTFVGGLKHLPIRYSVR
>CYP108B1_Mycobacterium smegmatis MC2 155 2689261027
MSTPVINDAARVLAEPRAYADEPRLHAALAELRSQTPVAYVDVPGYYPFWAITKHADVMAIERDNELFINAPR
PMLITKEKDDLAKANLAAGGGIRTLIHMDDPLHRDIRKIGADWFRPKAMRALKERVDELAKIYVDKLVEKGPE
CDFVQEVAVNYPLYVILSLLGLPESDFDRMLKLTQELFGNDDDEMGRGSSAEELNAVILDFFNYFTELTADRR
ANPTEDLASAIANAKLNGEYLNDVDCLSYYVIVASAGHDTTSAAISGGLLALTENQDQLARLKADMSLMPLAT
EEIIRWSAPVKEFMRTATRDTEVRGVPIKEGESVLLSYVSANRDEEIFENADKFDVGRDPNKHLSFGYGVHFC
LGAALARMEINSFFTELIPRLESIELAGDPEFMATTFVGGLKHLPIRYSVR
>CYP108B1_Mycobacterium smegmatis MC2 51 2586874974
MSTPVINDAARVLAEPRAYADEPRLHAALAELRSQTPVAYVDVPGYYPFWAITKHADVMAIERDNELFINAPR
PMLITKEKDDLAKANLAAGGGIRTLIHMDDPLHRDIRKIGADWFRPKAMRALKERVDELAKIYVDKLVEKGPE
CDFVQEVAVNYPLYVILSLLGLPESDFDRMLKLTQELFGNDDDEMGRGSSAEELNAVILDFFNYFTELTADRR
ANPTEDLASAIANAKLNGEYLNDVDCLSYYVIVASAGHDTTSAAISGGLLALTENQDQLARLKADMSLMPLAT
EEIIRWSAPVKEFMRTATRDTEVRGVPIKEGESVLLSYVSANRDEEIFENADKFDVGRDPNKHLSFGYGVHFC
LGAALARMEINSFFTELIPRLESIELAGDPEFMATTFVGGLKHLPIRYSVR
>CYP108B1_Mycobacterium smegmatis INHR1 2689416411
MSTPVINDAARVLAEPRAYADEPRLHAALAELRSQTPVAYVDVPGYYPFWAITKHADVMAIERDNELFINAPR
PMLITKEKDDLAKANLAAGGGIRTLIHMDDPLHRDIRKIGADWFRPKAMRALKERVDELAKIYVDKLVEKGPE
CDFVQEVAVNYPLYVILSLLGLPESDFDRMLKLTQELFGNDDDEMGRGSSAEELNAVILDFFNYFTELTADRR
ANPTEDLASAIANAKLNGEYLNDVDCLSYYVIVASAGHDTTSAAISGGLLALTENQDQLARLKADMSLMPLAT

EEIIRWSAPVKEFMRTATRDTEVRGVPIKEGESVLLSYVSANRDEEIFENADKFDVGRDPNKHLSFGYGVHFC
LGAALARMEINSFFTELIPRLESIELAGDPEFMATTFVGGLKHLPIRYSVR
>CYP108B2_Mycobacterium smegmatis INHR2 2687282852
MSTPTMDDAAKALADPTAYADDARLHEALARLRAENPVAWVDQAPYRPFWAITKHADIMAIERANDLWLSAPR
PLLATAEADDLGRSQQEMGIGLRTLIHMDDPHHRKVRAIGADWFRPKAMRELKVRVDELARIYVDKMREIGPE
CDFVTDIAVNFPLYVILSLLGLPEEDFGRMHMLTQEMFGGDDDEYKRGTTVEEQMAVLTDFFNYFSALTNSRR
ENPTDDLASAIANGRVDGELMSDMDTLSYYVIVASAGHDTTKDAISGGLHALIENPGELARLKADPGLMGTAV
EEMIRWSTPVKEFMRTAAEDTEVRGVPIAKGESVYLAYVSGNRDEEVFTDPFRFDVGRDPNKHLAFGYGVHFC
LGAALARMEMNSLFSELLPRLDSIELAGEPELSATTFVGGLKHLPIRYSIR
>CYP108B2_Mycobacterium smegmatis str. MC2 155 639739965
MSTPTMDDAAKALADPTAYADDARLHEALARLRAENPVAWVDQAPYRPFWAITKHADIMAIERANDLWLSAPR
PLLATAEADDLGRSQQEMGIGLRTLIHMDDPHHRKVRAIGADWFRPKAMRELKVRVDELARIYVDKMREIGPE
CDFVTDIAVNFPLYVILSLLGLPEEDFGRMHMLTQEMFGGDDDEYKRGTTVEEQMAVLTDFFNYFSALTNSRR
ENPTDDLASAIANGRVDGELMSDMDTLSYYVIVASAGHDTTKDAISGGLHALIENPGELARLKADPGLMGTAV
EEMIRWSTPVKEFMRTAAEDTEVRGVPIAKGESVYLAYVSGNRDEEVFTDPFRFDVGRDPNKHLAFGYGVHFC
LGAALARMEMNSLFSELLPRLDSIELAGEPELSATTFVGGLKHLPIRYSIR
>CYP108B2_Mycobacterium smegmatis MC2 155 2518775752
MDDAAKALADPTAYADDARLHEALARLRAENPVAWVDQAPYRPFWAITKHADIMAIERANDLWLSAPRPLLAT
AEADDLGRSQQEMGIGLRTLIHMDDPHHRKVRAIGADWFRPKAMRELKVRVDELARIYVDKMREIGPECDFVT
DIAVNFPLYVILSLLGLPEEDFGRMHMLTQEMFGGDDDEYKRGTTVEEQMAVLTDFFNYFSALTNSRRENPTD
DLASAIANGRVDGELMSDMDTLSYYVIVASAGHDTTKDAISGGLHALIENPGELARLKADPGLMGTAVEEMIR
WSTPVKEFMRTAAEDTEVRGVPIAKGESVYLAYVSGNRDEEVFTDPFRFDVGRDPNKHLAFGYGVHFCLGAAL
ARMEMNSLFSELLPRLDSIELAGEPELSATTFVGGLKHLPIRYSIR
>CYP108B2_Mycobacterium smegmatis MC2 155 2689261026
MSTPTMDDAAKALADPTAYADDARLHEALARLRAENPVAWVDQAPYRPFWAITKHADIMAIERANDLWLSAPR
PLLATAEADDLGRSQQEMGIGLRTLIHMDDPHHRKVRAIGADWFRPKAMRELKVRVDELARIYVDKMREIGPE
CDFVTDIAVNFPLYVILSLLGLPEEDFGRMHMLTQEMFGGDDDEYKRGTTVEEQMAVLTDFFNYFSALTNSRR
ENPTDDLASAIANGRVDGELMSDMDTLSYYVIVASAGHDTTKDAISGGLHALIENPGELARLKADPGLMGTAV
EEMIRWSTPVKEFMRTAAEDTEVRGVPIAKGESVYLAYVSGNRDEEVFTDPFRFDVGRDPNKHLAFGYGVHFC
LGAALARMEMNSLFSELLPRLDSIELAGEPELSATTFVGGLKHLPIRYSIR
>CYP108B2_Mycobacterium smegmatis MC2 51 2586874975
MSTPTMDDAAKALADPTAYADDARLHEALARLRAENPVAWVDQAPYRPFWAITKHADIMAIERANDLWLSAPR
PLLATAEADDLGRSQQEMGIGLRTLIHMDDPHHRKVRAIGADWFRPKAMRELKVRVDELARIYVDKMREIGPE
CDFVTDIAVNFPLYVILSLLGLPEEDFGRMHMLTQEMFGGDDDEYKRGTTVEEQMAVLTDFFNYFSALTNSRR
ENPTDDLASAIANGRVDGELMSDMDTLSYYVIVASAGHDTTKDAISGGLHALIENPGELARLKADPGLMGTAV
EEMIRWSTPVKEFMRTAAEDTEVRGVPIAKGESVYLAYVSGNRDEEVFTDPFRFDVGRDPNKHLAFGYGVHFC
LGAALARMEMNSLFSELLPRLDSIELAGEPELSATTFVGGLKHLPIRYSIR
>CYP108B2_Mycobacterium smegmatis INHR1 2689416410
MSTPTMDDAAKALADPTAYADDARLHEALARLRAENPVAWVDQAPYRPFWAITKHADIMAIERANDLWLSAPR
PLLATAEADDLGRSQQEMGIGLRTLIHMDDPHHRKVRAIGADWFRPKAMRELKVRVDELARIYVDKMREIGPE
CDFVTDIAVNFPLYVILSLLGLPEEDFGRMHMLTQEMFGGDDDEYKRGTTVEEQMAVLTDFFNYFSALTNSRR
ENPTDDLASAIANGRVDGELMSDMDTLSYYVIVASAGHDTTKDAISGGLHALIENPGELARLKADPGLMGTAV
EEMIRWSTPVKEFMRTAAEDTEVRGVPIAKGESVYLAYVSGNRDEEVFTDPFRFDVGRDPNKHLAFGYGVHFC
LGAALARMEMNSLFSELLPRLDSIELAGEPELSATTFVGGLKHLPIRYSIR
>CYP108B3_Mycobacterium smegmatis INHR2 2687283647
MTARTIDDAAKVFAMPSAYTDEAKFHEALTHLRVNAPVSWVDVPPYRPFWAITRYADIMAIERANDLFTNSPR
PVLMTAEEDEQQAAVGISTLIHMDDPQHRVIRAIGADWFRPKAMRALKIRVDELAKIHVDKMVAAGGECDFVQ
EITVNYPLYVIMSLLGIPEADFPLMLKLTQELFGNKDDEYQRSADEGDSMAALLEMFQYFTELTASRRANPTD
DLASAIANATVNGEPLNDIETVSYYAIVAAAGHDTTSATISGGMLALLEHPDQLERLRNDPSLMGTATEEMIR
WVTPVKAFMRTAATDTVVRDVPIAAGESLLLAYPSGNRDEEVFTDPFRFDVGRDPNKHVAFGYGVHFCLGAAL
ARMEINSFFAELIPRLESIELTGSPRHTATTFVGGLKHLPVRYALR
>CYP108B3_Mycobacterium smegmatis str. MC2 155 639740764
MTARTIDDAAKVFAMPSAYTDEAKFHEALTHLRVNAPVSWVDVPPYRPFWAITRYADIMAIERANDLFTNSPR
PVLMTAEEDEQQAAVGISTLIHMDDPQHRVIRAIGADWFRPKAMRALKIRVDELAKIHVDKMVAAGGECDFVQ
EITVNYPLYVIMSLLGIPEADFPLMLKLTQELFGNKDDEYQRSADEGDSMAALLEMFQYFTELTASRRANPTD
DLASAIANATVNGEPLNDIETVSYYAIVAAAGHDTTSATISGGMLALLEHPDQLERLRNDPSLMGTATEEMIR
WVTPVKAFMRTAATDTVVRDVPIAAGESLLLAYPSGNRDEEVFTDPFRFDVGRDPNKHVAFGYGVHFCLGAAL
ARMEINSFFAELIPRLESIELTGSPRHTATTFVGGLKHLPVRYALR

>CYP108B3_Mycobacterium smegmatis MC2 155 2518776558
MTARTIDDAAKVFAMPSAYTDEAKFHEALTHLRVNAPVSWVDVPPYRPFWAITRYADIMAIERANDLFTNSPR
PVLMTAEEDEQQAAVGISTLIHMDDPQHRVIRAIGADWFRPKAMRALKIRVDELAKIHVDKMVAAGGECDFVQ
EITVNYPLYVIMSLLGIPEADFPLMLKLTQELFGNKDDEYQRSADEGDSMAALLEMFQYFTELTASRRANPTD
DLASAIANATVNGEPLNDIETVSYYAIVAAAGHDTTSATISGGMLALLEHPDQLERLRNDPSLMGTATEEMIR
WVTPVKAFMRTAATDTVVRDVPIAAGESLLLAYPSGNRDEEVFTDPFRFDVGRDPNKHVAFGYGVHFCLGAAL
ARMEINSFFAELIPRLESIELTGSPRHTATTFVGGLKHLPVRYALR
>CYP108B3_Mycobacterium smegmatis MC2 155 2689261821
MTARTIDDAAKVFAMPSAYTDEAKFHEALTHLRVNAPVSWVDVPPYRPFWAITRYADIMAIERANDLFTNSPR
PVLMTAEEDEQQAAVGISTLIHMDDPQHRVIRAIGADWFRPKAMRALKIRVDELAKIHVDKMVAAGGECDFVQ
EITVNYPLYVIMSLLGIPEADFPLMLKLTQELFGNKDDEYQRSADEGDSMAALLEMFQYFTELTASRRANPTD
DLASAIANATVNGEPLNDIETVSYYAIVAAAGHDTTSATISGGMLALLEHPDQLERLRNDPSLMGTATEEMIR
WVTPVKAFMRTAATDTVVRDVPIAAGESLLLAYPSGNRDEEVFTDPFRFDVGRDPNKHVAFGYGVHFCLGAAL
ARMEINSFFAELIPRLESIELTGSPRHTATTFVGGLKHLPVRYALR
>CYP108B3_Mycobacterium smegmatis MC2 51 2586874070
MTARTIDDAAKVFAMPSAYTDEAKFHEALTHLRVNAPVSWVDVPPYRPFWAITRYADIMAIERANDLFTNSPR
PVLMTAEEDEQQAAVGISTLIHMDDPQHRVIRAIGADWFRPKAMRALKIRVDELAKIHVDKMVAAGGECDFVQ
EITVNYPLYVIMSLLGIPEADFPLMLKLTQELFGNKDDEYQRSADEGDSMAALLEMFQYFTELTASRRANPTD
DLASAIANATVNGEPLNDIETVSYYAIVAAAGHDTTSATISGGMLALLEHPDQLERLRNDPSLMGTATEEMIR
WVTPVKAFMRTAATDTVVRDVPIAAGESLLLAYPSGNRDEEVFTDPFRFDVGRDPNKHVAFGYGVHFCLGAAL
ARMEINSFFAELIPRLESIELTGSPRHTATTFVGGLKHLPVRYALR
>CYP108B3_Mycobacterium smegmatis INHR1 2689417205
MTARTIDDAAKVFAMPSAYTDEAKFHEALTHLRVNAPVSWVDVPPYRPFWAITRYADIMAIERANDLFTNSPR
PVLMTAEEDEQQAAVGISTLIHMDDPQHRVIRAIGADWFRPKAMRALKIRVDELAKIHVDKMVAAGGECDFVQ
EITVNYPLYVIMSLLGIPEADFPLMLKLTQELFGNKDDEYQRSADEGDSMAALLEMFQYFTELTASRRANPTD
DLASAIANATVNGEPLNDIETVSYYAIVAAAGHDTTSATISGGMLALLEHPDQLERLRNDPSLMGTATEEMIR
WVTPVKAFMRTAATDTVVRDVPIAAGESLLLAYPSGNRDEEVFTDPFRFDVGRDPNKHVAFGYGVHFCLGAAL
ARMEINSFFAELIPRLESIELTGSPRHTATTFVGGLKHLPVRYALR
>CYP108B4_Mycobacterium marinum M 641719242
MSAPTMDEAALLADPLAYTDETRLHAALTRLRAQAPVALVDVPNYRRFWAITRHADVMDIERDNTLFTNWPRP
VLATVAGDELQAAAGVRTLIHLDDPQHRVARAIGADWFRPKAMRALKVRVDELAKNYVDAMMAAGPECDFVQQ
IAVNYPLYVIMSLLGLPEADFPRMLKLTQELFGSDDSEFKRGSTSEDQLPALLDMFGYFNAVTTARREHPTED
LASAIANARIDGEPLSDIDTVSYYLIVATAGHDTTSATISGGLHALIEHPDQLARLQADPALIPLATEEMIRW
VTPVKQFMRTASRDTVVRGIPIAAGESVLLSYVSANRDEEVFDQPFRFDIGREPNKHVAFGYGVHFCMGAALA
RMEVGSFFTELLPRLKSIELSGDPQFTATTFVGGLKHLPVSYSLV
>CYP108B4_Mycobacterium marinum Europe 2543278741
MTAPTMDEAALLADPLAYTDETRLHAALTRLRAQAPVALVDVPNYRRFWAITRHADVMDIERDNTLFTNWPRP
VLATVAGDELQAAAGVRTLIHLDDPQHRVARAIGADWFRPKAMRALKVRVDELAKNYVDAMMAAGPECDFVQQ
IAVNYPLCVIMSLLGLPEADFPRMLKLTQELFGSDDSEFKRGSTSEDQLPALLDMFGYFNAVTTARREHPTED
LASAIANARIDGEPLSDIDTVSYYLIVATAGHDTTSATISGGLHALIEHPDQLARLQADPALIPLATEEMIRW
VTPVKQFMRTASRDTVVRGIPIAAGESVLLSYVSANRDEEVFDQPFRFDIGREPNKHVAFGYGVHFCMGAALA
RMEVGSFFTELLPRLKSIELSGDPQFTATTFVGGLKHLPVSYSLV
>CYP108B4_Mycobacterium marinum MB2 2546370451
MSAPTMDEAALLADPLAYTDETRLHAALTRLRAQAPVALVDVPNYRRFWAITRHADVMDIERDNTLFTNWPRP
VLATVAGDELQAAAGVRTLIHLDDPQHRVARAIGADWFRPKAMRALKVRVDELAKNYVDAMMAAGPECDFVQQ
IAVNYPLYVIMSLLGLPEADFPRMLKLTQELFGSDDSEFKRGSTSEDQLPALLDMFGYFNAVTTARREHPTAD
LASAIANARIDGEPLSDIDTVSYYLIVATAGHDTTSATISGGLHALIEHPDQLARLQADPALMSLATEEMIRW
VTPVKQFMRTASSDTVVRGIPIAAGESVLLSYVSANRDEEVFDQPFRFDIGREPNKHVAFGYGVHFCMGAALA
RMEVVSFFTELLPRLKSIELSGDPQFTATTFVGGLKHLPVSYSLM
>CYP108B4_Mycobacterium ulcerans Agy99 642570441
MSAPTMDEAALLADPLAYTDETRLHAALTRLRAQAPVALVDVPNYRRFWAITRHADVMDIERDNTLFTNWPRP
VLATVAGDELQAAAGVRTLIHLDDPQHRVARAIGADWFRPKAMRALKVRVDELAKNYVDAMMAAGPECDFVQQ
IAVNYPLYVIMSLLGLPEADFPRMLKLTQELFGSDDSEFKRGSTSEDQLPALLDMFGYFNAVTTARRAHPTAD
LASAIANARIDGEPLSDIDTVSYYLIVATAGHDTTSATISGGLHALIEHPDQLARLQADPALMSLATEEMIRW
VTPVKQFMRTASSDTVVRGIPIAAGESVLLSYVSANRDEEVFDQPFRFDIGREPNKHVAFGYGVHFCMGAALA
RMEVASFFTELLPRLKSIELSGDPQFTATTFVGGLKRLPVSYSLM
>CYP108B4_Mycobacterium ulcerans Harvey 2567106384

MSAPTMDEAALLADPLAYTDETRLHAALTRLRAQAPVALVDVPNYRRFWAITRHADVMDIERDNTLFTNWPRP
VLATVAGDELQAAAGVRTLIHLDDPQHRVARAIGADWFRPKAMRALKVRVDELAKNYVDAMMAAGPECDFVQQ
IAVNYPLYVIMSLLGLPEADFPRMLKLTQELFGSDDSEFKRGSTSEDQLPALLDMFGYFNAVTTARRAHPTAD
LASAIANARIDGEPLSDIDTVSYYLIVATAGHDTTSATISGGLHALIEHPDQLARLQADPALMSLATEEMIRW
VTPVKQFMRTASSDTVVRGIPIAAGESVLLSYVSANRDEEVFDQPFRFDIGREPNKHVAFGYGVHFCMGAALA
RMEVASFFTELLPRLKSIELSGDPQFTATTFVGGLKRLPVSYSLM
>CYP108B5_Mycobacterium sp. MAC_011194_8550 2582207152
MSTTTMDEAAKLLADPMAYTDEQRLHAALTHLRANAPVSWVEVPNYKPFWAITKHADVMDIERENMLFTNWPR
PVLTTAEGDEMQAAAGVRTLIHMDDPQHRVVRAIGSDWFRPKAMRALKVRVDELAKIYVDKMLAAGPECDFVQ
EVAVNYPLYVIMSLLGLPEADFPRMLKLTQELFGSDDSEFKRGSSNEDQLPALLDMFGYFNGVTAARREHPTE
DLASAIANARVDGEPLSDIDTVSYYLIVATAGHDTTSATISGGLQALIENPDQLQRLRDNLDLMPLATEEMIR
WVTPVKEFMRTAAKDTVVRGVPIAAGESVLLSYVSANRDEDVFDEPFRFDVGRDPNKHLAFGYGVHFCMGAAL
ARMEVNSFFTELLPRLKSIELTGDPELVATTFVGGLKHLPVRYSLA
>CYP108B5_Mycobacterium sp. MAC_080597_8934 2576976436
MSTTTMDEAAKLLADPMAYTDEQRLHAALTHLRANAPVSWVEVPNYKPFWAITKHADVMDIERENMLFTNWPR
PVLTTAEGDEMQAAAGVRTLIHMDDPQHRVVRAIGSDWFRPKAMRALKVRVDELAKIYVDKMLAAGPECDFVQ
EVAVNYPLYVIMSLLGLPEADFPRMLKLTQELFGSDDSEFKRGSSNEDQLPALLDMFGYFNGVTAARREHPTE
DLASAIANARVDGEPLSDIDTVSYYLIVATAGHDTTSATISGGLQALIENPDQLQRLRDNLDLMPLATEEMIR
WVTPVKEFMRTAAKDTVVRGVPIAAGESVLLSYVSANRDEDVFDEPFRFDVGRDPNKHLAFGYGVHFCMGAAL
ARMEVNSFFTELLPRLKSIELTGDPELVATTFVGGLKHLPVRYSLA
>CYP108B5_Mycobacterium avium 05-4293 2581912223
MSTTTMDEAAKLLADPMAYTDEQRLHAALTHLRANAPVSWVEVPNYKPFWAITKHADVMDIERENMLFTNWPR
PVLTTAEGDEMQAAAGVRTLIHMDDPQHRVVRAIGSDWFRPKAMRALKVRVDELAKIYVDKMLAAGPECDFVQ
EVAVNYPLYVIMSLLGLPEADFPRMLKLTQELFGSDDSEFKRGSSNEDQLPALLDMFGYFNGVTAARREHPTE
DLASAIANARVDGEPLSDIDTVSYYLIVATAGHDTTSATISGGLQALIENPDQLQRLRDNLDLMPLATEEMIR
WVTPVKEFMRTAAKDTVVRGVPIAAGESVLLSYVSANRDEDVFDEPFRFDVGRDPNKHLAFGYGVHFCMGAAL
ARMEVNSFFTELLPRLKSIELTGDPELVATTFVGGLKHLPVRYSLA
>CYP108B5_Mycobacterium avium 104 639734963
MSTTTMDEAAKLLADPMAYTDEQRLHAALTHLRANAPVSWVEVPNYKPFWAITKHADVMDIERENMLFTNWPR
PVLTTAEGDEMQAAAGVRTLIHMDDPQHRVVRAIGSDWFRPKAMRALKVRVDELAKIYVDKMLAAGPECDFVQ
EVAVNYPLYVIMSLLGLPEADFPRMLKLTQELFGSDDSEFKRGSSNEDQLPALLDMFGYFNGVTAARREHPTE
DLASAIANARVDGEPLSDIDTVSYYLIVATAGHDTTSATISGGLQALIENPDQLQRLRDNLDLMPLATEEMIR
WVTPVKEFMRTAAKDTVVRGVPIAAGESVLLSYVSANRDEDVFDEPFRFDVGRDPNKHLAFGYGVHFCMGAAL
ARMEVNSFFTELLPRLKSIELTGDPELVATTFVGGLKHLPVRYSLA
>CYP108B5_Mycobacterium avium 10-5560 2576191064
MSTTTMDEAAKLLADPMAYTDEQRLHAALTHLRANAPVSWVEVPNYKPFWAITKHADVMDIERENMLFTNWPR
PVLTTAEGDEMQAAAGVRTLIHMDDPQHRVVRAIGSDWFRPKAMRALKVRVDELAKIYVDKMLAAGPECDFVQ
EVAVNYPLYVIMSLLGLPEADFPRMLKLTQELFGSDDSEFKRGSSNEDQLPALLDMFGYFNGVTAARREHPTE
DLASAIANARVDGEPLSDIDTVSYYLIVATAGHDTTSATISGGLQALIENPDQLQRLRDNLDLMPLATEEMIR
WVTPVKEFMRTAAKDTVVRGVPIAAGESVLLSYVSANRDEDVFDEPFRFDVGRDPNKHLAFGYGVHFCMGAAL
ARMEVNSFFTELLPRLKSIELTGDPELVATTFVGGLKHLPVRYSLA
>CYP108B5_Mycobacterium avium avium 11-4751 2581175983
MSTTTMDEAAKLLADPMAYTDEQRLHAALTHLRANAPVSWVEVPNYKPFWAITKHADVMDIERENMLFTNWPR
PVLTTAEGDEMQAAAGVRTLIHMDDPQHRVVRAIGSDWFRPKAMRALKVRVDELAKIYVDKMLAAGPECDFVQ
EVAVNYPLYVIMSLLGLPEADFPRMLKLTQELFGSDDSEFKRGSSNEDQLPALLDMFGYFNGVTAARREHPTE
DLASAIANARVDGEPLSDIDTVSYYLIVATAGHDTTSATISGGLQALIENPDQLQRLRDNLDLMPLATEEMIR
WVTPVKEFMRTAAKDTVVRGVPIAAGESVLLSYVSANRDEDVFDEPFRFDVGRDPNKHLAFGYGVHFCMGAAL
ARMEVNSFFTELLPRLKSIELTGDPELVATTFVGGLKHLPVRYSLA
>CYP108B5_Mycobacterium avium avium 3388 2581396598
MDEAAKLLADPMAYTDEQRLHAALTHLRANAPVSWVEVPNYKPFWAITKHADVMDIERENMLFTNWPRPVLTT
AEGDEMQAAAGVRTLIHMDDPQHRVVRAIGSDWFRPKAMRALKVRVDELAKIYVDKMLAAGPECDFVQEVAVN
YPLYVIMSLLGLPEADFPRMLKLTQELFGSDDSEFKRGSSNEDQLPALLDMFGYFNGVTAARREHPTEDLASA
IANARVDGEPLSDIDTVSYYLIVATAGHDTTSATISGGLQALIENPDQLQRLRDNLDLMPLATEEMIRWVTPV
KEFMRTAAKDTVVRGVPIAAGESVLLSYVSANRDEDVFDEPFRFDVGRDPNKHLAFGYGVHFCMGAALARMEV
NSFFTELLPRLKSIELTGDPELVATTFVGGLKHLPVRYSLA
>CYP108B5_Mycobacterium avium paratuberculosis S397 2534965343
MSTTTMDEAAKLLADPMAYTDEQRLHAALTHLRANAPVSWVEVPNYKPFWAITKHADVMDIERENMLFTNWPR
PVLTTAEGDEMQAAAGVRTLIHMDDPQHRVVRAIGSDWFRPKAMRALKVRVDELAKIYVDKMLAAGPECDFVQ

EVAVNYPLYVIMSLLGLPEADFPRMLKLTQELFGSDDSEFKRGSSNEDQLPALLDMFGYFNGVTAARREHPTE
DLASAIANARVDGEPLSDIDTVSYYLIVATAGHDTTSATISGGLQALIENPDQLQRLRDNLDLMPLATEEMIR
WVTPVKEFMRTAAKDTVVRGVPIAAGESVLLSYVSANRDEDVFDEPFRFDVGRDPNKHLAFGYGVHFCMGAAL
ARMEVNSFFTELLPRLKSIELTGDPELVATTFVGGLKHLPVRYSLA
>CYP108B5_Mycobacterium avium paratuberculosis MAP4 2555734624
MSTTTMDEAAKLLADPMAYTDEQRLHAALTHLRANAPVSWVEVPNYKPFWAITKHADVMDIERENMLFTNWPR
PVLTTAEGDEMQAAAGVRTLIHMDDPQHRVVRAIGSDWFRPKAMRALKVRVDELAKIYVDKMLAAGPECDFVQ
EVAVNYPLYVIMSLLGLPEADFPRMLKLTQELFGSDDSEFKRGSSNEDQLPALLDMFGYFNGVTAARREHPTE
DLASAIANARVDGEPLSDIDTVSYYLIVATAGHDTTSATISGGLQALIENPDQLQRLRDNLDLMPLATEEMIR
WVTPVKEFMRTAAKDTVVRGVPIAAGESVLLSYVSANRDEDVFDEPFRFDVGRDPNKHLAFGYGVHFCMGAAL
ARMEVNSFFTELLPRLKSIELTGDPELVATTFVGGLKHLPVRYSLA
>CYP108B5_Mycobacterium avium subsp. paratuberculosis K-10 637135339
MSTTTMDEAAKLLADPMAYTDEQRLHAALTHLRANAPVSWVEVPNYKPFWAITKHADVMDIERENMLFTNWPR
PVLTTAEGDEMQAAAGVRTLIHMDDPQHRVVRAIGSDWFRPKAMRALKVRVDELAKIYVDKMLAAGPECDFVQ
EVAVNYPLYVIMSLLGLPEADFPRMLKLTQELFGSDDSEFKRGSSNEDQLPALLDMFGYFNGVTAARREHPTE
DLASAIANARVDGEPLSDIDTVSYYLIVATAGHDTTSATISGGLQALIENPDQLQRLRDNLDLMPLATEEMIR
WVTPVKEFMRTAAKDTVVRGVPIAAGESVLLSYVSANRDEDVFDEPFRFDVGRDPNKHLAFGYGVHFCMGAAL
ARMEVNSFFTELLPRLKSIELTGDPELVATTFVGGLKHLPVRYSLA
>CYP108B5_Mycobacterium avium paratuberculosis 10-8425 2592478779
MSTTTMDEAAKLLADPMAYTDEQRLHAALTHLRANAPVSWVEVPNYKPFWAITKHADVMDIERENMLFTNWPR
PVLTTAEGDEMQAAAGVRTLIHMDDPQHRVVRAIGSDWFRPKAMRALKVRVDELAKIYVDKMLAAGPECDFVQ
EVAVNYPLYVIMSLLGLPEADFPRMLKLTQELFGSDDSEFKRGSSNEDQLPALLDMFGYFNGVTAARREHPTE
DLASAIANARVDGEPLSDIDTVSYYLIVATAGHDTTSATISGGLQALIENPDQLQRLRDNLDLMPLATEEMIR
WVTPVKEFMRTAAKDTVVRGVPIAAGESVLLSYVSANRDEDVFDEPFRFDVGRDPNKHLAFGYGVHFCMGAAL
ARMEVNSFFTELLPRLKSIELTGDPELVATTFVGGLKHLPVRYSLA
>CYP108B5_Mycobacterium avium paratuberculosis 11-1786 2592474263
MSTTTMDEAAKLLADPMAYTDEQRLHAALTHLRANAPVSWVEVPNYKPFWAITKHADVMDIERENMLFTNWPR
PVLTTAEGDEMQAAAGVRTLIHMDDPQHRVVRAIGSDWFRPKAMRALKVRVDELAKIYVDKMLAAGPECDFVQ
EVAVNYPLYVIMSLLGLPEADFPRMLKLTQELFGSDDSEFKRGSSNEDQLPALLDMFGYFNGVTAARREHPTE
DLASAIANARVDGEPLSDIDTVSYYLIVATAGHDTTSATISGGLQALIENPDQLQRLRDNLDLMPLATEEMIR
WVTPVKEFMRTAAKDTVVRGVPIAAGESVLLSYVSANRDEDVFDEPFRFDVGRDPNKHLAFGYGVHFCMGAAL
ARMEVNSFFTELLPRLKSIELTGDPELVATTFVGGLKHLPVRYSLA
>CYP108B5_Mycobacterium avium paratuberculosis 10-5975 2576282184
MSTTTMDEAAKLLADPMAYTDEQRLHAALTHLRANAPVSWVEVPNYKPFWAITKHADVMDIERENMLFTNWPR
PVLTTAEGDEMQAAAGVRTLIHMDDPQHRVVRAIGSDWFRPKAMRALKVRVDELAKIYVDKMLAAGPECDFVQ
EVAVNYPLYVIMSLLGLPEADFPRMLKLTQELFGSDDSEFKRGSSNEDQLPALLDMFGYFNGVTAARREHPTE
DLASAIANARVDGEPLSDIDTVSYYLIVATAGHDTTSATISGGLQALIENPDQLQRLRDNLDLMPLATEEMIR
WVTPVKEFMRTAAKDTVVRGVPIAAGESVLLSYVSANRDEDVFDEPFRFDVGRDPNKHLAFGYGVHFCMGAAL
ARMEVNSFFTELLPRLKSIELTGDPELVATTFVGGLKHLPVRYSLA
>CYP108B5_Mycobacterium avium MAV_120809_2495 2580740736
MDEAAKLLADPMAYTDEQRLHAALTHLRANAPVSWVEVPNYKPFWAITKHADVMDIERENMLFTNWPRPVLTT
AEGDEMQAAAGVRTLIHMDDPQHRVVRAIGSDWFRPKAMRALKVRVDELAKIYVDKMLAAGPECDFVQEVAVN
YPLYVIMSLLGLPEADFPRMLKLTQELFGSDDSEFKRGSSNEDQLPALLDMFGYFNGVTAARREHPTEDLASA
IANARVDGEPLSDIDTVSYYLIVATAGHDTTSATISGGLQALIENPDQLQRLRDNLDLMPLATEEMIRWVTPV
KEFMRTAAKDTVVRGVPIAAGESVLLSYVSANRDEDVFDEPFRFDVGRDPNKHLAFGYGVHFCMGAALARMEV
NSFFTELLPRLKSIELTGDPELVATTFVGGLKHLPVRYSLA
>CYP108B5_Mycobacterium avium MAV_120709_2344 2582386927
MSTTTMDEAAKLLADPMAYTDEQRLHAALTHLRANAPVSWVEVPNYKPFWAITKHADVMDIERENMLFTNWPR
PVLTTAEGDEMQAAAGVRTLIHMDDPQHRVVRAIGSDWFRPKAMRALKVRVDELAKIYVDKMLAAGPECDFVQ
EVAVNYPLYVIMSLLGLPEADFPRMLKLTQELFGSDDSEFKRGSSNEDQLPALLDMFGYFNGVTAARREHPTE
DLASAIANARVDGEPLSDIDTVSYYLIVATAGHDTTSATISGGLQALIENPDQLQRLRDNLDLMPLATEEMIR
WVTPVKEFMRTAAKDTVVRGVPIAAGESVLLSYVSANRDEDVFDEPFRFDVGRDPNKHLAFGYGVHFCMGAAL
ARMEVNSFFTELLPRLKSIELTGDPELVATTFVGGLKHLPVRYSLA
>CYP108B5_Mycobacterium avium hominissuis MAH 2721 2573740807
MPLATEEMIRWVTPVKEFMRTAAKDTVVRGVPIAAGESVLLSYVSANRDEDVFDEPFRFDVGRDPNKHLAFGY
GVHFCMGAALARMEVNSFFTELLPRLKSIELTGDPELVATTFVAVSSTCRCGIRWRESGSGRHRCVIATLPVE
VWQ
>CYP108B5_Mycobacterium avium hominissuis 10-5606 2582115687

MSTTTMDEAAKLLADPMAYTDEQRLHAALTHLRANAPVSWVEVPNYKPFWAITKHADVMDIERENMLFTNWPR
PVLTTAEGDEMQAAAGVRTLIHMDDPQHRVVRAIGSDWFRPKAMRALKVRVDELAKIYVDKMLAAGPECDFVQ
EVAVNYPLYVIMSLLGLPEADFPRMLKLTQELFGSDDSEFKRGSSNEDQLPALLDMFGYFNGVTAARREHPTE
DLASAIANARVDGEPLSDIDTVSYYLIVATAGHDTTSATISGGLQALIENPDQLQRLRDNLDLMPLATEEMIR
WVTPVKEFMRTAAKDTVVRGVPIAAGESVLLSYVSANRDEDVFDEPFRFDVGRDPNKHLAFGYGVHFCMGAAL
ARMEVNSFFTELLPRLKSIELTGDPELVATTFVGGLKHLPVRYSLA
>CYP108B5_Mycobacterium avium 101 2580004971
MDEAAKLLADPMAYTDEQRLHAALTHLRANAPVSWVEVPNYKPFWAITKHADVMDIERENMLFTNWPRPVLTT
AEGDEMQAAAGVRTLIHMDDPQHRVVRAIGSDWFRPKAMRALKVRVDELAKIYVDKMLAAGPECDFVQEVAVN
YPLYVIMSLLGLPEADFPRMLKLTQELFGSDDSEFKRGSSNEDQLPALLDMFGYFNGVTAARREHPTEDLASA
IANARVDGEPLSDIDTVSYYLIVATAGHDTTSATISGGLQALIENPDQLQRLRDNLDLMPLATEEMIRWVTPV
KEFMRTAAKDTVVRGVPIAAGESVLLSYVSANRDEDVFDEPFRFDVGRDPNKHLAFGYGVHFCMGAALARMEV
NSFFTELLPRLKSIELTGDPELVATTFVGGLKHLPVRYSLA
>CYP108B5_Mycobacterium avium 100 2580973274
MDEAAKLLADPMAYTDEQRLHAALTHLRANAPVSWVEVPNYKPFWAITKHADVMDIERENMLFTNWPRPVLTT
AEGDEMQAAAGVRTLIHMDDPQHRVVRAIGSDWFRPKAMRALKVRVDELAKIYVDKMLAAGPECDFVQEVAVN
YPLYVIMSLLGLPEADFPRMLKLTQELFGSDDSEFKRGSSNEDQLPALLDMFGYFNGVTAARREHPTEDLASA
IANARVDGEPLSDIDTVSYYLIVATAGHDTTSATISGGLQALIENPDQLQRLRDNLDLMPLATEEMIRWVTPV
KEFMRTAAKDTVVRGVPIAAGESVLLSYVSANRDEDVFDEPFRFDVGRDPNKHLAFGYGVHFCMGAALARMEV
NSFFTELLPRLKSIELTGDPELVATTFVGGLKHLPVRYSLA
>CYP108B5_Mycobacterium avium subsp. avium ATCC 25291 645424287
MSTTTMDEAAKLLADPMAYTDEQRLHAALTHLRANAPVSWVEVPNYKPFWAITKHADVMDIERENMLFTNWPR
PVLTTAEGDEMQAAAGVRTLIHMDDPQHRVVRAIGSDWFRPKAMRALKVRVDELAKIYVDKMLAAGPECDFVQ
EVAVNYPLYVIMSLLGLPEADFPRMLKLTQELFGSDDSEFKRGSSNEDQLPALLDMFGYFNGVTAARREHPTE
DLASAIANARVDGEPLSDIDTVSYYLIVATAGHDTTSATISGGLQALIENPDQLQRLRDNLDLMPLATEEMIR
WVTPVKEFMRTAAKDTVVRGVPIAAGESVLLSYVSANRDEDVFDEPFRFDVGRDPNKHLAFGYGVHFCMGAAL
ARMEVNSFFTELLPRLKSIELTGDPELVATTFVGGLKHLPVRYSLA
>CYP108B5_Mycobacterium avium 11-0986 2583771822
MSTTTMDEAAKLLADPMAYTDEQRLHAALTHLRANAPVSWVEVPNYKPFWAITKHADVMDIERENMLFTNWPR
PVLTTAEGDEMQAAAGVRTLIHMDDPQHRVVRAIGSDWFRPKAMRALKVRVDELAKIYVDKMLAAGPECDFVQ
EVAVNYPLYVIMSLLGLPEADFPRMLKLTQELFGSDDSEFKRGSSNEDQLPALLDMFGYFNGVTAARREHPTE
DLASAIANARVDGEPLSDIDTVSYYLIVATAGHDTTSATISGGLQALIENPDQLQRLRDNLDLMPLATEEMIR
WVTPVKEFMRTAAKDTVVRGVPIAAGESVLLSYVSANRDEDVFDDPFRFDVGRDPNKHLAFGYGVHFCMGAAL
ARMEVNSFFTELLPRLKSIELTGDPELVATTFVGGLKHLPVRYSLA
>CYP108B5_Mycobacterium avium 2285 (R) 2567081050
MSTTTMDEAAKLLADPMAYTDEQRLHAALTHLRANAPVSWVEVPNYKPFWAITKHADVMDIERENMLFTNWPR
PVLTTAEGDEMQAAAGVRTLIHMDDPQHRVVRAIGSDWFRPKAMRALKVRVDELAKIYVDKMLAAGPECDFVQ
EVAVNYPLYVIMSLLGLPEADFPRMLKLTQELFGSDDSEFKRGSSNEDQLPALLDMFGYFNGVTAARREHPTE
DLASAIANARVDGEPLSDIDTVSYYLIVATAGHDTTSATISGGLQALIENPDQLQRLRDNLDLMPLATEEMIR
WVTPVKEFMRTAAKDTVVRGVPIAAGESVLLSYVSANRDEDVFDEPFCFDVGRDPNKHLAFGYGVHFCMGAAL
ARMEVNSFFTELLPRLKSIELTGDPELVATTFVGGLKHLPVRYSLA
>CYP108B5_Mycobacterium avium paratuberculosis Pt164 2548544604
VSTTTMDEAAKLLADPMAYTDEQRLHAALTHLRANAPVSWVEVPNYKPFWAITKHADVMDIERENMLFTNWPR
PVLTTAEGDEMQAAAGVRTLIHMDDPQHRVVRAIGSDWFRPKAMRALKVRVDELAKIYVDKMLAAGPECDFVQ
EVAVNYPLYVIMSLLGLPEADFPRMLKLTQELFGSDDSEFKRGSSNEDQLPALLDMFGYFNGVTAARREHPTE
DLASAIANARVDGEPLSDIDTVSYYLIVATAGHDTTSATISGGLQALIENPDQLQRLRDNLDLMPLATEEMIR
WVTPVKEFMRTAAKDTVVRGVPIAAGESVLLSYVSANRDEDVFDEPFRFDVGRDPNKHLAFGYGVHFCMGAAL
ARMEVNSFFTELLPRLKSIELTGDPELVATTFVGGLKHLPVRYSLA
>CYP108B5_Mycobacterium avium paratuberculosis Pt155 2548537568
VSTTTMDEAAKLLADPMAYTDEQRLHAALTHLRANAPVSWVEVPNYKPFWAITKHADVMDIERENMLFTNWPR
PVLTTAEGDEMQAAAGVRTLIHMDDPQHRVVRAIGSDWFRPKAMRALKVRVDELAKIYVDKMLAAGPECDFVQ
EVAVNYPLYVIMSLLGLPEADFPRMLKLTQELFGSDDSEFKRGSSNEDQLPALLDMFGYFNGVTAARREHPTE
DLASAIANARVDGEPLSDIDTVSYYLIVATAGHDTTSATISGGLQALIENPDQLQRLRDNLDLMPLATEEMIR
WVTPVKEFMRTAAKDTVVRGVPIAAGESVLLSYVSANRDEDVFDEPFRFDVGRDPNKHLAFGYGVHFCMGAAL
ARMEVNSFFTELLPRLKSIELTGDPELVATTFVGGLKHLPVRYSLA
>CYP108B5_Mycobacterium avium paratuberculosis Pt154 2548536493
VSTTTMDEAAKLLADPMAYTDEQRLHAALTHLRANAPVSWVEVPNYKPFWAITKHADVMDIERENMLFTNWPR
PVLTTAEGDEMQAAAGVRTLIHMDDPQHRVVRAIGSDWFRPKAMRALKVRVDELAKIYVDKMLAAGPECDFVQ

EVAVNYPLYVIMSLLGLPEADFPRMLKLTQELFGSDDSEFKRGSSNEDQLPALLDMFGYFNGVTAARREHPTE
DLASAIANARVDGEPLSDIDTVSYYLIVATAGHDTTSATISGGLQALIENPDQLQRLRDNLDLMPLATEEMIR
WVTPVKEFMRTAAKDTVVRGVPIAAGESVLLSYVSANRDEDVFDEPFRFDVGRDPNKHLAFGYGVHFCMGAAL
ARMEVNSFFTELLPRLKSIELTGDPELVATTFVGGLKHLPVRYSLA
>CYP108B5_Mycobacterium avium paratuberculosis Pt146 2548528542
VSTTTMDEAAKLLADPMAYTDEQRLHAALTHLRANAPVSWVEVPNYKPFWAITKHADVMDIERENMLFTNWPR
PVLTTAEGDEMQAAAGVRTLIHMDDPQHRVVRAIGSDWFRPKAMRALKVRVDELAKIYVDKMLAAGPECDFVQ
EVAVNYPLYVIMSLLGLPEADFPRMLKLTQELFGSDDSEFKRGSSNEDQLPALLDMFGYFNGVTAARREHPTE
DLASAIANARVDGEPLSDIDTVSYYLIVATAGHDTTSATISGGLQALIENPDQLQRLRDNLDLMPLATEEMIR
WVTPVKEFMRTAAKDTVVRGVPIAAGESVLLSYVSANRDEDVFDEPFRFDVGRDPNKHLAFGYGVHFCMGAAL
ARMEVNSFFTELLPRLKSIELTGDPELVATTFVGGLKHLPVRYSLA
>CYP108B5_Mycobacterium avium paratuberculosis Pt145 2548522300
VSTTTMDEAAKLLADPMAYTDEQRLHAALTHLRANAPVSWVEVPNYKPFWAITKHADVMDIERENMLFTNWPR
PVLTTAEGDEMQAAAGVRTLIHMDDPQHRVVRAIGSDWFRPKAMRALKVRVDELAKIYVDKMLAAGPECDFVQ
EVAVNYPLYVIMSLLGLPEADFPRMLKLTQELFGSDDSEFKRGSSNEDQLPALLDMFGYFNGVTAARREHPTE
DLASAIANARVDGEPLSDIDTVSYYLIVATAGHDTTSATISGGLQALIENPDQLQRLRDNLDLMPLATEEMIR
WVTPVKEFMRTAAKDTVVRGVPIAAGESVLLSYVSANRDEDVFDEPFRFDVGRDPNKHLAFGYGVHFCMGAAL
ARMEVNSFFTELLPRLKSIELTGDPELVATTFVGGLKHLPVRYSLA
>CYP108B5_Mycobacterium avium paratuberculosis Pt144 2548517280
VSTTTMDEAAKLLADPMAYTDEQRLHAALTHLRANAPVSWVEVPNYKPFWAITKHADVMDIERENMLFTNWPR
PVLTTAEGDEMQAAAGVRTLIHMDDPQHRVVRAIGSDWFRPKAMRALKVRVDELAKIYVDKMLAAGPECDFVQ
EVAVNYPLYVIMSLLGLPEADFPRMLKLTQELFGSDDSEFKRGSSNEDQLPALLDMFGYFNGVTAARREHPTE
DLASAIANARVDGEPLSDIDTVSYYLIVATAGHDTTSATISGGLQALIENPDQLQRLRDNLDLMPLATEEMIR
WVTPVKEFMRTAAKDTVVRGVPIAAGESVLLSYVSANRDEDVFDEPFRFDVGRDPNKHLAFGYGVHFCMGAAL
ARMEVNSFFTELLPRLKSIELTGDPELVATTFVGGLKHLPVRYSLA
>CYP108B5_Mycobacterium avium paratuberculosis Pt139 2548513478
VSTTTMDEAAKLLADPMAYTDEQRLHAALTHLRANAPVSWVEVPNYKPFWAITKHADVMDIERENMLFTNWPR
PVLTTAEGDEMQAAAGVRTLIHMDDPQHRVVRAIGSDWFRPKAMRALKVRVDELAKIYVDKMLAAGPECDFVQ
EVAVNYPLYVIMSLLGLPEADFPRMLKLTQELFGSDDSEFKRGSSNEDQLPALLDMFGYFNGVTAARREHPTE
DLASAIANARVDGEPLSDIDTVSYYLIVATAGHDTTSATISGGLQALIENPDQLQRLRDNLDLMPLATEEMIR
WVTPVKEFMRTAAKDTVVRGVPIAAGESVLLSYVSANRDEDVFDEPFRFDVGRDPNKHLAFGYGVHFCMGAAL
ARMEVNSFFTELLPRLKSIELTGDPELVATTFVGGLKHLPVRYSLA
>CYP108B5_Mycobacterium avium paratuberculosis MAP/TANUVAS/TN/India/2008
2719421422
VSTTTMDEAAKLLADPMAYTDEQRLHAALTHLRANAPVSWVEVPNYKPFWAITKHADVMDIERENMLFTNWPR
PVLTTAEGDEMQAAAGVRTLIHMDDPQHRVVRAIGSDWFRPKAMRALKVRVDELAKIYVDKMLAAGPECDFVQ
EVAVNYPLYVIMSLLGLPEADFPRMLKLTQELFGSDDSEFKRGSSNEDQLPALLDMFGYFNGVTAARREHPTE
DLASAIANARVDGEPLSDIDTVSYYLIVATAGHDTTSATISGGLQALIENPDQLQRLRDNLDLMPLATEEMIR
WVTPVKEFMRTAAKDTVVRGVPIAAGESVLLSYVSANRDEDVFDEPFRFDVGRDPNKHLAFGYGVHFCMGAAL
ARMEVNSFFTELLPRLKSIELTGDPELVATTFVGGLKHLPVRYSLA
>CYP108B5_Mycobacterium avium paratuberculosis M. ap E93 2689464518
VSTTTMDEAAKLLADPMAYTDEQRLHAALTHLRANAPVSWVEVPNYKPFWAITKHADVMDIERENMLFTNWPR
PVLTTAEGDEMQAAAGVRTLIHMDDPQHRVVRAIGSDWFRPKAMRALKVRVDELAKIYVDKMLAAGPECDFVQ
EVAVNYPLYVIMSLLGLPEADFPRMLKLTQELFGSDDSEFKRGSSNEDQLPALLDMFGYFNGVTAARREHPTE
DLASAIANARVDGEPLSDIDTVSYYLIVATAGHDTTSATISGGLQALIENPDQLQRLRDNLDLMPLATEEMIR
WVTPVKEFMRTAAKDTVVRGVPIAAGESVLLSYVSANRDEDVFDEPFRFDVGRDPNKHLAFGYGVHFCMGAAL
ARMEVNSFFTELLPRLKSIELTGDPELVATTFVGGLKHLPVRYSLA
>CYP108B5_Mycobacterium avium paratuberculosis JTC 1285 2549372375
VSTTTMDEAAKLLADPMAYTDEQRLHAALTHLRANAPVSWVEVPNYKPFWAITKHADVMDIERENMLFTNWPR
PVLTTAEGDEMQAAAGVRTLIHMDDPQHRVVRAIGSDWFRPKAMRALKVRVDELAKIYVDKMLAAGPECDFVQ
EVAVNYPLYVIMSLLGLPEADFPRMLKLTQELFGSDDSEFKRGSSNEDQLPALLDMFGYFNGVTAARREHPTE
DLASAIANARVDGEPLSDIDTVSYYLIVATAGHDTTSATISGGLQALIENPDQLQRLRDNLDLMPLATEEMIR
WVTPVKEFMRTAAKDTVVRGVPIAAGESVLLSYVSANRDEDVFDEPFRFDVGRDPNKHLAFGYGVHFCMGAAL
ARMEVNSFFTELLPRLKSIELTGDPELVATTFVGGLKHLPVRYSLA
>CYP108B5_Mycobacterium avium paratuberculosis JQ6 2549395537
VSTTTMDEAAKLLADPMAYTDEQRLHAALTHLRANAPVSWVEVPNYKPFWAITKHADVMDIERENMLFTNWPR
PVLTTAEGDEMQAAAGVRTLIHMDDPQHRVVRAIGSDWFRPKAMRALKVRVDELAKIYVDKMLAAGPECDFVQ
EVAVNYPLYVIMSLLGLPEADFPRMLKLTQELFGSDDSEFKRGSSNEDQLPALLDMFGYFNGVTAARREHPTE

DLASAIANARVDGEPLSDIDTVSYYLIVATAGHDTTSATISGGLQALIENPDQLQRLRDNLDLMPLATEEMIR
WVTPVKEFMRTAAKDTVVRGVPIAAGESVLLSYVSANRDEDVFDEPFRFDVGRDPNKHLAFGYGVHFCMGAAL
ARMEVNSFFTELLPRLKSIELTGDPELVATTFVGGLKHLPVRYSLA
>CYP108B5_Mycobacterium avium paratuberculosis JQ5 2549359135
VSTTTMDEAAKLLADPMAYTDEQRLHAALTHLRANAPVSWVEVPNYKPFWAITKHADVMDIERENMLFTNWPR
PVLTTAEGDEMQAAAGVRTLIHMDDPQHRVVRAIGSDWFRPKAMRALKVRVDELAKIYVDKMLAAGPECDFVQ
EVAVNYPLYVIMSLLGLPEADFPRMLKLTQELFGSDDSEFKRGSSNEDQLPALLDMFGYFNGVTAARREHPTE
DLASAIANARVDGEPLSDIDTVSYYLIVATAGHDTTSATISGGLQALIENPDQLQRLRDNLDLMPLATEEMIR
WVTPVKEFMRTAAKDTVVRGVPIAAGESVLLSYVSANRDEDVFDEPFRFDVGRDPNKHLAFGYGVHFCMGAAL
ARMEVNSFFTELLPRLKSIELTGDPELVATTFVGGLKHLPVRYSLA
>CYP108B5_Mycobacterium avium paratuberculosis Env 210 2549380280
VSTTTMDEAAKLLADPMAYTDEQRLHAALTHLRANAPVSWVEVPNYKPFWAITKHADVMDIERENMLFTNWPR
PVLTTAEGDEMQAAAGVRTLIHMDDPQHRVVRAIGSDWFRPKAMRALKVRVDELAKIYVDKMLAAGPECDFVQ
EVAVNYPLYVIMSLLGLPEADFPRMLKLTQELFGSDDSEFKRGSSNEDQLPALLDMFGYFNGVTAARREHPTE
DLASAIANARVDGEPLSDIDTVSYYLIVATAGHDTTSATISGGLQALIENPDQLQRLRDNLDLMPLATEEMIR
WVTPVKEFMRTAAKDTVVRGVPIAAGESVLLSYVSANRDEDVFDEPFRFDVGRDPNKHLAFGYGVHFCMGAAL
ARMEVNSFFTELLPRLKSIELTGDPELVATTFVGGLKHLPVRYSLA
>CYP108B5_Mycobacterium avium paratuberculosis E1 2688291910
VSTTTMDEAAKLLADPMAYTDEQRLHAALTHLRANAPVSWVEVPNYKPFWAITKHADVMDIERENMLFTNWPR
PVLTTAEGDEMQAAAGVRTLIHMDDPQHRVVRAIGSDWFRPKAMRALKVRVDELAKIYVDKMLAAGPECDFVQ
EVAVNYPLYVIMSLLGLPEADFPRMLKLTQELFGSDDSEFKRGSSNEDQLPALLDMFGYFNGVTAARREHPTE
DLASAIANARVDGEPLSDIDTVSYYLIVATAGHDTTSATISGGLQALIENPDQLQRLRDNLDLMPLATEEMIR
WVTPVKEFMRTAAKDTVVRGVPIAAGESVLLSYVSANRDEDVFDEPFRFDVGRDPNKHLAFGYGVHFCMGAAL
ARMEVNSFFTELLPRLKSIELTGDPELVATTFVGGLKHLPVRYSLA
>CYP108B5_Mycobacterium avium paratuberculosis DT 3 2549375760
VSTTTMDEAAKLLADPMAYTDEQRLHAALTHLRANAPVSWVEVPNYKPFWAITKHADVMDIERENMLFTNWPR
PVLTTAEGDEMQAAAGVRTLIHMDDPQHRVVRAIGSDWFRPKAMRALKVRVDELAKIYVDKMLAAGPECDFVQ
EVAVNYPLYVIMSLLGLPEADFPRMLKLTQELFGSDDSEFKRGSSNEDQLPALLDMFGYFNGVTAARREHPTE
DLASAIANARVDGEPLSDIDTVSYYLIVATAGHDTTSATISGGLQALIENPDQLQRLRDNLDLMPLATEEMIR
WVTPVKEFMRTAAKDTVVRGVPIAAGESVLLSYVSANRDEDVFDEPFRFDVGRDPNKHLAFGYGVHFCMGAAL
ARMEVNSFFTELLPRLKSIELTGDPELVATTFVGGLKHLPVRYSLA
>CYP108B5_Mycobacterium avium paratuberculosis CLIJ644 2547367509
VSTTTMDEAAKLLADPMAYTDEQRLHAALTHLRANAPVSWVEVPNYKPFWAITKHADVMDIERENMLFTNWPR
PVLTTAEGDEMQAAAGVRTLIHMDDPQHRVVRAIGSDWFRPKAMRALKVRVDELAKIYVDKMLAAGPECDFVQ
EVAVNYPLYVIMSLLGLPEADFPRMLKLTQELFGSDDSEFKRGSSNEDQLPALLDMFGYFNGVTAARREHPTE
DLASAIANARVDGEPLSDIDTVSYYLIVATAGHDTTSATISGGLQALIENPDQLQRLRDNLDLMPLATEEMIR
WVTPVKEFMRTAAKDTVVRGVPIAAGESVLLSYVSANRDEDVFDEPFRFDVGRDPNKHLAFGYGVHFCMGAAL
ARMEVNSFFTELLPRLKSIELTGDPELVATTFVGGLKHLPVRYSLA
>CYP108B5_Mycobacterium avium paratuberculosis CLIJ623 2548575217
VSTTTMDEAAKLLADPMAYTDEQRLHAALTHLRANAPVSWVEVPNYKPFWAITKHADVMDIERENMLFTNWPR
PVLTTAEGDEMQAAAGVRTLIHMDDPQHRVVRAIGSDWFRPKAMRALKVRVDELAKIYVDKMLAAGPECDFVQ
EVAVNYPLYVIMSLLGLPEADFPRMLKLTQELFGSDDSEFKRGSSNEDQLPALLDMFGYFNGVTAARREHPTE
DLASAIANARVDGEPLSDIDTVSYYLIVATAGHDTTSATISGGLQALIENPDQLQRLRDNLDLMPLATEEMIR
WVTPVKEFMRTAAKDTVVRGVPIAAGESVLLSYVSANRDEDVFDEPFRFDVGRDPNKHLAFGYGVHFCMGAAL
ARMEVNSFFTELLPRLKSIELTGDPELVATTFVGGLKHLPVRYSLA
>CYP108B5_Mycobacterium avium paratuberculosis CLIJ361 2548595190
VSTTTMDEAAKLLADPMAYTDEQRLHAALTHLRANAPVSWVEVPNYKPFWAITKHADVMDIERENMLFTNWPR
PVLTTAEGDEMQAAAGVRTLIHMDDPQHRVVRAIGSDWFRPKAMRALKVRVDELAKIYVDKMLAAGPECDFVQ
EVAVNYPLYVIMSLLGLPEADFPRMLKLTQELFGSDDSEFKRGSSNEDQLPALLDMFGYFNGVTAARREHPTE
DLASAIANARVDGEPLSDIDTVSYYLIVATAGHDTTSATISGGLQALIENPDQLQRLRDNLDLMPLATEEMIR
WVTPVKEFMRTAAKDTVVRGVPIAAGESVLLSYVSANRDEDVFDEPFRFDVGRDPNKHLAFGYGVHFCMGAAL
ARMEVNSFFTELLPRLKSIELTGDPELVATTFVGGLKHLPVRYSLA
>CYP108B5_Mycobacterium avium paratuberculosis ATCC 19698 2548999771
VSTTTMDEAAKLLADPMAYTDEQRLHAALTHLRANAPVSWVEVPNYKPFWAITKHADVMDIERENMLFTNWPR
PVLTTAEGDEMQAAAGVRTLIHMDDPQHRVVRAIGSDWFRPKAMRALKVRVDELAKIYVDKMLAAGPECDFVQ
EVAVNYPLYVIMSLLGLPEADFPRMLKLTQELFGSDDSEFKRGSSNEDQLPALLDMFGYFNGVTAARREHPTE
DLASAIANARVDGEPLSDIDTVSYYLIVATAGHDTTSATISGGLQALIENPDQLQRLRDNLDLMPLATEEMIR

WVTPVKEFMRTAAKDTVVRGVPIAAGESVLLSYVSANRDEDVFDEPFRFDVGRDPNKHLAFGYGVHFCMGAAL
ARMEVNSFFTELLPRLKSIELTGDPELVATTFVGGLKHLPVRYSLA
>CYP108B5_Mycobacterium avium paratuberculosis 1281 2549364626
VSTTTMDEAAKLLADPMAYTDEQRLHAALTHLRANAPVSWVEVPNYKPFWAITKHADVMDIERENMLFTNWPR
PVLTTAEGDEMQAAAGVRTLIHMDDPQHRVVRAIGSDWFRPKAMRALKVRVDELAKIYVDKMLAAGPECDFVQ
EVAVNYPLYVIMSLLGLPEADFPRMLKLTQELFGSDDSEFKRGSSNEDQLPALLDMFGYFNGVTAARREHPTE
DLASAIANARVDGEPLSDIDTVSYYLIVATAGHDTTSATISGGLQALIENPDQLQRLRDNLDLMPLATEEMIR
WVTPVKEFMRTAAKDTVVRGVPIAAGESVLLSYVSANRDEDVFDEPFRFDVGRDPNKHLAFGYGVHFCMGAAL
ARMEVNSFFTELLPRLKSIELTGDPELVATTFVGGLKHLPVRYSLA
>CYP108B5_Mycobacterium avium paratuberculosis 4B 2550739587
VSTTTMDEAAKLLADPMAYTDEQRLHAALTHLRANAPVSWVEVPNYKPFWAITKHADVMDIERENMLFTNWPR
PVLTTAEGDEMQAAAGVRTLIHMDDPQHRVVRAIGSDWFRPKAMRALKVRVDELAKIYVDKMLAAGPECDFVQ
EVAVNYPLYVIMSLLGLPEADFPRMLKLTQELFGSDDSEFKRGSSNEDQLPALLDMFGYFNGVTAARREHPTE
DLASAIANARVDGEPLSDIDTVSYYLIVATAGHDTTSATISGGLQALIENPDQLQRLRDNLDLMPLATEEMIR
WVTPVKEFMRTAAKDTVVRGVPIAAGESVLLSYVSANRDEDVFDEPFRFDVGRDPNKHLAFGYGVHFCMGAAL
ARMEVNSFFTELLPRLKSIELTGDPELVATTFVGGLKHLPVRYSLA
>CYP108B5_Mycobacterium avium hominissuis MAH 27-1 2581110904
VSTTTMDEAAKLLADPMAYTDEQRLHAALTHLRANAPVSWVEVPNYKPFWAITKHADVMDIERENMLFTNWPR
PVLTTAEGDEMQAAAGVRTLIHMDDPQHRVVRAIGSDWFRPKAMRALKVRVDELAKIYVDKMLAAGPECDFVQ
EVAVNYPLYVIMSLLGLPEADFPRMLKLTQELFGSDDSEFKRGSSNEDQLPALLDMFGYFNGVTAARREHPTE
DLASAIANARVDGEPLSDIDTVSYYLIVATAGHDTTSATISGGLQALIENPDQLQRLRDNLDLMPLATEEMIR
WVTPVKEFMRTAAKDTVVRGVPIAAGESVLLSYVSANRDEDVFDEPFRFDVGRDPNKHLAFGYGVHFCMGAAL
ARMEVNSFFTELLPRLKSIELTGDPELVATTFVGGLKHLPVRYSLA
>CYP108B5_Mycobacterium avium hominissuis MAH-P-9060-06 2725096071
VSTTTMDEAAKLLADPMAYTDEQRLHAALTHLRANAPVSWVEVPNYKPFWAITKHADVMDIERENMLFTNWPR
PVLTTAEGDEMQAAAGVRTLIHMDDPQHRVVRAIGSDWFRPKAMRALKVRVDELAKIYVDKMLAAGPECDFVQ
EVAVNYPLYVIMSLLGLPEADFPRMLKLTQELFGSDDSEFKRGSSNEDQLPALLDMFGYFNGVTAARREHPTE
DLASAIANARVDGEPLSDIDTVSYYLIVATAGHDTTSATISGGLQALIENPDQLQRLRDNLDLMPLATEEMIR
WVTPVKEFMRTAAKDTVVRGVPIAAGESVLLSYVSANRDEDVFDEPFRFDVGRDPNKHLAFGYGVHFCMGAAL
ARMEVNSFFTELLPRLKSIELTGDPELVATTFVGGLKHLPVRYSLA
>CYP108B5_Mycobacterium avium hominissuis MAH-P-8933-06 2725100852
VSTTTMDEAAKLLADPMAYTDEQRLHAALTHLRANAPVSWVEVPNYKPFWAITKHADVMDIERENMLFTNWPR
PVLTTAEGDEMQAAAGVRTLIHMDDPQHRVVRAIGSDWFRPKAMRALKVRVDELAKIYVDKMLAAGPECDFVQ
EVAVNYPLYVIMSLLGLPEADFPRMLKLTQELFGSDDSEFKRGSSNEDQLPALLDMFGYFNGVTAARREHPTE
DLASAIANARVDGEPLSDIDTVSYYLIVATAGHDTTSATISGGLQALIENPDQLQRLRDNLDLMPLATEEMIR
WVTPVKEFMRTAAKDTVVRGVPIAAGESVLLSYVSANRDEDVFDEPFRFDVGRDPNKHLAFGYGVHFCMGAAL
ARMEVNSFFTELLPRLKSIELTGDPELVATTFVGGLKHLPVRYSLA
>CYP108B5_Mycobacterium avium hominissuis MAHP-883 2725146486
VSTTTMDEAAKLLADPMAYTDEQRLHAALTHLRANAPVSWVEVPNYKPFWAITKHADVMDIERENMLFTNWPR
PVLTTAEGDEMQAAAGVRTLIHMDDPQHRVVRAIGSDWFRPKAMRALKVRVDELAKIYVDKMLAAGPECDFVQ
EVAVNYPLYVIMSLLGLPEADFPRMLKLTQELFGSDDSEFKRGSSNEDQLPALLDMFGYFNGVTAARREHPTE
DLASAIANARVDGEPLSDIDTVSYYLIVATAGHDTTSATISGGLQALIENPDQLQRLRDNLDLMPLATEEMIR
WVTPVKEFMRTAAKDTVVRGVPIAAGESVLLSYVSANRDEDVFDEPFRFDVGRDPNKHLAFGYGVHFCMGAAL
ARMEVNSFFTELLPRLKSIELTGDPELVATTFVGGLKHLPVRYSLA
>CYP108B5_Mycobacterium avium hominissuis MAH-P-772-08 2725125509
VSTTTMDEAAKLLADPMAYTDEQRLHAALTHLRANAPVSWVEVPNYKPFWAITKHADVMDIERENMLFTNWPR
PVLTTAEGDEMQAAAGVRTLIHMDDPQHRVVRAIGSDWFRPKAMRALKVRVDELAKIYVDKMLAAGPECDFVQ
EVAVNYPLYVIMSLLGLPEADFPRMLKLTQELFGSDDSEFKRGSSNEDQLPALLDMFGYFNGVTAARREHPTE
DLASAIANARVDGEPLSDIDTVSYYLIVATAGHDTTSATISGGLQALIENPDQLQRLRDNLDLMPLATEEMIR
WVTPVKEFMRTAAKDTVVRGVPIAAGESVLLSYVSANRDEDVFDEPFRFDVGRDPNKHLAFGYGVHFCMGAAL
ARMEVNSFFTELLPRLKSIELTGDPELVATTFVGGLKHLPVRYSLA
>CYP108B5_Mycobacterium avium hominissuis MAH-P-7673-04 2725155954
VSTTTMDEAAKLLADPMAYTDEQRLHAALTHLRANAPVSWVEVPNYKPFWAITKHADVMDIERENMLFTNWPR
PVLTTAEGDEMQAAAGVRTLIHMDDPQHRVVRAIGSDWFRPKAMRALKVRVDELAKIYVDKMLAAGPECDFVQ
EVAVNYPLYVIMSLLGLPEADFPRMLKLTQELFGSDDSEFKRGSSNEDQLPALLDMFGYFNGVTAARREHPTE
DLASAIANARVDGEPLSDIDTVSYYLIVATAGHDTTSATISGGLQALIENPDQLQRLRDNLDLMPLATEEMIR
WVTPVKEFMRTAAKDTVVRGVPIAAGESVLLSYVSANRDEDVFDEPFRFDVGRDPNKHLAFGYGVHFCMGAAL
ARMEVNSFFTELLPRLKSIELTGDPELVATTFVGGLKHLPVRYSLA

>CYP108B5_Mycobacterium avium hominissuis MAH-P-709-08 2725120214
VSTTTMDEAAKLLADPMAYTDEQRLHAALTHLRANAPVSWVEVPNYKPFWAITKHADVMDIERENMLFTNWPR
PVLTTAEGDEMQAAAGVRTLIHMDDPQHRVVRAIGSDWFRPKAMRALKVRVDELAKIYVDKMLAAGPECDFVQ
EVAVNYPLYVIMSLLGLPEADFPRMLKLTQELFGSDDSEFKRGSSNEDQLPALLDMFGYFNGVTAARREHPTE
DLASAIANARVDGEPLSDIDTVSYYLIVATAGHDTTSATISGGLQALIENPDQLQRLRDNLDLMPLATEEMIR
WVTPVKEFMRTAAKDTVVRGVPIAAGESVLLSYVSANRDEDVFDEPFRFDVGRDPNKHLAFGYGVHFCMGAAL
ARMEVNSFFTELLPRLKSIELTGDPELVATTFVGGLKHLPVRYSLA
>CYP108B5_Mycobacterium avium hominissuis MAH-P-589-08 2685182573
VSTTTMDEAAKLLADPMAYTDEQRLHAALTHLRANAPVSWVEVPNYKPFWAITKHADVMDIERENMLFTNWPR
PVLTTAEGDEMQAAAGVRTLIHMDDPQHRVVRAIGSDWFRPKAMRALKVRVDELAKIYVDKMLAAGPECDFVQ
EVAVNYPLYVIMSLLGLPEADFPRMLKLTQELFGSDDSEFKRGSSNEDQLPALLDMFGYFNGVTAARREHPTE
DLASAIANARVDGEPLSDIDTVSYYLIVATAGHDTTSATISGGLQALIENPDQLQRLRDNLDLMPLATEEMIR
WVTPVKEFMRTAAKDTVVRGVPIAAGESVLLSYVSANRDEDVFDEPFRFDVGRDPNKHLAFGYGVHFCMGAAL
ARMEVNSFFTELLPRLKSIELTGDPELVATTFVGGLKHLPVRYSLA
>CYP108B5_Mycobacterium avium hominissuis MAH-P-528-08 2725117775
VSTTTMDEAAKLLADPMAYTDEQRLHAALTHLRANAPVSWVEVPNYKPFWAITKHADVMDIERENMLFTNWPR
PVLTTAEGDEMQAAAGVRTLIHMDDPQHRVVRAIGSDWFRPKAMRALKVRVDELAKIYVDKMLAAGPECDFVQ
EVAVNYPLYVIMSLLGLPEADFPRMLKLTQELFGSDDSEFKRGSSNEDQLPALLDMFGYFNGVTAARREHPTE
DLASAIANARVDGEPLSDIDTVSYYLIVATAGHDTTSATISGGLQALIENPDQLQRLRDNLDLMPLATEEMIR
WVTPVKEFMRTAAKDTVVRGVPIAAGESVLLSYVSANRDEDVFDEPFRFDVGRDPNKHLAFGYGVHFCMGAAL
ARMEVNSFFTELLPRLKSIELTGDPELVATTFVGGLKHLPVRYSLA
>CYP108B5_Mycobacterium avium hominissuis MAH-P-4023-08 2725140913
VSTTTMDEAAKLLADPMAYTDEQRLHAALTHLRANAPVSWVEVPNYKPFWAITKHADVMDIERENMLFTNWPR
PVLTTAEGDEMQAAAGVRTLIHMDDPQHRVVRAIGSDWFRPKAMRALKVRVDELAKIYVDKMLAAGPECDFVQ
EVAVNYPLYVIMSLLGLPEADFPRMLKLTQELFGSDDSEFKRGSSNEDQLPALLDMFGYFNGVTAARREHPTE
DLASAIANARVDGEPLSDIDTVSYYLIVATAGHDTTSATISGGLQALIENPDQLQRLRDNLDLMPLATEEMIR
WVTPVKEFMRTAAKDTVVRGVPIAAGESVLLSYVSANRDEDVFDEPFRFDVGRDPNKHLAFGYGVHFCMGAAL
ARMEVNSFFTELLPRLKSIELTGDPELVATTFVGGLKHLPVRYSLA
>CYP108B5_Mycobacterium avium hominissuis MAH-P-3449-08 2725135793
VSTTTMDEAAKLLADPMAYTDEQRLHAALTHLRANAPVSWVEVPNYKPFWAITKHADVMDIERENMLFTNWPR
PVLTTAEGDEMQAAAGVRTLIHMDDPQHRVVRAIGSDWFRPKAMRALKVRVDELAKIYVDKMLAAGPECDFVQ
EVAVNYPLYVIMSLLGLPEADFPRMLKLTQELFGSDDSEFKRGSSNEDQLPALLDMFGYFNGVTAARREHPTE
DLASAIANARVDGEPLSDIDTVSYYLIVATAGHDTTSATISGGLQALIENPDQLQRLRDNLDLMPLATEEMIR
WVTPVKEFMRTAAKDTVVRGVPIAAGESVLLSYVSANRDEDVFDEPFRFDVGRDPNKHLAFGYGVHFCMGAAL
ARMEVNSFFTELLPRLKSIELTGDPELVATTFVGGLKHLPVRYSLA
>CYP108B5_Mycobacterium avium hominissuis MAH-P-2014-08 2725135417
VSTTTMDEAAKLLADPMAYTDEQRLHAALTHLRANAPVSWVEVPNYKPFWAITKHADVMDIERENMLFTNWPR
PVLTTAEGDEMQAAAGVRTLIHMDDPQHRVVRAIGSDWFRPKAMRALKVRVDELAKIYVDKMLAAGPECDFVQ
EVAVNYPLYVIMSLLGLPEADFPRMLKLTQELFGSDDSEFKRGSSNEDQLPALLDMFGYFNGVTAARREHPTE
DLASAIANARVDGEPLSDIDTVSYYLIVATAGHDTTSATISGGLQALIENPDQLQRLRDNLDLMPLATEEMIR
WVTPVKEFMRTAAKDTVVRGVPIAAGESVLLSYVSANRDEDVFDEPFRFDVGRDPNKHLAFGYGVHFCMGAAL
ARMEVNSFFTELLPRLKSIELTGDPELVATTFVGGLKHLPVRYSLA
>CYP108B5_Mycobacterium avium hominissuis MAH-P-11082-03 2725058817
VSTTTMDEAAKLLADPMAYTDEQRLHAALTHLRANAPVSWVEVPNYKPFWAITKHADVMDIERENMLFTNWPR
PVLTTAEGDEMQAAAGVRTLIHMDDPQHRVVRAIGSDWFRPKAMRALKVRVDELAKIYVDKMLAAGPECDFVQ
EVAVNYPLYVIMSLLGLPEADFPRMLKLTQELFGSDDSEFKRGSSNEDQLPALLDMFGYFNGVTAARREHPTE
DLASAIANARVDGEPLSDIDTVSYYLIVATAGHDTTSATISGGLQALIENPDQLQRLRDNLDLMPLATEEMIR
WVTPVKEFMRTAAKDTVVRGVPIAAGESVLLSYVSANRDEDVFDEPFRFDVGRDPNKHLAFGYGVHFCMGAAL
ARMEVNSFFTELLPRLKSIELTGDPELVATTFVGGLKHLPVRYSLA
>CYP108B5_Mycobacterium avium hominissuis MAH-P-10203-06 2678749472
VSTTTMDEAAKLLADPMAYTDEQRLHAALTHLRANAPVSWVEVPNYKPFWAITKHADVMDIERENMLFTNWPR
PVLTTAEGDEMQAAAGVRTLIHMDDPQHRVVRAIGSDWFRPKAMRALKVRVDELAKIYVDKMLAAGPECDFVQ
EVAVNYPLYVIMSLLGLPEADFPRMLKLTQELFGSDDSEFKRGSSNEDQLPALLDMFGYFNGVTAARREHPTE
DLASAIANARVDGEPLSDIDTVSYYLIVATAGHDTTSATISGGLQALIENPDQLQRLRDNLDLMPLATEEMIR
WVTPVKEFMRTAAKDTVVRGVPIAAGESVLLSYVSANRDEDVFDEPFRFDVGRDPNKHLAFGYGVHFCMGAAL
ARMEVNSFFTELLPRLKSIELTGDPELVATTFVGGLKHLPVRYSLA
>CYP108B5_Mycobacterium avium hominissuis MAH-P-10091-06 2678124232

VSTTTMDEAAKLLADPMAYTDEQRLHAALTHLRANAPVSWVEVPNYKPFWAITKHADVMDIERENMLFTNWPR
PVLTTAEGDEMQAAAGVRTLIHMDDPQHRVVRAIGSDWFRPKAMRALKVRVDELAKIYVDKMLAAGPECDFVQ
EVAVNYPLYVIMSLLGLPEADFPRMLKLTQELFGSDDSEFKRGSSNEDQLPALLDMFGYFNGVTAARREHPTE
DLASAIANARVDGEPLSDIDTVSYYLIVATAGHDTTSATISGGLQALIENPDQLQRLRDNLDLMPLATEEMIR
WVTPVKEFMRTAAKDTVVRGVPIAAGESVLLSYVSANRDEDVFDEPFRFDVGRDPNKHLAFGYGVHFCMGAAL
ARMEVNSFFTELLPRLKSIELTGDPELVATTFVGGLKHLPVRYSLA
>CYP108B5_Mycobacterium avium hominissuis MAHP-10058-06 2678801070
VSTTTMDEAAKLLADPMAYTDEQRLHAALTHLRANAPVSWVEVPNYKPFWAITKHADVMDIERENMLFTNWPR
PVLTTAEGDEMQAAAGVRTLIHMDDPQHRVVRAIGSDWFRPKAMRALKVRVDELAKIYVDKMLAAGPECDFVQ
EVAVNYPLYVIMSLLGLPEADFPRMLKLTQELFGSDDSEFKRGSSNEDQLPALLDMFGYFNGVTAARREHPTE
DLASAIANARVDGEPLSDIDTVSYYLIVATAGHDTTSATISGGLQALIENPDQLQRLRDNLDLMPLATEEMIR
WVTPVKEFMRTAAKDTVVRGVPIAAGESVLLSYVSANRDEDVFDEPFRFDVGRDPNKHLAFGYGVHFCMGAAL
ARMEVNSFFTELLPRLKSIELTGDPELVATTFVGGLKHLPVRYSLA
>CYP108B5_Mycobacterium avium hominissuis MAH-P-0913 2725076779
VSTTTMDEAAKLLADPMAYTDEQRLHAALTHLRANAPVSWVEVPNYKPFWAITKHADVMDIERENMLFTNWPR
PVLTTAEGDEMQAAAGVRTLIHMDDPQHRVVRAIGSDWFRPKAMRALKVRVDELAKIYVDKMLAAGPECDFVQ
EVAVNYPLYVIMSLLGLPEADFPRMLKLTQELFGSDDSEFKRGSSNEDQLPALLDMFGYFNGVTAARREHPTE
DLASAIANARVDGEPLSDIDTVSYYLIVATAGHDTTSATISGGLQALIENPDQLQRLRDNLDLMPLATEEMIR
WVTPVKEFMRTAAKDTVVRGVPIAAGESVLLSYVSANRDEDVFDEPFRFDVGRDPNKHLAFGYGVHFCMGAAL
ARMEVNSFFTELLPRLKSIELTGDPELVATTFVGGLKHLPVRYSLA
>CYP108B5_Mycobacterium avium hominissuis MAHP-04-13 2725070388
VSTTTMDEAAKLLADPMAYTDEQRLHAALTHLRANAPVSWVEVPNYKPFWAITKHADVMDIERENMLFTNWPR
PVLTTAEGDEMQAAAGVRTLIHMDDPQHRVVRAIGSDWFRPKAMRALKVRVDELAKIYVDKMLAAGPECDFVQ
EVAVNYPLYVIMSLLGLPEADFPRMLKLTQELFGSDDSEFKRGSSNEDQLPALLDMFGYFNGVTAARREHPTE
DLASAIANARVDGEPLSDIDTVSYYLIVATAGHDTTSATISGGLQALIENPDQLQRLRDNLDLMPLATEEMIR
WVTPVKEFMRTAAKDTVVRGVPIAAGESVLLSYVSANRDEDVFDEPFRFDVGRDPNKHLAFGYGVHFCMGAAL
ARMEVNSFFTELLPRLKSIELTGDPELVATTFVGGLKHLPVRYSLA
>CYP108B5_Mycobacterium avium hominissuis MAH-E-96-2 2724975341
VSTTTMDEAAKLLADPMAYTDEQRLHAALTHLRANAPVSWVEVPNYKPFWAITKHADVMDIERENMLFTNWPR
PVLTTAEGDEMQAAAGVRTLIHMDDPQHRVVRAIGSDWFRPKAMRALKVRVDELAKIYVDKMLAAGPECDFVQ
EVAVNYPLYVIMSLLGLPEADFPRMLKLTQELFGSDDSEFKRGSSNEDQLPALLDMFGYFNGVTAARREHPTE
DLASAIANARVDGEPLSDIDTVSYYLIVATAGHDTTSATISGGLQALIENPDQLQRLRDNLDLMPLATEEMIR
WVTPVKEFMRTAAKDTVVRGVPIAAGESVLLSYVSANRDEDVFDEPFRFDVGRDPNKHLAFGYGVHFCMGAAL
ARMEVNSFFTELLPRLKSIELTGDPELVATTFVGGLKHLPVRYSLA
>CYP108B5_Mycobacterium avium hominissuis MAH-E-89-1 2724954665
VSTTTMDEAAKLLADPMAYTDEQRLHAALTHLRANAPVSWVEVPNYKPFWAITKHADVMDIERENMLFTNWPR
PVLTTAEGDEMQAAAGVRTLIHMDDPQHRVVRAIGSDWFRPKAMRALKVRVDELAKIYVDKMLAAGPECDFVQ
EVAVNYPLYVIMSLLGLPEADFPRMLKLTQELFGSDDSEFKRGSSNEDQLPALLDMFGYFNGVTAARREHPTE
DLASAIANARVDGEPLSDIDTVSYYLIVATAGHDTTSATISGGLQALIENPDQLQRLRDNLDLMPLATEEMIR
WVTPVKEFMRTAAKDTVVRGVPIAAGESVLLSYVSANRDEDVFDEPFRFDVGRDPNKHLAFGYGVHFCMGAAL
ARMEVNSFFTELLPRLKSIELTGDPELVATTFVGGLKHLPVRYSLA
>CYP108B5_Mycobacterium avium hominissuis MAH-E-82-7 2725029619
VSTTTMDEAAKLLADPMAYTDEQRLHAALTHLRANAPVSWVEVPNYKPFWAITKHADVMDIERENMLFTNWPR
PVLTTAEGDEMQAAAGVRTLIHMDDPQHRVVRAIGSDWFRPKAMRALKVRVDELAKIYVDKMLAAGPECDFVQ
EVAVNYPLYVIMSLLGLPEADFPRMLKLTQELFGSDDSEFKRGSSNEDQLPALLDMFGYFNGVTAARREHPTE
DLASAIANARVDGEPLSDIDTVSYYLIVATAGHDTTSATISGGLQALIENPDQLQRLRDNLDLMPLATEEMIR
WVTPVKEFMRTAAKDTVVRGVPIAAGESVLLSYVSANRDEDVFDEPFRFDVGRDPNKHLAFGYGVHFCMGAAL
ARMEVNSFFTELLPRLKSIELTGDPELVATTFVGGLKHLPVRYSLA
>CYP108B5_Mycobacterium avium hominissuis MAH-E-88-1 2724948979
VSTTTMDEAAKLLADPMAYTDEQRLHAALTHLRANAPVSWVEVPNYKPFWAITKHADVMDIERENMLFTNWPR
PVLTTAEGDEMQAAAGVRTLIHMDDPQHRVVRAIGSDWFRPKAMRALKVRVDELAKIYVDKMLAAGPECDFVQ
EVAVNYPLYVIMSLLGLPEADFPRMLKLTQELFGSDDSEFKRGSSNEDQLPALLDMFGYFNGVTAARREHPTE
DLASAIANARVDGEPLSDIDTVSYYLIVATAGHDTTSATISGGLQALIENPDQLQRLRDNLDLMPLATEEMIR
WVTPVKEFMRTAAKDTVVRGVPIAAGESVLLSYVSANRDEDVFDEPFRFDVGRDPNKHLAFGYGVHFCMGAAL
ARMEVNSFFTELLPRLKSIELTGDPELVATTFVGGLKHLPVRYSLA
>CYP108B5_Mycobacterium avium hominissuis MAH-E-83-1 2725032962
VSTTTMDEAAKLLADPMAYTDEQRLHAALTHLRANAPVSWVEVPNYKPFWAITKHADVMDIERENMLFTNWPR
PVLTTAEGDEMQAAAGVRTLIHMDDPQHRVVRAIGSDWFRPKAMRALKVRVDELAKIYVDKMLAAGPECDFVQ

EVAVNYPLYVIMSLLGLPEADFPRMLKLTQELFGSDDSEFKRGSSNEDQLPALLDMFGYFNGVTAARREHPTE
DLASAIANARVDGEPLSDIDTVSYYLIVATAGHDTTSATISGGLQALIENPDQLQRLRDNLDLMPLATEEMIR
WVTPVKEFMRTAAKDTVVRGVPIAAGESVLLSYVSANRDEDVFDEPFRFDVGRDPNKHLAFGYGVHFCMGAAL
ARMEVNSFFTELLPRLKSIELTGDPELVATTFVGGLKHLPVRYSLA
>CYP108B5_Mycobacterium avium hominissuis MAH-E-63-1 2724969881
VSTTTMDEAAKLLADPMAYTDEQRLHAALTHLRANAPVSWVEVPNYKPFWAITKHADVMDIERENMLFTNWPR
PVLTTAEGDEMQAAAGVRTLIHMDDPQHRVVRAIGSDWFRPKAMRALKVRVDELAKIYVDKMLAAGPECDFVQ
EVAVNYPLYVIMSLLGLPEADFPRMLKLTQELFGSDDSEFKRGSSNEDQLPALLDMFGYFNGVTAARREHPTE
DLASAIANARVDGEPLSDIDTVSYYLIVATAGHDTTSATISGGLQALIENPDQLQRLRDNLDLMPLATEEMIR
WVTPVKEFMRTAAKDTVVRGVPIAAGESVLLSYVSANRDEDVFDEPFRFDVGRDPNKHLAFGYGVHFCMGAAL
ARMEVNSFFTELLPRLKSIELTGDPELVATTFVGGLKHLPVRYSLA
>CYP108B5_Mycobacterium avium hominissuis MAH-E-61-1y 2725042883
VSTTTMDEAAKLLADPMAYTDEQRLHAALTHLRANAPVSWVEVPNYKPFWAITKHADVMDIERENMLFTNWPR
PVLTTAEGDEMQAAAGVRTLIHMDDPQHRVVRAIGSDWFRPKAMRALKVRVDELAKIYVDKMLAAGPECDFVQ
EVAVNYPLYVIMSLLGLPEADFPRMLKLTQELFGSDDSEFKRGSSNEDQLPALLDMFGYFNGVTAARREHPTE
DLASAIANARVDGEPLSDIDTVSYYLIVATAGHDTTSATISGGLQALIENPDQLQRLRDNLDLMPLATEEMIR
WVTPVKEFMRTAAKDTVVRGVPIAAGESVLLSYVSANRDEDVFDEPFRFDVGRDPNKHLAFGYGVHFCMGAAL
ARMEVNSFFTELLPRLKSIELTGDPELVATTFVGGLKHLPVRYSLA
>CYP108B5_Mycobacterium avium hominissuis MAH-E-61-1t 2725037807
VSTTTMDEAAKLLADPMAYTDEQRLHAALTHLRANAPVSWVEVPNYKPFWAITKHADVMDIERENMLFTNWPR
PVLTTAEGDEMQAAAGVRTLIHMDDPQHRVVRAIGSDWFRPKAMRALKVRVDELAKIYVDKMLAAGPECDFVQ
EVAVNYPLYVIMSLLGLPEADFPRMLKLTQELFGSDDSEFKRGSSNEDQLPALLDMFGYFNGVTAARREHPTE
DLASAIANARVDGEPLSDIDTVSYYLIVATAGHDTTSATISGGLQALIENPDQLQRLRDNLDLMPLATEEMIR
WVTPVKEFMRTAAKDTVVRGVPIAAGESVLLSYVSANRDEDVFDEPFRFDVGRDPNKHLAFGYGVHFCMGAAL
ARMEVNSFFTELLPRLKSIELTGDPELVATTFVGGLKHLPVRYSLA
>CYP108B5_Mycobacterium avium hominissuis MAH-E-57-3 2724967904
VSTTTMDEAAKLLADPMAYTDEQRLHAALTHLRANAPVSWVEVPNYKPFWAITKHADVMDIERENMLFTNWPR
PVLTTAEGDEMQAAAGVRTLIHMDDPQHRVVRAIGSDWFRPKAMRALKVRVDELAKIYVDKMLAAGPECDFVQ
EVAVNYPLYVIMSLLGLPEADFPRMLKLTQELFGSDDSEFKRGSSNEDQLPALLDMFGYFNGVTAARREHPTE
DLASAIANARVDGEPLSDIDTVSYYLIVATAGHDTTSATISGGLQALIENPDQLQRLRDNLDLMPLATEEMIR
WVTPVKEFMRTAAKDTVVRGVPIAAGESVLLSYVSANRDEDVFDEPFRFDVGRDPNKHLAFGYGVHFCMGAAL
ARMEVNSFFTELLPRLKSIELTGDPELVATTFVGGLKHLPVRYSLA
>CYP108B5_Mycobacterium avium hominissuis MAH-E-3044 2725109210
VSTTTMDEAAKLLADPMAYTDEQRLHAALTHLRANAPVSWVEVPNYKPFWAITKHADVMDIERENMLFTNWPR
PVLTTAEGDEMQAAAGVRTLIHMDDPQHRVVRAIGSDWFRPKAMRALKVRVDELAKIYVDKMLAAGPECDFVQ
EVAVNYPLYVIMSLLGLPEADFPRMLKLTQELFGSDDSEFKRGSSNEDQLPALLDMFGYFNGVTAARREHPTE
DLASAIANARVDGEPLSDIDTVSYYLIVATAGHDTTSATISGGLQALIENPDQLQRLRDNLDLMPLATEEMIR
WVTPVKEFMRTAAKDTVVRGVPIAAGESVLLSYVSANRDEDVFDEPFRFDVGRDPNKHLAFGYGVHFCMGAAL
ARMEVNSFFTELLPRLKSIELTGDPELVATTFVGGLKHLPVRYSLA
>CYP108B5_Mycobacterium avium hominissuis MAH-E-2514 2725106023
VSTTTMDEAAKLLADPMAYTDEQRLHAALTHLRANAPVSWVEVPNYKPFWAITKHADVMDIERENMLFTNWPR
PVLTTAEGDEMQAAAGVRTLIHMDDPQHRVVRAIGSDWFRPKAMRALKVRVDELAKIYVDKMLAAGPECDFVQ
EVAVNYPLYVIMSLLGLPEADFPRMLKLTQELFGSDDSEFKRGSSNEDQLPALLDMFGYFNGVTAARREHPTE
DLASAIANARVDGEPLSDIDTVSYYLIVATAGHDTTSATISGGLQALIENPDQLQRLRDNLDLMPLATEEMIR
WVTPVKEFMRTAAKDTVVRGVPIAAGESVLLSYVSANRDEDVFDEPFRFDVGRDPNKHLAFGYGVHFCMGAAL
ARMEVNSFFTELLPRLKSIELTGDPELVATTFVGGLKHLPVRYSLA
>CYP108B5_Mycobacterium avium hominissuis MAHE-22-1 2724959625
VSTTTMDEAAKLLADPMAYTDEQRLHAALTHLRANAPVSWVEVPNYKPFWAITKHADVMDIERENMLFTNWPR
PVLTTAEGDEMQAAAGVRTLIHMDDPQHRVVRAIGSDWFRPKAMRALKVRVDELAKIYVDKMLAAGPECDFVQ
EVAVNYPLYVIMSLLGLPEADFPRMLKLTQELFGSDDSEFKRGSSNEDQLPALLDMFGYFNGVTAARREHPTE
DLASAIANARVDGEPLSDIDTVSYYLIVATAGHDTTSATISGGLQALIENPDQLQRLRDNLDLMPLATEEMIR
WVTPVKEFMRTAAKDTVVRGVPIAAGESVLLSYVSANRDEDVFDEPFRFDVGRDPNKHLAFGYGVHFCMGAAL
ARMEVNSFFTELLPRLKSIELTGDPELVATTFVGGLKHLPVRYSLA
>CYP108B5_Mycobacterium avium hominissuis MAH-E-149-7 2725065570
VSTTTMDEAAKLLADPMAYTDEQRLHAALTHLRANAPVSWVEVPNYKPFWAITKHADVMDIERENMLFTNWPR
PVLTTAEGDEMQAAAGVRTLIHMDDPQHRVVRAIGSDWFRPKAMRALKVRVDELAKIYVDKMLAAGPECDFVQ
EVAVNYPLYVIMSLLGLPEADFPRMLKLTQELFGSDDSEFKRGSSNEDQLPALLDMFGYFNGVTAARREHPTE
DLASAIANARVDGEPLSDIDTVSYYLIVATAGHDTTSATISGGLQALIENPDQLQRLRDNLDLMPLATEEMIR

WVTPVKEFMRTAAKDTVVRGVPIAAGESVLLSYVSANRDEDVFDEPFRFDVGRDPNKHLAFGYGVHFCMGAAL
ARMEVNSFFTELLPRLKSIELTGDPELVATTFVGGLKHLPVRYSLA
>CYP108B5_Mycobacterium avium hominissuis MAH-E-149-3 2725060756
VSTTTMDEAAKLLADPMAYTDEQRLHAALTHLRANAPVSWVEVPNYKPFWAITKHADVMDIERENMLFTNWPR
PVLTTAEGDEMQAAAGVRTLIHMDDPQHRVVRAIGSDWFRPKAMRALKVRVDELAKIYVDKMLAAGPECDFVQ
EVAVNYPLYVIMSLLGLPEADFPRMLKLTQELFGSDDSEFKRGSSNEDQLPALLDMFGYFNGVTAARREHPTE
DLASAIANARVDGEPLSDIDTVSYYLIVATAGHDTTSATISGGLQALIENPDQLQRLRDNLDLMPLATEEMIR
WVTPVKEFMRTAAKDTVVRGVPIAAGESVLLSYVSANRDEDVFDEPFRFDVGRDPNKHLAFGYGVHFCMGAAL
ARMEVNSFFTELLPRLKSIELTGDPELVATTFVGGLKHLPVRYSLA
>CYP108B5_Mycobacterium avium hominissuis MAH-E-149-2 2725047046
VSTTTMDEAAKLLADPMAYTDEQRLHAALTHLRANAPVSWVEVPNYKPFWAITKHADVMDIERENMLFTNWPR
PVLTTAEGDEMQAAAGVRTLIHMDDPQHRVVRAIGSDWFRPKAMRALKVRVDELAKIYVDKMLAAGPECDFVQ
EVAVNYPLYVIMSLLGLPEADFPRMLKLTQELFGSDDSEFKRGSSNEDQLPALLDMFGYFNGVTAARREHPTE
DLASAIANARVDGEPLSDIDTVSYYLIVATAGHDTTSATISGGLQALIENPDQLQRLRDNLDLMPLATEEMIR
WVTPVKEFMRTAAKDTVVRGVPIAAGESVLLSYVSANRDEDVFDEPFRFDVGRDPNKHLAFGYGVHFCMGAAL
ARMEVNSFFTELLPRLKSIELTGDPELVATTFVGGLKHLPVRYSLA
>CYP108B5_Mycobacterium avium hominissuis MAH-E-118 2678563431
VSTTTMDEAAKLLADPMAYTDEQRLHAALTHLRANAPVSWVEVPNYKPFWAITKHADVMDIERENMLFTNWPR
PVLTTAEGDEMQAAAGVRTLIHMDDPQHRVVRAIGSDWFRPKAMRALKVRVDELAKIYVDKMLAAGPECDFVQ
EVAVNYPLYVIMSLLGLPEADFPRMLKLTQELFGSDDSEFKRGSSNEDQLPALLDMFGYFNGVTAARREHPTE
DLASAIANARVDGEPLSDIDTVSYYLIVATAGHDTTSATISGGLQALIENPDQLQRLRDNLDLMPLATEEMIR
WVTPVKEFMRTAAKDTVVRGVPIAAGESVLLSYVSANRDEDVFDEPFRFDVGRDPNKHLAFGYGVHFCMGAAL
ARMEVNSFFTELLPRLKSIELTGDPELVATTFVGGLKHLPVRYSLA
>CYP108B5_Mycobacterium avium hominissuis MAH-E-108 2724988683
VSTTTMDEAAKLLADPMAYTDEQRLHAALTHLRANAPVSWVEVPNYKPFWAITKHADVMDIERENMLFTNWPR
PVLTTAEGDEMQAAAGVRTLIHMDDPQHRVVRAIGSDWFRPKAMRALKVRVDELAKIYVDKMLAAGPECDFVQ
EVAVNYPLYVIMSLLGLPEADFPRMLKLTQELFGSDDSEFKRGSSNEDQLPALLDMFGYFNGVTAARREHPTE
DLASAIANARVDGEPLSDIDTVSYYLIVATAGHDTTSATISGGLQALIENPDQLQRLRDNLDLMPLATEEMIR
WVTPVKEFMRTAAKDTVVRGVPIAAGESVLLSYVSANRDEDVFDEPFRFDVGRDPNKHLAFGYGVHFCMGAAL
ARMEVNSFFTELLPRLKSIELTGDPELVATTFVGGLKHLPVRYSLA
>CYP108B5_Mycobacterium avium hominissuis MAH-E-106 2678352236
VSTTTMDEAAKLLADPMAYTDEQRLHAALTHLRANAPVSWVEVPNYKPFWAITKHADVMDIERENMLFTNWPR
PVLTTAEGDEMQAAAGVRTLIHMDDPQHRVVRAIGSDWFRPKAMRALKVRVDELAKIYVDKMLAAGPECDFVQ
EVAVNYPLYVIMSLLGLPEADFPRMLKLTQELFGSDDSEFKRGSSNEDQLPALLDMFGYFNGVTAARREHPTE
DLASAIANARVDGEPLSDIDTVSYYLIVATAGHDTTSATISGGLQALIENPDQLQRLRDNLDLMPLATEEMIR
WVTPVKEFMRTAAKDTVVRGVPIAAGESVLLSYVSANRDEDVFDEPFRFDVGRDPNKHLAFGYGVHFCMGAAL
ARMEVNSFFTELLPRLKSIELTGDPELVATTFVGGLKHLPVRYSLA
>CYP108B5_Mycobacterium avium hominissuis MAH-E-104-1 2724984667
VSTTTMDEAAKLLADPMAYTDEQRLHAALTHLRANAPVSWVEVPNYKPFWAITKHADVMDIERENMLFTNWPR
PVLTTAEGDEMQAAAGVRTLIHMDDPQHRVVRAIGSDWFRPKAMRALKVRVDELAKIYVDKMLAAGPECDFVQ
EVAVNYPLYVIMSLLGLPEADFPRMLKLTQELFGSDDSEFKRGSSNEDQLPALLDMFGYFNGVTAARREHPTE
DLASAIANARVDGEPLSDIDTVSYYLIVATAGHDTTSATISGGLQALIENPDQLQRLRDNLDLMPLATEEMIR
WVTPVKEFMRTAAKDTVVRGVPIAAGESVLLSYVSANRDEDVFDEPFRFDVGRDPNKHLAFGYGVHFCMGAAL
ARMEVNSFFTELLPRLKSIELTGDPELVATTFVGGLKHLPVRYSLA
>CYP108B5_Mycobacterium avium hominissuis MAH-E-101-6 2724979529
VSTTTMDEAAKLLADPMAYTDEQRLHAALTHLRANAPVSWVEVPNYKPFWAITKHADVMDIERENMLFTNWPR
PVLTTAEGDEMQAAAGVRTLIHMDDPQHRVVRAIGSDWFRPKAMRALKVRVDELAKIYVDKMLAAGPECDFVQ
EVAVNYPLYVIMSLLGLPEADFPRMLKLTQELFGSDDSEFKRGSSNEDQLPALLDMFGYFNGVTAARREHPTE
DLASAIANARVDGEPLSDIDTVSYYLIVATAGHDTTSATISGGLQALIENPDQLQRLRDNLDLMPLATEEMIR
WVTPVKEFMRTAAKDTVVRGVPIAAGESVLLSYVSANRDEDVFDEPFRFDVGRDPNKHLAFGYGVHFCMGAAL
ARMEVNSFFTELLPRLKSIELTGDPELVATTFVGGLKHLPVRYSLA
>CYP108B5_Mycobacterium avium hominissuis 10-4249 2580524461
MSTTTMDEAAKLLADPMAYTDEQRLHAALTHLRANAPVSWVEVPNYKPFWAITKHADVMDIERENMLFTNWPR
PVLTTAEGDEMQAAAGVRTLIHMDDPQHRVVRAIGSDWFRPKAMRALKVRVDELAKIYVDKMLAVGPECDFVQ
EVAVNYPLYVIMSLLGLPEADFPRMLKLTQELFGSDDSEFKRGSSNEDQLPALLDMFGYFNGVTAARREHPTE
DLASAIANARVDGEPLSDIDTVSYYLIVATAGHDTTSATISGGLQALIENPDQLQRLRDNLDLMPLATEEMIR
WVTPVKEFMRTAAKDTVVRGVPIAAGESVLLSYVSANRDEDVFDEPFRFDVGRDPNKHLAFGYGVHFCMGAAL
ARMEVNSFFTELLPRLKSIELTGDPELVATTFVGGLKHLPVRYSLA

>CYP108B5_Mycobacterium avium avium DT 78 2549384609
VSTTTMDEAAKLLADPMAYTDEQRLHAALTHLRANAPVSWVEVPNYKPFWAITKHADVMDIERENMLFTNWPR
PVLTTAEGDEMQAAAGVRTLIHMDDPQHRVVRAIGSDWFRPKAMRALKVRVDELAKIYVDKMLAAGPECDFVQ
EVAVNYPLYVIMSLLGLPEADFPRMLKLTQELFGSDDSEFKRGSSNEDQLPALLDMFGYFNGVTAARREHPTE
DLASAIANARVDGEPLSDIDTVSYYLIVATAGHDTTSATISGGLQALIENPDQLQRLRDNLDLMPLATEEMIR
WVTPVKEFMRTAAKDTVVRGVPIAAGESVLLSYVSANRDEDVFDEPFRFDVGRDPNKHLAFGYGVHFCMGAAL
ARMEVNSFFTELLPRLKSIELTGDPELVATTFVGGLKHLPVRYSLA
>CYP108B5_Mycobacterium avium avium A5 2580782719
MDEAAKLLADPMAYTDEQRLHAALTHLRANAPVSWVEVPNYKPFWAITKHADVMDIERENMLFTNWPRPVLTT
AEGDEMQAAAGVRTLIHMDDPQHRVVRAIGSDWFRPKAMRALKVRVDELAKIYVDKMLAVGPECDFVQEVAVN
YPLYVIMSLLGLPEADFPRMLKLTQELFGSDDSEFKRGSSNEDQLPALLDMFGYFNGVTAARREHPTEDLASA
IANARVDGEPLSDIDTVSYYLIVATAGHDTTSATISGGLQALIENPDQLQRLRDNLDLMPLATEEMIRWVTPV
KEFMRTAAKDTVVRGVPIAAGESVLLSYVSANRDEDVFDEPFRFDVGRDPNKHLAFGYGVHFCMGAALARMEV
NSFFTELLPRLKSIELTGDPELVATTFVGGLKHLPVRYSLA
>CYP108B5_Mycobacterium avium 2285 (R) 2629822793
VSTTTMDEAAKLLADPMAYTDEQRLHAALTHLRANAPVSWVEVPNYKPFWAITKHADVMDIERENMLFTNWPR
PVLTTAEGDEMQAAAGVRTLIHMDDPQHRVVRAIGSDWFRPKAMRALKVRVDELAKIYVDKMLAAGPECDFVQ
EVAVNYPLYVIMSLLGLPEADFPRMLKLTQELFGSDDSEFKRGSSNEDQLPALLDMFGYFNGVTAARREHPTE
DLASAIANARVDGEPLSDIDTVSYYLIVATAGHDTTSATISGGLQALIENPDQLQRLRDNLDLMPLATEEMIR
WVTPVKEFMRTAAKDTVVRGVPIAAGESVLLSYVSANRDEDVFDEPFCFDVGRDPNKHLAFGYGVHFCMGAAL
ARMEVNSFFTELLPRLKSIELTGDPELVATTFVGGLKHLPVRYSLA
>CYP108B5_Mycobacterium avium 2285 (S) 2647943992
VSTTTMDEAAKLLADPMAYTDEQRLHAALTHLRANAPVSWVEVPNYKPFWAITKHADVMDIERENMLFTNWPR
PVLTTAEGDEMQAAAGVRTLIHMDDPQHRVVRAIGSDWFRPKAMRALKVRVDELAKIYVDKMLAAGPECDFVQ
EVAVNYPLYVIMSLLGLPEADFPRMLKLTQELFGSDDSEFKRGSSNEDQLPALLDMFGYFNGVTAARREHPTE
DLASAIANARVDGEPLSDIDTVSYYLIVATAGHDTTSATISGGLQALIENPDQLQRLRDNLDLMPLATEEMIR
WVTPVKEFMRTAAKDTVVRGVPIAAGESVLLSYVSANRDEDVFDEPFCFDVGRDPNKHLAFGYGVHFCMGAAL
ARMEVNSFFTELLPRLKSIELTGDPELVATTFVGGLKHLPVRYSLA
>CYP108B5_Mycobacterium avium avium DJO-44271 2628525636
VSTTTMDEAAKLLADPMAYTDEQRLHAALTHLRANAPVSWVEVPNYKPFWAITKHADVMDIERENMLFTNWPR
PVLTTAEGDEMQAAAGVRTLIHMDDPQHRVVRAIGSDWFRPKAMRALKVRVDELAKIYVDKMLAAGPECDFVQ
EVAVNYPLYVIMSLLGLPEADFPRMLKLTQELFGSDDSEFKRGSSNEDQLPALLDMFGYFNGVTAARREHPTE
DLASAIANARVDGEPLSDIDTVSYYLIVATAGHDTTSATISGGLQALIENPDQLQRLRDNLDLMPLATEEMIR
WVTPVKEFMRTAAKDTVVRGVPIAAGESVLLSYVSANRDEDVFDEPFCFDVGRDPNKHLAFGYGVHFCMGAAL
ARMEVNSFFTELLPRLKSIELTGDPELVATTFVGGLKHLPVRYSLA
>CYP108B5_Mycobacterium avium hominissuis MAH 2721 2573740805
VSTTTMDEAAKLLADPMAYTDEQRLHAALTHLRANAPVSWVEVPNYKPFWAITKHADVMDIERENMLFTNWPR
PVLTTAEGDEMQAAAGVRTLIHMDDPQHRVVRAIGSDWFRPKAMRA
>CYP108B5_Mycobacterium tuberculosis XTB13-223 2606413725
VSTTTMDEAAKLLADPMAYTDEQRLHAALTHLRANAPVSWVEVPNYKPFWAITKHADVMDIERENMLFTNWPR
PVLTTAEGDEMQAAAGVRTLIHMDDPQHRVVRAIGSDWFRPKAMRALKVRVDELAKIYVDKMLAAGPECDFVQ
EVAVNYPLYVIMSLLGLPEADFPRMLKLTQELFGSDDSEFKRGSSNEDQLPALLDMFGYFNGVTAARREHPTE
DLASAIANARVDGEPLSDIDTVSYYLIVATAGHDTTSATISGGLQALIENPDQLQRLRDNLDLMPLATEEMIR
WVTPVKEFMRTAAKDTVVRGVPIAAGESVLLSYVSANRDEDVFDEPFRFDVGRDPNKHLAFGYGVHFCMGAAL
ARMEVNSFFTELLPRLKSIELTGDPELVATTFVGGLKHLPVRYSLA
>CYP108B7_Mycobacterium austroafricanum DSM 44191 2649758297
MSVRIADEAAGVFADPSAYADEARLHAAMTHLRAHAPVSWVEVPGYNPFWAIAKHADIMAVERDNLVFTNSPR
PVLTTAEGDAQHEAMGISTLIHLDDPQHRKVRAIGADWFRPKAMRALKVRVDELARTFVDQMYERGGECDFVQ
EVAVNFPLYVIMSLLGIPESDFQRMLTYTQELFGNDDAELQRGESMEERGLALFDMFTYFNEITSARRAQPTE
DLASAIANARIDGAPLSDIDTVSYYLIVATAGHDTTSATISGGLQALIENPDQLQRLQQNPGLMPLAVEEMIR
WVTPVKEFMRTAQQDAEVRGVKIAAGESVLLSYPSGNRDEDVFTDPFRFDVGRDPNKHVAFGYGVHFCLGAAL
ARMEINSFFTELLPRLKSVELAGRPEHIATIFVGGLKHLPIRYSLTR
>CYP108N1_Mycobacterium rhodesiae JS60 2507061625
MTTTQQADTAILPEEIARRIVLPEGHADLAALHEAYKWMRHNMPVGKAVVAGYDPLWLVSKYADIQEVEGQPE
IFSAGGGAKPGSHNPILTNQAGDEFTKQLLGGSLRLMDALPFLDPPEHTTVKNIAFDWFKPIQLKKWEDQIRA
LANESIDKLKGVSAGDKQIDLVDDWALGFPLHVVMTLFGVPAEDEPRMMALTQEFFGTADPEHQRDDVEPLSP
EAMAQQFANTVQDFYGYFDTLIEDRRANPRDDLATLISVATTPDGDYWPTAYAYGWFMAICTAGHDTTSATLA
GTLQQLALHPDVLARIRADMTLVPNLIQEGLRYVAPVKHFMRRADADYELNGQLIKAGDRVMPLFQSACRDED

LFTDPDDFNIDRKPNNHMAFGFGGHTCVGQHLAKLELRVMFEQLLPRLDAIEVLGPGSVTHTNFVGGLKHLPA
RVTVV
>CYP108N2_Mycobacterium phlei RIVM601174 2523144778
MTTTQPVAQAILPDEIARKIVLPEGHADLDSLHAAYRWMRQNMPVGKAVVDGFDELWLVAKHADILQVEGMPE
VFAAGGGPDHPGSHNPILQNQAGDQFMKNTVGTVRIMDALPYMDGAEHSAMKAIAFDYFKPSRLAKLEDQIRQ
LARESIDKLKKVSEQGEIDLVTDWALGFPLHVIMSLFGVPPEDEPLMMKLTQEFFGTADPEHQRDDIEAMTPE
AMAQQFAASFQEFCGYFENLLQDRRANPRDDLASIIAQARRADGEYWPASFAYGWFMQVCTAGHDTTSSTLAG
TLKALAEHPDVLARVKADLSLVPKMVVEGVRYVAPVKHFMRIALTDTTLGGQTIKAGERVMPLFQSGCRDEDL
FENPDVFSLDRENNAHLGFGFGPHTCVGQHLAKLELRIMFEELLPRLESIEITGPGSLTPTNFVGGLKHLPAR
VKIS
>CYP108N2_Mycobacterium hassiacum DSM 44199 2533736942
MTTTQPVAQAILPDEIARKIVLPEGHADLDSLHAAYRWMRQNMPVGKAVVDGFDELWLVAKHADILQVEGMPE
VFAAGGGPDHPGSHNPILQNQAGDQFMKNTVGTVRIMDALPYMDGAEHSAMKAIAFDYFKPSRVAKLEDQIRQ
LARASIDKLKKVSEQGEIDLVTDWALGFPLHVIMSLFGVPPEDEPLMMKLTQEFFGTADPEYQRDDIEAMTPE
AMAQQFVASFQEFCGYFENLLQDRRANPRDDLATIIAQARRADGEYWPASFAYGWFMQVCTAGHDTTSSTLAG
TLKALAEHPDVLARVKADLSLVPKMVVEAVRYVAPVKHFMRIALTDTTLGGQTIKAGERVMPLFQSGCRDEDL
FENPDVFSLDRENNSHLGFGFGPHTCVGQHLAKLELRIMFEELLPRLESIEITGPGSLTPTNFVGGLKHLPAR
VKIS
>CYP121A1_Mycobacterium africanum 25 2688150487
MTATVLLEVPFSARGDRIPDAVAELRTREPIRKVRTITGAEAWLVSSYALCTQVLEDRRFSMKETAAAGAPRL
NALTVPPEVVNNMGNIADAGLRKAVMKAITPKAPGLEQFLRDTANSLLDNLITEGAPADLRNDFADPLATALH
CKVLGIPQEDGPKLFRSLSIAFMSSADPIPAAKINWDRDIEYMAGILENPNITTGLMGELSRLRKDPAYSHVS
DELFATIGVTFFGAGVISTGSFLTTALISLIQRPQLRNLLHEKPELIPAGVEELLRINLSFADGLPRLATADI
>CYP121A1_Mycobacterium africanum 25 2688150488
VLVRKGELVLVLLEGANFDPEHFPNPGSIELDRPNPTSHLAFGRGQHFCPGSALGRRHAQIGIEALLKKMPGV
DLAVPIDQLVWRTRFQRRIPERLPVLW
>CYP121A1_Mycobacterium africanum K85 2584197051
MTATVLLEVPFSARGDRIPDAVAELRTREPIRKVRTITGAEAWLVSSYALCTQVLEDRRFSMKETAAAGAPRL
NALTVPPEVVNNMGNIADAGLRKAVMKAITPKAPGLEQFLRDTANSLLDNLITEGAPADLRNDFADPLATALH
CKVLGIPQEDGPKLFRSLSIAFMSSADPIPAAKINWDRDIEYMAGILENPNITTGLMGELSRLRKDPAYSHVS
DELFATIGVTFFGAGVISTGSFLTTALISLIQRPQLRNLLHEKPELIPAGVEELLRINLSFADGLPRLATADI
QVGDVLVRKGELVLVLLEGANFDPEHFPNPGSIELDRPNPTSHLAFGRGQHFCPGSALGRRHAQIGIEALLKK
MPGVDLAVPIDQLVWRTRFQRRIPERLPVLW
>CYP121A1_Mycobacterium tuberculosis K85 646019325
MTATVLLEVPFSARGDRIPDAVAELRTREPIRKVRTITGAEAWLVSSYALCTQVLEDRRFSMKETAAAGAPRL
NALTVPPEVVNNMGNIADAGLRKAVMKAITPKAPGLEQFLRDTANSLLDNLITEGAPADLRNDFADPLATALH
CKVLGIPQEDGPKLFRSLSIAFMSSADPIPAAKINWDRDIEYMAGILENPNITTGLMGELSRLRKDPAYSHVS
DELFATIGVTFFGAGVISTGSFLTTALISLIQRPQLRNLLHEKPELIPAGVEELLRINLSFADGLPRLATADI
QVGDVLVRKGELVLVLLEGANFDPEHFPNPGSIELDRPNPTSHLAFGRGQHFCPGSALGRRHAQIGIEALLKK
MPGVDLAVPIDQLVWRTRFQRRIPERLPVLW
>CYP121A1_Mycobacterium africanum MAL010070 2583720424
MTATVLLEVPFSARGDRIPDAVAELRTREPIRKVRTITGAEAWLVSSYALCTQVLEDRRFSMKETAAAGAPRL
NALTVPPEVVNNMGNIADAGLRKAVMKAITPKAPGLEQFLRDTANSLLDNLITEGAPADLRNDFADPLATALH
CKVLGIPQEDGPKLFRSLSIAFMSSADPIPAAKINWDRDIEYMAGILENPNITTGLMGELSRLRKDPAYSHVS
DELFATIGVTFFGAGVISTGSFLTTALISLIQRPQLRNLLHEKPELIPAGVEELLRINLSFADGLPRLATADI
QVGDVLVRKGELVLVLLEGANFDPEHFPNPGSIELDRPNPTSHLAFGRGQHFCPGSALGRRHAQIGIEALLKK
MPGVDLAVPIDQLVWRTRFQRRIPERLPVLW
>CYP121A1_Mycobacterium africanum MAL010079 2580939789
MTATVLLEVPFSARGDRIPDAVAELRTREPIRKVRTITGAEAWLVSSYALCTQVLEDRRFSMKETAAAGAPRL
NALTVPPEVVNNMGNIADAGLRKAVMKAITPKAPGLEQFLRDTANSLLDNLITEGAPADLRNDFADPLATALH
CKVLGIPQEDGPKLFRSLSIAFMSSADPIPAAKINWDRDIEYMAGILENPNITTGLMGELSRLRKDPAYSHVS
DELFATIGVTFFGAGVISTGSFLTTALISLIQRPQLRNLLHEKPELIPAGVEELLRINLSFADGLPRLATADI
QVGDVLVRKGELVLVLLEGANFDPEHFPNPGSIELDRPNPTSHLAFGRGQHFCPGSALGRRHAQIGIEALLKK
MPGVDLAVPIDQLVWRTRFQRRIPERLPVLW
>CYP121A1_Mycobacterium africanum MAL010074 2582416070
MTATVLLEVPFSARGDRIPDAVAELRTREPIRKVRTITGAEAWLVSSYALCTQVLEDRRFSMKETAAAGAPRL
NALTVPPEVVNNMGNIADAGLRKAVMKAITPKAPGLEQFLRDTANSLLDNLITEGAPADLRNDFADPLATALH
CKVLGIPQEDGPKLFRSLSIAFMSSADPIPAAKINWDRDIEYMAGILENPNITTGLMGELSRLRKDPAYSHVS

DELFATIGVTFFGAGVISTGSFLTTALISLIQRPQLRNLLHEKPELIPAGVEELLRINLSFADGLPRLATADI
QVGDVLVRKGELVLVLLEGANFDPEHFPNPGSIELDRPNPTSHLAFGRGQHFCPGSALGRRHAQIGIEALLKK
MPGVDLAVPIDQLVWRTRFQRRIPERLPVLW
>CYP121A1_Mycobacterium africanum MAL010071 2582018776
MTATVLLEVPFSARGDRIPDAVAELRTREPIRKVRTITGAEAWLVSSYALCTQVLEDRRFSMKETAAAGAPRL
NALTVPPEVVNNMGNIADAGLRKAVMKAITPKAPGLEQFLRDTANSLLDNLITEGAPADLRNDFADPLATALH
CKVLGIPQEDGPKLFRSLSIAFMSSADPIPAAKINWDRDIEYMAGILENPNITTGLMGELSRLRKDPAYSHVS
DELFATIGVTFFGAGVISTGSFLTTALISLIQRPQLRNLLHEKPELIPAGVEELLRINLSFADGLPRLATADI
QVGDVLVRKGELVLVLLEGANFDPEHFPNPGSIELDRPNPTSHLAFGRGQHFCPGSALGRRHAQIGIEALLKK
MPGVDLAVPIDQLVWRTRFQRRIPERLPVLW
>CYP121A1_Mycobacterium africanum MAL010081 2580668486
MTATVLLEVPFSARGDRIPDAVAELRTREPIRKVRTITGAEAWLVSSYALCTQVLEDRRFSMKETAAAGAPRL
NALTVPPEVVNNMGNIADAGLRKAVMKAITPKAPGLEQFLRDTANSLLDNLITEGAPADLRNDFADPLATALH
CKVLGIPQEDGPKLFRSLSIAFMSSADPIPAAKINWDRDIEYMAGILENPNITTGLMGELSRLRKDPAYSHVS
DELFATIGVTFFGAGVISTGSFLTTALISLIQRPQLRNLLHEKPELIPAGVEELLRINLSFADGLPRLATADI
>CYP121A1_Mycobacterium africanum MAL010084 2581511494
MTATVLLEVPFSARGDRIPDAVAELRTREPIRKVRTITGAEAWLVSSYALCTQVLEDRRFSMKETAAAGAPRL
NALTVPPEVVNNMGNIADAGLRKAVMKAITPKAPGLEQFLRDTANSLLDNLITEGAPADLRNDFADPLATALH
CKVLGIPQEDGPKLFRSLSIAFMSSADPIPAAKINWDRDIEYMAGILENPNITTGLMGELSRLRKDPAYSHVS
DELFATIGVTFFGAGVISTGSFLTTALISLIQRPQLRNLLHEKPELIPAGVEELLRINLSFADGLPRLATADI
>CYP121A1_Mycobacterium africanum MAL010099 2583724460
MTATVLLEVPFSARGDRIPDAVAELRTREPIRKVRTITGAEAWLVSSYALCTQVLEDRRFSMKETAAAGAPRL
NALTVPPEVVNNMGNIADAGLRKAVMKAITPKAPGLEQFLRDTANSLLDNLITEGAPADLRNDFADPLATALH
CKVLGIPQEDGPKLFRSLSIAFMSSADPIPAAKINWDRDIEYMAGILENPNITTGLMGELSRLRKDPAYSHVS
DELFATIGVTFFGAGVISTGSFLTTALISLIQRPQLRNLLHEKPELIPAGVEELLRINLSFADGLPRLATADI
QVGDVLVRKGELVLVLLEGANFDPEHFPNPGSIELDRPNPTSHLAFGRGQHFCPGSALGRRHAQIGIEALLKK
MPGVDLAVPIDQLVWRTRFQRRIPERLPVLW
>CYP121A1_Mycobacterium africanum MAL010100 2574969009
MTATVLLEVPFSARGDRIPDAVAELRTREPIRKVRTITGAEAWLVSSYALCTQVLEDRRFSMKETAAAGAPRL
NALTVPPEVVNNMGNIADAGLRKAVMKAITPKAPGLEQFLRDTANSLLDNLITEGAPADLRNDFADPLATALH
CKVLGIPQEDGPKLFRSLSIAFMSSADPIPAAKINWDRDIEYMAGILENPNITTGLMGELSRLRKDPAYSHVS
DELFATIGVTFFGAGVISTGSFLTTALISLIQRPQLRNLLHEKPELIPAGVEELLRINLSFADGLPRLATADI
QVGDVLVRKGELVLVLLEGANFDPEHFPNPGSIELDRPNPTSHLAFGRGQHFCPGSALGRRHAQIGIEALLKK
MPGVDLAVPIDQLVWRTRFQRRIPERLPVLW
>CYP121A1_Mycobacterium africanum MAL010102 2577175801
MTATVLLEVPFSARGDRIPDAVAELRTREPIRKVRTITGAEAWLVSSYALCTQVLEDRRFSMKETAAAGAPRL
NALTVPPEVVNNMGNIADAGLRKAVMKAITPKAPGLEQFLRDTANSLLDNLITEGAPADLRNDFADPLATALH
CKVLGIPQEDGPKLFRSLSIAFMSSADPIPAAKINWDRDIEYMAGILENPNITTGLMGELSRLRKDPAYSHVS
DELFATIGVTFFGAGVISTGSFLTTALISLIQRPQLRNLLHEKPELIPAGVEELLRINLSFADGLPRLATADI
QVGDVLVRKGELVLVLLEGANFDPEHFPNPGSIELDRPNPTSHLAFGRGQHFCPGSALGRRHAQIGIEALLKK
MPGVDLAVPIDQLVWRTRFQRRIPERLPVLW
>CYP121A1_Mycobacterium africanum MAL010111 2575939587
MTATVLLEVPFSARGDRIPDAVAELRTREPIRKVRTITGAEAWLVSSYALCTQVLEDRRFSMKETAAAGAPRL
NALTVPPEVVNNMGNIADAGLRKAVMKAITPKAPGLEQFLRDTANSLLDNLITEGAPADLRNDFADPLATALH
CKVLGIPQEDGPKLFRSLSIAFMSSADPIPAAKINWDRDIEYMAGILENPNITTGLMGELSRLRKDPAYSHVS
DELFATIGVTFFGAGVISTGSFLTTALISLIQRPQLRNLLHEKPELIPAGVEELLRINLSFADGLPRLATADI
QVGDVLVRKGELVLVLLEGANFDPEHFPNPGSIELDRPNPTSHLAFGRGQHFCPGSALGRRHAQIGIEALLKK
MPGVDLAVPIDQLVWRTRFQRRIPERLPVLW
>CYP121A1_Mycobacterium africanum MAL010112 2575199210
MTATVLLEVPFSARGDRIPDAVAELRTREPIRKVRTITGAEAWLVSSYALCTQVLEDRRFSMKETAAAGAPRL
NALTVPPEVVNNMGNIADAGLRKAVMKAITPKAPGLEQFLRDTANSLLDNLITEGAPADLRNDFADPLATALH
CKVLGIPQEDGPKLFRSLSIAFMSSADPIPAAKINWDRDIEYMAGILENPNITTGLMGELSRLRKDPAYSHVS
DELFATIGVTFFGAGVISTGSFLTTALISLIQRPQLRNLLHEKPELIPAGVEELLRINLSFADGLPRLATADI
QVGDVLVRKGELVLVLLEGANFDPEHFPNPGSIELDRPNPTSHLAFGRGQHFCPGSALGRRHAQIGIEALLKK
MPGVDLAVPIDQLVWRTRFQRRIPERLPVLW
>CYP121A1_Mycobacterium africanum MAL010118 2581870401
MTATVLLEVPFSARGDRIPDAVAELRTREPIRKVRTITGAEAWLVSSYALCTQVLEDRRFSMKETAAAGAPRL
NALTVPPEVVNNMGNIADAGLRKAVMKAITPKAPGLEQFLRDTANSLLDNLITEGAPADLRNDFADPLATALH

CKVLGIPQEDGPKLFRSLSIAFMSSADPIPAAKINWDRDIEYMAGILENPNITTGLMGELSRLRKDPAYSHVS
DELFATIGVTFFGAGVISTGSFLTTALISLIQRPQLRNLLHEKPELIPAGVEELLRINLSFADGLPRLATADI
QVGDVLVRKGELVLVLLEGANFDPEHFPNPGSIELDRPNPTSHLAFGRGQHFCPGSALGRRHAQIGIEALLKK
MPGVDLAVPIDQLVWRTRFQRRIPERLPVLW
>CYP121A1_Mycobacterium africanum MAL010120 2580366721
MTATVLLEVPFSARGDRIPDAVAELRTREPIRKVRTITGAEAWLVSSYALCTQVLEDRRFSMKETAAAGAPRL
NALTVPPEVVNNMGNIADAGLRKAVMKAITPKAPGLEQFLRDTANSLLDNLITEGAPADLRNDFADPLATALH
CKVLGIPQEDGPKLFRSLSIAFMSSADPIPAAKINWDRDIEYMAGILENPNITTGLMGELSRLRKDPAYSHVS
DELFATIGVTFFGAGVISTGSFLTTALISLIQRPQLRNLLHEKPELIPAGVEELLRINLSFADGLPRLATADI
QVGDVLVRKGELVLVLLEGANFDPEHFPNPGSIELDRPNPTSHLAFGRGQHFCPGSALGRRHAQIGIEALLKK
MPGVDLAVPIDQLVWRTRFQRRIPERLPVLW
>CYP121A1_Mycobacterium africanum MAL010123 2581808312
MTATVLLEVPFSARGDRIPDAVAELRTREPIRKVRTITGAEAWLVSSYALCTQVLEDRRFSMKETAAAGAPRL
NALTVPPEVVNNMGNIADAGLRKAVMKAITPKAPGLEQFLRDTANSLLDNLITEGAPADLRNDFADPLATALH
CKVLGIPQEDGPKLFRSLSIAFMSSADPIPAAKINWDRDIEYMAGILENPNITTGLMGELSRLRKDPAYSHVS
DELFATIGVTFFGAGVISTGSFLTTALISLIQRPQLRNLLHEKPELIPAGVEELLRINLSFADGLPRLATADI
QVGDVLVRKGELVLVLLEGANFDPEHFPNPGSIELDRPNPTSHLAFGRGQHFCPGSALGRRHAQIGIEALLKK
MPGVDLAVPIDQLVWRTRFQRRIPERLPVLW
>CYP121A1_Mycobacterium africanum MAL010128 2582002516
MTATVLLEVPFSARGDRIPDAVAELRTREPIRKVRTITGAEAWLVSSYALCTQVLEDRRFSMKETAAAGAPRL
NALTVPPEVVNNMGNIADAGLRKAVMKAITPKAPGLEQFLRDTANSLLDNLITEGAPADLRNDFADPLATALH
CKVLGIPQEDGPKLFRSLSIAFMSSADPIPAAKINWDRDIEYMAGILENPNITTGLMGELSRLRKDPAYSHVS
DELFATIGVTFFGAGVISTGSFLTTALISLIQRPQLRNLLHEKPELIPAGVEELLRINLSFADGLPRLATADI
QVGDVLVRKGELVLVLLEGANFDPEHFPNPGSIELDRPNPTSHLAFGRGQHFCPGSALGRRHAQIGIEALLKK
MPGVDLAVPIDQLVWRTRFQRRIPERLPVLW
>CYP121A1_Mycobacterium africanum MAL010129 2581902018
MTATVLLEVPFSARGDRIPDAVAELRTREPIRKVRTITGAEAWLVSSYALCTQVLEDRRFSMKETAAAGAPRL
NALTVPPEVVNNMGNIADAGLRKAVMKAITPKAPGLEQFLRDTANSLLDNLITEGAPADLRNDFADPLATALH
CKVLGIPQEDGPKLFRSLSIAFMSSADPIPAAKINWDRDIEYMAGILENPNITTGLMGELSRLRKDPAYSHVS
DELFATIGVTFFGAGVISTGSFLTTALISLIQRPQLRNLLHEKPELIPAGVEELLRINLSFADGLPRLATADI
QVGDVLVRKGELVLVLLEGANFDPEHFPNPGSIELDRPNPTSHLAFGRGQHFCPGSALGRRHAQIGIEALLKK
MPGVDLAVPIDQLVWRTRFQRRIPERLPVLW
>CYP121A1_Mycobacterium africanum MAL010131 2581376412
MTATVLLEVPFSARGDRIPDAVAELRTREPIRKVRTITGAEAWLVSSYALCTQVLEDRRFSMKETAAAGAPRL
NALTVPPEVVNNMGNIADAGLRKAVMKAITPKAPGLEQFLRDTANSLLDNLITEGAPADLRNDFADPLATALH
CKVLGIPQEDGPKLFRSLSIAFMSSADPIPAAKINWDRDIEYMAGILENPNITTGLMGELSRLRKDPAYSHVS
DELFATIGVTFFGAGVISTGSFLTTALISLIQRPQLRNLLHEKPELIPAGVEELLRINLSFADGLPRLATADI
QVGDVLVRKGELVLVLLEGANFDPEHFPNPGSIELDRPNPTSHLAFGRGQHFCPGSALGRRHAQIGIEALLKK
MPGVDLAVPIDQLVWRTRFQRRIPERLPVLW
>CYP121A1_Mycobacterium africanum MAL010136 2583728530
MTATVLLEVPFSARGDRIPDAVAELRTREPIRKVRTITGAEAWLVSSYALCTQVLEDRRFSMKETAAAGAPRL
NALTVPPEVVNNMGNIADAGLRKAVMKAITPKAPGLEQFLRDTANSLLDNLITEGAPADLRNDFADPLATALH
CKVLGIPQEDGPKLFRSLSIAFMSSADPIPAAKINWDRDIEYMAGILENPNITTGLMGELSRLRKDPAYSHVS
DELFATIGVTFFGAGVISTGSFLTTALISLIQRPQLRNLLHEKPELIPAGVEELLRINLSFADGLPRLATADI
QVGDVLVRKGELVLVLLEGANFDPEHFPNPGSIELDRPNPTSHLAFGRGQHFCPGSALGRRHAQIGIEALLKK
MPGVDLAVPIDQLVWRTRFQRRIPERLPVLW
>CYP121A1_Mycobacterium africanum MAL010137 2575705842
MTATVLLEVPFSARGDRIPDAVAELRTREPIRKVRTITGAEAWLVSSYALCTQVLEDRRFSMKETAAAGAPRL
NALTVPPEVVNNMGNIADAGLRKAVMKAITPKAPGLEQFLRDTANSLLDNLITEGAPADLRNDFADPLATALH
CKVLGIPQEDGPKLFRSLSIAFMSSADPIPAAKINWDRDIEYMAGILENPNITTGLMGELSRLRKDPAYSHVS
DELFATIGVTFFGAGVISTGSFLTTALISLIQRPQLRNLLHEKPELIPAGVEELLRINLSFADGLPRLATADI
QVGDVLVRKGELVLVLLEGANFDPEHFPNPGSIELDRPNPTSHLAFGRGQHFCPGSALGRRHAQIGIEALLKK
MPGVDLAVPIDQLVWRTRFQRRIPERLPVLW
>CYP121A1_Mycobacterium africanum MAL020107 2581931365
MTATVLLEVPFSARGDRIPDAVAELRTREPIRKVRTITGAEAWLVSSYALCTQVLEDRRFSMKETAAAGAPRL
NALTVPPEVVNNMGNIADAGLRKAVMKAITPKAPGLEQFLRDTANSLLDNLITEGAPADLRNDFADPLATALH
CKVLGIPQEDGPKLFRSLSIAFMSSADPIPAAKINWDRDIEYMAGILENPNITTGLMGELSRLRKDPAYSHVS
DELFATIGVTFFGAGVISTGSFLTTALISLIQRPQLRNLLHEKPELIPAGVEELLRINLSFADGLPRLATADI

>CYP121A1_Mycobacterium africanum MAL020130 2583736625
MTATVLLEVPFSARGDRIPDAVAELRTREPIRKVRTITGAEAWLVSSYALCTQVLEDRRFSMKETAAAGAPRL
NALTVPPEVVNNMGNIADAGLRKAVMKAITPKAPGLEQFLRDTANSLLDNLITEGAPADLRNDFADPLATALH
CKVLGIPQEDGPKLFRSLSIAFMSSADPIPAAKINWDRDIEYMAGILENPNITTGLMGELSRLRKDPAYSHVS
DELFATIGVTFFGAGVISTGSFLTTALISLIQRPQLRNLLHEKPELIPAGVEELLRINLSFADGLPRLATADI
QVGDVLVRKGELVLVLLEGANFDPEHFPNPGSIELDRPNPTSHLAFGRGQHFCPGSALGRRHAQIGIEALLKK
MPGVDLAVPIDQLVWRTRFQRRIPERLPVLW
>CYP121A1_Mycobacterium africanum MAL020135 2583732577
MTATVLLEVPFSARGDRIPDAVAELRTREPIRKVRTITGAEAWLVSSYALCTQVLEDRRFSMKETAAAGAPRL
NALTVPPEVVNNMGNIADAGLRKAVMKAITPKAPGLEQFLRDTANSLLDNLITEGAPADLRNDFADPLATALH
CKVLGIPQEDGPKLFRSLSIAFMSSADPIPAAKINWDRDIEYMAGILENPNITTGLMGELSRLRKDPAYSHVS
DELFATIGVTFFGAGVISTGSFLTTALISLIQRPQLRNLLHEKPELIPAGVEELLRINLSFADGLPRLATADI
QVGDVLVRKGELVLVLLEGANFDPEHFPNPGSIELDRPNPTSHLAFGRGQHFCPGSALGRRHAQIGIEALLKK
MPGVDLAVPIDQLVWRTRFQRRIPERLPVLW
>CYP121A1_Mycobacterium africanum MAL020148 2581564129
MTATVLLEVPFSARGDRIPDAVAELRTREPIRKVRTITGAEAWLVSSYALCTQVLEDRRFSMKETAAAGAPRL
NALTVPPEVVNNMGNIADAGLRKAVMKAITPKAPGLEQFLRDTANSLLDNLITEGAPADLRNDFADPLATALH
CKVLGIPQEDGPKLFRSLSIAFMSSADPIPAAKINWDRDIEYMAGILENPNITTGLMGELSRLRKDPAYSHVS
DELFATIGVTFFGAGVISTGSFLTTALISLIQRPQLRNLLHEKPELIPAGVEELLRINLSFADGLPRLATADI
>CYP121A1_Mycobacterium africanum MAL020173 2581367176
MTATVLLEVPFSARGDRIPDAVAELRTREPIRKVRTITGAEAWLVSSYALCTQVLEDRRFSMKETAAAGAPRL
NALTVPPEVVNNMGNIADAGLRKAVMKAITPKAPGLEQFLRDTANSLLDNLITEGAPADLRNDFADPLATALH
CKVLGIPQEDGPKLFRSLSIAFMSSADPIPAAKINWDRDIEYMAGILENPNITTGLMGELSRLRKDPAYSHVS
DELFATIGVTFFGAGVISTGSFLTTALISLIQRPQLRNLLHEKPELIPAGVEELLRINLSFADGLPRLATADI
QVGDVLVRKGELVLVLLEGANFDPEHFPNPGSIELDRPNPTSHLAFGRGQHFCPGSALGRRHAQIGIEALLKK
MPGVDLAVPIDQLVWRTRFQRRIPERLPVLW
>CYP121A1_Mycobacterium africanum MAL020176 2583738967
MTATVLLEVPFSARGDRIPDAVAELRTREPIRKVRTITGAEAWLVSSYALCTQVLEDRRFSMKETAAAGAPRL
NALTVPPEVVNNMGNIADAGLRKAVMKAITPKAPGLEQFLRDTANSLLDNLITEGAPADLRNDFADPLATALH
CKVLGIPQEDGPKLFRSLSIAFMSSADPIPAAKINWDRDIEYMAGILENPNITTGLMGELSRLRKDPAYSHVS
DELFATIGVTFFGAGVISTGSFLTTALISLIQRPQLRNLLHEKPELIPAGVEELLRINLSFADGLPRLATADI
QVGDVLVRKGELVLVLLEGANFDPEHFPNPGSIELDRPNPTSHLAFGRGQHFCPGSALGRRHAQIGIEALLKK
MPGVDLAVPIDQLVWRTRFQRRIPERLPVLW
>CYP121A1_Mycobacterium africanum MAL020185 2580468414
MTATVLLEVPFSARGDRIPDAVAELRTREPIRKVRTITGAEAWLVSSYALCTQVLEDRRFSMKETAAAGAPRL
NALTVPPEVVNNMGNIADAGLRKAVMKAITPKAPGLEQFLRDTANSLLDNLITEGAPADLRNDFADPLATALH
CKVLGIPQEDGPKLFRSLSIAFMSSADPIPAAKINWDRDIEYMAGILENPNITTGLMGELSRLRKDPAYSHVS
DELFATIGVTFFGAGVISTGSFLTTALISLIQRPQLRNLLHEKPELIPAGVEELLRINLSFADGLPRLATADI
QVGDVLVRKGELVLVLLEGANFDPEHFPNPGSIELDRPNPTSHLAFGRGQHFCPGSALGRRHAQIGIEALLKK
MPGVDLAVPIDQLVWRTRFQRRIPERLPVLW
>CYP121A1_Mycobacterium africanum UT307 2687242995
MTATVLLEVPFSARGDRIPDAVAELRTREPIRKVRTITGAEAWLVSSYALCTQVLEDRRFSMKETAAAGAPRL
NALTVPPEVVNNMGNIADAGLRKAVMKAITPKAPGLEQFLRDTANSLLDNLITEGAPADLRNDFADPLATALH
CKVLGIPQEDGPKLFRSLSIAFMSSADPIPAAKINWDRDIEYMAGILENPNITTGLMGELSRLRKDPAYSHVS
DELFATIGVTFFGAGVISTGSFLTTALISLIQRPQLRNLLHEKPELIPAGVEELLRINLSFADGLPRLATADI
QVGDVLVRKGELVLVLLEGANFDPEHFPNPGSIELDRPNPTSHLAFGRGQHFCPGSALGRRHAQIGIEALLKK
MPGVDLAVPIDQLVWRTRFQRRIPERLPVLW
>CYP121A1_Mycobacterium bovis 1595 2638477570
MTATVLLEVPFSARGDRIPDAVAELRTREPIRKVRTITGAEAWLVSSYALCTQVLEDRRFSMKETAAAGAPRL
NALTVPPEVVNNMGNIADAGLRKAVMKAITPKAPGLEQFLRDTANSLLDNLITEGAPADLRNDFADPLATALH
CKVLGIPQEDGPKLFRSLSIAFMSSADPIPAAKINWDRDIEYMAGILENPNITTGLMGELSRLRKDPAYSHVS
DELFATIGVTFFGAGVISTGSFLTTALISLIQRPQLRNLLHEKPELIPAGVEELLRINLSFADGLPRLATADI
QVGDVLVRKGELVLVLLEGANFDPEHFPNPGSIELDRPNPTSHLAFGRGQHFCPGSALGRRHAQIGIEALLKK
MPGVDLAVPIDQLVWRTRFQRRIPERLPVLW
>CYP121A1_Mycobacterium bovis 30 2687958132
MTATVLLEVPFSARGDRIPDAVAELRTREPIRKVRTITGAEAWLVSSYALCTQVLEDRRFSMKETAAAGAPRL
NALTVPPEVVNNMGNIADAGLRKAVMKAITPKAPGLEQFLRDTANSLLDNLITEGAPADLRNDFADPLATALH
CKVLGIPQEDGPKLFRSLSIAFMSSADPIPAAKINWDRDIEYMAGILENPNITTGLMGELSRLRKDPAYSHVS

DELFATIGVTFFGAGVISTGSFLTTALISLIQRPQLRNLLHEKPELIPAGVEELLRINLSFADGLPRLATADI
QVGDVLVRKGELVLVLLEGANFDPEHFPNPGSIELDRPNPTSHLAFGRGQHFCPGSALGRRHAQIGIEALLKK
MPGVDLAVPIDQLVWRTRFQRRIPERLPVLW
>CYP121A1_Mycobacterium bovis AF 2122/97 2638123624
MTATVLLEVPFSARGDRIPDAVAELRTREPIRKVRTITGAEAWLVSSYALCTQVLEDRRFSMKETAAAGAPRL
NALTVPPEVVNNMGNIADAGLRKAVMKAITPKAPGLEQFLRDTANSLLDNLITEGAPADLRNDFADPLATALH
CKVLGIPQEDGPKLFRSLSIAFMSSADPIPAAKINWDRDIEYMAGILENPNITTGLMGELSRLRKDPAYSHVS
DELFATIGVTFFGAGVISTGSFLTTALISLIQRPQLRNLLHEKPELIPAGVEELLRINLSFADGLPRLATADI
QVGDVLVRKGELVLVLLEGANFDPEHFPNPGSIELDRPNPTSHLAFGRGQHFCPGSALGRRHAQIGIEALLKK
MPGVDLAVPIDQLVWRTRFQRRIPERLPVLW
>CYP121A1_Mycobacterium bovis AF2122/97 637139639
MTATVLLEVPFSARGDRIPDAVAELRTREPIRKVRTITGAEAWLVSSYALCTQVLEDRRFSMKETAAAGAPRL
NALTVPPEVVNNMGNIADAGLRKAVMKAITPKAPGLEQFLRDTANSLLDNLITEGAPADLRNDFADPLATALH
CKVLGIPQEDGPKLFRSLSIAFMSSADPIPAAKINWDRDIEYMAGILENPNITTGLMGELSRLRKDPAYSHVS
DELFATIGVTFFGAGVISTGSFLTTALISLIQRPQLRNLLHEKPELIPAGVEELLRINLSFADGLPRLATADI
QVGDVLVRKGELVLVLLEGANFDPEHFPNPGSIELDRPNPTSHLAFGRGQHFCPGSALGRRHAQIGIEALLKK
MPGVDLAVPIDQLVWRTRFQRRIPERLPVLW
>CYP121A1_Mycobacterium bovis B-3222 2652352738
MTATVLLEVPFSARGDRIPDAVAELRTREPIRKVRTITGAEAWLVSSYALCTQVLEDRRFSMKETAAAGAPRL
NALTVPPEVVNNMGNIADAGLRKAVMKAITPKAPGLEQFLRDTANSLLDNLITEGAPADLRNDFADPLATALH
CKVLGIPQEDGPKLFRSLSIAFMSSADPIPAAKINWDRDIEYMAGILENPNITTGLMGELSRLRKDPAYSHVS
DELFATIGVTFFGAGVISTGSFLTTALISLIQRPQLRNLLHEKPELIPAGVEELLRINLSFADGLPRLATADI
QVGDVLVRKGELVLVLLEGANFDPEHFPNPGSIELDRPNPTSHLAFGRGQHFCPGSALGRRHAQIGIEALLKK
MPGVDLAVPIDQLVWRTRFQRRIPERLPVLW
>CYP121A1_Mycobacterium bovis BCG 2646607764
MTATVLLEVPFSARGDRIPDAVAELRTREPIRKVRTITGAEAWLVSSYALCTQVLEDRRFSMKETAAAGAPRL
NALTVPPEVVNNMGNIADAGLRKAVMKAITPKAPGLEQFLRDTANSLLDNLITEGAPADLRNDFADPLATALH
CKVLGIPQEDGPKLFRSLSIAFMSSADPIPAAKINWDRDIEYMAGILENPNITTGLMGELSRLRKDPAYSHVS
DELFATIGVTFFGAGVISTGSFLTTALISLIQRPQLRNLLHEKPELIPAGVEELLRINLSFADGLPRLATADI
QVGDVLVRKGELVLVLLEGANFDPEHFPNPGSIELDRPNPTSHLAFGRGQHFCPGSALGRRHAQIGIEALLKK
MPGVDLAVPIDQLVWRTRFQRRIPERLPVLW
>CYP121A1_Mycobacterium bovis BCG 26 2687967893
MGNIADAGLRKAVMKAITPKAPGLEQFLRDTANSLLDNLITEGAPADLRNDFADPLATALHCKVLGIPQEDGP
KLFRSLSIAFMSSADPIPAAKINWDRDIEYMAGILENPNITTGLMGELSRLRKDPAYSHVSDELFATIGVTFF
GAGVISTGSFLTTALISLIQRPQLRNLLHEKPELIPAGVEELLRINLSFADGLPRLATADIQVGDVLVRKGEL
VLVLLEGANFDPEHFPNPGSIELDRPNPTSHLAFGRGQHFCPGSALGRRHAQIGIEALLKKMPGVDLAVPIDQ
LVWRTRFQRRIPERLPVLW
>CYP121A1_Mycobacterium bovis BCG 3281 (unscreened) 2646008776
MTATVLLEVPFSARGDRIPDAVAELRTREPIRKVRTITGAEAWLVSSYALCTQVLEDRRFSMKETAAAGAPRL
NALTVPPEVVNNMGNIADAGLRKAVMKAITPKAPGLEQFLRDTANSLLDNLITEGAPADLRNDFADPLATALH
CKVLGIPQEDGPKLFRSLSIAFMSSADPIPAAKINWDRDIEYMAGILENPNITTGLMGELSRLRKDPAYSHVS
DELFATIGVTFFGAGVISTGSFLTTALISLIQRPQLRNLLHEKPELIPAGVEELLRINLSFADGLPRLATADI
QVGDVLVRKGELVLVLLEGANFDPEHFPNPGSIELDRPNPTSHLAFGRGQHFCPGSALGRRHAQIGIEALLKK
MPGVDLAVPIDQLVWRTRFQRRIPERLPVLW
>CYP121A1_Mycobacterium bovis BCG Birkhaug 2695557053
MTATVLLEVPFSARGDRIPDAVAELRTREPIRKVRTITGAEAWLVSSYALCTQVLEDRRFSMKETAAAGAPRL
NALTVPPEVVNNMGNIADAGLRKAVMKAITPKAPGLEQFLRDTANSLLDNLITEGAPADLRNDFADPLATALH
CKVLGIPQEDGPKLFRSLSIAFMSSADPIPAAKINWDRDIEYMAGILENPNITTGLMGELSRLRKDPAYSHVS
DELFATIGVTFFGAGVISTGSFLTTALISLIQRPQLRNLLHEKPELIPAGVEELLRINLSFADGLPRLATADI
QVGDVLVRKGELVLVLLEGANFDPEHFPNPGSIELDRPNPTSHLAFGRGQHFCPGSALGRRHAQIGIEALLKK
MPGVDLAVPIDQLVWRTRFQRRIPERLPVLW
>CYP121A1_Mycobacterium bovis BCG China 2547305807
MTATVLLEVPFSARGDRIPDAVAELRTREPIRKVRTITGAEAWLVSSYALCTQVLEDRRFSMKETAAAGAPRL
NALTVPPEVVNNMGNIADAGLRKAVMKAITPKAPGLEQFLRDTANSLLDNLITEGAPADLRNDFADPLATALH
CKVLGIPQEDGPKLFRSLSIAFMSSADPIPAAKINWDRDIEYMAGILENPNITTGLMGELSRLRKDPAYSHVS
DELFATIGVTFFGAGVISTGSFLTTALISLIQRPQLRNLLHEKPELIPAGVEELLRINLSFADGLPRLATADI
QVGDVLVRKGELVLVLLEGANFDPEHFPNPGSIELDRPNPTSHLAFGRGQHFCPGSALGRRHAQIGIEALLKK
MPGVDLAVPIDQLVWRTRFQRRIPERLPVLW

>CYP121A1_Mycobacterium bovis BCG China 2695503122
MTATVLLEVPFSARGDRIPDAVAELRTREPIRKVRTITGAEAWLVSSYALCTQVLEDRRFSMKETAAAGAPRL
NALTVPPEVVNNMGNIADAGLRKAVMKAITPKAPGLEQFLRDTANSLLDNLITEGAPADLRNDFADPLATALH
CKVLGIPQEDGPKLFRSLSIAFMSSADPIPAAKINWDRDIEYMAGILENPNITTGLMGELSRLRKDPAYSHVS
DELFATIGVTFFGAGVISTGSFLTTALISLIQRPQLRNLLHEKPELIPAGVEELLRINLSFADGLPRLATADI
QVGDVLVRKGELVLVLLEGANFDPEHFPNPGSIELDRPNPTSHLAFGRGQHFCPGSALGRRHAQIGIEALLKK
MPGVDLAVPIDQLVWRTRFQRRIPERLPVLW
>CYP121A1_Mycobacterium bovis BCG Connaught 2695543269
MTATVLLEVPFSARGDRIPDAVAELRTREPIRKVRTITGAEAWLVSSYALCTQVLEDRRFSMKETAAAGAPRL
NALTVPPEVVNNMGNIADAGLRKAVMKAITPKAPGLEQFLRDTANSLLDNLITEGAPADLRNDFADPLATALH
CKVLGIPQEDGPKLFRSLSIAFMSSADPIPAAKINWDRDIEYMAGILENPNITTGLMGELSRLRKDPAYSHVS
DELFATIGVTFFGAGVISTGSFLTTALISLIQRPQLRNLLHEKPELIPAGVEELLRINLSFADGLPRLATADI
QVGDVLVRKGELVLVLLEGANFDPEHFPNPGSIELDRPNPTSHLAFGRGQHFCPGSALGRRHAQIGIEALLKK
MPGVDLAVPIDQLVWRTRFQRRIPERLPVLW
>CYP121A1_Mycobacterium bovis BCG Danish 2695499069
MTATVLLEVPFSARGDRIPDAVAELRTREPIRKVRTITGAEAWLVSSYALCTQVLEDRRFSMKETAAAGAPRL
NALTVPPEVVNNMGNIADAGLRKAVMKAITPKAPGLEQFLRDTANSLLDNLITEGAPADLRNDFADPLATALH
CKVLGIPQEDGPKLFRSLSIAFMSSADPIPAAKINWDRDIEYMAGILENPNITTGLMGELSRLRKDPAYSHVS
DELFATIGVTFFGAGVISTGSFLTTALISLIQRPQLRNLLHEKPELIPAGVEELLRINLSFADGLPRLATADI
QVGDVLVRKGELVLVLLEGANFDPEHFPNPGSIELDRPNPTSHLAFGRGQHFCPGSALGRRHAQIGIEALLKK
MPGVDLAVPIDQLVWRTRFQRRIPERLPVLW
>CYP121A1_Mycobacterium bovis BCG Frappier 2695529920
MTATVLLEVPFSARGDRIPDAVAELRTREPIRKVRTITGAEAWLVSSYALCTQVLEDRRFSMKETAAAGAPRL
NALTVPPEVVNNMGNIADAGLRKAVMKAITPKAPGLEQFLRDTANSLLDNLITEGAPADLRNDFADPLATALH
CKVLGIPQEDGPKLFRSLSIAFMSSADPIPAAKINWDRDIEYMAGILENPNITTGLMGELSRLRKDPAYSHVS
DELFATIGVTFFGAGVISTGSFLTTALISLIQRPQLRNLLHEKPELIPAGVEELLRINLSFADGLPRLATADI
QVGDVLVRKGELVLVLLEGANFDPEHFPNPGSIELDRPNPTSHLAFGRGQHFCPGSALGRRHAQIGIEALLKK
MPGVDLAVPIDQLVWRTRFQRRIPERLPVLW
>CYP121A1_Mycobacterium bovis BCG Glaxo 2600679472
MTATVLLEVPFSARGDRIPDAVAELRTREPIRKVRTITGAEAWLVSSYALCTQVLEDRRFSMKETAAAGAPRL
NALTVPPEVVNNMGNIADAGLRKAVMKAITPKAPGLEQFLRDTANSLLDNLITEGAPADLRNDFADPLATALH
CKVLGIPQEDGPKLFRSLSIAFMSSADPIPAAKINWDRDIEYMAGILENPNITTGLMGELSRLRKDPAYSHVS
DELFATIGVTFFGAGVISTGSFLTTALISLIQRPQLRNLLHEKPELIPAGVEELLRINLSFADGLPRLATADI
QVGDVLVRKGELVLVLLEGANFDPEHFPNPGSIELDRPNPTSHLAFGRGQHFCPGSALGRRHAQIGIEALLKK
MPGVDLAVPIDQLVWRTRFQRRIPERLPVLW
>CYP121A1_Mycobacterium bovis BCG Glaxo 2695533965
MTATVLLEVPFSARGDRIPDAVAELRTREPIRKVRTITGAEAWLVSSYALCTQVLEDRRFSMKETAAAGAPRL
NALTVPPEVVNNMGNIADAGLRKAVMKAITPKAPGLEQFLRDTANSLLDNLITEGAPADLRNDFADPLATALH
CKVLGIPQEDGPKLFRSLSIAFMSSADPIPAAKINWDRDIEYMAGILENPNITTGLMGELSRLRKDPAYSHVS
DELFATIGVTFFGAGVISTGSFLTTALISLIQRPQLRNLLHEKPELIPAGVEELLRINLSFADGLPRLATADI
QVGDVLVRKGELVLVLLEGANFDPEHFPNPGSIELDRPNPTSHLAFGRGQHFCPGSALGRRHAQIGIEALLKK
MPGVDLAVPIDQLVWRTRFQRRIPERLPVLW
>CYP121A1_Mycobacterium bovis BCG Glaxo 2600679472
MTATVLLEVPFSARGDRIPDAVAELRTREPIRKVRTITGAEAWLVSSYALCTQVLEDRRFSMKETAAAGAPRL
NALTVPPEVVNNMGNIADAGLRKAVMKAITPKAPGLEQFLRDTANSLLDNLITEGAPADLRNDFADPLATALH
CKVLGIPQEDGPKLFRSLSIAFMSSADPIPAAKINWDRDIEYMAGILENPNITTGLMGELSRLRKDPAYSHVS
DELFATIGVTFFGAGVISTGSFLTTALISLIQRPQLRNLLHEKPELIPAGVEELLRINLSFADGLPRLATADI
QVGDVLVRKGELVLVLLEGANFDPEHFPNPGSIELDRPNPTSHLAFGRGQHFCPGSALGRRHAQIGIEALLKK
MPGVDLAVPIDQLVWRTRFQRRIPERLPVLW
>CYP121A1_Mycobacterium bovis BCG Grappier 2600683724
MTATVLLEVPFSARGDRIPDAVAELRTREPIRKVRTITGAEAWLVSSYALCTQVLEDRRFSMKETAAAGAPRL
NALTVPPEVVNNMGNIADAGLRKAVMKAITPKAPGLEQFLRDTANSLLDNLITEGAPADLRNDFADPLATALH
CKVLGIPQEDGPKLFRSLSIAFMSSADPIPAAKINWDRDIEYMAGILENPNITTGLMGELSRLRKDPAYSHVS
DELFATIGVTFFGAGVISTGSFLTTALISLIQRPQLRNLLHEKPELIPAGVEELLRINLSFADGLPRLATADI
QVGDVLVRKGELVLVLLEGANFDPEHFPNPGSIELDRPNPTSHLAFGRGQHFCPGSALGRRHAQIGIEALLKK
MPGVDLAVPIDQLVWRTRFQRRIPERLPVLW
>CYP121A1_Mycobacterium bovis BCG Japan 2695643549

MTATVLLEVPFSARGDRIPDAVAELRTREPIRKVRTITGAEAWLVSSYALCTQVLEDRRFSMKETAAAGAPRL
NALTVPPEVVNNMGNIADAGLRKAVMKAITPKAPGLEQFLRDTANSLLDNLITEGAPADLRNDFADPLATALH
CKVLGIPQEDGPKLFRSLSIAFMSSADPIPAAKINWDRDIEYMAGILENPNITTGLMGELSRLRKDPAYSHVS
DELFATIGVTFFGAGVISTGSFLTTALISLIQRPQLRNLLHEKPELIPAGVEELLRINLSFADGLPRLATADI
QVGDVLVRKGELVLVLLEGANFDPEHFPNPGSIELDRPNPTSHLAFGRGQHFCPGSALGRRHAQIGIEALLKK
MPGVDLAVPIDQLVWRTRFQRRIPERLPVLW
>CYP121A1_Mycobacterium bovis BCG Korea 1168P 2540804454
MTATVLLEVPFSARGDRIPDAVAELRTREPIRKVRTITGAEAWLVSSYALCTQVLEDRRFSMKETAAAGAPRL
NALTVPPEVVNNMGNIADAGLRKAVMKAITPKAPGLEQFLRDTANSLLDNLITEGAPADLRNDFADPLATALH
CKVLGIPQEDGPKLFRSLSIAFMSSADPIPAAKINWDRDIEYMAGILENPNITTGLMGELSRLRKDPAYSHVS
DELFATIGVTFFGAGVISTGSFLTTALISLIQRPQLRNLLHEKPELIPAGVEELLRINLSFADGLPRLATADI
QVGDVLVRKGELVLVLLEGANFDPEHFPNPGSIELDRPNPTSHLAFGRGQHFCPGSALGRRHAQIGIEALLKK
MPGVDLAVPIDQLVWRTRFQRRIPERLPVLW
>CYP121A1_Mycobacterium bovis BCG Mexico 2511811882
MTATVLLEVPFSARGDRIPDAVAELRTREPIRKVRTITGAEAWLVSSYALCTQVLEDRRFSMKETAAAGAPRL
NALTVPPEVVNNMGNIADAGLRKAVMKAITPKAPGLEQFLRDTANSLLDNLITEGAPADLRNDFADPLATALH
CKVLGIPQEDGPKLFRSLSIAFMSSADPIPAAKINWDRDIEYMAGILENPNITTGLMGELSRLRKDPAYSHVS
DELFATIGVTFFGAGVISTGSFLTTALISLIQRPQLRNLLHEKPELIPAGVEELLRINLSFADGLPRLATADI
QVGDVLVRKGELVLVLLEGANFDPEHFPNPGSIELDRPNPTSHLAFGRGQHFCPGSALGRRHAQIGIEALLKK
MPGVDLAVPIDQLVWRTRFQRRIPERLPVLW
>CYP121A1_Mycobacterium bovis BCG Moreau 2600457110
MTATVLLEVPFSARGDRIPDAVAELRTREPIRKVRTITGAEAWLVSSYALCTQVLEDRRFSMKETAAAGAPRL
NALTVPPEVVNNMGNIADAGLRKAVMKAITPKAPGLEQFLRDTANSLLDNLITEGAPADLRNDFADPLATALH
CKVLGIPQEDGPKLFRSLSIAFMSSADPIPAAKINWDRDIEYMAGILENPNITTGLMGELSRLRKDPAYSHVS
DELFATIGVTFFGAGVISTGSFLTTALISLIQRPQLRNLLHEKPELIPAGVEELLRINLSFADGLPRLATADI
QVGDVLVRKGELVLVLLEGANFDPEHFPNPGSIELDRPNPTSHLAFGRGQHFCPGSALGRRHAQIGIEALLKK
MPGVDLAVPIDQLVWRTRFQRRIPERLPVLW
>CYP121A1_Mycobacterium bovis BCG Moreau 2695511259
MTATVLLEVPFSARGDRIPDAVAELRTREPIRKVRTITGAEAWLVSSYALCTQVLEDRRFSMKETAAAGAPRL
NALTVPPEVVNNMGNIADAGLRKAVMKAITPKAPGLEQFLRDTANSLLDNLITEGAPADLRNDFADPLATALH
CKVLGIPQEDGPKLFRSLSIAFMSSADPIPAAKINWDRDIEYMAGILENPNITTGLMGELSRLRKDPAYSHVS
DELFATIGVTFFGAGVISTGSFLTTALISLIQRPQLRNLLHEKPELIPAGVEELLRINLSFADGLPRLATADI
QVGDVLVRKGELVLVLLEGANFDPEHFPNPGSIELDRPNPTSHLAFGRGQHFCPGSALGRRHAQIGIEALLKK
MPGVDLAVPIDQLVWRTRFQRRIPERLPVLW
>CYP121A1_Mycobacterium bovis BCG Moreau RDJ 2620700323
MTATVLLEVPFSARGDRIPDAVAELRTREPIRKVRTITGAEAWLVSSYALCTQVLEDRRFSMKETAAAGAPRL
NALTVPPEVVNNMGNIADAGLRKAVMKAITPKAPGLEQFLRDTANSLLDNLITEGAPADLRNDFADPLATALH
CKVLGIPQEDGPKLFRSLSIAFMSSADPIPAAKINWDRDIEYMAGILENPNITTGLMGELSRLRKDPAYSHVS
DELFATIGVTFFGAGVISTGSFLTTALISLIQRPQLRNLLHEKPELIPAGVEELLRINLSFADGLPRLATADI
QVGDVLVRKGELVLVLLEGANFDPEHFPNPGSIELDRPNPTSHLAFGRGQHFCPGSALGRRHAQIGIEALLKK
MPGVDLAVPIDQLVWRTRFQRRIPERLPVLW
>CYP121A1_Mycobacterium bovis BCG Pasteur 2695566750
MTATVLLEVPFSARGDRIPDAVAELRTREPIRKVRTITGAEAWLVSSYALCTQVLEDRRFSMKETAAAGAPRL
NALTVPPEVVNNMGNIADAGLRKAVMKAITPKAPGLEQFLRDTANSLLDNLITEGAPADLRNDFADPLATALH
CKVLGIPQEDGPKLFRSLSIAFMSSADPIPAAKINWDRDIEYMAGILENPNITTGLMGELSRLRKDPAYSHVS
DELFATIGVTFFGAGVISTGSFLTTALISLIQRPQLRNLLHEKPELIPAGVEELLRINLSFADGLPRLATADI
QVGDVLVRKGELVLVLLEGANFDPEHFPNPGSIELDRPNPTSHLAFGRGQHFCPGSALGRRHAQIGIEALLKK
MPGVDLAVPIDQLVWRTRFQRRIPERLPVLW
>CYP121A1_Mycobacterium bovis BCG str. Pasteur 1173P2 639831204
MTATVLLEVPFSARGDRIPDAVAELRTREPIRKVRTITGAEAWLVSSYALCTQVLEDRRFSMKETAAAGAPRL
NALTVPPEVVNNMGNIADAGLRKAVMKAITPKAPGLEQFLRDTANSLLDNLITEGAPADLRNDFADPLATALH
CKVLGIPQEDGPKLFRSLSIAFMSSADPIPAAKINWDRDIEYMAGILENPNITTGLMGELSRLRKDPAYSHVS
DELFATIGVTFFGAGVISTGSFLTTALISLIQRPQLRNLLHEKPELIPAGVEELLRINLSFADGLPRLATADI
QVGDVLVRKGELVLVLLEGANFDPEHFPNPGSIELDRPNPTSHLAFGRGQHFCPGSALGRRHAQIGIEALLKK
MPGVDLAVPIDQLVWRTRFQRRIPERLPVLW
>CYP121A1_Mycobacterium bovis BCG Phipps 2600667897
MTATVLLEVPFSARGDRIPDAVAELRTREPIRKVRTITGAEAWLVSSYALCTQVLEDRRFSMKETAAAGAPRL
NALTVPPEVVNNMGNIADAGLRKAVMKAITPKAPGLEQFLRDTANSLLDNLITEGAPADLRNDFADPLATALH

CKVLGIPQEDGPKLFRSLSIAFMSSADPIPAAKINWDRDIEYMAGILENPNITTGLMGELSRLRKDPAYSHVS
DELFATIGVTFFGAGVISTGSFLTTALISLIQRPQLRNLLHEKPELIPAGVEELLRINLSFADGLPRLATADI
QVGDVLVRKGELVLVLLEGANFDPEHFPNPGSIELDRPNPTSHLAFGRGQHFCPGSALGRRHAQIGIEALLKK
MPGVDLAVPIDQLVWRTRFQRRIPERLPVLW
>CYP121A1_Mycobacterium bovis BCG Phipps 2695495017
MTATVLLEVPFSARGDRIPDAVAELRTREPIRKVRTITGAEAWLVSSYALCTQVLEDRRFSMKETAAAGAPRL
NALTVPPEVVNNMGNIADAGLRKAVMKAITPKAPGLEQFLRDTANSLLDNLITEGAPADLRNDFADPLATALH
CKVLGIPQEDGPKLFRSLSIAFMSSADPIPAAKINWDRDIEYMAGILENPNITTGLMGELSRLRKDPAYSHVS
DELFATIGVTFFGAGVISTGSFLTTALISLIQRPQLRNLLHEKPELIPAGVEELLRINLSFADGLPRLATADI
QVGDVLVRKGELVLVLLEGANFDPEHFPNPGSIELDRPNPTSHLAFGRGQHFCPGSALGRRHAQIGIEALLKK
MPGVDLAVPIDQLVWRTRFQRRIPERLPVLW
>CYP121A1_Mycobacterium bovis BCG Prague 2600675256
MTATVLLEVPFSARGDRIPDAVAELRTREPIRKVRTITGAEAWLVSSYALCTQVLEDRRFSMKETAAAGAPRL
NALTVPPEVVNNMGNIADAGLRKAVMKAITPKAPGLEQFLRDTANSLLDNLITEGAPADLRNDFADPLATALH
CKVLGIPQEDGPKLFRSLSIAFMSSADPIPAAKINWDRDIEYMAGILENPNITTGLMGELSRLRKDPAYSHVS
DELFATIGVTFFGAGVISTGSFLTTALISLIQRPQLRNLLHEKPELIPAGVEELLRINLSFADGLPRLATADI
QVGDVLVRKGELVLVLLEGANFDPEHFPNPGSIELDRPNPTSHLAFGRGQHFCPGSALGRRHAQIGIEALLKK
MPGVDLAVPIDQLVWRTRFQRRIPERLPVLW
>CYP121A1_Mycobacterium bovis BCG Prague 2695507230
MTATVLLEVPFSARGDRIPDAVAELRTREPIRKVRTITGAEAWLVSSYALCTQVLEDRRFSMKETAAAGAPRL
NALTVPPEVVNNMGNIADAGLRKAVMKAITPKAPGLEQFLRDTANSLLDNLITEGAPADLRNDFADPLATALH
CKVLGIPQEDGPKLFRSLSIAFMSSADPIPAAKINWDRDIEYMAGILENPNITTGLMGELSRLRKDPAYSHVS
DELFATIGVTFFGAGVISTGSFLTTALISLIQRPQLRNLLHEKPELIPAGVEELLRINLSFADGLPRLATADI
QVGDVLVRKGELVLVLLEGANFDPEHFPNPGSIELDRPNPTSHLAFGRGQHFCPGSALGRRHAQIGIEALLKK
MPGVDLAVPIDQLVWRTRFQRRIPERLPVLW
>CYP121A1_Mycobacterium bovis BCG Russia 2695647620
MTATVLLEVPFSARGDRIPDAVAELRTREPIRKVRTITGAEAWLVSSYALCTQVLEDRRFSMKETAAAGAPRL
NALTVPPEVVNNMGNIADAGLRKAVMKAITPKAPGLEQFLRDTANSLLDNLITEGAPADLRNDFADPLATALH
CKVLGIPQEDGPKLFRSLSIAFMSSADPIPAAKINWDRDIEYMAGILENPNITTGLMGELSRLRKDPAYSHVS
DELFATIGVTFFGAGVISTGSFLTTALISLIQRPQLRNLLHEKPELIPAGVEELLRINLSFADGLPRLATADI
QVGDVLVRKGELVLVLLEGANFDPEHFPNPGSIELDRPNPTSHLAFGRGQHFCPGSALGRRHAQIGIEALLKK
MPGVDLAVPIDQLVWRTRFQRRIPERLPVLW
>CYP121A1_Mycobacterium bovis BCG S4 2715053569
MTATVLLEVPFSARGDRIPDAVAELRTREPIRKVRTITGAEAWLVSSYALCTQVLEDRRFSMKETAAAGAPRL
NALTVPPEVVNNMGNIADAGLRKAVMKAITPKAPGLEQFLRDTANSLLDNLITEGAPADLRNDFADPLATALH
CKVLGIPQEDGPKLFRSLSIAFMSSADPIPAAKINWDRDIEYMAGILENPNITTGLMGELSRLRKDPAYSHVS
DELFATIGVTFFGAGVISTGSFLTTALISLIQRPQLRNLLHEKPELIPAGVEELLRINLSFADGLPRLATADI
QVGDVLVRKGELVLVLLEGANFDPEHFPNPGSIELDRPNPTSHLAFGRGQHFCPGSALGRRHAQIGIEALLKK
MPGVDLAVPIDQLVWRTRFQRRIPERLPVLW
>CYP121A1_Mycobacterium bovis BCG Sweden 2600670638
MTATVLLEVPFSARGDRIPDAVAELRTREPIRKVRTITGAEAWLVSSYALCTQVLEDRRFSMKETAAAGAPRL
NALTVPPEVVNNMGNIADAGLRKAVMKAITPKAPGLEQFLRDTANSLLDNLITEGAPADLRNDFADPLATALH
CKVLGIPQEDGPKLFRSLSIAFMSSADPIPAAKINWDRDIEYMAGILENPNITTGLMGELSRLRKDPAYSHVS
DELFATIGVTFFGAGVISTGSFLTTALISLIQRPQLRNLLHEKPELIPAGVEELLRINLSFADGLPRLATADI
QVGDVLVRKGELVLVLLEGANFDPEHFPNPGSIELDRPNPTSHLAFGRGQHFCPGSALGRRHAQIGIEALLKK
MPGVDLAVPIDQLVWRTRFQRRIPERLPVLW
>CYP121A1_Mycobacterium bovis BCG Sweden 2695654365
MTATVLLEVPFSARGDRIPDAVAELRTREPIRKVRTITGAEAWLVSSYALCTQVLEDRRFSMKETAAAGAPRL
NALTVPPEVVNNMGNIADAGLRKAVMKAITPKAPGLEQFLRDTANSLLDNLITEGAPADLRNDFADPLATALH
CKVLGIPQEDGPKLFRSLSIAFMSSADPIPAAKINWDRDIEYMAGILENPNITTGLMGELSRLRKDPAYSHVS
DELFATIGVTFFGAGVISTGSFLTTALISLIQRPQLRNLLHEKPELIPAGVEELLRINLSFADGLPRLATADI
QVGDVLVRKGELVLVLLEGANFDPEHFPNPGSIELDRPNPTSHLAFGRGQHFCPGSALGRRHAQIGIEALLKK
MPGVDLAVPIDQLVWRTRFQRRIPERLPVLW
>CYP121A1_Mycobacterium bovis BCG Tice 2695633057
MTATVLLEVPFSARGDRIPDAVAELRTREPIRKVRTITGAEAWLVSSYALCTQVLEDRRFSMKETAAAGAPRL
NALTVPPEVVNNMGNIADAGLRKAVMKAITPKAPGLEQFLRDTANSLLDNLITEGAPADLRNDFADPLATALH
CKVLGIPQEDGPKLFRSLSIAFMSSADPIPAAKINWDRDIEYMAGILENPNITTGLMGELSRLRKDPAYSHVS
DELFATIGVTFFGAGVISTGSFLTTALISLIQRPQLRNLLHEKPELIPAGVEELLRINLSFADGLPRLATADI

QVGDVLVRKGELVLVLLEGANFDPEHFPNPGSIELDRPNPTSHLAFGRGQHFCPGSALGRRHAQIGIEALLKK
MPGVDLAVPIDQLVWRTRFQRRIPERLPVLW
>CYP121A1_Mycobacterium bovis BCG str. Tokyo 172 643735112
MTATVLLEVPFSARGDRIPDAVAELRTREPIRKVRTITGAEAWLVSSYALCTQVLEDRRFSMKETAAAGAPRL
NALTVPPEVVNNMGNIADAGLRKAVMKAITPKAPGLEQFLRDTANSLLDNLITEGAPADLRNDFADPLATALH
CKVLGIPQEDGPKLFRSLSIAFMSSADPIPAAKINWDRDIEYMAGILENPNITTGLMGELSRLRKDPAYSHVS
DELFATIGVTFFGAGVISTGSFLTTALISLIQRPQLRNLLHEKPELIPAGVEELLRINLSFADGLPRLATADI
QVGDVLVRKGELVLVLLEGANFDPEHFPNPGSIELDRPNPTSHLAFGRGQHFCPGSALGRRHAQIGIEALLKK
MPGVDLAVPIDQLVWRTRFQRRIPERLPVLW
>CYP121A1_Mycobacterium bovis BCG Tokyo 172 2686792489
MTATVLLEVPFSARGDRIPDAVAELRTREPIRKVRTITGAEAWLVSSYALCTQVLEDRRFSMKETAAAGAPRL
NALTVPPEVVNNMGNIADAGLRKAVMKAITPKAPGLEQFLRDTANSLLDNLITEGAPADLRNDFADPLATALH
CKVLGIPQEDGPKLFRSLSIAFMSSADPIPAAKINWDRDIEYMAGILENPNITTGLMGELSRLRKDPAYSHVS
DELFATIGVTFFGAGVISTGSFLTTALISLIQRPQLRNLLHEKPELIPAGVEELLRINLSFADGLPRLATADI
QVGDVLVRKGELVLVLLEGANFDPEHFPNPGSIELDRPNPTSHLAFGRGQHFCPGSALGRRHAQIGIEALLKK
MPGVDLAVPIDQLVWRTRFQRRIPERLPVLW
>CYP121A1_Mycobacterium bovis BCG-1 2687572115
MTATVLLEVPFSARGDRIPDAVAELRTREPIRKVRTITGAEAWLVSSYALCTQVLEDRRFSMKETAAAGAPRL
NALTVPPEVVNNMGNIADAGLRKAVMKAITPKAPGLEQFLRDTANSLLDNLITEGAPADLRNDFADPLATALH
CKVLGIPQEDGPKLFRSLSIAFMSSADPIPAAKINWDRDIEYMAGILENPNITTGLMGELSRLRKDPAYSHVS
DELFATIGVTFFGAGVISTGSFLTTALISLIQRPQLRNLLHEKPELIPAGVEELLRINLSFADGLPRLATADI
QVGDVLVRKGELVLVLLEGANFDPEHFPNPGSIELDRPNPTSHLAFGRGQHFCPGSALGRRHAQIGIEALLKK
MPGVDLAVPIDQLVWRTRFQRRIPERLPVLW
>CYP121A1_Mycobacterium bovis BCG-1 (Russia) 2688651137
MTATVLLEVPFSARGDRIPDAVAELRTREPIRKVRTITGAEAWLVSSYALCTQVLEDRRFSMKETAAAGAPRL
NALTVPPEVVNNMGNIADAGLRKAVMKAITPKAPGLEQFLRDTANSLLDNLITEGAPADLRNDFADPLATALH
CKVLGIPQEDGPKLFRSLSIAFMSSADPIPAAKINWDRDIEYMAGILENPNITTGLMGELSRLRKDPAYSHVS
DELFATIGVTFFGAGVISTGSFLTTALISLIQRPQLRNLLHEKPELIPAGVEELLRINLSFADGLPRLATADI
QVGDVLVRKGELVLVLLEGANFDPEHFPNPGSIELDRPNPTSHLAFGRGQHFCPGSALGRRHAQIGIEALLKK
MPGVDLAVPIDQLVWRTRFQRRIPERLPVLW
>CYP121A1_Mycobacterium bovis BCG-Denmark TMC 1010 ATCC 35733 2547310375
MTATVLLEVPFSARGDRIPDAVAELRTREPIRKVRTITGAEAWLVSSYALCTQVLEDRRFSMKETAAAGAPRL
NALTVPPEVVNNMGNIADAGLRKAVMKAITPKAPGLEQFLRDTANSLLDNLITEGAPADLRNDFADPLATALH
CKVLGIPQEDGPKLFRSLSIAFMSSADPIPAAKINWDRDIEYMAGILENPNITTGLMGELSRLRKDPAYSHVS
DELFATIGVTFFGAGVISTGSFLTTALISLIQRPQLRNLLHEKPELIPAGVEELLRINLSFADGLPRLATADI
QVGDVLVRKGELVLVLLEGANFDPEHFPNPGSIELDRPNPTSHLAFGRGQHFCPGSALGRRHAQIGIEALLKK
MPGVDLAVPIDQLVWRTRFQRRIPERLPVLW
>CYP121A1_Mycobacterium bovis BCG-Russia TMC 1022 ATCC 35740 2547318842
MTATVLLEVPFSARGDRIPDAVAELRTREPIRKVRTITGAEAWLVSSYALCTQVLEDRRFSMKETAAAGAPRL
NALTVPPEVVNNMGNIADAGLRKAVMKAITPKAPGLEQFLRDTANSLLDNLITEGAPADLRNDFADPLATALH
CKVLGIPQEDGPKLFRSLSIAFMSSADPIPAAKINWDRDIEYMAGILENPNITTGLMGELSRLRKDPAYSHVS
DELFATIGVTFFGAGVISTGSFLTTALISLIQRPQLRNLLHEKPELIPAGVEELLRINLSFADGLPRLATADI
QVGDVLVRKGELVLVLLEGANFDPEHFPNPGSIELDRPNPTSHLAFGRGQHFCPGSALGRRHAQIGIEALLKK
MPGVDLAVPIDQLVWRTRFQRRIPERLPVLW
>CYP121A1_Mycobacterium bovis BCG-Tice TMC 1028 ATCC 35743 2547315533
MTATVLLEVPFSARGDRIPDAVAELRTREPIRKVRTITGAEAWLVSSYALCTQVLEDRRFSMKETAAAGAPRL
NALTVPPEVVNNMGNIADAGLRKAVMKAITPKAPGLEQFLRDTANSLLDNLITEGAPADLRNDFADPLATALH
CKVLGIPQEDGPKLFRSLSIAFMSSADPIPAAKINWDRDIEYMAGILENPNITTGLMGELSRLRKDPAYSHVS
DELFATIGVTFFGAGVISTGSFLTTALISLIQRPQLRNLLHEKPELIPAGVEELLRINLSFADGLPRLATADI
QVGDVLVRKGELVLVLLEGANFDPEHFPNPGSIELDRPNPTSHLAFGRGQHFCPGSALGRRHAQIGIEALLKK
MPGVDLAVPIDQLVWRTRFQRRIPERLPVLW
>CYP121A1_Mycobacterium bovis Bz 31150 2581586258
MTATVLLEVPFSARGDRIPDAVAELRTREPIRKVRTITGAEAWLVSSYALCTQVLEDRRFSMKETAAAGAPRL
NALTVPPEVVNNMGNIADAGLRKAVMKAITPKAPGLEQFLRDTANSLLDNLITEGAPADLRNDFADPLATALH
CKVLGIPQEDGPKLFRSLSIAFMSSADPIPAAKINWDRDIEYMAGILENPNITTGLMGELSRLRKDPAYSHVS
DELFATIGVTFFGAGVISTGSFLTTALISLIQRPQLRNLLHEKPELIPAGVEELLRINLSFADGLPRLATADI
QVGDVLVRKGELVLVLLEGANFDPEHFPNPGSIELDRPNPTSHLAFGRGQHFCPGSALGRRHAQIGIEALLKK
MPGVDLAVPIDQLVWRTRFQRRIPERLPVLW

>CYP121A1_Mycobacterium bovis D 4155 2580745050
MTATVLLEVPFSARGDRIPDAVAELRTREPIRKVRTITGAEAWLVSSYALCTQVLEDRRFSMKETAAAGAPRL
NALTVPPEVVNNMGNIADAGLRKAVMKAITPKAPGLEQFLRDTANSLLDNLITEGAPADLRNDFADPLATALH
CKVLGIPQEDGPKLFRSLSIAFMSSADPIPAAKINWDRDIEYMAGILENPNITTGLMGELSRLRKDPAYSHVS
DELFATIGVTFFGAGVISTGSFLTTALISLIQRPQLRNLLHEKPELIPAGVEELLRINLSFADGLPRLATADI
QVGDVLVRKGELVLVLLEGANFDPEHFPNPGSIELDRPNPTSHLAFGRGQHFCPGSALGRRHAQIGIEALLKK
MPGVDLAVPIDQLVWRTRFQRRIPERLPVLW
>CYP121A1_Mycobacterium bovis Kc 32216 2584105781
MTATVLLEVPFSARGDRIPDAVAELRTREPIRKVRTITGAEAWLVSSYALCTQVLEDRRFSMKETAAAGAPRL
NALTVPPEVVNNMGNIADAGLRKAVMKAITPKAPGLEQFLRDTANSLLDNLITEGAPADLRNDFADPLATALH
CKVLGIPQEDGPKLFRSLSIAFMSSADPIPAAKINWDRDIEYMAGILENPNITTGLMGELSRLRKDPAYSHVS
DELFATIGVTFFGAGVISTGSFLTTALISLIQRPQLRNLLHEKPELIPAGVEELLRINLSFADGLPRLATADI
QVGDVLVRKGELVLVLLEGANFDPEHFPNPGSIELDRPNPTSHLAFGRGQHFCPGSALGRRHAQIGIEALLKK
MPGVDLAVPIDQLVWRTRFQRRIPERLPVLW
>CYP121A1_Mycobacterium bovis Kc 9614 2580124466
MTATVLLEVPFSARGDRIPDAVAELRTREPIRKVRTITGAEAWLVSSYALCTQVLEDRRFSMKETAAAGAPRL
NALTVPPEVVNNMGNIADAGLRKAVMKAITPKAPGLEQFLRDTANSLLDNLITEGAPADLRNDFADPLATALH
CKVLGIPQEDGPKLFRSLSIAFMSSADPIPAAKINWDRDIEYMAGILENPNITTGLMGELSRLRKDPAYSHVS
DELFATIGVTFFGAGVISTGSFLTTALISLIQRPQLRNLLHEKPELIPAGVEELLRINLSFADGLPRLATADI
QVGDVLVRKGELVLVLLEGANFDPEHFPNPGSIELDRPNPTSHLAFGRGQHFCPGSALGRRHAQIGIEALLKK
MPGVDLAVPIDQLVWRTRFQRRIPERLPVLW
>CYP121A1_Mycobacterium bovis MAL010093 2583745909
MTATVLLEVPFSARGDRIPDAVAELRTREPIRKVRTITGAEAWLVSSYALCTQVLEDRRFSMKETAAAGAPRL
NALTVPPEVVNNMGNIADAGLRKAVMKAITPKAPGLEQFLRDTANSLLDNLITEGAPADLRNDFADPLATALH
CKVLGIPQEDGPKLFRSLSIAFMSSADPIPAAKINWDRDIEYMAGILENPNITTGLMGELSRLRKDPAYSHVS
DELFATIGVTFFGAGVISTGSFLTTALISLIQRPQLRNLLHEKPELIPAGVEELLRINLSFADGLPRLATADI
QVGDVLVRKGELVLVLLEGANFDPEHFPNPGSIELDRPNPTSHLAFGRGQHFCPGSALGRRHAQIGIEALLKK
MPGVDLAVPIDQLVWRTRFQRRIPERLPVLW
>CYP121A1_Mycobacterium bovis MB1 2652514096
MTATVLLEVPFSARGDRIPDAVAELRTREPIRKVRTITGAEAWLVSSYALCTQVLEDRRFSMKETAAAGAPRL
NALTVPPEVVNNMGNIADAGLRKAVMKAITPKAPGLEQFLRDTANSLLDNLITEGAPADLRNDFADPLATALH
CKVLGIPQEDGPKLFRSLSIAFMSSADPIPAAKINWDRDIEYMAGILENPNITTGLMGELSRLRKDPAYSHVS
DELFATIGVTFFGAGVISTGSFLTTALISLIQRPQLRNLLHEKPELIPAGVEELLRINLSFADGLPRLATADI
QVGDVLVRKGELVLVLLEGANFDPEHFPNPGSIELDRPNPTSHLAFGRGQHFCPGSALGRRHAQIGIEALLKK
MPGVDLAVPIDQLVWRTRFQRRIPERLPVLW
>CYP121A1_Mycobacterium bovis MB3 2641311500
MTATVLLEVPFSARGDRIPDAVAELRTREPIRKVRTITGAEAWLVSSYALCTQVLEDRRFSMKETAAAGAPRL
NALTVPPEVVNNMGNIADAGLRKAVMKAITPKAPGLEQFLRDTANSLLDNLITEGAPADLRNDFADPLATALH
CKVLGIPQEDGPKLFRSLSIAFMSSADPIPAAKINWDRDIEYMAGILENPNITTGLMGELSRLRKDPAYSHVS
DELFATIGVTFFGAGVISTGSFLTTALISLIQRPQLRNLLHEKPELIPAGVEELLRINLSFADGLPRLATADI
QVGDVLVRKGELVLVLLEGANFDPEHFPNPGSIELDRPNPTSHLAFGRGQHFCPGSALGRRHAQIGIEALLKK
MPGVDLAVPIDQLVWRTRFQRRIPERLPVLW
>CYP121A1_Mycobacterium bovis MB4 2707489337
MTATVLLEVPFSARGDRIPDAVAELRTREPIRKVRTITGAEAWLVSSYALCTQVLEDRRFSMKETAAAGAPRL
NALTVPPEVVNNMGNIADAGLRKAVMKAITPKAPGLEQFLRDTANSLLDNLITEGAPADLRNDFADPLATALH
CKVLGIPQEDGPKLFRSLSIAFMSSADPIPAAKINWDRDIEYMAGILENPNITTGLMGELSRLRKDPAYSHVS
DELFATIGVTFFGAGVISTGSFLTTALISLIQRPQLRNLLHEKPELIPAGVEELLRINLSFADGLPRLATADI
QVGDVLVRKGELVLVLLEGANFDPEHFPNPGSIELDRPNPTSHLAFGRGQHFCPGSALGRRHAQIGIEALLKK
MPGVDLAVPIDQLVWRTRFQRRIPERLPVLW
>CYP121A1_Mycobacterium bovis MbURU-001 2647879056
MTATVLLEVPFSARGDRIPDAVAELRTREPIRKVRTITGAEAWLVSSYALCTQVLEDRRFSMKETAAAGAPRL
NALTVPPEVVNNMGNIADAGLRKAVMKAITPKAPGLEQFLRDTANSLLDNLITEGAPADLRNDFADPLATALH
CKVLGIPQEDGPKLFRSLSIAFMSSADPIPAAKINWDRDIEYMAGILENPNITTGLMGELSRLRKDPAYSHVS
DELFATIGVTFFGAGVISTGSFLTTALISLIQRPQLRNLLHEKPELIPAGVEELLRINLSFADGLPRLATADI
QVGDVLVRKGELVLVLLEGANFDPEHFPNPGSIELDRPNPTSHLAFGRGQHFCPGSALGRRHAQIGIEALLKK
MPGVDLAVPIDQLVWRTRFQRRIPERLPVLW
>CYP121A1_Mycobacterium bovis Mr 4387 2580769514

MTATVLLEVPFSARGDRIPDAVAELRTREPIRKVRTITGAEAWLVSSYALCTQVLEDRRFSMKETAAAGAPRL
NALTVPPEVVNNMGNIADAGLRKAVMKAITPKAPGLEQFLRDTANSLLDNLITEGAPADLRNDFADPLATALH
CKVLGIPQEDGPKLFRSLSIAFMSSADPIPAAKINWDRDIEYMAGILENPNITTGLMGELSRLRKDPAYSHVS
DELFATIGVTFFGAGVISTGSFLTTALISLIQRPQLRNLLHEKPELIPAGVEELLRINLSFADGLPRLATADI
QVGDVLVRKGELVLVLLEGANFDPEHFPNPGSIELDRPNPTSHLAFGRGQHFCPGSALGRRHAQIGIEALLKK
MPGVDLAVPIDQLVWRTRFQRRIPERLPVLW
>CYP121A1_Mycobacterium bovis Wt 21231 2584004514
MTATVLLEVPFSARGDRIPDAVAELRTREPIRKVRTITGAEAWLVSSYALCTQVLEDRRFSMKETAAAGAPRL
NALTVPPEVVNNMGNIADAGLRKAVMKAITPKAPGLEQFLRDTANSLLDNLITEGAPADLRNDFADPLATALH
CKVLGIPQEDGPKLFRSLSIAFMSSADPIPAAKINWDRDIEYMAGILENPNITTGLMGELSRLRKDPAYSHVS
DELFATIGVTFFGAGVISTGSFLTTALISLIQRPQLRNLLHEKPELIPAGVEELLRINLSFADGLPRLATADI
QVGDVLVRKGELVLVLLEGANFDPEHFPNPGSIELDRPNPTSHLAFGRGQHFCPGSALGRRHAQIGIEALLKK
MPGVDLAVPIDQLVWRTRFQRRIPERLPVLW
>CYP121A1_Mycobacterium bovis Wt 21419 2581356886
MTATVLLEVPFSARGDRIPDAVAELRTREPIRKVRTITGAEAWLVSSYALCTQVLEDRRFSMKETAAAGAPRL
NALTVPPEVVNNMGNIADAGLRKAVMKAITPKAPGLEQFLRDTANSLLDNLITEGAPADLRNDFADPLATALH
CKVLGIPQEDGPKLFRSLSIAFMSSADPIPAAKINWDRDIEYMAGILENPNITTGLMGELSRLRKDPAYSHVS
DELFATIGVTFFGAGVISTGSFLTTALISLIQRPQLRNLLHEKPELIPAGVEELLRINLSFADGLPRLATADI
QVGDVLVRKGELVLVLLEGANFDPEHFPNPGSIELDRPNPTSHLAFGRGQHFCPGSALGRRHAQIGIEALLKK
MPGVDLAVPIDQLVWRTRFQRRIPERLPVLW
>CYP121A1_Mycobacterium caprae Allgaeu 2730172005
MTATVLLEVPFSARGDRIPDAVAELRTREPIRKVRTITGAEAWLVSSYALCTQVLEDRRFSMKETAAAGAPRL
NALTVPPEVVNNMGNIADAGLRKAVMKAITPKAPGLEQFLRDTANSLLDNLITEGAPADLRNDFADPLATALH
CKVLGIPQEDGPKLFRSLSIAFMSSADPIPAAKINWDRDIEYMAGILENPNITTGLMGELSRLRKDPAYSHVS
DELFATIGVTFFGAGVISTGSFLTTALISLIQRPQLRNLLHEKPELIPAGVEELLRINLSFADGLPRLATADI
QVGDVLVRKGELVLVLLEGANFDPEHFPNPGSIELDRPNPTSHLAFGRGQHFCPGSALGRRHAQIGIEALLKK
MPGVDLAVPIDQLVWRTRFQRRIPERLPVLW
>CYP121A1_Mycobacterium caprae MB2 2699765607
MTATVLLEVPFSARGDRIPDAVAELRTREPIRKVRTITGAEAWLVSSYALCTQVLEDRRFSMKETAAAGAPRL
NALTVPPEVVNNMGNIADAGLRKAVMKAITPKAPGLEQFLRDTANSLLDNLITEGAPADLRNDFADPLATALH
CKVLGIPQEDGPKLFRSLSIAFMSSADPIPAAKINWDRDIEYMAGILENPNITTGLMGELSRLRKDPAYSHVS
DELFATIGVTFFGAGVISTGSFLTTALISLIQRPQLRNLLHEKPELIPAGVEELLRINLSFADGLPRLATADI
QVGDVLVRKGELVLVLLEGANFDPEHFPNPGSIELDRPNPTSHLAFGRGQHFCPGSALGRRHAQIGIEALLKK
MPGVDLAVPIDQLVWRTRFQRRIPERLPVLW
>CYP121A1_Mycobacterium tuberculosis 001_13432 2716307950
MTATVLLEVPFSARGDRIPDAVAELRTREPIRKVRTITGAEAWLVSSYALCTQVLEDRRFSMKETAAAGAPRL
NALTVPPEVVNNMGNIADAGLRKAVMKAITPKAPGLEQFLRDTANSLLDNLITEGAPADLRNDFADPLATALH
CKVLGIPQEDGPKLFRSLSIAFMSSADPIPAAKINWDRDIEYMAGILENPNITTGLMGELSRLRKDPAYSHVS
DELFATIGVTFFGAGVISTGSFLTTALISLIQRPQLRNLLHEKPELIPAGVEELLRINLSFADGLPRLATADI
QVGDVLVRKGELVLVLLEGANFDPEHFPNPGSIELDRPNPTSHLAFGRGQHFCPGSALGRRHAQIGIEALLKK
MPGVDLAVPIDQLVWRTRFQRRIPERLPVLW
>CYP121A1_Mycobacterium tuberculosis 10060 2631864560
MTATVLLEVPFSARGDRIPDAVAELRTREPIRKVRTITGAEAWLVSSYALCTQVLEDRRFSMKETAAAGAPRL
NALTVPPEVVNNMGNIADAGLRKAVMKAITPKAPGLEQFLRDTANSLLDNLITEGAPADLRNDFADPLATALH
CKVLGIPQEDGPKLFRSLSIAFMSSADPIPAAKINWDRDIEYMAGILENPNITTGLMGELSRLRKDPAYSHVS
DELFATIGVTFFGAGVISTGSFLTTALISLIQRPQLRNLLHEKPELIPAGVEELLRINLSFADGLPRLATADI
QVGDVLVRKGELVLVLLEGANFDPEHFPNPGSIELDRPNPTSHLAFGRGQHFCPGSALGRRHAQIGIEALLKK
MPGVDLAVPIDQLVWRTRFQRRIPERLPVLW
>CYP121A1_Mycobacterium tuberculosis 10217 2621698763
MTATVLLEVPFSARGDRIPDAVAELRTREPIRKVRTITGAEAWLVSSYALCTQVLEDRRFSMKETAAAGAPRL
NALTVPPEVVNNMGNIADAGLRKAVMKAITPKAPGLEQFLRDTANSLLDNLITEGAPADLRNDFADPLATALH
CKVLGIPQEDGPKLFRSLSIAFMSSADPIPAAKINWDRDIEYMAGILENPNITTGLMGELSRLRKDPAYSHVS
DELFATIGVTFFGAGVISTGSFLTTALISLIQRPQLRNLLHEKPELIPAGVEELLRINLSFADGLPRLATADI
QVGDVLVRKGELVLVLLEGANFDPEHFPNPGSIELDRPNPTSHLAFGRGQHFCPGSALGRRHAQIGIEALLKK
MPGVDLAVPIDQLVWRTRFQRRIPERLPVLW
>CYP121A1_Mycobacterium tuberculosis 1173CS 2576560943
MTATVLLEVPFSARGDRIPDAVAELRTREPIRKVRTITGAEAWLVSSYALCTQVLEDRRFSMKETAAAGAPRL
NALTVPPEVVNNMGNIADAGLRKAVMKAITPKAPGLEQFLRDTANSLLDNLITEGAPADLRNDFADPLATALH

CKVLGIPQEDGPKLFRSLSIAFMSSADPIPAAKINWDRDIEYMAGILENPNITTGLMGELSRLRKDPAYSHVS
DELFATIGVTFFGAGVISTGSFLTTALISLIQRPQLRNLLHEKPELIPAGVEELLRINLSFADGLPRLATADI
QVGDVLVRKGELVLVLLEGANFDPEHFPNPGSIELDRPNPTSHLAFGRGQHFCPGSALGRRHAQIGIEALLKK
MPGVDLAVPIDQLVWRTRFQRRIPERLPVLW
>CYP121A1_Mycobacterium tuberculosis 1200172 2701676292
MTATVLLEVPFSARGDRIPDAVAELRTREPIRKVRTITGAEAWLVSSYALCTQVLEDRRFSMKETAAAGAPRL
NALTVPPEVVNNMGNIADAGLRKAVMKAITPKAPGLEQFLRDTANSLLDNLITEGAPADLRNDFADPLATALH
CKVLGIPQEDGPKLFRSLSIAFMSSADPIPAAKINWDRDIEYMAGILENPNITTGLMGELSRLRKDPAYSHVS
DELFATIGVTFFGAGVISTGSFLTTALISLIQRPQLRNLLHEKPELIPAGVEELLRINLSFADGLPRLATADI
QVGDVLVRKGELVLVLLEGANFDPEHFPNPGSIELDRPNPTSHLAFGRGQHFCPGSALGRRHAQIGIEALLKK
MPGVDLAVPIDQLVWRTRFQRRIPERLPVLW
>CYP121A1_Mycobacterium tuberculosis 1200782 2659059950
MTATVLLEVPFSARGDRIPDAVAELRTREPIRKVRTITGAEAWLVSSYALCTQVLEDRRFSMKETAAAGAPRL
NALTVPPEVVNNMGNIADAGLRKAVMKAITPKAPGLEQFLRDTANSLLDNLITEGAPADLRNDFADPLATALH
CKVLGIPQEDGPKLFRSLSIAFMSSADPIPAAKINWDRDIEYMAGILENPNITTGLMGELSRLRKDPAYSHVS
DELFATIGVTFFGAGVISTGSFLTTALISLIQRPQLRNLLHEKPELIPAGVEELLRINLSFADGLPRLATADI
QVGDVLVRKGELVLVLLEGANFDPEHFPNPGSIELDRPNPTSHLAFGRGQHFCPGSALGRRHAQIGIEALLKK
MPGVDLAVPIDQLVWRTRFQRRIPERLPVLW
>CYP121A1_Mycobacterium tuberculosis 1300039 2666672983
MTATVLLEVPFSARGDRIPDAVAELRTREPIRKVRTITGAEAWLVSSYALCTQVLEDRRFSMKETAAAGAPRL
NALTVPPEVVNNMGNIADAGLRKAVMKAITPKAPGLEQFLRDTANSLLDNLITEGAPADLRNDFADPLATALH
CKVLGIPQEDGPKLFRSLSIAFMSSADPIPAAKINWDRDIEYMAGILENPNITTGLMGELSRLRKDPAYSHVS
DELFATIGVTFFGAGVISTGSFLTTALISLIQRPQLRNLLHEKPELIPAGVEELLRINLSFADGLPRLATADI
QVGDVLVRKGELVLVLLEGANFDPEHFPNPGSIELDRPNPTSHLAFGRGQHFCPGSALGRRHAQIGIEALLKK
MPGVDLAVPIDQLVWRTRFQRRIPERLPVLW
>CYP121A1_Mycobacterium tuberculosis 1301087 2670244684
MTATVLLEVPFSARGDRIPDAVAELRTREPIRKVRTITGAEAWLVSSYALCTQVLEDRRFSMKETAAAGAPRL
NALTVPPEVVNNMGNIADAGLRKAVMKAITPKAPGLEQFLRDTANSLLDNLITEGAPADLRNDFADPLATALH
CKVLGIPQEDGPKLFRSLSIAFMSSADPIPAAKINWDRDIEYMAGILENPNITTGLMGELSRLRKDPAYSHVS
DELFATIGVTFFGAGVISTGSFLTTALISLIQRPQLRNLLHEKPELIPAGVEELLRINLSFADGLPRLATADI
QVGDVLVRKGELVLVLLEGANFDPEHFPNPGSIELDRPNPTSHLAFGRGQHFCPGSALGRRHAQIGIEALLKK
MPGVDLAVPIDQLVWRTRFQRRIPERLPVLW
>CYP121A1_Mycobacterium tuberculosis 1301088 2669624553
MTATVLLEVPFSARGDRIPDAVAELRTREPIRKVRTITGAEAWLVSSYALCTQVLEDRRFSMKETAAAGAPRL
NALTVPPEVVNNMGNIADAGLRKAVMKAITPKAPGLEQFLRDTANSLLDNLITEGAPADLRNDFADPLATALH
CKVLGIPQEDGPKLFRSLSIAFMSSADPIPAAKINWDRDIEYMAGILENPNITTGLMGELSRLRKDPAYSHVS
DELFATIGVTFFGAGVISTGSFLTTALISLIQRPQLRNLLHEKPELIPAGVEELLRINLSFADGLPRLATADI
QVGDVLVRKGELVLVLLEGANFDPEHFPNPGSIELDRPNPTSHLAFGRGQHFCPGSALGRRHAQIGIEALLKK
MPGVDLAVPIDQLVWRTRFQRRIPERLPVLW
>CYP121A1_Mycobacterium tuberculosis 13-3935 2651521225
EQFLRDTANSLLDNLITEGAPADLRNDFADPLATALHCKVLGIPQEDGPKLFRSLSIAFMSSADPIPAAKINW
DRDIEYMAGILENPNITTGLMGELSRLRKDPAYSHVSDELFATIGVTFFGAGVISTGSFLTTALISLIQRPQL
RNLLHEKPELIPAGVEELLRINLSFADGLPRLATADIQVGDVLVRKGELVLVLLEGANFDPEHFPNPGSIELD
RPNPTSHLAFGRGQHFCPGSALGRRHAQIGIEALLKKMPGVDLAVPIDQLVWRTRFQRRIPERLPVLW
>CYP121A1_Mycobacterium tuberculosis 1429BH 2606487833
MTATVLLEVPFSARGDRIPDAVAELRTREPIRKVRTITGAEAWLVSSYALCTQVLEDRRFSMKETAAAGAPRL
NALTVPPEVVNNMGNIADAGLRKAVMKAITPKAPGLEQFLRDTANSLLDNLITEGAPADLRNDFADPLATALH
CKVLGIPQEDGPKLFRSLSIAFMSSADPIPAAKINWDRDIEYMAGILENPNITTGLMGELSRLRKDPAYSHVS
DELFATIGVTFFGAGVISTGSFLTTALISLIQRPQLRNLLHEKPELIPAGVEELLRINLSFADGLPRLATADI
QVGDVLVRKGELVLVLLEGANFDPEHFPNPGSIELDRPNPTSHLAFGRGQHFCPGSALGRRHAQIGIEALLKK
MPGVDLAVPIDQLVWRTRFQRRIPERLPVLW
>CYP121A1_Mycobacterium tuberculosis 1430BH 2621760970
MTATVLLEVPFSARGDRIPDAVAELRTREPIRKVRTITGAEAWLVSSYALCTQVLEDRRFSMKETAAAGAPRL
NALTVPPEVVNNMGNIADAGLRKAVMKAITPKAPGLEQFLRDTANSLLDNLITEGAPADLRNDFADPLATALH
CKVLGIPQEDGPKLFRSLSIAFMSSADPIPAAKINWDRDIEYMAGILENPNITTGLMGELSRLRKDPAYSHVS
DELFATIGVTFFGAGVISTGSFLTTALISLIQRPQLRNLLHEKPELIPAGVEELLRINLSFADGLPRLATADI
QVGDVLVRKGELVLVLLEGANFDPEHFPNPGSIELDRPNPTSHLAFGRGQHFCPGSALGRRHAQIGIEALLKK
MPGVDLAVPIDQLVWRTRFQRRIPERLPVLW

>CYP121A1_Mycobacterium tuberculosis 1500108 2671006418
MTATVLLEVPFSARGDRIPDAVAELRTREPIRKVRTITGAEAWLVSSYALCTQVLEDRRFSMKETAAAGAPRL
NALTVPPEVVNNMGNIADAGLRKAVMKAITPKAPGLEQFLRDTANSLLDNLITEGAPADLRNDFADPLATALH
CKVLGIPQEDGPKLFRSLSIAFMSSADPIPAAKINWDRDIEYMAGILENPNITTGLMGELSRLRKDPAYSHVS
DELFATIGVTFFGAGVISTGSFLTTALISLIQRPQLRNLLHEKPELIPAGVEELLRINLSFADGLPRLATADI
QVGDVLVRKGELVLVLLEGANFDPEHFPNPGSIELDRPNPTSHLAFGRGQHFCPGSALGRRHAQIGIEALLKK
MPGVDLAVPIDQLVWRTRFQRRIPERLPVLW
>CYP121A1_Mycobacterium tuberculosis 1500176 2665600945
MTATVLLEVPFSARGDRIPDAVAELRTREPIRKVRTITGAEAWLVSSYALCTQVLEDRRFSMKETAAAGAPRL
NALTVPPEVVNNMGNIADAGLRKAVMKAITPKAPGLEQFLRDTANSLLDNLITEGAPADLRNDFADPLATALH
CKVLGIPQEDGPKLFRSLSIAFMSSADPIPAAKINWDRDIEYMAGILENPNITTGLMGELSRLRKDPAYSHVS
DELFATIGVTFFGAGVISTGSFLTTALISLIQRPQLRNLLHEKPELIPAGVEELLRINLSFADGLPRLATADI
QVGDVLVRKGELVLVLLEGANFDPEHFPNPGSIELDRPNPTSHLAFGRGQHFCPGSALGRRHAQIGIEALLKK
MPGVDLAVPIDQLVWRTRFQRRIPERLPVLW
>CYP121A1_Mycobacterium tuberculosis 1500524 2663439817
MTATVLLEVPFSARGDRIPDAVAELRTREPIRKVRTITGAEAWLVSSYALCTQVLEDRRFSMKETAAAGAPRL
NALTVPPEVVNNMGNIADAGLRKAVMKAITPKAPGLEQFLRDTANSLLDNLITEGAPADLRNDFADPLATALH
CKVLGIPQEDGPKLFRSLSIAFMSSADPIPAAKINWDRDIEYMAGILENPNITTGLMGELSRLRKDPAYSHVS
DELFATIGVTFFGAGVISTGSFLTTALISLIQRPQLRNLLHEKPELIPAGVEELLRINLSFADGLPRLATADI
QVGDVLVRKGELVLVLLEGANFDPEHFPNPGSIELDRPNPTSHLAFGRGQHFCPGSALGRRHAQIGIEALLKK
MPGVDLAVPIDQLVWRTRFQRRIPERLPVLW
>CYP121A1_Mycobacterium tuberculosis 1615 2577953223
MTATVLLEVPFSARGDRIPDAVAELRTREPIRKVRTITGAEAWLVSSYALCTQVLEDRRFSMKETAAAGAPRL
NALTVPPEVVNNMGNIADAGLRKAVMKAITPKAPGLEQFLRDTANSLLDNLITEGAPADLRNDFADPLATALH
CKVLGIPQEDGPKLFRSLSIAFMSSADPIPAAKINWDRDIEYMAGILENPNITTGLMGELSRLRKDPAYSHVS
DELFATIGVTFFGAGVISTGSFLTTALISLIQRPQLRNLLHEKPELIPAGVEELLRINLSFADGLPRLATADI
QVGDVLVRKGELVLVLLEGANFDPEHFPNPGSIELDRPNPTSHLAFGRGQHFCPGSALGRRHAQIGIEALLKK
MPGVDLAVPIDQLVWRTRFQRRIPERLPVLW
>CYP121A1_Mycobacterium tuberculosis 16954 2606379837
MTATVLLEVPFSARGDRIPDAVAELRTREPIRKVRTITGAEAWLVSSYALCTQVLEDRRFSMKETAAAGAPRL
NALTVPPEVVNNMGNIADAGLRKAVMKAITPKAPGLEQFLRDTANSLLDNLITEGAPADLRNDFADPLATALH
CKVLGIPQEDGPKLFRSLSIAFMSSADPIPAAKINWDRDIEYMAGILENPNITTGLMGELSRLRKDPAYSHVS
DELFATIGVTFFGAGVISTGSFLTTALISLIQRPQLRNLLHEKPELIPAGVEELLRINLSFADGLPRLATADI
QVGDVLVRKGELVLVLLEGANFDPEHFPNPGSIELDRPNPTSHLAFGRGQHFCPGSALGRRHAQIGIEALLKK
MPGVDLAVPIDQLVWRTRFQRRIPERLPVLW
>CYP121A1_Mycobacterium tuberculosis 16955 2575108176
MTATVLLEVPFSARGDRIPDAVAELRTREPIRKVRTITGAEAWLVSSYALCTQVLEDRRFSMKETAAAGAPRL
NALTVPPEVVNNMGNIADAGLRKAVMKAITPKAPGLEQFLRDTANSLLDNLITEGAPADLRNDFADPLATALH
CKVLGIPQEDGPKLFRSLSIAFMSSADPIPAAKINWDRDIEYMAGILENPNITTGLMGELSRLRKDPAYSHVS
DELFATIGVTFFGAGVISTGSFLTTALISLIQRPQLRNLLHEKPELIPAGVEELLRINLSFADGLPRLATADI
QVGDVLVRKGELVLVLLEGANFDPEHFPNPGSIELDRPNPTSHLAFGRGQHFCPGSALGRRHAQIGIEALLKK
MPGVDLAVPIDQLVWRTRFQRRIPERLPVLW
>CYP121A1_Mycobacterium tuberculosis 1700302 2661053203
MTATVLLEVPFSARGDRIPDAVAELRTREPIRKVRTITGAEAWLVSSYALCTQVLEDRRFSMKETAAAGAPRL
NALTVPPEVVNNMGNIADAGLRKAVMKAITPKAPGLEQFLRDTANSLLDNLITEGAPADLRNDFADPLATALH
CKVLGIPQEDGPKLFRSLSIAFMSSADPIPAAKINWDRDIEYMAGILENPNITTGLMGELSRLRKDPAYSHVS
DELFATIGVTFFGAGVISTGSFLTTALISLIQRPQLRNLLHEKPELIPAGVEELLRINLSFADGLPRLATADI
QVGDVLVRKGELVLVLLEGANFDPEHFPNPGSIELDRPNPTSHLAFGRGQHFCPGSALGRRHAQIGIEALLKK
MPGVDLAVPIDQLVWRTRFQRRIPERLPVLW
>CYP121A1_Mycobacterium tuberculosis 1700938 2691678361
MTATVLLEVPFSARGDRIPDAVAELRTREPIRKVRTITGAEAWLVSSYALCTQVLEDRRFSMKETAAAGAPRL
NALTVPPEVVNNMGNIADAGLRKAVMKAITPKAPGLEQFLRDTANSLLDNLITEGAPADLRNDFADPLATALH
CKVLGIPQEDGPKLFRSLSIAFMSSADPIPAAKINWDRDIEYMAGILENPNITTGLMGELSRLRKDPAYSHVS
DELFATIGVTFFGAGVISTGSFLTTALISLIQRPQLRNLLHEKPELIPAGVEELLRINLSFADGLPRLATADI
QVGDVLVRKGELVLVLLEGANFDPEHFPNPGSIELDRPNPTSHLAFGRGQHFCPGSALGRRHAQIGIEALLKK
MPGVDLAVPIDQLVWRTRFQRRIPERLPVLW
>CYP121A1_Mycobacterium tuberculosis 200105 2678009457

MTATVLLEVPFSARGDRIPDAVAELRTREPIRKVRTITGAEAWLVSSYALCTQVLEDRRFSMKETAAAGAPRL
NALTVPPEVVNNMGNIADAGLRKAVMKAITPKAPGLEQFLRDTANSLLDNLITEGAPADLRNDFADPLATALH
CKVLGIPQEDGPKLFRSLSIAFMSSADPIPAAKINWDRDIEYMAGILENPNITTGLMGELSRLRKDPAYSHVS
DELFATIGVTFFGAGVISTGSFLTTALISLIQRPQLRNLLHEKPELIPAGVEELLRINLSFADGLPRLATADI
QVGDVLVRKGELVLVLLEGANFDPEHFPNPGSIELDRPNPTSHLAFGRGQHFCPGSALGRRHAQIGIEALLKK
MPGVDLAVPIDQLVWRTRFQRRIPERLPVLW
>CYP121A1_Mycobacterium tuberculosis 200127 2682898110
MTATVLLEVPFSARGDRIPDAVAELRTREPIRKVRTITGAEAWLVSSYALCTQVLEDRRFSMKETAAAGAPRL
NALTVPPEVVNNMGNIADAGLRKAVMKAITPKAPGLEQFLRDTANSLLDNLITEGAPADLRNDFADPLATALH
CKVLGIPQEDGPKLFRSLSIAFMSSADPIPAAKINWDRDIEYMAGILENPNITTGLMGELSRLRKDPAYSHVS
DELFATIGVTFFGAGVISTGSFLTTALISLIQRPQLRNLLHEKPELIPAGVEELLRINLSFADGLPRLATADI
QVGDVLVRKGELVLVLLEGANFDPEHFPNPGSIELDRPNPTSHLAFGRGQHFCPGSALGRRHAQIGIEALLKK
MPGVDLAVPIDQLVWRTRFQRRIPERLPVLW
>CYP121A1_Mycobacterium tuberculosis 200135 2695840984
MTATVLLEVPFSARGDRIPDAVAELRTREPIRKVRTITGAEAWLVSSYALCTQVLEDRRFSMKETAAAGAPRL
NALTVPPEVVNNMGNIADAGLRKAVMKAITPKAPGLEQFLRDTANSLLDNLITEGAPADLRNDFADPLATALH
CKVLGIPQEDGPKLFRSLSIAFMSSADPIPAAKINWDRDIEYMAGILENPNITTGLMGELSRLRKDPAYSHVS
DELFATIGVTFFGAGVISTGSFLTTALISLIQRPQLRNLLHEKPELIPAGVEELLRINLSFADGLPRLATADI
QVGDVLVRKGELVLVLLEGANFDPEHFPNPGSIELDRPNPTSHLAFGRGQHFCPGSALGRRHAQIGIEALLKK
MPGVDLAVPIDQLVWRTRFQRRIPERLPVLW
>CYP121A1_Mycobacterium tuberculosis 200253 2668688289
MTATVLLEVPFSARGDRIPDAVAELRTREPIRKVRTITGAEAWLVSSYALCTQVLEDRRFSMKETAAAGAPRL
NALTVPPEVVNNMGNIADAGLRKAVMKAITPKAPGLEQFLRDTANSLLDNLITEGAPADLRNDFADPLATALH
CKVLGIPQEDGPKLFRSLSIAFMSSADPIPAAKINWDRDIEYMAGILENPNITTGLMGELSRLRKDPAYSHVS
DELFATIGVTFFGAGVISTGSFLTTALISLIQRPQLRNLLHEKPELIPAGVEELLRINLSFADGLPRLATADI
QVGDVLVRKGELVLVLLEGANFDPEHFPNPGSIELDRPNPTSHLAFGRGQHFCPGSALGRRHAQIGIEALLKK
MPGVDLAVPIDQLVWRTRFQRRIPERLPVLW
>CYP121A1_Mycobacterium tuberculosis 200353 2661495531
MTATVLLEVPFSARGDRIPDAVAELRTREPIRKVRTITGAEAWLVSSYALCTQVLEDRRFSMKETAAAGAPRL
NALTVPPEVVNNMGNIADAGLRKAVMKAITPKAPGLEQFLRDTANSLLDNLITEGAPADLRNDFADPLATALH
CKVLGIPQEDGPKLFRSLSIAFMSSADPIPAAKINWDRDIEYMAGILENPNITTGLMGELSRLRKDPAYSHVS
DELFATIGVTFFGAGVISTGSFLTTALISLIQRPQLRNLLHEKPELIPAGVEELLRINLSFADGLPRLATADI
QVGDVLVRKGELVLVLLEGANFDPEHFPNPGSIELDRPNPTSHLAFGRGQHFCPGSALGRRHAQIGIEALLKK
MPGVDLAVPIDQLVWRTRFQRRIPERLPVLW
>CYP121A1_Mycobacterium tuberculosis 200470 2659552637
MTATVLLEVPFSARGDRIPDAVAELRTREPIRKVRTITGAEAWLVSSYALCTQVLEDRRFSMKETAAAGAPRL
NALTVPPEVVNNMGNIADAGLRKAVMKAITPKAPGLEQFLRDTANSLLDNLITEGAPADLRNDFADPLATALH
CKVLGIPQEDGPKLFRSLSIAFMSSADPIPAAKINWDRDIEYMAGILENPNITTGLMGELSRLRKDPAYSHVS
DELFATIGVTFFGAGVISTGSFLTTALISLIQRPQLRNLLHEKPELIPAGVEELLRINLSFADGLPRLATADI
QVGDVLVRKGELVLVLLEGANFDPEHFPNPGSIELDRPNPTSHLAFGRGQHFCPGSALGRRHAQIGIEALLKK
MPGVDLAVPIDQLVWRTRFQRRIPERLPVLW
>CYP121A1_Mycobacterium tuberculosis 200625 2675456536
MTATVLLEVPFSARGDRIPDAVAELRTREPIRKVRTITGAEAWLVSSYALCTQVLEDRRFSMKETAAAGAPRL
NALTVPPEVVNNMGNIADAGLRKAVMKAITPKAPGLEQFLRDTANSLLDNLITEGAPADLRNDFADPLATALH
CKVLGIPQEDGPKLFRSLSIAFMSSADPIPAAKINWDRDIEYMAGILENPNITTGLMGELSRLRKDPAYSHVS
DELFATIGVTFFGAGVISTGSFLTTALISLIQRPQLRNLLHEKPELIPAGVEELLRINLSFADGLPRLATADI
QVGDVLVRKGELVLVLLEGANFDPEHFPNPGSIELDRPNPTSHLAFGRGQHFCPGSALGRRHAQIGIEALLKK
MPGVDLAVPIDQLVWRTRFQRRIPERLPVLW
>CYP121A1_Mycobacterium tuberculosis 201009 2693263273
MTATVLLEVPFSARGDRIPDAVAELRTREPIRKVRTITGAEAWLVSSYALCTQVLEDRRFSMKETAAAGAPRL
NALTVPPEVVNNMGNIADAGLRKAVMKAITPKAPGLEQFLRDTANSLLDNLITEGAPADLRNDFADPLATALH
CKVLGIPQEDGPKLFRSLSIAFMSSADPIPAAKINWDRDIEYMAGILENPNITTGLMGELSRLRKDPAYSHVS
DELFATIGVTFFGAGVISTGSFLTTALISLIQRPQLRNLLHEKPELIPAGVEELLRINLSFADGLPRLATADI
QVGDVLVRKGELVLVLLEGANFDPEHFPNPGSIELDRPNPTSHLAFGRGQHFCPGSALGRRHAQIGIEALLKK
MPGVDLAVPIDQLVWRTRFQRRIPERLPVLW
>CYP121A1_Mycobacterium tuberculosis 201113 2666292446
MTATVLLEVPFSARGDRIPDAVAELRTREPIRKVRTITGAEAWLVSSYALCTQVLEDRRFSMKETAAAGAPRL
NALTVPPEVVNNMGNIADAGLRKAVMKAITPKAPGLEQFLRDTANSLLDNLITEGAPADLRNDFADPLATALH

CKVLGIPQEDGPKLFRSLSIAFMSSADPIPAAKINWDRDIEYMAGILENPNITTGLMGELSRLRKDPAYSHVS
DELFATIGVTFFGAGVISTGSFLTTALISLIQRPQLRNLLHEKPELIPAGVEELLRINLSFADGLPRLATADI
QVGDVLVRKGELVLVLLEGANFDPEHFPNPGSIELDRPNPTSHLAFGRGQHFCPGSALGRRHAQIGIEALLKK
MPGVDLAVPIDQLVWRTRFQRRIPERLPVLW
>CYP121A1_Mycobacterium tuberculosis 201150 2682903954
MTATVLLEVPFSARGDRIPDAVAELRTREPIRKVRTITGAEAWLVSSYALCTQVLEDRRFSMKETAAAGAPRL
NALTVPPEVVNNMGNIADAGLRKAVMKAITPKAPGLEQFLRDTANSLLDNLITEGAPADLRNDFADPLATALH
CKVLGIPQEDGPKLFRSLSIAFMSSADPIPAAKINWDRDIEYMAGILENPNITTGLMGELSRLRKDPAYSHVS
DELFATIGVTFFGAGVISTGSFLTTALISLIQRPQLRNLLHEKPELIPAGVEELLRINLSFADGLPRLATADI
QVGDVLVRKGELVLVLLEGANFDPEHFPNPGSIELDRPNPTSHLAFGRGQHFCPGSALGRRHAQIGIEALLKK
MPGVDLAVPIDQLVWRTRFQRRIPERLPVLW
>CYP121A1_Mycobacterium tuberculosis 201314 2665808653
MTATVLLEVPFSARGDRIPDAVAELRTREPIRKVRTITGAEAWLVSSYALCTQVLEDRRFSMKETAAAGAPRL
NALTVPPEVVNNMGNIADAGLRKAVMKAITPKAPGLEQFLRDTANSLLDNLITEGAPADLRNDFADPLATALH
CKVLGIPQEDGPKLFRSLSIAFMSSADPIPAAKINWDRDIEYMAGILENPNITTGLMGELSRLRKDPAYSHVS
DELFATIGVTFFGAGVISTGSFLTTALISLIQRPQLRNLLHEKPELIPAGVEELLRINLSFADGLPRLATADI
QVGDVLVRKGELVLVLLEGANFDPEHFPNPGSIELDRPNPTSHLAFGRGQHFCPGSALGRRHAQIGIEALLKK
MPGVDLAVPIDQLVWRTRFQRRIPERLPVLW
>CYP121A1_Mycobacterium tuberculosis 201366 2660483785
MTATVLLEVPFSARGDRIPDAVAELRTREPIRKVRTITGAEAWLVSSYALCTQVLEDRRFSMKETAAAGAPRL
NALTVPPEVVNNMGNIADAGLRKAVMKAITPKAPGLEQFLRDTANSLLDNLITEGAPADLRNDFADPLATALH
CKVLGIPQEDGPKLFRSLSIAFMSSADPIPAAKINWDRDIEYMAGILENPNITTGLMGELSRLRKDPAYSHVS
DELFATIGVTFFGAGVISTGSFLTTALISLIQRPQLRNLLHEKPELIPAGVEELLRINLSFADGLPRLATADI
QVGDVLVRKGELVLVLLEGANFDPEHFPNPGSIELDRPNPTSHLAFGRGQHFCPGSALGRRHAQIGIEALLKK
MPGVDLAVPIDQLVWRTRFQRRIPERLPVLW
>CYP121A1_Mycobacterium tuberculosis 201404 2673358440
MTATVLLEVPFSARGDRIPDAVAELRTREPIRKVRTITGAEAWLVSSYALCTQVLEDRRFSMKETAAAGAPRL
NALTVPPEVVNNMGNIADAGLRKAVMKAITPKAPGLEQFLRDTANSLLDNLITEGAPADLRNDFADPLATALH
CKVLGIPQEDGPKLFRSLSIAFMSSADPIPAAKINWDRDIEYMAGILENPNITTGLMGELSRLRKDPAYSHVS
DELFATIGVTFFGAGVISTGSFLTTALISLIQRPQLRNLLHEKPELIPAGVEELLRINLSFADGLPRLATADI
QVGDVLVRKGELVLVLLEGANFDPEHFPNPGSIELDRPNPTSHLAFGRGQHFCPGSALGRRHAQIGIEALLKK
MPGVDLAVPIDQLVWRTRFQRRIPERLPVLW
>CYP121A1_Mycobacterium tuberculosis 201457 2658055734
MTATVLLEVPFSARGDRIPDAVAELRTREPIRKVRTITGAEAWLVSSYALCTQVLEDRRFSMKETAAAGAPRL
NALTVPPEVVNNMGNIADAGLRKAVMKAITPKAPGLEQFLRDTANSLLDNLITEGAPADLRNDFADPLATALH
CKVLGIPQEDGPKLFRSLSIAFMSSADPIPAAKINWDRDIEYMAGILENPNITTGLMGELSRLRKDPAYSHVS
DELFATIGVTFFGAGVISTGSFLTTALISLIQRPQLRNLLHEKPELIPAGVEELLRINLSFADGLPRLATADI
QVGDVLVRKGELVLVLLEGANFDPEHFPNPGSIELDRPNPTSHLAFGRGQHFCPGSALGRRHAQIGIEALLKK
MPGVDLAVPIDQLVWRTRFQRRIPERLPVLW
>CYP121A1_Mycobacterium tuberculosis 2089HD 2621752628
MTATVLLEVPFSARGDRIPDAVAELRTREPIRKVRTITGAEAWLVSSYALCTQVLEDRRFSMKETAAAGAPRL
NALTVPPEVVNNMGNIADAGLRKAVMKAITPKAPGLEQFLRDTANSLLDNLITEGAPADLRNDFADPLATALH
CKVLGIPQEDGPKLFRSLSIAFMSSADPIPAAKINWDRDIEYMAGILENPNITTGLMGELSRLRKDPAYSHVS
DELFATIGVTFFGAGVISTGSFLTTALISLIQRPQLRNLLHEKPELIPAGVEELLRINLSFADGLPRLATADI
QVGDVLVRKGELVLVLLEGANFDPEHFPNPGSIELDRPNPTSHLAFGRGQHFCPGSALGRRHAQIGIEALLKK
MPGVDLAVPIDQLVWRTRFQRRIPERLPVLW
>CYP121A1_Mycobacterium tuberculosis 2091HD 2584726674
MTATVLLEVPFSARGDRIPDAVAELRTREPIRKVRTITGAEAWLVSSYALCTQVLEDRRFSMKETAAAGAPRL
NALTVPPEVVNNMGNIADAGLRKAVMKAITPKAPGLEQFLRDTANSLLDNLITEGAPADLRNDFADPLATALH
CKVLGIPQEDGPKLFRSLSIAFMSSADPIPAAKINWDRDIEYMAGILENPNITTGLMGELSRLRKDPAYSHVS
DELFATIGVTFFGAGVISTGSFLTTALISLIQRPQLRNLLHEKPELIPAGVEELLRINLSFADGLPRLATADI
QVGDVLVRKGELVLVLLEGANFDPEHFPNPGSIELDRPNPTSHLAFGRGQHFCPGSALGRRHAQIGIEALLKK
MPGVDLAVPIDQLVWRTRFQRRIPERLPVLW
>CYP121A1_Mycobacterium tuberculosis 2092HD 2635592851
MTATVLLEVPFSARGDRIPDAVAELRTREPIRKVRTITGAEAWLVSSYALCTQVLEDRRFSMKETAAAGAPRL
NALTVPPEVVNNMGNIADAGLRKAVMKAITPKAPGLEQFLRDTANSLLDNLITEGAPADLRNDFADPLATALH
CKVLGIPQEDGPKLFRSLSIAFMSSADPIPAAKINWDRDIEYMAGILENPNITTGLMGELSRLRKDPAYSHVS
DELFATIGVTFFGAGVISTGSFLTTALISLIQRPQLRNLLHEKPELIPAGVEELLRINLSFADGLPRLATADI

QVGDVLVRKGELVLVLLEGANFDPEHFPNPGSIELDRPNPTSHLAFGRGQHFCPGSALGRRHAQIGIEALLKK
MPGVDLAVPIDQLVWRTRFQRRIPERLPVLW
>CYP121A1_Mycobacterium tuberculosis 2097HD 2606398798
MTATVLLEVPFSARGDRIPDAVAELRTREPIRKVRTITGAEAWLVSSYALCTQVLEDRRFSMKETAAAGAPRL
NALTVPPEVVNNMGNIADAGLRKAVMKAITPKAPGLEQFLRDTANSLLDNLITEGAPADLRNDFADPLATALH
CKVLGIPQEDGPKLFRSLSIAFMSSADPIPAAKINWDRDIEYMAGILENPNITTGLMGELSRLRKDPAYSHVS
DELFATIGVTFFGAGVISTGSFLTTALISLIQRPQLRNLLHEKPELIPAGVEELLRINLSFADGLPRLATADI
QVGDVLVRKGELVLVLLEGANFDPEHFPNPGSIELDRPNPTSHLAFGRGQHFCPGSALGRRHAQIGIEALLKK
MPGVDLAVPIDQLVWRTRFQRRIPERLPVLW
>CYP121A1_Mycobacterium tuberculosis 2099HD 2606393996
MTATVLLEVPFSARGDRIPDAVAELRTREPIRKVRTITGAEAWLVSSYALCTQVLEDRRFSMKETAAAGAPRL
NALTVPPEVVNNMGNIADAGLRKAVMKAITPKAPGLEQFLRDTANSLLDNLITEGAPADLRNDFADPLATALH
CKVLGIPQEDGPKLFRSLSIAFMSSADPIPAAKINWDRDIEYMAGILENPNITTGLMGELSRLRKDPAYSHVS
DELFATIGVTFFGAGVISTGSFLTTALISLIQRPQLRNLLHEKPELIPAGVEELLRINLSFADGLPRLATADI
QVGDVLVRKGELVLVLLEGANFDPEHFPNPGSIELDRPNPTSHLAFGRGQHFCPGSALGRRHAQIGIEALLKK
MPGVDLAVPIDQLVWRTRFQRRIPERLPVLW
>CYP121A1_Mycobacterium tuberculosis 2100HD 2606478632
MTATVLLEVPFSARGDRIPDAVAELRTREPIRKVRTITGAEAWLVSSYALCTQVLEDRRFSMKETAAAGAPRL
NALTVPPEVVNNMGNIADAGLRKAVMKAITPKAPGLEQFLRDTANSLLDNLITEGAPADLRNDFADPLATALH
CKVLGIPQEDGPKLFRSLSIAFMSSADPIPAAKINWDRDIEYMAGILENPNITTGLMGELSRLRKDPAYSHVS
DELFATIGVTFFGAGVISTGSFLTTALISLIQRPQLRNLLHEKPELIPAGVEELLRINLSFADGLPRLATADI
QVGDVLVRKGELVLVLLEGANFDPEHFPNPGSIELDRPNPTSHLAFGRGQHFCPGSALGRRHAQIGIEALLKK
MPGVDLAVPIDQLVWRTRFQRRIPERLPVLW
>CYP121A1_Mycobacterium tuberculosis 2104HD 2606382489
MTATVLLEVPFSARGDRIPDAVAELRTREPIRKVRTITGAEAWLVSSYALCTQVLEDRRFSMKETAAAGAPRL
NALTVPPEVVNNMGNIADAGLRKAVMKAITPKAPGLEQFLRDTANSLLDNLITEGAPADLRNDFADPLATALH
CKVLGIPQEDGPKLFRSLSIAFMSSADPIPAAKINWDRDIEYMAGILENPNITTGLMGELSRLRKDPAYSHVS
DELFATIGVTFFGAGVISTGSFLTTALISLIQRPQLRNLLHEKPELIPAGVEELLRINLSFADGLPRLATADI
QVGDVLVRKGELVLVLLEGANFDPEHFPNPGSIELDRPNPTSHLAFGRGQHFCPGSALGRRHAQIGIEALLKK
MPGVDLAVPIDQLVWRTRFQRRIPERLPVLW
>CYP121A1_Mycobacterium tuberculosis 2196MM 2621746050
MTATVLLEVPFSARGDRIPDAVAELRTREPIRKVRTITGAEAWLVSSYALCTQVLEDRRFSMKETAAAGAPRL
NALTVPPEVVNNMGNIADAGLRKAVMKAITPKAPGLEQFLRDTANSLLDNLITEGAPADLRNDFADPLATALH
CKVLGIPQEDGPKLFRSLSIAFMSSADPIPAAKINWDRDIEYMAGILENPNITTGLMGELSRLRKDPAYSHVS
DELFATIGVTFFGAGVISTGSFLTTALISLIQRPQLRNLLHEKPELIPAGVEELLRINLSFADGLPRLATADI
QVGDVLVRKGELVLVLLEGANFDPEHFPNPGSIELDRPNPTSHLAFGRGQHFCPGSALGRRHAQIGIEALLKK
MPGVDLAVPIDQLVWRTRFQRRIPERLPVLW
>CYP121A1_Mycobacterium tuberculosis 22103 2688928211
MTATVLLEVPFSARGDRIPDAVAELRTREPIRKVRTITGAEAWLVSSYALCTQVLEDRRFSMKETAAAGAPRL
NALTVPPEVVNNMGNIADAGLRKAVMKAITPKAPGLEQFLRDTANSLLDNLITEGAPADLRNDFADPLATALH
CKVLGIPQEDGPKLFRSLSIAFMSSADPIPAAKINWDRDIEYMAGILENPNITTGLMGELSRLRKDPAYSHVS
DELFATIGVTFFGAGVISTGSFLTTALISLIQRPQLRNLLHEKPELIPAGVEELLRINLSFADGLPRLATADI
QVGDVLVRKGELVLVLLEGANFDPEHFPNPGSIELDRPNPTSHLAFGRGQHFCPGSALGRRHAQIGIEALLKK
MPGVDLAVPIDQLVWRTRFQRRIPERLPVLW
>CYP121A1_Mycobacterium tuberculosis 22115 2688943119
MTATVLLEVPFSARGDRIPDAVAELRTREPIRKVRTITGAEAWLVSSYALCTQVLEDRRFSMKETAAAGAPRL
NALTVPPEVVNNMGNIADAGLRKAVMKAITPKAPGLEQFLRDTANSLLDNLITEGAPADLRNDFADPLATALH
CKVLGIPQEDGPKLFRSLSIAFMSSADPIPAAKINWDRDIEYMAGILENPNITTGLMGELSRLRKDPAYSHVS
DELFATIGVTFFGAGVISTGSFLTTALISLIQRPQLRNLLHEKPELIPAGVEELLRINLSFADGLPRLATADI
QVGDVLVRKGELVLVLLEGANFDPEHFPNPGSIELDRPNPTSHLAFGRGQHFCPGSALGRRHAQIGIEALLKK
MPGVDLAVPIDQLVWRTRFQRRIPERLPVLW
>CYP121A1_Mycobacterium tuberculosis 2228BH 2621731848
MTATVLLEVPFSARGDRIPDAVAELRTREPIRKVRTITGAEAWLVSSYALCTQVLEDRRFSMKETAAAGAPRL
NALTVPPEVVNNMGNIADAGLRKAVMKAITPKAPGLEQFLRDTANSLLDNLITEGAPADLRNDFADPLATALH
CKVLGIPQEDGPKLFRSLSIAFMSSADPIPAAKINWDRDIEYMAGILENPNITTGLMGELSRLRKDPAYSHVS
DELFATIGVTFFGAGVISTGSFLTTALISLIQRPQLRNLLHEKPELIPAGVEELLRINLSFADGLPRLATADI
QVGDVLVRKGELVLVLLEGANFDPEHFPNPGSIELDRPNPTSHLAFGRGQHFCPGSALGRRHAQIGIEALLKK
MPGVDLAVPIDQLVWRTRFQRRIPERLPVLW

>CYP121A1_Mycobacterium tuberculosis 2229BH 2633600335
MTATVLLEVPFSARGDRIPDAVAELRTREPIRKVRTITGAEAWLVSSYALCTQVLEDRRFSMKETAAAGAPRL
NALTVPPEVVNNMGNIADAGLRKAVMKAITPKAPGLEQFLRDTANSLLDNLITEGAPADLRNDFADPLATALH
CKVLGIPQEDGPKLFRSLSIAFMSSADPIPAAKINWDRDIEYMAGILENPNITTGLMGELSRLRKDPAYSHVS
DELFATIGVTFFGAGVISTGSFLTTALISLIQRPQLRNLLHEKPELIPAGVEELLRINLSFADGLPRLATADI
QVGDVLVRKGELVLVLLEGANFDPEHFPNPGSIELDRPNPTSHLAFGRGQHFCPGSALGRRHAQIGIEALLKK
MPGVDLAVPIDQLVWRTRFQRRIPERLPVLW
>CYP121A1_Mycobacterium tuberculosis 2230BH 2621730400
MTATVLLEVPFSARGDRIPDAVAELRTREPIRKVRTITGAEAWLVSSYALCTQVLEDRRFSMKETAAAGAPRL
NALTVPPEVVNNMGNIADAGLRKAVMKAITPKAPGLEQFLRDTANSLLDNLITEGAPADLRNDFADPLATALH
CKVLGIPQEDGPKLFRSLSIAFMSSADPIPAAKINWDRDIEYMAGILENPNITTGLMGELSRLRKDPAYSHVS
DELFATIGVTFFGAGVISTGSFLTTALISLIQRPQLRNLLHEKPELIPAGVEELLRINLSFADGLPRLATADI
QVGDVLVRKGELVLVLLEGANFDPEHFPNPGSIELDRPNPTSHLAFGRGQHFCPGSALGRRHAQIGIEALLKK
MPGVDLAVPIDQLVWRTRFQRRIPERLPVLW
>CYP121A1_Mycobacterium tuberculosis 2231BH 2647567109
MTATVLLEVPFSARGDRIPDAVAELRTREPIRKVRTITGAEAWLVSSYALCTQVLEDRRFSMKETAAAGAPRL
NALTVPPEVVNNMGNIADAGLRKAVMKAITPKAPGLEQFLRDTANSLLDNLITEGAPADLRNDFADPLATALH
CKVLGIPQEDGPKLFRSLSIAFMSSADPIPAAKINWDRDIEYMAGILENPNITTGLMGELSRLRKDPAYSHVS
DELFATIGVTFFGAGVISTGSFLTTALISLIQRPQLRNLLHEKPELIPAGVEELLRINLSFADGLPRLATADI
QVGDVLVRKGELVLVLLEGANFDPEHFPNPGSIELDRPNPTSHLAFGRGQHFCPGSALGRRHAQIGIEALLKK
MPGVDLAVPIDQLVWRTRFQRRIPERLPVLW
>CYP121A1_Mycobacterium tuberculosis 2233BH 2621719292
MTATVLLEVPFSARGDRIPDAVAELRTREPIRKVRTITGAEAWLVSSYALCTQVLEDRRFSMKETAAAGAPRL
NALTVPPEVVNNMGNIADAGLRKAVMKAITPKAPGLEQFLRDTANSLLDNLITEGAPADLRNDFADPLATALH
CKVLGIPQEDGPKLFRSLSIAFMSSADPIPAAKINWDRDIEYMAGILENPNITTGLMGELSRLRKDPAYSHVS
DELFATIGVTFFGAGVISTGSFLTTALISLIQRPQLRNLLHEKPELIPAGVEELLRINLSFADGLPRLATADI
QVGDVLVRKGELVLVLLEGANFDPEHFPNPGSIELDRPNPTSHLAFGRGQHFCPGSALGRRHAQIGIEALLKK
MPGVDLAVPIDQLVWRTRFQRRIPERLPVLW
>CYP121A1_Mycobacterium tuberculosis 2482AR 2606390019
MTATVLLEVPFSARGDRIPDAVAELRTREPIRKVRTITGAEAWLVSSYALCTQVLEDRRFSMKETAAAGAPRL
NALTVPPEVVNNMGNIADAGLRKAVMKAITPKAPGLEQFLRDTANSLLDNLITEGAPADLRNDFADPLATALH
CKVLGIPQEDGPKLFRSLSIAFMSSADPIPAAKINWDRDIEYMAGILENPNITTGLMGELSRLRKDPAYSHVS
DELFATIGVTFFGAGVISTGSFLTTALISLIQRPQLRNLLHEKPELIPAGVEELLRINLSFADGLPRLATADI
QVGDVLVRKGELVLVLLEGANFDPEHFPNPGSIELDRPNPTSHLAFGRGQHFCPGSALGRRHAQIGIEALLKK
MPGVDLAVPIDQLVWRTRFQRRIPERLPVLW
>CYP121A1_Mycobacterium tuberculosis 2483AR 2575162880
MTATVLLEVPFSARGDRIPDAVAELRTREPIRKVRTITGAEAWLVSSYALCTQVLEDRRFSMKETAAAGAPRL
NALTVPPEVVNNMGNIADAGLRKAVMKAITPKAPGLEQFLRDTANSLLDNLITEGAPADLRNDFADPLATALH
CKVLGIPQEDGPKLFRSLSIAFMSSADPIPAAKINWDRDIEYMAGILENPNITTGLMGELSRLRKDPAYSHVS
DELFATIGVTFFGAGVISTGSFLTTALISLIQRPQLRNLLHEKPELIPAGVEELLRINLSFADGLPRLATADI
QVGDVLVRKGELVLVLLEGANFDPEHFPNPGSIELDRPNPTSHLAFGRGQHFCPGSALGRRHAQIGIEALLKK
MPGVDLAVPIDQLVWRTRFQRRIPERLPVLW
>CYP121A1_Mycobacterium tuberculosis 2484AR 2606386209
MTATVLLEVPFSARGDRIPDAVAELRTREPIRKVRTITGAEAWLVSSYALCTQVLEDRRFSMKETAAAGAPRL
NALTVPPEVVNNMGNIADAGLRKAVMKAITPKAPGLEQFLRDTANSLLDNLITEGAPADLRNDFADPLATALH
CKVLGIPQEDGPKLFRSLSIAFMSSADPIPAAKINWDRDIEYMAGILENPNITTGLMGELSRLRKDPAYSHVS
DELFATIGVTFFGAGVISTGSFLTTALISLIQRPQLRNLLHEKPELIPAGVEELLRINLSFADGLPRLATADI
QVGDVLVRKGELVLVLLEGANFDPEHFPNPGSIELDRPNPTSHLAFGRGQHFCPGSALGRRHAQIGIEALLKK
MPGVDLAVPIDQLVWRTRFQRRIPERLPVLW
>CYP121A1_Mycobacterium tuberculosis 2485AR 2606474046
MTATVLLEVPFSARGDRIPDAVAELRTREPIRKVRTITGAEAWLVSSYALCTQVLEDRRFSMKETAAAGAPRL
NALTVPPEVVNNMGNIADAGLRKAVMKAITPKAPGLEQFLRDTANSLLDNLITEGAPADLRNDFADPLATALH
CKVLGIPQEDGPKLFRSLSIAFMSSADPIPAAKINWDRDIEYMAGILENPNITTGLMGELSRLRKDPAYSHVS
DELFATIGVTFFGAGVISTGSFLTTALISLIQRPQLRNLLHEKPELIPAGVEELLRINLSFADGLPRLATADI
QVGDVLVRKGELVLVLLEGANFDPEHFPNPGSIELDRPNPTSHLAFGRGQHFCPGSALGRRHAQIGIEALLKK
MPGVDLAVPIDQLVWRTRFQRRIPERLPVLW
>CYP121A1_Mycobacterium tuberculosis 2541MS 2576374288

MTATVLLEVPFSARGDRIPDAVAELRTREPIRKVRTITGAEAWLVSSYALCTQVLEDRRFSMKETAAAGAPRL
NALTVPPEVVNNMGNIADAGLRKAVMKAITPKAPGLEQFLRDTANSLLDNLITEGAPADLRNDFADPLATALH
CKVLGIPQEDGPKLFRSLSIAFMSSADPIPAAKINWDRDIEYMAGILENPNITTGLMGELSRLRKDPAYSHVS
DELFATIGVTFFGAGVISTGSFLTTALISLIQRPQLRNLLHEKPELIPAGVEELLRINLSFADGLPRLATADI
QVGDVLVRKGELVLVLLEGANFDPEHFPNPGSIELDRPNPTSHLAFGRGQHFCPGSALGRRHAQIGIEALLKK
MPGVDLAVPIDQLVWRTRFQRRIPERLPVLW
>CYP121A1_Mycobacterium tuberculosis 2542MS 2630525353
MTATVLLEVPFSARGDRIPDAVAELRTREPIRKVRTITGAEAWLVSSYALCTQVLEDRRFSMKETAAAGAPRL
NALTVPPEVVNNMGNIADAGLRKAVMKAITPKAPGLEQFLRDTANSLLDNLITEGAPADLRNDFADPLATALH
CKVLGIPQEDGPKLFRSLSIAFMSSADPIPAAKINWDRDIEYMAGILENPNITTGLMGELSRLRKDPAYSHVS
DELFATIGVTFFGAGVISTGSFLTTALISLIQRPQLRNLLHEKPELIPAGVEELLRINLSFADGLPRLATADI
QVGDVLVRKGELVLVLLEGANFDPEHFPNPGSIELDRPNPTSHLAFGRGQHFCPGSALGRRHAQIGIEALLKK
MPGVDLAVPIDQLVWRTRFQRRIPERLPVLW
>CYP121A1_Mycobacterium tuberculosis 2543MS 2606482671
MTATVLLEVPFSARGDRIPDAVAELRTREPIRKVRTITGAEAWLVSSYALCTQVLEDRRFSMKETAAAGAPRL
NALTVPPEVVNNMGNIADAGLRKAVMKAITPKAPGLEQFLRDTANSLLDNLITEGAPADLRNDFADPLATALH
CKVLGIPQEDGPKLFRSLSIAFMSSADPIPAAKINWDRDIEYMAGILENPNITTGLMGELSRLRKDPAYSHVS
DELFATIGVTFFGAGVISTGSFLTTALISLIQRPQLRNLLHEKPELIPAGVEELLRINLSFADGLPRLATADI
QVGDVLVRKGELVLVLLEGANFDPEHFPNPGSIELDRPNPTSHLAFGRGQHFCPGSALGRRHAQIGIEALLKK
MPGVDLAVPIDQLVWRTRFQRRIPERLPVLW
>CYP121A1_Mycobacterium tuberculosis 26105 2688178345
MTATVLLEVPFSARGDRIPDAVAELRTREPIRKVRTITGAEAWLVSSYALCTQVLEDRRFSMKETAAAGAPRL
NALTVPPEVVNNMGNIADAGLRKAVMKAITPKAPGLEQFLRDTANSLLDNLITEGAPADLRNDFADPLATALH
CKVLGIPQEDGPKLFRSLSIAFMSSADPIPAAKINWDRDIEYMAGILENPNITTGLMGELSRLRKDPAYSHVS
DELFATIGVTFFGAGVISTGSFLTTALISLIQRPQLRNLLHEKPELIPAGVEELLRINLSFADGLPRLATADI
QVGDVLVRKGELVLVLLEGANFDPEHFPNPGSIELDRPNPTSHLAFGRGQHFCPGSALGRRHAQIGIEALLKK
MPGVDLAVPIDQLVWRTRFQRRIPERLPVLW
>CYP121A1_Mycobacterium tuberculosis 2995AB 2635706378
MTATVLLEVPFSARGDRIPDAVAELRTREPIRKVRTITGAEAWLVSSYALCTQVLEDRRFSMKETAAAGAPRL
NALTVPPEVVNNMGNIADAGLRKAVMKAITPKAPGLEQFLRDTANSLLDNLITEGAPADLRNDFADPLATALH
CKVLGIPQEDGPKLFRSLSIAFMSSADPIPAAKINWDRDIEYMAGILENPNITTGLMGELSRLRKDPAYSHVS
DELFATIGVTFFGAGVISTGSFLTTALISLIQRPQLRNLLHEKPELIPAGVEELLRINLSFADGLPRLATADI
QVGDVLVRKGELVLVLLEGANFDPEHFPNPGSIELDRPNPTSHLAFGRGQHFCPGSALGRRHAQIGIEALLKK
MPGVDLAVPIDQLVWRTRFQRRIPERLPVLW
>CYP121A1_Mycobacterium tuberculosis 300239 2659963387
MTATVLLEVPFSARGDRIPDAVAELRTREPIRKVRTITGAEAWLVSSYALCTQVLEDRRFSMKETAAAGAPRL
NALTVPPEVVNNMGNIADAGLRKAVMKAITPKAPGLEQFLRDTANSLLDNLITEGAPADLRNDFADPLATALH
CKVLGIPQEDGPKLFRSLSIAFMSSADPIPAAKINWDRDIEYMAGILENPNITTGLMGELSRLRKDPAYSHVS
DELFATIGVTFFGAGVISTGSFLTTALISLIQRPQLRNLLHEKPELIPAGVEELLRINLSFADGLPRLATADI
QVGDVLVRKGELVLVLLEGANFDPEHFPNPGSIELDRPNPTSHLAFGRGQHFCPGSALGRRHAQIGIEALLKK
MPGVDLAVPIDQLVWRTRFQRRIPERLPVLW
>CYP121A1_Mycobacterium tuberculosis 300659 2668868903
MTATVLLEVPFSARGDRIPDAVAELRTREPIRKVRTITGAEAWLVSSYALCTQVLEDRRFSMKETAAAGAPRL
NALTVPPEVVNNMGNIADAGLRKAVMKAITPKAPGLEQFLRDTANSLLDNLITEGAPADLRNDFADPLATALH
CKVLGIPQEDGPKLFRSLSIAFMSSADPIPAAKINWDRDIEYMAGILENPNITTGLMGELSRLRKDPAYSHVS
DELFATIGVTFFGAGVISTGSFLTTALISLIQRPQLRNLLHEKPELIPAGVEELLRINLSFADGLPRLATADI
QVGDVLVRKGELVLVLLEGANFDPEHFPNPGSIELDRPNPTSHLAFGRGQHFCPGSALGRRHAQIGIEALLKK
MPGVDLAVPIDQLVWRTRFQRRIPERLPVLW
>CYP121A1_Mycobacterium tuberculosis 300879 2693685988
MTATVLLEVPFSARGDRIPDAVAELRTREPIRKVRTITGAEAWLVSSYALCTQVLEDRRFSMKETAAAGAPRL
NALTVPPEVVNNMGNIADAGLRKAVMKAITPKAPGLEQFLRDTANSLLDNLITEGAPADLRNDFADPLATALH
CKVLGIPQEDGPKLFRSLSIAFMSSADPIPAAKINWDRDIEYMAGILENPNITTGLMGELSRLRKDPAYSHVS
DELFATIGVTFFGAGVISTGSFLTTALISLIQRPQLRNLLHEKPELIPAGVEELLRINLSFADGLPRLATADI
QVGDVLVRKGELVLVLLEGANFDPEHFPNPGSIELDRPNPTSHLAFGRGQHFCPGSALGRRHAQIGIEALLKK
MPGVDLAVPIDQLVWRTRFQRRIPERLPVLW
>CYP121A1_Mycobacterium tuberculosis 301310 2669035214
MTATVLLEVPFSARGDRIPDAVAELRTREPIRKVRTITGAEAWLVSSYALCTQVLEDRRFSMKETAAAGAPRL
NALTVPPEVVNNMGNIADAGLRKAVMKAITPKAPGLEQFLRDTANSLLDNLITEGAPADLRNDFADPLATALH

CKVLGIPQEDGPKLFRSLSIAFMSSADPIPAAKINWDRDIEYMAGILENPNITTGLMGELSRLRKDPAYSHVS
DELFATIGVTFFGAGVISTGSFLTTALISLIQRPQLRNLLHEKPELIPAGVEELLRINLSFADGLPRLATADI
QVGDVLVRKGELVLVLLEGANFDPEHFPNPGSIELDRPNPTSHLAFGRGQHFCPGSALGRRHAQIGIEALLKK
MPGVDLAVPIDQLVWRTRFQRRIPERLPVLW
>CYP121A1_Mycobacterium tuberculosis 301347 2663321010
MTATVLLEVPFSARGDRIPDAVAELRTREPIRKVRTITGAEAWLVSSYALCTQVLEDRRFSMKETAAAGAPRL
NALTVPPEVVNNMGNIADAGLRKAVMKAITPKAPGLEQFLRDTANSLLDNLITEGAPADLRNDFADPLATALH
CKVLGIPQEDGPKLFRSLSIAFMSSADPIPAAKINWDRDIEYMAGILENPNITTGLMGELSRLRKDPAYSHVS
DELFATIGVTFFGAGVISTGSFLTTALISLIQRPQLRNLLHEKPELIPAGVEELLRINLSFADGLPRLATADI
QVGDVLVRKGELVLVLLEGANFDPEHFPNPGSIELDRPNPTSHLAFGRGQHFCPGSALGRRHAQIGIEALLKK
MPGVDLAVPIDQLVWRTRFQRRIPERLPVLW
>CYP121A1_Mycobacterium tuberculosis 301645 2697026403
MTATVLLEVPFSARGDRIPDAVAELRTREPIRKVRTITGAEAWLVSSYALCTQVLEDRRFSMKETAAAGAPRL
NALTVPPEVVNNMGNIADAGLRKAVMKAITPKAPGLEQFLRDTANSLLDNLITEGAPADLRNDFADPLATALH
CKVLGIPQEDGPKLFRSLSIAFMSSADPIPAAKINWDRDIEYMAGILENPNITTGLMGELSRLRKDPAYSHVS
DELFATIGVTFFGAGVISTGSFLTTALISLIQRPQLRNLLHEKPELIPAGVEELLRINLSFADGLPRLATADI
QVGDVLVRKGELVLVLLEGANFDPEHFPNPGSIELDRPNPTSHLAFGRGQHFCPGSALGRRHAQIGIEALLKK
MPGVDLAVPIDQLVWRTRFQRRIPERLPVLW
>CYP121A1_Mycobacterium tuberculosis 302203 2701647625
MTATVLLEVPFSARGDRIPDAVAELRTREPIRKVRTITGAEAWLVSSYALCTQVLEDRRFSMKETAAAGAPRL
NALTVPPEVVNNMGNIADAGLRKAVMKAITPKAPGLEQFLRDTANSLLDNLITEGAPADLRNDFADPLATALH
CKVLGIPQEDGPKLFRSLSIAFMSSADPIPAAKINWDRDIEYMAGILENPNITTGLMGELSRLRKDPAYSHVS
DELFATIGVTFFGAGVISTGSFLTTALISLIQRPQLRNLLHEKPELIPAGVEELLRINLSFADGLPRLATADI
QVGDVLVRKGELVLVLLEGANFDPEHFPNPGSIELDRPNPTSHLAFGRGQHFCPGSALGRRHAQIGIEALLKK
MPGVDLAVPIDQLVWRTRFQRRIPERLPVLW
>CYP121A1_Mycobacterium tuberculosis 302226 2660590827
MTATVLLEVPFSARGDRIPDAVAELRTREPIRKVRTITGAEAWLVSSYALCTQVLEDRRFSMKETAAAGAPRL
NALTVPPEVVNNMGNIADAGLRKAVMKAITPKAPGLEQFLRDTANSLLDNLITEGAPADLRNDFADPLATALH
CKVLGIPQEDGPKLFRSLSIAFMSSADPIPAAKINWDRDIEYMAGILENPNITTGLMGELSRLRKDPAYSHVS
DELFATIGVTFFGAGVISTGSFLTTALISLIQRPQLRNLLHEKPELIPAGVEELLRINLSFADGLPRLATADI
QVGDVLVRKGELVLVLLEGANFDPEHFPNPGSIELDRPNPTSHLAFGRGQHFCPGSALGRRHAQIGIEALLKK
MPGVDLAVPIDQLVWRTRFQRRIPERLPVLW
>CYP121A1_Mycobacterium tuberculosis 302235 2697889777
MTATVLLEVPFSARGDRIPDAVAELRTREPIRKVRTITGAEAWLVSSYALCTQVLEDRRFSMKETAAAGAPRL
NALTVPPEVVNNMGNIADAGLRKAVMKAITPKAPGLEQFLRDTANSLLDNLITEGAPADLRNDFADPLATALH
CKVLGIPQEDGPKLFRSLSIAFMSSADPIPAAKINWDRDIEYMAGILENPNITTGLMGELSRLRKDPAYSHVS
DELFATIGVTFFGAGVISTGSFLTTALISLIQRPQLRNLLHEKPELIPAGVEELLRINLSFADGLPRLATADI
QVGDVLVRKGELVLVLLEGANFDPEHFPNPGSIELDRPNPTSHLAFGRGQHFCPGSALGRRHAQIGIEALLKK
MPGVDLAVPIDQLVWRTRFQRRIPERLPVLW
>CYP121A1_Mycobacterium tuberculosis 302258 2700040771
MTATVLLEVPFSARGDRIPDAVAELRTREPIRKVRTITGAEAWLVSSYALCTQVLEDRRFSMKETAAAGAPRL
NALTVPPEVVNNMGNIADAGLRKAVMKAITPKAPGLEQFLRDTANSLLDNLITEGAPADLRNDFADPLATALH
CKVLGIPQEDGPKLFRSLSIAFMSSADPIPAAKINWDRDIEYMAGILENPNITTGLMGELSRLRKDPAYSHVS
DELFATIGVTFFGAGVISTGSFLTTALISLIQRPQLRNLLHEKPELIPAGVEELLRINLSFADGLPRLATADI
QVGDVLVRKGELVLVLLEGANFDPEHFPNPGSIELDRPNPTSHLAFGRGQHFCPGSALGRRHAQIGIEALLKK
MPGVDLAVPIDQLVWRTRFQRRIPERLPVLW
>CYP121A1_Mycobacterium tuberculosis 302434 2670953593
MTATVLLEVPFSARGDRIPDAVAELRTREPIRKVRTITGAEAWLVSSYALCTQVLEDRRFSMKETAAAGAPRL
NALTVPPEVVNNMGNIADAGLRKAVMKAITPKAPGLEQFLRDTANSLLDNLITEGAPADLRNDFADPLATALH
CKVLGIPQEDGPKLFRSLSIAFMSSADPIPAAKINWDRDIEYMAGILENPNITTGLMGELSRLRKDPAYSHVS
DELFATIGVTFFGAGVISTGSFLTTALISLIQRPQLRNLLHEKPELIPAGVEELLRINLSFADGLPRLATADI
QVGDVLVRKGELVLVLLEGANFDPEHFPNPGSIELDRPNPTSHLAFGRGQHFCPGSALGRRHAQIGIEALLKK
MPGVDLAVPIDQLVWRTRFQRRIPERLPVLW
>CYP121A1_Mycobacterium tuberculosis 3280CJ 2575654449
MTATVLLEVPFSARGDRIPDAVAELRTREPIRKVRTITGAEAWLVSSYALCTQVLEDRRFSMKETAAAGAPRL
NALTVPPEVVNNMGNIADAGLRKAVMKAITPKAPGLEQFLRDTANSLLDNLITEGAPADLRNDFADPLATALH
CKVLGIPQEDGPKLFRSLSIAFMSSADPIPAAKINWDRDIEYMAGILENPNITTGLMGELSRLRKDPAYSHVS
DELFATIGVTFFGAGVISTGSFLTTALISLIQRPQLRNLLHEKPELIPAGVEELLRINLSFADGLPRLATADI

QVGDVLVRKGELVLVLLEGANFDPEHFPNPGSIELDRPNPTSHLAFGRGQHFCPGSALGRRHAQIGIEALLKK
MPGVDLAVPIDQLVWRTRFQRRIPERLPVLW
>CYP121A1_Mycobacterium tuberculosis 3499MM 2584873530
MTATVLLEVPFSARGDRIPDAVAELRTREPIRKVRTITGAEAWLVSSYALCTQVLEDRRFSMKETAAAGAPRL
NALTVPPEVVNNMGNIADAGLRKAVMKAITPKAPGLEQFLRDTANSLLDNLITEGAPADLRNDFADPLATALH
CKVLGIPQEDGPKLFRSLSIAFMSSADPIPAAKINWDRDIEYMAGILENPNITTGLMGELSRLRKDPAYSHVS
DELFATIGVTFFGAGVISTGSFLTTALISLIQRPQLRNLLHEKPELIPAGVEELLRINLSFADGLPRLATADI
QVGDVLVRKGELVLVLLEGANFDPEHFPNPGSIELDRPNPTSHLAFGRGQHFCPGSALGRRHAQIGIEALLKK
MPGVDLAVPIDQLVWRTRFQRRIPERLPVLW
>CYP121A1_Mycobacterium tuberculosis 36361 2603456177
MTATVLLEVPFSARGDRIPDAVAELRTREPIRKVRTITGAEAWLVSSYALCTQVLEDRRFSMKETAAAGAPRL
NALTVPPEVVNNMGNIADAGLRKAVMKAITPKAPGLEQFLRDTANSLLDNLITEGAPADLRNDFADPLATALH
CKVLGIPQEDGPKLFRSLSIAFMSSADPIPAAKINWDRDIEYMAGILENPNITTGLMGELSRLRKDPAYSHVS
DELFATIGVTFFGAGVISTGSFLTTALISLIQRPQLRNLLHEKPELIPAGVEELLRINLSFADGLPRLATADI
QVGDVLVRKGELVLVLLEGANFDPEHFPNPGSIELDRPNPTSHLAFGRGQHFCPGSALGRRHAQIGIEALLKK
MPGVDLAVPIDQLVWRTRFQRRIPERLPVLW
>CYP121A1_Mycobacterium tuberculosis 37004 2688158552
MTATVLLEVPFSARGDRIPDAVAELRTREPIRKVRTITGAEAWLVSSYALCTQVLEDRRFSMKETAAAGAPRL
NALTVPPEVVNNMGNIADAGLRKAVMKAITPKAPGLEQFLRDTANSLLDNLITEGAPADLRNDFADPLATALH
CKVLGIPQEDGPKLFRSLSIAFMSSADPIPAAKINWDRDIEYMAGILENPNITTGLMGELSRLRKDPAYSHVS
DELFATIGVTFFGAGVISTGSFLTTALISLIQRPQLRNLLHEKPELIPAGVEELLRINLSFADGLPRLATADI
QVGDVLVRKGELVLVLLEGANFDPEHFPNPGSIELDRPNPTSHLAFGRGQHFCPGSALGRRHAQIGIEALLKK
MPGVDLAVPIDQLVWRTRFQRRIPERLPVLW
>CYP121A1_Mycobacterium tuberculosis 400260 2670882089
MTATVLLEVPFSARGDRIPDAVAELRTREPIRKVRTITGAEAWLVSSYALCTQVLEDRRFSMKETAAAGAPRL
NALTVPPEVVNNMGNIADAGLRKAVMKAITPKAPGLEQFLRDTANSLLDNLITEGAPADLRNDFADPLATALH
CKVLGIPQEDGPKLFRSLSIAFMSSADPIPAAKINWDRDIEYMAGILENPNITTGLMGELSRLRKDPAYSHVS
DELFATIGVTFFGAGVISTGSFLTTALISLIQRPQLRNLLHEKPELIPAGVEELLRINLSFADGLPRLATADI
QVGDVLVRKGELVLVLLEGANFDPEHFPNPGSIELDRPNPTSHLAFGRGQHFCPGSALGRRHAQIGIEALLKK
MPGVDLAVPIDQLVWRTRFQRRIPERLPVLW
>CYP121A1_Mycobacterium tuberculosis 400285 2662949371
MTATVLLEVPFSARGDRIPDAVAELRTREPIRKVRTITGAEAWLVSSYALCTQVLEDRRFSMKETAAAGAPRL
NALTVPPEVVNNMGNIADAGLRKAVMKAITPKAPGLEQFLRDTANSLLDNLITEGAPADLRNDFADPLATALH
CKVLGIPQEDGPKLFRSLSIAFMSSADPIPAAKINWDRDIEYMAGILENPNITTGLMGELSRLRKDPAYSHVS
DELFATIGVTFFGAGVISTGSFLTTALISLIQRPQLRNLLHEKPELIPAGVEELLRINLSFADGLPRLATADI
QVGDVLVRKGELVLVLLEGANFDPEHFPNPGSIELDRPNPTSHLAFGRGQHFCPGSALGRRHAQIGIEALLKK
MPGVDLAVPIDQLVWRTRFQRRIPERLPVLW
>CYP121A1_Mycobacterium tuberculosis 400541 2669074584
MTATVLLEVPFSARGDRIPDAVAELRTREPIRKVRTITGAEAWLVSSYALCTQVLEDRRFSMKETAAAGAPRL
NALTVPPEVVNNMGNIADAGLRKAVMKAITPKAPGLEQFLRDTANSLLDNLITEGAPADLRNDFADPLATALH
CKVLGIPQEDGPKLFRSLSIAFMSSADPIPAAKINWDRDIEYMAGILENPNITTGLMGELSRLRKDPAYSHVS
DELFATIGVTFFGAGVISTGSFLTTALISLIQRPQLRNLLHEKPELIPAGVEELLRINLSFADGLPRLATADI
QVGDVLVRKGELVLVLLEGANFDPEHFPNPGSIELDRPNPTSHLAFGRGQHFCPGSALGRRHAQIGIEALLKK
MPGVDLAVPIDQLVWRTRFQRRIPERLPVLW
>CYP121A1_Mycobacterium tuberculosis 400554 2661358641
MTATVLLEVPFSARGDRIPDAVAELRTREPIRKVRTITGAEAWLVSSYALCTQVLEDRRFSMKETAAAGAPRL
NALTVPPEVVNNMGNIADAGLRKAVMKAITPKAPGLEQFLRDTANSLLDNLITEGAPADLRNDFADPLATALH
CKVLGIPQEDGPKLFRSLSIAFMSSADPIPAAKINWDRDIEYMAGILENPNITTGLMGELSRLRKDPAYSHVS
DELFATIGVTFFGAGVISTGSFLTTALISLIQRPQLRNLLHEKPELIPAGVEELLRINLSFADGLPRLATADI
QVGDVLVRKGELVLVLLEGANFDPEHFPNPGSIELDRPNPTSHLAFGRGQHFCPGSALGRRHAQIGIEALLKK
MPGVDLAVPIDQLVWRTRFQRRIPERLPVLW
>CYP121A1_Mycobacterium tuberculosis 400673 2677272500
MTATVLLEVPFSARGDRIPDAVAELRTREPIRKVRTITGAEAWLVSSYALCTQVLEDRRFSMKETAAAGAPRL
NALTVPPEVVNNMGNIADAGLRKAVMKAITPKAPGLEQFLRDTANSLLDNLITEGAPADLRNDFADPLATALH
CKVLGIPQEDGPKLFRSLSIAFMSSADPIPAAKINWDRDIEYMAGILENPNITTGLMGELSRLRKDPAYSHVS
DELFATIGVTFFGAGVISTGSFLTTALISLIQRPQLRNLLHEKPELIPAGVEELLRINLSFADGLPRLATADI
QVGDVLVRKGELVLVLLEGANFDPEHFPNPGSIELDRPNPTSHLAFGRGQHFCPGSALGRRHAQIGIEALLKK
MPGVDLAVPIDQLVWRTRFQRRIPERLPVLW

>CYP121A1_Mycobacterium tuberculosis 400711 2659515404
MTATVLLEVPFSARGDRIPDAVAELRTREPIRKVRTITGAEAWLVSSYALCTQVLEDRRFSMKETAAAGAPRL
NALTVPPEVVNNMGNIADAGLRKAVMKAITPKAPGLEQFLRDTANSLLDNLITEGAPADLRNDFADPLATALH
CKVLGIPQEDGPKLFRSLSIAFMSSADPIPAAKINWDRDIEYMAGILENPNITTGLMGELSRLRKDPAYSHVS
DELFATIGVTFFGAGVISTGSFLTTALISLIQRPQLRNLLHEKPELIPAGVEELLRINLSFADGLPRLATADI
QVGDVLVRKGELVLVLLEGANFDPEHFPNPGSIELDRPNPTSHLAFGRGQHFCPGSALGRRHAQIGIEALLKK
MPGVDLAVPIDQLVWRTRFQRRIPERLPVLW
>CYP121A1_Mycobacterium tuberculosis 400739 2669994879
MTATVLLEVPFSARGDRIPDAVAELRTREPIRKVRTITGAEAWLVSSYALCTQVLEDRRFSMKETAAAGAPRL
NALTVPPEVVNNMGNIADAGLRKAVMKAITPKAPGLEQFLRDTANSLLDNLITEGAPADLRNDFADPLATALH
CKVLGIPQEDGPKLFRSLSIAFMSSADPIPAAKINWDRDIEYMAGILENPNITTGLMGELSRLRKDPAYSHVS
DELFATIGVTFFGAGVISTGSFLTTALISLIQRPQLRNLLHEKPELIPAGVEELLRINLSFADGLPRLATADI
QVGDVLVRKGELVLVLLEGANFDPEHFPNPGSIELDRPNPTSHLAFGRGQHFCPGSALGRRHAQIGIEALLKK
MPGVDLAVPIDQLVWRTRFQRRIPERLPVLW
>CYP121A1_Mycobacterium tuberculosis 400834 2661870645
MTATVLLEVPFSARGDRIPDAVAELRTREPIRKVRTITGAEAWLVSSYALCTQVLEDRRFSMKETAAAGAPRL
NALTVPPEVVNNMGNIADAGLRKAVMKAITPKAPGLEQFLRDTANSLLDNLITEGAPADLRNDFADPLATALH
CKVLGIPQEDGPKLFRSLSIAFMSSADPIPAAKINWDRDIEYMAGILENPNITTGLMGELSRLRKDPAYSHVS
DELFATIGVTFFGAGVISTGSFLTTALISLIQRPQLRNLLHEKPELIPAGVEELLRINLSFADGLPRLATADI
QVGDVLVRKGELVLVLLEGANFDPEHFPNPGSIELDRPNPTSHLAFGRGQHFCPGSALGRRHAQIGIEALLKK
MPGVDLAVPIDQLVWRTRFQRRIPERLPVLW
>CYP121A1_Mycobacterium tuberculosis 401063 2661644266
MTATVLLEVPFSARGDRIPDAVAELRTREPIRKVRTITGAEAWLVSSYALCTQVLEDRRFSMKETAAAGAPRL
NALTVPPEVVNNMGNIADAGLRKAVMKAITPKAPGLEQFLRDTANSLLDNLITEGAPADLRNDFADPLATALH
CKVLGIPQEDGPKLFRSLSIAFMSSADPIPAAKINWDRDIEYMAGILENPNITTGLMGELSRLRKDPAYSHVS
DELFATIGVTFFGAGVISTGSFLTTALISLIQRPQLRNLLHEKPELIPAGVEELLRINLSFADGLPRLATADI
QVGDVLVRKGELVLVLLEGANFDPEHFPNPGSIELDRPNPTSHLAFGRGQHFCPGSALGRRHAQIGIEALLKK
MPGVDLAVPIDQLVWRTRFQRRIPERLPVLW
>CYP121A1_Mycobacterium tuberculosis 401107 2696797034
MTATVLLEVPFSARGDRIPDAVAELRTREPIRKVRTITGAEAWLVSSYALCTQVLEDRRFSMKETAAAGAPRL
NALTVPPEVVNNMGNIADAGLRKAVMKAITPKAPGLEQFLRDTANSLLDNLITEGAPADLRNDFADPLATALH
CKVLGIPQEDGPKLFRSLSIAFMSSADPIPAAKINWDRDIEYMAGILENPNITTGLMGELSRLRKDPAYSHVS
DELFATIGVTFFGAGVISTGSFLTTALISLIQRPQLRNLLHEKPELIPAGVEELLRINLSFADGLPRLATADI
QVGDVLVRKGELVLVLLEGANFDPEHFPNPGSIELDRPNPTSHLAFGRGQHFCPGSALGRRHAQIGIEALLKK
MPGVDLAVPIDQLVWRTRFQRRIPERLPVLW
>CYP121A1_Mycobacterium tuberculosis 401212 2666215606
MTATVLLEVPFSARGDRIPDAVAELRTREPIRKVRTITGAEAWLVSSYALCTQVLEDRRFSMKETAAAGAPRL
NALTVPPEVVNNMGNIADAGLRKAVMKAITPKAPGLEQFLRDTANSLLDNLITEGAPADLRNDFADPLATALH
CKVLGIPQEDGPKLFRSLSIAFMSSADPIPAAKINWDRDIEYMAGILENPNITTGLMGELSRLRKDPAYSHVS
DELFATIGVTFFGAGVISTGSFLTTALISLIQRPQLRNLLHEKPELIPAGVEELLRINLSFADGLPRLATADI
QVGDVLVRKGELVLVLLEGANFDPEHFPNPGSIELDRPNPTSHLAFGRGQHFCPGSALGRRHAQIGIEALLKK
MPGVDLAVPIDQLVWRTRFQRRIPERLPVLW
>CYP121A1_Mycobacterium tuberculosis 401391 2661518247
MTATVLLEVPFSARGDRIPDAVAELRTREPIRKVRTITGAEAWLVSSYALCTQVLEDRRFSMKETAAAGAPRL
NALTVPPEVVNNMGNIADAGLRKAVMKAITPKAPGLEQFLRDTANSLLDNLITEGAPADLRNDFADPLATALH
CKVLGIPQEDGPKLFRSLSIAFMSSADPIPAAKINWDRDIEYMAGILENPNITTGLMGELSRLRKDPAYSHVS
DELFATIGVTFFGAGVISTGSFLTTALISLIQRPQLRNLLHEKPELIPAGVEELLRINLSFADGLPRLATADI
QVGDVLVRKGELVLVLLEGANFDPEHFPNPGSIELDRPNPTSHLAFGRGQHFCPGSALGRRHAQIGIEALLKK
MPGVDLAVPIDQLVWRTRFQRRIPERLPVLW
>CYP121A1_Mycobacterium tuberculosis 401539 2665355081
MTATVLLEVPFSARGDRIPDAVAELRTREPIRKVRTITGAEAWLVSSYALCTQVLEDRRFSMKETAAAGAPRL
NALTVPPEVVNNMGNIADAGLRKAVMKAITPKAPGLEQFLRDTANSLLDNLITEGAPADLRNDFADPLATALH
CKVLGIPQEDGPKLFRSLSIAFMSSADPIPAAKINWDRDIEYMAGILENPNITTGLMGELSRLRKDPAYSHVS
DELFATIGVTFFGAGVISTGSFLTTALISLIQRPQLRNLLHEKPELIPAGVEELLRINLSFADGLPRLATADI
QVGDVLVRKGELVLVLLEGANFDPEHFPNPGSIELDRPNPTSHLAFGRGQHFCPGSALGRRHAQIGIEALLKK
MPGVDLAVPIDQLVWRTRFQRRIPERLPVLW
>CYP121A1_Mycobacterium tuberculosis 401555 2701692870

MTATVLLEVPFSARGDRIPDAVAELRTREPIRKVRTITGAEAWLVSSYALCTQVLEDRRFSMKETAAAGAPRL
NALTVPPEVVNNMGNIADAGLRKAVMKAITPKAPGLEQFLRDTANSLLDNLITEGAPADLRNDFADPLATALH
CKVLGIPQEDGPKLFRSLSIAFMSSADPIPAAKINWDRDIEYMAGILENPNITTGLMGELSRLRKDPAYSHVS
DELFATIGVTFFGAGVISTGSFLTTALISLIQRPQLRNLLHEKPELIPAGVEELLRINLSFADGLPRLATADI
QVGDVLVRKGELVLVLLEGANFDPEHFPNPGSIELDRPNPTSHLAFGRGQHFCPGSALGRRHAQIGIEALLKK
MPGVDLAVPIDQLVWRTRFQRRIPERLPVLW
>CYP121A1_Mycobacterium tuberculosis 401787 2668758681
MTATVLLEVPFSARGDRIPDAVAELRTREPIRKVRTITGAEAWLVSSYALCTQVLEDRRFSMKETAAAGAPRL
NALTVPPEVVNNMGNIADAGLRKAVMKAITPKAPGLEQFLRDTANSLLDNLITEGAPADLRNDFADPLATALH
CKVLGIPQEDGPKLFRSLSIAFMSSADPIPAAKINWDRDIEYMAGILENPNITTGLMGELSRLRKDPAYSHVS
DELFATIGVTFFGAGVISTGSFLTTALISLIQRPQLRNLLHEKPELIPAGVEELLRINLSFADGLPRLATADI
QVGDVLVRKGELVLVLLEGANFDPEHFPNPGSIELDRPNPTSHLAFGRGQHFCPGSALGRRHAQIGIEALLKK
MPGVDLAVPIDQLVWRTRFQRRIPERLPVLW
>CYP121A1_Mycobacterium tuberculosis 402106 2659853188
MTATVLLEVPFSARGDRIPDAVAELRTREPIRKVRTITGAEAWLVSSYALCTQVLEDRRFSMKETAAAGAPRL
NALTVPPEVVNNMGNIADAGLRKAVMKAITPKAPGLEQFLRDTANSLLDNLITEGAPADLRNDFADPLATALH
CKVLGIPQEDGPKLFRSLSIAFMSSADPIPAAKINWDRDIEYMAGILENPNITTGLMGELSRLRKDPAYSHVS
DELFATIGVTFFGAGVISTGSFLTTALISLIQRPQLRNLLHEKPELIPAGVEELLRINLSFADGLPRLATADI
QVGDVLVRKGELVLVLLEGANFDPEHFPNPGSIELDRPNPTSHLAFGRGQHFCPGSALGRRHAQIGIEALLKK
MPGVDLAVPIDQLVWRTRFQRRIPERLPVLW
>CYP121A1_Mycobacterium tuberculosis 402138 2662945335
MTATVLLEVPFSARGDRIPDAVAELRTREPIRKVRTITGAEAWLVSSYALCTQVLEDRRFSMKETAAAGAPRL
NALTVPPEVVNNMGNIADAGLRKAVMKAITPKAPGLEQFLRDTANSLLDNLITEGAPADLRNDFADPLATALH
CKVLGIPQEDGPKLFRSLSIAFMSSADPIPAAKINWDRDIEYMAGILENPNITTGLMGELSRLRKDPAYSHVS
DELFATIGVTFFGAGVISTGSFLTTALISLIQRPQLRNLLHEKPELIPAGVEELLRINLSFADGLPRLATADI
QVGDVLVRKGELVLVLLEGANFDPEHFPNPGSIELDRPNPTSHLAFGRGQHFCPGSALGRRHAQIGIEALLKK
MPGVDLAVPIDQLVWRTRFQRRIPERLPVLW
>CYP121A1_Mycobacterium tuberculosis 402267 2692005559
MTATVLLEVPFSARGDRIPDAVAELRTREPIRKVRTITGAEAWLVSSYALCTQVLEDRRFSMKETAAAGAPRL
NALTVPPEVVNNMGNIADAGLRKAVMKAITPKAPGLEQFLRDTANSLLDNLITEGAPADLRNDFADPLATALH
CKVLGIPQEDGPKLFRSLSIAFMSSADPIPAAKINWDRDIEYMAGILENPNITTGLMGELSRLRKDPAYSHVS
DELFATIGVTFFGAGVISTGSFLTTALISLIQRPQLRNLLHEKPELIPAGVEELLRINLSFADGLPRLATADI
QVGDVLVRKGELVLVLLEGANFDPEHFPNPGSIELDRPNPTSHLAFGRGQHFCPGSALGRRHAQIGIEALLKK
MPGVDLAVPIDQLVWRTRFQRRIPERLPVLW
>CYP121A1_Mycobacterium tuberculosis 402321 2701655392
MTATVLLEVPFSARGDRIPDAVAELRTREPIRKVRTITGAEAWLVSSYALCTQVLEDRRFSMKETAAAGAPRL
NALTVPPEVVNNMGNIADAGLRKAVMKAITPKAPGLEQFLRDTANSLLDNLITEGAPADLRNDFADPLATALH
CKVLGIPQEDGPKLFRSLSIAFMSSADPIPAAKINWDRDIEYMAGILENPNITTGLMGELSRLRKDPAYSHVS
DELFATIGVTFFGAGVISTGSFLTTALISLIQRPQLRNLLHEKPELIPAGVEELLRINLSFADGLPRLATADI
QVGDVLVRKGELVLVLLEGANFDPEHFPNPGSIELDRPNPTSHLAFGRGQHFCPGSALGRRHAQIGIEALLKK
MPGVDLAVPIDQLVWRTRFQRRIPERLPVLW
>CYP121A1_Mycobacterium tuberculosis 402495 2665903185
MTATVLLEVPFSARGDRIPDAVAELRTREPIRKVRTITGAEAWLVSSYALCTQVLEDRRFSMKETAAAGAPRL
NALTVPPEVVNNMGNIADAGLRKAVMKAITPKAPGLEQFLRDTANSLLDNLITEGAPADLRNDFADPLATALH
CKVLGIPQEDGPKLFRSLSIAFMSSADPIPAAKINWDRDIEYMAGILENPNITTGLMGELSRLRKDPAYSHVS
DELFATIGVTFFGAGVISTGSFLTTALISLIQRPQLRNLLHEKPELIPAGVEELLRINLSFADGLPRLATADI
QVGDVLVRKGELVLVLLEGANFDPEHFPNPGSIELDRPNPTSHLAFGRGQHFCPGSALGRRHAQIGIEALLKK
MPGVDLAVPIDQLVWRTRFQRRIPERLPVLW
>CYP121A1_Mycobacterium tuberculosis 402538 2701666546
MTATVLLEVPFSARGDRIPDAVAELRTREPIRKVRTITGAEAWLVSSYALCTQVLEDRRFSMKETAAAGAPRL
NALTVPPEVVNNMGNIADAGLRKAVMKAITPKAPGLEQFLRDTANSLLDNLITEGAPADLRNDFADPLATALH
CKVLGIPQEDGPKLFRSLSIAFMSSADPIPAAKINWDRDIEYMAGILENPNITTGLMGELSRLRKDPAYSHVS
DELFATIGVTFFGAGVISTGSFLTTALISLIQRPQLRNLLHEKPELIPAGVEELLRINLSFADGLPRLATADI
QVGDVLVRKGELVLVLLEGANFDPEHFPNPGSIELDRPNPTSHLAFGRGQHFCPGSALGRRHAQIGIEALLKK
MPGVDLAVPIDQLVWRTRFQRRIPERLPVLW
>CYP121A1_Mycobacterium tuberculosis 402542 2678024377
MTATVLLEVPFSARGDRIPDAVAELRTREPIRKVRTITGAEAWLVSSYALCTQVLEDRRFSMKETAAAGAPRL
NALTVPPEVVNNMGNIADAGLRKAVMKAITPKAPGLEQFLRDTANSLLDNLITEGAPADLRNDFADPLATALH

CKVLGIPQEDGPKLFRSLSIAFMSSADPIPAAKINWDRDIEYMAGILENPNITTGLMGELSRLRKDPAYSHVS
DELFATIGVTFFGAGVISTGSFLTTALISLIQRPQLRNLLHEKPELIPAGVEELLRINLSFADGLPRLATADI
QVGDVLVRKGELVLVLLEGANFDPEHFPNPGSIELDRPNPTSHLAFGRGQHFCPGSALGRRHAQIGIEALLKK
MPGVDLAVPIDQLVWRTRFQRRIPERLPVLW
>CYP121A1_Mycobacterium tuberculosis 402568 2665026031
MTATVLLEVPFSARGDRIPDAVAELRTREPIRKVRTITGAEAWLVSSYALCTQVLEDRRFSMKETAAAGAPRL
NALTVPPEVVNNMGNIADAGLRKAVMKAITPKAPGLEQFLRDTANSLLDNLITEGAPADLRNDFADPLATALH
CKVLGIPQEDGPKLFRSLSIAFMSSADPIPAAKINWDRDIEYMAGILENPNITTGLMGELSRLRKDPAYSHVS
DELFATIGVTFFGAGVISTGSFLTTALISLIQRPQLRNLLHEKPELIPAGVEELLRINLSFADGLPRLATADI
QVGDVLVRKGELVLVLLEGANFDPEHFPNPGSIELDRPNPTSHLAFGRGQHFCPGSALGRRHAQIGIEALLKK
MPGVDLAVPIDQLVWRTRFQRRIPERLPVLW
>CYP121A1_Mycobacterium tuberculosis 402713 2701671879
MTATVLLEVPFSARGDRIPDAVAELRTREPIRKVRTITGAEAWLVSSYALCTQVLEDRRFSMKETAAAGAPRL
NALTVPPEVVNNMGNIADAGLRKAVMKAITPKAPGLEQFLRDTANSLLDNLITEGAPADLRNDFADPLATALH
CKVLGIPQEDGPKLFRSLSIAFMSSADPIPAAKINWDRDIEYMAGILENPNITTGLMGELSRLRKDPAYSHVS
DELFATIGVTFFGAGVISTGSFLTTALISLIQRPQLRNLLHEKPELIPAGVEELLRINLSFADGLPRLATADI
QVGDVLVRKGELVLVLLEGANFDPEHFPNPGSIELDRPNPTSHLAFGRGQHFCPGSALGRRHAQIGIEALLKK
MPGVDLAVPIDQLVWRTRFQRRIPERLPVLW
>CYP121A1_Mycobacterium tuberculosis 43-13838 2702446133
MTATVLLEVPFSARGDRIPDAVAELRTREPIRKVRTITGAEAWLVSSYALCTQVLEDRRFSMKETAAAGAPRL
NALTVPPEVVNNMGNIADAGLRKAVMKAITPKAPGLEQFLRDTANSLLDNLITEGAPADLRNDFADPLATALH
CKVLGIPQEDGPKLFRSLSIAFMSSADPIPAAKINWDRDIEYMAGILENPNITTGLMGELSRLRKDPAYSHVS
DELFATIGVTFFGAGVISTGSFLTTALISLIQRPQLRNLLHEKPELIPAGVEELLRINLSFADGLPRLATADI
QVGDVLVRKGELVLVLLEGANFDPEHFPNPGSIELDRPNPTSHLAFGRGQHFCPGSALGRRHAQIGIEALLKK
MPGVDLAVPIDQLVWRTRFQRRIPERLPVLW
>CYP121A1_Mycobacterium tuberculosis 43-16836 2603129325
MTATVLLEVPFSARGDRIPDAVAELRTREPIRKVRTITGAEAWLVSSYALCTQVLEDRRFSMKETAAAGAPRL
NALTVPPEVVNNMGNIADAGLRKAVMKAITPKAPGLEQFLRDTANSLLDNLITEGAPADLRNDFADPLATALH
CKVLGIPQEDGPKLFRSLSIAFMSSADPIPAAKINWDRDIEYMAGILENPNITTGLMGELSRLRKDPAYSHVS
DELFATIGVTFFGAGVISTGSFLTTALISLIQRPQLRNLLHEKPELIPAGVEELLRINLSFADGLPRLATADI
QVGDVLVRKGELVLVLLEGANFDPEHFPNPGSIELDRPNPTSHLAFGRGQHFCPGSALGRRHAQIGIEALLKK
MPGVDLAVPIDQLVWRTRFQRRIPERLPVLW
>CYP121A1_Mycobacterium tuberculosis 44503 2584612434
MTATVLLEVPFSARGDRIPDAVAELRTREPIRKVRTITGAEAWLVSSYALCTQVLEDRRFSMKETAAAGAPRL
NALTVPPEVVNNMGNIADAGLRKAVMKAITPKAPGLEQFLRDTANSLLDNLITEGAPADLRNDFADPLATALH
CKVLGIPQEDGPKLFRSLSIAFMSSADPIPAAKINWDRDIEYMAGILENPNITTGLMGELSRLRKDPAYSHVS
DELFATIGVTFFGAGVISTGSFLTTALISLIQRPQLRNLLHEKPELIPAGVEELLRINLSFADGLPRLATADI
QVGDVLVRKGELVLVLLEGANFDPEHFPNPGSIELDRPNPTSHLAFGRGQHFCPGSALGRRHAQIGIEALLKK
MPGVDLAVPIDQLVWRTRFQRRIPERLPVLW
>CYP121A1_Mycobacterium tuberculosis 46-5069 2702448980
MTATVLLEVPFSARGDRIPDAVAELRTREPIRKVRTITGAEAWLVSSYALCTQVLEDRRFSMKETAAAGAPRL
NALTVPPEVVNNMGNIADAGLRKAVMKAITPKAPGLEQFLRDTANSLLDNLITEGAPADLRNDFADPLATALH
CKVLGIPQEDGPKLFRSLSIAFMSSADPIPAAKINWDRDIEYMAGILENPNITTGLMGELSRLRKDPAYSHVS
DELFATIGVTFFGAGVISTGSFLTTALISLIQRPQLRNLLHEKPELIPAGVEELLRINLSFADGLPRLATADI
QVGDVLVRKGELVLVLLEGANFDPEHFPNPGSIELDRPNPTSHLAFGRGQHFCPGSALGRRHAQIGIEALLKK
MPGVDLAVPIDQLVWRTRFQRRIPERLPVLW
>CYP121A1_Mycobacterium tuberculosis 51628 2584637392
MTATVLLEVPFSARGDRIPDAVAELRTREPIRKVRTITGAEAWLVSSYALCTQVLEDRRFSMKETAAAGAPRL
NALTVPPEVVNNMGNIADAGLRKAVMKAITPKAPGLEQFLRDTANSLLDNLITEGAPADLRNDFADPLATALH
CKVLGIPQEDGPKLFRSLSIAFMSSADPIPAAKINWDRDIEYMAGILENPNITTGLMGELSRLRKDPAYSHVS
DELFATIGVTFFGAGVISTGSFLTTALISLIQRPQLRNLLHEKPELIPAGVEELLRINLSFADGLPRLATADI
QVGDVLVRKGELVLVLLEGANFDPEHFPNPGSIELDRPNPTSHLAFGRGQHFCPGSALGRRHAQIGIEALLKK
MPGVDLAVPIDQLVWRTRFQRRIPERLPVLW
>CYP121A1_Mycobacterium tuberculosis 6605 2621681850
MTATVLLEVPFSARGDRIPDAVAELRTREPIRKVRTITGAEAWLVSSYALCTQVLEDRRFSMKETAAAGAPRL
NALTVPPEVVNNMGNIADAGLRKAVMKAITPKAPGLEQFLRDTANSLLDNLITEGAPADLRNDFADPLATALH
CKVLGIPQEDGPKLFRSLSIAFMSSADPIPAAKINWDRDIEYMAGILENPNITTGLMGELSRLRKDPAYSHVS
DELFATIGVTFFGAGVISTGSFLTTALISLIQRPQLRNLLHEKPELIPAGVEELLRINLSFADGLPRLATADI

QVGDVLVRKGELVLVLLEGANFDPEHFPNPGSIELDRPNPTSHLAFGRGQHFCPGSALGRRHAQIGIEALLKK
MPGVDLAVPIDQLVWRTRFQRRIPERLPVLW
>CYP121A1_Mycobacterium tuberculosis 6638 2621704672
MTATVLLEVPFSARGDRIPDAVAELRTREPIRKVRTITGAEAWLVSSYALCTQVLEDRRFSMKETAAAGAPRL
NALTVPPEVVNNMGNIADAGLRKAVMKAITPKAPGLEQFLRDTANSLLDNLITEGAPADLRNDFADPLATALH
CKVLGIPQEDGPKLFRSLSIAFMSSADPIPAAKINWDRDIEYMAGILENPNITTGLMGELSRLRKDPAYSHVS
DELFATIGVTFFGAGVISTGSFLTTALISLIQRPQLRNLLHEKPELIPAGVEELLRINLSFADGLPRLATADI
QVGDVLVRKGELVLVLLEGANFDPEHFPNPGSIELDRPNPTSHLAFGRGQHFCPGSALGRRHAQIGIEALLKK
MPGVDLAVPIDQLVWRTRFQRRIPERLPVLW
>CYP121A1_Mycobacterium tuberculosis 7057 2621687011
MTATVLLEVPFSARGDRIPDAVAELRTREPIRKVRTITGAEAWLVSSYALCTQVLEDRRFSMKETAAAGAPRL
NALTVPPEVVNNMGNIADAGLRKAVMKAITPKAPGLEQFLRDTANSLLDNLITEGAPADLRNDFADPLATALH
CKVLGIPQEDGPKLFRSLSIAFMSSADPIPAAKINWDRDIEYMAGILENPNITTGLMGELSRLRKDPAYSHVS
DELFATIGVTFFGAGVISTGSFLTTALISLIQRPQLRNLLHEKPELIPAGVEELLRINLSFADGLPRLATADI
QVGDVLVRKGELVLVLLEGANFDPEHFPNPGSIELDRPNPTSHLAFGRGQHFCPGSALGRRHAQIGIEALLKK
MPGVDLAVPIDQLVWRTRFQRRIPERLPVLW
>CYP121A1_Mycobacterium tuberculosis 8442 2606472688
MTATVLLEVPFSARGDRIPDAVAELRTREPIRKVRTITGAEAWLVSSYALCTQVLEDRRFSMKETAAAGAPRL
NALTVPPEVVNNMGNIADAGLRKAVMKAITPKAPGLEQFLRDTANSLLDNLITEGAPADLRNDFADPLATALH
CKVLGIPQEDGPKLFRSLSIAFMSSADPIPAAKINWDRDIEYMAGILENPNITTGLMGELSRLRKDPAYSHVS
DELFATIGVTFFGAGVISTGSFLTTALISLIQRPQLRNLLHEKPELIPAGVEELLRINLSFADGLPRLATADI
QVGDVLVRKGELVLVLLEGANFDPEHFPNPGSIELDRPNPTSHLAFGRGQHFCPGSALGRRHAQIGIEALLKK
MPGVDLAVPIDQLVWRTRFQRRIPERLPVLW
>CYP121A1_Mycobacterium tuberculosis 9542 2621678528
MTATVLLEVPFSARGDRIPDAVAELRTREPIRKVRTITGAEAWLVSSYALCTQVLEDRRFSMKETAAAGAPRL
NALTVPPEVVNNMGNIADAGLRKAVMKAITPKAPGLEQFLRDTANSLLDNLITEGAPADLRNDFADPLATALH
CKVLGIPQEDGPKLFRSLSIAFMSSADPIPAAKINWDRDIEYMAGILENPNITTGLMGELSRLRKDPAYSHVS
DELFATIGVTFFGAGVISTGSFLTTALISLIQRPQLRNLLHEKPELIPAGVEELLRINLSFADGLPRLATADI
QVGDVLVRKGELVLVLLEGANFDPEHFPNPGSIELDRPNPTSHLAFGRGQHFCPGSALGRRHAQIGIEALLKK
MPGVDLAVPIDQLVWRTRFQRRIPERLPVLW
>CYP121A1_Mycobacterium tuberculosis 96121 2671957361
MTATVLLEVPFSARGDRIPDAVAELRTREPIRKVRTITGAEAWLVSSYALCTQVLEDRRFSMKETAAAGAPRL
NALTVPPEVVNNMGNIADAGLRKAVMKAITPKAPGLEQFLRDTANSLLDNLITEGAPADLRNDFADPLATALH
CKVLGIPQEDGPKLFRSLSIAFMSSADPIPAAKINWDRDIEYMAGILENPNITTGLMGELSRLRKDPAYSHVS
DELFATIGVTFFGAGVISTGSFLTTALISLIQRPQLRNLLHEKPELIPAGVEELLRINLSFADGLPRLATADI
QVGDVLVRKGELVLVLLEGANFDPEHFPNPGSIELDRPNPTSHLAFGRGQHFCPGSALGRRHAQIGIEALLKK
MPGVDLAVPIDQLVWRTRFQRRIPERLPVLW
>CYP121A1_Mycobacterium tuberculosis '98-R604 INH-RIF-EM' sequence
645192386
MTATVLLEVPFSARGDRIPDAVAELRTREPIRKVRTITGAEAWLVSSYALCTQVLEDRRFSMKETAAAGAPRL
NALTVPPEVVNNMGNIADAGLRKAVMKAITPKAPGLEQFLRDTANSLLDNLITEGAPADLRNDFADPLATALH
CKVLGIPQEDGPKLFRSLSIAFMSSADPIPAAKINWDRDIEYMAGILENPNITTGLMGELSRLRKDPAYSHVS
DELFATIGVTFFGAGVISTGSFLTTALISLIQRPQLRNLLHEKPELIPAGVEELLRINLSFADGLPRLATADI
QVGDVLVRKGELVLVLLEGANFDPEHFPNPGSIELDRPNPTSHLAFGRGQHFCPGSALGRRHAQIGIEALLKK
MPGVDLAVPIDQLVWRTRFQRRIPERLPVLW
>CYP121A1_Mycobacterium tuberculosis 9956 2621707610
MTATVLLEVPFSARGDRIPDAVAELRTREPIRKVRTITGAEAWLVSSYALCTQVLEDRRFSMKETAAAGAPRL
NALTVPPEVVNNMGNIADAGLRKAVMKAITPKAPGLEQFLRDTANSLLDNLITEGAPADLRNDFADPLATALH
CKVLGIPQEDGPKLFRSLSIAFMSSADPIPAAKINWDRDIEYMAGILENPNITTGLMGELSRLRKDPAYSHVS
DELFATIGVTFFGAGVISTGSFLTTALISLIQRPQLRNLLHEKPELIPAGVEELLRINLSFADGLPRLATADI
QVGDVLVRKGELVLVLLEGANFDPEHFPNPGSIELDRPNPTSHLAFGRGQHFCPGSALGRRHAQIGIEALLKK
MPGVDLAVPIDQLVWRTRFQRRIPERLPVLW
>CYP121A1_Mycobacterium tuberculosis A00301419 2706604204
MTATVLLEVPFSARGDRIPDAVAELRTREPIRKVRTITGAEAWLVSSYALCTQVLEDRRFSMKETAAAGAPRL
NALTVPPEVVNNMGNIADAGLRKAVMKAITPKAPGLEQFLRDTANSLLDNLITEGAPADLRNDFADPLATALH
CKVLGIPQEDGPKLFRSLSIAFMSSADPIPAAKINWDRDIEYMAGILENPNITTGLMGELSRLRKDPAYSHVS
DELFATIGVTFFGAGVISTGSFLTTALISLIQRPQLRNLLHEKPELIPAGVEELLRINLSFADGLPRLATADI

QVGDVLVRKGELVLVLLEGANFDPEHFPNPGSIELDRPNPTSHLAFGRGQHFCPGSALGRRHAQIGIEALLKK
MPGVDLAVPIDQLVWRTRFQRRIPERLPVLW
>CYP121A1_Mycobacterium tuberculosis A00400237 2677314976
MTATVLLEVPFSARGDRIPDAVAELRTREPIRKVRTITGAEAWLVSSYALCTQVLEDRRFSMKETAAAGAPRL
NALTVPPEVVNNMGNIADAGLRKAVMKAITPKAPGLEQFLRDTANSLLDNLITEGAPADLRNDFADPLATALH
CKVLGIPQEDGPKLFRSLSIAFMSSADPIPAAKINWDRDIEYMAGILENPNITTGLMGELSRLRKDPAYSHVS
DELFATIGVTFFGAGVISTGSFLTTALISLIQRPQLRNLLHEKPELIPAGVEELLRINLSFADGLPRLATADI
QVGDVLVRKGELVLVLLEGANFDPEHFPNPGSIELDRPNPTSHLAFGRGQHFCPGSALGRRHAQIGIEALLKK
MPGVDLAVPIDQLVWRTRFQRRIPERLPVLW
>CYP121A1_Mycobacterium tuberculosis A00400885 2673246095
MTATVLLEVPFSARGDRIPDAVAELRTREPIRKVRTITGAEAWLVSSYALCTQVLEDRRFSMKETAAAGAPRL
NALTVPPEVVNNMGNIADAGLRKAVMKAITPKAPGLEQFLRDTANSLLDNLITEGAPADLRNDFADPLATALH
CKVLGIPQEDGPKLFRSLSIAFMSSADPIPAAKINWDRDIEYMAGILENPNITTGLMGELSRLRKDPAYSHVS
DELFATIGVTFFGAGVISTGSFLTTALISLIQRPQLRNLLHEKPELIPAGVEELLRINLSFADGLPRLATADI
QVGDVLVRKGELVLVLLEGANFDPEHFPNPGSIELDRPNPTSHLAFGRGQHFCPGSALGRRHAQIGIEALLKK
MPGVDLAVPIDQLVWRTRFQRRIPERLPVLW
>CYP121A1_Mycobacterium tuberculosis A00401093 2706593588
MTATVLLEVPFSARGDRIPDAVAELRTREPIRKVRTITGAEAWLVSSYALCTQVLEDRRFSMKETAAAGAPRL
NALTVPPEVVNNMGNIADAGLRKAVMKAITPKAPGLEQFLRDTANSLLDNLITEGAPADLRNDFADPLATALH
CKVLGIPQEDGPKLFRSLSIAFMSSADPIPAAKINWDRDIEYMAGILENPNITTGLMGELSRLRKDPAYSHVS
DELFATIGVTFFGAGVISTGSFLTTALISLIQRPQLRNLLHEKPELIPAGVEELLRINLSFADGLPRLATADI
QVGDVLVRKGELVLVLLEGANFDPEHFPNPGSIELDRPNPTSHLAFGRGQHFCPGSALGRRHAQIGIEALLKK
MPGVDLAVPIDQLVWRTRFQRRIPERLPVLW
>CYP121A1_Mycobacterium tuberculosis A00401371 2683851306
MTATVLLEVPFSARGDRIPDAVAELRTREPIRKVRTITGAEAWLVSSYALCTQVLEDRRFSMKETAAAGAPRL
NALTVPPEVVNNMGNIADAGLRKAVMKAITPKAPGLEQFLRDTANSLLDNLITEGAPADLRNDFADPLATALH
CKVLGIPQEDGPKLFRSLSIAFMSSADPIPAAKINWDRDIEYMAGILENPNITTGLMGELSRLRKDPAYSHVS
DELFATIGVTFFGAGVISTGSFLTTALISLIQRPQLRNLLHEKPELIPAGVEELLRINLSFADGLPRLATADI
QVGDVLVRKGELVLVLLEGANFDPEHFPNPGSIELDRPNPTSHLAFGRGQHFCPGSALGRRHAQIGIEALLKK
MPGVDLAVPIDQLVWRTRFQRRIPERLPVLW
>CYP121A1_Mycobacterium tuberculosis A00500149 2703463502
MTATVLLEVPFSARGDRIPDAVAELRTREPIRKVRTITGAEAWLVSSYALCTQVLEDRRFSMKETAAAGAPRL
NALTVPPEVVNNMGNIADAGLRKAVMKAITPKAPGLEQFLRDTANSLLDNLITEGAPADLRNDFADPLATALH
CKVLGIPQEDGPKLFRSLSIAFMSSADPIPAAKINWDRDIEYMAGILENPNITTGLMGELSRLRKDPAYSHVS
DELFATIGVTFFGAGVISTGSFLTTALISLIQRPQLRNLLHEKPELIPAGVEELLRINLSFADGLPRLATADI
QVGDVLVRKGELVLVLLEGANFDPEHFPNPGSIELDRPNPTSHLAFGRGQHFCPGSALGRRHAQIGIEALLKK
MPGVDLAVPIDQLVWRTRFQRRIPERLPVLW
>CYP121A1_Mycobacterium tuberculosis A09900565 2674125806
MTATVLLEVPFSARGDRIPDAVAELRTREPIRKVRTITGAEAWLVSSYALCTQVLEDRRFSMKETAAAGAPRL
NALTVPPEVVNNMGNIADAGLRKAVMKAITPKAPGLEQFLRDTANSLLDNLITEGAPADLRNDFADPLATALH
CKVLGIPQEDGPKLFRSLSIAFMSSADPIPAAKINWDRDIEYMAGILENPNITTGLMGELSRLRKDPAYSHVS
DELFATIGVTFFGAGVISTGSFLTTALISLIQRPQLRNLLHEKPELIPAGVEELLRINLSFADGLPRLATADI
QVGDVLVRKGELVLVLLEGANFDPEHFPNPGSIELDRPNPTSHLAFGRGQHFCPGSALGRRHAQIGIEALLKK
MPGVDLAVPIDQLVWRTRFQRRIPERLPVLW
>CYP121A1_Mycobacterium tuberculosis A09901284 2706560680
MTATVLLEVPFSARGDRIPDAVAELRTREPIRKVRTITGAEAWLVSSYALCTQVLEDRRFSMKETAAAGAPRL
NALTVPPEVVNNMGNIADAGLRKAVMKAITPKAPGLEQFLRDTANSLLDNLITEGAPADLRNDFADPLATALH
CKVLGIPQEDGPKLFRSLSIAFMSSADPIPAAKINWDRDIEYMAGILENPNITTGLMGELSRLRKDPAYSHVS
DELFATIGVTFFGAGVISTGSFLTTALISLIQRPQLRNLLHEKPELIPAGVEELLRINLSFADGLPRLATADI
QVGDVLVRKGELVLVLLEGANFDPEHFPNPGSIELDRPNPTSHLAFGRGQHFCPGSALGRRHAQIGIEALLKK
MPGVDLAVPIDQLVWRTRFQRRIPERLPVLW
>CYP121A1_Mycobacterium tuberculosis A2 2715348928
MTATVLLEVPFSARGDRIPDAVAELRTREPIRKVRTITGAEAWLVSSYALCTQVLEDRRFSMKETAAAGAPRL
NALTVPPEVVNNMGNIADAGLRKAVMKAITPKAPGLEQFLRDTANSLLDNLITEGAPADLRNDFADPLATALH
CKVLGIPQEDGPKLFRSLSIAFMSSADPIPAAKINWDRDIEYMAGILENPNITTGLMGELSRLRKDPAYSHVS
DELFATIGVTFFGAGVISTGSFLTTALISLIQRPQLRNLLHEKPELIPAGVEELLRINLSFADGLPRLATADI
QVGDVLVRKGELVLVLLEGANFDPEHFPNPGSIELDRPNPTSHLAFGRGQHFCPGSALGRRHAQIGIEALLKK
MPGVDLAVPIDQLVWRTRFQRRIPERLPVLW

>CYP121A1_Mycobacterium tuberculosis A4 2715370176
MTATVLLEVPFSARGDRIPDAVAELRTREPIRKVRTITGAEAWLVSSYALCTQVLEDRRFSMKETAAAGAPRL
NALTVPPEVVNNMGNIADAGLRKAVMKAITPKAPGLEQFLRDTANSLLDNLITEGAPADLRNDFADPLATALH
CKVLGIPQEDGPKLFRSLSIAFMSSADPIPAAKINWDRDIEYMAGILENPNITTGLMGELSRLRKDPAYSHVS
DELFATIGVTFFGAGVISTGSFLTTALISLIQRPQLRNLLHEKPELIPAGVEELLRINLSFADGLPRLATADI
QVGDVLVRKGELVLVLLEGANFDPEHFPNPGSIELDRPNPTSHLAFGRGQHFCPGSALGRRHAQIGIEALLKK
MPGVDLAVPIDQLVWRTRFQRRIPERLPVLW
>CYP121A1_Mycobacterium tuberculosis A70011_1 2662930611
MTATVLLEVPFSARGDRIPDAVAELRTREPIRKVRTITGAEAWLVSSYALCTQVLEDRRFSMKETAAAGAPRL
NALTVPPEVVNNMGNIADAGLRKAVMKAITPKAPGLEQFLRDTANSLLDNLITEGAPADLRNDFADPLATALH
CKVLGIPQEDGPKLFRSLSIAFMSSADPIPAAKINWDRDIEYMAGILENPNITTGLMGELSRLRKDPAYSHVS
DELFATIGVTFFGAGVISTGSFLTTALISLIQRPQLRNLLHEKPELIPAGVEELLRINLSFADGLPRLATADI
QVGDVLVRKGELVLVLLEGANFDPEHFPNPGSIELDRPNPTSHLAFGRGQHFCPGSALGRRHAQIGIEALLKK
MPGVDLAVPIDQLVWRTRFQRRIPERLPVLW
>CYP121A1_Mycobacterium tuberculosis A70011_2 2694047010
MTATVLLEVPFSARGDRIPDAVAELRTREPIRKVRTITGAEAWLVSSYALCTQVLEDRRFSMKETAAAGAPRL
NALTVPPEVVNNMGNIADAGLRKAVMKAITPKAPGLEQFLRDTANSLLDNLITEGAPADLRNDFADPLATALH
CKVLGIPQEDGPKLFRSLSIAFMSSADPIPAAKINWDRDIEYMAGILENPNITTGLMGELSRLRKDPAYSHVS
DELFATIGVTFFGAGVISTGSFLTTALISLIQRPQLRNLLHEKPELIPAGVEELLRINLSFADGLPRLATADI
QVGDVLVRKGELVLVLLEGANFDPEHFPNPGSIELDRPNPTSHLAFGRGQHFCPGSALGRRHAQIGIEALLKK
MPGVDLAVPIDQLVWRTRFQRRIPERLPVLW
>CYP121A1_Mycobacterium tuberculosis A70011_3 2694009814
MTATVLLEVPFSARGDRIPDAVAELRTREPIRKVRTITGAEAWLVSSYALCTQVLEDRRFSMKETAAAGAPRL
NALTVPPEVVNNMGNIADAGLRKAVMKAITPKAPGLEQFLRDTANSLLDNLITEGAPADLRNDFADPLATALH
CKVLGIPQEDGPKLFRSLSIAFMSSADPIPAAKINWDRDIEYMAGILENPNITTGLMGELSRLRKDPAYSHVS
DELFATIGVTFFGAGVISTGSFLTTALISLIQRPQLRNLLHEKPELIPAGVEELLRINLSFADGLPRLATADI
QVGDVLVRKGELVLVLLEGANFDPEHFPNPGSIELDRPNPTSHLAFGRGQHFCPGSALGRRHAQIGIEALLKK
MPGVDLAVPIDQLVWRTRFQRRIPERLPVLW
>CYP121A1_Mycobacterium tuberculosis A70011_4 2659397894
MTATVLLEVPFSARGDRIPDAVAELRTREPIRKVRTITGAEAWLVSSYALCTQVLEDRRFSMKETAAAGAPRL
NALTVPPEVVNNMGNIADAGLRKAVMKAITPKAPGLEQFLRDTANSLLDNLITEGAPADLRNDFADPLATALH
CKVLGIPQEDGPKLFRSLSIAFMSSADPIPAAKINWDRDIEYMAGILENPNITTGLMGELSRLRKDPAYSHVS
DELFATIGVTFFGAGVISTGSFLTTALISLIQRPQLRNLLHEKPELIPAGVEELLRINLSFADGLPRLATADI
QVGDVLVRKGELVLVLLEGANFDPEHFPNPGSIELDRPNPTSHLAFGRGQHFCPGSALGRRHAQIGIEALLKK
MPGVDLAVPIDQLVWRTRFQRRIPERLPVLW
>CYP121A1_Mycobacterium tuberculosis A70011_5 2670884290
MTATVLLEVPFSARGDRIPDAVAELRTREPIRKVRTITGAEAWLVSSYALCTQVLEDRRFSMKETAAAGAPRL
NALTVPPEVVNNMGNIADAGLRKAVMKAITPKAPGLEQFLRDTANSLLDNLITEGAPADLRNDFADPLATALH
CKVLGIPQEDGPKLFRSLSIAFMSSADPIPAAKINWDRDIEYMAGILENPNITTGLMGELSRLRKDPAYSHVS
DELFATIGVTFFGAGVISTGSFLTTALISLIQRPQLRNLLHEKPELIPAGVEELLRINLSFADGLPRLATADI
QVGDVLVRKGELVLVLLEGANFDPEHFPNPGSIELDRPNPTSHLAFGRGQHFCPGSALGRRHAQIGIEALLKK
MPGVDLAVPIDQLVWRTRFQRRIPERLPVLW
>CYP121A1_Mycobacterium tuberculosis A70011_6 2670910643
MTATVLLEVPFSARGDRIPDAVAELRTREPIRKVRTITGAEAWLVSSYALCTQVLEDRRFSMKETAAAGAPRL
NALTVPPEVVNNMGNIADAGLRKAVMKAITPKAPGLEQFLRDTANSLLDNLITEGAPADLRNDFADPLATALH
CKVLGIPQEDGPKLFRSLSIAFMSSADPIPAAKINWDRDIEYMAGILENPNITTGLMGELSRLRKDPAYSHVS
DELFATIGVTFFGAGVISTGSFLTTALISLIQRPQLRNLLHEKPELIPAGVEELLRINLSFADGLPRLATADI
QVGDVLVRKGELVLVLLEGANFDPEHFPNPGSIELDRPNPTSHLAFGRGQHFCPGSALGRRHAQIGIEALLKK
MPGVDLAVPIDQLVWRTRFQRRIPERLPVLW
>CYP121A1_Mycobacterium tuberculosis A70012 2695717033
MTATVLLEVPFSARGDRIPDAVAELRTREPIRKVRTITGAEAWLVSSYALCTQVLEDRRFSMKETAAAGAPRL
NALTVPPEVVNNMGNIADAGLRKAVMKAITPKAPGLEQFLRDTANSLLDNLITEGAPADLRNDFADPLATALH
CKVLGIPQEDGPKLFRSLSIAFMSSADPIPAAKINWDRDIEYMAGILENPNITTGLMGELSRLRKDPAYSHVS
DELFATIGVTFFGAGVISTGSFLTTALISLIQRPQLRNLLHEKPELIPAGVEELLRINLSFADGLPRLATADI
QVGDVLVRKGELVLVLLEGANFDPEHFPNPGSIELDRPNPTSHLAFGRGQHFCPGSALGRRHAQIGIEALLKK
MPGVDLAVPIDQLVWRTRFQRRIPERLPVLW
>CYP121A1_Mycobacterium tuberculosis A70067_1 2693750150

MTATVLLEVPFSARGDRIPDAVAELRTREPIRKVRTITGAEAWLVSSYALCTQVLEDRRFSMKETAAAGAPRL
NALTVPPEVVNNMGNIADAGLRKAVMKAITPKAPGLEQFLRDTANSLLDNLITEGAPADLRNDFADPLATALH
CKVLGIPQEDGPKLFRSLSIAFMSSADPIPAAKINWDRDIEYMAGILENPNITTGLMGELSRLRKDPAYSHVS
DELFATIGVTFFGAGVISTGSFLTTALISLIQRPQLRNLLHEKPELIPAGVEELLRINLSFADGLPRLATADI
QVGDVLVRKGELVLVLLEGANFDPEHFPNPGSIELDRPNPTSHLAFGRGQHFCPGSALGRRHAQIGIEALLKK
MPGVDLAVPIDQLVWRTRFQRRIPERLPVLW
>CYP121A1_Mycobacterium tuberculosis A70067_2 2696874071
MTATVLLEVPFSARGDRIPDAVAELRTREPIRKVRTITGAEAWLVSSYALCTQVLEDRRFSMKETAAAGAPRL
NALTVPPEVVNNMGNIADAGLRKAVMKAITPKAPGLEQFLRDTANSLLDNLITEGAPADLRNDFADPLATALH
CKVLGIPQEDGPKLFRSLSIAFMSSADPIPAAKINWDRDIEYMAGILENPNITTGLMGELSRLRKDPAYSHVS
DELFATIGVTFFGAGVISTGSFLTTALISLIQRPQLRNLLHEKPELIPAGVEELLRINLSFADGLPRLATADI
QVGDVLVRKGELVLVLLEGANFDPEHFPNPGSIELDRPNPTSHLAFGRGQHFCPGSALGRRHAQIGIEALLKK
MPGVDLAVPIDQLVWRTRFQRRIPERLPVLW
>CYP121A1_Mycobacterium tuberculosis A70086 2691916080
MTATVLLEVPFSARGDRIPDAVAELRTREPIRKVRTITGAEAWLVSSYALCTQVLEDRRFSMKETAAAGAPRL
NALTVPPEVVNNMGNIADAGLRKAVMKAITPKAPGLEQFLRDTANSLLDNLITEGAPADLRNDFADPLATALH
CKVLGIPQEDGPKLFRSLSIAFMSSADPIPAAKINWDRDIEYMAGILENPNITTGLMGELSRLRKDPAYSHVS
DELFATIGVTFFGAGVISTGSFLTTALISLIQRPQLRNLLHEKPELIPAGVEELLRINLSFADGLPRLATADI
QVGDVLVRKGELVLVLLEGANFDPEHFPNPGSIELDRPNPTSHLAFGRGQHFCPGSALGRRHAQIGIEALLKK
MPGVDLAVPIDQLVWRTRFQRRIPERLPVLW
>CYP121A1_Mycobacterium tuberculosis A70088 2670438988
MTATVLLEVPFSARGDRIPDAVAELRTREPIRKVRTITGAEAWLVSSYALCTQVLEDRRFSMKETAAAGAPRL
NALTVPPEVVNNMGNIADAGLRKAVMKAITPKAPGLEQFLRDTANSLLDNLITEGAPADLRNDFADPLATALH
CKVLGIPQEDGPKLFRSLSIAFMSSADPIPAAKINWDRDIEYMAGILENPNITTGLMGELSRLRKDPAYSHVS
DELFATIGVTFFGAGVISTGSFLTTALISLIQRPQLRNLLHEKPELIPAGVEELLRINLSFADGLPRLATADI
QVGDVLVRKGELVLVLLEGANFDPEHFPNPGSIELDRPNPTSHLAFGRGQHFCPGSALGRRHAQIGIEALLKK
MPGVDLAVPIDQLVWRTRFQRRIPERLPVLW
>CYP121A1_Mycobacterium tuberculosis A70136_13046 2677462795
MTATVLLEVPFSARGDRIPDAVAELRTREPIRKVRTITGAEAWLVSSYALCTQVLEDRRFSMKETAAAGAPRL
NALTVPPEVVNNMGNIADAGLRKAVMKAITPKAPGLEQFLRDTANSLLDNLITEGAPADLRNDFADPLATALH
CKVLGIPQEDGPKLFRSLSIAFMSSADPIPAAKINWDRDIEYMAGILENPNITTGLMGELSRLRKDPAYSHVS
DELFATIGVTFFGAGVISTGSFLTTALISLIQRPQLRNLLHEKPELIPAGVEELLRINLSFADGLPRLATADI
QVGDVLVRKGELVLVLLEGANFDPEHFPNPGSIELDRPNPTSHLAFGRGQHFCPGSALGRRHAQIGIEALLKK
MPGVDLAVPIDQLVWRTRFQRRIPERLPVLW
>CYP121A1_Mycobacterium tuberculosis A70136_15354 2698040354
MTATVLLEVPFSARGDRIPDAVAELRTREPIRKVRTITGAEAWLVSSYALCTQVLEDRRFSMKETAAAGAPRL
NALTVPPEVVNNMGNIADAGLRKAVMKAITPKAPGLEQFLRDTANSLLDNLITEGAPADLRNDFADPLATALH
CKVLGIPQEDGPKLFRSLSIAFMSSADPIPAAKINWDRDIEYMAGILENPNITTGLMGELSRLRKDPAYSHVS
DELFATIGVTFFGAGVISTGSFLTTALISLIQRPQLRNLLHEKPELIPAGVEELLRINLSFADGLPRLATADI
QVGDVLVRKGELVLVLLEGANFDPEHFPNPGSIELDRPNPTSHLAFGRGQHFCPGSALGRRHAQIGIEALLKK
MPGVDLAVPIDQLVWRTRFQRRIPERLPVLW
>CYP121A1_Mycobacterium tuberculosis A70136_8962 2669855101
MTATVLLEVPFSARGDRIPDAVAELRTREPIRKVRTITGAEAWLVSSYALCTQVLEDRRFSMKETAAAGAPRL
NALTVPPEVVNNMGNIADAGLRKAVMKAITPKAPGLEQFLRDTANSLLDNLITEGAPADLRNDFADPLATALH
CKVLGIPQEDGPKLFRSLSIAFMSSADPIPAAKINWDRDIEYMAGILENPNITTGLMGELSRLRKDPAYSHVS
DELFATIGVTFFGAGVISTGSFLTTALISLIQRPQLRNLLHEKPELIPAGVEELLRINLSFADGLPRLATADI
QVGDVLVRKGELVLVLLEGANFDPEHFPNPGSIELDRPNPTSHLAFGRGQHFCPGSALGRRHAQIGIEALLKK
MPGVDLAVPIDQLVWRTRFQRRIPERLPVLW
>CYP121A1_Mycobacterium tuberculosis A70144_1 2662241210
MTATVLLEVPFSARGDRIPDAVAELRTREPIRKVRTITGAEAWLVSSYALCTQVLEDRRFSMKETAAAGAPRL
NALTVPPEVVNNMGNIADAGLRKAVMKAITPKAPGLEQFLRDTANSLLDNLITEGAPADLRNDFADPLATALH
CKVLGIPQEDGPKLFRSLSIAFMSSADPIPAAKINWDRDIEYMAGILENPNITTGLMGELSRLRKDPAYSHVS
DELFATIGVTFFGAGVISTGSFLTTALISLIQRPQLRNLLHEKPELIPAGVEELLRINLSFADGLPRLATADI
QVGDVLVRKGELVLVLLEGANFDPEHFPNPGSIELDRPNPTSHLAFGRGQHFCPGSALGRRHAQIGIEALLKK
MPGVDLAVPIDQLVWRTRFQRRIPERLPVLW
>CYP121A1_Mycobacterium tuberculosis A70144_2 2695921941
MTATVLLEVPFSARGDRIPDAVAELRTREPIRKVRTITGAEAWLVSSYALCTQVLEDRRFSMKETAAAGAPRL
NALTVPPEVVNNMGNIADAGLRKAVMKAITPKAPGLEQFLRDTANSLLDNLITEGAPADLRNDFADPLATALH

CKVLGIPQEDGPKLFRSLSIAFMSSADPIPAAKINWDRDIEYMAGILENPNITTGLMGELSRLRKDPAYSHVS
DELFATIGVTFFGAGVISTGSFLTTALISLIQRPQLRNLLHEKPELIPAGVEELLRINLSFADGLPRLATADI
QVGDVLVRKGELVLVLLEGANFDPEHFPNPGSIELDRPNPTSHLAFGRGQHFCPGSALGRRHAQIGIEALLKK
MPGVDLAVPIDQLVWRTRFQRRIPERLPVLW
>CYP121A1_Mycobacterium tuberculosis A70196 2694243532
MTATVLLEVPFSARGDRIPDAVAELRTREPIRKVRTITGAEAWLVSSYALCTQVLEDRRFSMKETAAAGAPRL
NALTVPPEVVNNMGNIADAGLRKAVMKAITPKAPGLEQFLRDTANSLLDNLITEGAPADLRNDFADPLATALH
CKVLGIPQEDGPKLFRSLSIAFMSSADPIPAAKINWDRDIEYMAGILENPNITTGLMGELSRLRKDPAYSHVS
DELFATIGVTFFGAGVISTGSFLTTALISLIQRPQLRNLLHEKPELIPAGVEELLRINLSFADGLPRLATADI
QVGDVLVRKGELVLVLLEGANFDPEHFPNPGSIELDRPNPTSHLAFGRGQHFCPGSALGRRHAQIGIEALLKK
MPGVDLAVPIDQLVWRTRFQRRIPERLPVLW
>CYP121A1_Mycobacterium tuberculosis A70170 2673858270
MTATVLLEVPFSARGDRIPDAVAELRTREPIRKVRTITGAEAWLVSSYALCTQVLEDRRFSMKETAAAGAPRL
NALTVPPEVVNNMGNIADAGLRKAVMKAITPKAPGLEQFLRDTANSLLDNLITEGAPADLRNDFADPLATALH
CKVLGIPQEDGPKLFRSLSIAFMSSADPIPAAKINWDRDIEYMAGILENPNITTGLMGELSRLRKDPAYSHVS
DELFATIGVTFFGAGVISTGSFLTTALISLIQRPQLRNLLHEKPELIPAGVEELLRINLSFADGLPRLATADI
QVGDVLVRKGELVLVLLEGANFDPEHFPNPGSIELDRPNPTSHLAFGRGQHFCPGSALGRRHAQIGIEALLKK
MPGVDLAVPIDQLVWRTRFQRRIPERLPVLW
>CYP121A1_Mycobacterium tuberculosis A70250 2693433637
MTATVLLEVPFSARGDRIPDAVAELRTREPIRKVRTITGAEAWLVSSYALCTQVLEDRRFSMKETAAAGAPRL
NALTVPPEVVNNMGNIADAGLRKAVMKAITPKAPGLEQFLRDTANSLLDNLITEGAPADLRNDFADPLATALH
CKVLGIPQEDGPKLFRSLSIAFMSSADPIPAAKINWDRDIEYMAGILENPNITTGLMGELSRLRKDPAYSHVS
DELFATIGVTFFGAGVISTGSFLTTALISLIQRPQLRNLLHEKPELIPAGVEELLRINLSFADGLPRLATADI
QVGDVLVRKGELVLVLLEGANFDPEHFPNPGSIELDRPNPTSHLAFGRGQHFCPGSALGRRHAQIGIEALLKK
MPGVDLAVPIDQLVWRTRFQRRIPERLPVLW
>CYP121A1_Mycobacterium tuberculosis A70260 2673922698
MTATVLLEVPFSARGDRIPDAVAELRTREPIRKVRTITGAEAWLVSSYALCTQVLEDRRFSMKETAAAGAPRL
NALTVPPEVVNNMGNIADAGLRKAVMKAITPKAPGLEQFLRDTANSLLDNLITEGAPADLRNDFADPLATALH
CKVLGIPQEDGPKLFRSLSIAFMSSADPIPAAKINWDRDIEYMAGILENPNITTGLMGELSRLRKDPAYSHVS
DELFATIGVTFFGAGVISTGSFLTTALISLIQRPQLRNLLHEKPELIPAGVEELLRINLSFADGLPRLATADI
QVGDVLVRKGELVLVLLEGANFDPEHFPNPGSIELDRPNPTSHLAFGRGQHFCPGSALGRRHAQIGIEALLKK
MPGVDLAVPIDQLVWRTRFQRRIPERLPVLW
>CYP121A1_Mycobacterium tuberculosis A70280 2665656305
MTATVLLEVPFSARGDRIPDAVAELRTREPIRKVRTITGAEAWLVSSYALCTQVLEDRRFSMKETAAAGAPRL
NALTVPPEVVNNMGNIADAGLRKAVMKAITPKAPGLEQFLRDTANSLLDNLITEGAPADLRNDFADPLATALH
CKVLGIPQEDGPKLFRSLSIAFMSSADPIPAAKINWDRDIEYMAGILENPNITTGLMGELSRLRKDPAYSHVS
DELFATIGVTFFGAGVISTGSFLTTALISLIQRPQLRNLLHEKPELIPAGVEELLRINLSFADGLPRLATADI
QVGDVLVRKGELVLVLLEGANFDPEHFPNPGSIELDRPNPTSHLAFGRGQHFCPGSALGRRHAQIGIEALLKK
MPGVDLAVPIDQLVWRTRFQRRIPERLPVLW
>CYP121A1_Mycobacterium tuberculosis A70376 2659218025
MTATVLLEVPFSARGDRIPDAVAELRTREPIRKVRTITGAEAWLVSSYALCTQVLEDRRFSMKETAAAGAPRL
NALTVPPEVVNNMGNIADAGLRKAVMKAITPKAPGLEQFLRDTANSLLDNLITEGAPADLRNDFADPLATALH
CKVLGIPQEDGPKLFRSLSIAFMSSADPIPAAKINWDRDIEYMAGILENPNITTGLMGELSRLRKDPAYSHVS
DELFATIGVTFFGAGVISTGSFLTTALISLIQRPQLRNLLHEKPELIPAGVEELLRINLSFADGLPRLATADI
QVGDVLVRKGELVLVLLEGANFDPEHFPNPGSIELDRPNPTSHLAFGRGQHFCPGSALGRRHAQIGIEALLKK
MPGVDLAVPIDQLVWRTRFQRRIPERLPVLW
>CYP121A1_Mycobacterium tuberculosis A70329 2659261044
MTATVLLEVPFSARGDRIPDAVAELRTREPIRKVRTITGAEAWLVSSYALCTQVLEDRRFSMKETAAAGAPRL
NALTVPPEVVNNMGNIADAGLRKAVMKAITPKAPGLEQFLRDTANSLLDNLITEGAPADLRNDFADPLATALH
CKVLGIPQEDGPKLFRSLSIAFMSSADPIPAAKINWDRDIEYMAGILENPNITTGLMGELSRLRKDPAYSHVS
DELFATIGVTFFGAGVISTGSFLTTALISLIQRPQLRNLLHEKPELIPAGVEELLRINLSFADGLPRLATADI
QVGDVLVRKGELVLVLLEGANFDPEHFPNPGSIELDRPNPTSHLAFGRGQHFCPGSALGRRHAQIGIEALLKK
MPGVDLAVPIDQLVWRTRFQRRIPERLPVLW
>CYP121A1_Mycobacterium tuberculosis A70387 2702063424
MTATVLLEVPFSARGDRIPDAVAELRTREPIRKVRTITGAEAWLVSSYALCTQVLEDRRFSMKETAAAGAPRL
NALTVPPEVVNNMGNIADAGLRKAVMKAITPKAPGLEQFLRDTANSLLDNLITEGAPADLRNDFADPLATALH
CKVLGIPQEDGPKLFRSLSIAFMSSADPIPAAKINWDRDIEYMAGILENPNITTGLMGELSRLRKDPAYSHVS
DELFATIGVTFFGAGVISTGSFLTTALISLIQRPQLRNLLHEKPELIPAGVEELLRINLSFADGLPRLATADI

QVGDVLVRKGELVLVLLEGANFDPEHFPNPGSIELDRPNPTSHLAFGRGQHFCPGSALGRRHAQIGIEALLKK
MPGVDLAVPIDQLVWRTRFQRRIPERLPVLW
>CYP121A1_Mycobacterium tuberculosis A70416 2702120913
MTATVLLEVPFSARGDRIPDAVAELRTREPIRKVRTITGAEAWLVSSYALCTQVLEDRRFSMKETAAAGAPRL
NALTVPPEVVNNMGNIADAGLRKAVMKAITPKAPGLEQFLRDTANSLLDNLITEGAPADLRNDFADPLATALH
CKVLGIPQEDGPKLFRSLSIAFMSSADPIPAAKINWDRDIEYMAGILENPNITTGLMGELSRLRKDPAYSHVS
DELFATIGVTFFGAGVISTGSFLTTALISLIQRPQLRNLLHEKPELIPAGVEELLRINLSFADGLPRLATADI
QVGDVLVRKGELVLVLLEGANFDPEHFPNPGSIELDRPNPTSHLAFGRGQHFCPGSALGRRHAQIGIEALLKK
MPGVDLAVPIDQLVWRTRFQRRIPERLPVLW
>CYP121A1_Mycobacterium tuberculosis A70428 2663814638
MTATVLLEVPFSARGDRIPDAVAELRTREPIRKVRTITGAEAWLVSSYALCTQVLEDRRFSMKETAAAGAPRL
NALTVPPEVVNNMGNIADAGLRKAVMKAITPKAPGLEQFLRDTANSLLDNLITEGAPADLRNDFADPLATALH
CKVLGIPQEDGPKLFRSLSIAFMSSADPIPAAKINWDRDIEYMAGILENPNITTGLMGELSRLRKDPAYSHVS
DELFATIGVTFFGAGVISTGSFLTTALISLIQRPQLRNLLHEKPELIPAGVEELLRINLSFADGLPRLATADI
QVGDVLVRKGELVLVLLEGANFDPEHFPNPGSIELDRPNPTSHLAFGRGQHFCPGSALGRRHAQIGIEALLKK
MPGVDLAVPIDQLVWRTRFQRRIPERLPVLW
>CYP121A1_Mycobacterium tuberculosis A70441 2673862068
MTATVLLEVPFSARGDRIPDAVAELRTREPIRKVRTITGAEAWLVSSYALCTQVLEDRRFSMKETAAAGAPRL
NALTVPPEVVNNMGNIADAGLRKAVMKAITPKAPGLEQFLRDTANSLLDNLITEGAPADLRNDFADPLATALH
CKVLGIPQEDGPKLFRSLSIAFMSSADPIPAAKINWDRDIEYMAGILENPNITTGLMGELSRLRKDPAYSHVS
DELFATIGVTFFGAGVISTGSFLTTALISLIQRPQLRNLLHEKPELIPAGVEELLRINLSFADGLPRLATADI
QVGDVLVRKGELVLVLLEGANFDPEHFPNPGSIELDRPNPTSHLAFGRGQHFCPGSALGRRHAQIGIEALLKK
MPGVDLAVPIDQLVWRTRFQRRIPERLPVLW
>CYP121A1_Mycobacterium tuberculosis A70448 2696812320
MTATVLLEVPFSARGDRIPDAVAELRTREPIRKVRTITGAEAWLVSSYALCTQVLEDRRFSMKETAAAGAPRL
NALTVPPEVVNNMGNIADAGLRKAVMKAITPKAPGLEQFLRDTANSLLDNLITEGAPADLRNDFADPLATALH
CKVLGIPQEDGPKLFRSLSIAFMSSADPIPAAKINWDRDIEYMAGILENPNITTGLMGELSRLRKDPAYSHVS
DELFATIGVTFFGAGVISTGSFLTTALISLIQRPQLRNLLHEKPELIPAGVEELLRINLSFADGLPRLATADI
QVGDVLVRKGELVLVLLEGANFDPEHFPNPGSIELDRPNPTSHLAFGRGQHFCPGSALGRRHAQIGIEALLKK
MPGVDLAVPIDQLVWRTRFQRRIPERLPVLW
>CYP121A1_Mycobacterium tuberculosis A70451 2696136234
MTATVLLEVPFSARGDRIPDAVAELRTREPIRKVRTITGAEAWLVSSYALCTQVLEDRRFSMKETAAAGAPRL
NALTVPPEVVNNMGNIADAGLRKAVMKAITPKAPGLEQFLRDTANSLLDNLITEGAPADLRNDFADPLATALH
CKVLGIPQEDGPKLFRSLSIAFMSSADPIPAAKINWDRDIEYMAGILENPNITTGLMGELSRLRKDPAYSHVS
DELFATIGVTFFGAGVISTGSFLTTALISLIQRPQLRNLLHEKPELIPAGVEELLRINLSFADGLPRLATADI
QVGDVLVRKGELVLVLLEGANFDPEHFPNPGSIELDRPNPTSHLAFGRGQHFCPGSALGRRHAQIGIEALLKK
MPGVDLAVPIDQLVWRTRFQRRIPERLPVLW
>CYP121A1_Mycobacterium tuberculosis A70458 2697713494
MTATVLLEVPFSARGDRIPDAVAELRTREPIRKVRTITGAEAWLVSSYALCTQVLEDRRFSMKETAAAGAPRL
NALTVPPEVVNNMGNIADAGLRKAVMKAITPKAPGLEQFLRDTANSLLDNLITEGAPADLRNDFADPLATALH
CKVLGIPQEDGPKLFRSLSIAFMSSADPIPAAKINWDRDIEYMAGILENPNITTGLMGELSRLRKDPAYSHVS
DELFATIGVTFFGAGVISTGSFLTTALISLIQRPQLRNLLHEKPELIPAGVEELLRINLSFADGLPRLATADI
QVGDVLVRKGELVLVLLEGANFDPEHFPNPGSIELDRPNPTSHLAFGRGQHFCPGSALGRRHAQIGIEALLKK
MPGVDLAVPIDQLVWRTRFQRRIPERLPVLW
>CYP121A1_Mycobacterium tuberculosis A70480 2669724656
MTATVLLEVPFSARGDRIPDAVAELRTREPIRKVRTITGAEAWLVSSYALCTQVLEDRRFSMKETAAAGAPRL
NALTVPPEVVNNMGNIADAGLRKAVMKAITPKAPGLEQFLRDTANSLLDNLITEGAPADLRNDFADPLATALH
CKVLGIPQEDGPKLFRSLSIAFMSSADPIPAAKINWDRDIEYMAGILENPNITTGLMGELSRLRKDPAYSHVS
DELFATIGVTFFGAGVISTGSFLTTALISLIQRPQLRNLLHEKPELIPAGVEELLRINLSFADGLPRLATADI
QVGDVLVRKGELVLVLLEGANFDPEHFPNPGSIELDRPNPTSHLAFGRGQHFCPGSALGRRHAQIGIEALLKK
MPGVDLAVPIDQLVWRTRFQRRIPERLPVLW
>CYP121A1_Mycobacterium tuberculosis A70490 2666326549
MTATVLLEVPFSARGDRIPDAVAELRTREPIRKVRTITGAEAWLVSSYALCTQVLEDRRFSMKETAAAGAPRL
NALTVPPEVVNNMGNIADAGLRKAVMKAITPKAPGLEQFLRDTANSLLDNLITEGAPADLRNDFADPLATALH
CKVLGIPQEDGPKLFRSLSIAFMSSADPIPAAKINWDRDIEYMAGILENPNITTGLMGELSRLRKDPAYSHVS
DELFATIGVTFFGAGVISTGSFLTTALISLIQRPQLRNLLHEKPELIPAGVEELLRINLSFADGLPRLATADI
QVGDVLVRKGELVLVLLEGANFDPEHFPNPGSIELDRPNPTSHLAFGRGQHFCPGSALGRRHAQIGIEALLKK
MPGVDLAVPIDQLVWRTRFQRRIPERLPVLW

>CYP121A1_Mycobacterium tuberculosis A70501 2665692427
MTATVLLEVPFSARGDRIPDAVAELRTREPIRKVRTITGAEAWLVSSYALCTQVLEDRRFSMKETAAAGAPRL
NALTVPPEVVNNMGNIADAGLRKAVMKAITPKAPGLEQFLRDTANSLLDNLITEGAPADLRNDFADPLATALH
CKVLGIPQEDGPKLFRSLSIAFMSSADPIPAAKINWDRDIEYMAGILENPNITTGLMGELSRLRKDPAYSHVS
DELFATIGVTFFGAGVISTGSFLTTALISLIQRPQLRNLLHEKPELIPAGVEELLRINLSFADGLPRLATADI
QVGDVLVRKGELVLVLLEGANFDPEHFPNPGSIELDRPNPTSHLAFGRGQHFCPGSALGRRHAQIGIEALLKK
MPGVDLAVPIDQLVWRTRFQRRIPERLPVLW
>CYP121A1_Mycobacterium tuberculosis A70547 2659475074
MTATVLLEVPFSARGDRIPDAVAELRTREPIRKVRTITGAEAWLVSSYALCTQVLEDRRFSMKETAAAGAPRL
NALTVPPEVVNNMGNIADAGLRKAVMKAITPKAPGLEQFLRDTANSLLDNLITEGAPADLRNDFADPLATALH
CKVLGIPQEDGPKLFRSLSIAFMSSADPIPAAKINWDRDIEYMAGILENPNITTGLMGELSRLRKDPAYSHVS
DELFATIGVTFFGAGVISTGSFLTTALISLIQRPQLRNLLHEKPELIPAGVEELLRINLSFADGLPRLATADI
QVGDVLVRKGELVLVLLEGANFDPEHFPNPGSIELDRPNPTSHLAFGRGQHFCPGSALGRRHAQIGIEALLKK
MPGVDLAVPIDQLVWRTRFQRRIPERLPVLW
>CYP121A1_Mycobacterium tuberculosis A70555 2668796034
MTATVLLEVPFSARGDRIPDAVAELRTREPIRKVRTITGAEAWLVSSYALCTQVLEDRRFSMKETAAAGAPRL
NALTVPPEVVNNMGNIADAGLRKAVMKAITPKAPGLEQFLRDTANSLLDNLITEGAPADLRNDFADPLATALH
CKVLGIPQEDGPKLFRSLSIAFMSSADPIPAAKINWDRDIEYMAGILENPNITTGLMGELSRLRKDPAYSHVS
DELFATIGVTFFGAGVISTGSFLTTALISLIQRPQLRNLLHEKPELIPAGVEELLRINLSFADGLPRLATADI
QVGDVLVRKGELVLVLLEGANFDPEHFPNPGSIELDRPNPTSHLAFGRGQHFCPGSALGRRHAQIGIEALLKK
MPGVDLAVPIDQLVWRTRFQRRIPERLPVLW
>CYP121A1_Mycobacterium tuberculosis A70582 2657987652
MTATVLLEVPFSARGDRIPDAVAELRTREPIRKVRTITGAEAWLVSSYALCTQVLEDRRFSMKETAAAGAPRL
NALTVPPEVVNNMGNIADAGLRKAVMKAITPKAPGLEQFLRDTANSLLDNLITEGAPADLRNDFADPLATALH
CKVLGIPQEDGPKLFRSLSIAFMSSADPIPAAKINWDRDIEYMAGILENPNITTGLMGELSRLRKDPAYSHVS
DELFATIGVTFFGAGVISTGSFLTTALISLIQRPQLRNLLHEKPELIPAGVEELLRINLSFADGLPRLATADI
QVGDVLVRKGELVLVLLEGANFDPEHFPNPGSIELDRPNPTSHLAFGRGQHFCPGSALGRRHAQIGIEALLKK
MPGVDLAVPIDQLVWRTRFQRRIPERLPVLW
>CYP121A1_Mycobacterium tuberculosis A70596 2668926327
MTATVLLEVPFSARGDRIPDAVAELRTREPIRKVRTITGAEAWLVSSYALCTQVLEDRRFSMKETAAAGAPRL
NALTVPPEVVNNMGNIADAGLRKAVMKAITPKAPGLEQFLRDTANSLLDNLITEGAPADLRNDFADPLATALH
CKVLGIPQEDGPKLFRSLSIAFMSSADPIPAAKINWDRDIEYMAGILENPNITTGLMGELSRLRKDPAYSHVS
DELFATIGVTFFGAGVISTGSFLTTALISLIQRPQLRNLLHEKPELIPAGVEELLRINLSFADGLPRLATADI
QVGDVLVRKGELVLVLLEGANFDPEHFPNPGSIELDRPNPTSHLAFGRGQHFCPGSALGRRHAQIGIEALLKK
MPGVDLAVPIDQLVWRTRFQRRIPERLPVLW
>CYP121A1_Mycobacterium tuberculosis A70620 2702087481
MTATVLLEVPFSARGDRIPDAVAELRTREPIRKVRTITGAEAWLVSSYALCTQVLEDRRFSMKETAAAGAPRL
NALTVPPEVVNNMGNIADAGLRKAVMKAITPKAPGLEQFLRDTANSLLDNLITEGAPADLRNDFADPLATALH
CKVLGIPQEDGPKLFRSLSIAFMSSADPIPAAKINWDRDIEYMAGILENPNITTGLMGELSRLRKDPAYSHVS
DELFATIGVTFFGAGVISTGSFLTTALISLIQRPQLRNLLHEKPELIPAGVEELLRINLSFADGLPRLATADI
QVGDVLVRKGELVLVLLEGANFDPEHFPNPGSIELDRPNPTSHLAFGRGQHFCPGSALGRRHAQIGIEALLKK
MPGVDLAVPIDQLVWRTRFQRRIPERLPVLW
>CYP121A1_Mycobacterium tuberculosis A70645 2666243877
MTATVLLEVPFSARGDRIPDAVAELRTREPIRKVRTITGAEAWLVSSYALCTQVLEDRRFSMKETAAAGAPRL
NALTVPPEVVNNMGNIADAGLRKAVMKAITPKAPGLEQFLRDTANSLLDNLITEGAPADLRNDFADPLATALH
CKVLGIPQEDGPKLFRSLSIAFMSSADPIPAAKINWDRDIEYMAGILENPNITTGLMGELSRLRKDPAYSHVS
DELFATIGVTFFGAGVISTGSFLTTALISLIQRPQLRNLLHEKPELIPAGVEELLRINLSFADGLPRLATADI
QVGDVLVRKGELVLVLLEGANFDPEHFPNPGSIELDRPNPTSHLAFGRGQHFCPGSALGRRHAQIGIEALLKK
MPGVDLAVPIDQLVWRTRFQRRIPERLPVLW
>CYP121A1_Mycobacterium tuberculosis A70655 2670570222
MTATVLLEVPFSARGDRIPDAVAELRTREPIRKVRTITGAEAWLVSSYALCTQVLEDRRFSMKETAAAGAPRL
NALTVPPEVVNNMGNIADAGLRKAVMKAITPKAPGLEQFLRDTANSLLDNLITEGAPADLRNDFADPLATALH
CKVLGIPQEDGPKLFRSLSIAFMSSADPIPAAKINWDRDIEYMAGILENPNITTGLMGELSRLRKDPAYSHVS
DELFATIGVTFFGAGVISTGSFLTTALISLIQRPQLRNLLHEKPELIPAGVEELLRINLSFADGLPRLATADI
QVGDVLVRKGELVLVLLEGANFDPEHFPNPGSIELDRPNPTSHLAFGRGQHFCPGSALGRRHAQIGIEALLKK
MPGVDLAVPIDQLVWRTRFQRRIPERLPVLW
>CYP121A1_Mycobacterium tuberculosis A70659 2694443150

MTATVLLEVPFSARGDRIPDAVAELRTREPIRKVRTITGAEAWLVSSYALCTQVLEDRRFSMKETAAAGAPRL
NALTVPPEVVNNMGNIADAGLRKAVMKAITPKAPGLEQFLRDTANSLLDNLITEGAPADLRNDFADPLATALH
CKVLGIPQEDGPKLFRSLSIAFMSSADPIPAAKINWDRDIEYMAGILENPNITTGLMGELSRLRKDPAYSHVS
DELFATIGVTFFGAGVISTGSFLTTALISLIQRPQLRNLLHEKPELIPAGVEELLRINLSFADGLPRLATADI
QVGDVLVRKGELVLVLLEGANFDPEHFPNPGSIELDRPNPTSHLAFGRGQHFCPGSALGRRHAQIGIEALLKK
MPGVDLAVPIDQLVWRTRFQRRIPERLPVLW
>CYP121A1_Mycobacterium tuberculosis A70661 2661732649
MTATVLLEVPFSARGDRIPDAVAELRTREPIRKVRTITGAEAWLVSSYALCTQVLEDRRFSMKETAAAGAPRL
NALTVPPEVVNNMGNIADAGLRKAVMKAITPKAPGLEQFLRDTANSLLDNLITEGAPADLRNDFADPLATALH
CKVLGIPQEDGPKLFRSLSIAFMSSADPIPAAKINWDRDIEYMAGILENPNITTGLMGELSRLRKDPAYSHVS
DELFATIGVTFFGAGVISTGSFLTTALISLIQRPQLRNLLHEKPELIPAGVEELLRINLSFADGLPRLATADI
QVGDVLVRKGELVLVLLEGANFDPEHFPNPGSIELDRPNPTSHLAFGRGQHFCPGSALGRRHAQIGIEALLKK
MPGVDLAVPIDQLVWRTRFQRRIPERLPVLW
>CYP121A1_Mycobacterium tuberculosis A70730 2697938305
MTATVLLEVPFSARGDRIPDAVAELRTREPIRKVRTITGAEAWLVSSYALCTQVLEDRRFSMKETAAAGAPRL
NALTVPPEVVNNMGNIADAGLRKAVMKAITPKAPGLEQFLRDTANSLLDNLITEGAPADLRNDFADPLATALH
CKVLGIPQEDGPKLFRSLSIAFMSSADPIPAAKINWDRDIEYMAGILENPNITTGLMGELSRLRKDPAYSHVS
DELFATIGVTFFGAGVISTGSFLTTALISLIQRPQLRNLLHEKPELIPAGVEELLRINLSFADGLPRLATADI
QVGDVLVRKGELVLVLLEGANFDPEHFPNPGSIELDRPNPTSHLAFGRGQHFCPGSALGRRHAQIGIEALLKK
MPGVDLAVPIDQLVWRTRFQRRIPERLPVLW
>CYP121A1_Mycobacterium tuberculosis A70757 2670579520
MTATVLLEVPFSARGDRIPDAVAELRTREPIRKVRTITGAEAWLVSSYALCTQVLEDRRFSMKETAAAGAPRL
NALTVPPEVVNNMGNIADAGLRKAVMKAITPKAPGLEQFLRDTANSLLDNLITEGAPADLRNDFADPLATALH
CKVLGIPQEDGPKLFRSLSIAFMSSADPIPAAKINWDRDIEYMAGILENPNITTGLMGELSRLRKDPAYSHVS
DELFATIGVTFFGAGVISTGSFLTTALISLIQRPQLRNLLHEKPELIPAGVEELLRINLSFADGLPRLATADI
QVGDVLVRKGELVLVLLEGANFDPEHFPNPGSIELDRPNPTSHLAFGRGQHFCPGSALGRRHAQIGIEALLKK
MPGVDLAVPIDQLVWRTRFQRRIPERLPVLW
>CYP121A1_Mycobacterium tuberculosis A70762 2672114967
MTATVLLEVPFSARGDRIPDAVAELRTREPIRKVRTITGAEAWLVSSYALCTQVLEDRRFSMKETAAAGAPRL
NALTVPPEVVNNMGNIADAGLRKAVMKAITPKAPGLEQFLRDTANSLLDNLITEGAPADLRNDFADPLATALH
CKVLGIPQEDGPKLFRSLSIAFMSSADPIPAAKINWDRDIEYMAGILENPNITTGLMGELSRLRKDPAYSHVS
DELFATIGVTFFGAGVISTGSFLTTALISLIQRPQLRNLLHEKPELIPAGVEELLRINLSFADGLPRLATADI
QVGDVLVRKGELVLVLLEGANFDPEHFPNPGSIELDRPNPTSHLAFGRGQHFCPGSALGRRHAQIGIEALLKK
MPGVDLAVPIDQLVWRTRFQRRIPERLPVLW
>CYP121A1_Mycobacterium tuberculosis A70763_1 2659945456
MTATVLLEVPFSARGDRIPDAVAELRTREPIRKVRTITGAEAWLVSSYALCTQVLEDRRFSMKETAAAGAPRL
NALTVPPEVVNNMGNIADAGLRKAVMKAITPKAPGLEQFLRDTANSLLDNLITEGAPADLRNDFADPLATALH
CKVLGIPQEDGPKLFRSLSIAFMSSADPIPAAKINWDRDIEYMAGILENPNITTGLMGELSRLRKDPAYSHVS
DELFATIGVTFFGAGVISTGSFLTTALISLIQRPQLRNLLHEKPELIPAGVEELLRINLSFADGLPRLATADI
QVGDVLVRKGELVLVLLEGANFDPEHFPNPGSIELDRPNPTSHLAFGRGQHFCPGSALGRRHAQIGIEALLKK
MPGVDLAVPIDQLVWRTRFQRRIPERLPVLW
>CYP121A1_Mycobacterium tuberculosis A70763_2 2659039835
MTATVLLEVPFSARGDRIPDAVAELRTREPIRKVRTITGAEAWLVSSYALCTQVLEDRRFSMKETAAAGAPRL
NALTVPPEVVNNMGNIADAGLRKAVMKAITPKAPGLEQFLRDTANSLLDNLITEGAPADLRNDFADPLATALH
CKVLGIPQEDGPKLFRSLSIAFMSSADPIPAAKINWDRDIEYMAGILENPNITTGLMGELSRLRKDPAYSHVS
DELFATIGVTFFGAGVISTGSFLTTALISLIQRPQLRNLLHEKPELIPAGVEELLRINLSFADGLPRLATADI
QVGDVLVRKGELVLVLLEGANFDPEHFPNPGSIELDRPNPTSHLAFGRGQHFCPGSALGRRHAQIGIEALLKK
MPGVDLAVPIDQLVWRTRFQRRIPERLPVLW
>CYP121A1_Mycobacterium tuberculosis A70769 2659134121
MTATVLLEVPFSARGDRIPDAVAELRTREPIRKVRTITGAEAWLVSSYALCTQVLEDRRFSMKETAAAGAPRL
NALTVPPEVVNNMGNIADAGLRKAVMKAITPKAPGLEQFLRDTANSLLDNLITEGAPADLRNDFADPLATALH
CKVLGIPQEDGPKLFRSLSIAFMSSADPIPAAKINWDRDIEYMAGILENPNITTGLMGELSRLRKDPAYSHVS
DELFATIGVTFFGAGVISTGSFLTTALISLIQRPQLRNLLHEKPELIPAGVEELLRINLSFADGLPRLATADI
QVGDVLVRKGELVLVLLEGANFDPEHFPNPGSIELDRPNPTSHLAFGRGQHFCPGSALGRRHAQIGIEALLKK
MPGVDLAVPIDQLVWRTRFQRRIPERLPVLW
>CYP121A1_Mycobacterium tuberculosis A70780 2682776317
MTATVLLEVPFSARGDRIPDAVAELRTREPIRKVRTITGAEAWLVSSYALCTQVLEDRRFSMKETAAAGAPRL
NALTVPPEVVNNMGNIADAGLRKAVMKAITPKAPGLEQFLRDTANSLLDNLITEGAPADLRNDFADPLATALH

CKVLGIPQEDGPKLFRSLSIAFMSSADPIPAAKINWDRDIEYMAGILENPNITTGLMGELSRLRKDPAYSHVS
DELFATIGVTFFGAGVISTGSFLTTALISLIQRPQLRNLLHEKPELIPAGVEELLRINLSFADGLPRLATADI
QVGDVLVRKGELVLVLLEGANFDPEHFPNPGSIELDRPNPTSHLAFGRGQHFCPGSALGRRHAQIGIEALLKK
MPGVDLAVPIDQLVWRTRFQRRIPERLPVLW
>CYP121A1_Mycobacterium tuberculosis A70785 2693128265
MTATVLLEVPFSARGDRIPDAVAELRTREPIRKVRTITGAEAWLVSSYALCTQVLEDRRFSMKETAAAGAPRL
NALTVPPEVVNNMGNIADAGLRKAVMKAITPKAPGLEQFLRDTANSLLDNLITEGAPADLRNDFADPLATALH
CKVLGIPQEDGPKLFRSLSIAFMSSADPIPAAKINWDRDIEYMAGILENPNITTGLMGELSRLRKDPAYSHVS
DELFATIGVTFFGAGVISTGSFLTTALISLIQRPQLRNLLHEKPELIPAGVEELLRINLSFADGLPRLATADI
QVGDVLVRKGELVLVLLEGANFDPEHFPNPGSIELDRPNPTSHLAFGRGQHFCPGSALGRRHAQIGIEALLKK
MPGVDLAVPIDQLVWRTRFQRRIPERLPVLW
>CYP121A1_Mycobacterium tuberculosis Aethiop_vetus_229 2746212293
MTATVLLEVPFSARGDRIPDAVAELRTREPIRKVRTITGAEAWLVSSYALCTQVLEDRRFSMKETAAAGAPRL
NALTVPPEVVNNMGNIADAGLRKAVMKAITPKAPGLEQFLRDTANSLLDNLITEGAPADLRNDFADPLATALH
CKVLGIPQEDGPKLFRSLSIAFMSSADPIPAAKINWDRDIEYMAGILENPNITTGLMGELSRLRKDPAYSHVS
DELFATIGVTFFGAGVISTGSFLTTALISLIQRPQLRNLLHEKPELIPAGVEELLRINLSFADGLPRLATADI
QVGDVLVRKGELVLVLLEGANFDPEHFPNPGSIELDRPNPTSHLAFGRGQHFCPGSALGRRHAQIGIEALLKK
MPGVDLAVPIDQLVWRTRFQRRIPERLPVLW
>CYP121A1_Mycobacterium tuberculosis Aethiop_vetus_230 2742636551
MTATVLLEVPFSARGDRIPDAVAELRTREPIRKVRTITGAEAWLVSSYALCTQVLEDRRFSMKETAAAGAPRL
NALTVPPEVVNNMGNIADAGLRKAVMKAITPKAPGLEQFLRDTANSLLDNLITEGAPADLRNDFADPLATALH
CKVLGIPQEDGPKLFRSLSIAFMSSADPIPAAKINWDRDIEYMAGILENPNITTGLMGELSRLRKDPAYSHVS
DELFATIGVTFFGAGVISTGSFLTTALISLIQRPQLRNLLHEKPELIPAGVEELLRINLSFADGLPRLATADI
QVGDVLVRKGELVLVLLEGANFDPEHFPNPGSIELDRPNPTSHLAFGRGQHFCPGSALGRRHAQIGIEALLKK
MPGVDLAVPIDQLVWRTRFQRRIPERLPVLW
>CYP121A1_Mycobacterium tuberculosis Aethiop_vetus_232 2746224250
MTATVLLEVPFSARGDRIPDAVAELRTREPIRKVRTITGAEAWLVSSYALCTQVLEDRRFSMKETAAAGAPRL
NALTVPPEVVNNMGNIADAGLRKAVMKAITPKAPGLEQFLRDTANSLLDNLITEGAPADLRNDFADPLATALH
CKVLGIPQEDGPKLFRSLSIAFMSSADPIPAAKINWDRDIEYMAGILENPNITTGLMGELSRLRKDPAYSHVS
DELFATIGVTFFGAGVISTGSFLTTALISLIQRPQLRNLLHEKPELIPAGVEELLRINLSFADGLPRLATADI
QVGDVLVRKGELVLVLLEGANFDPEHFPNPGSIELDRPNPTSHLAFGRGQHFCPGSALGRRHAQIGIEALLKK
MPGVDLAVPIDQLVWRTRFQRRIPERLPVLW
>CYP121A1_Mycobacterium tuberculosis Aethiop_vetus_233 2746225189
MTATVLLEVPFSARGDRIPDAVAELRTREPIRKVRTITGAEAWLVSSYALCTQVLEDRRFSMKETAAAGAPRL
NALTVPPEVVNNMGNIADAGLRKAVMKAITPKAPGLEQFLRDTANSLLDNLITEGAPADLRNDFADPLATALH
CKVLGIPQEDGPKLFRSLSIAFMSSADPIPAAKINWDRDIEYMAGILENPNITTGLMGELSRLRKDPAYSHVS
DELFATIGVTFFGAGVISTGSFLTTALISLIQRPQLRNLLHEKPELIPAGVEELLRINLSFADGLPRLATADI
QVGDVLVRKGELVLVLLEGANFDPEHFPNPGSIELDRPNPTSHLAFGRGQHFCPGSALGRRHAQIGIEALLKK
MPGVDLAVPIDQLVWRTRFQRRIPERLPVLW
>CYP121A1_Mycobacterium tuberculosis Aethiop_vetus_234 2746216971
MTATVLLEVPFSARGDRIPDAVAELRTREPIRKVRTITGAEAWLVSSYALCTQVLEDRRFSMKETAAAGAPRL
NALTVPPEVVNNMGNIADAGLRKAVMKAITPKAPGLEQFLRDTANSLLDNLITEGAPADLRNDFADPLATALH
CKVLGIPQEDGPKLFRSLSIAFMSSADPIPAAKINWDRDIEYMAGILENPNITTGLMGELSRLRKDPAYSHVS
DELFATIGVTFFGAGVISTGSFLTTALISLIQRPQLRNLLHEKPELIPAGVEELLRINLSFADGLPRLATADI
QVGDVLVRKGELVLVLLEGANFDPEHFPNPGSIELDRPNPTSHLAFGRGQHFCPGSALGRRHAQIGIEALLKK
MPGVDLAVPIDQLVWRTRFQRRIPERLPVLW
>CYP121A1_Mycobacterium tuberculosis AH_14271 2716313659
MTATVLLEVPFSARGDRIPDAVAELRTREPIRKVRTITGAEAWLVSSYALCTQVLEDRRFSMKETAAAGAPRL
NALTVPPEVVNNMGNIADAGLRKAVMKAITPKAPGLEQFLRDTANSLLDNLITEGAPADLRNDFADPLATALH
CKVLGIPQEDGPKLFRSLSIAFMSSADPIPAAKINWDRDIEYMAGILENPNITTGLMGELSRLRKDPAYSHVS
DELFATIGVTFFGAGVISTGSFLTTALISLIQRPQLRNLLHEKPELIPAGVEELLRINLSFADGLPRLATADI
QVGDVLVRKGELVLVLLEGANFDPEHFPNPGSIELDRPNPTSHLAFGRGQHFCPGSALGRRHAQIGIEALLKK
MPGVDLAVPIDQLVWRTRFQRRIPERLPVLW
>CYP121A1_Mycobacterium tuberculosis AH26_26663 2714488342
MTATVLLEVPFSARGDRIPDAVAELRTREPIRKVRTITGAEAWLVSSYALCTQVLEDRRFSMKETAAAGAPRL
NALTVPPEVVNNMGNIADAGLRKAVMKAITPKAPGLEQFLRDTANSLLDNLITEGAPADLRNDFADPLATALH
CKVLGIPQEDGPKLFRSLSIAFMSSADPIPAAKINWDRDIEYMAGILENPNITTGLMGELSRLRKDPAYSHVS
DELFATIGVTFFGAGVISTGSFLTTALISLIQRPQLRNLLHEKPELIPAGVEELLRINLSFADGLPRLATADI

QVGDVLVRKGELVLVLLEGANFDPEHFPNPGSIELDRPNPTSHLAFGRGQHFCPGSALGRRHAQIGIEALLKK
MPGVDLAVPIDQLVWRTRFQRRIPERLPVLW
>CYP121A1_Mycobacterium tuberculosis AH26_28866 2724803731
MTATVLLEVPFSARGDRIPDAVAELRTREPIRKVRTITGAEAWLVSSYALCTQVLEDRRFSMKETAAAGAPRL
NALTVPPEVVNNMGNIADAGLRKAVMKAITPKAPGLEQFLRDTANSLLDNLITEGAPADLRNDFADPLATALH
CKVLGIPQEDGPKLFRSLSIAFMSSADPIPAAKINWDRDIEYMAGILENPNITTGLMGELSRLRKDPAYSHVS
DELFATIGVTFFGAGVISTGSFLTTALISLIQRPQLRNLLHEKPELIPAGVEELLRINLSFADGLPRLATADI
QVGDVLVRKGELVLVLLEGANFDPEHFPNPGSIELDRPNPTSHLAFGRGQHFCPGSALGRRHAQIGIEALLKK
MPGVDLAVPIDQLVWRTRFQRRIPERLPVLW
>CYP121A1_Mycobacterium tuberculosis BE_10225 2714475784
MTATVLLEVPFSARGDRIPDAVAELRTREPIRKVRTITGAEAWLVSSYALCTQVLEDRRFSMKETAAAGAPRL
NALTVPPEVVNNMGNIADAGLRKAVMKAITPKAPGLEQFLRDTANSLLDNLITEGAPADLRNDFADPLATALH
CKVLGIPQEDGPKLFRSLSIAFMSSADPIPAAKINWDRDIEYMAGILENPNITTGLMGELSRLRKDPAYSHVS
DELFATIGVTFFGAGVISTGSFLTTALISLIQRPQLRNLLHEKPELIPAGVEELLRINLSFADGLPRLATADI
QVGDVLVRKGELVLVLLEGANFDPEHFPNPGSIELDRPNPTSHLAFGRGQHFCPGSALGRRHAQIGIEALLKK
MPGVDLAVPIDQLVWRTRFQRRIPERLPVLW
>CYP121A1_Mycobacterium tuberculosis BE_116771 2724792605
MTATVLLEVPFSARGDRIPDAVAELRTREPIRKVRTITGAEAWLVSSYALCTQVLEDRRFSMKETAAAGAPRL
NALTVPPEVVNNMGNIADAGLRKAVMKAITPKAPGLEQFLRDTANSLLDNLITEGAPADLRNDFADPLATALH
CKVLGIPQEDGPKLFRSLSIAFMSSADPIPAAKINWDRDIEYMAGILENPNITTGLMGELSRLRKDPAYSHVS
DELFATIGVTFFGAGVISTGSFLTTALISLIQRPQLRNLLHEKPELIPAGVEELLRINLSFADGLPRLATADI
QVGDVLVRKGELVLVLLEGANFDPEHFPNPGSIELDRPNPTSHLAFGRGQHFCPGSALGRRHAQIGIEALLKK
MPGVDLAVPIDQLVWRTRFQRRIPERLPVLW
>CYP121A1_Mycobacterium tuberculosis BE_13443 2677588790
MTATVLLEVPFSARGDRIPDAVAELRTREPIRKVRTITGAEAWLVSSYALCTQVLEDRRFSMKETAAAGAPRL
NALTVPPEVVNNMGNIADAGLRKAVMKAITPKAPGLEQFLRDTANSLLDNLITEGAPADLRNDFADPLATALH
CKVLGIPQEDGPKLFRSLSIAFMSSADPIPAAKINWDRDIEYMAGILENPNITTGLMGELSRLRKDPAYSHVS
DELFATIGVTFFGAGVISTGSFLTTALISLIQRPQLRNLLHEKPELIPAGVEELLRINLSFADGLPRLATADI
QVGDVLVRKGELVLVLLEGANFDPEHFPNPGSIELDRPNPTSHLAFGRGQHFCPGSALGRRHAQIGIEALLKK
MPGVDLAVPIDQLVWRTRFQRRIPERLPVLW
>CYP121A1_Mycobacterium tuberculosis BE_14248 2716316540
MTATVLLEVPFSARGDRIPDAVAELRTREPIRKVRTITGAEAWLVSSYALCTQVLEDRRFSMKETAAAGAPRL
NALTVPPEVVNNMGNIADAGLRKAVMKAITPKAPGLEQFLRDTANSLLDNLITEGAPADLRNDFADPLATALH
CKVLGIPQEDGPKLFRSLSIAFMSSADPIPAAKINWDRDIEYMAGILENPNITTGLMGELSRLRKDPAYSHVS
DELFATIGVTFFGAGVISTGSFLTTALISLIQRPQLRNLLHEKPELIPAGVEELLRINLSFADGLPRLATADI
QVGDVLVRKGELVLVLLEGANFDPEHFPNPGSIELDRPNPTSHLAFGRGQHFCPGSALGRRHAQIGIEALLKK
MPGVDLAVPIDQLVWRTRFQRRIPERLPVLW
>CYP121A1_Mycobacterium tuberculosis BE_7556 2675442624
MTATVLLEVPFSARGDRIPDAVAELRTREPIRKVRTITGAEAWLVSSYALCTQVLEDRRFSMKETAAAGAPRL
NALTVPPEVVNNMGNIADAGLRKAVMKAITPKAPGLEQFLRDTANSLLDNLITEGAPADLRNDFADPLATALH
CKVLGIPQEDGPKLFRSLSIAFMSSADPIPAAKINWDRDIEYMAGILENPNITTGLMGELSRLRKDPAYSHVS
DELFATIGVTFFGAGVISTGSFLTTALISLIQRPQLRNLLHEKPELIPAGVEELLRINLSFADGLPRLATADI
QVGDVLVRKGELVLVLLEGANFDPEHFPNPGSIELDRPNPTSHLAFGRGQHFCPGSALGRRHAQIGIEALLKK
MPGVDLAVPIDQLVWRTRFQRRIPERLPVLW
>CYP121A1_Mycobacterium tuberculosis BE3_11657 2716303387
MTATVLLEVPFSARGDRIPDAVAELRTREPIRKVRTITGAEAWLVSSYALCTQVLEDRRFSMKETAAAGAPRL
NALTVPPEVVNNMGNIADAGLRKAVMKAITPKAPGLEQFLRDTANSLLDNLITEGAPADLRNDFADPLATALH
CKVLGIPQEDGPKLFRSLSIAFMSSADPIPAAKINWDRDIEYMAGILENPNITTGLMGELSRLRKDPAYSHVS
DELFATIGVTFFGAGVISTGSFLTTALISLIQRPQLRNLLHEKPELIPAGVEELLRINLSFADGLPRLATADI
QVGDVLVRKGELVLVLLEGANFDPEHFPNPGSIELDRPNPTSHLAFGRGQHFCPGSALGRRHAQIGIEALLKK
MPGVDLAVPIDQLVWRTRFQRRIPERLPVLW
>CYP121A1_Mycobacterium tuberculosis BTB03-012 2577076610
MTATVLLEVPFSARGDRIPDAVAELRTREPIRKVRTITGAEAWLVSSYALCTQVLEDRRFSMKETAAAGAPRL
NALTVPPEVVNNMGNIADAGLRKAVMKAITPKAPGLEQFLRDTANSLLDNLITEGAPADLRNDFADPLATALH
CKVLGIPQEDGPKLFRSLSIAFMSSADPIPAAKINWDRDIEYMAGILENPNITTGLMGELSRLRKDPAYSHVS
DELFATIGVTFFGAGVISTGSFLTTALISLIQRPQLRNLLHEKPELIPAGVEELLRINLSFADGLPRLATADI
QVGDVLVRKGELVLVLLEGANFDPEHFPNPGSIELDRPNPTSHLAFGRGQHFCPGSALGRRHAQIGIEALLKK
MPGVDLAVPIDQLVWRTRFQRRIPERLPVLW

>CYP121A1_Mycobacterium tuberculosis BTB03-108 2604886153
MTATVLLEVPFSARGDRIPDAVAELRTREPIRKVRTITGAEAWLVSSYALCTQVLEDRRFSMKETAAAGAPRL
NALTVPPEVVNNMGNIADAGLRKAVMKAITPKAPGLEQFLRDTANSLLDNLITEGAPADLRNDFADPLATALH
CKVLGIPQEDGPKLFRSLSIAFMSSADPIPAAKINWDRDIEYMAGILENPNITTGLMGELSRLRKDPAYSHVS
DELFATIGVTFFGAGVISTGSFLTTALISLIQRPQLRNLLHEKPELIPAGVEELLRINLSFADGLPRLATADI
QVGDVLVRKGELVLVLLEGANFDPEHFPNPGSIELDRPNPTSHLAFGRGQHFCPGSALGRRHAQIGIEALLKK
MPGVDLAVPIDQLVWRTRFQRRIPERLPVLW
>CYP121A1_Mycobacterium tuberculosis BTB03-144 2576948343
MTATVLLEVPFSARGDRIPDAVAELRTREPIRKVRTITGAEAWLVSSYALCTQVLEDRRFSMKETAAAGAPRL
NALTVPPEVVNNMGNIADAGLRKAVMKAITPKAPGLEQFLRDTANSLLDNLITEGAPADLRNDFADPLATALH
CKVLGIPQEDGPKLFRSLSIAFMSSADPIPAAKINWDRDIEYMAGILENPNITTGLMGELSRLRKDPAYSHVS
DELFATIGVTFFGAGVISTGSFLTTALISLIQRPQLRNLLHEKPELIPAGVEELLRINLSFADGLPRLATADI
QVGDVLVRKGELVLVLLEGANFDPEHFPNPGSIELDRPNPTSHLAFGRGQHFCPGSALGRRHAQIGIEALLKK
MPGVDLAVPIDQLVWRTRFQRRIPERLPVLW
>CYP121A1_Mycobacterium tuberculosis BTB03-168 2605245294
MTATVLLEVPFSARGDRIPDAVAELRTREPIRKVRTITGAEAWLVSSYALCTQVLEDRRFSMKETAAAGAPRL
NALTVPPEVVNNMGNIADAGLRKAVMKAITPKAPGLEQFLRDTANSLLDNLITEGAPADLRNDFADPLATALH
CKVLGIPQEDGPKLFRSLSIAFMSSADPIPAAKINWDRDIEYMAGILENPNITTGLMGELSRLRKDPAYSHVS
DELFATIGVTFFGAGVISTGSFLTTALISLIQRPQLRNLLHEKPELIPAGVEELLRINLSFADGLPRLATADI
QVGDVLVRKGELVLVLLEGANFDPEHFPNPGSIELDRPNPTSHLAFGRGQHFCPGSALGRRHAQIGIEALLKK
MPGVDLAVPIDQLVWRTRFQRRIPERLPVLW
>CYP121A1_Mycobacterium tuberculosis BTB03-169 2604517075
MTATVLLEVPFSARGDRIPDAVAELRTREPIRKVRTITGAEAWLVSSYALCTQVLEDRRFSMKETAAAGAPRL
NALTVPPEVVNNMGNIADAGLRKAVMKAITPKAPGLEQFLRDTANSLLDNLITEGAPADLRNDFADPLATALH
CKVLGIPQEDGPKLFRSLSIAFMSSADPIPAAKINWDRDIEYMAGILENPNITTGLMGELSRLRKDPAYSHVS
DELFATIGVTFFGAGVISTGSFLTTALISLIQRPQLRNLLHEKPELIPAGVEELLRINLSFADGLPRLATADI
QVGDVLVRKGELVLVLLEGANFDPEHFPNPGSIELDRPNPTSHLAFGRGQHFCPGSALGRRHAQIGIEALLKK
MPGVDLAVPIDQLVWRTRFQRRIPERLPVLW
>CYP121A1_Mycobacterium tuberculosis BTB04-001 2604521152
MTATVLLEVPFSARGDRIPDAVAELRTREPIRKVRTITGAEAWLVSSYALCTQVLEDRRFSMKETAAAGAPRL
NALTVPPEVVNNMGNIADAGLRKAVMKAITPKAPGLEQFLRDTANSLLDNLITEGAPADLRNDFADPLATALH
CKVLGIPQEDGPKLFRSLSIAFMSSADPIPAAKINWDRDIEYMAGILENPNITTGLMGELSRLRKDPAYSHVS
DELFATIGVTFFGAGVISTGSFLTTALISLIQRPQLRNLLHEKPELIPAGVEELLRINLSFADGLPRLATADI
QVGDVLVRKGELVLVLLEGANFDPEHFPNPGSIELDRPNPTSHLAFGRGQHFCPGSALGRRHAQIGIEALLKK
MPGVDLAVPIDQLVWRTRFQRRIPERLPVLW
>CYP121A1_Mycobacterium tuberculosis BTB04-097 2605249398
MTATVLLEVPFSARGDRIPDAVAELRTREPIRKVRTITGAEAWLVSSYALCTQVLEDRRFSMKETAAAGAPRL
NALTVPPEVVNNMGNIADAGLRKAVMKAITPKAPGLEQFLRDTANSLLDNLITEGAPADLRNDFADPLATALH
CKVLGIPQEDGPKLFRSLSIAFMSSADPIPAAKINWDRDIEYMAGILENPNITTGLMGELSRLRKDPAYSHVS
DELFATIGVTFFGAGVISTGSFLTTALISLIQRPQLRNLLHEKPELIPAGVEELLRINLSFADGLPRLATADI
QVGDVLVRKGELVLVLLEGANFDPEHFPNPGSIELDRPNPTSHLAFGRGQHFCPGSALGRRHAQIGIEALLKK
MPGVDLAVPIDQLVWRTRFQRRIPERLPVLW
>CYP121A1_Mycobacterium tuberculosis BTB04-120 2604175527
MTATVLLEVPFSARGDRIPDAVAELRTREPIRKVRTITGAEAWLVSSYALCTQVLEDRRFSMKETAAAGAPRL
NALTVPPEVVNNMGNIADAGLRKAVMKAITPKAPGLEQFLRDTANSLLDNLITEGAPADLRNDFADPLATALH
CKVLGIPQEDGPKLFRSLSIAFMSSADPIPAAKINWDRDIEYMAGILENPNITTGLMGELSRLRKDPAYSHVS
DELFATIGVTFFGAGVISTGSFLTTALISLIQRPQLRNLLHEKPELIPAGVEELLRINLSFADGLPRLATADI
QVGDVLVRKGELVLVLLEGANFDPEHFPNPGSIELDRPNPTSHLAFGRGQHFCPGSALGRRHAQIGIEALLKK
MPGVDLAVPIDQLVWRTRFQRRIPERLPVLW
>CYP121A1_Mycobacterium tuberculosis BTB04-128 2603477936
MTATVLLEVPFSARGDRIPDAVAELRTREPIRKVRTITGAEAWLVSSYALCTQVLEDRRFSMKETAAAGAPRL
NALTVPPEVVNNMGNIADAGLRKAVMKAITPKAPGLEQFLRDTANSLLDNLITEGAPADLRNDFADPLATALH
CKVLGIPQEDGPKLFRSLSIAFMSSADPIPAAKINWDRDIEYMAGILENPNITTGLMGELSRLRKDPAYSHVS
DELFATIGVTFFGAGVISTGSFLTTALISLIQRPQLRNLLHEKPELIPAGVEELLRINLSFADGLPRLATADI
QVGDVLVRKGELVLVLLEGANFDPEHFPNPGSIELDRPNPTSHLAFGRGQHFCPGSALGRRHAQIGIEALLKK
MPGVDLAVPIDQLVWRTRFQRRIPERLPVLW
>CYP121A1_Mycobacterium tuberculosis BTB04-172 2604525269

MTATVLLEVPFSARGDRIPDAVAELRTREPIRKVRTITGAEAWLVSSYALCTQVLEDRRFSMKETAAAGAPRL
NALTVPPEVVNNMGNIADAGLRKAVMKAITPKAPGLEQFLRDTANSLLDNLITEGAPADLRNDFADPLATALH
CKVLGIPQEDGPKLFRSLSIAFMSSADPIPAAKINWDRDIEYMAGILENPNITTGLMGELSRLRKDPAYSHVS
DELFATIGVTFFGAGVISTGSFLTTALISLIQRPQLRNLLHEKPELIPAGVEELLRINLSFADGLPRLATADI
QVGDVLVRKGELVLVLLEGANFDPEHFPNPGSIELDRPNPTSHLAFGRGQHFCPGSALGRRHAQIGIEALLKK
MPGVDLAVPIDQLVWRTRFQRRIPERLPVLW
>CYP121A1_Mycobacterium tuberculosis BTB04-388 2604528587
MTATVLLEVPFSARGDRIPDAVAELRTREPIRKVRTITGAEAWLVSSYALCTQVLEDRRFSMKETAAAGAPRL
NALTVPPEVVNNMGNIADAGLRKAVMKAITPKAPGLEQFLRDTANSLLDNLITEGAPADLRNDFADPLATALH
CKVLGIPQEDGPKLFRSLSIAFMSSADPIPAAKINWDRDIEYMAGILENPNITTGLMGELSRLRKDPAYSHVS
DELFATIGVTFFGAGVISTGSFLTTALISLIQRPQLRNLLHEKPELIPAGVEELLRINLSFADGLPRLATADI
QVGDVLVRKGELVLVLLEGANFDPEHFPNPGSIELDRPNPTSHLAFGRGQHFCPGSALGRRHAQIGIEALLKK
MPGVDLAVPIDQLVWRTRFQRRIPERLPVLW
>CYP121A1_Mycobacterium tuberculosis BTB05-001 2603309220
MTATVLLEVPFSARGDRIPDAVAELRTREPIRKVRTITGAEAWLVSSYALCTQVLEDRRFSMKETAAAGAPRL
NALTVPPEVVNNMGNIADAGLRKAVMKAITPKAPGLEQFLRDTANSLLDNLITEGAPADLRNDFADPLATALH
CKVLGIPQEDGPKLFRSLSIAFMSSADPIPAAKINWDRDIEYMAGILENPNITTGLMGELSRLRKDPAYSHVS
DELFATIGVTFFGAGVISTGSFLTTALISLIQRPQLRNLLHEKPELIPAGVEELLRINLSFADGLPRLATADI
QVGDVLVRKGELVLVLLEGANFDPEHFPNPGSIELDRPNPTSHLAFGRGQHFCPGSALGRRHAQIGIEALLKK
MPGVDLAVPIDQLVWRTRFQRRIPERLPVLW
>CYP121A1_Mycobacterium tuberculosis BTB05-013 2576565031
MTATVLLEVPFSARGDRIPDAVAELRTREPIRKVRTITGAEAWLVSSYALCTQVLEDRRFSMKETAAAGAPRL
NALTVPPEVVNNMGNIADAGLRKAVMKAITPKAPGLEQFLRDTANSLLDNLITEGAPADLRNDFADPLATALH
CKVLGIPQEDGPKLFRSLSIAFMSSADPIPAAKINWDRDIEYMAGILENPNITTGLMGELSRLRKDPAYSHVS
DELFATIGVTFFGAGVISTGSFLTTALISLIQRPQLRNLLHEKPELIPAGVEELLRINLSFADGLPRLATADI
QVGDVLVRKGELVLVLLEGANFDPEHFPNPGSIELDRPNPTSHLAFGRGQHFCPGSALGRRHAQIGIEALLKK
MPGVDLAVPIDQLVWRTRFQRRIPERLPVLW
>CYP121A1_Mycobacterium tuberculosis BTB05-285 2577186070
MTATVLLEVPFSARGDRIPDAVAELRTREPIRKVRTITGAEAWLVSSYALCTQVLEDRRFSMKETAAAGAPRL
NALTVPPEVVNNMGNIADAGLRKAVMKAITPKAPGLEQFLRDTANSLLDNLITEGAPADLRNDFADPLATALH
CKVLGIPQEDGPKLFRSLSIAFMSSADPIPAAKINWDRDIEYMAGILENPNITTGLMGELSRLRKDPAYSHVS
DELFATIGVTFFGAGVISTGSFLTTALISLIQRPQLRNLLHEKPELIPAGVEELLRINLSFADGLPRLATADI
QVGDVLVRKGELVLVLLEGANFDPEHFPNPGSIELDRPNPTSHLAFGRGQHFCPGSALGRRHAQIGIEALLKK
MPGVDLAVPIDQLVWRTRFQRRIPERLPVLW
>CYP121A1_Mycobacterium tuberculosis BTB05-348 2605253090
MTATVLLEVPFSARGDRIPDAVAELRTREPIRKVRTITGAEAWLVSSYALCTQVLEDRRFSMKETAAAGAPRL
NALTVPPEVVNNMGNIADAGLRKAVMKAITPKAPGLEQFLRDTANSLLDNLITEGAPADLRNDFADPLATALH
CKVLGIPQEDGPKLFRSLSIAFMSSADPIPAAKINWDRDIEYMAGILENPNITTGLMGELSRLRKDPAYSHVS
DELFATIGVTFFGAGVISTGSFLTTALISLIQRPQLRNLLHEKPELIPAGVEELLRINLSFADGLPRLATADI
QVGDVLVRKGELVLVLLEGANFDPEHFPNPGSIELDRPNPTSHLAFGRGQHFCPGSALGRRHAQIGIEALLKK
MPGVDLAVPIDQLVWRTRFQRRIPERLPVLW
>CYP121A1_Mycobacterium tuberculosis BTB05-481 2603311473
MTATVLLEVPFSARGDRIPDAVAELRTREPIRKVRTITGAEAWLVSSYALCTQVLEDRRFSMKETAAAGAPRL
NALTVPPEVVNNMGNIADAGLRKAVMKAITPKAPGLEQFLRDTANSLLDNLITEGAPADLRNDFADPLATALH
CKVLGIPQEDGPKLFRSLSIAFMSSADPIPAAKINWDRDIEYMAGILENPNITTGLMGELSRLRKDPAYSHVS
DELFATIGVTFFGAGVISTGSFLTTALISLIQRPQLRNLLHEKPELIPAGVEELLRINLSFADGLPRLATADI
QVGDVLVRKGELVLVLLEGANFDPEHFPNPGSIELDRPNPTSHLAFGRGQHFCPGSALGRRHAQIGIEALLKK
MPGVDLAVPIDQLVWRTRFQRRIPERLPVLW
>CYP121A1_Mycobacterium tuberculosis BTB05-552 2548033852
MTATVLLEVPFSARGDRIPDAVAELRTREPIRKVRTITGAEAWLVSSYALCTQVLEDRRFSMKETAAAGAPRL
NALTVPPEVVNNMGNIADAGLRKAVMKAITPKAPGLEQFLRDTANSLLDNLITEGAPADLRNDFADPLATALH
CKVLGIPQEDGPKLFRSLSIAFMSSADPIPAAKINWDRDIEYMAGILENPNITTGLMGELSRLRKDPAYSHVS
DELFATIGVTFFGAGVISTGSFLTTALISLIQRPQLRNLLHEKPELIPAGVEELLRINLSFADGLPRLATADI
QVGDVLVRKGELVLVLLEGANFDPEHFPNPGSIELDRPNPTSHLAFGRGQHFCPGSALGRRHAQIGIEALLKK
MPGVDLAVPIDQLVWRTRFQRRIPERLPVLW
>CYP121A1_Mycobacterium tuberculosis BTB05-559 2548038089
MTATVLLEVPFSARGDRIPDAVAELRTREPIRKVRTITGAEAWLVSSYALCTQVLEDRRFSMKETAAAGAPRL
NALTVPPEVVNNMGNIADAGLRKAVMKAITPKAPGLEQFLRDTANSLLDNLITEGAPADLRNDFADPLATALH

CKVLGIPQEDGPKLFRSLSIAFMSSADPIPAAKINWDRDIEYMAGILENPNITTGLMGELSRLRKDPAYSHVS
DELFATIGVTFFGAGVISTGSFLTTALISLIQRPQLRNLLHEKPELIPAGVEELLRINLSFADGLPRLATADI
QVGDVLVRKGELVLVLLEGANFDPEHFPNPGSIELDRPNPTSHLAFGRGQHFCPGSALGRRHAQIGIEALLKK
MPGVDLAVPIDQLVWRTRFQRRIPERLPVLW
>CYP121A1_Mycobacterium tuberculosis BTB05-660 2604533523
MTATVLLEVPFSARGDRIPDAVAELRTREPIRKVRTITGAEAWLVSSYALCTQVLEDRRFSMKETAAAGAPRL
NALTVPPEVVNNMGNIADAGLRKAVMKAITPKAPGLEQFLRDTANSLLDNLITEGAPADLRNDFADPLATALH
CKVLGIPQEDGPKLFRSLSIAFMSSADPIPAAKINWDRDIEYMAGILENPNITTGLMGELSRLRKDPAYSHVS
DELFATIGVTFFGAGVISTGSFLTTALISLIQRPQLRNLLHEKPELIPAGVEELLRINLSFADGLPRLATADI
QVGDVLVRKGELVLVLLEGANFDPEHFPNPGSIELDRPNPTSHLAFGRGQHFCPGSALGRRHAQIGIEALLKK
MPGVDLAVPIDQLVWRTRFQRRIPERLPVLW
>CYP121A1_Mycobacterium tuberculosis BTB06-001 2578107830
MTATVLLEVPFSARGDRIPDAVAELRTREPIRKVRTITGAEAWLVSSYALCTQVLEDRRFSMKETAAAGAPRL
NALTVPPEVVNNMGNIADAGLRKAVMKAITPKAPGLEQFLRDTANSLLDNLITEGAPADLRNDFADPLATALH
CKVLGIPQEDGPKLFRSLSIAFMSSADPIPAAKINWDRDIEYMAGILENPNITTGLMGELSRLRKDPAYSHVS
DELFATIGVTFFGAGVISTGSFLTTALISLIQRPQLRNLLHEKPELIPAGVEELLRINLSFADGLPRLATADI
QVGDVLVRKGELVLVLLEGANFDPEHFPNPGSIELDRPNPTSHLAFGRGQHFCPGSALGRRHAQIGIEALLKK
MPGVDLAVPIDQLVWRTRFQRRIPERLPVLW
>CYP121A1_Mycobacterium tuberculosis BTB06-467 2604890604
MTATVLLEVPFSARGDRIPDAVAELRTREPIRKVRTITGAEAWLVSSYALCTQVLEDRRFSMKETAAAGAPRL
NALTVPPEVVNNMGNIADAGLRKAVMKAITPKAPGLEQFLRDTANSLLDNLITEGAPADLRNDFADPLATALH
CKVLGIPQEDGPKLFRSLSIAFMSSADPIPAAKINWDRDIEYMAGILENPNITTGLMGELSRLRKDPAYSHVS
DELFATIGVTFFGAGVISTGSFLTTALISLIQRPQLRNLLHEKPELIPAGVEELLRINLSFADGLPRLATADI
QVGDVLVRKGELVLVLLEGANFDPEHFPNPGSIELDRPNPTSHLAFGRGQHFCPGSALGRRHAQIGIEALLKK
MPGVDLAVPIDQLVWRTRFQRRIPERLPVLW
>CYP121A1_Mycobacterium tuberculosis BTB06-494 2605795541
MTATVLLEVPFSARGDRIPDAVAELRTREPIRKVRTITGAEAWLVSSYALCTQVLEDRRFSMKETAAAGAPRL
NALTVPPEVVNNMGNIADAGLRKAVMKAITPKAPGLEQFLRDTANSLLDNLITEGAPADLRNDFADPLATALH
CKVLGIPQEDGPKLFRSLSIAFMSSADPIPAAKINWDRDIEYMAGILENPNITTGLMGELSRLRKDPAYSHVS
DELFATIGVTFFGAGVISTGSFLTTALISLIQRPQLRNLLHEKPELIPAGVEELLRINLSFADGLPRLATADI
QVGDVLVRKGELVLVLLEGANFDPEHFPNPGSIELDRPNPTSHLAFGRGQHFCPGSALGRRHAQIGIEALLKK
MPGVDLAVPIDQLVWRTRFQRRIPERLPVLW
>CYP121A1_Mycobacterium tuberculosis BTB07-123 2604717938
MTATVLLEVPFSARGDRIPDAVAELRTREPIRKVRTITGAEAWLVSSYALCTQVLEDRRFSMKETAAAGAPRL
NALTVPPEVVNNMGNIADAGLRKAVMKAITPKAPGLEQFLRDTANSLLDNLITEGAPADLRNDFADPLATALH
CKVLGIPQEDGPKLFRSLSIAFMSSADPIPAAKINWDRDIEYMAGILENPNITTGLMGELSRLRKDPAYSHVS
DELFATIGVTFFGAGVISTGSFLTTALISLIQRPQLRNLLHEKPELIPAGVEELLRINLSFADGLPRLATADI
QVGDVLVRKGELVLVLLEGANFDPEHFPNPGSIELDRPNPTSHLAFGRGQHFCPGSALGRRHAQIGIEALLKK
MPGVDLAVPIDQLVWRTRFQRRIPERLPVLW
>CYP121A1_Mycobacterium tuberculosis BTB07-070 2605073149
MTATVLLEVPFSARGDRIPDAVAELRTREPIRKVRTITGAEAWLVSSYALCTQVLEDRRFSMKETAAAGAPRL
NALTVPPEVVNNMGNIADAGLRKAVMKAITPKAPGLEQFLRDTANSLLDNLITEGAPADLRNDFADPLATALH
CKVLGIPQEDGPKLFRSLSIAFMSSADPIPAAKINWDRDIEYMAGILENPNITTGLMGELSRLRKDPAYSHVS
DELFATIGVTFFGAGVISTGSFLTTALISLIQRPQLRNLLHEKPELIPAGVEELLRINLSFADGLPRLATADI
QVGDVLVRKGELVLVLLEGANFDPEHFPNPGSIELDRPNPTSHLAFGRGQHFCPGSALGRRHAQIGIEALLKK
MPGVDLAVPIDQLVWRTRFQRRIPERLPVLW
>CYP121A1_Mycobacterium tuberculosis BTB07-034 2577455581
MTATVLLEVPFSARGDRIPDAVAELRTREPIRKVRTITGAEAWLVSSYALCTQVLEDRRFSMKETAAAGAPRL
NALTVPPEVVNNMGNIADAGLRKAVMKAITPKAPGLEQFLRDTANSLLDNLITEGAPADLRNDFADPLATALH
CKVLGIPQEDGPKLFRSLSIAFMSSADPIPAAKINWDRDIEYMAGILENPNITTGLMGELSRLRKDPAYSHVS
DELFATIGVTFFGAGVISTGSFLTTALISLIQRPQLRNLLHEKPELIPAGVEELLRINLSFADGLPRLATADI
QVGDVLVRKGELVLVLLEGANFDPEHFPNPGSIELDRPNPTSHLAFGRGQHFCPGSALGRRHAQIGIEALLKK
MPGVDLAVPIDQLVWRTRFQRRIPERLPVLW
>CYP121A1_Mycobacterium tuberculosis BTB07-126 2603461467
MTATVLLEVPFSARGDRIPDAVAELRTREPIRKVRTITGAEAWLVSSYALCTQVLEDRRFSMKETAAAGAPRL
NALTVPPEVVNNMGNIADAGLRKAVMKAITPKAPGLEQFLRDTANSLLDNLITEGAPADLRNDFADPLATALH
CKVLGIPQEDGPKLFRSLSIAFMSSADPIPAAKINWDRDIEYMAGILENPNITTGLMGELSRLRKDPAYSHVS
DELFATIGVTFFGAGVISTGSFLTTALISLIQRPQLRNLLHEKPELIPAGVEELLRINLSFADGLPRLATADI

QVGDVLVRKGELVLVLLEGANFDPEHFPNPGSIELDRPNPTSHLAFGRGQHFCPGSALGRRHAQIGIEALLKK
MPGVDLAVPIDQLVWRTRFQRRIPERLPVLW
>CYP121A1_Mycobacterium tuberculosis BTB07-206 2574694331
MTATVLLEVPFSARGDRIPDAVAELRTREPIRKVRTITGAEAWLVSSYALCTQVLEDRRFSMKETAAAGAPRL
NALTVPPEVVNNMGNIADAGLRKAVMKAITPKAPGLEQFLRDTANSLLDNLITEGAPADLRNDFADPLATALH
CKVLGIPQEDGPKLFRSLSIAFMSSADPIPAAKINWDRDIEYMAGILENPNITTGLMGELSRLRKDPAYSHVS
DELFATIGVTFFGAGVISTGSFLTTALISLIQRPQLRNLLHEKPELIPAGVEELLRINLSFADGLPRLATADI
QVGDVLVRKGELVLVLLEGANFDPEHFPNPGSIELDRPNPTSHLAFGRGQHFCPGSALGRRHAQIGIEALLKK
MPGVDLAVPIDQLVWRTRFQRRIPERLPVLW
>CYP121A1_Mycobacterium tuberculosis BTB07-243 2603466093
MTATVLLEVPFSARGDRIPDAVAELRTREPIRKVRTITGAEAWLVSSYALCTQVLEDRRFSMKETAAAGAPRL
NALTVPPEVVNNMGNIADAGLRKAVMKAITPKAPGLEQFLRDTANSLLDNLITEGAPADLRNDFADPLATALH
CKVLGIPQEDGPKLFRSLSIAFMSSADPIPAAKINWDRDIEYMAGILENPNITTGLMGELSRLRKDPAYSHVS
DELFATIGVTFFGAGVISTGSFLTTALISLIQRPQLRNLLHEKPELIPAGVEELLRINLSFADGLPRLATADI
QVGDVLVRKGELVLVLLEGANFDPEHFPNPGSIELDRPNPTSHLAFGRGQHFCPGSALGRRHAQIGIEALLKK
MPGVDLAVPIDQLVWRTRFQRRIPERLPVLW
>CYP121A1_Mycobacterium tuberculosis BTB07-246 2584679202
MTATVLLEVPFSARGDRIPDAVAELRTREPIRKVRTITGAEAWLVSSYALCTQVLEDRRFSMKETAAAGAPRL
NALTVPPEVVNNMGNIADAGLRKAVMKAITPKAPGLEQFLRDTANSLLDNLITEGAPADLRNDFADPLATALH
CKVLGIPQEDGPKLFRSLSIAFMSSADPIPAAKINWDRDIEYMAGILENPNITTGLMGELSRLRKDPAYSHVS
DELFATIGVTFFGAGVISTGSFLTTALISLIQRPQLRNLLHEKPELIPAGVEELLRINLSFADGLPRLATADI
QVGDVLVRKGELVLVLLEGANFDPEHFPNPGSIELDRPNPTSHLAFGRGQHFCPGSALGRRHAQIGIEALLKK
MPGVDLAVPIDQLVWRTRFQRRIPERLPVLW
>CYP121A1_Mycobacterium tuberculosis BTB07-254 2584990767
MTATVLLEVPFSARGDRIPDAVAELRTREPIRKVRTITGAEAWLVSSYALCTQVLEDRRFSMKETAAAGAPRL
NALTVPPEVVNNMGNIADAGLRKAVMKAITPKAPGLEQFLRDTANSLLDNLITEGAPADLRNDFADPLATALH
CKVLGIPQEDGPKLFRSLSIAFMSSADPIPAAKINWDRDIEYMAGILENPNITTGLMGELSRLRKDPAYSHVS
DELFATIGVTFFGAGVISTGSFLTTALISLIQRPQLRNLLHEKPELIPAGVEELLRINLSFADGLPRLATADI
QVGDVLVRKGELVLVLLEGANFDPEHFPNPGSIELDRPNPTSHLAFGRGQHFCPGSALGRRHAQIGIEALLKK
MPGVDLAVPIDQLVWRTRFQRRIPERLPVLW
>CYP121A1_Mycobacterium tuberculosis BTB07-261 2605081367
MTATVLLEVPFSARGDRIPDAVAELRTREPIRKVRTITGAEAWLVSSYALCTQVLEDRRFSMKETAAAGAPRL
NALTVPPEVVNNMGNIADAGLRKAVMKAITPKAPGLEQFLRDTANSLLDNLITEGAPADLRNDFADPLATALH
CKVLGIPQEDGPKLFRSLSIAFMSSADPIPAAKINWDRDIEYMAGILENPNITTGLMGELSRLRKDPAYSHVS
DELFATIGVTFFGAGVISTGSFLTTALISLIQRPQLRNLLHEKPELIPAGVEELLRINLSFADGLPRLATADI
QVGDVLVRKGELVLVLLEGANFDPEHFPNPGSIELDRPNPTSHLAFGRGQHFCPGSALGRRHAQIGIEALLKK
MPGVDLAVPIDQLVWRTRFQRRIPERLPVLW
>CYP121A1_Mycobacterium tuberculosis BTB07-283 2604365614
MTATVLLEVPFSARGDRIPDAVAELRTREPIRKVRTITGAEAWLVSSYALCTQVLEDRRFSMKETAAAGAPRL
NALTVPPEVVNNMGNIADAGLRKAVMKAITPKAPGLEQFLRDTANSLLDNLITEGAPADLRNDFADPLATALH
CKVLGIPQEDGPKLFRSLSIAFMSSADPIPAAKINWDRDIEYMAGILENPNITTGLMGELSRLRKDPAYSHVS
DELFATIGVTFFGAGVISTGSFLTTALISLIQRPQLRNLLHEKPELIPAGVEELLRINLSFADGLPRLATADI
QVGDVLVRKGELVLVLLEGANFDPEHFPNPGSIELDRPNPTSHLAFGRGQHFCPGSALGRRHAQIGIEALLKK
MPGVDLAVPIDQLVWRTRFQRRIPERLPVLW
>CYP121A1_Mycobacterium tuberculosis BTB07-275 2604360520
MTATVLLEVPFSARGDRIPDAVAELRTREPIRKVRTITGAEAWLVSSYALCTQVLEDRRFSMKETAAAGAPRL
NALTVPPEVVNNMGNIADAGLRKAVMKAITPKAPGLEQFLRDTANSLLDNLITEGAPADLRNDFADPLATALH
CKVLGIPQEDGPKLFRSLSIAFMSSADPIPAAKINWDRDIEYMAGILENPNITTGLMGELSRLRKDPAYSHVS
DELFATIGVTFFGAGVISTGSFLTTALISLIQRPQLRNLLHEKPELIPAGVEELLRINLSFADGLPRLATADI
QVGDVLVRKGELVLVLLEGANFDPEHFPNPGSIELDRPNPTSHLAFGRGQHFCPGSALGRRHAQIGIEALLKK
MPGVDLAVPIDQLVWRTRFQRRIPERLPVLW
>CYP121A1_Mycobacterium tuberculosis BTB07-299 2603160906
MTATVLLEVPFSARGDRIPDAVAELRTREPIRKVRTITGAEAWLVSSYALCTQVLEDRRFSMKETAAAGAPRL
NALTVPPEVVNNMGNIADAGLRKAVMKAITPKAPGLEQFLRDTANSLLDNLITEGAPADLRNDFADPLATALH
CKVLGIPQEDGPKLFRSLSIAFMSSADPIPAAKINWDRDIEYMAGILENPNITTGLMGELSRLRKDPAYSHVS
DELFATIGVTFFGAGVISTGSFLTTALISLIQRPQLRNLLHEKPELIPAGVEELLRINLSFADGLPRLATADI
QVGDVLVRKGELVLVLLEGANFDPEHFPNPGSIELDRPNPTSHLAFGRGQHFCPGSALGRRHAQIGIEALLKK
MPGVDLAVPIDQLVWRTRFQRRIPERLPVLW

>CYP121A1_Mycobacterium tuberculosis BTB07-300 2604557423
MTATVLLEVPFSARGDRIPDAVAELRTREPIRKVRTITGAEAWLVSSYALCTQVLEDRRFSMKETAAAGAPRL
NALTVPPEVVNNMGNIADAGLRKAVMKAITPKAPGLEQFLRDTANSLLDNLITEGAPADLRNDFADPLATALH
CKVLGIPQEDGPKLFRSLSIAFMSSADPIPAAKINWDRDIEYMAGILENPNITTGLMGELSRLRKDPAYSHVS
DELFATIGVTFFGAGVISTGSFLTTALISLIQRPQLRNLLHEKPELIPAGVEELLRINLSFADGLPRLATADI
QVGDVLVRKGELVLVLLEGANFDPEHFPNPGSIELDRPNPTSHLAFGRGQHFCPGSALGRRHAQIGIEALLKK
MPGVDLAVPIDQLVWRTRFQRRIPERLPVLW
>CYP121A1_Mycobacterium tuberculosis BTB07-325 2578098386
MTATVLLEVPFSARGDRIPDAVAELRTREPIRKVRTITGAEAWLVSSYALCTQVLEDRRFSMKETAAAGAPRL
NALTVPPEVVNNMGNIADAGLRKAVMKAITPKAPGLEQFLRDTANSLLDNLITEGAPADLRNDFADPLATALH
CKVLGIPQEDGPKLFRSLSIAFMSSADPIPAAKINWDRDIEYMAGILENPNITTGLMGELSRLRKDPAYSHVS
DELFATIGVTFFGAGVISTGSFLTTALISLIQRPQLRNLLHEKPELIPAGVEELLRINLSFADGLPRLATADI
QVGDVLVRKGELVLVLLEGANFDPEHFPNPGSIELDRPNPTSHLAFGRGQHFCPGSALGRRHAQIGIEALLKK
MPGVDLAVPIDQLVWRTRFQRRIPERLPVLW
>CYP121A1_Mycobacterium tuberculosis BTB07-327 2605284008
MTATVLLEVPFSARGDRIPDAVAELRTREPIRKVRTITGAEAWLVSSYALCTQVLEDRRFSMKETAAAGAPRL
NALTVPPEVVNNMGNIADAGLRKAVMKAITPKAPGLEQFLRDTANSLLDNLITEGAPADLRNDFADPLATALH
CKVLGIPQEDGPKLFRSLSIAFMSSADPIPAAKINWDRDIEYMAGILENPNITTGLMGELSRLRKDPAYSHVS
DELFATIGVTFFGAGVISTGSFLTTALISLIQRPQLRNLLHEKPELIPAGVEELLRINLSFADGLPRLATADI
QVGDVLVRKGELVLVLLEGANFDPEHFPNPGSIELDRPNPTSHLAFGRGQHFCPGSALGRRHAQIGIEALLKK
MPGVDLAVPIDQLVWRTRFQRRIPERLPVLW
>CYP121A1_Mycobacterium tuberculosis BTB07-354 2605634897
MTATVLLEVPFSARGDRIPDAVAELRTREPIRKVRTITGAEAWLVSSYALCTQVLEDRRFSMKETAAAGAPRL
NALTVPPEVVNNMGNIADAGLRKAVMKAITPKAPGLEQFLRDTANSLLDNLITEGAPADLRNDFADPLATALH
CKVLGIPQEDGPKLFRSLSIAFMSSADPIPAAKINWDRDIEYMAGILENPNITTGLMGELSRLRKDPAYSHVS
DELFATIGVTFFGAGVISTGSFLTTALISLIQRPQLRNLLHEKPELIPAGVEELLRINLSFADGLPRLATADI
QVGDVLVRKGELVLVLLEGANFDPEHFPNPGSIELDRPNPTSHLAFGRGQHFCPGSALGRRHAQIGIEALLKK
MPGVDLAVPIDQLVWRTRFQRRIPERLPVLW
>CYP121A1_Mycobacterium tuberculosis BTB08-001 2603494904
MTATVLLEVPFSARGDRIPDAVAELRTREPIRKVRTITGAEAWLVSSYALCTQVLEDRRFSMKETAAAGAPRL
NALTVPPEVVNNMGNIADAGLRKAVMKAITPKAPGLEQFLRDTANSLLDNLITEGAPADLRNDFADPLATALH
CKVLGIPQEDGPKLFRSLSIAFMSSADPIPAAKINWDRDIEYMAGILENPNITTGLMGELSRLRKDPAYSHVS
DELFATIGVTFFGAGVISTGSFLTTALISLIQRPQLRNLLHEKPELIPAGVEELLRINLSFADGLPRLATADI
QVGDVLVRKGELVLVLLEGANFDPEHFPNPGSIELDRPNPTSHLAFGRGQHFCPGSALGRRHAQIGIEALLKK
MPGVDLAVPIDQLVWRTRFQRRIPERLPVLW
>CYP121A1_Mycobacterium tuberculosis BTB08-014 2603317142
MTATVLLEVPFSARGDRIPDAVAELRTREPIRKVRTITGAEAWLVSSYALCTQVLEDRRFSMKETAAAGAPRL
NALTVPPEVVNNMGNIADAGLRKAVMKAITPKAPGLEQFLRDTANSLLDNLITEGAPADLRNDFADPLATALH
CKVLGIPQEDGPKLFRSLSIAFMSSADPIPAAKINWDRDIEYMAGILENPNITTGLMGELSRLRKDPAYSHVS
DELFATIGVTFFGAGVISTGSFLTTALISLIQRPQLRNLLHEKPELIPAGVEELLRINLSFADGLPRLATADI
QVGDVLVRKGELVLVLLEGANFDPEHFPNPGSIELDRPNPTSHLAFGRGQHFCPGSALGRRHAQIGIEALLKK
MPGVDLAVPIDQLVWRTRFQRRIPERLPVLW
>CYP121A1_Mycobacterium tuberculosis BTB08-022 2577660420
MTATVLLEVPFSARGDRIPDAVAELRTREPIRKVRTITGAEAWLVSSYALCTQVLEDRRFSMKETAAAGAPRL
NALTVPPEVVNNMGNIADAGLRKAVMKAITPKAPGLEQFLRDTANSLLDNLITEGAPADLRNDFADPLATALH
CKVLGIPQEDGPKLFRSLSIAFMSSADPIPAAKINWDRDIEYMAGILENPNITTGLMGELSRLRKDPAYSHVS
DELFATIGVTFFGAGVISTGSFLTTALISLIQRPQLRNLLHEKPELIPAGVEELLRINLSFADGLPRLATADI
QVGDVLVRKGELVLVLLEGANFDPEHFPNPGSIELDRPNPTSHLAFGRGQHFCPGSALGRRHAQIGIEALLKK
MPGVDLAVPIDQLVWRTRFQRRIPERLPVLW
>CYP121A1_Mycobacterium tuberculosis BTB08-043 2603320724
MTATVLLEVPFSARGDRIPDAVAELRTREPIRKVRTITGAEAWLVSSYALCTQVLEDRRFSMKETAAAGAPRL
NALTVPPEVVNNMGNIADAGLRKAVMKAITPKAPGLEQFLRDTANSLLDNLITEGAPADLRNDFADPLATALH
CKVLGIPQEDGPKLFRSLSIAFMSSADPIPAAKINWDRDIEYMAGILENPNITTGLMGELSRLRKDPAYSHVS
DELFATIGVTFFGAGVISTGSFLTTALISLIQRPQLRNLLHEKPELIPAGVEELLRINLSFADGLPRLATADI
QVGDVLVRKGELVLVLLEGANFDPEHFPNPGSIELDRPNPTSHLAFGRGQHFCPGSALGRRHAQIGIEALLKK
MPGVDLAVPIDQLVWRTRFQRRIPERLPVLW
>CYP121A1_Mycobacterium tuberculosis BTB08-072 2603325567

MTATVLLEVPFSARGDRIPDAVAELRTREPIRKVRTITGAEAWLVSSYALCTQVLEDRRFSMKETAAAGAPRL
NALTVPPEVVNNMGNIADAGLRKAVMKAITPKAPGLEQFLRDTANSLLDNLITEGAPADLRNDFADPLATALH
CKVLGIPQEDGPKLFRSLSIAFMSSADPIPAAKINWDRDIEYMAGILENPNITTGLMGELSRLRKDPAYSHVS
DELFATIGVTFFGAGVISTGSFLTTALISLIQRPQLRNLLHEKPELIPAGVEELLRINLSFADGLPRLATADI
QVGDVLVRKGELVLVLLEGANFDPEHFPNPGSIELDRPNPTSHLAFGRGQHFCPGSALGRRHAQIGIEALLKK
MPGVDLAVPIDQLVWRTRFQRRIPERLPVLW
>CYP121A1_Mycobacterium tuberculosis BTB08-283 2604565750
MTATVLLEVPFSARGDRIPDAVAELRTREPIRKVRTITGAEAWLVSSYALCTQVLEDRRFSMKETAAAGAPRL
NALTVPPEVVNNMGNIADAGLRKAVMKAITPKAPGLEQFLRDTANSLLDNLITEGAPADLRNDFADPLATALH
CKVLGIPQEDGPKLFRSLSIAFMSSADPIPAAKINWDRDIEYMAGILENPNITTGLMGELSRLRKDPAYSHVS
DELFATIGVTFFGAGVISTGSFLTTALISLIQRPQLRNLLHEKPELIPAGVEELLRINLSFADGLPRLATADI
QVGDVLVRKGELVLVLLEGANFDPEHFPNPGSIELDRPNPTSHLAFGRGQHFCPGSALGRRHAQIGIEALLKK
MPGVDLAVPIDQLVWRTRFQRRIPERLPVLW
>CYP121A1_Mycobacterium tuberculosis BTB08-288 2604211884
MTATVLLEVPFSARGDRIPDAVAELRTREPIRKVRTITGAEAWLVSSYALCTQVLEDRRFSMKETAAAGAPRL
NALTVPPEVVNNMGNIADAGLRKAVMKAITPKAPGLEQFLRDTANSLLDNLITEGAPADLRNDFADPLATALH
CKVLGIPQEDGPKLFRSLSIAFMSSADPIPAAKINWDRDIEYMAGILENPNITTGLMGELSRLRKDPAYSHVS
DELFATIGVTFFGAGVISTGSFLTTALISLIQRPQLRNLLHEKPELIPAGVEELLRINLSFADGLPRLATADI
QVGDVLVRKGELVLVLLEGANFDPEHFPNPGSIELDRPNPTSHLAFGRGQHFCPGSALGRRHAQIGIEALLKK
MPGVDLAVPIDQLVWRTRFQRRIPERLPVLW
>CYP121A1_Mycobacterium tuberculosis BTB08-356 2605637098
MTATVLLEVPFSARGDRIPDAVAELRTREPIRKVRTITGAEAWLVSSYALCTQVLEDRRFSMKETAAAGAPRL
NALTVPPEVVNNMGNIADAGLRKAVMKAITPKAPGLEQFLRDTANSLLDNLITEGAPADLRNDFADPLATALH
CKVLGIPQEDGPKLFRSLSIAFMSSADPIPAAKINWDRDIEYMAGILENPNITTGLMGELSRLRKDPAYSHVS
DELFATIGVTFFGAGVISTGSFLTTALISLIQRPQLRNLLHEKPELIPAGVEELLRINLSFADGLPRLATADI
QVGDVLVRKGELVLVLLEGANFDPEHFPNPGSIELDRPNPTSHLAFGRGQHFCPGSALGRRHAQIGIEALLKK
MPGVDLAVPIDQLVWRTRFQRRIPERLPVLW
>CYP121A1_Mycobacterium tuberculosis BTB08-309 2604939192
MTATVLLEVPFSARGDRIPDAVAELRTREPIRKVRTITGAEAWLVSSYALCTQVLEDRRFSMKETAAAGAPRL
NALTVPPEVVNNMGNIADAGLRKAVMKAITPKAPGLEQFLRDTANSLLDNLITEGAPADLRNDFADPLATALH
CKVLGIPQEDGPKLFRSLSIAFMSSADPIPAAKINWDRDIEYMAGILENPNITTGLMGELSRLRKDPAYSHVS
DELFATIGVTFFGAGVISTGSFLTTALISLIQRPQLRNLLHEKPELIPAGVEELLRINLSFADGLPRLATADI
QVGDVLVRKGELVLVLLEGANFDPEHFPNPGSIELDRPNPTSHLAFGRGQHFCPGSALGRRHAQIGIEALLKK
MPGVDLAVPIDQLVWRTRFQRRIPERLPVLW
>CYP121A1_Mycobacterium tuberculosis BTB08-362 2575157701
MTATVLLEVPFSARGDRIPDAVAELRTREPIRKVRTITGAEAWLVSSYALCTQVLEDRRFSMKETAAAGAPRL
NALTVPPEVVNNMGNIADAGLRKAVMKAITPKAPGLEQFLRDTANSLLDNLITEGAPADLRNDFADPLATALH
CKVLGIPQEDGPKLFRSLSIAFMSSADPIPAAKINWDRDIEYMAGILENPNITTGLMGELSRLRKDPAYSHVS
DELFATIGVTFFGAGVISTGSFLTTALISLIQRPQLRNLLHEKPELIPAGVEELLRINLSFADGLPRLATADI
QVGDVLVRKGELVLVLLEGANFDPEHFPNPGSIELDRPNPTSHLAFGRGQHFCPGSALGRRHAQIGIEALLKK
MPGVDLAVPIDQLVWRTRFQRRIPERLPVLW
>CYP121A1_Mycobacterium tuberculosis BTB08-394 2603329771
MTATVLLEVPFSARGDRIPDAVAELRTREPIRKVRTITGAEAWLVSSYALCTQVLEDRRFSMKETAAAGAPRL
NALTVPPEVVNNMGNIADAGLRKAVMKAITPKAPGLEQFLRDTANSLLDNLITEGAPADLRNDFADPLATALH
CKVLGIPQEDGPKLFRSLSIAFMSSADPIPAAKINWDRDIEYMAGILENPNITTGLMGELSRLRKDPAYSHVS
DELFATIGVTFFGAGVISTGSFLTTALISLIQRPQLRNLLHEKPELIPAGVEELLRINLSFADGLPRLATADI
QVGDVLVRKGELVLVLLEGANFDPEHFPNPGSIELDRPNPTSHLAFGRGQHFCPGSALGRRHAQIGIEALLKK
MPGVDLAVPIDQLVWRTRFQRRIPERLPVLW
>CYP121A1_Mycobacterium tuberculosis BTB08-395 2603333895
MTATVLLEVPFSARGDRIPDAVAELRTREPIRKVRTITGAEAWLVSSYALCTQVLEDRRFSMKETAAAGAPRL
NALTVPPEVVNNMGNIADAGLRKAVMKAITPKAPGLEQFLRDTANSLLDNLITEGAPADLRNDFADPLATALH
CKVLGIPQEDGPKLFRSLSIAFMSSADPIPAAKINWDRDIEYMAGILENPNITTGLMGELSRLRKDPAYSHVS
DELFATIGVTFFGAGVISTGSFLTTALISLIQRPQLRNLLHEKPELIPAGVEELLRINLSFADGLPRLATADI
QVGDVLVRKGELVLVLLEGANFDPEHFPNPGSIELDRPNPTSHLAFGRGQHFCPGSALGRRHAQIGIEALLKK
MPGVDLAVPIDQLVWRTRFQRRIPERLPVLW
>CYP121A1_Mycobacterium tuberculosis BTB09-001 2604569785
MTATVLLEVPFSARGDRIPDAVAELRTREPIRKVRTITGAEAWLVSSYALCTQVLEDRRFSMKETAAAGAPRL
NALTVPPEVVNNMGNIADAGLRKAVMKAITPKAPGLEQFLRDTANSLLDNLITEGAPADLRNDFADPLATALH

CKVLGIPQEDGPKLFRSLSIAFMSSADPIPAAKINWDRDIEYMAGILENPNITTGLMGELSRLRKDPAYSHVS
DELFATIGVTFFGAGVISTGSFLTTALISLIQRPQLRNLLHEKPELIPAGVEELLRINLSFADGLPRLATADI
QVGDVLVRKGELVLVLLEGANFDPEHFPNPGSIELDRPNPTSHLAFGRGQHFCPGSALGRRHAQIGIEALLKK
MPGVDLAVPIDQLVWRTRFQRRIPERLPVLW
>CYP121A1_Mycobacterium tuberculosis BTB09-151 2603498210
MTATVLLEVPFSARGDRIPDAVAELRTREPIRKVRTITGAEAWLVSSYALCTQVLEDRRFSMKETAAAGAPRL
NALTVPPEVVNNMGNIADAGLRKAVMKAITPKAPGLEQFLRDTANSLLDNLITEGAPADLRNDFADPLATALH
CKVLGIPQEDGPKLFRSLSIAFMSSADPIPAAKINWDRDIEYMAGILENPNITTGLMGELSRLRKDPAYSHVS
DELFATIGVTFFGAGVISTGSFLTTALISLIQRPQLRNLLHEKPELIPAGVEELLRINLSFADGLPRLATADI
QVGDVLVRKGELVLVLLEGANFDPEHFPNPGSIELDRPNPTSHLAFGRGQHFCPGSALGRRHAQIGIEALLKK
MPGVDLAVPIDQLVWRTRFQRRIPERLPVLW
>CYP121A1_Mycobacterium tuberculosis BTB09-453 2604215925
MTATVLLEVPFSARGDRIPDAVAELRTREPIRKVRTITGAEAWLVSSYALCTQVLEDRRFSMKETAAAGAPRL
NALTVPPEVVNNMGNIADAGLRKAVMKAITPKAPGLEQFLRDTANSLLDNLITEGAPADLRNDFADPLATALH
CKVLGIPQEDGPKLFRSLSIAFMSSADPIPAAKINWDRDIEYMAGILENPNITTGLMGELSRLRKDPAYSHVS
DELFATIGVTFFGAGVISTGSFLTTALISLIQRPQLRNLLHEKPELIPAGVEELLRINLSFADGLPRLATADI
QVGDVLVRKGELVLVLLEGANFDPEHFPNPGSIELDRPNPTSHLAFGRGQHFCPGSALGRRHAQIGIEALLKK
MPGVDLAVPIDQLVWRTRFQRRIPERLPVLW
>CYP121A1_Mycobacterium tuberculosis BTB09-553 2603510549
MTATVLLEVPFSARGDRIPDAVAELRTREPIRKVRTITGAEAWLVSSYALCTQVLEDRRFSMKETAAAGAPRL
NALTVPPEVVNNMGNIADAGLRKAVMKAITPKAPGLEQFLRDTANSLLDNLITEGAPADLRNDFADPLATALH
CKVLGIPQEDGPKLFRSLSIAFMSSADPIPAAKINWDRDIEYMAGILENPNITTGLMGELSRLRKDPAYSHVS
DELFATIGVTFFGAGVISTGSFLTTALISLIQRPQLRNLLHEKPELIPAGVEELLRINLSFADGLPRLATADI
QVGDVLVRKGELVLVLLEGANFDPEHFPNPGSIELDRPNPTSHLAFGRGQHFCPGSALGRRHAQIGIEALLKK
MPGVDLAVPIDQLVWRTRFQRRIPERLPVLW
>CYP121A1_Mycobacterium tuberculosis BTB09-554 2605290693
MTATVLLEVPFSARGDRIPDAVAELRTREPIRKVRTITGAEAWLVSSYALCTQVLEDRRFSMKETAAAGAPRL
NALTVPPEVVNNMGNIADAGLRKAVMKAITPKAPGLEQFLRDTANSLLDNLITEGAPADLRNDFADPLATALH
CKVLGIPQEDGPKLFRSLSIAFMSSADPIPAAKINWDRDIEYMAGILENPNITTGLMGELSRLRKDPAYSHVS
DELFATIGVTFFGAGVISTGSFLTTALISLIQRPQLRNLLHEKPELIPAGVEELLRINLSFADGLPRLATADI
QVGDVLVRKGELVLVLLEGANFDPEHFPNPGSIELDRPNPTSHLAFGRGQHFCPGSALGRRHAQIGIEALLKK
MPGVDLAVPIDQLVWRTRFQRRIPERLPVLW
>CYP121A1_Mycobacterium tuberculosis BTB10-001 2605647286
MTATVLLEVPFSARGDRIPDAVAELRTREPIRKVRTITGAEAWLVSSYALCTQVLEDRRFSMKETAAAGAPRL
NALTVPPEVVNNMGNIADAGLRKAVMKAITPKAPGLEQFLRDTANSLLDNLITEGAPADLRNDFADPLATALH
CKVLGIPQEDGPKLFRSLSIAFMSSADPIPAAKINWDRDIEYMAGILENPNITTGLMGELSRLRKDPAYSHVS
DELFATIGVTFFGAGVISTGSFLTTALISLIQRPQLRNLLHEKPELIPAGVEELLRINLSFADGLPRLATADI
QVGDVLVRKGELVLVLLEGANFDPEHFPNPGSIELDRPNPTSHLAFGRGQHFCPGSALGRRHAQIGIEALLKK
MPGVDLAVPIDQLVWRTRFQRRIPERLPVLW
>CYP121A1_Mycobacterium tuberculosis BTB09-565 2578062971
MTATVLLEVPFSARGDRIPDAVAELRTREPIRKVRTITGAEAWLVSSYALCTQVLEDRRFSMKETAAAGAPRL
NALTVPPEVVNNMGNIADAGLRKAVMKAITPKAPGLEQFLRDTANSLLDNLITEGAPADLRNDFADPLATALH
CKVLGIPQEDGPKLFRSLSIAFMSSADPIPAAKINWDRDIEYMAGILENPNITTGLMGELSRLRKDPAYSHVS
DELFATIGVTFFGAGVISTGSFLTTALISLIQRPQLRNLLHEKPELIPAGVEELLRINLSFADGLPRLATADI
QVGDVLVRKGELVLVLLEGANFDPEHFPNPGSIELDRPNPTSHLAFGRGQHFCPGSALGRRHAQIGIEALLKK
MPGVDLAVPIDQLVWRTRFQRRIPERLPVLW
>CYP121A1_Mycobacterium tuberculosis BTB10-005 2603338004
MTATVLLEVPFSARGDRIPDAVAELRTREPIRKVRTITGAEAWLVSSYALCTQVLEDRRFSMKETAAAGAPRL
NALTVPPEVVNNMGNIADAGLRKAVMKAITPKAPGLEQFLRDTANSLLDNLITEGAPADLRNDFADPLATALH
CKVLGIPQEDGPKLFRSLSIAFMSSADPIPAAKINWDRDIEYMAGILENPNITTGLMGELSRLRKDPAYSHVS
DELFATIGVTFFGAGVISTGSFLTTALISLIQRPQLRNLLHEKPELIPAGVEELLRINLSFADGLPRLATADI
QVGDVLVRKGELVLVLLEGANFDPEHFPNPGSIELDRPNPTSHLAFGRGQHFCPGSALGRRHAQIGIEALLKK
MPGVDLAVPIDQLVWRTRFQRRIPERLPVLW
>CYP121A1_Mycobacterium tuberculosis BTB10-092 2605296404
MTATVLLEVPFSARGDRIPDAVAELRTREPIRKVRTITGAEAWLVSSYALCTQVLEDRRFSMKETAAAGAPRL
NALTVPPEVVNNMGNIADAGLRKAVMKAITPKAPGLEQFLRDTANSLLDNLITEGAPADLRNDFADPLATALH
CKVLGIPQEDGPKLFRSLSIAFMSSADPIPAAKINWDRDIEYMAGILENPNITTGLMGELSRLRKDPAYSHVS
DELFATIGVTFFGAGVISTGSFLTTALISLIQRPQLRNLLHEKPELIPAGVEELLRINLSFADGLPRLATADI

QVGDVLVRKGELVLVLLEGANFDPEHFPNPGSIELDRPNPTSHLAFGRGQHFCPGSALGRRHAQIGIEALLKK
MPGVDLAVPIDQLVWRTRFQRRIPERLPVLW
>CYP121A1_Mycobacterium tuberculosis BTB10-120 2605651390
MTATVLLEVPFSARGDRIPDAVAELRTREPIRKVRTITGAEAWLVSSYALCTQVLEDRRFSMKETAAAGAPRL
NALTVPPEVVNNMGNIADAGLRKAVMKAITPKAPGLEQFLRDTANSLLDNLITEGAPADLRNDFADPLATALH
CKVLGIPQEDGPKLFRSLSIAFMSSADPIPAAKINWDRDIEYMAGILENPNITTGLMGELSRLRKDPAYSHVS
DELFATIGVTFFGAGVISTGSFLTTALISLIQRPQLRNLLHEKPELIPAGVEELLRINLSFADGLPRLATADI
QVGDVLVRKGELVLVLLEGANFDPEHFPNPGSIELDRPNPTSHLAFGRGQHFCPGSALGRRHAQIGIEALLKK
MPGVDLAVPIDQLVWRTRFQRRIPERLPVLW
>CYP121A1_Mycobacterium tuberculosis BTB10-142 2603881618
MTATVLLEVPFSARGDRIPDAVAELRTREPIRKVRTITGAEAWLVSSYALCTQVLEDRRFSMKETAAAGAPRL
NALTVPPEVVNNMGNIADAGLRKAVMKAITPKAPGLEQFLRDTANSLLDNLITEGAPADLRNDFADPLATALH
CKVLGIPQEDGPKLFRSLSIAFMSSADPIPAAKINWDRDIEYMAGILENPNITTGLMGELSRLRKDPAYSHVS
DELFATIGVTFFGAGVISTGSFLTTALISLIQRPQLRNLLHEKPELIPAGVEELLRINLSFADGLPRLATADI
QVGDVLVRKGELVLVLLEGANFDPEHFPNPGSIELDRPNPTSHLAFGRGQHFCPGSALGRRHAQIGIEALLKK
MPGVDLAVPIDQLVWRTRFQRRIPERLPVLW
>CYP121A1_Mycobacterium tuberculosis BTB10-253 2604582217
MTATVLLEVPFSARGDRIPDAVAELRTREPIRKVRTITGAEAWLVSSYALCTQVLEDRRFSMKETAAAGAPRL
NALTVPPEVVNNMGNIADAGLRKAVMKAITPKAPGLEQFLRDTANSLLDNLITEGAPADLRNDFADPLATALH
CKVLGIPQEDGPKLFRSLSIAFMSSADPIPAAKINWDRDIEYMAGILENPNITTGLMGELSRLRKDPAYSHVS
DELFATIGVTFFGAGVISTGSFLTTALISLIQRPQLRNLLHEKPELIPAGVEELLRINLSFADGLPRLATADI
QVGDVLVRKGELVLVLLEGANFDPEHFPNPGSIELDRPNPTSHLAFGRGQHFCPGSALGRRHAQIGIEALLKK
MPGVDLAVPIDQLVWRTRFQRRIPERLPVLW
>CYP121A1_Mycobacterium tuberculosis BTB10-277 2604586359
MTATVLLEVPFSARGDRIPDAVAELRTREPIRKVRTITGAEAWLVSSYALCTQVLEDRRFSMKETAAAGAPRL
NALTVPPEVVNNMGNIADAGLRKAVMKAITPKAPGLEQFLRDTANSLLDNLITEGAPADLRNDFADPLATALH
CKVLGIPQEDGPKLFRSLSIAFMSSADPIPAAKINWDRDIEYMAGILENPNITTGLMGELSRLRKDPAYSHVS
DELFATIGVTFFGAGVISTGSFLTTALISLIQRPQLRNLLHEKPELIPAGVEELLRINLSFADGLPRLATADI
QVGDVLVRKGELVLVLLEGANFDPEHFPNPGSIELDRPNPTSHLAFGRGQHFCPGSALGRRHAQIGIEALLKK
MPGVDLAVPIDQLVWRTRFQRRIPERLPVLW
>CYP121A1_Mycobacterium tuberculosis BTB10-295 2603341813
MTATVLLEVPFSARGDRIPDAVAELRTREPIRKVRTITGAEAWLVSSYALCTQVLEDRRFSMKETAAAGAPRL
NALTVPPEVVNNMGNIADAGLRKAVMKAITPKAPGLEQFLRDTANSLLDNLITEGAPADLRNDFADPLATALH
CKVLGIPQEDGPKLFRSLSIAFMSSADPIPAAKINWDRDIEYMAGILENPNITTGLMGELSRLRKDPAYSHVS
DELFATIGVTFFGAGVISTGSFLTTALISLIQRPQLRNLLHEKPELIPAGVEELLRINLSFADGLPRLATADI
QVGDVLVRKGELVLVLLEGANFDPEHFPNPGSIELDRPNPTSHLAFGRGQHFCPGSALGRRHAQIGIEALLKK
MPGVDLAVPIDQLVWRTRFQRRIPERLPVLW
>CYP121A1_Mycobacterium tuberculosis BTB10-305 2604965106
MTATVLLEVPFSARGDRIPDAVAELRTREPIRKVRTITGAEAWLVSSYALCTQVLEDRRFSMKETAAAGAPRL
NALTVPPEVVNNMGNIADAGLRKAVMKAITPKAPGLEQFLRDTANSLLDNLITEGAPADLRNDFADPLATALH
CKVLGIPQEDGPKLFRSLSIAFMSSADPIPAAKINWDRDIEYMAGILENPNITTGLMGELSRLRKDPAYSHVS
DELFATIGVTFFGAGVISTGSFLTTALISLIQRPQLRNLLHEKPELIPAGVEELLRINLSFADGLPRLATADI
QVGDVLVRKGELVLVLLEGANFDPEHFPNPGSIELDRPNPTSHLAFGRGQHFCPGSALGRRHAQIGIEALLKK
MPGVDLAVPIDQLVWRTRFQRRIPERLPVLW
>CYP121A1_Mycobacterium tuberculosis BTB10-308 2576294197
MTATVLLEVPFSARGDRIPDAVAELRTREPIRKVRTITGAEAWLVSSYALCTQVLEDRRFSMKETAAAGAPRL
NALTVPPEVVNNMGNIADAGLRKAVMKAITPKAPGLEQFLRDTANSLLDNLITEGAPADLRNDFADPLATALH
CKVLGIPQEDGPKLFRSLSIAFMSSADPIPAAKINWDRDIEYMAGILENPNITTGLMGELSRLRKDPAYSHVS
DELFATIGVTFFGAGVISTGSFLTTALISLIQRPQLRNLLHEKPELIPAGVEELLRINLSFADGLPRLATADI
QVGDVLVRKGELVLVLLEGANFDPEHFPNPGSIELDRPNPTSHLAFGRGQHFCPGSALGRRHAQIGIEALLKK
MPGVDLAVPIDQLVWRTRFQRRIPERLPVLW
>CYP121A1_Mycobacterium tuberculosis BTB10-367 2604590474
MTATVLLEVPFSARGDRIPDAVAELRTREPIRKVRTITGAEAWLVSSYALCTQVLEDRRFSMKETAAAGAPRL
NALTVPPEVVNNMGNIADAGLRKAVMKAITPKAPGLEQFLRDTANSLLDNLITEGAPADLRNDFADPLATALH
CKVLGIPQEDGPKLFRSLSIAFMSSADPIPAAKINWDRDIEYMAGILENPNITTGLMGELSRLRKDPAYSHVS
DELFATIGVTFFGAGVISTGSFLTTALISLIQRPQLRNLLHEKPELIPAGVEELLRINLSFADGLPRLATADI
QVGDVLVRKGELVLVLLEGANFDPEHFPNPGSIELDRPNPTSHLAFGRGQHFCPGSALGRRHAQIGIEALLKK
MPGVDLAVPIDQLVWRTRFQRRIPERLPVLW

>CYP121A1_Mycobacterium tuberculosis BTB10-406 2605304618
MTATVLLEVPFSARGDRIPDAVAELRTREPIRKVRTITGAEAWLVSSYALCTQVLEDRRFSMKETAAAGAPRL
NALTVPPEVVNNMGNIADAGLRKAVMKAITPKAPGLEQFLRDTANSLLDNLITEGAPADLRNDFADPLATALH
CKVLGIPQEDGPKLFRSLSIAFMSSADPIPAAKINWDRDIEYMAGILENPNITTGLMGELSRLRKDPAYSHVS
DELFATIGVTFFGAGVISTGSFLTTALISLIQRPQLRNLLHEKPELIPAGVEELLRINLSFADGLPRLATADI
QVGDVLVRKGELVLVLLEGANFDPEHFPNPGSIELDRPNPTSHLAFGRGQHFCPGSALGRRHAQIGIEALLKK
MPGVDLAVPIDQLVWRTRFQRRIPERLPVLW
>CYP121A1_Mycobacterium tuberculosis BTB10-422 2605307212
MTATVLLEVPFSARGDRIPDAVAELRTREPIRKVRTITGAEAWLVSSYALCTQVLEDRRFSMKETAAAGAPRL
NALTVPPEVVNNMGNIADAGLRKAVMKAITPKAPGLEQFLRDTANSLLDNLITEGAPADLRNDFADPLATALH
CKVLGIPQEDGPKLFRSLSIAFMSSADPIPAAKINWDRDIEYMAGILENPNITTGLMGELSRLRKDPAYSHVS
DELFATIGVTFFGAGVISTGSFLTTALISLIQRPQLRNLLHEKPELIPAGVEELLRINLSFADGLPRLATADI
QVGDVLVRKGELVLVLLEGANFDPEHFPNPGSIELDRPNPTSHLAFGRGQHFCPGSALGRRHAQIGIEALLKK
MPGVDLAVPIDQLVWRTRFQRRIPERLPVLW
>CYP121A1_Mycobacterium tuberculosis BTB10-426 2603886023
MTATVLLEVPFSARGDRIPDAVAELRTREPIRKVRTITGAEAWLVSSYALCTQVLEDRRFSMKETAAAGAPRL
NALTVPPEVVNNMGNIADAGLRKAVMKAITPKAPGLEQFLRDTANSLLDNLITEGAPADLRNDFADPLATALH
CKVLGIPQEDGPKLFRSLSIAFMSSADPIPAAKINWDRDIEYMAGILENPNITTGLMGELSRLRKDPAYSHVS
DELFATIGVTFFGAGVISTGSFLTTALISLIQRPQLRNLLHEKPELIPAGVEELLRINLSFADGLPRLATADI
QVGDVLVRKGELVLVLLEGANFDPEHFPNPGSIELDRPNPTSHLAFGRGQHFCPGSALGRRHAQIGIEALLKK
MPGVDLAVPIDQLVWRTRFQRRIPERLPVLW
>CYP121A1_Mycobacterium tuberculosis BTB10-487 2576967964
MTATVLLEVPFSARGDRIPDAVAELRTREPIRKVRTITGAEAWLVSSYALCTQVLEDRRFSMKETAAAGAPRL
NALTVPPEVVNNMGNIADAGLRKAVMKAITPKAPGLEQFLRDTANSLLDNLITEGAPADLRNDFADPLATALH
CKVLGIPQEDGPKLFRSLSIAFMSSADPIPAAKINWDRDIEYMAGILENPNITTGLMGELSRLRKDPAYSHVS
DELFATIGVTFFGAGVISTGSFLTTALISLIQRPQLRNLLHEKPELIPAGVEELLRINLSFADGLPRLATADI
QVGDVLVRKGELVLVLLEGANFDPEHFPNPGSIELDRPNPTSHLAFGRGQHFCPGSALGRRHAQIGIEALLKK
MPGVDLAVPIDQLVWRTRFQRRIPERLPVLW
>CYP121A1_Mycobacterium tuberculosis BTB11-001 2575521366
MTATVLLEVPFSARGDRIPDAVAELRTREPIRKVRTITGAEAWLVSSYALCTQVLEDRRFSMKETAAAGAPRL
NALTVPPEVVNNMGNIADAGLRKAVMKAITPKAPGLEQFLRDTANSLLDNLITEGAPADLRNDFADPLATALH
CKVLGIPQEDGPKLFRSLSIAFMSSADPIPAAKINWDRDIEYMAGILENPNITTGLMGELSRLRKDPAYSHVS
DELFATIGVTFFGAGVISTGSFLTTALISLIQRPQLRNLLHEKPELIPAGVEELLRINLSFADGLPRLATADI
QVGDVLVRKGELVLVLLEGANFDPEHFPNPGSIELDRPNPTSHLAFGRGQHFCPGSALGRRHAQIGIEALLKK
MPGVDLAVPIDQLVWRTRFQRRIPERLPVLW
>CYP121A1_Mycobacterium tuberculosis BTB11-047 2604221670
MTATVLLEVPFSARGDRIPDAVAELRTREPIRKVRTITGAEAWLVSSYALCTQVLEDRRFSMKETAAAGAPRL
NALTVPPEVVNNMGNIADAGLRKAVMKAITPKAPGLEQFLRDTANSLLDNLITEGAPADLRNDFADPLATALH
CKVLGIPQEDGPKLFRSLSIAFMSSADPIPAAKINWDRDIEYMAGILENPNITTGLMGELSRLRKDPAYSHVS
DELFATIGVTFFGAGVISTGSFLTTALISLIQRPQLRNLLHEKPELIPAGVEELLRINLSFADGLPRLATADI
QVGDVLVRKGELVLVLLEGANFDPEHFPNPGSIELDRPNPTSHLAFGRGQHFCPGSALGRRHAQIGIEALLKK
MPGVDLAVPIDQLVWRTRFQRRIPERLPVLW
>CYP121A1_Mycobacterium tuberculosis BTB11-112 2604225923
MTATVLLEVPFSARGDRIPDAVAELRTREPIRKVRTITGAEAWLVSSYALCTQVLEDRRFSMKETAAAGAPRL
NALTVPPEVVNNMGNIADAGLRKAVMKAITPKAPGLEQFLRDTANSLLDNLITEGAPADLRNDFADPLATALH
CKVLGIPQEDGPKLFRSLSIAFMSSADPIPAAKINWDRDIEYMAGILENPNITTGLMGELSRLRKDPAYSHVS
DELFATIGVTFFGAGVISTGSFLTTALISLIQRPQLRNLLHEKPELIPAGVEELLRINLSFADGLPRLATADI
QVGDVLVRKGELVLVLLEGANFDPEHFPNPGSIELDRPNPTSHLAFGRGQHFCPGSALGRRHAQIGIEALLKK
MPGVDLAVPIDQLVWRTRFQRRIPERLPVLW
>CYP121A1_Mycobacterium tuberculosis BTB11-133 2604228647
MTATVLLEVPFSARGDRIPDAVAELRTREPIRKVRTITGAEAWLVSSYALCTQVLEDRRFSMKETAAAGAPRL
NALTVPPEVVNNMGNIADAGLRKAVMKAITPKAPGLEQFLRDTANSLLDNLITEGAPADLRNDFADPLATALH
CKVLGIPQEDGPKLFRSLSIAFMSSADPIPAAKINWDRDIEYMAGILENPNITTGLMGELSRLRKDPAYSHVS
DELFATIGVTFFGAGVISTGSFLTTALISLIQRPQLRNLLHEKPELIPAGVEELLRINLSFADGLPRLATADI
QVGDVLVRKGELVLVLLEGANFDPEHFPNPGSIELDRPNPTSHLAFGRGQHFCPGSALGRRHAQIGIEALLKK
MPGVDLAVPIDQLVWRTRFQRRIPERLPVLW
>CYP121A1_Mycobacterium tuberculosis BTB11-160 2603890145

MTATVLLEVPFSARGDRIPDAVAELRTREPIRKVRTITGAEAWLVSSYALCTQVLEDRRFSMKETAAAGAPRL
NALTVPPEVVNNMGNIADAGLRKAVMKAITPKAPGLEQFLRDTANSLLDNLITEGAPADLRNDFADPLATALH
CKVLGIPQEDGPKLFRSLSIAFMSSADPIPAAKINWDRDIEYMAGILENPNITTGLMGELSRLRKDPAYSHVS
DELFATIGVTFFGAGVISTGSFLTTALISLIQRPQLRNLLHEKPELIPAGVEELLRINLSFADGLPRLATADI
QVGDVLVRKGELVLVLLEGANFDPEHFPNPGSIELDRPNPTSHLAFGRGQHFCPGSALGRRHAQIGIEALLKK
MPGVDLAVPIDQLVWRTRFQRRIPERLPVLW
>CYP121A1_Mycobacterium tuberculosis BTB11-192 2605655212
MTATVLLEVPFSARGDRIPDAVAELRTREPIRKVRTITGAEAWLVSSYALCTQVLEDRRFSMKETAAAGAPRL
NALTVPPEVVNNMGNIADAGLRKAVMKAITPKAPGLEQFLRDTANSLLDNLITEGAPADLRNDFADPLATALH
CKVLGIPQEDGPKLFRSLSIAFMSSADPIPAAKINWDRDIEYMAGILENPNITTGLMGELSRLRKDPAYSHVS
DELFATIGVTFFGAGVISTGSFLTTALISLIQRPQLRNLLHEKPELIPAGVEELLRINLSFADGLPRLATADI
QVGDVLVRKGELVLVLLEGANFDPEHFPNPGSIELDRPNPTSHLAFGRGQHFCPGSALGRRHAQIGIEALLKK
MPGVDLAVPIDQLVWRTRFQRRIPERLPVLW
>CYP121A1_Mycobacterium tuberculosis BTB11-195 2604234277
MTATVLLEVPFSARGDRIPDAVAELRTREPIRKVRTITGAEAWLVSSYALCTQVLEDRRFSMKETAAAGAPRL
NALTVPPEVVNNMGNIADAGLRKAVMKAITPKAPGLEQFLRDTANSLLDNLITEGAPADLRNDFADPLATALH
CKVLGIPQEDGPKLFRSLSIAFMSSADPIPAAKINWDRDIEYMAGILENPNITTGLMGELSRLRKDPAYSHVS
DELFATIGVTFFGAGVISTGSFLTTALISLIQRPQLRNLLHEKPELIPAGVEELLRINLSFADGLPRLATADI
QVGDVLVRKGELVLVLLEGANFDPEHFPNPGSIELDRPNPTSHLAFGRGQHFCPGSALGRRHAQIGIEALLKK
MPGVDLAVPIDQLVWRTRFQRRIPERLPVLW
>CYP121A1_Mycobacterium tuberculosis BTB11-207 2605659645
MTATVLLEVPFSARGDRIPDAVAELRTREPIRKVRTITGAEAWLVSSYALCTQVLEDRRFSMKETAAAGAPRL
NALTVPPEVVNNMGNIADAGLRKAVMKAITPKAPGLEQFLRDTANSLLDNLITEGAPADLRNDFADPLATALH
CKVLGIPQEDGPKLFRSLSIAFMSSADPIPAAKINWDRDIEYMAGILENPNITTGLMGELSRLRKDPAYSHVS
DELFATIGVTFFGAGVISTGSFLTTALISLIQRPQLRNLLHEKPELIPAGVEELLRINLSFADGLPRLATADI
QVGDVLVRKGELVLVLLEGANFDPEHFPNPGSIELDRPNPTSHLAFGRGQHFCPGSALGRRHAQIGIEALLKK
MPGVDLAVPIDQLVWRTRFQRRIPERLPVLW
>CYP121A1_Mycobacterium tuberculosis BTB11-210 2604594613
MTATVLLEVPFSARGDRIPDAVAELRTREPIRKVRTITGAEAWLVSSYALCTQVLEDRRFSMKETAAAGAPRL
NALTVPPEVVNNMGNIADAGLRKAVMKAITPKAPGLEQFLRDTANSLLDNLITEGAPADLRNDFADPLATALH
CKVLGIPQEDGPKLFRSLSIAFMSSADPIPAAKINWDRDIEYMAGILENPNITTGLMGELSRLRKDPAYSHVS
DELFATIGVTFFGAGVISTGSFLTTALISLIQRPQLRNLLHEKPELIPAGVEELLRINLSFADGLPRLATADI
QVGDVLVRKGELVLVLLEGANFDPEHFPNPGSIELDRPNPTSHLAFGRGQHFCPGSALGRRHAQIGIEALLKK
MPGVDLAVPIDQLVWRTRFQRRIPERLPVLW
>CYP121A1_Mycobacterium tuberculosis BTB11-214 2576718237
MTATVLLEVPFSARGDRIPDAVAELRTREPIRKVRTITGAEAWLVSSYALCTQVLEDRRFSMKETAAAGAPRL
NALTVPPEVVNNMGNIADAGLRKAVMKAITPKAPGLEQFLRDTANSLLDNLITEGAPADLRNDFADPLATALH
CKVLGIPQEDGPKLFRSLSIAFMSSADPIPAAKINWDRDIEYMAGILENPNITTGLMGELSRLRKDPAYSHVS
DELFATIGVTFFGAGVISTGSFLTTALISLIQRPQLRNLLHEKPELIPAGVEELLRINLSFADGLPRLATADI
QVGDVLVRKGELVLVLLEGANFDPEHFPNPGSIELDRPNPTSHLAFGRGQHFCPGSALGRRHAQIGIEALLKK
MPGVDLAVPIDQLVWRTRFQRRIPERLPVLW
>CYP121A1_Mycobacterium tuberculosis BTB11-236 2578171631
MTATVLLEVPFSARGDRIPDAVAELRTREPIRKVRTITGAEAWLVSSYALCTQVLEDRRFSMKETAAAGAPRL
NALTVPPEVVNNMGNIADAGLRKAVMKAITPKAPGLEQFLRDTANSLLDNLITEGAPADLRNDFADPLATALH
CKVLGIPQEDGPKLFRSLSIAFMSSADPIPAAKINWDRDIEYMAGILENPNITTGLMGELSRLRKDPAYSHVS
DELFATIGVTFFGAGVISTGSFLTTALISLIQRPQLRNLLHEKPELIPAGVEELLRINLSFADGLPRLATADI
QVGDVLVRKGELVLVLLEGANFDPEHFPNPGSIELDRPNPTSHLAFGRGQHFCPGSALGRRHAQIGIEALLKK
MPGVDLAVPIDQLVWRTRFQRRIPERLPVLW
>CYP121A1_Mycobacterium tuberculosis BTB11-250 2603894233
MTATVLLEVPFSARGDRIPDAVAELRTREPIRKVRTITGAEAWLVSSYALCTQVLEDRRFSMKETAAAGAPRL
NALTVPPEVVNNMGNIADAGLRKAVMKAITPKAPGLEQFLRDTANSLLDNLITEGAPADLRNDFADPLATALH
CKVLGIPQEDGPKLFRSLSIAFMSSADPIPAAKINWDRDIEYMAGILENPNITTGLMGELSRLRKDPAYSHVS
DELFATIGVTFFGAGVISTGSFLTTALISLIQRPQLRNLLHEKPELIPAGVEELLRINLSFADGLPRLATADI
QVGDVLVRKGELVLVLLEGANFDPEHFPNPGSIELDRPNPTSHLAFGRGQHFCPGSALGRRHAQIGIEALLKK
MPGVDLAVPIDQLVWRTRFQRRIPERLPVLW
>CYP121A1_Mycobacterium tuberculosis BTB11-343 2575648517
MTATVLLEVPFSARGDRIPDAVAELRTREPIRKVRTITGAEAWLVSSYALCTQVLEDRRFSMKETAAAGAPRL
NALTVPPEVVNNMGNIADAGLRKAVMKAITPKAPGLEQFLRDTANSLLDNLITEGAPADLRNDFADPLATALH

CKVLGIPQEDGPKLFRSLSIAFMSSADPIPAAKINWDRDIEYMAGILENPNITTGLMGELSRLRKDPAYSHVS
DELFATIGVTFFGAGVISTGSFLTTALISLIQRPQLRNLLHEKPELIPAGVEELLRINLSFADGLPRLATADI
QVGDVLVRKGELVLVLLEGANFDPEHFPNPGSIELDRPNPTSHLAFGRGQHFCPGSALGRRHAQIGIEALLKK
MPGVDLAVPIDQLVWRTRFQRRIPERLPVLW
>CYP121A1_Mycobacterium tuberculosis BTB12-046 2604602529
MTATVLLEVPFSARGDRIPDAVAELRTREPIRKVRTITGAEAWLVSSYALCTQVLEDRRFSMKETAAAGAPRL
NALTVPPEVVNNMGNIADAGLRKAVMKAITPKAPGLEQFLRDTANSLLDNLITEGAPADLRNDFADPLATALH
CKVLGIPQEDGPKLFRSLSIAFMSSADPIPAAKINWDRDIEYMAGILENPNITTGLMGELSRLRKDPAYSHVS
DELFATIGVTFFGAGVISTGSFLTTALISLIQRPQLRNLLHEKPELIPAGVEELLRINLSFADGLPRLATADI
QVGDVLVRKGELVLVLLEGANFDPEHFPNPGSIELDRPNPTSHLAFGRGQHFCPGSALGRRHAQIGIEALLKK
MPGVDLAVPIDQLVWRTRFQRRIPERLPVLW
>CYP121A1_Mycobacterium tuberculosis BTB12-054 2603898333
MTATVLLEVPFSARGDRIPDAVAELRTREPIRKVRTITGAEAWLVSSYALCTQVLEDRRFSMKETAAAGAPRL
NALTVPPEVVNNMGNIADAGLRKAVMKAITPKAPGLEQFLRDTANSLLDNLITEGAPADLRNDFADPLATALH
CKVLGIPQEDGPKLFRSLSIAFMSSADPIPAAKINWDRDIEYMAGILENPNITTGLMGELSRLRKDPAYSHVS
DELFATIGVTFFGAGVISTGSFLTTALISLIQRPQLRNLLHEKPELIPAGVEELLRINLSFADGLPRLATADI
QVGDVLVRKGELVLVLLEGANFDPEHFPNPGSIELDRPNPTSHLAFGRGQHFCPGSALGRRHAQIGIEALLKK
MPGVDLAVPIDQLVWRTRFQRRIPERLPVLW
>CYP121A1_Mycobacterium tuberculosis BTB12-162 2604608199
MTATVLLEVPFSARGDRIPDAVAELRTREPIRKVRTITGAEAWLVSSYALCTQVLEDRRFSMKETAAAGAPRL
NALTVPPEVVNNMGNIADAGLRKAVMKAITPKAPGLEQFLRDTANSLLDNLITEGAPADLRNDFADPLATALH
CKVLGIPQEDGPKLFRSLSIAFMSSADPIPAAKINWDRDIEYMAGILENPNITTGLMGELSRLRKDPAYSHVS
DELFATIGVTFFGAGVISTGSFLTTALISLIQRPQLRNLLHEKPELIPAGVEELLRINLSFADGLPRLATADI
QVGDVLVRKGELVLVLLEGANFDPEHFPNPGSIELDRPNPTSHLAFGRGQHFCPGSALGRRHAQIGIEALLKK
MPGVDLAVPIDQLVWRTRFQRRIPERLPVLW
>CYP121A1_Mycobacterium tuberculosis BTB12-206 2584891311
MTATVLLEVPFSARGDRIPDAVAELRTREPIRKVRTITGAEAWLVSSYALCTQVLEDRRFSMKETAAAGAPRL
NALTVPPEVVNNMGNIADAGLRKAVMKAITPKAPGLEQFLRDTANSLLDNLITEGAPADLRNDFADPLATALH
CKVLGIPQEDGPKLFRSLSIAFMSSADPIPAAKINWDRDIEYMAGILENPNITTGLMGELSRLRKDPAYSHVS
DELFATIGVTFFGAGVISTGSFLTTALISLIQRPQLRNLLHEKPELIPAGVEELLRINLSFADGLPRLATADI
QVGDVLVRKGELVLVLLEGANFDPEHFPNPGSIELDRPNPTSHLAFGRGQHFCPGSALGRRHAQIGIEALLKK
MPGVDLAVPIDQLVWRTRFQRRIPERLPVLW
>CYP121A1_Mycobacterium tuberculosis BTB12-294 2605668362
MTATVLLEVPFSARGDRIPDAVAELRTREPIRKVRTITGAEAWLVSSYALCTQVLEDRRFSMKETAAAGAPRL
NALTVPPEVVNNMGNIADAGLRKAVMKAITPKAPGLEQFLRDTANSLLDNLITEGAPADLRNDFADPLATALH
CKVLGIPQEDGPKLFRSLSIAFMSSADPIPAAKINWDRDIEYMAGILENPNITTGLMGELSRLRKDPAYSHVS
DELFATIGVTFFGAGVISTGSFLTTALISLIQRPQLRNLLHEKPELIPAGVEELLRINLSFADGLPRLATADI
QVGDVLVRKGELVLVLLEGANFDPEHFPNPGSIELDRPNPTSHLAFGRGQHFCPGSALGRRHAQIGIEALLKK
MPGVDLAVPIDQLVWRTRFQRRIPERLPVLW
>CYP121A1_Mycobacterium tuberculosis BTB12-304 2603177367
MTATVLLEVPFSARGDRIPDAVAELRTREPIRKVRTITGAEAWLVSSYALCTQVLEDRRFSMKETAAAGAPRL
NALTVPPEVVNNMGNIADAGLRKAVMKAITPKAPGLEQFLRDTANSLLDNLITEGAPADLRNDFADPLATALH
CKVLGIPQEDGPKLFRSLSIAFMSSADPIPAAKINWDRDIEYMAGILENPNITTGLMGELSRLRKDPAYSHVS
DELFATIGVTFFGAGVISTGSFLTTALISLIQRPQLRNLLHEKPELIPAGVEELLRINLSFADGLPRLATADI
QVGDVLVRKGELVLVLLEGANFDPEHFPNPGSIELDRPNPTSHLAFGRGQHFCPGSALGRRHAQIGIEALLKK
MPGVDLAVPIDQLVWRTRFQRRIPERLPVLW
>CYP121A1_Mycobacterium tuberculosis BTB12-313 2603358559
MTATVLLEVPFSARGDRIPDAVAELRTREPIRKVRTITGAEAWLVSSYALCTQVLEDRRFSMKETAAAGAPRL
NALTVPPEVVNNMGNIADAGLRKAVMKAITPKAPGLEQFLRDTANSLLDNLITEGAPADLRNDFADPLATALH
CKVLGIPQEDGPKLFRSLSIAFMSSADPIPAAKINWDRDIEYMAGILENPNITTGLMGELSRLRKDPAYSHVS
DELFATIGVTFFGAGVISTGSFLTTALISLIQRPQLRNLLHEKPELIPAGVEELLRINLSFADGLPRLATADI
QVGDVLVRKGELVLVLLEGANFDPEHFPNPGSIELDRPNPTSHLAFGRGQHFCPGSALGRRHAQIGIEALLKK
MPGVDLAVPIDQLVWRTRFQRRIPERLPVLW
>CYP121A1_Mycobacterium tuberculosis BTB12-315 2604969506
MTATVLLEVPFSARGDRIPDAVAELRTREPIRKVRTITGAEAWLVSSYALCTQVLEDRRFSMKETAAAGAPRL
NALTVPPEVVNNMGNIADAGLRKAVMKAITPKAPGLEQFLRDTANSLLDNLITEGAPADLRNDFADPLATALH
CKVLGIPQEDGPKLFRSLSIAFMSSADPIPAAKINWDRDIEYMAGILENPNITTGLMGELSRLRKDPAYSHVS
DELFATIGVTFFGAGVISTGSFLTTALISLIQRPQLRNLLHEKPELIPAGVEELLRINLSFADGLPRLATADI

QVGDVLVRKGELVLVLLEGANFDPEHFPNPGSIELDRPNPTSHLAFGRGQHFCPGSALGRRHAQIGIEALLKK
MPGVDLAVPIDQLVWRTRFQRRIPERLPVLW
>CYP121A1_Mycobacterium tuberculosis BTB12-400 2576683422
MTATVLLEVPFSARGDRIPDAVAELRTREPIRKVRTITGAEAWLVSSYALCTQVLEDRRFSMKETAAAGAPRL
NALTVPPEVVNNMGNIADAGLRKAVMKAITPKAPGLEQFLRDTANSLLDNLITEGAPADLRNDFADPLATALH
CKVLGIPQEDGPKLFRSLSIAFMSSADPIPAAKINWDRDIEYMAGILENPNITTGLMGELSRLRKDPAYSHVS
DELFATIGVTFFGAGVISTGSFLTTALISLIQRPQLRNLLHEKPELIPAGVEELLRINLSFADGLPRLATADI
QVGDVLVRKGELVLVLLEGANFDPEHFPNPGSIELDRPNPTSHLAFGRGQHFCPGSALGRRHAQIGIEALLKK
MPGVDLAVPIDQLVWRTRFQRRIPERLPVLW
>CYP121A1_Mycobacterium tuberculosis BTB13-001 2603362702
MTATVLLEVPFSARGDRIPDAVAELRTREPIRKVRTITGAEAWLVSSYALCTQVLEDRRFSMKETAAAGAPRL
NALTVPPEVVNNMGNIADAGLRKAVMKAITPKAPGLEQFLRDTANSLLDNLITEGAPADLRNDFADPLATALH
CKVLGIPQEDGPKLFRSLSIAFMSSADPIPAAKINWDRDIEYMAGILENPNITTGLMGELSRLRKDPAYSHVS
DELFATIGVTFFGAGVISTGSFLTTALISLIQRPQLRNLLHEKPELIPAGVEELLRINLSFADGLPRLATADI
QVGDVLVRKGELVLVLLEGANFDPEHFPNPGSIELDRPNPTSHLAFGRGQHFCPGSALGRRHAQIGIEALLKK
MPGVDLAVPIDQLVWRTRFQRRIPERLPVLW
>CYP121A1_Mycobacterium tuberculosis BTB13-156 2603187233
MTATVLLEVPFSARGDRIPDAVAELRTREPIRKVRTITGAEAWLVSSYALCTQVLEDRRFSMKETAAAGAPRL
NALTVPPEVVNNMGNIADAGLRKAVMKAITPKAPGLEQFLRDTANSLLDNLITEGAPADLRNDFADPLATALH
CKVLGIPQEDGPKLFRSLSIAFMSSADPIPAAKINWDRDIEYMAGILENPNITTGLMGELSRLRKDPAYSHVS
DELFATIGVTFFGAGVISTGSFLTTALISLIQRPQLRNLLHEKPELIPAGVEELLRINLSFADGLPRLATADI
QVGDVLVRKGELVLVLLEGANFDPEHFPNPGSIELDRPNPTSHLAFGRGQHFCPGSALGRRHAQIGIEALLKK
MPGVDLAVPIDQLVWRTRFQRRIPERLPVLW
>CYP121A1_Mycobacterium tuberculosis BTB13-206 2584748638
MTATVLLEVPFSARGDRIPDAVAELRTREPIRKVRTITGAEAWLVSSYALCTQVLEDRRFSMKETAAAGAPRL
NALTVPPEVVNNMGNIADAGLRKAVMKAITPKAPGLEQFLRDTANSLLDNLITEGAPADLRNDFADPLATALH
CKVLGIPQEDGPKLFRSLSIAFMSSADPIPAAKINWDRDIEYMAGILENPNITTGLMGELSRLRKDPAYSHVS
DELFATIGVTFFGAGVISTGSFLTTALISLIQRPQLRNLLHEKPELIPAGVEELLRINLSFADGLPRLATADI
QVGDVLVRKGELVLVLLEGANFDPEHFPNPGSIELDRPNPTSHLAFGRGQHFCPGSALGRRHAQIGIEALLKK
MPGVDLAVPIDQLVWRTRFQRRIPERLPVLW
>CYP121A1_Mycobacterium tuberculosis BTB13-222 2576431743
MTATVLLEVPFSARGDRIPDAVAELRTREPIRKVRTITGAEAWLVSSYALCTQVLEDRRFSMKETAAAGAPRL
NALTVPPEVVNNMGNIADAGLRKAVMKAITPKAPGLEQFLRDTANSLLDNLITEGAPADLRNDFADPLATALH
CKVLGIPQEDGPKLFRSLSIAFMSSADPIPAAKINWDRDIEYMAGILENPNITTGLMGELSRLRKDPAYSHVS
DELFATIGVTFFGAGVISTGSFLTTALISLIQRPQLRNLLHEKPELIPAGVEELLRINLSFADGLPRLATADI
QVGDVLVRKGELVLVLLEGANFDPEHFPNPGSIELDRPNPTSHLAFGRGQHFCPGSALGRRHAQIGIEALLKK
MPGVDLAVPIDQLVWRTRFQRRIPERLPVLW
>CYP121A1_Mycobacterium tuberculosis C unfinished sequence 638727521
MTATVLLEVPFSARGDRIPDAVAELRTREPIRKVRTITGAEAWLVSSYALCTQVLEDRRFSMKETAAAGAPRL
NALTVPPEVVNNMGNIADAGLRKAVMKAITPKAPGLEQFLRDTANSLLDNLITEGAPADLRNDFADPLATALH
CKVLGIPQEDGPKLFRSLSIAFMSSADPIPAAKINWDRDIEYMAGILENPNITTGLMGELSRLRKDPAYSHVS
DELFATIGVTFFGAGVISTGSFLTTALISLIQRPQLRNLLHEKPELIPAGVEELLRINLSFADGLPRLATADI
QVGDVLVRKGELVLVLLEGANFDPEHFPNPGSIELDRPNPTSHLAFGRGQHFCPGSALGRRHAQIGIEALLKK
MPGVDLAVPIDQLVWRTRFQRRIPERLPVLW
>CYP121A1_Mycobacterium tuberculosis C_10367 2675218108
MTATVLLEVPFSARGDRIPDAVAELRTREPIRKVRTITGAEAWLVSSYALCTQVLEDRRFSMKETAAAGAPRL
NALTVPPEVVNNMGNIADAGLRKAVMKAITPKAPGLEQFLRDTANSLLDNLITEGAPADLRNDFADPLATALH
CKVLGIPQEDGPKLFRSLSIAFMSSADPIPAAKINWDRDIEYMAGILENPNITTGLMGELSRLRKDPAYSHVS
DELFATIGVTFFGAGVISTGSFLTTALISLIQRPQLRNLLHEKPELIPAGVEELLRINLSFADGLPRLATADI
QVGDVLVRKGELVLVLLEGANFDPEHFPNPGSIELDRPNPTSHLAFGRGQHFCPGSALGRRHAQIGIEALLKK
MPGVDLAVPIDQLVWRTRFQRRIPERLPVLW
>CYP121A1_Mycobacterium tuberculosis C_14229 2677392221
MTATVLLEVPFSARGDRIPDAVAELRTREPIRKVRTITGAEAWLVSSYALCTQVLEDRRFSMKETAAAGAPRL
NALTVPPEVVNNMGNIADAGLRKAVMKAITPKAPGLEQFLRDTANSLLDNLITEGAPADLRNDFADPLATALH
CKVLGIPQEDGPKLFRSLSIAFMSSADPIPAAKINWDRDIEYMAGILENPNITTGLMGELSRLRKDPAYSHVS
DELFATIGVTFFGAGVISTGSFLTTALISLIQRPQLRNLLHEKPELIPAGVEELLRINLSFADGLPRLATADI
QVGDVLVRKGELVLVLLEGANFDPEHFPNPGSIELDRPNPTSHLAFGRGQHFCPGSALGRRHAQIGIEALLKK
MPGVDLAVPIDQLVWRTRFQRRIPERLPVLW

>CYP121A1_Mycobacterium tuberculosis C_913 2724808696
MTATVLLEVPFSARGDRIPDAVAELRTREPIRKVRTITGAEAWLVSSYALCTQVLEDRRFSMKETAAAGAPRL
NALTVPPEVVNNMGNIADAGLRKAVMKAITPKAPGLEQFLRDTANSLLDNLITEGAPADLRNDFADPLATALH
CKVLGIPQEDGPKLFRSLSIAFMSSADPIPAAKINWDRDIEYMAGILENPNITTGLMGELSRLRKDPAYSHVS
DELFATIGVTFFGAGVISTGSFLTTALISLIQRPQLRNLLHEKPELIPAGVEELLRINLSFADGLPRLATADI
QVGDVLVRKGELVLVLLEGANFDPEHFPNPGSIELDRPNPTSHLAFGRGQHFCPGSALGRRHAQIGIEALLKK
MPGVDLAVPIDQLVWRTRFQRRIPERLPVLW
>CYP121A1_Mycobacterium tuberculosis C00401055 2703451315
MTATVLLEVPFSARGDRIPDAVAELRTREPIRKVRTITGAEAWLVSSYALCTQVLEDRRFSMKETAAAGAPRL
NALTVPPEVVNNMGNIADAGLRKAVMKAITPKAPGLEQFLRDTANSLLDNLITEGAPADLRNDFADPLATALH
CKVLGIPQEDGPKLFRSLSIAFMSSADPIPAAKINWDRDIEYMAGILENPNITTGLMGELSRLRKDPAYSHVS
DELFATIGVTFFGAGVISTGSFLTTALISLIQRPQLRNLLHEKPELIPAGVEELLRINLSFADGLPRLATADI
QVGDVLVRKGELVLVLLEGANFDPEHFPNPGSIELDRPNPTSHLAFGRGQHFCPGSALGRRHAQIGIEALLKK
MPGVDLAVPIDQLVWRTRFQRRIPERLPVLW
>CYP121A1_Mycobacterium tuberculosis C09400866 2703445008
MTATVLLEVPFSARGDRIPDAVAELRTREPIRKVRTITGAEAWLVSSYALCTQVLEDRRFSMKETAAAGAPRL
NALTVPPEVVNNMGNIADAGLRKAVMKAITPKAPGLEQFLRDTANSLLDNLITEGAPADLRNDFADPLATALH
CKVLGIPQEDGPKLFRSLSIAFMSSADPIPAAKINWDRDIEYMAGILENPNITTGLMGELSRLRKDPAYSHVS
DELFATIGVTFFGAGVISTGSFLTTALISLIQRPQLRNLLHEKPELIPAGVEELLRINLSFADGLPRLATADI
QVGDVLVRKGELVLVLLEGANFDPEHFPNPGSIELDRPNPTSHLAFGRGQHFCPGSALGRRHAQIGIEALLKK
MPGVDLAVPIDQLVWRTRFQRRIPERLPVLW
>CYP121A1_Mycobacterium tuberculosis C09601061 2699439882
MTATVLLEVPFSARGDRIPDAVAELRTREPIRKVRTITGAEAWLVSSYALCTQVLEDRRFSMKETAAAGAPRL
NALTVPPEVVNNMGNIADAGLRKAVMKAITPKAPGLEQFLRDTANSLLDNLITEGAPADLRNDFADPLATALH
CKVLGIPQEDGPKLFRSLSIAFMSSADPIPAAKINWDRDIEYMAGILENPNITTGLMGELSRLRKDPAYSHVS
DELFATIGVTFFGAGVISTGSFLTTALISLIQRPQLRNLLHEKPELIPAGVEELLRINLSFADGLPRLATADI
QVGDVLVRKGELVLVLLEGANFDPEHFPNPGSIELDRPNPTSHLAFGRGQHFCPGSALGRRHAQIGIEALLKK
MPGVDLAVPIDQLVWRTRFQRRIPERLPVLW
>CYP121A1_Mycobacterium tuberculosis C130 2685358732
MTATVLLEVPFSARGDRIPDAVAELRTREPIRKVRTITGAEAWLVSSYALCTQVLEDRRFSMKETAAAGAPRL
NALTVPPEVVNNMGNIADAGLRKAVMKAITPKAPGLEQFLRDTANSLLDNLITEGAPADLRNDFADPLATALH
CKVLGIPQEDGPKLFRSLSIAFMSSADPIPAAKINWDRDIEYMAGILENPNITTGLMGELSRLRKDPAYSHVS
DELFATIGVTFFGAGVISTGSFLTTALISLIQRPQLRNLLHEKPELIPAGVEELLRINLSFADGLPRLATADI
QVGDVLVRKGELVLVLLEGANFDPEHFPNPGSIELDRPNPTSHLAFGRGQHFCPGSALGRRHAQIGIEALLKK
MPGVDLAVPIDQLVWRTRFQRRIPERLPVLW
>CYP121A1_Mycobacterium tuberculosis C2 2622030995
MTATVLLEVPFSARGDRIPDAVAELRTREPIRKVRTITGAEAWLVSSYALCTQVLEDRRFSMKETAAAGAPRL
NALTVPPEVVNNMGNIADAGLRKAVMKAITPKAPGLEQFLRDTANSLLDNLITEGAPADLRNDFADPLATALH
CKVLGIPQEDGPKLFRSLSIAFMSSADPIPAAKINWDRDIEYMAGILENPNITTGLMGELSRLRKDPAYSHVS
DELFATIGVTFFGAGVISTGSFLTTALISLIQRPQLRNLLHEKPELIPAGVEELLRINLSFADGLPRLATADI
QVGDVLVRKGELVLVLLEGANFDPEHFPNPGSIELDRPNPTSHLAFGRGQHFCPGSALGRRHAQIGIEALLKK
MPGVDLAVPIDQLVWRTRFQRRIPERLPVLW
>CYP121A1_Mycobacterium tuberculosis C2 2622247906
MTATVLLEVPFSARGDRIPDAVAELRTREPIRKVRTITGAEAWLVSSYALCTQVLEDRRFSMKETAAAGAPRL
NALTVPPEVVNNMGNIADAGLRKAVMKAITPKAPGLEQFLRDTANSLLDNLITEGAPADLRNDFADPLATALH
CKVLGIPQEDGPKLFRSLSIAFMSSADPIPAAKINWDRDIEYMAGILENPNITTGLMGELSRLRKDPAYSHVS
DELFATIGVTFFGAGVISTGSFLTTALISLIQRPQLRNLLHEKPELIPAGVEELLRINLSFADGLPRLATADI
QVGDVLVRKGELVLVLLEGANFDPEHFPNPGSIELDRPNPTSHLAFGRGQHFCPGSALGRRHAQIGIEALLKK
MPGVDLAVPIDQLVWRTRFQRRIPERLPVLW
>CYP121A1_Mycobacterium tuberculosis C24_20545 2714480319
MTATVLLEVPFSARGDRIPDAVAELRTREPIRKVRTITGAEAWLVSSYALCTQVLEDRRFSMKETAAAGAPRL
NALTVPPEVVNNMGNIADAGLRKAVMKAITPKAPGLEQFLRDTANSLLDNLITEGAPADLRNDFADPLATALH
CKVLGIPQEDGPKLFRSLSIAFMSSADPIPAAKINWDRDIEYMAGILENPNITTGLMGELSRLRKDPAYSHVS
DELFATIGVTFFGAGVISTGSFLTTALISLIQRPQLRNLLHEKPELIPAGVEELLRINLSFADGLPRLATADI
QVGDVLVRKGELVLVLLEGANFDPEHFPNPGSIELDRPNPTSHLAFGRGQHFCPGSALGRRHAQIGIEALLKK
MPGVDLAVPIDQLVWRTRFQRRIPERLPVLW
>CYP121A1_Mycobacterium tuberculosis C28_9319 2714484178

MTATVLLEVPFSARGDRIPDAVAELRTREPIRKVRTITGAEAWLVSSYALCTQVLEDRRFSMKETAAAGAPRL
NALTVPPEVVNNMGNIADAGLRKAVMKAITPKAPGLEQFLRDTANSLLDNLITEGAPADLRNDFADPLATALH
CKVLGIPQEDGPKLFRSLSIAFMSSADPIPAAKINWDRDIEYMAGILENPNITTGLMGELSRLRKDPAYSHVS
DELFATIGVTFFGAGVISTGSFLTTALISLIQRPQLRNLLHEKPELIPAGVEELLRINLSFADGLPRLATADI
QVGDVLVRKGELVLVLLEGANFDPEHFPNPGSIELDRPNPTSHLAFGRGQHFCPGSALGRRHAQIGIEALLKK
MPGVDLAVPIDQLVWRTRFQRRIPERLPVLW
>CYP121A1_Mycobacterium tuberculosis C28_9904 2714495334
MTATVLLEVPFSARGDRIPDAVAELRTREPIRKVRTITGAEAWLVSSYALCTQVLEDRRFSMKETAAAGAPRL
NALTVPPEVVNNMGNIADAGLRKAVMKAITPKAPGLEQFLRDTANSLLDNLITEGAPADLRNDFADPLATALH
CKVLGIPQEDGPKLFRSLSIAFMSSADPIPAAKINWDRDIEYMAGILENPNITTGLMGELSRLRKDPAYSHVS
DELFATIGVTFFGAGVISTGSFLTTALISLIQRPQLRNLLHEKPELIPAGVEELLRINLSFADGLPRLATADI
QVGDVLVRKGELVLVLLEGANFDPEHFPNPGSIELDRPNPTSHLAFGRGQHFCPGSALGRRHAQIGIEALLKK
MPGVDLAVPIDQLVWRTRFQRRIPERLPVLW
>CYP121A1_Mycobacterium tuberculosis C30_19588 2716321032
MTATVLLEVPFSARGDRIPDAVAELRTREPIRKVRTITGAEAWLVSSYALCTQVLEDRRFSMKETAAAGAPRL
NALTVPPEVVNNMGNIADAGLRKAVMKAITPKAPGLEQFLRDTANSLLDNLITEGAPADLRNDFADPLATALH
CKVLGIPQEDGPKLFRSLSIAFMSSADPIPAAKINWDRDIEYMAGILENPNITTGLMGELSRLRKDPAYSHVS
DELFATIGVTFFGAGVISTGSFLTTALISLIQRPQLRNLLHEKPELIPAGVEELLRINLSFADGLPRLATADI
QVGDVLVRKGELVLVLLEGANFDPEHFPNPGSIELDRPNPTSHLAFGRGQHFCPGSALGRRHAQIGIEALLKK
MPGVDLAVPIDQLVWRTRFQRRIPERLPVLW
>CYP121A1_Mycobacterium tuberculosis C34_13853 2716330432
MTATVLLEVPFSARGDRIPDAVAELRTREPIRKVRTITGAEAWLVSSYALCTQVLEDRRFSMKETAAAGAPRL
NALTVPPEVVNNMGNIADAGLRKAVMKAITPKAPGLEQFLRDTANSLLDNLITEGAPADLRNDFADPLATALH
CKVLGIPQEDGPKLFRSLSIAFMSSADPIPAAKINWDRDIEYMAGILENPNITTGLMGELSRLRKDPAYSHVS
DELFATIGVTFFGAGVISTGSFLTTALISLIQRPQLRNLLHEKPELIPAGVEELLRINLSFADGLPRLATADI
QVGDVLVRKGELVLVLLEGANFDPEHFPNPGSIELDRPNPTSHLAFGRGQHFCPGSALGRRHAQIGIEALLKK
MPGVDLAVPIDQLVWRTRFQRRIPERLPVLW
>CYP121A1_Mycobacterium tuberculosis C4_16679 2716345533
MTATVLLEVPFSARGDRIPDAVAELRTREPIRKVRTITGAEAWLVSSYALCTQVLEDRRFSMKETAAAGAPRL
NALTVPPEVVNNMGNIADAGLRKAVMKAITPKAPGLEQFLRDTANSLLDNLITEGAPADLRNDFADPLATALH
CKVLGIPQEDGPKLFRSLSIAFMSSADPIPAAKINWDRDIEYMAGILENPNITTGLMGELSRLRKDPAYSHVS
DELFATIGVTFFGAGVISTGSFLTTALISLIQRPQLRNLLHEKPELIPAGVEELLRINLSFADGLPRLATADI
QVGDVLVRKGELVLVLLEGANFDPEHFPNPGSIELDRPNPTSHLAFGRGQHFCPGSALGRRHAQIGIEALLKK
MPGVDLAVPIDQLVWRTRFQRRIPERLPVLW
>CYP121A1_Mycobacterium tuberculosis C49_20090 2716349549
MTATVLLEVPFSARGDRIPDAVAELRTREPIRKVRTITGAEAWLVSSYALCTQVLEDRRFSMKETAAAGAPRL
NALTVPPEVVNNMGNIADAGLRKAVMKAITPKAPGLEQFLRDTANSLLDNLITEGAPADLRNDFADPLATALH
CKVLGIPQEDGPKLFRSLSIAFMSSADPIPAAKINWDRDIEYMAGILENPNITTGLMGELSRLRKDPAYSHVS
DELFATIGVTFFGAGVISTGSFLTTALISLIQRPQLRNLLHEKPELIPAGVEELLRINLSFADGLPRLATADI
QVGDVLVRKGELVLVLLEGANFDPEHFPNPGSIELDRPNPTSHLAFGRGQHFCPGSALGRRHAQIGIEALLKK
MPGVDLAVPIDQLVWRTRFQRRIPERLPVLW
>CYP121A1_Mycobacterium tuberculosis CAS/NITR204 2546202683
MTATVLLEVPFSARGDRIPDAVAELRTREPIRKVRTITGAEAWLVSSYALCTQVLEDRRFSMKETAAAGAPRL
NALTVPPEVVNNMGNIADAGLRKAVMKAITPKAPGLEQFLRDTANSLLDNLITEGAPADLRNDFADPLATALH
CKVLGIPQEDGPKLFRSLSIAFMSSADPIPAAKINWDRDIEYMAGILENPNITTGLMGELSRLRKDPAYSHVS
DELFATIGVTFFGAGVISTGSFLTTALISLIQRPQLRNLLHEKPELIPAGVEELLRINLSFADGLPRLATADI
QVGDVLVRKGELVLVLLEGANFDPEHFPNPGSIELDRPNPTSHLAFGRGQHFCPGSALGRRHAQIGIEALLKK
MPGVDLAVPIDQLVWRTRFQRRIPERLPVLW
>CYP121A1_Mycobacterium tuberculosis CDC1551 637096686
MTATVLLEVPFSARGDRIPDAVAELRTREPIRKVRTITGAEAWLVSSYALCTQVLEDRRFSMKETAAAGAPRL
NALTVPPEVVNNMGNIADAGLRKAVMKAITPKAPGLEQFLRDTANSLLDNLITEGAPADLRNDFADPLATALH
CKVLGIPQEDGPKLFRSLSIAFMSSADPIPAAKINWDRDIEYMAGILENPNITTGLMGELSRLRKDPAYSHVS
DELFATIGVTFFGAGVISTGSFLTTALISLIQRPQLRNLLHEKPELIPAGVEELLRINLSFADGLPRLATADI
QVGDVLVRKGELVLVLLEGANFDPEHFPNPGSIELDRPNPTSHLAFGRGQHFCPGSALGRRHAQIGIEALLKK
MPGVDLAVPIDQLVWRTRFQRRIPERLPVLW
>CYP121A1_Mycobacterium tuberculosis cdc1551 2605055760
MTATVLLEVPFSARGDRIPDAVAELRTREPIRKVRTITGAEAWLVSSYALCTQVLEDRRFSMKETAAAGAPRL
NALTVPPEVVNNMGNIADAGLRKAVMKAITPKAPGLEQFLRDTANSLLDNLITEGAPADLRNDFADPLATALH

CKVLGIPQEDGPKLFRSLSIAFMSSADPIPAAKINWDRDIEYMAGILENPNITTGLMGELSRLRKDPAYSHVS
DELFATIGVTFFGAGVISTGSFLTTALISLIQRPQLRNLLHEKPELIPAGVEELLRINLSFADGLPRLATADI
QVGDVLVRKGELVLVLLEGANFDPEHFPNPGSIELDRPNPTSHLAFGRGQHFCPGSALGRRHAQIGIEALLKK
MPGVDLAVPIDQLVWRTRFQRRIPERLPVLW
>CYP121A1_Mycobacterium tuberculosis CDC1551A 2537735936
MTATVLLEVPFSARGDRIPDAVAELRTREPIRKVRTITGAEAWLVSSYALCTQVLEDRRFSMKETAAAGAPRL
NALTVPPEVVNNMGNIADAGLRKAVMKAITPKAPGLEQFLRDTANSLLDNLITEGAPADLRNDFADPLATALH
CKVLGIPQEDGPKLFRSLSIAFMSSADPIPAAKINWDRDIEYMAGILENPNITTGLMGELSRLRKDPAYSHVS
DELFATIGVTFFGAGVISTGSFLTTALISLIQRPQLRNLLHEKPELIPAGVEELLRINLSFADGLPRLATADI
QVGDVLVRKGELVLVLLEGANFDPEHFPNPGSIELDRPNPTSHLAFGRGQHFCPGSALGRRHAQIGIEALLKK
MPGVDLAVPIDQLVWRTRFQRRIPERLPVLW
>CYP121A1_Mycobacterium tuberculosis CPHL_A 2577093746
MTATVLLEVPFSARGDRIPDAVAELRTREPIRKVRTITGAEAWLVSSYALCTQVLEDRRFSMKETAAAGAPRL
NALTVPPEVVNNMGNIADAGLRKAVMKAITPKAPGLEQFLRDTANSLLDNLITEGAPADLRNDFADPLATALH
CKVLGIPQEDGPKLFRSLSIAFMSSADPIPAAKINWDRDIEYMAGILENPNITTGLMGELSRLRKDPAYSHVS
DELFATIGVTFFGAGVISTGSFLTTALISLIQRPQLRNLLHEKPELIPAGVEELLRINLSFADGLPRLATADI
QVGDVLVRKGELVLVLLEGANFDPEHFPNPGSIELDRPNPTSHLAFGRGQHFCPGSALGRRHAQIGIEALLKK
MPGVDLAVPIDQLVWRTRFQRRIPERLPVLW
>CYP121A1_Mycobacterium tuberculosis CPHL_A 646015088
MTATVLLEVPFSARGDRIPDAVAELRTREPIRKVRTITGAEAWLVSSYALCTQVLEDRRFSMKETAAAGAPRL
NALTVPPEVVNNMGNIADAGLRKAVMKAITPKAPGLEQFLRDTANSLLDNLITEGAPADLRNDFADPLATALH
CKVLGIPQEDGPKLFRSLSIAFMSSADPIPAAKINWDRDIEYMAGILENPNITTGLMGELSRLRKDPAYSHVS
DELFATIGVTFFGAGVISTGSFLTTALISLIQRPQLRNLLHEKPELIPAGVEELLRINLSFADGLPRLATADI
QVGDVLVRKGELVLVLLEGANFDPEHFPNPGSIELDRPNPTSHLAFGRGQHFCPGSALGRRHAQIGIEALLKK
MPGVDLAVPIDQLVWRTRFQRRIPERLPVLW
>CYP121A1_Mycobacterium tuberculosis CSF3053 2702452097
MTATVLLEVPFSARGDRIPDAVAELRTREPIRKVRTITGAEAWLVSSYALCTQVLEDRRFSMKETAAAGAPRL
NALTVPPEVVNNMGNIADAGLRKAVMKAITPKAPGLEQFLRDTANSLLDNLITEGAPADLRNDFADPLATALH
CKVLGIPQEDGPKLFRSLSIAFMSSADPIPAAKINWDRDIEYMAGILENPNITTGLMGELSRLRKDPAYSHVS
DELFATIGVTFFGAGVISTGSFLTTALISLIQRPQLRNLLHEKPELIPAGVEELLRINLSFADGLPRLATADI
QVGDVLVRKGELVLVLLEGANFDPEHFPNPGSIELDRPNPTSHLAFGRGQHFCPGSALGRRHAQIGIEALLKK
MPGVDLAVPIDQLVWRTRFQRRIPERLPVLW
>CYP121A1_Mycobacterium tuberculosis CTRI-2 2511736685
MTATVLLEVPFSARGDRIPDAVAELRTREPIRKVRTITGAEAWLVSSYALCTQVLEDRRFSMKETAAAGAPRL
NALTVPPEVVNNMGNIADAGLRKAVMKAITPKAPGLEQFLRDTANSLLDNLITEGAPADLRNDFADPLATALH
CKVLGIPQEDGPKLFRSLSIAFMSSADPIPAAKINWDRDIEYMAGILENPNITTGLMGELSRLRKDPAYSHVS
DELFATIGVTFFGAGVISTGSFLTTALISLIQRPQLRNLLHEKPELIPAGVEELLRINLSFADGLPRLATADI
QVGDVLVRKGELVLVLLEGANFDPEHFPNPGSIELDRPNPTSHLAFGRGQHFCPGSALGRRHAQIGIEALLKK
MPGVDLAVPIDQLVWRTRFQRRIPERLPVLW
>CYP121A1_Mycobacterium tuberculosis D00000071 2675427477
MTATVLLEVPFSARGDRIPDAVAELRTREPIRKVRTITGAEAWLVSSYALCTQVLEDRRFSMKETAAAGAPRL
NALTVPPEVVNNMGNIADAGLRKAVMKAITPKAPGLEQFLRDTANSLLDNLITEGAPADLRNDFADPLATALH
CKVLGIPQEDGPKLFRSLSIAFMSSADPIPAAKINWDRDIEYMAGILENPNITTGLMGELSRLRKDPAYSHVS
DELFATIGVTFFGAGVISTGSFLTTALISLIQRPQLRNLLHEKPELIPAGVEELLRINLSFADGLPRLATADI
QVGDVLVRKGELVLVLLEGANFDPEHFPNPGSIELDRPNPTSHLAFGRGQHFCPGSALGRRHAQIGIEALLKK
MPGVDLAVPIDQLVWRTRFQRRIPERLPVLW
>CYP121A1_Mycobacterium tuberculosis D00000538 2672977609
MTATVLLEVPFSARGDRIPDAVAELRTREPIRKVRTITGAEAWLVSSYALCTQVLEDRRFSMKETAAAGAPRL
NALTVPPEVVNNMGNIADAGLRKAVMKAITPKAPGLEQFLRDTANSLLDNLITEGAPADLRNDFADPLATALH
CKVLGIPQEDGPKLFRSLSIAFMSSADPIPAAKINWDRDIEYMAGILENPNITTGLMGELSRLRKDPAYSHVS
DELFATIGVTFFGAGVISTGSFLTTALISLIQRPQLRNLLHEKPELIPAGVEELLRINLSFADGLPRLATADI
QVGDVLVRKGELVLVLLEGANFDPEHFPNPGSIELDRPNPTSHLAFGRGQHFCPGSALGRRHAQIGIEALLKK
MPGVDLAVPIDQLVWRTRFQRRIPERLPVLW
>CYP121A1_Mycobacterium tuberculosis D00501624 2703465359
MTATVLLEVPFSARGDRIPDAVAELRTREPIRKVRTITGAEAWLVSSYALCTQVLEDRRFSMKETAAAGAPRL
NALTVPPEVVNNMGNIADAGLRKAVMKAITPKAPGLEQFLRDTANSLLDNLITEGAPADLRNDFADPLATALH
CKVLGIPQEDGPKLFRSLSIAFMSSADPIPAAKINWDRDIEYMAGILENPNITTGLMGELSRLRKDPAYSHVS
DELFATIGVTFFGAGVISTGSFLTTALISLIQRPQLRNLLHEKPELIPAGVEELLRINLSFADGLPRLATADI

QVGDVLVRKGELVLVLLEGANFDPEHFPNPGSIELDRPNPTSHLAFGRGQHFCPGSALGRRHAQIGIEALLKK
MPGVDLAVPIDQLVWRTRFQRRIPERLPVLW
>CYP121A1_Mycobacterium tuberculosis D00700688 2703457890
MTATVLLEVPFSARGDRIPDAVAELRTREPIRKVRTITGAEAWLVSSYALCTQVLEDRRFSMKETAAAGAPRL
NALTVPPEVVNNMGNIADAGLRKAVMKAITPKAPGLEQFLRDTANSLLDNLITEGAPADLRNDFADPLATALH
CKVLGIPQEDGPKLFRSLSIAFMSSADPIPAAKINWDRDIEYMAGILENPNITTGLMGELSRLRKDPAYSHVS
DELFATIGVTFFGAGVISTGSFLTTALISLIQRPQLRNLLHEKPELIPAGVEELLRINLSFADGLPRLATADI
QVGDVLVRKGELVLVLLEGANFDPEHFPNPGSIELDRPNPTSHLAFGRGQHFCPGSALGRRHAQIGIEALLKK
MPGVDLAVPIDQLVWRTRFQRRIPERLPVLW
>CYP121A1_Mycobacterium tuberculosis DK9897 2721365630
MTATVLLEVPFSARGDRIPDAVAELRTREPIRKVRTITGAEAWLVSSYALCTQVLEDRRFSMKETAAAGAPRL
NALTVPPEVVNNMGNIADAGLRKAVMKAITPKAPGLEQFLRDTANSLLDNLITEGAPADLRNDFADPLATALH
CKVLGIPQEDGPKLFRSLSIAFMSSADPIPAAKINWDRDIEYMAGILENPNITTGLMGELSRLRKDPAYSHVS
DELFATIGVTFFGAGVISTGSFLTTALISLIQRPQLRNLLHEKPELIPAGVEELLRINLSFADGLPRLATADI
QVGDVLVRKGELVLVLLEGANFDPEHFPNPGSIELDRPNPTSHLAFGRGQHFCPGSALGRRHAQIGIEALLKK
MPGVDLAVPIDQLVWRTRFQRRIPERLPVLW
>CYP121A1_Mycobacterium tuberculosis E09700455 2674937825
MTATVLLEVPFSARGDRIPDAVAELRTREPIRKVRTITGAEAWLVSSYALCTQVLEDRRFSMKETAAAGAPRL
NALTVPPEVVNNMGNIADAGLRKAVMKAITPKAPGLEQFLRDTANSLLDNLITEGAPADLRNDFADPLATALH
CKVLGIPQEDGPKLFRSLSIAFMSSADPIPAAKINWDRDIEYMAGILENPNITTGLMGELSRLRKDPAYSHVS
DELFATIGVTFFGAGVISTGSFLTTALISLIQRPQLRNLLHEKPELIPAGVEELLRINLSFADGLPRLATADI
QVGDVLVRKGELVLVLLEGANFDPEHFPNPGSIELDRPNPTSHLAFGRGQHFCPGSALGRRHAQIGIEALLKK
MPGVDLAVPIDQLVWRTRFQRRIPERLPVLW
>CYP121A1_Mycobacterium tuberculosis E09701106 2703431200
MTATVLLEVPFSARGDRIPDAVAELRTREPIRKVRTITGAEAWLVSSYALCTQVLEDRRFSMKETAAAGAPRL
NALTVPPEVVNNMGNIADAGLRKAVMKAITPKAPGLEQFLRDTANSLLDNLITEGAPADLRNDFADPLATALH
CKVLGIPQEDGPKLFRSLSIAFMSSADPIPAAKINWDRDIEYMAGILENPNITTGLMGELSRLRKDPAYSHVS
DELFATIGVTFFGAGVISTGSFLTTALISLIQRPQLRNLLHEKPELIPAGVEELLRINLSFADGLPRLATADI
QVGDVLVRKGELVLVLLEGANFDPEHFPNPGSIELDRPNPTSHLAFGRGQHFCPGSALGRRHAQIGIEALLKK
MPGVDLAVPIDQLVWRTRFQRRIPERLPVLW
>CYP121A1_Mycobacterium tuberculosis E09701191 2703441484
MTATVLLEVPFSARGDRIPDAVAELRTREPIRKVRTITGAEAWLVSSYALCTQVLEDRRFSMKETAAAGAPRL
NALTVPPEVVNNMGNIADAGLRKAVMKAITPKAPGLEQFLRDTANSLLDNLITEGAPADLRNDFADPLATALH
CKVLGIPQEDGPKLFRSLSIAFMSSADPIPAAKINWDRDIEYMAGILENPNITTGLMGELSRLRKDPAYSHVS
DELFATIGVTFFGAGVISTGSFLTTALISLIQRPQLRNLLHEKPELIPAGVEELLRINLSFADGLPRLATADI
QVGDVLVRKGELVLVLLEGANFDPEHFPNPGSIELDRPNPTSHLAFGRGQHFCPGSALGRRHAQIGIEALLKK
MPGVDLAVPIDQLVWRTRFQRRIPERLPVLW
>CYP121A1_Mycobacterium tuberculosis E09701891 2706580484
MTATVLLEVPFSARGDRIPDAVAELRTREPIRKVRTITGAEAWLVSSYALCTQVLEDRRFSMKETAAAGAPRL
NALTVPPEVVNNMGNIADAGLRKAVMKAITPKAPGLEQFLRDTANSLLDNLITEGAPADLRNDFADPLATALH
CKVLGIPQEDGPKLFRSLSIAFMSSADPIPAAKINWDRDIEYMAGILENPNITTGLMGELSRLRKDPAYSHVS
DELFATIGVTFFGAGVISTGSFLTTALISLIQRPQLRNLLHEKPELIPAGVEELLRINLSFADGLPRLATADI
QVGDVLVRKGELVLVLLEGANFDPEHFPNPGSIELDRPNPTSHLAFGRGQHFCPGSALGRRHAQIGIEALLKK
MPGVDLAVPIDQLVWRTRFQRRIPERLPVLW
>CYP121A1_Mycobacterium tuberculosis E09801032 2699039532
MTATVLLEVPFSARGDRIPDAVAELRTREPIRKVRTITGAEAWLVSSYALCTQVLEDRRFSMKETAAAGAPRL
NALTVPPEVVNNMGNIADAGLRKAVMKAITPKAPGLEQFLRDTANSLLDNLITEGAPADLRNDFADPLATALH
CKVLGIPQEDGPKLFRSLSIAFMSSADPIPAAKINWDRDIEYMAGILENPNITTGLMGELSRLRKDPAYSHVS
DELFATIGVTFFGAGVISTGSFLTTALISLIQRPQLRNLLHEKPELIPAGVEELLRINLSFADGLPRLATADI
QVGDVLVRKGELVLVLLEGANFDPEHFPNPGSIELDRPNPTSHLAFGRGQHFCPGSALGRRHAQIGIEALLKK
MPGVDLAVPIDQLVWRTRFQRRIPERLPVLW
>CYP121A1_Mycobacterium tuberculosis EAI/OSDD271 2554690953
MTATVLLEVPFSARGDRIPDAVAELRTREPIRKVRTITGAEAWLVSSYALCTQVLEDRRFSMKETAAAGAPRL
NALTVPPEVVNNMGNIADAGLRKAVMKAITPKAPGLEQFLRDTANSLLDNLITEGAPADLRNDFADPLATALH
CKVLGIPQEDGPKLFRSLSIAFMSSADPIPAAKINWDRDIEYMAGILENPNITTGLMGELSRLRKDPAYSHVS
DELFATIGVTFFGAGVISTGSFLTTALISLIQRPQLRNLLHEKPELIPAGVEELLRINLSFADGLPRLATADI
QVGDVLVRKGELVLVLLEGANFDPEHFPNPGSIELDRPNPTSHLAFGRGQHFCPGSALGRRHAQIGIEALLKK
MPGVDLAVPIDQLVWRTRFQRRIPERLPVLW

>CYP121A1_Mycobacterium tuberculosis EAI5 2555961073
MTATVLLEVPFSARGDRIPDAVAELRTREPIRKVRTITGAEAWLVSSYALCTQVLEDRRFSMKETAAAGAPRL
NALTVPPEVVNNMGNIADAGLRKAVMKAITPKAPGLEQFLRDTANSLLDNLITEGAPADLRNDFADPLATALH
CKVLGIPQEDGPKLFRSLSIAFMSSADPIPAAKINWDRDIEYMAGILENPNITTGLMGELSRLRKDPAYSHVS
DELFATIGVTFFGAGVISTGSFLTTALISLIQRPQLRNLLHEKPELIPAGVEELLRINLSFADGLPRLATADI
QVGDVLVRKGELVLVLLEGANFDPEHFPNPGSIELDRPNPTSHLAFGRGQHFCPGSALGRRHAQIGIEALLKK
MPGVDLAVPIDQLVWRTRFQRRIPERLPVLW
>CYP121A1_Mycobacterium tuberculosis EAI5/NITR206 2546206766
MTATVLLEVPFSARGDRIPDAVAELRTREPIRKVRTITGAEAWLVSSYALCTQVLEDRRFSMKETAAAGAPRL
NALTVPPEVVNNMGNIADAGLRKAVMKAITPKAPGLEQFLRDTANSLLDNLITEGAPADLRNDFADPLATALH
CKVLGIPQEDGPKLFRSLSIAFMSSADPIPAAKINWDRDIEYMAGILENPNITTGLMGELSRLRKDPAYSHVS
DELFATIGVTFFGAGVISTGSFLTTALISLIQRPQLRNLLHEKPELIPAGVEELLRINLSFADGLPRLATADI
QVGDVLVRKGELVLVLLEGANFDPEHFPNPGSIELDRPNPTSHLAFGRGQHFCPGSALGRRHAQIGIEALLKK
MPGVDLAVPIDQLVWRTRFQRRIPERLPVLW
>CYP121A1_Mycobacterium tuberculosis EAS054 2621964180
MTATVLLEVPFSARGDRIPDAVAELRTREPIRKVRTITGAEAWLVSSYALCTQVLEDRRFSMKETAAAGAPRL
NALTVPPEVVNNMGNIADAGLRKAVMKAITPKAPGLEQFLRDTANSLLDNLITEGAPADLRNDFADPLATALH
CKVLGIPQEDGPKLFRSLSIAFMSSADPIPAAKINWDRDIEYMAGILENPNITTGLMGELSRLRKDPAYSHVS
DELFATIGVTFFGAGVISTGSFLTTALISLIQRPQLRNLLHEKPELIPAGVEELLRINLSFADGLPRLATADI
QVGDVLVRKGELVLVLLEGANFDPEHFPNPGSIELDRPNPTSHLAFGRGQHFCPGSALGRRHAQIGIEALLKK
MPGVDLAVPIDQLVWRTRFQRRIPERLPVLW
>CYP121A1_Mycobacterium tuberculosis EAS054 unfinished sequence 643032441
MTATVLLEVPFSARGDRIPDAVAELRTREPIRKVRTITGAEAWLVSSYALCTQVLEDRRFSMKETAAAGAPRL
NALTVPPEVVNNMGNIADAGLRKAVMKAITPKAPGLEQFLRDTANSLLDNLITEGAPADLRNDFADPLATALH
CKVLGIPQEDGPKLFRSLSIAFMSSADPIPAAKINWDRDIEYMAGILENPNITTGLMGELSRLRKDPAYSHVS
DELFATIGVTFFGAGVISTGSFLTTALISLIQRPQLRNLLHEKPELIPAGVEELLRINLSFADGLPRLATADI
QVGDVLVRKGELVLVLLEGANFDPEHFPNPGSIELDRPNPTSHLAFGRGQHFCPGSALGRRHAQIGIEALLKK
MPGVDLAVPIDQLVWRTRFQRRIPERLPVLW
>CYP121A1_Mycobacterium tuberculosis East African-Indian NZ3MDR1
2633043887
MTATVLLEVPFSARGDRIPDAVAELRTREPIRKVRTITGAEAWLVSSYALCTQVLEDRRFSMKETAAAGAPRL
NALTVPPEVVNNMGNIADAGLRKAVMKAITPKAPGLEQFLRDTANSLLDNLITEGAPADLRNDFADPLATALH
CKVLGIPQEDGPKLFRSLSIAFMSSADPIPAAKINWDRDIEYMAGILENPNITTGLMGELSRLRKDPAYSHVS
DELFATIGVTFFGAGVISTGSFLTTALISLIQRPQLRNLLHEKPELIPAGVEELLRINLSFADGLPRLATADI
QVGDVLVRKGELVLVLLEGANFDPEHFPNPGSIELDRPNPTSHLAFGRGQHFCPGSALGRRHAQIGIEALLKK
MPGVDLAVPIDQLVWRTRFQRRIPERLPVLW
>CYP121A1_Mycobacterium tuberculosis F1 2688963645
MTATVLLEVPFSARGDRIPDAVAELRTREPIRKVRTITGAEAWLVSSYALCTQVLEDRRFSMKETAAAGAPRL
NALTVPPEVVNNMGNIADAGLRKAVMKAITPKAPGLEQFLRDTANSLLDNLITEGAPADLRNDFADPLATALH
CKVLGIPQEDGPKLFRSLSIAFMSSADPIPAAKINWDRDIEYMAGILENPNITTGLMGELSRLRKDPAYSHVS
DELFATIGVTFFGAGVISTGSFLTTALISLIQRPQLRNLLHEKPELIPAGVEELLRINLSFADGLPRLATADI
QVGDVLVRKGELVLVLLEGANFDPEHFPNPGSIELDRPNPTSHLAFGRGQHFCPGSALGRRHAQIGIEALLKK
MPGVDLAVPIDQLVWRTRFQRRIPERLPVLW
>CYP121A1_Mycobacterium tuberculosis F11 2603383285
MTATVLLEVPFSARGDRIPDAVAELRTREPIRKVRTITGAEAWLVSSYALCTQVLEDRRFSMKETAAAGAPRL
NALTVPPEVVNNMGNIADAGLRKAVMKAITPKAPGLEQFLRDTANSLLDNLITEGAPADLRNDFADPLATALH
CKVLGIPQEDGPKLFRSLSIAFMSSADPIPAAKINWDRDIEYMAGILENPNITTGLMGELSRLRKDPAYSHVS
DELFATIGVTFFGAGVISTGSFLTTALISLIQRPQLRNLLHEKPELIPAGVEELLRINLSFADGLPRLATADI
QVGDVLVRKGELVLVLLEGANFDPEHFPNPGSIELDRPNPTSHLAFGRGQHFCPGSALGRRHAQIGIEALLKK
MPGVDLAVPIDQLVWRTRFQRRIPERLPVLW
>CYP121A1_Mycobacterium tuberculosis F11 640607058
MTATVLLEVPFSARGDRIPDAVAELRTREPIRKVRTITGAEAWLVSSYALCTQVLEDRRFSMKETAAAGAPRL
NALTVPPEVVNNMGNIADAGLRKAVMKAITPKAPGLEQFLRDTANSLLDNLITEGAPADLRNDFADPLATALH
CKVLGIPQEDGPKLFRSLSIAFMSSADPIPAAKINWDRDIEYMAGILENPNITTGLMGELSRLRKDPAYSHVS
DELFATIGVTFFGAGVISTGSFLTTALISLIQRPQLRNLLHEKPELIPAGVEELLRINLSFADGLPRLATADI
QVGDVLVRKGELVLVLLEGANFDPEHFPNPGSIELDRPNPTSHLAFGRGQHFCPGSALGRRHAQIGIEALLKK
MPGVDLAVPIDQLVWRTRFQRRIPERLPVLW
>CYP121A1_Mycobacterium tuberculosis F28 2688938872

MTATVLLEVPFSARGDRIPDAVAELRTREPIRKVRTITGAEAWLVSSYALCTQVLEDRRFSMKETAAAGAPRL
NALTVPPEVVNNMGNIADAGLRKAVMKAITPKAPGLEQFLRDTANSLLDNLITEGAPADLRNDFADPLATALH
CKVLGIPQEDGPKLFRSLSIAFMSSADPIPAAKINWDRDIEYMAGILENPNITTGLMGELSRLRKDPAYSHVS
DELFATIGVTFFGAGVISTGSFLTTALISLIQRPQLRNLLHEKPELIPAGVEELLRINLSFADGLPRLATADI
QVGDVLVRKGELVLVLLEGANFDPEHFPNPGSIELDRPNPTSHLAFGRGQHFCPGSALGRRHAQIGIEALLKK
MPGVDLAVPIDQLVWRTRFQRRIPERLPVLW
>CYP121A1_Mycobacterium tuberculosis G00000978 2678225464
MTATVLLEVPFSARGDRIPDAVAELRTREPIRKVRTITGAEAWLVSSYALCTQVLEDRRFSMKETAAAGAPRL
NALTVPPEVVNNMGNIADAGLRKAVMKAITPKAPGLEQFLRDTANSLLDNLITEGAPADLRNDFADPLATALH
CKVLGIPQEDGPKLFRSLSIAFMSSADPIPAAKINWDRDIEYMAGILENPNITTGLMGELSRLRKDPAYSHVS
DELFATIGVTFFGAGVISTGSFLTTALISLIQRPQLRNLLHEKPELIPAGVEELLRINLSFADGLPRLATADI
QVGDVLVRKGELVLVLLEGANFDPEHFPNPGSIELDRPNPTSHLAFGRGQHFCPGSALGRRHAQIGIEALLKK
MPGVDLAVPIDQLVWRTRFQRRIPERLPVLW
>CYP121A1_Mycobacterium tuberculosis G00001233 2692053554
MTATVLLEVPFSARGDRIPDAVAELRTREPIRKVRTITGAEAWLVSSYALCTQVLEDRRFSMKETAAAGAPRL
NALTVPPEVVNNMGNIADAGLRKAVMKAITPKAPGLEQFLRDTANSLLDNLITEGAPADLRNDFADPLATALH
CKVLGIPQEDGPKLFRSLSIAFMSSADPIPAAKINWDRDIEYMAGILENPNITTGLMGELSRLRKDPAYSHVS
DELFATIGVTFFGAGVISTGSFLTTALISLIQRPQLRNLLHEKPELIPAGVEELLRINLSFADGLPRLATADI
QVGDVLVRKGELVLVLLEGANFDPEHFPNPGSIELDRPNPTSHLAFGRGQHFCPGSALGRRHAQIGIEALLKK
MPGVDLAVPIDQLVWRTRFQRRIPERLPVLW
>CYP121A1_Mycobacterium tuberculosis G00101715 2697047923
MTATVLLEVPFSARGDRIPDAVAELRTREPIRKVRTITGAEAWLVSSYALCTQVLEDRRFSMKETAAAGAPRL
NALTVPPEVVNNMGNIADAGLRKAVMKAITPKAPGLEQFLRDTANSLLDNLITEGAPADLRNDFADPLATALH
CKVLGIPQEDGPKLFRSLSIAFMSSADPIPAAKINWDRDIEYMAGILENPNITTGLMGELSRLRKDPAYSHVS
DELFATIGVTFFGAGVISTGSFLTTALISLIQRPQLRNLLHEKPELIPAGVEELLRINLSFADGLPRLATADI
QVGDVLVRKGELVLVLLEGANFDPEHFPNPGSIELDRPNPTSHLAFGRGQHFCPGSALGRRHAQIGIEALLKK
MPGVDLAVPIDQLVWRTRFQRRIPERLPVLW
>CYP121A1_Mycobacterium tuberculosis G09801536 2703623769
MTATVLLEVPFSARGDRIPDAVAELRTREPIRKVRTITGAEAWLVSSYALCTQVLEDRRFSMKETAAAGAPRL
NALTVPPEVVNNMGNIADAGLRKAVMKAITPKAPGLEQFLRDTANSLLDNLITEGAPADLRNDFADPLATALH
CKVLGIPQEDGPKLFRSLSIAFMSSADPIPAAKINWDRDIEYMAGILENPNITTGLMGELSRLRKDPAYSHVS
DELFATIGVTFFGAGVISTGSFLTTALISLIQRPQLRNLLHEKPELIPAGVEELLRINLSFADGLPRLATADI
QVGDVLVRKGELVLVLLEGANFDPEHFPNPGSIELDRPNPTSHLAFGRGQHFCPGSALGRRHAQIGIEALLKK
MPGVDLAVPIDQLVWRTRFQRRIPERLPVLW
>CYP121A1_Mycobacterium tuberculosis G09901357 2706555470
MTATVLLEVPFSARGDRIPDAVAELRTREPIRKVRTITGAEAWLVSSYALCTQVLEDRRFSMKETAAAGAPRL
NALTVPPEVVNNMGNIADAGLRKAVMKAITPKAPGLEQFLRDTANSLLDNLITEGAPADLRNDFADPLATALH
CKVLGIPQEDGPKLFRSLSIAFMSSADPIPAAKINWDRDIEYMAGILENPNITTGLMGELSRLRKDPAYSHVS
DELFATIGVTFFGAGVISTGSFLTTALISLIQRPQLRNLLHEKPELIPAGVEELLRINLSFADGLPRLATADI
QVGDVLVRKGELVLVLLEGANFDPEHFPNPGSIELDRPNPTSHLAFGRGQHFCPGSALGRRHAQIGIEALLKK
MPGVDLAVPIDQLVWRTRFQRRIPERLPVLW
>CYP121A1_Mycobacterium tuberculosis GM 1503 2577893853
MTATVLLEVPFSARGDRIPDAVAELRTREPIRKVRTITGAEAWLVSSYALCTQVLEDRRFSMKETAAAGAPRL
NALTVPPEVVNNMGNIADAGLRKAVMKAITPKAPGLEQFLRDTANSLLDNLITEGAPADLRNDFADPLATALH
CKVLGIPQEDGPKLFRSLSIAFMSSADPIPAAKINWDRDIEYMAGILENPNITTGLMGELSRLRKDPAYSHVS
DELFATIGVTFFGAGVISTGSFLTTALISLIQRPQLRNLLHEKPELIPAGVEELLRINLSFADGLPRLATADI
QVGDVLVRKGELVLVLLEGANFDPEHFPNPGSIELDRPNPTSHLAFGRGQHFCPGSALGRRHAQIGIEALLKK
MPGVDLAVPIDQLVWRTRFQRRIPERLPVLW
>CYP121A1_Mycobacterium tuberculosis GM 1503 unfinished sequence
643045712
MTATVLLEVPFSARGDRIPDAVAELRTREPIRKVRTITGAEAWLVSSYALCTQVLEDRRFSMKETAAAGAPRL
NALTVPPEVVNNMGNIADAGLRKAVMKAITPKAPGLEQFLRDTANSLLDNLITEGAPADLRNDFADPLATALH
CKVLGIPQEDGPKLFRSLSIAFMSSADPIPAAKINWDRDIEYMAGILENPNITTGLMGELSRLRKDPAYSHVS
DELFATIGVTFFGAGVISTGSFLTTALISLIQRPQLRNLLHEKPELIPAGVEELLRINLSFADGLPRLATADI
QVGDVLVRKGELVLVLLEGANFDPEHFPNPGSIELDRPNPTSHLAFGRGQHFCPGSALGRRHAQIGIEALLKK
MPGVDLAVPIDQLVWRTRFQRRIPERLPVLW
>CYP121A1_Mycobacterium tuberculosis GuangZ0019 2546454201

MTATVLLEVPFSARGDRIPDAVAELRTREPIRKVRTITGAEAWLVSSYALCTQVLEDRRFSMKETAAAGAPRL
NALTVPPEVVNNMGNIADAGLRKAVMKAITPKAPGLEQFLRDTANSLLDNLITEGAPADLRNDFADPLATALH
CKVLGIPQEDGPKLFRSLSIAFMSSADPIPAAKINWDRDIEYMAGILENPNITTGLMGELSRLRKDPAYSHVS
DELFATIGVTFFGAGVISTGSFLTTALISLIQRPQLRNLLHEKPELIPAGVEELLRINLSFADGLPRLATADI
QVGDVLVRKGELVLVLLEGANFDPEHFPNPGSIELDRPNPTSHLAFGRGQHFCPGSALGRRHAQIGIEALLKK
MPGVDLAVPIDQLVWRTRFQRRIPERLPVLW
>CYP121A1_Mycobacterium tuberculosis H_13559 2716354010
MTATVLLEVPFSARGDRIPDAVAELRTREPIRKVRTITGAEAWLVSSYALCTQVLEDRRFSMKETAAAGAPRL
NALTVPPEVVNNMGNIADAGLRKAVMKAITPKAPGLEQFLRDTANSLLDNLITEGAPADLRNDFADPLATALH
CKVLGIPQEDGPKLFRSLSIAFMSSADPIPAAKINWDRDIEYMAGILENPNITTGLMGELSRLRKDPAYSHVS
DELFATIGVTFFGAGVISTGSFLTTALISLIQRPQLRNLLHEKPELIPAGVEELLRINLSFADGLPRLATADI
QVGDVLVRKGELVLVLLEGANFDPEHFPNPGSIELDRPNPTSHLAFGRGQHFCPGSALGRRHAQIGIEALLKK
MPGVDLAVPIDQLVWRTRFQRRIPERLPVLW
>CYP121A1_Mycobacterium tuberculosis H_13571 2716358542
MTATVLLEVPFSARGDRIPDAVAELRTREPIRKVRTITGAEAWLVSSYALCTQVLEDRRFSMKETAAAGAPRL
NALTVPPEVVNNMGNIADAGLRKAVMKAITPKAPGLEQFLRDTANSLLDNLITEGAPADLRNDFADPLATALH
CKVLGIPQEDGPKLFRSLSIAFMSSADPIPAAKINWDRDIEYMAGILENPNITTGLMGELSRLRKDPAYSHVS
DELFATIGVTFFGAGVISTGSFLTTALISLIQRPQLRNLLHEKPELIPAGVEELLRINLSFADGLPRLATADI
QVGDVLVRKGELVLVLLEGANFDPEHFPNPGSIELDRPNPTSHLAFGRGQHFCPGSALGRRHAQIGIEALLKK
MPGVDLAVPIDQLVWRTRFQRRIPERLPVLW
>CYP121A1_Mycobacterium tuberculosis H_7300 2678876410
MTATVLLEVPFSARGDRIPDAVAELRTREPIRKVRTITGAEAWLVSSYALCTQVLEDRRFSMKETAAAGAPRL
NALTVPPEVVNNMGNIADAGLRKAVMKAITPKAPGLEQFLRDTANSLLDNLITEGAPADLRNDFADPLATALH
CKVLGIPQEDGPKLFRSLSIAFMSSADPIPAAKINWDRDIEYMAGILENPNITTGLMGELSRLRKDPAYSHVS
DELFATIGVTFFGAGVISTGSFLTTALISLIQRPQLRNLLHEKPELIPAGVEELLRINLSFADGLPRLATADI
QVGDVLVRKGELVLVLLEGANFDPEHFPNPGSIELDRPNPTSHLAFGRGQHFCPGSALGRRHAQIGIEALLKK
MPGVDLAVPIDQLVWRTRFQRRIPERLPVLW
>CYP121A1_Mycobacterium tuberculosis H1249 2622252818
MTATVLLEVPFSARGDRIPDAVAELRTREPIRKVRTITGAEAWLVSSYALCTQVLEDRRFSMKETAAAGAPRL
NALTVPPEVVNNMGNIADAGLRKAVMKAITPKAPGLEQFLRDTANSLLDNLITEGAPADLRNDFADPLATALH
CKVLGIPQEDGPKLFRSLSIAFMSSADPIPAAKINWDRDIEYMAGILENPNITTGLMGELSRLRKDPAYSHVS
DELFATIGVTFFGAGVISTGSFLTTALISLIQRPQLRNLLHEKPELIPAGVEELLRINLSFADGLPRLATADI
QVGDVLVRKGELVLVLLEGANFDPEHFPNPGSIELDRPNPTSHLAFGRGQHFCPGSALGRRHAQIGIEALLKK
MPGVDLAVPIDQLVWRTRFQRRIPERLPVLW
>CYP121A1_Mycobacterium tuberculosis H1578 2574790860
MTATVLLEVPFSARGDRIPDAVAELRTREPIRKVRTITGAEAWLVSSYALCTQVLEDRRFSMKETAAAGAPRL
NALTVPPEVVNNMGNIADAGLRKAVMKAITPKAPGLEQFLRDTANSLLDNLITEGAPADLRNDFADPLATALH
CKVLGIPQEDGPKLFRSLSIAFMSSADPIPAAKINWDRDIEYMAGILENPNITTGLMGELSRLRKDPAYSHVS
DELFATIGVTFFGAGVISTGSFLTTALISLIQRPQLRNLLHEKPELIPAGVEELLRINLSFADGLPRLATADI
QVGDVLVRKGELVLVLLEGANFDPEHFPNPGSIELDRPNPTSHLAFGRGQHFCPGSALGRRHAQIGIEALLKK
MPGVDLAVPIDQLVWRTRFQRRIPERLPVLW
>CYP121A1_Mycobacterium tuberculosis H1580 2622259586
MTATVLLEVPFSARGDRIPDAVAELRTREPIRKVRTITGAEAWLVSSYALCTQVLEDRRFSMKETAAAGAPRL
NALTVPPEVVNNMGNIADAGLRKAVMKAITPKAPGLEQFLRDTANSLLDNLITEGAPADLRNDFADPLATALH
CKVLGIPQEDGPKLFRSLSIAFMSSADPIPAAKINWDRDIEYMAGILENPNITTGLMGELSRLRKDPAYSHVS
DELFATIGVTFFGAGVISTGSFLTTALISLIQRPQLRNLLHEKPELIPAGVEELLRINLSFADGLPRLATADI
QVGDVLVRKGELVLVLLEGANFDPEHFPNPGSIELDRPNPTSHLAFGRGQHFCPGSALGRRHAQIGIEALLKK
MPGVDLAVPIDQLVWRTRFQRRIPERLPVLW
>CYP121A1_Mycobacterium tuberculosis H1589 2622263901
MTATVLLEVPFSARGDRIPDAVAELRTREPIRKVRTITGAEAWLVSSYALCTQVLEDRRFSMKETAAAGAPRL
NALTVPPEVVNNMGNIADAGLRKAVMKAITPKAPGLEQFLRDTANSLLDNLITEGAPADLRNDFADPLATALH
CKVLGIPQEDGPKLFRSLSIAFMSSADPIPAAKINWDRDIEYMAGILENPNITTGLMGELSRLRKDPAYSHVS
DELFATIGVTFFGAGVISTGSFLTTALISLIQRPQLRNLLHEKPELIPAGVEELLRINLSFADGLPRLATADI
QVGDVLVRKGELVLVLLEGANFDPEHFPNPGSIELDRPNPTSHLAFGRGQHFCPGSALGRRHAQIGIEALLKK
MPGVDLAVPIDQLVWRTRFQRRIPERLPVLW
>CYP121A1_Mycobacterium tuberculosis H1964 2646371023
MTATVLLEVPFSARGDRIPDAVAELRTREPIRKVRTITGAEAWLVSSYALCTQVLEDRRFSMKETAAAGAPRL
NALTVPPEVVNNMGNIADAGLRKAVMKAITPKAPGLEQFLRDTANSLLDNLITEGAPADLRNDFADPLATALH

CKVLGIPQEDGPKLFRSLSIAFMSSADPIPAAKINWDRDIEYMAGILENPNITTGLMGELSRLRKDPAYSHVS
DELFATIGVTFFGAGVISTGSFLTTALISLIQRPQLRNLLHEKPELIPAGVEELLRINLSFADGLPRLATADI
QVGDVLVRKGELVLVLLEGANFDPEHFPNPGSIELDRPNPTSHLAFGRGQHFCPGSALGRRHAQIGIEALLKK
MPGVDLAVPIDQLVWRTRFQRRIPERLPVLW
>CYP121A1_Mycobacterium tuberculosis H1996 2622273140
MTATVLLEVPFSARGDRIPDAVAELRTREPIRKVRTITGAEAWLVSSYALCTQVLEDRRFSMKETAAAGAPRL
NALTVPPEVVNNMGNIADAGLRKAVMKAITPKAPGLEQFLRDTANSLLDNLITEGAPADLRNDFADPLATALH
CKVLGIPQEDGPKLFRSLSIAFMSSADPIPAAKINWDRDIEYMAGILENPNITTGLMGELSRLRKDPAYSHVS
DELFATIGVTFFGAGVISTGSFLTTALISLIQRPQLRNLLHEKPELIPAGVEELLRINLSFADGLPRLATADI
QVGDVLVRKGELVLVLLEGANFDPEHFPNPGSIELDRPNPTSHLAFGRGQHFCPGSALGRRHAQIGIEALLKK
MPGVDLAVPIDQLVWRTRFQRRIPERLPVLW
>CYP121A1_Mycobacterium tuberculosis H2101 2622275975
MTATVLLEVPFSARGDRIPDAVAELRTREPIRKVRTITGAEAWLVSSYALCTQVLEDRRFSMKETAAAGAPRL
NALTVPPEVVNNMGNIADAGLRKAVMKAITPKAPGLEQFLRDTANSLLDNLITEGAPADLRNDFADPLATALH
CKVLGIPQEDGPKLFRSLSIAFMSSADPIPAAKINWDRDIEYMAGILENPNITTGLMGELSRLRKDPAYSHVS
DELFATIGVTFFGAGVISTGSFLTTALISLIQRPQLRNLLHEKPELIPAGVEELLRINLSFADGLPRLATADI
QVGDVLVRKGELVLVLLEGANFDPEHFPNPGSIELDRPNPTSHLAFGRGQHFCPGSALGRRHAQIGIEALLKK
MPGVDLAVPIDQLVWRTRFQRRIPERLPVLW
>CYP121A1_Mycobacterium tuberculosis H2102 2622279890
MTATVLLEVPFSARGDRIPDAVAELRTREPIRKVRTITGAEAWLVSSYALCTQVLEDRRFSMKETAAAGAPRL
NALTVPPEVVNNMGNIADAGLRKAVMKAITPKAPGLEQFLRDTANSLLDNLITEGAPADLRNDFADPLATALH
CKVLGIPQEDGPKLFRSLSIAFMSSADPIPAAKINWDRDIEYMAGILENPNITTGLMGELSRLRKDPAYSHVS
DELFATIGVTFFGAGVISTGSFLTTALISLIQRPQLRNLLHEKPELIPAGVEELLRINLSFADGLPRLATADI
QVGDVLVRKGELVLVLLEGANFDPEHFPNPGSIELDRPNPTSHLAFGRGQHFCPGSALGRRHAQIGIEALLKK
MPGVDLAVPIDQLVWRTRFQRRIPERLPVLW
>CYP121A1_Mycobacterium tuberculosis H2264 2622283981
MTATVLLEVPFSARGDRIPDAVAELRTREPIRKVRTITGAEAWLVSSYALCTQVLEDRRFSMKETAAAGAPRL
NALTVPPEVVNNMGNIADAGLRKAVMKAITPKAPGLEQFLRDTANSLLDNLITEGAPADLRNDFADPLATALH
CKVLGIPQEDGPKLFRSLSIAFMSSADPIPAAKINWDRDIEYMAGILENPNITTGLMGELSRLRKDPAYSHVS
DELFATIGVTFFGAGVISTGSFLTTALISLIQRPQLRNLLHEKPELIPAGVEELLRINLSFADGLPRLATADI
QVGDVLVRKGELVLVLLEGANFDPEHFPNPGSIELDRPNPTSHLAFGRGQHFCPGSALGRRHAQIGIEALLKK
MPGVDLAVPIDQLVWRTRFQRRIPERLPVLW
>CYP121A1_Mycobacterium tuberculosis H2375 2647411145
MTATVLLEVPFSARGDRIPDAVAELRTREPIRKVRTITGAEAWLVSSYALCTQVLEDRRFSMKETAAAGAPRL
NALTVPPEVVNNMGNIADAGLRKAVMKAITPKAPGLEQFLRDTANSLLDNLITEGAPADLRNDFADPLATALH
CKVLGIPQEDGPKLFRSLSIAFMSSADPIPAAKINWDRDIEYMAGILENPNITTGLMGELSRLRKDPAYSHVS
DELFATIGVTFFGAGVISTGSFLTTALISLIQRPQLRNLLHEKPELIPAGVEELLRINLSFADGLPRLATADI
QVGDVLVRKGELVLVLLEGANFDPEHFPNPGSIELDRPNPTSHLAFGRGQHFCPGSALGRRHAQIGIEALLKK
MPGVDLAVPIDQLVWRTRFQRRIPERLPVLW
>CYP121A1_Mycobacterium tuberculosis H2438 2584964033
MTATVLLEVPFSARGDRIPDAVAELRTREPIRKVRTITGAEAWLVSSYALCTQVLEDRRFSMKETAAAGAPRL
NALTVPPEVVNNMGNIADAGLRKAVMKAITPKAPGLEQFLRDTANSLLDNLITEGAPADLRNDFADPLATALH
CKVLGIPQEDGPKLFRSLSIAFMSSADPIPAAKINWDRDIEYMAGILENPNITTGLMGELSRLRKDPAYSHVS
DELFATIGVTFFGAGVISTGSFLTTALISLIQRPQLRNLLHEKPELIPAGVEELLRINLSFADGLPRLATADI
QVGDVLVRKGELVLVLLEGANFDPEHFPNPGSIELDRPNPTSHLAFGRGQHFCPGSALGRRHAQIGIEALLKK
MPGVDLAVPIDQLVWRTRFQRRIPERLPVLW
>CYP121A1_Mycobacterium tuberculosis H2463 2622292578
MTATVLLEVPFSARGDRIPDAVAELRTREPIRKVRTITGAEAWLVSSYALCTQVLEDRRFSMKETAAAGAPRL
NALTVPPEVVNNMGNIADAGLRKAVMKAITPKAPGLEQFLRDTANSLLDNLITEGAPADLRNDFADPLATALH
CKVLGIPQEDGPKLFRSLSIAFMSSADPIPAAKINWDRDIEYMAGILENPNITTGLMGELSRLRKDPAYSHVS
DELFATIGVTFFGAGVISTGSFLTTALISLIQRPQLRNLLHEKPELIPAGVEELLRINLSFADGLPRLATADI
QVGDVLVRKGELVLVLLEGANFDPEHFPNPGSIELDRPNPTSHLAFGRGQHFCPGSALGRRHAQIGIEALLKK
MPGVDLAVPIDQLVWRTRFQRRIPERLPVLW
>CYP121A1_Mycobacterium tuberculosis H2754 2622296629
MTATVLLEVPFSARGDRIPDAVAELRTREPIRKVRTITGAEAWLVSSYALCTQVLEDRRFSMKETAAAGAPRL
NALTVPPEVVNNMGNIADAGLRKAVMKAITPKAPGLEQFLRDTANSLLDNLITEGAPADLRNDFADPLATALH
CKVLGIPQEDGPKLFRSLSIAFMSSADPIPAAKINWDRDIEYMAGILENPNITTGLMGELSRLRKDPAYSHVS
DELFATIGVTFFGAGVISTGSFLTTALISLIQRPQLRNLLHEKPELIPAGVEELLRINLSFADGLPRLATADI

QVGDVLVRKGELVLVLLEGANFDPEHFPNPGSIELDRPNPTSHLAFGRGQHFCPGSALGRRHAQIGIEALLKK
MPGVDLAVPIDQLVWRTRFQRRIPERLPVLW
>CYP121A1_Mycobacterium tuberculosis H2760 2622300885
MTATVLLEVPFSARGDRIPDAVAELRTREPIRKVRTITGAEAWLVSSYALCTQVLEDRRFSMKETAAAGAPRL
NALTVPPEVVNNMGNIADAGLRKAVMKAITPKAPGLEQFLRDTANSLLDNLITEGAPADLRNDFADPLATALH
CKVLGIPQEDGPKLFRSLSIAFMSSADPIPAAKINWDRDIEYMAGILENPNITTGLMGELSRLRKDPAYSHVS
DELFATIGVTFFGAGVISTGSFLTTALISLIQRPQLRNLLHEKPELIPAGVEELLRINLSFADGLPRLATADI
QVGDVLVRKGELVLVLLEGANFDPEHFPNPGSIELDRPNPTSHLAFGRGQHFCPGSALGRRHAQIGIEALLKK
MPGVDLAVPIDQLVWRTRFQRRIPERLPVLW
>CYP121A1_Mycobacterium tuberculosis H3005 2622310776
MTATVLLEVPFSARGDRIPDAVAELRTREPIRKVRTITGAEAWLVSSYALCTQVLEDRRFSMKETAAAGAPRL
NALTVPPEVVNNMGNIADAGLRKAVMKAITPKAPGLEQFLRDTANSLLDNLITEGAPADLRNDFADPLATALH
CKVLGIPQEDGPKLFRSLSIAFMSSADPIPAAKINWDRDIEYMAGILENPNITTGLMGELSRLRKDPAYSHVS
DELFATIGVTFFGAGVISTGSFLTTALISLIQRPQLRNLLHEKPELIPAGVEELLRINLSFADGLPRLATADI
QVGDVLVRKGELVLVLLEGANFDPEHFPNPGSIELDRPNPTSHLAFGRGQHFCPGSALGRRHAQIGIEALLKK
MPGVDLAVPIDQLVWRTRFQRRIPERLPVLW
>CYP121A1_Mycobacterium tuberculosis H3033 2622313073
MTATVLLEVPFSARGDRIPDAVAELRTREPIRKVRTITGAEAWLVSSYALCTQVLEDRRFSMKETAAAGAPRL
NALTVPPEVVNNMGNIADAGLRKAVMKAITPKAPGLEQFLRDTANSLLDNLITEGAPADLRNDFADPLATALH
CKVLGIPQEDGPKLFRSLSIAFMSSADPIPAAKINWDRDIEYMAGILENPNITTGLMGELSRLRKDPAYSHVS
DELFATIGVTFFGAGVISTGSFLTTALISLIQRPQLRNLLHEKPELIPAGVEELLRINLSFADGLPRLATADI
QVGDVLVRKGELVLVLLEGANFDPEHFPNPGSIELDRPNPTSHLAFGRGQHFCPGSALGRRHAQIGIEALLKK
MPGVDLAVPIDQLVWRTRFQRRIPERLPVLW
>CYP121A1_Mycobacterium tuberculosis H3094 2622317443
MTATVLLEVPFSARGDRIPDAVAELRTREPIRKVRTITGAEAWLVSSYALCTQVLEDRRFSMKETAAAGAPRL
NALTVPPEVVNNMGNIADAGLRKAVMKAITPKAPGLEQFLRDTANSLLDNLITEGAPADLRNDFADPLATALH
CKVLGIPQEDGPKLFRSLSIAFMSSADPIPAAKINWDRDIEYMAGILENPNITTGLMGELSRLRKDPAYSHVS
DELFATIGVTFFGAGVISTGSFLTTALISLIQRPQLRNLLHEKPELIPAGVEELLRINLSFADGLPRLATADI
QVGDVLVRKGELVLVLLEGANFDPEHFPNPGSIELDRPNPTSHLAFGRGQHFCPGSALGRRHAQIGIEALLKK
MPGVDLAVPIDQLVWRTRFQRRIPERLPVLW
>CYP121A1_Mycobacterium tuberculosis H3361 2575023709
MTATVLLEVPFSARGDRIPDAVAELRTREPIRKVRTITGAEAWLVSSYALCTQVLEDRRFSMKETAAAGAPRL
NALTVPPEVVNNMGNIADAGLRKAVMKAITPKAPGLEQFLRDTANSLLDNLITEGAPADLRNDFADPLATALH
CKVLGIPQEDGPKLFRSLSIAFMSSADPIPAAKINWDRDIEYMAGILENPNITTGLMGELSRLRKDPAYSHVS
DELFATIGVTFFGAGVISTGSFLTTALISLIQRPQLRNLLHEKPELIPAGVEELLRINLSFADGLPRLATADI
QVGDVLVRKGELVLVLLEGANFDPEHFPNPGSIELDRPNPTSHLAFGRGQHFCPGSALGRRHAQIGIEALLKK
MPGVDLAVPIDQLVWRTRFQRRIPERLPVLW
>CYP121A1_Mycobacterium tuberculosis H3367 2622326777
MTATVLLEVPFSARGDRIPDAVAELRTREPIRKVRTITGAEAWLVSSYALCTQVLEDRRFSMKETAAAGAPRL
NALTVPPEVVNNMGNIADAGLRKAVMKAITPKAPGLEQFLRDTANSLLDNLITEGAPADLRNDFADPLATALH
CKVLGIPQEDGPKLFRSLSIAFMSSADPIPAAKINWDRDIEYMAGILENPNITTGLMGELSRLRKDPAYSHVS
DELFATIGVTFFGAGVISTGSFLTTALISLIQRPQLRNLLHEKPELIPAGVEELLRINLSFADGLPRLATADI
QVGDVLVRKGELVLVLLEGANFDPEHFPNPGSIELDRPNPTSHLAFGRGQHFCPGSALGRRHAQIGIEALLKK
MPGVDLAVPIDQLVWRTRFQRRIPERLPVLW
>CYP121A1_Mycobacterium tuberculosis H37Ra 640603021
MTATVLLEVPFSARGDRIPDAVAELRTREPIRKVRTITGAEAWLVSSYALCTQVLEDRRFSMKETAAAGAPRL
NALTVPPEVVNNMGNIADAGLRKAVMKAITPKAPGLEQFLRDTANSLLDNLITEGAPADLRNDFADPLATALH
CKVLGIPQEDGPKLFRSLSIAFMSSADPIPAAKINWDRDIEYMAGILENPNITTGLMGELSRLRKDPAYSHVS
DELFATIGVTFFGAGVISTGSFLTTALISLIQRPQLRNLLHEKPELIPAGVEELLRINLSFADGLPRLATADI
QVGDVLVRKGELVLVLLEGANFDPEHFPNPGSIELDRPNPTSHLAFGRGQHFCPGSALGRRHAQIGIEALLKK
MPGVDLAVPIDQLVWRTRFQRRIPERLPVLW
>CYP121A1_Mycobacterium tuberculosis H37Rv 2604656591
MTATVLLEVPFSARGDRIPDAVAELRTREPIRKVRTITGAEAWLVSSYALCTQVLEDRRFSMKETAAAGAPRL
NALTVPPEVVNNMGNIADAGLRKAVMKAITPKAPGLEQFLRDTANSLLDNLITEGAPADLRNDFADPLATALH
CKVLGIPQEDGPKLFRSLSIAFMSSADPIPAAKINWDRDIEYMAGILENPNITTGLMGELSRLRKDPAYSHVS
DELFATIGVTFFGAGVISTGSFLTTALISLIQRPQLRNLLHEKPELIPAGVEELLRINLSFADGLPRLATADI
QVGDVLVRKGELVLVLLEGANFDPEHFPNPGSIELDRPNPTSHLAFGRGQHFCPGSALGRRHAQIGIEALLKK
MPGVDLAVPIDQLVWRTRFQRRIPERLPVLW

>CYP121A1_Mycobacterium tuberculosis H37Rv 637027501
MTATVLLEVPFSARGDRIPDAVAELRTREPIRKVRTITGAEAWLVSSYALCTQVLEDRRFSMKETAAAGAPRL
NALTVPPEVVNNMGNIADAGLRKAVMKAITPKAPGLEQFLRDTANSLLDNLITEGAPADLRNDFADPLATALH
CKVLGIPQEDGPKLFRSLSIAFMSSADPIPAAKINWDRDIEYMAGILENPNITTGLMGELSRLRKDPAYSHVS
DELFATIGVTFFGAGVISTGSFLTTALISLIQRPQLRNLLHEKPELIPAGVEELLRINLSFADGLPRLATADI
QVGDVLVRKGELVLVLLEGANFDPEHFPNPGSIELDRPNPTSHLAFGRGQHFCPGSALGRRHAQIGIEALLKK
MPGVDLAVPIDQLVWRTRFQRRIPERLPVLW
>CYP121A1_Mycobacterium tuberculosis H37Rv 2527057539
MTATVLLEVPFSARGDRIPDAVAELRTREPIRKVRTITGAEAWLVSSYALCTQVLEDRRFSMKETAAAGAPRL
NALTVPPEVVNNMGNIADAGLRKAVMKAITPKAPGLEQFLRDTANSLLDNLITEGAPADLRNDFADPLATALH
CKVLGIPQEDGPKLFRSLSIAFMSSADPIPAAKINWDRDIEYMAGILENPNITTGLMGELSRLRKDPAYSHVS
DELFATIGVTFFGAGVISTGSFLTTALISLIQRPQLRNLLHEKPELIPAGVEELLRINLSFADGLPRLATADI
QVGDVLVRKGELVLVLLEGANFDPEHFPNPGSIELDRPNPTSHLAFGRGQHFCPGSALGRRHAQIGIEALLKK
MPGVDLAVPIDQLVWRTRFQRRIPERLPVLW
>CYP121A1_Mycobacterium tuberculosis H37Rv 2633791008
MTATVLLEVPFSARGDRIPDAVAELRTREPIRKVRTITGAEAWLVSSYALCTQVLEDRRFSMKETAAAGAPRL
NALTVPPEVVNNMGNIADAGLRKAVMKAITPKAPGLEQFLRDTANSLLDNLITEGAPADLRNDFADPLATALH
CKVLGIPQEDGPKLFRSLSIAFMSSADPIPAAKINWDRDIEYMAGILENPNITTGLMGELSRLRKDPAYSHVS
DELFATIGVTFFGAGVISTGSFLTTALISLIQRPQLRNLLHEKPELIPAGVEELLRINLSFADGLPRLATADI
QVGDVLVRKGELVLVLLEGANFDPEHFPNPGSIELDRPNPTSHLAFGRGQHFCPGSALGRRHAQIGIEALLKK
MPGVDLAVPIDQLVWRTRFQRRIPERLPVLW
>CYP121A1_Mycobacterium tuberculosis H37RvAE 2603096249
MTATVLLEVPFSARGDRIPDAVAELRTREPIRKVRTITGAEAWLVSSYALCTQVLEDRRFSMKETAAAGAPRL
NALTVPPEVVNNMGNIADAGLRKAVMKAITPKAPGLEQFLRDTANSLLDNLITEGAPADLRNDFADPLATALH
CKVLGIPQEDGPKLFRSLSIAFMSSADPIPAAKINWDRDIEYMAGILENPNITTGLMGELSRLRKDPAYSHVS
DELFATIGVTFFGAGVISTGSFLTTALISLIQRPQLRNLLHEKPELIPAGVEELLRINLSFADGLPRLATADI
QVGDVLVRKGELVLVLLEGANFDPEHFPNPGSIELDRPNPTSHLAFGRGQHFCPGSALGRRHAQIGIEALLKK
MPGVDLAVPIDQLVWRTRFQRRIPERLPVLW
>CYP121A1_Mycobacterium tuberculosis H37RvCO 2547164854
MTATVLLEVPFSARGDRIPDAVAELRTREPIRKVRTITGAEAWLVSSYALCTQVLEDRRFSMKETAAAGAPRL
NALTVPPEVVNNMGNIADAGLRKAVMKAITPKAPGLEQFLRDTANSLLDNLITEGAPADLRNDFADPLATALH
CKVLGIPQEDGPKLFRSLSIAFMSSADPIPAAKINWDRDIEYMAGILENPNITTGLMGELSRLRKDPAYSHVS
DELFATIGVTFFGAGVISTGSFLTTALISLIQRPQLRNLLHEKPELIPAGVEELLRINLSFADGLPRLATADI
QVGDVLVRKGELVLVLLEGANFDPEHFPNPGSIELDRPNPTSHLAFGRGQHFCPGSALGRRHAQIGIEALLKK
MPGVDLAVPIDQLVWRTRFQRRIPERLPVLW
>CYP121A1_Mycobacterium tuberculosis H37RvLP 2603100401
MTATVLLEVPFSARGDRIPDAVAELRTREPIRKVRTITGAEAWLVSSYALCTQVLEDRRFSMKETAAAGAPRL
NALTVPPEVVNNMGNIADAGLRKAVMKAITPKAPGLEQFLRDTANSLLDNLITEGAPADLRNDFADPLATALH
CKVLGIPQEDGPKLFRSLSIAFMSSADPIPAAKINWDRDIEYMAGILENPNITTGLMGELSRLRKDPAYSHVS
DELFATIGVTFFGAGVISTGSFLTTALISLIQRPQLRNLLHEKPELIPAGVEELLRINLSFADGLPRLATADI
QVGDVLVRKGELVLVLLEGANFDPEHFPNPGSIELDRPNPTSHLAFGRGQHFCPGSALGRRHAQIGIEALLKK
MPGVDLAVPIDQLVWRTRFQRRIPERLPVLW
>CYP121A1_Mycobacterium tuberculosis H37RvMA 2603104592
MTATVLLEVPFSARGDRIPDAVAELRTREPIRKVRTITGAEAWLVSSYALCTQVLEDRRFSMKETAAAGAPRL
NALTVPPEVVNNMGNIADAGLRKAVMKAITPKAPGLEQFLRDTANSLLDNLITEGAPADLRNDFADPLATALH
CKVLGIPQEDGPKLFRSLSIAFMSSADPIPAAKINWDRDIEYMAGILENPNITTGLMGELSRLRKDPAYSHVS
DELFATIGVTFFGAGVISTGSFLTTALISLIQRPQLRNLLHEKPELIPAGVEELLRINLSFADGLPRLATADI
QVGDVLVRKGELVLVLLEGANFDPEHFPNPGSIELDRPNPTSHLAFGRGQHFCPGSALGRRHAQIGIEALLKK
MPGVDLAVPIDQLVWRTRFQRRIPERLPVLW
>CYP121A1_Mycobacterium tuberculosis H37RvSiena 2647321601
MTATVLLEVPFSARGDRIPDAVAELRTREPIRKVRTITGAEAWLVSSYALCTQVLEDRRFSMKETAAAGAPRL
NALTVPPEVVNNMGNIADAGLRKAVMKAITPKAPGLEQFLRDTANSLLDNLITEGAPADLRNDFADPLATALH
CKVLGIPQEDGPKLFRSLSIAFMSSADPIPAAKINWDRDIEYMAGILENPNITTGLMGELSRLRKDPAYSHVS
DELFATIGVTFFGAGVISTGSFLTTALISLIQRPQLRNLLHEKPELIPAGVEELLRINLSFADGLPRLATADI
QVGDVLVRKGELVLVLLEGANFDPEHFPNPGSIELDRPNPTSHLAFGRGQHFCPGSALGRRHAQIGIEALLKK
MPGVDLAVPIDQLVWRTRFQRRIPERLPVLW
>CYP121A1_Mycobacterium tuberculosis H3986 2575491203

MTATVLLEVPFSARGDRIPDAVAELRTREPIRKVRTITGAEAWLVSSYALCTQVLEDRRFSMKETAAAGAPRL
NALTVPPEVVNNMGNIADAGLRKAVMKAITPKAPGLEQFLRDTANSLLDNLITEGAPADLRNDFADPLATALH
CKVLGIPQEDGPKLFRSLSIAFMSSADPIPAAKINWDRDIEYMAGILENPNITTGLMGELSRLRKDPAYSHVS
DELFATIGVTFFGAGVISTGSFLTTALISLIQRPQLRNLLHEKPELIPAGVEELLRINLSFADGLPRLATADI
QVGDVLVRKGELVLVLLEGANFDPEHFPNPGSIELDRPNPTSHLAFGRGQHFCPGSALGRRHAQIGIEALLKK
MPGVDLAVPIDQLVWRTRFQRRIPERLPVLW
>CYP121A1_Mycobacterium tuberculosis H55_24991 2716365352
MTATVLLEVPFSARGDRIPDAVAELRTREPIRKVRTITGAEAWLVSSYALCTQVLEDRRFSMKETAAAGAPRL
NALTVPPEVVNNMGNIADAGLRKAVMKAITPKAPGLEQFLRDTANSLLDNLITEGAPADLRNDFADPLATALH
CKVLGIPQEDGPKLFRSLSIAFMSSADPIPAAKINWDRDIEYMAGILENPNITTGLMGELSRLRKDPAYSHVS
DELFATIGVTFFGAGVISTGSFLTTALISLIQRPQLRNLLHEKPELIPAGVEELLRINLSFADGLPRLATADI
QVGDVLVRKGELVLVLLEGANFDPEHFPNPGSIELDRPNPTSHLAFGRGQHFCPGSALGRRHAQIGIEALLKK
MPGVDLAVPIDQLVWRTRFQRRIPERLPVLW
>CYP121A1_Mycobacterium tuberculosis H6_10443 2716366558
MTATVLLEVPFSARGDRIPDAVAELRTREPIRKVRTITGAEAWLVSSYALCTQVLEDRRFSMKETAAAGAPRL
NALTVPPEVVNNMGNIADAGLRKAVMKAITPKAPGLEQFLRDTANSLLDNLITEGAPADLRNDFADPLATALH
CKVLGIPQEDGPKLFRSLSIAFMSSADPIPAAKINWDRDIEYMAGILENPNITTGLMGELSRLRKDPAYSHVS
DELFATIGVTFFGAGVISTGSFLTTALISLIQRPQLRNLLHEKPELIPAGVEELLRINLSFADGLPRLATADI
QVGDVLVRKGELVLVLLEGANFDPEHFPNPGSIELDRPNPTSHLAFGRGQHFCPGSALGRRHAQIGIEALLKK
MPGVDLAVPIDQLVWRTRFQRRIPERLPVLW
>CYP121A1_Mycobacterium tuberculosis H6_12226 2716371073
MTATVLLEVPFSARGDRIPDAVAELRTREPIRKVRTITGAEAWLVSSYALCTQVLEDRRFSMKETAAAGAPRL
NALTVPPEVVNNMGNIADAGLRKAVMKAITPKAPGLEQFLRDTANSLLDNLITEGAPADLRNDFADPLATALH
CKVLGIPQEDGPKLFRSLSIAFMSSADPIPAAKINWDRDIEYMAGILENPNITTGLMGELSRLRKDPAYSHVS
DELFATIGVTFFGAGVISTGSFLTTALISLIQRPQLRNLLHEKPELIPAGVEELLRINLSFADGLPRLATADI
QVGDVLVRKGELVLVLLEGANFDPEHFPNPGSIELDRPNPTSHLAFGRGQHFCPGSALGRRHAQIGIEALLKK
MPGVDLAVPIDQLVWRTRFQRRIPERLPVLW
>CYP121A1_Mycobacterium tuberculosis H6_7420 2716299347
MTATVLLEVPFSARGDRIPDAVAELRTREPIRKVRTITGAEAWLVSSYALCTQVLEDRRFSMKETAAAGAPRL
NALTVPPEVVNNMGNIADAGLRKAVMKAITPKAPGLEQFLRDTANSLLDNLITEGAPADLRNDFADPLATALH
CKVLGIPQEDGPKLFRSLSIAFMSSADPIPAAKINWDRDIEYMAGILENPNITTGLMGELSRLRKDPAYSHVS
DELFATIGVTFFGAGVISTGSFLTTALISLIQRPQLRNLLHEKPELIPAGVEELLRINLSFADGLPRLATADI
QVGDVLVRKGELVLVLLEGANFDPEHFPNPGSIELDRPNPTSHLAFGRGQHFCPGSALGRRHAQIGIEALLKK
MPGVDLAVPIDQLVWRTRFQRRIPERLPVLW
>CYP121A1_Mycobacterium tuberculosis I_15762 2716378155
MTATVLLEVPFSARGDRIPDAVAELRTREPIRKVRTITGAEAWLVSSYALCTQVLEDRRFSMKETAAAGAPRL
NALTVPPEVVNNMGNIADAGLRKAVMKAITPKAPGLEQFLRDTANSLLDNLITEGAPADLRNDFADPLATALH
CKVLGIPQEDGPKLFRSLSIAFMSSADPIPAAKINWDRDIEYMAGILENPNITTGLMGELSRLRKDPAYSHVS
DELFATIGVTFFGAGVISTGSFLTTALISLIQRPQLRNLLHEKPELIPAGVEELLRINLSFADGLPRLATADI
QVGDVLVRKGELVLVLLEGANFDPEHFPNPGSIELDRPNPTSHLAFGRGQHFCPGSALGRRHAQIGIEALLKK
MPGVDLAVPIDQLVWRTRFQRRIPERLPVLW
>CYP121A1_Mycobacterium tuberculosis I0002353-6 2722600530
MTATVLLEVPFSARGDRIPDAVAELRTREPIRKVRTITGAEAWLVSSYALCTQVLEDRRFSMKETAAAGAPRL
NALTVPPEVVNNMGNIADAGLRKAVMKAITPKAPGLEQFLRDTANSLLDNLITEGAPADLRNDFADPLATALH
CKVLGIPQEDGPKLFRSLSIAFMSSADPIPAAKINWDRDIEYMAGILENPNITTGLMGELSRLRKDPAYSHVS
DELFATIGVTFFGAGVISTGSFLTTALISLIQRPQLRNLLHEKPELIPAGVEELLRINLSFADGLPRLATADI
QVGDVLVRKGELVLVLLEGANFDPEHFPNPGSIELDRPNPTSHLAFGRGQHFCPGSALGRRHAQIGIEALLKK
MPGVDLAVPIDQLVWRTRFQRRIPERLPVLW
>CYP121A1_Mycobacterium tuberculosis I0002801-4 2720706788
MTATVLLEVPFSARGDRIPDAVAELRTREPIRKVRTITGAEAWLVSSYALCTQVLEDRRFSMKETAAAGAPRL
NALTVPPEVVNNMGNIADAGLRKAVMKAITPKAPGLEQFLRDTANSLLDNLITEGAPADLRNDFADPLATALH
CKVLGIPQEDGPKLFRSLSIAFMSSADPIPAAKINWDRDIEYMAGILENPNITTGLMGELSRLRKDPAYSHVS
DELFATIGVTFFGAGVISTGSFLTTALISLIQRPQLRNLLHEKPELIPAGVEELLRINLSFADGLPRLATADI
QVGDVLVRKGELVLVLLEGANFDPEHFPNPGSIELDRPNPTSHLAFGRGQHFCPGSALGRRHAQIGIEALLKK
MPGVDLAVPIDQLVWRTRFQRRIPERLPVLW
>CYP121A1_Mycobacterium tuberculosis I0004000-1 2720702684
MTATVLLEVPFSARGDRIPDAVAELRTREPIRKVRTITGAEAWLVSSYALCTQVLEDRRFSMKETAAAGAPRL
NALTVPPEVVNNMGNIADAGLRKAVMKAITPKAPGLEQFLRDTANSLLDNLITEGAPADLRNDFADPLATALH

CKVLGIPQEDGPKLFRSLSIAFMSSADPIPAAKINWDRDIEYMAGILENPNITTGLMGELSRLRKDPAYSHVS
DELFATIGVTFFGAGVISTGSFLTTALISLIQRPQLRNLLHEKPELIPAGVEELLRINLSFADGLPRLATADI
QVGDVLVRKGELVLVLLEGANFDPEHFPNPGSIELDRPNPTSHLAFGRGQHFCPGSALGRRHAQIGIEALLKK
MPGVDLAVPIDQLVWRTRFQRRIPERLPVLW
>CYP121A1_Mycobacterium tuberculosis I0004241-1 2721357394
MTATVLLEVPFSARGDRIPDAVAELRTREPIRKVRTITGAEAWLVSSYALCTQVLEDRRFSMKETAAAGAPRL
NALTVPPEVVNNMGNIADAGLRKAVMKAITPKAPGLEQFLRDTANSLLDNLITEGAPADLRNDFADPLATALH
CKVLGIPQEDGPKLFRSLSIAFMSSADPIPAAKINWDRDIEYMAGILENPNITTGLMGELSRLRKDPAYSHVS
DELFATIGVTFFGAGVISTGSFLTTALISLIQRPQLRNLLHEKPELIPAGVEELLRINLSFADGLPRLATADI
QVGDVLVRKGELVLVLLEGANFDPEHFPNPGSIELDRPNPTSHLAFGRGQHFCPGSALGRRHAQIGIEALLKK
MPGVDLAVPIDQLVWRTRFQRRIPERLPVLW
>CYP121A1_Mycobacterium tuberculosis INS_MDR 2621350170
MTATVLLEVPFSARGDRIPDAVAELRTREPIRKVRTITGAEAWLVSSYALCTQVLEDRRFSMKETAAAGAPRL
NALTVPPEVVNNMGNIADAGLRKAVMKAITPKAPGLEQFLRDTANSLLDNLITEGAPADLRNDFADPLATALH
CKVLGIPQEDGPKLFRSLSIAFMSSADPIPAAKINWDRDIEYMAGILENPNITTGLMGELSRLRKDPAYSHVS
DELFATIGVTFFGAGVISTGSFLTTALISLIQRPQLRNLLHEKPELIPAGVEELLRINLSFADGLPRLATADI
QVGDVLVRKGELVLVLLEGANFDPEHFPNPGSIELDRPNPTSHLAFGRGQHFCPGSALGRRHAQIGIEALLKK
MPGVDLAVPIDQLVWRTRFQRRIPERLPVLW
>CYP121A1_Mycobacterium tuberculosis INS_SEN 2621354494
MTATVLLEVPFSARGDRIPDAVAELRTREPIRKVRTITGAEAWLVSSYALCTQVLEDRRFSMKETAAAGAPRL
NALTVPPEVVNNMGNIADAGLRKAVMKAITPKAPGLEQFLRDTANSLLDNLITEGAPADLRNDFADPLATALH
CKVLGIPQEDGPKLFRSLSIAFMSSADPIPAAKINWDRDIEYMAGILENPNITTGLMGELSRLRKDPAYSHVS
DELFATIGVTFFGAGVISTGSFLTTALISLIQRPQLRNLLHEKPELIPAGVEELLRINLSFADGLPRLATADI
QVGDVLVRKGELVLVLLEGANFDPEHFPNPGSIELDRPNPTSHLAFGRGQHFCPGSALGRRHAQIGIEALLKK
MPGVDLAVPIDQLVWRTRFQRRIPERLPVLW
>CYP121A1_Mycobacterium tuberculosis INS_XDR 2573561345
MTATVLLEVPFSARGDRIPDAVAELRTREPIRKVRTITGAEAWLVSSYALCTQVLEDRRFSMKETAAAGAPRL
NALTVPPEVVNNMGNIADAGLRKAVMKAITPKAPGLEQFLRDTANSLLDNLITEGAPADLRNDFADPLATALH
CKVLGIPQEDGPKLFRSLSIAFMSSADPIPAAKINWDRDIEYMAGILENPNITTGLMGELSRLRKDPAYSHVS
DELFATIGVTFFGAGVISTGSFLTTALISLIQRPQLRNLLHEKPELIPAGVEELLRINLSFADGLPRLATADI
QVGDVLVRKGELVLVLLEGANFDPEHFPNPGSIELDRPNPTSHLAFGRGQHFCPGSALGRRHAQIGIEALLKK
MPGVDLAVPIDQLVWRTRFQRRIPERLPVLW
>CYP121A1_Mycobacterium tuberculosis J09400698 2675372412
EQFLRDTANSLLDNLITEGAPADLRNDFADPLATALHCKVLGIPQEDGPKLFRSLSIAFMSSADPIPAAKINW
DRDIEYMAGILENPNITTGLMGELSRLRKDPAYSHVSDELFATIGVTFFGAGVISTGSFLTTALISLIQRPQL
RNLLHEKPELIPAGVEELLRINLSFADGLPRLATADIQVGDVLVRKGELVLVLLEGANFDPEHFPNPGSIELD
RPNPTSHLAFGRGQHFCPGSALGRRHAQIGIEALLKKMPGVDLAVPIDQLVWRTRFQRRIPERLPVLW
>CYP121A1_Mycobacterium tuberculosis J09401337 2697651817
MTATVLLEVPFSARGDRIPDAVAELRTREPIRKVRTITGAEAWLVSSYALCTQVLEDRRFSMKETAAAGAPRL
NALTVPPEVVNNMGNIADAGLRKAVMKAITPKAPGLEQFLRDTANSLLDNLITEGAPADLRNDFADPLATALH
CKVLGIPQEDGPKLFRSLSIAFMSSADPIPAAKINWDRDIEYMAGILENPNITTGLMGELSRLRKDPAYSHVS
DELFATIGVTFFGAGVISTGSFLTTALISLIQRPQLRNLLHEKPELIPAGVEELLRINLSFADGLPRLATADI
QVGDVLVRKGELVLVLLEGANFDPEHFPNPGSIELDRPNPTSHLAFGRGQHFCPGSALGRRHAQIGIEALLKK
MPGVDLAVPIDQLVWRTRFQRRIPERLPVLW
>CYP121A1_Mycobacterium tuberculosis J09402203 2673323867
MTATVLLEVPFSARGDRIPDAVAELRTREPIRKVRTITGAEAWLVSSYALCTQVLEDRRFSMKETAAAGAPRL
NALTVPPEVVNNMGNIADAGLRKAVMKAITPKAPGLEQFLRDTANSLLDNLITEGAPADLRNDFADPLATALH
CKVLGIPQEDGPKLFRSLSIAFMSSADPIPAAKINWDRDIEYMAGILENPNITTGLMGELSRLRKDPAYSHVS
DELFATIGVTFFGAGVISTGSFLTTALISLIQRPQLRNLLHEKPELIPAGVEELLRINLSFADGLPRLATADI
QVGDVLVRKGELVLVLLEGANFDPEHFPNPGSIELDRPNPTSHLAFGRGQHFCPGSALGRRHAQIGIEALLKK
MPGVDLAVPIDQLVWRTRFQRRIPERLPVLW
>CYP121A1_Mycobacterium tuberculosis J09500385 2703484252
MTATVLLEVPFSARGDRIPDAVAELRTREPIRKVRTITGAEAWLVSSYALCTQVLEDRRFSMKETAAAGAPRL
NALTVPPEVVNNMGNIADAGLRKAVMKAITPKAPGLEQFLRDTANSLLDNLITEGAPADLRNDFADPLATALH
CKVLGIPQEDGPKLFRSLSIAFMSSADPIPAAKINWDRDIEYMAGILENPNITTGLMGELSRLRKDPAYSHVS
DELFATIGVTFFGAGVISTGSFLTTALISLIQRPQLRNLLHEKPELIPAGVEELLRINLSFADGLPRLATADI
QVGDVLVRKGELVLVLLEGANFDPEHFPNPGSIELDRPNPTSHLAFGRGQHFCPGSALGRRHAQIGIEALLKK
MPGVDLAVPIDQLVWRTRFQRRIPERLPVLW

>CYP121A1_Mycobacterium tuberculosis J09500392 2703486389
MTATVLLEVPFSARGDRIPDAVAELRTREPIRKVRTITGAEAWLVSSYALCTQVLEDRRFSMKETAAAGAPRL
NALTVPPEVVNNMGNIADAGLRKAVMKAITPKAPGLEQFLRDTANSLLDNLITEGAPADLRNDFADPLATALH
CKVLGIPQEDGPKLFRSLSIAFMSSADPIPAAKINWDRDIEYMAGILENPNITTGLMGELSRLRKDPAYSHVS
DELFATIGVTFFGAGVISTGSFLTTALISLIQRPQLRNLLHEKPELIPAGVEELLRINLSFADGLPRLATADI
QVGDVLVRKGELVLVLLEGANFDPEHFPNPGSIELDRPNPTSHLAFGRGQHFCPGSALGRRHAQIGIEALLKK
MPGVDLAVPIDQLVWRTRFQRRIPERLPVLW
>CYP121A1_Mycobacterium tuberculosis J09700837 2706609337
MTATVLLEVPFSARGDRIPDAVAELRTREPIRKVRTITGAEAWLVSSYALCTQVLEDRRFSMKETAAAGAPRL
NALTVPPEVVNNMGNIADAGLRKAVMKAITPKAPGLEQFLRDTANSLLDNLITEGAPADLRNDFADPLATALH
CKVLGIPQEDGPKLFRSLSIAFMSSADPIPAAKINWDRDIEYMAGILENPNITTGLMGELSRLRKDPAYSHVS
DELFATIGVTFFGAGVISTGSFLTTALISLIQRPQLRNLLHEKPELIPAGVEELLRINLSFADGLPRLATADI
QVGDVLVRKGELVLVLLEGANFDPEHFPNPGSIELDRPNPTSHLAFGRGQHFCPGSALGRRHAQIGIEALLKK
MPGVDLAVPIDQLVWRTRFQRRIPERLPVLW
>CYP121A1_Mycobacterium tuberculosis J09701231 2703496933
MTATVLLEVPFSARGDRIPDAVAELRTREPIRKVRTITGAEAWLVSSYALCTQVLEDRRFSMKETAAAGAPRL
NALTVPPEVVNNMGNIADAGLRKAVMKAITPKAPGLEQFLRDTANSLLDNLITEGAPADLRNDFADPLATALH
CKVLGIPQEDGPKLFRSLSIAFMSSADPIPAAKINWDRDIEYMAGILENPNITTGLMGELSRLRKDPAYSHVS
DELFATIGVTFFGAGVISTGSFLTTALISLIQRPQLRNLLHEKPELIPAGVEELLRINLSFADGLPRLATADI
QVGDVLVRKGELVLVLLEGANFDPEHFPNPGSIELDRPNPTSHLAFGRGQHFCPGSALGRRHAQIGIEALLKK
MPGVDLAVPIDQLVWRTRFQRRIPERLPVLW
>CYP121A1_Mycobacterium tuberculosis J09701362 2706572476
MTATVLLEVPFSARGDRIPDAVAELRTREPIRKVRTITGAEAWLVSSYALCTQVLEDRRFSMKETAAAGAPRL
NALTVPPEVVNNMGNIADAGLRKAVMKAITPKAPGLEQFLRDTANSLLDNLITEGAPADLRNDFADPLATALH
CKVLGIPQEDGPKLFRSLSIAFMSSADPIPAAKINWDRDIEYMAGILENPNITTGLMGELSRLRKDPAYSHVS
DELFATIGVTFFGAGVISTGSFLTTALISLIQRPQLRNLLHEKPELIPAGVEELLRINLSFADGLPRLATADI
QVGDVLVRKGELVLVLLEGANFDPEHFPNPGSIELDRPNPTSHLAFGRGQHFCPGSALGRRHAQIGIEALLKK
MPGVDLAVPIDQLVWRTRFQRRIPERLPVLW
>CYP121A1_Mycobacterium tuberculosis J09701593 2696834075
MTATVLLEVPFSARGDRIPDAVAELRTREPIRKVRTITGAEAWLVSSYALCTQVLEDRRFSMKETAAAGAPRL
NALTVPPEVVNNMGNIADAGLRKAVMKAITPKAPGLEQFLRDTANSLLDNLITEGAPADLRNDFADPLATALH
CKVLGIPQEDGPKLFRSLSIAFMSSADPIPAAKINWDRDIEYMAGILENPNITTGLMGELSRLRKDPAYSHVS
DELFATIGVTFFGAGVISTGSFLTTALISLIQRPQLRNLLHEKPELIPAGVEELLRINLSFADGLPRLATADI
QVGDVLVRKGELVLVLLEGANFDPEHFPNPGSIELDRPNPTSHLAFGRGQHFCPGSALGRRHAQIGIEALLKK
MPGVDLAVPIDQLVWRTRFQRRIPERLPVLW
>CYP121A1_Mycobacterium tuberculosis J09701920 2703480504
MTATVLLEVPFSARGDRIPDAVAELRTREPIRKVRTITGAEAWLVSSYALCTQVLEDRRFSMKETAAAGAPRL
NALTVPPEVVNNMGNIADAGLRKAVMKAITPKAPGLEQFLRDTANSLLDNLITEGAPADLRNDFADPLATALH
CKVLGIPQEDGPKLFRSLSIAFMSSADPIPAAKINWDRDIEYMAGILENPNITTGLMGELSRLRKDPAYSHVS
DELFATIGVTFFGAGVISTGSFLTTALISLIQRPQLRNLLHEKPELIPAGVEELLRINLSFADGLPRLATADI
QVGDVLVRKGELVLVLLEGANFDPEHFPNPGSIELDRPNPTSHLAFGRGQHFCPGSALGRRHAQIGIEALLKK
MPGVDLAVPIDQLVWRTRFQRRIPERLPVLW
>CYP121A1_Mycobacterium tuberculosis J09800554 2693204360
MTATVLLEVPFSARGDRIPDAVAELRTREPIRKVRTITGAEAWLVSSYALCTQVLEDRRFSMKETAAAGAPRL
NALTVPPEVVNNMGNIADAGLRKAVMKAITPKAPGLEQFLRDTANSLLDNLITEGAPADLRNDFADPLATALH
CKVLGIPQEDGPKLFRSLSIAFMSSADPIPAAKINWDRDIEYMAGILENPNITTGLMGELSRLRKDPAYSHVS
DELFATIGVTFFGAGVISTGSFLTTALISLIQRPQLRNLLHEKPELIPAGVEELLRINLSFADGLPRLATADI
QVGDVLVRKGELVLVLLEGANFDPEHFPNPGSIELDRPNPTSHLAFGRGQHFCPGSALGRRHAQIGIEALLKK
MPGVDLAVPIDQLVWRTRFQRRIPERLPVLW
>CYP121A1_Mycobacterium tuberculosis J09800628 2673569556
MTATVLLEVPFSARGDRIPDAVAELRTREPIRKVRTITGAEAWLVSSYALCTQVLEDRRFSMKETAAAGAPRL
NALTVPPEVVNNMGNIADAGLRKAVMKAITPKAPGLEQFLRDTANSLLDNLITEGAPADLRNDFADPLATALH
CKVLGIPQEDGPKLFRSLSIAFMSSADPIPAAKINWDRDIEYMAGILENPNITTGLMGELSRLRKDPAYSHVS
DELFATIGVTFFGAGVISTGSFLTTALISLIQRPQLRNLLHEKPELIPAGVEELLRINLSFADGLPRLATADI
QVGDVLVRKGELVLVLLEGANFDPEHFPNPGSIELDRPNPTSHLAFGRGQHFCPGSALGRRHAQIGIEALLKK
MPGVDLAVPIDQLVWRTRFQRRIPERLPVLW
>CYP121A1_Mycobacterium tuberculosis J09900137 2703506867

MTATVLLEVPFSARGDRIPDAVAELRTREPIRKVRTITGAEAWLVSSYALCTQVLEDRRFSMKETAAAGAPRL
NALTVPPEVVNNMGNIADAGLRKAVMKAITPKAPGLEQFLRDTANSLLDNLITEGAPADLRNDFADPLATALH
CKVLGIPQEDGPKLFRSLSIAFMSSADPIPAAKINWDRDIEYMAGILENPNITTGLMGELSRLRKDPAYSHVS
DELFATIGVTFFGAGVISTGSFLTTALISLIQRPQLRNLLHEKPELIPAGVEELLRINLSFADGLPRLATADI
QVGDVLVRKGELVLVLLEGANFDPEHFPNPGSIELDRPNPTSHLAFGRGQHFCPGSALGRRHAQIGIEALLKK
MPGVDLAVPIDQLVWRTRFQRRIPERLPVLW
>CYP121A1_Mycobacterium tuberculosis J09900221 2683846950
MTATVLLEVPFSARGDRIPDAVAELRTREPIRKVRTITGAEAWLVSSYALCTQVLEDRRFSMKETAAAGAPRL
NALTVPPEVVNNMGNIADAGLRKAVMKAITPKAPGLEQFLRDTANSLLDNLITEGAPADLRNDFADPLATALH
CKVLGIPQEDGPKLFRSLSIAFMSSADPIPAAKINWDRDIEYMAGILENPNITTGLMGELSRLRKDPAYSHVS
DELFATIGVTFFGAGVISTGSFLTTALISLIQRPQLRNLLHEKPELIPAGVEELLRINLSFADGLPRLATADI
QVGDVLVRKGELVLVLLEGANFDPEHFPNPGSIELDRPNPTSHLAFGRGQHFCPGSALGRRHAQIGIEALLKK
MPGVDLAVPIDQLVWRTRFQRRIPERLPVLW
>CYP121A1_Mycobacterium tuberculosis J09900583 2696307723
MTATVLLEVPFSARGDRIPDAVAELRTREPIRKVRTITGAEAWLVSSYALCTQVLEDRRFSMKETAAAGAPRL
NALTVPPEVVNNMGNIADAGLRKAVMKAITPKAPGLEQFLRDTANSLLDNLITEGAPADLRNDFADPLATALH
CKVLGIPQEDGPKLFRSLSIAFMSSADPIPAAKINWDRDIEYMAGILENPNITTGLMGELSRLRKDPAYSHVS
DELFATIGVTFFGAGVISTGSFLTTALISLIQRPQLRNLLHEKPELIPAGVEELLRINLSFADGLPRLATADI
QVGDVLVRKGELVLVLLEGANFDPEHFPNPGSIELDRPNPTSHLAFGRGQHFCPGSALGRRHAQIGIEALLKK
MPGVDLAVPIDQLVWRTRFQRRIPERLPVLW
>CYP121A1_Mycobacterium tuberculosis J09902073 2675871402
MTATVLLEVPFSARGDRIPDAVAELRTREPIRKVRTITGAEAWLVSSYALCTQVLEDRRFSMKETAAAGAPRL
NALTVPPEVVNNMGNIADAGLRKAVMKAITPKAPGLEQFLRDTANSLLDNLITEGAPADLRNDFADPLATALH
CKVLGIPQEDGPKLFRSLSIAFMSSADPIPAAKINWDRDIEYMAGILENPNITTGLMGELSRLRKDPAYSHVS
DELFATIGVTFFGAGVISTGSFLTTALISLIQRPQLRNLLHEKPELIPAGVEELLRINLSFADGLPRLATADI
QVGDVLVRKGELVLVLLEGANFDPEHFPNPGSIELDRPNPTSHLAFGRGQHFCPGSALGRRHAQIGIEALLKK
MPGVDLAVPIDQLVWRTRFQRRIPERLPVLW
>CYP121A1_Mycobacterium tuberculosis K00101539 2706619885
MTATVLLEVPFSARGDRIPDAVAELRTREPIRKVRTITGAEAWLVSSYALCTQVLEDRRFSMKETAAAGAPRL
NALTVPPEVVNNMGNIADAGLRKAVMKAITPKAPGLEQFLRDTANSLLDNLITEGAPADLRNDFADPLATALH
CKVLGIPQEDGPKLFRSLSIAFMSSADPIPAAKINWDRDIEYMAGILENPNITTGLMGELSRLRKDPAYSHVS
DELFATIGVTFFGAGVISTGSFLTTALISLIQRPQLRNLLHEKPELIPAGVEELLRINLSFADGLPRLATADI
QVGDVLVRKGELVLVLLEGANFDPEHFPNPGSIELDRPNPTSHLAFGRGQHFCPGSALGRRHAQIGIEALLKK
MPGVDLAVPIDQLVWRTRFQRRIPERLPVLW
>CYP121A1_Mycobacterium tuberculosis K00200539 2706589761
MTATVLLEVPFSARGDRIPDAVAELRTREPIRKVRTITGAEAWLVSSYALCTQVLEDRRFSMKETAAAGAPRL
NALTVPPEVVNNMGNIADAGLRKAVMKAITPKAPGLEQFLRDTANSLLDNLITEGAPADLRNDFADPLATALH
CKVLGIPQEDGPKLFRSLSIAFMSSADPIPAAKINWDRDIEYMAGILENPNITTGLMGELSRLRKDPAYSHVS
DELFATIGVTFFGAGVISTGSFLTTALISLIQRPQLRNLLHEKPELIPAGVEELLRINLSFADGLPRLATADI
QVGDVLVRKGELVLVLLEGANFDPEHFPNPGSIELDRPNPTSHLAFGRGQHFCPGSALGRRHAQIGIEALLKK
MPGVDLAVPIDQLVWRTRFQRRIPERLPVLW
>CYP121A1_Mycobacterium tuberculosis K00201415 2703669376
MTATVLLEVPFSARGDRIPDAVAELRTREPIRKVRTITGAEAWLVSSYALCTQVLEDRRFSMKETAAAGAPRL
NALTVPPEVVNNMGNIADAGLRKAVMKAITPKAPGLEQFLRDTANSLLDNLITEGAPADLRNDFADPLATALH
CKVLGIPQEDGPKLFRSLSIAFMSSADPIPAAKINWDRDIEYMAGILENPNITTGLMGELSRLRKDPAYSHVS
DELFATIGVTFFGAGVISTGSFLTTALISLIQRPQLRNLLHEKPELIPAGVEELLRINLSFADGLPRLATADI
QVGDVLVRKGELVLVLLEGANFDPEHFPNPGSIELDRPNPTSHLAFGRGQHFCPGSALGRRHAQIGIEALLKK
MPGVDLAVPIDQLVWRTRFQRRIPERLPVLW
>CYP121A1_Mycobacterium tuberculosis K00500041 2683879924
MTATVLLEVPFSARGDRIPDAVAELRTREPIRKVRTITGAEAWLVSSYALCTQVLEDRRFSMKETAAAGAPRL
NALTVPPEVVNNMGNIADAGLRKAVMKAITPKAPGLEQFLRDTANSLLDNLITEGAPADLRNDFADPLATALH
CKVLGIPQEDGPKLFRSLSIAFMSSADPIPAAKINWDRDIEYMAGILENPNITTGLMGELSRLRKDPAYSHVS
DELFATIGVTFFGAGVISTGSFLTTALISLIQRPQLRNLLHEKPELIPAGVEELLRINLSFADGLPRLATADI
QVGDVLVRKGELVLVLLEGANFDPEHFPNPGSIELDRPNPTSHLAFGRGQHFCPGSALGRRHAQIGIEALLKK
MPGVDLAVPIDQLVWRTRFQRRIPERLPVLW
>CYP121A1_Mycobacterium tuberculosis K00500133 2696815235
MTATVLLEVPFSARGDRIPDAVAELRTREPIRKVRTITGAEAWLVSSYALCTQVLEDRRFSMKETAAAGAPRL
NALTVPPEVVNNMGNIADAGLRKAVMKAITPKAPGLEQFLRDTANSLLDNLITEGAPADLRNDFADPLATALH

CKVLGIPQEDGPKLFRSLSIAFMSSADPIPAAKINWDRDIEYMAGILENPNITTGLMGELSRLRKDPAYSHVS
DELFATIGVTFFGAGVISTGSFLTTALISLIQRPQLRNLLHEKPELIPAGVEELLRINLSFADGLPRLATADI
QVGDVLVRKGELVLVLLEGANFDPEHFPNPGSIELDRPNPTSHLAFGRGQHFCPGSALGRRHAQIGIEALLKK
MPGVDLAVPIDQLVWRTRFQRRIPERLPVLW
>CYP121A1_Mycobacterium tuberculosis K00700186 2693220732
MTATVLLEVPFSARGDRIPDAVAELRTREPIRKVRTITGAEAWLVSSYALCTQVLEDRRFSMKETAAAGAPRL
NALTVPPEVVNNMGNIADAGLRKAVMKAITPKAPGLEQFLRDTANSLLDNLITEGAPADLRNDFADPLATALH
CKVLGIPQEDGPKLFRSLSIAFMSSADPIPAAKINWDRDIEYMAGILENPNITTGLMGELSRLRKDPAYSHVS
DELFATIGVTFFGAGVISTGSFLTTALISLIQRPQLRNLLHEKPELIPAGVEELLRINLSFADGLPRLATADI
QVGDVLVRKGELVLVLLEGANFDPEHFPNPGSIELDRPNPTSHLAFGRGQHFCPGSALGRRHAQIGIEALLKK
MPGVDLAVPIDQLVWRTRFQRRIPERLPVLW
>CYP121A1_Mycobacterium tuberculosis K09600911 2699415125
MTATVLLEVPFSARGDRIPDAVAELRTREPIRKVRTITGAEAWLVSSYALCTQVLEDRRFSMKETAAAGAPRL
NALTVPPEVVNNMGNIADAGLRKAVMKAITPKAPGLEQFLRDTANSLLDNLITEGAPADLRNDFADPLATALH
CKVLGIPQEDGPKLFRSLSIAFMSSADPIPAAKINWDRDIEYMAGILENPNITTGLMGELSRLRKDPAYSHVS
DELFATIGVTFFGAGVISTGSFLTTALISLIQRPQLRNLLHEKPELIPAGVEELLRINLSFADGLPRLATADI
QVGDVLVRKGELVLVLLEGANFDPEHFPNPGSIELDRPNPTSHLAFGRGQHFCPGSALGRRHAQIGIEALLKK
MPGVDLAVPIDQLVWRTRFQRRIPERLPVLW
>CYP121A1_Mycobacterium tuberculosis K09700503 2703630778
MTATVLLEVPFSARGDRIPDAVAELRTREPIRKVRTITGAEAWLVSSYALCTQVLEDRRFSMKETAAAGAPRL
NALTVPPEVVNNMGNIADAGLRKAVMKAITPKAPGLEQFLRDTANSLLDNLITEGAPADLRNDFADPLATALH
CKVLGIPQEDGPKLFRSLSIAFMSSADPIPAAKINWDRDIEYMAGILENPNITTGLMGELSRLRKDPAYSHVS
DELFATIGVTFFGAGVISTGSFLTTALISLIQRPQLRNLLHEKPELIPAGVEELLRINLSFADGLPRLATADI
QVGDVLVRKGELVLVLLEGANFDPEHFPNPGSIELDRPNPTSHLAFGRGQHFCPGSALGRRHAQIGIEALLKK
MPGVDLAVPIDQLVWRTRFQRRIPERLPVLW
>CYP121A1_Mycobacterium tuberculosis K09900646 2703647886
MTATVLLEVPFSARGDRIPDAVAELRTREPIRKVRTITGAEAWLVSSYALCTQVLEDRRFSMKETAAAGAPRL
NALTVPPEVVNNMGNIADAGLRKAVMKAITPKAPGLEQFLRDTANSLLDNLITEGAPADLRNDFADPLATALH
CKVLGIPQEDGPKLFRSLSIAFMSSADPIPAAKINWDRDIEYMAGILENPNITTGLMGELSRLRKDPAYSHVS
DELFATIGVTFFGAGVISTGSFLTTALISLIQRPQLRNLLHEKPELIPAGVEELLRINLSFADGLPRLATADI
QVGDVLVRKGELVLVLLEGANFDPEHFPNPGSIELDRPNPTSHLAFGRGQHFCPGSALGRRHAQIGIEALLKK
MPGVDLAVPIDQLVWRTRFQRRIPERLPVLW
>CYP121A1_Mycobacterium tuberculosis K09901478 2675142080
MTATVLLEVPFSARGDRIPDAVAELRTREPIRKVRTITGAEAWLVSSYALCTQVLEDRRFSMKETAAAGAPRL
NALTVPPEVVNNMGNIADAGLRKAVMKAITPKAPGLEQFLRDTANSLLDNLITEGAPADLRNDFADPLATALH
CKVLGIPQEDGPKLFRSLSIAFMSSADPIPAAKINWDRDIEYMAGILENPNITTGLMGELSRLRKDPAYSHVS
DELFATIGVTFFGAGVISTGSFLTTALISLIQRPQLRNLLHEKPELIPAGVEELLRINLSFADGLPRLATADI
QVGDVLVRKGELVLVLLEGANFDPEHFPNPGSIELDRPNPTSHLAFGRGQHFCPGSALGRRHAQIGIEALLKK
MPGVDLAVPIDQLVWRTRFQRRIPERLPVLW
>CYP121A1_Mycobacterium tuberculosis K09901972 2703663987
MTATVLLEVPFSARGDRIPDAVAELRTREPIRKVRTITGAEAWLVSSYALCTQVLEDRRFSMKETAAAGAPRL
NALTVPPEVVNNMGNIADAGLRKAVMKAITPKAPGLEQFLRDTANSLLDNLITEGAPADLRNDFADPLATALH
CKVLGIPQEDGPKLFRSLSIAFMSSADPIPAAKINWDRDIEYMAGILENPNITTGLMGELSRLRKDPAYSHVS
DELFATIGVTFFGAGVISTGSFLTTALISLIQRPQLRNLLHEKPELIPAGVEELLRINLSFADGLPRLATADI
QVGDVLVRKGELVLVLLEGANFDPEHFPNPGSIELDRPNPTSHLAFGRGQHFCPGSALGRRHAQIGIEALLKK
MPGVDLAVPIDQLVWRTRFQRRIPERLPVLW
>CYP121A1_Mycobacterium tuberculosis KI_19771 2716379513
MTATVLLEVPFSARGDRIPDAVAELRTREPIRKVRTITGAEAWLVSSYALCTQVLEDRRFSMKETAAAGAPRL
NALTVPPEVVNNMGNIADAGLRKAVMKAITPKAPGLEQFLRDTANSLLDNLITEGAPADLRNDFADPLATALH
CKVLGIPQEDGPKLFRSLSIAFMSSADPIPAAKINWDRDIEYMAGILENPNITTGLMGELSRLRKDPAYSHVS
DELFATIGVTFFGAGVISTGSFLTTALISLIQRPQLRNLLHEKPELIPAGVEELLRINLSFADGLPRLATADI
QVGDVLVRKGELVLVLLEGANFDPEHFPNPGSIELDRPNPTSHLAFGRGQHFCPGSALGRRHAQIGIEALLKK
MPGVDLAVPIDQLVWRTRFQRRIPERLPVLW
>CYP121A1_Mycobacterium tuberculosis KI_19771 2716379580
MTATVLLEVPFSARGDRIPDAVAELRTREPIRKVRTITGAEAWLVSSYALCTQVLEDRRFSMKETAAAGAPRL
NALTVPPEVVNNMGNIADAGLRKAVMKAITPKAPGLEQFLRDTANSLLDNLITEGAPADLRNDFADPLATALH
CKVLGIPQEDGPKLFRSLSIAFMSSADPIPAAKINWDRDIEYMAGILENPNITTGLMGELSRLRKDPAYSHVS
DELFATIGVTFFGAGVISTGSFLTTALISLIQRPQLRNLLHEKPELIPAGVEELLRINLSFADGLPRLATADI

QVGDVLVRKGELVLVLLEGANFDPEHFPNPGSIELDRPNPTSHLAFGRGQHFCPGSALGRRHAQIGIEALLKK
MPGVDLAVPIDQLVWRTRFQRRIPERLPVLW
>CYP121A1_Mycobacterium tuberculosis KT-0040 2575965457
MTATVLLEVPFSARGDRIPDAVAELRTREPIRKVRTITGAEAWLVSSYALCTQVLEDRRFSMKETAAAGAPRL
NALTVPPEVVNNMGNIADAGLRKAVMKAITPKAPGLEQFLRDTANSLLDNLITEGAPADLRNDFADPLATALH
CKVLGIPQEDGPKLFRSLSIAFMSSADPIPAAKINWDRDIEYMAGILENPNITTGLMGELSRLRKDPAYSHVS
DELFATIGVTFFGAGVISTGSFLTTALISLIQRPQLRNLLHEKPELIPAGVEELLRINLSFADGLPRLATADI
QVGDVLVRKGELVLVLLEGANFDPEHFPNPGSIELDRPNPTSHLAFGRGQHFCPGSALGRRHAQIGIEALLKK
MPGVDLAVPIDQLVWRTRFQRRIPERLPVLW
>CYP121A1_Mycobacterium tuberculosis KT-0056 2576714016
MTATVLLEVPFSARGDRIPDAVAELRTREPIRKVRTITGAEAWLVSSYALCTQVLEDRRFSMKETAAAGAPRL
NALTVPPEVVNNMGNIADAGLRKAVMKAITPKAPGLEQFLRDTANSLLDNLITEGAPADLRNDFADPLATALH
CKVLGIPQEDGPKLFRSLSIAFMSSADPIPAAKINWDRDIEYMAGILENPNITTGLMGELSRLRKDPAYSHVS
DELFATIGVTFFGAGVISTGSFLTTALISLIQRPQLRNLLHEKPELIPAGVEELLRINLSFADGLPRLATADI
QVGDVLVRKGELVLVLLEGANFDPEHFPNPGSIELDRPNPTSHLAFGRGQHFCPGSALGRRHAQIGIEALLKK
MPGVDLAVPIDQLVWRTRFQRRIPERLPVLW
>CYP121A1_Mycobacterium tuberculosis KT-0181 2651508598
MTATVLLEVPFSARGDRIPDAVAELRTREPIRKVRTITGAEAWLVSSYALCTQVLEDRRFSMKETAAAGAPRL
NALTVPPEVVNNMGNIADAGLRKAVMKAITPKAPGLEQFLRDTANSLLDNLITEGAPADLRNDFADPLATALH
CKVLGIPQEDGPKLFRSLSIAFMSSADPIPAAKINWDRDIEYMAGILENPNITTGLMGELSRLRKDPAYSHVS
DELFATIGVTFFGAGVISTGSFLTTALISLIQRPQLRNLLHEKPELIPAGVEELLRINLSFADGLPRLATADI
QVGDVLVRKGELVLVLLEGANFDPEHFPNPGSIELDRPNPTSHLAFGRGQHFCPGSALGRRHAQIGIEALLKK
MPGVDLAVPIDQLVWRTRFQRRIPERLPVLW
>CYP121A1_Mycobacterium tuberculosis KT-0187 2637196584
MTATVLLEVPFSARGDRIPDAVAELRTREPIRKVRTITGAEAWLVSSYALCTQVLEDRRFSMKETAAAGAPRL
NALTVPPEVVNNMGNIADAGLRKAVMKAITPKAPGLEQFLRDTANSLLDNLITEGAPADLRNDFADPLATALH
CKVLGIPQEDGPKLFRSLSIAFMSSADPIPAAKINWDRDIEYMAGILENPNITTGLMGELSRLRKDPAYSHVS
DELFATIGVTFFGAGVISTGSFLTTALISLIQRPQLRNLLHEKPELIPAGVEELLRINLSFADGLPRLATADI
QVGDVLVRKGELVLVLLEGANFDPEHFPNPGSIELDRPNPTSHLAFGRGQHFCPGSALGRRHAQIGIEALLKK
MPGVDLAVPIDQLVWRTRFQRRIPERLPVLW
>CYP121A1_Mycobacterium tuberculosis KT-0192 2636427473
MTATVLLEVPFSARGDRIPDAVAELRTREPIRKVRTITGAEAWLVSSYALCTQVLEDRRFSMKETAAAGAPRL
NALTVPPEVVNNMGNIADAGLRKAVMKAITPKAPGLEQFLRDTANSLLDNLITEGAPADLRNDFADPLATALH
CKVLGIPQEDGPKLFRSLSIAFMSSADPIPAAKINWDRDIEYMAGILENPNITTGLMGELSRLRKDPAYSHVS
DELFATIGVTFFGAGVISTGSFLTTALISLIQRPQLRNLLHEKPELIPAGVEELLRINLSFADGLPRLATADI
QVGDVLVRKGELVLVLLEGANFDPEHFPNPGSIELDRPNPTSHLAFGRGQHFCPGSALGRRHAQIGIEALLKK
MPGVDLAVPIDQLVWRTRFQRRIPERLPVLW
>CYP121A1_Mycobacterium tuberculosis KT-0194 2631398014
MTATVLLEVPFSARGDRIPDAVAELRTREPIRKVRTITGAEAWLVSSYALCTQVLEDRRFSMKETAAAGAPRL
NALTVPPEVVNNMGNIADAGLRKAVMKAITPKAPGLEQFLRDTANSLLDNLITEGAPADLRNDFADPLATALH
CKVLGIPQEDGPKLFRSLSIAFMSSADPIPAAKINWDRDIEYMAGILENPNITTGLMGELSRLRKDPAYSHVS
DELFATIGVTFFGAGVISTGSFLTTALISLIQRPQLRNLLHEKPELIPAGVEELLRINLSFADGLPRLATADI
QVGDVLVRKGELVLVLLEGANFDPEHFPNPGSIELDRPNPTSHLAFGRGQHFCPGSALGRRHAQIGIEALLKK
MPGVDLAVPIDQLVWRTRFQRRIPERLPVLW
>CYP121A1_Mycobacterium tuberculosis KT-0199 2632697477
MTATVLLEVPFSARGDRIPDAVAELRTREPIRKVRTITGAEAWLVSSYALCTQVLEDRRFSMKETAAAGAPRL
NALTVPPEVVNNMGNIADAGLRKAVMKAITPKAPGLEQFLRDTANSLLDNLITEGAPADLRNDFADPLATALH
CKVLGIPQEDGPKLFRSLSIAFMSSADPIPAAKINWDRDIEYMAGILENPNITTGLMGELSRLRKDPAYSHVS
DELFATIGVTFFGAGVISTGSFLTTALISLIQRPQLRNLLHEKPELIPAGVEELLRINLSFADGLPRLATADI
QVGDVLVRKGELVLVLLEGANFDPEHFPNPGSIELDRPNPTSHLAFGRGQHFCPGSALGRRHAQIGIEALLKK
MPGVDLAVPIDQLVWRTRFQRRIPERLPVLW
>CYP121A1_Mycobacterium tuberculosis KT-0204 2633024382
MTATVLLEVPFSARGDRIPDAVAELRTREPIRKVRTITGAEAWLVSSYALCTQVLEDRRFSMKETAAAGAPRL
NALTVPPEVVNNMGNIADAGLRKAVMKAITPKAPGLEQFLRDTANSLLDNLITEGAPADLRNDFADPLATALH
CKVLGIPQEDGPKLFRSLSIAFMSSADPIPAAKINWDRDIEYMAGILENPNITTGLMGELSRLRKDPAYSHVS
DELFATIGVTFFGAGVISTGSFLTTALISLIQRPQLRNLLHEKPELIPAGVEELLRINLSFADGLPRLATADI
QVGDVLVRKGELVLVLLEGANFDPEHFPNPGSIELDRPNPTSHLAFGRGQHFCPGSALGRRHAQIGIEALLKK
MPGVDLAVPIDQLVWRTRFQRRIPERLPVLW

>CYP121A1_Mycobacterium tuberculosis KZN 1435 644879443
MTATVLLEVPFSARGDRIPDAVAELRTREPIRKVRTITGAEAWLVSSYALCTQVLEDRRFSMKETAAAGAPRL
NALTVPPEVVNNMGNIADAGLRKAVMKAITPKAPGLEQFLRDTANSLLDNLITEGAPADLRNDFADPLATALH
CKVLGIPQEDGPKLFRSLSIAFMSSADPIPAAKINWDRDIEYMAGILENPNITTGLMGELSRLRKDPAYSHVS
DELFATIGVTFFGAGVISTGSFLTTALISLIQRPQLRNLLHEKPELIPAGVEELLRINLSFADGLPRLATADI
QVGDVLVRKGELVLVLLEGANFDPEHFPNPGSIELDRPNPTSHLAFGRGQHFCPGSALGRRHAQIGIEALLKK
MPGVDLAVPIDQLVWRTRFQRRIPERLPVLW
>CYP121A1_Mycobacterium tuberculosis KZN 4207 NZ_ACVS02000035 647086961
MTATVLLEVPFSARGDRIPDAVAELRTREPIRKVRTITGAEAWLVSSYALCTQVLEDRRFSMKETAAAGAPRL
NALTVPPEVVNNMGNIADAGLRKAVMKAITPKAPGLEQFLRDTANSLLDNLITEGAPADLRNDFADPLATALH
CKVLGIPQEDGPKLFRSLSIAFMSSADPIPAAKINWDRDIEYMAGILENPNITTGLMGELSRLRKDPAYSHVS
DELFATIGVTFFGAGVISTGSFLTTALISLIQRPQLRNLLHEKPELIPAGVEELLRINLSFADGLPRLATADI
QVGDVLVRKGELVLVLLEGANFDPEHFPNPGSIELDRPNPTSHLAFGRGQHFCPGSALGRRHAQIGIEALLKK
MPGVDLAVPIDQLVWRTRFQRRIPERLPVLW
>CYP121A1_Mycobacterium tuberculosis KZN 4207 (DS) 2511552689
MTATVLLEVPFSARGDRIPDAVAELRTREPIRKVRTITGAEAWLVSSYALCTQVLEDRRFSMKETAAAGAPRL
NALTVPPEVVNNMGNIADAGLRKAVMKAITPKAPGLEQFLRDTANSLLDNLITEGAPADLRNDFADPLATALH
CKVLGIPQEDGPKLFRSLSIAFMSSADPIPAAKINWDRDIEYMAGILENPNITTGLMGELSRLRKDPAYSHVS
DELFATIGVTFFGAGVISTGSFLTTALISLIQRPQLRNLLHEKPELIPAGVEELLRINLSFADGLPRLATADI
QVGDVLVRKGELVLVLLEGANFDPEHFPNPGSIELDRPNPTSHLAFGRGQHFCPGSALGRRHAQIGIEALLKK
MPGVDLAVPIDQLVWRTRFQRRIPERLPVLW
>CYP121A1_Mycobacterium tuberculosis KZN R506 NZ_ACVU02000049 648336643
MTATVLLEVPFSARGDRIPDAVAELRTREPIRKVRTITGAEAWLVSSYALCTQVLEDRRFSMKETAAAGAPRL
NALTVPPEVVNNMGNIADAGLRKAVMKAITPKAPGLEQFLRDTANSLLDNLITEGAPADLRNDFADPLATALH
CKVLGIPQEDGPKLFRSLSIAFMSSADPIPAAKINWDRDIEYMAGILENPNITTGLMGELSRLRKDPAYSHVS
DELFATIGVTFFGAGVISTGSFLTTALISLIQRPQLRNLLHEKPELIPAGVEELLRINLSFADGLPRLATADI
QVGDVLVRKGELVLVLLEGANFDPEHFPNPGSIELDRPNPTSHLAFGRGQHFCPGSALGRRHAQIGIEALLKK
MPGVDLAVPIDQLVWRTRFQRRIPERLPVLW
>CYP121A1_Mycobacterium tuberculosis KZN V2475 NZ_ACVT02000054 647091172
MTATVLLEVPFSARGDRIPDAVAELRTREPIRKVRTITGAEAWLVSSYALCTQVLEDRRFSMKETAAAGAPRL
NALTVPPEVVNNMGNIADAGLRKAVMKAITPKAPGLEQFLRDTANSLLDNLITEGAPADLRNDFADPLATALH
CKVLGIPQEDGPKLFRSLSIAFMSSADPIPAAKINWDRDIEYMAGILENPNITTGLMGELSRLRKDPAYSHVS
DELFATIGVTFFGAGVISTGSFLTTALISLIQRPQLRNLLHEKPELIPAGVEELLRINLSFADGLPRLATADI
QVGDVLVRKGELVLVLLEGANFDPEHFPNPGSIELDRPNPTSHLAFGRGQHFCPGSALGRRHAQIGIEALLKK
MPGVDLAVPIDQLVWRTRFQRRIPERLPVLW
>CYP121A1_Mycobacterium tuberculosis L00201493 2703662725
MTATVLLEVPFSARGDRIPDAVAELRTREPIRKVRTITGAEAWLVSSYALCTQVLEDRRFSMKETAAAGAPRL
NALTVPPEVVNNMGNIADAGLRKAVMKAITPKAPGLEQFLRDTANSLLDNLITEGAPADLRNDFADPLATALH
CKVLGIPQEDGPKLFRSLSIAFMSSADPIPAAKINWDRDIEYMAGILENPNITTGLMGELSRLRKDPAYSHVS
DELFATIGVTFFGAGVISTGSFLTTALISLIQRPQLRNLLHEKPELIPAGVEELLRINLSFADGLPRLATADI
QVGDVLVRKGELVLVLLEGANFDPEHFPNPGSIELDRPNPTSHLAFGRGQHFCPGSALGRRHAQIGIEALLKK
MPGVDLAVPIDQLVWRTRFQRRIPERLPVLW
>CYP121A1_Mycobacterium tuberculosis L00401786 2703679508
MTATVLLEVPFSARGDRIPDAVAELRTREPIRKVRTITGAEAWLVSSYALCTQVLEDRRFSMKETAAAGAPRL
NALTVPPEVVNNMGNIADAGLRKAVMKAITPKAPGLEQFLRDTANSLLDNLITEGAPADLRNDFADPLATALH
CKVLGIPQEDGPKLFRSLSIAFMSSADPIPAAKINWDRDIEYMAGILENPNITTGLMGELSRLRKDPAYSHVS
DELFATIGVTFFGAGVISTGSFLTTALISLIQRPQLRNLLHEKPELIPAGVEELLRINLSFADGLPRLATADI
QVGDVLVRKGELVLVLLEGANFDPEHFPNPGSIELDRPNPTSHLAFGRGQHFCPGSALGRRHAQIGIEALLKK
MPGVDLAVPIDQLVWRTRFQRRIPERLPVLW
>CYP121A1_Mycobacterium tuberculosis M00018020 2693105666
MTATVLLEVPFSARGDRIPDAVAELRTREPIRKVRTITGAEAWLVSSYALCTQVLEDRRFSMKETAAAGAPRL
NALTVPPEVVNNMGNIADAGLRKAVMKAITPKAPGLEQFLRDTANSLLDNLITEGAPADLRNDFADPLATALH
CKVLGIPQEDGPKLFRSLSIAFMSSADPIPAAKINWDRDIEYMAGILENPNITTGLMGELSRLRKDPAYSHVS
DELFATIGVTFFGAGVISTGSFLTTALISLIQRPQLRNLLHEKPELIPAGVEELLRINLSFADGLPRLATADI
QVGDVLVRKGELVLVLLEGANFDPEHFPNPGSIELDRPNPTSHLAFGRGQHFCPGSALGRRHAQIGIEALLKK
MPGVDLAVPIDQLVWRTRFQRRIPERLPVLW
>CYP121A1_Mycobacterium tuberculosis M0002959-6 2720401964

MTATVLLEVPFSARGDRIPDAVAELRTREPIRKVRTITGAEAWLVSSYALCTQVLEDRRFSMKETAAAGAPRL
NALTVPPEVVNNMGNIADAGLRKAVMKAITPKAPGLEQFLRDTANSLLDNLITEGAPADLRNDFADPLATALH
CKVLGIPQEDGPKLFRSLSIAFMSSADPIPAAKINWDRDIEYMAGILENPNITTGLMGELSRLRKDPAYSHVS
DELFATIGVTFFGAGVISTGSFLTTALISLIQRPQLRNLLHEKPELIPAGVEELLRINLSFADGLPRLATADI
QVGDVLVRKGELVLVLLEGANFDPEHFPNPGSIELDRPNPTSHLAFGRGQHFCPGSALGRRHAQIGIEALLKK
MPGVDLAVPIDQLVWRTRFQRRIPERLPVLW
>CYP121A1_Mycobacterium tuberculosis M00102058 2706546549
MTATVLLEVPFSARGDRIPDAVAELRTREPIRKVRTITGAEAWLVSSYALCTQVLEDRRFSMKETAAAGAPRL
NALTVPPEVVNNMGNIADAGLRKAVMKAITPKAPGLEQFLRDTANSLLDNLITEGAPADLRNDFADPLATALH
CKVLGIPQEDGPKLFRSLSIAFMSSADPIPAAKINWDRDIEYMAGILENPNITTGLMGELSRLRKDPAYSHVS
DELFATIGVTFFGAGVISTGSFLTTALISLIQRPQLRNLLHEKPELIPAGVEELLRINLSFADGLPRLATADI
QVGDVLVRKGELVLVLLEGANFDPEHFPNPGSIELDRPNPTSHLAFGRGQHFCPGSALGRRHAQIGIEALLKK
MPGVDLAVPIDQLVWRTRFQRRIPERLPVLW
>CYP121A1_Mycobacterium tuberculosis M0018684-2 2730248501
MTATVLLEVPFSARGDRIPDAVAELRTREPIRKVRTITGAEAWLVSSYALCTQVLEDRRFSMKETAAAGAPRL
NALTVPPEVVNNMGNIADAGLRKAVMKAITPKAPGLEQFLRDTANSLLDNLITEGAPADLRNDFADPLATALH
CKVLGIPQEDGPKLFRSLSIAFMSSADPIPAAKINWDRDIEYMAGILENPNITTGLMGELSRLRKDPAYSHVS
DELFATIGVTFFGAGVISTGSFLTTALISLIQRPQLRNLLHEKPELIPAGVEELLRINLSFADGLPRLATADI
QVGDVLVRKGELVLVLLEGANFDPEHFPNPGSIELDRPNPTSHLAFGRGQHFCPGSALGRRHAQIGIEALLKK
MPGVDLAVPIDQLVWRTRFQRRIPERLPVLW
>CYP121A1_Mycobacterium tuberculosis M09400328 2703675032
MTATVLLEVPFSARGDRIPDAVAELRTREPIRKVRTITGAEAWLVSSYALCTQVLEDRRFSMKETAAAGAPRL
NALTVPPEVVNNMGNIADAGLRKAVMKAITPKAPGLEQFLRDTANSLLDNLITEGAPADLRNDFADPLATALH
CKVLGIPQEDGPKLFRSLSIAFMSSADPIPAAKINWDRDIEYMAGILENPNITTGLMGELSRLRKDPAYSHVS
DELFATIGVTFFGAGVISTGSFLTTALISLIQRPQLRNLLHEKPELIPAGVEELLRINLSFADGLPRLATADI
QVGDVLVRKGELVLVLLEGANFDPEHFPNPGSIELDRPNPTSHLAFGRGQHFCPGSALGRRHAQIGIEALLKK
MPGVDLAVPIDQLVWRTRFQRRIPERLPVLW
>CYP121A1_Mycobacterium tuberculosis M09400374 2699618745
MTATVLLEVPFSARGDRIPDAVAELRTREPIRKVRTITGAEAWLVSSYALCTQVLEDRRFSMKETAAAGAPRL
NALTVPPEVVNNMGNIADAGLRKAVMKAITPKAPGLEQFLRDTANSLLDNLITEGAPADLRNDFADPLATALH
CKVLGIPQEDGPKLFRSLSIAFMSSADPIPAAKINWDRDIEYMAGILENPNITTGLMGELSRLRKDPAYSHVS
DELFATIGVTFFGAGVISTGSFLTTALISLIQRPQLRNLLHEKPELIPAGVEELLRINLSFADGLPRLATADI
QVGDVLVRKGELVLVLLEGANFDPEHFPNPGSIELDRPNPTSHLAFGRGQHFCPGSALGRRHAQIGIEALLKK
MPGVDLAVPIDQLVWRTRFQRRIPERLPVLW
>CYP121A1_Mycobacterium tuberculosis M09400397 2697407138
MTATVLLEVPFSARGDRIPDAVAELRTREPIRKVRTITGAEAWLVSSYALCTQVLEDRRFSMKETAAAGAPRL
NALTVPPEVVNNMGNIADAGLRKAVMKAITPKAPGLEQFLRDTANSLLDNLITEGAPADLRNDFADPLATALH
CKVLGIPQEDGPKLFRSLSIAFMSSADPIPAAKINWDRDIEYMAGILENPNITTGLMGELSRLRKDPAYSHVS
DELFATIGVTFFGAGVISTGSFLTTALISLIQRPQLRNLLHEKPELIPAGVEELLRINLSFADGLPRLATADI
QVGDVLVRKGELVLVLLEGANFDPEHFPNPGSIELDRPNPTSHLAFGRGQHFCPGSALGRRHAQIGIEALLKK
MPGVDLAVPIDQLVWRTRFQRRIPERLPVLW
>CYP121A1_Mycobacterium tuberculosis M09400937 2703682241
MTATVLLEVPFSARGDRIPDAVAELRTREPIRKVRTITGAEAWLVSSYALCTQVLEDRRFSMKETAAAGAPRL
NALTVPPEVVNNMGNIADAGLRKAVMKAITPKAPGLEQFLRDTANSLLDNLITEGAPADLRNDFADPLATALH
CKVLGIPQEDGPKLFRSLSIAFMSSADPIPAAKINWDRDIEYMAGILENPNITTGLMGELSRLRKDPAYSHVS
DELFATIGVTFFGAGVISTGSFLTTALISLIQRPQLRNLLHEKPELIPAGVEELLRINLSFADGLPRLATADI
QVGDVLVRKGELVLVLLEGANFDPEHFPNPGSIELDRPNPTSHLAFGRGQHFCPGSALGRRHAQIGIEALLKK
MPGVDLAVPIDQLVWRTRFQRRIPERLPVLW
>CYP121A1_Mycobacterium tuberculosis M09401471 2703642472
MTATVLLEVPFSARGDRIPDAVAELRTREPIRKVRTITGAEAWLVSSYALCTQVLEDRRFSMKETAAAGAPRL
NALTVPPEVVNNMGNIADAGLRKAVMKAITPKAPGLEQFLRDTANSLLDNLITEGAPADLRNDFADPLATALH
CKVLGIPQEDGPKLFRSLSIAFMSSADPIPAAKINWDRDIEYMAGILENPNITTGLMGELSRLRKDPAYSHVS
DELFATIGVTFFGAGVISTGSFLTTALISLIQRPQLRNLLHEKPELIPAGVEELLRINLSFADGLPRLATADI
QVGDVLVRKGELVLVLLEGANFDPEHFPNPGSIELDRPNPTSHLAFGRGQHFCPGSALGRRHAQIGIEALLKK
MPGVDLAVPIDQLVWRTRFQRRIPERLPVLW
>CYP121A1_Mycobacterium tuberculosis M09402043 2697733746
MTATVLLEVPFSARGDRIPDAVAELRTREPIRKVRTITGAEAWLVSSYALCTQVLEDRRFSMKETAAAGAPRL
NALTVPPEVVNNMGNIADAGLRKAVMKAITPKAPGLEQFLRDTANSLLDNLITEGAPADLRNDFADPLATALH

CKVLGIPQEDGPKLFRSLSIAFMSSADPIPAAKINWDRDIEYMAGILENPNITTGLMGELSRLRKDPAYSHVS
DELFATIGVTFFGAGVISTGSFLTTALISLIQRPQLRNLLHEKPELIPAGVEELLRINLSFADGLPRLATADI
QVGDVLVRKGELVLVLLEGANFDPEHFPNPGSIELDRPNPTSHLAFGRGQHFCPGSALGRRHAQIGIEALLKK
MPGVDLAVPIDQLVWRTRFQRRIPERLPVLW
>CYP121A1_Mycobacterium tuberculosis M09500081 2703584887
MTATVLLEVPFSARGDRIPDAVAELRTREPIRKVRTITGAEAWLVSSYALCTQVLEDRRFSMKETAAAGAPRL
NALTVPPEVVNNMGNIADAGLRKAVMKAITPKAPGLEQFLRDTANSLLDNLITEGAPADLRNDFADPLATALH
CKVLGIPQEDGPKLFRSLSIAFMSSADPIPAAKINWDRDIEYMAGILENPNITTGLMGELSRLRKDPAYSHVS
DELFATIGVTFFGAGVISTGSFLTTALISLIQRPQLRNLLHEKPELIPAGVEELLRINLSFADGLPRLATADI
QVGDVLVRKGELVLVLLEGANFDPEHFPNPGSIELDRPNPTSHLAFGRGQHFCPGSALGRRHAQIGIEALLKK
MPGVDLAVPIDQLVWRTRFQRRIPERLPVLW
>CYP121A1_Mycobacterium tuberculosis M09502139 2703655728
MTATVLLEVPFSARGDRIPDAVAELRTREPIRKVRTITGAEAWLVSSYALCTQVLEDRRFSMKETAAAGAPRL
NALTVPPEVVNNMGNIADAGLRKAVMKAITPKAPGLEQFLRDTANSLLDNLITEGAPADLRNDFADPLATALH
CKVLGIPQEDGPKLFRSLSIAFMSSADPIPAAKINWDRDIEYMAGILENPNITTGLMGELSRLRKDPAYSHVS
DELFATIGVTFFGAGVISTGSFLTTALISLIQRPQLRNLLHEKPELIPAGVEELLRINLSFADGLPRLATADI
QVGDVLVRKGELVLVLLEGANFDPEHFPNPGSIELDRPNPTSHLAFGRGQHFCPGSALGRRHAQIGIEALLKK
MPGVDLAVPIDQLVWRTRFQRRIPERLPVLW
>CYP121A1_Mycobacterium tuberculosis M1004 2577270076
MTATVLLEVPFSARGDRIPDAVAELRTREPIRKVRTITGAEAWLVSSYALCTQVLEDRRFSMKETAAAGAPRL
NALTVPPEVVNNMGNIADAGLRKAVMKAITPKAPGLEQFLRDTANSLLDNLITEGAPADLRNDFADPLATALH
CKVLGIPQEDGPKLFRSLSIAFMSSADPIPAAKINWDRDIEYMAGILENPNITTGLMGELSRLRKDPAYSHVS
DELFATIGVTFFGAGVISTGSFLTTALISLIQRPQLRNLLHEKPELIPAGVEELLRINLSFADGLPRLATADI
QVGDVLVRKGELVLVLLEGANFDPEHFPNPGSIELDRPNPTSHLAFGRGQHFCPGSALGRRHAQIGIEALLKK
MPGVDLAVPIDQLVWRTRFQRRIPERLPVLW
>CYP121A1_Mycobacterium tuberculosis M1007 2576554577
MTATVLLEVPFSARGDRIPDAVAELRTREPIRKVRTITGAEAWLVSSYALCTQVLEDRRFSMKETAAAGAPRL
NALTVPPEVVNNMGNIADAGLRKAVMKAITPKAPGLEQFLRDTANSLLDNLITEGAPADLRNDFADPLATALH
CKVLGIPQEDGPKLFRSLSIAFMSSADPIPAAKINWDRDIEYMAGILENPNITTGLMGELSRLRKDPAYSHVS
DELFATIGVTFFGAGVISTGSFLTTALISLIQRPQLRNLLHEKPELIPAGVEELLRINLSFADGLPRLATADI
QVGDVLVRKGELVLVLLEGANFDPEHFPNPGSIELDRPNPTSHLAFGRGQHFCPGSALGRRHAQIGIEALLKK
MPGVDLAVPIDQLVWRTRFQRRIPERLPVLW
>CYP121A1_Mycobacterium tuberculosis M1008 2576567594
MTATVLLEVPFSARGDRIPDAVAELRTREPIRKVRTITGAEAWLVSSYALCTQVLEDRRFSMKETAAAGAPRL
NALTVPPEVVNNMGNIADAGLRKAVMKAITPKAPGLEQFLRDTANSLLDNLITEGAPADLRNDFADPLATALH
CKVLGIPQEDGPKLFRSLSIAFMSSADPIPAAKINWDRDIEYMAGILENPNITTGLMGELSRLRKDPAYSHVS
DELFATIGVTFFGAGVISTGSFLTTALISLIQRPQLRNLLHEKPELIPAGVEELLRINLSFADGLPRLATADI
QVGDVLVRKGELVLVLLEGANFDPEHFPNPGSIELDRPNPTSHLAFGRGQHFCPGSALGRRHAQIGIEALLKK
MPGVDLAVPIDQLVWRTRFQRRIPERLPVLW
>CYP121A1_Mycobacterium tuberculosis M1009 2622330743
MTATVLLEVPFSARGDRIPDAVAELRTREPIRKVRTITGAEAWLVSSYALCTQVLEDRRFSMKETAAAGAPRL
NALTVPPEVVNNMGNIADAGLRKAVMKAITPKAPGLEQFLRDTANSLLDNLITEGAPADLRNDFADPLATALH
CKVLGIPQEDGPKLFRSLSIAFMSSADPIPAAKINWDRDIEYMAGILENPNITTGLMGELSRLRKDPAYSHVS
DELFATIGVTFFGAGVISTGSFLTTALISLIQRPQLRNLLHEKPELIPAGVEELLRINLSFADGLPRLATADI
QVGDVLVRKGELVLVLLEGANFDPEHFPNPGSIELDRPNPTSHLAFGRGQHFCPGSALGRRHAQIGIEALLKK
MPGVDLAVPIDQLVWRTRFQRRIPERLPVLW
>CYP121A1_Mycobacterium tuberculosis M1010 2622334859
MTATVLLEVPFSARGDRIPDAVAELRTREPIRKVRTITGAEAWLVSSYALCTQVLEDRRFSMKETAAAGAPRL
NALTVPPEVVNNMGNIADAGLRKAVMKAITPKAPGLEQFLRDTANSLLDNLITEGAPADLRNDFADPLATALH
CKVLGIPQEDGPKLFRSLSIAFMSSADPIPAAKINWDRDIEYMAGILENPNITTGLMGELSRLRKDPAYSHVS
DELFATIGVTFFGAGVISTGSFLTTALISLIQRPQLRNLLHEKPELIPAGVEELLRINLSFADGLPRLATADI
QVGDVLVRKGELVLVLLEGANFDPEHFPNPGSIELDRPNPTSHLAFGRGQHFCPGSALGRRHAQIGIEALLKK
MPGVDLAVPIDQLVWRTRFQRRIPERLPVLW
>CYP121A1_Mycobacterium tuberculosis M1011 2622339507
MTATVLLEVPFSARGDRIPDAVAELRTREPIRKVRTITGAEAWLVSSYALCTQVLEDRRFSMKETAAAGAPRL
NALTVPPEVVNNMGNIADAGLRKAVMKAITPKAPGLEQFLRDTANSLLDNLITEGAPADLRNDFADPLATALH
CKVLGIPQEDGPKLFRSLSIAFMSSADPIPAAKINWDRDIEYMAGILENPNITTGLMGELSRLRKDPAYSHVS
DELFATIGVTFFGAGVISTGSFLTTALISLIQRPQLRNLLHEKPELIPAGVEELLRINLSFADGLPRLATADI

QVGDVLVRKGELVLVLLEGANFDPEHFPNPGSIELDRPNPTSHLAFGRGQHFCPGSALGRRHAQIGIEALLKK
MPGVDLAVPIDQLVWRTRFQRRIPERLPVLW
>CYP121A1_Mycobacterium tuberculosis M1012 2622341602
MTATVLLEVPFSARGDRIPDAVAELRTREPIRKVRTITGAEAWLVSSYALCTQVLEDRRFSMKETAAAGAPRL
NALTVPPEVVNNMGNIADAGLRKAVMKAITPKAPGLEQFLRDTANSLLDNLITEGAPADLRNDFADPLATALH
CKVLGIPQEDGPKLFRSLSIAFMSSADPIPAAKINWDRDIEYMAGILENPNITTGLMGELSRLRKDPAYSHVS
DELFATIGVTFFGAGVISTGSFLTTALISLIQRPQLRNLLHEKPELIPAGVEELLRINLSFADGLPRLATADI
QVGDVLVRKGELVLVLLEGANFDPEHFPNPGSIELDRPNPTSHLAFGRGQHFCPGSALGRRHAQIGIEALLKK
MPGVDLAVPIDQLVWRTRFQRRIPERLPVLW
>CYP121A1_Mycobacterium tuberculosis M1017 2577883724
MTATVLLEVPFSARGDRIPDAVAELRTREPIRKVRTITGAEAWLVSSYALCTQVLEDRRFSMKETAAAGAPRL
NALTVPPEVVNNMGNIADAGLRKAVMKAITPKAPGLEQFLRDTANSLLDNLITEGAPADLRNDFADPLATALH
CKVLGIPQEDGPKLFRSLSIAFMSSADPIPAAKINWDRDIEYMAGILENPNITTGLMGELSRLRKDPAYSHVS
DELFATIGVTFFGAGVISTGSFLTTALISLIQRPQLRNLLHEKPELIPAGVEELLRINLSFADGLPRLATADI
QVGDVLVRKGELVLVLLEGANFDPEHFPNPGSIELDRPNPTSHLAFGRGQHFCPGSALGRRHAQIGIEALLKK
MPGVDLAVPIDQLVWRTRFQRRIPERLPVLW
>CYP121A1_Mycobacterium tuberculosis M1021 2622346930
MTATVLLEVPFSARGDRIPDAVAELRTREPIRKVRTITGAEAWLVSSYALCTQVLEDRRFSMKETAAAGAPRL
NALTVPPEVVNNMGNIADAGLRKAVMKAITPKAPGLEQFLRDTANSLLDNLITEGAPADLRNDFADPLATALH
CKVLGIPQEDGPKLFRSLSIAFMSSADPIPAAKINWDRDIEYMAGILENPNITTGLMGELSRLRKDPAYSHVS
DELFATIGVTFFGAGVISTGSFLTTALISLIQRPQLRNLLHEKPELIPAGVEELLRINLSFADGLPRLATADI
QVGDVLVRKGELVLVLLEGANFDPEHFPNPGSIELDRPNPTSHLAFGRGQHFCPGSALGRRHAQIGIEALLKK
MPGVDLAVPIDQLVWRTRFQRRIPERLPVLW
>CYP121A1_Mycobacterium tuberculosis M1022 2622351343
MTATVLLEVPFSARGDRIPDAVAELRTREPIRKVRTITGAEAWLVSSYALCTQVLEDRRFSMKETAAAGAPRL
NALTVPPEVVNNMGNIADAGLRKAVMKAITPKAPGLEQFLRDTANSLLDNLITEGAPADLRNDFADPLATALH
CKVLGIPQEDGPKLFRSLSIAFMSSADPIPAAKINWDRDIEYMAGILENPNITTGLMGELSRLRKDPAYSHVS
DELFATIGVTFFGAGVISTGSFLTTALISLIQRPQLRNLLHEKPELIPAGVEELLRINLSFADGLPRLATADI
QVGDVLVRKGELVLVLLEGANFDPEHFPNPGSIELDRPNPTSHLAFGRGQHFCPGSALGRRHAQIGIEALLKK
MPGVDLAVPIDQLVWRTRFQRRIPERLPVLW
>CYP121A1_Mycobacterium tuberculosis M1023 2622355442
MTATVLLEVPFSARGDRIPDAVAELRTREPIRKVRTITGAEAWLVSSYALCTQVLEDRRFSMKETAAAGAPRL
NALTVPPEVVNNMGNIADAGLRKAVMKAITPKAPGLEQFLRDTANSLLDNLITEGAPADLRNDFADPLATALH
CKVLGIPQEDGPKLFRSLSIAFMSSADPIPAAKINWDRDIEYMAGILENPNITTGLMGELSRLRKDPAYSHVS
DELFATIGVTFFGAGVISTGSFLTTALISLIQRPQLRNLLHEKPELIPAGVEELLRINLSFADGLPRLATADI
QVGDVLVRKGELVLVLLEGANFDPEHFPNPGSIELDRPNPTSHLAFGRGQHFCPGSALGRRHAQIGIEALLKK
MPGVDLAVPIDQLVWRTRFQRRIPERLPVLW
>CYP121A1_Mycobacterium tuberculosis M1024 2622787765
MTATVLLEVPFSARGDRIPDAVAELRTREPIRKVRTITGAEAWLVSSYALCTQVLEDRRFSMKETAAAGAPRL
NALTVPPEVVNNMGNIADAGLRKAVMKAITPKAPGLEQFLRDTANSLLDNLITEGAPADLRNDFADPLATALH
CKVLGIPQEDGPKLFRSLSIAFMSSADPIPAAKINWDRDIEYMAGILENPNITTGLMGELSRLRKDPAYSHVS
DELFATIGVTFFGAGVISTGSFLTTALISLIQRPQLRNLLHEKPELIPAGVEELLRINLSFADGLPRLATADI
QVGDVLVRKGELVLVLLEGANFDPEHFPNPGSIELDRPNPTSHLAFGRGQHFCPGSALGRRHAQIGIEALLKK
MPGVDLAVPIDQLVWRTRFQRRIPERLPVLW
>CYP121A1_Mycobacterium tuberculosis M1025 2578156446
MTATVLLEVPFSARGDRIPDAVAELRTREPIRKVRTITGAEAWLVSSYALCTQVLEDRRFSMKETAAAGAPRL
NALTVPPEVVNNMGNIADAGLRKAVMKAITPKAPGLEQFLRDTANSLLDNLITEGAPADLRNDFADPLATALH
CKVLGIPQEDGPKLFRSLSIAFMSSADPIPAAKINWDRDIEYMAGILENPNITTGLMGELSRLRKDPAYSHVS
DELFATIGVTFFGAGVISTGSFLTTALISLIQRPQLRNLLHEKPELIPAGVEELLRINLSFADGLPRLATADI
QVGDVLVRKGELVLVLLEGANFDPEHFPNPGSIELDRPNPTSHLAFGRGQHFCPGSALGRRHAQIGIEALLKK
MPGVDLAVPIDQLVWRTRFQRRIPERLPVLW
>CYP121A1_Mycobacterium tuberculosis M1026 2622359256
MTATVLLEVPFSARGDRIPDAVAELRTREPIRKVRTITGAEAWLVSSYALCTQVLEDRRFSMKETAAAGAPRL
NALTVPPEVVNNMGNIADAGLRKAVMKAITPKAPGLEQFLRDTANSLLDNLITEGAPADLRNDFADPLATALH
CKVLGIPQEDGPKLFRSLSIAFMSSADPIPAAKINWDRDIEYMAGILENPNITTGLMGELSRLRKDPAYSHVS
DELFATIGVTFFGAGVISTGSFLTTALISLIQRPQLRNLLHEKPELIPAGVEELLRINLSFADGLPRLATADI
QVGDVLVRKGELVLVLLEGANFDPEHFPNPGSIELDRPNPTSHLAFGRGQHFCPGSALGRRHAQIGIEALLKK
MPGVDLAVPIDQLVWRTRFQRRIPERLPVLW

>CYP121A1_Mycobacterium tuberculosis M1029 2622362627
MTATVLLEVPFSARGDRIPDAVAELRTREPIRKVRTITGAEAWLVSSYALCTQVLEDRRFSMKETAAAGAPRL
NALTVPPEVVNNMGNIADAGLRKAVMKAITPKAPGLEQFLRDTANSLLDNLITEGAPADLRNDFADPLATALH
CKVLGIPQEDGPKLFRSLSIAFMSSADPIPAAKINWDRDIEYMAGILENPNITTGLMGELSRLRKDPAYSHVS
DELFATIGVTFFGAGVISTGSFLTTALISLIQRPQLRNLLHEKPELIPAGVEELLRINLSFADGLPRLATADI
QVGDVLVRKGELVLVLLEGANFDPEHFPNPGSIELDRPNPTSHLAFGRGQHFCPGSALGRRHAQIGIEALLKK
MPGVDLAVPIDQLVWRTRFQRRIPERLPVLW
>CYP121A1_Mycobacterium tuberculosis M1030 2622368315
MSYVAAEPGVLISPTDDLQSPRSAPAAHDENADGITGGTRDDSAPNSRFQLGRRIPEATAQEGFLVRPFTQQC
QIIHTEGDHAVIGVSPGNSYFSRQRLRDLGLWGLTNFDRVDFVYTDVHVAESYEALGGSAIEARRKAVKNIRG
VRAKITTTVNELDPAGARLCVRPMSEFQSNEAYRELHADLLTRLKDDEDLRAVCQDLVRRFLSTKVGPRQGGD
GYSRAGVHGLHLRRGPAIPRHTCDSRSAVVVELLPPITAPRRNALRPRIGTTGIAQSRPRHCYPGWEPRRMTA
TVLLEVPFSARGDRIPDAVAELRTREPIRKVRTITGAEAWLVSSYALCTQVLEDRRFSMKETAAAGAPRLNAL
TVPPEVVNNMGNIADAGLRKAVMKAITPKAPGLEQFLRDTANSLLDNLITEGAPADLRNDFADPLATALHCKV
LGIPQEDGPKLFRSLSIAFMSSADPIPAAKINWDRDIEYMAGILENPNITTGLMGELSRLRKDPAYSHVSDEL
FATIGVTFFGAGVISTGSFLTTALISLIQRPQLRNLLHEKPELIPAGVEELLRINLSFADGLPRLATADIQVG
DVLVRKGELVLVLLEGANFDPEHFPNPGSIELDRPNPTSHLAFGRGQHFCPGSALGRRHAQIGIEALLKKMPG
VDLAVPIDQLVWRTRFQRRIPERLPVLW
>CYP121A1_Mycobacterium tuberculosis M1034 2622369793
MTATVLLEVPFSARGDRIPDAVAELRTREPIRKVRTITGAEAWLVSSYALCTQVLEDRRFSMKETAAAGAPRL
NALTVPPEVVNNMGNIADAGLRKAVMKAITPKAPGLEQFLRDTANSLLDNLITEGAPADLRNDFADPLATALH
CKVLGIPQEDGPKLFRSLSIAFMSSADPIPAAKINWDRDIEYMAGILENPNITTGLMGELSRLRKDPAYSHVS
DELFATIGVTFFGAGVISTGSFLTTALISLIQRPQLRNLLHEKPELIPAGVEELLRINLSFADGLPRLATADI
QVGDVLVRKGELVLVLLEGANFDPEHFPNPGSIELDRPNPTSHLAFGRGQHFCPGSALGRRHAQIGIEALLKK
MPGVDLAVPIDQLVWRTRFQRRIPERLPVLW
>CYP121A1_Mycobacterium tuberculosis M1210 2622376104
MTATVLLEVPFSARGDRIPDAVAELRTREPIRKVRTITGAEAWLVSSYALCTQVLEDRRFSMKETAAAGAPRL
NALTVPPEVVNNMGNIADAGLRKAVMKAITPKAPGLEQFLRDTANSLLDNLITEGAPADLRNDFADPLATALH
CKVLGIPQEDGPKLFRSLSIAFMSSADPIPAAKINWDRDIEYMAGILENPNITTGLMGELSRLRKDPAYSHVS
DELFATIGVTFFGAGVISTGSFLTTALISLIQRPQLRNLLHEKPELIPAGVEELLRINLSFADGLPRLATADI
QVGDVLVRKGELVLVLLEGANFDPEHFPNPGSIELDRPNPTSHLAFGRGQHFCPGSALGRRHAQIGIEALLKK
MPGVDLAVPIDQLVWRTRFQRRIPERLPVLW
>CYP121A1_Mycobacterium tuberculosis M1212 2622380226
MTATVLLEVPFSARGDRIPDAVAELRTREPIRKVRTITGAEAWLVSSYALCTQVLEDRRFSMKETAAAGAPRL
NALTVPPEVVNNMGNIADAGLRKAVMKAITPKAPGLEQFLRDTANSLLDNLITEGAPADLRNDFADPLATALH
CKVLGIPQEDGPKLFRSLSIAFMSSADPIPAAKINWDRDIEYMAGILENPNITTGLMGELSRLRKDPAYSHVS
DELFATIGVTFFGAGVISTGSFLTTALISLIQRPQLRNLLHEKPELIPAGVEELLRINLSFADGLPRLATADI
QVGDVLVRKGELVLVLLEGANFDPEHFPNPGSIELDRPNPTSHLAFGRGQHFCPGSALGRRHAQIGIEALLKK
MPGVDLAVPIDQLVWRTRFQRRIPERLPVLW
>CYP121A1_Mycobacterium tuberculosis M1213 2575561973
MTATVLLEVPFSARGDRIPDAVAELRTREPIRKVRTITGAEAWLVSSYALCTQVLEDRRFSMKETAAAGAPRL
NALTVPPEVVNNMGNIADAGLRKAVMKAITPKAPGLEQFLRDTANSLLDNLITEGAPADLRNDFADPLATALH
CKVLGIPQEDGPKLFRSLSIAFMSSADPIPAAKINWDRDIEYMAGILENPNITTGLMGELSRLRKDPAYSHVS
DELFATIGVTFFGAGVISTGSFLTTALISLIQRPQLRNLLHEKPELIPAGVEELLRINLSFADGLPRLATADI
QVGDVLVRKGELVLVLLEGANFDPEHFPNPGSIELDRPNPTSHLAFGRGQHFCPGSALGRRHAQIGIEALLKK
MPGVDLAVPIDQLVWRTRFQRRIPERLPVLW
>CYP121A1_Mycobacterium tuberculosis M1215 2622382741
MTATVLLEVPFSARGDRIPDAVAELRTREPIRKVRTITGAEAWLVSSYALCTQVLEDRRFSMKETAAAGAPRL
NALTVPPEVVNNMGNIADAGLRKAVMKAITPKAPGLEQFLRDTANSLLDNLITEGAPADLRNDFADPLATALH
CKVLGIPQEDGPKLFRSLSIAFMSSADPIPAAKINWDRDIEYMAGILENPNITTGLMGELSRLRKDPAYSHVS
DELFATIGVTFFGAGVISTGSFLTTALISLIQRPQLRNLLHEKPELIPAGVEELLRINLSFADGLPRLATADI
QVGDVLVRKGELVLVLLEGANFDPEHFPNPGSIELDRPNPTSHLAFGRGQHFCPGSALGRRHAQIGIEALLKK
MPGVDLAVPIDQLVWRTRFQRRIPERLPVLW
>CYP121A1_Mycobacterium tuberculosis M1221 2575516298
MTATVLLEVPFSARGDRIPDAVAELRTREPIRKVRTITGAEAWLVSSYALCTQVLEDRRFSMKETAAAGAPRL
NALTVPPEVVNNMGNIADAGLRKAVMKAITPKAPGLEQFLRDTANSLLDNLITEGAPADLRNDFADPLATALH
CKVLGIPQEDGPKLFRSLSIAFMSSADPIPAAKINWDRDIEYMAGILENPNITTGLMGELSRLRKDPAYSHVS
DELFATIGVTFFGAGVISTGSFLTTALISLIQRPQLRNLLHEKPELIPAGVEELLRINLSFADGLPRLATADI

QVGDVLVRKGELVLVLLEGANFDPEHFPNPGSIELDRPNPTSHLAFGRGQHFCPGSALGRRHAQIGIEALLKK
MPGVDLAVPIDQLVWRTRFQRRIPERLPVLW
>CYP121A1_Mycobacterium tuberculosis M1232 2622386902
MTATVLLEVPFSARGDRIPDAVAELRTREPIRKVRTITGAEAWLVSSYALCTQVLEDRRFSMKETAAAGAPRL
NALTVPPEVVNNMGNIADAGLRKAVMKAITPKAPGLEQFLRDTANSLLDNLITEGAPADLRNDFADPLATALH
CKVLGIPQEDGPKLFRSLSIAFMSSADPIPAAKINWDRDIEYMAGILENPNITTGLMGELSRLRKDPAYSHVS
DELFATIGVTFFGAGVISTGSFLTTALISLIQRPQLRNLLHEKPELIPAGVEELLRINLSFADGLPRLATADI
QVGDVLVRKGELVLVLLEGANFDPEHFPNPGSIELDRPNPTSHLAFGRGQHFCPGSALGRRHAQIGIEALLKK
MPGVDLAVPIDQLVWRTRFQRRIPERLPVLW
>CYP121A1_Mycobacterium tuberculosis M1233 2575665028
MTATVLLEVPFSARGDRIPDAVAELRTREPIRKVRTITGAEAWLVSSYALCTQVLEDRRFSMKETAAAGAPRL
NALTVPPEVVNNMGNIADAGLRKAVMKAITPKAPGLEQFLRDTANSLLDNLITEGAPADLRNDFADPLATALH
CKVLGIPQEDGPKLFRSLSIAFMSSADPIPAAKINWDRDIEYMAGILENPNITTGLMGELSRLRKDPAYSHVS
DELFATIGVTFFGAGVISTGSFLTTALISLIQRPQLRNLLHEKPELIPAGVEELLRINLSFADGLPRLATADI
QVGDVLVRKGELVLVLLEGANFDPEHFPNPGSIELDRPNPTSHLAFGRGQHFCPGSALGRRHAQIGIEALLKK
MPGVDLAVPIDQLVWRTRFQRRIPERLPVLW
>CYP121A1_Mycobacterium tuberculosis M1253 2622396681
MTATVLLEVPFSARGDRIPDAVAELRTREPIRKVRTITGAEAWLVSSYALCTQVLEDRRFSMKETAAAGAPRL
NALTVPPEVVNNMGNIADAGLRKAVMKAITPKAPGLEQFLRDTANSLLDNLITEGAPADLRNDFADPLATALH
CKVLGIPQEDGPKLFRSLSIAFMSSADPIPAAKINWDRDIEYMAGILENPNITTGLMGELSRLRKDPAYSHVS
DELFATIGVTFFGAGVISTGSFLTTALISLIQRPQLRNLLHEKPELIPAGVEELLRINLSFADGLPRLATADI
QVGDVLVRKGELVLVLLEGANFDPEHFPNPGSIELDRPNPTSHLAFGRGQHFCPGSALGRRHAQIGIEALLKK
MPGVDLAVPIDQLVWRTRFQRRIPERLPVLW
>CYP121A1_Mycobacterium tuberculosis M1270 2622400811
MTATVLLEVPFSARGDRIPDAVAELRTREPIRKVRTITGAEAWLVSSYALCTQVLEDRRFSMKETAAAGAPRL
NALTVPPEVVNNMGNIADAGLRKAVMKAITPKAPGLEQFLRDTANSLLDNLITEGAPADLRNDFADPLATALH
CKVLGIPQEDGPKLFRSLSIAFMSSADPIPAAKINWDRDIEYMAGILENPNITTGLMGELSRLRKDPAYSHVS
DELFATIGVTFFGAGVISTGSFLTTALISLIQRPQLRNLLHEKPELIPAGVEELLRINLSFADGLPRLATADI
QVGDVLVRKGELVLVLLEGANFDPEHFPNPGSIELDRPNPTSHLAFGRGQHFCPGSALGRRHAQIGIEALLKK
MPGVDLAVPIDQLVWRTRFQRRIPERLPVLW
>CYP121A1_Mycobacterium tuberculosis M1271 2622403400
MTATVLLEVPFSARGDRIPDAVAELRTREPIRKVRTITGAEAWLVSSYALCTQVLEDRRFSMKETAAAGAPRL
NALTVPPEVVNNMGNIADAGLRKAVMKAITPKAPGLEQFLRDTANSLLDNLITEGAPADLRNDFADPLATALH
CKVLGIPQEDGPKLFRSLSIAFMSSADPIPAAKINWDRDIEYMAGILENPNITTGLMGELSRLRKDPAYSHVS
DELFATIGVTFFGAGVISTGSFLTTALISLIQRPQLRNLLHEKPELIPAGVEELLRINLSFADGLPRLATADI
QVGDVLVRKGELVLVLLEGANFDPEHFPNPGSIELDRPNPTSHLAFGRGQHFCPGSALGRRHAQIGIEALLKK
MPGVDLAVPIDQLVWRTRFQRRIPERLPVLW
>CYP121A1_Mycobacterium tuberculosis M1272 2622409047
MTATVLLEVPFSARGDRIPDAVAELRTREPIRKVRTITGAEAWLVSSYALCTQVLEDRRFSMKETAAAGAPRL
NALTVPPEVVNNMGNIADAGLRKAVMKAITPKAPGLEQFLRDTANSLLDNLITEGAPADLRNDFADPLATALH
CKVLGIPQEDGPKLFRSLSIAFMSSADPIPAAKINWDRDIEYMAGILENPNITTGLMGELSRLRKDPAYSHVS
DELFATIGVTFFGAGVISTGSFLTTALISLIQRPQLRNLLHEKPELIPAGVEELLRINLSFADGLPRLATADI
QVGDVLVRKGELVLVLLEGANFDPEHFPNPGSIELDRPNPTSHLAFGRGQHFCPGSALGRRHAQIGIEALLKK
MPGVDLAVPIDQLVWRTRFQRRIPERLPVLW
>CYP121A1_Mycobacterium tuberculosis M1274 2622416045
MTATVLLEVPFSARGDRIPDAVAELRTREPIRKVRTITGAEAWLVSSYALCTQVLEDRRFSMKETAAAGAPRL
NALTVPPEVVNNMGNIADAGLRKAVMKAITPKAPGLEQFLRDTANSLLDNLITEGAPADLRNDFADPLATALH
CKVLGIPQEDGPKLFRSLSIAFMSSADPIPAAKINWDRDIEYMAGILENPNITTGLMGELSRLRKDPAYSHVS
DELFATIGVTFFGAGVISTGSFLTTALISLIQRPQLRNLLHEKPELIPAGVEELLRINLSFADGLPRLATADI
QVGDVLVRKGELVLVLLEGANFDPEHFPNPGSIELDRPNPTSHLAFGRGQHFCPGSALGRRHAQIGIEALLKK
MPGVDLAVPIDQLVWRTRFQRRIPERLPVLW
>CYP121A1_Mycobacterium tuberculosis M1283 2577804128
MTATVLLEVPFSARGDRIPDAVAELRTREPIRKVRTITGAEAWLVSSYALCTQVLEDRRFSMKETAAAGAPRL
NALTVPPEVVNNMGNIADAGLRKAVMKAITPKAPGLEQFLRDTANSLLDNLITEGAPADLRNDFADPLATALH
CKVLGIPQEDGPKLFRSLSIAFMSSADPIPAAKINWDRDIEYMAGILENPNITTGLMGELSRLRKDPAYSHVS
DELFATIGVTFFGAGVISTGSFLTTALISLIQRPQLRNLLHEKPELIPAGVEELLRINLSFADGLPRLATADI
QVGDVLVRKGELVLVLLEGANFDPEHFPNPGSIELDRPNPTSHLAFGRGQHFCPGSALGRRHAQIGIEALLKK
MPGVDLAVPIDQLVWRTRFQRRIPERLPVLW

>CYP121A1_Mycobacterium tuberculosis M1287 2622419894
MTATVLLEVPFSARGDRIPDAVAELRTREPIRKVRTITGAEAWLVSSYALCTQVLEDRRFSMKETAAAGAPRL
NALTVPPEVVNNMGNIADAGLRKAVMKAITPKAPGLEQFLRDTANSLLDNLITEGAPADLRNDFADPLATALH
CKVLGIPQEDGPKLFRSLSIAFMSSADPIPAAKINWDRDIEYMAGILENPNITTGLMGELSRLRKDPAYSHVS
DELFATIGVTFFGAGVISTGSFLTTALISLIQRPQLRNLLHEKPELIPAGVEELLRINLSFADGLPRLATADI
QVGDVLVRKGELVLVLLEGANFDPEHFPNPGSIELDRPNPTSHLAFGRGQHFCPGSALGRRHAQIGIEALLKK
MPGVDLAVPIDQLVWRTRFQRRIPERLPVLW
>CYP121A1_Mycobacterium tuberculosis M1288 2622425519
MTATVLLEVPFSARGDRIPDAVAELRTREPIRKVRTITGAEAWLVSSYALCTQVLEDRRFSMKETAAAGAPRL
NALTVPPEVVNNMGNIADAGLRKAVMKAITPKAPGLEQFLRDTANSLLDNLITEGAPADLRNDFADPLATALH
CKVLGIPQEDGPKLFRSLSIAFMSSADPIPAAKINWDRDIEYMAGILENPNITTGLMGELSRLRKDPAYSHVS
DELFATIGVTFFGAGVISTGSFLTTALISLIQRPQLRNLLHEKPELIPAGVEELLRINLSFADGLPRLATADI
QVGDVLVRKGELVLVLLEGANFDPEHFPNPGSIELDRPNPTSHLAFGRGQHFCPGSALGRRHAQIGIEALLKK
MPGVDLAVPIDQLVWRTRFQRRIPERLPVLW
>CYP121A1_Mycobacterium tuberculosis M1292 2622428471
MTATVLLEVPFSARGDRIPDAVAELRTREPIRKVRTITGAEAWLVSSYALCTQVLEDRRFSMKETAAAGAPRL
NALTVPPEVVNNMGNIADAGLRKAVMKAITPKAPGLEQFLRDTANSLLDNLITEGAPADLRNDFADPLATALH
CKVLGIPQEDGPKLFRSLSIAFMSSADPIPAAKINWDRDIEYMAGILENPNITTGLMGELSRLRKDPAYSHVS
DELFATIGVTFFGAGVISTGSFLTTALISLIQRPQLRNLLHEKPELIPAGVEELLRINLSFADGLPRLATADI
QVGDVLVRKGELVLVLLEGANFDPEHFPNPGSIELDRPNPTSHLAFGRGQHFCPGSALGRRHAQIGIEALLKK
MPGVDLAVPIDQLVWRTRFQRRIPERLPVLW
>CYP121A1_Mycobacterium tuberculosis M1295 2622437924
MTATVLLEVPFSARGDRIPDAVAELRTREPIRKVRTITGAEAWLVSSYALCTQVLEDRRFSMKETAAAGAPRL
NALTVPPEVVNNMGNIADAGLRKAVMKAITPKAPGLEQFLRDTANSLLDNLITEGAPADLRNDFADPLATALH
CKVLGIPQEDGPKLFRSLSIAFMSSADPIPAAKINWDRDIEYMAGILENPNITTGLMGELSRLRKDPAYSHVS
DELFATIGVTFFGAGVISTGSFLTTALISLIQRPQLRNLLHEKPELIPAGVEELLRINLSFADGLPRLATADI
QVGDVLVRKGELVLVLLEGANFDPEHFPNPGSIELDRPNPTSHLAFGRGQHFCPGSALGRRHAQIGIEALLKK
MPGVDLAVPIDQLVWRTRFQRRIPERLPVLW
>CYP121A1_Mycobacterium tuberculosis M13 2622440955
MTATVLLEVPFSARGDRIPDAVAELRTREPIRKVRTITGAEAWLVSSYALCTQVLEDRRFSMKETAAAGAPRL
NALTVPPEVVNNMGNIADAGLRKAVMKAITPKAPGLEQFLRDTANSLLDNLITEGAPADLRNDFADPLATALH
CKVLGIPQEDGPKLFRSLSIAFMSSADPIPAAKINWDRDIEYMAGILENPNITTGLMGELSRLRKDPAYSHVS
DELFATIGVTFFGAGVISTGSFLTTALISLIQRPQLRNLLHEKPELIPAGVEELLRINLSFADGLPRLATADI
QVGDVLVRKGELVLVLLEGANFDPEHFPNPGSIELDRPNPTSHLAFGRGQHFCPGSALGRRHAQIGIEALLKK
MPGVDLAVPIDQLVWRTRFQRRIPERLPVLW
>CYP121A1_Mycobacterium tuberculosis M1300 2622446176
MTATVLLEVPFSARGDRIPDAVAELRTREPIRKVRTITGAEAWLVSSYALCTQVLEDRRFSMKETAAAGAPRL
NALTVPPEVVNNMGNIADAGLRKAVMKAITPKAPGLEQFLRDTANSLLDNLITEGAPADLRNDFADPLATALH
CKVLGIPQEDGPKLFRSLSIAFMSSADPIPAAKINWDRDIEYMAGILENPNITTGLMGELSRLRKDPAYSHVS
DELFATIGVTFFGAGVISTGSFLTTALISLIQRPQLRNLLHEKPELIPAGVEELLRINLSFADGLPRLATADI
QVGDVLVRKGELVLVLLEGANFDPEHFPNPGSIELDRPNPTSHLAFGRGQHFCPGSALGRRHAQIGIEALLKK
MPGVDLAVPIDQLVWRTRFQRRIPERLPVLW
>CYP121A1_Mycobacterium tuberculosis M1304 2622450288
MTATVLLEVPFSARGDRIPDAVAELRTREPIRKVRTITGAEAWLVSSYALCTQVLEDRRFSMKETAAAGAPRL
NALTVPPEVVNNMGNIADAGLRKAVMKAITPKAPGLEQFLRDTANSLLDNLITEGAPADLRNDFADPLATALH
CKVLGIPQEDGPKLFRSLSIAFMSSADPIPAAKINWDRDIEYMAGILENPNITTGLMGELSRLRKDPAYSHVS
DELFATIGVTFFGAGVISTGSFLTTALISLIQRPQLRNLLHEKPELIPAGVEELLRINLSFADGLPRLATADI
QVGDVLVRKGELVLVLLEGANFDPEHFPNPGSIELDRPNPTSHLAFGRGQHFCPGSALGRRHAQIGIEALLKK
MPGVDLAVPIDQLVWRTRFQRRIPERLPVLW
>CYP121A1_Mycobacterium tuberculosis M1307 2641869353
MTATVLLEVPFSARGDRIPDAVAELRTREPIRKVRTITGAEAWLVSSYALCTQVLEDRRFSMKETAAAGAPRL
NALTVPPEVVNNMGNIADAGLRKAVMKAITPKAPGLEQFLRDTANSLLDNLITEGAPADLRNDFADPLATALH
CKVLGIPQEDGPKLFRSLSIAFMSSADPIPAAKINWDRDIEYMAGILENPNITTGLMGELSRLRKDPAYSHVS
DELFATIGVTFFGAGVISTGSFLTTALISLIQRPQLRNLLHEKPELIPAGVEELLRINLSFADGLPRLATADI
QVGDVLVRKGELVLVLLEGANFDPEHFPNPGSIELDRPNPTSHLAFGRGQHFCPGSALGRRHAQIGIEALLKK
MPGVDLAVPIDQLVWRTRFQRRIPERLPVLW
>CYP121A1_Mycobacterium tuberculosis M1309 2622454420

MTATVLLEVPFSARGDRIPDAVAELRTREPIRKVRTITGAEAWLVSSYALCTQVLEDRRFSMKETAAAGAPRL
NALTVPPEVVNNMGNIADAGLRKAVMKAITPKAPGLEQFLRDTANSLLDNLITEGAPADLRNDFADPLATALH
CKVLGIPQEDGPKLFRSLSIAFMSSADPIPAAKINWDRDIEYMAGILENPNITTGLMGELSRLRKDPAYSHVS
DELFATIGVTFFGAGVISTGSFLTTALISLIQRPQLRNLLHEKPELIPAGVEELLRINLSFADGLPRLATADI
QVGDVLVRKGELVLVLLEGANFDPEHFPNPGSIELDRPNPTSHLAFGRGQHFCPGSALGRRHAQIGIEALLKK
MPGVDLAVPIDQLVWRTRFQRRIPERLPVLW
>CYP121A1_Mycobacterium tuberculosis M1310 2622457017
MTATVLLEVPFSARGDRIPDAVAELRTREPIRKVRTITGAEAWLVSSYALCTQVLEDRRFSMKETAAAGAPRL
NALTVPPEVVNNMGNIADAGLRKAVMKAITPKAPGLEQFLRDTANSLLDNLITEGAPADLRNDFADPLATALH
CKVLGIPQEDGPKLFRSLSIAFMSSADPIPAAKINWDRDIEYMAGILENPNITTGLMGELSRLRKDPAYSHVS
DELFATIGVTFFGAGVISTGSFLTTALISLIQRPQLRNLLHEKPELIPAGVEELLRINLSFADGLPRLATADI
QVGDVLVRKGELVLVLLEGANFDPEHFPNPGSIELDRPNPTSHLAFGRGQHFCPGSALGRRHAQIGIEALLKK
MPGVDLAVPIDQLVWRTRFQRRIPERLPVLW
>CYP121A1_Mycobacterium tuberculosis M1315 2622462347
MTATVLLEVPFSARGDRIPDAVAELRTREPIRKVRTITGAEAWLVSSYALCTQVLEDRRFSMKETAAAGAPRL
NALTVPPEVVNNMGNIADAGLRKAVMKAITPKAPGLEQFLRDTANSLLDNLITEGAPADLRNDFADPLATALH
CKVLGIPQEDGPKLFRSLSIAFMSSADPIPAAKINWDRDIEYMAGILENPNITTGLMGELSRLRKDPAYSHVS
DELFATIGVTFFGAGVISTGSFLTTALISLIQRPQLRNLLHEKPELIPAGVEELLRINLSFADGLPRLATADI
QVGDVLVRKGELVLVLLEGANFDPEHFPNPGSIELDRPNPTSHLAFGRGQHFCPGSALGRRHAQIGIEALLKK
MPGVDLAVPIDQLVWRTRFQRRIPERLPVLW
>CYP121A1_Mycobacterium tuberculosis M1317 2622466748
MTATVLLEVPFSARGDRIPDAVAELRTREPIRKVRTITGAEAWLVSSYALCTQVLEDRRFSMKETAAAGAPRL
NALTVPPEVVNNMGNIADAGLRKAVMKAITPKAPGLEQFLRDTANSLLDNLITEGAPADLRNDFADPLATALH
CKVLGIPQEDGPKLFRSLSIAFMSSADPIPAAKINWDRDIEYMAGILENPNITTGLMGELSRLRKDPAYSHVS
DELFATIGVTFFGAGVISTGSFLTTALISLIQRPQLRNLLHEKPELIPAGVEELLRINLSFADGLPRLATADI
QVGDVLVRKGELVLVLLEGANFDPEHFPNPGSIELDRPNPTSHLAFGRGQHFCPGSALGRRHAQIGIEALLKK
MPGVDLAVPIDQLVWRTRFQRRIPERLPVLW
>CYP121A1_Mycobacterium tuberculosis M1319 2622470844
MTATVLLEVPFSARGDRIPDAVAELRTREPIRKVRTITGAEAWLVSSYALCTQVLEDRRFSMKETAAAGAPRL
NALTVPPEVVNNMGNIADAGLRKAVMKAITPKAPGLEQFLRDTANSLLDNLITEGAPADLRNDFADPLATALH
CKVLGIPQEDGPKLFRSLSIAFMSSADPIPAAKINWDRDIEYMAGILENPNITTGLMGELSRLRKDPAYSHVS
DELFATIGVTFFGAGVISTGSFLTTALISLIQRPQLRNLLHEKPELIPAGVEELLRINLSFADGLPRLATADI
QVGDVLVRKGELVLVLLEGANFDPEHFPNPGSIELDRPNPTSHLAFGRGQHFCPGSALGRRHAQIGIEALLKK
MPGVDLAVPIDQLVWRTRFQRRIPERLPVLW
>CYP121A1_Mycobacterium tuberculosis M1324 2622474949
MTATVLLEVPFSARGDRIPDAVAELRTREPIRKVRTITGAEAWLVSSYALCTQVLEDRRFSMKETAAAGAPRL
NALTVPPEVVNNMGNIADAGLRKAVMKAITPKAPGLEQFLRDTANSLLDNLITEGAPADLRNDFADPLATALH
CKVLGIPQEDGPKLFRSLSIAFMSSADPIPAAKINWDRDIEYMAGILENPNITTGLMGELSRLRKDPAYSHVS
DELFATIGVTFFGAGVISTGSFLTTALISLIQRPQLRNLLHEKPELIPAGVEELLRINLSFADGLPRLATADI
QVGDVLVRKGELVLVLLEGANFDPEHFPNPGSIELDRPNPTSHLAFGRGQHFCPGSALGRRHAQIGIEALLKK
MPGVDLAVPIDQLVWRTRFQRRIPERLPVLW
>CYP121A1_Mycobacterium tuberculosis M1325 2622478776
MTATVLLEVPFSARGDRIPDAVAELRTREPIRKVRTITGAEAWLVSSYALCTQVLEDRRFSMKETAAAGAPRL
NALTVPPEVVNNMGNIADAGLRKAVMKAITPKAPGLEQFLRDTANSLLDNLITEGAPADLRNDFADPLATALH
CKVLGIPQEDGPKLFRSLSIAFMSSADPIPAAKINWDRDIEYMAGILENPNITTGLMGELSRLRKDPAYSHVS
DELFATIGVTFFGAGVISTGSFLTTALISLIQRPQLRNLLHEKPELIPAGVEELLRINLSFADGLPRLATADI
QVGDVLVRKGELVLVLLEGANFDPEHFPNPGSIELDRPNPTSHLAFGRGQHFCPGSALGRRHAQIGIEALLKK
MPGVDLAVPIDQLVWRTRFQRRIPERLPVLW
>CYP121A1_Mycobacterium tuberculosis M1340 2584620237
MTATVLLEVPFSARGDRIPDAVAELRTREPIRKVRTITGAEAWLVSSYALCTQVLEDRRFSMKETAAAGAPRL
NALTVPPEVVNNMGNIADAGLRKAVMKAITPKAPGLEQFLRDTANSLLDNLITEGAPADLRNDFADPLATALH
CKVLGIPQEDGPKLFRSLSIAFMSSADPIPAAKINWDRDIEYMAGILENPNITTGLMGELSRLRKDPAYSHVS
DELFATIGVTFFGAGVISTGSFLTTALISLIQRPQLRNLLHEKPELIPAGVEELLRINLSFADGLPRLATADI
QVGDVLVRKGELVLVLLEGANFDPEHFPNPGSIELDRPNPTSHLAFGRGQHFCPGSALGRRHAQIGIEALLKK
MPGVDLAVPIDQLVWRTRFQRRIPERLPVLW
>CYP121A1_Mycobacterium tuberculosis M1373 2622482903
MTATVLLEVPFSARGDRIPDAVAELRTREPIRKVRTITGAEAWLVSSYALCTQVLEDRRFSMKETAAAGAPRL
NALTVPPEVVNNMGNIADAGLRKAVMKAITPKAPGLEQFLRDTANSLLDNLITEGAPADLRNDFADPLATALH

CKVLGIPQEDGPKLFRSLSIAFMSSADPIPAAKINWDRDIEYMAGILENPNITTGLMGELSRLRKDPAYSHVS
DELFATIGVTFFGAGVISTGSFLTTALISLIQRPQLRNLLHEKPELIPAGVEELLRINLSFADGLPRLATADI
QVGDVLVRKGELVLVLLEGANFDPEHFPNPGSIELDRPNPTSHLAFGRGQHFCPGSALGRRHAQIGIEALLKK
MPGVDLAVPIDQLVWRTRFQRRIPERLPVLW
>CYP121A1_Mycobacterium tuberculosis M1381 2622491452
MTATVLLEVPFSARGDRIPDAVAELRTREPIRKVRTITGAEAWLVSSYALCTQVLEDRRFSMKETAAAGAPRL
NALTVPPEVVNNMGNIADAGLRKAVMKAITPKAPGLEQFLRDTANSLLDNLITEGAPADLRNDFADPLATALH
CKVLGIPQEDGPKLFRSLSIAFMSSADPIPAAKINWDRDIEYMAGILENPNITTGLMGELSRLRKDPAYSHVS
DELFATIGVTFFGAGVISTGSFLTTALISLIQRPQLRNLLHEKPELIPAGVEELLRINLSFADGLPRLATADI
QVGDVLVRKGELVLVLLEGANFDPEHFPNPGSIELDRPNPTSHLAFGRGQHFCPGSALGRRHAQIGIEALLKK
MPGVDLAVPIDQLVWRTRFQRRIPERLPVLW
>CYP121A1_Mycobacterium tuberculosis M1384 2622495951
MTATVLLEVPFSARGDRIPDAVAELRTREPIRKVRTITGAEAWLVSSYALCTQVLEDRRFSMKETAAAGAPRL
NALTVPPEVVNNMGNIADAGLRKAVMKAITPKAPGLEQFLRDTANSLLDNLITEGAPADLRNDFADPLATALH
CKVLGIPQEDGPKLFRSLSIAFMSSADPIPAAKINWDRDIEYMAGILENPNITTGLMGELSRLRKDPAYSHVS
DELFATIGVTFFGAGVISTGSFLTTALISLIQRPQLRNLLHEKPELIPAGVEELLRINLSFADGLPRLATADI
QVGDVLVRKGELVLVLLEGANFDPEHFPNPGSIELDRPNPTSHLAFGRGQHFCPGSALGRRHAQIGIEALLKK
MPGVDLAVPIDQLVWRTRFQRRIPERLPVLW
>CYP121A1_Mycobacterium tuberculosis M1389 2622498912
MTATVLLEVPFSARGDRIPDAVAELRTREPIRKVRTITGAEAWLVSSYALCTQVLEDRRFSMKETAAAGAPRL
NALTVPPEVVNNMGNIADAGLRKAVMKAITPKAPGLEQFLRDTANSLLDNLITEGAPADLRNDFADPLATALH
CKVLGIPQEDGPKLFRSLSIAFMSSADPIPAAKINWDRDIEYMAGILENPNITTGLMGELSRLRKDPAYSHVS
DELFATIGVTFFGAGVISTGSFLTTALISLIQRPQLRNLLHEKPELIPAGVEELLRINLSFADGLPRLATADI
QVGDVLVRKGELVLVLLEGANFDPEHFPNPGSIELDRPNPTSHLAFGRGQHFCPGSALGRRHAQIGIEALLKK
MPGVDLAVPIDQLVWRTRFQRRIPERLPVLW
>CYP121A1_Mycobacterium tuberculosis M1392 2622793452
MTATVLLEVPFSARGDRIPDAVAELRTREPIRKVRTITGAEAWLVSSYALCTQVLEDRRFSMKETAAAGAPRL
NALTVPPEVVNNMGNIADAGLRKAVMKAITPKAPGLEQFLRDTANSLLDNLITEGAPADLRNDFADPLATALH
CKVLGIPQEDGPKLFRSLSIAFMSSADPIPAAKINWDRDIEYMAGILENPNITTGLMGELSRLRKDPAYSHVS
DELFATIGVTFFGAGVISTGSFLTTALISLIQRPQLRNLLHEKPELIPAGVEELLRINLSFADGLPRLATADI
QVGDVLVRKGELVLVLLEGANFDPEHFPNPGSIELDRPNPTSHLAFGRGQHFCPGSALGRRHAQIGIEALLKK
MPGVDLAVPIDQLVWRTRFQRRIPERLPVLW
>CYP121A1_Mycobacterium tuberculosis M1393 2622503813
MTATVLLEVPFSARGDRIPDAVAELRTREPIRKVRTITGAEAWLVSSYALCTQVLEDRRFSMKETAAAGAPRL
NALTVPPEVVNNMGNIADAGLRKAVMKAITPKAPGLEQFLRDTANSLLDNLITEGAPADLRNDFADPLATALH
CKVLGIPQEDGPKLFRSLSIAFMSSADPIPAAKINWDRDIEYMAGILENPNITTGLMGELSRLRKDPAYSHVS
DELFATIGVTFFGAGVISTGSFLTTALISLIQRPQLRNLLHEKPELIPAGVEELLRINLSFADGLPRLATADI
QVGDVLVRKGELVLVLLEGANFDPEHFPNPGSIELDRPNPTSHLAFGRGQHFCPGSALGRRHAQIGIEALLKK
MPGVDLAVPIDQLVWRTRFQRRIPERLPVLW
>CYP121A1_Mycobacterium tuberculosis M1400 2622511757
MTATVLLEVPFSARGDRIPDAVAELRTREPIRKVRTITGAEAWLVSSYALCTQVLEDRRFSMKETAAAGAPRL
NALTVPPEVVNNMGNIADAGLRKAVMKAITPKAPGLEQFLRDTANSLLDNLITEGAPADLRNDFADPLATALH
CKVLGIPQEDGPKLFRSLSIAFMSSADPIPAAKINWDRDIEYMAGILENPNITTGLMGELSRLRKDPAYSHVS
DELFATIGVTFFGAGVISTGSFLTTALISLIQRPQLRNLLHEKPELIPAGVEELLRINLSFADGLPRLATADI
QVGDVLVRKGELVLVLLEGANFDPEHFPNPGSIELDRPNPTSHLAFGRGQHFCPGSALGRRHAQIGIEALLKK
MPGVDLAVPIDQLVWRTRFQRRIPERLPVLW
>CYP121A1_Mycobacterium tuberculosis M1402 2622514755
MTATVLLEVPFSARGDRIPDAVAELRTREPIRKVRTITGAEAWLVSSYALCTQVLEDRRFSMKETAAAGAPRL
NALTVPPEVVNNMGNIADAGLRKAVMKAITPKAPGLEQFLRDTANSLLDNLITEGAPADLRNDFADPLATALH
CKVLGIPQEDGPKLFRSLSIAFMSSADPIPAAKINWDRDIEYMAGILENPNITTGLMGELSRLRKDPAYSHVS
DELFATIGVTFFGAGVISTGSFLTTALISLIQRPQLRNLLHEKPELIPAGVEELLRINLSFADGLPRLATADI
QVGDVLVRKGELVLVLLEGANFDPEHFPNPGSIELDRPNPTSHLAFGRGQHFCPGSALGRRHAQIGIEALLKK
MPGVDLAVPIDQLVWRTRFQRRIPERLPVLW
>CYP121A1_Mycobacterium tuberculosis M1404 2622519980
MTATVLLEVPFSARGDRIPDAVAELRTREPIRKVRTITGAEAWLVSSYALCTQVLEDRRFSMKETAAAGAPRL
NALTVPPEVVNNMGNIADAGLRKAVMKAITPKAPGLEQFLRDTANSLLDNLITEGAPADLRNDFADPLATALH
CKVLGIPQEDGPKLFRSLSIAFMSSADPIPAAKINWDRDIEYMAGILENPNITTGLMGELSRLRKDPAYSHVS
DELFATIGVTFFGAGVISTGSFLTTALISLIQRPQLRNLLHEKPELIPAGVEELLRINLSFADGLPRLATADI

QVGDVLVRKGELVLVLLEGANFDPEHFPNPGSIELDRPNPTSHLAFGRGQHFCPGSALGRRHAQIGIEALLKK
MPGVDLAVPIDQLVWRTRFQRRIPERLPVLW
>CYP121A1_Mycobacterium tuberculosis M1410 2622524089
MTATVLLEVPFSARGDRIPDAVAELRTREPIRKVRTITGAEAWLVSSYALCTQVLEDRRFSMKETAAAGAPRL
NALTVPPEVVNNMGNIADAGLRKAVMKAITPKAPGLEQFLRDTANSLLDNLITEGAPADLRNDFADPLATALH
CKVLGIPQEDGPKLFRSLSIAFMSSADPIPAAKINWDRDIEYMAGILENPNITTGLMGELSRLRKDPAYSHVS
DELFATIGVTFFGAGVISTGSFLTTALISLIQRPQLRNLLHEKPELIPAGVEELLRINLSFADGLPRLATADI
QVGDVLVRKGELVLVLLEGANFDPEHFPNPGSIELDRPNPTSHLAFGRGQHFCPGSALGRRHAQIGIEALLKK
MPGVDLAVPIDQLVWRTRFQRRIPERLPVLW
>CYP121A1_Mycobacterium tuberculosis M1415 2584983690
MTATVLLEVPFSARGDRIPDAVAELRTREPIRKVRTITGAEAWLVSSYALCTQVLEDRRFSMKETAAAGAPRL
NALTVPPEVVNNMGNIADAGLRKAVMKAITPKAPGLEQFLRDTANSLLDNLITEGAPADLRNDFADPLATALH
CKVLGIPQEDGPKLFRSLSIAFMSSADPIPAAKINWDRDIEYMAGILENPNITTGLMGELSRLRKDPAYSHVS
DELFATIGVTFFGAGVISTGSFLTTALISLIQRPQLRNLLHEKPELIPAGVEELLRINLSFADGLPRLATADI
QVGDVLVRKGELVLVLLEGANFDPEHFPNPGSIELDRPNPTSHLAFGRGQHFCPGSALGRRHAQIGIEALLKK
MPGVDLAVPIDQLVWRTRFQRRIPERLPVLW
>CYP121A1_Mycobacterium tuberculosis M1416 2622528504
MTATVLLEVPFSARGDRIPDAVAELRTREPIRKVRTITGAEAWLVSSYALCTQVLEDRRFSMKETAAAGAPRL
NALTVPPEVVNNMGNIADAGLRKAVMKAITPKAPGLEQFLRDTANSLLDNLITEGAPADLRNDFADPLATALH
CKVLGIPQEDGPKLFRSLSIAFMSSADPIPAAKINWDRDIEYMAGILENPNITTGLMGELSRLRKDPAYSHVS
DELFATIGVTFFGAGVISTGSFLTTALISLIQRPQLRNLLHEKPELIPAGVEELLRINLSFADGLPRLATADI
QVGDVLVRKGELVLVLLEGANFDPEHFPNPGSIELDRPNPTSHLAFGRGQHFCPGSALGRRHAQIGIEALLKK
MPGVDLAVPIDQLVWRTRFQRRIPERLPVLW
>CYP121A1_Mycobacterium tuberculosis M1417 2622532597
MTATVLLEVPFSARGDRIPDAVAELRTREPIRKVRTITGAEAWLVSSYALCTQVLEDRRFSMKETAAAGAPRL
NALTVPPEVVNNMGNIADAGLRKAVMKAITPKAPGLEQFLRDTANSLLDNLITEGAPADLRNDFADPLATALH
CKVLGIPQEDGPKLFRSLSIAFMSSADPIPAAKINWDRDIEYMAGILENPNITTGLMGELSRLRKDPAYSHVS
DELFATIGVTFFGAGVISTGSFLTTALISLIQRPQLRNLLHEKPELIPAGVEELLRINLSFADGLPRLATADI
QVGDVLVRKGELVLVLLEGANFDPEHFPNPGSIELDRPNPTSHLAFGRGQHFCPGSALGRRHAQIGIEALLKK
MPGVDLAVPIDQLVWRTRFQRRIPERLPVLW
>CYP121A1_Mycobacterium tuberculosis M1418 2577010234
MTATVLLEVPFSARGDRIPDAVAELRTREPIRKVRTITGAEAWLVSSYALCTQVLEDRRFSMKETAAAGAPRL
NALTVPPEVVNNMGNIADAGLRKAVMKAITPKAPGLEQFLRDTANSLLDNLITEGAPADLRNDFADPLATALH
CKVLGIPQEDGPKLFRSLSIAFMSSADPIPAAKINWDRDIEYMAGILENPNITTGLMGELSRLRKDPAYSHVS
DELFATIGVTFFGAGVISTGSFLTTALISLIQRPQLRNLLHEKPELIPAGVEELLRINLSFADGLPRLATADI
QVGDVLVRKGELVLVLLEGANFDPEHFPNPGSIELDRPNPTSHLAFGRGQHFCPGSALGRRHAQIGIEALLKK
MPGVDLAVPIDQLVWRTRFQRRIPERLPVLW
>CYP121A1_Mycobacterium tuberculosis M1422 2622536697
MTATVLLEVPFSARGDRIPDAVAELRTREPIRKVRTITGAEAWLVSSYALCTQVLEDRRFSMKETAAAGAPRL
NALTVPPEVVNNMGNIADAGLRKAVMKAITPKAPGLEQFLRDTANSLLDNLITEGAPADLRNDFADPLATALH
CKVLGIPQEDGPKLFRSLSIAFMSSADPIPAAKINWDRDIEYMAGILENPNITTGLMGELSRLRKDPAYSHVS
DELFATIGVTFFGAGVISTGSFLTTALISLIQRPQLRNLLHEKPELIPAGVEELLRINLSFADGLPRLATADI
QVGDVLVRKGELVLVLLEGANFDPEHFPNPGSIELDRPNPTSHLAFGRGQHFCPGSALGRRHAQIGIEALLKK
MPGVDLAVPIDQLVWRTRFQRRIPERLPVLW
>CYP121A1_Mycobacterium tuberculosis M1424 2622540110
MTATVLLEVPFSARGDRIPDAVAELRTREPIRKVRTITGAEAWLVSSYALCTQVLEDRRFSMKETAAAGAPRL
NALTVPPEVVNNMGNIADAGLRKAVMKAITPKAPGLEQFLRDTANSLLDNLITEGAPADLRNDFADPLATALH
CKVLGIPQEDGPKLFRSLSIAFMSSADPIPAAKINWDRDIEYMAGILENPNITTGLMGELSRLRKDPAYSHVS
DELFATIGVTFFGAGVISTGSFLTTALISLIQRPQLRNLLHEKPELIPAGVEELLRINLSFADGLPRLATADI
QVGDVLVRKGELVLVLLEGANFDPEHFPNPGSIELDRPNPTSHLAFGRGQHFCPGSALGRRHAQIGIEALLKK
MPGVDLAVPIDQLVWRTRFQRRIPERLPVLW
>CYP121A1_Mycobacterium tuberculosis M1425 2622544919
MTATVLLEVPFSARGDRIPDAVAELRTREPIRKVRTITGAEAWLVSSYALCTQVLEDRRFSMKETAAAGAPRL
NALTVPPEVVNNMGNIADAGLRKAVMKAITPKAPGLEQFLRDTANSLLDNLITEGAPADLRNDFADPLATALH
CKVLGIPQEDGPKLFRSLSIAFMSSADPIPAAKINWDRDIEYMAGILENPNITTGLMGELSRLRKDPAYSHVS
DELFATIGVTFFGAGVISTGSFLTTALISLIQRPQLRNLLHEKPELIPAGVEELLRINLSFADGLPRLATADI
QVGDVLVRKGELVLVLLEGANFDPEHFPNPGSIELDRPNPTSHLAFGRGQHFCPGSALGRRHAQIGIEALLKK
MPGVDLAVPIDQLVWRTRFQRRIPERLPVLW

>CYP121A1_Mycobacterium tuberculosis M1429 2622547739
MTATVLLEVPFSARGDRIPDAVAELRTREPIRKVRTITGAEAWLVSSYALCTQVLEDRRFSMKETAAAGAPRL
NALTVPPEVVNNMGNIADAGLRKAVMKAITPKAPGLEQFLRDTANSLLDNLITEGAPADLRNDFADPLATALH
CKVLGIPQEDGPKLFRSLSIAFMSSADPIPAAKINWDRDIEYMAGILENPNITTGLMGELSRLRKDPAYSHVS
DELFATIGVTFFGAGVISTGSFLTTALISLIQRPQLRNLLHEKPELIPAGVEELLRINLSFADGLPRLATADI
QVGDVLVRKGELVLVLLEGANFDPEHFPNPGSIELDRPNPTSHLAFGRGQHFCPGSALGRRHAQIGIEALLKK
MPGVDLAVPIDQLVWRTRFQRRIPERLPVLW
>CYP121A1_Mycobacterium tuberculosis M1431 2622553159
MTATVLLEVPFSARGDRIPDAVAELRTREPIRKVRTITGAEAWLVSSYALCTQVLEDRRFSMKETAAAGAPRL
NALTVPPEVVNNMGNIADAGLRKAVMKAITPKAPGLEQFLRDTANSLLDNLITEGAPADLRNDFADPLATALH
CKVLGIPQEDGPKLFRSLSIAFMSSADPIPAAKINWDRDIEYMAGILENPNITTGLMGELSRLRKDPAYSHVS
DELFATIGVTFFGAGVISTGSFLTTALISLIQRPQLRNLLHEKPELIPAGVEELLRINLSFADGLPRLATADI
QVGDVLVRKGELVLVLLEGANFDPEHFPNPGSIELDRPNPTSHLAFGRGQHFCPGSALGRRHAQIGIEALLKK
MPGVDLAVPIDQLVWRTRFQRRIPERLPVLW
>CYP121A1_Mycobacterium tuberculosis M1438 2574663909
MTATVLLEVPFSARGDRIPDAVAELRTREPIRKVRTITGAEAWLVSSYALCTQVLEDRRFSMKETAAAGAPRL
NALTVPPEVVNNMGNIADAGLRKAVMKAITPKAPGLEQFLRDTANSLLDNLITEGAPADLRNDFADPLATALH
CKVLGIPQEDGPKLFRSLSIAFMSSADPIPAAKINWDRDIEYMAGILENPNITTGLMGELSRLRKDPAYSHVS
DELFATIGVTFFGAGVISTGSFLTTALISLIQRPQLRNLLHEKPELIPAGVEELLRINLSFADGLPRLATADI
QVGDVLVRKGELVLVLLEGANFDPEHFPNPGSIELDRPNPTSHLAFGRGQHFCPGSALGRRHAQIGIEALLKK
MPGVDLAVPIDQLVWRTRFQRRIPERLPVLW
>CYP121A1_Mycobacterium tuberculosis M1439 2622557280
MTATVLLEVPFSARGDRIPDAVAELRTREPIRKVRTITGAEAWLVSSYALCTQVLEDRRFSMKETAAAGAPRL
NALTVPPEVVNNMGNIADAGLRKAVMKAITPKAPGLEQFLRDTANSLLDNLITEGAPADLRNDFADPLATALH
CKVLGIPQEDGPKLFRSLSIAFMSSADPIPAAKINWDRDIEYMAGILENPNITTGLMGELSRLRKDPAYSHVS
DELFATIGVTFFGAGVISTGSFLTTALISLIQRPQLRNLLHEKPELIPAGVEELLRINLSFADGLPRLATADI
QVGDVLVRKGELVLVLLEGANFDPEHFPNPGSIELDRPNPTSHLAFGRGQHFCPGSALGRRHAQIGIEALLKK
MPGVDLAVPIDQLVWRTRFQRRIPERLPVLW
>CYP121A1_Mycobacterium tuberculosis M1441 2622561383
MTATVLLEVPFSARGDRIPDAVAELRTREPIRKVRTITGAEAWLVSSYALCTQVLEDRRFSMKETAAAGAPRL
NALTVPPEVVNNMGNIADAGLRKAVMKAITPKAPGLEQFLRDTANSLLDNLITEGAPADLRNDFADPLATALH
CKVLGIPQEDGPKLFRSLSIAFMSSADPIPAAKINWDRDIEYMAGILENPNITTGLMGELSRLRKDPAYSHVS
DELFATIGVTFFGAGVISTGSFLTTALISLIQRPQLRNLLHEKPELIPAGVEELLRINLSFADGLPRLATADI
QVGDVLVRKGELVLVLLEGANFDPEHFPNPGSIELDRPNPTSHLAFGRGQHFCPGSALGRRHAQIGIEALLKK
MPGVDLAVPIDQLVWRTRFQRRIPERLPVLW
>CYP121A1_Mycobacterium tuberculosis M1444 2576016377
MTATVLLEVPFSARGDRIPDAVAELRTREPIRKVRTITGAEAWLVSSYALCTQVLEDRRFSMKETAAAGAPRL
NALTVPPEVVNNMGNIADAGLRKAVMKAITPKAPGLEQFLRDTANSLLDNLITEGAPADLRNDFADPLATALH
CKVLGIPQEDGPKLFRSLSIAFMSSADPIPAAKINWDRDIEYMAGILENPNITTGLMGELSRLRKDPAYSHVS
DELFATIGVTFFGAGVISTGSFLTTALISLIQRPQLRNLLHEKPELIPAGVEELLRINLSFADGLPRLATADI
QVGDVLVRKGELVLVLLEGANFDPEHFPNPGSIELDRPNPTSHLAFGRGQHFCPGSALGRRHAQIGIEALLKK
MPGVDLAVPIDQLVWRTRFQRRIPERLPVLW
>CYP121A1_Mycobacterium tuberculosis M1449 2575363133
MTATVLLEVPFSARGDRIPDAVAELRTREPIRKVRTITGAEAWLVSSYALCTQVLEDRRFSMKETAAAGAPRL
NALTVPPEVVNNMGNIADAGLRKAVMKAITPKAPGLEQFLRDTANSLLDNLITEGAPADLRNDFADPLATALH
CKVLGIPQEDGPKLFRSLSIAFMSSADPIPAAKINWDRDIEYMAGILENPNITTGLMGELSRLRKDPAYSHVS
DELFATIGVTFFGAGVISTGSFLTTALISLIQRPQLRNLLHEKPELIPAGVEELLRINLSFADGLPRLATADI
QVGDVLVRKGELVLVLLEGANFDPEHFPNPGSIELDRPNPTSHLAFGRGQHFCPGSALGRRHAQIGIEALLKK
MPGVDLAVPIDQLVWRTRFQRRIPERLPVLW
>CYP121A1_Mycobacterium tuberculosis M1452 2622569301
MTATVLLEVPFSARGDRIPDAVAELRTREPIRKVRTITGAEAWLVSSYALCTQVLEDRRFSMKETAAAGAPRL
NALTVPPEVVNNMGNIADAGLRKAVMKAITPKAPGLEQFLRDTANSLLDNLITEGAPADLRNDFADPLATALH
CKVLGIPQEDGPKLFRSLSIAFMSSADPIPAAKINWDRDIEYMAGILENPNITTGLMGELSRLRKDPAYSHVS
DELFATIGVTFFGAGVISTGSFLTTALISLIQRPQLRNLLHEKPELIPAGVEELLRINLSFADGLPRLATADI
QVGDVLVRKGELVLVLLEGANFDPEHFPNPGSIELDRPNPTSHLAFGRGQHFCPGSALGRRHAQIGIEALLKK
MPGVDLAVPIDQLVWRTRFQRRIPERLPVLW
>CYP121A1_Mycobacterium tuberculosis M1458 2622577501

MTATVLLEVPFSARGDRIPDAVAELRTREPIRKVRTITGAEAWLVSSYALCTQVLEDRRFSMKETAAAGAPRL
NALTVPPEVVNNMGNIADAGLRKAVMKAITPKAPGLEQFLRDTANSLLDNLITEGAPADLRNDFADPLATALH
CKVLGIPQEDGPKLFRSLSIAFMSSADPIPAAKINWDRDIEYMAGILENPNITTGLMGELSRLRKDPAYSHVS
DELFATIGVTFFGAGVISTGSFLTTALISLIQRPQLRNLLHEKPELIPAGVEELLRINLSFADGLPRLATADI
QVGDVLVRKGELVLVLLEGANFDPEHFPNPGSIELDRPNPTSHLAFGRGQHFCPGSALGRRHAQIGIEALLKK
MPGVDLAVPIDQLVWRTRFQRRIPERLPVLW
>CYP121A1_Mycobacterium tuberculosis M1459 2622581630
MTATVLLEVPFSARGDRIPDAVAELRTREPIRKVRTITGAEAWLVSSYALCTQVLEDRRFSMKETAAAGAPRL
NALTVPPEVVNNMGNIADAGLRKAVMKAITPKAPGLEQFLRDTANSLLDNLITEGAPADLRNDFADPLATALH
CKVLGIPQEDGPKLFRSLSIAFMSSADPIPAAKINWDRDIEYMAGILENPNITTGLMGELSRLRKDPAYSHVS
DELFATIGVTFFGAGVISTGSFLTTALISLIQRPQLRNLLHEKPELIPAGVEELLRINLSFADGLPRLATADI
QVGDVLVRKGELVLVLLEGANFDPEHFPNPGSIELDRPNPTSHLAFGRGQHFCPGSALGRRHAQIGIEALLKK
MPGVDLAVPIDQLVWRTRFQRRIPERLPVLW
>CYP121A1_Mycobacterium tuberculosis M1461 2622586058
MTATVLLEVPFSARGDRIPDAVAELRTREPIRKVRTITGAEAWLVSSYALCTQVLEDRRFSMKETAAAGAPRL
NALTVPPEVVNNMGNIADAGLRKAVMKAITPKAPGLEQFLRDTANSLLDNLITEGAPADLRNDFADPLATALH
CKVLGIPQEDGPKLFRSLSIAFMSSADPIPAAKINWDRDIEYMAGILENPNITTGLMGELSRLRKDPAYSHVS
DELFATIGVTFFGAGVISTGSFLTTALISLIQRPQLRNLLHEKPELIPAGVEELLRINLSFADGLPRLATADI
QVGDVLVRKGELVLVLLEGANFDPEHFPNPGSIELDRPNPTSHLAFGRGQHFCPGSALGRRHAQIGIEALLKK
MPGVDLAVPIDQLVWRTRFQRRIPERLPVLW
>CYP121A1_Mycobacterium tuberculosis M1475 2584770001
MTATVLLEVPFSARGDRIPDAVAELRTREPIRKVRTITGAEAWLVSSYALCTQVLEDRRFSMKETAAAGAPRL
NALTVPPEVVNNMGNIADAGLRKAVMKAITPKAPGLEQFLRDTANSLLDNLITEGAPADLRNDFADPLATALH
CKVLGIPQEDGPKLFRSLSIAFMSSADPIPAAKINWDRDIEYMAGILENPNITTGLMGELSRLRKDPAYSHVS
DELFATIGVTFFGAGVISTGSFLTTALISLIQRPQLRNLLHEKPELIPAGVEELLRINLSFADGLPRLATADI
QVGDVLVRKGELVLVLLEGANFDPEHFPNPGSIELDRPNPTSHLAFGRGQHFCPGSALGRRHAQIGIEALLKK
MPGVDLAVPIDQLVWRTRFQRRIPERLPVLW
>CYP121A1_Mycobacterium tuberculosis M1481 2575253017
MTATVLLEVPFSARGDRIPDAVAELRTREPIRKVRTITGAEAWLVSSYALCTQVLEDRRFSMKETAAAGAPRL
NALTVPPEVVNNMGNIADAGLRKAVMKAITPKAPGLEQFLRDTANSLLDNLITEGAPADLRNDFADPLATALH
CKVLGIPQEDGPKLFRSLSIAFMSSADPIPAAKINWDRDIEYMAGILENPNITTGLMGELSRLRKDPAYSHVS
DELFATIGVTFFGAGVISTGSFLTTALISLIQRPQLRNLLHEKPELIPAGVEELLRINLSFADGLPRLATADI
QVGDVLVRKGELVLVLLEGANFDPEHFPNPGSIELDRPNPTSHLAFGRGQHFCPGSALGRRHAQIGIEALLKK
MPGVDLAVPIDQLVWRTRFQRRIPERLPVLW
>CYP121A1_Mycobacterium tuberculosis M1488 2622588936
MTATVLLEVPFSARGDRIPDAVAELRTREPIRKVRTITGAEAWLVSSYALCTQVLEDRRFSMKETAAAGAPRL
NALTVPPEVVNNMGNIADAGLRKAVMKAITPKAPGLEQFLRDTANSLLDNLITEGAPADLRNDFADPLATALH
CKVLGIPQEDGPKLFRSLSIAFMSSADPIPAAKINWDRDIEYMAGILENPNITTGLMGELSRLRKDPAYSHVS
DELFATIGVTFFGAGVISTGSFLTTALISLIQRPQLRNLLHEKPELIPAGVEELLRINLSFADGLPRLATADI
QVGDVLVRKGELVLVLLEGANFDPEHFPNPGSIELDRPNPTSHLAFGRGQHFCPGSALGRRHAQIGIEALLKK
MPGVDLAVPIDQLVWRTRFQRRIPERLPVLW
>CYP121A1_Mycobacterium tuberculosis M1520 2622594317
MTATVLLEVPFSARGDRIPDAVAELRTREPIRKVRTITGAEAWLVSSYALCTQVLEDRRFSMKETAAAGAPRL
NALTVPPEVVNNMGNIADAGLRKAVMKAITPKAPGLEQFLRDTANSLLDNLITEGAPADLRNDFADPLATALH
CKVLGIPQEDGPKLFRSLSIAFMSSADPIPAAKINWDRDIEYMAGILENPNITTGLMGELSRLRKDPAYSHVS
DELFATIGVTFFGAGVISTGSFLTTALISLIQRPQLRNLLHEKPELIPAGVEELLRINLSFADGLPRLATADI
QVGDVLVRKGELVLVLLEGANFDPEHFPNPGSIELDRPNPTSHLAFGRGQHFCPGSALGRRHAQIGIEALLKK
MPGVDLAVPIDQLVWRTRFQRRIPERLPVLW
>CYP121A1_Mycobacterium tuberculosis M1533 2622598124
MTATVLLEVPFSARGDRIPDAVAELRTREPIRKVRTITGAEAWLVSSYALCTQVLEDRRFSMKETAAAGAPRL
NALTVPPEVVNNMGNIADAGLRKAVMKAITPKAPGLEQFLRDTANSLLDNLITEGAPADLRNDFADPLATALH
CKVLGIPQEDGPKLFRSLSIAFMSSADPIPAAKINWDRDIEYMAGILENPNITTGLMGELSRLRKDPAYSHVS
DELFATIGVTFFGAGVISTGSFLTTALISLIQRPQLRNLLHEKPELIPAGVEELLRINLSFADGLPRLATADI
QVGDVLVRKGELVLVLLEGANFDPEHFPNPGSIELDRPNPTSHLAFGRGQHFCPGSALGRRHAQIGIEALLKK
MPGVDLAVPIDQLVWRTRFQRRIPERLPVLW
>CYP121A1_Mycobacterium tuberculosis M1545 2622602534
MTATVLLEVPFSARGDRIPDAVAELRTREPIRKVRTITGAEAWLVSSYALCTQVLEDRRFSMKETAAAGAPRL
NALTVPPEVVNNMGNIADAGLRKAVMKAITPKAPGLEQFLRDTANSLLDNLITEGAPADLRNDFADPLATALH

CKVLGIPQEDGPKLFRSLSIAFMSSADPIPAAKINWDRDIEYMAGILENPNITTGLMGELSRLRKDPAYSHVS
DELFATIGVTFFGAGVISTGSFLTTALISLIQRPQLRNLLHEKPELIPAGVEELLRINLSFADGLPRLATADI
QVGDVLVRKGELVLVLLEGANFDPEHFPNPGSIELDRPNPTSHLAFGRGQHFCPGSALGRRHAQIGIEALLKK
MPGVDLAVPIDQLVWRTRFQRRIPERLPVLW
>CYP121A1_Mycobacterium tuberculosis M1559 2575794568
MTATVLLEVPFSARGDRIPDAVAELRTREPIRKVRTITGAEAWLVSSYALCTQVLEDRRFSMKETAAAGAPRL
NALTVPPEVVNNMGNIADAGLRKAVMKAITPKAPGLEQFLRDTANSLLDNLITEGAPADLRNDFADPLATALH
CKVLGIPQEDGPKLFRSLSIAFMSSADPIPAAKINWDRDIEYMAGILENPNITTGLMGELSRLRKDPAYSHVS
DELFATIGVTFFGAGVISTGSFLTTALISLIQRPQLRNLLHEKPELIPAGVEELLRINLSFADGLPRLATADI
QVGDVLVRKGELVLVLLEGANFDPEHFPNPGSIELDRPNPTSHLAFGRGQHFCPGSALGRRHAQIGIEALLKK
MPGVDLAVPIDQLVWRTRFQRRIPERLPVLW
>CYP121A1_Mycobacterium tuberculosis M1560 2622605628
MTATVLLEVPFSARGDRIPDAVAELRTREPIRKVRTITGAEAWLVSSYALCTQVLEDRRFSMKETAAAGAPRL
NALTVPPEVVNNMGNIADAGLRKAVMKAITPKAPGLEQFLRDTANSLLDNLITEGAPADLRNDFADPLATALH
CKVLGIPQEDGPKLFRSLSIAFMSSADPIPAAKINWDRDIEYMAGILENPNITTGLMGELSRLRKDPAYSHVS
DELFATIGVTFFGAGVISTGSFLTTALISLIQRPQLRNLLHEKPELIPAGVEELLRINLSFADGLPRLATADI
QVGDVLVRKGELVLVLLEGANFDPEHFPNPGSIELDRPNPTSHLAFGRGQHFCPGSALGRRHAQIGIEALLKK
MPGVDLAVPIDQLVWRTRFQRRIPERLPVLW
>CYP121A1_Mycobacterium tuberculosis M1570 2622610236
MTATVLLEVPFSARGDRIPDAVAELRTREPIRKVRTITGAEAWLVSSYALCTQVLEDRRFSMKETAAAGAPRL
NALTVPPEVVNNMGNIADAGLRKAVMKAITPKAPGLEQFLRDTANSLLDNLITEGAPADLRNDFADPLATALH
CKVLGIPQEDGPKLFRSLSIAFMSSADPIPAAKINWDRDIEYMAGILENPNITTGLMGELSRLRKDPAYSHVS
DELFATIGVTFFGAGVISTGSFLTTALISLIQRPQLRNLLHEKPELIPAGVEELLRINLSFADGLPRLATADI
QVGDVLVRKGELVLVLLEGANFDPEHFPNPGSIELDRPNPTSHLAFGRGQHFCPGSALGRRHAQIGIEALLKK
MPGVDLAVPIDQLVWRTRFQRRIPERLPVLW
>CYP121A1_Mycobacterium tuberculosis M1702 2622622847
MTATVLLEVPFSARGDRIPDAVAELRTREPIRKVRTITGAEAWLVSSYALCTQVLEDRRFSMKETAAAGAPRL
NALTVPPEVVNNMGNIADAGLRKAVMKAITPKAPGLEQFLRDTANSLLDNLITEGAPADLRNDFADPLATALH
CKVLGIPQEDGPKLFRSLSIAFMSSADPIPAAKINWDRDIEYMAGILENPNITTGLMGELSRLRKDPAYSHVS
DELFATIGVTFFGAGVISTGSFLTTALISLIQRPQLRNLLHEKPELIPAGVEELLRINLSFADGLPRLATADI
QVGDVLVRKGELVLVLLEGANFDPEHFPNPGSIELDRPNPTSHLAFGRGQHFCPGSALGRRHAQIGIEALLKK
MPGVDLAVPIDQLVWRTRFQRRIPERLPVLW
>CYP121A1_Mycobacterium tuberculosis M1703 2584730932
MTATVLLEVPFSARGDRIPDAVAELRTREPIRKVRTITGAEAWLVSSYALCTQVLEDRRFSMKETAAAGAPRL
NALTVPPEVVNNMGNIADAGLRKAVMKAITPKAPGLEQFLRDTANSLLDNLITEGAPADLRNDFADPLATALH
CKVLGIPQEDGPKLFRSLSIAFMSSADPIPAAKINWDRDIEYMAGILENPNITTGLMGELSRLRKDPAYSHVS
DELFATIGVTFFGAGVISTGSFLTTALISLIQRPQLRNLLHEKPELIPAGVEELLRINLSFADGLPRLATADI
QVGDVLVRKGELVLVLLEGANFDPEHFPNPGSIELDRPNPTSHLAFGRGQHFCPGSALGRRHAQIGIEALLKK
MPGVDLAVPIDQLVWRTRFQRRIPERLPVLW
>CYP121A1_Mycobacterium tuberculosis M1704 2622626221
MTATVLLEVPFSARGDRIPDAVAELRTREPIRKVRTITGAEAWLVSSYALCTQVLEDRRFSMKETAAAGAPRL
NALTVPPEVVNNMGNIADAGLRKAVMKAITPKAPGLEQFLRDTANSLLDNLITEGAPADLRNDFADPLATALH
CKVLGIPQEDGPKLFRSLSIAFMSSADPIPAAKINWDRDIEYMAGILENPNITTGLMGELSRLRKDPAYSHVS
DELFATIGVTFFGAGVISTGSFLTTALISLIQRPQLRNLLHEKPELIPAGVEELLRINLSFADGLPRLATADI
QVGDVLVRKGELVLVLLEGANFDPEHFPNPGSIELDRPNPTSHLAFGRGQHFCPGSALGRRHAQIGIEALLKK
MPGVDLAVPIDQLVWRTRFQRRIPERLPVLW
>CYP121A1_Mycobacterium tuberculosis M1726 2622630912
MTATVLLEVPFSARGDRIPDAVAELRTREPIRKVRTITGAEAWLVSSYALCTQVLEDRRFSMKETAAAGAPRL
NALTVPPEVVNNMGNIADAGLRKAVMKAITPKAPGLEQFLRDTANSLLDNLITEGAPADLRNDFADPLATALH
CKVLGIPQEDGPKLFRSLSIAFMSSADPIPAAKINWDRDIEYMAGILENPNITTGLMGELSRLRKDPAYSHVS
DELFATIGVTFFGAGVISTGSFLTTALISLIQRPQLRNLLHEKPELIPAGVEELLRINLSFADGLPRLATADI
QVGDVLVRKGELVLVLLEGANFDPEHFPNPGSIELDRPNPTSHLAFGRGQHFCPGSALGRRHAQIGIEALLKK
MPGVDLAVPIDQLVWRTRFQRRIPERLPVLW
>CYP121A1_Mycobacterium tuberculosis M1734 2622639240
MTATVLLEVPFSARGDRIPDAVAELRTREPIRKVRTITGAEAWLVSSYALCTQVLEDRRFSMKETAAAGAPRL
NALTVPPEVVNNMGNIADAGLRKAVMKAITPKAPGLEQFLRDTANSLLDNLITEGAPADLRNDFADPLATALH
CKVLGIPQEDGPKLFRSLSIAFMSSADPIPAAKINWDRDIEYMAGILENPNITTGLMGELSRLRKDPAYSHVS
DELFATIGVTFFGAGVISTGSFLTTALISLIQRPQLRNLLHEKPELIPAGVEELLRINLSFADGLPRLATADI

QVGDVLVRKGELVLVLLEGANFDPEHFPNPGSIELDRPNPTSHLAFGRGQHFCPGSALGRRHAQIGIEALLKK
MPGVDLAVPIDQLVWRTRFQRRIPERLPVLW
>CYP121A1_Mycobacterium tuberculosis M1759 2630119362
MTATVLLEVPFSARGDRIPDAVAELRTREPIRKVRTITGAEAWLVSSYALCTQVLEDRRFSMKETAAAGAPRL
NALTVPPEVVNNMGNIADAGLRKAVMKAITPKAPGLEQFLRDTANSLLDNLITEGAPADLRNDFADPLATALH
CKVLGIPQEDGPKLFRSLSIAFMSSADPIPAAKINWDRDIEYMAGILENPNITTGLMGELSRLRKDPAYSHVS
DELFATIGVTFFGAGVISTGSFLTTALISLIQRPQLRNLLHEKPELIPAGVEELLRINLSFADGLPRLATADI
QVGDVLVRKGELVLVLLEGANFDPEHFPNPGSIELDRPNPTSHLAFGRGQHFCPGSALGRRHAQIGIEALLKK
MPGVDLAVPIDQLVWRTRFQRRIPERLPVLW
>CYP121A1_Mycobacterium tuberculosis M1762 2574901636
MTATVLLEVPFSARGDRIPDAVAELRTREPIRKVRTITGAEAWLVSSYALCTQVLEDRRFSMKETAAAGAPRL
NALTVPPEVVNNMGNIADAGLRKAVMKAITPKAPGLEQFLRDTANSLLDNLITEGAPADLRNDFADPLATALH
CKVLGIPQEDGPKLFRSLSIAFMSSADPIPAAKINWDRDIEYMAGILENPNITTGLMGELSRLRKDPAYSHVS
DELFATIGVTFFGAGVISTGSFLTTALISLIQRPQLRNLLHEKPELIPAGVEELLRINLSFADGLPRLATADI
QVGDVLVRKGELVLVLLEGANFDPEHFPNPGSIELDRPNPTSHLAFGRGQHFCPGSALGRRHAQIGIEALLKK
MPGVDLAVPIDQLVWRTRFQRRIPERLPVLW
>CYP121A1_Mycobacterium tuberculosis M1780 2622643751
MTATVLLEVPFSARGDRIPDAVAELRTREPIRKVRTITGAEAWLVSSYALCTQVLEDRRFSMKETAAAGAPRL
NALTVPPEVVNNMGNIADAGLRKAVMKAITPKAPGLEQFLRDTANSLLDNLITEGAPADLRNDFADPLATALH
CKVLGIPQEDGPKLFRSLSIAFMSSADPIPAAKINWDRDIEYMAGILENPNITTGLMGELSRLRKDPAYSHVS
DELFATIGVTFFGAGVISTGSFLTTALISLIQRPQLRNLLHEKPELIPAGVEELLRINLSFADGLPRLATADI
QVGDVLVRKGELVLVLLEGANFDPEHFPNPGSIELDRPNPTSHLAFGRGQHFCPGSALGRRHAQIGIEALLKK
MPGVDLAVPIDQLVWRTRFQRRIPERLPVLW
>CYP121A1_Mycobacterium tuberculosis M1787 2579817338
MTATVLLEVPFSARGDRIPDAVAELRTREPIRKVRTITGAEAWLVSSYALCTQVLEDRRFSMKETAAAGAPRL
NALTVPPEVVNNMGNIADAGLRKAVMKAITPKAPGLEQFLRDTANSLLDNLITEGAPADLRNDFADPLATALH
CKVLGIPQEDGPKLFRSLSIAFMSSADPIPAAKINWDRDIEYMAGILENPNITTGLMGELSRLRKDPAYSHVS
DELFATIGVTFFGAGVISTGSFLTTALISLIQRPQLRNLLHEKPELIPAGVEELLRINLSFADGLPRLATADI
QVGDVLVRKGELVLVLLEGANFDPEHFPNPGSIELDRPNPTSHLAFGRGQHFCPGSALGRRHAQIGIEALLKK
MPGVDLAVPIDQLVWRTRFQRRIPERLPVLW
>CYP121A1_Mycobacterium tuberculosis M1816 2622651151
MTATVLLEVPFSARGDRIPDAVAELRTREPIRKVRTITGAEAWLVSSYALCTQVLEDRRFSMKETAAAGAPRL
NALTVPPEVVNNMGNIADAGLRKAVMKAITPKAPGLEQFLRDTANSLLDNLITEGAPADLRNDFADPLATALH
CKVLGIPQEDGPKLFRSLSIAFMSSADPIPAAKINWDRDIEYMAGILENPNITTGLMGELSRLRKDPAYSHVS
DELFATIGVTFFGAGVISTGSFLTTALISLIQRPQLRNLLHEKPELIPAGVEELLRINLSFADGLPRLATADI
QVGDVLVRKGELVLVLLEGANFDPEHFPNPGSIELDRPNPTSHLAFGRGQHFCPGSALGRRHAQIGIEALLKK
MPGVDLAVPIDQLVWRTRFQRRIPERLPVLW
>CYP121A1_Mycobacterium tuberculosis M1893 2622660642
MTATVLLEVPFSARGDRIPDAVAELRTREPIRKVRTITGAEAWLVSSYALCTQVLEDRRFSMKETAAAGAPRL
NALTVPPEVVNNMGNIADAGLRKAVMKAITPKAPGLEQFLRDTANSLLDNLITEGAPADLRNDFADPLATALH
CKVLGIPQEDGPKLFRSLSIAFMSSADPIPAAKINWDRDIEYMAGILENPNITTGLMGELSRLRKDPAYSHVS
DELFATIGVTFFGAGVISTGSFLTTALISLIQRPQLRNLLHEKPELIPAGVEELLRINLSFADGLPRLATADI
QVGDVLVRKGELVLVLLEGANFDPEHFPNPGSIELDRPNPTSHLAFGRGQHFCPGSALGRRHAQIGIEALLKK
MPGVDLAVPIDQLVWRTRFQRRIPERLPVLW
>CYP121A1_Mycobacterium tuberculosis M1913 2576928897
MTATVLLEVPFSARGDRIPDAVAELRTREPIRKVRTITGAEAWLVSSYALCTQVLEDRRFSMKETAAAGAPRL
NALTVPPEVVNNMGNIADAGLRKAVMKAITPKAPGLEQFLRDTANSLLDNLITEGAPADLRNDFADPLATALH
CKVLGIPQEDGPKLFRSLSIAFMSSADPIPAAKINWDRDIEYMAGILENPNITTGLMGELSRLRKDPAYSHVS
DELFATIGVTFFGAGVISTGSFLTTALISLIQRPQLRNLLHEKPELIPAGVEELLRINLSFADGLPRLATADI
QVGDVLVRKGELVLVLLEGANFDPEHFPNPGSIELDRPNPTSHLAFGRGQHFCPGSALGRRHAQIGIEALLKK
MPGVDLAVPIDQLVWRTRFQRRIPERLPVLW
>CYP121A1_Mycobacterium tuberculosis M1928 2622676337
MTATVLLEVPFSARGDRIPDAVAELRTREPIRKVRTITGAEAWLVSSYALCTQVLEDRRFSMKETAAAGAPRL
NALTVPPEVVNNMGNIADAGLRKAVMKAITPKAPGLEQFLRDTANSLLDNLITEGAPADLRNDFADPLATALH
CKVLGIPQEDGPKLFRSLSIAFMSSADPIPAAKINWDRDIEYMAGILENPNITTGLMGELSRLRKDPAYSHVS
DELFATIGVTFFGAGVISTGSFLTTALISLIQRPQLRNLLHEKPELIPAGVEELLRINLSFADGLPRLATADI
QVGDVLVRKGELVLVLLEGANFDPEHFPNPGSIELDRPNPTSHLAFGRGQHFCPGSALGRRHAQIGIEALLKK
MPGVDLAVPIDQLVWRTRFQRRIPERLPVLW

>CYP121A1_Mycobacterium tuberculosis M1929 2622680471
MTATVLLEVPFSARGDRIPDAVAELRTREPIRKVRTITGAEAWLVSSYALCTQVLEDRRFSMKETAAAGAPRL
NALTVPPEVVNNMGNIADAGLRKAVMKAITPKAPGLEQFLRDTANSLLDNLITEGAPADLRNDFADPLATALH
CKVLGIPQEDGPKLFRSLSIAFMSSADPIPAAKINWDRDIEYMAGILENPNITTGLMGELSRLRKDPAYSHVS
DELFATIGVTFFGAGVISTGSFLTTALISLIQRPQLRNLLHEKPELIPAGVEELLRINLSFADGLPRLATADI
QVGDVLVRKGELVLVLLEGANFDPEHFPNPGSIELDRPNPTSHLAFGRGQHFCPGSALGRRHAQIGIEALLKK
MPGVDLAVPIDQLVWRTRFQRRIPERLPVLW
>CYP121A1_Mycobacterium tuberculosis M1948 2622684444
MTATVLLEVPFSARGDRIPDAVAELRTREPIRKVRTITGAEAWLVSSYALCTQVLEDRRFSMKETAAAGAPRL
NALTVPPEVVNNMGNIADAGLRKAVMKAITPKAPGLEQFLRDTANSLLDNLITEGAPADLRNDFADPLATALH
CKVLGIPQEDGPKLFRSLSIAFMSSADPIPAAKINWDRDIEYMAGILENPNITTGLMGELSRLRKDPAYSHVS
DELFATIGVTFFGAGVISTGSFLTTALISLIQRPQLRNLLHEKPELIPAGVEELLRINLSFADGLPRLATADI
QVGDVLVRKGELVLVLLEGANFDPEHFPNPGSIELDRPNPTSHLAFGRGQHFCPGSALGRRHAQIGIEALLKK
MPGVDLAVPIDQLVWRTRFQRRIPERLPVLW
>CYP121A1_Mycobacterium tuberculosis M1955 2622689725
MTATVLLEVPFSARGDRIPDAVAELRTREPIRKVRTITGAEAWLVSSYALCTQVLEDRRFSMKETAAAGAPRL
NALTVPPEVVNNMGNIADAGLRKAVMKAITPKAPGLEQFLRDTANSLLDNLITEGAPADLRNDFADPLATALH
CKVLGIPQEDGPKLFRSLSIAFMSSADPIPAAKINWDRDIEYMAGILENPNITTGLMGELSRLRKDPAYSHVS
DELFATIGVTFFGAGVISTGSFLTTALISLIQRPQLRNLLHEKPELIPAGVEELLRINLSFADGLPRLATADI
QVGDVLVRKGELVLVLLEGANFDPEHFPNPGSIELDRPNPTSHLAFGRGQHFCPGSALGRRHAQIGIEALLKK
MPGVDLAVPIDQLVWRTRFQRRIPERLPVLW
>CYP121A1_Mycobacterium tuberculosis M1959 2622694483
MTATVLLEVPFSARGDRIPDAVAELRTREPIRKVRTITGAEAWLVSSYALCTQVLEDRRFSMKETAAAGAPRL
NALTVPPEVVNNMGNIADAGLRKAVMKAITPKAPGLEQFLRDTANSLLDNLITEGAPADLRNDFADPLATALH
CKVLGIPQEDGPKLFRSLSIAFMSSADPIPAAKINWDRDIEYMAGILENPNITTGLMGELSRLRKDPAYSHVS
DELFATIGVTFFGAGVISTGSFLTTALISLIQRPQLRNLLHEKPELIPAGVEELLRINLSFADGLPRLATADI
QVGDVLVRKGELVLVLLEGANFDPEHFPNPGSIELDRPNPTSHLAFGRGQHFCPGSALGRRHAQIGIEALLKK
MPGVDLAVPIDQLVWRTRFQRRIPERLPVLW
>CYP121A1_Mycobacterium tuberculosis M1961 2584690482
MTATVLLEVPFSARGDRIPDAVAELRTREPIRKVRTITGAEAWLVSSYALCTQVLEDRRFSMKETAAAGAPRL
NALTVPPEVVNNMGNIADAGLRKAVMKAITPKAPGLEQFLRDTANSLLDNLITEGAPADLRNDFADPLATALH
CKVLGIPQEDGPKLFRSLSIAFMSSADPIPAAKINWDRDIEYMAGILENPNITTGLMGELSRLRKDPAYSHVS
DELFATIGVTFFGAGVISTGSFLTTALISLIQRPQLRNLLHEKPELIPAGVEELLRINLSFADGLPRLATADI
QVGDVLVRKGELVLVLLEGANFDPEHFPNPGSIELDRPNPTSHLAFGRGQHFCPGSALGRRHAQIGIEALLKK
MPGVDLAVPIDQLVWRTRFQRRIPERLPVLW
>CYP121A1_Mycobacterium tuberculosis M1967 2622697039
MTATVLLEVPFSARGDRIPDAVAELRTREPIRKVRTITGAEAWLVSSYALCTQVLEDRRFSMKETAAAGAPRL
NALTVPPEVVNNMGNIADAGLRKAVMKAITPKAPGLEQFLRDTANSLLDNLITEGAPADLRNDFADPLATALH
CKVLGIPQEDGPKLFRSLSIAFMSSADPIPAAKINWDRDIEYMAGILENPNITTGLMGELSRLRKDPAYSHVS
DELFATIGVTFFGAGVISTGSFLTTALISLIQRPQLRNLLHEKPELIPAGVEELLRINLSFADGLPRLATADI
QVGDVLVRKGELVLVLLEGANFDPEHFPNPGSIELDRPNPTSHLAFGRGQHFCPGSALGRRHAQIGIEALLKK
MPGVDLAVPIDQLVWRTRFQRRIPERLPVLW
>CYP121A1_Mycobacterium tuberculosis M1976 2622704474
MTATVLLEVPFSARGDRIPDAVAELRTREPIRKVRTITGAEAWLVSSYALCTQVLEDRRFSMKETAAAGAPRL
NALTVPPEVVNNMGNIADAGLRKAVMKAITPKAPGLEQFLRDTANSLLDNLITEGAPADLRNDFADPLATALH
CKVLGIPQEDGPKLFRSLSIAFMSSADPIPAAKINWDRDIEYMAGILENPNITTGLMGELSRLRKDPAYSHVS
DELFATIGVTFFGAGVISTGSFLTTALISLIQRPQLRNLLHEKPELIPAGVEELLRINLSFADGLPRLATADI
QVGDVLVRKGELVLVLLEGANFDPEHFPNPGSIELDRPNPTSHLAFGRGQHFCPGSALGRRHAQIGIEALLKK
MPGVDLAVPIDQLVWRTRFQRRIPERLPVLW
>CYP121A1_Mycobacterium tuberculosis M1978 2578087308
MTATVLLEVPFSARGDRIPDAVAELRTREPIRKVRTITGAEAWLVSSYALCTQVLEDRRFSMKETAAAGAPRL
NALTVPPEVVNNMGNIADAGLRKAVMKAITPKAPGLEQFLRDTANSLLDNLITEGAPADLRNDFADPLATALH
CKVLGIPQEDGPKLFRSLSIAFMSSADPIPAAKINWDRDIEYMAGILENPNITTGLMGELSRLRKDPAYSHVS
DELFATIGVTFFGAGVISTGSFLTTALISLIQRPQLRNLLHEKPELIPAGVEELLRINLSFADGLPRLATADI
QVGDVLVRKGELVLVLLEGANFDPEHFPNPGSIELDRPNPTSHLAFGRGQHFCPGSALGRRHAQIGIEALLKK
MPGVDLAVPIDQLVWRTRFQRRIPERLPVLW
>CYP121A1_Mycobacterium tuberculosis M1979 2622709498

MTATVLLEVPFSARGDRIPDAVAELRTREPIRKVRTITGAEAWLVSSYALCTQVLEDRRFSMKETAAAGAPRL
NALTVPPEVVNNMGNIADAGLRKAVMKAITPKAPGLEQFLRDTANSLLDNLITEGAPADLRNDFADPLATALH
CKVLGIPQEDGPKLFRSLSIAFMSSADPIPAAKINWDRDIEYMAGILENPNITTGLMGELSRLRKDPAYSHVS
DELFATIGVTFFGAGVISTGSFLTTALISLIQRPQLRNLLHEKPELIPAGVEELLRINLSFADGLPRLATADI
QVGDVLVRKGELVLVLLEGANFDPEHFPNPGSIELDRPNPTSHLAFGRGQHFCPGSALGRRHAQIGIEALLKK
MPGVDLAVPIDQLVWRTRFQRRIPERLPVLW
>CYP121A1_Mycobacterium tuberculosis M2003 2622715270
MTATVLLEVPFSARGDRIPDAVAELRTREPIRKVRTITGAEAWLVSSYALCTQVLEDRRFSMKETAAAGAPRL
NALTVPPEVVNNMGNIADAGLRKAVMKAITPKAPGLEQFLRDTANSLLDNLITEGAPADLRNDFADPLATALH
CKVLGIPQEDGPKLFRSLSIAFMSSADPIPAAKINWDRDIEYMAGILENPNITTGLMGELSRLRKDPAYSHVS
DELFATIGVTFFGAGVISTGSFLTTALISLIQRPQLRNLLHEKPELIPAGVEELLRINLSFADGLPRLATADI
QVGDVLVRKGELVLVLLEGANFDPEHFPNPGSIELDRPNPTSHLAFGRGQHFCPGSALGRRHAQIGIEALLKK
MPGVDLAVPIDQLVWRTRFQRRIPERLPVLW
>CYP121A1_Mycobacterium tuberculosis M2006 2577305992
MTATVLLEVPFSARGDRIPDAVAELRTREPIRKVRTITGAEAWLVSSYALCTQVLEDRRFSMKETAAAGAPRL
NALTVPPEVVNNMGNIADAGLRKAVMKAITPKAPGLEQFLRDTANSLLDNLITEGAPADLRNDFADPLATALH
CKVLGIPQEDGPKLFRSLSIAFMSSADPIPAAKINWDRDIEYMAGILENPNITTGLMGELSRLRKDPAYSHVS
DELFATIGVTFFGAGVISTGSFLTTALISLIQRPQLRNLLHEKPELIPAGVEELLRINLSFADGLPRLATADI
QVGDVLVRKGELVLVLLEGANFDPEHFPNPGSIELDRPNPTSHLAFGRGQHFCPGSALGRRHAQIGIEALLKK
MPGVDLAVPIDQLVWRTRFQRRIPERLPVLW
>CYP121A1_Mycobacterium tuberculosis M2007 2622797038
MTATVLLEVPFSARGDRIPDAVAELRTREPIRKVRTITGAEAWLVSSYALCTQVLEDRRFSMKETAAAGAPRL
NALTVPPEVVNNMGNIADAGLRKAVMKAITPKAPGLEQFLRDTANSLLDNLITEGAPADLRNDFADPLATALH
CKVLGIPQEDGPKLFRSLSIAFMSSADPIPAAKINWDRDIEYMAGILENPNITTGLMGELSRLRKDPAYSHVS
DELFATIGVTFFGAGVISTGSFLTTALISLIQRPQLRNLLHEKPELIPAGVEELLRINLSFADGLPRLATADI
QVGDVLVRKGELVLVLLEGANFDPEHFPNPGSIELDRPNPTSHLAFGRGQHFCPGSALGRRHAQIGIEALLKK
MPGVDLAVPIDQLVWRTRFQRRIPERLPVLW
>CYP121A1_Mycobacterium tuberculosis M2010 2622801917
MTATVLLEVPFSARGDRIPDAVAELRTREPIRKVRTITGAEAWLVSSYALCTQVLEDRRFSMKETAAAGAPRL
NALTVPPEVVNNMGNIADAGLRKAVMKAITPKAPGLEQFLRDTANSLLDNLITEGAPADLRNDFADPLATALH
CKVLGIPQEDGPKLFRSLSIAFMSSADPIPAAKINWDRDIEYMAGILENPNITTGLMGELSRLRKDPAYSHVS
DELFATIGVTFFGAGVISTGSFLTTALISLIQRPQLRNLLHEKPELIPAGVEELLRINLSFADGLPRLATADI
QVGDVLVRKGELVLVLLEGANFDPEHFPNPGSIELDRPNPTSHLAFGRGQHFCPGSALGRRHAQIGIEALLKK
MPGVDLAVPIDQLVWRTRFQRRIPERLPVLW
>CYP121A1_Mycobacterium tuberculosis M2020 2622805055
MTATVLLEVPFSARGDRIPDAVAELRTREPIRKVRTITGAEAWLVSSYALCTQVLEDRRFSMKETAAAGAPRL
NALTVPPEVVNNMGNIADAGLRKAVMKAITPKAPGLEQFLRDTANSLLDNLITEGAPADLRNDFADPLATALH
CKVLGIPQEDGPKLFRSLSIAFMSSADPIPAAKINWDRDIEYMAGILENPNITTGLMGELSRLRKDPAYSHVS
DELFATIGVTFFGAGVISTGSFLTTALISLIQRPQLRNLLHEKPELIPAGVEELLRINLSFADGLPRLATADI
QVGDVLVRKGELVLVLLEGANFDPEHFPNPGSIELDRPNPTSHLAFGRGQHFCPGSALGRRHAQIGIEALLKK
MPGVDLAVPIDQLVWRTRFQRRIPERLPVLW
>CYP121A1_Mycobacterium tuberculosis M2021 2622809244
MTATVLLEVPFSARGDRIPDAVAELRTREPIRKVRTITGAEAWLVSSYALCTQVLEDRRFSMKETAAAGAPRL
NALTVPPEVVNNMGNIADAGLRKAVMKAITPKAPGLEQFLRDTANSLLDNLITEGAPADLRNDFADPLATALH
CKVLGIPQEDGPKLFRSLSIAFMSSADPIPAAKINWDRDIEYMAGILENPNITTGLMGELSRLRKDPAYSHVS
DELFATIGVTFFGAGVISTGSFLTTALISLIQRPQLRNLLHEKPELIPAGVEELLRINLSFADGLPRLATADI
QVGDVLVRKGELVLVLLEGANFDPEHFPNPGSIELDRPNPTSHLAFGRGQHFCPGSALGRRHAQIGIEALLKK
MPGVDLAVPIDQLVWRTRFQRRIPERLPVLW
>CYP121A1_Mycobacterium tuberculosis M2032 (re-annotation Oct 2015)
2639514615
MTATVLLEVPFSARGDRIPDAVAELRTREPIRKVRTITGAEAWLVSSYALCTQVLEDRRFSMKETAAAGAPRL
NALTVPPEVVNNMGNIADAGLRKAVMKAITPKAPGLEQFLRDTANSLLDNLITEGAPADLRNDFADPLATALH
CKVLGIPQEDGPKLFRSLSIAFMSSADPIPAAKINWDRDIEYMAGILENPNITTGLMGELSRLRKDPAYSHVS
DELFATIGVTFFGAGVISTGSFLTTALISLIQRPQLRNLLHEKPELIPAGVEELLRINLSFADGLPRLATADI
QVGDVLVRKGELVLVLLEGANFDPEHFPNPGSIELDRPNPTSHLAFGRGQHFCPGSALGRRHAQIGIEALLKK
MPGVDLAVPIDQLVWRTRFQRRIPERLPVLW
>CYP121A1_Mycobacterium tuberculosis M2113 2577068745

MTATVLLEVPFSARGDRIPDAVAELRTREPIRKVRTITGAEAWLVSSYALCTQVLEDRRFSMKETAAAGAPRL
NALTVPPEVVNNMGNIADAGLRKAVMKAITPKAPGLEQFLRDTANSLLDNLITEGAPADLRNDFADPLATALH
CKVLGIPQEDGPKLFRSLSIAFMSSADPIPAAKINWDRDIEYMAGILENPNITTGLMGELSRLRKDPAYSHVS
DELFATIGVTFFGAGVISTGSFLTTALISLIQRPQLRNLLHEKPELIPAGVEELLRINLSFADGLPRLATADI
QVGDVLVRKGELVLVLLEGANFDPEHFPNPGSIELDRPNPTSHLAFGRGQHFCPGSALGRRHAQIGIEALLKK
MPGVDLAVPIDQLVWRTRFQRRIPERLPVLW
>CYP121A1_Mycobacterium tuberculosis M2116 2578033737
MTATVLLEVPFSARGDRIPDAVAELRTREPIRKVRTITGAEAWLVSSYALCTQVLEDRRFSMKETAAAGAPRL
NALTVPPEVVNNMGNIADAGLRKAVMKAITPKAPGLEQFLRDTANSLLDNLITEGAPADLRNDFADPLATALH
CKVLGIPQEDGPKLFRSLSIAFMSSADPIPAAKINWDRDIEYMAGILENPNITTGLMGELSRLRKDPAYSHVS
DELFATIGVTFFGAGVISTGSFLTTALISLIQRPQLRNLLHEKPELIPAGVEELLRINLSFADGLPRLATADI
QVGDVLVRKGELVLVLLEGANFDPEHFPNPGSIELDRPNPTSHLAFGRGQHFCPGSALGRRHAQIGIEALLKK
MPGVDLAVPIDQLVWRTRFQRRIPERLPVLW
>CYP121A1_Mycobacterium tuberculosis M2128 2577613840
MTATVLLEVPFSARGDRIPDAVAELRTREPIRKVRTITGAEAWLVSSYALCTQVLEDRRFSMKETAAAGAPRL
NALTVPPEVVNNMGNIADAGLRKAVMKAITPKAPGLEQFLRDTANSLLDNLITEGAPADLRNDFADPLATALH
CKVLGIPQEDGPKLFRSLSIAFMSSADPIPAAKINWDRDIEYMAGILENPNITTGLMGELSRLRKDPAYSHVS
DELFATIGVTFFGAGVISTGSFLTTALISLIQRPQLRNLLHEKPELIPAGVEELLRINLSFADGLPRLATADI
QVGDVLVRKGELVLVLLEGANFDPEHFPNPGSIELDRPNPTSHLAFGRGQHFCPGSALGRRHAQIGIEALLKK
MPGVDLAVPIDQLVWRTRFQRRIPERLPVLW
>CYP121A1_Mycobacterium tuberculosis M2129 2584995469
MTATVLLEVPFSARGDRIPDAVAELRTREPIRKVRTITGAEAWLVSSYALCTQVLEDRRFSMKETAAAGAPRL
NALTVPPEVVNNMGNIADAGLRKAVMKAITPKAPGLEQFLRDTANSLLDNLITEGAPADLRNDFADPLATALH
CKVLGIPQEDGPKLFRSLSIAFMSSADPIPAAKINWDRDIEYMAGILENPNITTGLMGELSRLRKDPAYSHVS
DELFATIGVTFFGAGVISTGSFLTTALISLIQRPQLRNLLHEKPELIPAGVEELLRINLSFADGLPRLATADI
QVGDVLVRKGELVLVLLEGANFDPEHFPNPGSIELDRPNPTSHLAFGRGQHFCPGSALGRRHAQIGIEALLKK
MPGVDLAVPIDQLVWRTRFQRRIPERLPVLW
>CYP121A1_Mycobacterium tuberculosis M2134 2637399280
MTATVLLEVPFSARGDRIPDAVAELRTREPIRKVRTITGAEAWLVSSYALCTQVLEDRRFSMKETAAAGAPRL
NALTVPPEVVNNMGNIADAGLRKAVMKAITPKAPGLEQFLRDTANSLLDNLITEGAPADLRNDFADPLATALH
CKVLGIPQEDGPKLFRSLSIAFMSSADPIPAAKINWDRDIEYMAGILENPNITTGLMGELSRLRKDPAYSHVS
DELFATIGVTFFGAGVISTGSFLTTALISLIQRPQLRNLLHEKPELIPAGVEELLRINLSFADGLPRLATADI
QVGDVLVRKGELVLVLLEGANFDPEHFPNPGSIELDRPNPTSHLAFGRGQHFCPGSALGRRHAQIGIEALLKK
MPGVDLAVPIDQLVWRTRFQRRIPERLPVLW
>CYP121A1_Mycobacterium tuberculosis M2136 2575986361
MTATVLLEVPFSARGDRIPDAVAELRTREPIRKVRTITGAEAWLVSSYALCTQVLEDRRFSMKETAAAGAPRL
NALTVPPEVVNNMGNIADAGLRKAVMKAITPKAPGLEQFLRDTANSLLDNLITEGAPADLRNDFADPLATALH
CKVLGIPQEDGPKLFRSLSIAFMSSADPIPAAKINWDRDIEYMAGILENPNITTGLMGELSRLRKDPAYSHVS
DELFATIGVTFFGAGVISTGSFLTTALISLIQRPQLRNLLHEKPELIPAGVEELLRINLSFADGLPRLATADI
QVGDVLVRKGELVLVLLEGANFDPEHFPNPGSIELDRPNPTSHLAFGRGQHFCPGSALGRRHAQIGIEALLKK
MPGVDLAVPIDQLVWRTRFQRRIPERLPVLW
>CYP121A1_Mycobacterium tuberculosis M2137 2577499321
MTATVLLEVPFSARGDRIPDAVAELRTREPIRKVRTITGAEAWLVSSYALCTQVLEDRRFSMKETAAAGAPRL
NALTVPPEVVNNMGNIADAGLRKAVMKAITPKAPGLEQFLRDTANSLLDNLITEGAPADLRNDFADPLATALH
CKVLGIPQEDGPKLFRSLSIAFMSSADPIPAAKINWDRDIEYMAGILENPNITTGLMGELSRLRKDPAYSHVS
DELFATIGVTFFGAGVISTGSFLTTALISLIQRPQLRNLLHEKPELIPAGVEELLRINLSFADGLPRLATADI
QVGDVLVRKGELVLVLLEGANFDPEHFPNPGSIELDRPNPTSHLAFGRGQHFCPGSALGRRHAQIGIEALLKK
MPGVDLAVPIDQLVWRTRFQRRIPERLPVLW
>CYP121A1_Mycobacterium tuberculosis M2138 2651638216
MTATVLLEVPFSARGDRIPDAVAELRTREPIRKVRTITGAEAWLVSSYALCTQVLEDRRFSMKETAAAGAPRL
NALTVPPEVVNNMGNIADAGLRKAVMKAITPKAPGLEQFLRDTANSLLDNLITEGAPADLRNDFADPLATALH
CKVLGIPQEDGPKLFRSLSIAFMSSADPIPAAKINWDRDIEYMAGILENPNITTGLMGELSRLRKDPAYSHVS
DELFATIGVTFFGAGVISTGSFLTTALISLIQRPQLRNLLHEKPELIPAGVEELLRINLSFADGLPRLATADI
QVGDVLVRKGELVLVLLEGANFDPEHFPNPGSIELDRPNPTSHLAFGRGQHFCPGSALGRRHAQIGIEALLKK
MPGVDLAVPIDQLVWRTRFQRRIPERLPVLW
>CYP121A1_Mycobacterium tuberculosis M2139 2635730493
MTATVLLEVPFSARGDRIPDAVAELRTREPIRKVRTITGAEAWLVSSYALCTQVLEDRRFSMKETAAAGAPRL
NALTVPPEVVNNMGNIADAGLRKAVMKAITPKAPGLEQFLRDTANSLLDNLITEGAPADLRNDFADPLATALH

CKVLGIPQEDGPKLFRSLSIAFMSSADPIPAAKINWDRDIEYMAGILENPNITTGLMGELSRLRKDPAYSHVS
DELFATIGVTFFGAGVISTGSFLTTALISLIQRPQLRNLLHEKPELIPAGVEELLRINLSFADGLPRLATADI
QVGDVLVRKGELVLVLLEGANFDPEHFPNPGSIELDRPNPTSHLAFGRGQHFCPGSALGRRHAQIGIEALLKK
MPGVDLAVPIDQLVWRTRFQRRIPERLPVLW
>CYP121A1_Mycobacterium tuberculosis M2140 2630874657
MTATVLLEVPFSARGDRIPDAVAELRTREPIRKVRTITGAEAWLVSSYALCTQVLEDRRFSMKETAAAGAPRL
NALTVPPEVVNNMGNIADAGLRKAVMKAITPKAPGLEQFLRDTANSLLDNLITEGAPADLRNDFADPLATALH
CKVLGIPQEDGPKLFRSLSIAFMSSADPIPAAKINWDRDIEYMAGILENPNITTGLMGELSRLRKDPAYSHVS
DELFATIGVTFFGAGVISTGSFLTTALISLIQRPQLRNLLHEKPELIPAGVEELLRINLSFADGLPRLATADI
QVGDVLVRKGELVLVLLEGANFDPEHFPNPGSIELDRPNPTSHLAFGRGQHFCPGSALGRRHAQIGIEALLKK
MPGVDLAVPIDQLVWRTRFQRRIPERLPVLW
>CYP121A1_Mycobacterium tuberculosis M2141 2633452752
MTATVLLEVPFSARGDRIPDAVAELRTREPIRKVRTITGAEAWLVSSYALCTQVLEDRRFSMKETAAAGAPRL
NALTVPPEVVNNMGNIADAGLRKAVMKAITPKAPGLEQFLRDTANSLLDNLITEGAPADLRNDFADPLATALH
CKVLGIPQEDGPKLFRSLSIAFMSSADPIPAAKINWDRDIEYMAGILENPNITTGLMGELSRLRKDPAYSHVS
DELFATIGVTFFGAGVISTGSFLTTALISLIQRPQLRNLLHEKPELIPAGVEELLRINLSFADGLPRLATADI
QVGDVLVRKGELVLVLLEGANFDPEHFPNPGSIELDRPNPTSHLAFGRGQHFCPGSALGRRHAQIGIEALLKK
MPGVDLAVPIDQLVWRTRFQRRIPERLPVLW
>CYP121A1_Mycobacterium tuberculosis M2142 2637756491
MTATVLLEVPFSARGDRIPDAVAELRTREPIRKVRTITGAEAWLVSSYALCTQVLEDRRFSMKETAAAGAPRL
NALTVPPEVVNNMGNIADAGLRKAVMKAITPKAPGLEQFLRDTANSLLDNLITEGAPADLRNDFADPLATALH
CKVLGIPQEDGPKLFRSLSIAFMSSADPIPAAKINWDRDIEYMAGILENPNITTGLMGELSRLRKDPAYSHVS
DELFATIGVTFFGAGVISTGSFLTTALISLIQRPQLRNLLHEKPELIPAGVEELLRINLSFADGLPRLATADI
QVGDVLVRKGELVLVLLEGANFDPEHFPNPGSIELDRPNPTSHLAFGRGQHFCPGSALGRRHAQIGIEALLKK
MPGVDLAVPIDQLVWRTRFQRRIPERLPVLW
>CYP121A1_Mycobacterium tuberculosis M2144 2646084528
MTATVLLEVPFSARGDRIPDAVAELRTREPIRKVRTITGAEAWLVSSYALCTQVLEDRRFSMKETAAAGAPRL
NALTVPPEVVNNMGNIADAGLRKAVMKAITPKAPGLEQFLRDTANSLLDNLITEGAPADLRNDFADPLATALH
CKVLGIPQEDGPKLFRSLSIAFMSSADPIPAAKINWDRDIEYMAGILENPNITTGLMGELSRLRKDPAYSHVS
DELFATIGVTFFGAGVISTGSFLTTALISLIQRPQLRNLLHEKPELIPAGVEELLRINLSFADGLPRLATADI
QVGDVLVRKGELVLVLLEGANFDPEHFPNPGSIELDRPNPTSHLAFGRGQHFCPGSALGRRHAQIGIEALLKK
MPGVDLAVPIDQLVWRTRFQRRIPERLPVLW
>CYP121A1_Mycobacterium tuberculosis M2192 2631900571
MTATVLLEVPFSARGDRIPDAVAELRTREPIRKVRTITGAEAWLVSSYALCTQVLEDRRFSMKETAAAGAPRL
NALTVPPEVVNNMGNIADAGLRKAVMKAITPKAPGLEQFLRDTANSLLDNLITEGAPADLRNDFADPLATALH
CKVLGIPQEDGPKLFRSLSIAFMSSADPIPAAKINWDRDIEYMAGILENPNITTGLMGELSRLRKDPAYSHVS
DELFATIGVTFFGAGVISTGSFLTTALISLIQRPQLRNLLHEKPELIPAGVEELLRINLSFADGLPRLATADI
QVGDVLVRKGELVLVLLEGANFDPEHFPNPGSIELDRPNPTSHLAFGRGQHFCPGSALGRRHAQIGIEALLKK
MPGVDLAVPIDQLVWRTRFQRRIPERLPVLW
>CYP121A1_Mycobacterium tuberculosis M2193 2631473315
MTATVLLEVPFSARGDRIPDAVAELRTREPIRKVRTITGAEAWLVSSYALCTQVLEDRRFSMKETAAAGAPRL
NALTVPPEVVNNMGNIADAGLRKAVMKAITPKAPGLEQFLRDTANSLLDNLITEGAPADLRNDFADPLATALH
CKVLGIPQEDGPKLFRSLSIAFMSSADPIPAAKINWDRDIEYMAGILENPNITTGLMGELSRLRKDPAYSHVS
DELFATIGVTFFGAGVISTGSFLTTALISLIQRPQLRNLLHEKPELIPAGVEELLRINLSFADGLPRLATADI
QVGDVLVRKGELVLVLLEGANFDPEHFPNPGSIELDRPNPTSHLAFGRGQHFCPGSALGRRHAQIGIEALLKK
MPGVDLAVPIDQLVWRTRFQRRIPERLPVLW
>CYP121A1_Mycobacterium tuberculosis M2198 2628351488
MTATVLLEVPFSARGDRIPDAVAELRTREPIRKVRTITGAEAWLVSSYALCTQVLEDRRFSMKETAAAGAPRL
NALTVPPEVVNNMGNIADAGLRKAVMKAITPKAPGLEQFLRDTANSLLDNLITEGAPADLRNDFADPLATALH
CKVLGIPQEDGPKLFRSLSIAFMSSADPIPAAKINWDRDIEYMAGILENPNITTGLMGELSRLRKDPAYSHVS
DELFATIGVTFFGAGVISTGSFLTTALISLIQRPQLRNLLHEKPELIPAGVEELLRINLSFADGLPRLATADI
QVGDVLVRKGELVLVLLEGANFDPEHFPNPGSIELDRPNPTSHLAFGRGQHFCPGSALGRRHAQIGIEALLKK
MPGVDLAVPIDQLVWRTRFQRRIPERLPVLW
>CYP121A1_Mycobacterium tuberculosis M2199 2632048300
MTATVLLEVPFSARGDRIPDAVAELRTREPIRKVRTITGAEAWLVSSYALCTQVLEDRRFSMKETAAAGAPRL
NALTVPPEVVNNMGNIADAGLRKAVMKAITPKAPGLEQFLRDTANSLLDNLITEGAPADLRNDFADPLATALH
CKVLGIPQEDGPKLFRSLSIAFMSSADPIPAAKINWDRDIEYMAGILENPNITTGLMGELSRLRKDPAYSHVS
DELFATIGVTFFGAGVISTGSFLTTALISLIQRPQLRNLLHEKPELIPAGVEELLRINLSFADGLPRLATADI

QVGDVLVRKGELVLVLLEGANFDPEHFPNPGSIELDRPNPTSHLAFGRGQHFCPGSALGRRHAQIGIEALLKK
MPGVDLAVPIDQLVWRTRFQRRIPERLPVLW
>CYP121A1_Mycobacterium tuberculosis M2203 2584649884
MTATVLLEVPFSARGDRIPDAVAELRTREPIRKVRTITGAEAWLVSSYALCTQVLEDRRFSMKETAAAGAPRL
NALTVPPEVVNNMGNIADAGLRKAVMKAITPKAPGLEQFLRDTANSLLDNLITEGAPADLRNDFADPLATALH
CKVLGIPQEDGPKLFRSLSIAFMSSADPIPAAKINWDRDIEYMAGILENPNITTGLMGELSRLRKDPAYSHVS
DELFATIGVTFFGAGVISTGSFLTTALISLIQRPQLRNLLHEKPELIPAGVEELLRINLSFADGLPRLATADI
QVGDVLVRKGELVLVLLEGANFDPEHFPNPGSIELDRPNPTSHLAFGRGQHFCPGSALGRRHAQIGIEALLKK
MPGVDLAVPIDQLVWRTRFQRRIPERLPVLW
>CYP121A1_Mycobacterium tuberculosis M2206 2635613118
MTATVLLEVPFSARGDRIPDAVAELRTREPIRKVRTITGAEAWLVSSYALCTQVLEDRRFSMKETAAAGAPRL
NALTVPPEVVNNMGNIADAGLRKAVMKAITPKAPGLEQFLRDTANSLLDNLITEGAPADLRNDFADPLATALH
CKVLGIPQEDGPKLFRSLSIAFMSSADPIPAAKINWDRDIEYMAGILENPNITTGLMGELSRLRKDPAYSHVS
DELFATIGVTFFGAGVISTGSFLTTALISLIQRPQLRNLLHEKPELIPAGVEELLRINLSFADGLPRLATADI
QVGDVLVRKGELVLVLLEGANFDPEHFPNPGSIELDRPNPTSHLAFGRGQHFCPGSALGRRHAQIGIEALLKK
MPGVDLAVPIDQLVWRTRFQRRIPERLPVLW
>CYP121A1_Mycobacterium tuberculosis M2211 2630023364
MTATVLLEVPFSARGDRIPDAVAELRTREPIRKVRTITGAEAWLVSSYALCTQVLEDRRFSMKETAAAGAPRL
NALTVPPEVVNNMGNIADAGLRKAVMKAITPKAPGLEQFLRDTANSLLDNLITEGAPADLRNDFADPLATALH
CKVLGIPQEDGPKLFRSLSIAFMSSADPIPAAKINWDRDIEYMAGILENPNITTGLMGELSRLRKDPAYSHVS
DELFATIGVTFFGAGVISTGSFLTTALISLIQRPQLRNLLHEKPELIPAGVEELLRINLSFADGLPRLATADI
QVGDVLVRKGELVLVLLEGANFDPEHFPNPGSIELDRPNPTSHLAFGRGQHFCPGSALGRRHAQIGIEALLKK
MPGVDLAVPIDQLVWRTRFQRRIPERLPVLW
>CYP121A1_Mycobacterium tuberculosis M2218 2628255190
MTATVLLEVPFSARGDRIPDAVAELRTREPIRKVRTITGAEAWLVSSYALCTQVLEDRRFSMKETAAAGAPRL
NALTVPPEVVNNMGNIADAGLRKAVMKAITPKAPGLEQFLRDTANSLLDNLITEGAPADLRNDFADPLATALH
CKVLGIPQEDGPKLFRSLSIAFMSSADPIPAAKINWDRDIEYMAGILENPNITTGLMGELSRLRKDPAYSHVS
DELFATIGVTFFGAGVISTGSFLTTALISLIQRPQLRNLLHEKPELIPAGVEELLRINLSFADGLPRLATADI
QVGDVLVRKGELVLVLLEGANFDPEHFPNPGSIELDRPNPTSHLAFGRGQHFCPGSALGRRHAQIGIEALLKK
MPGVDLAVPIDQLVWRTRFQRRIPERLPVLW
>CYP121A1_Mycobacterium tuberculosis M2248 2577218913
MTATVLLEVPFSARGDRIPDAVAELRTREPIRKVRTITGAEAWLVSSYALCTQVLEDRRFSMKETAAAGAPRL
NALTVPPEVVNNMGNIADAGLRKAVMKAITPKAPGLEQFLRDTANSLLDNLITEGAPADLRNDFADPLATALH
CKVLGIPQEDGPKLFRSLSIAFMSSADPIPAAKINWDRDIEYMAGILENPNITTGLMGELSRLRKDPAYSHVS
DELFATIGVTFFGAGVISTGSFLTTALISLIQRPQLRNLLHEKPELIPAGVEELLRINLSFADGLPRLATADI
QVGDVLVRKGELVLVLLEGANFDPEHFPNPGSIELDRPNPTSHLAFGRGQHFCPGSALGRRHAQIGIEALLKK
MPGVDLAVPIDQLVWRTRFQRRIPERLPVLW
>CYP121A1_Mycobacterium tuberculosis M2249 2577918772
MTATVLLEVPFSARGDRIPDAVAELRTREPIRKVRTITGAEAWLVSSYALCTQVLEDRRFSMKETAAAGAPRL
NALTVPPEVVNNMGNIADAGLRKAVMKAITPKAPGLEQFLRDTANSLLDNLITEGAPADLRNDFADPLATALH
CKVLGIPQEDGPKLFRSLSIAFMSSADPIPAAKINWDRDIEYMAGILENPNITTGLMGELSRLRKDPAYSHVS
DELFATIGVTFFGAGVISTGSFLTTALISLIQRPQLRNLLHEKPELIPAGVEELLRINLSFADGLPRLATADI
QVGDVLVRKGELVLVLLEGANFDPEHFPNPGSIELDRPNPTSHLAFGRGQHFCPGSALGRRHAQIGIEALLKK
MPGVDLAVPIDQLVWRTRFQRRIPERLPVLW
>CYP121A1_Mycobacterium tuberculosis M2259 2628939107
MTATVLLEVPFSARGDRIPDAVAELRTREPIRKVRTITGAEAWLVSSYALCTQVLEDRRFSMKETAAAGAPRL
NALTVPPEVVNNMGNIADAGLRKAVMKAITPKAPGLEQFLRDTANSLLDNLITEGAPADLRNDFADPLATALH
CKVLGIPQEDGPKLFRSLSIAFMSSADPIPAAKINWDRDIEYMAGILENPNITTGLMGELSRLRKDPAYSHVS
DELFATIGVTFFGAGVISTGSFLTTALISLIQRPQLRNLLHEKPELIPAGVEELLRINLSFADGLPRLATADI
QVGDVLVRKGELVLVLLEGANFDPEHFPNPGSIELDRPNPTSHLAFGRGQHFCPGSALGRRHAQIGIEALLKK
MPGVDLAVPIDQLVWRTRFQRRIPERLPVLW
>CYP121A1_Mycobacterium tuberculosis M2276 2633231211
MTATVLLEVPFSARGDRIPDAVAELRTREPIRKVRTITGAEAWLVSSYALCTQVLEDRRFSMKETAAAGAPRL
NALTVPPEVVNNMGNIADAGLRKAVMKAITPKAPGLEQFLRDTANSLLDNLITEGAPADLRNDFADPLATALH
CKVLGIPQEDGPKLFRSLSIAFMSSADPIPAAKINWDRDIEYMAGILENPNITTGLMGELSRLRKDPAYSHVS
DELFATIGVTFFGAGVISTGSFLTTALISLIQRPQLRNLLHEKPELIPAGVEELLRINLSFADGLPRLATADI
QVGDVLVRKGELVLVLLEGANFDPEHFPNPGSIELDRPNPTSHLAFGRGQHFCPGSALGRRHAQIGIEALLKK
MPGVDLAVPIDQLVWRTRFQRRIPERLPVLW

>CYP121A1_Mycobacterium tuberculosis M2277 2636830325
MTATVLLEVPFSARGDRIPDAVAELRTREPIRKVRTITGAEAWLVSSYALCTQVLEDRRFSMKETAAAGAPRL
NALTVPPEVVNNMGNIADAGLRKAVMKAITPKAPGLEQFLRDTANSLLDNLITEGAPADLRNDFADPLATALH
CKVLGIPQEDGPKLFRSLSIAFMSSADPIPAAKINWDRDIEYMAGILENPNITTGLMGELSRLRKDPAYSHVS
DELFATIGVTFFGAGVISTGSFLTTALISLIQRPQLRNLLHEKPELIPAGVEELLRINLSFADGLPRLATADI
QVGDVLVRKGELVLVLLEGANFDPEHFPNPGSIELDRPNPTSHLAFGRGQHFCPGSALGRRHAQIGIEALLKK
MPGVDLAVPIDQLVWRTRFQRRIPERLPVLW
>CYP121A1_Mycobacterium tuberculosis M2278 2641738649
MTATVLLEVPFSARGDRIPDAVAELRTREPIRKVRTITGAEAWLVSSYALCTQVLEDRRFSMKETAAAGAPRL
NALTVPPEVVNNMGNIADAGLRKAVMKAITPKAPGLEQFLRDTANSLLDNLITEGAPADLRNDFADPLATALH
CKVLGIPQEDGPKLFRSLSIAFMSSADPIPAAKINWDRDIEYMAGILENPNITTGLMGELSRLRKDPAYSHVS
DELFATIGVTFFGAGVISTGSFLTTALISLIQRPQLRNLLHEKPELIPAGVEELLRINLSFADGLPRLATADI
QVGDVLVRKGELVLVLLEGANFDPEHFPNPGSIELDRPNPTSHLAFGRGQHFCPGSALGRRHAQIGIEALLKK
MPGVDLAVPIDQLVWRTRFQRRIPERLPVLW
>CYP121A1_Mycobacterium tuberculosis M2323 2629768326
MTATVLLEVPFSARGDRIPDAVAELRTREPIRKVRTITGAEAWLVSSYALCTQVLEDRRFSMKETAAAGAPRL
NALTVPPEVVNNMGNIADAGLRKAVMKAITPKAPGLEQFLRDTANSLLDNLITEGAPADLRNDFADPLATALH
CKVLGIPQEDGPKLFRSLSIAFMSSADPIPAAKINWDRDIEYMAGILENPNITTGLMGELSRLRKDPAYSHVS
DELFATIGVTFFGAGVISTGSFLTTALISLIQRPQLRNLLHEKPELIPAGVEELLRINLSFADGLPRLATADI
QVGDVLVRKGELVLVLLEGANFDPEHFPNPGSIELDRPNPTSHLAFGRGQHFCPGSALGRRHAQIGIEALLKK
MPGVDLAVPIDQLVWRTRFQRRIPERLPVLW
>CYP121A1_Mycobacterium tuberculosis M2324 2629228846
MTATVLLEVPFSARGDRIPDAVAELRTREPIRKVRTITGAEAWLVSSYALCTQVLEDRRFSMKETAAAGAPRL
NALTVPPEVVNNMGNIADAGLRKAVMKAITPKAPGLEQFLRDTANSLLDNLITEGAPADLRNDFADPLATALH
CKVLGIPQEDGPKLFRSLSIAFMSSADPIPAAKINWDRDIEYMAGILENPNITTGLMGELSRLRKDPAYSHVS
DELFATIGVTFFGAGVISTGSFLTTALISLIQRPQLRNLLHEKPELIPAGVEELLRINLSFADGLPRLATADI
QVGDVLVRKGELVLVLLEGANFDPEHFPNPGSIELDRPNPTSHLAFGRGQHFCPGSALGRRHAQIGIEALLKK
MPGVDLAVPIDQLVWRTRFQRRIPERLPVLW
>CYP121A1_Mycobacterium tuberculosis M2342 2637499730
MTATVLLEVPFSARGDRIPDAVAELRTREPIRKVRTITGAEAWLVSSYALCTQVLEDRRFSMKETAAAGAPRL
NALTVPPEVVNNMGNIADAGLRKAVMKAITPKAPGLEQFLRDTANSLLDNLITEGAPADLRNDFADPLATALH
CKVLGIPQEDGPKLFRSLSIAFMSSADPIPAAKINWDRDIEYMAGILENPNITTGLMGELSRLRKDPAYSHVS
DELFATIGVTFFGAGVISTGSFLTTALISLIQRPQLRNLLHEKPELIPAGVEELLRINLSFADGLPRLATADI
QVGDVLVRKGELVLVLLEGANFDPEHFPNPGSIELDRPNPTSHLAFGRGQHFCPGSALGRRHAQIGIEALLKK
MPGVDLAVPIDQLVWRTRFQRRIPERLPVLW
>CYP121A1_Mycobacterium tuberculosis M2343 2575346333
MTATVLLEVPFSARGDRIPDAVAELRTREPIRKVRTITGAEAWLVSSYALCTQVLEDRRFSMKETAAAGAPRL
NALTVPPEVVNNMGNIADAGLRKAVMKAITPKAPGLEQFLRDTANSLLDNLITEGAPADLRNDFADPLATALH
CKVLGIPQEDGPKLFRSLSIAFMSSADPIPAAKINWDRDIEYMAGILENPNITTGLMGELSRLRKDPAYSHVS
DELFATIGVTFFGAGVISTGSFLTTALISLIQRPQLRNLLHEKPELIPAGVEELLRINLSFADGLPRLATADI
QVGDVLVRKGELVLVLLEGANFDPEHFPNPGSIELDRPNPTSHLAFGRGQHFCPGSALGRRHAQIGIEALLKK
MPGVDLAVPIDQLVWRTRFQRRIPERLPVLW
>CYP121A1_Mycobacterium tuberculosis M2346 2584927100
MTATVLLEVPFSARGDRIPDAVAELRTREPIRKVRTITGAEAWLVSSYALCTQVLEDRRFSMKETAAAGAPRL
NALTVPPEVVNNMGNIADAGLRKAVMKAITPKAPGLEQFLRDTANSLLDNLITEGAPADLRNDFADPLATALH
CKVLGIPQEDGPKLFRSLSIAFMSSADPIPAAKINWDRDIEYMAGILENPNITTGLMGELSRLRKDPAYSHVS
DELFATIGVTFFGAGVISTGSFLTTALISLIQRPQLRNLLHEKPELIPAGVEELLRINLSFADGLPRLATADI
QVGDVLVRKGELVLVLLEGANFDPEHFPNPGSIELDRPNPTSHLAFGRGQHFCPGSALGRRHAQIGIEALLKK
MPGVDLAVPIDQLVWRTRFQRRIPERLPVLW
>CYP121A1_Mycobacterium tuberculosis M2350 2629406406
MTATVLLEVPFSARGDRIPDAVAELRTREPIRKVRTITGAEAWLVSSYALCTQVLEDRRFSMKETAAAGAPRL
NALTVPPEVVNNMGNIADAGLRKAVMKAITPKAPGLEQFLRDTANSLLDNLITEGAPADLRNDFADPLATALH
CKVLGIPQEDGPKLFRSLSIAFMSSADPIPAAKINWDRDIEYMAGILENPNITTGLMGELSRLRKDPAYSHVS
DELFATIGVTFFGAGVISTGSFLTTALISLIQRPQLRNLLHEKPELIPAGVEELLRINLSFADGLPRLATADI
QVGDVLVRKGELVLVLLEGANFDPEHFPNPGSIELDRPNPTSHLAFGRGQHFCPGSALGRRHAQIGIEALLKK
MPGVDLAVPIDQLVWRTRFQRRIPERLPVLW
>CYP121A1_Mycobacterium tuberculosis M2352 2631505405

MTATVLLEVPFSARGDRIPDAVAELRTREPIRKVRTITGAEAWLVSSYALCTQVLEDRRFSMKETAAAGAPRL
NALTVPPEVVNNMGNIADAGLRKAVMKAITPKAPGLEQFLRDTANSLLDNLITEGAPADLRNDFADPLATALH
CKVLGIPQEDGPKLFRSLSIAFMSSADPIPAAKINWDRDIEYMAGILENPNITTGLMGELSRLRKDPAYSHVS
DELFATIGVTFFGAGVISTGSFLTTALISLIQRPQLRNLLHEKPELIPAGVEELLRINLSFADGLPRLATADI
QVGDVLVRKGELVLVLLEGANFDPEHFPNPGSIELDRPNPTSHLAFGRGQHFCPGSALGRRHAQIGIEALLKK
MPGVDLAVPIDQLVWRTRFQRRIPERLPVLW
>CYP121A1_Mycobacterium tuberculosis M2376 2642109455
MTATVLLEVPFSARGDRIPDAVAELRTREPIRKVRTITGAEAWLVSSYALCTQVLEDRRFSMKETAAAGAPRL
NALTVPPEVVNNMGNIADAGLRKAVMKAITPKAPGLEQFLRDTANSLLDNLITEGAPADLRNDFADPLATALH
CKVLGIPQEDGPKLFRSLSIAFMSSADPIPAAKINWDRDIEYMAGILENPNITTGLMGELSRLRKDPAYSHVS
DELFATIGVTFFGAGVISTGSFLTTALISLIQRPQLRNLLHEKPELIPAGVEELLRINLSFADGLPRLATADI
QVGDVLVRKGELVLVLLEGANFDPEHFPNPGSIELDRPNPTSHLAFGRGQHFCPGSALGRRHAQIGIEALLKK
MPGVDLAVPIDQLVWRTRFQRRIPERLPVLW
>CYP121A1_Mycobacterium tuberculosis M2384 (re-annotation Oct 2015)
2639251389
MTATVLLEVPFSARGDRIPDAVAELRTREPIRKVRTITGAEAWLVSSYALCTQVLEDRRFSMKETAAAGAPRL
NALTVPPEVVNNMGNIADAGLRKAVMKAITPKAPGLEQFLRDTANSLLDNLITEGAPADLRNDFADPLATALH
CKVLGIPQEDGPKLFRSLSIAFMSSADPIPAAKINWDRDIEYMAGILENPNITTGLMGELSRLRKDPAYSHVS
DELFATIGVTFFGAGVISTGSFLTTALISLIQRPQLRNLLHEKPELIPAGVEELLRINLSFADGLPRLATADI
QVGDVLVRKGELVLVLLEGANFDPEHFPNPGSIELDRPNPTSHLAFGRGQHFCPGSALGRRHAQIGIEALLKK
MPGVDLAVPIDQLVWRTRFQRRIPERLPVLW
>CYP121A1_Mycobacterium tuberculosis M2402 2574854297
MTATVLLEVPFSARGDRIPDAVAELRTREPIRKVRTITGAEAWLVSSYALCTQVLEDRRFSMKETAAAGAPRL
NALTVPPEVVNNMGNIADAGLRKAVMKAITPKAPGLEQFLRDTANSLLDNLITEGAPADLRNDFADPLATALH
CKVLGIPQEDGPKLFRSLSIAFMSSADPIPAAKINWDRDIEYMAGILENPNITTGLMGELSRLRKDPAYSHVS
DELFATIGVTFFGAGVISTGSFLTTALISLIQRPQLRNLLHEKPELIPAGVEELLRINLSFADGLPRLATADI
QVGDVLVRKGELVLVLLEGANFDPEHFPNPGSIELDRPNPTSHLAFGRGQHFCPGSALGRRHAQIGIEALLKK
MPGVDLAVPIDQLVWRTRFQRRIPERLPVLW
>CYP121A1_Mycobacterium tuberculosis M2416 2574832982
MTATVLLEVPFSARGDRIPDAVAELRTREPIRKVRTITGAEAWLVSSYALCTQVLEDRRFSMKETAAAGAPRL
NALTVPPEVVNNMGNIADAGLRKAVMKAITPKAPGLEQFLRDTANSLLDNLITEGAPADLRNDFADPLATALH
CKVLGIPQEDGPKLFRSLSIAFMSSADPIPAAKINWDRDIEYMAGILENPNITTGLMGELSRLRKDPAYSHVS
DELFATIGVTFFGAGVISTGSFLTTALISLIQRPQLRNLLHEKPELIPAGVEELLRINLSFADGLPRLATADI
QVGDVLVRKGELVLVLLEGANFDPEHFPNPGSIELDRPNPTSHLAFGRGQHFCPGSALGRRHAQIGIEALLKK
MPGVDLAVPIDQLVWRTRFQRRIPERLPVLW
>CYP121A1_Mycobacterium tuberculosis M2435 2633802328
MTATVLLEVPFSARGDRIPDAVAELRTREPIRKVRTITGAEAWLVSSYALCTQVLEDRRFSMKETAAAGAPRL
NALTVPPEVVNNMGNIADAGLRKAVMKAITPKAPGLEQFLRDTANSLLDNLITEGAPADLRNDFADPLATALH
CKVLGIPQEDGPKLFRSLSIAFMSSADPIPAAKINWDRDIEYMAGILENPNITTGLMGELSRLRKDPAYSHVS
DELFATIGVTFFGAGVISTGSFLTTALISLIQRPQLRNLLHEKPELIPAGVEELLRINLSFADGLPRLATADI
QVGDVLVRKGELVLVLLEGANFDPEHFPNPGSIELDRPNPTSHLAFGRGQHFCPGSALGRRHAQIGIEALLKK
MPGVDLAVPIDQLVWRTRFQRRIPERLPVLW
>CYP121A1_Mycobacterium tuberculosis M2440 2637262563
MTATVLLEVPFSARGDRIPDAVAELRTREPIRKVRTITGAEAWLVSSYALCTQVLEDRRFSMKETAAAGAPRL
NALTVPPEVVNNMGNIADAGLRKAVMKAITPKAPGLEQFLRDTANSLLDNLITEGAPADLRNDFADPLATALH
CKVLGIPQEDGPKLFRSLSIAFMSSADPIPAAKINWDRDIEYMAGILENPNITTGLMGELSRLRKDPAYSHVS
DELFATIGVTFFGAGVISTGSFLTTALISLIQRPQLRNLLHEKPELIPAGVEELLRINLSFADGLPRLATADI
QVGDVLVRKGELVLVLLEGANFDPEHFPNPGSIELDRPNPTSHLAFGRGQHFCPGSALGRRHAQIGIEALLKK
MPGVDLAVPIDQLVWRTRFQRRIPERLPVLW
>CYP121A1_Mycobacterium tuberculosis M2479 2577722369
MTATVLLEVPFSARGDRIPDAVAELRTREPIRKVRTITGAEAWLVSSYALCTQVLEDRRFSMKETAAAGAPRL
NALTVPPEVVNNMGNIADAGLRKAVMKAITPKAPGLEQFLRDTANSLLDNLITEGAPADLRNDFADPLATALH
CKVLGIPQEDGPKLFRSLSIAFMSSADPIPAAKINWDRDIEYMAGILENPNITTGLMGELSRLRKDPAYSHVS
DELFATIGVTFFGAGVISTGSFLTTALISLIQRPQLRNLLHEKPELIPAGVEELLRINLSFADGLPRLATADI
QVGDVLVRKGELVLVLLEGANFDPEHFPNPGSIELDRPNPTSHLAFGRGQHFCPGSALGRRHAQIGIEALLKK
MPGVDLAVPIDQLVWRTRFQRRIPERLPVLW
>CYP121A1_Mycobacterium tuberculosis M2508 2577235717

MTATVLLEVPFSARGDRIPDAVAELRTREPIRKVRTITGAEAWLVSSYALCTQVLEDRRFSMKETAAAGAPRL
NALTVPPEVVNNMGNIADAGLRKAVMKAITPKAPGLEQFLRDTANSLLDNLITEGAPADLRNDFADPLATALH
CKVLGIPQEDGPKLFRSLSIAFMSSADPIPAAKINWDRDIEYMAGILENPNITTGLMGELSRLRKDPAYSHVS
DELFATIGVTFFGAGVISTGSFLTTALISLIQRPQLRNLLHEKPELIPAGVEELLRINLSFADGLPRLATADI
QVGDVLVRKGELVLVLLEGANFDPEHFPNPGSIELDRPNPTSHLAFGRGQHFCPGSALGRRHAQIGIEALLKK
MPGVDLAVPIDQLVWRTRFQRRIPERLPVLW
>CYP121A1_Mycobacterium tuberculosis M2828 2630584150
MTATVLLEVPFSARGDRIPDAVAELRTREPIRKVRTITGAEAWLVSSYALCTQVLEDRRFSMKETAAAGAPRL
NALTVPPEVVNNMGNIADAGLRKAVMKAITPKAPGLEQFLRDTANSLLDNLITEGAPADLRNDFADPLATALH
CKVLGIPQEDGPKLFRSLSIAFMSSADPIPAAKINWDRDIEYMAGILENPNITTGLMGELSRLRKDPAYSHVS
DELFATIGVTFFGAGVISTGSFLTTALISLIQRPQLRNLLHEKPELIPAGVEELLRINLSFADGLPRLATADI
QVGDVLVRKGELVLVLLEGANFDPEHFPNPGSIELDRPNPTSHLAFGRGQHFCPGSALGRRHAQIGIEALLKK
MPGVDLAVPIDQLVWRTRFQRRIPERLPVLW
>CYP121A1_Mycobacterium tuberculosis M984 2637249897
MTATVLLEVPFSARGDRIPDAVAELRTREPIRKVRTITGAEAWLVSSYALCTQVLEDRRFSMKETAAAGAPRL
NALTVPPEVVNNMGNIADAGLRKAVMKAITPKAPGLEQFLRDTANSLLDNLITEGAPADLRNDFADPLATALH
CKVLGIPQEDGPKLFRSLSIAFMSSADPIPAAKINWDRDIEYMAGILENPNITTGLMGELSRLRKDPAYSHVS
DELFATIGVTFFGAGVISTGSFLTTALISLIQRPQLRNLLHEKPELIPAGVEELLRINLSFADGLPRLATADI
QVGDVLVRKGELVLVLLEGANFDPEHFPNPGSIELDRPNPTSHLAFGRGQHFCPGSALGRRHAQIGIEALLKK
MPGVDLAVPIDQLVWRTRFQRRIPERLPVLW
>CYP121A1_Mycobacterium tuberculosis M987 2631483670
MTATVLLEVPFSARGDRIPDAVAELRTREPIRKVRTITGAEAWLVSSYALCTQVLEDRRFSMKETAAAGAPRL
NALTVPPEVVNNMGNIADAGLRKAVMKAITPKAPGLEQFLRDTANSLLDNLITEGAPADLRNDFADPLATALH
CKVLGIPQEDGPKLFRSLSIAFMSSADPIPAAKINWDRDIEYMAGILENPNITTGLMGELSRLRKDPAYSHVS
DELFATIGVTFFGAGVISTGSFLTTALISLIQRPQLRNLLHEKPELIPAGVEELLRINLSFADGLPRLATADI
QVGDVLVRKGELVLVLLEGANFDPEHFPNPGSIELDRPNPTSHLAFGRGQHFCPGSALGRRHAQIGIEALLKK
MPGVDLAVPIDQLVWRTRFQRRIPERLPVLW
>CYP121A1_Mycobacterium tuberculosis M991 2629955725
MTATVLLEVPFSARGDRIPDAVAELRTREPIRKVRTITGAEAWLVSSYALCTQVLEDRRFSMKETAAAGAPRL
NALTVPPEVVNNMGNIADAGLRKAVMKAITPKAPGLEQFLRDTANSLLDNLITEGAPADLRNDFADPLATALH
CKVLGIPQEDGPKLFRSLSIAFMSSADPIPAAKINWDRDIEYMAGILENPNITTGLMGELSRLRKDPAYSHVS
DELFATIGVTFFGAGVISTGSFLTTALISLIQRPQLRNLLHEKPELIPAGVEELLRINLSFADGLPRLATADI
QVGDVLVRKGELVLVLLEGANFDPEHFPNPGSIELDRPNPTSHLAFGRGQHFCPGSALGRRHAQIGIEALLKK
MPGVDLAVPIDQLVWRTRFQRRIPERLPVLW
>CYP121A1_Mycobacterium tuberculosis M992 2635645933
MTATVLLEVPFSARGDRIPDAVAELRTREPIRKVRTITGAEAWLVSSYALCTQVLEDRRFSMKETAAAGAPRL
NALTVPPEVVNNMGNIADAGLRKAVMKAITPKAPGLEQFLRDTANSLLDNLITEGAPADLRNDFADPLATALH
CKVLGIPQEDGPKLFRSLSIAFMSSADPIPAAKINWDRDIEYMAGILENPNITTGLMGELSRLRKDPAYSHVS
DELFATIGVTFFGAGVISTGSFLTTALISLIQRPQLRNLLHEKPELIPAGVEELLRINLSFADGLPRLATADI
QVGDVLVRKGELVLVLLEGANFDPEHFPNPGSIELDRPNPTSHLAFGRGQHFCPGSALGRRHAQIGIEALLKK
MPGVDLAVPIDQLVWRTRFQRRIPERLPVLW
>CYP121A1_Mycobacterium tuberculosis M995 2579809112
MTATVLLEVPFSARGDRIPDAVAELRTREPIRKVRTITGAEAWLVSSYALCTQVLEDRRFSMKETAAAGAPRL
NALTVPPEVVNNMGNIADAGLRKAVMKAITPKAPGLEQFLRDTANSLLDNLITEGAPADLRNDFADPLATALH
CKVLGIPQEDGPKLFRSLSIAFMSSADPIPAAKINWDRDIEYMAGILENPNITTGLMGELSRLRKDPAYSHVS
DELFATIGVTFFGAGVISTGSFLTTALISLIQRPQLRNLLHEKPELIPAGVEELLRINLSFADGLPRLATADI
QVGDVLVRKGELVLVLLEGANFDPEHFPNPGSIELDRPNPTSHLAFGRGQHFCPGSALGRRHAQIGIEALLKK
MPGVDLAVPIDQLVWRTRFQRRIPERLPVLW
>CYP121A1_Mycobacterium tuberculosis MAL010075 2621977781
MTATVLLEVPFSARGDRIPDAVAELRTREPIRKVRTITGAEAWLVSSYALCTQVLEDRRFSMKETAAAGAPRL
NALTVPPEVVNNMGNIADAGLRKAVMKAITPKAPGLEQFLRDTANSLLDNLITEGAPADLRNDFADPLATALH
CKVLGIPQEDGPKLFRSLSIAFMSSADPIPAAKINWDRDIEYMAGILENPNITTGLMGELSRLRKDPAYSHVS
DELFATIGVTFFGAGVISTGSFLTTALISLIQRPQLRNLLHEKPELIPAGVEELLRINLSFADGLPRLATADI
QVGDVLVRKGELVLVLLEGANFDPEHFPNPGSIELDRPNPTSHLAFGRGQHFCPGSALGRRHAQIGIEALLKK
MPGVDLAVPIDQLVWRTRFQRRIPERLPVLW
>CYP121A1_Mycobacterium tuberculosis MAL010077 2621982201
MTATVLLEVPFSARGDRIPDAVAELRTREPIRKVRTITGAEAWLVSSYALCTQVLEDRRFSMKETAAAGAPRL
NALTVPPEVVNNMGNIADAGLRKAVMKAITPKAPGLEQFLRDTANSLLDNLITEGAPADLRNDFADPLATALH

CKVLGIPQEDGPKLFRSLSIAFMSSADPIPAAKINWDRDIEYMAGILENPNITTGLMGELSRLRKDPAYSHVS
DELFATIGVTFFGAGVISTGSFLTTALISLIQRPQLRNLLHEKPELIPAGVEELLRINLSFADGLPRLATADI
QVGDVLVRKGELVLVLLEGANFDPEHFPNPGSIELDRPNPTSHLAFGRGQHFCPGSALGRRHAQIGIEALLKK
MPGVDLAVPIDQLVWRTRFQRRIPERLPVLW
>CYP121A1_Mycobacterium tuberculosis MAL010103 2576151178
MTATVLLEVPFSARGDRIPDAVAELRTREPIRKVRTITGAEAWLVSSYALCTQVLEDRRFSMKETAAAGAPRL
NALTVPPEVVNNMGNIADAGLRKAVMKAITPKAPGLEQFLRDTANSLLDNLITEGAPADLRNDFADPLATALH
CKVLGIPQEDGPKLFRSLSIAFMSSADPIPAAKINWDRDIEYMAGILENPNITTGLMGELSRLRKDPAYSHVS
DELFATIGVTFFGAGVISTGSFLTTALISLIQRPQLRNLLHEKPELIPAGVEELLRINLSFADGLPRLATADI
QVGDVLVRKGELVLVLLEGANFDPEHFPNPGSIELDRPNPTSHLAFGRGQHFCPGSALGRRHAQIGIEALLKK
MPGVDLAVPIDQLVWRTRFQRRIPERLPVLW
>CYP121A1_Mycobacterium tuberculosis MAL010109 2576457616
MTATVLLEVPFSARGDRIPDAVAELRTREPIRKVRTITGAEAWLVSSYALCTQVLEDRRFSMKETAAAGAPRL
NALTVPPEVVNNMGNIADAGLRKAVMKAITPKAPGLEQFLRDTANSLLDNLITEGAPADLRNDFADPLATALH
CKVLGIPQEDGPKLFRSLSIAFMSSADPIPAAKINWDRDIEYMAGILENPNITTGLMGELSRLRKDPAYSHVS
DELFATIGVTFFGAGVISTGSFLTTALISLIQRPQLRNLLHEKPELIPAGVEELLRINLSFADGLPRLATADI
QVGDVLVRKGELVLVLLEGANFDPEHFPNPGSIELDRPNPTSHLAFGRGQHFCPGSALGRRHAQIGIEALLKK
MPGVDLAVPIDQLVWRTRFQRRIPERLPVLW
>CYP121A1_Mycobacterium tuberculosis MAL020120 2577195999
MTATVLLEVPFSARGDRIPDAVAELRTREPIRKVRTITGAEAWLVSSYALCTQVLEDRRFSMKETAAAGAPRL
NALTVPPEVVNNMGNIADAGLRKAVMKAITPKAPGLEQFLRDTANSLLDNLITEGAPADLRNDFADPLATALH
CKVLGIPQEDGPKLFRSLSIAFMSSADPIPAAKINWDRDIEYMAGILENPNITTGLMGELSRLRKDPAYSHVS
DELFATIGVTFFGAGVISTGSFLTTALISLIQRPQLRNLLHEKPELIPAGVEELLRINLSFADGLPRLATADI
QVGDVLVRKGELVLVLLEGANFDPEHFPNPGSIELDRPNPTSHLAFGRGQHFCPGSALGRRHAQIGIEALLKK
MPGVDLAVPIDQLVWRTRFQRRIPERLPVLW
>CYP121A1_Mycobacterium tuberculosis MAL020131 2574753557
MTATVLLEVPFSARGDRIPDAVAELRTREPIRKVRTITGAEAWLVSSYALCTQVLEDRRFSMKETAAAGAPRL
NALTVPPEVVNNMGNIADAGLRKAVMKAITPKAPGLEQFLRDTANSLLDNLITEGAPADLRNDFADPLATALH
CKVLGIPQEDGPKLFRSLSIAFMSSADPIPAAKINWDRDIEYMAGILENPNITTGLMGELSRLRKDPAYSHVS
DELFATIGVTFFGAGVISTGSFLTTALISLIQRPQLRNLLHEKPELIPAGVEELLRINLSFADGLPRLATADI
QVGDVLVRKGELVLVLLEGANFDPEHFPNPGSIELDRPNPTSHLAFGRGQHFCPGSALGRRHAQIGIEALLKK
MPGVDLAVPIDQLVWRTRFQRRIPERLPVLW
>CYP121A1_Mycobacterium tuberculosis MAL020139 2621990128
MTATVLLEVPFSARGDRIPDAVAELRTREPIRKVRTITGAEAWLVSSYALCTQVLEDRRFSMKETAAAGAPRL
NALTVPPEVVNNMGNIADAGLRKAVMKAITPKAPGLEQFLRDTANSLLDNLITEGAPADLRNDFADPLATALH
CKVLGIPQEDGPKLFRSLSIAFMSSADPIPAAKINWDRDIEYMAGILENPNITTGLMGELSRLRKDPAYSHVS
DELFATIGVTFFGAGVISTGSFLTTALISLIQRPQLRNLLHEKPELIPAGVEELLRINLSFADGLPRLATADI
QVGDVLVRKGELVLVLLEGANFDPEHFPNPGSIELDRPNPTSHLAFGRGQHFCPGSALGRRHAQIGIEALLKK
MPGVDLAVPIDQLVWRTRFQRRIPERLPVLW
>CYP121A1_Mycobacterium tuberculosis MAL020144 2576703661
MTATVLLEVPFSARGDRIPDAVAELRTREPIRKVRTITGAEAWLVSSYALCTQVLEDRRFSMKETAAAGAPRL
NALTVPPEVVNNMGNIADAGLRKAVMKAITPKAPGLEQFLRDTANSLLDNLITEGAPADLRNDFADPLATALH
CKVLGIPQEDGPKLFRSLSIAFMSSADPIPAAKINWDRDIEYMAGILENPNITTGLMGELSRLRKDPAYSHVS
DELFATIGVTFFGAGVISTGSFLTTALISLIQRPQLRNLLHEKPELIPAGVEELLRINLSFADGLPRLATADI
QVGDVLVRKGELVLVLLEGANFDPEHFPNPGSIELDRPNPTSHLAFGRGQHFCPGSALGRRHAQIGIEALLKK
MPGVDLAVPIDQLVWRTRFQRRIPERLPVLW
>CYP121A1_Mycobacterium tuberculosis MAL020145 2584763889
MTATVLLEVPFSARGDRIPDAVAELRTREPIRKVRTITGAEAWLVSSYALCTQVLEDRRFSMKETAAAGAPRL
NALTVPPEVVNNMGNIADAGLRKAVMKAITPKAPGLEQFLRDTANSLLDNLITEGAPADLRNDFADPLATALH
CKVLGIPQEDGPKLFRSLSIAFMSSADPIPAAKINWDRDIEYMAGILENPNITTGLMGELSRLRKDPAYSHVS
DELFATIGVTFFGAGVISTGSFLTTALISLIQRPQLRNLLHEKPELIPAGVEELLRINLSFADGLPRLATADI
QVGDVLVRKGELVLVLLEGANFDPEHFPNPGSIELDRPNPTSHLAFGRGQHFCPGSALGRRHAQIGIEALLKK
MPGVDLAVPIDQLVWRTRFQRRIPERLPVLW
>CYP121A1_Mycobacterium tuberculosis MAL020151 2621992791
MTATVLLEVPFSARGDRIPDAVAELRTREPIRKVRTITGAEAWLVSSYALCTQVLEDRRFSMKETAAAGAPRL
NALTVPPEVVNNMGNIADAGLRKAVMKAITPKAPGLEQFLRDTANSLLDNLITEGAPADLRNDFADPLATALH
CKVLGIPQEDGPKLFRSLSIAFMSSADPIPAAKINWDRDIEYMAGILENPNITTGLMGELSRLRKDPAYSHVS
DELFATIGVTFFGAGVISTGSFLTTALISLIQRPQLRNLLHEKPELIPAGVEELLRINLSFADGLPRLATADI

QVGDVLVRKGELVLVLLEGANFDPEHFPNPGSIELDRPNPTSHLAFGRGQHFCPGSALGRRHAQIGIEALLKK
MPGVDLAVPIDQLVWRTRFQRRIPERLPVLW
>CYP121A1_Mycobacterium tuberculosis MAL020162 2575533067
MTATVLLEVPFSARGDRIPDAVAELRTREPIRKVRTITGAEAWLVSSYALCTQVLEDRRFSMKETAAAGAPRL
NALTVPPEVVNNMGNIADAGLRKAVMKAITPKAPGLEQFLRDTANSLLDNLITEGAPADLRNDFADPLATALH
CKVLGIPQEDGPKLFRSLSIAFMSSADPIPAAKINWDRDIEYMAGILENPNITTGLMGELSRLRKDPAYSHVS
DELFATIGVTFFGAGVISTGSFLTTALISLIQRPQLRNLLHEKPELIPAGVEELLRINLSFADGLPRLATADI
QVGDVLVRKGELVLVLLEGANFDPEHFPNPGSIELDRPNPTSHLAFGRGQHFCPGSALGRRHAQIGIEALLKK
MPGVDLAVPIDQLVWRTRFQRRIPERLPVLW
>CYP121A1_Mycobacterium tuberculosis MAL020179 2576602354
MTATVLLEVPFSARGDRIPDAVAELRTREPIRKVRTITGAEAWLVSSYALCTQVLEDRRFSMKETAAAGAPRL
NALTVPPEVVNNMGNIADAGLRKAVMKAITPKAPGLEQFLRDTANSLLDNLITEGAPADLRNDFADPLATALH
CKVLGIPQEDGPKLFRSLSIAFMSSADPIPAAKINWDRDIEYMAGILENPNITTGLMGELSRLRKDPAYSHVS
DELFATIGVTFFGAGVISTGSFLTTALISLIQRPQLRNLLHEKPELIPAGVEELLRINLSFADGLPRLATADI
QVGDVLVRKGELVLVLLEGANFDPEHFPNPGSIELDRPNPTSHLAFGRGQHFCPGSALGRRHAQIGIEALLKK
MPGVDLAVPIDQLVWRTRFQRRIPERLPVLW
>CYP121A1_Mycobacterium tuberculosis MAL020181 2574683113
MTATVLLEVPFSARGDRIPDAVAELRTREPIRKVRTITGAEAWLVSSYALCTQVLEDRRFSMKETAAAGAPRL
NALTVPPEVVNNMGNIADAGLRKAVMKAITPKAPGLEQFLRDTANSLLDNLITEGAPADLRNDFADPLATALH
CKVLGIPQEDGPKLFRSLSIAFMSSADPIPAAKINWDRDIEYMAGILENPNITTGLMGELSRLRKDPAYSHVS
DELFATIGVTFFGAGVISTGSFLTTALISLIQRPQLRNLLHEKPELIPAGVEELLRINLSFADGLPRLATADI
QVGDVLVRKGELVLVLLEGANFDPEHFPNPGSIELDRPNPTSHLAFGRGQHFCPGSALGRRHAQIGIEALLKK
MPGVDLAVPIDQLVWRTRFQRRIPERLPVLW
>CYP121A1_Mycobacterium tuberculosis MD13744 2628656257
MTATVLLEVPFSARGDRIPDAVAELRTREPIRKVRTITGAEAWLVSSYALCTQVLEDRRFSMKETAAAGAPRL
NALTVPPEVVNNMGNIADAGLRKAVMKAITPKAPGLEQFLRDTANSLLDNLITEGAPADLRNDFADPLATALH
CKVLGIPQEDGPKLFRSLSIAFMSSADPIPAAKINWDRDIEYMAGILENPNITTGLMGELSRLRKDPAYSHVS
DELFATIGVTFFGAGVISTGSFLTTALISLIQRPQLRNLLHEKPELIPAGVEELLRINLSFADGLPRLATADI
QVGDVLVRKGELVLVLLEGANFDPEHFPNPGSIELDRPNPTSHLAFGRGQHFCPGSALGRRHAQIGIEALLKK
MPGVDLAVPIDQLVWRTRFQRRIPERLPVLW
>CYP121A1_Mycobacterium tuberculosis MD13874 2622192027
MTATVLLEVPFSARGDRIPDAVAELRTREPIRKVRTITGAEAWLVSSYALCTQVLEDRRFSMKETAAAGAPRL
NALTVPPEVVNNMGNIADAGLRKAVMKAITPKAPGLEQFLRDTANSLLDNLITEGAPADLRNDFADPLATALH
CKVLGIPQEDGPKLFRSLSIAFMSSADPIPAAKINWDRDIEYMAGILENPNITTGLMGELSRLRKDPAYSHVS
DELFATIGVTFFGAGVISTGSFLTTALISLIQRPQLRNLLHEKPELIPAGVEELLRINLSFADGLPRLATADI
QVGDVLVRKGELVLVLLEGANFDPEHFPNPGSIELDRPNPTSHLAFGRGQHFCPGSALGRRHAQIGIEALLKK
MPGVDLAVPIDQLVWRTRFQRRIPERLPVLW
>CYP121A1_Mycobacterium tuberculosis MD13878 2575053058
MTATVLLEVPFSARGDRIPDAVAELRTREPIRKVRTITGAEAWLVSSYALCTQVLEDRRFSMKETAAAGAPRL
NALTVPPEVVNNMGNIADAGLRKAVMKAITPKAPGLEQFLRDTANSLLDNLITEGAPADLRNDFADPLATALH
CKVLGIPQEDGPKLFRSLSIAFMSSADPIPAAKINWDRDIEYMAGILENPNITTGLMGELSRLRKDPAYSHVS
DELFATIGVTFFGAGVISTGSFLTTALISLIQRPQLRNLLHEKPELIPAGVEELLRINLSFADGLPRLATADI
QVGDVLVRKGELVLVLLEGANFDPEHFPNPGSIELDRPNPTSHLAFGRGQHFCPGSALGRRHAQIGIEALLKK
MPGVDLAVPIDQLVWRTRFQRRIPERLPVLW
>CYP121A1_Mycobacterium tuberculosis MD14002 2622208741
MTATVLLEVPFSARGDRIPDAVAELRTREPIRKVRTITGAEAWLVSSYALCTQVLEDRRFSMKETAAAGAPRL
NALTVPPEVVNNMGNIADAGLRKAVMKAITPKAPGLEQFLRDTANSLLDNLITEGAPADLRNDFADPLATALH
CKVLGIPQEDGPKLFRSLSIAFMSSADPIPAAKINWDRDIEYMAGILENPNITTGLMGELSRLRKDPAYSHVS
DELFATIGVTFFGAGVISTGSFLTTALISLIQRPQLRNLLHEKPELIPAGVEELLRINLSFADGLPRLATADI
QVGDVLVRKGELVLVLLEGANFDPEHFPNPGSIELDRPNPTSHLAFGRGQHFCPGSALGRRHAQIGIEALLKK
MPGVDLAVPIDQLVWRTRFQRRIPERLPVLW
>CYP121A1_Mycobacterium tuberculosis MD14435 2577825317
MTATVLLEVPFSARGDRIPDAVAELRTREPIRKVRTITGAEAWLVSSYALCTQVLEDRRFSMKETAAAGAPRL
NALTVPPEVVNNMGNIADAGLRKAVMKAITPKAPGLEQFLRDTANSLLDNLITEGAPADLRNDFADPLATALH
CKVLGIPQEDGPKLFRSLSIAFMSSADPIPAAKINWDRDIEYMAGILENPNITTGLMGELSRLRKDPAYSHVS
DELFATIGVTFFGAGVISTGSFLTTALISLIQRPQLRNLLHEKPELIPAGVEELLRINLSFADGLPRLATADI
QVGDVLVRKGELVLVLLEGANFDPEHFPNPGSIELDRPNPTSHLAFGRGQHFCPGSALGRRHAQIGIEALLKK
MPGVDLAVPIDQLVWRTRFQRRIPERLPVLW

>CYP121A1_Mycobacterium tuberculosis MD14621 2622197689
MTATVLLEVPFSARGDRIPDAVAELRTREPIRKVRTITGAEAWLVSSYALCTQVLEDRRFSMKETAAAGAPRL
NALTVPPEVVNNMGNIADAGLRKAVMKAITPKAPGLEQFLRDTANSLLDNLITEGAPADLRNDFADPLATALH
CKVLGIPQEDGPKLFRSLSIAFMSSADPIPAAKINWDRDIEYMAGILENPNITTGLMGELSRLRKDPAYSHVS
DELFATIGVTFFGAGVISTGSFLTTALISLIQRPQLRNLLHEKPELIPAGVEELLRINLSFADGLPRLATADI
QVGDVLVRKGELVLVLLEGANFDPEHFPNPGSIELDRPNPTSHLAFGRGQHFCPGSALGRRHAQIGIEALLKK
MPGVDLAVPIDQLVWRTRFQRRIPERLPVLW
>CYP121A1_Mycobacterium tuberculosis MD14720 2622216958
MTATVLLEVPFSARGDRIPDAVAELRTREPIRKVRTITGAEAWLVSSYALCTQVLEDRRFSMKETAAAGAPRL
NALTVPPEVVNNMGNIADAGLRKAVMKAITPKAPGLEQFLRDTANSLLDNLITEGAPADLRNDFADPLATALH
CKVLGIPQEDGPKLFRSLSIAFMSSADPIPAAKINWDRDIEYMAGILENPNITTGLMGELSRLRKDPAYSHVS
DELFATIGVTFFGAGVISTGSFLTTALISLIQRPQLRNLLHEKPELIPAGVEELLRINLSFADGLPRLATADI
QVGDVLVRKGELVLVLLEGANFDPEHFPNPGSIELDRPNPTSHLAFGRGQHFCPGSALGRRHAQIGIEALLKK
MPGVDLAVPIDQLVWRTRFQRRIPERLPVLW
>CYP121A1_Mycobacterium tuberculosis MD14825 2632256631
MTATVLLEVPFSARGDRIPDAVAELRTREPIRKVRTITGAEAWLVSSYALCTQVLEDRRFSMKETAAAGAPRL
NALTVPPEVVNNMGNIADAGLRKAVMKAITPKAPGLEQFLRDTANSLLDNLITEGAPADLRNDFADPLATALH
CKVLGIPQEDGPKLFRSLSIAFMSSADPIPAAKINWDRDIEYMAGILENPNITTGLMGELSRLRKDPAYSHVS
DELFATIGVTFFGAGVISTGSFLTTALISLIQRPQLRNLLHEKPELIPAGVEELLRINLSFADGLPRLATADI
QVGDVLVRKGELVLVLLEGANFDPEHFPNPGSIELDRPNPTSHLAFGRGQHFCPGSALGRRHAQIGIEALLKK
MPGVDLAVPIDQLVWRTRFQRRIPERLPVLW
>CYP121A1_Mycobacterium tuberculosis MD14839 2636803765
MTATVLLEVPFSARGDRIPDAVAELRTREPIRKVRTITGAEAWLVSSYALCTQVLEDRRFSMKETAAAGAPRL
NALTVPPEVVNNMGNIADAGLRKAVMKAITPKAPGLEQFLRDTANSLLDNLITEGAPADLRNDFADPLATALH
CKVLGIPQEDGPKLFRSLSIAFMSSADPIPAAKINWDRDIEYMAGILENPNITTGLMGELSRLRKDPAYSHVS
DELFATIGVTFFGAGVISTGSFLTTALISLIQRPQLRNLLHEKPELIPAGVEELLRINLSFADGLPRLATADI
QVGDVLVRKGELVLVLLEGANFDPEHFPNPGSIELDRPNPTSHLAFGRGQHFCPGSALGRRHAQIGIEALLKK
MPGVDLAVPIDQLVWRTRFQRRIPERLPVLW
>CYP121A1_Mycobacterium tuberculosis MD14844 2622184798
MTATVLLEVPFSARGDRIPDAVAELRTREPIRKVRTITGAEAWLVSSYALCTQVLEDRRFSMKETAAAGAPRL
NALTVPPEVVNNMGNIADAGLRKAVMKAITPKAPGLEQFLRDTANSLLDNLITEGAPADLRNDFADPLATALH
CKVLGIPQEDGPKLFRSLSIAFMSSADPIPAAKINWDRDIEYMAGILENPNITTGLMGELSRLRKDPAYSHVS
DELFATIGVTFFGAGVISTGSFLTTALISLIQRPQLRNLLHEKPELIPAGVEELLRINLSFADGLPRLATADI
QVGDVLVRKGELVLVLLEGANFDPEHFPNPGSIELDRPNPTSHLAFGRGQHFCPGSALGRRHAQIGIEALLKK
MPGVDLAVPIDQLVWRTRFQRRIPERLPVLW
>CYP121A1_Mycobacterium tuberculosis MD14847 2622189436
MTATVLLEVPFSARGDRIPDAVAELRTREPIRKVRTITGAEAWLVSSYALCTQVLEDRRFSMKETAAAGAPRL
NALTVPPEVVNNMGNIADAGLRKAVMKAITPKAPGLEQFLRDTANSLLDNLITEGAPADLRNDFADPLATALH
CKVLGIPQEDGPKLFRSLSIAFMSSADPIPAAKINWDRDIEYMAGILENPNITTGLMGELSRLRKDPAYSHVS
DELFATIGVTFFGAGVISTGSFLTTALISLIQRPQLRNLLHEKPELIPAGVEELLRINLSFADGLPRLATADI
QVGDVLVRKGELVLVLLEGANFDPEHFPNPGSIELDRPNPTSHLAFGRGQHFCPGSALGRRHAQIGIEALLKK
MPGVDLAVPIDQLVWRTRFQRRIPERLPVLW
>CYP121A1_Mycobacterium tuberculosis MD15212 2577519963
MTATVLLEVPFSARGDRIPDAVAELRTREPIRKVRTITGAEAWLVSSYALCTQVLEDRRFSMKETAAAGAPRL
NALTVPPEVVNNMGNIADAGLRKAVMKAITPKAPGLEQFLRDTANSLLDNLITEGAPADLRNDFADPLATALH
CKVLGIPQEDGPKLFRSLSIAFMSSADPIPAAKINWDRDIEYMAGILENPNITTGLMGELSRLRKDPAYSHVS
DELFATIGVTFFGAGVISTGSFLTTALISLIQRPQLRNLLHEKPELIPAGVEELLRINLSFADGLPRLATADI
QVGDVLVRKGELVLVLLEGANFDPEHFPNPGSIELDRPNPTSHLAFGRGQHFCPGSALGRRHAQIGIEALLKK
MPGVDLAVPIDQLVWRTRFQRRIPERLPVLW
>CYP121A1_Mycobacterium tuberculosis MD15226 2622142184
MTATVLLEVPFSARGDRIPDAVAELRTREPIRKVRTITGAEAWLVSSYALCTQVLEDRRFSMKETAAAGAPRL
NALTVPPEVVNNMGNIADAGLRKAVMKAITPKAPGLEQFLRDTANSLLDNLITEGAPADLRNDFADPLATALH
CKVLGIPQEDGPKLFRSLSIAFMSSADPIPAAKINWDRDIEYMAGILENPNITTGLMGELSRLRKDPAYSHVS
DELFATIGVTFFGAGVISTGSFLTTALISLIQRPQLRNLLHEKPELIPAGVEELLRINLSFADGLPRLATADI
QVGDVLVRKGELVLVLLEGANFDPEHFPNPGSIELDRPNPTSHLAFGRGQHFCPGSALGRRHAQIGIEALLKK
MPGVDLAVPIDQLVWRTRFQRRIPERLPVLW
>CYP121A1_Mycobacterium tuberculosis MD15430 2622160767

MTATVLLEVPFSARGDRIPDAVAELRTREPIRKVRTITGAEAWLVSSYALCTQVLEDRRFSMKETAAAGAPRL
NALTVPPEVVNNMGNIADAGLRKAVMKAITPKAPGLEQFLRDTANSLLDNLITEGAPADLRNDFADPLATALH
CKVLGIPQEDGPKLFRSLSIAFMSSADPIPAAKINWDRDIEYMAGILENPNITTGLMGELSRLRKDPAYSHVS
DELFATIGVTFFGAGVISTGSFLTTALISLIQRPQLRNLLHEKPELIPAGVEELLRINLSFADGLPRLATADI
QVGDVLVRKGELVLVLLEGANFDPEHFPNPGSIELDRPNPTSHLAFGRGQHFCPGSALGRRHAQIGIEALLKK
MPGVDLAVPIDQLVWRTRFQRRIPERLPVLW
>CYP121A1_Mycobacterium tuberculosis MD15584 2622229734
MTATVLLEVPFSARGDRIPDAVAELRTREPIRKVRTITGAEAWLVSSYALCTQVLEDRRFSMKETAAAGAPRL
NALTVPPEVVNNMGNIADAGLRKAVMKAITPKAPGLEQFLRDTANSLLDNLITEGAPADLRNDFADPLATALH
CKVLGIPQEDGPKLFRSLSIAFMSSADPIPAAKINWDRDIEYMAGILENPNITTGLMGELSRLRKDPAYSHVS
DELFATIGVTFFGAGVISTGSFLTTALISLIQRPQLRNLLHEKPELIPAGVEELLRINLSFADGLPRLATADI
QVGDVLVRKGELVLVLLEGANFDPEHFPNPGSIELDRPNPTSHLAFGRGQHFCPGSALGRRHAQIGIEALLKK
MPGVDLAVPIDQLVWRTRFQRRIPERLPVLW
>CYP121A1_Mycobacterium tuberculosis MD15751 2634008953
MTATVLLEVPFSARGDRIPDAVAELRTREPIRKVRTITGAEAWLVSSYALCTQVLEDRRFSMKETAAAGAPRL
NALTVPPEVVNNMGNIADAGLRKAVMKAITPKAPGLEQFLRDTANSLLDNLITEGAPADLRNDFADPLATALH
CKVLGIPQEDGPKLFRSLSIAFMSSADPIPAAKINWDRDIEYMAGILENPNITTGLMGELSRLRKDPAYSHVS
DELFATIGVTFFGAGVISTGSFLTTALISLIQRPQLRNLLHEKPELIPAGVEELLRINLSFADGLPRLATADI
QVGDVLVRKGELVLVLLEGANFDPEHFPNPGSIELDRPNPTSHLAFGRGQHFCPGSALGRRHAQIGIEALLKK
MPGVDLAVPIDQLVWRTRFQRRIPERLPVLW
>CYP121A1_Mycobacterium tuberculosis MD15762 2622242367
MTATVLLEVPFSARGDRIPDAVAELRTREPIRKVRTITGAEAWLVSSYALCTQVLEDRRFSMKETAAAGAPRL
NALTVPPEVVNNMGNIADAGLRKAVMKAITPKAPGLEQFLRDTANSLLDNLITEGAPADLRNDFADPLATALH
CKVLGIPQEDGPKLFRSLSIAFMSSADPIPAAKINWDRDIEYMAGILENPNITTGLMGELSRLRKDPAYSHVS
DELFATIGVTFFGAGVISTGSFLTTALISLIQRPQLRNLLHEKPELIPAGVEELLRINLSFADGLPRLATADI
QVGDVLVRKGELVLVLLEGANFDPEHFPNPGSIELDRPNPTSHLAFGRGQHFCPGSALGRRHAQIGIEALLKK
MPGVDLAVPIDQLVWRTRFQRRIPERLPVLW
>CYP121A1_Mycobacterium tuberculosis MD15765 2622239423
MTATVLLEVPFSARGDRIPDAVAELRTREPIRKVRTITGAEAWLVSSYALCTQVLEDRRFSMKETAAAGAPRL
NALTVPPEVVNNMGNIADAGLRKAVMKAITPKAPGLEQFLRDTANSLLDNLITEGAPADLRNDFADPLATALH
CKVLGIPQEDGPKLFRSLSIAFMSSADPIPAAKINWDRDIEYMAGILENPNITTGLMGELSRLRKDPAYSHVS
DELFATIGVTFFGAGVISTGSFLTTALISLIQRPQLRNLLHEKPELIPAGVEELLRINLSFADGLPRLATADI
QVGDVLVRKGELVLVLLEGANFDPEHFPNPGSIELDRPNPTSHLAFGRGQHFCPGSALGRRHAQIGIEALLKK
MPGVDLAVPIDQLVWRTRFQRRIPERLPVLW
>CYP121A1_Mycobacterium tuberculosis MD15766 2622138406
MTATVLLEVPFSARGDRIPDAVAELRTREPIRKVRTITGAEAWLVSSYALCTQVLEDRRFSMKETAAAGAPRL
NALTVPPEVVNNMGNIADAGLRKAVMKAITPKAPGLEQFLRDTANSLLDNLITEGAPADLRNDFADPLATALH
CKVLGIPQEDGPKLFRSLSIAFMSSADPIPAAKINWDRDIEYMAGILENPNITTGLMGELSRLRKDPAYSHVS
DELFATIGVTFFGAGVISTGSFLTTALISLIQRPQLRNLLHEKPELIPAGVEELLRINLSFADGLPRLATADI
QVGDVLVRKGELVLVLLEGANFDPEHFPNPGSIELDRPNPTSHLAFGRGQHFCPGSALGRRHAQIGIEALLKK
MPGVDLAVPIDQLVWRTRFQRRIPERLPVLW
>CYP121A1_Mycobacterium tuberculosis MD15774 2630814339
MTATVLLEVPFSARGDRIPDAVAELRTREPIRKVRTITGAEAWLVSSYALCTQVLEDRRFSMKETAAAGAPRL
NALTVPPEVVNNMGNIADAGLRKAVMKAITPKAPGLEQFLRDTANSLLDNLITEGAPADLRNDFADPLATALH
CKVLGIPQEDGPKLFRSLSIAFMSSADPIPAAKINWDRDIEYMAGILENPNITTGLMGELSRLRKDPAYSHVS
DELFATIGVTFFGAGVISTGSFLTTALISLIQRPQLRNLLHEKPELIPAGVEELLRINLSFADGLPRLATADI
QVGDVLVRKGELVLVLLEGANFDPEHFPNPGSIELDRPNPTSHLAFGRGQHFCPGSALGRRHAQIGIEALLKK
MPGVDLAVPIDQLVWRTRFQRRIPERLPVLW
>CYP121A1_Mycobacterium tuberculosis MD15958 2636729638
MTATVLLEVPFSARGDRIPDAVAELRTREPIRKVRTITGAEAWLVSSYALCTQVLEDRRFSMKETAAAGAPRL
NALTVPPEVVNNMGNIADAGLRKAVMKAITPKAPGLEQFLRDTANSLLDNLITEGAPADLRNDFADPLATALH
CKVLGIPQEDGPKLFRSLSIAFMSSADPIPAAKINWDRDIEYMAGILENPNITTGLMGELSRLRKDPAYSHVS
DELFATIGVTFFGAGVISTGSFLTTALISLIQRPQLRNLLHEKPELIPAGVEELLRINLSFADGLPRLATADI
QVGDVLVRKGELVLVLLEGANFDPEHFPNPGSIELDRPNPTSHLAFGRGQHFCPGSALGRRHAQIGIEALLKK
MPGVDLAVPIDQLVWRTRFQRRIPERLPVLW
>CYP121A1_Mycobacterium tuberculosis MD15963 2622174351
MTATVLLEVPFSARGDRIPDAVAELRTREPIRKVRTITGAEAWLVSSYALCTQVLEDRRFSMKETAAAGAPRL
NALTVPPEVVNNMGNIADAGLRKAVMKAITPKAPGLEQFLRDTANSLLDNLITEGAPADLRNDFADPLATALH

CKVLGIPQEDGPKLFRSLSIAFMSSADPIPAAKINWDRDIEYMAGILENPNITTGLMGELSRLRKDPAYSHVS
DELFATIGVTFFGAGVISTGSFLTTALISLIQRPQLRNLLHEKPELIPAGVEELLRINLSFADGLPRLATADI
QVGDVLVRKGELVLVLLEGANFDPEHFPNPGSIELDRPNPTSHLAFGRGQHFCPGSALGRRHAQIGIEALLKK
MPGVDLAVPIDQLVWRTRFQRRIPERLPVLW
>CYP121A1_Mycobacterium tuberculosis MD15973 2622207616
MTATVLLEVPFSARGDRIPDAVAELRTREPIRKVRTITGAEAWLVSSYALCTQVLEDRRFSMKETAAAGAPRL
NALTVPPEVVNNMGNIADAGLRKAVMKAITPKAPGLEQFLRDTANSLLDNLITEGAPADLRNDFADPLATALH
CKVLGIPQEDGPKLFRSLSIAFMSSADPIPAAKINWDRDIEYMAGILENPNITTGLMGELSRLRKDPAYSHVS
DELFATIGVTFFGAGVISTGSFLTTALISLIQRPQLRNLLHEKPELIPAGVEELLRINLSFADGLPRLATADI
QVGDVLVRKGELVLVLLEGANFDPEHFPNPGSIELDRPNPTSHLAFGRGQHFCPGSALGRRHAQIGIEALLKK
MPGVDLAVPIDQLVWRTRFQRRIPERLPVLW
>CYP121A1_Mycobacterium tuberculosis MD15974 2577199334
MTATVLLEVPFSARGDRIPDAVAELRTREPIRKVRTITGAEAWLVSSYALCTQVLEDRRFSMKETAAAGAPRL
NALTVPPEVVNNMGNIADAGLRKAVMKAITPKAPGLEQFLRDTANSLLDNLITEGAPADLRNDFADPLATALH
CKVLGIPQEDGPKLFRSLSIAFMSSADPIPAAKINWDRDIEYMAGILENPNITTGLMGELSRLRKDPAYSHVS
DELFATIGVTFFGAGVISTGSFLTTALISLIQRPQLRNLLHEKPELIPAGVEELLRINLSFADGLPRLATADI
QVGDVLVRKGELVLVLLEGANFDPEHFPNPGSIELDRPNPTSHLAFGRGQHFCPGSALGRRHAQIGIEALLKK
MPGVDLAVPIDQLVWRTRFQRRIPERLPVLW
>CYP121A1_Mycobacterium tuberculosis MD15977 2574563131
MTATVLLEVPFSARGDRIPDAVAELRTREPIRKVRTITGAEAWLVSSYALCTQVLEDRRFSMKETAAAGAPRL
NALTVPPEVVNNMGNIADAGLRKAVMKAITPKAPGLEQFLRDTANSLLDNLITEGAPADLRNDFADPLATALH
CKVLGIPQEDGPKLFRSLSIAFMSSADPIPAAKINWDRDIEYMAGILENPNITTGLMGELSRLRKDPAYSHVS
DELFATIGVTFFGAGVISTGSFLTTALISLIQRPQLRNLLHEKPELIPAGVEELLRINLSFADGLPRLATADI
QVGDVLVRKGELVLVLLEGANFDPEHFPNPGSIELDRPNPTSHLAFGRGQHFCPGSALGRRHAQIGIEALLKK
MPGVDLAVPIDQLVWRTRFQRRIPERLPVLW
>CYP121A1_Mycobacterium tuberculosis MD16261 2622098033
MTATVLLEVPFSARGDRIPDAVAELRTREPIRKVRTITGAEAWLVSSYALCTQVLEDRRFSMKETAAAGAPRL
NALTVPPEVVNNMGNIADAGLRKAVMKAITPKAPGLEQFLRDTANSLLDNLITEGAPADLRNDFADPLATALH
CKVLGIPQEDGPKLFRSLSIAFMSSADPIPAAKINWDRDIEYMAGILENPNITTGLMGELSRLRKDPAYSHVS
DELFATIGVTFFGAGVISTGSFLTTALISLIQRPQLRNLLHEKPELIPAGVEELLRINLSFADGLPRLATADI
QVGDVLVRKGELVLVLLEGANFDPEHFPNPGSIELDRPNPTSHLAFGRGQHFCPGSALGRRHAQIGIEALLKK
MPGVDLAVPIDQLVWRTRFQRRIPERLPVLW
>CYP121A1_Mycobacterium tuberculosis MD16265 2577422996
MTATVLLEVPFSARGDRIPDAVAELRTREPIRKVRTITGAEAWLVSSYALCTQVLEDRRFSMKETAAAGAPRL
NALTVPPEVVNNMGNIADAGLRKAVMKAITPKAPGLEQFLRDTANSLLDNLITEGAPADLRNDFADPLATALH
CKVLGIPQEDGPKLFRSLSIAFMSSADPIPAAKINWDRDIEYMAGILENPNITTGLMGELSRLRKDPAYSHVS
DELFATIGVTFFGAGVISTGSFLTTALISLIQRPQLRNLLHEKPELIPAGVEELLRINLSFADGLPRLATADI
QVGDVLVRKGELVLVLLEGANFDPEHFPNPGSIELDRPNPTSHLAFGRGQHFCPGSALGRRHAQIGIEALLKK
MPGVDLAVPIDQLVWRTRFQRRIPERLPVLW
>CYP121A1_Mycobacterium tuberculosis MD16277 2576174034
MTATVLLEVPFSARGDRIPDAVAELRTREPIRKVRTITGAEAWLVSSYALCTQVLEDRRFSMKETAAAGAPRL
NALTVPPEVVNNMGNIADAGLRKAVMKAITPKAPGLEQFLRDTANSLLDNLITEGAPADLRNDFADPLATALH
CKVLGIPQEDGPKLFRSLSIAFMSSADPIPAAKINWDRDIEYMAGILENPNITTGLMGELSRLRKDPAYSHVS
DELFATIGVTFFGAGVISTGSFLTTALISLIQRPQLRNLLHEKPELIPAGVEELLRINLSFADGLPRLATADI
QVGDVLVRKGELVLVLLEGANFDPEHFPNPGSIELDRPNPTSHLAFGRGQHFCPGSALGRRHAQIGIEALLKK
MPGVDLAVPIDQLVWRTRFQRRIPERLPVLW
>CYP121A1_Mycobacterium tuberculosis MD16553 2574586129
MTATVLLEVPFSARGDRIPDAVAELRTREPIRKVRTITGAEAWLVSSYALCTQVLEDRRFSMKETAAAGAPRL
NALTVPPEVVNNMGNIADAGLRKAVMKAITPKAPGLEQFLRDTANSLLDNLITEGAPADLRNDFADPLATALH
CKVLGIPQEDGPKLFRSLSIAFMSSADPIPAAKINWDRDIEYMAGILENPNITTGLMGELSRLRKDPAYSHVS
DELFATIGVTFFGAGVISTGSFLTTALISLIQRPQLRNLLHEKPELIPAGVEELLRINLSFADGLPRLATADI
QVGDVLVRKGELVLVLLEGANFDPEHFPNPGSIELDRPNPTSHLAFGRGQHFCPGSALGRRHAQIGIEALLKK
MPGVDLAVPIDQLVWRTRFQRRIPERLPVLW
>CYP121A1_Mycobacterium tuberculosis MD16728 2577697930
MTATVLLEVPFSARGDRIPDAVAELRTREPIRKVRTITGAEAWLVSSYALCTQVLEDRRFSMKETAAAGAPRL
NALTVPPEVVNNMGNIADAGLRKAVMKAITPKAPGLEQFLRDTANSLLDNLITEGAPADLRNDFADPLATALH
CKVLGIPQEDGPKLFRSLSIAFMSSADPIPAAKINWDRDIEYMAGILENPNITTGLMGELSRLRKDPAYSHVS
DELFATIGVTFFGAGVISTGSFLTTALISLIQRPQLRNLLHEKPELIPAGVEELLRINLSFADGLPRLATADI

QVGDVLVRKGELVLVLLEGANFDPEHFPNPGSIELDRPNPTSHLAFGRGQHFCPGSALGRRHAQIGIEALLKK
MPGVDLAVPIDQLVWRTRFQRRIPERLPVLW
>CYP121A1_Mycobacterium tuberculosis MD16573 2622227001
MTATVLLEVPFSARGDRIPDAVAELRTREPIRKVRTITGAEAWLVSSYALCTQVLEDRRFSMKETAAAGAPRL
NALTVPPEVVNNMGNIADAGLRKAVMKAITPKAPGLEQFLRDTANSLLDNLITEGAPADLRNDFADPLATALH
CKVLGIPQEDGPKLFRSLSIAFMSSADPIPAAKINWDRDIEYMAGILENPNITTGLMGELSRLRKDPAYSHVS
DELFATIGVTFFGAGVISTGSFLTTALISLIQRPQLRNLLHEKPELIPAGVEELLRINLSFADGLPRLATADI
QVGDVLVRKGELVLVLLEGANFDPEHFPNPGSIELDRPNPTSHLAFGRGQHFCPGSALGRRHAQIGIEALLKK
MPGVDLAVPIDQLVWRTRFQRRIPERLPVLW
>CYP121A1_Mycobacterium tuberculosis MD16765 2622131951
MTATVLLEVPFSARGDRIPDAVAELRTREPIRKVRTITGAEAWLVSSYALCTQVLEDRRFSMKETAAAGAPRL
NALTVPPEVVNNMGNIADAGLRKAVMKAITPKAPGLEQFLRDTANSLLDNLITEGAPADLRNDFADPLATALH
CKVLGIPQEDGPKLFRSLSIAFMSSADPIPAAKINWDRDIEYMAGILENPNITTGLMGELSRLRKDPAYSHVS
DELFATIGVTFFGAGVISTGSFLTTALISLIQRPQLRNLLHEKPELIPAGVEELLRINLSFADGLPRLATADI
QVGDVLVRKGELVLVLLEGANFDPEHFPNPGSIELDRPNPTSHLAFGRGQHFCPGSALGRRHAQIGIEALLKK
MPGVDLAVPIDQLVWRTRFQRRIPERLPVLW
>CYP121A1_Mycobacterium tuberculosis MD16775 2575988578
MTATVLLEVPFSARGDRIPDAVAELRTREPIRKVRTITGAEAWLVSSYALCTQVLEDRRFSMKETAAAGAPRL
NALTVPPEVVNNMGNIADAGLRKAVMKAITPKAPGLEQFLRDTANSLLDNLITEGAPADLRNDFADPLATALH
CKVLGIPQEDGPKLFRSLSIAFMSSADPIPAAKINWDRDIEYMAGILENPNITTGLMGELSRLRKDPAYSHVS
DELFATIGVTFFGAGVISTGSFLTTALISLIQRPQLRNLLHEKPELIPAGVEELLRINLSFADGLPRLATADI
QVGDVLVRKGELVLVLLEGANFDPEHFPNPGSIELDRPNPTSHLAFGRGQHFCPGSALGRRHAQIGIEALLKK
MPGVDLAVPIDQLVWRTRFQRRIPERLPVLW
>CYP121A1_Mycobacterium tuberculosis MD17240 2622124728
MTATVLLEVPFSARGDRIPDAVAELRTREPIRKVRTITGAEAWLVSSYALCTQVLEDRRFSMKETAAAGAPRL
NALTVPPEVVNNMGNIADAGLRKAVMKAITPKAPGLEQFLRDTANSLLDNLITEGAPADLRNDFADPLATALH
CKVLGIPQEDGPKLFRSLSIAFMSSADPIPAAKINWDRDIEYMAGILENPNITTGLMGELSRLRKDPAYSHVS
DELFATIGVTFFGAGVISTGSFLTTALISLIQRPQLRNLLHEKPELIPAGVEELLRINLSFADGLPRLATADI
QVGDVLVRKGELVLVLLEGANFDPEHFPNPGSIELDRPNPTSHLAFGRGQHFCPGSALGRRHAQIGIEALLKK
MPGVDLAVPIDQLVWRTRFQRRIPERLPVLW
>CYP121A1_Mycobacterium tuberculosis MD17503 2622112033
MTATVLLEVPFSARGDRIPDAVAELRTREPIRKVRTITGAEAWLVSSYALCTQVLEDRRFSMKETAAAGAPRL
NALTVPPEVVNNMGNIADAGLRKAVMKAITPKAPGLEQFLRDTANSLLDNLITEGAPADLRNDFADPLATALH
CKVLGIPQEDGPKLFRSLSIAFMSSADPIPAAKINWDRDIEYMAGILENPNITTGLMGELSRLRKDPAYSHVS
DELFATIGVTFFGAGVISTGSFLTTALISLIQRPQLRNLLHEKPELIPAGVEELLRINLSFADGLPRLATADI
QVGDVLVRKGELVLVLLEGANFDPEHFPNPGSIELDRPNPTSHLAFGRGQHFCPGSALGRRHAQIGIEALLKK
MPGVDLAVPIDQLVWRTRFQRRIPERLPVLW
>CYP121A1_Mycobacterium tuberculosis MD17517 2577633845
MTATVLLEVPFSARGDRIPDAVAELRTREPIRKVRTITGAEAWLVSSYALCTQVLEDRRFSMKETAAAGAPRL
NALTVPPEVVNNMGNIADAGLRKAVMKAITPKAPGLEQFLRDTANSLLDNLITEGAPADLRNDFADPLATALH
CKVLGIPQEDGPKLFRSLSIAFMSSADPIPAAKINWDRDIEYMAGILENPNITTGLMGELSRLRKDPAYSHVS
DELFATIGVTFFGAGVISTGSFLTTALISLIQRPQLRNLLHEKPELIPAGVEELLRINLSFADGLPRLATADI
QVGDVLVRKGELVLVLLEGANFDPEHFPNPGSIELDRPNPTSHLAFGRGQHFCPGSALGRRHAQIGIEALLKK
MPGVDLAVPIDQLVWRTRFQRRIPERLPVLW
>CYP121A1_Mycobacterium tuberculosis MD17613 2622117375
MTATVLLEVPFSARGDRIPDAVAELRTREPIRKVRTITGAEAWLVSSYALCTQVLEDRRFSMKETAAAGAPRL
NALTVPPEVVNNMGNIADAGLRKAVMKAITPKAPGLEQFLRDTANSLLDNLITEGAPADLRNDFADPLATALH
CKVLGIPQEDGPKLFRSLSIAFMSSADPIPAAKINWDRDIEYMAGILENPNITTGLMGELSRLRKDPAYSHVS
DELFATIGVTFFGAGVISTGSFLTTALISLIQRPQLRNLLHEKPELIPAGVEELLRINLSFADGLPRLATADI
QVGDVLVRKGELVLVLLEGANFDPEHFPNPGSIELDRPNPTSHLAFGRGQHFCPGSALGRRHAQIGIEALLKK
MPGVDLAVPIDQLVWRTRFQRRIPERLPVLW
>CYP121A1_Mycobacterium tuberculosis MD17615 2584812285
MTATVLLEVPFSARGDRIPDAVAELRTREPIRKVRTITGAEAWLVSSYALCTQVLEDRRFSMKETAAAGAPRL
NALTVPPEVVNNMGNIADAGLRKAVMKAITPKAPGLEQFLRDTANSLLDNLITEGAPADLRNDFADPLATALH
CKVLGIPQEDGPKLFRSLSIAFMSSADPIPAAKINWDRDIEYMAGILENPNITTGLMGELSRLRKDPAYSHVS
DELFATIGVTFFGAGVISTGSFLTTALISLIQRPQLRNLLHEKPELIPAGVEELLRINLSFADGLPRLATADI
QVGDVLVRKGELVLVLLEGANFDPEHFPNPGSIELDRPNPTSHLAFGRGQHFCPGSALGRRHAQIGIEALLKK
MPGVDLAVPIDQLVWRTRFQRRIPERLPVLW

>CYP121A1_Mycobacterium tuberculosis MD17646 2649127951
MTATVLLEVPFSARGDRIPDAVAELRTREPIRKVRTITGAEAWLVSSYALCTQVLEDRRFSMKETAAAGAPRL
NALTVPPEVVNNMGNIADAGLRKAVMKAITPKAPGLEQFLRDTANSLLDNLITEGAPADLRNDFADPLATALH
CKVLGIPQEDGPKLFRSLSIAFMSSADPIPAAKINWDRDIEYMAGILENPNITTGLMGELSRLRKDPAYSHVS
DELFATIGVTFFGAGVISTGSFLTTALISLIQRPQLRNLLHEKPELIPAGVEELLRINLSFADGLPRLATADI
QVGDVLVRKGELVLVLLEGANFDPEHFPNPGSIELDRPNPTSHLAFGRGQHFCPGSALGRRHAQIGIEALLKK
MPGVDLAVPIDQLVWRTRFQRRIPERLPVLW
>CYP121A1_Mycobacterium tuberculosis MD17656 2622133669
MTATVLLEVPFSARGDRIPDAVAELRTREPIRKVRTITGAEAWLVSSYALCTQVLEDRRFSMKETAAAGAPRL
NALTVPPEVVNNMGNIADAGLRKAVMKAITPKAPGLEQFLRDTANSLLDNLITEGAPADLRNDFADPLATALH
CKVLGIPQEDGPKLFRSLSIAFMSSADPIPAAKINWDRDIEYMAGILENPNITTGLMGELSRLRKDPAYSHVS
DELFATIGVTFFGAGVISTGSFLTTALISLIQRPQLRNLLHEKPELIPAGVEELLRINLSFADGLPRLATADI
QVGDVLVRKGELVLVLLEGANFDPEHFPNPGSIELDRPNPTSHLAFGRGQHFCPGSALGRRHAQIGIEALLKK
MPGVDLAVPIDQLVWRTRFQRRIPERLPVLW
>CYP121A1_Mycobacterium tuberculosis MD17888 2575084949
MTATVLLEVPFSARGDRIPDAVAELRTREPIRKVRTITGAEAWLVSSYALCTQVLEDRRFSMKETAAAGAPRL
NALTVPPEVVNNMGNIADAGLRKAVMKAITPKAPGLEQFLRDTANSLLDNLITEGAPADLRNDFADPLATALH
CKVLGIPQEDGPKLFRSLSIAFMSSADPIPAAKINWDRDIEYMAGILENPNITTGLMGELSRLRKDPAYSHVS
DELFATIGVTFFGAGVISTGSFLTTALISLIQRPQLRNLLHEKPELIPAGVEELLRINLSFADGLPRLATADI
QVGDVLVRKGELVLVLLEGANFDPEHFPNPGSIELDRPNPTSHLAFGRGQHFCPGSALGRRHAQIGIEALLKK
MPGVDLAVPIDQLVWRTRFQRRIPERLPVLW
>CYP121A1_Mycobacterium tuberculosis MD17902 2575139708
MTATVLLEVPFSARGDRIPDAVAELRTREPIRKVRTITGAEAWLVSSYALCTQVLEDRRFSMKETAAAGAPRL
NALTVPPEVVNNMGNIADAGLRKAVMKAITPKAPGLEQFLRDTANSLLDNLITEGAPADLRNDFADPLATALH
CKVLGIPQEDGPKLFRSLSIAFMSSADPIPAAKINWDRDIEYMAGILENPNITTGLMGELSRLRKDPAYSHVS
DELFATIGVTFFGAGVISTGSFLTTALISLIQRPQLRNLLHEKPELIPAGVEELLRINLSFADGLPRLATADI
QVGDVLVRKGELVLVLLEGANFDPEHFPNPGSIELDRPNPTSHLAFGRGQHFCPGSALGRRHAQIGIEALLKK
MPGVDLAVPIDQLVWRTRFQRRIPERLPVLW
>CYP121A1_Mycobacterium tuberculosis MD17903 2622075654
MTATVLLEVPFSARGDRIPDAVAELRTREPIRKVRTITGAEAWLVSSYALCTQVLEDRRFSMKETAAAGAPRL
NALTVPPEVVNNMGNIADAGLRKAVMKAITPKAPGLEQFLRDTANSLLDNLITEGAPADLRNDFADPLATALH
CKVLGIPQEDGPKLFRSLSIAFMSSADPIPAAKINWDRDIEYMAGILENPNITTGLMGELSRLRKDPAYSHVS
DELFATIGVTFFGAGVISTGSFLTTALISLIQRPQLRNLLHEKPELIPAGVEELLRINLSFADGLPRLATADI
QVGDVLVRKGELVLVLLEGANFDPEHFPNPGSIELDRPNPTSHLAFGRGQHFCPGSALGRRHAQIGIEALLKK
MPGVDLAVPIDQLVWRTRFQRRIPERLPVLW
>CYP121A1_Mycobacterium tuberculosis MD17904 2622006816
MTATVLLEVPFSARGDRIPDAVAELRTREPIRKVRTITGAEAWLVSSYALCTQVLEDRRFSMKETAAAGAPRL
NALTVPPEVVNNMGNIADAGLRKAVMKAITPKAPGLEQFLRDTANSLLDNLITEGAPADLRNDFADPLATALH
CKVLGIPQEDGPKLFRSLSIAFMSSADPIPAAKINWDRDIEYMAGILENPNITTGLMGELSRLRKDPAYSHVS
DELFATIGVTFFGAGVISTGSFLTTALISLIQRPQLRNLLHEKPELIPAGVEELLRINLSFADGLPRLATADI
QVGDVLVRKGELVLVLLEGANFDPEHFPNPGSIELDRPNPTSHLAFGRGQHFCPGSALGRRHAQIGIEALLKK
MPGVDLAVPIDQLVWRTRFQRRIPERLPVLW
>CYP121A1_Mycobacterium tuberculosis MD18090 2622089862
MTATVLLEVPFSARGDRIPDAVAELRTREPIRKVRTITGAEAWLVSSYALCTQVLEDRRFSMKETAAAGAPRL
NALTVPPEVVNNMGNIADAGLRKAVMKAITPKAPGLEQFLRDTANSLLDNLITEGAPADLRNDFADPLATALH
CKVLGIPQEDGPKLFRSLSIAFMSSADPIPAAKINWDRDIEYMAGILENPNITTGLMGELSRLRKDPAYSHVS
DELFATIGVTFFGAGVISTGSFLTTALISLIQRPQLRNLLHEKPELIPAGVEELLRINLSFADGLPRLATADI
QVGDVLVRKGELVLVLLEGANFDPEHFPNPGSIELDRPNPTSHLAFGRGQHFCPGSALGRRHAQIGIEALLKK
MPGVDLAVPIDQLVWRTRFQRRIPERLPVLW
>CYP121A1_Mycobacterium tuberculosis MD18112 2622072884
MTATVLLEVPFSARGDRIPDAVAELRTREPIRKVRTITGAEAWLVSSYALCTQVLEDRRFSMKETAAAGAPRL
NALTVPPEVVNNMGNIADAGLRKAVMKAITPKAPGLEQFLRDTANSLLDNLITEGAPADLRNDFADPLATALH
CKVLGIPQEDGPKLFRSLSIAFMSSADPIPAAKINWDRDIEYMAGILENPNITTGLMGELSRLRKDPAYSHVS
DELFATIGVTFFGAGVISTGSFLTTALISLIQRPQLRNLLHEKPELIPAGVEELLRINLSFADGLPRLATADI
QVGDVLVRKGELVLVLLEGANFDPEHFPNPGSIELDRPNPTSHLAFGRGQHFCPGSALGRRHAQIGIEALLKK
MPGVDLAVPIDQLVWRTRFQRRIPERLPVLW
>CYP121A1_Mycobacterium tuberculosis MD18116 2622087107

MTATVLLEVPFSARGDRIPDAVAELRTREPIRKVRTITGAEAWLVSSYALCTQVLEDRRFSMKETAAAGAPRL
NALTVPPEVVNNMGNIADAGLRKAVMKAITPKAPGLEQFLRDTANSLLDNLITEGAPADLRNDFADPLATALH
CKVLGIPQEDGPKLFRSLSIAFMSSADPIPAAKINWDRDIEYMAGILENPNITTGLMGELSRLRKDPAYSHVS
DELFATIGVTFFGAGVISTGSFLTTALISLIQRPQLRNLLHEKPELIPAGVEELLRINLSFADGLPRLATADI
QVGDVLVRKGELVLVLLEGANFDPEHFPNPGSIELDRPNPTSHLAFGRGQHFCPGSALGRRHAQIGIEALLKK
MPGVDLAVPIDQLVWRTRFQRRIPERLPVLW
>CYP121A1_Mycobacterium tuberculosis MD18314 2622092505
MTATVLLEVPFSARGDRIPDAVAELRTREPIRKVRTITGAEAWLVSSYALCTQVLEDRRFSMKETAAAGAPRL
NALTVPPEVVNNMGNIADAGLRKAVMKAITPKAPGLEQFLRDTANSLLDNLITEGAPADLRNDFADPLATALH
CKVLGIPQEDGPKLFRSLSIAFMSSADPIPAAKINWDRDIEYMAGILENPNITTGLMGELSRLRKDPAYSHVS
DELFATIGVTFFGAGVISTGSFLTTALISLIQRPQLRNLLHEKPELIPAGVEELLRINLSFADGLPRLATADI
QVGDVLVRKGELVLVLLEGANFDPEHFPNPGSIELDRPNPTSHLAFGRGQHFCPGSALGRRHAQIGIEALLKK
MPGVDLAVPIDQLVWRTRFQRRIPERLPVLW
>CYP121A1_Mycobacterium tuberculosis MD18320 2622009601
MTATVLLEVPFSARGDRIPDAVAELRTREPIRKVRTITGAEAWLVSSYALCTQVLEDRRFSMKETAAAGAPRL
NALTVPPEVVNNMGNIADAGLRKAVMKAITPKAPGLEQFLRDTANSLLDNLITEGAPADLRNDFADPLATALH
CKVLGIPQEDGPKLFRSLSIAFMSSADPIPAAKINWDRDIEYMAGILENPNITTGLMGELSRLRKDPAYSHVS
DELFATIGVTFFGAGVISTGSFLTTALISLIQRPQLRNLLHEKPELIPAGVEELLRINLSFADGLPRLATADI
QVGDVLVRKGELVLVLLEGANFDPEHFPNPGSIELDRPNPTSHLAFGRGQHFCPGSALGRRHAQIGIEALLKK
MPGVDLAVPIDQLVWRTRFQRRIPERLPVLW
>CYP121A1_Mycobacterium tuberculosis MD18472 2622052514
MTATVLLEVPFSARGDRIPDAVAELRTREPIRKVRTITGAEAWLVSSYALCTQVLEDRRFSMKETAAAGAPRL
NALTVPPEVVNNMGNIADAGLRKAVMKAITPKAPGLEQFLRDTANSLLDNLITEGAPADLRNDFADPLATALH
CKVLGIPQEDGPKLFRSLSIAFMSSADPIPAAKINWDRDIEYMAGILENPNITTGLMGELSRLRKDPAYSHVS
DELFATIGVTFFGAGVISTGSFLTTALISLIQRPQLRNLLHEKPELIPAGVEELLRINLSFADGLPRLATADI
QVGDVLVRKGELVLVLLEGANFDPEHFPNPGSIELDRPNPTSHLAFGRGQHFCPGSALGRRHAQIGIEALLKK
MPGVDLAVPIDQLVWRTRFQRRIPERLPVLW
>CYP121A1_Mycobacterium tuberculosis MD18478 2577879840
MTATVLLEVPFSARGDRIPDAVAELRTREPIRKVRTITGAEAWLVSSYALCTQVLEDRRFSMKETAAAGAPRL
NALTVPPEVVNNMGNIADAGLRKAVMKAITPKAPGLEQFLRDTANSLLDNLITEGAPADLRNDFADPLATALH
CKVLGIPQEDGPKLFRSLSIAFMSSADPIPAAKINWDRDIEYMAGILENPNITTGLMGELSRLRKDPAYSHVS
DELFATIGVTFFGAGVISTGSFLTTALISLIQRPQLRNLLHEKPELIPAGVEELLRINLSFADGLPRLATADI
QVGDVLVRKGELVLVLLEGANFDPEHFPNPGSIELDRPNPTSHLAFGRGQHFCPGSALGRRHAQIGIEALLKK
MPGVDLAVPIDQLVWRTRFQRRIPERLPVLW
>CYP121A1_Mycobacterium tuberculosis MD18486 2622013777
MTATVLLEVPFSARGDRIPDAVAELRTREPIRKVRTITGAEAWLVSSYALCTQVLEDRRFSMKETAAAGAPRL
NALTVPPEVVNNMGNIADAGLRKAVMKAITPKAPGLEQFLRDTANSLLDNLITEGAPADLRNDFADPLATALH
CKVLGIPQEDGPKLFRSLSIAFMSSADPIPAAKINWDRDIEYMAGILENPNITTGLMGELSRLRKDPAYSHVS
DELFATIGVTFFGAGVISTGSFLTTALISLIQRPQLRNLLHEKPELIPAGVEELLRINLSFADGLPRLATADI
QVGDVLVRKGELVLVLLEGANFDPEHFPNPGSIELDRPNPTSHLAFGRGQHFCPGSALGRRHAQIGIEALLKK
MPGVDLAVPIDQLVWRTRFQRRIPERLPVLW
>CYP121A1_Mycobacterium tuberculosis MD18498 2574987499
MTATVLLEVPFSARGDRIPDAVAELRTREPIRKVRTITGAEAWLVSSYALCTQVLEDRRFSMKETAAAGAPRL
NALTVPPEVVNNMGNIADAGLRKAVMKAITPKAPGLEQFLRDTANSLLDNLITEGAPADLRNDFADPLATALH
CKVLGIPQEDGPKLFRSLSIAFMSSADPIPAAKINWDRDIEYMAGILENPNITTGLMGELSRLRKDPAYSHVS
DELFATIGVTFFGAGVISTGSFLTTALISLIQRPQLRNLLHEKPELIPAGVEELLRINLSFADGLPRLATADI
QVGDVLVRKGELVLVLLEGANFDPEHFPNPGSIELDRPNPTSHLAFGRGQHFCPGSALGRRHAQIGIEALLKK
MPGVDLAVPIDQLVWRTRFQRRIPERLPVLW
>CYP121A1_Mycobacterium tuberculosis MD19051 2622059954
MTATVLLEVPFSARGDRIPDAVAELRTREPIRKVRTITGAEAWLVSSYALCTQVLEDRRFSMKETAAAGAPRL
NALTVPPEVVNNMGNIADAGLRKAVMKAITPKAPGLEQFLRDTANSLLDNLITEGAPADLRNDFADPLATALH
CKVLGIPQEDGPKLFRSLSIAFMSSADPIPAAKINWDRDIEYMAGILENPNITTGLMGELSRLRKDPAYSHVS
DELFATIGVTFFGAGVISTGSFLTTALISLIQRPQLRNLLHEKPELIPAGVEELLRINLSFADGLPRLATADI
QVGDVLVRKGELVLVLLEGANFDPEHFPNPGSIELDRPNPTSHLAFGRGQHFCPGSALGRRHAQIGIEALLKK
MPGVDLAVPIDQLVWRTRFQRRIPERLPVLW
>CYP121A1_Mycobacterium tuberculosis MD19339 2622043548
MTATVLLEVPFSARGDRIPDAVAELRTREPIRKVRTITGAEAWLVSSYALCTQVLEDRRFSMKETAAAGAPRL
NALTVPPEVVNNMGNIADAGLRKAVMKAITPKAPGLEQFLRDTANSLLDNLITEGAPADLRNDFADPLATALH

CKVLGIPQEDGPKLFRSLSIAFMSSADPIPAAKINWDRDIEYMAGILENPNITTGLMGELSRLRKDPAYSHVS
DELFATIGVTFFGAGVISTGSFLTTALISLIQRPQLRNLLHEKPELIPAGVEELLRINLSFADGLPRLATADI
QVGDVLVRKGELVLVLLEGANFDPEHFPNPGSIELDRPNPTSHLAFGRGQHFCPGSALGRRHAQIGIEALLKK
MPGVDLAVPIDQLVWRTRFQRRIPERLPVLW
>CYP121A1_Mycobacterium tuberculosis MD19591 2622049217
MTATVLLEVPFSARGDRIPDAVAELRTREPIRKVRTITGAEAWLVSSYALCTQVLEDRRFSMKETAAAGAPRL
NALTVPPEVVNNMGNIADAGLRKAVMKAITPKAPGLEQFLRDTANSLLDNLITEGAPADLRNDFADPLATALH
CKVLGIPQEDGPKLFRSLSIAFMSSADPIPAAKINWDRDIEYMAGILENPNITTGLMGELSRLRKDPAYSHVS
DELFATIGVTFFGAGVISTGSFLTTALISLIQRPQLRNLLHEKPELIPAGVEELLRINLSFADGLPRLATADI
QVGDVLVRKGELVLVLLEGANFDPEHFPNPGSIELDRPNPTSHLAFGRGQHFCPGSALGRRHAQIGIEALLKK
MPGVDLAVPIDQLVWRTRFQRRIPERLPVLW
>CYP121A1_Mycobacterium tuberculosis MD19693 2622021873
MTATVLLEVPFSARGDRIPDAVAELRTREPIRKVRTITGAEAWLVSSYALCTQVLEDRRFSMKETAAAGAPRL
NALTVPPEVVNNMGNIADAGLRKAVMKAITPKAPGLEQFLRDTANSLLDNLITEGAPADLRNDFADPLATALH
CKVLGIPQEDGPKLFRSLSIAFMSSADPIPAAKINWDRDIEYMAGILENPNITTGLMGELSRLRKDPAYSHVS
DELFATIGVTFFGAGVISTGSFLTTALISLIQRPQLRNLLHEKPELIPAGVEELLRINLSFADGLPRLATADI
QVGDVLVRKGELVLVLLEGANFDPEHFPNPGSIELDRPNPTSHLAFGRGQHFCPGSALGRRHAQIGIEALLKK
MPGVDLAVPIDQLVWRTRFQRRIPERLPVLW
>CYP121A1_Mycobacterium tuberculosis MD20344 2577509344
MTATVLLEVPFSARGDRIPDAVAELRTREPIRKVRTITGAEAWLVSSYALCTQVLEDRRFSMKETAAAGAPRL
NALTVPPEVVNNMGNIADAGLRKAVMKAITPKAPGLEQFLRDTANSLLDNLITEGAPADLRNDFADPLATALH
CKVLGIPQEDGPKLFRSLSIAFMSSADPIPAAKINWDRDIEYMAGILENPNITTGLMGELSRLRKDPAYSHVS
DELFATIGVTFFGAGVISTGSFLTTALISLIQRPQLRNLLHEKPELIPAGVEELLRINLSFADGLPRLATADI
QVGDVLVRKGELVLVLLEGANFDPEHFPNPGSIELDRPNPTSHLAFGRGQHFCPGSALGRRHAQIGIEALLKK
MPGVDLAVPIDQLVWRTRFQRRIPERLPVLW
>CYP121A1_Mycobacterium tuberculosis MD20845 2622038486
MTATVLLEVPFSARGDRIPDAVAELRTREPIRKVRTITGAEAWLVSSYALCTQVLEDRRFSMKETAAAGAPRL
NALTVPPEVVNNMGNIADAGLRKAVMKAITPKAPGLEQFLRDTANSLLDNLITEGAPADLRNDFADPLATALH
CKVLGIPQEDGPKLFRSLSIAFMSSADPIPAAKINWDRDIEYMAGILENPNITTGLMGELSRLRKDPAYSHVS
DELFATIGVTFFGAGVISTGSFLTTALISLIQRPQLRNLLHEKPELIPAGVEELLRINLSFADGLPRLATADI
QVGDVLVRKGELVLVLLEGANFDPEHFPNPGSIELDRPNPTSHLAFGRGQHFCPGSALGRRHAQIGIEALLKK
MPGVDLAVPIDQLVWRTRFQRRIPERLPVLW
>CYP121A1_Mycobacterium tuberculosis MT433 2646890050
MTATVLLEVPFSARGDRIPDAVAELRTREPIRKVRTITGAEAWLVSSYALCTQVLEDRRFSMKETAAAGAPRL
NALTVPPEVVNNMGNIADAGLRKAVMKAITPKAPGLEQFLRDTANSLLDNLITEGAPADLRNDFADPLATALH
CKVLGIPQEDGPKLFRSLSIAFMSSADPIPAAKINWDRDIEYMAGILENPNITTGLMGELSRLRKDPAYSHVS
DELFATIGVTFFGAGVISTGSFLTTALISLIQRPQLRNLLHEKPELIPAGVEELLRINLSFADGLPRLATADI
QVGDVLVRKGELVLVLLEGANFDPEHFPNPGSIELDRPNPTSHLAFGRGQHFCPGSALGRRHAQIGIEALLKK
MPGVDLAVPIDQLVWRTRFQRRIPERLPVLW
>CYP121A1_Mycobacterium tuberculosis MTB-476 2573573508
MTATVLLEVPFSARGDRIPDAVAELRTREPIRKVRTITGAEAWLVSSYALCTQVLEDRRFSMKETAAAGAPRL
NALTVPPEVVNNMGNIADAGLRKAVMKAITPKAPGLEQFLRDTANSLLDNLITEGAPADLRNDFADPLATALH
CKVLGIPQEDGPKLFRSLSIAFMSSADPIPAAKINWDRDIEYMAGILENPNITTGLMGELSRLRKDPAYSHVS
DELFATIGVTFFGAGVISTGSFLTTALISLIQRPQLRNLLHEKPELIPAGVEELLRINLSFADGLPRLATADI
QVGDVLVRKGELVLVLLEGANFDPEHFPNPGSIELDRPNPTSHLAFGRGQHFCPGSALGRRHAQIGIEALLKK
MPGVDLAVPIDQLVWRTRFQRRIPERLPVLW
>CYP121A1_Mycobacterium tuberculosis MTB-489 2621356222
MTATVLLEVPFSARGDRIPDAVAELRTREPIRKVRTITGAEAWLVSSYALCTQVLEDRRFSMKETAAAGAPRL
NALTVPPEVVNNMGNIADAGLRKAVMKAITPKAPGLEQFLRDTANSLLDNLITEGAPADLRNDFADPLATALH
CKVLGIPQEDGPKLFRSLSIAFMSSADPIPAAKINWDRDIEYMAGILENPNITTGLMGELSRLRKDPAYSHVS
DELFATIGVTFFGAGVISTGSFLTTALISLIQRPQLRNLLHEKPELIPAGVEELLRINLSFADGLPRLATADI
QVGDVLVRKGELVLVLLEGANFDPEHFPNPGSIELDRPNPTSHLAFGRGQHFCPGSALGRRHAQIGIEALLKK
MPGVDLAVPIDQLVWRTRFQRRIPERLPVLW
>CYP121A1_Mycobacterium tuberculosis MTBR1/09 2667026756
MTATVLLEVPFSARGDRIPDAVAELRTREPIRKVRTITGAEAWLVSSYALCTQVLEDRRFSMKETAAAGAPRL
NALTVPPEVVNNMGNIADAGLRKAVMKAITPKAPGLEQFLRDTANSLLDNLITEGAPADLRNDFADPLATALH
CKVLGIPQEDGPKLFRSLSIAFMSSADPIPAAKINWDRDIEYMAGILENPNITTGLMGELSRLRKDPAYSHVS
DELFATIGVTFFGAGVISTGSFLTTALISLIQRPQLRNLLHEKPELIPAGVEELLRINLSFADGLPRLATADI

QVGDVLVRKGELVLVLLEGANFDPEHFPNPGSIELDRPNPTSHLAFGRGQHFCPGSALGRRHAQIGIEALLKK
MPGVDLAVPIDQLVWRTRFQRRIPERLPVLW
>CYP121A1_Mycobacterium tuberculosis MtURU-002 2630828065
MTATVLLEVPFSARGDRIPDAVAELRTREPIRKVRTITGAEAWLVSSYALCTQVLEDRRFSMKETAAAGAPRL
NALTVPPEVVNNMGNIADAGLRKAVMKAITPKAPGLEQFLRDTANSLLDNLITEGAPADLRNDFADPLATALH
CKVLGIPQEDGPKLFRSLSIAFMSSADPIPAAKINWDRDIEYMAGILENPNITTGLMGELSRLRKDPAYSHVS
DELFATIGVTFFGAGVISTGSFLTTALISLIQRPQLRNLLHEKPELIPAGVEELLRINLSFADGLPRLATADI
QVGDVLVRKGELVLVLLEGANFDPEHFPNPGSIELDRPNPTSHLAFGRGQHFCPGSALGRRHAQIGIEALLKK
MPGVDLAVPIDQLVWRTRFQRRIPERLPVLW
>CYP121A1_Mycobacterium tuberculosis N00000064 2699010850
MTATVLLEVPFSARGDRIPDAVAELRTREPIRKVRTITGAEAWLVSSYALCTQVLEDRRFSMKETAAAGAPRL
NALTVPPEVVNNMGNIADAGLRKAVMKAITPKAPGLEQFLRDTANSLLDNLITEGAPADLRNDFADPLATALH
CKVLGIPQEDGPKLFRSLSIAFMSSADPIPAAKINWDRDIEYMAGILENPNITTGLMGELSRLRKDPAYSHVS
DELFATIGVTFFGAGVISTGSFLTTALISLIQRPQLRNLLHEKPELIPAGVEELLRINLSFADGLPRLATADI
QVGDVLVRKGELVLVLLEGANFDPEHFPNPGSIELDRPNPTSHLAFGRGQHFCPGSALGRRHAQIGIEALLKK
MPGVDLAVPIDQLVWRTRFQRRIPERLPVLW
>CYP121A1_Mycobacterium tuberculosis N00400624 2703602178
MTATVLLEVPFSARGDRIPDAVAELRTREPIRKVRTITGAEAWLVSSYALCTQVLEDRRFSMKETAAAGAPRL
NALTVPPEVVNNMGNIADAGLRKAVMKAITPKAPGLEQFLRDTANSLLDNLITEGAPADLRNDFADPLATALH
CKVLGIPQEDGPKLFRSLSIAFMSSADPIPAAKINWDRDIEYMAGILENPNITTGLMGELSRLRKDPAYSHVS
DELFATIGVTFFGAGVISTGSFLTTALISLIQRPQLRNLLHEKPELIPAGVEELLRINLSFADGLPRLATADI
QVGDVLVRKGELVLVLLEGANFDPEHFPNPGSIELDRPNPTSHLAFGRGQHFCPGSALGRRHAQIGIEALLKK
MPGVDLAVPIDQLVWRTRFQRRIPERLPVLW
>CYP121A1_Mycobacterium tuberculosis N00400960 2698902129
MTATVLLEVPFSARGDRIPDAVAELRTREPIRKVRTITGAEAWLVSSYALCTQVLEDRRFSMKETAAAGAPRL
NALTVPPEVVNNMGNIADAGLRKAVMKAITPKAPGLEQFLRDTANSLLDNLITEGAPADLRNDFADPLATALH
CKVLGIPQEDGPKLFRSLSIAFMSSADPIPAAKINWDRDIEYMAGILENPNITTGLMGELSRLRKDPAYSHVS
DELFATIGVTFFGAGVISTGSFLTTALISLIQRPQLRNLLHEKPELIPAGVEELLRINLSFADGLPRLATADI
QVGDVLVRKGELVLVLLEGANFDPEHFPNPGSIELDRPNPTSHLAFGRGQHFCPGSALGRRHAQIGIEALLKK
MPGVDLAVPIDQLVWRTRFQRRIPERLPVLW
>CYP121A1_Mycobacterium tuberculosis N09900612 2699155851
MTATVLLEVPFSARGDRIPDAVAELRTREPIRKVRTITGAEAWLVSSYALCTQVLEDRRFSMKETAAAGAPRL
NALTVPPEVVNNMGNIADAGLRKAVMKAITPKAPGLEQFLRDTANSLLDNLITEGAPADLRNDFADPLATALH
CKVLGIPQEDGPKLFRSLSIAFMSSADPIPAAKINWDRDIEYMAGILENPNITTGLMGELSRLRKDPAYSHVS
DELFATIGVTFFGAGVISTGSFLTTALISLIQRPQLRNLLHEKPELIPAGVEELLRINLSFADGLPRLATADI
QVGDVLVRKGELVLVLLEGANFDPEHFPNPGSIELDRPNPTSHLAFGRGQHFCPGSALGRRHAQIGIEALLKK
MPGVDLAVPIDQLVWRTRFQRRIPERLPVLW
>CYP121A1_Mycobacterium tuberculosis N09900855 2695857904
MTATVLLEVPFSARGDRIPDAVAELRTREPIRKVRTITGAEAWLVSSYALCTQVLEDRRFSMKETAAAGAPRL
NALTVPPEVVNNMGNIADAGLRKAVMKAITPKAPGLEQFLRDTANSLLDNLITEGAPADLRNDFADPLATALH
CKVLGIPQEDGPKLFRSLSIAFMSSADPIPAAKINWDRDIEYMAGILENPNITTGLMGELSRLRKDPAYSHVS
DELFATIGVTFFGAGVISTGSFLTTALISLIQRPQLRNLLHEKPELIPAGVEELLRINLSFADGLPRLATADI
QVGDVLVRKGELVLVLLEGANFDPEHFPNPGSIELDRPNPTSHLAFGRGQHFCPGSALGRRHAQIGIEALLKK
MPGVDLAVPIDQLVWRTRFQRRIPERLPVLW
>CYP121A1_Mycobacterium tuberculosis N09901607 2703610633
MTATVLLEVPFSARGDRIPDAVAELRTREPIRKVRTITGAEAWLVSSYALCTQVLEDRRFSMKETAAAGAPRL
NALTVPPEVVNNMGNIADAGLRKAVMKAITPKAPGLEQFLRDTANSLLDNLITEGAPADLRNDFADPLATALH
CKVLGIPQEDGPKLFRSLSIAFMSSADPIPAAKINWDRDIEYMAGILENPNITTGLMGELSRLRKDPAYSHVS
DELFATIGVTFFGAGVISTGSFLTTALISLIQRPQLRNLLHEKPELIPAGVEELLRINLSFADGLPRLATADI
QVGDVLVRKGELVLVLLEGANFDPEHFPNPGSIELDRPNPTSHLAFGRGQHFCPGSALGRRHAQIGIEALLKK
MPGVDLAVPIDQLVWRTRFQRRIPERLPVLW
>CYP121A1_Mycobacterium tuberculosis N09901829 2706575972
MTATVLLEVPFSARGDRIPDAVAELRTREPIRKVRTITGAEAWLVSSYALCTQVLEDRRFSMKETAAAGAPRL
NALTVPPEVVNNMGNIADAGLRKAVMKAITPKAPGLEQFLRDTANSLLDNLITEGAPADLRNDFADPLATALH
CKVLGIPQEDGPKLFRSLSIAFMSSADPIPAAKINWDRDIEYMAGILENPNITTGLMGELSRLRKDPAYSHVS
DELFATIGVTFFGAGVISTGSFLTTALISLIQRPQLRNLLHEKPELIPAGVEELLRINLSFADGLPRLATADI
QVGDVLVRKGELVLVLLEGANFDPEHFPNPGSIELDRPNPTSHLAFGRGQHFCPGSALGRRHAQIGIEALLKK
MPGVDLAVPIDQLVWRTRFQRRIPERLPVLW

>CYP121A1_Mycobacterium tuberculosis NA-A0008 2551858110
MTATVLLEVPFSARGDRIPDAVAELRTREPIRKVRTITGAEAWLVSSYALCTQVLEDRRFSMKETAAAGAPRL
NALTVPPEVVNNMGNIADAGLRKAVMKAITPKAPGLEQFLRDTANSLLDNLITEGAPADLRNDFADPLATALH
CKVLGIPQEDGPKLFRSLSIAFMSSADPIPAAKINWDRDIEYMAGILENPNITTGLMGELSRLRKDPAYSHVS
DELFATIGVTFFGAGVISTGSFLTTALISLIQRPQLRNLLHEKPELIPAGVEELLRINLSFADGLPRLATADI
QVGDVLVRKGELVLVLLEGANFDPEHFPNPGSIELDRPNPTSHLAFGRGQHFCPGSALGRRHAQIGIEALLKK
MPGVDLAVPIDQLVWRTRFQRRIPERLPVLW
>CYP121A1_Mycobacterium tuberculosis NA-A0009 2551812899
MTATVLLEVPFSARGDRIPDAVAELRTREPIRKVRTITGAEAWLVSSYALCTQVLEDRRFSMKETAAAGAPRL
NALTVPPEVVNNMGNIADAGLRKAVMKAITPKAPGLEQFLRDTANSLLDNLITEGAPADLRNDFADPLATALH
CKVLGIPQEDGPKLFRSLSIAFMSSADPIPAAKINWDRDIEYMAGILENPNITTGLMGELSRLRKDPAYSHVS
DELFATIGVTFFGAGVISTGSFLTTALISLIQRPQLRNLLHEKPELIPAGVEELLRINLSFADGLPRLATADI
QVGDVLVRKGELVLVLLEGANFDPEHFPNPGSIELDRPNPTSHLAFGRGQHFCPGSALGRRHAQIGIEALLKK
MPGVDLAVPIDQLVWRTRFQRRIPERLPVLW
>CYP121A1_Mycobacterium tuberculosis NRITLD14 2577922942
MTATVLLEVPFSARGDRIPDAVAELRTREPIRKVRTITGAEAWLVSSYALCTQVLEDRRFSMKETAAAGAPRL
NALTVPPEVVNNMGNIADAGLRKAVMKAITPKAPGLEQFLRDTANSLLDNLITEGAPADLRNDFADPLATALH
CKVLGIPQEDGPKLFRSLSIAFMSSADPIPAAKINWDRDIEYMAGILENPNITTGLMGELSRLRKDPAYSHVS
DELFATIGVTFFGAGVISTGSFLTTALISLIQRPQLRNLLHEKPELIPAGVEELLRINLSFADGLPRLATADI
QVGDVLVRKGELVLVLLEGANFDPEHFPNPGSIELDRPNPTSHLAFGRGQHFCPGSALGRRHAQIGIEALLKK
MPGVDLAVPIDQLVWRTRFQRRIPERLPVLW
>CYP121A1_Mycobacterium tuberculosis NRITLD17 2633873266
MTATVLLEVPFSARGDRIPDAVAELRTREPIRKVRTITGAEAWLVSSYALCTQVLEDRRFSMKETAAAGAPRL
NALTVPPEVVNNMGNIADAGLRKAVMKAITPKAPGLEQFLRDTANSLLDNLITEGAPADLRNDFADPLATALH
CKVLGIPQEDGPKLFRSLSIAFMSSADPIPAAKINWDRDIEYMAGILENPNITTGLMGELSRLRKDPAYSHVS
DELFATIGVTFFGAGVISTGSFLTTALISLIQRPQLRNLLHEKPELIPAGVEELLRINLSFADGLPRLATADI
QVGDVLVRKGELVLVLLEGANFDPEHFPNPGSIELDRPNPTSHLAFGRGQHFCPGSALGRRHAQIGIEALLKK
MPGVDLAVPIDQLVWRTRFQRRIPERLPVLW
>CYP121A1_Mycobacterium tuberculosis NRITLD20 2635662989
MTATVLLEVPFSARGDRIPDAVAELRTREPIRKVRTITGAEAWLVSSYALCTQVLEDRRFSMKETAAAGAPRL
NALTVPPEVVNNMGNIADAGLRKAVMKAITPKAPGLEQFLRDTANSLLDNLITEGAPADLRNDFADPLATALH
CKVLGIPQEDGPKLFRSLSIAFMSSADPIPAAKINWDRDIEYMAGILENPNITTGLMGELSRLRKDPAYSHVS
DELFATIGVTFFGAGVISTGSFLTTALISLIQRPQLRNLLHEKPELIPAGVEELLRINLSFADGLPRLATADI
QVGDVLVRKGELVLVLLEGANFDPEHFPNPGSIELDRPNPTSHLAFGRGQHFCPGSALGRRHAQIGIEALLKK
MPGVDLAVPIDQLVWRTRFQRRIPERLPVLW
>CYP121A1_Mycobacterium tuberculosis NRITLD28 2655076973
MTATVLLEVPFSARGDRIPDAVAELRTREPIRKVRTITGAEAWLVSSYALCTQVLEDRRFSMKETAAAGAPRL
NALTVPPEVVNNMGNIADAGLRKAVMKAITPKAPGLEQFLRDTANSLLDNLITEGAPADLRNDFADPLATALH
CKVLGIPQEDGPKLFRSLSIAFMSSADPIPAAKINWDRDIEYMAGILENPNITTGLMGELSRLRKDPAYSHVS
DELFATIGVTFFGAGVISTGSFLTTALISLIQRPQLRNLLHEKPELIPAGVEELLRINLSFADGLPRLATADI
QVGDVLVRKGELVLVLLEGANFDPEHFPNPGSIELDRPNPTSHLAFGRGQHFCPGSALGRRHAQIGIEALLKK
MPGVDLAVPIDQLVWRTRFQRRIPERLPVLW
>CYP121A1_Mycobacterium tuberculosis NRITLD31 2635570161
MTATVLLEVPFSARGDRIPDAVAELRTREPIRKVRTITGAEAWLVSSYALCTQVLEDRRFSMKETAAAGAPRL
NALTVPPEVVNNMGNIADAGLRKAVMKAITPKAPGLEQFLRDTANSLLDNLITEGAPADLRNDFADPLATALH
CKVLGIPQEDGPKLFRSLSIAFMSSADPIPAAKINWDRDIEYMAGILENPNITTGLMGELSRLRKDPAYSHVS
DELFATIGVTFFGAGVISTGSFLTTALISLIQRPQLRNLLHEKPELIPAGVEELLRINLSFADGLPRLATADI
QVGDVLVRKGELVLVLLEGANFDPEHFPNPGSIELDRPNPTSHLAFGRGQHFCPGSALGRRHAQIGIEALLKK
MPGVDLAVPIDQLVWRTRFQRRIPERLPVLW
>CYP121A1_Mycobacterium tuberculosis NRITLD33 2633751131
MTATVLLEVPFSARGDRIPDAVAELRTREPIRKVRTITGAEAWLVSSYALCTQVLEDRRFSMKETAAAGAPRL
NALTVPPEVVNNMGNIADAGLRKAVMKAITPKAPGLEQFLRDTANSLLDNLITEGAPADLRNDFADPLATALH
CKVLGIPQEDGPKLFRSLSIAFMSSADPIPAAKINWDRDIEYMAGILENPNITTGLMGELSRLRKDPAYSHVS
DELFATIGVTFFGAGVISTGSFLTTALISLIQRPQLRNLLHEKPELIPAGVEELLRINLSFADGLPRLATADI
QVGDVLVRKGELVLVLLEGANFDPEHFPNPGSIELDRPNPTSHLAFGRGQHFCPGSALGRRHAQIGIEALLKK
MPGVDLAVPIDQLVWRTRFQRRIPERLPVLW
>CYP121A1_Mycobacterium tuberculosis NRITLD34 2630803996

MTATVLLEVPFSARGDRIPDAVAELRTREPIRKVRTITGAEAWLVSSYALCTQVLEDRRFSMKETAAAGAPRL
NALTVPPEVVNNMGNIADAGLRKAVMKAITPKAPGLEQFLRDTANSLLDNLITEGAPADLRNDFADPLATALH
CKVLGIPQEDGPKLFRSLSIAFMSSADPIPAAKINWDRDIEYMAGILENPNITTGLMGELSRLRKDPAYSHVS
DELFATIGVTFFGAGVISTGSFLTTALISLIQRPQLRNLLHEKPELIPAGVEELLRINLSFADGLPRLATADI
QVGDVLVRKGELVLVLLEGANFDPEHFPNPGSIELDRPNPTSHLAFGRGQHFCPGSALGRRHAQIGIEALLKK
MPGVDLAVPIDQLVWRTRFQRRIPERLPVLW
>CYP121A1_Mycobacterium tuberculosis NRITLD44 2575709316
MTATVLLEVPFSARGDRIPDAVAELRTREPIRKVRTITGAEAWLVSSYALCTQVLEDRRFSMKETAAAGAPRL
NALTVPPEVVNNMGNIADAGLRKAVMKAITPKAPGLEQFLRDTANSLLDNLITEGAPADLRNDFADPLATALH
CKVLGIPQEDGPKLFRSLSIAFMSSADPIPAAKINWDRDIEYMAGILENPNITTGLMGELSRLRKDPAYSHVS
DELFATIGVTFFGAGVISTGSFLTTALISLIQRPQLRNLLHEKPELIPAGVEELLRINLSFADGLPRLATADI
QVGDVLVRKGELVLVLLEGANFDPEHFPNPGSIELDRPNPTSHLAFGRGQHFCPGSALGRRHAQIGIEALLKK
MPGVDLAVPIDQLVWRTRFQRRIPERLPVLW
>CYP121A1_Mycobacterium tuberculosis NRITLD45 2633493200
MTATVLLEVPFSARGDRIPDAVAELRTREPIRKVRTITGAEAWLVSSYALCTQVLEDRRFSMKETAAAGAPRL
NALTVPPEVVNNMGNIADAGLRKAVMKAITPKAPGLEQFLRDTANSLLDNLITEGAPADLRNDFADPLATALH
CKVLGIPQEDGPKLFRSLSIAFMSSADPIPAAKINWDRDIEYMAGILENPNITTGLMGELSRLRKDPAYSHVS
DELFATIGVTFFGAGVISTGSFLTTALISLIQRPQLRNLLHEKPELIPAGVEELLRINLSFADGLPRLATADI
QVGDVLVRKGELVLVLLEGANFDPEHFPNPGSIELDRPNPTSHLAFGRGQHFCPGSALGRRHAQIGIEALLKK
MPGVDLAVPIDQLVWRTRFQRRIPERLPVLW
>CYP121A1_Mycobacterium tuberculosis NRITLD53 2632105818
MTATVLLEVPFSARGDRIPDAVAELRTREPIRKVRTITGAEAWLVSSYALCTQVLEDRRFSMKETAAAGAPRL
NALTVPPEVVNNMGNIADAGLRKAVMKAITPKAPGLEQFLRDTANSLLDNLITEGAPADLRNDFADPLATALH
CKVLGIPQEDGPKLFRSLSIAFMSSADPIPAAKINWDRDIEYMAGILENPNITTGLMGELSRLRKDPAYSHVS
DELFATIGVTFFGAGVISTGSFLTTALISLIQRPQLRNLLHEKPELIPAGVEELLRINLSFADGLPRL
>CYP121A1_Mycobacterium tuberculosis NRITLD54 2632338692
MTATVLLEVPFSARGDRIPDAVAELRTREPIRKVRTITGAEAWLVSSYALCTQVLEDRRFSMKETAAAGAPRL
NALTVPPEVVNNMGNIADAGLRKAVMKAITPKAPGLEQFLRDTANSLLDNLITEGAPADLRNDFADPLATALH
CKVLGIPQEDGPKLFRSLSIAFMSSADPIPAAKINWDRDIEYMAGILENPNITTGLMGELSRLRKDPAYSHVS
DELFATIGVTFFGAGVISTGSFLTTALISLIQRPQLRNLLHEKPELIPAGVEELLRINLSFADGLPRLATADI
QVGDVLVRKGELVLVLLEGANFDPEHFPNPGSIELDRPNPTSHLAFGRGQHFCPGSALGRRHAQIGIEALLKK
MPGVDLAVPIDQLVWRTRFQRRIPERLPVLW
>CYP121A1_Mycobacterium tuberculosis NRITLD57 2575385691
MTATVLLEVPFSARGDRIPDAVAELRTREPIRKVRTITGAEAWLVSSYALCTQVLEDRRFSMKETAAAGAPRL
NALTVPPEVVNNMGNIADAGLRKAVMKAITPKAPGLEQFLRDTANSLLDNLITEGAPADLRNDFADPLATALH
CKVLGIPQEDGPKLFRSLSIAFMSSADPIPAAKINWDRDIEYMAGILENPNITTGLMGELSRLRKDPAYSHVS
DELFATIGVTFFGAGVISTGSFLTTALISLIQRPQLRNLLHEKPELIPAGVEELLRINLSFADGLPRLATADI
QVGDVLVRKGELVLVLLEGANFDPEHFPNPGSIELDRPNPTSHLAFGRGQHFCPGSALGRRHAQIGIEALLKK
MPGVDLAVPIDQLVWRTRFQRRIPERLPVLW
>CYP121A1_Mycobacterium tuberculosis NRITLD60 2628776693
MTATVLLEVPFSARGDRIPDAVAELRTREPIRKVRTITGAEAWLVSSYALCTQVLEDRRFSMKETAAAGAPRL
NALTVPPEVVNNMGNIADAGLRKAVMKAITPKAPGLEQFLRDTANSLLDNLITEGAPADLRNDFADPLATALH
CKVLGIPQEDGPKLFRSLSIAFMSSADPIPAAKINWDRDIEYMAGILENPNITTGLMGELSRLRKDPAYSHVS
DELFATIGVTFFGAGVISTGSFLTTALISLIQRPQLRNLLHEKPELIPAGVEELLRINLSFADGLPRLATADI
QVGDVLVRKGELVLVLLEGANFDPEHFPNPGSIELDRPNPTSHLAFGRGQHFCPGSALGRRHAQIGIEALLKK
MPGVDLAVPIDQLVWRTRFQRRIPERLPVLW
>CYP121A1_Mycobacterium tuberculosis NZ494 2746196691
MTATVLLEVPFSARGDRIPDAVAELRTREPIRKVRTITGAEAWLVSSYALCTQVLEDRRFSMKETAAAGAPRL
NALTVPPEVVNNMGNIADAGLRKAVMKAITPKAPGLEQFLRDTANSLLDNLITEGAPADLRNDFADPLATALH
CKVLGIPQEDGPKLFRSLSIAFMSSADPIPAAKINWDRDIEYMAGILENPNITTGLMGELSRLRKDPAYSHVS
DELFATIGVTFFGAGVISTGSFLTTALISLIQRPQLRNLLHEKPELIPAGVEELLRINLSFADGLPRLATADI
QVGDVLVRKGELVLVLLEGANFDPEHFPNPGSIELDRPNPTSHLAFGRGQHFCPGSALGRRHAQIGIEALLKK
MPGVDLAVPIDQLVWRTRFQRRIPERLPVLW
>CYP121A1_Mycobacterium tuberculosis O00100977 2692178497
MTATVLLEVPFSARGDRIPDAVAELRTREPIRKVRTITGAEAWLVSSYALCTQVLEDRRFSMKETAAAGAPRL
NALTVPPEVVNNMGNIADAGLRKAVMKAITPKAPGLEQFLRDTANSLLDNLITEGAPADLRNDFADPLATALH
CKVLGIPQEDGPKLFRSLSIAFMSSADPIPAAKINWDRDIEYMAGILENPNITTGLMGELSRLRKDPAYSHVS
DELFATIGVTFFGAGVISTGSFLTTALISLIQRPQLRNLLHEKPELIPAGVEELLRINLSFADGLPRLATADI

QVGDVLVRKGELVLVLLEGANFDPEHFPNPGSIELDRPNPTSHLAFGRGQHFCPGSALGRRHAQIGIEALLKK
MPGVDLAVPIDQLVWRTRFQRRIPERLPVLW
>CYP121A1_Mycobacterium tuberculosis O00200127 2671983896
MTATVLLEVPFSARGDRIPDAVAELRTREPIRKVRTITGAEAWLVSSYALCTQVLEDRRFSMKETAAAGAPRL
NALTVPPEVVNNMGNIADAGLRKAVMKAITPKAPGLEQFLRDTANSLLDNLITEGAPADLRNDFADPLATALH
CKVLGIPQEDGPKLFRSLSIAFMSSADPIPAAKINWDRDIEYMAGILENPNITTGLMGELSRLRKDPAYSHVS
DELFATIGVTFFGAGVISTGSFLTTALISLIQRPQLRNLLHEKPELIPAGVEELLRINLSFADGLPRLATADI
QVGDVLVRKGELVLVLLEGANFDPEHFPNPGSIELDRPNPTSHLAFGRGQHFCPGSALGRRHAQIGIEALLKK
MPGVDLAVPIDQLVWRTRFQRRIPERLPVLW
>CYP121A1_Mycobacterium tuberculosis O00401640 2673979543
MTATVLLEVPFSARGDRIPDAVAELRTREPIRKVRTITGAEAWLVSSYALCTQVLEDRRFSMKETAAAGAPRL
NALTVPPEVVNNMGNIADAGLRKAVMKAITPKAPGLEQFLRDTANSLLDNLITEGAPADLRNDFADPLATALH
CKVLGIPQEDGPKLFRSLSIAFMSSADPIPAAKINWDRDIEYMAGILENPNITTGLMGELSRLRKDPAYSHVS
DELFATIGVTFFGAGVISTGSFLTTALISLIQRPQLRNLLHEKPELIPAGVEELLRINLSFADGLPRLATADI
QVGDVLVRKGELVLVLLEGANFDPEHFPNPGSIELDRPNPTSHLAFGRGQHFCPGSALGRRHAQIGIEALLKK
MPGVDLAVPIDQLVWRTRFQRRIPERLPVLW
>CYP121A1_Mycobacterium tuberculosis O09401777 2697085891
MTATVLLEVPFSARGDRIPDAVAELRTREPIRKVRTITGAEAWLVSSYALCTQVLEDRRFSMKETAAAGAPRL
NALTVPPEVVNNMGNIADAGLRKAVMKAITPKAPGLEQFLRDTANSLLDNLITEGAPADLRNDFADPLATALH
CKVLGIPQEDGPKLFRSLSIAFMSSADPIPAAKINWDRDIEYMAGILENPNITTGLMGELSRLRKDPAYSHVS
DELFATIGVTFFGAGVISTGSFLTTALISLIQRPQLRNLLHEKPELIPAGVEELLRINLSFADGLPRLATADI
QVGDVLVRKGELVLVLLEGANFDPEHFPNPGSIELDRPNPTSHLAFGRGQHFCPGSALGRRHAQIGIEALLKK
MPGVDLAVPIDQLVWRTRFQRRIPERLPVLW
>CYP121A1_Mycobacterium tuberculosis O09501089 2698764935
MTATVLLEVPFSARGDRIPDAVAELRTREPIRKVRTITGAEAWLVSSYALCTQVLEDRRFSMKETAAAGAPRL
NALTVPPEVVNNMGNIADAGLRKAVMKAITPKAPGLEQFLRDTANSLLDNLITEGAPADLRNDFADPLATALH
CKVLGIPQEDGPKLFRSLSIAFMSSADPIPAAKINWDRDIEYMAGILENPNITTGLMGELSRLRKDPAYSHVS
DELFATIGVTFFGAGVISTGSFLTTALISLIQRPQLRNLLHEKPELIPAGVEELLRINLSFADGLPRLATADI
QVGDVLVRKGELVLVLLEGANFDPEHFPNPGSIELDRPNPTSHLAFGRGQHFCPGSALGRRHAQIGIEALLKK
MPGVDLAVPIDQLVWRTRFQRRIPERLPVLW
>CYP121A1_Mycobacterium tuberculosis O09501127 2694331730
MTATVLLEVPFSARGDRIPDAVAELRTREPIRKVRTITGAEAWLVSSYALCTQVLEDRRFSMKETAAAGAPRL
NALTVPPEVVNNMGNIADAGLRKAVMKAITPKAPGLEQFLRDTANSLLDNLITEGAPADLRNDFADPLATALH
CKVLGIPQEDGPKLFRSLSIAFMSSADPIPAAKINWDRDIEYMAGILENPNITTGLMGELSRLRKDPAYSHVS
DELFATIGVTFFGAGVISTGSFLTTALISLIQRPQLRNLLHEKPELIPAGVEELLRINLSFADGLPRLATADI
QVGDVLVRKGELVLVLLEGANFDPEHFPNPGSIELDRPNPTSHLAFGRGQHFCPGSALGRRHAQIGIEALLKK
MPGVDLAVPIDQLVWRTRFQRRIPERLPVLW
>CYP121A1_Mycobacterium tuberculosis O09700898 2703511821
MTATVLLEVPFSARGDRIPDAVAELRTREPIRKVRTITGAEAWLVSSYALCTQVLEDRRFSMKETAAAGAPRL
NALTVPPEVVNNMGNIADAGLRKAVMKAITPKAPGLEQFLRDTANSLLDNLITEGAPADLRNDFADPLATALH
CKVLGIPQEDGPKLFRSLSIAFMSSADPIPAAKINWDRDIEYMAGILENPNITTGLMGELSRLRKDPAYSHVS
DELFATIGVTFFGAGVISTGSFLTTALISLIQRPQLRNLLHEKPELIPAGVEELLRINLSFADGLPRLATADI
QVGDVLVRKGELVLVLLEGANFDPEHFPNPGSIELDRPNPTSHLAFGRGQHFCPGSALGRRHAQIGIEALLKK
MPGVDLAVPIDQLVWRTRFQRRIPERLPVLW
>CYP121A1_Mycobacterium tuberculosis O09700920 2675698410
MTATVLLEVPFSARGDRIPDAVAELRTREPIRKVRTITGAEAWLVSSYALCTQVLEDRRFSMKETAAAGAPRL
NALTVPPEVVNNMGNIADAGLRKAVMKAITPKAPGLEQFLRDTANSLLDNLITEGAPADLRNDFADPLATALH
CKVLGIPQEDGPKLFRSLSIAFMSSADPIPAAKINWDRDIEYMAGILENPNITTGLMGELSRLRKDPAYSHVS
DELFATIGVTFFGAGVISTGSFLTTALISLIQRPQLRNLLHEKPELIPAGVEELLRINLSFADGLPRLATADI
QVGDVLVRKGELVLVLLEGANFDPEHFPNPGSIELDRPNPTSHLAFGRGQHFCPGSALGRRHAQIGIEALLKK
MPGVDLAVPIDQLVWRTRFQRRIPERLPVLW
>CYP121A1_Mycobacterium tuberculosis O09701563 2696016958
MTATVLLEVPFSARGDRIPDAVAELRTREPIRKVRTITGAEAWLVSSYALCTQVLEDRRFSMKETAAAGAPRL
NALTVPPEVVNNMGNIADAGLRKAVMKAITPKAPGLEQFLRDTANSLLDNLITEGAPADLRNDFADPLATALH
CKVLGIPQEDGPKLFRSLSIAFMSSADPIPAAKINWDRDIEYMAGILENPNITTGLMGELSRLRKDPAYSHVS
DELFATIGVTFFGAGVISTGSFLTTALISLIQRPQLRNLLHEKPELIPAGVEELLRINLSFADGLPRLATADI
QVGDVLVRKGELVLVLLEGANFDPEHFPNPGSIELDRPNPTSHLAFGRGQHFCPGSALGRRHAQIGIEALLKK
MPGVDLAVPIDQLVWRTRFQRRIPERLPVLW

>CYP121A1_Mycobacterium tuberculosis O09801157 2706552771
MTATVLLEVPFSARGDRIPDAVAELRTREPIRKVRTITGAEAWLVSSYALCTQVLEDRRFSMKETAAAGAPRL
NALTVPPEVVNNMGNIADAGLRKAVMKAITPKAPGLEQFLRDTANSLLDNLITEGAPADLRNDFADPLATALH
CKVLGIPQEDGPKLFRSLSIAFMSSADPIPAAKINWDRDIEYMAGILENPNITTGLMGELSRLRKDPAYSHVS
DELFATIGVTFFGAGVISTGSFLTTALISLIQRPQLRNLLHEKPELIPAGVEELLRINLSFADGLPRLATADI
QVGDVLVRKGELVLVLLEGANFDPEHFPNPGSIELDRPNPTSHLAFGRGQHFCPGSALGRRHAQIGIEALLKK
MPGVDLAVPIDQLVWRTRFQRRIPERLPVLW
>CYP121A1_Mycobacterium tuberculosis O09801359 2696573543
MTATVLLEVPFSARGDRIPDAVAELRTREPIRKVRTITGAEAWLVSSYALCTQVLEDRRFSMKETAAAGAPRL
NALTVPPEVVNNMGNIADAGLRKAVMKAITPKAPGLEQFLRDTANSLLDNLITEGAPADLRNDFADPLATALH
CKVLGIPQEDGPKLFRSLSIAFMSSADPIPAAKINWDRDIEYMAGILENPNITTGLMGELSRLRKDPAYSHVS
DELFATIGVTFFGAGVISTGSFLTTALISLIQRPQLRNLLHEKPELIPAGVEELLRINLSFADGLPRLATADI
QVGDVLVRKGELVLVLLEGANFDPEHFPNPGSIELDRPNPTSHLAFGRGQHFCPGSALGRRHAQIGIEALLKK
MPGVDLAVPIDQLVWRTRFQRRIPERLPVLW
>CYP121A1_Mycobacterium tuberculosis OFXR-19 2576865289
MTATVLLEVPFSARGDRIPDAVAELRTREPIRKVRTITGAEAWLVSSYALCTQVLEDRRFSMKETAAAGAPRL
NALTVPPEVVNNMGNIADAGLRKAVMKAITPKAPGLEQFLRDTANSLLDNLITEGAPADLRNDFADPLATALH
CKVLGIPQEDGPKLFRSLSIAFMSSADPIPAAKINWDRDIEYMAGILENPNITTGLMGELSRLRKDPAYSHVS
DELFATIGVTFFGAGVISTGSFLTTALISLIQRPQLRNLLHEKPELIPAGVEELLRINLSFADGLPRLATADI
QVGDVLVRKGELVLVLLEGANFDPEHFPNPGSIELDRPNPTSHLAFGRGQHFCPGSALGRRHAQIGIEALLKK
MPGVDLAVPIDQLVWRTRFQRRIPERLPVLW
>CYP121A1_Mycobacterium tuberculosis OFXR-27 2603148811
MTATVLLEVPFSARGDRIPDAVAELRTREPIRKVRTITGAEAWLVSSYALCTQVLEDRRFSMKETAAAGAPRL
NALTVPPEVVNNMGNIADAGLRKAVMKAITPKAPGLEQFLRDTANSLLDNLITEGAPADLRNDFADPLATALH
CKVLGIPQEDGPKLFRSLSIAFMSSADPIPAAKINWDRDIEYMAGILENPNITTGLMGELSRLRKDPAYSHVS
DELFATIGVTFFGAGVISTGSFLTTALISLIQRPQLRNLLHEKPELIPAGVEELLRINLSFADGLPRLATADI
QVGDVLVRKGELVLVLLEGANFDPEHFPNPGSIELDRPNPTSHLAFGRGQHFCPGSALGRRHAQIGIEALLKK
MPGVDLAVPIDQLVWRTRFQRRIPERLPVLW
>CYP121A1_Mycobacterium tuberculosis OFXR-28 2604339473
MTATVLLEVPFSARGDRIPDAVAELRTREPIRKVRTITGAEAWLVSSYALCTQVLEDRRFSMKETAAAGAPRL
NALTVPPEVVNNMGNIADAGLRKAVMKAITPKAPGLEQFLRDTANSLLDNLITEGAPADLRNDFADPLATALH
CKVLGIPQEDGPKLFRSLSIAFMSSADPIPAAKINWDRDIEYMAGILENPNITTGLMGELSRLRKDPAYSHVS
DELFATIGVTFFGAGVISTGSFLTTALISLIQRPQLRNLLHEKPELIPAGVEELLRINLSFADGLPRLATADI
QVGDVLVRKGELVLVLLEGANFDPEHFPNPGSIELDRPNPTSHLAFGRGQHFCPGSALGRRHAQIGIEALLKK
MPGVDLAVPIDQLVWRTRFQRRIPERLPVLW
>CYP121A1_Mycobacterium tuberculosis OFXR-3 2576470588
MTATVLLEVPFSARGDRIPDAVAELRTREPIRKVRTITGAEAWLVSSYALCTQVLEDRRFSMKETAAAGAPRL
NALTVPPEVVNNMGNIADAGLRKAVMKAITPKAPGLEQFLRDTANSLLDNLITEGAPADLRNDFADPLATALH
CKVLGIPQEDGPKLFRSLSIAFMSSADPIPAAKINWDRDIEYMAGILENPNITTGLMGELSRLRKDPAYSHVS
DELFATIGVTFFGAGVISTGSFLTTALISLIQRPQLRNLLHEKPELIPAGVEELLRINLSFADGLPRLATADI
QVGDVLVRKGELVLVLLEGANFDPEHFPNPGSIELDRPNPTSHLAFGRGQHFCPGSALGRRHAQIGIEALLKK
MPGVDLAVPIDQLVWRTRFQRRIPERLPVLW
>CYP121A1_Mycobacterium tuberculosis OFXR-33 2577373969
MTATVLLEVPFSARGDRIPDAVAELRTREPIRKVRTITGAEAWLVSSYALCTQVLEDRRFSMKETAAAGAPRL
NALTVPPEVVNNMGNIADAGLRKAVMKAITPKAPGLEQFLRDTANSLLDNLITEGAPADLRNDFADPLATALH
CKVLGIPQEDGPKLFRSLSIAFMSSADPIPAAKINWDRDIEYMAGILENPNITTGLMGELSRLRKDPAYSHVS
DELFATIGVTFFGAGVISTGSFLTTALISLIQRPQLRNLLHEKPELIPAGVEELLRINLSFADGLPRLATADI
QVGDVLVRKGELVLVLLEGANFDPEHFPNPGSIELDRPNPTSHLAFGRGQHFCPGSALGRRHAQIGIEALLKK
MPGVDLAVPIDQLVWRTRFQRRIPERLPVLW
>CYP121A1_Mycobacterium tuberculosis OFXR-6 2575324793
MTATVLLEVPFSARGDRIPDAVAELRTREPIRKVRTITGAEAWLVSSYALCTQVLEDRRFSMKETAAAGAPRL
NALTVPPEVVNNMGNIADAGLRKAVMKAITPKAPGLEQFLRDTANSLLDNLITEGAPADLRNDFADPLATALH
CKVLGIPQEDGPKLFRSLSIAFMSSADPIPAAKINWDRDIEYMAGILENPNITTGLMGELSRLRKDPAYSHVS
DELFATIGVTFFGAGVISTGSFLTTALISLIQRPQLRNLLHEKPELIPAGVEELLRINLSFADGLPRLATADI
QVGDVLVRKGELVLVLLEGANFDPEHFPNPGSIELDRPNPTSHLAFGRGQHFCPGSALGRRHAQIGIEALLKK
MPGVDLAVPIDQLVWRTRFQRRIPERLPVLW
>CYP121A1_Mycobacterium tuberculosis OFXR-7 2575619870

MTATVLLEVPFSARGDRIPDAVAELRTREPIRKVRTITGAEAWLVSSYALCTQVLEDRRFSMKETAAAGAPRL
NALTVPPEVVNNMGNIADAGLRKAVMKAITPKAPGLEQFLRDTANSLLDNLITEGAPADLRNDFADPLATALH
CKVLGIPQEDGPKLFRSLSIAFMSSADPIPAAKINWDRDIEYMAGILENPNITTGLMGELSRLRKDPAYSHVS
DELFATIGVTFFGAGVISTGSFLTTALISLIQRPQLRNLLHEKPELIPAGVEELLRINLSFADGLPRLATADI
QVGDVLVRKGELVLVLLEGANFDPEHFPNPGSIELDRPNPTSHLAFGRGQHFCPGSALGRRHAQIGIEALLKK
MPGVDLAVPIDQLVWRTRFQRRIPERLPVLW
>CYP121A1_Mycobacterium tuberculosis OSDD493 2603142804
MTATVLLEVPFSARGDRIPDAVAELRTREPIRKVRTITGAEAWLVSSYALCTQVLEDRRFSMKETAAAGAPRL
NALTVPPEVVNNMGNIADAGLRKAVMKAITPKAPGLEQFLRDTANSLLDNLITEGAPADLRNDFADPLATALH
CKVLGIPQEDGPKLFRSLSIAFMSSADPIPAAKINWDRDIEYMAGILENPNITTGLMGELSRLRKDPAYSHVS
DELFATIGVTFFGAGVISTGSFLTTALISLIQRPQLRNLLHEKPELIPAGVEELLRINLSFADGLPRLATADI
QVGDVLVRKGELVLVLLEGANFDPEHFPNPGSIELDRPNPTSHLAFGRGQHFCPGSALGRRHAQIGIEALLKK
MPGVDLAVPIDQLVWRTRFQRRIPERLPVLW
>CYP121A1_Mycobacterium tuberculosis OSDD504 2549411117
MTATVLLEVPFSARGDRIPDAVAELRTREPIRKVRTITGAEAWLVSSYALCTQVLEDRRFSMKETAAAGAPRL
NALTVPPEVVNNMGNIADAGLRKAVMKAITPKAPGLEQFLRDTANSLLDNLITEGAPADLRNDFADPLATALH
CKVLGIPQEDGPKLFRSLSIAFMSSADPIPAAKINWDRDIEYMAGILENPNITTGLMGELSRLRKDPAYSHVS
DELFATIGVTFFGAGVISTGSFLTTALISLIQRPQLRNLLHEKPELIPAGVEELLRINLSFADGLPRLATADI
QVGDVLVRKGELVLVLLEGANFDPEHFPNPGSIELDRPNPTSHLAFGRGQHFCPGSALGRRHAQIGIEALLKK
MPGVDLAVPIDQLVWRTRFQRRIPERLPVLW
>CYP121A1_Mycobacterium tuberculosis OSDD518 2549416255
MTATVLLEVPFSARGDRIPDAVAELRTREPIRKVRTITGAEAWLVSSYALCTQVLEDRRFSMKETAAAGAPRL
NALTVPPEVVNNMGNIADAGLRKAVMKAITPKAPGLEQFLRDTANSLLDNLITEGAPADLRNDFADPLATALH
CKVLGIPQEDGPKLFRSLSIAFMSSADPIPAAKINWDRDIEYMAGILENPNITTGLMGELSRLRKDPAYSHVS
DELFATIGVTFFGAGVISTGSFLTTALISLIQRPQLRNLLHEKPELIPAGVEELLRINLSFADGLPRLATADI
QVGDVLVRKGELVLVLLEGANFDPEHFPNPGSIELDRPNPTSHLAFGRGQHFCPGSALGRRHAQIGIEALLKK
MPGVDLAVPIDQLVWRTRFQRRIPERLPVLW
>CYP121A1_Mycobacterium tuberculosis PanR0201 2555579505
MTATVLLEVPFSARGDRIPDAVAELRTREPIRKVRTITGAEAWLVSSYALCTQVLEDRRFSMKETAAAGAPRL
NALTVPPEVVNNMGNIADAGLRKAVMKAITPKAPGLEQFLRDTANSLLDNLITEGAPADLRNDFADPLATALH
CKVLGIPQEDGPKLFRSLSIAFMSSADPIPAAKINWDRDIEYMAGILENPNITTGLMGELSRLRKDPAYSHVS
DELFATIGVTFFGAGVISTGSFLTTALISLIQRPQLRNLLHEKPELIPAGVEELLRINLSFADGLPRLATADI
QVGDVLVRKGELVLVLLEGANFDPEHFPNPGSIELDRPNPTSHLAFGRGQHFCPGSALGRRHAQIGIEALLKK
MPGVDLAVPIDQLVWRTRFQRRIPERLPVLW
>CYP121A1_Mycobacterium tuberculosis PanR0202 2598068047
MTATVLLEVPFSARGDRIPDAVAELRTREPIRKVRTITGAEAWLVSSYALCTQVLEDRRFSMKETAAAGAPRL
NALTVPPEVVNNMGNIADAGLRKAVMKAITPKAPGLEQFLRDTANSLLDNLITEGAPADLRNDFADPLATALH
CKVLGIPQEDGPKLFRSLSIAFMSSADPIPAAKINWDRDIEYMAGILENPNITTGLMGELSRLRKDPAYSHVS
DELFATIGVTFFGAGVISTGSFLTTALISLIQRPQLRNLLHEKPELIPAGVEELLRINLSFADGLPRLATADI
QVGDVLVRKGELVLVLLEGANFDPEHFPNPGSIELDRPNPTSHLAFGRGQHFCPGSALGRRHAQIGIEALLKK
MPGVDLAVPIDQLVWRTRFQRRIPERLPVLW
>CYP121A1_Mycobacterium tuberculosis PanR0203 2555149130
MTATVLLEVPFSARGDRIPDAVAELRTREPIRKVRTITGAEAWLVSSYALCTQVLEDRRFSMKETAAAGAPRL
NALTVPPEVVNNMGNIADAGLRKAVMKAITPKAPGLEQFLRDTANSLLDNLITEGAPADLRNDFADPLATALH
CKVLGIPQEDGPKLFRSLSIAFMSSADPIPAAKINWDRDIEYMAGILENPNITTGLMGELSRLRKDPAYSHVS
DELFATIGVTFFGAGVISTGSFLTTALISLIQRPQLRNLLHEKPELIPAGVEELLRINLSFADGLPRLATADI
QVGDVLVRKGELVLVLLEGANFDPEHFPNPGSIELDRPNPTSHLAFGRGQHFCPGSALGRRHAQIGIEALLKK
MPGVDLAVPIDQLVWRTRFQRRIPERLPVLW
>CYP121A1_Mycobacterium tuberculosis PanR0205 2555140751
MTATVLLEVPFSARGDRIPDAVAELRTREPIRKVRTITGAEAWLVSSYALCTQVLEDRRFSMKETAAAGAPRL
NALTVPPEVVNNMGNIADAGLRKAVMKAITPKAPGLEQFLRDTANSLLDNLITEGAPADLRNDFADPLATALH
CKVLGIPQEDGPKLFRSLSIAFMSSADPIPAAKINWDRDIEYMAGILENPNITTGLMGELSRLRKDPAYSHVS
DELFATIGVTFFGAGVISTGSFLTTALISLIQRPQLRNLLHEKPELIPAGVEELLRINLSFADGLPRLATADI
QVGDVLVRKGELVLVLLEGANFDPEHFPNPGSIELDRPNPTSHLAFGRGQHFCPGSALGRRHAQIGIEALLKK
MPGVDLAVPIDQLVWRTRFQRRIPERLPVLW
>CYP121A1_Mycobacterium tuberculosis PanR0207 2555160928
MTATVLLEVPFSARGDRIPDAVAELRTREPIRKVRTITGAEAWLVSSYALCTQVLEDRRFSMKETAAAGAPRL
NALTVPPEVVNNMGNIADAGLRKAVMKAITPKAPGLEQFLRDTANSLLDNLITEGAPADLRNDFADPLATALH

CKVLGIPQEDGPKLFRSLSIAFMSSADPIPAAKINWDRDIEYMAGILENPNITTGLMGELSRLRKDPAYSHVS
DELFATIGVTFFGAGVISTGSFLTTALISLIQRPQLRNLLHEKPELIPAGVEELLRINLSFADGLPRLATADI
QVGDVLVRKGELVLVLLEGANFDPEHFPNPGSIELDRPNPTSHLAFGRGQHFCPGSALGRRHAQIGIEALLKK
MPGVDLAVPIDQLVWRTRFQRRIPERLPVLW
>CYP121A1_Mycobacterium tuberculosis PanR0208 2555153352
MTATVLLEVPFSARGDRIPDAVAELRTREPIRKVRTITGAEAWLVSSYALCTQVLEDRRFSMKETAAAGAPRL
NALTVPPEVVNNMGNIADAGLRKAVMKAITPKAPGLEQFLRDTANSLLDNLITEGAPADLRNDFADPLATALH
CKVLGIPQEDGPKLFRSLSIAFMSSADPIPAAKINWDRDIEYMAGILENPNITTGLMGELSRLRKDPAYSHVS
DELFATIGVTFFGAGVISTGSFLTTALISLIQRPQLRNLLHEKPELIPAGVEELLRINLSFADGLPRLATADI
QVGDVLVRKGELVLVLLEGANFDPEHFPNPGSIELDRPNPTSHLAFGRGQHFCPGSALGRRHAQIGIEALLKK
MPGVDLAVPIDQLVWRTRFQRRIPERLPVLW
>CYP121A1_Mycobacterium tuberculosis PanR0209 2555165090
MTATVLLEVPFSARGDRIPDAVAELRTREPIRKVRTITGAEAWLVSSYALCTQVLEDRRFSMKETAAAGAPRL
NALTVPPEVVNNMGNIADAGLRKAVMKAITPKAPGLEQFLRDTANSLLDNLITEGAPADLRNDFADPLATALH
CKVLGIPQEDGPKLFRSLSIAFMSSADPIPAAKINWDRDIEYMAGILENPNITTGLMGELSRLRKDPAYSHVS
DELFATIGVTFFGAGVISTGSFLTTALISLIQRPQLRNLLHEKPELIPAGVEELLRINLSFADGLPRLATADI
QVGDVLVRKGELVLVLLEGANFDPEHFPNPGSIELDRPNPTSHLAFGRGQHFCPGSALGRRHAQIGIEALLKK
MPGVDLAVPIDQLVWRTRFQRRIPERLPVLW
>CYP121A1_Mycobacterium tuberculosis PanR0304 2555278762
MTATVLLEVPFSARGDRIPDAVAELRTREPIRKVRTITGAEAWLVSSYALCTQVLEDRRFSMKETAAAGAPRL
NALTVPPEVVNNMGNIADAGLRKAVMKAITPKAPGLEQFLRDTANSLLDNLITEGAPADLRNDFADPLATALH
CKVLGIPQEDGPKLFRSLSIAFMSSADPIPAAKINWDRDIEYMAGILENPNITTGLMGELSRLRKDPAYSHVS
DELFATIGVTFFGAGVISTGSFLTTALISLIQRPQLRNLLHEKPELIPAGVEELLRINLSFADGLPRLATADI
QVGDVLVRKGELVLVLLEGANFDPEHFPNPGSIELDRPNPTSHLAFGRGQHFCPGSALGRRHAQIGIEALLKK
MPGVDLAVPIDQLVWRTRFQRRIPERLPVLW
>CYP121A1_Mycobacterium tuberculosis PanR0306 2555304139
MTATVLLEVPFSARGDRIPDAVAELRTREPIRKVRTITGAEAWLVSSYALCTQVLEDRRFSMKETAAAGAPRL
NALTVPPEVVNNMGNIADAGLRKAVMKAITPKAPGLEQFLRDTANSLLDNLITEGAPADLRNDFADPLATALH
CKVLGIPQEDGPKLFRSLSIAFMSSADPIPAAKINWDRDIEYMAGILENPNITTGLMGELSRLRKDPAYSHVS
DELFATIGVTFFGAGVISTGSFLTTALISLIQRPQLRNLLHEKPELIPAGVEELLRINLSFADGLPRLATADI
QVGDVLVRKGELVLVLLEGANFDPEHFPNPGSIELDRPNPTSHLAFGRGQHFCPGSALGRRHAQIGIEALLKK
MPGVDLAVPIDQLVWRTRFQRRIPERLPVLW
>CYP121A1_Mycobacterium tuberculosis PanR0307 2555291560
MTATVLLEVPFSARGDRIPDAVAELRTREPIRKVRTITGAEAWLVSSYALCTQVLEDRRFSMKETAAAGAPRL
NALTVPPEVVNNMGNIADAGLRKAVMKAITPKAPGLEQFLRDTANSLLDNLITEGAPADLRNDFADPLATALH
CKVLGIPQEDGPKLFRSLSIAFMSSADPIPAAKINWDRDIEYMAGILENPNITTGLMGELSRLRKDPAYSHVS
DELFATIGVTFFGAGVISTGSFLTTALISLIQRPQLRNLLHEKPELIPAGVEELLRINLSFADGLPRLATADI
QVGDVLVRKGELVLVLLEGANFDPEHFPNPGSIELDRPNPTSHLAFGRGQHFCPGSALGRRHAQIGIEALLKK
MPGVDLAVPIDQLVWRTRFQRRIPERLPVLW
>CYP121A1_Mycobacterium tuberculosis PanR0308 2555274519
MTATVLLEVPFSARGDRIPDAVAELRTREPIRKVRTITGAEAWLVSSYALCTQVLEDRRFSMKETAAAGAPRL
NALTVPPEVVNNMGNIADAGLRKAVMKAITPKAPGLEQFLRDTANSLLDNLITEGAPADLRNDFADPLATALH
CKVLGIPQEDGPKLFRSLSIAFMSSADPIPAAKINWDRDIEYMAGILENPNITTGLMGELSRLRKDPAYSHVS
DELFATIGVTFFGAGVISTGSFLTTALISLIQRPQLRNLLHEKPELIPAGVEELLRINLSFADGLPRLATADI
QVGDVLVRKGELVLVLLEGANFDPEHFPNPGSIELDRPNPTSHLAFGRGQHFCPGSALGRRHAQIGIEALLKK
MPGVDLAVPIDQLVWRTRFQRRIPERLPVLW
>CYP121A1_Mycobacterium tuberculosis PanR0309 2555295733
MTATVLLEVPFSARGDRIPDAVAELRTREPIRKVRTITGAEAWLVSSYALCTQVLEDRRFSMKETAAAGAPRL
NALTVPPEVVNNMGNIADAGLRKAVMKAITPKAPGLEQFLRDTANSLLDNLITEGAPADLRNDFADPLATALH
CKVLGIPQEDGPKLFRSLSIAFMSSADPIPAAKINWDRDIEYMAGILENPNITTGLMGELSRLRKDPAYSHVS
DELFATIGVTFFGAGVISTGSFLTTALISLIQRPQLRNLLHEKPELIPAGVEELLRINLSFADGLPRLATADI
QVGDVLVRKGELVLVLLEGANFDPEHFPNPGSIELDRPNPTSHLAFGRGQHFCPGSALGRRHAQIGIEALLKK
MPGVDLAVPIDQLVWRTRFQRRIPERLPVLW
>CYP121A1_Mycobacterium tuberculosis PanR0311 2555287283
MTATVLLEVPFSARGDRIPDAVAELRTREPIRKVRTITGAEAWLVSSYALCTQVLEDRRFSMKETAAAGAPRL
NALTVPPEVVNNMGNIADAGLRKAVMKAITPKAPGLEQFLRDTANSLLDNLITEGAPADLRNDFADPLATALH
CKVLGIPQEDGPKLFRSLSIAFMSSADPIPAAKINWDRDIEYMAGILENPNITTGLMGELSRLRKDPAYSHVS
DELFATIGVTFFGAGVISTGSFLTTALISLIQRPQLRNLLHEKPELIPAGVEELLRINLSFADGLPRLATADI

QVGDVLVRKGELVLVLLEGANFDPEHFPNPGSIELDRPNPTSHLAFGRGQHFCPGSALGRRHAQIGIEALLKK
MPGVDLAVPIDQLVWRTRFQRRIPERLPVLW
>CYP121A1_Mycobacterium tuberculosis PanR0313 2555346741
MTATVLLEVPFSARGDRIPDAVAELRTREPIRKVRTITGAEAWLVSSYALCTQVLEDRRFSMKETAAAGAPRL
NALTVPPEVVNNMGNIADAGLRKAVMKAITPKAPGLEQFLRDTANSLLDNLITEGAPADLRNDFADPLATALH
CKVLGIPQEDGPKLFRSLSIAFMSSADPIPAAKINWDRDIEYMAGILENPNITTGLMGELSRLRKDPAYSHVS
DELFATIGVTFFGAGVISTGSFLTTALISLIQRPQLRNLLHEKPELIPAGVEELLRINLSFADGLPRLATADI
QVGDVLVRKGELVLVLLEGANFDPEHFPNPGSIELDRPNPTSHLAFGRGQHFCPGSALGRRHAQIGIEALLKK
MPGVDLAVPIDQLVWRTRFQRRIPERLPVLW
>CYP121A1_Mycobacterium tuberculosis PanR0314 2555308349
MTATVLLEVPFSARGDRIPDAVAELRTREPIRKVRTITGAEAWLVSSYALCTQVLEDRRFSMKETAAAGAPRL
NALTVPPEVVNNMGNIADAGLRKAVMKAITPKAPGLEQFLRDTANSLLDNLITEGAPADLRNDFADPLATALH
CKVLGIPQEDGPKLFRSLSIAFMSSADPIPAAKINWDRDIEYMAGILENPNITTGLMGELSRLRKDPAYSHVS
DELFATIGVTFFGAGVISTGSFLTTALISLIQRPQLRNLLHEKPELIPAGVEELLRINLSFADGLPRLATADI
QVGDVLVRKGELVLVLLEGANFDPEHFPNPGSIELDRPNPTSHLAFGRGQHFCPGSALGRRHAQIGIEALLKK
MPGVDLAVPIDQLVWRTRFQRRIPERLPVLW
>CYP121A1_Mycobacterium tuberculosis PanR0317 2555561501
MTATVLLEVPFSARGDRIPDAVAELRTREPIRKVRTITGAEAWLVSSYALCTQVLEDRRFSMKETAAAGAPRL
NALTVPPEVVNNMGNIADAGLRKAVMKAITPKAPGLEQFLRDTANSLLDNLITEGAPADLRNDFADPLATALH
CKVLGIPQEDGPKLFRSLSIAFMSSADPIPAAKINWDRDIEYMAGILENPNITTGLMGELSRLRKDPAYSHVS
DELFATIGVTFFGAGVISTGSFLTTALISLIQRPQLRNLLHEKPELIPAGVEELLRINLSFADGLPRLATADI
QVGDVLVRKGELVLVLLEGANFDPEHFPNPGSIELDRPNPTSHLAFGRGQHFCPGSALGRRHAQIGIEALLKK
MPGVDLAVPIDQLVWRTRFQRRIPERLPVLW
>CYP121A1_Mycobacterium tuberculosis PanR0401 2555600476
MTATVLLEVPFSARGDRIPDAVAELRTREPIRKVRTITGAEAWLVSSYALCTQVLEDRRFSMKETAAAGAPRL
NALTVPPEVVNNMGNIADAGLRKAVMKAITPKAPGLEQFLRDTANSLLDNLITEGAPADLRNDFADPLATALH
CKVLGIPQEDGPKLFRSLSIAFMSSADPIPAAKINWDRDIEYMAGILENPNITTGLMGELSRLRKDPAYSHVS
DELFATIGVTFFGAGVISTGSFLTTALISLIQRPQLRNLLHEKPELIPAGVEELLRINLSFADGLPRLATADI
QVGDVLVRKGELVLVLLEGANFDPEHFPNPGSIELDRPNPTSHLAFGRGQHFCPGSALGRRHAQIGIEALLKK
MPGVDLAVPIDQLVWRTRFQRRIPERLPVLW
>CYP121A1_Mycobacterium tuberculosis PanR0402 2555552950
MTATVLLEVPFSARGDRIPDAVAELRTREPIRKVRTITGAEAWLVSSYALCTQVLEDRRFSMKETAAAGAPRL
NALTVPPEVVNNMGNIADAGLRKAVMKAITPKAPGLEQFLRDTANSLLDNLITEGAPADLRNDFADPLATALH
CKVLGIPQEDGPKLFRSLSIAFMSSADPIPAAKINWDRDIEYMAGILENPNITTGLMGELSRLRKDPAYSHVS
DELFATIGVTFFGAGVISTGSFLTTALISLIQRPQLRNLLHEKPELIPAGVEELLRINLSFADGLPRLATADI
QVGDVLVRKGELVLVLLEGANFDPEHFPNPGSIELDRPNPTSHLAFGRGQHFCPGSALGRRHAQIGIEALLKK
MPGVDLAVPIDQLVWRTRFQRRIPERLPVLW
>CYP121A1_Mycobacterium bovis AN5 2655181024
MTATVLLEVPFSARGDRIPDAVAELRTREPIRKVRTITGAEAWLVSSYALCTQVLEDRRFSMKETAAAGAPRL
NALTVPPEVVNNMGNIADAGLRKAVMKAITPKAPGLEQFLRDTANSLLDNLITEGAPADLRNDFADPLATALH
CKVLGIPQEDGPKLFRSLSIAFMSSADPIPAAKINWDRDIEYMAGILENPNITTGLMGELSRLRKDPAYSHVS
DELFATIGVTFFGAGVISTGSFLTTALISLIQRPQLRNLLHEKPELIPAGVEKLLRINLSFADGLPRLATADI
QVGDVLVRKGELVLVLLEGANFDPEHFPNPGSIELDRPNPTSHLAFGRGQHFCPGSALGRRHAQIGIEALLKK
MPGVDLAVPIDQLVWRTRFQRRIPERLPVLW
>CYP121A1_Mycobacterium tuberculosis BTB08-183 2604561417
MTATVLLEVPFSARGDRIPDAVAELRTREPIRKVRTITGAEAWLVSSYALCTQVLEDRRFSMKETAAAGAPRL
NALTVPPEVVNNMGNIADAGLRKAVMKAITPKAPGLEQFLRDTANSLLDNLITEGAPADLRNDFADPLATALH
CKVLGIPQEDGPKLFRSLSIAFMSSADPIPAAKINWDRDIEYMAGILENPNITTGLMGELSRLRKDPAYSHVS
DELFATIGVTFFGAGVISTGSFLTTALISLIQRPQLRNLLHEKPELIPAGVEELLRINLSFADGLPRLATADI
QVGDVLVRKGELVLVLLEGANFDPEYFPNPGSIELDRPNPTSHLAFGRGQHFCPGSALGRRHAQIGIEALLKK
MPGVDLAVPIDQLVWRTRFQRRIPERLPVLW
>CYP121A1_Mycobacterium tuberculosis 02_1987 2577464750
MTATVLLEVPFSARGDRIPDAVAELRTREPIRKVRTITGAEAWLVSSYALCTQVLEDRRFSMKETAAAGAPRL
NALTVPPEVVNNMGNIADAGLRKAVMKAITPKAPGLEQFLRDTANSLLDNLITEGAPADLRNDFADPLATALH
CKVLGIPQEDGPKLFRSLSIAFMSSADPIPAAKINWDRDIEYMAGILENPNITTGLMGELSRLRKDPAYSHVS
DELFATIGVTFFGAGVISTGSFLTTALISLIQRPQLRNLLHEKPELIPAGVEELLRINLSFADGLPRLATADI
QVGDVLVRKGELVLVLLEGANFDPEYFPNPGSIELDRPNPTSHLAFGRGQHFCPGSALGRRHAQIGIEALLKK
MPGVDLAVPIDQLVWRTRFQRRIPERLPVLW

>CYP121A1_Mycobacterium tuberculosis 02_1987 unfinished sequence 643023299
MTATVLLEVPFSARGDRIPDAVAELRTREPIRKVRTITGAEAWLVSSYALCTQVLEDRRFSMKETAAAGAPRL
NALTVPPEVVNNMGNIADAGLRKAVMKAITPKAPGLEQFLRDTANSLLDNLITEGAPADLRNDFADPLATALH
CKVLGIPQEDGPKLFRSLSIAFMSSADPIPAAKINWDRDIEYMAGILENPNITTGLMGELSRLRKDPAYSHVS
DELFATIGVTFFGAGVISTGSFLTTALISLIQRPQLRNLLHEKPELIPAGVEELLRINLSFADGLPRLATADI
QVGDVLVRKGELVLVLLEGANFDPEYFPNPGSIELDRPNPTSHLAFGRGQHFCPGSALGRRHAQIGIEALLKK
MPGVDLAVPIDQLVWRTRFQRRIPERLPVLW
>CYP121A1_Mycobacterium tuberculosis 0A005DS 2720373035
MTATVLLEVPFSARGDRIPDAVAELRTREPIRKVRTITGAEAWLVSSYALCTQVLEDRRFSMKETAAAGAPRL
NALTVPPEVVNNMGNIADAGLRKAVMKAITPKAPGLEQFLRDTANSLLDNLITEGAPADLRNDFADPLATALH
CKVLGIPQEDGPKLFRSLSIAFMSSADPIPAAKINWDRDIEYMAGILENPNITTGLMGELSRLRKDPAYSHVS
DELFATIGVTFFGAGVISTGSFLTTALISLIQRPQLRNLLHEKPELIPAGVEELLRINLSFADGLPRLATADI
QVGDVLVRKGELVLVLLEGANFDPEYFPNPGSIELDRPNPTSHLAFGRGQHFCPGSALGRRHAQIGIEALLKK
MPGVDLAVPIDQLVWRTRFQRRIPERLPVLW
>CYP121A1_Mycobacterium tuberculosis 0A012DS 2718845603
MTATVLLEVPFSARGDRIPDAVAELRTREPIRKVRTITGAEAWLVSSYALCTQVLEDRRFSMKETAAAGAPRL
NALTVPPEVVNNMGNIADAGLRKAVMKAITPKAPGLEQFLRDTANSLLDNLITEGAPADLRNDFADPLATALH
CKVLGIPQEDGPKLFRSLSIAFMSSADPIPAAKINWDRDIEYMAGILENPNITTGLMGELSRLRKDPAYSHVS
DELFATIGVTFFGAGVISTGSFLTTALISLIQRPQLRNLLHEKPELIPAGVEELLRINLSFADGLPRLATADI
QVGDVLVRKGELVLVLLEGANFDPEYFPNPGSIELDRPNPTSHLAFGRGQHFCPGSALGRRHAQIGIEALLKK
MPGVDLAVPIDQLVWRTRFQRRIPERLPVLW
>CYP121A1_Mycobacterium tuberculosis 0A029DS 2722763213
MTATVLLEVPFSARGDRIPDAVAELRTREPIRKVRTITGAEAWLVSSYALCTQVLEDRRFSMKETAAAGAPRL
NALTVPPEVVNNMGNIADAGLRKAVMKAITPKAPGLEQFLRDTANSLLDNLITEGAPADLRNDFADPLATALH
CKVLGIPQEDGPKLFRSLSIAFMSSADPIPAAKINWDRDIEYMAGILENPNITTGLMGELSRLRKDPAYSHVS
DELFATIGVTFFGAGVISTGSFLTTALISLIQRPQLRNLLHEKPELIPAGVEELLRINLSFADGLPRLATADI
QVGDVLVRKGELVLVLLEGANFDPEYFPNPGSIELDRPNPTSHLAFGRGQHFCPGSALGRRHAQIGIEALLKK
MPGVDLAVPIDQLVWRTRFQRRIPERLPVLW
>CYP121A1_Mycobacterium tuberculosis 0A033DS 2730275423
MTATVLLEVPFSARGDRIPDAVAELRTREPIRKVRTITGAEAWLVSSYALCTQVLEDRRFSMKETAAAGAPRL
NALTVPPEVVNNMGNIADAGLRKAVMKAITPKAPGLEQFLRDTANSLLDNLITEGAPADLRNDFADPLATALH
CKVLGIPQEDGPKLFRSLSIAFMSSADPIPAAKINWDRDIEYMAGILENPNITTGLMGELSRLRKDPAYSHVS
DELFATIGVTFFGAGVISTGSFLTTALISLIQRPQLRNLLHEKPELIPAGVEELLRINLSFADGLPRLATADI
QVGDVLVRKGELVLVLLEGANFDPEYFPNPGSIELDRPNPTSHLAFGRGQHFCPGSALGRRHAQIGIEALLKK
MPGVDLAVPIDQLVWRTRFQRRIPERLPVLW
>CYP121A1_Mycobacterium tuberculosis 0A036DS 2720569024
MTATVLLEVPFSARGDRIPDAVAELRTREPIRKVRTITGAEAWLVSSYALCTQVLEDRRFSMKETAAAGAPRL
NALTVPPEVVNNMGNIADAGLRKAVMKAITPKAPGLEQFLRDTANSLLDNLITEGAPADLRNDFADPLATALH
CKVLGIPQEDGPKLFRSLSIAFMSSADPIPAAKINWDRDIEYMAGILENPNITTGLMGELSRLRKDPAYSHVS
DELFATIGVTFFGAGVISTGSFLTTALISLIQRPQLRNLLHEKPELIPAGVEELLRINLSFADGLPRLATADI
QVGDVLVRKGELVLVLLEGANFDPEYFPNPGSIELDRPNPTSHLAFGRGQHFCPGSALGRRHAQIGIEALLKK
MPGVDLAVPIDQLVWRTRFQRRIPERLPVLW
>CYP121A1_Mycobacterium tuberculosis 0A087DS 2723410401
MTATVLLEVPFSARGDRIPDAVAELRTREPIRKVRTITGAEAWLVSSYALCTQVLEDRRFSMKETAAAGAPRL
NALTVPPEVVNNMGNIADAGLRKAVMKAITPKAPGLEQFLRDTANSLLDNLITEGAPADLRNDFADPLATALH
CKVLGIPQEDGPKLFRSLSIAFMSSADPIPAAKINWDRDIEYMAGILENPNITTGLMGELSRLRKDPAYSHVS
DELFATIGVTFFGAGVISTGSFLTTALISLIQRPQLRNLLHEKPELIPAGVEELLRINLSFADGLPRLATADI
QVGDVLVRKGELVLVLLEGANFDPEYFPNPGSIELDRPNPTSHLAFGRGQHFCPGSALGRRHAQIGIEALLKK
MPGVDLAVPIDQLVWRTRFQRRIPERLPVLW
>CYP121A1_Mycobacterium tuberculosis 0A092DS 2720279068
MTATVLLEVPFSARGDRIPDAVAELRTREPIRKVRTITGAEAWLVSSYALCTQVLEDRRFSMKETAAAGAPRL
NALTVPPEVVNNMGNIADAGLRKAVMKAITPKAPGLEQFLRDTANSLLDNLITEGAPADLRNDFADPLATALH
CKVLGIPQEDGPKLFRSLSIAFMSSADPIPAAKINWDRDIEYMAGILENPNITTGLMGELSRLRKDPAYSHVS
DELFATIGVTFFGAGVISTGSFLTTALISLIQRPQLRNLLHEKPELIPAGVEELLRINLSFADGLPRLATADI
QVGDVLVRKGELVLVLLEGANFDPEYFPNPGSIELDRPNPTSHLAFGRGQHFCPGSALGRRHAQIGIEALLKK
MPGVDLAVPIDQLVWRTRFQRRIPERLPVLW
>CYP121A1_Mycobacterium tuberculosis 0A093DS 2719234832

MTATVLLEVPFSARGDRIPDAVAELRTREPIRKVRTITGAEAWLVSSYALCTQVLEDRRFSMKETAAAGAPRL
NALTVPPEVVNNMGNIADAGLRKAVMKAITPKAPGLEQFLRDTANSLLDNLITEGAPADLRNDFADPLATALH
CKVLGIPQEDGPKLFRSLSIAFMSSADPIPAAKINWDRDIEYMAGILENPNITTGLMGELSRLRKDPAYSHVS
DELFATIGVTFFGAGVISTGSFLTTALISLIQRPQLRNLLHEKPELIPAGVEELLRINLSFADGLPRLATADI
QVGDVLVRKGELVLVLLEGANFDPEYFPNPGSIELDRPNPTSHLAFGRGQHFCPGSALGRRHAQIGIEALLKK
MPGVDLAVPIDQLVWRTRFQRRIPERLPVLW
>CYP121A1_Mycobacterium tuberculosis 0A094DS 2719252743
MTATVLLEVPFSARGDRIPDAVAELRTREPIRKVRTITGAEAWLVSSYALCTQVLEDRRFSMKETAAAGAPRL
NALTVPPEVVNNMGNIADAGLRKAVMKAITPKAPGLEQFLRDTANSLLDNLITEGAPADLRNDFADPLATALH
CKVLGIPQEDGPKLFRSLSIAFMSSADPIPAAKINWDRDIEYMAGILENPNITTGLMGELSRLRKDPAYSHVS
DELFATIGVTFFGAGVISTGSFLTTALISLIQRPQLRNLLHEKPELIPAGVEELLRINLSFADGLPRLATADI
QVGDVLVRKGELVLVLLEGANFDPEYFPNPGSIELDRPNPTSHLAFGRGQHFCPGSALGRRHAQIGIEALLKK
MPGVDLAVPIDQLVWRTRFQRRIPERLPVLW
>CYP121A1_Mycobacterium tuberculosis 0A115DS 2723662337
MTATVLLEVPFSARGDRIPDAVAELRTREPIRKVRTITGAEAWLVSSYALCTQVLEDRRFSMKETAAAGAPRL
NALTVPPEVVNNMGNIADAGLRKAVMKAITPKAPGLEQFLRDTANSLLDNLITEGAPADLRNDFADPLATALH
CKVLGIPQEDGPKLFRSLSIAFMSSADPIPAAKINWDRDIEYMAGILENPNITTGLMGELSRLRKDPAYSHVS
DELFATIGVTFFGAGVISTGSFLTTALISLIQRPQLRNLLHEKPELIPAGVEELLRINLSFADGLPRLATADI
QVGDVLVRKGELVLVLLEGANFDPEYFPNPGSIELDRPNPTSHLAFGRGQHFCPGSALGRRHAQIGIEALLKK
MPGVDLAVPIDQLVWRTRFQRRIPERLPVLW
>CYP121A1_Mycobacterium tuberculosis 0A117DS 2719114285
MTATVLLEVPFSARGDRIPDAVAELRTREPIRKVRTITGAEAWLVSSYALCTQVLEDRRFSMKETAAAGAPRL
NALTVPPEVVNNMGNIADAGLRKAVMKAITPKAPGLEQFLRDTANSLLDNLITEGAPADLRNDFADPLATALH
CKVLGIPQEDGPKLFRSLSIAFMSSADPIPAAKINWDRDIEYMAGILENPNITTGLMGELSRLRKDPAYSHVS
DELFATIGVTFFGAGVISTGSFLTTALISLIQRPQLRNLLHEKPELIPAGVEELLRINLSFADGLPRLATADI
QVGDVLVRKGELVLVLLEGANFDPEYFPNPGSIELDRPNPTSHLAFGRGQHFCPGSALGRRHAQIGIEALLKK
MPGVDLAVPIDQLVWRTRFQRRIPERLPVLW
>CYP121A1_Mycobacterium tuberculosis 0B049XDR 2720261552
MTATVLLEVPFSARGDRIPDAVAELRTREPIRKVRTITGAEAWLVSSYALCTQVLEDRRFSMKETAAAGAPRL
NALTVPPEVVNNMGNIADAGLRKAVMKAITPKAPGLEQFLRDTANSLLDNLITEGAPADLRNDFADPLATALH
CKVLGIPQEDGPKLFRSLSIAFMSSADPIPAAKINWDRDIEYMAGILENPNITTGLMGELSRLRKDPAYSHVS
DELFATIGVTFFGAGVISTGSFLTTALISLIQRPQLRNLLHEKPELIPAGVEELLRINLSFADGLPRLATADI
QVGDVLVRKGELVLVLLEGANFDPEYFPNPGSIELDRPNPTSHLAFGRGQHFCPGSALGRRHAQIGIEALLKK
MPGVDLAVPIDQLVWRTRFQRRIPERLPVLW
>CYP121A1_Mycobacterium tuberculosis 0B070XDR 2720985768
MTATVLLEVPFSARGDRIPDAVAELRTREPIRKVRTITGAEAWLVSSYALCTQVLEDRRFSMKETAAAGAPRL
NALTVPPEVVNNMGNIADAGLRKAVMKAITPKAPGLEQFLRDTANSLLDNLITEGAPADLRNDFADPLATALH
CKVLGIPQEDGPKLFRSLSIAFMSSADPIPAAKINWDRDIEYMAGILENPNITTGLMGELSRLRKDPAYSHVS
DELFATIGVTFFGAGVISTGSFLTTALISLIQRPQLRNLLHEKPELIPAGVEELLRINLSFADGLPRLATADI
QVGDVLVRKGELVLVLLEGANFDPEYFPNPGSIELDRPNPTSHLAFGRGQHFCPGSALGRRHAQIGIEALLKK
MPGVDLAVPIDQLVWRTRFQRRIPERLPVLW
>CYP121A1_Mycobacterium tuberculosis 0B076XDR 2720065830
MTATVLLEVPFSARGDRIPDAVAELRTREPIRKVRTITGAEAWLVSSYALCTQVLEDRRFSMKETAAAGAPRL
NALTVPPEVVNNMGNIADAGLRKAVMKAITPKAPGLEQFLRDTANSLLDNLITEGAPADLRNDFADPLATALH
CKVLGIPQEDGPKLFRSLSIAFMSSADPIPAAKINWDRDIEYMAGILENPNITTGLMGELSRLRKDPAYSHVS
DELFATIGVTFFGAGVISTGSFLTTALISLIQRPQLRNLLHEKPELIPAGVEELLRINLSFADGLPRLATADI
QVGDVLVRKGELVLVLLEGANFDPEYFPNPGSIELDRPNPTSHLAFGRGQHFCPGSALGRRHAQIGIEALLKK
MPGVDLAVPIDQLVWRTRFQRRIPERLPVLW
>CYP121A1_Mycobacterium tuberculosis 0B123ND 2720990079
MTATVLLEVPFSARGDRIPDAVAELRTREPIRKVRTITGAEAWLVSSYALCTQVLEDRRFSMKETAAAGAPRL
NALTVPPEVVNNMGNIADAGLRKAVMKAITPKAPGLEQFLRDTANSLLDNLITEGAPADLRNDFADPLATALH
CKVLGIPQEDGPKLFRSLSIAFMSSADPIPAAKINWDRDIEYMAGILENPNITTGLMGELSRLRKDPAYSHVS
DELFATIGVTFFGAGVISTGSFLTTALISLIQRPQLRNLLHEKPELIPAGVEELLRINLSFADGLPRLATADI
QVGDVLVRKGELVLVLLEGANFDPEYFPNPGSIELDRPNPTSHLAFGRGQHFCPGSALGRRHAQIGIEALLKK
MPGVDLAVPIDQLVWRTRFQRRIPERLPVLW
>CYP121A1_Mycobacterium tuberculosis 0B169XDR 2720994693
MTATVLLEVPFSARGDRIPDAVAELRTREPIRKVRTITGAEAWLVSSYALCTQVLEDRRFSMKETAAAGAPRL
NALTVPPEVVNNMGNIADAGLRKAVMKAITPKAPGLEQFLRDTANSLLDNLITEGAPADLRNDFADPLATALH

CKVLGIPQEDGPKLFRSLSIAFMSSADPIPAAKINWDRDIEYMAGILENPNITTGLMGELSRLRKDPAYSHVS
DELFATIGVTFFGAGVISTGSFLTTALISLIQRPQLRNLLHEKPELIPAGVEELLRINLSFADGLPRLATADI
QVGDVLVRKGELVLVLLEGANFDPEYFPNPGSIELDRPNPTSHLAFGRGQHFCPGSALGRRHAQIGIEALLKK
MPGVDLAVPIDQLVWRTRFQRRIPERLPVLW
>CYP121A1_Mycobacterium tuberculosis 0B218DS 2720186680
MTATVLLEVPFSARGDRIPDAVAELRTREPIRKVRTITGAEAWLVSSYALCTQVLEDRRFSMKETAAAGAPRL
NALTVPPEVVNNMGNIADAGLRKAVMKAITPKAPGLEQFLRDTANSLLDNLITEGAPADLRNDFADPLATALH
CKVLGIPQEDGPKLFRSLSIAFMSSADPIPAAKINWDRDIEYMAGILENPNITTGLMGELSRLRKDPAYSHVS
DELFATIGVTFFGAGVISTGSFLTTALISLIQRPQLRNLLHEKPELIPAGVEELLRINLSFADGLPRLATADI
QVGDVLVRKGELVLVLLEGANFDPEYFPNPGSIELDRPNPTSHLAFGRGQHFCPGSALGRRHAQIGIEALLKK
MPGVDLAVPIDQLVWRTRFQRRIPERLPVLW
>CYP121A1_Mycobacterium tuberculosis 0B222DS 2723520186
MTATVLLEVPFSARGDRIPDAVAELRTREPIRKVRTITGAEAWLVSSYALCTQVLEDRRFSMKETAAAGAPRL
NALTVPPEVVNNMGNIADAGLRKAVMKAITPKAPGLEQFLRDTANSLLDNLITEGAPADLRNDFADPLATALH
CKVLGIPQEDGPKLFRSLSIAFMSSADPIPAAKINWDRDIEYMAGILENPNITTGLMGELSRLRKDPAYSHVS
DELFATIGVTFFGAGVISTGSFLTTALISLIQRPQLRNLLHEKPELIPAGVEELLRINLSFADGLPRLATADI
QVGDVLVRKGELVLVLLEGANFDPEYFPNPGSIELDRPNPTSHLAFGRGQHFCPGSALGRRHAQIGIEALLKK
MPGVDLAVPIDQLVWRTRFQRRIPERLPVLW
>CYP121A1_Mycobacterium tuberculosis 0B228DS 2720266698
MTATVLLEVPFSARGDRIPDAVAELRTREPIRKVRTITGAEAWLVSSYALCTQVLEDRRFSMKETAAAGAPRL
NALTVPPEVVNNMGNIADAGLRKAVMKAITPKAPGLEQFLRDTANSLLDNLITEGAPADLRNDFADPLATALH
CKVLGIPQEDGPKLFRSLSIAFMSSADPIPAAKINWDRDIEYMAGILENPNITTGLMGELSRLRKDPAYSHVS
DELFATIGVTFFGAGVISTGSFLTTALISLIQRPQLRNLLHEKPELIPAGVEELLRINLSFADGLPRLATADI
QVGDVLVRKGELVLVLLEGANFDPEYFPNPGSIELDRPNPTSHLAFGRGQHFCPGSALGRRHAQIGIEALLKK
MPGVDLAVPIDQLVWRTRFQRRIPERLPVLW
>CYP121A1_Mycobacterium tuberculosis 0B229DS 2721102362
MTATVLLEVPFSARGDRIPDAVAELRTREPIRKVRTITGAEAWLVSSYALCTQVLEDRRFSMKETAAAGAPRL
NALTVPPEVVNNMGNIADAGLRKAVMKAITPKAPGLEQFLRDTANSLLDNLITEGAPADLRNDFADPLATALH
CKVLGIPQEDGPKLFRSLSIAFMSSADPIPAAKINWDRDIEYMAGILENPNITTGLMGELSRLRKDPAYSHVS
DELFATIGVTFFGAGVISTGSFLTTALISLIQRPQLRNLLHEKPELIPAGVEELLRINLSFADGLPRLATADI
QVGDVLVRKGELVLVLLEGANFDPEYFPNPGSIELDRPNPTSHLAFGRGQHFCPGSALGRRHAQIGIEALLKK
MPGVDLAVPIDQLVWRTRFQRRIPERLPVLW
>CYP121A1_Mycobacterium tuberculosis 0B259XDR 2718550948
MTATVLLEVPFSARGDRIPDAVAELRTREPIRKVRTITGAEAWLVSSYALCTQVLEDRRFSMKETAAAGAPRL
NALTVPPEVVNNMGNIADAGLRKAVMKAITPKAPGLEQFLRDTANSLLDNLITEGAPADLRNDFADPLATALH
CKVLGIPQEDGPKLFRSLSIAFMSSADPIPAAKINWDRDIEYMAGILENPNITTGLMGELSRLRKDPAYSHVS
DELFATIGVTFFGAGVISTGSFLTTALISLIQRPQLRNLLHEKPELIPAGVEELLRINLSFADGLPRLATADI
QVGDVLVRKGELVLVLLEGANFDPEYFPNPGSIELDRPNPTSHLAFGRGQHFCPGSALGRRHAQIGIEALLKK
MPGVDLAVPIDQLVWRTRFQRRIPERLPVLW
>CYP121A1_Mycobacterium tuberculosis 0B329XDR 2719142687
MTATVLLEVPFSARGDRIPDAVAELRTREPIRKVRTITGAEAWLVSSYALCTQVLEDRRFSMKETAAAGAPRL
NALTVPPEVVNNMGNIADAGLRKAVMKAITPKAPGLEQFLRDTANSLLDNLITEGAPADLRNDFADPLATALH
CKVLGIPQEDGPKLFRSLSIAFMSSADPIPAAKINWDRDIEYMAGILENPNITTGLMGELSRLRKDPAYSHVS
DELFATIGVTFFGAGVISTGSFLTTALISLIQRPQLRNLLHEKPELIPAGVEELLRINLSFADGLPRLATADI
QVGDVLVRKGELVLVLLEGANFDPEYFPNPGSIELDRPNPTSHLAFGRGQHFCPGSALGRRHAQIGIEALLKK
MPGVDLAVPIDQLVWRTRFQRRIPERLPVLW
>CYP121A1_Mycobacterium tuberculosis 12974 2621695743
MTATVLLEVPFSARGDRIPDAVAELRTREPIRKVRTITGAEAWLVSSYALCTQVLEDRRFSMKETAAAGAPRL
NALTVPPEVVNNMGNIADAGLRKAVMKAITPKAPGLEQFLRDTANSLLDNLITEGAPADLRNDFADPLATALH
CKVLGIPQEDGPKLFRSLSIAFMSSADPIPAAKINWDRDIEYMAGILENPNITTGLMGELSRLRKDPAYSHVS
DELFATIGVTFFGAGVISTGSFLTTALISLIQRPQLRNLLHEKPELIPAGVEELLRINLSFADGLPRLATADI
QVGDVLVRKGELVLVLLEGANFDPEYFPNPGSIELDRPNPTSHLAFGRGQHFCPGSALGRRHAQIGIEALLKK
MPGVDLAVPIDQLVWRTRFQRRIPERLPVLW
>CYP121A1_Mycobacterium tuberculosis 1300037 2692715896
MTATVLLEVPFSARGDRIPDAVAELRTREPIRKVRTITGAEAWLVSSYALCTQVLEDRRFSMKETAAAGAPRL
NALTVPPEVVNNMGNIADAGLRKAVMKAITPKAPGLEQFLRDTANSLLDNLITEGAPADLRNDFADPLATALH
CKVLGIPQEDGPKLFRSLSIAFMSSADPIPAAKINWDRDIEYMAGILENPNITTGLMGELSRLRKDPAYSHVS
DELFATIGVTFFGAGVISTGSFLTTALISLIQRPQLRNLLHEKPELIPAGVEELLRINLSFADGLPRLATADI

QVGDVLVRKGELVLVLLEGANFDPEYFPNPGSIELDRPNPTSHLAFGRGQHFCPGSALGRRHAQIGIEALLKK
MPGVDLAVPIDQLVWRTRFQRRIPERLPVLW
>CYP121A1_Mycobacterium tuberculosis 1300325 2659691284
MTATVLLEVPFSARGDRIPDAVAELRTREPIRKVRTITGAEAWLVSSYALCTQVLEDRRFSMKETAAAGAPRL
NALTVPPEVVNNMGNIADAGLRKAVMKAITPKAPGLEQFLRDTANSLLDNLITEGAPADLRNDFADPLATALH
CKVLGIPQEDGPKLFRSLSIAFMSSADPIPAAKINWDRDIEYMAGILENPNITTGLMGELSRLRKDPAYSHVS
DELFATIGVTFFGAGVISTGSFLTTALISLIQRPQLRNLLHEKPELIPAGVEELLRINLSFADGLPRLATADI
QVGDVLVRKGELVLVLLEGANFDPEYFPNPGSIELDRPNPTSHLAFGRGQHFCPGSALGRRHAQIGIEALLKK
MPGVDLAVPIDQLVWRTRFQRRIPERLPVLW
>CYP121A1_Mycobacterium tuberculosis 13-2459 2641678911
MTATVLLEVPFSARGDRIPDAVAELRTREPIRKVRTITGAEAWLVSSYALCTQVLEDRRFSMKETAAAGAPRL
NALTVPPEVVNNMGNIADAGLRKAVMKAITPKAPGLEQFLRDTANSLLDNLITEGAPADLRNDFADPLATALH
CKVLGIPQEDGPKLFRSLSIAFMSSADPIPAAKINWDRDIEYMAGILENPNITTGLMGELSRLRKDPAYSHVS
DELFATIGVTFFGAGVISTGSFLTTALISLIQRPQLRNLLHEKPELIPAGVEELLRINLSFADGLPRLATADI
QVGDVLVRKGELVLVLLEGANFDPEYFPNPGSIELDRPNPTSHLAFGRGQHFCPGSALGRRHAQIGIEALLKK
MPGVDLAVPIDQLVWRTRFQRRIPERLPVLW
>CYP121A1_Mycobacterium tuberculosis 13-4152 2635743043
MTATVLLEVPFSARGDRIPDAVAELRTREPIRKVRTITGAEAWLVSSYALCTQVLEDRRFSMKETAAAGAPRL
NALTVPPEVVNNMGNIADAGLRKAVMKAITPKAPGLEQFLRDTANSLLDNLITEGAPADLRNDFADPLATALH
CKVLGIPQEDGPKLFRSLSIAFMSSADPIPAAKINWDRDIEYMAGILENPNITTGLMGELSRLRKDPAYSHVS
DELFATIGVTFFGAGVISTGSFLTTALISLIQRPQLRNLLHEKPELIPAGVEELLRINLSFADGLPRLATADI
QVGDVLVRKGELVLVLLEGANFDPEYFPNPGSIELDRPNPTSHLAFGRGQHFCPGSALGRRHAQIGIEALLKK
MPGVDLAVPIDQLVWRTRFQRRIPERLPVLW
>CYP121A1_Mycobacterium tuberculosis 1458 2722808897
MTATVLLEVPFSARGDRIPDAVAELRTREPIRKVRTITGAEAWLVSSYALCTQVLEDRRFSMKETAAAGAPRL
NALTVPPEVVNNMGNIADAGLRKAVMKAITPKAPGLEQFLRDTANSLLDNLITEGAPADLRNDFADPLATALH
CKVLGIPQEDGPKLFRSLSIAFMSSADPIPAAKINWDRDIEYMAGILENPNITTGLMGELSRLRKDPAYSHVS
DELFATIGVTFFGAGVISTGSFLTTALISLIQRPQLRNLLHEKPELIPAGVEELLRINLSFADGLPRLATADI
QVGDVLVRKGELVLVLLEGANFDPEYFPNPGSIELDRPNPTSHLAFGRGQHFCPGSALGRRHAQIGIEALLKK
MPGVDLAVPIDQLVWRTRFQRRIPERLPVLW
>CYP121A1_Mycobacterium tuberculosis 1500034 2658282835
MTATVLLEVPFSARGDRIPDAVAELRTREPIRKVRTITGAEAWLVSSYALCTQVLEDRRFSMKETAAAGAPRL
NALTVPPEVVNNMGNIADAGLRKAVMKAITPKAPGLEQFLRDTANSLLDNLITEGAPADLRNDFADPLATALH
CKVLGIPQEDGPKLFRSLSIAFMSSADPIPAAKINWDRDIEYMAGILENPNITTGLMGELSRLRKDPAYSHVS
DELFATIGVTFFGAGVISTGSFLTTALISLIQRPQLRNLLHEKPELIPAGVEELLRINLSFADGLPRLATADI
QVGDVLVRKGELVLVLLEGANFDPEYFPNPGSIELDRPNPTSHLAFGRGQHFCPGSALGRRHAQIGIEALLKK
MPGVDLAVPIDQLVWRTRFQRRIPERLPVLW
>CYP121A1_Mycobacterium tuberculosis 1500283 2658888643
MTATVLLEVPFSARGDRIPDAVAELRTREPIRKVRTITGAEAWLVSSYALCTQVLEDRRFSMKETAAAGAPRL
NALTVPPEVVNNMGNIADAGLRKAVMKAITPKAPGLEQFLRDTANSLLDNLITEGAPADLRNDFADPLATALH
CKVLGIPQEDGPKLFRSLSIAFMSSADPIPAAKINWDRDIEYMAGILENPNITTGLMGELSRLRKDPAYSHVS
DELFATIGVTFFGAGVISTGSFLTTALISLIQRPQLRNLLHEKPELIPAGVEELLRINLSFADGLPRLATADI
QVGDVLVRKGELVLVLLEGANFDPEYFPNPGSIELDRPNPTSHLAFGRGQHFCPGSALGRRHAQIGIEALLKK
MPGVDLAVPIDQLVWRTRFQRRIPERLPVLW
>CYP121A1_Mycobacterium tuberculosis 1700089 2667082832
MTATVLLEVPFSARGDRIPDAVAELRTREPIRKVRTITGAEAWLVSSYALCTQVLEDRRFSMKETAAAGAPRL
NALTVPPEVVNNMGNIADAGLRKAVMKAITPKAPGLEQFLRDTANSLLDNLITEGAPADLRNDFADPLATALH
CKVLGIPQEDGPKLFRSLSIAFMSSADPIPAAKINWDRDIEYMAGILENPNITTGLMGELSRLRKDPAYSHVS
DELFATIGVTFFGAGVISTGSFLTTALISLIQRPQLRNLLHEKPELIPAGVEELLRINLSFADGLPRLATADI
QVGDVLVRKGELVLVLLEGANFDPEYFPNPGSIELDRPNPTSHLAFGRGQHFCPGSALGRRHAQIGIEALLKK
MPGVDLAVPIDQLVWRTRFQRRIPERLPVLW
>CYP121A1_Mycobacterium tuberculosis 1700284 2702164282
MTATVLLEVPFSARGDRIPDAVAELRTREPIRKVRTITGAEAWLVSSYALCTQVLEDRRFSMKETAAAGAPRL
NALTVPPEVVNNMGNIADAGLRKAVMKAITPKAPGLEQFLRDTANSLLDNLITEGAPADLRNDFADPLATALH
CKVLGIPQEDGPKLFRSLSIAFMSSADPIPAAKINWDRDIEYMAGILENPNITTGLMGELSRLRKDPAYSHVS
DELFATIGVTFFGAGVISTGSFLTTALISLIQRPQLRNLLHEKPELIPAGVEELLRINLSFADGLPRLATADI
QVGDVLVRKGELVLVLLEGANFDPEYFPNPGSIELDRPNPTSHLAFGRGQHFCPGSALGRRHAQIGIEALLKK
MPGVDLAVPIDQLVWRTRFQRRIPERLPVLW

>CYP121A1_Mycobacterium tuberculosis 1700745 2660523606
MTATVLLEVPFSARGDRIPDAVAELRTREPIRKVRTITGAEAWLVSSYALCTQVLEDRRFSMKETAAAGAPRL
NALTVPPEVVNNMGNIADAGLRKAVMKAITPKAPGLEQFLRDTANSLLDNLITEGAPADLRNDFADPLATALH
CKVLGIPQEDGPKLFRSLSIAFMSSADPIPAAKINWDRDIEYMAGILENPNITTGLMGELSRLRKDPAYSHVS
DELFATIGVTFFGAGVISTGSFLTTALISLIQRPQLRNLLHEKPELIPAGVEELLRINLSFADGLPRLATADI
QVGDVLVRKGELVLVLLEGANFDPEYFPNPGSIELDRPNPTSHLAFGRGQHFCPGSALGRRHAQIGIEALLKK
MPGVDLAVPIDQLVWRTRFQRRIPERLPVLW
>CYP121A1_Mycobacterium tuberculosis 1700893 2659619423
MTATVLLEVPFSARGDRIPDAVAELRTREPIRKVRTITGAEAWLVSSYALCTQVLEDRRFSMKETAAAGAPRL
NALTVPPEVVNNMGNIADAGLRKAVMKAITPKAPGLEQFLRDTANSLLDNLITEGAPADLRNDFADPLATALH
CKVLGIPQEDGPKLFRSLSIAFMSSADPIPAAKINWDRDIEYMAGILENPNITTGLMGELSRLRKDPAYSHVS
DELFATIGVTFFGAGVISTGSFLTTALISLIQRPQLRNLLHEKPELIPAGVEELLRINLSFADGLPRLATADI
QVGDVLVRKGELVLVLLEGANFDPEYFPNPGSIELDRPNPTSHLAFGRGQHFCPGSALGRRHAQIGIEALLKK
MPGVDLAVPIDQLVWRTRFQRRIPERLPVLW
>CYP121A1_Mycobacterium tuberculosis 18b 2640957617
MGLNTAIATRVNGTPPPEVPIADIELGSLDFWALDDDVRDGAFATLRREAPISFWPTIELPGFVAGNGHWALT
KYDDVFYASRHPDIFSSYPNITINDQTPELAEYFGSMIVLDDPRHQRLRSIVSRAFTPKVVARIEAAVRDRAH
RLVSSMIANNPDRQADLVSELAGPLPLQIICDMMGIPKADHQRIFHWTNVILGFGDPDLATDFDEFMQVSADI
GAYATALAEDRRVNHHDDLTSSLVEAEVDGERLSSREIASFFILLVVAGNETTRNAITHGVLALSRYPEQRDR
WWSDFDGLAPTAVEEIVRWASPVVYMRRTLTQDIELRGTKMAAGDKVSLWYCSANRDESKFADPWTFDLARNP
NPHLGFGGGGAHFCLGANLARREIRVAFDELRRQMPDVVATEEPARLLSQFIHGIKTLPVTWS
>CYP121A1_Mycobacterium tuberculosis 200066 2693441612
MTATVLLEVPFSARGDRIPDAVAELRTREPIRKVRTITGAEAWLVSSYALCTQVLEDRRFSMKETAAAGAPRL
NALTVPPEVVNNMGNIADAGLRKAVMKAITPKAPGLEQFLRDTANSLLDNLITEGAPADLRNDFADPLATALH
CKVLGIPQEDGPKLFRSLSIAFMSSADPIPAAKINWDRDIEYMAGILENPNITTGLMGELSRLRKDPAYSHVS
DELFATIGVTFFGAGVISTGSFLTTALISLIQRPQLRNLLHEKPELIPAGVEELLRINLSFADGLPRLATADI
QVGDVLVRKGELVLVLLEGANFDPEYFPNPGSIELDRPNPTSHLAFGRGQHFCPGSALGRRHAQIGIEALLKK
MPGVDLAVPIDQLVWRTRFQRRIPERLPVLW
>CYP121A1_Mycobacterium tuberculosis 200541 2691881132
MTATVLLEVPFSARGDRIPDAVAELRTREPIRKVRTITGAEAWLVSSYALCTQVLEDRRFSMKETAAAGAPRL
NALTVPPEVVNNMGNIADAGLRKAVMKAITPKAPGLEQFLRDTANSLLDNLITEGAPADLRNDFADPLATALH
CKVLGIPQEDGPKLFRSLSIAFMSSADPIPAAKINWDRDIEYMAGILENPNITTGLMGELSRLRKDPAYSHVS
DELFATIGVTFFGAGVISTGSFLTTALISLIQRPQLRNLLHEKPELIPAGVEELLRINLSFADGLPRLATADI
QVGDVLVRKGELVLVLLEGANFDPEYFPNPGSIELDRPNPTSHLAFGRGQHFCPGSALGRRHAQIGIEALLKK
MPGVDLAVPIDQLVWRTRFQRRIPERLPVLW
>CYP121A1_Mycobacterium tuberculosis 201035 2662920744
MTATVLLEVPFSARGDRIPDAVAELRTREPIRKVRTITGAEAWLVSSYALCTQVLEDRRFSMKETAAAGAPRL
NALTVPPEVVNNMGNIADAGLRKAVMKAITPKAPGLEQFLRDTANSLLDNLITEGAPADLRNDFADPLATALH
CKVLGIPQEDGPKLFRSLSIAFMSSADPIPAAKINWDRDIEYMAGILENPNITTGLMGELSRLRKDPAYSHVS
DELFATIGVTFFGAGVISTGSFLTTALISLIQRPQLRNLLHEKPELIPAGVEELLRINLSFADGLPRLATADI
QVGDVLVRKGELVLVLLEGANFDPEYFPNPGSIELDRPNPTSHLAFGRGQHFCPGSALGRRHAQIGIEALLKK
MPGVDLAVPIDQLVWRTRFQRRIPERLPVLW
>CYP121A1_Mycobacterium tuberculosis 201127 2702147904
MTATVLLEVPFSARGDRIPDAVAELRTREPIRKVRTITGAEAWLVSSYALCTQVLEDRRFSMKETAAAGAPRL
NALTVPPEVVNNMGNIADAGLRKAVMKAITPKAPGLEQFLRDTANSLLDNLITEGAPADLRNDFADPLATALH
CKVLGIPQEDGPKLFRSLSIAFMSSADPIPAAKINWDRDIEYMAGILENPNITTGLMGELSRLRKDPAYSHVS
DELFATIGVTFFGAGVISTGSFLTTALISLIQRPQLRNLLHEKPELIPAGVEELLRINLSFADGLPRLATADI
QVGDVLVRKGELVLVLLEGANFDPEYFPNPGSIELDRPNPTSHLAFGRGQHFCPGSALGRRHAQIGIEALLKK
MPGVDLAVPIDQLVWRTRFQRRIPERLPVLW
>CYP121A1_Mycobacterium tuberculosis 2242 2687551225
MTATVLLEVPFSARGDRIPDAVAELRTREPIRKVRTITGAEAWLVSSYALCTQVLEDRRFSMKETAAAGAPRL
NALTVPPEVVNNMGNIADAGLRKAVMKAITPKAPGLEQFLRDTANSLLDNLITEGAPADLRNDFADPLATALH
CKVLGIPQEDGPKLFRSLSIAFMSSADPIPAAKINWDRDIEYMAGILENPNITTGLMGELSRLRKDPAYSHVS
DELFATIGVTFFGAGVISTGSFLTTALISLIQRPQLRNLLHEKPELIPAGVEELLRINLSFADGLPRLATADI
QVGDVLVRKGELVLVLLEGANFDPEYFPNPGSIELDRPNPTSHLAFGRGQHFCPGSALGRRHAQIGIEALLKK
MPGVDLAVPIDQLVWRTRFQRRIPERLPVLW
>CYP121A1_Mycobacterium tuberculosis 2279 2686590687

MTATVLLEVPFSARGDRIPDAVAELRTREPIRKVRTITGAEAWLVSSYALCTQVLEDRRFSMKETAAAGAPRL
NALTVPPEVVNNMGNIADAGLRKAVMKAITPKAPGLEQFLRDTANSLLDNLITEGAPADLRNDFADPLATALH
CKVLGIPQEDGPKLFRSLSIAFMSSADPIPAAKINWDRDIEYMAGILENPNITTGLMGELSRLRKDPAYSHVS
DELFATIGVTFFGAGVISTGSFLTTALISLIQRPQLRNLLHEKPELIPAGVEELLRINLSFADGLPRLATADI
QVGDVLVRKGELVLVLLEGANFDPEYFPNPGSIELDRPNPTSHLAFGRGQHFCPGSALGRRHAQIGIEALLKK
MPGVDLAVPIDQLVWRTRFQRRIPERLPVLW
>CYP121A1_Mycobacterium tuberculosis 2489 2621716654
MTATVLLEVPFSARGDRIPDAVAELRTREPIRKVRTITGAEAWLVSSYALCTQVLEDRRFSMKETAAAGAPRL
NALTVPPEVVNNMGNIADAGLRKAVMKAITPKAPGLEQFLRDTANSLLDNLITEGAPADLRNDFADPLATALH
CKVLGIPQEDGPKLFRSLSIAFMSSADPIPAAKINWDRDIEYMAGILENPNITTGLMGELSRLRKDPAYSHVS
DELFATIGVTFFGAGVISTGSFLTTALISLIQRPQLRNLLHEKPELIPAGVEELLRINLSFADGLPRLATADI
QVGDVLVRKGELVLVLLEGANFDPEYFPNPGSIELDRPNPTSHLAFGRGQHFCPGSALGRRHAQIGIEALLKK
MPGVDLAVPIDQLVWRTRFQRRIPERLPVLW
>CYP121A1_Mycobacterium tuberculosis 300191 2669248089
MTATVLLEVPFSARGDRIPDAVAELRTREPIRKVRTITGAEAWLVSSYALCTQVLEDRRFSMKETAAAGAPRL
NALTVPPEVVNNMGNIADAGLRKAVMKAITPKAPGLEQFLRDTANSLLDNLITEGAPADLRNDFADPLATALH
CKVLGIPQEDGPKLFRSLSIAFMSSADPIPAAKINWDRDIEYMAGILENPNITTGLMGELSRLRKDPAYSHVS
DELFATIGVTFFGAGVISTGSFLTTALISLIQRPQLRNLLHEKPELIPAGVEELLRINLSFADGLPRLATADI
QVGDVLVRKGELVLVLLEGANFDPEYFPNPGSIELDRPNPTSHLAFGRGQHFCPGSALGRRHAQIGIEALLKK
MPGVDLAVPIDQLVWRTRFQRRIPERLPVLW
>CYP121A1_Mycobacterium tuberculosis 300234 2673506835
MTATVLLEVPFSARGDRIPDAVAELRTREPIRKVRTITGAEAWLVSSYALCTQVLEDRRFSMKETAAAGAPRL
NALTVPPEVVNNMGNIADAGLRKAVMKAITPKAPGLEQFLRDTANSLLDNLITEGAPADLRNDFADPLATALH
CKVLGIPQEDGPKLFRSLSIAFMSSADPIPAAKINWDRDIEYMAGILENPNITTGLMGELSRLRKDPAYSHVS
DELFATIGVTFFGAGVISTGSFLTTALISLIQRPQLRNLLHEKPELIPAGVEELLRINLSFADGLPRLATADI
QVGDVLVRKGELVLVLLEGANFDPEYFPNPGSIELDRPNPTSHLAFGRGQHFCPGSALGRRHAQIGIEALLKK
MPGVDLAVPIDQLVWRTRFQRRIPERLPVLW
>CYP121A1_Mycobacterium tuberculosis 300260 2665927182
MTATVLLEVPFSARGDRIPDAVAELRTREPIRKVRTITGAEAWLVSSYALCTQVLEDRRFSMKETAAAGAPRL
NALTVPPEVVNNMGNIADAGLRKAVMKAITPKAPGLEQFLRDTANSLLDNLITEGAPADLRNDFADPLATALH
CKVLGIPQEDGPKLFRSLSIAFMSSADPIPAAKINWDRDIEYMAGILENPNITTGLMGELSRLRKDPAYSHVS
DELFATIGVTFFGAGVISTGSFLTTALISLIQRPQLRNLLHEKPELIPAGVEELLRINLSFADGLPRLATADI
QVGDVLVRKGELVLVLLEGANFDPEYFPNPGSIELDRPNPTSHLAFGRGQHFCPGSALGRRHAQIGIEALLKK
MPGVDLAVPIDQLVWRTRFQRRIPERLPVLW
>CYP121A1_Mycobacterium tuberculosis 300798 2666225012
MTATVLLEVPFSARGDRIPDAVAELRTREPIRKVRTITGAEAWLVSSYALCTQVLEDRRFSMKETAAAGAPRL
NALTVPPEVVNNMGNIADAGLRKAVMKAITPKAPGLEQFLRDTANSLLDNLITEGAPADLRNDFADPLATALH
CKVLGIPQEDGPKLFRSLSIAFMSSADPIPAAKINWDRDIEYMAGILENPNITTGLMGELSRLRKDPAYSHVS
DELFATIGVTFFGAGVISTGSFLTTALISLIQRPQLRNLLHEKPELIPAGVEELLRINLSFADGLPRLATADI
QVGDVLVRKGELVLVLLEGANFDPEYFPNPGSIELDRPNPTSHLAFGRGQHFCPGSALGRRHAQIGIEALLKK
MPGVDLAVPIDQLVWRTRFQRRIPERLPVLW
>CYP121A1_Mycobacterium tuberculosis 301092 2682908214
MTATVLLEVPFSARGDRIPDAVAELRTREPIRKVRTITGAEAWLVSSYALCTQVLEDRRFSMKETAAAGAPRL
NALTVPPEVVNNMGNIADAGLRKAVMKAITPKAPGLEQFLRDTANSLLDNLITEGAPADLRNDFADPLATALH
CKVLGIPQEDGPKLFRSLSIAFMSSADPIPAAKINWDRDIEYMAGILENPNITTGLMGELSRLRKDPAYSHVS
DELFATIGVTFFGAGVISTGSFLTTALISLIQRPQLRNLLHEKPELIPAGVEELLRINLSFADGLPRLATADI
QVGDVLVRKGELVLVLLEGANFDPEYFPNPGSIELDRPNPTSHLAFGRGQHFCPGSALGRRHAQIGIEALLKK
MPGVDLAVPIDQLVWRTRFQRRIPERLPVLW
>CYP121A1_Mycobacterium tuberculosis 301725 2697159158
MTATVLLEVPFSARGDRIPDAVAELRTREPIRKVRTITGAEAWLVSSYALCTQVLEDRRFSMKETAAAGAPRL
NALTVPPEVVNNMGNIADAGLRKAVMKAITPKAPGLEQFLRDTANSLLDNLITEGAPADLRNDFADPLATALH
CKVLGIPQEDGPKLFRSLSIAFMSSADPIPAAKINWDRDIEYMAGILENPNITTGLMGELSRLRKDPAYSHVS
DELFATIGVTFFGAGVISTGSFLTTALISLIQRPQLRNLLHEKPELIPAGVEELLRINLSFADGLPRLATADI
QVGDVLVRKGELVLVLLEGANFDPEYFPNPGSIELDRPNPTSHLAFGRGQHFCPGSALGRRHAQIGIEALLKK
MPGVDLAVPIDQLVWRTRFQRRIPERLPVLW
>CYP121A1_Mycobacterium tuberculosis 301747 2669899405
MTATVLLEVPFSARGDRIPDAVAELRTREPIRKVRTITGAEAWLVSSYALCTQVLEDRRFSMKETAAAGAPRL
NALTVPPEVVNNMGNIADAGLRKAVMKAITPKAPGLEQFLRDTANSLLDNLITEGAPADLRNDFADPLATALH

CKVLGIPQEDGPKLFRSLSIAFMSSADPIPAAKINWDRDIEYMAGILENPNITTGLMGELSRLRKDPAYSHVS
DELFATIGVTFFGAGVISTGSFLTTALISLIQRPQLRNLLHEKPELIPAGVEELLRINLSFADGLPRLATADI
QVGDVLVRKGELVLVLLEGANFDPEYFPNPGSIELDRPNPTSHLAFGRGQHFCPGSALGRRHAQIGIEALLKK
MPGVDLAVPIDQLVWRTRFQRRIPERLPVLW
>CYP121A1_Mycobacterium tuberculosis 301816 2673021308
MTATVLLEVPFSARGDRIPDAVAELRTREPIRKVRTITGAEAWLVSSYALCTQVLEDRRFSMKETAAAGAPRL
NALTVPPEVVNNMGNIADAGLRKAVMKAITPKAPGLEQFLRDTANSLLDNLITEGAPADLRNDFADPLATALH
CKVLGIPQEDGPKLFRSLSIAFMSSADPIPAAKINWDRDIEYMAGILENPNITTGLMGELSRLRKDPAYSHVS
DELFATIGVTFFGAGVISTGSFLTTALISLIQRPQLRNLLHEKPELIPAGVEELLRINLSFADGLPRLATADI
QVGDVLVRKGELVLVLLEGANFDPEYFPNPGSIELDRPNPTSHLAFGRGQHFCPGSALGRRHAQIGIEALLKK
MPGVDLAVPIDQLVWRTRFQRRIPERLPVLW
>CYP121A1_Mycobacterium tuberculosis 302174 2661455776
MTATVLLEVPFSARGDRIPDAVAELRTREPIRKVRTITGAEAWLVSSYALCTQVLEDRRFSMKETAAAGAPRL
NALTVPPEVVNNMGNIADAGLRKAVMKAITPKAPGLEQFLRDTANSLLDNLITEGAPADLRNDFADPLATALH
CKVLGIPQEDGPKLFRSLSIAFMSSADPIPAAKINWDRDIEYMAGILENPNITTGLMGELSRLRKDPAYSHVS
DELFATIGVTFFGAGVISTGSFLTTALISLIQRPQLRNLLHEKPELIPAGVEELLRINLSFADGLPRLATADI
QVGDVLVRKGELVLVLLEGANFDPEYFPNPGSIELDRPNPTSHLAFGRGQHFCPGSALGRRHAQIGIEALLKK
MPGVDLAVPIDQLVWRTRFQRRIPERLPVLW
>CYP121A1_Mycobacterium tuberculosis 302350 2692569999
MTATVLLEVPFSARGDRIPDAVAELRTREPIRKVRTITGAEAWLVSSYALCTQVLEDRRFSMKETAAAGAPRL
NALTVPPEVVNNMGNIADAGLRKAVMKAITPKAPGLEQFLRDTANSLLDNLITEGAPADLRNDFADPLATALH
CKVLGIPQEDGPKLFRSLSIAFMSSADPIPAAKINWDRDIEYMAGILENPNITTGLMGELSRLRKDPAYSHVS
DELFATIGVTFFGAGVISTGSFLTTALISLIQRPQLRNLLHEKPELIPAGVEELLRINLSFADGLPRLATADI
QVGDVLVRKGELVLVLLEGANFDPEYFPNPGSIELDRPNPTSHLAFGRGQHFCPGSALGRRHAQIGIEALLKK
MPGVDLAVPIDQLVWRTRFQRRIPERLPVLW
>CYP121A1_Mycobacterium tuberculosis 302642 2694440333
MTATVLLEVPFSARGDRIPDAVAELRTREPIRKVRTITGAEAWLVSSYALCTQVLEDRRFSMKETAAAGAPRL
NALTVPPEVVNNMGNIADAGLRKAVMKAITPKAPGLEQFLRDTANSLLDNLITEGAPADLRNDFADPLATALH
CKVLGIPQEDGPKLFRSLSIAFMSSADPIPAAKINWDRDIEYMAGILENPNITTGLMGELSRLRKDPAYSHVS
DELFATIGVTFFGAGVISTGSFLTTALISLIQRPQLRNLLHEKPELIPAGVEELLRINLSFADGLPRLATADI
QVGDVLVRKGELVLVLLEGANFDPEYFPNPGSIELDRPNPTSHLAFGRGQHFCPGSALGRRHAQIGIEALLKK
MPGVDLAVPIDQLVWRTRFQRRIPERLPVLW
>CYP121A1_Mycobacterium tuberculosis 38088 2604025448
MTATVLLEVPFSARGDRIPDAVAELRTREPIRKVRTITGAEAWLVSSYALCTQVLEDRRFSMKETAAAGAPRL
NALTVPPEVVNNMGNIADAGLRKAVMKAITPKAPGLEQFLRDTANSLLDNLITEGAPADLRNDFADPLATALH
CKVLGIPQEDGPKLFRSLSIAFMSSADPIPAAKINWDRDIEYMAGILENPNITTGLMGELSRLRKDPAYSHVS
DELFATIGVTFFGAGVISTGSFLTTALISLIQRPQLRNLLHEKPELIPAGVEELLRINLSFADGLPRLATADI
QVGDVLVRKGELVLVLLEGANFDPEYFPNPGSIELDRPNPTSHLAFGRGQHFCPGSALGRRHAQIGIEALLKK
MPGVDLAVPIDQLVWRTRFQRRIPERLPVLW
>CYP121A1_Mycobacterium tuberculosis 38765 2605419443
MTATVLLEVPFSARGDRIPDAVAELRTREPIRKVRTITGAEAWLVSSYALCTQVLEDRRFSMKETAAAGAPRL
NALTVPPEVVNNMGNIADAGLRKAVMKAITPKAPGLEQFLRDTANSLLDNLITEGAPADLRNDFADPLATALH
CKVLGIPQEDGPKLFRSLSIAFMSSADPIPAAKINWDRDIEYMAGILENPNITTGLMGELSRLRKDPAYSHVS
DELFATIGVTFFGAGVISTGSFLTTALISLIQRPQLRNLLHEKPELIPAGVEELLRINLSFADGLPRLATADI
QVGDVLVRKGELVLVLLEGANFDPEYFPNPGSIELDRPNPTSHLAFGRGQHFCPGSALGRRHAQIGIEALLKK
MPGVDLAVPIDQLVWRTRFQRRIPERLPVLW
>CYP121A1_Mycobacterium tuberculosis 400044 2659627604
MTATVLLEVPFSARGDRIPDAVAELRTREPIRKVRTITGAEAWLVSSYALCTQVLEDRRFSMKETAAAGAPRL
NALTVPPEVVNNMGNIADAGLRKAVMKAITPKAPGLEQFLRDTANSLLDNLITEGAPADLRNDFADPLATALH
CKVLGIPQEDGPKLFRSLSIAFMSSADPIPAAKINWDRDIEYMAGILENPNITTGLMGELSRLRKDPAYSHVS
DELFATIGVTFFGAGVISTGSFLTTALISLIQRPQLRNLLHEKPELIPAGVEELLRINLSFADGLPRLATADI
QVGDVLVRKGELVLVLLEGANFDPEYFPNPGSIELDRPNPTSHLAFGRGQHFCPGSALGRRHAQIGIEALLKK
MPGVDLAVPIDQLVWRTRFQRRIPERLPVLW
>CYP121A1_Mycobacterium tuberculosis 400491 2665697071
MTATVLLEVPFSARGDRIPDAVAELRTREPIRKVRTITGAEAWLVSSYALCTQVLEDRRFSMKETAAAGAPRL
NALTVPPEVVNNMGNIADAGLRKAVMKAITPKAPGLEQFLRDTANSLLDNLITEGAPADLRNDFADPLATALH
CKVLGIPQEDGPKLFRSLSIAFMSSADPIPAAKINWDRDIEYMAGILENPNITTGLMGELSRLRKDPAYSHVS
DELFATIGVTFFGAGVISTGSFLTTALISLIQRPQLRNLLHEKPELIPAGVEELLRINLSFADGLPRLATADI

QVGDVLVRKGELVLVLLEGANFDPEYFPNPGSIELDRPNPTSHLAFGRGQHFCPGSALGRRHAQIGIEALLKK
MPGVDLAVPIDQLVWRTRFQRRIPERLPVLW
>CYP121A1_Mycobacterium tuberculosis 400731 2702143708
MTATVLLEVPFSARGDRIPDAVAELRTREPIRKVRTITGAEAWLVSSYALCTQVLEDRRFSMKETAAAGAPRL
NALTVPPEVVNNMGNIADAGLRKAVMKAITPKAPGLEQFLRDTANSLLDNLITEGAPADLRNDFADPLATALH
CKVLGIPQEDGPKLFRSLSIAFMSSADPIPAAKINWDRDIEYMAGILENPNITTGLMGELSRLRKDPAYSHVS
DELFATIGVTFFGAGVISTGSFLTTALISLIQRPQLRNLLHEKPELIPAGVEELLRINLSFADGLPRLATADI
QVGDVLVRKGELVLVLLEGANFDPEYFPNPGSIELDRPNPTSHLAFGRGQHFCPGSALGRRHAQIGIEALLKK
MPGVDLAVPIDQLVWRTRFQRRIPERLPVLW
>CYP121A1_Mycobacterium tuberculosis 400958 2657561938
MTATVLLEVPFSARGDRIPDAVAELRTREPIRKVRTITGAEAWLVSSYALCTQVLEDRRFSMKETAAAGAPRL
NALTVPPEVVNNMGNIADAGLRKAVMKAITPKAPGLEQFLRDTANSLLDNLITEGAPADLRNDFADPLATALH
CKVLGIPQEDGPKLFRSLSIAFMSSADPIPAAKINWDRDIEYMAGILENPNITTGLMGELSRLRKDPAYSHVS
DELFATIGVTFFGAGVISTGSFLTTALISLIQRPQLRNLLHEKPELIPAGVEELLRINLSFADGLPRLATADI
QVGDVLVRKGELVLVLLEGANFDPEYFPNPGSIELDRPNPTSHLAFGRGQHFCPGSALGRRHAQIGIEALLKK
MPGVDLAVPIDQLVWRTRFQRRIPERLPVLW
>CYP121A1_Mycobacterium tuberculosis 400968 2665483565
MTATVLLEVPFSARGDRIPDAVAELRTREPIRKVRTITGAEAWLVSSYALCTQVLEDRRFSMKETAAAGAPRL
NALTVPPEVVNNMGNIADAGLRKAVMKAITPKAPGLEQFLRDTANSLLDNLITEGAPADLRNDFADPLATALH
CKVLGIPQEDGPKLFRSLSIAFMSSADPIPAAKINWDRDIEYMAGILENPNITTGLMGELSRLRKDPAYSHVS
DELFATIGVTFFGAGVISTGSFLTTALISLIQRPQLRNLLHEKPELIPAGVEELLRINLSFADGLPRLATADI
QVGDVLVRKGELVLVLLEGANFDPEYFPNPGSIELDRPNPTSHLAFGRGQHFCPGSALGRRHAQIGIEALLKK
MPGVDLAVPIDQLVWRTRFQRRIPERLPVLW
>CYP121A1_Mycobacterium tuberculosis 401206 2702152172
MTATVLLEVPFSARGDRIPDAVAELRTREPIRKVRTITGAEAWLVSSYALCTQVLEDRRFSMKETAAAGAPRL
NALTVPPEVVNNMGNIADAGLRKAVMKAITPKAPGLEQFLRDTANSLLDNLITEGAPADLRNDFADPLATALH
CKVLGIPQEDGPKLFRSLSIAFMSSADPIPAAKINWDRDIEYMAGILENPNITTGLMGELSRLRKDPAYSHVS
DELFATIGVTFFGAGVISTGSFLTTALISLIQRPQLRNLLHEKPELIPAGVEELLRINLSFADGLPRLATADI
QVGDVLVRKGELVLVLLEGANFDPEYFPNPGSIELDRPNPTSHLAFGRGQHFCPGSALGRRHAQIGIEALLKK
MPGVDLAVPIDQLVWRTRFQRRIPERLPVLW
>CYP121A1_Mycobacterium tuberculosis 401243 2702167665
MTATVLLEVPFSARGDRIPDAVAELRTREPIRKVRTITGAEAWLVSSYALCTQVLEDRRFSMKETAAAGAPRL
NALTVPPEVVNNMGNIADAGLRKAVMKAITPKAPGLEQFLRDTANSLLDNLITEGAPADLRNDFADPLATALH
CKVLGIPQEDGPKLFRSLSIAFMSSADPIPAAKINWDRDIEYMAGILENPNITTGLMGELSRLRKDPAYSHVS
DELFATIGVTFFGAGVISTGSFLTTALISLIQRPQLRNLLHEKPELIPAGVEELLRINLSFADGLPRLATADI
QVGDVLVRKGELVLVLLEGANFDPEYFPNPGSIELDRPNPTSHLAFGRGQHFCPGSALGRRHAQIGIEALLKK
MPGVDLAVPIDQLVWRTRFQRRIPERLPVLW
>CYP121A1_Mycobacterium tuberculosis 401325 2657825772
MTATVLLEVPFSARGDRIPDAVAELRTREPIRKVRTITGAEAWLVSSYALCTQVLEDRRFSMKETAAAGAPRL
NALTVPPEVVNNMGNIADAGLRKAVMKAITPKAPGLEQFLRDTANSLLDNLITEGAPADLRNDFADPLATALH
CKVLGIPQEDGPKLFRSLSIAFMSSADPIPAAKINWDRDIEYMAGILENPNITTGLMGELSRLRKDPAYSHVS
DELFATIGVTFFGAGVISTGSFLTTALISLIQRPQLRNLLHEKPELIPAGVEELLRINLSFADGLPRLATADI
QVGDVLVRKGELVLVLLEGANFDPEYFPNPGSIELDRPNPTSHLAFGRGQHFCPGSALGRRHAQIGIEALLKK
MPGVDLAVPIDQLVWRTRFQRRIPERLPVLW
>CYP121A1_Mycobacterium tuberculosis 401594 2701696418
MTATVLLEVPFSARGDRIPDAVAELRTREPIRKVRTITGAEAWLVSSYALCTQVLEDRRFSMKETAAAGAPRL
NALTVPPEVVNNMGNIADAGLRKAVMKAITPKAPGLEQFLRDTANSLLDNLITEGAPADLRNDFADPLATALH
CKVLGIPQEDGPKLFRSLSIAFMSSADPIPAAKINWDRDIEYMAGILENPNITTGLMGELSRLRKDPAYSHVS
DELFATIGVTFFGAGVISTGSFLTTALISLIQRPQLRNLLHEKPELIPAGVEELLRINLSFADGLPRLATADI
QVGDVLVRKGELVLVLLEGANFDPEYFPNPGSIELDRPNPTSHLAFGRGQHFCPGSALGRRHAQIGIEALLKK
MPGVDLAVPIDQLVWRTRFQRRIPERLPVLW
>CYP121A1_Mycobacterium tuberculosis 402206 2657631754
MTATVLLEVPFSARGDRIPDAVAELRTREPIRKVRTITGAEAWLVSSYALCTQVLEDRRFSMKETAAAGAPRL
NALTVPPEVVNNMGNIADAGLRKAVMKAITPKAPGLEQFLRDTANSLLDNLITEGAPADLRNDFADPLATALH
CKVLGIPQEDGPKLFRSLSIAFMSSADPIPAAKINWDRDIEYMAGILENPNITTGLMGELSRLRKDPAYSHVS
DELFATIGVTFFGAGVISTGSFLTTALISLIQRPQLRNLLHEKPELIPAGVEELLRINLSFADGLPRLATADI
QVGDVLVRKGELVLVLLEGANFDPEYFPNPGSIELDRPNPTSHLAFGRGQHFCPGSALGRRHAQIGIEALLKK
MPGVDLAVPIDQLVWRTRFQRRIPERLPVLW

>CYP121A1_Mycobacterium tuberculosis 402248 2659774215
MTATVLLEVPFSARGDRIPDAVAELRTREPIRKVRTITGAEAWLVSSYALCTQVLEDRRFSMKETAAAGAPRL
NALTVPPEVVNNMGNIADAGLRKAVMKAITPKAPGLEQFLRDTANSLLDNLITEGAPADLRNDFADPLATALH
CKVLGIPQEDGPKLFRSLSIAFMSSADPIPAAKINWDRDIEYMAGILENPNITTGLMGELSRLRKDPAYSHVS
DELFATIGVTFFGAGVISTGSFLTTALISLIQRPQLRNLLHEKPELIPAGVEELLRINLSFADGLPRLATADI
QVGDVLVRKGELVLVLLEGANFDPEYFPNPGSIELDRPNPTSHLAFGRGQHFCPGSALGRRHAQIGIEALLKK
MPGVDLAVPIDQLVWRTRFQRRIPERLPVLW
>CYP121A1_Mycobacterium tuberculosis 402251 2701656990
MTATVLLEVPFSARGDRIPDAVAELRTREPIRKVRTITGAEAWLVSSYALCTQVLEDRRFSMKETAAAGAPRL
NALTVPPEVVNNMGNIADAGLRKAVMKAITPKAPGLEQFLRDTANSLLDNLITEGAPADLRNDFADPLATALH
CKVLGIPQEDGPKLFRSLSIAFMSSADPIPAAKINWDRDIEYMAGILENPNITTGLMGELSRLRKDPAYSHVS
DELFATIGVTFFGAGVISTGSFLTTALISLIQRPQLRNLLHEKPELIPAGVEELLRINLSFADGLPRLATADI
QVGDVLVRKGELVLVLLEGANFDPEYFPNPGSIELDRPNPTSHLAFGRGQHFCPGSALGRRHAQIGIEALLKK
MPGVDLAVPIDQLVWRTRFQRRIPERLPVLW
>CYP121A1_Mycobacterium tuberculosis 402431 2657781652
MTATVLLEVPFSARGDRIPDAVAELRTREPIRKVRTITGAEAWLVSSYALCTQVLEDRRFSMKETAAAGAPRL
NALTVPPEVVNNMGNIADAGLRKAVMKAITPKAPGLEQFLRDTANSLLDNLITEGAPADLRNDFADPLATALH
CKVLGIPQEDGPKLFRSLSIAFMSSADPIPAAKINWDRDIEYMAGILENPNITTGLMGELSRLRKDPAYSHVS
DELFATIGVTFFGAGVISTGSFLTTALISLIQRPQLRNLLHEKPELIPAGVEELLRINLSFADGLPRLATADI
QVGDVLVRKGELVLVLLEGANFDPEYFPNPGSIELDRPNPTSHLAFGRGQHFCPGSALGRRHAQIGIEALLKK
MPGVDLAVPIDQLVWRTRFQRRIPERLPVLW
>CYP121A1_Mycobacterium tuberculosis 402629 2658123624
MTATVLLEVPFSARGDRIPDAVAELRTREPIRKVRTITGAEAWLVSSYALCTQVLEDRRFSMKETAAAGAPRL
NALTVPPEVVNNMGNIADAGLRKAVMKAITPKAPGLEQFLRDTANSLLDNLITEGAPADLRNDFADPLATALH
CKVLGIPQEDGPKLFRSLSIAFMSSADPIPAAKINWDRDIEYMAGILENPNITTGLMGELSRLRKDPAYSHVS
DELFATIGVTFFGAGVISTGSFLTTALISLIQRPQLRNLLHEKPELIPAGVEELLRINLSFADGLPRLATADI
QVGDVLVRKGELVLVLLEGANFDPEYFPNPGSIELDRPNPTSHLAFGRGQHFCPGSALGRRHAQIGIEALLKK
MPGVDLAVPIDQLVWRTRFQRRIPERLPVLW
>CYP121A1_Mycobacterium tuberculosis 402797 2701680032
MTATVLLEVPFSARGDRIPDAVAELRTREPIRKVRTITGAEAWLVSSYALCTQVLEDRRFSMKETAAAGAPRL
NALTVPPEVVNNMGNIADAGLRKAVMKAITPKAPGLEQFLRDTANSLLDNLITEGAPADLRNDFADPLATALH
CKVLGIPQEDGPKLFRSLSIAFMSSADPIPAAKINWDRDIEYMAGILENPNITTGLMGELSRLRKDPAYSHVS
DELFATIGVTFFGAGVISTGSFLTTALISLIQRPQLRNLLHEKPELIPAGVEELLRINLSFADGLPRLATADI
QVGDVLVRKGELVLVLLEGANFDPEYFPNPGSIELDRPNPTSHLAFGRGQHFCPGSALGRRHAQIGIEALLKK
MPGVDLAVPIDQLVWRTRFQRRIPERLPVLW
>CYP121A1_Mycobacterium tuberculosis 49-02 2687653637
MTATVLLEVPFSARGDRIPDAVAELRTREPIRKVRTITGAEAWLVSSYALCTQVLEDRRFSMKETAAAGAPRL
NALTVPPEVVNNMGNIADAGLRKAVMKAITPKAPGLEQFLRDTANSLLDNLITEGAPADLRNDFADPLATALH
CKVLGIPQEDGPKLFRSLSIAFMSSADPIPAAKINWDRDIEYMAGILENPNITTGLMGELSRLRKDPAYSHVS
DELFATIGVTFFGAGVISTGSFLTTALISLIQRPQLRNLLHEKPELIPAGVEELLRINLSFADGLPRLATADI
QVGDVLVRKGELVLVLLEGANFDPEYFPNPGSIELDRPNPTSHLAFGRGQHFCPGSALGRRHAQIGIEALLKK
MPGVDLAVPIDQLVWRTRFQRRIPERLPVLW
>CYP121A1_Mycobacterium tuberculosis 49375 2577431953
MTATVLLEVPFSARGDRIPDAVAELRTREPIRKVRTITGAEAWLVSSYALCTQVLEDRRFSMKETAAAGAPRL
NALTVPPEVVNNMGNIADAGLRKAVMKAITPKAPGLEQFLRDTANSLLDNLITEGAPADLRNDFADPLATALH
CKVLGIPQEDGPKLFRSLSIAFMSSADPIPAAKINWDRDIEYMAGILENPNITTGLMGELSRLRKDPAYSHVS
DELFATIGVTFFGAGVISTGSFLTTALISLIQRPQLRNLLHEKPELIPAGVEELLRINLSFADGLPRLATADI
QVGDVLVRKGELVLVLLEGANFDPEYFPNPGSIELDRPNPTSHLAFGRGQHFCPGSALGRRHAQIGIEALLKK
MPGVDLAVPIDQLVWRTRFQRRIPERLPVLW
>CYP121A1_Mycobacterium tuberculosis 6A024XDR 2720080551
MTATVLLEVPFSARGDRIPDAVAELRTREPIRKVRTITGAEAWLVSSYALCTQVLEDRRFSMKETAAAGAPRL
NALTVPPEVVNNMGNIADAGLRKAVMKAITPKAPGLEQFLRDTANSLLDNLITEGAPADLRNDFADPLATALH
CKVLGIPQEDGPKLFRSLSIAFMSSADPIPAAKINWDRDIEYMAGILENPNITTGLMGELSRLRKDPAYSHVS
DELFATIGVTFFGAGVISTGSFLTTALISLIQRPQLRNLLHEKPELIPAGVEELLRINLSFADGLPRLATADI
QVGDVLVRKGELVLVLLEGANFDPEYFPNPGSIELDRPNPTSHLAFGRGQHFCPGSALGRRHAQIGIEALLKK
MPGVDLAVPIDQLVWRTRFQRRIPERLPVLW
>CYP121A1_Mycobacterium tuberculosis 7837 2621690881

MTATVLLEVPFSARGDRIPDAVAELRTREPIRKVRTITGAEAWLVSSYALCTQVLEDRRFSMKETAAAGAPRL
NALTVPPEVVNNMGNIADAGLRKAVMKAITPKAPGLEQFLRDTANSLLDNLITEGAPADLRNDFADPLATALH
CKVLGIPQEDGPKLFRSLSIAFMSSADPIPAAKINWDRDIEYMAGILENPNITTGLMGELSRLRKDPAYSHVS
DELFATIGVTFFGAGVISTGSFLTTALISLIQRPQLRNLLHEKPELIPAGVEELLRINLSFADGLPRLATADI
QVGDVLVRKGELVLVLLEGANFDPEYFPNPGSIELDRPNPTSHLAFGRGQHFCPGSALGRRHAQIGIEALLKK
MPGVDLAVPIDQLVWRTRFQRRIPERLPVLW
>CYP121A1_Mycobacterium tuberculosis 94_M4241A unfinished sequence
643019683
MTATVLLEVPFSARGDRIPDAVAELRTREPIRKVRTITGAEAWLVSSYALCTQVLEDRRFSMKETAAAGAPRL
NALTVPPEVVNNMGNIADAGLRKAVMKAITPKAPGLEQFLRDTANSLLDNLITEGAPADLRNDFADPLATALH
CKVLGIPQEDGPKLFRSLSIAFMSSADPIPAAKINWDRDIEYMAGILENPNITTGLMGELSRLRKDPAYSHVS
DELFATIGVTFFGAGVISTGSFLTTALISLIQRPQLRNLLHEKPELIPAGVEELLRINLSFADGLPRLATADI
QVGDVLVRKGELVLVLLEGANFDPEYFPNPGSIELDRPNPTSHLAFGRGQHFCPGSALGRRHAQIGIEALLKK
MPGVDLAVPIDQLVWRTRFQRRIPERLPVLW
>CYP121A1_Mycobacterium tuberculosis 94_M4241A 2621959731
MTATVLLEVPFSARGDRIPDAVAELRTREPIRKVRTITGAEAWLVSSYALCTQVLEDRRFSMKETAAAGAPRL
NALTVPPEVVNNMGNIADAGLRKAVMKAITPKAPGLEQFLRDTANSLLDNLITEGAPADLRNDFADPLATALH
CKVLGIPQEDGPKLFRSLSIAFMSSADPIPAAKINWDRDIEYMAGILENPNITTGLMGELSRLRKDPAYSHVS
DELFATIGVTFFGAGVISTGSFLTTALISLIQRPQLRNLLHEKPELIPAGVEELLRINLSFADGLPRLATADI
QVGDVLVRKGELVLVLLEGANFDPEYFPNPGSIELDRPNPTSHLAFGRGQHFCPGSALGRRHAQIGIEALLKK
MPGVDLAVPIDQLVWRTRFQRRIPERLPVLW
>CYP121A1_Mycobacterium tuberculosis 96075 2687622068
MTATVLLEVPFSARGDRIPDAVAELRTREPIRKVRTITGAEAWLVSSYALCTQVLEDRRFSMKETAAAGAPRL
NALTVPPEVVNNMGNIADAGLRKAVMKAITPKAPGLEQFLRDTANSLLDNLITEGAPADLRNDFADPLATALH
CKVLGIPQEDGPKLFRSLSIAFMSSADPIPAAKINWDRDIEYMAGILENPNITTGLMGELSRLRKDPAYSHVS
DELFATIGVTFFGAGVISTGSFLTTALISLIQRPQLRNLLHEKPELIPAGVEELLRINLSFADGLPRLATADI
QVGDVLVRKGELVLVLLEGANFDPEYFPNPGSIELDRPNPTSHLAFGRGQHFCPGSALGRRHAQIGIEALLKK
MPGVDLAVPIDQLVWRTRFQRRIPERLPVLW
>CYP121A1_Mycobacterium tuberculosis B9741 2745703110
MTATVLLEVPFSARGDRIPDAVAELRTREPIRKVRTITGAEAWLVSSYALCTQVLEDRRFSMKETAAAGAPRL
NALTVPPEVVNNMGNIADAGLRKAVMKAITPKAPGLEQFLRDTANSLLDNLITEGAPADLRNDFADPLATALH
CKVLGIPQEDGPKLFRSLSIAFMSSADPIPAAKINWDRDIEYMAGILENPNITTGLMGELSRLRKDPAYSHVS
DELFATIGVTFFGAGVISTGSFLTTALISLIQRPQLRNLLHEKPELIPAGVEELLRINLSFADGLPRLATADI
QVGDVLVRKGELVLVLLEGANFDPEYFPNPGSIELDRPNPTSHLAFGRGQHFCPGSALGRRHAQIGIEALLKK
MPGVDLAVPIDQLVWRTRFQRRIPERLPVLW
>CYP121A1_Mycobacterium tuberculosis Beijing 2730186958
MTATVLLEVPFSARGDRIPDAVAELRTREPIRKVRTITGAEAWLVSSYALCTQVLEDRRFSMKETAAAGAPRL
NALTVPPEVVNNMGNIADAGLRKAVMKAITPKAPGLEQFLRDTANSLLDNLITEGAPADLRNDFADPLATALH
CKVLGIPQEDGPKLFRSLSIAFMSSADPIPAAKINWDRDIEYMAGILENPNITTGLMGELSRLRKDPAYSHVS
DELFATIGVTFFGAGVISTGSFLTTALISLIQRPQLRNLLHEKPELIPAGVEELLRINLSFADGLPRLATADI
QVGDVLVRKGELVLVLLEGANFDPEYFPNPGSIELDRPNPTSHLAFGRGQHFCPGSALGRRHAQIGIEALLKK
MPGVDLAVPIDQLVWRTRFQRRIPERLPVLW
>CYP121A1_Mycobacterium tuberculosis Beijing 2712474355
MTATVLLEVPFSARGDRIPDAVAELRTREPIRKVRTITGAEAWLVSSYALCTQVLEDRRFSMKETAAAGAPRL
NALTVPPEVVNNMGNIADAGLRKAVMKAITPKAPGLEQFLRDTANSLLDNLITEGAPADLRNDFADPLATALH
CKVLGIPQEDGPKLFRSLSIAFMSSADPIPAAKINWDRDIEYMAGILENPNITTGLMGELSRLRKDPAYSHVS
DELFATIGVTFFGAGVISTGSFLTTALISLIQRPQLRNLLHEKPELIPAGVEELLRINLSFADGLPRLATADI
QVGDVLVRKGELVLVLLEGANFDPEYFPNPGSIELDRPNPTSHLAFGRGQHFCPGSALGRRHAQIGIEALLKK
MPGVDLAVPIDQLVWRTRFQRRIPERLPVLW
>CYP121A1_Mycobacterium tuberculosis Beijing/NITR203 2546188767
MTATVLLEVPFSARGDRIPDAVAELRTREPIRKVRTITGAEAWLVSSYALCTQVLEDRRFSMKETAAAGAPRL
NALTVPPEVVNNMGNIADAGLRKAVMKAITPKAPGLEQFLRDTANSLLDNLITEGAPADLRNDFADPLATALH
CKVLGIPQEDGPKLFRSLSIAFMSSADPIPAAKINWDRDIEYMAGILENPNITTGLMGELSRLRKDPAYSHVS
DELFATIGVTFFGAGVISTGSFLTTALISLIQRPQLRNLLHEKPELIPAGVEELLRINLSFADGLPRLATADI
QVGDVLVRKGELVLVLLEGANFDPEYFPNPGSIELDRPNPTSHLAFGRGQHFCPGSALGRRHAQIGIEALLKK
MPGVDLAVPIDQLVWRTRFQRRIPERLPVLW
>CYP121A1_Mycobacterium tuberculosis 323 2647009131

MTATVLLEVPFSARGDRIPDAVAELRTREPIRKVRTITGAEAWLVSSYALCTQVLEDRRFSMKETAAAGAPRL
NALTVPPEVVNNMGNIADAGLRKAVMKAITPKAPGLEQFLRDTANSLLDNLITEGAPADLRNDFADPLATALH
CKVLGIPQEDGPKLFRSLSIAFMSSADPIPAAKINWDRDIEYMAGILENPNITTGLMGELSRLRKDPAYSHVS
DELFATIGVTFFGAGVISTGSFLTTALISLIQRPQLRNLLHEKPELIPAGVEELLRINLSFADGLPRLATADI
QVGDVLVRKGELVLVLLEGANFDPEYFPNPGSIELDRPNPTSHLAFGRGQHFCPGSALGRRHAQIGIEALLKK
MPGVDLAVPIDQLVWRTRFQRRIPERLPVLW
>CYP121A1_Mycobacterium tuberculosis BS1 2559164148
MTATVLLEVPFSARGDRIPDAVAELRTREPIRKVRTITGAEAWLVSSYALCTQVLEDRRFSMKETAAAGAPRL
NALTVPPEVVNNMGNIADAGLRKAVMKAITPKAPGLEQFLRDTANSLLDNLITEGAPADLRNDFADPLATALH
CKVLGIPQEDGPKLFRSLSIAFMSSADPIPAAKINWDRDIEYMAGILENPNITTGLMGELSRLRKDPAYSHVS
DELFATIGVTFFGAGVISTGSFLTTALISLIQRPQLRNLLHEKPELIPAGVEELLRINLSFADGLPRLATADI
QVGDVLVRKGELVLVLLEGANFDPEYFPNPGSIELDRPNPTSHLAFGRGQHFCPGSALGRRHAQIGIEALLKK
MPGVDLAVPIDQLVWRTRFQRRIPERLPVLW
>CYP121A1_Mycobacterium tuberculosis BT2 2566259661
MTATVLLEVPFSARGDRIPDAVAELRTREPIRKVRTITGAEAWLVSSYALCTQVLEDRRFSMKETAAAGAPRL
NALTVPPEVVNNMGNIADAGLRKAVMKAITPKAPGLEQFLRDTANSLLDNLITEGAPADLRNDFADPLATALH
CKVLGIPQEDGPKLFRSLSIAFMSSADPIPAAKINWDRDIEYMAGILENPNITTGLMGELSRLRKDPAYSHVS
DELFATIGVTFFGAGVISTGSFLTTALISLIQRPQLRNLLHEKPELIPAGVEELLRINLSFADGLPRLATADI
QVGDVLVRKGELVLVLLEGANFDPEYFPNPGSIELDRPNPTSHLAFGRGQHFCPGSALGRRHAQIGIEALLKK
MPGVDLAVPIDQLVWRTRFQRRIPERLPVLW
>CYP121A1_Mycobacterium tuberculosis BT1 2688582661
MTATVLLEVPFSARGDRIPDAVAELRTREPIRKVRTITGAEAWLVSSYALCTQVLEDRRFSMKETAAAGAPRL
NALTVPPEVVNNMGNIADAGLRKAVMKAITPKAPGLEQFLRDTANSLLDNLITEGAPADLRNDFADPLATALH
CKVLGIPQEDGPKLFRSLSIAFMSSADPIPAAKINWDRDIEYMAGILENPNITTGLMGELSRLRKDPAYSHVS
DELFATIGVTFFGAGVISTGSFLTTALISLIQRPQLRNLLHEKPELIPAGVEELLRINLSFADGLPRLATADI
QVGDVLVRKGELVLVLLEGANFDPEYFPNPGSIELDRPNPTSHLAFGRGQHFCPGSALGRRHAQIGIEALLKK
MPGVDLAVPIDQLVWRTRFQRRIPERLPVLW
>CYP121A1_Mycobacterium tuberculosis BTB03-142 2603300973
MTATVLLEVPFSARGDRIPDAVAELRTREPIRKVRTITGAEAWLVSSYALCTQVLEDRRFSMKETAAAGAPRL
NALTVPPEVVNNMGNIADAGLRKAVMKAITPKAPGLEQFLRDTANSLLDNLITEGAPADLRNDFADPLATALH
CKVLGIPQEDGPKLFRSLSIAFMSSADPIPAAKINWDRDIEYMAGILENPNITTGLMGELSRLRKDPAYSHVS
DELFATIGVTFFGAGVISTGSFLTTALISLIQRPQLRNLLHEKPELIPAGVEELLRINLSFADGLPRLATADI
QVGDVLVRKGELVLVLLEGANFDPEYFPNPGSIELDRPNPTSHLAFGRGQHFCPGSALGRRHAQIGIEALLKK
MPGVDLAVPIDQLVWRTRFQRRIPERLPVLW
>CYP121A1_Mycobacterium tuberculosis BTB03-143 2576645524
MTATVLLEVPFSARGDRIPDAVAELRTREPIRKVRTITGAEAWLVSSYALCTQVLEDRRFSMKETAAAGAPRL
NALTVPPEVVNNMGNIADAGLRKAVMKAITPKAPGLEQFLRDTANSLLDNLITEGAPADLRNDFADPLATALH
CKVLGIPQEDGPKLFRSLSIAFMSSADPIPAAKINWDRDIEYMAGILENPNITTGLMGELSRLRKDPAYSHVS
DELFATIGVTFFGAGVISTGSFLTTALISLIQRPQLRNLLHEKPELIPAGVEELLRINLSFADGLPRLATADI
QVGDVLVRKGELVLVLLEGANFDPEYFPNPGSIELDRPNPTSHLAFGRGQHFCPGSALGRRHAQIGIEALLKK
MPGVDLAVPIDQLVWRTRFQRRIPERLPVLW
>CYP121A1_Mycobacterium tuberculosis BTB04-011 2604172202
MTATVLLEVPFSARGDRIPDAVAELRTREPIRKVRTITGAEAWLVSSYALCTQVLEDRRFSMKETAAAGAPRL
NALTVPPEVVNNMGNIADAGLRKAVMKAITPKAPGLEQFLRDTANSLLDNLITEGAPADLRNDFADPLATALH
CKVLGIPQEDGPKLFRSLSIAFMSSADPIPAAKINWDRDIEYMAGILENPNITTGLMGELSRLRKDPAYSHVS
DELFATIGVTFFGAGVISTGSFLTTALISLIQRPQLRNLLHEKPELIPAGVEELLRINLSFADGLPRLATADI
QVGDVLVRKGELVLVLLEGANFDPEYFPNPGSIELDRPNPTSHLAFGRGQHFCPGSALGRRHAQIGIEALLKK
MPGVDLAVPIDQLVWRTRFQRRIPERLPVLW
>CYP121A1_Mycobacterium tuberculosis BTB04-158 2605588821
MTATVLLEVPFSARGDRIPDAVAELRTREPIRKVRTITGAEAWLVSSYALCTQVLEDRRFSMKETAAAGAPRL
NALTVPPEVVNNMGNIADAGLRKAVMKAITPKAPGLEQFLRDTANSLLDNLITEGAPADLRNDFADPLATALH
CKVLGIPQEDGPKLFRSLSIAFMSSADPIPAAKINWDRDIEYMAGILENPNITTGLMGELSRLRKDPAYSHVS
DELFATIGVTFFGAGVISTGSFLTTALISLIQRPQLRNLLHEKPELIPAGVEELLRINLSFADGLPRLATADI
QVGDVLVRKGELVLVLLEGANFDPEYFPNPGSIELDRPNPTSHLAFGRGQHFCPGSALGRRHAQIGIEALLKK
MPGVDLAVPIDQLVWRTRFQRRIPERLPVLW
>CYP121A1_Mycobacterium tuberculosis BTB04-416 2603304837
MTATVLLEVPFSARGDRIPDAVAELRTREPIRKVRTITGAEAWLVSSYALCTQVLEDRRFSMKETAAAGAPRL
NALTVPPEVVNNMGNIADAGLRKAVMKAITPKAPGLEQFLRDTANSLLDNLITEGAPADLRNDFADPLATALH

CKVLGIPQEDGPKLFRSLSIAFMSSADPIPAAKINWDRDIEYMAGILENPNITTGLMGELSRLRKDPAYSHVS
DELFATIGVTFFGAGVISTGSFLTTALISLIQRPQLRNLLHEKPELIPAGVEELLRINLSFADGLPRLATADI
QVGDVLVRKGELVLVLLEGANFDPEYFPNPGSIELDRPNPTSHLAFGRGQHFCPGSALGRRHAQIGIEALLKK
MPGVDLAVPIDQLVWRTRFQRRIPERLPVLW
>CYP121A1_Mycobacterium tuberculosis BTB04-452 2576009820
MTATVLLEVPFSARGDRIPDAVAELRTREPIRKVRTITGAEAWLVSSYALCTQVLEDRRFSMKETAAAGAPRL
NALTVPPEVVNNMGNIADAGLRKAVMKAITPKAPGLEQFLRDTANSLLDNLITEGAPADLRNDFADPLATALH
CKVLGIPQEDGPKLFRSLSIAFMSSADPIPAAKINWDRDIEYMAGILENPNITTGLMGELSRLRKDPAYSHVS
DELFATIGVTFFGAGVISTGSFLTTALISLIQRPQLRNLLHEKPELIPAGVEELLRINLSFADGLPRLATADI
QVGDVLVRKGELVLVLLEGANFDPEYFPNPGSIELDRPNPTSHLAFGRGQHFCPGSALGRRHAQIGIEALLKK
MPGVDLAVPIDQLVWRTRFQRRIPERLPVLW
>CYP121A1_Mycobacterium tuberculosis BTB05-650 2604179220
MTATVLLEVPFSARGDRIPDAVAELRTREPIRKVRTITGAEAWLVSSYALCTQVLEDRRFSMKETAAAGAPRL
NALTVPPEVVNNMGNIADAGLRKAVMKAITPKAPGLEQFLRDTANSLLDNLITEGAPADLRNDFADPLATALH
CKVLGIPQEDGPKLFRSLSIAFMSSADPIPAAKINWDRDIEYMAGILENPNITTGLMGELSRLRKDPAYSHVS
DELFATIGVTFFGAGVISTGSFLTTALISLIQRPQLRNLLHEKPELIPAGVEELLRINLSFADGLPRLATADI
QVGDVLVRKGELVLVLLEGANFDPEYFPNPGSIELDRPNPTSHLAFGRGQHFCPGSALGRRHAQIGIEALLKK
MPGVDLAVPIDQLVWRTRFQRRIPERLPVLW
>CYP121A1_Mycobacterium tuberculosis BTB06-340 2603482062
MTATVLLEVPFSARGDRIPDAVAELRTREPIRKVRTITGAEAWLVSSYALCTQVLEDRRFSMKETAAAGAPRL
NALTVPPEVVNNMGNIADAGLRKAVMKAITPKAPGLEQFLRDTANSLLDNLITEGAPADLRNDFADPLATALH
CKVLGIPQEDGPKLFRSLSIAFMSSADPIPAAKINWDRDIEYMAGILENPNITTGLMGELSRLRKDPAYSHVS
DELFATIGVTFFGAGVISTGSFLTTALISLIQRPQLRNLLHEKPELIPAGVEELLRINLSFADGLPRLATADI
QVGDVLVRKGELVLVLLEGANFDPEYFPNPGSIELDRPNPTSHLAFGRGQHFCPGSALGRRHAQIGIEALLKK
MPGVDLAVPIDQLVWRTRFQRRIPERLPVLW
>CYP121A1_Mycobacterium tuberculosis BTB07-001 2604357372
MTATVLLEVPFSARGDRIPDAVAELRTREPIRKVRTITGAEAWLVSSYALCTQVLEDRRFSMKETAAAGAPRL
NALTVPPEVVNNMGNIADAGLRKAVMKAITPKAPGLEQFLRDTANSLLDNLITEGAPADLRNDFADPLATALH
CKVLGIPQEDGPKLFRSLSIAFMSSADPIPAAKINWDRDIEYMAGILENPNFTTGLMGELSRLRKDPAYSHVS
DELFATIGVTFFGAGVISTGSFLTTALISLIQRPQLRNLLHEKPELIPAGVEELLRINLSFADGLPRLATADI
QVGDVLVRKGELVLVLLEGANFDPEHFPNPGSIELDRPNPTSHLAFGRGQHFCPGSALGRRHAQIGIEALLKK
MPGVDLAVPIDQLVWRTRFQRRIPERLPVLW
>CYP121A1_Mycobacterium tuberculosis BTB07-170 2605077786
MTATVLLEVPFSARGDRIPDAVAELRTREPIRKVRTITGAEAWLVSSYALCTQVLEDRRFSMKETAAAGAPRL
NALTVPPEVVNNMGNIADAGLRKAVMKAITPKAPGLEQFLRDTANSLLDNLITEGAPADLRNDFADPLATALH
CKVLGIPQEDGPKLFRSLSIAFMSSADPIPAAKINWDRDIEYMAGILENPNITTGLMGELSRLRKDPAYSHVS
DELFATIGVTFFGAGVISTGSFLTTALISLIQRPQLRNLLHEKPELIPAGVEELLRINLSFADGLPRLATADI
QVGDVLVRKGELVLVLLEGANFDPEYFPNPGSIELDRPNPTSHLAFGRGQHFCPGSALGRRHAQIGIEALLKK
MPGVDLAVPIDQLVWRTRFQRRIPERLPVLW
>CYP121A1_Mycobacterium tuberculosis BTB07-234 2605800585
MTATVLLEVPFSARGDRIPDAVAELRTREPIRKVRTITGAEAWLVSSYALCTQVLEDRRFSMKETAAAGAPRL
NALTVPPEVVNNMGNIADAGLRKAVMKAITPKAPGLEQFLRDTANSLLDNLITEGAPADLRNDFADPLATALH
CKVLGIPQEDGPKLFRSLSIAFMSSADPIPAAKINWDRDIEYMAGILENPNITTGLMGELSRLRKDPAYSHVS
DELFATIGVTFFGAGVISTGSFLTTALISLIQRPQLRNLLHEKPELIPAGVEELLRINLSFADGLPRLATADI
QVGDVLVRKGELVLVLLEGANFDPEYFPNPGSIELDRPNPTSHLAFGRGQHFCPGSALGRRHAQIGIEALLKK
MPGVDLAVPIDQLVWRTRFQRRIPERLPVLW
>CYP121A1_Mycobacterium tuberculosis BTB07-314 2604936268
MTATVLLEVPFSARGDRIPDAVAELRTREPIRKVRTITGAEAWLVSSYALCTQVLEDRRFSMKETAAAGAPRL
NALTVPPEVVNNMGNIADAGLRKAVMKAITPKAPGLEQFLRDTANSLLDNLITEGAPADLRNDFADPLATALH
CKVLGIPQEDGPKLFRSLSIAFMSSADPIPAAKINWDRDIEYMAGILENPNITTGLMGELSRLRKDPAYSHVS
DELFATIGVTFFGAGVISTGSFLTTALISLIQRPQLRNLLHEKPELIPAGVEELLRINLSFADGLPRLATADI
QVGDVLVRKGELVLVLLEGANFDPEYFPNPGSIELDRPNPTSHLAFGRGQHFCPGSALGRRHAQIGIEALLKK
MPGVDLAVPIDQLVWRTRFQRRIPERLPVLW
>CYP121A1_Mycobacterium tuberculosis BTB07-323 2603486178
MTATVLLEVPFSARGDRIPDAVAELRTREPIRKVRTITGAEAWLVSSYALCTQVLEDRRFSMKETAAAGAPRL
NALTVPPEVVNNMGNIADAGLRKAVMKAITPKAPGLEQFLRDTANSLLDNLITEGAPADLRNDFADPLATALH
CKVLGIPQEDGPKLFRSLSIAFMSSADPIPAAKINWDRDIEYMAGILENPNITTGLMGELSRLRKDPAYSHVS
DELFATIGVTFFGAGVISTGSFLTTALISLIQRPQLRNLLHEKPELIPAGVEELLRINLSFADGLPRLATADI

QVGDVLVRKGELVLVLLEGANFDPEYFPNPGSIELDRPNPTSHLAFGRGQHFCPGSALGRRHAQIGIEALLKK
MPGVDLAVPIDQLVWRTRFQRRIPERLPVLW
>CYP121A1_Mycobacterium tuberculosis BTB07-324 2603490279
MTATVLLEVPFSARGDRIPDAVAELRTREPIRKVRTITGAEAWLVSSYALCTQVLEDRRFSMKETAAAGAPRL
NALTVPPEVVNNMGNIADAGLRKAVMKAITPKAPGLEQFLRDTANSLLDNLITEGAPADLRNDFADPLATALH
CKVLGIPQEDGPKLFRSLSIAFMSSADPIPAAKINWDRDIEYMAGILENPNITTGLMGELSRLRKDPAYSHVS
DELFATIGVTFFGAGVISTGSFLTTALISLIQRPQLRNLLHEKPELIPAGVEELLRINLSFADGLPRLATADI
QVGDVLVRKGELVLVLLEGANFDPEYFPNPGSIELDRPNPTSHLAFGRGQHFCPGSALGRRHAQIGIEALLKK
MPGVDLAVPIDQLVWRTRFQRRIPERLPVLW
>CYP121A1_Mycobacterium tuberculosis BTB08-093 2604209618
MTATVLLEVPFSARGDRIPDAVAELRTREPIRKVRTITGAEAWLVSSYALCTQVLEDRRFSMKETAAAGAPRL
NALTVPPEVVNNMGNIADAGLRKAVMKAITPKAPGLEQFLRDTANSLLDNLITEGAPADLRNDFADPLATALH
CKVLGIPQEDGPKLFRSLSIAFMSSADPIPAAKINWDRDIEYMAGILENPNITTGLMGELSRLRKDPAYSHVS
DELFATIGVTFFGAGVISTGSFLTTALISLIQRPQLRNLLHEKPELIPAGVEELLRINLSFADGLPRLATADI
QVGDVLVRKGELVLVLLEGANFDPEYFPNPGSIELDRPNPTSHLAFGRGQHFCPGSALGRRHAQIGIEALLKK
MPGVDLAVPIDQLVWRTRFQRRIPERLPVLW
>CYP121A1_Mycobacterium tuberculosis BTB08-148 2576610581
MTATVLLEVPFSARGDRIPDAVAELRTREPIRKVRTITGAEAWLVSSYALCTQVLEDRRFSMKETAAAGAPRL
NALTVPPEVVNNMGNIADAGLRKAVMKAITPKAPGLEQFLRDTANSLLDNLITEGAPTDLRNDFADPLATALH
CKVLGIPQEDGPKLFRSLSIAFMSSADPIPAAKINWDRDIEYMAGILENPNITTGLMGELSRLRKDPAYSHVS
DELFATIGVTFFGAGVISTGSFLTTALISLIQRPQLRNLLHEKPELIPAGVEELLRINLSFADGLPRLATADI
QVGDVLVRKGELVLVLLEGANFDPEHFPNPGSIELDRPNPTSHLAFGRGQHFCPGSALGRRHAQIGIEALLKK
MPGVDLAVPIDQLVWRTRFQRRIPERLPVLW
>CYP121A1_Mycobacterium tuberculosis BTB08-183 2604561417
MTATVLLEVPFSARGDRIPDAVAELRTREPIRKVRTITGAEAWLVSSYALCTQVLEDRRFSMKETAAAGAPRL
NALTVPPEVVNNMGNIADAGLRKAVMKAITPKAPGLEQFLRDTANSLLDNLITEGAPADLRNDFADPLATALH
CKVLGIPQEDGPKLFRSLSIAFMSSADPIPAAKINWDRDIEYMAGILENPNITTGLMGELSRLRKDPAYSHVS
DELFATIGVTFFGAGVISTGSFLTTALISLIQRPQLRNLLHEKPELIPAGVEELLRINLSFADGLPRLATADI
QVGDVLVRKGELVLVLLEGANFDPEYFPNPGSIELDRPNPTSHLAFGRGQHFCPGSALGRRHAQIGIEALLKK
MPGVDLAVPIDQLVWRTRFQRRIPERLPVLW
>CYP121A1_Mycobacterium tuberculosis BTB08-221 2605288127
MTATVLLEVPFSARGDRIPDAVAELRTREPIRKVRTITGAEAWLVSSYALCTQVLEDRRFSMKETAAAGAPRL
NALTVPPEVVNNMGNIADAGLRKAVMKAITPKAPGLEQFLRDTANSLLDNLITEGAPADLRNDFADPLATALH
CKVLGIPQEDGPKLFRSLSIAFMSSADPIPAAKINWDRDIEYMAGILENPNITTGLMGELSRLRKDPAYSHVS
DELFATIGVTFFGAGVISTGSFLTTALISLIQRPQLRNLLHEKPELIPAGVEELLRINLSFADGLPRLATADI
QVGDVLVRKGELVLVLLEGANFDPEYFPNPGSIELDRPNPTSHLAFGRGQHFCPGSALGRRHAQIGIEALLKK
MPGVDLAVPIDQLVWRTRFQRRIPERLPVLW
>CYP121A1_Mycobacterium tuberculosis BTB09-036 2604943253
MTATVLLEVPFSARGDRIPDAVAELRTREPIRKVRTITGAEAWLVSSYALCTQVLEDRRFSMKETAAAGAPRL
NALTVPPEVVNNMGNIADAGLRKAVMKAITPKAPGLEQFLRDTANSLLDNLITEGAPADLRNDFADPLATALH
CKVLGIPQEDGPKLFRSLSIAFMSSADPIPAAKINWDRDIEYMAGILENPNITTGLMGELSRLRKDPAYSHVS
DELFATIGVTFFGAGVISTGSFLTTALISLIQRPQLRNLLHEKPELIPAGVEELLRINLSFADGLPRLATADI
QVGDVLVRKGELVLVLLEGANFDPEHFPNPGSIELDRPNPTSHLAFGRGQHFCPGSALGRRHAQIGIEALLKK
MPGVDLTVPIDQLVWRTRFQRRIPERLPVLW
>CYP121A1_Mycobacterium tuberculosis BTB09-058 2578111905
MTATVLLEVPFSARGDRIPDAVAELRTREPIRKVRTITGAEAWLVSSYALCTQVLEDRRFSMKETAAAGAPRL
NALTVPPEVVNNMGNIADAGLRKAVMKAITPKAPGLEQFLRDTANSLLDNLITEGAPADLRNDFADPLATALH
CKVLGIPQEDGPKLFRSLSIAFMSSADPIPAAKINWDRDIEYMAGILENPNITTGLMGELSRLRKDPAYSHVS
DELFATIGVTFFGAGVISTGSFLTTALISLIQRPQLRNLLHEKPELIPAGVEELLRINLSFADGLPRLATADI
QVGDVLVRKGELVLVLLEGANFDPEYFPNPGSIELDRPNPTSHLAFGRGQHFCPGSALGRRHAQIGIEALLKK
MPGVDLAVPIDQLVWRTRFQRRIPERLPVLW
>CYP121A1_Mycobacterium tuberculosis BTB09-085 2603164794
MTATVLLEVPFSARGDRIPDAVAELRTREPIRKVRTITGAEAWLVSSYALCTQVLEDRRFSMKETAAAGAPRL
NALTVPPEVVNNMGNIADAGLRKAVMKAITPKAPGLEQFLRDTANSLLDNLITEGAPADLRNDFADPLATALH
CKVLGIPQEDGPKLFRSLSIAFMSSADPIPAAKINWDRDIEYMAGILENPNITTGLMGELSRLRKDPAYSHVS
DELFATIGVTFFGAGVISTGSFLTTALISLIQRPQLRNLLHEKPELIPAGVEELLRINLSFADGLPRLATADI
QVGDVLVRKGELVLVLLEGANFDPEYFPNPGSIELDRPNPTSHLAFGRGQHFCPGSALGRRHAQIGIEALLKK
MPGVDLAVPIDQLVWRTRFQRRIPERLPVLW

>CYP121A1_Mycobacterium tuberculosis BTB09-086 2605643149
MTATVLLEVPFSARGDRIPDAVAELRTREPIRKVRTITGAEAWLVSSYALCTQVLEDRRFSMKETAAAGAPRL
NALTVPPEVVNNMGNIADAGLRKAVMKAITPKAPGLEQFLRDTANSLLDNLITEGAPADLRNDFADPLATALH
CKVLGIPQEDGPKLFRSLSIAFMSSADPIPAAKINWDRDIEYMAGILENPNITTGLMGELSRLRKDPAYSHVS
DELFATIGVTFFGAGVISTGSFLTTALISLIQRPQLRNLLHEKPELIPAGVEELLRINLSFADGLPRLATADI
QVGDVLVRKGELVLVLLEGANFDPEYFPNPGSIELDRPNPTSHLAFGRGQHFCPGSALGRRHAQIGIEALLKK
MPGVDLAVPIDQLVWRTRFQRRIPERLPVLW
>CYP121A1_Mycobacterium tuberculosis BTB09-091 2604948965
MTATVLLEVPFSARGDRIPDAVAELRTREPIRKVRTITGAEAWLVSSYALCTQVLEDRRFSMKETAAAGAPRL
NALTVPPEVVNNMGNIADAGLRKAVMKAITPKAPGLEQFLRDTANSLLDNLITEGAPADLRNDFADPLATALH
CKVLGIPQEDGPKLFRSLSIAFMSSADPIPAAKINWDRDIEYMAGILENPNITTGLMGELSRLRKDPAYSHVS
DELFATIGVTFFGAGVISTGSFLTTALISLIQRPQLRNLLHEKPELIPAGVEELLRINLSFADGLPRLATADI
QVGDVLVRKGELVLVLLEGANFDPEYFPNPGSIELDRPNPTSHLAFGRGQHFCPGSALGRRHAQIGIEALLKK
MPGVDLAVPIDQLVWRTRFQRRIPERLPVLW
>CYP121A1_Mycobacterium tuberculosis BTB09-157 2603502636
MTATVLLEVPFSARGDRIPDAVAELRTREPIRKVRTITGAEAWLVSSYALCTQVLEDRRFSMKETAAAGAPRL
NALTVPPEVVNNMGNIADAGLRKAVMKAITPKAPGLEQFLRDTANSLLDNLITEGAPADLRNDFADPLATALH
CKVLGIPQEDGPKLFRSLSIAFMSSADPIPAAKINWDRDIEYMAGILENPNITTGLMGELSRLRKDPAYSHVS
DELFATIGVTFFGAGVISTGSFLTTALISLIQRPQLRNLLHEKPELIPAGVEELLRINLSFADGLPRLATADI
QVGDVLVRKGELVLVLLEGANFDPEYFPNPGSIELDRPNPTSHLAFGRGQHFCPGSALGRRHAQIGIEALLKK
MPGVDLAVPIDQLVWRTRFQRRIPERLPVLW
>CYP121A1_Mycobacterium tuberculosis BTB09-184 2604953093
MTATVLLEVPFSARGDRIPDAVAELRTREPIRKVRTITGAEAWLVSSYALCTQVLEDRRFSMKETAAAGAPRL
NALTVPPEVVNNMGNIADAGLRKAVMKAITPKAPGLEQFLRDTANSLLDNLITEGAPADLRNDFADPLATALH
CKVLGIPQEDGPKLFRSLSIAFMSSADPIPAAKINWDRDIEYMAGILENPNITTGLMGELSRLRKDPAYSHVS
DELFATIGVTFFGAGVISTGSFLTTALISLIQRPQLRNLLHEKPELIPAGVEELLRINLSFADGLPRLATADI
QVGDVLVRKGELVLVLLEGANFDPEYFPNPGSIELDRPNPTSHLAFGRGQHFCPGSALGRRHAQIGIEALLKK
MPGVDLAVPIDQLVWRTRFQRRIPERLPVLW
>CYP121A1_Mycobacterium tuberculosis BTB09-230 2604956814
MTATVLLEVPFSARGDRIPDAVAELRTREPIRKVRTITGAEAWLVSSYALCTQVLEDRRFSMKETAAAGAPRL
NALTVPPEVVNNMGNIADAGLRKAVMKAITPKAPGLEQFLRDTANSLLDNLITEGAPTDLRNDFADPLATALH
CKVLGIPQEDGPKLFRSLSIAFMSSADPIPAAKINWDRDIEYMAGILENPNITTGLMGELSRLRKDPAYSHVS
DELFATIGVTFFGAGVISTGSFLTTALISLIQRPQLRNLLHEKPELIPAGVEELLRINLSFADGLPRLATADI
QVGDVLVRKGELVLVLLEGANFDPEHFPNPGSIELDRPNPTSHLAFGRGQHFCPGSALGRRHAQIGIEALLKK
MPGVDLAVPIDQLVWRTRFQRRIPERLPVLW
>CYP121A1_Mycobacterium tuberculosis BTB09-377 2604960487
MTATVLLEVPFSARGDRIPDAVAELRTREPIRKVRTITGAEAWLVSSYALCTQVLEDRRFSMKETAAAGAPRL
NALTVPPEVVNNMGNIADAGLRKAVMKAITPKAPGLEQFLRDTANSLLDNLITEGAPADLRNDFADPLATALH
CKVLGIPQEDGPKLFRSLSIAFMSSADPIPAAKINWDRDIEYMAGILENPNITTGLMGELSRLRKDPAYSHVS
DELFATIGVTFFGAGVISTGSFLTTALISLIQRPQLRNLLHEKPELIPAGVEELLRINLSFADGLPRLATADI
QVGDVLVRKGELVLVLLEGANFDPEYFPNPGSIELDRPNPTSHLAFGRGQHFCPGSALGRRHAQIGIEALLKK
MPGVDLAVPIDQLVWRTRFQRRIPERLPVLW
>CYP121A1_Mycobacterium tuberculosis BTB09-382 2603506442
MTATVLLEVPFSARGDRIPDAVAELRTREPIRKVRTITGAEAWLVSSYALCTQVLEDRRFSMKETAAAGAPRL
NALTVPPEVVNNMGNIADAGLRKAVMKAITPKAPGLEQFLRDTANSLLDNLITEGAPADLRNDFADPLATALH
CKVLGIPQEDGPKLFRSLSIAFMSSADPIPAAKINWDRDIEYMAGILENPNITTGLMGELSHLRKDPAYSHVS
DELFATIGVTFFGAGVISTGSFLTTALISLIQRPQLRNLLHEKPELIPAGVEELLRINLSFADGLPRLATADI
QVGDVLVRKGELVLVLLEGANFDPEHFPNPGSIELDRPNPTSHLAFGRGQHFCPGSALGRRHAQIGIEALLKK
MPGVDLAVPIDQLVWRTRFQRRIPERLPVLW
>CYP121A1_Mycobacterium tuberculosis BTB09-485 2604572740
MTATVLLEVPFSARGDRIPDAVAELRTREPIRKVRTITGAEAWLVSSYALCTQVLEDRRFSMKETAAAGAPRL
NALTVPPEVVNNMGNIADAGLRKAVMKAITPKAPGLEQFLRDTANSLLDNLITEGAPADLRNDFADPLATALH
CKVLGIPQEDGPKLFRSLSIAFMSSADPIPAAKINWDRDIEYMAGILENPNITTGLMGELSRLRKDPAYSHVS
DELFATIGVTFFGAGVISTGSFLTTALISLIQRPQLRNLLHEKPELIPAGVEELLRINLSFADGLPRLATADI
QVGDVLVRKGELVLVLLEGANFDPEYFPNPGSIELDRPNPTSHLAFGRGQHFCPGSALGRRHAQIGIEALLKK
MPGVDLAVPIDQLVWRTRFQRRIPERLPVLW
>CYP121A1_Mycobacterium tuberculosis BTB10-097 2604578119

MTATVLLEVPFSARGDRIPDAVAELRTREPIRKVRTITGAEAWLVSSYALCTQVLEDRRFSMKETAAAGAPRL
NALTVPPEVVNNMGNIADAGLRKAVMKAITPKAPGLEQFLRDTANSLLDNLITEGAPADLRNDFADPLATALH
CKVLGIPQEDGPKLFRSLSIAFMSSADPIPAAKINWDRDIEYMAGILENPNITTGLMGELSRLRKDPAYSHVS
DELFATIGVTFFGAGVISTGSFLTTALISLIQRPQLRNLLHEKPELIPAGVEELLRINLSFADGLPRLATADI
QVGDVLVRKGELVLVLLEGANFDPEYFPNPGSIELDRPNPTSHLAFGRGQHFCPGSALGRRHAQIGIEALLKK
MPGVDLAVPIDQLVWRTRFQRRIPERLPVLW
>CYP121A1_Mycobacterium tuberculosis BTB10-257 2605300518
MTATVLLEVPFSARGDRIPDAVAELRTREPIRKVRTITGAEAWLVSSYALCTQVLEDRRFSMKETAAAGAPRL
NALTVPPEVVNNMGNIADAGLRKAVMKAITPKAPGLEQFLRDTANSLLDNLITEGAPADLRNDFADPLATALH
CKVLGIPQEDGPKLFRSLSIAFMSSADPIPAAKINWDRDIEYMAGILENPNITTGLMGELSRLRKDPAYSHVS
DELFATIGVTFFGAGVISTGSFLTTALISLIQRPQLRNLLHEKPELIPAGVEELLRINLSFADGLPRLATADI
QVGDVLVRKGELVLVLLEGANFDPEYFPNPGSIELDRPNPTSHLAFGRGQHFCPGSALGRRHAQIGIEALLKK
MPGVDLAVPIDQLVWRTRFQRRIPERLPVLW
>CYP121A1_Mycobacterium tuberculosis BTB10-258 2603168856
MTATVLLEVPFSARGDRIPDAVAELRTREPIRKVRTITGAEAWLVSSYALCTQVLEDRRFSMKETAAAGAPRL
NALTVPPEVVNNMGNIADAGLRKAVMKAITPKAPGLEQFLRDTANSLLDNLITEGAPADLRNDFADPLATALH
CKVLGIPQEDGPKLFRSLSIAFMSSADPIPAAKINWDRDIEYMAGILENPNITTGLMGELSRLRKDPAYSHVS
DELFATIGVTFFGAGVISTGSFLTTALISLIQRPQLRNLLHEKPELIPAGVEELLRINLSFADGLPRLATADI
QVGDVLVRKGELVLVLLEGANFDPEYFPNPGSIELDRPNPTSHLAFGRGQHFCPGSALGRRHAQIGIEALLKK
MPGVDLAVPIDQLVWRTRFQRRIPERLPVLW
>CYP121A1_Mycobacterium tuberculosis BTB10-357 2584630012
MTATVLLEVPFSARGDRIPDAVAELRTREPIRKVRTITGAEAWLVSSYALCTQVLEDRRFSMKETAAAGAPRL
NALTVPPEVVNNMGNIADAGLRKAVMKAITPKAPGLEQFLRDTANSLLDNLITEGAPADLRNDFADPLATALH
CKVLGIPQEDGPKLFRSLSIAFMSSADPIPAAKINWDRDIEYMAGILENPNITTGLMGELSRLRKDPAYSHVS
DELFATIGVTFFGAGVISTGSFLTTALISLIQRPQLRNLLHEKPELIPAGVEELLRINLSFADGLPRLATADI
QVGDVLVRKGELVLVLLEGANFDPEYFPNPGSIELDRPNPTSHLAFGRGQHFCPGSALGRRHAQIGIEALLKK
MPGVDLAVPIDQLVWRTRFQRRIPERLPVLW
>CYP121A1_Mycobacterium tuberculosis 2242 2687551225
MTATVLLEVPFSARGDRIPDAVAELRTREPIRKVRTITGAEAWLVSSYALCTQVLEDRRFSMKETAAAGAPRL
NALTVPPEVVNNMGNIADAGLRKAVMKAITPKAPGLEQFLRDTANSLLDNLITEGAPADLRNDFADPLATALH
CKVLGIPQEDGPKLFRSLSIAFMSSADPIPAAKINWDRDIEYMAGILENPNITTGLMGELSRLRKDPAYSHVS
DELFATIGVTFFGAGVISTGSFLTTALISLIQRPQLRNLLHEKPELIPAGVEELLRINLSFADGLPRLATADI
QVGDVLVRKGELVLVLLEGANFDPEYFPNPGSIELDRPNPTSHLAFGRGQHFCPGSALGRRHAQIGIEALLKK
MPGVDLAVPIDQLVWRTRFQRRIPERLPVLW
>CYP121A1_Mycobacterium tuberculosis BTB10-472 2605312409
MTATVLLEVPFSARGDRIPDAVAELRTREPIRKVRTITGAEAWLVSSYALCTQVLEDRRFSMKETAAAGAPRL
NALTVPPEVVNNMGNIADAGLRKAVMKAITPKAPGLEQFLRDTANSLLDNLITEGAPADLRNDFADPLATALH
CKVLGIPQEDGPKLFRSLSIAFMSSADPIPAAKINWDRDIEYMAGILENPNITTGLMGELSRLRKDPAYSHVS
DELFATIGVTFFGAGVISTGSFLTTALISLIQRPQLRNLLHEKPELIPAGVEELLRINLSFADGLPRLATADI
QVGDVLVRKGELVLVLLEGANFDPEYFPNPGSIELDRPNPTSHLAFGRGQHFCPGSALGRRHAQIGIEALLKK
MPGVDLAVPIDQLVWRTRFQRRIPERLPVLW
>CYP121A1_Mycobacterium tuberculosis BTB11-139 2603350309
MTATVLLEVPFSARGDRIPDAVAELRTREPIRKVRTITGAEAWLVSSYALCTQVLEDRRFSMKETAAAGAPRL
NALTVPPEVVNNMGNIADAGLRKAVMKAITPKAPGLEQFLRDTANSLLDNLITEGAPADLRNDFADPLATALH
CKVLGIPQEDGPKLFRSLSIAFMSSADPIPAAKINWDRDIEYMAGILENPNITTGLMGELSRLRKDPAYSHVS
DELFATIGVTFFGAGVISTGSFLTTALISLIQRPQLRNLLHEKPELIPAGVEELLRINLSFADGLPRLATADI
QVGDVLVRKGELVLVLLEGANFDPEYFPNPGSIELDRPNPTSHLAFGRGQHFCPGSALGRRHAQIGIEALLKK
MPGVDLAVPIDQLVWRTRFQRRIPERLPVLW
>CYP121A1_Mycobacterium tuberculosis BTB11-289 2604239940
MTATVLLEVPFSARGDRIPDAVAELRTREPIRKVRTITGAEAWLVSSYALCTQVLEDRRFSMKETAAAGAPRL
NALTVPPEVVNNMGNIADAGLRKAVMKAITPKAPGLEQFLRDTANSLLDNLITEGAPADLRNDFADPLATALH
CKVLGIPQEDGPKLFRSLSIAFMSSADPIPAAKINWDRDIEYMAGILENPNITTGLMGELSRLRKDPAYSHVS
DELFATIGVTFFGAGVISTGSFLTTALISLIQRPQLRNLLHEKPELIPAGVEELLRINLSFADGLPRLATADI
QVGDVLVRKGELVLVLLEGANFDPEYFPNPGSIELDRPNPTSHLAFGRGQHFCPGSALGRRHAQIGIEALLKK
MPGVDLAVPIDQLVWRTRFQRRIPERLPVLW
>CYP121A1_Mycobacterium tuberculosis BTB11-299 2604242515
MTATVLLEVPFSARGDRIPDAVAELRTREPIRKVRTITGAEAWLVSSYALCTQVLEDRRFSMKETAAAGAPRL
NALTVPPEVVNNMGNIADAGLRKAVMKAITPKAPGLEQFLRDTANSLLDNLITEGAPADLRNDFADPLATALH

CKVLGIPQEDGPKLFRSLSIAFMSSADPIPAAKINWDRDIEYMAGILENPNITTGLMGELSRLRKDPAYSHVS
DELFATIGVTFFGAGVISTGSFLTTALISLIQRPQLRNLLHEKPELIPAGVEELLRINLSFADGLPRLATADI
QVGDVLVRKGELVLVLLEGANFDPEYFPNPGSIELDRPNPTSHLAFGRGQHFCPGSALGRRHAQIGIEALLKK
MPGVDLAVPIDQLVWRTRFQRRIPERLPVLW
>CYP121A1_Mycobacterium tuberculosis BTB11-300 2605663785
MTATVLLEVPFSARGDRIPDAVAELRTREPIRKVRTITGAEAWLVSSYALCTQVLEDRRFSMKETAAAGAPRL
NALTVPPEVVNNMGNIADAGLRKAVMKAITPKAPGLEQFLRDTANSLLDNLITEGAPADLRNDFADPLATALH
CKVLGIPQEDGPKLFRSLSIAFMSSADPIPAAKINWDRDIEYMAGILENPNITTGLMGELSRLRKDPAYSHVS
DELFATIGVTFFGAGVISTGSFLTTALISLIQRPQLRNLLHEKPELIPAGVEELLRINLSFADGLPRLATADI
QVGDVLVRKGELVLVLLEGANFDPEYFPNPGSIELDRPNPTSHLAFGRGQHFCPGSALGRRHAQIGIEALLKK
MPGVDLAVPIDQLVWRTRFQRRIPERLPVLW
>CYP121A1_Mycobacterium tuberculosis BTB11-371 2604597333
MTATVLLEVPFSARGDRIPDAVAELRTREPIRKVRTITGAEAWLVSSYALCTQVLEDRRFSMKETAAAGAPRL
NALTVPPEVVNNMGNIADAGLRKAVMKAITPKAPGLEQFLRDTANSLLDNLITEGAPADLRNDFADPLATALH
CKVLGIPQEDGPKLFRSLSIAFMSSADPIPAAKINWDRDIEYMAGILENPNITTGLMGELSRLRKDPAYSHVS
DELFATIGVTFFGAGVISTGSFLTTALISLIQRPQLRNLLHEKPELIPAGVEELLRINLSFADGLPRLATADI
QVGDVLVRKGELVLVLLEGANFDPEYFPNPGSIELDRPNPTSHLAFGRGQHFCPGSALGRRHAQIGIEALLKK
MPGVDLAVPIDQLVWRTRFQRRIPERLPVLW
>CYP121A1_Mycobacterium tuberculosis BTB12-001 2575016822
MTATVLLEVPFSARGDRIPDAVAELRTREPIRKVRTITGAEAWLVSSYALCTQVLEDRRFSMKETAAAGAPRL
NALTVPPEVVNNMGNIADAGLRKAVMKAITPKAPGLEQFLRDTANSLLDNLITEGAPADLRNDFADPLATALH
CKVLGIPQEDGPKLFRSLSIAFMSSADPIPAAKINWDRDIEYMAGILENPNITTGLMGELSRLRKDPAYSHVS
DELFATIGVTFFGAGVISTGSFLTTALISLIQRPQLRNLLHEKPELIPAGVEELLRINLSFADGLPRLATADI
QVGDVLVRKGELVLVLLEGANFDPEYFPNPGSIELDRPNPTSHLAFGRGQHFCPGSALGRRHAQIGIEALLKK
MPGVDLAVPIDQLVWRTRFQRRIPERLPVLW
>CYP121A1_Mycobacterium tuberculosis BTB12-002 2603354426
MTATVLLEVPFSARGDRIPDAVAELRTREPIRKVRTITGAEAWLVSSYALCTQVLEDRRFSMKETAAAGAPRL
NALTVPPEVVNNMGNIADAGLRKAVMKAITPKAPGLEQFLRDTANSLLDNLITEGAPADLRNDFADPLATALH
CKVLGIPQEDGPKLFRSLSIAFMSSADPIPAAKINWDRDIEYMAGILENPNITTGLMGELSRLRKDPAYSHVS
DELFATIGVTFFGAGVISTGSFLTTALISLIQRPQLRNLLHEKPELIPAGVEELLRINLSFADGLPRLATADI
QVGDVLVRKGELVLVLLEGANFDPEYFPNPGSIELDRPNPTSHLAFGRGQHFCPGSALGRRHAQIGIEALLKK
MPGVDLAVPIDQLVWRTRFQRRIPERLPVLW
>CYP121A1_Mycobacterium tuberculosis BTB12-094 2603902440
MTATVLLEVPFSARGDRIPDAVAELRTREPIRKVRTITGAEAWLVSSYALCTQVLEDRRFSMKETAAAGAPRL
NALTVPPEVVNNMGNIADAGLRKAVMKAITPKAPGLEQFLRDTANSLLDNLITEGAPADLRNDFADPLATALH
CKVLGIPQEDGPKLFRSLSIAFMSSADPIPAAKINWDRDIEYMAGILENPNITTGLMGELSRLRKDPAYSHVS
DELFATIGVTFFGAGVISTGSFLTTALISLIQRPQLRNLLHEKPELIPAGVEELLRINLSFADGLPRLATADI
QVGDVLVRKGELVLVLLEGANFDPEYFPNPGSIELDRPNPTSHLAFGRGQHFCPGSALGRRHAQIGIEALLKK
MPGVDLAVPIDQLVWRTRFQRRIPERLPVLW
>CYP121A1_Mycobacterium tuberculosis BTB12-211 2603173267
MTATVLLEVPFSARGDRIPDAVAELRTREPIRKVRTITGAEAWLVSSYALCTQVLEDRRFSMKETAAAGAPRL
NALTVPPEVVNNMGNIADAGLRKAVMKAITPKAPGLEQFLRDTANSLLDNLITEGAPADLRNDFADPLATALH
CKVLGIPQEDGPKLFRSLSIAFMSSADPIPAAKINWDRDIEYMAGILENPNITTGLMGELSRLRKDPAYSHVS
DELFATIGVTFFGAGVISTGSFLTTALISLIQRPQLRNLLHEKPELIPAGVEELLRINLSFADGLPRLATADI
QVGDVLVRKGELVLVLLEGANFDPEYFPNPGSIELDRPNPTSHLAFGRGQHFCPGSALGRRHAQIGIEALLKK
MPGVDLAVPIDQLVWRTRFQRRIPERLPVLW
>CYP121A1_Mycobacterium tuberculosis BTB12-305 2603906565
MTATVLLEVPFSARGDRIPDAVAELRTREPIRKVRTITGAEAWLVSSYALCTQVLEDRRFSMKETAAAGAPRL
NALTVPPEVVNNMGNIADAGLRKAVMKAITPKAPGLEQFLRDTANSLLDNLITEGAPADLRNDFADPLATALH
CKVLGIPQEDGPKLFRSLSIAFMSSADPIPAAKINWDRDIEYMAGILENPNITTGLMGELSRLRKDPAYSHVS
DELFATIGVTFFGAGVISTGSFLTTALISLIQRPQLRNLLHEKPELIPAGVEELLRINLSFADGLPRLATADI
QVGDVLVRKGELVLVLLEGANFDPEYFPNPGSIELDRPNPTSHLAFGRGQHFCPGSALGRRHAQIGIEALLKK
MPGVDLAVPIDQLVWRTRFQRRIPERLPVLW
>CYP121A1_Mycobacterium tuberculosis BTB12-314 2577060977
MTATVLLEVPFSARGDRIPDAVAELRTREPIRKVRTITGAEAWLVSSYALCTQVLEDRRFSMKETAAAGAPRL
NALTVPPEVVNNMGNIADAGLRKAVMKAITPKAPGLEQFLRDTANSLLDNLITEGAPADLRNDFADPLATALH
CKVLGIPQEDGPKLFRSLSIAFMSSADPIPAAKINWDRDIEYMAGILENPNITTGLMGELSRLRKDPAYSHVS
DELFATIGVTFFGAGVISTGSFLTTALISLIQRPQLRNLLHEKPELIPAGVEELLRINLSFADGLPRLATADI

QVGDVLVRKGELVLVLLEGANFDPEYFPNPGSIELDRPNPTSHLAFGRGQHFCPGSALGRRHAQIGIEALLKK
MPGVDLAVPIDQLVWRTRFQRRIPERLPVLW
>CYP121A1_Mycobacterium tuberculosis BTB12-384 2584971843
MTATVLLEVPFSARGDRIPDAVAELRTREPIRKVRTITGAEAWLVSSYALCTQVLEDRRFSMKETAAAGAPRL
NALTVPPEVVNNMGNIADAGLRKAVMKAITPKAPGLEQFLRDTANSLLDNLITEGAPADLRNDFADPLATALH
CKVLGIPQEDGPKLFRSLSIAFMSSADPIPAAKINWDRDIEYMAGILENPNITTGLMGELSRLRKDPAYSHVS
DELFATIGVTFFGAGVISTGSFLTTALISLIQRPQLRNLLHEKPELIPAGVEELLRINLSFADGLPRLATADI
QVGDVLVRKGELVLVLLEGANFDPEYFPNPGSIELDRPNPTSHLAFGRGQHFCPGSALGRRHAQIGIEALLKK
MPGVDLAVPIDQLVWRTRFQRRIPERLPVLW
>CYP121A1_Mycobacterium tuberculosis BTB12-449 2577099020
MTATVLLEVPFSARGDRIPDAVAELRTREPIRKVRTITGAEAWLVSSYALCTQVLEDRRFSMKETAAAGAPRL
NALTVPPEVVNNMGNIADAGLRKAVMKAITPKAPGLEQFLRDTANSLLDNLITEGAPADLRNDFADPLATALH
CKVLGIPQEDGPKLFRSLSIAFMSSADPIPAAKINWDRDIEYMAGILENPNITTGLMGELSRLRKDPAYSHVS
DELFATIGVTFFGAGVISTGSFLTTALISLIQRPQLRNLLHEKPELIPAGVEELLRINLSFADGLPRLATADI
QVGDVLVRKGELVLVLLEGANFDPEYFPNPGSIELDRPNPTSHLAFGRGQHFCPGSALGRRHAQIGIEALLKK
MPGVDLAVPIDQLVWRTRFQRRIPERLPVLW
>CYP121A1_Mycobacterium tuberculosis BTB13-063 2576197139
MTATVLLEVPFSARGDRIPDAVAELRTREPIRKVRTITGAEAWLVSSYALCTQVLEDRRFSMKETAAAGAPRL
NALTVPPEVVNNMGNIADAGLRKAVMKAITPKAPGLEQFLRDTANSLLDNLITEGAPADLRNDFADPLATALH
CKVLGIPQEDGPKLFRSLSIAFMSSADPIPAAKINWDRDIEYMAGILENPNITTGLMGELSRLRKDPAYSHVS
DELFATIGVTFFGAGVISTGSFLTTALISLIQRPQLRNLLHEKPELIPAGVEELLRINLSFADGLPRLATADI
QVGDVLVRKGELVLVLLEGANFDPEYFPNPGSIELDRPNPTSHLAFGRGQHFCPGSALGRRHAQIGIEALLKK
MPGVDLAVPIDQLVWRTRFQRRIPERLPVLW
>CYP121A1_Mycobacterium tuberculosis BTB13-089 2603366822
MTATVLLEVPFSARGDRIPDAVAELRTREPIRKVRTITGAEAWLVSSYALCTQVLEDRRFSMKETAAAGAPRL
NALTVPPEVVNNMGNIADAGLRKAVMKAITPKAPGLEQFLRDTANSLLDNLITEGAPADLRNDFADPLATALH
CKVLGIPQEDGPKLFRSLSIAFMSSADPIPAAKINWDRDIEYMAGILENPNITTGLMGELSRLRKDPAYSHVS
DELFATIGVTFFGAGVISTGSFLTTALISLIQRPQLRNLLHEKPELIPAGVEELLRINLSFADGLPRLATADI
QVGDVLVRKGELVLVLLEGANFDPEYFPNPGSIELDRPNPTSHLAFGRGQHFCPGSALGRRHAQIGIEALLKK
MPGVDLAVPIDQLVWRTRFQRRIPERLPVLW
>CYP121A1_Mycobacterium tuberculosis BTB13-127 2603180783
MTATVLLEVPFSARGDRIPDAVAELRTREPIRKVRTITGAEAWLVSSYALCTQVLEDRRFSMKETAAAGAPRL
NALTVPPEVVNNMGNIADAGLRKAVMKAITPKAPGLEQFLRDTANSLLDNLITEGAPADLRNDFADPLATALH
CKVLGIPQEDGPKLFRSLSIAFMSSADPIPAAKINWDRDIEYMAGILENPNITTGLMGELSRLRKDPAYSHVS
DELFATIGVTFFGAGVISTGSFLTTALISLIQRPQLRNLLHEKPELIPAGVEELLRINLSFADGLPRLATADI
QVGDVLVRKGELVLVLLEGANFDPEYFPNPGSIELDRPNPTSHLAFGRGQHFCPGSALGRRHAQIGIEALLKK
MPGVDLAVPIDQLVWRTRFQRRIPERLPVLW
>CYP121A1_Mycobacterium tuberculosis BTB13-128 2574881707
MTATVLLEVPFSARGDRIPDAVAELRTREPIRKVRTITGAEAWLVSSYALCTQVLEDRRFSMKETAAAGAPRL
NALTVPPEVVNNMGNIADAGLRKAVMKAITPKAPGLEQFLRDTANSLLDNLITEGAPADLRNDFADPLATALH
CKVLGIPQEDGPKLFRSLSIAFMSSADPIPAAKINWDRDIEYMAGILENPNITTGLMGELSRLRKDPAYSHVS
DELFATIGVTFFGAGVISTGSFLTTALISLIQRPQLRNLLHEKPELIPAGVEELLRINLSFADGLPRLATADI
QVGDVLVRKGELVLVLLEGANFDPEYFPNPGSIELDRPNPTSHLAFGRGQHFCPGSALGRRHAQIGIEALLKK
MPGVDLAVPIDQLVWRTRFQRRIPERLPVLW
>CYP121A1_Mycobacterium tuberculosis BTB13-276 2603189724
MTATVLLEVPFSARGDRIPDAVAELRTREPIRKVRTITGAEAWLVSSYALCTQVLEDRRFSMKETAAAGAPRL
NALTVPPEVVNNMGNIADAGLRKAVMKAITPKAPGLEQFLRDTANSLLDNLITEGAPADLRNDFADPLATALH
CKVLGIPQEDGPKLFRSLSIAFMSSADPIPAAKINWDRDIEYMAGILENPNITTGLMGELSRLRKDPAYSHVS
DELFATIGVTFFGAGVISTGSFLTTALISLIQRPQLRNLLHEKPELIPAGVEELLRINLSFADGLPRLATADI
QVGDVLVRKGELVLVLLEGANFDPEYFPNPGSIELDRPNPTSHLAFGRGQHFCPGSALGRRHAQIGIEALLKK
MPGVDLAVPIDQLVWRTRFQRRIPERLPVLW
>CYP121A1_Mycobacterium tuberculosis CCDC5079 651084995
MTATVLLEVPFSARGDRIPDAVAELRTREPIRKVRTITGAEAWLVSSYALCTQVLEDRRFSMKETAAAGAPRL
NALTVPPEVVNNMGNIADAGLRKAVMKAITPKAPGLEQFLRDTANSLLDNLITEGAPADLRNDFADPLATALH
CKVLGIPQEDGPKLFRSLSIAFMSSADPIPAAKINWDRDIEYMAGILENPNITTGLMGELSRLRKDPAYSHVS
DELFATIGVTFFGAGVISTGSFLTTALISLIQRPQLRNLLHEKPELIPAGVEELLRINLSFADGLPRLATADI
QVGDVLVRKGELVLVLLEGANFDPEYFPNPGSIELDRPNPTSHLAFGRGQHFCPGSALGRRHAQIGIEALLKK
MPGVDLAVPIDQLVWRTRFQRRIPERLPVLW

>CYP121A1_Mycobacterium tuberculosis CCDC5079 2688525027
MTATVLLEVPFSARGDRIPDAVAELRTREPIRKVRTITGAEAWLVSSYALCTQVLEDRRFSMKETAAAGAPRL
NALTVPPEVVNNMGNIADAGLRKAVMKAITPKAPGLEQFLRDTANSLLDNLITEGAPADLRNDFADPLATALH
CKVLGIPQEDGPKLFRSLSIAFMSSADPIPAAKINWDRDIEYMAGILENPNITTGLMGELSRLRKDPAYSHVS
DELFATIGVTFFGAGVISTGSFLTTALISLIQRPQLRNLLHEKPELIPAGVEELLRINLSFADGLPRLATADI
QVGDVLVRKGELVLVLLEGANFDPEYFPNPGSIELDRPNPTSHLAFGRGQHFCPGSALGRRHAQIGIEALLKK
MPGVDLAVPIDQLVWRTRFQRRIPERLPVLW
>CYP121A1_Mycobacterium tuberculosis CCDC5180 651088662
MTATVLLEVPFSARGDRIPDAVAELRTREPIRKVRTITGAEAWLVSSYALCTQVLEDRRFSMKETAAAGAPRL
NALTVPPEVVNNMGNIADAGLRKAVMKAITPKAPGLEQFLRDTANSLLDNLITEGAPADLRNDFADPLATALH
CKVLGIPQEDGPKLFRSLSIAFMSSADPIPAAKINWDRDIEYMAGILENPNITTGLMGELSRLRKDPAYSHVS
DELFATIGVTFFGAGVISTGSFLTTALISLIQRPQLRNLLHEKPELIPAGVEELLRINLSFADGLPRLATADI
QVGDVLVRKGELVLVLLEGANFDPEYFPNPGSIELDRPNPTSHLAFGRGQHFCPGSALGRRHAQIGIEALLKK
MPGVDLAVPIDQLVWRTRFQRRIPERLPVLW
>CYP121A1_Mycobacterium tuberculosis CCDC5180 2687610282
MTATVLLEVPFSARGDRIPDAVAELRTREPIRKVRTITGAEAWLVSSYALCTQVLEDRRFSMKETAAAGAPRL
NALTVPPEVVNNMGNIADAGLRKAVMKAITPKAPGLEQFLRDTANSLLDNLITEGAPADLRNDFADPLATALH
CKVLGIPQEDGPKLFRSLSIAFMSSADPIPAAKINWDRDIEYMAGILENPNITTGLMGELSRLRKDPAYSHVS
DELFATIGVTFFGAGVISTGSFLTTALISLIQRPQLRNLLHEKPELIPAGVEELLRINLSFADGLPRLATADI
QVGDVLVRKGELVLVLLEGANFDPEYFPNPGSIELDRPNPTSHLAFGRGQHFCPGSALGRRHAQIGIEALLKK
MPGVDLAVPIDQLVWRTRFQRRIPERLPVLW
>CYP121A1_Mycobacterium tuberculosis CTRI-4 2547881412
MTATVLLEVPFSARGDRIPDAVAELRTREPIRKVRTITGAEAWLVSSYALCTQVLEDRRFSMKETAAAGAPRL
NALTVPPEVVNNMGNIADAGLRKAVMKAITPKAPGLEQFLRDTANSLLDNLITEGAPADLRNDFADPLATALH
CKVLGIPQEDGPKLFRSLSIAFMSSADPIPAAKINWDRDIEYMAGILENPNITTGLMGELSRLRKDPAYSHVS
DELFATIGVTFFGAGVISTGSFLTTALISLIQRPQLRNLLHEKPELIPAGVEELLRINLSFADGLPRLATADI
QVGDVLVRKGELVLVLLEGANFDPEYFPNPGSIELDRPNPTSHLAFGRGQHFCPGSALGRRHAQIGIEALLKK
MPGVDLAVPIDQLVWRTRFQRRIPERLPVLW
>CYP121A1_Mycobacterium tuberculosis CWCFVRF MDRTB 670 2621957384
MTATVLLEVPFSARGDRIPDAVAELRTREPIRKVRTITGAEAWLVSSYALCTQVLEDRRFSMKETAAAGAPRL
NALTVPPEVVNNMGNIADAGLRKAVMKAITPKAPGLEQFLRDTANSLLDNLITEGAPADLRNDFADPLATALH
CKVLGIPQEDGPKLFRSLSIAFMSSADPIPAAKINWDRDIEYMAGILENPNITTGLMGELSRLRKDPAYSHVS
DELFATIGVTFFGAGVISTGSFLTTALISLIQRPQLRNLLHEKPELIPAGVEELLRINLSFADGLPRLATADI
QVGDVLVRKGELVLVLLEGANFDPEYFPNPGSIELDRPNPTSHLAFGRGQHFCPGSALGRRHAQIGIEALLKK
MPGVDLAVPIDQLVWRTRFQRRIPERLPVLW
>CYP121A1_Mycobacterium tuberculosis E186hv 2633778699
MTATVLLEVPFSARGDRIPDAVAELRTREPIRKVRTITGAEAWLVSSYALCTQVLEDRRFSMKETAAAGAPRL
NALTVPPEVVNNMGNIADAGLRKAVMKAITPKAPGLEQFLRDTANSLLDNLITEGAPADLRNDFADPLATALH
CKVLGIPQEDGPKLFRSLSIAFMSSADPIPAAKINWDRDIEYMAGILENPNITTGLMGELSRLRKDPAYSHVS
DELFATIGVTFFGAGVISTGSFLTTALISLIQRPQLRNLLHEKPELIPAGVEELLRINLSFADGLPRLATADI
QVGDVLVRKGELVLVLLEGANFDPEYFPNPGSIELDRPNPTSHLAFGRGQHFCPGSALGRRHAQIGIEALLKK
MPGVDLAVPIDQLVWRTRFQRRIPERLPVLW
>CYP121A1_Mycobacterium tuberculosis EE0502463 2674541884
MTATVLLEVPFSARGDRIPDAVAELRTREPIRKVRTITGAEAWLVSSYALCTQVLEDRRFSMKETAAAGAPRL
NALTVPPEVVNNMGNIADAGLRKAVMKAITPKAPGLEQFLRDTANSLLDNLITEGAPADLRNDFADPLATALH
CKVLGIPQEDGPKLFRSLSIAFMSSADPIPAAKINWDRDIEYMAGILENPNITTGLMGELSRLRKDPAYSHVS
DELFATIGVTFFGAGVISTGSFLTTALISLIQRPQLRNLLHEKPELIPAGVEELLRINLSFADGLPRLATADI
QVGDVLVRKGELVLVLLEGANFDPEYFPNPGSIELDRPNPTSHLAFGRGQHFCPGSALGRRHAQIGIEALLKK
MPGVDLAVPIDQLVWRTRFQRRIPERLPVLW
>CYP121A1_Mycobacterium tuberculosis EE0503811 2683838986
MTATVLLEVPFSARGDRIPDAVAELRTREPIRKVRTITGAEAWLVSSYALCTQVLEDRRFSMKETAAAGAPRL
NALTVPPEVVNNMGNIADAGLRKAVMKAITPKAPGLEQFLRDTANSLLDNLITEGAPADLRNDFADPLATALH
CKVLGIPQEDGPKLFRSLSIAFMSSADPIPAAKINWDRDIEYMAGILENPNITTGLMGELSRLRKDPAYSHVS
DELFATIGVTFFGAGVISTGSFLTTALISLIQRPQLRNLLHEKPELIPAGVEELLRINLSFADGLPRLATADI
QVGDVLVRKGELVLVLLEGANFDPEYFPNPGSIELDRPNPTSHLAFGRGQHFCPGSALGRRHAQIGIEALLKK
MPGVDLAVPIDQLVWRTRFQRRIPERLPVLW
>CYP121A1_Mycobacterium tuberculosis EE0506178 2683844011

MTATVLLEVPFSARGDRIPDAVAELRTREPIRKVRTITGAEAWLVSSYALCTQVLEDRRFSMKETAAAGAPRL
NALTVPPEVVNNMGNIADAGLRKAVMKAITPKAPGLEQFLRDTANSLLDNLITEGAPADLRNDFADPLATALH
CKVLGIPQEDGPKLFRSLSIAFMSSADPIPAAKINWDRDIEYMAGILENPNITTGLMGELSRLRKDPAYSHVS
DELFATIGVTFFGAGVISTGSFLTTALISLIQRPQLRNLLHEKPELIPAGVEELLRINLSFADGLPRLATADI
QVGDVLVRKGELVLVLLEGANFDPEYFPNPGSIELDRPNPTSHLAFGRGQHFCPGSALGRRHAQIGIEALLKK
MPGVDLAVPIDQLVWRTRFQRRIPERLPVLW
>CYP121A1_Mycobacterium tuberculosis EE0506784 2683834414
MTATVLLEVPFSARGDRIPDAVAELRTREPIRKVRTITGAEAWLVSSYALCTQVLEDRRFSMKETAAAGAPRL
NALTVPPEVVNNMGNIADAGLRKAVMKAITPKAPGLEQFLRDTANSLLDNLITEGAPADLRNDFADPLATALH
CKVLGIPQEDGPKLFRSLSIAFMSSADPIPAAKINWDRDIEYMAGILENPNITTGLMGELSRLRKDPAYSHVS
DELFATIGVTFFGAGVISTGSFLTTALISLIQRPQLRNLLHEKPELIPAGVEELLRINLSFADGLPRLATADI
QVGDVLVRKGELVLVLLEGANFDPEYFPNPGSIELDRPNPTSHLAFGRGQHFCPGSALGRRHAQIGIEALLKK
MPGVDLAVPIDQLVWRTRFQRRIPERLPVLW
>CYP121A1_Mycobacterium tuberculosis FJ05194 2545499226
MTATVLLEVPFSARGDRIPDAVAELRTREPIRKVRTITGAEAWLVSSYALCTQVLEDRRFSMKETAAAGAPRL
NALTVPPEVVNNMGNIADAGLRKAVMKAITPKAPGLEQFLRDTANSLLDNLITEGAPADLRNDFADPLATALH
CKVLGIPQEDGPKLFRSLSIAFMSSADPIPAAKINWDRDIEYMAGILENPNITTGLMGELSRLRKDPAYSHVS
DELFATIGVTFFGAGVISTGSFLTTALISLIQRPQLRNLLHEKPELIPAGVEELLRINLSFADGLPRLATADI
QVGDVLVRKGELVLVLLEGANFDPEYFPNPGSIELDRPNPTSHLAFGRGQHFCPGSALGRRHAQIGIEALLKK
MPGVDLAVPIDQLVWRTRFQRRIPERLPVLW
>CYP121A1_Mycobacterium tuberculosis G-12-005 2577283035
MTATVLLEVPFSARGDRIPDAVAELRTREPIRKVRTITGAEAWLVSSYALCTQVLEDRRFSMKETAAAGAPRL
NALTVPPEVVNNMGNIADAGLRKAVMKAITPKAPGLEQFLRDTANSLLDNLITEGAPADLRNDFADPLATALH
CKVLGIPQEDGPKLFRSLSIAFMSSADPIPAAKINWDRDIEYMAGILENPNITTGLMGELSRLRKDPAYSHVS
DELFATIGVTFFGAGVISTGSFLTTALISLIQRPQLRNLLHEKPELIPAGVEELLRINLSFADGLPRLATADI
QVGDVLVRKGELVLVLLEGANFDPEYFPNPGSIELDRPNPTSHLAFGRGQHFCPGSALGRRHAQIGIEALLKK
MPGVDLAVPIDQLVWRTRFQRRIPERLPVLW
>CYP121A1_Mycobacterium tuberculosis H1329 2622255620
MTATVLLEVPFSARGDRIPDAVAELRTREPIRKVRTITGAEAWLVSSYALCTQVLEDRRFSMKETAAAGAPRL
NALTVPPEVVNNMGNIADAGLRKAVMKAITPKAPGLEQFLRDTANSLLDNLITEGAPADLRNDFADPLATALH
CKVLGIPQEDGPKLFRSLSIAFMSSADPIPAAKINWDRDIEYMAGILENPNITTGLMGELSRLRKDPAYSHVS
DELFATIGVTFFGAGVISTGSFLTTALISLIQRPQLRNLLHEKPELIPAGVEELLRINLSFADGLPRLATADI
QVGDVLVRKGELVLVLLEGANFDPEYFPNPGSIELDRPNPTSHLAFGRGQHFCPGSALGRRHAQIGIEALLKK
MPGVDLAVPIDQLVWRTRFQRRIPERLPVLW
>CYP121A1_Mycobacterium tuberculosis H1856 2622267730
MTATVLLEVPFSARGDRIPDAVAELRTREPIRKVRTITGAEAWLVSSYALCTQVLEDRRFSMKETAAAGAPRL
NALTVPPEVVNNMGNIADAGLRKAVMKAITPKAPGLEQFLRDTANSLLDNLITEGAPADLRNDFADPLATALH
CKVLGIPQEDGPKLFRSLSIAFMSSADPIPAAKINWDRDIEYMAGILENPNITTGLMGELSRLRKDPAYSHVS
DELFATIGVTFFGAGVISTGSFLTTALISLIQRPQLRNLLHEKPELIPAGVEELLRINLSFADGLPRLATADI
QVGDVLVRKGELVLVLLEGANFDPEYFPNPGSIELDRPNPTSHLAFGRGQHFCPGSALGRRHAQIGIEALLKK
MPGVDLAVPIDQLVWRTRFQRRIPERLPVLW
>CYP121A1_Mycobacterium tuberculosis H2300 2622290161
MTATVLLEVPFSARGDRIPDAVAELRTREPIRKVRTITGAEAWLVSSYALCTQVLEDRRFSMKETAAAGAPRL
NALTVPPEVVNNMGNIADAGLRKAVMKAITPKAPGLEQFLRDTANSLLDNLITEGAPADLRNDFADPLATALH
CKVLGIPQEDGPKLFRSLSIAFMSSADPIPAAKINWDRDIEYMAGILENPNITTGLMGELSRLRKDPAYSHVS
DELFATIGVTFFGAGVISTGSFLTTALISLIQRPQLRNLLHEKPELIPAGVEELLRINLSFADGLPRLATADI
QVGDVLVRKGELVLVLLEGANFDPEYFPNPGSIELDRPNPTSHLAFGRGQHFCPGSALGRRHAQIGIEALLKK
MPGVDLAVPIDQLVWRTRFQRRIPERLPVLW
>CYP121A1_Mycobacterium tuberculosis H2398 2577685280
MTATVLLEVPFSARGDRIPDAVAELRTREPIRKVRTITGAEAWLVSSYALCTQVLEDRRFSMKETAAAGAPRL
NALTVPPEVVNNMGNIADAGLRKAVMKAITPKAPGLEQFLRDTANSLLDNLITEGAPADLRNDLADPLATALH
CKVLGIPQEDGPKLFRSLSIAFMSSADPIPAAKINWDRDIEYMAGILENPNITTGLMGELSRLRKDPAYSHVS
DELFATIGVTFFGAGVISTGSFLTTALISLIQRPQLRNLLHEKPELIPAGVEELLRINLSFADGLPRLATADI
QVGDVLVRKGELVLVLLEGANFDPEHFPNPGSIELDRPNPTSHLAFGRGQHFCPGSALGRRHAQIGIEALLKK
MPGVDLAVPIDQLVWRTRFQRRIPERLPVLW
>CYP121A1_Mycobacterium tuberculosis H2581 2576392967
MTATVLLEVPFSARGDRIPDAVAELRTREPIRKVRTITGAEAWLVSSYALCTQVLEDRRFSMKETAAAGAPRL
NALTVPPEVVNNMGNIADAGLRKAVMKAITPKAPGLEQFLRDTANSLLDNLITEGAPADLRNDFADPLATALH

CKVLGIPQEDGPKLFRSLSIAFMSSADPIPAAKINWDRDIEYMAGILENPNITTGLMGELSRLRKDPAYSHVS
DELFATIGVTFFGAGVISTGSFLTTALISLIQRPQLRNLLHEKPELIPAGVEELLRINLSFADGLPRLATADI
QVGDVLVRKGELVLVLLEGANFDPEYFPNPGSIELDRPNPTSHLAFGRGQHFCPGSALGRRHAQIGIEALLKK
MPGVDLAVPIDQLVWRTRFQRRIPERLPVLW
>CYP121A1_Mycobacterium tuberculosis H3360 2622321313
MTATVLLEVPFSARGDRIPDAVAELRTREPIRKVRTITGAEAWLVSSYALCTQVLEDRRFSMKETAAAGAPRL
NALTVPPEVVNNMGNIADAGLRKAVMKAITPKAPGLEQFLRDTANSLLDNLITEGAPADLRNDFADPLATALH
CKVLGIPQEDGPKLFRSLSIAFMSSADPIPAAKINWDRDIEYMAGILENPNITTGLMGELSRLRKDPAYSHVS
DELFATIGVTFFGAGVISTGSFLTTALISLIQRPQLRNLLHEKPELIPAGVEELLRINLSFADGLPRLATADI
QVGDVLVRKGELVLVLLEGANFDPEYFPNPGSIELDRPNPTSHLAFGRGQHFCPGSALGRRHAQIGIEALLKK
MPGVDLAVPIDQLVWRTRFQRRIPERLPVLW
>CYP121A1_Mycobacterium tuberculosis HM 2621347400
MTATVLLEVPFSARGDRIPDAVAELRTREPIRKVRTITGAEAWLVSSYALCTQVLEDRRFSMKETAAAGAPRL
NALTVPPEVVNNMGNIADAGLRKAVMKAITPKAPGLEQFLRDTANSLLDNLITEGAPADLRNDFADPLATALH
CKVLGIPQEDGPKLFRSLSIAFMSSADPIPAAKINWDRDIEYMAGILENPNITTGLMGELSRLRKDPAYSHVS
DELFATIGVTFFGAGVISTGSFLTTALISLIQRPQLRNLSHEKPELIPAGVEELLRINLSFADGLPRLATADI
QVGDVLVRKGELVLVLLEGANFDPEHFPNPGSIELDRPNPTSHLAFGRGQHFCPGSALGRRHAQIGIEALLKK
MPGVDLAVPIDQLVWRTRFQRRIPERLPVLW
>CYP121A1_Mycobacterium tuberculosis HN878 2547960421
MTATVLLEVPFSARGDRIPDAVAELRTREPIRKVRTITGAEAWLVSSYALCTQVLEDRRFSMKETAAAGAPRL
NALTVPPEVVNNMGNIADAGLRKAVMKAITPKAPGLEQFLRDTANSLLDNLITEGAPADLRNDFADPLATALH
CKVLGIPQEDGPKLFRSLSIAFMSSADPIPAAKINWDRDIEYMAGILENPNITTGLMGELSRLRKDPAYSHVS
DELFATIGVTFFGAGVISTGSFLTTALISLIQRPQLRNLLHEKPELIPAGVEELLRINLSFADGLPRLATADI
QVGDVLVRKGELVLVLLEGANFDPEYFPNPGSIELDRPNPTSHLAFGRGQHFCPGSALGRRHAQIGIEALLKK
MPGVDLAVPIDQLVWRTRFQRRIPERLPVLW
>CYP121A1_Mycobacterium tuberculosis IEXDR1 2628596940
MTATVLLEVPFSARGDRIPDAVAELRTREPIRKVRTITGAEAWLVSSYALCTQVLEDRRFSMKETAAAGAPRL
NALTVPPEVVNNMGNIADAGLRKAVMKAITPKAPGLEQFLRDTANSLLDNLITEGAPADLRNDFADPLATALH
CKVLGIPQEDGPKLFRSLSIAFMSSADPIPAAKINWDRDIEYMAGILENPNITTGLMGELSRLRKDPAYSHVS
DELFATIGVTFFGAGVISTGSFLTTALISLIQRPQLRNLLHEKPELIPAGVEELLRINLSFADGLPRLATADI
QVGDVLVRKGELVLVLLEGANFDPEYFPNPGSIELDRPNPTSHLAFGRGQHFCPGSALGRRHAQIGIEALLKK
MPGVDLAVPIDQLVWRTRFQRRIPERLPVLW
>CYP121A1_Mycobacterium tuberculosis KT-0001 2578083108
MTATVLLEVPFSARGDRIPDAVAELRTREPIRKVRTITGAEAWLVSSYALCTQVLEDRRFSMKETAAAGAPRL
NALTVPPEVVNNMGNIADAGLRKAVMKAITPKAPGLEQFLRDTANSLLDNLITEGAPADLRNDFADPLATALH
CKVLGIPQEDGPKLFRSLSIAFMSSADPIPAAKINWDRDIEYMAGILENPNITTGLMGELSRLRKDPAYSHVS
DELFATIGVTFFGAGVISTGSFLTTALISLIQRPQLRNLLHEKPELIPAGVEELLRINLSFADGLPRLATADI
QVGDVLVRKGELVLVLLEGANFDPEYFPNPGSIELDRPNPTSHLAFGRGQHFCPGSALGRRHAQIGIEALLKK
MPGVDLAVPIDQLVWRTRFQRRIPERLPVLW
>CYP121A1_Mycobacterium tuberculosis KT-0003 2576200396
MTATVLLEVPFSARGDRIPDAVAELRTREPIRKVRTITGAEAWLVSSYALCTQVLEDRRFSMKETAAAGAPRL
NALTVPPEVVNNMGNIADAGLRKAVMKAITPKAPGLEQFLRDTANSLLDNLITEGAPADLRNDFADPLATALH
CKVLGIPQEDGPKLFRSLSIAFMSSADPIPAAKINWDRDIEYMAGILENPNITTGLMGELSRLRKDPAYSHVS
DELFATIGVTFFGAGVISTGSFLTTALISLIQRPQLRNLLHEKPELIPAGVEELLRINLSFADGLPRLATADI
QVGDVLVRKGELVLVLLEGANFDPEYFPNPGSIELDRPNPTSHLAFGRGQHFCPGSALGRRHAQIGIEALLKK
MPGVDLAVPIDQLVWRTRFQRRIPERLPVLW
>CYP121A1_Mycobacterium tuberculosis KT-0004 2575256123
MTATVLLEVPFSARGDRIPDAVAELRTREPIRKVRTITGAEAWLVSSYALCTQVLEDRRFSMKETAAAGAPRL
NALTVPPEVVNNMGNIADAGLRKAVMKAITPKAPGLEQFLRDTANSLLDNLITEGAPADLRNDFADPLATALH
CKVLGIPQEDGPKLFRSLSIAFMSSADPIPAAKINWDRDIEYMAGILENPNITTGLMGELSRLRKDPAYSHVS
DELFATIGVTFFGAGVISTGSFLTTALISLIQRPQLRNLLHEKPELIPAGVEELLRINLSFADGLPRLATADI
QVGDVLVRKGELVLVLLEGANFDPEYFPNPGSIELDRPNPTSHLAFGRGQHFCPGSALGRRHAQIGIEALLKK
MPGVDLAVPIDQLVWRTRFQRRIPERLPVLW
>CYP121A1_Mycobacterium tuberculosis KT-0005 2653109963
MTATVLLEVPFSARGDRIPDAVAELRTREPIRKVRTITGAEAWLVSSYALCTQVLEDRRFSMKETAAAGAPRL
NALTVPPEVVNNMGNIADAGLRKAVMKAITPKAPGLEQFLRDTANSLLDNLITEGAPADLRNDFADPLATALH
CKVLGIPQEDGPKLFRSLSIAFMSSADPIPAAKINWDRDIEYMAGILENPNITTGLMGELSRLRKDPAYSHVS
DELFATIGVTFFGAGVISTGSFLTTALISLIQRPQLRNLLHEKPELIPAGVEELLRINLSFADGLPRLATADI

QVGDVLVRKGELVLVLLEGANFDPEYFPNPGSIELDRPNPTSHLAFGRGQHFCPGSALGRRHAQIGIEALLKK
MPGVDLAVPIDQLVWRTRFQRRIPERLPVLW
>CYP121A1_Mycobacterium tuberculosis KT-0010 2604609992
MTATVLLEVPFSARGDRIPDAVAELRTREPIRKVRTITGAEAWLVSSYALCTQVLEDRRFSMKETAAAGAPRL
NALTVPPEVVNNMGNIADAGLRKAVMKAITPKAPGLEQFLRDTANSLLDNLITEGAPADLRNDFADPLATALH
CKVLGIPQEDGPKLFRSLSIAFMSSADPIPAAKINWDRDIEYMAGILENPNITTGLMGELSRLRKDPAYSHVS
DELFATIGVTFFGAGVISTGSFLTTALISLIQRPQLRNLLHEKPELIPAGVEELLRINLSFADGLPRLATADI
QVGDVLVRKGELVLVLLEGANFDPEYFPNPGSIELDRPNPTSHLAFGRGQHFCPGSALGRRHAQIGIEALLKK
MPGVDLAVPIDQLVWRTRFQRRIPERLPVLW
>CYP121A1_Mycobacterium tuberculosis KT-0011 2584911716
MTATVLLEVPFSARGDRIPDAVAELRTREPIRKVRTITGAEAWLVSSYALCTQVLEDRRFSMKETAAAGAPRL
NALTVPPEVVNNMGNIADAGLRKAVMKAITPKAPGLEQFLRDTANSLLDNLITEGAPADLRNDFADPLATALH
CKVLGIPQEDGPKLFRSLSIAFMSSADPIPAAKINWDRDIEYMAGILENPNITTGLMGELSRLRKDPAYSHVS
DELFATIGVTFFGAGVISTGSFLTTALISLIQRPQLRNLLHEKPELIPAGVEELLRINLSFADGLPRLATADI
QVGDVLVRKGELVLVLLEGANFDPEYFPNPGSIELDRPNPTSHLAFGRGQHFCPGSALGRRHAQIGIEALLKK
MPGVDLAVPIDQLVWRTRFQRRIPERLPVLW
>CYP121A1_Mycobacterium tuberculosis KT-0012 2605315155
MTATVLLEVPFSARGDRIPDAVAELRTREPIRKVRTITGAEAWLVSSYALCTQVLEDRRFSMKETAAAGAPRL
NALTVPPEVVNNMGNIADAGLRKAVMKAITPKAPGLEQFLRDTANSLLDNLITEGAPADLRNDFADPLATALH
CKVLGIPQEDGPKLFRSLSIAFMSSADPIPAAKINWDRDIEYMAGILENPNITTGLMGELSRLRKDPAYSHVS
DELFATIGVTFFGAGVISTGSFLTTALISLIQRPQLRNLLHEKPELIPAGVEELLRINLSFADGLPRLATADI
QVGDVLVRKGELVLVLLEGANFDPEYFPNPGSIELDRPNPTSHLAFGRGQHFCPGSALGRRHAQIGIEALLKK
MPGVDLAVPIDQLVWRTRFQRRIPERLPVLW
>CYP121A1_Mycobacterium tuberculosis KT-0018 2604974680
MTATVLLEVPFSARGDRIPDAVAELRTREPIRKVRTITGAEAWLVSSYALCTQVLEDRRFSMKETAAAGAPRL
NALTVPPEVVNNMGNIADAGLRKAVMKAITPKAPGLEQFLRDTANSLLDNLITEGAPADLRNDFADPLATALH
CKVLGIPQEDGPKLFRSLSIAFMSSADPIPAAKINWDRDIEYMAGILENPNITTGLMGELSRLRKDPAYSHVS
DELFATIGVTFFGAGVISTGSFLTTALISLIQRPQLRNLLHEKPELIPAGVEELLRINLSFADGLPRLATADI
QVGDVLVRKGELVLVLLEGANFDPEYFPNPGSIELDRPNPTSHLAFGRGQHFCPGSALGRRHAQIGIEALLKK
MPGVDLAVPIDQLVWRTRFQRRIPERLPVLW
>CYP121A1_Mycobacterium tuberculosis KT-0019 2584848120
MTATVLLEVPFSARGDRIPDAVAELRTREPIRKVRTITGAEAWLVSSYALCTQVLEDRRFSMKETAAAGAPRL
NALTVPPEVVNNMGNIADAGLRKAVMKAITPKAPGLEQFLRDTANSLLDNLITEGAPADLRNDFADPLATALH
CKVLGIPQEDGPKLFRSLSIAFMSSADPIPAAKINWDRDIEYMAGILENPNITTGLMGELSRLRKDPAYSHVS
DELFATIGVTFFGAGVISTGSFLTTALISLIQRPQLRNLLHEKPELIPAGVEELLRINLSFADGLPRLATADI
QVGDVLVRKGELVLVLLEGANFDPEYFPNPGSIELDRPNPTSHLAFGRGQHFCPGSALGRRHAQIGIEALLKK
MPGVDLAVPIDQLVWRTRFQRRIPERLPVLW
>CYP121A1_Mycobacterium tuberculosis KT-0020 2604246610
MTATVLLEVPFSARGDRIPDAVAELRTREPIRKVRTITGAEAWLVSSYALCTQVLEDRRFSMKETAAAGAPRL
NALTVPPEVVNNMGNIADAGLRKAVMKAITPKAPGLEQFLRDTANSLLDNLITEGAPADLRNDFADPLATALH
CKVLGIPQEDGPKLFRSLSIAFMSSADPIPAAKINWDRDIEYMAGILENPNITTGLMGELSRLRKDPAYSHVS
DELFATIGVTFFGAGVISTGSFLTTALISLIQRPQLRNLLHEKPELIPAGVEELLRINLSFADGLPRLATADI
QVGDVLVRKGELVLVLLEGANFDPEYFPNPGSIELDRPNPTSHLAFGRGQHFCPGSALGRRHAQIGIEALLKK
MPGVDLAVPIDQLVWRTRFQRRIPERLPVLW
>CYP121A1_Mycobacterium tuberculosis KT-0024 2577110762
MTATVLLEVPFSARGDRIPDAVAELRTREPIRKVRTITGAEAWLVSSYALCTQVLEDRRFSMKETAAAGAPRL
NALTVPPEVVNNMGNIADAGLRKAVMKAITPKAPGLEQFLRDTANSLLDNLITEGAPADLRNDFADPLATALH
CKVLGIPQEDGPKLFRSLSIAFMSSADPIPAAKINWDRDIEYMAGILENPNITTGLMGELSRLRKDPAYSHVS
DELFATIGVTFFGAGVISTGSFLTTALISLIQRPQLRNLLHEKPELIPAGVEELLRINLSFADGLPRLATADI
QVGDVLVRKGELVLVLLEGANFDPEYFPNPGSIELDRPNPTSHLAFGRGQHFCPGSALGRRHAQIGIEALLKK
MPGVDLAVPIDQLVWRTRFQRRIPERLPVLW
>CYP121A1_Mycobacterium tuberculosis KT-0025 2603369854
MTATVLLEVPFSARGDRIPDAVAELRTREPIRKVRTITGAEAWLVSSYALCTQVLEDRRFSMKETAAAGAPRL
NALTVPPEVVNNMGNIADAGLRKAVMKAITPKAPGLEQFLRDTANSLLDNLITEGAPADLRNDFADPLATALH
CKVLGIPQEDGPKLFRSLSIAFMSSADPIPAAKINWDRDIEYMAGILENPNITTGLMGELSRLRKDPAYSHVS
DELFATIGVTFFGAGVISTGSFLTTALISLIQRPQLRNLLHEKPELIPAGVEELLRINLSFADGLPRLATADI
QVGDVLVRKGELVLVLLEGANFDPEYFPNPGSIELDRPNPTSHLAFGRGQHFCPGSALGRRHAQIGIEALLKK
MPGVDLAVPIDQLVWRTRFQRRIPERLPVLW

>CYP121A1_Mycobacterium tuberculosis KT-0029 2604249883
MTATVLLEVPFSARGDRIPDAVAELRTREPIRKVRTITGAEAWLVSSYALCTQVLEDRRFSMKETAAAGAPRL
NALTVPPEVVNNMGNIADAGLRKAVMKAITPKAPGLEQFLRDTANSLLDNLITEGAPADLRNDFADPLATALH
CKVLGIPQEDGPKLFRSLSIAFMSSADPIPAAKINWDRDIEYMAGILENPNITTGLMGELSRLRKDPAYSHVS
DELFATIGVTFFGAGVISTGSFLTTALISLIQRPQLRNLLHEKPELIPAGVEELLRINLSFADGLPRLATADI
QVGDVLVRKGELVLVLLEGANFDPEYFPNPGSIELDRPNPTSHLAFGRGQHFCPGSALGRRHAQIGIEALLKK
MPGVDLAVPIDQLVWRTRFQRRIPERLPVLW
>CYP121A1_Mycobacterium tuberculosis KT-0031 2604978744
MTATVLLEVPFSARGDRIPDAVAELRTREPIRKVRTITGAEAWLVSSYALCTQVLEDRRFSMKETAAAGAPRL
NALTVPPEVVNNMGNIADAGLRKAVMKAITPKAPGLEQFLRDTANSLLDNLITEGAPADLRNDFADPLATALH
CKVLGIPQEDGPKLFRSLSIAFMSSADPIPAAKINWDRDIEYMAGILENPNITTGLMGELSRLRKDPAYSHVS
DELFATIGVTFFGAGVISTGSFLTTALISLIQRPQLRNLLHEKPELIPAGVEELLRINLSFADGLPRLATADI
QVGDVLVRKGELVLVLLEGANFDPEYFPNPGSIELDRPNPTSHLAFGRGQHFCPGSALGRRHAQIGIEALLKK
MPGVDLAVPIDQLVWRTRFQRRIPERLPVLW
>CYP121A1_Mycobacterium tuberculosis KT-0032 2604615076
MTATVLLEVPFSARGDRIPDAVAELRTREPIRKVRTITGAEAWLVSSYALCTQVLEDRRFSMKETAAAGAPRL
NALTVPPEVVNNMGNIADAGLRKAVMKAITPKAPGLEQFLRDTANSLLDNLITEGAPADLRNDFADPLATALH
CKVLGIPQEDGPKLFRSLSIAFMSSADPIPAAKINWDRDIEYMAGILENPNITTGLMGELSRLRKDPAYSHVS
DELFATIGVTFFGAGVISTGSFLTTALISLIQRPQLRNLLHEKPELIPAGVEELLRINLSFADGLPRLATADI
QVGDVLVRKGELVLVLLEGANFDPEYFPNPGSIELDRPNPTSHLAFGRGQHFCPGSALGRRHAQIGIEALLKK
MPGVDLAVPIDQLVWRTRFQRRIPERLPVLW
>CYP121A1_Mycobacterium tuberculosis KT-0033 2603192954
MTATVLLEVPFSARGDRIPDAVAELRTREPIRKVRTITGAEAWLVSSYALCTQVLEDRRFSMKETAAAGAPRL
NALTVPPEVVNNMGNIADAGLRKAVMKAITPKAPGLEQFLRDTANSLLDNLITEGAPADLRNDFADPLATALH
CKVLGIPQEDGPKLFRSLSIAFMSSADPIPAAKINWDRDIEYMAGILENPNITTGLMGELSRLRKDPAYSHVS
DELFATIGVTFFGAGVISTGSFLTTALISLIQRPQLRNLLHEKPELIPAGVEELLRINLSFADGLPRLATADI
QVGDVLVRKGELVLVLLEGANFDPEYFPNPGSIELDRPNPTSHLAFGRGQHFCPGSALGRRHAQIGIEALLKK
MPGVDLAVPIDQLVWRTRFQRRIPERLPVLW
>CYP121A1_Mycobacterium tuberculosis KT-0034 2575871541
MTATVLLEVPFSARGDRIPDAVAELRTREPIRKVRTITGAEAWLVSSYALCTQVLEDRRFSMKETAAAGAPRL
NALTVPPEVVNNMGNIADAGLRKAVMKAITPKAPGLEQFLRDTANSLLDNLITEGAPADLRNDFADPLATALH
CKVLGIPQEDGPKLFRSLSIAFMSSADPIPAAKINWDRDIEYMAGILENPNITTGLMGELSRLRKDPAYSHVS
DELFATIGVTFFGAGVISTGSFLTTALISLIQRPQLRNLLHEKPELIPAGVEELLRINLSFADGLPRLATADI
QVGDVLVRKGELVLVLLEGANFDPEYFPNPGSIELDRPNPTSHLAFGRGQHFCPGSALGRRHAQIGIEALLKK
MPGVDLAVPIDQLVWRTRFQRRIPERLPVLW
>CYP121A1_Mycobacterium tuberculosis KT-0027 2577997758
MTATVLLEVPFSARGDRIPDAVAELRTREPIRKVRTITGAEAWLVSSYALCTQVLEDRRFSMKETAAAGAPRL
NALTVPPEVVNNMGNIADAGLRKAVMKAITPKAPGLEQFLRDTANSLLDNLITEGAPADLRNDFADPLATALH
CKVLGIPQEDGPKLFRSLSIAFMSSADPIPAAKINWDRDIEYMAGILENPNITTGLMGELSRLRKDPAYSHVS
DELFATIGVTFFGAGVISTGSFLTTALISLIQRPQLRNLLHEKPELIPAGVEELLRINLSFADGLPRLATADI
QVGDVLVRKGELVLVLLEGANFDPEYFPNPGSIELDRPNPTSHLAFGRGQHFCPGSALGRRHAQIGIEALLKK
MPGVDLAVPIDQLVWRTRFQRRIPERLPVLW
>CYP121A1_Mycobacterium tuberculosis KT-0037 2576523534
MTATVLLEVPFSARGDRIPDAVAELRTREPIRKVRTITGAEAWLVSSYALCTQVLEDRRFSMKETAAAGAPRL
NALTVPPEVVNNMGNIADAGLRKAVMKAITPKAPGLEQFLRDTANSLLDNLITEGAPADLRNDFADPLATALH
CKVLGIPQEDGPKLFRSLSIAFMSSADPIPAAKINWDRDIEYMAGILENPNITTGLMGELSRLRKDPAYSHVS
DELFATIGVTFFGAGVISTGSFLTTALISLIQRPQLRNLLHEKPELIPAGVEELLRINLSFADGLPRLATADI
QVGDVLVRKGELVLVLLEGANFDPEYFPNPGSIELDRPNPTSHLAFGRGQHFCPGSALGRRHAQIGIEALLKK
MPGVDLAVPIDQLVWRTRFQRRIPERLPVLW
>CYP121A1_Mycobacterium tuberculosis KT-0041 2584682031
MTATVLLEVPFSARGDRIPDAVAELRTREPIRKVRTITGAEAWLVSSYALCTQVLEDRRFSMKETAAAGAPRL
NALTVPPEVVNNMGNIADAGLRKAVMKAITPKAPGLEQFLRDTANSLLDNLITEGAPADLRNDFADPLATALH
CKVLGIPQEDGPKLFRSLSIAFMSSADPIPAAKINWDRDIEYMAGILENPNITTGLMGELSRLRKDPAYSHVS
DELFATIGVTFFGAGVISTGSFLTTALISLIQRPQLRNLLHEKPELIPAGVEELLRINLSFADGLPRLATADI
QVGDVLVRKGELVLVLLEGANFDPEYFPNPGSIELDRPNPTSHLAFGRGQHFCPGSALGRRHAQIGIEALLKK
MPGVDLAVPIDQLVWRTRFQRRIPERLPVLW
>CYP121A1_Mycobacterium tuberculosis KT-0042 2574948432

MTATVLLEVPFSARGDRIPDAVAELRTREPIRKVRTITGAEAWLVSSYALCTQVLEDRRFSMKETAAAGAPRL
NALTVPPEVVNNMGNIADAGLRKAVMKAITPKAPGLEQFLRDTANSLLDNLITEGAPADLRNDFADPLATALH
CKVLGIPQEDGPKLFRSLSIAFMSSADPIPAAKINWDRDIEYMAGILENPNITTGLMGELSRLRKDPAYSHVS
DELFATIGVTFFGAGVISTGSFLTTALISLIQRPQLRNLLHEKPELIPAGVEELLRINLSFADGLPRLATADI
QVGDVLVRKGELVLVLLEGANFDPEYFPNPGSIELDRPNPTSHLAFGRGQHFCPGSALGRRHAQIGIEALLKK
MPGVDLAVPIDQLVWRTRFQRRIPERLPVLW
>CYP121A1_Mycobacterium tuberculosis KT-0051 2575094967
MTATVLLEVPFSARGDRIPDAVAELRTREPIRKVRTITGAEAWLVSSYALCTQVLEDRRFSMKETAAAGAPRL
NALTVPPEVVNNMGNIADAGLRKAVMKAITPKAPGLEQFLRDTANSLLDNLITEGAPADLRNDFADPLATALH
CKVLGIPQEDGPKLFRSLSIAFMSSADPIPAAKINWDRDIEYMAGILENPNITTGLMGELSRLRKDPAYSHVS
DELFATIGVTFFGAGVISTGSFLTTALISLIQRPQLRNLLHEKPELIPAGVEELLRINLSFADGLPRLATADI
QVGDVLVRKGELVLVLLEGANFDPEYFPNPGSIELDRPNPTSHLAFGRGQHFCPGSALGRRHAQIGIEALLKK
MPGVDLAVPIDQLVWRTRFQRRIPERLPVLW
>CYP121A1_Mycobacterium tuberculosis KT-0057 2584722635
MTATVLLEVPFSARGDRIPDAVAELRTREPIRKVRTITGAEAWLVSSYALCTQVLEDRRFSMKETAAAGAPRL
NALTVPPEVVNNMGNIADAGLRKAVMKAITPKAPGLEQFLRDTANSLLDNLITEGAPADLRNDFADPLATALH
CKVLGIPQEDGPKLFRSLSIAFMSSADPIPAAKINWDRDIEYMAGILENPNITTGLMGELSRLRKDPAYSHVS
DELFATIGVTFFGAGVISTGSFLTTALISLIQRPQLRNLLHEKPELIPAGVEELLRINLSFADGLPRLATADI
QVGDVLVRKGELVLVLLEGANFDPEYFPNPGSIELDRPNPTSHLAFGRGQHFCPGSALGRRHAQIGIEALLKK
MPGVDLAVPIDQLVWRTRFQRRIPERLPVLW
>CYP121A1_Mycobacterium tuberculosis KT-0060 2604618934
MTATVLLEVPFSARGDRIPDAVAELRTREPIRKVRTITGAEAWLVSSYALCTQVLEDRRFSMKETAAAGAPRL
NALTVPPEVVNNMGNIADAGLRKAVMKAITPKAPGLEQFLRDTANSLLDNLITEGAPADLRNDFADPLATALH
CKVLGIPQEDGPKLFRSLSIAFMSSADPIPAAKINWDRDIEYMAGILENPNITTGLMGELSRLRKDPAYSHVS
DELFATIGVTFFGAGVISTGSFLTTALISLIQRPQLRNLLHEKPELIPAGVEELLRINLSFADGLPRLATADI
QVGDVLVRKGELVLVLLEGANFDPEYFPNPGSIELDRPNPTSHLAFGRGQHFCPGSALGRRHAQIGIEALLKK
MPGVDLAVPIDQLVWRTRFQRRIPERLPVLW
>CYP121A1_Mycobacterium tuberculosis KT-0067 2574706305
MTATVLLEVPFSARGDRIPDAVAELRTREPIRKVRTITGAEAWLVSSYALCTQVLEDRRFSMKETAAAGAPRL
NALTVPPEVVNNMGNIADAGLRKAVMKAITPKAPGLEQFLRDTANSLLDNLITEGAPADLRNDFADPLATALH
CKVLGIPQEDGPKLFRSLSIAFMSSADPIPAAKINWDRDIEYMAGILENPNITTGLMGELSRLRKDPAYSHVS
DELFATIGVTFFGAGVISTGSFLTTALISLIQRPQLRNLLHEKPELIPAGVEELLRINLSFADGLPRLATADI
QVGDVLVRKGELVLVLLEGANFDPEYFPNPGSIELDRPNPTSHLAFGRGQHFCPGSALGRRHAQIGIEALLKK
MPGVDLAVPIDQLVWRTRFQRRIPERLPVLW
>CYP121A1_Mycobacterium tuberculosis KT-0069 2574912837
MTATVLLEVPFSARGDRIPDAVAELRTREPIRKVRTITGAEAWLVSSYALCTQVLEDRRFSMKETAAAGAPRL
NALTVPPEVVNNMGNIADAGLRKAVMKAITPKAPGLEQFLRDTANSLLDNLITEGAPADLRNDFADPLATALH
CKVLGIPQEDGPKLFRSLSIAFMSSADPIPAAKINWDRDIEYMAGILENPNITTGLMGELSRLRKDPAYSHVS
DELFATIGVTFFGAGVISTGSFLTTALISLIQRPQLRNLLHEKPELIPAGVEELLRINLSFADGLPRLATADI
QVGDVLVRKGELVLVLLEGANFDPEYFPNPGSIELDRPNPTSHLAFGRGQHFCPGSALGRRHAQIGIEALLKK
MPGVDLAVPIDQLVWRTRFQRRIPERLPVLW
>CYP121A1_Mycobacterium tuberculosis KT-0070 2577656020
MTATVLLEVPFSARGDRIPDAVAELRTREPIRKVRTITGAEAWLVSSYALCTQVLEDRRFSMKETAAAGAPRL
NALTVPPEVVNNMGNIADAGLRKAVMKAITPKAPGLEQFLRDTANSLLDNLITEGAPADLRNDFADPLATALH
CKVLGIPQEDGPKLFRSLSIAFMSSADPIPAAKINWDRDIEYMAGILENPNITTGLMGELSRLRKDPAYSHVS
DELFATIGVTFFGAGVISTGSFLTTALISLIQRPQLRNLLHEKPELIPAGVEELLRINLSFADGLPRLATADI
QVGDVLVRKGELVLVLLEGANFDPEYFPNPGSIELDRPNPTSHLAFGRGQHFCPGSALGRRHAQIGIEALLKK
MPGVDLAVPIDQLVWRTRFQRRIPERLPVLW
>CYP121A1_Mycobacterium tuberculosis KT-0072 2576101895
MTATVLLEVPFSARGDRIPDAVAELRTREPIRKVRTITGAEAWLVSSYALCTQVLEDRRFSMKETAAAGAPRL
NALTVPPEVVNNMGNIADAGLRKAVMKAITPKAPGLEQFLRDTANSLLDNLITEGAPADLRNDFADPLATALH
CKVLGIPQEDGPKLFRSLSIAFMSSADPIPAAKINWDRDIEYMAGILENPNITTGLMGELSRLRKDPAYSHVS
DELFATIGVTFFGAGVISTGSFLTTALISLIQRPQLRNLLHEKPELIPAGVEELLRINLSFADGLPRLATADI
QVGDVLVRKGELVLVLLEGANFDPEYFPNPGSIELDRPNPTSHLAFGRGQHFCPGSALGRRHAQIGIEALLKK
MPGVDLAVPIDQLVWRTRFQRRIPERLPVLW
>CYP121A1_Mycobacterium tuberculosis KT-0081 2605673006
MTATVLLEVPFSARGDRIPDAVAELRTREPIRKVRTITGAEAWLVSSYALCTQVLEDRRFSMKETAAAGAPRL
NALTVPPEVVNNMGNIADAGLRKAVMKAITPKAPGLEQFLRDTANSLLDNLITEGAPADLRNDFADPLATALH

CKVLGIPQEDGPKLFRSLSIAFMSSADPIPAAKINWDRDIEYMAGILENPNITTGLMGELSRLRKDPAYSHVS
DELFATIGVTFFGAGVISTGSFLTTALISLIQRPQLRNLLHEKPELIPAGVEELLRINLSFADGLPRLATADI
QVGDVLVRKGELVLVLLEGANFDPEYFPNPGSIELDRPNPTSHLAFGRGQHFCPGSALGRRHAQIGIEALLKK
MPGVDLAVPIDQLVWRTRFQRRIPERLPVLW
>CYP121A1_Mycobacterium tuberculosis KT-0082 2604982820
MTATVLLEVPFSARGDRIPDAVAELRTREPIRKVRTITGAEAWLVSSYALCTQVLEDRRFSMKETAAAGAPRL
NALTVPPEVVNNMGNIADAGLRKAVMKAITPKAPGLEQFLRDTANSLLDNLITEGAPADLRNDFADPLATALH
CKVLGIPQEDGPKLFRSLSIAFMSSADPIPAAKINWDRDIEYMAGILENPNITTGLMGELSRLRKDPAYSHVS
DELFATIGVTFFGAGVISTGSFLTTALISLIQRPQLRNLLHEKPELIPAGVEELLRINLSFADGLPRLATADI
QVGDVLVRKGELVLVLLEGANFDPEYFPNPGSIELDRPNPTSHLAFGRGQHFCPGSALGRRHAQIGIEALLKK
MPGVDLAVPIDQLVWRTRFQRRIPERLPVLW
>CYP121A1_Mycobacterium tuberculosis KT-0086 2574780958
MTATVLLEVPFSARGDRIPDAVAELRTREPIRKVRTITGAEAWLVSSYALCTQVLEDRRFSMKETAAAGAPRL
NALTVPPEVVNNMGNIADAGLRKAVMKAITPKAPGLEQFLRDTANSLLDNLITEGAPADLRNDFADPLATALH
CKVLGIPQEDGPKLFRSLSIAFMSSADPIPAAKINWDRDIEYMAGILENPNITTGLMGELSRLRKDPAYSHVS
DELFATIGVTFFGAGVISTGSFLTTALISLIQRPQLRNLLHEKPELIPAGVEELLRINLSFADGLPRLATADI
QVGDVLVRKGELVLVLLEGANFDPEYFPNPGSIELDRPNPTSHLAFGRGQHFCPGSALGRRHAQIGIEALLKK
MPGVDLAVPIDQLVWRTRFQRRIPERLPVLW
>CYP121A1_Mycobacterium tuberculosis KT-0087 2574836605
MTATVLLEVPFSARGDRIPDAVAELRTREPIRKVRTITGAEAWLVSSYALCTQVLEDRRFSMKETAAAGAPRL
NALTVPPEVVNNMGNIADAGLRKAVMKAITPKAPGLEQFLRDTANSLLDNLITEGAPADLRNDFADPLATALH
CKVLGIPQEDGPKLFRSLSIAFMSSADPIPAAKINWDRDIEYMAGILENPNITTGLMGELSRLRKDPAYSHVS
DELFATIGVTFFGAGVISTGSFLTTALISLIQRPQLRNLLHEKPELIPAGVEELLRINLSFADGLPRLATADI
QVGDVLVRKGELVLVLLEGANFDPEYFPNPGSIELDRPNPTSHLAFGRGQHFCPGSALGRRHAQIGIEALLKK
MPGVDLAVPIDQLVWRTRFQRRIPERLPVLW
>CYP121A1_Mycobacterium tuberculosis KT-0091 2575468110
MTATVLLEVPFSARGDRIPDAVAELRTREPIRKVRTITGAEAWLVSSYALCTQVLEDRRFSMKETAAAGAPRL
NALTVPPEVVNNMGNIADAGLRKAVMKAITPKAPGLEQFLRDTANSLLDNLITEGAPADLRNDFADPLATALH
CKVLGIPQEDGPKLFRSLSIAFMSSADPIPAAKINWDRDIEYMAGILENPNITTGLMGELSRLRKDPAYSHVS
DELFATIGVTFFGAGVISTGSFLTTALISLIQRPQLRNLLHEKPELIPAGVEELLRINLSFADGLPRLATADI
QVGDVLVRKGELVLVLLEGANFDPEYFPNPGSIELDRPNPTSHLAFGRGQHFCPGSALGRRHAQIGIEALLKK
MPGVDLAVPIDQLVWRTRFQRRIPERLPVLW
>CYP121A1_Mycobacterium tuberculosis KT-0096 2578189168
MTATVLLEVPFSARGDRIPDAVAELRTREPIRKVRTITGAEAWLVSSYALCTQVLEDRRFSMKETAAAGAPRL
NALTVPPEVVNNMGNIADAGLRKAVMKAITPKAPGLEQFLRDTANSLLDNLITEGAPADLRNDFADPLATALH
CKVLGIPQEDGPKLFRSLSIAFMSSADPIPAAKINWDRDIEYMAGILENPNITTGLMGELSRLRKDPAYSHVS
DELFATIGVTFFGAGVISTGSFLTTALISLIQRPQLRNLLHEKPELIPAGVEELLRINLSFADGLPRLATADI
QVGDVLVRKGELVLVLLEGANFDPEYFPNPGSIELDRPNPTSHLAFGRGQHFCPGSALGRRHAQIGIEALLKK
MPGVDLAVPIDQLVWRTRFQRRIPERLPVLW
>CYP121A1_Mycobacterium tuberculosis KT-0098 2584880036
MTATVLLEVPFSARGDRIPDAVAELRTREPIRKVRTITGAEAWLVSSYALCTQVLEDRRFSMKETAAAGAPRL
NALTVPPEVVNNMGNIADAGLRKAVMKAITPKAPGLEQFLRDTANSLLDNLITEGAPADLRNDFADPLATALH
CKVLGIPQEDGPKLFRSLSIAFMSSADPIPAAKINWDRDIEYMAGILENPNITTGLMGELSRLRKDPAYSHVS
DELFATIGVTFFGAGVISTGSFLTTALISLIQRPQLRNLLHEKPELIPAGVEELLRINLSFADGLPRLATADI
QVGDVLVRKGELVLVLLEGANFDPEYFPNPGSIELDRPNPTSHLAFGRGQHFCPGSALGRRHAQIGIEALLKK
MPGVDLAVPIDQLVWRTRFQRRIPERLPVLW
>CYP121A1_Mycobacterium tuberculosis KT-0100 2577734106
MTATVLLEVPFSARGDRIPDAVAELRTREPIRKVRTITGAEAWLVSSYALCTQVLEDRRFSMKETAAAGAPRL
NALTVPPEVVNNMGNIADAGLRKAVMKAITPKAPGLEQFLRDTANSLLDNLITEGAPADLRNDFADPLATALH
CKVLGIPQEDGPKLFRSLSIAFMSSADPIPAAKINWDRDIEYMAGILENPNITTGLMGELSRLRKDPAYSHVS
DELFATIGVTFFGAGVISTGSFLTTALISLIQRPQLRNLLHEKPELIPAGVEELLRINLSFADGLPRLATADI
QVGDVLVRKGELVLVLLEGANFDPEYFPNPGSIELDRPNPTSHLAFGRGQHFCPGSALGRRHAQIGIEALLKK
MPGVDLAVPIDQLVWRTRFQRRIPERLPVLW
>CYP121A1_Mycobacterium tuberculosis KT-0108 2575419467
MTATVLLEVPFSARGDRIPDAVAELRTREPIRKVRTITGAEAWLVSSYALCTQVLEDRRFSMKETAAAGAPRL
NALTVPPEVVNNMGNIADAGLRKAVMKAITPKAPGLEQFLRDTANSLLDNLITEGAPADLRNDFADPLATALH
CKVLGIPQEDGPKLFRSLSIAFMSSADPIPAAKINWDRDIEYMAGILENPNITTGLMGELSRLRKDPAYSHVS
DELFATIGVTFFGAGVISTGSFLTTALISLIQRPQLRNLLHEKPELIPAGVEELLRINLSFADGLPRLATADI

QVGDVLVRKGELVLVLLEGANFDPEYFPNPGSIELDRPNPTSHLAFGRGQHFCPGSALGRRHAQIGIEALLKK
MPGVDLAVPIDQLVWRTRFQRRIPERLPVLW
>CYP121A1_Mycobacterium tuberculosis KT-0109 2584998010
MTATVLLEVPFSARGDRIPDAVAELRTREPIRKVRTITGAEAWLVSSYALCTQVLEDRRFSMKETAAAGAPRL
NALTVPPEVVNNMGNIADAGLRKAVMKAITPKAPGLEQFLRDTANSLLDNLITEGAPADLRNDFADPLATALH
CKVLGIPQEDGPKLFRSLSIAFMSSADPIPAAKINWDRDIEYMAGILENPNITTGLMGELSRLRKDPAYSHVS
DELFATIGVTFFGAGVISTGSFLTTALISLIQRPQLRNLLHEKPELIPAGVEELLRINLSFADGLPRLATADI
QVGDVLVRKGELVLVLLEGANFDPEYFPNPGSIELDRPNPTSHLAFGRGQHFCPGSALGRRHAQIGIEALLKK
MPGVDLAVPIDQLVWRTRFQRRIPERLPVLW
>CYP121A1_Mycobacterium tuberculosis KT-0110 2576252233
MTATVLLEVPFSARGDRIPDAVAELRTREPIRKVRTITGAEAWLVSSYALCTQVLEDRRFSMKETAAAGAPRL
NALTVPPEVVNNMGNIADAGLRKAVMKAITPKAPGLEQFLRDTANSLLDNLITEGAPADLRNDFADPLATALH
CKVLGIPQEDGPKLFRSLSIAFMSSADPIPAAKINWDRDIEYMAGILENPNITTGLMGELSRLRKDPAYSHVS
DELFATIGVTFFGAGVISTGSFLTTALISLIQRPQLRNLLHEKPELIPAGVEELLRINLSFADGLPRLATADI
QVGDVLVRKGELVLVLLEGANFDPEYFPNPGSIELDRPNPTSHLAFGRGQHFCPGSALGRRHAQIGIEALLKK
MPGVDLAVPIDQLVWRTRFQRRIPERLPVLW
>CYP121A1_Mycobacterium tuberculosis KT-0133 2638408046
MTATVLLEVPFSARGDRIPDAVAELRTREPIRKVRTITGAEAWLVSSYALCTQVLEDRRFSMKETAAAGAPRL
NALTVPPEVVNNMGNIADAGLRKAVMKAITPKAPGLEQFLRDTANSLLDNLITEGAPADLRNDFADPLATALH
CKVLGIPQEDGPKLFRSLSIAFMSSADPIPAAKINWDRDIEYMAGILENPNITTGLMGELSRLRKDPAYSHVS
DELFATIGVTFFGAGVISTGSFLTTALISLIQRPQLRNLLHEKPELIPAGVEELLRINLSFADGLPRLATADI
QVGDVLVRKGELVLVLLEGANFDPEYFPNPGSIELDRPNPTSHLAFGRGQHFCPGSALGRRHAQIGIEALLKK
MPGVDLAVPIDQLVWRTRFQRRIPERLPVLW
>CYP121A1_Mycobacterium tuberculosis KT-0140 2634025579
MTATVLLEVPFSARGDRIPDAVAELRTREPIRKVRTITGAEAWLVSSYALCTQVLEDRRFSMKETAAAGAPRL
NALTVPPEVVNNMGNIADAGLRKAVMKAITPKAPGLEQFLRDTANSLLDNLITEGAPADLRNDFADPLATALH
CKVLGIPQEDGPKLFRSLSIAFMSSADPIPAAKINWDRDIEYMAGILENPNITTGLMGELSRLRKDPAYSHVS
DELFATIGVTFFGAGVISTGSFLTTALISLIQRPQLRNLLHEKPELIPAGVEELLRINLSFADGLPRLATADI
QVGDVLVRKGELVLVLLEGANFDPEYFPNPGSIELDRPNPTSHLAFGRGQHFCPGSALGRRHAQIGIEALLKK
MPGVDLAVPIDQLVWRTRFQRRIPERLPVLW
>CYP121A1_Mycobacterium tuberculosis KT-0182 2637787196
MTATVLLEVPFSARGDRIPDAVAELRTREPIRKVRTITGAEAWLVSSYALCTQVLEDRRFSMKETAAAGAPRL
NALTVPPEVVNNMGNIADAGLRKAVMKAITPKAPGLEQFLRDTANSLLDNLITEGAPADLRNDFADPLATALH
CKVLGIPQEDGPKLFRSLSIAFMSSADPIPAAKINWDRDIEYMAGILENPNITTGLMGELSRLRKDPAYSHVS
DELFATIGVTFFGAGVISTGSFLTTALISLIQRPQLRNLLHEKPELIPAGVEELLRINLSFADGLPRLATADI
QVGDVLVRKGELVLVLLEGANFDPEYFPNPGSIELDRPNPTSHLAFGRGQHFCPGSALGRRHAQIGIEALLKK
MPGVDLAVPIDQLVWRTRFQRRIPERLPVLW
>CYP121A1_Mycobacterium tuberculosis KT-0183 2651183646
MTATVLLEVPFSARGDRIPDAVAELRTREPIRKVRTITGAEAWLVSSYALCTQVLEDRRFSMKETAAAGAPRL
NALTVPPEVVNNMGNIADAGLRKAVMKAITPKAPGLEQFLRDTANSLLDNLITEGAPADLRNDFADPLATALH
CKVLGIPQEDGPKLFRSLSIAFMSSADPIPAAKINWDRDIEYMAGILENPNITTGLMGELSRLRKDPAYSHVS
DELFATIGVTFFGAGVISTGSFLTTALISLIQRPQLRNLLHEKPELIPAGVEELLRINLSFADGLPRLATADI
QVGDVLVRKGELVLVLLEGANFDPEYFPNPGSIELDRPNPTSHLAFGRGQHFCPGSALGRRHAQIGIEALLKK
MPGVDLAVPIDQLVWRTRFQRRIPERLPVLW
>CYP121A1_Mycobacterium tuberculosis KT-0184 2633866673
MTATVLLEVPFSARGDRIPDAVAELRTREPIRKVRTITGAEAWLVSSYALCTQVLEDRRFSMKETAAAGAPRL
NALTVPPEVVNNMGNIADAGLRKAVMKAITPKAPGLEQFLRDTANSLLDNLITEGAPADLRNDFADPLATALH
CKVLGIPQEDGPKLFRSLSIAFMSSADPIPAAKINWDRDIEYMAGILENPNITTGLMGELSRLRKDPAYSHVS
DELFATIGVTFFGAGVISTGSFLTTALISLIQRPQLRNLLHEKPELIPAGVEELLRINLSFADGLPRLATADI
QVGDVLVRKGELVLVLLEGANFDPEYFPNPGSIELDRPNPTSHLAFGRGQHFCPGSALGRRHAQIGIEALLKK
MPGVDLAVPIDQLVWRTRFQRRIPERLPVLW
>CYP121A1_Mycobacterium tuberculosis KT-0185 2648916707
MTATVLLEVPFSARGDRIPDAVAELRTREPIRKVRTITGAEAWLVSSYALCTQVLEDRRFSMKETAAAGAPRL
NALTVPPEVVNNMGNIADAGLRKAVMKAITPKAPGLEQFLRDTANSLLDNLITEGAPADLRNDFADPLATALH
CKVLGIPQEDGPKLFRSLSIAFMSSADPIPAAKINWDRDIEYMAGILENPNITTGLMGELSRLRKDPAYSHVS
DELFATIGVTFFGAGVISTGSFLTTALISLIQRPQLRNLLHEKPELIPAGVEELLRINLSFADGLPRLATADI
QVGDVLVRKGELVLVLLEGANFDPEYFPNPGSIELDRPNPTSHLAFGRGQHFCPGSALGRRHAQIGIEALLKK
MPGVDLAVPIDQLVWRTRFQRRIPERLPVLW

>CYP121A1_Mycobacterium tuberculosis KT-0186 2637695721
MTATVLLEVPFSARGDRIPDAVAELRTREPIRKVRTITGAEAWLVSSYALCTQVLEDRRFSMKETAAAGAPRL
NALTVPPEVVNNMGNIADAGLRKAVMKAITPKAPGLEQFLRDTANSLLDNLITEGAPADLRNDFADPLATALH
CKVLGIPQEDGPKLFRSLSIAFMSSADPIPAAKINWDRDIEYMAGILENPNITTGLMGELSRLRKDPAYSHVS
DELFATIGVTFFGAGVISTGSFLTTALISLIQRPQLRNLLHEKPELIPAGVEELLRINLSFADGLPRLATADI
QVGDVLVRKGELVLVLLEGANFDPEYFPNPGSIELDRPNPTSHLAFGRGQHFCPGSALGRRHAQIGIEALLKK
MPGVDLAVPIDQLVWRTRFQRRIPERLPVLW
>CYP121A1_Mycobacterium tuberculosis KT-0188 2638161505
MTATVLLEVPFSARGDRIPDAVAELRTREPIRKVRTITGAEAWLVSSYALCTQVLEDRRFSMKETAAAGAPRL
NALTVPPEVVNNMGNIADAGLRKAVMKAITPKAPGLEQFLRDTANSLLDNLITEGAPADLRNDFADPLATALH
CKVLGIPQEDGPKLFRSLSIAFMSSADPIPAAKINWDRDIEYMAGILENPNITTGLMGELSRLRKDPAYSHVS
DELFATIGVTFFGAGVISTGSFLTTALISLIQRPQLRNLLHEKPELIPAGVEELLRINLSFADGLPRLATADI
QVGDVLVRKGELVLVLLEGANFDPEYFPNPGSIELDRPNPTSHLAFGRGQHFCPGSALGRRHAQIGIEALLKK
MPGVDLAVPIDQLVWRTRFQRRIPERLPVLW
>CYP121A1_Mycobacterium tuberculosis KT-0189 2649392175
MTATVLLEVPFSARGDRIPDAVAELRTREPIRKVRTITGAEAWLVSSYALCTQVLEDRRFSMKETAAAGAPRL
NALTVPPEVVNNMGNIADAGLRKAVMKAITPKAPGLEQFLRDTANSLLDNLITEGAPADLRNDFADPLATALH
CKVLGIPQEDGPKLFRSLSIAFMSSADPIPAAKINWDRDIEYMAGILENPNITTGLMGELSRLRKDPAYSHVS
DELFATIGVTFFGAGVISTGSFLTTALISLIQRPQLRNLLHEKPELIPAGVEELLRINLSFADGLPRLATADI
QVGDVLVRKGELVLVLLEGANFDPEYFPNPGSIELDRPNPTSHLAFGRGQHFCPGSALGRRHAQIGIEALLKK
MPGVDLAVPIDQLVWRTRFQRRIPERLPVLW
>CYP121A1_Mycobacterium tuberculosis KT-0190 2640473058
MTATVLLEVPFSARGDRIPDAVAELRTREPIRKVRTITGAEAWLVSSYALCTQVLEDRRFSMKETAAAGAPRL
NALTVPPEVVNNMGNIADAGLRKAVMKAITPKAPGLEQFLRDTANSLLDNLITEGAPADLRNDFADPLATALH
CKVLGIPQEDGPKLFRSLSIAFMSSADPIPAAKINWDRDIEYMAGILENPNITTGLMGELSRLRKDPAYSHVS
DELFATIGVTFFGAGVISTGSFLTTALISLIQRPQLRNLLHEKPELIPAGVEELLRINLSFADGLPRLATADI
QVGDVLVRKGELVLVLLEGANFDPEYFPNPGSIELDRPNPTSHLAFGRGQHFCPGSALGRRHAQIGIEALLKK
MPGVDLAVPIDQLVWRTRFQRRIPERLPVLW
>CYP121A1_Mycobacterium tuberculosis KT-0191 2633813052
MTATVLLEVPFSARGDRIPDAVAELRTREPIRKVRTITGAEAWLVSSYALCTQVLEDRRFSMKETAAAGAPRL
NALTVPPEVVNNMGNIADAGLRKAVMKAITPKAPGLEQFLRDTANSLLDNLITEGAPADLRNDFADPLATALH
CKVLGIPQEDGPKLFRSLSIAFMSSADPIPAAKINWDRDIEYMAGILENPNITTGLMGELSRLRKDPAYSHVS
DELFATIGVTFFGAGVISTGSFLTTALISLIQRPQLRNLLHEKPELIPAGVEELLRINLSFADGLPRLATADI
QVGDVLVRKGELVLVLLEGANFDPEYFPNPGSIELDRPNPTSHLAFGRGQHFCPGSALGRRHAQIGIEALLKK
MPGVDLAVPIDQLVWRTRFQRRIPERLPVLW
>CYP121A1_Mycobacterium tuberculosis KT-0193 2633034967
MTATVLLEVPFSARGDRIPDAVAELRTREPIRKVRTITGAEAWLVSSYALCTQVLEDRRFSMKETAAAGAPRL
NALTVPPEVVNNMGNIADAGLRKAVMKAITPKAPGLEQFLRDTANSLLDNLITEGAPADLRNDFADPLATALH
CKVLGIPQEDGPKLFRSLSIAFMSSADPIPAAKINWDRDIEYMAGILENPNITTGLMGELSRLRKDPAYSHVS
DELFATIGVTFFGAGVISTGSFLTTALISLIQRPQLRNLLHEKPELIPAGVEELLRINLSFADGLPRLATADI
QVGDVLVRKGELVLVLLEGANFDPEYFPNPGSIELDRPNPTSHLAFGRGQHFCPGSALGRRHAQIGIEALLKK
MPGVDLAVPIDQLVWRTRFQRRIPERLPVLW
>CYP121A1_Mycobacterium tuberculosis KT-0196 2652493297
MTATVLLEVPFSARGDRIPDAVAELRTREPIRKVRTITGAEAWLVSSYALCTQVLEDRRFSMKETAAAGAPRL
NALTVPPEVVNNMGNIADAGLRKAVMKAITPKAPGLEQFLRDTANSLLDNLITEGAPADLRNDFADPLATALH
CKVLGIPQEDGPKLFRSLSIAFMSSADPIPAAKINWDRDIEYMAGILENPNITTGLMGELSRLRKDPAYSHVS
DELFATIGVTFFGAGVISTGSFLTTALISLIQRPQLRNLLHEKPELIPAGVEELLRINLSFADGLPRLATADI
QVGDVLVRKGELVLVLLEGANFDPEYFPNPGSIELDRPNPTSHLAFGRGQHFCPGSALGRRHAQIGIEALLKK
MPGVDLAVPIDQLVWRTRFQRRIPERLPVLW
>CYP121A1_Mycobacterium tuberculosis KT-0197 2631950232
MTATVLLEVPFSARGDRIPDAVAELRTREPIRKVRTITGAEAWLVSSYALCTQVLEDRRFSMKETAAAGAPRL
NALTVPPEVVNNMGNIADAGLRKAVMKAITPKAPGLEQFLRDTANSLLDNLITEGAPADLRNDFADPLATALH
CKVLGIPQEDGPKLFRSLSIAFMSSADPIPAAKINWDRDIEYMAGILENPNITTGLMGELSRLRKDPAYSHVS
DELFATIGVTFFGAGVISTGSFLTTALISLIQRPQLRNLLHEKPELIPAGVEELLRINLSFADGLPRLATADI
QVGDVLVRKGELVLVLLEGANFDPEYFPNPGSIELDRPNPTSHLAFGRGQHFCPGSALGRRHAQIGIEALLKK
MPGVDLAVPIDQLVWRTRFQRRIPERLPVLW
>CYP121A1_Mycobacterium tuberculosis KT-0198 2635488314

MTATVLLEVPFSARGDRIPDAVAELRTREPIRKVRTITGAEAWLVSSYALCTQVLEDRRFSMKETAAAGAPRL
NALTVPPEVVNNMGNIADAGLRKAVMKAITPKAPGLEQFLRDTANSLLDNLITEGAPADLRNDFADPLATALH
CKVLGIPQEDGPKLFRSLSIAFMSSADPIPAAKINWDRDIEYMAGILENPNITTGLMGELSRLRKDPAYSHVS
DELFATIGVTFFGAGVISTGSFLTTALISLIQRPQLRNLLHEKPELIPAGVEELLRINLSFADGLPRLATADI
QVGDVLVRKGELVLVLLEGANFDPEYFPNPGSIELDRPNPTSHLAFGRGQHFCPGSALGRRHAQIGIEALLKK
MPGVDLAVPIDQLVWRTRFQRRIPERLPVLW
>CYP121A1_Mycobacterium tuberculosis KT-0200 2633380298
MTATVLLEVPFSARGDRIPDAVAELRTREPIRKVRTITGAEAWLVSSYALCTQVLEDRRFSMKETAAAGAPRL
NALTVPPEVVNNMGNIADAGLRKAVMKAITPKAPGLEQFLRDTANSLLDNLITEGAPADLRNDFADPLATALH
CKVLGIPQEDGPKLFRSLSIAFMSSADPIPAAKINWDRDIEYMAGILENPNITTGLMGELSRLRKDPAYSHVS
DELFATIGVTFFGAGVISTGSFLTTALISLIQRPQLRNLLHEKPELIPAGVEELLRINLSFADGLPRLATADI
QVGDVLVRKGELVLVLLEGANFDPEYFPNPGSIELDRPNPTSHLAFGRGQHFCPGSALGRRHAQIGIEALLKK
MPGVDLAVPIDQLVWRTRFQRRIPERLPVLW
>CYP121A1_Mycobacterium tuberculosis KT-0201 2640537530
MTATVLLEVPFSARGDRIPDAVAELRTREPIRKVRTITGAEAWLVSSYALCTQVLEDRRFSMKETAAAGAPRL
NALTVPPEVVNNMGNIADAGLRKAVMKAITPKAPGLEQFLRDTANSLLDNLITEGAPADLRNDFADPLATALH
CKVLGIPQEDGPKLFRSLSIAFMSSADPIPAAKINWDRDIEYMAGILENPNITTGLMGELSRLRKDPAYSHVS
DELFATIGVTFFGAGVISTGSFLTTALISLIQRPQLRNLLHEKPELIPAGVEELLRINLSFADGLPRLATADI
QVGDVLVRKGELVLVLLEGANFDPEYFPNPGSIELDRPNPTSHLAFGRGQHFCPGSALGRRHAQIGIEALLKK
MPGVDLAVPIDQLVWRTRFQRRIPERLPVLW
>CYP121A1_Mycobacterium tuberculosis KT-0202 2632682659
MTATVLLEVPFSARGDRIPDAVAELRTREPIRKVRTITGAEAWLVSSYALCTQVLEDRRFSMKETAAAGAPRL
NALTVPPEVVNNMGNIADAGLRKAVMKAITPKAPGLEQFLRDTANSLLDNLITEGAPADLRNDFADPLATALH
CKVLGIPQEDGPKLFRSLSIAFMSSADPIPAAKINWDRDIEYMAGILENPNITTGLMGELSRLRKDPAYSHVS
DELFATIGVTFFGAGVISTGSFLTTALISLIQRPQLRNLLHEKPELIPAGVEELLRINLSFADGLPRLATADI
QVGDVLVRKGELVLVLLEGANFDPEYFPNPGSIELDRPNPTSHLAFGRGQHFCPGSALGRRHAQIGIEALLKK
MPGVDLAVPIDQLVWRTRFQRRIPERLPVLW
>CYP121A1_Mycobacterium tuberculosis KT-0203 2636465997
MTATVLLEVPFSARGDRIPDAVAELRTREPIRKVRTITGAEAWLVSSYALCTQVLEDRRFSMKETAAAGAPRL
NALTVPPEVVNNMGNIADAGLRKAVMKAITPKAPGLEQFLRDTANSLLDNLITEGAPADLRNDFADPLATALH
CKVLGIPQEDGPKLFRSLSIAFMSSADPIPAAKINWDRDIEYMAGILENPNITTGLMGELSRLRKDPAYSHVS
DELFATIGVTFFGAGVISTGSFLTTALISLIQRPQLRNLLHEKPELIPAGVEELLRINLSFADGLPRLATADI
QVGDVLVRKGELVLVLLEGANFDPEYFPNPGSIELDRPNPTSHLAFGRGQHFCPGSALGRRHAQIGIEALLKK
MPGVDLAVPIDQLVWRTRFQRRIPERLPVLW
>CYP121A1_Mycobacterium tuberculosis L_13621 2716383830
MTATVLLEVPFSARGDRIPDAVAELRTREPIRKVRTITGAEAWLVSSYALCTQVLEDRRFSMKETAAAGAPRL
NALTVPPEVVNNMGNIADAGLRKAVMKAITSKAPGLEQFLRDTANSLLDNLITEGAPADLRNDFADPLATALH
CKVLGIPQEDGPKLFRSLSIAFMSSADPIPAAKINWDRDIEYMAGILENPNITTGLMGELSRLRKDPAYSHVS
DELFATIGVTFFGAGVISTGSFLTTALISLIQRPQLRNLLHEKPELIPAGVEELLRINLSFADGLPRLATADI
QVGDVLVRKGELVLVLLEGANFDPEHFPNPGSIELDRPNPTSHLAFGRGQHFCPGSALGRRHAQIGIEALLKK
MPGVDLAVPIDQLVWRTRFQRRIPERLPVLW
>CYP121A1_Mycobacterium tuberculosis M1236 2622393011
MTATVLLEVPFSARGDRIPDAVAELRTREPIRKVRTITGAEAWLVSSYALCTQVLEDRRFSMKETAAAGAPRL
NALTVPPEVVNNMGNIADAGLRKAVMKAITPKAPGLEQFLRDTANSLLDNLITEGAPADLRNDFADPLATALH
CKVLGIPQEDGPKLFRSLSIAFMSSADPIPAAKINWDRDIEYMAGILENPNITTGLMGELSRLRKDPAYSHVS
DELFATIGVTFFGAGVISTGSFLTTALISLIQRPQLRNLLHEKPELIPAGVEELLRINLSFADGLPRLATADI
QVGDVLVRKGELVLVLLEGATFDPEHFPNPGSIELDRPNPTSHLAFGRGQHFCPGSALGRRHAQIGIEALLKK
MPGVDLAVPIDQLVWRTRFQRRIPERLPVLW
>CYP121A1_Mycobacterium tuberculosis M1273 2622413141
MTATVLLEVPFSARGDRIPDAVAELRTREPIRKVRTITGAEAWLVSSYALCTQVLEDRRFSMKETAAAGAPRL
NALTVPPEVVNNMGNIADAGLRKAVMKAITPKAPGLEQFLRDTANSLLDNLITEGAPADLRNDFADPLATALH
CKVLGIPQEDGPKLFRSLSIAFMSSADPIPAAKINWDRDIEYMAGILENPNITTGLMGELSRLRKDPAYSHVS
NELFATIGVTFFGAGVISTGSFLTTALISLIQRPQLRNLLHEKPELIPAGVEELLRINLSFADGLPRLATADI
QVGDVLVRKGELVLVLLEGANFDPEHFPNPGSIELDRPNPTSHLAFGRGQHFCPGSALGRRHAQIGIEALLKK
MPGVDLAVPIDQLVWRTRFQRRIPERLPVLW
>CYP121A1_Mycobacterium tuberculosis M1294 2622432799
MTATVLLEVPFSARGDRIPDAVAELRTREPIRKVRTITGAEAWLVSSYALCTQVLEDRRFSMKETAAAGAPRL
NALTVPPEVVNNMGNIADAGLRKAVMKAITPKAPGLEQFLRDTANSLLDNLITEGAPADLRNDFADPLATALH

CKVLGIPQEDGPKLFRSLSIAFMSSADPIPAAKINWDRDIEYMAGILENPNITTGLMGELSRLRKDPAYSHVS
DELFATIGVTFFGAGVISTGSFLTTALISLIQRPQLRNLLHEKPELIPAGVEELLRINLSFADGLPRLATADI
QVGDVLVRKGELVLVLLEGANFDPEHFPNPGSIELDRPNPTSHLAFGRGQHFCPGSALGRRHAQIGIEALLKK
MRGVDLAVPIDQLVWRTRFQRRIPERLPVLW
>CYP121A1_Mycobacterium tuberculosis M1374 2622487345
MTATVLLEVPFSARGDRIPDAVAELRTREPIRKVRTITGAEAWLVSSYALCTQVLEDRRFSMKETAAAGAPRL
NALTVPPEVVNNMGNIADAGLRKAVMKAITPKAPGLEQFLRDTANSLLDNLITEGAPADLRNDFADPLATALH
CKVLGIPQEDGPKLFRSLSIAFMSSADPIPAAKINWDRDIEYMAGILENPNITTGLMGELSRLRKDPAYSHVS
DELFATIGVTFFGAGVISTGSFLTTALISLIQRPQLRNLLHEKPELIPAGVEELLRINLSFADGLPRLATADI
QVGDVLVRKGELVLVLLEGANFDPEHFPNPGSIELDRPNPTSHLAFGRGQHFCPGSALGRRHAQIGIEALLKK
MPGVDLTVPIDQLVWRTRFQRRIPERLPVLW
>CYP121A1_Mycobacterium tuberculosis M1397 2622507200
MTATVLLEVPFSARGDRIPDAVAELRTREPIRKVRTITGAEAWLVSSYALCTQVLEDRRFSMKETAAAGAPRL
NALTVPPEVVNNMGNIADAGLRKAVMKAITPKAPGLEQFLRDTANSLLDNLITEGAPADLRNDFADPLATALH
CKVLGIPQEDGPKLFRSLSIAFMSSADPIPAAKINWDRDIEYMAGILENPNITTGLMGELSRLRKDPAYSHVS
DELFATIGVTFFGAGVISTGSFLTTALISLIQRPQLRNLLHEKPELIPAGVEELLRINLSFADGLPRLATADI
QVGDVLVRKGELVLVLLEGANFDPEYFPNPGSIELDRPNPTSHLAFGRGQHFCPGSALGRRHAQIGIEALLKK
MPGVDLAVPIDQLVWRTRFQRRIPERLPVLW
>CYP121A1_Mycobacterium tuberculosis M1448 2622565503
MTATVLLEVPFSARGDRIPDAVAELRTREPIRKVRTITGAEAWLVSSYALCTQVLEDRRFSMKETAAAGAPRL
NALTVPPEVVNNMGNIADAGLRKAVMKAITPKAPGLEQFLRDTANSLLDNLITEGAPADLRNDFADPLATALH
CKVLGIPQEDGPKLFRSLSIAFMSSADPIPAAKINWDRDIEYMAGILENPNITTGLMGELSRLRKDPAYSHVS
DELFATIGVTFFGAGVISTGSFLTTALISLIQRPQLRNLLHEKPELIPAGVEELLRINLSFADGLPRLATADI
QVGDVLVRKGELVLVLLEGANFDPEHFPNPGSIELDRPNPTSHLAFGRGQHFCPGSALGRRHAQIGIEALLKK
MPGVDLAVPIDQLVWGTRFQRRIPERLPVLW
>CYP121A1_Mycobacterium tuberculosis M1456 2622573395
MTATVLLEVPFSARGDRIPDAVAELRTREPIRKVRTITGAEAWLVSSYALCTQVLEDRRFSMKETAAAGAPRL
NALTVPPEVVNNMGNIADAGLRKAVMKAITPKAPGLEQFLRDTANSLLDNLITEGAPADLRNDFADPLATALH
CKVLGIPQEDGPKLFRSLSIAFMSSADPIPAAKINWDRDIEYMAGILENPNITTGLMGELSRLRKDPAYSHVS
NELFATIGVTFFGAGVISTGSFLTTALISLIQRPQLRNLLHEKPELIPAGVEELLRINLSFADGLPRLATADI
QVGDVLVRKGELVLVLLEGANFDPEHFPNPGSIELDRPNPTSHLAFGRGQHFCPGSALGRRHAQIGIEALLKK
MPGVDLAVPIDQLVWRTRFQRRIPERLPVLW
>CYP121A1_Mycobacterium tuberculosis M1615 2622614335
MTATVLLEVPFSARGDRIPDAVAELRTREPIRKVRTITGAEAWLVSSYALCTQVLEDRRFSMKETAAAGAPRL
NALTVPPEVVNNMGNIADAGLRKAVMKAITPKAPGLEQFLRDTANSLLDNLITEGAPADLRNDFADPLATALH
CKVLGIPQEDGPKLFRSLSIAFMSSADPIPAAKINWDRDIEYMAGILENPNITTGLMGELSRLRKDPAYSHVS
DELFATIGVTFFGAGVISTGSFLTTALISLIQRPQLRNLLHEKPELIPAGVEELLRINLSFADGLPRLATADI
QVGDVLVRKGELVLVLLEGANFDPEYFPNPGSIELDRPNPTSHLAFGRGQHFCPGSALGRRHAQIGIEALLKK
MPGVDLAVPIDQLVWRTRFQRRIPERLPVLW
>CYP121A1_Mycobacterium tuberculosis M1638 2622617993
MTATVLLEVPFSARGDRIPDAVAELRTREPIRKVRTITGAEAWLVSSYALCTQVLEDRRFSMKETAAAGAPRL
NALTVPPEVVNNMGNIADAGLRKAVMKAITPKAPGLEQFLRDTANSLLDNLITEGAPADLRNDFADPLATALH
CKVLGIPQEDGPKLFRSLSIAFMSSADPIPAAKINWDRDIEYMAGILENPNITTGLMGELSRLRKDPAYSHVS
DELFATIGVTFFGAGVISTGSFLTTALISLIQRPQLRNLLHEKPELIPAGVEELLRINLSFADGLPRLATADI
QVGDVLVRKGELVLVLLEGANFDPEHFPNPGSIELDRPNPTSHLAFGRGQHFCPGSALGRRHAQIGIEALLKK
MRGVDLAVPIDQLVWRTRFQRRIPERLPVLW
>CYP121A1_Mycobacterium tuberculosis M1700 2584914280
MTATVLLEVPFSARGDRIPDAVAELRTREPIRKVRTITGAEAWLVSSYALCTQVLEDRRFSMKETAAAGAPRL
NALTVPPEVVNNMGNIADAGLRKAVMKAITPKAPGLEQFLRDTANSLLDNLITEGAPADLRNDFADPLATALH
CKVLGIPQEDGPKLFRSLSIAFMSSADPIPAAKINWDRDIEYMAGILENPNITTGLMGELSRLRKDPAYSHVS
DELFATIGVTFFGAGVISTGSFLTTALISLIQRPQLRNLLHEKPELIPAGVEELLRINLSFADGLPRLATADI
QVGDVLVRKGELVLVLLEGANFDPEYFPNPGSIELDRPNPTSHLAFGRGQHFCPGSALGRRHAQIGIEALLKK
MPGVDLAVPIDQLVWRTRFQRRIPERLPVLW
>CYP121A1_Mycobacterium tuberculosis M1728 2622634876
MTATVLLEVPFSARGDRIPDAVAELRTREPIRKVRTITGAEAWLVSSYALCTQVLEDRRFSMKETAAAGAPRL
NALTVPPEVVNNMGNIADAGLRKAVMKAITPKAPGLEQFLRDTANSLLDNLITEGAPADLRNDFADPLATALH
CKVLGIPQEDGPKLFRSLSIAFMSSADPIPAAKINWDRDIEYMAGILENPNITTGLMGELSRLRKDPAYSHVS
DELFATIGVTFFGAGVISTGSFLTTALISLIQRPQLRNLLHEKPELIPAGVEELLRINLSFADGLPRLATADI

QVGDVLVRKGELVLVLLEGANFDPEYFPNPGSIELDRPNPTSHLAFGRGQHFCPGSALGRRHAQIGIEALLKK
MPGVDLAVPIDQLVWRTRFQRRIPERLPVLW
>CYP121A1_Mycobacterium tuberculosis M1782 2622649125
MTATVLLEVPFSARGDRIPDAVAELRTREPIRKVRTITGAEAWLVSSYALCTQVLEDRRFSMKETAAAGAPRL
NALTVPPEVVNNMGNIADAGLRKAVMKAITPKAPGLEQFLRDTANSLLDNLITEGAPADLRNDFADPLATALH
CKVLGIPQEDGPKLFRSLSIAFMSSADPIPAAKINWDRDIEYMAGILENPNITTGLMGELSRLRKDPAYSHVS
DELFATIGVTFFGAGVISTGSFLTTALISLIQRPQLRNLLHEKPELIPAGVEELLRINLSFADGLPRLATADI
QVGDVLVRKGELVLVLLEGANFDPEYFPNPGSIELDRPNPTSHLAFGRGQHFCPGSALGRRHAQIGIEALLKK
MPGVDLAVPIDQLVWRTRFQRRIPERLPVLW
>CYP121A1_Mycobacterium tuberculosis M1822 2622655856
MTATVLLEVPFSARGDRIPDAVAELRTREPIRKVRTITGAEAWLVSSYALCTQVLEDRRFSMKETAAAGAPRL
NALTVPPEVVNNMGNIADAGLRKAVMKAITPKAPGLEQFLRDTANSLLDNLITEGAPADLRNDFADPLATALH
CKVLGIPQEDGPKLFRSLSIAFMSSADPIPAAKINWDRDIEYMAGILENPNITTGLMGELSRLRKDPAYSHVS
DELFATIGVTFFGAGVISTGSFLTTALISLIQRPQLRNLLHEKPELIPAGVEELLRINLSFADGLPRLATADI
QVGDVLVRKGELVLVLLEGANFDPEYFPNPGSIELDRPNPTSHLAFGRGQHFCPGSALGRRHAQIGIEALLKK
MPGVDLAVPIDQLVWRTRFQRRIPERLPVLW
>CYP121A1_Mycobacterium tuberculosis M1848 2574773556
MTATVLLEVPFSARGDRIPDAVAELRTREPIRKVRTITGAEAWLVSSYALCTQVLEDRRFSMKETAAAGAPRL
NALTVPPEVVNNMGNIADAGLRKAVMKAITPKAPGLEQFLRDTANSLLDNLITEGAPADLRNDFADPLATALH
CKVLGIPQEDGPKLFRSLSIAFMSSADPIPAAKINWDRDIEYMAGILENPNITTGLMGELSRLRKDPAYSHVS
DELFATIGVTFFGAGVISTGSFLTTALISLIQRPQLRNLLHEKPELIPAGVEELLRINLSFADGLPRLATADI
QVGDVLVRKGELVLVLLEGANFDPEYFPNPGSIELDRPNPTSHLAFGRGQHFCPGSALGRRHAQIGIEALLKK
MPGVDLAVPIDQLVWRTRFQRRIPERLPVLW
>CYP121A1_Mycobacterium tuberculosis M1895 2622663819
MTATVLLEVPFSARGDRIPDAVAELRTREPIRKVRTITGAEAWLVSSYALCTQVLEDRRFSMKETAAAGAPRL
NALTVPPEVVNNMGNIADAGLRKAVMKAITPKAPGLEQFLRDTANSLLDNLITEGAPADLRNDFADPLATALH
CKVLGIPQEDGPKLFRSLSIAFMSSADPIPAAKINWDRDIEYMAGILENPNITTGLMGELSRLRKDPAYSHVS
DELFATIGVTFFGAGVISTGSFLTTALISLIQRPQLRNLLHEKPELIPAGVEELLRINLSFADGLPRLATADI
QVGDVLVRKGELVLVLLEGANFDPEYFPNPGSIELDRPNPTSHLAFGRGQHFCPGSALGRRHAQIGIEALLKK
MPGVDLAVPIDQLVWRTRFQRRIPERLPVLW
>CYP121A1_Mycobacterium tuberculosis M1906 2576123776
MTATVLLEVPFSARGDRIPDAVAELRTREPIRKVRTITGAEAWLVSSYALCTQVLEDRRFSMKETAAAGAPRL
NALTVPPEVVNNMGNIADAGLRKAVMKAITPKAPGLEQFLRDTANSLLDNLITEGAPTDLRNDFADPLATALH
CKVLGIPQEDGPKLFRSLSIAFMSSADPIPAAKINWDRDIEYMAGILENPNITTGLMGELSRLRKDPAYSHVS
DELFATIGVTFFGAGVISTGSFLTTALISLIQRPQLRNLLHEKPELIPAGVEELLRINLSFADGLPRLATADI
QVGDVLVRKGELVLVLLEGANFDPEHFPNPGSIELDRPNPTSHLAFGRGQHFCPGSALGRRHAQIGIEALLKK
MPGVDLAVPIDQLVWRTRFQRRIPERLPVLW
>CYP121A1_Mycobacterium tuberculosis M1914 2622667955
MTATVLLEVPFSARGDRIPDAVAELRTREPIRKVRTITGAEAWLVSSYALCTQVLEDRRFSMKETAAAGAPRL
NALTVPPEVVNNMGNIADAGLRKAVMKAITPKAPGLEQFLRDTANSLLDNLITEGAPADLRNDFADPLATALH
CKVLGIPQEDGPKLFRSLSIAFMSSADPIPAAKINWDRDIEYMAGILENPNITTGLMGELSRLRKDPAYSHVS
DELFATIGVTFFGAGVISTGSFLTTALISLIQRPQLRNLLHEKPELIPAGVEELLRINLSFADGLPRLATADI
QVGDVLVRKGELVLVLLEGANFDPEYFPNPGSIELDRPNPTSHLAFGRGQHFCPGSALGRRHAQIGIEALLKK
MPGVDLAVPIDQLVWRTRFQRRIPERLPVLW
>CYP121A1_Mycobacterium tuberculosis M1926 2622672259
MTATVLLEVPFSARGDRIPDAVAELRTREPIRKVRTITGAEAWLVSSYALCTQVLEDRRFSMKETAAAGAPRL
NALTVPPEVVNNMGNIADAGLRKAVMKAITPKAPGLEQFLRDTANSLLDNLITEGAPADLRNDFADPLATALH
CKVLGIPQEDGPKLFRSLSIAFMSSADPIPAAKINWDRDIEYMAGILENPNITTGLMGELSRLRKDPAYSHVS
DELFATIGVTFFGAGVISTGSFLTTALISLIQRPQLRNLLHEKPELIPAGVEELLRINLSFADGLPRLATADI
QVGDVLVRKGELVLVLLEGANFDPEYFPNPGSIELDRPNPTSHLAFGRGQHFCPGSALGRRHAQIGIEALLKK
MPGVDLAVPIDQLVWRTRFQRRIPERLPVLW
>CYP121A1_Mycobacterium tuberculosis M1932 2577253600
MTATVLLEVPFSARGDRIPDAVAELRTREPIRKVRTITGAEAWLVSSYALCTQVLEDRRFSMKETAAAGAPRL
NALTVPPEVVNNMGNIADAGLRKAVMKAITPKAPGLEQFLRDTANSLLDNLITEGAPADLRNDFADPLATALH
CKVLGIPQEDGPKLFRSLSIAFMSSADPIPAAKINWDRDIEYMAGILENPNITTGLMGELSRLRKDPAYSHVS
DELFATIGVTFFGAGVISTGSFLTTALISLIQRPQLRNLLHEKPELIPAGVEELLRINLSFADGLPRLATADI
QVGDVLVRKGELVLVLLEGANFDPEYFPNPGSIELDRPNPTSHLAFGRGQHFCPGSALGRRHAQIGIEALLKK
MPGVDLAVPIDQLVWRTRFQRRIPERLPVLW

>CYP121A1_Mycobacterium tuberculosis M1956 2584634458
MTATVLLEVPFSARGDRIPDAVAELRTREPIRKVRTITGAEAWLVSSYALCTQVLEDRRFSMKETAAAGAPRL
NALTVPPEVVNNMGNIADAGLRKAVMKAITPKAPGLEQFLRDTANSLLDNLITEGAPADLRNDFADPLATALH
CKVLGIPQEDGPKLFRSLSIAFMSSADPIPAAKINWDRDIEYMAGILENPNITTGLMGELSRLRKDPAYSHVS
DELFATIGVTFFGAGVISTGSFLTTALISLIQRPQLRNLLHEKPELIPAGVEELLRINLSFADGLPRLATADI
QVGDVLVRKGELVLVLLEGANFDPEYFPNPGSIELDRPNPTSHLAFGRGQHFCPGSALGRRHAQIGIEALLKK
MPGVDLAVPIDQLVWRTRFQRRIPERLPVLW
>CYP121A1_Mycobacterium tuberculosis M1973 2622701009
MTATVLLEVPFSARGDRIPDAVAELRTREPIRKVRTITGAEAWLVSSYALCTQVLEDRRFSMKETAAAGAPRL
NALTVPPEVVNNMGNIADAGLRKAVMKAITPKAPGLEQFLRDTANSLLDNLITEGAPADLRNDFADPLATALH
CKVLGIPQEDGPKLFRSLSIAFMSSADPIPAAKINWDRDIEYMAGILENPNITTGLMGELSRLRKDPAYSHVS
DELFATIGVTFFGAGVISTGSFLTTALISLIQRPQLRNLLHEKPELIPAGVEELLRINLSFADGLPRLATADI
QVGDVLVRKGELVLVLLEGANFDPEYFPNPGSIELDRPNPTSHLAFGRGQHFCPGSALGRRHAQIGIEALLKK
MPGVDLAVPIDQLVWRTRFQRRIPERLPVLW
>CYP121A1_Mycobacterium tuberculosis M2084 2629499967
MTATVLLEVPFSARGDRIPDAVAELRTREPIRKVRTITGAEAWLVSSYALCTQVLEDRRFSMKETAAAGAPRL
NALTVPPEVVNNMGNIADAGLRKAVMKAITPKAPGLEQFLRDTANSLLDNLITEGAPADLRNDFADPLATALH
CKVLGIPQEDGPKLFRSLSIAFMSSADPIPAAKINWDRDIEYMAGILENPNITTGLMGELSRLRKDPAYSHVS
DELFATIGVTFFGAGVISTGSFLTTALISLIQRPQLRNLLHEKPELIPAGVEELLRINLSFADGLPRLATADI
QVGDVLVRKGELVLVLLEGANFDPEYFPNPGSIELDRPNPTSHLAFGRGQHFCPGSALGRRHAQIGIEALLKK
MPGVDLAVPIDQLVWRTRFQRRIPERLPVLW
>CYP121A1_Mycobacterium tuberculosis M2085 2577906638
MTATVLLEVPFSARGDRIPDAVAELRTREPIRKVRTITGAEAWLVSSYALCTQVLEDRRFSMKETAAAGAPRL
NALTVPPEVVNNMGNIADAGLRKAVMKAITPKAPGLEQFLRDTANSLLDNLITEGAPADLRNDFADPLATALH
CKVLGIPQEDGPKLFRSLSIAFMSSADPIPAAKINWDRDIEYMAGILENPNITTGLMGELSRLRKDPAYSHVS
DELFATIGVTFFGAGVISTGSFLTTALISLIQRPQLRNLLHEKPELIPAGVEELLRINLSFADGLPRLATADI
QVGDVLVRKGELVLVLLEGANFDPEYFPNPGSIELDRPNPTSHLAFGRGQHFCPGSALGRRHAQIGIEALLKK
MPGVDLAVPIDQLVWRTRFQRRIPERLPVLW
>CYP121A1_Mycobacterium tuberculosis M2086 2632817153
MTATVLLEVPFSARGDRIPDAVAELRTREPIRKVRTITGAEAWLVSSYALCTQVLEDRRFSMKETAAAGAPRL
NALTVPPEVVNNMGNIADAGLRKAVMKAITPKAPGLEQFLRDTANSLLDNLITEGAPADLRNDFADPLATALH
CKVLGIPQEDGPKLFRSLSIAFMSSADPIPAAKINWDRDIEYMAGILENPNITTGLMGELSRLRKDPAYSHVS
DELFATIGVTFFGAGVISTGSFLTTALISLIQRPQLRNLLHEKPELIPAGVEELLRINLSFADGLPRLATADI
QVGDVLVRKGELVLVLLEGANFDPEYFPNPGSIELDRPNPTSHLAFGRGQHFCPGSALGRRHAQIGIEALLKK
MPGVDLAVPIDQLVWRTRFQRRIPERLPVLW
>CYP121A1_Mycobacterium tuberculosis M2130 2633916035
MTATVLLEVPFSARGDRIPDAVAELRTREPIRKVRTITGAEAWLVSSYALCTQVLEDRRFSMKETAAAGAPRL
NALTVPPEVVNNMGNIADAGLRKAVMKAITPKAPGLEQFLRDTANSLLDNLITEGAPADLRNDFADPLATALH
CKVLGIPQEDGPKLFRSLSIAFMSSADPIPAAKINWDRDIEYMAGILENPNITTGLMGELSRLRKDPAYSHVS
DELFATIGVTFFGAGVISTGSFLTTALISLIQRPQLRNLLHEKPELIPAGVEELLRINLSFADGLPRLATADI
QVGDVLVRKGELVLVLLEGANFDPEYFPNPGSIELDRPNPTSHLAFGRGQHFCPGSALGRRHAQIGIEALLKK
MPGVDLAVPIDQLVWRTRFQRRIPERLPVLW
>CYP121A1_Mycobacterium tuberculosis M2131 2576732435
MTATVLLEVPFSARGDRIPDAVAELRTREPIRKVRTITGAEAWLVSSYALCTQVLEDRRFSMKETAAAGAPRL
NALTVPPEVVNNMGNIADAGLRKAVMKAITPKAPGLEQFLRDTANSLLDNLITEGAPADLRNDFADPLATALH
CKVLGIPQEDGPKLFRSLSIAFMSSADPIPAAKINWDRDIEYMAGILENPNITTGLMGELSRLRKDPAYSHVS
DELFATIGVTFFGAGVISTGSFLTTALISLIQRPQLRNLLHEKPELIPAGVEELLRINLSFADGLPRLATADI
QVGDVLVRKGELVLVLLEGANFDPEYFPNPGSIELDRPNPTSHLAFGRGQHFCPGSALGRRHAQIGIEALLKK
MPGVDLAVPIDQLVWRTRFQRRIPERLPVLW
>CYP121A1_Mycobacterium tuberculosis M2145 2638527336
MTATVLLEVPFSARGDRIPDAVAELRTREPIRKVRTITGAEAWLVSSYALCTQVLEDRRFSMKETAAAGAPRL
NALTVPPEVVNNMGNIADAGLRKAVMKAITPKAPGLEQFLRDTANSLLDNLITEGAPADLRNDFADPLATALH
CKVLGIPQEDGPKLFRSLSIAFMSSADPIPAAKINWDRDIEYMAGILENPNITTGLMGELSRLRKDPAYSHVS
DELFATIDVTFFGAGVISTGSFLTTALISLIQRPQLRNLLHEKPELIPAGVEELLRINLSFADGLPRLATADI
QVGDVLVRKGELVLVLLEGANFDPEHFPNPGSIELDRPNPTSHLAFGRGQHFCPGSALGRRHAQIGIEALLKK
MPGVDLAVPIDQLVWRTRFQRRIPERLPVLW
>CYP121A1_Mycobacterium tuberculosis M2191 2642222978

MTATVLLEVPFSARGDRIPDAVAELRTREPIRKVRTITGAEAWLVSSYALCTQVLEDRRFSMKETAAAGAPRL
NALTVPPEVVNNMGNIADAGLRKAVMKAITPKAPGLEQFLRDTANSLLDNLITEGAPADLRNDFADPLATALH
CKVLGIPQEDGPKLFRSLSIAFMSSADPIPAAKINWDRDIEYMAGILENPNITTGLMGELSRLRKDPAYSHVS
DELFATIGVIFFGAGVISTGSFLTTALISLIQRPQLRNLLHEKPELIPAGVEELLRINLSFADGLPRLATADI
QVGDVLVRKGELVLVLLEGANFDPEHFPNPGSIELDRPNPTSHLAFGRGQHFCPGSALGRRHAQIGIEALLKK
MPGVDLAVPIDQLVWRTRFQRRIPERLPVLW
>CYP121A1_Mycobacterium tuberculosis M2204 2638693142
MTATVLLEVPFSARGDRIPDAVAELRTREPIRKVRTITGAEAWLVSSYALCTQVLEDRRFSMKETAAAGAPRL
NALTVPPEVVNNMGNIADAGLRKAVMKAITPKAPGLEQFLRDTANSLLDNLITEGAPADLRNDFADPLATALH
CKVLGIPQEDGPKLFRSLSIAFMSSADPIPAAKINWDRDIEYMAGILENPNITTGLMGELSRLRKDPAYSHVS
DELFATIGVTFFGAGVISTGSFLTTALISLIQRPQLRNLLHEKPELIPAGVEELLRINLSFADGLPRLATADI
QVGDVLVRKGELVLVLLEGANFDPEYFPNPGSIELDRPNPTSHLAFGRGQHFCPGSALGRRHAQIGIEALLKK
MPGVDLAVPIDQLVWRTRFQRRIPERLPVLW
>CYP121A1_Mycobacterium tuberculosis M2360 2630254255
MTATVLLEVPFSARGDRIPDAVAELRTREPIRKVRTITGAEAWLVSSYALCTQVLEDRRFSMKETAAAGAPRL
NALTVPPEVVNNMGNIADAGLRKAVMKAITPKAPGLEQFLRDTANSLLDNLITEGAPADLRNDFADPLATALH
CKVLGIPQEDGPKLFRSLSIAFMSSADPIPAAKINWDRDIEYMAGILENPNITTGLMGELSRLRKDPAYSHVS
DELFATIGVTFFGAGVISTGSFLTTALISLIQRPQLRNLLHEKPELIPAGVEELLRINLSFADGLPRLATADI
QVGDVLVRKGELVLVLLEGANFDPEYFPNPGSIELDRPNPTSHLAFGRGQHFCPGSALGRRHAQIGIEALLKK
MPGVDLAVPIDQLVWRTRFQRRIPERLPVLW
>CYP121A1_Mycobacterium tuberculosis M2361 2630885836
MTATVLLEVPFSARGDRIPDAVAELRTREPIRKVRTITGAEAWLVSSYALCTQVLEDRRFSMKETAAAGAPRL
NALTVPPEVVNNMGNIADAGLRKAVMKAITPKAPGLEQFLRDTANSLLDNLITEGAPADLRNDFADPLATALH
CKVLGIPQEDGPKLFRSLSIAFMSSADPIPAAKINWDRDIEYMAGILENPNITTGLMGELSRLRKDPAYSHVS
DELFATIGVTFFGAGVISTGSFLTTALISLIQRPQLRNLLHEKPELIPAGVEELLRINLSFADGLPRLATADI
QVGDVLVRKGELVLVLLEGANFDPEYFPNPGSIELDRPNPTSHLAFGRGQHFCPGSALGRRHAQIGIEALLKK
MPGVDLAVPIDQLVWRTRFQRRIPERLPVLW
>CYP121A1_Mycobacterium tuberculosis M2442 2638658961
MTATVLLEVPFSARGDRIPDAVAELRTREPIRKVRTITGAEAWLVSSYALCTQVLEDRRFSMKETAAAGAPRL
NALTVPPEVVNNMGNIADAGLRKAVMKAITPKAPGLEQFLRDTANSLLDNLITEGAPADLRNDFADPLATALH
CKVLGIPQEDGPKLFRSLSIAFMSSADPIPAAKINWDRDIEYMAGILENPNITTGLMGELSRLRKDPAYSHVS
DELFATIGVTFFGAGVISTGSFLTTALISLIQRPQLRNLLHEKPELIPAGVEELLRINLSFADGLPRLATADI
QVGDVLVRKGELVLVLLEGANFDPEHFPNPGSIELDRPNPTSHLAFGRDQHFCPGSALGRRHAQIGIEALLKK
MPGVDLAVPIDQLVWRTRFQRRIPERLPVLW
>CYP121A1_Mycobacterium tuberculosis M2459 2641353345
MTATVLLEVPFSARGDRIPDAVAELRTREPIRKVRTITGAEAWLVSSYALCTQVLEDRRFSMKETAAAGAPRL
NALTVPPEVVNNMGNIADAGLRKAVMKAITPKAPGLEQFLRDTANSLLDNLITEGAPADLRNDFADPLATALH
CKVLGIPQEDGPKLFRSLSIAFMSSADPIPAAKINWDRDIEYMAGILENPNITTGLMGELSRLRKDPAYSHVS
DELFATIGVTFFGAGVISTGSFLTTALISLIQRPQLRNLLHEKPELIPAGVEELLRINLSFADGLPRLATADI
QVGDVLVRKGELVLVLLEGANFDPEYFPNPGSIELDRPNPTSHLAFGRGQHFCPGSALGRRHAQIGIEALLKK
MPGVDLAVPIDQLVWRTRFQRRIPERLPVLW
>CYP121A1_Mycobacterium tuberculosis M990 2633030510
MTATVLLEVPFSARGDRIPDAVAELRTREPIRKVRTITGAEAWLVSSYALCTQVLEDRRFSMKETAAAGAPRL
NALTVPPEVVNNMGNIADAGLRKAVMKAITPKAPGLEQFLRDTANSLLDNLITEGAPADLRNDFADPLATALH
CKVLGIPQEDGPKLFRSLSIAFMSSADPIPAAKINWDRDIEYMAGILENPNITTGLMGELSRLRKDPAYSHVS
DELFATIGVTFFGAGVISTGSFLTTALISLIQRPHLRNLLHEKPELIPAGVEELLRINLSFADGLPRLATADI
QVGDVLVRKGELVLVLLEGANFDPEHFPNPGSIELDRPNPTSHLAFGRGQHFCPGSALGRRHAQIGIEALLKK
MPGVDLAVPIDQLVWRTRFQRRIPERLPVLW
>CYP121A1_Mycobacterium tuberculosis MAL010119 2621985068
MTATVLLEVPFSARGDRIPDAVAELRTREPIRKVRTITGAEAWLVSSYALCTQVLEDRRFSMKETAAAGAPRL
NALTVPPEVVNNMGNIADAGLRKAVMKAITPKAPGLEQFLRDTANSLLDNLITEGAPADLRNDFADPLATALH
CKVLGIPQEDGPKLFRSLSIAFMSSADPIPAAKINWDRDIEYMAGILENPNITTGLMGELSRLRKDPAYSHVS
DELFATIGVTFFGAGVISTGSFLTTALISLIQRPQLRNLLHEKPELIPAGVEELLRINLSFADGLPRLATADI
QVGDVLVRKGELVLVLLEGANFDPEYFPNPGSIELDRPNPTSHLAFGRGQHFCPGSALGRRHAQIGIEALLKK
MPGVDLAVPIDQLVWRTRFQRRIPERLPVLW
>CYP121A1_Mycobacterium tuberculosis MAL020165 2621999324
MTATVLLEVPFSARGDRIPDAVAELRTREPIRKVRTITGAEAWLVSSYALCTQVLEDRRFSMKETAAAGAPRL
NALTVPPEVVNNMGNIADAGLRKAVMKAITPKAPGLEQFLRDTANSLLDNLITEGAPADLRNDFADPLATALH

CKVLGIPQEDGPKLFRSLSIAFMSSADPIPAAKINWDRDIEYMAGILENPNITTGLMGELSRLRKDPAYSHVS
DELFATIGVTFFGAGVISTGSFLTTALISLIQRPQLRNLLHEKPELIPAGVEELLRINLSFADGLPRLATADI
QVGDVLVRKGELVLVLLEGANFDPEYFPNPGSIELDRPNPTSHLAFGRGQHFCPGSALGRRHAQIGIEALLKK
MPGVDLAVPIDQLVWRTRFQRRIPERLPVLW
>CYP121A1_Mycobacterium tuberculosis Manu ancestor SIT523 2648997106
MTATVLLEVPFSARGDRIPDAVAELRTREPIRKVRTITGAEAWLVSSYALCTQVLEDRRFSMKETAAAGAPRL
NALTVPPEVVNNMGNIADAGLRKAVMKAITPKAPGLEQFLRDTANSLLDNLITEGAPADLRNDFADPLATALH
CKVLGIPQEDGPKLFRSLSIAFMSSADPIPAAKINWDRDIEYMAGILENPNITTGLMGELSRLRKDPAYSHVS
DELFATIGVTFFGAGVISTGSFLTTALISLIQRPQLRNLLHEKPELIPAGVEELLRINLSFADGLPRLATADI
QVGDVLVRKGELVLVLLEGANFDPEYFPNPGSIELDRPNPTSHLAFGRGQHFCPGSALGRRHAQIGIEALLKK
MPGVDLAVPIDQLVWRTRFQRRIPERLPVLW
>CYP121A1_Mycobacterium tuberculosis MD14620 2622180352
MTATVLLEVPFSARGDRIPDAVAELRTREPIRKVRTITGAEAWLVSSYALCTQVLEDRRFSMKETAAAGAPRL
NALTVPPEVVNNMGNIADAGLRKAVMKAITPKAPGLEQFLRDTANSLLDNLITEGAPADLRNDFADPLATALH
CKVLGIPQEDGPKLFRSLSIAFMSSADPIPAAKINWDRDIEYMAGILENPNITTGLMGELSRLRKDPAYSHVS
DELFATIGVTFFGAGVISTGSFLTTALISLIQRPQLRNLLHEKPELIPAGVEELLRINLSFADGLPRLATADI
QVGDVLVRKGELVLVLLEGANFDPEYFPNPGSIELDRPNPTSHLAFGRGQHFCPGSALGRRHAQIGIEALLKK
MPGVDLAVPIDQLVWRTRFQRRIPERLPVLW
>CYP121A1_Mycobacterium tuberculosis MD14709 2622213695
MTATVLLEVPFSARGDRIPDAVAELRTREPIRKVRTITGAEAWLVSSYALCTQVLEDRRFSMKETAAAGAPRL
NALTVPPEVVNNMGNIADAGLRKAVMKAITPKAPGLEQFLRDTANSLLDNLITEGAPADLRNDFADPLATALH
CKVLGIPQEDGPKLFRSLSIAFMSSADPIPAAKINWDRDIEYMAGILENPNITTGLMGELSRLRKDPAYSHVS
DELFATIGVTFFGAGVISTGSFLTTALISLIQRPQLRNLLHEKPELIPAGVEELLRINLSFADGLPRLATADI
QVGDVLVRKGELVLVLLEGANFDPEYFPNPGSIELDRPNPTSHLAFGRGQHFCPGSALGRRHAQIGIEALLKK
MPGVDLAVPIDQLVWRTRFQRRIPERLPVLW
>CYP121A1_Mycobacterium tuberculosis MD14832 2622201279
MTATVLLEVPFSARGDRIPDAVAELRTREPIRKVRTITGAEAWLVSSYALCTQVLEDRRFSMKETAAAGAPRL
NALTVPPEVVNNMGNIADAGLRKAVMKAITPKAPGLEQFLRDTANSLLDNLITEGAPADLRNDFADPLATALH
CKVLGIPQEDGPKLFRSLSIAFMSSADPIPAAKINWDRDIEYMAGILENPNITTGLMGELSRLRKDPAYSHVS
DELFATIGVTFFGAGVISTGSFLTTALISLIQRPQLRNLLHEKPELIPAGVEELLRINLSFADGLPRLATADI
QVGDVLVRKGELVLVLLEGANFDPEYFPNPGSIELDRPNPTSHLAFGRGQHFCPGSALGRRHAQIGIEALLKK
MPGVDLAVPIDQLVWRTRFQRRIPERLPVLW
>CYP121A1_Mycobacterium tuberculosis MD15050 2584947459
MTATVLLEVPFSARGDRIPDAVAELRTREPIRKVRTITGAEAWLVSSYALCTQVLEDRRFSMKETAAAGAPRL
NALTVPPEVVNNMGNIADAGLRKAVMKAITPKAPGLEQFLRDTANSLLDNLITEGAPADLRNDFADPLATALH
CKVLGIPQEDGPKLFRSLSIAFMSSADPIPAAKINWDRDIEYMAGILENPNITTGLMGELSRLRKDPAYSHVS
DELFATIGVTFFGAGVISTGSFLTTALISLIQRPQLRNLLHEKPELIPAGVEELLRINLSFADGLPRLATADI
QVGDVLVRKGELVLVLLEGANFDPEYFPNPGSIELDRPNPTSHLAFGRGQHFCPGSALGRRHAQIGIEALLKK
MPGVDLAVPIDQLVWRTRFQRRIPERLPVLW
>CYP121A1_Mycobacterium tuberculosis MD15594 2622080603
MTATVLLEVPFSARGDRIPDAVAELRTREPIRKVRTITGAEAWLVSSYALCTQVLEDRRFSMKETAAAGAPRL
NALTVPPEVVNNMGNIADAGLRKAVMKAITPKAPGLEQFLRDTANSLLDNLITEGAPADLRNDFADPLATALH
CKVLGIPQEDGPKLFRSLSIAFMSSADPIPAAKINWDRDIEYMAGILENPNITTGLMGELSRLRKDPAYSHVS
DELFATIGVTFFGAGVISTGSFLTTALISLIQRPQLRNLLHEKPELIPAGVEELLRINLSFADGLPRLATADI
QVGDVLVRKGELVLVLLEGANFDPEYFPNPGSIELDRPNPTSHLAFGRGQHFCPGSALGRRHAQIGIEALLKK
MPGVDLAVPIDQLVWRTRFQRRIPERLPVLW
>CYP121A1_Mycobacterium tuberculosis MD15597 2584950271
MTATVLLEVPFSARGDRIPDAVAELRTREPIRKVRTITGAEAWLVSSYALCTQVLEDRRFSMKETAAAGAPRL
NALTVPPEVVNNMGNIADAGLRKAVMKAITPKAPGLEQFLRDTANSLLDNLITEGAPADLRNDFADPLATALH
CKVLGIPQEDGPKLFRSLSIAFMSSADPIPAAKINWDRDIEYMAGILENPNITTGLMGELSRLRKDPAYSHVS
DELFATIGVTFFGAGVISTGSFLTTALISLIQRPQLRNLLHEKPELIPAGVEELLRINLSFADGLPRLATADI
QVGDVLVRKGELVLVLLEGANFDPEYFPNPGSIELDRPNPTSHLAFGRGQHFCPGSALGRRHAQIGIEALLKK
MPGVDLAVPIDQLVWRTRFQRRIPERLPVLW
>CYP121A1_Mycobacterium tuberculosis MD15758 2622234581
MTATVLLEVPFSARGDRIPDAVAELRTREPIRKVRTITGAEAWLVSSYALCTQVLEDRRFSMKETAAAGAPRL
NALTVPPEVVNNMGNIADAGLRKAVMKAITPKAPGLEQFLRDTANSLLDNLITEGAPADLRNDFADPLATALH
CKVLGIPQEDGPKLFRSLSIAFMSSADPIPAAKINWDRDIEYMAGILENPNITTGLMGELSRLRKDPAYSHVS
DELFATIGVTFFGAGVISTGSFLTTALISLIQRPQLRNLLHEKPELIPAGVEELLRINLSFADGLPRLATADI

QVGDVLVRKGELVLVLLEGANFDPEYFPNPGSIELDRPNPTSHLAFGRGQHFCPGSALGRRHAQIGIEALLKK
MPGVDLAVPIDQLVWRTRFQRRIPERLPVLW
>CYP121A1_Mycobacterium tuberculosis MD15770 2622169653
MTATVLLEVPFSARGDRIPDAVAELRTREPIRKVRTITGAEAWLVSSYALCTQVLEDRRFSMKETAAAGAPRL
NALTVPPEVVNNMGNIADAGLRKAVMKAITPKAPGLEQFLRDTANSLLDNLITEGAPADLRNDFADPLATALH
CKVLGIPQEDGPKLFRSLSIAFMSSADPIPAAKINWDRDIEYMAGILENPNITTGLMGELSRLRKDPAYSHVS
DELFATIGVTFFGAGVISTGSFLTTALISLIQRPQLRNLLHEKPELIPAGVEELLRINLSFADGLPRLATADI
QVGDVLVRKGELVLVLLEGANFDPEYFPNPGSIELDRPNPTSHLAFGRGQHFCPGSALGRRHAQIGIEALLKK
MPGVDLAVPIDQLVWRTRFQRRIPERLPVLW
>CYP121A1_Mycobacterium tuberculosis MD15855 2577447938
MTATVLLEVPFSARGDRIPDAVAELRTREPIRKVRTITGAEAWLVSSYALCTQVLEDRRFSMKETAAAGAPRL
NALTVPPEVVNNMGNIADAGLRKAVMKAITPKAPGLEQFLRDTANSLLDNLITEGAPADLRNDFADPLATALH
CKVLGIPQEDGPKLFRSLSIAFMSSADPIPAAKINWDRDIEYMAGILENPNITTGLMGELSRLRKDPAYSHVS
DELFATIGVTFFGAGVISTGSFLTTALISLIQRPQLRNLLHEKPELIPAGVEELLRINLSFADGLPRLATADI
QVGDVLVRKGELVLVLLEGANFDPEYFPNPGSIELDRPNPTSHLAFGRGQHFCPGSALGRRHAQIGIEALLKK
MPGVDLAVPIDQLVWRTRFQRRIPERLPVLW
>CYP121A1_Mycobacterium tuberculosis MD16257 2622175747
MTATVLLEVPFSARGDRIPDAVAELRTREPIRKVRTITGAEAWLVSSYALCTQVLEDRRFSMKETAAAGAPRL
NALTVPPEVVNNMGNIADAGLRKAVMKAITPKAPGLEQFLRDTANSLLDNLITEGAPADLRNDFADPLATALH
CKVLGIPQEDGPKLFRSLSIAFMSSADPIPAAKINWDRDIEYMAGILENPNITTGLMGELSRLRKDPAYSHVS
DELFATIGVTFFGAGVISTGSFLTTALISLIQRPQLRNLLHEKPELIPAGVEELLRINLSFADGLPRLATADI
QVGDVLVRKGELVLVLLEGANFDPEYFPNPGSIELDRPNPTSHLAFGRGQHFCPGSALGRRHAQIGIEALLKK
MPGVDLAVPIDQLVWRTRFQRRIPERLPVLW
>CYP121A1_Mycobacterium tuberculosis MD16555 2584869764
MTATVLLEVPFSARGDRIPDAVAELRTREPIRKVRTITGAEAWLVSSYALCTQVLEDRRFSMKETAAAGAPRL
NALTVPPEVVNNMGNIADAGLRKAVMKAITPKAPGLEQFLRDTANSLLDNLITEGAPADLRNDFADPLATALH
CKVLGIPQEDGPKLFRSLSIAFMSSADPIPAAKINWDRDIEYMAGILENPNITTGLMGELSRLRKDPAYSHVS
DELFATIGVTFFGAGVISTGSFLTTALISLIQRPQLRNLLHEKPELIPAGVEELLRINLSFADGLPRLATADI
QVGDVLVRKGELVLVLLEGANFDPEYFPNPGSIELDRPNPTSHLAFGRGQHFCPGSALGRRHAQIGIEALLKK
MPGVDLAVPIDQLVWRTRFQRRIPERLPVLW
>CYP121A1_Mycobacterium tuberculosis MD16568 2622147178
MTATVLLEVPFSARGDRIPDAVAELRTREPIRKVRTITGAEAWLVSSYALCTQVLEDRRFSMKETAAAGAPRL
NALTVPPEVVNNMGNIADAGLRKAVMKAITPKAPGLEQFLRDTANSLLDNLITEGAPADLRNDFADPLATALH
CKVLGIPQEDGPKLFRSLSIAFMSSADPIPAAKINWDRDIEYMAGILENPNITTGLMGELSRLRKDPAYSHVS
DELFATIGVTFFGAGVISTGSFLTTALISLIQRPQLRNLLHEKPELIPAGVEELLRINLSFADGLPRLATADI
QVGDVLVRKGELVLVLLEGANFDPEYFPNPGSIELDRPNPTSHLAFGRGQHFCPGSALGRRHAQIGIEALLKK
MPGVDLAVPIDQLVWRTRFQRRIPERLPVLW
>CYP121A1_Mycobacterium tuberculosis MD16571 2622151360
MTATVLLEVPFSARGDRIPDAVAELRTREPIRKVRTITGAEAWLVSSYALCTQVLEDRRFSMKETAAAGAPRL
NALTVPPEVVNNMGNIADAGLRKAVMKAITPKAPGLEQFLRDTANSLLDNLITEGAPADLRNDFADPLATALH
CKVLGIPQEDGPKLFRSLSIAFMSSADPIPAAKINWDRDIEYMAGILENPNITTGLMGELSRLRKDPAYSHVS
DELFATIGVTFFGAGVISTGSFLTTALISLIQRPQLRNLLHEKPELIPAGVEELLRINLSFADGLPRLATADI
QVGDVLVRKGELVLVLLEGANFDPEYFPNPGSIELDRPNPTSHLAFGRGQHFCPGSALGRRHAQIGIEALLKK
MPGVDLAVPIDQLVWRTRFQRRIPERLPVLW
>CYP121A1_Mycobacterium tuberculosis MD16577 2584931223
MTATVLLEVPFSARGDRIPDAVAELRTREPIRKVRTITGAEAWLVSSYALCTQVLEDRRFSMKETAAAGAPRL
NALTVPPEVVNNMGNIADAGLRKAVMKAITPKAPGLEQFLRDTANSLLDNLITEGAPADLRNDFADPLATALH
CKVLGIPQEDGPKLFRSLSIAFMSSADPIPAAKINWDRDIEYMAGILENPNITTGLMGELSRLRKDPAYSHVS
DELFATIGVTFFGAGVISTGSFLTTALISLIQRPQLRNLLHEKPELIPAGVEELLRINLSFADGLPRLATADI
QVGDVLVRKGELVLVLLEGANFDPEYFPNPGSIELDRPNPTSHLAFGRGQHFCPGSALGRRHAQIGIEALLKK
MPGVDLAVPIDQLVWRTRFQRRIPERLPVLW
>CYP121A1_Mycobacterium tuberculosis MD16732 2622101104
MTATVLLEVPFSARGDRIPDAVAELRTREPIRKVRTITGAEAWLVSSYALCTQVLEDRRFSMKETAAAGAPRL
NALTVPPEVVNNMGNIADAGLRKAVMKAITPKAPGLEQFLRDTANSLLDNLITEGAPADLRNDFADPLATALH
CKVLGIPQEDGPKLFRSLSIAFMSSADPIPAAKINWDRDIEYMAGILENPNITTGLMGELSRLRKDPAYSHVS
DELFATIGVTFFGAGVISTGSFLTTALISLIQRPQLRNLLHEKPELIPAGVEELLRINLSFADGLPRLATADI
QVGDVLVRKGELVLVLLEGANFDPEYFPNPGSIELDRPNPTSHLAFGRGQHFCPGSALGRRHAQIGIEALLKK
MPGVDLAVPIDQLVWRTRFQRRIPERLPVLW

>CYP121A1_Mycobacterium tuberculosis MD16764 2622065947
MTATVLLEVPFSARGDRIPDAVAELRTREPIRKVRTITGAEAWLVSSYALCTQVLEDRRFSMKETAAAGAPRL
NALTVPPEVVNNMGNIADAGLRKAVMKAITPKAPGLEQFLRDTANSLLDNLITEGAPADLRNDFADPLATALH
CKVLGIPQEDGPKLFRSLSIAFMSSADPIPAAKINWDRDIEYMAGILENPNITTGLMGELSRLRKDPAYSHVS
DELFATIGVTFFGAGVISTGSFLTTALISLIQRPQLRNLLHEKPELIPAGVEELLRINLSFADGLPRLATADI
QVGDVLVRKGELVLVLLEGANFDPEYFPNPGSIELDRPNPTSHLAFGRGQHFCPGSALGRRHAQIGIEALLKK
MPGVDLAVPIDQLVWRTRFQRRIPERLPVLW
>CYP121A1_Mycobacterium tuberculosis MD17006 2622156745
MTATVLLEVPFSARGDRIPDAVAELRTREPIRKVRTITGAEAWLVSSYALCTQVLEDRRFSMKETAAAGAPRL
NALTVPPEVVNNMGNIADAGLRKAVMKAITPKAPGLEQFLRDTANSLLDNLITEGAPADLRNDFADPLATALH
CKVLGIPQEDGPKLFRSLSIAFMSSADPIPAAKINWDRDIEYMAGILENPNITTGLMGELSRLRKDPAYSHVS
DELFATIGVTFFGAGVISTGSFLTTALISLIQRPQLRNLLHEKPELIPAGVEELLRINLSFADGLPRLATADI
QVGDVLVRKGELVLVLLEGANFDPEYFPNPGSIELDRPNPTSHLAFGRGQHFCPGSALGRRHAQIGIEALLKK
MPGVDLAVPIDQLVWRTRFQRRIPERLPVLW
>CYP121A1_Mycobacterium tuberculosis MD17021 2622107278
MTATVLLEVPFSARGDRIPDAVAELRTREPIRKVRTITGAEAWLVSSYALCTQVLEDRRFSMKETAAAGAPRL
NALTVPPEVVNNMGNIADAGLRKAVMKAITPKAPGLEQFLRDTANSLLDNLITEGAPADLRNDFADPLATALH
CKVLGIPQEDGPKLFRSLSIAFMSSADPIPAAKINWDRDIEYMAGILENPNITTGLMGELSRLRKDPAYSHVS
DELFATIGVTFFGAGVISTGSFLTTALISLIQRPQLRNLLHEKPELIPAGVEELLRINLSFADGLPRLATADI
QVGDVLVRKGELVLVLLEGANFDPEYFPNPGSIELDRPNPTSHLAFGRGQHFCPGSALGRRHAQIGIEALLKK
MPGVDLAVPIDQLVWRTRFQRRIPERLPVLW
>CYP121A1_Mycobacterium tuberculosis MD17232 2622165562
MTATVLLEVPFSARGDRIPDAVAELRTREPIRKVRTITGAEAWLVSSYALCTQVLEDRRFSMKETAAAGAPRL
NALTVPPEVVNNMGNIADAGLRKAVMKAITPKAPGLEQFLRDTANSLLDNLITEGAPADLRNDFADPLATALH
CKVLGIPQEDGPKLFRSLSIAFMSSADPIPAAKINWDRDIEYMAGILENPNITTGLMGELSRLRKDPAYSHVS
DELFATIGVTFFGAGVISTGSFLTTALISLIQRPQLRNLLHEKPELIPAGVEELLRINLSFADGLPRLATADI
QVGDVLVRKGELVLVLLEGANFDPEYFPNPGSIELDRPNPTSHLAFGRGQHFCPGSALGRRHAQIGIEALLKK
MPGVDLAVPIDQLVWRTRFQRRIPERLPVLW
>CYP121A1_Mycobacterium tuberculosis MD17614 2630668023
MTATVLLEVPFSARGDRIPDAVAELRTREPIRKVRTITGAEAWLVSSYALCTQVLEDRRFSMKETAAAGAPRL
NALTVPPEVVNNMGNIADAGLRKAVMKAITPKAPGLEQFLRDTANSLLDNLITEGAPADLRNDFADPLATALH
CKVLGIPQEDGPKLFRSLSIAFMSSADPIPAAKINWDRDIEYMAGILENPNITTGLMGELSRLRKDPAYSHVS
DELFATIGVTFFGAGVISTGSFLTTALISLIQRPQLRNLLHEKPELIPAGVEELLRINLSFADGLPRLATADI
QVGDVLVRKGELVLVLLEGANFDPEYFPNPGSIELDRPNPTSHLAFGRGQHFCPGSALGRRHAQIGIEALLKK
MPGVDLAVPIDQLVWRTRFQRRIPERLPVLW
>CYP121A1_Mycobacterium tuberculosis MD17647 2622115755
MTATVLLEVPFSARGDRIPDAVAELRTREPIRKVRTITGAEAWLVSSYALCTQVLEDRRFSMKETAAAGAPRL
NALTVPPEVVNNMGNIADAGLRKAVMKAITPKAPGLEQFLRDTANSLLDNLITEGAPADLRNDFADPLATALH
CKVLGIPQEDGPKLFRSLSIAFMSSADPIPAAKINWDRDIEYMAGILENPNITTGLMGELSRLRKDPAYSHVS
DELFATIGVTFFGAGVISTGSFLTTALISLIQRPQLRNLLHEKPELIPAGVEELLRINLSFADGLPRLATADI
QVGDVLVRKGELVLVLLEGANFDPEYFPNPGSIELDRPNPTSHLAFGRGQHFCPGSALGRRHAQIGIEALLKK
MPGVDLAVPIDQLVWRTRFQRRIPERLPVLW
>CYP121A1_Mycobacterium tuberculosis MD17748 2622035081
MTATVLLEVPFSARGDRIPDAVAELRTREPIRKVRTITGAEAWLVSSYALCTQVLEDRRFSMKETAAAGAPRL
NALTVPPEVVNNMGNIADAGLRKAVMKAITPKAPGLEQFLRDTANSLLDNLITEGAPADLRNDFADPLATALH
CKVLGIPQEDGPKLFRSLSIAFMSSADPIPAAKINWDRDIEYMAGILENPNITTGLMGELSRLRKDPAYSHVS
DELFATIGVTFFGAGVISTGSFLTTALISLIQRPQLRNLLHEKPELIPAGVEELLRINLSFADGLPRLATADI
QVGDVLVRKGELVLVLLEGANFDPEYFPNPGSIELDRPNPTSHLAFGRGQHFCPGSALGRRHAQIGIEALLKK
MPGVDLAVPIDQLVWRTRFQRRIPERLPVLW
>CYP121A1_Mycobacterium tuberculosis MD17749 2622128134
MTATVLLEVPFSARGDRIPDAVAELRTREPIRKVRTITGAEAWLVSSYALCTQVLEDRRFSMKETAAAGAPRL
NALTVPPEVVNNMGNIADAGLRKAVMKAITPKAPGLEQFLRDTANSLLDNLITEGAPADLRNDFADPLATALH
CKVLGIPQEDGPKLFRSLSIAFMSSADPIPAAKINWDRDIEYMAGILENPNITTGLMGELSRLRKDPAYSHVS
DELFATIGVTFFGAGVISTGSFLTTALISLIQRPQLRNLLHEKPELIPAGVEELLRINLSFADGLPRLATADI
QVGDVLVRKGELVLVLLEGANFDPEYFPNPGSIELDRPNPTSHLAFGRGQHFCPGSALGRRHAQIGIEALLKK
MPGVDLAVPIDQLVWRTRFQRRIPERLPVLW
>CYP121A1_Mycobacterium tuberculosis MD17890 2633505871

MTATVLLEVPFSARGDRIPDAVAELRTREPIRKVRTITGAEAWLVSSYALCTQVLEDRRFSMKETAAAGAPRL
NALTVPPEVVNNMGNIADAGLRKAVMKAITPKAPGLEQFLRDTANSLLDNLITEGAPADLRNDFADPLATALH
CKVLGIPQEDGPKLFRSLSIAFMSSADPIPAAKINWDRDIEYMAGILENPNITTGLMGELSRLRKDPAYSHVS
DELFATIGVTFFGAGVISTGSFLTTALISLIQRPQLRNLLHEKPELIPAGVEELLRINLSFADGLPRLATADI
QVGDVLVRKGELVLVLLEGANFDPEYFPNPGSIELDRPNPTSHLAFGRGQHFCPGSALGRRHAQIGIEALLKK
MPGVDLAVPIDQLVWRTRFQRRIPERLPVLW
>CYP121A1_Mycobacterium tuberculosis MD17898 2622001926
MTATVLLEVPFSARGDRIPDAVAELRTREPIRKVRTITGAEAWLVSSYALCTQVLEDRRFSMKETAAAGAPRL
NALTVPPEVVNNMGNIADAGLRKAVMKAITPKAPGLEQFLRDTANSLLDNLITEGAPADLRNDFADPLATALH
CKVLGIPQEDGPKLFRSLSIAFMSSADPIPAAKINWDRDIEYMAGILENPNITTGLMGELSRLRKDPAYSHVS
DELFATIGVTFFGAGVISTGSFLTTALISLIQRPQLRNLLHEKPELIPAGVEELLRINLSFADGLPRLATADI
QVGDVLVRKGELVLVLLEGANFDPEYFPNPGSIELDRPNPTSHLAFGRGQHFCPGSALGRRHAQIGIEALLKK
MPGVDLAVPIDQLVWRTRFQRRIPERLPVLW
>CYP121A1_Mycobacterium tuberculosis MD17901 2631968720
MTATVLLEVPFSARGDRIPDAVAELRTREPIRKVRTITGAEAWLVSSYALCTQVLEDRRFSMKETAAAGAPRL
NALTVPPEVVNNMGNIADAGLRKAVMKAITPKAPGLEQFLRDTANSLLDNLITEGAPADLRNDFADPLATALH
CKVLGIPQEDGPKLFRSLSIAFMSSADPIPAAKINWDRDIEYMAGILENPNITTGLMGELSRLRKDPAYSHVS
DELFATIGVTFFGAGVISTGSFLTTALISLIQRPQLRNLLHEKPELIPAGVEELLRINLSFADGLPRLATADI
QVGDVLVRKGELVLVLLEGANFDPEYFPNPGSIELDRPNPTSHLAFGRGQHFCPGSALGRRHAQIGIEALLKK
MPGVDLAVPIDQLVWRTRFQRRIPERLPVLW
>CYP121A1_Mycobacterium tuberculosis MD17973 2622068359
MTATVLLEVPFSARGDRIPDAVAELRTREPIRKVRTITGAEAWLVSSYALCTQVLEDRRFSMKETAAAGAPRL
NALTVPPEVVNNMGNIADAGLRKAVMKAITPKAPGLEQFLRDTANSLLDNLITEGAPADLRNDFADPLATALH
CKVLGIPQEDGPKLFRSLSIAFMSSADPIPAAKINWDRDIEYMAGILENPNITTGLMGELSRLRKDPAYSHVS
DELFATIGVTFFGAGVISTGSFLTTALISLIQRPQLRNLLHEKPELIPAGVEELLRINLSFADGLPRLATADI
QVGDVLVRKGELVLVLLEGANFDPEYFPNPGSIELDRPNPTSHLAFGRGQHFCPGSALGRRHAQIGIEALLKK
MPGVDLAVPIDQLVWRTRFQRRIPERLPVLW
>CYP121A1_Mycobacterium tuberculosis MD18096 2576273997
MTATVLLEVPFSARGDRIPDAVAELRTREPIRKVRTITGAEAWLVSSYALCTQVLEDRRFSMKETAAAGAPRL
NALTVPPEVVNNMGNIADAGLRKAVMKAITPKAPGLEQFLRDTANSLLDNLITEGAPADLRNDFADPLATALH
CKVLGIPQEDGPKLFRSLSIAFMSSADPIPAAKINWDRDIEYMAGILENPNITTGLMGELSRLRKDPAYSHVS
DELFATIGVTFFGAGVISTGSFLTTALISLIQRPQLRNLLHEKPELIPAGVEELLRINLSFADGLPRLATADI
QVGDVLVRKGELVLVLLEGANFDPEYFPNPGSIELDRPNPTSHLAFGRGQHFCPGSALGRRHAQIGIEALLKK
MPGVDLAVPIDQLVWRTRFQRRIPERLPVLW
>CYP121A1_Mycobacterium tuberculosis MD18115 2631906161
MTATVLLEVPFSARGDRIPDAVAELRTREPIRKVRTITGAEAWLVSSYALCTQVLEDRRFSMKETAAAGAPRL
NALTVPPEVVNNMGNIADAGLRKAVMKAITPKAPGLEQFLRDTANSLLDNLITEGAPADLRNDFADPLATALH
CKVLGIPQEDGPKLFRSLSIAFMSSADPIPAAKINWDRDIEYMAGILENPNITTGLMGELSRLRKDPAYSHVS
DELFATIGVTFFGAGVISTGSFLTTALISLIQRPQLRNLLHEKPELIPAGVEELLRINLSFADGLPRLATADI
QVGDVLVRKGELVLVLLEGANFDPEYFPNPGSIELDRPNPTSHLAFGRGQHFCPGSALGRRHAQIGIEALLKK
MPGVDLAVPIDQLVWRTRFQRRIPERLPVLW
>CYP121A1_Mycobacterium tuberculosis MD18489 2622018095
MTATVLLEVPFSARGDRIPDAVAELRTREPIRKVRTITGAEAWLVSSYALCTQVLEDRRFSMKETAAAGAPRL
NALTVPPEVVNNMGNIADAGLRKAVMKAITPKAPGLEQFLRDTANSLLDNLITEGAPADLRNDFADPLATALH
CKVLGIPQEDGPKLFRSLSIAFMSSADPIPAAKINWDRDIEYMAGILENPNITTGLMGELSRLRKDPAYSHVS
DELFATIGVTFFGAGVISTGSFLTTALISLIQRPQLRNLLHEKPELIPAGVEELLRINLSFADGLPRLATADI
QVGDVLVRKGELVLVLLEGANFDPEYFPNPGSIELDRPNPTSHLAFGRGQHFCPGSALGRRHAQIGIEALLKK
MPGVDLAVPIDQLVWRTRFQRRIPERLPVLW
>CYP121A1_Mycobacterium tuberculosis MD19042 2622026725
MTATVLLEVPFSARGDRIPDAVAELRTREPIRKVRTITGAEAWLVSSYALCTQVLEDRRFSMKETAAAGAPRL
NALTVPPEVVNNMGNIADAGLRKAVMKAITPKAPGLEQFLRDTANSLLDNLITEGAPADLRNDFADPLATALH
CKVLGIPQEDGPKLFRSLSIAFMSSADPIPAAKINWDRDIEYMAGILENPNITTGLMGELSRLRKDPAYSHVS
DELFATIGVTFFGAGVISTGSFLTTALISLIQRPQLRNLLHEKPELIPAGVEELLRINLSFADGLPRLATADI
QVGDVLVRKGELVLVLLEGANFDPEYFPNPGSIELDRPNPTSHLAFGRGQHFCPGSALGRRHAQIGIEALLKK
MPGVDLAVPIDQLVWRTRFQRRIPERLPVLW
>CYP121A1_Mycobacterium tuberculosis MD19043 2574762491
MTATVLLEVPFSARGDRIPDAVAELRTREPIRKVRTITGAEAWLVSSYALCTQVLEDRRFSMKETAAAGAPRL
NALTVPPEVVNNMGNIADAGLRKAVMKAITPKAPGLEQFLRDTANSLLDNLITEGAPADLRNDFADPLATALH

CKVLGIPQEDGPKLFRSLSIAFMSSADPIPAAKINWDRDIEYMAGILENPNITTGLMGELSRLRKDPAYSHVS
DELFATIGVTFFGAGVISTGSFLTTALISLIQRPQLRNLLHEKPELIPAGVEELLRINLSFADGLPRLATADI
QVGDVLVRKGELVLVLLEGANFDPEYFPNPGSIELDRPNPTSHLAFGRGQHFCPGSALGRRHAQIGIEALLKK
MPGVDLAVPIDQLVWRTRFQRRIPERLPVLW
>CYP121A1_Mycobacterium tuberculosis MD19323 2622057596
MTATVLLEVPFSARGDRIPDAVAELRTREPIRKVRTITGAEAWLVSSYALCTQVLEDRRFSMKETAAAGAPRL
NALTVPPEVVNNMGNIADAGLRKAVMKAITPKAPGLEQFLRDTANSLLDNLITEGAPADLRNDFADPLATALH
CKVLGIPQEDGPKLFRSLSIAFMSSADPIPAAKINWDRDIEYMAGILENPNITTGLMGELSRLRKDPAYSHVS
DELFATIGVTFFGAGVISTGSFLTTALISLIQRPQLRNLLHEKPELIPAGVEELLRINLSFADGLPRLATADI
QVGDVLVRKGELVLVLLEGANFDPEYFPNPGSIELDRPNPTSHLAFGRGQHFCPGSALGRRHAQIGIEALLKK
MPGVDLAVPIDQLVWRTRFQRRIPERLPVLW
>CYP121A1_Mycobacterium tuberculosis MD19964 2575187519
MTATVLLEVPFSARGDRIPDAVAELRTREPIRKVRTITGAEAWLVSSYALCTQVLEDRRFSMKETAAAGAPRL
NALTVPPEVVNNMGNIADAGLRKAVMKAITPKAPGLEQFLRDTANSLLDNLITEGAPADLRNDFADPLATALH
CKVLGIPQEDGPKLFRSLSIAFMSSADPIPAAKINWDRDIEYMAGILENPNITTGLMGELSRLRKDPAYSHVS
DELFATIGVTFFGAGVISTGSFLTTALISLIQRPQLRNLLHEKPELIPAGVEELLRINLSFADGLPRLATADI
QVGDVLVRKGELVLVLLEGANFDPEYFPNPGSIELDRPNPTSHLAFGRGQHFCPGSALGRRHAQIGIEALLKK
MPGVDLAVPIDQLVWRTRFQRRIPERLPVLW
>CYP121A1_Mycobacterium tuberculosis Mtb194 2606423801
MTATVLLEVPFSARGDRIPDAVAELRTREPIRKVRTITGAEAWLVSSYALCTQVLEDRRFSMKETAAAGAPRL
NALTVPPEVVNNMGNIADAGLRKAVMKAITPKAPGLEQFLRDTANSLLDNLITEGAPADLRNDFADPLATALH
CKVLGIPQEDGPKLFRSLSIAFMSSADPIPAAKINWDRDIEYMAGILENPNITTGLMGELSRLRKDPAYSHVS
DELFATIGVTFFGAGVISTGSFLTTALISLIQRPQLRNLLHEKPELIPAGVEELLRINLSFADGLPRLATADI
QVGDVLVRKGELVLVLLEGANFDPEYFPNPGSIELDRPNPTSHLAFGRGQHFCPGSALGRRHAQIGIEALLKK
MPGVDLAVPIDQLVWRTRFQRRIPERLPVLW
>CYP121A1_Mycobacterium tuberculosis Mtb293 2645903081
MTATVLLEVPFSARGDRIPDAVAELRTREPIRKVRTITGAEAWLVSSYALCTQVLEDRRFSMKETAAAGAPRL
NALTVPPEVVNNMGNIADAGLRKAVMKAITPKAPGLEQFLRDTANSLLDNLITEGAPADLRNDFADPLATALH
CKVLGIPQEDGPKLFRSLSIAFMSSADPIPAAKINWDRDIEYMAGILENPNITTGLMGELSRLRKDPAYSHVS
DELFATIGVTFFGAGVISTGSFLTTALISLIQRPQLRNLLHEKPELIPAGVEELLRINLSFADGLPRLATADI
QVGDVLVRKGELVLVLLEGANFDPEYFPNPGSIELDRPNPTSHLAFGRGQHFCPGSALGRRHAQIGIEALLKK
MPGVDLAVPIDQLVWRTRFQRRIPERLPVLW
>CYP121A1_Mycobacterium tuberculosis Mtb43 2654752959
MTATVLLEVPFSARGDRIPDAVAELRTREPIRKVRTITGAEAWLVSSYALCTQVLEDRRFSMKETAAAGAPRL
NALTVPPEVVNNMGNIADAGLRKAVMKAITPKAPGLEQFLRDTANSLLDNLITEGAPADLRNDFADPLATALH
CKVLGIPQEDGPKLFRSLSIAFMSSADPIPAAKINWDRDIEYMAGILENPNITTGLMGELSRLRKDPAYSHVS
DELFATIGVTFFGAGVISTGSFLTTALISLIQRPQLRNLLHEKPELIPAGVEELLRINLSFADGLPRLATADI
QVGDVLVRKGELVLVLLEGANFDPEYFPNPGSIELDRPNPTSHLAFGRGQHFCPGSALGRRHAQIGIEALLKK
MPGVDLAVPIDQLVWRTRFQRRIPERLPVLW
>CYP121A1_Mycobacterium tuberculosis Mtb526 2641992202
MTATVLLEVPFSARGDRIPDAVAELRTREPIRKVRTITGAEAWLVSSYALCTQVLEDRRFSMKETAAAGAPRL
NALTVPPEVVNNMGNIADAGLRKAVMKAITPKAPGLEQFLRDTANSLLDNLITEGAPADLRNDFADPLATALH
CKVLGIPQEDGPKLFRSLSIAFMSSADPIPAAKINWDRDIEYMAGILENPNITTGLMGELSRLRKDPAYSHVS
DELFATIGVTFFGAGVISTGSFLTTALISLIQRPQLRNLLHEKPELIPAGVEELLRINLSFADGLPRLATADI
QVGDVLVRKGELVLVLLEGANFDPEYFPNPGSIELDRPNPTSHLAFGRGQHFCPGSALGRRHAQIGIEALLKK
MPGVDLAVPIDQLVWRTRFQRRIPERLPVLW
>CYP121A1_Mycobacterium tuberculosis Mtb562 2649310295
MTATVLLEVPFSARGDRIPDAVAELRTREPIRKVRTITGAEAWLVSSYALCTQVLEDRRFSMKETAAAGAPRL
NALTVPPEVVNNMGNIADAGLRKAVMKAITPKAPGLEQFLRDTANSLLDNLITEGAPADLRNDFADPLATALH
CKVLGIPQEDGPKLFRSLSIAFMSSADPIPAAKINWDRDIEYMAGILENPNITTGLMGELSRLRKDPAYSHVS
DELFATIGVTFFGAGVISTGSFLTTALISLIQRPQLRNLLHEKPELIPAGVEELLRINLSFADGLPRLATADI
QVGDVLVRKGELVLVLLEGANFDPEYFPNPGSIELDRPNPTSHLAFGRGQHFCPGSALGRRHAQIGIEALLKK
MPGVDLAVPIDQLVWRTRFQRRIPERLPVLW
>CYP121A1_Mycobacterium tuberculosis Mtb940 2606428145
MTATVLLEVPFSARGDRIPDAVAELRTREPIRKVRTITGAEAWLVSSYALCTQVLEDRRFSMKETAAAGAPRL
NALTVPPEVVNNMGNIADAGLRKAVMKAITPKAPGLEQFLRDTANSLLDNLITEGAPADLRNDFADPLATALH
CKVLGIPQEDGPKLFRSLSIAFMSSADPIPAAKINWDRDIEYMAGILENPNITTGLMGELSRLRKDPAYSHVS
DELFATIGVTFFGAGVISTGSFLTTALISLIQRPQLRNLLHEKPELIPAGVEELLRINLSFADGLPRLATADI

QVGDVLVRKGELVLVLLEGANFDPEYFPNPGSIELDRPNPTSHLAFGRGQHFCPGSALGRRHAQIGIEALLKK
MPGVDLAVPIDQLVWRTRFQRRIPERLPVLW
>CYP121A1_Mycobacterium tuberculosis Mtb984 2647498970
MTATVLLEVPFSARGDRIPDAVAELRTREPIRKVRTITGAEAWLVSSYALCTQVLEDRRFSMKETAAAGAPRL
NALTVPPEVVNNMGNIADAGLRKAVMKAITPKAPGLEQFLRDTANSLLDNLITEGAPADLRNDFADPLATALH
CKVLGIPQEDGPKLFRSLSIAFMSSADPIPAAKINWDRDIEYMAGILENPNITTGLMGELSRLRKDPAYSHVS
DELFATIGVTFFGAGVISTGSFLTTALISLIQRPQLRNLLHEKPELIPAGVEELLRINLSFADGLPRLATADI
QVGDVLVRKGELVLVLLEGANFDPEYFPNPGSIELDRPNPTSHLAFGRGQHFCPGSALGRRHAQIGIEALLKK
MPGVDLAVPIDQLVWRTRFQRRIPERLPVLW
>CYP121A1_Mycobacterium tuberculosis MTBR2/09 2660404033
MTATVLLEVPFSARGDRIPDAVAELRTREPIRKVRTITGAEAWLVSSYALCTQVLEDRRFSMKETAAAGAPRL
NALTVPPEVVNNMGNIADAGLRKAVMKAITPKAPGLEQFLRDTANSLLDNLITEGAPADLRNDFADPLATALH
CKVLGIPQEDGPKLFRSLSIAFMSSADPIPAAKINWDRDIEYMAGILENPNITTGLMGELSRLRKDPAYSHVS
DELFATIGVTFFGAGVISTGSFLTTALISLIQRPQLRNLLHEKPELIPAGVEELLRINLSFADGLPRLATADI
QVGDVLVRKGELVLVLLEGANFDPEYFPNPGSIELDRPNPTSHLAFGRGQHFCPGSALGRRHAQIGIEALLKK
MPGVDLAVPIDQLVWRTRFQRRIPERLPVLW
>CYP121A1_Mycobacterium tuberculosis MTBR3/09 2703074301
MTATVLLEVPFSARGDRIPDAVAELRTREPIRKVRTITGAEAWLVSSYALCTQVLEDRRFSMKETAAAGAPRL
NALTVPPEVVNNMGNIADAGLRKAVMKAITPKAPGLEQFLRDTANSLLDNLITEGAPADLRNDFADPLATALH
CKVLGIPQEDGPKLFRSLSIAFMSSADPIPAAKINWDRDIEYMAGILENPNITTGLMGELSRLRKDPAYSHVS
DELFATIGVTFFGAGVISTGSFLTTALISLIQRPQLRNLLHEKPELIPAGVEELLRINLSFADGLPRLATADI
QVGDVLVRKGELVLVLLEGANFDPEYFPNPGSIELDRPNPTSHLAFGRGQHFCPGSALGRRHAQIGIEALLKK
MPGVDLAVPIDQLVWRTRFQRRIPERLPVLW
>CYP121A1_Mycobacterium tuberculosis N00001996 2706568116
MTATVLLEVPFSARGDRIPDAVAELRTREPIRKVRTITGAEAWLVSSYALCTQVLEDRRFSMKETAAAGAPRL
NALTVPPEVVNNMGNIADAGLRKAVMKAITPKAPGLEQFLRDTANSLLDNLITEGAPADLRNDFADPLATALH
CKVLGIPQEDGPKLFRSLSIAFMSSADPIPAAKINWDRDIEYMAGILENPNITTGLMGELSRLRKDPAYSHVS
DELFATIGVTFFGAGVISTGSFLTTALISLIQRPQLRNLLHEKPELIPAGVEELLRINLSFADGLPRLATADI
QVGDVLVRKGELVLVLLEGANFDPEYFPNPGSIELDRPNPTSHLAFGRGQHFCPGSALGRRHAQIGIEALLKK
MPGVDLAVPIDQLVWRTRFQRRIPERLPVLW
>CYP121A1_Mycobacterium tuberculosis N00200616 2693857464
MTATVLLEVPFSARGDRIPDAVAELRTREPIRKVRTITGAEAWLVSSYALCTQVLEDRRFSMKETAAAGAPRL
NALTVPPEVVNNMGNIADAGLRKAVMKAITPKAPGLEQFLRDTANSLLDNLITEGAPADLRNDFADPLATALH
CKVLGIPQEDGPKLFRSLSIAFMSSADPIPAAKINWDRDIEYMAGILENPNITTGLMGELSRLRKDPAYSHVS
DELFATIGVTFFGAGVISTGSFLTTALISLIQRPQLRNLLHEKPELIPAGVEELLRINLSFADGLPRLATADI
QVGDVLVRKGELVLVLLEGANFDPEYFPNPGSIELDRPNPTSHLAFGRGQHFCPGSALGRRHAQIGIEALLKK
MPGVDLAVPIDQLVWRTRFQRRIPERLPVLW
>CYP121A1_Mycobacterium tuberculosis N00302086 2683865317
MTATVLLEVPFSARGDRIPDAVAELRTREPIRKVRTITGAEAWLVSSYALCTQVLEDRRFSMKETAAAGAPRL
NALTVPPEVVNNMGNIADAGLRKAVMKAITPKAPGLEQFLRDTANSLLDNLITEGAPADLRNDFADPLATALH
CKVLGIPQEDGPKLFRSLSIAFMSSADPIPAAKINWDRDIEYMAGILENPNITTGLMGELSRLRKDPAYSHVS
DELFATIGVTFFGAGVISTGSFLTTALISLIQRPQLRNLLHEKPELIPAGVEELLRINLSFADGLPRLATADI
QVGDVLVRKGELVLVLLEGANFDPEYFPNPGSIELDRPNPTSHLAFGRGQHFCPGSALGRRHAQIGIEALLKK
MPGVDLAVPIDQLVWRTRFQRRIPERLPVLW
>CYP121A1_Mycobacterium tuberculosis N00401131 2683869424
MTATVLLEVPFSARGDRIPDAVAELRTREPIRKVRTITGAEAWLVSSYALCTQVLEDRRFSMKETAAAGAPRL
NALTVPPEVVNNMGNIADAGLRKAVMKAITPKAPGLEQFLRDTANSLLDNLITEGAPADLRNDFADPLATALH
CKVLGIPQEDGPKLFRSLSIAFMSSADPIPAAKINWDRDIEYMAGILENPNITTGLMGELSRLRKDPAYSHVS
DELFATIGVTFFGAGVISTGSFLTTALISLIQRPQLRNLLHEKPELIPAGVEELLRINLSFADGLPRLATADI
QVGDVLVRKGELVLVLLEGANFDPEYFPNPGSIELDRPNPTSHLAFGRGQHFCPGSALGRRHAQIGIEALLKK
MPGVDLAVPIDQLVWRTRFQRRIPERLPVLW
>CYP121A1_Mycobacterium tuberculosis N00500759 2692377946
MTATVLLEVPFSARGDRIPDAVAELRTREPIRKVRTITGAEAWLVSSYALCTQVLEDRRFSMKETAAAGAPRL
NALTVPPEVVNNMGNIADAGLRKAVMKAITPKAPGLEQFLRDTANSLLDNLITEGAPADLRNDFADPLATALH
CKVLGIPQEDGPKLFRSLSIAFMSSADPIPAAKINWDRDIEYMAGILENPNITTGLMGELSRLRKDPAYSHVS
DELFATIGVTFFGAGVISTGSFLTTALISLIQRPQLRNLLHEKPELIPAGVEELLRINLSFADGLPRLATADI
QVGDVLVRKGELVLVLLEGANFDPEYFPNPGSIELDRPNPTSHLAFGRGQHFCPGSALGRRHAQIGIEALLKK
MPGVDLAVPIDQLVWRTRFQRRIPERLPVLW

>CYP121A1_Mycobacterium tuberculosis N00501578 2697271745
MTATVLLEVPFSARGDRIPDAVAELRTREPIRKVRTITGAEAWLVSSYALCTQVLEDRRFSMKETAAAGAPRL
NALTVPPEVVNNMGNIADAGLRKAVMKAITPKAPGLEQFLRDTANSLLDNLITEGAPADLRNDFADPLATALH
CKVLGIPQEDGPKLFRSLSIAFMSSADPIPAAKINWDRDIEYMAGILENPNITTGLMGELSRLRKDPAYSHVS
DELFATIGVTFFGAGVISTGSFLTTALISLIQRPQLRNLLHEKPELIPAGVEELLRINLSFADGLPRLATADI
QVGDVLVRKGELVLVLLEGANFDPEYFPNPGSIELDRPNPTSHLAFGRGQHFCPGSALGRRHAQIGIEALLKK
MPGVDLAVPIDQLVWRTRFQRRIPERLPVLW
>CYP121A1_Mycobacterium tuberculosis N00501741 2672452941
MTATVLLEVPFSARGDRIPDAVAELRTREPIRKVRTITGAEAWLVSSYALCTQVLEDRRFSMKETAAAGAPRL
NALTVPPEVVNNMGNIADAGLRKAVMKAITPKAPGLEQFLRDTANSLLDNLITEGAPADLRNDFADPLATALH
CKVLGIPQEDGPKLFRSLSIAFMSSADPIPAAKINWDRDIEYMAGILENPNITTGLMGELSRLRKDPAYSHVS
DELFATIGVTFFGAGVISTGSFLTTALISLIQRPQLRNLLHEKPELIPAGVEELLRINLSFADGLPRLATADI
QVGDVLVRKGELVLVLLEGANFDPEYFPNPGSIELDRPNPTSHLAFGRGQHFCPGSALGRRHAQIGIEALLKK
MPGVDLAVPIDQLVWRTRFQRRIPERLPVLW
>CYP121A1_Mycobacterium tuberculosis N00701874 2697195766
MTATVLLEVPFSARGDRIPDAVAELRTREPIRKVRTITGAEAWLVSSYALCTQVLEDRRFSMKETAAAGAPRL
NALTVPPEVVNNMGNIADAGLRKAVMKAITPKAPGLEQFLRDTANSLLDNLITEGAPADLRNDFADPLATALH
CKVLGIPQEDGPKLFRSLSIAFMSSADPIPAAKINWDRDIEYMAGILENPNITTGLMGELSRLRKDPAYSHVS
DELFATIGVTFFGAGVISTGSFLTTALISLIQRPQLRNLLHEKPELIPAGVEELLRINLSFADGLPRLATADI
QVGDVLVRKGELVLVLLEGANFDPEYFPNPGSIELDRPNPTSHLAFGRGQHFCPGSALGRRHAQIGIEALLKK
MPGVDLAVPIDQLVWRTRFQRRIPERLPVLW
>CYP121A1_Mycobacterium tuberculosis N00800620 2696316620
MTATVLLEVPFSARGDRIPDAVAELRTREPIRKVRTITGAEAWLVSSYALCTQVLEDRRFSMKETAAAGAPRL
NALTVPPEVVNNMGNIADAGLRKAVMKAITPKAPGLEQFLRDTANSLLDNLITEGAPADLRNDFADPLATALH
CKVLGIPQEDGPKLFRSLSIAFMSSADPIPAAKINWDRDIEYMAGILENPNITTGLMGELSRLRKDPAYSHVS
DELFATIGVTFFGAGVISTGSFLTTALISLIQRPQLRNLLHEKPELIPAGVEELLRINLSFADGLPRLATADI
QVGDVLVRKGELVLVLLEGANFDPEYFPNPGSIELDRPNPTSHLAFGRGQHFCPGSALGRRHAQIGIEALLKK
MPGVDLAVPIDQLVWRTRFQRRIPERLPVLW
>CYP121A1_Mycobacterium tuberculosis N00800922 2694131085
MTATVLLEVPFSARGDRIPDAVAELRTREPIRKVRTITGAEAWLVSSYALCTQVLEDRRFSMKETAAAGAPRL
NALTVPPEVVNNMGNIADAGLRKAVMKAITPKAPGLEQFLRDTANSLLDNLITEGAPADLRNDFADPLATALH
CKVLGIPQEDGPKLFRSLSIAFMSSADPIPAAKINWDRDIEYMAGILENPNITTGLMGELSRLRKDPAYSHVS
DELFATIGVTFFGAGVISTGSFLTTALISLIQRPQLRNLLHEKPELIPAGVEELLRINLSFADGLPRLATADI
QVGDVLVRKGELVLVLLEGANFDPEYFPNPGSIELDRPNPTSHLAFGRGQHFCPGSALGRRHAQIGIEALLKK
MPGVDLAVPIDQLVWRTRFQRRIPERLPVLW
>CYP121A1_Mycobacterium tuberculosis N09501026 2706615266
MTATVLLEVPFSARGDRIPDAVAELRTREPIRKVRTITGAEAWLVSSYALCTQVLEDRRFSMKETAAAGAPRL
NALTVPPEVVNNMGNIADAGLRKAVMKAITPKAPGLEQFLRDTANSLLDNLITEGAPADLRNDFADPLATALH
CKVLGIPQEDGPKLFRSLSIAFMSSADPIPAAKINWDRDIEYMAGILENPNITTGLMGELSRLRKDPAYSHVS
DELFATIGVTFFGAGVISTGSFLTTALISLIQRPQLRNLLHEKPELIPAGVEELLRINLSFADGLPRLATADI
QVGDVLVRKGELVLVLLEGANFDPEYFPNPGSIELDRPNPTSHLAFGRGQHFCPGSALGRRHAQIGIEALLKK
MPGVDLAVPIDQLVWRTRFQRRIPERLPVLW
>CYP121A1_Mycobacterium tuberculosis NCGM2209 2549402447
MTATVLLEVPFSARGDRIPDAVAELRTREPIRKVRTITGAEAWLVSSYALCTQVLEDRRFSMKETAAAGAPRL
NALTVPPEVVNNMGNIADAGLRKAVMKAITPKAPGLEQFLRDTANSLLDNLITEGAPADLRNDFADPLATALH
CKVLGIPQEDGPKLFRSLSIAFMSSADPIPAAKINWDRDIEYMAGILENPNITTGLMGELSRLRKDPAYSHVS
DELFATIGVTFFGAGVISTGSFLTTALISLIQRPQLRNLLHEKPELIPAGVEELLRINLSFADGLPRLATADI
QVGDVLVRKGELVLVLLEGANFDPEYFPNPGSIELDRPNPTSHLAFGRGQHFCPGSALGRRHAQIGIEALLKK
MPGVDLAVPIDQLVWRTRFQRRIPERLPVLW
>CYP121A1_Mycobacterium tuberculosis NRITLD09 2584831363
MTATVLLEVPFSARGDRIPDAVAELRTREPIRKVRTITGAEAWLVSSYALCTQVLEDRRFSMKETAAAGAPRL
NALTVPPEVVNNMGNIADAGLRKAVMKAITPKAPGLEQFLRDTANSLLDNLITEGAPADLRNDFADPLATALH
CKVLGIPQEDGPKLFRSLSIAFMSSADPIPAAKINWDRDIEYMAGILENPNITTGLMGELSRLRKDPAYSHVS
DELFATIGVTFFGAGVISTGSFLTTALISLIQRPQLRNLLHEKPELIPAGVEELLRINLSFADGLPRLATADI
QVGDVLVRKGELVLVLLEGANFDPEYFPNPGSIELDRPNPTSHLAFGRGQHFCPGSALGRRHAQIGIEALLKK
MPGVDLAVPIDQLVWRTRFQRRIPERLPVLW
>CYP121A1_Mycobacterium tuberculosis NRITLD10 2638346545

MTATVLLEVPFSARGDRIPDAVAELRTREPIRKVRTITGAEAWLVSSYALCTQVLEDRRFSMKETAAAGAPRL
NALTVPPEVVNNMGNIADAGLRKAVMKAITPKAPGLEQFLRDTANSLLDNLITEGAPADLRNDFADPLATALH
CKVLGIPQEDGPKLFRSLSIAFMSSADPIPAAKINWDRDIEYMAGILENPNITTGLMGELSRLRKDPAYSHVS
DELFATIGVTFFGAGVISTGSFLTTALISLIQRPQLRNLLHEKPELIPAGVEELLRINLSFADGLPRLATADI
QVGDVLVRKGELVLVLLEGANFDPEYFPNPGSIELDRPNPTSHLAFGRGQHFCPGSALGRRHAQIGIEALLKK
MPGVDLAVPIDQLVWRTRFQRRIPERLPVLW
>CYP121A1_Mycobacterium tuberculosis NRITLD11 2638495011
MTATVLLEVPFSARGDRIPDAVAELRTREPIRKVRTITGAEAWLVSSYALCTQVLEDRRFSMKETAAAGAPRL
NALTVPPEVVNNMGNIADAGLRKAVMKAITPKAPGLEQFLRDTANSLLDNLITEGAPADLRNDFADPLATALH
CKVLGIPQEDGPKLFRSLSIAFMSSADPIPAAKINWDRDIEYMAGILENPNITTGLMGELSRLRKDPAYSHVS
DELFATIGVTFFGAGVISTGSFLTTALISLIQRPQLRNLLHEKPELIPAGVEELLRINLSFADGLPRLATADI
QVGDVLVRKGELVLVLLEGANFDPEYFPNPGSIELDRPNPTSHLAFGRGQHFCPGSALGRRHAQIGIEALLKK
MPGVDLAVPIDQLVWRTRFQRRIPERLPVLW
>CYP121A1_Mycobacterium tuberculosis NRITLD12 2574656825
MTATVLLEVPFSARGDRIPDAVAELRTREPIRKVRTITGAEAWLVSSYALCTQVLEDRRFSMKETAAAGAPRL
NALTVPPEVVNNMGNIADAGLRKAVMKAITPKAPGLEQFLRDTANSLLDNLITEGAPADLRNDFADPLATALH
CKVLGIPQEDGPKLFRSLSIAFMSSADPIPAAKINWDRDIEYMAGILENPNITTGLMGELSRLRKDPAYSHVS
DELFATIGVTFFGAGVISTGSFLTTALISLIQRPQLRNLLHEKPELIPAGVEELLRINLSFADGLPRLATADI
QVGDVLVRKGELVLVLLEGANFDPEYFPNPGSIELDRPNPTSHLAFGRGQHFCPGSALGRRHAQIGIEALLKK
MPGVDLAVPIDQLVWRTRFQRRIPERLPVLW
>CYP121A1_Mycobacterium tuberculosis NRITLD13 2641030245
MTATVLLEVPFSARGDRIPDAVAELRTREPIRKVRTITGAEAWLVSSYALCTQVLEDRRFSMKETAAAGAPRL
NALTVPPEVVNNMGNIADAGLRKAVMKAITPKAPGLEQFLRDTANSLLDNLITEGAPADLRNDFADPLATALH
CKVLGIPQEDGPKLFRSLSIAFMSSADPIPAAKINWDRDIEYMAGILENPNITTGLMGELSRLRKDPAYSHVS
DELFATIGVTFFGAGVISTGSFLTTALISLIQRPQLRNLLHEKPELIPAGVEELLRINLSFADGLPRLATADI
QVGDVLVRKGELVLVLLEGANFDPEYFPNPGSIELDRPNPTSHLAFGRGQHFCPGSALGRRHAQIGIEALLKK
MPGVDLAVPIDQLVWRTRFQRRIPERLPVLW
>CYP121A1_Mycobacterium tuberculosis NRITLD15 2578093250
MTATVLLEVPFSARGDRIPDAVAELRTREPIRKVRTITGAEAWLVSSYALCTQVLEDRRFSMKETAAAGAPRL
NALTVPPEVVNNMGNIADAGLRKAVMKAITPKAPGLEQFLRDTANSLLDNLITEGAPADLRNDFADPLATALH
CKVLGIPQEDGPKLFRSLSIAFMSSADPIPAAKINWDRDIEYMAGILENPNITTGLMGELSRLRKDPAYSHVS
DELFATIGVTFFGAGVISTGSFLTTALISLIQRPQLRNLLHEKPELIPAGVEELLRINLSFADGLPRLATADI
QVGDVLVRKGELVLVLLEGANFDPEYFPNPGSIELDRPNPTSHLAFGRGQHFCPGSALGRRHAQIGIEALLKK
MPGVDLAVPIDQLVWRTRFQRRIPERLPVLW
>CYP121A1_Mycobacterium tuberculosis NRITLD16 2635674495
MTATVLLEVPFSARGDRIPDAVAELRTREPIRKVRTITGAEAWLVSSYALCTQVLEDRRFSMKETAAAGAPRL
NALTVPPEVVNNMGNIADAGLRKAVMKAITPKAPGLEQFLRDTANSLLDNLITEGAPADLRNDFADPLATALH
CKVLGIPQEDGPKLFRSLSIAFMSSADPIPAAKINWDRDIEYMAGILENPNITTGLMGELSRLRKDPAYSHVS
DELFATIGVTFFGAGVISTGSFLTTALISLIQRPQLRNLLHEKPELIPAGVEELLRINLSFADGLPRLATADI
QVGDVLVRKGELVLVLLEGANFDPEYFPNPGSIELDRPNPTSHLAFGRGQHFCPGSALGRRHAQIGIEALLKK
MPGVDLAVPIDQLVWRTRFQRRIPERLPVLW
>CYP121A1_Mycobacterium tuberculosis NRITLD18 2655328750
MTATVLLEVPFSARGDRIPDAVAELRTREPIRKVRTITGAEAWLVSSYALCTQVLEDRRFSMKETAAAGAPRL
NALTVPPEVVNNMGNIADAGLRKAVMKAITPKAPGLEQFLRDTANSLLDNLITEGAPADLRNDFADPLATALH
CKVLGIPQEDGPKLFRSLSIAFMSSADPIPAAKINWDRDIEYMAGILENPNITTGLMGELSRLRKDPAYSHVS
DELFATIGVTFFGAGVISTGSFLTTALISLIQRPQLRNLLHEKPELIPAGVEELLRINLSFADGLPRLATADI
QVGDVLVRKGELVLVLLEGANFDPEYFPNPGSIELDRPNPTSHLAFGRGQHFCPGSALGRRHAQIGIEALLKK
MPGVDLAVPIDQLVWRTRFQRRIPERLPVLW
>CYP121A1_Mycobacterium tuberculosis NRITLD19 2637460471
MTATVLLEVPFSARGDRIPDAVAELRTREPIRKVRTITGAEAWLVSSYALCTQVLEDRRFSMKETAAAGAPRL
NALTVPPEVVNNMGNIADAGLRKAVMKAITPKAPGLEQFLRDTANSLLDNLITEGAPADLRNDFADPLATALH
CKVLGIPQEDGPKLFRSLSIAFMSSADPIPAAKINWDRDIEYMAGILENPNITTGLMGELSRLRKDPAYSHVS
DELFATIGVTFFGAGVISTGSFLTTALISLIQRPQLRNLLHEKPELIPAGVEELLRINLSFADGLPRLATADI
QVGDVLVRKGELVLVLLEGANFDPEYFPNPGSIELDRPNPTSHLAFGRGQHFCPGSALGRRHAQIGIEALLKK
MPGVDLAVPIDQLVWRTRFQRRIPERLPVLW
>CYP121A1_Mycobacterium tuberculosis NRITLD21 2630535932
MTATVLLEVPFSARGDRIPDAVAELRTREPIRKVRTITGAEAWLVSSYALCTQVLEDRRFSMKETAAAGAPRL
NALTVPPEVVNNMGNIADAGLRKAVMKAITPKAPGLEQFLRDTANSLLDNLITEGAPADLRNDFADPLATALH

CKVLGIPQEDGPKLFRSLSIAFMSSADPIPAAKINWDRDIEYMAGILENPNITTGLMGELSRLRKDPAYSHVS
DELFATIGVTFFGAGVISTGSFLTTALISLIQRPQLRNLLHEKPELIPAGVEELLRINLSFADGLPRLATADI
QVGDVLVRKGELVLVLLEGANFDPEYFPNPGSIELDRPNPTSHLAFGRGQHFCPGSALGRRHAQIGIEALLKK
MPGVDLAVPIDQLVWRTRFQRRIPERLPVLW
>CYP121A1_Mycobacterium tuberculosis NRITLD37 2647423600
MTATVLLEVPFSARGDRIPDAVAELRTREPIRKVRTITGAEAWLVSSYALCTQVLEDRRFSMKETAAAGAPRL
NALTVPPEVVNNMGNIADAGLRKAVMKAITPKAPGLEQFLRDTANSLLDNLITEGAPADLRNDFADPLATALH
CKVLGIPQEDGPKLFRSLSIAFMSSADPIPAAKINWDRDIEYMAGILENPNITTGLMGELSRLRKDPAYSHVS
DELFATIGVTFFGAGVISTGSFLTTALISLIQRPQLRNLLHEKPELIPAGVEELLRINLSFADGLPRLATADI
QVGDVLVRKGELVLVLLEGANFDPEYFPNPGSIELDRPNPTSHLAFGRGQHFCPGSALGRRHAQIGIEALLKK
MPGVDLAVPIDQLVWRTRFQRRIPERLPVLW
>CYP121A1_Mycobacterium tuberculosis NRITLD38 2652371740
MTATVLLEVPFSARGDRIPDAVAELRTREPIRKVRTITGAEAWLVSSYALCTQVLEDRRFSMKETAAAGAPRL
NALTVPPEVVNNMGNIADAGLRKAVMKAITPKAPGLEQFLRDTANSLLDNLITEGAPADLRNDFADPLATALH
CKVLGIPQEDGPKLFRSLSIAFMSSADPIPAAKINWDRDIEYMAGILENPNITTGLMGELSRLRKDPAYSHVS
DELFATIGVTFFGAGVISTGSFLTTALISLIQRPQLRNLLHEKPELIPAGVEELLRINLSFADGLPRLATADI
QVGDVLVRKGELVLVLLEGANFDPEYFPNPGSIELDRPNPTSHLAFGRGQHFCPGSALGRRHAQIGIEALLKK
MPGVDLAVPIDQLVWRTRFQRRIPERLPVLW
>CYP121A1_Mycobacterium tuberculosis NRITLD47 2632799375
MTATVLLEVPFSARGDRIPDAVAELRTREPIRKVRTITGAEAWLVSSYALCTQVLEDRRFSMKETAAAGAPRL
NALTVPPEVVNNMGNIADAGLRKAVMKAITPKAPGLEQFLRDTANSLLDNLITEGAPADLRNDFADPLATALH
CKVLGIPQEDGPKLFRSLSIAFMSSADPIPAAKINWDRDIEYMAGILENPNITTGLMGELSRLRKDPAYSHVS
DELFATIGVTFFGAGVISTGSFLTTALISLIQRPQLRNLLHEKPELIPAGVEELLRINLSFADGLPRLATADI
QVGDVLVRKGELVLVLLEGANFDPEYFPNPGSIELDRPNPTSHLAFGRGQHFCPGSALGRRHAQIGIEALLKK
MPGVDLAVPIDQLVWRTRFQRRIPERLPVLW
>CYP121A1_Mycobacterium tuberculosis NRITLD49 2642025692
MTATVLLEVPFSARGDRIPDAVAELRTREPIRKVRTITGAEAWLVSSYALCTQVLEDRRFSMKETAAAGAPRL
NALTVPPEVVNNMGNIADAGLRKAVMKAITPKAPGLEQFLRDTANSLLDNLITEGAPADLRNDFADPLATALH
CKVLGIPQEDGPKLFRSLSIAFMSSADPIPAAKINWDRDIEYMAGILENPNITTGLMGELSRLRKDPAYSHVS
DELFATIGVTFFGAGVISTGSFLTTALISLIQRPQLRNLLHEKPELIPAGVEELLRINLSFADGLPRLATADI
QVGDVLVRKGELVLVLLEGANFDPEYFPNPGSIELDRPNPTSHLAFGRGQHFCPGSALGRRHAQIGIEALLKK
MPGVDLAVPIDQLVWRTRFQRRIPERLPVLW
>CYP121A1_Mycobacterium tuberculosis NRITLD50 2630496350
MTATVLLEVPFSARGDRIPDAVAELRTREPIRKVRTITGAEAWLVSSYALCTQVLEDRRFSMKETAAAGAPRL
NALTVPPEVVNNMGNIADAGLRKAVMKAITPKAPGLEQFLRDTANSLLDNLITEGAPADLRNDFADPLATALH
CKVLGIPQEDGPKLFRSLSIAFMSSADPIPAAKINWDRDIEYMAGILENPNITTGLMGELSRLRKDPAYSHVS
DELFATIGVTFFGAGVISTGSFLTTALISLIQRPQLRNLLHEKPELIPAGVEELLRINLSFADGLPRLATADI
QVGDVLVRKGELVLVLLEGANFDPEYFPNPGSIELDRPNPTSHLAFGRGQHFCPGSALGRRHAQIGIEALLKK
MPGVDLAVPIDQLVWRTRFQRRIPERLPVLW
>CYP121A1_Mycobacterium tuberculosis NRITLD51 2648299686
MTATVLLEVPFSARGDRIPDAVAELRTREPIRKVRTITGAEAWLVSSYALCTQVLEDRRFSMKETAAAGAPRL
NALTVPPEVVNNMGNIADAGLRKAVMKAITPKAPGLEQFLRDTANSLLDNLITEGAPADLRNDFADPLATALH
CKVLGIPQEDGPKLFRSLSIAFMSSADPIPAAKINWDRDIEYMAGILENPNITTGLMGELSRLRKDPAYSHVS
DELFATIGVTFFGAGVISTGSFLTTALISLIQRPQLRNLLHEKPELIPAGVEELLRINLSFADGLPRLATADI
QVGDVLVRKGELVLVLLEGANFDPEYFPNPGSIELDRPNPTSHLAFGRGQHFCPGSALGRRHAQIGIEALLKK
MPGVDLAVPIDQLVWRTRFQRRIPERLPVLW
>CYP121A1_Mycobacterium tuberculosis NRITLD55 2629964922
MTATVLLEVPFSARGDRIPDAVAELRTREPIRKVRTITGAEAWLVSSYALCTQVLEDRRFSMKETAAAGAPRL
NALTVPPEVVNNMGNIADAGLRKAVMKAITPKAPGLEQFLRDTANSLLDNLITEGAPADLRNDFADPLATALH
CKVLGIPQEDGPKLFRSLSIAFMSSADPIPAAKINWDRDIEYMAGILENPNITTGLMGELSRLRKDPAYSHVS
DELFATIGVTFFGAGVISTGSFLTTALISLIQRPQLRNLLHEKPELIPAGVEELLRINLSFADGLPRLATADI
QVGDVLVRKGELVLVLLEGANFDPEYFPNPGSIELDRPNPTSHLAFGRGQHFCPGSALGRRHAQIGIEALLKK
MPGVDLAVPIDQLVWRTRFQRRIPERLPVLW
>CYP121A1_Mycobacterium tuberculosis NRITLD56 2577470924
MTATVLLEVPFSARGDRIPDAVAELRTREPIRKVRTITGAEAWLVSSYALCTQVLEDRRFSMKETAAAGAPRL
NALTVPPEVVNNMGNIADAGLRKAVMKAITPKAPGLEQFLRDTANSLLDNLITEGAPADLRNDFADPLATALH
CKVLGIPQEDGPKLFRSLSIAFMSSADPIPAAKINWDRDIEYMAGILENPNITTGLMGELSRLRKDPAYSHVS
DELFATIGVTFFGAGVISTGSFLTTALISLIQRPQLRNLLHEKPELIPAGVEELLRINLSFADGLPRLATADI

QVGDVLVRKGELVLVLLEGANFDPEYFPNPGSIELDRPNPTSHLAFGRGQHFCPGSALGRRHAQIGIEALLKK
MPGVDLAVPIDQLVWRTRFQRRIPERLPVLW
>CYP121A1_Mycobacterium tuberculosis NRITLD59 2632422770
MTATVLLEVPFSARGDRIPDAVAELRTREPIRKVRTITGAEAWLVSSYALCTQVLEDRRFSMKETAAAGAPRL
NALTVPPEVVNNMGNIADAGLRKAVMKAITPKAPGLEQFLRDTANSLLDNLITEGAPADLRNDFADPLATALH
CKVLGIPQEDGPKLFRSLSIAFMSSADPIPAAKINWDRDIEYMAGILENPNITTGLMGELSRLRKDPAYSHVS
DELFATIGVTFFGAGVISTGSFLTTALISLIQRPQLRNLLHEKPELIPAGVEELLRINLSFADGLPRLATADI
QVGDVLVRKGELVLVLLEGANFDPEYFPNPGSIELDRPNPTSHLAFGRGQHFCPGSALGRRHAQIGIEALLKK
MPGVDLAVPIDQLVWRTRFQRRIPERLPVLW
>CYP121A1_Mycobacterium tuberculosis NZXDR1 2628821473
MTATVLLEVPFSARGDRIPDAVAELRTREPIRKVRTITGAEAWLVSSYALCTQVLEDRRFSMKETAAAGAPRL
NALTVPPEVVNNMGNIADAGLRKAVMKAITPKAPGLEQFLRDTANSLLDNLITEGAPADLRNDFADPLATALH
CKVLGIPQEDGPKLFRSLSIAFMSSADPIPAAKINWDRDIEYMAGILENPNITTGLMGELSRLRKDPAYSHVS
DELFATIGVTFFGAGVISTGSFLTTALISLIQRPQLRNLLHEKPELIPAGVEELLRINLSFADGLPRLATADI
QVGDVLVRKGELVLVLLEGANFDPEYFPNPGSIELDRPNPTSHLAFGRGQHFCPGSALGRRHAQIGIEALLKK
MPGVDLAVPIDQLVWRTRFQRRIPERLPVLW
>CYP121A1_Mycobacterium tuberculosis OFXR-10 2578230042
MTATVLLEVPFSARGDRIPDAVAELRTREPIRKVRTITGAEAWLVSSYALCTQVLEDRRFSMKETAAAGAPRL
NALTVPPEVVNNMGNIADAGLRKAVMKAITPKAPGLEQFLRDTANSLLDNLITEGAPADLRNDFADPLATALH
CKVLGIPQEDGPKLFRSLSIAFMSSADPIPAAKINWDRDIEYMAGILENPNITTGLMGELSRLRKDPAYSHVS
DELFATIGVTFFGAGVISTGSFLTTALISLIQRPQLRNLLHEKPELIPAGVEELLRINLSFADGLPRLATADI
QVGDVLVRKGELVLVLLEGANFDPEYFPNPGSIELDRPNPTSHLAFGRGQHFCPGSALGRRHAQIGIEALLKK
MPGVDLAVPIDQLVWRTRFQRRIPERLPVLW
>CYP121A1_Mycobacterium tuberculosis OFXR-13 2576534289
MTATVLLEVPFSARGDRIPDAVAELRTREPIRKVRTITGAEAWLVSSYALCTQVLEDRRFSMKETAAAGAPRL
NALTVPPEVVNNMGNIADAGLRKAVMKAITPKAPGLEQFLRDTANSLLDNLITEGAPADLRNDFADPLATALH
CKVLGIPQEDGPKLFRSLSIAFMSSADPIPAAKINWDRDIEYMAGILENPNITTGLMGELSRLRKDPAYSHVS
DELFATIGVTFFGAGVISTGSFLTTALISLIQRPQLRNLLHEKPELIPAGVEELLRINLSFADGLPRLATADI
QVGDVLVRKGELVLVLLEGANFDPEYFPNPGSIELDRPNPTSHLAFGRGQHFCPGSALGRRHAQIGIEALLKK
MPGVDLAVPIDQLVWRTRFQRRIPERLPVLW
>CYP121A1_Mycobacterium tuberculosis OFXR-15 2576477717
MTATVLLEVPFSARGDRIPDAVAELRTREPIRKVRTITGAEAWLVSSYALCTQVLEDRRFSMKETAAAGAPRL
NALTVPPEVVNNMGNIADAGLRKAVMKAITPKAPGLEQFLRDTANSLLDNLITEGAPADLRNDFADPLATALH
CKVLGIPQEDGPKLFRSLSIAFMSSADPIPAAKINWDRDIEYMAGILENPNITTGLMGELSRLRKDPAYSHVS
DELFATIGVTFFGAGVISTGSFLTTALISLIQRPQLRNLLHEKPELIPAGVEELLRINLSFADGLPRLATADI
QVGDVLVRKGELVLVLLEGANFDPEYFPNPGSIELDRPNPTSHLAFGRGQHFCPGSALGRRHAQIGIEALLKK
MPGVDLAVPIDQLVWRTRFQRRIPERLPVLW
>CYP121A1_Mycobacterium tuberculosis OFXR-26 2576999526
MTATVLLEVPFSARGDRIPDAVAELRTREPIRKVRTITGAEAWLVSSYALCTQVLEDRRFSMKETAAAGAPRL
NALTVPPEVVNNMGNIADAGLRKAVMKAITPKAPGLEQFLRDTANSLLDNLITEGAPADLRNDFADPLATALH
CKVLGIPQEDGPKLFRSLSIAFMSSADPIPAAKINWDRDIEYMAGILENPNITTGLMGELSRLRKDPAYSHVS
DELFATIGVTFFGAGVISTGSFLTTALISLIQRPQLRNLLHEKPELIPAGVEELLRINLSFADGLPRLATADI
QVGDVLVRKGELVLVLLEGANFDPEYFPNPGSIELDRPNPTSHLAFGRGQHFCPGSALGRRHAQIGIEALLKK
MPGVDLAVPIDQLVWRTRFQRRIPERLPVLW
>CYP121A1_Mycobacterium tuberculosis OFXR-8 2575464904
MTATVLLEVPFSARGDRIPDAVAELRTREPIRKVRTITGAEAWLVSSYALCTQVLEDRRFSMKETAAAGAPRL
NALTVPPEVVNNMGNIADAGLRKAVMKAITPKAPGLEQFLRDTANSLLDNLITEGAPADLRNDFADPLATALH
CKVLGIPQEDGPKLFRSLSIAFMSSADPIPAAKINWDRDIEYMAGILENPNITTGLMGELSRLRKDPAYSHVS
DELFATIGVTFFGAGVISTGSFLTTALISLIQRPQLRNLLHEKPELIPAGVEELLRINLSFADGLPRLATADI
QVGDVLVRKGELVLVLLEGANFDPEYFPNPGSIELDRPNPTSHLAFGRGQHFCPGSALGRRHAQIGIEALLKK
MPGVDLAVPIDQLVWRTRFQRRIPERLPVLW
>CYP121A1_Mycobacterium tuberculosis OM-V02_005 2570709321
MTATVLLEVPFSARGDRIPDAVAELRTREPIRKVRTITGAEAWLVSSYALCTQVLEDRRFSMKETAAAGAPRL
NALTVPPEVVNNMGNIADAGLRKAVMKAITPKAPGLEQFLRDTANSLLDNLITEGAPADLRNDFADPLATALH
CKVLGIPQEDGPKLFRSLSIAFMSSADPIPAAKINWDRDIEYMAGILENPNITTGLMGELSRLRKDPAYSHVS
DELFATIGVTFFGAGVISTGSFLTTALISLIQRPQLRNLLHEKPELIPAGVEELLRINLSFADGLPRLATADI
QVGDVLVRKGELVLVLLEGANFDPEYFPNPGSIELDRPNPTSHLAFGRGQHFCPGSALGRRHAQIGIEALLKK
MPGVDLAVPIDQLVWRTRFQRRIPERLPVLW

>CYP121A1_Mycobacterium tuberculosis OSDD071 2549405958
MTATVLLEVPFSARGDRIPDAVAELRTREPIRKVRTITGAEAWLVSSYALCTQVLEDRRFSMKETAAAGAPRL
NALTVPPEVVNNMGNIADAGLRKAVMKAITPKAPGLEQFLRDTANSLVDNLITEGAPADLRNDFADPLATALH
CKVLGIPQEDGPKLFRSLSIAFMSSADPIPAAKINWDRDIEYMAGILENPNITTGLMGELSRLRKDPAYSHVS
DELFATIGVTFFGAGVISTGSFLTTALISLIQRPQLRNLLHEKPELIPAGVEELLRINLSFADGLPRLATADI
QVGDVLVRKGELVLVLLEGANFDPEHFPNPGSIELDRPNPTSHLAFGRGQHFCPGSALGRRHAQIGIEALLKK
MPGVDLAVPIDQLVWRTRFQRRIPERLPVLW
>CYP121A1_Mycobacterium tuberculosis P00301553 2697962198
MTATVLLEVPFSARGDRIPDAVAELRTREPIRKVRTITGAEAWLVSSYALCTQVLEDRRFSMKETAAAGAPRL
NALTVPPEVVNNMGNIADAGLRKAVMKAITPKAPGLEQFLRDTANSLLDNLITEGAPADLRNDFADPLATALH
CKVLGIPQEDGPKLFRSLSIAFMSSADPIPAAKINWDRDIEYMAGILENPNITTGLMGELSRLRKDPAYSHVS
DELFATIGVTFFGAGVISTGSFLTTALISLIQRPQLRNLLHEKPELIPAGVEELLRINLSFADGLPRLATADI
QVGDVLVRKGELVLVLLEGANFDPEYFPNPGSIELDRPNPTSHLAFGRGQHFCPGSALGRRHAQIGIEALLKK
MPGVDLAVPIDQLVWRTRFQRRIPERLPVLW
>CYP121A1_Mycobacterium tuberculosis P00401210 2674504667
MTATVLLEVPFSARGDRIPDAVAELRTREPIRKVRTITGAEAWLVSSYALCTQVLEDRRFSMKETAAAGAPRL
NALTVPPEVVNNMGNIADAGLRKAVMKAITPKAPGLEQFLRDTANSLLDNLITEGAPADLRNDFADPLATALH
CKVLGIPQEDGPKLFRSLSIAFMSSADPIPAAKINWDRDIEYMAGILENPNITTGLMGELSRLRKDPAYSHVS
DELFATIGVTFFGAGVISTGSFLTTALISLIQRPQLRNLLHEKPELIPAGVEELLRINLSFADGLPRLATADI
QVGDVLVRKGELVLVLLEGANFDPEYFPNPGSIELDRPNPTSHLAFGRGQHFCPGSALGRRHAQIGIEALLKK
MPGVDLAVPIDQLVWRTRFQRRIPERLPVLW
>CYP121A1_Mycobacterium tuberculosis P00401720 2692508602
MTATVLLEVPFSARGDRIPDAVAELRTREPIRKVRTITGAEAWLVSSYALCTQVLEDRRFSMKETAAAGAPRL
NALTVPPEVVNNMGNIADAGLRKAVMKAITPKAPGLEQFLRDTANSLLDNLITEGAPADLRNDFADPLATALH
CKVLGIPQEDGPKLFRSLSIAFMSSADPIPAAKINWDRDIEYMAGILENPNITTGLMGELSRLRKDPAYSHVS
DELFATIGVTFFGAGVISTGSFLTTALISLIQRPQLRNLLHEKPELIPAGVEELLRINLSFADGLPRLATADI
QVGDVLVRKGELVLVLLEGANFDPEYFPNPGSIELDRPNPTSHLAFGRGQHFCPGSALGRRHAQIGIEALLKK
MPGVDLAVPIDQLVWRTRFQRRIPERLPVLW
>CYP121A1_Mycobacterium tuberculosis P00601463 2697654100
MTATVLLEVPFSARGDRIPDAVAELRTREPIRKVRTITGAEAWLVSSYALCTQVLEDRRFSMKETAAAGAPRL
NALTVPPEVVNNMGNIADAGLRKAVMKAITPKAPGLEQFLRDTANSLLDNLITEGAPADLRNDFADPLATALH
CKVLGIPQEDGPKLFRSLSIAFMSSADPIPAAKINWDRDIEYMAGILENPNITTGLMGELSRLRKDPAYSHVS
DELFATIGVTFFGAGVISTGSFLTTALISLIQRPQLRNLLHEKPELIPAGVEELLRINLSFADGLPRLATADI
QVGDVLVRKGELVLVLLEGANFDPEYFPNPGSIELDRPNPTSHLAFGRGQHFCPGSALGRRHAQIGIEALLKK
MPGVDLAVPIDQLVWRTRFQRRIPERLPVLW
>CYP121A1_Mycobacterium tuberculosis P09501076 2698016801
MTATVLLEVPFSARGDRIPDAVAELRTREPIRKVRTITGAEAWLVSSYALCTQVLEDRRFSMKETAAAGAPRL
NALTVPPEVVNNMGNIADAGLRKAVMKAITPKAPGLEQFLRDTANSLLDNLITEGAPADLRNDFADPLATALH
CKVLGIPQEDGPKLFRSLSIAFMSSADPIPAAKINWDRDIEYMAGILENPNITTGLMGELSRLRKDPAYSHVS
DELFATIGVTFFGAGVISTGSFLTTALISLIQRPQLRNLLHEKPELIPAGVEELLRINLSFADGLPRLATADI
QVGDVLVRKGELVLVLLEGANFDPEYFPNPGSIELDRPNPTSHLAFGRGQHFCPGSALGRRHAQIGIEALLKK
MPGVDLAVPIDQLVWRTRFQRRIPERLPVLW
>CYP121A1_Mycobacterium tuberculosis P09501164 2699227722
MTATVLLEVPFSARGDRIPDAVAELRTREPIRKVRTITGAEAWLVSSYALCTQVLEDRRFSMKETAAAGAPRL
NALTVPPEVVNNMGNIADAGLRKAVMKAITPKAPGLEQFLRDTANSLLDNLITEGAPADLRNDFADPLATALH
CKVLGIPQEDGPKLFRSLSIAFMSSADPIPAAKINWDRDIEYMAGILENPNITTGLMGELSRLRKDPAYSHVS
DELFATIGVTFFGAGVISTGSFLTTALISLIQRPQLRNLLHEKPELIPAGVEELLRINLSFADGLPRLATADI
QVGDVLVRKGELVLVLLEGANFDPEYFPNPGSIELDRPNPTSHLAFGRGQHFCPGSALGRRHAQIGIEALLKK
MPGVDLAVPIDQLVWRTRFQRRIPERLPVLW
>CYP121A1_Mycobacterium tuberculosis P09501258 2673318081
MTATVLLEVPFSARGDRIPDAVAELRTREPIRKVRTITGAEAWLVSSYALCTQVLEDRRFSMKETAAAGAPRL
NALTVPPEVVNNMGNIADAGLRKAVMKAITPKAPGLEQFLRDTANSLLDNLITEGAPADLRNDFADPLATALH
CKVLGIPQEDGPKLFRSLSIAFMSSADPIPAAKINWDRDIEYMAGILENPNITTGLMGELSRLRKDPAYSHVS
DELFATIGVTFFGAGVISTGSFLTTALISLIQRPQLRNLLHEKPELIPAGVEELLRINLSFADGLPRLATADI
QVGDVLVRKGELVLVLLEGANFDPEYFPNPGSIELDRPNPTSHLAFGRGQHFCPGSALGRRHAQIGIEALLKK
MPGVDLAVPIDQLVWRTRFQRRIPERLPVLW
>CYP121A1_Mycobacterium tuberculosis P09501329 2692822647

MTATVLLEVPFSARGDRIPDAVAELRTREPIRKVRTITGAEAWLVSSYALCTQVLEDRRFSMKETAAAGAPRL
NALTVPPEVVNNMGNIADAGLRKAVMKAITPKAPGLEQFLRDTANSLLDNLITEGAPADLRNDFADPLATALH
CKVLGIPQEDGPKLFRSLSIAFMSSADPIPAAKINWDRDIEYMAGILENPNITTGLMGELSRLRKDPAYSHVS
DELFATIGVTFFGAGVISTGSFLTTALISLIQRPQLRNLLHEKPELIPAGVEELLRINLSFADGLPRLATADI
QVGDVLVRKGELVLVLLEGANFDPEYFPNPGSIELDRPNPTSHLAFGRGQHFCPGSALGRRHAQIGIEALLKK
MPGVDLAVPIDQLVWRTRFQRRIPERLPVLW
>CYP121A1_Mycobacterium tuberculosis P09501583 2698981652
MTATVLLEVPFSARGDRIPDAVAELRTREPIRKVRTITGAEAWLVSSYALCTQVLEDRRFSMKETAAAGAPRL
NALTVPPEVVNNMGNIADAGLRKAVMKAITPKAPGLEQFLRDTANSLLDNLITEGAPADLRNDFADPLATALH
CKVLGIPQEDGPKLFRSLSIAFMSSADPIPAAKINWDRDIEYMAGILENPNITTGLMGELSRLRKDPAYSHVS
DELFATIGVTFFGAGVISTGSFLTTALISLIQRPQLRNLLHEKPELIPAGVEELLRINLSFADGLPRLATADI
QVGDVLVRKGELVLVLLEGANFDPEYFPNPGSIELDRPNPTSHLAFGRGQHFCPGSALGRRHAQIGIEALLKK
MPGVDLAVPIDQLVWRTRFQRRIPERLPVLW
>CYP121A1_Mycobacterium tuberculosis P09501896 2694505349
MTATVLLEVPFSARGDRIPDAVAELRTREPIRKVRTITGAEAWLVSSYALCTQVLEDRRFSMKETAAAGAPRL
NALTVPPEVVNNMGNIADAGLRKAVMKAITPKAPGLEQFLRDTANSLLDNLITEGAPADLRNDFADPLATALH
CKVLGIPQEDGPKLFRSLSIAFMSSADPIPAAKINWDRDIEYMAGILENPNITTGLMGELSRLRKDPAYSHVS
DELFATIGVTFFGAGVISTGSFLTTALISLIQRPQLRNLLHEKPELIPAGVEELLRINLSFADGLPRLATADI
QVGDVLVRKGELVLVLLEGANFDPEYFPNPGSIELDRPNPTSHLAFGRGQHFCPGSALGRRHAQIGIEALLKK
MPGVDLAVPIDQLVWRTRFQRRIPERLPVLW
>CYP121A1_Mycobacterium tuberculosis P09502221 2678551514
MTATVLLEVPFSARGDRIPDAVAELRTREPIRKVRTITGAEAWLVSSYALCTQVLEDRRFSMKETAAAGAPRL
NALTVPPEVVNNMGNIADAGLRKAVMKAITPKAPGLEQFLRDTANSLLDNLITEGAPADLRNDFADPLATALH
CKVLGIPQEDGPKLFRSLSIAFMSSADPIPAAKINWDRDIEYMAGILENPNITTGLMGELSRLRKDPAYSHVS
DELFATIGVTFFGAGVISTGSFLTTALISLIQRPQLRNLLHEKPELIPAGVEELLRINLSFADGLPRLATADI
QVGDVLVRKGELVLVLLEGANFDPEYFPNPGSIELDRPNPTSHLAFGRGQHFCPGSALGRRHAQIGIEALLKK
MPGVDLAVPIDQLVWRTRFQRRIPERLPVLW
>CYP121A1_Mycobacterium tuberculosis P09600222 2690929838
MTATVLLEVPFSARGDRIPDAVAELRTREPIRKVRTITGAEAWLVSSYALCTQVLEDRRFSMKETAAAGAPRL
NALTVPPEVVNNMGNIADAGLRKAVMKAITPKAPGLEQFLRDTANSLLDNLITEGAPADLRNDFADPLATALH
CKVLGIPQEDGPKLFRSLSIAFMSSADPIPAAKINWDRDIEYMAGILENPNITTGLMGELSRLRKDPAYSHVS
DELFATIGVTFFGAGVISTGSFLTTALISLIQRPQLRNLLHEKPELIPAGVEELLRINLSFADGLPRLATADI
QVGDVLVRKGELVLVLLEGANFDPEYFPNPGSIELDRPNPTSHLAFGRGQHFCPGSALGRRHAQIGIEALLKK
MPGVDLAVPIDQLVWRTRFQRRIPERLPVLW
>CYP121A1_Mycobacterium tuberculosis P09701236 2692980339
MTATVLLEVPFSARGDRIPDAVAELRTREPIRKVRTITGAEAWLVSSYALCTQVLEDRRFSMKETAAAGAPRL
NALTVPPEVVNNMGNIADAGLRKAVMKAITPKAPGLEQFLRDTANSLLDNLITEGAPADLRNDFADPLATALH
CKVLGIPQEDGPKLFRSLSIAFMSSADPIPAAKINWDRDIEYMAGILENPNITTGLMGELSRLRKDPAYSHVS
DELFATIGVTFFGAGVISTGSFLTTALISLIQRPQLRNLLHEKPELIPAGVEELLRINLSFADGLPRLATADI
QVGDVLVRKGELVLVLLEGANFDPEYFPNPGSIELDRPNPTSHLAFGRGQHFCPGSALGRRHAQIGIEALLKK
MPGVDLAVPIDQLVWRTRFQRRIPERLPVLW
>CYP121A1_Mycobacterium tuberculosis PanR0301 2555283023
MTATVLLEVPFSARGDRIPDAVAELRTREPIRKVRTITGAEAWLVSSYALCTQVLEDRRFSMKETAAAGAPRL
NALTVPPEVVNNMGNIADAGLRKAVMKAITPKAPGLEQFLRDTANSLLDNLITEGAPADLRNDFADPLATALH
CKVLGIPQEDGPKLFRSLSIAFMSSADPIPAAKINWDRDIEYMAGILENPNITTGLMGELSRLRKDPAYSHVS
DELFATIGVTFFGAGVISTGSFLTTALISLIQRPQLRNLLPEKPELIPAGVEELLRINLSFADGLPRLATADI
QVGDVLVRKGELVLVLLEGANFDPEHFPNPGSIELDRPNPTSHLAFGRGQHFCPGSALGRRHAQIGIEALLKK
MPGVDLAVPIDQLVWRTRFQRRIPERLPVLW
>CYP121A1_Mycobacterium tuberculosis PanR0316 2555329988
MTATVLLEVPFSARGDRIPDAVAELRTREPIRKVRTITGAEAWLVSSYALCTQVLEDRRFSMKETAAAGAPRL
NALTVPPEVVNNMGNIADAGLRKAVMKAITPKAPGLEQFLRDTANSLLDNLITEGAPADLRNDFADPLATALH
CKVLGIPQEDGPKLFRSLSIAFMSSADPIPAAKINWDRDIEYMAGILENPNITTGLMGELSRLRKDPAYSHVS
DELFATIGVTFFGAGVISTGSFLTTALISLIQRPQLRNLLPEKPELIPAGVEELLRINLSFADGLPRLATADI
QVGDVLVRKGELVLVLLEGANFDPEHFPNPGSIELDRPNPTSHLAFGRGQHFCPGSALGRRHAQIGIEALLKK
MPGVDLAVPIDQLVWRTRFQRRIPERLPVLW
>CYP121A1_Mycobacterium tuberculosis H37Ra unfinished sequence 641813884
MHCATQVLEDRRFSMKETAAAGAPRLNALTVPPEVVNNMGNIADAGLRKAVMKAITPKAPGLEQFLRDTANSL
LDNLITEGAPADLRNDFADPLATALHCKVLGIPQEDGPKLFRSLSIAFMSSADPIPAAKINWDRDIEYMAGIL

ENPNITTGLMGELSRLRKDPAYSHVSDELFATIGVTFFGAGVISTGSFLTTALISLIQRPQLRNLLHEKPELI
PAGVEELLRINLSFADGLPRLATADIQVGDVLVRKGELVLVLLEGANFDPEHFPNPGSIELDRPNPTSHLAFG
RGQHFCPGSALGRRHAQIGIEALLKKMPGVDLAVPIDQLVWRTRFQRRIPERLPVLW
>CYP121A1_Mycobacterium tuberculosis 0B026XDR 2720191370
MGNIADAGLRKAVMKAITPKAPGLEQFLRDTANSLLDNLITEGAPADLRNDFADPLATALHCKVLGIPQEDGP
KLFRSLSIAFMSSADPIPAAKINWDRDIEYMAGILENPNITTGLMGELSRLRKDPAYSHVSDELFATIGVTFF
GAGVISTGSFLTTALISLIQRPQLRNLLHEKPELIPAGVEELLRINLSFADGLPRLATADIQVGDVLVRKGEL
VLVLLEGANFDPEYFPNPGSIELDRPNPTSHLAFGRGQHFCPGSALGRRHAQIGIEALLKKMPGVDLAVPIDQ
LVWRTRFQRRIPERLPVLW
>CYP121A1_Mycobacterium canettii CIPT 140010059 651039619
MTATVLLEVPFSARGDRIPDAVAELRTREPIRKVRTITGAEAWLVSSYALCTQVLEDRRFSMKETAAAGAPRL
NALTVPPEVVNNMGNIADAGLRKAVMKAITPKAPGLEQFLRDTANSLLDNLITEGAPADLRNDFADPLATALH
CKVLGIPQEDGPKLFRSLSIAFMSSADPIPAAKINWDRDIEYMAGILENPNITTGLMGELGRLRKDPAYSHVS
DELFATIGVTFFGAGVISTGSFLTTALISLIQRPQLRNLLHEKPELIPAGVEELLRINLSFADGLPRLATADI
QVGDVLVRKGELVLVLLEGANFDPEHFPNPGSIELDRPNPTSHLAFGRGQHFCPGSALGRRHAQIGIEALLKK
MPDVDLAVPIDQLVWRTRFQRRIPERLPVLW
>CYP121A1_Mycobacterium canettii CIPT 140060008 2541578689
MTATVLLEVPFSARGDRIPDAVAELRTREPIRKVRTVTGAEAWLVSSYALCTQVLEDRRFSMKETAAAGAPRL
NALTVPPEVVNNMGNIADAGLRKAVMKAITPKAPGLEQFLRDTANSLLDNLITEGAPADLRNDFADPLATALH
CKVLGIPQEDGPKLFRSLSIAFMSSADPIPAAKINWDRDIEYMAGILENPNITTGLMGELGRLRKDPAYSHVS
DELFATIGVTFFGAGVISTGSFLTTALISLIQRPQLRNLLHEKPELIPAGVEELLRINLSFADGLPRLATADI
QVGDVLVRKGELVLVLLEGANFDPEHFPNPGSIELDRPNPTSHLAFGRGQHFCPGSALGRRHAQIGIEALLKK
MPGVDLAVPIDQLVWRTRFQRRIPERLPVLW
>CYP121A1_Mycobacterium canettii CIPT 140070002 2566981594
MTATVLLEVPFSARGDRIPDAVAELRTREPIRKVRTVTGAEAWLVSSYALCTQVLEDRRFSMKETAAAGAPRL
NALTVPPEVVNNMGNIADAGLRKAVMKAITPKAPGLEQFLRDTANSLLDNLITEGAPADLRNDFADPLATALH
CKVLGIPQEDGPKLFRSLSIAFMSSADPIPAAKINWDRDIEYMAGILENPNITTGLMGELGRLRKDPAYSHVS
DELFATIGVTFFGAGVISTGSFLTTALISLIQRPQLRNLLHEKPELIPAGVEELLRINLSFADGLPRLATADI
QVGDVLVRKGELVLVLLEGANFDPEHFPNPGSIELDRPNPTSHLAFGRGQHFCPGSALGRRHAQIGIEALLKK
MPGVDLAVPIDQLVWRTRFQRRIPERLPVLW
>CYP121A1_Mycobacterium canettii CIPT 140070005 2566985868
MTATVLLEVPFSARGDRIPDAVAELRTREPIRKVRTVTGAEAWLVSSYALCTQVLEDRRFSMKETAAAGAPRL
NALTVPPEVVNNMGNIADAGLRKAVMKAITPKAPGLEQFLRDTANSLLDNLITEGAPADLRNDFADPLATALH
CKVLGIPQEDGPKLFRSLSIAFMSSADPIPAAKINWDRDIEYMAGILENPNITTGLMGELGRLRKDPAYSHVS
DELFATIGVTFFGAGVISTGSFLTTALISLIQRPQLRNLLHEKPELIPAGVEELLRINLSFADGLPRLATADI
QVGDVLVRKGELVLVLLEGANFDPEHFPNPGSIELDRPNPTSHLAFGRGQHFCPGSALGRRHAQIGIEALLKK
MPGVDLAVPIDQLVWRTRFQRRIPERLPVLW
>CYP121A1_Mycobacterium canettii CIPT 140070007 2566973032
MTATVLLEVPFSARGDRIPDAVAELRTREPIRKVRTVTGAEAWLVSSYALCTQVLEDRRFSMKETAAAGAPRL
NALTVPPEVVNNMGNIADAGLRKAVMKAITPKAPGLEQFLRDTANSLLDNLITEGAPADLRNDFADPLATALH
CKVLGIPQEDGPKLFRSLSIAFMSSADPIPAAKINWDRDIEYMAGILENPNITTGLMGELGRLRKDPAYSHVS
DELFATIGVTFFGAGVISTGSFLTTALISLIQRPQLRNLLHEKPELIPAGVEELLRINLSFADGLPRLATADI
QVGDVLVRKGELVLVLLEGANFDPEHFPNPGSIELDRPNPTSHLAFGRGQHFCPGSALGRRHAQIGIEALLKK
MPGVDLAVPIDQLVWRTRFQRRIPERLPVLW
>CYP121A1_Mycobacterium canettii CIPT 140070008 2541570452
MTATVLLEVPFSARGDRIPDAVAELRTREPIRKVRTVTGAEAWLVSSYALCTQVLEDRRFSMKETAAAGAPRL
NALTVPPEVVNNMGNIADAGLRKAVMKAITPKAPGLEQFLRDTANSLLDNLITEGAPADLRNDFADPLATALH
CKVLGIPQEDGPKLFRSLSIAFMSSADPIPAAKINWDRDIEYMAGILENPNITTGLMGELGRLRKDPAYSHVS
DELFATIGVTFFGAGVISTGSFLTTALISLIQRPQLRNLLHEKPELIPAGVEELLRINLSFADGLPRLATADI
QVGDVLVRKGELVLVLLEGANFDPEHFPNPGSIELDRPNPTSHLAFGRGQHFCPGSALGRRHAQIGIEALLKK
MPGVDLAVPIDQLVWRTRFQRRIPERLPVLW
>CYP121A1_Mycobacterium canettii CIPT 140070013 2566977306
MTATVLLEVPFSARGDRIPDAVAELRTREPIRKVRTVTGAEAWLVSSYALCTQVLEDRRFSMKETAAAGAPRL
NALTVPPEVVNNMGNIADAGLRKAVMKAITPKAPGLEQFLRDTANSLLDNLITEGAPADLRNDFADPLATALH
CKVLGIPQEDGPKLFRSLSIAFMSSADPIPAAKINWDRDIEYMAGILENPNITTGLMGELGRLRKDPAYSHVS
DELFATIGVTFFGAGVISTGSFLTTALISLIQRPQLRNLLHEKPELIPAGVEELLRINLSFADGLPRLATADI
QVGDVLVRKGELVLVLLEGANFDPEHFPNPGSIELDRPNPTSHLAFGRGQHFCPGSALGRRHAQIGIEALLKK
MPGVDLAVPIDQLVWRTRFQRRIPERLPVLW

>CYP121A1_Mycobacterium canettii CIPT 140070017 2541574567
MTATVLLEVPFSARGDRIPDAVAELRTREPIRKVRTVTGAEAWLVSSYALCTQVLEDRRFSMKETAAAGAPRL
NALTVPPEVVNNMGNIADAGLRKAVMKAITPKAPGLEQFLRDTANSLLDNLITEGAPADLRNDFADPLATALH
CKVLGIPQEDGPKLFRSLSIAFMSSADPIPAAKINWDRDIEYMAGILENPNITTGLMGELGRLRKDPAYSHVS
DELFATIGVTFFGAGVISTGSFLTTALISLIQRPQLRNLLHEKPELIPAGVEELLRINLSFADGLPRLATADI
QVGDVLVRKGELVLVLLEGANFDPEHFPNPGSIELDRPNPTSHLAFGRGQHFCPGSALGRRHAQIGIEALLKK
MPGVDLAVPIDQLVWRTRFQRRIPERLPVLW
>CYP121A1_Mycobacterium tuberculosis 1034 2621340531
MTATVLLEVPFSARGDRIPDAVAELRTREPIRKVRTITGAEAWLVSSYALCTQVLEDRRFSMKETAAAGAPRL
NALTVPPEVVNNMGNIADAGLRKAVMKAITPKAPGLEQFLRDTANSLLDNLITEGAPADLRNDFADPLATALH
CKVLGIPQEDGPKLFRSLSIAFMSSADPIPAAKINWDRDIEYMAGILENPNITTGLMGELSRLRKDPAYSHVS
DELFATIGVTFFGAGVISTGSFLTTALISLIQRPQLRNLLHEKPELIPAGVEGLLRINLSFADGLPRLATADI
QVGDVLVRKGELVLVLLEGANFDPEYFPNPGSIELDRPNPTSHLAFGRGQHFCPGSALGRRHAQIGIEALLKK
MPGVDLAVPIDQLVWRTRFQRRIPERLPVLW
>CYP121A1_Mycobacterium tuberculosis 1034 2621340531
MTATVLLEVPFSARGDRIPDAVAELRTREPIRKVRTITGAEAWLVSSYALCTQVLEDRRFSMKETAAAGAPRL
NALTVPPEVVNNMGNIADAGLRKAVMKAITPKAPGLEQFLRDTANSLLDNLITEGAPADLRNDFADPLATALH
CKVLGIPQEDGPKLFRSLSIAFMSSADPIPAAKINWDRDIEYMAGILENPNITTGLMGELSRLRKDPAYSHVS
DELFATIGVTFFGAGVISTGSFLTTALISLIQRPQLRNLLHEKPELIPAGVEGLLRINLSFADGLPRLATADI
QVGDVLVRKGELVLVLLEGANFDPEYFPNPGSIELDRPNPTSHLAFGRGQHFCPGSALGRRHAQIGIEALLKK
MPGVDLAVPIDQLVWRTRFQRRIPERLPVLW
>CYP121A1_Mycobacterium tuberculosis 210 NZ_ADAB01000044 647206459
MTATVLLEVPFSARGDRIPDAVAELRTREPIRKVRTITGAEAWLVSSYALCTQVLEDRRFSMKETAAAGAPRL
NALTVPPEVVNNMGNIADAGLRKAVMKAITPKAPGLEQFLRDTANSLLDNLITEGAPADLRNDFADPLATALH
CKVLGIPQEDGPKLFRSLSIAFMSSADPIPAAKINWDRDIEYMAGILENPNITTGLMGELSRLRKDPAYSHVS
DELFATIGVTFFGAGVISTGSFLTTALISLIQRPQLRNLLHEKPELIPAGVEELLRINLSFADGLPRLATADI
QVGDVLVRKGELVLVLLEGANFDPEYFPNPGSIELDRPNPTSHLAFGRGQHFCPGSALGRRHAQIGIEALLKK
MPGVDLAVPIDQLVWRTRFQRRHPRTPSGALVGFRKLTRAITARFGKRWDRTISTLHRPKALPST
>CYP121A1_Mycobacterium tuberculosis BTB11-027 2603346213
MTATVLLEVPFSARGDRIPDAVAELRTREPIRKVRTITGAEAWLVSSYALYTQVLEDRRFSMKETAAAGAPRL
NALTVPPEVVNNMGNIADAGLRKAVMKAITPKAPGLEQFLRDTANSLLDNLITEGAPADLRNDFADPLATALH
CKVLGIPQEDGPKLFRSLSIAFMSSADPIPAAKINWDRDIEYMAGILENPNITTGLMGELSRLRKDPAYSHVS
DELFATIGVTFFGAGVISTGSFLTTALISLIQRPQLRNLLHEKPELIPAGVEELLRINLSFADGLPRLATADI
QVGDVLVRKGELVLVLLEGANFDPEYFPNPGSIELDRPNPTSHLAFGRGQHFCPGSALGRRHAQIGIEALLKK
MPGVDLAVPIDQLVWRTRFQRRIPERLPVLW
>CYP121A1_Mycobacterium tuberculosis C53_20899 2678507759
MTATVLLEVPFSARGDRIPDAVAELRTREPIRKVRTITGAEAWLVSSYALCTQVLEDRRFSMKETAAAGAPRL
NALTVPPEVVNNMGNIADAGLRKAVMKAITPKAPGLEQFLRDTANSLLDNLITEGAPADLRNDFADPLATALH
CKVLGIPQEDGPKLFRSLSIAFMSSADPIPAAKINWDRDIEYMAGILENPNITTGLMGELSRLRKDPAYSHVS
DELFATIGVTFFGAGVISTGSFLTTALISLIQRPQLRNLLHEKPELIPAGVEELLRINLSFADGLPRLATADI
QVGDVLVRKGELVLVLLEGANFDPEHFPNPGSIELDRPNPTSHLAFGRGQHFCPGSALGRRHAQIGIEALLKT
MPGVDLAVPIDQLVWRTRFQRRIPEHLPVLW
>CYP121A1_Mycobacterium tuberculosis KT-0026 2633894857
MTATVLLEVPFSARGDRIPDAVAELRTREPIRKVRTITGAEAWLVSSYALCTQVLEDRRFSMKETAAAGAPRL
NALTVPPEVVNNMGNIADAGLRKAVMKAITPKAPGLEQFLRDTANSLLDNLITEGAPADLRNDFADPLATALH
CKVLGIPQEDGPKLFRSLSIAFMSSADPIPAAKINWDRDIEYMAGILENPNITTGLMGELSRLRKDPAYSHVS
DELFATIGVTFFGAGVISTGSFLTTALISLIQRPQLRNLLHEKPELIPAGVEDLLRINLSFADGLPRLATADI
QVGDVLVRKGELVLVLLEGANFDPEYFPNPGSIELDRPNPTSHLAFGRGQHFCPGSALGRRHAQIGIEALLKK
MPGVDLAVPIDQLVWRTRFQRRIPERLPVLW
>CYP121A1_Mycobacterium tuberculosis KT-0195 2632821967
MTATVLLEVPFSARGDRIPDAVAELRTREPIRKVRTITGAEAWLVSSYALCTQVLEDRRFSMKETAAAGAPRL
NALTVPPEVVNNMGNIADAGLRKAVMKAITPKAPGLEQFLRDTANSLLDNLITEGAPADLRNDFADPLATALH
CKVLGIPQEDGPKLFRSLSIAFMSSADPIPAAKINWDRDIEYMAGILENANITTGLMGELSRLRKDPAYSHVS
DELFATIGVTFFGAGVISTGSFLTTALISLIQRPQLRNLLHEKPELIPAGVEELLRINLSFADGLPRLATADI
QVGDVLVRKGELVLVLLEGANFDPEYFPNPGSIELDRPNPTSHLAFGRGQHFCPGSALGRRHAQIGIEALLKK
MPGVDLAVPIDQLVWRTRFQRRIPERLPVLW
>CYP121A1_Mycobacterium tuberculosis O09501596 2675610192

MTATVLLEVPFSARGDRIPDASAELRPREPIRKVRTITGAEAWLVSSYALCTQVLEDRRFSMKETAAAGAPRL
NALTVPPEVVNNMGNIADAGLRKAVMKAITPKAPGLEQFLRDTANSLLDNLITEGAPADLRNDFADPLATALH
CKVLGIPQEDGPKLFRSLSIAFMSSADPIPAAKINWDRDIEYMAGILENPNITTGLMGELSRLRKDPAYSHVS
DELFATIGVTFFGAGVISTGSFLTTALISLIQRPQLRNLLHEKPELIPAGVEELLRINLSFADGLPRLATADI
QVGDVLVRKGELVLVLLEGANFDPEHFPNPGSIELDRPNPTSHLAFGRGQHFCPGSALGRRHAQIGIEALLKK
MPGVDLAVPIDQLVWRTRFQRRIPERLPVLW
>CYP121A1_Mycobacterium canettii CIPT 140070010 2540555216
MTATVLLEVPFSARGDRIPDAVAELRTREPIRKVRTVTGAEAWLVSSYALCTQVLEDRRFSMKETAAAGAPRL
NALTVPPEVVNNMGNIADAGLRKAVMKAITPKAPGLEQFLRDTANSLLDNLITEGAPADLRNDFADPLATALH
CKVLGIPQEDGPKLFRSLSIAFMSSADPIPAAKINWDRDIEYMAGILENPNITTGLMGELGRLRKDPAYSHVS
DELFATIGVTFFGAGVISTGSFLTTALISLIQRPQLRNLLHEKPQLIPAGVEELLRINLSFADGLPRLATADI
QVGDVLVRKGELVLVLLEGANFDPEHFPNPGSIELDRPNPTSHLAFGRGQHFCPGSALGRRHAQIGIEALLKK
MPGVDLAVPIDQLVWRTRFQRRIPERLPVLW
>CYP121A1_Mycobacterium tuberculosis NRITLD53 2632107265
KGELVLVLLEGANFDPEYFPNPGSIELDRPNPTSHLAFGRGQHFCPGSALGRRHAQIGIEALLKKMPGVDLAV
PIDQLVWRTRFQRRIPERLPVLW
>CYP121A1_Mycobacterium tuberculosis H3004 2622304390
MTATVLLEVPFSARGDRIPDAVAELRTREPIRKVRTITGAEAWLVSSYALCTQVLEDRRFSMKETAAAGAPRL
NALTVPPEVVNNMGNIADAGLRKAVMKAITPKAPGLEQFLRDTANSLLDNLITEGAPADLRNDFADPLATALH
CKVLGIPQEDGPKLFRSSADPIPAAKINWDRDIEYMAGILENPNITTGLMGELSRLRKDPAYSHVSDELFATI
GVTFFGAGVISTGSFLTTALISLIQRPQLRNLLHEKPELIPAGVEELLRINLSFADGLPRLATADIQVGDVLV
RKGELVLVLLEGANFDPEHFPNPGSIELDRPNPTSHLAFGRGQHFCPGSALGRRHAQIGIEALLKKMPGVDLA
VPIDQLVWRTRFQRRIPERLPVLW
>CYP121A1_Mycobacterium tuberculosis PanR0403 2555544213
MTATVLLEVPFSARGDRIPDAVAELRTREPIRKVRTITGAEAWLVSSYALCTQVLEDRRFSMKETAAAGAPRL
NALTVPPEVVNNMGNIADAGLRKAVMKAITPKAPGLEQFLRDTANSLLDNLITEGAPADLRNDFADPLATALH
CKVLGIPQEDGPKLFRSLSIAFMSSADPIPAAKINWDRDIEYMAGILENPNITTGLMGELSRLRKDPAYSHVS
DELFATIGVTFFGAGVISTGSFLTTALISLIQRPQLRNLLHEKPELIPAGVEELLRINLSFADGLPRLATADI
QVGDVLVRKGELVLVLLEGANFDPEHFPNPGSIELDRPNPTSHLAFGRGQHFCPGSALGRRHAQIGIEALLKK
MPGVDLAVPIDQLVWRTRFQRRIPERLPVLW
>CYP121A1_Mycobacterium tuberculosis PanR0404 2555557181
MTATVLLEVPFSARGDRIPDAVAELRTREPIRKVRTITGAEAWLVSSYALCTQVLEDRRFSMKETAAAGAPRL
NALTVPPEVVNNMGNIADAGLRKAVMKAITPKAPGLEQFLRDTANSLLDNLITEGAPADLRNDFADPLATALH
CKVLGIPQEDGPKLFRSLSIAFMSSADPIPAAKINWDRDIEYMAGILENPNITTGLMGELSRLRKDPAYSHVS
DELFATIGVTFFGAGVISTGSFLTTALISLIQRPQLRNLLHEKPELIPAGVEELLRINLSFADGLPRLATADI
QVGDVLVRKGELVLVLLEGANFDPEHFPNPGSIELDRPNPTSHLAFGRGQHFCPGSALGRRHAQIGIEALLKK
MPGVDLAVPIDQLVWRTRFQRRIPERLPVLW
>CYP121A1_Mycobacterium tuberculosis PanR0405 2555516667
MTATVLLEVPFSARGDRIPDAVAELRTREPIRKVRTITGAEAWLVSSYALCTQVLEDRRFSMKETAAAGAPRL
NALTVPPEVVNNMGNIADAGLRKAVMKAITPKAPGLEQFLRDTANSLLDNLITEGAPADLRNDFADPLATALH
CKVLGIPQEDGPKLFRSLSIAFMSSADPIPAAKINWDRDIEYMAGILENPNITTGLMGELSRLRKDPAYSHVS
DELFATIGVTFFGAGVISTGSFLTTALISLIQRPQLRNLLHEKPELIPAGVEELLRINLSFADGLPRLATADI
QVGDVLVRKGELVLVLLEGANFDPEHFPNPGSIELDRPNPTSHLAFGRGQHFCPGSALGRRHAQIGIEALLKK
MPGVDLAVPIDQLVWRTRFQRRIPERLPVLW
>CYP121A1_Mycobacterium tuberculosis PanR0407 2555548605
MTATVLLEVPFSARGDRIPDAVAELRTREPIRKVRTITGAEAWLVSSYALCTQVLEDRRFSMKETAAAGAPRL
NALTVPPEVVNNMGNIADAGLRKAVMKAITPKAPGLEQFLRDTANSLLDNLITEGAPADLRNDFADPLATALH
CKVLGIPQEDGPKLFRSLSIAFMSSADPIPAAKINWDRDIEYMAGILENPNITTGLMGELSRLRKDPAYSHVS
DELFATIGVTFFGAGVISTGSFLTTALISLIQRPQLRNLLHEKPELIPAGVEELLRINLSFADGLPRLATADI
QVGDVLVRKGELVLVLLEGANFDPEHFPNPGSIELDRPNPTSHLAFGRGQHFCPGSALGRRHAQIGIEALLKK
MPGVDLAVPIDQLVWRTRFQRRIPERLPVLW
>CYP121A1_Mycobacterium tuberculosis PanR0409 2555587859
MTATVLLEVPFSARGDRIPDAVAELRTREPIRKVRTITGAEAWLVSSYALCTQVLEDRRFSMKETAAAGAPRL
NALTVPPEVVNNMGNIADAGLRKAVMKAITPKAPGLEQFLRDTANSLLDNLITEGAPADLRNDFADPLATALH
CKVLGIPQEDGPKLFRSLSIAFMSSADPIPAAKINWDRDIEYMAGILENPNITTGLMGELSRLRKDPAYSHVS
DELFATIGVTFFGAGVISTGSFLTTALISLIQRPQLRNLLHEKPELIPAGVEELLRINLSFADGLPRLATADI
QVGDVLVRKGELVLVLLEGANFDPEHFPNPGSIELDRPNPTSHLAFGRGQHFCPGSALGRRHAQIGIEALLKK
MPGVDLAVPIDQLVWRTRFQRRIPERLPVLW

>CYP121A1_Mycobacterium tuberculosis PanR0410 2555583684
MTATVLLEVPFSARGDRIPDAVAELRTREPIRKVRTITGAEAWLVSSYALCTQVLEDRRFSMKETAAAGAPRL
NALTVPPEVVNNMGNIADAGLRKAVMKAITPKAPGLEQFLRDTANSLLDNLITEGAPADLRNDFADPLATALH
CKVLGIPQEDGPKLFRSLSIAFMSSADPIPAAKINWDRDIEYMAGILENPNITTGLMGELSRLRKDPAYSHVS
DELFATIGVTFFGAGVISTGSFLTTALISLIQRPQLRNLLHEKPELIPAGVEELLRINLSFADGLPRLATADI
QVGDVLVRKGELVLVLLEGANFDPEHFPNPGSIELDRPNPTSHLAFGRGQHFCPGSALGRRHAQIGIEALLKK
MPGVDLAVPIDQLVWRTRFQRRIPERLPVLW
>CYP121A1_Mycobacterium tuberculosis PanR0411 2555520836
MTATVLLEVPFSARGDRIPDAVAELRTREPIRKVRTITGAEAWLVSSYALCTQVLEDRRFSMKETAAAGAPRL
NALTVPPEVVNNMGNIADAGLRKAVMKAITPKAPGLEQFLRDTANSLLDNLITEGAPADLRNDFADPLATALH
CKVLGIPQEDGPKLFRSLSIAFMSSADPIPAAKINWDRDIEYMAGILENPNITTGLMGELSRLRKDPAYSHVS
DELFATIGVTFFGAGVISTGSFLTTALISLIQRPQLRNLLHEKPELIPAGVEELLRINLSFADGLPRLATADI
QVGDVLVRKGELVLVLLEGANFDPEHFPNPGSIELDRPNPTSHLAFGRGQHFCPGSALGRRHAQIGIEALLKK
MPGVDLAVPIDQLVWRTRFQRRIPERLPVLW
>CYP121A1_Mycobacterium tuberculosis PanR0412 2555592053
MTATVLLEVPFSARGDRIPDAVAELRTREPIRKVRTITGAEAWLVSSYALCTQVLEDRRFSMKETAAAGAPRL
NALTVPPEVVNNMGNIADAGLRKAVMKAITPKAPGLEQFLRDTANSLLDNLITEGAPADLRNDFADPLATALH
CKVLGIPQEDGPKLFRSLSIAFMSSADPIPAAKINWDRDIEYMAGILENPNITTGLMGELSRLRKDPAYSHVS
DELFATIGVTFFGAGVISTGSFLTTALISLIQRPQLRNLLHEKPELIPAGVEELLRINLSFADGLPRLATADI
QVGDVLVRKGELVLVLLEGANFDPEHFPNPGSIELDRPNPTSHLAFGRGQHFCPGSALGRRHAQIGIEALLKK
MPGVDLAVPIDQLVWRTRFQRRIPERLPVLW
>CYP121A1_Mycobacterium tuberculosis PanR0501 2555334195
MTATVLLEVPFSARGDRIPDAVAELRTREPIRKVRTITGAEAWLVSSYALCTQVLEDRRFSMKETAAAGAPRL
NALTVPPEVVNNMGNIADAGLRKAVMKAITPKAPGLEQFLRDTANSLLDNLITEGAPADLRNDFADPLATALH
CKVLGIPQEDGPKLFRSLSIAFMSSADPIPAAKINWDRDIEYMAGILENPNITTGLMGELSRLRKDPAYSHVS
DELFATIGVTFFGAGVISTGSFLTTALISLIQRPQLRNLLHEKPELIPAGVEELLRINLSFADGLPRLATADI
QVGDVLVRKGELVLVLLEGANFDPEHFPNPGSIELDRPNPTSHLAFGRGQHFCPGSALGRRHAQIGIEALLKK
MPGVDLAVPIDQLVWRTRFQRRIPERLPVLW
>CYP121A1_Mycobacterium tuberculosis PanR0503 2555350943
MTATVLLEVPFSARGDRIPDAVAELRTREPIRKVRTITGAEAWLVSSYALCTQVLEDRRFSMKETAAAGAPRL
NALTVPPEVVNNMGNIADAGLRKAVMKAITPKAPGLEQFLRDTANSLLDNLITEGAPADLRNDFADPLATALH
CKVLGIPQEDGPKLFRSLSIAFMSSADPIPAAKINWDRDIEYMAGILENPNITTGLMGELSRLRKDPAYSHVS
DELFATIGVTFFGAGVISTGSFLTTALISLIQRPQLRNLLHEKPELIPAGVEELLRINLSFADGLPRLATADI
QVGDVLVRKGELVLVLLEGANFDPEHFPNPGSIELDRPNPTSHLAFGRGQHFCPGSALGRRHAQIGIEALLKK
MPGVDLAVPIDQLVWRTRFQRRIPERLPVLW
>CYP121A1_Mycobacterium tuberculosis PanR0505 2555355173
MTATVLLEVPFSARGDRIPDAVAELRTREPIRKVRTITGAEAWLVSSYALCTQVLEDRRFSMKETAAAGAPRL
NALTVPPEVVNNMGNIADAGLRKAVMKAITPKAPGLEQFLRDTANSLLDNLITEGAPADLRNDFADPLATALH
CKVLGIPQEDGPKLFRSLSIAFMSSADPIPAAKINWDRDIEYMAGILENPNITTGLMGELSRLRKDPAYSHVS
DELFATIGVTFFGAGVISTGSFLTTALISLIQRPQLRNLLHEKPELIPAGVEELLRINLSFADGLPRLATADI
QVGDVLVRKGELVLVLLEGANFDPEHFPNPGSIELDRPNPTSHLAFGRGQHFCPGSALGRRHAQIGIEALLKK
MPGVDLAVPIDQLVWRTRFQRRIPERLPVLW
>CYP121A1_Mycobacterium tuberculosis PanR0601 2555321379
MTATVLLEVPFSARGDRIPDAVAELRTREPIRKVRTITGAEAWLVSSYALCTQVLEDRRFSMKETAAAGAPRL
NALTVPPEVVNNMGNIADAGLRKAVMKAITPKAPGLEQFLRDTANSLLDNLITEGAPADLRNDFADPLATALH
CKVLGIPQEDGPKLFRSLSIAFMSSADPIPAAKINWDRDIEYMAGILENPNITTGLMGELSRLRKDPAYSHVS
DELFATIGVTFFGAGVISTGSFLTTALISLIQRPQLRNLLHEKPELIPAGVEELLRINLSFADGLPRLATADI
QVGDVLVRKGELVLVLLEGANFDPEHFPNPGSIELDRPNPTSHLAFGRGQHFCPGSALGRRHAQIGIEALLKK
MPGVDLAVPIDQLVWRTRFQRRIPERLPVLW
>CYP121A1_Mycobacterium tuberculosis PanR0602 2555338405
MTATVLLEVPFSARGDRIPDAVAELRTREPIRKVRTITGAEAWLVSSYALCTQVLEDRRFSMKETAAAGAPRL
NALTVPPEVVNNMGNIADAGLRKAVMKAITPKAPGLEQFLRDTANSLLDNLITEGAPADLRNDFADPLATALH
CKVLGIPQEDGPKLFRSLSIAFMSSADPIPAAKINWDRDIEYMAGILENPNITTGLMGELSRLRKDPAYSHVS
DELFATIGVTFFGAGVISTGSFLTTALISLIQRPQLRNLLHEKPELIPAGVEELLRINLSFADGLPRLATADI
QVGDVLVRKGELVLVLLEGANFDPEHFPNPGSIELDRPNPTSHLAFGRGQHFCPGSALGRRHAQIGIEALLKK
MPGVDLAVPIDQLVWRTRFQRRIPERLPVLW
>CYP121A1_Mycobacterium tuberculosis PanR0603 2555316883

MTATVLLEVPFSARGDRIPDAVAELRTREPIRKVRTITGAEAWLVSSYALCTQVLEDRRFSMKETAAAGAPRL
NALTVPPEVVNNMGNIADAGLRKAVMKAITPKAPGLEQFLRDTANSLLDNLITEGAPADLRNDFADPLATALH
CKVLGIPQEDGPKLFRSLSIAFMSSADPIPAAKINWDRDIEYMAGILENPNITTGLMGELSRLRKDPAYSHVS
DELFATIGVTFFGAGVISTGSFLTTALISLIQRPQLRNLLHEKPELIPAGVEELLRINLSFADGLPRLATADI
QVGDVLVRKGELVLVLLEGANFDPEHFPNPGSIELDRPNPTSHLAFGRGQHFCPGSALGRRHAQIGIEALLKK
MPGVDLAVPIDQLVWRTRFQRRIPERLPVLW
>CYP121A1_Mycobacterium tuberculosis PanR0604 2555325787
MTATVLLEVPFSARGDRIPDAVAELRTREPIRKVRTITGAEAWLVSSYALCTQVLEDRRFSMKETAAAGAPRL
NALTVPPEVVNNMGNIADAGLRKAVMKAITPKAPGLEQFLRDTANSLLDNLITEGAPADLRNDFADPLATALH
CKVLGIPQEDGPKLFRSLSIAFMSSADPIPAAKINWDRDIEYMAGILENPNITTGLMGELSRLRKDPAYSHVS
DELFATIGVTFFGAGVISTGSFLTTALISLIQRPQLRNLLHEKPELIPAGVEELLRINLSFADGLPRLATADI
QVGDVLVRKGELVLVLLEGANFDPEHFPNPGSIELDRPNPTSHLAFGRGQHFCPGSALGRRHAQIGIEALLKK
MPGVDLAVPIDQLVWRTRFQRRIPERLPVLW
>CYP121A1_Mycobacterium tuberculosis PanR0607 2555409833
MTATVLLEVPFSARGDRIPDAVAELRTREPIRKVRTITGAEAWLVSSYALCTQVLEDRRFSMKETAAAGAPRL
NALTVPPEVVNNMGNIADAGLRKAVMKAITPKAPGLEQFLRDTANSLLDNLITEGAPADLRNDFADPLATALH
CKVLGIPQEDGPKLFRSLSIAFMSSADPIPAAKINWDRDIEYMAGILENPNITTGLMGELSRLRKDPAYSHVS
DELFATIGVTFFGAGVISTGSFLTTALISLIQRPQLRNLLHEKPELIPAGVEELLRINLSFADGLPRLATADI
QVGDVLVRKGELVLVLLEGANFDPEHFPNPGSIELDRPNPTSHLAFGRGQHFCPGSALGRRHAQIGIEALLKK
MPGVDLAVPIDQLVWRTRFQRRIPERLPVLW
>CYP121A1_Mycobacterium tuberculosis PanR0609 2555405656
MTATVLLEVPFSARGDRIPDAVAELRTREPIRKVRTITGAEAWLVSSYALCTQVLEDRRFSMKETAAAGAPRL
NALTVPPEVVNNMGNIADAGLRKAVMKAITPKAPGLEQFLRDTANSLLDNLITEGAPADLRNDFADPLATALH
CKVLGIPQEDGPKLFRSLSIAFMSSADPIPAAKINWDRDIEYMAGILENPNITTGLMGELSRLRKDPAYSHVS
DELFATIGVTFFGAGVISTGSFLTTALISLIQRPQLRNLLHEKPELIPAGVEELLRINLSFADGLPRLATADI
QVGDVLVRKGELVLVLLEGANFDPEHFPNPGSIELDRPNPTSHLAFGRGQHFCPGSALGRRHAQIGIEALLKK
MPGVDLAVPIDQLVWRTRFQRRIPERLPVLW
>CYP121A1_Mycobacterium tuberculosis PanR0610 2555397239
MTATVLLEVPFSARGDRIPDAVAELRTREPIRKVRTITGAEAWLVSSYALCTQVLEDRRFSMKETAAAGAPRL
NALTVPPEVVNNMGNIADAGLRKAVMKAITPKAPGLEQFLRDTANSLLDNLITEGAPADLRNDFADPLATALH
CKVLGIPQEDGPKLFRSLSIAFMSSADPIPAAKINWDRDIEYMAGILENPNITTGLMGELSRLRKDPAYSHVS
DELFATIGVTFFGAGVISTGSFLTTALISLIQRPQLRNLLHEKPELIPAGVEELLRINLSFADGLPRLATADI
QVGDVLVRKGELVLVLLEGANFDPEHFPNPGSIELDRPNPTSHLAFGRGQHFCPGSALGRRHAQIGIEALLKK
MPGVDLAVPIDQLVWRTRFQRRIPERLPVLW
>CYP121A1_Mycobacterium tuberculosis PanR0611 2555414045
MTATVLLEVPFSARGDRIPDAVAELRTREPIRKVRTITGAEAWLVSSYALCTQVLEDRRFSMKETAAAGAPRL
NALTVPPEVVNNMGNIADAGLRKAVMKAITPKAPGLEQFLRDTANSLLDNLITEGAPADLRNDFADPLATALH
CKVLGIPQEDGPKLFRSLSIAFMSSADPIPAAKINWDRDIEYMAGILENPNITTGLMGELSRLRKDPAYSHVS
DELFATIGVTFFGAGVISTGSFLTTALISLIQRPQLRNLLHEKPELIPAGVEELLRINLSFADGLPRLATADI
QVGDVLVRKGELVLVLLEGANFDPEHFPNPGSIELDRPNPTSHLAFGRGQHFCPGSALGRRHAQIGIEALLKK
MPGVDLAVPIDQLVWRTRFQRRIPERLPVLW
>CYP121A1_Mycobacterium tuberculosis PanR0702 2555418279
MTATVLLEVPFSARGDRIPDAVAELRTREPIRKVRTITGAEAWLVSSYALCTQVLEDRRFSMKETAAAGAPRL
NALTVPPEVVNNMGNIADAGLRKAVMKAITPKAPGLEQFLRDTANSLLDNLITEGAPADLRNDFADPLATALH
CKVLGIPQEDGPKLFRSLSIAFMSSADPIPAAKINWDRDIEYMAGILENPNITTGLMGELSRLRKDPAYSHVS
DELFATIGVTFFGAGVISTGSFLTTALISLIQRPQLRNLLHEKPELIPAGVEELLRINLSFADGLPRLATADI
QVGDVLVRKGELVLVLLEGANFDPEHFPNPGSIELDRPNPTSHLAFGRGQHFCPGSALGRRHAQIGIEALLKK
MPGVDLAVPIDQLVWRTRFQRRIPERLPVLW
>CYP121A1_Mycobacterium tuberculosis PanR0704 2555529198
MTATVLLEVPFSARGDRIPDAVAELRTREPIRKVRTITGAEAWLVSSYALCTQVLEDRRFSMKETAAAGAPRL
NALTVPPEVVNNMGNIADAGLRKAVMKAITPKAPGLEQFLRDTANSLLDNLITEGAPADLRNDFADPLATALH
CKVLGIPQEDGPKLFRSLSIAFMSSADPIPAAKINWDRDIEYMAGILENPNITTGLMGELSRLRKDPAYSHVS
DELFATIGVTFFGAGVISTGSFLTTALISLIQRPQLRNLLHEKPELIPAGVEELLRINLSFADGLPRLATADI
QVGDVLVRKGELVLVLLEGANFDPEHFPNPGSIELDRPNPTSHLAFGRGQHFCPGSALGRRHAQIGIEALLKK
MPGVDLAVPIDQLVWRTRFQRRIPERLPVLW
>CYP121A1_Mycobacterium tuberculosis PanR0707 2555388802
MTATVLLEVPFSARGDRIPDAVAELRTREPIRKVRTITGAEAWLVSSYALCTQVLEDRRFSMKETAAAGAPRL
NALTVPPEVVNNMGNIADAGLRKAVMKAITPKAPGLEQFLRDTANSLLDNLITEGAPADLRNDFADPLATALH

CKVLGIPQEDGPKLFRSLSIAFMSSADPIPAAKINWDRDIEYMAGILENPNITTGLMGELSRLRKDPAYSHVS
DELFATIGVTFFGAGVISTGSFLTTALISLIQRPQLRNLLHEKPELIPAGVEELLRINLSFADGLPRLATADI
QVGDVLVRKGELVLVLLEGANFDPEHFPNPGSIELDRPNPTSHLAFGRGQHFCPGSALGRRHAQIGIEALLKK
MPGVDLAVPIDQLVWRTRFQRRIPERLPVLW
>CYP121A1_Mycobacterium tuberculosis PanR0708 2555372005
MTATVLLEVPFSARGDRIPDAVAELRTREPIRKVRTITGAEAWLVSSYALCTQVLEDRRFSMKETAAAGAPRL
NALTVPPEVVNNMGNIADAGLRKAVMKAITPKAPGLEQFLRDTANSLLDNLITEGAPADLRNDFADPLATALH
CKVLGIPQEDGPKLFRSLSIAFMSSADPIPAAKINWDRDIEYMAGILENPNITTGLMGELSRLRKDPAYSHVS
DELFATIGVTFFGAGVISTGSFLTTALISLIQRPQLRNLLHEKPELIPAGVEELLRINLSFADGLPRLATADI
QVGDVLVRKGELVLVLLEGANFDPEHFPNPGSIELDRPNPTSHLAFGRGQHFCPGSALGRRHAQIGIEALLKK
MPGVDLAVPIDQLVWRTRFQRRIPERLPVLW
>CYP121A1_Mycobacterium tuberculosis PanR0802 2555525022
MTATVLLEVPFSARGDRIPDAVAELRTREPIRKVRTITGAEAWLVSSYALCTQVLEDRRFSMKETAAAGAPRL
NALTVPPEVVNNMGNIADAGLRKAVMKAITPKAPGLEQFLRDTANSLLDNLITEGAPADLRNDFADPLATALH
CKVLGIPQEDGPKLFRSLSIAFMSSADPIPAAKINWDRDIEYMAGILENPNITTGLMGELSRLRKDPAYSHVS
DELFATIGVTFFGAGVISTGSFLTTALISLIQRPQLRNLLHEKPELIPAGVEELLRINLSFADGLPRLATADI
QVGDVLVRKGELVLVLLEGANFDPEHFPNPGSIELDRPNPTSHLAFGRGQHFCPGSALGRRHAQIGIEALLKK
MPGVDLAVPIDQLVWRTRFQRRIPERLPVLW
>CYP121A1_Mycobacterium tuberculosis PanR0803 2555367848
MTATVLLEVPFSARGDRIPDAVAELRTREPIRKVRTITGAEAWLVSSYALCTQVLEDRRFSMKETAAAGAPRL
NALTVPPEVVNNMGNIADAGLRKAVMKAITPKAPGLEQFLRDTANSLLDNLITEGAPADLRNDFADPLATALH
CKVLGIPQEDGPKLFRSLSIAFMSSADPIPAAKINWDRDIEYMAGILENPNITTGLMGELSRLRKDPAYSHVS
DELFATIGVTFFGAGVISTGSFLTTALISLIQRPQLRNLLHEKPELIPAGVEELLRINLSFADGLPRLATADI
QVGDVLVRKGELVLVLLEGANFDPEHFPNPGSIELDRPNPTSHLAFGRGQHFCPGSALGRRHAQIGIEALLKK
MPGVDLAVPIDQLVWRTRFQRRIPERLPVLW
>CYP121A1_Mycobacterium tuberculosis PanR0804 2555393032
MTATVLLEVPFSARGDRIPDAVAELRTREPIRKVRTITGAEAWLVSSYALCTQVLEDRRFSMKETAAAGAPRL
NALTVPPEVVNNMGNIADAGLRKAVMKAITPKAPGLEQFLRDTANSLLDNLITEGAPADLRNDFADPLATALH
CKVLGIPQEDGPKLFRSLSIAFMSSADPIPAAKINWDRDIEYMAGILENPNITTGLMGELSRLRKDPAYSHVS
DELFATIGVTFFGAGVISTGSFLTTALISLIQRPQLRNLLHEKPELIPAGVEELLRINLSFADGLPRLATADI
QVGDVLVRKGELVLVLLEGANFDPEHFPNPGSIELDRPNPTSHLAFGRGQHFCPGSALGRRHAQIGIEALLKK
MPGVDLAVPIDQLVWRTRFQRRIPERLPVLW
>CYP121A1_Mycobacterium tuberculosis PanR0805 2555363646
MTATVLLEVPFSARGDRIPDAVAELRTREPIRKVRTITGAEAWLVSSYALCTQVLEDRRFSMKETAAAGAPRL
NALTVPPEVVNNMGNIADAGLRKAVMKAITPKAPGLEQFLRDTANSLLDNLITEGAPADLRNDFADPLATALH
CKVLGIPQEDGPKLFRSLSIAFMSSADPIPAAKINWDRDIEYMAGILENPNITTGLMGELSRLRKDPAYSHVS
DELFATIGVTFFGAGVISTGSFLTTALISLIQRPQLRNLLHEKPELIPAGVEELLRINLSFADGLPRLATADI
QVGDVLVRKGELVLVLLEGANFDPEHFPNPGSIELDRPNPTSHLAFGRGQHFCPGSALGRRHAQIGIEALLKK
MPGVDLAVPIDQLVWRTRFQRRIPERLPVLW
>CYP121A1_Mycobacterium tuberculosis PanR0903 2555376145
MTATVLLEVPFSARGDRIPDAVAELRTREPIRKVRTITGAEAWLVSSYALCTQVLEDRRFSMKETAAAGAPRL
NALTVPPEVVNNMGNIADAGLRKAVMKAITPKAPGLEQFLRDTANSLLDNLITEGAPADLRNDFADPLATALH
CKVLGIPQEDGPKLFRSLSIAFMSSADPIPAAKINWDRDIEYMAGILENPNITTGLMGELSRLRKDPAYSHVS
DELFATIGVTFFGAGVISTGSFLTTALISLIQRPQLRNLLHEKPELIPAGVEELLRINLSFADGLPRLATADI
QVGDVLVRKGELVLVLLEGANFDPEHFPNPGSIELDRPNPTSHLAFGRGQHFCPGSALGRRHAQIGIEALLKK
MPGVDLAVPIDQLVWRTRFQRRIPERLPVLW
>CYP121A1_Mycobacterium tuberculosis PanR0904 2555443303
MTATVLLEVPFSARGDRIPDAVAELRTREPIRKVRTITGAEAWLVSSYALCTQVLEDRRFSMKETAAAGAPRL
NALTVPPEVVNNMGNIADAGLRKAVMKAITPKAPGLEQFLRDTANSLLDNLITEGAPADLRNDFADPLATALH
CKVLGIPQEDGPKLFRSLSIAFMSSADPIPAAKINWDRDIEYMAGILENPNITTGLMGELSRLRKDPAYSHVS
DELFATIGVTFFGAGVISTGSFLTTALISLIQRPQLRNLLHEKPELIPAGVEELLRINLSFADGLPRLATADI
QVGDVLVRKGELVLVLLEGANFDPEHFPNPGSIELDRPNPTSHLAFGRGQHFCPGSALGRRHAQIGIEALLKK
MPGVDLAVPIDQLVWRTRFQRRIPERLPVLW
>CYP121A1_Mycobacterium tuberculosis PanR0906 2555447473
MTATVLLEVPFSARGDRIPDAVAELRTREPIRKVRTITGAEAWLVSSYALCTQVLEDRRFSMKETAAAGAPRL
NALTVPPEVVNNMGNIADAGLRKAVMKAITPKAPGLEQFLRDTANSLLDNLITEGAPADLRNDFADPLATALH
CKVLGIPQEDGPKLFRSLSIAFMSSADPIPAAKINWDRDIEYMAGILENPNITTGLMGELSRLRKDPAYSHVS
DELFATIGVTFFGAGVISTGSFLTTALISLIQRPQLRNLLHEKPELIPAGVEELLRINLSFADGLPRLATADI

QVGDVLVRKGELVLVLLEGANFDPEHFPNPGSIELDRPNPTSHLAFGRGQHFCPGSALGRRHAQIGIEALLKK
MPGVDLAVPIDQLVWRTRFQRRIPERLPVLW
>CYP121A1_Mycobacterium tuberculosis PanR0908 2555422525
MTATVLLEVPFSARGDRIPDAVAELRTREPIRKVRTITGAEAWLVSSYALCTQVLEDRRFSMKETAAAGAPRL
NALTVPPEVVNNMGNIADAGLRKAVMKAITPKAPGLEQFLRDTANSLLDNLITEGAPADLRNDFADPLATALH
CKVLGIPQEDGPKLFRSLSIAFMSSADPIPAAKINWDRDIEYMAGILENPNITTGLMGELSRLRKDPAYSHVS
DELFATIGVTFFGAGVISTGSFLTTALISLIQRPQLRNLLHEKPELIPAGVEELLRINLSFADGLPRLATADI
QVGDVLVRKGELVLVLLEGANFDPEHFPNPGSIELDRPNPTSHLAFGRGQHFCPGSALGRRHAQIGIEALLKK
MPGVDLAVPIDQLVWRTRFQRRIPERLPVLW
>CYP121A1_Mycobacterium tuberculosis PanR0909 2555430761
MTATVLLEVPFSARGDRIPDAVAELRTREPIRKVRTITGAEAWLVSSYALCTQVLEDRRFSMKETAAAGAPRL
NALTVPPEVVNNMGNIADAGLRKAVMKAITPKAPGLEQFLRDTANSLLDNLITEGAPADLRNDFADPLATALH
CKVLGIPQEDGPKLFRSLSIAFMSSADPIPAAKINWDRDIEYMAGILENPNITTGLMGELSRLRKDPAYSHVS
DELFATIGVTFFGAGVISTGSFLTTALISLIQRPQLRNLLHEKPELIPAGVEELLRINLSFADGLPRLATADI
QVGDVLVRKGELVLVLLEGANFDPEHFPNPGSIELDRPNPTSHLAFGRGQHFCPGSALGRRHAQIGIEALLKK
MPGVDLAVPIDQLVWRTRFQRRIPERLPVLW
>CYP121A1_Mycobacterium tuberculosis PanR1005 2555596218
MTATVLLEVPFSARGDRIPDAVAELRTREPIRKVRTITGAEAWLVSSYALCTQVLEDRRFSMKETAAAGAPRL
NALTVPPEVVNNMGNIADAGLRKAVMKAITPKAPGLEQFLRDTANSLLDNLITEGAPADLRNDFADPLATALH
CKVLGIPQEDGPKLFRSLSIAFMSSADPIPAAKINWDRDIEYMAGILENPNITTGLMGELSRLRKDPAYSHVS
DELFATIGVTFFGAGVISTGSFLTTALISLIQRPQLRNLLHEKPELIPAGVEELLRINLSFADGLPRLATADI
QVGDVLVRKGELVLVLLEGANFDPEHFPNPGSIELDRPNPTSHLAFGRGQHFCPGSALGRRHAQIGIEALLKK
MPGVDLAVPIDQLVWRTRFQRRIPERLPVLW
>CYP121A1_Mycobacterium tuberculosis PanR1006 2555451661
MTATVLLEVPFSARGDRIPDAVAELRTREPIRKVRTITGAEAWLVSSYALCTQVLEDRRFSMKETAAAGAPRL
NALTVPPEVVNNMGNIADAGLRKAVMKAITPKAPGLEQFLRDTANSLLDNLITEGAPADLRNDFADPLATALH
CKVLGIPQEDGPKLFRSLSIAFMSSADPIPAAKINWDRDIEYMAGILENPNITTGLMGELSRLRKDPAYSHVS
DELFATIGVTFFGAGVISTGSFLTTALISLIQRPQLRNLLHEKPELIPAGVEELLRINLSFADGLPRLATADI
QVGDVLVRKGELVLVLLEGANFDPEHFPNPGSIELDRPNPTSHLAFGRGQHFCPGSALGRRHAQIGIEALLKK
MPGVDLAVPIDQLVWRTRFQRRIPERLPVLW
>CYP121A1_Mycobacterium tuberculosis PanR1007 2555434918
MTATVLLEVPFSARGDRIPDAVAELRTREPIRKVRTITGAEAWLVSSYALCTQVLEDRRFSMKETAAAGAPRL
NALTVPPEVVNNMGNIADAGLRKAVMKAITPKAPGLEQFLRDTANSLLDNLITEGAPADLRNDFADPLATALH
CKVLGIPQEDGPKLFRSLSIAFMSSADPIPAAKINWDRDIEYMAGILENPNITTGLMGELSRLRKDPAYSHVS
DELFATIGVTFFGAGVISTGSFLTTALISLIQRPQLRNLLHEKPELIPAGVEELLRINLSFADGLPRLATADI
QVGDVLVRKGELVLVLLEGANFDPEHFPNPGSIELDRPNPTSHLAFGRGQHFCPGSALGRRHAQIGIEALLKK
MPGVDLAVPIDQLVWRTRFQRRIPERLPVLW
>CYP121A1_Mycobacterium tuberculosis PanR1101 2555439099
MTATVLLEVPFSARGDRIPDAVAELRTREPIRKVRTITGAEAWLVSSYALCTQVLEDRRFSMKETAAAGAPRL
NALTVPPEVVNNMGNIADAGLRKAVMKAITPKAPGLEQFLRDTANSLLDNLITEGAPADLRNDFADPLATALH
CKVLGIPQEDGPKLFRSLSIAFMSSADPIPAAKINWDRDIEYMAGILENPNITTGLMGELSRLRKDPAYSHVS
DELFATIGVTFFGAGVISTGSFLTTALISLIQRPQLRNLLHEKPELIPAGVEELLRINLSFADGLPRLATADI
QVGDVLVRKGELVLVLLEGANFDPEHFPNPGSIELDRPNPTSHLAFGRGQHFCPGSALGRRHAQIGIEALLKK
MPGVDLAVPIDQLVWRTRFQRRIPERLPVLW
>CYP121A1_Mycobacterium tuberculosis PR05 2554700740
MTATVLLEVPFSARGDRIPDAVAELRTREPIRKVRTITGAEAWLVSSYALCTQVLEDRRFSMKETAAAGAPRL
NALTVPPEVVNNMGNIADAGLRKAVMKAITPKAPGLEQFLRDTANSLLDNLITEGAPADLRNDFADPLATALH
CKVLGIPQEDGPKLFRSLSIAFMSSADPIPAAKINWDRDIEYMAGILENPNITTGLMGELSRLRKDPAYSHVS
DELFATIGVTFFGAGVISTGSFLTTALISLIQRPQLRNLLHEKPELIPAGVEELLRINLSFADGLPRLATADI
QVGDVLVRKGELVLVLLEGANFDPEHFPNPGSIELDRPNPTSHLAFGRGQHFCPGSALGRRHAQIGIEALLKK
MPGVDLAVPIDQLVWRTRFQRRIPERLPVLW
>CYP121A1_Mycobacterium tuberculosis R00301759 2703549670
MTATVLLEVPFSARGDRIPDAVAELRTREPIRKVRTITGAEAWLVSSYALCTQVLEDRRFSMKETAAAGAPRL
NALTVPPEVVNNMGNIADAGLRKAVMKAITPKAPGLEQFLRDTANSLLDNLITEGAPADLRNDFADPLATALH
CKVLGIPQEDGPKLFRSLSIAFMSSADPIPAAKINWDRDIEYMAGILENPNITTGLMGELSRLRKDPAYSHVS
DELFATIGVTFFGAGVISTGSFLTTALISLIQRPQLRNLLHEKPELIPAGVEELLRINLSFADGLPRLATADI
QVGDVLVRKGELVLVLLEGANFDPEHFPNPGSIELDRPNPTSHLAFGRGQHFCPGSALGRRHAQIGIEALLKK
MPGVDLAVPIDQLVWRTRFQRRIPERLPVLW

>CYP121A1_Mycobacterium tuberculosis R00401166 2703534003
MTATVLLEVPFSARGDRIPDAVAELRTREPIRKVRTITGAEAWLVSSYALCTQVLEDRRFSMKETAAAGAPRL
NALTVPPEVVNNMGNIADAGLRKAVMKAITPKAPGLEQFLRDTANSLLDNLITEGAPADLRNDFADPLATALH
CKVLGIPQEDGPKLFRSLSIAFMSSADPIPAAKINWDRDIEYMAGILENPNITTGLMGELSRLRKDPAYSHVS
DELFATIGVTFFGAGVISTGSFLTTALISLIQRPQLRNLLHEKPELIPAGVEELLRINLSFADGLPRLATADI
QVGDVLVRKGELVLVLLEGANFDPEHFPNPGSIELDRPNPTSHLAFGRGQHFCPGSALGRRHAQIGIEALLKK
MPGVDLAVPIDQLVWRTRFQRRIPERLPVLW
>CYP121A1_Mycobacterium tuberculosis R09401256 2675743364
MTATVLLEVPFSARGDRIPDAVAELRTREPIRKVRTITGAEAWLVSSYALCTQVLEDRRFSMKETAAAGAPRL
NALTVPPEVVNNMGNIADAGLRKAVMKAITPKAPGLEQFLRDTANSLLDNLITEGAPADLRNDFADPLATALH
CKVLGIPQEDGPKLFRSLSIAFMSSADPIPAAKINWDRDIEYMAGILENPNITTGLMGELSRLRKDPAYSHVS
DELFATIGVTFFGAGVISTGSFLTTALISLIQRPQLRNLLHEKPELIPAGVEELLRINLSFADGLPRLATADI
QVGDVLVRKGELVLVLLEGANFDPEHFPNPGSIELDRPNPTSHLAFGRGQHFCPGSALGRRHAQIGIEALLKK
MPGVDLAVPIDQLVWRTRFQRRIPERLPVLW
>CYP121A1_Mycobacterium tuberculosis R09401839 2696526778
MTATVLLEVPFSARGDRIPDAVAELRTREPIRKVRTITGAEAWLVSSYALCTQVLEDRRFSMKETAAAGAPRL
NALTVPPEVVNNMGNIADAGLRKAVMKAITPKAPGLEQFLRDTANSLLDNLITEGAPADLRNDFADPLATALH
CKVLGIPQEDGPKLFRSLSIAFMSSADPIPAAKINWDRDIEYMAGILENPNITTGLMGELSRLRKDPAYSHVS
DELFATIGVTFFGAGVISTGSFLTTALISLIQRPQLRNLLHEKPELIPAGVEELLRINLSFADGLPRLATADI
QVGDVLVRKGELVLVLLEGANFDPEHFPNPGSIELDRPNPTSHLAFGRGQHFCPGSALGRRHAQIGIEALLKK
MPGVDLAVPIDQLVWRTRFQRRIPERLPVLW
>CYP121A1_Mycobacterium tuberculosis R09401899 2694456577
MTATVLLEVPFSARGDRIPDAVAELRTREPIRKVRTITGAEAWLVSSYALCTQVLEDRRFSMKETAAAGAPRL
NALTVPPEVVNNMGNIADAGLRKAVMKAITPKAPGLEQFLRDTANSLLDNLITEGAPADLRNDFADPLATALH
CKVLGIPQEDGPKLFRSLSIAFMSSADPIPAAKINWDRDIEYMAGILENPNITTGLMGELSRLRKDPAYSHVS
DELFATIGVTFFGAGVISTGSFLTTALISLIQRPQLRNLLHEKPELIPAGVEELLRINLSFADGLPRLATADI
QVGDVLVRKGELVLVLLEGANFDPEHFPNPGSIELDRPNPTSHLAFGRGQHFCPGSALGRRHAQIGIEALLKK
MPGVDLAVPIDQLVWRTRFQRRIPERLPVLW
>CYP121A1_Mycobacterium tuberculosis R09402296 2694335948
MTATVLLEVPFSARGDRIPDAVAELRTREPIRKVRTITGAEAWLVSSYALCTQVLEDRRFSMKETAAAGAPRL
NALTVPPEVVNNMGNIADAGLRKAVMKAITPKAPGLEQFLRDTANSLLDNLITEGAPADLRNDFADPLATALH
CKVLGIPQEDGPKLFRSLSIAFMSSADPIPAAKINWDRDIEYMAGILENPNITTGLMGELSRLRKDPAYSHVS
DELFATIGVTFFGAGVISTGSFLTTALISLIQRPQLRNLLHEKPELIPAGVEELLRINLSFADGLPRLATADI
QVGDVLVRKGELVLVLLEGANFDPEHFPNPGSIELDRPNPTSHLAFGRGQHFCPGSALGRRHAQIGIEALLKK
MPGVDLAVPIDQLVWRTRFQRRIPERLPVLW
>CYP121A1_Mycobacterium tuberculosis R09500613 2703561916
MTATVLLEVPFSARGDRIPDAVAELRTREPIRKVRTITGAEAWLVSSYALCTQVLEDRRFSMKETAAAGAPRL
NALTVPPEVVNNMGNIADAGLRKAVMKAITPKAPGLEQFLRDTANSLLDNLITEGAPADLRNDFADPLATALH
CKVLGIPQEDGPKLFRSLSIAFMSSADPIPAAKINWDRDIEYMAGILENPNITTGLMGELSRLRKDPAYSHVS
DELFATIGVTFFGAGVISTGSFLTTALISLIQRPQLRNLLHEKPELIPAGVEELLRINLSFADGLPRLATADI
QVGDVLVRKGELVLVLLEGANFDPEHFPNPGSIELDRPNPTSHLAFGRGQHFCPGSALGRRHAQIGIEALLKK
MPGVDLAVPIDQLVWRTRFQRRIPERLPVLW
>CYP121A1_Mycobacterium tuberculosis R09500614 2692671500
MTATVLLEVPFSARGDRIPDAVAELRTREPIRKVRTITGAEAWLVSSYALCTQVLEDRRFSMKETAAAGAPRL
NALTVPPEVVNNMGNIADAGLRKAVMKAITPKAPGLEQFLRDTANSLLDNLITEGAPADLRNDFADPLATALH
CKVLGIPQEDGPKLFRSLSIAFMSSADPIPAAKINWDRDIEYMAGILENPNITTGLMGELSRLRKDPAYSHVS
DELFATIGVTFFGAGVISTGSFLTTALISLIQRPQLRNLLHEKPELIPAGVEELLRINLSFADGLPRLATADI
QVGDVLVRKGELVLVLLEGANFDPEHFPNPGSIELDRPNPTSHLAFGRGQHFCPGSALGRRHAQIGIEALLKK
MPGVDLAVPIDQLVWRTRFQRRIPERLPVLW
>CYP121A1_Mycobacterium tuberculosis R09601315 2703554020
MTATVLLEVPFSARGDRIPDAVAELRTREPIRKVRTITGAEAWLVSSYALCTQVLEDRRFSMKETAAAGAPRL
NALTVPPEVVNNMGNIADAGLRKAVMKAITPKAPGLEQFLRDTANSLLDNLITEGAPADLRNDFADPLATALH
CKVLGIPQEDGPKLFRSLSIAFMSSADPIPAAKINWDRDIEYMAGILENPNITTGLMGELSRLRKDPAYSHVS
DELFATIGVTFFGAGVISTGSFLTTALISLIQRPQLRNLLHEKPELIPAGVEELLRINLSFADGLPRLATADI
QVGDVLVRKGELVLVLLEGANFDPEHFPNPGSIELDRPNPTSHLAFGRGQHFCPGSALGRRHAQIGIEALLKK
MPGVDLAVPIDQLVWRTRFQRRIPERLPVLW
>CYP121A1_Mycobacterium tuberculosis R09801836 2696869435

MTATVLLEVPFSARGDRIPDAVAELRTREPIRKVRTITGAEAWLVSSYALCTQVLEDRRFSMKETAAAGAPRL
NALTVPPEVVNNMGNIADAGLRKAVMKAITPKAPGLEQFLRDTANSLLDNLITEGAPADLRNDFADPLATALH
CKVLGIPQEDGPKLFRSLSIAFMSSADPIPAAKINWDRDIEYMAGILENPNITTGLMGELSRLRKDPAYSHVS
DELFATIGVTFFGAGVISTGSFLTTALISLIQRPQLRNLLHEKPELIPAGVEELLRINLSFADGLPRLATADI
QVGDVLVRKGELVLVLLEGANFDPEHFPNPGSIELDRPNPTSHLAFGRGQHFCPGSALGRRHAQIGIEALLKK
MPGVDLAVPIDQLVWRTRFQRRIPERLPVLW
>CYP121A1_Mycobacterium tuberculosis RGTB327 2512819809
MTATVLLEVPFSARGDRIPDAVAELRTREPIRKVRTITGAEAWLVSSYALCTQVLEDRRFSMKETAAAGAPRL
NALTVPPEVVNNMGNIADAGLRKAVMKAITPKAPGLEQFLRDTANSLLDNLITEGAPADLRNDFADPLATALH
CKVLGIPQEDGPKLFRSLSIAFMSSADPIPAAKINWDRDIEYMAGILENPNITTGLMGELSRLRKDPAYSHVS
DELFATIGVTFFGAGVISTGSFLTTALISLIQRPQLRNLLHEKPELIPAGVEELLRINLSFADGLPRLATADI
QVGDVLVRKGELVLVLLEGANFDPEHFPNPGSIELDRPNPTSHLAFGRGQHFCPGSALGRRHAQIGIEALLKK
MPGVDLAVPIDQLVWRTRFQRRIPERLPVLW
>CYP121A1_Mycobacterium tuberculosis S96-129 2548029555
MTATVLLEVPFSARGDRIPDAVAELRTREPIRKVRTITGAEAWLVSSYALCTQVLEDRRFSMKETAAAGAPRL
NALTVPPEVVNNMGNIADAGLRKAVMKAITPKAPGLEQFLRDTANSLLDNLITEGAPADLRNDFADPLATALH
CKVLGIPQEDGPKLFRSLSIAFMSSADPIPAAKINWDRDIEYMAGILENPNITTGLMGELSRLRKDPAYSHVS
DELFATIGVTFFGAGVISTGSFLTTALISLIQRPQLRNLLHEKPELIPAGVEELLRINLSFADGLPRLATADI
QVGDVLVRKGELVLVLLEGANFDPEHFPNPGSIELDRPNPTSHLAFGRGQHFCPGSALGRRHAQIGIEALLKK
MPGVDLAVPIDQLVWRTRFQRRIPERLPVLW
>CYP121A1_Mycobacterium tuberculosis SH1-874 2698758703
MTATVLLEVPFSARGDRIPDAVAELRTREPIRKVRTITGAEAWLVSSYALCTQVLEDRRFSMKETAAAGAPRL
NALTVPPEVVNNMGNIADAGLRKAVMKAITPKAPGLEQFLRDTANSLLDNLITEGAPADLRNDFADPLATALH
CKVLGIPQEDGPKLFRSLSIAFMSSADPIPAAKINWDRDIEYMAGILENPNITTGLMGELSRLRKDPAYSHVS
DELFATIGVTFFGAGVISTGSFLTTALISLIQRPQLRNLLHEKPELIPAGVEELLRINLSFADGLPRLATADI
QVGDVLVRKGELVLVLLEGANFDPEHFPNPGSIELDRPNPTSHLAFGRGQHFCPGSALGRRHAQIGIEALLKK
MPGVDLAVPIDQLVWRTRFQRRIPERLPVLW
>CYP121A1_Mycobacterium tuberculosis SH5-875 2697436816
MTATVLLEVPFSARGDRIPDAVAELRTREPIRKVRTITGAEAWLVSSYALCTQVLEDRRFSMKETAAAGAPRL
NALTVPPEVVNNMGNIADAGLRKAVMKAITPKAPGLEQFLRDTANSLLDNLITEGAPADLRNDFADPLATALH
CKVLGIPQEDGPKLFRSLSIAFMSSADPIPAAKINWDRDIEYMAGILENPNITTGLMGELSRLRKDPAYSHVS
DELFATIGVTFFGAGVISTGSFLTTALISLIQRPQLRNLLHEKPELIPAGVEELLRINLSFADGLPRLATADI
QVGDVLVRKGELVLVLLEGANFDPEHFPNPGSIELDRPNPTSHLAFGRGQHFCPGSALGRRHAQIGIEALLKK
MPGVDLAVPIDQLVWRTRFQRRIPERLPVLW
>CYP121A1_Mycobacterium tuberculosis SIT745/EAI1-MYS 2730909408
MTATVLLEVPFSARGDRIPDAVAELRTREPIRKVRTITGAEAWLVSSYALCTQVLEDRRFSMKETAAAGAPRL
NALTVPPEVVNNMGNIADAGLRKAVMKAITPKAPGLEQFLRDTANSLLDNLITEGAPADLRNDFADPLATALH
CKVLGIPQEDGPKLFRSLSIAFMSSADPIPAAKINWDRDIEYMAGILENPNITTGLMGELSRLRKDPAYSHVS
DELFATIGVTFFGAGVISTGSFLTTALISLIQRPQLRNLLHEKPELIPAGVEELLRINLSFADGLPRLATADI
QVGDVLVRKGELVLVLLEGANFDPEHFPNPGSIELDRPNPTSHLAFGRGQHFCPGSALGRRHAQIGIEALLKK
MPGVDLAVPIDQLVWRTRFQRRIPERLPVLW
>CYP121A1_Mycobacterium tuberculosis str. Kurono 2648144657
MTATVLLEVPFSARGDRIPDAVAELRTREPIRKVRTITGAEAWLVSSYALCTQVLEDRRFSMKETAAAGAPRL
NALTVPPEVVNNMGNIADAGLRKAVMKAITPKAPGLEQFLRDTANSLLDNLITEGAPADLRNDFADPLATALH
CKVLGIPQEDGPKLFRSLSIAFMSSADPIPAAKINWDRDIEYMAGILENPNITTGLMGELSRLRKDPAYSHVS
DELFATIGVTFFGAGVISTGSFLTTALISLIQRPQLRNLLHEKPELIPAGVEELLRINLSFADGLPRLATADI
QVGDVLVRKGELVLVLLEGANFDPEHFPNPGSIELDRPNPTSHLAFGRGQHFCPGSALGRRHAQIGIEALLKK
MPGVDLAVPIDQLVWRTRFQRRIPERLPVLW
>CYP121A1_Mycobacterium tuberculosis SUMu001 cont1.107 648443888
MTATVLLEVPFSARGDRIPDAVAELRTREPIRKVRTITGAEAWLVSSYALCTQVLEDRRFSMKETAAAGAPRL
NALTVPPEVVNNMGNIADAGLRKAVMKAITPKAPGLEQFLRDTANSLLDNLITEGAPADLRNDFADPLATALH
CKVLGIPQEDGPKLFRSLSIAFMSSADPIPAAKINWDRDIEYMAGILENPNITTGLMGELSRLRKDPAYSHVS
DELFATIGVTFFGAGVISTGSFLTTALISLIQRPQLRNLLHEKPELIPAGVEELLRINLSFADGLPRLATADI
QVGDVLVRKGELVLVLLEGANFDPEHFPNPGSIELDRPNPTSHLAFGRGQHFCPGSALGRRHAQIGIEALLKK
MPGVDLAVPIDQLVWRTRFQRRIPERLPVLW
>CYP121A1_Mycobacterium tuberculosis SUMu002 NZ_ADHR01000093 648447569
MTATVLLEVPFSARGDRIPDAVAELRTREPIRKVRTITGAEAWLVSSYALCTQVLEDRRFSMKETAAAGAPRL
NALTVPPEVVNNMGNIADAGLRKAVMKAITPKAPGLEQFLRDTANSLLDNLITEGAPADLRNDFADPLATALH

CKVLGIPQEDGPKLFRSLSIAFMSSADPIPAAKINWDRDIEYMAGILENPNITTGLMGELSRLRKDPAYSHVS
DELFATIGVTFFGAGVISTGSFLTTALISLIQRPQLRNLLHEKPELIPAGVEELLRINLSFADGLPRLATADI
QVGDVLVRKGELVLVLLEGANFDPEHFPNPGSIELDRPNPTSHLAFGRGQHFCPGSALGRRHAQIGIEALLKK
MPGVDLAVPIDQLVWRTRFQRRIPERLPVLW
>CYP121A1_Mycobacterium tuberculosis SUMu003 NZ_ADHS01000102 648452355
MTATVLLEVPFSARGDRIPDAVAELRTREPIRKVRTITGAEAWLVSSYALCTQVLEDRRFSMKETAAAGAPRL
NALTVPPEVVNNMGNIADAGLRKAVMKAITPKAPGLEQFLRDTANSLLDNLITEGAPADLRNDFADPLATALH
CKVLGIPQEDGPKLFRSLSIAFMSSADPIPAAKINWDRDIEYMAGILENPNITTGLMGELSRLRKDPAYSHVS
DELFATIGVTFFGAGVISTGSFLTTALISLIQRPQLRNLLHEKPELIPAGVEELLRINLSFADGLPRLATADI
QVGDVLVRKGELVLVLLEGANFDPEHFPNPGSIELDRPNPTSHLAFGRGQHFCPGSALGRRHAQIGIEALLKK
MPGVDLAVPIDQLVWRTRFQRRIPERLPVLW
>CYP121A1_Mycobacterium tuberculosis SUMu004 NZ_ADHT01000111 648456769
MTATVLLEVPFSARGDRIPDAVAELRTREPIRKVRTITGAEAWLVSSYALCTQVLEDRRFSMKETAAAGAPRL
NALTVPPEVVNNMGNIADAGLRKAVMKAITPKAPGLEQFLRDTANSLLDNLITEGAPADLRNDFADPLATALH
CKVLGIPQEDGPKLFRSLSIAFMSSADPIPAAKINWDRDIEYMAGILENPNITTGLMGELSRLRKDPAYSHVS
DELFATIGVTFFGAGVISTGSFLTTALISLIQRPQLRNLLHEKPELIPAGVEELLRINLSFADGLPRLATADI
QVGDVLVRKGELVLVLLEGANFDPEHFPNPGSIELDRPNPTSHLAFGRGQHFCPGSALGRRHAQIGIEALLKK
MPGVDLAVPIDQLVWRTRFQRRIPERLPVLW
>CYP121A1_Mycobacterium tuberculosis SUMu005 NZ_ADHU01000112 648461119
MTATVLLEVPFSARGDRIPDAVAELRTREPIRKVRTITGAEAWLVSSYALCTQVLEDRRFSMKETAAAGAPRL
NALTVPPEVVNNMGNIADAGLRKAVMKAITPKAPGLEQFLRDTANSLLDNLITEGAPADLRNDFADPLATALH
CKVLGIPQEDGPKLFRSLSIAFMSSADPIPAAKINWDRDIEYMAGILENPNITTGLMGELSRLRKDPAYSHVS
DELFATIGVTFFGAGVISTGSFLTTALISLIQRPQLRNLLHEKPELIPAGVEELLRINLSFADGLPRLATADI
QVGDVLVRKGELVLVLLEGANFDPEHFPNPGSIELDRPNPTSHLAFGRGQHFCPGSALGRRHAQIGIEALLKK
MPGVDLAVPIDQLVWRTRFQRRIPERLPVLW
>CYP121A1_Mycobacterium tuberculosis SUMu006 NZ_ADHV01000118 648465565
MTATVLLEVPFSARGDRIPDAVAELRTREPIRKVRTITGAEAWLVSSYALCTQVLEDRRFSMKETAAAGAPRL
NALTVPPEVVNNMGNIADAGLRKAVMKAITPKAPGLEQFLRDTANSLLDNLITEGAPADLRNDFADPLATALH
CKVLGIPQEDGPKLFRSLSIAFMSSADPIPAAKINWDRDIEYMAGILENPNITTGLMGELSRLRKDPAYSHVS
DELFATIGVTFFGAGVISTGSFLTTALISLIQRPQLRNLLHEKPELIPAGVEELLRINLSFADGLPRLATADI
QVGDVLVRKGELVLVLLEGANFDPEHFPNPGSIELDRPNPTSHLAFGRGQHFCPGSALGRRHAQIGIEALLKK
MPGVDLAVPIDQLVWRTRFQRRIPERLPVLW
>CYP121A1_Mycobacterium tuberculosis SUMu007 NZ_ADHW01000120 648470224
MTATVLLEVPFSARGDRIPDAVAELRTREPIRKVRTITGAEAWLVSSYALCTQVLEDRRFSMKETAAAGAPRL
NALTVPPEVVNNMGNIADAGLRKAVMKAITPKAPGLEQFLRDTANSLLDNLITEGAPADLRNDFADPLATALH
CKVLGIPQEDGPKLFRSLSIAFMSSADPIPAAKINWDRDIEYMAGILENPNITTGLMGELSRLRKDPAYSHVS
DELFATIGVTFFGAGVISTGSFLTTALISLIQRPQLRNLLHEKPELIPAGVEELLRINLSFADGLPRLATADI
QVGDVLVRKGELVLVLLEGANFDPEHFPNPGSIELDRPNPTSHLAFGRGQHFCPGSALGRRHAQIGIEALLKK
MPGVDLAVPIDQLVWRTRFQRRIPERLPVLW
>CYP121A1_Mycobacterium tuberculosis SUMu008 NZ_ADHX01000132 648474087
MTATVLLEVPFSARGDRIPDAVAELRTREPIRKVRTITGAEAWLVSSYALCTQVLEDRRFSMKETAAAGAPRL
NALTVPPEVVNNMGNIADAGLRKAVMKAITPKAPGLEQFLRDTANSLLDNLITEGAPADLRNDFADPLATALH
CKVLGIPQEDGPKLFRSLSIAFMSSADPIPAAKINWDRDIEYMAGILENPNITTGLMGELSRLRKDPAYSHVS
DELFATIGVTFFGAGVISTGSFLTTALISLIQRPQLRNLLHEKPELIPAGVEELLRINLSFADGLPRLATADI
QVGDVLVRKGELVLVLLEGANFDPEHFPNPGSIELDRPNPTSHLAFGRGQHFCPGSALGRRHAQIGIEALLKK
MPGVDLAVPIDQLVWRTRFQRRIPERLPVLW
>CYP121A1_Mycobacterium tuberculosis SUMu009 NZ_ADHY01000098 648477599
MTATVLLEVPFSARGDRIPDAVAELRTREPIRKVRTITGAEAWLVSSYALCTQVLEDRRFSMKETAAAGAPRL
NALTVPPEVVNNMGNIADAGLRKAVMKAITPKAPGLEQFLRDTANSLLDNLITEGAPADLRNDFADPLATALH
CKVLGIPQEDGPKLFRSLSIAFMSSADPIPAAKINWDRDIEYMAGILENPNITTGLMGELSRLRKDPAYSHVS
DELFATIGVTFFGAGVISTGSFLTTALISLIQRPQLRNLLHEKPELIPAGVEELLRINLSFADGLPRLATADI
QVGDVLVRKGELVLVLLEGANFDPEHFPNPGSIELDRPNPTSHLAFGRGQHFCPGSALGRRHAQIGIEALLKK
MPGVDLAVPIDQLVWRTRFQRRIPERLPVLW
>CYP121A1_Mycobacterium tuberculosis SUMu010 NZ_ADHZ01000101 648481874
MTATVLLEVPFSARGDRIPDAVAELRTREPIRKVRTITGAEAWLVSSYALCTQVLEDRRFSMKETAAAGAPRL
NALTVPPEVVNNMGNIADAGLRKAVMKAITPKAPGLEQFLRDTANSLLDNLITEGAPADLRNDFADPLATALH
CKVLGIPQEDGPKLFRSLSIAFMSSADPIPAAKINWDRDIEYMAGILENPNITTGLMGELSRLRKDPAYSHVS
DELFATIGVTFFGAGVISTGSFLTTALISLIQRPQLRNLLHEKPELIPAGVEELLRINLSFADGLPRLATADI

QVGDVLVRKGELVLVLLEGANFDPEHFPNPGSIELDRPNPTSHLAFGRGQHFCPGSALGRRHAQIGIEALLKK
MPGVDLAVPIDQLVWRTRFQRRIPERLPVLW
>CYP121A1_Mycobacterium tuberculosis SUMu012 NZ_ADIB01000184 648490718
MTATVLLEVPFSARGDRIPDAVAELRTREPIRKVRTITGAEAWLVSSYALCTQVLEDRRFSMKETAAAGAPRL
NALTVPPEVVNNMGNIADAGLRKAVMKAITPKAPGLEQFLRDTANSLLDNLITEGAPADLRNDFADPLATALH
CKVLGIPQEDGPKLFRSLSIAFMSSADPIPAAKINWDRDIEYMAGILENPNITTGLMGELSRLRKDPAYSHVS
DELFATIGVTFFGAGVISTGSFLTTALISLIQRPQLRNLLHEKPELIPAGVEELLRINLSFADGLPRLATADI
QVGDVLVRKGELVLVLLEGANFDPEHFPNPGSIELDRPNPTSHLAFGRGQHFCPGSALGRRHAQIGIEALLKK
MPGVDLAVPIDQLVWRTRFQRRIPERLPVLW
>CYP121A1_Mycobacterium tuberculosis T00200958 2672895734
MTATVLLEVPFSARGDRIPDAVAELRTREPIRKVRTITGAEAWLVSSYALCTQVLEDRRFSMKETAAAGAPRL
NALTVPPEVVNNMGNIADAGLRKAVMKAITPKAPGLEQFLRDTANSLLDNLITEGAPADLRNDFADPLATALH
CKVLGIPQEDGPKLFRSLSIAFMSSADPIPAAKINWDRDIEYMAGILENPNITTGLMGELSRLRKDPAYSHVS
DELFATIGVTFFGAGVISTGSFLTTALISLIQRPQLRNLLHEKPELIPAGVEELLRINLSFADGLPRLATADI
QVGDVLVRKGELVLVLLEGANFDPEHFPNPGSIELDRPNPTSHLAFGRGQHFCPGSALGRRHAQIGIEALLKK
MPGVDLAVPIDQLVWRTRFQRRIPERLPVLW
>CYP121A1_Mycobacterium tuberculosis T09601545 2703554882
MTATVLLEVPFSARGDRIPDAVAELRTREPIRKVRTITGAEAWLVSSYALCTQVLEDRRFSMKETAAAGAPRL
NALTVPPEVVNNMGNIADAGLRKAVMKAITPKAPGLEQFLRDTANSLLDNLITEGAPADLRNDFADPLATALH
CKVLGIPQEDGPKLFRSLSIAFMSSADPIPAAKINWDRDIEYMAGILENPNITTGLMGELSRLRKDPAYSHVS
DELFATIGVTFFGAGVISTGSFLTTALISLIQRPQLRNLLHEKPELIPAGVEELLRINLSFADGLPRLATADI
QVGDVLVRKGELVLVLLEGANFDPEHFPNPGSIELDRPNPTSHLAFGRGQHFCPGSALGRRHAQIGIEALLKK
MPGVDLAVPIDQLVWRTRFQRRIPERLPVLW
>CYP121A1_Mycobacterium tuberculosis T09800777 2703576844
MTATVLLEVPFSARGDRIPDAVAELRTREPIRKVRTITGAEAWLVSSYALCTQVLEDRRFSMKETAAAGAPRL
NALTVPPEVVNNMGNIADAGLRKAVMKAITPKAPGLEQFLRDTANSLLDNLITEGAPADLRNDFADPLATALH
CKVLGIPQEDGPKLFRSLSIAFMSSADPIPAAKINWDRDIEYMAGILENPNITTGLMGELSRLRKDPAYSHVS
DELFATIGVTFFGAGVISTGSFLTTALISLIQRPQLRNLLHEKPELIPAGVEELLRINLSFADGLPRLATADI
QVGDVLVRKGELVLVLLEGANFDPEHFPNPGSIELDRPNPTSHLAFGRGQHFCPGSALGRRHAQIGIEALLKK
MPGVDLAVPIDQLVWRTRFQRRIPERLPVLW
>CYP121A1_Mycobacterium tuberculosis T09800928 2703582648
MTATVLLEVPFSARGDRIPDAVAELRTREPIRKVRTITGAEAWLVSSYALCTQVLEDRRFSMKETAAAGAPRL
NALTVPPEVVNNMGNIADAGLRKAVMKAITPKAPGLEQFLRDTANSLLDNLITEGAPADLRNDFADPLATALH
CKVLGIPQEDGPKLFRSLSIAFMSSADPIPAAKINWDRDIEYMAGILENPNITTGLMGELSRLRKDPAYSHVS
DELFATIGVTFFGAGVISTGSFLTTALISLIQRPQLRNLLHEKPELIPAGVEELLRINLSFADGLPRLATADI
QVGDVLVRKGELVLVLLEGANFDPEHFPNPGSIELDRPNPTSHLAFGRGQHFCPGSALGRRHAQIGIEALLKK
MPGVDLAVPIDQLVWRTRFQRRIPERLPVLW
>CYP121A1_Mycobacterium tuberculosis T09900050 2674945314
MTATVLLEVPFSARGDRIPDAVAELRTREPIRKVRTITGAEAWLVSSYALCTQVLEDRRFSMKETAAAGAPRL
NALTVPPEVVNNMGNIADAGLRKAVMKAITPKAPGLEQFLRDTANSLLDNLITEGAPADLRNDFADPLATALH
CKVLGIPQEDGPKLFRSLSIAFMSSADPIPAAKINWDRDIEYMAGILENPNITTGLMGELSRLRKDPAYSHVS
DELFATIGVTFFGAGVISTGSFLTTALISLIQRPQLRNLLHEKPELIPAGVEELLRINLSFADGLPRLATADI
QVGDVLVRKGELVLVLLEGANFDPEHFPNPGSIELDRPNPTSHLAFGRGQHFCPGSALGRRHAQIGIEALLKK
MPGVDLAVPIDQLVWRTRFQRRIPERLPVLW
>CYP121A1_Mycobacterium tuberculosis T09900518 2674819926
MTATVLLEVPFSARGDRIPDAVAELRTREPIRKVRTITGAEAWLVSSYALCTQVLEDRRFSMKETAAAGAPRL
NALTVPPEVVNNMGNIADAGLRKAVMKAITPKAPGLEQFLRDTANSLLDNLITEGAPADLRNDFADPLATALH
CKVLGIPQEDGPKLFRSLSIAFMSSADPIPAAKINWDRDIEYMAGILENPNITTGLMGELSRLRKDPAYSHVS
DELFATIGVTFFGAGVISTGSFLTTALISLIQRPQLRNLLHEKPELIPAGVEELLRINLSFADGLPRLATADI
QVGDVLVRKGELVLVLLEGANFDPEHFPNPGSIELDRPNPTSHLAFGRGQHFCPGSALGRRHAQIGIEALLKK
MPGVDLAVPIDQLVWRTRFQRRIPERLPVLW
>CYP121A1_Mycobacterium tuberculosis T09901127 2703575412
MTATVLLEVPFSARGDRIPDAVAELRTREPIRKVRTITGAEAWLVSSYALCTQVLEDRRFSMKETAAAGAPRL
NALTVPPEVVNNMGNIADAGLRKAVMKAITPKAPGLEQFLRDTANSLLDNLITEGAPADLRNDFADPLATALH
CKVLGIPQEDGPKLFRSLSIAFMSSADPIPAAKINWDRDIEYMAGILENPNITTGLMGELSRLRKDPAYSHVS
DELFATIGVTFFGAGVISTGSFLTTALISLIQRPQLRNLLHEKPELIPAGVEELLRINLSFADGLPRLATADI
QVGDVLVRKGELVLVLLEGANFDPEHFPNPGSIELDRPNPTSHLAFGRGQHFCPGSALGRRHAQIGIEALLKK
MPGVDLAVPIDQLVWRTRFQRRIPERLPVLW

>CYP121A1_Mycobacterium tuberculosis T17 2621970327
MTATVLLEVPFSARGDRIPDAVAELRTREPIRKVRTITGAEAWLVSSYALCTQVLEDRRFSMKETAAAGAPRL
NALTVPPEVVNNMGNIADAGLRKAVMKAITPKAPGLEQFLRDTANSLLDNLITEGAPADLRNDFADPLATALH
CKVLGIPQEDGPKLFRSLSIAFMSSADPIPAAKINWDRDIEYMAGILENPNITTGLMGELSRLRKDPAYSHVS
DELFATIGVTFFGAGVISTGSFLTTALISLIQRPQLRNLLHEKPELIPAGVEELLRINLSFADGLPRLATADI
QVGDVLVRKGELVLVLLEGANFDPEHFPNPGSIELDRPNPTSHLAFGRGQHFCPGSALGRRHAQIGIEALLKK
MPGVDLAVPIDQLVWRTRFQRRIPERLPVLW
>CYP121A1_Mycobacterium tuberculosis T17 unfinished sequence 643050251
MTATVLLEVPFSARGDRIPDAVAELRTREPIRKVRTITGAEAWLVSSYALCTQVLEDRRFSMKETAAAGAPRL
NALTVPPEVVNNMGNIADAGLRKAVMKAITPKAPGLEQFLRDTANSLLDNLITEGAPADLRNDFADPLATALH
CKVLGIPQEDGPKLFRSLSIAFMSSADPIPAAKINWDRDIEYMAGILENPNITTGLMGELSRLRKDPAYSHVS
DELFATIGVTFFGAGVISTGSFLTTALISLIQRPQLRNLLHEKPELIPAGVEELLRINLSFADGLPRLATADI
QVGDVLVRKGELVLVLLEGANFDPEHFPNPGSIELDRPNPTSHLAFGRGQHFCPGSALGRRHAQIGIEALLKK
MPGVDLAVPIDQLVWRTRFQRRIPERLPVLW
>CYP121A1_Mycobacterium tuberculosis T46 2574757711
MTATVLLEVPFSARGDRIPDAVAELRTREPIRKVRTITGAEAWLVSSYALCTQVLEDRRFSMKETAAAGAPRL
NALTVPPEVVNNMGNIADAGLRKAVMKAITPKAPGLEQFLRDTANSLLDNLITEGAPADLRNDFADPLATALH
CKVLGIPQEDGPKLFRSLSIAFMSSADPIPAAKINWDRDIEYMAGILENPNITTGLMGELSRLRKDPAYSHVS
DELFATIGVTFFGAGVISTGSFLTTALISLIQRPQLRNLLHEKPELIPAGVEELLRINLSFADGLPRLATADI
QVGDVLVRKGELVLVLLEGANFDPEHFPNPGSIELDRPNPTSHLAFGRGQHFCPGSALGRRHAQIGIEALLKK
MPGVDLAVPIDQLVWRTRFQRRIPERLPVLW
>CYP121A1_Mycobacterium tuberculosis T46 646010906
MTATVLLEVPFSARGDRIPDAVAELRTREPIRKVRTITGAEAWLVSSYALCTQVLEDRRFSMKETAAAGAPRL
NALTVPPEVVNNMGNIADAGLRKAVMKAITPKAPGLEQFLRDTANSLLDNLITEGAPADLRNDFADPLATALH
CKVLGIPQEDGPKLFRSLSIAFMSSADPIPAAKINWDRDIEYMAGILENPNITTGLMGELSRLRKDPAYSHVS
DELFATIGVTFFGAGVISTGSFLTTALISLIQRPQLRNLLHEKPELIPAGVEELLRINLSFADGLPRLATADI
QVGDVLVRKGELVLVLLEGANFDPEHFPNPGSIELDRPNPTSHLAFGRGQHFCPGSALGRRHAQIGIEALLKK
MPGVDLAVPIDQLVWRTRFQRRIPERLPVLW
>CYP121A1_Mycobacterium tuberculosis T92 unfinished sequence 643028848
MTATVLLEVPFSARGDRIPDAVAELRTREPIRKVRTITGAEAWLVSSYALCTQVLEDRRFSMKETAAAGAPRL
NALTVPPEVVNNMGNIADAGLRKAVMKAITPKAPGLEQFLRDTANSLLDNLITEGAPADLRNDFADPLATALH
CKVLGIPQEDGPKLFRSLSIAFMSSADPIPAAKINWDRDIEYMAGILENPNITTGLMGELSRLRKDPAYSHVS
DELFATIGVTFFGAGVISTGSFLTTALISLIQRPQLRNLLHEKPELIPAGVEELLRINLSFADGLPRLATADI
QVGDVLVRKGELVLVLLEGANFDPEHFPNPGSIELDRPNPTSHLAFGRGQHFCPWISSRSPPRTDRHRSAVEK
DARRRPGCAHRPIGLAHPIPKTHPRTPSGALVGFRKLTRAITARFGKRWDRTISTLHRPKALPSTE
>CYP121A1_Mycobacterium tuberculosis T92 2584885168
MTATVLLEVPFSARGDRIPDAVAELRTREPIRKVRTITGAEAWLVSSYALCTQVLEDRRFSMKETAAAGAPRL
NALTVPPEVVNNMGNIADAGLRKAVMKAITPKAPGLEQFLRDTANSLLDNLITEGAPADLRNDFADPLATALH
CKVLGIPQEDGPKLFRSLSIAFMSSADPIPAAKINWDRDIEYMAGILENPNITTGLMGELSRLRKDPAYSHVS
DELFATIGVTFFGAGVISTGSFLTTALISLIQRPQLRNLLHEKPELIPAGVEELLRINLSFADGLPRLATADI
QVGDVLVRKGELVLVLLEGANFDPEHFPNPGSIELDRPNPTSHLAFGRGQHFCPGSALGRRHAQIGIEALLKK
MPGVDLAVPIDQLVWRTRFQRRIPERLPVLW
>CYP121A1_Mycobacterium tuberculosis TB_RSA07 2577873226
MTATVLLEVPFSARGDRIPDAVAELRTREPIRKVRTITGAEAWLVSSYALCTQVLEDRRFSMKETAAAGAPRL
NALTVPPEVVNNMGNIADAGLRKAVMKAITPKAPGLEQFLRDTANSLLDNLITEGAPADLRNDFADPLATALH
CKVLGIPQEDGPKLFRSLSIAFMSSADPIPAAKINWDRDIEYMAGILENPNITTGLMGELSRLRKDPAYSHVS
DELFATIGVTFFGAGVISTGSFLTTALISLIQRPQLRNLLHEKPELIPAGVEELLRINLSFADGLPRLATADI
QVGDVLVRKGELVLVLLEGANFDPEHFPNPGSIELDRPNPTSHLAFGRGQHFCPGSALGRRHAQIGIEALLKK
MPGVDLAVPIDQLVWRTRFQRRIPERLPVLW
>CYP121A1_Mycobacterium tuberculosis TB_RSA08 2603387087
MTATVLLEVPFSARGDRIPDAVAELRTREPIRKVRTITGAEAWLVSSYALCTQVLEDRRFSMKETAAAGAPRL
NALTVPPEVVNNMGNIADAGLRKAVMKAITPKAPGLEQFLRDTANSLLDNLITEGAPADLRNDFADPLATALH
CKVLGIPQEDGPKLFRSLSIAFMSSADPIPAAKINWDRDIEYMAGILENPNITTGLMGELSRLRKDPAYSHVS
DELFATIGVTFFGAGVISTGSFLTTALISLIQRPQLRNLLHEKPELIPAGVEELLRINLSFADGLPRLATADI
QVGDVLVRKGELVLVLLEGANFDPEHFPNPGSIELDRPNPTSHLAFGRGQHFCPGSALGRRHAQIGIEALLKK
MPGVDLAVPIDQLVWRTRFQRRIPERLPVLW
>CYP121A1_Mycobacterium tuberculosis TB_RSA100 2603391467

MTATVLLEVPFSARGDRIPDAVAELRTREPIRKVRTITGAEAWLVSSYALCTQVLEDRRFSMKETAAAGAPRL
NALTVPPEVVNNMGNIADAGLRKAVMKAITPKAPGLEQFLRDTANSLLDNLITEGAPADLRNDFADPLATALH
CKVLGIPQEDGPKLFRSLSIAFMSSADPIPAAKINWDRDIEYMAGILENPNITTGLMGELSRLRKDPAYSHVS
DELFATIGVTFFGAGVISTGSFLTTALISLIQRPQLRNLLHEKPELIPAGVEELLRINLSFADGLPRLATADI
QVGDVLVRKGELVLVLLEGANFDPEHFPNPGSIELDRPNPTSHLAFGRGQHFCPGSALGRRHAQIGIEALLKK
MPGVDLAVPIDQLVWRTRFQRRIPERLPVLW
>CYP121A1_Mycobacterium tuberculosis TB_RSA101 2605343644
MTATVLLEVPFSARGDRIPDAVAELRTREPIRKVRTITGAEAWLVSSYALCTQVLEDRRFSMKETAAAGAPRL
NALTVPPEVVNNMGNIADAGLRKAVMKAITPKAPGLEQFLRDTANSLLDNLITEGAPADLRNDFADPLATALH
CKVLGIPQEDGPKLFRSLSIAFMSSADPIPAAKINWDRDIEYMAGILENPNITTGLMGELSRLRKDPAYSHVS
DELFATIGVTFFGAGVISTGSFLTTALISLIQRPQLRNLLHEKPELIPAGVEELLRINLSFADGLPRLATADI
QVGDVLVRKGELVLVLLEGANFDPEHFPNPGSIELDRPNPTSHLAFGRGQHFCPGSALGRRHAQIGIEALLKK
MPGVDLAVPIDQLVWRTRFQRRIPERLPVLW
>CYP121A1_Mycobacterium tuberculosis TB_RSA102 2575863229
MTATVLLEVPFSARGDRIPDAVAELRTREPIRKVRTITGAEAWLVSSYALCTQVLEDRRFSMKETAAAGAPRL
NALTVPPEVVNNMGNIADAGLRKAVMKAITPKAPGLEQFLRDTANSLLDNLITEGAPADLRNDFADPLATALH
CKVLGIPQEDGPKLFRSLSIAFMSSADPIPAAKINWDRDIEYMAGILENPNITTGLMGELSRLRKDPAYSHVS
DELFATIGVTFFGAGVISTGSFLTTALISLIQRPQLRNLLHEKPELIPAGVEELLRINLSFADGLPRLATADI
QVGDVLVRKGELVLVLLEGANFDPEHFPNPGSIELDRPNPTSHLAFGRGQHFCPGSALGRRHAQIGIEALLKK
MPGVDLAVPIDQLVWRTRFQRRIPERLPVLW
>CYP121A1_Mycobacterium tuberculosis TB_RSA103 2605349609
MTATVLLEVPFSARGDRIPDAVAELRTREPIRKVRTITGAEAWLVSSYALCTQVLEDRRFSMKETAAAGAPRL
NALTVPPEVVNNMGNIADAGLRKAVMKAITPKAPGLEQFLRDTANSLLDNLITEGAPADLRNDFADPLATALH
CKVLGIPQEDGPKLFRSLSIAFMSSADPIPAAKINWDRDIEYMAGILENPNITTGLMGELSRLRKDPAYSHVS
DELFATIGVTFFGAGVISTGSFLTTALISLIQRPQLRNLLHEKPELIPAGVEELLRINLSFADGLPRLATADI
QVGDVLVRKGELVLVLLEGANFDPEHFPNPGSIELDRPNPTSHLAFGRGQHFCPGSALGRRHAQIGIEALLKK
MPGVDLAVPIDQLVWRTRFQRRIPERLPVLW
>CYP121A1_Mycobacterium tuberculosis TB_RSA104 2584803905
MTATVLLEVPFSARGDRIPDAVAELRTREPIRKVRTITGAEAWLVSSYALCTQVLEDRRFSMKETAAAGAPRL
NALTVPPEVVNNMGNIADAGLRKAVMKAITPKAPGLEQFLRDTANSLLDNLITEGAPADLRNDFADPLATALH
CKVLGIPQEDGPKLFRSLSIAFMSSADPIPAAKINWDRDIEYMAGILENPNITTGLMGELSRLRKDPAYSHVS
DELFATIGVTFFGAGVISTGSFLTTALISLIQRPQLRNLLHEKPELIPAGVEELLRINLSFADGLPRLATADI
QVGDVLVRKGELVLVLLEGANFDPEHFPNPGSIELDRPNPTSHLAFGRGQHFCPGSALGRRHAQIGIEALLKK
MPGVDLAVPIDQLVWRTRFQRRIPERLPVLW
>CYP121A1_Mycobacterium tuberculosis TB_RSA105 2603939216
MTATVLLEVPFSARGDRIPDAVAELRTREPIRKVRTITGAEAWLVSSYALCTQVLEDRRFSMKETAAAGAPRL
NALTVPPEVVNNMGNIADAGLRKAVMKAITPKAPGLEQFLRDTANSLLDNLITEGAPADLRNDFADPLATALH
CKVLGIPQEDGPKLFRSLSIAFMSSADPIPAAKINWDRDIEYMAGILENPNITTGLMGELSRLRKDPAYSHVS
DELFATIGVTFFGAGVISTGSFLTTALISLIQRPQLRNLLHEKPELIPAGVEELLRINLSFADGLPRLATADI
QVGDVLVRKGELVLVLLEGANFDPEHFPNPGSIELDRPNPTSHLAFGRGQHFCPGSALGRRHAQIGIEALLKK
MPGVDLAVPIDQLVWRTRFQRRIPERLPVLW
>CYP121A1_Mycobacterium tuberculosis TB_RSA106 2603943624
MTATVLLEVPFSARGDRIPDAVAELRTREPIRKVRTITGAEAWLVSSYALCTQVLEDRRFSMKETAAAGAPRL
NALTVPPEVVNNMGNIADAGLRKAVMKAITPKAPGLEQFLRDTANSLLDNLITEGAPADLRNDFADPLATALH
CKVLGIPQEDGPKLFRSLSIAFMSSADPIPAAKINWDRDIEYMAGILENPNITTGLMGELSRLRKDPAYSHVS
DELFATIGVTFFGAGVISTGSFLTTALISLIQRPQLRNLLHEKPELIPAGVEELLRINLSFADGLPRLATADI
QVGDVLVRKGELVLVLLEGANFDPEHFPNPGSIELDRPNPTSHLAFGRGQHFCPGSALGRRHAQIGIEALLKK
MPGVDLAVPIDQLVWRTRFQRRIPERLPVLW
>CYP121A1_Mycobacterium tuberculosis TB_RSA107 2584894423
MTATVLLEVPFSARGDRIPDAVAELRTREPIRKVRTITGAEAWLVSSYALCTQVLEDRRFSMKETAAAGAPRL
NALTVPPEVVNNMGNIADAGLRKAVMKAITPKAPGLEQFLRDTANSLLDNLITEGAPADLRNDFADPLATALH
CKVLGIPQEDGPKLFRSLSIAFMSSADPIPAAKINWDRDIEYMAGILENPNITTGLMGELSRLRKDPAYSHVS
DELFATIGVTFFGAGVISTGSFLTTALISLIQRPQLRNLLHEKPELIPAGVEELLRINLSFADGLPRLATADI
QVGDVLVRKGELVLVLLEGANFDPEHFPNPGSIELDRPNPTSHLAFGRGQHFCPGSALGRRHAQIGIEALLKK
MPGVDLAVPIDQLVWRTRFQRRIPERLPVLW
>CYP121A1_Mycobacterium tuberculosis TB_RSA108 2603947546
MTATVLLEVPFSARGDRIPDAVAELRTREPIRKVRTITGAEAWLVSSYALCTQVLEDRRFSMKETAAAGAPRL
NALTVPPEVVNNMGNIADAGLRKAVMKAITPKAPGLEQFLRDTANSLLDNLITEGAPADLRNDFADPLATALH

CKVLGIPQEDGPKLFRSLSIAFMSSADPIPAAKINWDRDIEYMAGILENPNITTGLMGELSRLRKDPAYSHVS
DELFATIGVTFFGAGVISTGSFLTTALISLIQRPQLRNLLHEKPELIPAGVEELLRINLSFADGLPRLATADI
QVGDVLVRKGELVLVLLEGANFDPEHFPNPGSIELDRPNPTSHLAFGRGQHFCPGSALGRRHAQIGIEALLKK
MPGVDLAVPIDQLVWRTRFQRRIPERLPVLW
>CYP121A1_Mycobacterium tuberculosis TB_RSA109 2605708893
MTATVLLEVPFSARGDRIPDAVAELRTREPIRKVRTITGAEAWLVSSYALCTQVLEDRRFSMKETAAAGAPRL
NALTVPPEVVNNMGNIADAGLRKAVMKAITPKAPGLEQFLRDTANSLLDNLITEGAPADLRNDFADPLATALH
CKVLGIPQEDGPKLFRSLSIAFMSSADPIPAAKINWDRDIEYMAGILENPNITTGLMGELSRLRKDPAYSHVS
DELFATIGVTFFGAGVISTGSFLTTALISLIQRPQLRNLLHEKPELIPAGVEELLRINLSFADGLPRLATADI
QVGDVLVRKGELVLVLLEGANFDPEHFPNPGSIELDRPNPTSHLAFGRGQHFCPGSALGRRHAQIGIEALLKK
MPGVDLAVPIDQLVWRTRFQRRIPERLPVLW
>CYP121A1_Mycobacterium tuberculosis TB_RSA110 2604291556
MTATVLLEVPFSARGDRIPDAVAELRTREPIRKVRTITGAEAWLVSSYALCTQVLEDRRFSMKETAAAGAPRL
NALTVPPEVVNNMGNIADAGLRKAVMKAITPKAPGLEQFLRDTANSLLDNLITEGAPADLRNDFADPLATALH
CKVLGIPQEDGPKLFRSLSIAFMSSADPIPAAKINWDRDIEYMAGILENPNITTGLMGELSRLRKDPAYSHVS
DELFATIGVTFFGAGVISTGSFLTTALISLIQRPQLRNLLHEKPELIPAGVEELLRINLSFADGLPRLATADI
QVGDVLVRKGELVLVLLEGANFDPEHFPNPGSIELDRPNPTSHLAFGRGQHFCPGSALGRRHAQIGIEALLKK
MPGVDLAVPIDQLVWRTRFQRRIPERLPVLW
>CYP121A1_Mycobacterium tuberculosis TB_RSA111 2574615246
MTATVLLEVPFSARGDRIPDAVAELRTREPIRKVRTITGAEAWLVSSYALCTQVLEDRRFSMKETAAAGAPRL
NALTVPPEVVNNMGNIADAGLRKAVMKAITPKAPGLEQFLRDTANSLLDNLITEGAPADLRNDFADPLATALH
CKVLGIPQEDGPKLFRSLSIAFMSSADPIPAAKINWDRDIEYMAGILENPNITTGLMGELSRLRKDPAYSHVS
DELFATIGVTFFGAGVISTGSFLTTALISLIQRPQLRNLLHEKPELIPAGVEELLRINLSFADGLPRLATADI
QVGDVLVRKGELVLVLLEGANFDPEHFPNPGSIELDRPNPTSHLAFGRGQHFCPGSALGRRHAQIGIEALLKK
MPGVDLAVPIDQLVWRTRFQRRIPERLPVLW
>CYP121A1_Mycobacterium tuberculosis TB_RSA113 2603949993
MTATVLLEVPFSARGDRIPDAVAELRTREPIRKVRTITGAEAWLVSSYALCTQVLEDRRFSMKETAAAGAPRL
NALTVPPEVVNNMGNIADAGLRKAVMKAITPKAPGLEQFLRDTANSLLDNLITEGAPADLRNDFADPLATALH
CKVLGIPQEDGPKLFRSLSIAFMSSADPIPAAKINWDRDIEYMAGILENPNITTGLMGELSRLRKDPAYSHVS
DELFATIGVTFFGAGVISTGSFLTTALISLIQRPQLRNLLHEKPELIPAGVEELLRINLSFADGLPRLATADI
QVGDVLVRKGELVLVLLEGANFDPEHFPNPGSIELDRPNPTSHLAFGRGQHFCPGSALGRRHAQIGIEALLKK
MPGVDLAVPIDQLVWRTRFQRRIPERLPVLW
>CYP121A1_Mycobacterium tuberculosis TB_RSA114 2603393406
MTATVLLEVPFSARGDRIPDAVAELRTREPIRKVRTITGAEAWLVSSYALCTQVLEDRRFSMKETAAAGAPRL
NALTVPPEVVNNMGNIADAGLRKAVMKAITPKAPGLEQFLRDTANSLLDNLITEGAPADLRNDFADPLATALH
CKVLGIPQEDGPKLFRSLSIAFMSSADPIPAAKINWDRDIEYMAGILENPNITTGLMGELSRLRKDPAYSHVS
DELFATIGVTFFGAGVISTGSFLTTALISLIQRPQLRNLLHEKPELIPAGVEELLRINLSFADGLPRLATADI
QVGDVLVRKGELVLVLLEGANFDPEHFPNPGSIELDRPNPTSHLAFGRGQHFCPGSALGRRHAQIGIEALLKK
MPGVDLAVPIDQLVWRTRFQRRIPERLPVLW
>CYP121A1_Mycobacterium tuberculosis TB_RSA121 2584706957
MTATVLLEVPFSARGDRIPDAVAELRTREPIRKVRTITGAEAWLVSSYALCTQVLEDRRFSMKETAAAGAPRL
NALTVPPEVVNNMGNIADAGLRKAVMKAITPKAPGLEQFLRDTANSLLDNLITEGAPADLRNDFADPLATALH
CKVLGIPQEDGPKLFRSLSIAFMSSADPIPAAKINWDRDIEYMAGILENPNITTGLMGELSRLRKDPAYSHVS
DELFATIGVTFFGAGVISTGSFLTTALISLIQRPQLRNLLHEKPELIPAGVEELLRINLSFADGLPRLATADI
QVGDVLVRKGELVLVLLEGANFDPEHFPNPGSIELDRPNPTSHLAFGRGQHFCPGSALGRRHAQIGIEALLKK
MPGVDLAVPIDQLVWRTRFQRRIPERLPVLW
>CYP121A1_Mycobacterium tuberculosis TB_RSA122 2604664813
MTATVLLEVPFSARGDRIPDAVAELRTREPIRKVRTITGAEAWLVSSYALCTQVLEDRRFSMKETAAAGAPRL
NALTVPPEVVNNMGNIADAGLRKAVMKAITPKAPGLEQFLRDTANSLLDNLITEGAPADLRNDFADPLATALH
CKVLGIPQEDGPKLFRSLSIAFMSSADPIPAAKINWDRDIEYMAGILENPNITTGLMGELSRLRKDPAYSHVS
DELFATIGVTFFGAGVISTGSFLTTALISLIQRPQLRNLLHEKPELIPAGVEELLRINLSFADGLPRLATADI
QVGDVLVRKGELVLVLLEGANFDPEHFPNPGSIELDRPNPTSHLAFGRGQHFCPGSALGRRHAQIGIEALLKK
MPGVDLAVPIDQLVWRTRFQRRIPERLPVLW
>CYP121A1_Mycobacterium tuberculosis TB_RSA123 2578003164
MTATVLLEVPFSARGDRIPDAVAELRTREPIRKVRTITGAEAWLVSSYALCTQVLEDRRFSMKETAAAGAPRL
NALTVPPEVVNNMGNIADAGLRKAVMKAITPKAPGLEQFLRDTANSLLDNLITEGAPADLRNDFADPLATALH
CKVLGIPQEDGPKLFRSLSIAFMSSADPIPAAKINWDRDIEYMAGILENPNITTGLMGELSRLRKDPAYSHVS
DELFATIGVTFFGAGVISTGSFLTTALISLIQRPQLRNLLHEKPELIPAGVEELLRINLSFADGLPRLATADI

QVGDVLVRKGELVLVLLEGANFDPEHFPNPGSIELDRPNPTSHLAFGRGQHFCPGSALGRRHAQIGIEALLKK
MPGVDLAVPIDQLVWRTRFQRRIPERLPVLW
>CYP121A1_Mycobacterium tuberculosis TB_RSA124 2578201051
MTATVLLEVPFSARGDRIPDAVAELRTREPIRKVRTITGAEAWLVSSYALCTQVLEDRRFSMKETAAAGAPRL
NALTVPPEVVNNMGNIADAGLRKAVMKAITPKAPGLEQFLRDTANSLLDNLITEGAPADLRNDFADPLATALH
CKVLGIPQEDGPKLFRSLSIAFMSSADPIPAAKINWDRDIEYMAGILENPNITTGLMGELSRLRKDPAYSHVS
DELFATIGVTFFGAGVISTGSFLTTALISLIQRPQLRNLLHEKPELIPAGVEELLRINLSFADGLPRLATADI
QVGDVLVRKGELVLVLLEGANFDPEHFPNPGSIELDRPNPTSHLAFGRGQHFCPGSALGRRHAQIGIEALLKK
MPGVDLAVPIDQLVWRTRFQRRIPERLPVLW
>CYP121A1_Mycobacterium tuberculosis TB_RSA125 2603243255
MTATVLLEVPFSARGDRIPDAVAELRTREPIRKVRTITGAEAWLVSSYALCTQVLEDRRFSMKETAAAGAPRL
NALTVPPEVVNNMGNIADAGLRKAVMKAITPKAPGLEQFLRDTANSLLDNLITEGAPADLRNDFADPLATALH
CKVLGIPQEDGPKLFRSLSIAFMSSADPIPAAKINWDRDIEYMAGILENPNITTGLMGELSRLRKDPAYSHVS
DELFATIGVTFFGAGVISTGSFLTTALISLIQRPQLRNLLHEKPELIPAGVEELLRINLSFADGLPRLATADI
QVGDVLVRKGELVLVLLEGANFDPEHFPNPGSIELDRPNPTSHLAFGRGQHFCPGSALGRRHAQIGIEALLKK
MPGVDLAVPIDQLVWRTRFQRRIPERLPVLW
>CYP121A1_Mycobacterium tuberculosis TB_RSA126 2605711670
MTATVLLEVPFSARGDRIPDAVAELRTREPIRKVRTITGAEAWLVSSYALCTQVLEDRRFSMKETAAAGAPRL
NALTVPPEVVNNMGNIADAGLRKAVMKAITPKAPGLEQFLRDTANSLLDNLITEGAPADLRNDFADPLATALH
CKVLGIPQEDGPKLFRSLSIAFMSSADPIPAAKINWDRDIEYMAGILENPNITTGLMGELSRLRKDPAYSHVS
DELFATIGVTFFGAGVISTGSFLTTALISLIQRPQLRNLLHEKPELIPAGVEELLRINLSFADGLPRLATADI
QVGDVLVRKGELVLVLLEGANFDPEHFPNPGSIELDRPNPTSHLAFGRGQHFCPGSALGRRHAQIGIEALLKK
MPGVDLAVPIDQLVWRTRFQRRIPERLPVLW
>CYP121A1_Mycobacterium tuberculosis TB_RSA127 2577551730
MTATVLLEVPFSARGDRIPDAVAELRTREPIRKVRTITGAEAWLVSSYALCTQVLEDRRFSMKETAAAGAPRL
NALTVPPEVVNNMGNIADAGLRKAVMKAITPKAPGLEQFLRDTANSLLDNLITEGAPADLRNDFADPLATALH
CKVLGIPQEDGPKLFRSLSIAFMSSADPIPAAKINWDRDIEYMAGILENPNITTGLMGELSRLRKDPAYSHVS
DELFATIGVTFFGAGVISTGSFLTTALISLIQRPQLRNLLHEKPELIPAGVEELLRINLSFADGLPRLATADI
QVGDVLVRKGELVLVLLEGANFDPEHFPNPGSIELDRPNPTSHLAFGRGQHFCPGSALGRRHAQIGIEALLKK
MPGVDLAVPIDQLVWRTRFQRRIPERLPVLW
>CYP121A1_Mycobacterium tuberculosis TB_RSA128 2603955915
MTATVLLEVPFSARGDRIPDAVAELRTREPIRKVRTITGAEAWLVSSYALCTQVLEDRRFSMKETAAAGAPRL
NALTVPPEVVNNMGNIADAGLRKAVMKAITPKAPGLEQFLRDTANSLLDNLITEGAPADLRNDFADPLATALH
CKVLGIPQEDGPKLFRSLSIAFMSSADPIPAAKINWDRDIEYMAGILENPNITTGLMGELSRLRKDPAYSHVS
DELFATIGVTFFGAGVISTGSFLTTALISLIQRPQLRNLLHEKPELIPAGVEELLRINLSFADGLPRLATADI
QVGDVLVRKGELVLVLLEGANFDPEHFPNPGSIELDRPNPTSHLAFGRGQHFCPGSALGRRHAQIGIEALLKK
MPGVDLAVPIDQLVWRTRFQRRIPERLPVLW
>CYP121A1_Mycobacterium tuberculosis TB_RSA129 2605717230
MTATVLLEVPFSARGDRIPDAVAELRTREPIRKVRTITGAEAWLVSSYALCTQVLEDRRFSMKETAAAGAPRL
NALTVPPEVVNNMGNIADAGLRKAVMKAITPKAPGLEQFLRDTANSLLDNLITEGAPADLRNDFADPLATALH
CKVLGIPQEDGPKLFRSLSIAFMSSADPIPAAKINWDRDIEYMAGILENPNITTGLMGELSRLRKDPAYSHVS
DELFATIGVTFFGAGVISTGSFLTTALISLIQRPQLRNLLHEKPELIPAGVEELLRINLSFADGLPRLATADI
QVGDVLVRKGELVLVLLEGANFDPEHFPNPGSIELDRPNPTSHLAFGRGQHFCPGSALGRRHAQIGIEALLKK
MPGVDLAVPIDQLVWRTRFQRRIPERLPVLW
>CYP121A1_Mycobacterium tuberculosis TB_RSA130 2605354036
MTATVLLEVPFSARGDRIPDAVAELRTREPIRKVRTITGAEAWLVSSYALCTQVLEDRRFSMKETAAAGAPRL
NALTVPPEVVNNMGNIADAGLRKAVMKAITPKAPGLEQFLRDTANSLLDNLITEGAPADLRNDFADPLATALH
CKVLGIPQEDGPKLFRSLSIAFMSSADPIPAAKINWDRDIEYMAGILENPNITTGLMGELSRLRKDPAYSHVS
DELFATIGVTFFGAGVISTGSFLTTALISLIQRPQLRNLLHEKPELIPAGVEELLRINLSFADGLPRLATADI
QVGDVLVRKGELVLVLLEGANFDPEHFPNPGSIELDRPNPTSHLAFGRGQHFCPGSALGRRHAQIGIEALLKK
MPGVDLAVPIDQLVWRTRFQRRIPERLPVLW
>CYP121A1_Mycobacterium tuberculosis TB_RSA131 2603403202
MTATVLLEVPFSARGDRIPDAVAELRTREPIRKVRTITGAEAWLVSSYALCTQVLEDRRFSMKETAAAGAPRL
NALTVPPEVVNNMGNIADAGLRKAVMKAITPKAPGLEQFLRDTANSLLDNLITEGAPADLRNDFADPLATALH
CKVLGIPQEDGPKLFRSLSIAFMSSADPIPAAKINWDRDIEYMAGILENPNITTGLMGELSRLRKDPAYSHVS
DELFATIGVTFFGAGVISTGSFLTTALISLIQRPQLRNLLHEKPELIPAGVEELLRINLSFADGLPRLATADI
QVGDVLVRKGELVLVLLEGANFDPEHFPNPGSIELDRPNPTSHLAFGRGQHFCPGSALGRRHAQIGIEALLKK
MPGVDLAVPIDQLVWRTRFQRRIPERLPVLW

>CYP121A1_Mycobacterium tuberculosis TB_RSA132 2577988879
MTATVLLEVPFSARGDRIPDAVAELRTREPIRKVRTITGAEAWLVSSYALCTQVLEDRRFSMKETAAAGAPRL
NALTVPPEVVNNMGNIADAGLRKAVMKAITPKAPGLEQFLRDTANSLLDNLITEGAPADLRNDFADPLATALH
CKVLGIPQEDGPKLFRSLSIAFMSSADPIPAAKINWDRDIEYMAGILENPNITTGLMGELSRLRKDPAYSHVS
DELFATIGVTFFGAGVISTGSFLTTALISLIQRPQLRNLLHEKPELIPAGVEELLRINLSFADGLPRLATADI
QVGDVLVRKGELVLVLLEGANFDPEHFPNPGSIELDRPNPTSHLAFGRGQHFCPGSALGRRHAQIGIEALLKK
MPGVDLAVPIDQLVWRTRFQRRIPERLPVLW
>CYP121A1_Mycobacterium tuberculosis TB_RSA133 2605356601
MTATVLLEVPFSARGDRIPDAVAELRTREPIRKVRTITGAEAWLVSSYALCTQVLEDRRFSMKETAAAGAPRL
NALTVPPEVVNNMGNIADAGLRKAVMKAITPKAPGLEQFLRDTANSLLDNLITEGAPADLRNDFADPLATALH
CKVLGIPQEDGPKLFRSLSIAFMSSADPIPAAKINWDRDIEYMAGILENPNITTGLMGELSRLRKDPAYSHVS
DELFATIGVTFFGAGVISTGSFLTTALISLIQRPQLRNLLHEKPELIPAGVEELLRINLSFADGLPRLATADI
QVGDVLVRKGELVLVLLEGANFDPEHFPNPGSIELDRPNPTSHLAFGRGQHFCPGSALGRRHAQIGIEALLKK
MPGVDLAVPIDQLVWRTRFQRRIPERLPVLW
>CYP121A1_Mycobacterium tuberculosis TB_RSA134 2575625427
MTATVLLEVPFSARGDRIPDAVAELRTREPIRKVRTITGAEAWLVSSYALCTQVLEDRRFSMKETAAAGAPRL
NALTVPPEVVNNMGNIADAGLRKAVMKAITPKAPGLEQFLRDTANSLLDNLITEGAPADLRNDFADPLATALH
CKVLGIPQEDGPKLFRSLSIAFMSSADPIPAAKINWDRDIEYMAGILENPNITTGLMGELSRLRKDPAYSHVS
DELFATIGVTFFGAGVISTGSFLTTALISLIQRPQLRNLLHEKPELIPAGVEELLRINLSFADGLPRLATADI
QVGDVLVRKGELVLVLLEGANFDPEHFPNPGSIELDRPNPTSHLAFGRGQHFCPGSALGRRHAQIGIEALLKK
MPGVDLAVPIDQLVWRTRFQRRIPERLPVLW
>CYP121A1_Mycobacterium tuberculosis TB_RSA135 2603957955
MTATVLLEVPFSARGDRIPDAVAELRTREPIRKVRTITGAEAWLVSSYALCTQVLEDRRFSMKETAAAGAPRL
NALTVPPEVVNNMGNIADAGLRKAVMKAITPKAPGLEQFLRDTANSLLDNLITEGAPADLRNDFADPLATALH
CKVLGIPQEDGPKLFRSLSIAFMSSADPIPAAKINWDRDIEYMAGILENPNITTGLMGELSRLRKDPAYSHVS
DELFATIGVTFFGAGVISTGSFLTTALISLIQRPQLRNLLHEKPELIPAGVEELLRINLSFADGLPRLATADI
QVGDVLVRKGELVLVLLEGANFDPEHFPNPGSIELDRPNPTSHLAFGRGQHFCPGSALGRRHAQIGIEALLKK
MPGVDLAVPIDQLVWRTRFQRRIPERLPVLW
>CYP121A1_Mycobacterium tuberculosis TB_RSA136 2576444237
MTATVLLEVPFSARGDRIPDAVAELRTREPIRKVRTITGAEAWLVSSYALCTQVLEDRRFSMKETAAAGAPRL
NALTVPPEVVNNMGNIADAGLRKAVMKAITPKAPGLEQFLRDTANSLLDNLITEGAPADLRNDFADPLATALH
CKVLGIPQEDGPKLFRSLSIAFMSSADPIPAAKINWDRDIEYMAGILENPNITTGLMGELSRLRKDPAYSHVS
DELFATIGVTFFGAGVISTGSFLTTALISLIQRPQLRNLLHEKPELIPAGVEELLRINLSFADGLPRLATADI
QVGDVLVRKGELVLVLLEGANFDPEHFPNPGSIELDRPNPTSHLAFGRGQHFCPGSALGRRHAQIGIEALLKK
MPGVDLAVPIDQLVWRTRFQRRIPERLPVLW
>CYP121A1_Mycobacterium tuberculosis TB_RSA137 2603963920
MTATVLLEVPFSARGDRIPDAVAELRTREPIRKVRTITGAEAWLVSSYALCTQVLEDRRFSMKETAAAGAPRL
NALTVPPEVVNNMGNIADAGLRKAVMKAITPKAPGLEQFLRDTANSLLDNLITEGAPADLRNDFADPLATALH
CKVLGIPQEDGPKLFRSLSIAFMSSADPIPAAKINWDRDIEYMAGILENPNITTGLMGELSRLRKDPAYSHVS
DELFATIGVTFFGAGVISTGSFLTTALISLIQRPQLRNLLHEKPELIPAGVEELLRINLSFADGLPRLATADI
QVGDVLVRKGELVLVLLEGANFDPEHFPNPGSIELDRPNPTSHLAFGRGQHFCPGSALGRRHAQIGIEALLKK
MPGVDLAVPIDQLVWRTRFQRRIPERLPVLW
>CYP121A1_Mycobacterium tuberculosis TB_RSA138 2577901589
MTATVLLEVPFSARGDRIPDAVAELRTREPIRKVRTITGAEAWLVSSYALCTQVLEDRRFSMKETAAAGAPRL
NALTVPPEVVNNMGNIADAGLRKAVMKAITPKAPGLEQFLRDTANSLLDNLITEGAPADLRNDFADPLATALH
CKVLGIPQEDGPKLFRSLSIAFMSSADPIPAAKINWDRDIEYMAGILENPNITTGLMGELSRLRKDPAYSHVS
DELFATIGVTFFGAGVISTGSFLTTALISLIQRPQLRNLLHEKPELIPAGVEELLRINLSFADGLPRLATADI
QVGDVLVRKGELVLVLLEGANFDPEHFPNPGSIELDRPNPTSHLAFGRGQHFCPGSALGRRHAQIGIEALLKK
MPGVDLAVPIDQLVWRTRFQRRIPERLPVLW
>CYP121A1_Mycobacterium tuberculosis TB_RSA139 2605361155
MTATVLLEVPFSARGDRIPDAVAELRTREPIRKVRTITGAEAWLVSSYALCTQVLEDRRFSMKETAAAGAPRL
NALTVPPEVVNNMGNIADAGLRKAVMKAITPKAPGLEQFLRDTANSLLDNLITEGAPADLRNDFADPLATALH
CKVLGIPQEDGPKLFRSLSIAFMSSADPIPAAKINWDRDIEYMAGILENPNITTGLMGELSRLRKDPAYSHVS
DELFATIGVTFFGAGVISTGSFLTTALISLIQRPQLRNLLHEKPELIPAGVEELLRINLSFADGLPRLATADI
QVGDVLVRKGELVLVLLEGANFDPEHFPNPGSIELDRPNPTSHLAFGRGQHFCPGSALGRRHAQIGIEALLKK
MPGVDLAVPIDQLVWRTRFQRRIPERLPVLW
>CYP121A1_Mycobacterium tuberculosis TB_RSA140 2576164469

MTATVLLEVPFSARGDRIPDAVAELRTREPIRKVRTITGAEAWLVSSYALCTQVLEDRRFSMKETAAAGAPRL
NALTVPPEVVNNMGNIADAGLRKAVMKAITPKAPGLEQFLRDTANSLLDNLITEGAPADLRNDFADPLATALH
CKVLGIPQEDGPKLFRSLSIAFMSSADPIPAAKINWDRDIEYMAGILENPNITTGLMGELSRLRKDPAYSHVS
DELFATIGVTFFGAGVISTGSFLTTALISLIQRPQLRNLLHEKPELIPAGVEELLRINLSFADGLPRLATADI
QVGDVLVRKGELVLVLLEGANFDPEHFPNPGSIELDRPNPTSHLAFGRGQHFCPGSALGRRHAQIGIEALLKK
MPGVDLAVPIDQLVWRTRFQRRIPERLPVLW
>CYP121A1_Mycobacterium tuberculosis TB_RSA141 2603469383
MTATVLLEVPFSARGDRIPDAVAELRTREPIRKVRTITGAEAWLVSSYALCTQVLEDRRFSMKETAAAGAPRL
NALTVPPEVVNNMGNIADAGLRKAVMKAITPKAPGLEQFLRDTANSLLDNLITEGAPADLRNDFADPLATALH
CKVLGIPQEDGPKLFRSLSIAFMSSADPIPAAKINWDRDIEYMAGILENPNITTGLMGELSRLRKDPAYSHVS
DELFATIGVTFFGAGVISTGSFLTTALISLIQRPQLRNLLHEKPELIPAGVEELLRINLSFADGLPRLATADI
QVGDVLVRKGELVLVLLEGANFDPEHFPNPGSIELDRPNPTSHLAFGRGQHFCPGSALGRRHAQIGIEALLKK
MPGVDLAVPIDQLVWRTRFQRRIPERLPVLW
>CYP121A1_Mycobacterium tuberculosis TB_RSA142 2603473781
MTATVLLEVPFSARGDRIPDAVAELRTREPIRKVRTITGAEAWLVSSYALCTQVLEDRRFSMKETAAAGAPRL
NALTVPPEVVNNMGNIADAGLRKAVMKAITPKAPGLEQFLRDTANSLLDNLITEGAPADLRNDFADPLATALH
CKVLGIPQEDGPKLFRSLSIAFMSSADPIPAAKINWDRDIEYMAGILENPNITTGLMGELSRLRKDPAYSHVS
DELFATIGVTFFGAGVISTGSFLTTALISLIQRPQLRNLLHEKPELIPAGVEELLRINLSFADGLPRLATADI
QVGDVLVRKGELVLVLLEGANFDPEHFPNPGSIELDRPNPTSHLAFGRGQHFCPGSALGRRHAQIGIEALLKK
MPGVDLAVPIDQLVWRTRFQRRIPERLPVLW
>CYP121A1_Mycobacterium tuberculosis TB_RSA143 2604428538
MTATVLLEVPFSARGDRIPDAVAELRTREPIRKVRTITGAEAWLVSSYALCTQVLEDRRFSMKETAAAGAPRL
NALTVPPEVVNNMGNIADAGLRKAVMKAITPKAPGLEQFLRDTANSLLDNLITEGAPADLRNDFADPLATALH
CKVLGIPQEDGPKLFRSLSIAFMSSADPIPAAKINWDRDIEYMAGILENPNITTGLMGELSRLRKDPAYSHVS
DELFATIGVTFFGAGVISTGSFLTTALISLIQRPQLRNLLHEKPELIPAGVEELLRINLSFADGLPRLATADI
QVGDVLVRKGELVLVLLEGANFDPEHFPNPGSIELDRPNPTSHLAFGRGQHFCPGSALGRRHAQIGIEALLKK
MPGVDLAVPIDQLVWRTRFQRRIPERLPVLW
>CYP121A1_Mycobacterium tuberculosis TB_RSA144 2604771225
MTATVLLEVPFSARGDRIPDAVAELRTREPIRKVRTITGAEAWLVSSYALCTQVLEDRRFSMKETAAAGAPRL
NALTVPPEVVNNMGNIADAGLRKAVMKAITPKAPGLEQFLRDTANSLLDNLITEGAPADLRNDFADPLATALH
CKVLGIPQEDGPKLFRSLSIAFMSSADPIPAAKINWDRDIEYMAGILENPNITTGLMGELSRLRKDPAYSHVS
DELFATIGVTFFGAGVISTGSFLTTALISLIQRPQLRNLLHEKPELIPAGVEELLRINLSFADGLPRLATADI
QVGDVLVRKGELVLVLLEGANFDPEHFPNPGSIELDRPNPTSHLAFGRGQHFCPGSALGRRHAQIGIEALLKK
MPGVDLAVPIDQLVWRTRFQRRIPERLPVLW
>CYP121A1_Mycobacterium tuberculosis TB_RSA145 2605010631
MTATVLLEVPFSARGDRIPDAVAELRTREPIRKVRTITGAEAWLVSSYALCTQVLEDRRFSMKETAAAGAPRL
NALTVPPEVVNNMGNIADAGLRKAVMKAITPKAPGLEQFLRDTANSLLDNLITEGAPADLRNDFADPLATALH
CKVLGIPQEDGPKLFRSLSIAFMSSADPIPAAKINWDRDIEYMAGILENPNITTGLMGELSRLRKDPAYSHVS
DELFATIGVTFFGAGVISTGSFLTTALISLIQRPQLRNLLHEKPELIPAGVEELLRINLSFADGLPRLATADI
QVGDVLVRKGELVLVLLEGANFDPEHFPNPGSIELDRPNPTSHLAFGRGQHFCPGSALGRRHAQIGIEALLKK
MPGVDLAVPIDQLVWRTRFQRRIPERLPVLW
>CYP121A1_Mycobacterium tuberculosis TB_RSA146 2604294127
MTATVLLEVPFSARGDRIPDAVAELRTREPIRKVRTITGAEAWLVSSYALCTQVLEDRRFSMKETAAAGAPRL
NALTVPPEVVNNMGNIADAGLRKAVMKAITPKAPGLEQFLRDTANSLLDNLITEGAPADLRNDFADPLATALH
CKVLGIPQEDGPKLFRSLSIAFMSSADPIPAAKINWDRDIEYMAGILENPNITTGLMGELSRLRKDPAYSHVS
DELFATIGVTFFGAGVISTGSFLTTALISLIQRPQLRNLLHEKPELIPAGVEELLRINLSFADGLPRLATADI
QVGDVLVRKGELVLVLLEGANFDPEHFPNPGSIELDRPNPTSHLAFGRGQHFCPGSALGRRHAQIGIEALLKK
MPGVDLAVPIDQLVWRTRFQRRIPERLPVLW
>CYP121A1_Mycobacterium tuberculosis TB_RSA147 2603249927
MTATVLLEVPFSARGDRIPDAVAELRTREPIRKVRTITGAEAWLVSSYALCTQVLEDRRFSMKETAAAGAPRL
NALTVPPEVVNNMGNIADAGLRKAVMKAITPKAPGLEQFLRDTANSLLDNLITEGAPADLRNDFADPLATALH
CKVLGIPQEDGPKLFRSLSIAFMSSADPIPAAKINWDRDIEYMAGILENPNITTGLMGELSRLRKDPAYSHVS
DELFATIGVTFFGAGVISTGSFLTTALISLIQRPQLRNLLHEKPELIPAGVEELLRINLSFADGLPRLATADI
QVGDVLVRKGELVLVLLEGANFDPEHFPNPGSIELDRPNPTSHLAFGRGQHFCPGSALGRRHAQIGIEALLKK
MPGVDLAVPIDQLVWRTRFQRRIPERLPVLW
>CYP121A1_Mycobacterium tuberculosis TB_RSA148 2575295964
MTATVLLEVPFSARGDRIPDAVAELRTREPIRKVRTITGAEAWLVSSYALCTQVLEDRRFSMKETAAAGAPRL
NALTVPPEVVNNMGNIADAGLRKAVMKAITPKAPGLEQFLRDTANSLLDNLITEGAPADLRNDFADPLATALH

CKVLGIPQEDGPKLFRSLSIAFMSSADPIPAAKINWDRDIEYMAGILENPNITTGLMGELSRLRKDPAYSHVS
DELFATIGVTFFGAGVISTGSFLTTALISLIQRPQLRNLLHEKPELIPAGVEELLRINLSFADGLPRLATADI
QVGDVLVRKGELVLVLLEGANFDPEHFPNPGSIELDRPNPTSHLAFGRGQHFCPGSALGRRHAQIGIEALLKK
MPGVDLAVPIDQLVWRTRFQRRIPERLPVLW
>CYP121A1_Mycobacterium tuberculosis TB_RSA149 2576981651
MTATVLLEVPFSARGDRIPDAVAELRTREPIRKVRTITGAEAWLVSSYALCTQVLEDRRFSMKETAAAGAPRL
NALTVPPEVVNNMGNIADAGLRKAVMKAITPKAPGLEQFLRDTANSLLDNLITEGAPADLRNDFADPLATALH
CKVLGIPQEDGPKLFRSLSIAFMSSADPIPAAKINWDRDIEYMAGILENPNITTGLMGELSRLRKDPAYSHVS
DELFATIGVTFFGAGVISTGSFLTTALISLIQRPQLRNLLHEKPELIPAGVEELLRINLSFADGLPRLATADI
QVGDVLVRKGELVLVLLEGANFDPEHFPNPGSIELDRPNPTSHLAFGRGQHFCPGSALGRRHAQIGIEALLKK
MPGVDLAVPIDQLVWRTRFQRRIPERLPVLW
>CYP121A1_Mycobacterium tuberculosis TB_RSA150 2605370477
MTATVLLEVPFSARGDRIPDAVAELRTREPIRKVRTITGAEAWLVSSYALCTQVLEDRRFSMKETAAAGAPRL
NALTVPPEVVNNMGNIADAGLRKAVMKAITPKAPGLEQFLRDTANSLLDNLITEGAPADLRNDFADPLATALH
CKVLGIPQEDGPKLFRSLSIAFMSSADPIPAAKINWDRDIEYMAGILENPNITTGLMGELSRLRKDPAYSHVS
DELFATIGVTFFGAGVISTGSFLTTALISLIQRPQLRNLLHEKPELIPAGVEELLRINLSFADGLPRLATADI
QVGDVLVRKGELVLVLLEGANFDPEHFPNPGSIELDRPNPTSHLAFGRGQHFCPGSALGRRHAQIGIEALLKK
MPGVDLAVPIDQLVWRTRFQRRIPERLPVLW
>CYP121A1_Mycobacterium tuberculosis TB_RSA151 2605012888
MTATVLLEVPFSARGDRIPDAVAELRTREPIRKVRTITGAEAWLVSSYALCTQVLEDRRFSMKETAAAGAPRL
NALTVPPEVVNNMGNIADAGLRKAVMKAITPKAPGLEQFLRDTANSLLDNLITEGAPADLRNDFADPLATALH
CKVLGIPQEDGPKLFRSLSIAFMSSADPIPAAKINWDRDIEYMAGILENPNITTGLMGELSRLRKDPAYSHVS
DELFATIGVTFFGAGVISTGSFLTTALISLIQRPQLRNLLHEKPELIPAGVEELLRINLSFADGLPRLATADI
QVGDVLVRKGELVLVLLEGANFDPEHFPNPGSIELDRPNPTSHLAFGRGQHFCPGSALGRRHAQIGIEALLKK
MPGVDLAVPIDQLVWRTRFQRRIPERLPVLW
>CYP121A1_Mycobacterium tuberculosis TB_RSA152 2603255020
MTATVLLEVPFSARGDRIPDAVAELRTREPIRKVRTITGAEAWLVSSYALCTQVLEDRRFSMKETAAAGAPRL
NALTVPPEVVNNMGNIADAGLRKAVMKAITPKAPGLEQFLRDTANSLLDNLITEGAPADLRNDFADPLATALH
CKVLGIPQEDGPKLFRSLSIAFMSSADPIPAAKINWDRDIEYMAGILENPNITTGLMGELSRLRKDPAYSHVS
DELFATIGVTFFGAGVISTGSFLTTALISLIQRPQLRNLLHEKPELIPAGVEELLRINLSFADGLPRLATADI
QVGDVLVRKGELVLVLLEGANFDPEHFPNPGSIELDRPNPTSHLAFGRGQHFCPGSALGRRHAQIGIEALLKK
MPGVDLAVPIDQLVWRTRFQRRIPERLPVLW
>CYP121A1_Mycobacterium tuberculosis TB_RSA153 2603406100
MTATVLLEVPFSARGDRIPDAVAELRTREPIRKVRTITGAEAWLVSSYALCTQVLEDRRFSMKETAAAGAPRL
NALTVPPEVVNNMGNIADAGLRKAVMKAITPKAPGLEQFLRDTANSLLDNLITEGAPADLRNDFADPLATALH
CKVLGIPQEDGPKLFRSLSIAFMSSADPIPAAKINWDRDIEYMAGILENPNITTGLMGELSRLRKDPAYSHVS
DELFATIGVTFFGAGVISTGSFLTTALISLIQRPQLRNLLHEKPELIPAGVEELLRINLSFADGLPRLATADI
QVGDVLVRKGELVLVLLEGANFDPEHFPNPGSIELDRPNPTSHLAFGRGQHFCPGSALGRRHAQIGIEALLKK
MPGVDLAVPIDQLVWRTRFQRRIPERLPVLW
>CYP121A1_Mycobacterium tuberculosis TB_RSA154 2605376197
MTATVLLEVPFSARGDRIPDAVAELRTREPIRKVRTITGAEAWLVSSYALCTQVLEDRRFSMKETAAAGAPRL
NALTVPPEVVNNMGNIADAGLRKAVMKAITPKAPGLEQFLRDTANSLLDNLITEGAPADLRNDFADPLATALH
CKVLGIPQEDGPKLFRSLSIAFMSSADPIPAAKINWDRDIEYMAGILENPNITTGLMGELSRLRKDPAYSHVS
DELFATIGVTFFGAGVISTGSFLTTALISLIQRPQLRNLLHEKPELIPAGVEELLRINLSFADGLPRLATADI
QVGDVLVRKGELVLVLLEGANFDPEHFPNPGSIELDRPNPTSHLAFGRGQHFCPGSALGRRHAQIGIEALLKK
MPGVDLAVPIDQLVWRTRFQRRIPERLPVLW
>CYP121A1_Mycobacterium tuberculosis TB_RSA155 2605016651
MTATVLLEVPFSARGDRIPDAVAELRTREPIRKVRTITGAEAWLVSSYALCTQVLEDRRFSMKETAAAGAPRL
NALTVPPEVVNNMGNIADAGLRKAVMKAITPKAPGLEQFLRDTANSLLDNLITEGAPADLRNDFADPLATALH
CKVLGIPQEDGPKLFRSLSIAFMSSADPIPAAKINWDRDIEYMAGILENPNITTGLMGELSRLRKDPAYSHVS
DELFATIGVTFFGAGVISTGSFLTTALISLIQRPQLRNLLHEKPELIPAGVEELLRINLSFADGLPRLATADI
QVGDVLVRKGELVLVLLEGANFDPEHFPNPGSIELDRPNPTSHLAFGRGQHFCPGSALGRRHAQIGIEALLKK
MPGVDLAVPIDQLVWRTRFQRRIPERLPVLW
>CYP121A1_Mycobacterium tuberculosis TB_RSA156 2605379240
MTATVLLEVPFSARGDRIPDAVAELRTREPIRKVRTITGAEAWLVSSYALCTQVLEDRRFSMKETAAAGAPRL
NALTVPPEVVNNMGNIADAGLRKAVMKAITPKAPGLEQFLRDTANSLLDNLITEGAPADLRNDFADPLATALH
CKVLGIPQEDGPKLFRSLSIAFMSSADPIPAAKINWDRDIEYMAGILENPNITTGLMGELSRLRKDPAYSHVS
DELFATIGVTFFGAGVISTGSFLTTALISLIQRPQLRNLLHEKPELIPAGVEELLRINLSFADGLPRLATADI

QVGDVLVRKGELVLVLLEGANFDPEHFPNPGSIELDRPNPTSHLAFGRGQHFCPGSALGRRHAQIGIEALLKK
MPGVDLAVPIDQLVWRTRFQRRIPERLPVLW
>CYP121A1_Mycobacterium tuberculosis TB_RSA157 2603412065
MTATVLLEVPFSARGDRIPDAVAELRTREPIRKVRTITGAEAWLVSSYALCTQVLEDRRFSMKETAAAGAPRL
NALTVPPEVVNNMGNIADAGLRKAVMKAITPKAPGLEQFLRDTANSLLDNLITEGAPADLRNDFADPLATALH
CKVLGIPQEDGPKLFRSLSIAFMSSADPIPAAKINWDRDIEYMAGILENPNITTGLMGELSRLRKDPAYSHVS
DELFATIGVTFFGAGVISTGSFLTTALISLIQRPQLRNLLHEKPELIPAGVEELLRINLSFADGLPRLATADI
QVGDVLVRKGELVLVLLEGANFDPEHFPNPGSIELDRPNPTSHLAFGRGQHFCPGSALGRRHAQIGIEALLKK
MPGVDLAVPIDQLVWRTRFQRRIPERLPVLW
>CYP121A1_Mycobacterium tuberculosis TB_RSA158 2605719574
MTATVLLEVPFSARGDRIPDAVAELRTREPIRKVRTITGAEAWLVSSYALCTQVLEDRRFSMKETAAAGAPRL
NALTVPPEVVNNMGNIADAGLRKAVMKAITPKAPGLEQFLRDTANSLLDNLITEGAPADLRNDFADPLATALH
CKVLGIPQEDGPKLFRSLSIAFMSSADPIPAAKINWDRDIEYMAGILENPNITTGLMGELSRLRKDPAYSHVS
DELFATIGVTFFGAGVISTGSFLTTALISLIQRPQLRNLLHEKPELIPAGVEELLRINLSFADGLPRLATADI
QVGDVLVRKGELVLVLLEGANFDPEHFPNPGSIELDRPNPTSHLAFGRGQHFCPGSALGRRHAQIGIEALLKK
MPGVDLAVPIDQLVWRTRFQRRIPERLPVLW
>CYP121A1_Mycobacterium tuberculosis TB_RSA159 2605022951
MTATVLLEVPFSARGDRIPDAVAELRTREPIRKVRTITGAEAWLVSSYALCTQVLEDRRFSMKETAAAGAPRL
NALTVPPEVVNNMGNIADAGLRKAVMKAITPKAPGLEQFLRDTANSLLDNLITEGAPADLRNDFADPLATALH
CKVLGIPQEDGPKLFRSLSIAFMSSADPIPAAKINWDRDIEYMAGILENPNITTGLMGELSRLRKDPAYSHVS
DELFATIGVTFFGAGVISTGSFLTTALISLIQRPQLRNLLHEKPELIPAGVEELLRINLSFADGLPRLATADI
QVGDVLVRKGELVLVLLEGANFDPEHFPNPGSIELDRPNPTSHLAFGRGQHFCPGSALGRRHAQIGIEALLKK
MPGVDLAVPIDQLVWRTRFQRRIPERLPVLW
>CYP121A1_Mycobacterium tuberculosis TB_RSA160 2604299945
MTATVLLEVPFSARGDRIPDAVAELRTREPIRKVRTITGAEAWLVSSYALCTQVLEDRRFSMKETAAAGAPRL
NALTVPPEVVNNMGNIADAGLRKAVMKAITPKAPGLEQFLRDTANSLLDNLITEGAPADLRNDFADPLATALH
CKVLGIPQEDGPKLFRSLSIAFMSSADPIPAAKINWDRDIEYMAGILENPNITTGLMGELSRLRKDPAYSHVS
DELFATIGVTFFGAGVISTGSFLTTALISLIQRPQLRNLLHEKPELIPAGVEELLRINLSFADGLPRLATADI
QVGDVLVRKGELVLVLLEGANFDPEHFPNPGSIELDRPNPTSHLAFGRGQHFCPGSALGRRHAQIGIEALLKK
MPGVDLAVPIDQLVWRTRFQRRIPERLPVLW
>CYP121A1_Mycobacterium tuberculosis TB_RSA161 2577567209
MTATVLLEVPFSARGDRIPDAVAELRTREPIRKVRTITGAEAWLVSSYALCTQVLEDRRFSMKETAAAGAPRL
NALTVPPEVVNNMGNIADAGLRKAVMKAITPKAPGLEQFLRDTANSLLDNLITEGAPADLRNDFADPLATALH
CKVLGIPQEDGPKLFRSLSIAFMSSADPIPAAKINWDRDIEYMAGILENPNITTGLMGELSRLRKDPAYSHVS
DELFATIGVTFFGAGVISTGSFLTTALISLIQRPQLRNLLHEKPELIPAGVEELLRINLSFADGLPRLATADI
QVGDVLVRKGELVLVLLEGANFDPEHFPNPGSIELDRPNPTSHLAFGRGQHFCPGSALGRRHAQIGIEALLKK
MPGVDLAVPIDQLVWRTRFQRRIPERLPVLW
>CYP121A1_Mycobacterium tuberculosis TB_RSA162 2604672150
MTATVLLEVPFSARGDRIPDAVAELRTREPIRKVRTITGAEAWLVSSYALCTQVLEDRRFSMKETAAAGAPRL
NALTVPPEVVNNMGNIADAGLRKAVMKAITPKAPGLEQFLRDTANSLLDNLITEGAPADLRNDFADPLATALH
CKVLGIPQEDGPKLFRSLSIAFMSSADPIPAAKINWDRDIEYMAGILENPNITTGLMGELSRLRKDPAYSHVS
DELFATIGVTFFGAGVISTGSFLTTALISLIQRPQLRNLLHEKPELIPAGVEELLRINLSFADGLPRLATADI
QVGDVLVRKGELVLVLLEGANFDPEHFPNPGSIELDRPNPTSHLAFGRGQHFCPGSALGRRHAQIGIEALLKK
MPGVDLAVPIDQLVWRTRFQRRIPERLPVLW
>CYP121A1_Mycobacterium tuberculosis TB_RSA163 2576882899
MTATVLLEVPFSARGDRIPDAVAELRTREPIRKVRTITGAEAWLVSSYALCTQVLEDRRFSMKETAAAGAPRL
NALTVPPEVVNNMGNIADAGLRKAVMKAITPKAPGLEQFLRDTANSLLDNLITEGAPADLRNDFADPLATALH
CKVLGIPQEDGPKLFRSLSIAFMSSADPIPAAKINWDRDIEYMAGILENPNITTGLMGELSRLRKDPAYSHVS
DELFATIGVTFFGAGVISTGSFLTTALISLIQRPQLRNLLHEKPELIPAGVEELLRINLSFADGLPRLATADI
QVGDVLVRKGELVLVLLEGANFDPEHFPNPGSIELDRPNPTSHLAFGRGQHFCPGSALGRRHAQIGIEALLKK
MPGVDLAVPIDQLVWRTRFQRRIPERLPVLW
>CYP121A1_Mycobacterium tuberculosis TB_RSA164 2605381550
MTATVLLEVPFSARGDRIPDAVAELRTREPIRKVRTITGAEAWLVSSYALCTQVLEDRRFSMKETAAAGAPRL
NALTVPPEVVNNMGNIADAGLRKAVMKAITPKAPGLEQFLRDTANSLLDNLITEGAPADLRNDFADPLATALH
CKVLGIPQEDGPKLFRSLSIAFMSSADPIPAAKINWDRDIEYMAGILENPNITTGLMGELSRLRKDPAYSHVS
DELFATIGVTFFGAGVISTGSFLTTALISLIQRPQLRNLLHEKPELIPAGVEELLRINLSFADGLPRLATADI
QVGDVLVRKGELVLVLLEGANFDPEHFPNPGSIELDRPNPTSHLAFGRGQHFCPGSALGRRHAQIGIEALLKK
MPGVDLAVPIDQLVWRTRFQRRIPERLPVLW

>CYP121A1_Mycobacterium tuberculosis TB_RSA167 2603968031
MTATVLLEVPFSARGDRIPDAVAELRTREPIRKVRTITGAEAWLVSSYALCTQVLEDRRFSMKETAAAGAPRL
NALTVPPEVVNNMGNIADAGLRKAVMKAITPKAPGLEQFLRDTANSLLDNLITEGAPADLRNDFADPLATALH
CKVLGIPQEDGPKLFRSLSIAFMSSADPIPAAKINWDRDIEYMAGILENPNITTGLMGELSRLRKDPAYSHVS
DELFATIGVTFFGAGVISTGSFLTTALISLIQRPQLRNLLHEKPELIPAGVEELLRINLSFADGLPRLATADI
QVGDVLVRKGELVLVLLEGANFDPEHFPNPGSIELDRPNPTSHLAFGRGQHFCPGSALGRRHAQIGIEALLKK
MPGVDLAVPIDQLVWRTRFQRRIPERLPVLW
>CYP121A1_Mycobacterium tuberculosis TB_RSA168 2605725565
MTATVLLEVPFSARGDRIPDAVAELRTREPIRKVRTITGAEAWLVSSYALCTQVLEDRRFSMKETAAAGAPRL
NALTVPPEVVNNMGNIADAGLRKAVMKAITPKAPGLEQFLRDTANSLLDNLITEGAPADLRNDFADPLATALH
CKVLGIPQEDGPKLFRSLSIAFMSSADPIPAAKINWDRDIEYMAGILENPNITTGLMGELSRLRKDPAYSHVS
DELFATIGVTFFGAGVISTGSFLTTALISLIQRPQLRNLLHEKPELIPAGVEELLRINLSFADGLPRLATADI
QVGDVLVRKGELVLVLLEGANFDPEHFPNPGSIELDRPNPTSHLAFGRGQHFCPGSALGRRHAQIGIEALLKK
MPGVDLAVPIDQLVWRTRFQRRIPERLPVLW
>CYP121A1_Mycobacterium tuberculosis TB_RSA169 2603258361
MTATVLLEVPFSARGDRIPDAVAELRTREPIRKVRTITGAEAWLVSSYALCTQVLEDRRFSMKETAAAGAPRL
NALTVPPEVVNNMGNIADAGLRKAVMKAITPKAPGLEQFLRDTANSLLDNLITEGAPADLRNDFADPLATALH
CKVLGIPQEDGPKLFRSLSIAFMSSADPIPAAKINWDRDIEYMAGILENPNITTGLMGELSRLRKDPAYSHVS
DELFATIGVTFFGAGVISTGSFLTTALISLIQRPQLRNLLHEKPELIPAGVEELLRINLSFADGLPRLATADI
QVGDVLVRKGELVLVLLEGANFDPEHFPNPGSIELDRPNPTSHLAFGRGQHFCPGSALGRRHAQIGIEALLKK
MPGVDLAVPIDQLVWRTRFQRRIPERLPVLW
>CYP121A1_Mycobacterium tuberculosis TB_RSA170 2605729364
MTATVLLEVPFSARGDRIPDAVAELRTREPIRKVRTITGAEAWLVSSYALCTQVLEDRRFSMKETAAAGAPRL
NALTVPPEVVNNMGNIADAGLRKAVMKAITPKAPGLEQFLRDTANSLLDNLITEGAPADLRNDFADPLATALH
CKVLGIPQEDGPKLFRSLSIAFMSSADPIPAAKINWDRDIEYMAGILENPNITTGLMGELSRLRKDPAYSHVS
DELFATIGVTFFGAGVISTGSFLTTALISLIQRPQLRNLLHEKPELIPAGVEELLRINLSFADGLPRLATADI
QVGDVLVRKGELVLVLLEGANFDPEHFPNPGSIELDRPNPTSHLAFGRGQHFCPGSALGRRHAQIGIEALLKK
MPGVDLAVPIDQLVWRTRFQRRIPERLPVLW
>CYP121A1_Mycobacterium tuberculosis TB_RSA171 2603414311
MTATVLLEVPFSARGDRIPDAVAELRTREPIRKVRTITGAEAWLVSSYALCTQVLEDRRFSMKETAAAGAPRL
NALTVPPEVVNNMGNIADAGLRKAVMKAITPKAPGLEQFLRDTANSLLDNLITEGAPADLRNDFADPLATALH
CKVLGIPQEDGPKLFRSLSIAFMSSADPIPAAKINWDRDIEYMAGILENPNITTGLMGELSRLRKDPAYSHVS
DELFATIGVTFFGAGVISTGSFLTTALISLIQRPQLRNLLHEKPELIPAGVEELLRINLSFADGLPRLATADI
QVGDVLVRKGELVLVLLEGANFDPEHFPNPGSIELDRPNPTSHLAFGRGQHFCPGSALGRRHAQIGIEALLKK
MPGVDLAVPIDQLVWRTRFQRRIPERLPVLW
>CYP121A1_Mycobacterium tuberculosis TB_RSA172 2604302349
MTATVLLEVPFSARGDRIPDAVAELRTREPIRKVRTITGAEAWLVSSYALCTQVLEDRRFSMKETAAAGAPRL
NALTVPPEVVNNMGNIADAGLRKAVMKAITPKAPGLEQFLRDTANSLLDNLITEGAPADLRNDFADPLATALH
CKVLGIPQEDGPKLFRSLSIAFMSSADPIPAAKINWDRDIEYMAGILENPNITTGLMGELSRLRKDPAYSHVS
DELFATIGVTFFGAGVISTGSFLTTALISLIQRPQLRNLLHEKPELIPAGVEELLRINLSFADGLPRLATADI
QVGDVLVRKGELVLVLLEGANFDPEHFPNPGSIELDRPNPTSHLAFGRGQHFCPGSALGRRHAQIGIEALLKK
MPGVDLAVPIDQLVWRTRFQRRIPERLPVLW
>CYP121A1_Mycobacterium tuberculosis TB_RSA179 2603976544
MTATVLLEVPFSARGDRIPDAVAELRTREPIRKVRTITGAEAWLVSSYALCTQVLEDRRFSMKETAAAGAPRL
NALTVPPEVVNNMGNIADAGLRKAVMKAITPKAPGLEQFLRDTANSLLDNLITEGAPADLRNDFADPLATALH
CKVLGIPQEDGPKLFRSLSIAFMSSADPIPAAKINWDRDIEYMAGILENPNITTGLMGELSRLRKDPAYSHVS
DELFATIGVTFFGAGVISTGSFLTTALISLIQRPQLRNLLHEKPELIPAGVEELLRINLSFADGLPRLATADI
QVGDVLVRKGELVLVLLEGANFDPEHFPNPGSIELDRPNPTSHLAFGRGQHFCPGSALGRRHAQIGIEALLKK
MPGVDLAVPIDQLVWRTRFQRRIPERLPVLW
>CYP121A1_Mycobacterium tuberculosis TB_RSA180 2603980654
MTATVLLEVPFSARGDRIPDAVAELRTREPIRKVRTITGAEAWLVSSYALCTQVLEDRRFSMKETAAAGAPRL
NALTVPPEVVNNMGNIADAGLRKAVMKAITPKAPGLEQFLRDTANSLLDNLITEGAPADLRNDFADPLATALH
CKVLGIPQEDGPKLFRSLSIAFMSSADPIPAAKINWDRDIEYMAGILENPNITTGLMGELSRLRKDPAYSHVS
DELFATIGVTFFGAGVISTGSFLTTALISLIQRPQLRNLLHEKPELIPAGVEELLRINLSFADGLPRLATADI
QVGDVLVRKGELVLVLLEGANFDPEHFPNPGSIELDRPNPTSHLAFGRGQHFCPGSALGRRHAQIGIEALLKK
MPGVDLAVPIDQLVWRTRFQRRIPERLPVLW
>CYP121A1_Mycobacterium tuberculosis TB_RSA181 2603419215

MTATVLLEVPFSARGDRIPDAVAELRTREPIRKVRTITGAEAWLVSSYALCTQVLEDRRFSMKETAAAGAPRL
NALTVPPEVVNNMGNIADAGLRKAVMKAITPKAPGLEQFLRDTANSLLDNLITEGAPADLRNDFADPLATALH
CKVLGIPQEDGPKLFRSLSIAFMSSADPIPAAKINWDRDIEYMAGILENPNITTGLMGELSRLRKDPAYSHVS
DELFATIGVTFFGAGVISTGSFLTTALISLIQRPQLRNLLHEKPELIPAGVEELLRINLSFADGLPRLATADI
QVGDVLVRKGELVLVLLEGANFDPEHFPNPGSIELDRPNPTSHLAFGRGQHFCPGSALGRRHAQIGIEALLKK
MPGVDLAVPIDQLVWRTRFQRRIPERLPVLW
>CYP121A1_Mycobacterium tuberculosis TB_RSA182 2604308329
MTATVLLEVPFSARGDRIPDAVAELRTREPIRKVRTITGAEAWLVSSYALCTQVLEDRRFSMKETAAAGAPRL
NALTVPPEVVNNMGNIADAGLRKAVMKAITPKAPGLEQFLRDTANSLLDNLITEGAPADLRNDFADPLATALH
CKVLGIPQEDGPKLFRSLSIAFMSSADPIPAAKINWDRDIEYMAGILENPNITTGLMGELSRLRKDPAYSHVS
DELFATIGVTFFGAGVISTGSFLTTALISLIQRPQLRNLLHEKPELIPAGVEELLRINLSFADGLPRLATADI
QVGDVLVRKGELVLVLLEGANFDPEHFPNPGSIELDRPNPTSHLAFGRGQHFCPGSALGRRHAQIGIEALLKK
MPGVDLAVPIDQLVWRTRFQRRIPERLPVLW
>CYP121A1_Mycobacterium tuberculosis TB_RSA184 2603266856
MTATVLLEVPFSARGDRIPDAVAELRTREPIRKVRTITGAEAWLVSSYALCTQVLEDRRFSMKETAAAGAPRL
NALTVPPEVVNNMGNIADAGLRKAVMKAITPKAPGLEQFLRDTANSLLDNLITEGAPADLRNDFADPLATALH
CKVLGIPQEDGPKLFRSLSIAFMSSADPIPAAKINWDRDIEYMAGILENPNITTGLMGELSRLRKDPAYSHVS
DELFATIGVTFFGAGVISTGSFLTTALISLIQRPQLRNLLHEKPELIPAGVEELLRINLSFADGLPRLATADI
QVGDVLVRKGELVLVLLEGANFDPEHFPNPGSIELDRPNPTSHLAFGRGQHFCPGSALGRRHAQIGIEALLKK
MPGVDLAVPIDQLVWRTRFQRRIPERLPVLW
>CYP121A1_Mycobacterium tuberculosis TB_RSA195 2576400345
MTATVLLEVPFSARGDRIPDAVAELRTREPIRKVRTITGAEAWLVSSYALCTQVLEDRRFSMKETAAAGAPRL
NALTVPPEVVNNMGNIADAGLRKAVMKAITPKAPGLEQFLRDTANSLLDNLITEGAPADLRNDFADPLATALH
CKVLGIPQEDGPKLFRSLSIAFMSSADPIPAAKINWDRDIEYMAGILENPNITTGLMGELSRLRKDPAYSHVS
DELFATIGVTFFGAGVISTGSFLTTALISLIQRPQLRNLLHEKPELIPAGVEELLRINLSFADGLPRLATADI
QVGDVLVRKGELVLVLLEGANFDPEHFPNPGSIELDRPNPTSHLAFGRGQHFCPGSALGRRHAQIGIEALLKK
MPGVDLAVPIDQLVWRTRFQRRIPERLPVLW
>CYP121A1_Mycobacterium tuberculosis TB_RSA199 2576060599
MTATVLLEVPFSARGDRIPDAVAELRTREPIRKVRTITGAEAWLVSSYALCTQVLEDRRFSMKETAAAGAPRL
NALTVPPEVVNNMGNIADAGLRKAVMKAITPKAPGLEQFLRDTANSLLDNLITEGAPADLRNDFADPLATALH
CKVLGIPQEDGPKLFRSLSIAFMSSADPIPAAKINWDRDIEYMAGILENPNITTGLMGELSRLRKDPAYSHVS
DELFATIGVTFFGAGVISTGSFLTTALISLIQRPQLRNLLHEKPELIPAGVEELLRINLSFADGLPRLATADI
QVGDVLVRKGELVLVLLEGANFDPEHFPNPGSIELDRPNPTSHLAFGRGQHFCPGSALGRRHAQIGIEALLKK
MPGVDLAVPIDQLVWRTRFQRRIPERLPVLW
>CYP121A1_Mycobacterium tuberculosis TB_RSA25 2584734325
MTATVLLEVPFSARGDRIPDAVAELRTREPIRKVRTITGAEAWLVSSYALCTQVLEDRRFSMKETAAAGAPRL
NALTVPPEVVNNMGNIADAGLRKAVMKAITPKAPGLEQFLRDTANSLLDNLITEGAPADLRNDFADPLATALH
CKVLGIPQEDGPKLFRSLSIAFMSSADPIPAAKINWDRDIEYMAGILENPNITTGLMGELSRLRKDPAYSHVS
DELFATIGVTFFGAGVISTGSFLTTALISLIQRPQLRNLLHEKPELIPAGVEELLRINLSFADGLPRLATADI
QVGDVLVRKGELVLVLLEGANFDPEHFPNPGSIELDRPNPTSHLAFGRGQHFCPGSALGRRHAQIGIEALLKK
MPGVDLAVPIDQLVWRTRFQRRIPERLPVLW
>CYP121A1_Mycobacterium tuberculosis TB_RSA26 2604324667
MTATVLLEVPFSARGDRIPDAVAELRTREPIRKVRTITGAEAWLVSSYALCTQVLEDRRFSMKETAAAGAPRL
NALTVPPEVVNNMGNIADAGLRKAVMKAITPKAPGLEQFLRDTANSLLDNLITEGAPADLRNDFADPLATALH
CKVLGIPQEDGPKLFRSLSIAFMSSADPIPAAKINWDRDIEYMAGILENPNITTGLMGELSRLRKDPAYSHVS
DELFATIGVTFFGAGVISTGSFLTTALISLIQRPQLRNLLHEKPELIPAGVEELLRINLSFADGLPRLATADI
QVGDVLVRKGELVLVLLEGANFDPEHFPNPGSIELDRPNPTSHLAFGRGQHFCPGSALGRRHAQIGIEALLKK
MPGVDLAVPIDQLVWRTRFQRRIPERLPVLW
>CYP121A1_Mycobacterium tuberculosis TB_RSA39 2604005113
MTATVLLEVPFSARGDRIPDAVAELRTREPIRKVRTITGAEAWLVSSYALCTQVLEDRRFSMKETAAAGAPRL
NALTVPPEVVNNMGNIADAGLRKAVMKAITPKAPGLEQFLRDTANSLLDNLITEGAPADLRNDFADPLATALH
CKVLGIPQEDGPKLFRSLSIAFMSSADPIPAAKINWDRDIEYMAGILENPNITTGLMGELSRLRKDPAYSHVS
DELFATIGVTFFGAGVISTGSFLTTALISLIQRPQLRNLLHEKPELIPAGVEELLRINLSFADGLPRLATADI
QVGDVLVRKGELVLVLLEGANFDPEHFPNPGSIELDRPNPTSHLAFGRGQHFCPGSALGRRHAQIGIEALLKK
MPGVDLAVPIDQLVWRTRFQRRIPERLPVLW
>CYP121A1_Mycobacterium tuberculosis TB_RSA40 2604007410
MTATVLLEVPFSARGDRIPDAVAELRTREPIRKVRTITGAEAWLVSSYALCTQVLEDRRFSMKETAAAGAPRL
NALTVPPEVVNNMGNIADAGLRKAVMKAITPKAPGLEQFLRDTANSLLDNLITEGAPADLRNDFADPLATALH

CKVLGIPQEDGPKLFRSLSIAFMSSADPIPAAKINWDRDIEYMAGILENPNITTGLMGELSRLRKDPAYSHVS
DELFATIGVTFFGAGVISTGSFLTTALISLIQRPQLRNLLHEKPELIPAGVEELLRINLSFADGLPRLATADI
QVGDVLVRKGELVLVLLEGANFDPEHFPNPGSIELDRPNPTSHLAFGRGQHFCPGSALGRRHAQIGIEALLKK
MPGVDLAVPIDQLVWRTRFQRRIPERLPVLW
>CYP121A1_Mycobacterium tuberculosis TB_RSA53 2603432679
MTATVLLEVPFSARGDRIPDAVAELRTREPIRKVRTITGAEAWLVSSYALCTQVLEDRRFSMKETAAAGAPRL
NALTVPPEVVNNMGNIADAGLRKAVMKAITPKAPGLEQFLRDTANSLLDNLITEGAPADLRNDFADPLATALH
CKVLGIPQEDGPKLFRSLSIAFMSSADPIPAAKINWDRDIEYMAGILENPNITTGLMGELSRLRKDPAYSHVS
DELFATIGVTFFGAGVISTGSFLTTALISLIQRPQLRNLLHEKPELIPAGVEELLRINLSFADGLPRLATADI
QVGDVLVRKGELVLVLLEGANFDPEHFPNPGSIELDRPNPTSHLAFGRGQHFCPGSALGRRHAQIGIEALLKK
MPGVDLAVPIDQLVWRTRFQRRIPERLPVLW
>CYP121A1_Mycobacterium tuberculosis TB_RSA54 2605752243
MTATVLLEVPFSARGDRIPDAVAELRTREPIRKVRTITGAEAWLVSSYALCTQVLEDRRFSMKETAAAGAPRL
NALTVPPEVVNNMGNIADAGLRKAVMKAITPKAPGLEQFLRDTANSLLDNLITEGAPADLRNDFADPLATALH
CKVLGIPQEDGPKLFRSLSIAFMSSADPIPAAKINWDRDIEYMAGILENPNITTGLMGELSRLRKDPAYSHVS
DELFATIGVTFFGAGVISTGSFLTTALISLIQRPQLRNLLHEKPELIPAGVEELLRINLSFADGLPRLATADI
QVGDVLVRKGELVLVLLEGANFDPEHFPNPGSIELDRPNPTSHLAFGRGQHFCPGSALGRRHAQIGIEALLKK
MPGVDLAVPIDQLVWRTRFQRRIPERLPVLW
>CYP121A1_Mycobacterium tuberculosis TB_RSA57 2605756643
MTATVLLEVPFSARGDRIPDAVAELRTREPIRKVRTITGAEAWLVSSYALCTQVLEDRRFSMKETAAAGAPRL
NALTVPPEVVNNMGNIADAGLRKAVMKAITPKAPGLEQFLRDTANSLLDNLITEGAPADLRNDFADPLATALH
CKVLGIPQEDGPKLFRSLSIAFMSSADPIPAAKINWDRDIEYMAGILENPNITTGLMGELSRLRKDPAYSHVS
DELFATIGVTFFGAGVISTGSFLTTALISLIQRPQLRNLLHEKPELIPAGVEELLRINLSFADGLPRLATADI
QVGDVLVRKGELVLVLLEGANFDPEHFPNPGSIELDRPNPTSHLAFGRGQHFCPGSALGRRHAQIGIEALLKK
MPGVDLAVPIDQLVWRTRFQRRIPERLPVLW
>CYP121A1_Mycobacterium tuberculosis TB_RSA58 2604335291
MTATVLLEVPFSARGDRIPDAVAELRTREPIRKVRTITGAEAWLVSSYALCTQVLEDRRFSMKETAAAGAPRL
NALTVPPEVVNNMGNIADAGLRKAVMKAITPKAPGLEQFLRDTANSLLDNLITEGAPADLRNDFADPLATALH
CKVLGIPQEDGPKLFRSLSIAFMSSADPIPAAKINWDRDIEYMAGILENPNITTGLMGELSRLRKDPAYSHVS
DELFATIGVTFFGAGVISTGSFLTTALISLIQRPQLRNLLHEKPELIPAGVEELLRINLSFADGLPRLATADI
QVGDVLVRKGELVLVLLEGANFDPEHFPNPGSIELDRPNPTSHLAFGRGQHFCPGSALGRRHAQIGIEALLKK
MPGVDLAVPIDQLVWRTRFQRRIPERLPVLW
>CYP121A1_Mycobacterium tuberculosis TB_RSA61 2605761037
MTATVLLEVPFSARGDRIPDAVAELRTREPIRKVRTITGAEAWLVSSYALCTQVLEDRRFSMKETAAAGAPRL
NALTVPPEVVNNMGNIADAGLRKAVMKAITPKAPGLEQFLRDTANSLLDNLITEGAPADLRNDFADPLATALH
CKVLGIPQEDGPKLFRSLSIAFMSSADPIPAAKINWDRDIEYMAGILENPNITTGLMGELSRLRKDPAYSHVS
DELFATIGVTFFGAGVISTGSFLTTALISLIQRPQLRNLLHEKPELIPAGVEELLRINLSFADGLPRLATADI
QVGDVLVRKGELVLVLLEGANFDPEHFPNPGSIELDRPNPTSHLAFGRGQHFCPGSALGRRHAQIGIEALLKK
MPGVDLAVPIDQLVWRTRFQRRIPERLPVLW
>CYP121A1_Mycobacterium tuberculosis TB_RSA62 2584903557
MTATVLLEVPFSARGDRIPDAVAELRTREPIRKVRTITGAEAWLVSSYALCTQVLEDRRFSMKETAAAGAPRL
NALTVPPEVVNNMGNIADAGLRKAVMKAITPKAPGLEQFLRDTANSLLDNLITEGAPADLRNDFADPLATALH
CKVLGIPQEDGPKLFRSLSIAFMSSADPIPAAKINWDRDIEYMAGILENPNITTGLMGELSRLRKDPAYSHVS
DELFATIGVTFFGAGVISTGSFLTTALISLIQRPQLRNLLHEKPELIPAGVEELLRINLSFADGLPRLATADI
QVGDVLVRKGELVLVLLEGANFDPEHFPNPGSIELDRPNPTSHLAFGRGQHFCPGSALGRRHAQIGIEALLKK
MPGVDLAVPIDQLVWRTRFQRRIPERLPVLW
>CYP121A1_Mycobacterium tuberculosis TB_RSA63 2605764800
MTATVLLEVPFSARGDRIPDAVAELRTREPIRKVRTITGAEAWLVSSYALCTQVLEDRRFSMKETAAAGAPRL
NALTVPPEVVNNMGNIADAGLRKAVMKAITPKAPGLEQFLRDTANSLLDNLITEGAPADLRNDFADPLATALH
CKVLGIPQEDGPKLFRSLSIAFMSSADPIPAAKINWDRDIEYMAGILENPNITTGLMGELSRLRKDPAYSHVS
DELFATIGVTFFGAGVISTGSFLTTALISLIQRPQLRNLLHEKPELIPAGVEELLRINLSFADGLPRLATADI
QVGDVLVRKGELVLVLLEGANFDPEHFPNPGSIELDRPNPTSHLAFGRGQHFCPGSALGRRHAQIGIEALLKK
MPGVDLAVPIDQLVWRTRFQRRIPERLPVLW
>CYP121A1_Mycobacterium tuberculosis TB_RSA64 2579802245
MTATVLLEVPFSARGDRIPDAVAELRTREPIRKVRTITGAEAWLVSSYALCTQVLEDRRFSMKETAAAGAPRL
NALTVPPEVVNNMGNIADAGLRKAVMKAITPKAPGLEQFLRDTANSLLDNLITEGAPADLRNDFADPLATALH
CKVLGIPQEDGPKLFRSLSIAFMSSADPIPAAKINWDRDIEYMAGILENPNITTGLMGELSRLRKDPAYSHVS
DELFATIGVTFFGAGVISTGSFLTTALISLIQRPQLRNLLHEKPELIPAGVEELLRINLSFADGLPRLATADI

QVGDVLVRKGELVLVLLEGANFDPEHFPNPGSIELDRPNPTSHLAFGRGQHFCPGSALGRRHAQIGIEALLKK
MPGVDLAVPIDQLVWRTRFQRRIPERLPVLW
>CYP121A1_Mycobacterium tuberculosis TB_RSA66 2576613631
MTATVLLEVPFSARGDRIPDAVAELRTREPIRKVRTITGAEAWLVSSYALCTQVLEDRRFSMKETAAAGAPRL
NALTVPPEVVNNMGNIADAGLRKAVMKAITPKAPGLEQFLRDTANSLLDNLITEGAPADLRNDFADPLATALH
CKVLGIPQEDGPKLFRSLSIAFMSSADPIPAAKINWDRDIEYMAGILENPNITTGLMGELSRLRKDPAYSHVS
DELFATIGVTFFGAGVISTGSFLTTALISLIQRPQLRNLLHEKPELIPAGVEELLRINLSFADGLPRLATADI
QVGDVLVRKGELVLVLLEGANFDPEHFPNPGSIELDRPNPTSHLAFGRGQHFCPGSALGRRHAQIGIEALLKK
MPGVDLAVPIDQLVWRTRFQRRIPERLPVLW
>CYP121A1_Mycobacterium tuberculosis TB_RSA67 2576052451
MTATVLLEVPFSARGDRIPDAVAELRTREPIRKVRTITGAEAWLVSSYALCTQVLEDRRFSMKETAAAGAPRL
NALTVPPEVVNNMGNIADAGLRKAVMKAITPKAPGLEQFLRDTANSLLDNLITEGAPADLRNDFADPLATALH
CKVLGIPQEDGPKLFRSLSIAFMSSADPIPAAKINWDRDIEYMAGILENPNITTGLMGELSRLRKDPAYSHVS
DELFATIGVTFFGAGVISTGSFLTTALISLIQRPQLRNLLHEKPELIPAGVEELLRINLSFADGLPRLATADI
QVGDVLVRKGELVLVLLEGANFDPEHFPNPGSIELDRPNPTSHLAFGRGQHFCPGSALGRRHAQIGIEALLKK
MPGVDLAVPIDQLVWRTRFQRRIPERLPVLW
>CYP121A1_Mycobacterium tuberculosis TB_RSA68 2574871779
MTATVLLEVPFSARGDRIPDAVAELRTREPIRKVRTITGAEAWLVSSYALCTQVLEDRRFSMKETAAAGAPRL
NALTVPPEVVNNMGNIADAGLRKAVMKAITPKAPGLEQFLRDTANSLLDNLITEGAPADLRNDFADPLATALH
CKVLGIPQEDGPKLFRSLSIAFMSSADPIPAAKINWDRDIEYMAGILENPNITTGLMGELSRLRKDPAYSHVS
DELFATIGVTFFGAGVISTGSFLTTALISLIQRPQLRNLLHEKPELIPAGVEELLRINLSFADGLPRLATADI
QVGDVLVRKGELVLVLLEGANFDPEHFPNPGSIELDRPNPTSHLAFGRGQHFCPGSALGRRHAQIGIEALLKK
MPGVDLAVPIDQLVWRTRFQRRIPERLPVLW
>CYP121A1_Mycobacterium tuberculosis TB_RSA69 2603436778
MTATVLLEVPFSARGDRIPDAVAELRTREPIRKVRTITGAEAWLVSSYALCTQVLEDRRFSMKETAAAGAPRL
NALTVPPEVVNNMGNIADAGLRKAVMKAITPKAPGLEQFLRDTANSLLDNLITEGAPADLRNDFADPLATALH
CKVLGIPQEDGPKLFRSLSIAFMSSADPIPAAKINWDRDIEYMAGILENPNITTGLMGELSRLRKDPAYSHVS
DELFATIGVTFFGAGVISTGSFLTTALISLIQRPQLRNLLHEKPELIPAGVEELLRINLSFADGLPRLATADI
QVGDVLVRKGELVLVLLEGANFDPEHFPNPGSIELDRPNPTSHLAFGRGQHFCPGSALGRRHAQIGIEALLKK
MPGVDLAVPIDQLVWRTRFQRRIPERLPVLW
>CYP121A1_Mycobacterium tuberculosis TB_RSA70 2577642543
MTATVLLEVPFSARGDRIPDAVAELRTREPIRKVRTITGAEAWLVSSYALCTQVLEDRRFSMKETAAAGAPRL
NALTVPPEVVNNMGNIADAGLRKAVMKAITPKAPGLEQFLRDTANSLLDNLITEGAPADLRNDFADPLATALH
CKVLGIPQEDGPKLFRSLSIAFMSSADPIPAAKINWDRDIEYMAGILENPNITTGLMGELSRLRKDPAYSHVS
DELFATIGVTFFGAGVISTGSFLTTALISLIQRPQLRNLLHEKPELIPAGVEELLRINLSFADGLPRLATADI
QVGDVLVRKGELVLVLLEGANFDPEHFPNPGSIELDRPNPTSHLAFGRGQHFCPGSALGRRHAQIGIEALLKK
MPGVDLAVPIDQLVWRTRFQRRIPERLPVLW
>CYP121A1_Mycobacterium tuberculosis TB_RSA71 2605409728
MTATVLLEVPFSARGDRIPDAVAELRTREPIRKVRTITGAEAWLVSSYALCTQVLEDRRFSMKETAAAGAPRL
NALTVPPEVVNNMGNIADAGLRKAVMKAITPKAPGLEQFLRDTANSLLDNLITEGAPADLRNDFADPLATALH
CKVLGIPQEDGPKLFRSLSIAFMSSADPIPAAKINWDRDIEYMAGILENPNITTGLMGELSRLRKDPAYSHVS
DELFATIGVTFFGAGVISTGSFLTTALISLIQRPQLRNLLHEKPELIPAGVEELLRINLSFADGLPRLATADI
QVGDVLVRKGELVLVLLEGANFDPEHFPNPGSIELDRPNPTSHLAFGRGQHFCPGSALGRRHAQIGIEALLKK
MPGVDLAVPIDQLVWRTRFQRRIPERLPVLW
>CYP121A1_Mycobacterium tuberculosis TB_RSA72 2605037851
MTATVLLEVPFSARGDRIPDAVAELRTREPIRKVRTITGAEAWLVSSYALCTQVLEDRRFSMKETAAAGAPRL
NALTVPPEVVNNMGNIADAGLRKAVMKAITPKAPGLEQFLRDTANSLLDNLITEGAPADLRNDFADPLATALH
CKVLGIPQEDGPKLFRSLSIAFMSSADPIPAAKINWDRDIEYMAGILENPNITTGLMGELSRLRKDPAYSHVS
DELFATIGVTFFGAGVISTGSFLTTALISLIQRPQLRNLLHEKPELIPAGVEELLRINLSFADGLPRLATADI
QVGDVLVRKGELVLVLLEGANFDPEHFPNPGSIELDRPNPTSHLAFGRGQHFCPGSALGRRHAQIGIEALLKK
MPGVDLAVPIDQLVWRTRFQRRIPERLPVLW
>CYP121A1_Mycobacterium tuberculosis TB_RSA73 2604700932
MTATVLLEVPFSARGDRIPDAVAELRTREPIRKVRTITGAEAWLVSSYALCTQVLEDRRFSMKETAAAGAPRL
NALTVPPEVVNNMGNIADAGLRKAVMKAITPKAPGLEQFLRDTANSLLDNLITEGAPADLRNDFADPLATALH
CKVLGIPQEDGPKLFRSLSIAFMSSADPIPAAKINWDRDIEYMAGILENPNITTGLMGELSRLRKDPAYSHVS
DELFATIGVTFFGAGVISTGSFLTTALISLIQRPQLRNLLHEKPELIPAGVEELLRINLSFADGLPRLATADI
QVGDVLVRKGELVLVLLEGANFDPEHFPNPGSIELDRPNPTSHLAFGRGQHFCPGSALGRRHAQIGIEALLKK
MPGVDLAVPIDQLVWRTRFQRRIPERLPVLW

>CYP121A1_Mycobacterium tuberculosis TB_RSA74 2578074402
MTATVLLEVPFSARGDRIPDAVAELRTREPIRKVRTITGAEAWLVSSYALCTQVLEDRRFSMKETAAAGAPRL
NALTVPPEVVNNMGNIADAGLRKAVMKAITPKAPGLEQFLRDTANSLLDNLITEGAPADLRNDFADPLATALH
CKVLGIPQEDGPKLFRSLSIAFMSSADPIPAAKINWDRDIEYMAGILENPNITTGLMGELSRLRKDPAYSHVS
DELFATIGVTFFGAGVISTGSFLTTALISLIQRPQLRNLLHEKPELIPAGVEELLRINLSFADGLPRLATADI
QVGDVLVRKGELVLVLLEGANFDPEHFPNPGSIELDRPNPTSHLAFGRGQHFCPGSALGRRHAQIGIEALLKK
MPGVDLAVPIDQLVWRTRFQRRIPERLPVLW
>CYP121A1_Mycobacterium tuberculosis TB_RSA75 2605770100
MTATVLLEVPFSARGDRIPDAVAELRTREPIRKVRTITGAEAWLVSSYALCTQVLEDRRFSMKETAAAGAPRL
NALTVPPEVVNNMGNIADAGLRKAVMKAITPKAPGLEQFLRDTANSLLDNLITEGAPADLRNDFADPLATALH
CKVLGIPQEDGPKLFRSLSIAFMSSADPIPAAKINWDRDIEYMAGILENPNITTGLMGELSRLRKDPAYSHVS
DELFATIGVTFFGAGVISTGSFLTTALISLIQRPQLRNLLHEKPELIPAGVEELLRINLSFADGLPRLATADI
QVGDVLVRKGELVLVLLEGANFDPEHFPNPGSIELDRPNPTSHLAFGRGQHFCPGSALGRRHAQIGIEALLKK
MPGVDLAVPIDQLVWRTRFQRRIPERLPVLW
>CYP121A1_Mycobacterium tuberculosis TB_RSA76 2576321990
MTATVLLEVPFSARGDRIPDAVAELRTREPIRKVRTITGAEAWLVSSYALCTQVLEDRRFSMKETAAAGAPRL
NALTVPPEVVNNMGNIADAGLRKAVMKAITPKAPGLEQFLRDTANSLLDNLITEGAPADLRNDFADPLATALH
CKVLGIPQEDGPKLFRSLSIAFMSSADPIPAAKINWDRDIEYMAGILENPNITTGLMGELSRLRKDPAYSHVS
DELFATIGVTFFGAGVISTGSFLTTALISLIQRPQLRNLLHEKPELIPAGVEELLRINLSFADGLPRLATADI
QVGDVLVRKGELVLVLLEGANFDPEHFPNPGSIELDRPNPTSHLAFGRGQHFCPGSALGRRHAQIGIEALLKK
MPGVDLAVPIDQLVWRTRFQRRIPERLPVLW
>CYP121A1_Mycobacterium tuberculosis TB_RSA77 2577204471
MTATVLLEVPFSARGDRIPDAVAELRTREPIRKVRTITGAEAWLVSSYALCTQVLEDRRFSMKETAAAGAPRL
NALTVPPEVVNNMGNIADAGLRKAVMKAITPKAPGLEQFLRDTANSLLDNLITEGAPADLRNDFADPLATALH
CKVLGIPQEDGPKLFRSLSIAFMSSADPIPAAKINWDRDIEYMAGILENPNITTGLMGELSRLRKDPAYSHVS
DELFATIGVTFFGAGVISTGSFLTTALISLIQRPQLRNLLHEKPELIPAGVEELLRINLSFADGLPRLATADI
QVGDVLVRKGELVLVLLEGANFDPEHFPNPGSIELDRPNPTSHLAFGRGQHFCPGSALGRRHAQIGIEALLKK
MPGVDLAVPIDQLVWRTRFQRRIPERLPVLW
>CYP121A1_Mycobacterium tuberculosis TB_RSA78 2578054007
MTATVLLEVPFSARGDRIPDAVAELRTREPIRKVRTITGAEAWLVSSYALCTQVLEDRRFSMKETAAAGAPRL
NALTVPPEVVNNMGNIADAGLRKAVMKAITPKAPGLEQFLRDTANSLLDNLITEGAPADLRNDFADPLATALH
CKVLGIPQEDGPKLFRSLSIAFMSSADPIPAAKINWDRDIEYMAGILENPNITTGLMGELSRLRKDPAYSHVS
DELFATIGVTFFGAGVISTGSFLTTALISLIQRPQLRNLLHEKPELIPAGVEELLRINLSFADGLPRLATADI
QVGDVLVRKGELVLVLLEGANFDPEHFPNPGSIELDRPNPTSHLAFGRGQHFCPGSALGRRHAQIGIEALLKK
MPGVDLAVPIDQLVWRTRFQRRIPERLPVLW
>CYP121A1_Mycobacterium tuberculosis TB_RSA79 2584923921
MTATVLLEVPFSARGDRIPDAVAELRTREPIRKVRTITGAEAWLVSSYALCTQVLEDRRFSMKETAAAGAPRL
NALTVPPEVVNNMGNIADAGLRKAVMKAITPKAPGLEQFLRDTANSLLDNLITEGAPADLRNDFADPLATALH
CKVLGIPQEDGPKLFRSLSIAFMSSADPIPAAKINWDRDIEYMAGILENPNITTGLMGELSRLRKDPAYSHVS
DELFATIGVTFFGAGVISTGSFLTTALISLIQRPQLRNLLHEKPELIPAGVEELLRINLSFADGLPRLATADI
QVGDVLVRKGELVLVLLEGANFDPEHFPNPGSIELDRPNPTSHLAFGRGQHFCPGSALGRRHAQIGIEALLKK
MPGVDLAVPIDQLVWRTRFQRRIPERLPVLW
>CYP121A1_Mycobacterium tuberculosis TB_RSA80 2605043499
MTATVLLEVPFSARGDRIPDAVAELRTREPIRKVRTITGAEAWLVSSYALCTQVLEDRRFSMKETAAAGAPRL
NALTVPPEVVNNMGNIADAGLRKAVMKAITPKAPGLEQFLRDTANSLLDNLITEGAPADLRNDFADPLATALH
CKVLGIPQEDGPKLFRSLSIAFMSSADPIPAAKINWDRDIEYMAGILENPNITTGLMGELSRLRKDPAYSHVS
DELFATIGVTFFGAGVISTGSFLTTALISLIQRPQLRNLLHEKPELIPAGVEELLRINLSFADGLPRLATADI
QVGDVLVRKGELVLVLLEGANFDPEHFPNPGSIELDRPNPTSHLAFGRGQHFCPGSALGRRHAQIGIEALLKK
MPGVDLAVPIDQLVWRTRFQRRIPERLPVLW
>CYP121A1_Mycobacterium tuberculosis TB_RSA81 2603288536
MTATVLLEVPFSARGDRIPDAVAELRTREPIRKVRTITGAEAWLVSSYALCTQVLEDRRFSMKETAAAGAPRL
NALTVPPEVVNNMGNIADAGLRKAVMKAITPKAPGLEQFLRDTANSLLDNLITEGAPADLRNDFADPLATALH
CKVLGIPQEDGPKLFRSLSIAFMSSADPIPAAKINWDRDIEYMAGILENPNITTGLMGELSRLRKDPAYSHVS
DELFATIGVTFFGAGVISTGSFLTTALISLIQRPQLRNLLHEKPELIPAGVEELLRINLSFADGLPRLATADI
QVGDVLVRKGELVLVLLEGANFDPEHFPNPGSIELDRPNPTSHLAFGRGQHFCPGSALGRRHAQIGIEALLKK
MPGVDLAVPIDQLVWRTRFQRRIPERLPVLW
>CYP121A1_Mycobacterium tuberculosis TB_RSA82 2576580581

MTATVLLEVPFSARGDRIPDAVAELRTREPIRKVRTITGAEAWLVSSYALCTQVLEDRRFSMKETAAAGAPRL
NALTVPPEVVNNMGNIADAGLRKAVMKAITPKAPGLEQFLRDTANSLLDNLITEGAPADLRNDFADPLATALH
CKVLGIPQEDGPKLFRSLSIAFMSSADPIPAAKINWDRDIEYMAGILENPNITTGLMGELSRLRKDPAYSHVS
DELFATIGVTFFGAGVISTGSFLTTALISLIQRPQLRNLLHEKPELIPAGVEELLRINLSFADGLPRLATADI
QVGDVLVRKGELVLVLLEGANFDPEHFPNPGSIELDRPNPTSHLAFGRGQHFCPGSALGRRHAQIGIEALLKK
MPGVDLAVPIDQLVWRTRFQRRIPERLPVLW
>CYP121A1_Mycobacterium tuberculosis TB_RSA83 2576158663
MTATVLLEVPFSARGDRIPDAVAELRTREPIRKVRTITGAEAWLVSSYALCTQVLEDRRFSMKETAAAGAPRL
NALTVPPEVVNNMGNIADAGLRKAVMKAITPKAPGLEQFLRDTANSLLDNLITEGAPADLRNDFADPLATALH
CKVLGIPQEDGPKLFRSLSIAFMSSADPIPAAKINWDRDIEYMAGILENPNITTGLMGELSRLRKDPAYSHVS
DELFATIGVTFFGAGVISTGSFLTTALISLIQRPQLRNLLHEKPELIPAGVEELLRINLSFADGLPRLATADI
QVGDVLVRKGELVLVLLEGANFDPEHFPNPGSIELDRPNPTSHLAFGRGQHFCPGSALGRRHAQIGIEALLKK
MPGVDLAVPIDQLVWRTRFQRRIPERLPVLW
>CYP121A1_Mycobacterium tuberculosis TB_RSA84 2603439337
MTATVLLEVPFSARGDRIPDAVAELRTREPIRKVRTITGAEAWLVSSYALCTQVLEDRRFSMKETAAAGAPRL
NALTVPPEVVNNMGNIADAGLRKAVMKAITPKAPGLEQFLRDTANSLLDNLITEGAPADLRNDFADPLATALH
CKVLGIPQEDGPKLFRSLSIAFMSSADPIPAAKINWDRDIEYMAGILENPNITTGLMGELSRLRKDPAYSHVS
DELFATIGVTFFGAGVISTGSFLTTALISLIQRPQLRNLLHEKPELIPAGVEELLRINLSFADGLPRLATADI
QVGDVLVRKGELVLVLLEGANFDPEHFPNPGSIELDRPNPTSHLAFGRGQHFCPGSALGRRHAQIGIEALLKK
MPGVDLAVPIDQLVWRTRFQRRIPERLPVLW
>CYP121A1_Mycobacterium tuberculosis TB_RSA87 2605047583
MTATVLLEVPFSARGDRIPDAVAELRTREPIRKVRTITGAEAWLVSSYALCTQVLEDRRFSMKETAAAGAPRL
NALTVPPEVVNNMGNIADAGLRKAVMKAITPKAPGLEQFLRDTANSLLDNLITEGAPADLRNDFADPLATALH
CKVLGIPQEDGPKLFRSLSIAFMSSADPIPAAKINWDRDIEYMAGILENPNITTGLMGELSRLRKDPAYSHVS
DELFATIGVTFFGAGVISTGSFLTTALISLIQRPQLRNLLHEKPELIPAGVEELLRINLSFADGLPRLATADI
QVGDVLVRKGELVLVLLEGANFDPEHFPNPGSIELDRPNPTSHLAFGRGQHFCPGSALGRRHAQIGIEALLKK
MPGVDLAVPIDQLVWRTRFQRRIPERLPVLW
>CYP121A1_Mycobacterium tuberculosis TB_RSA88 2604705866
MTATVLLEVPFSARGDRIPDAVAELRTREPIRKVRTITGAEAWLVSSYALCTQVLEDRRFSMKETAAAGAPRL
NALTVPPEVVNNMGNIADAGLRKAVMKAITPKAPGLEQFLRDTANSLLDNLITEGAPADLRNDFADPLATALH
CKVLGIPQEDGPKLFRSLSIAFMSSADPIPAAKINWDRDIEYMAGILENPNITTGLMGELSRLRKDPAYSHVS
DELFATIGVTFFGAGVISTGSFLTTALISLIQRPQLRNLLHEKPELIPAGVEELLRINLSFADGLPRLATADI
QVGDVLVRKGELVLVLLEGANFDPEHFPNPGSIELDRPNPTSHLAFGRGQHFCPGSALGRRHAQIGIEALLKK
MPGVDLAVPIDQLVWRTRFQRRIPERLPVLW
>CYP121A1_Mycobacterium tuberculosis TB_RSA89 2603443528
MTATVLLEVPFSARGDRIPDAVAELRTREPIRKVRTITGAEAWLVSSYALCTQVLEDRRFSMKETAAAGAPRL
NALTVPPEVVNNMGNIADAGLRKAVMKAITPKAPGLEQFLRDTANSLLDNLITEGAPADLRNDFADPLATALH
CKVLGIPQEDGPKLFRSLSIAFMSSADPIPAAKINWDRDIEYMAGILENPNITTGLMGELSRLRKDPAYSHVS
DELFATIGVTFFGAGVISTGSFLTTALISLIQRPQLRNLLHEKPELIPAGVEELLRINLSFADGLPRLATADI
QVGDVLVRKGELVLVLLEGANFDPEHFPNPGSIELDRPNPTSHLAFGRGQHFCPGSALGRRHAQIGIEALLKK
MPGVDLAVPIDQLVWRTRFQRRIPERLPVLW
>CYP121A1_Mycobacterium tuberculosis TB_RSA90 2575477984
MTATVLLEVPFSARGDRIPDAVAELRTREPIRKVRTITGAEAWLVSSYALCTQVLEDRRFSMKETAAAGAPRL
NALTVPPEVVNNMGNIADAGLRKAVMKAITPKAPGLEQFLRDTANSLLDNLITEGAPADLRNDFADPLATALH
CKVLGIPQEDGPKLFRSLSIAFMSSADPIPAAKINWDRDIEYMAGILENPNITTGLMGELSRLRKDPAYSHVS
DELFATIGVTFFGAGVISTGSFLTTALISLIQRPQLRNLLHEKPELIPAGVEELLRINLSFADGLPRLATADI
QVGDVLVRKGELVLVLLEGANFDPEHFPNPGSIELDRPNPTSHLAFGRGQHFCPGSALGRRHAQIGIEALLKK
MPGVDLAVPIDQLVWRTRFQRRIPERLPVLW
>CYP121A1_Mycobacterium tuberculosis TB_RSA91 2603449069
MTATVLLEVPFSARGDRIPDAVAELRTREPIRKVRTITGAEAWLVSSYALCTQVLEDRRFSMKETAAAGAPRL
NALTVPPEVVNNMGNIADAGLRKAVMKAITPKAPGLEQFLRDTANSLLDNLITEGAPADLRNDFADPLATALH
CKVLGIPQEDGPKLFRSLSIAFMSSADPIPAAKINWDRDIEYMAGILENPNITTGLMGELSRLRKDPAYSHVS
DELFATIGVTFFGAGVISTGSFLTTALISLIQRPQLRNLLHEKPELIPAGVEELLRINLSFADGLPRLATADI
QVGDVLVRKGELVLVLLEGANFDPEHFPNPGSIELDRPNPTSHLAFGRGQHFCPGSALGRRHAQIGIEALLKK
MPGVDLAVPIDQLVWRTRFQRRIPERLPVLW
>CYP121A1_Mycobacterium tuberculosis TB_RSA92 2605051683
MTATVLLEVPFSARGDRIPDAVAELRTREPIRKVRTITGAEAWLVSSYALCTQVLEDRRFSMKETAAAGAPRL
NALTVPPEVVNNMGNIADAGLRKAVMKAITPKAPGLEQFLRDTANSLLDNLITEGAPADLRNDFADPLATALH

CKVLGIPQEDGPKLFRSLSIAFMSSADPIPAAKINWDRDIEYMAGILENPNITTGLMGELSRLRKDPAYSHVS
DELFATIGVTFFGAGVISTGSFLTTALISLIQRPQLRNLLHEKPELIPAGVEELLRINLSFADGLPRLATADI
QVGDVLVRKGELVLVLLEGANFDPEHFPNPGSIELDRPNPTSHLAFGRGQHFCPGSALGRRHAQIGIEALLKK
MPGVDLAVPIDQLVWRTRFQRRIPERLPVLW
>CYP121A1_Mycobacterium tuberculosis TB_RSA93 2605412851
MTATVLLEVPFSARGDRIPDAVAELRTREPIRKVRTITGAEAWLVSSYALCTQVLEDRRFSMKETAAAGAPRL
NALTVPPEVVNNMGNIADAGLRKAVMKAITPKAPGLEQFLRDTANSLLDNLITEGAPADLRNDFADPLATALH
CKVLGIPQEDGPKLFRSLSIAFMSSADPIPAAKINWDRDIEYMAGILENPNITTGLMGELSRLRKDPAYSHVS
DELFATIGVTFFGAGVISTGSFLTTALISLIQRPQLRNLLHEKPELIPAGVEELLRINLSFADGLPRLATADI
QVGDVLVRKGELVLVLLEGANFDPEHFPNPGSIELDRPNPTSHLAFGRGQHFCPGSALGRRHAQIGIEALLKK
MPGVDLAVPIDQLVWRTRFQRRIPERLPVLW
>CYP121A1_Mycobacterium tuberculosis TB_RSA94 2605773224
MTATVLLEVPFSARGDRIPDAVAELRTREPIRKVRTITGAEAWLVSSYALCTQVLEDRRFSMKETAAAGAPRL
NALTVPPEVVNNMGNIADAGLRKAVMKAITPKAPGLEQFLRDTANSLLDNLITEGAPADLRNDFADPLATALH
CKVLGIPQEDGPKLFRSLSIAFMSSADPIPAAKINWDRDIEYMAGILENPNITTGLMGELSRLRKDPAYSHVS
DELFATIGVTFFGAGVISTGSFLTTALISLIQRPQLRNLLHEKPELIPAGVEELLRINLSFADGLPRLATADI
QVGDVLVRKGELVLVLLEGANFDPEHFPNPGSIELDRPNPTSHLAFGRGQHFCPGSALGRRHAQIGIEALLKK
MPGVDLAVPIDQLVWRTRFQRRIPERLPVLW
>CYP121A1_Mycobacterium tuberculosis TB_RSA95 2603294695
MTATVLLEVPFSARGDRIPDAVAELRTREPIRKVRTITGAEAWLVSSYALCTQVLEDRRFSMKETAAAGAPRL
NALTVPPEVVNNMGNIADAGLRKAVMKAITPKAPGLEQFLRDTANSLLDNLITEGAPADLRNDFADPLATALH
CKVLGIPQEDGPKLFRSLSIAFMSSADPIPAAKINWDRDIEYMAGILENPNITTGLMGELSRLRKDPAYSHVS
DELFATIGVTFFGAGVISTGSFLTTALISLIQRPQLRNLLHEKPELIPAGVEELLRINLSFADGLPRLATADI
QVGDVLVRKGELVLVLLEGANFDPEHFPNPGSIELDRPNPTSHLAFGRGQHFCPGSALGRRHAQIGIEALLKK
MPGVDLAVPIDQLVWRTRFQRRIPERLPVLW
>CYP121A1_Mycobacterium tuberculosis TB_RSA96 2574738693
MTATVLLEVPFSARGDRIPDAVAELRTREPIRKVRTITGAEAWLVSSYALCTQVLEDRRFSMKETAAAGAPRL
NALTVPPEVVNNMGNIADAGLRKAVMKAITPKAPGLEQFLRDTANSLLDNLITEGAPADLRNDFADPLATALH
CKVLGIPQEDGPKLFRSLSIAFMSSADPIPAAKINWDRDIEYMAGILENPNITTGLMGELSRLRKDPAYSHVS
DELFATIGVTFFGAGVISTGSFLTTALISLIQRPQLRNLLHEKPELIPAGVEELLRINLSFADGLPRLATADI
QVGDVLVRKGELVLVLLEGANFDPEHFPNPGSIELDRPNPTSHLAFGRGQHFCPGSALGRRHAQIGIEALLKK
MPGVDLAVPIDQLVWRTRFQRRIPERLPVLW
>CYP121A1_Mycobacterium tuberculosis TB_RSA98 2603453197
MTATVLLEVPFSARGDRIPDAVAELRTREPIRKVRTITGAEAWLVSSYALCTQVLEDRRFSMKETAAAGAPRL
NALTVPPEVVNNMGNIADAGLRKAVMKAITPKAPGLEQFLRDTANSLLDNLITEGAPADLRNDFADPLATALH
CKVLGIPQEDGPKLFRSLSIAFMSSADPIPAAKINWDRDIEYMAGILENPNITTGLMGELSRLRKDPAYSHVS
DELFATIGVTFFGAGVISTGSFLTTALISLIQRPQLRNLLHEKPELIPAGVEELLRINLSFADGLPRLATADI
QVGDVLVRKGELVLVLLEGANFDPEHFPNPGSIELDRPNPTSHLAFGRGQHFCPGSALGRRHAQIGIEALLKK
MPGVDLAVPIDQLVWRTRFQRRIPERLPVLW
>CYP121A1_Mycobacterium tuberculosis TB_RSA99 2575612465
MTATVLLEVPFSARGDRIPDAVAELRTREPIRKVRTITGAEAWLVSSYALCTQVLEDRRFSMKETAAAGAPRL
NALTVPPEVVNNMGNIADAGLRKAVMKAITPKAPGLEQFLRDTANSLLDNLITEGAPADLRNDFADPLATALH
CKVLGIPQEDGPKLFRSLSIAFMSSADPIPAAKINWDRDIEYMAGILENPNITTGLMGELSRLRKDPAYSHVS
DELFATIGVTFFGAGVISTGSFLTTALISLIQRPQLRNLLHEKPELIPAGVEELLRINLSFADGLPRLATADI
QVGDVLVRKGELVLVLLEGANFDPEHFPNPGSIELDRPNPTSHLAFGRGQHFCPGSALGRRHAQIGIEALLKK
MPGVDLAVPIDQLVWRTRFQRRIPERLPVLW
>CYP121A1_Mycobacterium tuberculosis TBR-152 2696496160
MTATVLLEVPFSARGDRIPDAVAELRTREPIRKVRTITGAEAWLVSSYALCTQVLEDRRFSMKETAAAGAPRL
NALTVPPEVVNNMGNIADAGLRKAVMKAITPKAPGLEQFLRDTANSLLDNLITEGAPADLRNDFADPLATALH
CKVLGIPQEDGPKLFRSLSIAFMSSADPIPAAKINWDRDIEYMAGILENPNITTGLMGELSRLRKDPAYSHVS
DELFATIGVTFFGAGVISTGSFLTTALISLIQRPQLRNLLHEKPELIPAGVEELLRINLSFADGLPRLATADI
QVGDVLVRKGELVLVLLEGANFDPEHFPNPGSIELDRPNPTSHLAFGRGQHFCPGSALGRRHAQIGIEALLKK
MPGVDLAVPIDQLVWRTRFQRRIPERLPVLW
>CYP121A1_Mycobacterium tuberculosis TBR-175 2663319422
MTATVLLEVPFSARGDRIPDAVAELRTREPIRKVRTITGAEAWLVSSYALCTQVLEDRRFSMKETAAAGAPRL
NALTVPPEVVNNMGNIADAGLRKAVMKAITPKAPGLEQFLRDTANSLLDNLITEGAPADLRNDFADPLATALH
CKVLGIPQEDGPKLFRSLSIAFMSSADPIPAAKINWDRDIEYMAGILENPNITTGLMGELSRLRKDPAYSHVS
DELFATIGVTFFGAGVISTGSFLTTALISLIQRPQLRNLLHEKPELIPAGVEELLRINLSFADGLPRLATADI

QVGDVLVRKGELVLVLLEGANFDPEHFPNPGSIELDRPNPTSHLAFGRGQHFCPGSALGRRHAQIGIEALLKK
MPGVDLAVPIDQLVWRTRFQRRIPERLPVLW
>CYP121A1_Mycobacterium tuberculosis TBR26 2577793360
MTATVLLEVPFSARGDRIPDAVAELRTREPIRKVRTITGAEAWLVSSYALCTQVLEDRRFSMKETAAAGAPRL
NALTVPPEVVNNMGNIADAGLRKAVMKAITPKAPGLEQFLRDTANSLLDNLITEGAPADLRNDFADPLATALH
CKVLGIPQEDGPKLFRSLSIAFMSSADPIPAAKINWDRDIEYMAGILENPNITTGLMGELSRLRKDPAYSHVS
DELFATIGVTFFGAGVISTGSFLTTALISLIQRPQLRNLLHEKPELIPAGVEELLRINLSFADGLPRLATADI
QVGDVLVRKGELVLVLLEGANFDPEHFPNPGSIELDRPNPTSHLAFGRGQHFCPGSALGRRHAQIGIEALLKK
MPGVDLAVPIDQLVWRTRFQRRIPERLPVLW
>CYP121A1_Mycobacterium tuberculosis TBR28 2575382321
MTATVLLEVPFSARGDRIPDAVAELRTREPIRKVRTITGAEAWLVSSYALCTQVLEDRRFSMKETAAAGAPRL
NALTVPPEVVNNMGNIADAGLRKAVMKAITPKAPGLEQFLRDTANSLLDNLITEGAPADLRNDFADPLATALH
CKVLGIPQEDGPKLFRSLSIAFMSSADPIPAAKINWDRDIEYMAGILENPNITTGLMGELSRLRKDPAYSHVS
DELFATIGVTFFGAGVISTGSFLTTALISLIQRPQLRNLLHEKPELIPAGVEELLRINLSFADGLPRLATADI
QVGDVLVRKGELVLVLLEGANFDPEHFPNPGSIELDRPNPTSHLAFGRGQHFCPGSALGRRHAQIGIEALLKK
MPGVDLAVPIDQLVWRTRFQRRIPERLPVLW
>CYP121A1_Mycobacterium tuberculosis TBR31 2574635670
MTATVLLEVPFSARGDRIPDAVAELRTREPIRKVRTITGAEAWLVSSYALCTQVLEDRRFSMKETAAAGAPRL
NALTVPPEVVNNMGNIADAGLRKAVMKAITPKAPGLEQFLRDTANSLLDNLITEGAPADLRNDFADPLATALH
CKVLGIPQEDGPKLFRSLSIAFMSSADPIPAAKINWDRDIEYMAGILENPNITTGLMGELSRLRKDPAYSHVS
DELFATIGVTFFGAGVISTGSFLTTALISLIQRPQLRNLLHEKPELIPAGVEELLRINLSFADGLPRLATADI
QVGDVLVRKGELVLVLLEGANFDPEHFPNPGSIELDRPNPTSHLAFGRGQHFCPGSALGRRHAQIGIEALLKK
MPGVDLAVPIDQLVWRTRFQRRIPERLPVLW
>CYP121A1_Mycobacterium tuberculosis TBR37 2576758370
MTATVLLEVPFSARGDRIPDAVAELRTREPIRKVRTITGAEAWLVSSYALCTQVLEDRRFSMKETAAAGAPRL
NALTVPPEVVNNMGNIADAGLRKAVMKAITPKAPGLEQFLRDTANSLLDNLITEGAPADLRNDFADPLATALH
CKVLGIPQEDGPKLFRSLSIAFMSSADPIPAAKINWDRDIEYMAGILENPNITTGLMGELSRLRKDPAYSHVS
DELFATIGVTFFGAGVISTGSFLTTALISLIQRPQLRNLLHEKPELIPAGVEELLRINLSFADGLPRLATADI
QVGDVLVRKGELVLVLLEGANFDPEHFPNPGSIELDRPNPTSHLAFGRGQHFCPGSALGRRHAQIGIEALLKK
MPGVDLAVPIDQLVWRTRFQRRIPERLPVLW
>CYP121A1_Mycobacterium tuberculosis TBR41 2575889214
MTATVLLEVPFSARGDRIPDAVAELRTREPIRKVRTITGAEAWLVSSYALCTQVLEDRRFSMKETAAAGAPRL
NALTVPPEVVNNMGNIADAGLRKAVMKAITPKAPGLEQFLRDTANSLLDNLITEGAPADLRNDFADPLATALH
CKVLGIPQEDGPKLFRSLSIAFMSSADPIPAAKINWDRDIEYMAGILENPNITTGLMGELSRLRKDPAYSHVS
DELFATIGVTFFGAGVISTGSFLTTALISLIQRPQLRNLLHEKPELIPAGVEELLRINLSFADGLPRLATADI
QVGDVLVRKGELVLVLLEGANFDPEHFPNPGSIELDRPNPTSHLAFGRGQHFCPGSALGRRHAQIGIEALLKK
MPGVDLAVPIDQLVWRTRFQRRIPERLPVLW
>CYP121A1_Mycobacterium tuberculosis TBR50 2576156157
MTATVLLEVPFSARGDRIPDAVAELRTREPIRKVRTITGAEAWLVSSYALCTQVLEDRRFSMKETAAAGAPRL
NALTVPPEVVNNMGNIADAGLRKAVMKAITPKAPGLEQFLRDTANSLLDNLITEGAPADLRNDFADPLATALH
CKVLGIPQEDGPKLFRSLSIAFMSSADPIPAAKINWDRDIEYMAGILENPNITTGLMGELSRLRKDPAYSHVS
DELFATIGVTFFGAGVISTGSFLTTALISLIQRPQLRNLLHEKPELIPAGVEELLRINLSFADGLPRLATADI
QVGDVLVRKGELVLVLLEGANFDPEHFPNPGSIELDRPNPTSHLAFGRGQHFCPGSALGRRHAQIGIEALLKK
MPGVDLAVPIDQLVWRTRFQRRIPERLPVLW
>CYP121A1_Mycobacterium tuberculosis TBR51 2584609707
MTATVLLEVPFSARGDRIPDAVAELRTREPIRKVRTITGAEAWLVSSYALCTQVLEDRRFSMKETAAAGAPRL
NALTVPPEVVNNMGNIADAGLRKAVMKAITPKAPGLEQFLRDTANSLLDNLITEGAPADLRNDFADPLATALH
CKVLGIPQEDGPKLFRSLSIAFMSSADPIPAAKINWDRDIEYMAGILENPNITTGLMGELSRLRKDPAYSHVS
DELFATIGVTFFGAGVISTGSFLTTALISLIQRPQLRNLLHEKPELIPAGVEELLRINLSFADGLPRLATADI
QVGDVLVRKGELVLVLLEGANFDPEHFPNPGSIELDRPNPTSHLAFGRGQHFCPGSALGRRHAQIGIEALLKK
MPGVDLAVPIDQLVWRTRFQRRIPERLPVLW
>CYP121A1_Mycobacterium tuberculosis TBR58 2574877219
MTATVLLEVPFSARGDRIPDAVAELRTREPIRKVRTITGAEAWLVSSYALCTQVLEDRRFSMKETAAAGAPRL
NALTVPPEVVNNMGNIADAGLRKAVMKAITPKAPGLEQFLRDTANSLLDNLITEGAPADLRNDFADPLATALH
CKVLGIPQEDGPKLFRSLSIAFMSSADPIPAAKINWDRDIEYMAGILENPNITTGLMGELSRLRKDPAYSHVS
DELFATIGVTFFGAGVISTGSFLTTALISLIQRPQLRNLLHEKPELIPAGVEELLRINLSFADGLPRLATADI
QVGDVLVRKGELVLVLLEGANFDPEHFPNPGSIELDRPNPTSHLAFGRGQHFCPGSALGRRHAQIGIEALLKK
MPGVDLAVPIDQLVWRTRFQRRIPERLPVLW

>CYP121A1_Mycobacterium tuberculosis TBR65 2574885670
MTATVLLEVPFSARGDRIPDAVAELRTREPIRKVRTITGAEAWLVSSYALCTQVLEDRRFSMKETAAAGAPRL
NALTVPPEVVNNMGNIADAGLRKAVMKAITPKAPGLEQFLRDTANSLLDNLITEGAPADLRNDFADPLATALH
CKVLGIPQEDGPKLFRSLSIAFMSSADPIPAAKINWDRDIEYMAGILENPNITTGLMGELSRLRKDPAYSHVS
DELFATIGVTFFGAGVISTGSFLTTALISLIQRPQLRNLLHEKPELIPAGVEELLRINLSFADGLPRLATADI
QVGDVLVRKGELVLVLLEGANFDPEHFPNPGSIELDRPNPTSHLAFGRGQHFCPGSALGRRHAQIGIEALLKK
MPGVDLAVPIDQLVWRTRFQRRIPERLPVLW
>CYP121A1_Mycobacterium tuberculosis TBR7 2575979045
MTATVLLEVPFSARGDRIPDAVAELRTREPIRKVRTITGAEAWLVSSYALCTQVLEDRRFSMKETAAAGAPRL
NALTVPPEVVNNMGNIADAGLRKAVMKAITPKAPGLEQFLRDTANSLLDNLITEGAPADLRNDFADPLATALH
CKVLGIPQEDGPKLFRSLSIAFMSSADPIPAAKINWDRDIEYMAGILENPNITTGLMGELSRLRKDPAYSHVS
DELFATIGVTFFGAGVISTGSFLTTALISLIQRPQLRNLLHEKPELIPAGVEELLRINLSFADGLPRLATADI
QVGDVLVRKGELVLVLLEGANFDPEHFPNPGSIELDRPNPTSHLAFGRGQHFCPGSALGRRHAQIGIEALLKK
MPGVDLAVPIDQLVWRTRFQRRIPERLPVLW
>CYP121A1_Mycobacterium tuberculosis TKK_02_0006 2592434645
MTATVLLEVPFSARGDRIPDAVAELRTREPIRKVRTITGAEAWLVSSYALCTQVLEDRRFSMKETAAAGAPRL
NALTVPPEVVNNMGNIADAGLRKAVMKAITPKAPGLEQFLRDTANSLLDNLITEGAPADLRNDFADPLATALH
CKVLGIPQEDGPKLFRSLSIAFMSSADPIPAAKINWDRDIEYMAGILENPNITTGLMGELSRLRKDPAYSHVS
DELFATIGVTFFGAGVISTGSFLTTALISLIQRPQLRNLLHEKPELIPAGVEELLRINLSFADGLPRLATADI
QVGDVLVRKGELVLVLLEGANFDPEHFPNPGSIELDRPNPTSHLAFGRGQHFCPGSALGRRHAQIGIEALLKK
MPGVDLAVPIDQLVWRTRFQRRIPERLPVLW
>CYP121A1_Mycobacterium tuberculosis TKK_02_0012 2592431031
MTATVLLEVPFSARGDRIPDAVAELRTREPIRKVRTITGAEAWLVSSYALCTQVLEDRRFSMKETAAAGAPRL
NALTVPPEVVNNMGNIADAGLRKAVMKAITPKAPGLEQFLRDTANSLLDNLITEGAPADLRNDFADPLATALH
CKVLGIPQEDGPKLFRSLSIAFMSSADPIPAAKINWDRDIEYMAGILENPNITTGLMGELSRLRKDPAYSHVS
DELFATIGVTFFGAGVISTGSFLTTALISLIQRPQLRNLLHEKPELIPAGVEELLRINLSFADGLPRLATADI
QVGDVLVRKGELVLVLLEGANFDPEHFPNPGSIELDRPNPTSHLAFGRGQHFCPGSALGRRHAQIGIEALLKK
MPGVDLAVPIDQLVWRTRFQRRIPERLPVLW
>CYP121A1_Mycobacterium tuberculosis TKK_02_0013 2592426960
MTATVLLEVPFSARGDRIPDAVAELRTREPIRKVRTITGAEAWLVSSYALCTQVLEDRRFSMKETAAAGAPRL
NALTVPPEVVNNMGNIADAGLRKAVMKAITPKAPGLEQFLRDTANSLLDNLITEGAPADLRNDFADPLATALH
CKVLGIPQEDGPKLFRSLSIAFMSSADPIPAAKINWDRDIEYMAGILENPNITTGLMGELSRLRKDPAYSHVS
DELFATIGVTFFGAGVISTGSFLTTALISLIQRPQLRNLLHEKPELIPAGVEELLRINLSFADGLPRLATADI
QVGDVLVRKGELVLVLLEGANFDPEHFPNPGSIELDRPNPTSHLAFGRGQHFCPGSALGRRHAQIGIEALLKK
MPGVDLAVPIDQLVWRTRFQRRIPERLPVLW
>CYP121A1_Mycobacterium tuberculosis TKK_02_0014 2584940164
MTATVLLEVPFSARGDRIPDAVAELRTREPIRKVRTITGAEAWLVSSYALCTQVLEDRRFSMKETAAAGAPRL
NALTVPPEVVNNMGNIADAGLRKAVMKAITPKAPGLEQFLRDTANSLLDNLITEGAPADLRNDFADPLATALH
CKVLGIPQEDGPKLFRSLSIAFMSSADPIPAAKINWDRDIEYMAGILENPNITTGLMGELSRLRKDPAYSHVS
DELFATIGVTFFGAGVISTGSFLTTALISLIQRPQLRNLLHEKPELIPAGVEELLRINLSFADGLPRLATADI
QVGDVLVRKGELVLVLLEGANFDPEHFPNPGSIELDRPNPTSHLAFGRGQHFCPGSALGRRHAQIGIEALLKK
MPGVDLAVPIDQLVWRTRFQRRIPERLPVLW
>CYP121A1_Mycobacterium tuberculosis TKK_02_0015 2574726760
MTATVLLEVPFSARGDRIPDAVAELRTREPIRKVRTITGAEAWLVSSYALCTQVLEDRRFSMKETAAAGAPRL
NALTVPPEVVNNMGNIADAGLRKAVMKAITPKAPGLEQFLRDTANSLLDNLITEGAPADLRNDFADPLATALH
CKVLGIPQEDGPKLFRSLSIAFMSSADPIPAAKINWDRDIEYMAGILENPNITTGLMGELSRLRKDPAYSHVS
DELFATIGVTFFGAGVISTGSFLTTALISLIQRPQLRNLLHEKPELIPAGVEELLRINLSFADGLPRLATADI
QVGDVLVRKGELVLVLLEGANFDPEHFPNPGSIELDRPNPTSHLAFGRGQHFCPGSALGRRHAQIGIEALLKK
MPGVDLAVPIDQLVWRTRFQRRIPERLPVLW
>CYP121A1_Mycobacterium tuberculosis TKK_02_0016 2592422886
MTATVLLEVPFSARGDRIPDAVAELRTREPIRKVRTITGAEAWLVSSYALCTQVLEDRRFSMKETAAAGAPRL
NALTVPPEVVNNMGNIADAGLRKAVMKAITPKAPGLEQFLRDTANSLLDNLITEGAPADLRNDFADPLATALH
CKVLGIPQEDGPKLFRSLSIAFMSSADPIPAAKINWDRDIEYMAGILENPNITTGLMGELSRLRKDPAYSHVS
DELFATIGVTFFGAGVISTGSFLTTALISLIQRPQLRNLLHEKPELIPAGVEELLRINLSFADGLPRLATADI
QVGDVLVRKGELVLVLLEGANFDPEHFPNPGSIELDRPNPTSHLAFGRGQHFCPGSALGRRHAQIGIEALLKK
MPGVDLAVPIDQLVWRTRFQRRIPERLPVLW
>CYP121A1_Mycobacterium tuberculosis TKK_02_0017 2592418029

MTATVLLEVPFSARGDRIPDAVAELRTREPIRKVRTITGAEAWLVSSYALCTQVLEDRRFSMKETAAAGAPRL
NALTVPPEVVNNMGNIADAGLRKAVMKAITPKAPGLEQFLRDTANSLLDNLITEGAPADLRNDFADPLATALH
CKVLGIPQEDGPKLFRSLSIAFMSSADPIPAAKINWDRDIEYMAGILENPNITTGLMGELSRLRKDPAYSHVS
DELFATIGVTFFGAGVISTGSFLTTALISLIQRPQLRNLLHEKPELIPAGVEELLRINLSFADGLPRLATADI
QVGDVLVRKGELVLVLLEGANFDPEHFPNPGSIELDRPNPTSHLAFGRGQHFCPGSALGRRHAQIGIEALLKK
MPGVDLAVPIDQLVWRTRFQRRIPERLPVLW
>CYP121A1_Mycobacterium tuberculosis TKK_02_0018 2592414836
MTATVLLEVPFSARGDRIPDAVAELRTREPIRKVRTITGAEAWLVSSYALCTQVLEDRRFSMKETAAAGAPRL
NALTVPPEVVNNMGNIADAGLRKAVMKAITPKAPGLEQFLRDTANSLLDNLITEGAPADLRNDFADPLATALH
CKVLGIPQEDGPKLFRSLSIAFMSSADPIPAAKINWDRDIEYMAGILENPNITTGLMGELSRLRKDPAYSHVS
DELFATIGVTFFGAGVISTGSFLTTALISLIQRPQLRNLLHEKPELIPAGVEELLRINLSFADGLPRLATADI
QVGDVLVRKGELVLVLLEGANFDPEHFPNPGSIELDRPNPTSHLAFGRGQHFCPGSALGRRHAQIGIEALLKK
MPGVDLAVPIDQLVWRTRFQRRIPERLPVLW
>CYP121A1_Mycobacterium tuberculosis TKK_02_0019 2575329973
MTATVLLEVPFSARGDRIPDAVAELRTREPIRKVRTITGAEAWLVSSYALCTQVLEDRRFSMKETAAAGAPRL
NALTVPPEVVNNMGNIADAGLRKAVMKAITPKAPGLEQFLRDTANSLLDNLITEGAPADLRNDFADPLATALH
CKVLGIPQEDGPKLFRSLSIAFMSSADPIPAAKINWDRDIEYMAGILENPNITTGLMGELSRLRKDPAYSHVS
DELFATIGVTFFGAGVISTGSFLTTALISLIQRPQLRNLLHEKPELIPAGVEELLRINLSFADGLPRLATADI
QVGDVLVRKGELVLVLLEGANFDPEHFPNPGSIELDRPNPTSHLAFGRGQHFCPGSALGRRHAQIGIEALLKK
MPGVDLAVPIDQLVWRTRFQRRIPERLPVLW
>CYP121A1_Mycobacterium tuberculosis TKK_02_0020 2592410668
MTATVLLEVPFSARGDRIPDAVAELRTREPIRKVRTITGAEAWLVSSYALCTQVLEDRRFSMKETAAAGAPRL
NALTVPPEVVNNMGNIADAGLRKAVMKAITPKAPGLEQFLRDTANSLLDNLITEGAPADLRNDFADPLATALH
CKVLGIPQEDGPKLFRSLSIAFMSSADPIPAAKINWDRDIEYMAGILENPNITTGLMGELSRLRKDPAYSHVS
DELFATIGVTFFGAGVISTGSFLTTALISLIQRPQLRNLLHEKPELIPAGVEELLRINLSFADGLPRLATADI
QVGDVLVRKGELVLVLLEGANFDPEHFPNPGSIELDRPNPTSHLAFGRGQHFCPGSALGRRHAQIGIEALLKK
MPGVDLAVPIDQLVWRTRFQRRIPERLPVLW
>CYP121A1_Mycobacterium tuberculosis TKK_02_0021 2592406598
MTATVLLEVPFSARGDRIPDAVAELRTREPIRKVRTITGAEAWLVSSYALCTQVLEDRRFSMKETAAAGAPRL
NALTVPPEVVNNMGNIADAGLRKAVMKAITPKAPGLEQFLRDTANSLLDNLITEGAPADLRNDFADPLATALH
CKVLGIPQEDGPKLFRSLSIAFMSSADPIPAAKINWDRDIEYMAGILENPNITTGLMGELSRLRKDPAYSHVS
DELFATIGVTFFGAGVISTGSFLTTALISLIQRPQLRNLLHEKPELIPAGVEELLRINLSFADGLPRLATADI
QVGDVLVRKGELVLVLLEGANFDPEHFPNPGSIELDRPNPTSHLAFGRGQHFCPGSALGRRHAQIGIEALLKK
MPGVDLAVPIDQLVWRTRFQRRIPERLPVLW
>CYP121A1_Mycobacterium tuberculosis TKK_02_0022 2592402460
MTATVLLEVPFSARGDRIPDAVAELRTREPIRKVRTITGAEAWLVSSYALCTQVLEDRRFSMKETAAAGAPRL
NALTVPPEVVNNMGNIADAGLRKAVMKAITPKAPGLEQFLRDTANSLLDNLITEGAPADLRNDFADPLATALH
CKVLGIPQEDGPKLFRSLSIAFMSSADPIPAAKINWDRDIEYMAGILENPNITTGLMGELSRLRKDPAYSHVS
DELFATIGVTFFGAGVISTGSFLTTALISLIQRPQLRNLLHEKPELIPAGVEELLRINLSFADGLPRLATADI
QVGDVLVRKGELVLVLLEGANFDPEHFPNPGSIELDRPNPTSHLAFGRGQHFCPGSALGRRHAQIGIEALLKK
MPGVDLAVPIDQLVWRTRFQRRIPERLPVLW
>CYP121A1_Mycobacterium tuberculosis TKK_02_0025 2592398522
MTATVLLEVPFSARGDRIPDAVAELRTREPIRKVRTITGAEAWLVSSYALCTQVLEDRRFSMKETAAAGAPRL
NALTVPPEVVNNMGNIADAGLRKAVMKAITPKAPGLEQFLRDTANSLLDNLITEGAPADLRNDFADPLATALH
CKVLGIPQEDGPKLFRSLSIAFMSSADPIPAAKINWDRDIEYMAGILENPNITTGLMGELSRLRKDPAYSHVS
DELFATIGVTFFGAGVISTGSFLTTALISLIQRPQLRNLLHEKPELIPAGVEELLRINLSFADGLPRLATADI
QVGDVLVRKGELVLVLLEGANFDPEHFPNPGSIELDRPNPTSHLAFGRGQHFCPGSALGRRHAQIGIEALLKK
MPGVDLAVPIDQLVWRTRFQRRIPERLPVLW
>CYP121A1_Mycobacterium tuberculosis TKK_02_0027 2592394171
MTATVLLEVPFSARGDRIPDAVAELRTREPIRKVRTITGAEAWLVSSYALCTQVLEDRRFSMKETAAAGAPRL
NALTVPPEVVNNMGNIADAGLRKAVMKAITPKAPGLEQFLRDTANSLLDNLITEGAPADLRNDFADPLATALH
CKVLGIPQEDGPKLFRSLSIAFMSSADPIPAAKINWDRDIEYMAGILENPNITTGLMGELSRLRKDPAYSHVS
DELFATIGVTFFGAGVISTGSFLTTALISLIQRPQLRNLLHEKPELIPAGVEELLRINLSFADGLPRLATADI
QVGDVLVRKGELVLVLLEGANFDPEHFPNPGSIELDRPNPTSHLAFGRGQHFCPGSALGRRHAQIGIEALLKK
MPGVDLAVPIDQLVWRTRFQRRIPERLPVLW
>CYP121A1_Mycobacterium tuberculosis TKK_02_0033 2592390338
MTATVLLEVPFSARGDRIPDAVAELRTREPIRKVRTITGAEAWLVSSYALCTQVLEDRRFSMKETAAAGAPRL
NALTVPPEVVNNMGNIADAGLRKAVMKAITPKAPGLEQFLRDTANSLLDNLITEGAPADLRNDFADPLATALH

CKVLGIPQEDGPKLFRSLSIAFMSSADPIPAAKINWDRDIEYMAGILENPNITTGLMGELSRLRKDPAYSHVS
DELFATIGVTFFGAGVISTGSFLTTALISLIQRPQLRNLLHEKPELIPAGVEELLRINLSFADGLPRLATADI
QVGDVLVRKGELVLVLLEGANFDPEHFPNPGSIELDRPNPTSHLAFGRGQHFCPGSALGRRHAQIGIEALLKK
MPGVDLAVPIDQLVWRTRFQRRIPERLPVLW
>CYP121A1_Mycobacterium tuberculosis TKK_02_0034 2592386231
MTATVLLEVPFSARGDRIPDAVAELRTREPIRKVRTITGAEAWLVSSYALCTQVLEDRRFSMKETAAAGAPRL
NALTVPPEVVNNMGNIADAGLRKAVMKAITPKAPGLEQFLRDTANSLLDNLITEGAPADLRNDFADPLATALH
CKVLGIPQEDGPKLFRSLSIAFMSSADPIPAAKINWDRDIEYMAGILENPNITTGLMGELSRLRKDPAYSHVS
DELFATIGVTFFGAGVISTGSFLTTALISLIQRPQLRNLLHEKPELIPAGVEELLRINLSFADGLPRLATADI
QVGDVLVRKGELVLVLLEGANFDPEHFPNPGSIELDRPNPTSHLAFGRGQHFCPGSALGRRHAQIGIEALLKK
MPGVDLAVPIDQLVWRTRFQRRIPERLPVLW
>CYP121A1_Mycobacterium tuberculosis TKK_02_0036 2592380136
MTATVLLEVPFSARGDRIPDAVAELRTREPIRKVRTITGAEAWLVSSYALCTQVLEDRRFSMKETAAAGAPRL
NALTVPPEVVNNMGNIADAGLRKAVMKAITPKAPGLEQFLRDTANSLLDNLITEGAPADLRNDFADPLATALH
CKVLGIPQEDGPKLFRSLSIAFMSSADPIPAAKINWDRDIEYMAGILENPNITTGLMGELSRLRKDPAYSHVS
DELFATIGVTFFGAGVISTGSFLTTALISLIQRPQLRNLLHEKPELIPAGVEELLRINLSFADGLPRLATADI
QVGDVLVRKGELVLVLLEGANFDPEHFPNPGSIELDRPNPTSHLAFGRGQHFCPGSALGRRHAQIGIEALLKK
MPGVDLAVPIDQLVWRTRFQRRIPERLPVLW
>CYP121A1_Mycobacterium tuberculosis TKK_02_0038 2577652221
MTATVLLEVPFSARGDRIPDAVAELRTREPIRKVRTITGAEAWLVSSYALCTQVLEDRRFSMKETAAAGAPRL
NALTVPPEVVNNMGNIADAGLRKAVMKAITPKAPGLEQFLRDTANSLLDNLITEGAPADLRNDFADPLATALH
CKVLGIPQEDGPKLFRSLSIAFMSSADPIPAAKINWDRDIEYMAGILENPNITTGLMGELSRLRKDPAYSHVS
DELFATIGVTFFGAGVISTGSFLTTALISLIQRPQLRNLLHEKPELIPAGVEELLRINLSFADGLPRLATADI
QVGDVLVRKGELVLVLLEGANFDPEHFPNPGSIELDRPNPTSHLAFGRGQHFCPGSALGRRHAQIGIEALLKK
MPGVDLAVPIDQLVWRTRFQRRIPERLPVLW
>CYP121A1_Mycobacterium tuberculosis TKK_02_0039 2592378091
MTATVLLEVPFSARGDRIPDAVAELRTREPIRKVRTITGAEAWLVSSYALCTQVLEDRRFSMKETAAAGAPRL
NALTVPPEVVNNMGNIADAGLRKAVMKAITPKAPGLEQFLRDTANSLLDNLITEGAPADLRNDFADPLATALH
CKVLGIPQEDGPKLFRSLSIAFMSSADPIPAAKINWDRDIEYMAGILENPNITTGLMGELSRLRKDPAYSHVS
DELFATIGVTFFGAGVISTGSFLTTALISLIQRPQLRNLLHEKPELIPAGVEELLRINLSFADGLPRLATADI
QVGDVLVRKGELVLVLLEGANFDPEHFPNPGSIELDRPNPTSHLAFGRGQHFCPGSALGRRHAQIGIEALLKK
MPGVDLAVPIDQLVWRTRFQRRIPERLPVLW
>CYP121A1_Mycobacterium tuberculosis TKK_02_0045 2576367041
MTATVLLEVPFSARGDRIPDAVAELRTREPIRKVRTITGAEAWLVSSYALCTQVLEDRRFSMKETAAAGAPRL
NALTVPPEVVNNMGNIADAGLRKAVMKAITPKAPGLEQFLRDTANSLLDNLITEGAPADLRNDFADPLATALH
CKVLGIPQEDGPKLFRSLSIAFMSSADPIPAAKINWDRDIEYMAGILENPNITTGLMGELSRLRKDPAYSHVS
DELFATIGVTFFGAGVISTGSFLTTALISLIQRPQLRNLLHEKPELIPAGVEELLRINLSFADGLPRLATADI
QVGDVLVRKGELVLVLLEGANFDPEHFPNPGSIELDRPNPTSHLAFGRGQHFCPGSALGRRHAQIGIEALLKK
MPGVDLAVPIDQLVWRTRFQRRIPERLPVLW
>CYP121A1_Mycobacterium tuberculosis TKK_02_0052 2592369858
MTATVLLEVPFSARGDRIPDAVAELRTREPIRKVRTITGAEAWLVSSYALCTQVLEDRRFSMKETAAAGAPRL
NALTVPPEVVNNMGNIADAGLRKAVMKAITPKAPGLEQFLRDTANSLLDNLITEGAPADLRNDFADPLATALH
CKVLGIPQEDGPKLFRSLSIAFMSSADPIPAAKINWDRDIEYMAGILENPNITTGLMGELSRLRKDPAYSHVS
DELFATIGVTFFGAGVISTGSFLTTALISLIQRPQLRNLLHEKPELIPAGVEELLRINLSFADGLPRLATADI
QVGDVLVRKGELVLVLLEGANFDPEHFPNPGSIELDRPNPTSHLAFGRGQHFCPGSALGRRHAQIGIEALLKK
MPGVDLAVPIDQLVWRTRFQRRIPERLPVLW
>CYP121A1_Mycobacterium tuberculosis TKK_02_0061 2592364915
MTATVLLEVPFSARGDRIPDAVAELRTREPIRKVRTITGAEAWLVSSYALCTQVLEDRRFSMKETAAAGAPRL
NALTVPPEVVNNMGNIADAGLRKAVMKAITPKAPGLEQFLRDTANSLLDNLITEGAPADLRNDFADPLATALH
CKVLGIPQEDGPKLFRSLSIAFMSSADPIPAAKINWDRDIEYMAGILENPNITTGLMGELSRLRKDPAYSHVS
DELFATIGVTFFGAGVISTGSFLTTALISLIQRPQLRNLLHEKPELIPAGVEELLRINLSFADGLPRLATADI
QVGDVLVRKGELVLVLLEGANFDPEHFPNPGSIELDRPNPTSHLAFGRGQHFCPGSALGRRHAQIGIEALLKK
MPGVDLAVPIDQLVWRTRFQRRIPERLPVLW
>CYP121A1_Mycobacterium tuberculosis TKK_02_0062 2592361860
MTATVLLEVPFSARGDRIPDAVAELRTREPIRKVRTITGAEAWLVSSYALCTQVLEDRRFSMKETAAAGAPRL
NALTVPPEVVNNMGNIADAGLRKAVMKAITPKAPGLEQFLRDTANSLLDNLITEGAPADLRNDFADPLATALH
CKVLGIPQEDGPKLFRSLSIAFMSSADPIPAAKINWDRDIEYMAGILENPNITTGLMGELSRLRKDPAYSHVS
DELFATIGVTFFGAGVISTGSFLTTALISLIQRPQLRNLLHEKPELIPAGVEELLRINLSFADGLPRLATADI

QVGDVLVRKGELVLVLLEGANFDPEHFPNPGSIELDRPNPTSHLAFGRGQHFCPGSALGRRHAQIGIEALLKK
MPGVDLAVPIDQLVWRTRFQRRIPERLPVLW
>CYP121A1_Mycobacterium tuberculosis TKK_02_0067 2592353657
MTATVLLEVPFSARGDRIPDAVAELRTREPIRKVRTITGAEAWLVSSYALCTQVLEDRRFSMKETAAAGAPRL
NALTVPPEVVNNMGNIADAGLRKAVMKAITPKAPGLEQFLRDTANSLLDNLITEGAPADLRNDFADPLATALH
CKVLGIPQEDGPKLFRSLSIAFMSSADPIPAAKINWDRDIEYMAGILENPNITTGLMGELSRLRKDPAYSHVS
DELFATIGVTFFGAGVISTGSFLTTALISLIQRPQLRNLLHEKPELIPAGVEELLRINLSFADGLPRLATADI
QVGDVLVRKGELVLVLLEGANFDPEHFPNPGSIELDRPNPTSHLAFGRGQHFCPGSALGRRHAQIGIEALLKK
MPGVDLAVPIDQLVWRTRFQRRIPERLPVLW
>CYP121A1_Mycobacterium tuberculosis TKK_02_0068 2592347606
MTATVLLEVPFSARGDRIPDAVAELRTREPIRKVRTITGAEAWLVSSYALCTQVLEDRRFSMKETAAAGAPRL
NALTVPPEVVNNMGNIADAGLRKAVMKAITPKAPGLEQFLRDTANSLLDNLITEGAPADLRNDFADPLATALH
CKVLGIPQEDGPKLFRSLSIAFMSSADPIPAAKINWDRDIEYMAGILENPNITTGLMGELSRLRKDPAYSHVS
DELFATIGVTFFGAGVISTGSFLTTALISLIQRPQLRNLLHEKPELIPAGVEELLRINLSFADGLPRLATADI
QVGDVLVRKGELVLVLLEGANFDPEHFPNPGSIELDRPNPTSHLAFGRGQHFCPGSALGRRHAQIGIEALLKK
MPGVDLAVPIDQLVWRTRFQRRIPERLPVLW
>CYP121A1_Mycobacterium tuberculosis TKK_02_0069 2592344808
MTATVLLEVPFSARGDRIPDAVAELRTREPIRKVRTITGAEAWLVSSYALCTQVLEDRRFSMKETAAAGAPRL
NALTVPPEVVNNMGNIADAGLRKAVMKAITPKAPGLEQFLRDTANSLLDNLITEGAPADLRNDFADPLATALH
CKVLGIPQEDGPKLFRSLSIAFMSSADPIPAAKINWDRDIEYMAGILENPNITTGLMGELSRLRKDPAYSHVS
DELFATIGVTFFGAGVISTGSFLTTALISLIQRPQLRNLLHEKPELIPAGVEELLRINLSFADGLPRLATADI
QVGDVLVRKGELVLVLLEGANFDPEHFPNPGSIELDRPNPTSHLAFGRGQHFCPGSALGRRHAQIGIEALLKK
MPGVDLAVPIDQLVWRTRFQRRIPERLPVLW
>CYP121A1_Mycobacterium tuberculosis TKK_02_0071 2574859560
MTATVLLEVPFSARGDRIPDAVAELRTREPIRKVRTITGAEAWLVSSYALCTQVLEDRRFSMKETAAAGAPRL
NALTVPPEVVNNMGNIADAGLRKAVMKAITPKAPGLEQFLRDTANSLLDNLITEGAPADLRNDFADPLATALH
CKVLGIPQEDGPKLFRSLSIAFMSSADPIPAAKINWDRDIEYMAGILENPNITTGLMGELSRLRKDPAYSHVS
DELFATIGVTFFGAGVISTGSFLTTALISLIQRPQLRNLLHEKPELIPAGVEELLRINLSFADGLPRLATADI
QVGDVLVRKGELVLVLLEGANFDPEHFPNPGSIELDRPNPTSHLAFGRGQHFCPGSALGRRHAQIGIEALLKK
MPGVDLAVPIDQLVWRTRFQRRIPERLPVLW
>CYP121A1_Mycobacterium tuberculosis TKK_02_0073 2592337167
MTATVLLEVPFSARGDRIPDAVAELRTREPIRKVRTITGAEAWLVSSYALCTQVLEDRRFSMKETAAAGAPRL
NALTVPPEVVNNMGNIADAGLRKAVMKAITPKAPGLEQFLRDTANSLLDNLITEGAPADLRNDFADPLATALH
CKVLGIPQEDGPKLFRSLSIAFMSSADPIPAAKINWDRDIEYMAGILENPNITTGLMGELSRLRKDPAYSHVS
DELFATIGVTFFGAGVISTGSFLTTALISLIQRPQLRNLLHEKPELIPAGVEELLRINLSFADGLPRLATADI
QVGDVLVRKGELVLVLLEGANFDPEHFPNPGSIELDRPNPTSHLAFGRGQHFCPGSALGRRHAQIGIEALLKK
MPGVDLAVPIDQLVWRTRFQRRIPERLPVLW
>CYP121A1_Mycobacterium tuberculosis TKK_02_0077 2592333243
MTATVLLEVPFSARGDRIPDAVAELRTREPIRKVRTITGAEAWLVSSYALCTQVLEDRRFSMKETAAAGAPRL
NALTVPPEVVNNMGNIADAGLRKAVMKAITPKAPGLEQFLRDTANSLLDNLITEGAPADLRNDFADPLATALH
CKVLGIPQEDGPKLFRSLSIAFMSSADPIPAAKINWDRDIEYMAGILENPNITTGLMGELSRLRKDPAYSHVS
DELFATIGVTFFGAGVISTGSFLTTALISLIQRPQLRNLLHEKPELIPAGVEELLRINLSFADGLPRLATADI
QVGDVLVRKGELVLVLLEGANFDPEHFPNPGSIELDRPNPTSHLAFGRGQHFCPGSALGRRHAQIGIEALLKK
MPGVDLAVPIDQLVWRTRFQRRIPERLPVLW
>CYP121A1_Mycobacterium tuberculosis TKK_03_0015 2649278030
MTATVLLEVPFSARGDRIPDAVAELRTREPIRKVRTITGAEAWLVSSYALCTQVLEDRRFSMKETAAAGAPRL
NALTVPPEVVNNMGNIADAGLRKAVMKAITPKAPGLEQFLRDTANSLLDNLITEGAPADLRNDFADPLATALH
CKVLGIPQEDGPKLFRSLSIAFMSSADPIPAAKINWDRDIEYMAGILENPNITTGLMGELSRLRKDPAYSHVS
DELFATIGVTFFGAGVISTGSFLTTALISLIQRPQLRNLLHEKPELIPAGVEELLRINLSFADGLPRLATADI
QVGDVLVRKGELVLVLLEGANFDPEHFPNPGSIELDRPNPTSHLAFGRGQHFCPGSALGRRHAQIGIEALLKK
MPGVDLAVPIDQLVWRTRFQRRIPERLPVLW
>CYP121A1_Mycobacterium tuberculosis TKK_03_0018 2592328662
MTATVLLEVPFSARGDRIPDAVAELRTREPIRKVRTITGAEAWLVSSYALCTQVLEDRRFSMKETAAAGAPRL
NALTVPPEVVNNMGNIADAGLRKAVMKAITPKAPGLEQFLRDTANSLLDNLITEGAPADLRNDFADPLATALH
CKVLGIPQEDGPKLFRSLSIAFMSSADPIPAAKINWDRDIEYMAGILENPNITTGLMGELSRLRKDPAYSHVS
DELFATIGVTFFGAGVISTGSFLTTALISLIQRPQLRNLLHEKPELIPAGVEELLRINLSFADGLPRLATADI
QVGDVLVRKGELVLVLLEGANFDPEHFPNPGSIELDRPNPTSHLAFGRGQHFCPGSALGRRHAQIGIEALLKK
MPGVDLAVPIDQLVWRTRFQRRIPERLPVLW

>CYP121A1_Mycobacterium tuberculosis TKK_03_0020 2592325051
MTATVLLEVPFSARGDRIPDAVAELRTREPIRKVRTITGAEAWLVSSYALCTQVLEDRRFSMKETAAAGAPRL
NALTVPPEVVNNMGNIADAGLRKAVMKAITPKAPGLEQFLRDTANSLLDNLITEGAPADLRNDFADPLATALH
CKVLGIPQEDGPKLFRSLSIAFMSSADPIPAAKINWDRDIEYMAGILENPNITTGLMGELSRLRKDPAYSHVS
DELFATIGVTFFGAGVISTGSFLTTALISLIQRPQLRNLLHEKPELIPAGVEELLRINLSFADGLPRLATADI
QVGDVLVRKGELVLVLLEGANFDPEHFPNPGSIELDRPNPTSHLAFGRGQHFCPGSALGRRHAQIGIEALLKK
MPGVDLAVPIDQLVWRTRFQRRIPERLPVLW
>CYP121A1_Mycobacterium tuberculosis TKK_03_0021 2633287636
MTATVLLEVPFSARGDRIPDAVAELRTREPIRKVRTITGAEAWLVSSYALCTQVLEDRRFSMKETAAAGAPRL
NALTVPPEVVNNMGNIADAGLRKAVMKAITPKAPGLEQFLRDTANSLLDNLITEGAPADLRNDFADPLATALH
CKVLGIPQEDGPKLFRSLSIAFMSSADPIPAAKINWDRDIEYMAGILENPNITTGLMGELSRLRKDPAYSHVS
DELFATIGVTFFGAGVISTGSFLTTALISLIQRPQLRNLLHEKPELIPAGVEELLRINLSFADGLPRLATADI
QVGDVLVRKGELVLVLLEGANFDPEHFPNPGSIELDRPNPTSHLAFGRGQHFCPGSALGRRHAQIGIEALLKK
MPGVDLAVPIDQLVWRTRFQRRIPERLPVLW
>CYP121A1_Mycobacterium tuberculosis TKK_03_0023 2641038745
MTATVLLEVPFSARGDRIPDAVAELRTREPIRKVRTITGAEAWLVSSYALCTQVLEDRRFSMKETAAAGAPRL
NALTVPPEVVNNMGNIADAGLRKAVMKAITPKAPGLEQFLRDTANSLLDNLITEGAPADLRNDFADPLATALH
CKVLGIPQEDGPKLFRSLSIAFMSSADPIPAAKINWDRDIEYMAGILENPNITTGLMGELSRLRKDPAYSHVS
DELFATIGVTFFGAGVISTGSFLTTALISLIQRPQLRNLLHEKPELIPAGVEELLRINLSFADGLPRLATADI
QVGDVLVRKGELVLVLLEGANFDPEHFPNPGSIELDRPNPTSHLAFGRGQHFCPGSALGRRHAQIGIEALLKK
MPGVDLAVPIDQLVWRTRFQRRIPERLPVLW
>CYP121A1_Mycobacterium tuberculosis TKK_03_0024 2577169119
MTATVLLEVPFSARGDRIPDAVAELRTREPIRKVRTITGAEAWLVSSYALCTQVLEDRRFSMKETAAAGAPRL
NALTVPPEVVNNMGNIADAGLRKAVMKAITPKAPGLEQFLRDTANSLLDNLITEGAPADLRNDFADPLATALH
CKVLGIPQEDGPKLFRSLSIAFMSSADPIPAAKINWDRDIEYMAGILENPNITTGLMGELSRLRKDPAYSHVS
DELFATIGVTFFGAGVISTGSFLTTALISLIQRPQLRNLLHEKPELIPAGVEELLRINLSFADGLPRLATADI
QVGDVLVRKGELVLVLLEGANFDPEHFPNPGSIELDRPNPTSHLAFGRGQHFCPGSALGRRHAQIGIEALLKK
MPGVDLAVPIDQLVWRTRFQRRIPERLPVLW
>CYP121A1_Mycobacterium tuberculosis TKK_03_0026 2574803879
MTATVLLEVPFSARGDRIPDAVAELRTREPIRKVRTITGAEAWLVSSYALCTQVLEDRRFSMKETAAAGAPRL
NALTVPPEVVNNMGNIADAGLRKAVMKAITPKAPGLEQFLRDTANSLLDNLITEGAPADLRNDFADPLATALH
CKVLGIPQEDGPKLFRSLSIAFMSSADPIPAAKINWDRDIEYMAGILENPNITTGLMGELSRLRKDPAYSHVS
DELFATIGVTFFGAGVISTGSFLTTALISLIQRPQLRNLLHEKPELIPAGVEELLRINLSFADGLPRLATADI
QVGDVLVRKGELVLVLLEGANFDPEHFPNPGSIELDRPNPTSHLAFGRGQHFCPGSALGRRHAQIGIEALLKK
MPGVDLAVPIDQLVWRTRFQRRIPERLPVLW
>CYP121A1_Mycobacterium tuberculosis TKK_03_0030 2592303897
MTATVLLEVPFSARGDRIPDAVAELRTREPIRKVRTITGAEAWLVSSYALCTQVLEDRRFSMKETAAAGAPRL
NALTVPPEVVNNMGNIADAGLRKAVMKAITPKAPGLEQFLRDTANSLLDNLITEGAPADLRNDFADPLATALH
CKVLGIPQEDGPKLFRSLSIAFMSSADPIPAAKINWDRDIEYMAGILENPNITTGLMGELSRLRKDPAYSHVS
DELFATIGVTFFGAGVISTGSFLTTALISLIQRPQLRNLLHEKPELIPAGVEELLRINLSFADGLPRLATADI
QVGDVLVRKGELVLVLLEGANFDPEHFPNPGSIELDRPNPTSHLAFGRGQHFCPGSALGRRHAQIGIEALLKK
MPGVDLAVPIDQLVWRTRFQRRIPERLPVLW
>CYP121A1_Mycobacterium tuberculosis TKK_03_0033 2592300595
MTATVLLEVPFSARGDRIPDAVAELRTREPIRKVRTITGAEAWLVSSYALCTQVLEDRRFSMKETAAAGAPRL
NALTVPPEVVNNMGNIADAGLRKAVMKAITPKAPGLEQFLRDTANSLLDNLITEGAPADLRNDFADPLATALH
CKVLGIPQEDGPKLFRSLSIAFMSSADPIPAAKINWDRDIEYMAGILENPNITTGLMGELSRLRKDPAYSHVS
DELFATIGVTFFGAGVISTGSFLTTALISLIQRPQLRNLLHEKPELIPAGVEELLRINLSFADGLPRLATADI
QVGDVLVRKGELVLVLLEGANFDPEHFPNPGSIELDRPNPTSHLAFGRGQHFCPGSALGRRHAQIGIEALLKK
MPGVDLAVPIDQLVWRTRFQRRIPERLPVLW
>CYP121A1_Mycobacterium tuberculosis TKK_03_0035 2635883331
MTATVLLEVPFSARGDRIPDAVAELRTREPIRKVRTITGAEAWLVSSYALCTQVLEDRRFSMKETAAAGAPRL
NALTVPPEVVNNMGNIADAGLRKAVMKAITPKAPGLEQFLRDTANSLLDNLITEGAPADLRNDFADPLATALH
CKVLGIPQEDGPKLFRSLSIAFMSSADPIPAAKINWDRDIEYMAGILENPNITTGLMGELSRLRKDPAYSHVS
DELFATIGVTFFGAGVISTGSFLTTALISLIQRPQLRNLLHEKPELIPAGVEELLRINLSFADGLPRLATADI
QVGDVLVRKGELVLVLLEGANFDPEHFPNPGSIELDRPNPTSHLAFGRGQHFCPGSALGRRHAQIGIEALLKK
MPGVDLAVPIDQLVWRTRFQRRIPERLPVLW
>CYP121A1_Mycobacterium tuberculosis TKK_03_0036 2592292119

MTATVLLEVPFSARGDRIPDAVAELRTREPIRKVRTITGAEAWLVSSYALCTQVLEDRRFSMKETAAAGAPRL
NALTVPPEVVNNMGNIADAGLRKAVMKAITPKAPGLEQFLRDTANSLLDNLITEGAPADLRNDFADPLATALH
CKVLGIPQEDGPKLFRSLSIAFMSSADPIPAAKINWDRDIEYMAGILENPNITTGLMGELSRLRKDPAYSHVS
DELFATIGVTFFGAGVISTGSFLTTALISLIQRPQLRNLLHEKPELIPAGVEELLRINLSFADGLPRLATADI
QVGDVLVRKGELVLVLLEGANFDPEHFPNPGSIELDRPNPTSHLAFGRGQHFCPGSALGRRHAQIGIEALLKK
MPGVDLAVPIDQLVWRTRFQRRIPERLPVLW
>CYP121A1_Mycobacterium tuberculosis TKK_03_0037 2652970977
MTATVLLEVPFSARGDRIPDAVAELRTREPIRKVRTITGAEAWLVSSYALCTQVLEDRRFSMKETAAAGAPRL
NALTVPPEVVNNMGNIADAGLRKAVMKAITPKAPGLEQFLRDTANSLLDNLITEGAPADLRNDFADPLATALH
CKVLGIPQEDGPKLFRSLSIAFMSSADPIPAAKINWDRDIEYMAGILENPNITTGLMGELSRLRKDPAYSHVS
DELFATIGVTFFGAGVISTGSFLTTALISLIQRPQLRNLLHEKPELIPAGVEELLRINLSFADGLPRLATADI
QVGDVLVRKGELVLVLLEGANFDPEHFPNPGSIELDRPNPTSHLAFGRGQHFCPGSALGRRHAQIGIEALLKK
MPGVDLAVPIDQLVWRTRFQRRIPERLPVLW
>CYP121A1_Mycobacterium tuberculosis TKK_03_0042 2575859447
MTATVLLEVPFSARGDRIPDAVAELRTREPIRKVRTITGAEAWLVSSYALCTQVLEDRRFSMKETAAAGAPRL
NALTVPPEVVNNMGNIADAGLRKAVMKAITPKAPGLEQFLRDTANSLLDNLITEGAPADLRNDFADPLATALH
CKVLGIPQEDGPKLFRSLSIAFMSSADPIPAAKINWDRDIEYMAGILENPNITTGLMGELSRLRKDPAYSHVS
DELFATIGVTFFGAGVISTGSFLTTALISLIQRPQLRNLLHEKPELIPAGVEELLRINLSFADGLPRLATADI
QVGDVLVRKGELVLVLLEGANFDPEHFPNPGSIELDRPNPTSHLAFGRGQHFCPGSALGRRHAQIGIEALLKK
MPGVDLAVPIDQLVWRTRFQRRIPERLPVLW
>CYP121A1_Mycobacterium tuberculosis TKK_03_0044 2629573614
MTATVLLEVPFSARGDRIPDAVAELRTREPIRKVRTITGAEAWLVSSYALCTQVLEDRRFSMKETAAAGAPRL
NALTVPPEVVNNMGNIADAGLRKAVMKAITPKAPGLEQFLRDTANSLLDNLITEGAPADLRNDFADPLATALH
CKVLGIPQEDGPKLFRSLSIAFMSSADPIPAAKINWDRDIEYMAGILENPNITTGLMGELSRLRKDPAYSHVS
DELFATIGVTFFGAGVISTGSFLTTALISLIQRPQLRNLLHEKPELIPAGVEELLRINLSFADGLPRLATADI
QVGDVLVRKGELVLVLLEGANFDPEHFPNPGSIELDRPNPTSHLAFGRGQHFCPGSALGRRHAQIGIEALLKK
MPGVDLAVPIDQLVWRTRFQRRIPERLPVLW
>CYP121A1_Mycobacterium tuberculosis TKK_03_0050 2648064777
MTATVLLEVPFSARGDRIPDAVAELRTREPIRKVRTITGAEAWLVSSYALCTQVLEDRRFSMKETAAAGAPRL
NALTVPPEVVNNMGNIADAGLRKAVMKAITPKAPGLEQFLRDTANSLLDNLITEGAPADLRNDFADPLATALH
CKVLGIPQEDGPKLFRSLSIAFMSSADPIPAAKINWDRDIEYMAGILENPNITTGLMGELSRLRKDPAYSHVS
DELFATIGVTFFGAGVISTGSFLTTALISLIQRPQLRNLLHEKPELIPAGVEELLRINLSFADGLPRLATADI
QVGDVLVRKGELVLVLLEGANFDPEHFPNPGSIELDRPNPTSHLAFGRGQHFCPGSALGRRHAQIGIEALLKK
MPGVDLAVPIDQLVWRTRFQRRIPERLPVLW
>CYP121A1_Mycobacterium tuberculosis TKK_03_0059 2584671036
MTATVLLEVPFSARGDRIPDAVAELRTREPIRKVRTITGAEAWLVSSYALCTQVLEDRRFSMKETAAAGAPRL
NALTVPPEVVNNMGNIADAGLRKAVMKAITPKAPGLEQFLRDTANSLLDNLITEGAPADLRNDFADPLATALH
CKVLGIPQEDGPKLFRSLSIAFMSSADPIPAAKINWDRDIEYMAGILENPNITTGLMGELSRLRKDPAYSHVS
DELFATIGVTFFGAGVISTGSFLTTALISLIQRPQLRNLLHEKPELIPAGVEELLRINLSFADGLPRLATADI
QVGDVLVRKGELVLVLLEGANFDPEHFPNPGSIELDRPNPTSHLAFGRGQHFCPGSALGRRHAQIGIEALLKK
MPGVDLAVPIDQLVWRTRFQRRIPERLPVLW
>CYP121A1_Mycobacterium tuberculosis TKK_03_0062 2640934173
MTATVLLEVPFSARGDRIPDAVAELRTREPIRKVRTITGAEAWLVSSYALCTQVLEDRRFSMKETAAAGAPRL
NALTVPPEVVNNMGNIADAGLRKAVMKAITPKAPGLEQFLRDTANSLLDNLITEGAPADLRNDFADPLATALH
CKVLGIPQEDGPKLFRSLSIAFMSSADPIPAAKINWDRDIEYMAGILENPNITTGLMGELSRLRKDPAYSHVS
DELFATIGVTFFGAGVISTGSFLTTALISLIQRPQLRNLLHEKPELIPAGVEELLRINLSFADGLPRLATADI
QVGDVLVRKGELVLVLLEGANFDPEHFPNPGSIELDRPNPTSHLAFGRGQHFCPGSALGRRHAQIGIEALLKK
MPGVDLAVPIDQLVWRTRFQRRIPERLPVLW
>CYP121A1_Mycobacterium tuberculosis TKK_03_0063 2630160792
MTATVLLEVPFSARGDRIPDAVAELRTREPIRKVRTITGAEAWLVSSYALCTQVLEDRRFSMKETAAAGAPRL
NALTVPPEVVNNMGNIADAGLRKAVMKAITPKAPGLEQFLRDTANSLLDNLITEGAPADLRNDFADPLATALH
CKVLGIPQEDGPKLFRSLSIAFMSSADPIPAAKINWDRDIEYMAGILENPNITTGLMGELSRLRKDPAYSHVS
DELFATIGVTFFGAGVISTGSFLTTALISLIQRPQLRNLLHEKPELIPAGVEELLRINLSFADGLPRLATADI
QVGDVLVRKGELVLVLLEGANFDPEHFPNPGSIELDRPNPTSHLAFGRGQHFCPGSALGRRHAQIGIEALLKK
MPGVDLAVPIDQLVWRTRFQRRIPERLPVLW
>CYP121A1_Mycobacterium tuberculosis TKK_03_0064 2647357784
MTATVLLEVPFSARGDRIPDAVAELRTREPIRKVRTITGAEAWLVSSYALCTQVLEDRRFSMKETAAAGAPRL
NALTVPPEVVNNMGNIADAGLRKAVMKAITPKAPGLEQFLRDTANSLLDNLITEGAPADLRNDFADPLATALH

CKVLGIPQEDGPKLFRSLSIAFMSSADPIPAAKINWDRDIEYMAGILENPNITTGLMGELSRLRKDPAYSHVS
DELFATIGVTFFGAGVISTGSFLTTALISLIQRPQLRNLLHEKPELIPAGVEELLRINLSFADGLPRLATADI
QVGDVLVRKGELVLVLLEGANFDPEHFPNPGSIELDRPNPTSHLAFGRGQHFCPGSALGRRHAQIGIEALLKK
MPGVDLAVPIDQLVWRTRFQRRIPERLPVLW
>CYP121A1_Mycobacterium tuberculosis TKK_03_0081 2592280024
MTATVLLEVPFSARGDRIPDAVAELRTREPIRKVRTITGAEAWLVSSYALCTQVLEDRRFSMKETAAAGAPRL
NALTVPPEVVNNMGNIADAGLRKAVMKAITPKAPGLEQFLRDTANSLLDNLITEGAPADLRNDFADPLATALH
CKVLGIPQEDGPKLFRSLSIAFMSSADPIPAAKINWDRDIEYMAGILENPNITTGLMGELSRLRKDPAYSHVS
DELFATIGVTFFGAGVISTGSFLTTALISLIQRPQLRNLLHEKPELIPAGVEELLRINLSFADGLPRLATADI
QVGDVLVRKGELVLVLLEGANFDPEHFPNPGSIELDRPNPTSHLAFGRGQHFCPGSALGRRHAQIGIEALLKK
MPGVDLAVPIDQLVWRTRFQRRIPERLPVLW
>CYP121A1_Mycobacterium tuberculosis TKK_03_0082 2592276097
MTATVLLEVPFSARGDRIPDAVAELRTREPIRKVRTITGAEAWLVSSYALCTQVLEDRRFSMKETAAAGAPRL
NALTVPPEVVNNMGNIADAGLRKAVMKAITPKAPGLEQFLRDTANSLLDNLITEGAPADLRNDFADPLATALH
CKVLGIPQEDGPKLFRSLSIAFMSSADPIPAAKINWDRDIEYMAGILENPNITTGLMGELSRLRKDPAYSHVS
DELFATIGVTFFGAGVISTGSFLTTALISLIQRPQLRNLLHEKPELIPAGVEELLRINLSFADGLPRLATADI
QVGDVLVRKGELVLVLLEGANFDPEHFPNPGSIELDRPNPTSHLAFGRGQHFCPGSALGRRHAQIGIEALLKK
MPGVDLAVPIDQLVWRTRFQRRIPERLPVLW
>CYP121A1_Mycobacterium tuberculosis TKK_03_0089 2642265833
MTATVLLEVPFSARGDRIPDAVAELRTREPIRKVRTITGAEAWLVSSYALCTQVLEDRRFSMKETAAAGAPRL
NALTVPPEVVNNMGNIADAGLRKAVMKAITPKAPGLEQFLRDTANSLLDNLITEGAPADLRNDFADPLATALH
CKVLGIPQEDGPKLFRSLSIAFMSSADPIPAAKINWDRDIEYMAGILENPNITTGLMGELSRLRKDPAYSHVS
DELFATIGVTFFGAGVISTGSFLTTALISLIQRPQLRNLLHEKPELIPAGVEELLRINLSFADGLPRLATADI
QVGDVLVRKGELVLVLLEGANFDPEHFPNPGSIELDRPNPTSHLAFGRGQHFCPGSALGRRHAQIGIEALLKK
MPGVDLAVPIDQLVWRTRFQRRIPERLPVLW
>CYP121A1_Mycobacterium tuberculosis TKK_03_0092 2637412067
MTATVLLEVPFSARGDRIPDAVAELRTREPIRKVRTITGAEAWLVSSYALCTQVLEDRRFSMKETAAAGAPRL
NALTVPPEVVNNMGNIADAGLRKAVMKAITPKAPGLEQFLRDTANSLLDNLITEGAPADLRNDFADPLATALH
CKVLGIPQEDGPKLFRSLSIAFMSSADPIPAAKINWDRDIEYMAGILENPNITTGLMGELSRLRKDPAYSHVS
DELFATIGVTFFGAGVISTGSFLTTALISLIQRPQLRNLLHEKPELIPAGVEELLRINLSFADGLPRLATADI
QVGDVLVRKGELVLVLLEGANFDPEHFPNPGSIELDRPNPTSHLAFGRGQHFCPGSALGRRHAQIGIEALLKK
MPGVDLAVPIDQLVWRTRFQRRIPERLPVLW
>CYP121A1_Mycobacterium tuberculosis TKK_03_0094 2578247185
MTATVLLEVPFSARGDRIPDAVAELRTREPIRKVRTITGAEAWLVSSYALCTQVLEDRRFSMKETAAAGAPRL
NALTVPPEVVNNMGNIADAGLRKAVMKAITPKAPGLEQFLRDTANSLLDNLITEGAPADLRNDFADPLATALH
CKVLGIPQEDGPKLFRSLSIAFMSSADPIPAAKINWDRDIEYMAGILENPNITTGLMGELSRLRKDPAYSHVS
DELFATIGVTFFGAGVISTGSFLTTALISLIQRPQLRNLLHEKPELIPAGVEELLRINLSFADGLPRLATADI
QVGDVLVRKGELVLVLLEGANFDPEHFPNPGSIELDRPNPTSHLAFGRGQHFCPGSALGRRHAQIGIEALLKK
MPGVDLAVPIDQLVWRTRFQRRIPERLPVLW
>CYP121A1_Mycobacterium tuberculosis TKK_03_0096 2578192385
MTATVLLEVPFSARGDRIPDAVAELRTREPIRKVRTITGAEAWLVSSYALCTQVLEDRRFSMKETAAAGAPRL
NALTVPPEVVNNMGNIADAGLRKAVMKAITPKAPGLEQFLRDTANSLLDNLITEGAPADLRNDFADPLATALH
CKVLGIPQEDGPKLFRSLSIAFMSSADPIPAAKINWDRDIEYMAGILENPNITTGLMGELSRLRKDPAYSHVS
DELFATIGVTFFGAGVISTGSFLTTALISLIQRPQLRNLLHEKPELIPAGVEELLRINLSFADGLPRLATADI
QVGDVLVRKGELVLVLLEGANFDPEHFPNPGSIELDRPNPTSHLAFGRGQHFCPGSALGRRHAQIGIEALLKK
MPGVDLAVPIDQLVWRTRFQRRIPERLPVLW
>CYP121A1_Mycobacterium tuberculosis TKK_03_0098 2647501579
MTATVLLEVPFSARGDRIPDAVAELRTREPIRKVRTITGAEAWLVSSYALCTQVLEDRRFSMKETAAAGAPRL
NALTVPPEVVNNMGNIADAGLRKAVMKAITPKAPGLEQFLRDTANSLLDNLITEGAPADLRNDFADPLATALH
CKVLGIPQEDGPKLFRSLSIAFMSSADPIPAAKINWDRDIEYMAGILENPNITTGLMGELSRLRKDPAYSHVS
DELFATIGVTFFGAGVISTGSFLTTALISLIQRPQLRNLLHEKPELIPAGVEELLRINLSFADGLPRLATADI
QVGDVLVRKGELVLVLLEGANFDPEHFPNPGSIELDRPNPTSHLAFGRGQHFCPGSALGRRHAQIGIEALLKK
MPGVDLAVPIDQLVWRTRFQRRIPERLPVLW
>CYP121A1_Mycobacterium tuberculosis TKK_03_0099 2575806795
MTATVLLEVPFSARGDRIPDAVAELRTREPIRKVRTITGAEAWLVSSYALCTQVLEDRRFSMKETAAAGAPRL
NALTVPPEVVNNMGNIADAGLRKAVMKAITPKAPGLEQFLRDTANSLLDNLITEGAPADLRNDFADPLATALH
CKVLGIPQEDGPKLFRSLSIAFMSSADPIPAAKINWDRDIEYMAGILENPNITTGLMGELSRLRKDPAYSHVS
DELFATIGVTFFGAGVISTGSFLTTALISLIQRPQLRNLLHEKPELIPAGVEELLRINLSFADGLPRLATADI

QVGDVLVRKGELVLVLLEGANFDPEHFPNPGSIELDRPNPTSHLAFGRGQHFCPGSALGRRHAQIGIEALLKK
MPGVDLAVPIDQLVWRTRFQRRIPERLPVLW
>CYP121A1_Mycobacterium tuberculosis TKK_03_0102 2632400171
MTATVLLEVPFSARGDRIPDAVAELRTREPIRKVRTITGAEAWLVSSYALCTQVLEDRRFSMKETAAAGAPRL
NALTVPPEVVNNMGNIADAGLRKAVMKAITPKAPGLEQFLRDTANSLLDNLITEGAPADLRNDFADPLATALH
CKVLGIPQEDGPKLFRSLSIAFMSSADPIPAAKINWDRDIEYMAGILENPNITTGLMGELSRLRKDPAYSHVS
DELFATIGVTFFGAGVISTGSFLTTALISLIQRPQLRNLLHEKPELIPAGVEELLRINLSFADGLPRLATADI
QVGDVLVRKGELVLVLLEGANFDPEHFPNPGSIELDRPNPTSHLAFGRGQHFCPGSALGRRHAQIGIEALLKK
MPGVDLAVPIDQLVWRTRFQRRIPERLPVLW
>CYP121A1_Mycobacterium tuberculosis TKK_03_0105 2637959934
MTATVLLEVPFSARGDRIPDAVAELRTREPIRKVRTITGAEAWLVSSYALCTQVLEDRRFSMKETAAAGAPRL
NALTVPPEVVNNMGNIADAGLRKAVMKAITPKAPGLEQFLRDTANSLLDNLITEGAPADLRNDFADPLATALH
CKVLGIPQEDGPKLFRSLSIAFMSSADPIPAAKINWDRDIEYMAGILENPNITTGLMGELSRLRKDPAYSHVS
DELFATIGVTFFGAGVISTGSFLTTALISLIQRPQLRNLLHEKPELIPAGVEELLRINLSFADGLPRLATADI
QVGDVLVRKGELVLVLLEGANFDPEHFPNPGSIELDRPNPTSHLAFGRGQHFCPGSALGRRHAQIGIEALLKK
MPGVDLAVPIDQLVWRTRFQRRIPERLPVLW
>CYP121A1_Mycobacterium tuberculosis TKK_03_0106 2629882014
MTATVLLEVPFSARGDRIPDAVAELRTREPIRKVRTITGAEAWLVSSYALCTQVLEDRRFSMKETAAAGAPRL
NALTVPPEVVNNMGNIADAGLRKAVMKAITPKAPGLEQFLRDTANSLLDNLITEGAPADLRNDFADPLATALH
CKVLGIPQEDGPKLFRSLSIAFMSSADPIPAAKINWDRDIEYMAGILENPNITTGLMGELSRLRKDPAYSHVS
DELFATIGVTFFGAGVISTGSFLTTALISLIQRPQLRNLLHEKPELIPAGVEELLRINLSFADGLPRLATADI
QVGDVLVRKGELVLVLLEGANFDPEHFPNPGSIELDRPNPTSHLAFGRGQHFCPGSALGRRHAQIGIEALLKK
MPGVDLAVPIDQLVWRTRFQRRIPERLPVLW
>CYP121A1_Mycobacterium tuberculosis TKK_03_0108 2628385357
MTATVLLEVPFSARGDRIPDAVAELRTREPIRKVRTITGAEAWLVSSYALCTQVLEDRRFSMKETAAAGAPRL
NALTVPPEVVNNMGNIADAGLRKAVMKAITPKAPGLEQFLRDTANSLLDNLITEGAPADLRNDFADPLATALH
CKVLGIPQEDGPKLFRSLSIAFMSSADPIPAAKINWDRDIEYMAGILENPNITTGLMGELSRLRKDPAYSHVS
DELFATIGVTFFGAGVISTGSFLTTALISLIQRPQLRNLLHEKPELIPAGVEELLRINLSFADGLPRLATADI
QVGDVLVRKGELVLVLLEGANFDPEHFPNPGSIELDRPNPTSHLAFGRGQHFCPGSALGRRHAQIGIEALLKK
MPGVDLAVPIDQLVWRTRFQRRIPERLPVLW
>CYP121A1_Mycobacterium tuberculosis TKK_03_0113 2637033020
MTATVLLEVPFSARGDRIPDAVAELRTREPIRKVRTITGAEAWLVSSYALCTQVLEDRRFSMKETAAAGAPRL
NALTVPPEVVNNMGNIADAGLRKAVMKAITPKAPGLEQFLRDTANSLLDNLITEGAPADLRNDFADPLATALH
CKVLGIPQEDGPKLFRSLSIAFMSSADPIPAAKINWDRDIEYMAGILENPNITTGLMGELSRLRKDPAYSHVS
DELFATIGVTFFGAGVISTGSFLTTALISLIQRPQLRNLLHEKPELIPAGVEELLRINLSFADGLPRLATADI
QVGDVLVRKGELVLVLLEGANFDPEHFPNPGSIELDRPNPTSHLAFGRGQHFCPGSALGRRHAQIGIEALLKK
MPGVDLAVPIDQLVWRTRFQRRIPERLPVLW
>CYP121A1_Mycobacterium tuberculosis TKK_03_0115 2638672338
MTATVLLEVPFSARGDRIPDAVAELRTREPIRKVRTITGAEAWLVSSYALCTQVLEDRRFSMKETAAAGAPRL
NALTVPPEVVNNMGNIADAGLRKAVMKAITPKAPGLEQFLRDTANSLLDNLITEGAPADLRNDFADPLATALH
CKVLGIPQEDGPKLFRSLSIAFMSSADPIPAAKINWDRDIEYMAGILENPNITTGLMGELSRLRKDPAYSHVS
DELFATIGVTFFGAGVISTGSFLTTALISLIQRPQLRNLLHEKPELIPAGVEELLRINLSFADGLPRLATADI
QVGDVLVRKGELVLVLLEGANFDPEHFPNPGSIELDRPNPTSHLAFGRGQHFCPGSALGRRHAQIGIEALLKK
MPGVDLAVPIDQLVWRTRFQRRIPERLPVLW
>CYP121A1_Mycobacterium tuberculosis TKK_03_0116 2576936967
MTATVLLEVPFSARGDRIPDAVAELRTREPIRKVRTITGAEAWLVSSYALCTQVLEDRRFSMKETAAAGAPRL
NALTVPPEVVNNMGNIADAGLRKAVMKAITPKAPGLEQFLRDTANSLLDNLITEGAPADLRNDFADPLATALH
CKVLGIPQEDGPKLFRSLSIAFMSSADPIPAAKINWDRDIEYMAGILENPNITTGLMGELSRLRKDPAYSHVS
DELFATIGVTFFGAGVISTGSFLTTALISLIQRPQLRNLLHEKPELIPAGVEELLRINLSFADGLPRLATADI
QVGDVLVRKGELVLVLLEGANFDPEHFPNPGSIELDRPNPTSHLAFGRGQHFCPGSALGRRHAQIGIEALLKK
MPGVDLAVPIDQLVWRTRFQRRIPERLPVLW
>CYP121A1_Mycobacterium tuberculosis TKK_03_0150 2631321254
MTATVLLEVPFSARGDRIPDAVAELRTREPIRKVRTITGAEAWLVSSYALCTQVLEDRRFSMKETAAAGAPRL
NALTVPPEVVNNMGNIADAGLRKAVMKAITPKAPGLEQFLRDTANSLLDNLITEGAPADLRNDFADPLATALH
CKVLGIPQEDGPKLFRSLSIAFMSSADPIPAAKINWDRDIEYMAGILENPNITTGLMGELSRLRKDPAYSHVS
DELFATIGVTFFGAGVISTGSFLTTALISLIQRPQLRNLLHEKPELIPAGVEELLRINLSFADGLPRLATADI
QVGDVLVRKGELVLVLLEGANFDPEHFPNPGSIELDRPNPTSHLAFGRGQHFCPGSALGRRHAQIGIEALLKK
MPGVDLAVPIDQLVWRTRFQRRIPERLPVLW

>CYP121A1_Mycobacterium tuberculosis TKK_03_0153 2633662823
MTATVLLEVPFSARGDRIPDAVAELRTREPIRKVRTITGAEAWLVSSYALCTQVLEDRRFSMKETAAAGAPRL
NALTVPPEVVNNMGNIADAGLRKAVMKAITPKAPGLEQFLRDTANSLLDNLITEGAPADLRNDFADPLATALH
CKVLGIPQEDGPKLFRSLSIAFMSSADPIPAAKINWDRDIEYMAGILENPNITTGLMGELSRLRKDPAYSHVS
DELFATIGVTFFGAGVISTGSFLTTALISLIQRPQLRNLLHEKPELIPAGVEELLRINLSFADGLPRLATADI
QVGDVLVRKGELVLVLLEGANFDPEHFPNPGSIELDRPNPTSHLAFGRGQHFCPGSALGRRHAQIGIEALLKK
MPGVDLAVPIDQLVWRTRFQRRIPERLPVLW
>CYP121A1_Mycobacterium tuberculosis TKK_03_0158 2576932908
MTATVLLEVPFSARGDRIPDAVAELRTREPIRKVRTITGAEAWLVSSYALCTQVLEDRRFSMKETAAAGAPRL
NALTVPPEVVNNMGNIADAGLRKAVMKAITPKAPGLEQFLRDTANSLLDNLITEGAPADLRNDFADPLATALH
CKVLGIPQEDGPKLFRSLSIAFMSSADPIPAAKINWDRDIEYMAGILENPNITTGLMGELSRLRKDPAYSHVS
DELFATIGVTFFGAGVISTGSFLTTALISLIQRPQLRNLLHEKPELIPAGVEELLRINLSFADGLPRLATADI
QVGDVLVRKGELVLVLLEGANFDPEHFPNPGSIELDRPNPTSHLAFGRGQHFCPGSALGRRHAQIGIEALLKK
MPGVDLAVPIDQLVWRTRFQRRIPERLPVLW
>CYP121A1_Mycobacterium tuberculosis TKK_03_0159 2637666356
MTATVLLEVPFSARGDRIPDAVAELRTREPIRKVRTITGAEAWLVSSYALCTQVLEDRRFSMKETAAAGAPRL
NALTVPPEVVNNMGNIADAGLRKAVMKAITPKAPGLEQFLRDTANSLLDNLITEGAPADLRNDFADPLATALH
CKVLGIPQEDGPKLFRSLSIAFMSSADPIPAAKINWDRDIEYMAGILENPNITTGLMGELSRLRKDPAYSHVS
DELFATIGVTFFGAGVISTGSFLTTALISLIQRPQLRNLLHEKPELIPAGVEELLRINLSFADGLPRLATADI
QVGDVLVRKGELVLVLLEGANFDPEHFPNPGSIELDRPNPTSHLAFGRGQHFCPGSALGRRHAQIGIEALLKK
MPGVDLAVPIDQLVWRTRFQRRIPERLPVLW
>CYP121A1_Mycobacterium tuberculosis TKK_04_0002 2584967627
MTATVLLEVPFSARGDRIPDAVAELRTREPIRKVRTITGAEAWLVSSYALCTQVLEDRRFSMKETAAAGAPRL
NALTVPPEVVNNMGNIADAGLRKAVMKAITPKAPGLEQFLRDTANSLLDNLITEGAPADLRNDFADPLATALH
CKVLGIPQEDGPKLFRSLSIAFMSSADPIPAAKINWDRDIEYMAGILENPNITTGLMGELSRLRKDPAYSHVS
DELFATIGVTFFGAGVISTGSFLTTALISLIQRPQLRNLLHEKPELIPAGVEELLRINLSFADGLPRLATADI
QVGDVLVRKGELVLVLLEGANFDPEHFPNPGSIELDRPNPTSHLAFGRGQHFCPGSALGRRHAQIGIEALLKK
MPGVDLAVPIDQLVWRTRFQRRIPERLPVLW
>CYP121A1_Mycobacterium tuberculosis TKK_04_0003 2576389547
MTATVLLEVPFSARGDRIPDAVAELRTREPIRKVRTITGAEAWLVSSYALCTQVLEDRRFSMKETAAAGAPRL
NALTVPPEVVNNMGNIADAGLRKAVMKAITPKAPGLEQFLRDTANSLLDNLITEGAPADLRNDFADPLATALH
CKVLGIPQEDGPKLFRSLSIAFMSSADPIPAAKINWDRDIEYMAGILENPNITTGLMGELSRLRKDPAYSHVS
DELFATIGVTFFGAGVISTGSFLTTALISLIQRPQLRNLLHEKPELIPAGVEELLRINLSFADGLPRLATADI
QVGDVLVRKGELVLVLLEGANFDPEHFPNPGSIELDRPNPTSHLAFGRGQHFCPGSALGRRHAQIGIEALLKK
MPGVDLAVPIDQLVWRTRFQRRIPERLPVLW
>CYP121A1_Mycobacterium tuberculosis TKK_04_0005 2592267925
MTATVLLEVPFSARGDRIPDAVAELRTREPIRKVRTITGAEAWLVSSYALCTQVLEDRRFSMKETAAAGAPRL
NALTVPPEVVNNMGNIADAGLRKAVMKAITPKAPGLEQFLRDTANSLLDNLITEGAPADLRNDFADPLATALH
CKVLGIPQEDGPKLFRSLSIAFMSSADPIPAAKINWDRDIEYMAGILENPNITTGLMGELSRLRKDPAYSHVS
DELFATIGVTFFGAGVISTGSFLTTALISLIQRPQLRNLLHEKPELIPAGVEELLRINLSFADGLPRLATADI
QVGDVLVRKGELVLVLLEGANFDPEHFPNPGSIELDRPNPTSHLAFGRGQHFCPGSALGRRHAQIGIEALLKK
MPGVDLAVPIDQLVWRTRFQRRIPERLPVLW
>CYP121A1_Mycobacterium tuberculosis TKK_04_0007 2592259768
MTATVLLEVPFSARGDRIPDAVAELRTREPIRKVRTITGAEAWLVSSYALCTQVLEDRRFSMKETAAAGAPRL
NALTVPPEVVNNMGNIADAGLRKAVMKAITPKAPGLEQFLRDTANSLLDNLITEGAPADLRNDFADPLATALH
CKVLGIPQEDGPKLFRSLSIAFMSSADPIPAAKINWDRDIEYMAGILENPNITTGLMGELSRLRKDPAYSHVS
DELFATIGVTFFGAGVISTGSFLTTALISLIQRPQLRNLLHEKPELIPAGVEELLRINLSFADGLPRLATADI
QVGDVLVRKGELVLVLLEGANFDPEHFPNPGSIELDRPNPTSHLAFGRGQHFCPGSALGRRHAQIGIEALLKK
MPGVDLAVPIDQLVWRTRFQRRIPERLPVLW
>CYP121A1_Mycobacterium tuberculosis TKK_04_0008 2592254793
MTATVLLEVPFSARGDRIPDAVAELRTREPIRKVRTITGAEAWLVSSYALCTQVLEDRRFSMKETAAAGAPRL
NALTVPPEVVNNMGNIADAGLRKAVMKAITPKAPGLEQFLRDTANSLLDNLITEGAPADLRNDFADPLATALH
CKVLGIPQEDGPKLFRSLSIAFMSSADPIPAAKINWDRDIEYMAGILENPNITTGLMGELSRLRKDPAYSHVS
DELFATIGVTFFGAGVISTGSFLTTALISLIQRPQLRNLLHEKPELIPAGVEELLRINLSFADGLPRLATADI
QVGDVLVRKGELVLVLLEGANFDPEHFPNPGSIELDRPNPTSHLAFGRGQHFCPGSALGRRHAQIGIEALLKK
MPGVDLAVPIDQLVWRTRFQRRIPERLPVLW
>CYP121A1_Mycobacterium tuberculosis TKK_04_0013 2592251567

MTATVLLEVPFSARGDRIPDAVAELRTREPIRKVRTITGAEAWLVSSYALCTQVLEDRRFSMKETAAAGAPRL
NALTVPPEVVNNMGNIADAGLRKAVMKAITPKAPGLEQFLRDTANSLLDNLITEGAPADLRNDFADPLATALH
CKVLGIPQEDGPKLFRSLSIAFMSSADPIPAAKINWDRDIEYMAGILENPNITTGLMGELSRLRKDPAYSHVS
DELFATIGVTFFGAGVISTGSFLTTALISLIQRPQLRNLLHEKPELIPAGVEELLRINLSFADGLPRLATADI
QVGDVLVRKGELVLVLLEGANFDPEHFPNPGSIELDRPNPTSHLAFGRGQHFCPGSALGRRHAQIGIEALLKK
MPGVDLAVPIDQLVWRTRFQRRIPERLPVLW
>CYP121A1_Mycobacterium tuberculosis TKK_04_0015 2592243435
MTATVLLEVPFSARGDRIPDAVAELRTREPIRKVRTITGAEAWLVSSYALCTQVLEDRRFSMKETAAAGAPRL
NALTVPPEVVNNMGNIADAGLRKAVMKAITPKAPGLEQFLRDTANSLLDNLITEGAPADLRNDFADPLATALH
CKVLGIPQEDGPKLFRSLSIAFMSSADPIPAAKINWDRDIEYMAGILENPNITTGLMGELSRLRKDPAYSHVS
DELFATIGVTFFGAGVISTGSFLTTALISLIQRPQLRNLLHEKPELIPAGVEELLRINLSFADGLPRLATADI
QVGDVLVRKGELVLVLLEGANFDPEHFPNPGSIELDRPNPTSHLAFGRGQHFCPGSALGRRHAQIGIEALLKK
MPGVDLAVPIDQLVWRTRFQRRIPERLPVLW
>CYP121A1_Mycobacterium tuberculosis TKK_04_0020 2592231200
MTATVLLEVPFSARGDRIPDAVAELRTREPIRKVRTITGAEAWLVSSYALCTQVLEDRRFSMKETAAAGAPRL
NALTVPPEVVNNMGNIADAGLRKAVMKAITPKAPGLEQFLRDTANSLLDNLITEGAPADLRNDFADPLATALH
CKVLGIPQEDGPKLFRSLSIAFMSSADPIPAAKINWDRDIEYMAGILENPNITTGLMGELSRLRKDPAYSHVS
DELFATIGVTFFGAGVISTGSFLTTALISLIQRPQLRNLLHEKPELIPAGVEELLRINLSFADGLPRLATADI
QVGDVLVRKGELVLVLLEGANFDPEHFPNPGSIELDRPNPTSHLAFGRGQHFCPGSALGRRHAQIGIEALLKK
MPGVDLAVPIDQLVWRTRFQRRIPERLPVLW
>CYP121A1_Mycobacterium tuberculosis TKK_04_0021 2575543463
MTATVLLEVPFSARGDRIPDAVAELRTREPIRKVRTITGAEAWLVSSYALCTQVLEDRRFSMKETAAAGAPRL
NALTVPPEVVNNMGNIADAGLRKAVMKAITPKAPGLEQFLRDTANSLLDNLITEGAPADLRNDFADPLATALH
CKVLGIPQEDGPKLFRSLSIAFMSSADPIPAAKINWDRDIEYMAGILENPNITTGLMGELSRLRKDPAYSHVS
DELFATIGVTFFGAGVISTGSFLTTALISLIQRPQLRNLLHEKPELIPAGVEELLRINLSFADGLPRLATADI
QVGDVLVRKGELVLVLLEGANFDPEHFPNPGSIELDRPNPTSHLAFGRGQHFCPGSALGRRHAQIGIEALLKK
MPGVDLAVPIDQLVWRTRFQRRIPERLPVLW
>CYP121A1_Mycobacterium tuberculosis TKK_04_0022 2592227122
MTATVLLEVPFSARGDRIPDAVAELRTREPIRKVRTITGAEAWLVSSYALCTQVLEDRRFSMKETAAAGAPRL
NALTVPPEVVNNMGNIADAGLRKAVMKAITPKAPGLEQFLRDTANSLLDNLITEGAPADLRNDFADPLATALH
CKVLGIPQEDGPKLFRSLSIAFMSSADPIPAAKINWDRDIEYMAGILENPNITTGLMGELSRLRKDPAYSHVS
DELFATIGVTFFGAGVISTGSFLTTALISLIQRPQLRNLLHEKPELIPAGVEELLRINLSFADGLPRLATADI
QVGDVLVRKGELVLVLLEGANFDPEHFPNPGSIELDRPNPTSHLAFGRGQHFCPGSALGRRHAQIGIEALLKK
MPGVDLAVPIDQLVWRTRFQRRIPERLPVLW
>CYP121A1_Mycobacterium tuberculosis TKK_04_0024 2576078466
MTATVLLEVPFSARGDRIPDAVAELRTREPIRKVRTITGAEAWLVSSYALCTQVLEDRRFSMKETAAAGAPRL
NALTVPPEVVNNMGNIADAGLRKAVMKAITPKAPGLEQFLRDTANSLLDNLITEGAPADLRNDFADPLATALH
CKVLGIPQEDGPKLFRSLSIAFMSSADPIPAAKINWDRDIEYMAGILENPNITTGLMGELSRLRKDPAYSHVS
DELFATIGVTFFGAGVISTGSFLTTALISLIQRPQLRNLLHEKPELIPAGVEELLRINLSFADGLPRLATADI
QVGDVLVRKGELVLVLLEGANFDPEHFPNPGSIELDRPNPTSHLAFGRGQHFCPGSALGRRHAQIGIEALLKK
MPGVDLAVPIDQLVWRTRFQRRIPERLPVLW
>CYP121A1_Mycobacterium tuberculosis TKK_04_0029 2592578370
MTATVLLEVPFSARGDRIPDAVAELRTREPIRKVRTITGAEAWLVSSYALCTQVLEDRRFSMKETAAAGAPRL
NALTVPPEVVNNMGNIADAGLRKAVMKAITPKAPGLEQFLRDTANSLLDNLITEGAPADLRNDFADPLATALH
CKVLGIPQEDGPKLFRSLSIAFMSSADPIPAAKINWDRDIEYMAGILENPNITTGLMGELSRLRKDPAYSHVS
DELFATIGVTFFGAGVISTGSFLTTALISLIQRPQLRNLLHEKPELIPAGVEELLRINLSFADGLPRLATADI
QVGDVLVRKGELVLVLLEGANFDPEHFPNPGSIELDRPNPTSHLAFGRGQHFCPGSALGRRHAQIGIEALLKK
MPGVDLAVPIDQLVWRTRFQRRIPERLPVLW
>CYP121A1_Mycobacterium tuberculosis TKK_04_0030 2592575270
MTATVLLEVPFSARGDRIPDAVAELRTREPIRKVRTITGAEAWLVSSYALCTQVLEDRRFSMKETAAAGAPRL
NALTVPPEVVNNMGNIADAGLRKAVMKAITPKAPGLEQFLRDTANSLLDNLITEGAPADLRNDFADPLATALH
CKVLGIPQEDGPKLFRSLSIAFMSSADPIPAAKINWDRDIEYMAGILENPNITTGLMGELSRLRKDPAYSHVS
DELFATIGVTFFGAGVISTGSFLTTALISLIQRPQLRNLLHEKPELIPAGVEELLRINLSFADGLPRLATADI
QVGDVLVRKGELVLVLLEGANFDPEHFPNPGSIELDRPNPTSHLAFGRGQHFCPGSALGRRHAQIGIEALLKK
MPGVDLAVPIDQLVWRTRFQRRIPERLPVLW
>CYP121A1_Mycobacterium tuberculosis TKK_04_0031 2592571194
MTATVLLEVPFSARGDRIPDAVAELRTREPIRKVRTITGAEAWLVSSYALCTQVLEDRRFSMKETAAAGAPRL
NALTVPPEVVNNMGNIADAGLRKAVMKAITPKAPGLEQFLRDTANSLLDNLITEGAPADLRNDFADPLATALH

CKVLGIPQEDGPKLFRSLSIAFMSSADPIPAAKINWDRDIEYMAGILENPNITTGLMGELSRLRKDPAYSHVS
DELFATIGVTFFGAGVISTGSFLTTALISLIQRPQLRNLLHEKPELIPAGVEELLRINLSFADGLPRLATADI
QVGDVLVRKGELVLVLLEGANFDPEHFPNPGSIELDRPNPTSHLAFGRGQHFCPGSALGRRHAQIGIEALLKK
MPGVDLAVPIDQLVWRTRFQRRIPERLPVLW
>CYP121A1_Mycobacterium tuberculosis TKK_04_0034 2592567110
MTATVLLEVPFSARGDRIPDAVAELRTREPIRKVRTITGAEAWLVSSYALCTQVLEDRRFSMKETAAAGAPRL
NALTVPPEVVNNMGNIADAGLRKAVMKAITPKAPGLEQFLRDTANSLLDNLITEGAPADLRNDFADPLATALH
CKVLGIPQEDGPKLFRSLSIAFMSSADPIPAAKINWDRDIEYMAGILENPNITTGLMGELSRLRKDPAYSHVS
DELFATIGVTFFGAGVISTGSFLTTALISLIQRPQLRNLLHEKPELIPAGVEELLRINLSFADGLPRLATADI
QVGDVLVRKGELVLVLLEGANFDPEHFPNPGSIELDRPNPTSHLAFGRGQHFCPGSALGRRHAQIGIEALLKK
MPGVDLAVPIDQLVWRTRFQRRIPERLPVLW
>CYP121A1_Mycobacterium tuberculosis TKK_04_0036 2592563023
MTATVLLEVPFSARGDRIPDAVAELRTREPIRKVRTITGAEAWLVSSYALCTQVLEDRRFSMKETAAAGAPRL
NALTVPPEVVNNMGNIADAGLRKAVMKAITPKAPGLEQFLRDTANSLLDNLITEGAPADLRNDFADPLATALH
CKVLGIPQEDGPKLFRSLSIAFMSSADPIPAAKINWDRDIEYMAGILENPNITTGLMGELSRLRKDPAYSHVS
DELFATIGVTFFGAGVISTGSFLTTALISLIQRPQLRNLLHEKPELIPAGVEELLRINLSFADGLPRLATADI
QVGDVLVRKGELVLVLLEGANFDPEHFPNPGSIELDRPNPTSHLAFGRGQHFCPGSALGRRHAQIGIEALLKK
MPGVDLAVPIDQLVWRTRFQRRIPERLPVLW
>CYP121A1_Mycobacterium tuberculosis TKK_04_0037 2592558948
MTATVLLEVPFSARGDRIPDAVAELRTREPIRKVRTITGAEAWLVSSYALCTQVLEDRRFSMKETAAAGAPRL
NALTVPPEVVNNMGNIADAGLRKAVMKAITPKAPGLEQFLRDTANSLLDNLITEGAPADLRNDFADPLATALH
CKVLGIPQEDGPKLFRSLSIAFMSSADPIPAAKINWDRDIEYMAGILENPNITTGLMGELSRLRKDPAYSHVS
DELFATIGVTFFGAGVISTGSFLTTALISLIQRPQLRNLLHEKPELIPAGVEELLRINLSFADGLPRLATADI
QVGDVLVRKGELVLVLLEGANFDPEHFPNPGSIELDRPNPTSHLAFGRGQHFCPGSALGRRHAQIGIEALLKK
MPGVDLAVPIDQLVWRTRFQRRIPERLPVLW
>CYP121A1_Mycobacterium tuberculosis TKK_04_0038 2584837597
MTATVLLEVPFSARGDRIPDAVAELRTREPIRKVRTITGAEAWLVSSYALCTQVLEDRRFSMKETAAAGAPRL
NALTVPPEVVNNMGNIADAGLRKAVMKAITPKAPGLEQFLRDTANSLLDNLITEGAPADLRNDFADPLATALH
CKVLGIPQEDGPKLFRSLSIAFMSSADPIPAAKINWDRDIEYMAGILENPNITTGLMGELSRLRKDPAYSHVS
DELFATIGVTFFGAGVISTGSFLTTALISLIQRPQLRNLLHEKPELIPAGVEELLRINLSFADGLPRLATADI
QVGDVLVRKGELVLVLLEGANFDPEHFPNPGSIELDRPNPTSHLAFGRGQHFCPGSALGRRHAQIGIEALLKK
MPGVDLAVPIDQLVWRTRFQRRIPERLPVLW
>CYP121A1_Mycobacterium tuberculosis TKK_04_0039 2592554118
MTATVLLEVPFSARGDRIPDAVAELRTREPIRKVRTITGAEAWLVSSYALCTQVLEDRRFSMKETAAAGAPRL
NALTVPPEVVNNMGNIADAGLRKAVMKAITPKAPGLEQFLRDTANSLLDNLITEGAPADLRNDFADPLATALH
CKVLGIPQEDGPKLFRSLSIAFMSSADPIPAAKINWDRDIEYMAGILENPNITTGLMGELSRLRKDPAYSHVS
DELFATIGVTFFGAGVISTGSFLTTALISLIQRPQLRNLLHEKPELIPAGVEELLRINLSFADGLPRLATADI
QVGDVLVRKGELVLVLLEGANFDPEHFPNPGSIELDRPNPTSHLAFGRGQHFCPGSALGRRHAQIGIEALLKK
MPGVDLAVPIDQLVWRTRFQRRIPERLPVLW
>CYP121A1_Mycobacterium tuberculosis TKK_04_0040 2576941067
MTATVLLEVPFSARGDRIPDAVAELRTREPIRKVRTITGAEAWLVSSYALCTQVLEDRRFSMKETAAAGAPRL
NALTVPPEVVNNMGNIADAGLRKAVMKAITPKAPGLEQFLRDTANSLLDNLITEGAPADLRNDFADPLATALH
CKVLGIPQEDGPKLFRSLSIAFMSSADPIPAAKINWDRDIEYMAGILENPNITTGLMGELSRLRKDPAYSHVS
DELFATIGVTFFGAGVISTGSFLTTALISLIQRPQLRNLLHEKPELIPAGVEELLRINLSFADGLPRLATADI
QVGDVLVRKGELVLVLLEGANFDPEHFPNPGSIELDRPNPTSHLAFGRGQHFCPGSALGRRHAQIGIEALLKK
MPGVDLAVPIDQLVWRTRFQRRIPERLPVLW
>CYP121A1_Mycobacterium tuberculosis TKK_04_0042 2584907335
MTATVLLEVPFSARGDRIPDAVAELRTREPIRKVRTITGAEAWLVSSYALCTQVLEDRRFSMKETAAAGAPRL
NALTVPPEVVNNMGNIADAGLRKAVMKAITPKAPGLEQFLRDTANSLLDNLITEGAPADLRNDFADPLATALH
CKVLGIPQEDGPKLFRSLSIAFMSSADPIPAAKINWDRDIEYMAGILENPNITTGLMGELSRLRKDPAYSHVS
DELFATIGVTFFGAGVISTGSFLTTALISLIQRPQLRNLLHEKPELIPAGVEELLRINLSFADGLPRLATADI
QVGDVLVRKGELVLVLLEGANFDPEHFPNPGSIELDRPNPTSHLAFGRGQHFCPGSALGRRHAQIGIEALLKK
MPGVDLAVPIDQLVWRTRFQRRIPERLPVLW
>CYP121A1_Mycobacterium tuberculosis TKK_04_0043 2592549990
MTATVLLEVPFSARGDRIPDAVAELRTREPIRKVRTITGAEAWLVSSYALCTQVLEDRRFSMKETAAAGAPRL
NALTVPPEVVNNMGNIADAGLRKAVMKAITPKAPGLEQFLRDTANSLLDNLITEGAPADLRNDFADPLATALH
CKVLGIPQEDGPKLFRSLSIAFMSSADPIPAAKINWDRDIEYMAGILENPNITTGLMGELSRLRKDPAYSHVS
DELFATIGVTFFGAGVISTGSFLTTALISLIQRPQLRNLLHEKPELIPAGVEELLRINLSFADGLPRLATADI

QVGDVLVRKGELVLVLLEGANFDPEHFPNPGSIELDRPNPTSHLAFGRGQHFCPGSALGRRHAQIGIEALLKK
MPGVDLAVPIDQLVWRTRFQRRIPERLPVLW
>CYP121A1_Mycobacterium tuberculosis TKK_04_0044 2575936306
MTATVLLEVPFSARGDRIPDAVAELRTREPIRKVRTITGAEAWLVSSYALCTQVLEDRRFSMKETAAAGAPRL
NALTVPPEVVNNMGNIADAGLRKAVMKAITPKAPGLEQFLRDTANSLLDNLITEGAPADLRNDFADPLATALH
CKVLGIPQEDGPKLFRSLSIAFMSSADPIPAAKINWDRDIEYMAGILENPNITTGLMGELSRLRKDPAYSHVS
DELFATIGVTFFGAGVISTGSFLTTALISLIQRPQLRNLLHEKPELIPAGVEELLRINLSFADGLPRLATADI
QVGDVLVRKGELVLVLLEGANFDPEHFPNPGSIELDRPNPTSHLAFGRGQHFCPGSALGRRHAQIGIEALLKK
MPGVDLAVPIDQLVWRTRFQRRIPERLPVLW
>CYP121A1_Mycobacterium tuberculosis TKK_04_0045 2584858698
MTATVLLEVPFSARGDRIPDAVAELRTREPIRKVRTITGAEAWLVSSYALCTQVLEDRRFSMKETAAAGAPRL
NALTVPPEVVNNMGNIADAGLRKAVMKAITPKAPGLEQFLRDTANSLLDNLITEGAPADLRNDFADPLATALH
CKVLGIPQEDGPKLFRSLSIAFMSSADPIPAAKINWDRDIEYMAGILENPNITTGLMGELSRLRKDPAYSHVS
DELFATIGVTFFGAGVISTGSFLTTALISLIQRPQLRNLLHEKPELIPAGVEELLRINLSFADGLPRLATADI
QVGDVLVRKGELVLVLLEGANFDPEHFPNPGSIELDRPNPTSHLAFGRGQHFCPGSALGRRHAQIGIEALLKK
MPGVDLAVPIDQLVWRTRFQRRIPERLPVLW
>CYP121A1_Mycobacterium tuberculosis TKK_04_0046 2592546739
MTATVLLEVPFSARGDRIPDAVAELRTREPIRKVRTITGAEAWLVSSYALCTQVLEDRRFSMKETAAAGAPRL
NALTVPPEVVNNMGNIADAGLRKAVMKAITPKAPGLEQFLRDTANSLLDNLITEGAPADLRNDFADPLATALH
CKVLGIPQEDGPKLFRSLSIAFMSSADPIPAAKINWDRDIEYMAGILENPNITTGLMGELSRLRKDPAYSHVS
DELFATIGVTFFGAGVISTGSFLTTALISLIQRPQLRNLLHEKPELIPAGVEELLRINLSFADGLPRLATADI
QVGDVLVRKGELVLVLLEGANFDPEHFPNPGSIELDRPNPTSHLAFGRGQHFCPGSALGRRHAQIGIEALLKK
MPGVDLAVPIDQLVWRTRFQRRIPERLPVLW
>CYP121A1_Mycobacterium tuberculosis TKK_04_0047 2592542659
MTATVLLEVPFSARGDRIPDAVAELRTREPIRKVRTITGAEAWLVSSYALCTQVLEDRRFSMKETAAAGAPRL
NALTVPPEVVNNMGNIADAGLRKAVMKAITPKAPGLEQFLRDTANSLLDNLITEGAPADLRNDFADPLATALH
CKVLGIPQEDGPKLFRSLSIAFMSSADPIPAAKINWDRDIEYMAGILENPNITTGLMGELSRLRKDPAYSHVS
DELFATIGVTFFGAGVISTGSFLTTALISLIQRPQLRNLLHEKPELIPAGVEELLRINLSFADGLPRLATADI
QVGDVLVRKGELVLVLLEGANFDPEHFPNPGSIELDRPNPTSHLAFGRGQHFCPGSALGRRHAQIGIEALLKK
MPGVDLAVPIDQLVWRTRFQRRIPERLPVLW
>CYP121A1_Mycobacterium tuberculosis TKK_04_0048 2592538500
MTATVLLEVPFSARGDRIPDAVAELRTREPIRKVRTITGAEAWLVSSYALCTQVLEDRRFSMKETAAAGAPRL
NALTVPPEVVNNMGNIADAGLRKAVMKAITPKAPGLEQFLRDTANSLLDNLITEGAPADLRNDFADPLATALH
CKVLGIPQEDGPKLFRSLSIAFMSSADPIPAAKINWDRDIEYMAGILENPNITTGLMGELSRLRKDPAYSHVS
DELFATIGVTFFGAGVISTGSFLTTALISLIQRPQLRNLLHEKPELIPAGVEELLRINLSFADGLPRLATADI
QVGDVLVRKGELVLVLLEGANFDPEHFPNPGSIELDRPNPTSHLAFGRGQHFCPGSALGRRHAQIGIEALLKK
MPGVDLAVPIDQLVWRTRFQRRIPERLPVLW
>CYP121A1_Mycobacterium tuberculosis TKK_04_0051 2576379587
MTATVLLEVPFSARGDRIPDAVAELRTREPIRKVRTITGAEAWLVSSYALCTQVLEDRRFSMKETAAAGAPRL
NALTVPPEVVNNMGNIADAGLRKAVMKAITPKAPGLEQFLRDTANSLLDNLITEGAPADLRNDFADPLATALH
CKVLGIPQEDGPKLFRSLSIAFMSSADPIPAAKINWDRDIEYMAGILENPNITTGLMGELSRLRKDPAYSHVS
DELFATIGVTFFGAGVISTGSFLTTALISLIQRPQLRNLLHEKPELIPAGVEELLRINLSFADGLPRLATADI
QVGDVLVRKGELVLVLLEGANFDPEHFPNPGSIELDRPNPTSHLAFGRGQHFCPGSALGRRHAQIGIEALLKK
MPGVDLAVPIDQLVWRTRFQRRIPERLPVLW
>CYP121A1_Mycobacterium tuberculosis TKK_04_0059 2630635372
MTATVLLEVPFSARGDRIPDAVAELRTREPIRKVRTITGAEAWLVSSYALCTQVLEDRRFSMKETAAAGAPRL
NALTVPPEVVNNMGNIADAGLRKAVMKAITPKAPGLEQFLRDTANSLLDNLITEGAPADLRNDFADPLATALH
CKVLGIPQEDGPKLFRSLSIAFMSSADPIPAAKINWDRDIEYMAGILENPNITTGLMGELSRLRKDPAYSHVS
DELFATIGVTFFGAGVISTGSFLTTALISLIQRPQLRNLLHEKPELIPAGVEELLRINLSFADGLPRLATADI
QVGDVLVRKGELVLVLLEGANFDPEHFPNPGSIELDRPNPTSHLAFGRGQHFCPGSALGRRHAQIGIEALLKK
MPGVDLAVPIDQLVWRTRFQRRIPERLPVLW
>CYP121A1_Mycobacterium tuberculosis TKK_04_0060 2584841669
MTATVLLEVPFSARGDRIPDAVAELRTREPIRKVRTITGAEAWLVSSYALCTQVLEDRRFSMKETAAAGAPRL
NALTVPPEVVNNMGNIADAGLRKAVMKAITPKAPGLEQFLRDTANSLLDNLITEGAPADLRNDFADPLATALH
CKVLGIPQEDGPKLFRSLSIAFMSSADPIPAAKINWDRDIEYMAGILENPNITTGLMGELSRLRKDPAYSHVS
DELFATIGVTFFGAGVISTGSFLTTALISLIQRPQLRNLLHEKPELIPAGVEELLRINLSFADGLPRLATADI
QVGDVLVRKGELVLVLLEGANFDPEHFPNPGSIELDRPNPTSHLAFGRGQHFCPGSALGRRHAQIGIEALLKK
MPGVDLAVPIDQLVWRTRFQRRIPERLPVLW

>CYP121A1_Mycobacterium tuberculosis TKK_04_0062 2633629019
MTATVLLEVPFSARGDRIPDAVAELRTREPIRKVRTITGAEAWLVSSYALCTQVLEDRRFSMKETAAAGAPRL
NALTVPPEVVNNMGNIADAGLRKAVMKAITPKAPGLEQFLRDTANSLLDNLITEGAPADLRNDFADPLATALH
CKVLGIPQEDGPKLFRSLSIAFMSSADPIPAAKINWDRDIEYMAGILENPNITTGLMGELSRLRKDPAYSHVS
DELFATIGVTFFGAGVISTGSFLTTALISLIQRPQLRNLLHEKPELIPAGVEELLRINLSFADGLPRLATADI
QVGDVLVRKGELVLVLLEGANFDPEHFPNPGSIELDRPNPTSHLAFGRGQHFCPGSALGRRHAQIGIEALLKK
MPGVDLAVPIDQLVWRTRFQRRIPERLPVLW
>CYP121A1_Mycobacterium tuberculosis TKK_04_0063 2637013264
MTATVLLEVPFSARGDRIPDAVAELRTREPIRKVRTITGAEAWLVSSYALCTQVLEDRRFSMKETAAAGAPRL
NALTVPPEVVNNMGNIADAGLRKAVMKAITPKAPGLEQFLRDTANSLLDNLITEGAPADLRNDFADPLATALH
CKVLGIPQEDGPKLFRSLSIAFMSSADPIPAAKINWDRDIEYMAGILENPNITTGLMGELSRLRKDPAYSHVS
DELFATIGVTFFGAGVISTGSFLTTALISLIQRPQLRNLLHEKPELIPAGVEELLRINLSFADGLPRLATADI
QVGDVLVRKGELVLVLLEGANFDPEHFPNPGSIELDRPNPTSHLAFGRGQHFCPGSALGRRHAQIGIEALLKK
MPGVDLAVPIDQLVWRTRFQRRIPERLPVLW
>CYP121A1_Mycobacterium tuberculosis TKK_04_0064 2575921062
MTATVLLEVPFSARGDRIPDAVAELRTREPIRKVRTITGAEAWLVSSYALCTQVLEDRRFSMKETAAAGAPRL
NALTVPPEVVNNMGNIADAGLRKAVMKAITPKAPGLEQFLRDTANSLLDNLITEGAPADLRNDFADPLATALH
CKVLGIPQEDGPKLFRSLSIAFMSSADPIPAAKINWDRDIEYMAGILENPNITTGLMGELSRLRKDPAYSHVS
DELFATIGVTFFGAGVISTGSFLTTALISLIQRPQLRNLLHEKPELIPAGVEELLRINLSFADGLPRLATADI
QVGDVLVRKGELVLVLLEGANFDPEHFPNPGSIELDRPNPTSHLAFGRGQHFCPGSALGRRHAQIGIEALLKK
MPGVDLAVPIDQLVWRTRFQRRIPERLPVLW
>CYP121A1_Mycobacterium tuberculosis TKK_04_0066 2584695476
MTATVLLEVPFSARGDRIPDAVAELRTREPIRKVRTITGAEAWLVSSYALCTQVLEDRRFSMKETAAAGAPRL
NALTVPPEVVNNMGNIADAGLRKAVMKAITPKAPGLEQFLRDTANSLLDNLITEGAPADLRNDFADPLATALH
CKVLGIPQEDGPKLFRSLSIAFMSSADPIPAAKINWDRDIEYMAGILENPNITTGLMGELSRLRKDPAYSHVS
DELFATIGVTFFGAGVISTGSFLTTALISLIQRPQLRNLLHEKPELIPAGVEELLRINLSFADGLPRLATADI
QVGDVLVRKGELVLVLLEGANFDPEHFPNPGSIELDRPNPTSHLAFGRGQHFCPGSALGRRHAQIGIEALLKK
MPGVDLAVPIDQLVWRTRFQRRIPERLPVLW
>CYP121A1_Mycobacterium tuberculosis TKK_04_0067 2584979880
MTATVLLEVPFSARGDRIPDAVAELRTREPIRKVRTITGAEAWLVSSYALCTQVLEDRRFSMKETAAAGAPRL
NALTVPPEVVNNMGNIADAGLRKAVMKAITPKAPGLEQFLRDTANSLLDNLITEGAPADLRNDFADPLATALH
CKVLGIPQEDGPKLFRSLSIAFMSSADPIPAAKINWDRDIEYMAGILENPNITTGLMGELSRLRKDPAYSHVS
DELFATIGVTFFGAGVISTGSFLTTALISLIQRPQLRNLLHEKPELIPAGVEELLRINLSFADGLPRLATADI
QVGDVLVRKGELVLVLLEGANFDPEHFPNPGSIELDRPNPTSHLAFGRGQHFCPGSALGRRHAQIGIEALLKK
MPGVDLAVPIDQLVWRTRFQRRIPERLPVLW
>CYP121A1_Mycobacterium tuberculosis TKK_04_0068 2649200947
MTATVLLEVPFSARGDRIPDAVAELRTREPIRKVRTITGAEAWLVSSYALCTQVLEDRRFSMKETAAAGAPRL
NALTVPPEVVNNMGNIADAGLRKAVMKAITPKAPGLEQFLRDTANSLLDNLITEGAPADLRNDFADPLATALH
CKVLGIPQEDGPKLFRSLSIAFMSSADPIPAAKINWDRDIEYMAGILENPNITTGLMGELSRLRKDPAYSHVS
DELFATIGVTFFGAGVISTGSFLTTALISLIQRPQLRNLLHEKPELIPAGVEELLRINLSFADGLPRLATADI
QVGDVLVRKGELVLVLLEGANFDPEHFPNPGSIELDRPNPTSHLAFGRGQHFCPGSALGRRHAQIGIEALLKK
MPGVDLAVPIDQLVWRTRFQRRIPERLPVLW
>CYP121A1_Mycobacterium tuberculosis TKK_04_0069 2647569721
MTATVLLEVPFSARGDRIPDAVAELRTREPIRKVRTITGAEAWLVSSYALCTQVLEDRRFSMKETAAAGAPRL
NALTVPPEVVNNMGNIADAGLRKAVMKAITPKAPGLEQFLRDTANSLLDNLITEGAPADLRNDFADPLATALH
CKVLGIPQEDGPKLFRSLSIAFMSSADPIPAAKINWDRDIEYMAGILENPNITTGLMGELSRLRKDPAYSHVS
DELFATIGVTFFGAGVISTGSFLTTALISLIQRPQLRNLLHEKPELIPAGVEELLRINLSFADGLPRLATADI
QVGDVLVRKGELVLVLLEGANFDPEHFPNPGSIELDRPNPTSHLAFGRGQHFCPGSALGRRHAQIGIEALLKK
MPGVDLAVPIDQLVWRTRFQRRIPERLPVLW
>CYP121A1_Mycobacterium tuberculosis TKK_04_0070 2629710370
MTATVLLEVPFSARGDRIPDAVAELRTREPIRKVRTITGAEAWLVSSYALCTQVLEDRRFSMKETAAAGAPRL
NALTVPPEVVNNMGNIADAGLRKAVMKAITPKAPGLEQFLRDTANSLLDNLITEGAPADLRNDFADPLATALH
CKVLGIPQEDGPKLFRSLSIAFMSSADPIPAAKINWDRDIEYMAGILENPNITTGLMGELSRLRKDPAYSHVS
DELFATIGVTFFGAGVISTGSFLTTALISLIQRPQLRNLLHEKPELIPAGVEELLRINLSFADGLPRLATADI
QVGDVLVRKGELVLVLLEGANFDPEHFPNPGSIELDRPNPTSHLAFGRGQHFCPGSALGRRHAQIGIEALLKK
MPGVDLAVPIDQLVWRTRFQRRIPERLPVLW
>CYP121A1_Mycobacterium tuberculosis TKK_04_0071 2631963717

MTATVLLEVPFSARGDRIPDAVAELRTREPIRKVRTITGAEAWLVSSYALCTQVLEDRRFSMKETAAAGAPRL
NALTVPPEVVNNMGNIADAGLRKAVMKAITPKAPGLEQFLRDTANSLLDNLITEGAPADLRNDFADPLATALH
CKVLGIPQEDGPKLFRSLSIAFMSSADPIPAAKINWDRDIEYMAGILENPNITTGLMGELSRLRKDPAYSHVS
DELFATIGVTFFGAGVISTGSFLTTALISLIQRPQLRNLLHEKPELIPAGVEELLRINLSFADGLPRLATADI
QVGDVLVRKGELVLVLLEGANFDPEHFPNPGSIELDRPNPTSHLAFGRGQHFCPGSALGRRHAQIGIEALLKK
MPGVDLAVPIDQLVWRTRFQRRIPERLPVLW
>CYP121A1_Mycobacterium tuberculosis TKK_04_0072 2584703721
MTATVLLEVPFSARGDRIPDAVAELRTREPIRKVRTITGAEAWLVSSYALCTQVLEDRRFSMKETAAAGAPRL
NALTVPPEVVNNMGNIADAGLRKAVMKAITPKAPGLEQFLRDTANSLLDNLITEGAPADLRNDFADPLATALH
CKVLGIPQEDGPKLFRSLSIAFMSSADPIPAAKINWDRDIEYMAGILENPNITTGLMGELSRLRKDPAYSHVS
DELFATIGVTFFGAGVISTGSFLTTALISLIQRPQLRNLLHEKPELIPAGVEELLRINLSFADGLPRLATADI
QVGDVLVRKGELVLVLLEGANFDPEHFPNPGSIELDRPNPTSHLAFGRGQHFCPGSALGRRHAQIGIEALLKK
MPGVDLAVPIDQLVWRTRFQRRIPERLPVLW
>CYP121A1_Mycobacterium tuberculosis TKK_04_0074 2632878138
MTATVLLEVPFSARGDRIPDAVAELRTREPIRKVRTITGAEAWLVSSYALCTQVLEDRRFSMKETAAAGAPRL
NALTVPPEVVNNMGNIADAGLRKAVMKAITPKAPGLEQFLRDTANSLLDNLITEGAPADLRNDFADPLATALH
CKVLGIPQEDGPKLFRSLSIAFMSSADPIPAAKINWDRDIEYMAGILENPNITTGLMGELSRLRKDPAYSHVS
DELFATIGVTFFGAGVISTGSFLTTALISLIQRPQLRNLLHEKPELIPAGVEELLRINLSFADGLPRLATADI
QVGDVLVRKGELVLVLLEGANFDPEHFPNPGSIELDRPNPTSHLAFGRGQHFCPGSALGRRHAQIGIEALLKK
MPGVDLAVPIDQLVWRTRFQRRIPERLPVLW
>CYP121A1_Mycobacterium tuberculosis TKK_04_0078 2630530923
MTATVLLEVPFSARGDRIPDAVAELRTREPIRKVRTITGAEAWLVSSYALCTQVLEDRRFSMKETAAAGAPRL
NALTVPPEVVNNMGNIADAGLRKAVMKAITPKAPGLEQFLRDTANSLLDNLITEGAPADLRNDFADPLATALH
CKVLGIPQEDGPKLFRSLSIAFMSSADPIPAAKINWDRDIEYMAGILENPNITTGLMGELSRLRKDPAYSHVS
DELFATIGVTFFGAGVISTGSFLTTALISLIQRPQLRNLLHEKPELIPAGVEELLRINLSFADGLPRLATADI
QVGDVLVRKGELVLVLLEGANFDPEHFPNPGSIELDRPNPTSHLAFGRGQHFCPGSALGRRHAQIGIEALLKK
MPGVDLAVPIDQLVWRTRFQRRIPERLPVLW
>CYP121A1_Mycobacterium tuberculosis TKK_04_0079 2638053618
MTATVLLEVPFSARGDRIPDAVAELRTREPIRKVRTITGAEAWLVSSYALCTQVLEDRRFSMKETAAAGAPRL
NALTVPPEVVNNMGNIADAGLRKAVMKAITPKAPGLEQFLRDTANSLLDNLITEGAPADLRNDFADPLATALH
CKVLGIPQEDGPKLFRSLSIAFMSSADPIPAAKINWDRDIEYMAGILENPNITTGLMGELSRLRKDPAYSHVS
DELFATIGVTFFGAGVISTGSFLTTALISLIQRPQLRNLLHEKPELIPAGVEELLRINLSFADGLPRLATADI
QVGDVLVRKGELVLVLLEGANFDPEHFPNPGSIELDRPNPTSHLAFGRGQHFCPGSALGRRHAQIGIEALLKK
MPGVDLAVPIDQLVWRTRFQRRIPERLPVLW
>CYP121A1_Mycobacterium tuberculosis TKK_04_0080 2574734645
MTATVLLEVPFSARGDRIPDAVAELRTREPIRKVRTITGAEAWLVSSYALCTQVLEDRRFSMKETAAAGAPRL
NALTVPPEVVNNMGNIADAGLRKAVMKAITPKAPGLEQFLRDTANSLLDNLITEGAPADLRNDFADPLATALH
CKVLGIPQEDGPKLFRSLSIAFMSSADPIPAAKINWDRDIEYMAGILENPNITTGLMGELSRLRKDPAYSHVS
DELFATIGVTFFGAGVISTGSFLTTALISLIQRPQLRNLLHEKPELIPAGVEELLRINLSFADGLPRLATADI
QVGDVLVRKGELVLVLLEGANFDPEHFPNPGSIELDRPNPTSHLAFGRGQHFCPGSALGRRHAQIGIEALLKK
MPGVDLAVPIDQLVWRTRFQRRIPERLPVLW
>CYP121A1_Mycobacterium tuberculosis TKK_04_0081 2630849226
MTATVLLEVPFSARGDRIPDAVAELRTREPIRKVRTITGAEAWLVSSYALCTQVLEDRRFSMKETAAAGAPRL
NALTVPPEVVNNMGNIADAGLRKAVMKAITPKAPGLEQFLRDTANSLLDNLITEGAPADLRNDFADPLATALH
CKVLGIPQEDGPKLFRSLSIAFMSSADPIPAAKINWDRDIEYMAGILENPNITTGLMGELSRLRKDPAYSHVS
DELFATIGVTFFGAGVISTGSFLTTALISLIQRPQLRNLLHEKPELIPAGVEELLRINLSFADGLPRLATADI
QVGDVLVRKGELVLVLLEGANFDPEHFPNPGSIELDRPNPTSHLAFGRGQHFCPGSALGRRHAQIGIEALLKK
MPGVDLAVPIDQLVWRTRFQRRIPERLPVLW
>CYP121A1_Mycobacterium tuberculosis TKK_04_0082 2584758591
MTATVLLEVPFSARGDRIPDAVAELRTREPIRKVRTITGAEAWLVSSYALCTQVLEDRRFSMKETAAAGAPRL
NALTVPPEVVNNMGNIADAGLRKAVMKAITPKAPGLEQFLRDTANSLLDNLITEGAPADLRNDFADPLATALH
CKVLGIPQEDGPKLFRSLSIAFMSSADPIPAAKINWDRDIEYMAGILENPNITTGLMGELSRLRKDPAYSHVS
DELFATIGVTFFGAGVISTGSFLTTALISLIQRPQLRNLLHEKPELIPAGVEELLRINLSFADGLPRLATADI
QVGDVLVRKGELVLVLLEGANFDPEHFPNPGSIELDRPNPTSHLAFGRGQHFCPGSALGRRHAQIGIEALLKK
MPGVDLAVPIDQLVWRTRFQRRIPERLPVLW
>CYP121A1_Mycobacterium tuberculosis TKK_04_0083 2629196084
MTATVLLEVPFSARGDRIPDAVAELRTREPIRKVRTITGAEAWLVSSYALCTQVLEDRRFSMKETAAAGAPRL
NALTVPPEVVNNMGNIADAGLRKAVMKAITPKAPGLEQFLRDTANSLLDNLITEGAPADLRNDFADPLATALH

CKVLGIPQEDGPKLFRSLSIAFMSSADPIPAAKINWDRDIEYMAGILENPNITTGLMGELSRLRKDPAYSHVS
DELFATIGVTFFGAGVISTGSFLTTALISLIQRPQLRNLLHEKPELIPAGVEELLRINLSFADGLPRLATADI
QVGDVLVRKGELVLVLLEGANFDPEHFPNPGSIELDRPNPTSHLAFGRGQHFCPGSALGRRHAQIGIEALLKK
MPGVDLAVPIDQLVWRTRFQRRIPERLPVLW
>CYP121A1_Mycobacterium tuberculosis TKK_04_0085 2629669688
MTATVLLEVPFSARGDRIPDAVAELRTREPIRKVRTITGAEAWLVSSYALCTQVLEDRRFSMKETAAAGAPRL
NALTVPPEVVNNMGNIADAGLRKAVMKAITPKAPGLEQFLRDTANSLLDNLITEGAPADLRNDFADPLATALH
CKVLGIPQEDGPKLFRSLSIAFMSSADPIPAAKINWDRDIEYMAGILENPNITTGLMGELSRLRKDPAYSHVS
DELFATIGVTFFGAGVISTGSFLTTALISLIQRPQLRNLLHEKPELIPAGVEELLRINLSFADGLPRLATADI
QVGDVLVRKGELVLVLLEGANFDPEHFPNPGSIELDRPNPTSHLAFGRGQHFCPGSALGRRHAQIGIEALLKK
MPGVDLAVPIDQLVWRTRFQRRIPERLPVLW
>CYP121A1_Mycobacterium tuberculosis TKK_04_0090 2628511711
MTATVLLEVPFSARGDRIPDAVAELRTREPIRKVRTITGAEAWLVSSYALCTQVLEDRRFSMKETAAAGAPRL
NALTVPPEVVNNMGNIADAGLRKAVMKAITPKAPGLEQFLRDTANSLLDNLITEGAPADLRNDFADPLATALH
CKVLGIPQEDGPKLFRSLSIAFMSSADPIPAAKINWDRDIEYMAGILENPNITTGLMGELSRLRKDPAYSHVS
DELFATIGVTFFGAGVISTGSFLTTALISLIQRPQLRNLLHEKPELIPAGVEELLRINLSFADGLPRLATADI
QVGDVLVRKGELVLVLLEGANFDPEHFPNPGSIELDRPNPTSHLAFGRGQHFCPGSALGRRHAQIGIEALLKK
MPGVDLAVPIDQLVWRTRFQRRIPERLPVLW
>CYP121A1_Mycobacterium tuberculosis TKK_04_0094 2584974943
MTATVLLEVPFSARGDRIPDAVAELRTREPIRKVRTITGAEAWLVSSYALCTQVLEDRRFSMKETAAAGAPRL
NALTVPPEVVNNMGNIADAGLRKAVMKAITPKAPGLEQFLRDTANSLLDNLITEGAPADLRNDFADPLATALH
CKVLGIPQEDGPKLFRSLSIAFMSSADPIPAAKINWDRDIEYMAGILENPNITTGLMGELSRLRKDPAYSHVS
DELFATIGVTFFGAGVISTGSFLTTALISLIQRPQLRNLLHEKPELIPAGVEELLRINLSFADGLPRLATADI
QVGDVLVRKGELVLVLLEGANFDPEHFPNPGSIELDRPNPTSHLAFGRGQHFCPGSALGRRHAQIGIEALLKK
MPGVDLAVPIDQLVWRTRFQRRIPERLPVLW
>CYP121A1_Mycobacterium tuberculosis TKK_04_0096 2633262939
MTATVLLEVPFSARGDRIPDAVAELRTREPIRKVRTITGAEAWLVSSYALCTQVLEDRRFSMKETAAAGAPRL
NALTVPPEVVNNMGNIADAGLRKAVMKAITPKAPGLEQFLRDTANSLLDNLITEGAPADLRNDFADPLATALH
CKVLGIPQEDGPKLFRSLSIAFMSSADPIPAAKINWDRDIEYMAGILENPNITTGLMGELSRLRKDPAYSHVS
DELFATIGVTFFGAGVISTGSFLTTALISLIQRPQLRNLLHEKPELIPAGVEELLRINLSFADGLPRLATADI
QVGDVLVRKGELVLVLLEGANFDPEHFPNPGSIELDRPNPTSHLAFGRGQHFCPGSALGRRHAQIGIEALLKK
MPGVDLAVPIDQLVWRTRFQRRIPERLPVLW
>CYP121A1_Mycobacterium tuberculosis TKK_04_0097 2629448175
MTATVLLEVPFSARGDRIPDAVAELRTREPIRKVRTITGAEAWLVSSYALCTQVLEDRRFSMKETAAAGAPRL
NALTVPPEVVNNMGNIADAGLRKAVMKAITPKAPGLEQFLRDTANSLLDNLITEGAPADLRNDFADPLATALH
CKVLGIPQEDGPKLFRSLSIAFMSSADPIPAAKINWDRDIEYMAGILENPNITTGLMGELSRLRKDPAYSHVS
DELFATIGVTFFGAGVISTGSFLTTALISLIQRPQLRNLLHEKPELIPAGVEELLRINLSFADGLPRLATADI
QVGDVLVRKGELVLVLLEGANFDPEHFPNPGSIELDRPNPTSHLAFGRGQHFCPGSALGRRHAQIGIEALLKK
MPGVDLAVPIDQLVWRTRFQRRIPERLPVLW
>CYP121A1_Mycobacterium tuberculosis TKK_04_0099 2628930687
MTATVLLEVPFSARGDRIPDAVAELRTREPIRKVRTITGAEAWLVSSYALCTQVLEDRRFSMKETAAAGAPRL
NALTVPPEVVNNMGNIADAGLRKAVMKAITPKAPGLEQFLRDTANSLLDNLITEGAPADLRNDFADPLATALH
CKVLGIPQEDGPKLFRSLSIAFMSSADPIPAAKINWDRDIEYMAGILENPNITTGLMGELSRLRKDPAYSHVS
DELFATIGVTFFGAGVISTGSFLTTALISLIQRPQLRNLLHEKPELIPAGVEELLRINLSFADGLPRLATADI
QVGDVLVRKGELVLVLLEGANFDPEHFPNPGSIELDRPNPTSHLAFGRGQHFCPGSALGRRHAQIGIEALLKK
MPGVDLAVPIDQLVWRTRFQRRIPERLPVLW
>CYP121A1_Mycobacterium tuberculosis TKK_04_0103 2576247782
MTATVLLEVPFSARGDRIPDAVAELRTREPIRKVRTITGAEAWLVSSYALCTQVLEDRRFSMKETAAAGAPRL
NALTVPPEVVNNMGNIADAGLRKAVMKAITPKAPGLEQFLRDTANSLLDNLITEGAPADLRNDFADPLATALH
CKVLGIPQEDGPKLFRSLSIAFMSSADPIPAAKINWDRDIEYMAGILENPNITTGLMGELSRLRKDPAYSHVS
DELFATIGVTFFGAGVISTGSFLTTALISLIQRPQLRNLLHEKPELIPAGVEELLRINLSFADGLPRLATADI
QVGDVLVRKGELVLVLLEGANFDPEHFPNPGSIELDRPNPTSHLAFGRGQHFCPGSALGRRHAQIGIEALLKK
MPGVDLAVPIDQLVWRTRFQRRIPERLPVLW
>CYP121A1_Mycobacterium tuberculosis TKK_04_0104 2636778557
MTATVLLEVPFSARGDRIPDAVAELRTREPIRKVRTITGAEAWLVSSYALCTQVLEDRRFSMKETAAAGAPRL
NALTVPPEVVNNMGNIADAGLRKAVMKAITPKAPGLEQFLRDTANSLLDNLITEGAPADLRNDFADPLATALH
CKVLGIPQEDGPKLFRSLSIAFMSSADPIPAAKINWDRDIEYMAGILENPNITTGLMGELSRLRKDPAYSHVS
DELFATIGVTFFGAGVISTGSFLTTALISLIQRPQLRNLLHEKPELIPAGVEELLRINLSFADGLPRLATADI

QVGDVLVRKGELVLVLLEGANFDPEHFPNPGSIELDRPNPTSHLAFGRGQHFCPGSALGRRHAQIGIEALLKK
MPGVDLAVPIDQLVWRTRFQRRIPERLPVLW
>CYP121A1_Mycobacterium tuberculosis TKK_04_0105 2632883343
MTATVLLEVPFSARGDRIPDAVAELRTREPIRKVRTITGAEAWLVSSYALCTQVLEDRRFSMKETAAAGAPRL
NALTVPPEVVNNMGNIADAGLRKAVMKAITPKAPGLEQFLRDTANSLLDNLITEGAPADLRNDFADPLATALH
CKVLGIPQEDGPKLFRSLSIAFMSSADPIPAAKINWDRDIEYMAGILENPNITTGLMGELSRLRKDPAYSHVS
DELFATIGVTFFGAGVISTGSFLTTALISLIQRPQLRNLLHEKPELIPAGVEELLRINLSFADGLPRLATADI
QVGDVLVRKGELVLVLLEGANFDPEHFPNPGSIELDRPNPTSHLAFGRGQHFCPGSALGRRHAQIGIEALLKK
MPGVDLAVPIDQLVWRTRFQRRIPERLPVLW
>CYP121A1_Mycobacterium tuberculosis TKK_04_0106 2640717187
MTATVLLEVPFSARGDRIPDAVAELRTREPIRKVRTITGAEAWLVSSYALCTQVLEDRRFSMKETAAAGAPRL
NALTVPPEVVNNMGNIADAGLRKAVMKAITPKAPGLEQFLRDTANSLLDNLITEGAPADLRNDFADPLATALH
CKVLGIPQEDGPKLFRSLSIAFMSSADPIPAAKINWDRDIEYMAGILENPNITTGLMGELSRLRKDPAYSHVS
DELFATIGVTFFGAGVISTGSFLTTALISLIQRPQLRNLLHEKPELIPAGVEELLRINLSFADGLPRLATADI
QVGDVLVRKGELVLVLLEGANFDPEHFPNPGSIELDRPNPTSHLAFGRGQHFCPGSALGRRHAQIGIEALLKK
MPGVDLAVPIDQLVWRTRFQRRIPERLPVLW
>CYP121A1_Mycobacterium tuberculosis TKK_04_0108 2576669585
MTATVLLEVPFSARGDRIPDAVAELRTREPIRKVRTITGAEAWLVSSYALCTQVLEDRRFSMKETAAAGAPRL
NALTVPPEVVNNMGNIADAGLRKAVMKAITPKAPGLEQFLRDTANSLLDNLITEGAPADLRNDFADPLATALH
CKVLGIPQEDGPKLFRSLSIAFMSSADPIPAAKINWDRDIEYMAGILENPNITTGLMGELSRLRKDPAYSHVS
DELFATIGVTFFGAGVISTGSFLTTALISLIQRPQLRNLLHEKPELIPAGVEELLRINLSFADGLPRLATADI
QVGDVLVRKGELVLVLLEGANFDPEHFPNPGSIELDRPNPTSHLAFGRGQHFCPGSALGRRHAQIGIEALLKK
MPGVDLAVPIDQLVWRTRFQRRIPERLPVLW
>CYP121A1_Mycobacterium tuberculosis TKK_04_0109 2628661987
MTATVLLEVPFSARGDRIPDAVAELRTREPIRKVRTITGAEAWLVSSYALCTQVLEDRRFSMKETAAAGAPRL
NALTVPPEVVNNMGNIADAGLRKAVMKAITPKAPGLEQFLRDTANSLLDNLITEGAPADLRNDFADPLATALH
CKVLGIPQEDGPKLFRSLSIAFMSSADPIPAAKINWDRDIEYMAGILENPNITTGLMGELSRLRKDPAYSHVS
DELFATIGVTFFGAGVISTGSFLTTALISLIQRPQLRNLLHEKPELIPAGVEELLRINLSFADGLPRLATADI
QVGDVLVRKGELVLVLLEGANFDPEHFPNPGSIELDRPNPTSHLAFGRGQHFCPGSALGRRHAQIGIEALLKK
MPGVDLAVPIDQLVWRTRFQRRIPERLPVLW
>CYP121A1_Mycobacterium tuberculosis TKK_04_0112 2631501410
MTATVLLEVPFSARGDRIPDAVAELRTREPIRKVRTITGAEAWLVSSYALCTQVLEDRRFSMKETAAAGAPRL
NALTVPPEVVNNMGNIADAGLRKAVMKAITPKAPGLEQFLRDTANSLLDNLITEGAPADLRNDFADPLATALH
CKVLGIPQEDGPKLFRSLSIAFMSSADPIPAAKINWDRDIEYMAGILENPNITTGLMGELSRLRKDPAYSHVS
DELFATIGVTFFGAGVISTGSFLTTALISLIQRPQLRNLLHEKPELIPAGVEELLRINLSFADGLPRLATADI
QVGDVLVRKGELVLVLLEGANFDPEHFPNPGSIELDRPNPTSHLAFGRGQHFCPGSALGRRHAQIGIEALLKK
MPGVDLAVPIDQLVWRTRFQRRIPERLPVLW
>CYP121A1_Mycobacterium tuberculosis TKK_04_0113 2630865645
MTATVLLEVPFSARGDRIPDAVAELRTREPIRKVRTITGAEAWLVSSYALCTQVLEDRRFSMKETAAAGAPRL
NALTVPPEVVNNMGNIADAGLRKAVMKAITPKAPGLEQFLRDTANSLLDNLITEGAPADLRNDFADPLATALH
CKVLGIPQEDGPKLFRSLSIAFMSSADPIPAAKINWDRDIEYMAGILENPNITTGLMGELSRLRKDPAYSHVS
DELFATIGVTFFGAGVISTGSFLTTALISLIQRPQLRNLLHEKPELIPAGVEELLRINLSFADGLPRLATADI
QVGDVLVRKGELVLVLLEGANFDPEHFPNPGSIELDRPNPTSHLAFGRGQHFCPGSALGRRHAQIGIEALLKK
MPGVDLAVPIDQLVWRTRFQRRIPERLPVLW
>CYP121A1_Mycobacterium tuberculosis TKK_04_0117 2576666346
MTATVLLEVPFSARGDRIPDAVAELRTREPIRKVRTITGAEAWLVSSYALCTQVLEDRRFSMKETAAAGAPRL
NALTVPPEVVNNMGNIADAGLRKAVMKAITPKAPGLEQFLRDTANSLLDNLITEGAPADLRNDFADPLATALH
CKVLGIPQEDGPKLFRSLSIAFMSSADPIPAAKINWDRDIEYMAGILENPNITTGLMGELSRLRKDPAYSHVS
DELFATIGVTFFGAGVISTGSFLTTALISLIQRPQLRNLLHEKPELIPAGVEELLRINLSFADGLPRLATADI
QVGDVLVRKGELVLVLLEGANFDPEHFPNPGSIELDRPNPTSHLAFGRGQHFCPGSALGRRHAQIGIEALLKK
MPGVDLAVPIDQLVWRTRFQRRIPERLPVLW
>CYP121A1_Mycobacterium tuberculosis TKK_04_0118 2646189196
MTATVLLEVPFSARGDRIPDAVAELRTREPIRKVRTITGAEAWLVSSYALCTQVLEDRRFSMKETAAAGAPRL
NALTVPPEVVNNMGNIADAGLRKAVMKAITPKAPGLEQFLRDTANSLLDNLITEGAPADLRNDFADPLATALH
CKVLGIPQEDGPKLFRSLSIAFMSSADPIPAAKINWDRDIEYMAGILENPNITTGLMGELSRLRKDPAYSHVS
DELFATIGVTFFGAGVISTGSFLTTALISLIQRPQLRNLLHEKPELIPAGVEELLRINLSFADGLPRLATADI
QVGDVLVRKGELVLVLLEGANFDPEHFPNPGSIELDRPNPTSHLAFGRGQHFCPGSALGRRHAQIGIEALLKK
MPGVDLAVPIDQLVWRTRFQRRIPERLPVLW

>CYP121A1_Mycobacterium tuberculosis TKK_04_0120 2574638910
MTATVLLEVPFSARGDRIPDAVAELRTREPIRKVRTITGAEAWLVSSYALCTQVLEDRRFSMKETAAAGAPRL
NALTVPPEVVNNMGNIADAGLRKAVMKAITPKAPGLEQFLRDTANSLLDNLITEGAPADLRNDFADPLATALH
CKVLGIPQEDGPKLFRSLSIAFMSSADPIPAAKINWDRDIEYMAGILENPNITTGLMGELSRLRKDPAYSHVS
DELFATIGVTFFGAGVISTGSFLTTALISLIQRPQLRNLLHEKPELIPAGVEELLRINLSFADGLPRLATADI
QVGDVLVRKGELVLVLLEGANFDPEHFPNPGSIELDRPNPTSHLAFGRGQHFCPGSALGRRHAQIGIEALLKK
MPGVDLAVPIDQLVWRTRFQRRIPERLPVLW
>CYP121A1_Mycobacterium tuberculosis TKK_04_0122 2630307500
MTATVLLEVPFSARGDRIPDAVAELRTREPIRKVRTITGAEAWLVSSYALCTQVLEDRRFSMKETAAAGAPRL
NALTVPPEVVNNMGNIADAGLRKAVMKAITPKAPGLEQFLRDTANSLLDNLITEGAPADLRNDFADPLATALH
CKVLGIPQEDGPKLFRSLSIAFMSSADPIPAAKINWDRDIEYMAGILENPNITTGLMGELSRLRKDPAYSHVS
DELFATIGVTFFGAGVISTGSFLTTALISLIQRPQLRNLLHEKPELIPAGVEELLRINLSFADGLPRLATADI
QVGDVLVRKGELVLVLLEGANFDPEHFPNPGSIELDRPNPTSHLAFGRGQHFCPGSALGRRHAQIGIEALLKK
MPGVDLAVPIDQLVWRTRFQRRIPERLPVLW
>CYP121A1_Mycobacterium tuberculosis TKK_04_0123 2630355249
MTATVLLEVPFSARGDRIPDAVAELRTREPIRKVRTITGAEAWLVSSYALCTQVLEDRRFSMKETAAAGAPRL
NALTVPPEVVNNMGNIADAGLRKAVMKAITPKAPGLEQFLRDTANSLLDNLITEGAPADLRNDFADPLATALH
CKVLGIPQEDGPKLFRSLSIAFMSSADPIPAAKINWDRDIEYMAGILENPNITTGLMGELSRLRKDPAYSHVS
DELFATIGVTFFGAGVISTGSFLTTALISLIQRPQLRNLLHEKPELIPAGVEELLRINLSFADGLPRLATADI
QVGDVLVRKGELVLVLLEGANFDPEHFPNPGSIELDRPNPTSHLAFGRGQHFCPGSALGRRHAQIGIEALLKK
MPGVDLAVPIDQLVWRTRFQRRIPERLPVLW
>CYP121A1_Mycobacterium tuberculosis TKK_04_0124 2656051490
MTATVLLEVPFSARGDRIPDAVAELRTREPIRKVRTITGAEAWLVSSYALCTQVLEDRRFSMKETAAAGAPRL
NALTVPPEVVNNMGNIADAGLRKAVMKAITPKAPGLEQFLRDTANSLLDNLITEGAPADLRNDFADPLATALH
CKVLGIPQEDGPKLFRSLSIAFMSSADPIPAAKINWDRDIEYMAGILENPNITTGLMGELSRLRKDPAYSHVS
DELFATIGVTFFGAGVISTGSFLTTALISLIQRPQLRNLLHEKPELIPAGVEELLRINLSFADGLPRLATADI
QVGDVLVRKGELVLVLLEGANFDPEHFPNPGSIELDRPNPTSHLAFGRGQHFCPGSALGRRHAQIGIEALLKK
MPGVDLAVPIDQLVWRTRFQRRIPERLPVLW
>CYP121A1_Mycobacterium tuberculosis TKK_04_0125 2652672083
MTATVLLEVPFSARGDRIPDAVAELRTREPIRKVRTITGAEAWLVSSYALCTQVLEDRRFSMKETAAAGAPRL
NALTVPPEVVNNMGNIADAGLRKAVMKAITPKAPGLEQFLRDTANSLLDNLITEGAPADLRNDFADPLATALH
CKVLGIPQEDGPKLFRSLSIAFMSSADPIPAAKINWDRDIEYMAGILENPNITTGLMGELSRLRKDPAYSHVS
DELFATIGVTFFGAGVISTGSFLTTALISLIQRPQLRNLLHEKPELIPAGVEELLRINLSFADGLPRLATADI
QVGDVLVRKGELVLVLLEGANFDPEHFPNPGSIELDRPNPTSHLAFGRGQHFCPGSALGRRHAQIGIEALLKK
MPGVDLAVPIDQLVWRTRFQRRIPERLPVLW
>CYP121A1_Mycobacterium tuberculosis TKK_04_0129 2584777042
MTATVLLEVPFSARGDRIPDAVAELRTREPIRKVRTITGAEAWLVSSYALCTQVLEDRRFSMKETAAAGAPRL
NALTVPPEVVNNMGNIADAGLRKAVMKAITPKAPGLEQFLRDTANSLLDNLITEGAPADLRNDFADPLATALH
CKVLGIPQEDGPKLFRSLSIAFMSSADPIPAAKINWDRDIEYMAGILENPNITTGLMGELSRLRKDPAYSHVS
DELFATIGVTFFGAGVISTGSFLTTALISLIQRPQLRNLLHEKPELIPAGVEELLRINLSFADGLPRLATADI
QVGDVLVRKGELVLVLLEGANFDPEHFPNPGSIELDRPNPTSHLAFGRGQHFCPGSALGRRHAQIGIEALLKK
MPGVDLAVPIDQLVWRTRFQRRIPERLPVLW
>CYP121A1_Mycobacterium tuberculosis TKK_04_0130 2632001966
MTATVLLEVPFSARGDRIPDAVAELRTREPIRKVRTITGAEAWLVSSYALCTQVLEDRRFSMKETAAAGAPRL
NALTVPPEVVNNMGNIADAGLRKAVMKAITPKAPGLEQFLRDTANSLLDNLITEGAPADLRNDFADPLATALH
CKVLGIPQEDGPKLFRSLSIAFMSSADPIPAAKINWDRDIEYMAGILENPNITTGLMGELSRLRKDPAYSHVS
DELFATIGVTFFGAGVISTGSFLTTALISLIQRPQLRNLLHEKPELIPAGVEELLRINLSFADGLPRLATADI
QVGDVLVRKGELVLVLLEGANFDPEHFPNPGSIELDRPNPTSHLAFGRGQHFCPGSALGRRHAQIGIEALLKK
MPGVDLAVPIDQLVWRTRFQRRIPERLPVLW
>CYP121A1_Mycobacterium tuberculosis TKK_04_0132 2577516686
MTATVLLEVPFSARGDRIPDAVAELRTREPIRKVRTITGAEAWLVSSYALCTQVLEDRRFSMKETAAAGAPRL
NALTVPPEVVNNMGNIADAGLRKAVMKAITPKAPGLEQFLRDTANSLLDNLITEGAPADLRNDFADPLATALH
CKVLGIPQEDGPKLFRSLSIAFMSSADPIPAAKINWDRDIEYMAGILENPNITTGLMGELSRLRKDPAYSHVS
DELFATIGVTFFGAGVISTGSFLTTALISLIQRPQLRNLLHEKPELIPAGVEELLRINLSFADGLPRLATADI
QVGDVLVRKGELVLVLLEGANFDPEHFPNPGSIELDRPNPTSHLAFGRGQHFCPGSALGRRHAQIGIEALLKK
MPGVDLAVPIDQLVWRTRFQRRIPERLPVLW
>CYP121A1_Mycobacterium tuberculosis TKK_04_0137 2621795768

MTATVLLEVPFSARGDRIPDAVAELRTREPIRKVRTITGAEAWLVSSYALCTQVLEDRRFSMKETAAAGAPRL
NALTVPPEVVNNMGNIADAGLRKAVMKAITPKAPGLEQFLRDTANSLLDNLITEGAPADLRNDFADPLATALH
CKVLGIPQEDGPKLFRSLSIAFMSSADPIPAAKINWDRDIEYMAGILENPNITTGLMGELSRLRKDPAYSHVS
DELFATIGVTFFGAGVISTGSFLTTALISLIQRPQLRNLLHEKPELIPAGVEELLRINLSFADGLPRLATADI
QVGDVLVRKGELVLVLLEGANFDPEHFPNPGSIELDRPNPTSHLAFGRGQHFCPGSALGRRHAQIGIEALLKK
MPGVDLAVPIDQLVWRTRFQRRIPERLPVLW
>CYP121A1_Mycobacterium tuberculosis TKK_04_0139 2621766315
MTATVLLEVPFSARGDRIPDAVAELRTREPIRKVRTITGAEAWLVSSYALCTQVLEDRRFSMKETAAAGAPRL
NALTVPPEVVNNMGNIADAGLRKAVMKAITPKAPGLEQFLRDTANSLLDNLITEGAPADLRNDFADPLATALH
CKVLGIPQEDGPKLFRSLSIAFMSSADPIPAAKINWDRDIEYMAGILENPNITTGLMGELSRLRKDPAYSHVS
DELFATIGVTFFGAGVISTGSFLTTALISLIQRPQLRNLLHEKPELIPAGVEELLRINLSFADGLPRLATADI
QVGDVLVRKGELVLVLLEGANFDPEHFPNPGSIELDRPNPTSHLAFGRGQHFCPGSALGRRHAQIGIEALLKK
MPGVDLAVPIDQLVWRTRFQRRIPERLPVLW
>CYP121A1_Mycobacterium tuberculosis TKK_04_0140 2576495121
MTATVLLEVPFSARGDRIPDAVAELRTREPIRKVRTITGAEAWLVSSYALCTQVLEDRRFSMKETAAAGAPRL
NALTVPPEVVNNMGNIADAGLRKAVMKAITPKAPGLEQFLRDTANSLLDNLITEGAPADLRNDFADPLATALH
CKVLGIPQEDGPKLFRSLSIAFMSSADPIPAAKINWDRDIEYMAGILENPNITTGLMGELSRLRKDPAYSHVS
DELFATIGVTFFGAGVISTGSFLTTALISLIQRPQLRNLLHEKPELIPAGVEELLRINLSFADGLPRLATADI
QVGDVLVRKGELVLVLLEGANFDPEHFPNPGSIELDRPNPTSHLAFGRGQHFCPGSALGRRHAQIGIEALLKK
MPGVDLAVPIDQLVWRTRFQRRIPERLPVLW
>CYP121A1_Mycobacterium tuberculosis TKK_04_0141 2621770178
MTATVLLEVPFSARGDRIPDAVAELRTREPIRKVRTITGAEAWLVSSYALCTQVLEDRRFSMKETAAAGAPRL
NALTVPPEVVNNMGNIADAGLRKAVMKAITPKAPGLEQFLRDTANSLLDNLITEGAPADLRNDFADPLATALH
CKVLGIPQEDGPKLFRSLSIAFMSSADPIPAAKINWDRDIEYMAGILENPNITTGLMGELSRLRKDPAYSHVS
DELFATIGVTFFGAGVISTGSFLTTALISLIQRPQLRNLLHEKPELIPAGVEELLRINLSFADGLPRLATADI
QVGDVLVRKGELVLVLLEGANFDPEHFPNPGSIELDRPNPTSHLAFGRGQHFCPGSALGRRHAQIGIEALLKK
MPGVDLAVPIDQLVWRTRFQRRIPERLPVLW
>CYP121A1_Mycobacterium tuberculosis TKK_04_0145 2621774610
MTATVLLEVPFSARGDRIPDAVAELRTREPIRKVRTITGAEAWLVSSYALCTQVLEDRRFSMKETAAAGAPRL
NALTVPPEVVNNMGNIADAGLRKAVMKAITPKAPGLEQFLRDTANSLLDNLITEGAPADLRNDFADPLATALH
CKVLGIPQEDGPKLFRSLSIAFMSSADPIPAAKINWDRDIEYMAGILENPNITTGLMGELSRLRKDPAYSHVS
DELFATIGVTFFGAGVISTGSFLTTALISLIQRPQLRNLLHEKPELIPAGVEELLRINLSFADGLPRLATADI
QVGDVLVRKGELVLVLLEGANFDPEHFPNPGSIELDRPNPTSHLAFGRGQHFCPGSALGRRHAQIGIEALLKK
MPGVDLAVPIDQLVWRTRFQRRIPERLPVLW
>CYP121A1_Mycobacterium tuberculosis TKK_04_0148 2621779502
MTATVLLEVPFSARGDRIPDAVAELRTREPIRKVRTITGAEAWLVSSYALCTQVLEDRRFSMKETAAAGAPRL
NALTVPPEVVNNMGNIADAGLRKAVMKAITPKAPGLEQFLRDTANSLLDNLITEGAPADLRNDFADPLATALH
CKVLGIPQEDGPKLFRSLSIAFMSSADPIPAAKINWDRDIEYMAGILENPNITTGLMGELSRLRKDPAYSHVS
DELFATIGVTFFGAGVISTGSFLTTALISLIQRPQLRNLLHEKPELIPAGVEELLRINLSFADGLPRLATADI
QVGDVLVRKGELVLVLLEGANFDPEHFPNPGSIELDRPNPTSHLAFGRGQHFCPGSALGRRHAQIGIEALLKK
MPGVDLAVPIDQLVWRTRFQRRIPERLPVLW
>CYP121A1_Mycobacterium tuberculosis TKK_04_0150 2621787612
MTATVLLEVPFSARGDRIPDAVAELRTREPIRKVRTITGAEAWLVSSYALCTQVLEDRRFSMKETAAAGAPRL
NALTVPPEVVNNMGNIADAGLRKAVMKAITPKAPGLEQFLRDTANSLLDNLITEGAPADLRNDFADPLATALH
CKVLGIPQEDGPKLFRSLSIAFMSSADPIPAAKINWDRDIEYMAGILENPNITTGLMGELSRLRKDPAYSHVS
DELFATIGVTFFGAGVISTGSFLTTALISLIQRPQLRNLLHEKPELIPAGVEELLRINLSFADGLPRLATADI
QVGDVLVRKGELVLVLLEGANFDPEHFPNPGSIELDRPNPTSHLAFGRGQHFCPGSALGRRHAQIGIEALLKK
MPGVDLAVPIDQLVWRTRFQRRIPERLPVLW
>CYP121A1_Mycobacterium tuberculosis TKK_04_0153 2621790192
[truncated: 473,532 more chars]
